# Severe COVID-19 Is Characterised by Perturbations in Plasma Amines Correlated with Immune Response Markers, and Linked to Inflammation and Oxidative Stress

**DOI:** 10.3390/metabo12070618

**Published:** 2022-07-02

**Authors:** Naama Karu, Alida Kindt, Adriaan J. van Gammeren, Anton A. M. Ermens, Amy C. Harms, Lutzen Portengen, Roel C. H. Vermeulen, Willem A. Dik, Anton W. Langerak, Vincent H. J. van der Velden, Thomas Hankemeier

**Affiliations:** 1 Metabolomics and Analytics Centre, Leiden Academic Centre for Drug Research, Leiden University, 2333 CC Leiden, The Netherlands; a.s.d.kindt@lacdr.leidenuniv.nl (A.K.); a.c.harms@lacdr.leidenuniv.nl (A.C.H.); 2 Department of Clinical Chemistry and Hematology, Amphia Hospital, 4818 CK Breda, The Netherlands; avangammeren@amphia.nl (A.J.v.G.); aamermens@gmail.com (A.A.M.E.); 3 Department of Population Health Sciences, Institute for Risk Assessment Sciences, University Utrecht, 3584 CK Utrecht, The Netherlands; l.portengen@uu.nl (L.P.); r.c.h.vermeulen@uu.nl (R.C.H.V.); 4 Laboratory Medical Immunology, Department of Immunology, Erasmus MC University Medical Center Rotterdam, 3015 GD Rotterdam, The Netherlands; w.dik@erasmusmc.nl (W.A.D.); a.langerak@erasmusmc.nl (A.W.L.); v.h.j.vandervelden@erasmusmc.nl (V.H.J.v.d.V.)

**Keywords:** SARS-CoV-2, COVID-19, amino acid, amine, metabolomics, cytokine, inflammation, oxidative stress

## Abstract

The COVID-19 pandemic raised a need to characterise the biochemical response to SARS-CoV-2 infection and find biological markers to identify therapeutic targets. In support of these aims, we applied a range of LC-MS platforms to analyse over 100 plasma samples from patients with varying COVID-19 severity and with detailed clinical information on inflammatory responses (>30 immune markers). The first publication in a series reports the results of quantitative LC-MS/MS profiling of 56 amino acids and derivatives. A comparison between samples taken from ICU and ward patients revealed a notable increase in ten post-translationally modified amino acids that correlated with markers indicative of an excessive immune response: TNF-alpha, neutrophils, markers for macrophage, and leukocyte activation. Severe patients also had increased kynurenine, positively correlated with CRP and cytokines that induce its production. ICU and ward patients with high IL-6 showed decreased levels of 22 immune-supporting and anti-oxidative amino acids and derivatives (e.g., glutathione, GABA). These negatively correlated with CRP and IL-6 and positively correlated with markers indicative of adaptive immune activation. Including corresponding alterations in convalescing ward patients, the overall metabolic picture of severe COVID-19 reflected enhanced metabolic demands to maintain cell proliferation and redox balance, alongside increased inflammation and oxidative stress.

## 1. Introduction

Severe acute respiratory syndrome coronavirus 2 (SARS-CoV-2) caused a worldwide pandemic following the first known cases in late 2019 in China. The mechanisms by which SARS-CoV-2 infects the human body have been established [1], yet a major observation is that the response to infection varies greatly [2,3]. A predisposition toward severe COVID-19 and mortality includes older age, male sex, and underlying metabolic conditions such as diabetes, obesity, hypertension, chronic kidney disease, cardiovascular and respiratory diseases [4,5,6]. The higher risk may stem from a compromised immune system, pre-existing endothelial dysfunction, an environment of chronic inflammation and oxidative stress [7,8], enhanced expression and activity of angiotensin-converting enzyme (ACE) 2, dyslipidemia, and lower cardiorespiratory fitness [9,10,11,12,13,14]. The disease progression is commonly described based on lung function (mainly acute respiratory distress syndrome, ARDS) and specific complications such as thrombosis or multi-organ dysfunction. Unfortunately, the turning point from mild pneumonia to critical illness is poorly defined, warranting efforts to characterise it and further improve the clinical response.

The immunopathology of COVID-19 allows for the monitoring of patient status, for example, via increases in the hepatic C-reactive protein (CRP), ferritin, D-dimer, and cytokines such as interleukin-6 (IL-6) [15], or via the depletion of lymphocytes and specifically CD4+T cells [1,16,17]. Complementary information on disease progression can be obtained from transcriptomics, proteomics, and metabolomics analyses (targeted or untargeted). These technologies collectively revealed perturbations in the innate immune response, energy metabolism, and metabolism of lipids, amino acids, aminosugars, purines, and nucleotides [18,19,20,21,22,23,24,25]. The metabolic alterations were related to the negative effects of inflammation and disruptions in energy production and hepatic function.

Here we investigated the relationship between amino acid metabolism and immune response in COVID-19 patients. We applied targeted metabolomics analysis of 56 amines in over 100 plasma samples taken in early 2020 from COVID-19 patients at varying disease states (95% of whom were not treated with corticosteroids). The quantified metabolites were further correlated with over 30 immune response markers (leukocytes, cytokines, and others) that have been obtained for the same cohort, indicating increased innate immune activation in severe COVID-19 [26]. The results of the current study underwent comprehensive biochemical interpretation to support future research on COVID-19 and promote early intervention or personalised treatment.

## 2. Results

### 2.1. Unsupervised Multivariate Analysis

**Figure 1:**
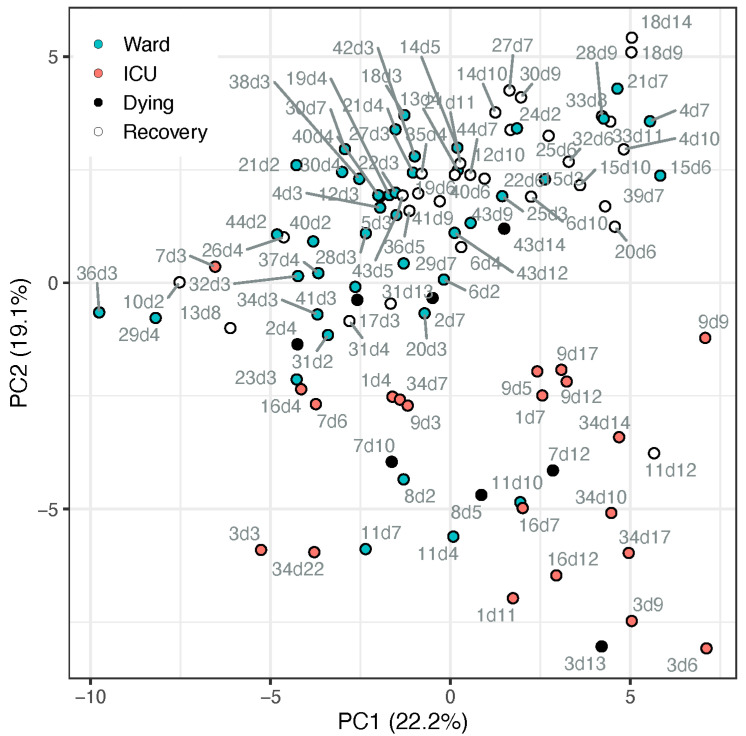
PCA scores plot of samples from patients admitted to ward (blue markers) or ICU (red markers), based on all metabolite data (cube-root transformed and Pareto-scaled). Data points of samples taken within a day of release from hospital (“recovery”) are described by open circle, and samples taken within 4 days of death are in black. Each data point is tagged with the patient ID and sample day (corresponding with Table S2). ICU patients #7 and #17 appeared among the ward patients, both on day 3 when they deteriorated and were transferred from ward to ICU. Ward patient #8 (*n* = 2) appeared among the ICU cluster, an elderly male with chronic kidney disease (CKD), the only known active smoker, who died after a week in the ward. Ward patient #11 appeared within the ICU cluster (*n* = 4), has metabolic syndrome and CKD, suffered kidney failure during the hospitalisation, yet recovered.

The metabolic profile of amino acids and derivatives was obtained from the COVID-19 patient cohort, summarised in Table 1. Utilising all 56 amines that passed the quality control process, we conducted a principal component analysis (PCA, Figure 1). The PCA demonstrated a partial separation by PC2 between samples taken from patients in the ward (suffering from pneumonia) and patients in the ICU (suffering from ARDS and other complications). Moreover, it shows that most recovering ward patients (white markers) were clustered further away from ICU patients, while most samples taken from ward patients up to 4 days before dying (black markers) appeared on the boundary between ward and ICU clusters or among the ICU samples. The loadings of the PCA (Table S7; Figure S1) indicate the metabolites directing these clusters, including, for example, higher tryptophan, serotonin, glutamine, and glutathione in ward patients and higher kynurenine, methionine sulfone, and cystathionine in ICU patients. To further assess the potential biomarkers, a univariate analysis was conducted.

### 2.2. Metabolic Markers Associated with Disease Severity

**Figure 2:**
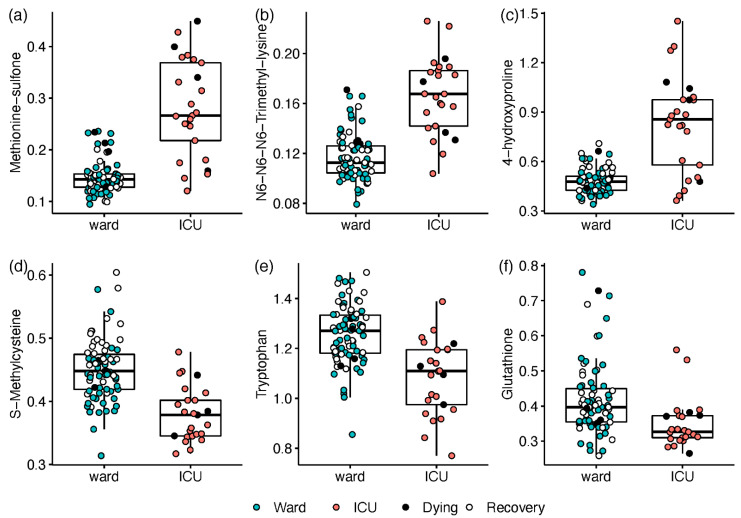
Box and whisker and scatter plots of metabolites differentiating between hospitalisation status: ICU (red) vs. ward (blue; open markers for recovering patients within 24 h of release). Black markers represent patients who died within 4 days. Prior to plotting, metabolite peak area ratios with internal standards were cube root-transformed. Metabolites: (**a**) methionine sulfone; (**b**) N6,N6,N6-trimethyllysine; (**c**) 4-hydroxyproline; (**d**) S-Methylcysteine; (**e**) tryptophan; (**f**) glutathione. The detailed results are in Table S8.

The disease severity at the time of sampling was first defined as the hospitalisation status (ICU; ward), and all samples were utilised in a univariate regression analysis. Compared with ward patients, ICU patients exhibited elevated levels of 14 amines and a decrease in 8 amines (*n* = 19 with Q < 0.05; see Table S8 and Document S2). The most prominent differences consisted of ICU-elevated markers of oxidative stress and inflammation (*n* = 12). Increases of 2.5- to 6.5-fold in ICU patients (Q ≤ 3.3 × 10^−8^) were led by the inflammation-related ratio kynurenine/tryptophan (Kyn/Trp) and post-translationally modified amino acids including methionine sulfone, N6,N6,N6-trimethyllysine, and 4-hydroxyproline (Figure 2). Milder increases of 50–70% in ICU patients were recorded for glycylglycine, glutamate, and proline, while decreases of 55–70% were observed for S-methylcysteine (Figure 2d), tryptophan (Figure 2e), and glutathione (Figure 2f).

Next, the measured plasma cytokine IL-6 was utilised as a proxy for COVID-19 severity, reflecting the extent of the inflammatory response [27,28,29,30]. Considering the literature and the distribution of IL-6 levels in our cohort, patient samples were grouped as follows (in pg/mL): normal (<8), *n* = 18; low (8–20), *n* = 34; medium (21–45), *n* = 32; high (>45) *n* = 19, noting that no levels were recorded above 500 pg/mL, classified as “hyper-inflammation” [27,31]. The above classification did not fully overlap with the severity classification by hospitalisation status, as about 25 samples from non-recovered ward patients had medium-high IL-6 levels (Figure S2). Regression analysis differentiating between pairs of IL-6 classes revealed 38 metabolites with significant differences between samples with high IL-6 levels and normal or low IL-6 (Table S9; Document S3). For most modified amino acids, the significant changes between normal + low classes and medium + high classes were statistically driven by the ICU class.

**Figure 3:**
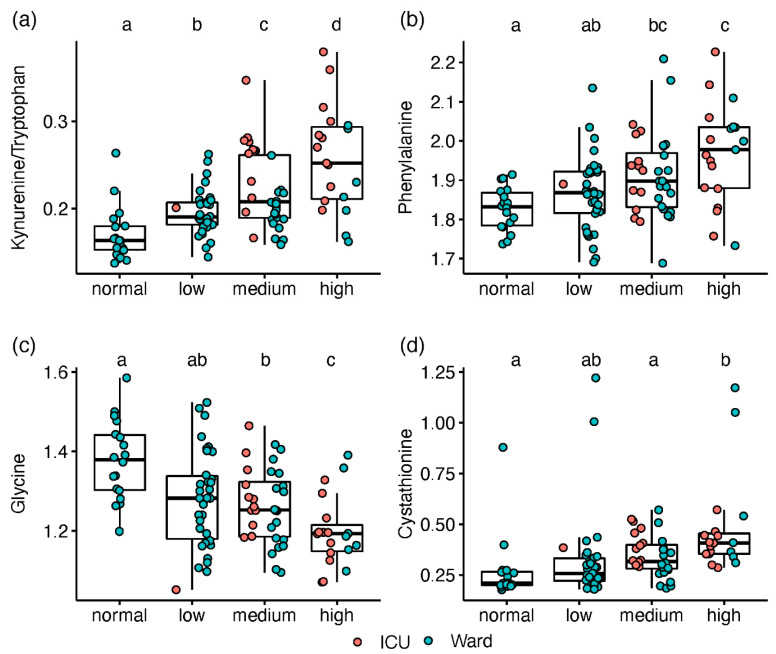
Box and whisker and scatter plots of metabolite levels, classified according to IL-6 groups (normal < 8 pg/mL, n = 18; low, 8–20, n = 34; medium, 21–45, n = 32; high, >45, n = 19). Metabolite peak area ratios with internal standards were cube root-transformed prior to plotting. Metabolites: (**a**) kynurenine/tryptophan; (**b**) phenylalanine; (**c**) glycine; (**d**); cystathionine. red markers represent patients in ICU, and blue markers are patients in ward. Significant differences between IL-6 classes (FDR Q < 0.1), based on linear regression, are indicated by dissimilar letters above each box plot. Detailed results are provided in Table S9.

A gradual change along the increased IL-6 levels was observed for kynurenine and Kyn/Trp (Figure 3a). Among the metabolites with no apparent impact on ICU status, phenylalanine was elevated with increased IL-6 (Figure 3b), while serine and glycine decreased (Figure 3c). Only patients with high IL-6 had lower gamma-aminobutyrate (GABA) levels or higher cystathionine (Figure 3d). Using the IL-6 stratification approach, we found 18 amines that were not significantly different between ICU and ward patients. Although directed by inflammation, this provides complementary biochemical information, assessing the disease state without the non-COVID-19 specific impact of ICU admission (i.e., due to strong antibiotics and extreme changes in feeding).

### 2.3. Paired Analysis in Non-Critical Patients

The infrequent availability of plasma samples at varying time points for some of the patients (Table S2) allowed limited longitudinal analysis or outcome prediction by baseline parameters. Nevertheless, sufficient samples taken from patients in the ward (but not in ICU) enabled the exploration of the metabolic changes during recovery from mild-moderate COVID-19. Two plasma samples were compared per patient (*n* = 16): one taken at the start of hospitalisation (days 1–4 since admission) and one at the recovery stage (up to a day before release from the hospital) with no less than 3 days in between.

**Figure 4:**
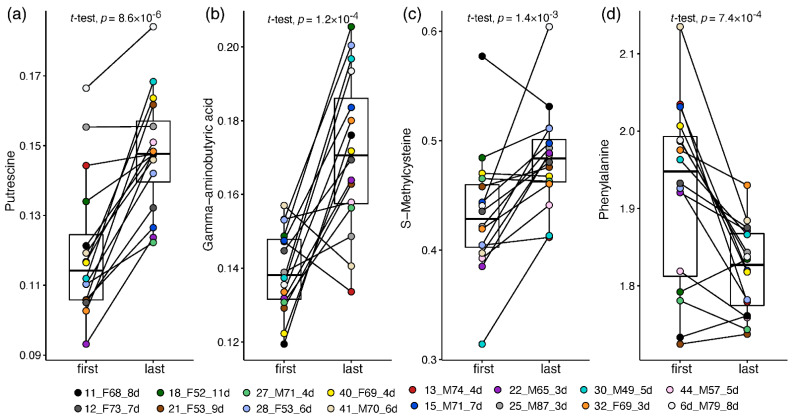
Box and whisker and scatter plots of paired changes of metabolite levels in COVID-19 ward patients. A line connects each patient’s paired samples, with the first time point being not more than 4 days from admission and last time point during the 24 h before release from hospital. Metabolite peak area ratios with internal standards were cube root-transformed. Metabolites: (**a**) putrescine; (**b**) GABA; (**c**) S-methylcysteine; (**d**) Phenylalanine. The legend shows the individual patient by marker colour, with indication of patient number, sex, age and number of days between time points. Patient information is provided in Table S2.

This approach enabled a patient-corrected analysis of metabolic changes and a more meaningful metabolite fold-change than that calculated in the non-paired analysis. Altogether, 33 plasma amines significantly changed by at least 20% towards the recovery of ward patients (Table S10; Document S4). Thirty amines increased in recovering patients, led by 1.7–2 fold increases in putrescine (Figure 4a), GABA (Figure 4b), O-acetylserine, and taurine. Milder increases of 20–65% towards recovery were recorded in 16 amines, including glycine, hydroxylysine, carnosine, and S-methylcysteine (Figure 4c; statistically driven by males). Owing to the small sample size, some metabolite changes were statistically driven by one sex (mostly females, increasing), while 14 alterations were observed in one sex only, stemming mainly from differences in baseline levels and from high within-sex variance. Only male patients showed a significant decrease of 25% in phenylalanine towards recovery (Figure 4d) and a two-fold decrease in cystathionine. Only female patients showed a 50% increase in glutathione and 20–75% in nine other amines, alongside a dramatic 7-fold decrease in glycylproline.

### 2.4. Correlation between Metabolites and Immune Response Markers

**Figure 5:**
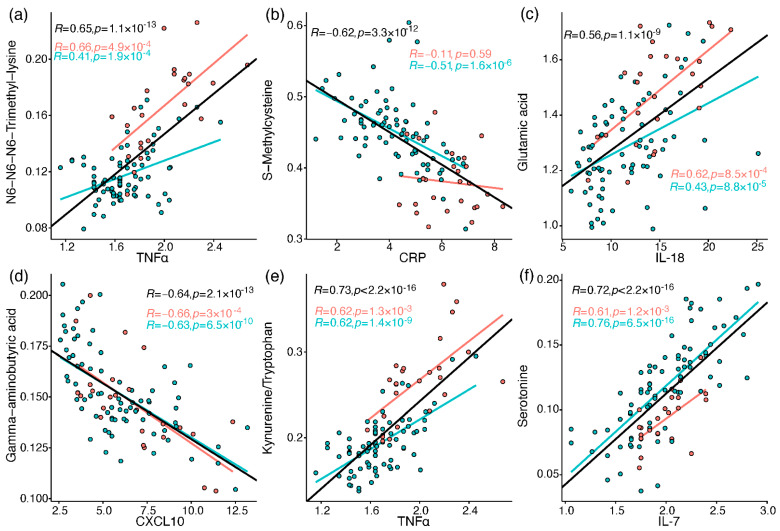
Pearson correlation between metabolites and immune response markers (cube root-transformed). Red markers are samples from ICU patients, and blue markers are from ward patients. The regression lines and Pearson R values and *p* values (uncorrected) are in black for all samples, red for ICU, and blue for ward. (**a**) N6,N6,N6-trimethyllysine vs. TNF-alpha; (**b**) S-methylcysteine vs. CRP; (**c**) glutamate vs. IL-18; (**d**) GABA vs. CXCL10; (**e**) kynurenine/tryptophan vs. TNF-alpha; (**f**) serotonin vs. IL-7.

The immune profiles of the cohort patients were characterised as reported previously by Schrijver et al. [26]. Briefly, neutrophils, CRP, IL-6, CCL2, CXCL10, and GM-CSF were elevated in baseline samples taken from patients who were admitted to ICU or died, compared to patients admitted to the ward (who also showed greater decline along the hospitalisation period in most of the elevated markers). To link the metabolic perturbations measured in the current study to relevant immune processes, Pearson correlations were calculated between all metabolites and 37 immune response markers, including different leukocytes, chemokines, cytokines, and others (Tables S11–S13). A correlation heatmap was generated for metabolites and selected immune markers (Figure S3), providing a snapshot of interesting correlations out of over 600 significant correlations (FDR-corrected). These correlations are embedded in the discussion, and some of the strong correlations (|R| > 0.55) are individually plotted in Figure 5 (see Document S5 for all plots).

## 3. Discussion

**Figure 6:**
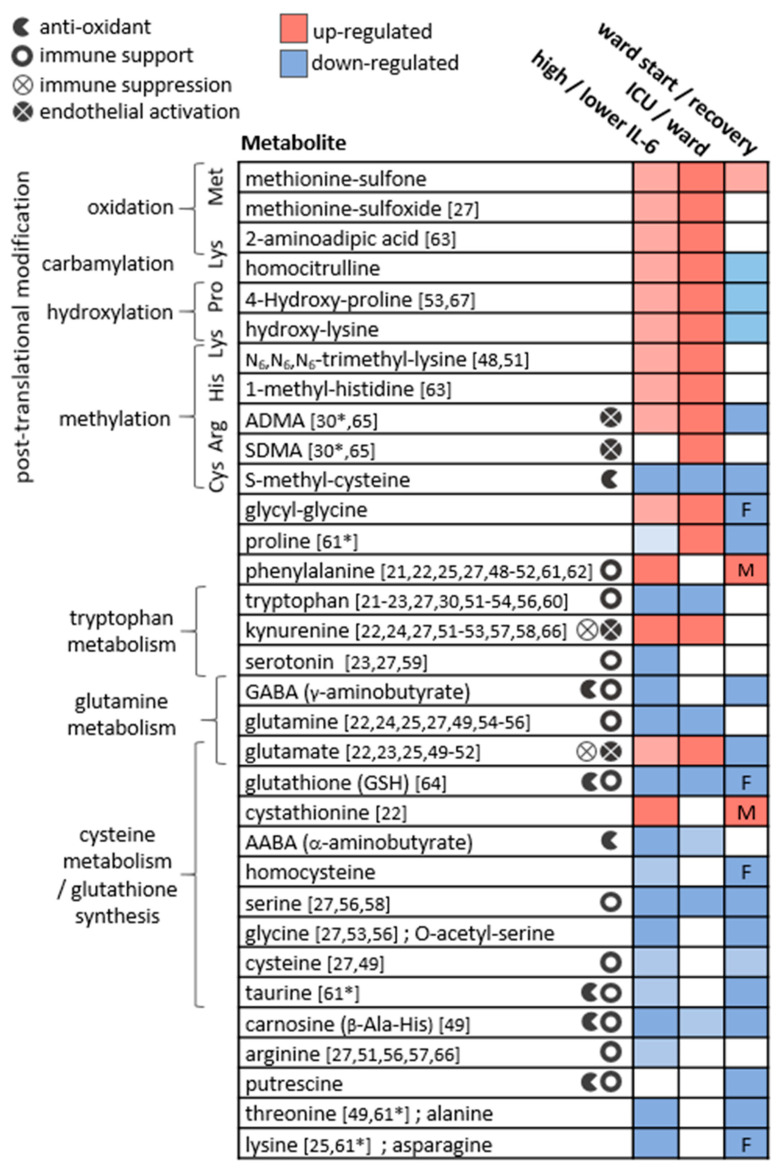
The main metabolic changes obtained for plasma amines in the three applied statistical analyses. The metabolites are clustered by their biochemical context, and icons indicate relevant biochemical activity. References indicate studies reporting similar results comparing patients at varying COVID-19 stages (* in recovering patients). Studies reporting significant results comparing COVID-19 patients to non-COVID-19 patients or to healthy controls were not cited here. In ward paired results F,M indicate significant findings in one sex only. ADMA and SDMA are asymmetric and symmetric dimethylarginine, respectively.

The presented study demonstrated consistent perturbations in the plasma amines profile of COVID-19 patients, as consolidated in Figure 6. 

**Figure 7:**
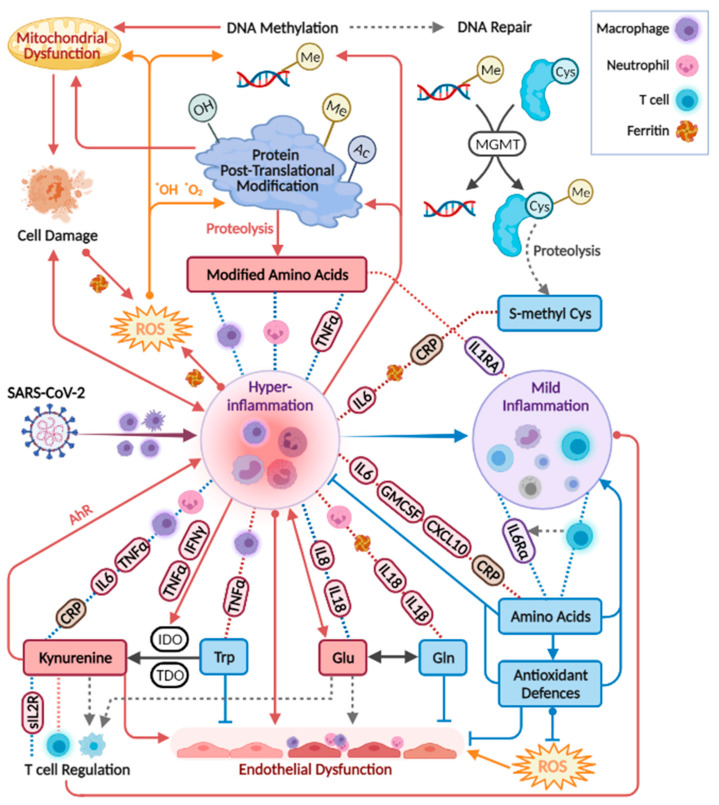
Biochemical processes and hypotheses (broken grey arrows) incorporating the study results. Red boxes indicate metabolites up-regulated in severe COVID-19, and blue boxes are generally down-regulated. Correlations appear in broken lines, blue (positive) or red (negative), including tags for specific immune markers, or icons of neutrophils (count), T cells (count), ferritin, or macrophage activation (CD206 and CD163; CCL2 (MCP1) that attract various immune cells). Tryptophan correlations are in addition to those indicated for amino acids. IDO, indoleamine 2,3-dioxygenase. TDO, Tryptophan 2,3-Deoxygenase. The figure was created with BioRender.com.

For metabolites including glutamine, glutamate, dimethylarginines, kynurenine, tryptophan, and phenylalanine, observations were in line with studies in patients with critical illness and sepsis [32,33,34,35,36,37,38,39,40], respiratory conditions (COPD, asthma, and ARDS) [41,42,43], bacterial pneumonia [44], and HIV-AIDS [45,46,47]. Similar differences in individual metabolites were reported in other studies comparing patients at varying COVID-19 stages, and those are indicated in Figure 6 per metabolite [21,22,23,24,25,27,30,48,49,50,51,52,53,54,55,56,57,58,59,60,61,62,63,64,65,66,67]. Although sporadic, the common findings highlight a consistency in the field despite the highly varied cohort characteristics, technical, and statistical approaches. Our study suffers from some limitations that are common among COVID-19 studies, including a small and imbalanced cohort, with sporadic time points that are not ideal for longitudinal analysis. Furthermore, we did not utilise a control group, for example, one consisting of patients with similar symptoms yet not infected by SARS-CoV2 [68]. Some COVID-19 metabolomics studies obtained significant findings based on comparisons with healthy individuals, and these were not highlighted here. We refrained from adding control samples from a separate cohort to avoid an expected technical bias. The above limitations warrant a follow-up study with a larger cohort. The metabolic map in Figure 7 illustrates established biochemical processes as well as generated hypotheses stemming from the results. The overall picture relates severe COVID-19 to the innate immune response (“hyper-inflammation”) and increased oxidative stress, as reflected by metabolites in red boxes. The metabolic profile of non-severe patients was related to the adaptive immune response (resolution of inflammation), with increased levels of immune-supportive and antioxidative amino acids and derivatives (in blue boxes).

### 3.1. Post-Translationally-Modified Amino Acids

The strongest and most consistent findings in this study included an array of modified amino acids that were significantly higher in ICU patients compared to ward patients and, as a result, also higher in patients with medium-high IL-6 levels. They are listed in Figure 6 alongside the specific post-translational modification of amino acid residue in a protein, which can then release the metabolite by proteolysis. In a disease state, older age, higher BMI, inflammation, and oxidative stress accelerate post-translational modifications. Such modifications lead not only to the malfunction of proteins but also have the ability to regulate many signalling reactions, including thrombosis [68]. Some modified amino acids, such as dimethylarginines, also inhibit nitric-oxide synthases (NOS) and are associated with endothelial dysfunction in COVID-19 [30,65]. The close link between the modified amino acids and hyper-inflammation is demonstrated in Figure 7 via multiple correlations with neutrophils, markers of macrophage and leukocyte activation (CD163, CD206, CCL2, sIL-2R), and TNF-alpha (e.g., trimethyllysine in Figure 5a). Modified amino acids were the only metabolites to show a negative association with IL-1 receptor antagonist (IL-1RA) that acts against the exacerbated inflammation induced by IL-1 and IL-6. Post-translational modifications and proteolysis also occur at a high rate in skeletal muscles; therefore, the detected metabolites could indicate muscle breakdown or the consumption of meat. The former is an inevitable ICU bias that should be addressed in a future study by the inclusion of a control group consisting of ICU patients who are COVID-19-free [69]. Nevertheless, the strong correlations we found between the modified amino acids and COVID-19-characterising inflammation markers support a production mechanism that is beyond muscle breakdown.

S-methylcysteine was the sole post-translational modification product that was strongly and consistently associated with better health status (Figure 6) and negatively correlated with CRP (Figure 5b), IL-6 and also ferritin, which promotes the formation of reactive oxygen species (ROS). S-methylcysteine is a powerful antioxidant, and its most common sources are from the ingestion of plants; therefore, diet and gut microbiota profile could contribute to its status as a strong marker of recovery (Figure 2d and Figure 4c). However, another hypothesis is outlined in Figure 7, suggesting that it may originate in the repair of methylated DNA by the enzyme MGMT (O6-methylguanine-DNA methyltransferase) [70,71].

### 3.2. Amino Acids and Derivatives

Amino acids are mainly viewed as building blocks that support the increased cellular demands in disease. However, they also regulate the innate and adaptive immune responses, especially via catabolism of tryptophan, phenylalanine, cysteine, glutamine, and arginine [72,73,74]. In our cohort, the only amino acids that were lower in ICU patients were tryptophan, serine, and glutamine, that aid in immune cell proliferation [75,76]. Close to 20 amino acids and derivatives decreased with increasing inflammation (indicated by IL-6) and also increased towards recovery in the ward (Figure 6). Such alterations could be non-specific to COVID-19; however, a study found lower levels of some of the above amino acids in hospitalised patients with COVID-19 compared to patients who tested negative for SARS-CoV2 [69]. Figure 7 groups the amino acids and derivatives together as related to the adaptive immune response, characterised by increased pro-resolving T cells, unlike the low levels typical of severe COVID-19 [1,16,17]. These metabolites showed positive correlations with T cells (especially CD4+) and the anti-inflammatory IL-6 receptor alpha (Figure S3). Most amino acids also negatively correlated with markers of innate immune response and hyper-inflammation, which characterise severe COVID-19 [26]: CRP, IL-6, the cytokine GM-CSF that promotes the activation of granulocytes (e.g., differentiate to macrophages) [53], and the chemokine CXCL10 (IP10) that attracts various immune cells [1].

A few exceptions to these trends are listed next. Phenylalanine accumulated in a worse health state, perhaps due to the excessive oxidation of a cofactor critical to its metabolism into tyrosine and catecholamines [33,77]. Glutamate also showed higher levels in severe patients, and similar to glutamine, exhibited a unique correlation pattern with immune response markers (see IL-18 in Figure 5c). Glutamate modulates immune cell development [78,79], and its excessive production was related to damage in endothelial cells [80,81,82]. The glutamine–glutamate conversion cycle is regulated by cellular demand and uptake [83] and was hypothesised to be re-programmed in host cells infected by SARS-CoV2 [84].

Tryptophan declined in patients in ICU and patients with higher IL-6 and was negatively correlated with an array of markers of hyper-inflammation, wider than any other amino acid (Figure S3). As expected, the exact opposite observations were recorded for its metabolite kynurenine and for the conversion ratio Kyn/Trp (e.g., with TNF-alpha in Figure 5e). The absolute Kyn/Trp values along the hospitalisation period (Figure S4) were related to disease severity, agreeing with reference levels and other COVID-19 studies [57,85]. Kynurenine acts as a pro-inflammatory mediator through binding to the aryl-hydrocarbon receptor (AhR) [86]. It also affects T cell differentiation [87] and contributes to endothelial activation and impaired microvascular reactivity [31,40]. Kynurenine was reported as a strong predictor of long hospitalisation or death in COVID-19 patients [21,66,88] and was elevated in hospitalised patients with COVID-19 compared to non-COVID-19 [69]. The same large cohort showed no difference in tryptophan levels between the hospitalised patients, yet lower tryptophan levels were a strong marker of fatality in patients with COVID-19 [69].

### 3.3. Amines and Antioxidative Defenses

The increased inflammation in patients with COVID-19 was accompanied by a decrease in amino acid derivatives that are part of the anti-oxidative and immune-regulating arsenal (see icons in Figure 6). Glutathione depletion was proposed to be a major contributor to COVID-19 severity and tissue and organ damage [89,90,91]. We found significantly lower glutathione levels in ICU patients and in patients with higher IL-6. Moreover, TNF-alpha (and BMI) negatively correlated with glutathione and showed a strong positive correlation with cystathionine. Glutathione production is controlled by the rate-limiting step catalysed by glutamate-cysteine ligase and enhanced by ROS (see the metabolic pathway in Figure S5). However, low glutathione levels can also result from the impaired metabolism of cystathionine into cysteine (by cystathionine-gamma-lyase), a common bottleneck in the pathway. The expression of cystathionine–gamma–lyase is enhanced by estrogen and protects from oxidative stress [92,93]. Interestingly, only in male ward patients, there was a decrease in cystathionine towards recovery, while only female ward patients recorded increased glutathione. Additional immune-supportive and anti-oxidative amines that decreased in worse disease states, include carnosine, taurine, putrescine, GABA (that exhibited a negative correlation with CXCL10, see Figure 5d), and serotonin. Apart from impaired regulation of immune cell function, the depletion of serotonin may worsen hypoxia in COVID-19 patients [94]. Serotonin is a pulmonary vasoconstrictor and a calcium-dependent activator of NOS in pulmonary endothelial cells [95], affecting the endothelial barrier permeability [96]. In our cohort, the lower serotonin strongly correlated with IL-7 (Figure 5f) that is uniquely expressed in thrombocytes [97], which also store substantial amounts of serotonin [95], perhaps merely indicating lower levels of thrombocytes.

Lower defences against inflammation, ROS and RNS may contribute to sex-dependent metabolic differences observed in recovering ward patients (Figure 6); however, the sample size is rather small to investigate this. The lower defences in males can stem from higher expression and activity of the inflammation and ROS-promoting ACE2 [13,14] and lower glutathione levels and activity compared to females [98]. In females, on the other hand, the activation of endothelial estrogen receptors induces NOS activity and promotes vascular health [12].

### 3.4. Biomarkers Implementation in COVID-19 Treatment

Various immunomodulatory strategies were suggested to reduce the “cytokine storm” in COVID-19 patients [99]. The main findings in our study align with additional therapeutic interventions targeting the metabolic perturbations related to inflammation and oxidative stress. To decrease kynurenine levels and the positive feedback loop with inflammation, key enzymes along the tryptophan–kynurenine pathway can be targeted by drugs [100], as demonstrated in SARS-CoV-2 ex vivo models leading to a reduction in cytokines [23]. Addressing glutamine depletion [41], nutritional supplementation in critical patients [32] and also in COVID-19 patients [101] was associated with a shorter hospitalisation period. Supplementation with carnosine was suggested in COVID-19 patients [102] due to its antioxidative properties [103], anti-inflammatory immune modulation [104], and structural potential to inhibit the binding of SARS-CoV2 spike to ACE2 receptor [105]. Other approaches supporting the redox state include nano-carriers that deliver antioxidant enzymes (such as MSR) to vascular cells [106], arginine supplementation [23], the ingestion of encapsulated glutathione (GSH) [91,107,108], or indirect increase in glutathione production via vitamin D supplementation [109]. Reduced ARDS incidence and death of COVID-19 patients were reported after treatment with Dapsone, an amino-sulfone analogue of methionine oxidation products that competes with the inflammasome [110]. The above studies showcase the potential of amine-related targets to support the immune system, reduce the severity of COVID-19 and improve the survival rates.

## 4. Materials and Methods

### 4.1. Cohort

The cohort consisted of 44 adults admitted to the regional Amphia hospital in Breda, the Netherlands, from 24 March 2020 to 14 April 2020. Table 1 summarises the key characteristics of the 44 patients and 103 collected blood samples (a more detailed summary is in Table S1).

Table S2 provides background information and hospitalisation details per patient, such as comorbidities, treatment, and outcome. All patients reported COVID-19-related complaints and tested positive for the SARS-CoV-2 by a PCR.

### 4.2. Samples

EDTA blood samples were collected in intervals of 3–4 days throughout the study, as detailed in Table S2 per patient. A small aliquot of the collected blood was immediately taken for flow cytometric immune profiling. The plasma was isolated from the remaining blood, aliquoted and stored at −20 °C until serological analysis or until transportation to the analytical chemistry laboratory, where they were kept at −80 °C until sub-aliquoting and LC-MS analysis.

### 4.3. Haematological and Serological Analysis

Flowcytometric leukocyte analysis and serological analysis of cytokines and soluble cell surface molecules have been reported previously by Schrijver; et al. [26]. All of the assays were performed according to the manufacturer’s protocol. The measured parameters, values, and units are detailed in Supplementary Table S3.

### 4.4. Plasma Amines Analysis

The analysis of amino acids and derivatives was conducted using LC-MS/MS following derivatisation by AccQ•Tag ^™^ kit purchased from Waters Corporation (Etten-Leur, The Netherlands). For ultra-high performance liquid chromatography (UPLC), an Agilent 1290 Infinity II system was used, equipped with a Waters AccQ-Tag Ultra C18 column (2.1 × 100 mm, 1.7 μm). The Mass Spectrometer was an AB SCIEX Qtrap 6500 triple-quadrupole with an electrospray ionisation (ESI) source. Analytes were detected in positive ionisation mode, using multiple reaction monitoring (MRM). Details of the sample preparation, the metabolic coverage, and analytical method are provided in Supplementary Document S1.

The acquired data were processed using the Sciex vendor software (MultiQuant v3.0.2). MRM peaks were integrated and further corrected to match internal standards. An in-house quality-control software (mzQual) was utilised to assess the analytical performance (based on study pooled QC replicates, blanks, and internal standards) and perform necessary corrections. A total of 69 metabolites were measured by the platform, of which 56 passed the strict quality rules, as examined by a data analysis expert, and utilised in the statistical analysis (see Document S1). The processed peak areas per metabolite and sample are deposited in Table S4. MS Excel was used for absolute quantitation of selected metabolites based on calibration curves (Table S5).

### 4.5. Statistical Analysis

All statistical analyses were performed in R, and graphs were plotted using the packages ggpubr and stats. All 56 metabolites presented zero missingness, and we could not identify clear outliers. Cytokine and immune marker data (*n* = 37) were analysed as provided (Table S3 [26]). All variables were cube root-transformed prior to statistical analyses. Differential analyses incorporating all samples were performed using linear regression correcting for age, sex and BMI, grouped by patient and weighted by the inverse number of observations per patient. The correlation between age, sex, and BMI and all variables is detailed in Supplementary Table S6. Paired analyses between two time points of the same patient were performed using a paired *t*-test assuming unequal variances. Metabolite fold-change values were calculated based on the untransformed data per patient in the paired *t*-test analysis or by dividing the medians of experimental classes in non-paired analysis. Pearson correlation analyses between metabolites and immune markers were conducted for all samples together and per hospitalisation status (ICU or ward), plotted as three regression lines to provide complementary information. The *p* values obtained in all tests were adjusted for multiple testing using the Benjamini–Hochberg method implemented in the *p*.adjust R function (v.4.0.3) and termed Q-values. The significance levels were defined as Q < 0.1. The corrections were for either the number of variables in univariate tests (*n* = 60, including four metabolic ratios) or for the number of unique correlations in the Pearson correlation tests (*n* = 2220).

## 5. Conclusions

The presented study provided detailed evidence of altered amino acid metabolism in severe COVID-19, tightly correlated with a multitude of immune response markers. We demonstrated a significant decrease in immune-supporting and antioxidative amines, accompanied by dramatic increases in post-translational modified amino acids. The latter cluster was linked to oxidative stress and hyper-inflammation and has not been highlighted elsewhere as such a strong group of biomarkers in critical COVID-19 patients. Although our results cannot provide clear mechanistic conclusions, they can be utilised to generate hypotheses to be studied in cell cultures or animal models, as well as in the treatment of COVID-19 patients. In conclusion, our study can assist in the ongoing global efforts toward a better understanding of the metabolic impact of COVID-19.

## Figures and Tables

**Table 1:** Selected characteristics of the COVID-19 patients in the metabolomics study *. Values are *n* (%) or median [full range]. See Tables S1 and S2 for further information.

	Patients (*n* = 44)	Samples (*n* = 103)
Age, years	73 [49–87]	71 [49–87]
Male (%)	30 (68%)	65 (63%)
BMI	27 [19–42]	
Diabetes and/or cardiovascular disease (CVD)	14 (32%)	
Chronic obstructive pulmonary disease (COPD)	8 (18%)	
Days with symptoms until hospitalisation	8 [1–19]	
Total hospitalisation days	7 [2–62]	
Admitted to ward	37 (84%)	78 (76%)
Admitted to ICU	7 (16%)	25 (24%)
Deceased	9 (20%)	
Treatment with chloroquine	35 (80%)	
Treatment with antibiotics	38 (86%)	
Treatment with corticosteroids	2 (5%)	
CRP, mg/L (normal < 10)		104.5 [3–577]
Lymphocytes, 109/L (normal 1.0–2.8)		0.95 [0.26–3.15]
Neutrophils, 109/L (normal 1.7–6.5)		6.36 [2.3–17.5]

* Information about comorbidities and medication (4 weeks pre-admission) is missing for 25% of patients (*n* = 12).

## Data Availability

All data utilised in the statistical analyses are available as part of the Supplementary files.

## Supplementary Materials

The following supporting information can be downloaded at: https://www.mdpi.com/article/10.3390/metabo12070618/s1, Figure S1: PCA loadings of all study samples, based on all metabolites; Figure S2: Box and whisker and scatter plots of absolute cytokine IL-6 values; Figure S3: Heatmap of Pearson correlations between metabolites and immune markers; Figure S4: Longitudinal trajectories of plasma kynurenine/tryptophan ratio; Figure S5: A simplified pathway map of cysteine and glutathione metabolism; Table S1: Demographics, health and treatment overview of the cohort patients; Table S2: Description of the cohort patients; Table S3: Immune markers measurement results; Table S4: Processed relative peak areas in the amine platform; Table S5: Calibration lines and derived absolute concentrations for selected amines; Table S6: Pearson correlation analysis between confounders and analysed variables; Table S7: Loading values of the PCA plot in Figure 1 and the loading plot in Figure S1; Table S8: Significance values for metabolites differentiating between ICU and ward; Table S9: Significance values for metabolites differentiating between IL-6 groups; Table S10: Paired *t*-tests comparing a first and last time point of selected ward patients; Table S11: Pearson correlation coefficients between all variables; Table S12: Pearson correlation *p* values between all variables; Table S13: Pearson correlation FDR Q values between metabolites and immune markers; Document S1: Amines platform metabolic coverage, analytical method and performance; Document S2: Box and scatter plots for all comparisons between ICU and ward patients; Document S3: Box and scatter plots for all comparisons between IL-6 classes; Document S4: Box and scatter plots for all paired comparisons between ward patients; Document S5: Pearson correlation plots between metabolites and immune markers;

## References

[1] Tay M.Z., Poh C.M., Rénia L., MacAry P.A., Ng L.F.P. (2020). The trinity of COVID-19: Immunity, inflammation and intervention. Nat. Rev. Immunol..

[2] Ayres J.S. (2020). A metabolic handbook for the COVID-19 pandemic. Nat. Metab..

[3] Kirby T. (2021). New variant of SARS-CoV-2 in UK causes surge of COVID-19. Lancet Respir. Med..

[4] Petrilli C.M., Jones S.A., Yang J., Rajagopalan H., O’Donnell L., Chernyak Y., Tobin K.A., Cerfolio R.J., Francois F., Horwitz L.I. (2020). Factors associated with hospital admission and critical illness among 5279 people with coronavirus disease 2019 in New York City: Prospective cohort study. BMJ.

[5] Palaiodimos L., Kokkinidis D.G., Li W., Karamanis D., Ognibene J., Arora S., Southern W.N., Mantzoros C.S. (2020). Severe obesity, increasing age and male sex are independently associated with worse in-hospital outcomes, and higher in-hospital mortality, in a cohort of patients with COVID-19 in the Bronx, New York. Metabolism.

[6] Zheng Z., Peng F., Xu B., Zhao J., Liu H., Peng J., Li Q., Jiang C., Zhou Y., Liu S. (2020). Risk factors of critical & mortal COVID-19 cases: A systematic literature review and meta-analysis. J. Infect..

[7] Dietz W., Santos-Burgoa C. (2020). Obesity and its implications for COVID-19 mortality. Obesity.

[8] Alberca R.W., Oliveira L.d.M., Branco A.C.C.C., Pereira N.Z., Sato M.N. (2020). Obesity as a risk factor for COVID-19: An overview. Crit. Rev. Food Sci. Nutr..

[9] Sattar N., McInnes I.B., McMurray J.J.V. (2020). Obesity Is a Risk Factor for Severe COVID-19 Infection. Circulation.

[10] Hariyanto T.I., Kurniawan A. (2020). Dyslipidemia is associated with severe coronavirus disease 2019 (COVID-19) infection. Diabetes Metab. Syndr. Clin. Res. Rev..

[11] Yanai H. (2020). Metabolic Syndrome and COVID-19. Cardiol. Res..

[12] Froldi G., Dorigo P. (2020). Endothelial dysfunction in Coronavirus disease 2019 (COVID-19): Gender and age influences. Med. Hypotheses.

[13] Salah H.M., Mehta J.L. (2021). Hypothesis: Sex-related differences in ACE2 activity may contribute to higher mortality in men versus women with COVID-19. J. Cardiovasc. Pharmacol. Ther..

[14] Gebhard C., Regitz-Zagrosek V., Neuhauser H.K., Morgan R., Klein S.L. (2020). Impact of sex and gender on COVID-19 outcomes in Europe. Biol. Sex Differ..

[15] Zeng F., Huang Y., Guo Y., Yin M., Chen X., Xiao L., Deng G. (2020). Association of inflammatory markers with the severity of COVID-19: A meta-analysis. Int. J. Infect. Dis..

[16] Pedersen S.F., Ho Y.-C. (2020). SARS-CoV-2: A storm is raging. J. Clin. Investig..

[17] Qin C., Zhou L., Hu Z., Zhang S., Yang S., Tao Y., Xie C., Ma K., Shang K., Wang W. (2020). Dysregulation of Immune Response in Patients With Coronavirus 2019 (COVID-19) in Wuhan, China. Clin. Infect. Dis..

[18] Xiong Y., Liu Y., Cao L., Wang D., Guo M., Jiang A., Guo D., Hu W., Yang J., Tang Z. (2020). Transcriptomic characteristics of bronchoalveolar lavage fluid and peripheral blood mononuclear cells in COVID-19 patients. Emerg. Microbes Infect..

[19] Gardinassi L.G., Souza C.O., Sales-Campos H., Fonseca S.G. (2020). Immune and metabolic signatures of COVID-19 revealed by transcriptomics data reuse. Front. Immunol..

[20] Tsoukalas D., Sarandi E., Georgaki S. (2021). The snapshot of metabolic health in evaluating micronutrient status, the risk of infection and clinical outcome of COVID-19. Clin. Nutr. ESPEN.

[21] Overmyer K.A., Shishkova E., Miller I.J., Balnis J., Bernstein M.N., Peters-Clarke T.M., Meyer J.G., Quan Q., Muehlbauer L.K., Trujillo E.A. (2021). Large-Scale Multi-omic Analysis of COVID-19 Severity. Cell Syst..

[22] Kimhofer T., Lodge S., Whiley L., Gray N., Loo R.L., Lawler N.G., Nitschke P., Bong S.-H., Morrison D.L., Begum S. (2020). Integrative modeling of quantitative plasma lipoprotein, metabolic, and amino acid data reveals a multiorgan pathological signature of SARS-CoV-2 infection. J. Proteome Res..

[23] Xiao N., Nie M., Pang H., Wang B., Hu J., Meng X., Li K., Ran X., Long Q., Deng H. (2021). Integrated cytokine and metabolite analysis reveals immunometabolic reprogramming in COVID-19 patients with therapeutic implications. Nat. Commun..

[24] Shen B., Yi X., Sun Y., Bi X., Du J., Zhang C., Quan S., Zhang F., Sun R., Qian L. (2020). Proteomic and metabolomic characterization of COVID-19 patient sera. Cell.

[25] Bruzzone C., Bizkarguenaga M., Gil-Redondo R., Diercks T., Arana E., García de Vicuña A., Seco M., Bosch A., Palazón A., San Juan I. (2020). SARS-CoV-2 Infection Dysregulates the Metabolomic and Lipidomic Profiles of Serum. iScience.

[26] Schrijver B., Assmann J.L., van Gammeren A.J., Vermeulen R.C., Portengen L., Heukels P., Langerak A.W., Dik W.A., van der Velden V.H., Ermens T.A. (2020). Extensive longitudinal immune profiling reveals sustained innate immune activaton in COVID-19 patients with unfavorable outcome. Eur. Cytokine Netw..

[27] Thomas T., Stefanoni D., Reisz J.A., Nemkov T., Bertolone L., Francis R.O., Hudson K.E., Zimring J.C., Hansen K.C., Hod E.A. (2020). COVID-19 infection alters kynurenine and fatty acid metabolism, correlating with IL-6 levels and renal status. JCI Insight.

[28] McElvaney O.J., Hobbs B.D., Qiao D., McElvaney O.F., Moll M., McEvoy N.L., Clarke J., O’Connor E., Walsh S., Cho M.H. (2020). A linear prognostic score based on the ratio of interleukin-6 to interleukin-10 predicts outcomes in COVID-19. EBioMedicine.

[29] Coperchini F., Chiovato L., Croce L., Magri F., Rotondi M. (2020). The cytokine storm in COVID-19: An overview of the involvement of the chemokine/chemokine-receptor system. Cytokine Growth Factor Rev..

[30] Jud P., Gressenberger P., Muster V., Avian A., Meinitzer A., Strohmaier H., Sourij H., Raggam R.B., Stradner M.H., Demel U. (2021). Evaluation of Endothelial Dysfunction and Inflammatory Vasculopathy After SARS-CoV-2 Infection-A Cross-Sectional Study. Front. Cardiovasc. Med..

[31] Leisman D.E., Deutschman C.S., Legrand M. (2020). Facing COVID-19 in the ICU: Vascular dysfunction, thrombosis, and dysregulated inflammation. Intensive Care Med..

[32] Wischmeyer P.E., Dhaliwal R., McCall M., Ziegler T.R., Heyland D.K. (2014). Parenteral glutamine supplementation in critical illness: A systematic review. Crit. Care.

[33] Ploder M., Neurauter G., Spittler A., Schroecksnadel K., Roth E., Fuchs D. (2008). Serum phenylalanine in patients post trauma and with sepsis correlate to neopterin concentrations. Amino Acids.

[34] Wang J., Sun Y., Teng S., Li K. (2020). Prediction of sepsis mortality using metabolite biomarkers in the blood: A meta-analysis of death-related pathways and prospective validation. BMC Med..

[35] Kauppi A.M., Edin A., Ziegler I., Mölling P., Sjöstedt A., Gylfe Å., Strålin K., Johansson A. (2016). Metabolites in blood for prediction of bacteremic sepsis in the emergency room. PLoS ONE.

[36] Mogensen K.M., Lasky-Su J., Rogers A.J., Baron R.M., Fredenburgh L.E., Rawn J., Robinson M.K., Massarro A., Choi A.M., Christopher K.B. (2017). Metabolites Associated With Malnutrition in the Intensive Care Unit Are Also Associated With 28-Day Mortality: A Prospective Cohort Study. J. Parenter. Enter. Nutr..

[37] Mortensen K.M., Itenov T.S., Haase N., Müller R.B., Ostrowski S.R., Johansson P.I., Olsen N.V., Perner A., Søe-Jensen P., Bestle M.H. (2016). High levels of methylarginines were associated with increased mortality in patients with severe sepsis. Shock. Inj. Inflamm. Sepsis Lab. Clin. Approaches.

[38] Koch A., Weiskirchen R., Bruensing J., Dückers H., Buendgens L., Kunze J., Matthes M., Luedde T., Trautwein C., Tacke F. (2013). Regulation and prognostic relevance of symmetric dimethylarginine serum concentrations in critical illness and sepsis. Mediat. Inflamm..

[39] O’Dwyer M.J., Dempsey F., Crowley V., Kelleher D.P., McManus R., Ryan T. (2006). Septic shock is correlated with asymmetrical dimethyl arginine levels, which may be influenced by a polymorphism in the dimethylarginine dimethylaminohydrolase II gene: A prospective observational study. Crit. Care.

[40] Darcy C.J., Davis J.S., Woodberry T., McNeil Y.R., Stephens D.P., Yeo T.W., Anstey N.M. (2011). An observational cohort study of the kynurenine to tryptophan ratio in sepsis: Association with impaired immune and microvascular function. PLoS ONE.

[41] Oliveira G.P., De Abreu M.G., Pelosi P., Rocco P.R.M. (2016). Exogenous Glutamine in Respiratory Diseases: Myth or Reality?. Nutrients.

[42] Stringer K.A., McKay R.T., Karnovsky A., Quémerais B., Lacy P. (2016). Metabolomics and its application to acute lung diseases. Front. Immunol..

[43] Stringer K.A., Serkova N.J., Karnovsky A., Guire K., Paine III R., Standiford T.J. (2011). Metabolic consequences of sepsis-induced acute lung injury revealed by plasma 1H-nuclear magnetic resonance quantitative metabolomics and computational analysis. Am. J. Physiol. -Lung Cell. Mol. Physiol..

[44] Vögeli A., Ottiger M., Meier M.A., Steuer C., Bernasconi L., Kulkarni P., Huber A., Christ-Crain M., Henzen C., Hoess C. (2017). Admission levels of asymmetric and symmetric dimethylarginine predict long-term outcome in patients with community-acquired pneumonia. Respir. Res..

[45] Eck H.-P., Frey H., Dröge W. (1989). Elevated plasma glutamate concentrations in HIV-1-infected patients may contribute to loss of macrophage and lymphocyte functions. Int. Immunol..

[46] Dröge W., Eck H.-P., Betzler M. (1987). Elevated plasma glutamate levels in colorectal carcinoma patients and in patients with acquired immunodeficiency syndrome (AIDS). Immunobiology.

[47] Dröge W., Eck H.-P., Betzler M., Schlag P., Drings P., Ebert W. (1988). Plasma glutamate concentration and lymphocyte activity. J. Cancer Res. Clin. Oncol..

[48] Wu J., Zhao M., Li C., Zhang Y., Wang D.W. (2021). The SARS-CoV-2 induced targeted amino acid profiling in patients at hospitalized and convalescent stage. Biosci. Rep..

[49] Páez-Franco J.C., Torres-Ruiz J., Sosa-Hernández V.A., Cervantes-Díaz R., Romero-Ramírez S., Pérez-Fragoso A., Meza-Sánchez D.E., Germán-Acacio J.M., Maravillas-Montero J.L., Mejía-Domínguez N.R. (2021). Metabolomics analysis reveals a modified amino acid metabolism that correlates with altered oxygen homeostasis in COVID-19 patients. Sci. Rep..

[50] Shi D., Yan R., Lv L., Jiang H., Lu Y., Sheng J., Xie J., Wu W., Xia J., Xu K. (2021). The serum metabolome of COVID-19 patients is distinctive and predictive. Metabolism.

[51] Danlos F.-X., Grajeda-Iglesias C., Durand S., Sauvat A., Roumier M., Cantin D., Colomba E., Rohmer J., Pommeret F., Baciarello G. (2021). Metabolomic analyses of COVID-19 patients unravel stage-dependent and prognostic biomarkers. Cell Death Dis..

[52] Lawler N.G., Gray N., Kimhofer T., Boughton B., Gay M., Yang R., Morillon A.-C., Chin S.-T., Ryan M., Begum S. (2021). Systemic Perturbations in Amine and Kynurenine Metabolism Associated with Acute SARS-CoV-2 Infection and Inflammatory Cytokine Responses. J. Proteome Res..

[53] Giron L.B., Dweep H., Yin X., Wang H., Damra M., Goldman A.R., Gorman N., Palmer C.S., Tang H.-Y., Shaikh M.W. (2021). Plasma Markers of Disrupted Gut Permeability in Severe COVID-19 Patients. Front. Immunol..

[54] Barberis E., Timo S., Amede E., Vanella V.V., Puricelli C., Cappellano G., Raineri D., Cittone M.G., Rizzi E., Pedrinelli A.R. (2020). Large-Scale Plasma Analysis Revealed New Mechanisms and Molecules Associated with the Host Response to SARS-CoV-2. Int. J. Mol. Sci..

[55] Doğan H.O., Şenol O., Bolat S., Yıldız Ş.N., Büyüktuna S.A., Sarıismailoğlu R., Doğan K., Hasbek M., Hekim N. (2020). Understanding the Pathophysiological Changes Via Untargated Metabolomics in COVID-19 Patients. J. Med. Virol..

[56] Rees C.A., Rostad C.A., Mantus G., Anderson E.J., Chahroudi A., Jaggi P., Wrammert J., Ochoa J.B., Ochoa A., Basu R.K. (2021). Altered amino acid profile in patients with SARS-CoV-2 infection. Proc. Natl. Acad. Sci. USA.

[57] Reizine F., Lesouhaitier M., Gregoire M., Pinceaux K., Gacouin A., Maamar A., Painvin B., Camus C., Le Tulzo Y., Tattevin P. (2021). SARS-CoV-2-induced ARDS associates with MDSC expansion, lymphocyte dysfunction, and arginine shortage. J. Clin. Immunol..

[58] Sindelar M., Stancliffe E., Schwaiger-Haber M., Anbukumar D.S., Adkins-Travis K., Goss C.W., O’Halloran J.A., Mudd P.A., Liu W.-C., Albrecht R.A. (2021). Longitudinal metabolomics of human plasma reveals prognostic markers of COVID-19 disease severity. Cell Rep. Med..

[59] Soria-Castro R., Meneses-Preza Y.G., Rodríguez-López G.M., Romero-Ramírez S., Sosa-Hernández V.A., Cervantes-Díaz R., Pérez-Fragoso A., Torres-Ruíz J.J., Gómez-Martín D., Campillo-Navarro M. (2021). Severe COVID-19 is marked by dysregulated serum levels of carboxypeptidase A3 and serotonin. J. Leukoc. Biol..

[60] Song J.-W., Lam S.M., Fan X., Cao W.-J., Wang S.-Y., Tian H., Chua G.H., Zhang C., Meng F.-P., Xu Z. (2020). Omics-driven systems interrogation of metabolic dysregulation in COVID-19 pathogenesis. Cell Metab..

[61] Acosta-Ampudia Y., Monsalve D.M., Rojas M., Rodríguez Y., Gallo J.E., Salazar-Uribe J.C., Santander M.J., Cala M.P., Zapata W., Zapata M.I. (2021). COVID-19 convalescent plasma composition and immunological effects in severe patients. J. Autoimmun..

[62] Lodge S., Nitschke P., Kimhofer T., Coudert J.D., Begum S., Bong S.-H., Richards T., Edgar D., Raby E., Spraul M. (2021). NMR Spectroscopic Windows on the Systemic Effects of SARS-CoV-2 Infection on Plasma Lipoproteins and Metabolites in Relation to Circulating Cytokines. J. Proteome Res..

[63] Borchers C., Richard V., Gaither C., Popp R., Chaplygina D., Brzhozovskiy A., Kononikhin A., Zahedi R., Nikolaev E. (2021). Early prediction of COVID-19 patient survival by targeted plasma multi-omics and machine learning. preprint.

[64] Karkhanei B., Talebi Ghane E., Mehri F. (2021). Evaluation of oxidative stress level: Total antioxidant capacity, total oxidant status and glutathione activity in patients with COVID-19. New Microbes New Infect..

[65] Hannemann J., Balfanz P., Schwedhelm E., Hartmann B., Ule J., Müller-Wieland D., Dahl E., Dreher M., Marx N., Böger R. (2021). Elevated serum SDMA and ADMA at hospital admission predict in-hospital mortality of COVID-19 patients. Sci. Rep..

[66] Roberts I., Wright Muelas M., Taylor J.M., Davison A.S., Xu Y., Grixti J.M., Gotts N., Sorokin A., Goodacre R., Kell D.B. (2022). Untargeted metabolomics of COVID-19 patient serum reveals potential prognostic markers of both severity and outcome. Metabolomics.

[67] Masoodi M., Peschka M., Schmiedel S., Haddad M., Frye M., Maas C., Lohse A., Huber S., Kirchhof P., Nofer J.-R. (2022). Disturbed lipid and amino acid metabolisms in COVID-19 patients. J. Mol. Med..

[68] Gu S.X., Stevens J.W., Lentz S.R. (2015). Regulation of thrombosis and vascular function by protein methionine oxidation. Blood J. Am. Soc. Hematol..

[69] D’Alessandro A., Thomas T., Akpan I.J., Reisz J.A., Cendali F.I., Gamboni F., Nemkov T., Thangaraju K., Katneni U., Tanaka K. (2021). Biological and Clinical Factors contributing to the Metabolic Heterogeneity of Hospitalized Patients with and without COVID-19. Cells.

[70] Fabregat A., Sidiropoulos K., Viteri G., Marin-Garcia P., Ping P., Stein L., D’Eustachio P., Hermjakob H. (2018). Reactome diagram viewer: Data structures and strategies to boost performance. Bioinformatics.

[71] Mitra S., Kaina B. (1993). Regulation of repair of alkylation damage in mammalian genomes. Prog. Nucleic Acid Res. Mol. Biol..

[72] McGaha T.L., Huang L., Lemos H., Metz R., Mautino M., Prendergast G.C., Mellor A.L. (2012). Amino acid catabolism: A pivotal regulator of innate and adaptive immunity. Immunol. Rev..

[73] Grohmann U., Mondanelli G., Belladonna M.L., Orabona C., Pallotta M.T., Iacono A., Puccetti P., Volpi C. (2017). Amino-acid sensing and degrading pathways in immune regulation. Cytokine Growth Factor Rev..

[74] Sikalidis A.K. (2015). Amino acids and immune response: A role for cysteine, glutamine, phenylalanine, tryptophan and arginine in T-cell function and cancer?. Pathol. Oncol. Res..

[75] Ma E.H., Bantug G., Griss T., Condotta S., Johnson R.M., Samborska B., Mainolfi N., Suri V., Guak H., Balmer M.L. (2017). Serine Is an Essential Metabolite for Effector T Cell Expansion. Cell Metab..

[76] Newsholme P. (2001). Why is L-glutamine metabolism important to cells of the immune system in health, postinjury, surgery or infection?. J. Nutr..

[77] Neurauter G., Schrocksnadel K., Scholl-Burgi S., Sperner-Unterweger B., Schubert C., Ledochowski M., Fuchs D. (2008). Chronic immune stimulation correlates with reduced phenylalanine turnover. Curr. Drug Metab..

[78] Pacheco R., Gallart T., Lluis C., Franco R. (2007). Role of glutamate on T-cell mediated immunity. J. Neuroimmunol..

[79] Gao M., Jin W., Qian Y., Ji L., Feng G., Sun J. (2011). Effect of N-methyl-D-aspartate receptor antagonist on T helper cell differentiation induced by phorbol-myristate-acetate and ionomycin. Cytokine.

[80] Nassar T., Bdeir K., Yarovoi S., Fanne R.A., Murciano J.-C., Idell S., Allen T.C., Cines D.B., Higazi A.A.-R. (2011). tPA regulates pulmonary vascular activity through NMDA receptors. Am. J. Physiol. Lung Cell. Mol. Physiol..

[81] Collard C.D., Park K.A., Montalto M.C., Alapati S., Buras J.A., Stahl G.L., Colgan S.P. (2002). Neutrophil-derived Glutamate Regulates Vascular Endothelial Barrier Function *. J. Biol. Chem..

[82] Blaylock R.L. (2021). Excitotoxicity (Immunoexcitotoxicity) as a Critical Component of the Cytokine Storm Reaction in Pulmonary Viral Infections, Including SARS-CoV-2. Int. J. Vaccine Theory Pract. Res..

[83] Cruzat V., Macedo Rogero M., Noel Keane K., Curi R., Newsholme P. (2018). Glutamine: Metabolism and immune function, supplementation and clinical translation. Nutrients.

[84] Bharadwaj S., Singh M., Kirtipal N., Kang S.G. (2021). SARS-CoV-2 and Glutamine: SARS-CoV-2 Triggered Pathogenesis via Metabolic Reprograming of Glutamine in Host Cells. Front. Mol. Biosci..

[85] Lionetto L., Ulivieri M., Capi M., De Bernardini D., Fazio F., Petrucca A., Pomes L.M., De Luca O., Gentile G., Casolla B. (2021). Increased kynurenine-to-tryptophan ratio in the serum of patients infected with SARS-CoV2: An observational cohort study. Biochim. Et Biophys. Acta (BBA)-Mol. Basis Dis..

[86] Mezrich J.D., Fechner J.H., Zhang X., Johnson B.P., Burlingham W.J., Bradfield C.A. (2010). An interaction between kynurenine and the aryl hydrocarbon receptor can generate regulatory T cells. J. Immunol..

[87] Fallarino F., Grohmann U., Vacca C., Bianchi R., Orabona C., Spreca A., Fioretti M., Puccetti P. (2002). T cell apoptosis by tryptophan catabolism. Cell Death Differ..

[88] Fraser D.D., Slessarev M., Martin C.M., Daley M., Patel M.A., Miller M.R., Patterson E.K., O’Gorman D.B., Gill S.E., Wishart D.S. (2020). Metabolomics Profiling of Critically Ill Coronavirus Disease 2019 Patients: Identification of Diagnostic and Prognostic Biomarkers. Crit. Care Explor..

[89] Khanfar A., Al Qaroot B. (2020). Could glutathione depletion be the Trojan horse of COVID-19 mortality?. Eur. Rev. Med. Pharmacol. Sci..

[90] Bartolini D., Stabile A.M., Bastianelli S., Giustarini D., Pierucci S., Busti C., Vacca C., Gidari A., Francisci D., Castronari R. (2021). SARS-CoV2 infection impairs the metabolism and redox function of cellular glutathione. Redox Biol..

[91] Polonikov A. (2020). Endogenous deficiency of glutathione as the most likely cause of serious manifestations and death in COVID-19 patients. ACS Infect. Dis..

[92] Zhu X., Tang Z., Cong B., Du J., Wang C., Wang L., Ni X., Lu J. (2013). Estrogens increase cystathionine-γ-lyase expression and decrease inflammation and oxidative stress in the myocardium of ovariectomized rats. Menopause.

[93] Lambertini E., Penolazzi L., Angelozzi M., Grassi F., Gambari L., Lisignoli G., De Bonis P., Cavallo M., Piva R. (2017). The expression of cystathionine gamma-lyase is regulated by estrogen receptor alpha in human osteoblasts. Oncotarget.

[94] Sen A. (2021). Does serotonin deficiency lead to anosmia, ageusia, dysfunctional chemesthesis and increased severity of illness in COVID-19?. Med. Hypotheses.

[95] Lyhne M.D., Kline J.A., Nielsen-Kudsk J.E., Andersen A. (2020). Pulmonary vasodilation in acute pulmonary embolism–a systematic review. Pulm. Circ..

[96] Majno G., Palade G. (1961). Studies on inflammation: I. The effect of histamine and serotonin on vascular permeability: An electron microscopic study. J. Cell Biol..

[97] Soslau G., Morgan D.A., Jaffe J.S., Brodsky I., Wang Y. (1997). Cytokine mRNA expression in human platelets and a megakaryocytic cell line and cytokine modulation of platelet function. Cytokine.

[98] Wang L., Ahn Y.J., Asmis R. (2020). Sexual dimorphism in glutathione metabolism and glutathione-dependent responses. Redox Biol..

[99] Bhaskar S., Sinha A., Banach M., Mittoo S., Weissert R., Kass J.S., Rajagopal S., Pai A.R., Kutty S. (2020). Cytokine storm in COVID-19—immunopathological mechanisms, clinical considerations, and therapeutic approaches: The REPROGRAM consortium position paper. Front. Immunol..

[100] Chen Y., Guillemin G.J. (2009). Kynurenine pathway metabolites in humans: Disease and healthy states. Int. J. Tryptophan Res..

[101] Cengiz M., Uysal B.B., Ikitimur H., Ozcan E., Islamoğlu M.S., Aktepe E., Yavuzer H., Yavuzer S. (2020). Effect of oral L-Glutamine supplementation on COVID-19 treatment. Clin. Nutr. Exp..

[102] Feehan J., de Courten M., Apostolopoulos V., de Courten B. (2021). Nutritional Interventions for COVID-19: A Role for Carnosine?. Nutrients.

[103] Xing L., Chee M.E., Zhang H., Zhang W., Mine Y. (2019). Carnosine—A natural bioactive dipeptide: Bioaccessibility, bioavailability and health benefits. J. Food Bioact..

[104] Fresta C.G., Fidilio A., Lazzarino G., Musso N., Grasso M., Merlo S., Amorini A.M., Bucolo C., Tavazzi B., Lazzarino G. (2020). Modulation of Pro-Oxidant and Pro-Inflammatory Activities of M1 Macrophages by the Natural Dipeptide Carnosine. Int. J. Mol. Sci..

[105] Saadah L.M., Deiab G.A.I.A., Al-Balas Q., Basheti I.A. (2020). Carnosine to Combat Novel Coronavirus (nCoV): Molecular Docking and Modeling to Cocrystallized Host Angiotensin-Converting Enzyme 2 (ACE2) and Viral Spike Protein. Molecules.

[106] Hood E.D., Chorny M., Greineder C.F., Alferiev I.S., Levy R.J., Muzykantov V.R. (2014). Endothelial targeting of nanocarriers loaded with antioxidant enzymes for protection against vascular oxidative stress and inflammation. Biomaterials.

[107] Guloyan V., Oganesian B., Baghdasaryan N., Yeh C., Singh M., Guilford F., Ting Y.-S., Venketaraman V. (2020). Glutathione supplementation as an adjunctive therapy in COVID-19. Antioxidants.

[108] Silvagno F., Vernone A., Pescarmona G.P. (2020). The role of glutathione in protecting against the severe inflammatory response triggered by COVID-19. Antioxidants.

[109] Lei G.-S., Zhang C., Cheng B.-H., Lee C.-H. (2017). Mechanisms of Action of Vitamin D as Supplemental Therapy for Pneumocystis Pneumonia. Antimicrob. Agents Chemother..

[110] Kanwar B., Lee C.J., Lee J.-H. (2021). Specific Treatment Exists for SARS-CoV-2 ARDS. Vaccines.

