# Supplementary material for: Severe COVID-19 Is Characterised by Perturbations in Plasma Amines Correlated with Immune Response Markers, and Linked to Inflammation and Oxidative Stress
Source: Metabolites. 2022 Jul 2;12(7):618. doi: 10.3390/metabo12070618 (PMC9321395; doi:10.3390/metabo12070618)
Supplement: Supplementary file 1 [file metabolites-12-00618-s001.zip › Document S3.pdf]

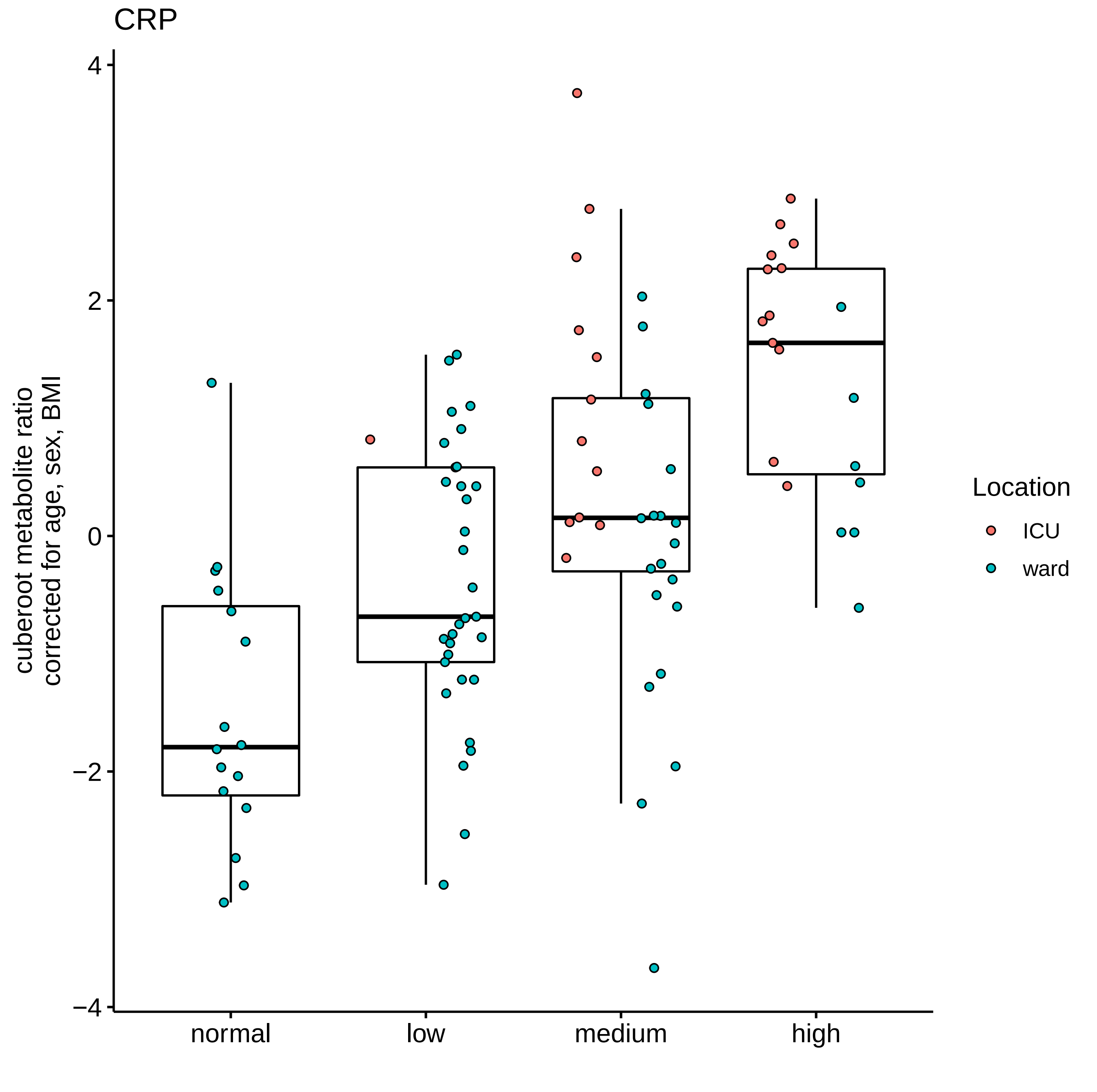

CRP
cuberoot metabolite ratio
corrected for age, sex, BMI
4
2
0
−2
−4
normal
low
medium
high
Location
ICU
ward

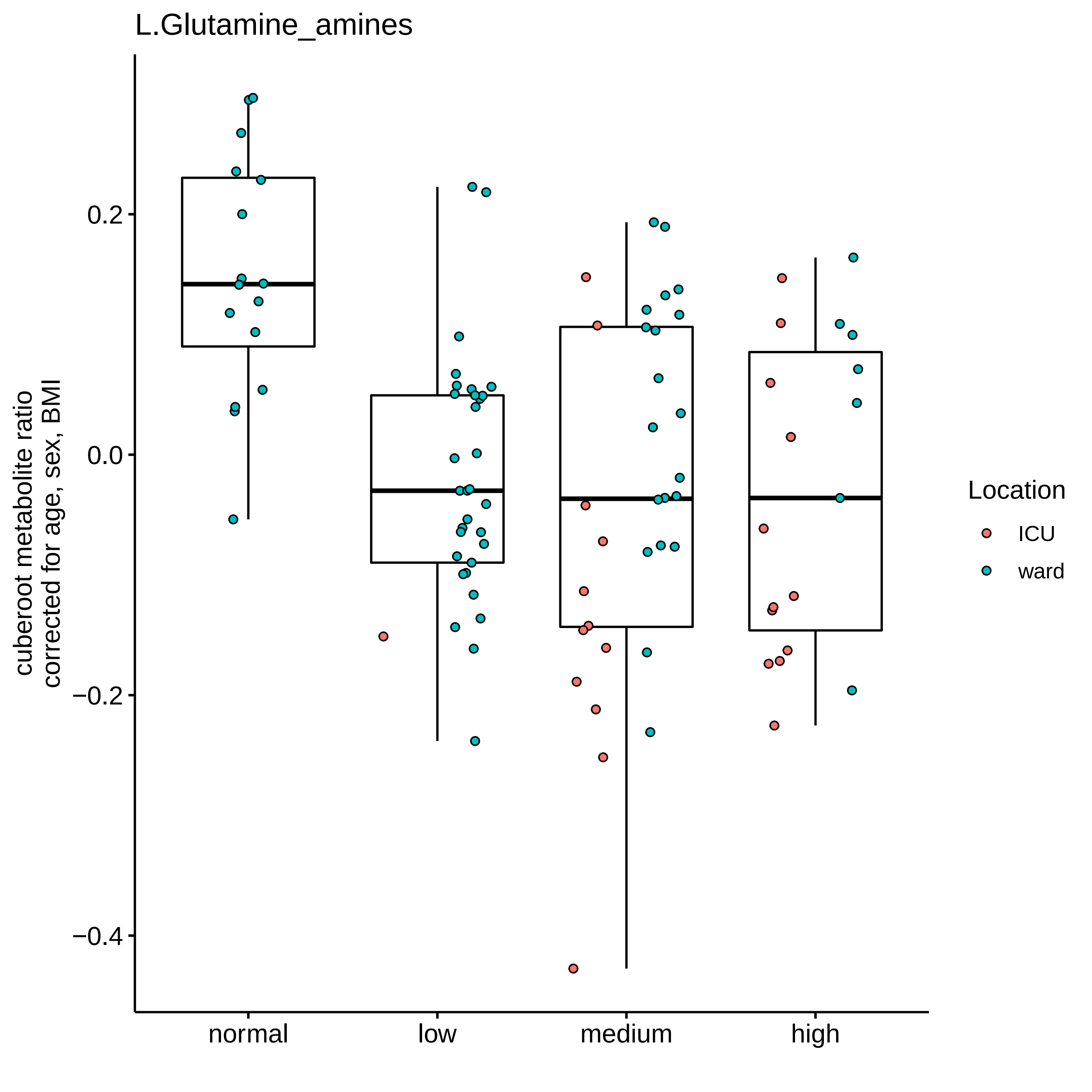

L.Glutamine_amines
cuberoot metabolite ratio
corrected for age, sex, BMI
0.2
0.0
−0.2
−0.4
normal
low
medium
high
Location
ICU
ward

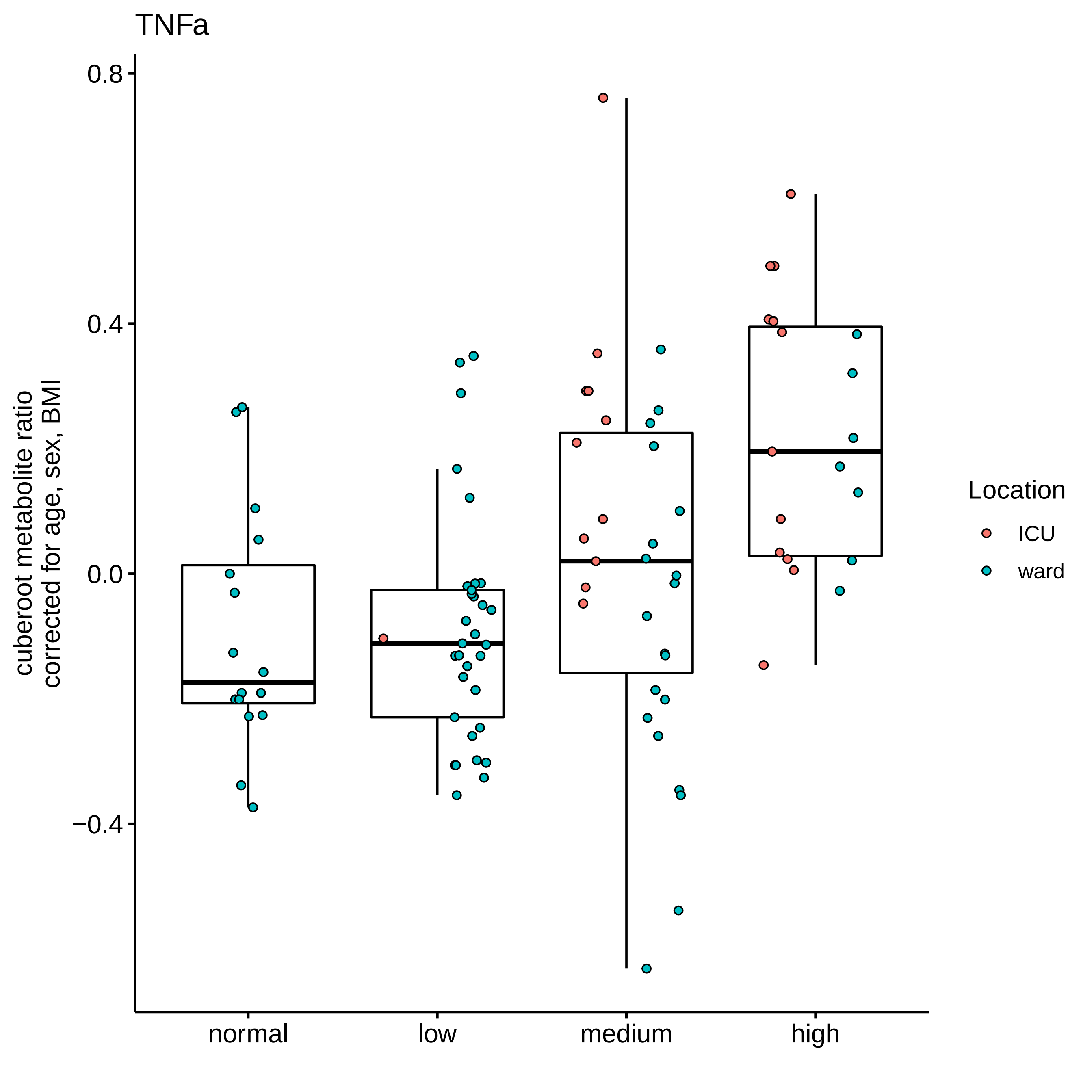
TNFa
0.8
0.4
0.0
−0.4
cuberoot metabolite ratio
corrected for age, sex, BMI
normal
low
medium
high
Location
ICU
ward

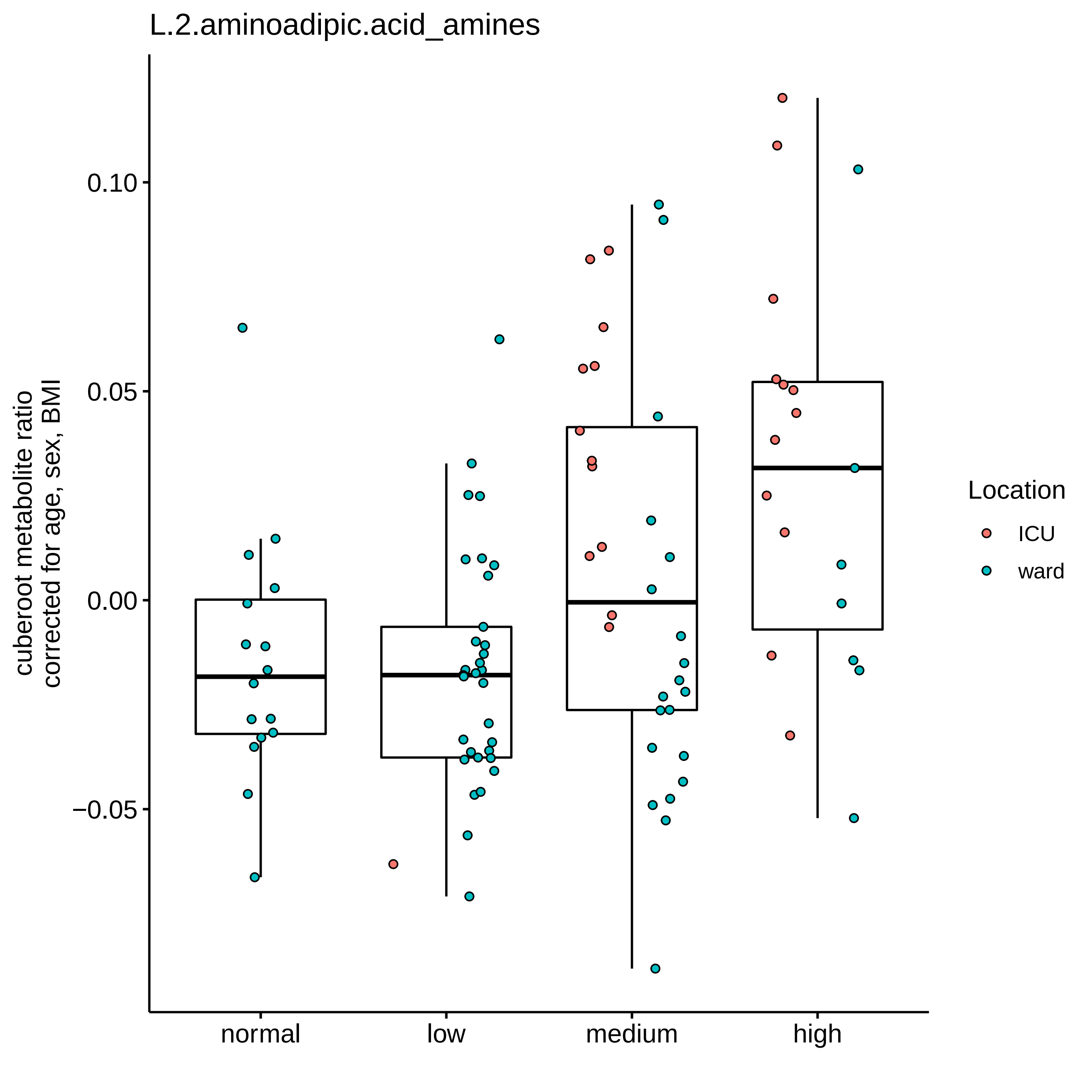
L.2.aminoadipic.acid_amines
cuberoot metabolite ratio
corrected for age, sex, BMI
0.10
0.05
0.00
−0.05
normal
low
medium
high
Location
ICU
ward

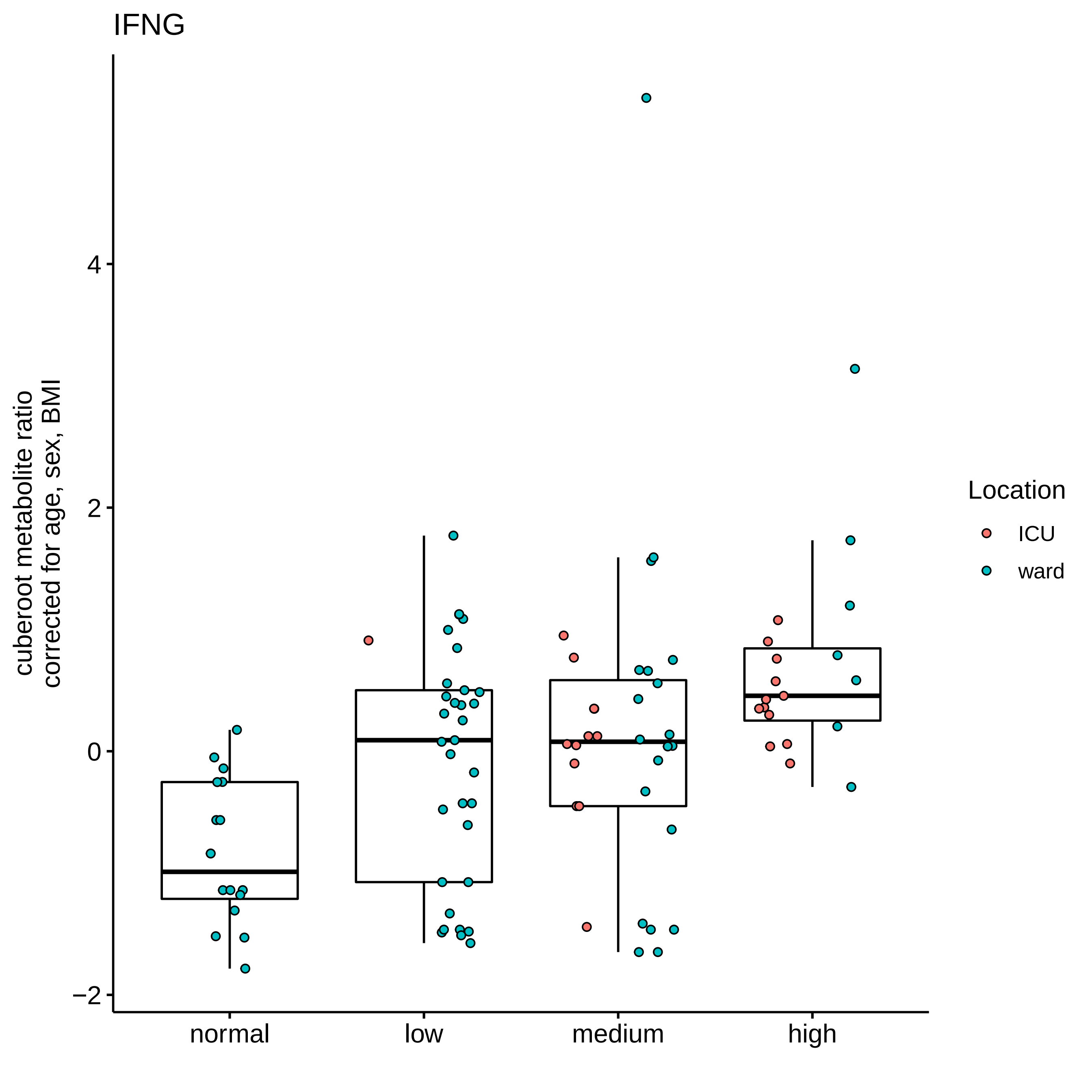

IFNG
cuberoot metabolite ratio
corrected for age, sex, BMI
4
2
0
−2
normal
low
medium
high
Location
ICU
ward

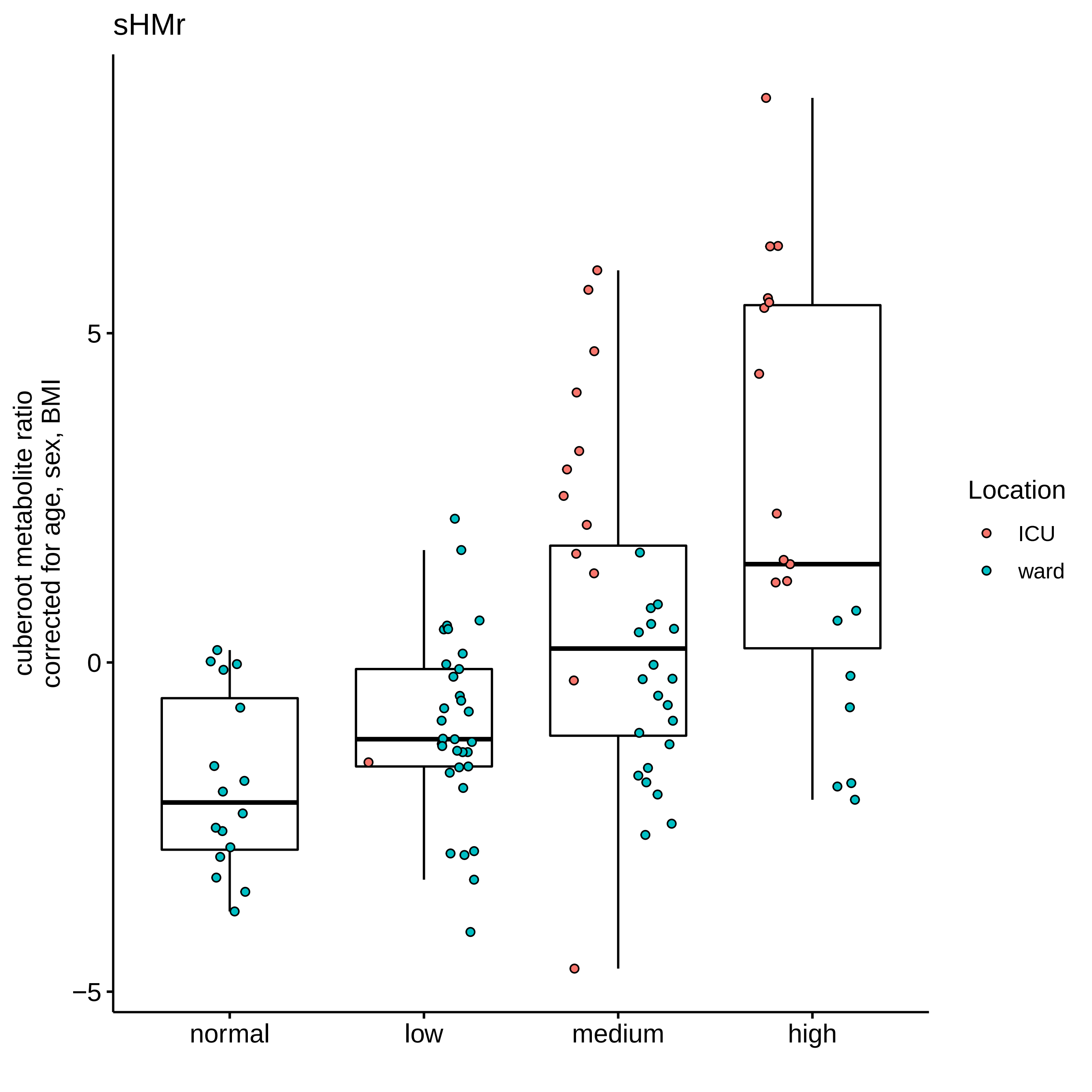

sHMr
cuberoot metabolite ratio
corrected for age, sex, BMI
5
0
−5
normal
low
medium
high
Location
ICU
ward

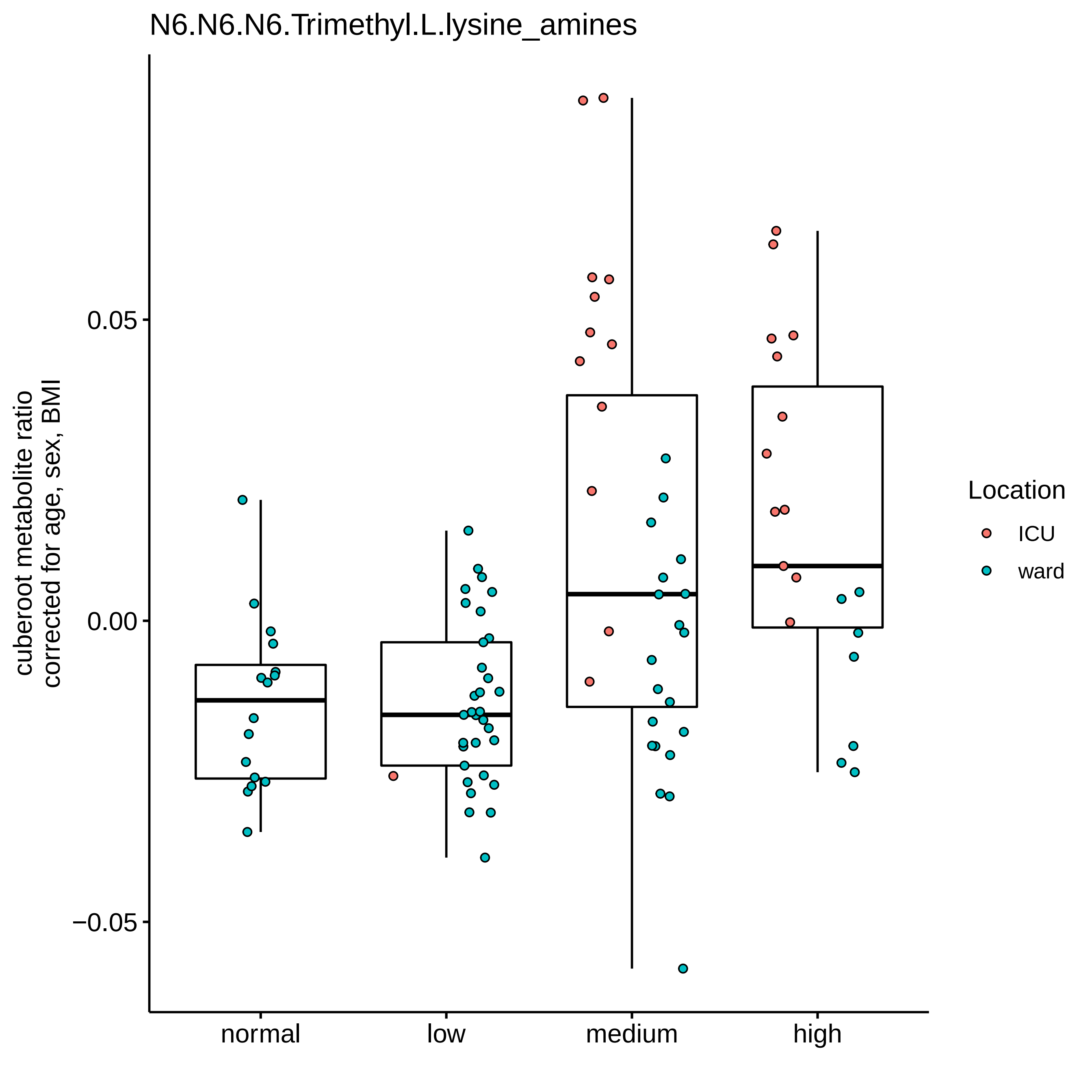
N6.N6.N6.Trimethyl.L.lysine_amines
cuberoot metabolite ratio
corrected for age, sex, BMI
0.05
0.00
−0.05
normal
low
medium
high
Location
ICU
ward

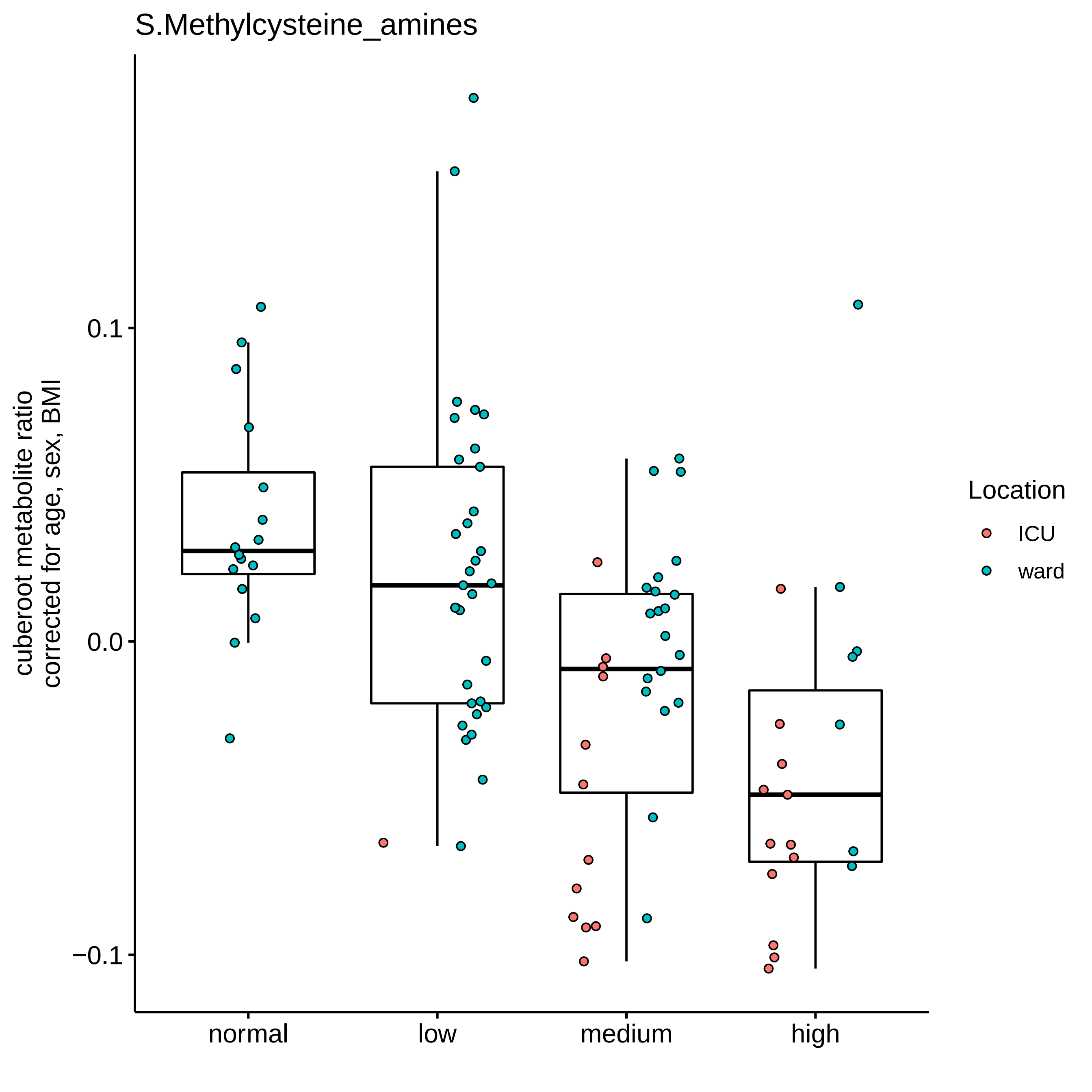
S.Methylcysteine_amines
cuberoot metabolite ratio
corrected for age, sex, BMI
0.1
0.0
−0.1
normal
low
medium
high
Location
ICU
ward

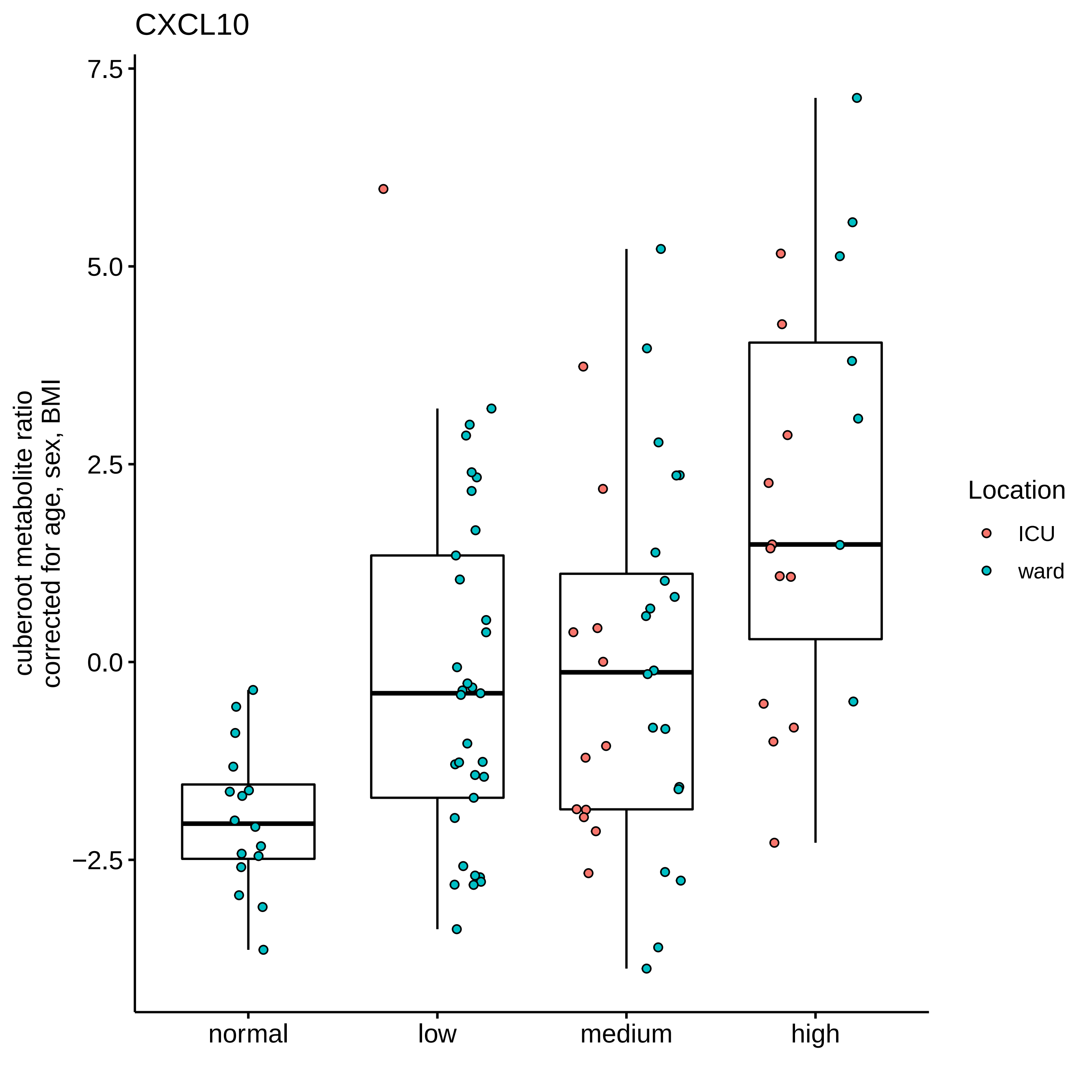
CXCL10
cuberoot metabolite ratio
corrected for age, sex, BMI
7.5
5.0
2.5
0.0
−2.5
normal
low
medium
high
Location
ICU
ward

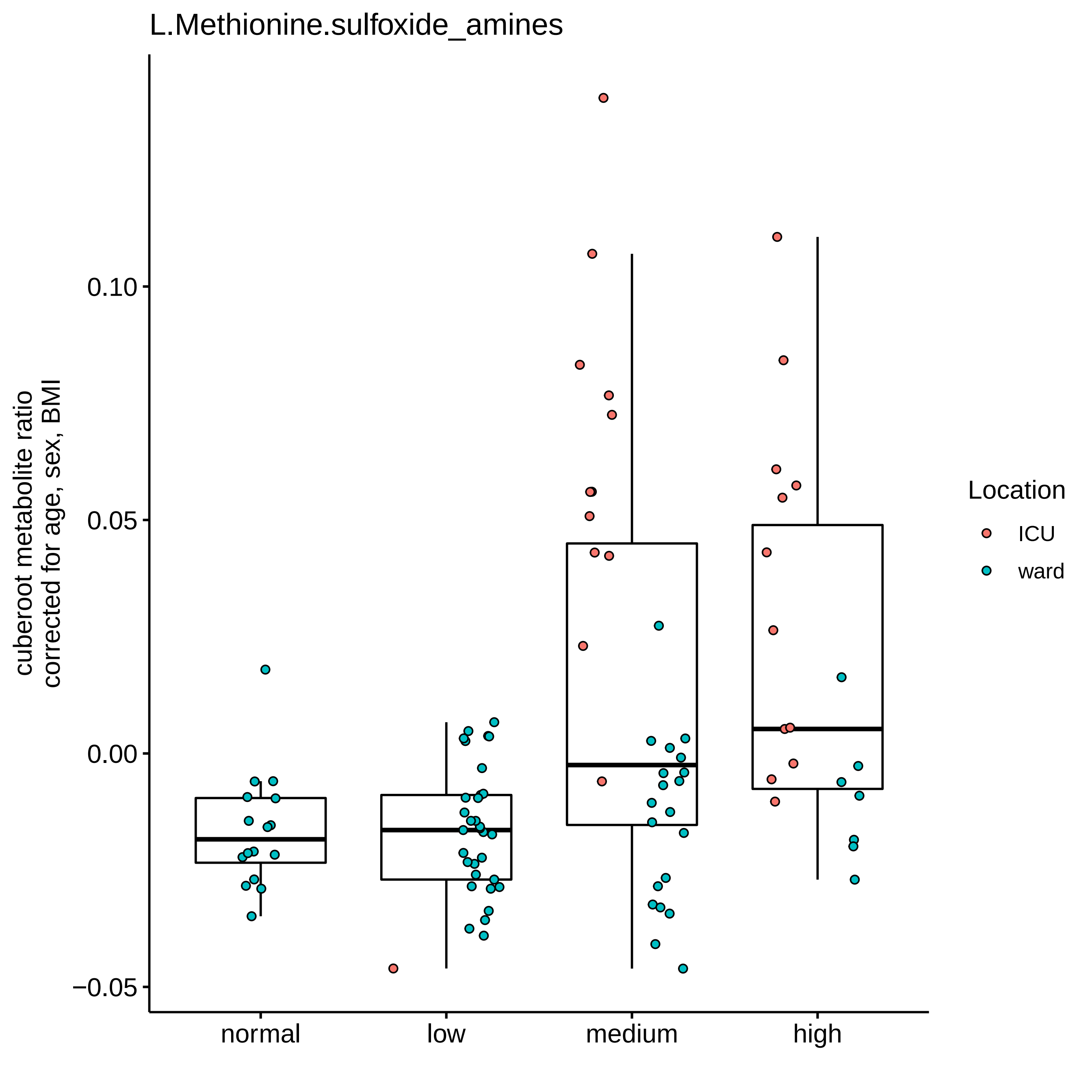

L.Methionine.sulfoxide_amines
cuberoot metabolite ratio
corrected for age, sex, BMI
0.10
0.05
0.00
−0.05
normal
low
medium
high
Location
ICU
ward

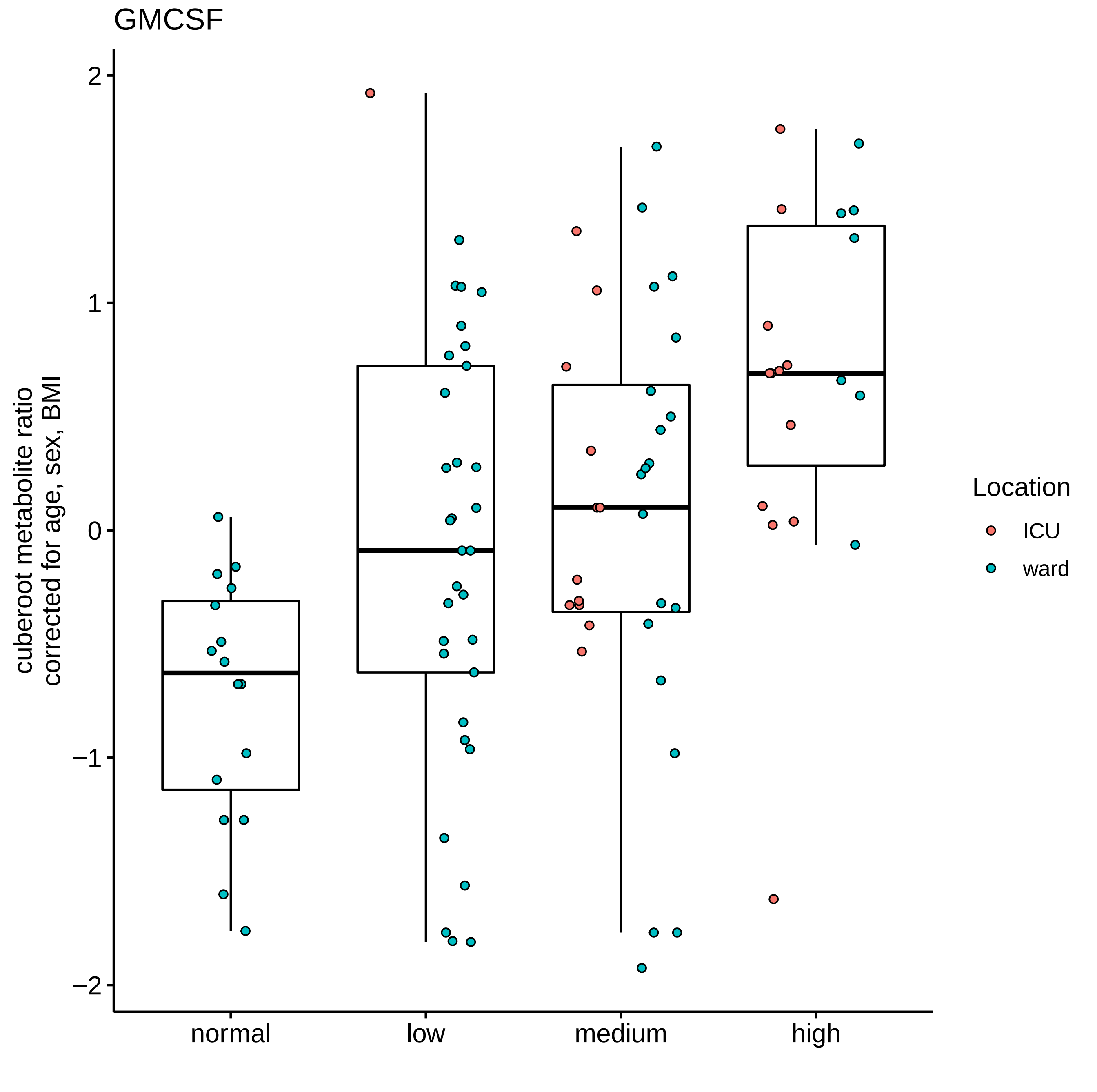
GMCSF
cuberoot metabolite ratio
corrected for age, sex, BMI
2
1
0
−1
−2
normal
low
medium
high
Location
ICU
ward

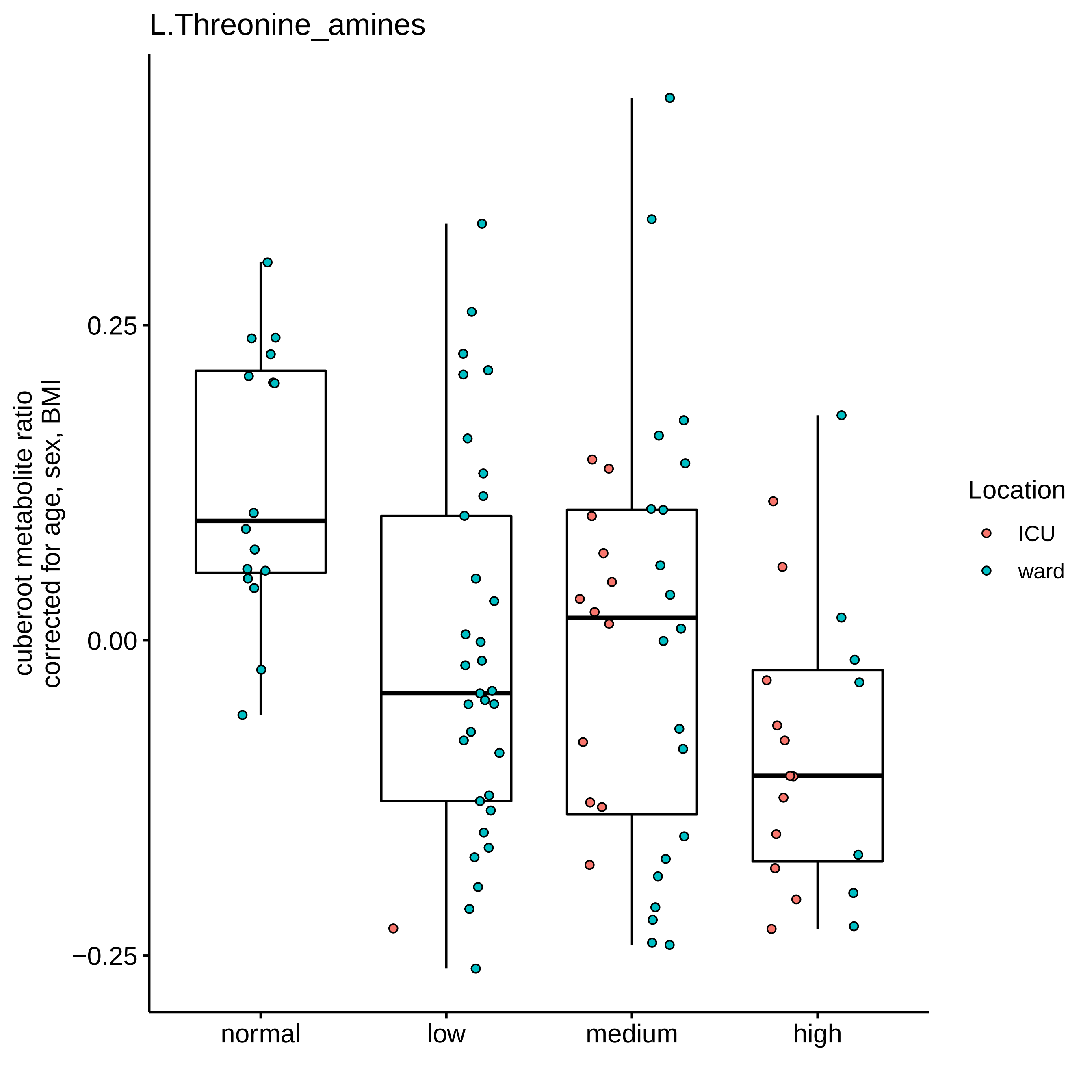

L.Threonine_amines
cuberoot metabolite ratio
corrected for age, sex, BMI
0.25
0.00
−0.25
normal
low
medium
high
Location
ICU
ward

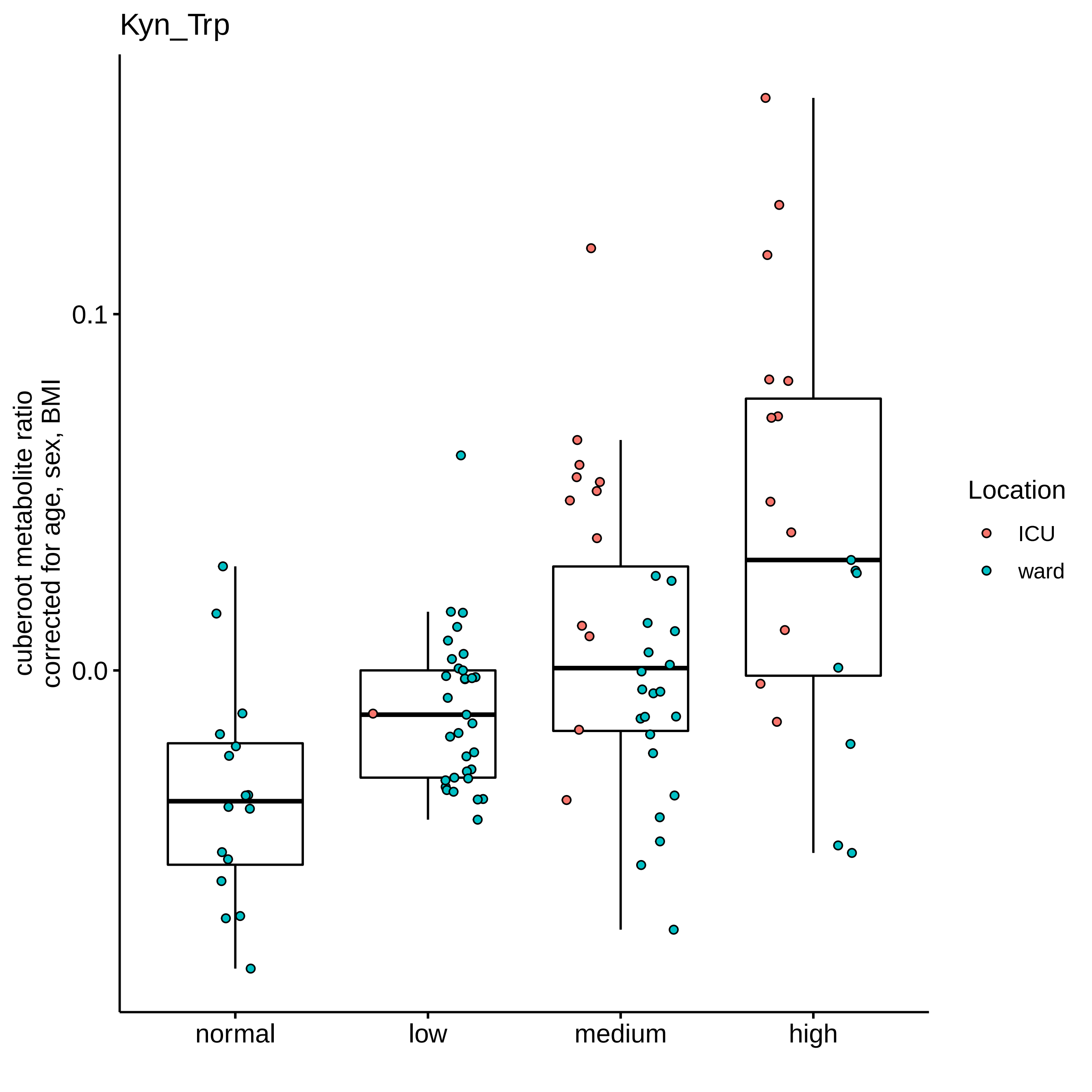

Kyn_Trp
cuberoot metabolite ratio
corrected for age, sex, BMI
0.1
0.0
normal
low
medium
high
Location
ICU
ward

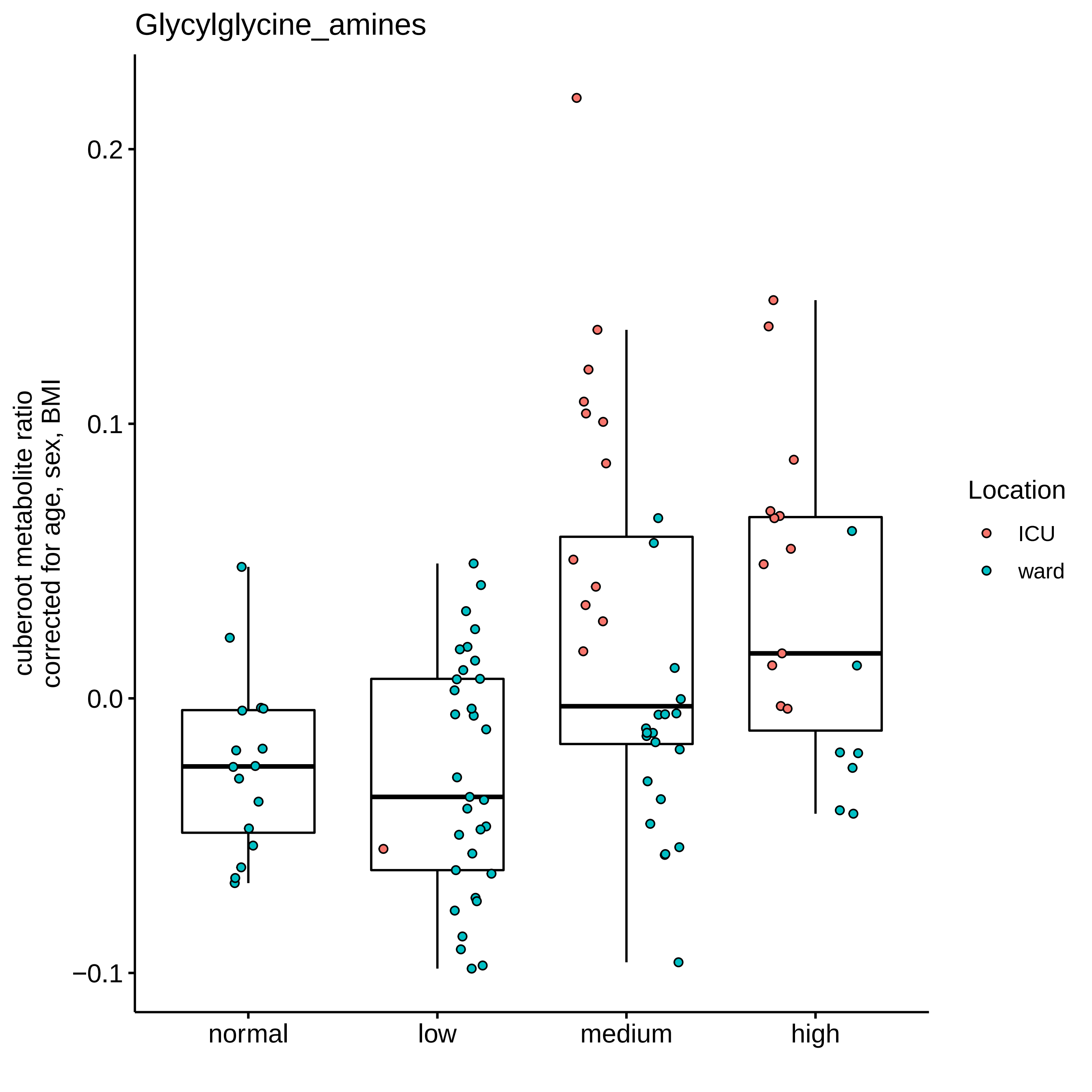
Glycylglycine_amines
cuberoot metabolite ratio
corrected for age, sex, BMI
0.2
0.1
0.0
−0.1
normal
low
medium
high
Location
ICU
ward

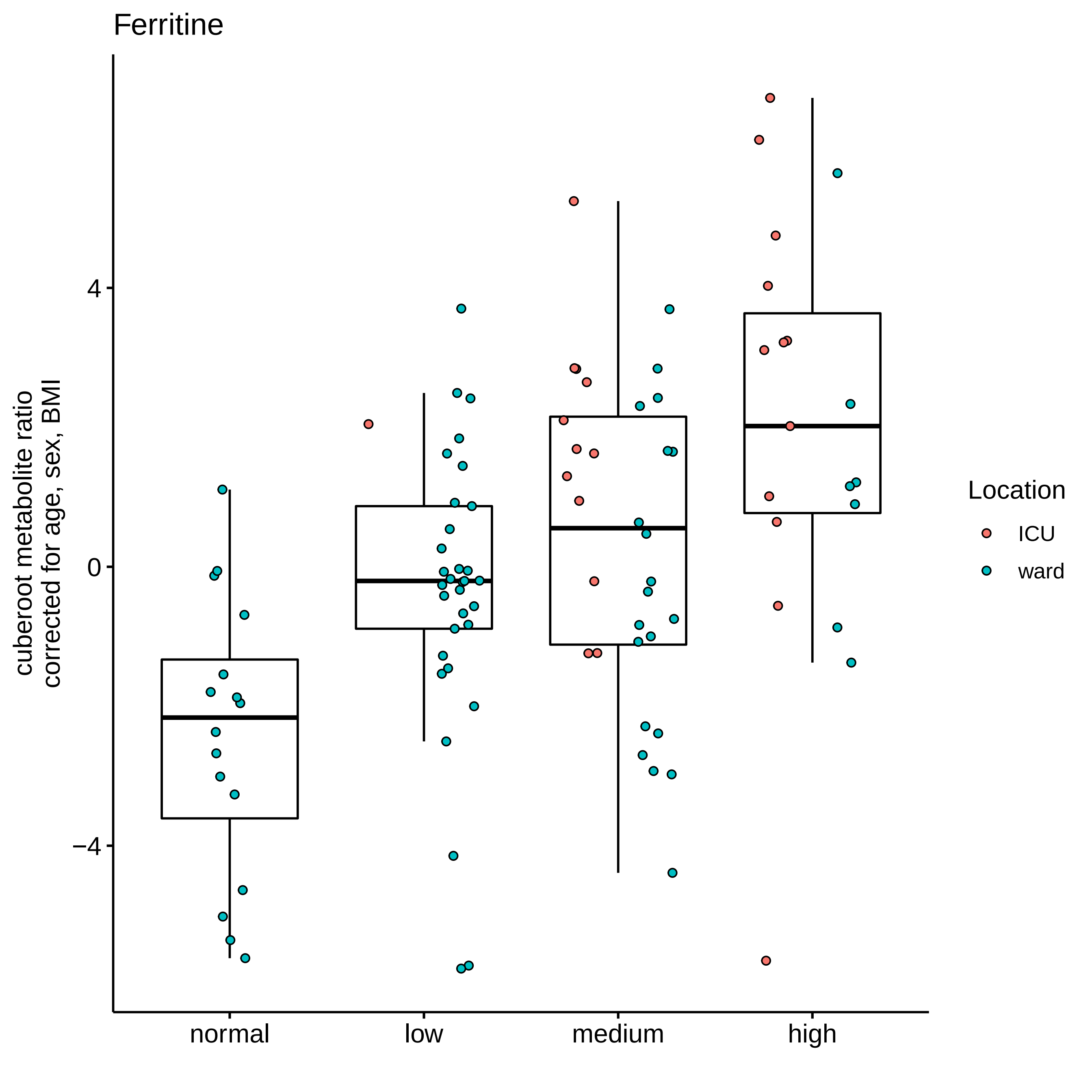
Ferritine
cuberoot metabolite ratio
corrected for age, sex, BMI
4
0
−4
normal
low
medium
high
Location
ICU
ward

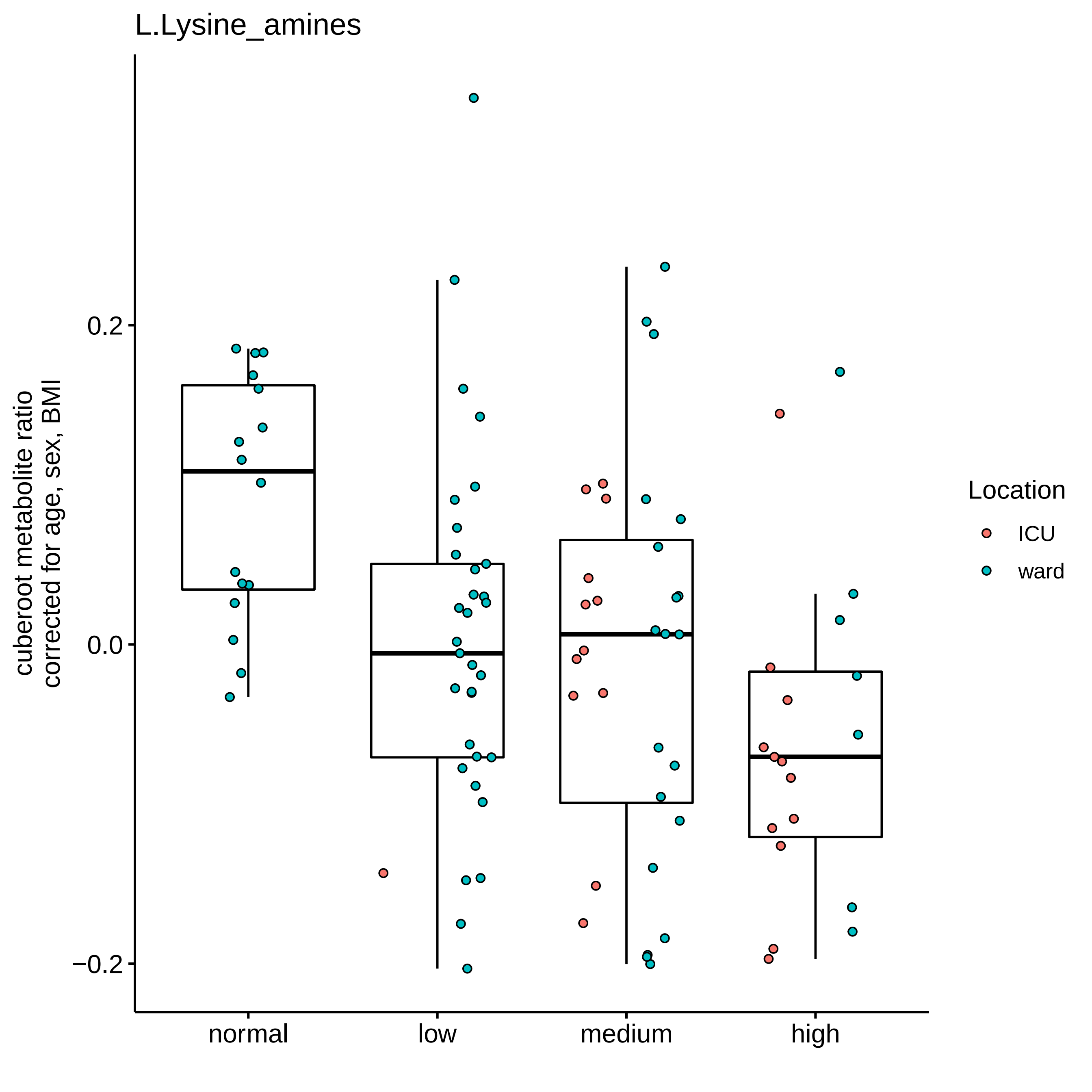

L.Lysine_amines
cuberoot metabolite ratio
corrected for age, sex, BMI
0.2
0.0
−0.2
normal
low
medium
high
Location
ICU
ward

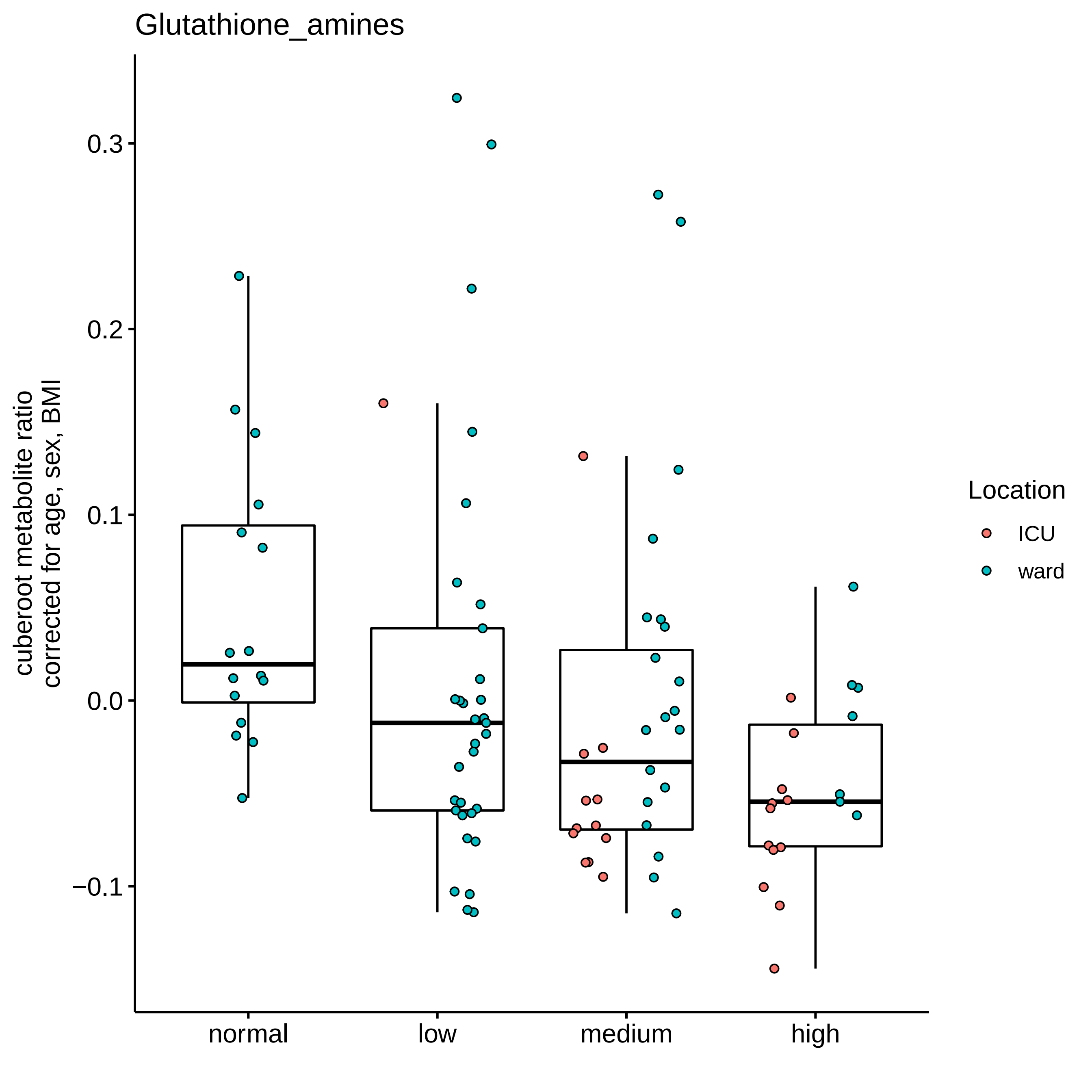

Glutathione_amines
cuberoot metabolite ratio
corrected for age, sex, BMI
0.3
0.2
0.1
0.0
−0.1
normal
low
medium
high
Location
ICU
ward

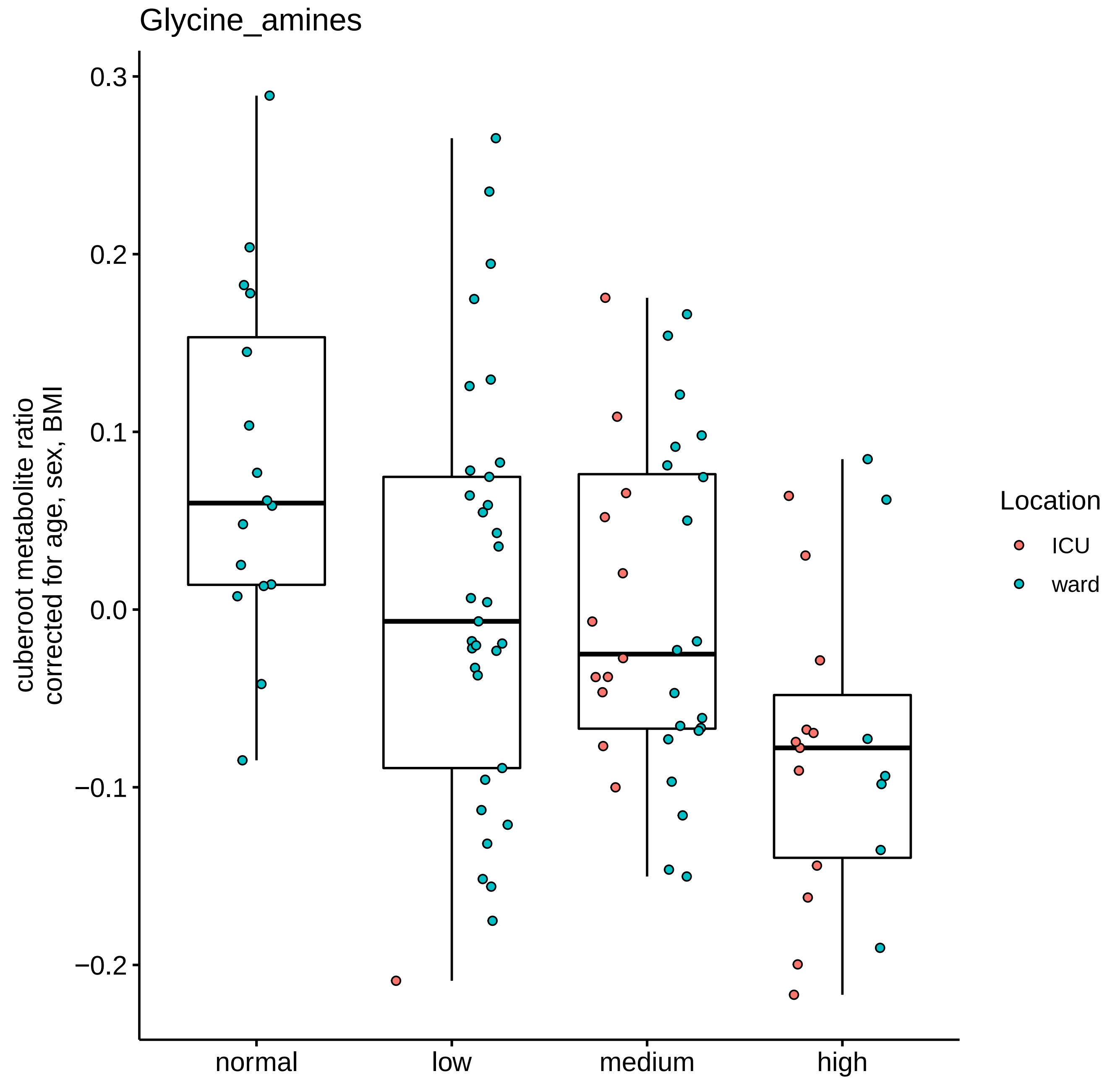
Glycine_amines
cuberoot metabolite ratio
corrected for age, sex, BMI
0.3
0.2
0.1
0.0
−0.1
−0.2
normal
low
medium
high
Location
ICU
ward

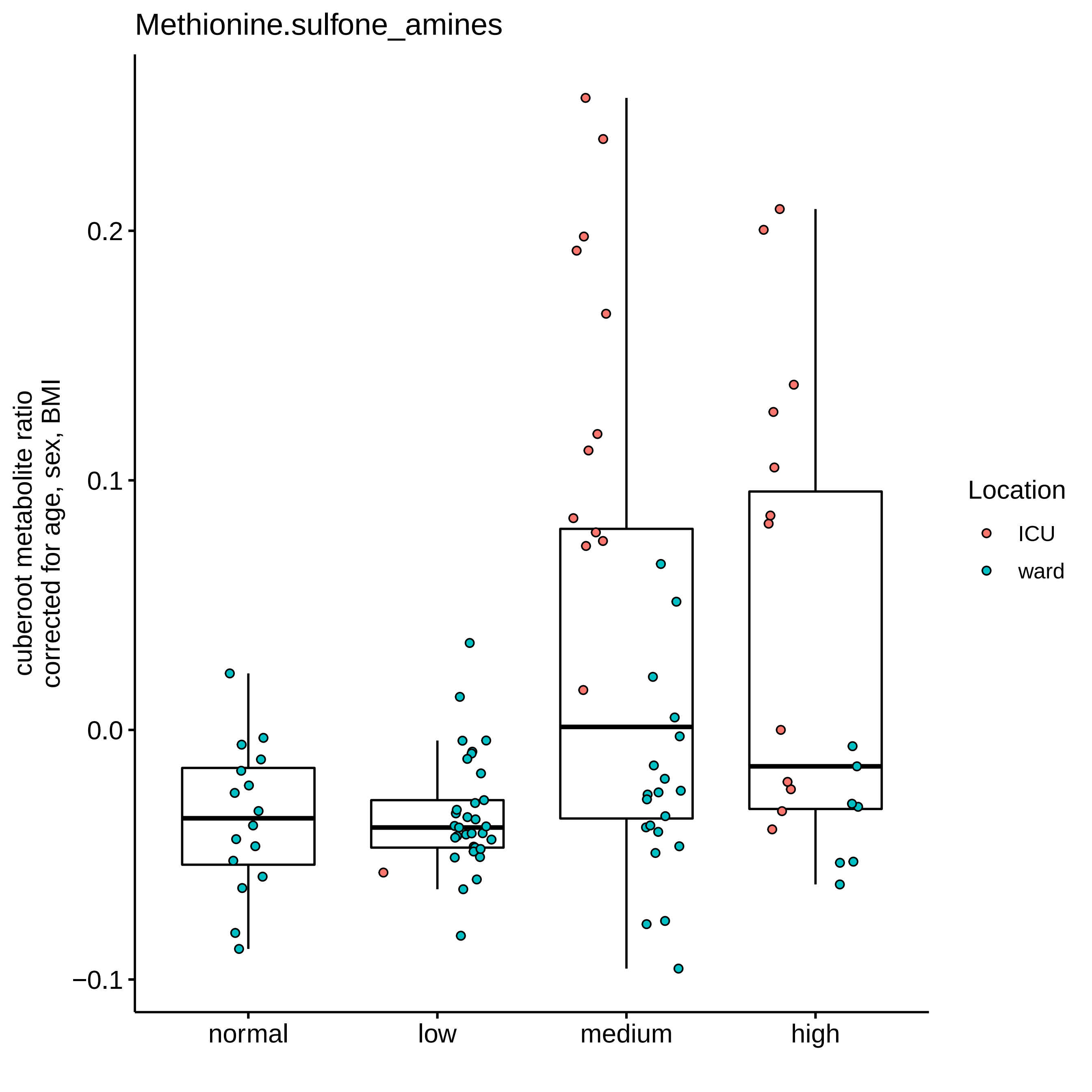

Methionine.sulfone_amines
cuberoot metabolite ratio
corrected for age, sex, BMI
0.2
0.1
0.0
−0.1
normal
low
medium
high
Location
ICU
ward

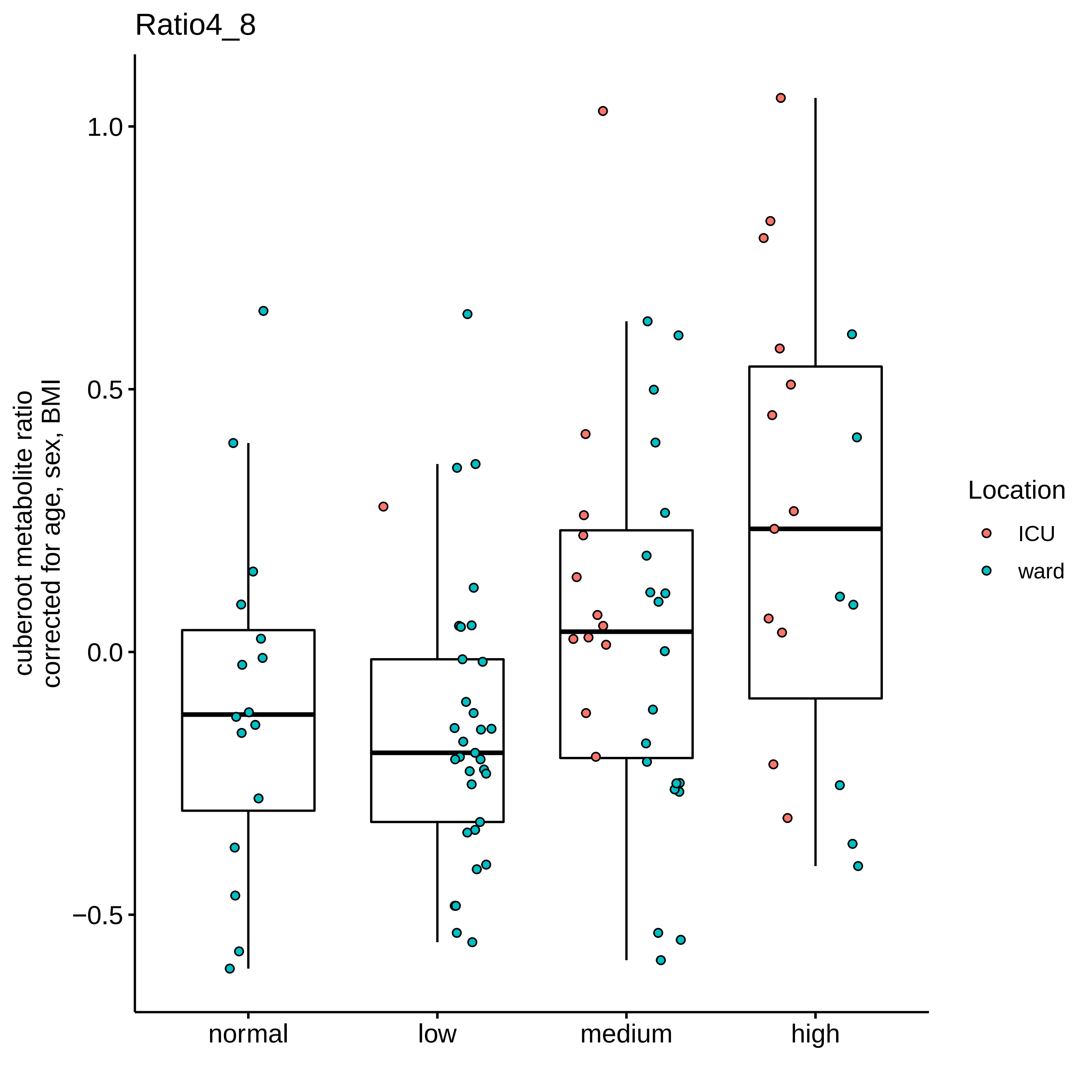
Ratio4_8
cuberoot metabolite ratio
corrected for age, sex, BMI
1.0
0.5
0.0
−0.5
normal
low
medium
high
Location
ICU
ward

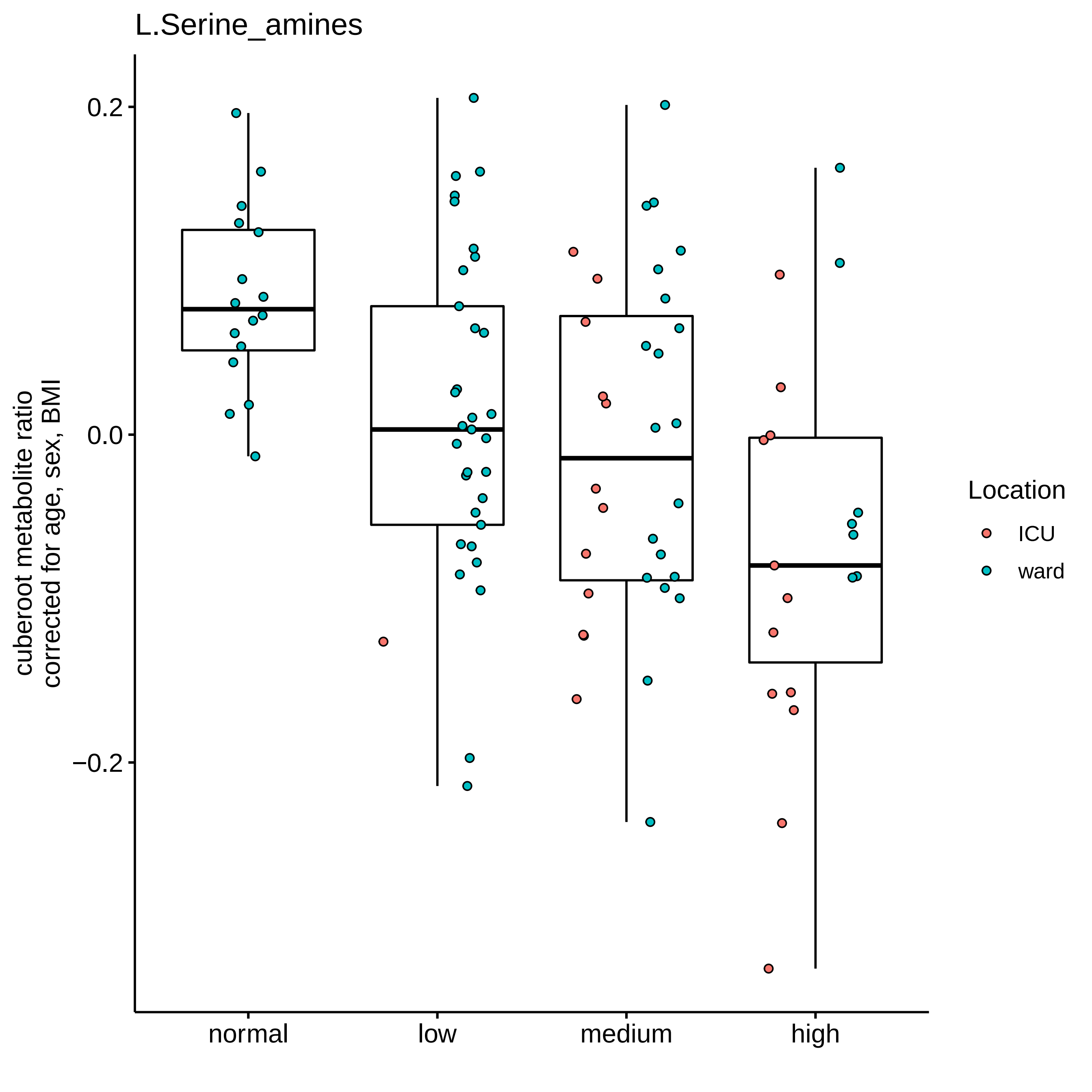

L.Serine_amines
cuberoot metabolite ratio
corrected for age, sex, BMI
0.2
0.0
−0.2
normal
low
medium
high
Location
ICU
ward

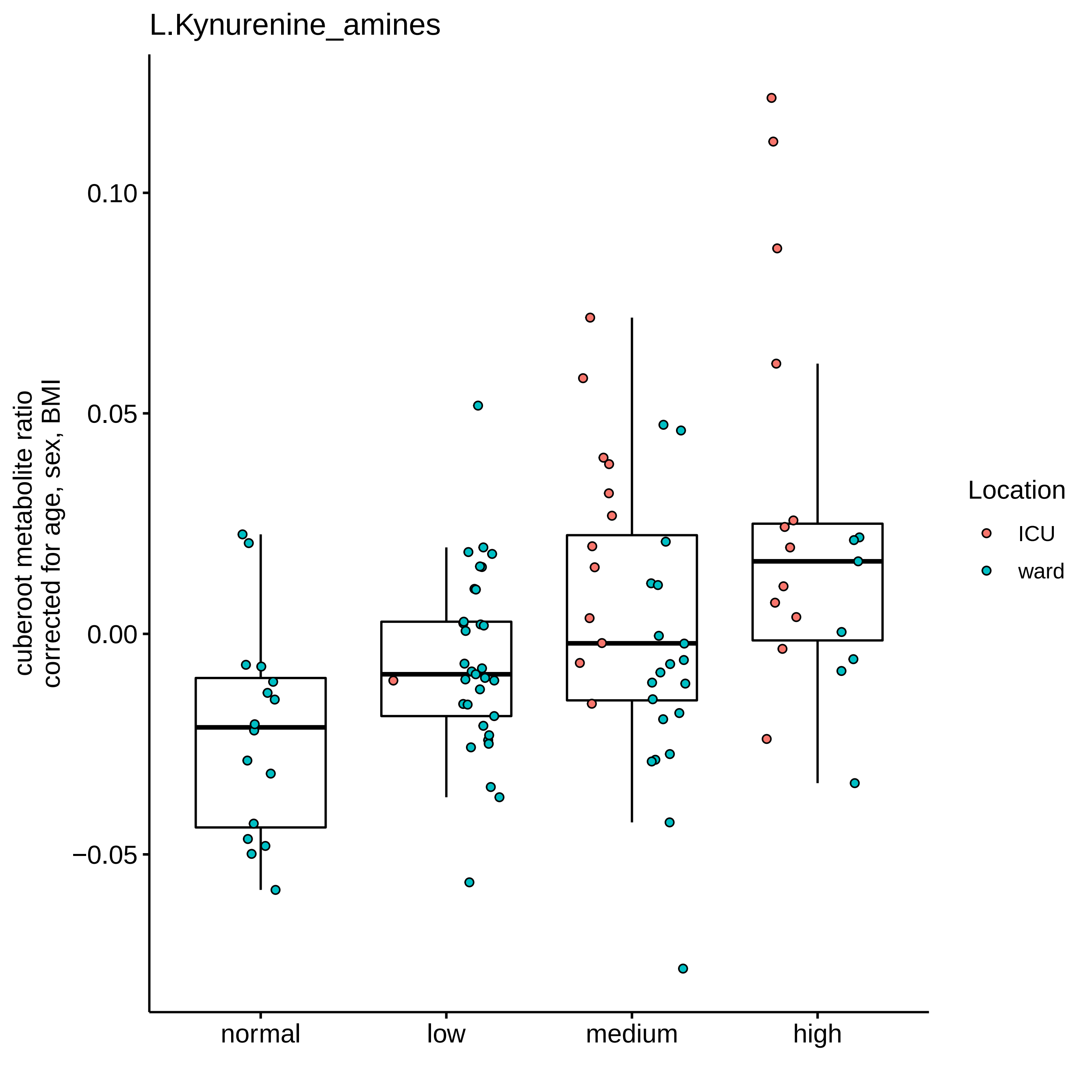

L.Kynurenine_amines
cuberoot metabolite ratio
corrected for age, sex, BMI
0.10
0.05
0.00
−0.05
normal
low
medium
high
Location
ICU
ward

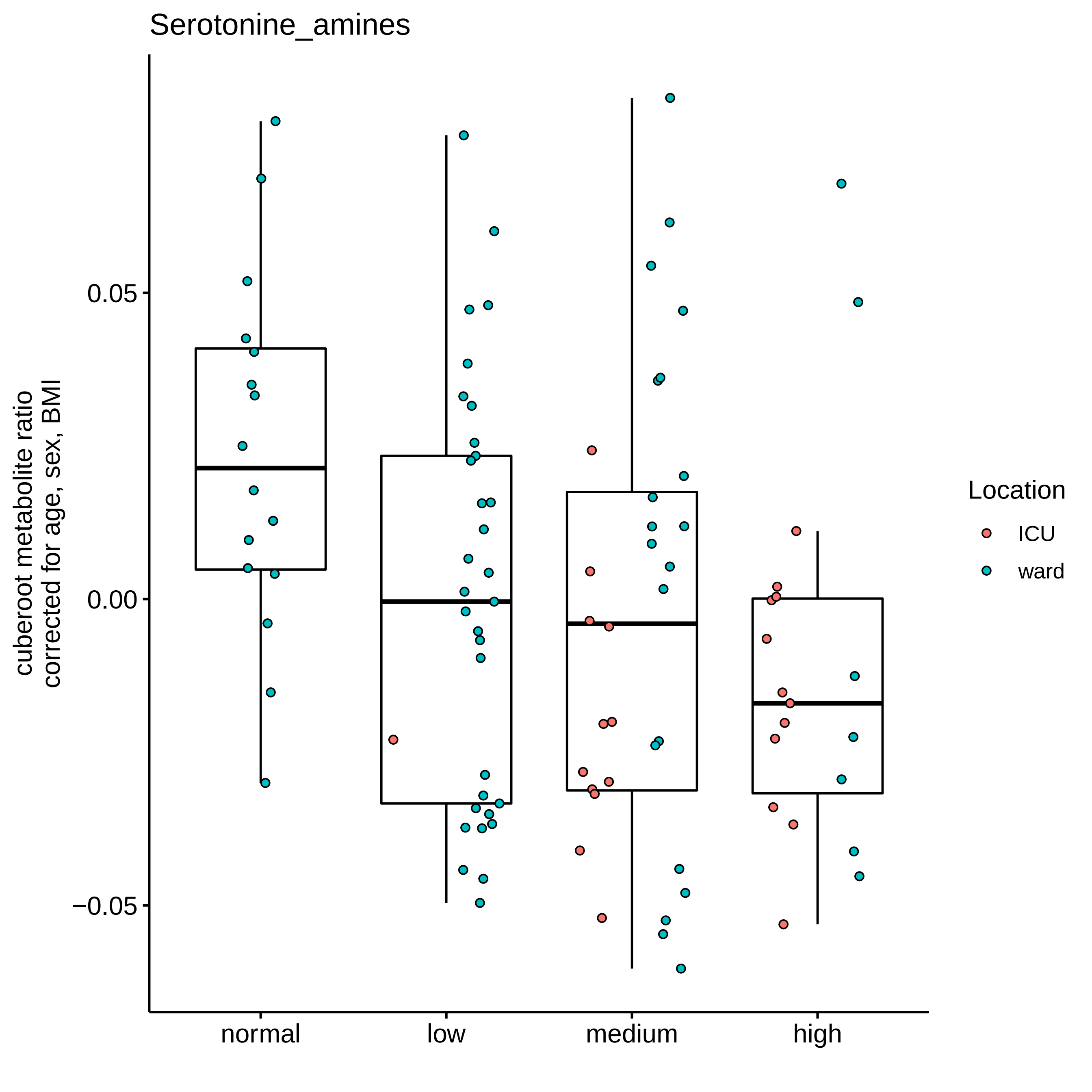
Serotonine_amines
cuberoot metabolite ratio
corrected for age, sex, BMI
0.05
0.00
−0.05
normal
low
medium
high
Location
ICU
ward

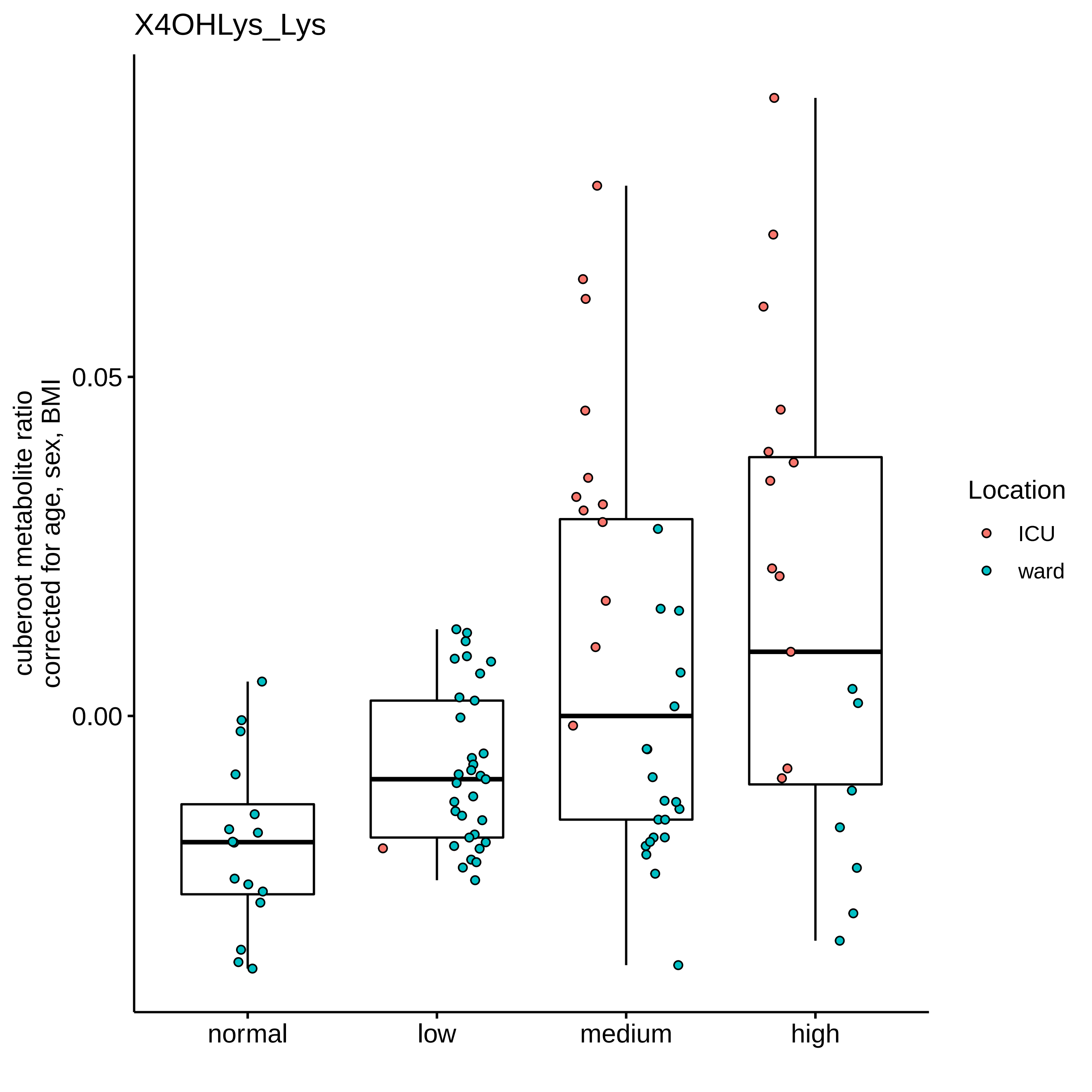
X4OHLys_Lys
cuberoot metabolite ratio
corrected for age, sex, BMI
0.05
0.00
normal
low
medium
high
Location
ICU
ward

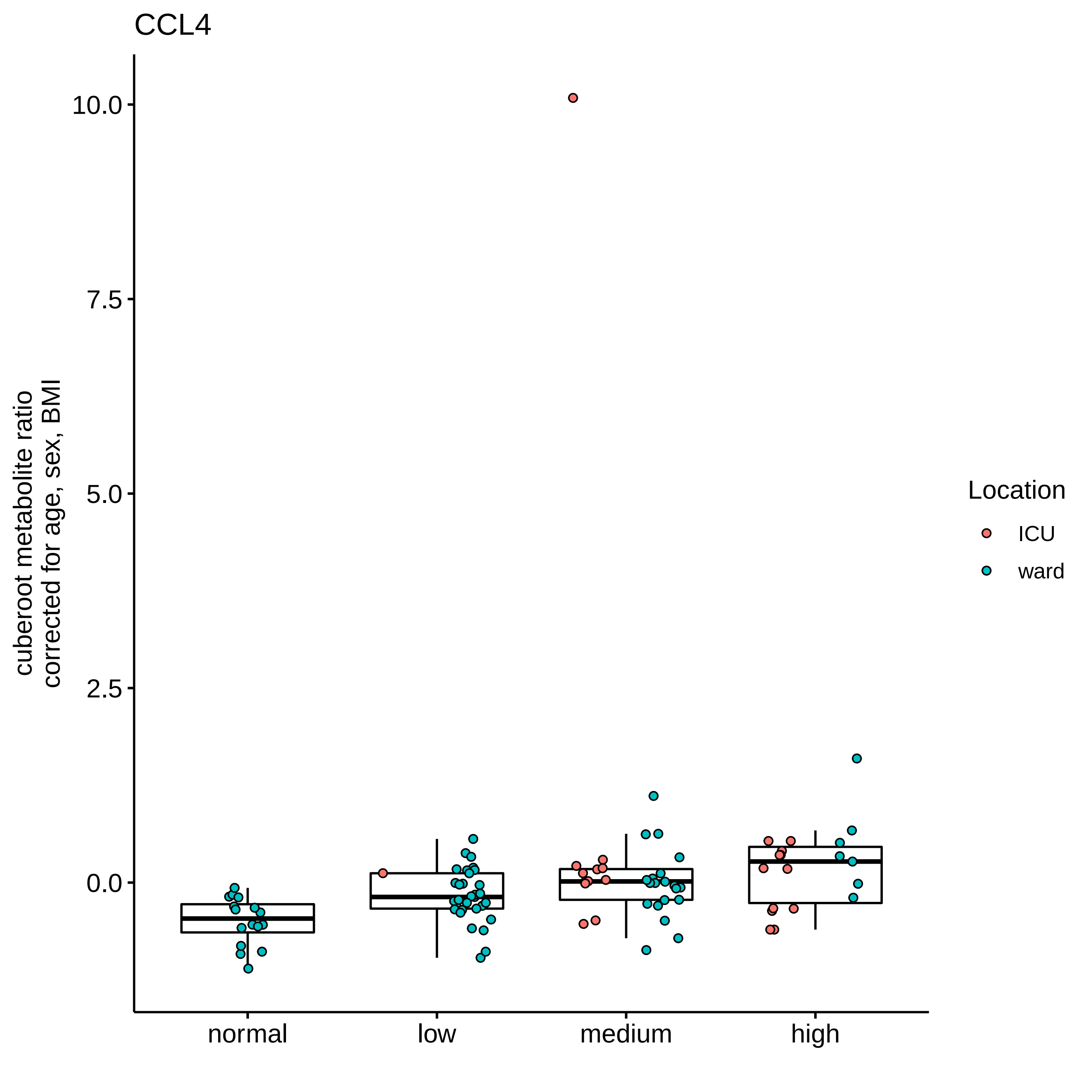

CCL4
cuberoot metabolite ratio
corrected for age, sex, BMI
10.0
7.5
5.0
2.5
0.0
normal
low
medium
high
Location
ICU
ward

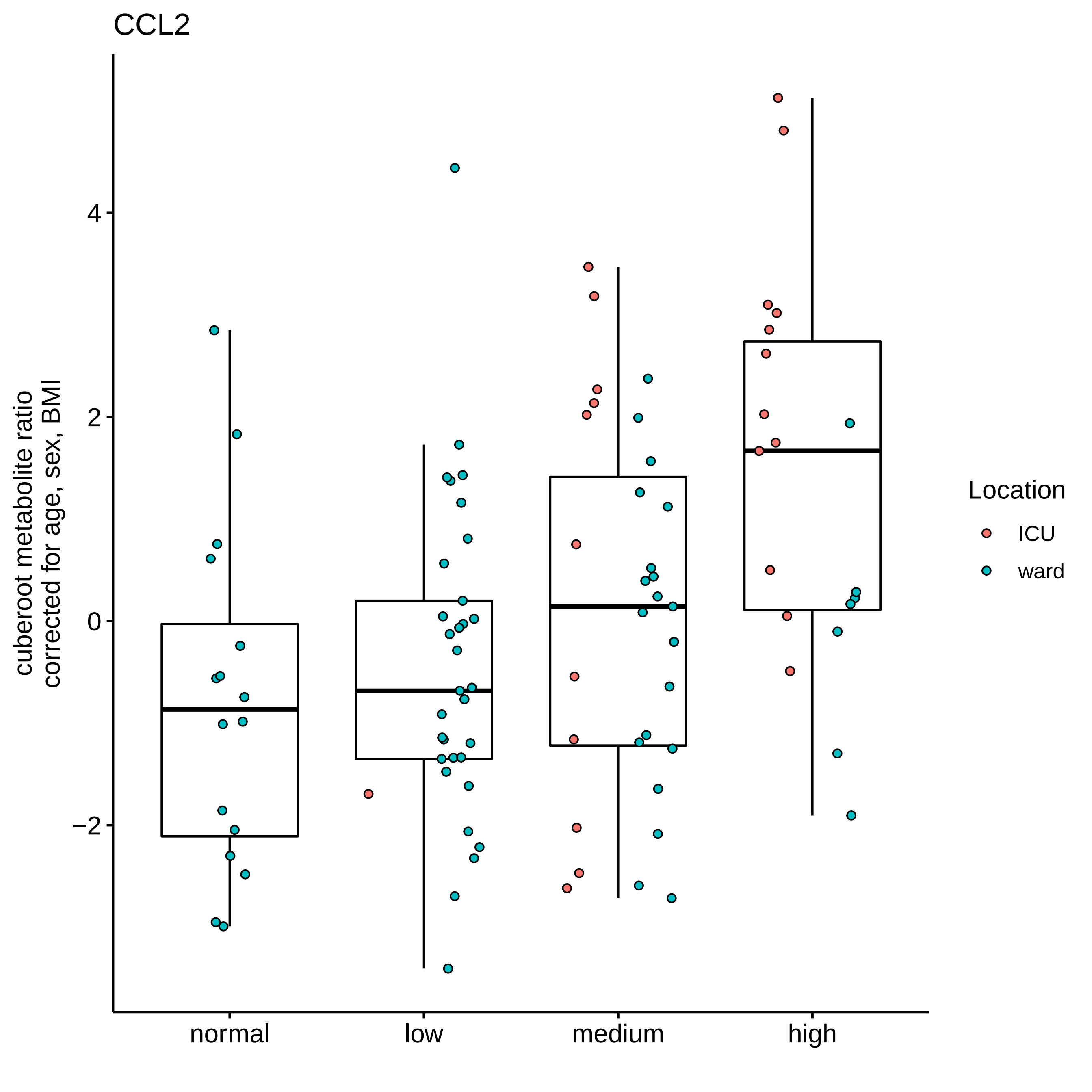
CCL2
cuberoot metabolite ratio
corrected for age, sex, BMI
4
2
0
−2
normal
low
medium
high
Location
ICU
ward

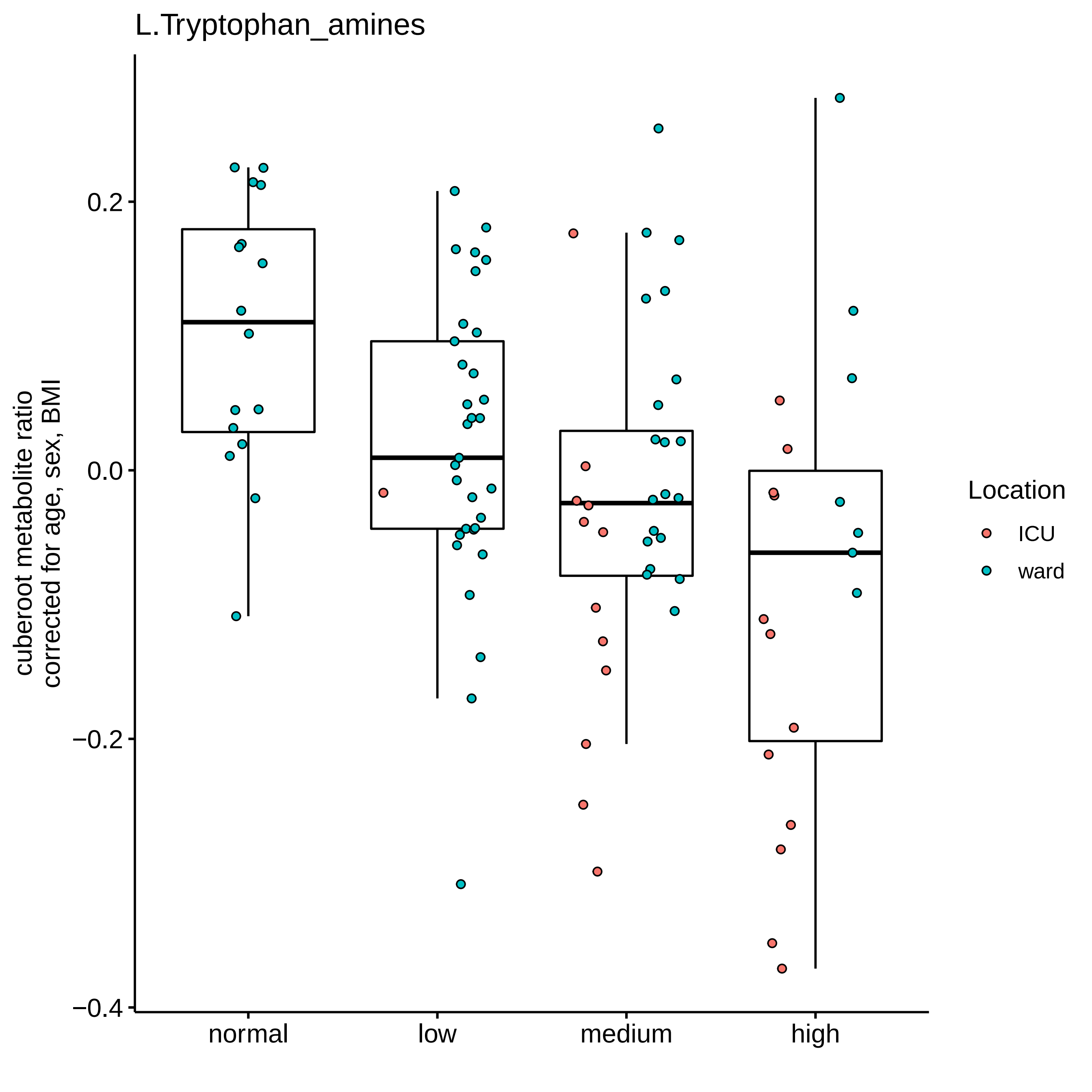

L.Tryptophan_amines
cuberoot metabolite ratio
corrected for age, sex, BMI
0.2
0.0
−0.2
−0.4
normal
low
medium
high
Location
ICU
ward

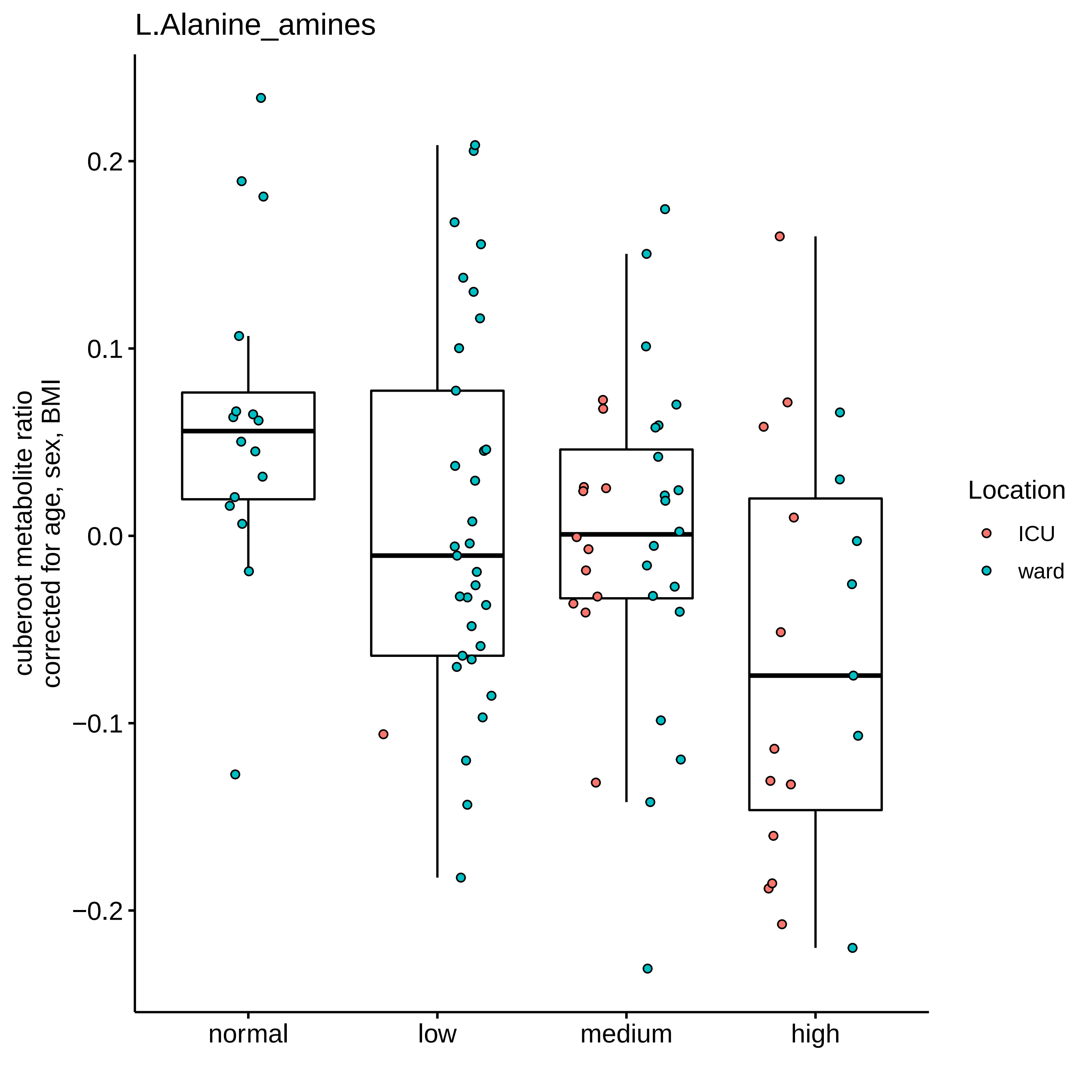

L.Alanine_amines
cuberoot metabolite ratio
corrected for age, sex, BMI
0.2
0.1
0.0
−0.1
−0.2
normal
low
medium
high
Location
ICU
ward

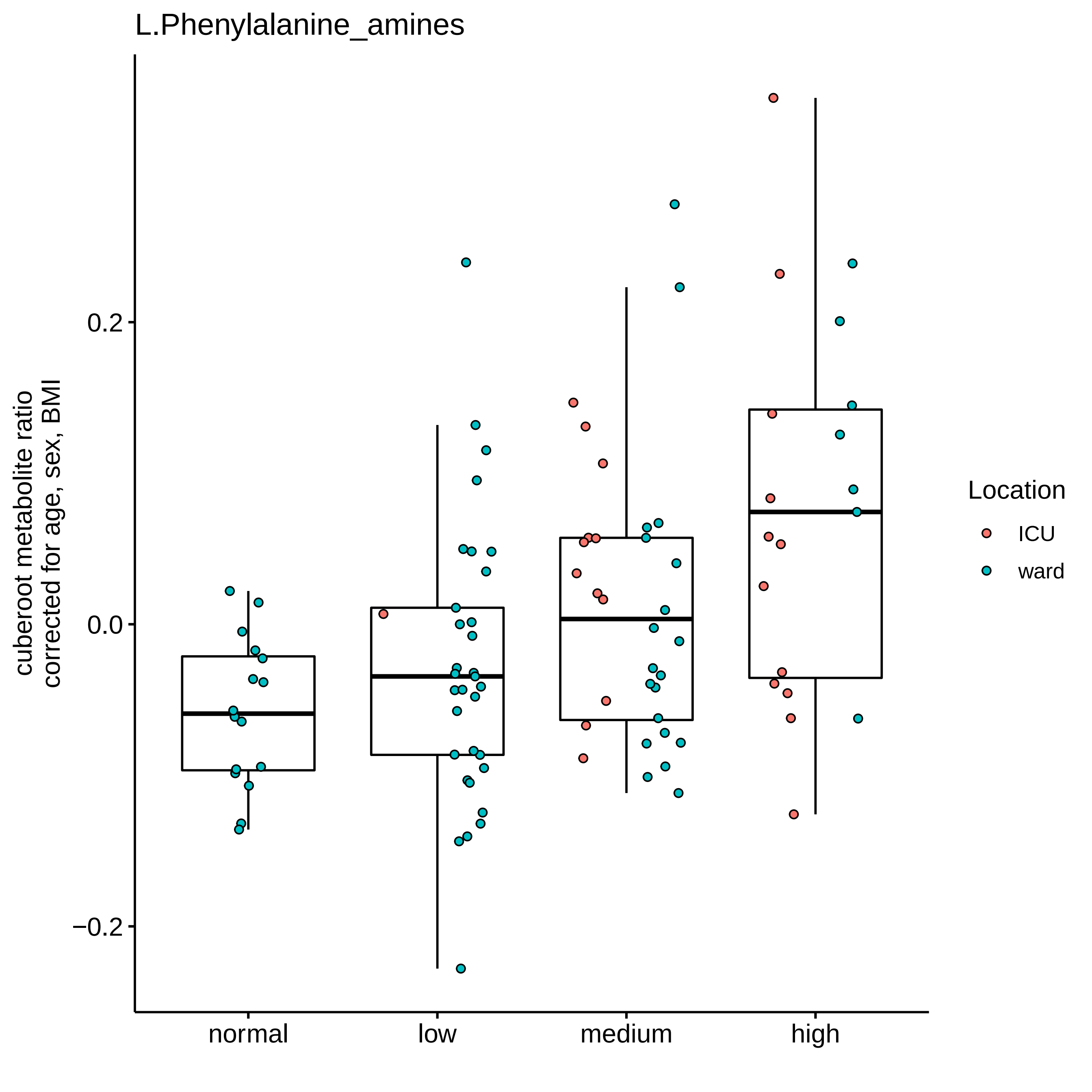
L.Phenylalanine_amines
cuberoot metabolite ratio
corrected for age, sex, BMI
0.2
0.0
−0.2
normal
low
medium
high
Location
ICU
ward

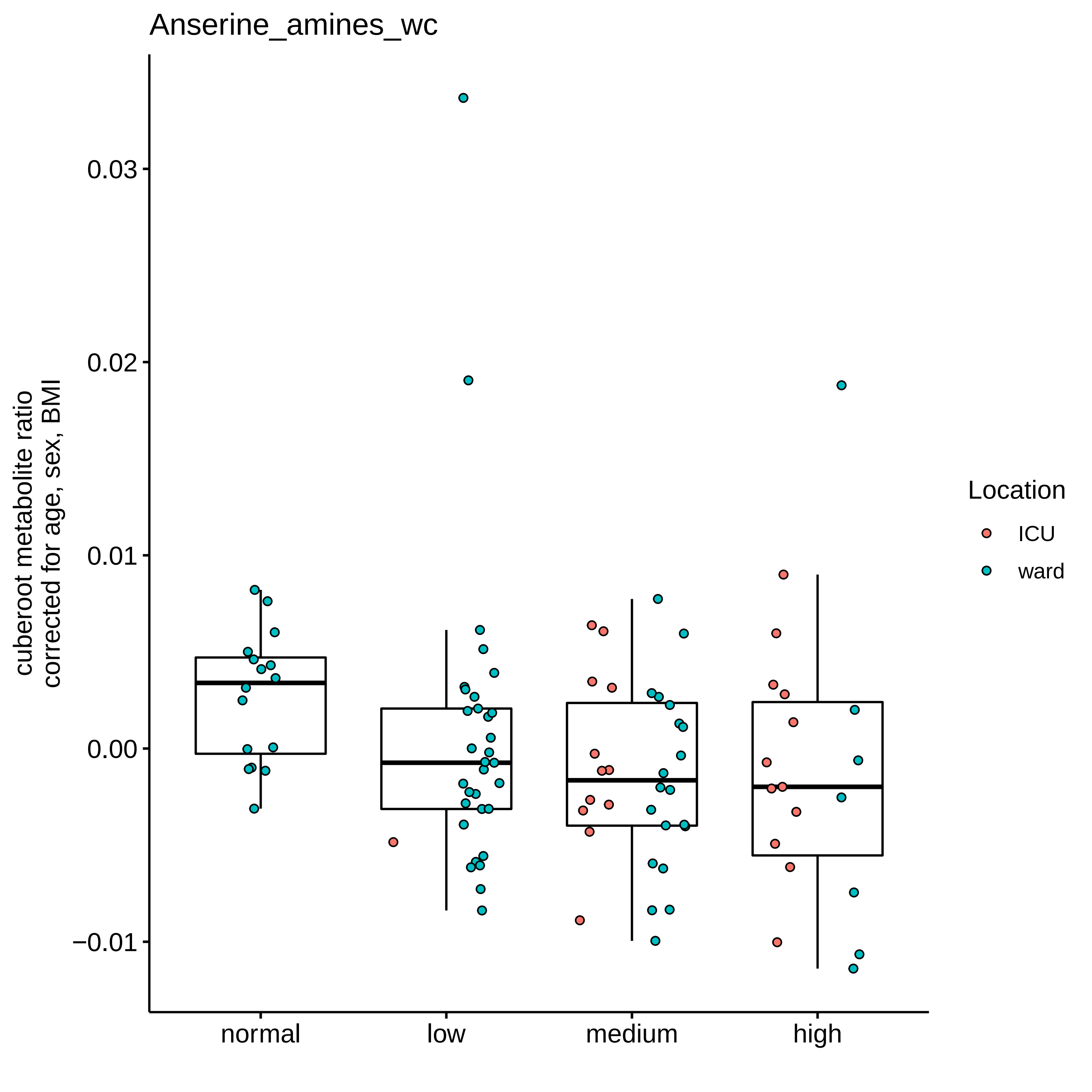

Anserine_amines_wc
cuberoot metabolite ratio
corrected for age, sex, BMI
0.03
0.02
0.01
0.00
−0.01
normal
low
medium
high
Location
ICU
ward

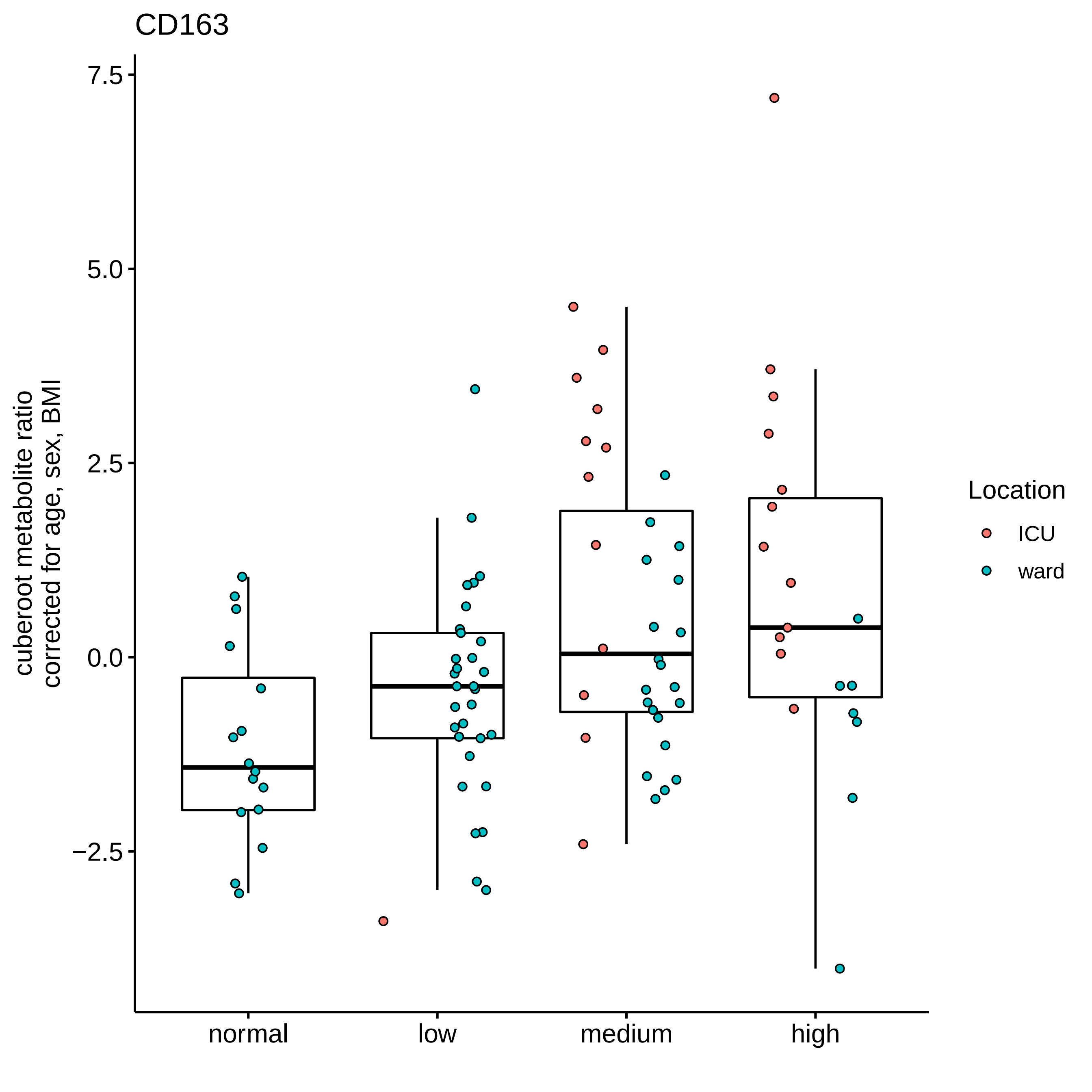
CD163
7.5
5.0
2.5
0.0
−2.5
cuberoot metabolite ratio
corrected for age, sex, BMI
normal
low
medium
high
Location
ICU
ward

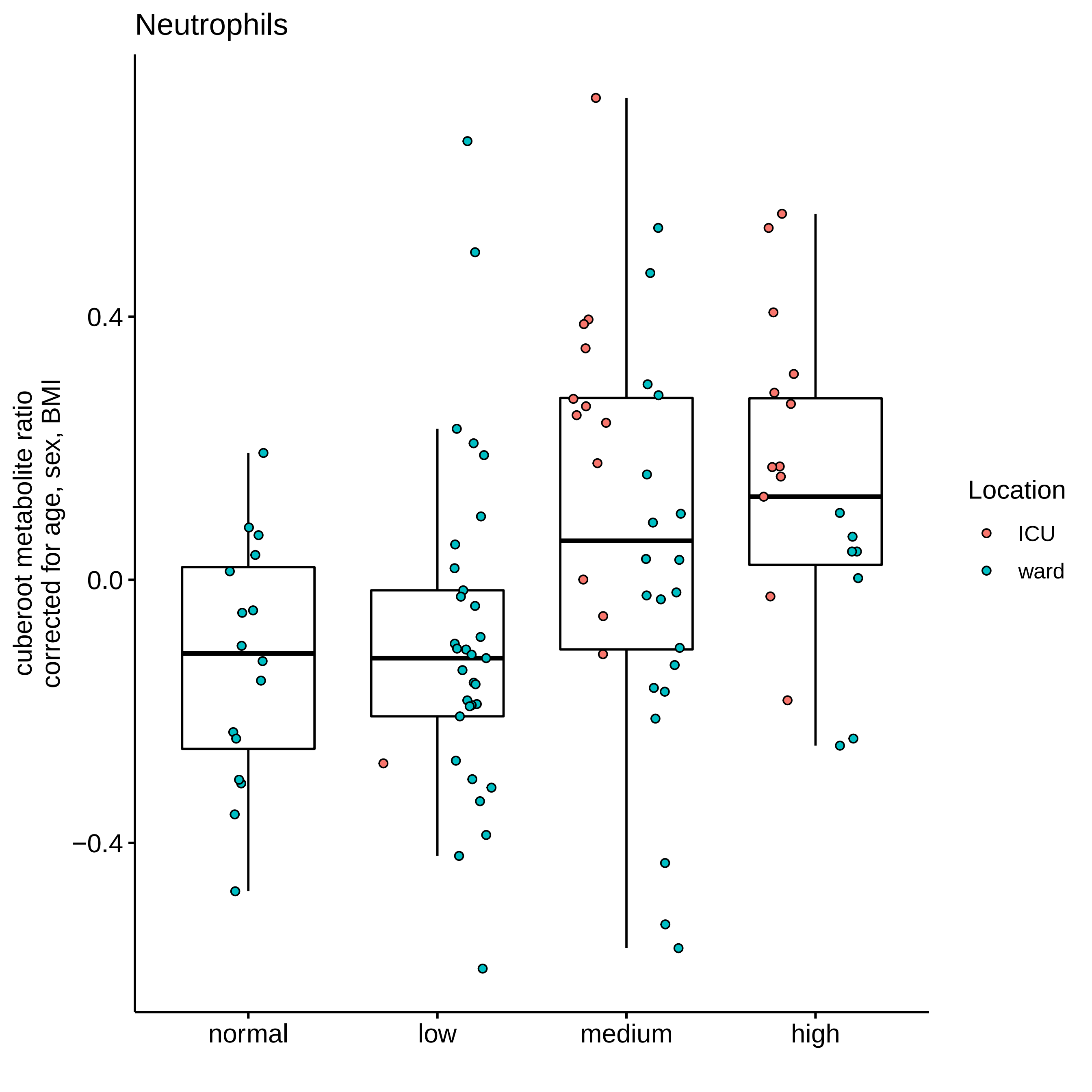
Neutrophils
cuberoot metabolite ratio
corrected for age, sex, BMI
0.4
0.0
−0.4
normal
low
medium
high
Location
ICU
ward

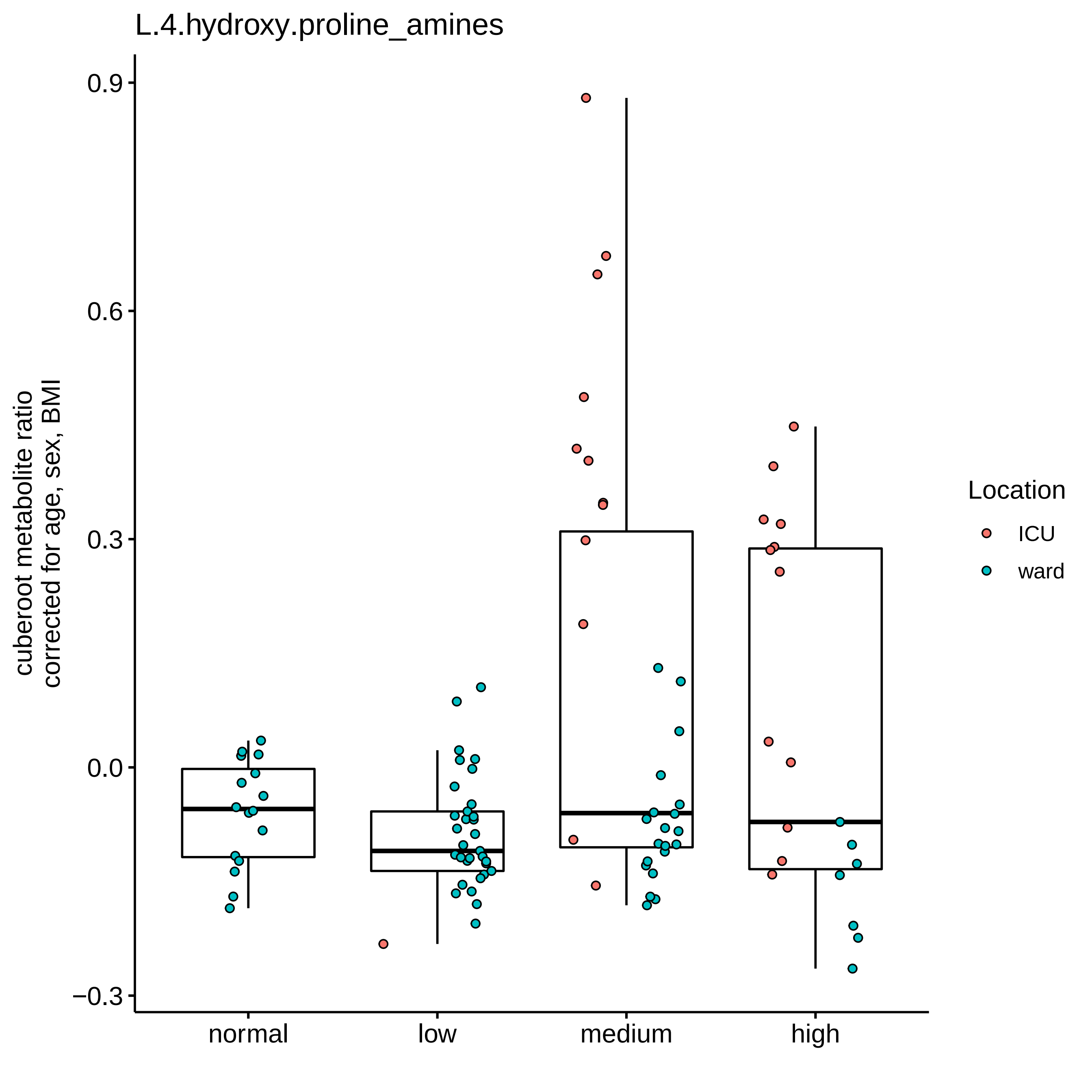

L.4.hydroxy.proline_amines
cuberoot metabolite ratio
corrected for age, sex, BMI
0.9
0.6
0.3
0.0
−0.3
normal
low
medium
high
Location
ICU
ward

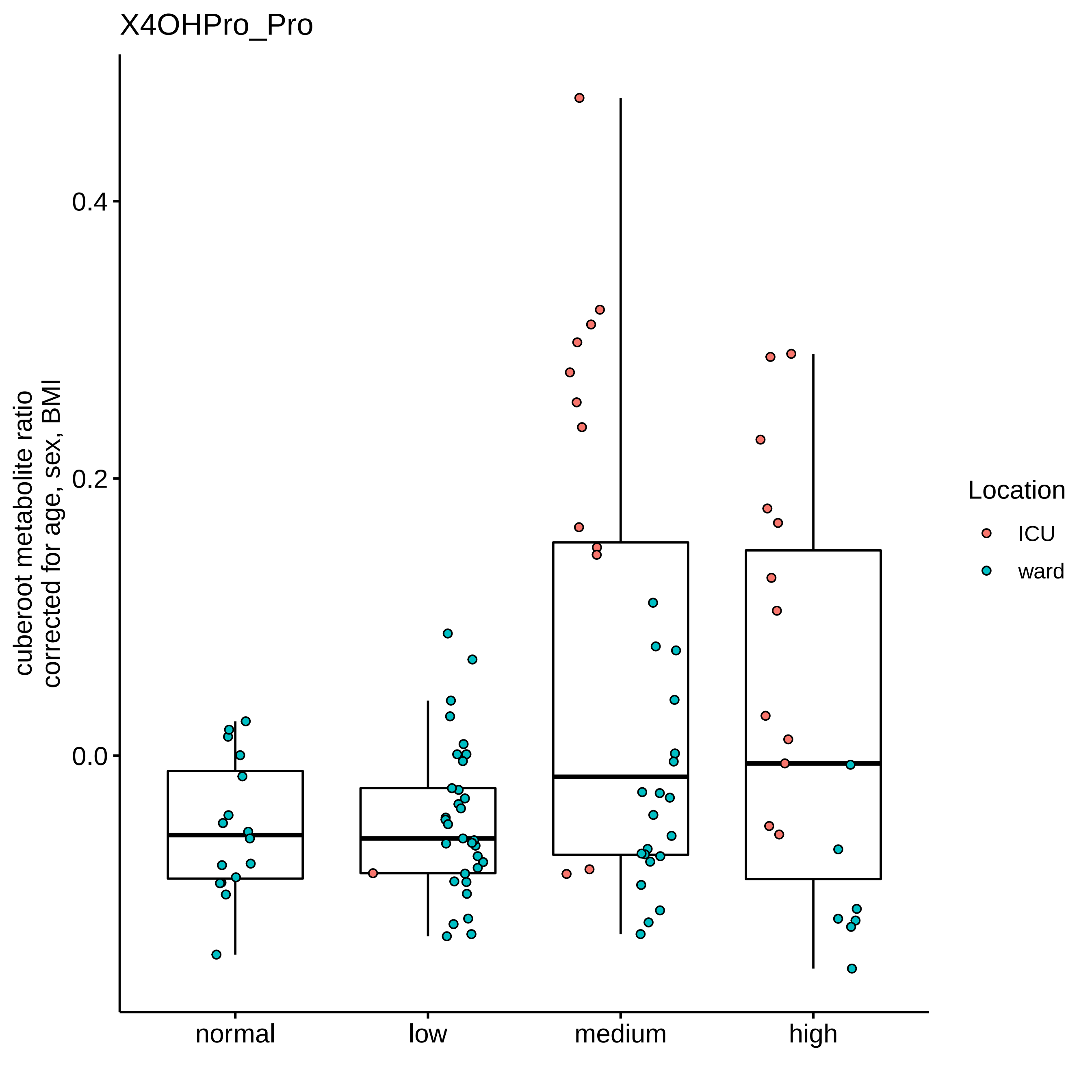

X4OHPro_Pro
cuberoot metabolite ratio
corrected for age, sex, BMI
0.4
0.2
0.0
normal
low
medium
high
Location
ICU
ward

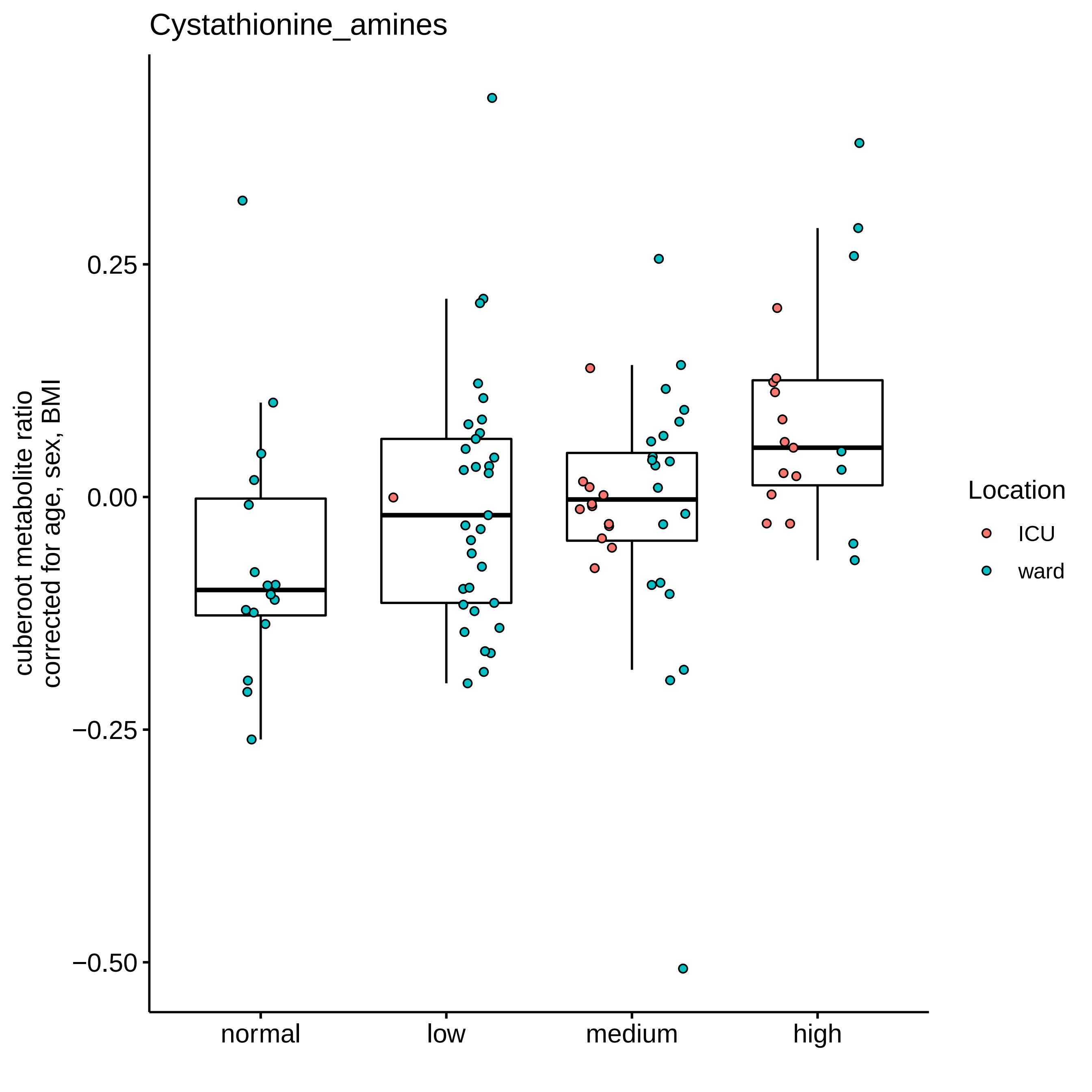
Cystathionine_amines
cuberoot metabolite ratio
corrected for age, sex, BMI
0.25
0.00
−0.25
−0.50
normal
low
medium
high
Location
ICU
ward

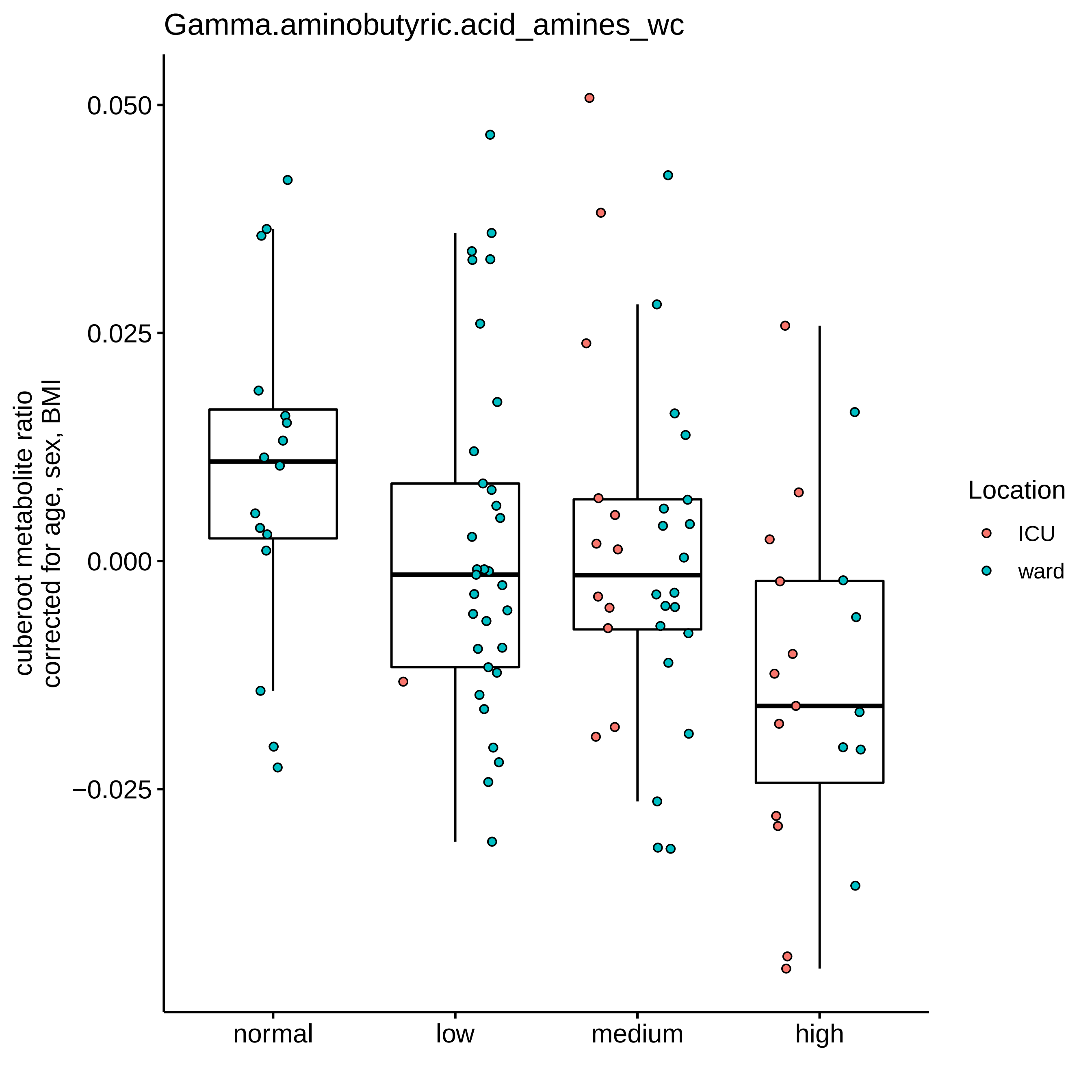

Gamma.aminobutyric.acid_amines_wc
cuberoot metabolite ratio
corrected for age, sex, BMI
0.050
0.025
0.000
−0.025
normal
low
medium
high
Location
ICU
ward

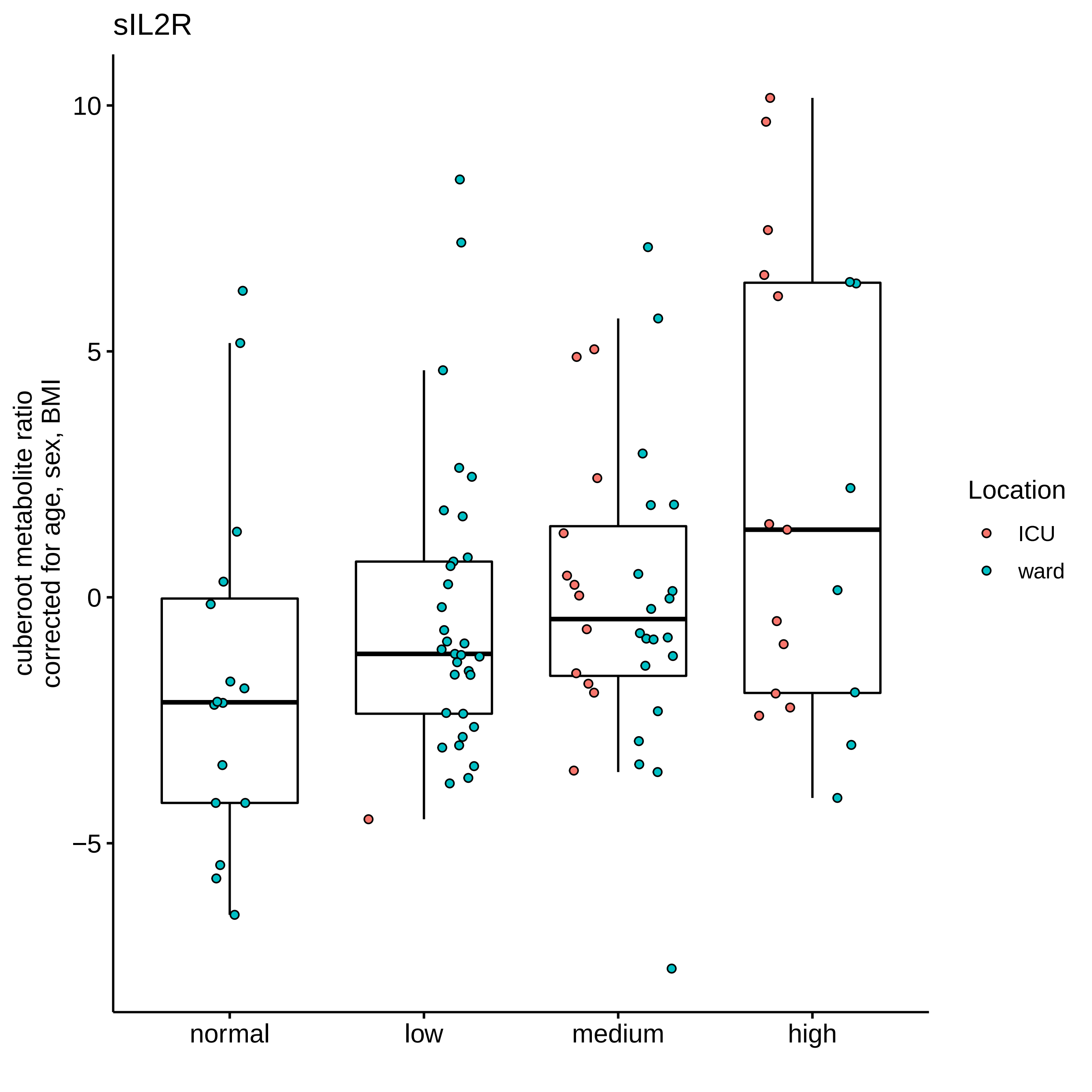

sIL2R
10
5
0
−5
cuberoot metabolite ratio
corrected for age, sex, BMI
normal
low
medium
high
Location
ICU
ward

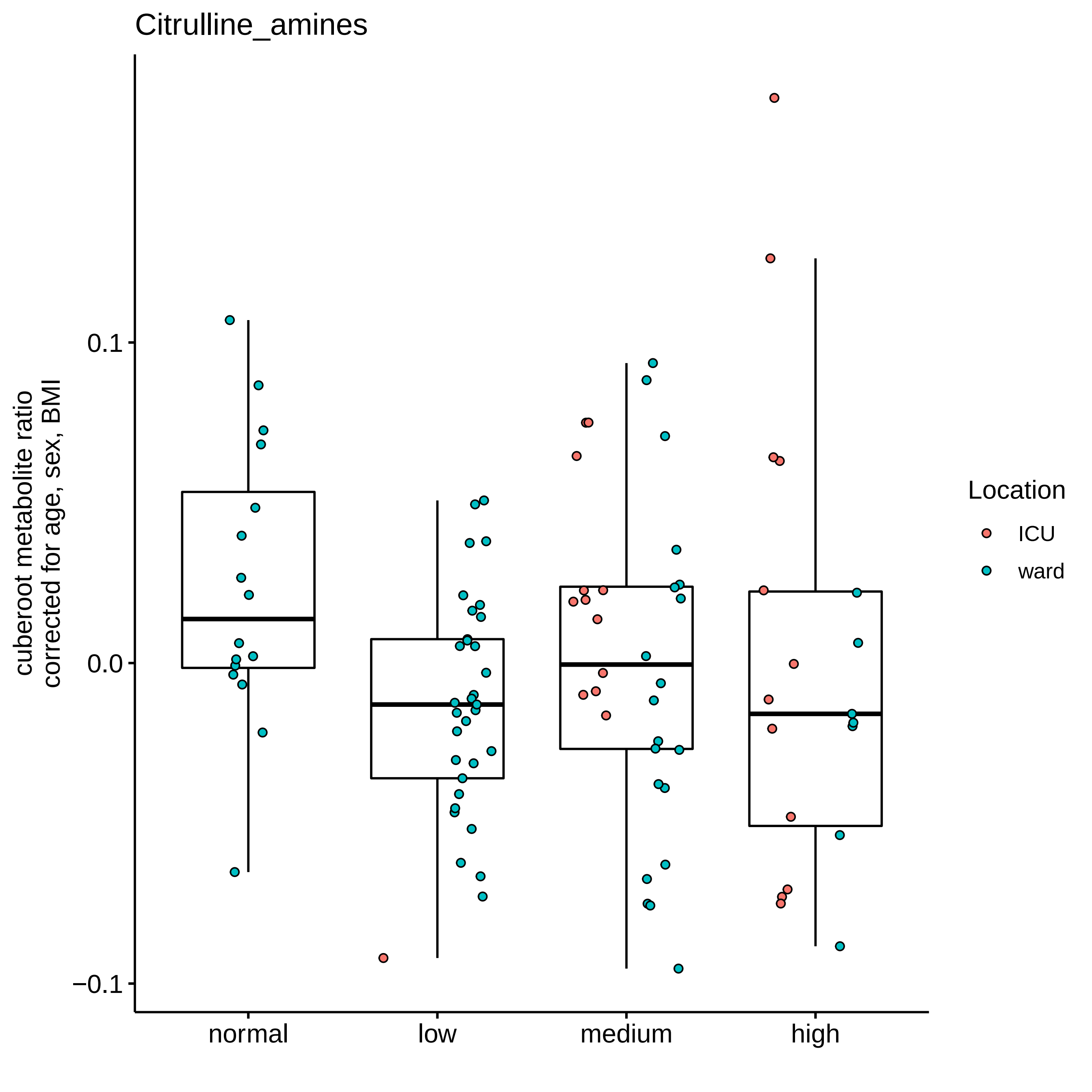

Citrulline_amines
cuberoot metabolite ratio
corrected for age, sex, BMI
0.1
0.0
−0.1
normal
low
medium
high
Location
ICU
ward

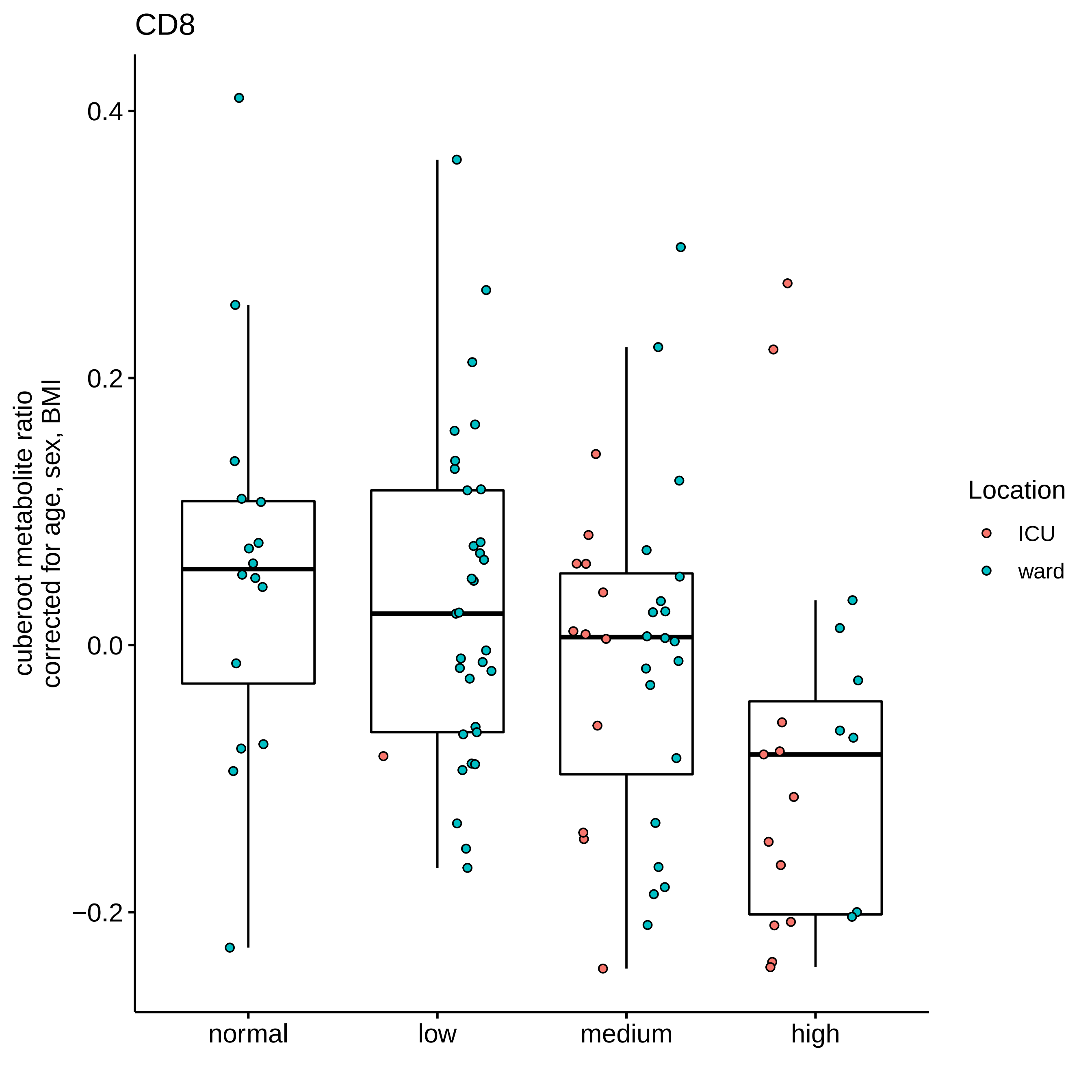
CD8
cuberoot metabolite ratio
corrected for age, sex, BMI
0.4
0.2
0.0
−0.2
normal
low
medium
high
Location
ICU
ward

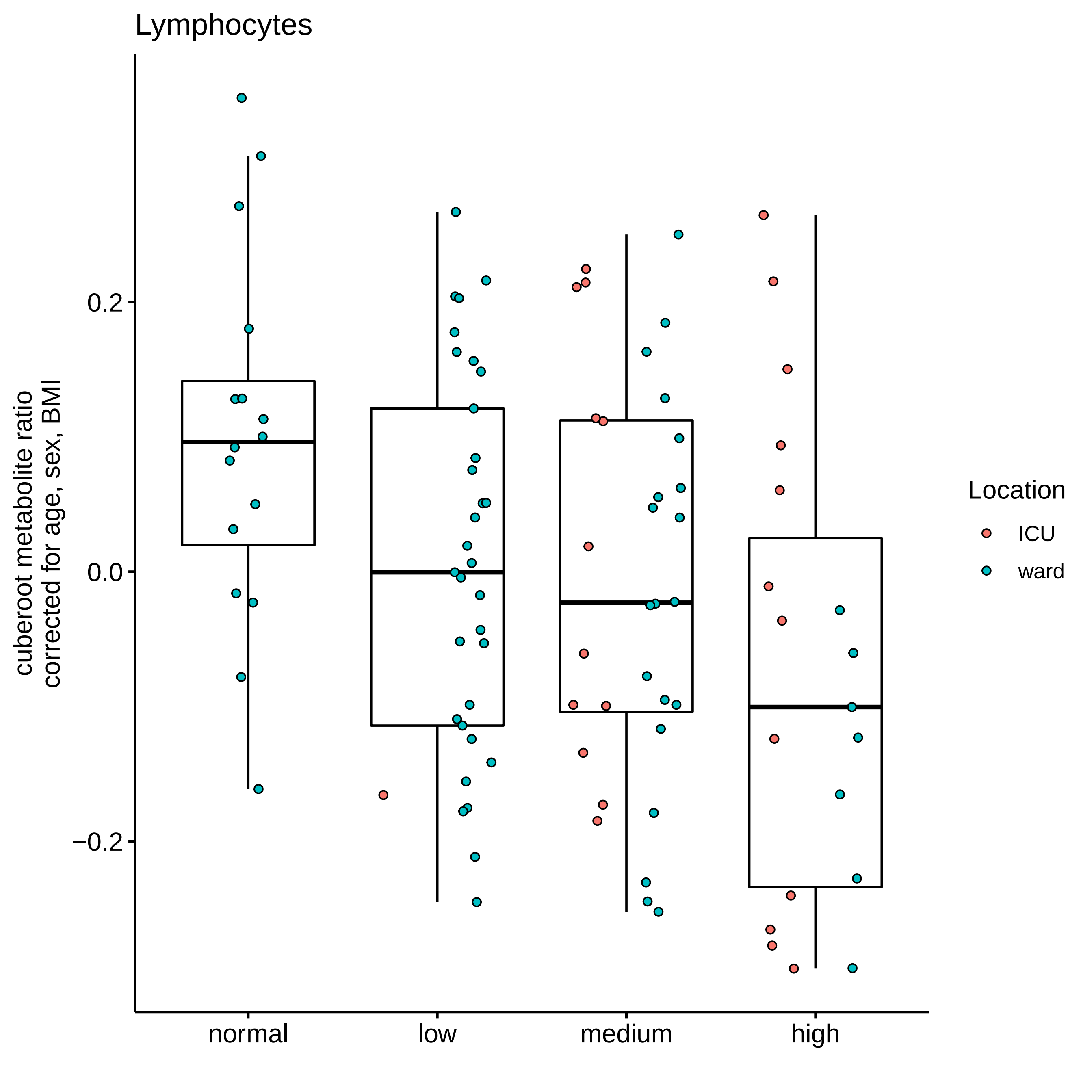

Lymphocytes
cuberoot metabolite ratio
corrected for age, sex, BMI
0.2
0.0
−0.2
normal
low
medium
high
Location
ICU
ward

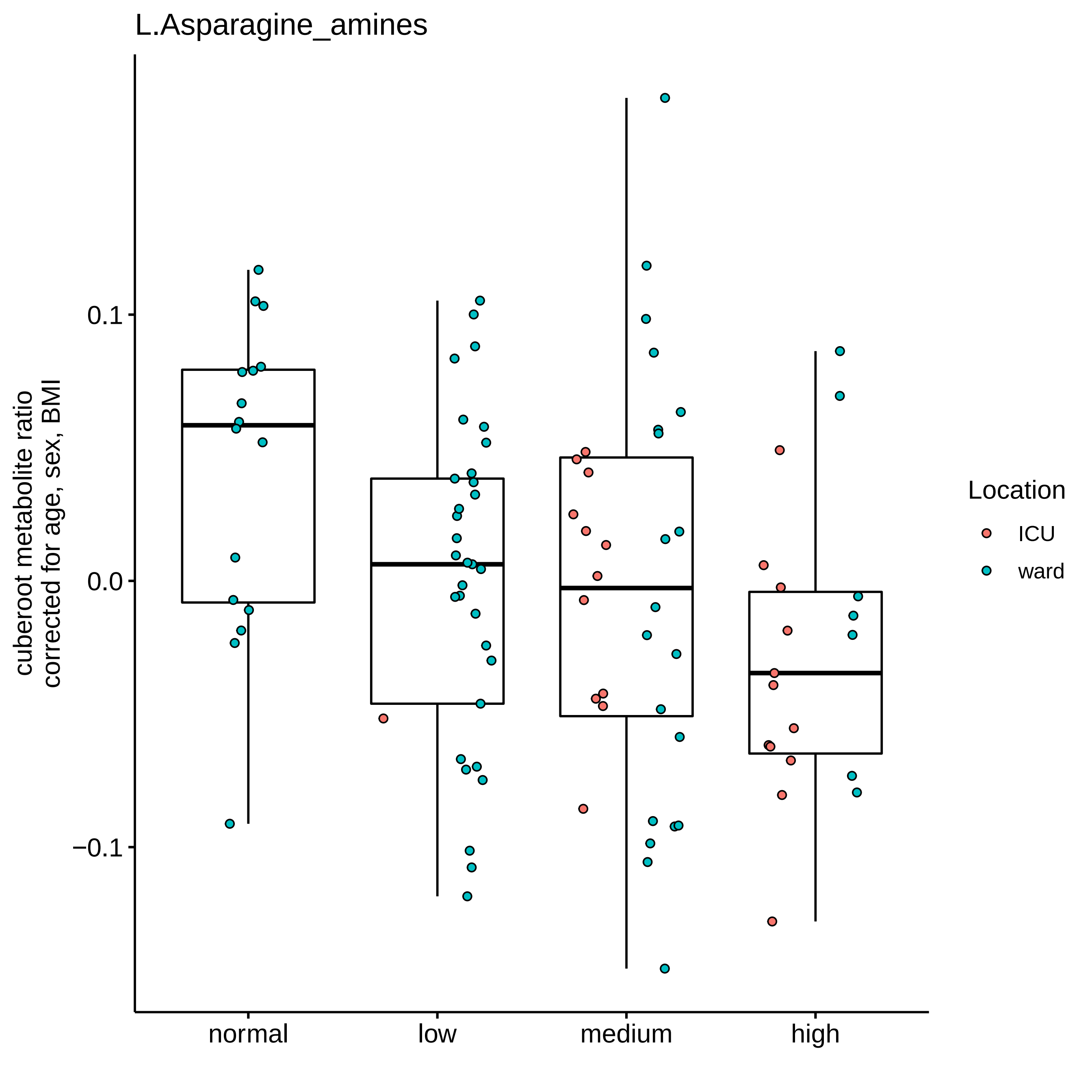

L.Asparagine_amines
cuberoot metabolite ratio
corrected for age, sex, BMI
0.1
0.0
−0.1
normal
low
medium
high
Location
ICU
ward

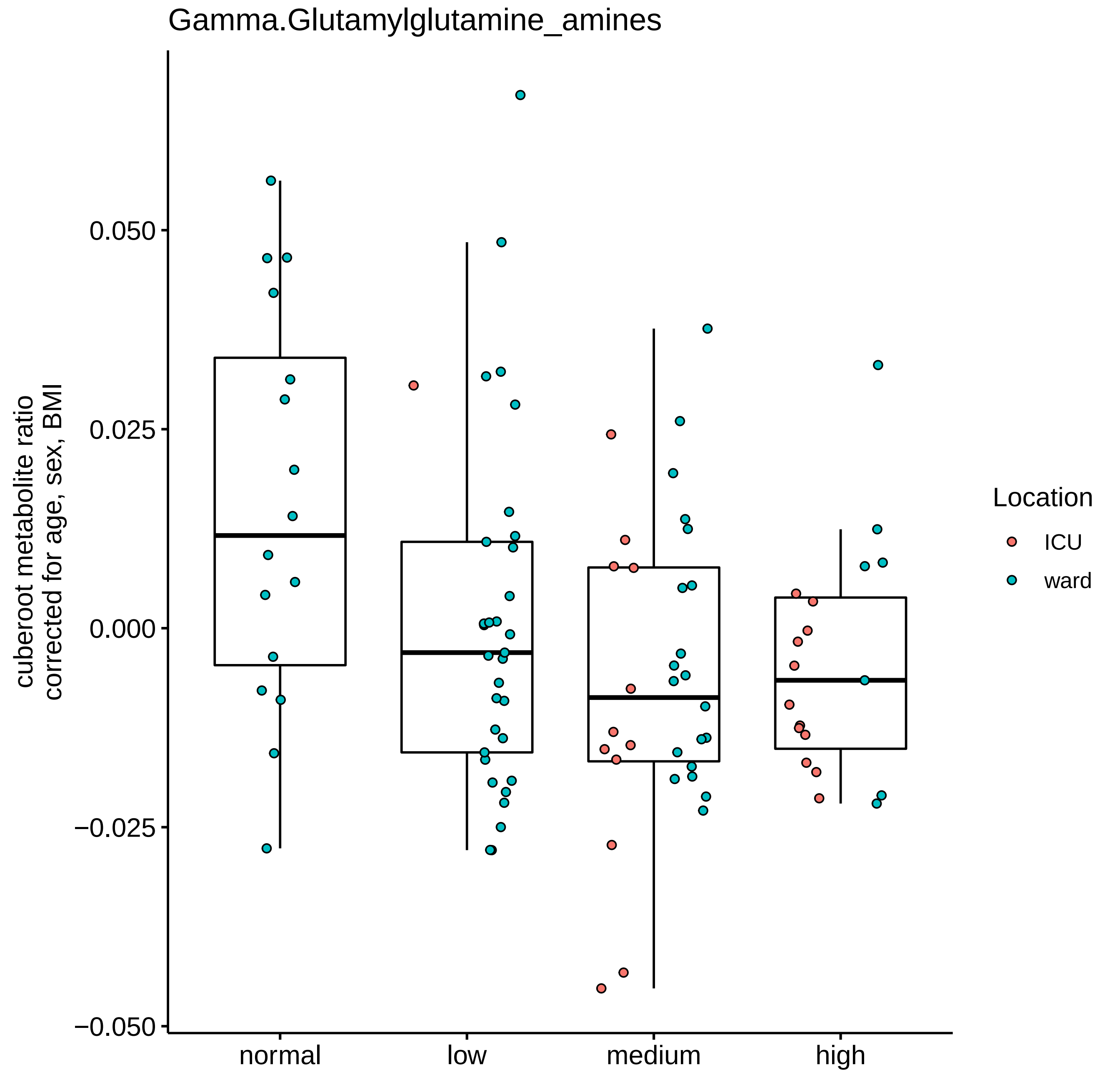

Gamma.Glutamylglutamine_amines
cuberoot metabolite ratio
corrected for age, sex, BMI
0.050
0.025
0.000
−0.025
−0.050
normal
low
medium
high
Location
ICU
ward

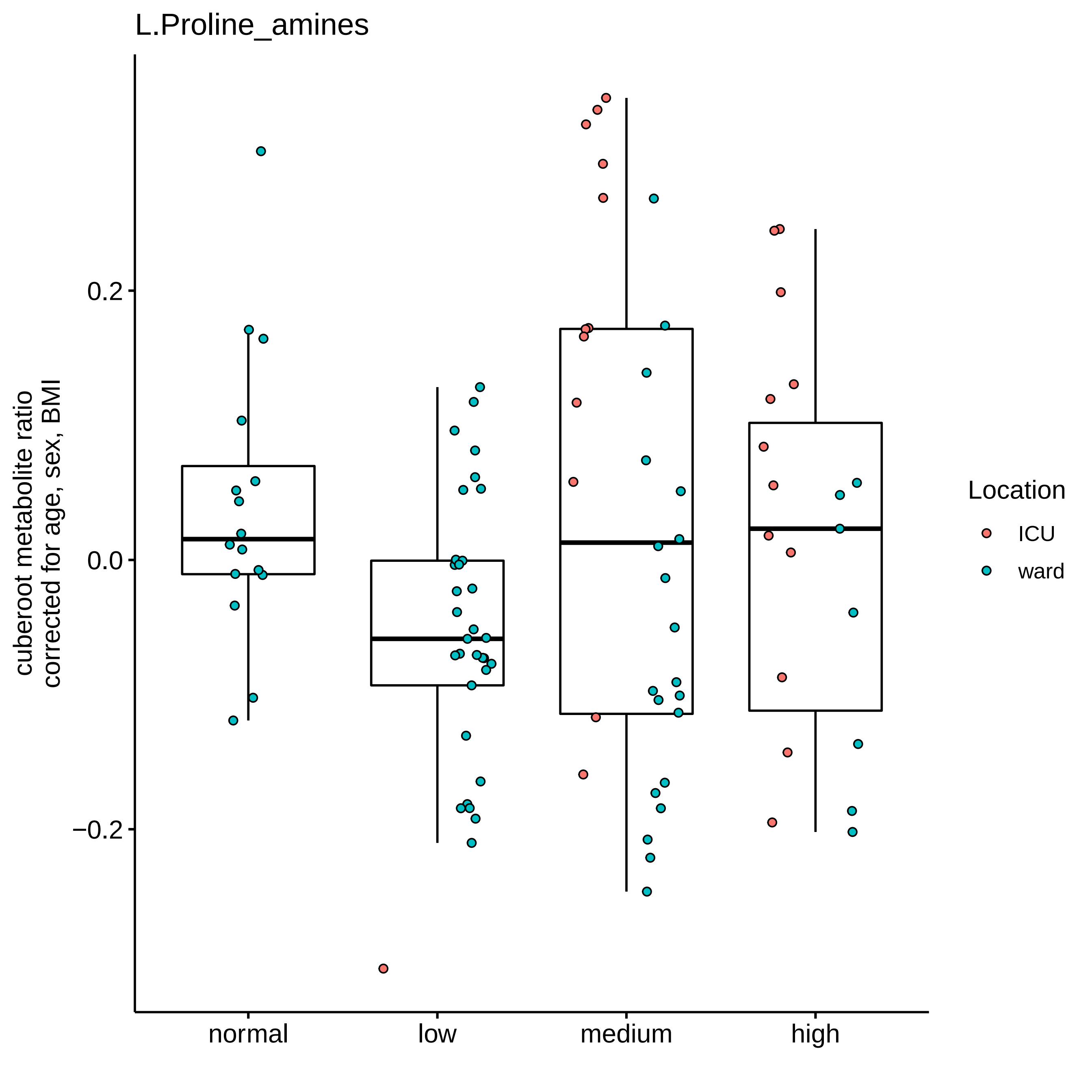
L.Proline_amines
cuberoot metabolite ratio
corrected for age, sex, BMI
0.2
0.0
−0.2
normal
low
medium
high
Location
ICU
ward

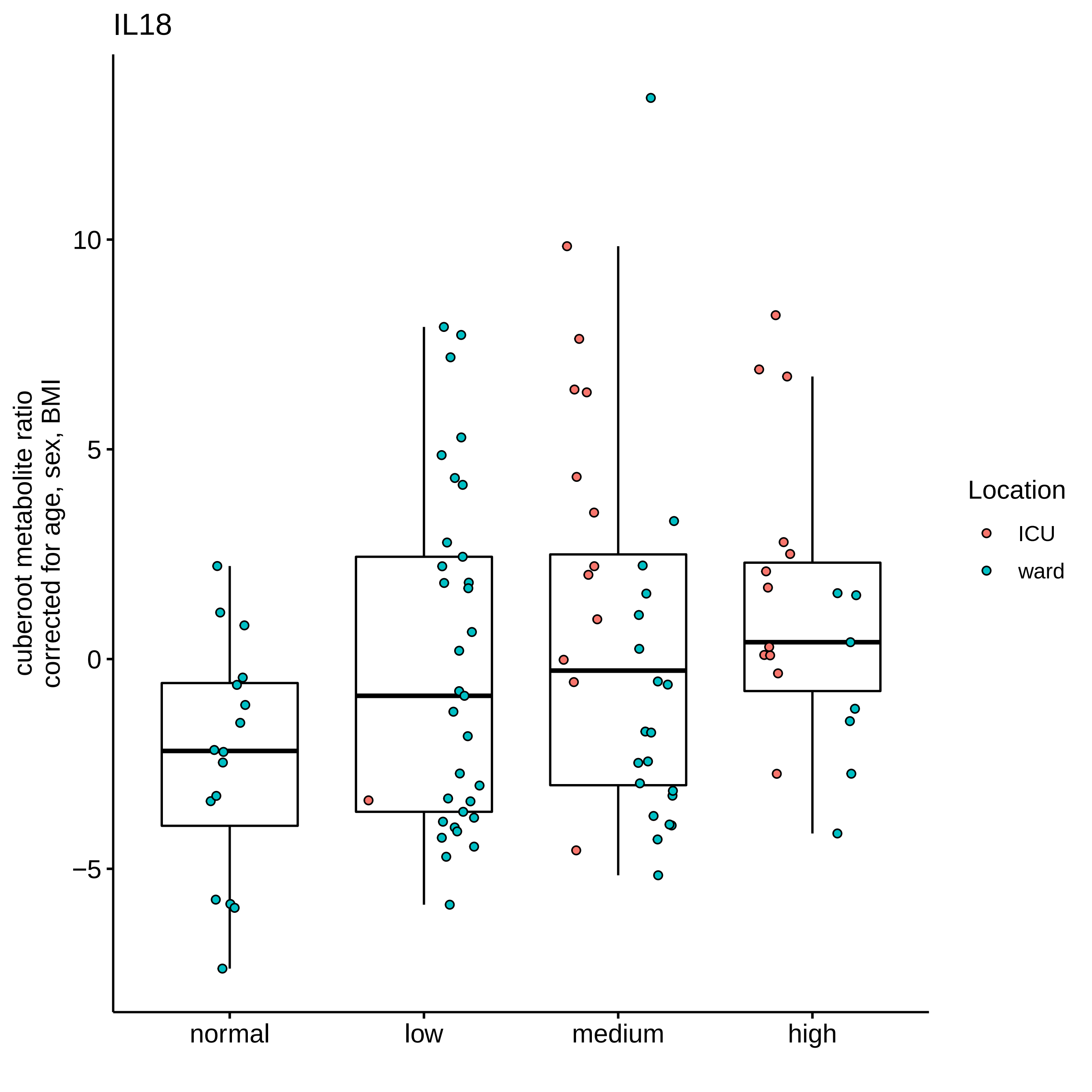

IL18
cuberoot metabolite ratio
corrected for age, sex, BMI
10
5
0
−5
normal
low
medium
high
Location
ICU
ward

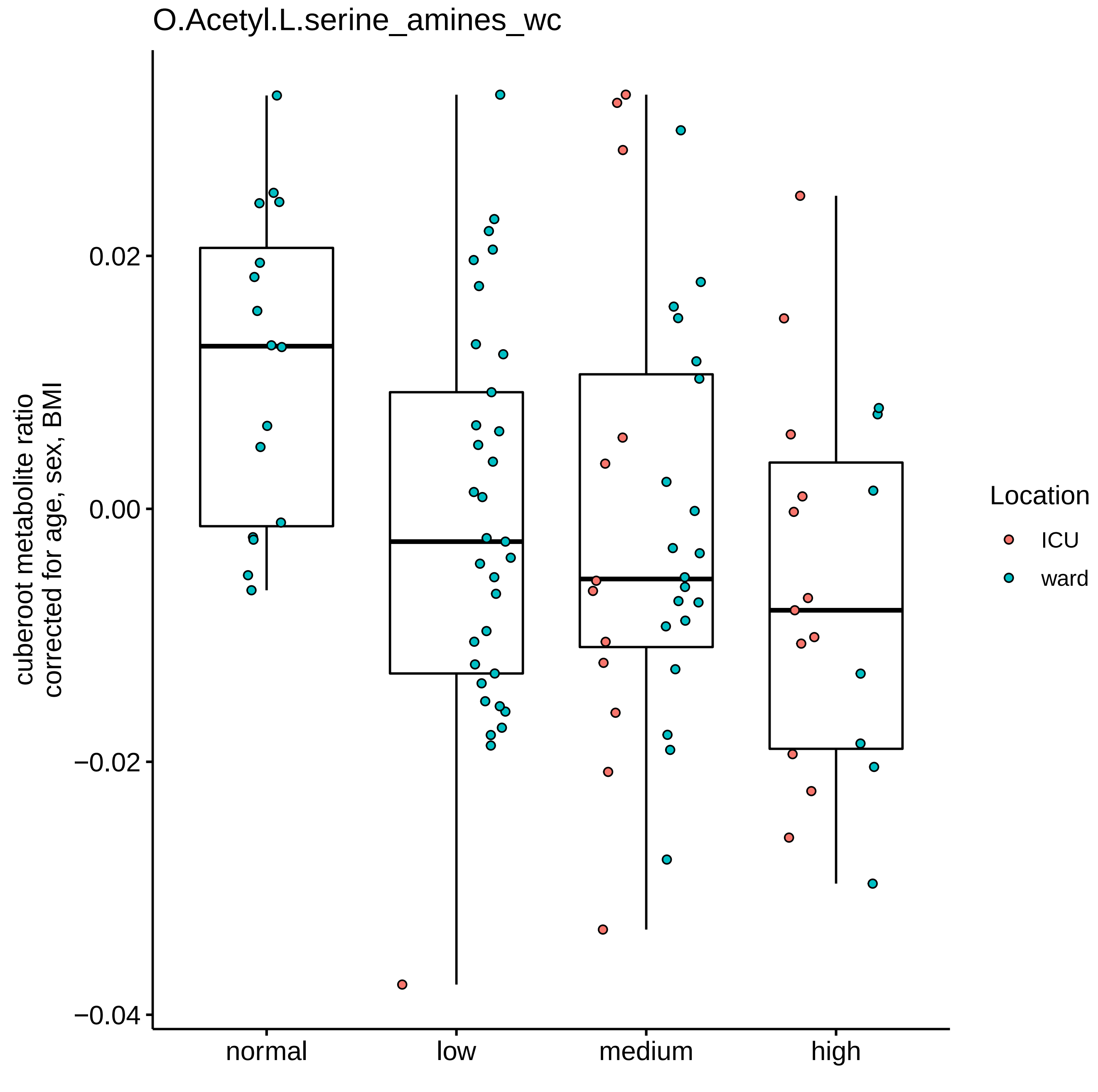
O.Acetyl.L.serine_amines_wc
cuberoot metabolite ratio
corrected for age, sex, BMI
0.02
0.00
−0.02
−0.04
normal
low
medium
high
Location
ICU
ward

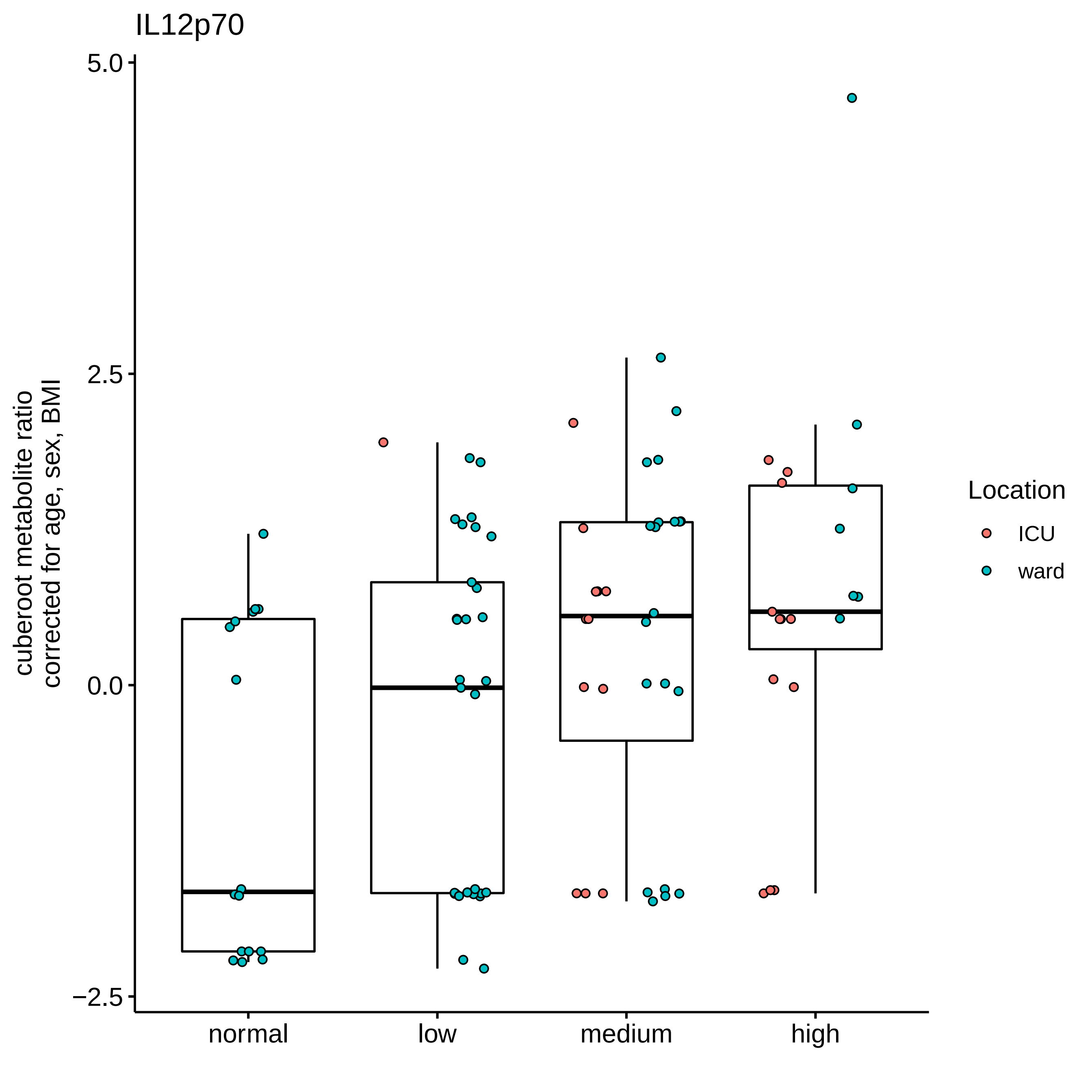

IL12p70
cuberoot metabolite ratio corrected for age, sex, BMI
5.0
2.5
0.0
−2.5
normal
low
medium
high
Location
ICU
ward

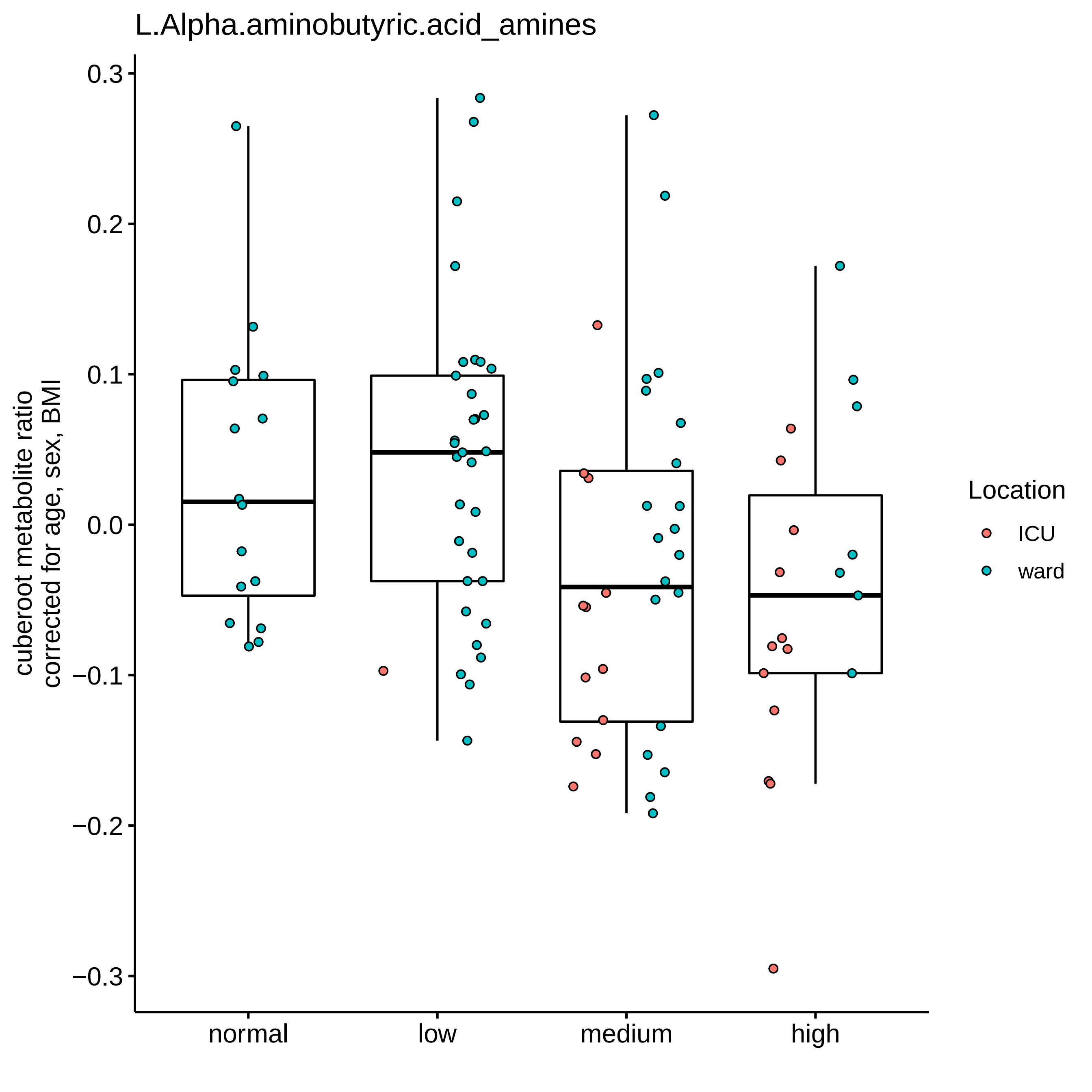
L.Alpha.aminobutyric.acid_amines
cuberoot metabolite ratio
corrected for age, sex, BMI
0.3
0.2
0.1
0.0
−0.1
−0.2
−0.3
normal
low
medium
high
Location
ICU
ward

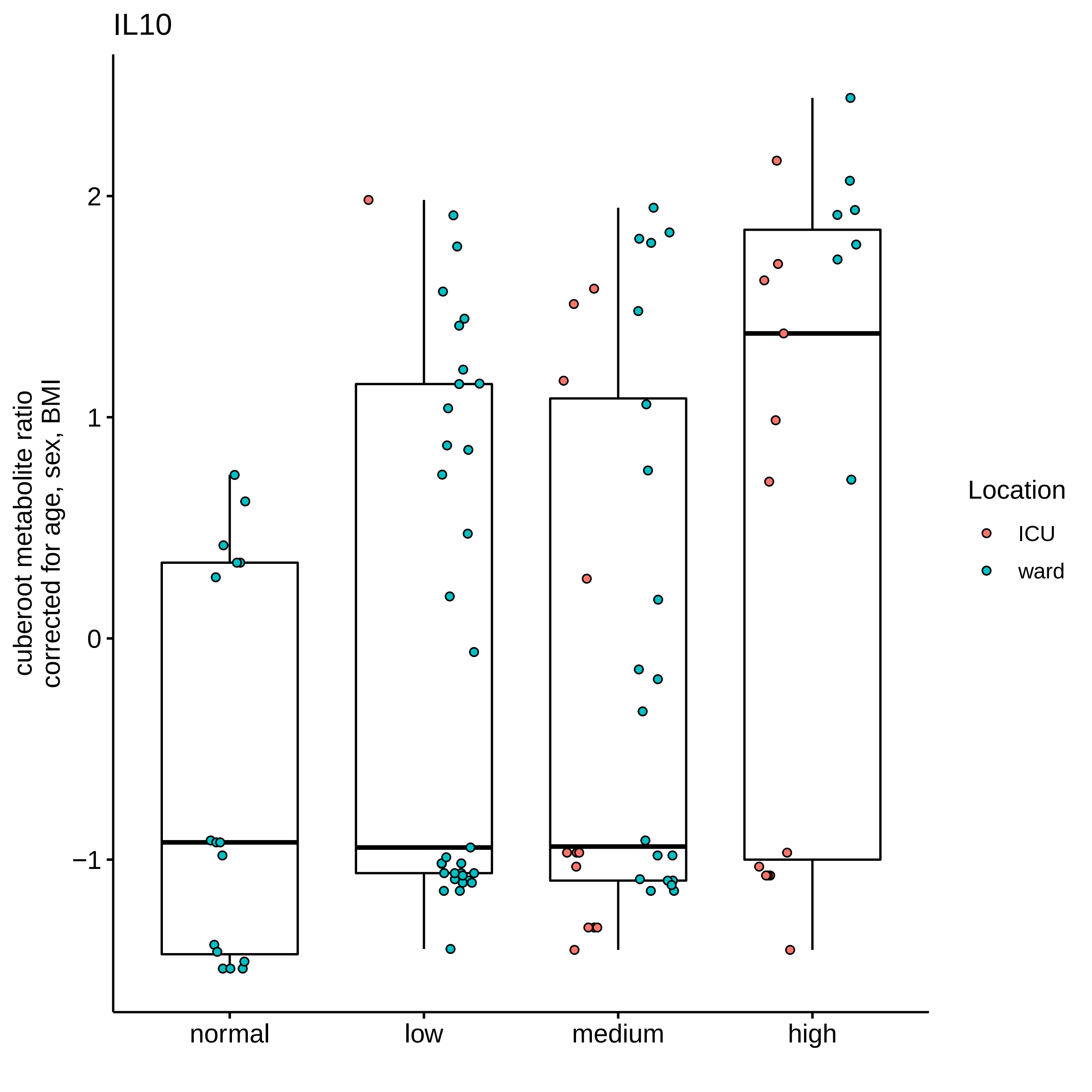
IL10
cuberoot metabolite ratio
corrected for age, sex, BMI
2
1
0
−1
normal
low
medium
high
Location
ICU
ward

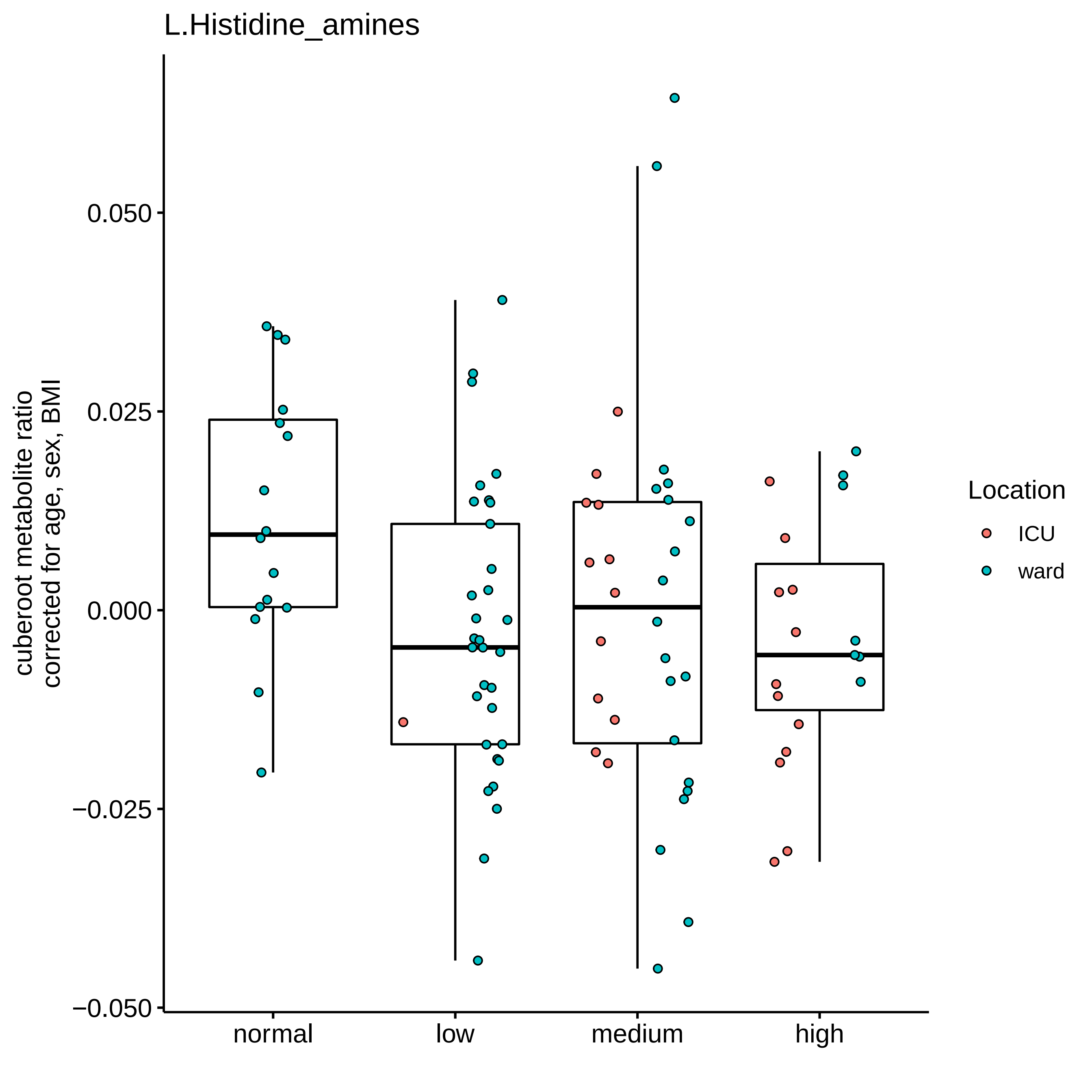

L.Histidine_amines
cuberoot metabolite ratio
corrected for age, sex, BMI
0.050
0.025
0.000
−0.025
−0.050
normal
low
medium
high
Location
ICU
ward

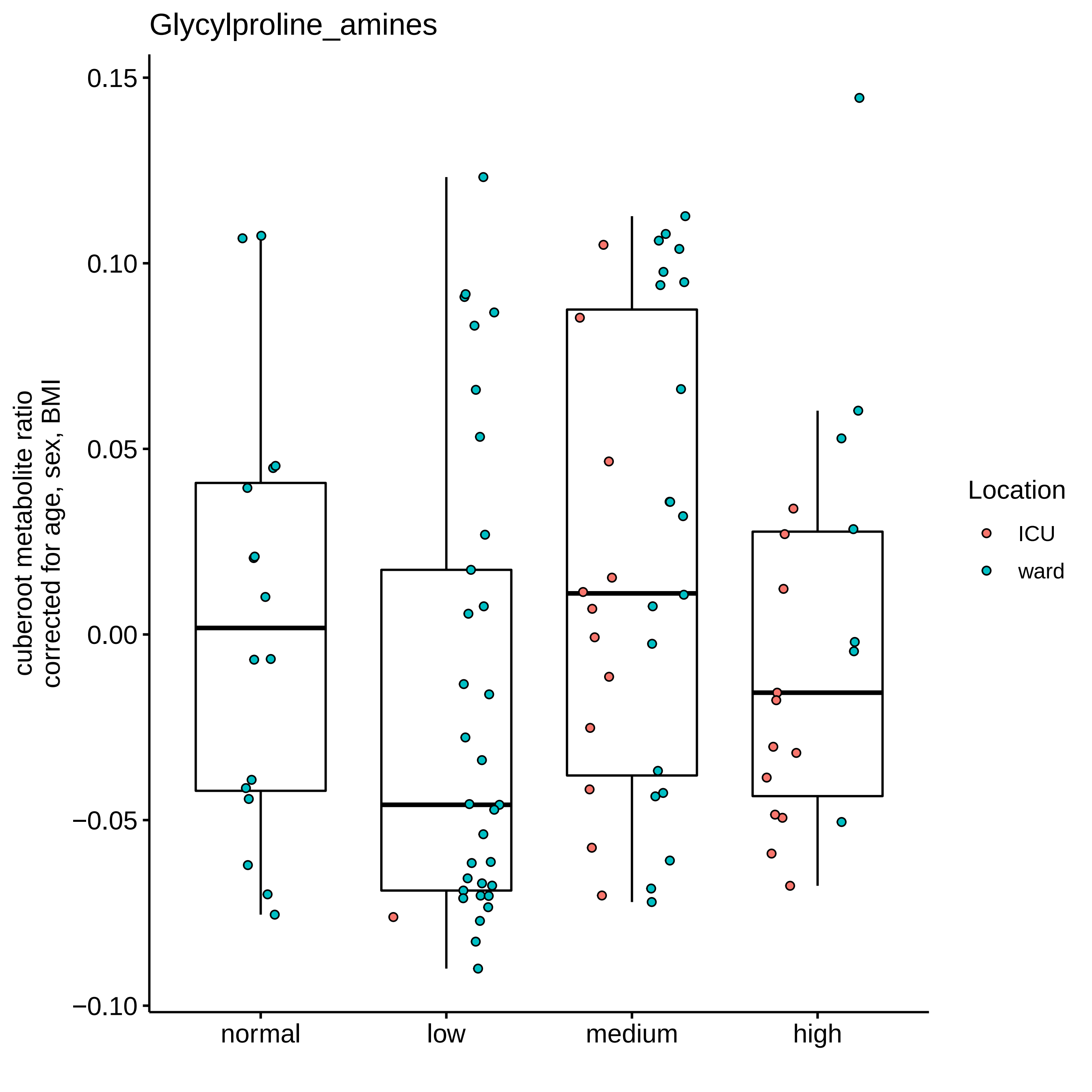

Glycylproline_amines
cuberoot metabolite ratio
corrected for age, sex, BMI
0.15
0.10
0.05
0.00
−0.05
−0.10
normal
low
medium
high
Location
ICU
ward

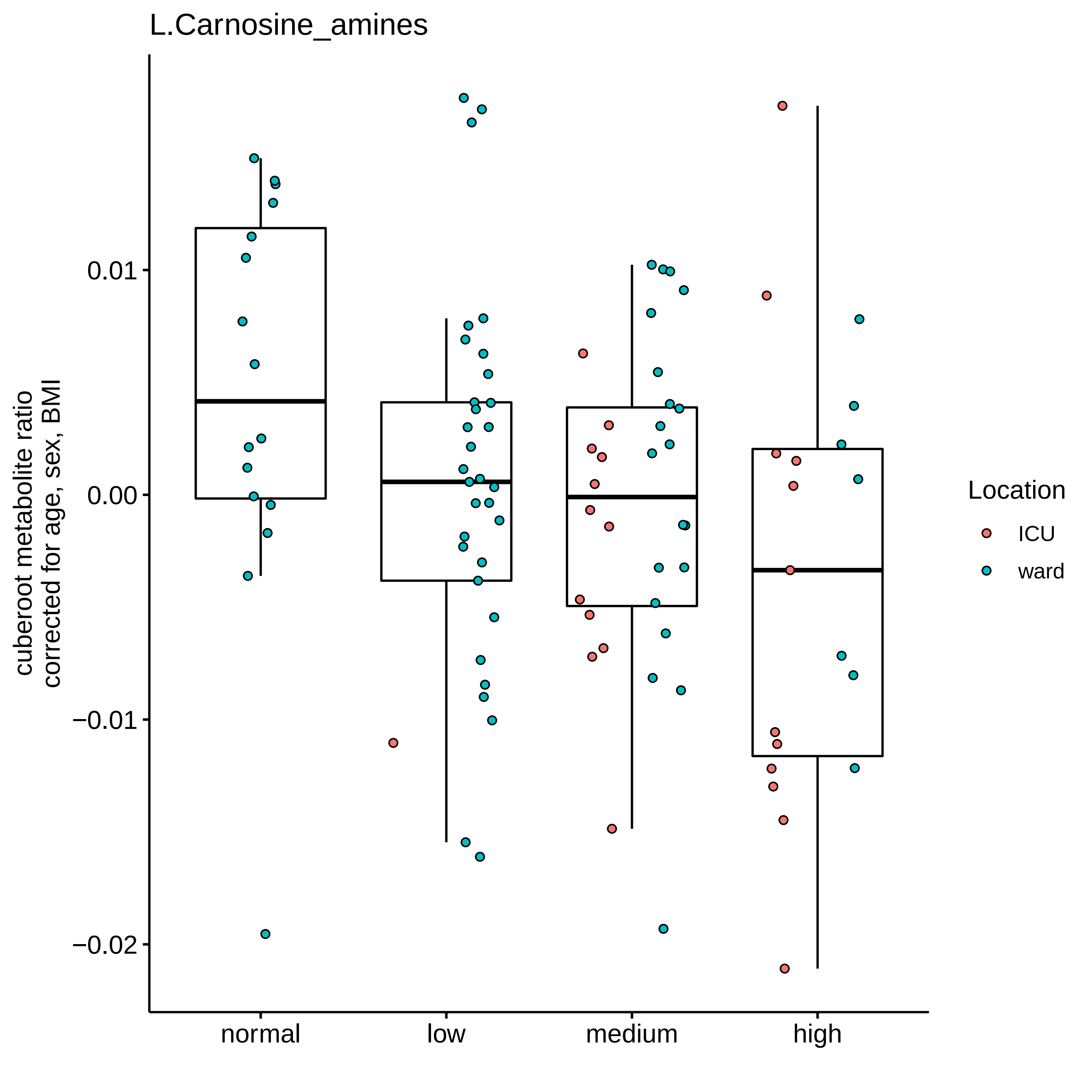

L.Carnosine_amines
cuberoot metabolite ratio
corrected for age, sex, BMI
0.01
0.00
−0.01
−0.02
normal
low
medium
high
Location
ICU
ward

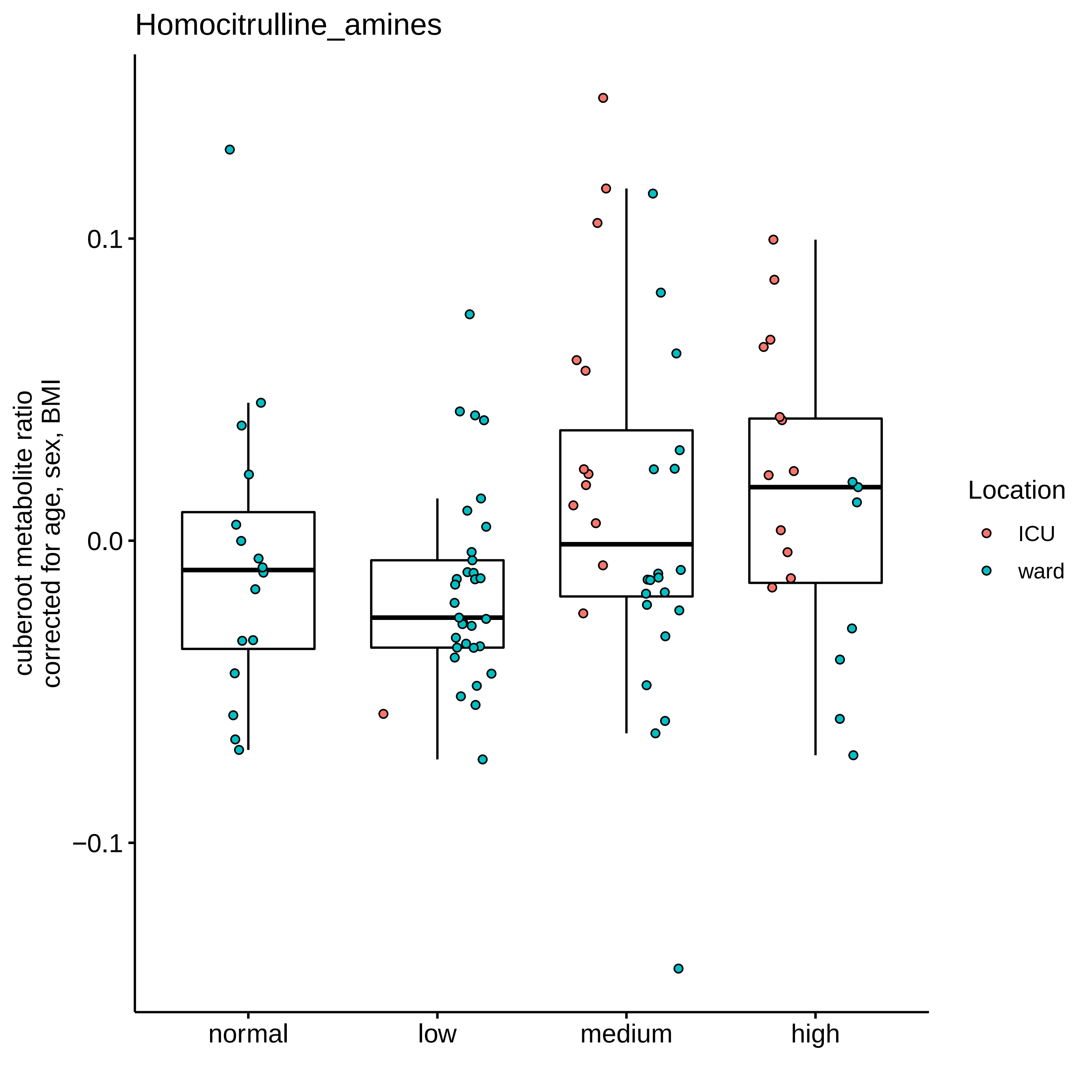
Homocitrulline_amines
cuberoot metabolite ratio
corrected for age, sex, BMI
0.1
0.0
−0.1
normal
low
medium
high
Location
ICU
ward

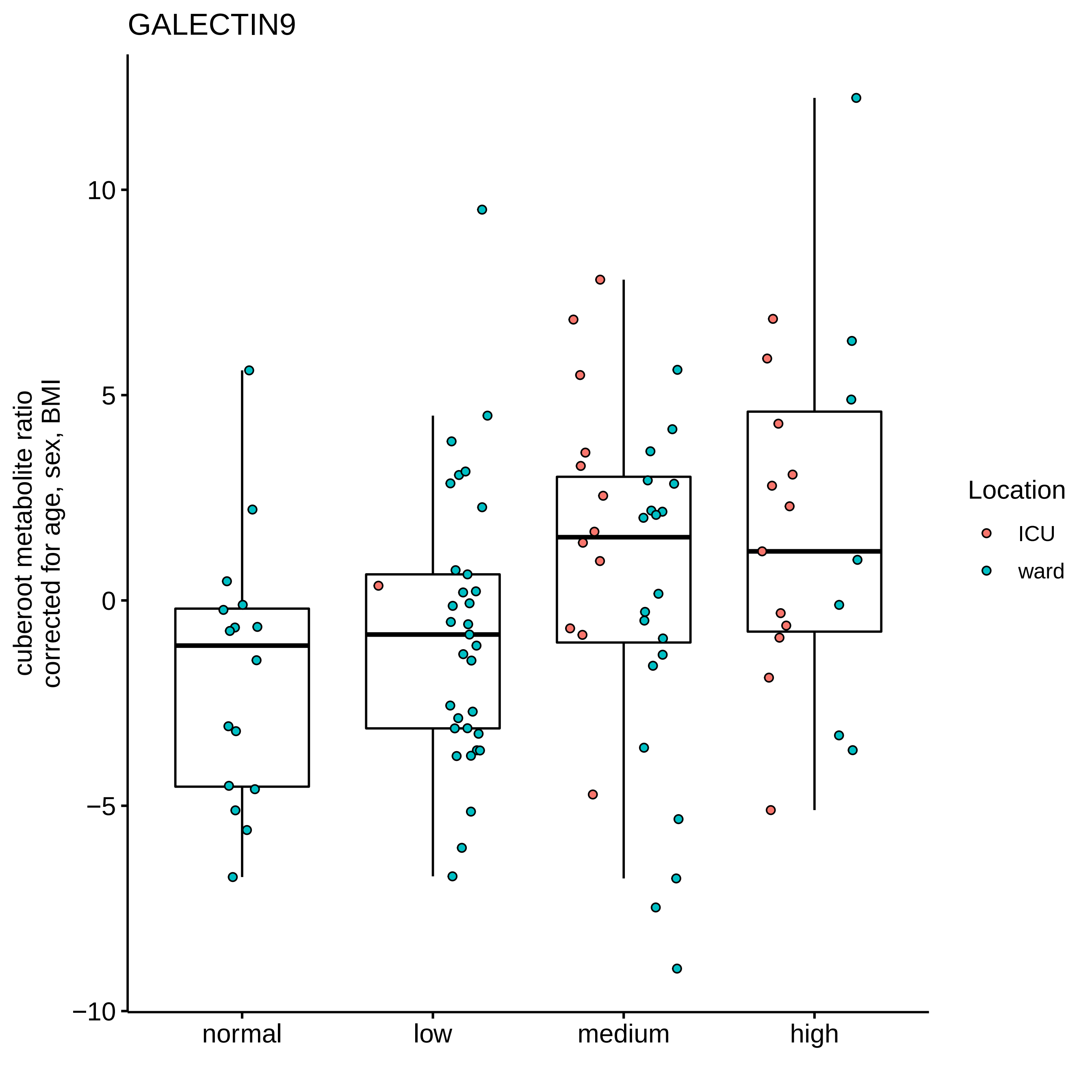
GALECTIN9
cuberoot metabolite ratio
corrected for age, sex, BMI
10
5
0
−5
−10
normal
low
medium
high
Location
ICU
ward

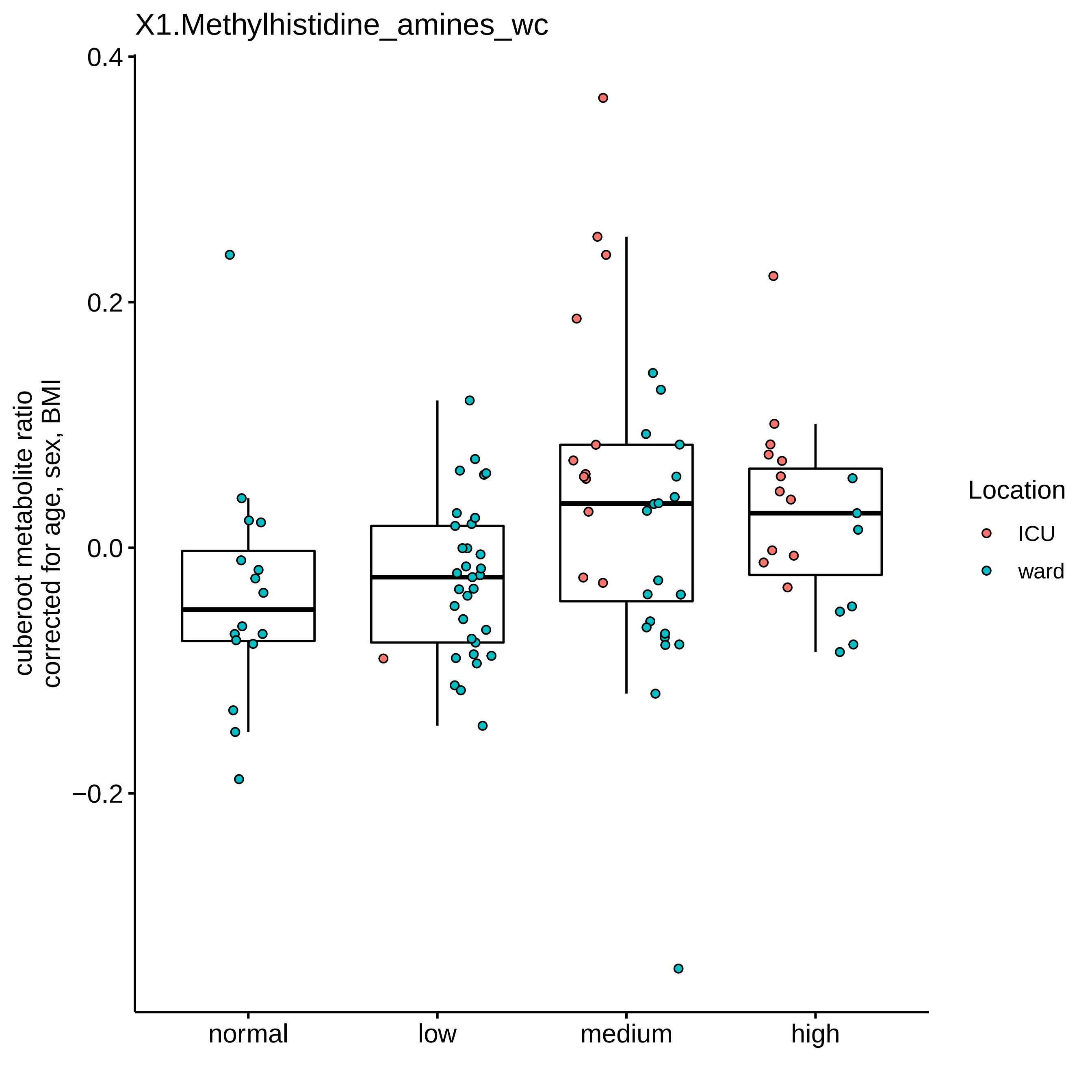

X1.Methylhistidine_amines_wc
cuberoot metabolite ratio
corrected for age, sex, BMI
0.4
0.2
0.0
−0.2
normal
low
medium
high
Location
ICU
ward

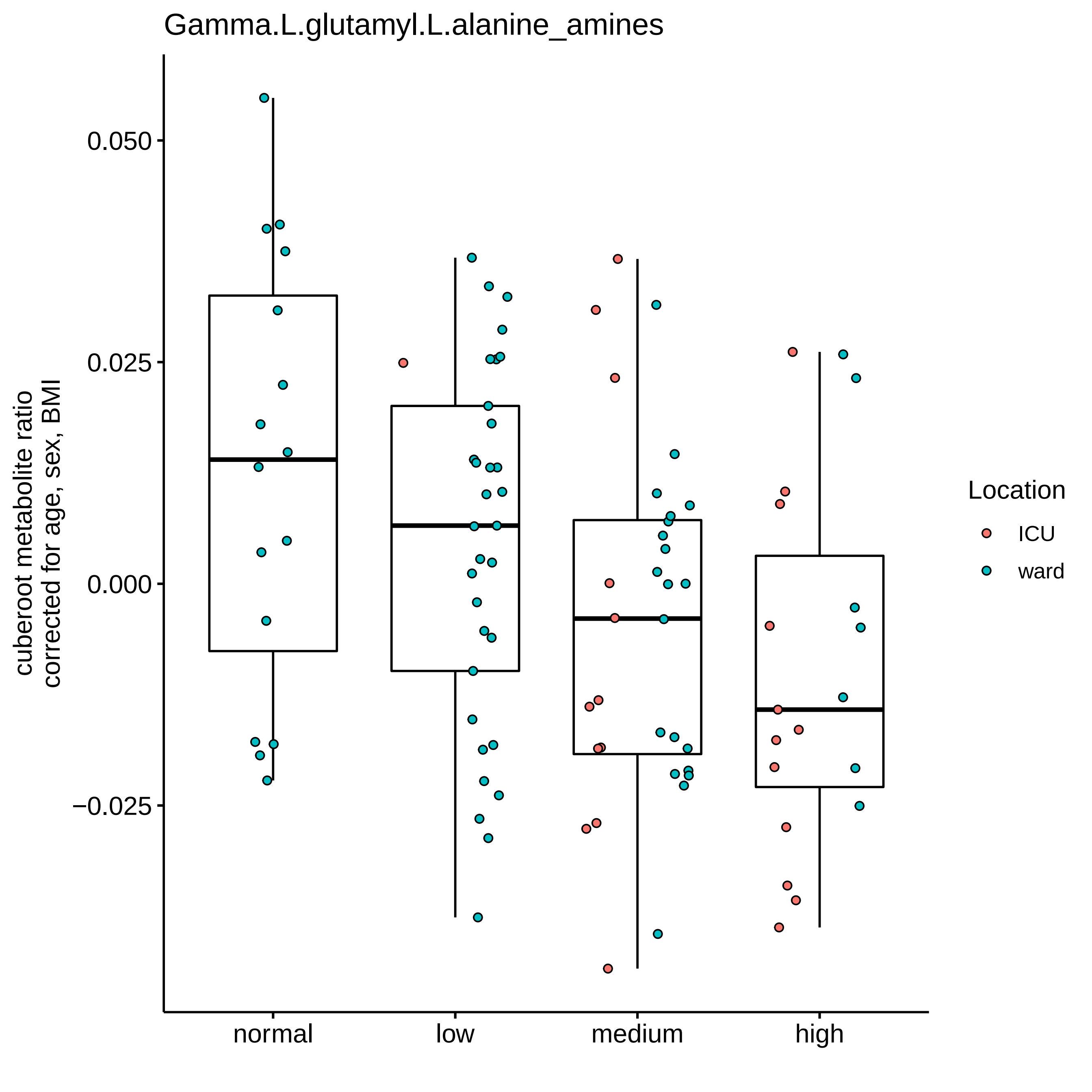
Gamma.L.glutamyl.L.alanine_amines
cuberoot metabolite ratio
corrected for age, sex, BMI
0.050
0.025
0.000
−0.025
normal
low
medium
high
Location
ICU
ward

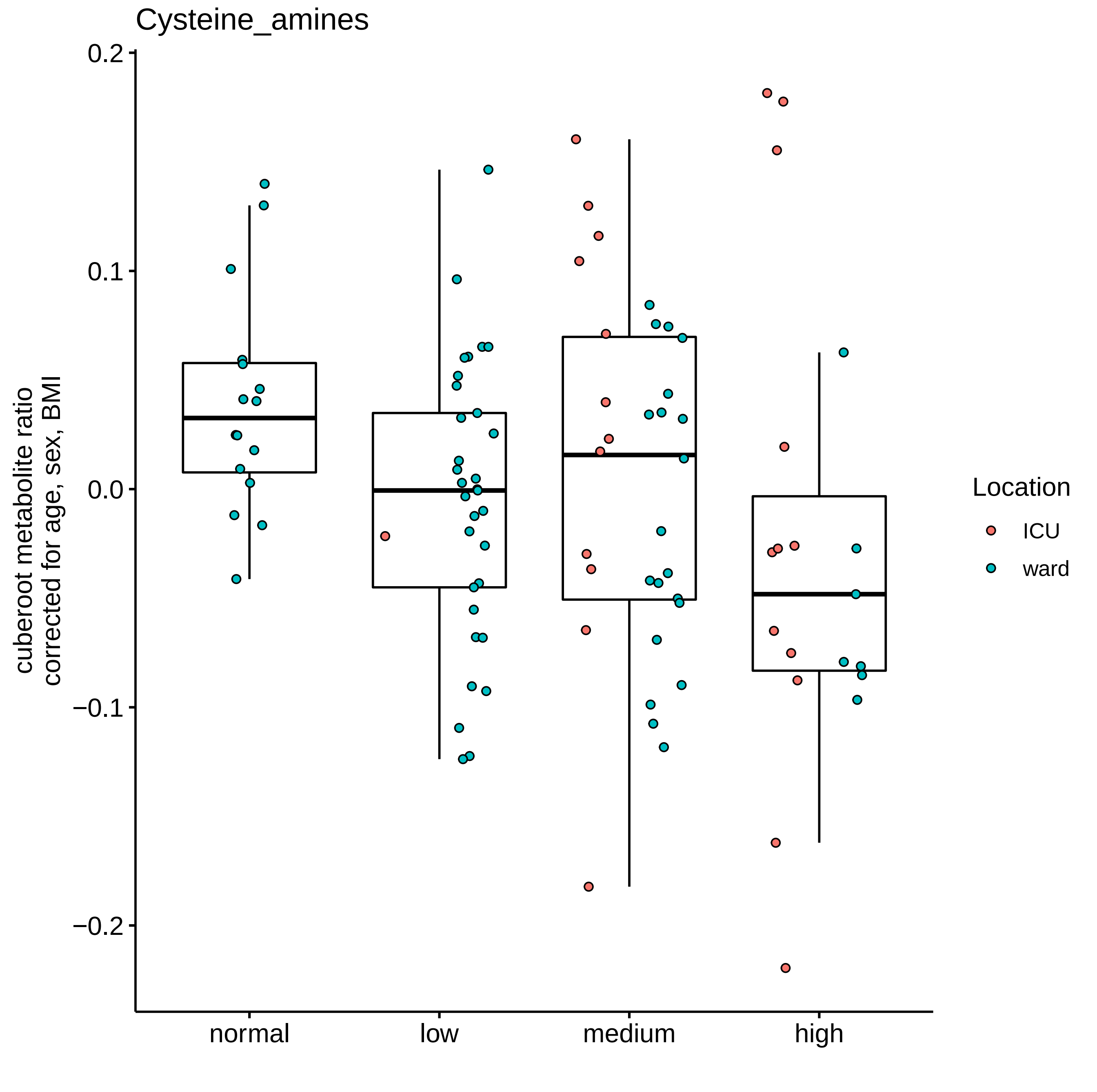
Cysteine_amines
cuberoot metabolite ratio
corrected for age, sex, BMI
0.2
0.1
0.0
−0.1
−0.2
normal
low
medium
high
Location
ICU
ward

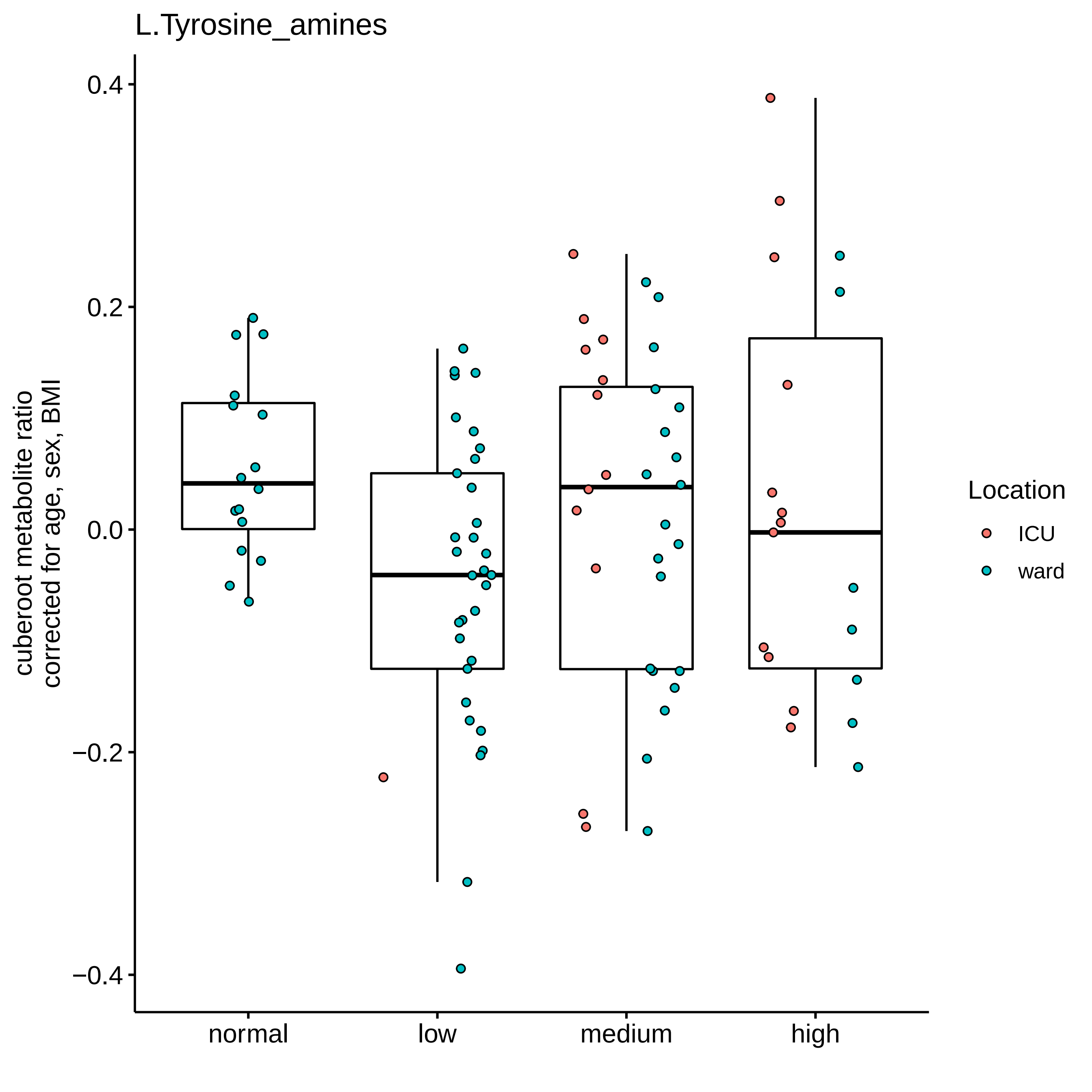
L.Tyrosine_amines
cuberoot metabolite ratio
corrected for age, sex, BMI
0.4
0.2
0.0
−0.2
−0.4
normal
low
medium
high
Location
ICU
ward

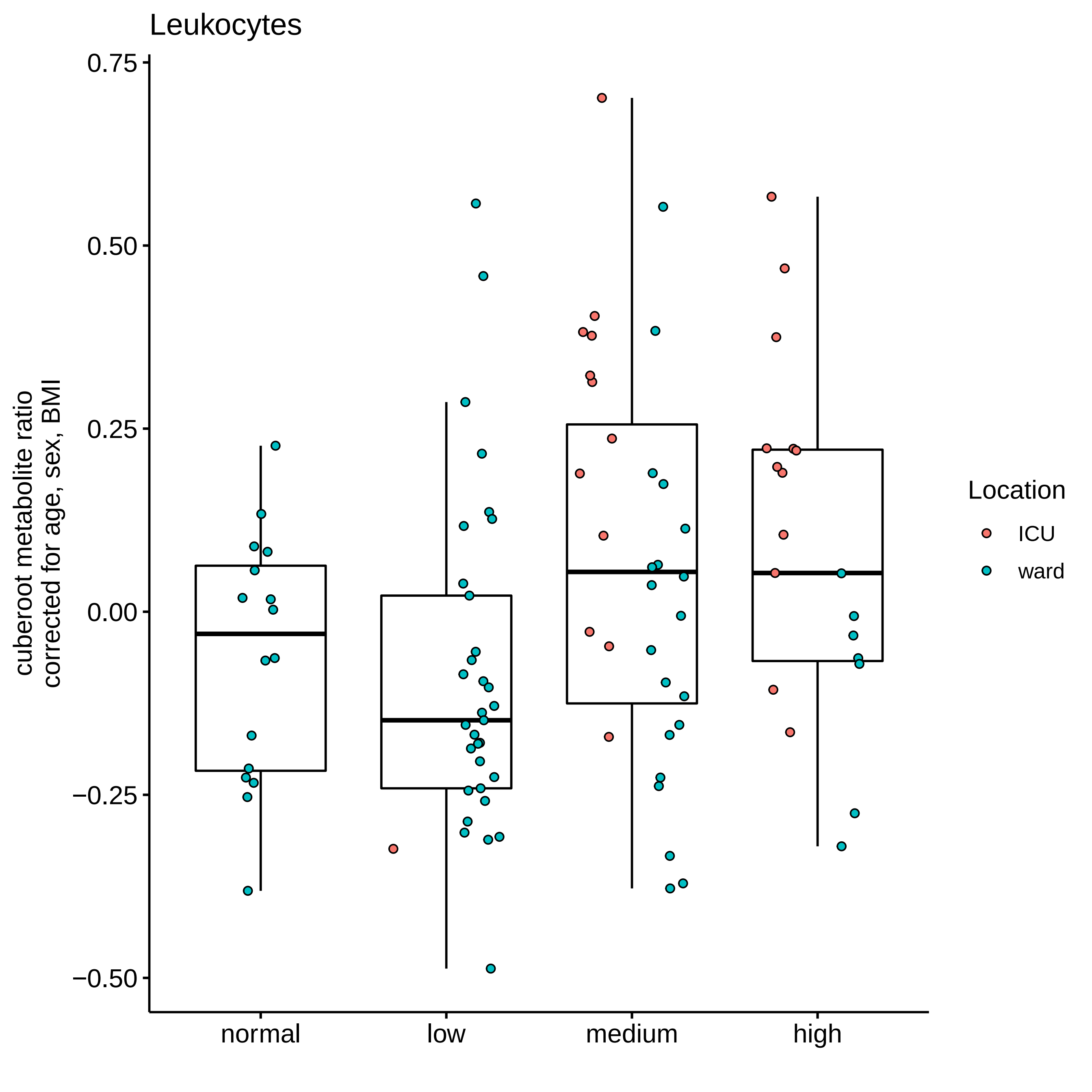

Leukocytes
cuberoot metabolite ratio
corrected for age, sex, BMI
0.75
0.50
0.25
0.00
−0.25
−0.50
normal
low
medium
high
Location
ICU
ward

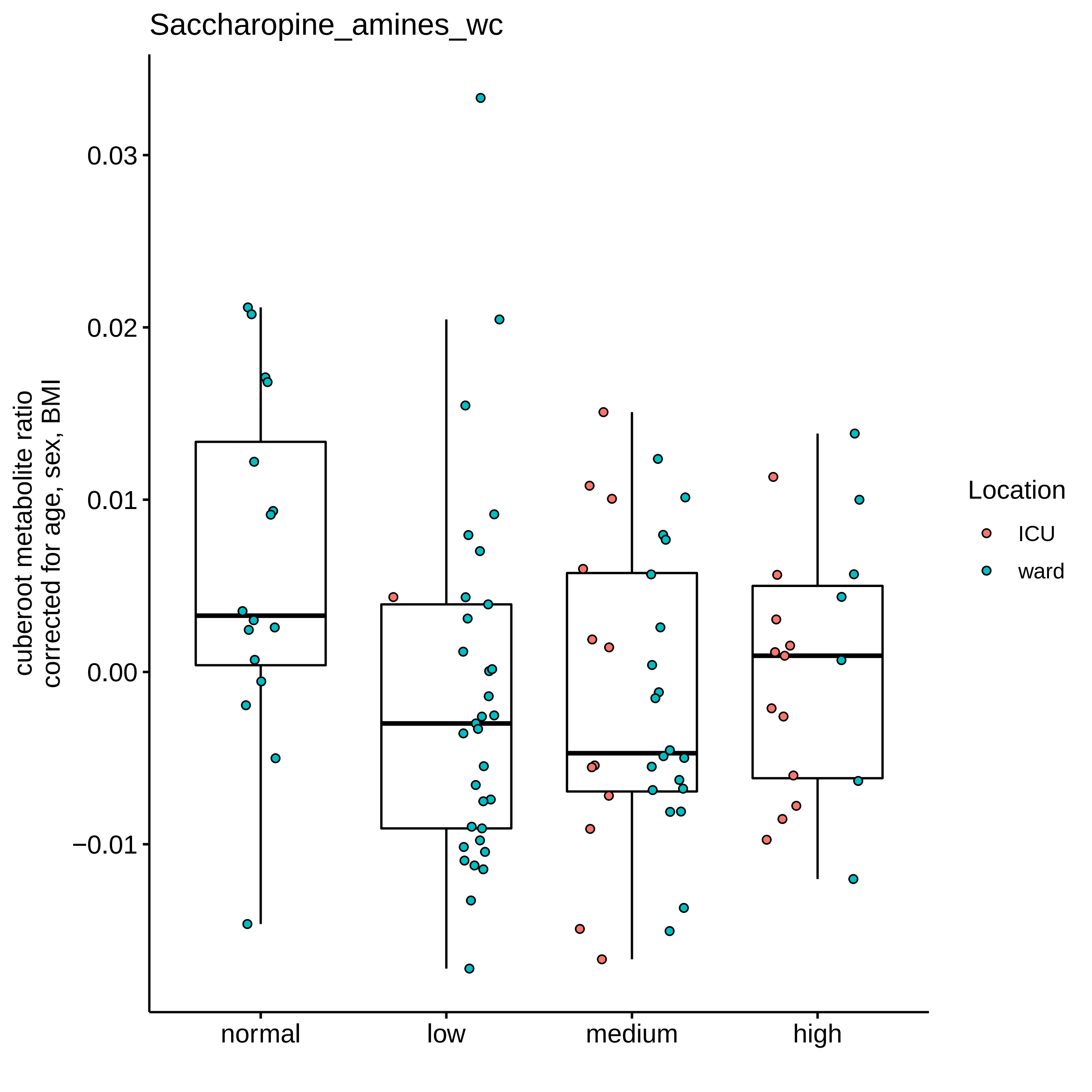

Saccharopine_amines_wc
cuberoot metabolite ratio
corrected for age, sex, BMI
0.03
0.02
0.01
0.00
−0.01
normal
low
medium
high
Location
ICU
ward

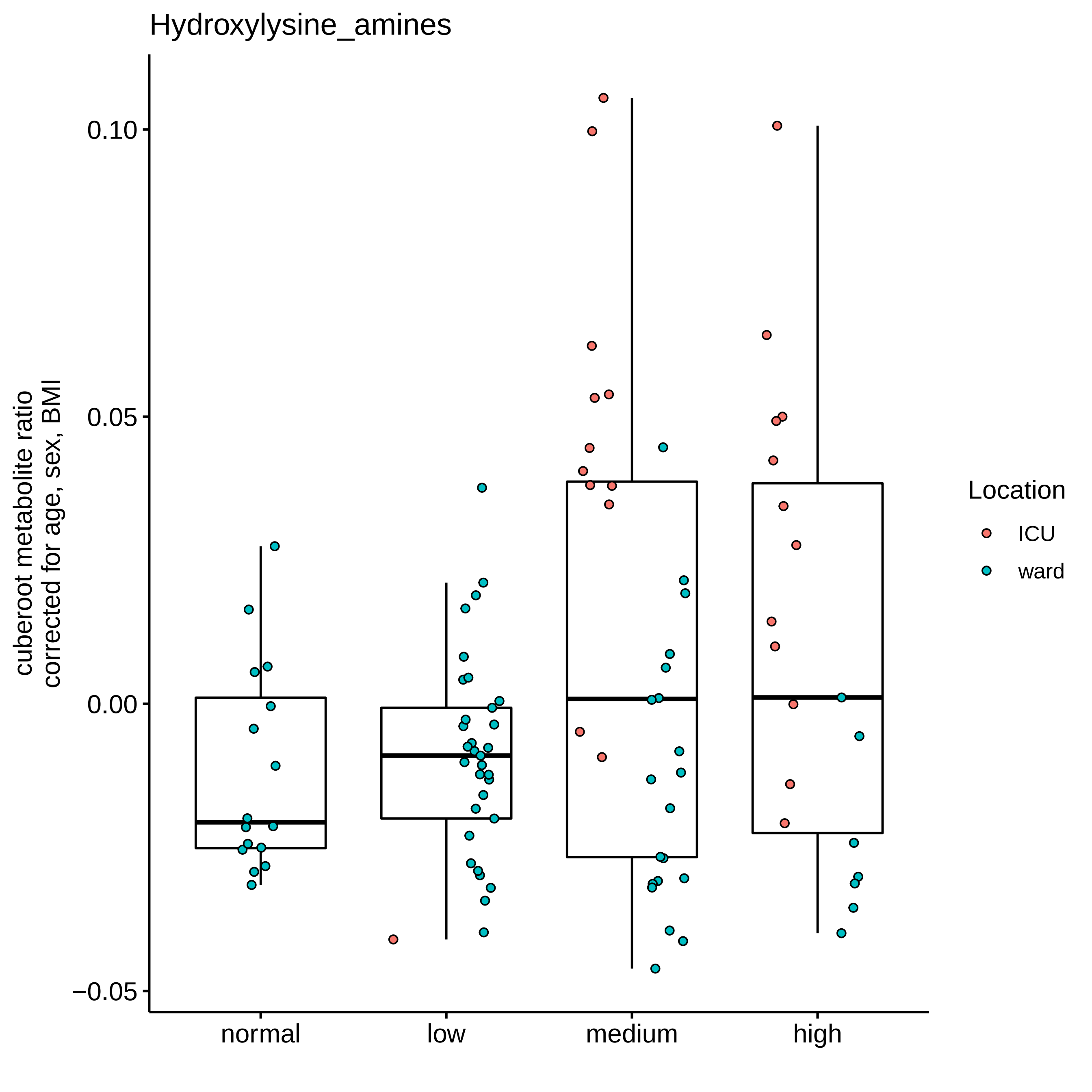

Hydroxylysine_amines
cuberoot metabolite ratio
corrected for age, sex, BMI
0.10
0.05
0.00
−0.05
normal
low
medium
high
Location
ICU
ward

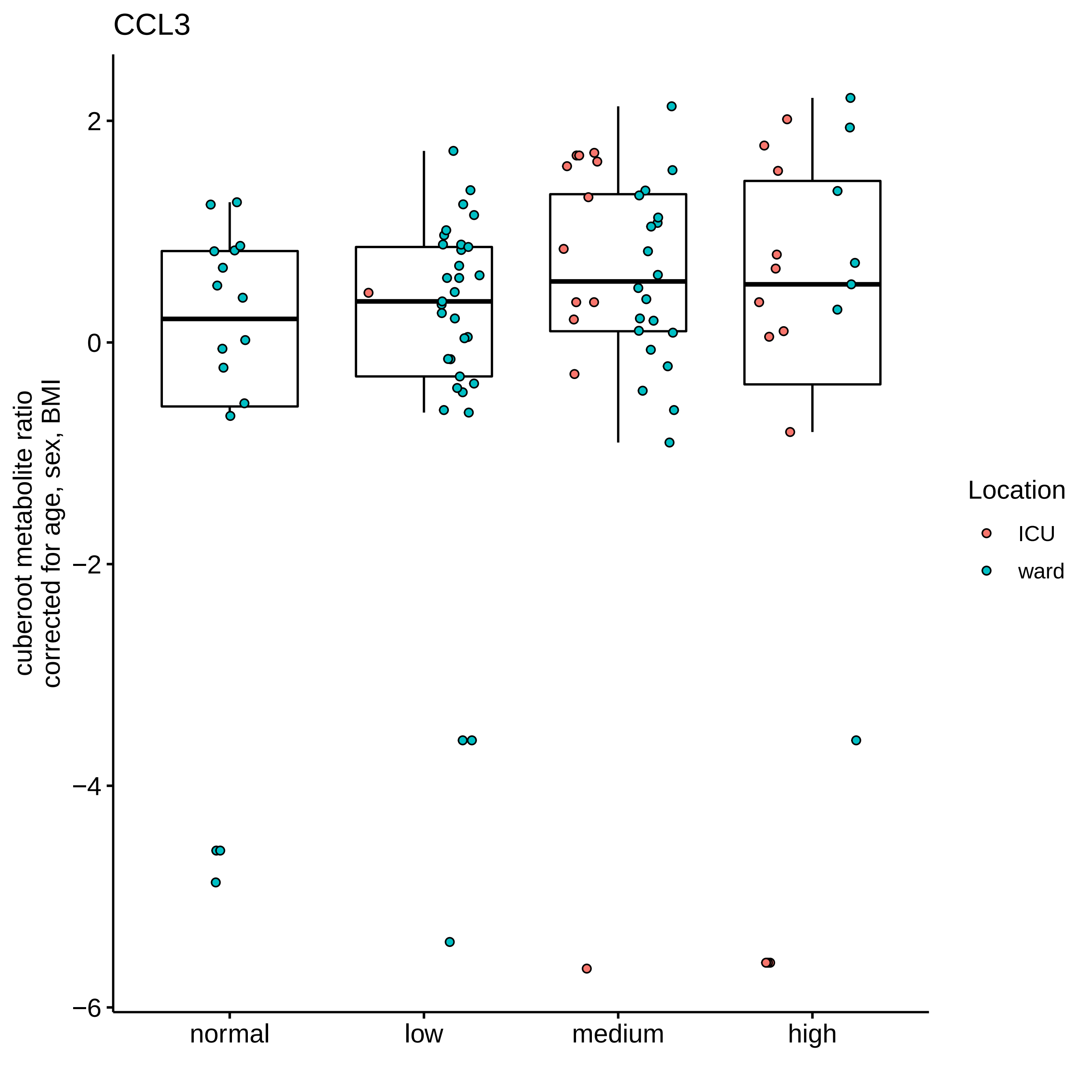
CCL3
cuberoot metabolite ratio
corrected for age, sex, BMI
2
0
−2
−4
−6
normal
low
medium
high
Location
ICU
ward

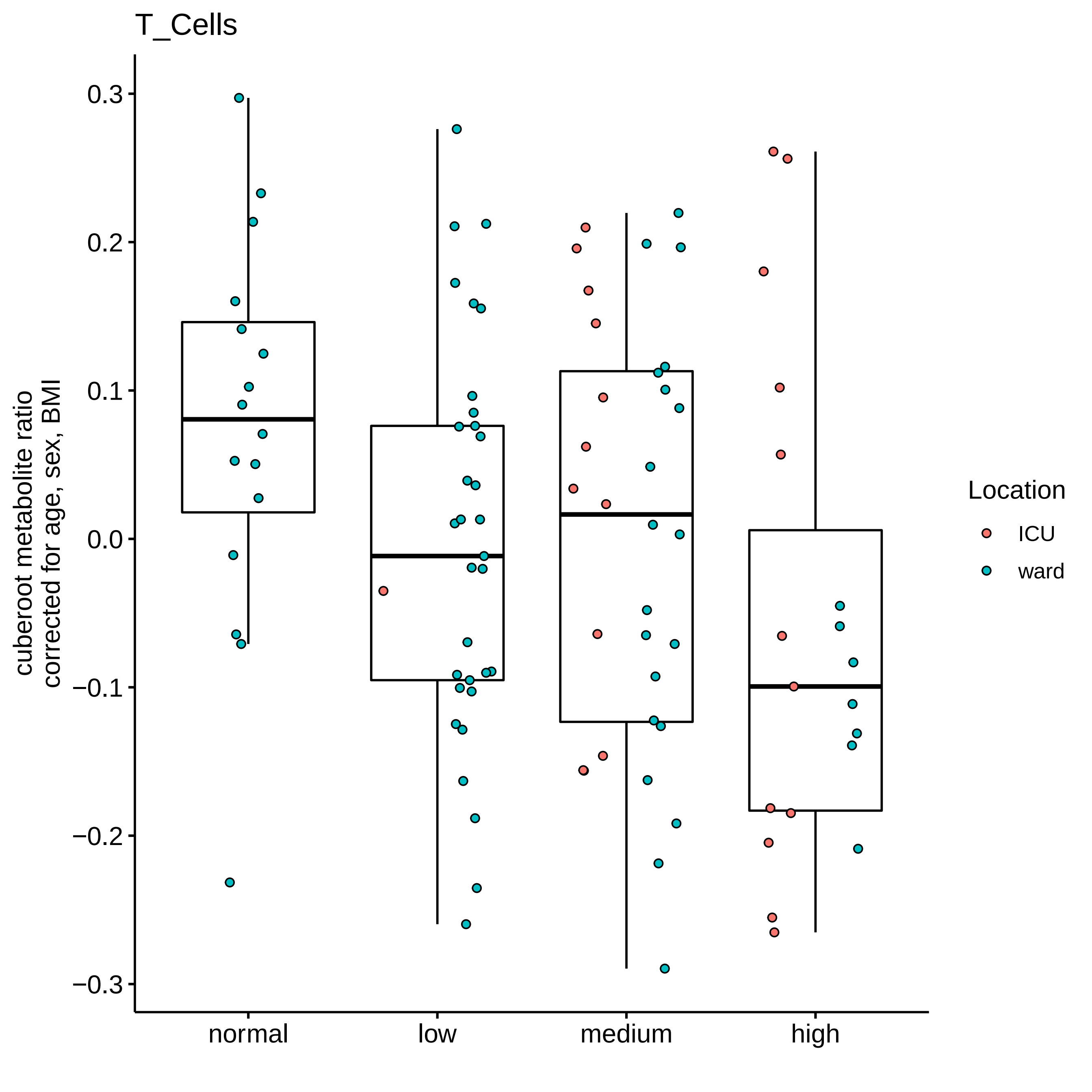

T_Cells
cuberoot metabolite ratio
corrected for age, sex, BMI
0.3
0.2
0.1
0.0
−0.1
−0.2
−0.3
normal
low
medium
high
Location
ICU
ward

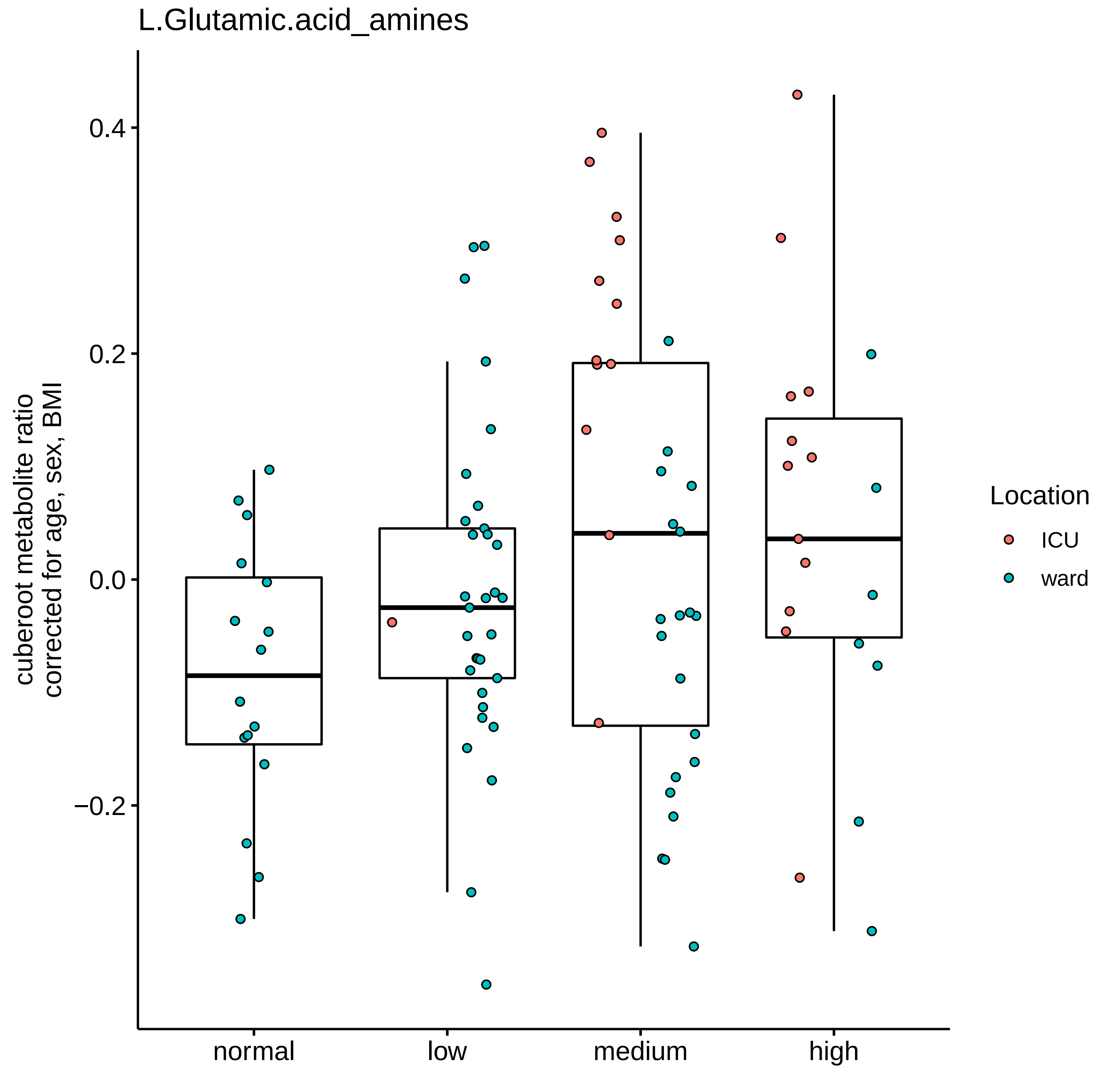

L.Glutamic.acid_amines
cuberoot metabolite ratio
corrected for age, sex, BMI
0.4
0.2
0.0
−0.2
normal
low
medium
high
Location
ICU
ward

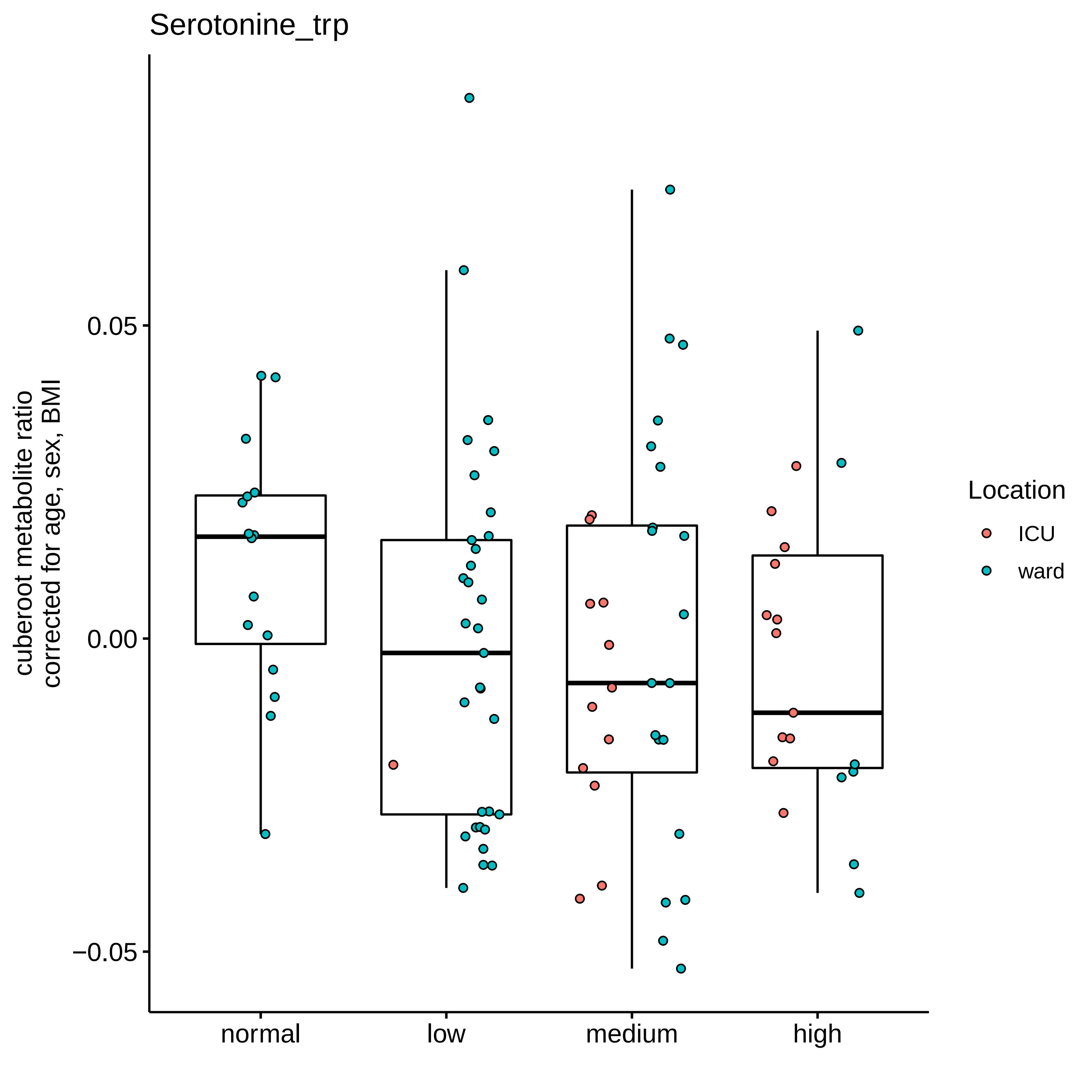
Serotonine_trp
cuberoot metabolite ratio
corrected for age, sex, BMI
0.05
0.00
−0.05
normal
low
medium
high
Location
ICU
ward

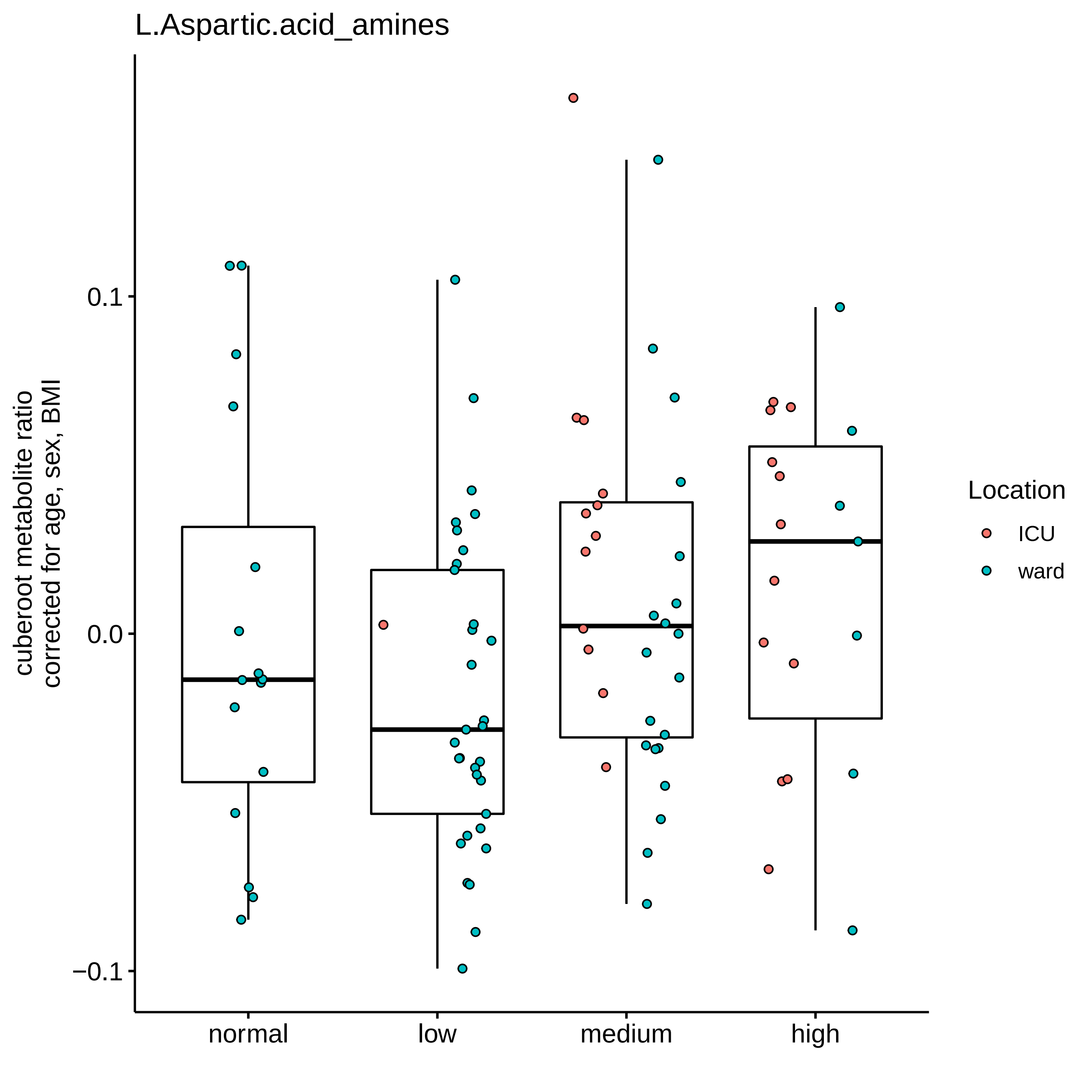

L.Aspartic.acid_amines
cuberoot metabolite ratio
corrected for age, sex, BMI
0.1
0.0
−0.1
normal
low
medium
high
Location
ICU
ward

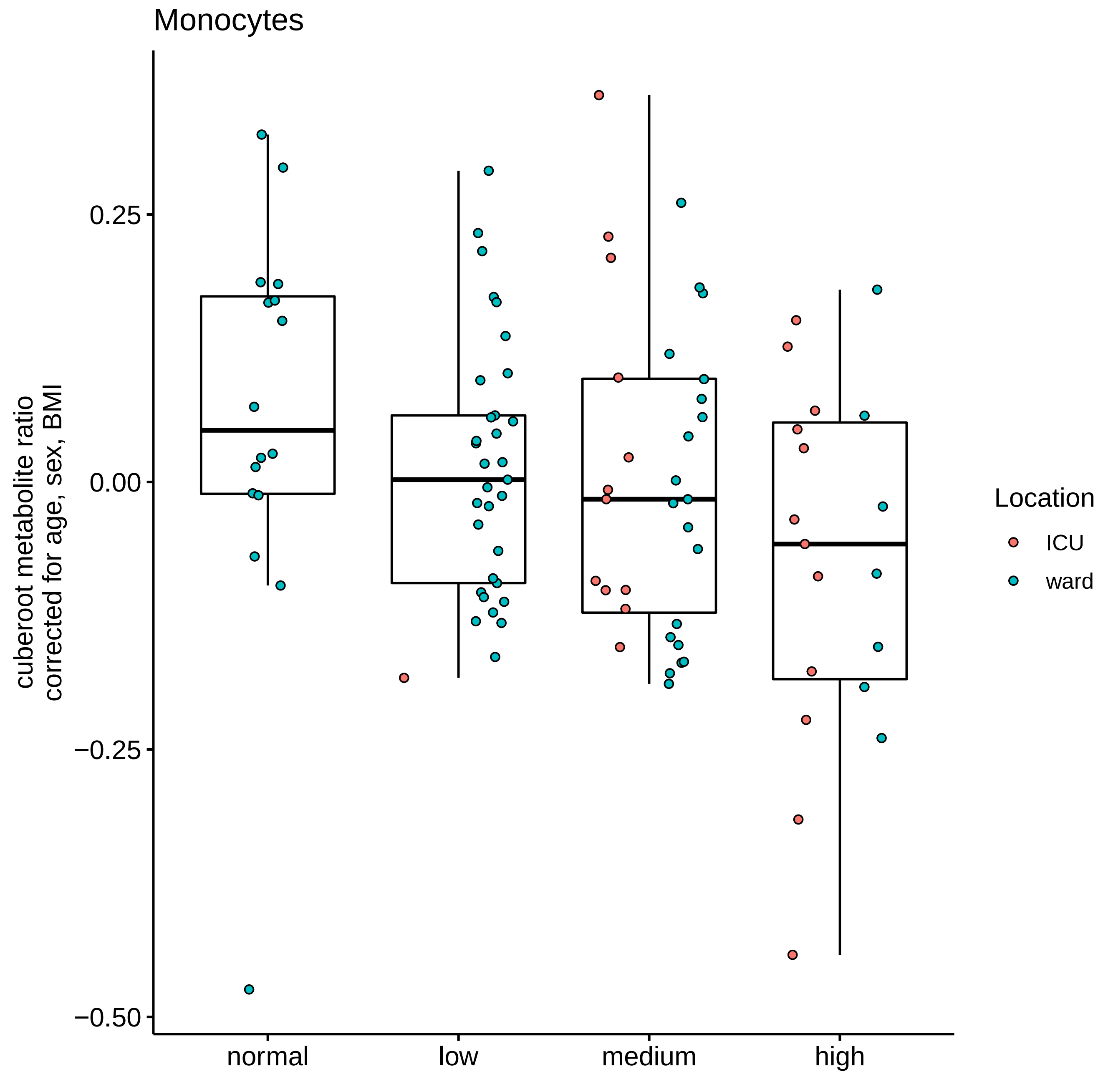

Monocytes
cuberoot metabolite ratio
corrected for age, sex, BMI
0.25
0.00
−0.25
−0.50
normal
low
medium
high
Location
ICU
ward

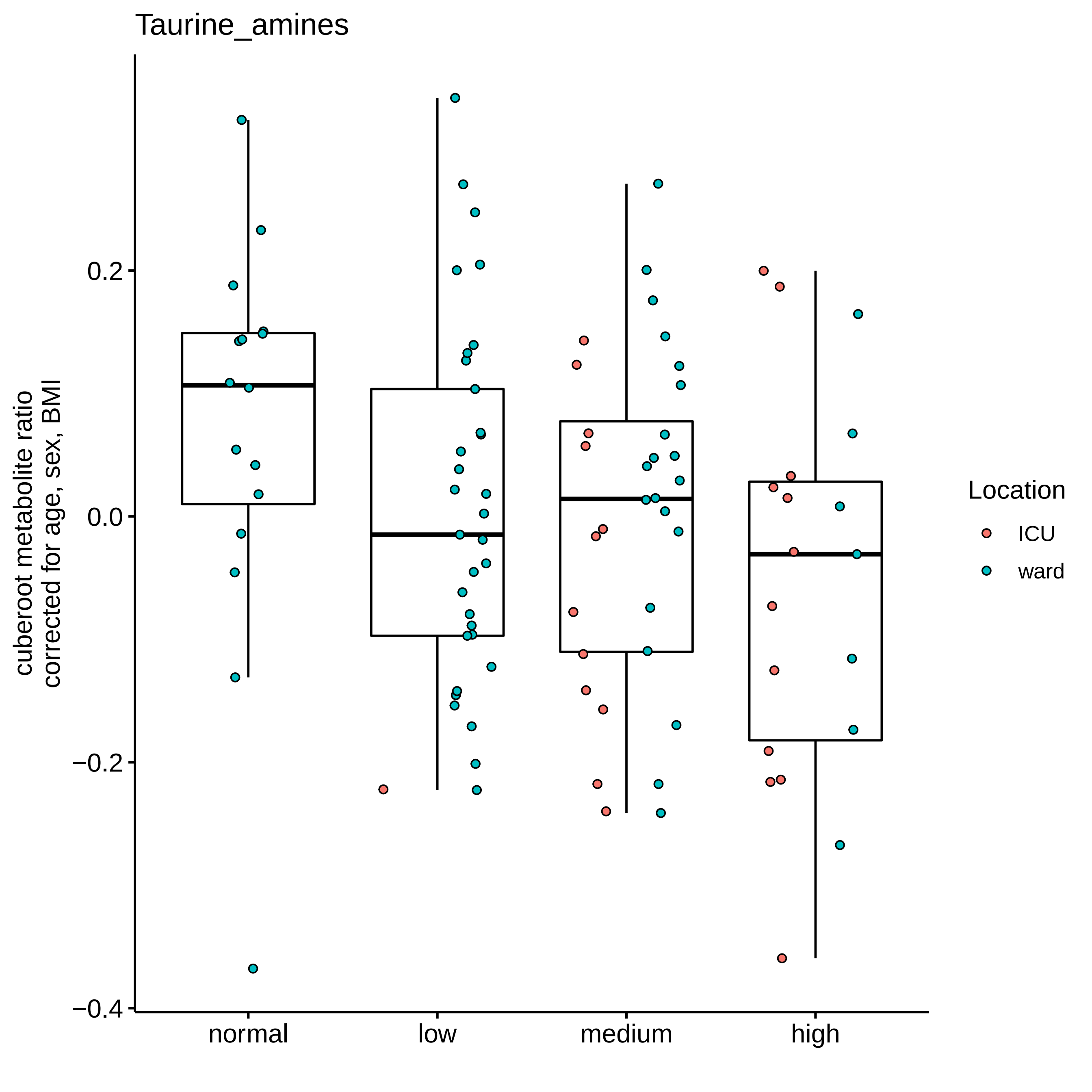

Taurine_amines
cuberoot metabolite ratio
corrected for age, sex, BMI
0.2
0.0
−0.2
−0.4
normal
low
medium
high
Location
ICU
ward

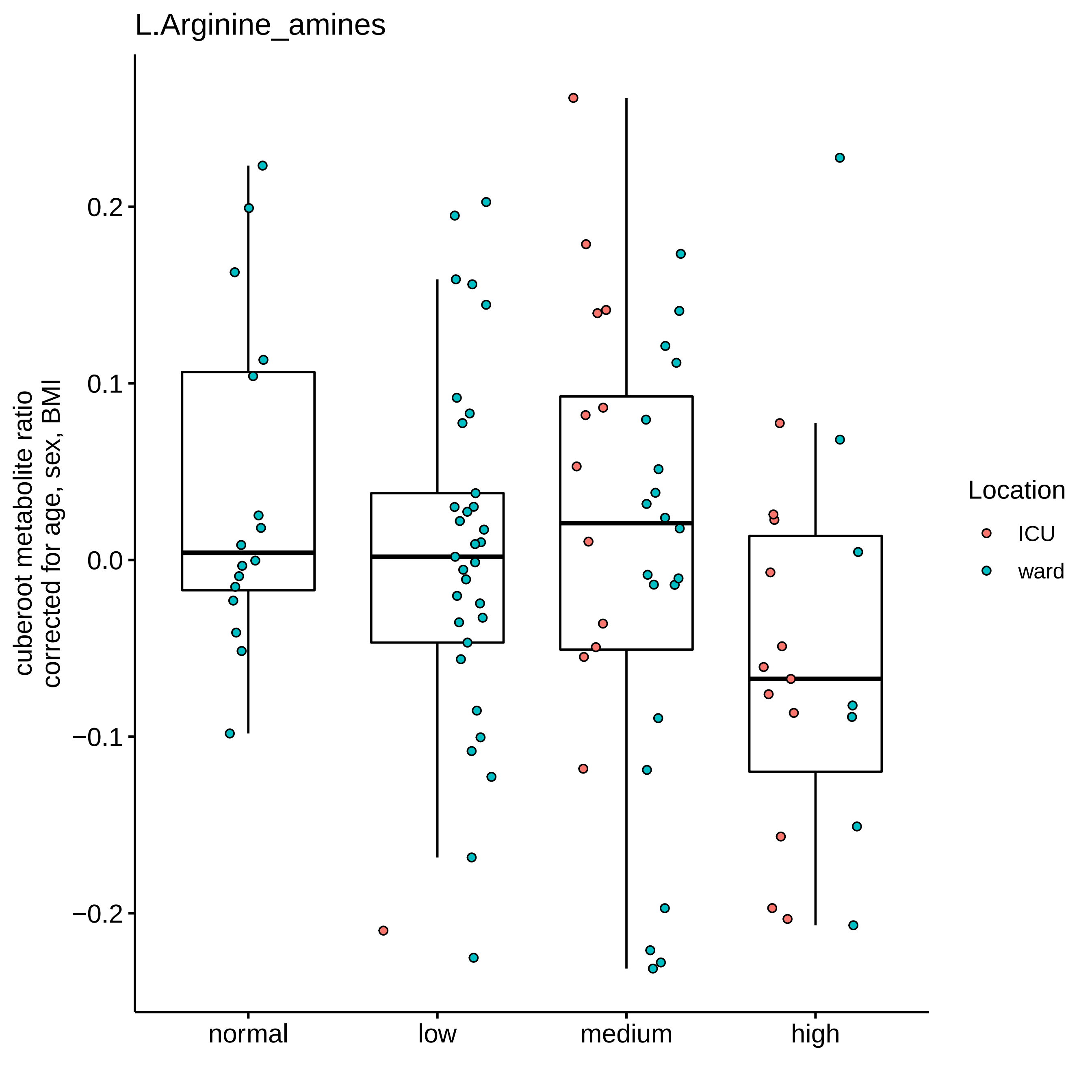
L.Arginine_amines
cuberoot metabolite ratio
corrected for age, sex, BMI
0.2
0.1
0.0
−0.1
−0.2
normal
low
medium
high
Location
ICU
ward

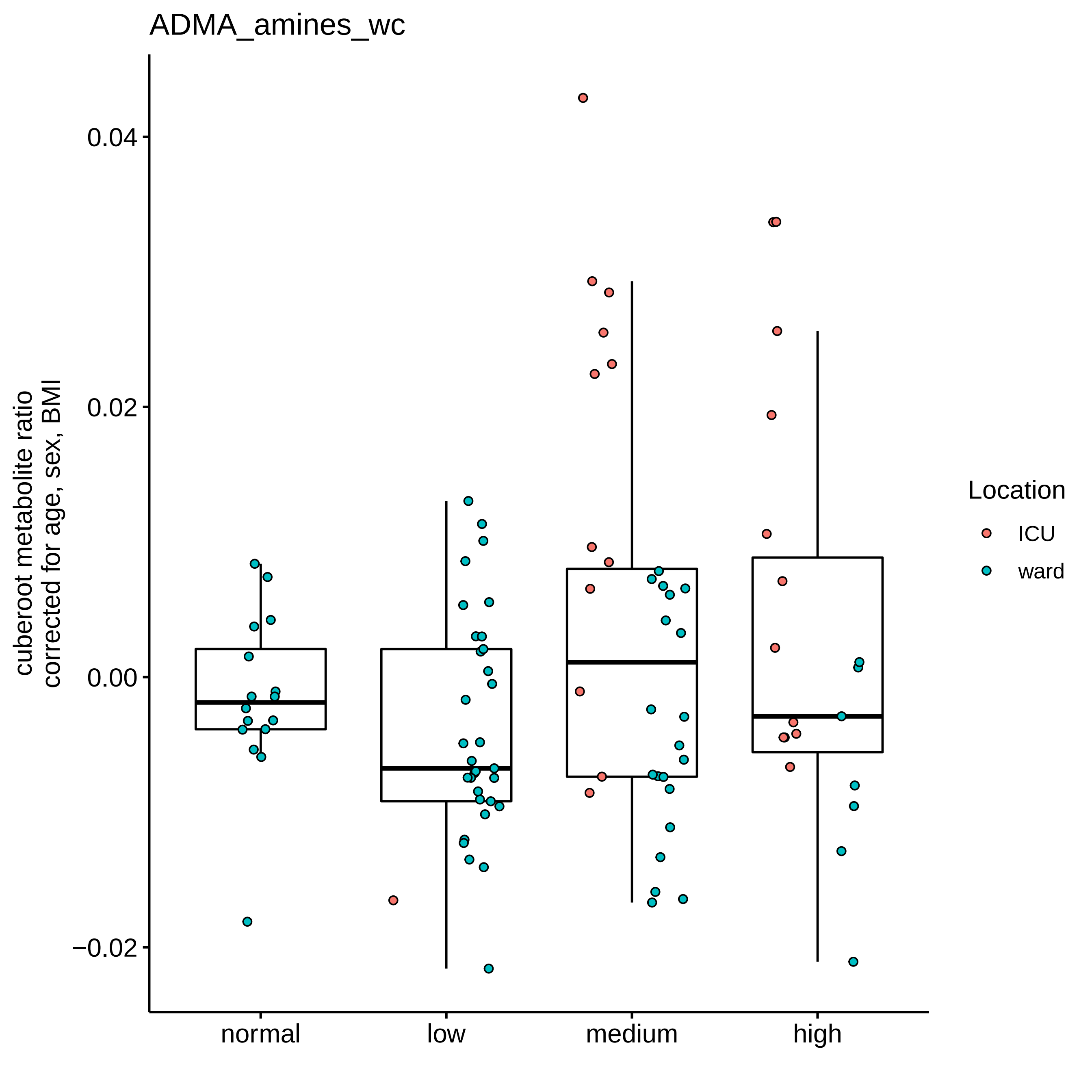
ADMA_amines_wc
cuberoot metabolite ratio
corrected for age, sex, BMI
0.04
0.02
0.00
−0.02
normal
low
medium
high
Location
ICU
ward

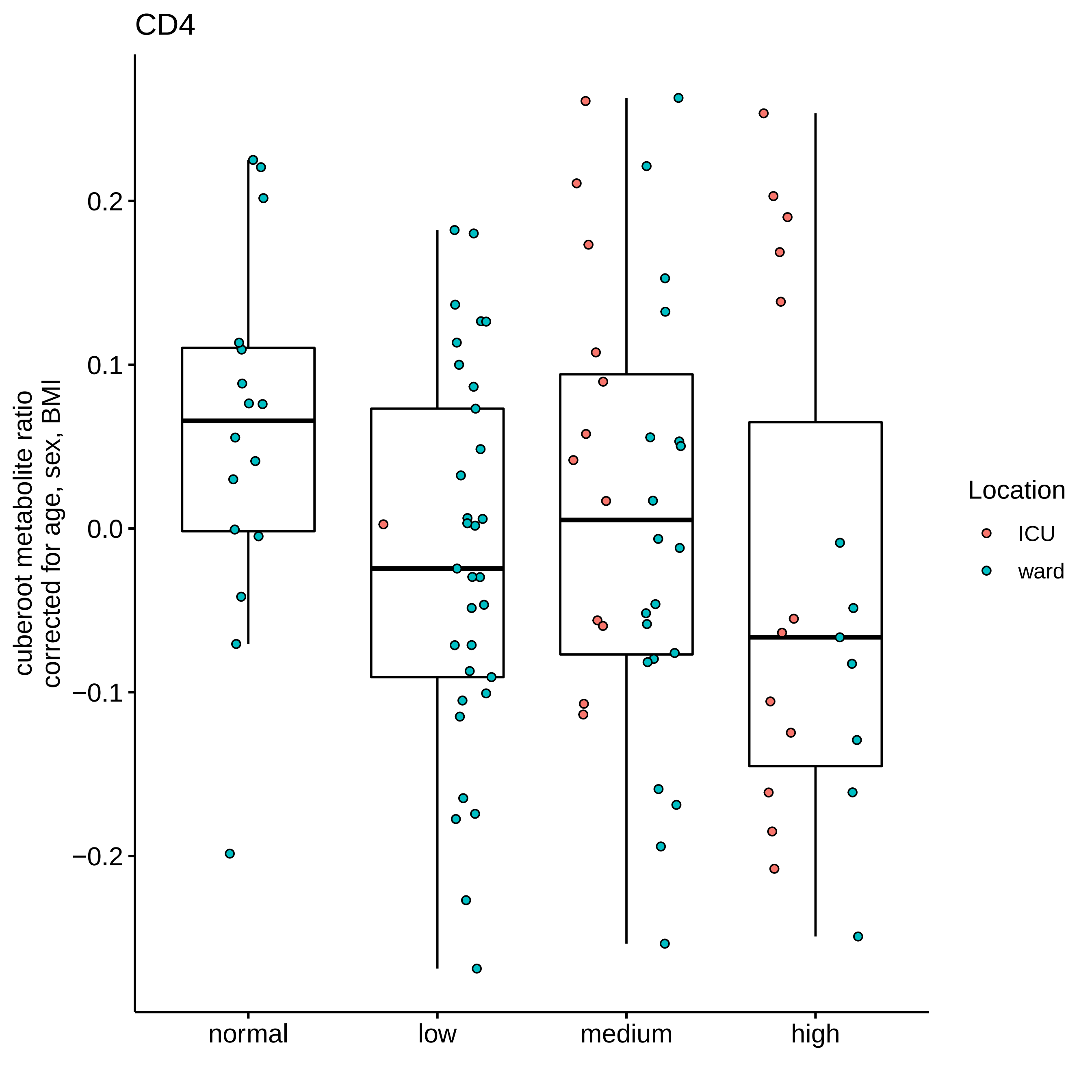

CD4
cuberoot metabolite ratio
corrected for age, sex, BMI
0.2
0.1
0.0
−0.1
−0.2
normal
low
medium
high
Location
ICU
ward

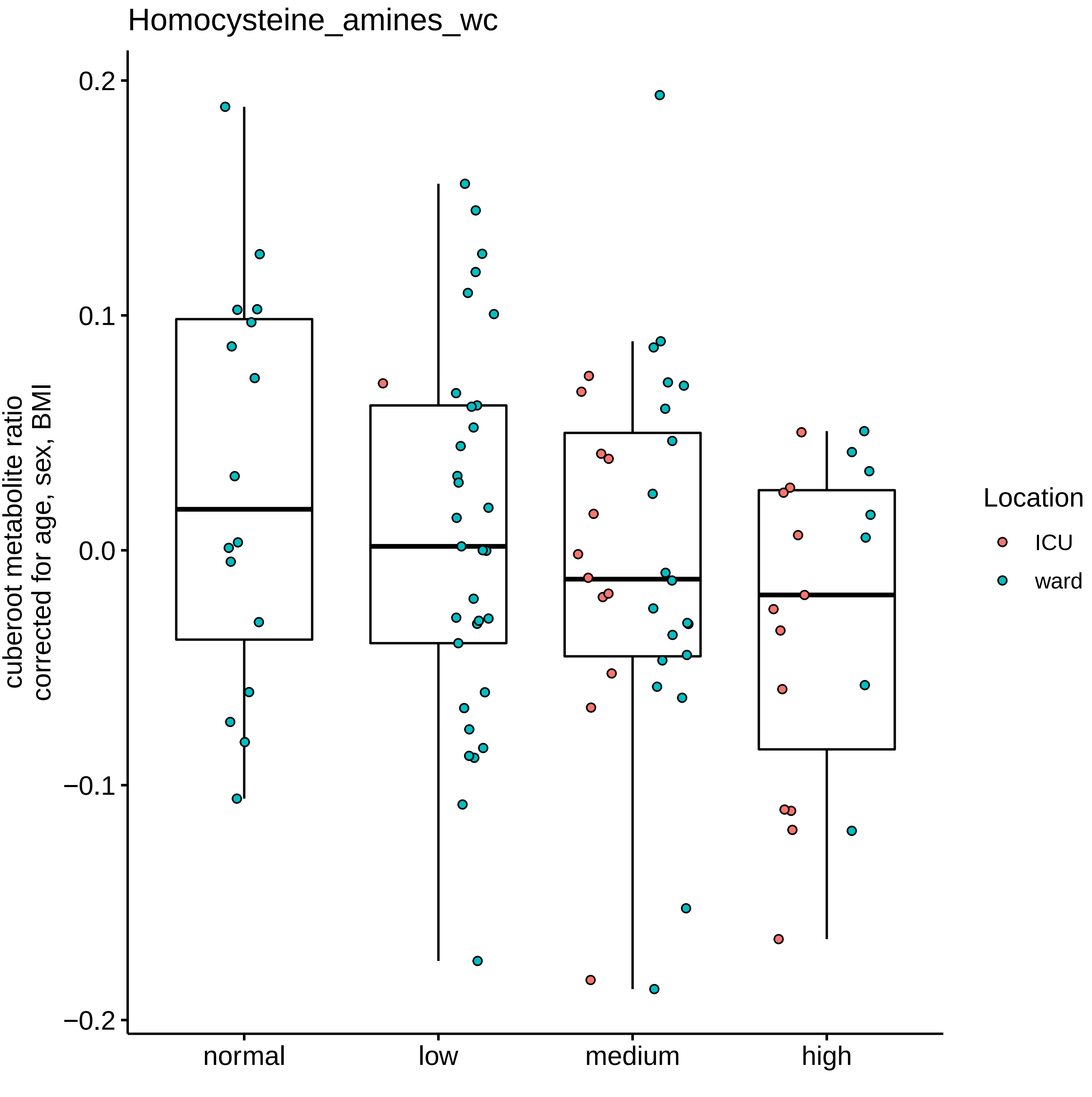

Homocysteine_amines_wc
cuberoot metabolite ratio
corrected for age, sex, BMI
0.2
0.1
0.0
−0.1
−0.2
normal
low
medium
high
Location
ICU
ward

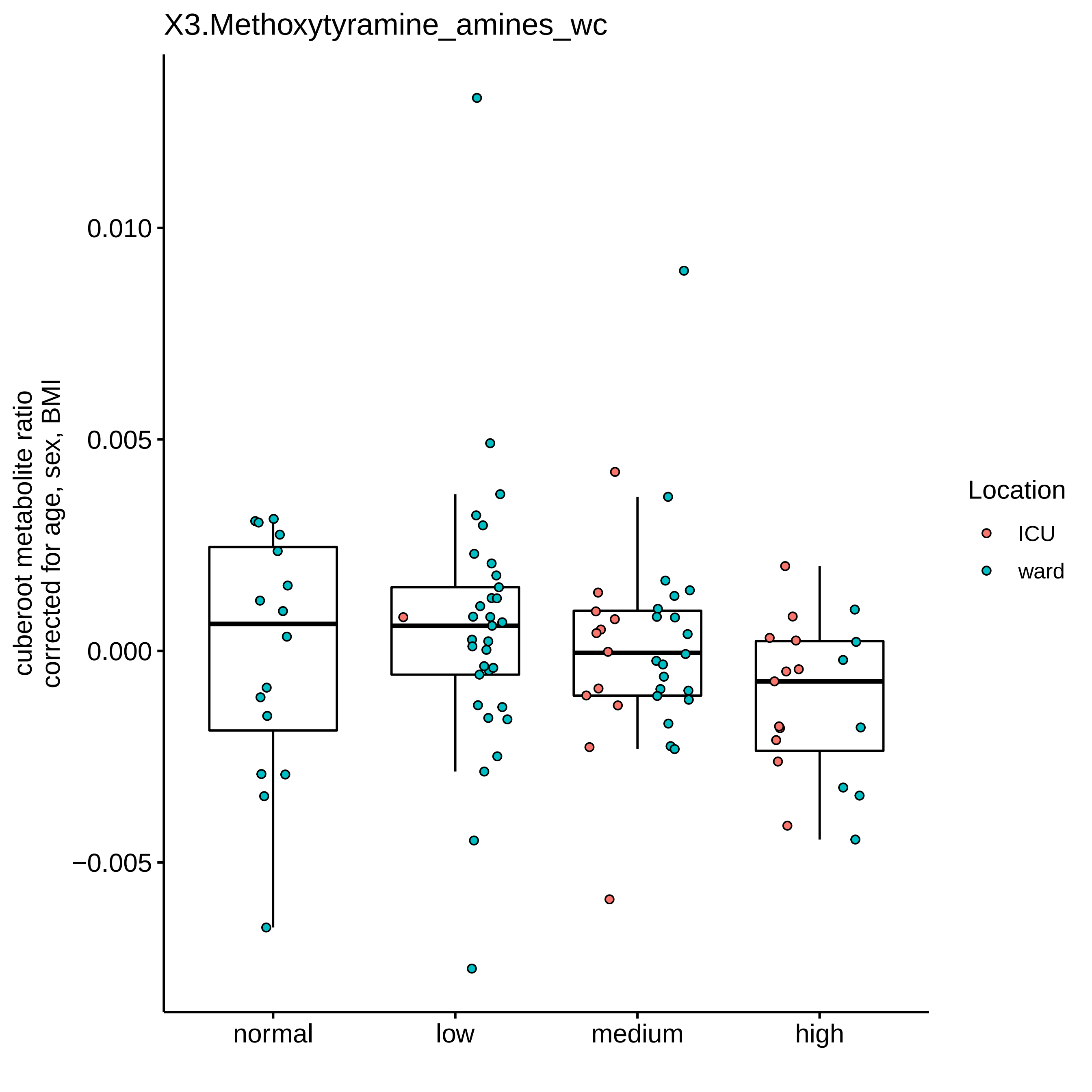

X3.Methoxytyramine_amines_wc
cuberoot metabolite ratio
corrected for age, sex, BMI
0.010
0.005
0.000
−0.005
normal
low
medium
high
Location
ICU
ward

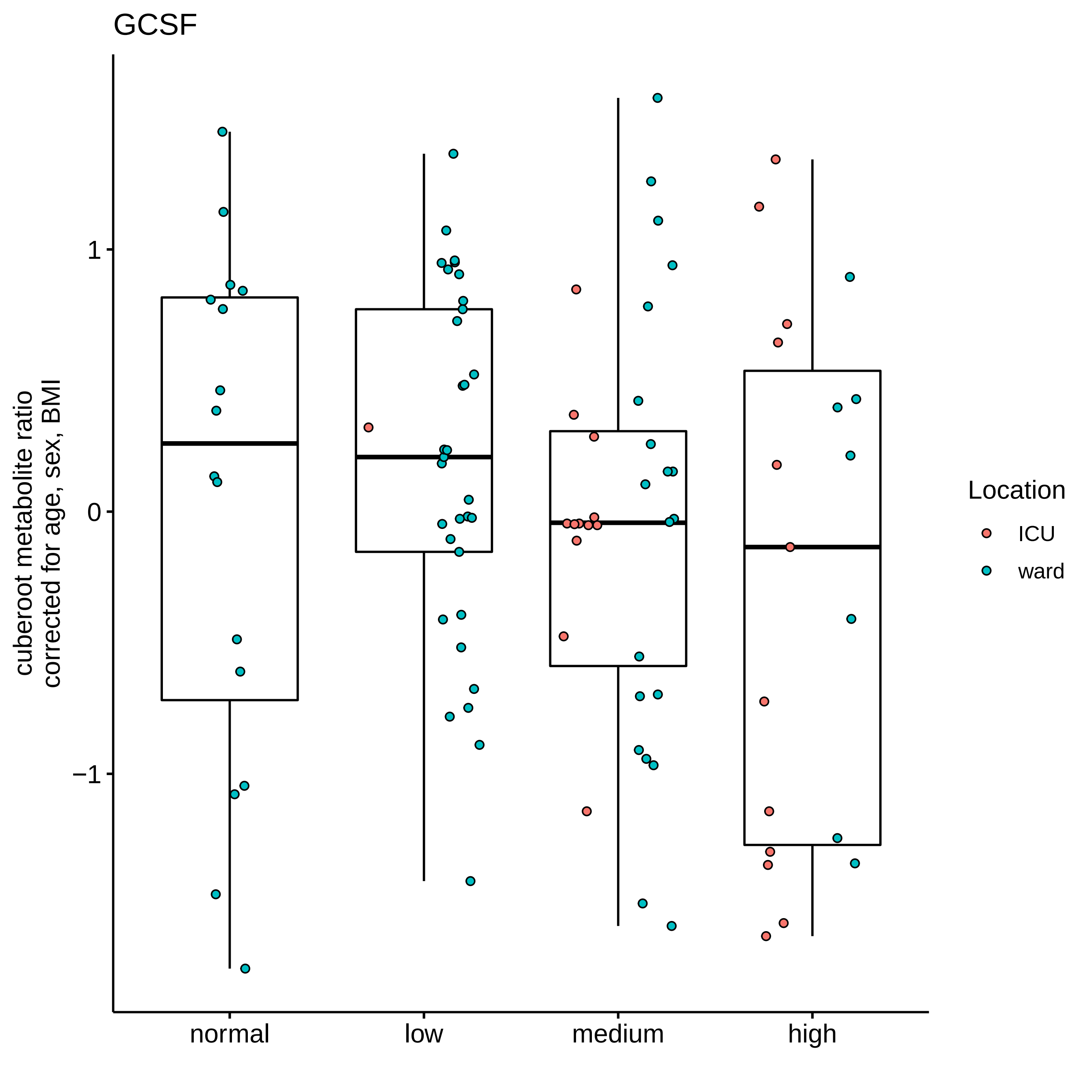
GCSF
cuberoot metabolite ratio
corrected for age, sex, BMI
1
0
−1
normal
low
medium
high
Location
ICU
ward

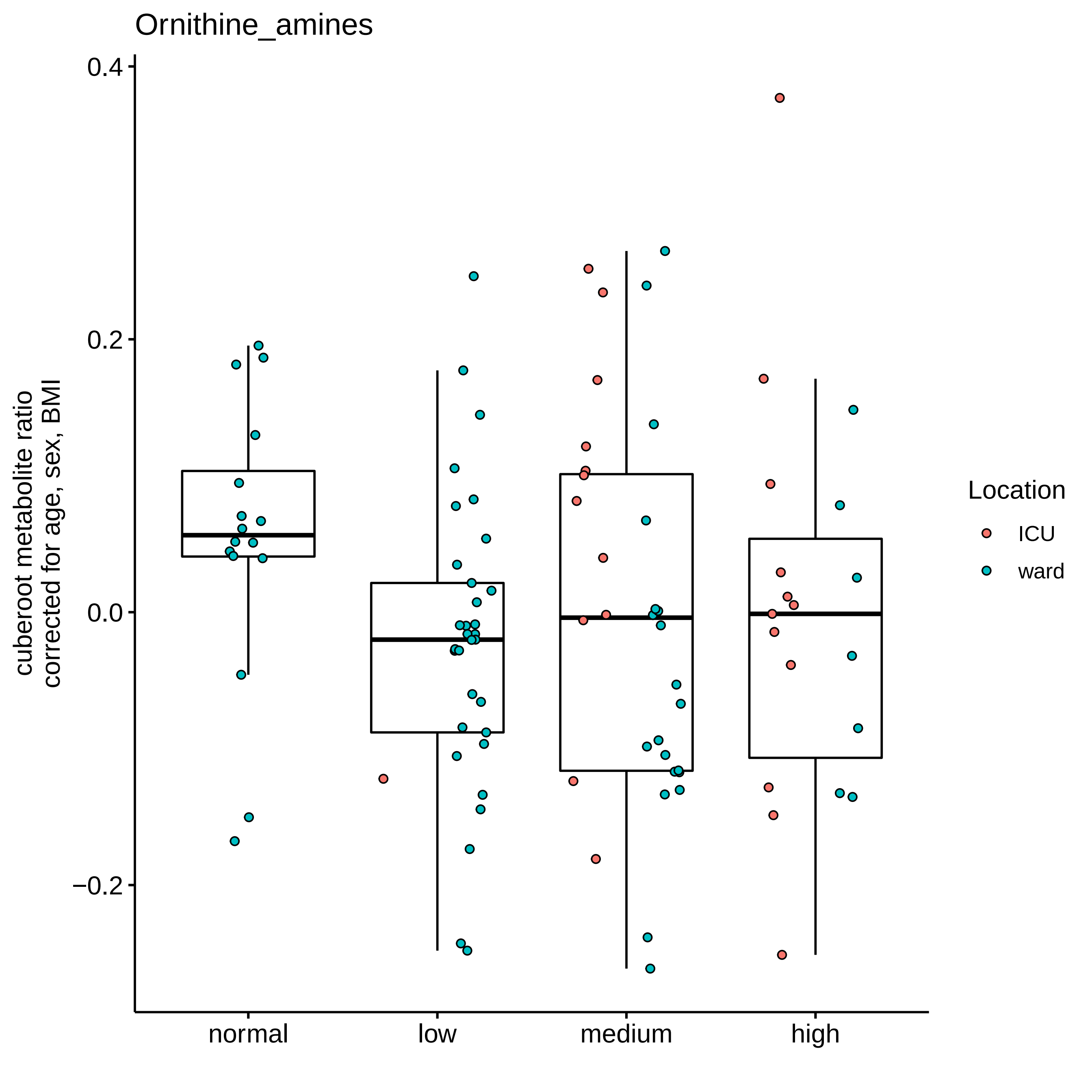
Ornithine_amines
cuberoot metabolite ratio
corrected for age, sex, BMI
0.4
0.2
0.0
−0.2
normal
low
medium
high
Location
ICU
ward

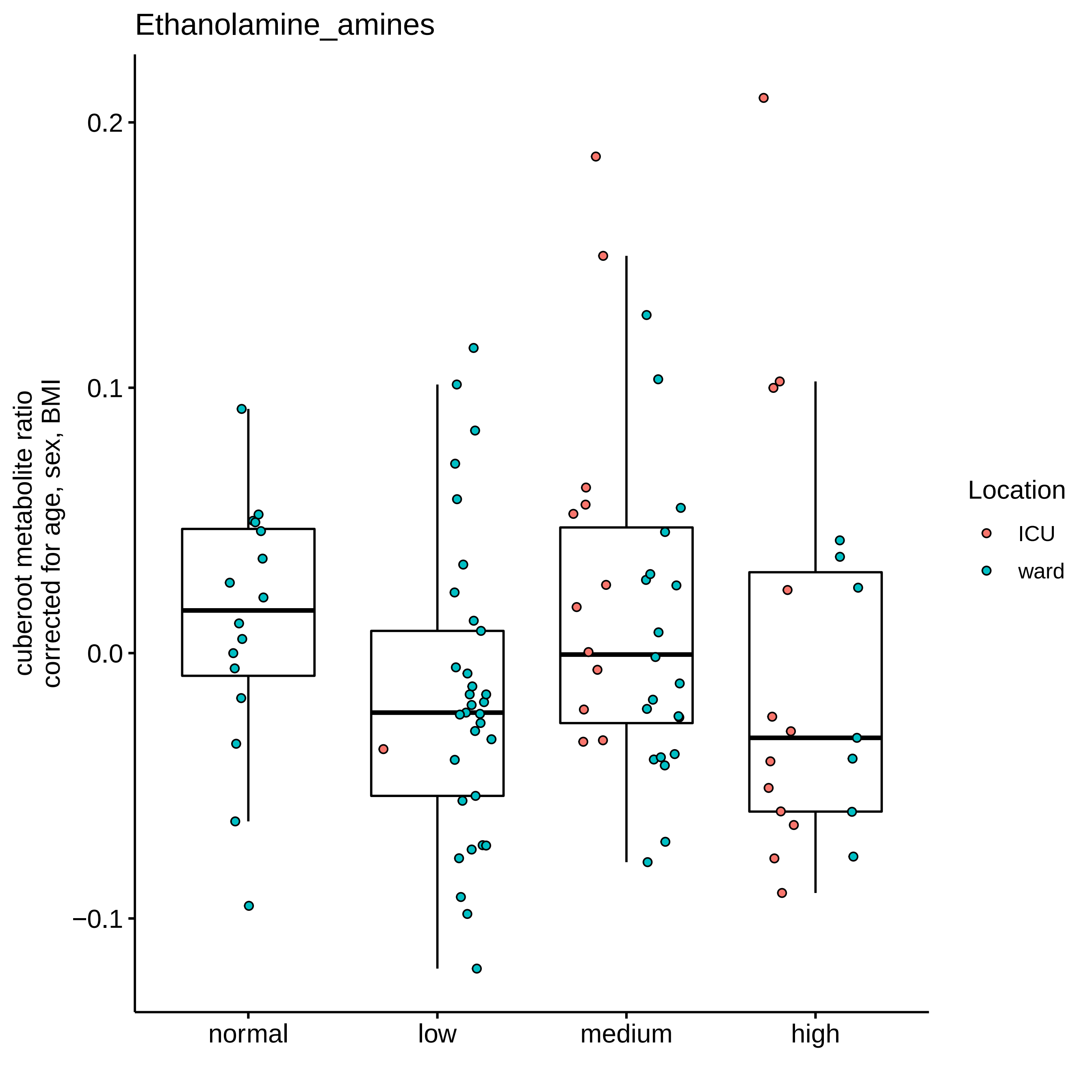

Ethanolamine_amines
cuberoot metabolite ratio
corrected for age, sex, BMI
0.2
0.1
0.0
−0.1
normal
low
medium
high
Location
ICU
ward

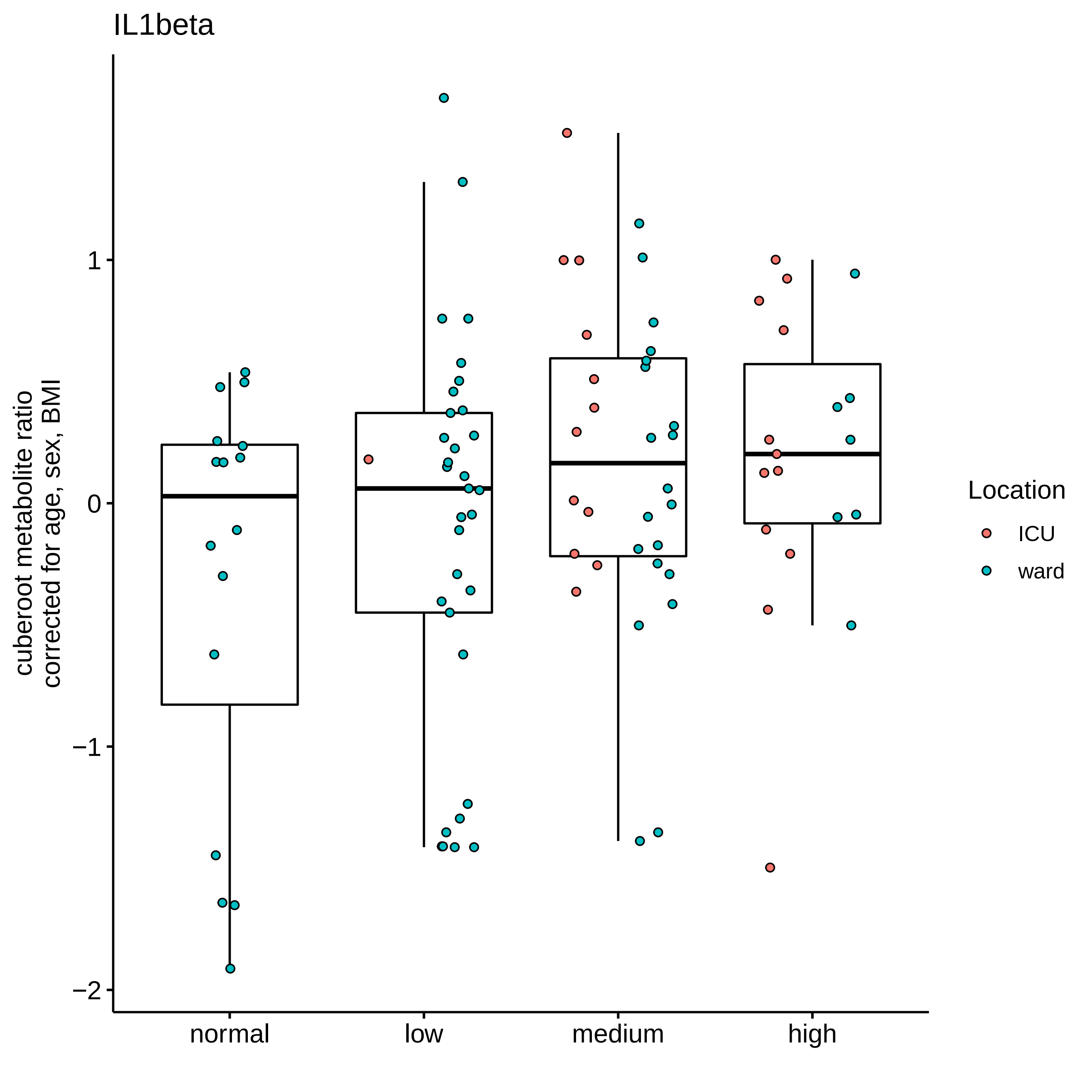
IL1beta
cuberoot metabolite ratio
corrected for age, sex, BMI
1
0
−1
−2
normal
low
medium
high
Location
ICU
ward

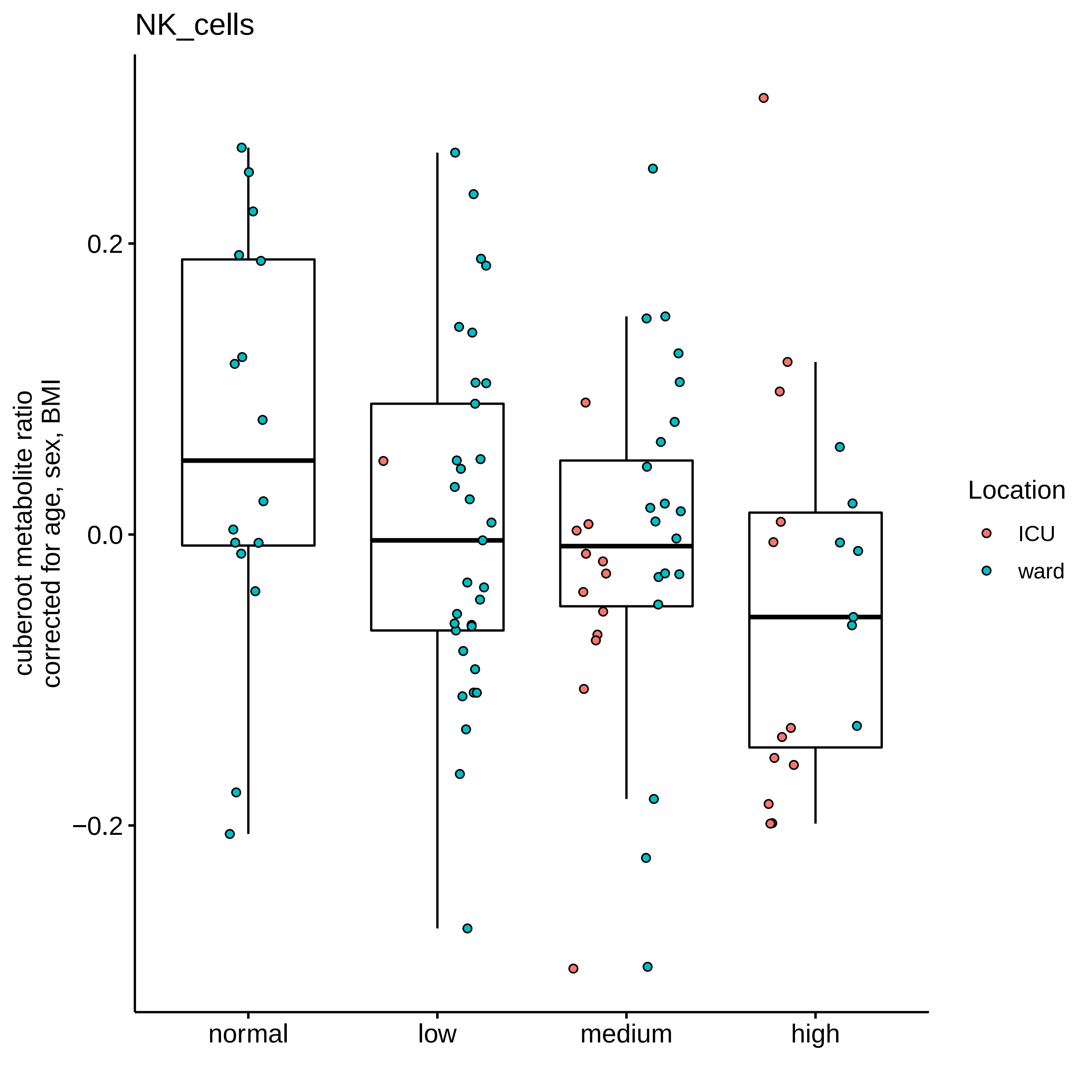
NK_cells
cuberoot metabolite ratio
corrected for age, sex, BMI
0.2
0.0
−0.2
normal
low
medium
high
Location
ICU
ward

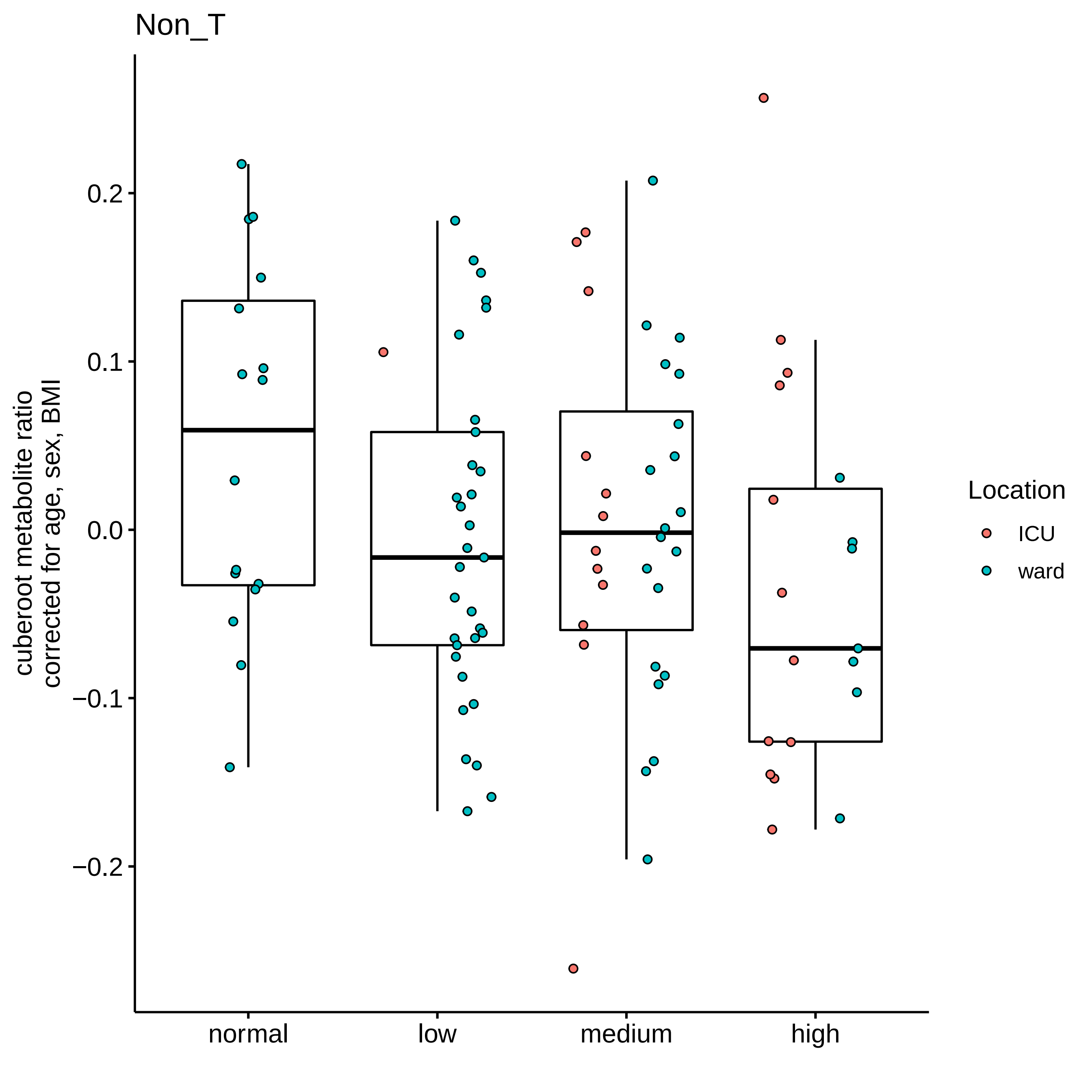
Non_T
cuberoot metabolite ratio
corrected for age, sex, BMI
0.2
0.1
0.0
−0.1
−0.2
normal
low
medium
high
Location
ICU
ward

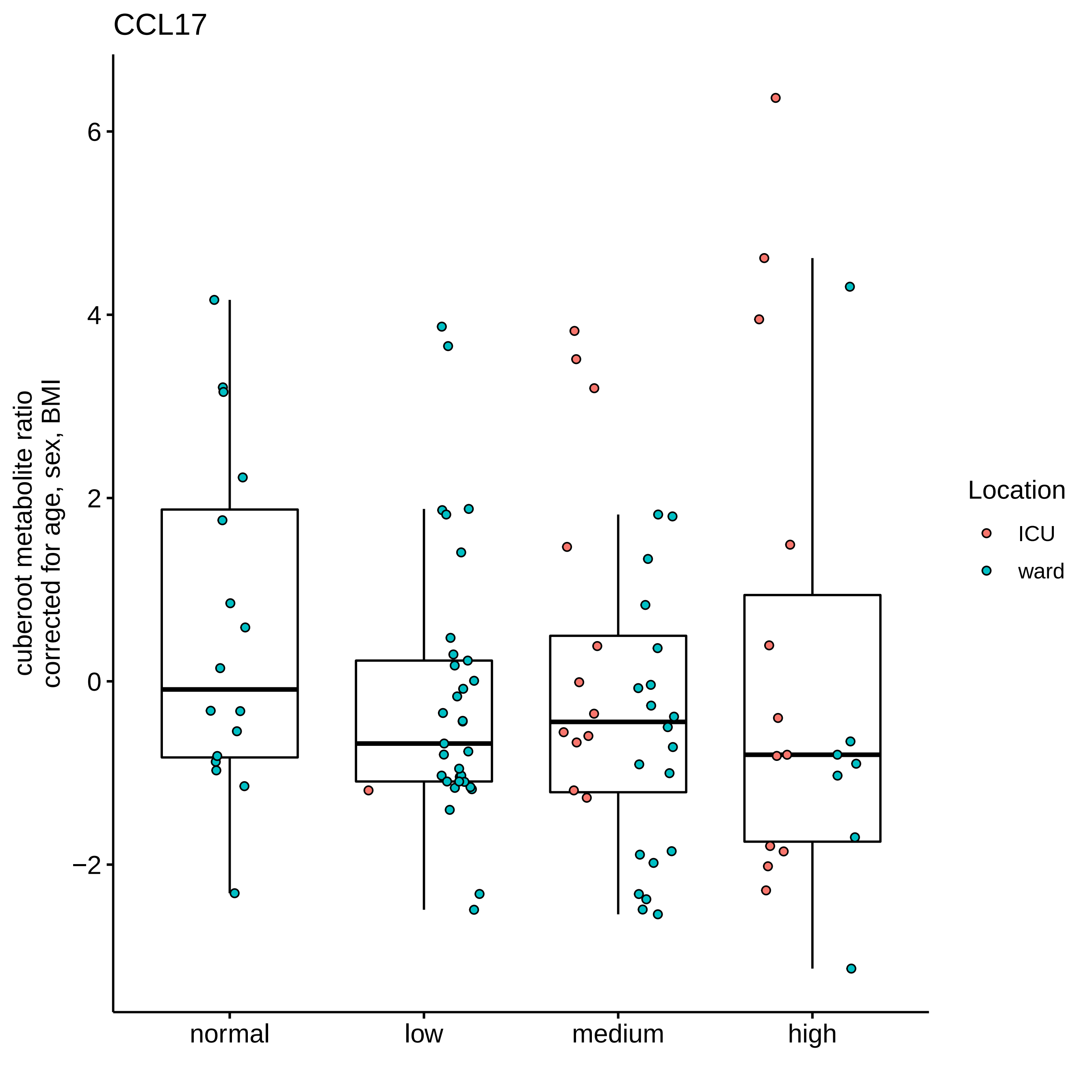

CCL17
cuberoot metabolite ratio
corrected for age, sex, BMI
6
4
2
0
−2
normal
low
medium
high
Location
ICU
ward

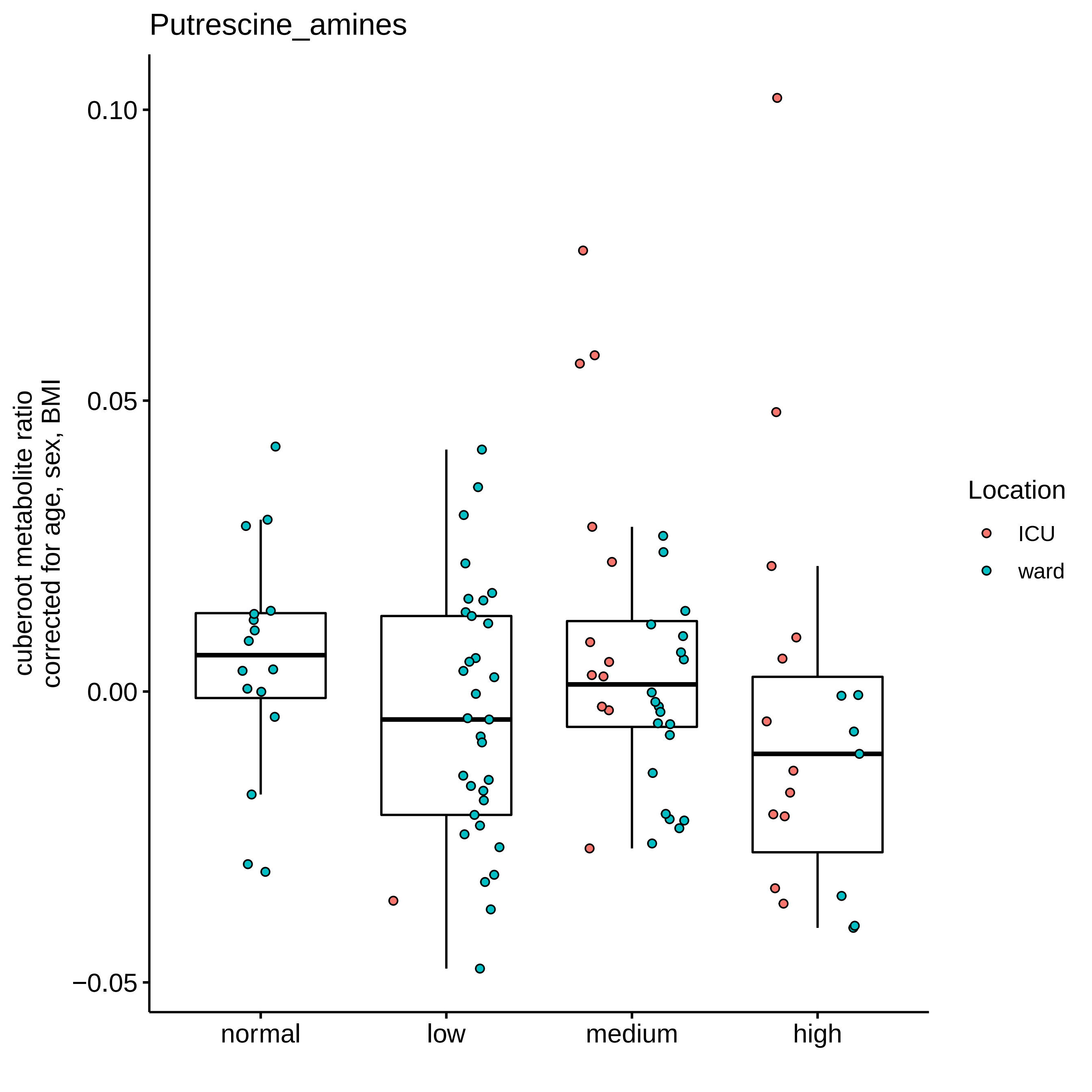
Putrescine_amines
cuberoot metabolite ratio
corrected for age, sex, BMI
0.10
0.05
0.00
−0.05
normal
low
medium
high
Location
ICU
ward

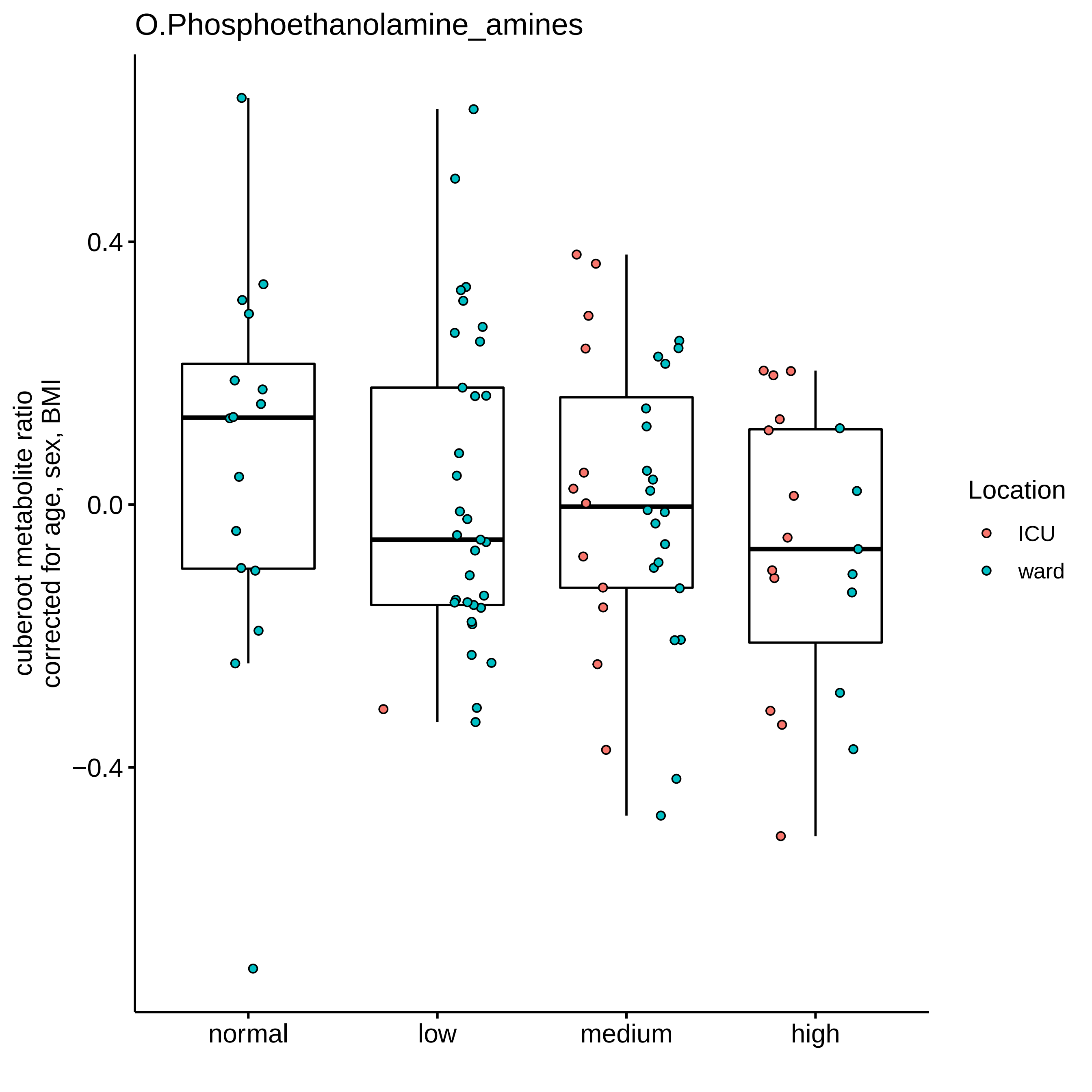

O.Phosphoethanolamine_amines
cuberoot metabolite ratio
corrected for age, sex, BMI
0.4
0.0
−0.4
normal
low
medium
high
Location
ICU
ward

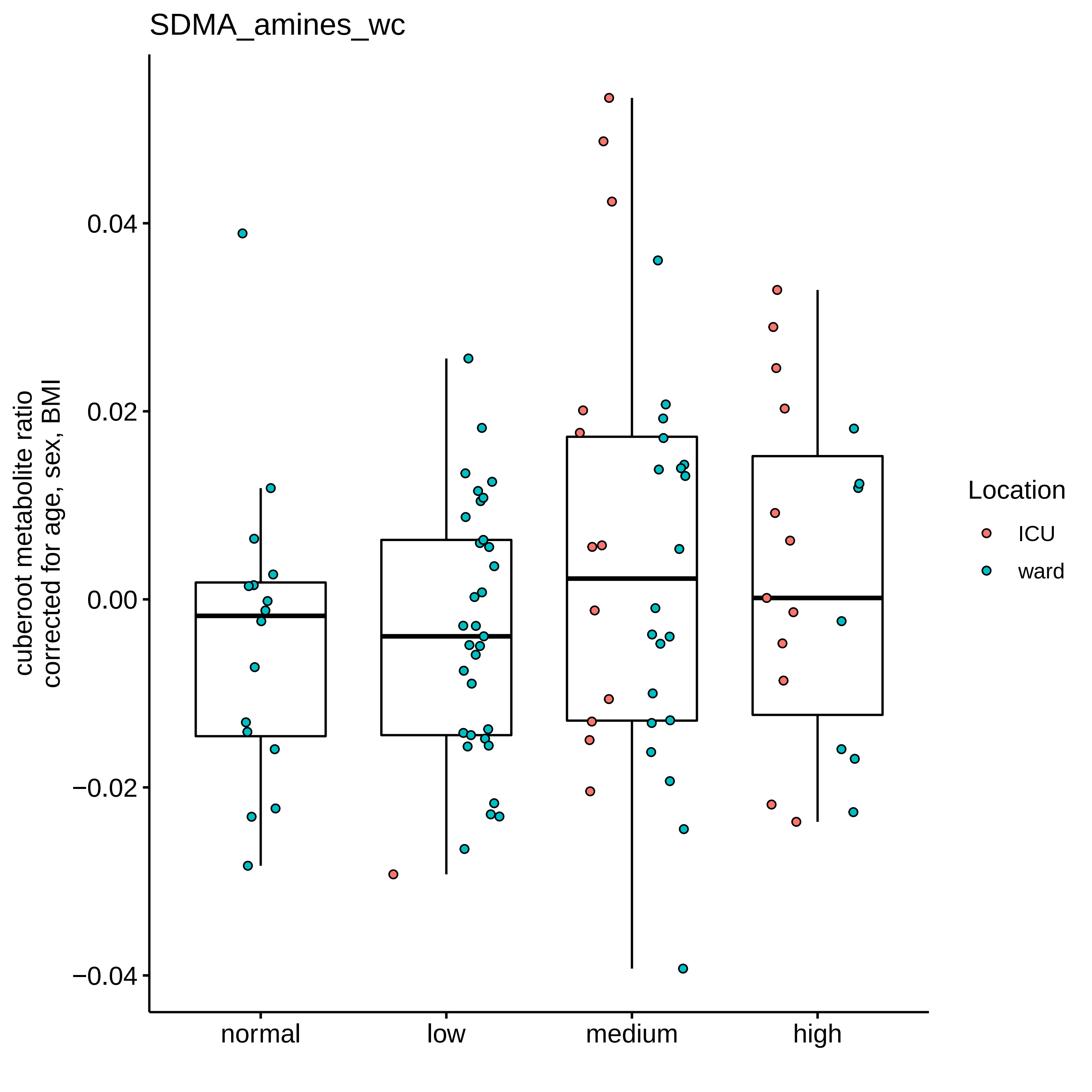

SDMA_amines_wc
cuberoot metabolite ratio
corrected for age, sex, BMI
0.04
0.02
0.00
−0.02
−0.04
normal
low
medium
high
Location
ICU
ward

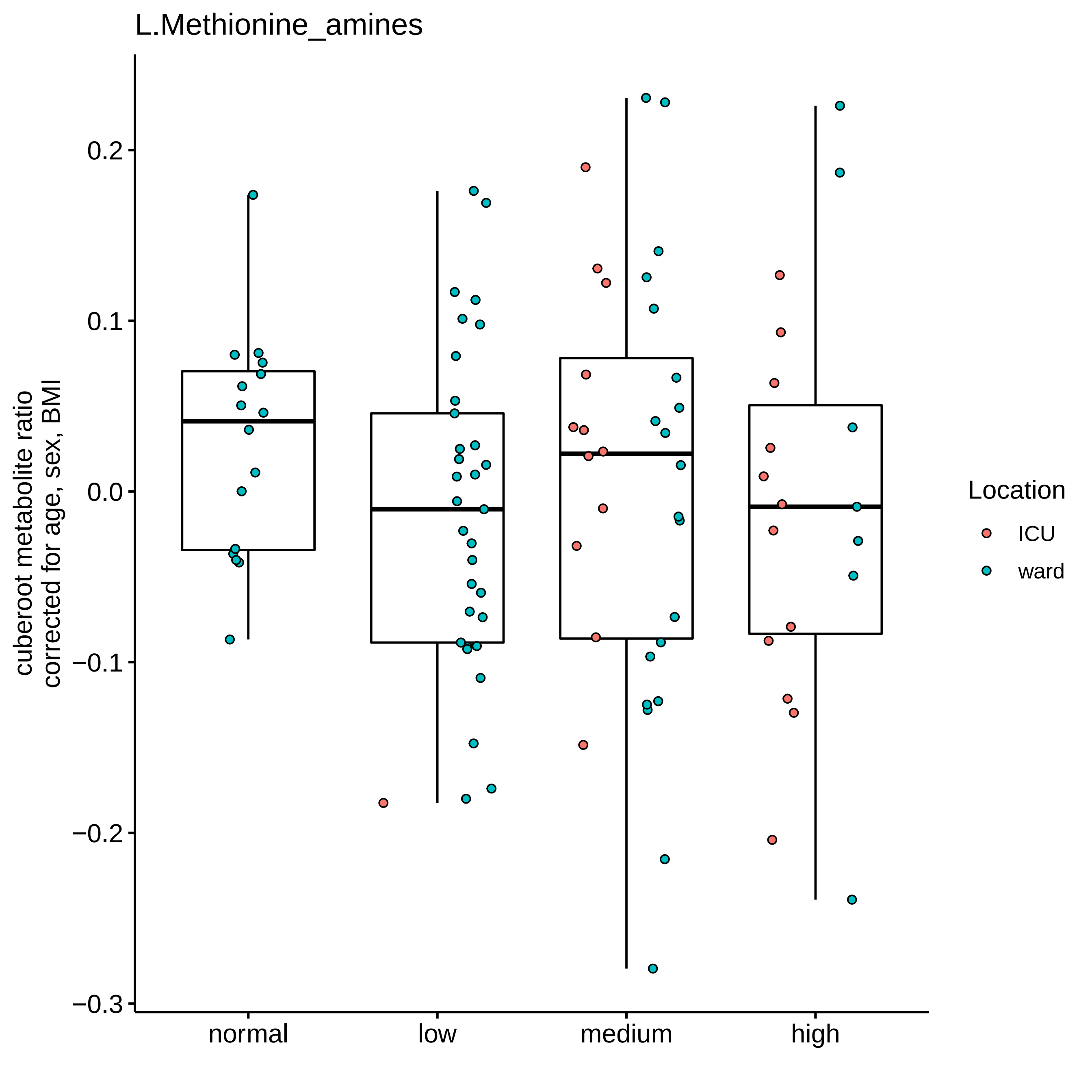

L.Methionine_amines
cuberoot metabolite ratio
corrected for age, sex, BMI
0.2
0.1
0.0
−0.1
−0.2
−0.3
normal
low
medium
high
Location
ICU
ward

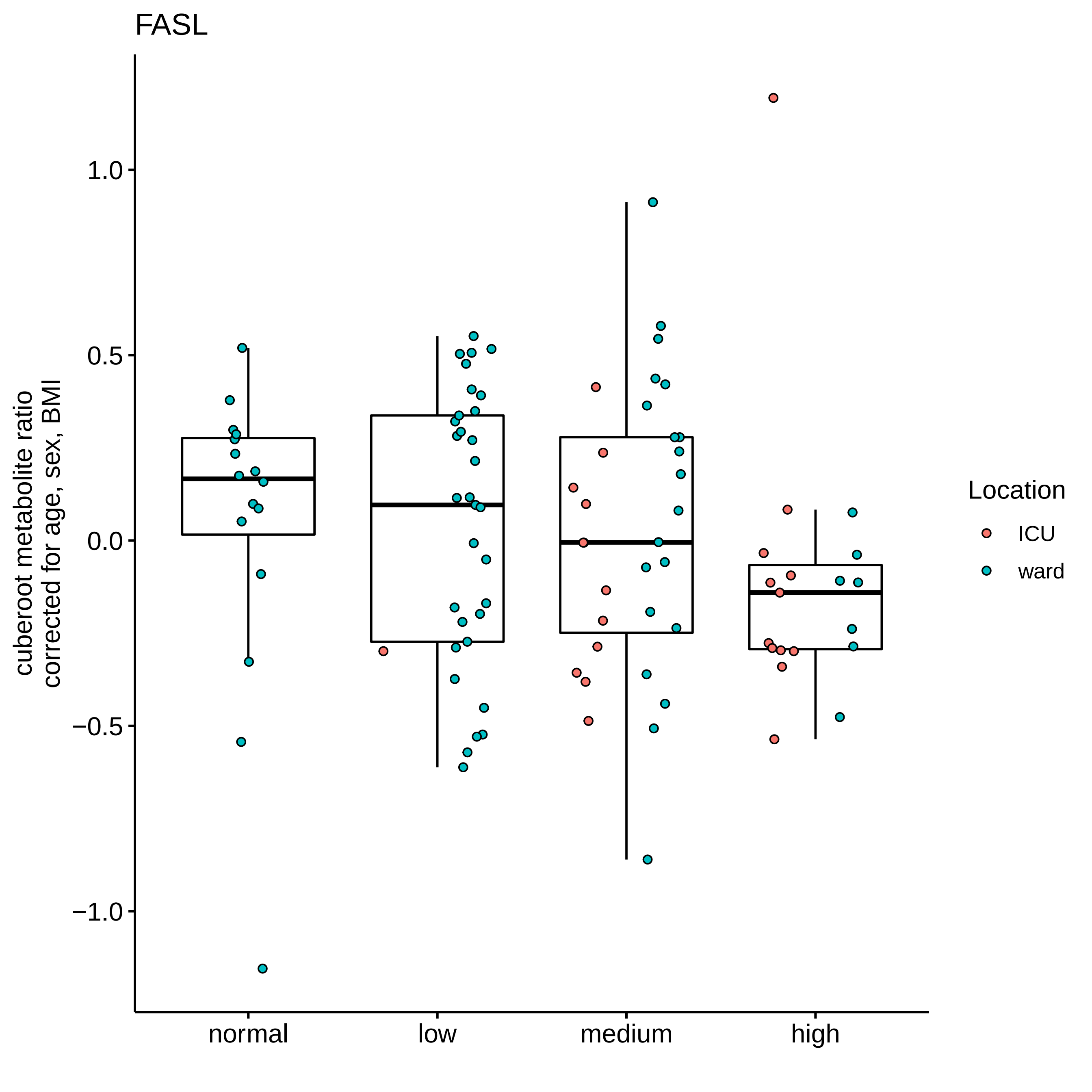
FASL
cuberoot metabolite ratio
corrected for age, sex, BMI
1.0
0.5
0.0
−0.5
−1.0
normal
low
medium
high
Location
ICU
ward

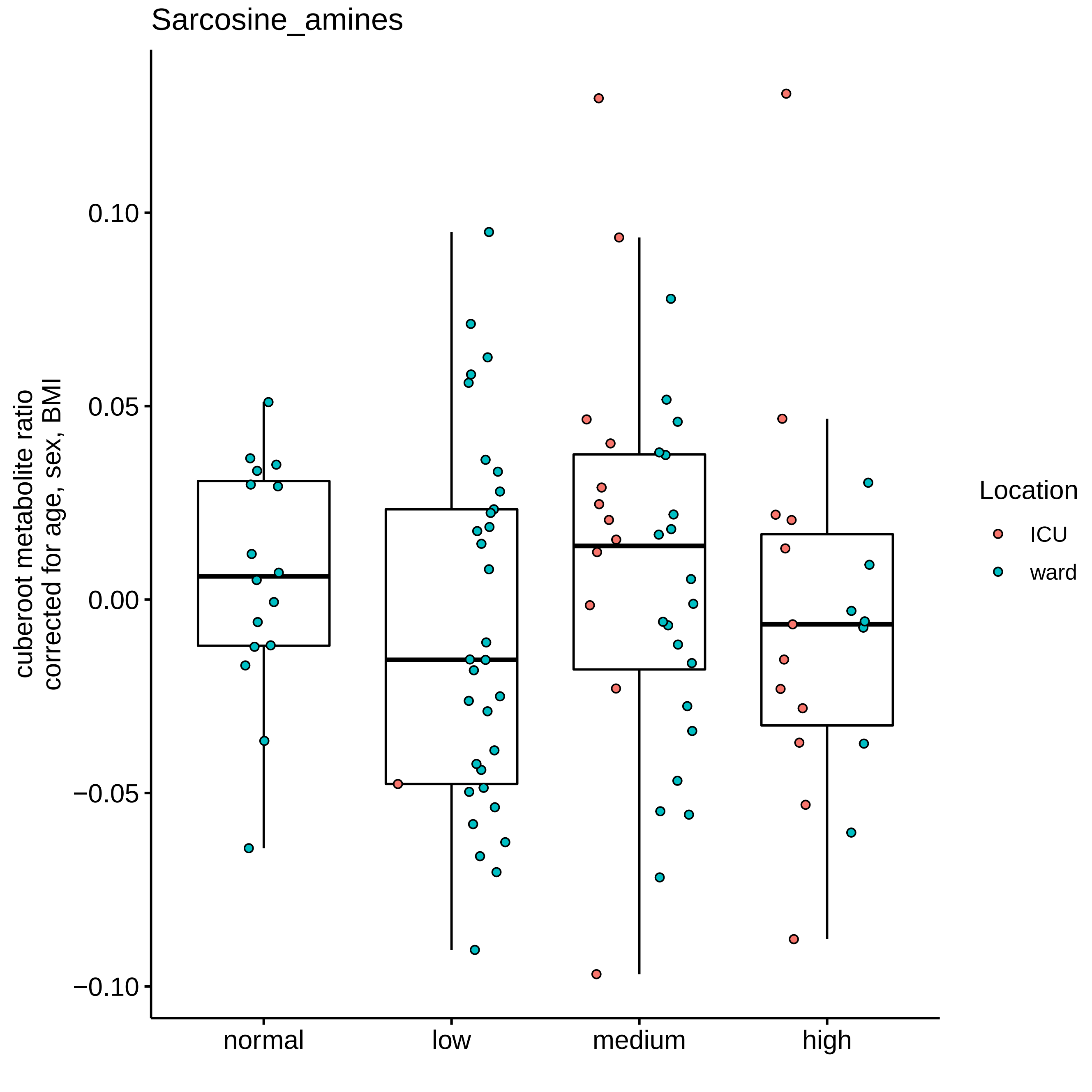

Sarcosine_amines
cuberoot metabolite ratio
corrected for age, sex, BMI
0.10
0.05
0.00
−0.05
−0.10
normal
low
medium
high
Location
ICU
ward

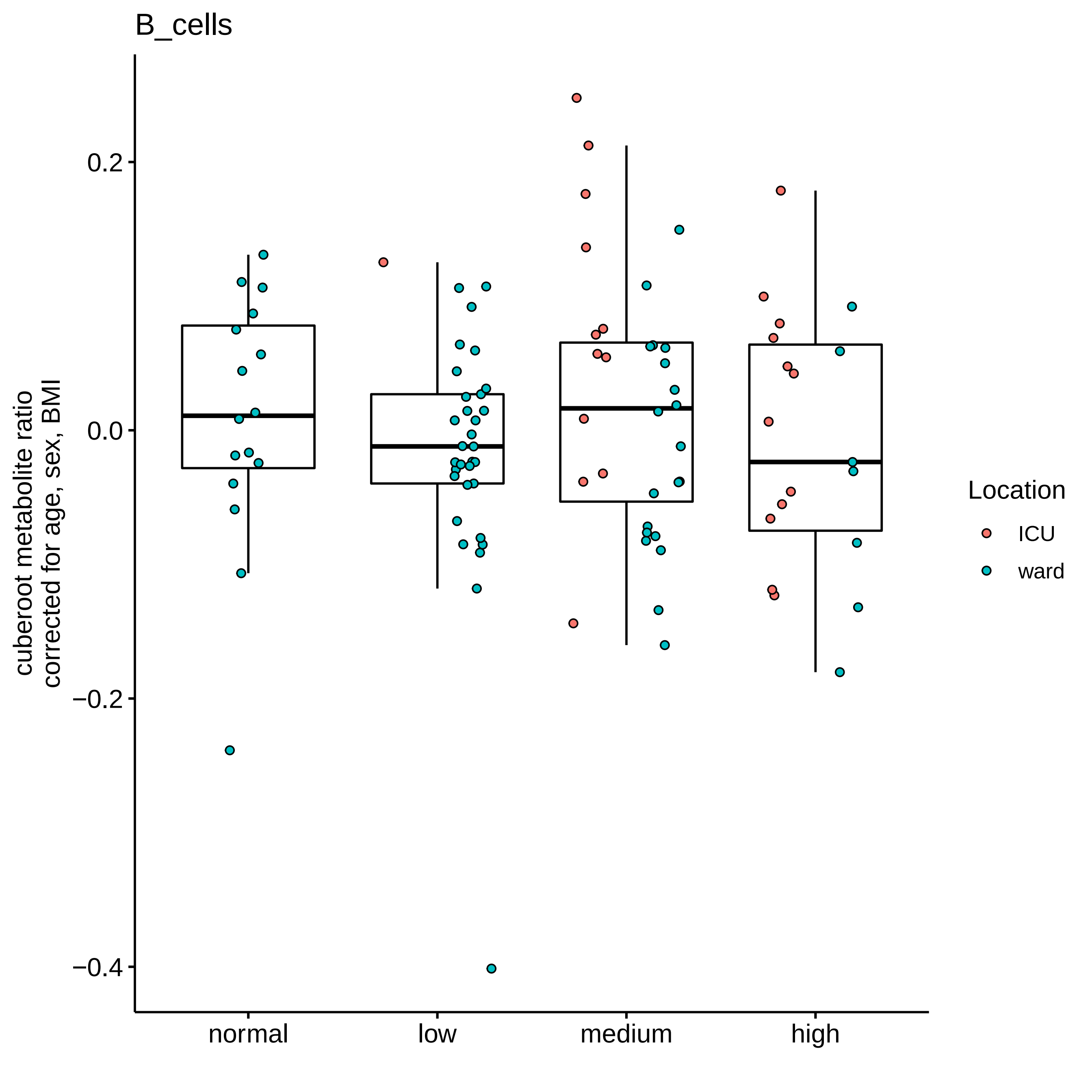

B_cells
cuberoot metabolite ratio
corrected for age, sex, BMI
0.2
0.0
−0.2
−0.4
normal
low
medium
high
Location
ICU
ward

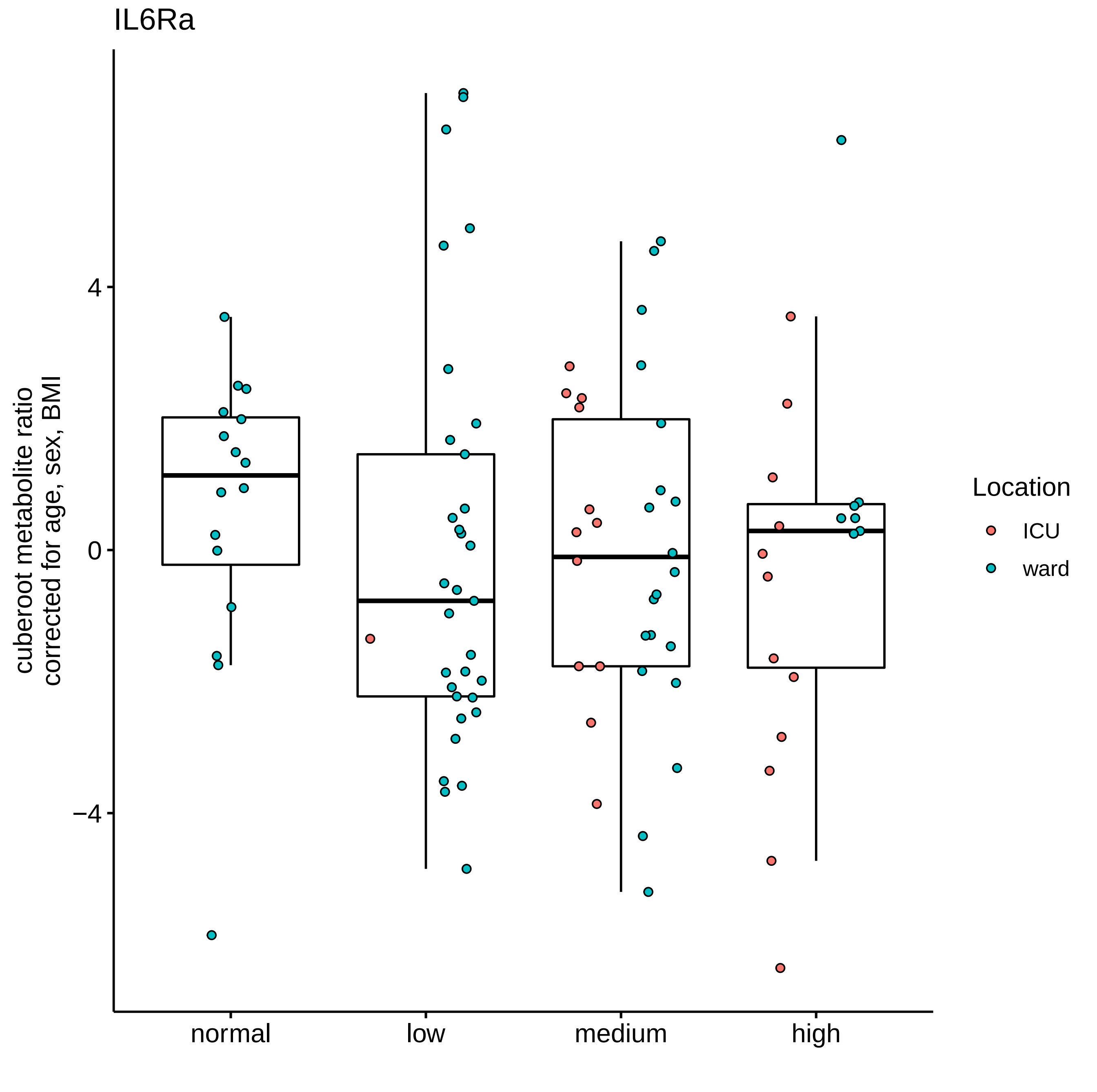
IL6Ra
cuberoot metabolite ratio
corrected for age, sex, BMI
4
0
−4
normal
low
medium
high
Location
ICU
ward

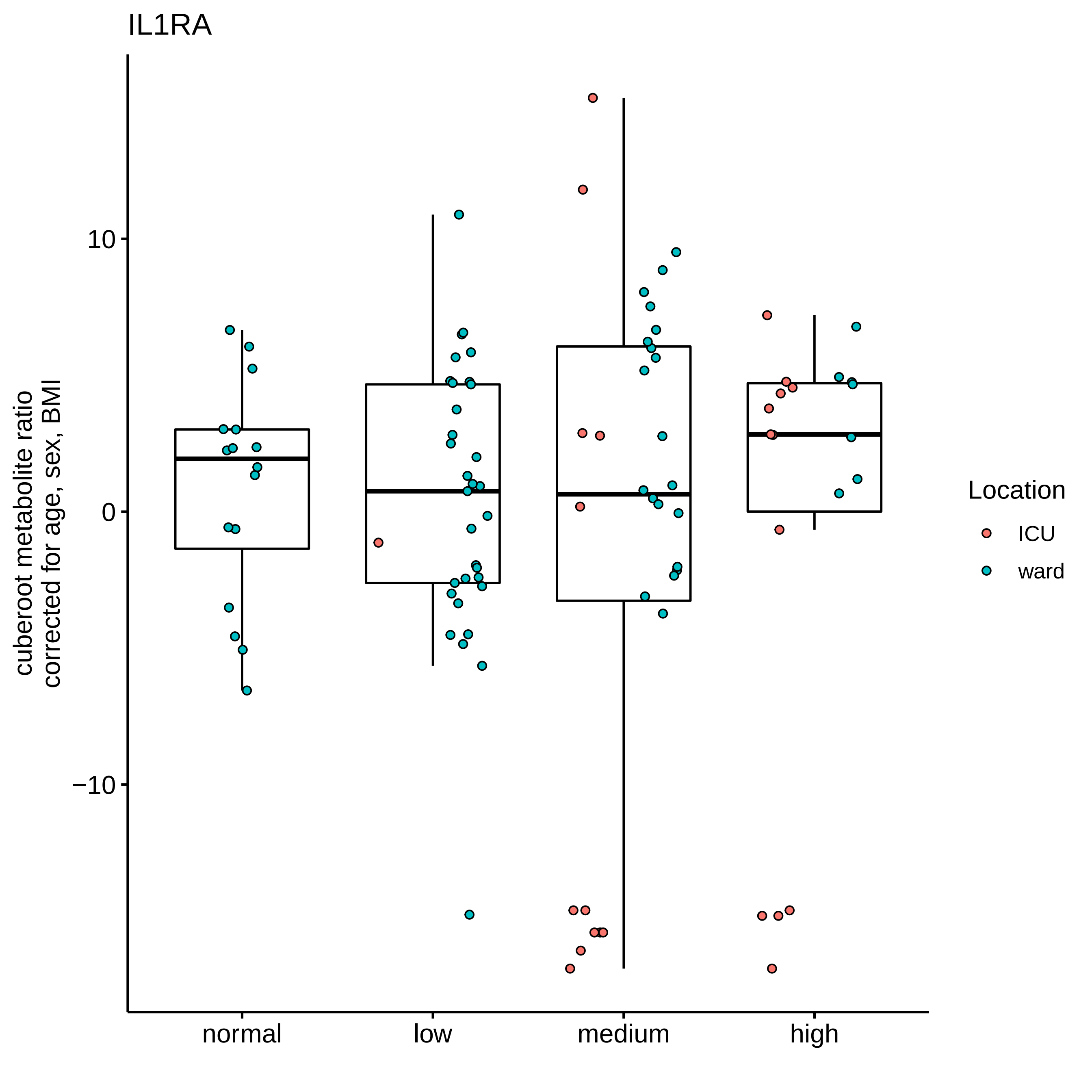
IL1RA
cuberoot metabolite ratio
corrected for age, sex, BMI
10
0
−10
normal
low
medium
high
Location
ICU
ward

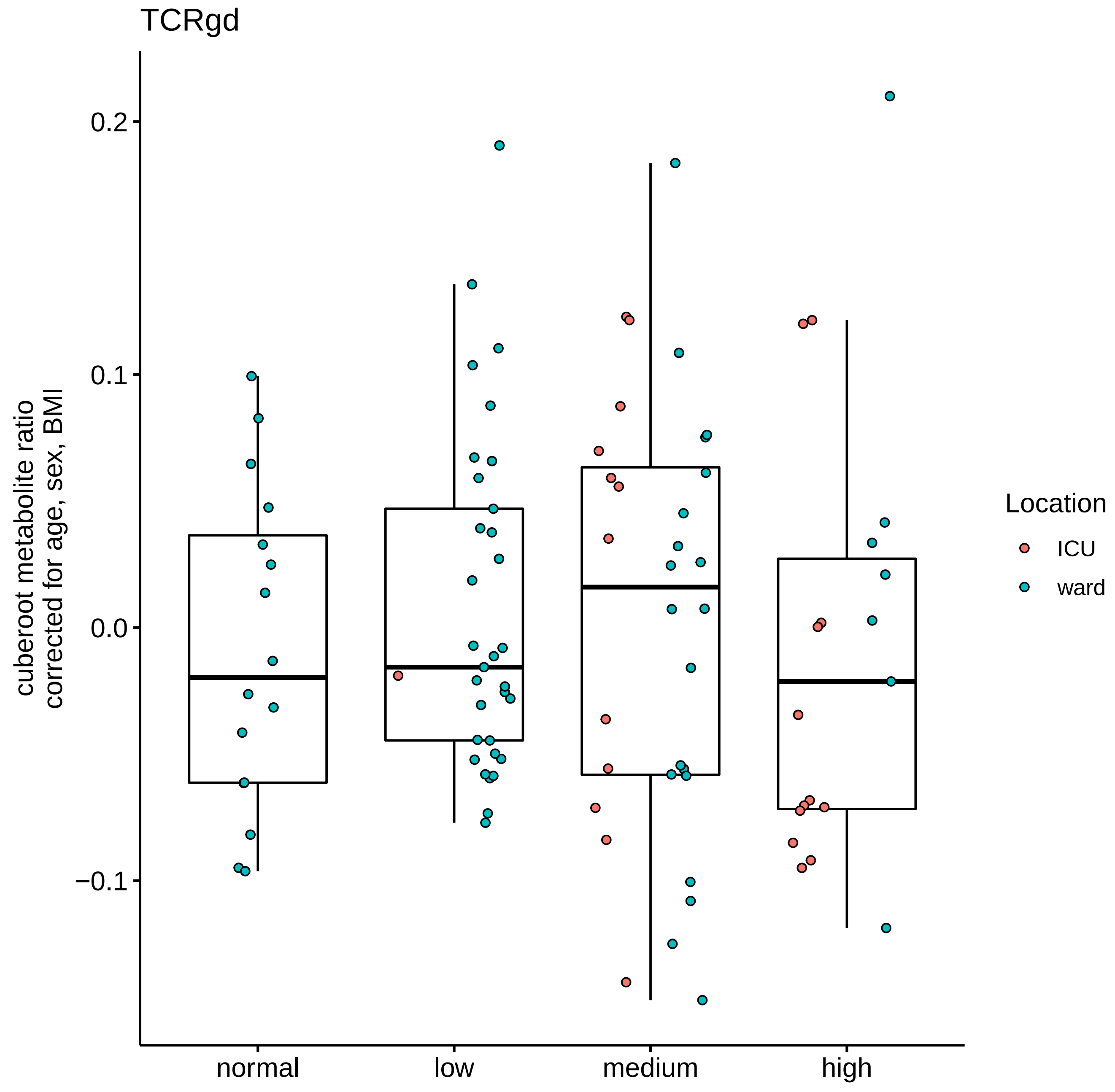

TCRgd
cuberoot metabolite ratio
corrected for age, sex, BMI
0.2
0.1
0.0
−0.1
normal
low
medium
high
Location
ICU
ward

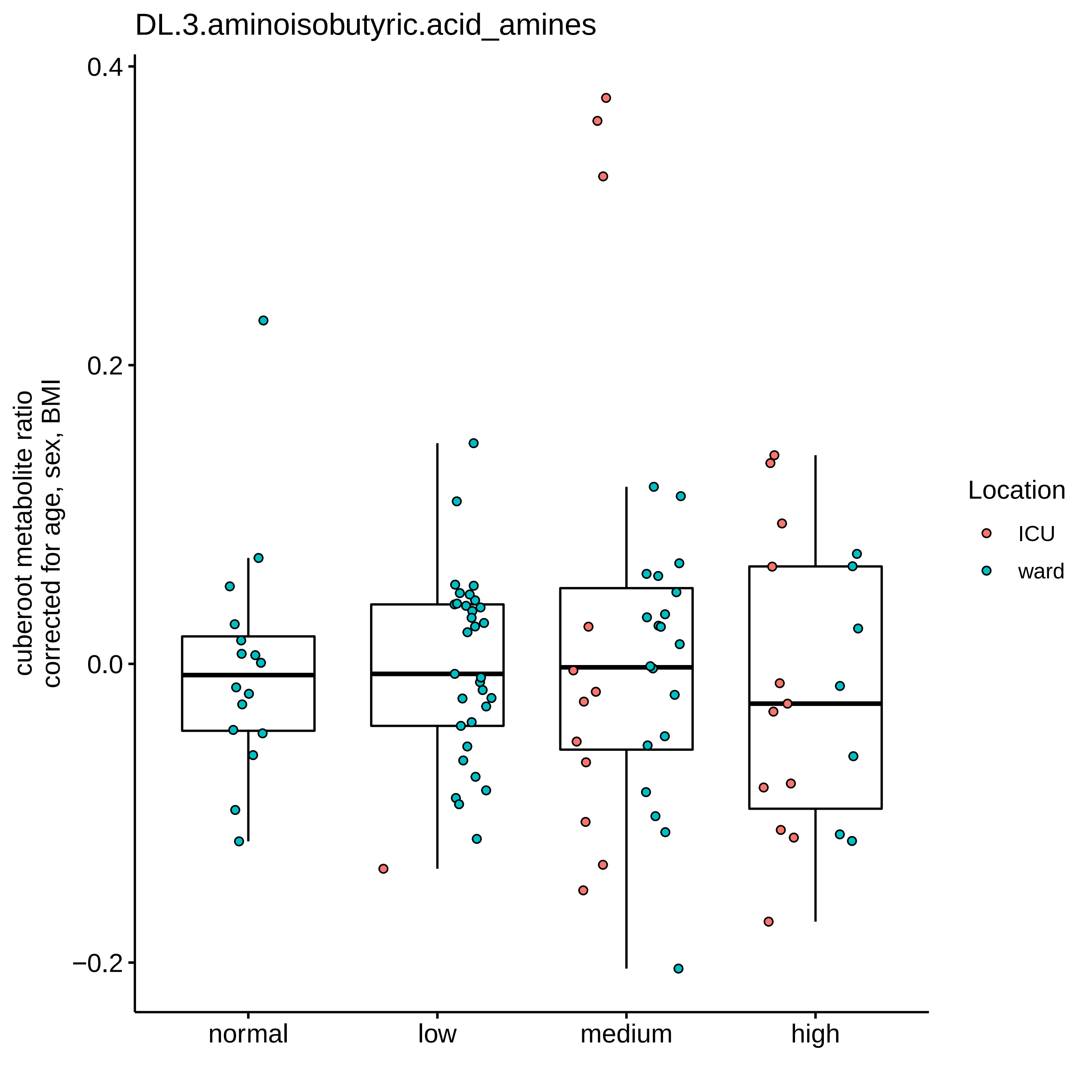

DL.3.aminoisobutyric.acid_amines
0.4
0.2
0.0
−0.2
cuberoot metabolite ratio
corrected for age, sex, BMI
normal
low
medium
high
Location
ICU
ward

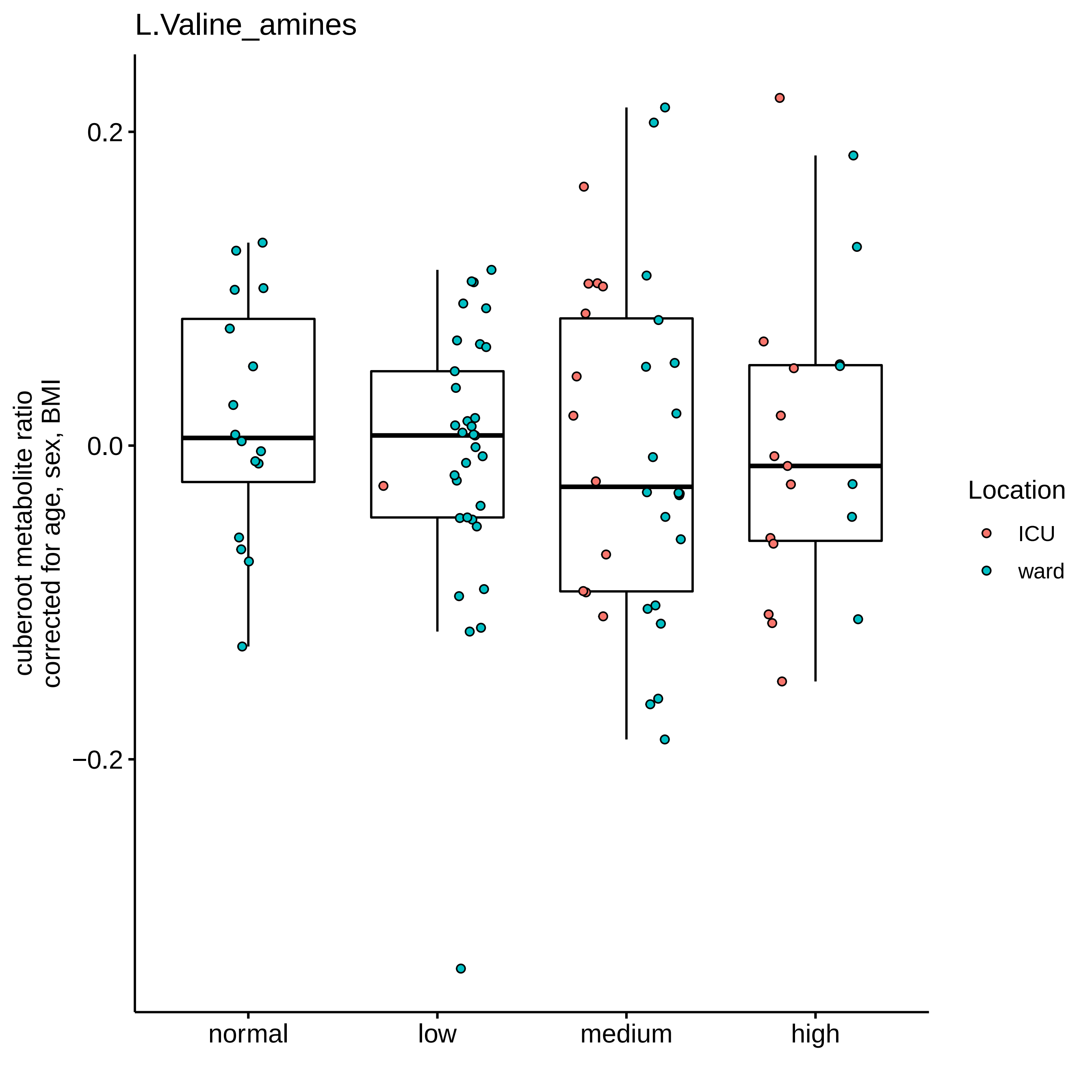

L.Valine_amines
cuberoot metabolite ratio
corrected for age, sex, BMI
0.2
0.0
−0.2
normal
low
medium
high
Location
ICU
ward

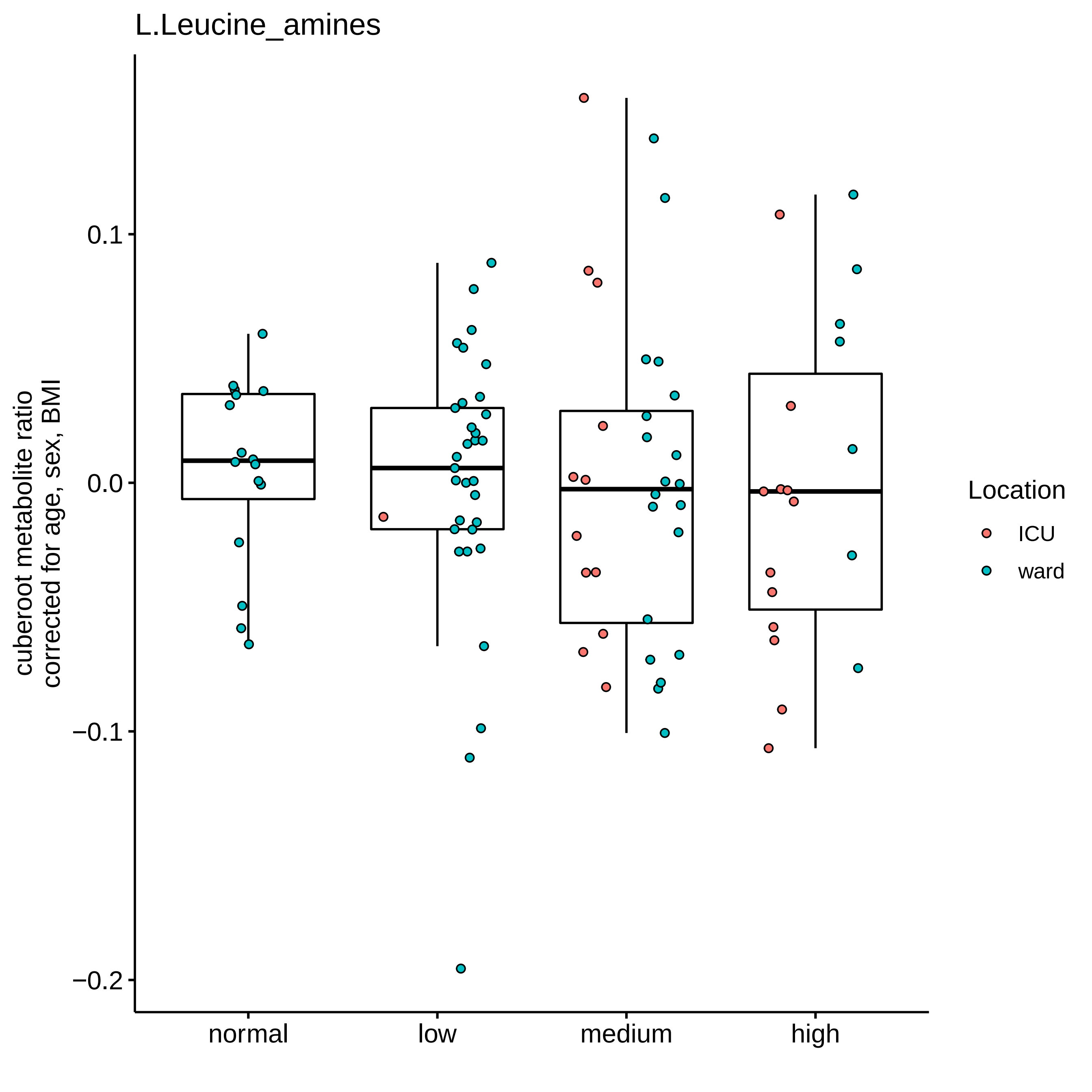

L.Leucine_amines
cuberoot metabolite ratio
corrected for age, sex, BMI
0.1
0.0
−0.1
−0.2
normal
low
medium
high
Location
ICU
ward

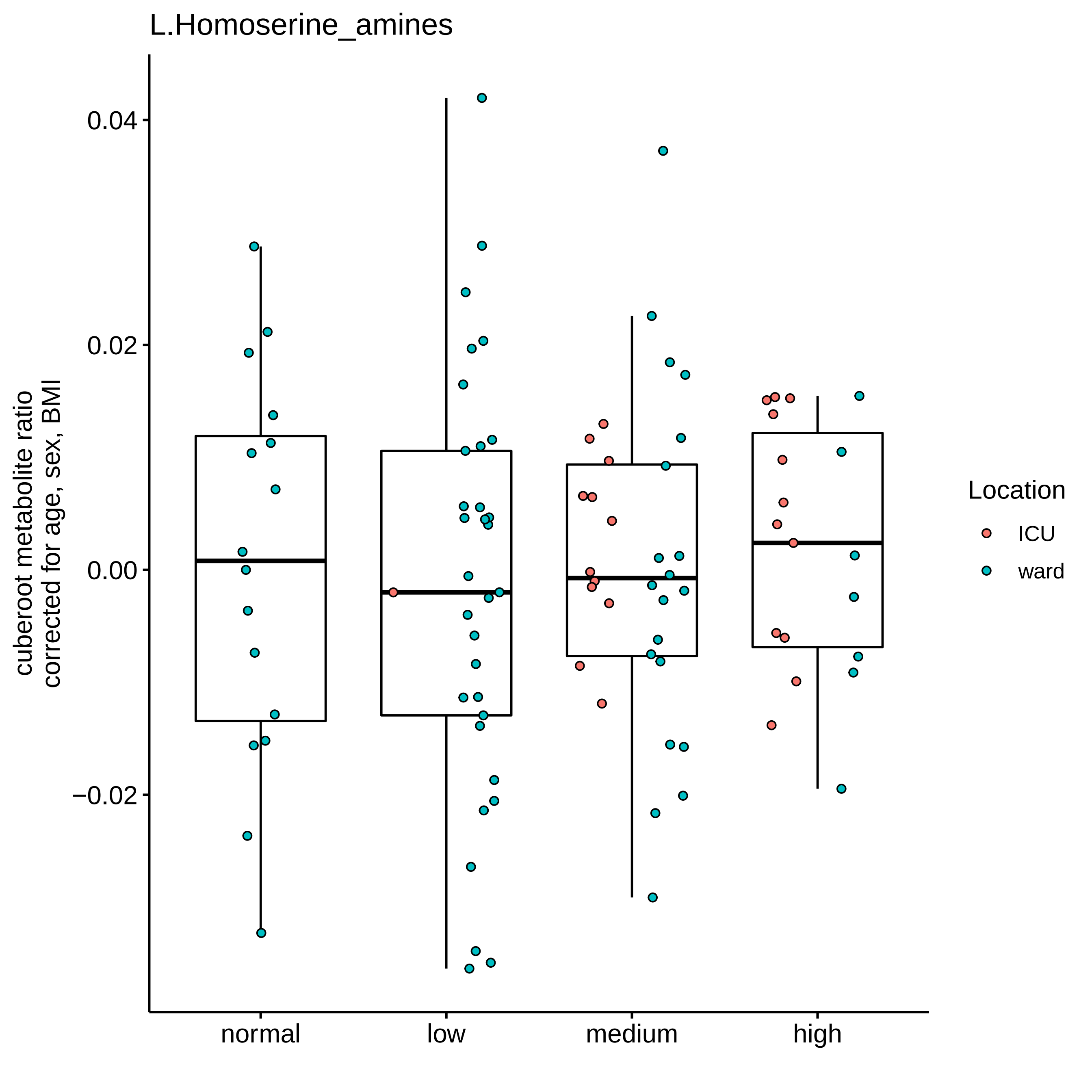

L.Homoserine_amines
cuberoot metabolite ratio
corrected for age, sex, BMI
0.04
0.02
0.00
−0.02
normal
low
medium
high
Location
ICU
ward

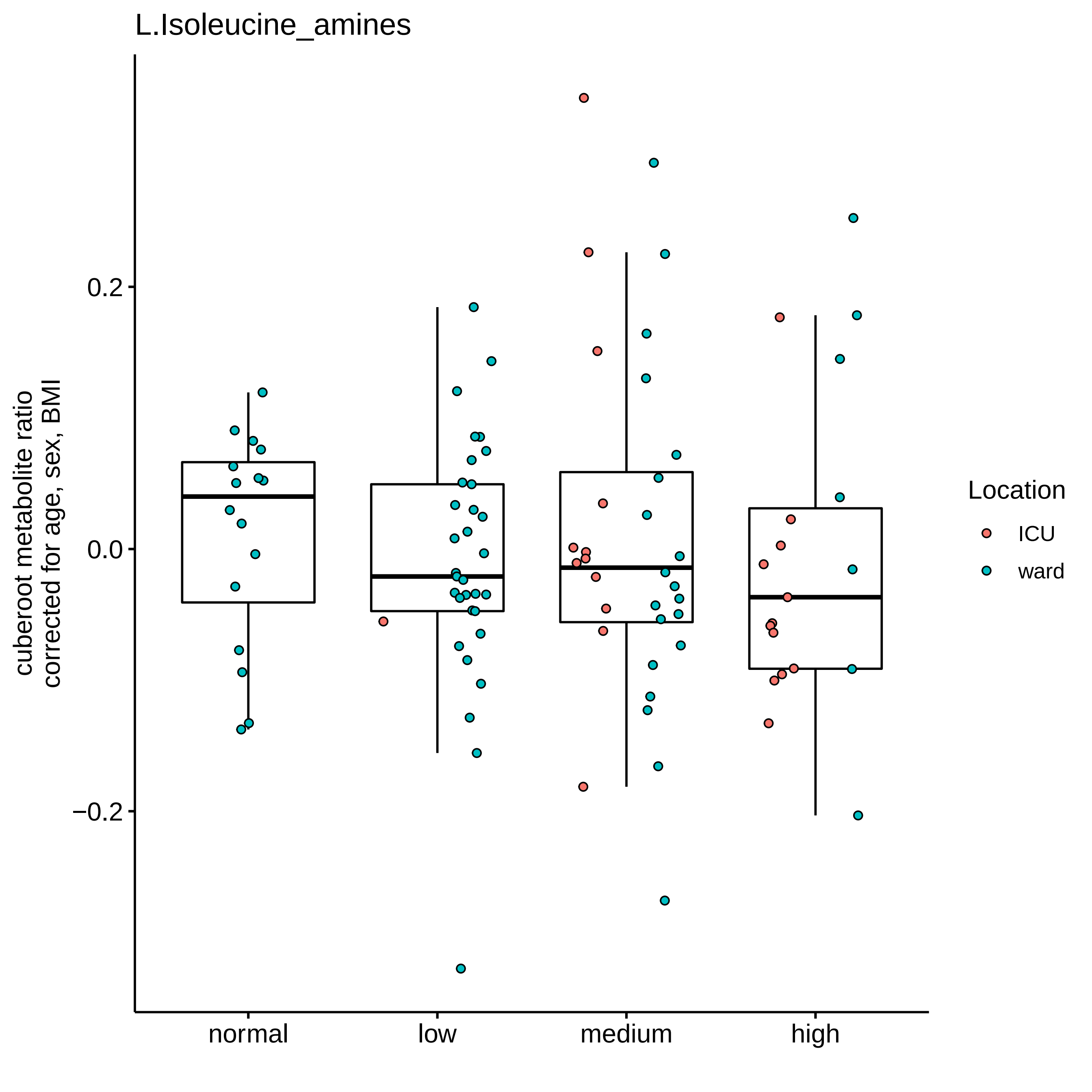
L.Isoleucine_amines
cuberoot metabolite ratio
corrected for age, sex, BMI
0.2
0.0
−0.2
normal
low
medium
high
Location
ICU
ward

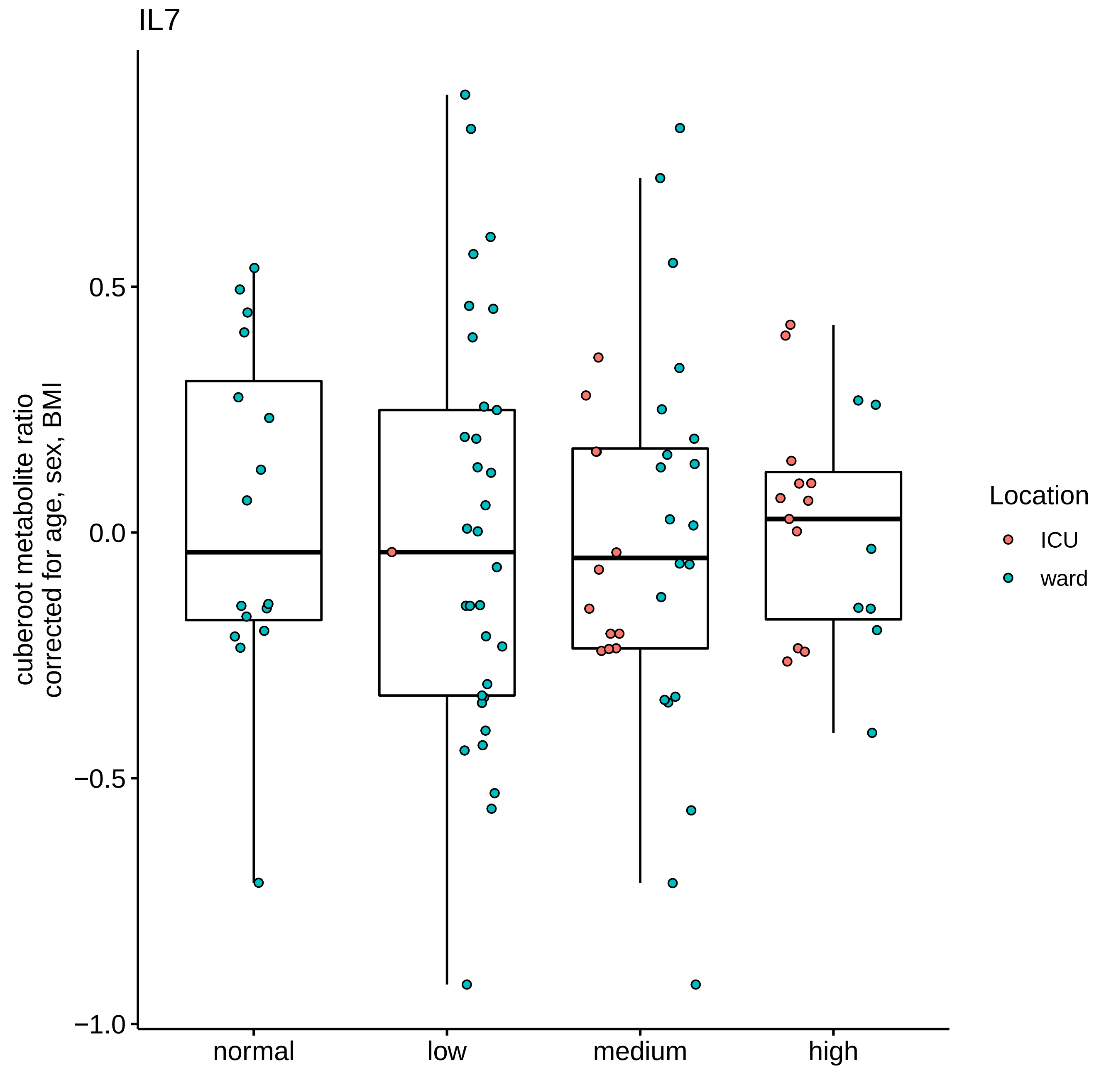
IL7
cuberoot metabolite ratio
corrected for age, sex, BMI
0.5
0.0
−0.5
−1.0
normal
low
medium
high
Location
ICU
ward

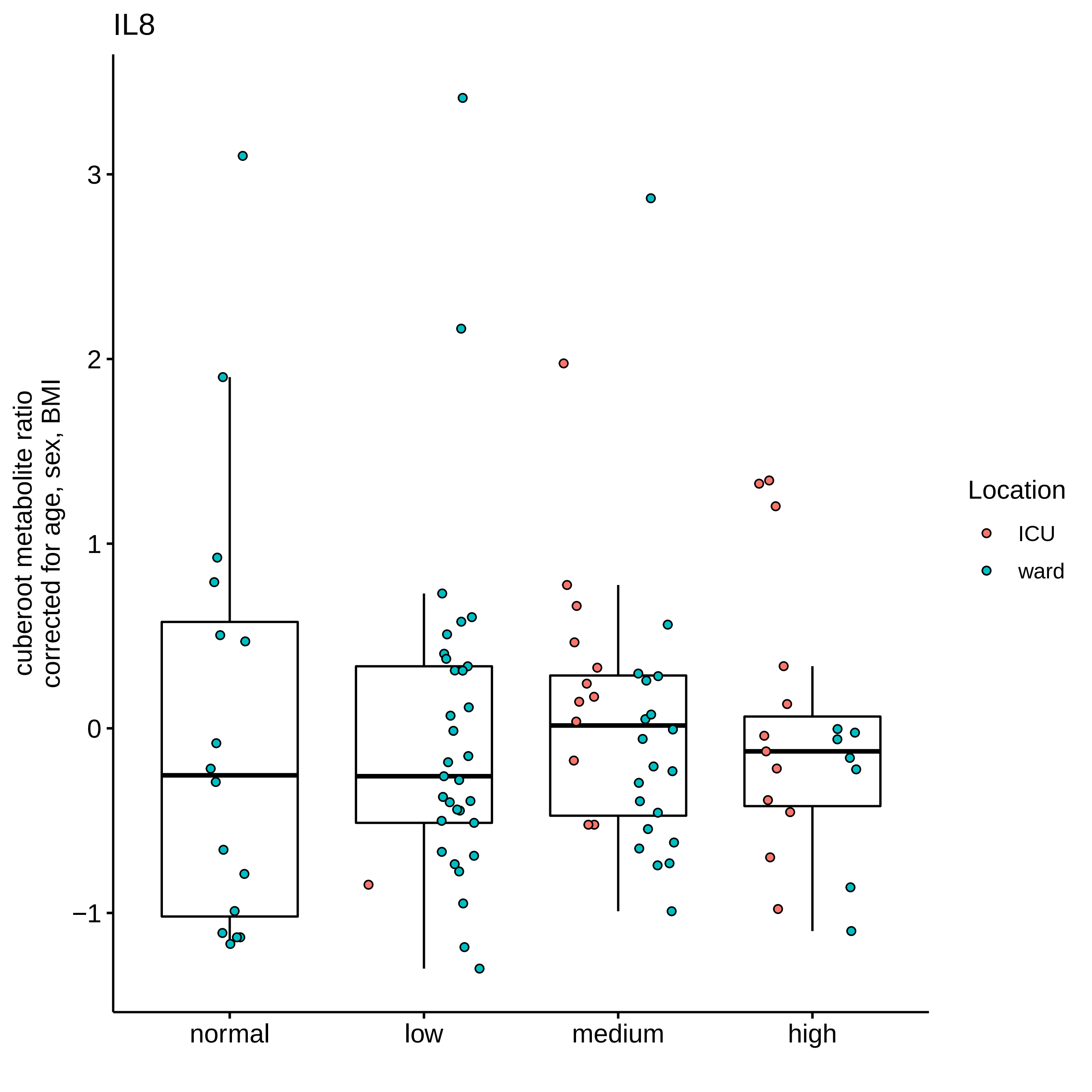
IL8
cuberoot metabolite ratio
corrected for age, sex, BMI
3
2
1
0
−1
normal
low
medium
high
Location
ICU
ward
