# Supplementary material for: Severe COVID-19 Is Characterised by Perturbations in Plasma Amines Correlated with Immune Response Markers, and Linked to Inflammation and Oxidative Stress
Source: Metabolites. 2022 Jul 2;12(7):618. doi: 10.3390/metabo12070618 (PMC9321395; doi:10.3390/metabo12070618)
Supplement: Supplementary file 1 [file metabolites-12-00618-s001.zip › Document S4.pdf]

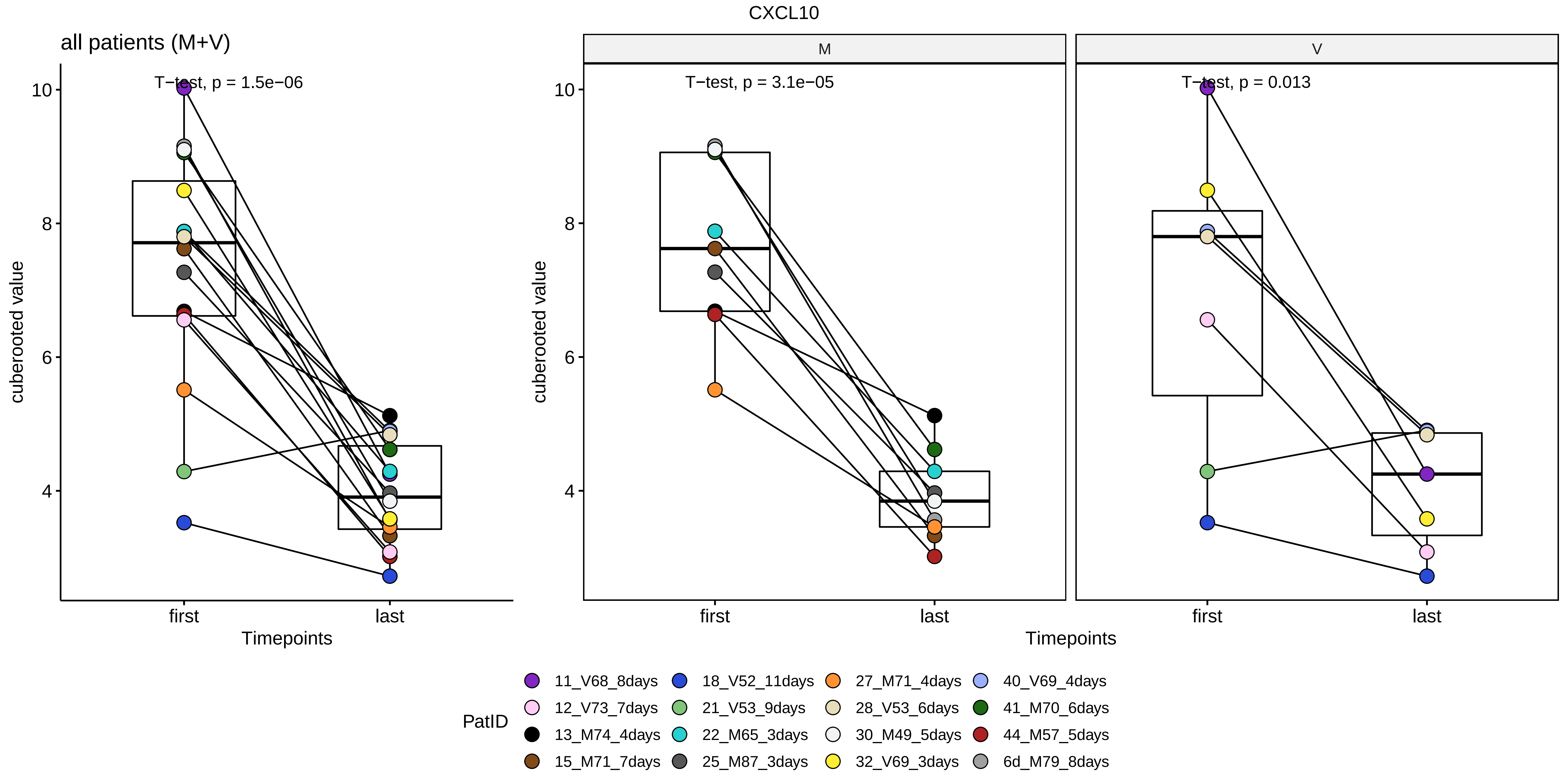
CXCL10
all patients (M+V)
T−test, p = 1.5e−06
M
T−test, p = 3.1e−05
V
T−test, p = 0.013
cuberooted value
10
8
6
4
first
last
Timepoints
PatID
11_V68_8days
12_V73_7days
13_M74_4days
15_M71_7days
18_V52_11days
21_V53_9days
22_M65_3days
25_M87_3days
27_M71_4days
28_V53_6days
30_M49_5days
32_V69_3days
40_V69_4days
41_M70_6days
44_M57_5days
6d_M79_8days

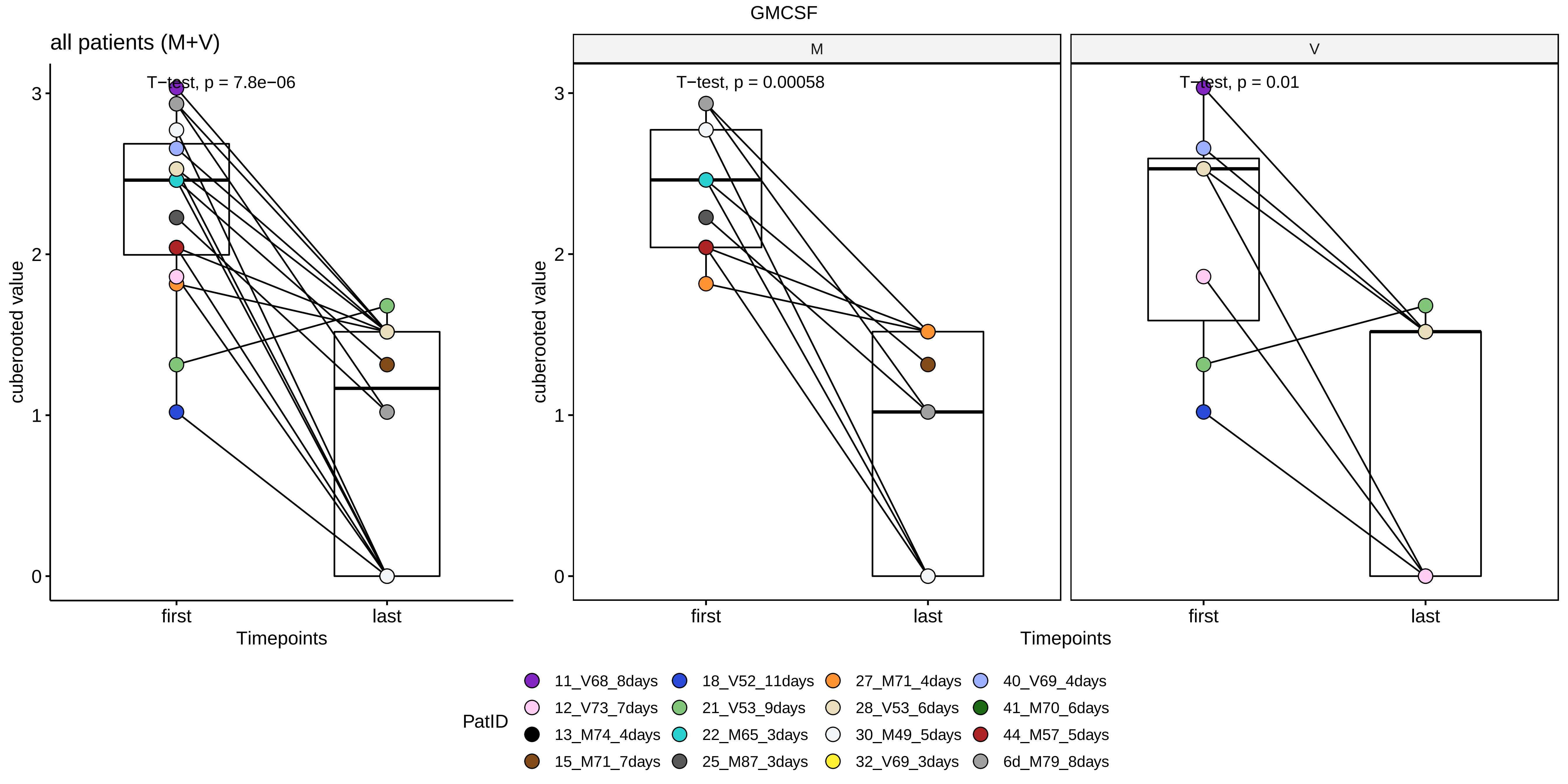
GMCSF
all patients (M+V)
T−test, p = 7.8e−06
M
T−test, p = 0.00058
V
T−test, p = 0.01
cuberooted value
0
1
2
3
first
last
Timepoints
PatID
11_V68_8days
12_V73_7days
13_M74_4days
15_M71_7days
18_V52_11days
21_V53_9days
22_M65_3days
25_M87_3days
27_M71_4days
28_V53_6days
30_M49_5days
32_V69_3days
40_V69_4days
41_M70_6days
44_M57_5days
6d_M79_8days

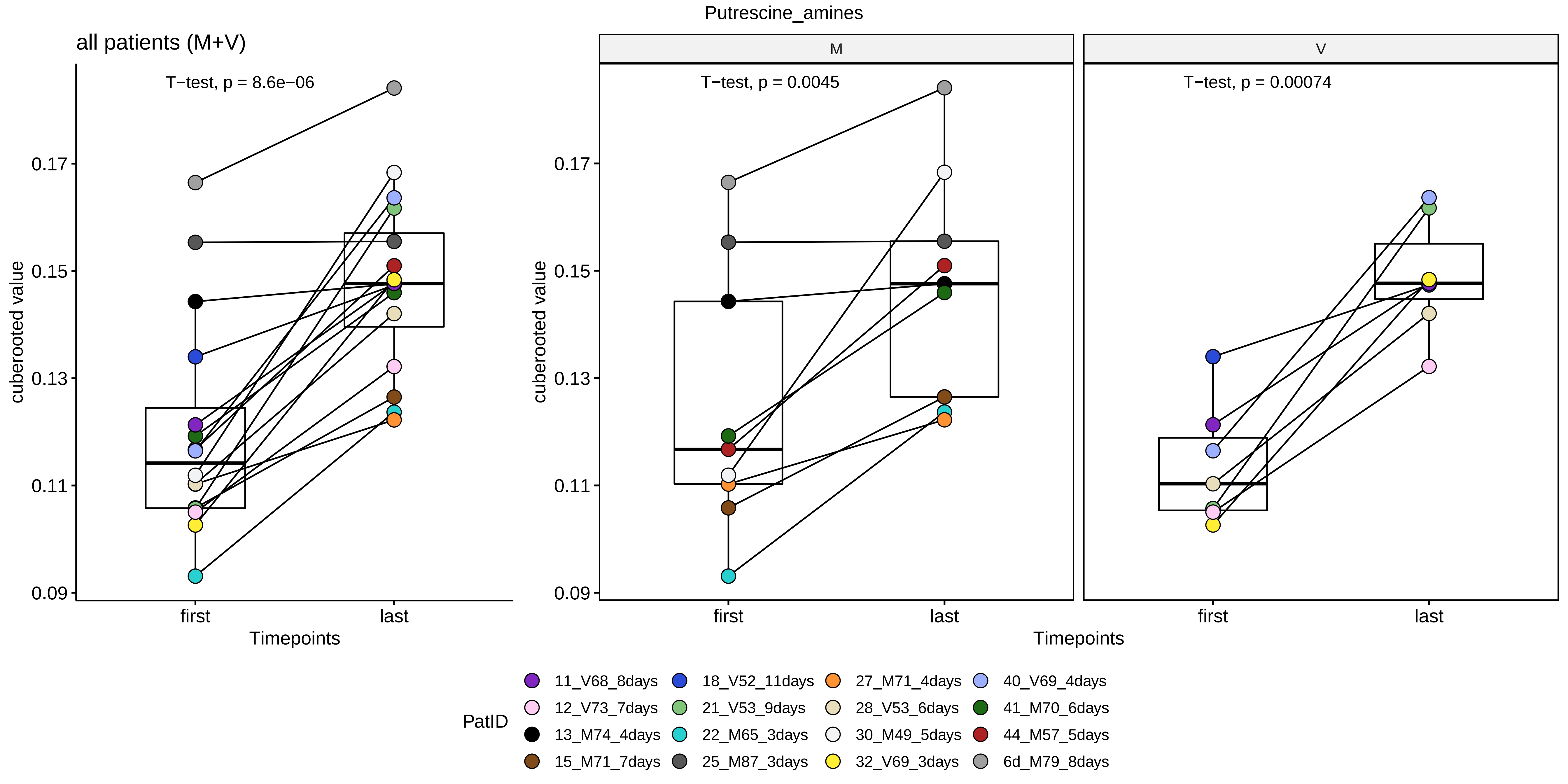
Putrescine_amines
all patients (M+V)
T−test, p = 8.6e−06
M
T−test, p = 0.0045
V
T−test, p = 0.00074
cuberooted value
0.17
0.15
0.13
0.11
0.09
first
last
Timepoints
PatID
11_V68_8days
12_V73_7days
13_M74_4days
15_M71_7days
18_V52_11days
21_V53_9days
22_M65_3days
25_M87_3days
27_M71_4days
28_V53_6days
30_M49_5days
32_V69_3days
40_V69_4days
41_M70_6days
44_M57_5days
6d_M79_8days

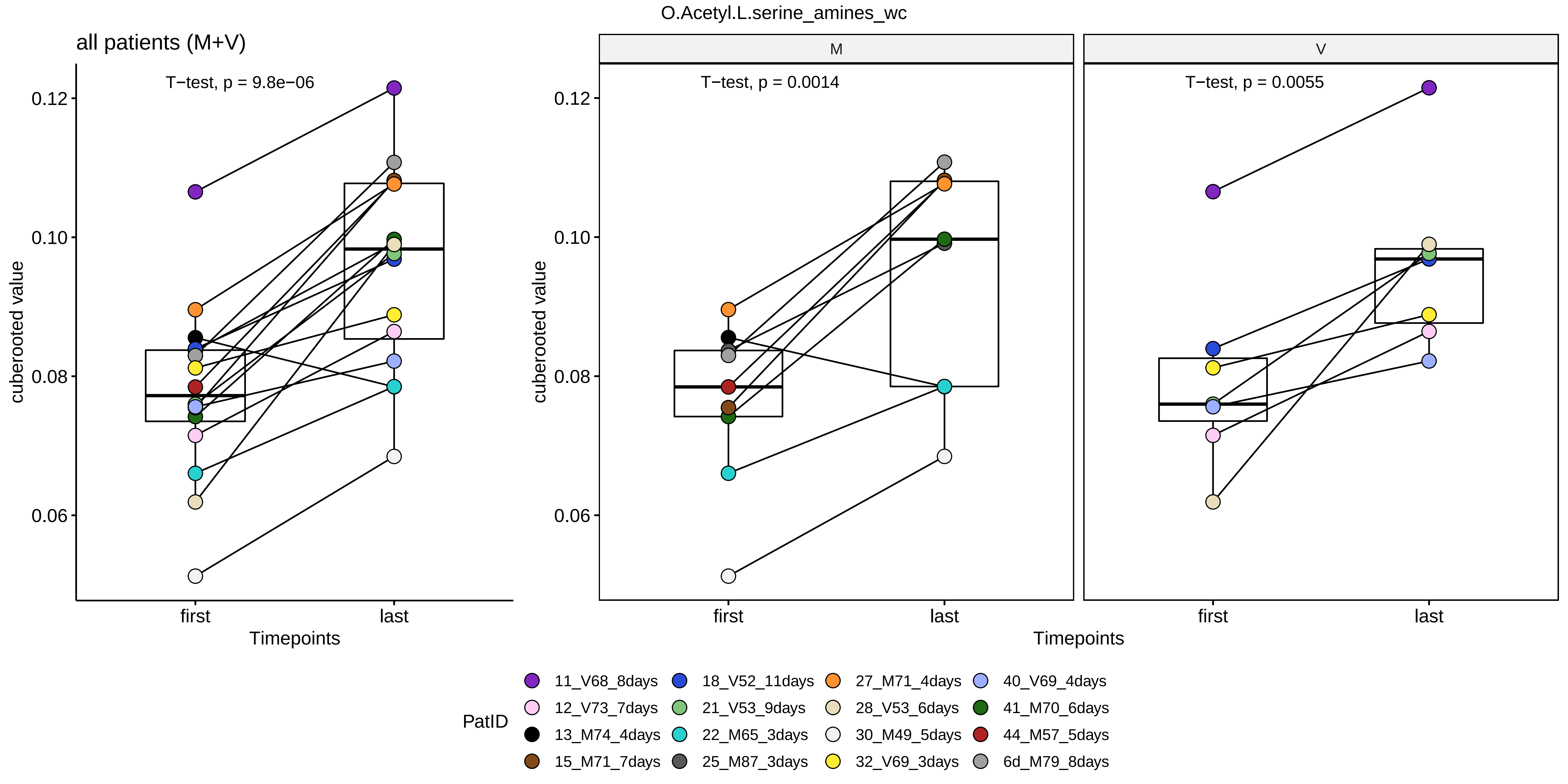
O.Acetyl.L.serine_amines_wc
all patients (M+V)
T−test, p = 9.8e−06
cuberooted value
0.12
0.10
0.08
0.06
first
last
Timepoints
M
T−test, p = 0.0014
V
T−test, p = 0.0055
PatID
11_V68_8days
12_V73_7days
13_M74_4days
15_M71_7days
18_V52_11days
21_V53_9days
22_M65_3days
25_M87_3days
27_M71_4days
28_V53_6days
30_M49_5days
32_V69_3days
40_V69_4days
41_M70_6days
44_M57_5days
6d_M79_8days

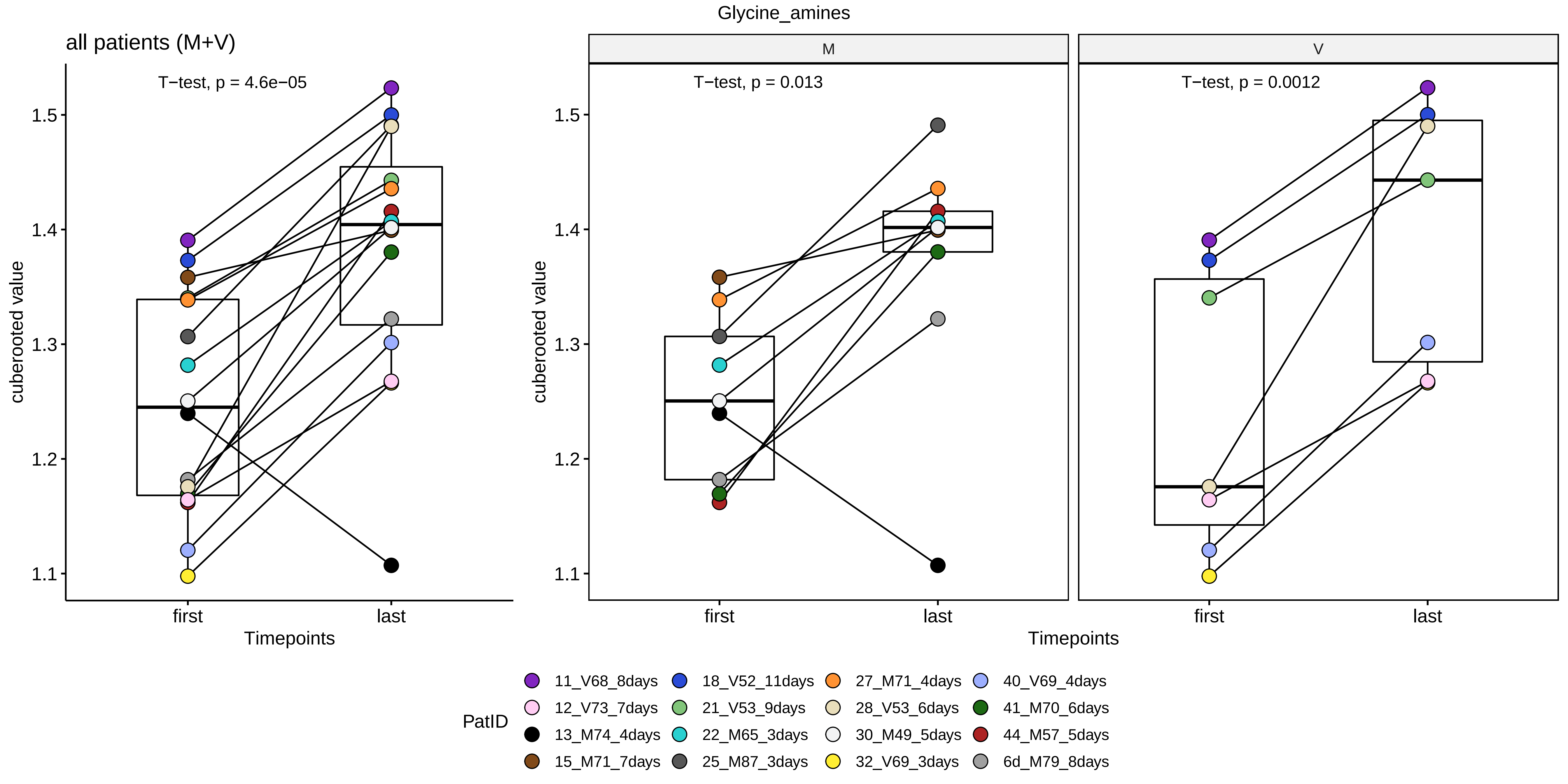
Glycine_amines
all patients (M+V)
T−test, p = 4.6e−05
M
T−test, p = 0.013
V
T−test, p = 0.0012
cuberooted value
1.5
1.4
1.3
1.2
1.1
first
last
Timepoints
PatID
11_V68_8days
12_V73_7days
13_M74_4days
15_M71_7days
18_V52_11days
21_V53_9days
22_M65_3days
25_M87_3days
27_M71_4days
28_V53_6days
30_M49_5days
32_V69_3days
40_V69_4days
41_M70_6days
44_M57_5days
6d_M79_8days

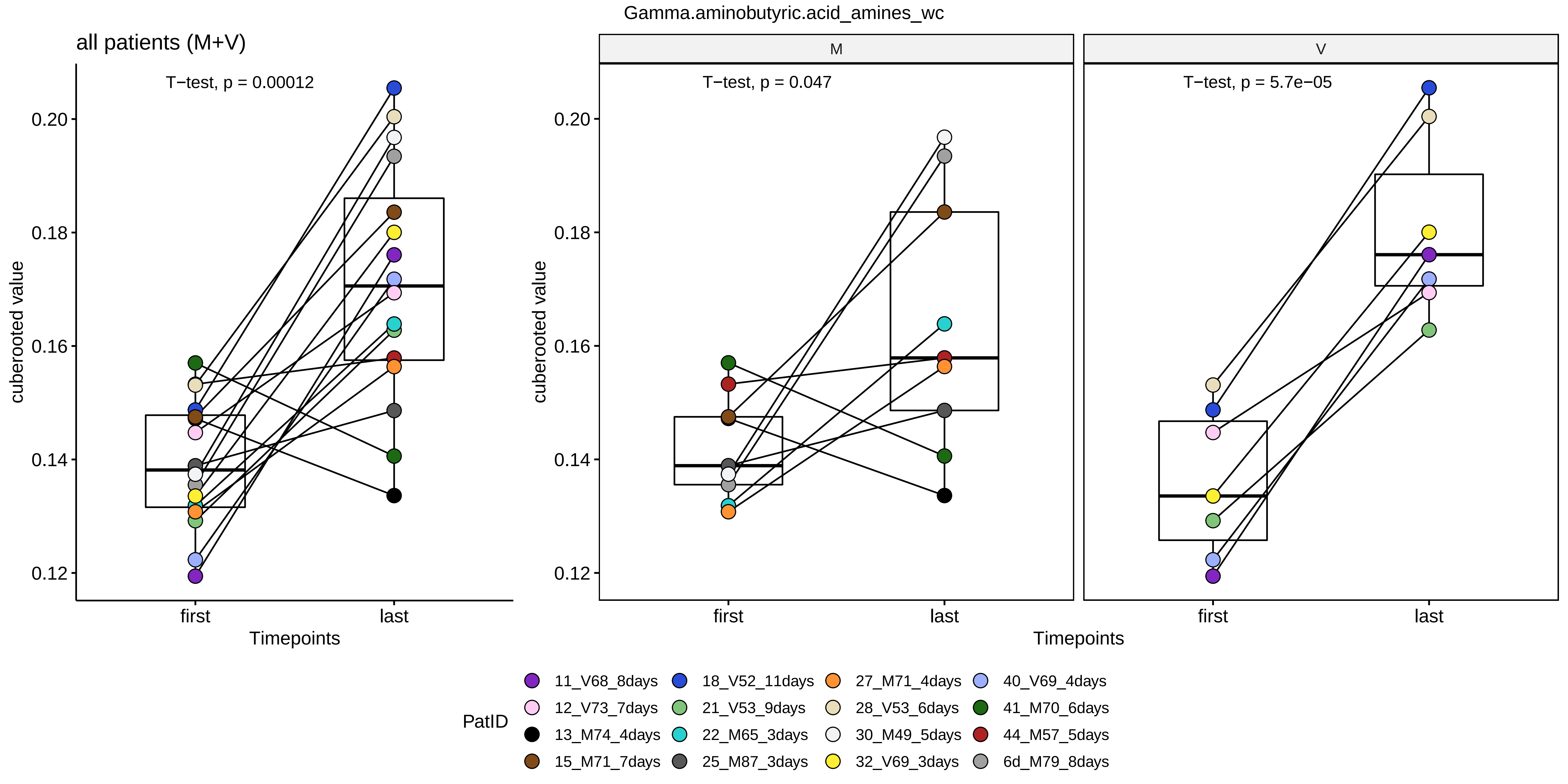

Gamma.aminobutyric.acid_amines_wc
all patients (M+V)
T−test, p = 0.00012
0.20
0.18
0.16
0.14
0.12
cuberooted value
first
last
Timepoints
M
T−test, p = 0.047
V
T−test, p = 5.7e−05
PatID
11_V68_8days
12_V73_7days
13_M74_4days
15_M71_7days
18_V52_11days
21_V53_9days
22_M65_3days
25_M87_3days
27_M71_4days
28_V53_6days
30_M49_5days
32_V69_3days
40_V69_4days
41_M70_6days
44_M57_5days
6d_M79_8days

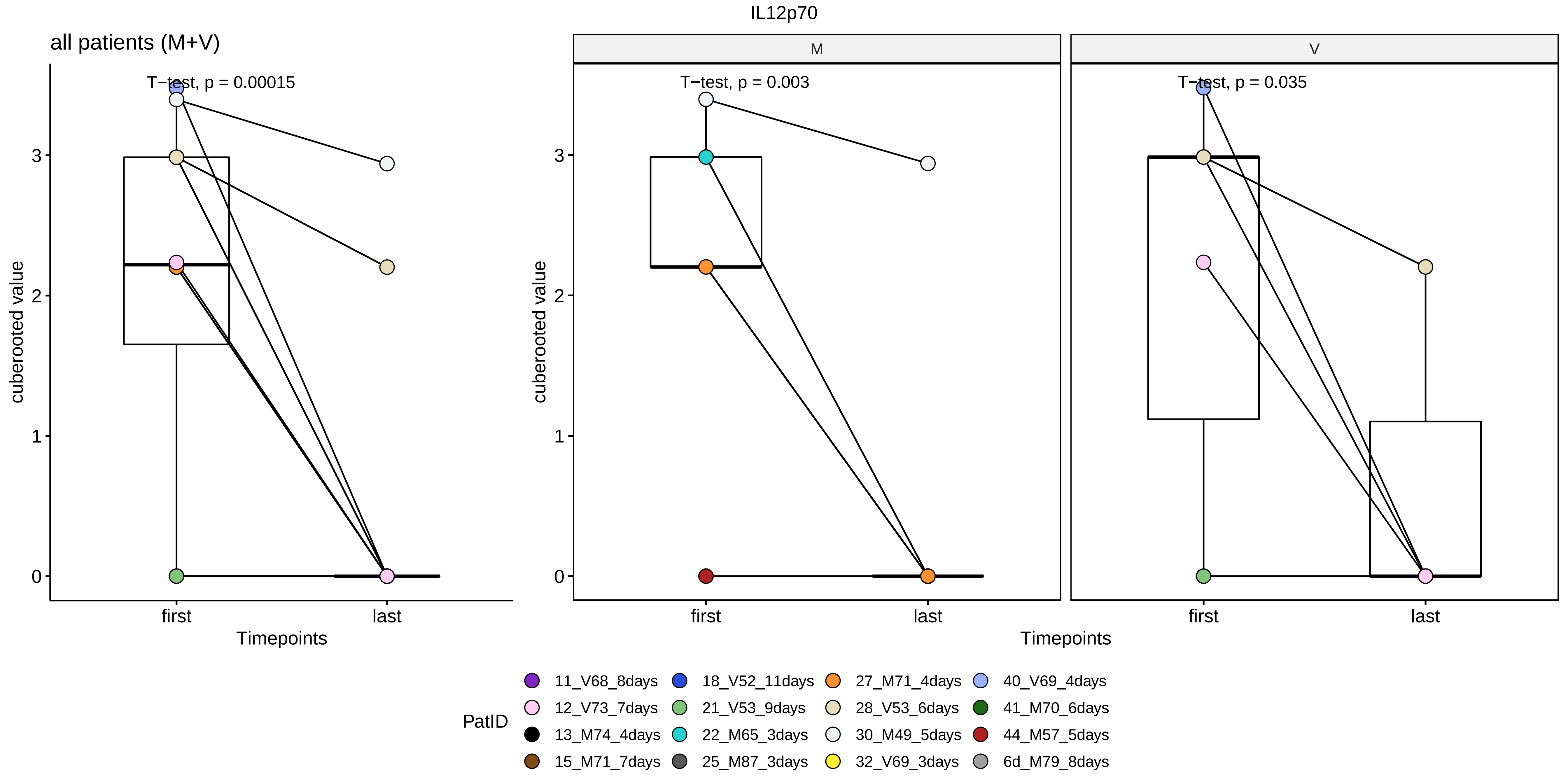
IL12p70
all patients (M+V)
T−test, p = 0.00015
cuberooted value
first
last
Timepoints
M
T−test, p = 0.003
V
T−test, p = 0.035
PatID
11_V68_8days
12_V73_7days
13_M74_4days
15_M71_7days
18_V52_11days
21_V53_9days
22_M65_3days
25_M87_3days
27_M71_4days
28_V53_6days
30_M49_5days
32_V69_3days
40_V69_4days
41_M70_6days
44_M57_5days
6d_M79_8days

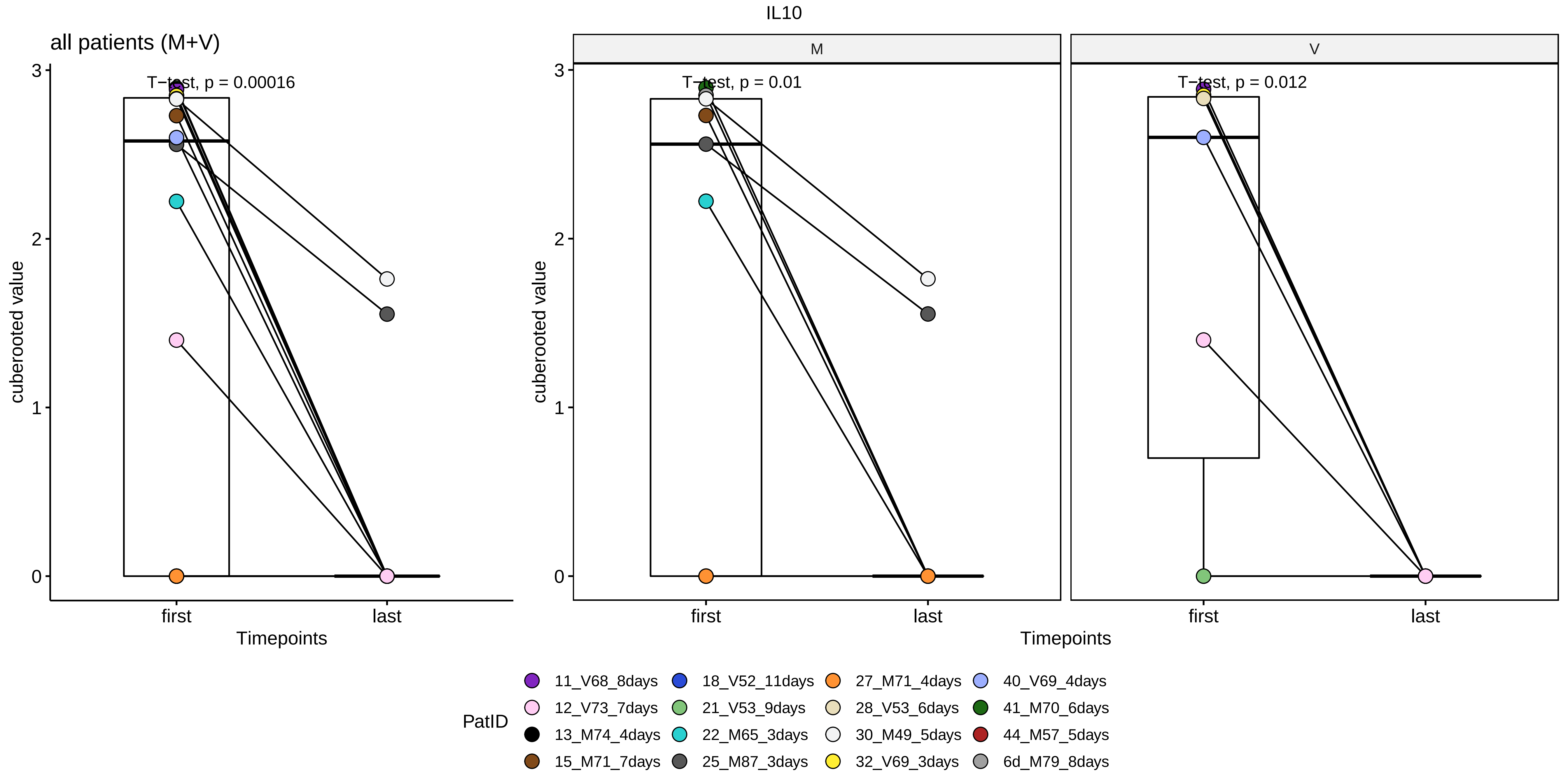

IL10
all patients (M+V)
T−test, p = 0.00016
M
T−test, p = 0.01
V
T−test, p = 0.012
cuberooted value
0
1
2
3
first
last
Timepoints
PatID
11_V68_8days
12_V73_7days
13_M74_4days
15_M71_7days
18_V52_11days
21_V53_9days
22_M65_3days
25_M87_3days
27_M71_4days
28_V53_6days
30_M49_5days
32_V69_3days
40_V69_4days
41_M70_6days
44_M57_5days
6d_M79_8days

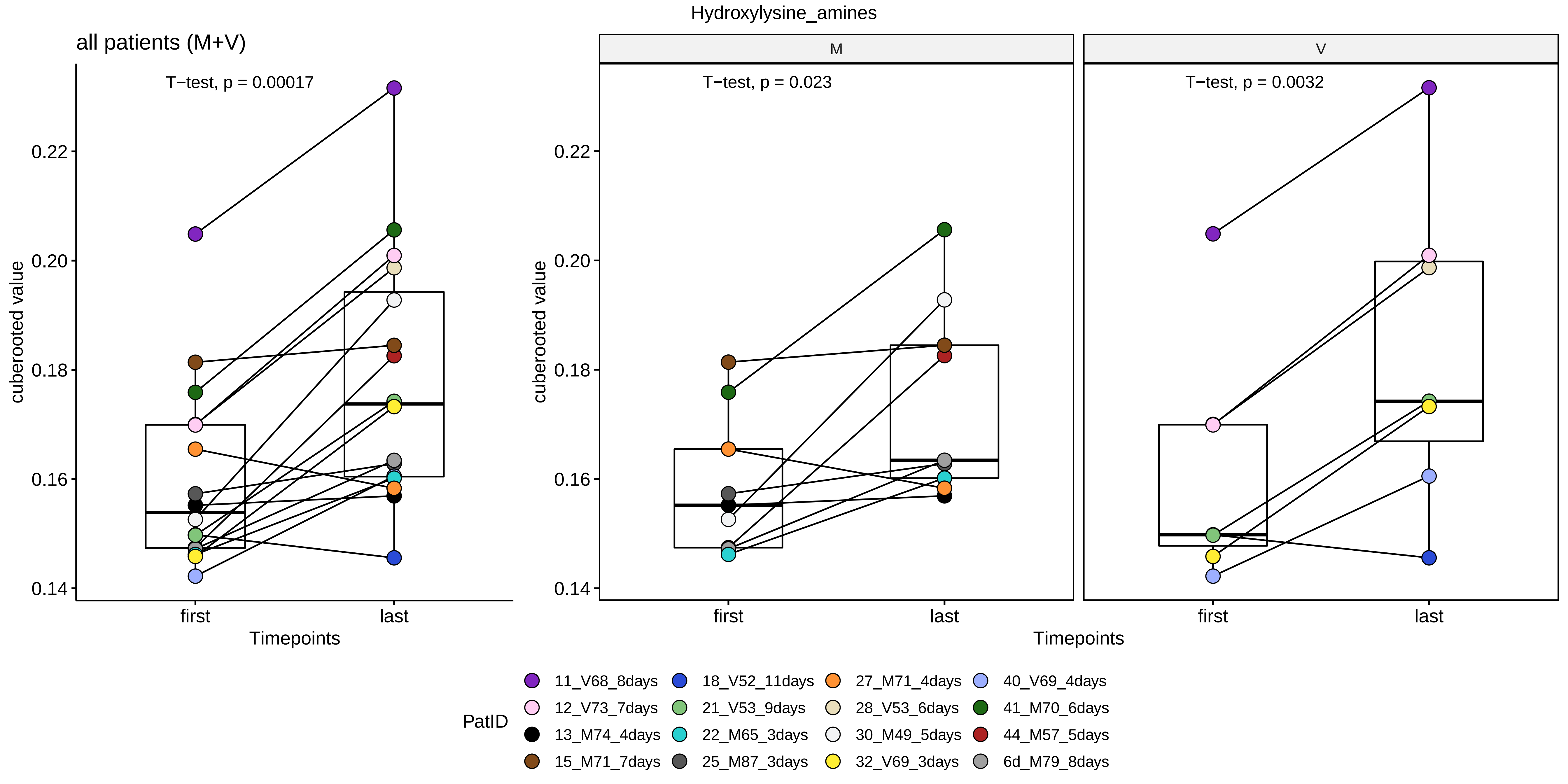

Hydroxylysine_amines
all patients (M+V)
T−test, p = 0.00017
M
T−test, p = 0.023
V
T−test, p = 0.0032
cuberooted value
0.22
0.20
0.18
0.16
0.14
first
last
Timepoints
PatID
11_V68_8days
12_V73_7days
13_M74_4days
15_M71_7days
18_V52_11days
21_V53_9days
22_M65_3days
25_M87_3days
27_M71_4days
28_V53_6days
30_M49_5days
32_V69_3days
40_V69_4days
41_M70_6days
44_M57_5days
6d_M79_8days

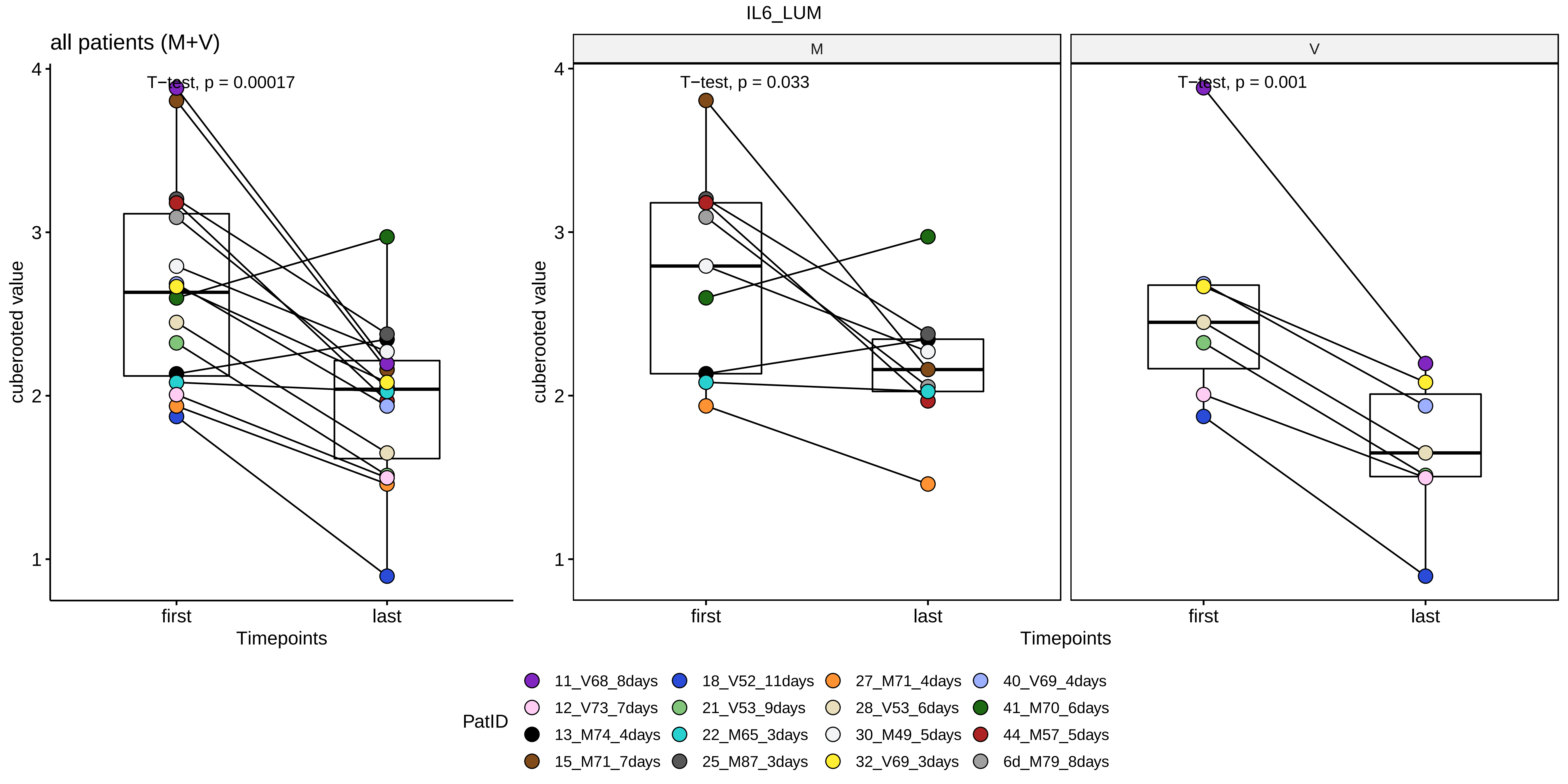

IL6_LUM
all patients (M+V)
T−test, p = 0.00017
M
V
T−test, p = 0.033
T−test, p = 0.001
cuberooted value
1
2
3
4
first
last
Timepoints
PatID
11_V68_8days
12_V73_7days
13_M74_4days
15_M71_7days
18_V52_11days
21_V53_9days
22_M65_3days
25_M87_3days
27_M71_4days
28_V53_6days
30_M49_5days
32_V69_3days
40_V69_4days
41_M70_6days
44_M57_5days
6d_M79_8days

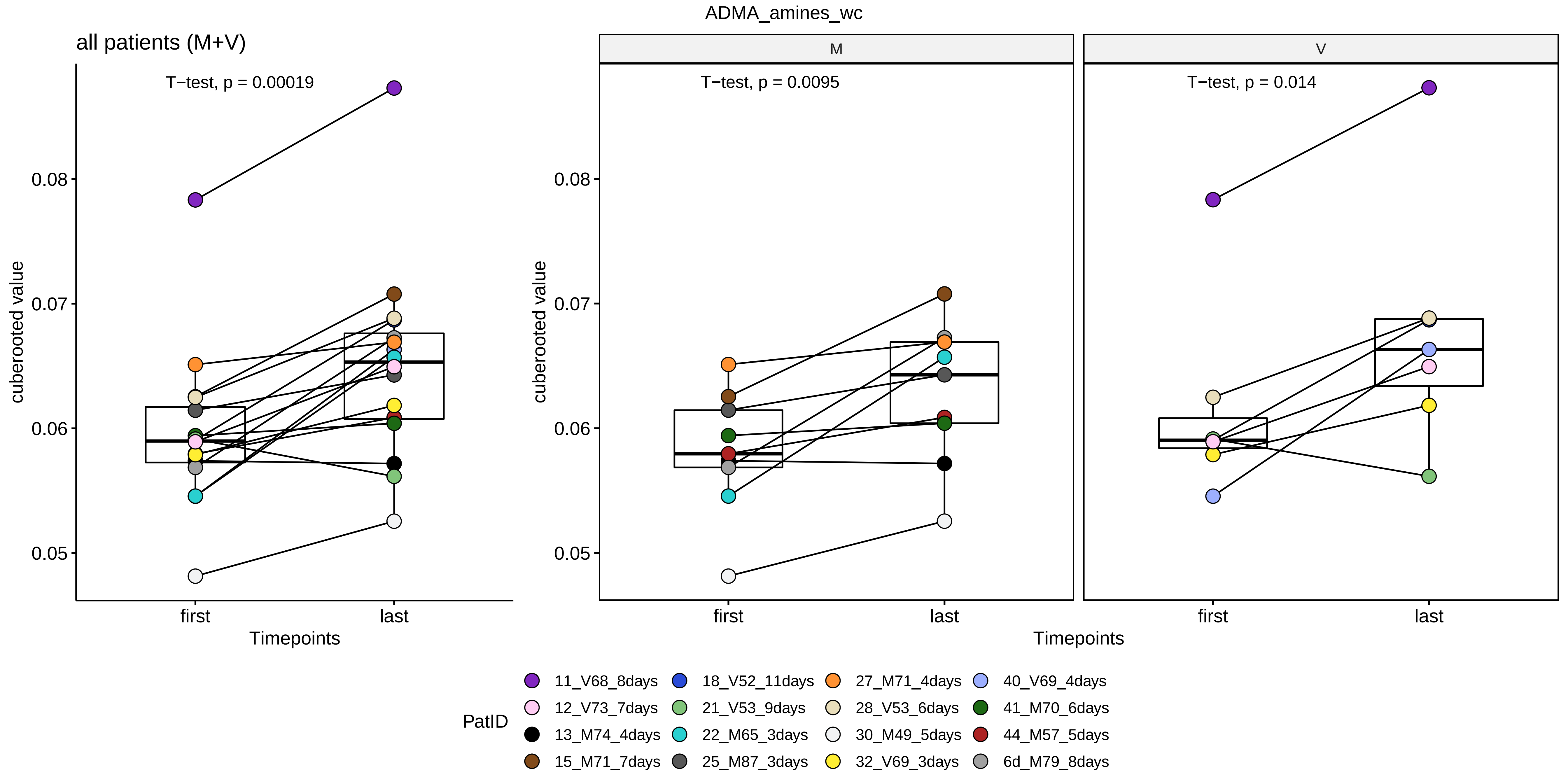
ADMA_amines_wc
all patients (M+V)
T−test, p = 0.00019
M
T−test, p = 0.0095
V
T−test, p = 0.014
cuberooted value
0.08
0.07
0.06
0.05
first
last
Timepoints
PatID
11_V68_8days
12_V73_7days
13_M74_4days
15_M71_7days
18_V52_11days
21_V53_9days
22_M65_3days
25_M87_3days
27_M71_4days
28_V53_6days
30_M49_5days
32_V69_3days
40_V69_4days
41_M70_6days
44_M57_5days
6d_M79_8days

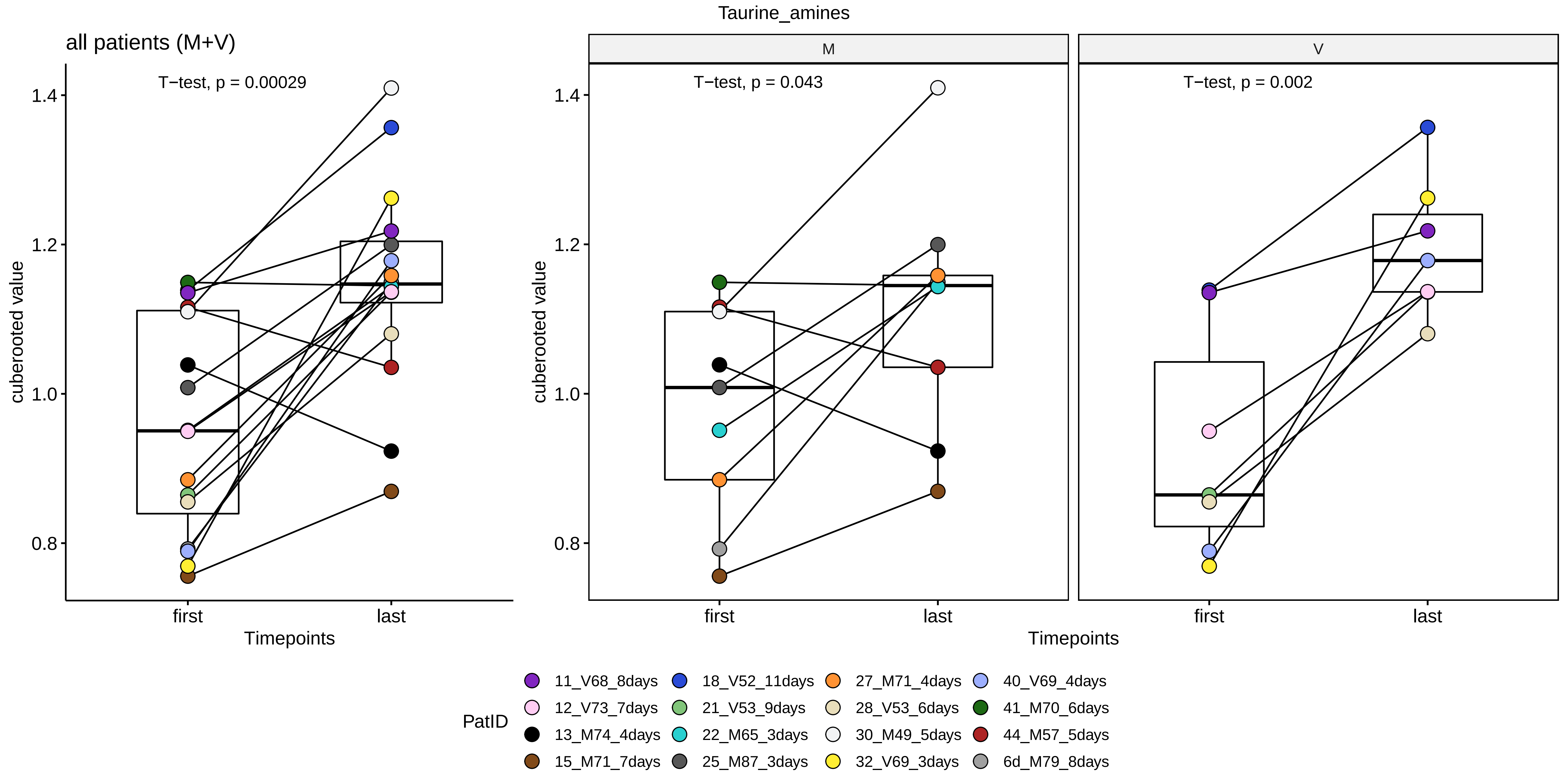
Taurine_amines
all patients (M+V)
T−test, p = 0.00029
M
T−test, p = 0.043
V
T−test, p = 0.002
cuberooted value
1.4
1.2
1.0
0.8
first
last
Timepoints
PatID
11_V68_8days
12_V73_7days
13_M74_4days
15_M71_7days
18_V52_11days
21_V53_9days
22_M65_3days
25_M87_3days
27_M71_4days
28_V53_6days
30_M49_5days
32_V69_3days
40_V69_4days
41_M70_6days
44_M57_5days
6d_M79_8days

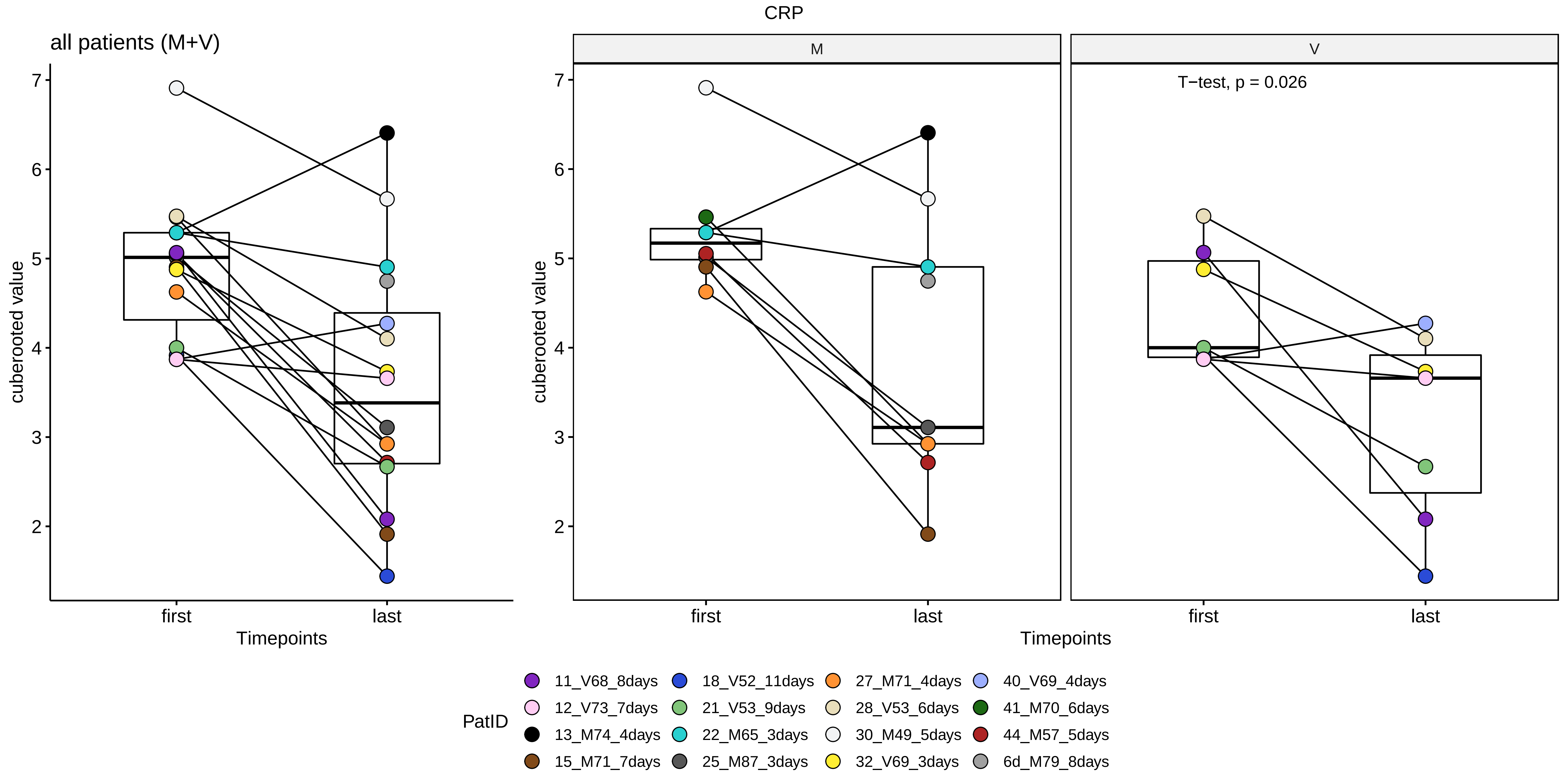

CRP
all patients (M+V)
M
V
T−test, p = 0.026
cuberooted value
Timepoints
first
last
PatID
11_V68_8days
12_V73_7days
13_M74_4days
15_M71_7days
18_V52_11days
21_V53_9days
22_M65_3days
25_M87_3days
27_M71_4days
28_V53_6days
30_M49_5days
32_V69_3days
40_V69_4days
41_M70_6days
44_M57_5days
6d_M79_8days

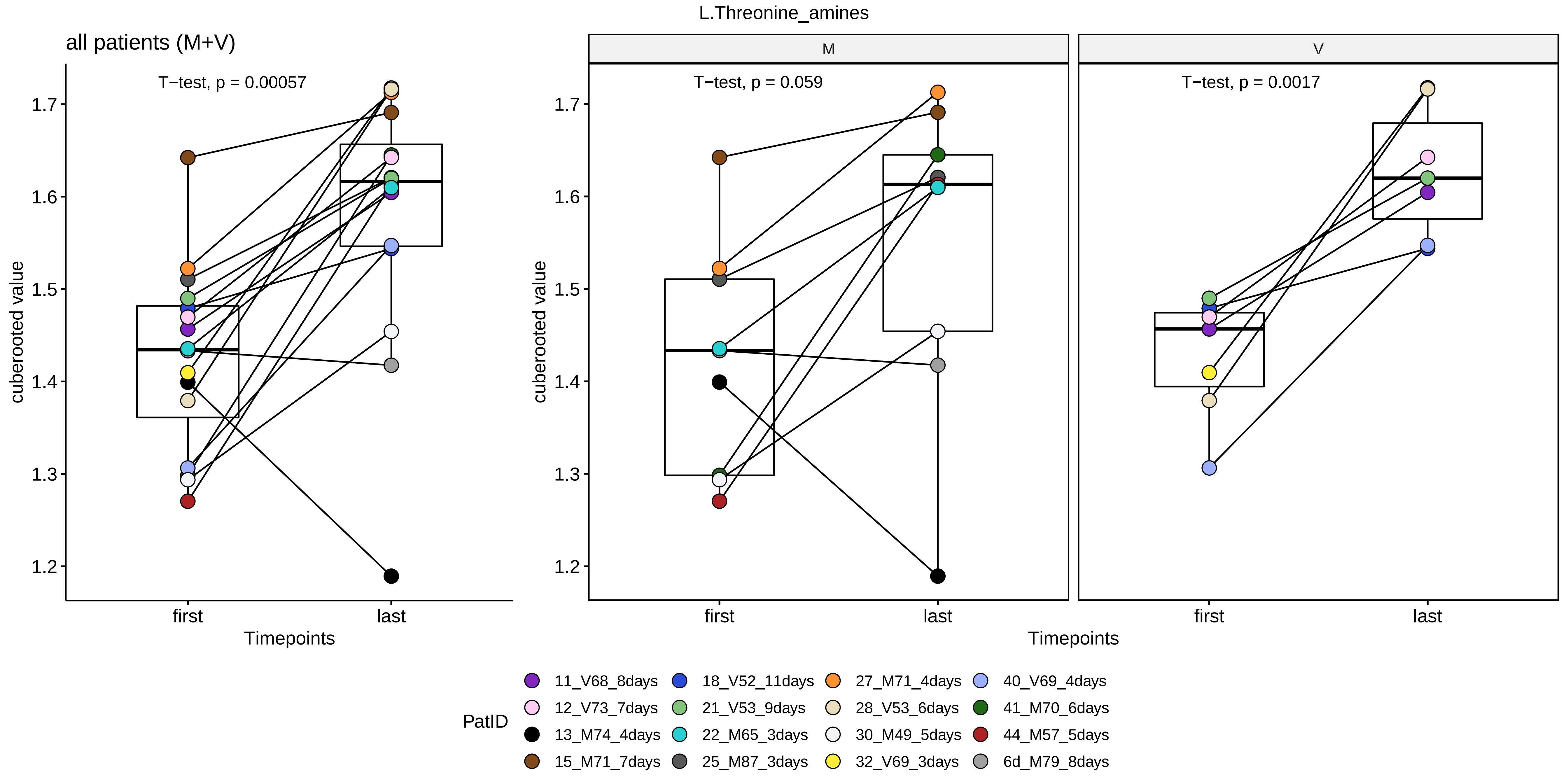
L.Threonine_amines
all patients (M+V)
T−test, p = 0.00057
M
T−test, p = 0.059
V
T−test, p = 0.0017
cuberooted value
1.2
1.3
1.4
1.5
1.6
1.7
first
last
Timepoints
PatID
11_V68_8days
12_V73_7days
13_M74_4days
15_M71_7days
18_V52_11days
21_V53_9days
22_M65_3days
25_M87_3days
27_M71_4days
28_V53_6days
30_M49_5days
32_V69_3days
40_V69_4days
41_M70_6days
44_M57_5days
6d_M79_8days

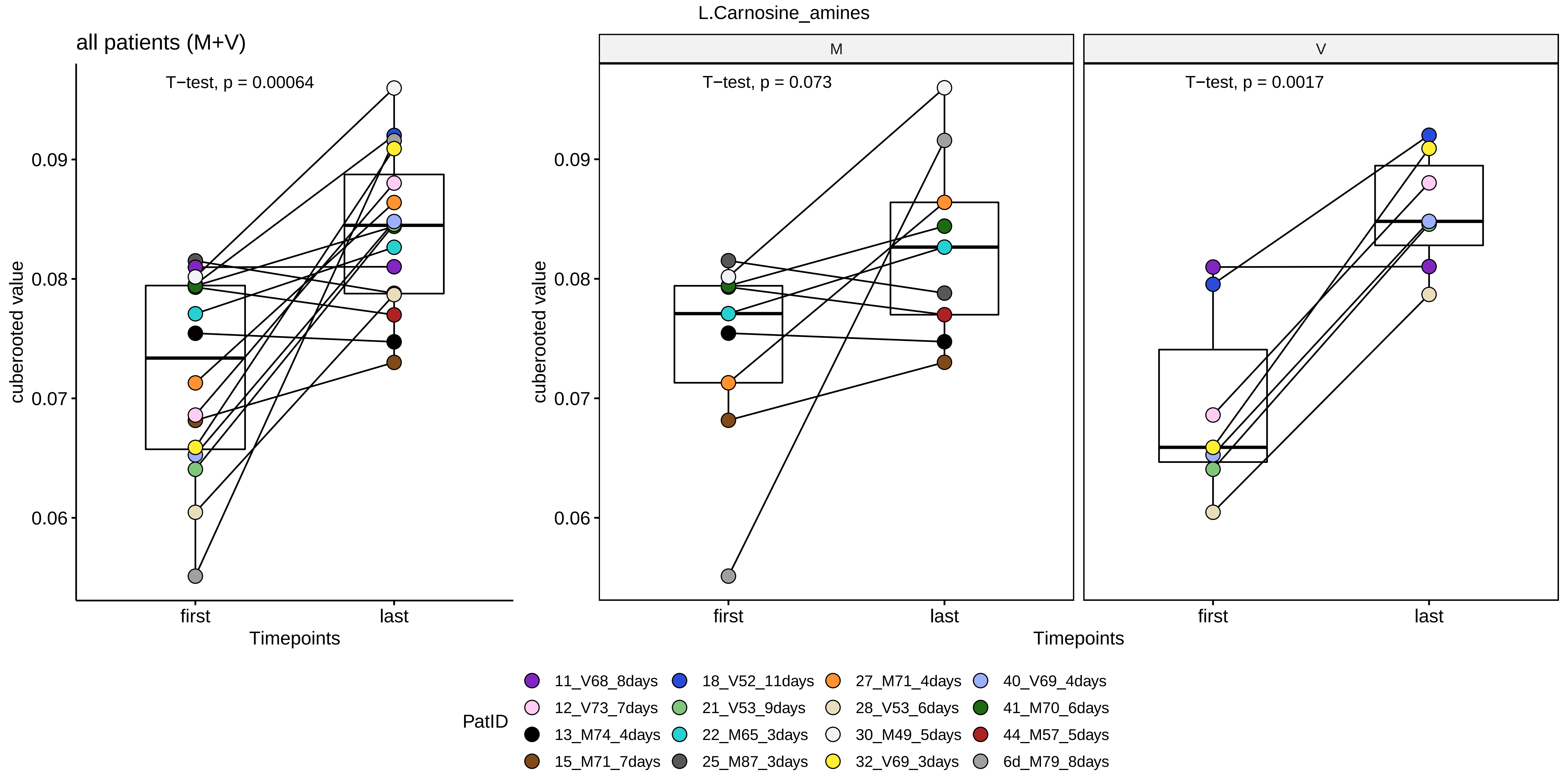

L.Carnosine_amines
all patients (M+V)
T−test, p = 0.00064
M
T−test, p = 0.073
V
T−test, p = 0.0017
cuberooted value
0.06
0.07
0.08
0.09
first
last
Timepoints
PatID
11_V68_8days
12_V73_7days
13_M74_4days
15_M71_7days
18_V52_11days
21_V53_9days
22_M65_3days
25_M87_3days
27_M71_4days
28_V53_6days
30_M49_5days
32_V69_3days
40_V69_4days
41_M70_6days
44_M57_5days
6d_M79_8days

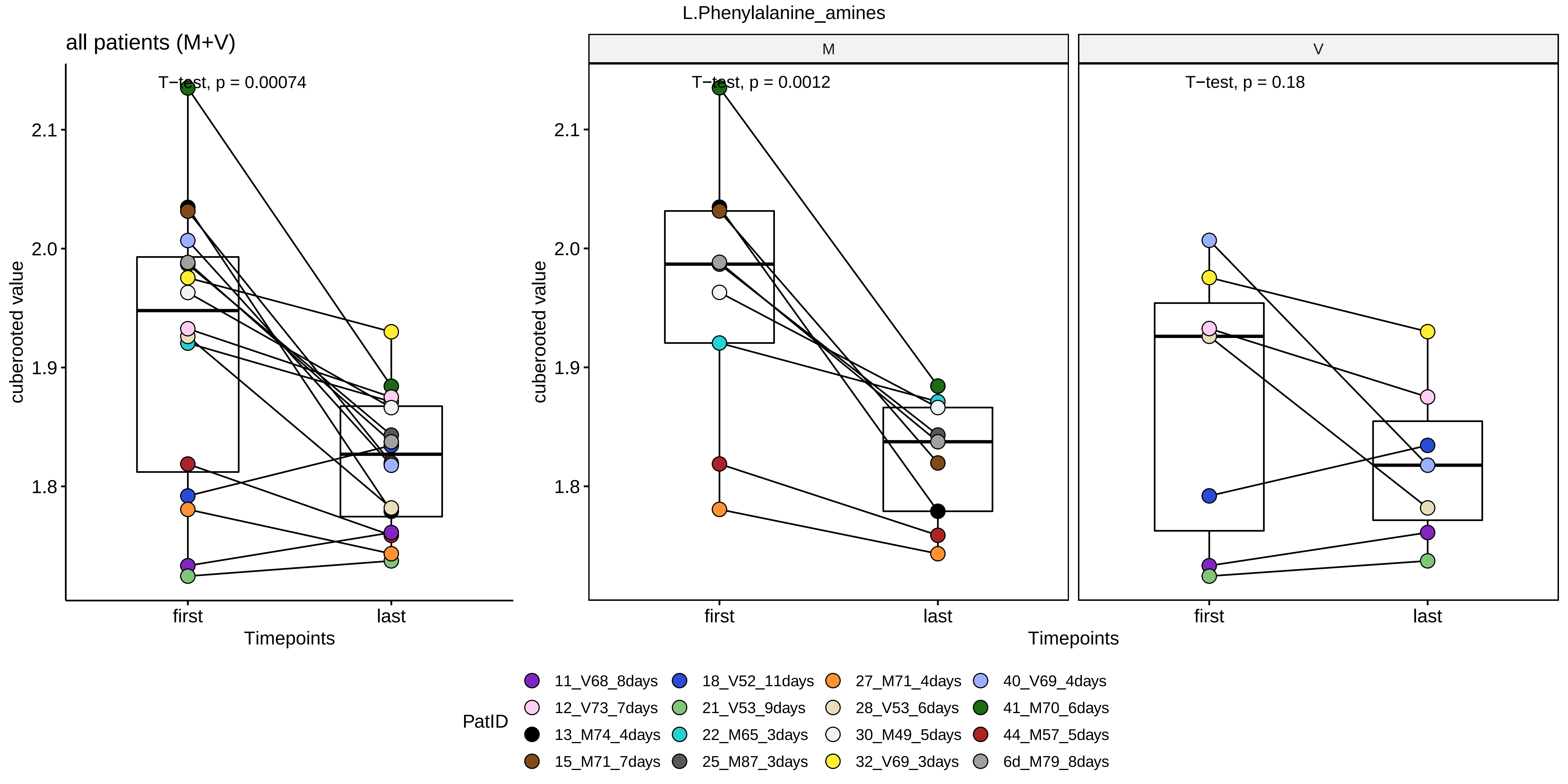

L.Phenylalanine_amines
all patients (M+V)
T−test, p = 0.00074
M
T−test, p = 0.0012
V
T−test, p = 0.18
cuberooted value
2.1
2.0
1.9
1.8
first
last
Timepoints
PatID
11_V68_8days
12_V73_7days
13_M74_4days
15_M71_7days
18_V52_11days
21_V53_9days
22_M65_3days
25_M87_3days
27_M71_4days
28_V53_6days
30_M49_5days
32_V69_3days
40_V69_4days
41_M70_6days
44_M57_5days
6d_M79_8days

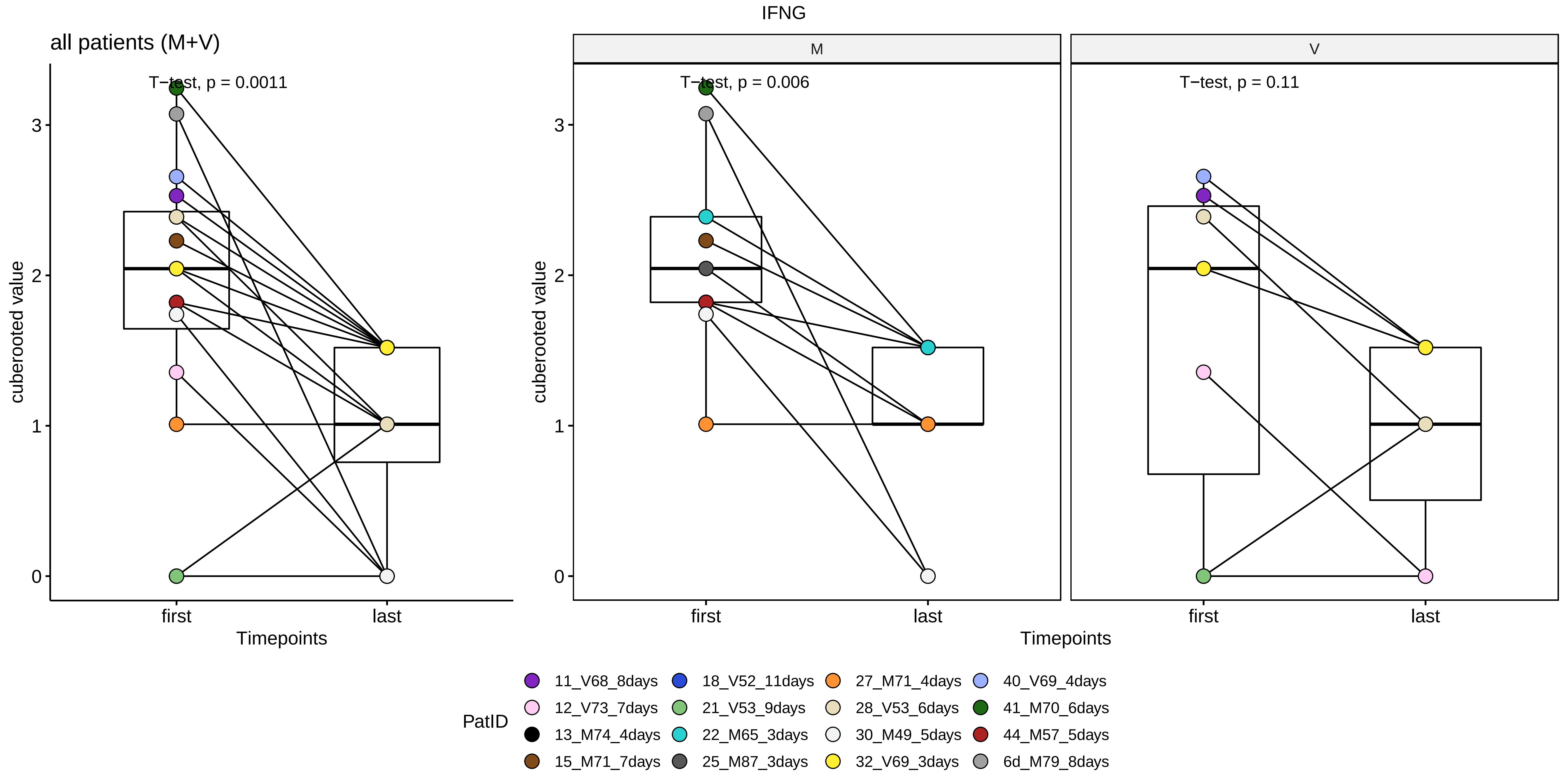
IFNG
all patients (M+V)
T−test, p = 0.0011
M
T−test, p = 0.006
V
T−test, p = 0.11
cuberooted value
Timepoints
first
last
PatID
11_V68_8days
12_V73_7days
13_M74_4days
15_M71_7days
18_V52_11days
21_V53_9days
22_M65_3days
25_M87_3days
27_M71_4days
28_V53_6days
30_M49_5days
32_V69_3days
40_V69_4days
41_M70_6days
44_M57_5days
6d_M79_8days

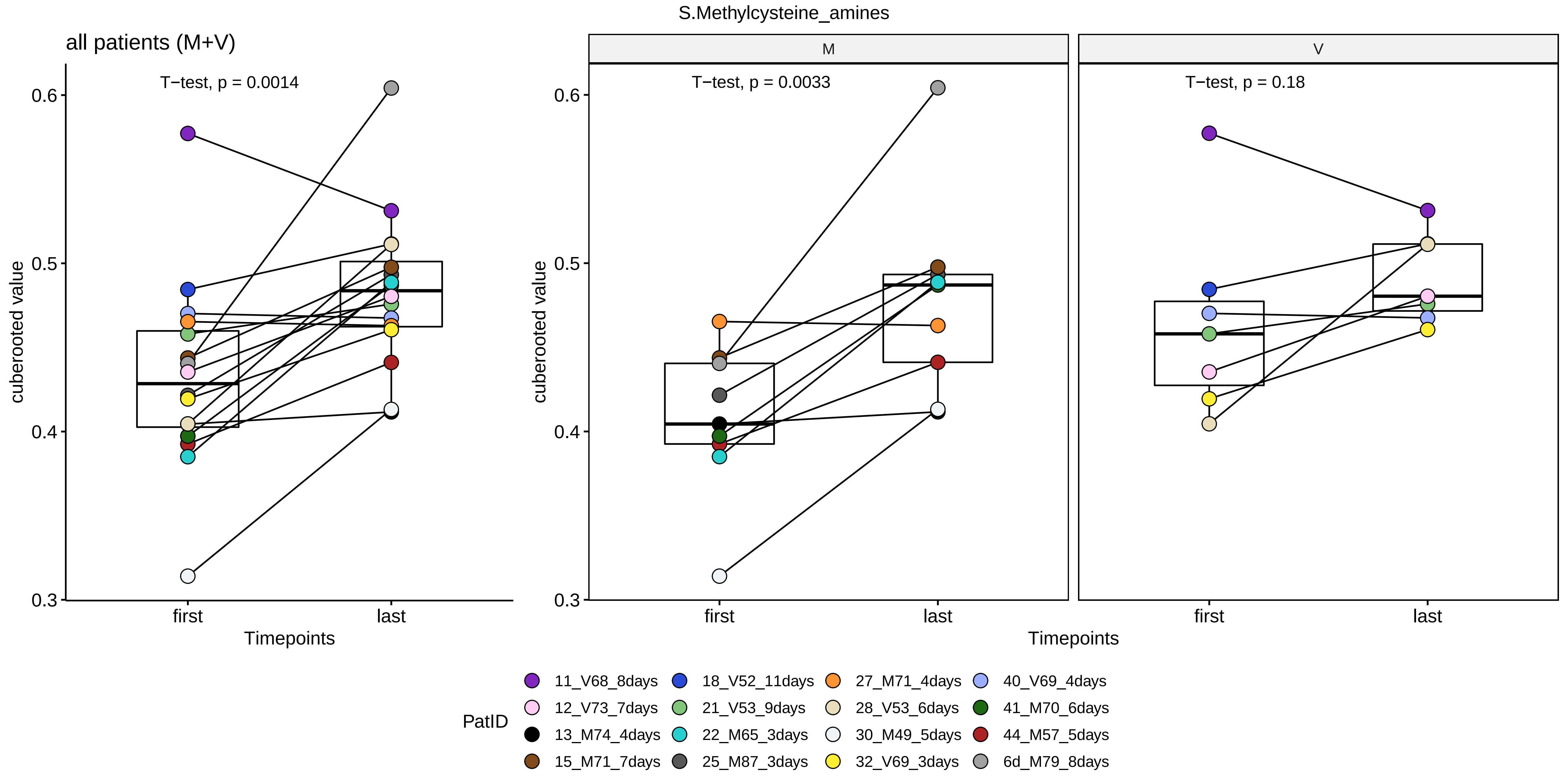

S.Methylcysteine_amines
all patients (M+V)
T−test, p = 0.0014
M
T−test, p = 0.0033
V
T−test, p = 0.18
cuberooted value
0.6
0.5
0.4
0.3
first
last
Timepoints
PatID
11_V68_8days
12_V73_7days
13_M74_4days
15_M71_7days
18_V52_11days
21_V53_9days
22_M65_3days
25_M87_3days
27_M71_4days
28_V53_6days
30_M49_5days
32_V69_3days
40_V69_4days
41_M70_6days
44_M57_5days
6d_M79_8days

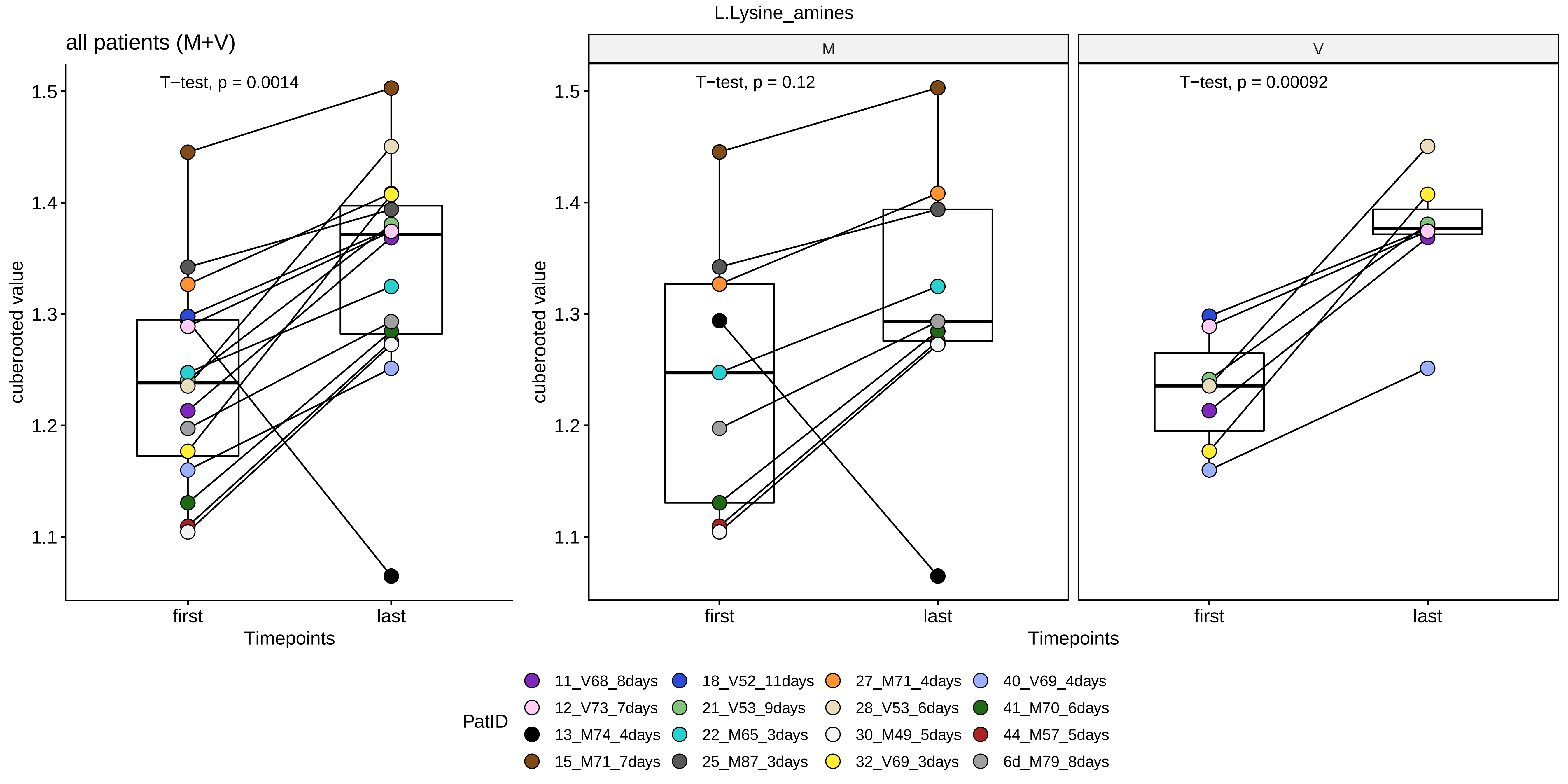

L.Lysine_amines
all patients (M+V)
T−test, p = 0.0014
M
T−test, p = 0.12
V
T−test, p = 0.00092
cuberooted value
1.5
1.4
1.3
1.2
1.1
first
last
Timepoints
PatID
11_V68_8days
12_V73_7days
13_M74_4days
15_M71_7days
18_V52_11days
21_V53_9days
22_M65_3days
25_M87_3days
27_M71_4days
28_V53_6days
30_M49_5days
32_V69_3days
40_V69_4days
41_M70_6days
44_M57_5days
6d_M79_8days

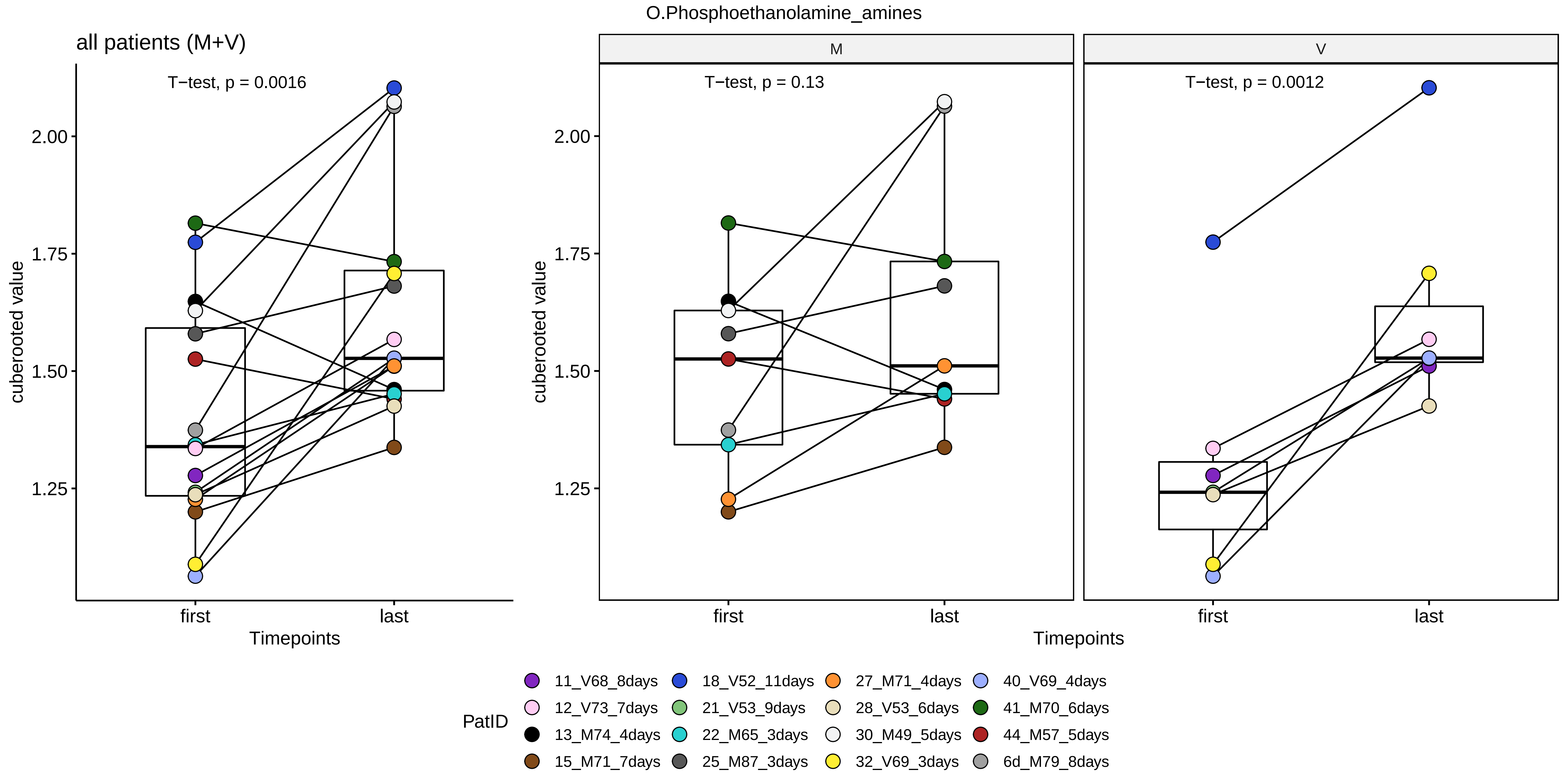

O.Phosphoethanolamine_amines
all patients (M+V)
T−test, p = 0.0016
M
T−test, p = 0.13
V
T−test, p = 0.0012
cuberooted value
2.00
1.75
1.50
1.25
first
last
Timepoints
PatID
11_V68_8days
12_V73_7days
13_M74_4days
15_M71_7days
18_V52_11days
21_V53_9days
22_M65_3days
25_M87_3days
27_M71_4days
28_V53_6days
30_M49_5days
32_V69_3days
40_V69_4days
41_M70_6days
44_M57_5days
6d_M79_8days

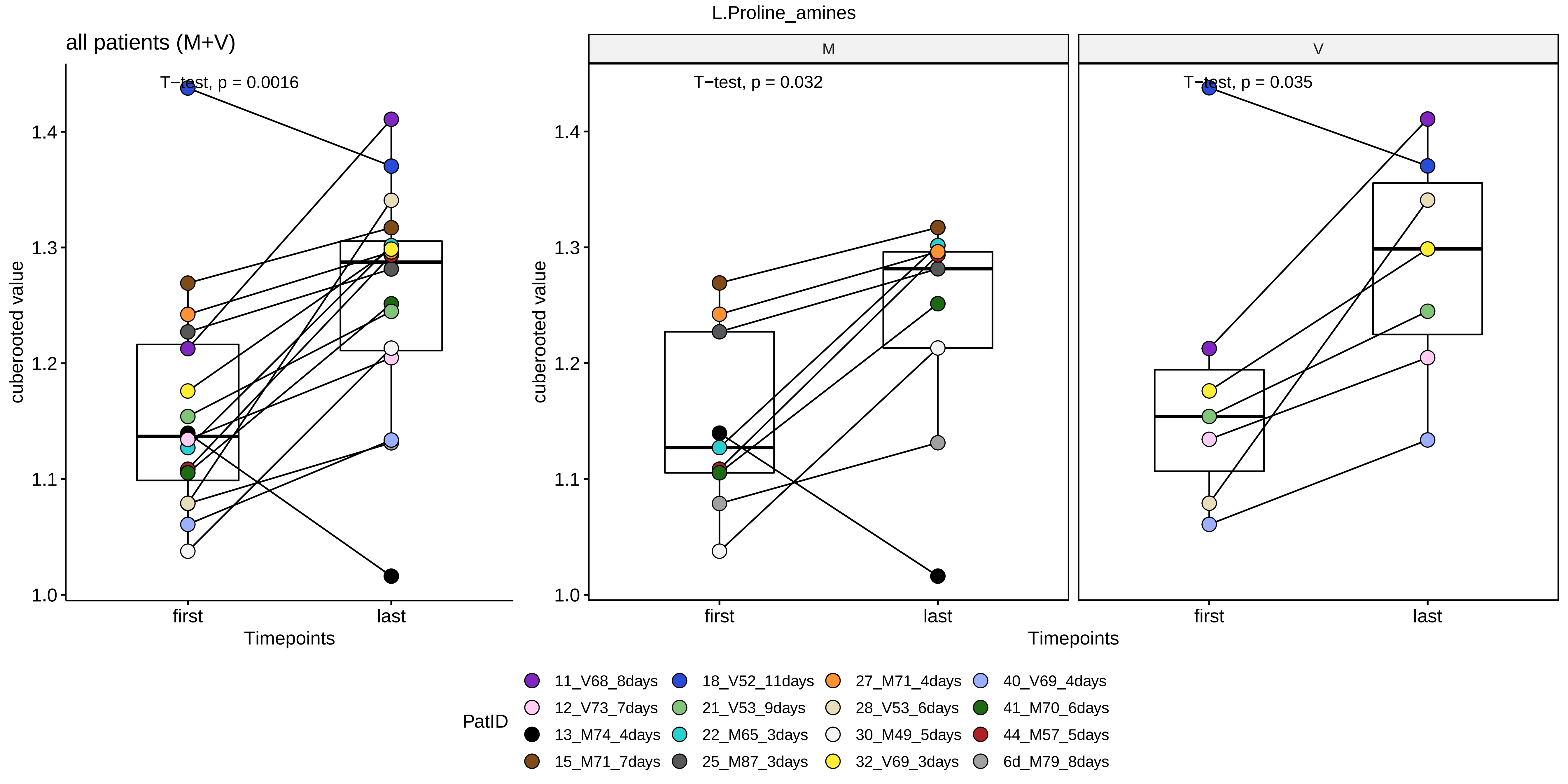

L.Proline_amines
all patients (M+V)
T−test, p = 0.0016
M
T−test, p = 0.032
V
T−test, p = 0.035
cuberooted value
1.0
1.1
1.2
1.3
1.4
first
last
Timepoints
PatID
11_V68_8days
12_V73_7days
13_M74_4days
15_M71_7days
18_V52_11days
21_V53_9days
22_M65_3days
25_M87_3days
27_M71_4days
28_V53_6days
30_M49_5days
32_V69_3days
40_V69_4days
41_M70_6days
44_M57_5days
6d_M79_8days

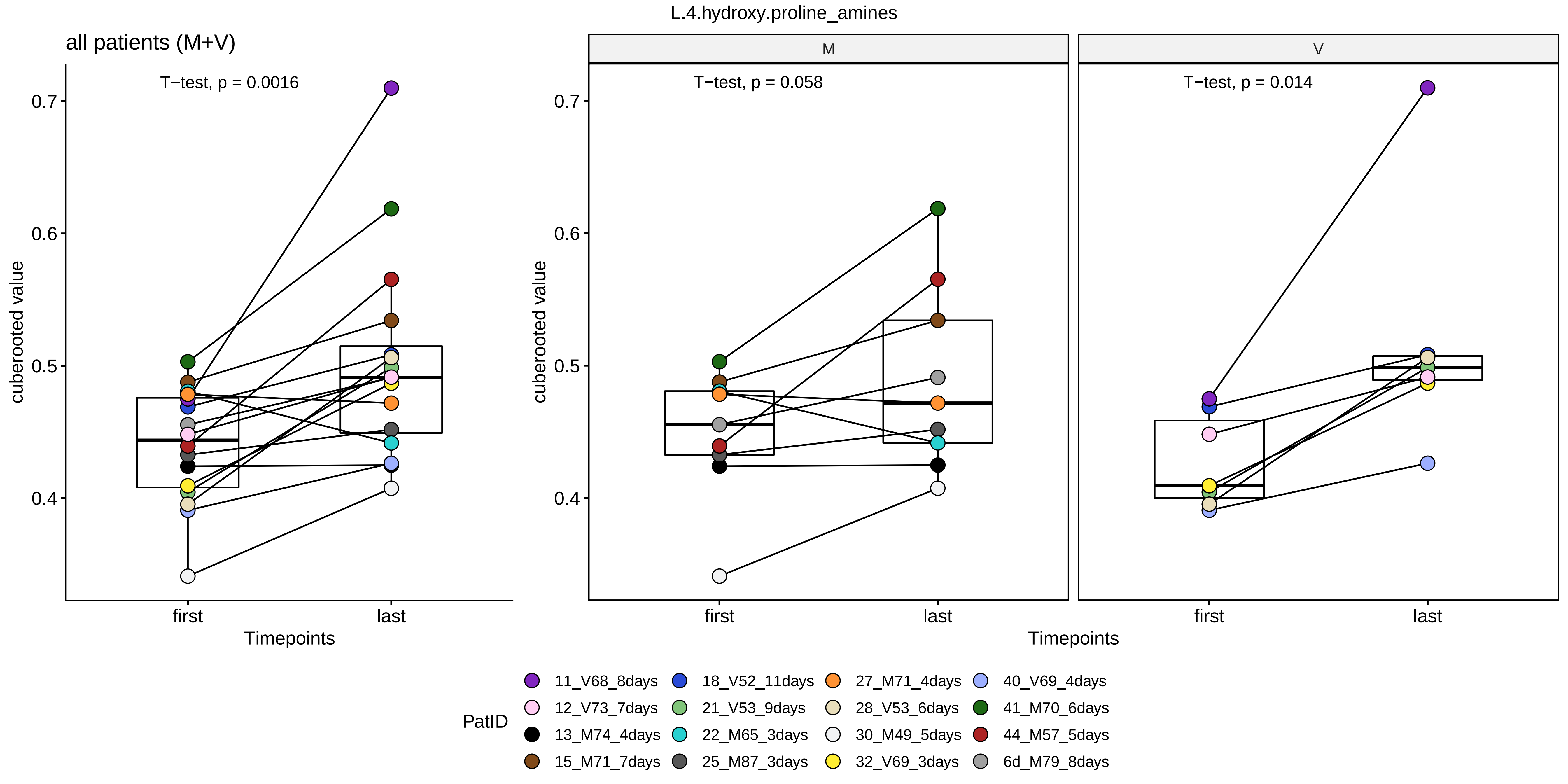

L.4.hydroxy.proline_amines
all patients (M+V)
T−test, p = 0.0016
M
T−test, p = 0.058
V
T−test, p = 0.014
cuberooted value
0.7
0.6
0.5
0.4
first
last
Timepoints
PatID
11_V68_8days
12_V73_7days
13_M74_4days
15_M71_7days
18_V52_11days
21_V53_9days
22_M65_3days
25_M87_3days
27_M71_4days
28_V53_6days
30_M49_5days
32_V69_3days
40_V69_4days
41_M70_6days
44_M57_5days
6d_M79_8days

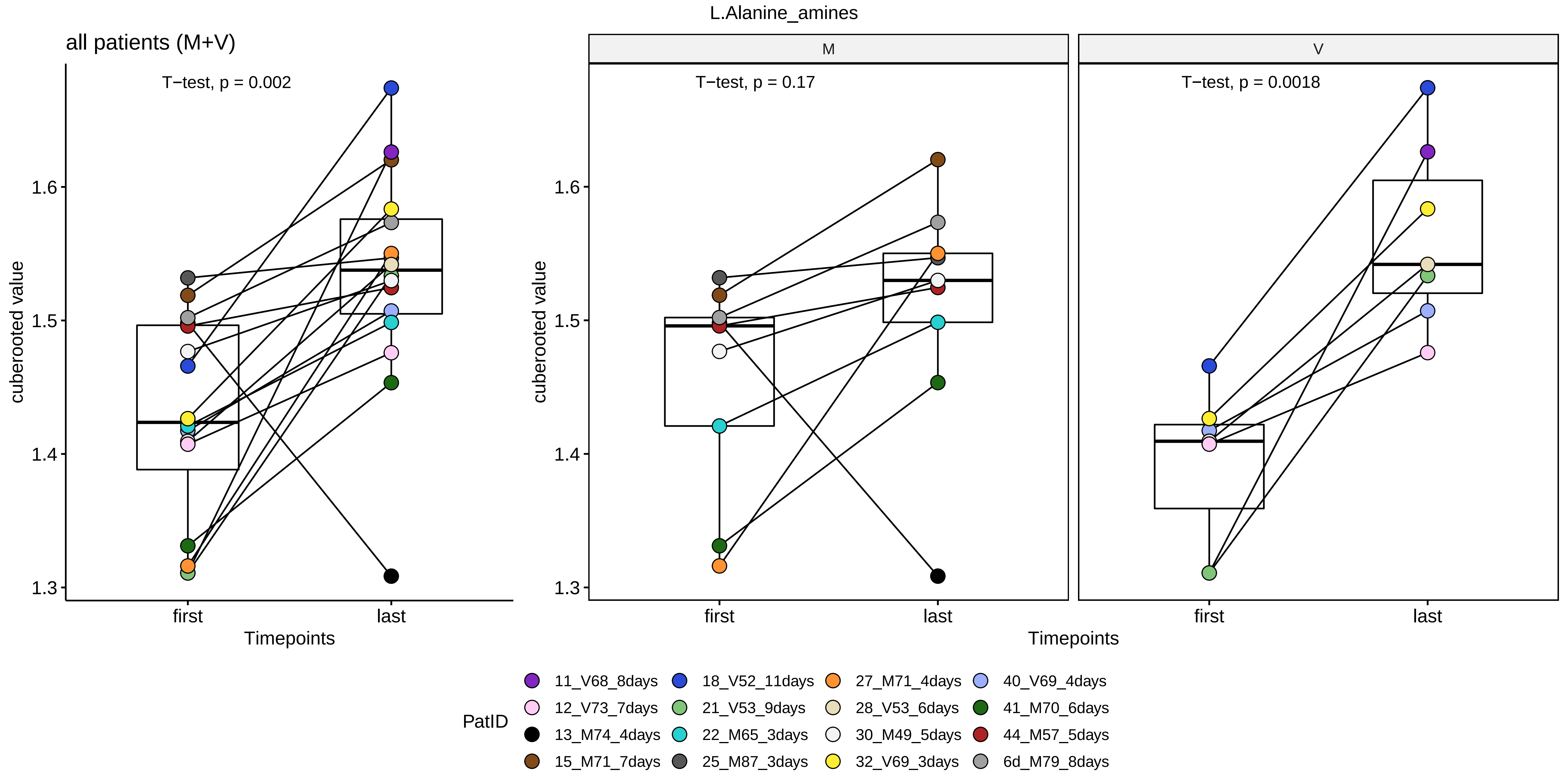
L.Alanine_amines
all patients (M+V)
T−test, p = 0.002
M
T−test, p = 0.17
V
T−test, p = 0.0018
cuberooted value
1.3
1.4
1.5
1.6
first
last
Timepoints
PatID
11_V68_8days
12_V73_7days
13_M74_4days
15_M71_7days
18_V52_11days
21_V53_9days
22_M65_3days
25_M87_3days
27_M71_4days
28_V53_6days
30_M49_5days
32_V69_3days
40_V69_4days
41_M70_6days
44_M57_5days
6d_M79_8days

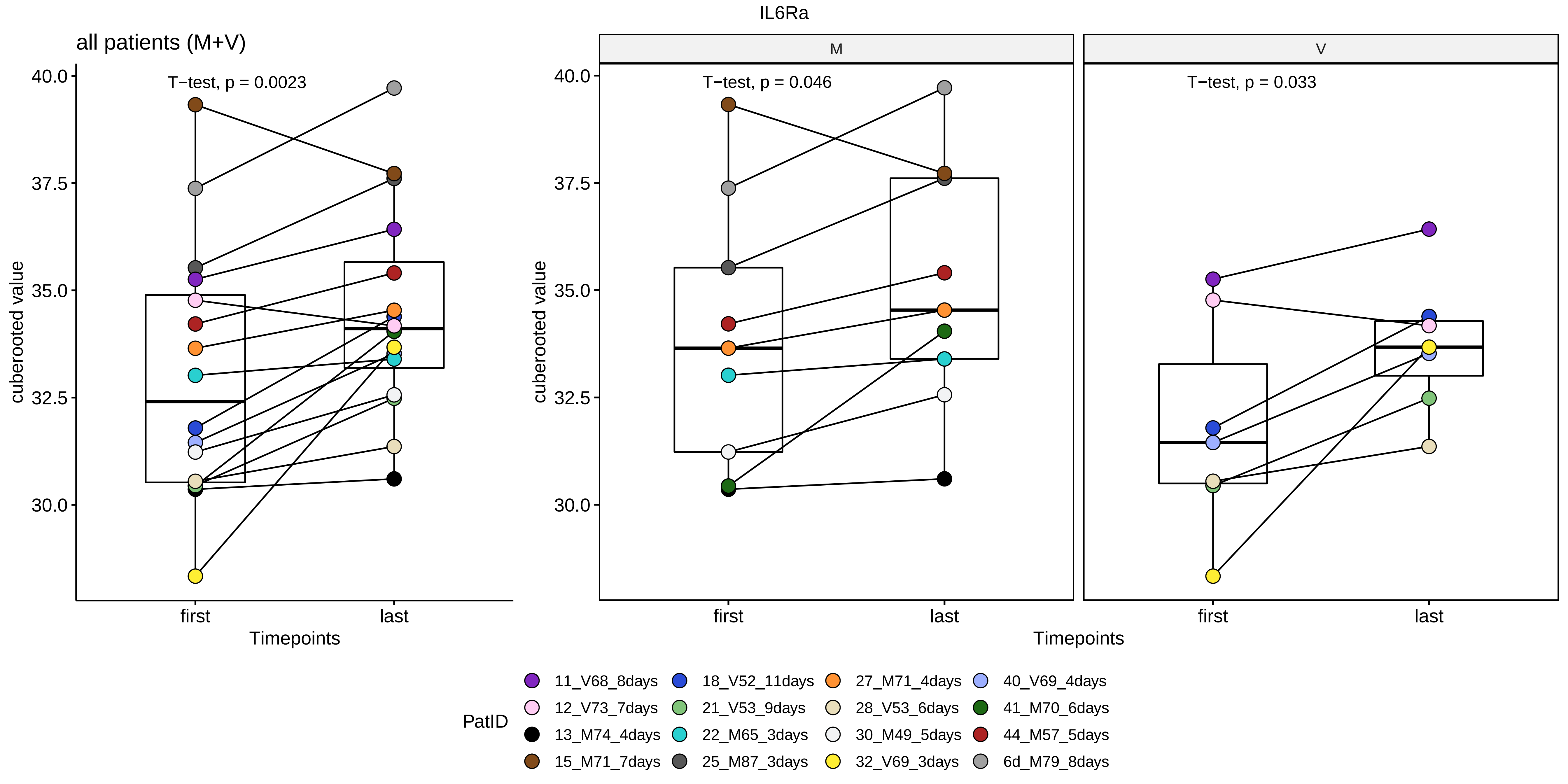

IL6Ra
all patients (M+V)
T−test, p = 0.0023
M
T−test, p = 0.046
V
T−test, p = 0.033
cuberooted value
40.0
37.5
35.0
32.5
30.0
first
last
Timepoints
PatID
11_V68_8days
12_V73_7days
13_M74_4days
15_M71_7days
18_V52_11days
21_V53_9days
22_M65_3days
25_M87_3days
27_M71_4days
28_V53_6days
30_M49_5days
32_V69_3days
40_V69_4days
41_M70_6days
44_M57_5days
6d_M79_8days

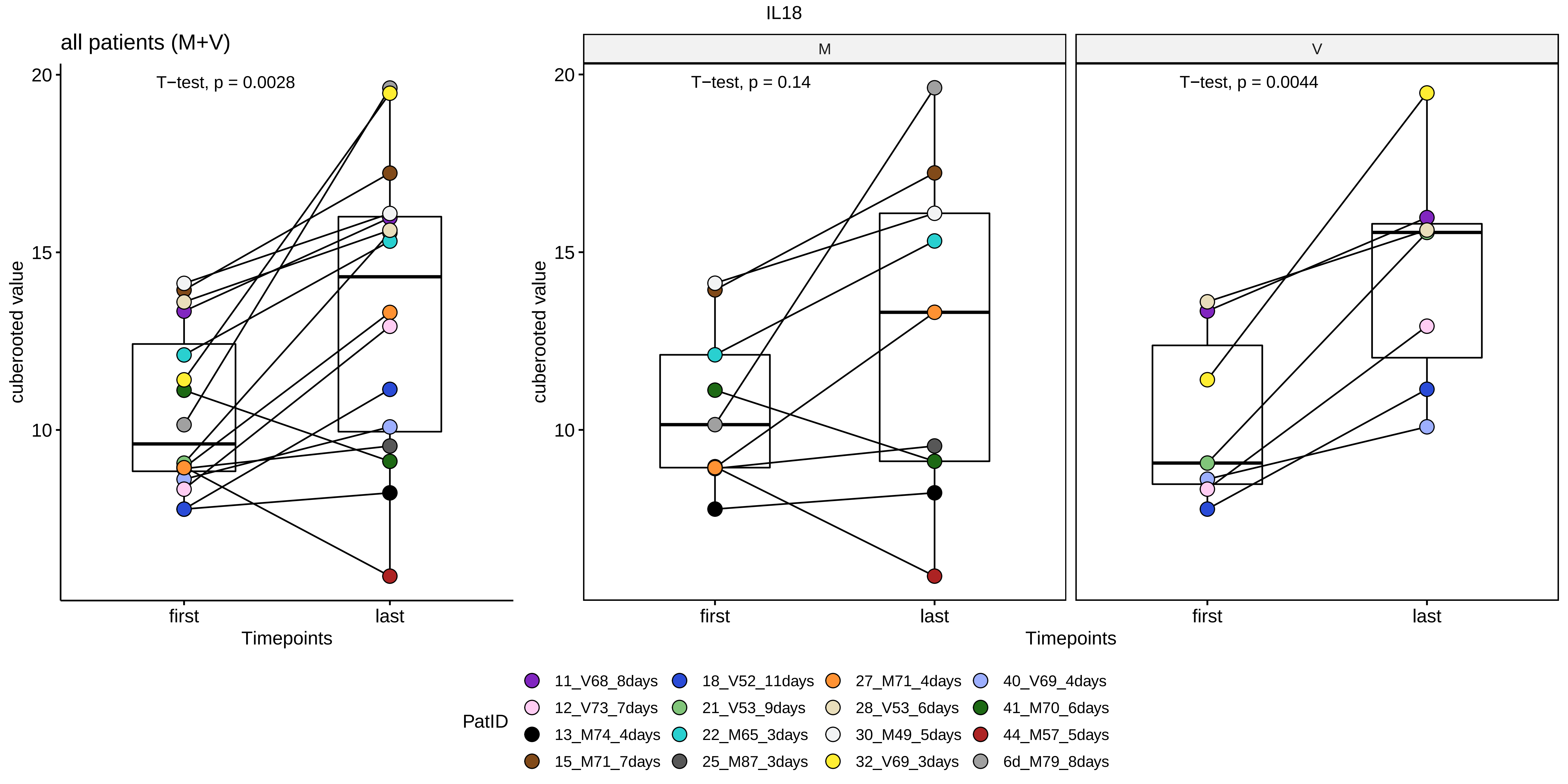
IL18
all patients (M+V)
T−test, p = 0.0028
M
T−test, p = 0.14
V
T−test, p = 0.0044
cuberooted value
20
15
10
first
last
Timepoints
PatID
11_V68_8days
12_V73_7days
13_M74_4days
15_M71_7days
18_V52_11days
21_V53_9days
22_M65_3days
25_M87_3days
27_M71_4days
28_V53_6days
30_M49_5days
32_V69_3days
40_V69_4days
41_M70_6days
44_M57_5days
6d_M79_8days

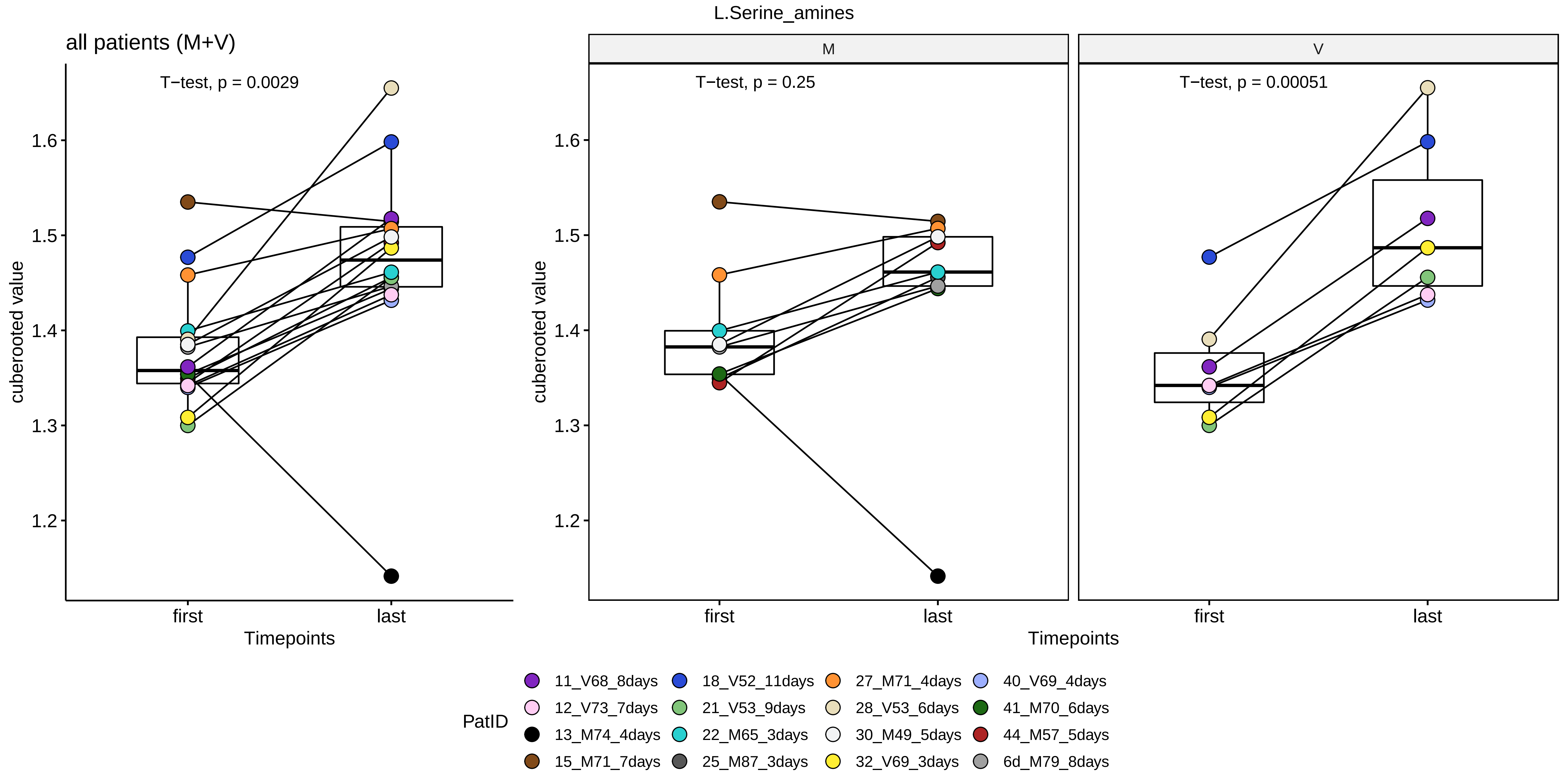
L.Serine_amines
all patients (M+V)
T−test, p = 0.0029
M
T−test, p = 0.25
V
T−test, p = 0.00051
cuberooted value
1.2
1.3
1.4
1.5
1.6
first
last
Timepoints
PatID
11_V68_8days
12_V73_7days
13_M74_4days
15_M71_7days
18_V52_11days
21_V53_9days
22_M65_3days
25_M87_3days
27_M71_4days
28_V53_6days
30_M49_5days
32_V69_3days
40_V69_4days
41_M70_6days
44_M57_5days
6d_M79_8days

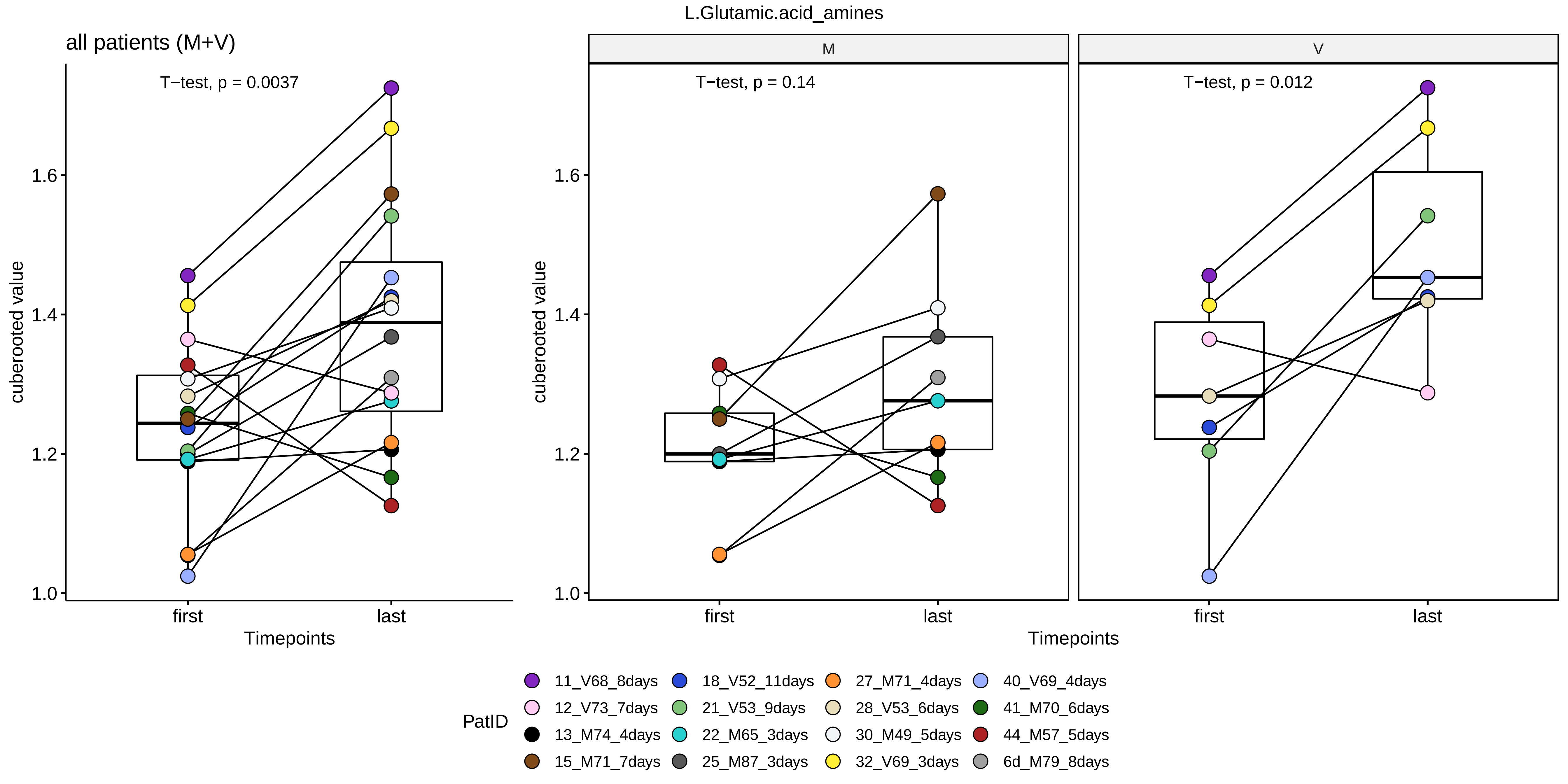

L.Glutamic.acid_amines
all patients (M+V)
T−test, p = 0.0037
M
T−test, p = 0.14
V
T−test, p = 0.012
cuberooted value
1.0
1.2
1.4
1.6
first
last
Timepoints
PatID
11_V68_8days
12_V73_7days
13_M74_4days
15_M71_7days
18_V52_11days
21_V53_9days
22_M65_3days
25_M87_3days
27_M71_4days
28_V53_6days
30_M49_5days
32_V69_3days
40_V69_4days
41_M70_6days
44_M57_5days
6d_M79_8days

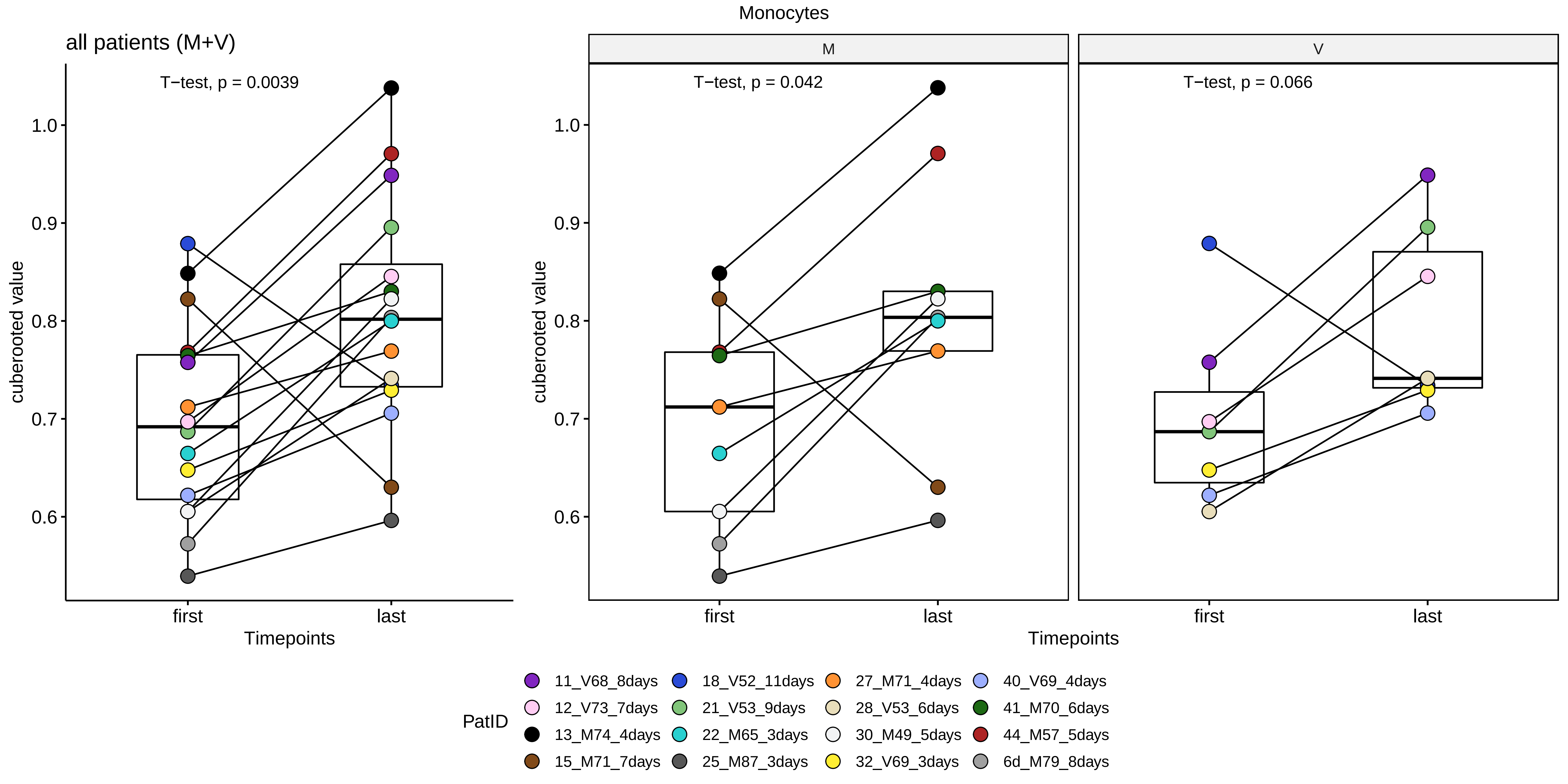

Monocytes
all patients (M+V)
T−test, p = 0.0039
M
T−test, p = 0.042
V
T−test, p = 0.066
cuberooted value
1.0
0.9
0.8
0.7
0.6
first
last
Timepoints
PatID
11_V68_8days
12_V73_7days
13_M74_4days
15_M71_7days
18_V52_11days
21_V53_9days
22_M65_3days
25_M87_3days
27_M71_4days
28_V53_6days
30_M49_5days
32_V69_3days
40_V69_4days
41_M70_6days
44_M57_5days
6d_M79_8days

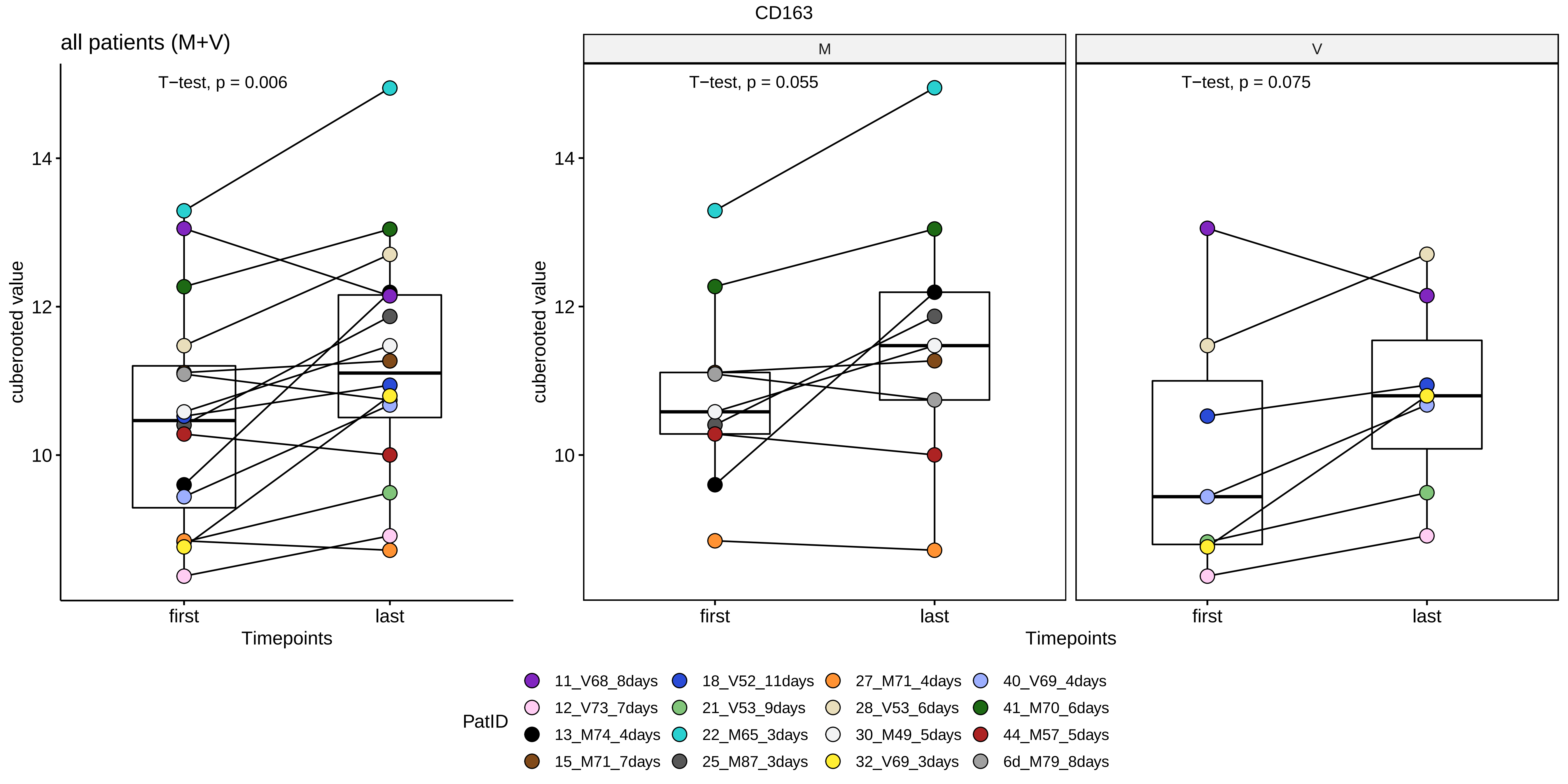

CD163
all patients (M+V)
T−test, p = 0.006
M
T−test, p = 0.055
V
T−test, p = 0.075
cuberooted value
14
12
10
first
last
Timepoints
PatID
11_V68_8days
12_V73_7days
13_M74_4days
15_M71_7days
18_V52_11days
21_V53_9days
22_M65_3days
25_M87_3days
27_M71_4days
28_V53_6days
30_M49_5days
32_V69_3days
40_V69_4days
41_M70_6days
44_M57_5days
6d_M79_8days

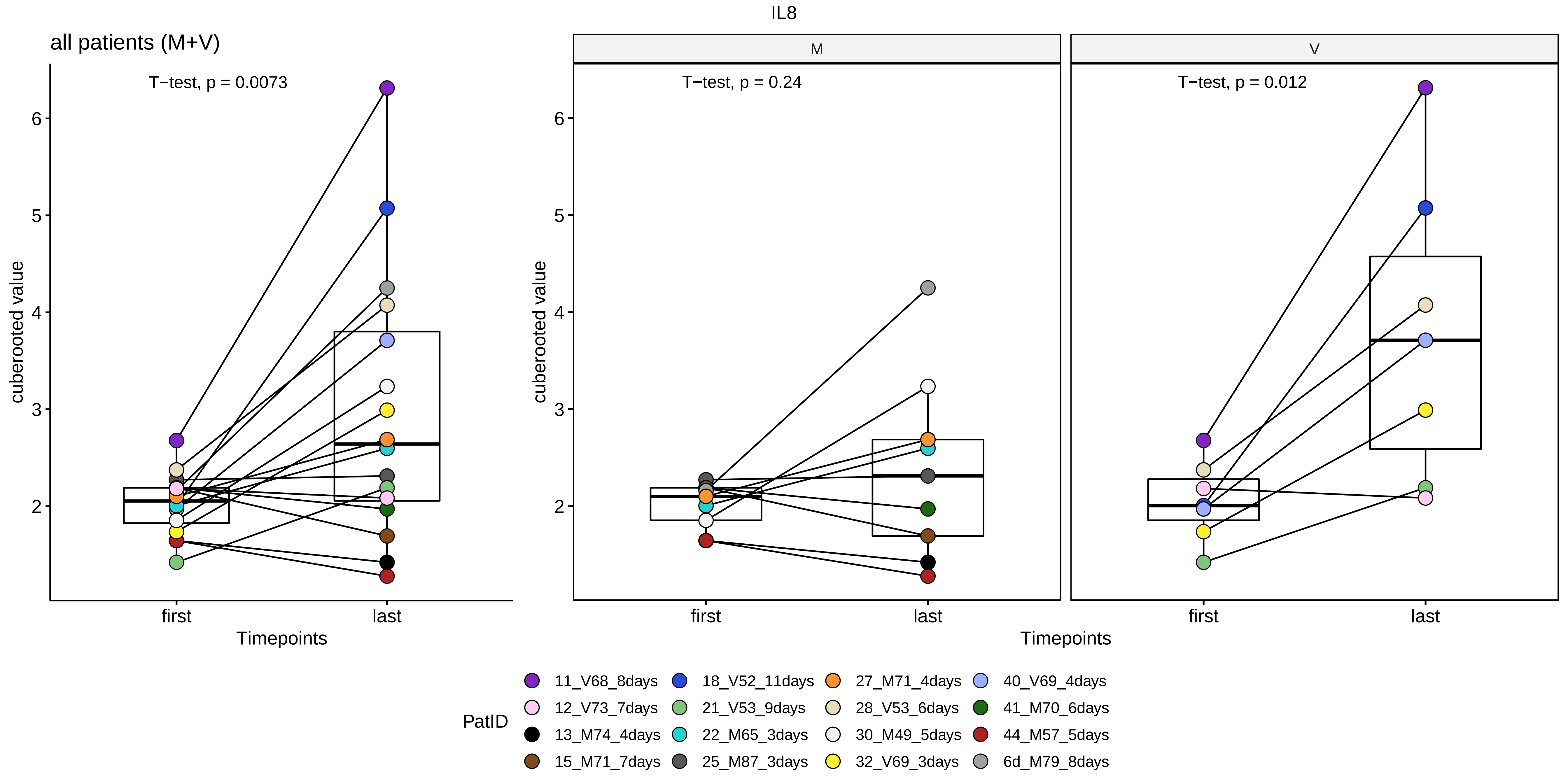
IL8
all patients (M+V)
T−test, p = 0.0073
M
T−test, p = 0.24
V
T−test, p = 0.012
cuberooted value
6
5
4
3
2
first
last
Timepoints
PatID
11_V68_8days
12_V73_7days
13_M74_4days
15_M71_7days
18_V52_11days
21_V53_9days
22_M65_3days
25_M87_3days
27_M71_4days
28_V53_6days
30_M49_5days
32_V69_3days
40_V69_4days
41_M70_6days
44_M57_5days
6d_M79_8days

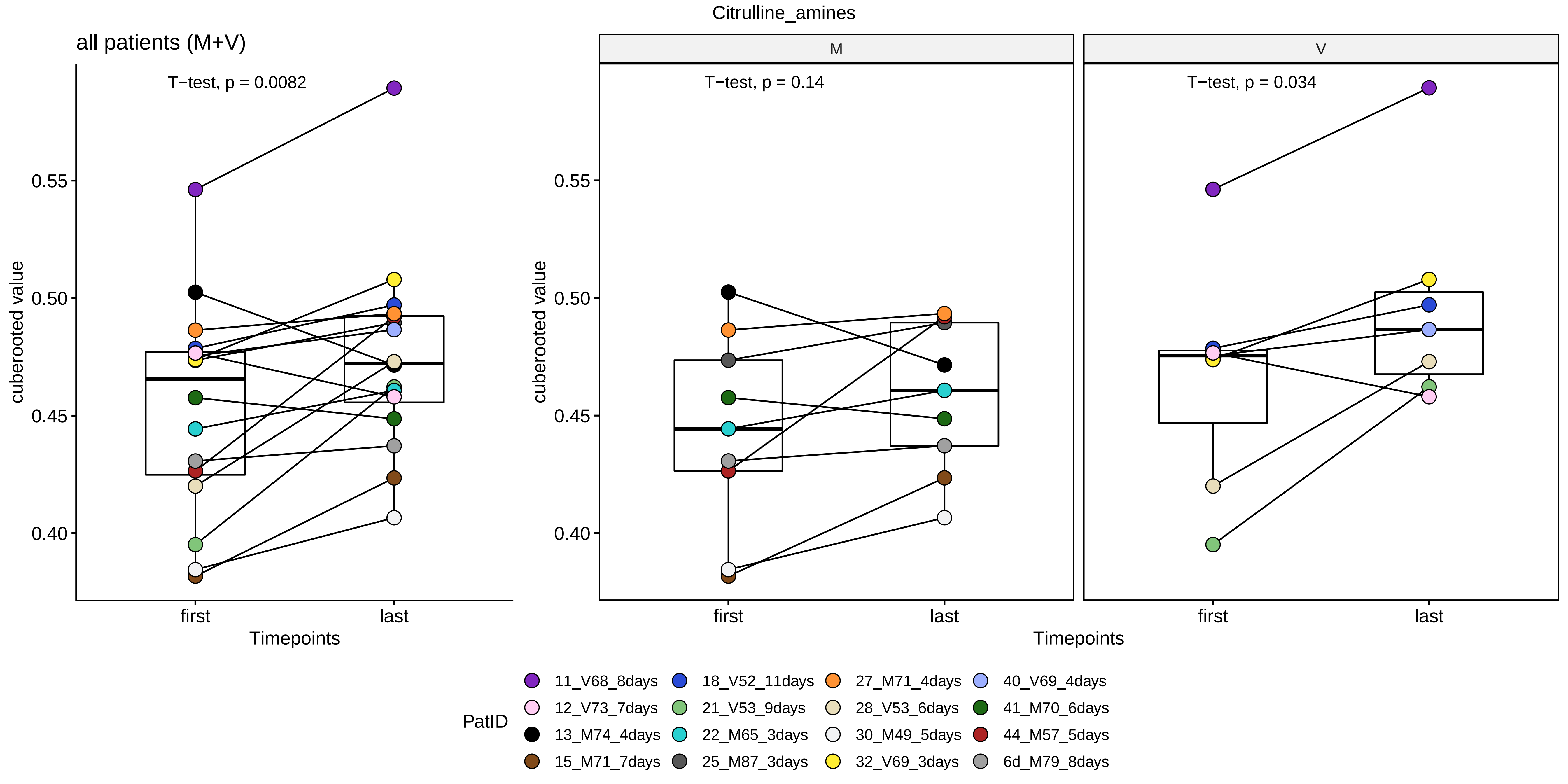
Citrulline_amines
all patients (M+V)
T−test, p = 0.0082
M
T−test, p = 0.14
V
T−test, p = 0.034
cuberooted value
0.55
0.50
0.45
0.40
first
last
Timepoints
PatID
11_V68_8days
12_V73_7days
13_M74_4days
15_M71_7days
18_V52_11days
21_V53_9days
22_M65_3days
25_M87_3days
27_M71_4days
28_V53_6days
30_M49_5days
32_V69_3days
40_V69_4days
41_M70_6days
44_M57_5days
6d_M79_8days

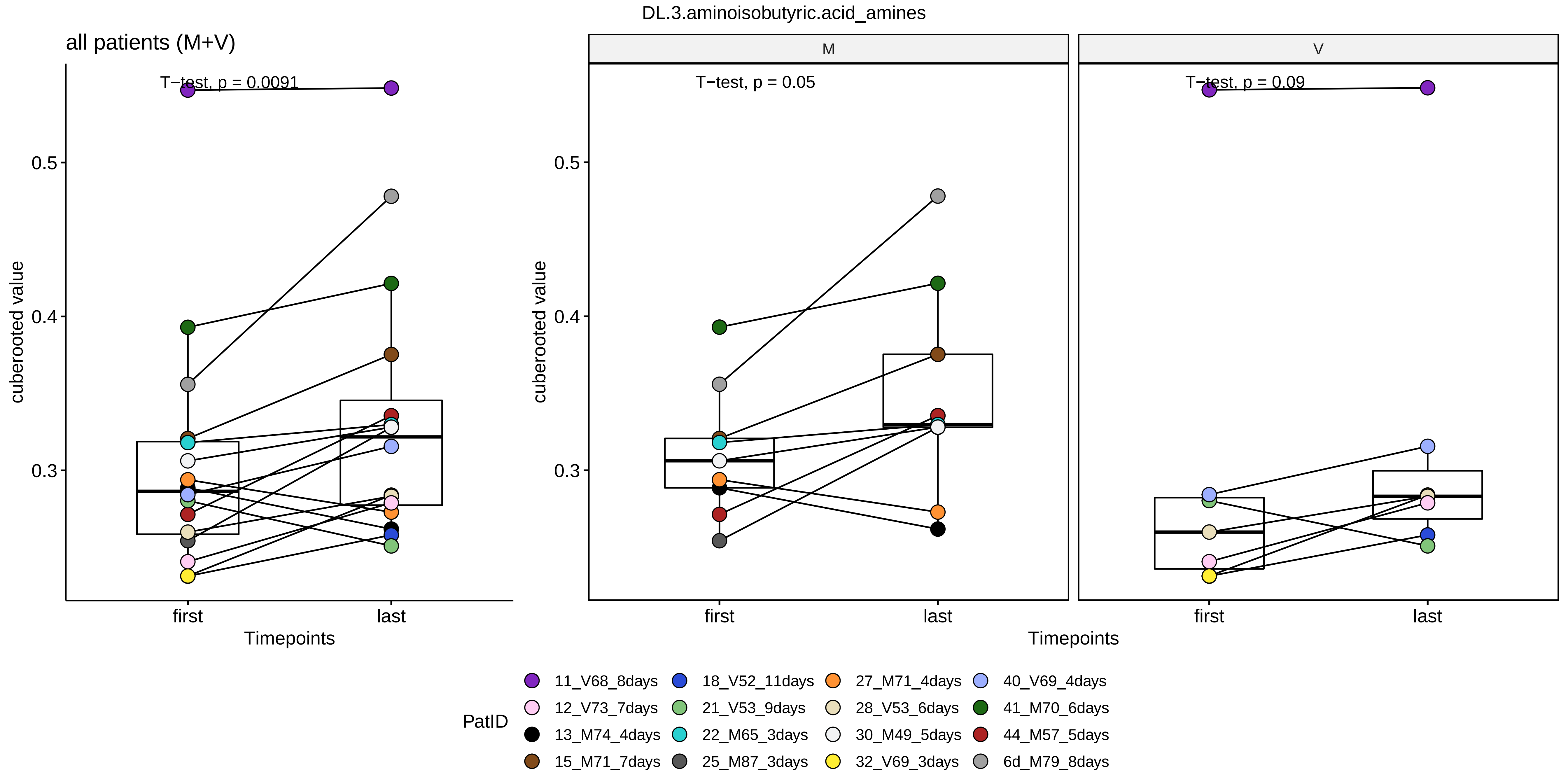
DL.3.aminoisobutyric.acid_amines
all patients (M+V)
T−test, p = 0.0091
M
T−test, p = 0.05
V
T−test, p = 0.09
cuberooted value
0.5
0.4
0.3
first
last
Timepoints
PatID
11_V68_8days
12_V73_7days
13_M74_4days
15_M71_7days
18_V52_11days
21_V53_9days
22_M65_3days
25_M87_3days
27_M71_4days
28_V53_6days
30_M49_5days
32_V69_3days
40_V69_4days
41_M70_6days
44_M57_5days
6d_M79_8days

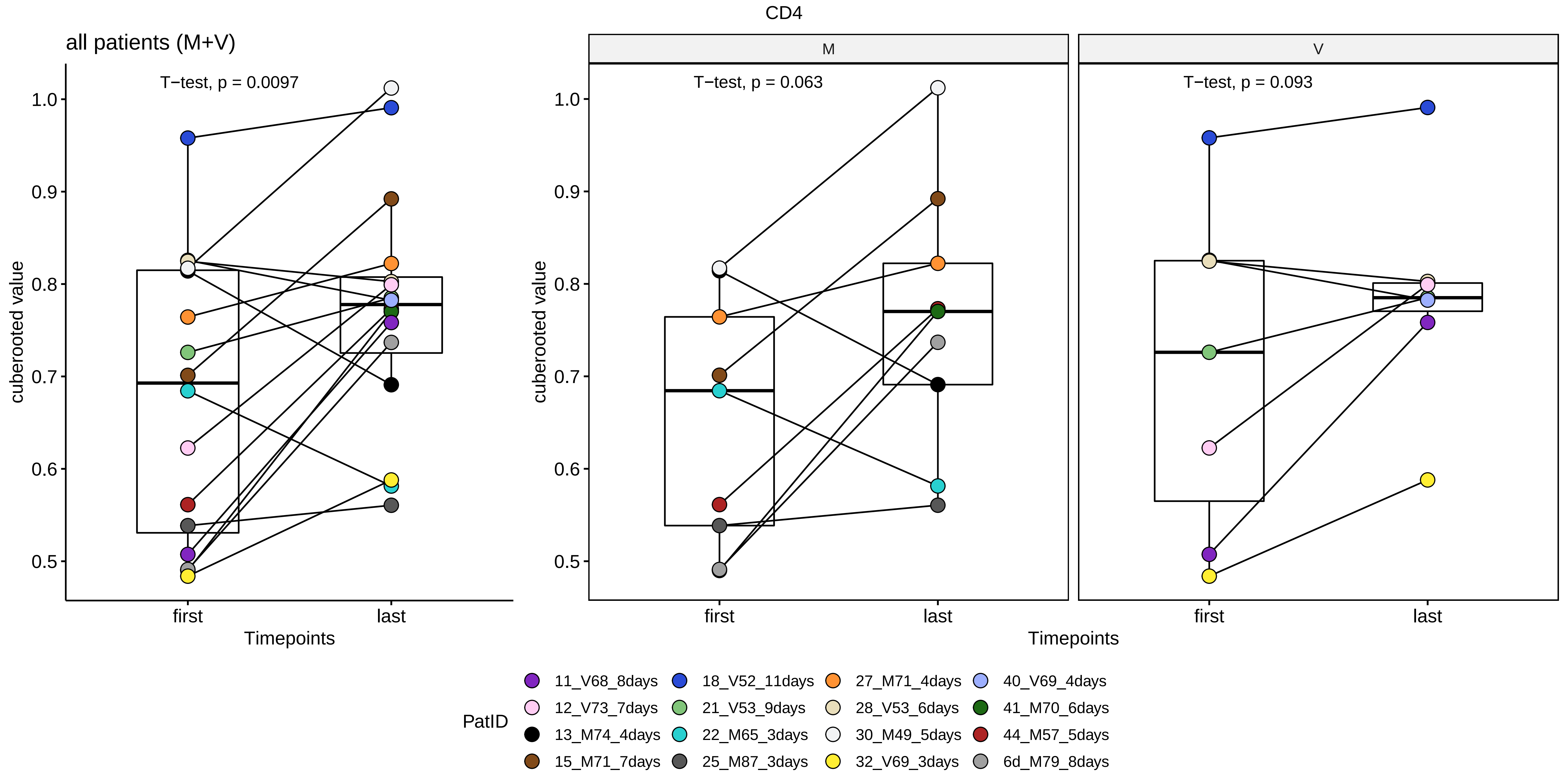

CD4
all patients (M+V)
T−test, p = 0.0097
M
T−test, p = 0.063
V
T−test, p = 0.093
cuberooted value
1.0
0.9
0.8
0.7
0.6
0.5
first
last
Timepoints
PatID
11_V68_8days
12_V73_7days
13_M74_4days
15_M71_7days
18_V52_11days
21_V53_9days
22_M65_3days
25_M87_3days
27_M71_4days
28_V53_6days
30_M49_5days
32_V69_3days
40_V69_4days
41_M70_6days
44_M57_5days
6d_M79_8days

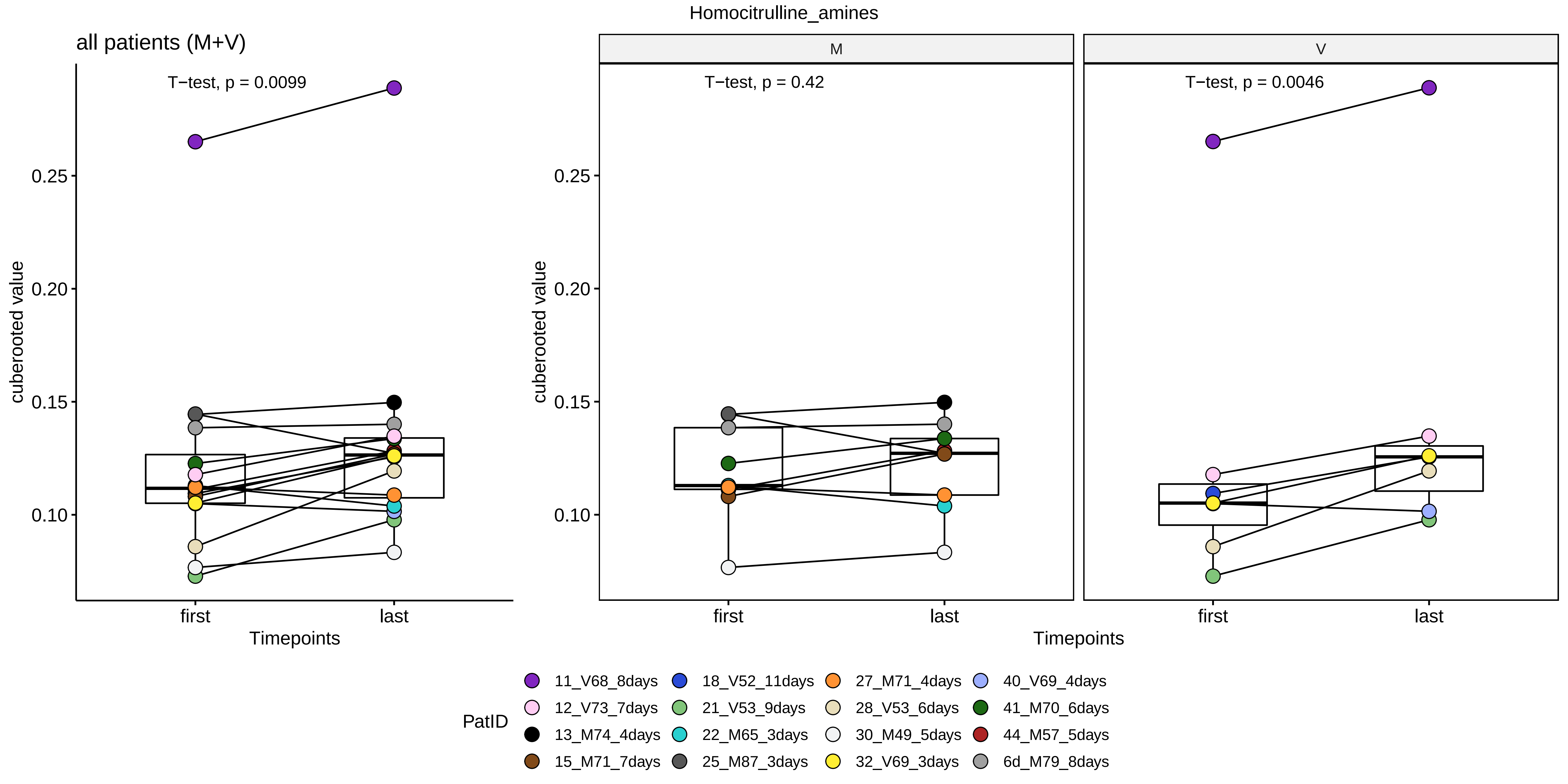

Homocitrulline_amines
all patients (M+V)
T−test, p = 0.0099
M
T−test, p = 0.42
V
T−test, p = 0.0046
cuberooted value
0.25
0.20
0.15
0.10
first
last
Timepoints
PatID
11_V68_8days
12_V73_7days
13_M74_4days
15_M71_7days
18_V52_11days
21_V53_9days
22_M65_3days
25_M87_3days
27_M71_4days
28_V53_6days
30_M49_5days
32_V69_3days
40_V69_4days
41_M70_6days
44_M57_5days
6d_M79_8days

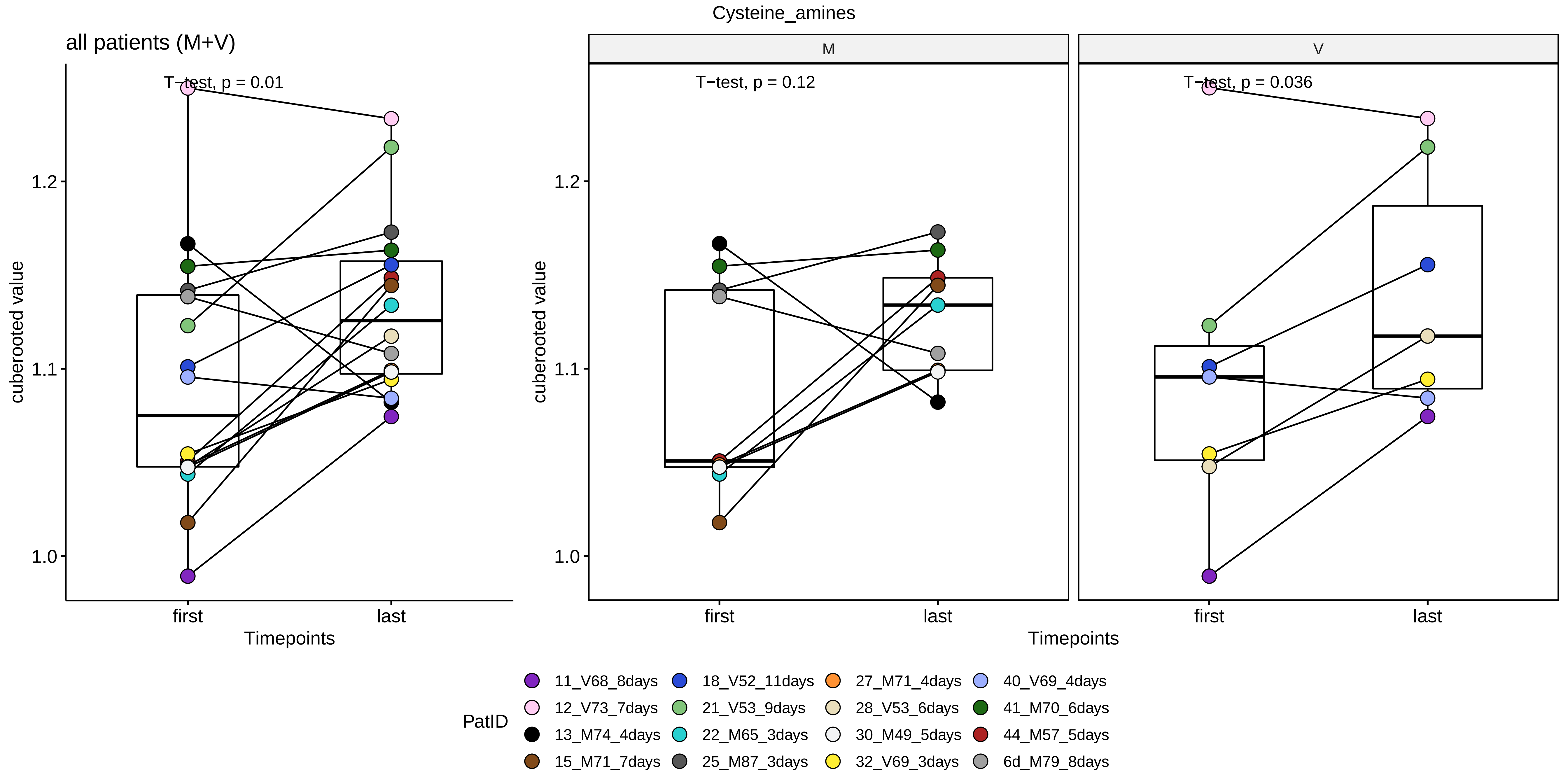

Cysteine_amines
all patients (M+V)
T−test, p = 0.01
M
T−test, p = 0.12
V
T−test, p = 0.036
cuberooted value
1.2
1.1
1.0
first
last
Timepoints
PatID
11_V68_8days
12_V73_7days
13_M74_4days
15_M71_7days
18_V52_11days
21_V53_9days
22_M65_3days
25_M87_3days
27_M71_4days
28_V53_6days
30_M49_5days
32_V69_3days
40_V69_4days
41_M70_6days
44_M57_5days
6d_M79_8days

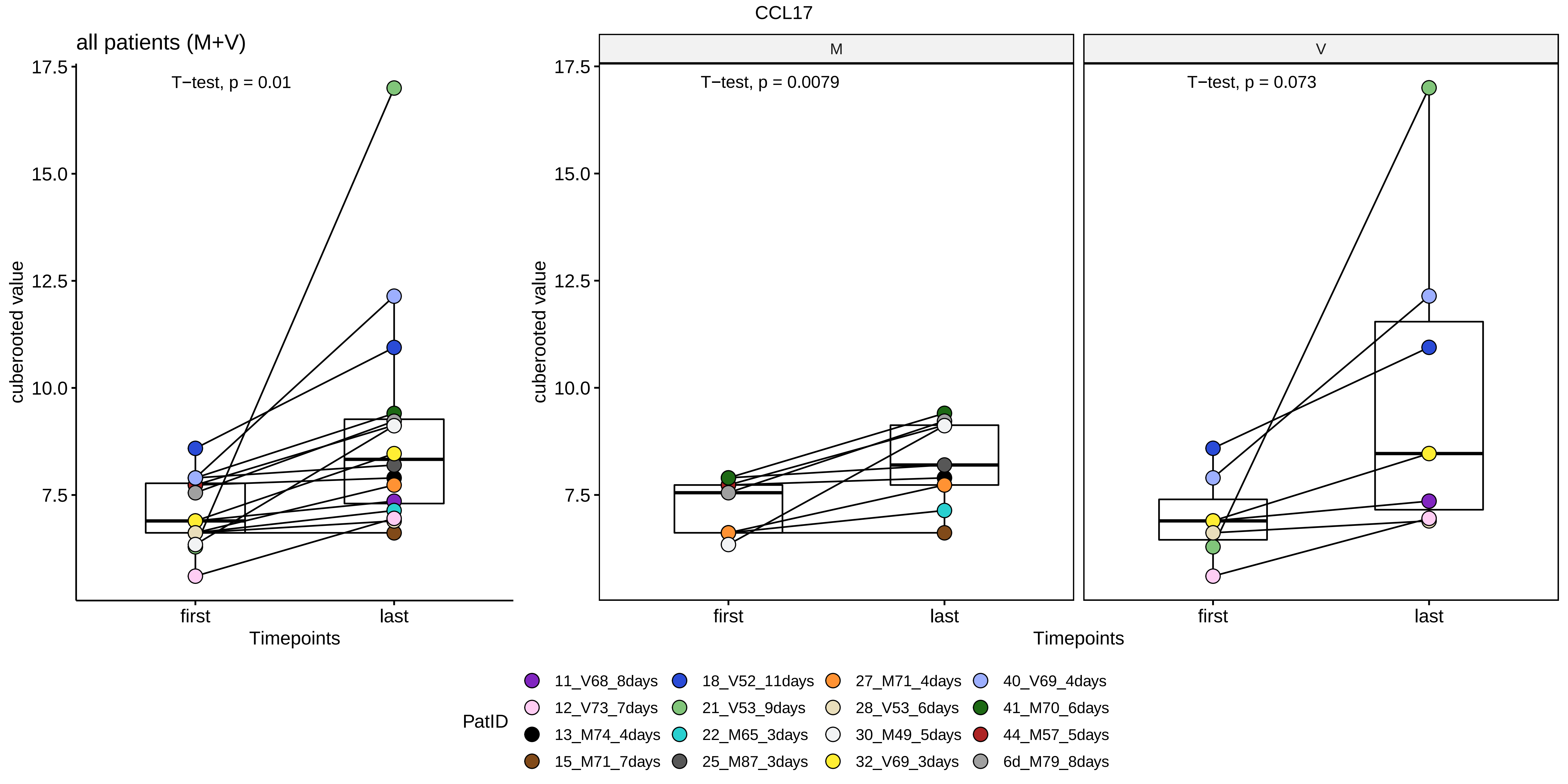
CCL17
all patients (M+V)
T−test, p = 0.01
M
T−test, p = 0.0079
V
T−test, p = 0.073
cuberooted value
17.5
15.0
12.5
10.0
7.5
first
last
Timepoints
PatID
11_V68_8days
12_V73_7days
13_M74_4days
15_M71_7days
18_V52_11days
21_V53_9days
22_M65_3days
25_M87_3days
27_M71_4days
28_V53_6days
30_M49_5days
32_V69_3days
40_V69_4days
41_M70_6days
44_M57_5days
6d_M79_8days

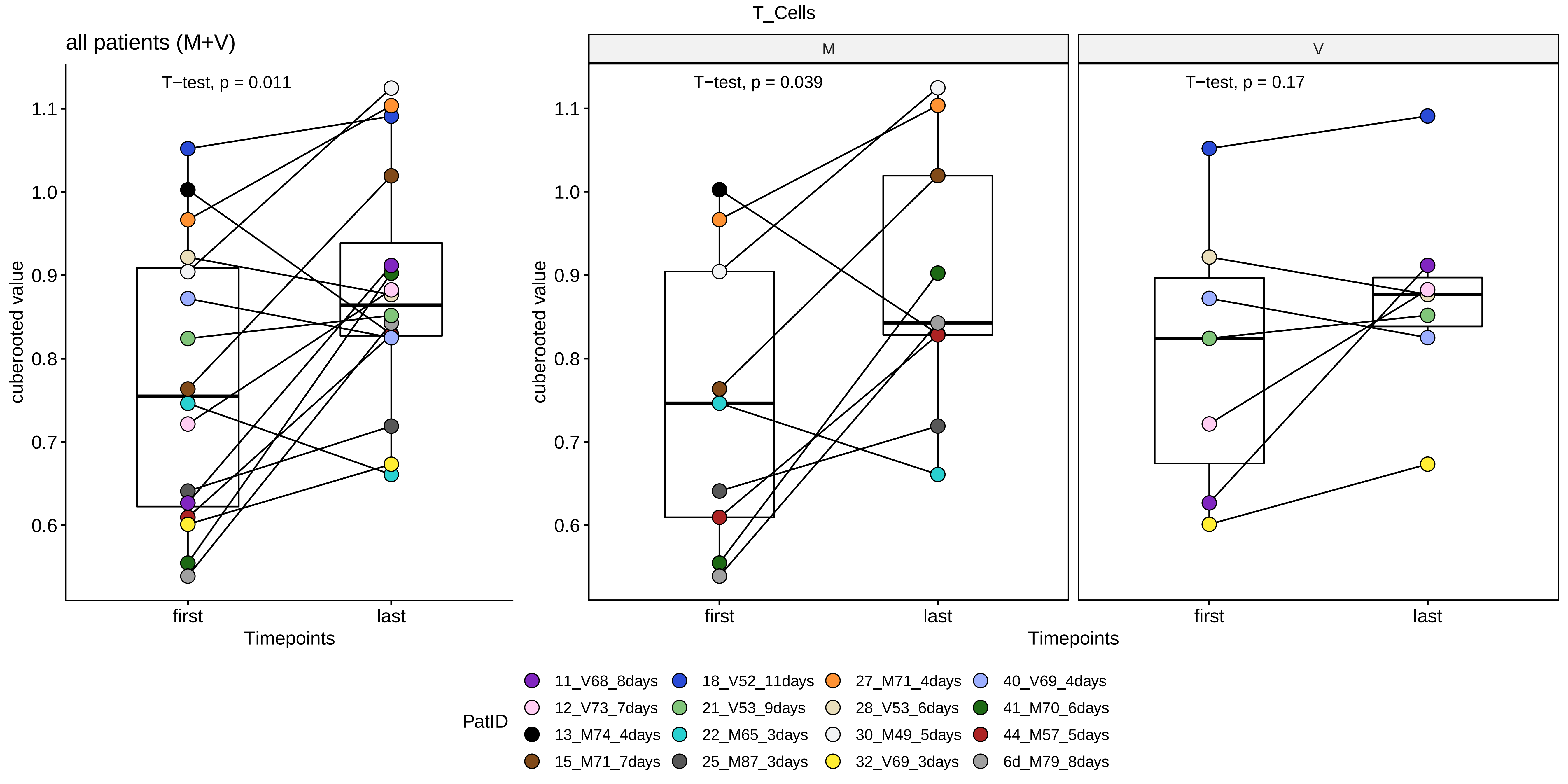

T_Cells
all patients (M+V)
T−test, p = 0.011
M
T−test, p = 0.039
V
T−test, p = 0.17
cuberooted value
1.1
1.0
0.9
0.8
0.7
0.6
first
last
Timepoints
PatID
11_V68_8days
12_V73_7days
13_M74_4days
15_M71_7days
18_V52_11days
21_V53_9days
22_M65_3days
25_M87_3days
27_M71_4days
28_V53_6days
30_M49_5days
32_V69_3days
40_V69_4days
41_M70_6days
44_M57_5days
6d_M79_8days

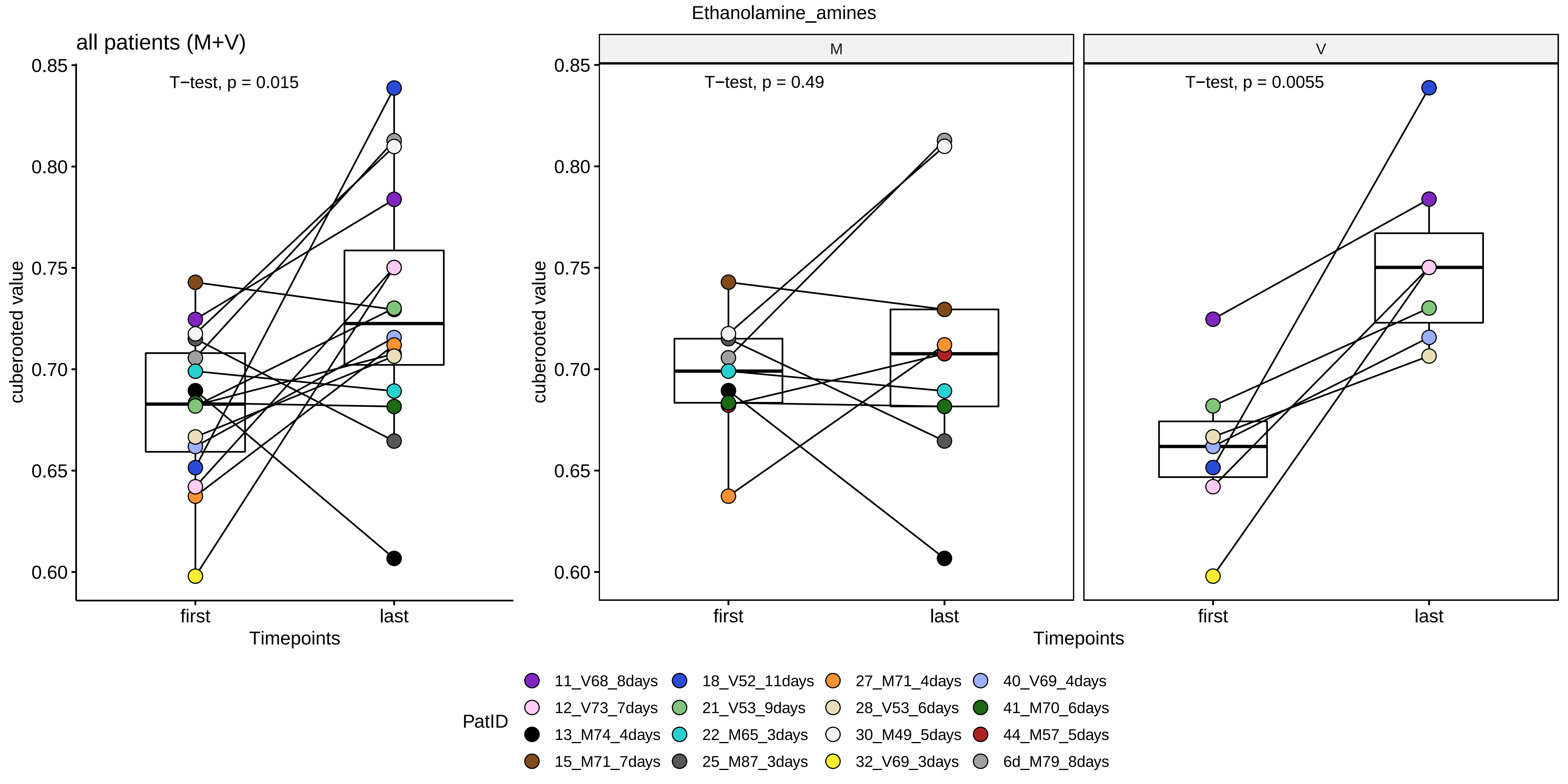

Ethanolamine_amines
all patients (M+V)
T−test, p = 0.015
M
T−test, p = 0.49
V
T−test, p = 0.0055
0.85
0.80
0.75
0.70
0.65
0.60
cuberooted value
first
last
Timepoints
PatID
11_V68_8days
12_V73_7days
13_M74_4days
15_M71_7days
18_V52_11days
21_V53_9days
22_M65_3days
25_M87_3days
27_M71_4days
28_V53_6days
30_M49_5days
32_V69_3days
40_V69_4days
41_M70_6days
44_M57_5days
6d_M79_8days

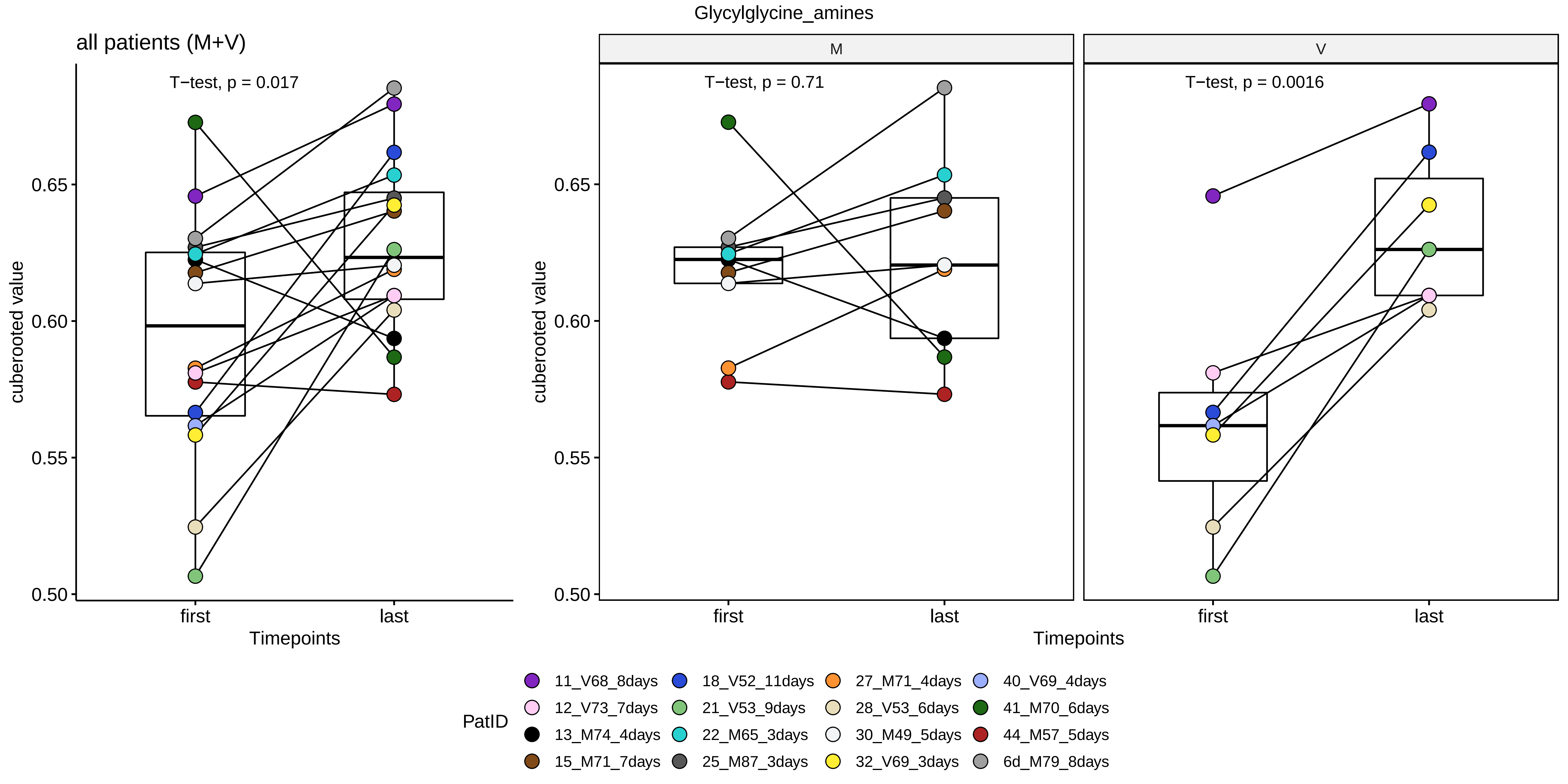

Glycylglycine_amines
all patients (M+V)
T−test, p = 0.017
M
T−test, p = 0.71
V
T−test, p = 0.0016
cuberooted value
0.65
0.60
0.55
0.50
first
last
Timepoints
PatID
11_V68_8days
12_V73_7days
13_M74_4days
15_M71_7days
18_V52_11days
21_V53_9days
22_M65_3days
25_M87_3days
27_M71_4days
28_V53_6days
30_M49_5days
32_V69_3days
40_V69_4days
41_M70_6days
44_M57_5days
6d_M79_8days

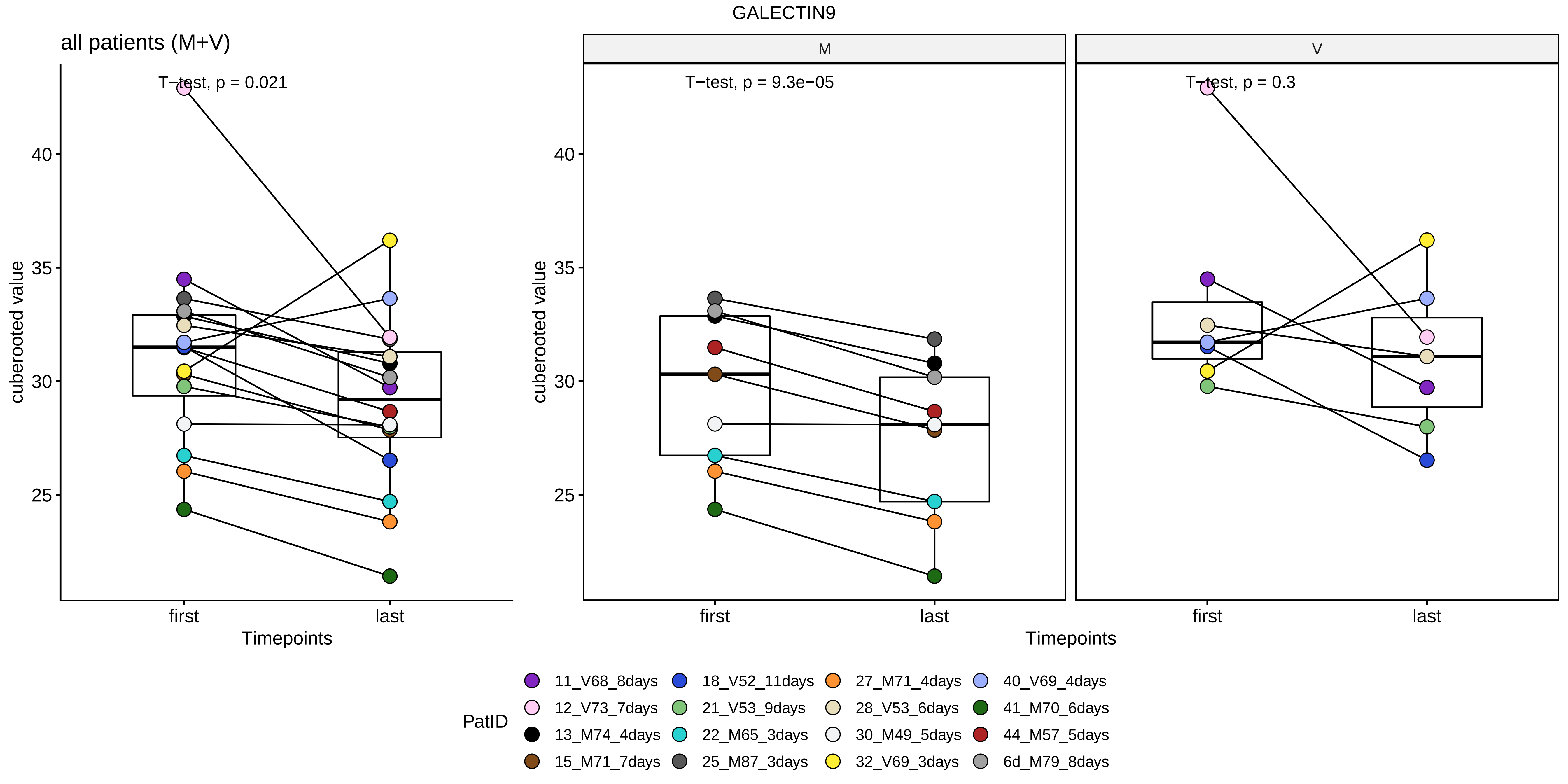
GALECTIN9
all patients (M+V)
T−test, p = 0.021
M
T−test, p = 9.3e−05
V
T−test, p = 0.3
cuberooted value
40
35
30
25
first
last
Timepoints
PatID
11_V68_8days
12_V73_7days
13_M74_4days
15_M71_7days
18_V52_11days
21_V53_9days
22_M65_3days
25_M87_3days
27_M71_4days
28_V53_6days
30_M49_5days
32_V69_3days
40_V69_4days
41_M70_6days
44_M57_5days
6d_M79_8days

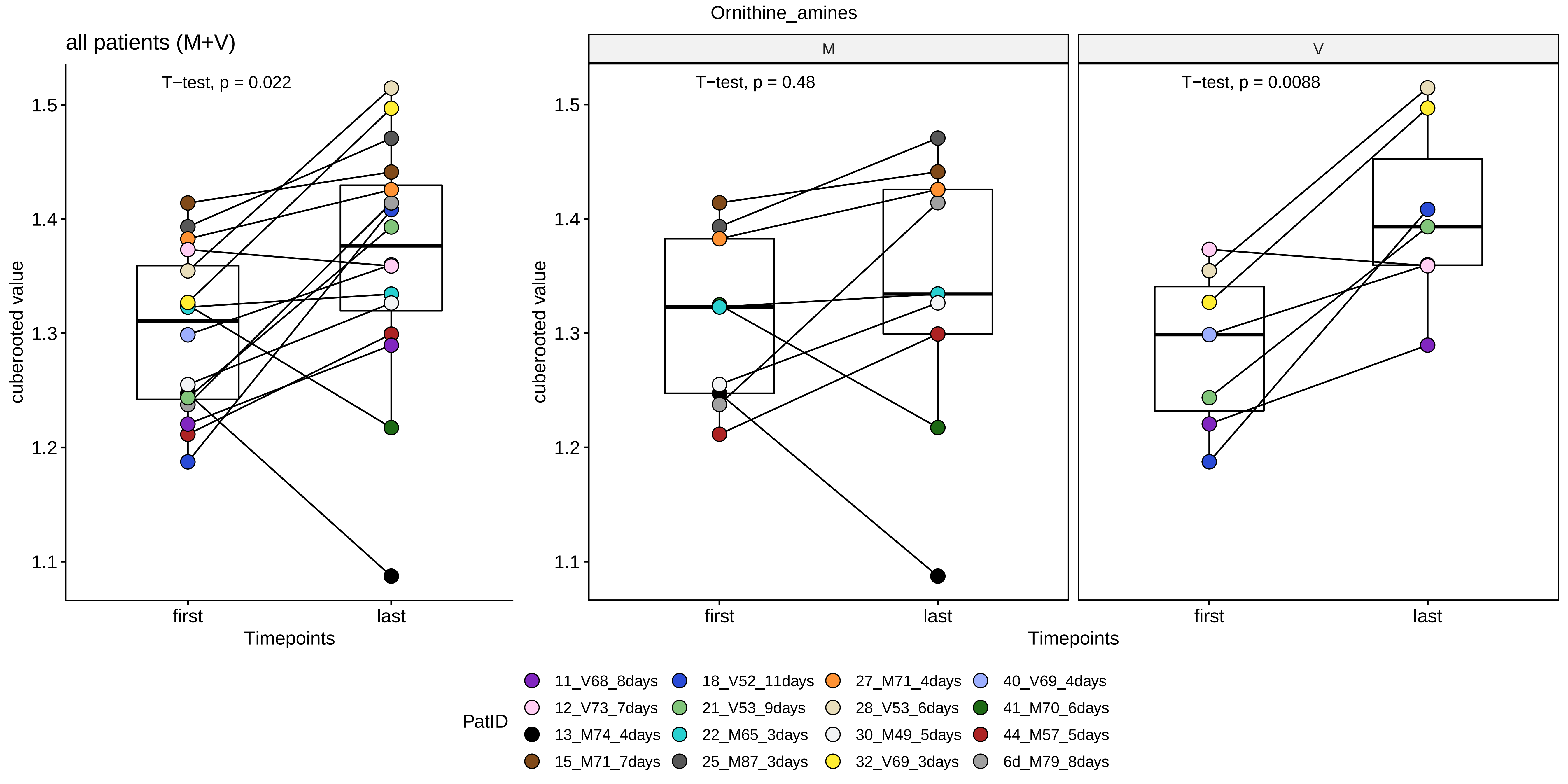

Ornithine_amines
all patients (M+V)
T−test, p = 0.022
M
T−test, p = 0.48
V
T−test, p = 0.0088
cuberooted value
1.1
1.2
1.3
1.4
1.5
first
last
Timepoints
PatID
11_V68_8days
12_V73_7days
13_M74_4days
15_M71_7days
18_V52_11days
21_V53_9days
22_M65_3days
25_M87_3days
27_M71_4days
28_V53_6days
30_M49_5days
32_V69_3days
40_V69_4days
41_M70_6days
44_M57_5days
6d_M79_8days

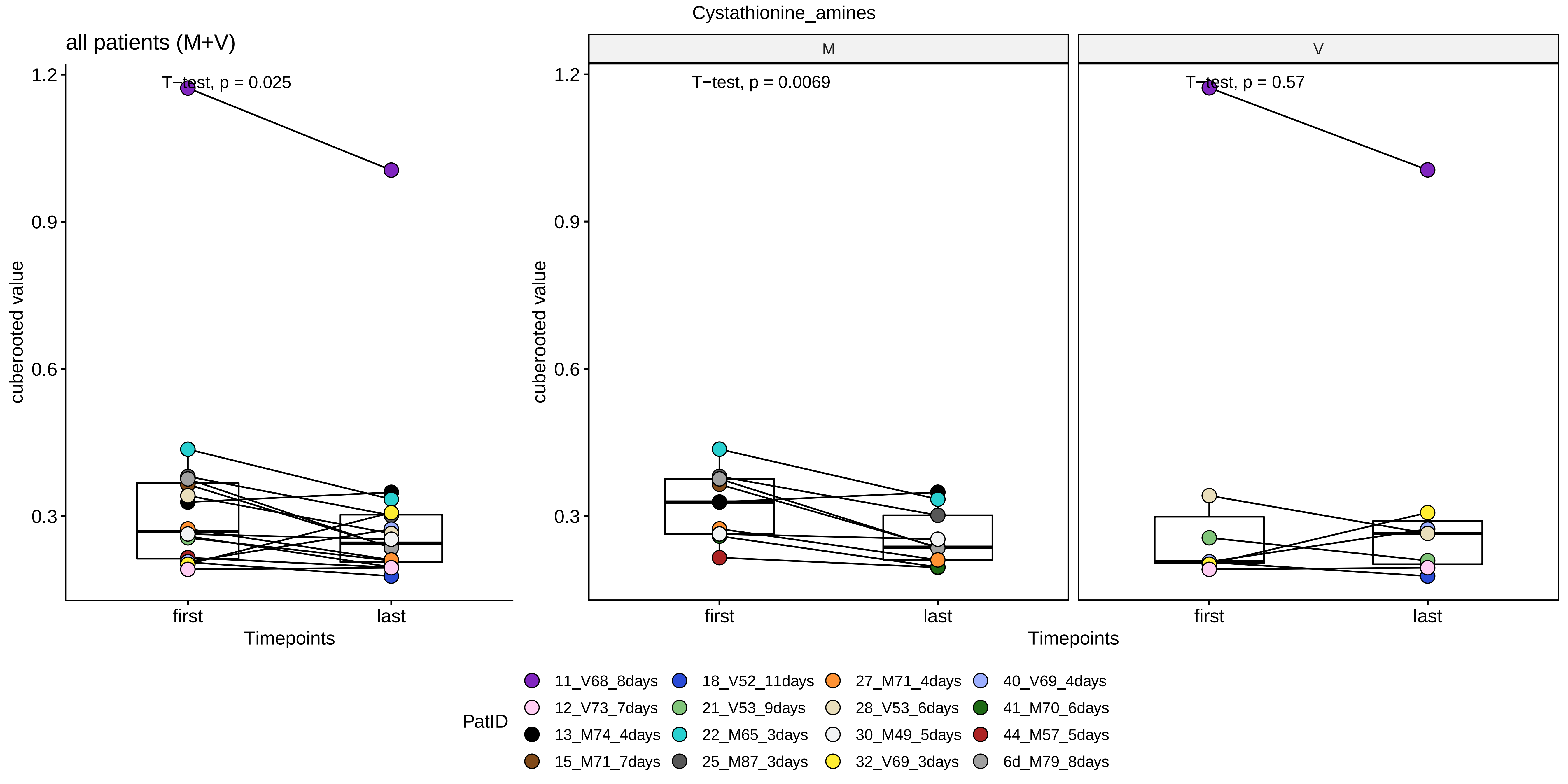

Cystathionine_amines
all patients (M+V)
T−test, p = 0.025
T−test, p = 0.0069
T−test, p = 0.57
M
V
1.2
0.9
0.6
0.3
cuberooted value
first
last
Timepoints
PatID
11_V68_8days
12_V73_7days
13_M74_4days
15_M71_7days
18_V52_11days
21_V53_9days
22_M65_3days
25_M87_3days
27_M71_4days
28_V53_6days
30_M49_5days
32_V69_3days
40_V69_4days
41_M70_6days
44_M57_5days
6d_M79_8days

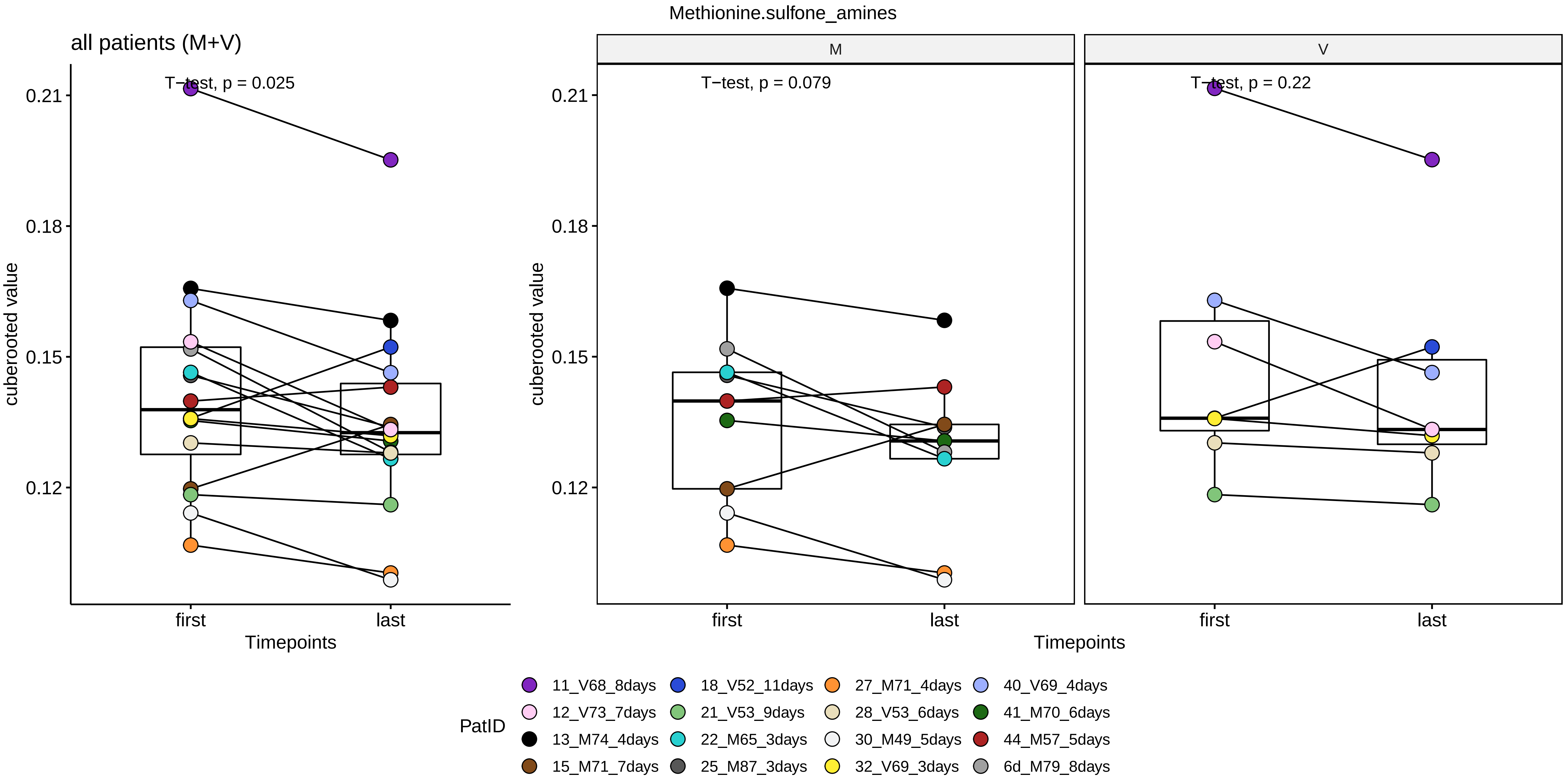
Methionine.sulfone_amines
all patients (M+V)
T−test, p = 0.025
M
T−test, p = 0.079
V
T−test, p = 0.22
0.21
0.18
0.15
0.12
cuberooted value
first
last
Timepoints
PatID
11_V68_8days
12_V73_7days
13_M74_4days
15_M71_7days
18_V52_11days
21_V53_9days
22_M65_3days
25_M87_3days
27_M71_4days
28_V53_6days
30_M49_5days
32_V69_3days
40_V69_4days
41_M70_6days
44_M57_5days
6d_M79_8days

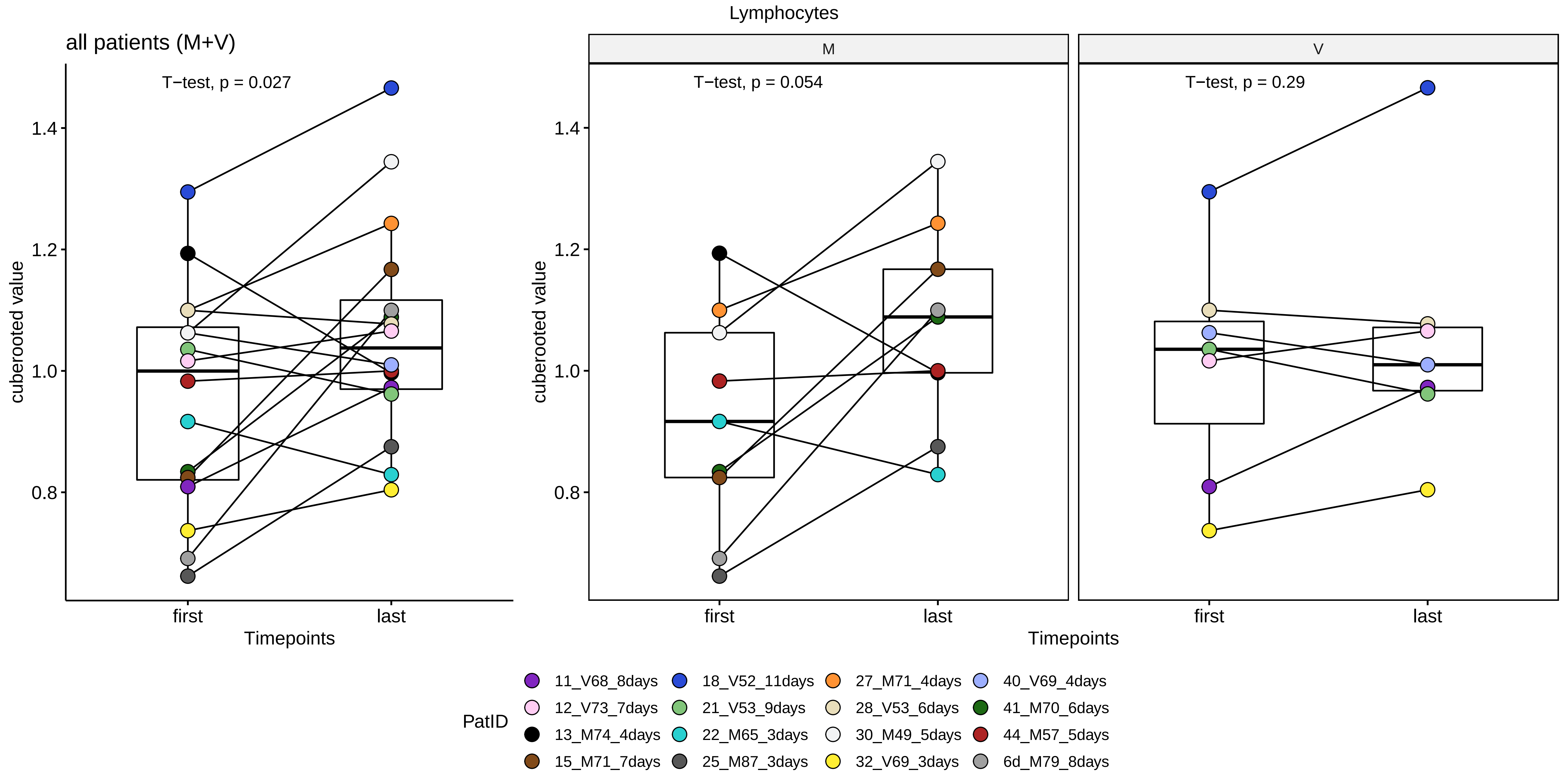
Lymphocytes
all patients (M+V)
T−test, p = 0.027
M
T−test, p = 0.054
V
T−test, p = 0.29
cuberooted value
1.4
1.2
1.0
0.8
first
last
Timepoints
PatID
11_V68_8days
12_V73_7days
13_M74_4days
15_M71_7days
18_V52_11days
21_V53_9days
22_M65_3days
25_M87_3days
27_M71_4days
28_V53_6days
30_M49_5days
32_V69_3days
40_V69_4days
41_M70_6days
44_M57_5days
6d_M79_8days

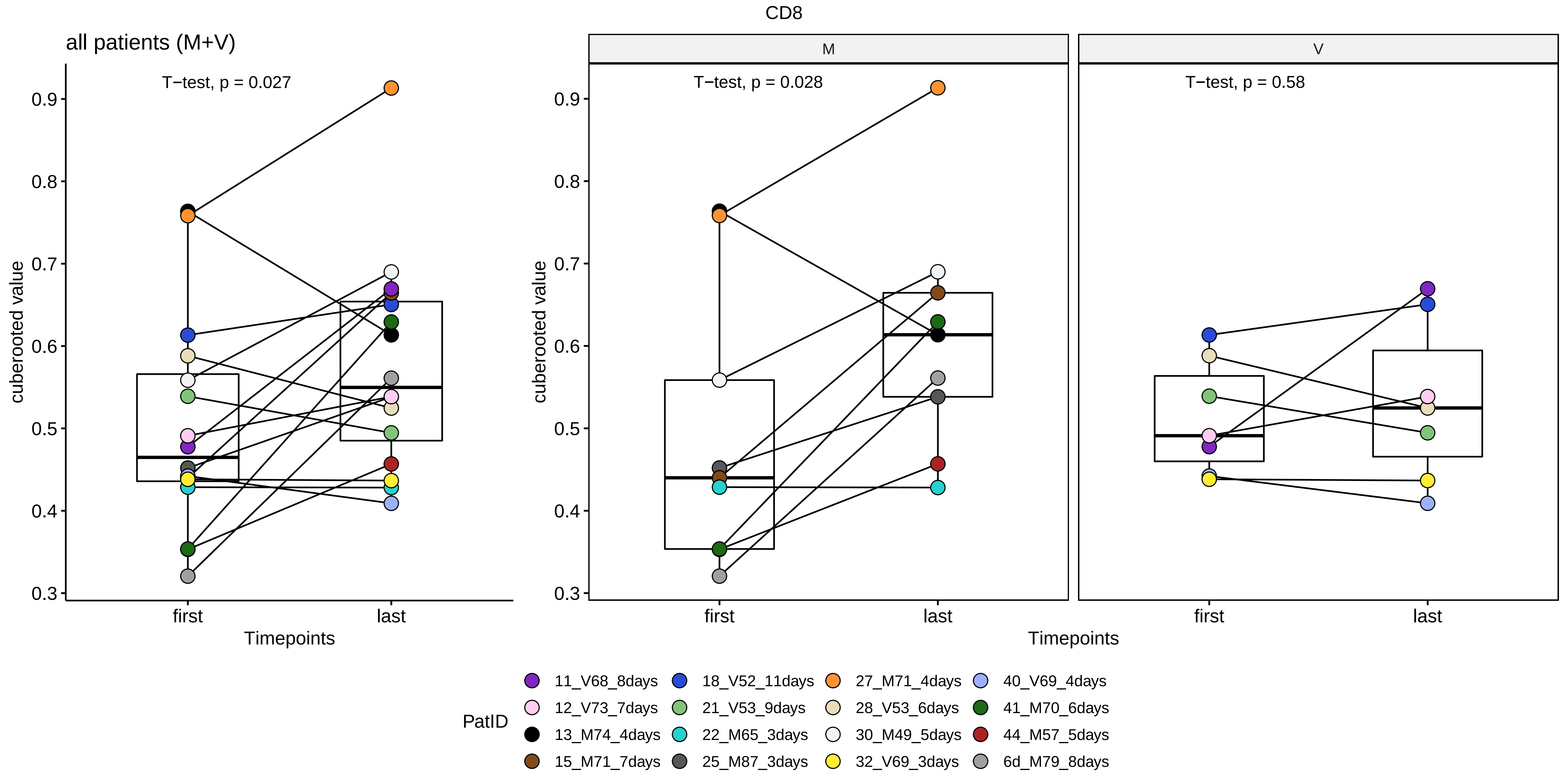

CD8
all patients (M+V)
T−test, p = 0.027
M
T−test, p = 0.028
V
T−test, p = 0.58
cuberooted value
0.3
0.4
0.5
0.6
0.7
0.8
0.9
first
last
Timepoints
PatID
11_V68_8days
12_V73_7days
13_M74_4days
15_M71_7days
18_V52_11days
21_V53_9days
22_M65_3days
25_M87_3days
27_M71_4days
28_V53_6days
30_M49_5days
32_V69_3days
40_V69_4days
41_M70_6days
44_M57_5days
6d_M79_8days

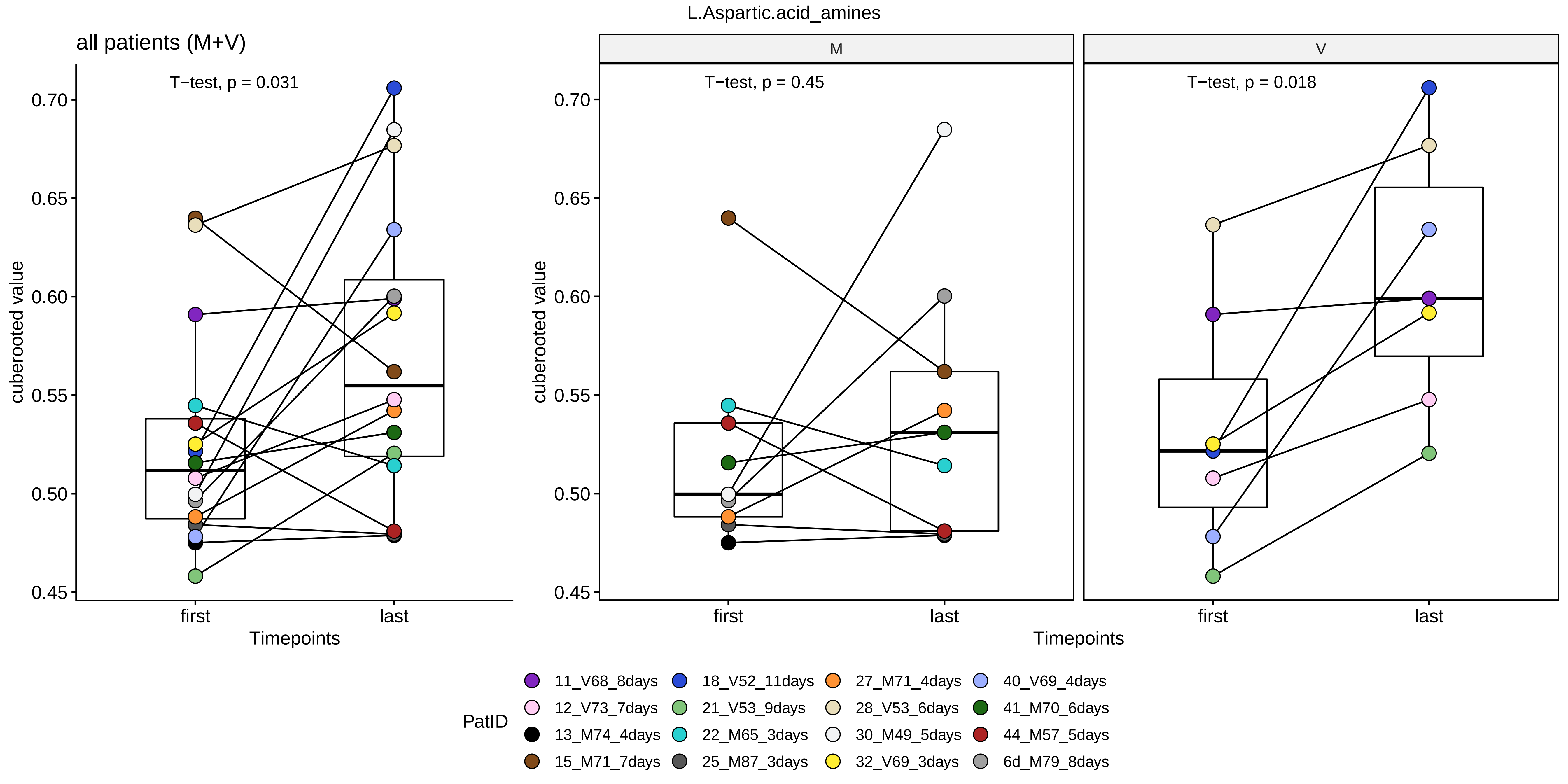

L.Aspartic.acid_amines
all patients (M+V)
T−test, p = 0.031
M
T−test, p = 0.45
V
T−test, p = 0.018
cuberooted value
0.45
0.50
0.55
0.60
0.65
0.70
first
last
Timepoints
PatID
11_V68_8days
12_V73_7days
13_M74_4days
15_M71_7days
18_V52_11days
21_V53_9days
22_M65_3days
25_M87_3days
27_M71_4days
28_V53_6days
30_M49_5days
32_V69_3days
40_V69_4days
41_M70_6days
44_M57_5days
6d_M79_8days

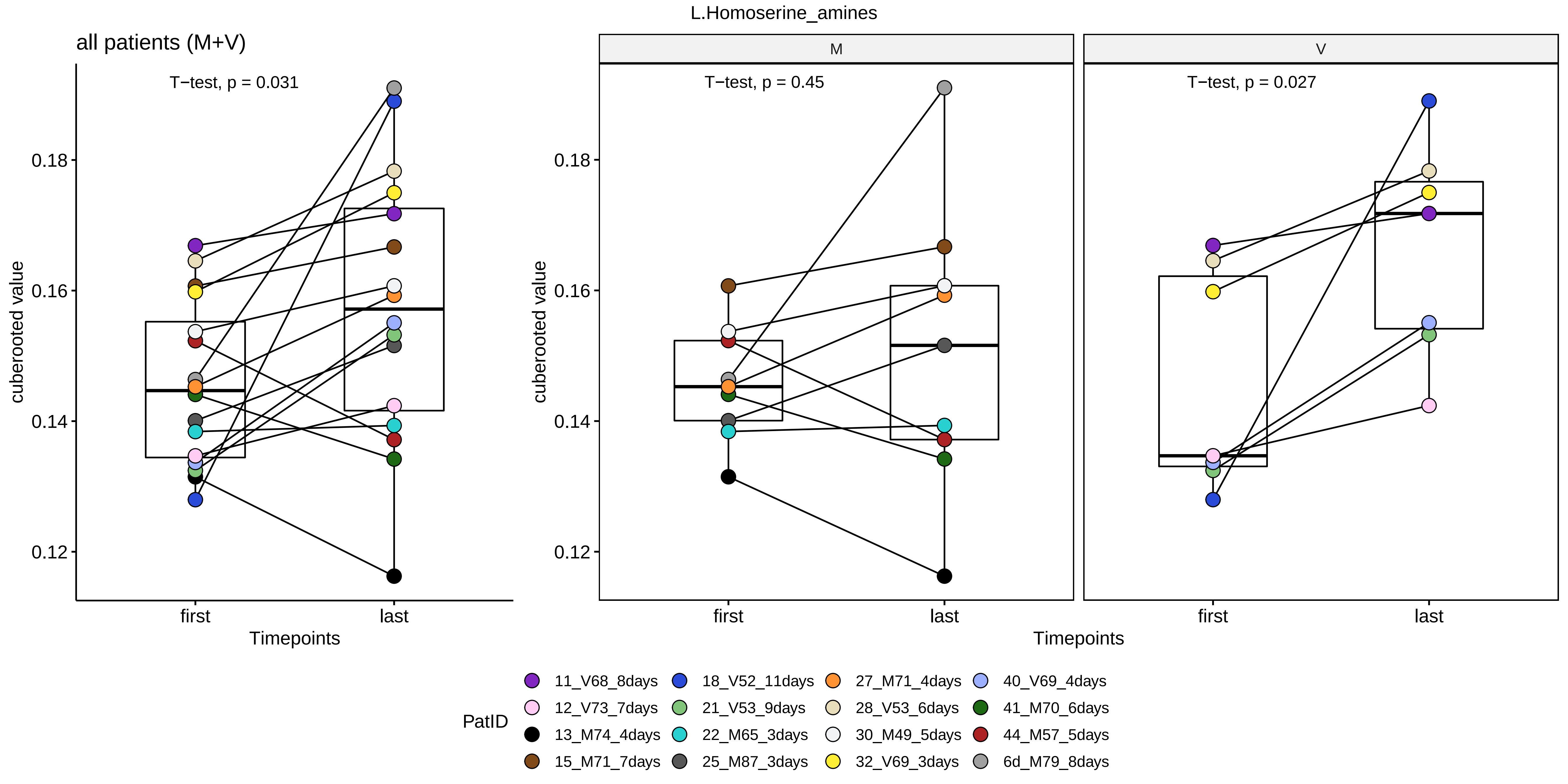

L.Homoserine_amines
all patients (M+V)
T−test, p = 0.031
M
T−test, p = 0.45
V
T−test, p = 0.027
cuberooted value
0.12
0.14
0.16
0.18
first
last
Timepoints
PatID
11_V68_8days
12_V73_7days
13_M74_4days
15_M71_7days
18_V52_11days
21_V53_9days
22_M65_3days
25_M87_3days
27_M71_4days
28_V53_6days
30_M49_5days
32_V69_3days
40_V69_4days
41_M70_6days
44_M57_5days
6d_M79_8days

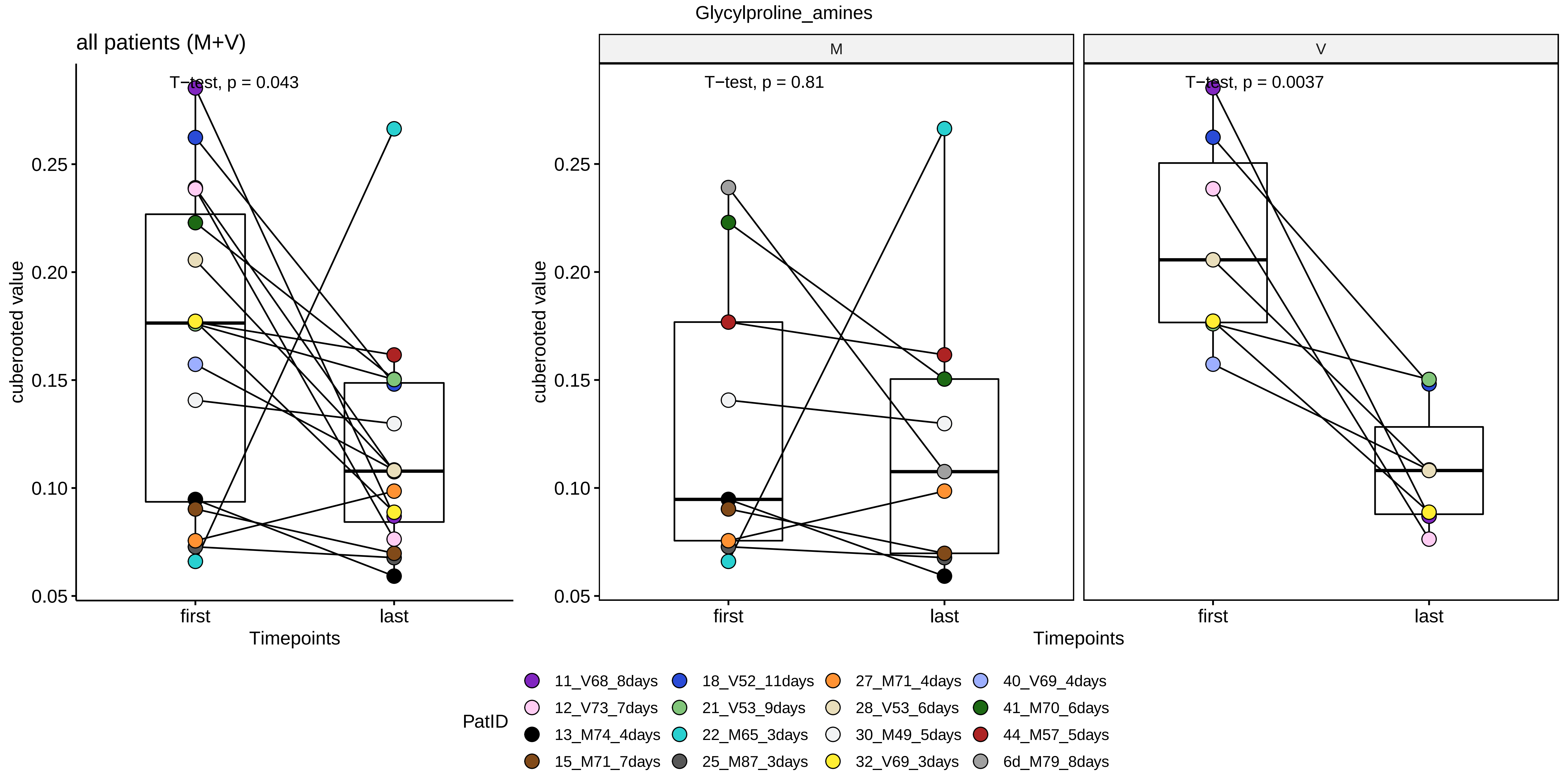

Glycylproline_amines
all patients (M+V)
T−test, p = 0.043
M
T−test, p = 0.81
V
T−test, p = 0.0037
cuberooted value
0.25
0.20
0.15
0.10
0.05
first
last
Timepoints
PatID
11_V68_8days
12_V73_7days
13_M74_4days
15_M71_7days
18_V52_11days
21_V53_9days
22_M65_3days
25_M87_3days
27_M71_4days
28_V53_6days
30_M49_5days
32_V69_3days
40_V69_4days
41_M70_6days
44_M57_5days
6d_M79_8days

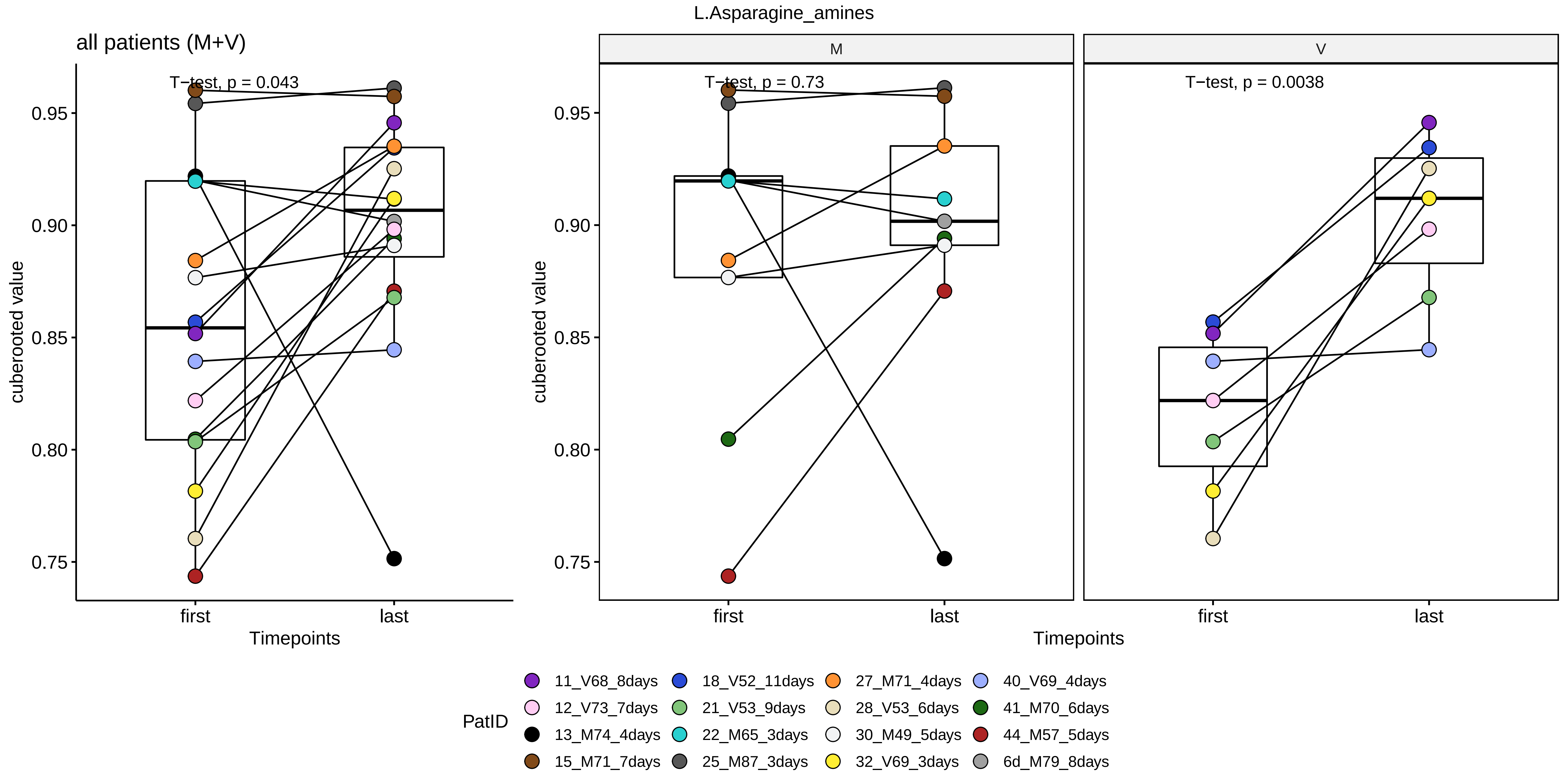

L.Asparagine_amines
all patients (M+V)
T−test, p = 0.043
M
T−test, p = 0.73
V
T−test, p = 0.0038
cuberooted value
0.95
0.90
0.85
0.80
0.75
first
last
Timepoints
PatID
11_V68_8days
12_V73_7days
13_M74_4days
15_M71_7days
18_V52_11days
21_V53_9days
22_M65_3days
25_M87_3days
27_M71_4days
28_V53_6days
30_M49_5days
32_V69_3days
40_V69_4days
41_M70_6days
44_M57_5days
6d_M79_8days

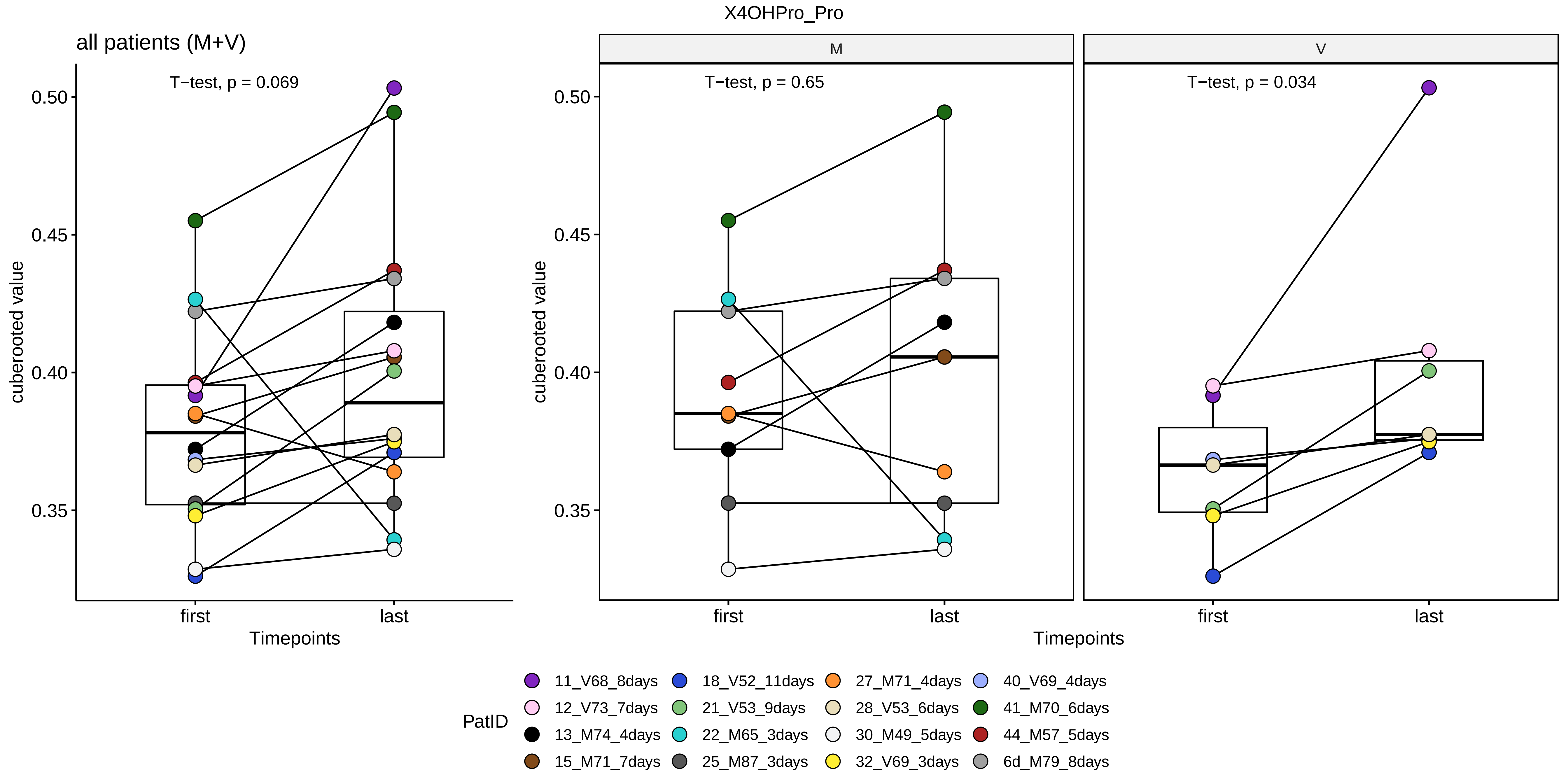

X4OHPro_Pro
all patients (M+V)
T−test, p = 0.069
M
T−test, p = 0.65
V
T−test, p = 0.034
0.50
0.45
0.40
0.35
cuberooted value
first
last
Timepoints
PatID
11_V68_8days
12_V73_7days
13_M74_4days
15_M71_7days
18_V52_11days
21_V53_9days
22_M65_3days
25_M87_3days
27_M71_4days
28_V53_6days
30_M49_5days
32_V69_3days
40_V69_4days
41_M70_6days
44_M57_5days
6d_M79_8days

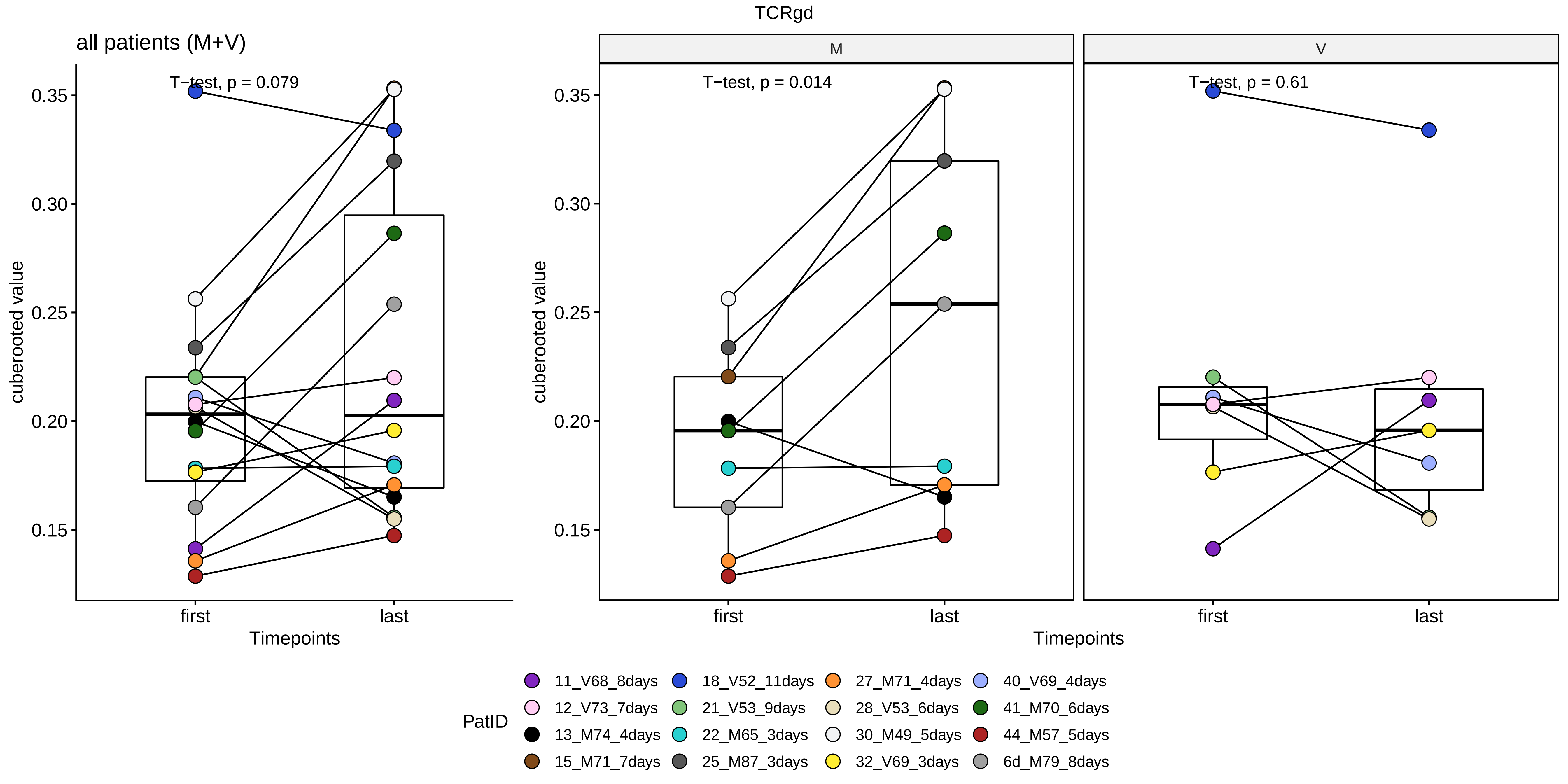

TCRgd
all patients (M+V)
T−test, p = 0.079
M
T−test, p = 0.014
V
T−test, p = 0.61
cuberooted value
0.35
0.30
0.25
0.20
0.15
first
last
Timepoints
PatID
11_V68_8days
12_V73_7days
13_M74_4days
15_M71_7days
18_V52_11days
21_V53_9days
22_M65_3days
25_M87_3days
27_M71_4days
28_V53_6days
30_M49_5days
32_V69_3days
40_V69_4days
41_M70_6days
44_M57_5days
6d_M79_8days

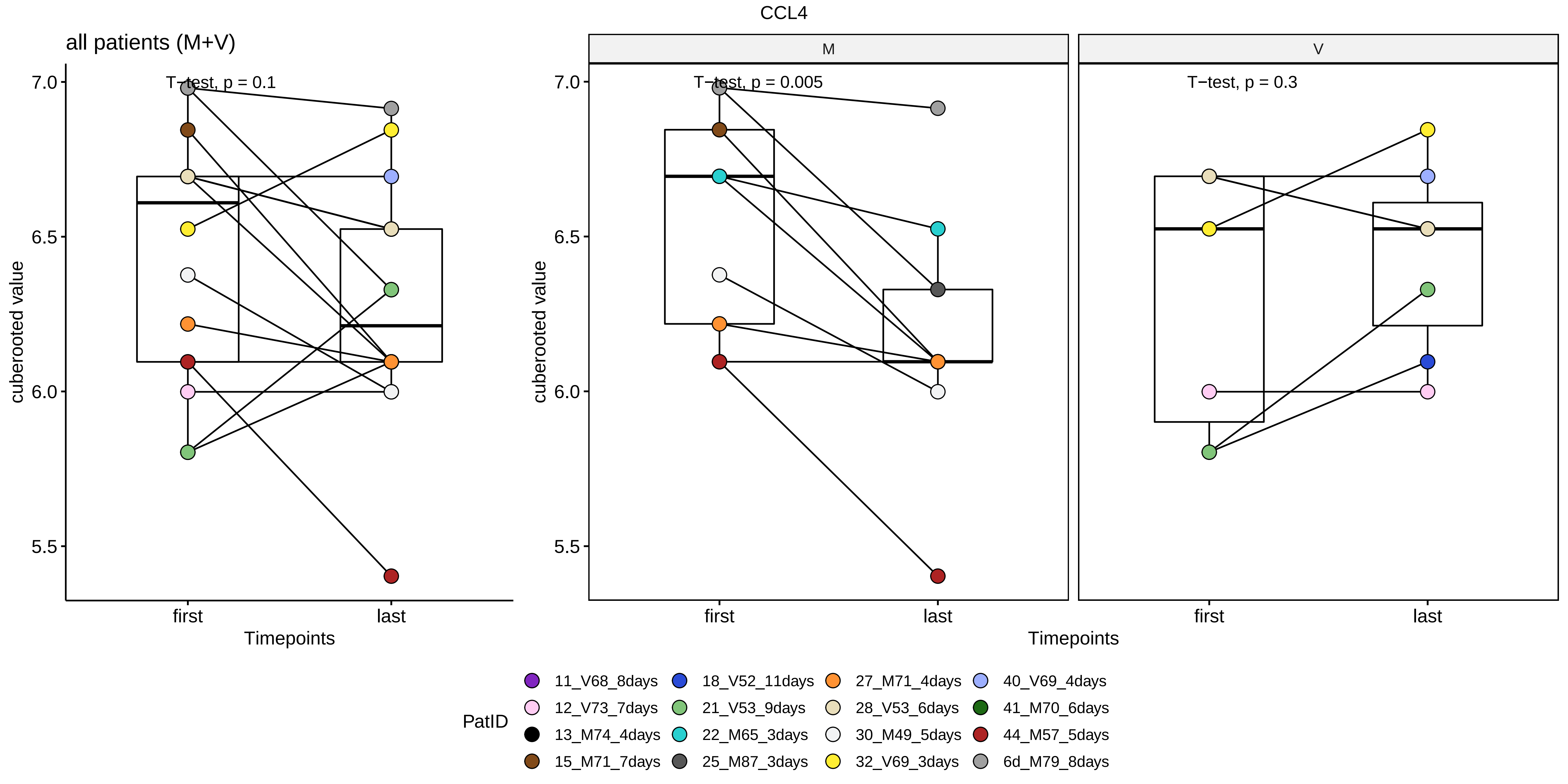
CCL4
all patients (M+V)
T−test, p = 0.1
M
T−test, p = 0.005
V
T−test, p = 0.3
cuberooted value
7.0
6.5
6.0
5.5
first
last
Timepoints
PatID
11_V68_8days
12_V73_7days
13_M74_4days
15_M71_7days
18_V52_11days
21_V53_9days
22_M65_3days
25_M87_3days
27_M71_4days
28_V53_6days
30_M49_5days
32_V69_3days
40_V69_4days
41_M70_6days
44_M57_5days
6d_M79_8days

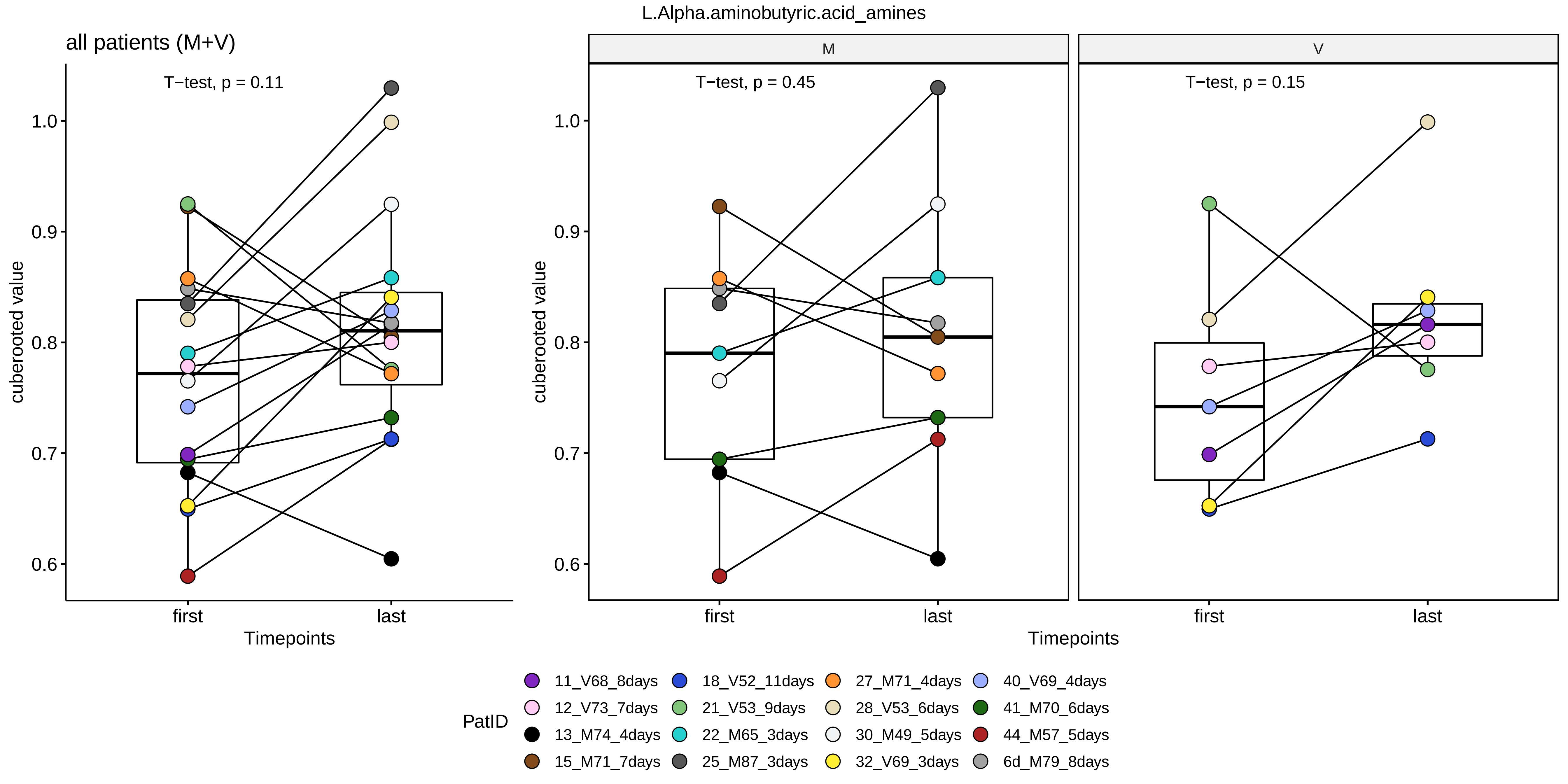

L.Alpha.aminobutyric.acid_amines
all patients (M+V)
T−test, p = 0.11
M
T−test, p = 0.45
V
T−test, p = 0.15
cuberooted value
1.0
0.9
0.8
0.7
0.6
first
last
Timepoints
PatID
11_V68_8days
12_V73_7days
13_M74_4days
15_M71_7days
18_V52_11days
21_V53_9days
22_M65_3days
25_M87_3days
27_M71_4days
28_V53_6days
30_M49_5days
32_V69_3days
40_V69_4days
41_M70_6days
44_M57_5days
6d_M79_8days

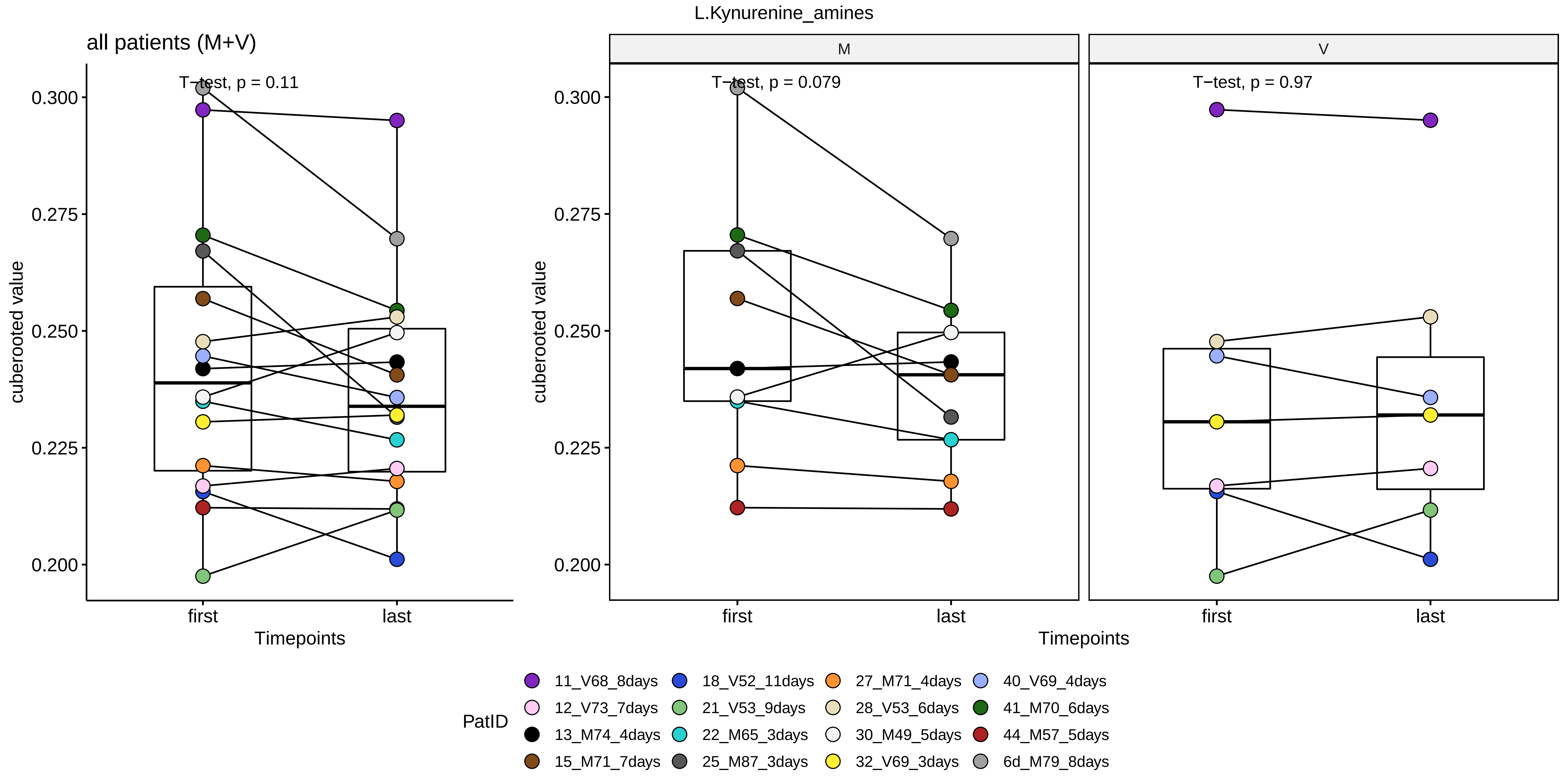
L.Kynurenine_amines
all patients (M+V)
T−test, p = 0.11
0.300
0.275
0.250
0.225
0.200
cuberooted value
first
last
Timepoints
M
T−test, p = 0.079
V
T−test, p = 0.97
PatID
11_V68_8days
12_V73_7days
13_M74_4days
15_M71_7days
18_V52_11days
21_V53_9days
22_M65_3days
25_M87_3days
27_M71_4days
28_V53_6days
30_M49_5days
32_V69_3days
40_V69_4days
41_M70_6days
44_M57_5days
6d_M79_8days

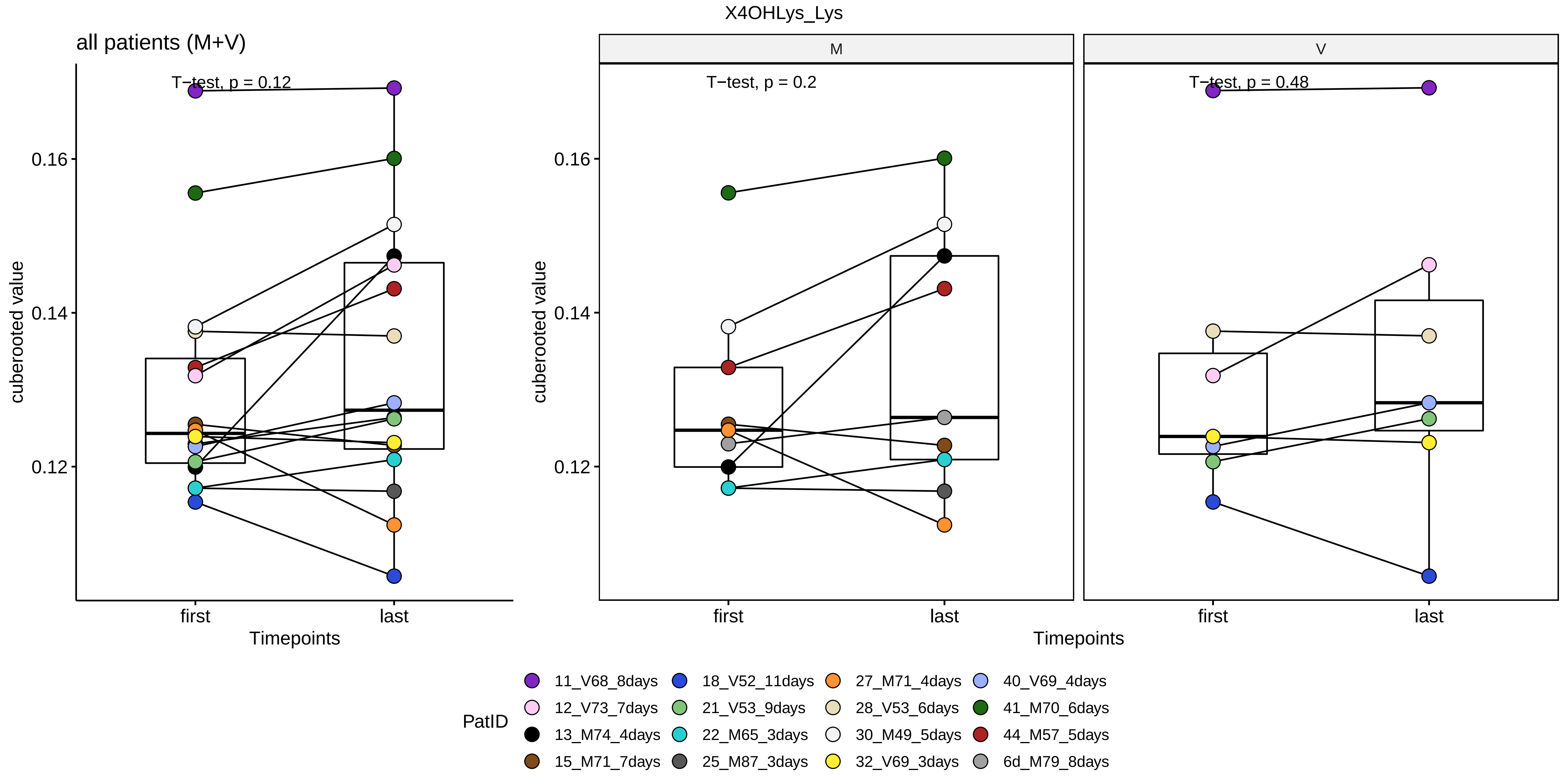

X4OHLys_Lys
all patients (M+V)
T−test, p = 0.12
M
T−test, p = 0.2
V
T−test, p = 0.48
cuberooted value
0.16
0.14
0.12
first
last
Timepoints
PatID
11_V68_8days
12_V73_7days
13_M74_4days
15_M71_7days
18_V52_11days
21_V53_9days
22_M65_3days
25_M87_3days
27_M71_4days
28_V53_6days
30_M49_5days
32_V69_3days
40_V69_4days
41_M70_6days
44_M57_5days
6d_M79_8days

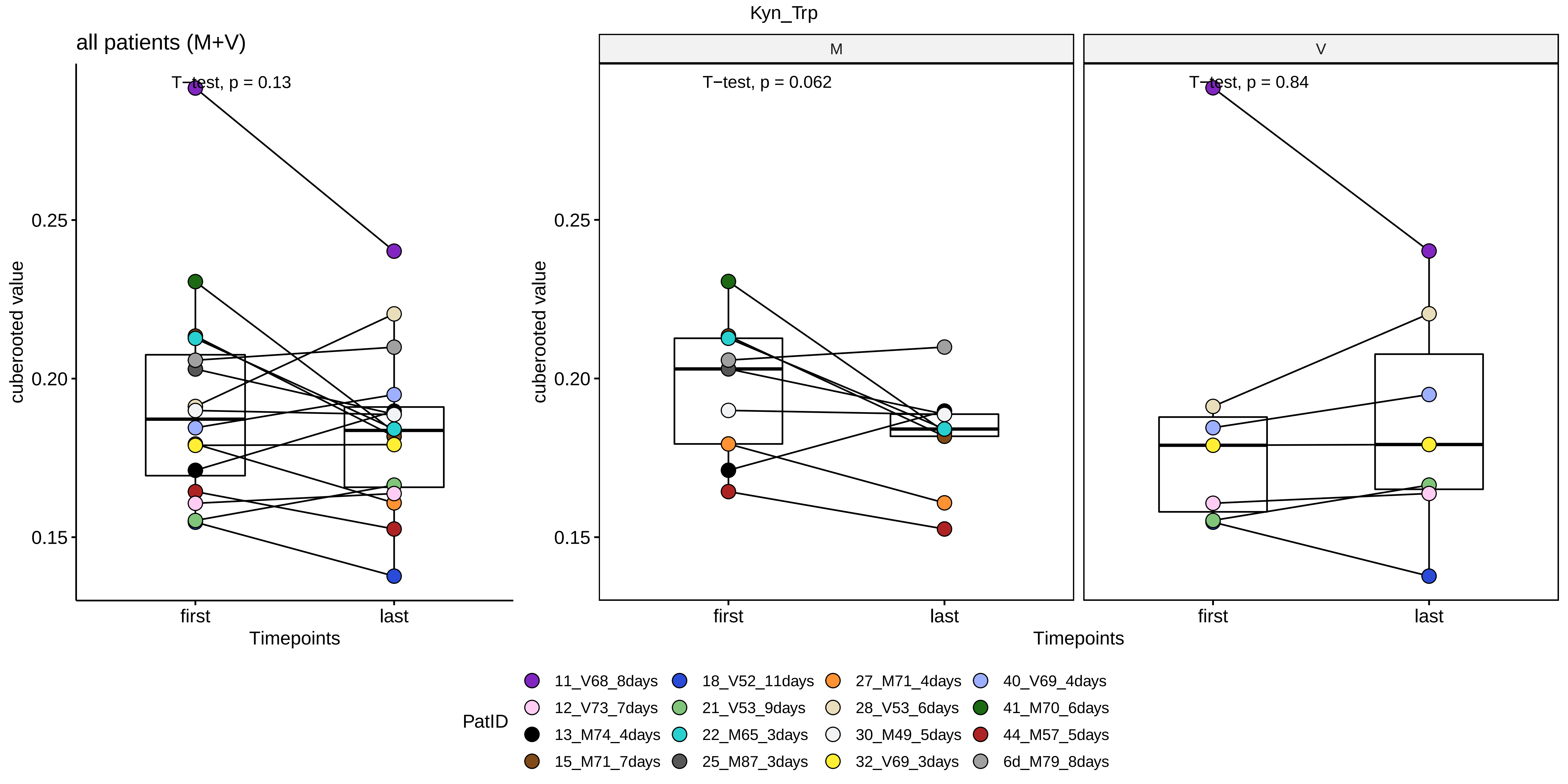

Kyn_Trp
all patients (M+V)
T−test, p = 0.13
cuberooted value
0.25
0.20
0.15
first
last
Timepoints
M
V
T−test, p = 0.062
T−test, p = 0.84
PatID
11_V68_8days
12_V73_7days
13_M74_4days
15_M71_7days
18_V52_11days
21_V53_9days
22_M65_3days
25_M87_3days
27_M71_4days
28_V53_6days
30_M49_5days
32_V69_3days
40_V69_4days
41_M70_6days
44_M57_5days
6d_M79_8days

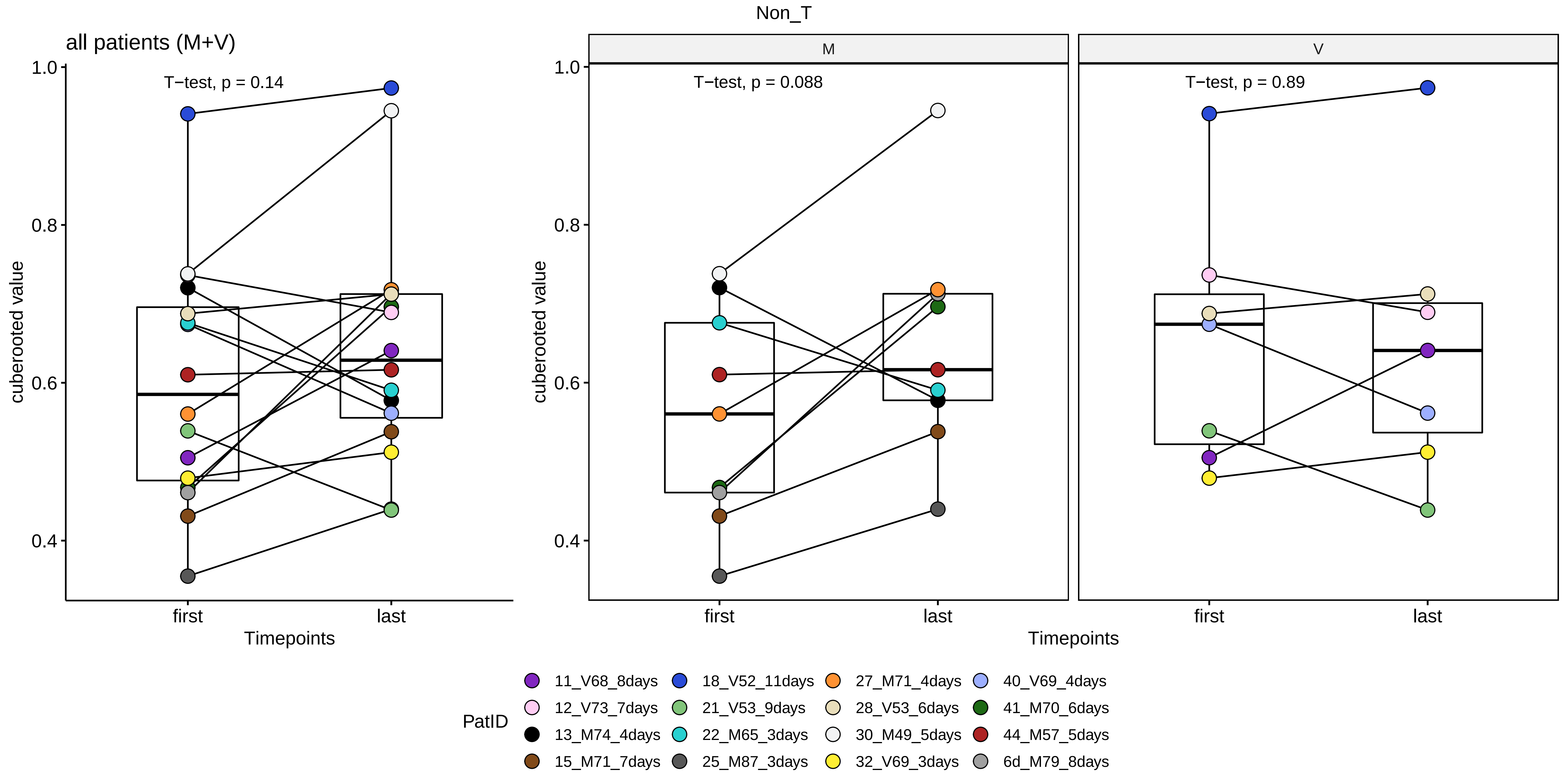

Non_T
all patients (M+V)
T−test, p = 0.14
M
T−test, p = 0.088
V
T−test, p = 0.89
cuberooted value
1.0
0.8
0.6
0.4
first
last
Timepoints
PatID
11_V68_8days
12_V73_7days
13_M74_4days
15_M71_7days
18_V52_11days
21_V53_9days
22_M65_3days
25_M87_3days
27_M71_4days
28_V53_6days
30_M49_5days
32_V69_3days
40_V69_4days
41_M70_6days
44_M57_5days
6d_M79_8days

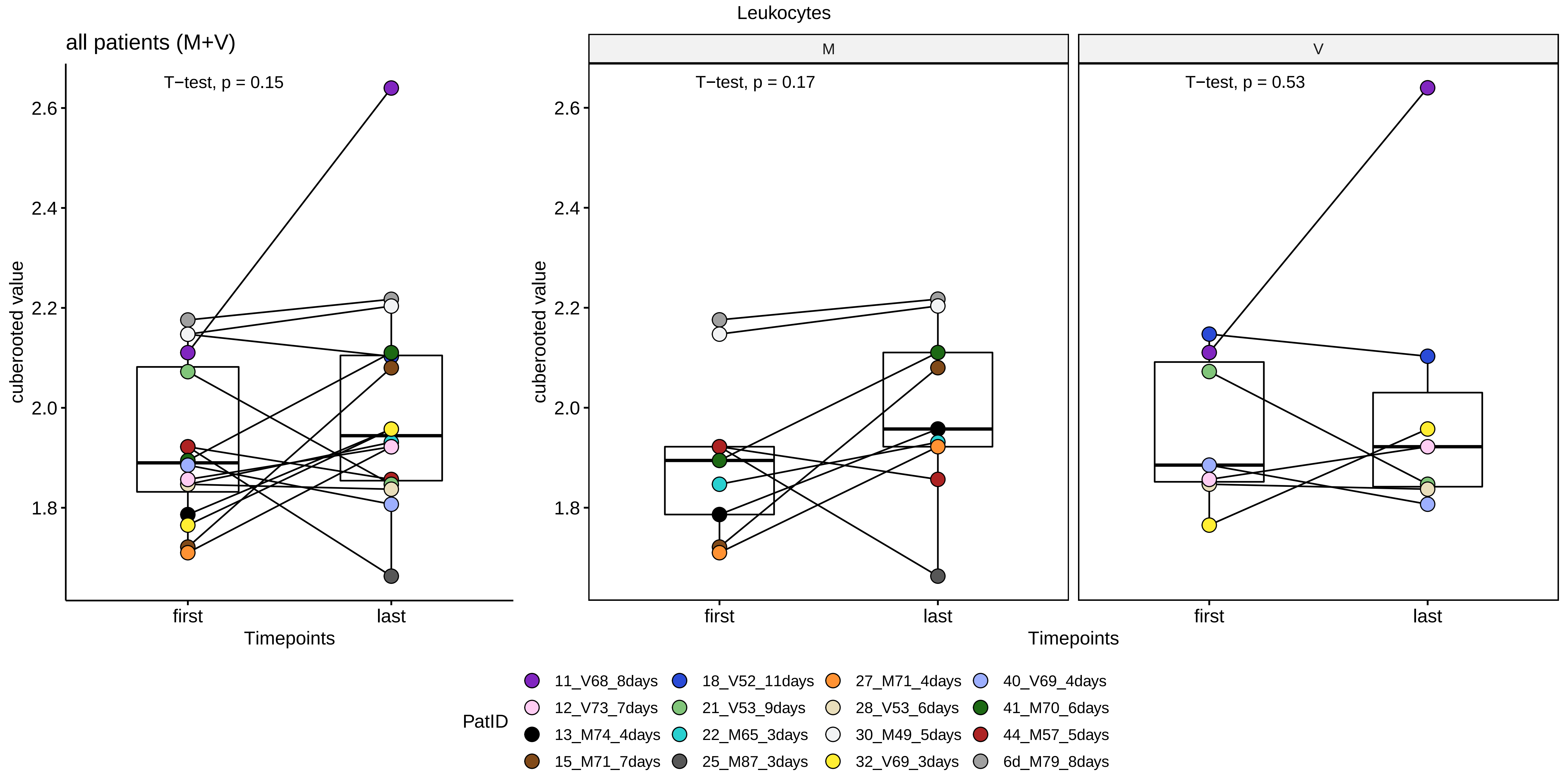

Leukocytes
all patients (M+V)
M
V
T−test, p = 0.15
T−test, p = 0.17
T−test, p = 0.53
cuberooted value
2.6
2.4
2.2
2.0
1.8
first
last
Timepoints
PatID
11_V68_8days
12_V73_7days
13_M74_4days
15_M71_7days
18_V52_11days
21_V53_9days
22_M65_3days
25_M87_3days
27_M71_4days
28_V53_6days
30_M49_5days
32_V69_3days
40_V69_4days
41_M70_6days
44_M57_5days
6d_M79_8days

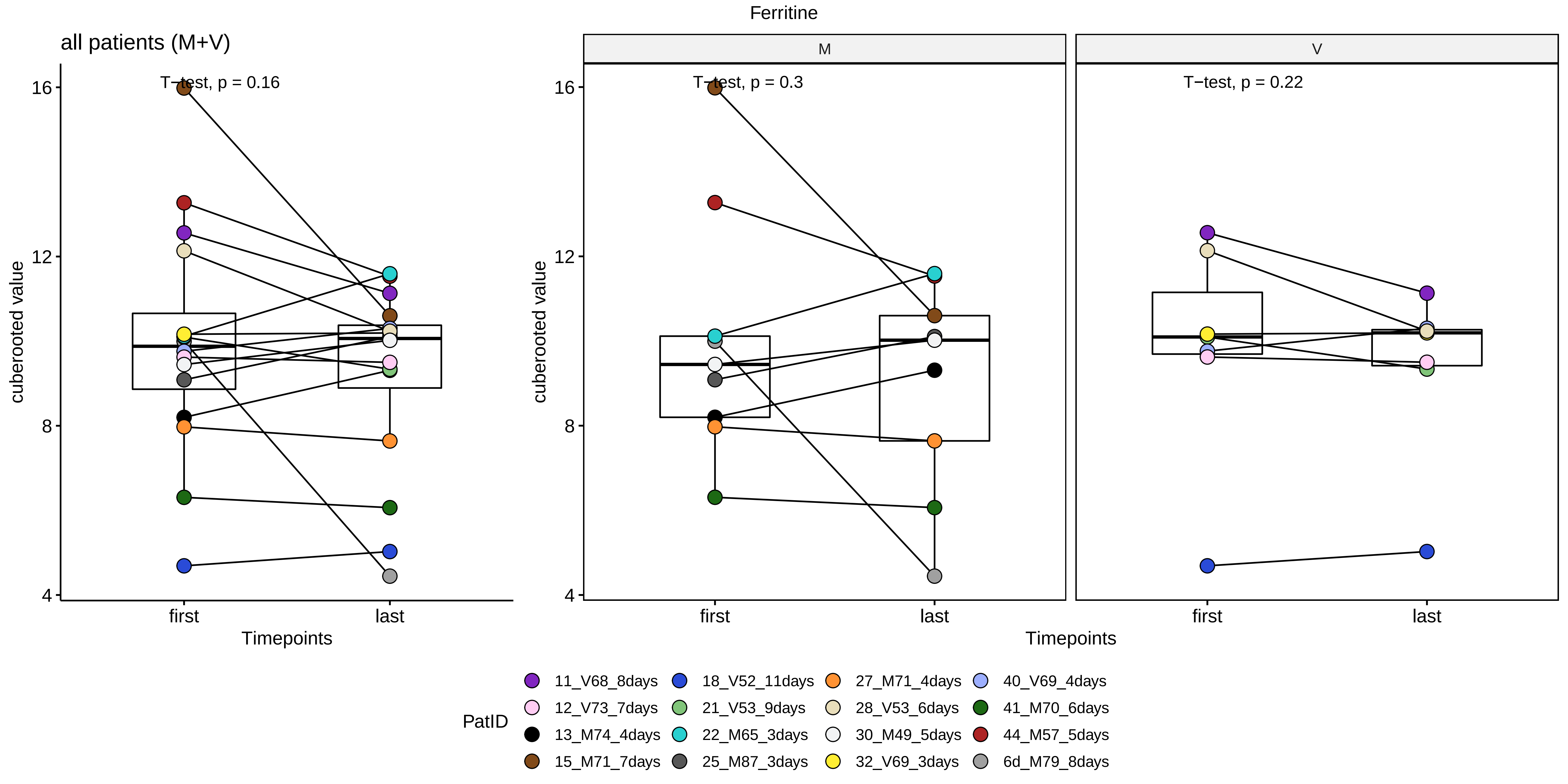

Ferritine
all patients (M+V)
M
V
T−test, p = 0.16
T−test, p = 0.3
T−test, p = 0.22
16
12
8
4
cuberooted value
first
last
Timepoints
PatID
11_V68_8days
12_V73_7days
13_M74_4days
15_M71_7days
18_V52_11days
21_V53_9days
22_M65_3days
25_M87_3days
27_M71_4days
28_V53_6days
30_M49_5days
32_V69_3days
40_V69_4days
41_M70_6days
44_M57_5days
6d_M79_8days

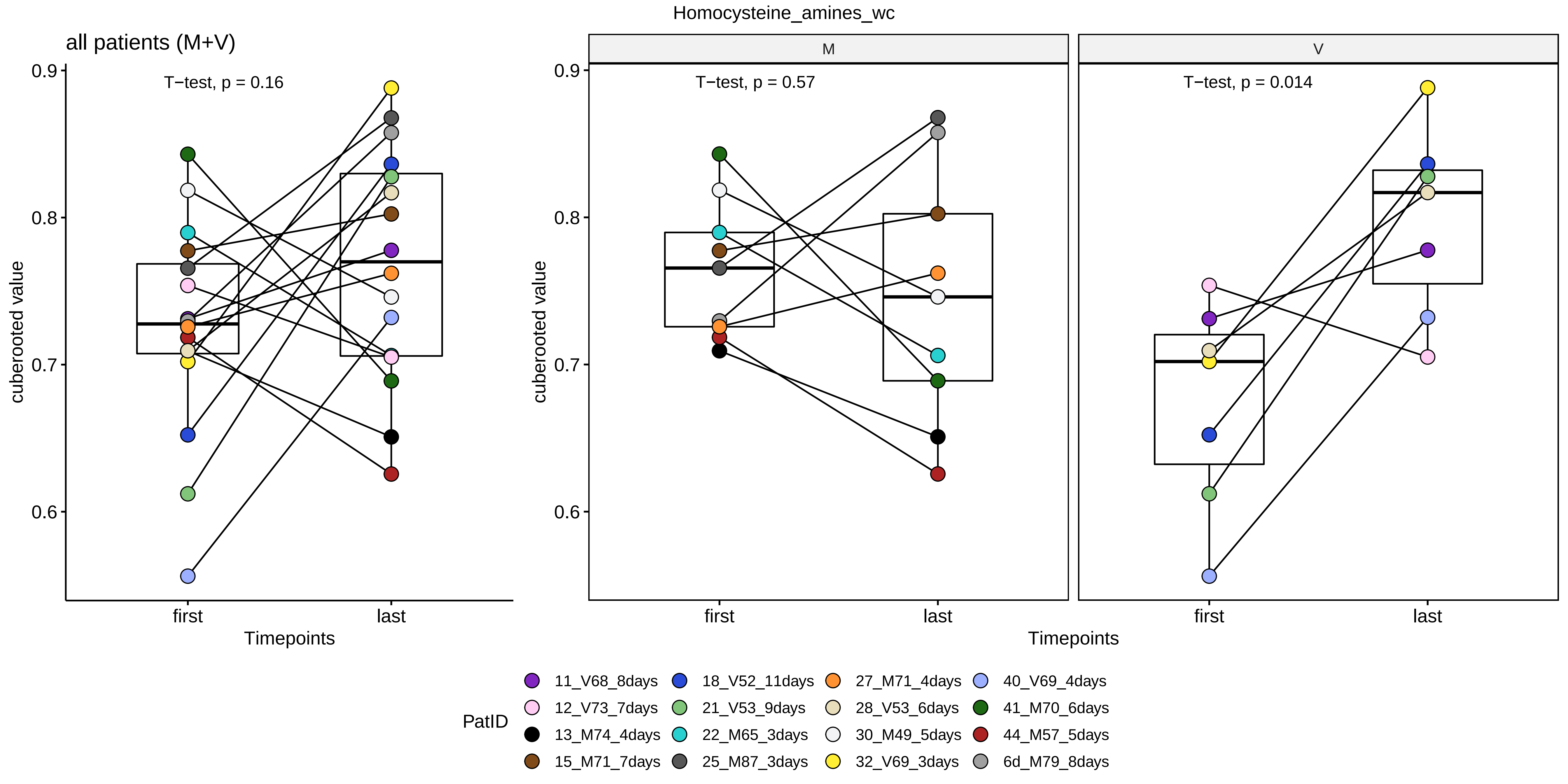

Homocysteine_amines_wc
all patients (M+V)
T−test, p = 0.16
M
T−test, p = 0.57
V
T−test, p = 0.014
cuberooted value
0.9
0.8
0.7
0.6
first
last
Timepoints
PatID
11_V68_8days
12_V73_7days
13_M74_4days
15_M71_7days
18_V52_11days
21_V53_9days
22_M65_3days
25_M87_3days
27_M71_4days
28_V53_6days
30_M49_5days
32_V69_3days
40_V69_4days
41_M70_6days
44_M57_5days
6d_M79_8days

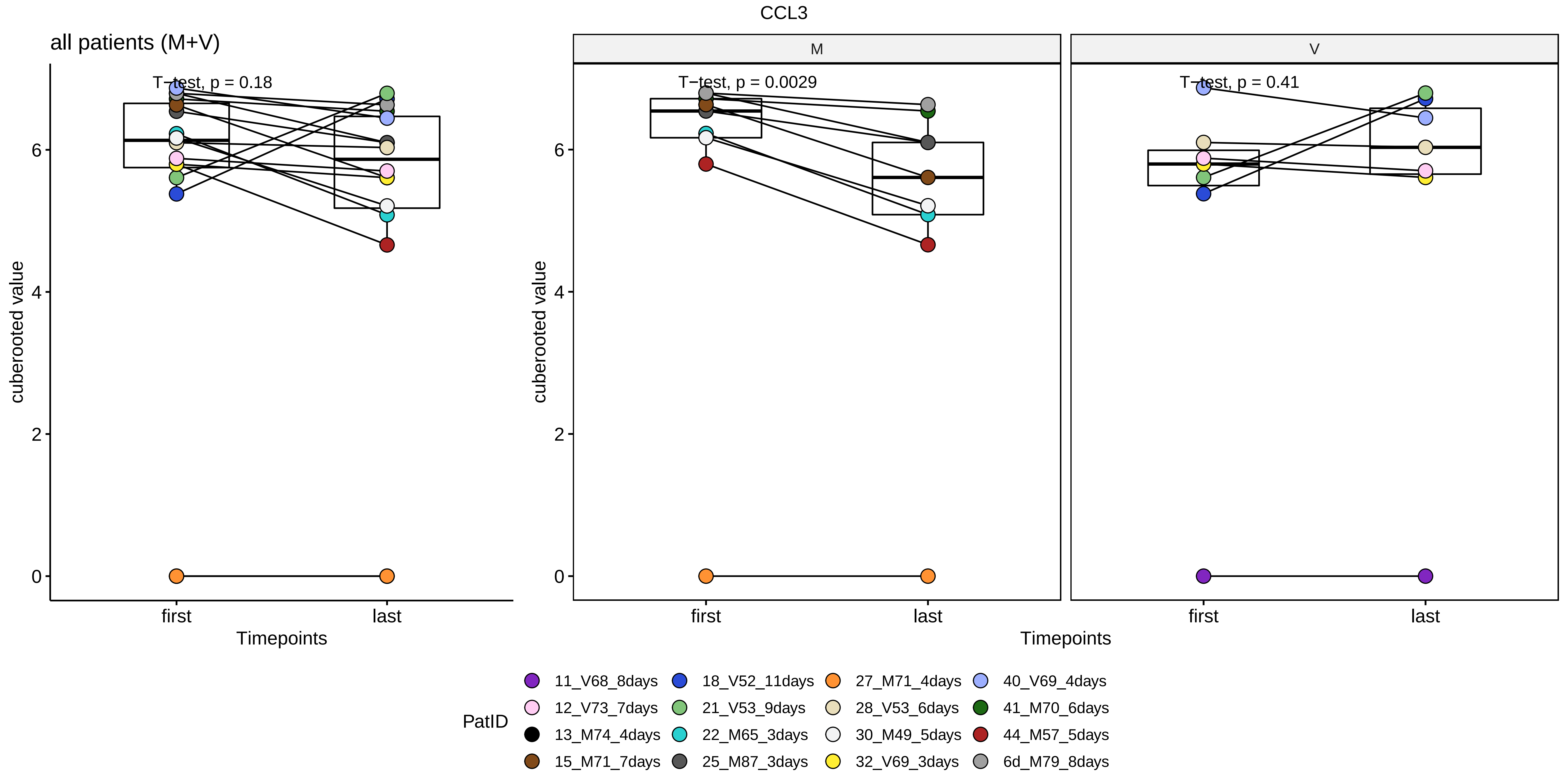

CCL3
all patients (M+V)
T−test, p = 0.18
M
T−test, p = 0.0029
V
T−test, p = 0.41
cuberooted value
0
2
4
6
first
last
Timepoints
PatID
11_V68_8days
12_V73_7days
13_M74_4days
15_M71_7days
18_V52_11days
21_V53_9days
22_M65_3days
25_M87_3days
27_M71_4days
28_V53_6days
30_M49_5days
32_V69_3days
40_V69_4days
41_M70_6days
44_M57_5days
6d_M79_8days

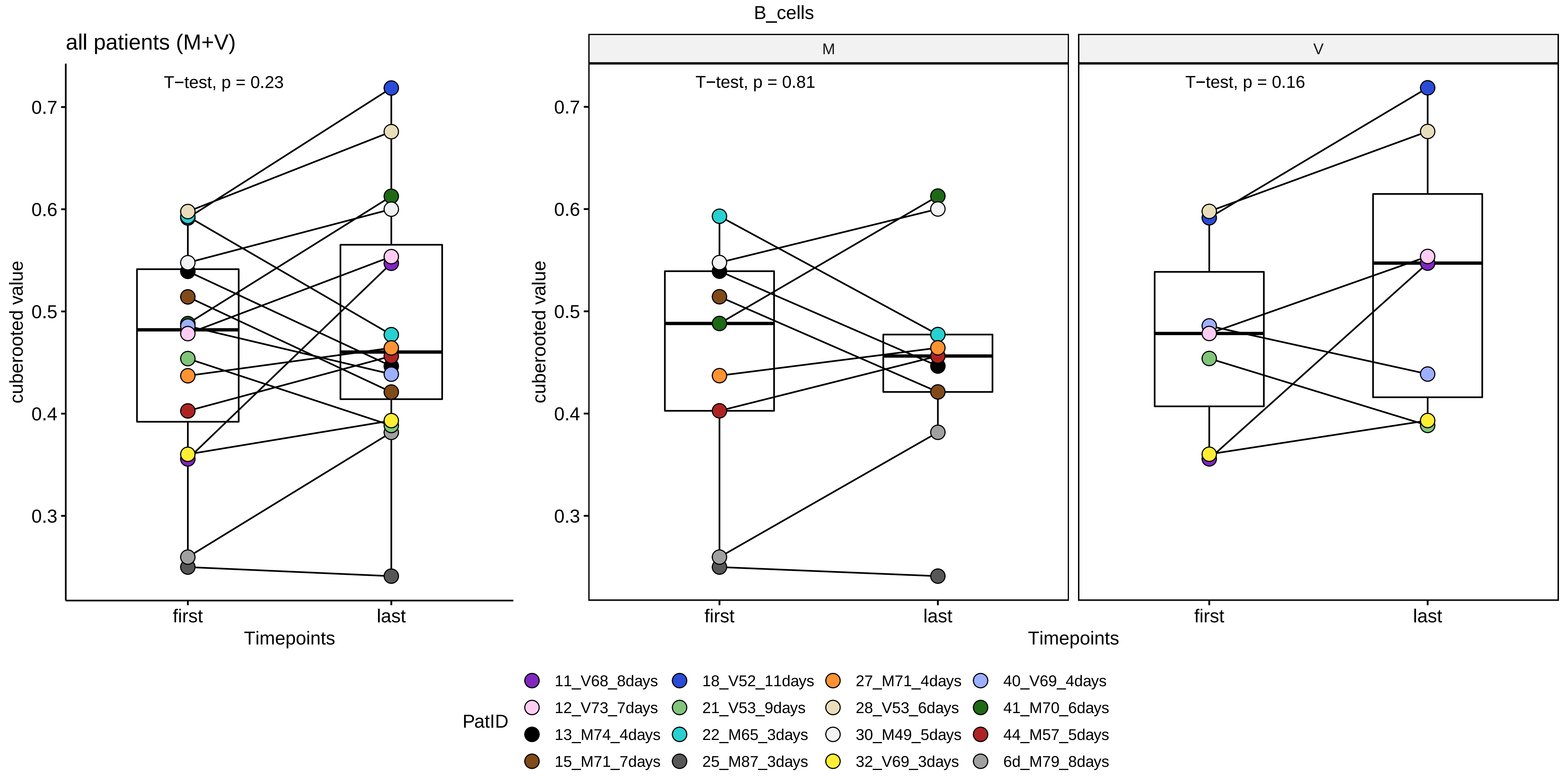
B_cells
all patients (M+V)
T−test, p = 0.23
M
T−test, p = 0.81
V
T−test, p = 0.16
cuberooted value
0.3
0.4
0.5
0.6
0.7
first
last
Timepoints
PatID
11_V68_8days
12_V73_7days
13_M74_4days
15_M71_7days
18_V52_11days
21_V53_9days
22_M65_3days
25_M87_3days
27_M71_4days
28_V53_6days
30_M49_5days
32_V69_3days
40_V69_4days
41_M70_6days
44_M57_5days
6d_M79_8days

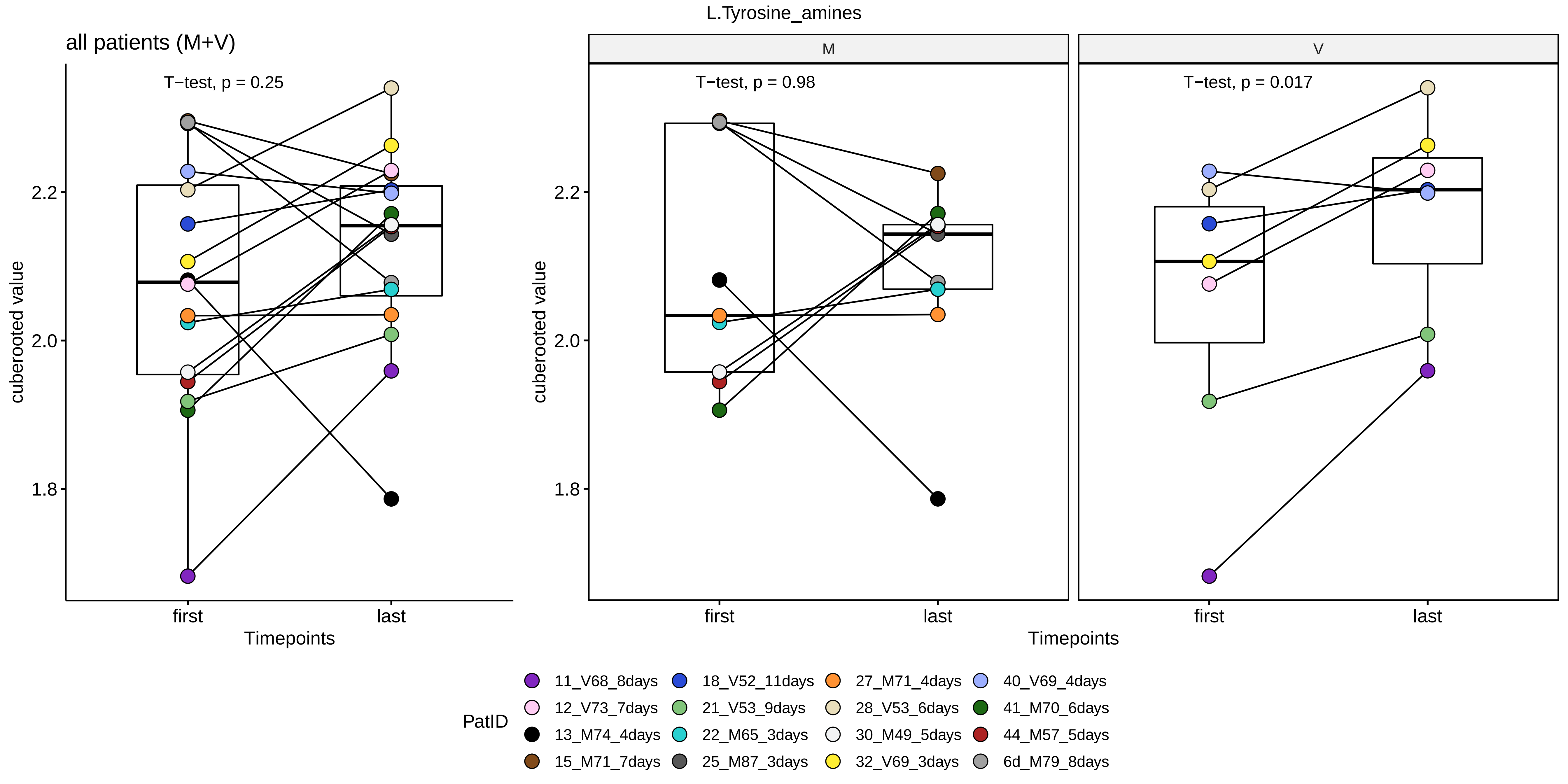

L.Tyrosine_amines
all patients (M+V)
T−test, p = 0.25
M
T−test, p = 0.98
V
T−test, p = 0.017
cuberooted value
2.2
2.0
1.8
first
last
Timepoints
PatID
11_V68_8days
12_V73_7days
13_M74_4days
15_M71_7days
18_V52_11days
21_V53_9days
22_M65_3days
25_M87_3days
27_M71_4days
28_V53_6days
30_M49_5days
32_V69_3days
40_V69_4days
41_M70_6days
44_M57_5days
6d_M79_8days

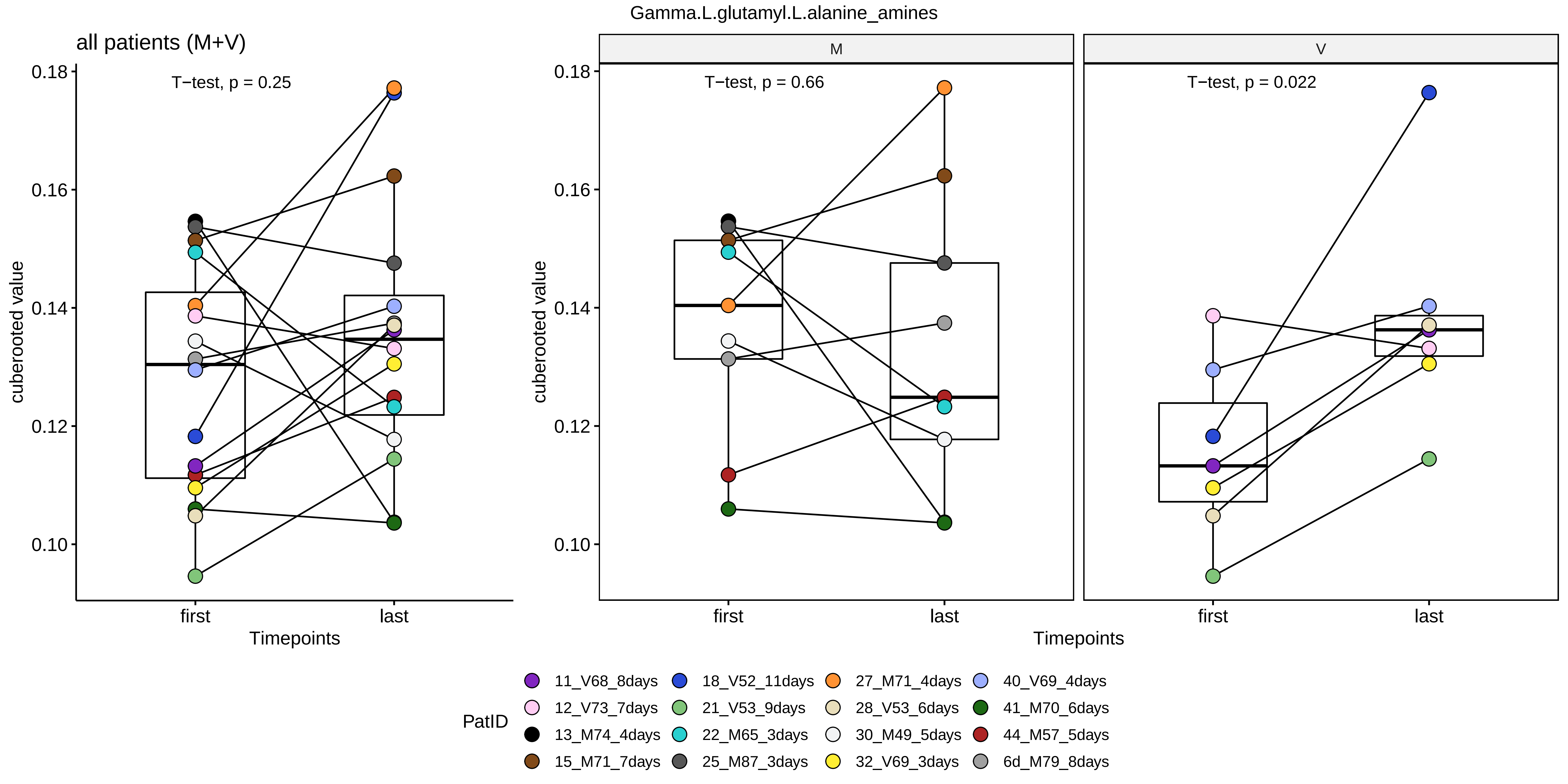
Gamma.L.glutamyl.L.alanine_amines
all patients (M+V)
T−test, p = 0.25
M
T−test, p = 0.66
V
T−test, p = 0.022
cuberooted value
0.18
0.16
0.14
0.12
0.10
first
last
Timepoints
PatID
11_V68_8days
12_V73_7days
13_M74_4days
15_M71_7days
18_V52_11days
21_V53_9days
22_M65_3days
25_M87_3days
27_M71_4days
28_V53_6days
30_M49_5days
32_V69_3days
40_V69_4days
41_M70_6days
44_M57_5days
6d_M79_8days

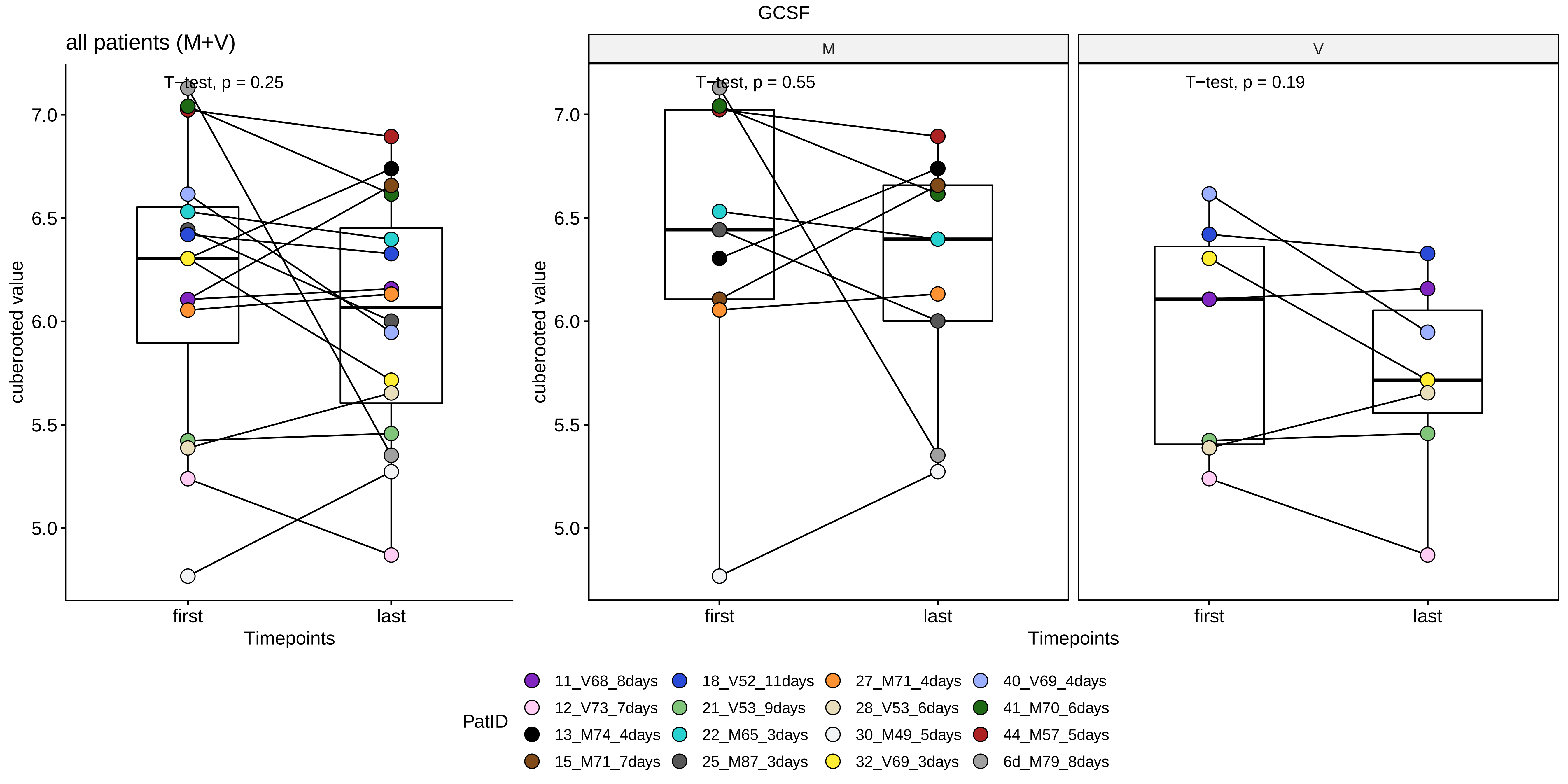
GCSF
all patients (M+V)
T−test, p = 0.25
M
T−test, p = 0.55
V
T−test, p = 0.19
cuberooted value
7.0
6.5
6.0
5.5
5.0
first
last
Timepoints
PatID
11_V68_8days
12_V73_7days
13_M74_4days
15_M71_7days
18_V52_11days
21_V53_9days
22_M65_3days
25_M87_3days
27_M71_4days
28_V53_6days
30_M49_5days
32_V69_3days
40_V69_4days
41_M70_6days
44_M57_5days
6d_M79_8days

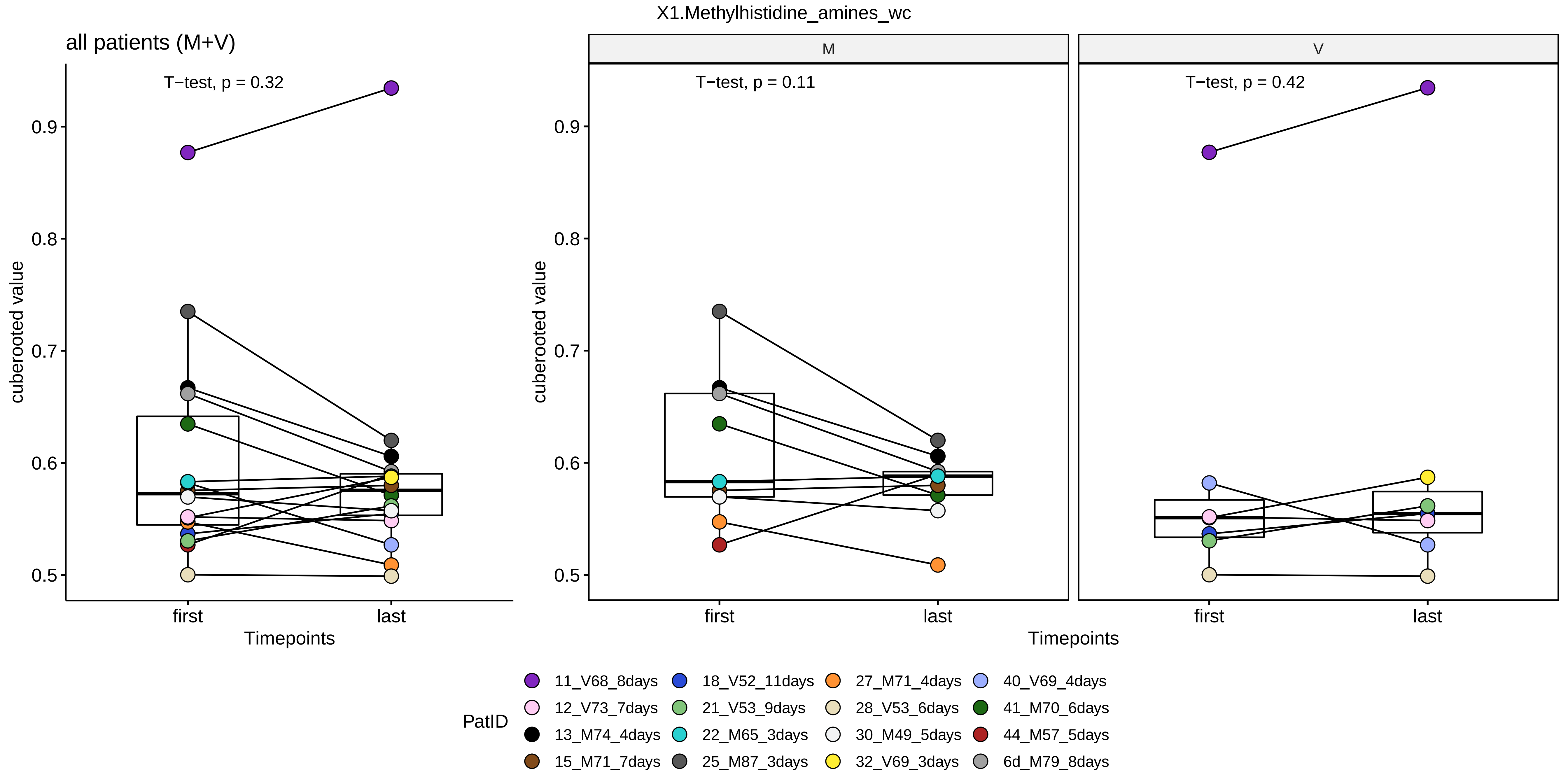

X1.Methylhistidine_amines_wc
all patients (M+V)
T−test, p = 0.32
M
T−test, p = 0.11
V
T−test, p = 0.42
cuberooted value
0.9
0.8
0.7
0.6
0.5
first
last
Timepoints
PatID
11_V68_8days
12_V73_7days
13_M74_4days
15_M71_7days
18_V52_11days
21_V53_9days
22_M65_3days
25_M87_3days
27_M71_4days
28_V53_6days
30_M49_5days
32_V69_3days
40_V69_4days
41_M70_6days
44_M57_5days
6d_M79_8days

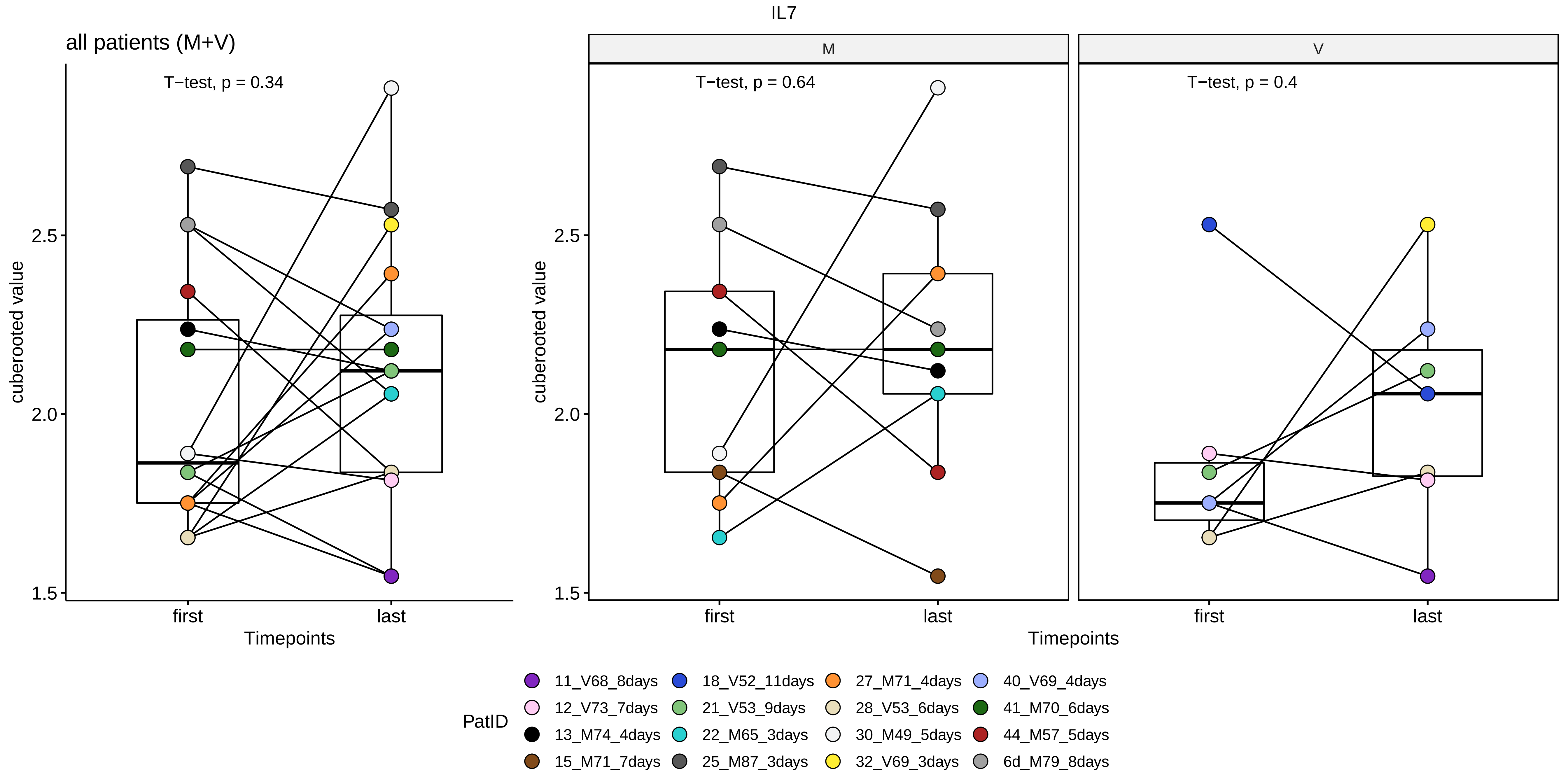
IL7
all patients (M+V)
T−test, p = 0.34
cuberooted value
2.5
2.0
1.5
first
last
Timepoints
M
T−test, p = 0.64
V
T−test, p = 0.4
PatID
11_V68_8days
12_V73_7days
13_M74_4days
15_M71_7days
18_V52_11days
21_V53_9days
22_M65_3days
25_M87_3days
27_M71_4days
28_V53_6days
30_M49_5days
32_V69_3days
40_V69_4days
41_M70_6days
44_M57_5days
6d_M79_8days

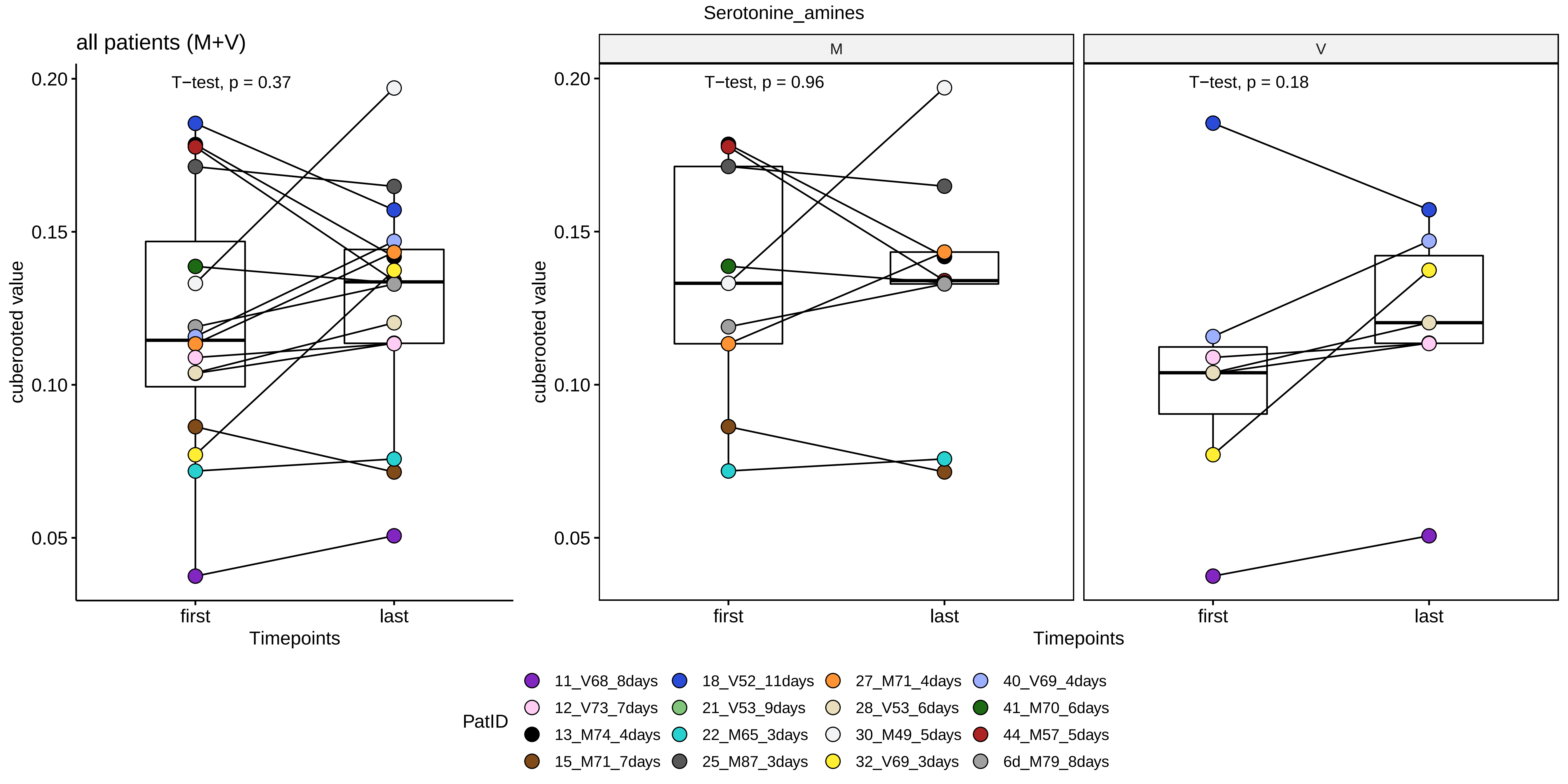
Serotonine_amines
all patients (M+V)
T−test, p = 0.37
M
T−test, p = 0.96
V
T−test, p = 0.18
cuberooted value
0.20
0.15
0.10
0.05
first
last
Timepoints
PatID
11_V68_8days
12_V73_7days
13_M74_4days
15_M71_7days
18_V52_11days
21_V53_9days
22_M65_3days
25_M87_3days
27_M71_4days
28_V53_6days
30_M49_5days
32_V69_3days
40_V69_4days
41_M70_6days
44_M57_5days
6d_M79_8days

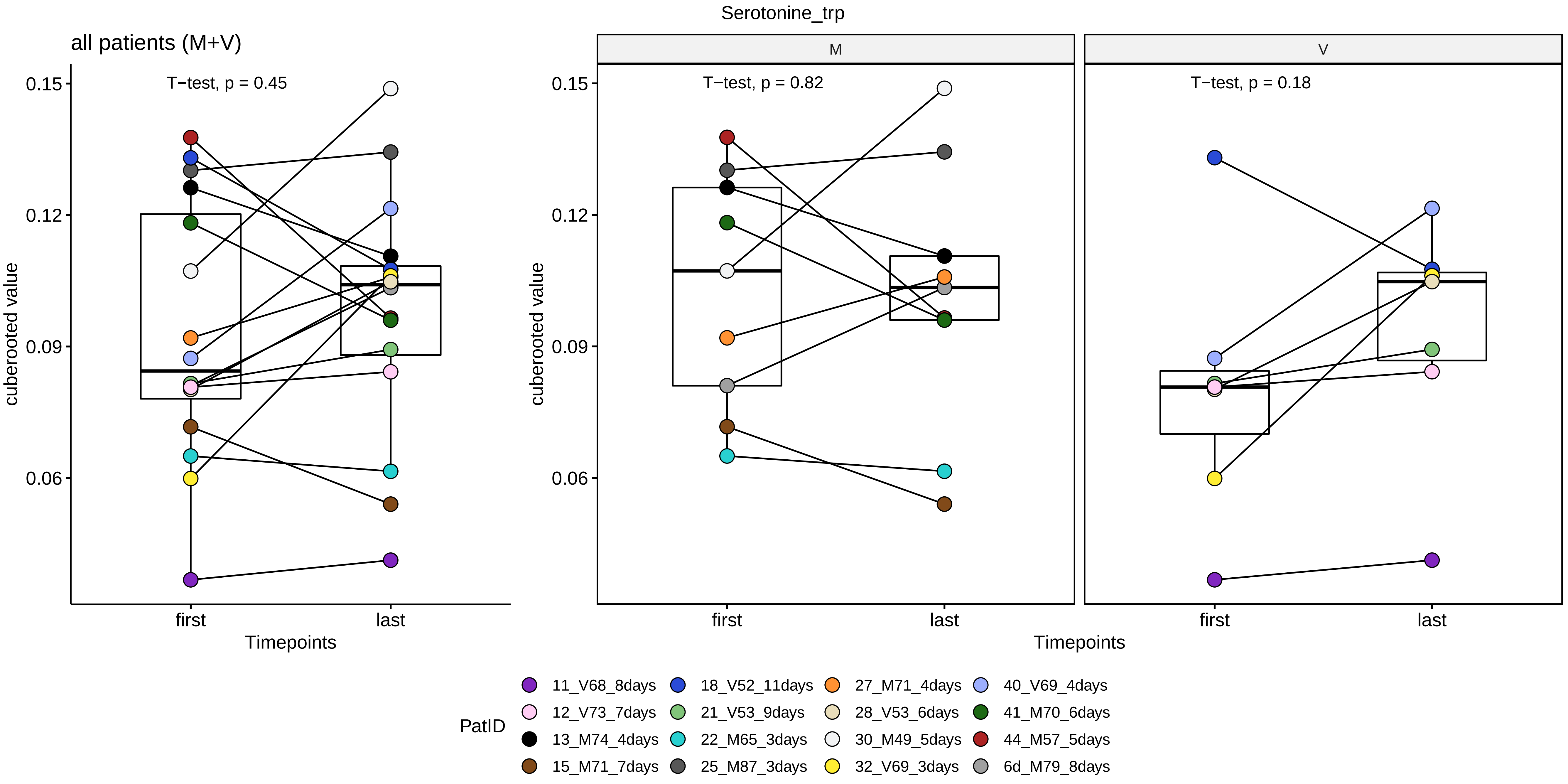

Serotonine_trp
all patients (M+V)
T−test, p = 0.45
M
T−test, p = 0.82
V
T−test, p = 0.18
0.15
0.12
0.09
0.06
cuberooted value
first
last
Timepoints
PatID
11_V68_8days
12_V73_7days
13_M74_4days
15_M71_7days
18_V52_11days
21_V53_9days
22_M65_3days
25_M87_3days
27_M71_4days
28_V53_6days
30_M49_5days
32_V69_3days
40_V69_4days
41_M70_6days
44_M57_5days
6d_M79_8days

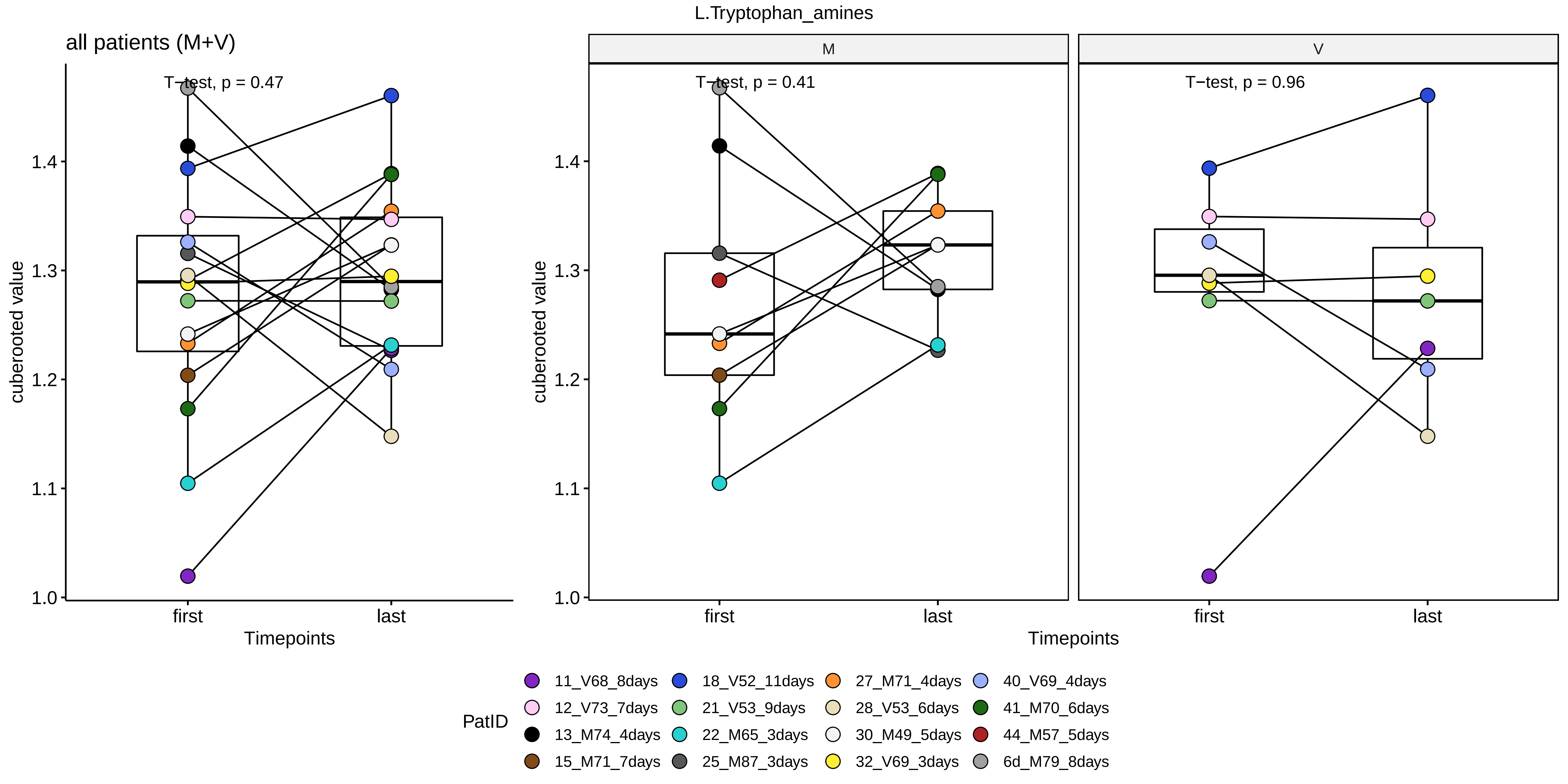

L.Tryptophan_amines
all patients (M+V)
T−test, p = 0.47
M
T−test, p = 0.41
V
T−test, p = 0.96
cuberooted value
1.0
1.1
1.2
1.3
1.4
first
last
Timepoints
PatID
11_V68_8days
12_V73_7days
13_M74_4days
15_M71_7days
18_V52_11days
21_V53_9days
22_M65_3days
25_M87_3days
27_M71_4days
28_V53_6days
30_M49_5days
32_V69_3days
40_V69_4days
41_M70_6days
44_M57_5days
6d_M79_8days

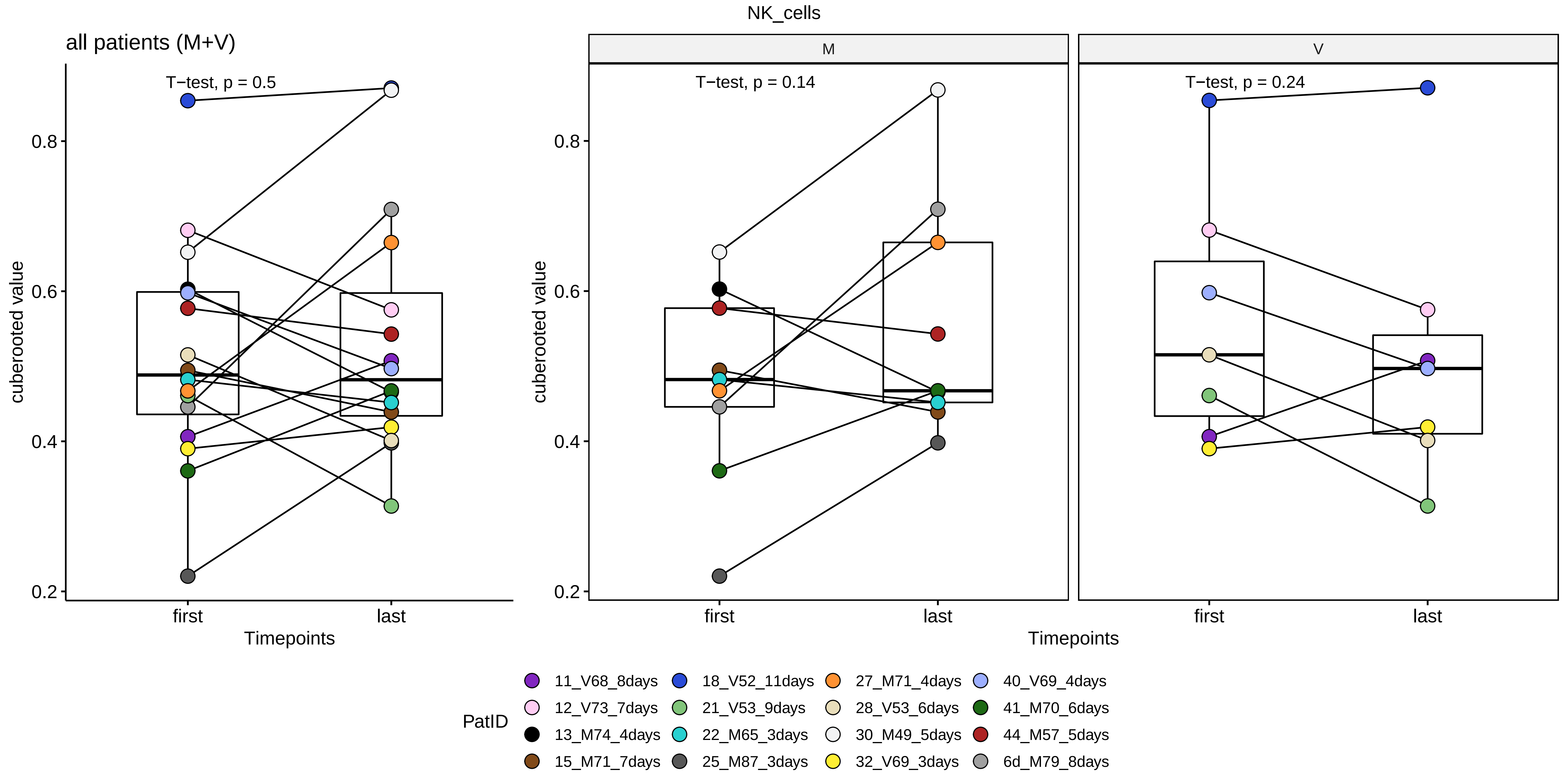
NK_cells
all patients (M+V)
T−test, p = 0.5
M
T−test, p = 0.14
V
T−test, p = 0.24
cuberooted value
0.2
0.4
0.6
0.8
first
last
Timepoints
PatID
11_V68_8days
12_V73_7days
13_M74_4days
15_M71_7days
18_V52_11days
21_V53_9days
22_M65_3days
25_M87_3days
27_M71_4days
28_V53_6days
30_M49_5days
32_V69_3days
40_V69_4days
41_M70_6days
44_M57_5days
6d_M79_8days

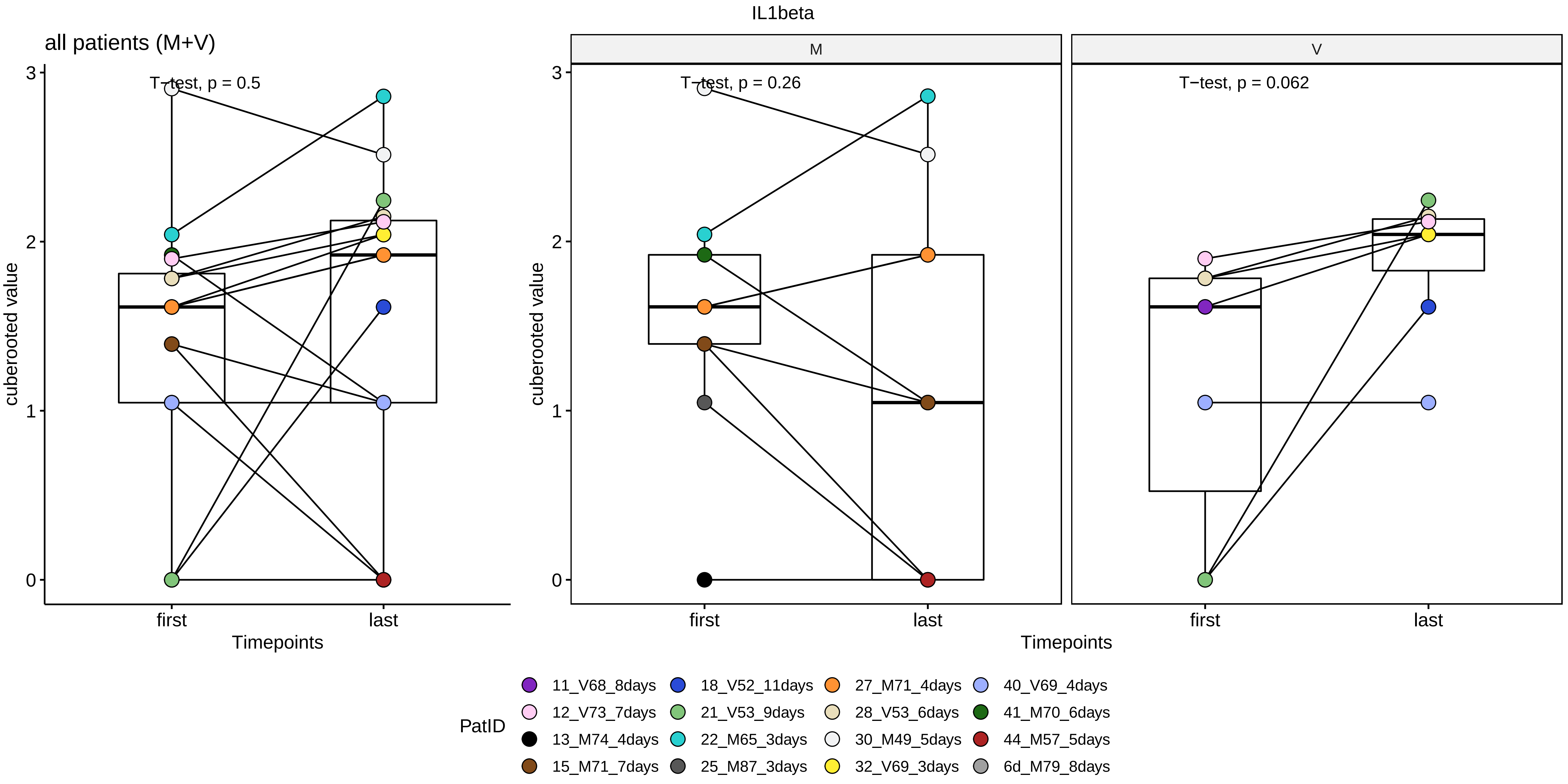
IL1beta
all patients (M+V)
T−test, p = 0.5
T−test, p = 0.26
T−test, p = 0.062
M
V
cuberooted value
0
1
2
3
first
last
Timepoints
PatID
11_V68_8days
12_V73_7days
13_M74_4days
15_M71_7days
18_V52_11days
21_V53_9days
22_M65_3days
25_M87_3days
27_M71_4days
28_V53_6days
30_M49_5days
32_V69_3days
40_V69_4days
41_M70_6days
44_M57_5days
6d_M79_8days

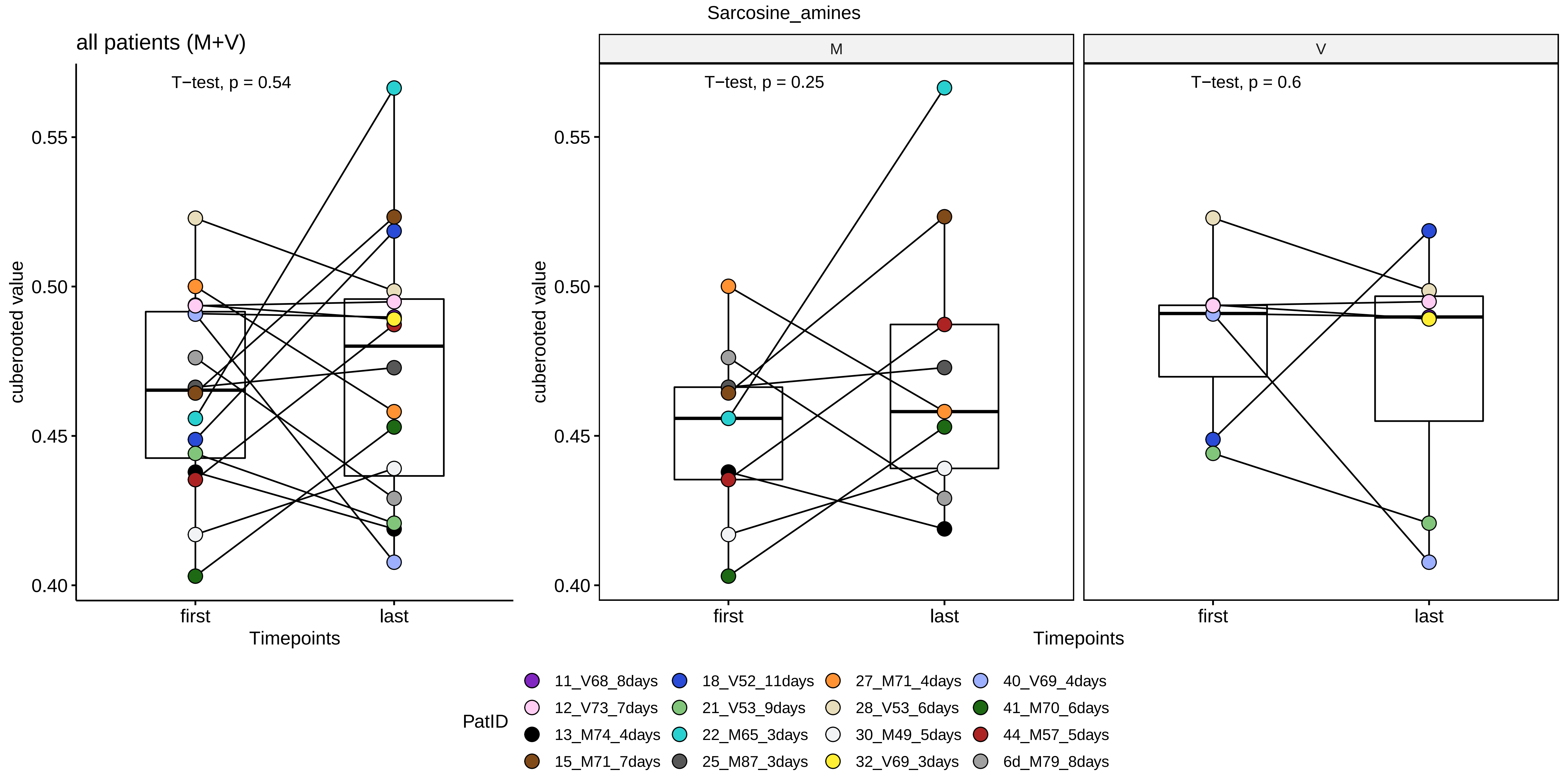

Sarcosine_amines
all patients (M+V)
T−test, p = 0.54
M
T−test, p = 0.25
V
T−test, p = 0.6
cuberooted value
0.40
0.45
0.50
0.55
first
last
Timepoints
PatID
11_V68_8days
12_V73_7days
13_M74_4days
15_M71_7days
18_V52_11days
21_V53_9days
22_M65_3days
25_M87_3days
27_M71_4days
28_V53_6days
30_M49_5days
32_V69_3days
40_V69_4days
41_M70_6days
44_M57_5days
6d_M79_8days

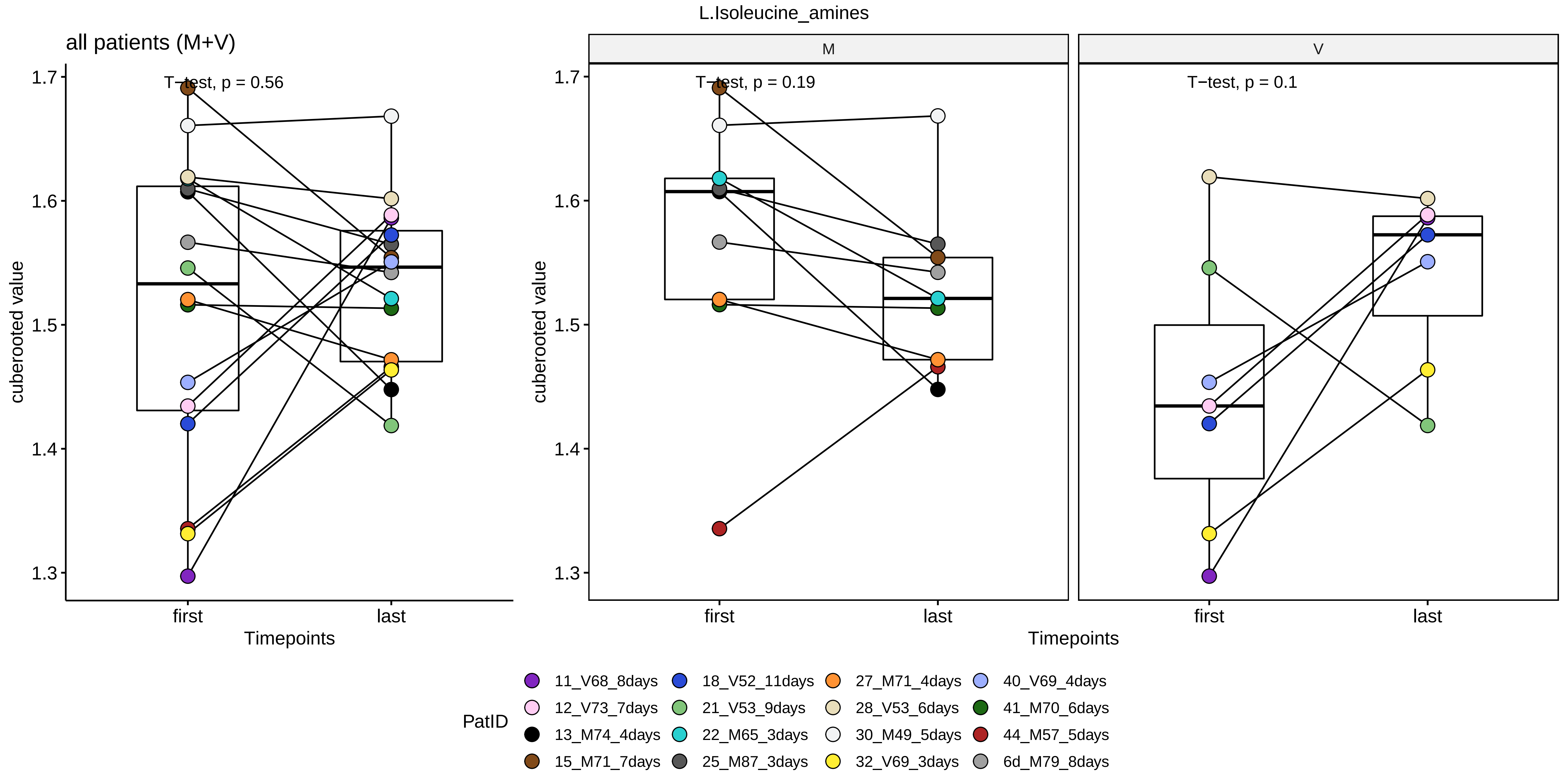

L.Isoleucine_amines
all patients (M+V)
T−test, p = 0.56
M
T−test, p = 0.19
V
T−test, p = 0.1
cuberooted value
1.7
1.6
1.5
1.4
1.3
first
last
Timepoints
PatID
11_V68_8days
12_V73_7days
13_M74_4days
15_M71_7days
18_V52_11days
21_V53_9days
22_M65_3days
25_M87_3days
27_M71_4days
28_V53_6days
30_M49_5days
32_V69_3days
40_V69_4days
41_M70_6days
44_M57_5days
6d_M79_8days

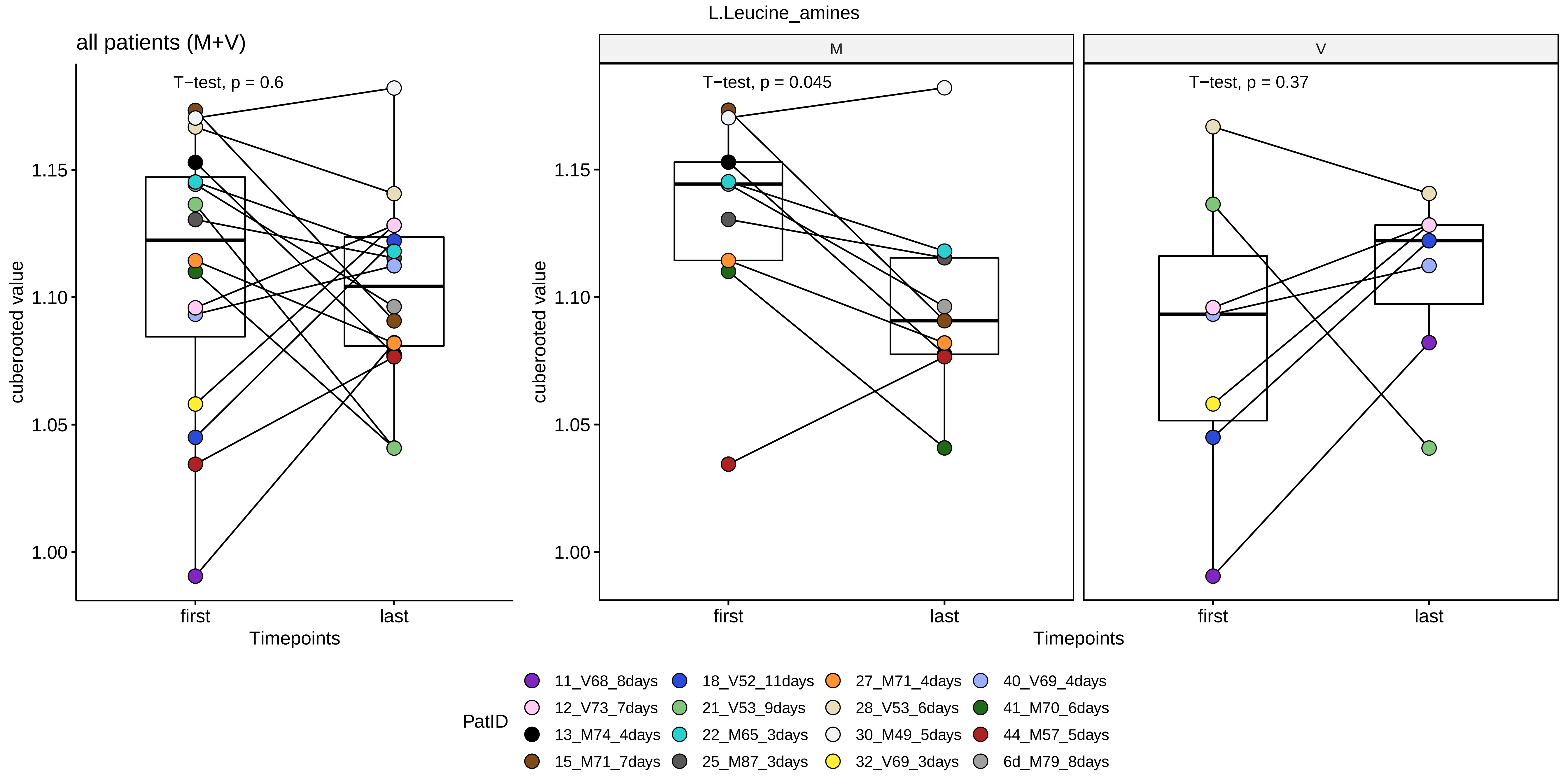

L.Leucine_amines
all patients (M+V)
T−test, p = 0.6
M
T−test, p = 0.045
V
T−test, p = 0.37
cuberooted value
1.15
1.10
1.05
1.00
first
last
Timepoints
PatID
11_V68_8days
12_V73_7days
13_M74_4days
15_M71_7days
18_V52_11days
21_V53_9days
22_M65_3days
25_M87_3days
27_M71_4days
28_V53_6days
30_M49_5days
32_V69_3days
40_V69_4days
41_M70_6days
44_M57_5days
6d_M79_8days

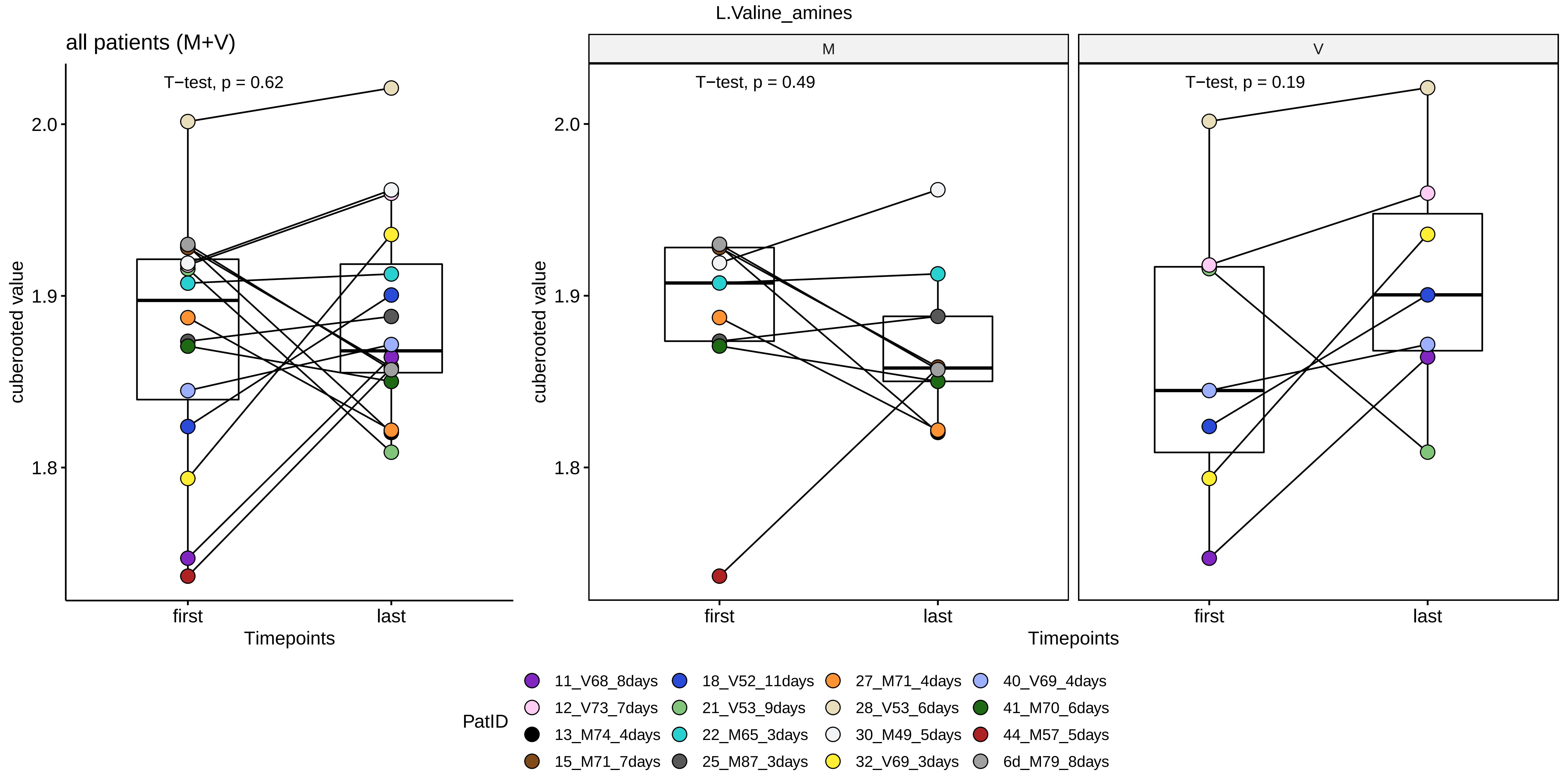
L.Valine_amines
all patients (M+V)
T−test, p = 0.62
M
T−test, p = 0.49
V
T−test, p = 0.19
cuberooted value
2.0
1.9
1.8
first
last
Timepoints
PatID
11_V68_8days
12_V73_7days
13_M74_4days
15_M71_7days
18_V52_11days
21_V53_9days
22_M65_3days
25_M87_3days
27_M71_4days
28_V53_6days
30_M49_5days
32_V69_3days
40_V69_4days
41_M70_6days
44_M57_5days
6d_M79_8days

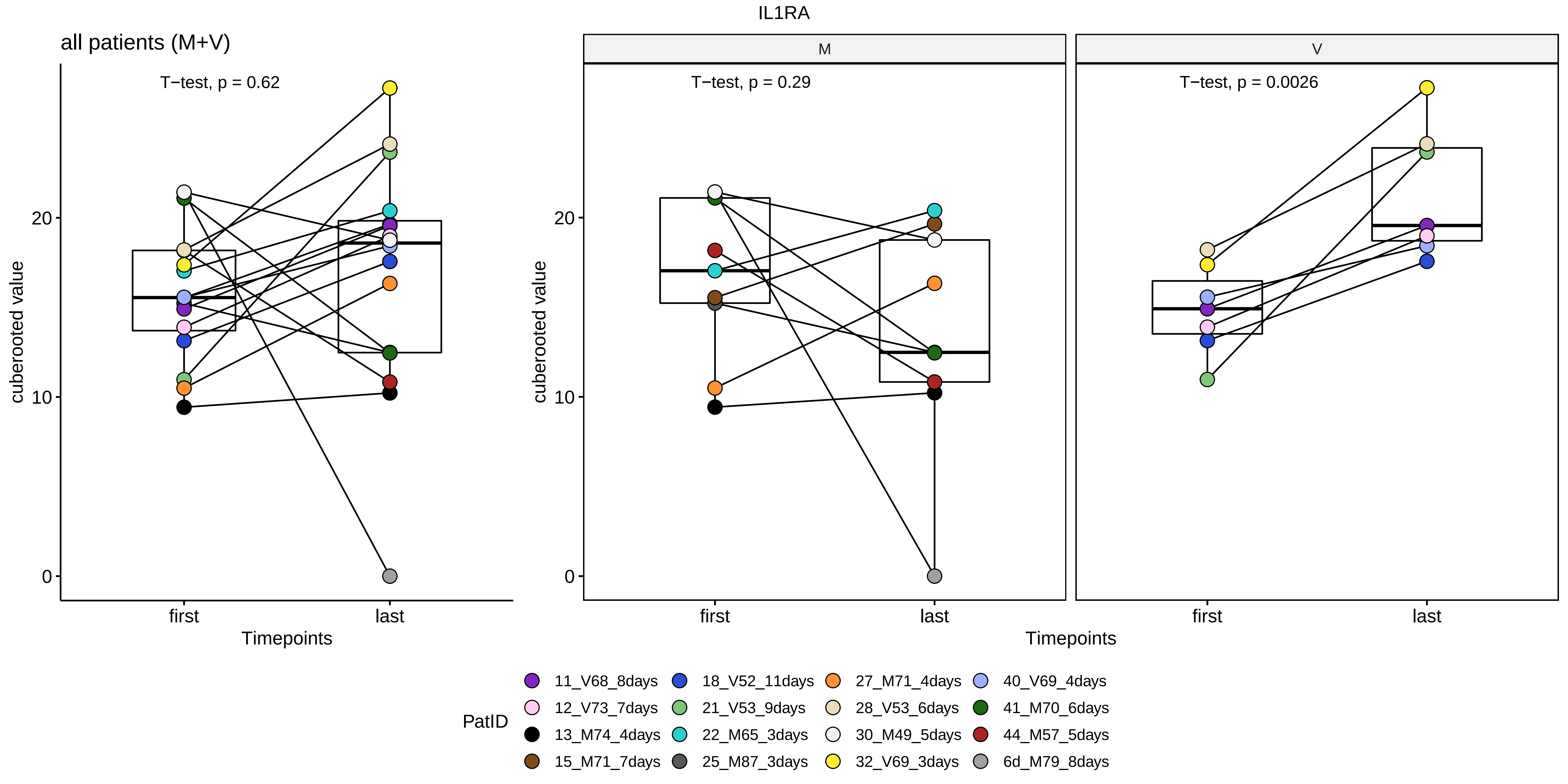
IL1RA
all patients (M+V)
T−test, p = 0.62
cuberooted value
Timepoints
first
last
M
T−test, p = 0.29
V
T−test, p = 0.0026
PatID
11_V68_8days
12_V73_7days
13_M74_4days
15_M71_7days
18_V52_11days
21_V53_9days
22_M65_3days
25_M87_3days
27_M71_4days
28_V53_6days
30_M49_5days
32_V69_3days
40_V69_4days
41_M70_6days
44_M57_5days
6d_M79_8days

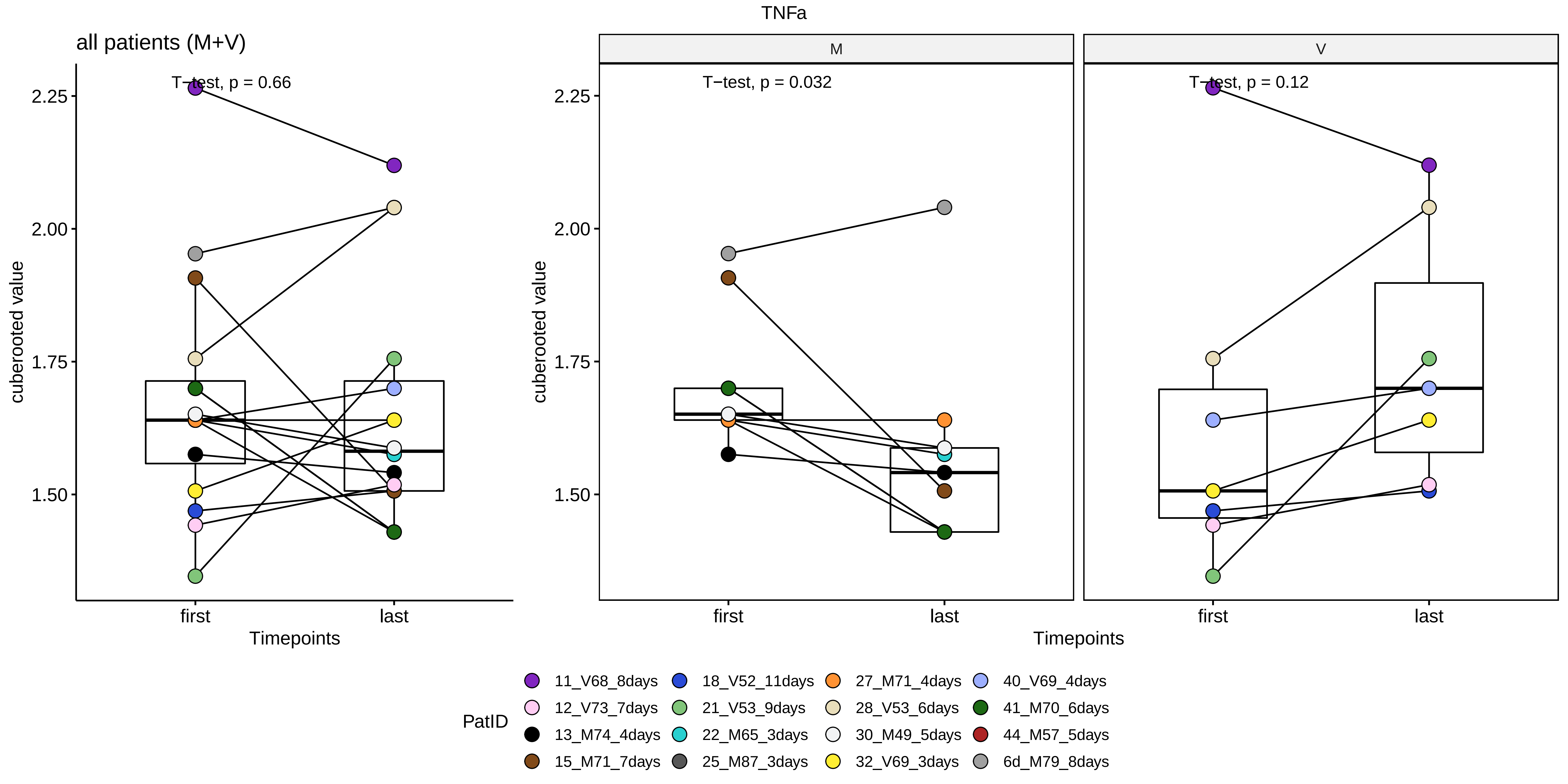
TNFa
all patients (M+V)
T−test, p = 0.66
cuberooted value
2.25
2.00
1.75
1.50
first
last
Timepoints
M
V
T−test, p = 0.032
T−test, p = 0.12
PatID
11_V68_8days
12_V73_7days
13_M74_4days
15_M71_7days
18_V52_11days
21_V53_9days
22_M65_3days
25_M87_3days
27_M71_4days
28_V53_6days
30_M49_5days
32_V69_3days
40_V69_4days
41_M70_6days
44_M57_5days
6d_M79_8days

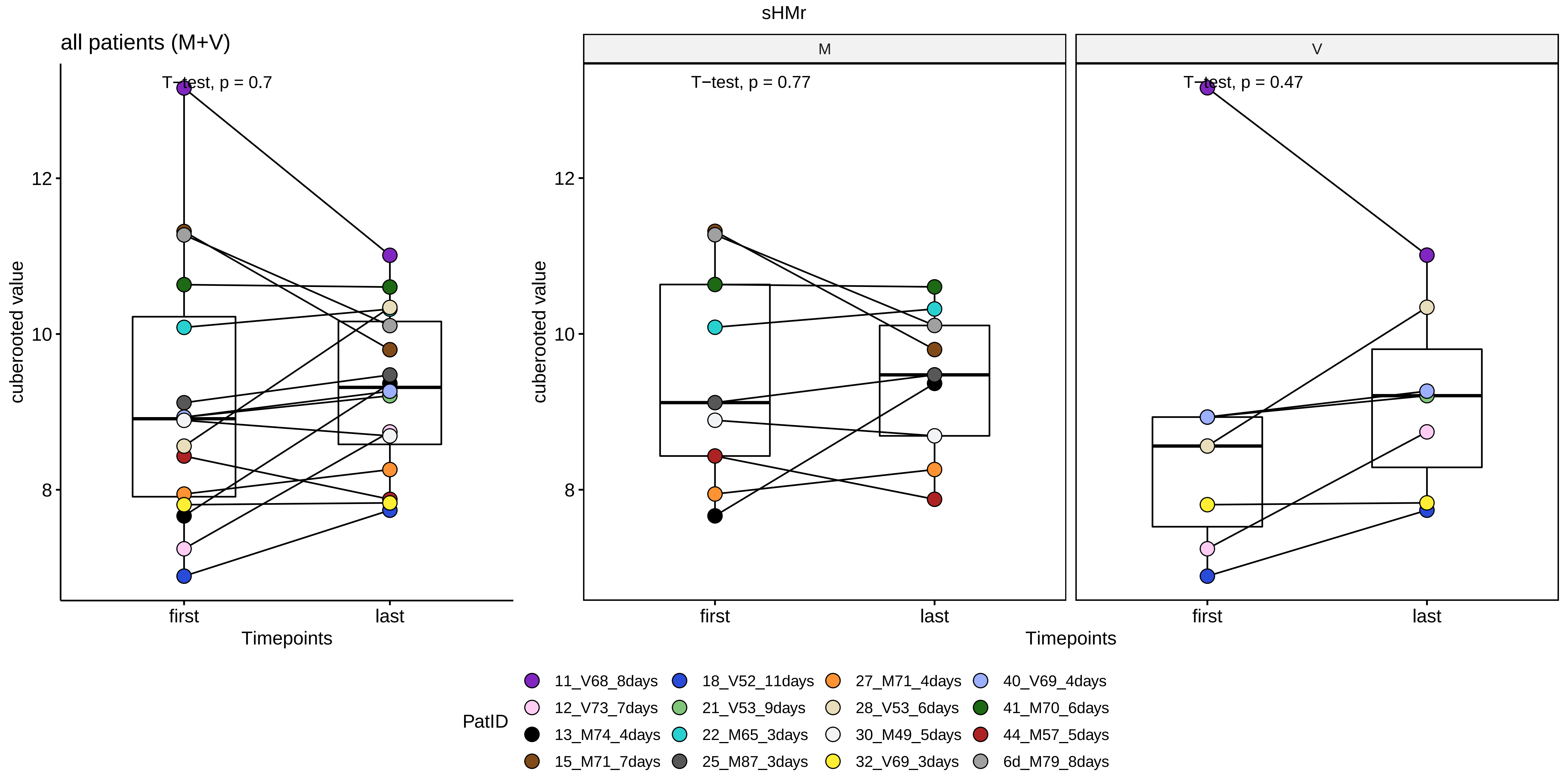

sHMr
all patients (M+V)
M
V
T−test, p = 0.7
T−test, p = 0.77
T−test, p = 0.47
cuberooted value
12
10
8
first
last
Timepoints
PatID
11_V68_8days
12_V73_7days
13_M74_4days
15_M71_7days
18_V52_11days
21_V53_9days
22_M65_3days
25_M87_3days
27_M71_4days
28_V53_6days
30_M49_5days
32_V69_3days
40_V69_4days
41_M70_6days
44_M57_5days
6d_M79_8days

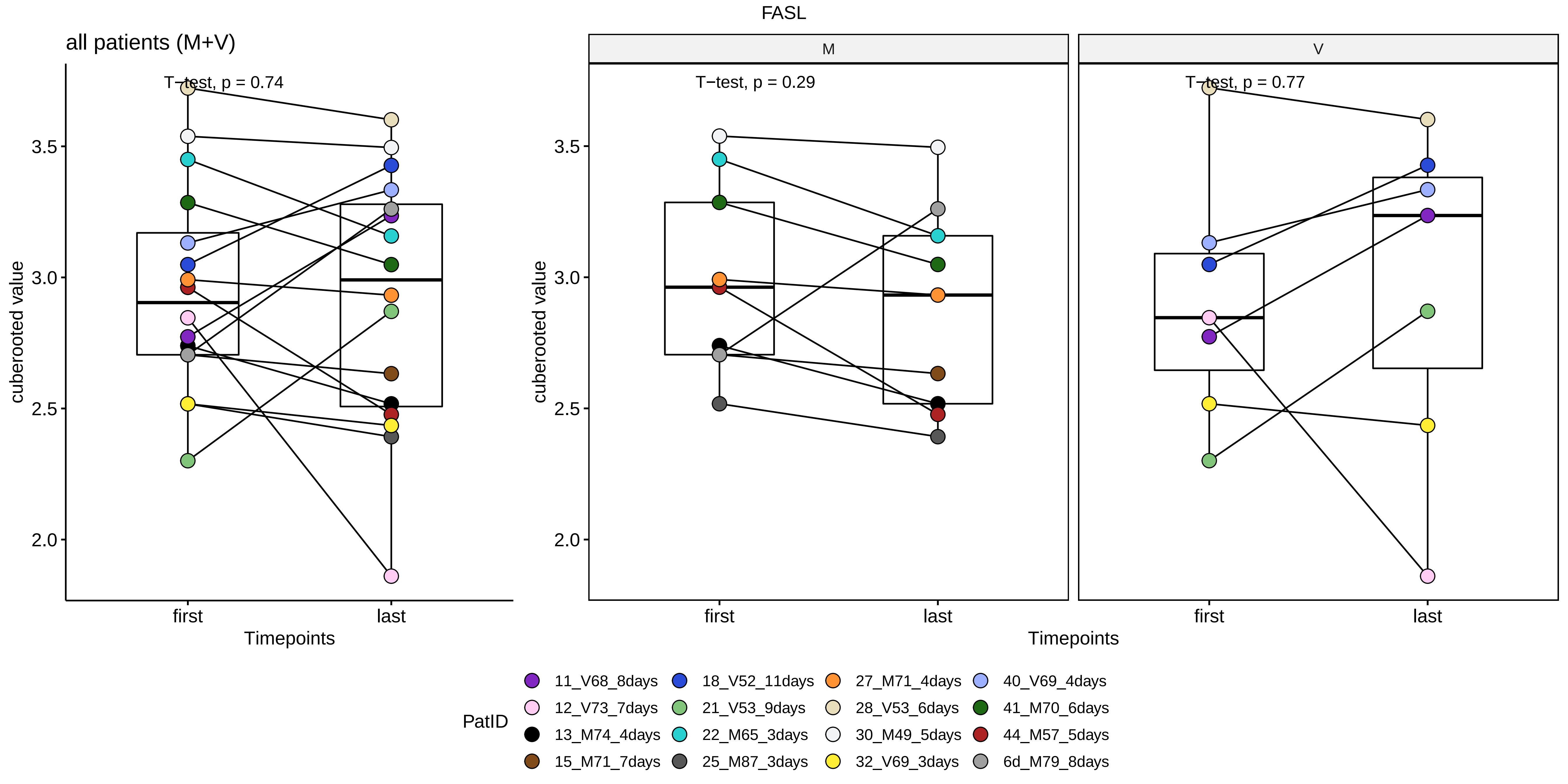
FASL
all patients (M+V)
T−test, p = 0.74
M
T−test, p = 0.29
V
T−test, p = 0.77
cuberooted value
3.5
3.0
2.5
2.0
first
last
Timepoints
PatID
11_V68_8days
12_V73_7days
13_M74_4days
15_M71_7days
18_V52_11days
21_V53_9days
22_M65_3days
25_M87_3days
27_M71_4days
28_V53_6days
30_M49_5days
32_V69_3days
40_V69_4days
41_M70_6days
44_M57_5days
6d_M79_8days

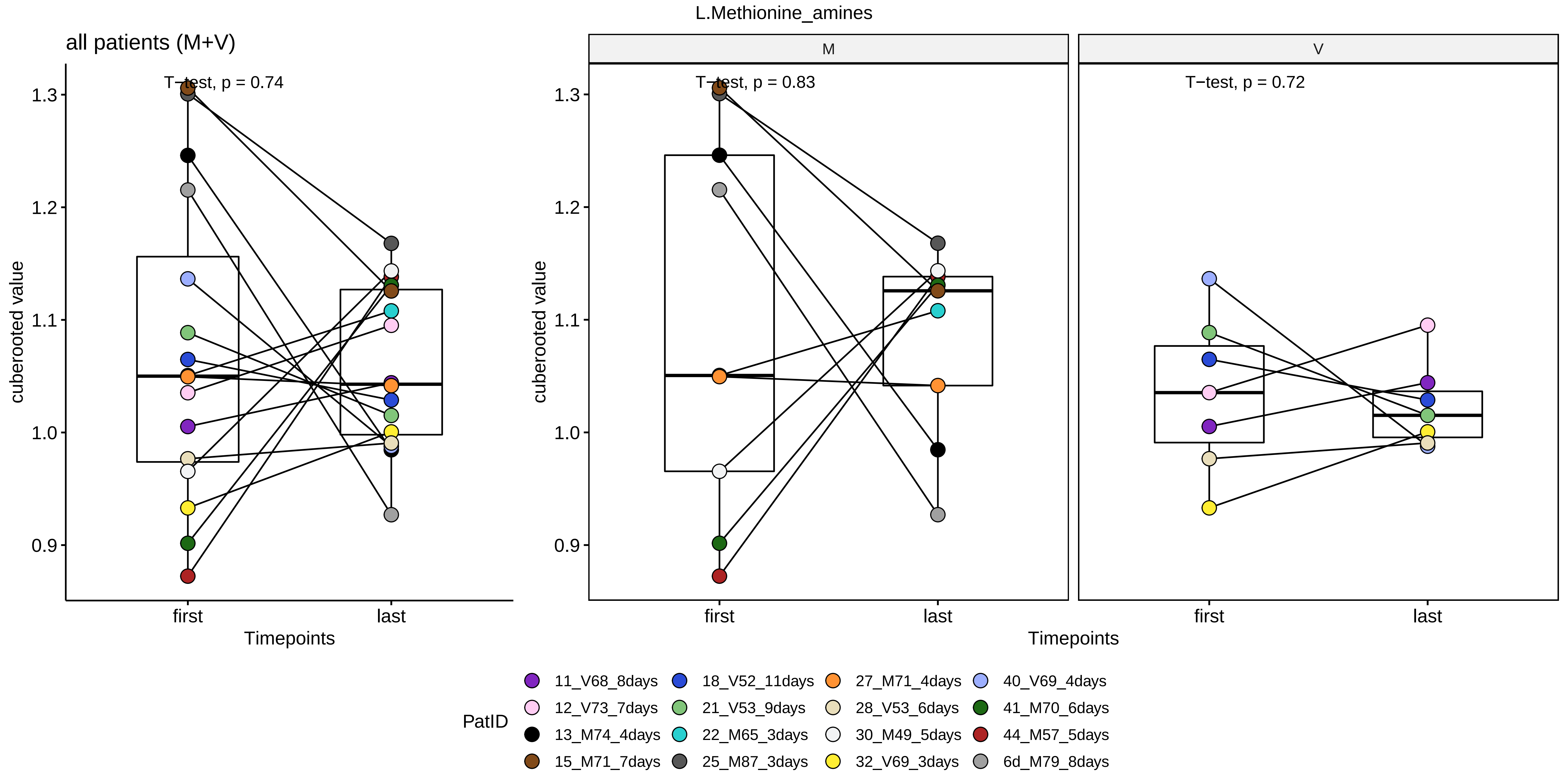
L.Methionine_amines
all patients (M+V)
T−test, p = 0.74
M
V
T−test, p = 0.83
T−test, p = 0.72
cuberooted value
1.3
1.2
1.1
1.0
0.9
first
last
Timepoints
PatID
11_V68_8days
12_V73_7days
13_M74_4days
15_M71_7days
18_V52_11days
21_V53_9days
22_M65_3days
25_M87_3days
27_M71_4days
28_V53_6days
30_M49_5days
32_V69_3days
40_V69_4days
41_M70_6days
44_M57_5days
6d_M79_8days

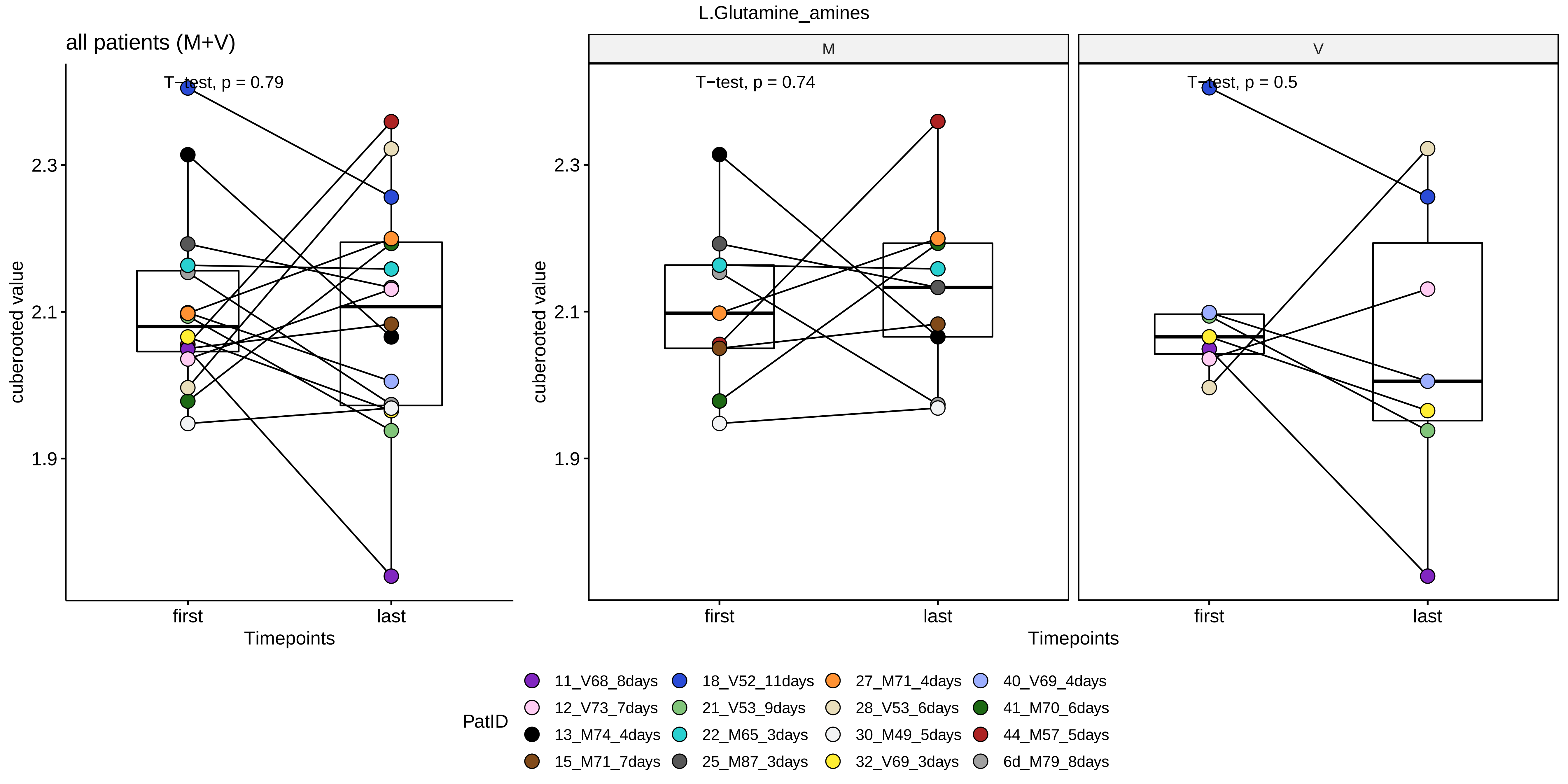

L.Glutamine_amines
all patients (M+V)
T−test, p = 0.79
M
T−test, p = 0.74
V
T−test, p = 0.5
cuberooted value
2.3
2.1
1.9
first
last
Timepoints
PatID
11_V68_8days
12_V73_7days
13_M74_4days
15_M71_7days
18_V52_11days
21_V53_9days
22_M65_3days
25_M87_3days
27_M71_4days
28_V53_6days
30_M49_5days
32_V69_3days
40_V69_4days
41_M70_6days
44_M57_5days
6d_M79_8days

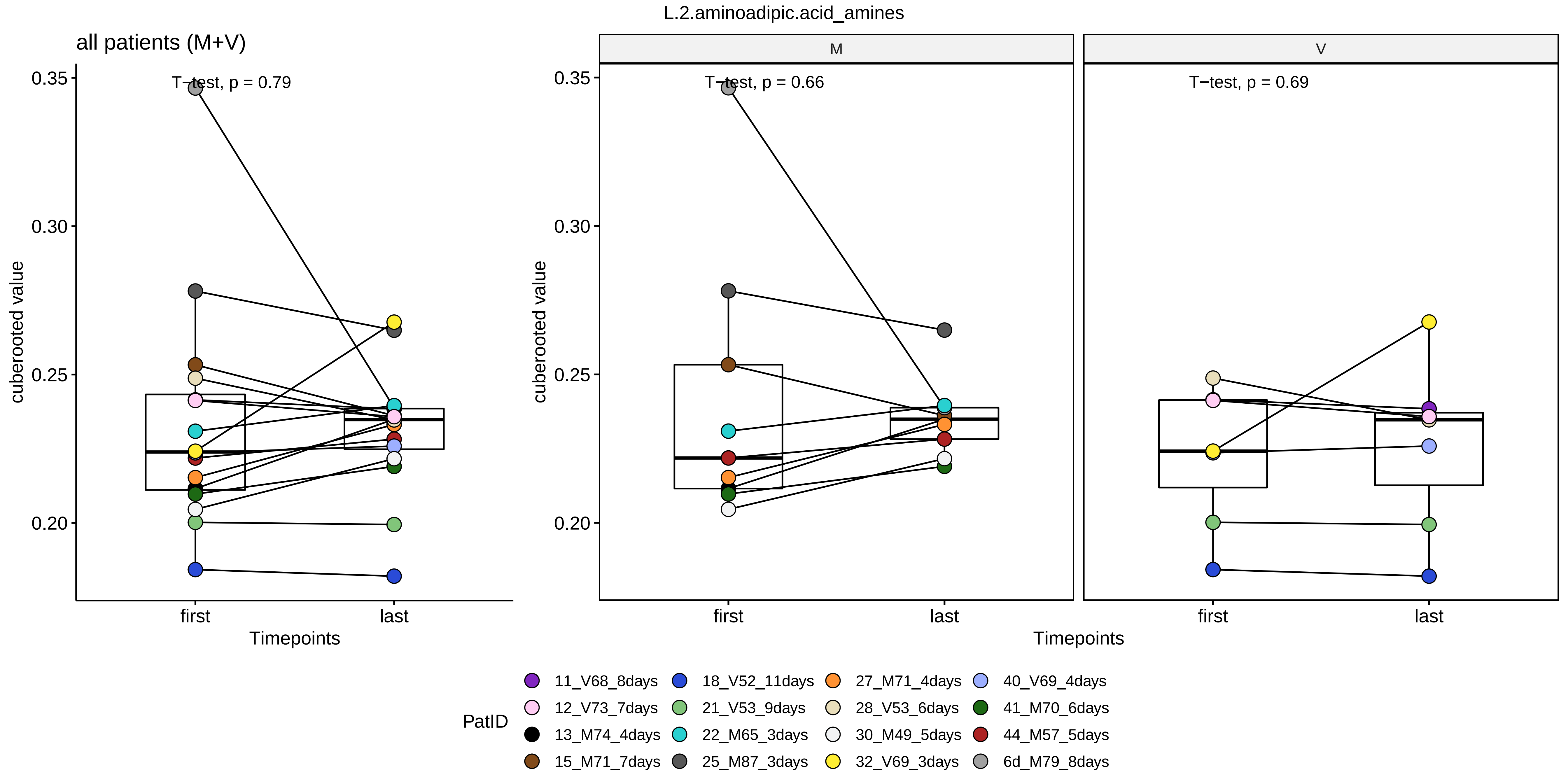

L.2.aminoadipic.acid_amines
all patients (M+V)
T−test, p = 0.79
cuberooted value
0.35
0.30
0.25
0.20
first
last
Timepoints
M
T−test, p = 0.66
V
T−test, p = 0.69
PatID
11_V68_8days
12_V73_7days
13_M74_4days
15_M71_7days
18_V52_11days
21_V53_9days
22_M65_3days
25_M87_3days
27_M71_4days
28_V53_6days
30_M49_5days
32_V69_3days
40_V69_4days
41_M70_6days
44_M57_5days
6d_M79_8days

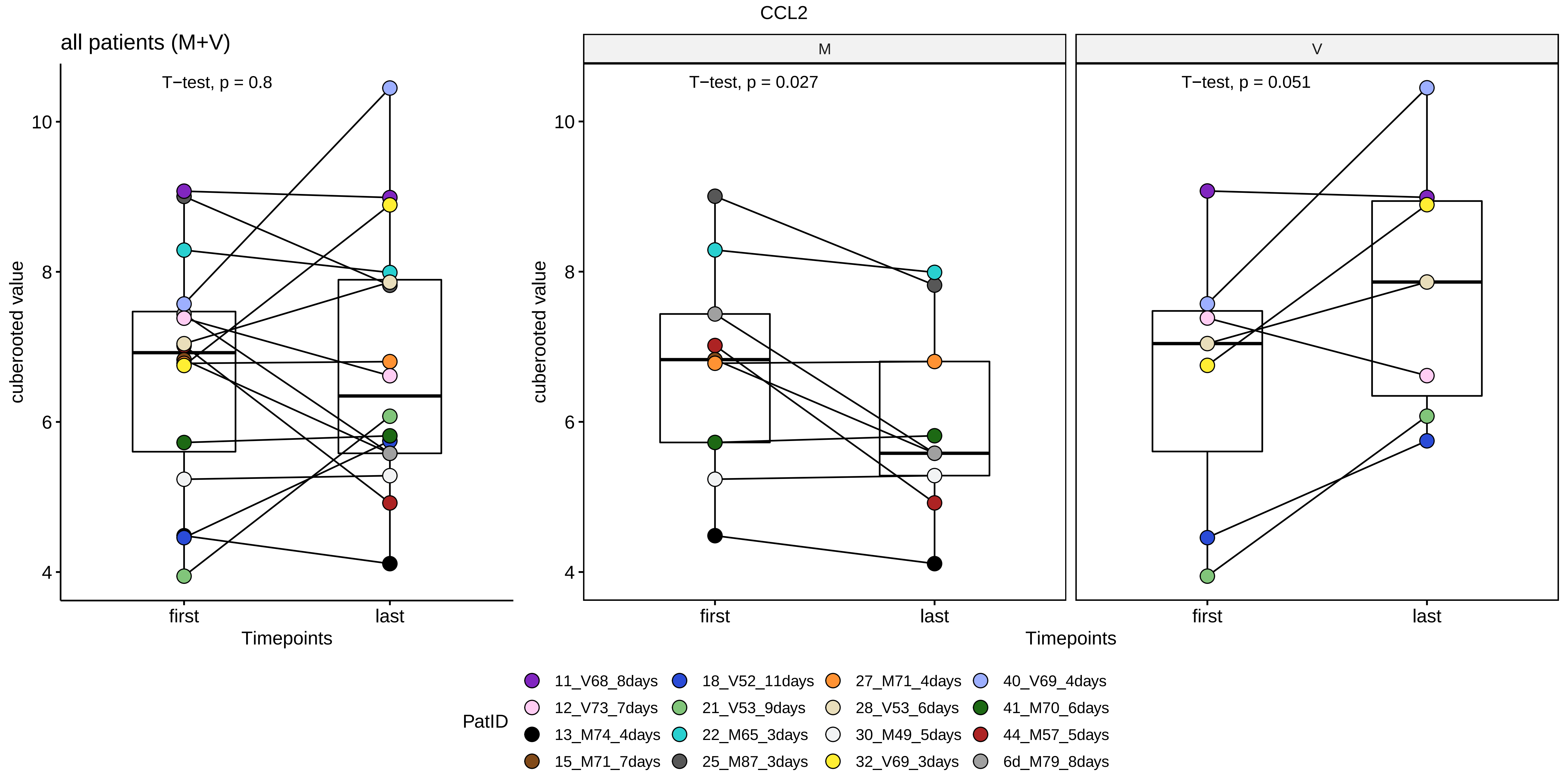

CCL2
all patients (M+V)
T−test, p = 0.8
M
T−test, p = 0.027
V
T−test, p = 0.051
cuberooted value
10
8
6
4
first
last
Timepoints
PatID
11_V68_8days
12_V73_7days
13_M74_4days
15_M71_7days
18_V52_11days
21_V53_9days
22_M65_3days
25_M87_3days
27_M71_4days
28_V53_6days
30_M49_5days
32_V69_3days
40_V69_4days
41_M70_6days
44_M57_5days
6d_M79_8days

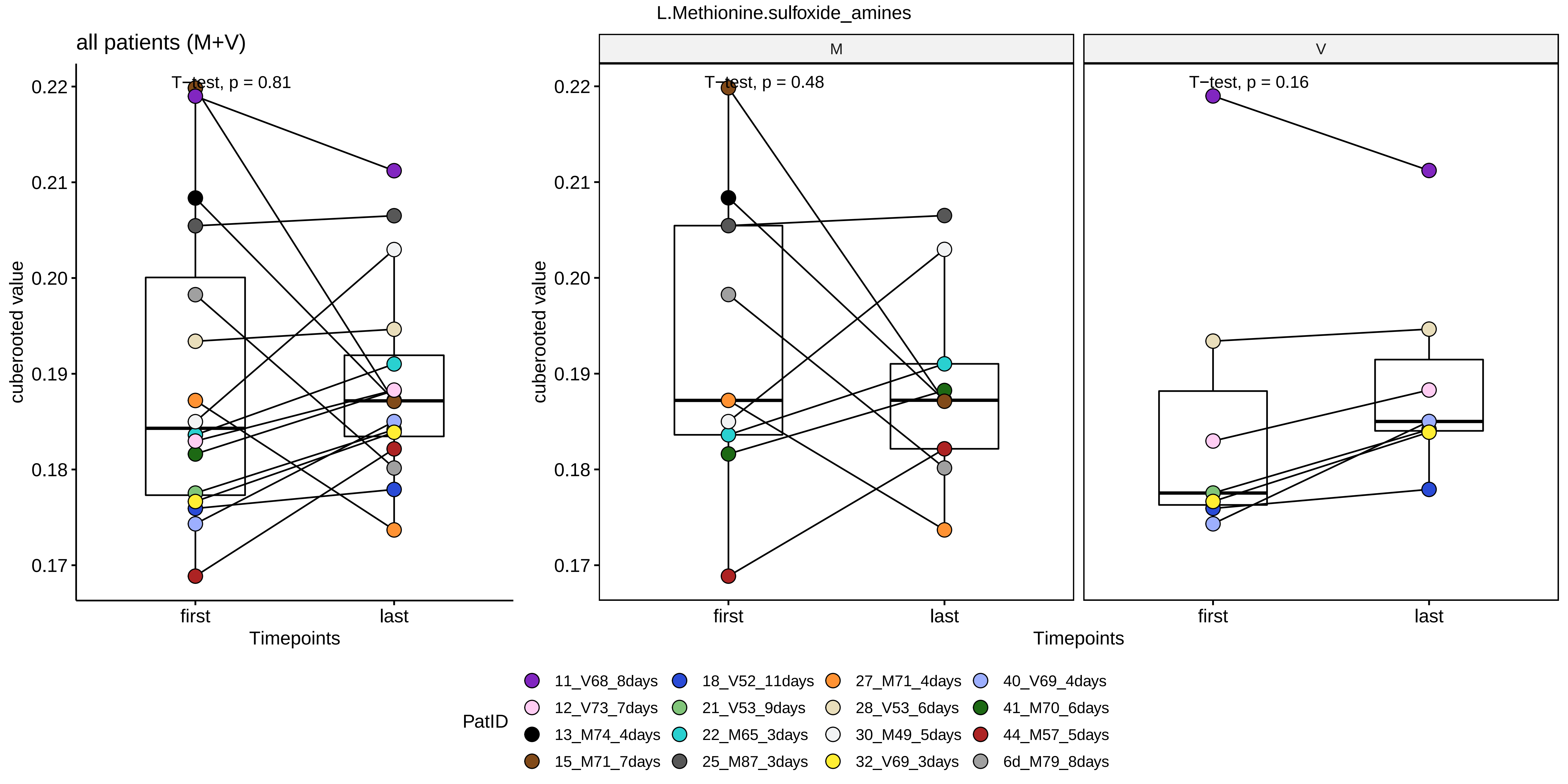

L.Methionine.sulfoxide_amines
all patients (M+V)
T−test, p = 0.81
M
T−test, p = 0.48
V
T−test, p = 0.16
cuberooted value
0.22
0.21
0.20
0.19
0.18
0.17
first
last
Timepoints
PatID
11_V68_8days
12_V73_7days
13_M74_4days
15_M71_7days
18_V52_11days
21_V53_9days
22_M65_3days
25_M87_3days
27_M71_4days
28_V53_6days
30_M49_5days
32_V69_3days
40_V69_4days
41_M70_6days
44_M57_5days
6d_M79_8days

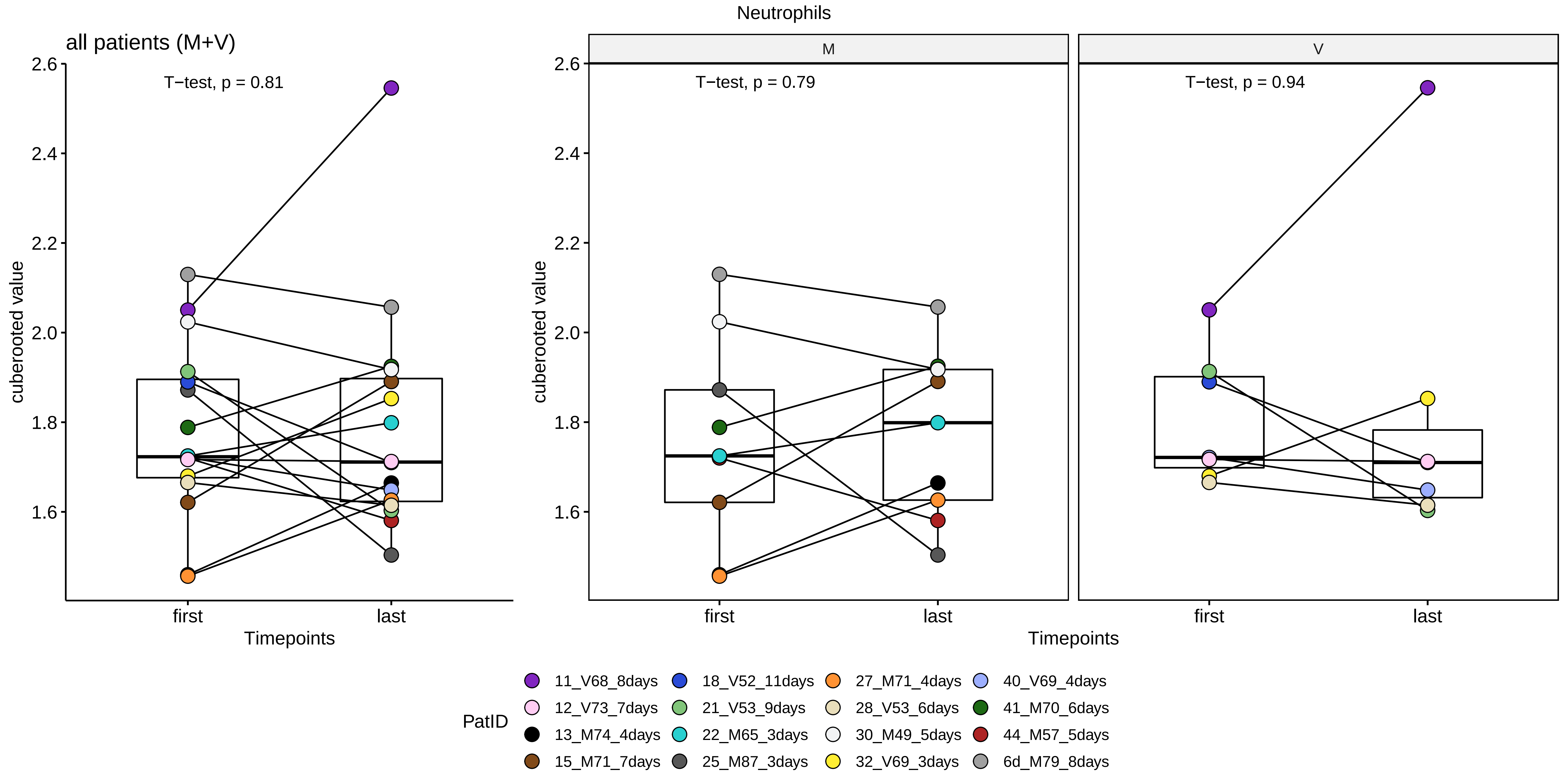
Neutrophils
all patients (M+V)
T−test, p = 0.81
M
T−test, p = 0.79
V
T−test, p = 0.94
cuberooted value
2.6
2.4
2.2
2.0
1.8
1.6
first
last
Timepoints
PatID
11_V68_8days
12_V73_7days
13_M74_4days
15_M71_7days
18_V52_11days
21_V53_9days
22_M65_3days
25_M87_3days
27_M71_4days
28_V53_6days
30_M49_5days
32_V69_3days
40_V69_4days
41_M70_6days
44_M57_5days
6d_M79_8days

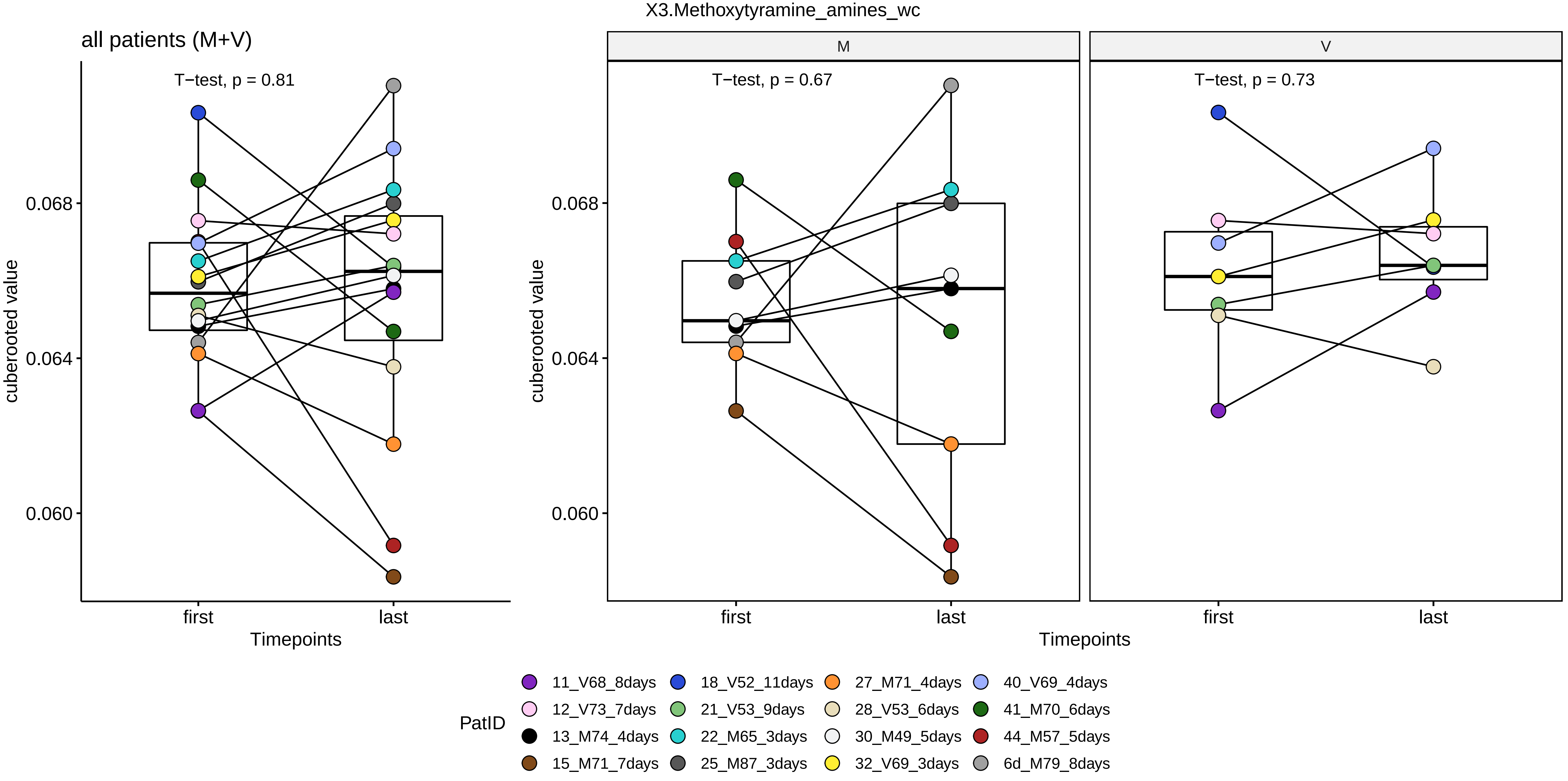

X3.Methoxytyramine_amines_wc
all patients (M+V)
T−test, p = 0.81
M
T−test, p = 0.67
V
T−test, p = 0.73
cuberooted value
0.068
0.064
0.060
first
last
Timepoints
PatID
11_V68_8days
12_V73_7days
13_M74_4days
15_M71_7days
18_V52_11days
21_V53_9days
22_M65_3days
25_M87_3days
27_M71_4days
28_V53_6days
30_M49_5days
32_V69_3days
40_V69_4days
41_M70_6days
44_M57_5days
6d_M79_8days

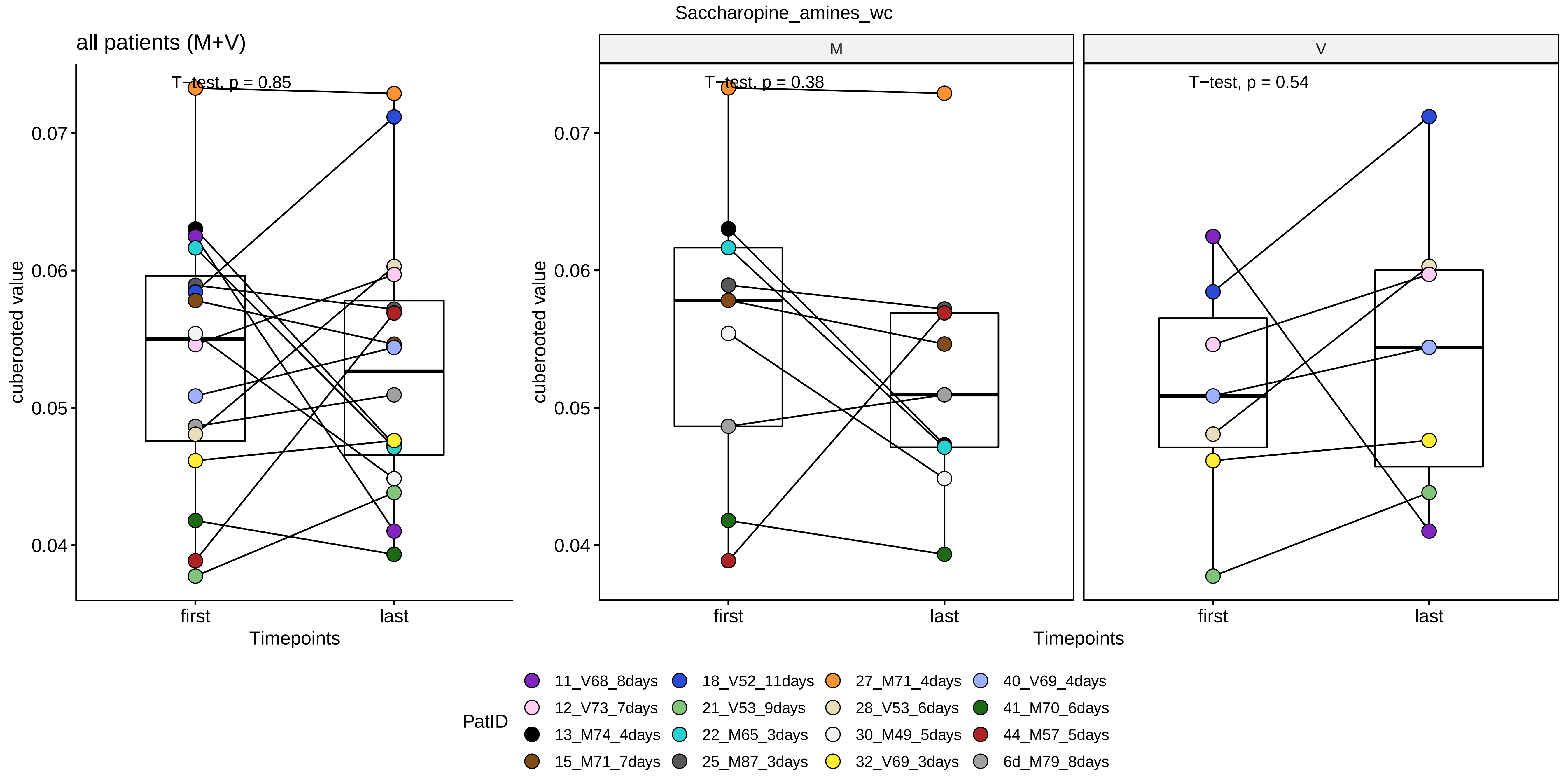
Saccharopine_amines_wc
all patients (M+V)
T−test, p = 0.85
M
T−test, p = 0.38
V
T−test, p = 0.54
cuberooted value
0.04
0.05
0.06
0.07
first
last
Timepoints
PatID
11_V68_8days
12_V73_7days
13_M74_4days
15_M71_7days
18_V52_11days
21_V53_9days
22_M65_3days
25_M87_3days
27_M71_4days
28_V53_6days
30_M49_5days
32_V69_3days
40_V69_4days
41_M70_6days
44_M57_5days
6d_M79_8days

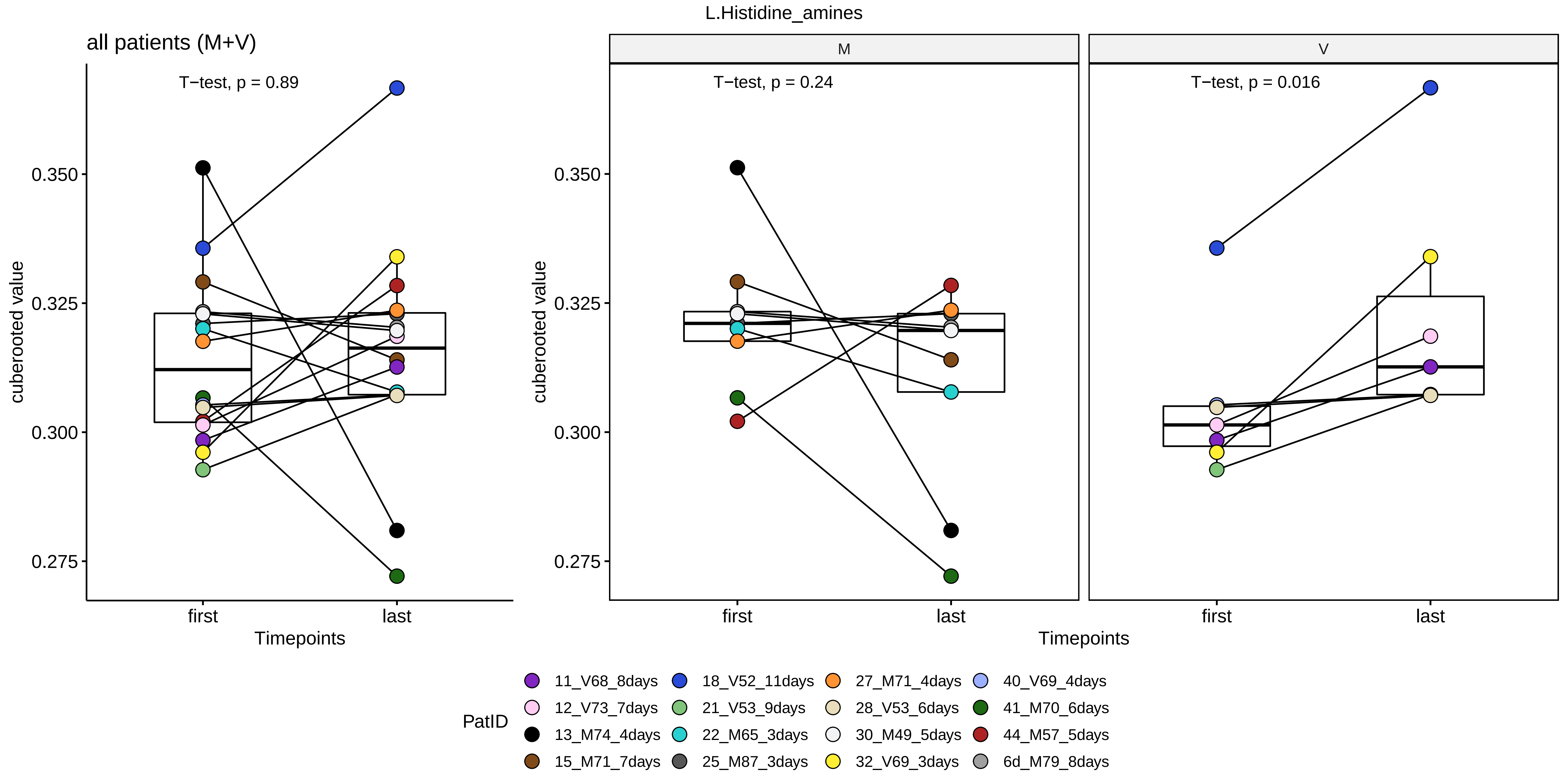

L.Histidine_amines
all patients (M+V)
T−test, p = 0.89
M
T−test, p = 0.24
V
T−test, p = 0.016
cuberooted value
0.350
0.325
0.300
0.275
first
last
Timepoints
PatID
11_V68_8days
12_V73_7days
13_M74_4days
15_M71_7days
18_V52_11days
21_V53_9days
22_M65_3days
25_M87_3days
27_M71_4days
28_V53_6days
30_M49_5days
32_V69_3days
40_V69_4days
41_M70_6days
44_M57_5days
6d_M79_8days

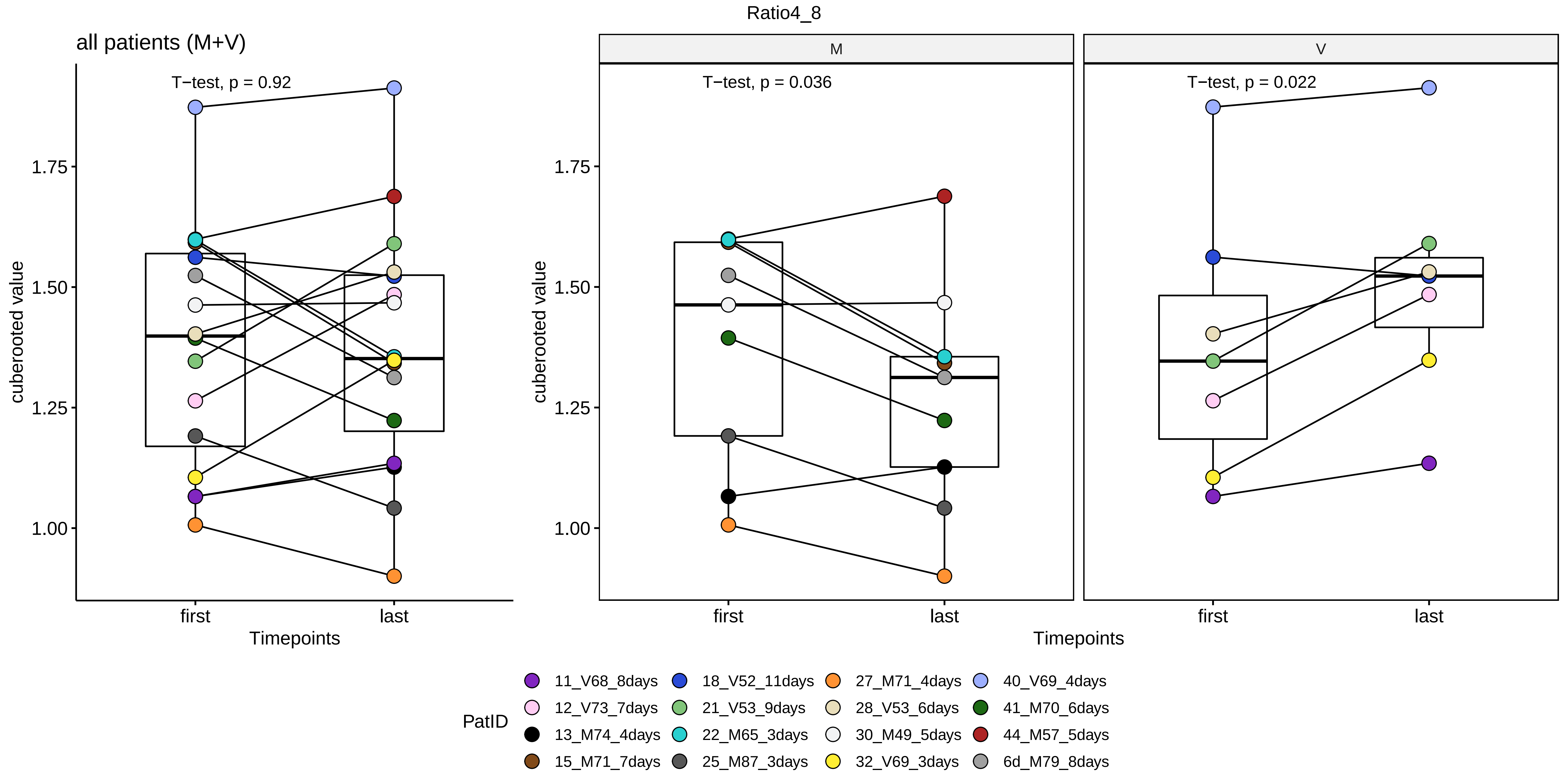

Ratio4_8
all patients (M+V)
T−test, p = 0.92
M
T−test, p = 0.036
V
T−test, p = 0.022
cuberooted value
1.75
1.50
1.25
1.00
first
last
Timepoints
PatID
11_V68_8days
12_V73_7days
13_M74_4days
15_M71_7days
18_V52_11days
21_V53_9days
22_M65_3days
25_M87_3days
27_M71_4days
28_V53_6days
30_M49_5days
32_V69_3days
40_V69_4days
41_M70_6days
44_M57_5days
6d_M79_8days

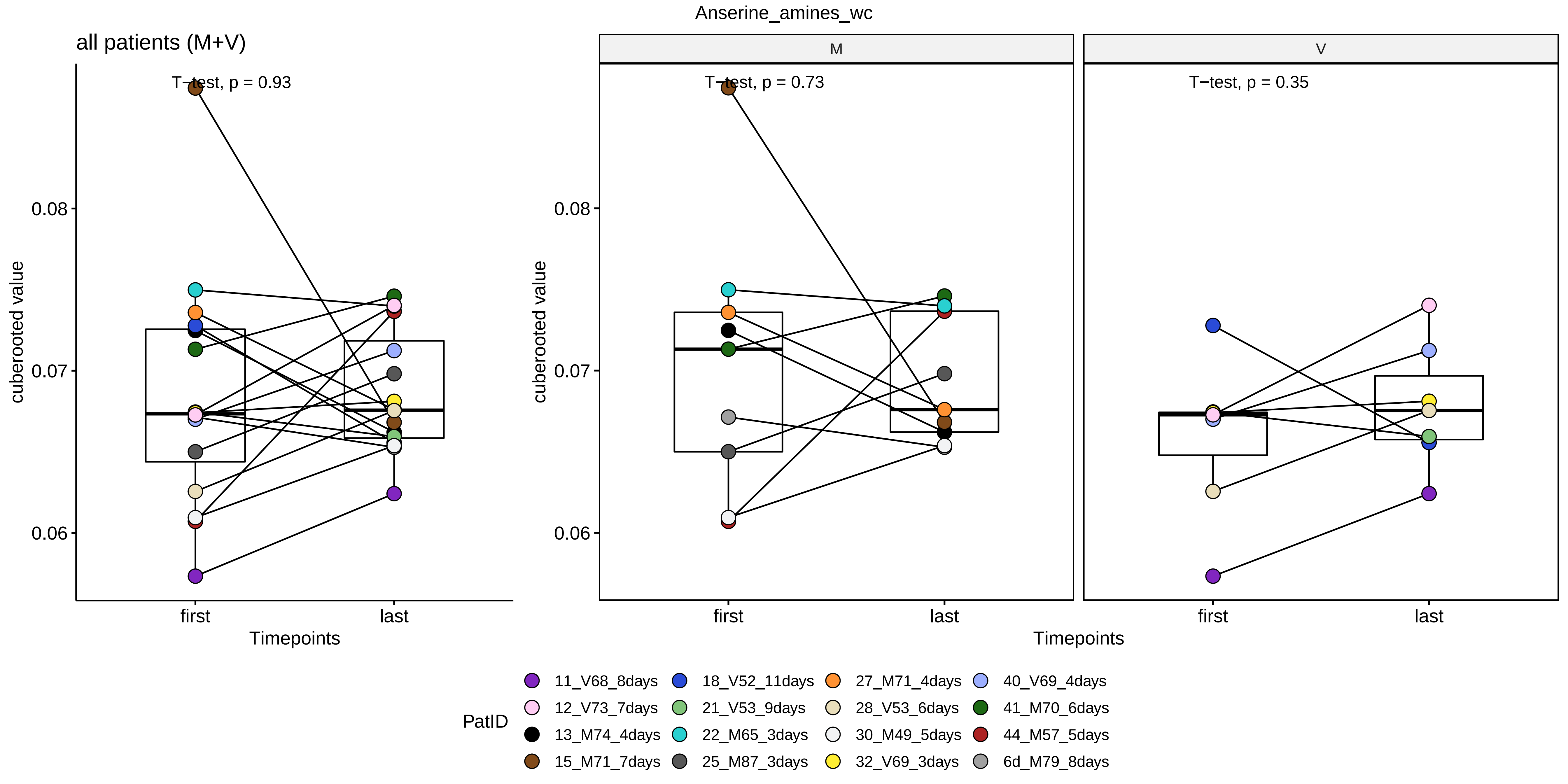

Anserine_amines_wc
all patients (M+V)
T−test, p = 0.93
cuberooted value
0.08
0.07
0.06
first
last
Timepoints
M
V
T−test, p = 0.73
T−test, p = 0.35
PatID
11_V68_8days
12_V73_7days
13_M74_4days
15_M71_7days
18_V52_11days
21_V53_9days
22_M65_3days
25_M87_3days
27_M71_4days
28_V53_6days
30_M49_5days
32_V69_3days
40_V69_4days
41_M70_6days
44_M57_5days
6d_M79_8days

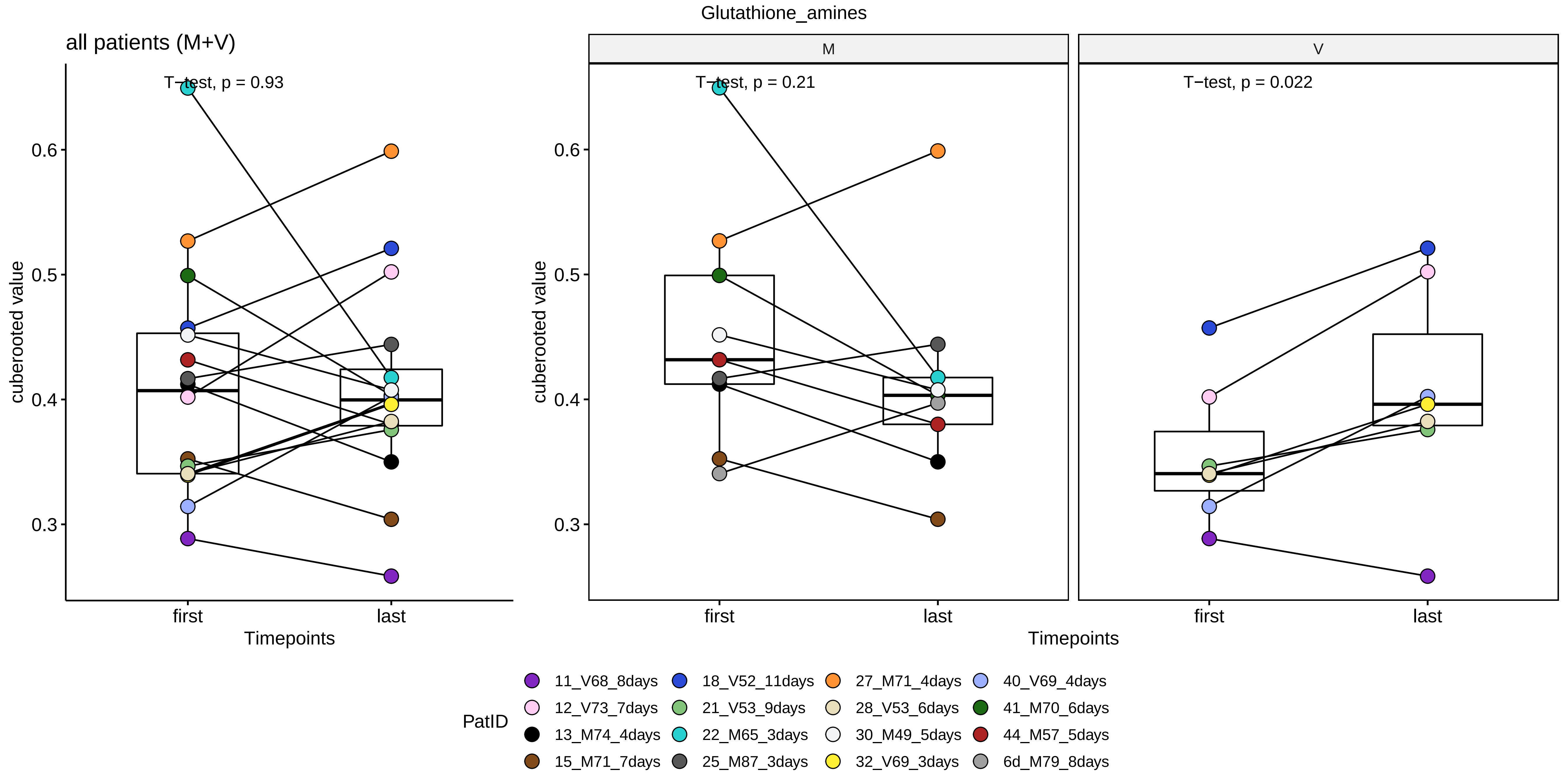

Glutathione_amines
all patients (M+V)
T−test, p = 0.93
cuberooted value
0.6
0.5
0.4
0.3
first
last
Timepoints
M
V
T−test, p = 0.21
T−test, p = 0.022
PatID
11_V68_8days
12_V73_7days
13_M74_4days
15_M71_7days
18_V52_11days
21_V53_9days
22_M65_3days
25_M87_3days
27_M71_4days
28_V53_6days
30_M49_5days
32_V69_3days
40_V69_4days
41_M70_6days
44_M57_5days
6d_M79_8days

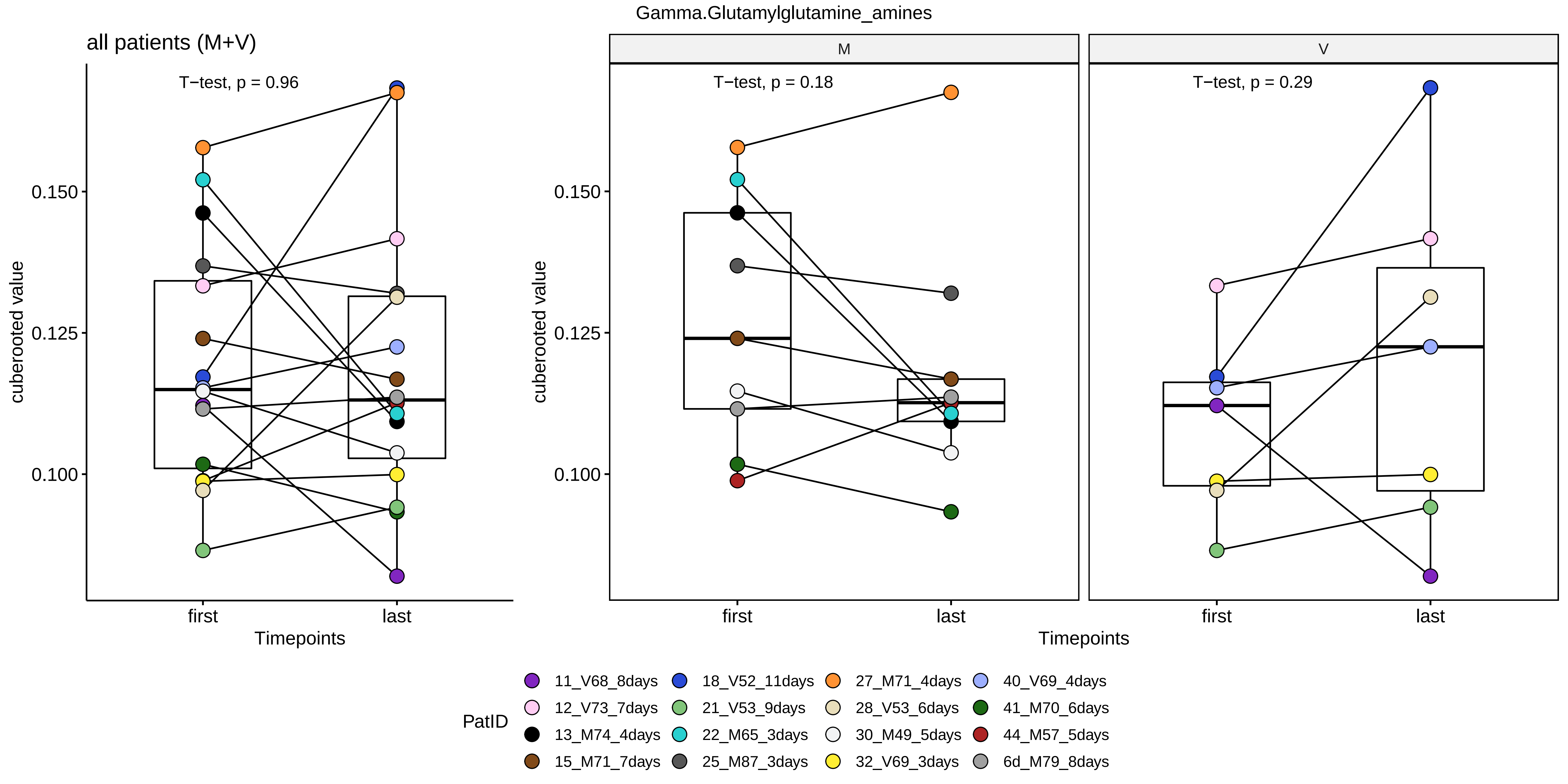
Gamma.Glutamylglutamine_amines
all patients (M+V)
T−test, p = 0.96
cuberooted value
0.150
0.125
0.100
first
last
Timepoints
M
T−test, p = 0.18
V
T−test, p = 0.29
PatID
11_V68_8days
12_V73_7days
13_M74_4days
15_M71_7days
18_V52_11days
21_V53_9days
22_M65_3days
25_M87_3days
27_M71_4days
28_V53_6days
30_M49_5days
32_V69_3days
40_V69_4days
41_M70_6days
44_M57_5days
6d_M79_8days

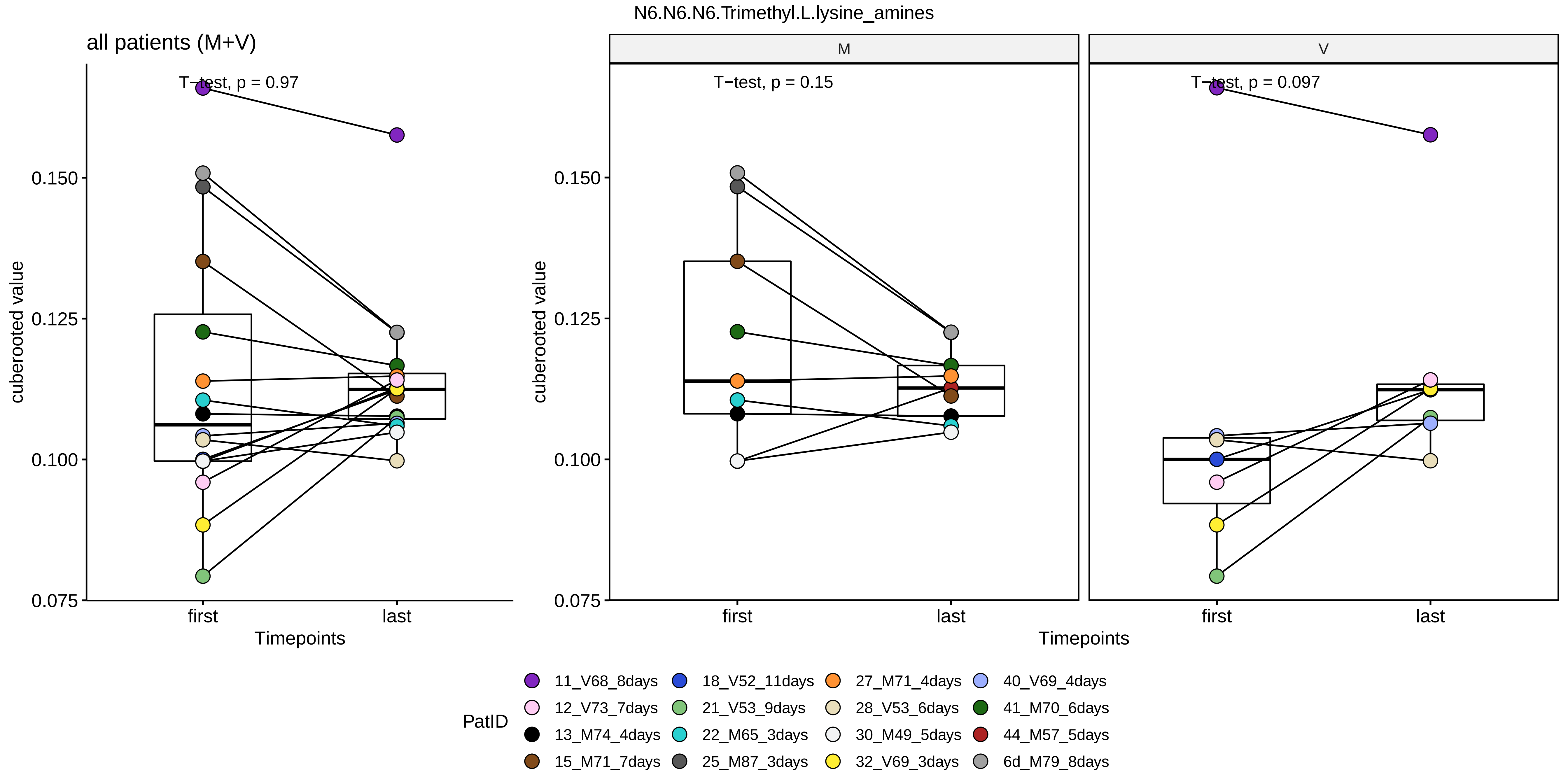

N6.N6.N6.Trimethyl.L.lysine_amines
all patients (M+V)
T−test, p = 0.97
M
T−test, p = 0.15
V
T−test, p = 0.097
cuberooted value
0.075
0.100
0.125
0.150
first
last
Timepoints
PatID
11_V68_8days
12_V73_7days
13_M74_4days
15_M71_7days
18_V52_11days
21_V53_9days
22_M65_3days
25_M87_3days
27_M71_4days
28_V53_6days
30_M49_5days
32_V69_3days
40_V69_4days
41_M70_6days
44_M57_5days
6d_M79_8days

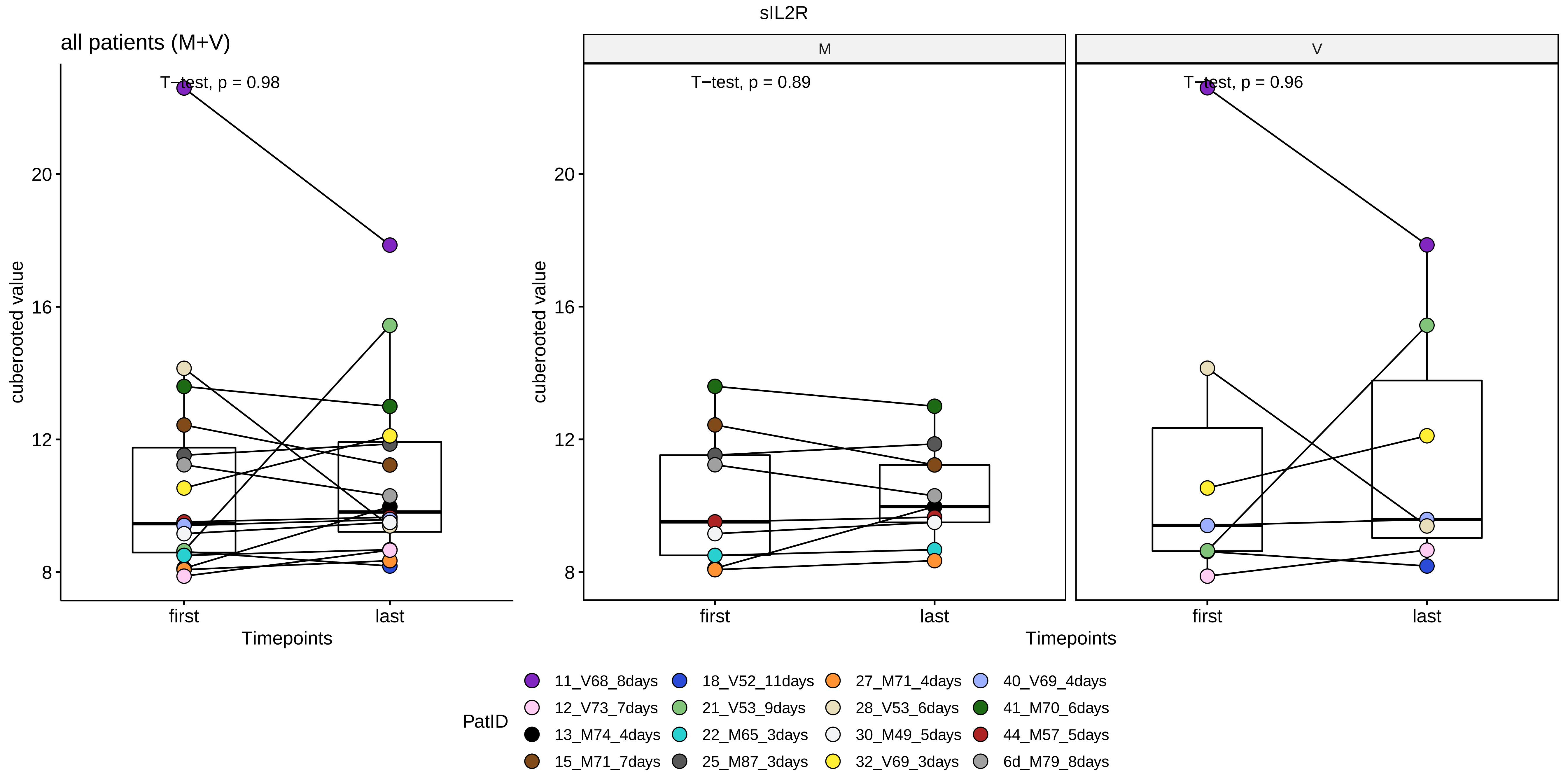

sIL2R
all patients (M+V)
M
V
T−test, p = 0.98
T−test, p = 0.89
T−test, p = 0.96
cuberooted value
8
12
16
20
first
last
Timepoints
PatID
11_V68_8days
12_V73_7days
13_M74_4days
15_M71_7days
18_V52_11days
21_V53_9days
22_M65_3days
25_M87_3days
27_M71_4days
28_V53_6days
30_M49_5days
32_V69_3days
40_V69_4days
41_M70_6days
44_M57_5days
6d_M79_8days

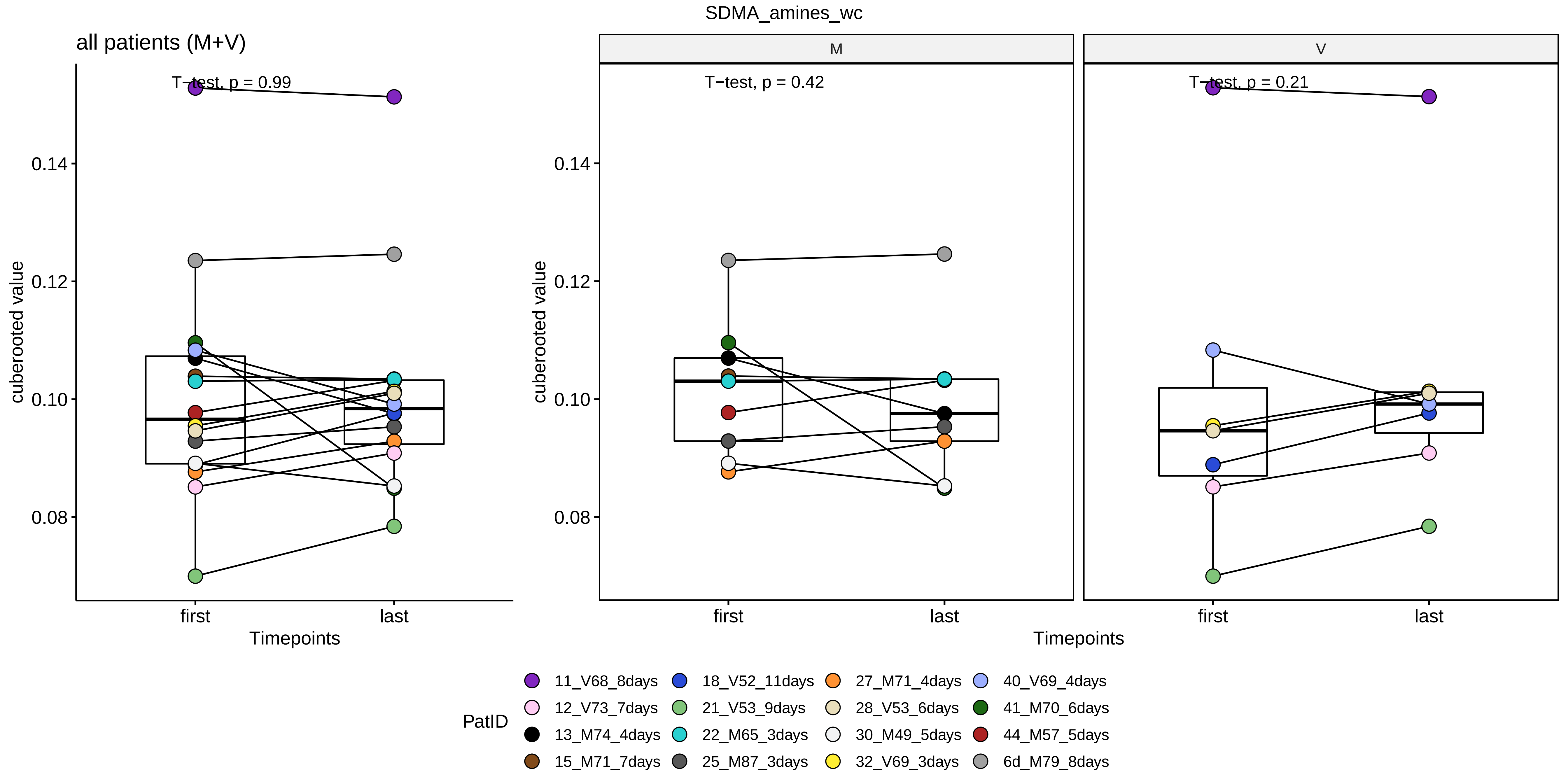

SDMA_amines_wc
all patients (M+V)
T−test, p = 0.99
M
T−test, p = 0.42
V
T−test, p = 0.21
cuberooted value
0.14
0.12
0.10
0.08
first
last
Timepoints
PatID
11_V68_8days
12_V73_7days
13_M74_4days
15_M71_7days
18_V52_11days
21_V53_9days
22_M65_3days
25_M87_3days
27_M71_4days
28_V53_6days
30_M49_5days
32_V69_3days
40_V69_4days
41_M70_6days
44_M57_5days
6d_M79_8days

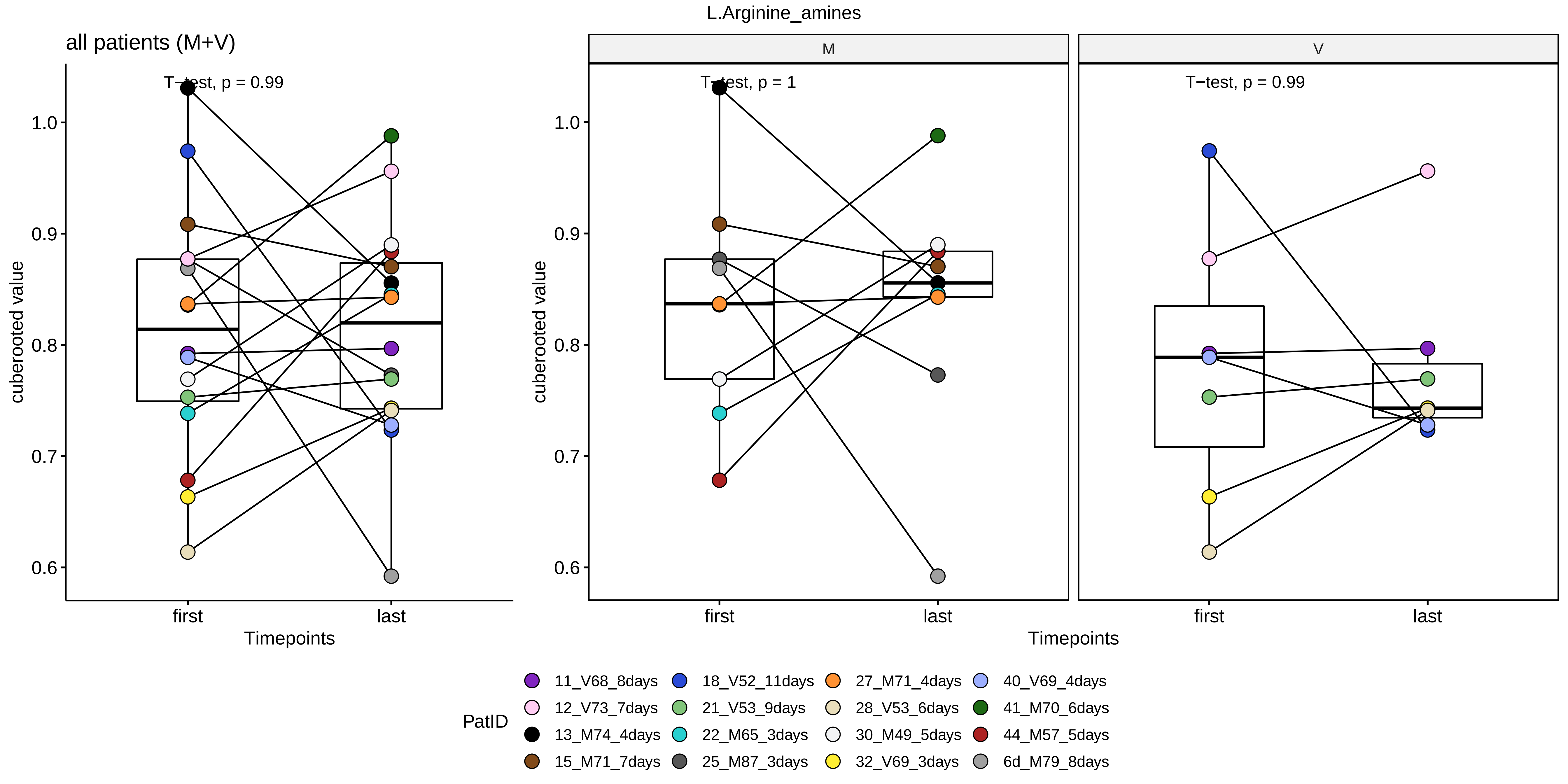

L.Arginine_amines
all patients (M+V)
T−test, p = 0.99
M
T−test, p = 1
V
T−test, p = 0.99
cuberooted value
1.0
0.9
0.8
0.7
0.6
first
last
Timepoints
PatID
11_V68_8days
12_V73_7days
13_M74_4days
15_M71_7days
18_V52_11days
21_V53_9days
22_M65_3days
25_M87_3days
27_M71_4days
28_V53_6days
30_M49_5days
32_V69_3days
40_V69_4days
41_M70_6days
44_M57_5days
6d_M79_8days
