# Supplementary material for: Severe COVID-19 Is Characterised by Perturbations in Plasma Amines Correlated with Immune Response Markers, and Linked to Inflammation and Oxidative Stress
Source: Metabolites. 2022 Jul 2;12(7):618. doi: 10.3390/metabo12070618 (PMC9321395; doi:10.3390/metabo12070618)
Supplement: Supplementary file 1 [file metabolites-12-00618-s001.zip › Supplementary figures.pptx]

## Slide 1
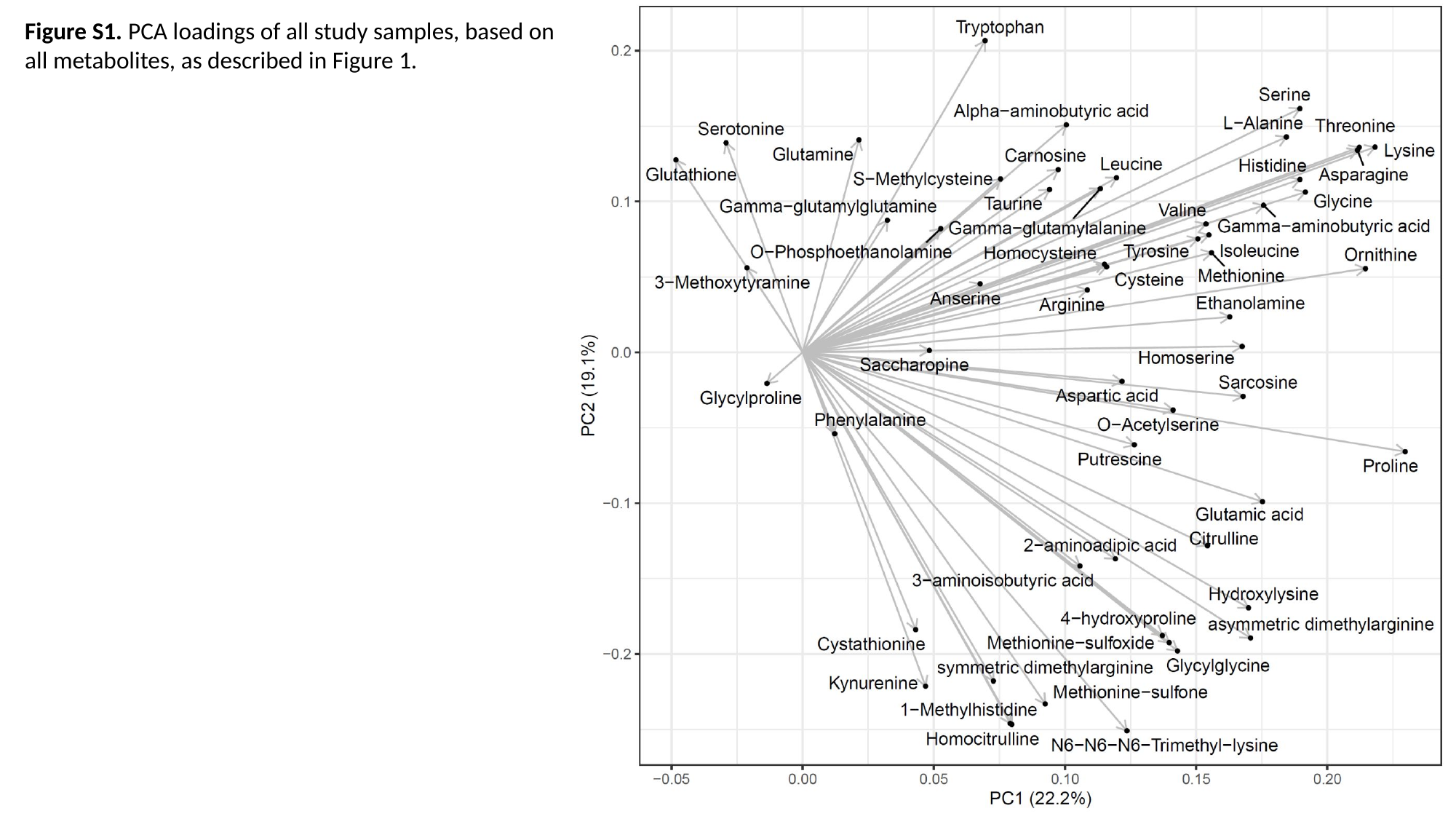

Figure S1. PCA loadings of all study samples, based on all metabolites, as described in Figure 1.

## Slide 2
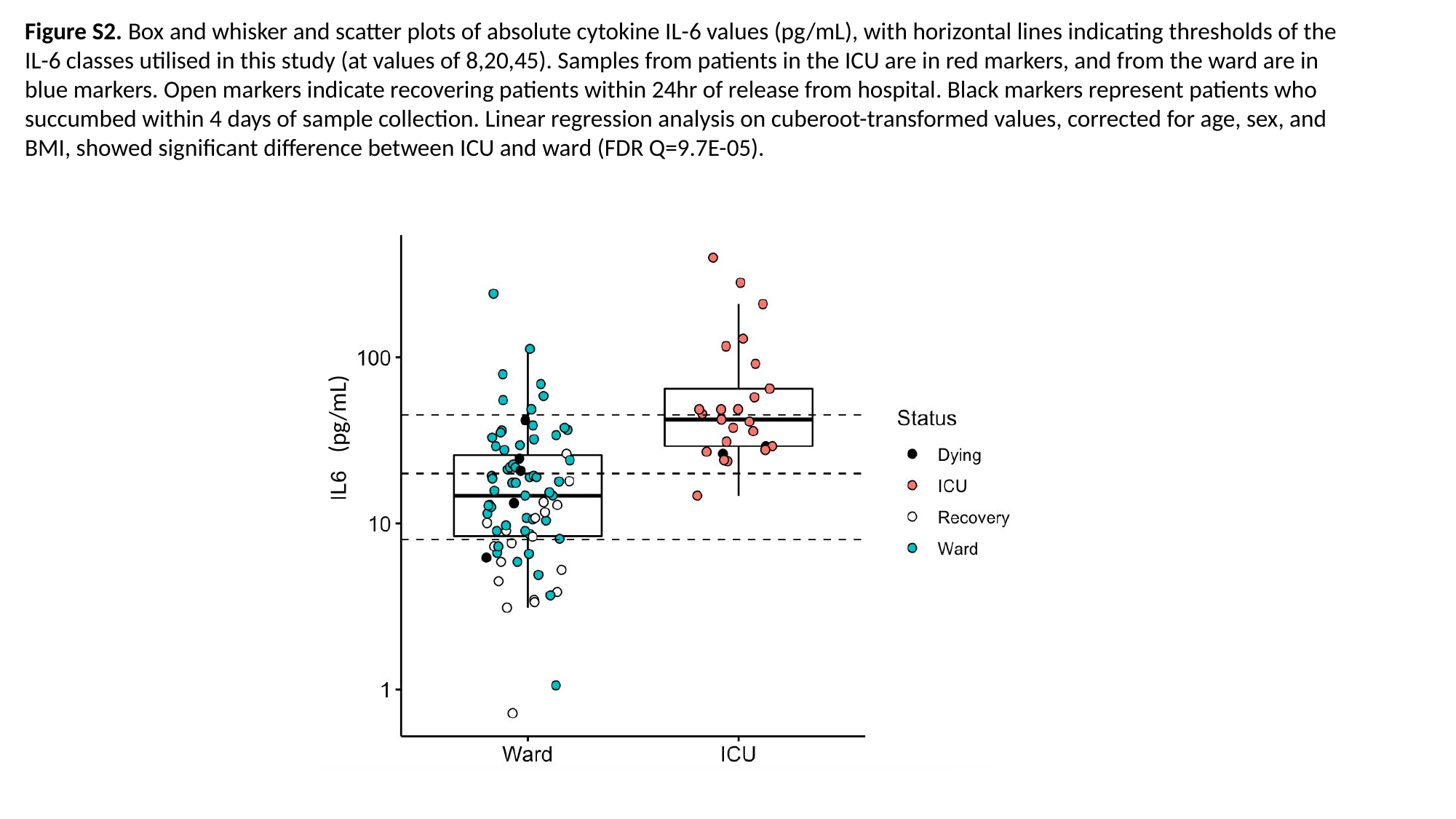

Figure S2. Box and whisker and scatter plots of absolute cytokine IL-6 values (pg/mL), with horizontal lines indicating thresholds of the IL-6 classes utilised in this study (at values of 8,20,45). Samples from patients in the ICU are in red markers, and from the ward are in blue markers. Open markers indicate recovering patients within 24hr of release from hospital. Black markers represent patients who succumbed within 4 days of sample collection. Linear regression analysis on cuberoot-transformed values, corrected for age, sex, and BMI, showed significant difference between ICU and ward (FDR Q=9.7E-05).
(pg/mL)

## Slide 3
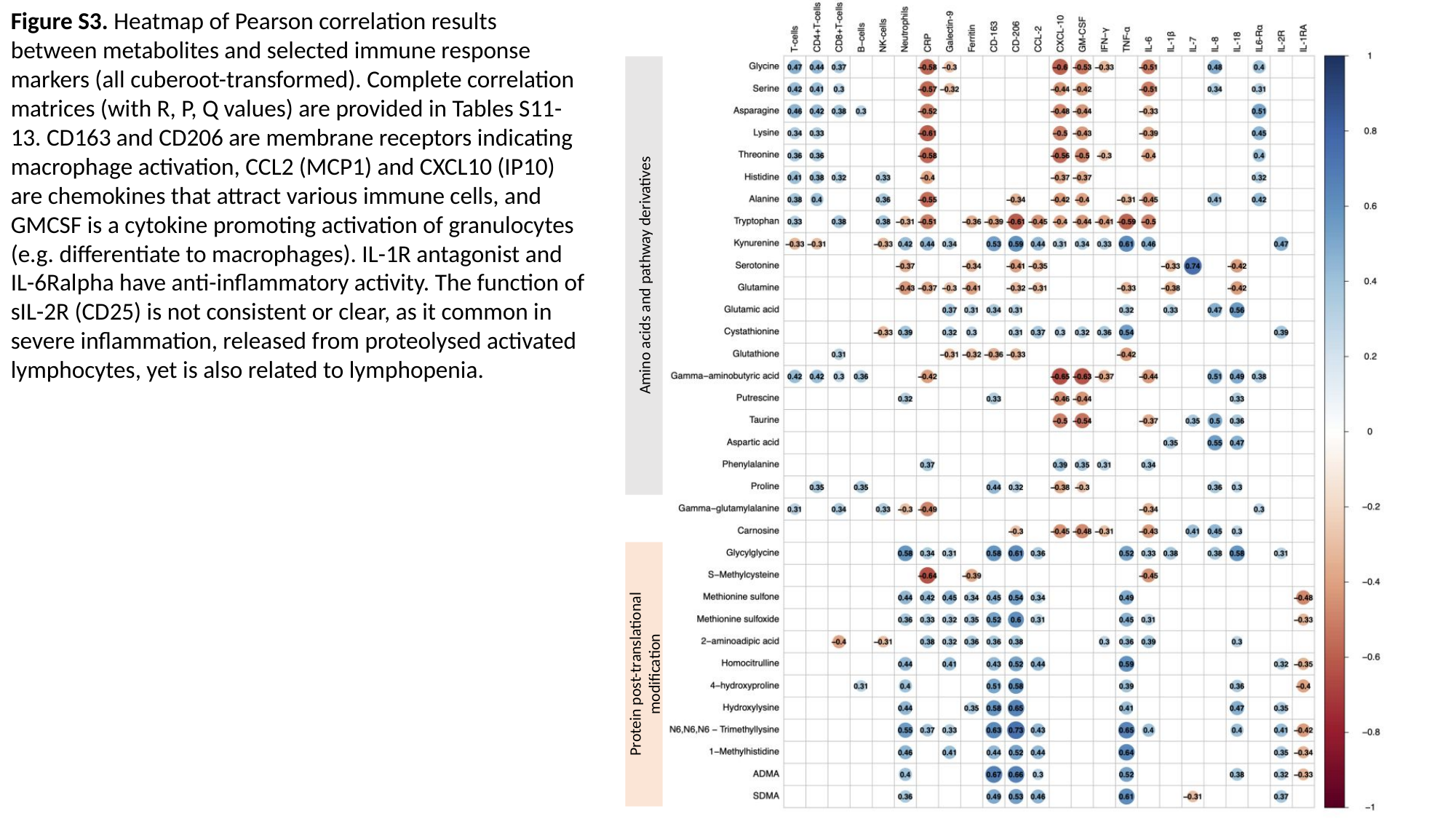

Figure S3. Heatmap of Pearson correlation results between metabolites and selected immune response markers (all cuberoot-transformed). Complete correlation matrices (with R, P, Q values) are provided in Tables S11-13. CD163 and CD206 are membrane receptors indicating macrophage activation, CCL2 (MCP1) and CXCL10 (IP10) are chemokines that attract various immune cells, and GMCSF is a cytokine promoting activation of granulocytes (e.g. differentiate to macrophages). IL-1R antagonist and IL-6Ralpha have anti-inflammatory activity. The function of sIL-2R (CD25) is not consistent or clear, as it common in severe inflammation, released from proteolysed activated lymphocytes, yet is also related to lymphopenia.
Amino acids and pathway derivatives
Protein post-translational modification

## Slide 4
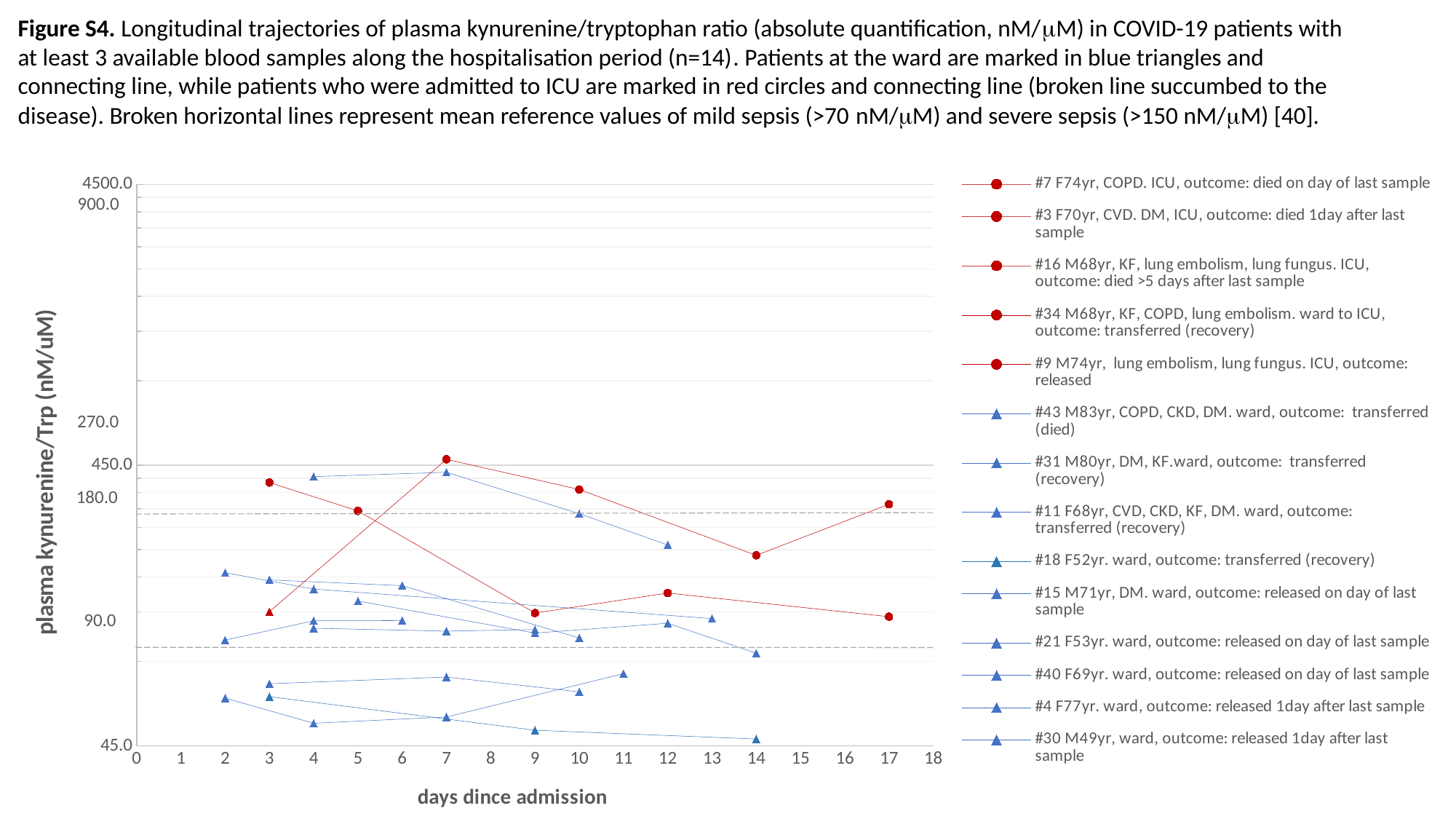

Figure S4. Longitudinal trajectories of plasma kynurenine/tryptophan ratio (absolute quantification, nM/μM) in COVID-19 patients with at least 3 available blood samples along the hospitalisation period (n=14). Patients at the ward are marked in blue triangles and connecting line, while patients who were admitted to ICU are marked in red circles and connecting line (broken line succumbed to the disease). Broken horizontal lines represent mean reference values of mild sepsis (>70 nM/μM) and severe sepsis (>150 nM/μM) [40].
### Chart
| Category | #7 F74yr, COPD. ICU, outcome: died on day of last sample | #3 F70yr, CVD. DM, ICU, outcome: died 1day after last sample | #16 M68yr, KF, lung embolism, lung fungus. ICU, outcome: died >5 days after last sample | #34 M68yr, KF, COPD, lung embolism. ward to ICU, outcome: transferred (recovery) | #9 M74yr, lung embolism, lung fungus. ICU, outcome: released | #43 M83yr, COPD, CKD, DM. ward, outcome: transferred (died) | #31 M80yr, DM, KF.ward, outcome: transferred (recovery) | #11 F68yr, CVD, CKD, KF, DM. ward, outcome: transferred (recovery) | #18 F52yr. ward, outcome: transferred (recovery) | #15 M71yr, DM. ward, outcome: released on day of last sample | #21 F53yr. ward, outcome: released on day of last sample | #40 F69yr. ward, outcome: released on day of last sample | #4 F77yr. ward, outcome: released 1day after last sample | #30 M49yr, ward, outcome: released 1day after last sample |
|---|---|---|---|---|---|---|---|---|---|---|---|---|---|---|900.0
270.0
180.0
90.0

## Slide 5
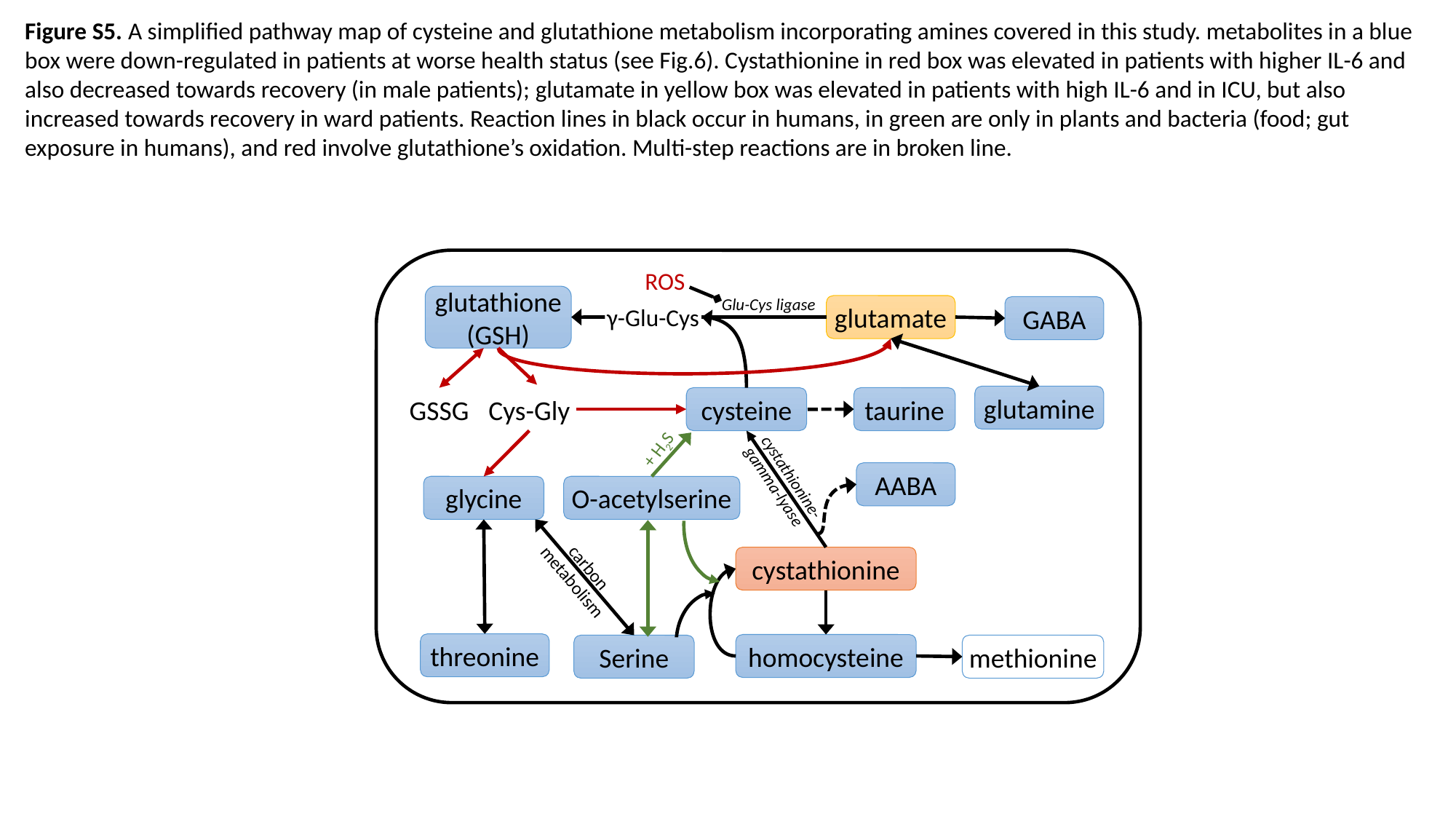

Figure S5. A simplified pathway map of cysteine and glutathione metabolism incorporating amines covered in this study. metabolites in a blue box were down-regulated in patients at worse health status (see Fig.6). Cystathionine in red box was elevated in patients with higher IL-6 and also decreased towards recovery (in male patients); glutamate in yellow box was elevated in patients with high IL-6 and in ICU, but also increased towards recovery in ward patients. Reaction lines in black occur in humans, in green are only in plants and bacteria (food; gut exposure in humans), and red involve glutathione’s oxidation. Multi-step reactions are in broken line.
ROS
glutathione (GSH)
Glu-Cys ligase
γ-Glu-Cys
glutamate
GABA
glutamine
GSSG
Cys-Gly
cysteine
taurine
+ H2S
AABA
cystathionine-gamma-lyase
glycine
O-acetylserine
cystathionine
carbon metabolism
threonine
homocysteine
Serine
methionine
