# Supplementary figures and images for: Severe COVID-19 Is Characterised by Perturbations in Plasma Amines Correlated with Immune Response Markers, and Linked to Inflammation and Oxidative Stress
Source: Metabolites. 2022 Jul 2;12(7):618. doi: 10.3390/metabo12070618 (PMC9321395; doi:10.3390/metabo12070618)

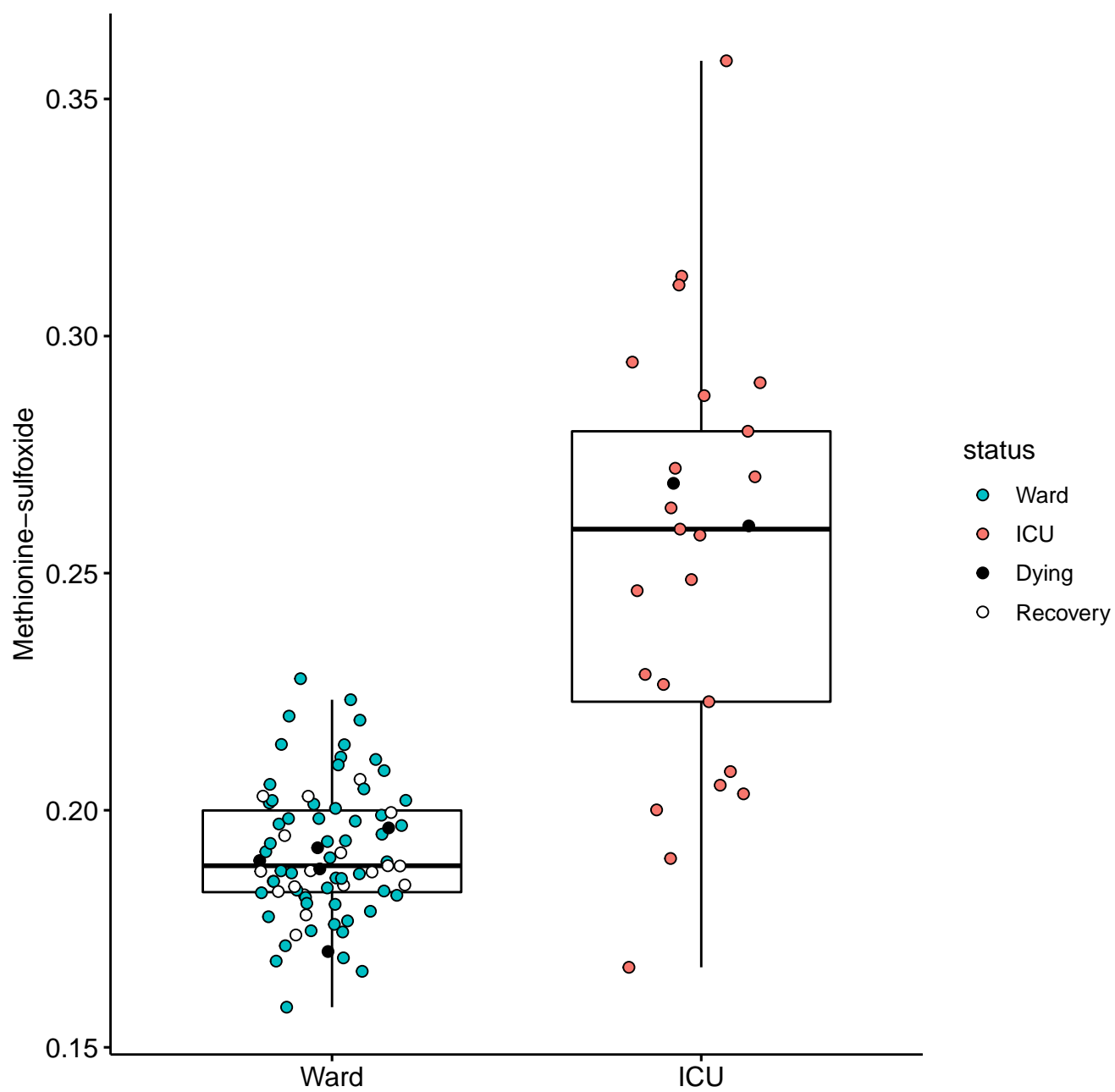

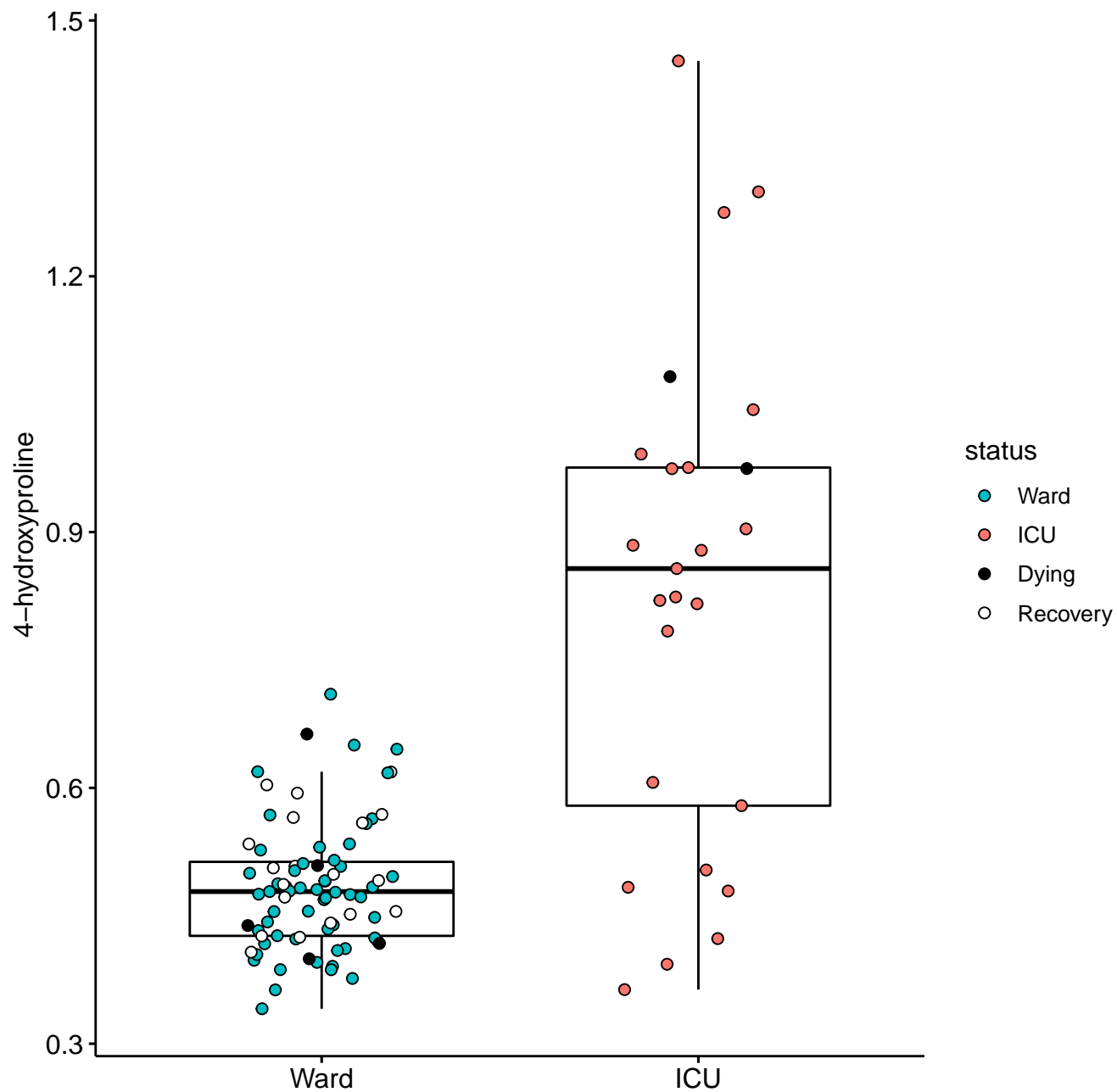

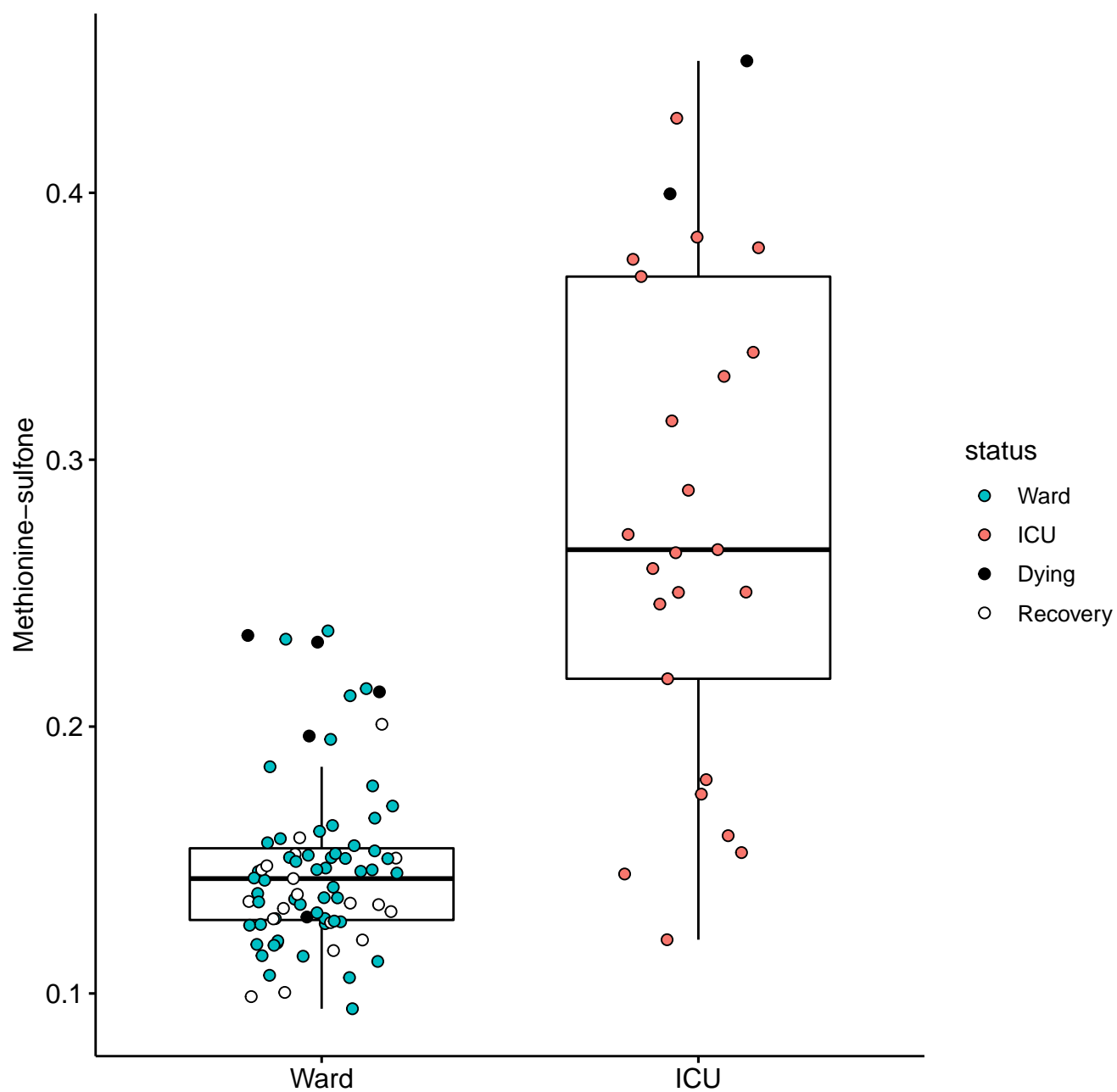

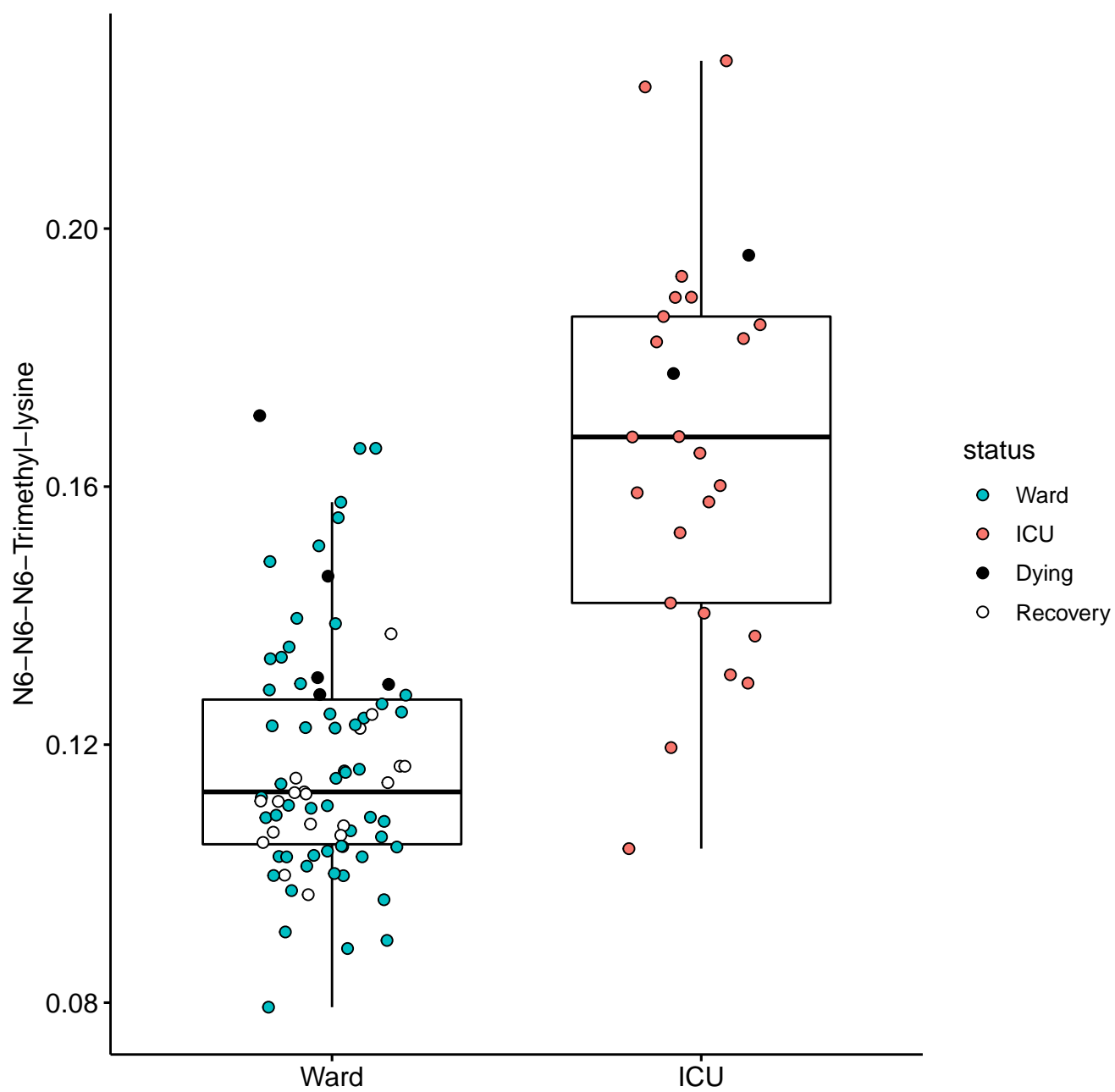

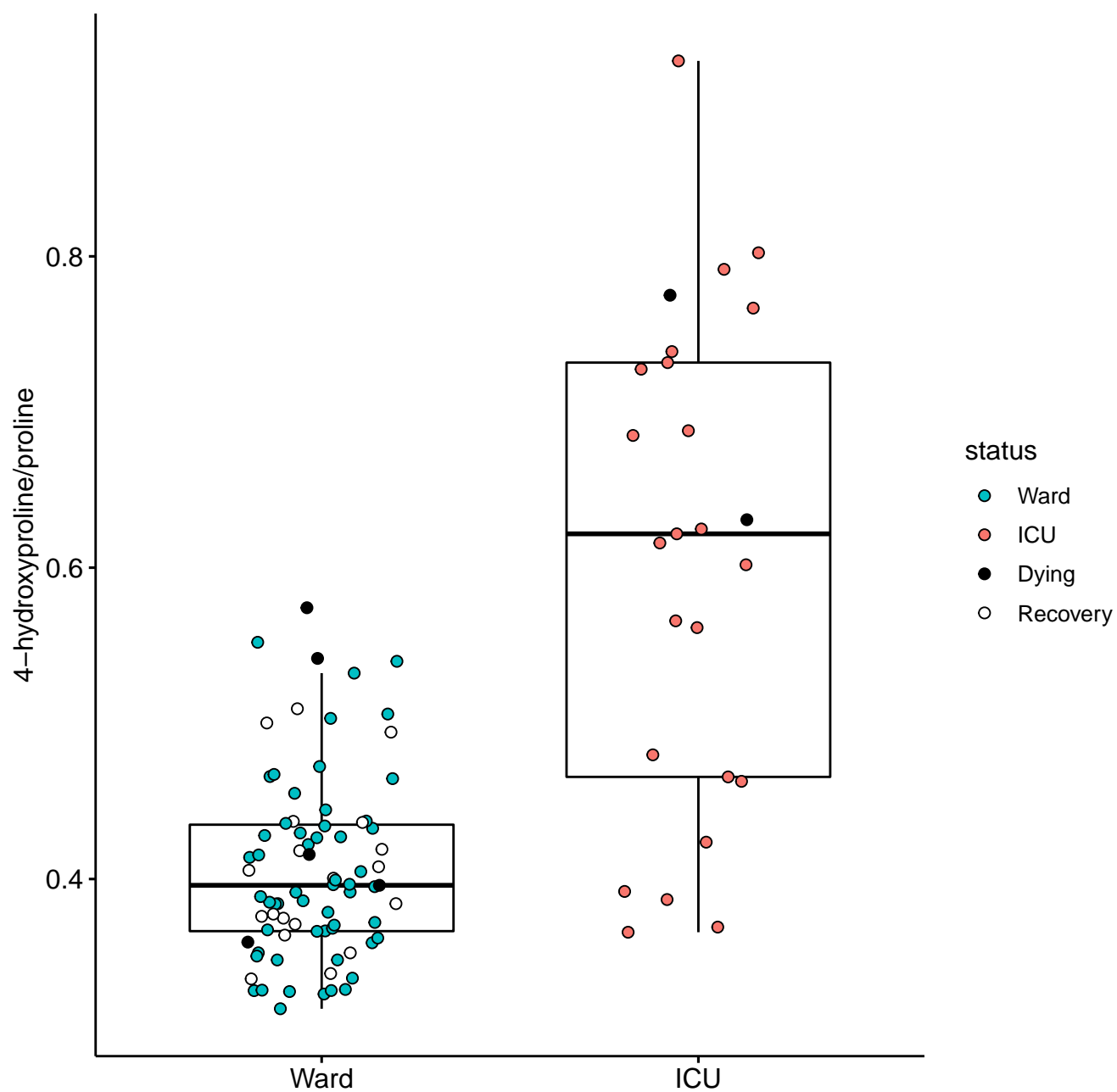

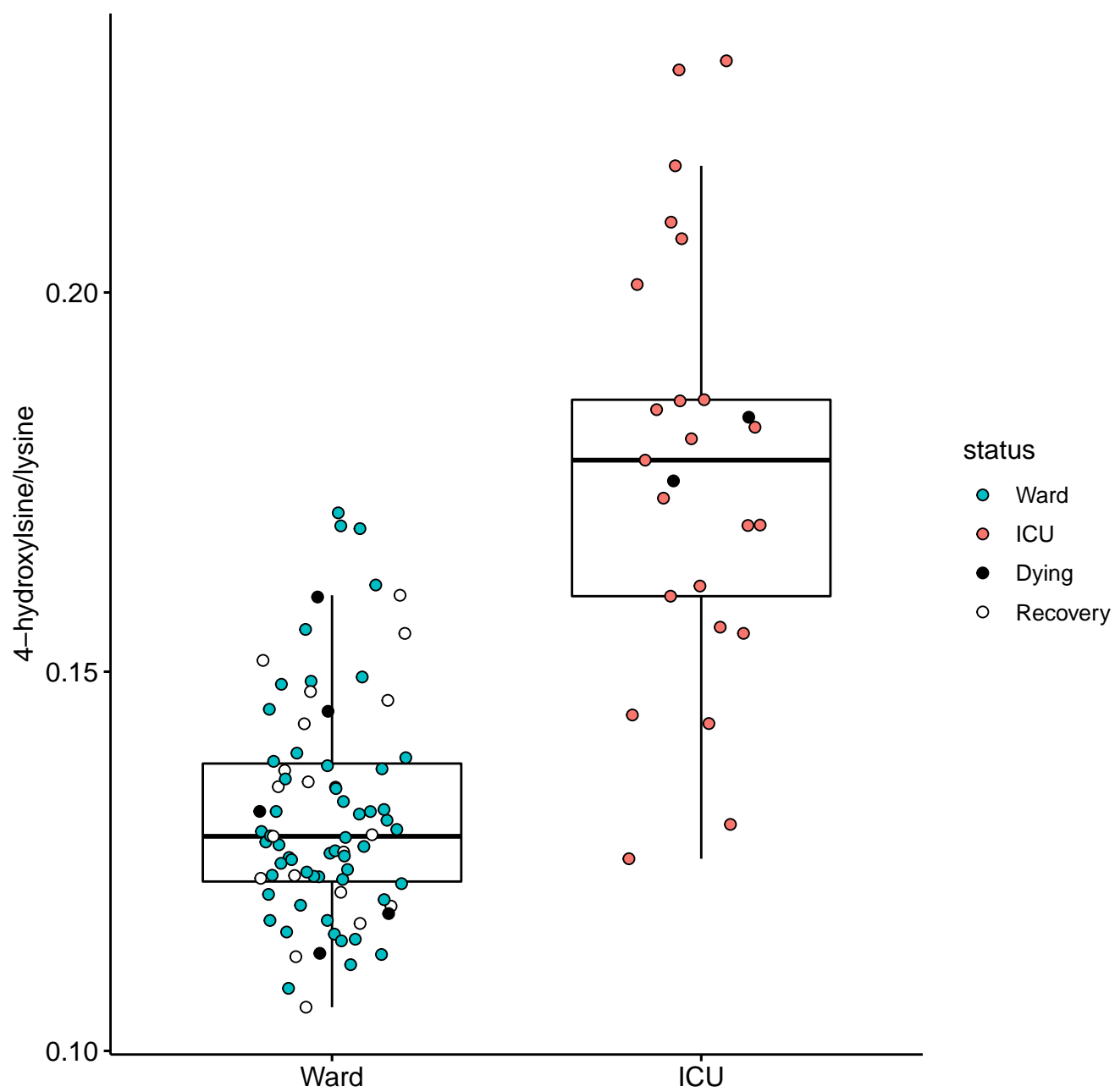

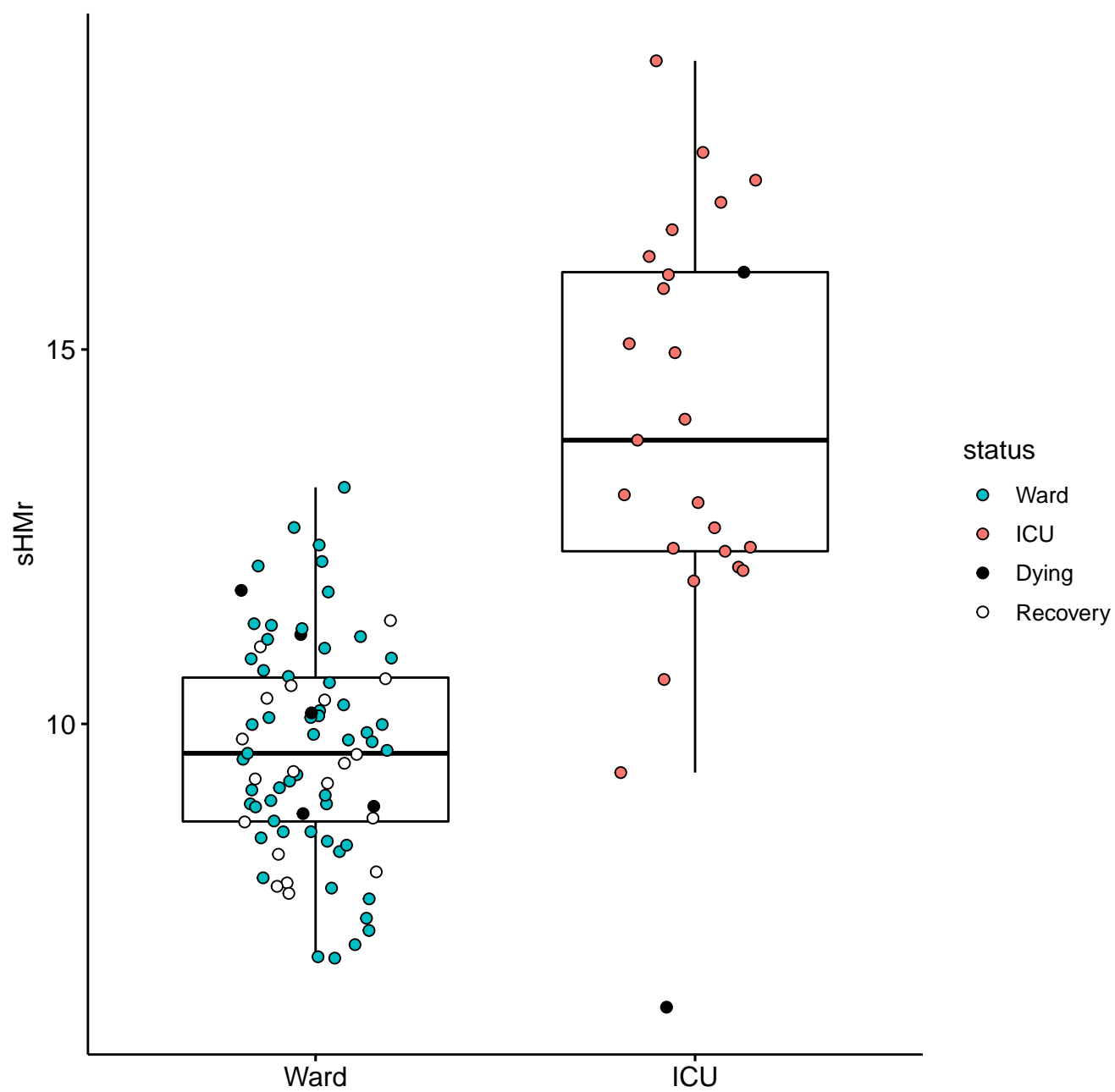

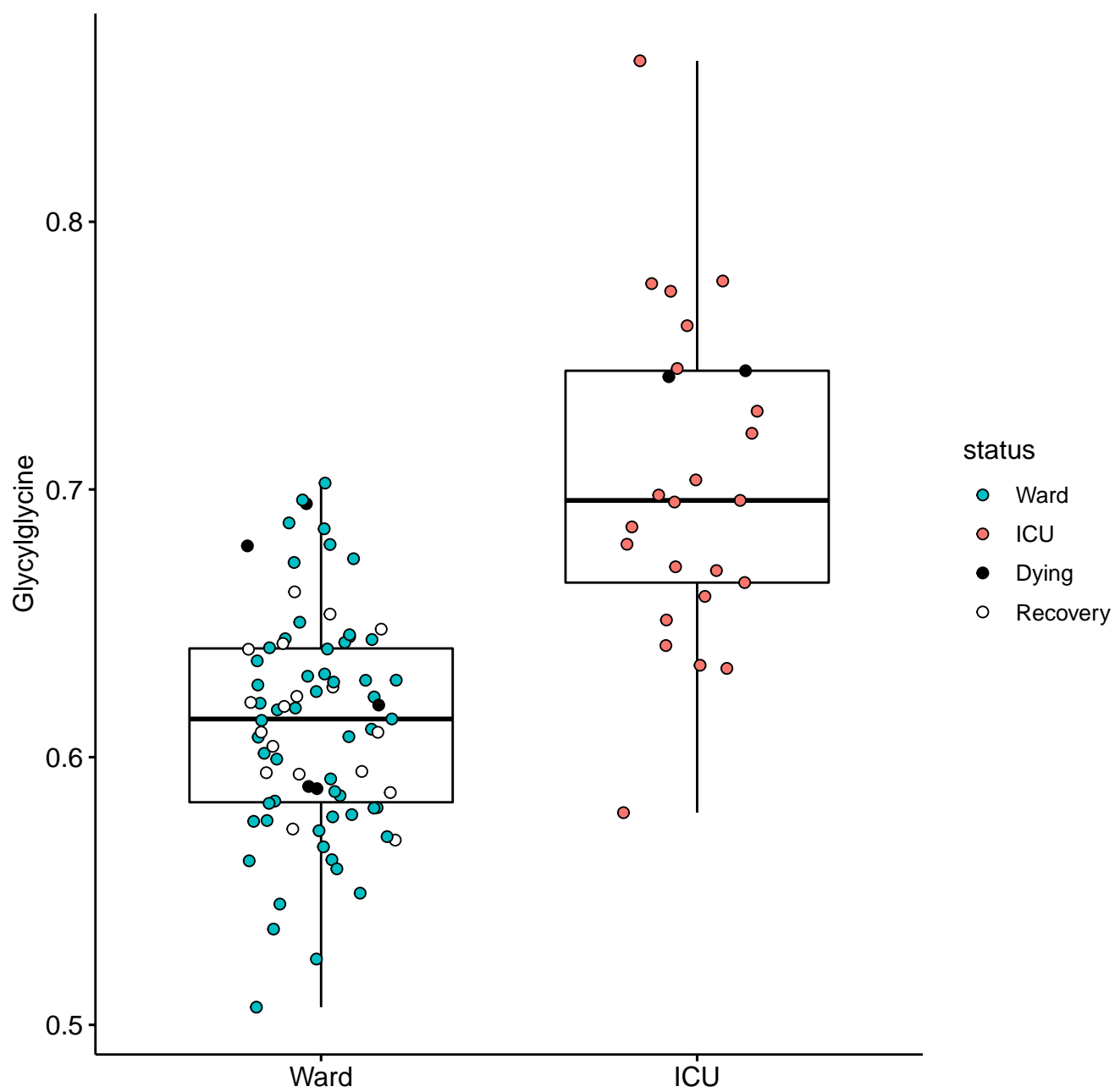

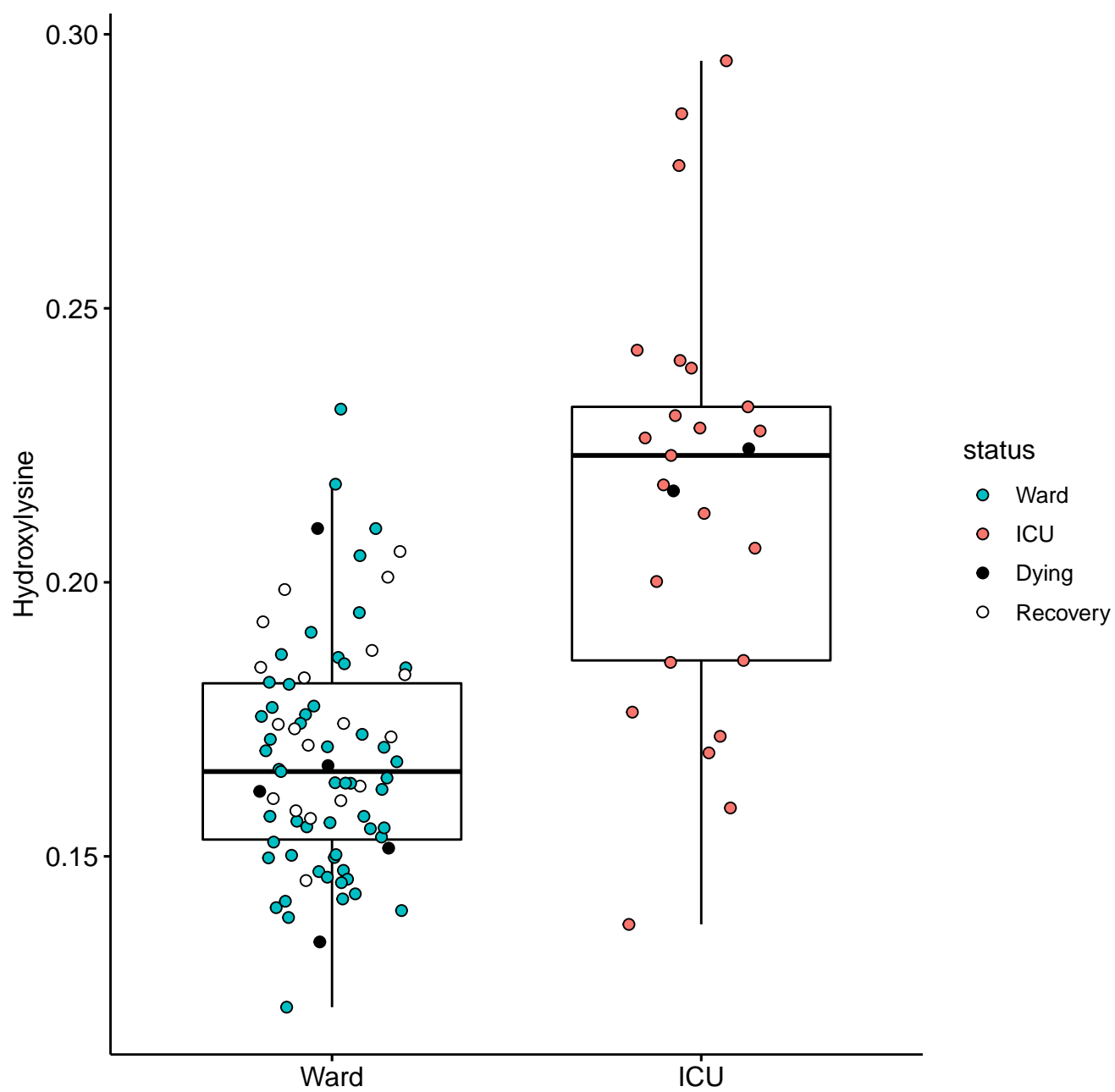

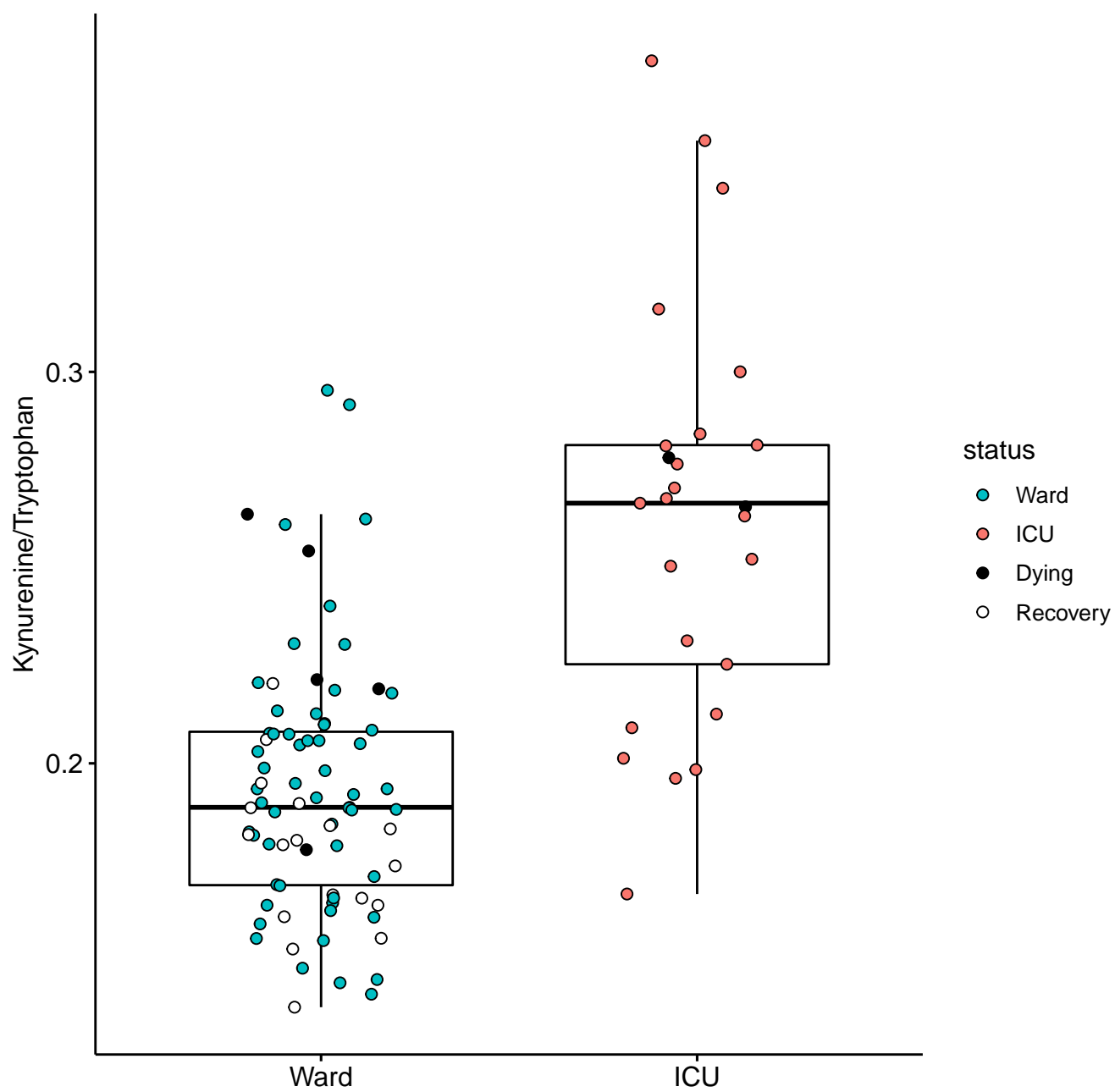

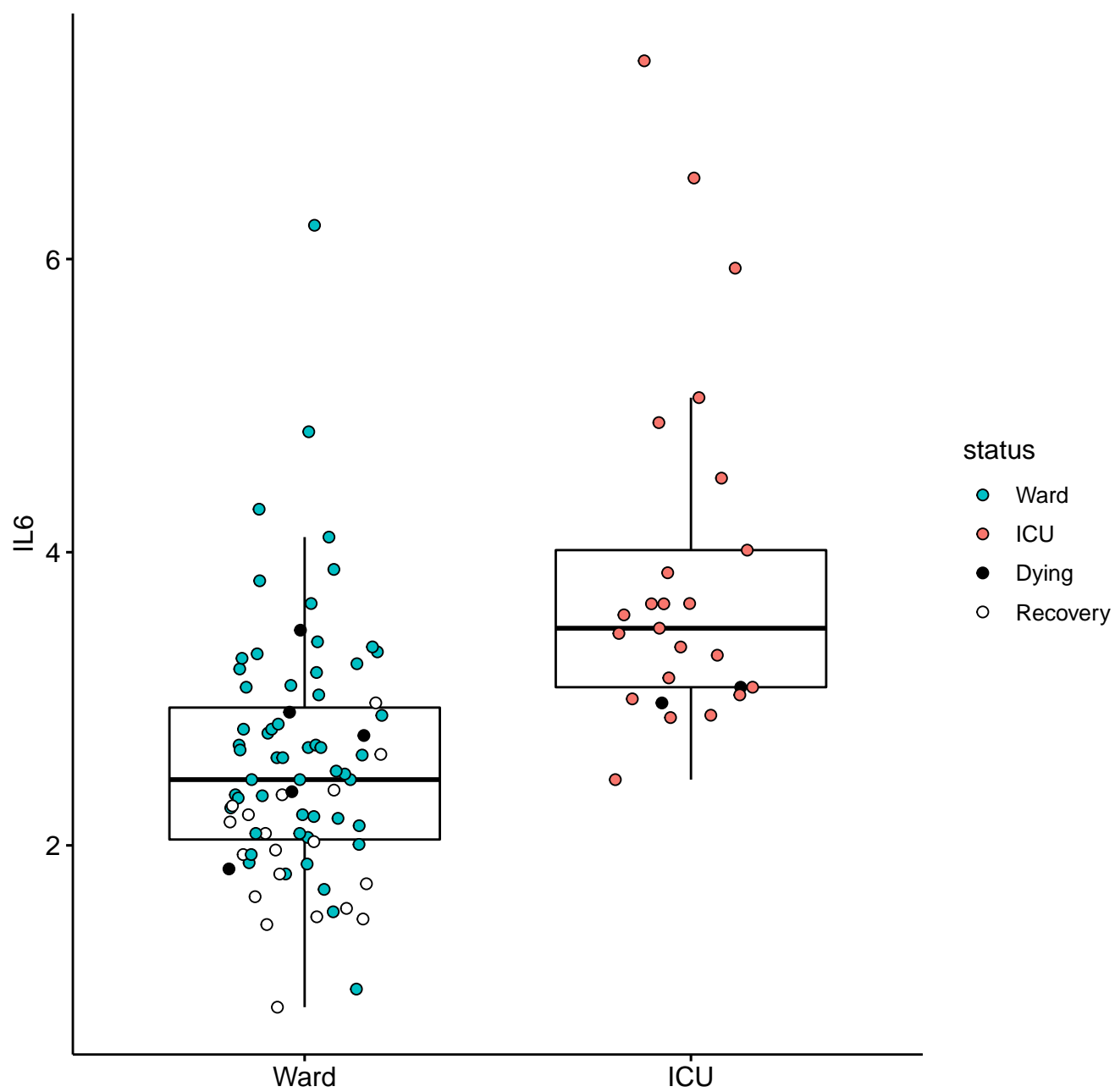

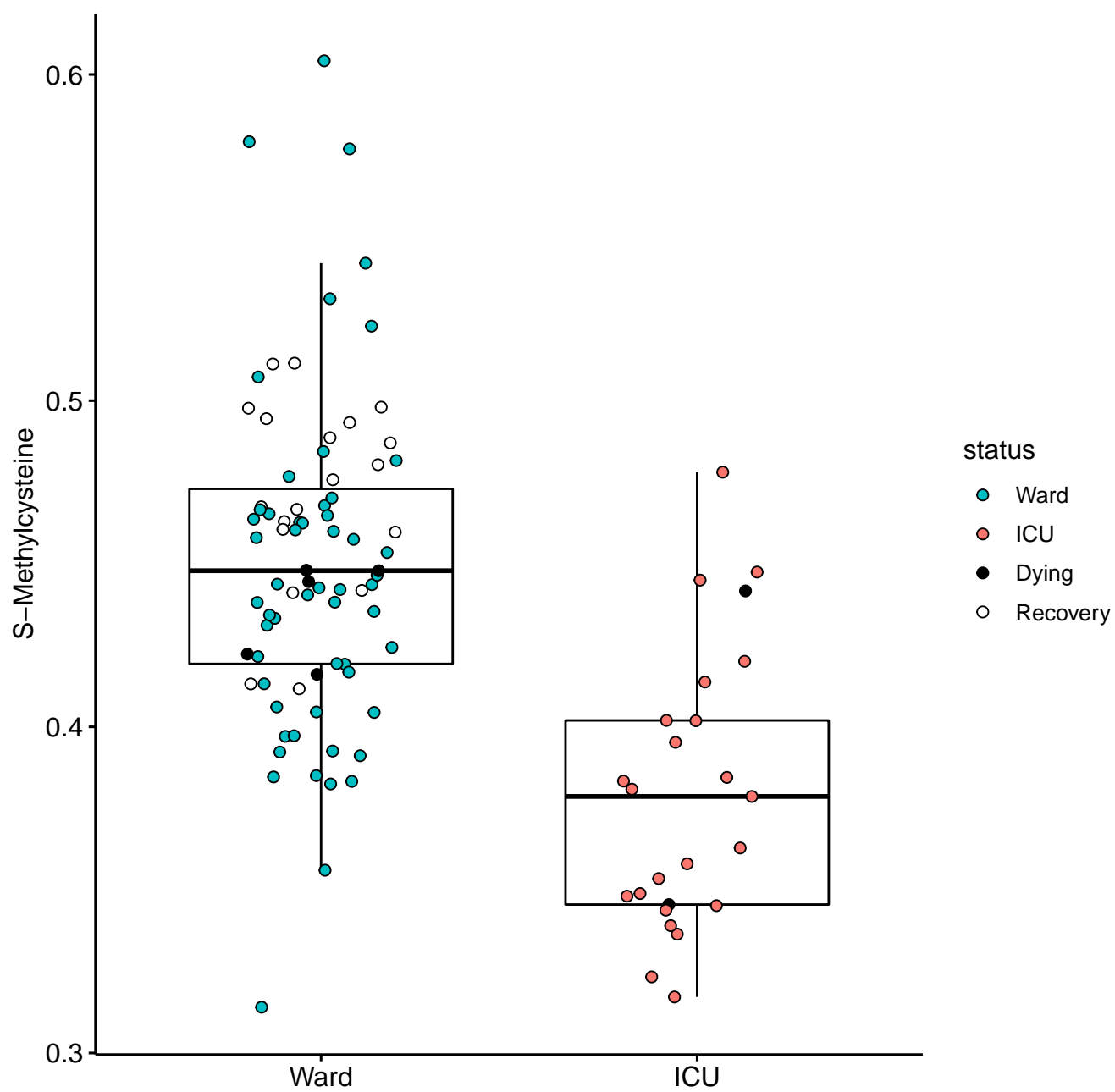

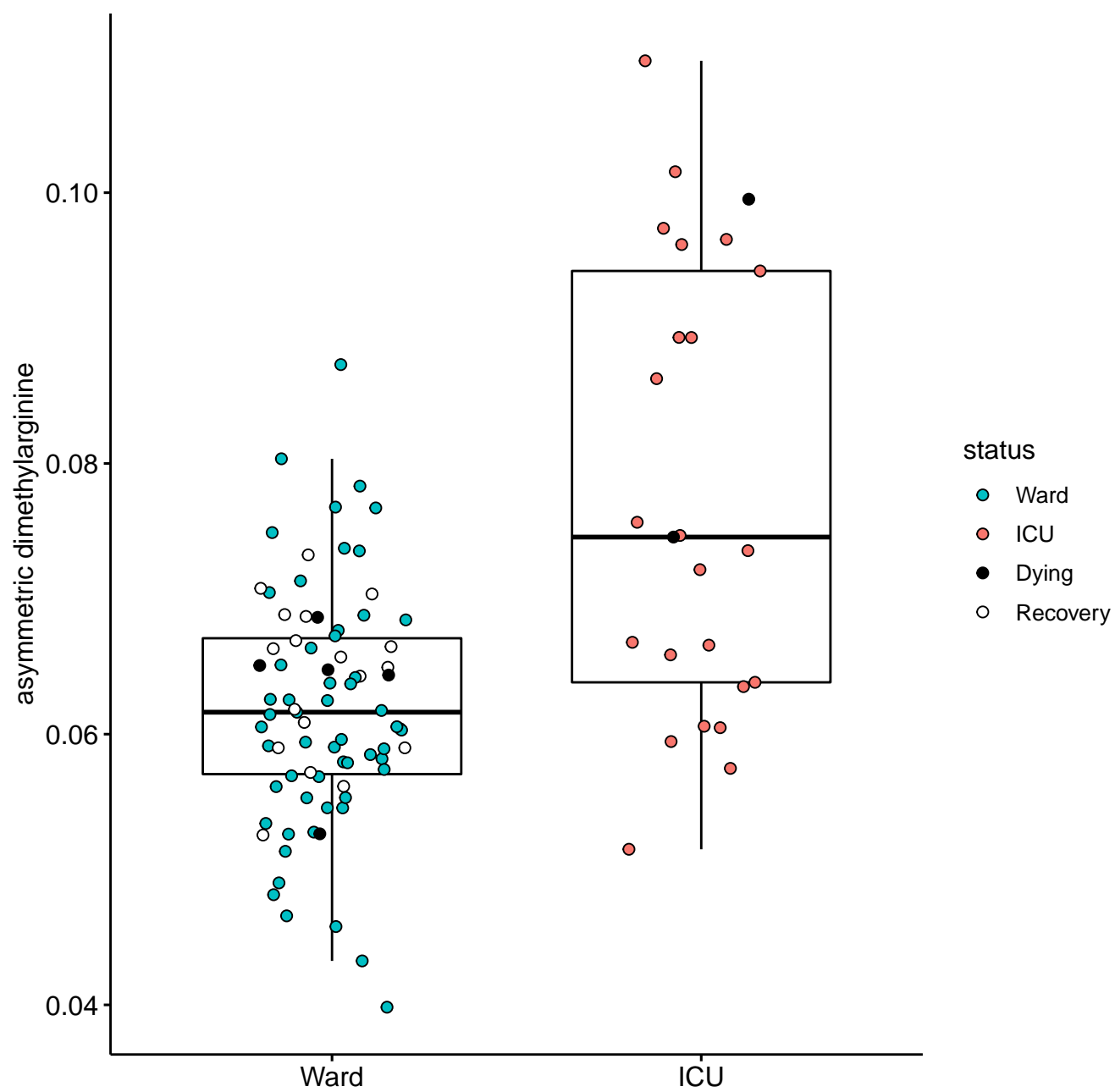

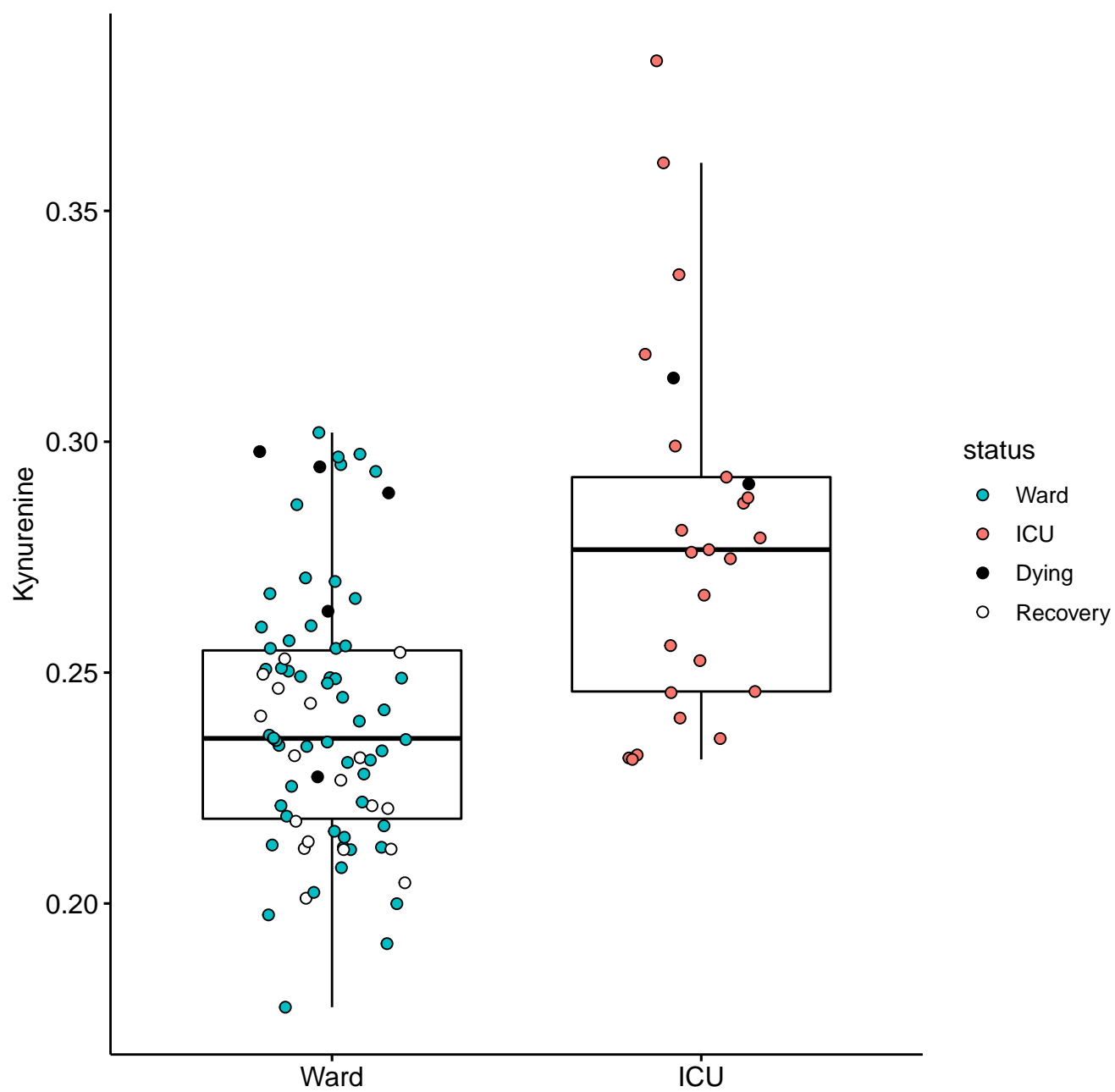

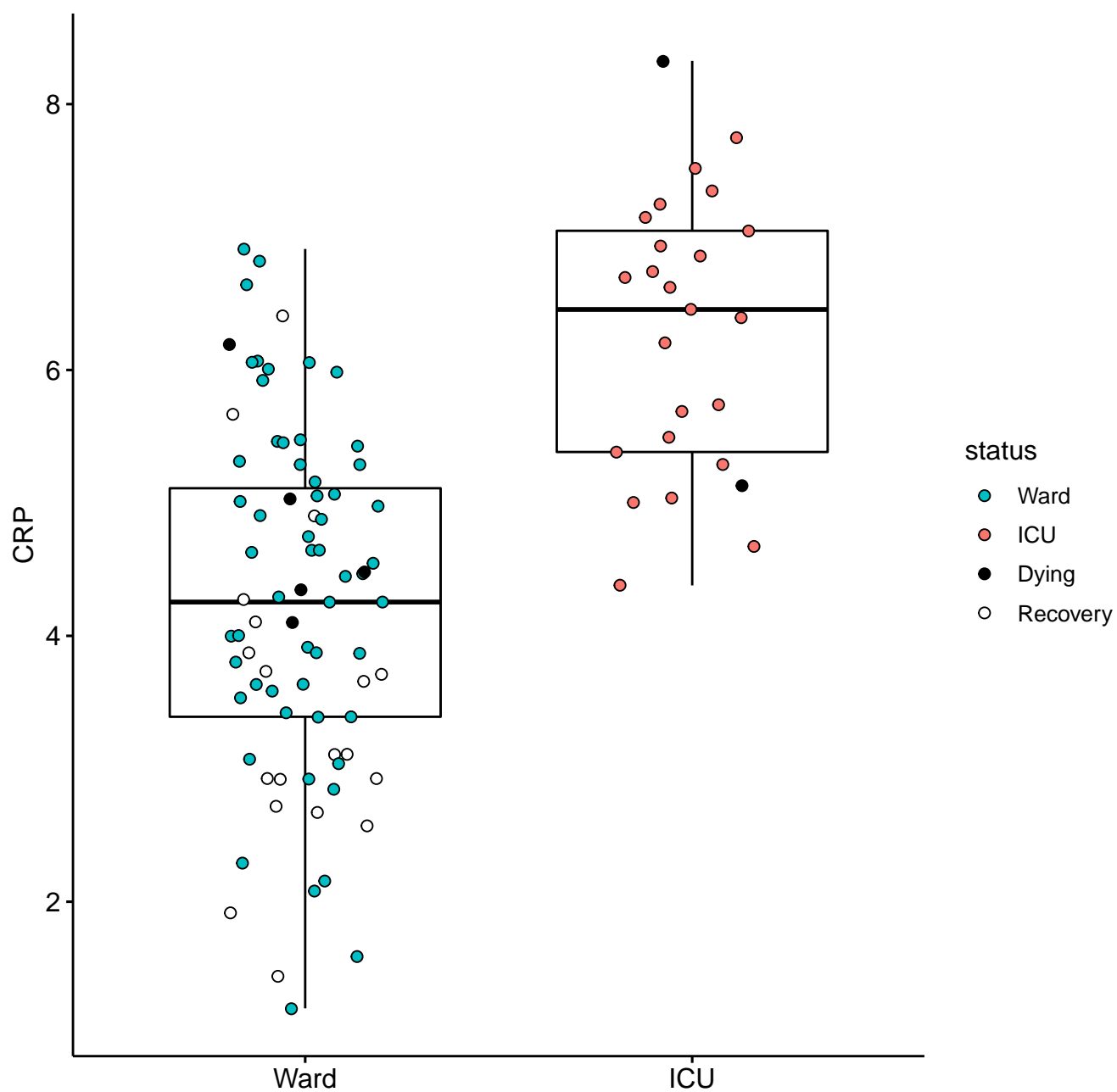

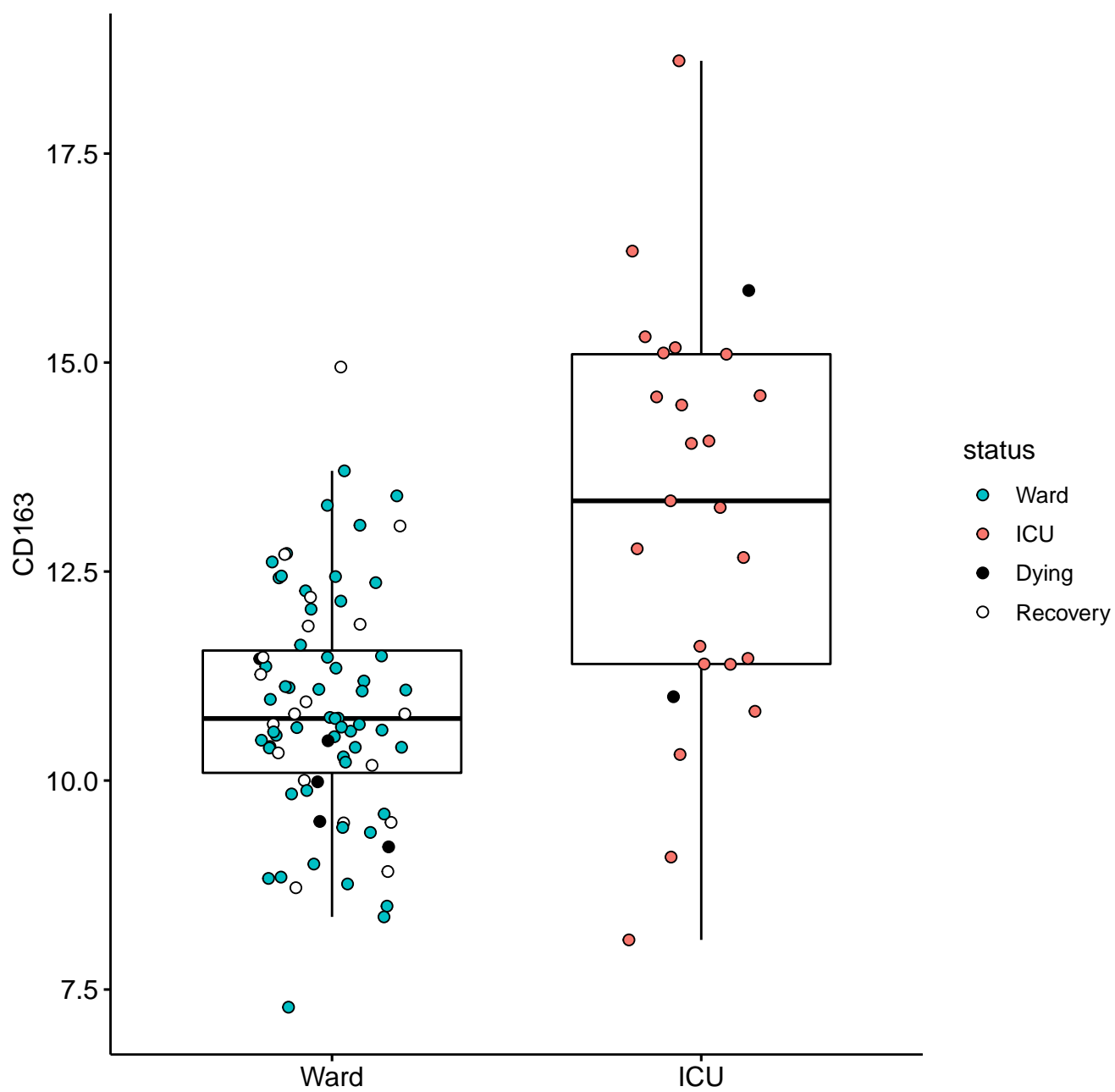

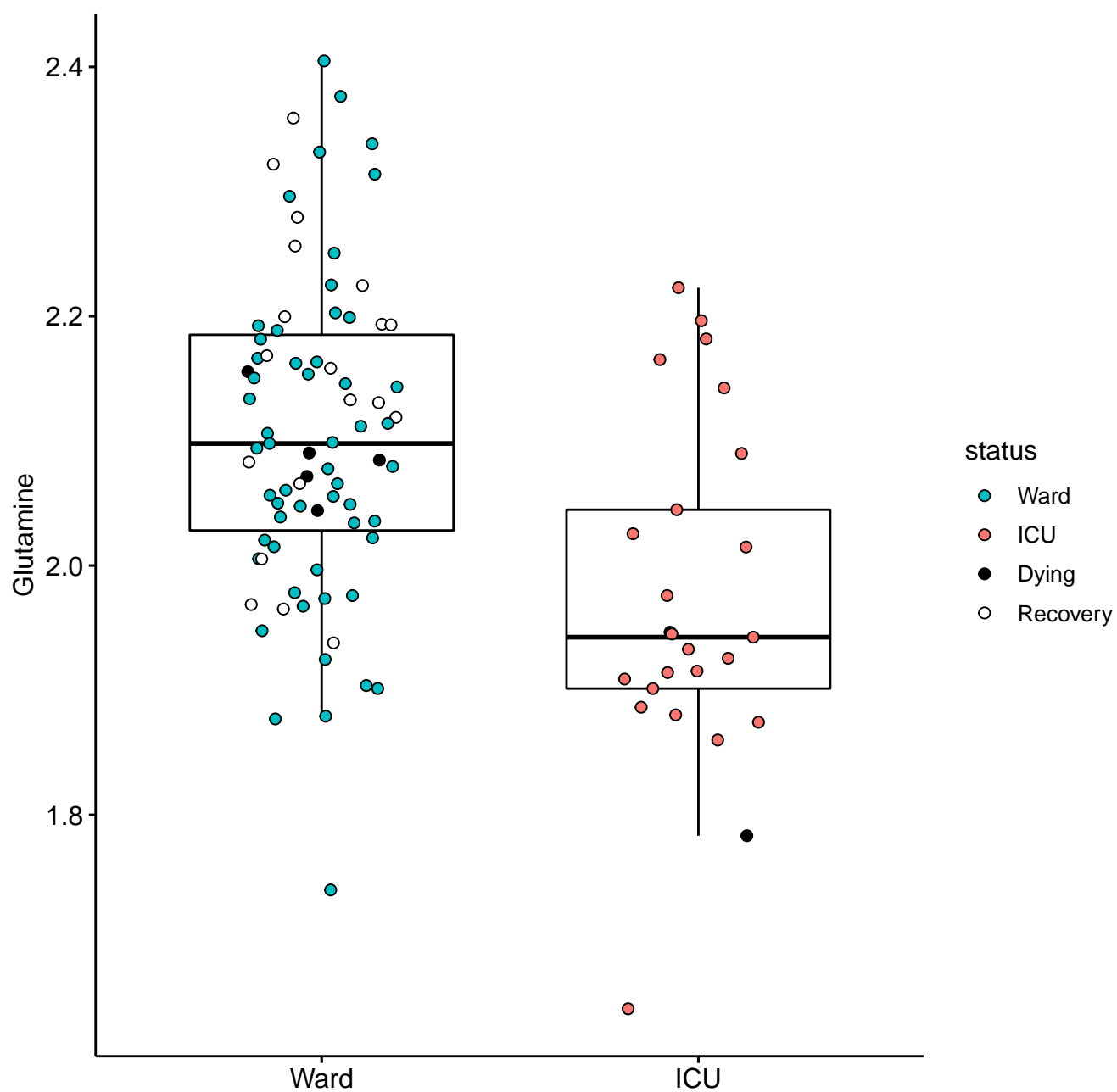

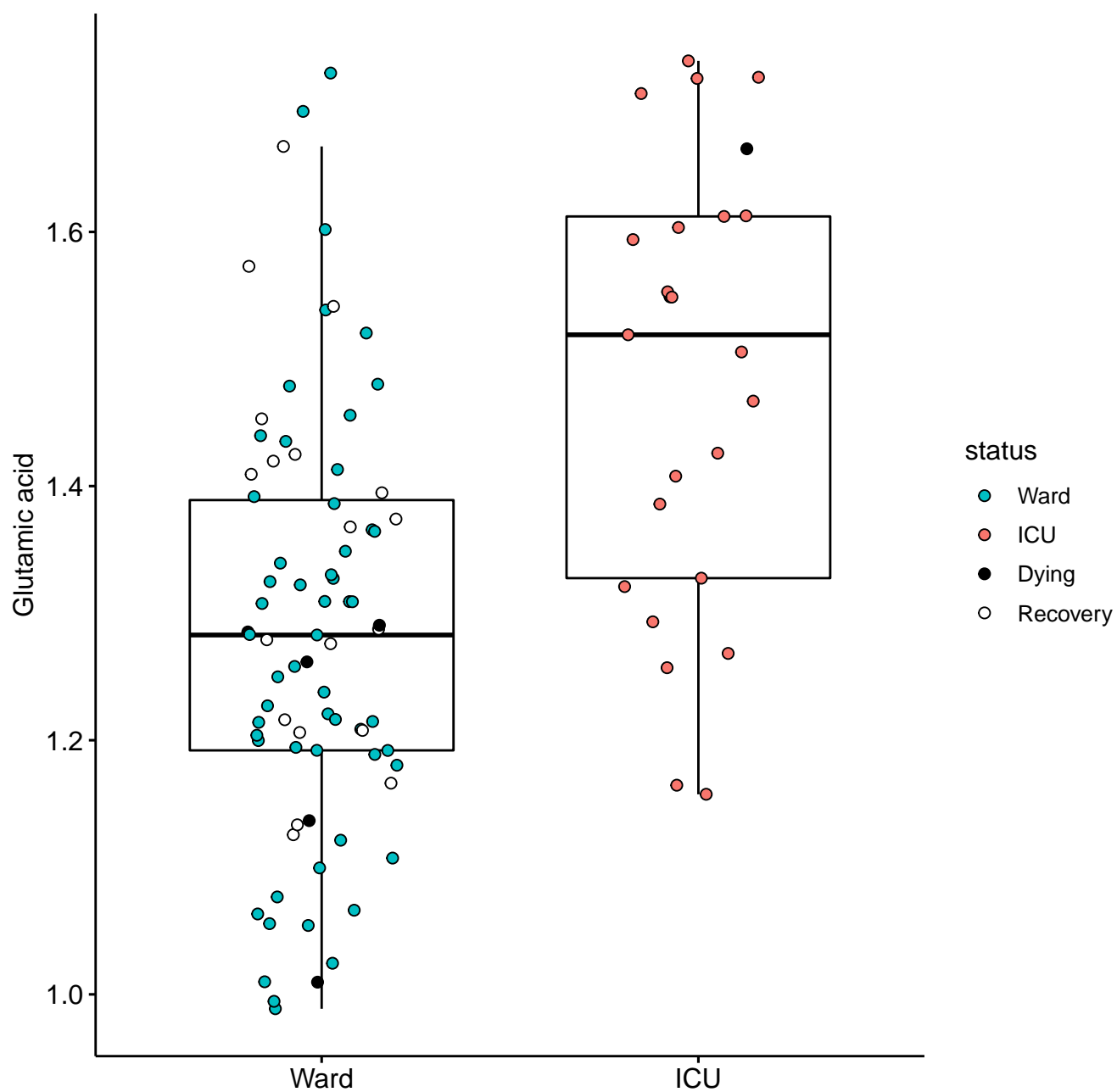

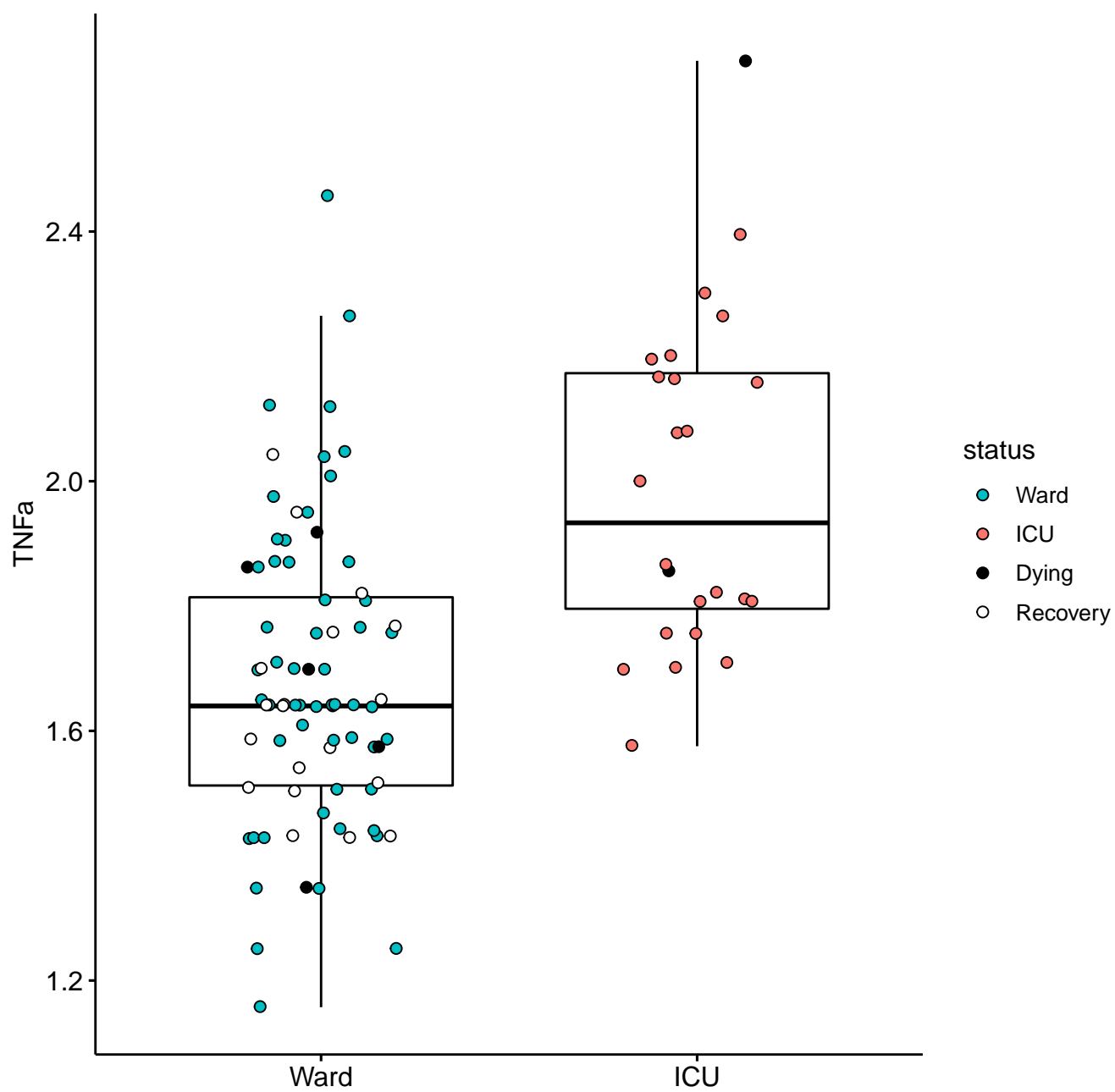

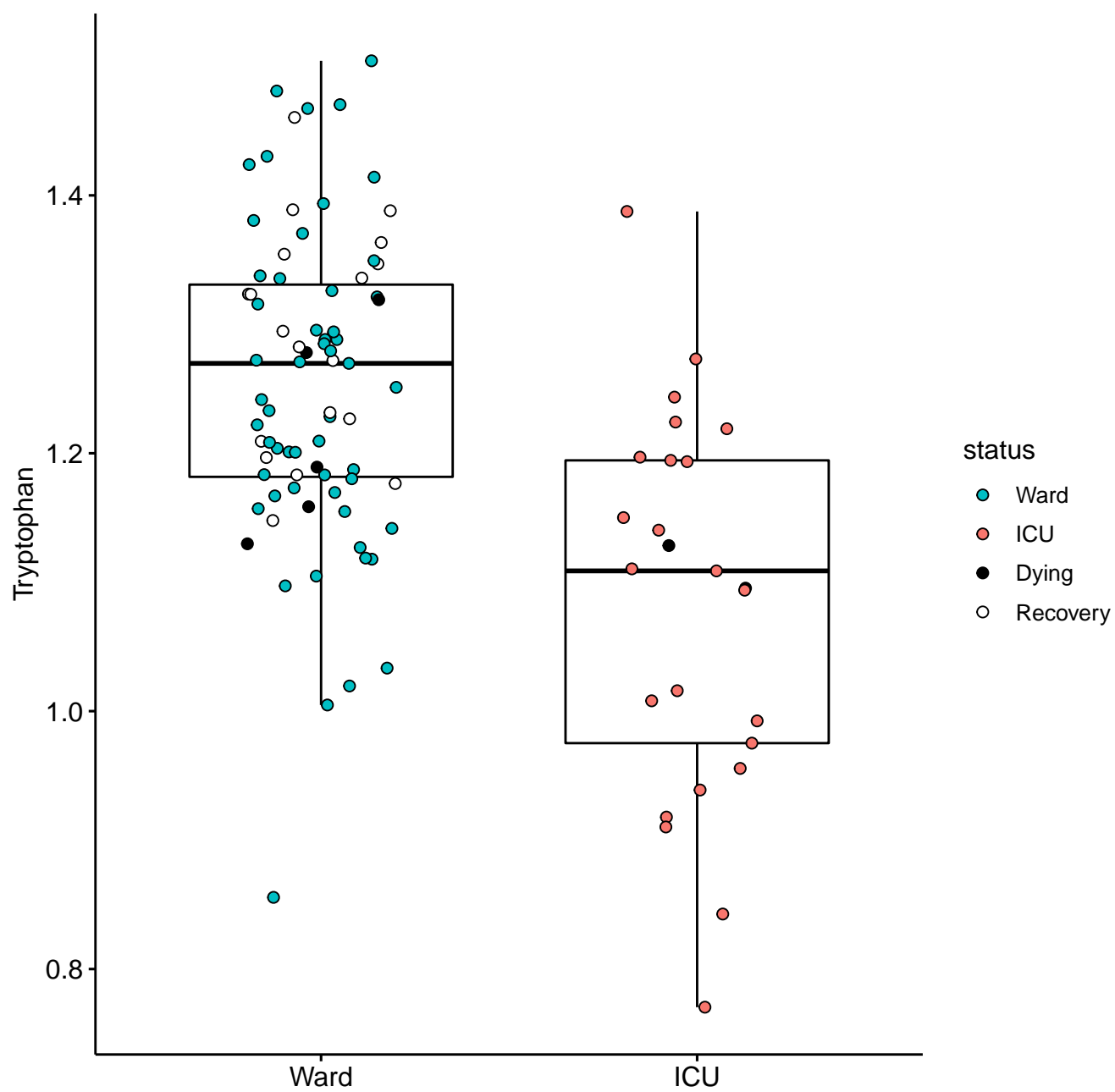

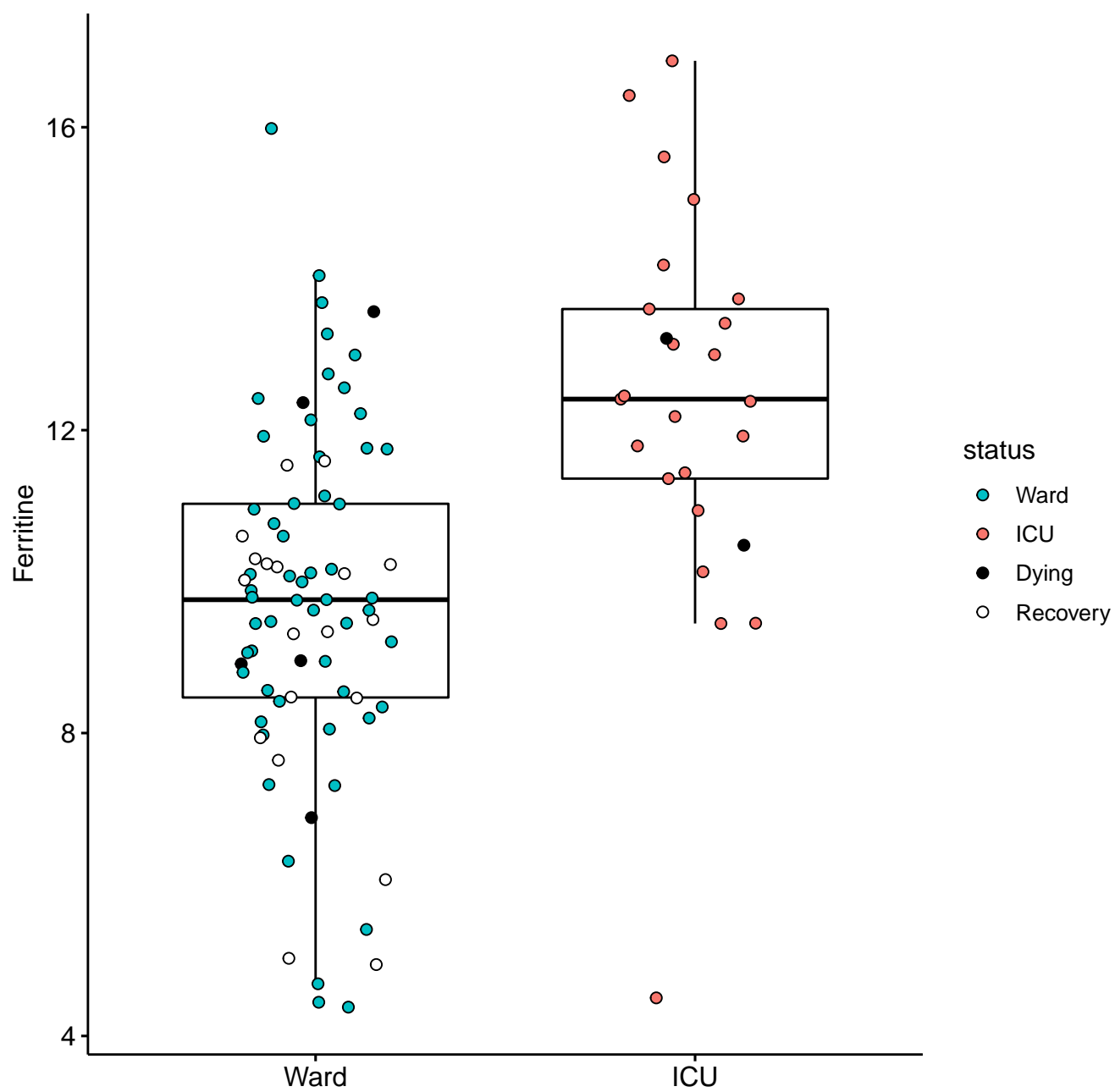

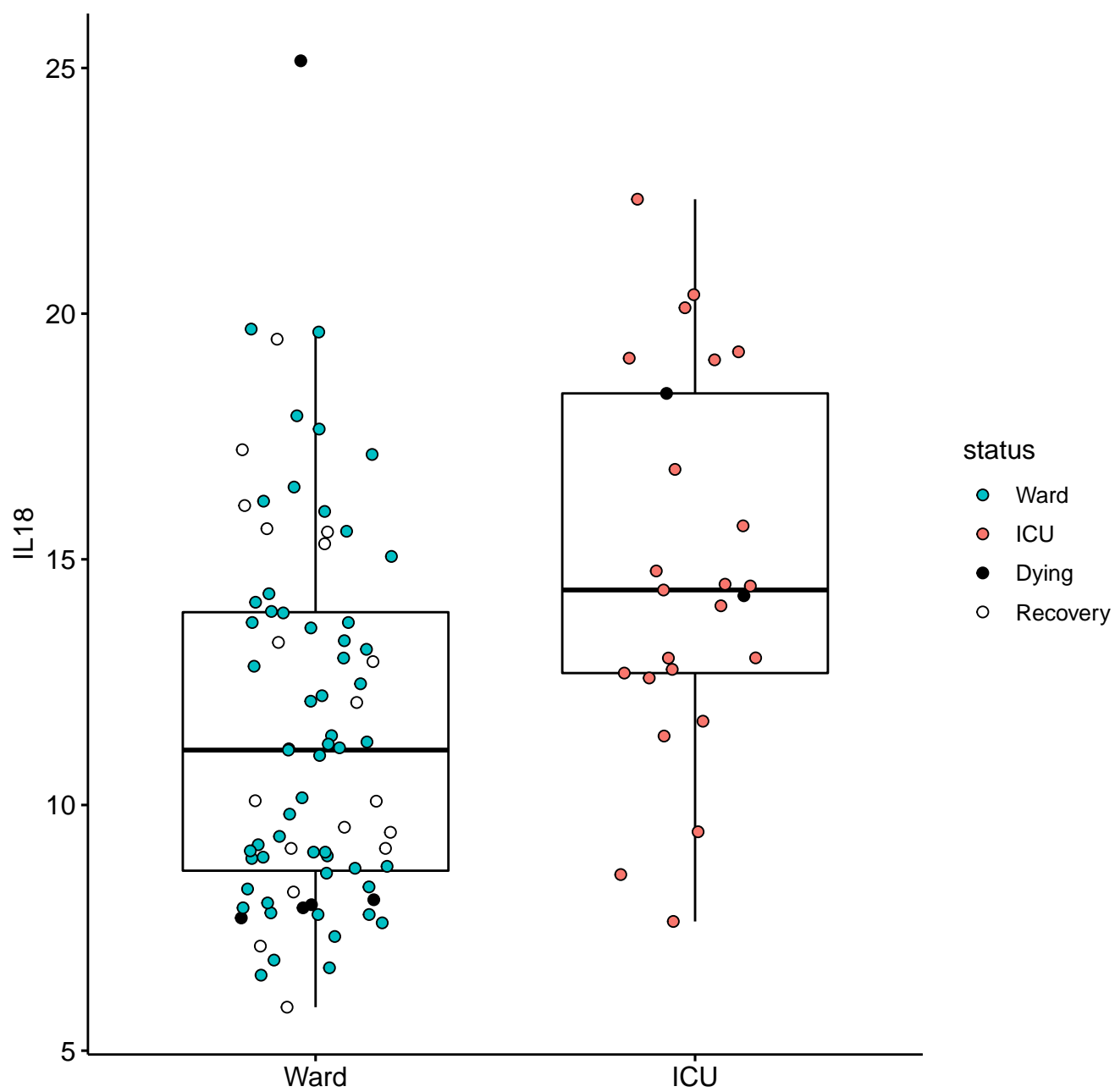

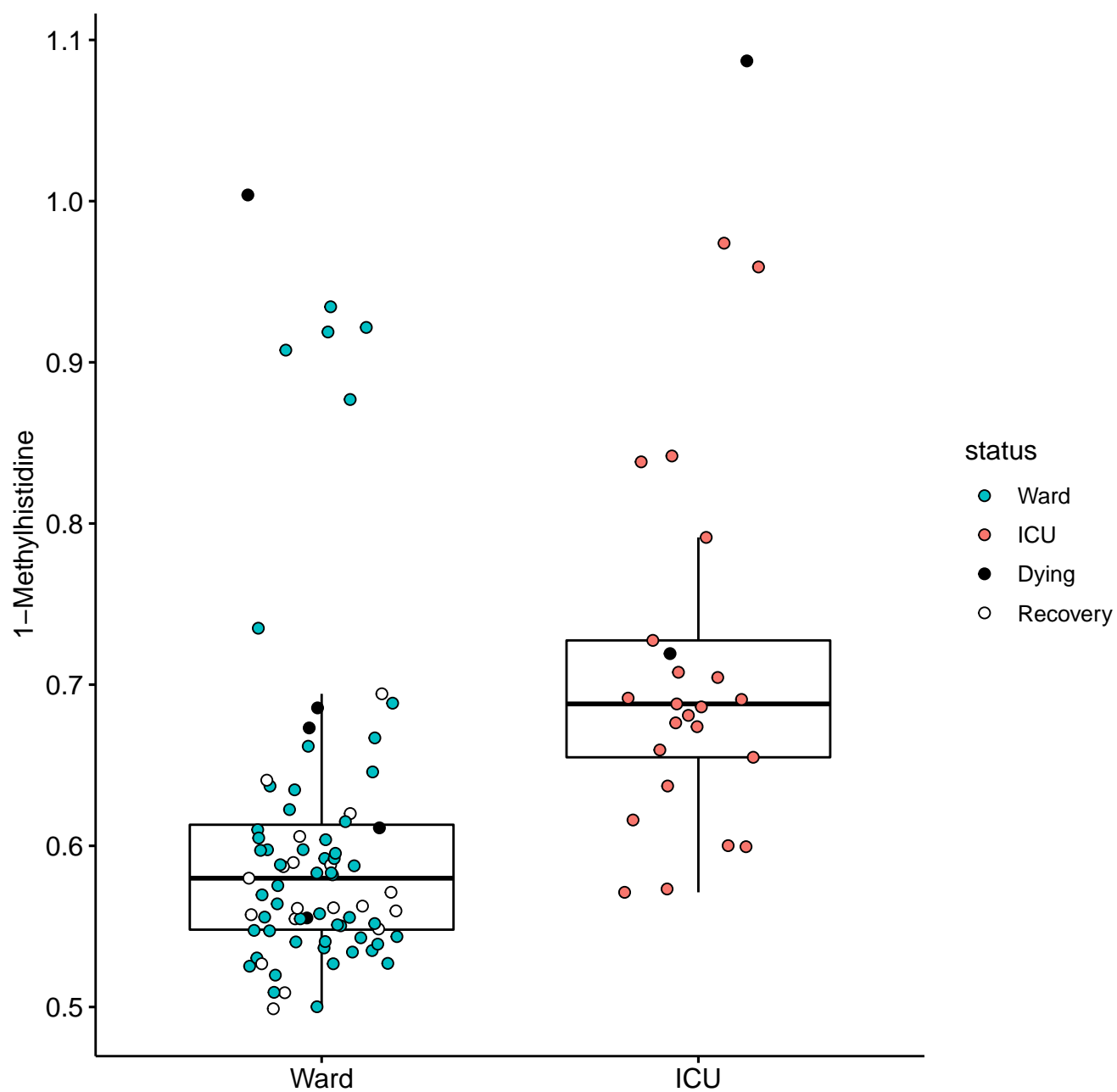

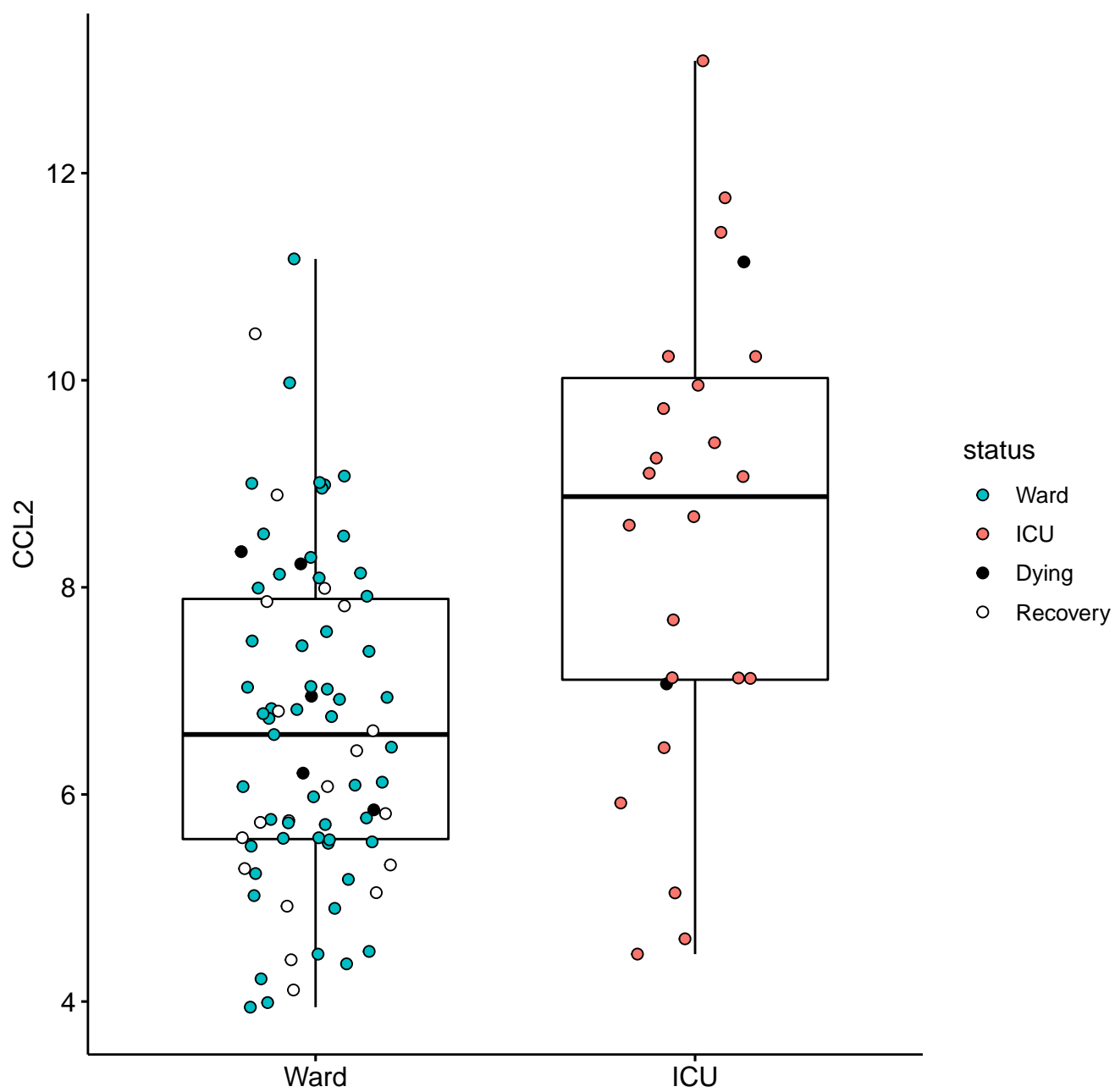

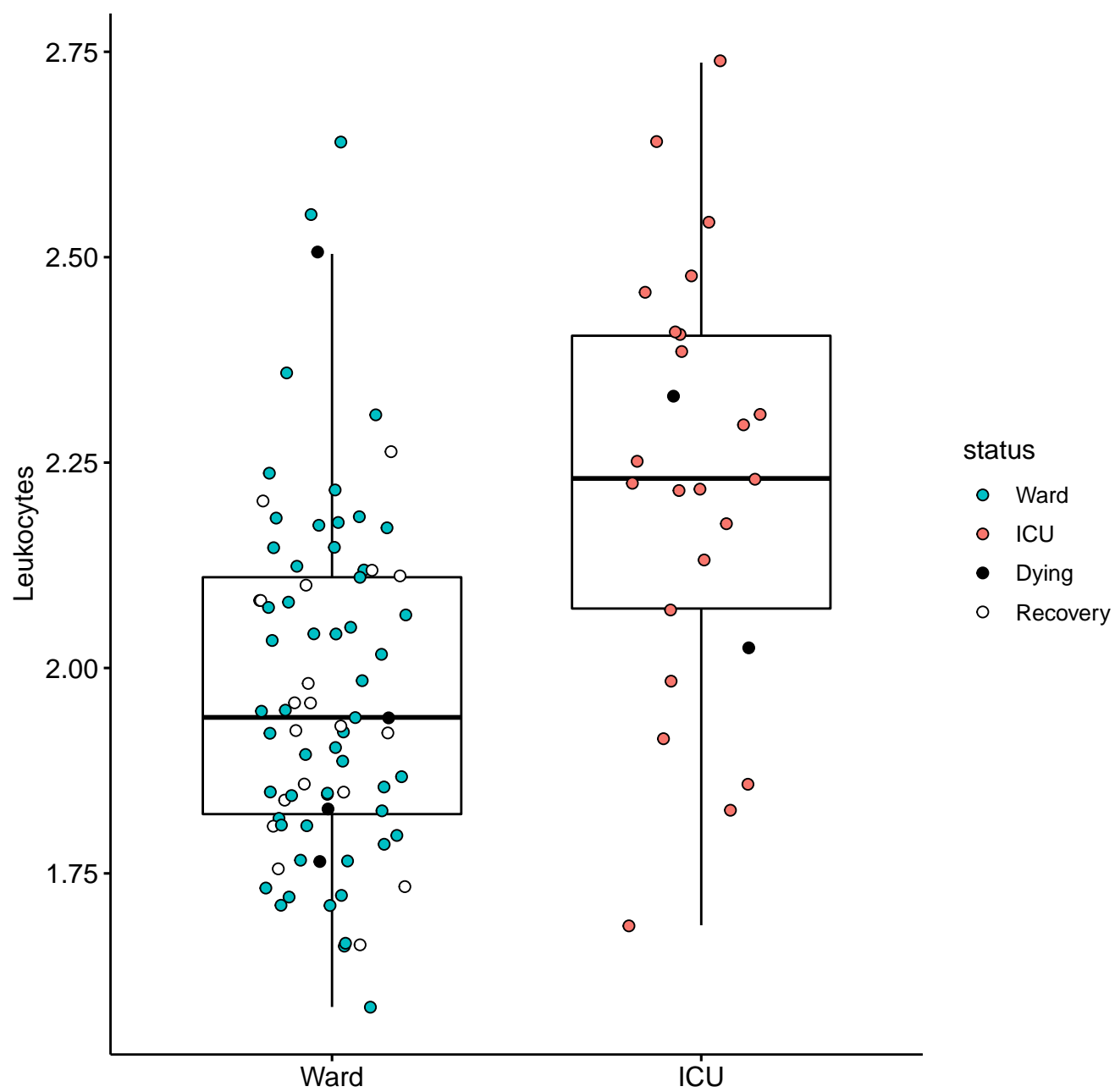

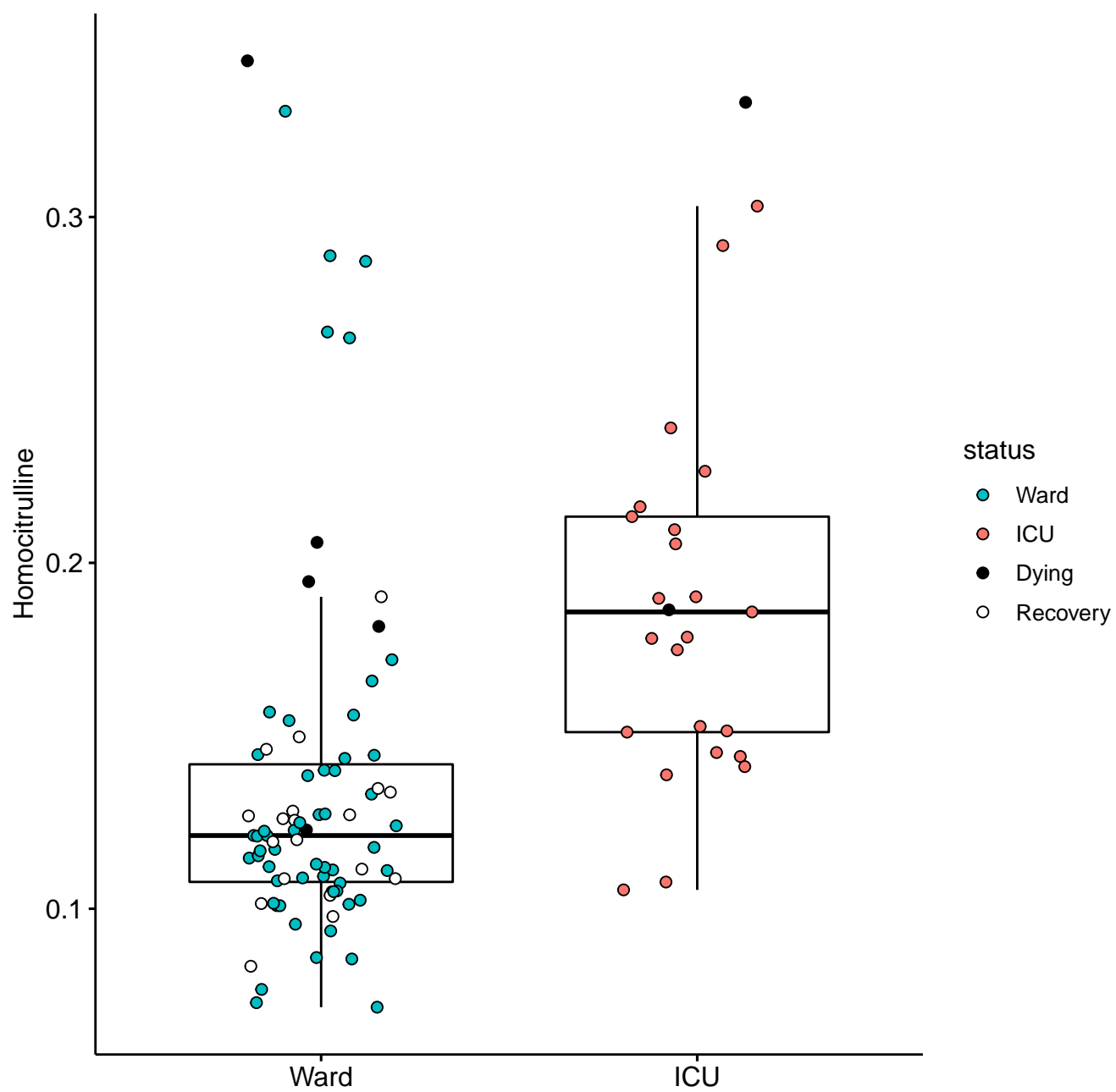

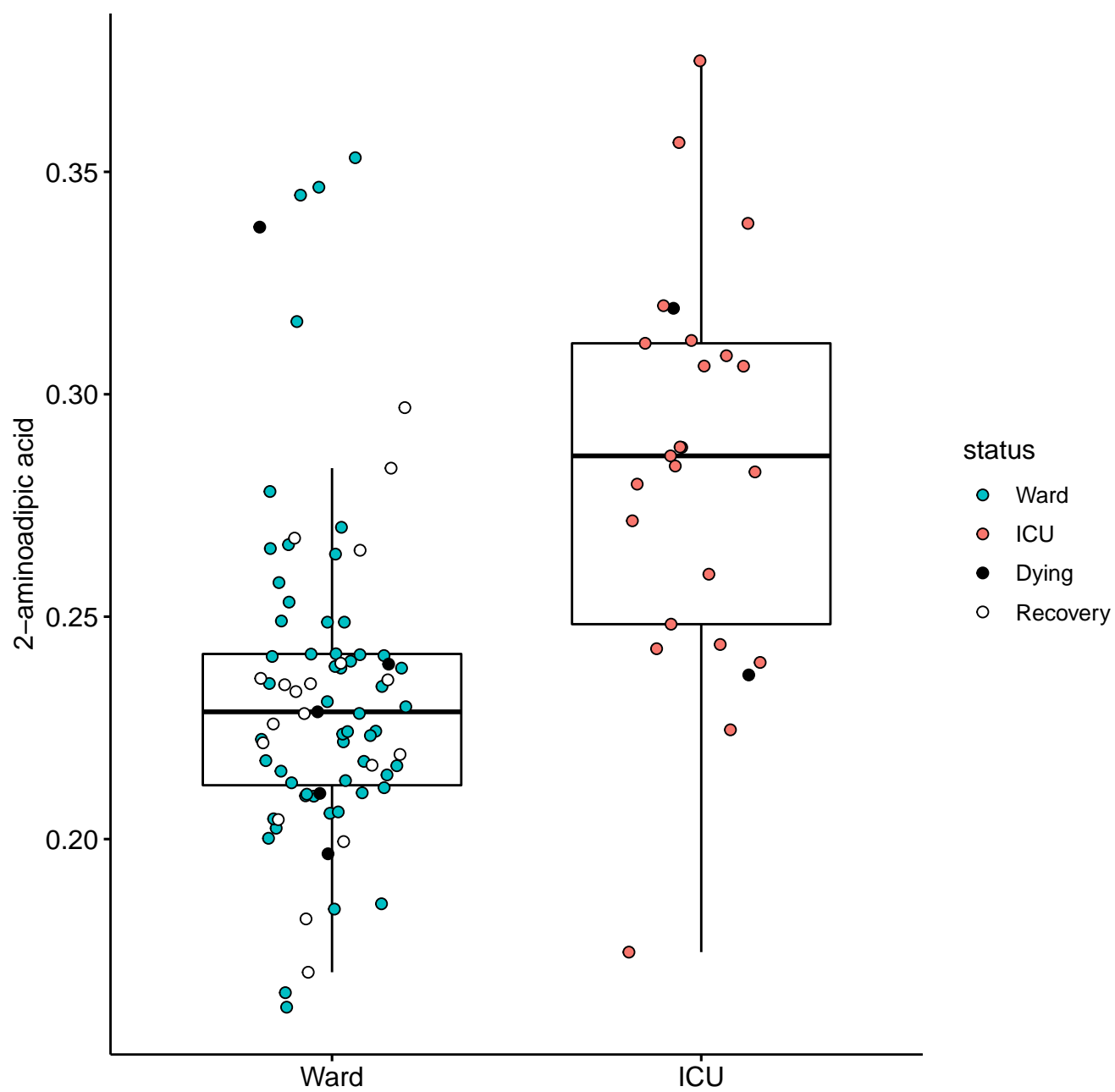

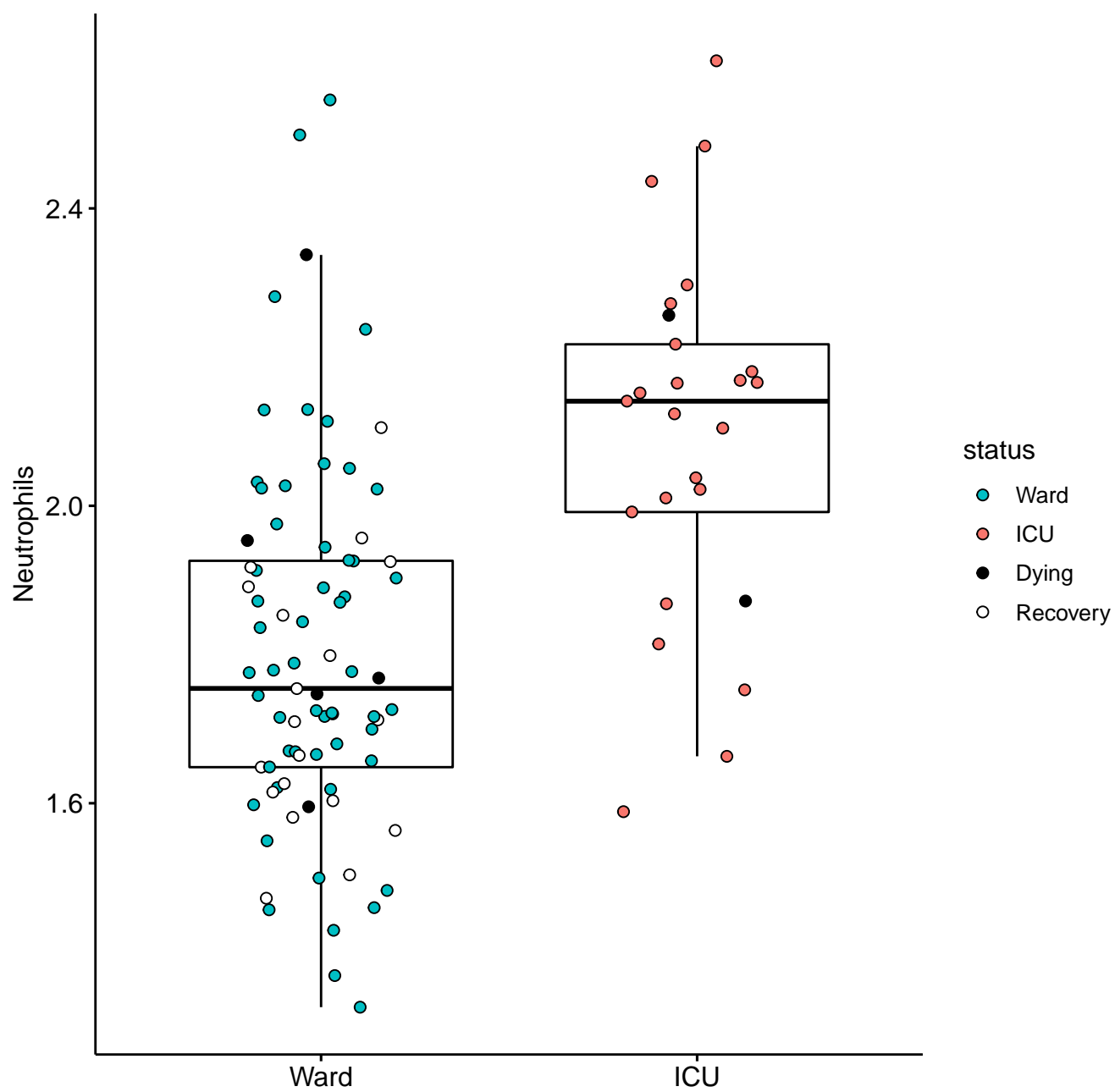

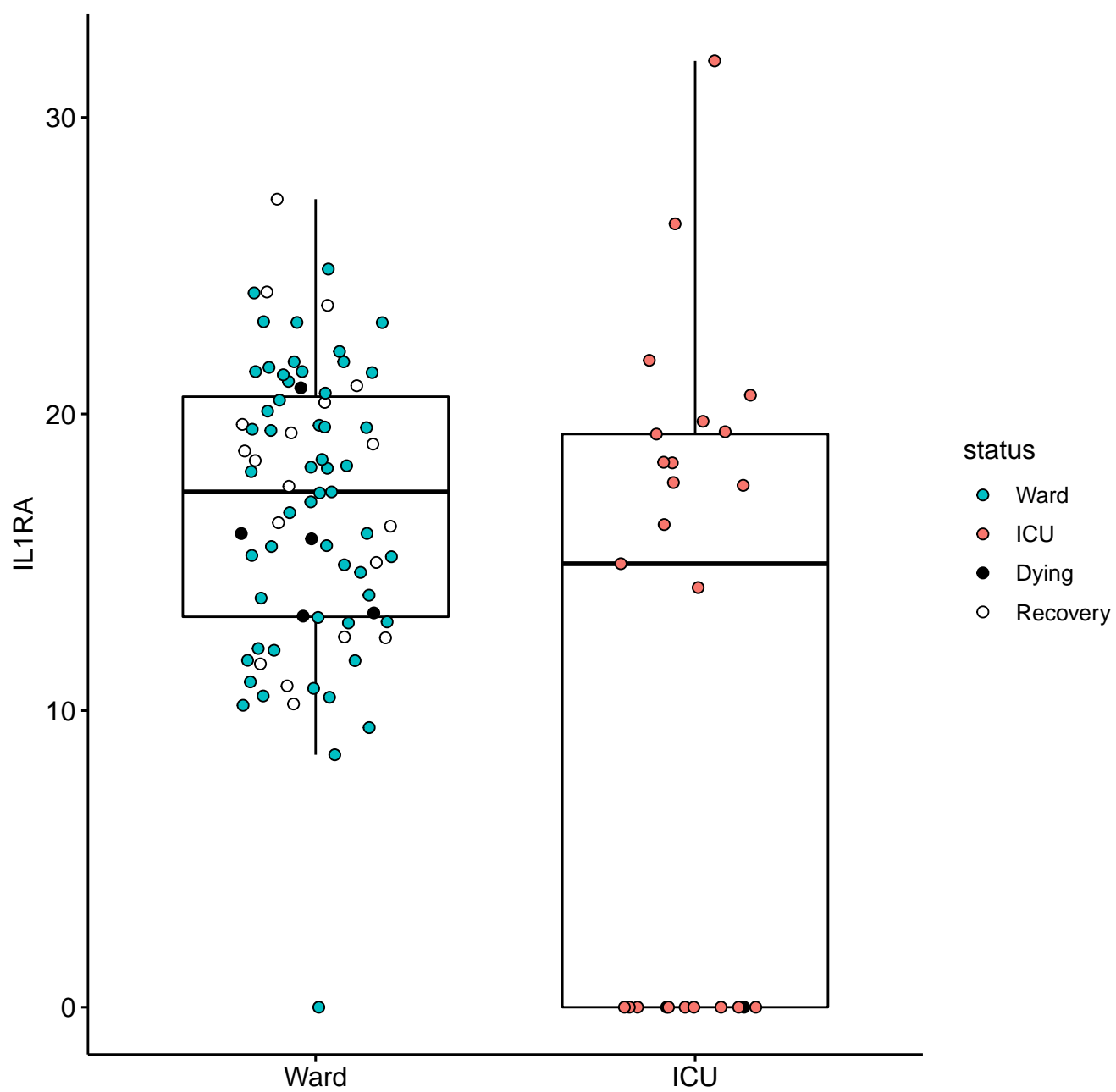

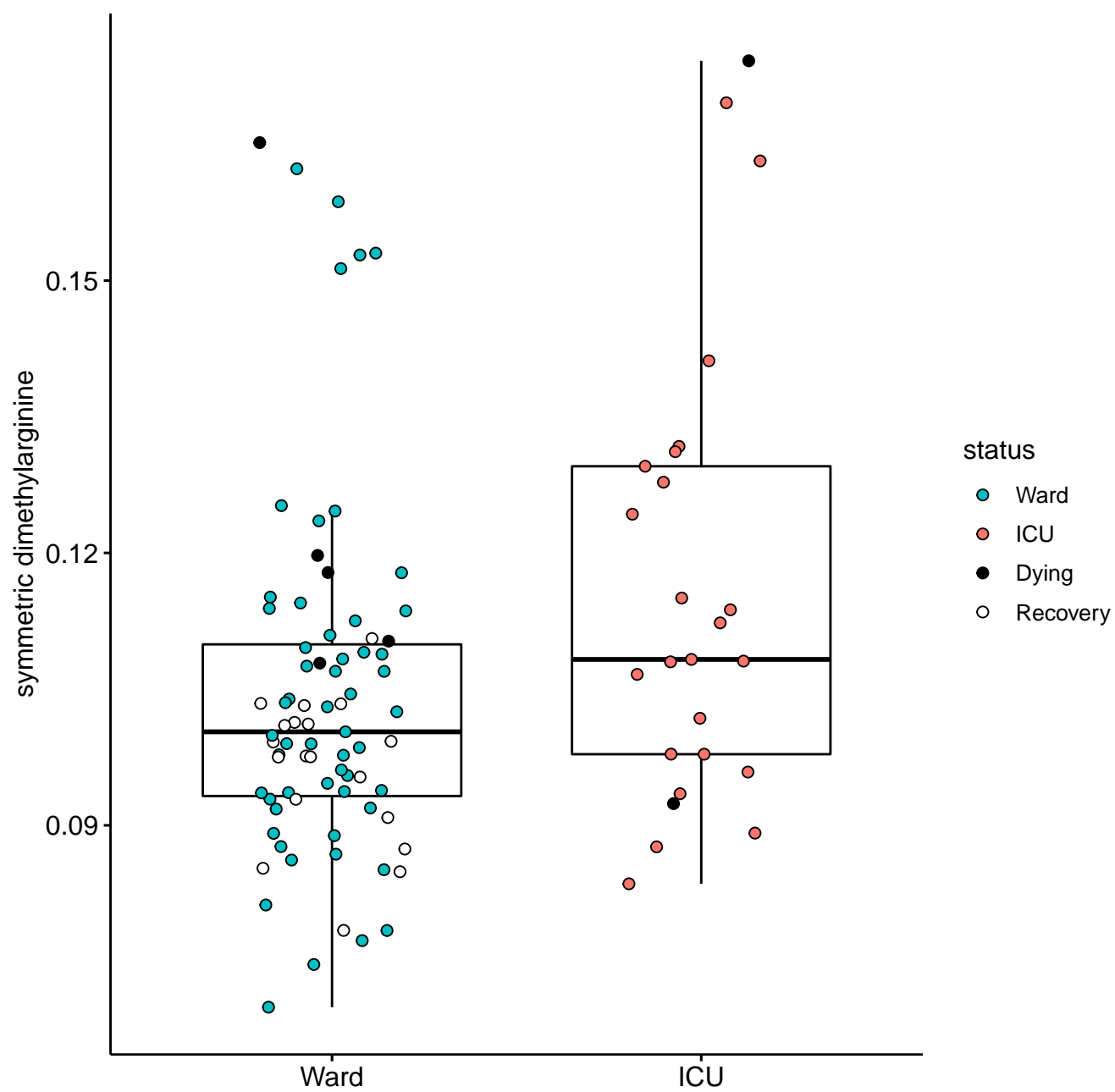

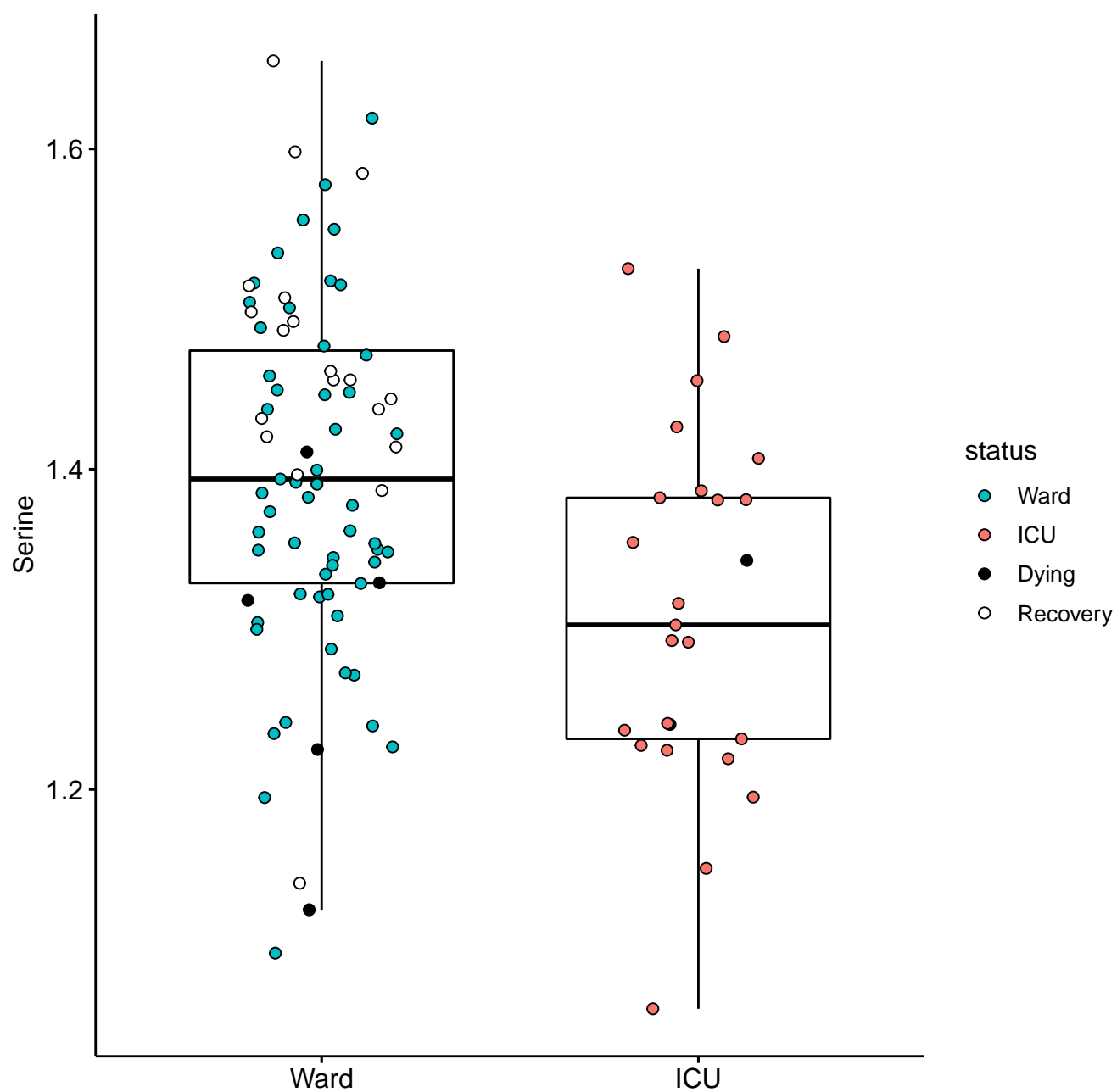

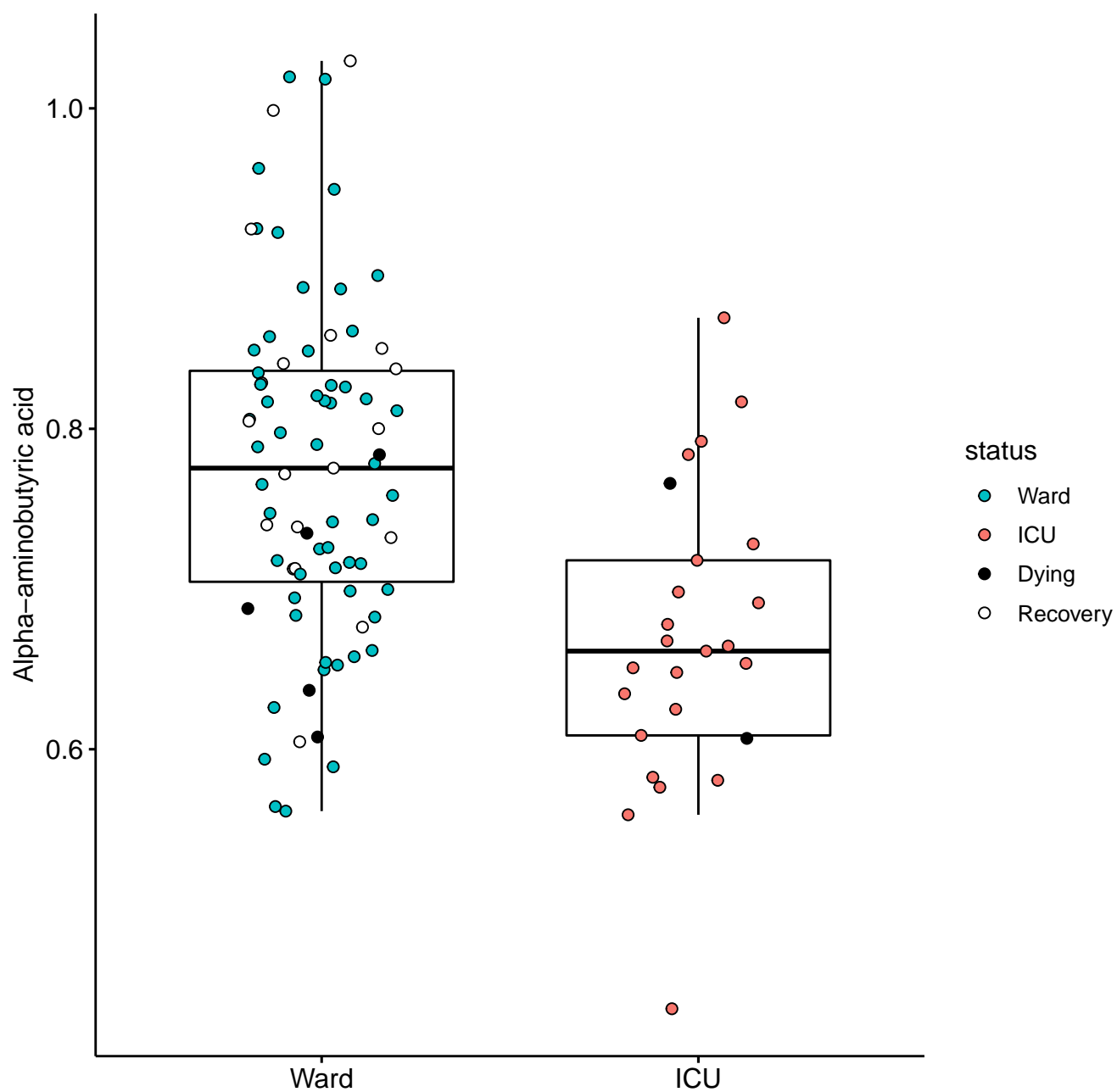

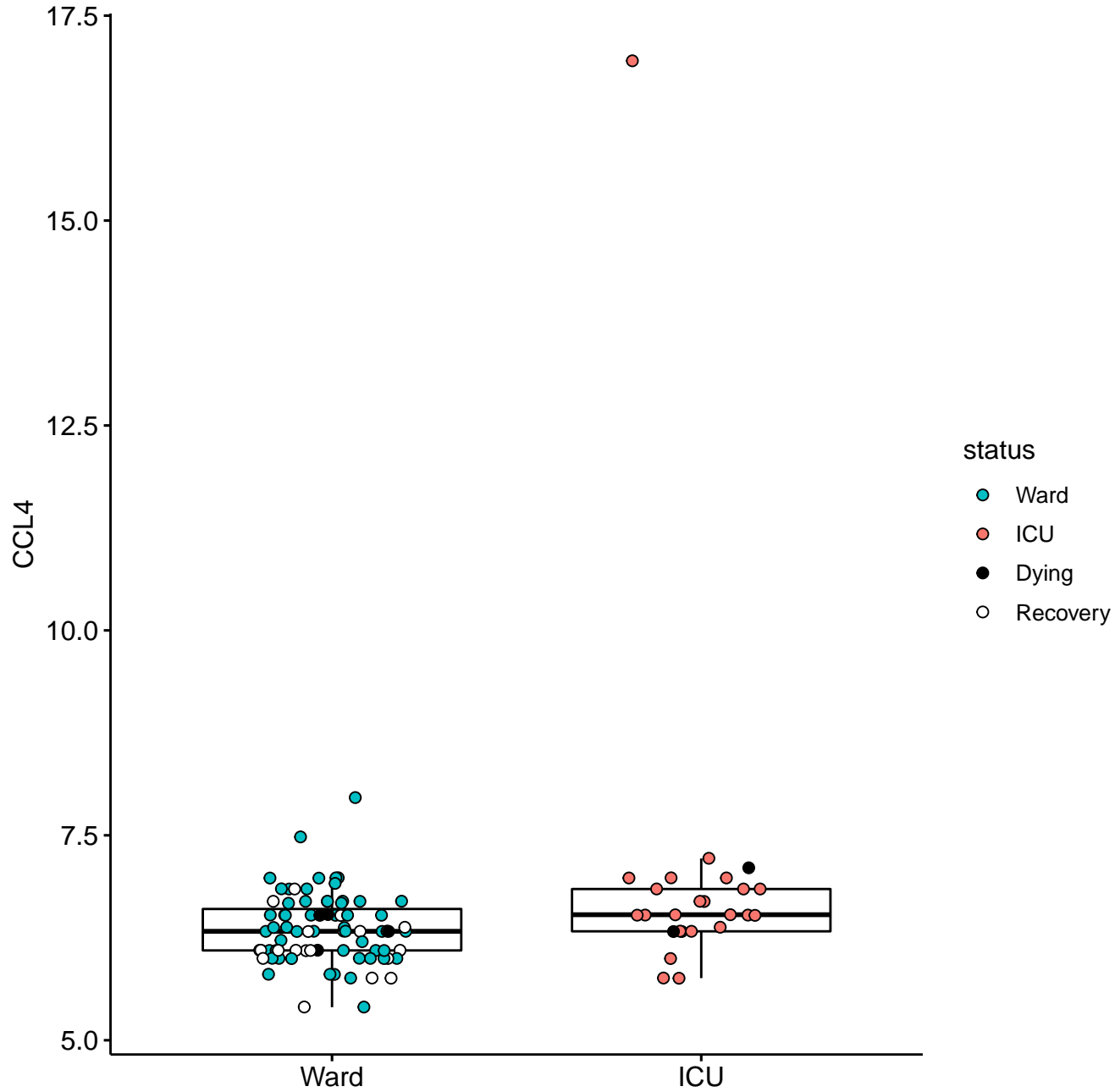

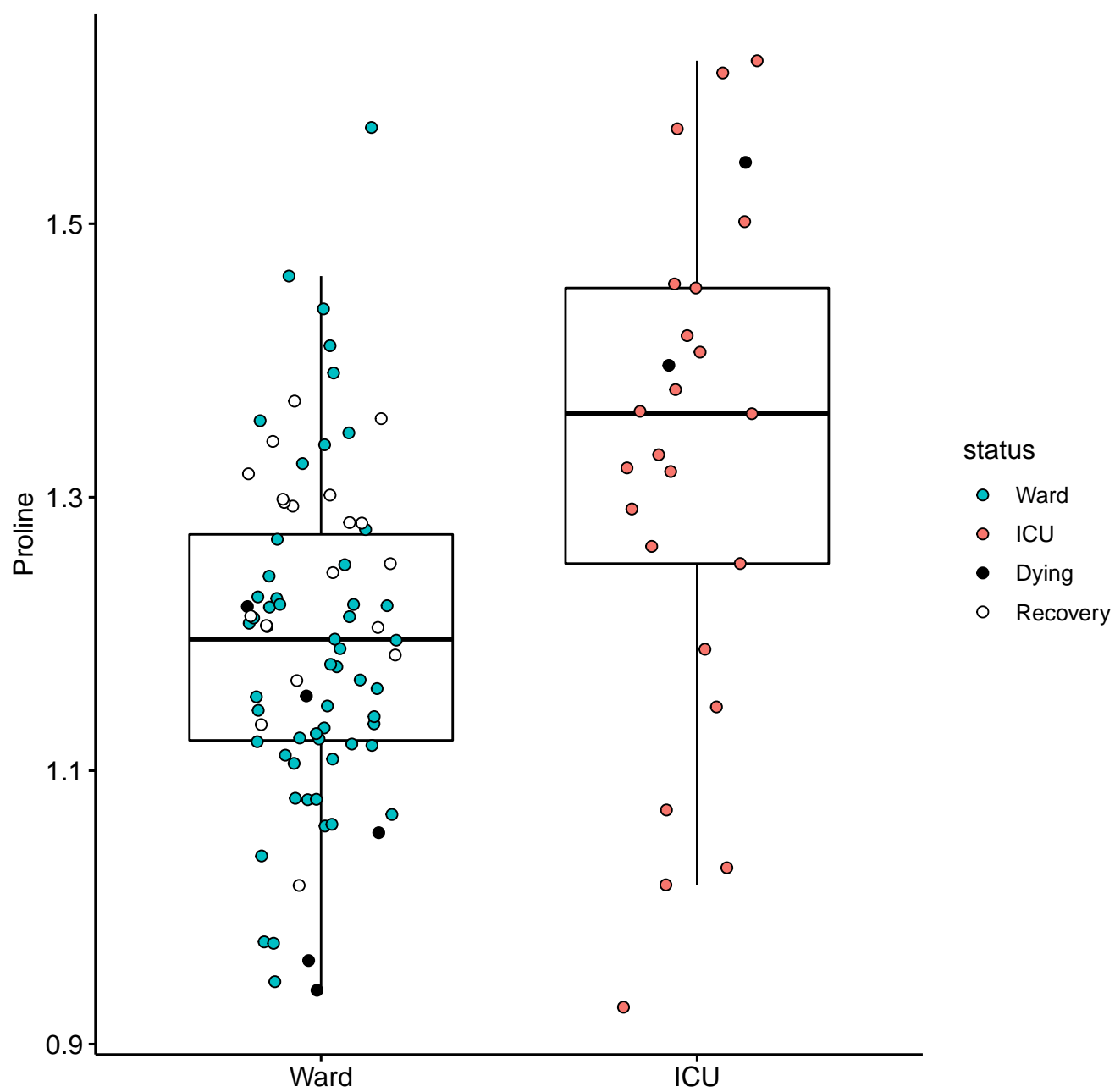

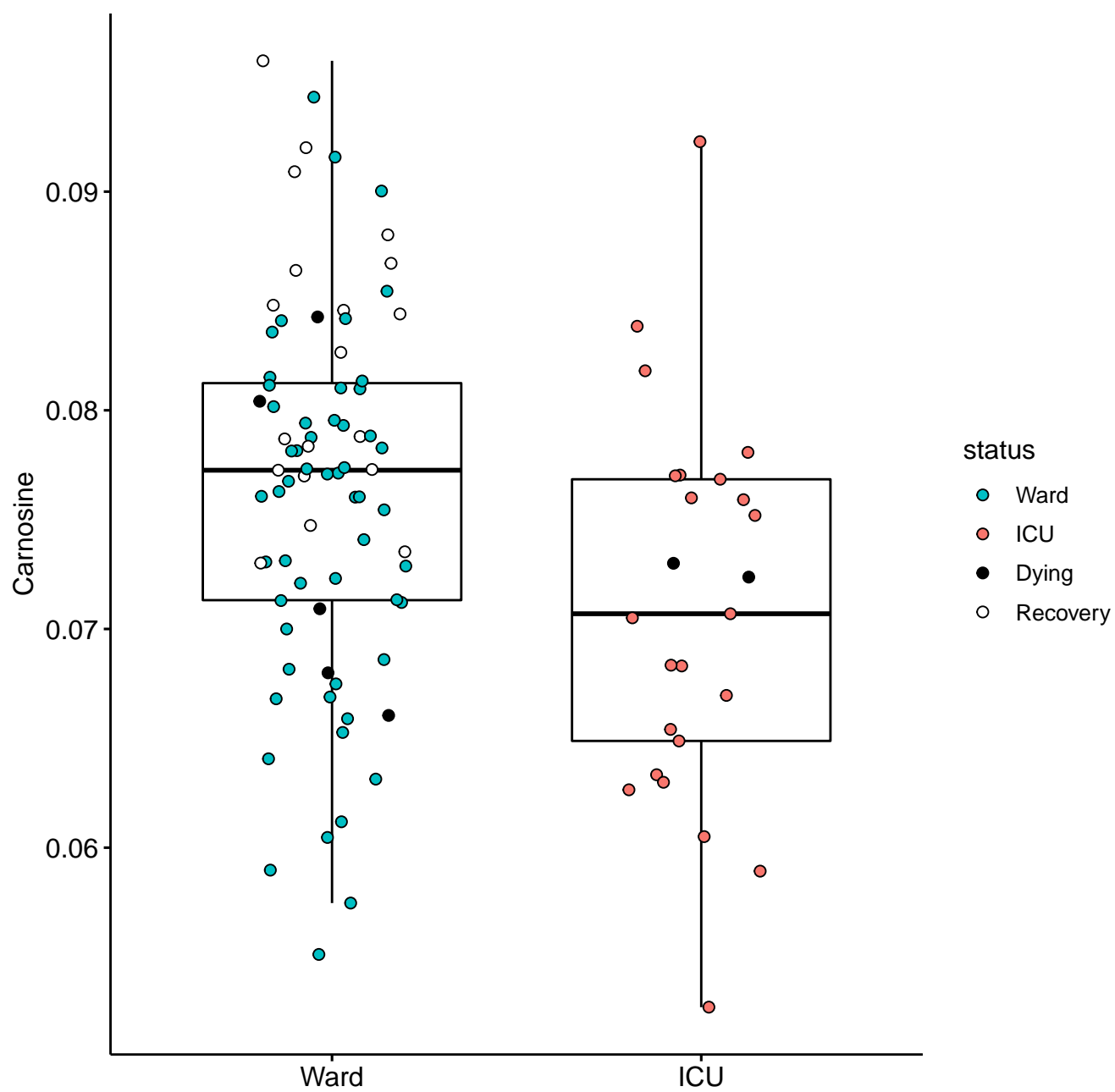

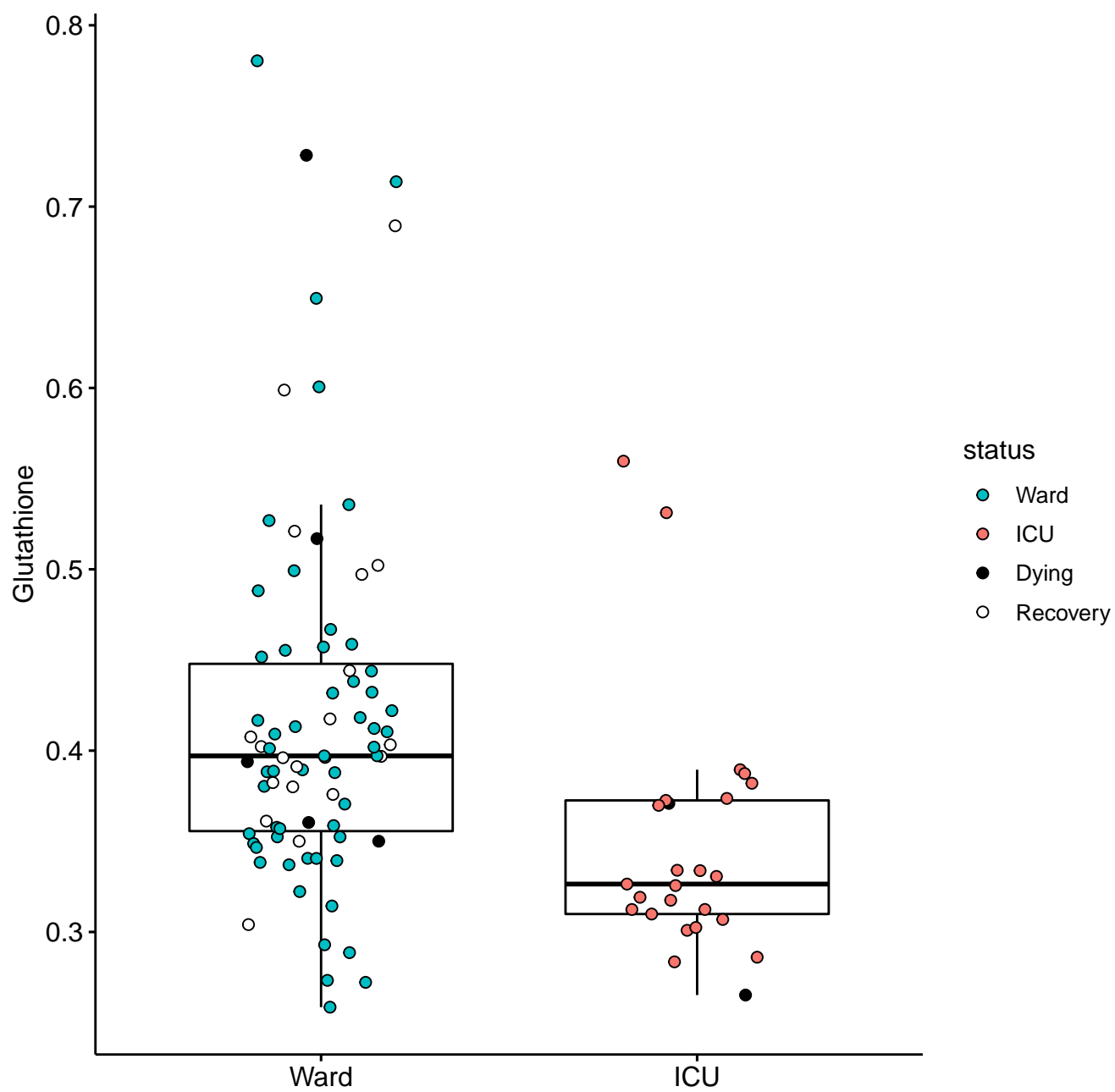

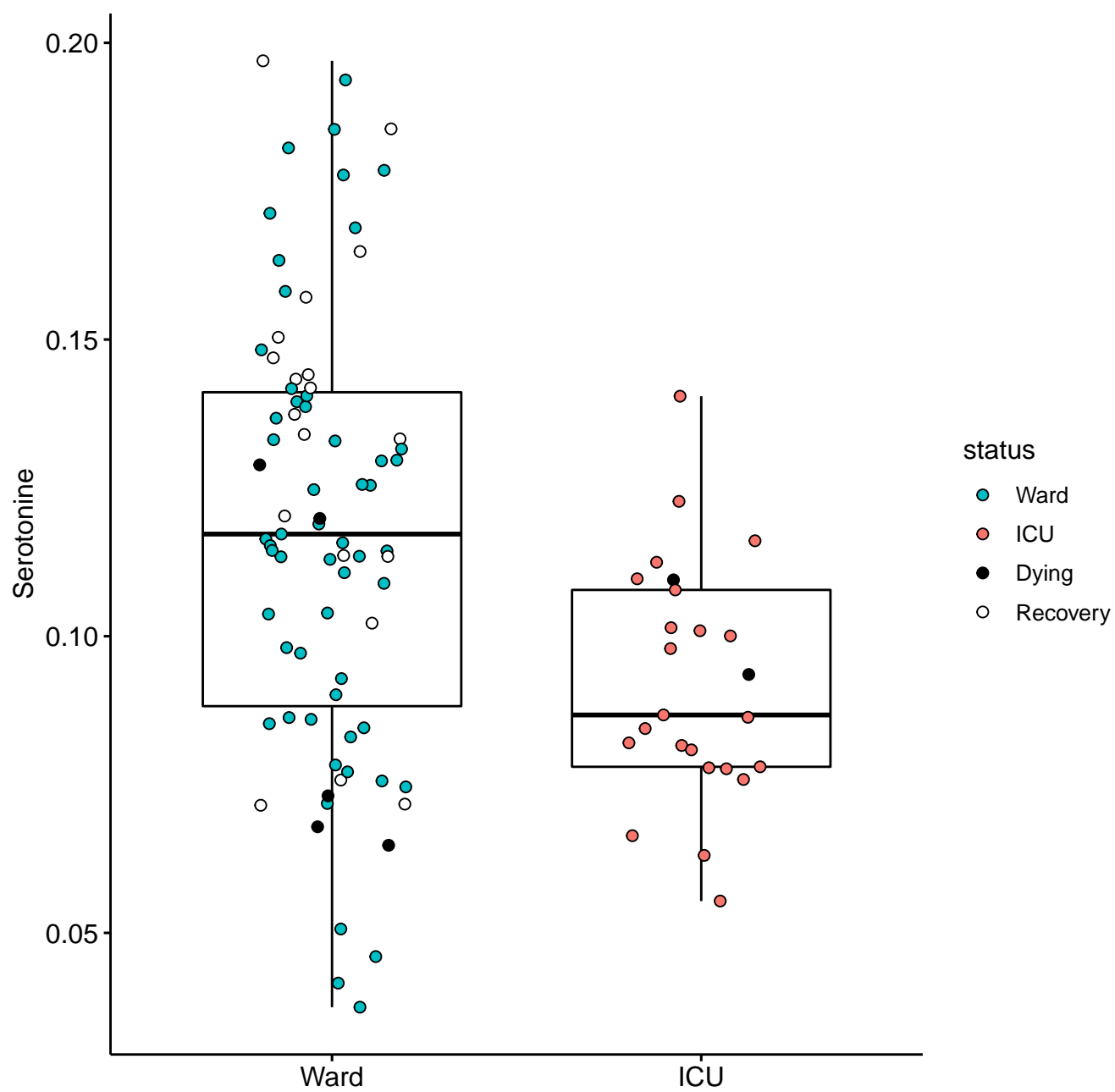

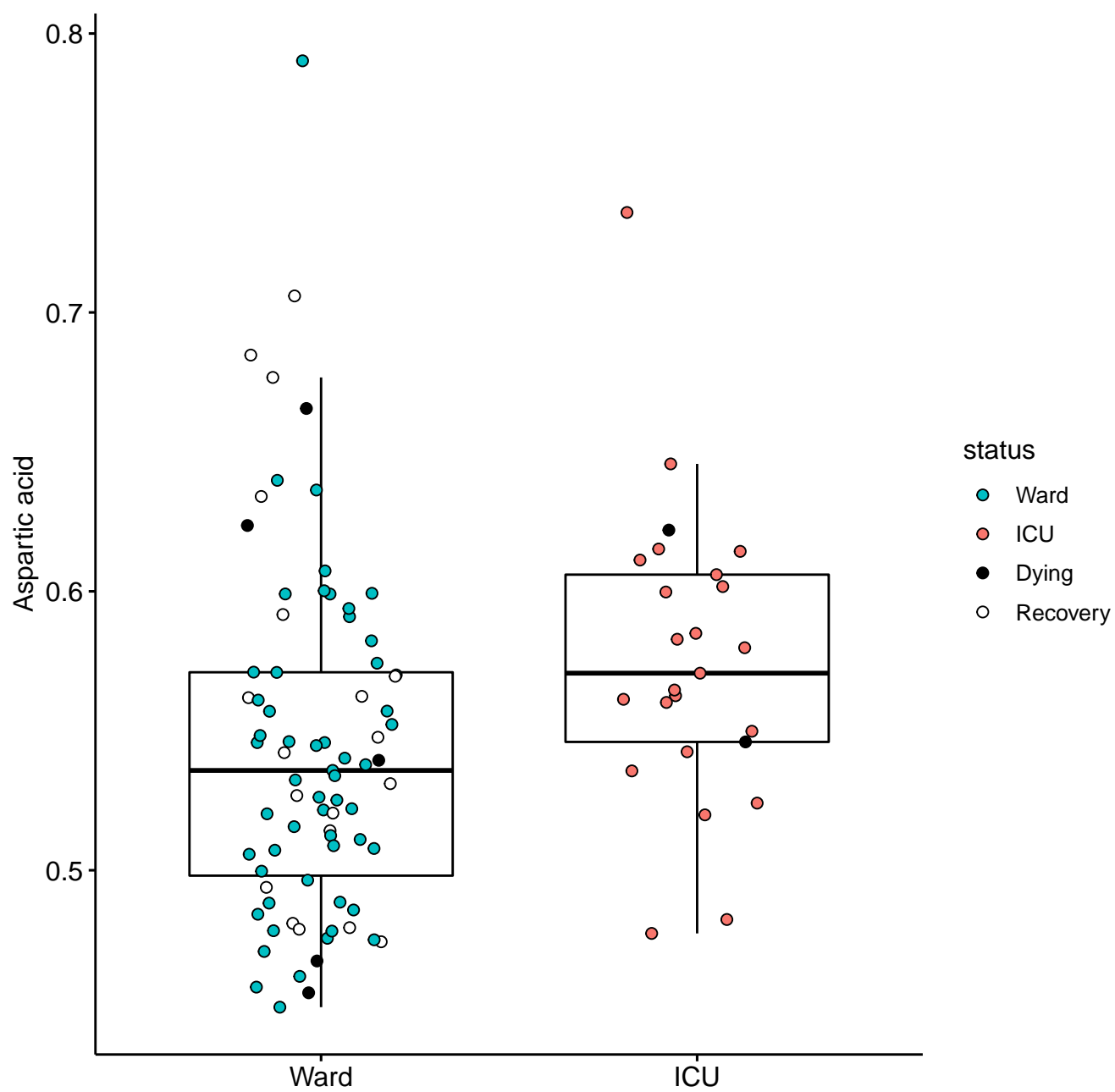

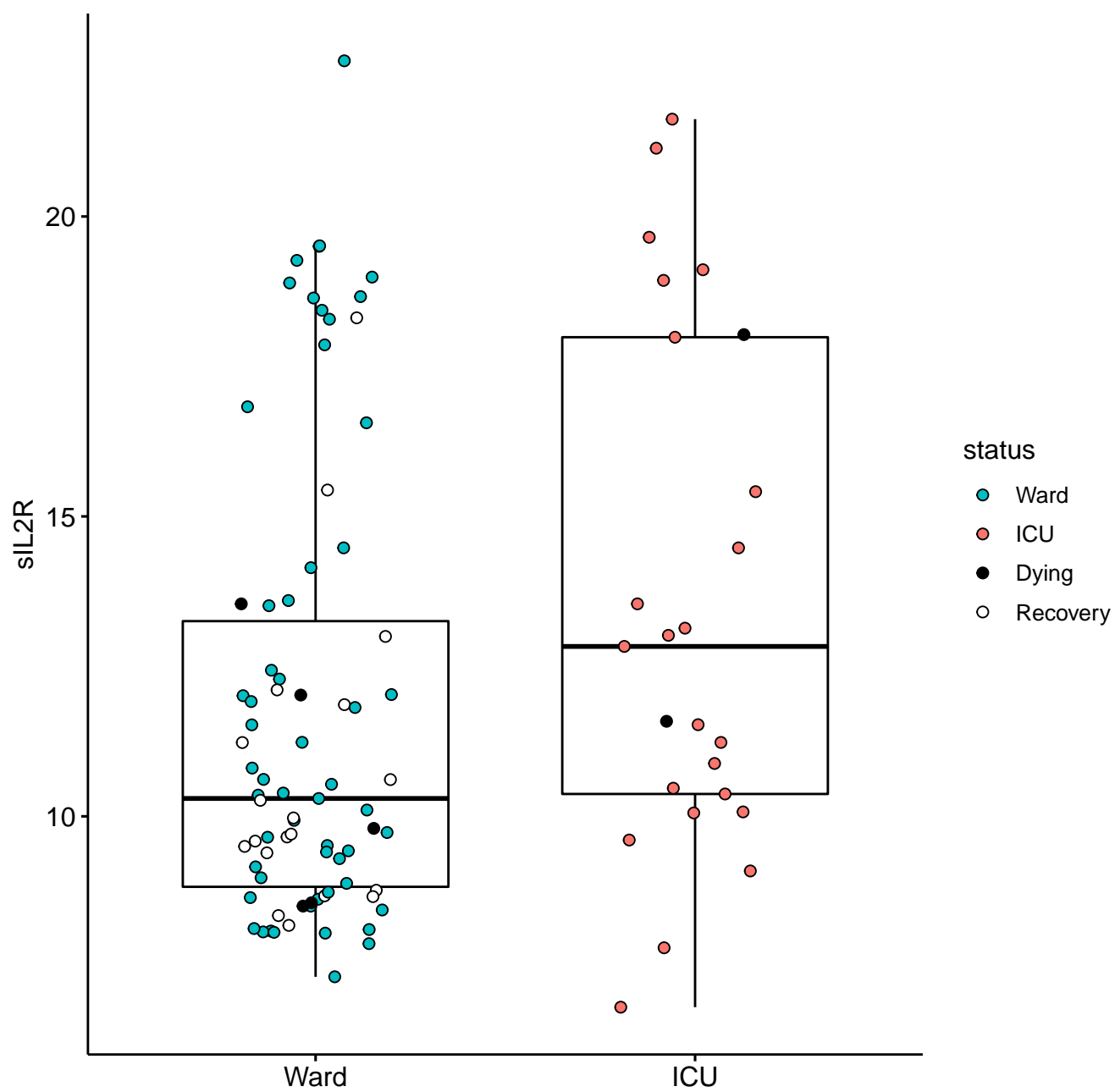

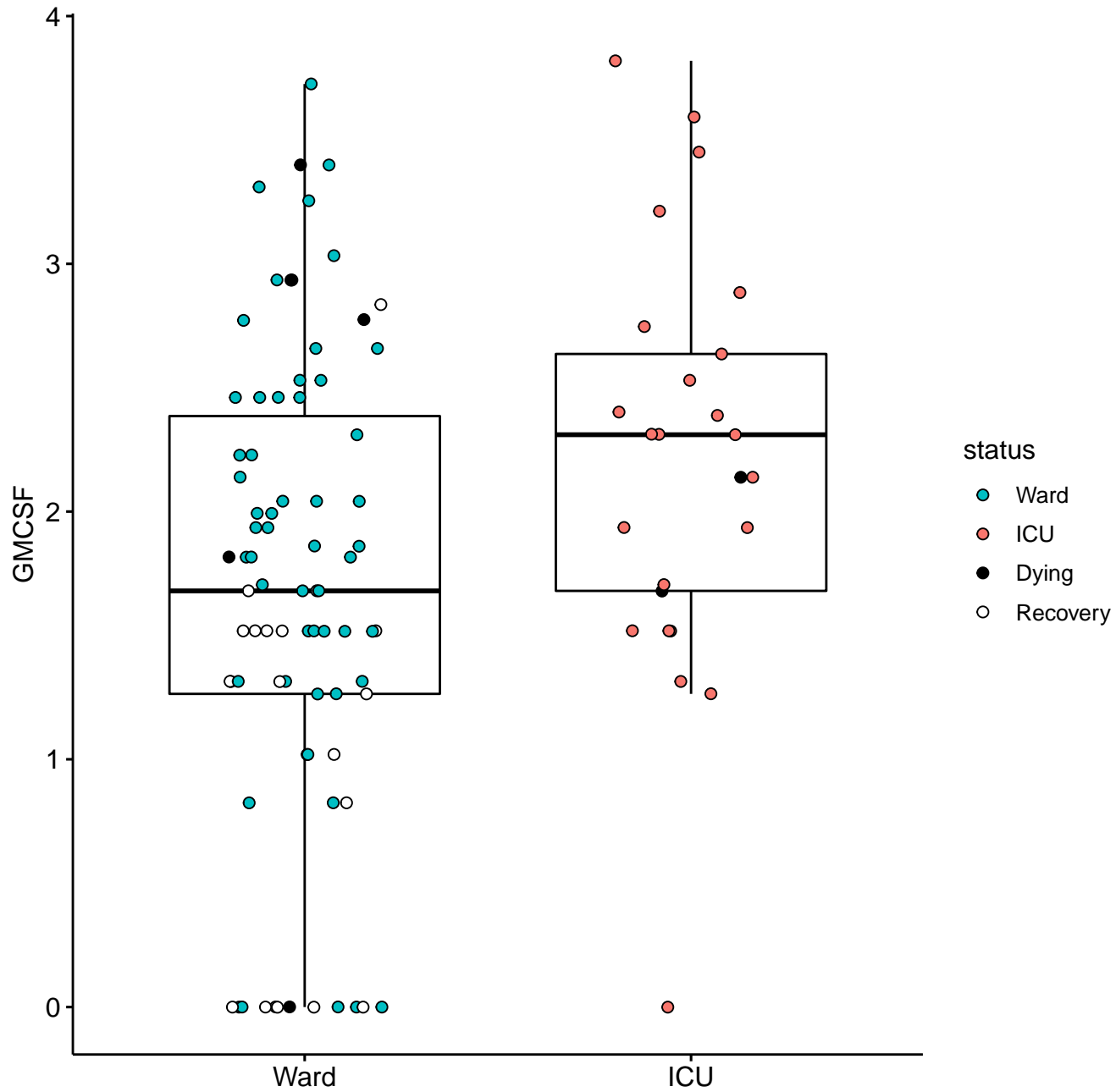

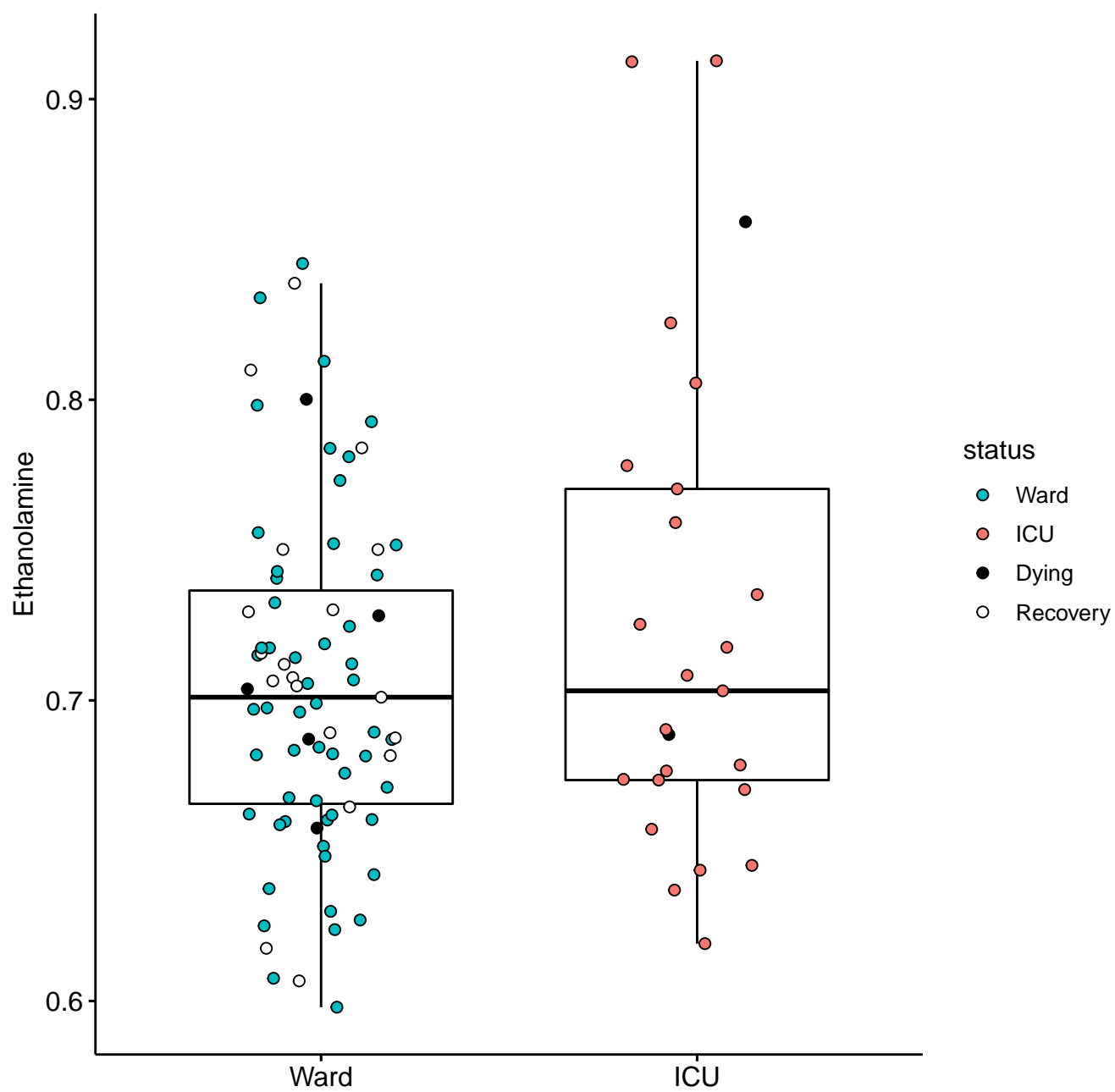

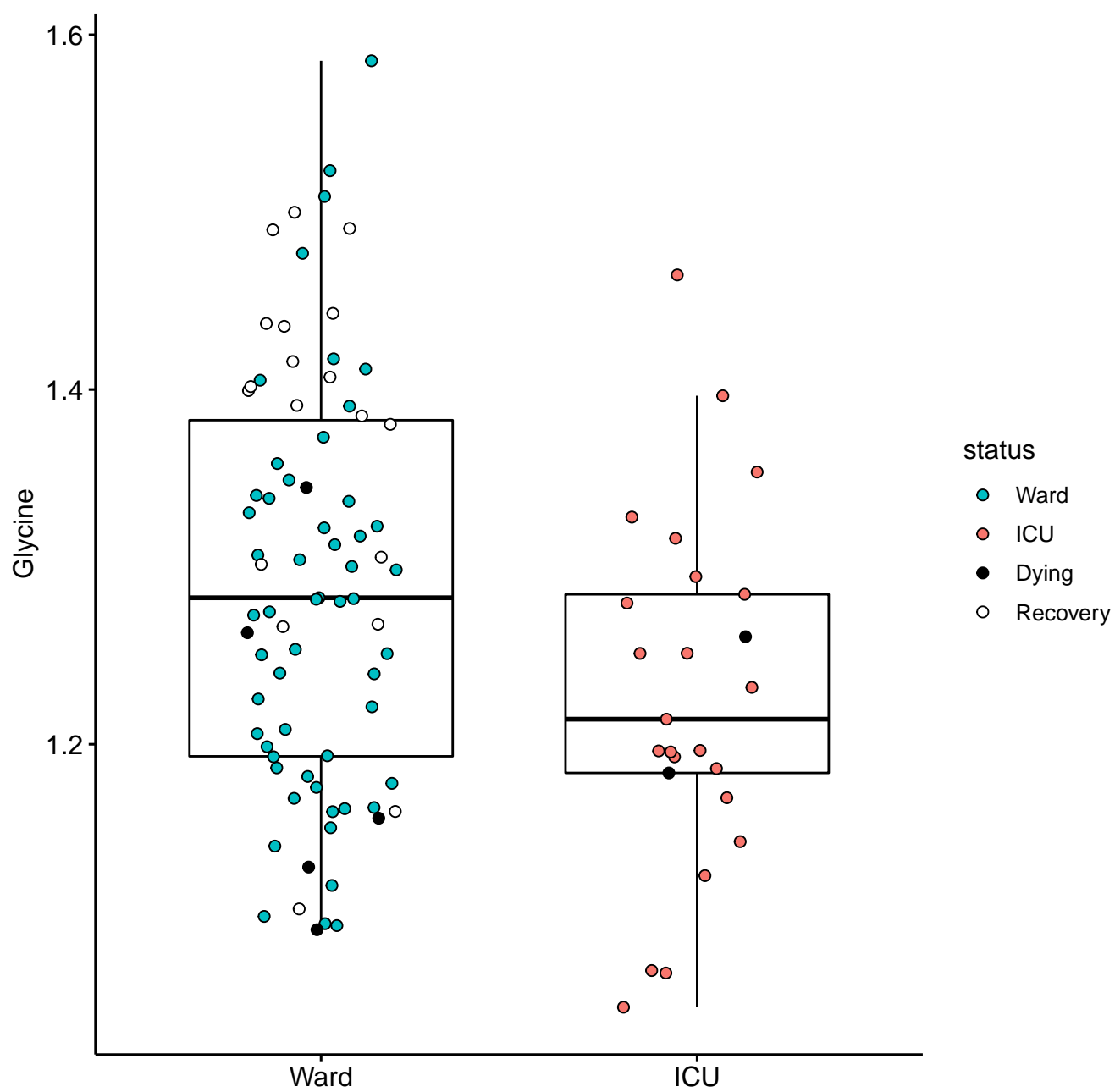

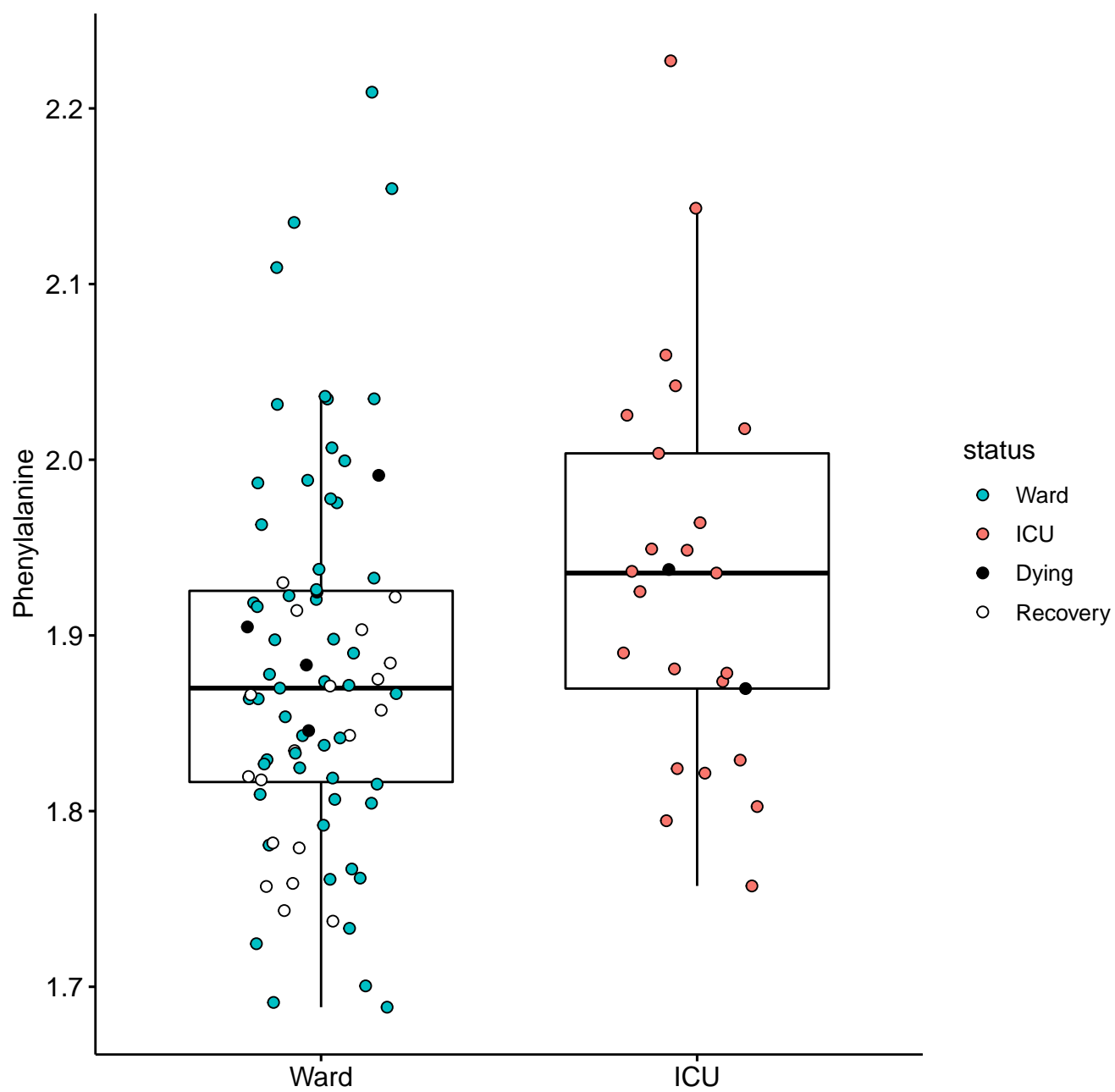

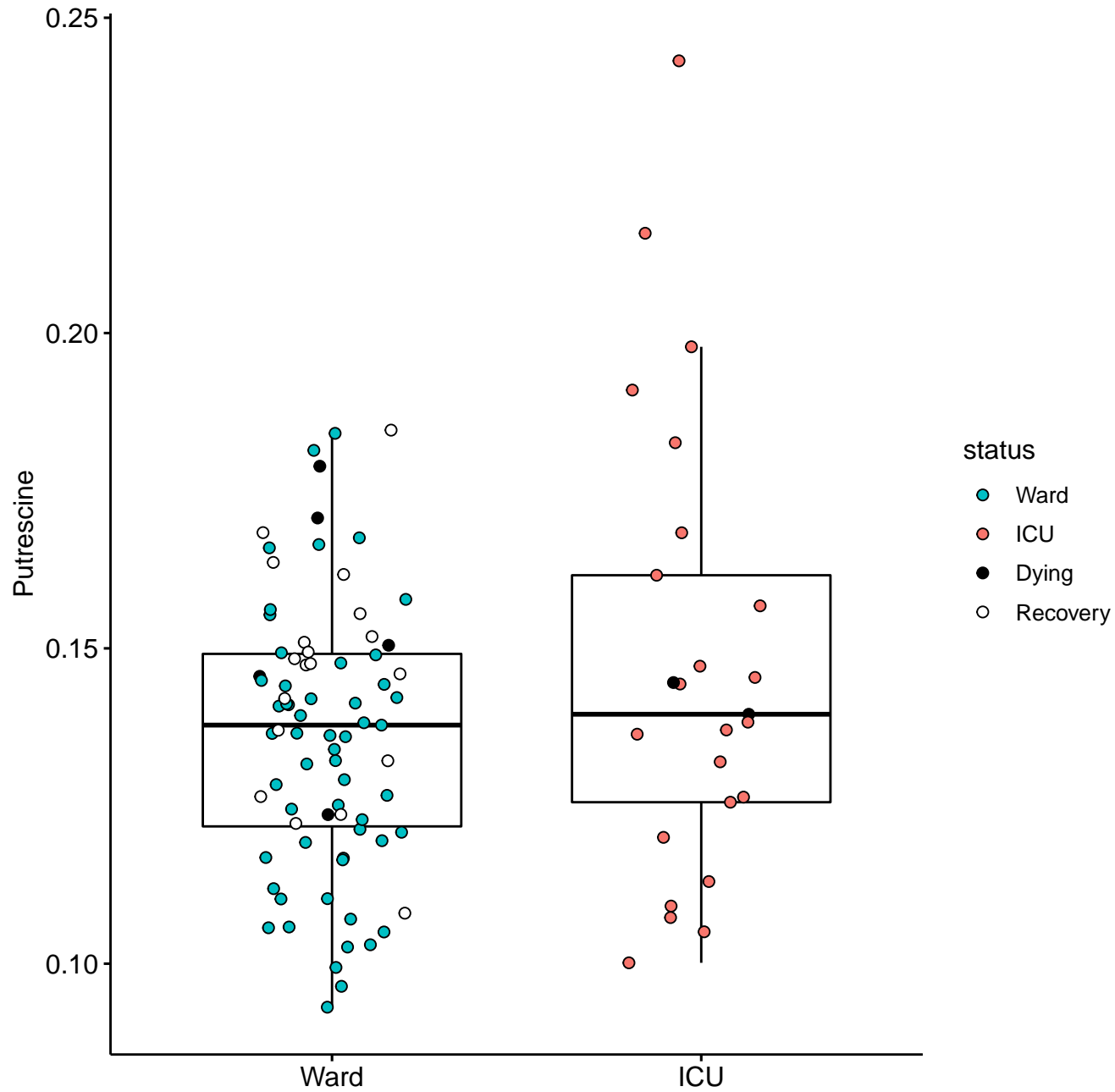

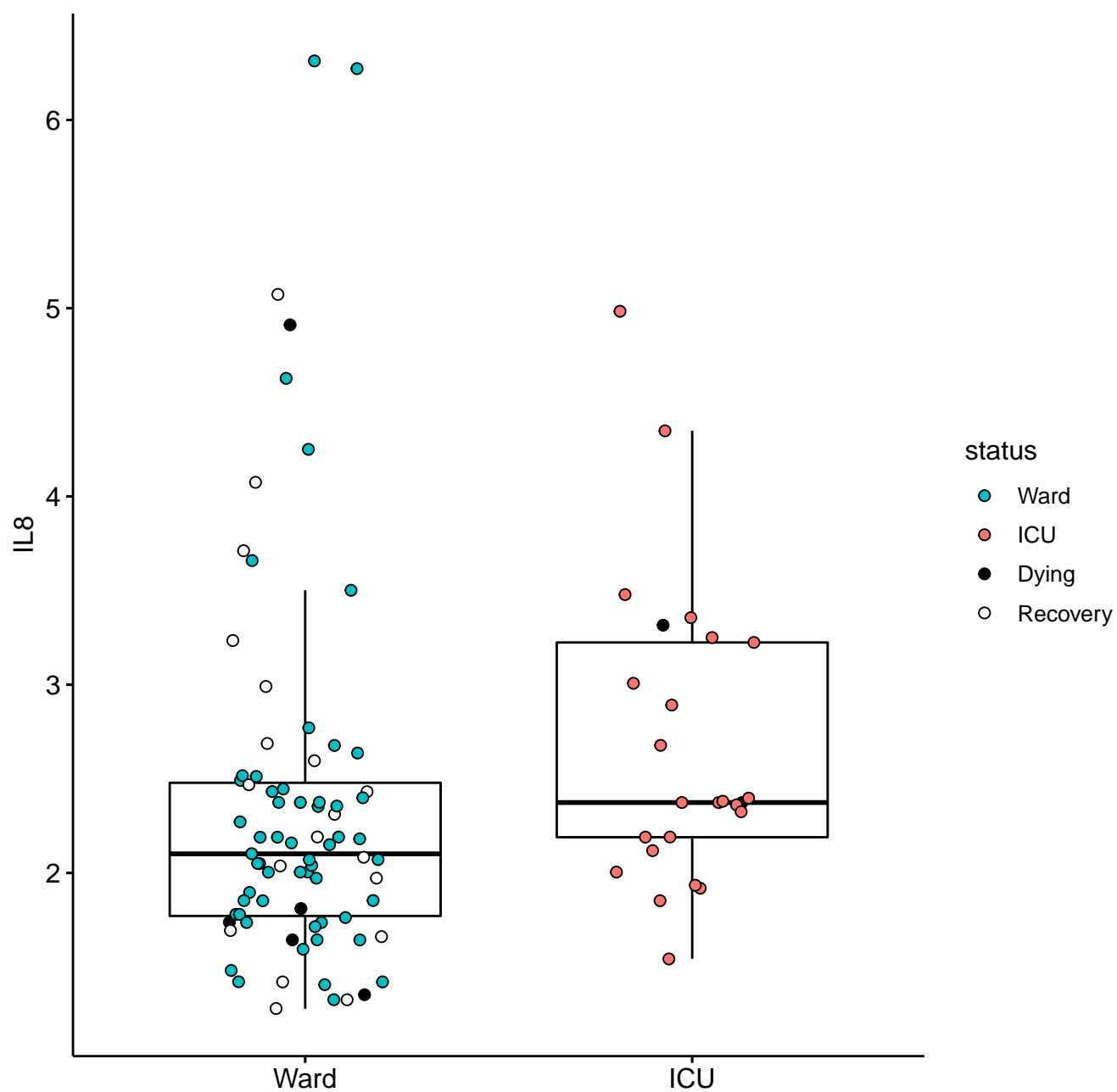

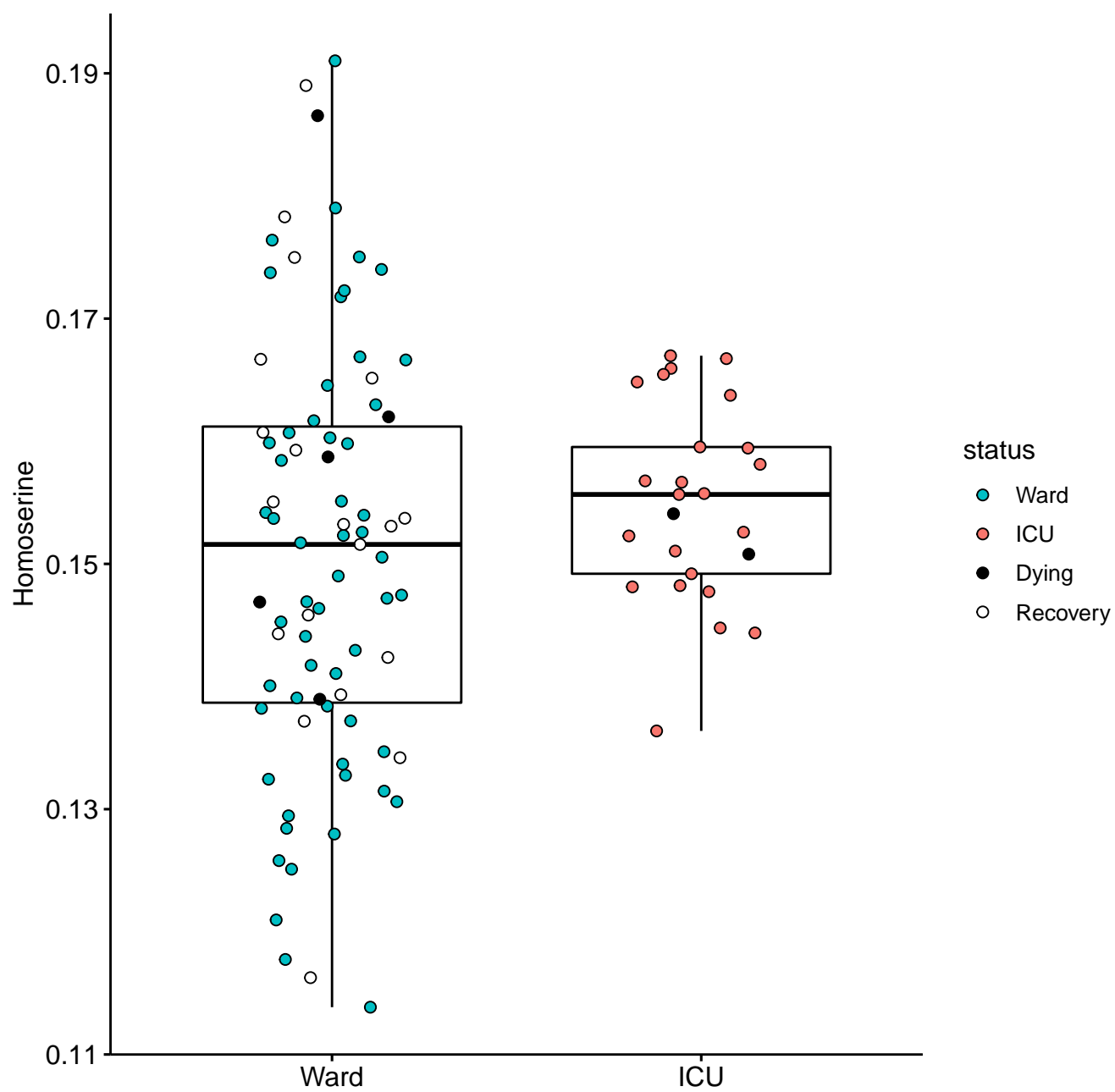

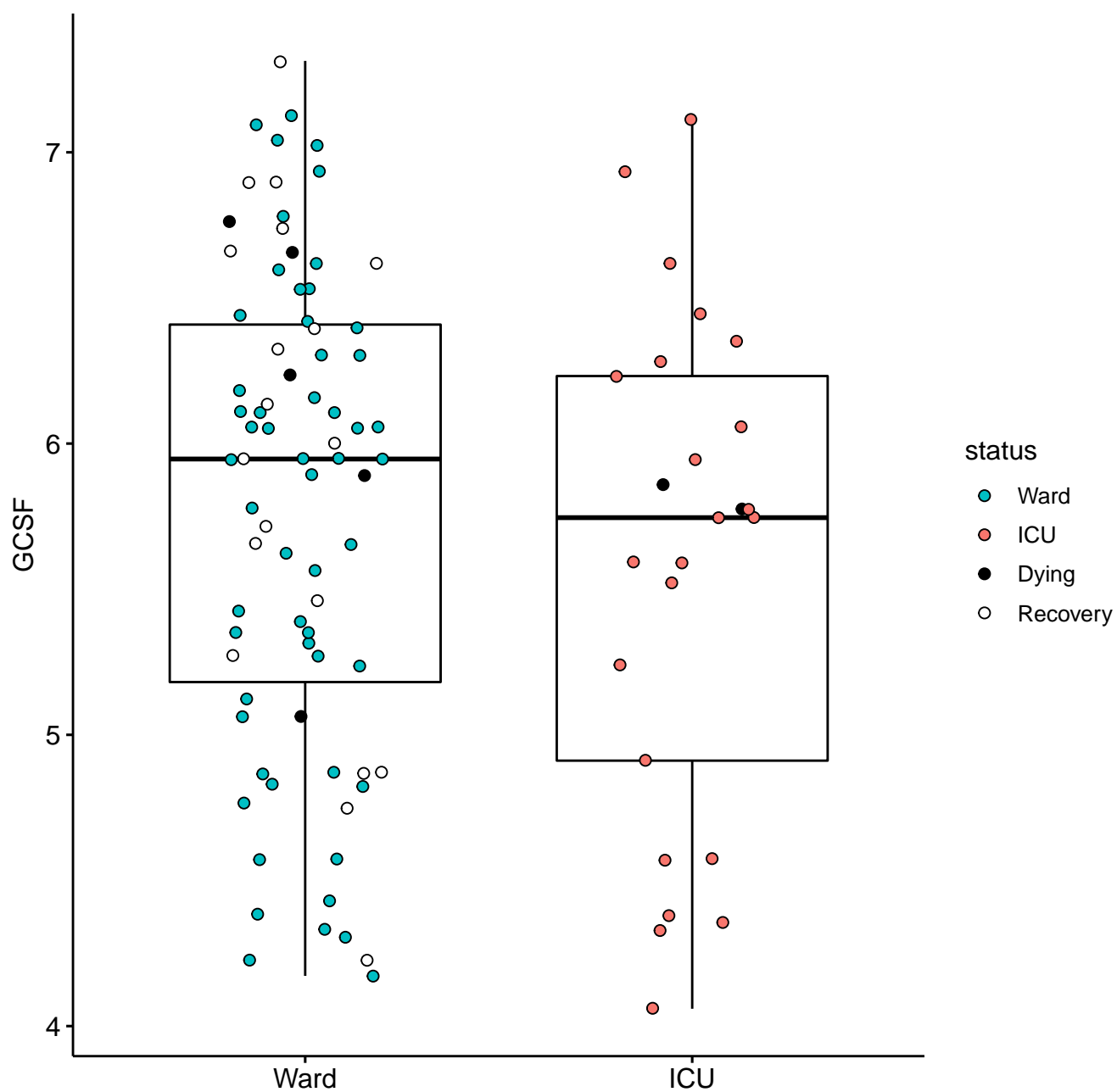

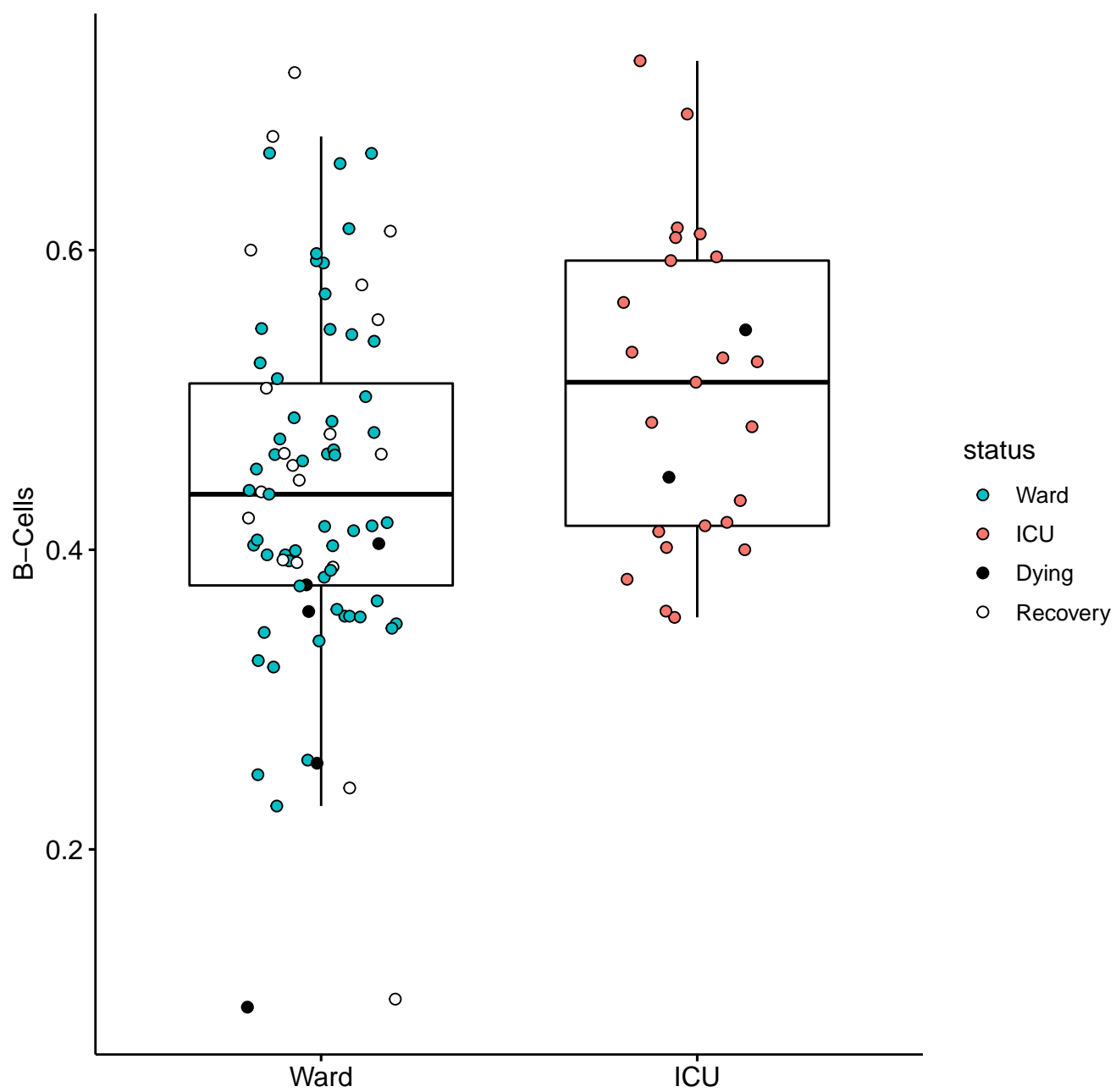

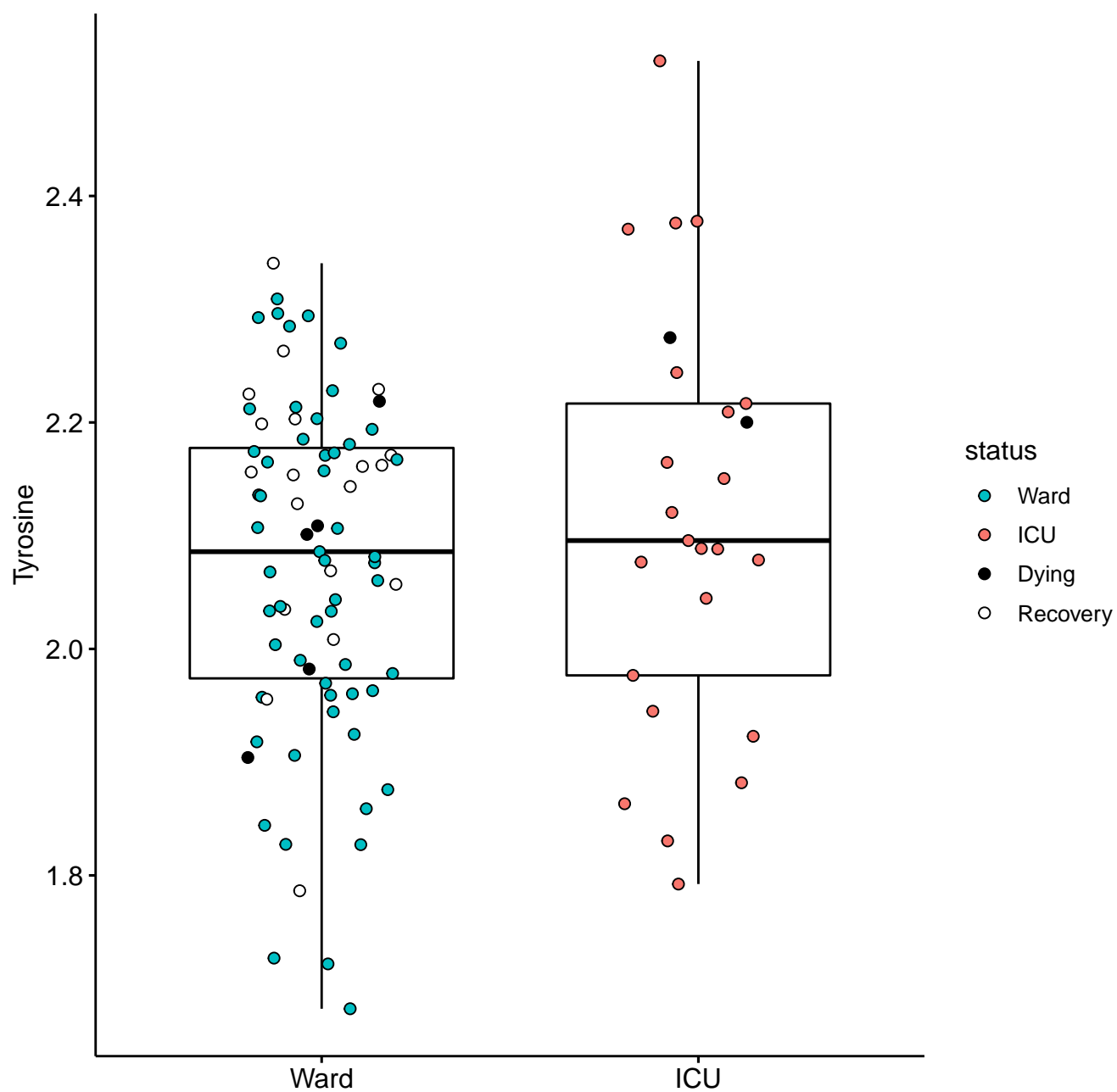

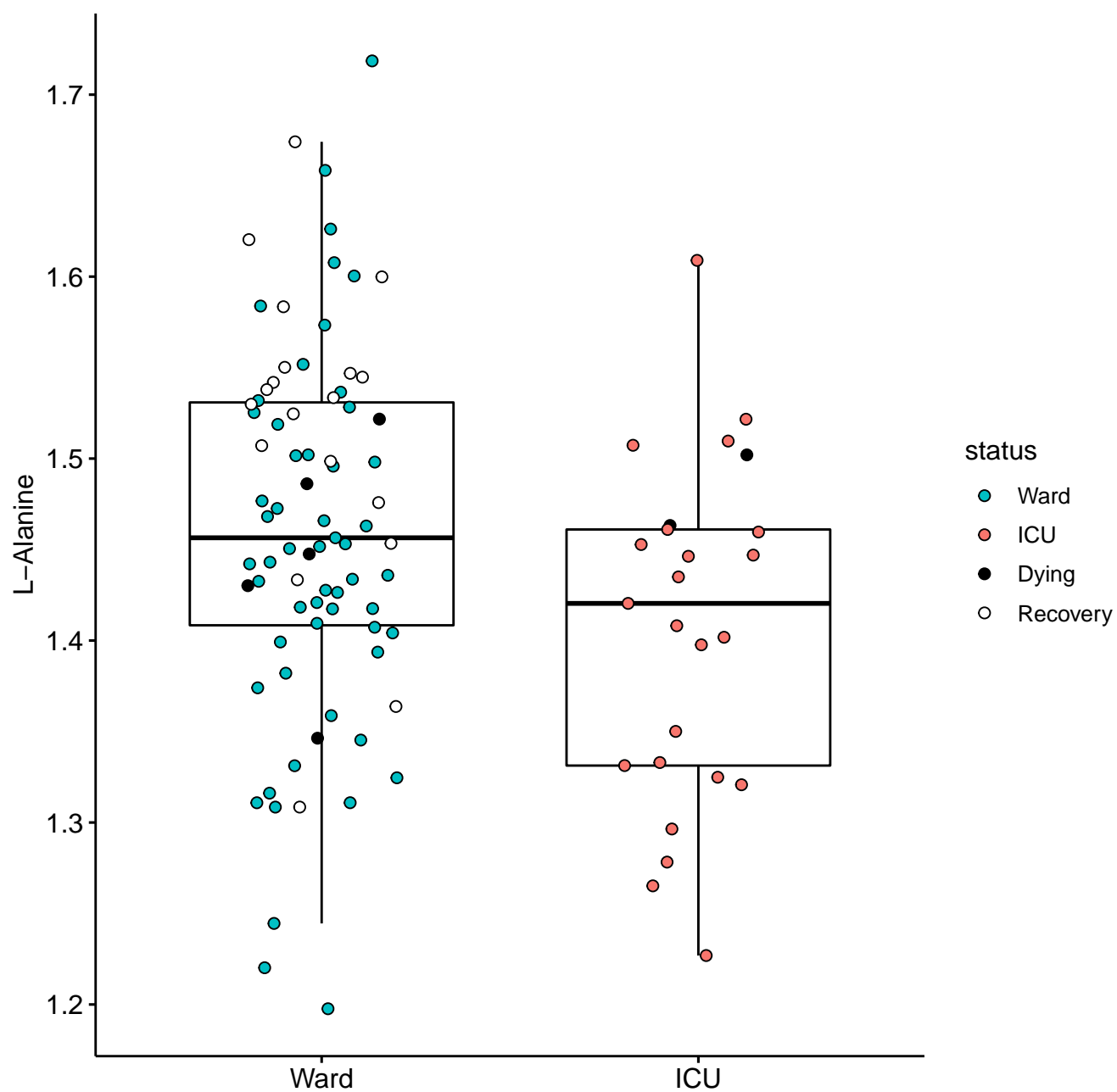

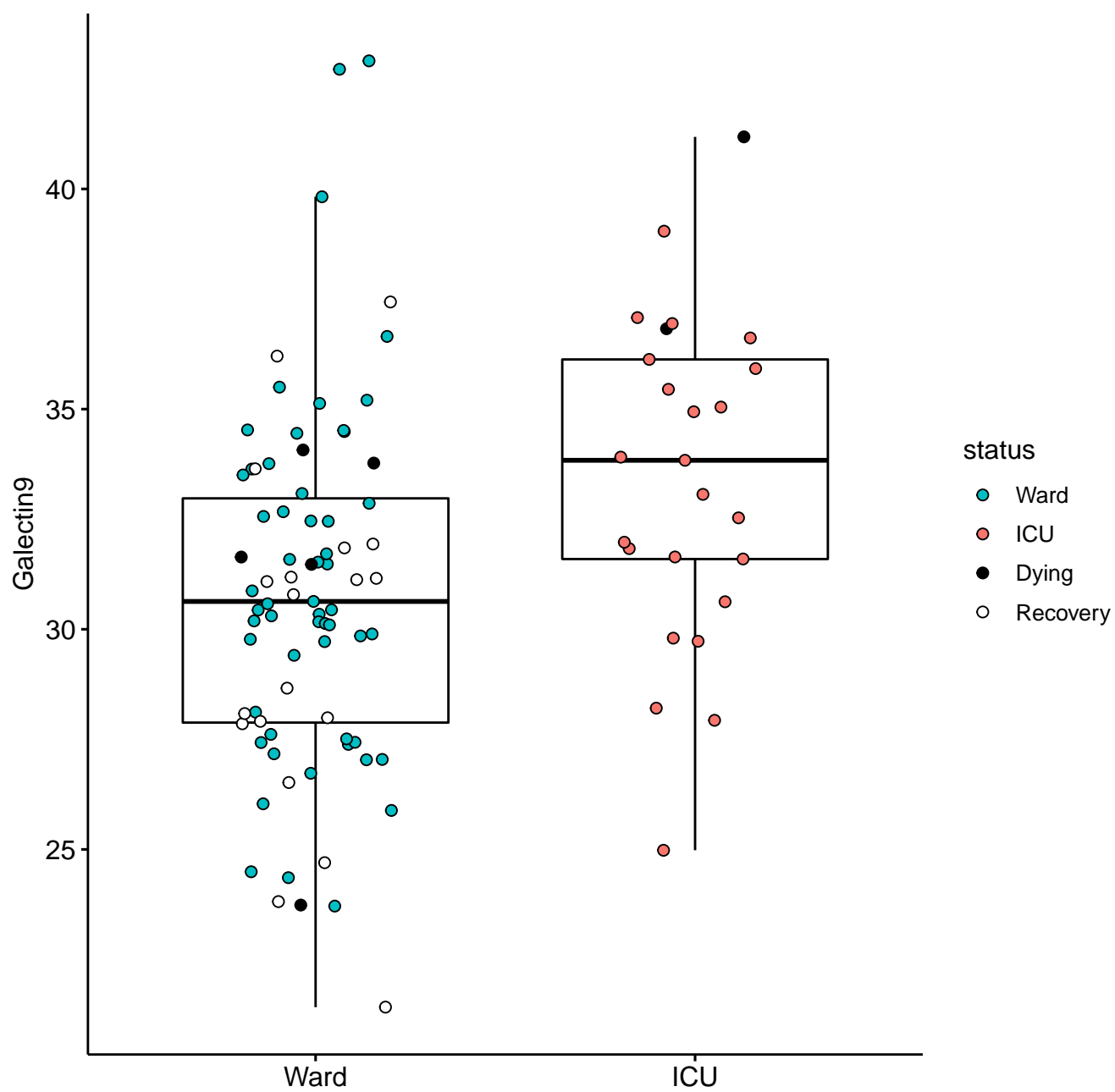

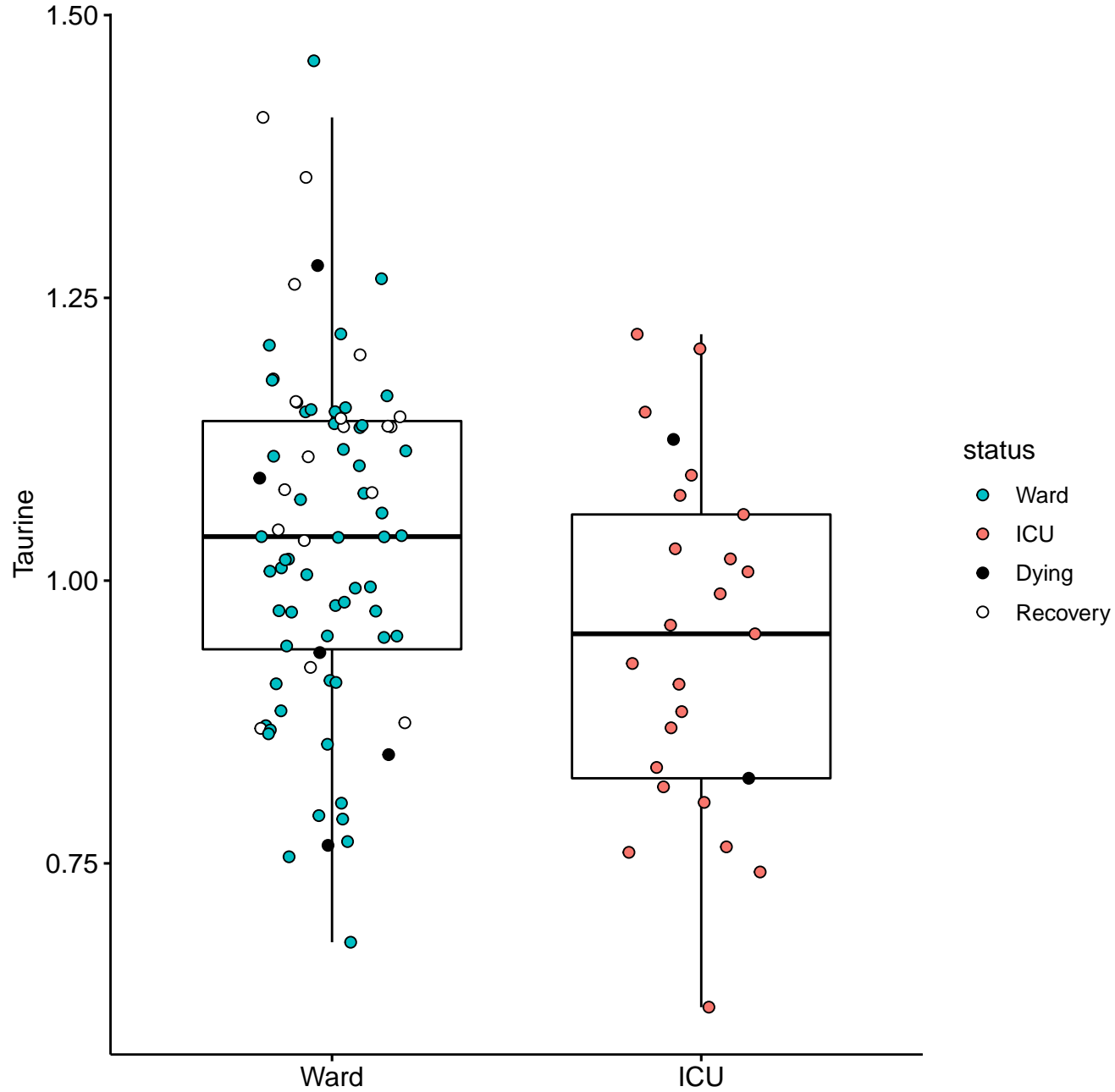

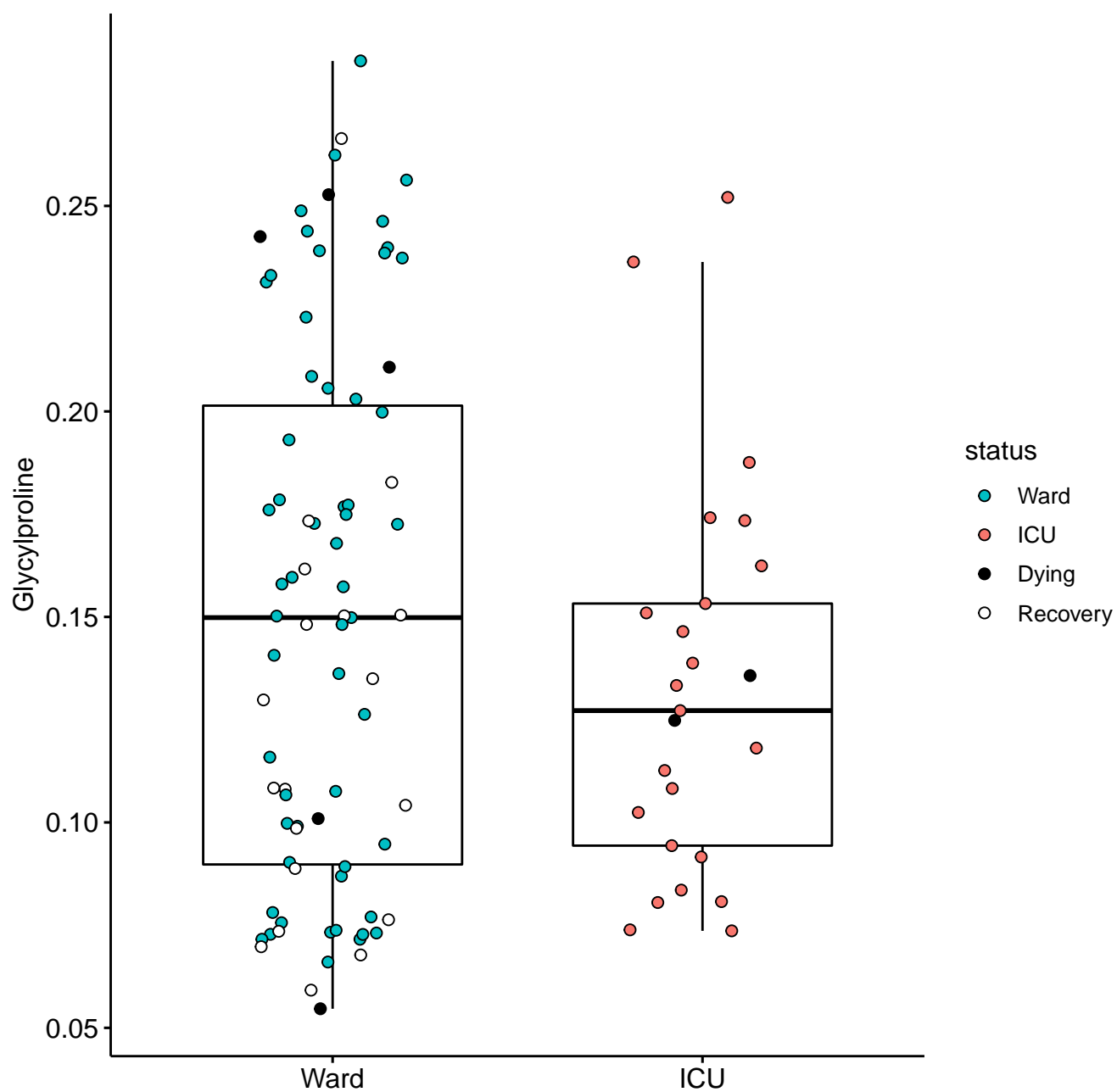

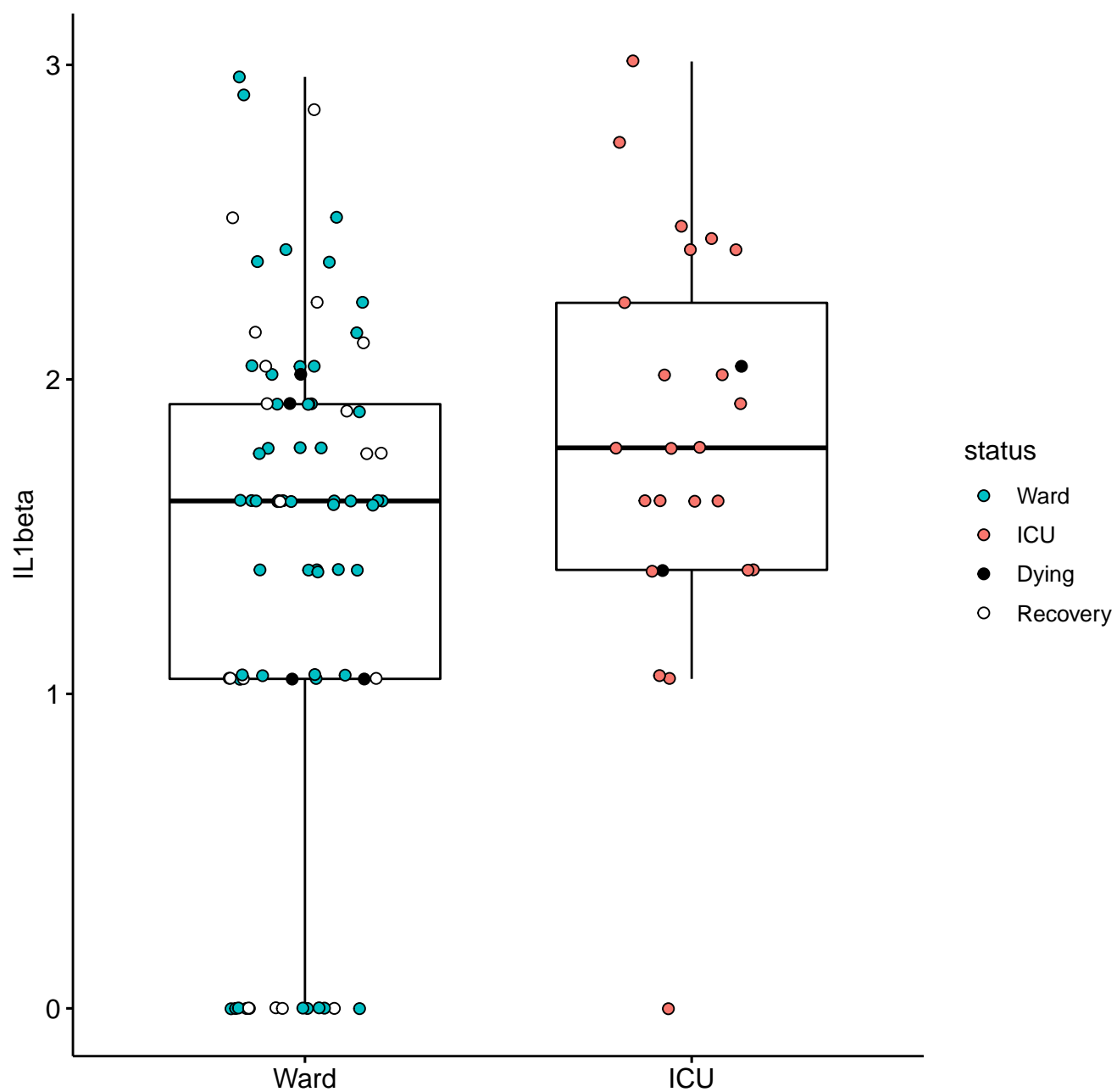

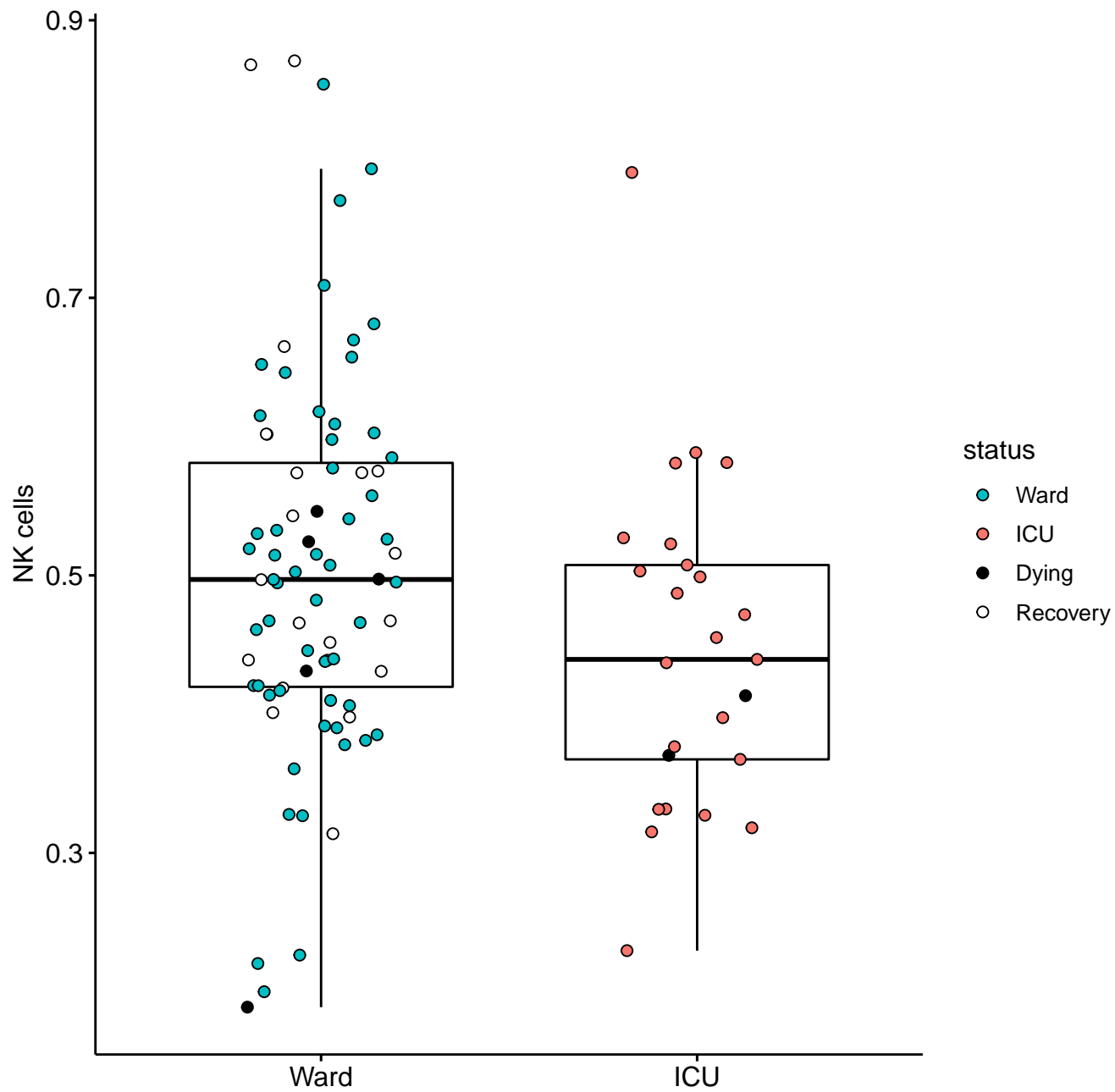

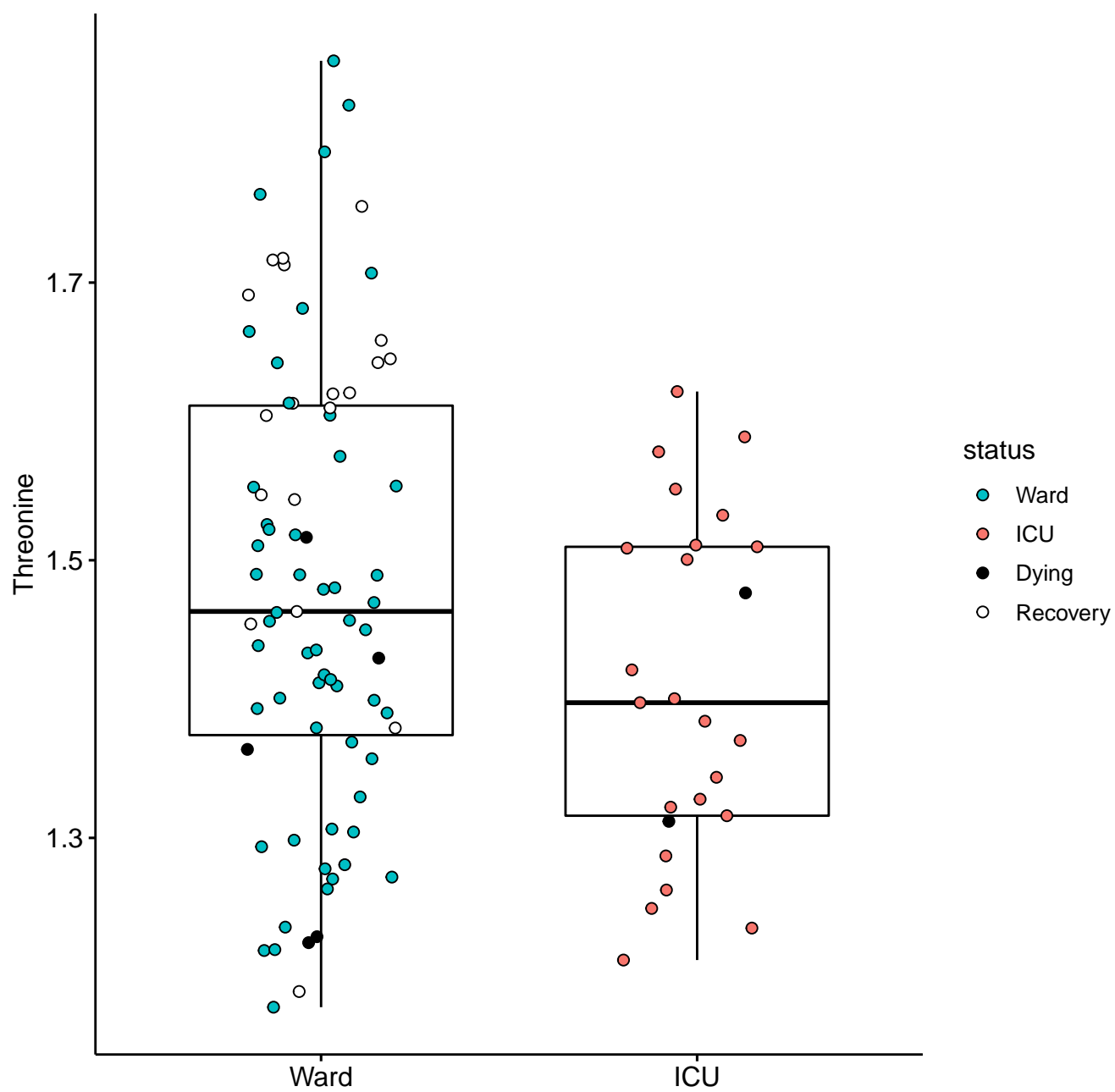

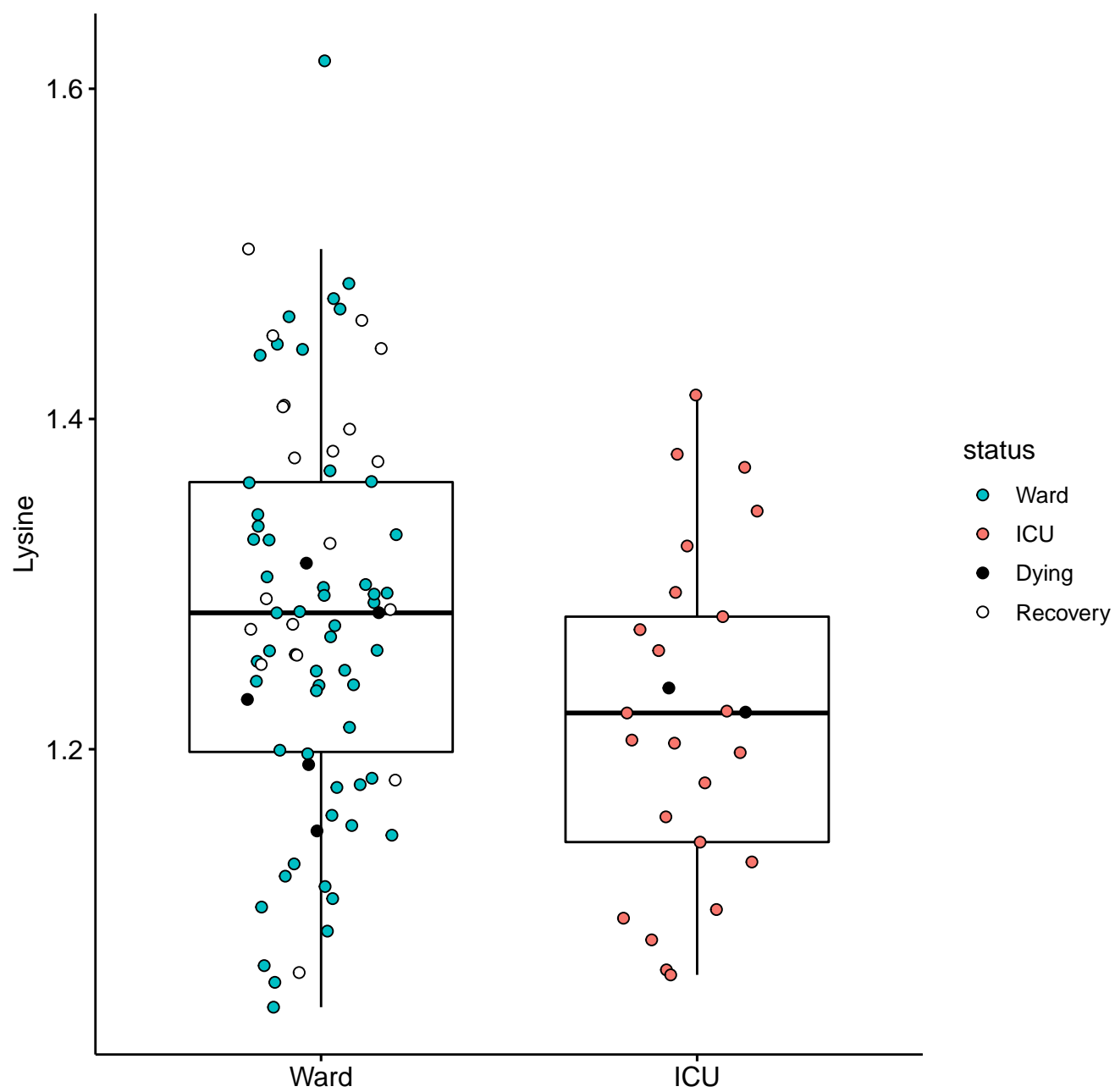

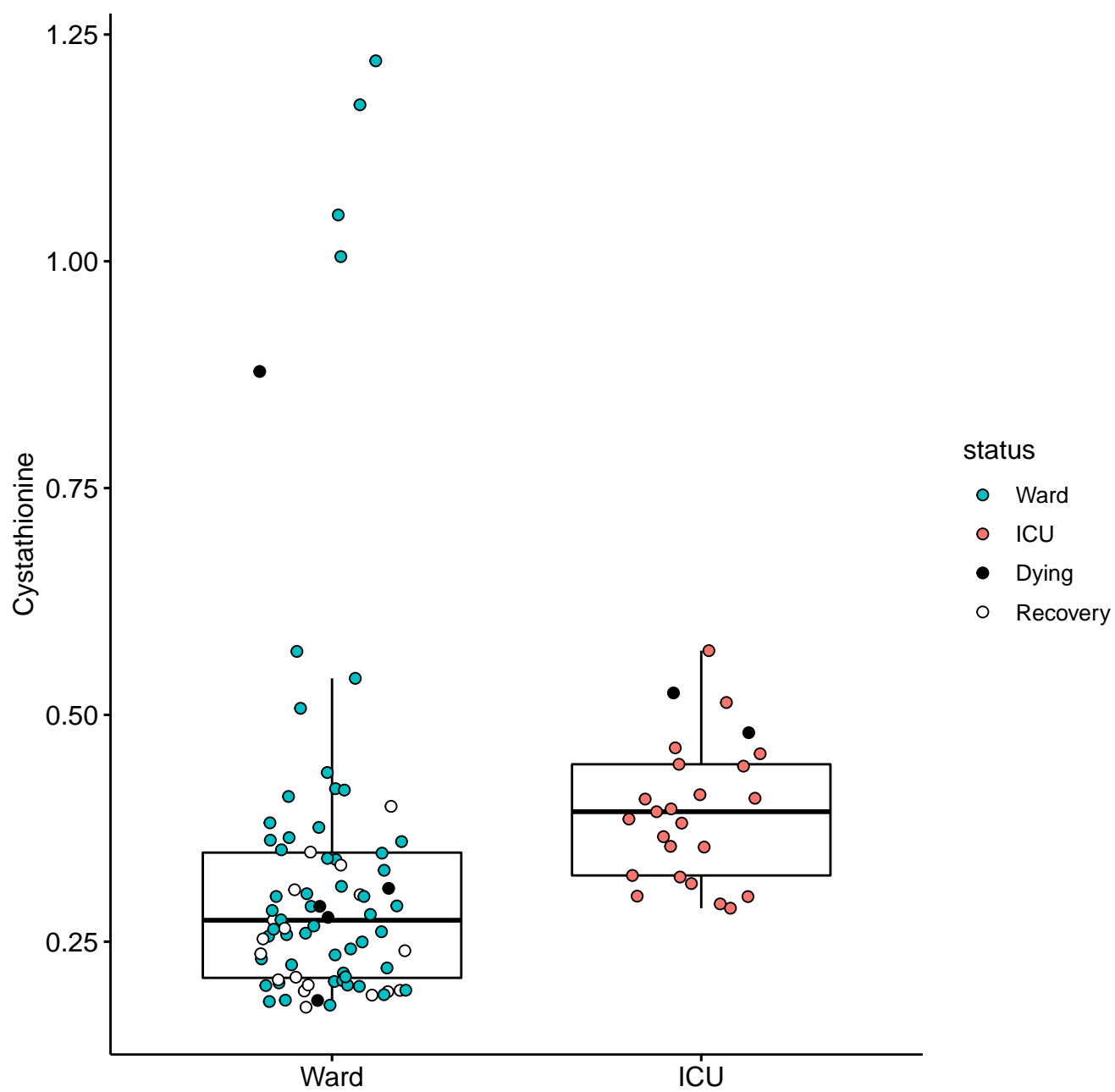

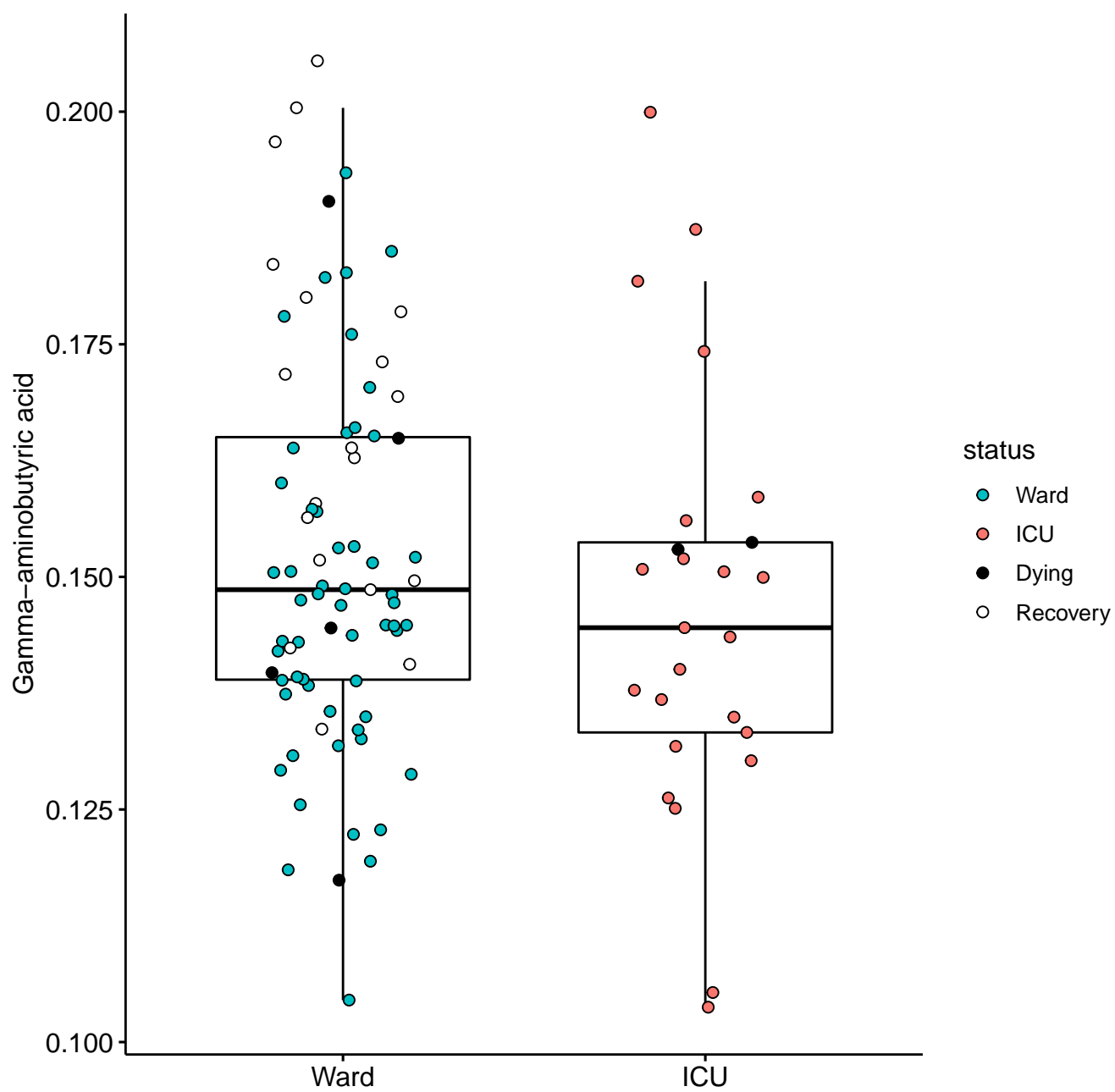

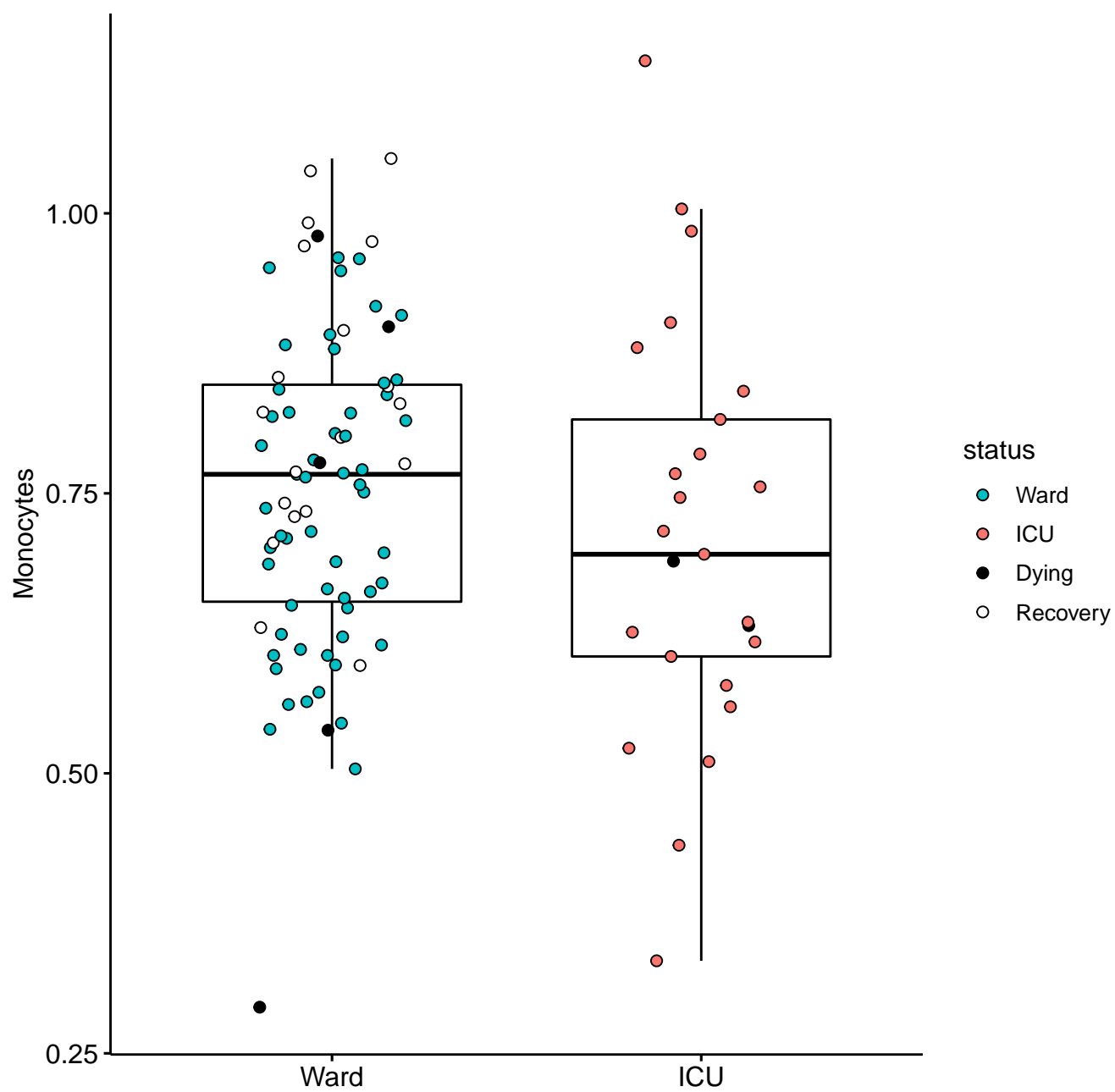

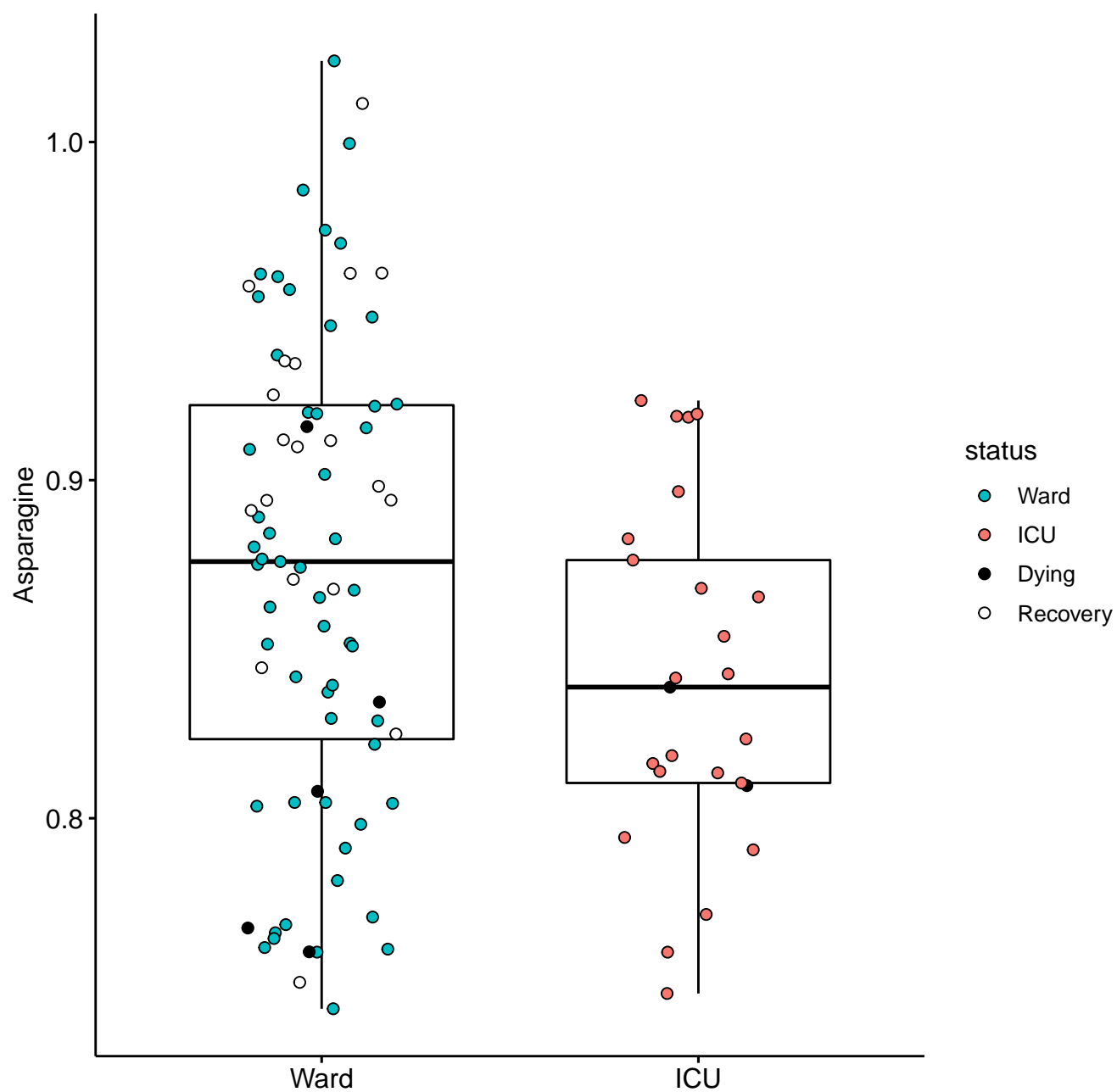

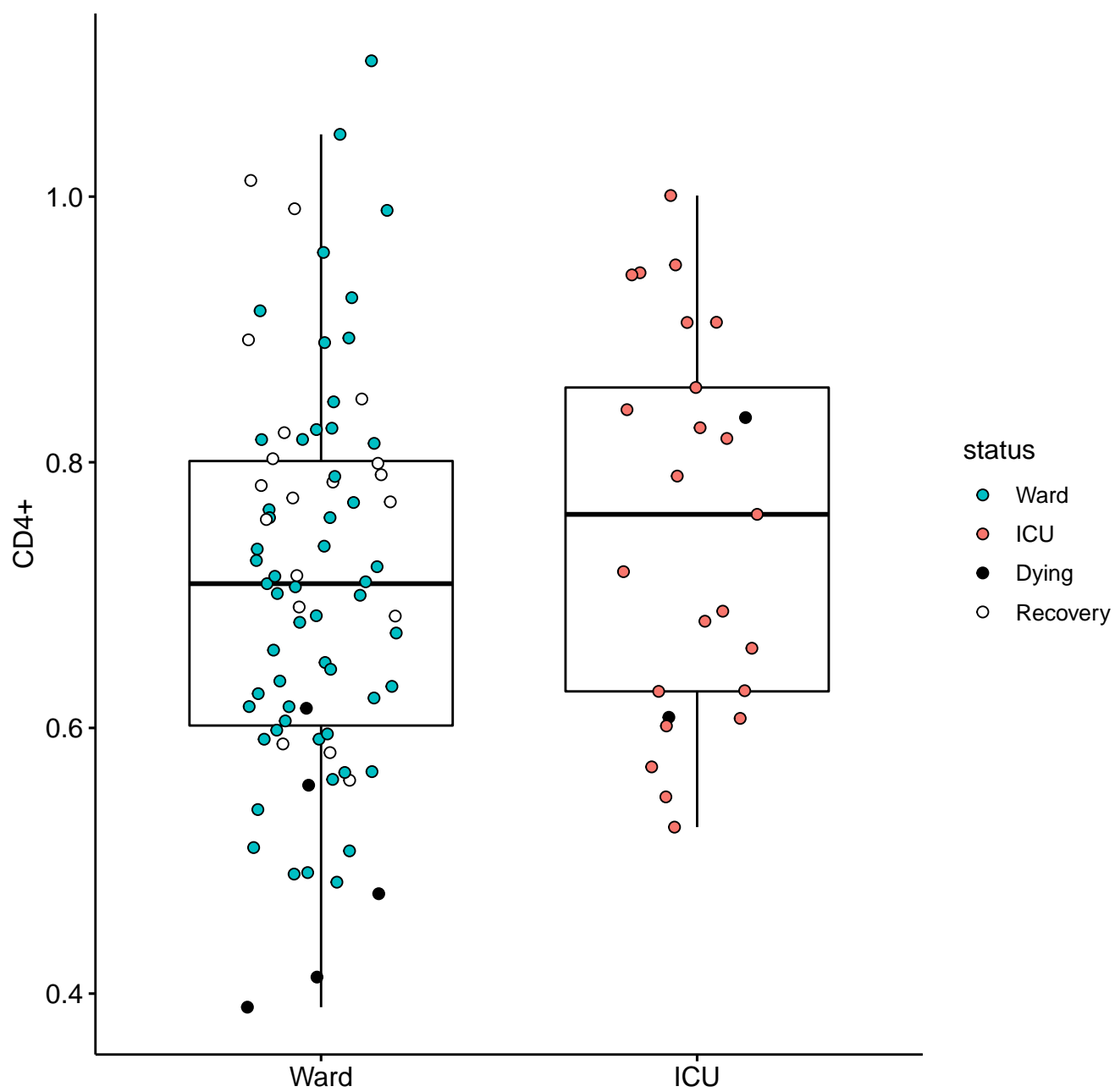

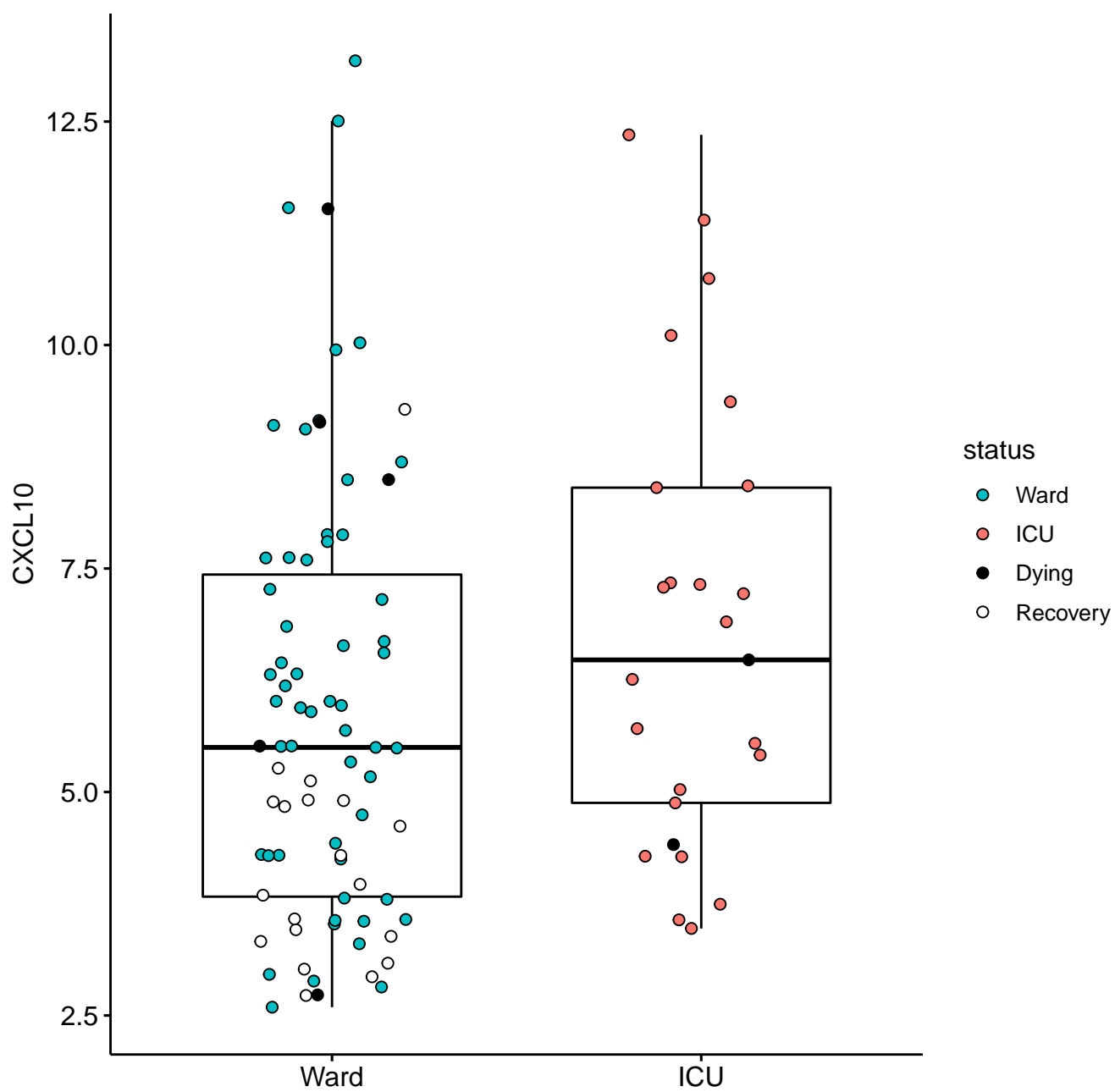

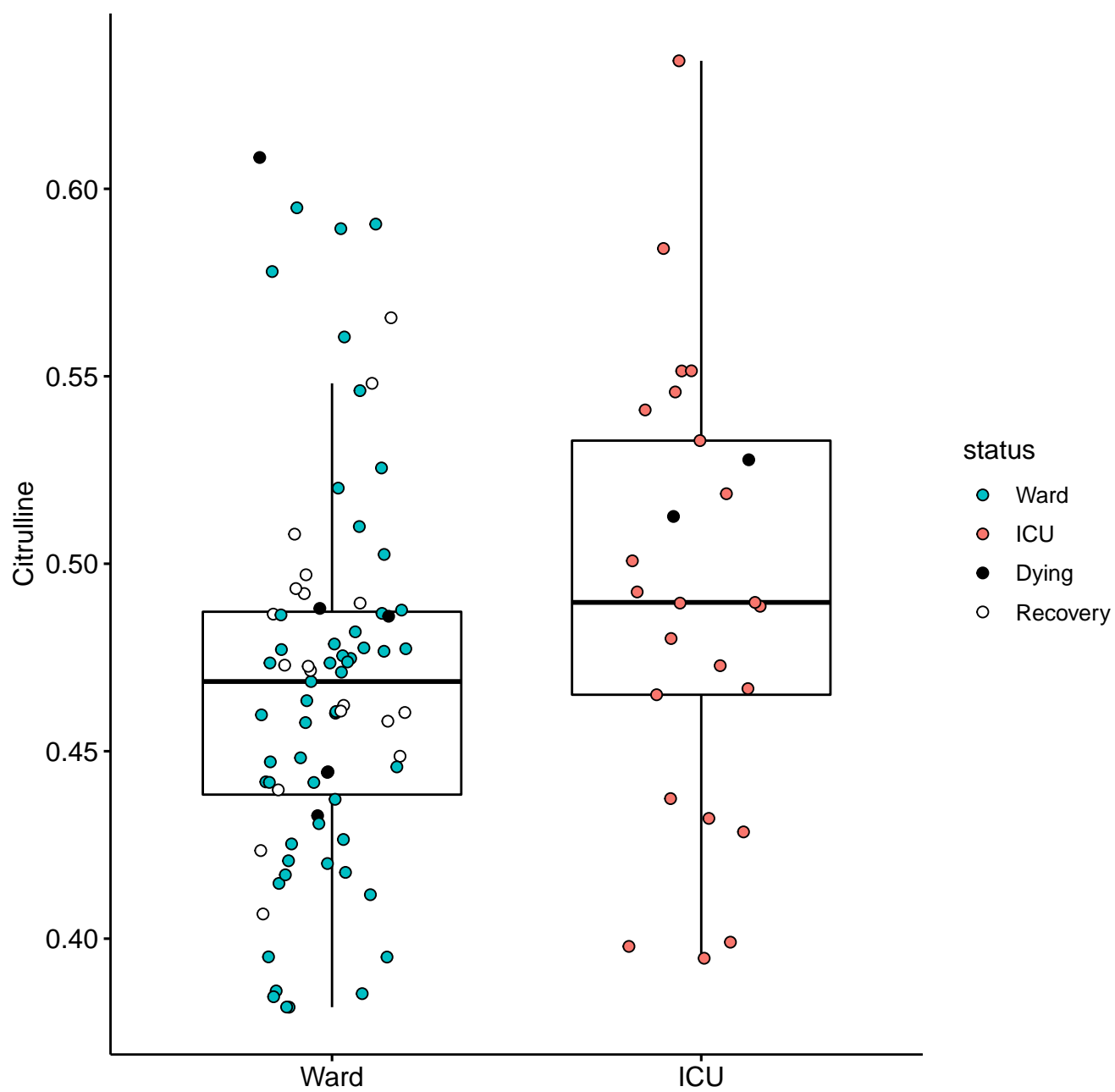

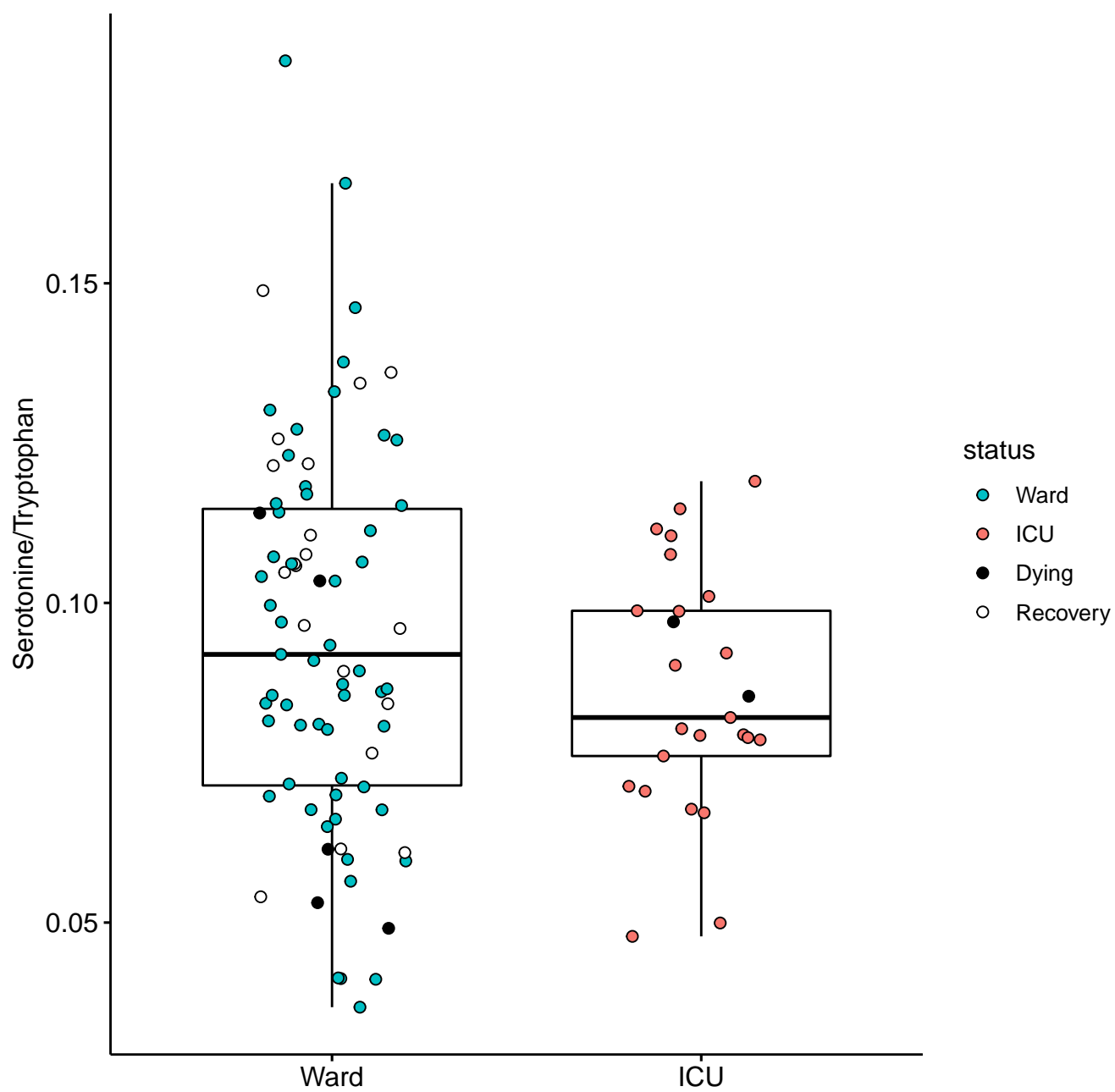

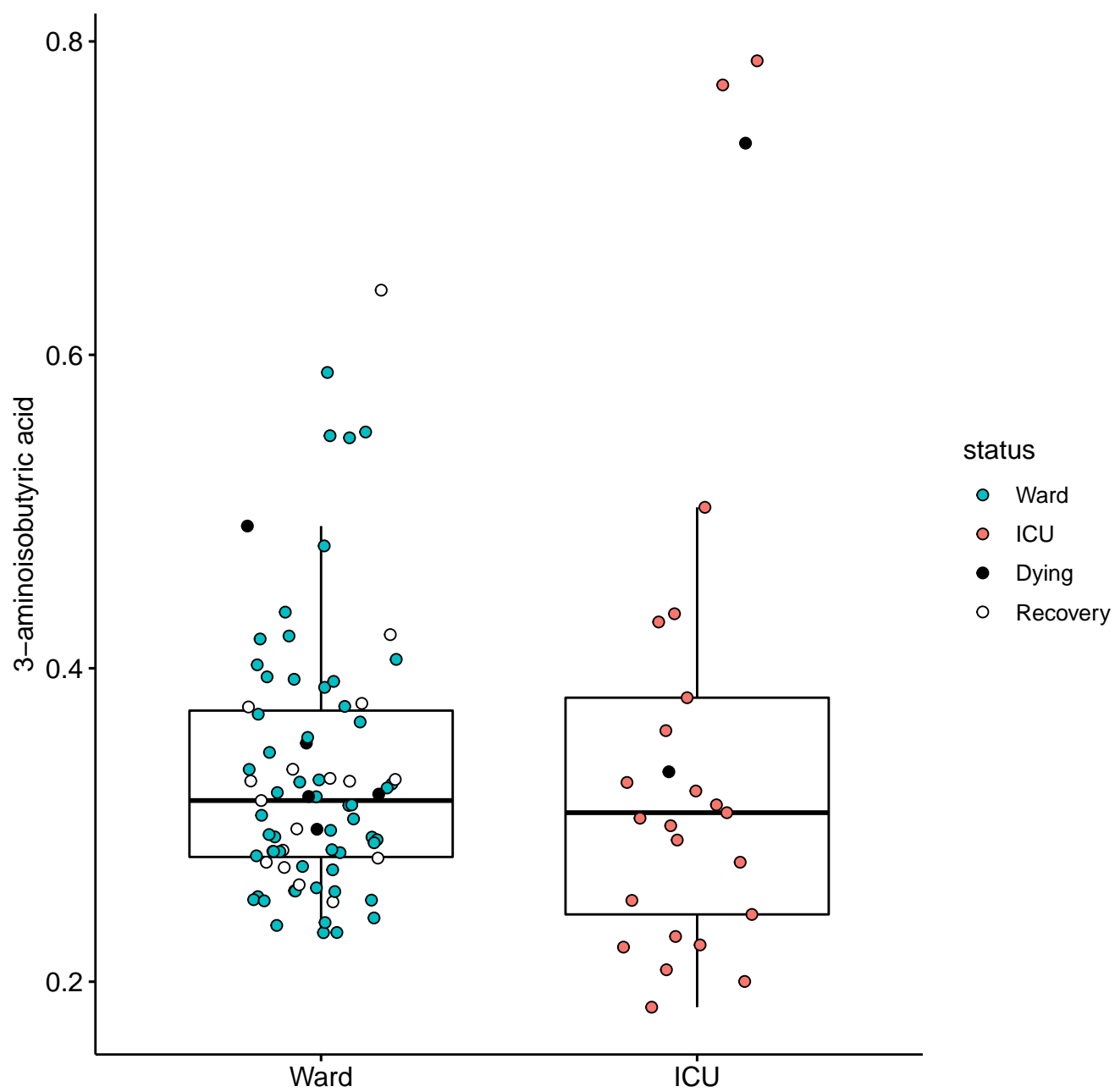

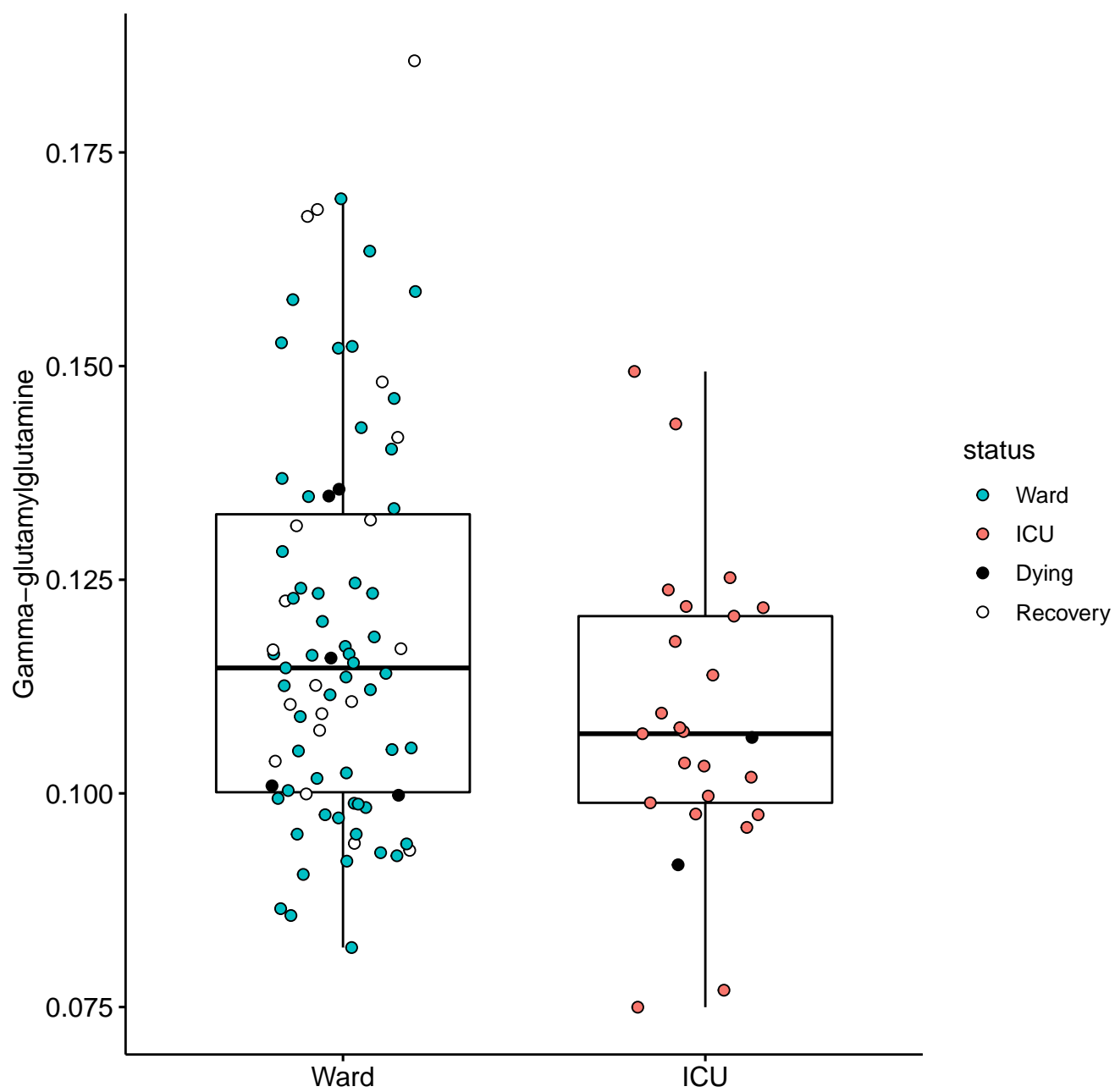

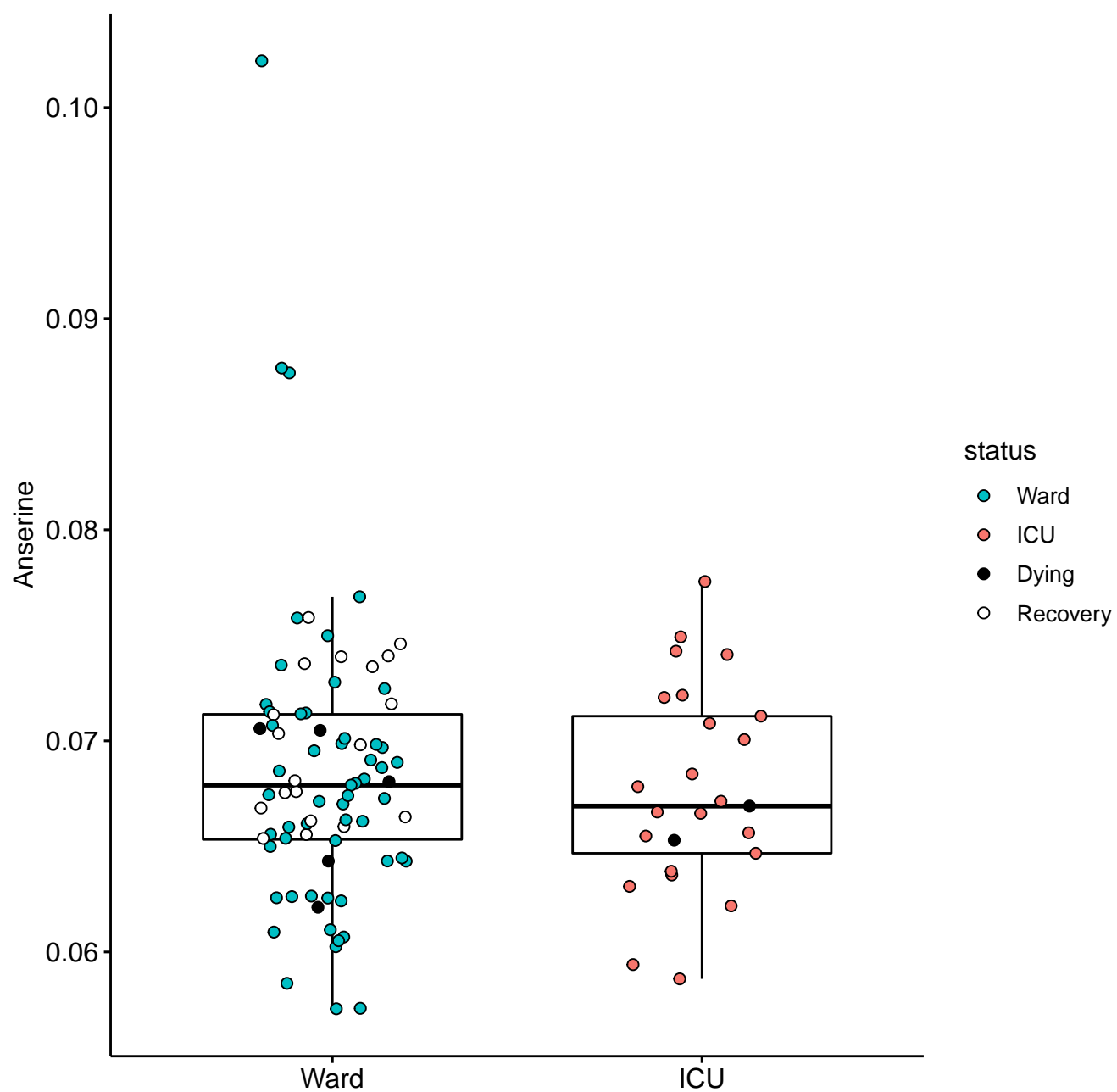

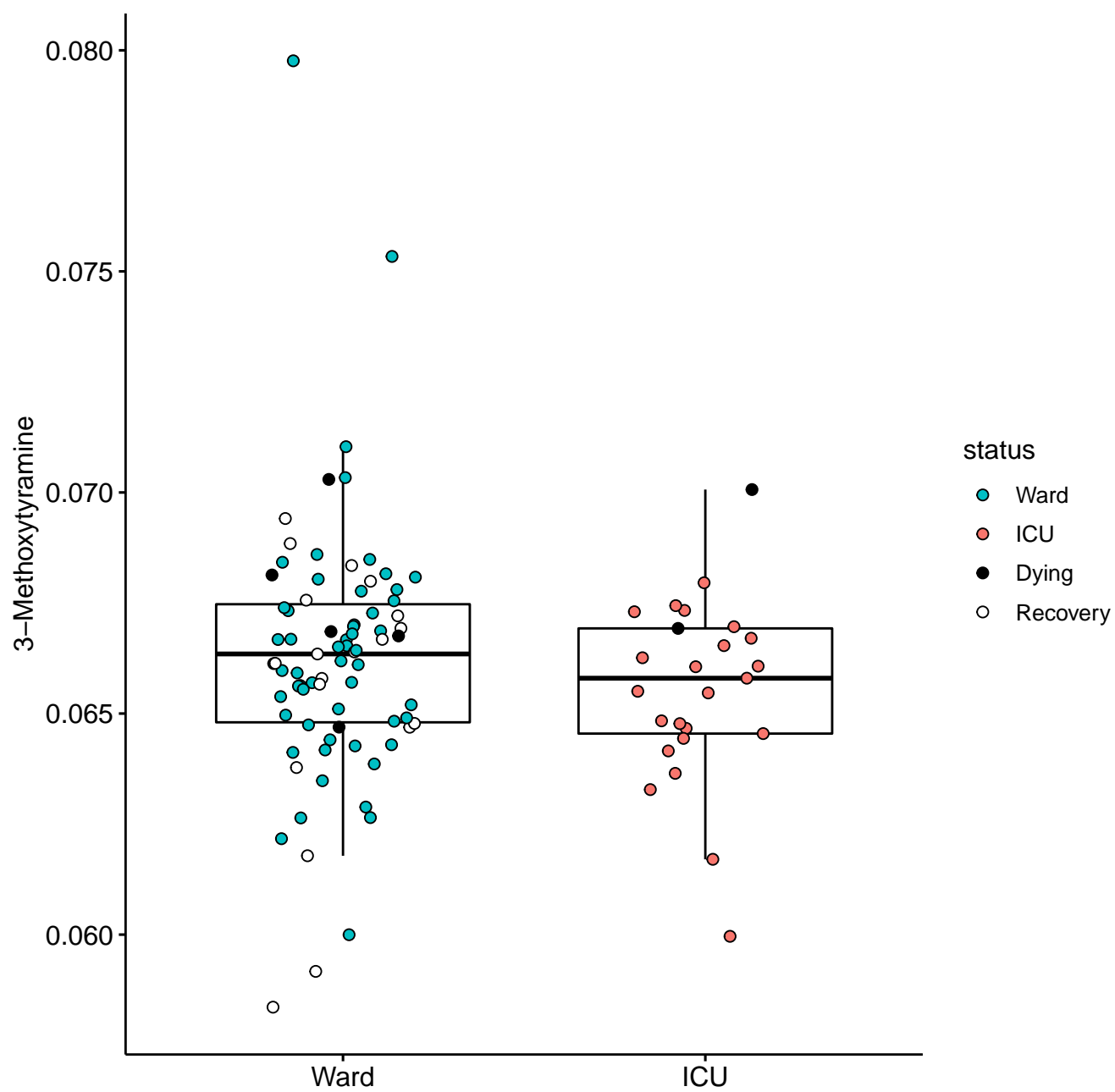

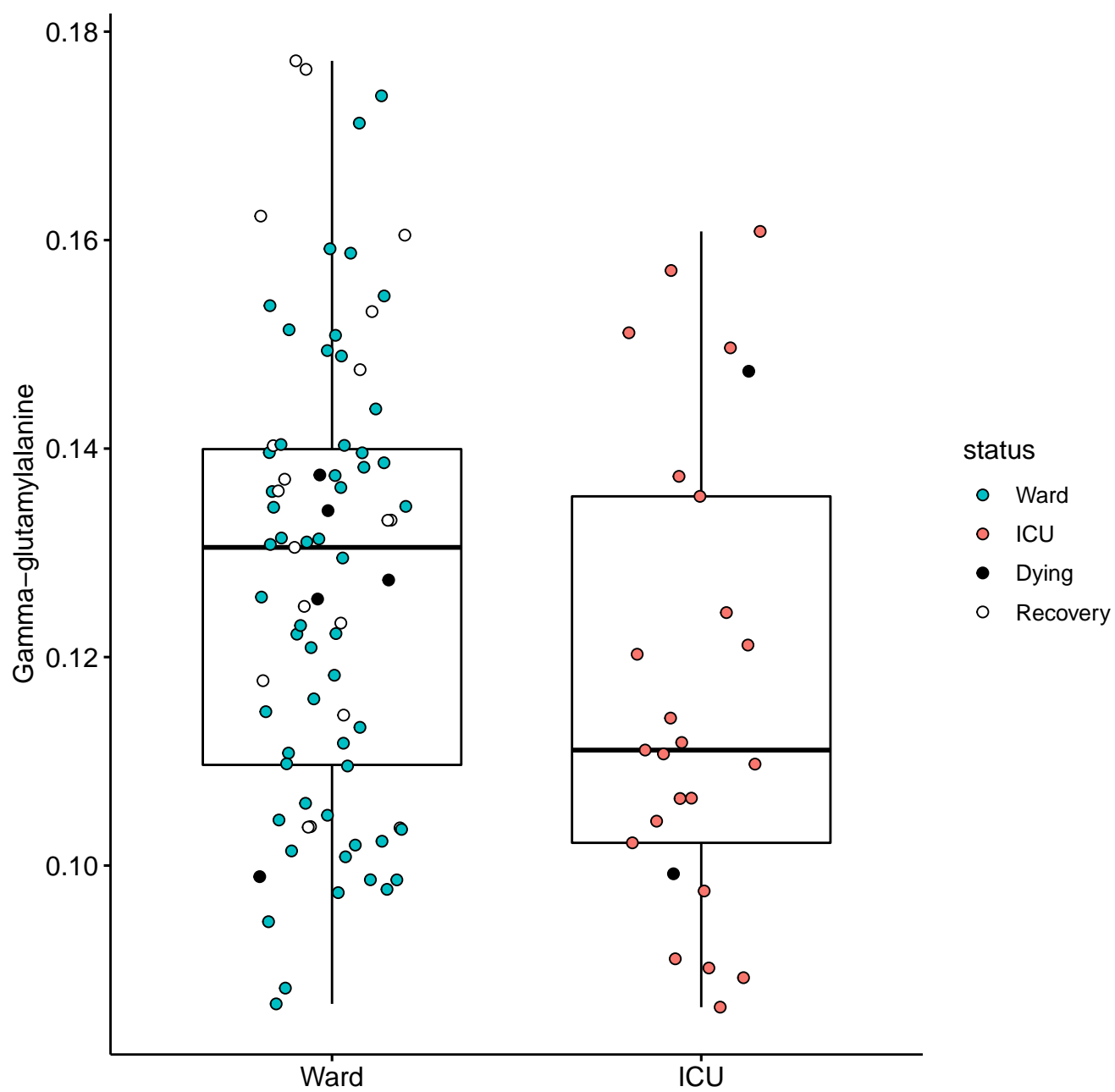

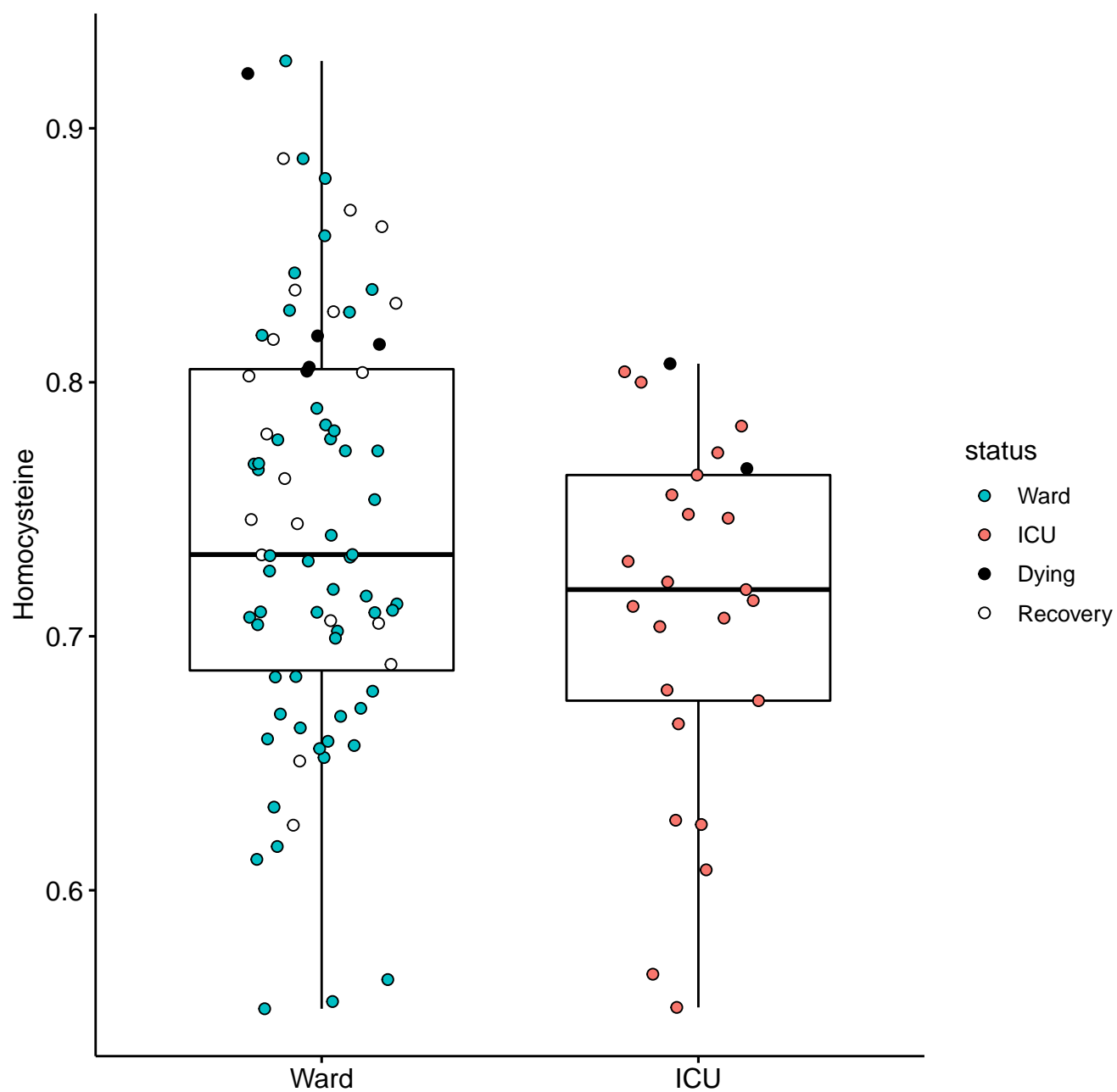

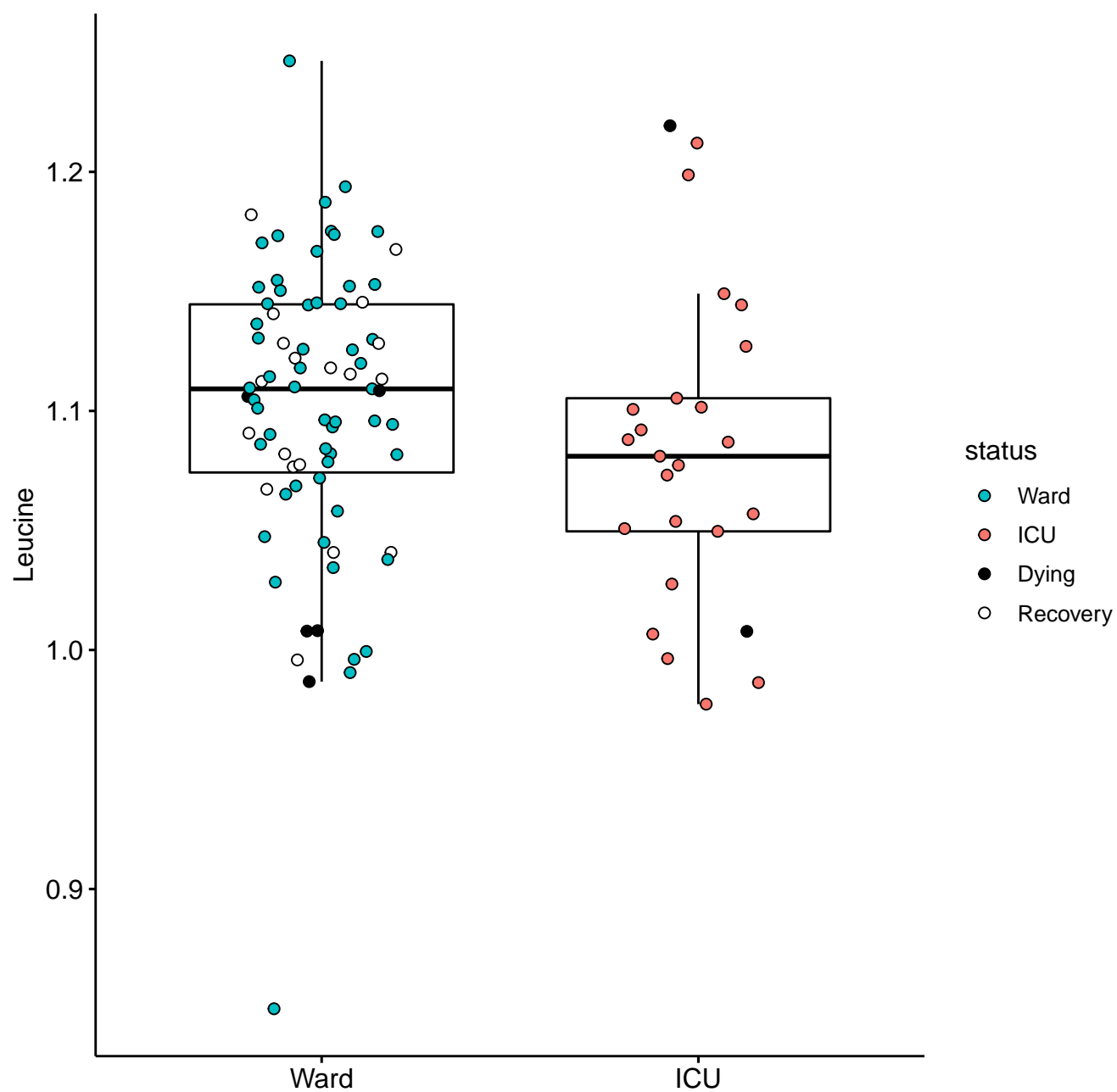

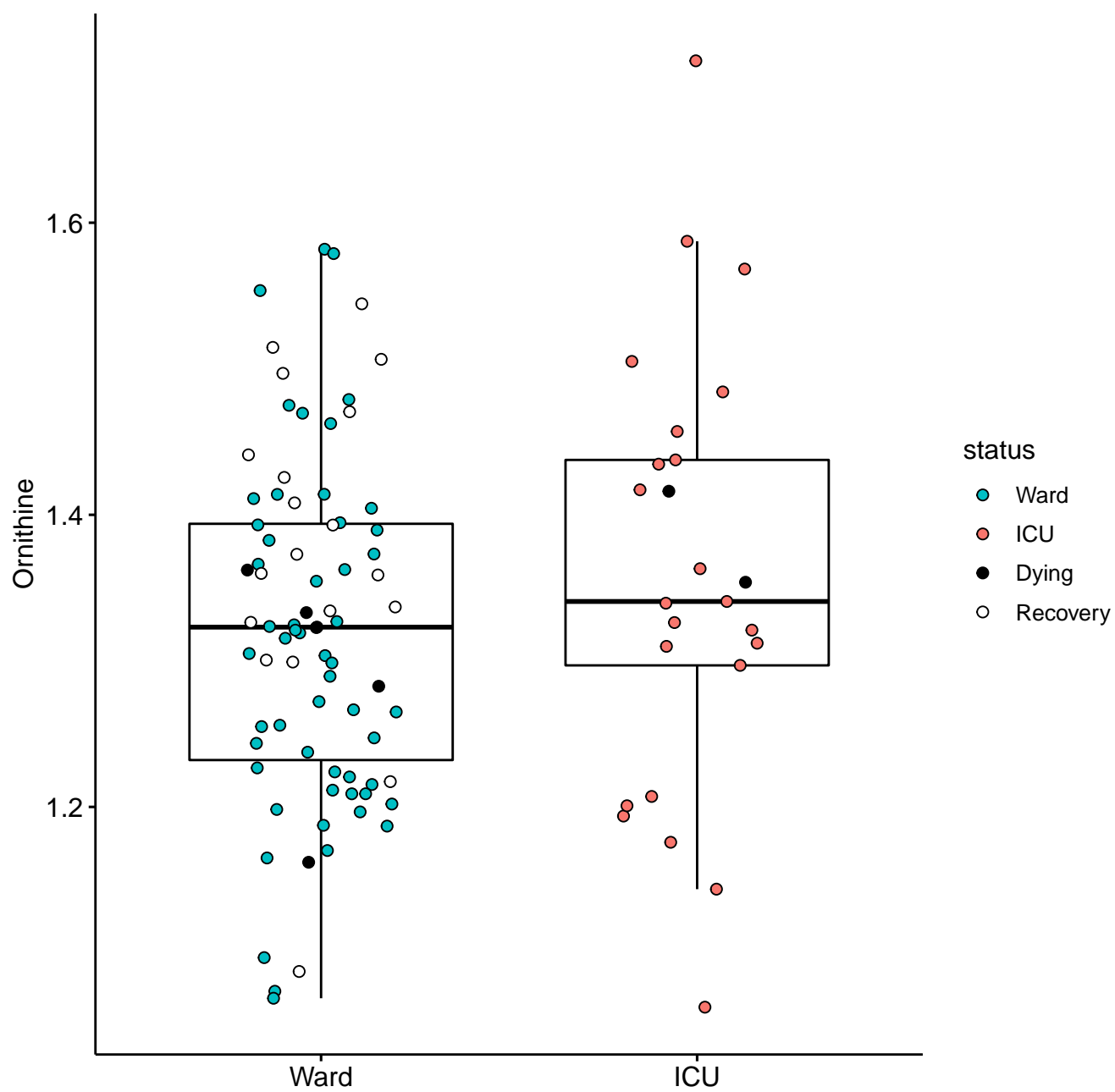

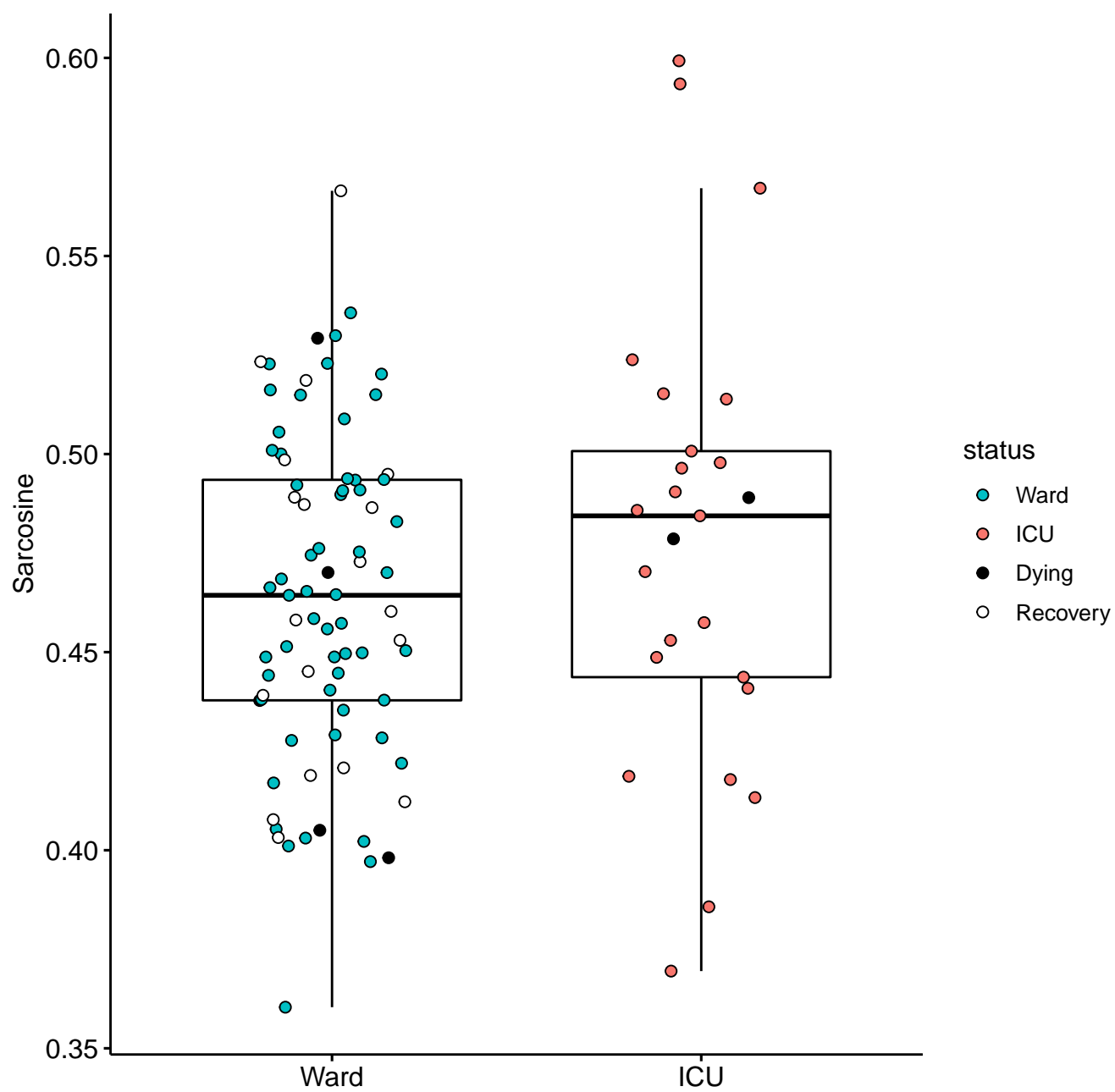

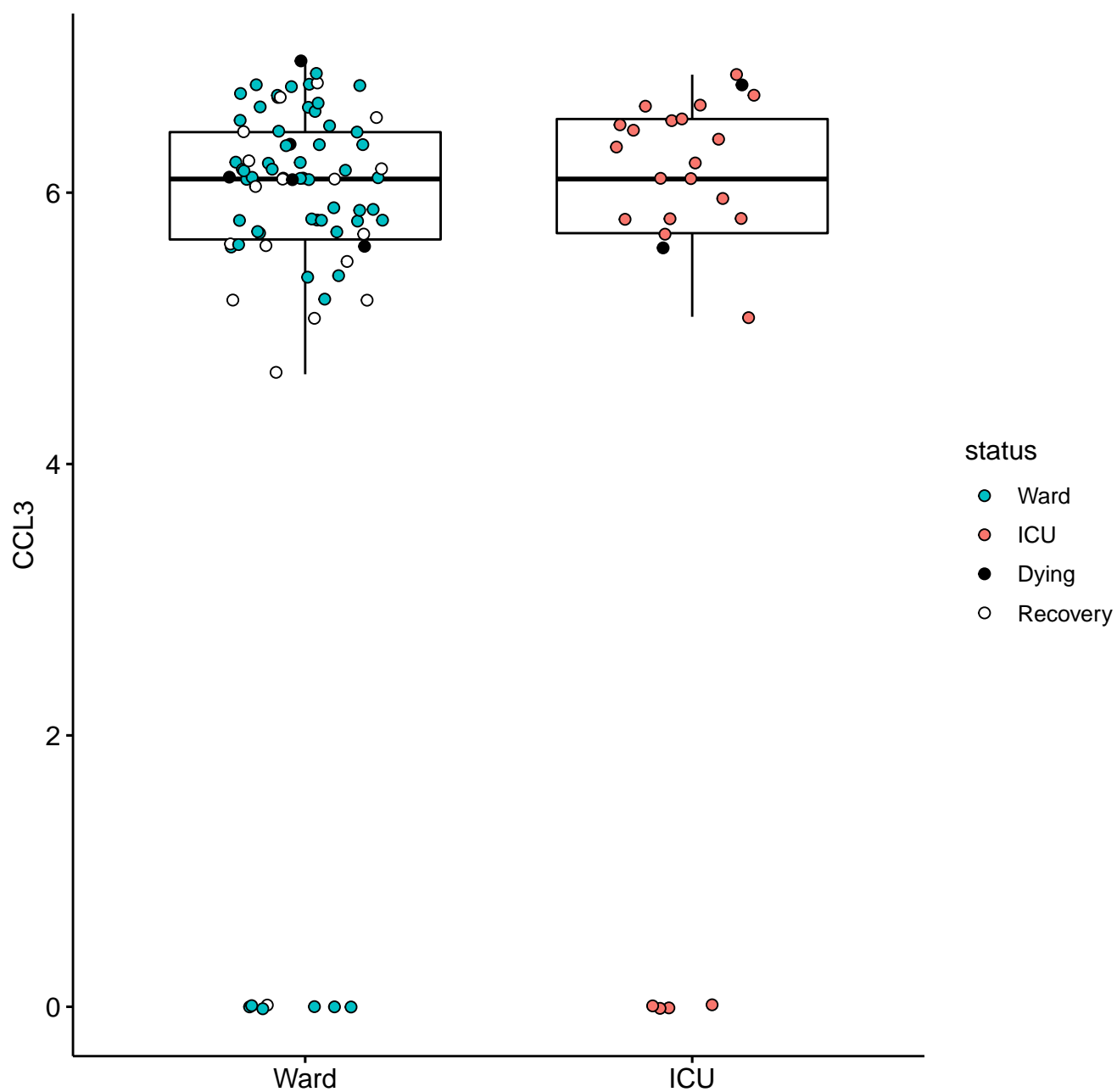

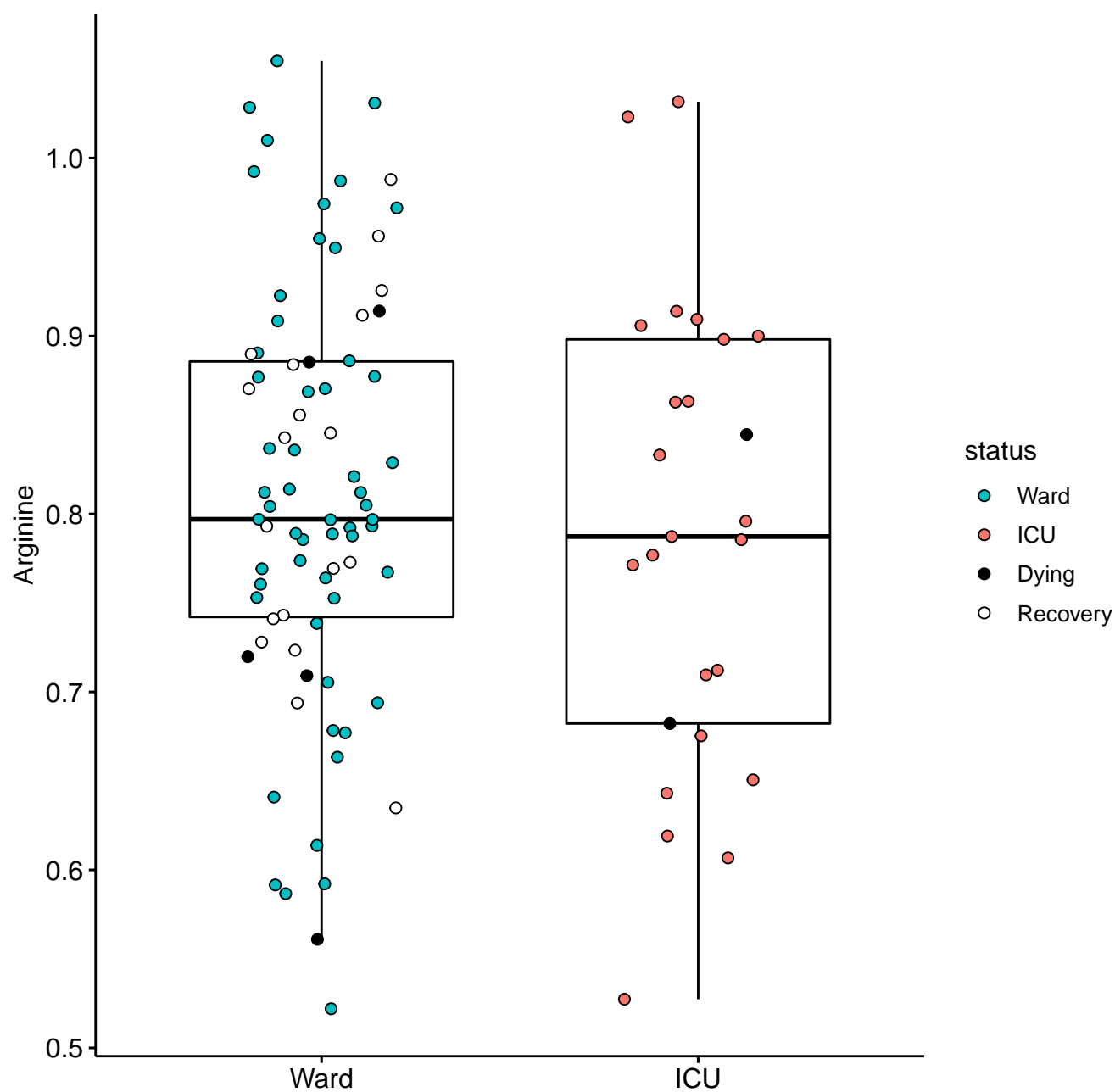

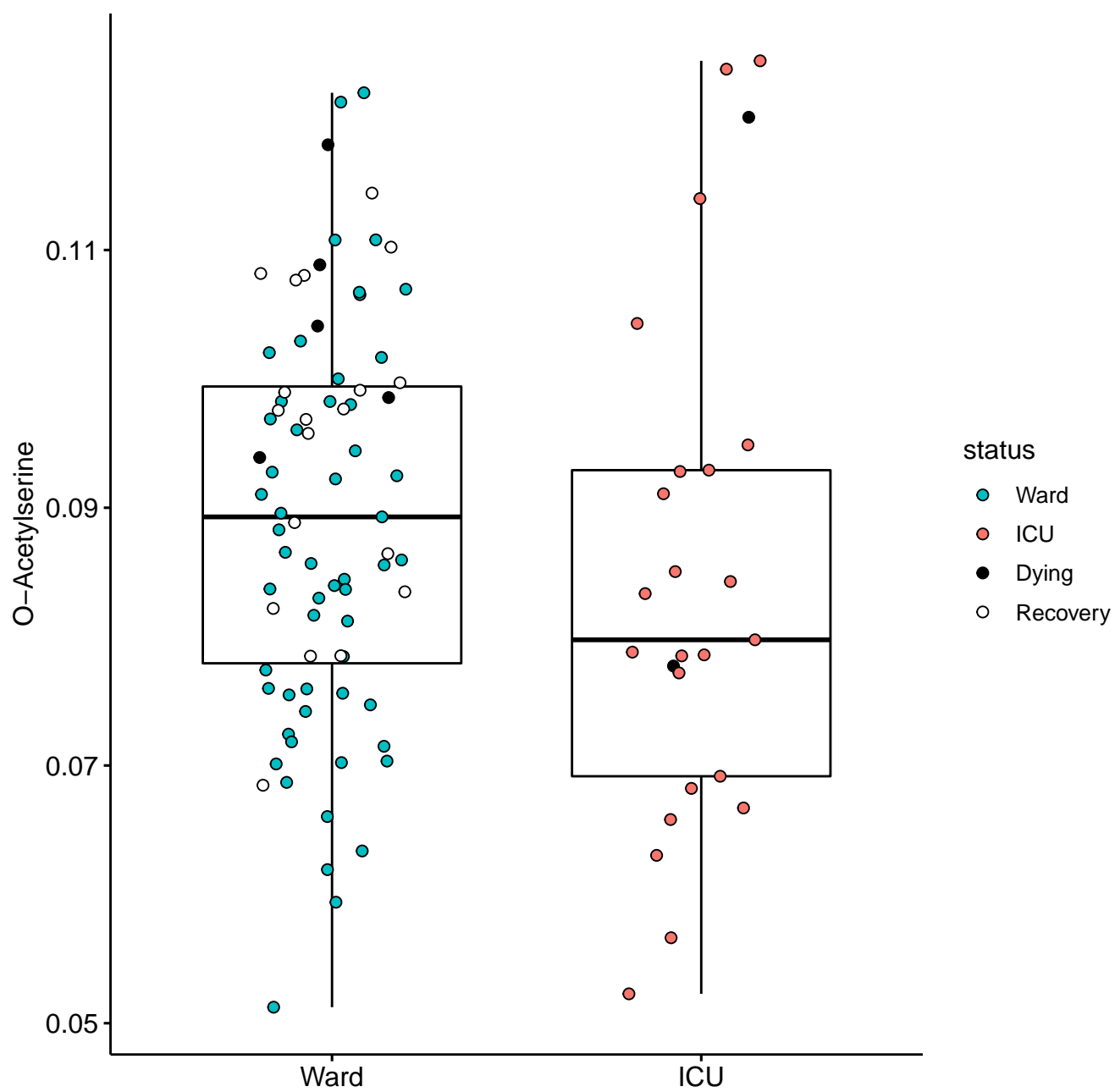

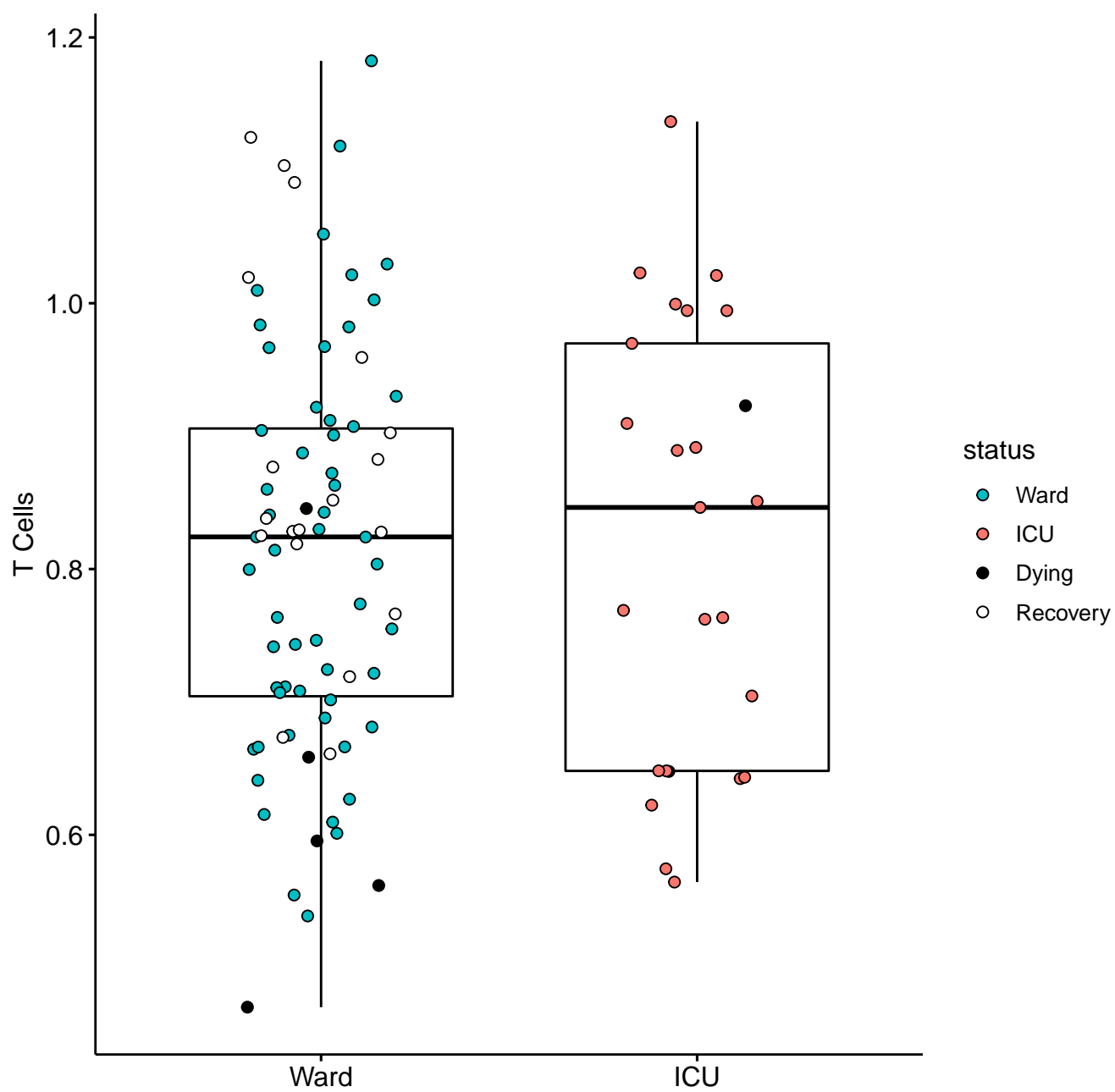



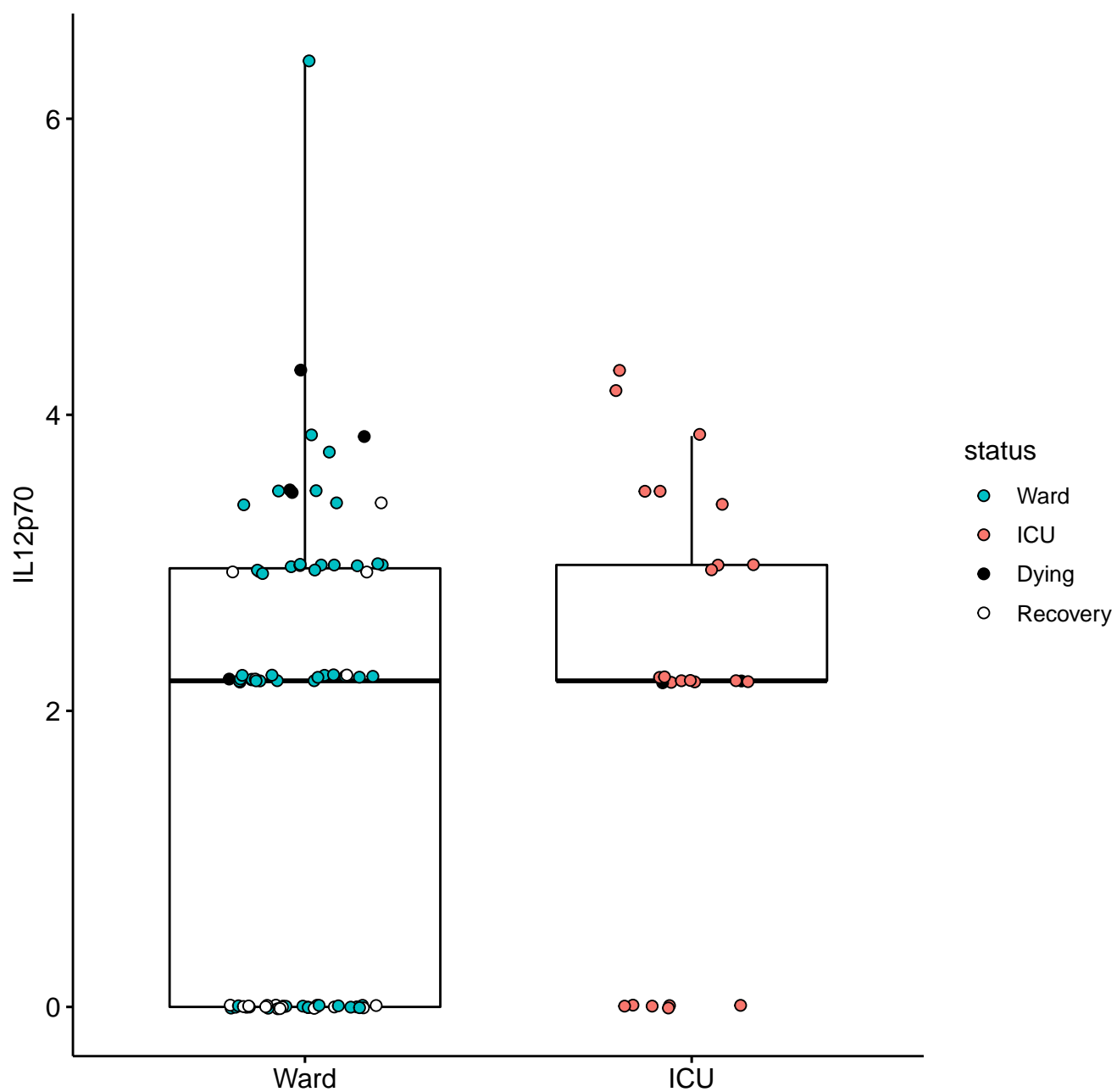

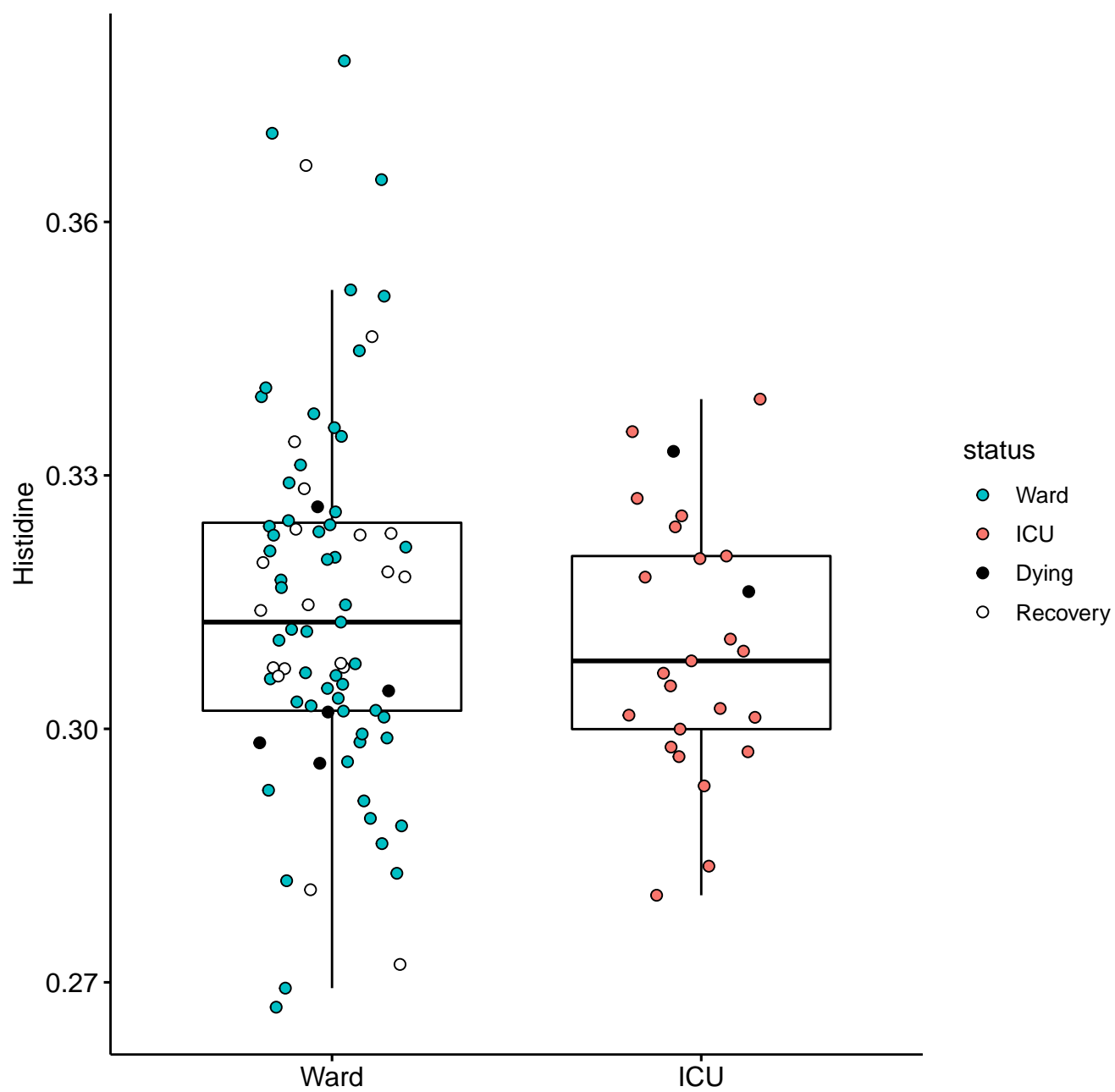

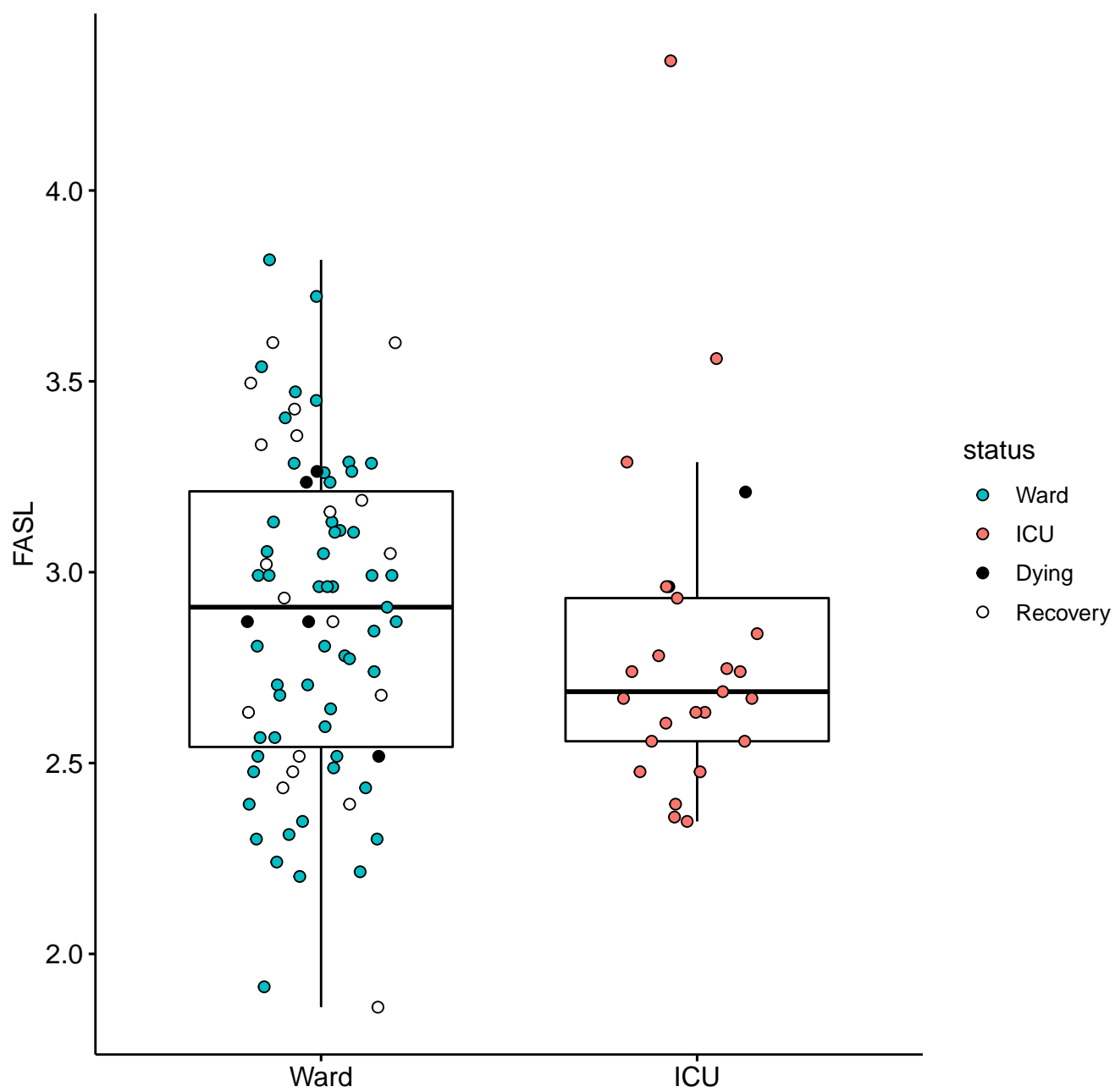

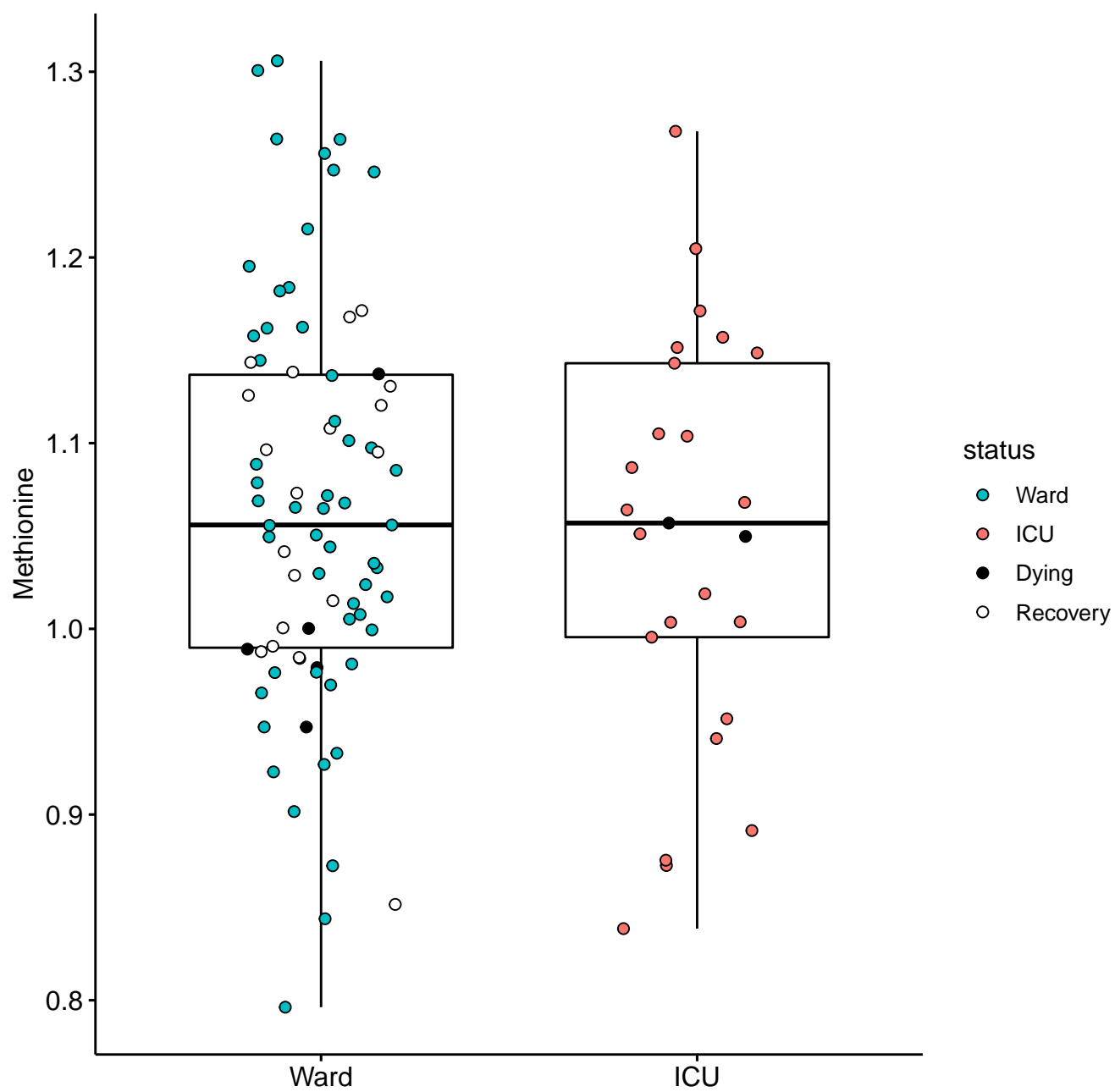

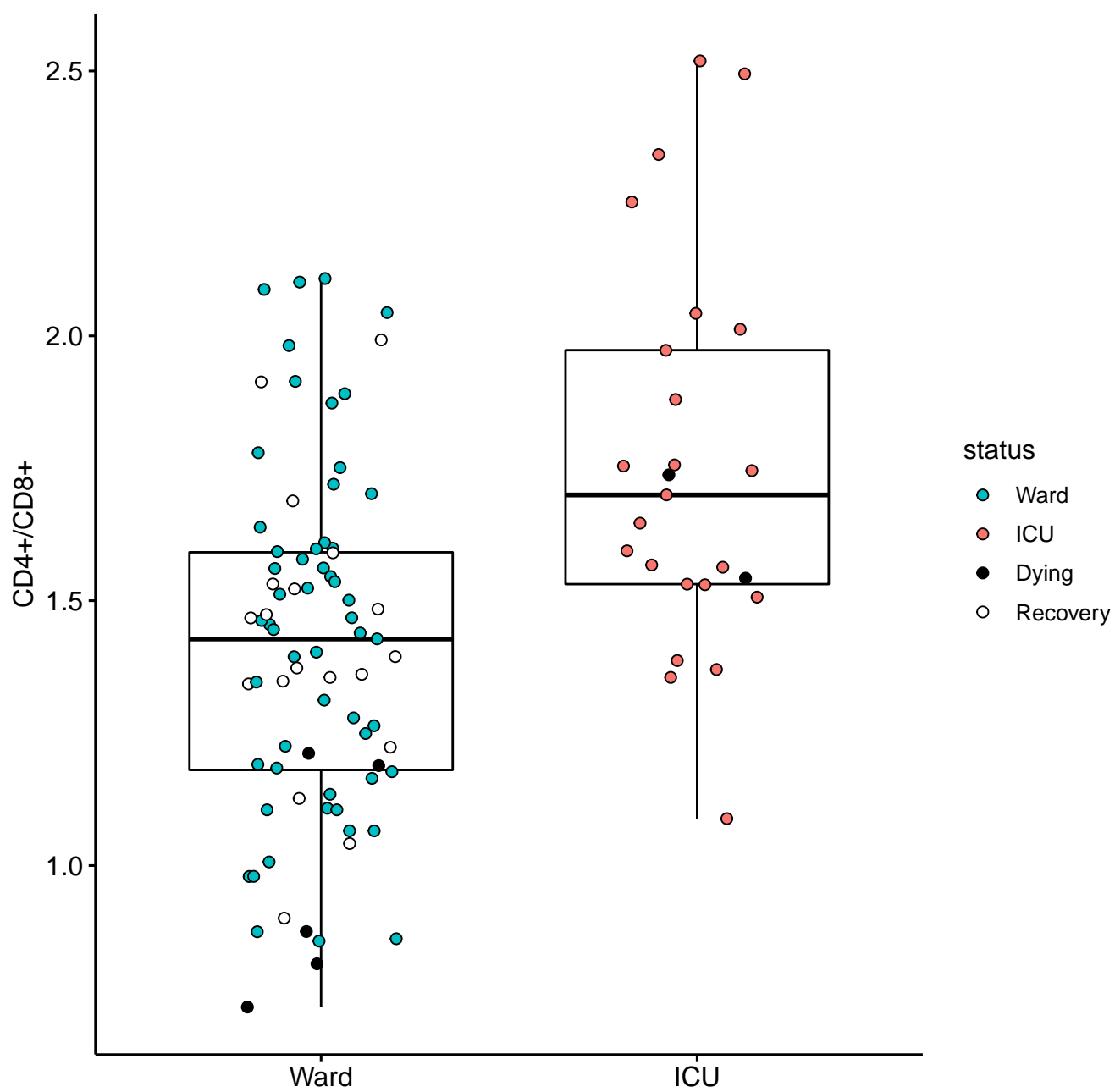

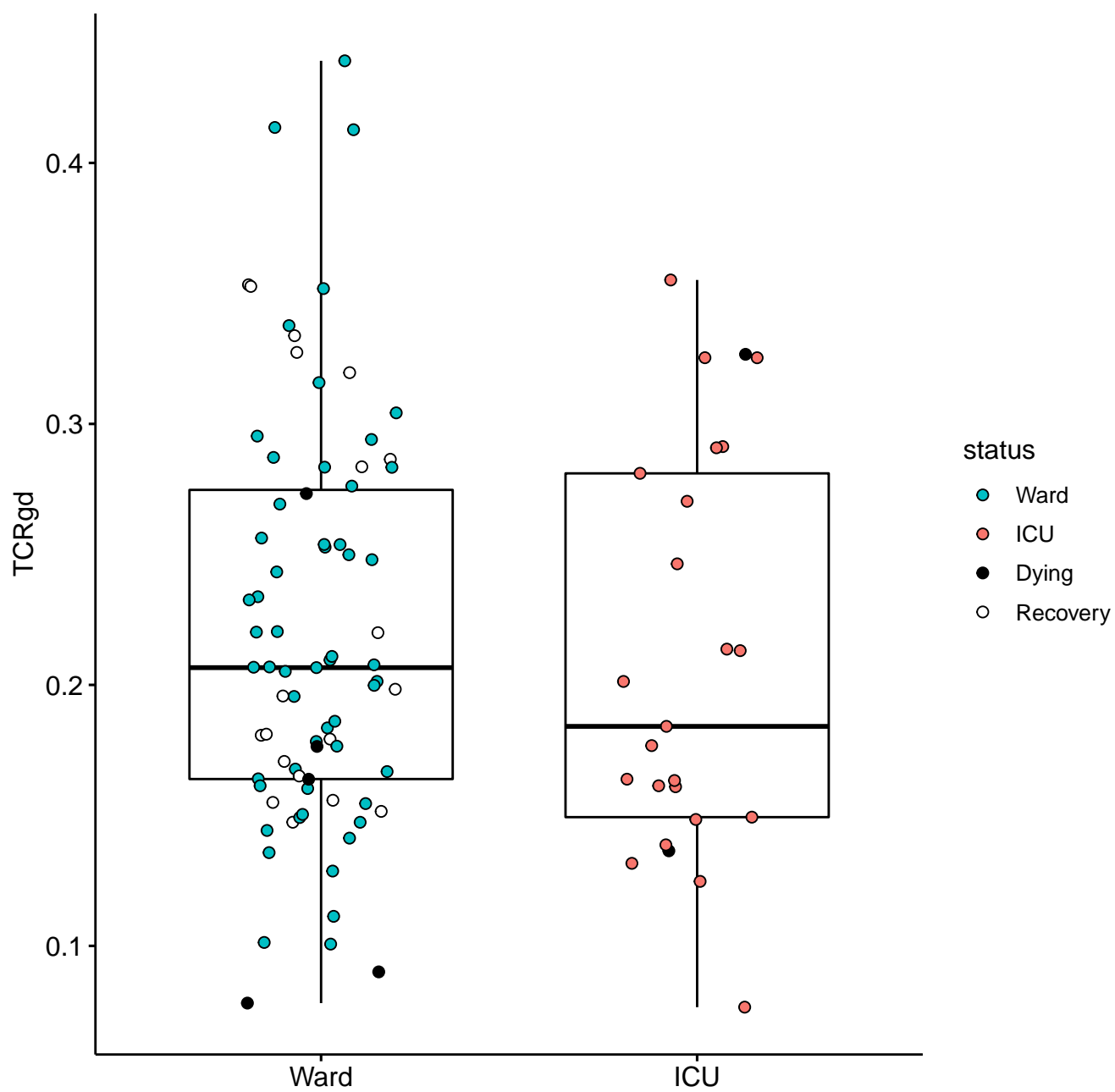

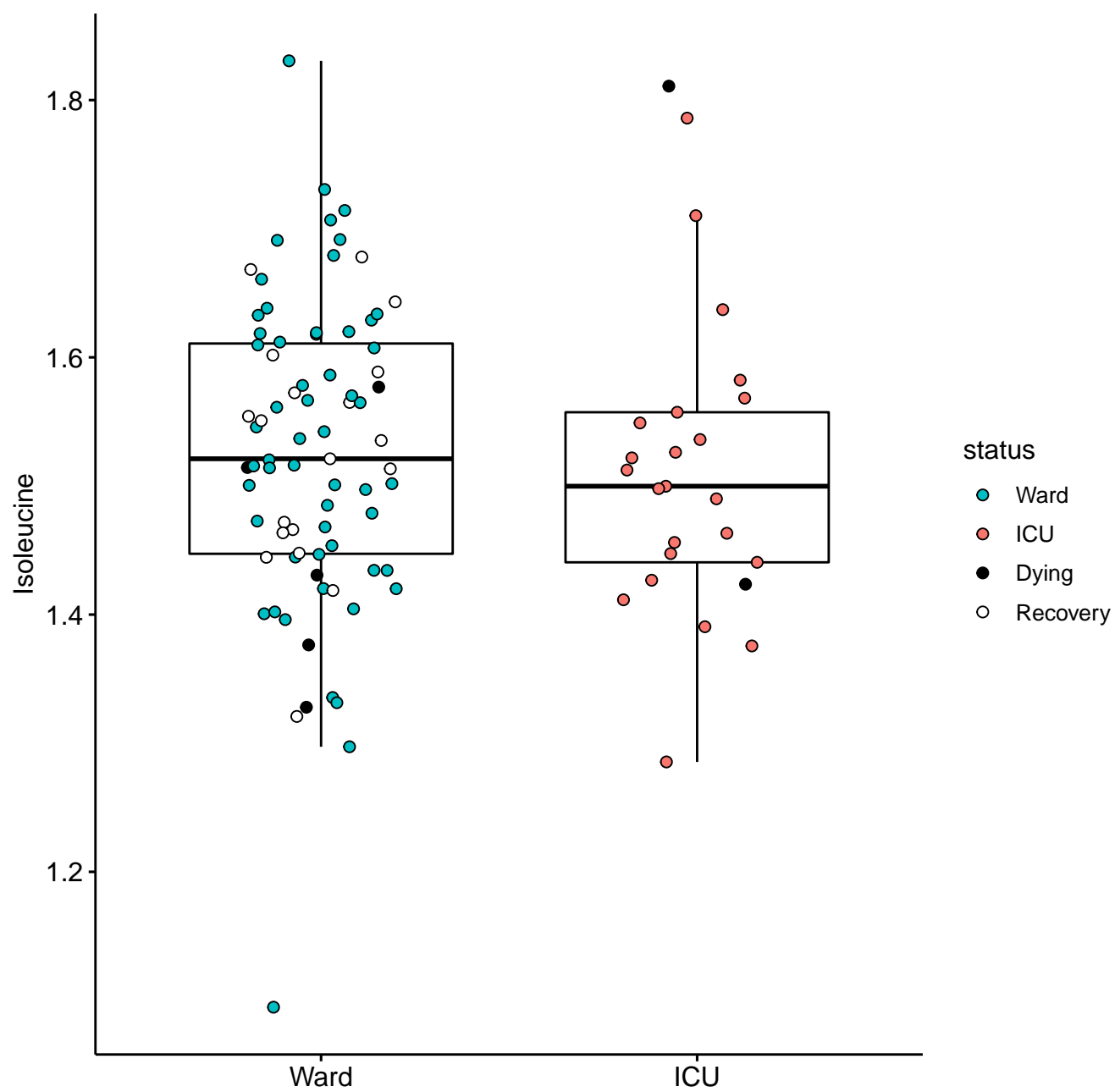

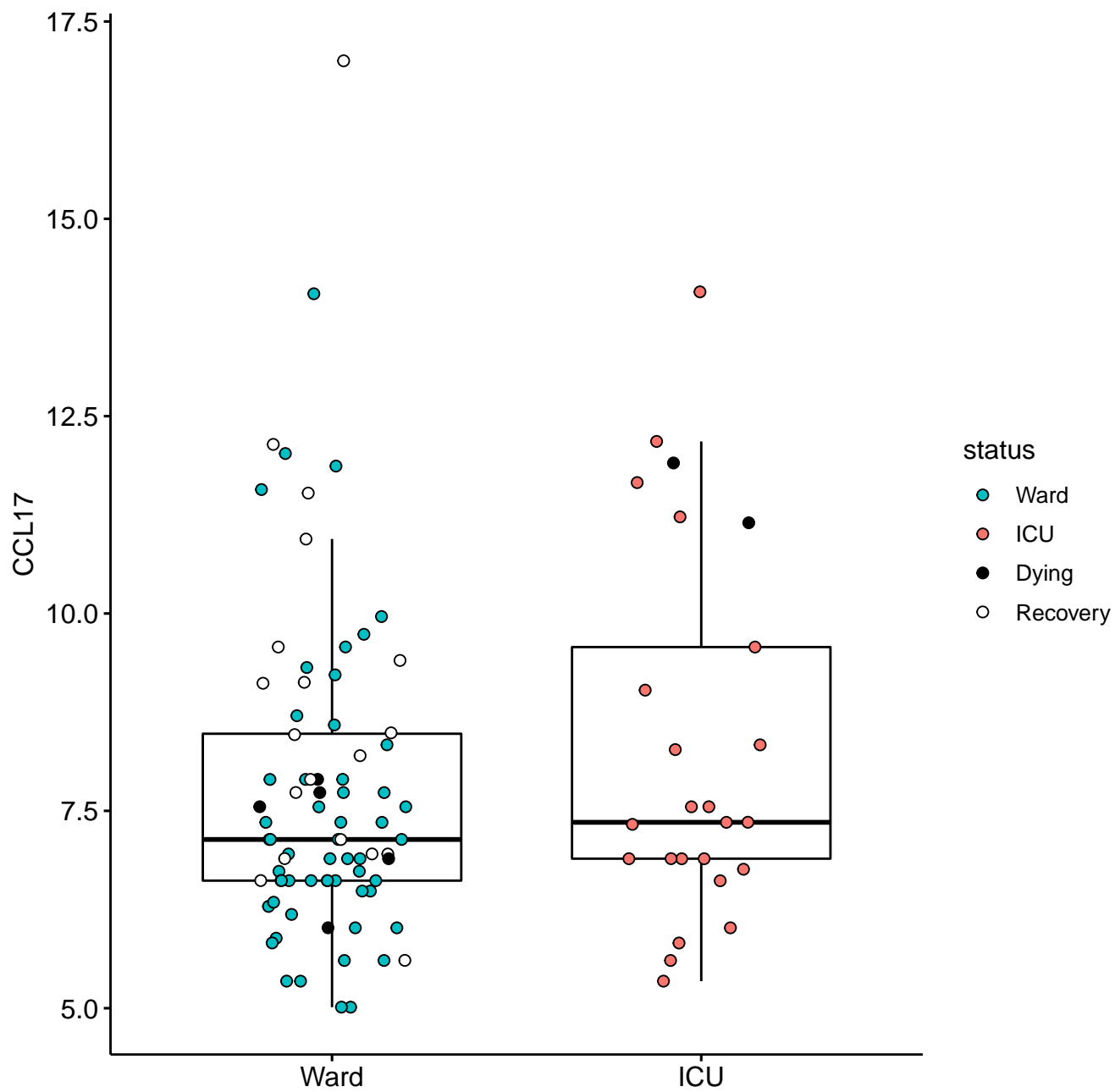

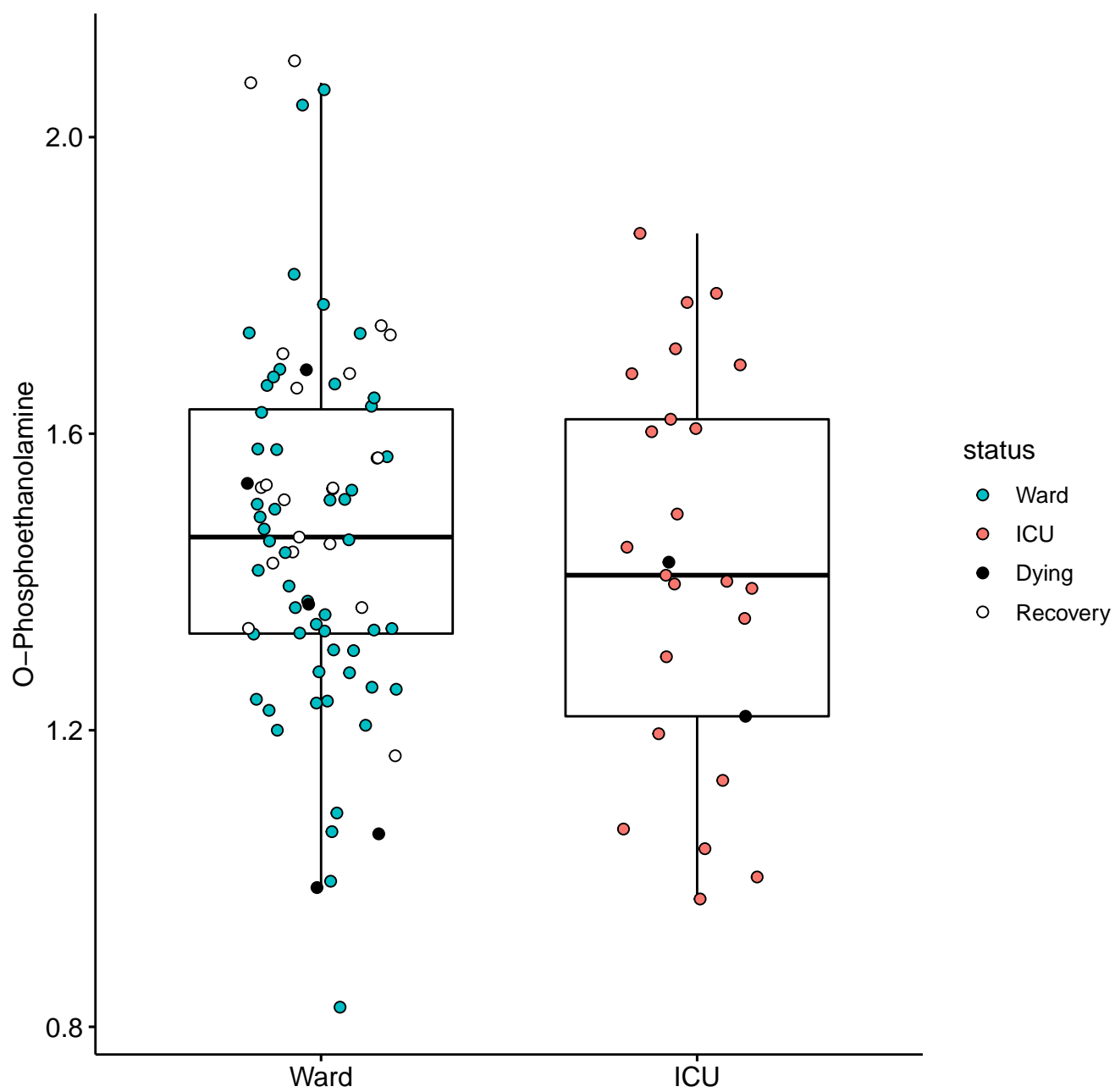

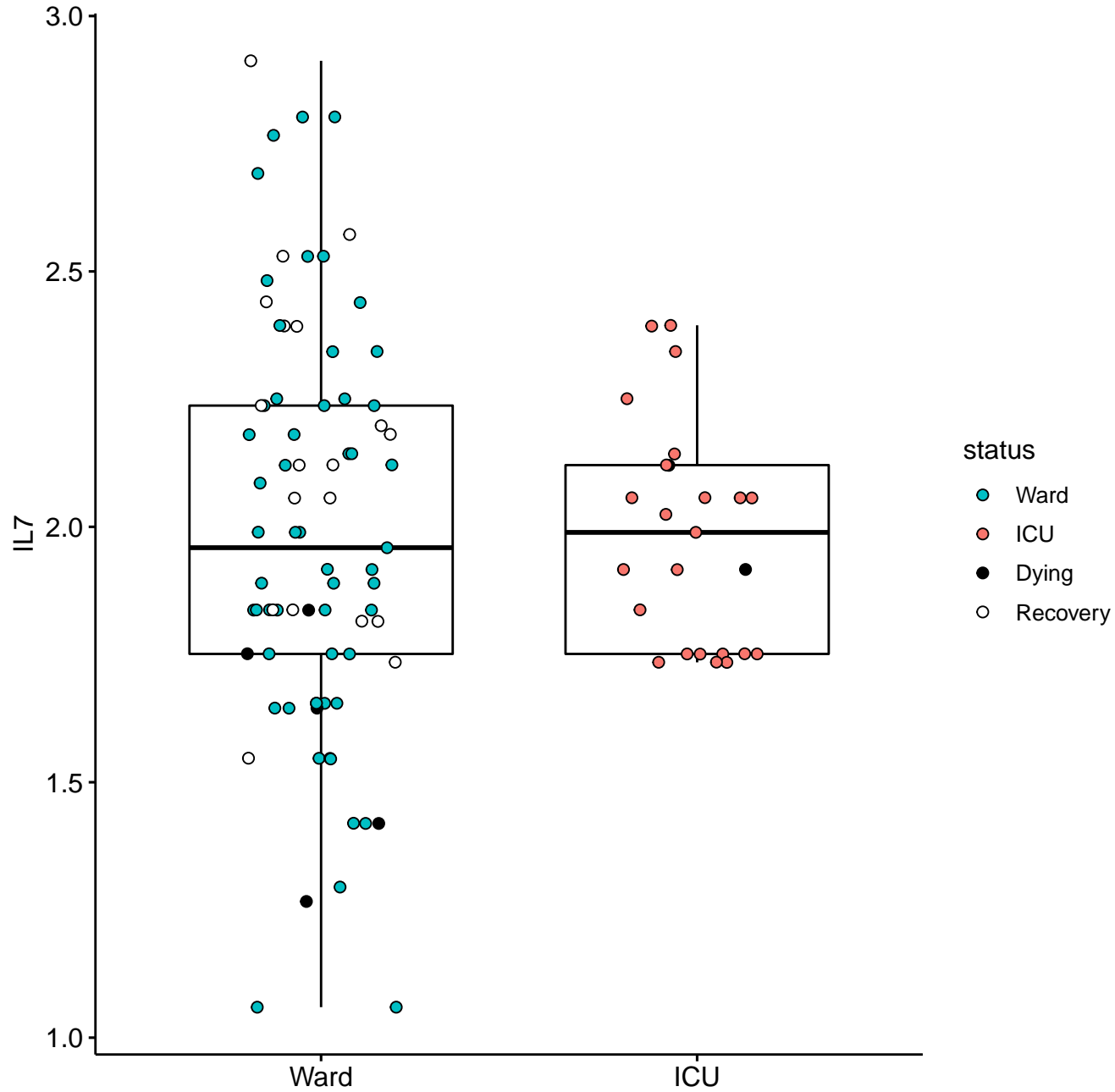

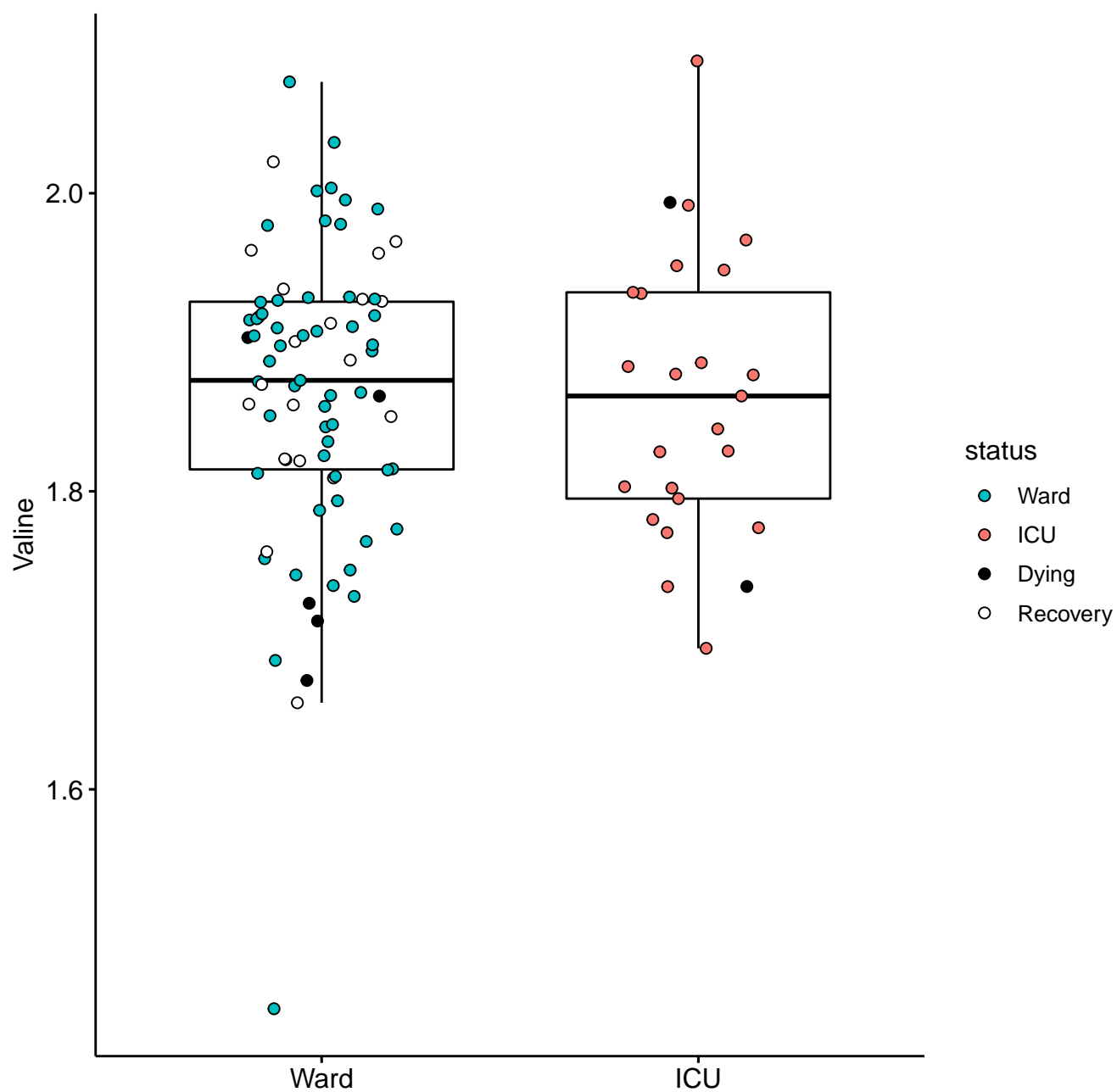

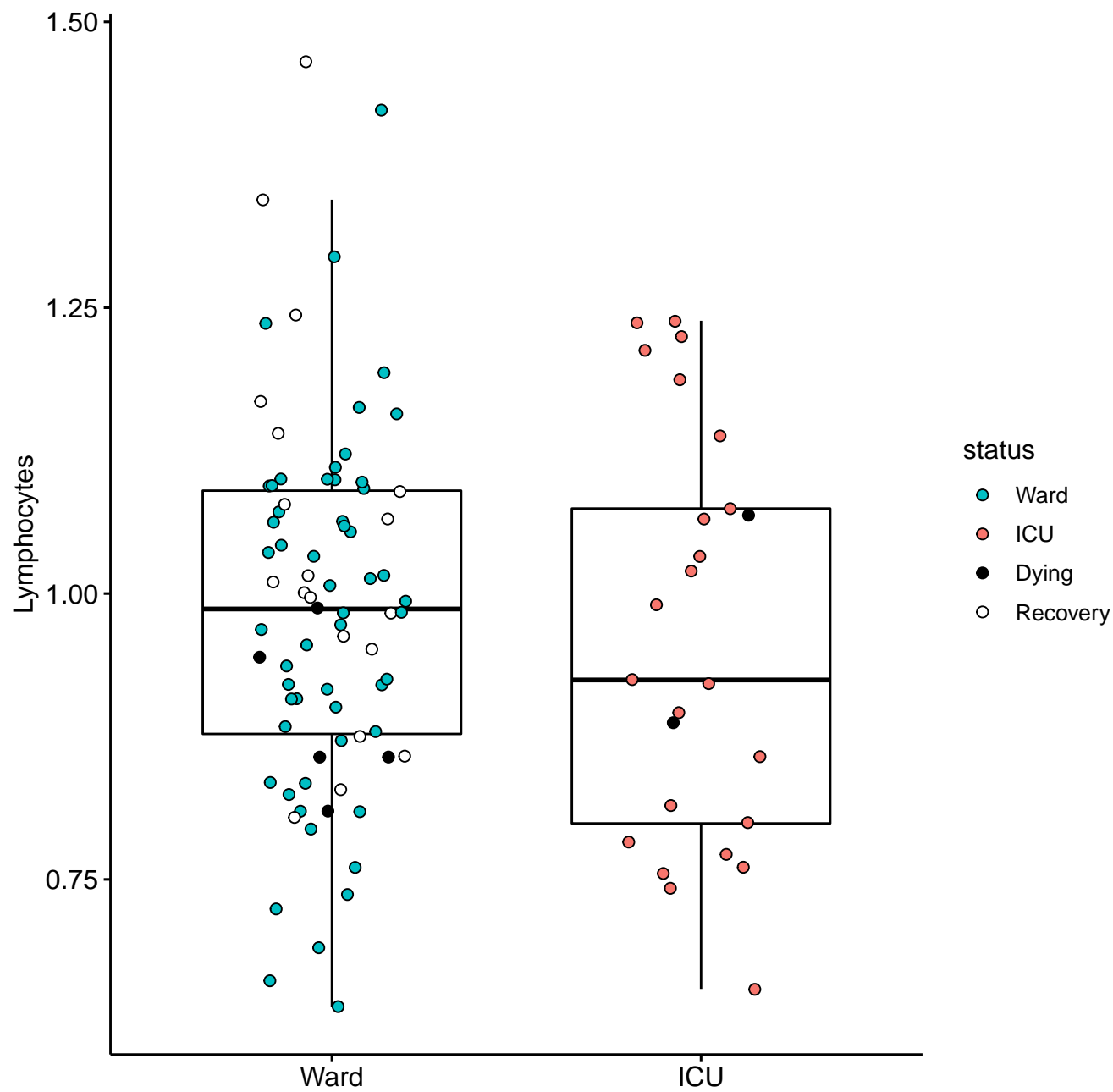

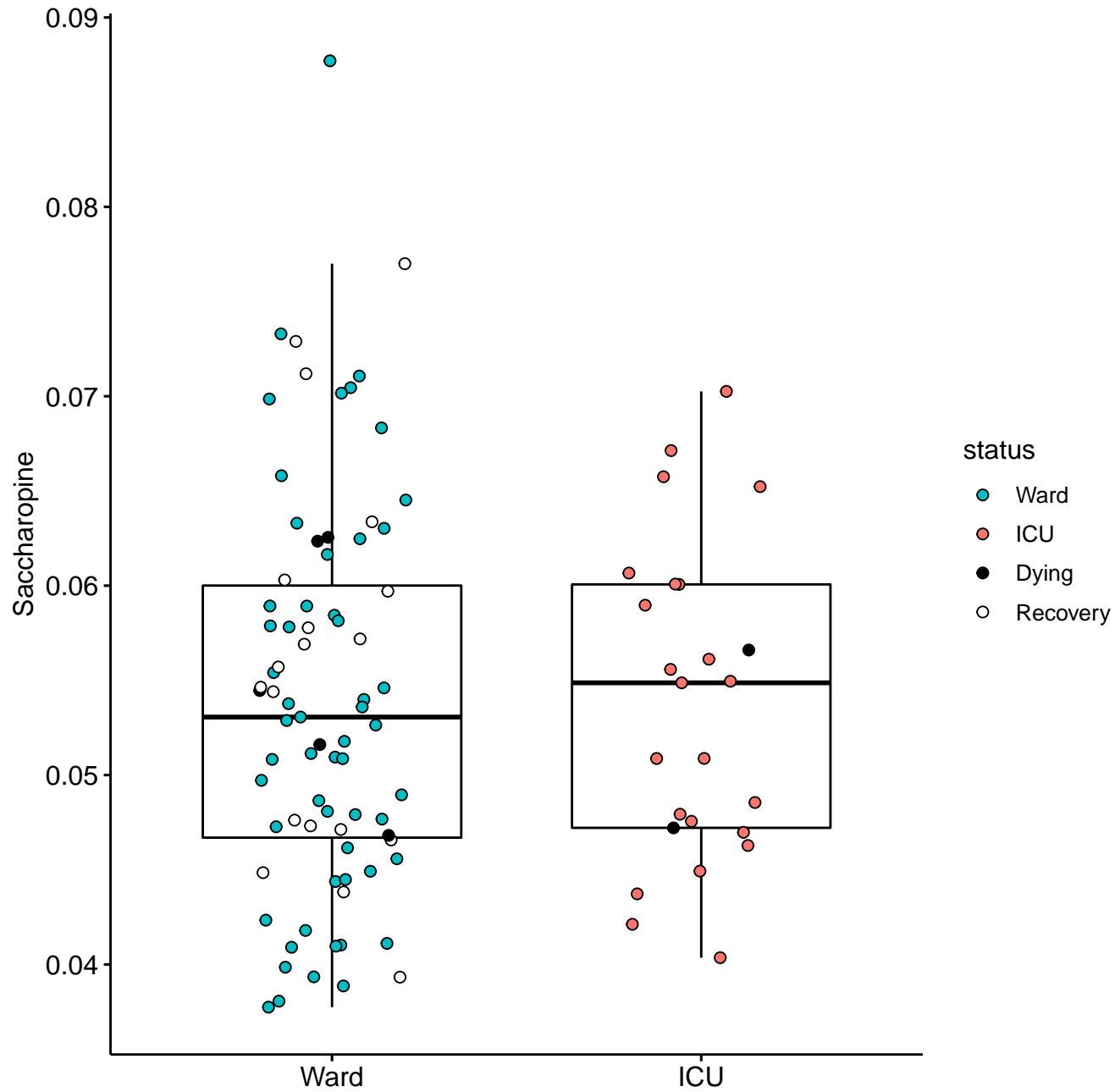

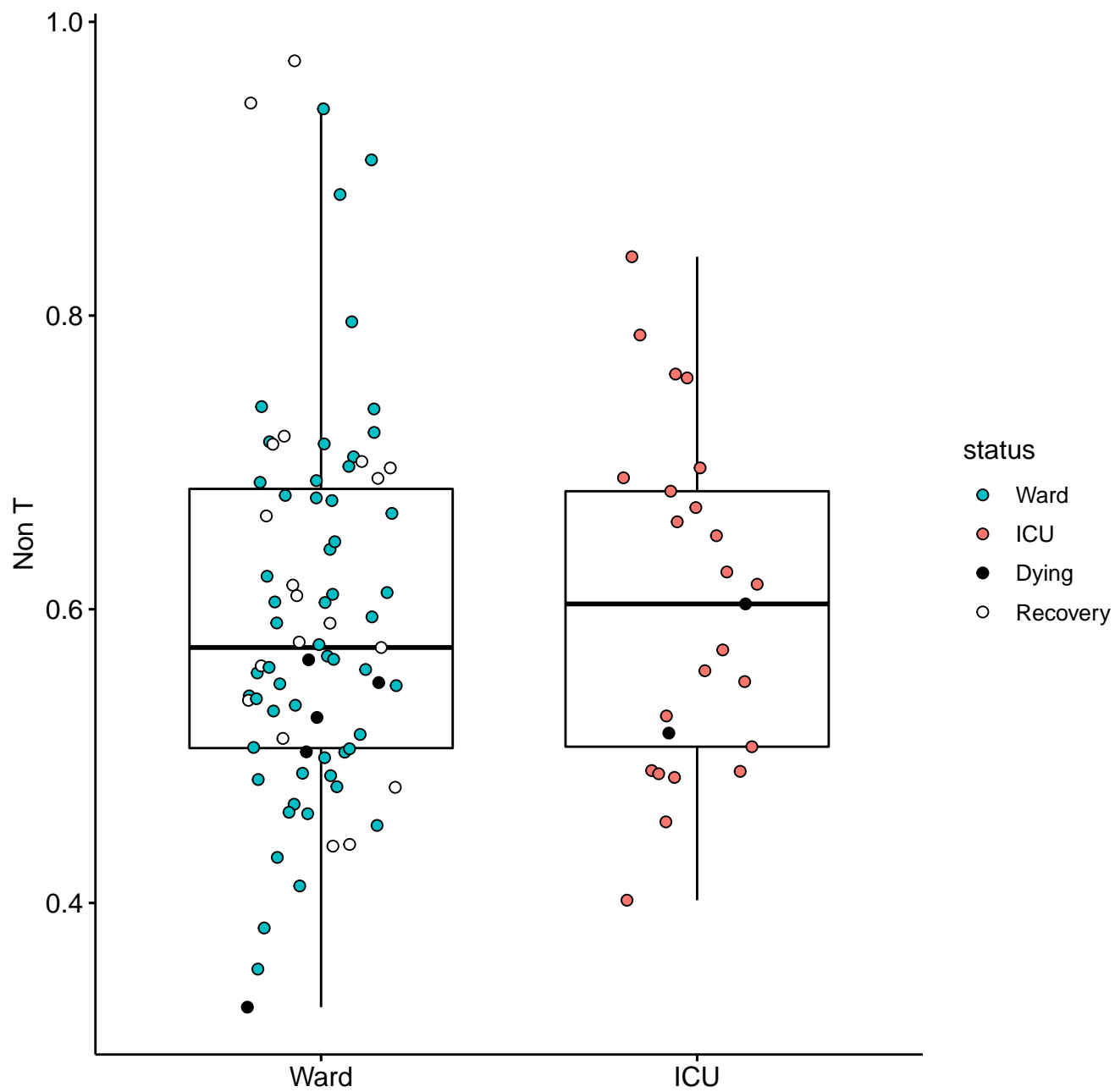

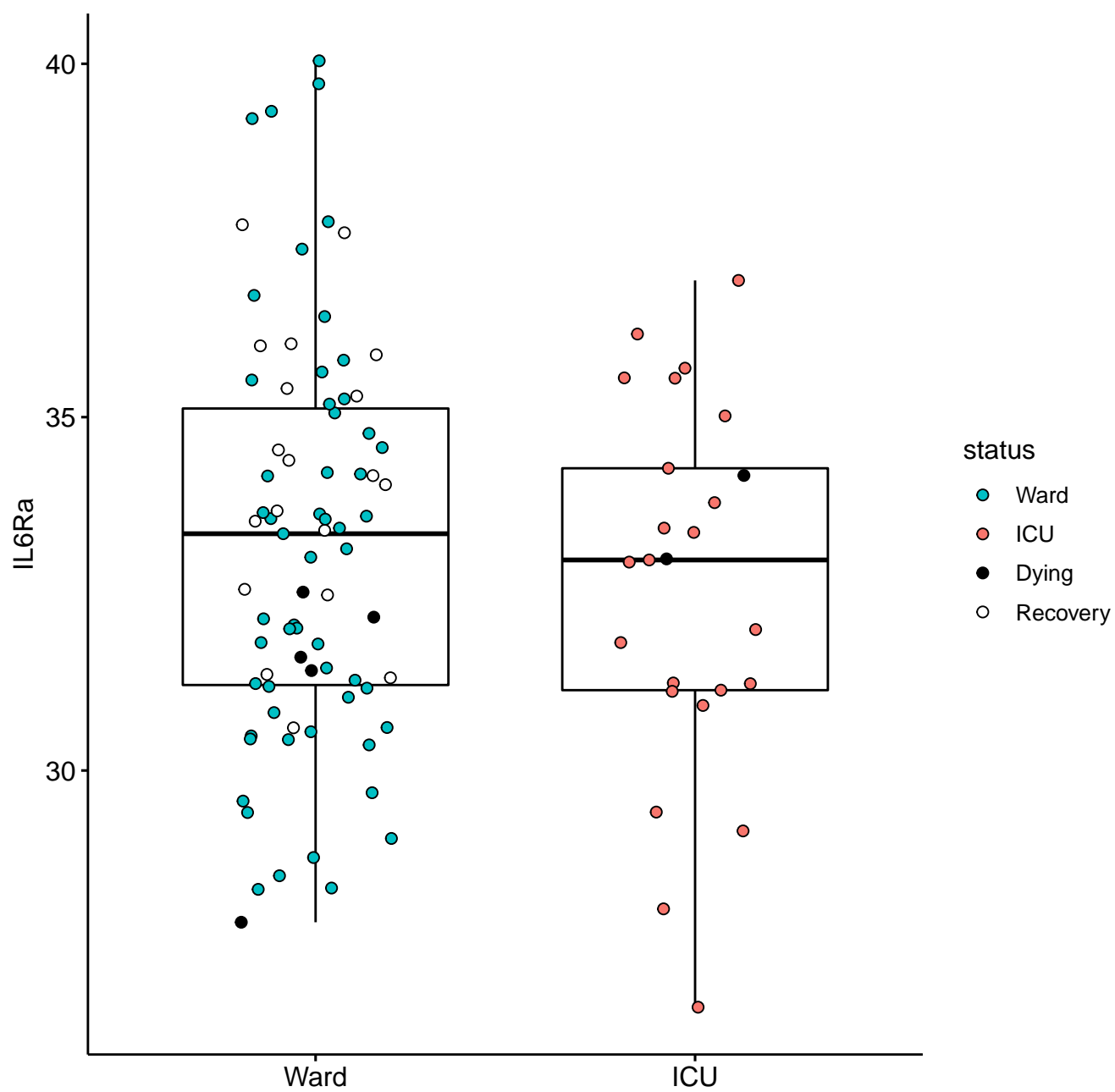

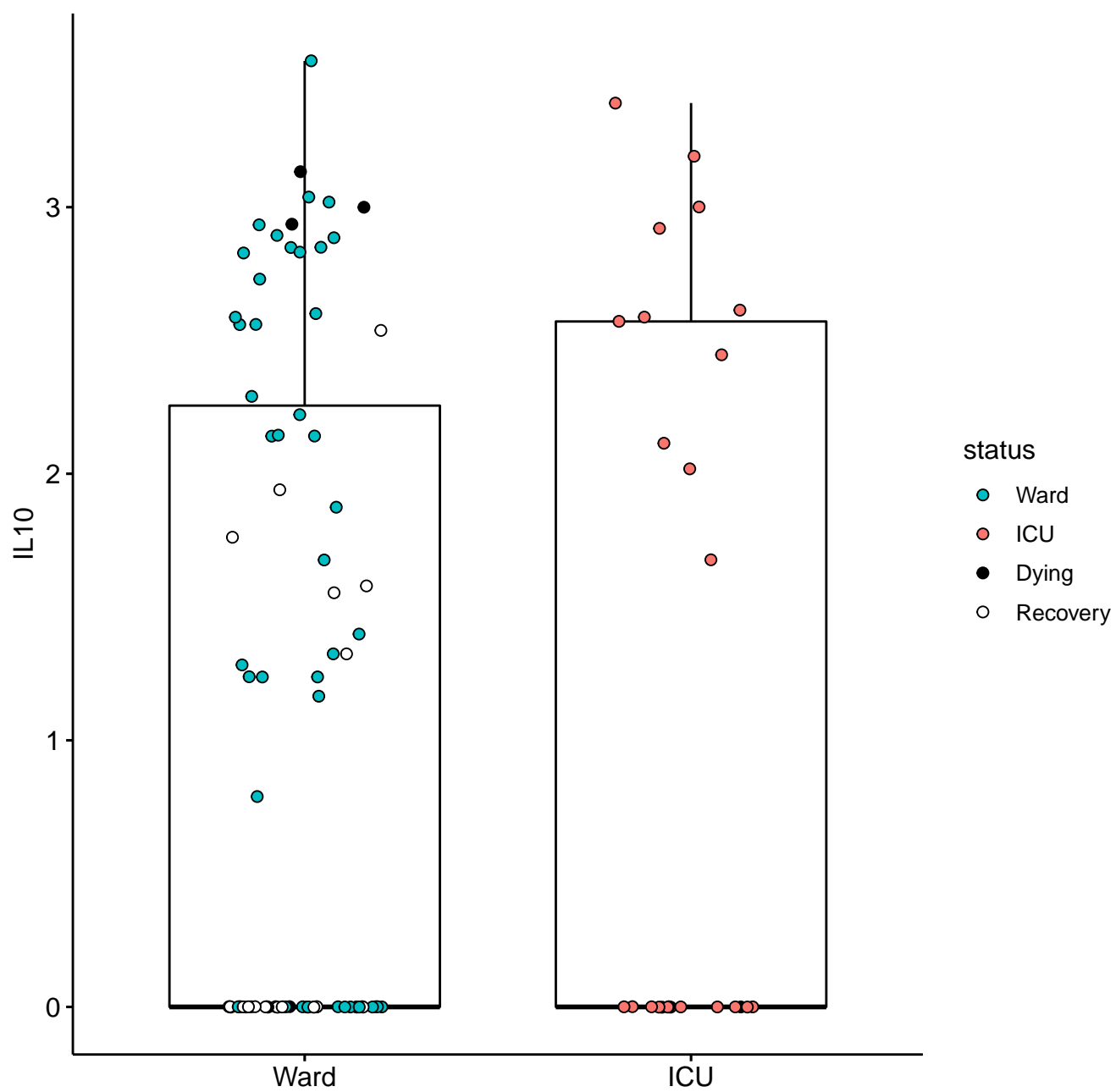

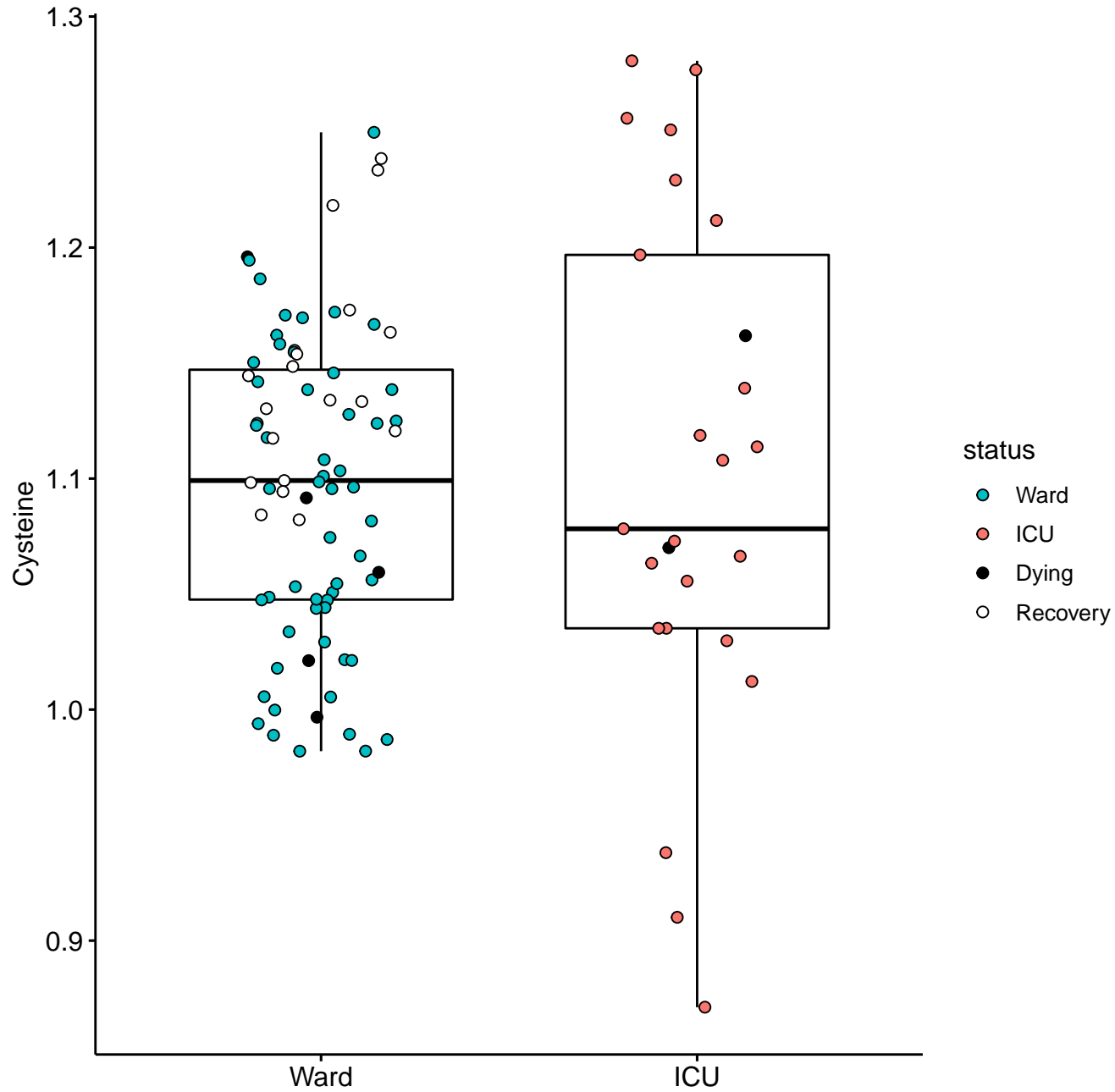

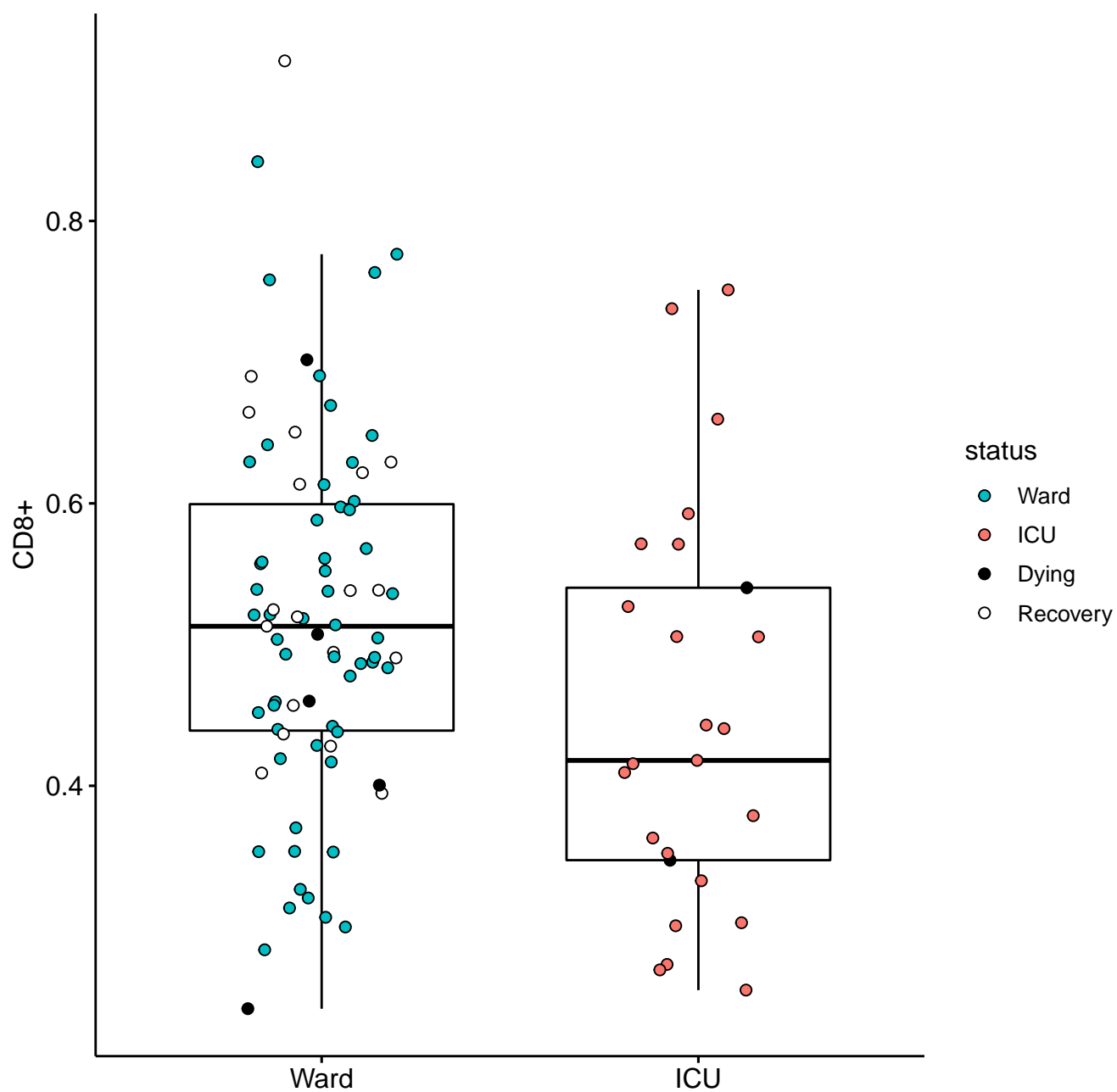

Supplement: Supplementary file 1 [file metabolites-12-00618-s001.zip › Document S2.pdf]
