# Supplementary material for: Severe COVID-19 Is Characterised by Perturbations in Plasma Amines Correlated with Immune Response Markers, and Linked to Inflammation and Oxidative Stress
Source: Metabolites. 2022 Jul 2;12(7):618. doi: 10.3390/metabo12070618 (PMC9321395; doi:10.3390/metabo12070618)
Supplement: Supplementary file 1 [file Document S5.pdf › Document S5.pdf]

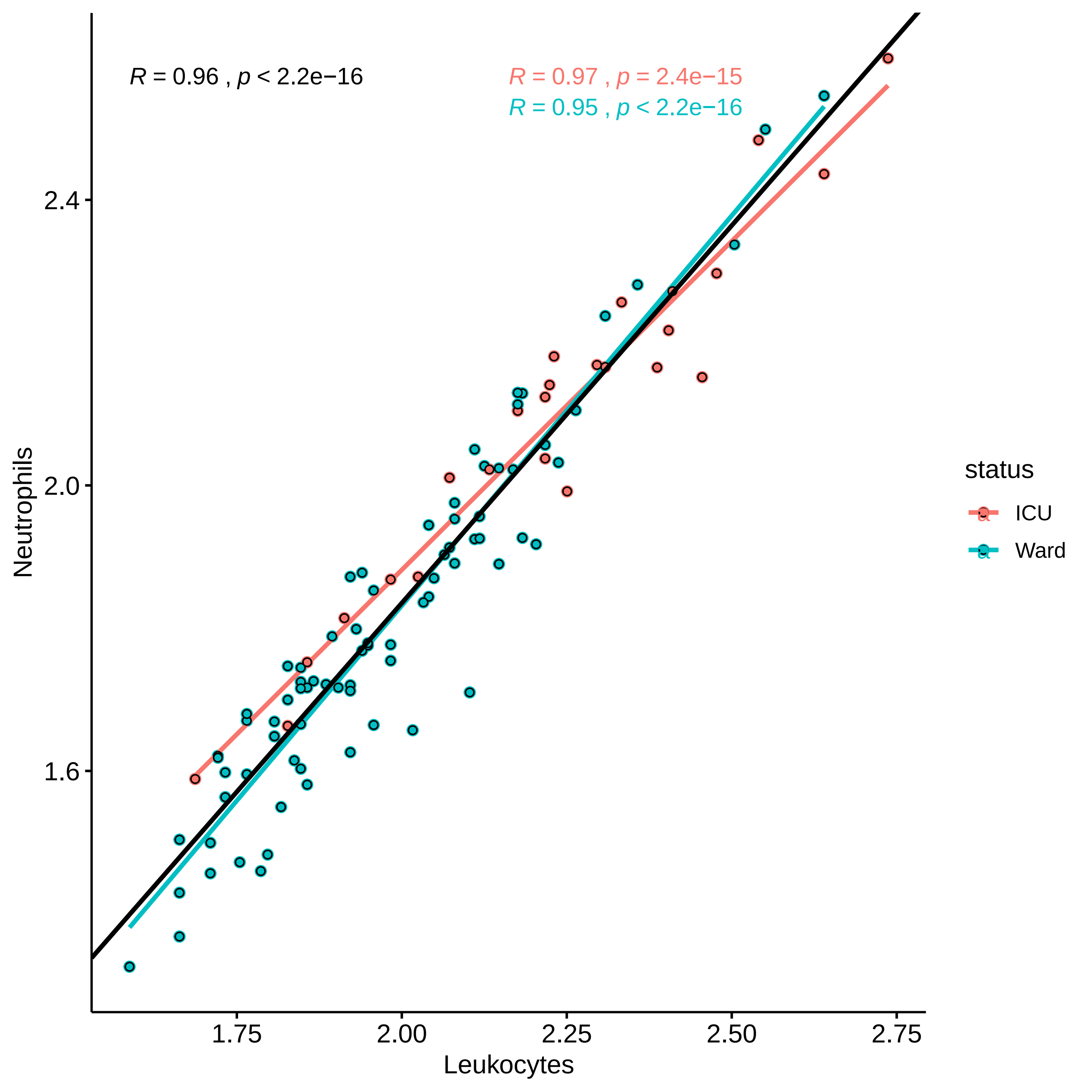

R = 0.96 , p < 2.2e−16
R = 0.97 , p = 2.4e−15
R = 0.95 , p < 2.2e−16
Neutrophils
2.4
2.0
1.6
1.75
2.00
2.25
2.50
2.75
Leukocytes
status
ICU
Ward

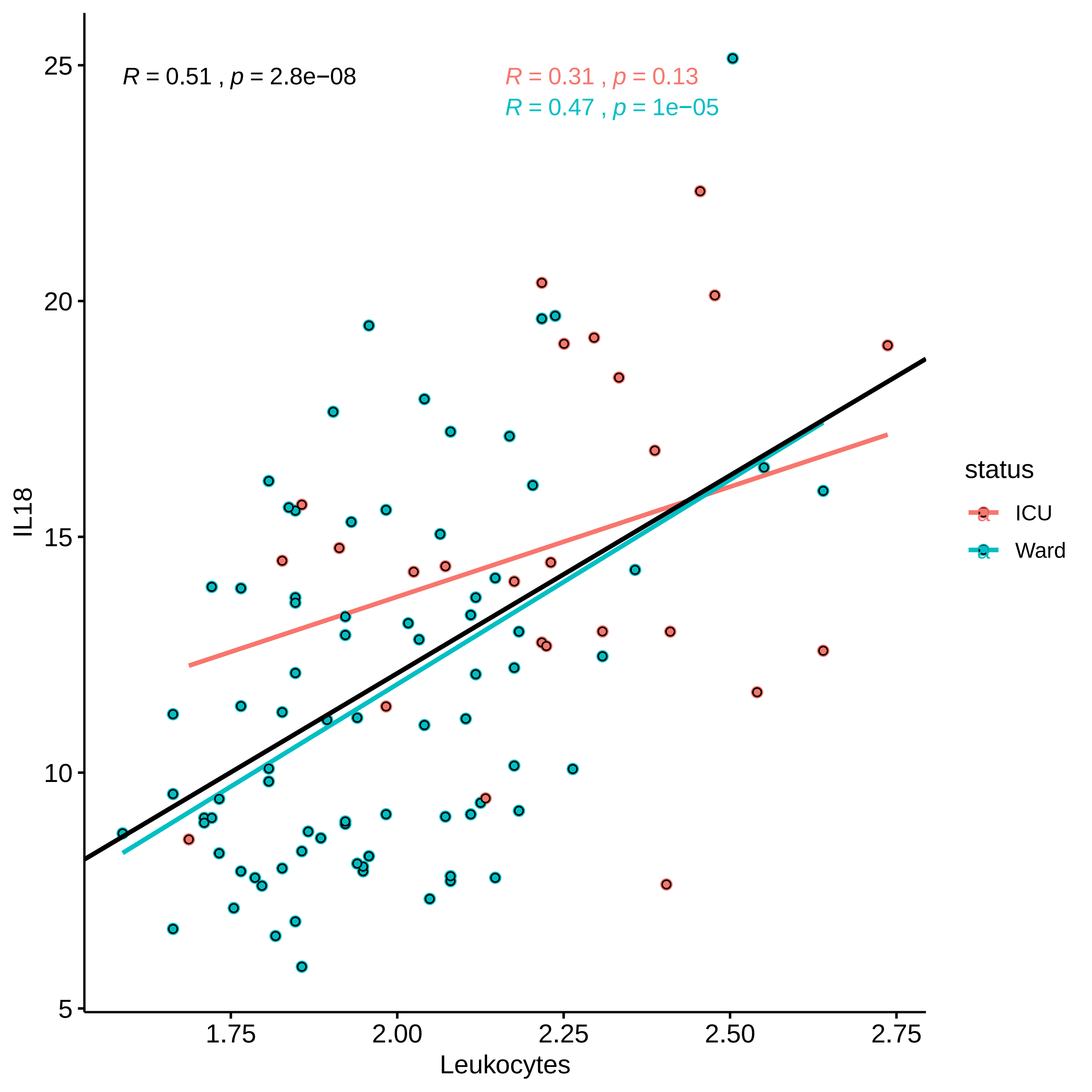

R = 0.51 , p = 2.8e−08
R = 0.31 , p = 0.13
R = 0.47 , p = 1e−05
IL18
25
20
15
10
5
1.75
2.00
2.25
2.50
2.75
Leukocytes
status
ICU
Ward

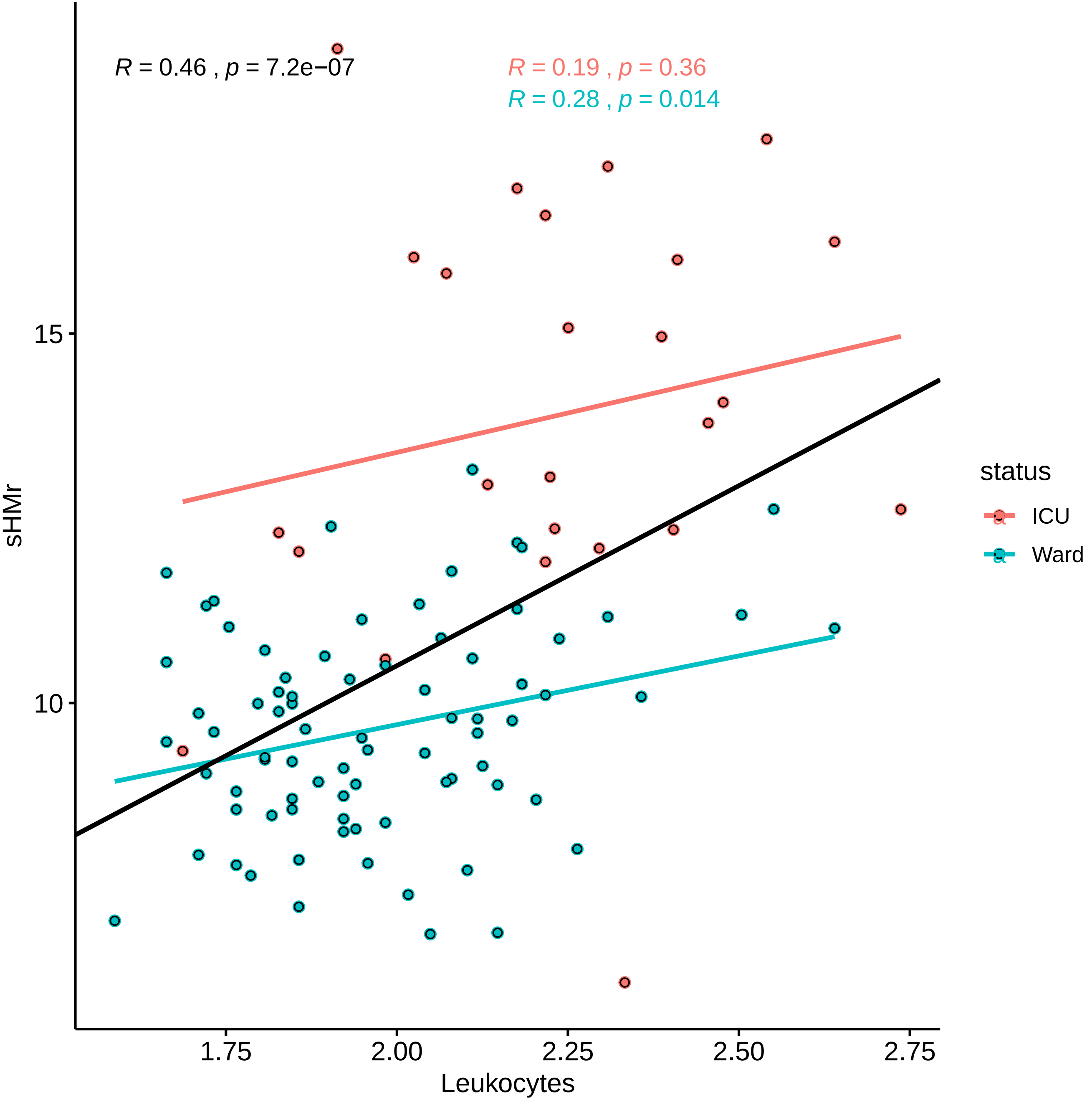
R = 0.46 , p = 7.2e−07
R = 0.19 , p = 0.36
R = 0.28 , p = 0.014
sHMr
15
10
Leukocytes
1.75
2.00
2.25
2.50
2.75
status
ICU
Ward

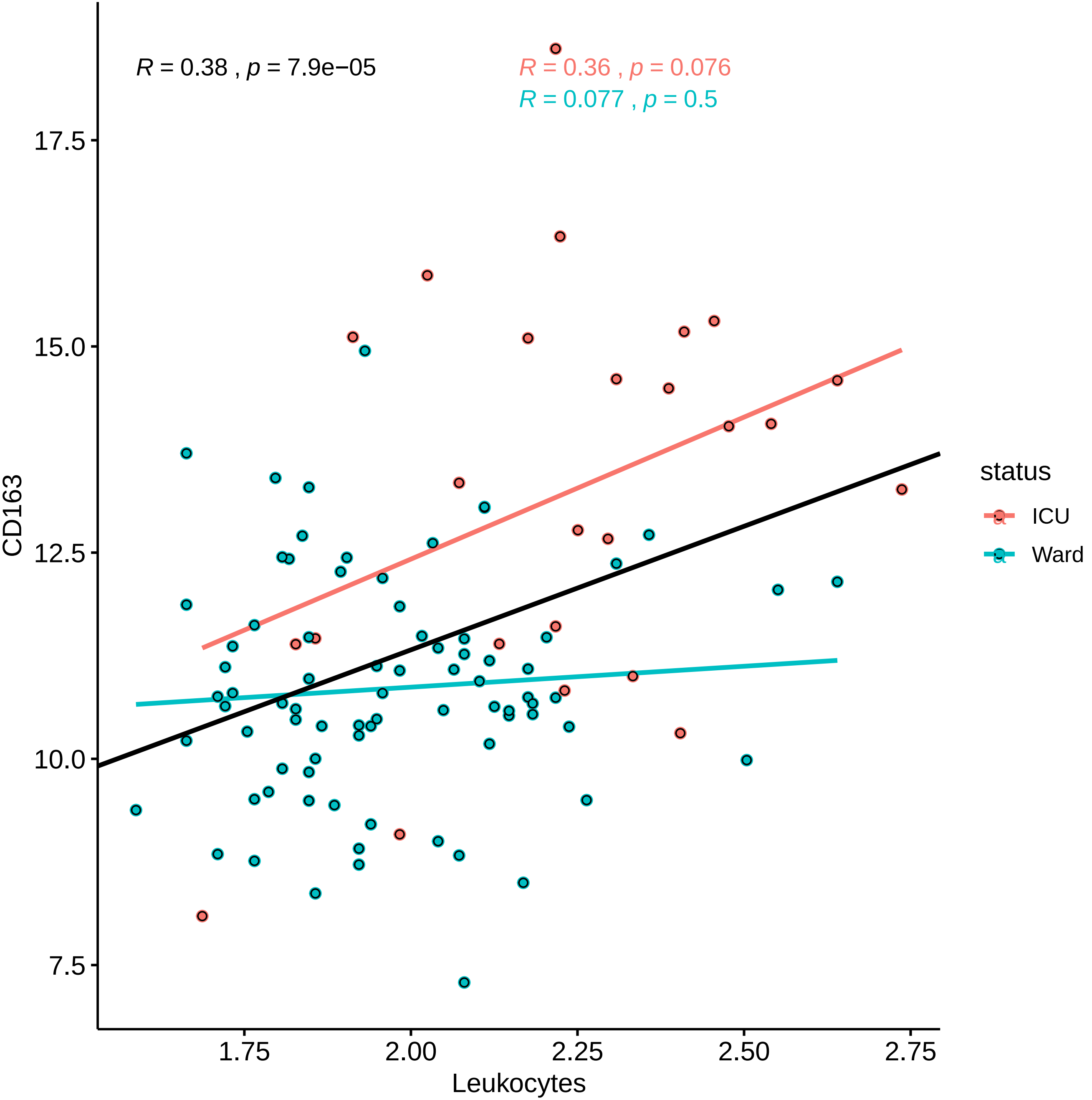

R = 0.38 , p = 7.9e−05
R = 0.36 , p = 0.076
R = 0.077 , p = 0.5
17.5
15.0
12.5
10.0
7.5
CD163
1.75
2.00
2.25
2.50
2.75
Leukocytes
status
ICU
Ward

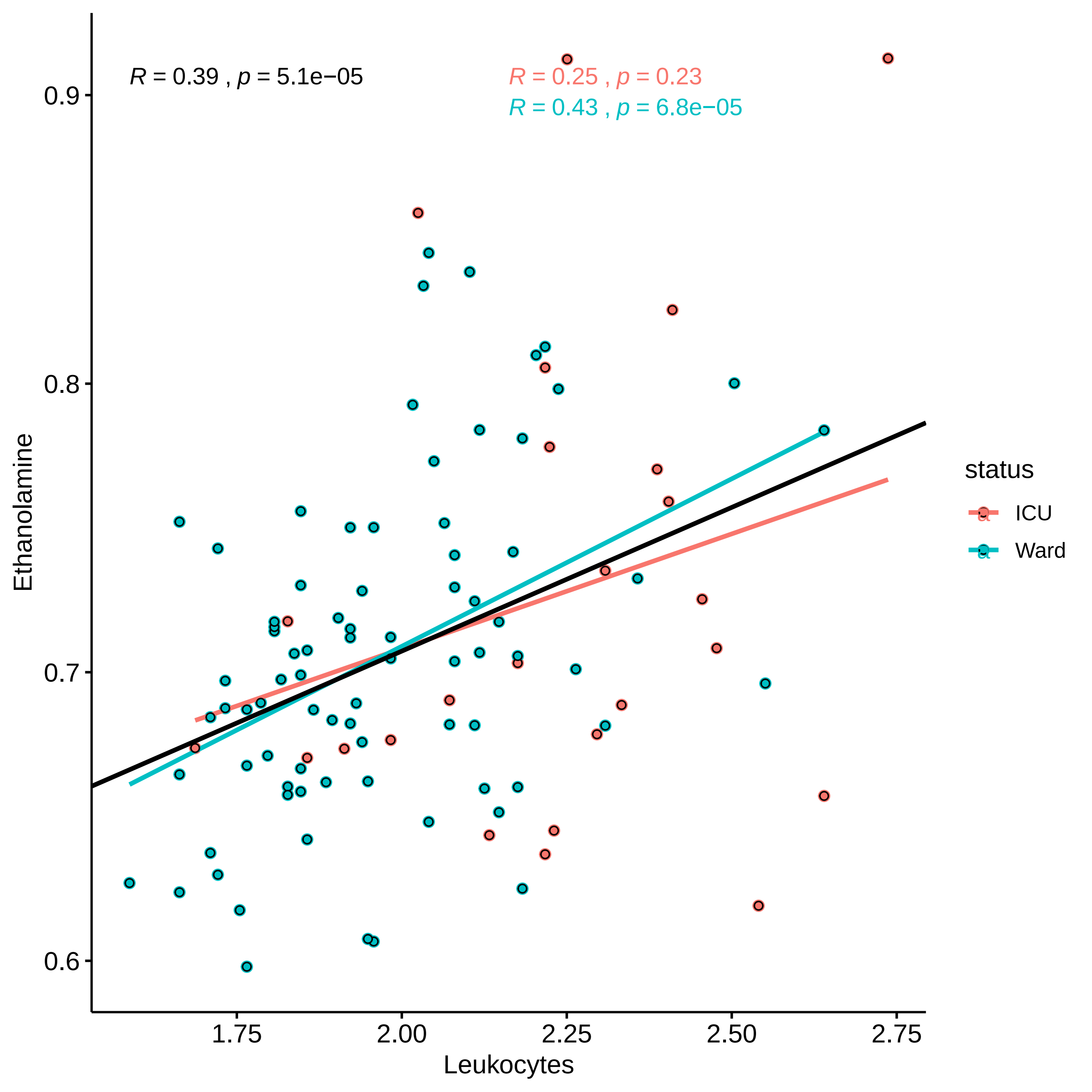

R = 0.39 , p = 5.1e−05
R = 0.25 , p = 0.23
R = 0.43 , p = 6.8e−05
0.9
0.8
0.7
0.6
Ethanolamine
1.75
2.00
2.25
2.50
2.75
Leukocytes
status
ICU
Ward

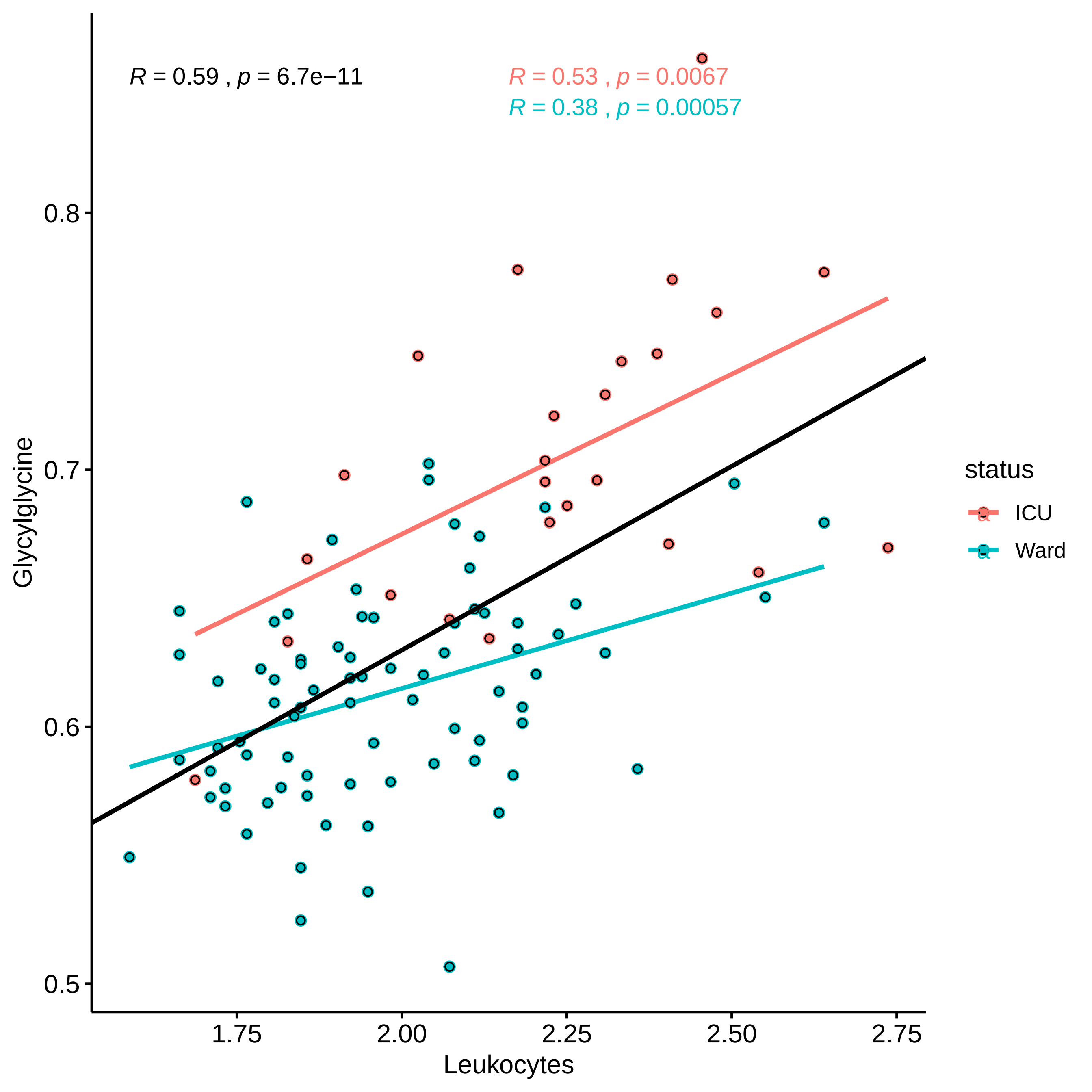

R = 0.59 , p = 6.7e−11
R = 0.53 , p = 0.0067
R = 0.38 , p = 0.00057
0.8
0.7
0.6
0.5
Glycylglycine
1.75
2.00
2.25
2.50
2.75
Leukocytes
status
ICU
Ward

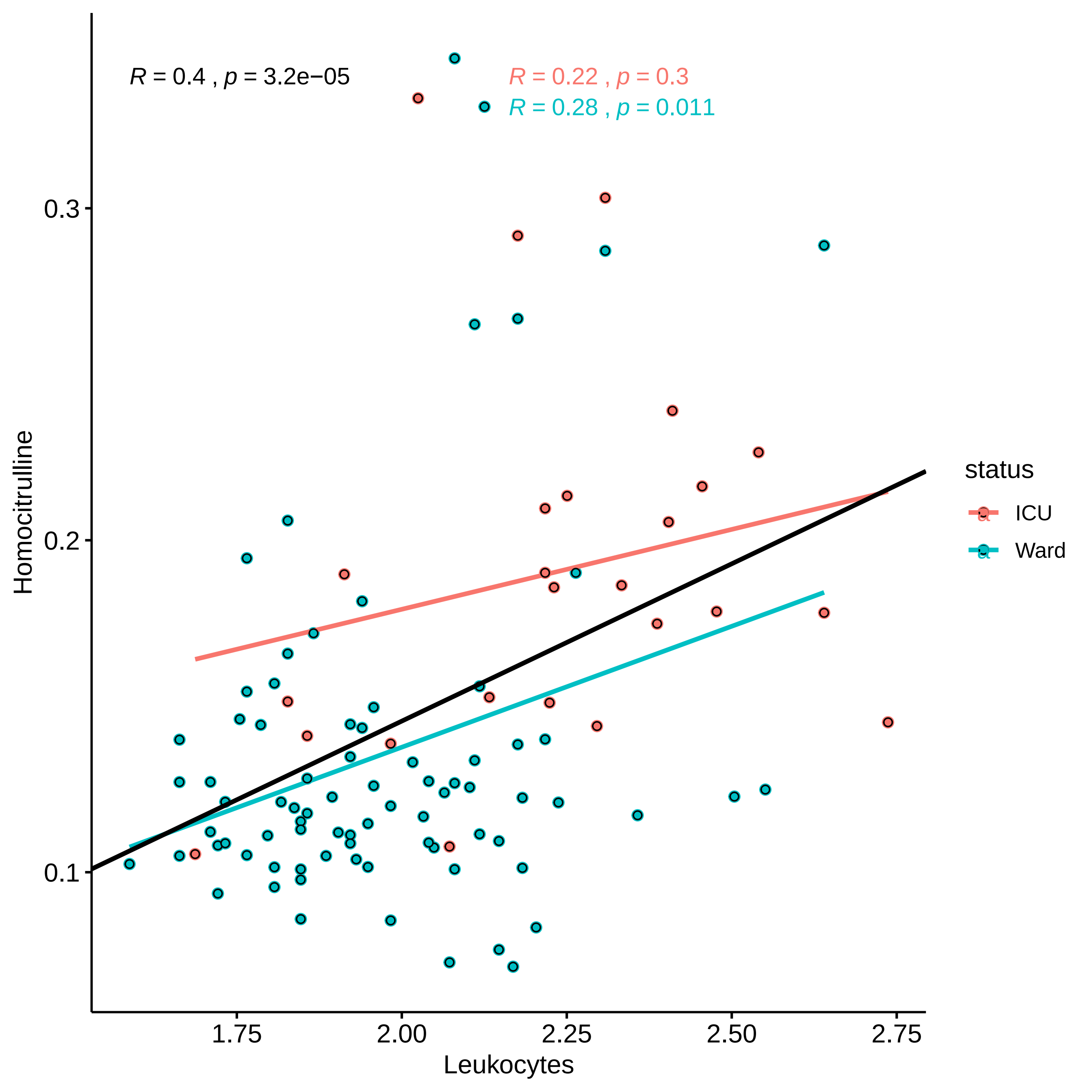

R = 0.4 , p = 3.2e−05
R = 0.22 , p = 0.3
R = 0.28 , p = 0.011
0.3
0.2
0.1
Homocitrulline
1.75
2.00
2.25
2.50
2.75
Leukocytes
status
ICU
Ward

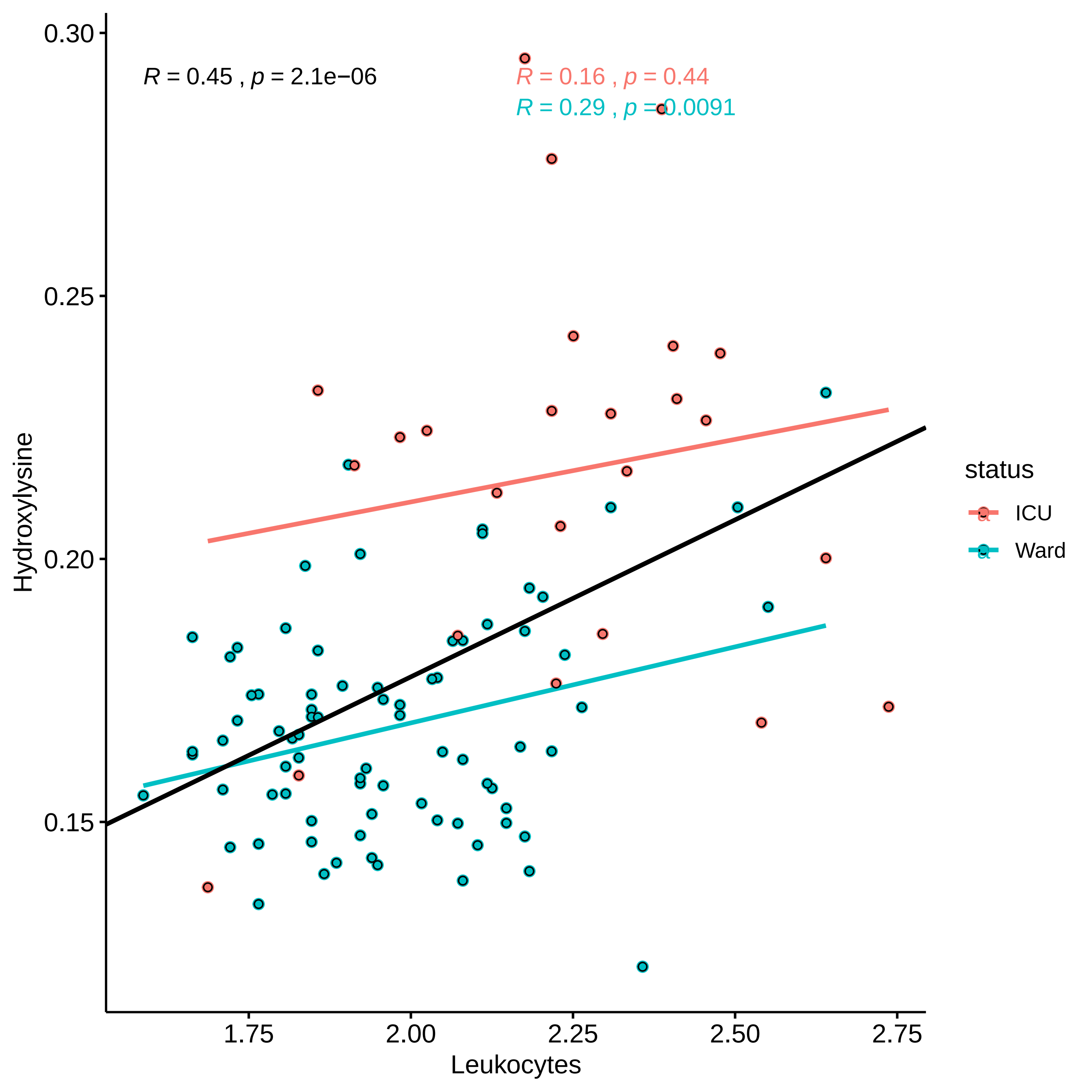

R = 0.45 , p = 2.1e−06
R = 0.16 , p = 0.44
R = 0.29 , p = 0.0091
0.30
0.25
0.20
0.15
Hydroxylysine
1.75
2.00
2.25
2.50
2.75
Leukocytes
status
ICU
Ward

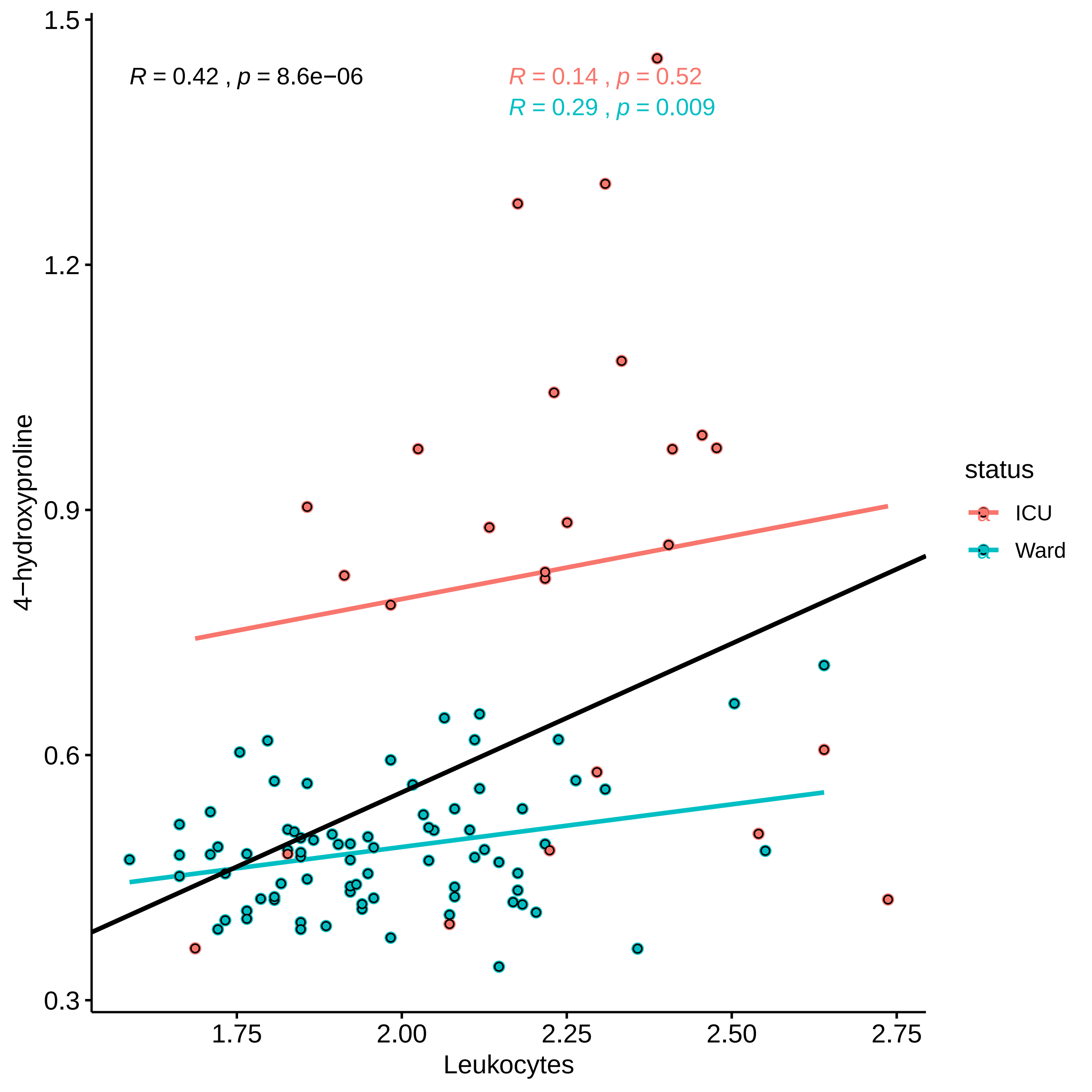

R = 0.42 , p = 8.6e−06
R = 0.14 , p = 0.52
R = 0.29 , p = 0.009
4−hydroxyproline
Leukocytes
1.5
1.2
0.9
0.6
0.3
1.75
2.00
2.25
2.50
2.75
status
ICU
Ward

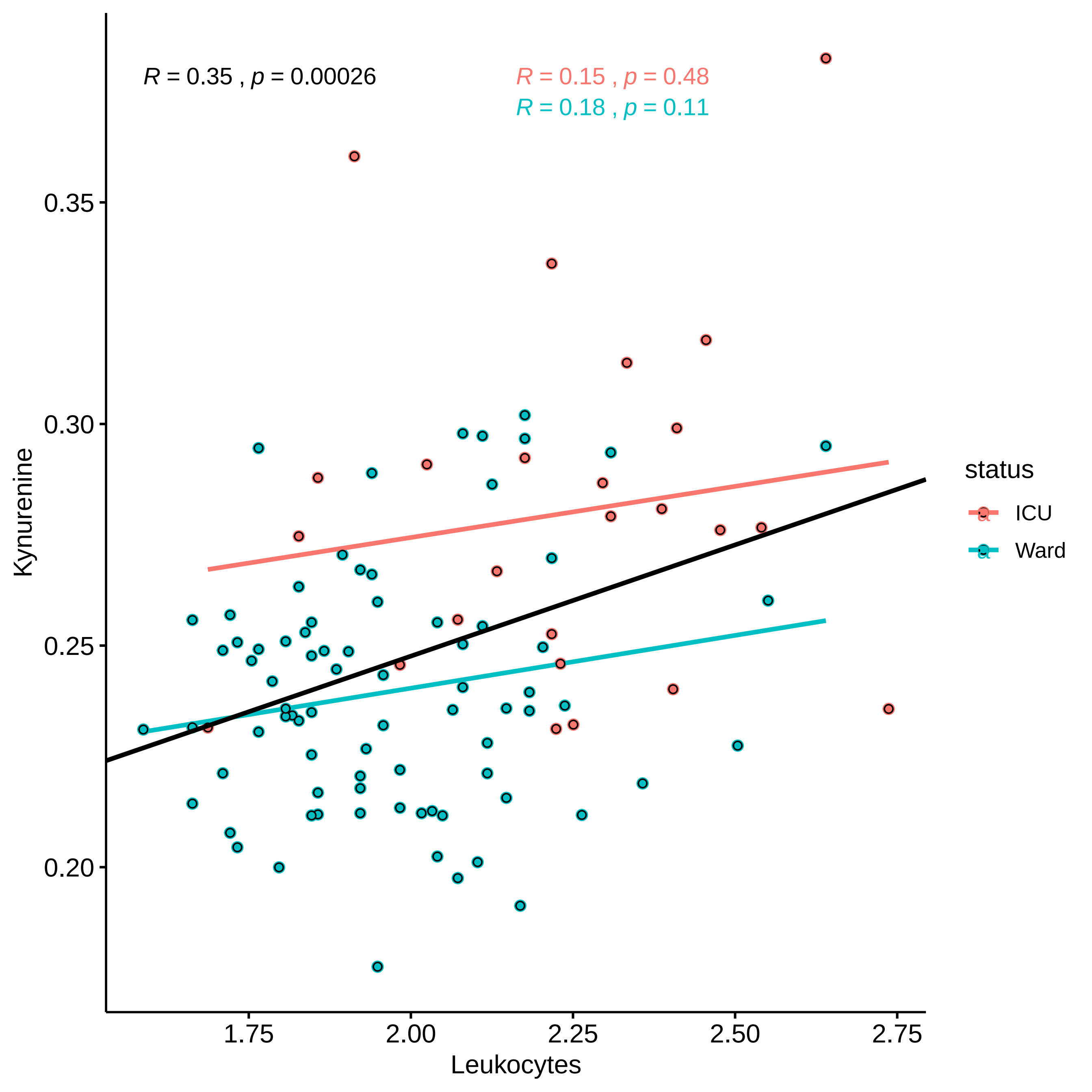

R = 0.35 , p = 0.00026
R = 0.15 , p = 0.48
R = 0.18 , p = 0.11
Kynurenine
0.35
0.30
0.25
0.20
1.75
2.00
2.25
2.50
2.75
Leukocytes
status
ICU
Ward

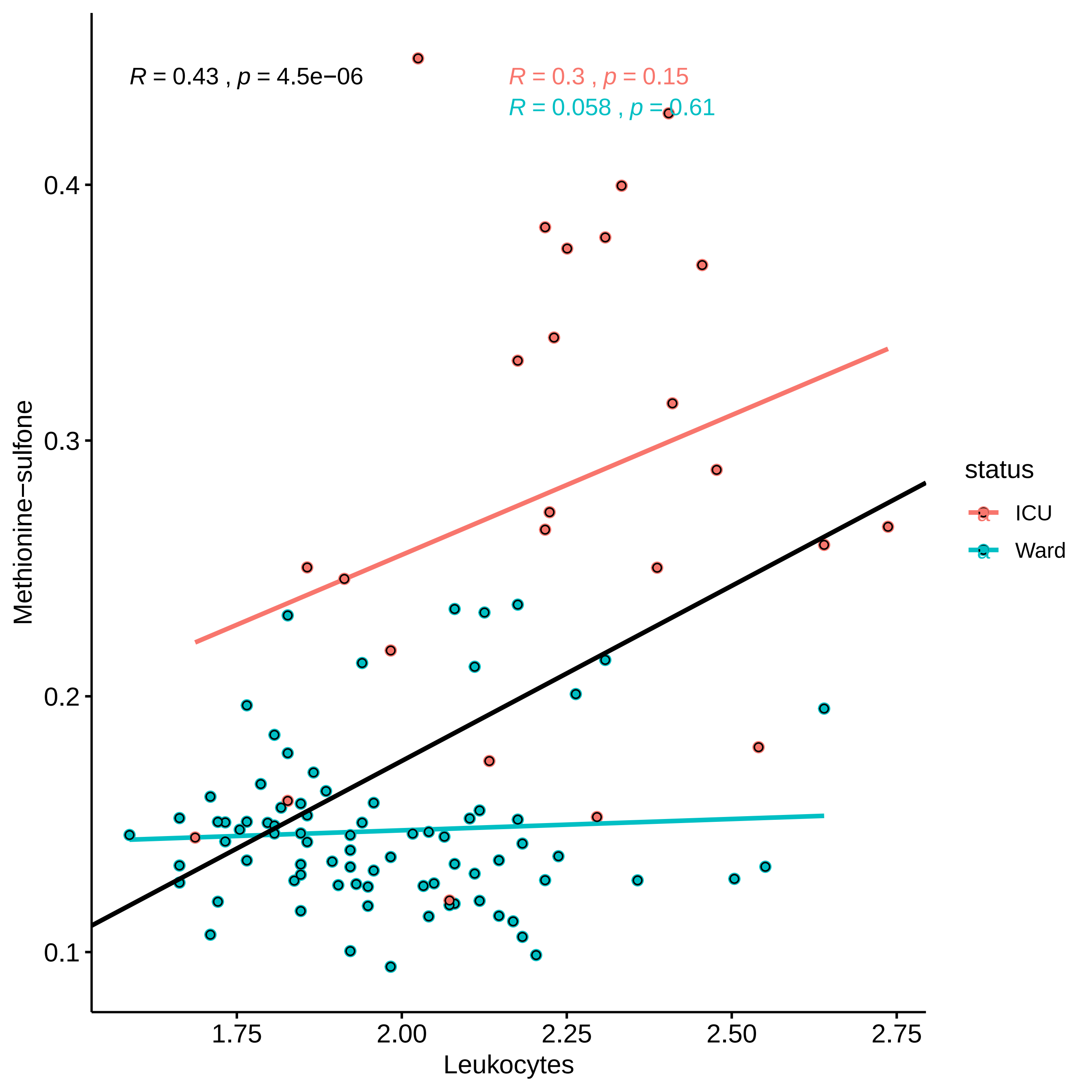

$R = 0.43$, $p = 4.5e-06$
$R = 0.3$, $p = 0.15$
$R = 0.058$, $p = 0.61$
0.4
0.3
0.2
0.1
Methionine−sulfone
1.75
2.00
2.25
2.50
2.75
Leukocytes
status
ICU
Ward

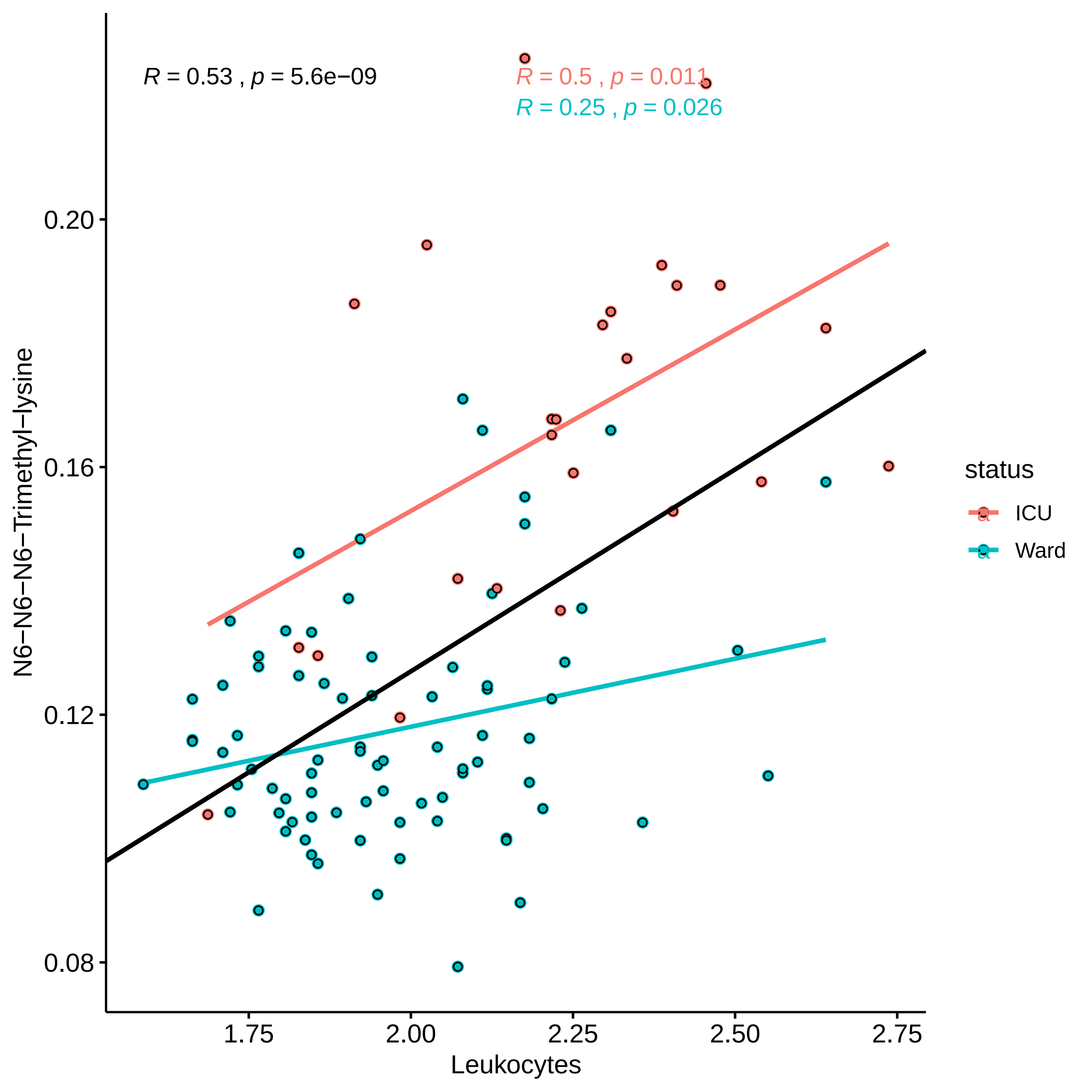

R = 0.53 , p = 5.6e−09
R = 0.5 , p = 0.011
R = 0.25 , p = 0.026
N6−N6−N6−Trimethyl−lysine
0.20
0.16
0.12
0.08
1.75
2.00
2.25
2.50
2.75
Leukocytes
status
ICU
Ward

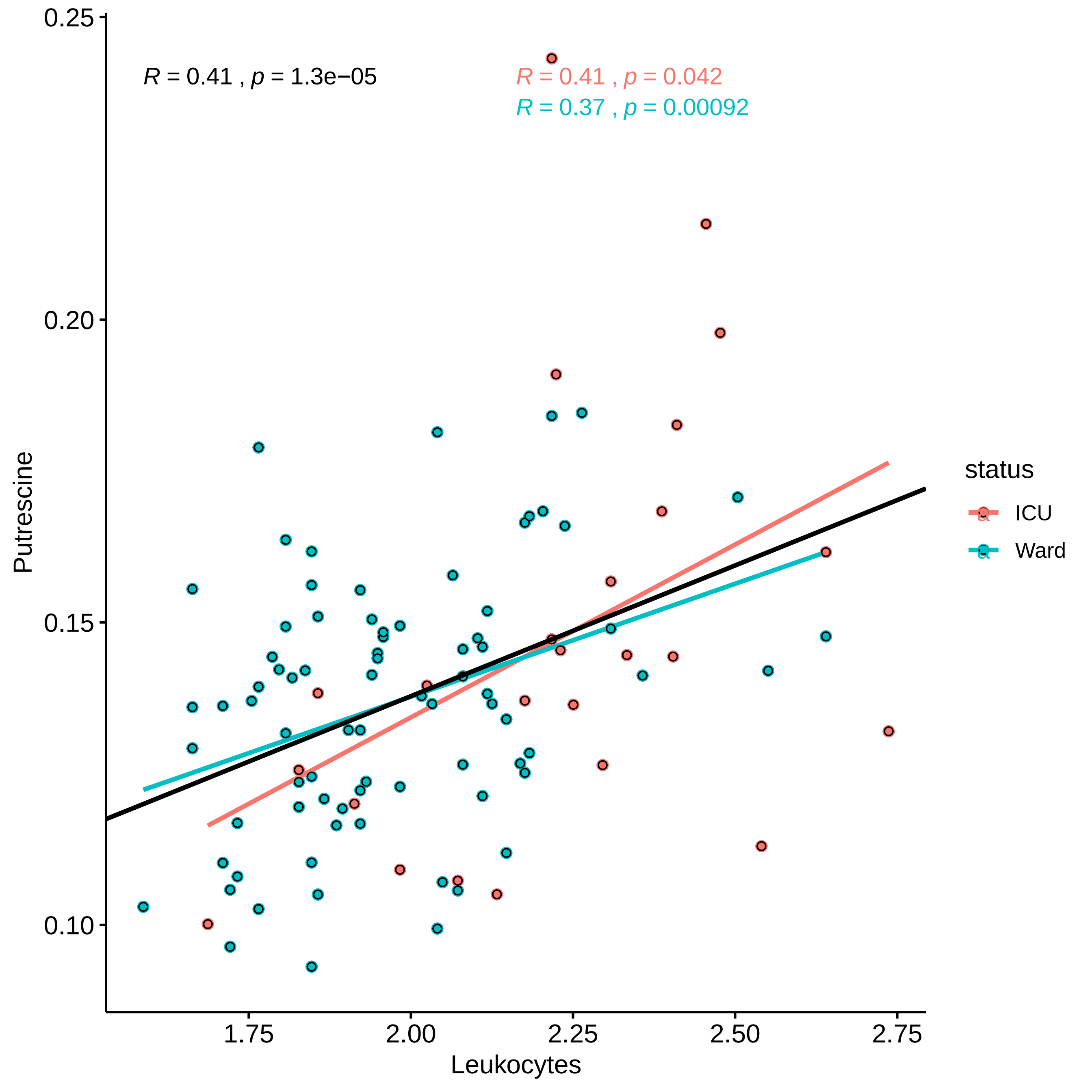
R = 0.41 , p = 1.3e−05
R = 0.41 , p = 0.042
R = 0.37 , p = 0.00092
0.25
0.20
0.15
0.10
Putrescine
1.75
2.00
2.25
2.50
2.75
Leukocytes
status
ICU
Ward

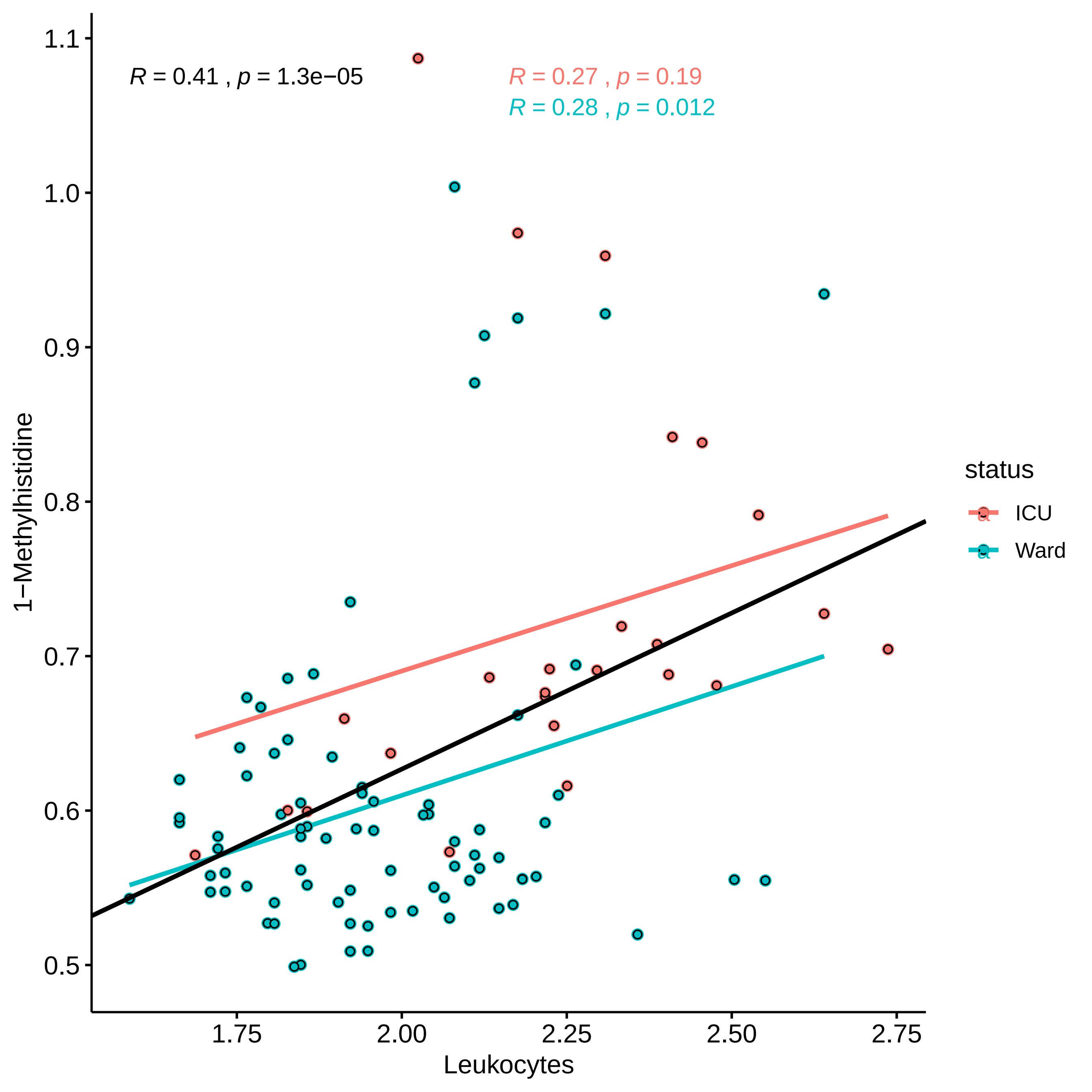

R = 0.41 , p = 1.3e−05
R = 0.27 , p = 0.19
R = 0.28 , p = 0.012
1−Methylhistidine
Leukocytes
1.1
1.0
0.9
0.8
0.7
0.6
0.5
1.75
2.00
2.25
2.50
2.75
status
ICU
Ward

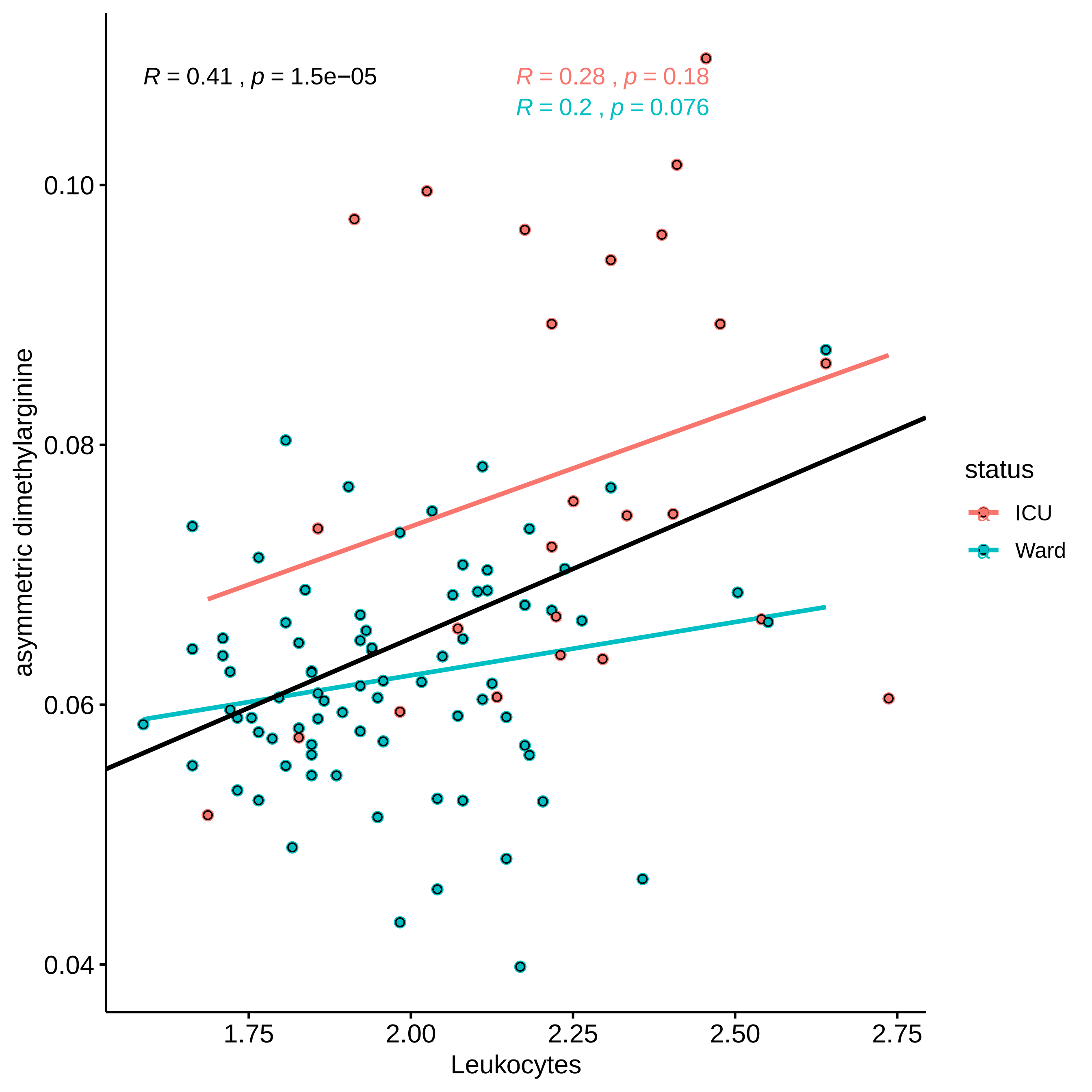

R = 0.41 , p = 1.5e−05
R = 0.28 , p = 0.18
R = 0.2 , p = 0.076
0.10
0.08
0.06
0.04
asymmetric dimethylarginine
1.75
2.00
2.25
2.50
2.75
Leukocytes
status
ICU
Ward

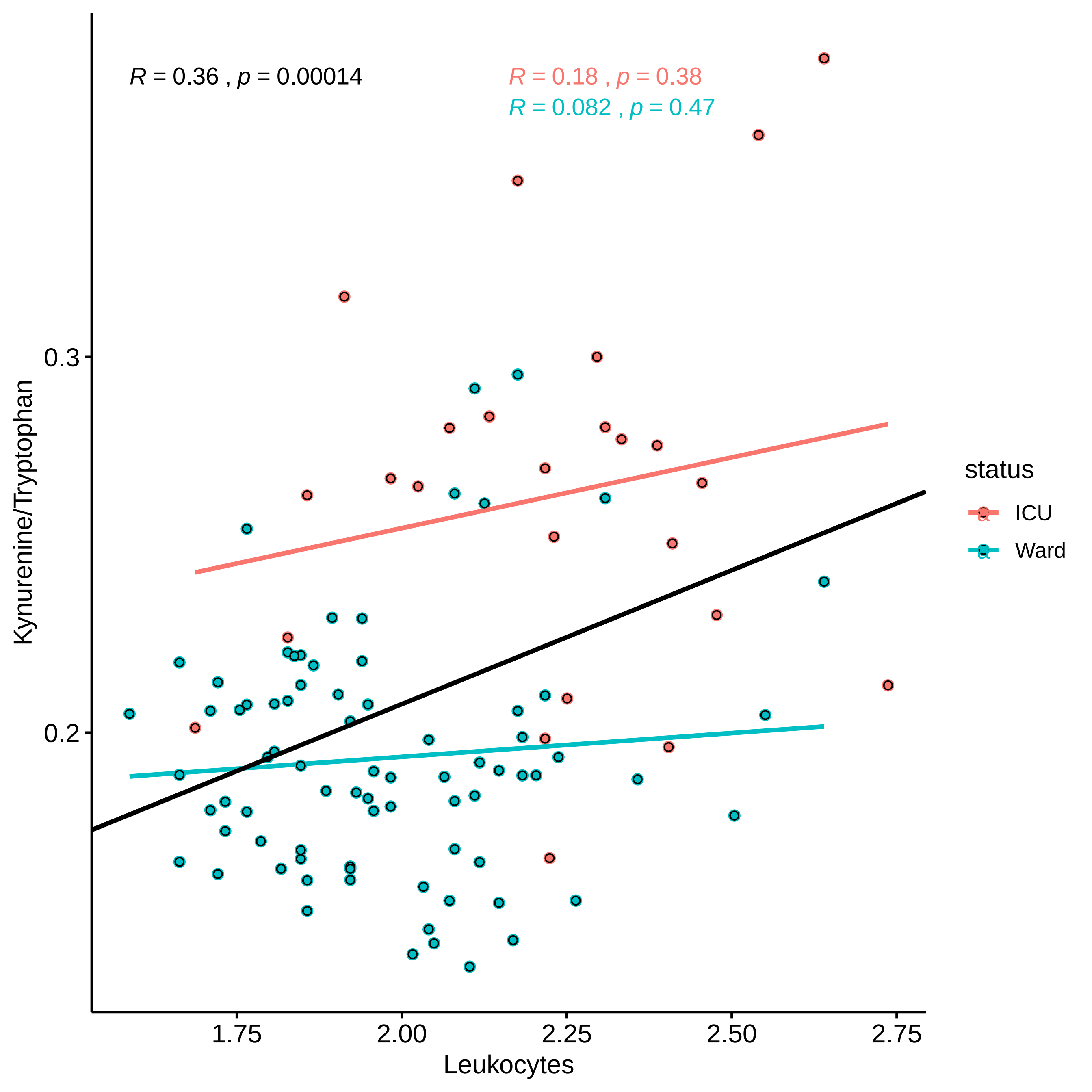
R = 0.36 , p = 0.00014
R = 0.18 , p = 0.38
R = 0.082 , p = 0.47
Kynurenine/Tryptophan
0.3
0.2
1.75
2.00
2.25
2.50
2.75
Leukocytes
status
ICU
Ward

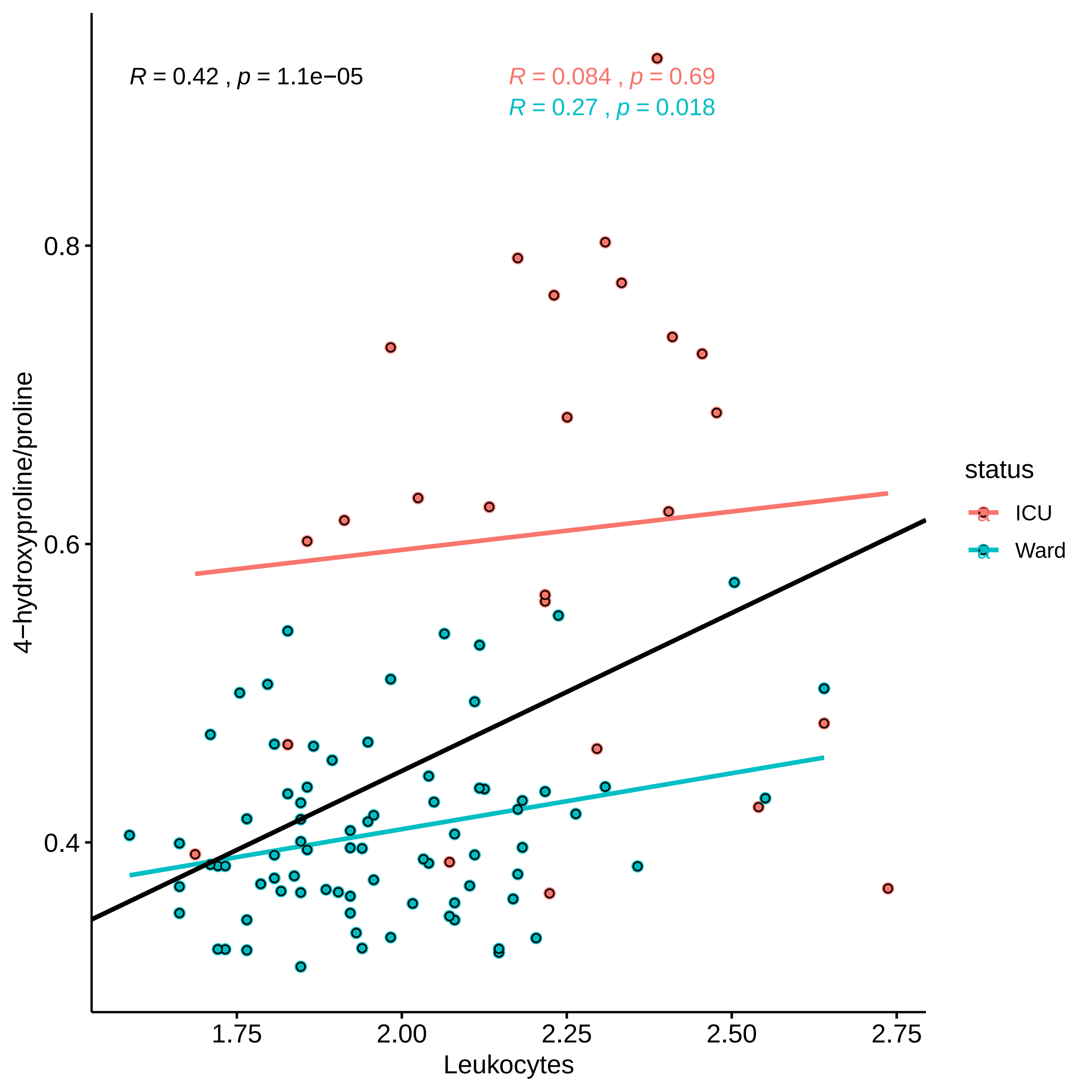

R = 0.42 , p = 1.1e−05
R = 0.084 , p = 0.69
R = 0.27 , p = 0.018
4−hydroxyproline/proline
0.4
0.6
0.8
1.75
2.00
2.25
2.50
2.75
Leukocytes
status
ICU
Ward

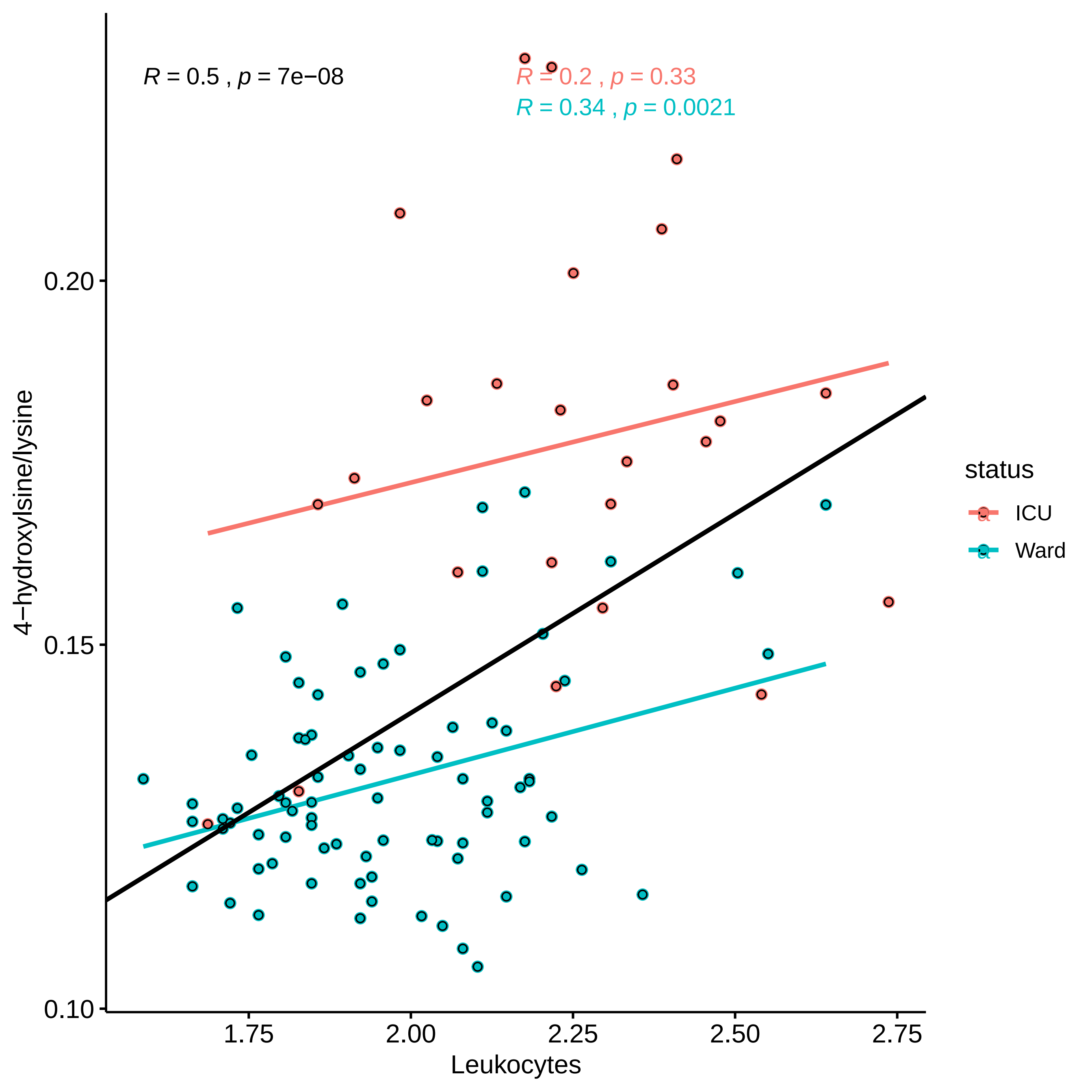

R = 0.5 , p = 7e−08
R = 0.2 , p = 0.33
R = 0.34 , p = 0.0021
4−hydroxylsine/lysine
0.20
0.15
0.10
Leukocytes
1.75
2.00
2.25
2.50
2.75
status
ICU
Ward

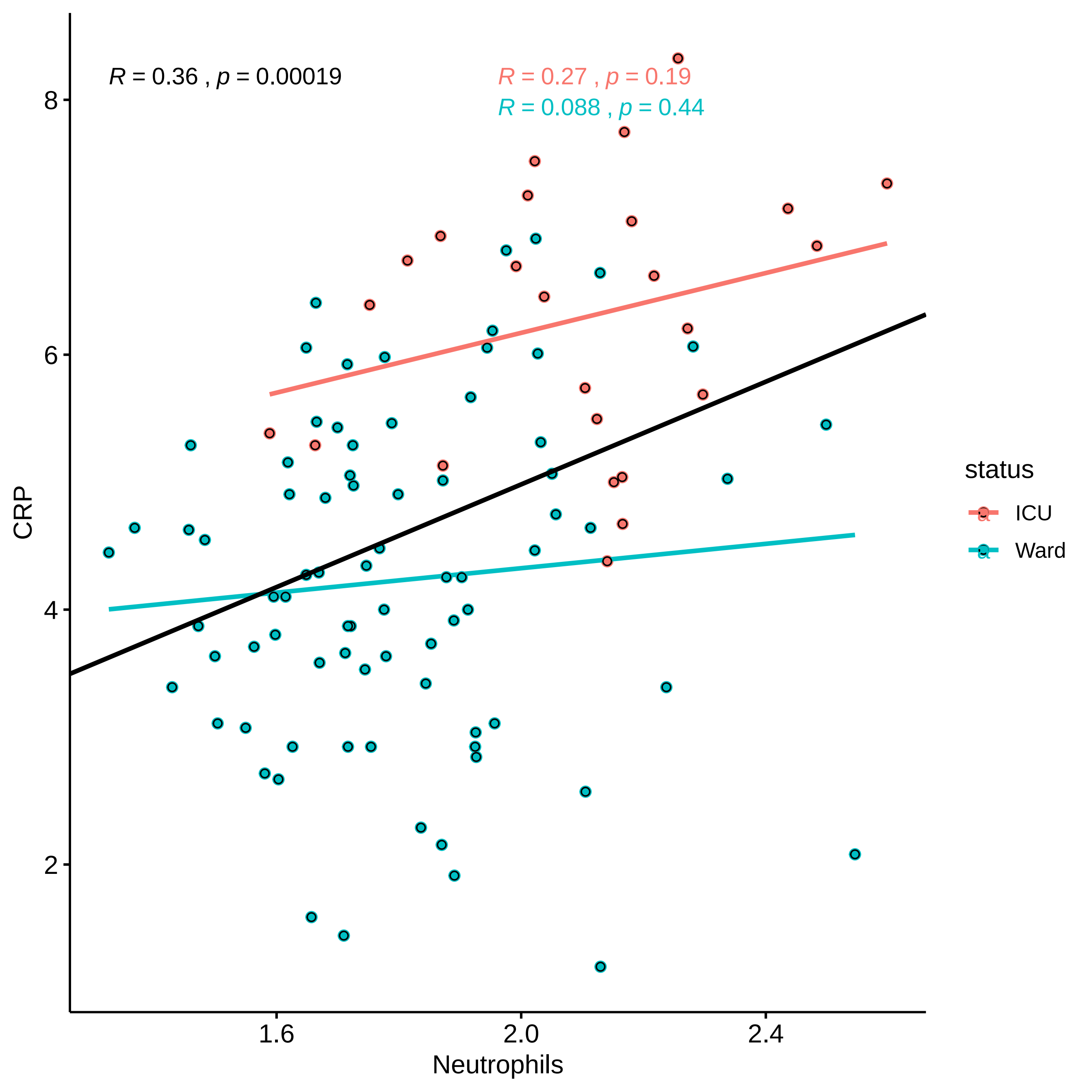

R = 0.36 , p = 0.00019
R = 0.27 , p = 0.19
R = 0.088 , p = 0.44
8
6
4
2
CRP
1.6
2.0
2.4
Neutrophils
status
ICU
Ward

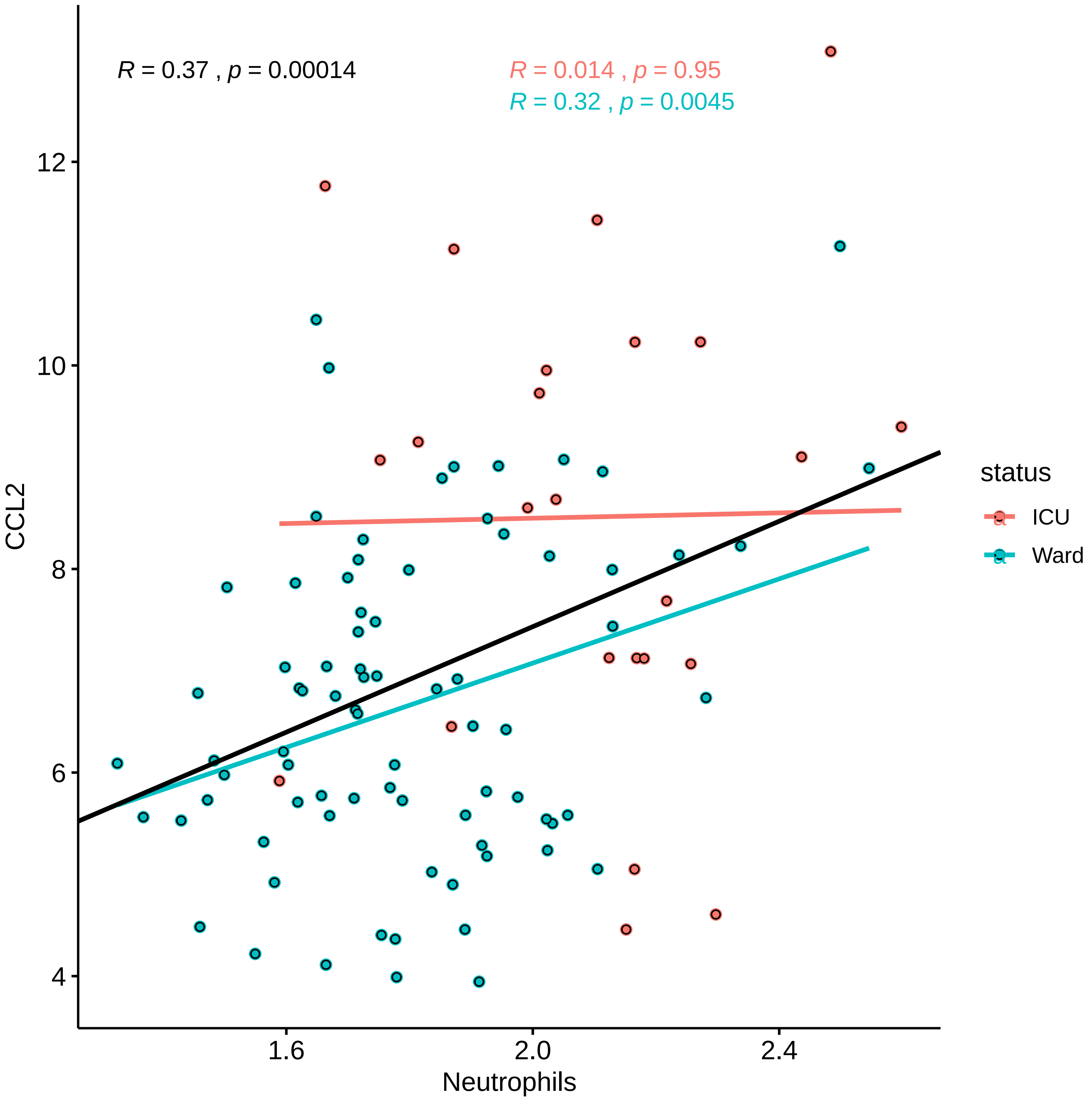

R = 0.37 , p = 0.00014
R = 0.014 , p = 0.95
R = 0.32 , p = 0.0045
CCL2
12
10
8
6
4
1.6
2.0
2.4
Neutrophils
status
ICU
Ward

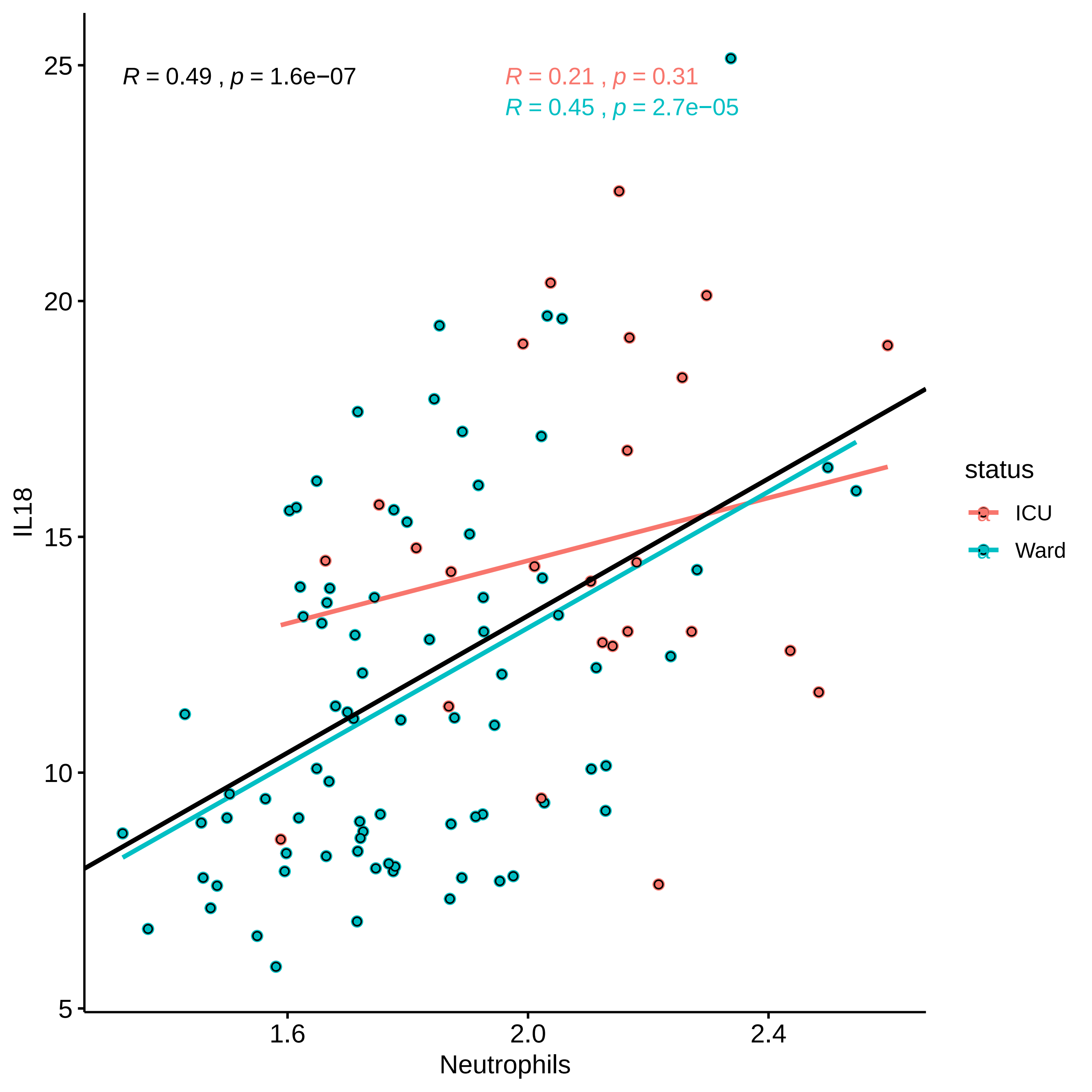

R = 0.49 , p = 1.6e−07
R = 0.21 , p = 0.31
R = 0.45 , p = 2.7e−05
IL18
25
20
15
10
5
1.6
2.0
2.4
Neutrophils
status
ICU
Ward

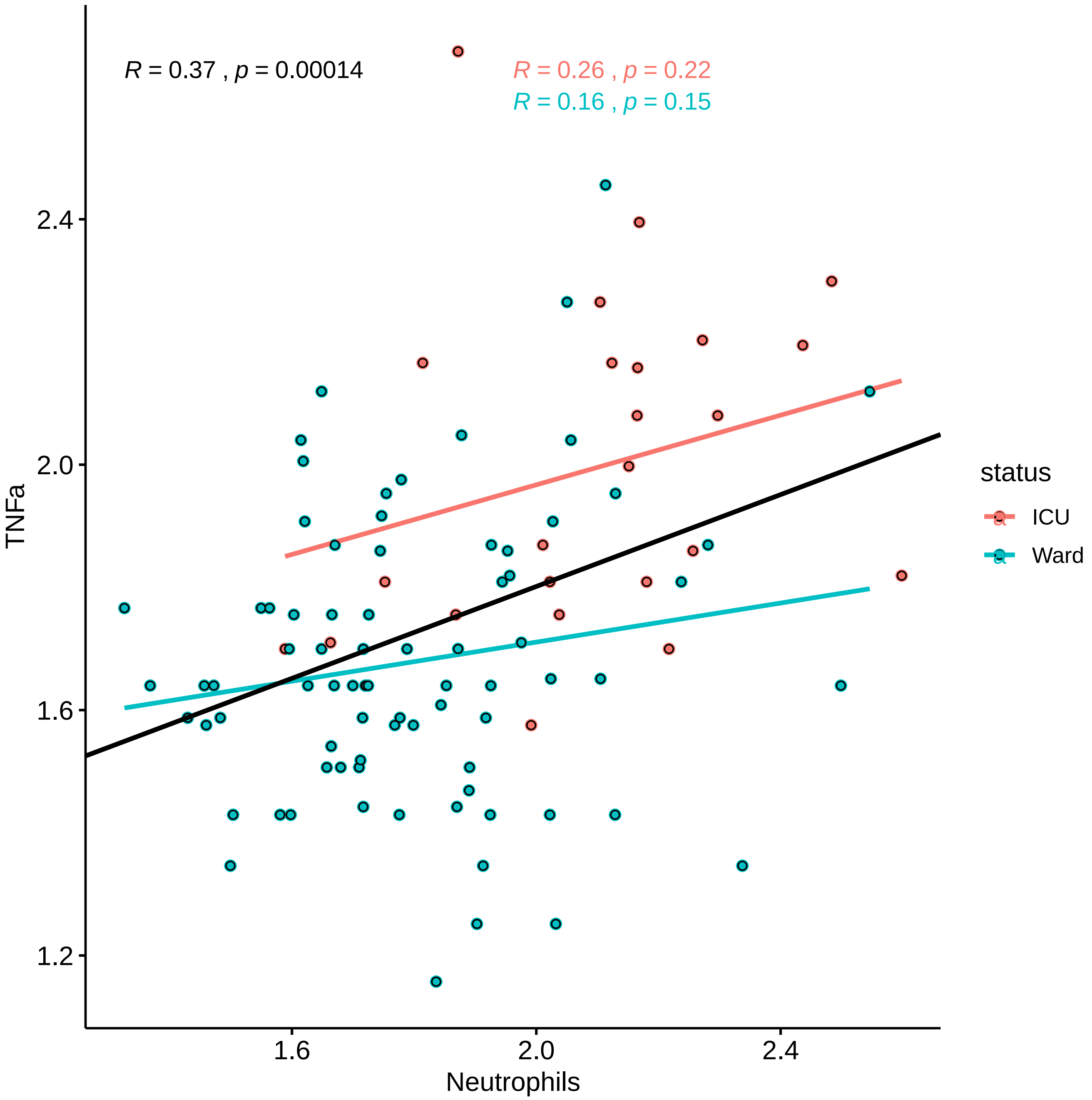
R = 0.37 , p = 0.00014
R = 0.26 , p = 0.22
R = 0.16 , p = 0.15
TNFa
2.4
2.0
1.6
1.2
1.6
2.0
2.4
Neutrophils
status
ICU
Ward

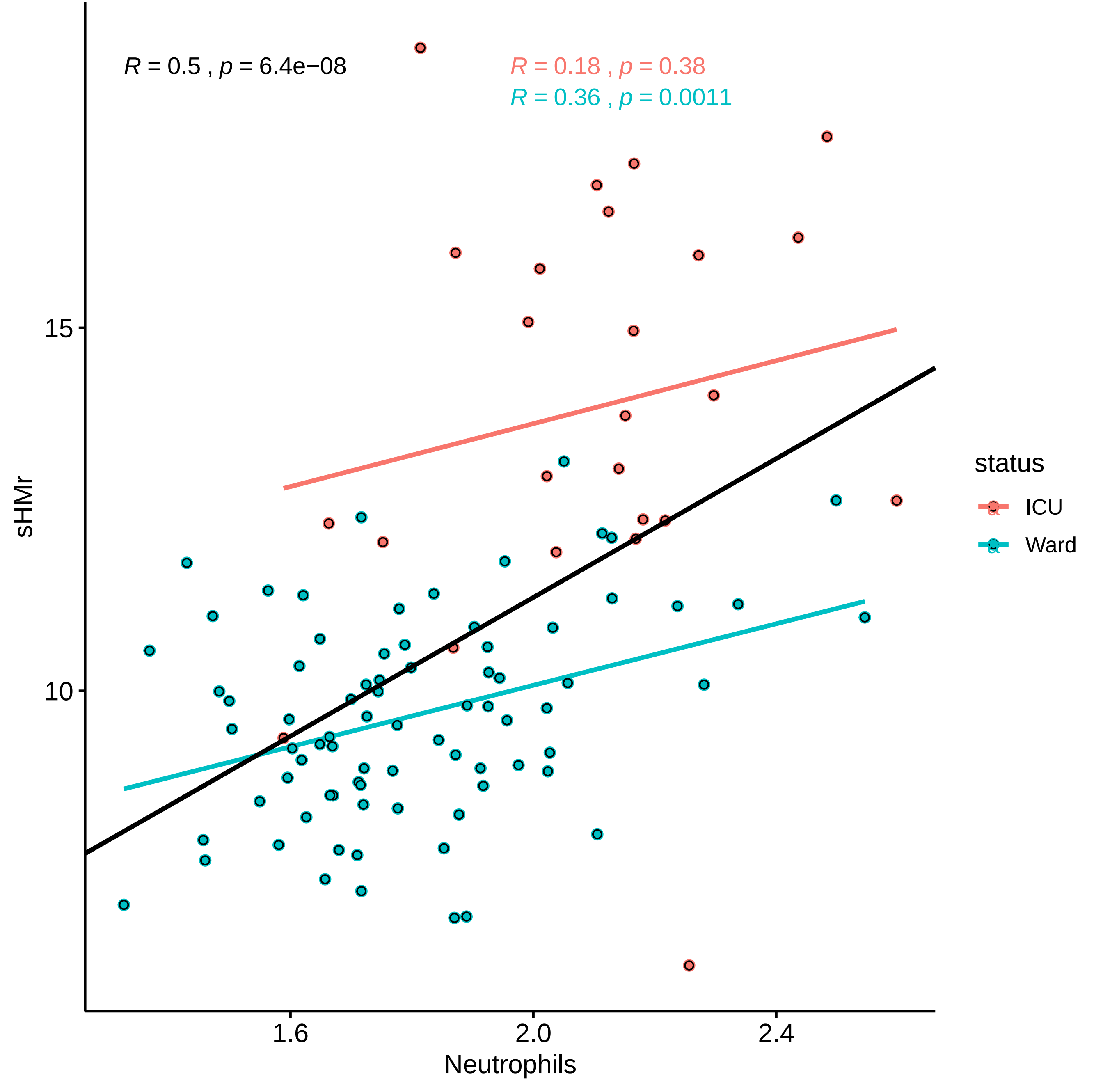

R = 0.5 , p = 6.4e−08
R = 0.18 , p = 0.38
R = 0.36 , p = 0.0011
sHMr
15
10
1.6
2.0
2.4
Neutrophils
status
ICU
Ward

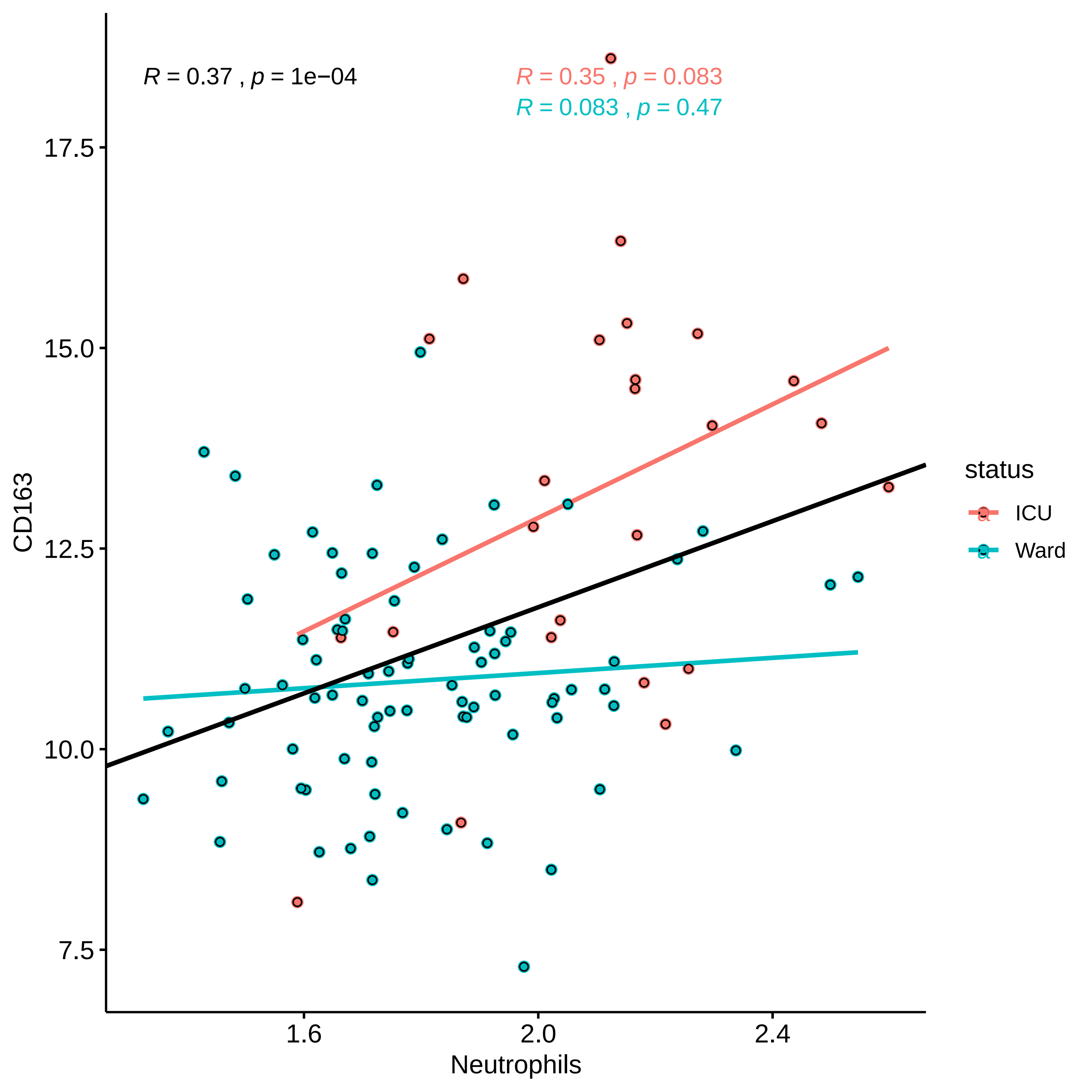

R = 0.37 , p = 1e−04
R = 0.35 , p = 0.083
R = 0.083 , p = 0.47
17.5
15.0
12.5
10.0
7.5
CD163
1.6
2.0
2.4
Neutrophils
status
ICU
Ward

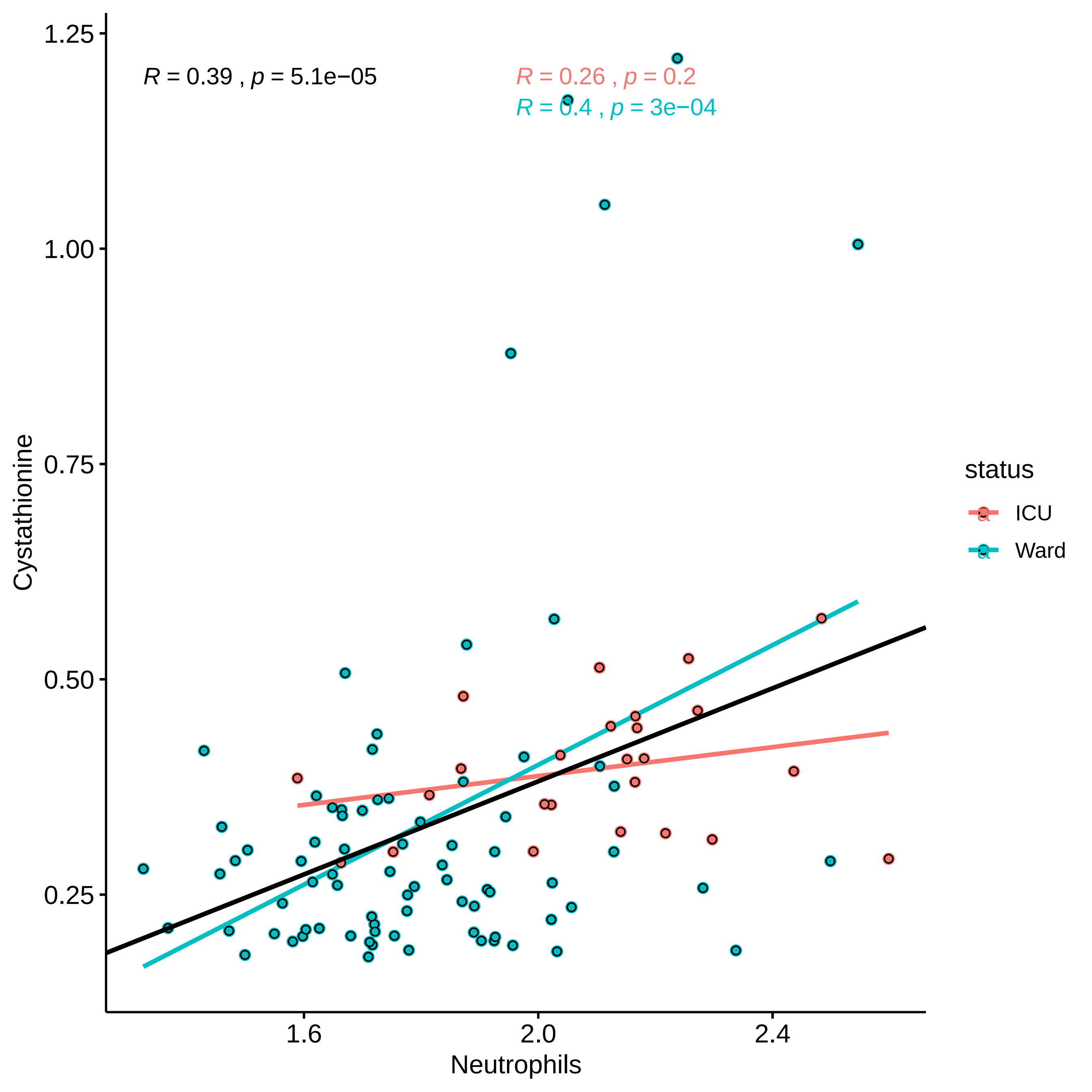

R = 0.39 , p = 5.1e−05
R = 0.26 , p = 0.2
R = 0.4 , p = 3e−04
1.25
1.00
0.75
0.50
0.25
Cystathionine
1.6
2.0
2.4
Neutrophils
status
ICU
Ward

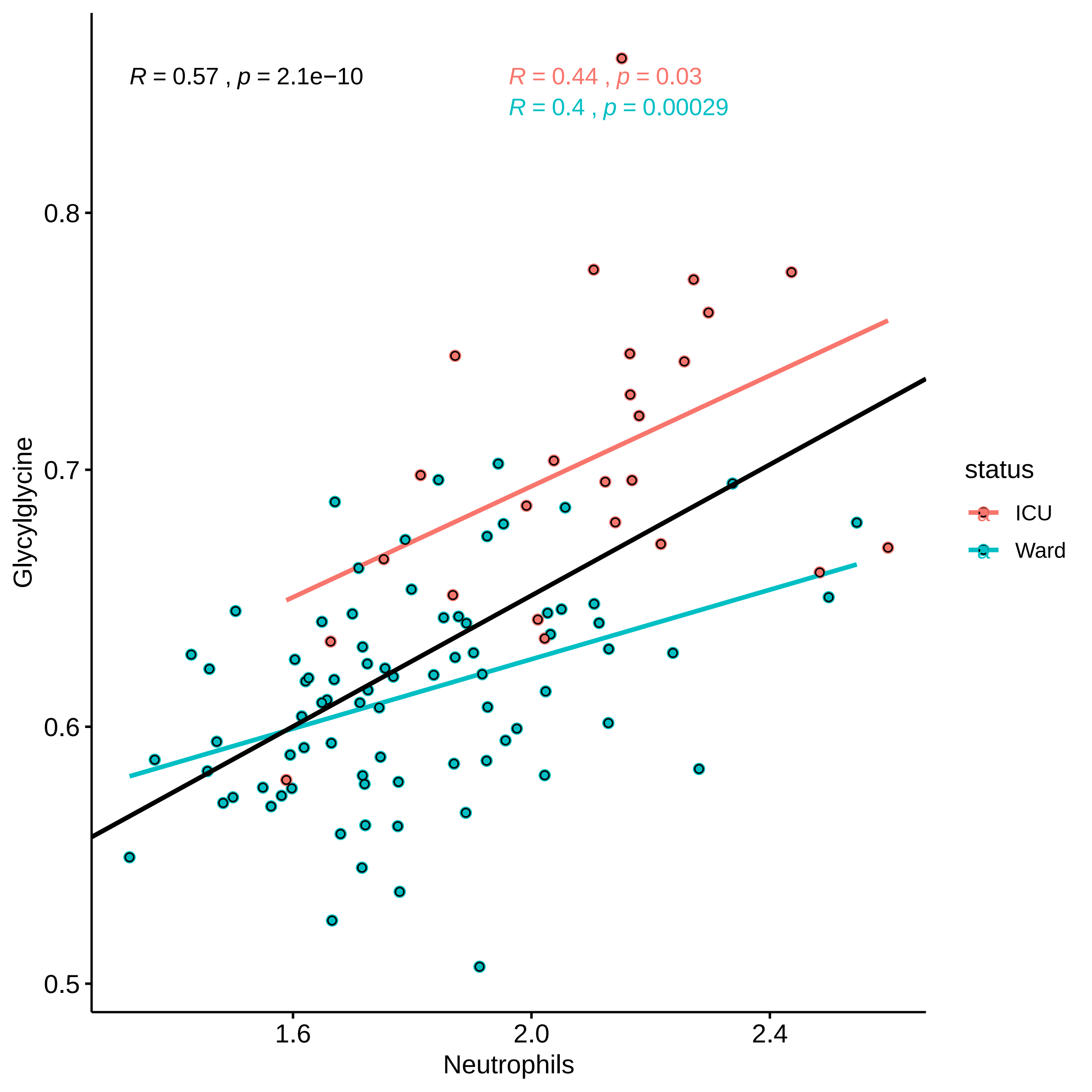

R = 0.57 , p = 2.1e−10
R = 0.44 , p = 0.03
R = 0.4 , p = 0.00029
0.8
0.7
0.6
0.5
Glycylglycine
1.6
2.0
2.4
Neutrophils
status
ICU
Ward

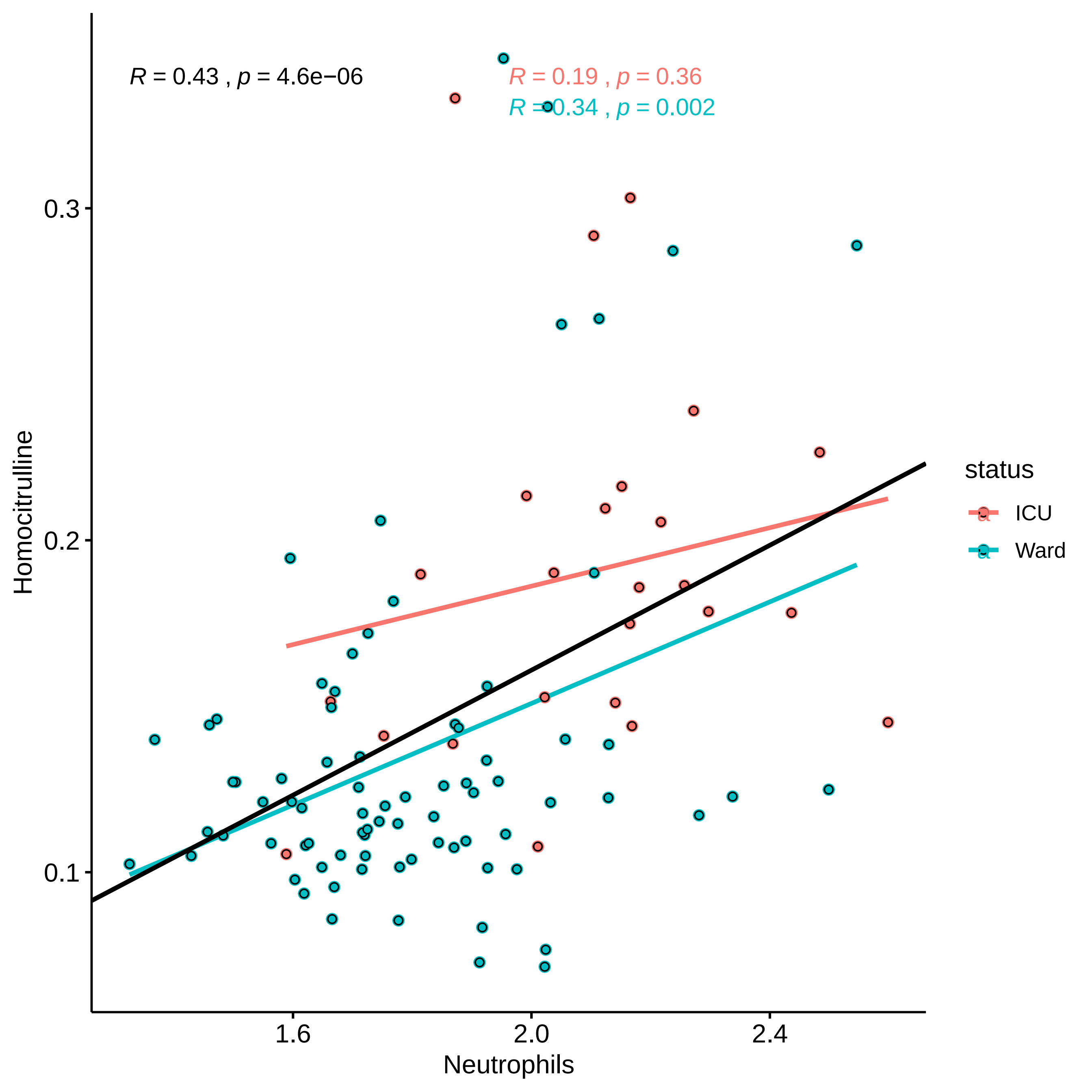

R = 0.43 , p = 4.6e−06
R = 0.19 , p = 0.36
R = 0.34 , p = 0.002
Homocitrulline
0.3
0.2
0.1
1.6
2.0
2.4
Neutrophils
status
ICU
Ward

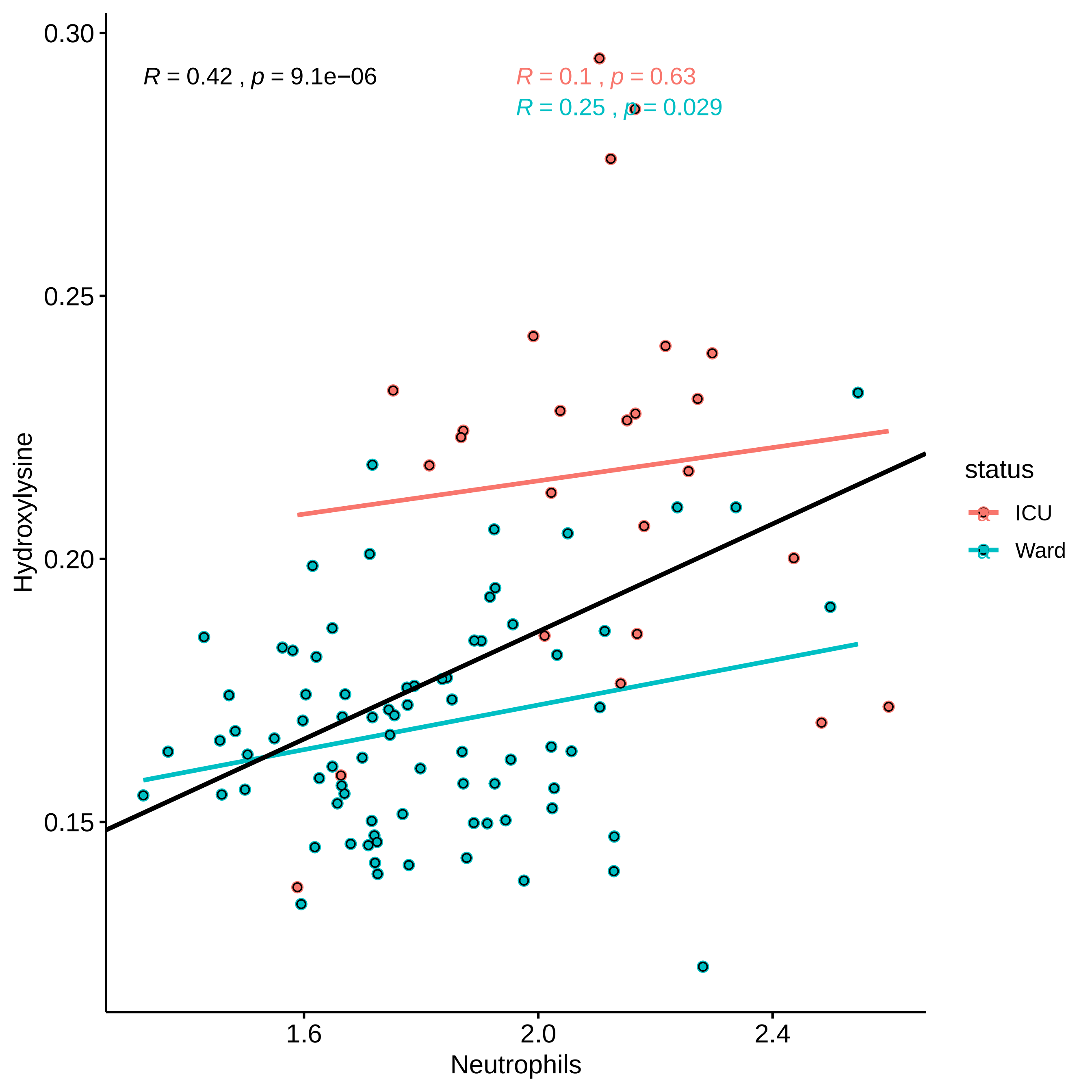

R = 0.42 , p = 9.1e−06
R = 0.1 , p = 0.63
R = 0.25 , p = 0.029
0.30
0.25
0.20
0.15
Hydroxylysine
1.6
2.0
2.4
Neutrophils
status
ICU
Ward

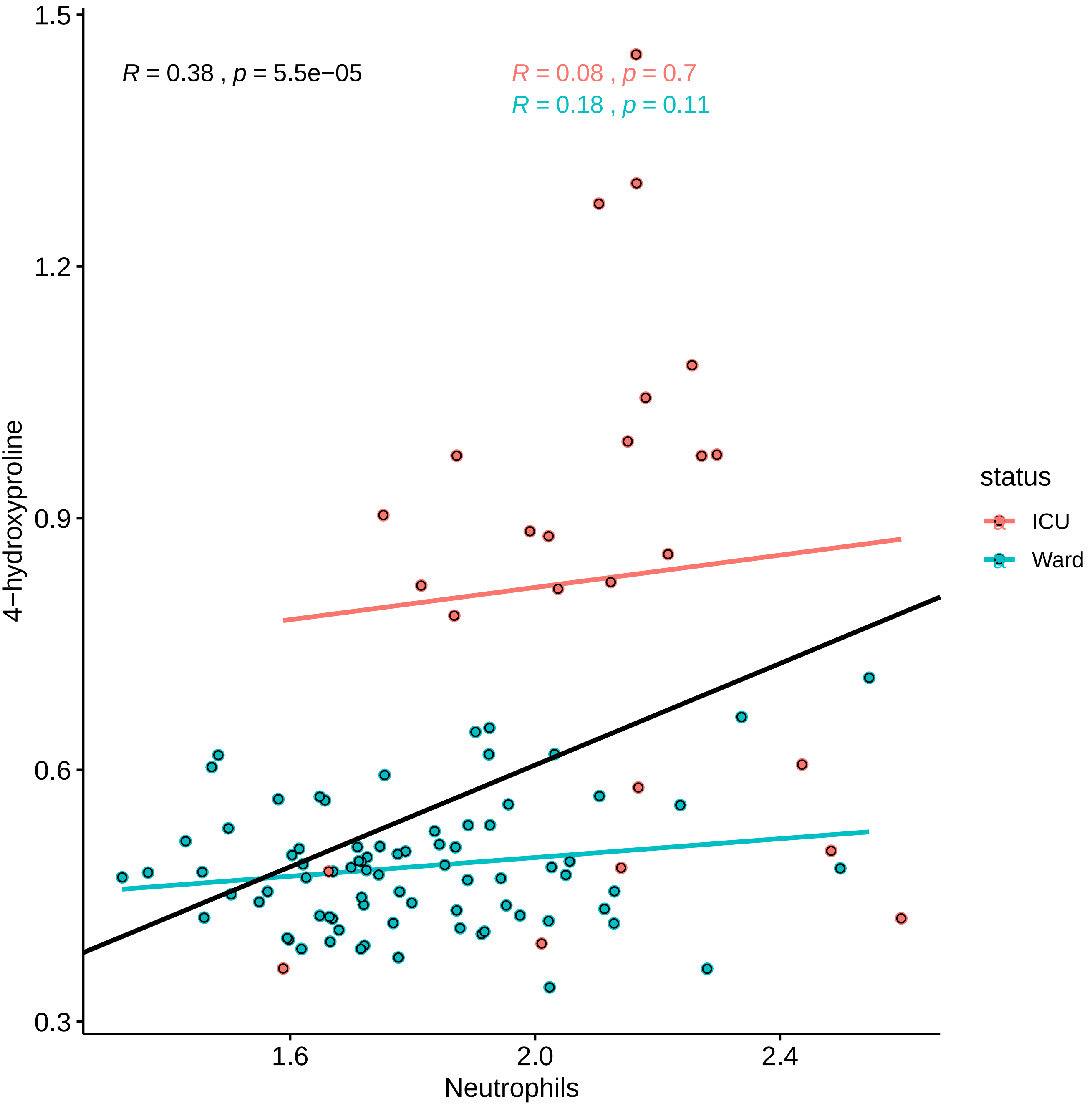

R = 0.38 , p = 5.5e−05
R = 0.08 , p = 0.7
R = 0.18 , p = 0.11
4−hydroxyproline
1.5
1.2
0.9
0.6
0.3
1.6
2.0
2.4
Neutrophils
status
ICU
Ward

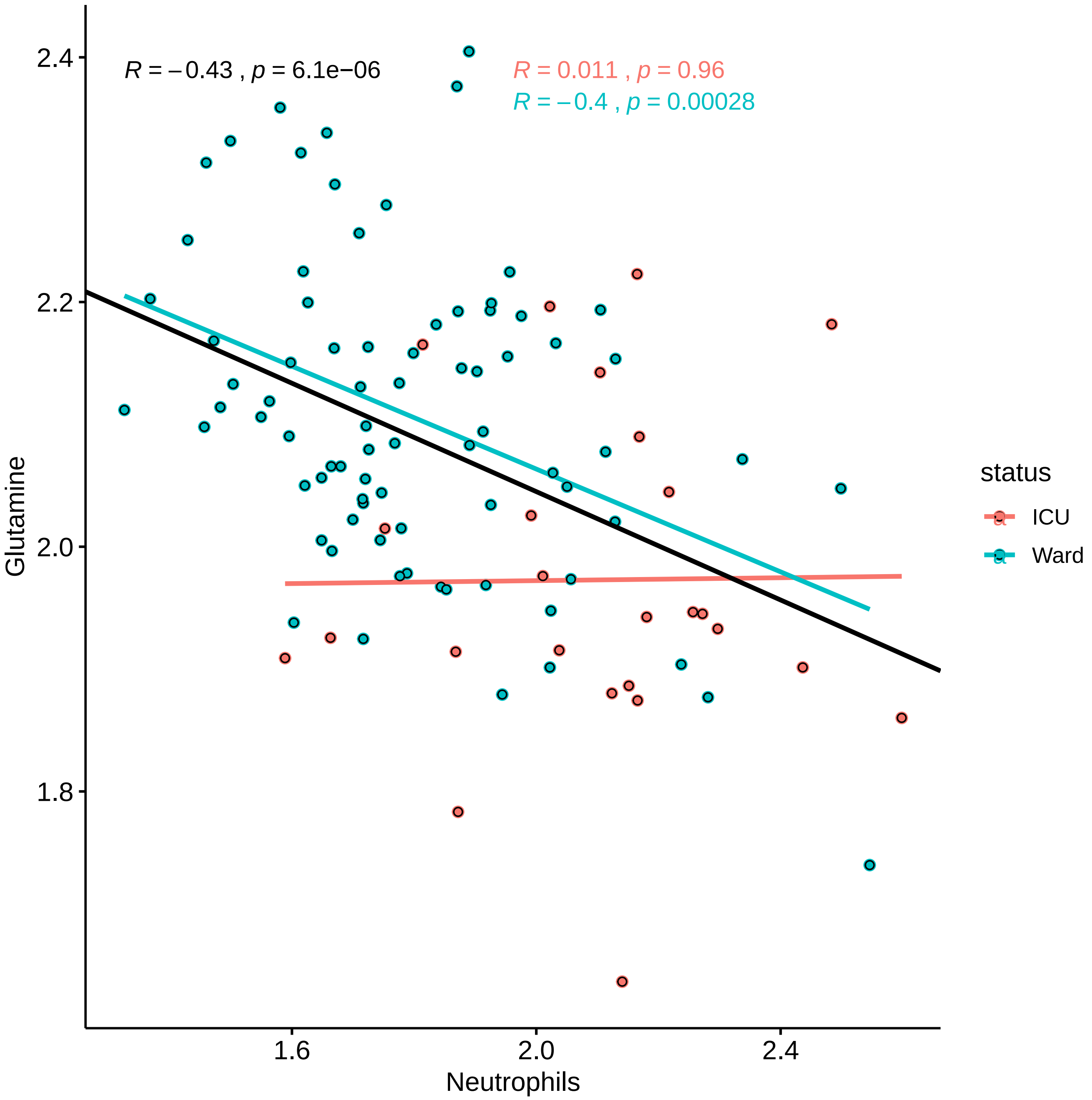

R = −0.43 , p = 6.1e−06
R = 0.011 , p = 0.96
R = −0.4 , p = 0.00028
2.4
2.2
2.0
1.8
Glutamine
1.6
2.0
2.4
Neutrophils
status
ICU
Ward

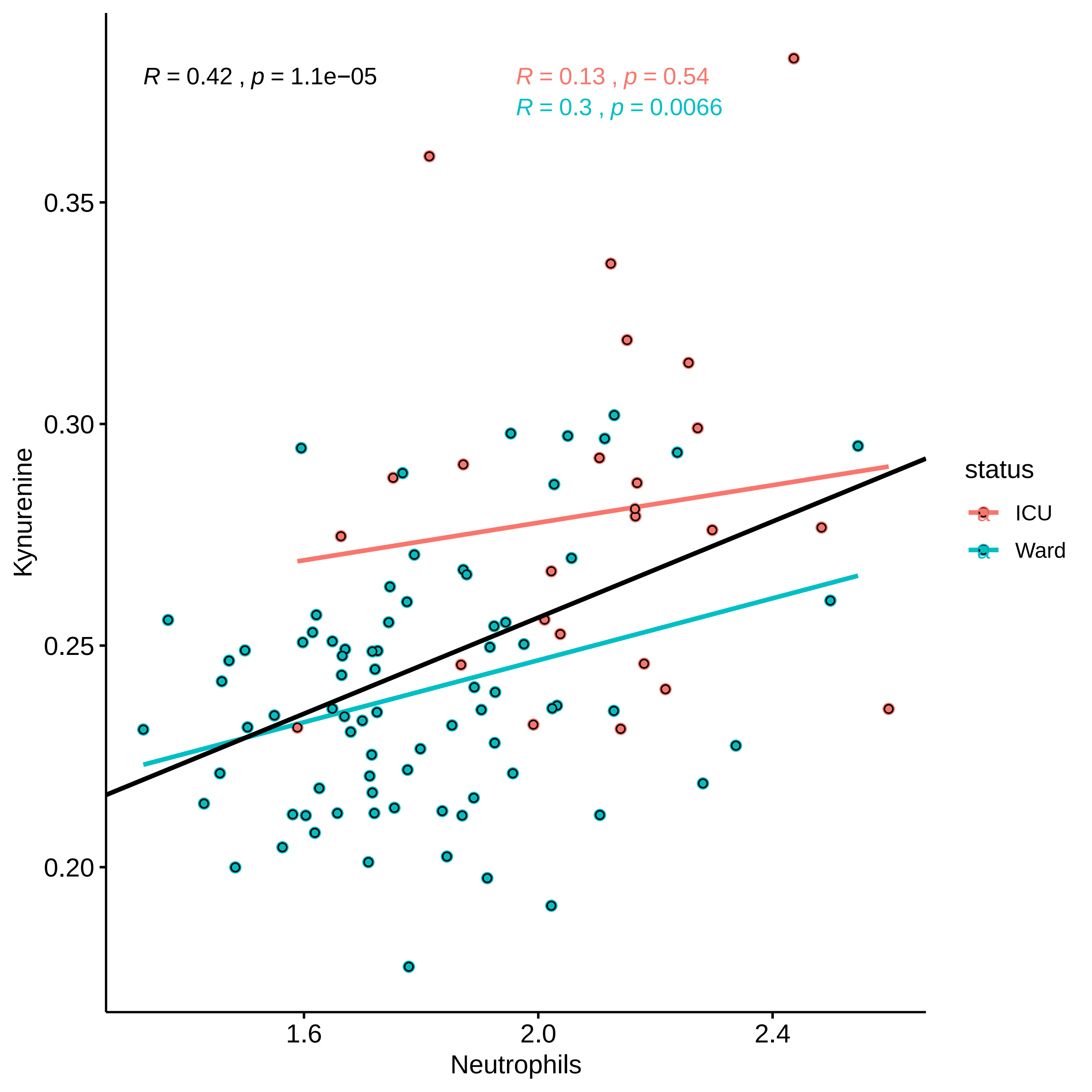

R = 0.42 , p = 1.1e−05
R = 0.13 , p = 0.54
R = 0.3 , p = 0.0066
Kynurenine
0.35
0.30
0.25
0.20
1.6
2.0
2.4
Neutrophils
status
ICU
Ward

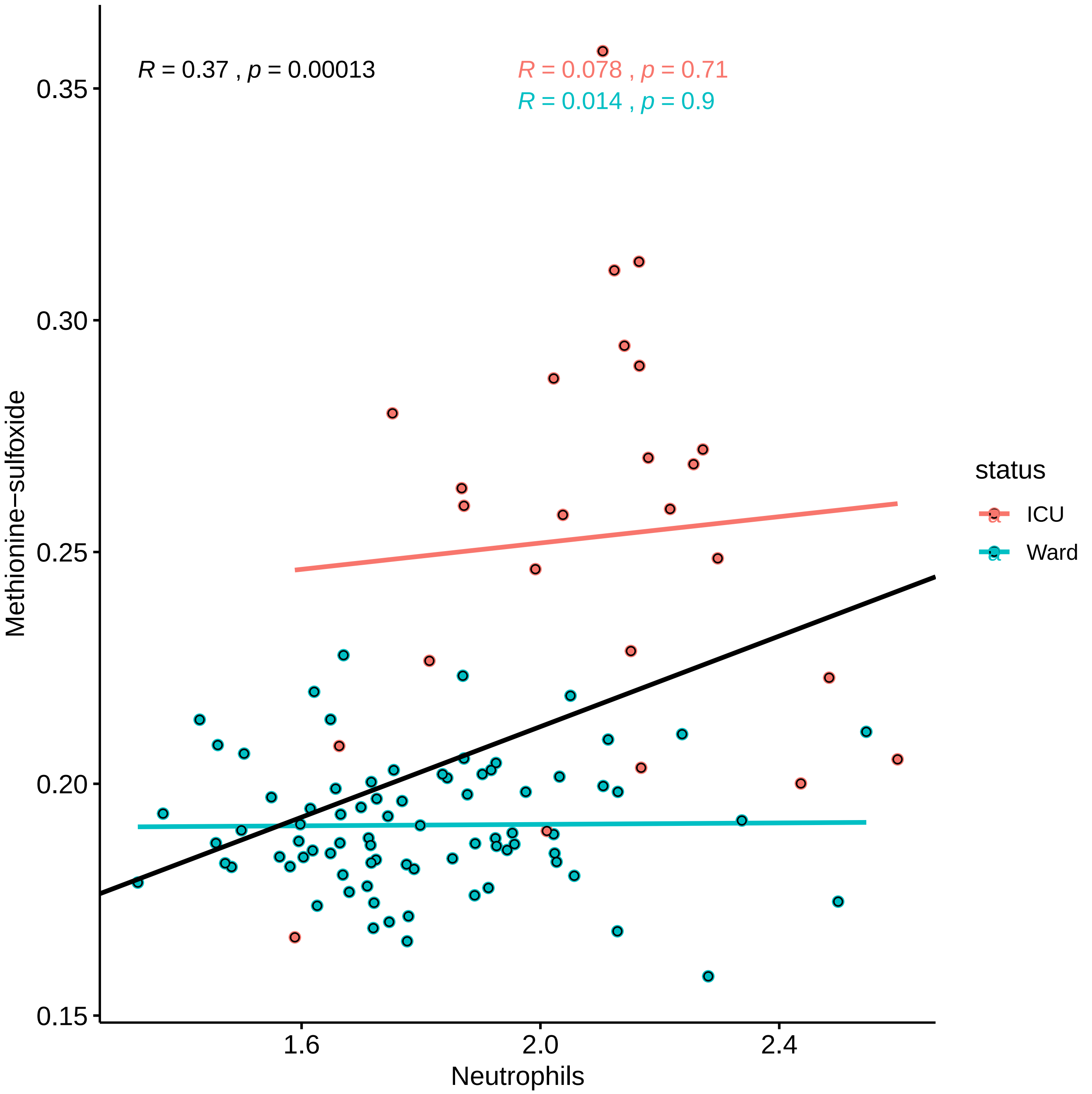

R = 0.37 , p = 0.00013
R = 0.078 , p = 0.71
R = 0.014 , p = 0.9
0.35
0.30
0.25
0.20
0.15
Methionine−sulfoxide
1.6
2.0
2.4
Neutrophils
status
ICU
Ward

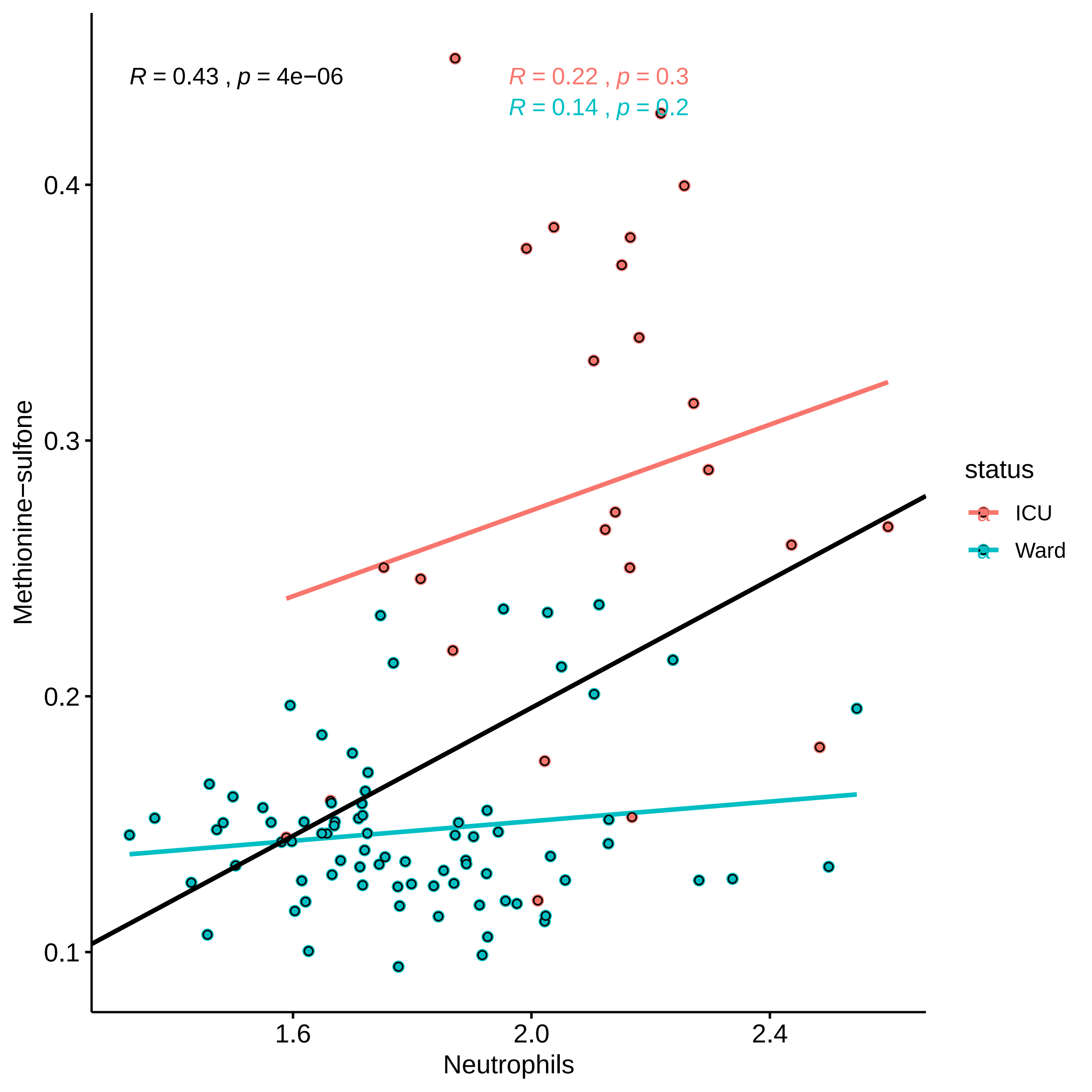

R = 0.43 , p = 4e−06
R = 0.22 , p = 0.3
R = 0.14 , p = 0.2
Methionine−sulfone
0.4
0.3
0.2
0.1
1.6
2.0
2.4
Neutrophils
status
ICU
Ward

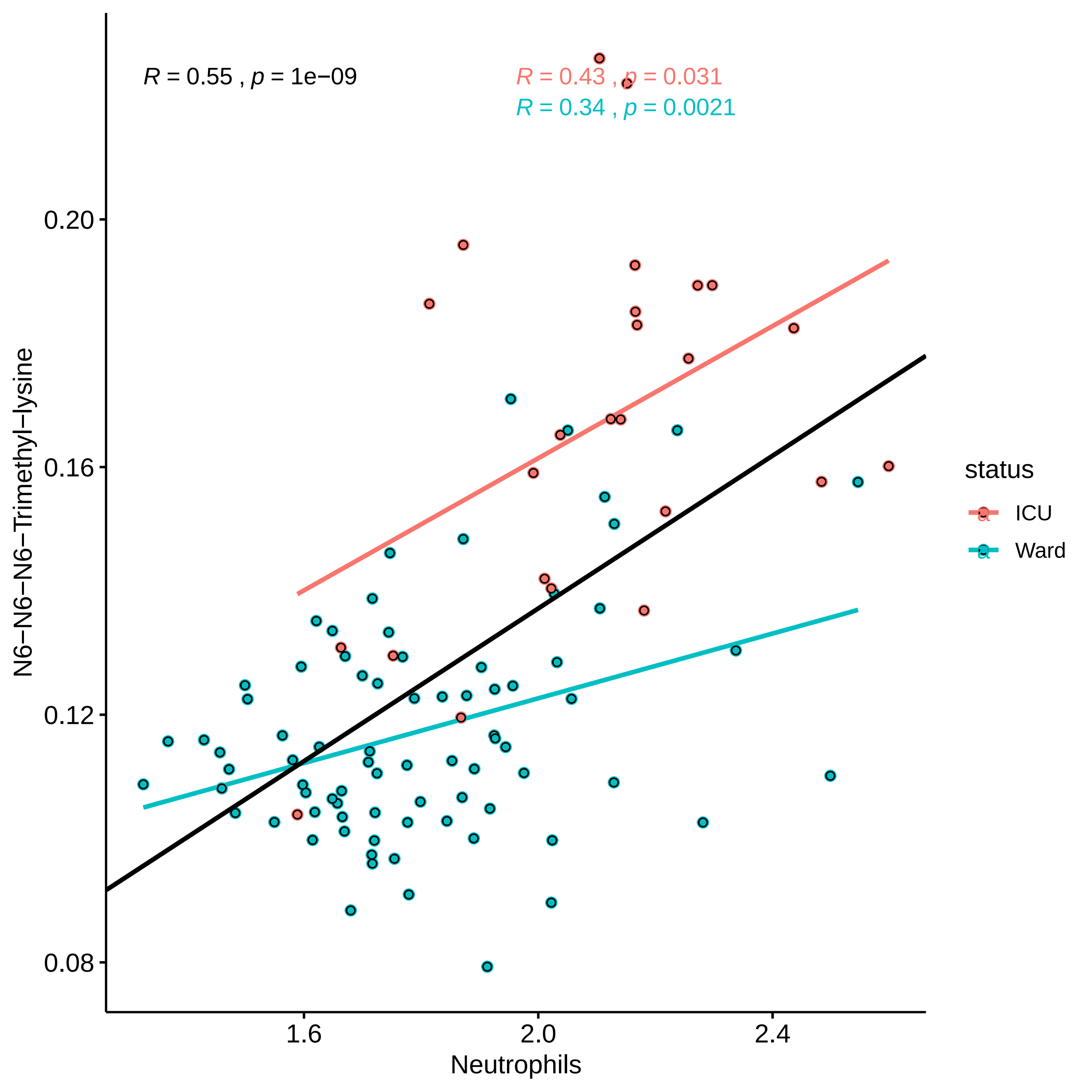

R = 0.55 , p = 1e−09
R = 0.43 , p = 0.031
R = 0.34 , p = 0.0021
N6−N6−N6−Trimethyl−lysine
0.20
0.16
0.12
0.08
1.6
2.0
2.4
Neutrophils
status
ICU
Ward

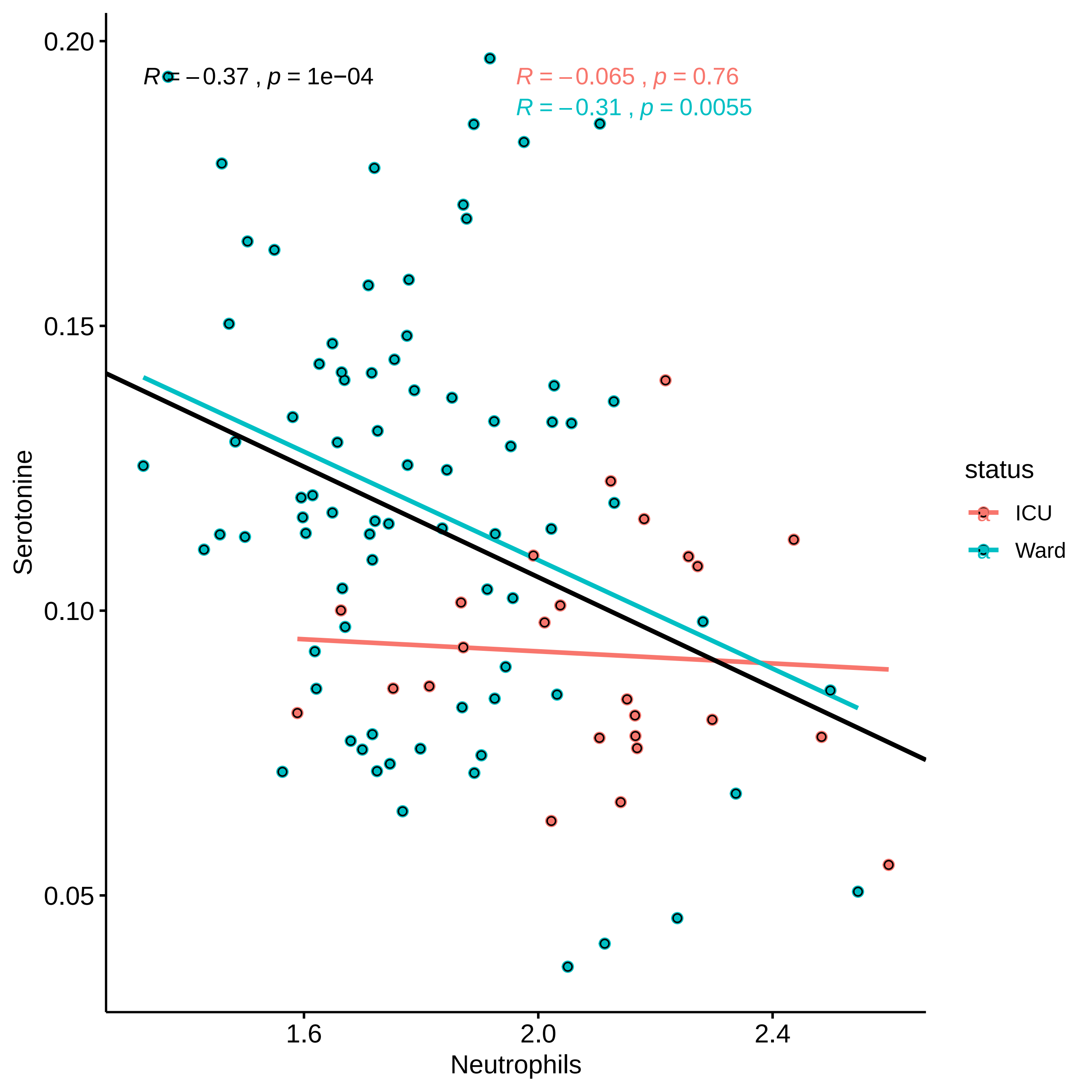

R = −0.37 , p = 1e−04
R = −0.065 , p = 0.76
R = −0.31 , p = 0.0055
0.20
0.15
0.10
0.05
Serotonine
1.6
2.0
2.4
Neutrophils
status
ICU
Ward

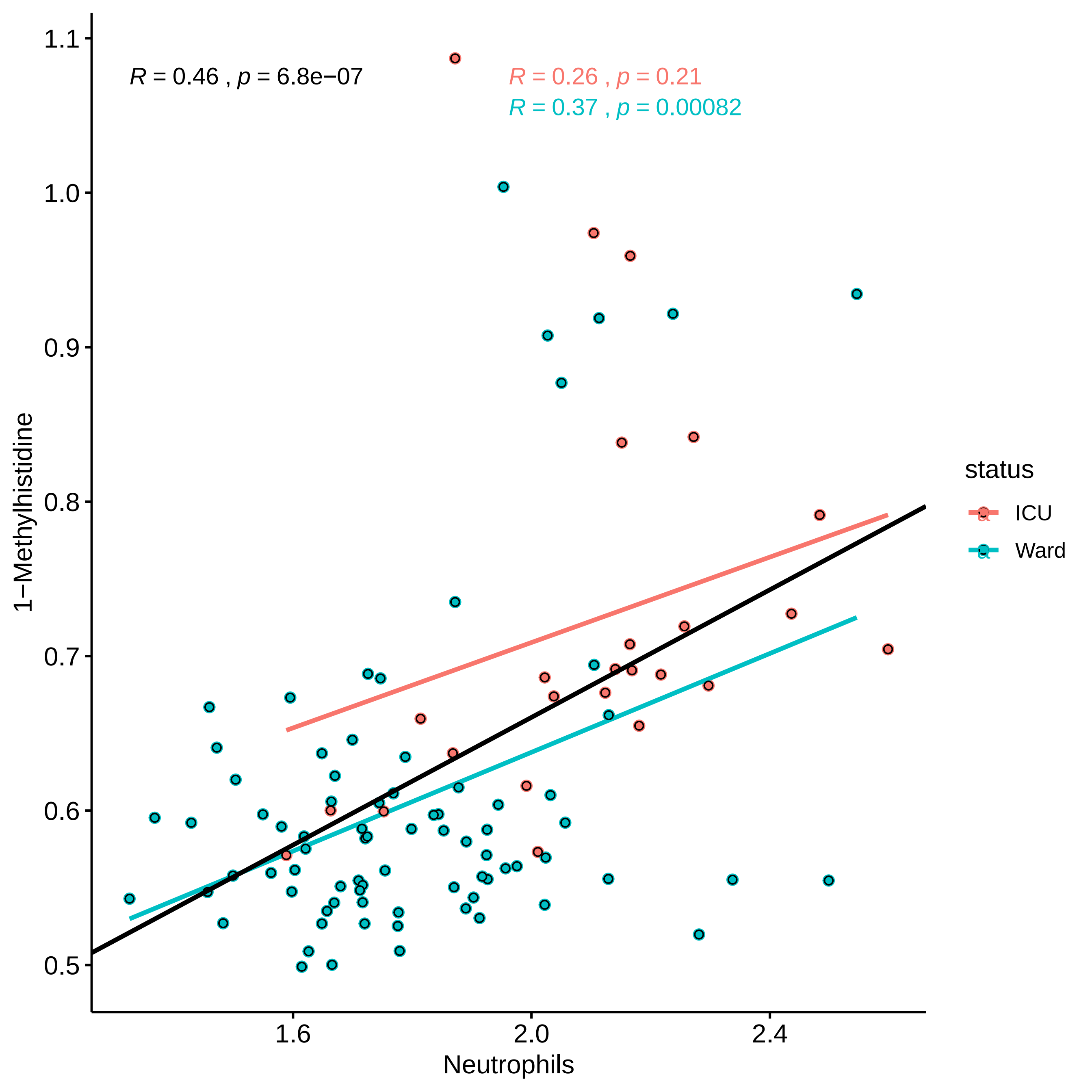
R = 0.46 , p = 6.8e−07
R = 0.26 , p = 0.21
R = 0.37 , p = 0.00082
1−Methylhistidine
1.1
1.0
0.9
0.8
0.7
0.6
0.5
1.6
2.0
2.4
Neutrophils
status
ICU
Ward

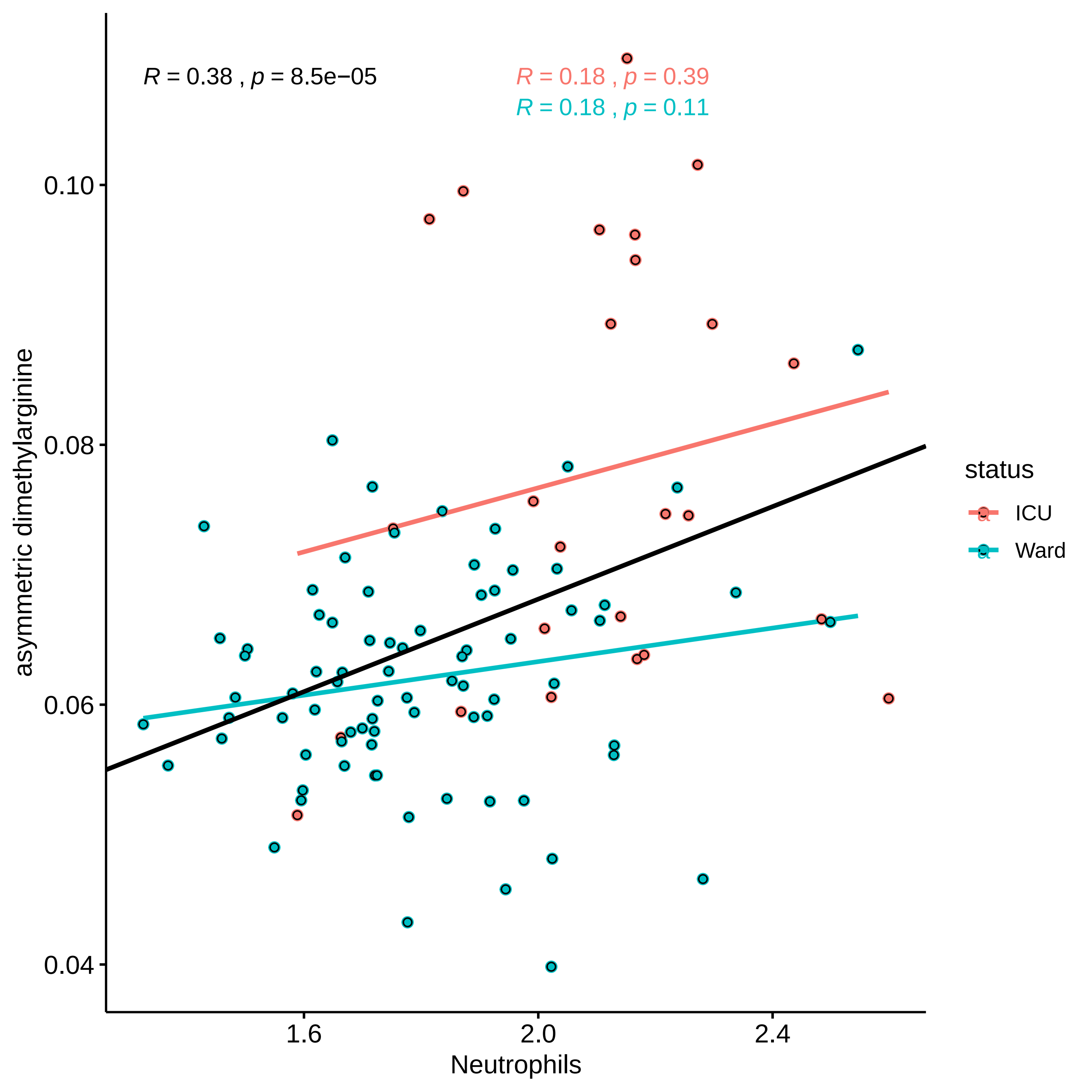

R = 0.38 , p = 8.5e−05
R = 0.18 , p = 0.39
R = 0.18 , p = 0.11
asymmetric dimethylarginine
0.10
0.08
0.06
0.04
1.6
2.0
2.4
Neutrophils
status
ICU
Ward

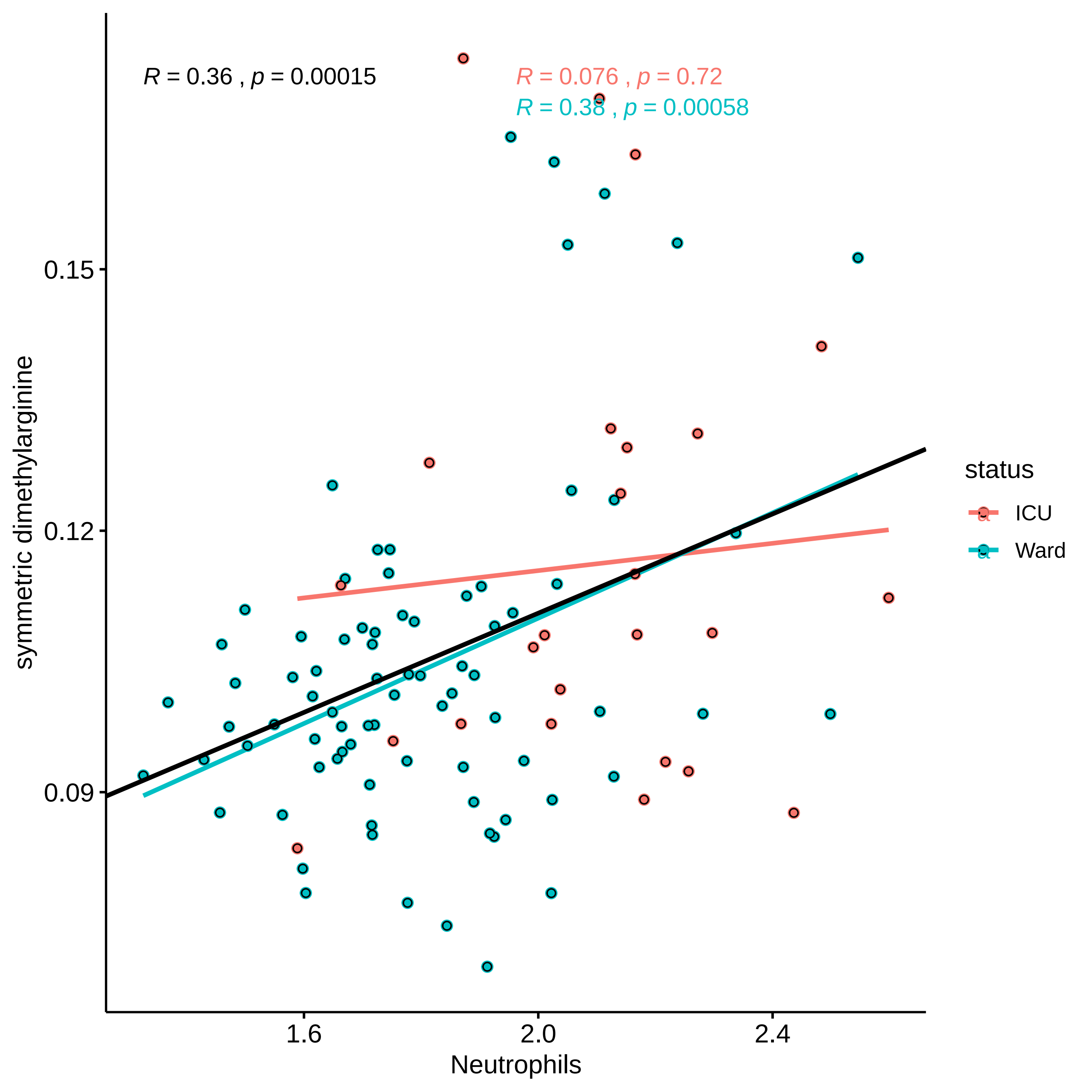
R = 0.36 , p = 0.00015
R = 0.076 , p = 0.72
R = 0.38 , p = 0.00058
symmetric dimethylarginine
0.15
0.12
0.09
1.6
2.0
2.4
Neutrophils
status
ICU
Ward

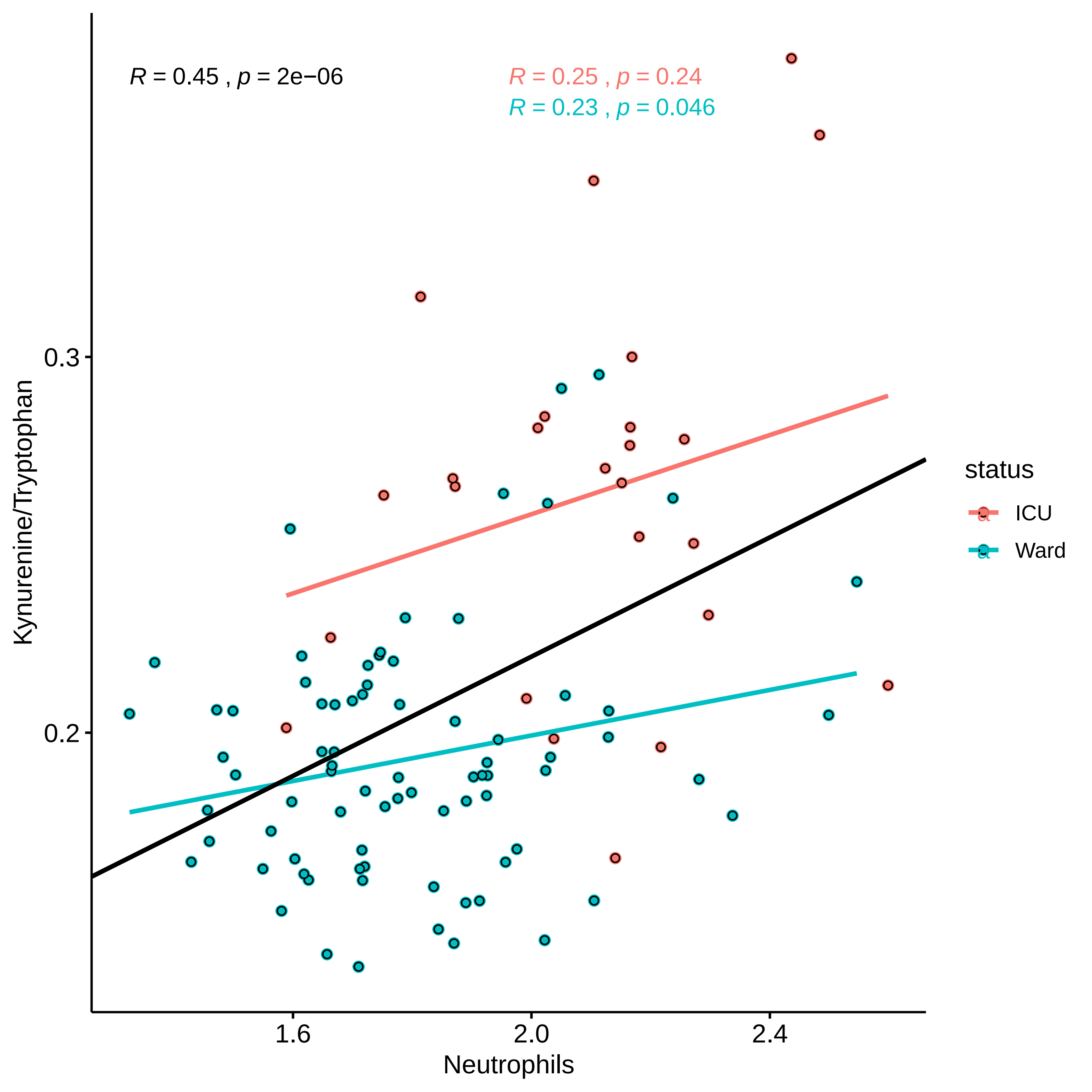
R = 0.45 , p = 2e−06
R = 0.25 , p = 0.24
R = 0.23 , p = 0.046
0.3
0.2
Kynurenine/Tryptophan
1.6
2.0
2.4
Neutrophils
status
ICU
Ward

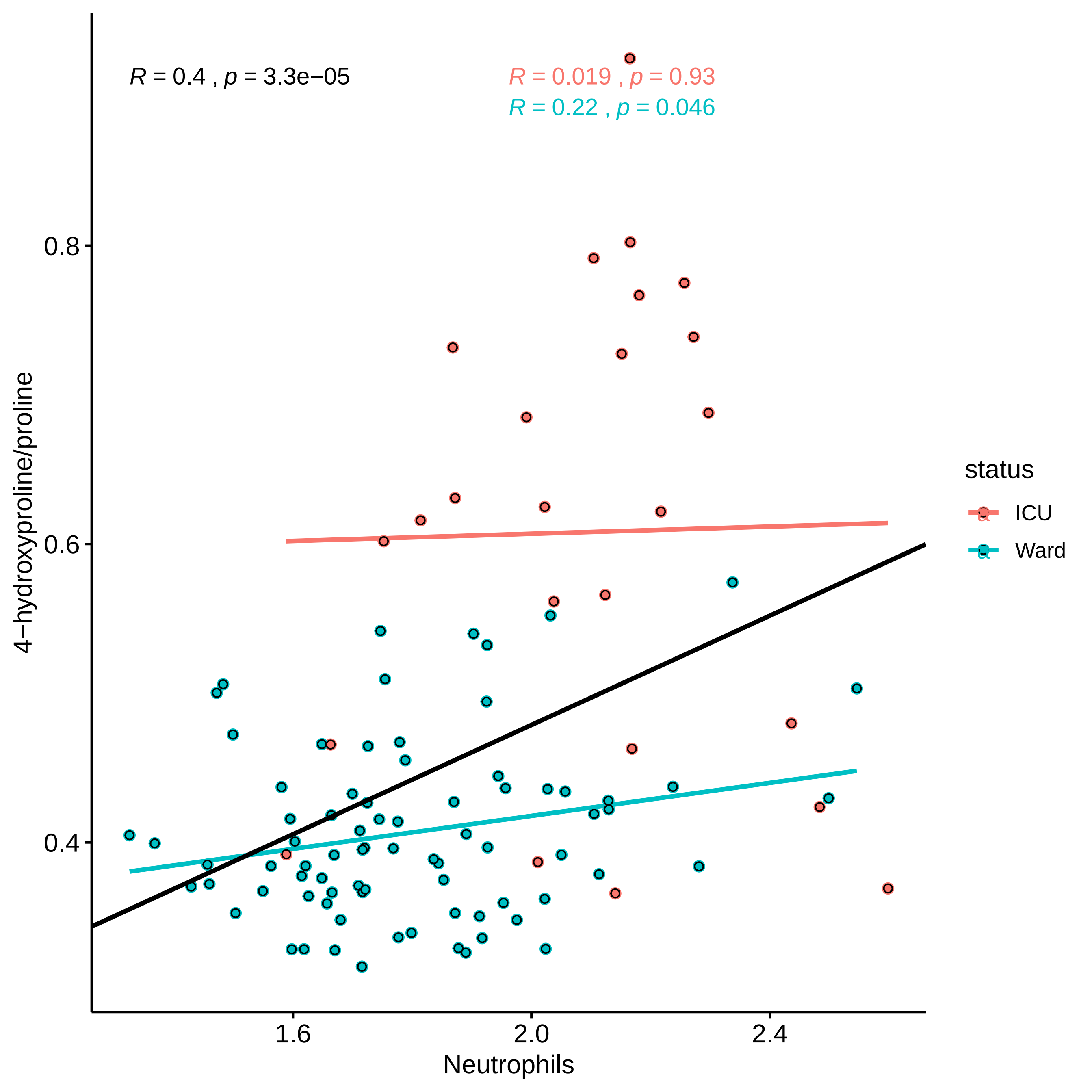

R = 0.4 , p = 3.3e−05
R = 0.019 , p = 0.93
R = 0.22 , p = 0.046
0.8
0.6
0.4
4−hydroxyproline/proline
1.6
2.0
2.4
Neutrophils
status
ICU
Ward

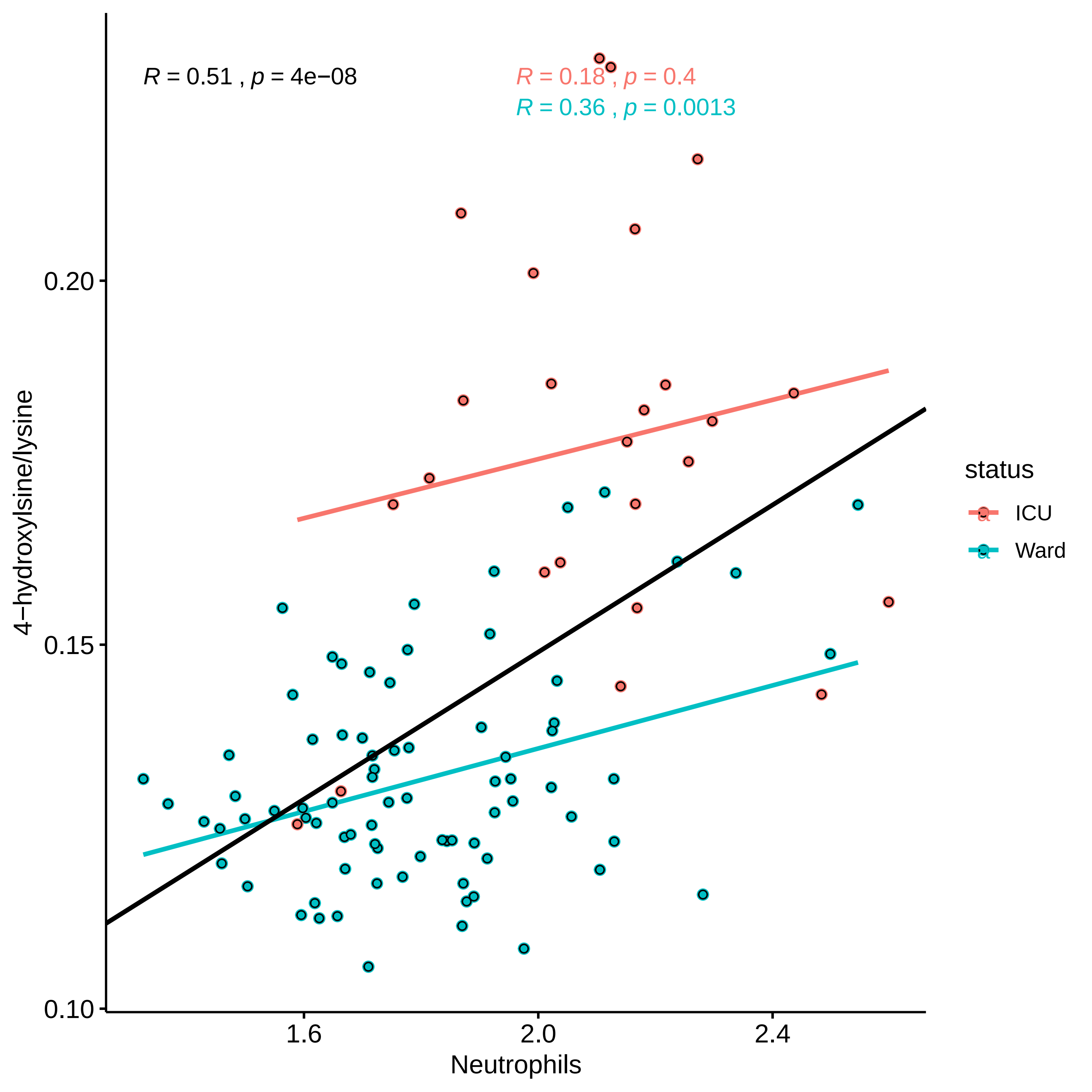

R = 0.51 , p = 4e−08
R = 0.18 , p = 0.4
R = 0.36 , p = 0.0013
4−hydroxylysine/lysine
0.20
0.15
0.10
1.6
2.0
2.4
Neutrophils
status
ICU
Ward

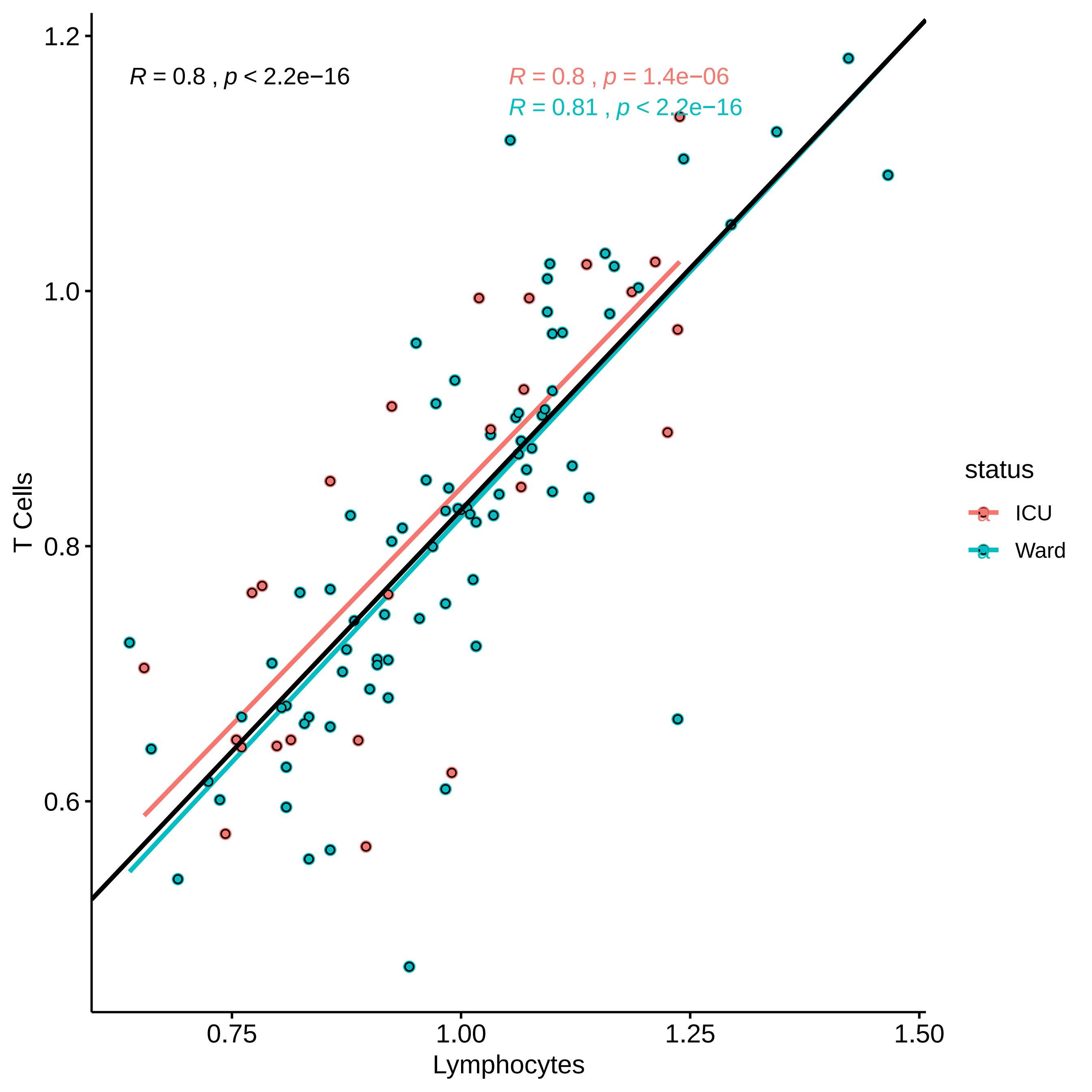

R = 0.8 , p < 2.2e−16
R = 0.8 , p = 1.4e−06
R = 0.81 , p < 2.2e−16
1.2
1.0
0.8
0.6
T Cells
0.75
1.00
1.25
1.50
Lymphocytes
status
ICU
Ward

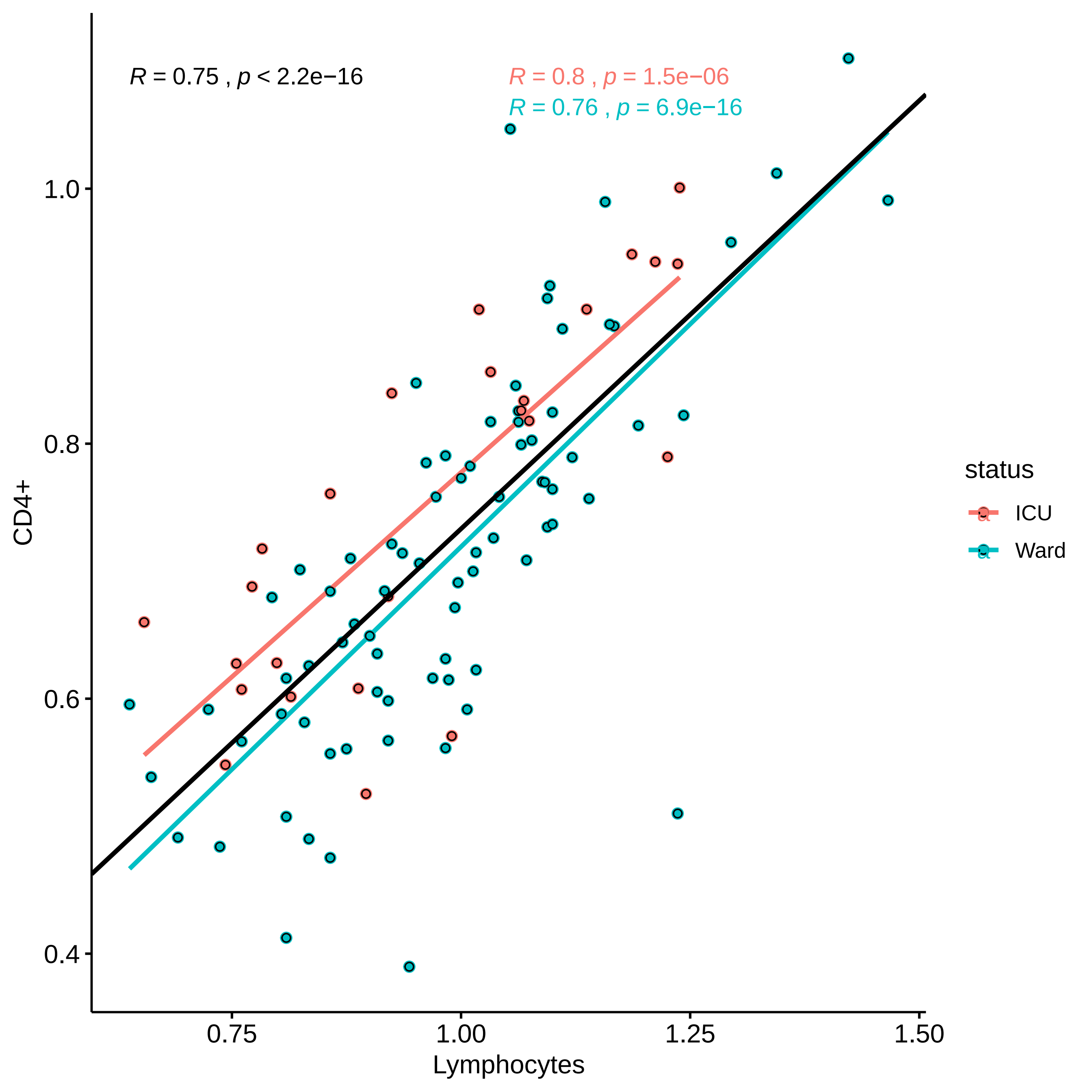

R = 0.75 , p < 2.2e−16
R = 0.8 , p = 1.5e−06
R = 0.76 , p = 6.9e−16
CD4+
1.0
0.8
0.6
0.4
0.75
1.00
1.25
1.50
Lymphocytes
status
ICU
Ward

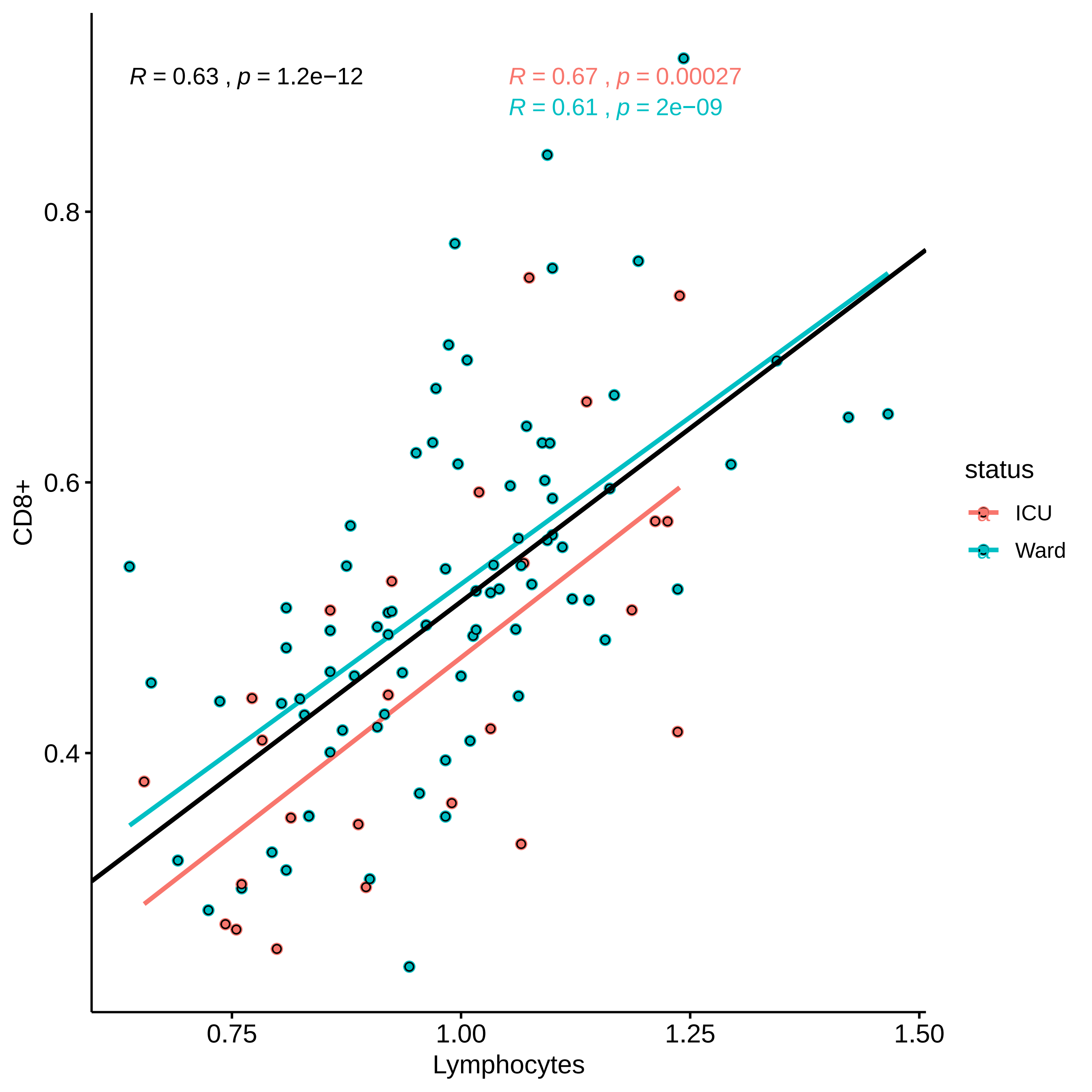

R = 0.63 , p = 1.2e−12
R = 0.67 , p = 0.00027
R = 0.61 , p = 2e−09
CD8+
0.8
0.6
0.4
0.75
1.00
1.25
1.50
Lymphocytes
status
ICU
Ward

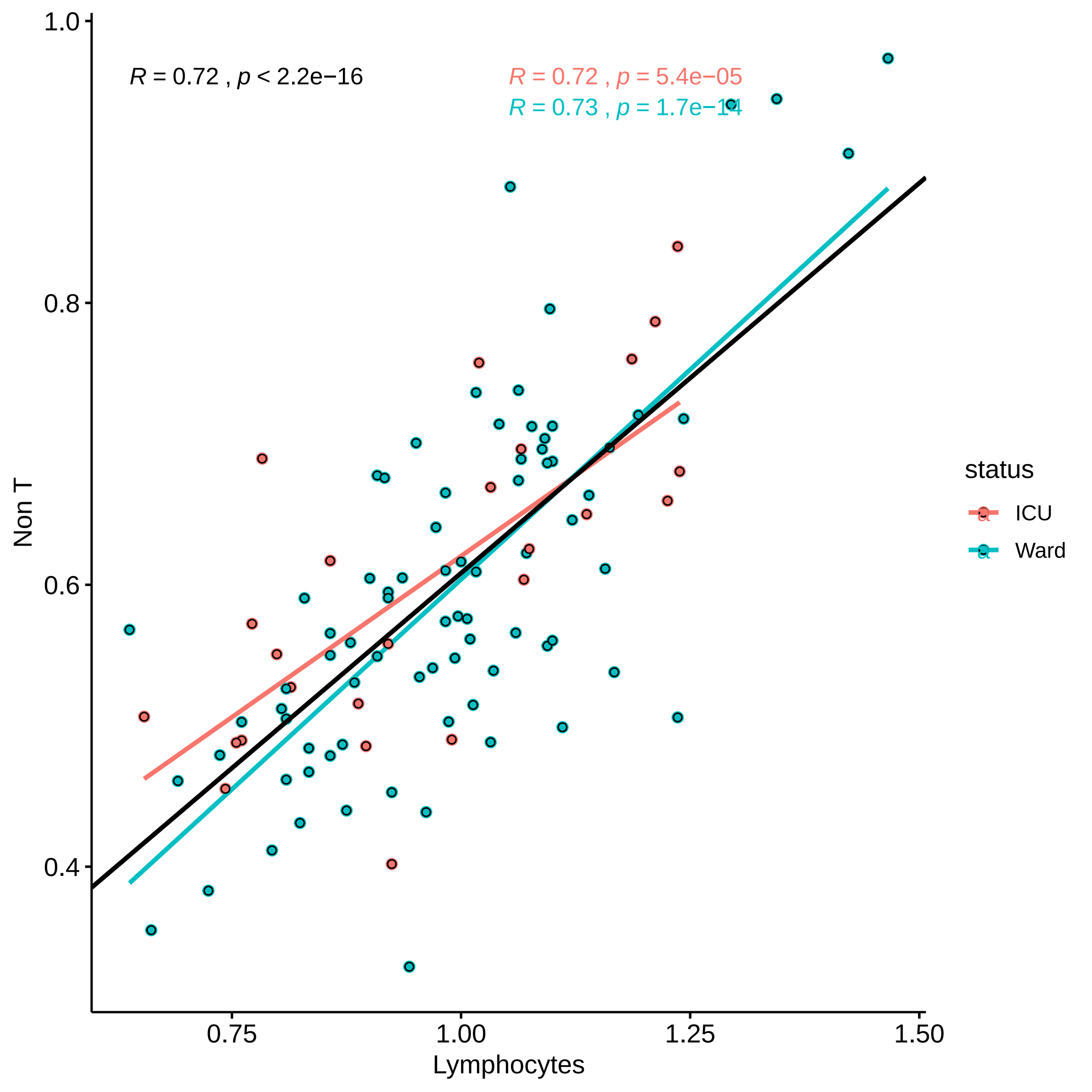

R = 0.72 , p < 2.2e−16
R = 0.72 , p = 5.4e−05
R = 0.73 , p = 1.7e−14
Non T
1.0
0.8
0.6
0.4
0.75
1.00
1.25
1.50
Lymphocytes
status
ICU
Ward

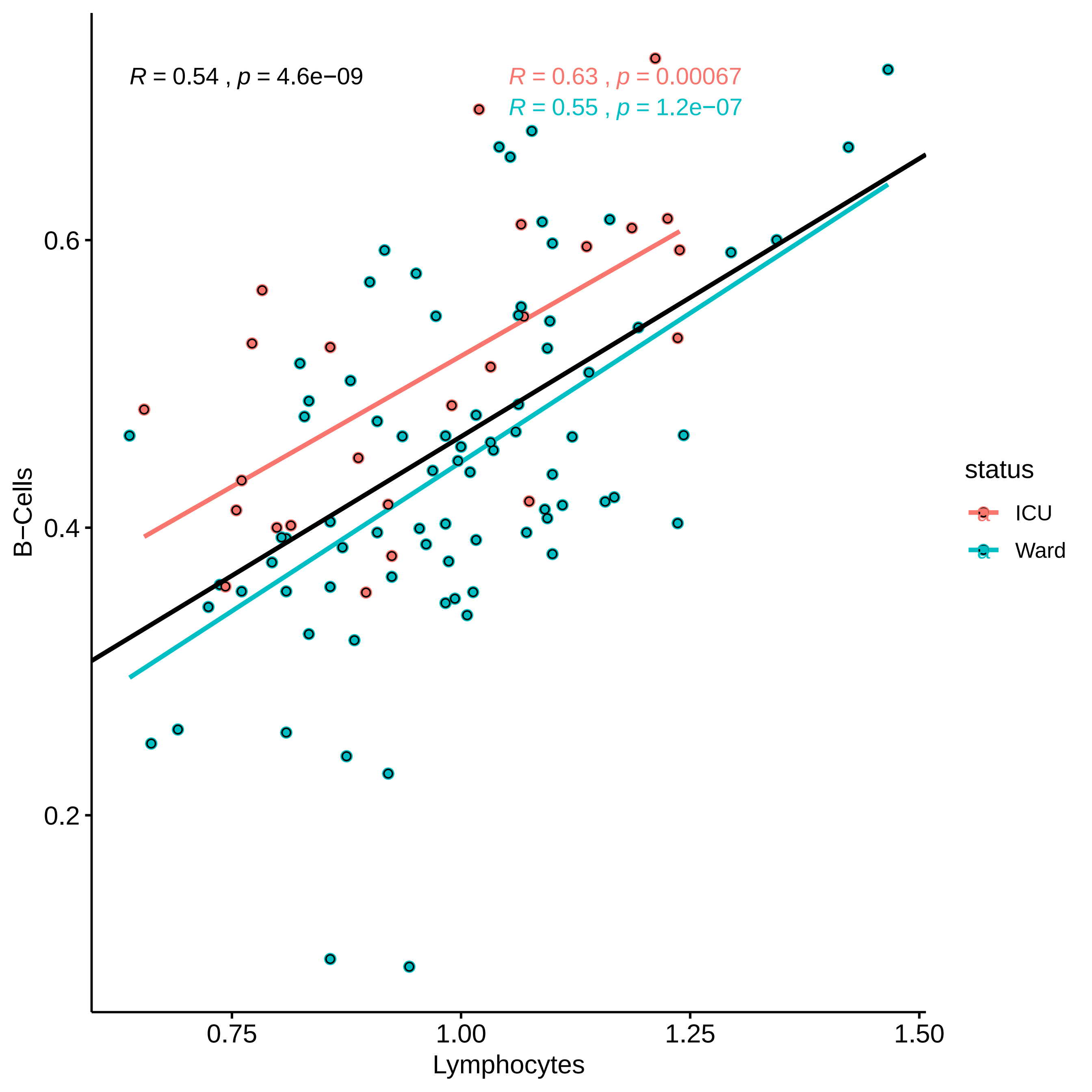

R = 0.54 , p = 4.6e−09
R = 0.63 , p = 0.00067
R = 0.55 , p = 1.2e−07
0.6
0.4
0.2
0.75
1.00
1.25
1.50
B−Cells
Lymphocytes
status
ICU
Ward

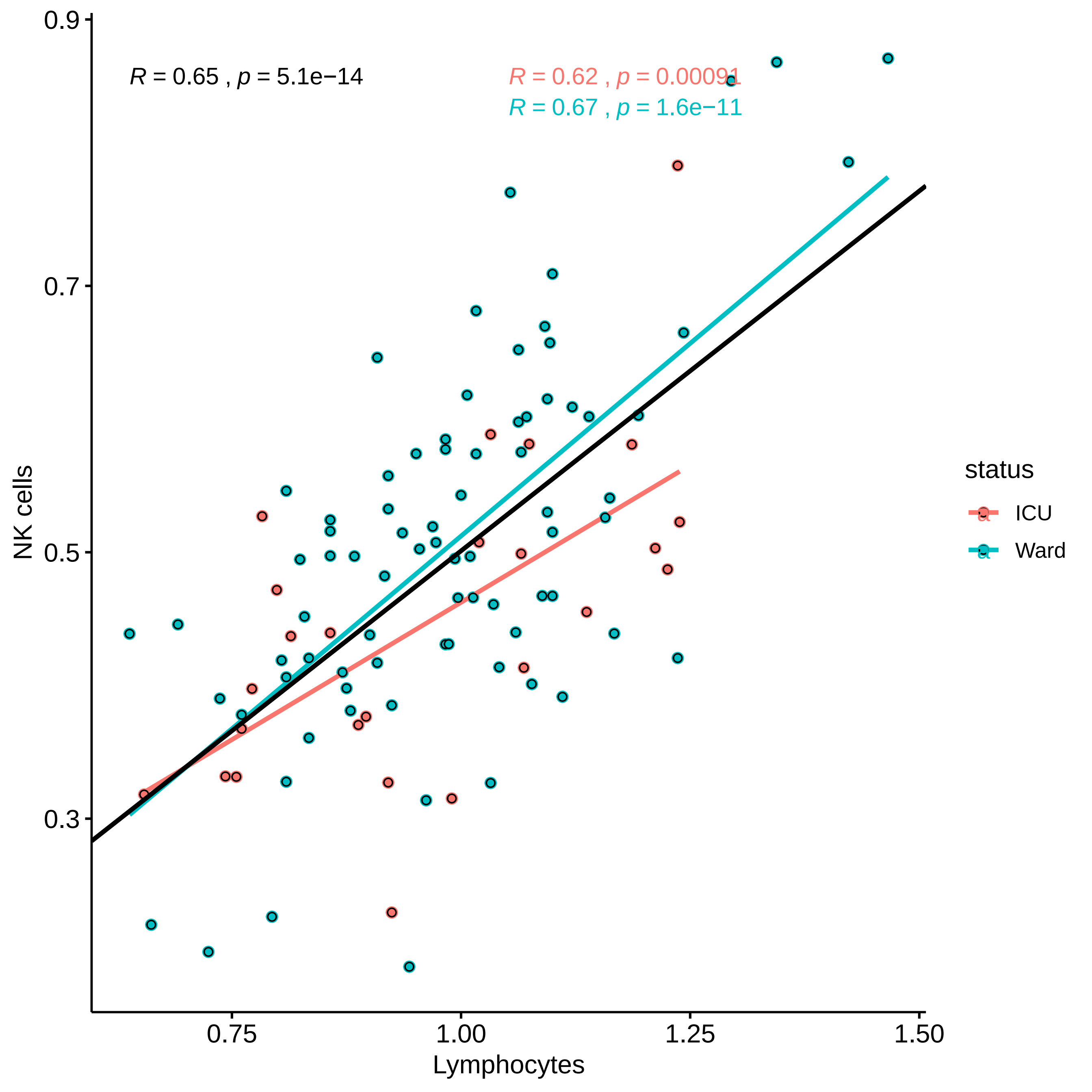

R = 0.65 , p = 5.1e−14
R = 0.62 , p = 0.00091
R = 0.67 , p = 1.6e−11
NK cells
0.9
0.7
0.5
0.3
0.75
1.00
1.25
1.50
Lymphocytes
status
ICU
Ward

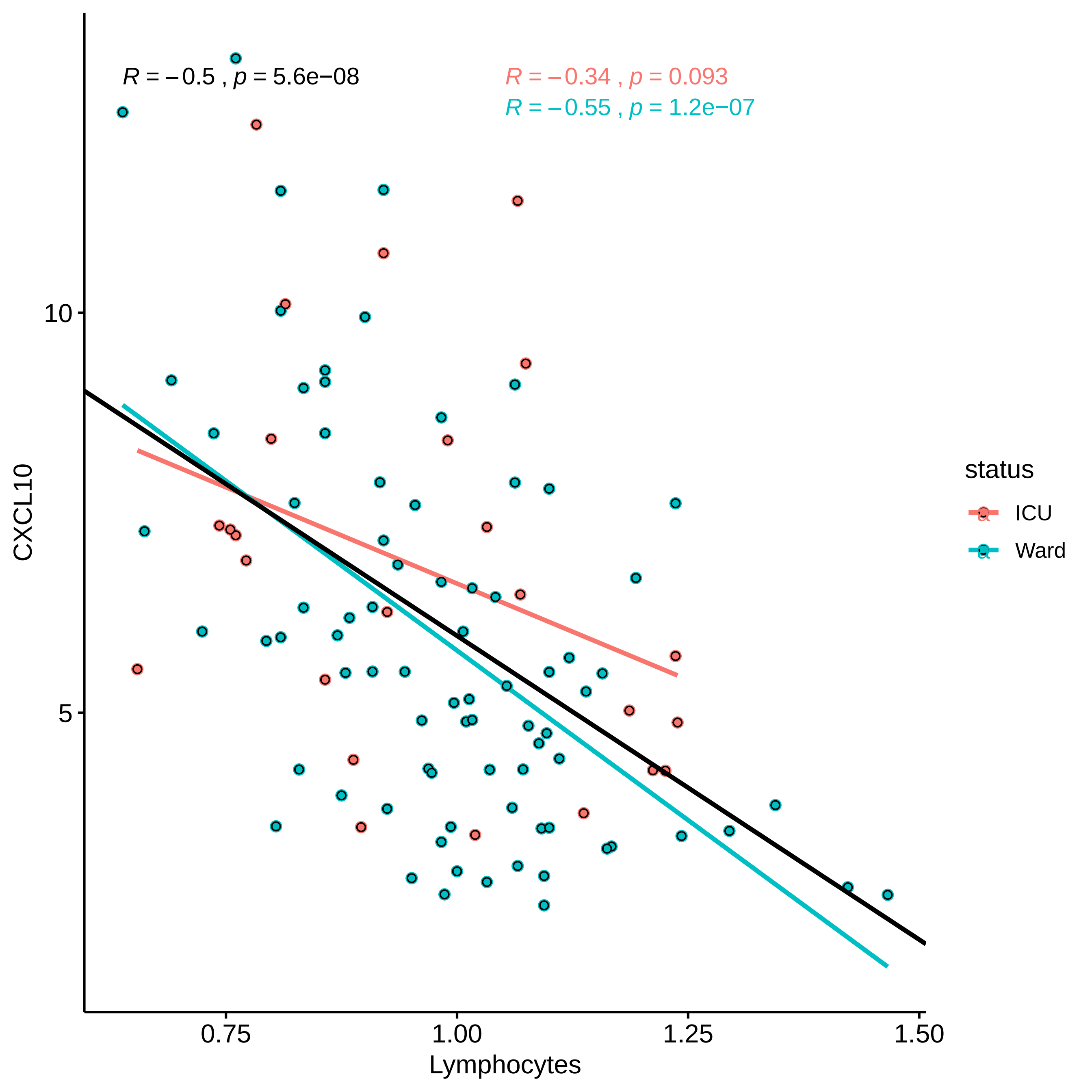

R = −0.5 , p = 5.6e−08
R = −0.34 , p = 0.093
R = −0.55 , p = 1.2e−07
CXCL10
10
5
0.75
1.00
1.25
1.50
Lymphocytes
status
ICU
Ward

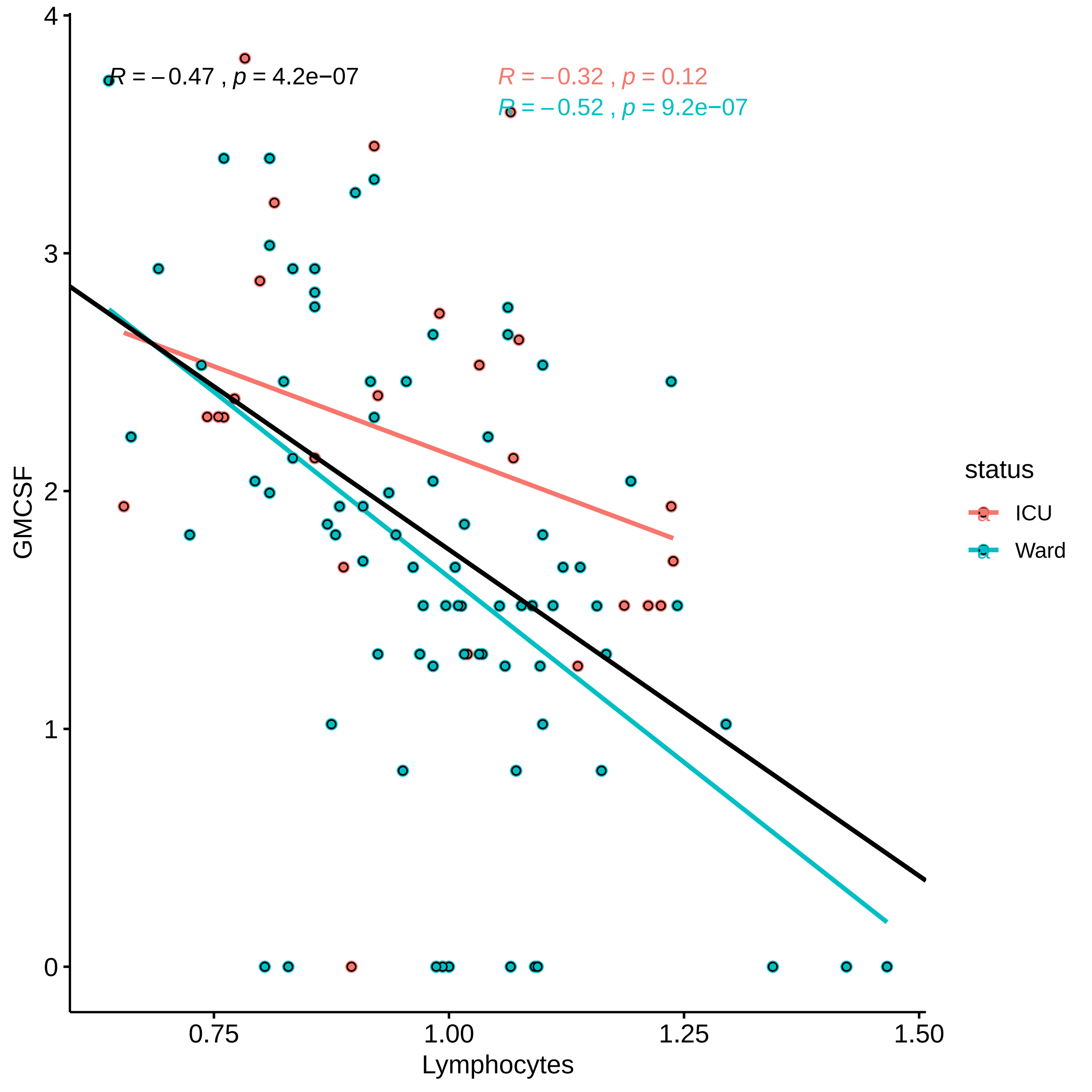

R = −0.47 , p = 4.2e−07
R = −0.32 , p = 0.12
R = −0.52 , p = 9.2e−07
GMCSF
Lymphocytes
0
1
2
3
4
0.75
1.00
1.25
1.50
status
ICU
Ward

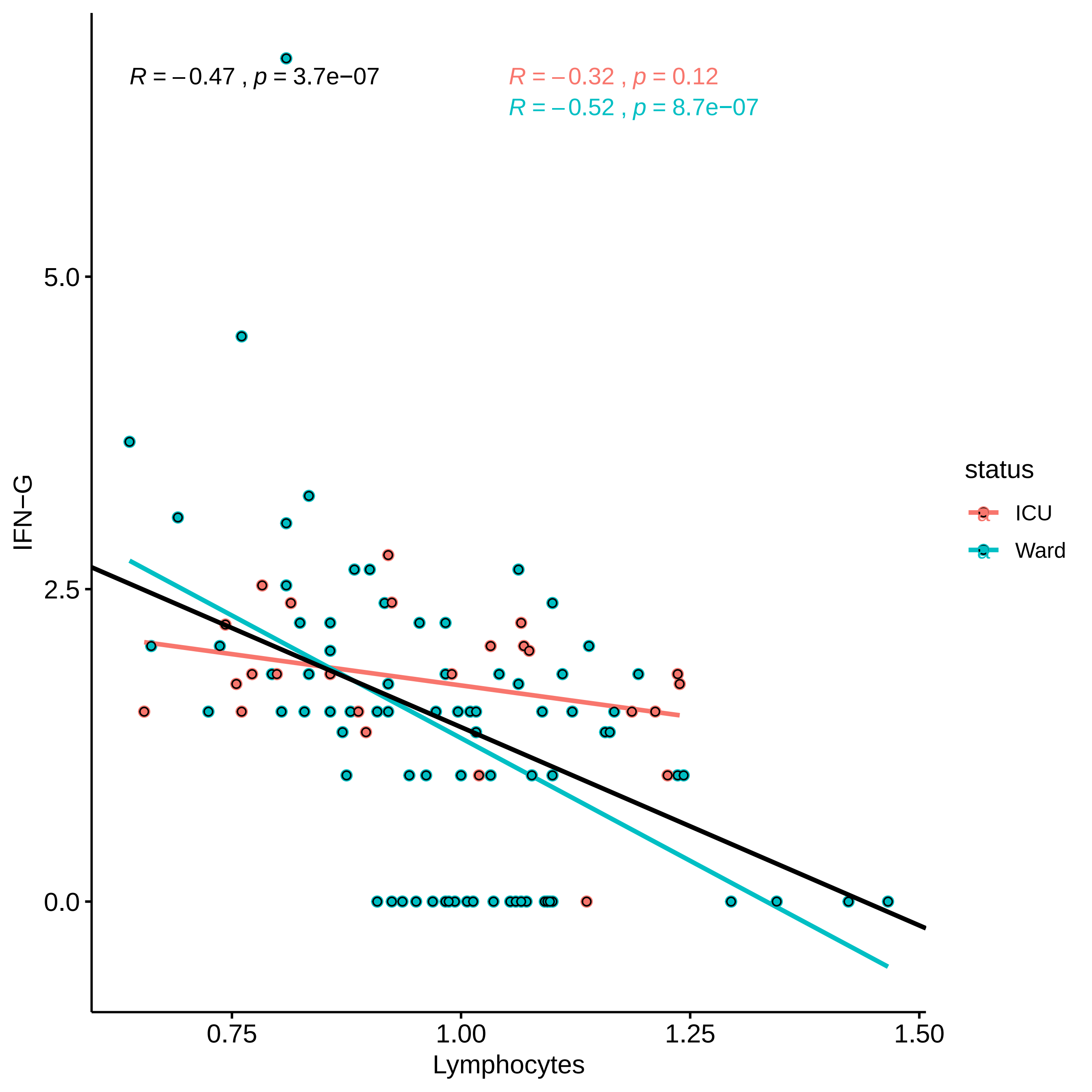
R = −0.47 , p = 3.7e−07
R = −0.32 , p = 0.12
R = −0.52 , p = 8.7e−07
IFN−G
Lymphocytes
status
ICU
Ward
0.0
2.5
5.0
0.75
1.00
1.25
1.50

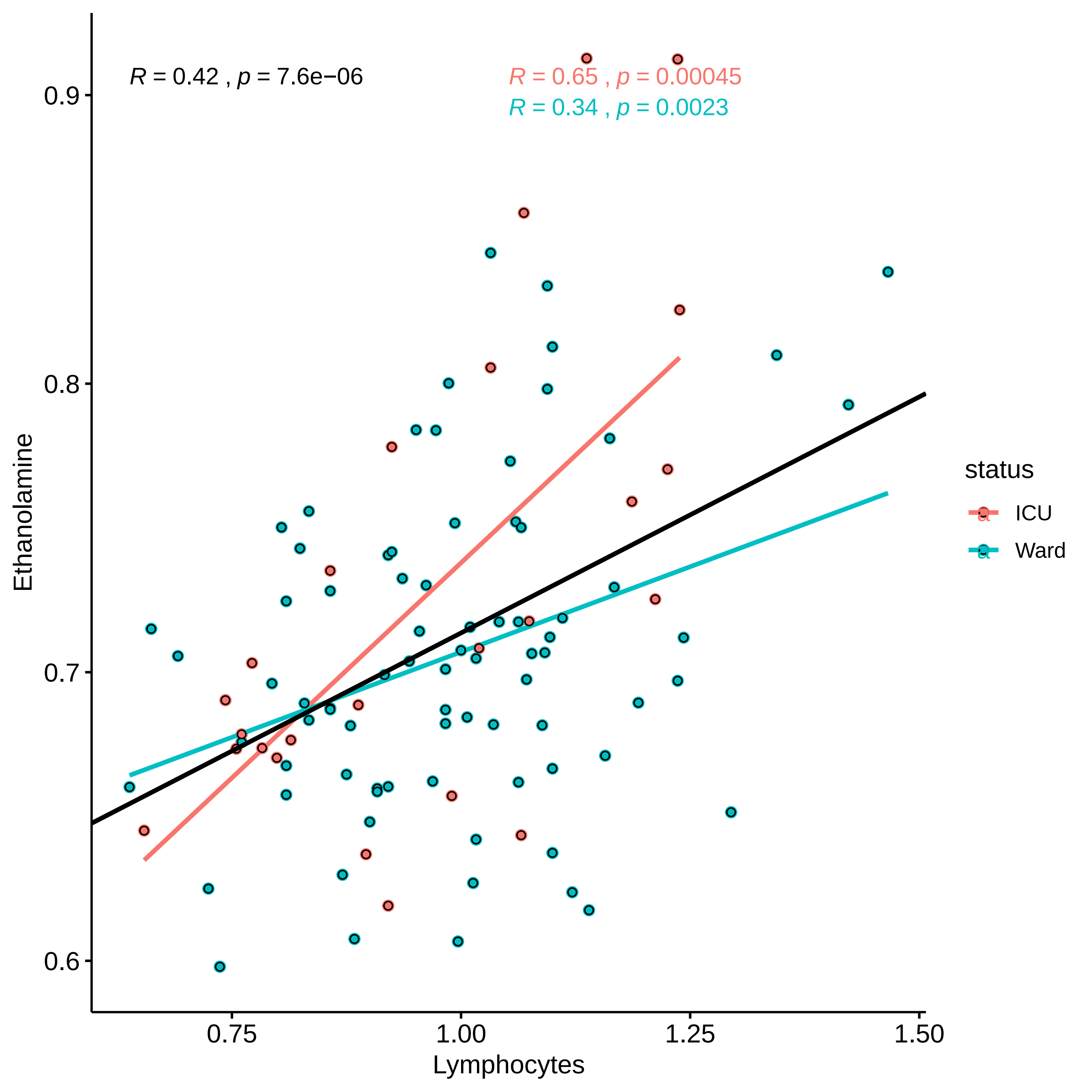

R = 0.42 , p = 7.6e−06
R = 0.65 , p = 0.00045
R = 0.34 , p = 0.0023
0.9
0.8
0.7
0.6
Ethanolamine
0.75
1.00
1.25
1.50
Lymphocytes
status
ICU
Ward

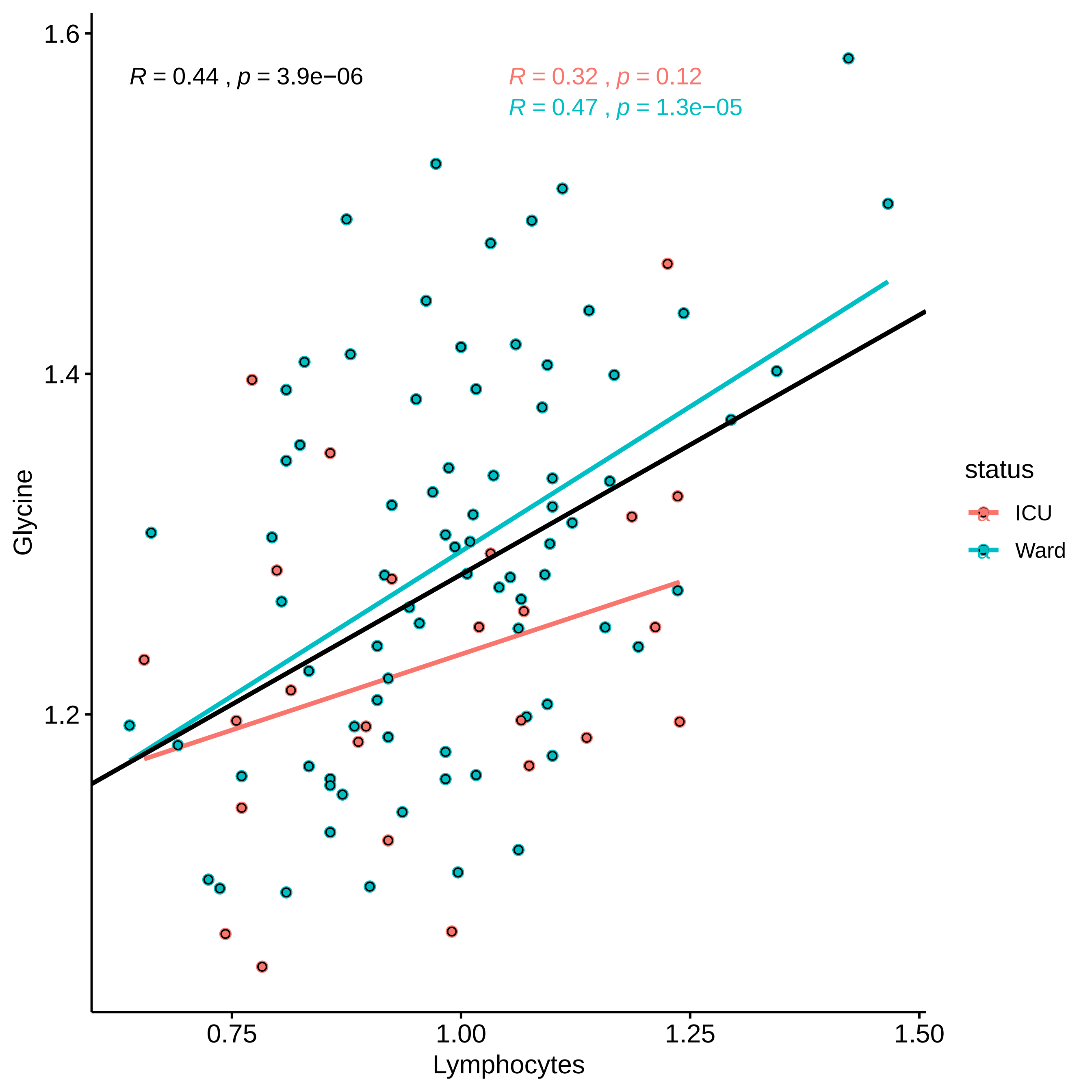

R = 0.44 , p = 3.9e−06
R = 0.32 , p = 0.12
R = 0.47 , p = 1.3e−05
Glycine
1.6
1.4
1.2
0.75
1.00
1.25
1.50
Lymphocytes
status
ICU
Ward

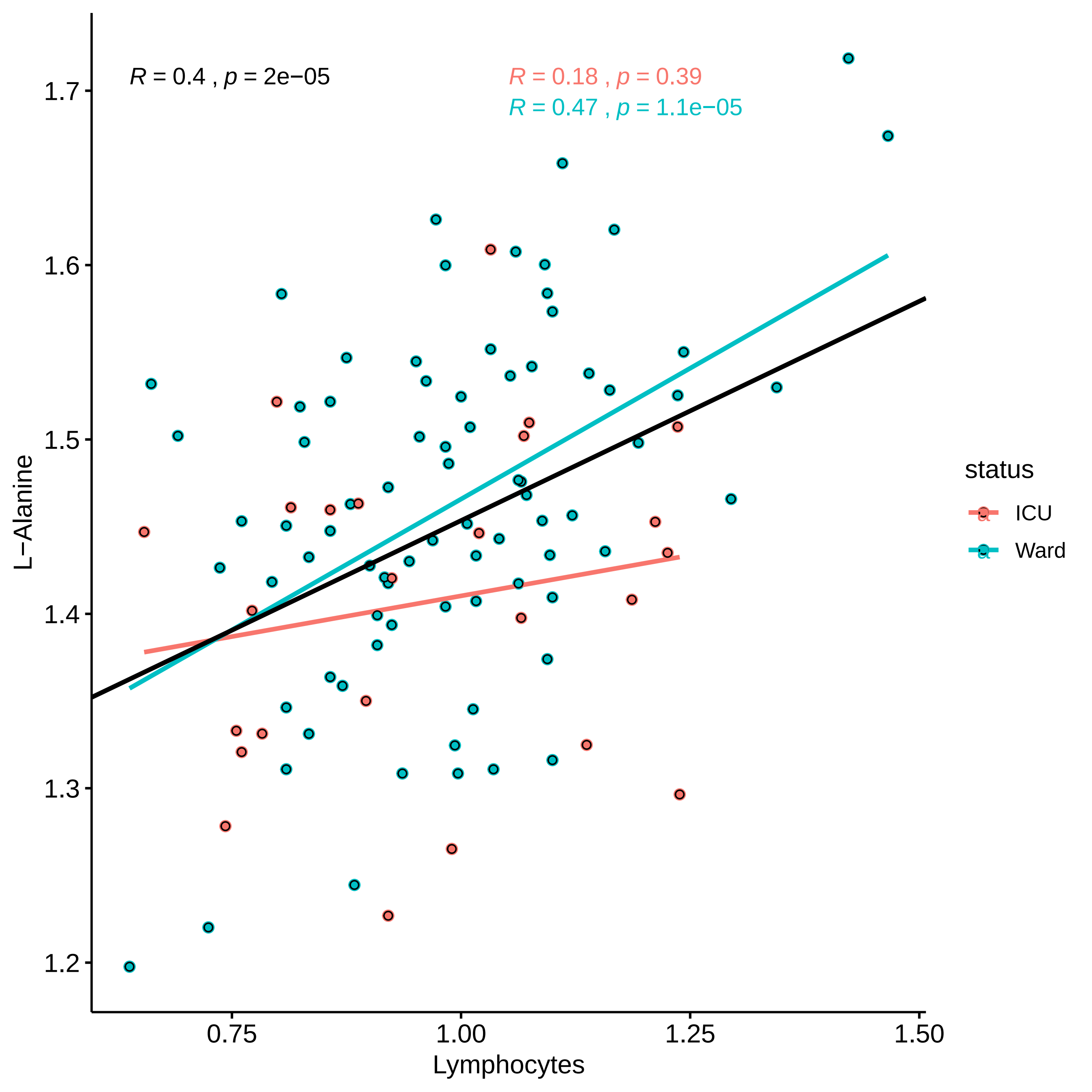

R = 0.4 , p = 2e−05
R = 0.18 , p = 0.39
R = 0.47 , p = 1.1e−05
L−Alanine
1.7
1.6
1.5
1.4
1.3
1.2
0.75
1.00
1.25
1.50
Lymphocytes
status
ICU
Ward

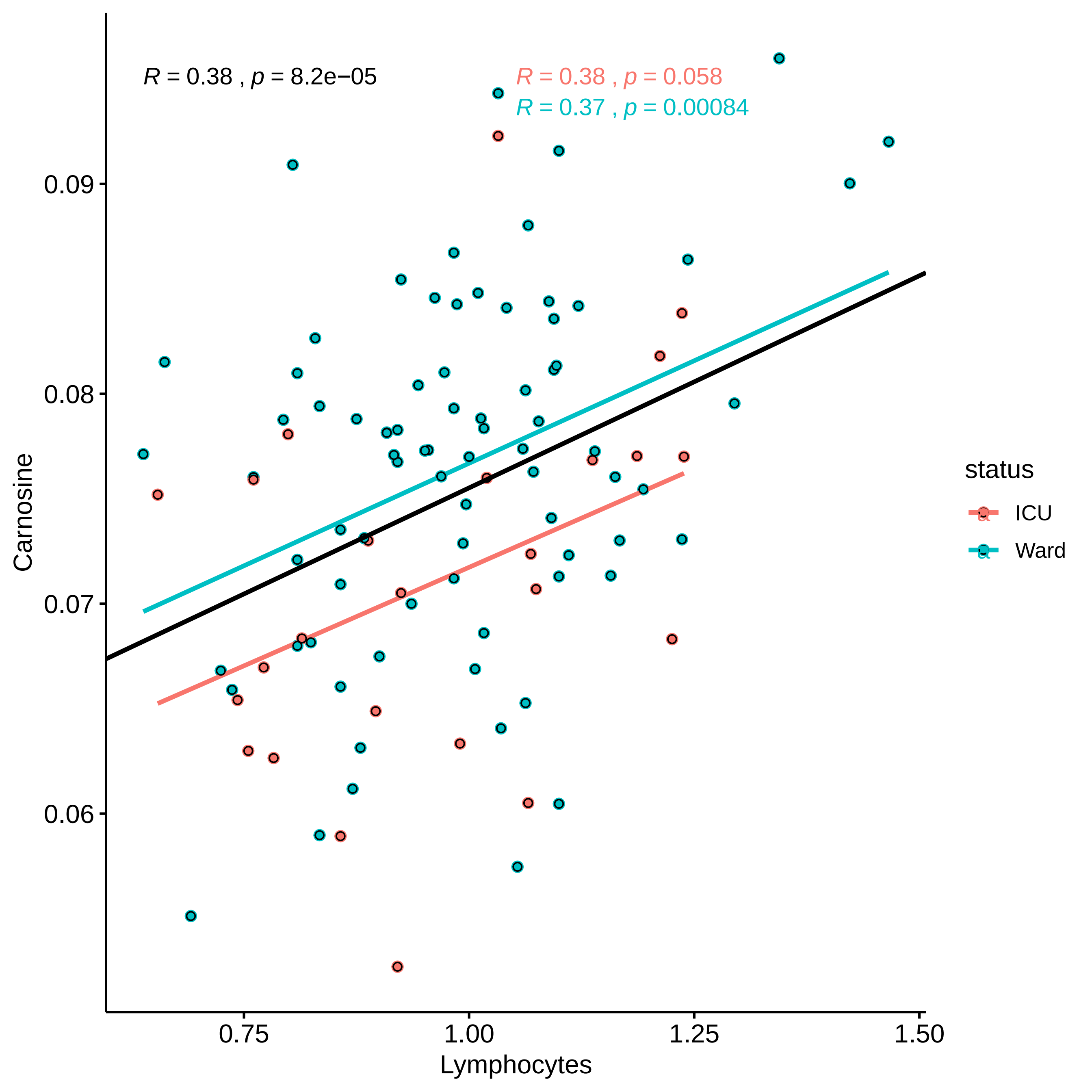
R = 0.38 , p = 8.2e−05
R = 0.38 , p = 0.058
R = 0.37 , p = 0.00084
0.09
0.08
0.07
0.06
Carnosine
0.75
1.00
1.25
1.50
Lymphocytes
status
ICU
Ward

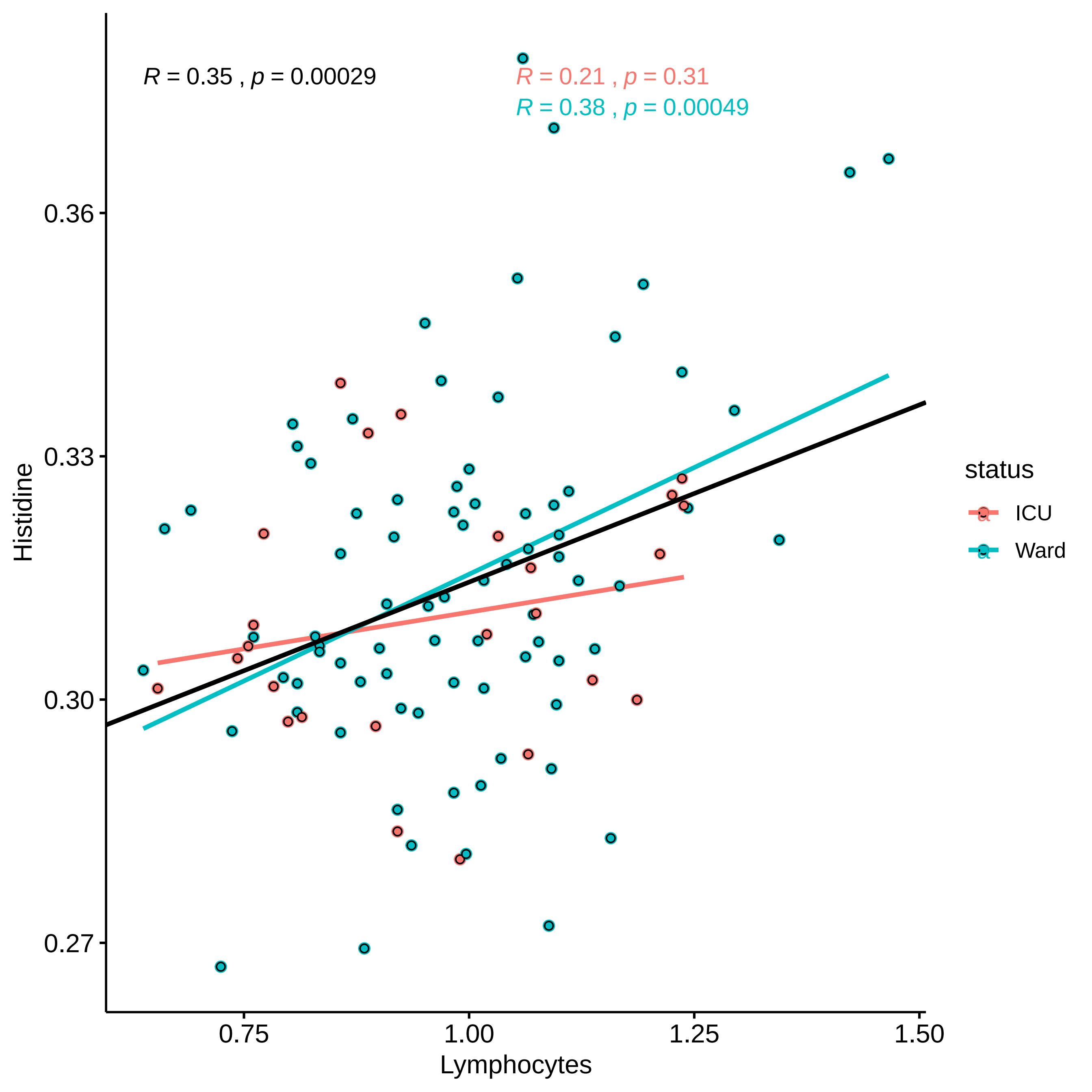
R = 0.35 , p = 0.00029
R = 0.21 , p = 0.31
R = 0.38 , p = 0.00049
Histidine
0.36
0.33
0.30
0.27
0.75
1.00
1.25
1.50
Lymphocytes
status
ICU
Ward

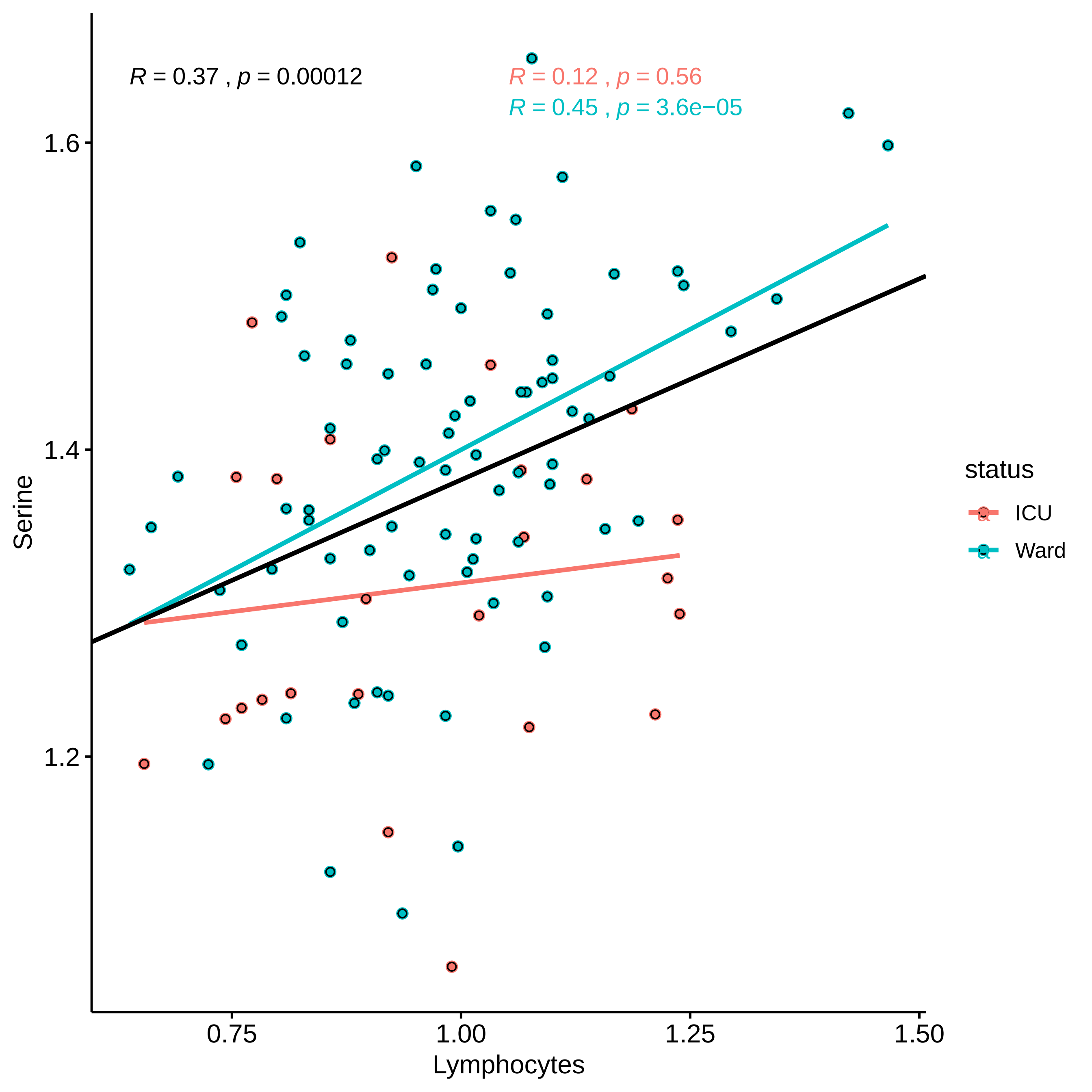

R = 0.37 , p = 0.00012
R = 0.12 , p = 0.56
R = 0.45 , p = 3.6e−05
Serine
1.2
1.4
1.6
0.75
1.00
1.25
1.50
Lymphocytes
status
ICU
Ward

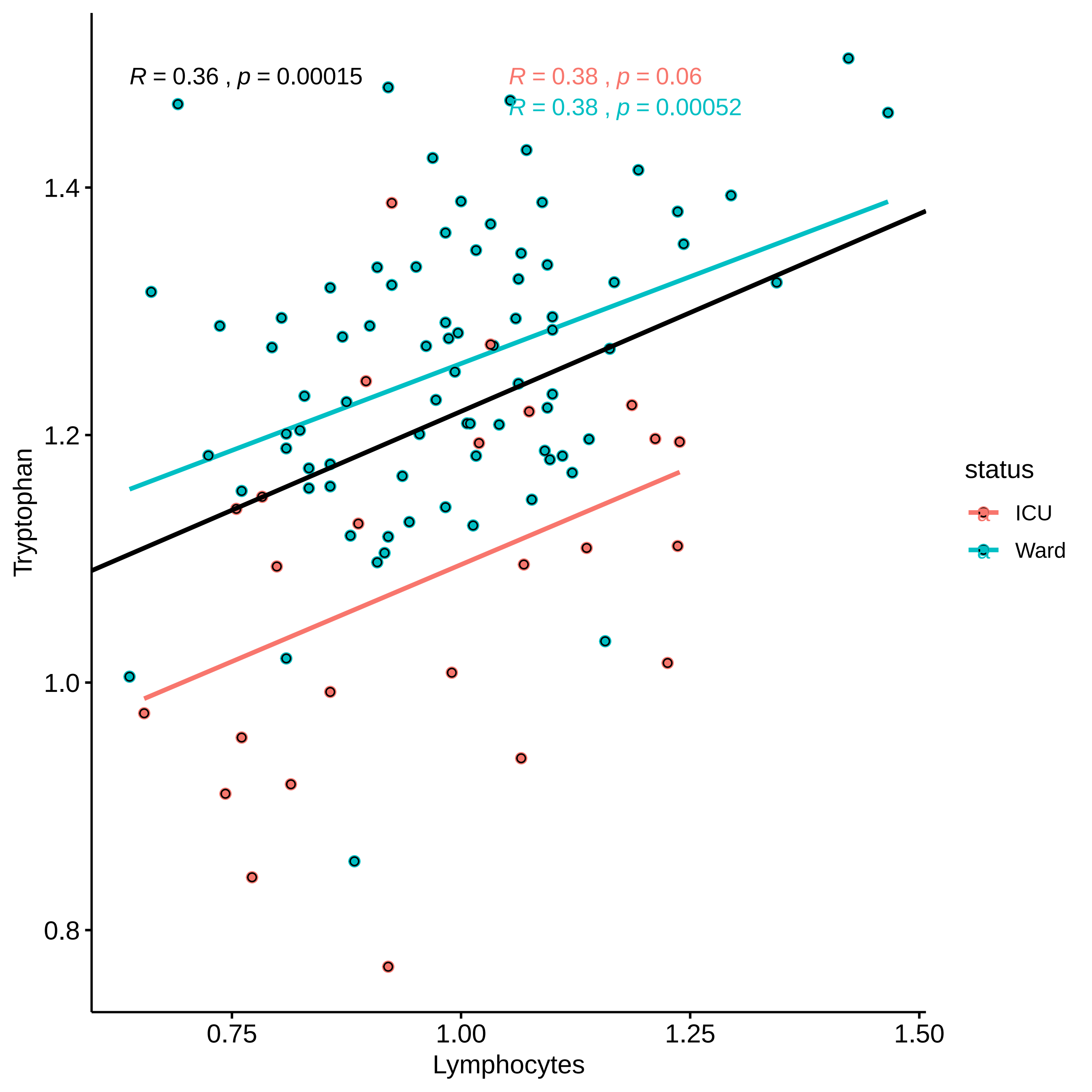

R = 0.36 , p = 0.00015
R = 0.38 , p = 0.06
R = 0.38 , p = 0.00052
Tryptophan
1.4
1.2
1.0
0.8
0.75
1.00
1.25
1.50
Lymphocytes
status
ICU
Ward

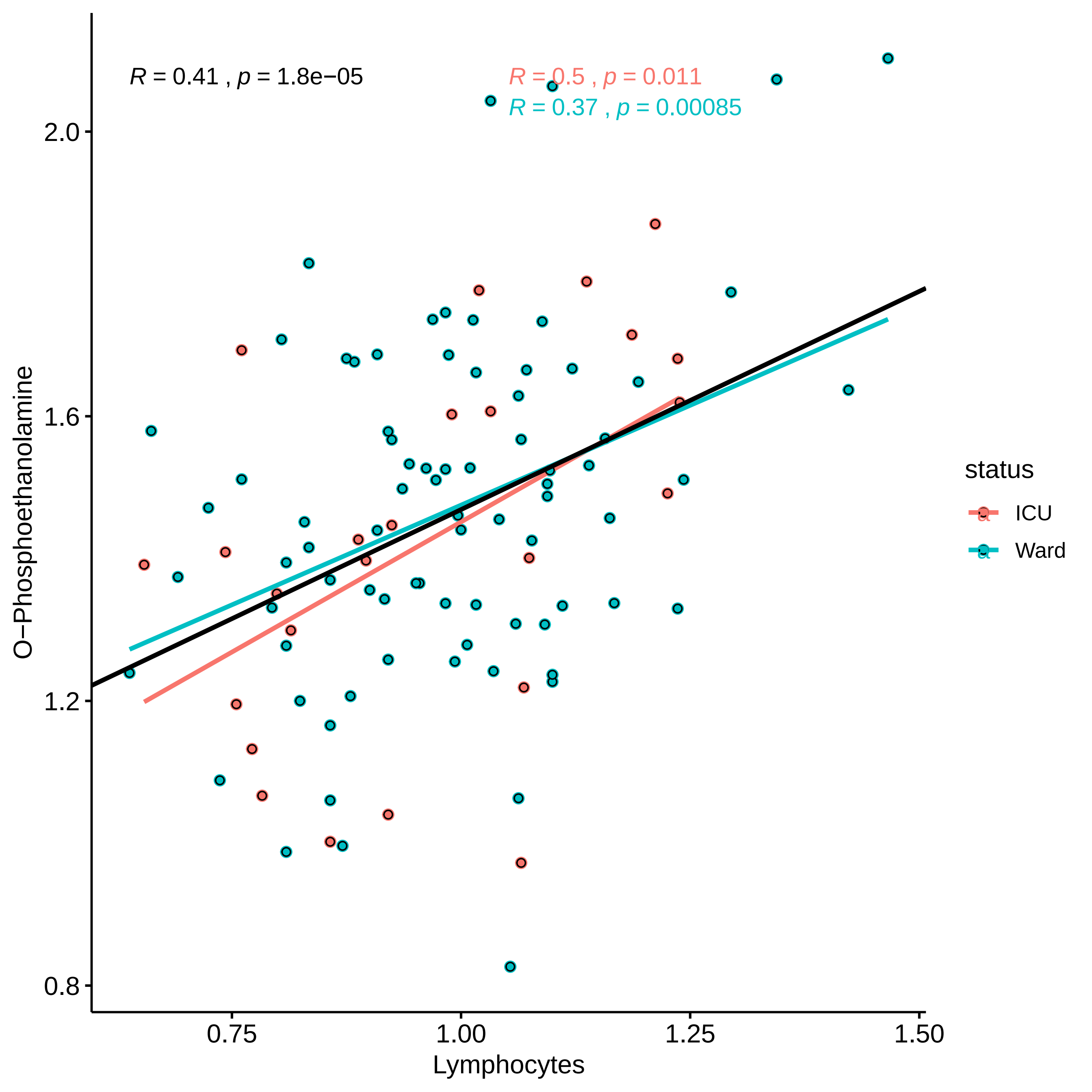
R = 0.41 , p = 1.8e−05
R = 0.5 , p = 0.011
R = 0.37 , p = 0.00085
O−Phosphoethanolamine
2.0
1.6
1.2
0.8
0.75
1.00
1.25
1.50
Lymphocytes
status
ICU
Ward

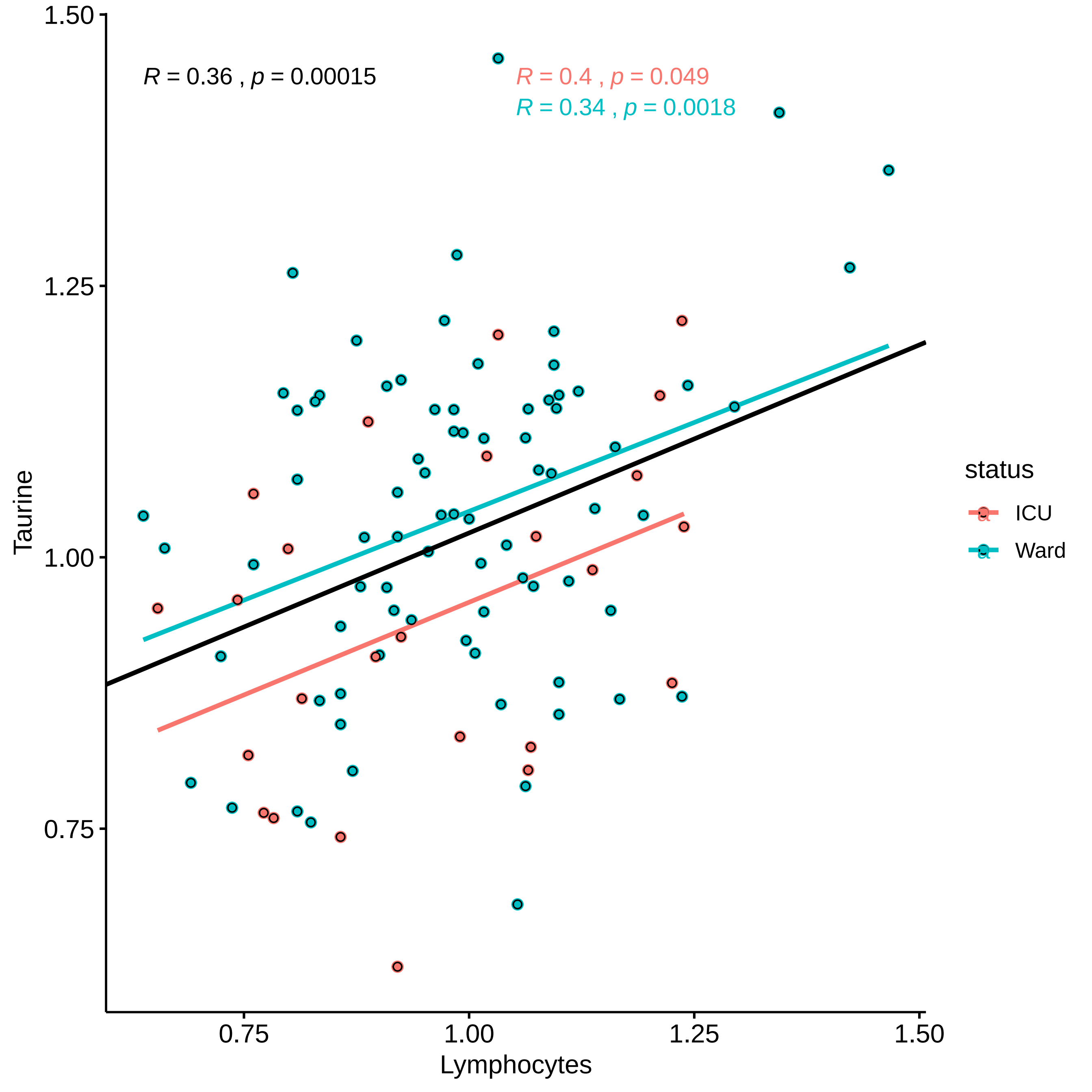
R = 0.36 , p = 0.00015
R = 0.4 , p = 0.049
R = 0.34 , p = 0.0018
1.50
1.25
1.00
0.75
Taurine
0.75
1.00
1.25
1.50
Lymphocytes
status
ICU
Ward

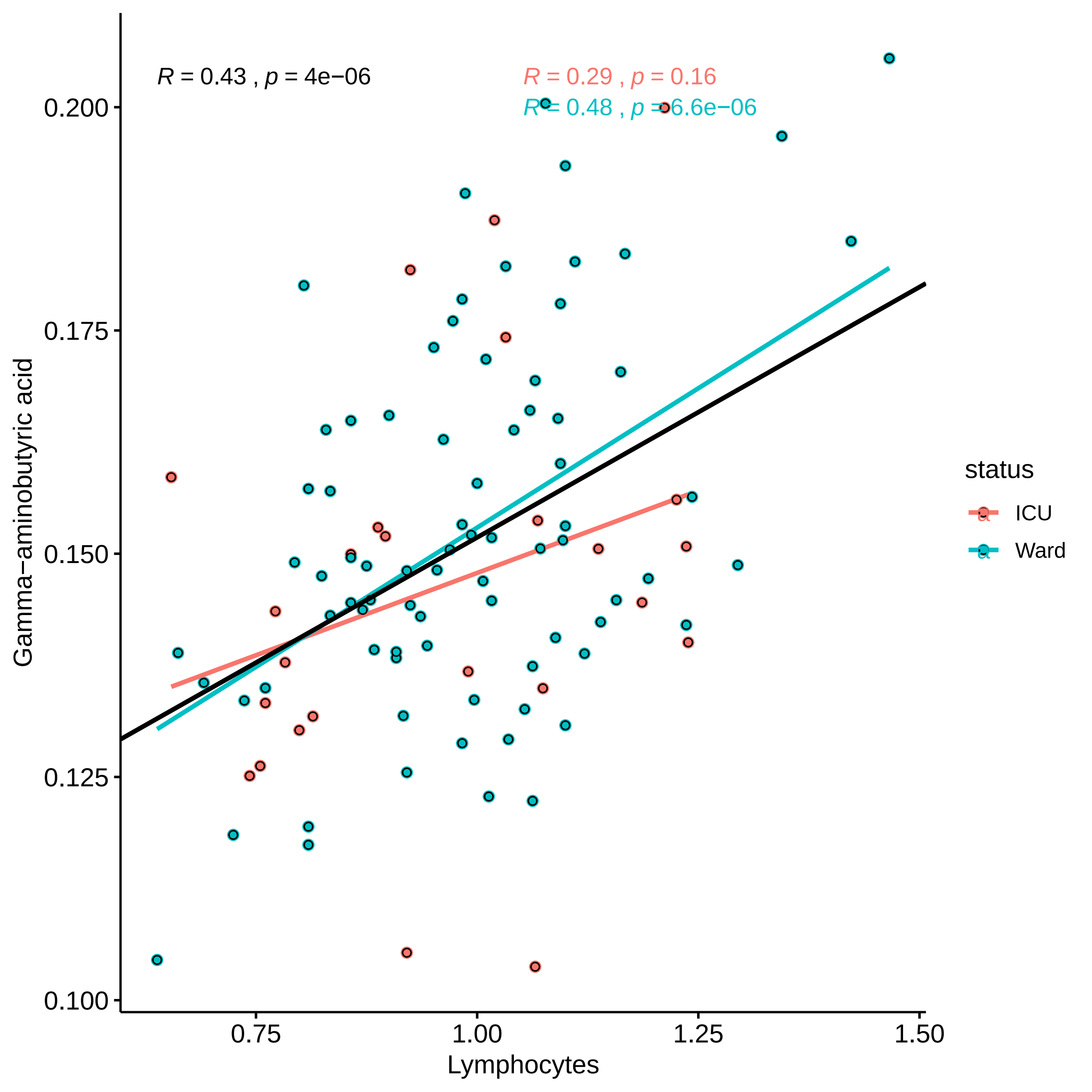

R = 0.43 , p = 4e−06
R = 0.29 , p = 0.16
R = 0.48 , p = 6.6e−06
Gamma−aminobutyric acid
0.200
0.175
0.150
0.125
0.100
0.75
1.00
1.25
1.50
Lymphocytes
status
ICU
Ward

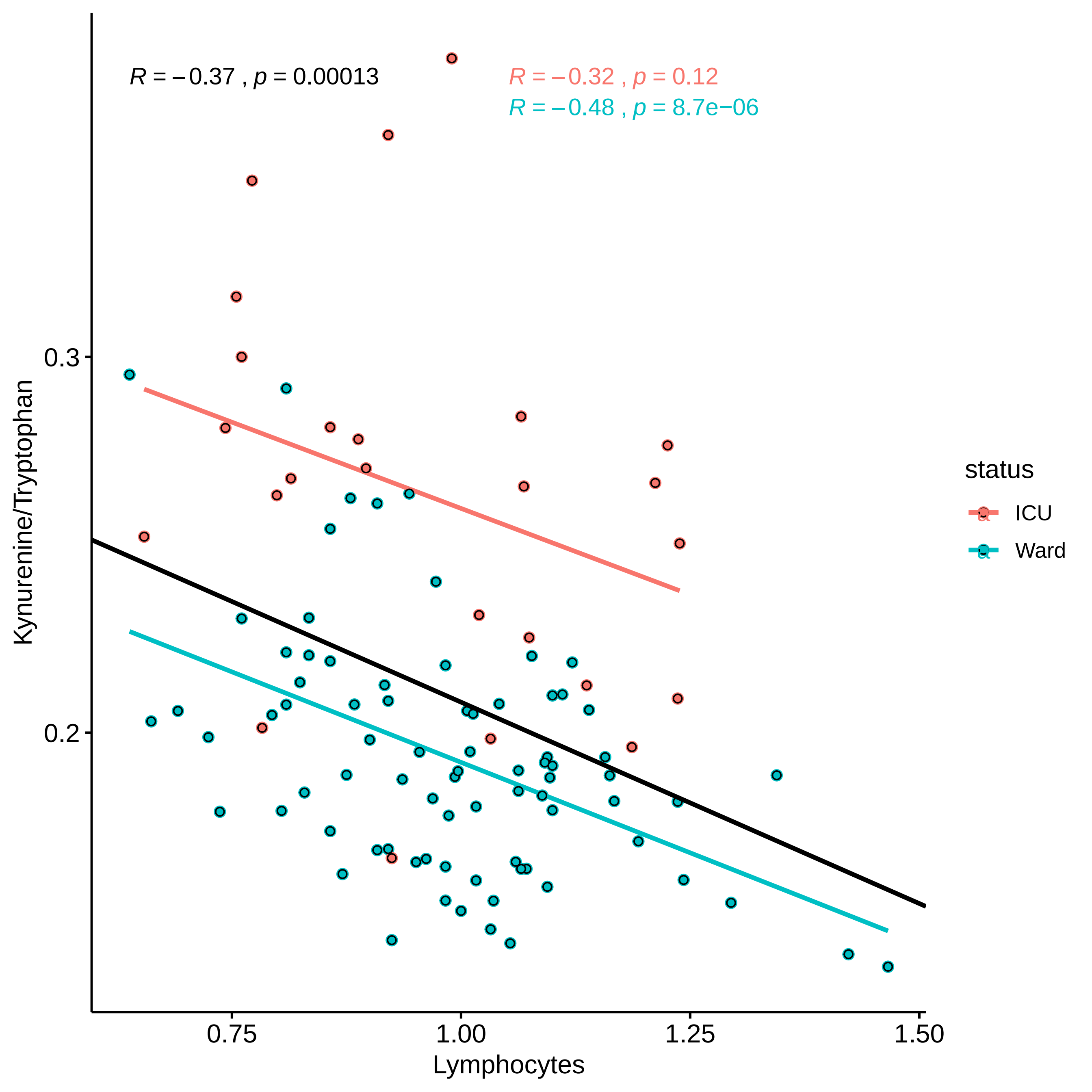

R = −0.37 , p = 0.00013
R = −0.32 , p = 0.12
R = −0.48 , p = 8.7e−06
Kynurenine/Tryptophan
0.3
0.2
0.75
1.00
1.25
1.50
Lymphocytes
status
ICU
Ward

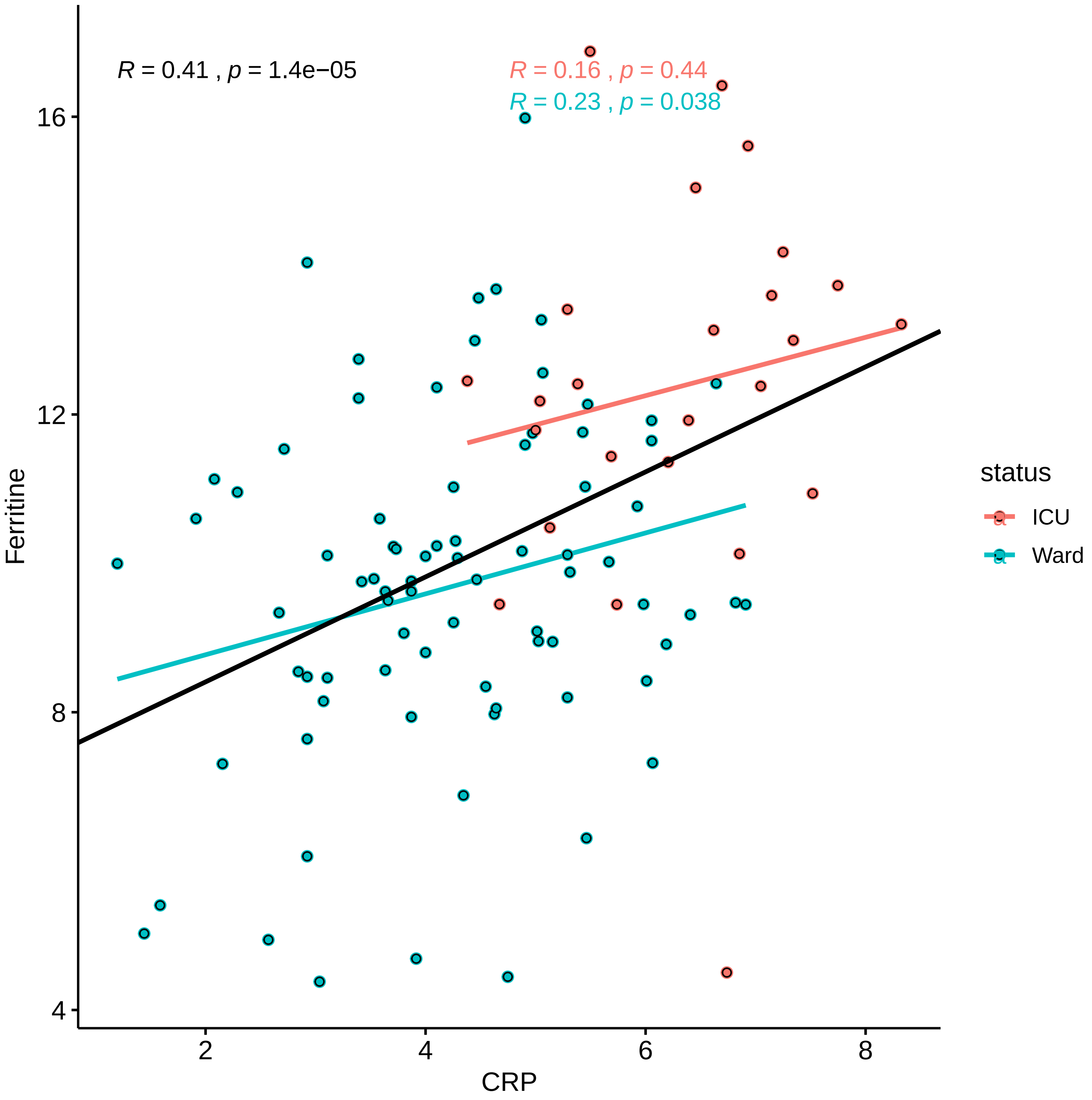

R = 0.41 , p = 1.4e−05
R = 0.16 , p = 0.44
R = 0.23 , p = 0.038
16
12
8
4
2
4
6
8
Ferritine
CRP
status
ICU
Ward

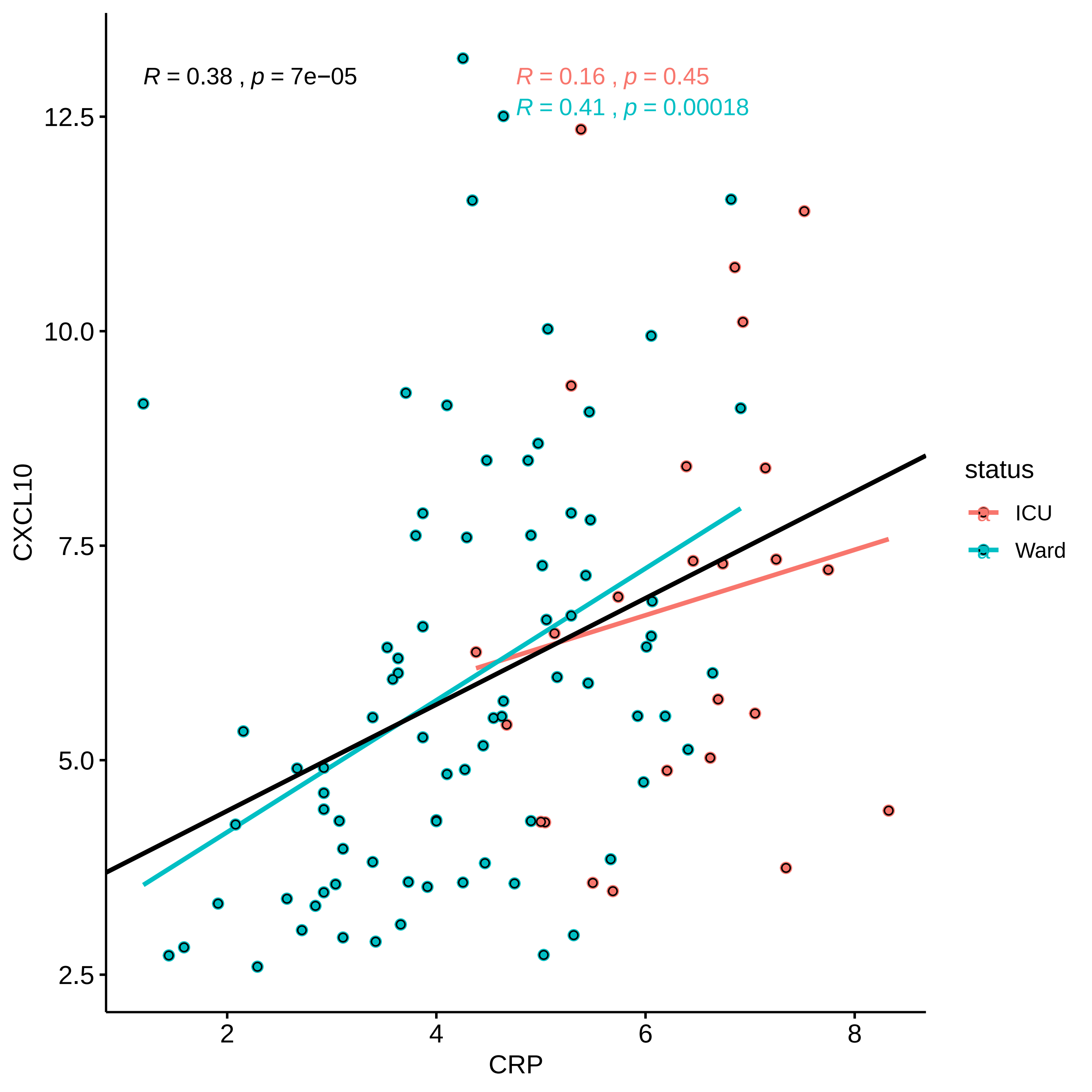
R = 0.38 , p = 7e−05
R = 0.16 , p = 0.45
R = 0.41 , p = 0.00018
CXCL10
12.5
10.0
7.5
5.0
2.5
2
4
6
8
CRP
status
ICU
Ward

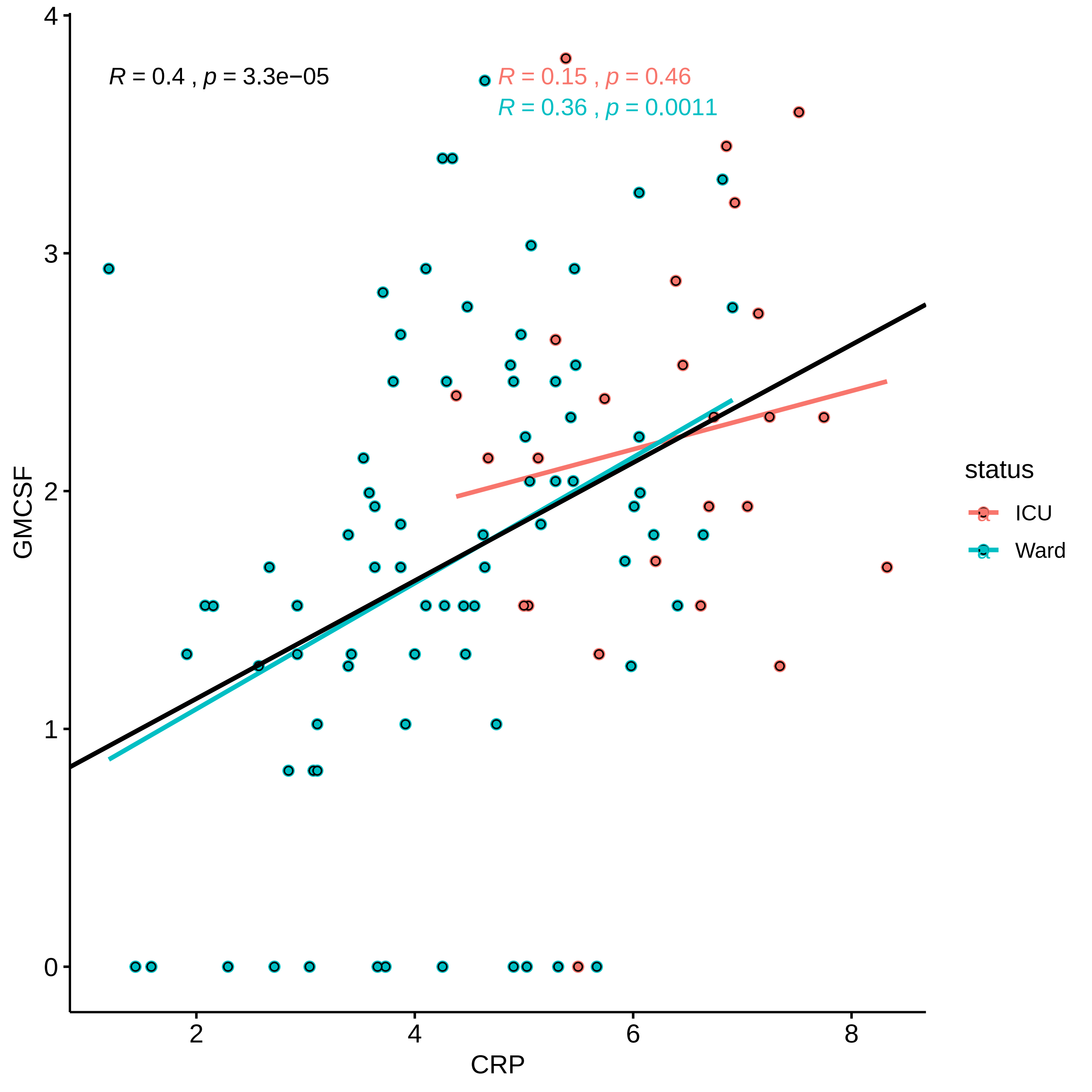

R = 0.4 , p = 3.3e−05
R = 0.15 , p = 0.46
R = 0.36 , p = 0.0011
GMCSF
CRP
status
ICU
Ward
0
1
2
3
4
2
4
6
8

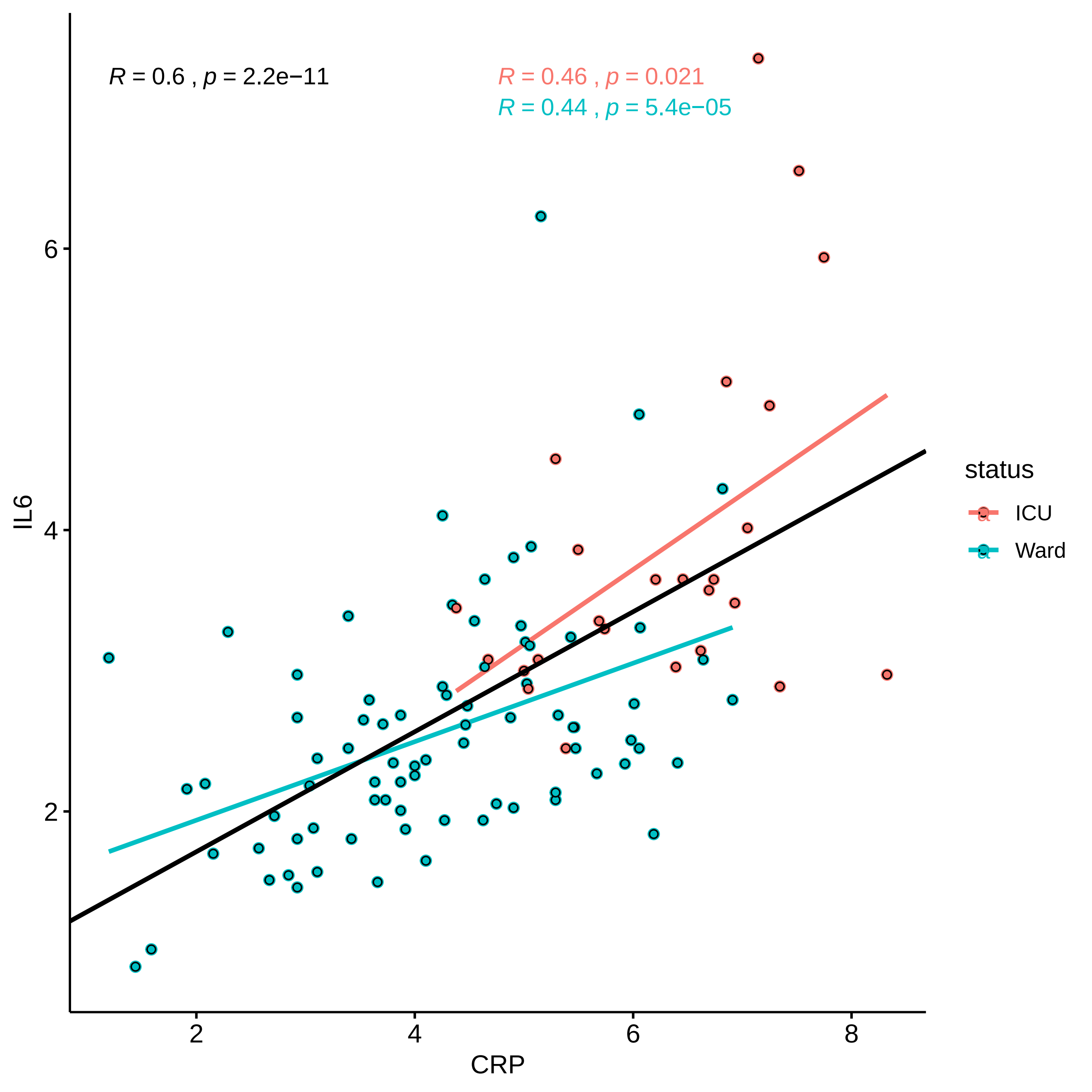

R = 0.6 , p = 2.2e−11
R = 0.46 , p = 0.021
R = 0.44 , p = 5.4e−05
IL6
6
4
2
2
4
6
8
CRP
status
ICU
Ward

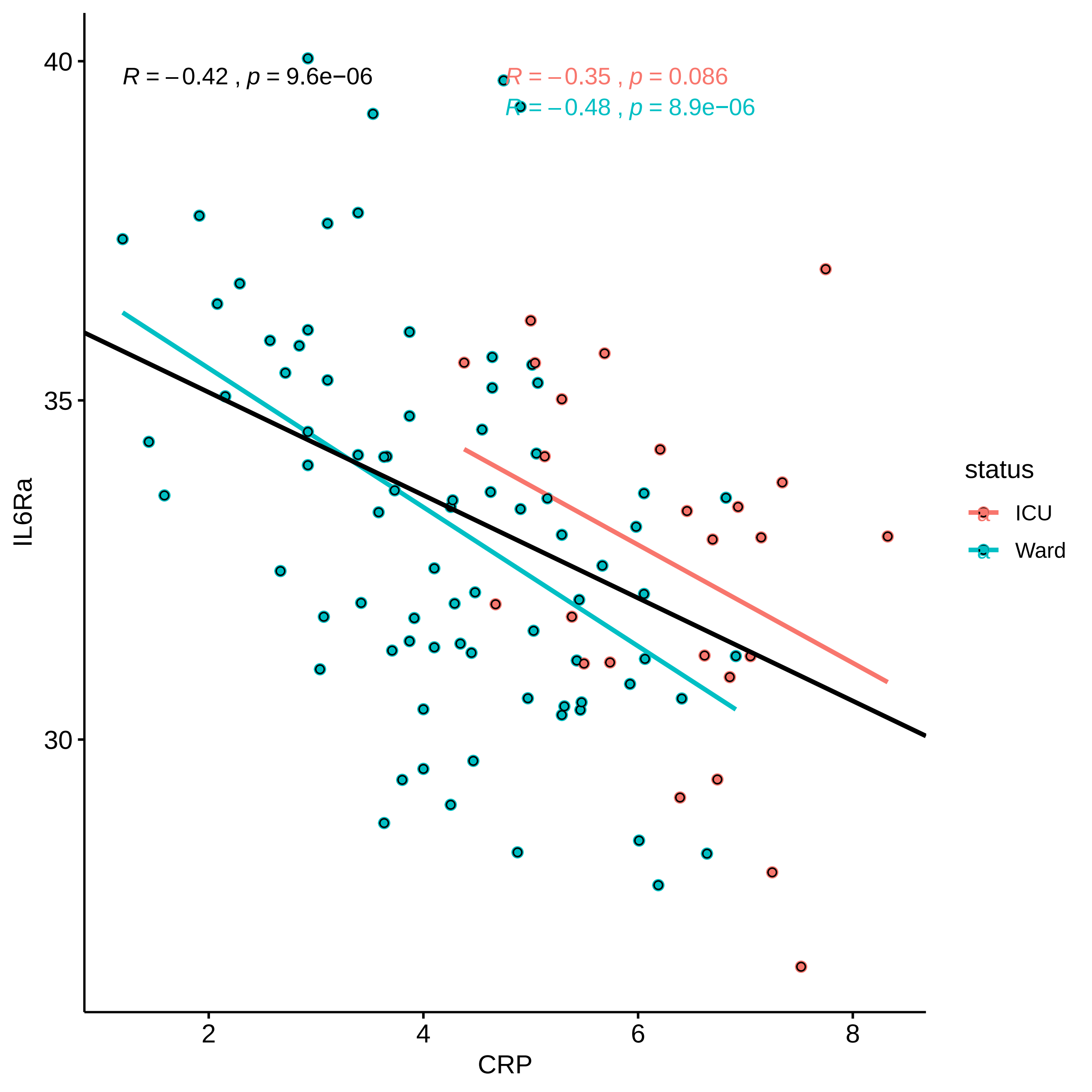

R = −0.42 , p = 9.6e−06
R = −0.35 , p = 0.086
R = −0.48 , p = 8.9e−06
40
35
30
IL6Ra
2
4
6
8
CRP
status
ICU
Ward

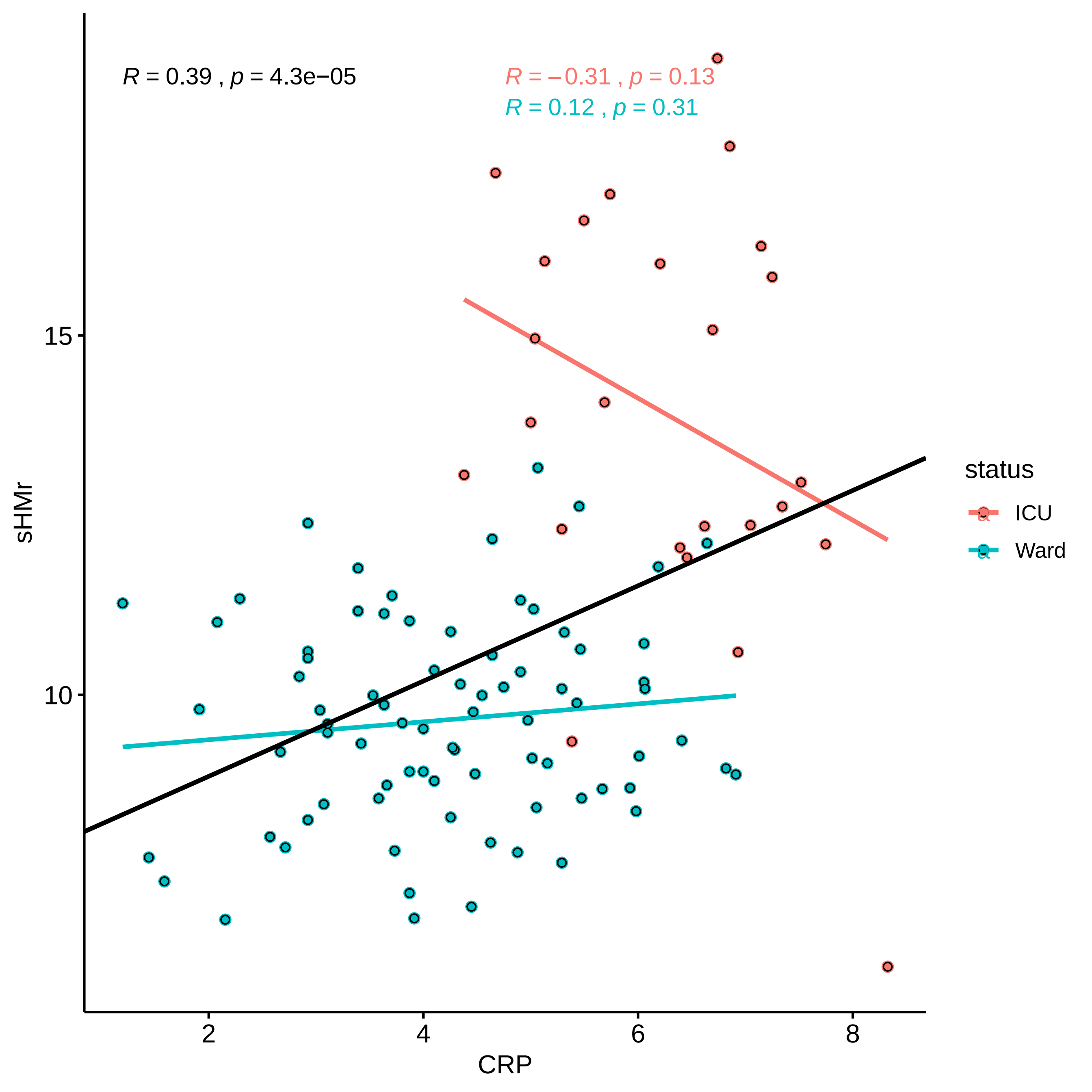

R = 0.39 , p = 4.3e−05
R = −0.31 , p = 0.13
R = 0.12 , p = 0.31
sHMr
15
10
2
4
6
8
CRP
status
ICU
Ward

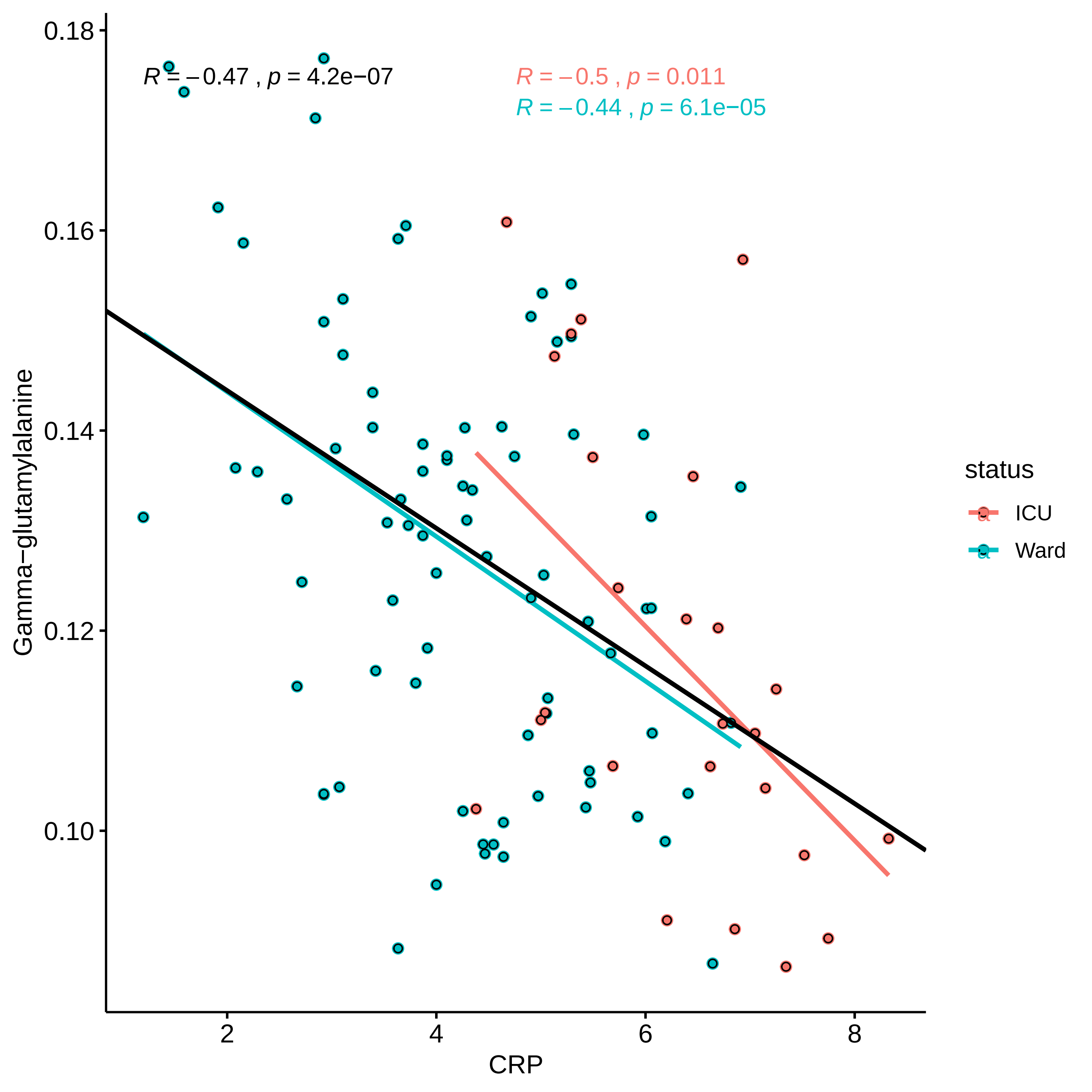

R = −0.47 , p = 4.2e−07
R = −0.5 , p = 0.011
R = −0.44 , p = 6.1e−05
Gamma−glutamylalanine
0.18
0.16
0.14
0.12
0.10
2
4
6
8
CRP
status
ICU
Ward

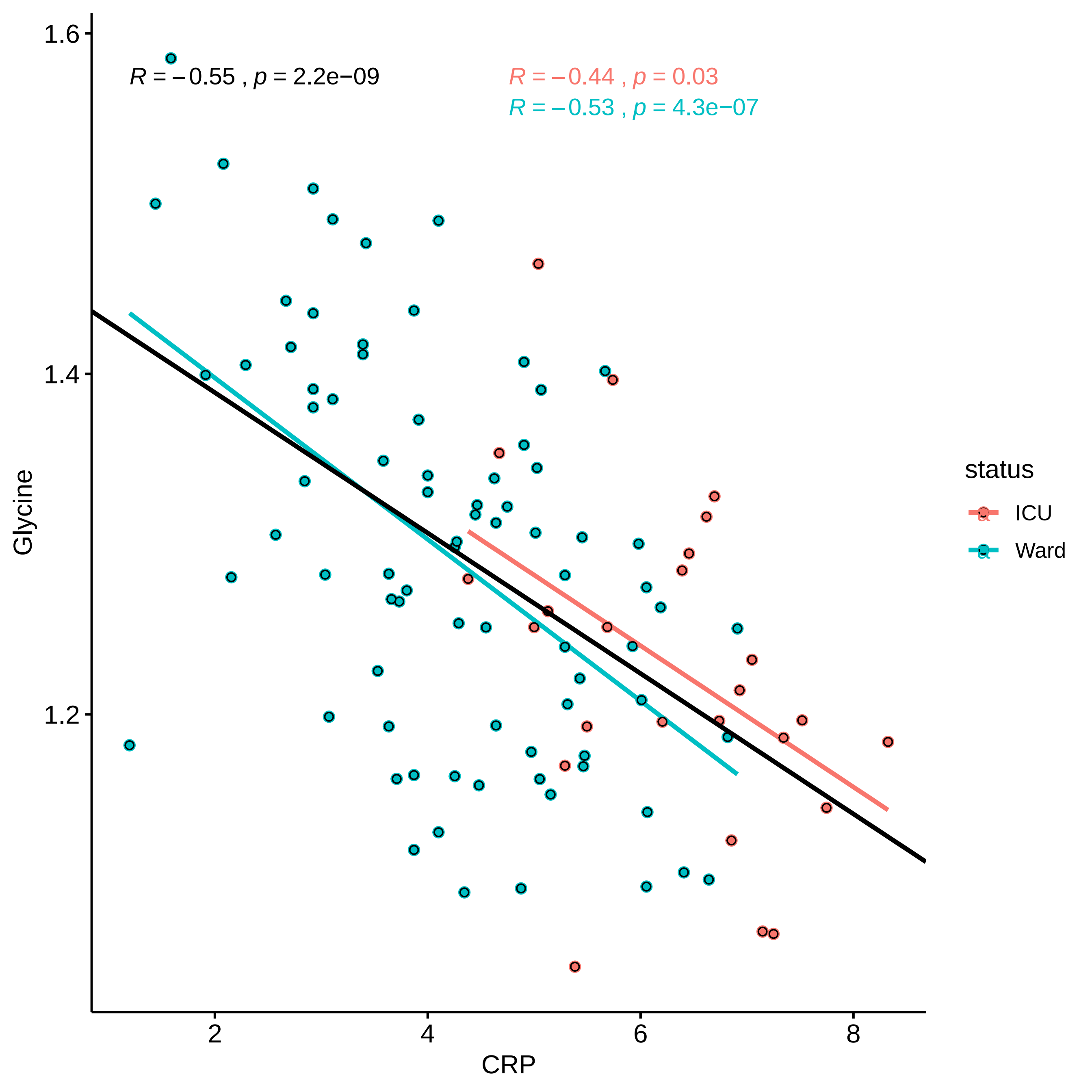

R = −0.55 , p = 2.2e−09
R = −0.44 , p = 0.03
R = −0.53 , p = 4.3e−07
1.6
1.4
1.2
Glycine
2
4
6
8
CRP
status
ICU
Ward

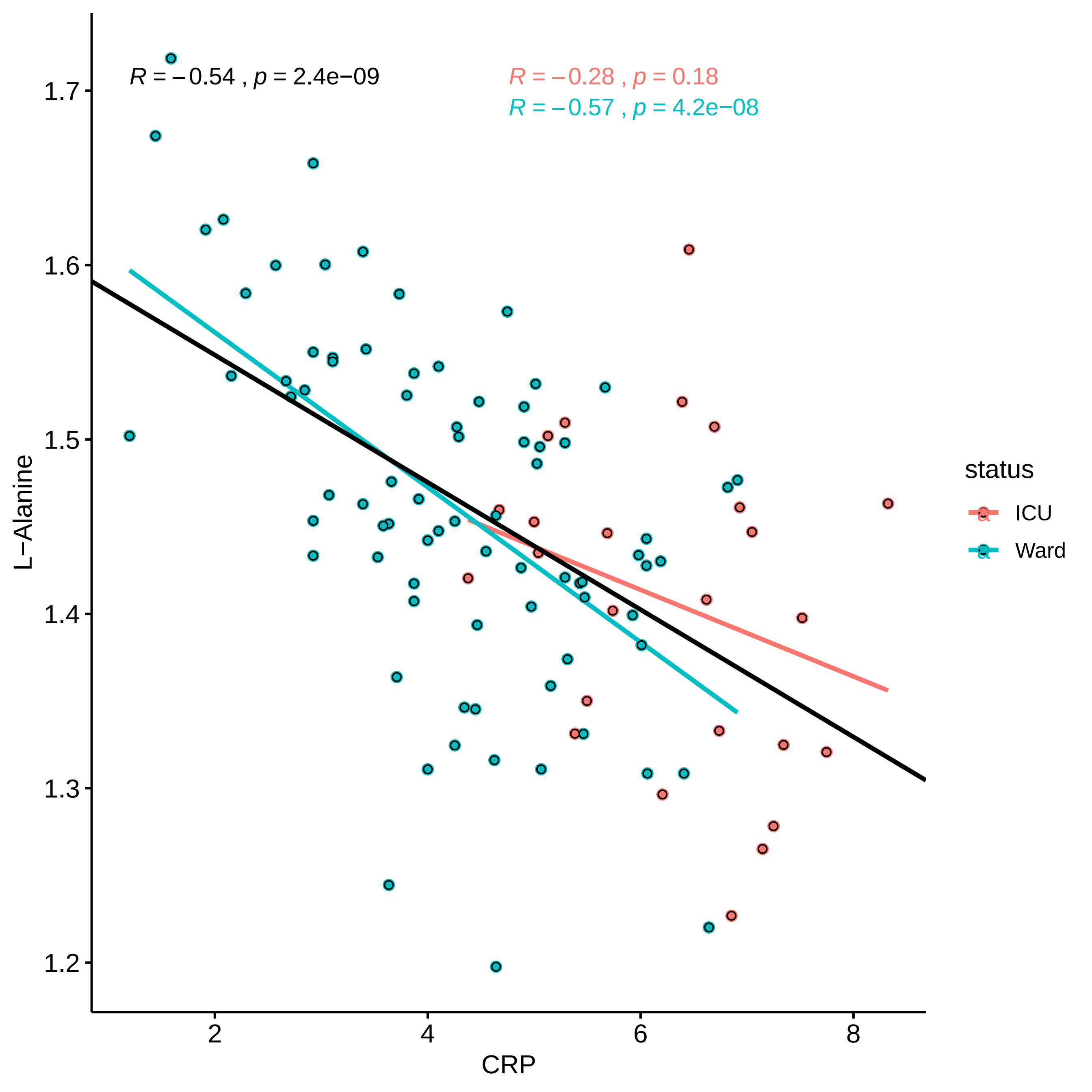

R = −0.54 , p = 2.4e−09
R = −0.28 , p = 0.18
R = −0.57 , p = 4.2e−08
L−Alanine
1.7
1.6
1.5
1.4
1.3
1.2
2
4
6
8
CRP
status
ICU
Ward

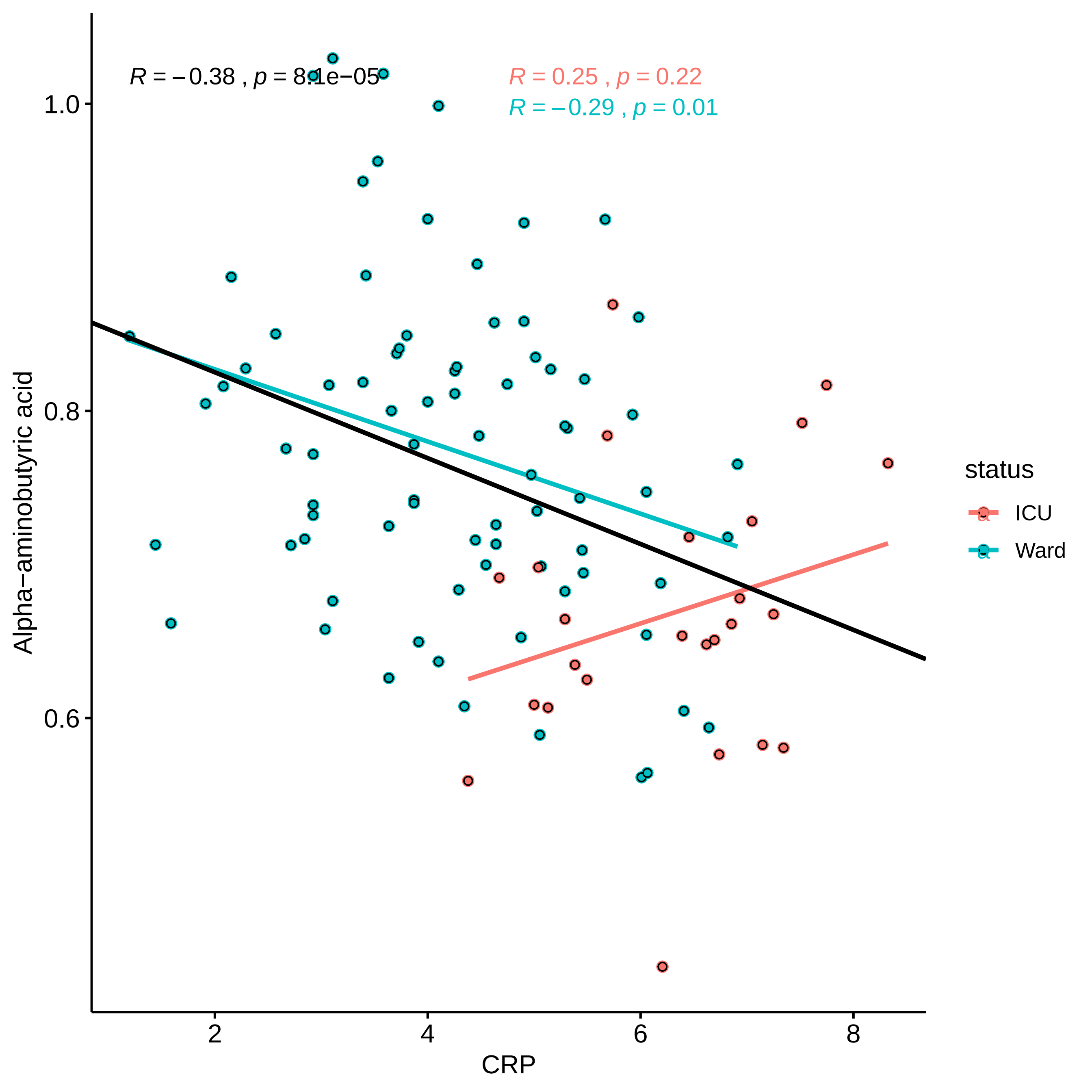

R = −0.38 , p = 8.1e−05
R = 0.25 , p = 0.22
R = −0.29 , p = 0.01
1.0
0.8
0.6
2
4
6
8
CRP
Alpha−aminobutyric acid
status
ICU
Ward

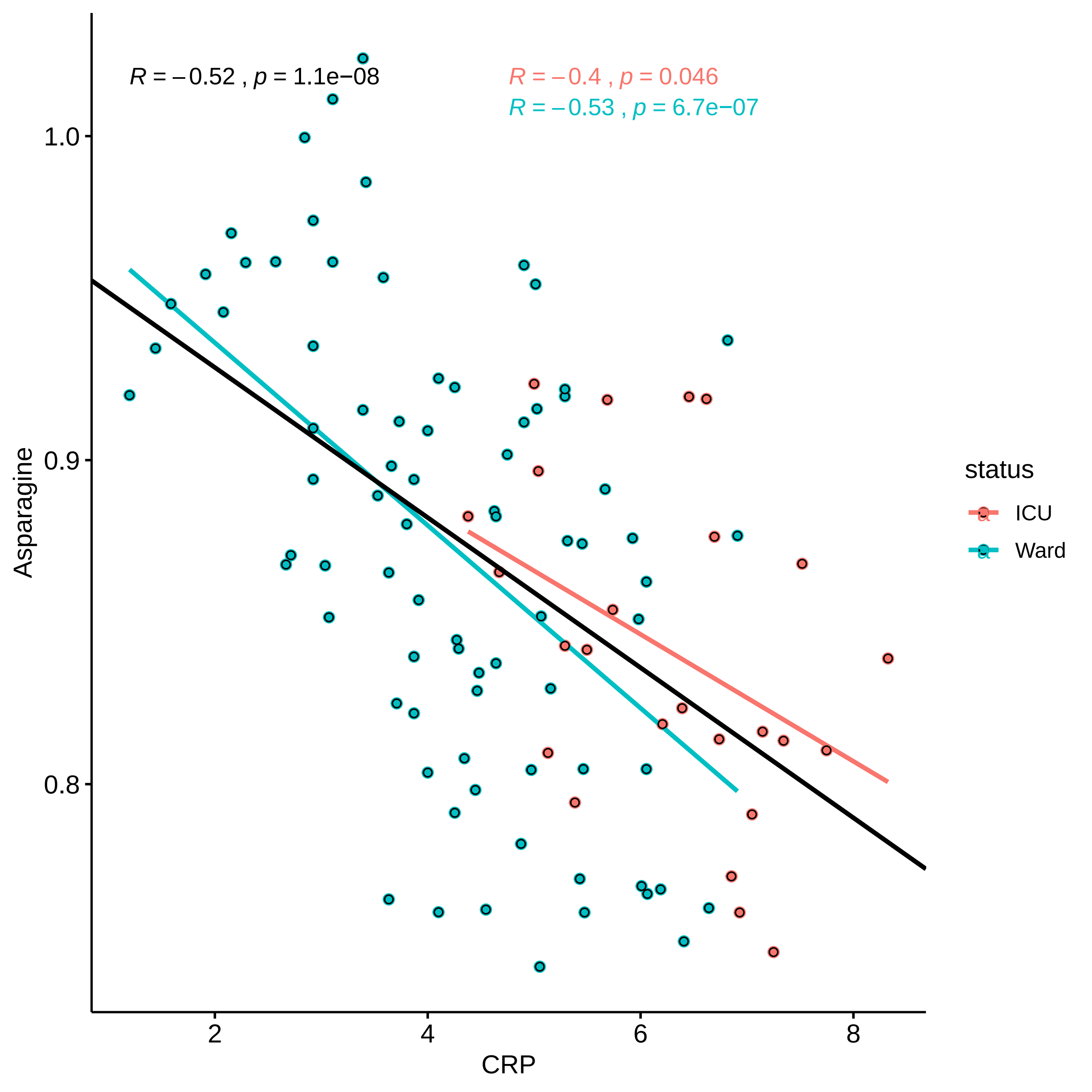

R = −0.52 , p = 1.1e−08
R = −0.4 , p = 0.046
R = −0.53 , p = 6.7e−07
1.0
0.9
0.8
Asparagine
2
4
6
8
CRP
status
ICU
Ward

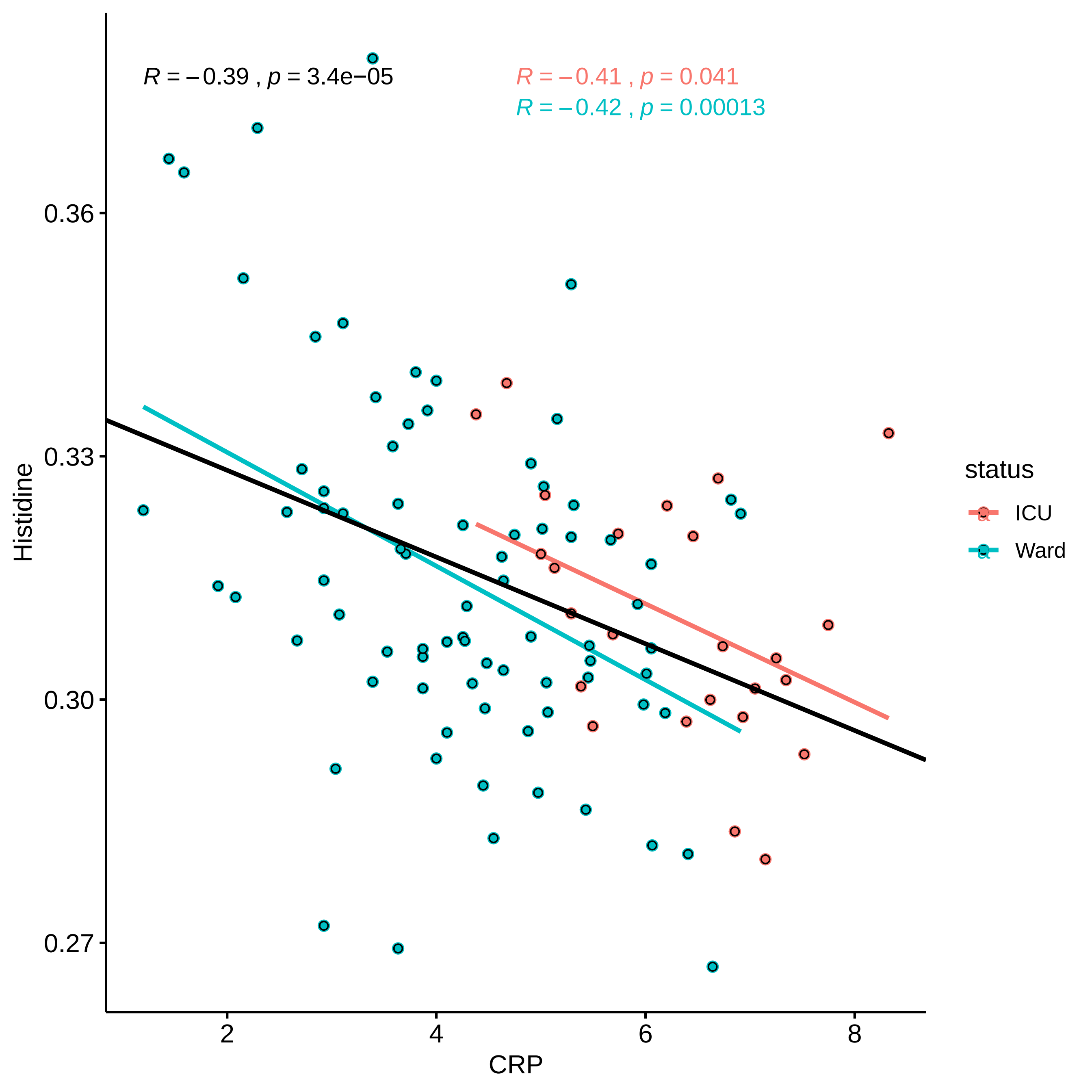

R = −0.39 , p = 3.4e−05
R = −0.41 , p = 0.041
R = −0.42 , p = 0.00013
Histidine
0.36
0.33
0.30
0.27
2
4
6
8
CRP
status
ICU
Ward

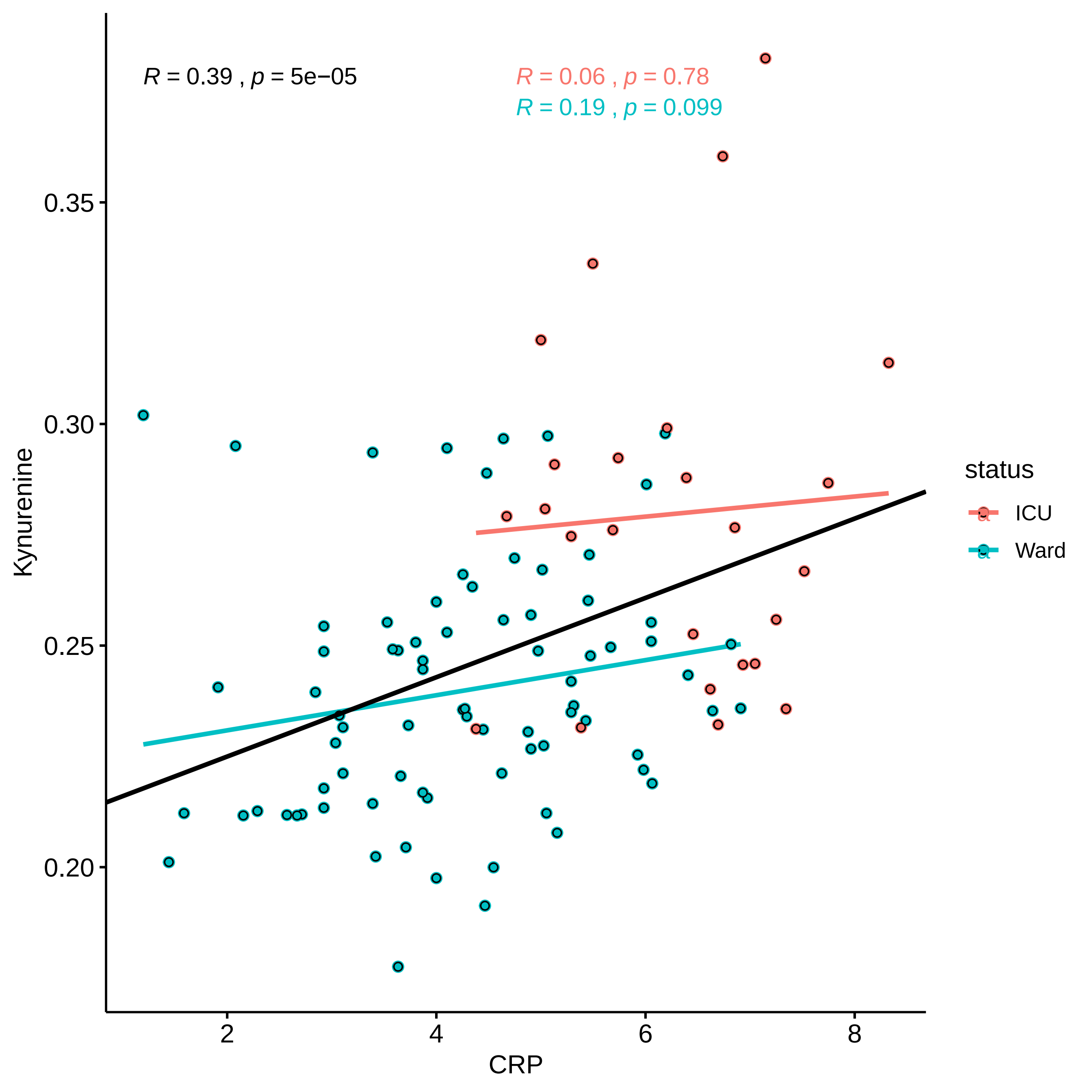

R = 0.39 , p = 5e−05
R = 0.06 , p = 0.78
R = 0.19 , p = 0.099
0.35
0.30
0.25
0.20
Kynurenine
2
4
6
8
CRP
status
ICU
Ward

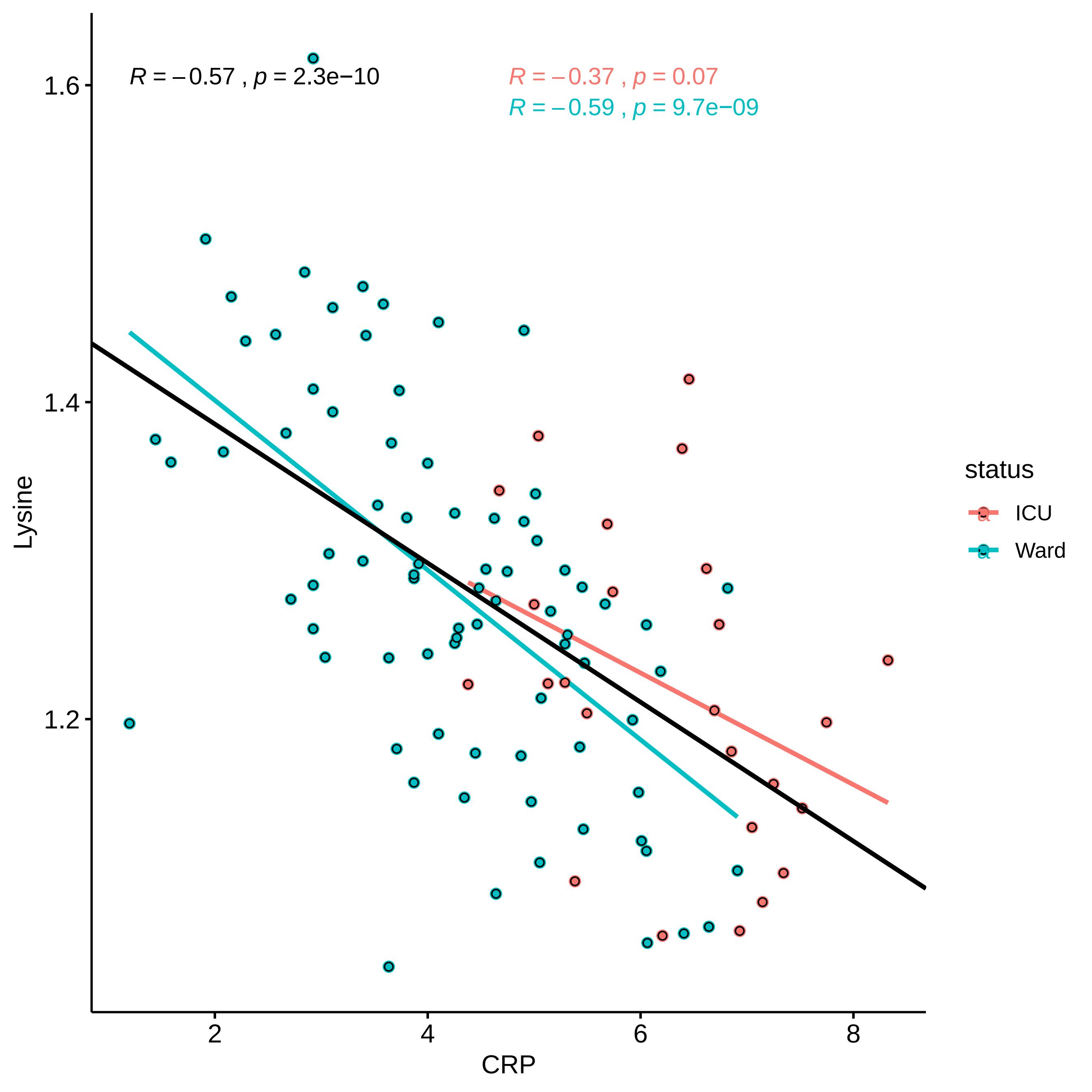

R = −0.57 , p = 2.3e−10
R = −0.37 , p = 0.07
R = −0.59 , p = 9.7e−09
Lysine
1.6
1.4
1.2
2
4
6
8
CRP
status
ICU
Ward

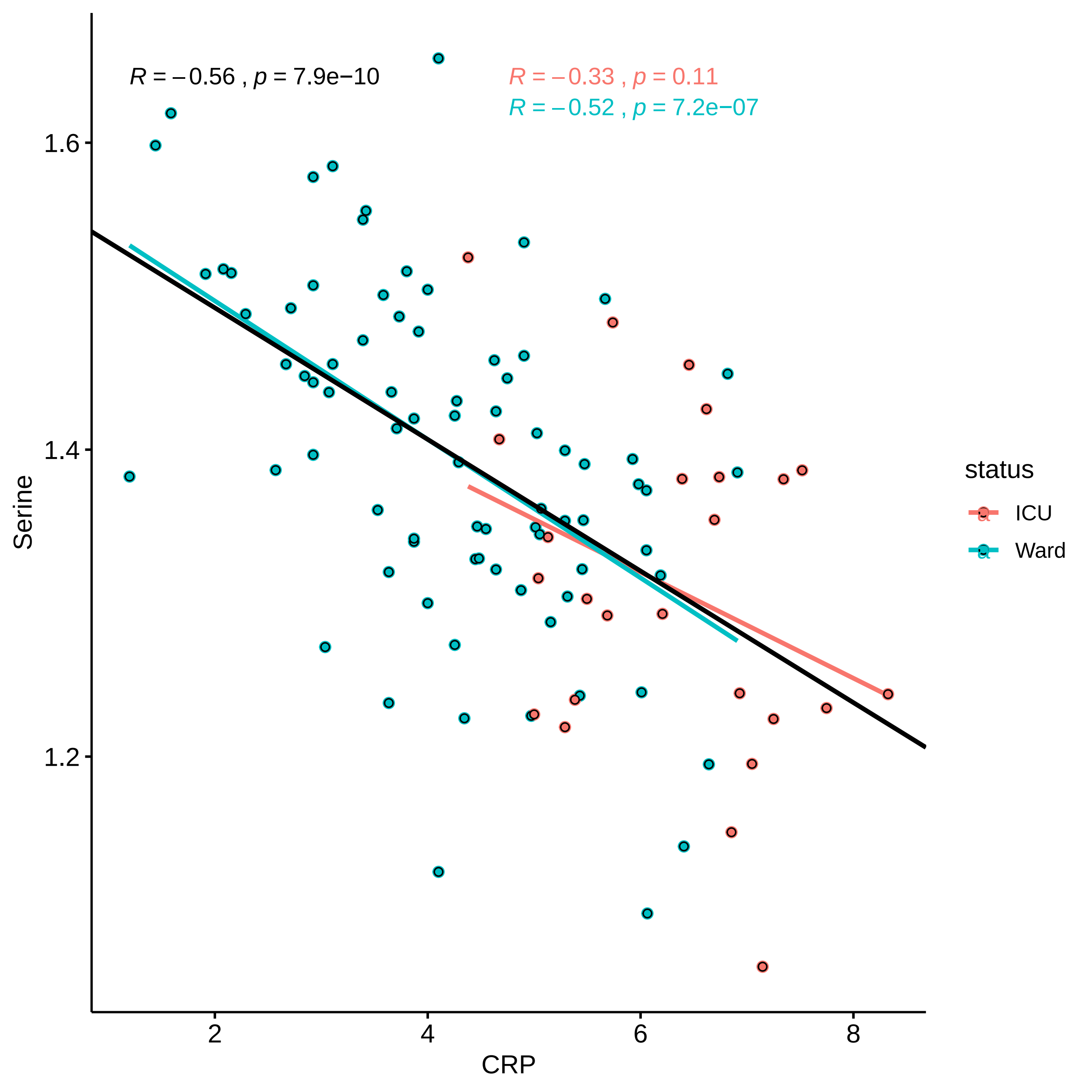

R = −0.56 , p = 7.9e−10
R = −0.33 , p = 0.11
R = −0.52 , p = 7.2e−07
1.6
1.4
1.2
Serine
2
4
6
8
CRP
status
ICU
Ward

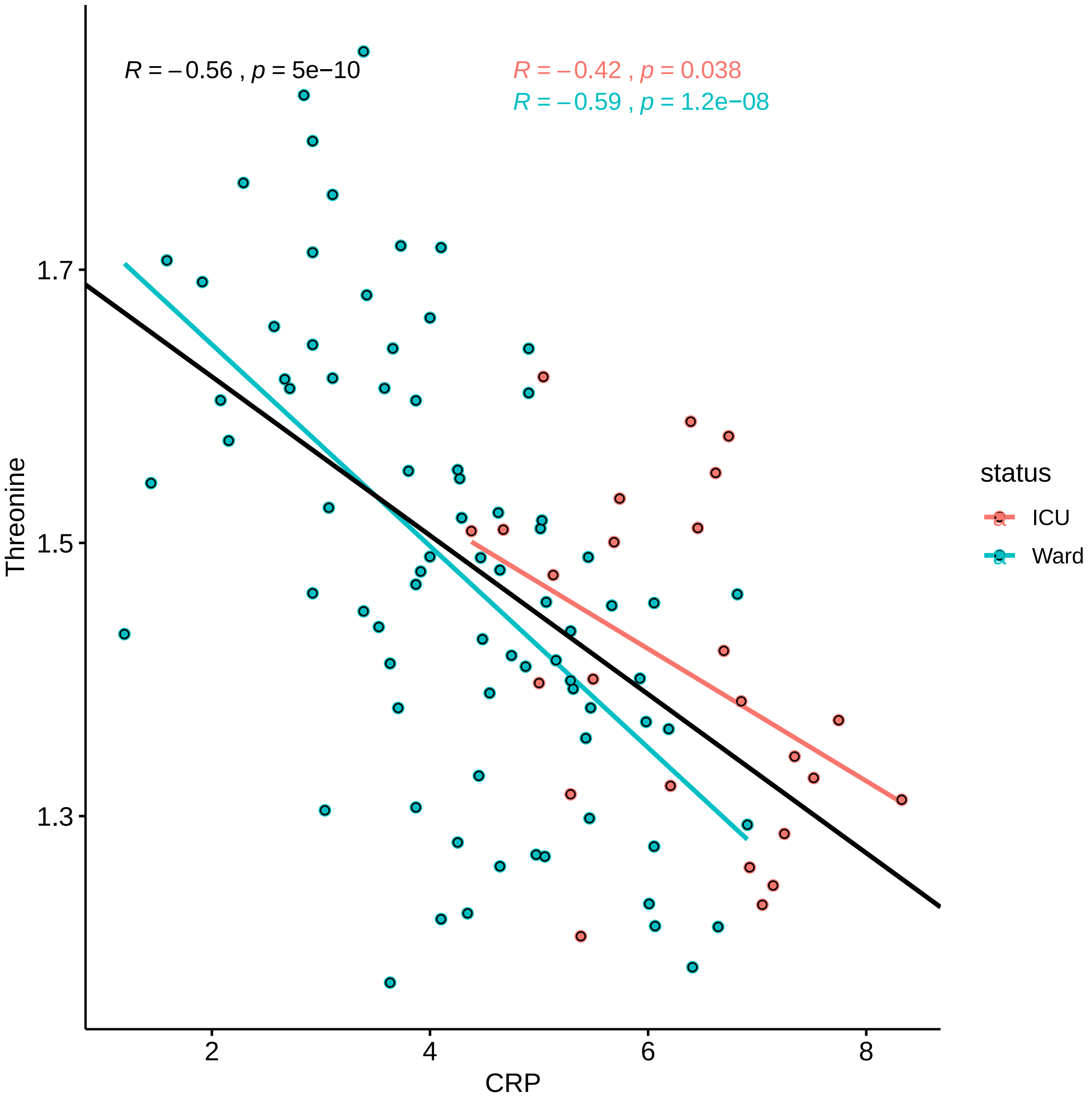

R = −0.56 , p = 5e−10
R = −0.42 , p = 0.038
R = −0.59 , p = 1.2e−08
Threonine
1.7
1.5
1.3
CRP
2
4
6
8
status
ICU
Ward

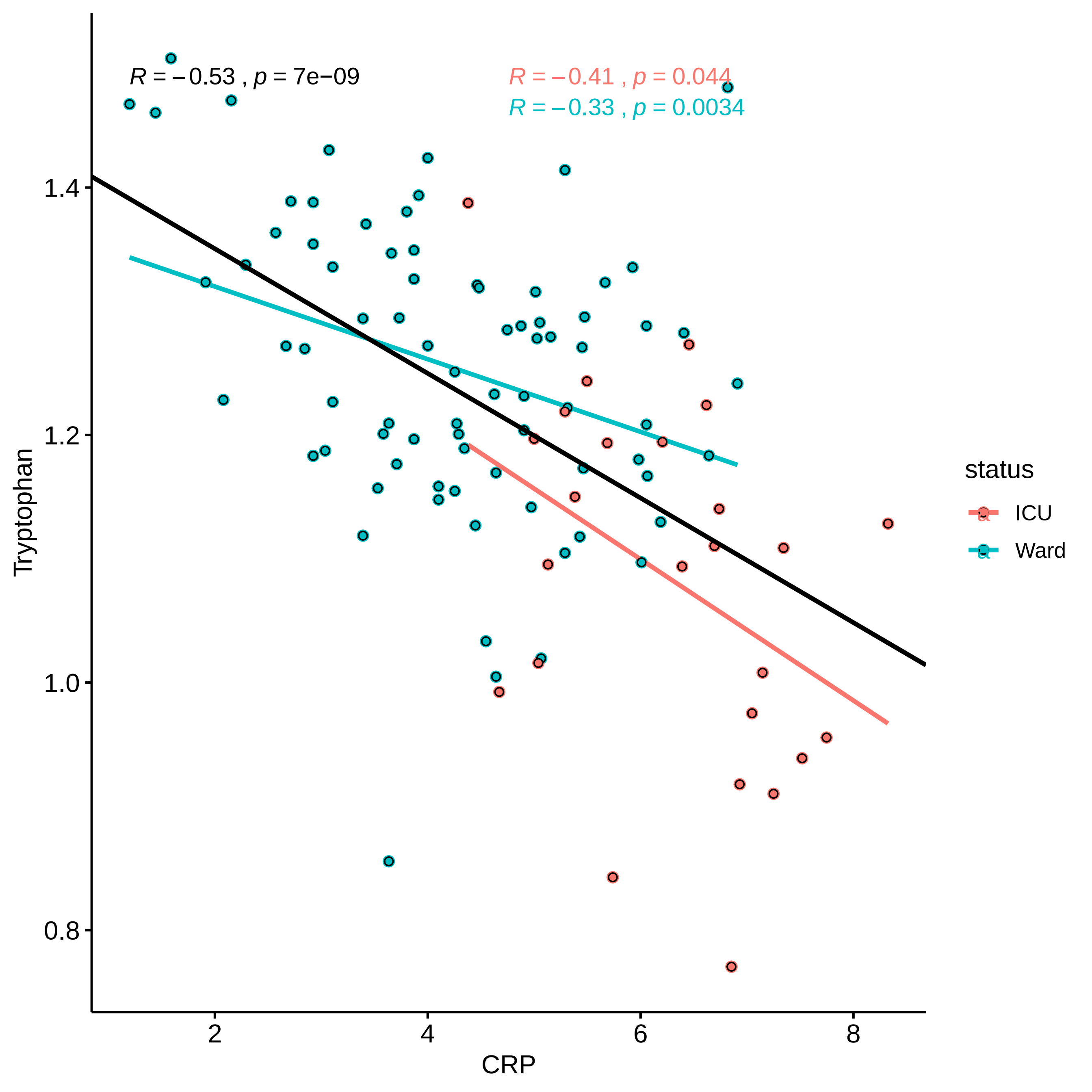

R = −0.53 , p = 7e−09
R = −0.41 , p = 0.044
R = −0.33 , p = 0.0034
Tryptophan
1.4
1.2
1.0
0.8
CRP
2
4
6
8
status
ICU
Ward

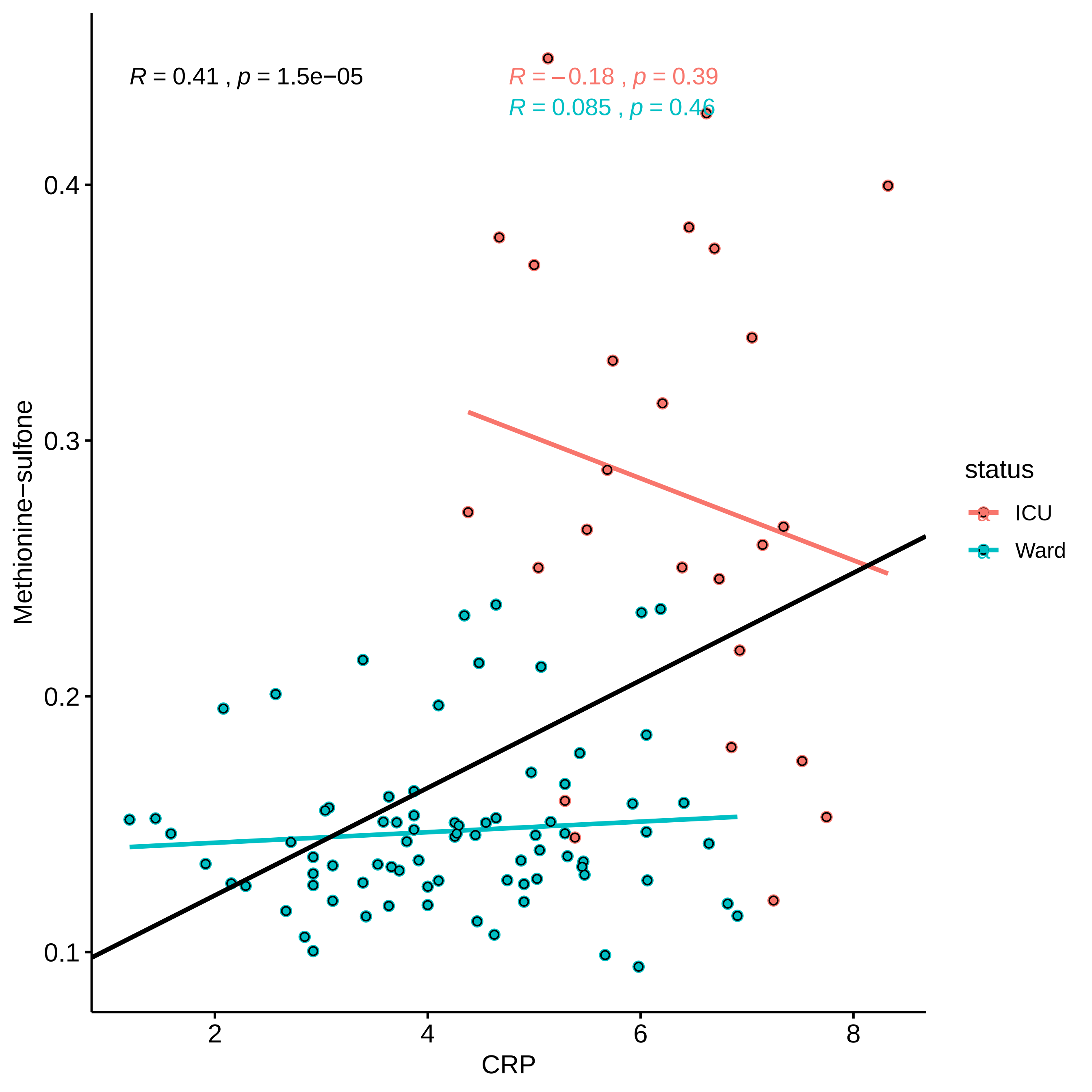

R = 0.41 , p = 1.5e−05
R = −0.18 , p = 0.39
R = 0.085 , p = 0.46
Methionine−sulfone
0.1
0.2
0.3
0.4
2
4
6
8
CRP
status
ICU
Ward

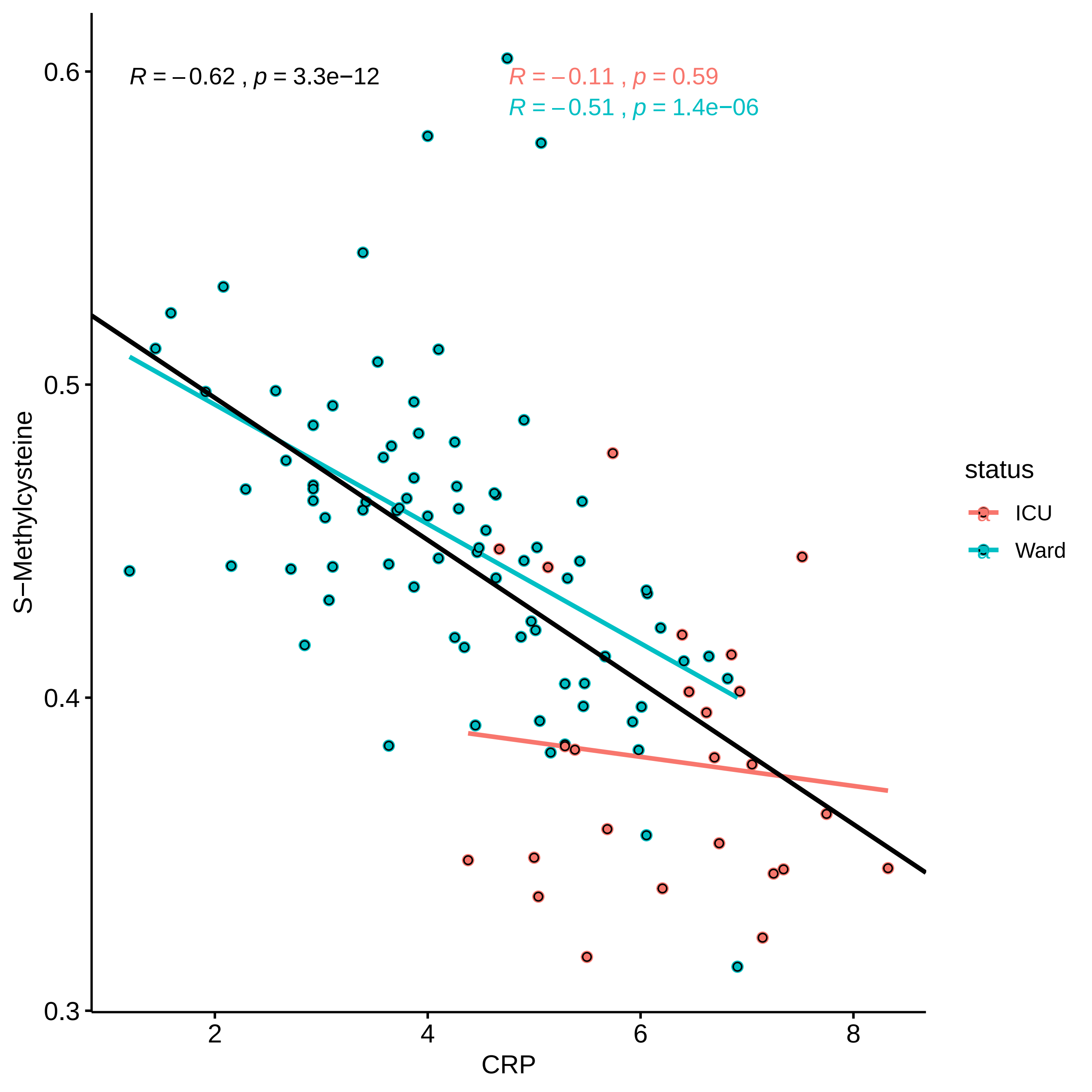

R = −0.62 , p = 3.3e−12
R = −0.11 , p = 0.59
R = −0.51 , p = 1.4e−06
S−Methylcysteine
0.6
0.5
0.4
0.3
2
4
6
8
CRP
status
ICU
Ward

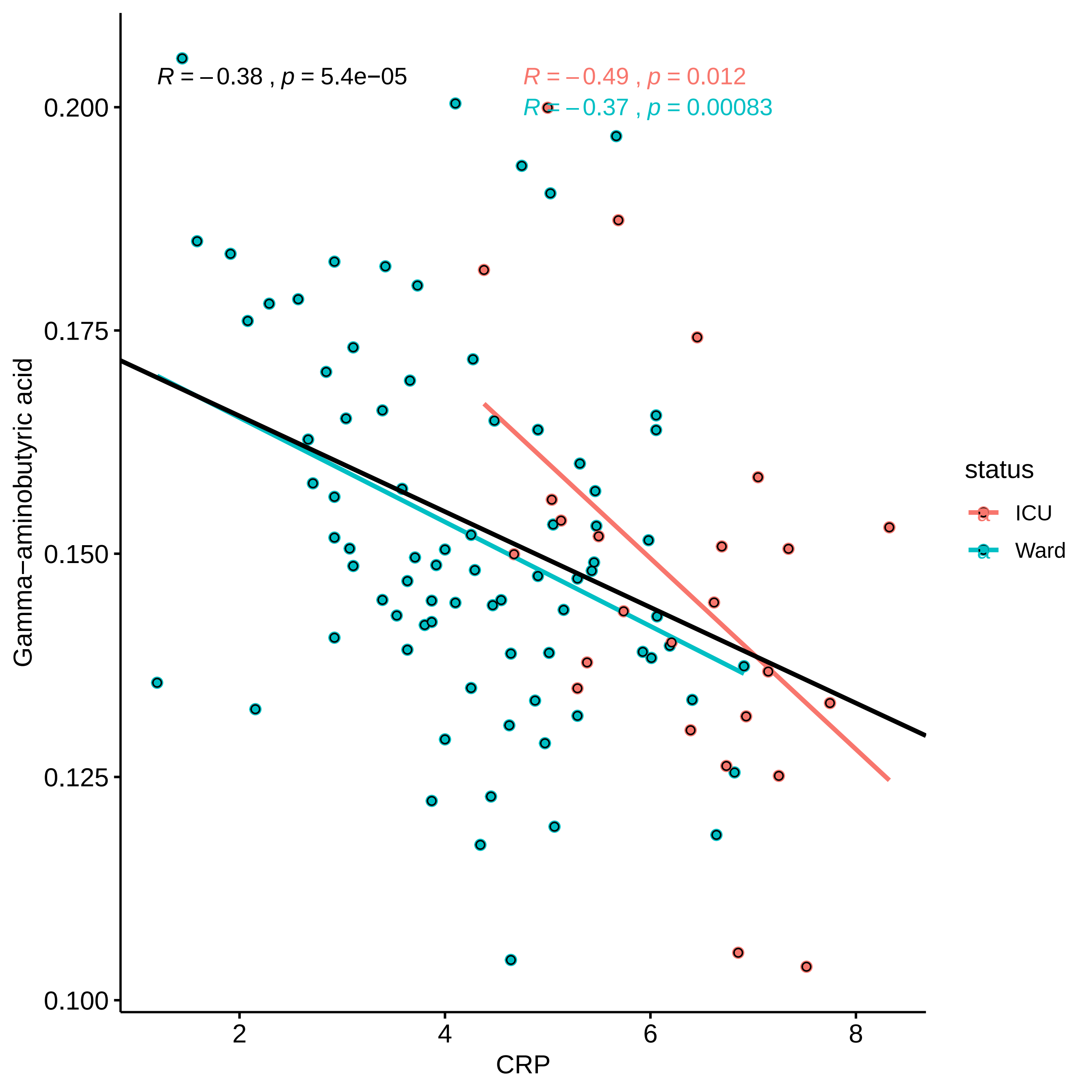

R = −0.38 , p = 5.4e−05
R = −0.49 , p = 0.012
R = −0.37 , p = 0.00083
0.200
0.175
0.150
0.125
0.100
Gamma−aminobutyric acid
2
4
6
8
CRP
status
ICU
Ward

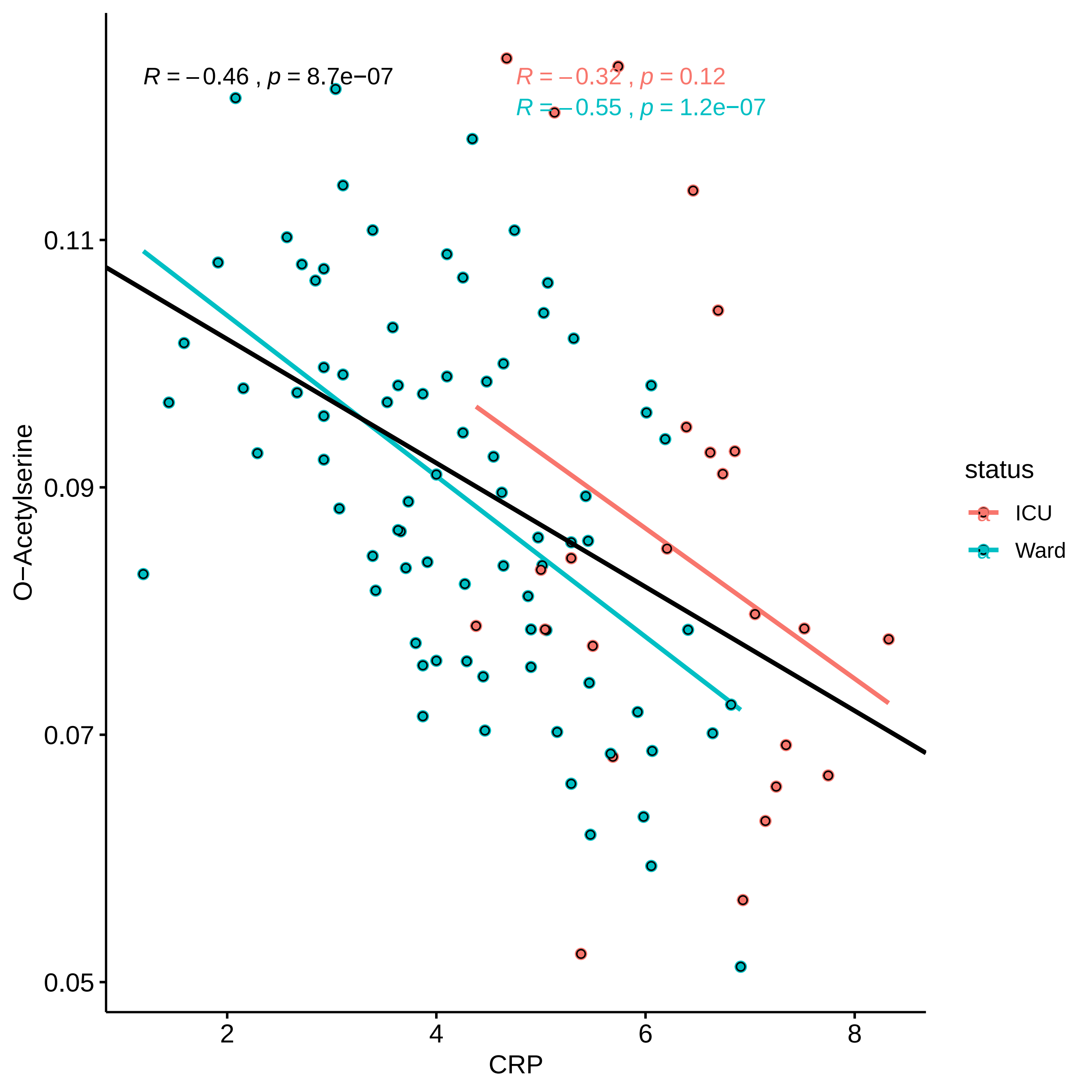

R = −0.46 , p = 8.7e−07
R = −0.32 , p = 0.12
R = −0.55 , p = 1.2e−07
O−Acetylserine
0.11
0.09
0.07
0.05
2
4
6
8
CRP
status
ICU
Ward

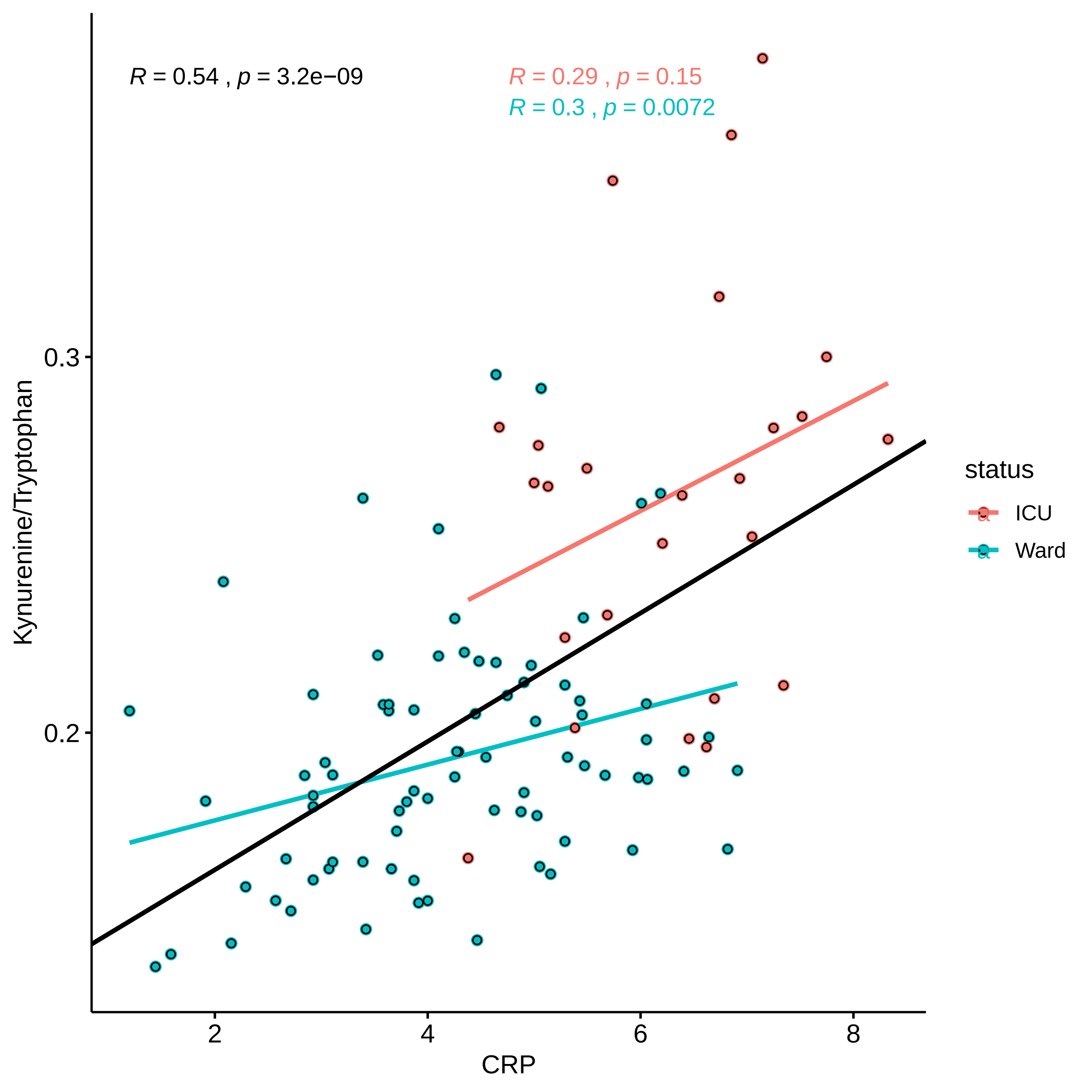

R = 0.54 , p = 3.2e−09
R = 0.29 , p = 0.15
R = 0.3 , p = 0.0072
Kynurenine/Tryptophan
0.3
0.2
2
4
6
8
CRP
status
ICU
Ward

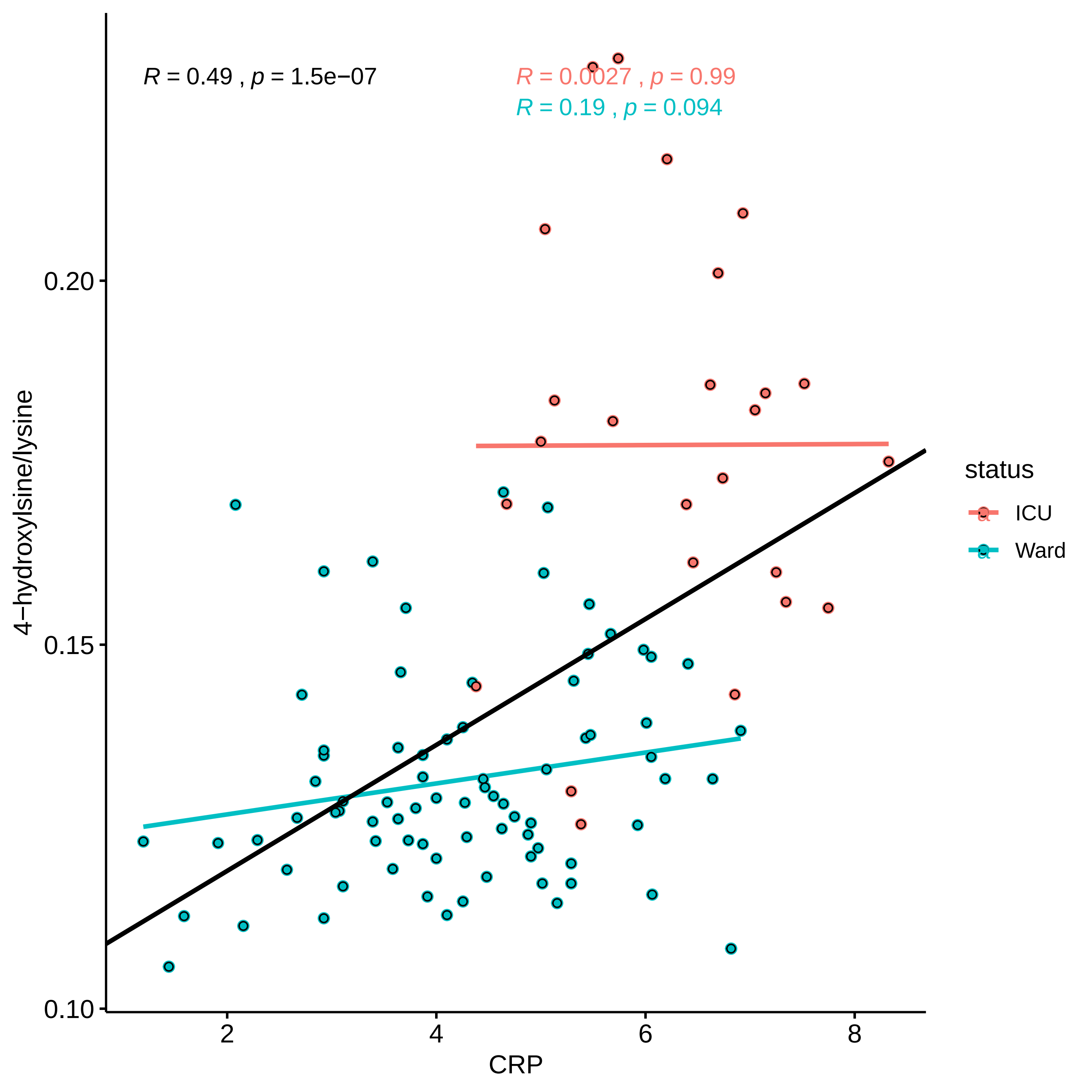

R = 0.49 , p = 1.5e−07
R = 0.0027 , p = 0.99
R = 0.19 , p = 0.094
4−hydroxylsine/lysine
0.20
0.15
0.10
2
4
6
8
CRP
status
ICU
Ward

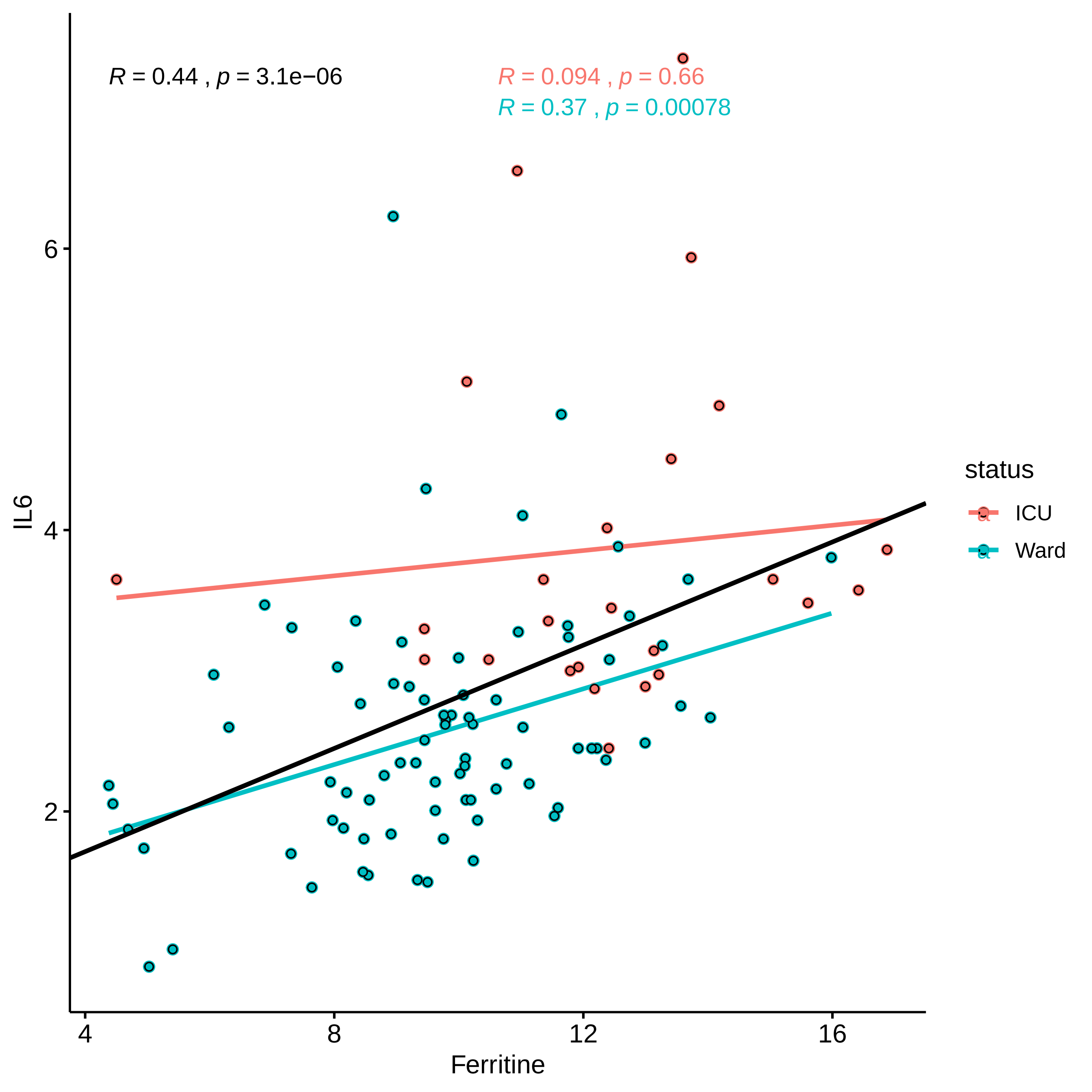

R = 0.44 , p = 3.1e−06
R = 0.094 , p = 0.66
R = 0.37 , p = 0.00078
IL6
6
4
2
4
8
12
16
Ferritine
status
ICU
Ward

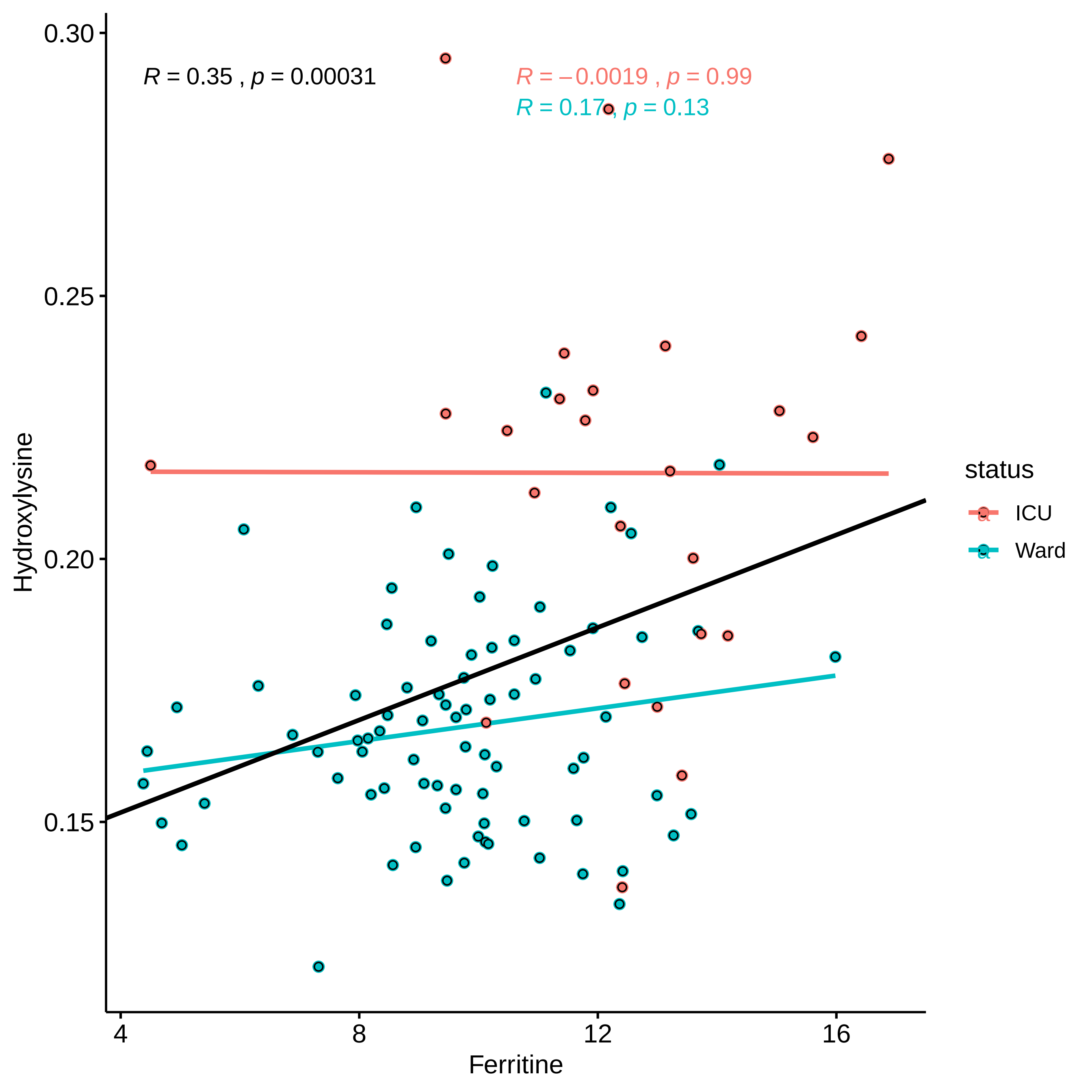

R = 0.35 , p = 0.00031
R = −0.0019 , p = 0.99
R = 0.17 , p = 0.13
Hydroxylysine
0.30
0.25
0.20
0.15
Ferritine
4
8
12
16
status
ICU
Ward

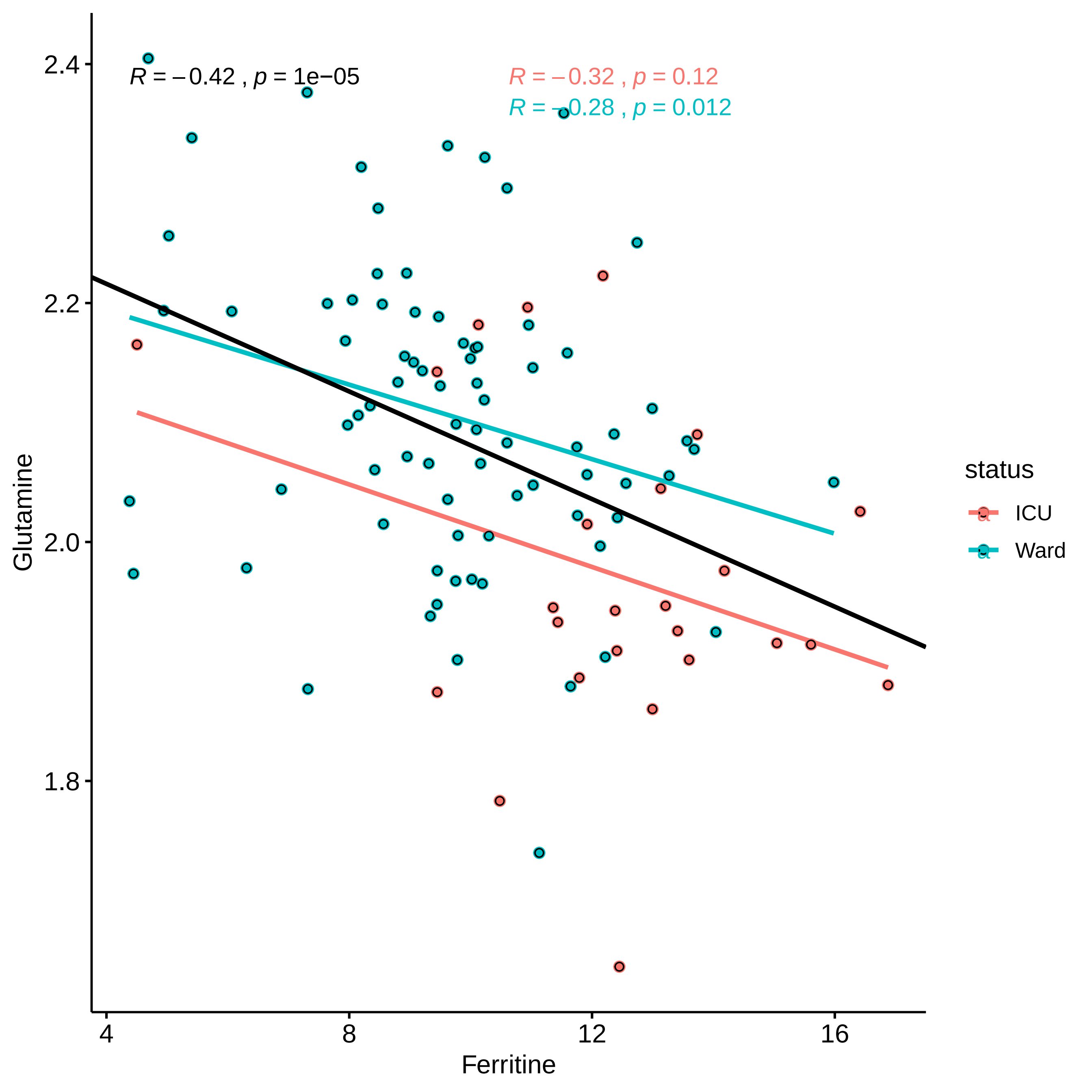

R = −0.42 , p = 1e−05
R = −0.32 , p = 0.12
R = −0.28 , p = 0.012
Glutamine
2.4
2.2
2.0
1.8
4
8
12
16
Ferritine
status
ICU
Ward

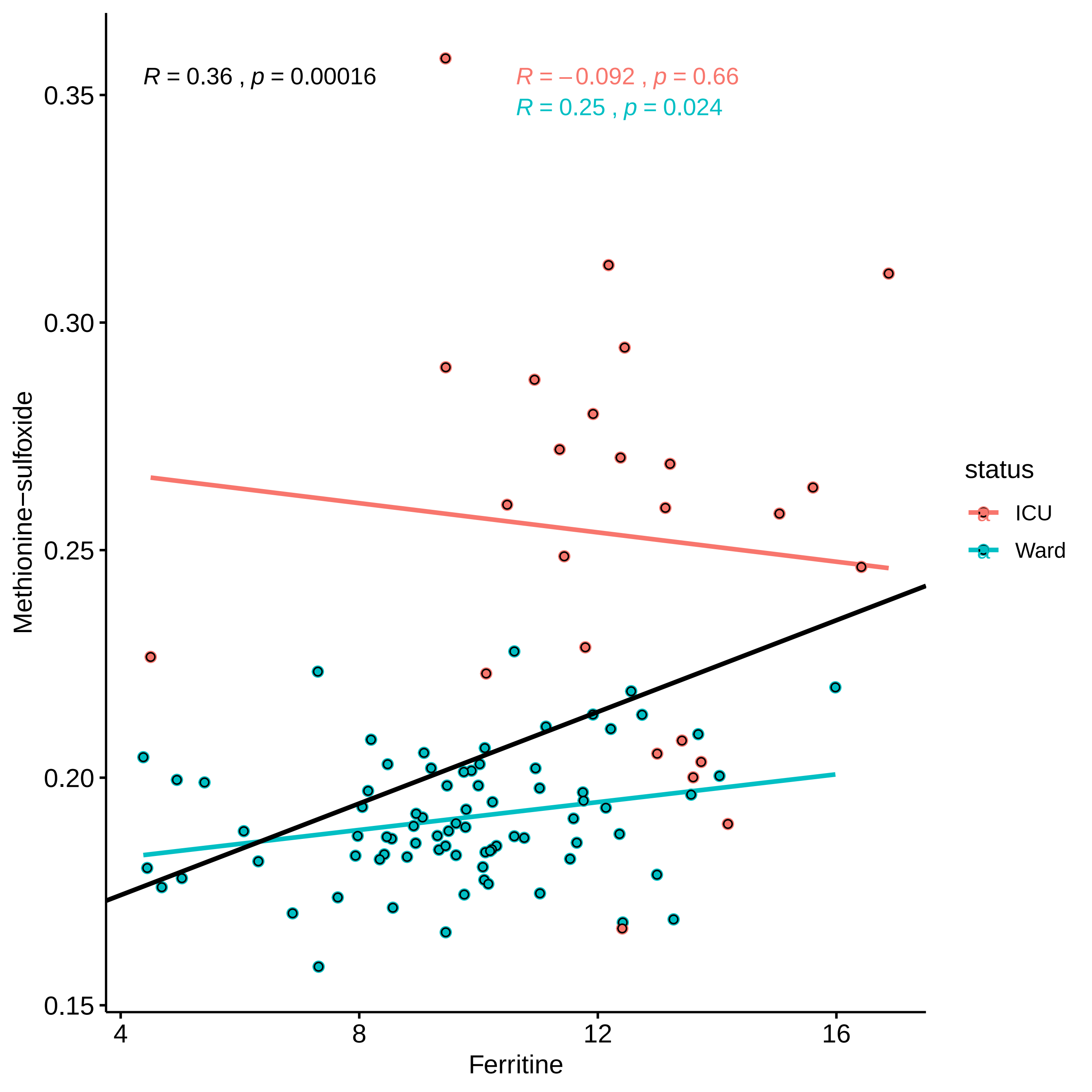

R = 0.36 , p = 0.00016
R = −0.092 , p = 0.66
R = 0.25 , p = 0.024
0.35
0.30
0.25
0.20
0.15
Methionine−sulfoxide
4
8
12
16
Ferritine
status
ICU
Ward

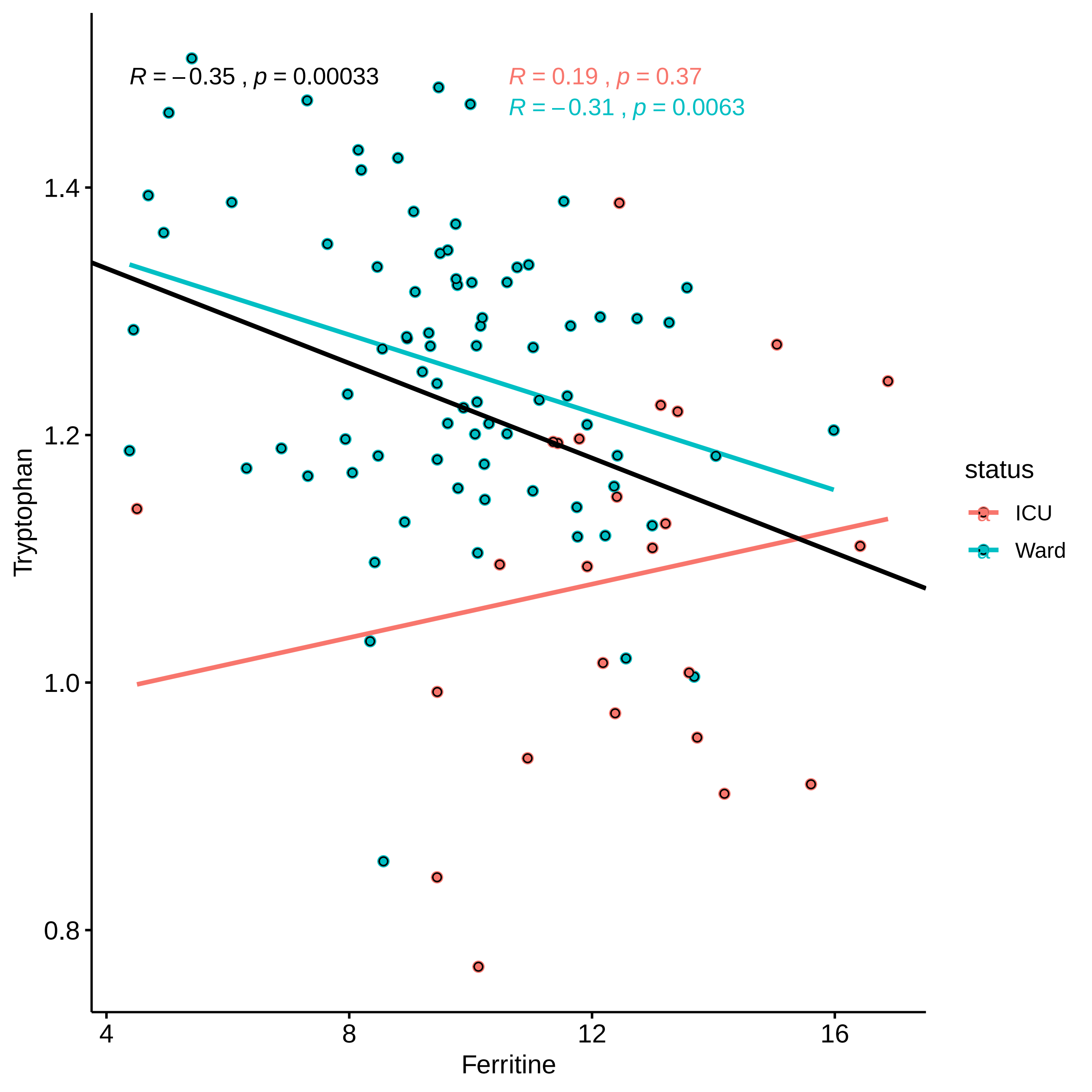

R = −0.35 , p = 0.00033
R = 0.19 , p = 0.37
R = −0.31 , p = 0.0063
Tryptophan
1.4
1.2
1.0
0.8
4
8
12
16
Ferritine
status
ICU
Ward

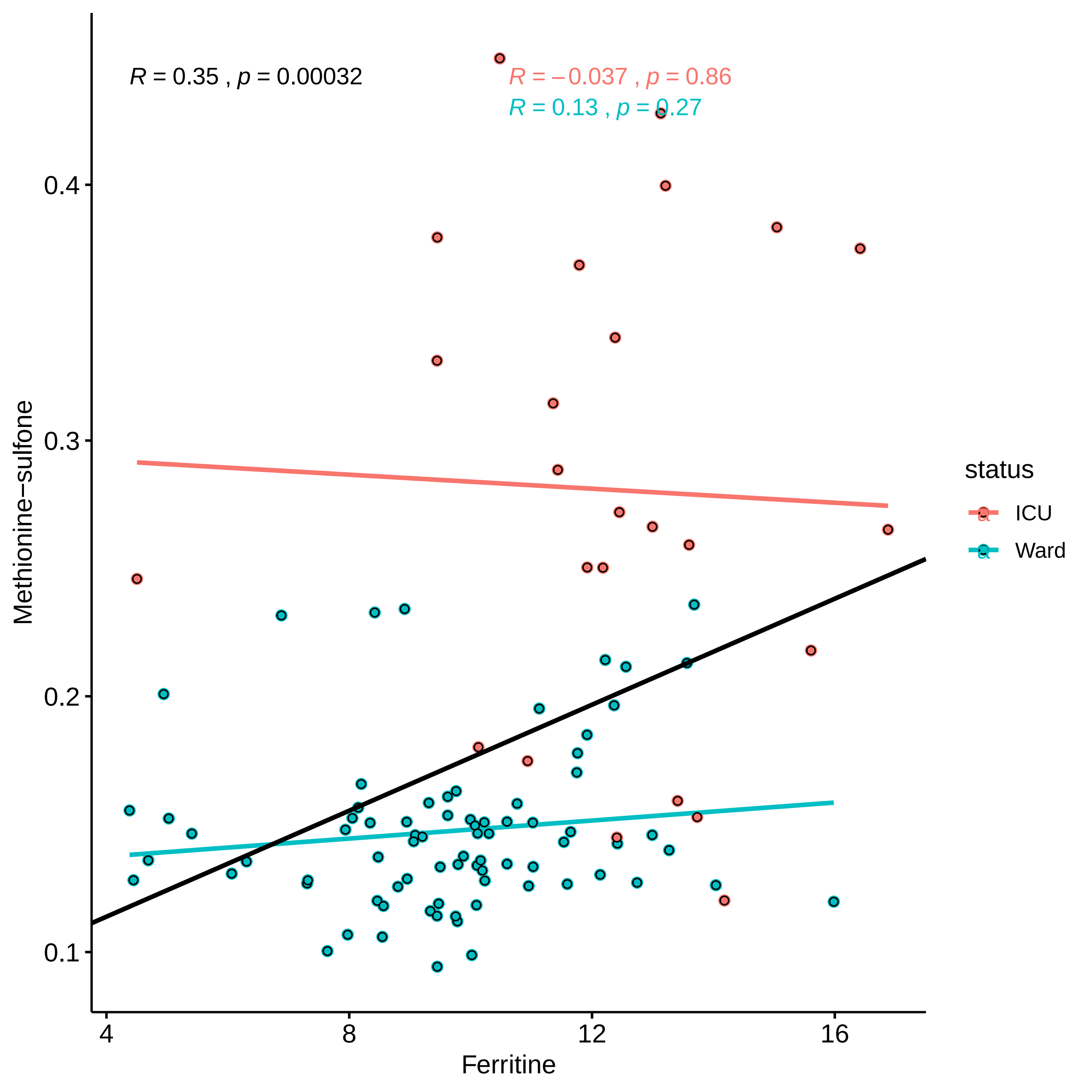

R = 0.35 , p = 0.00032
R = −0.037 , p = 0.86
R = 0.13 , p = 0.27
0.4
0.3
0.2
0.1
Methionine−sulfone
4
8
12
16
Ferritine
status
ICU
Ward

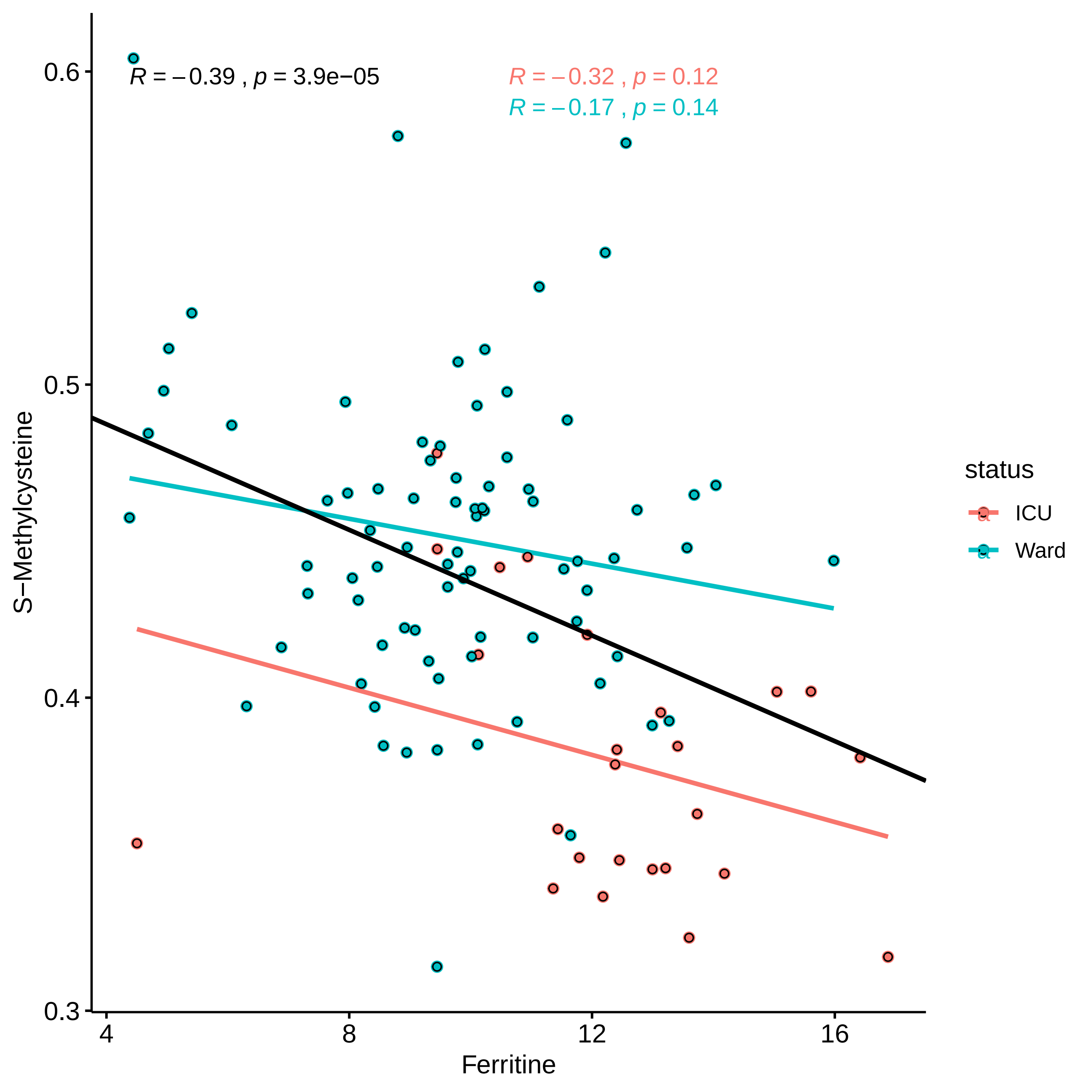

R = −0.39 , p = 3.9e−05
R = −0.32 , p = 0.12
R = −0.17 , p = 0.14
S−Methylcysteine
0.6
0.5
0.4
0.3
4
8
12
16
Ferritine
status
ICU
Ward

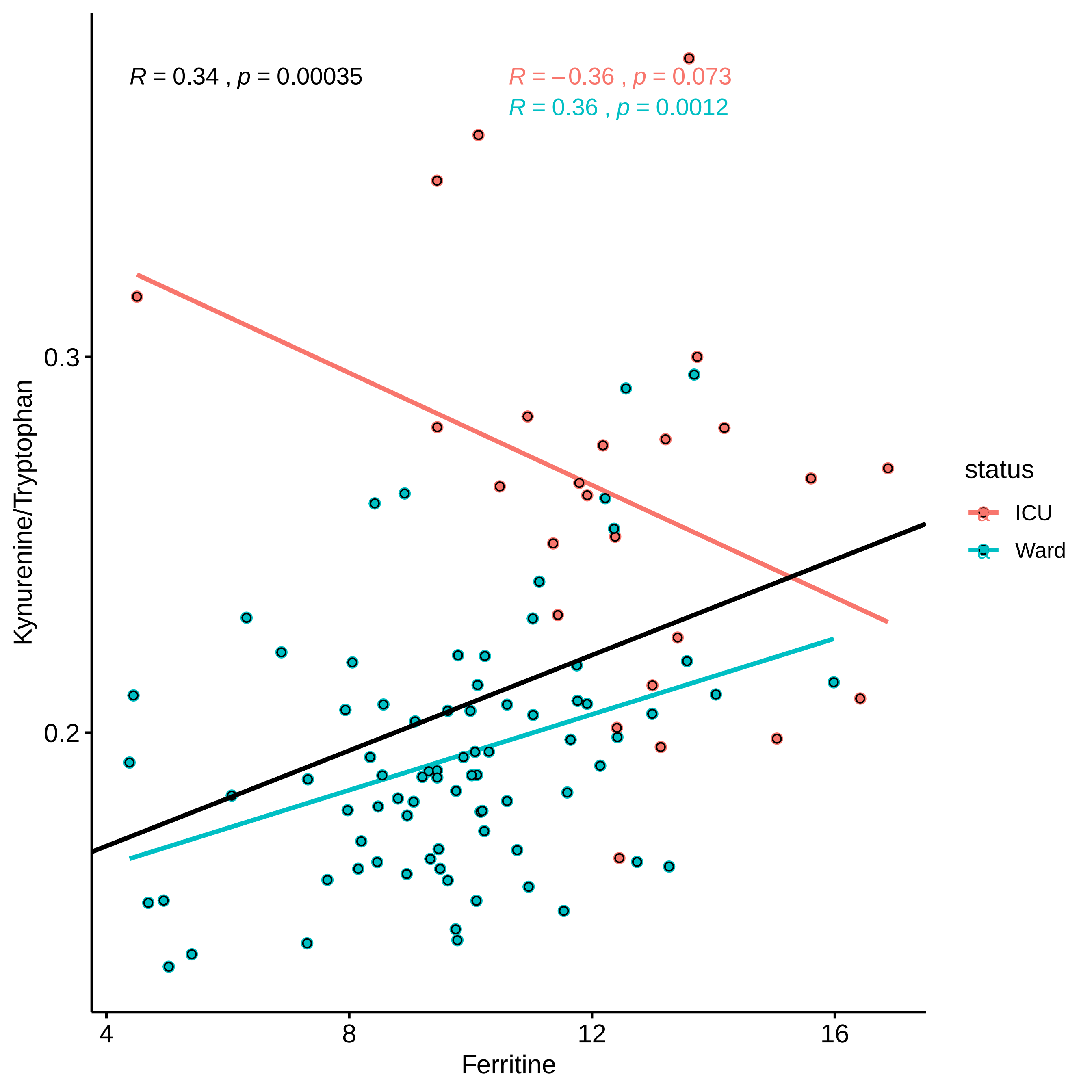

$R = 0.34$, $p = 0.00035$
$R = -0.36$, $p = 0.073$
$R = 0.36$, $p = 0.0012$
Kynurenine/Tryptophan
0.3
0.2
4
8
12
16
Ferritine
status
ICU
Ward

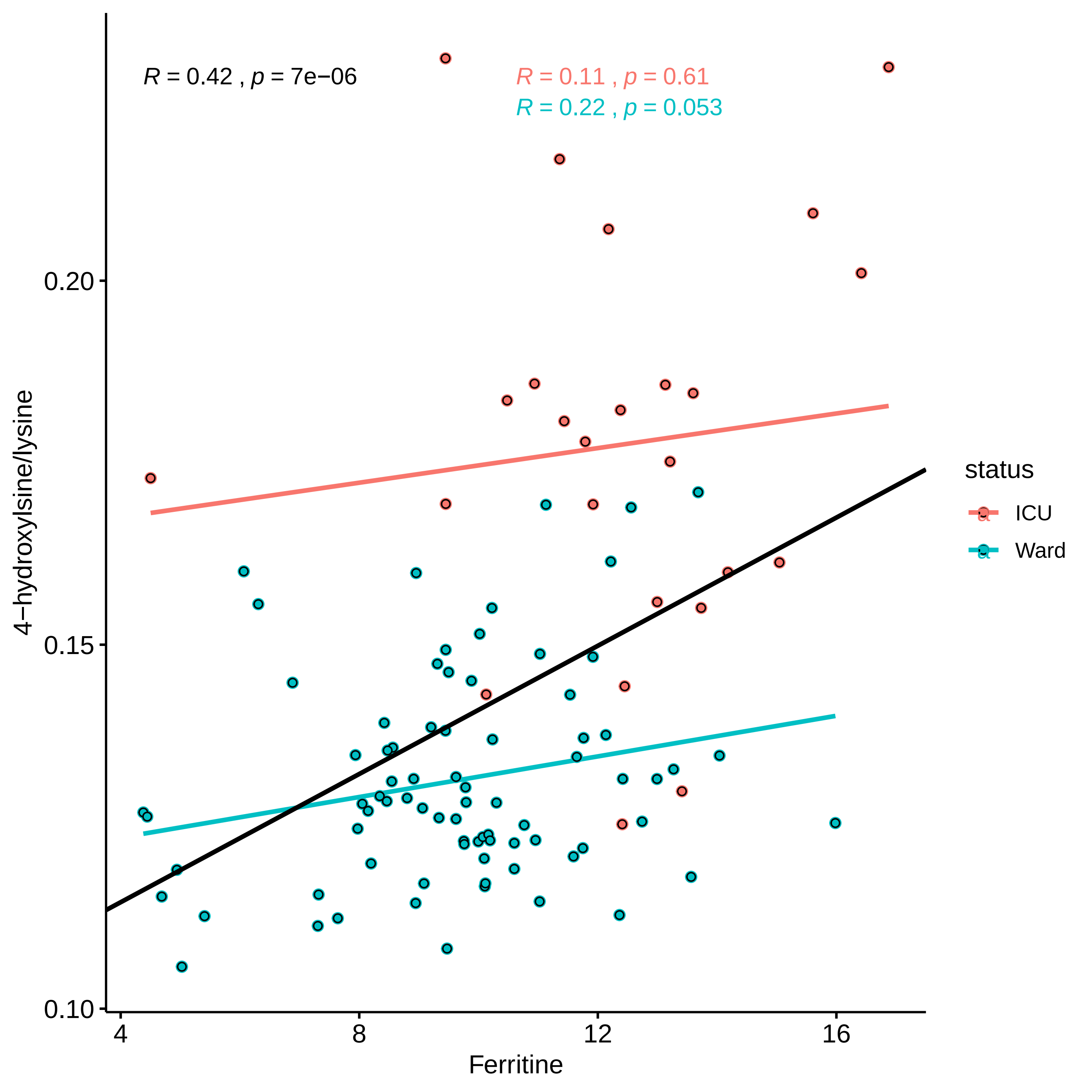

R = 0.42 , p = 7e−06
R = 0.11 , p = 0.61
R = 0.22 , p = 0.053
4−hydroxylsine/lysine
0.20
0.15
0.10
4
8
12
16
Ferritine
status
ICU
Ward

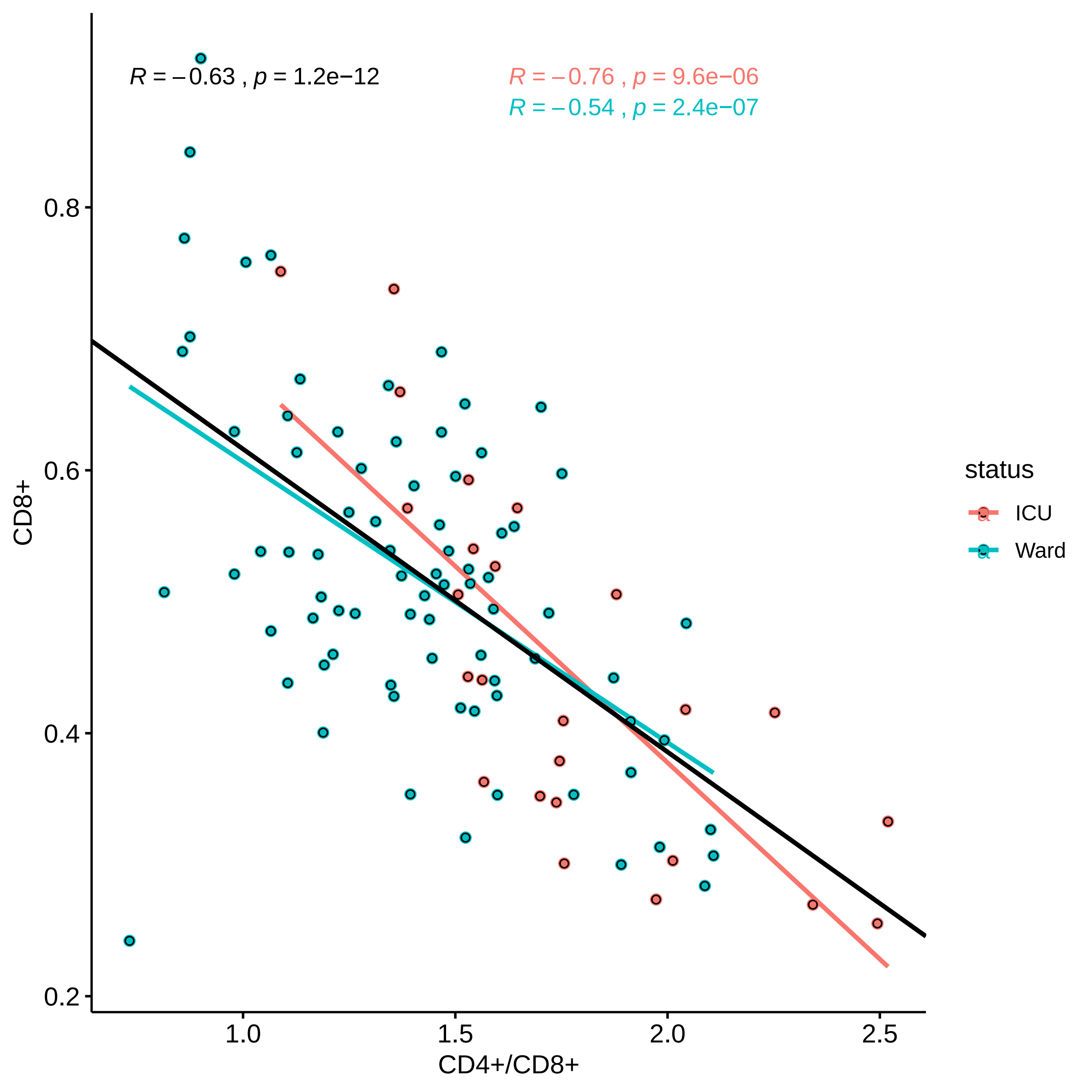

R = −0.63 , p = 1.2e−12
R = −0.76 , p = 9.6e−06
R = −0.54 , p = 2.4e−07
0.8
0.6
0.4
0.2
CD8+
1.0
1.5
2.0
2.5
CD4+/CD8+
status
ICU
Ward

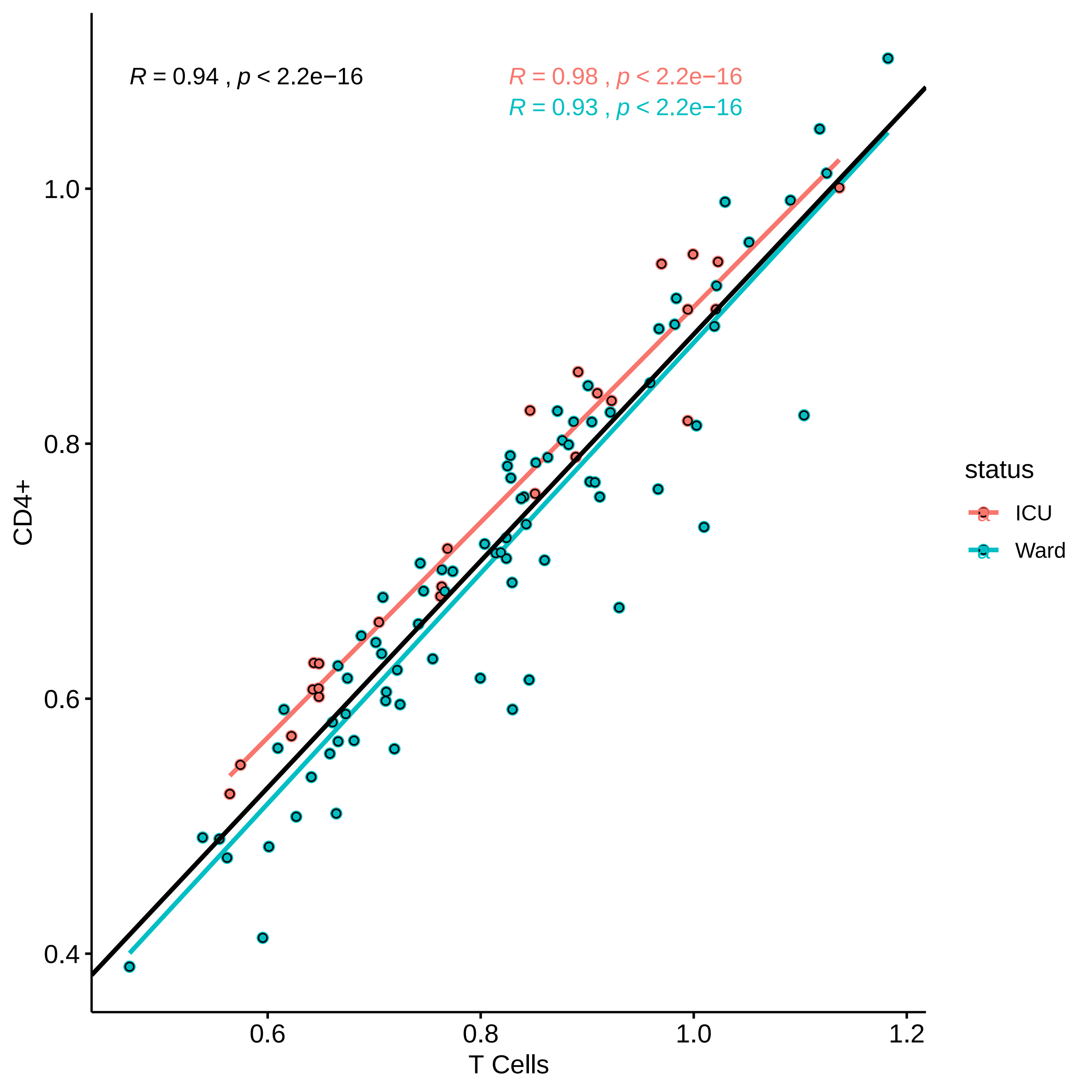

R = 0.94 , p < 2.2e−16
R = 0.98 , p < 2.2e−16
R = 0.93 , p < 2.2e−16
CD4+
1.0
0.8
0.6
0.4
0.6
0.8
1.0
1.2
T Cells
status
ICU
Ward

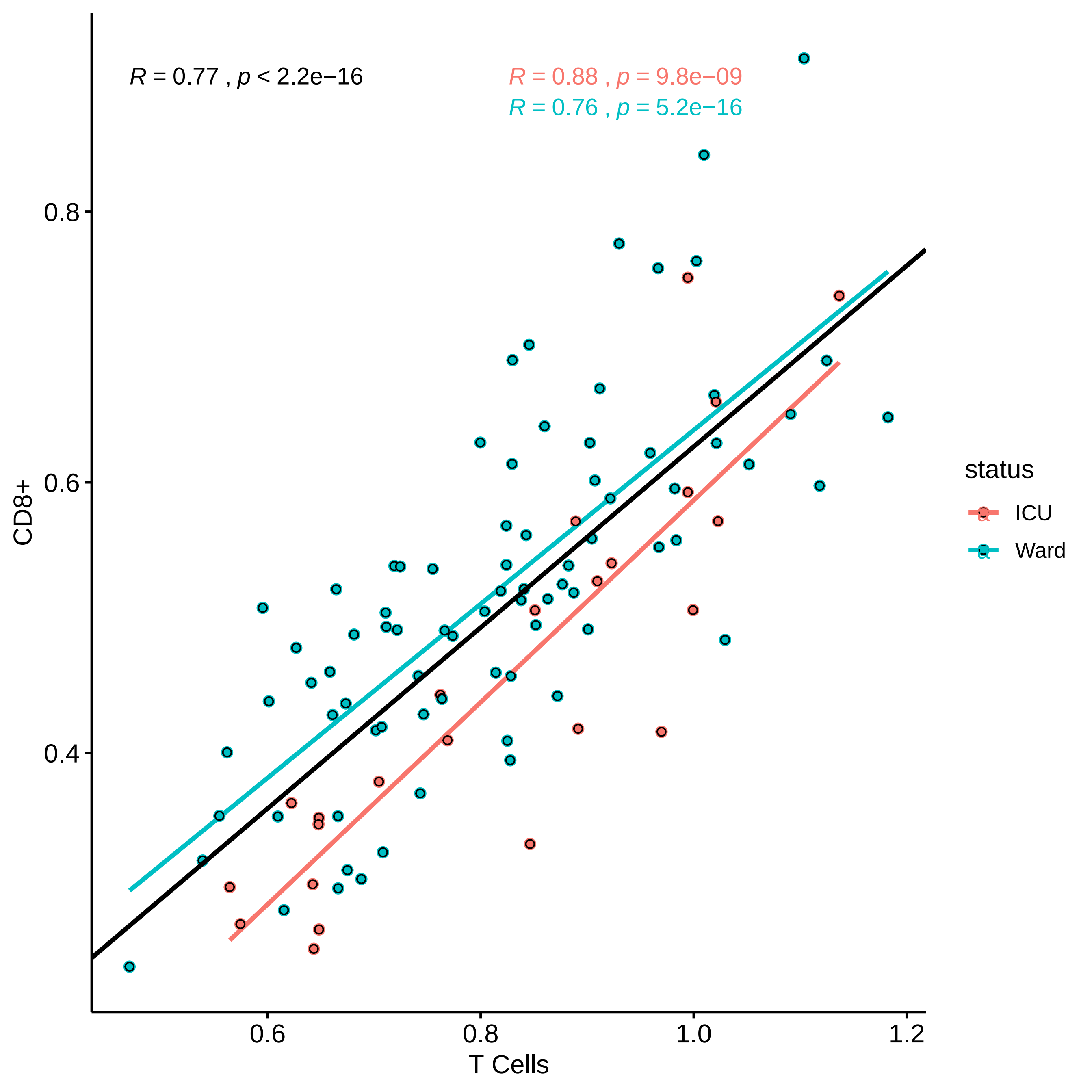

R = 0.77 , p < 2.2e−16
R = 0.88 , p = 9.8e−09
R = 0.76 , p = 5.2e−16
CD8+
T Cells
0.4
0.6
0.8
0.6
0.8
1.0
1.2
status
ICU
Ward

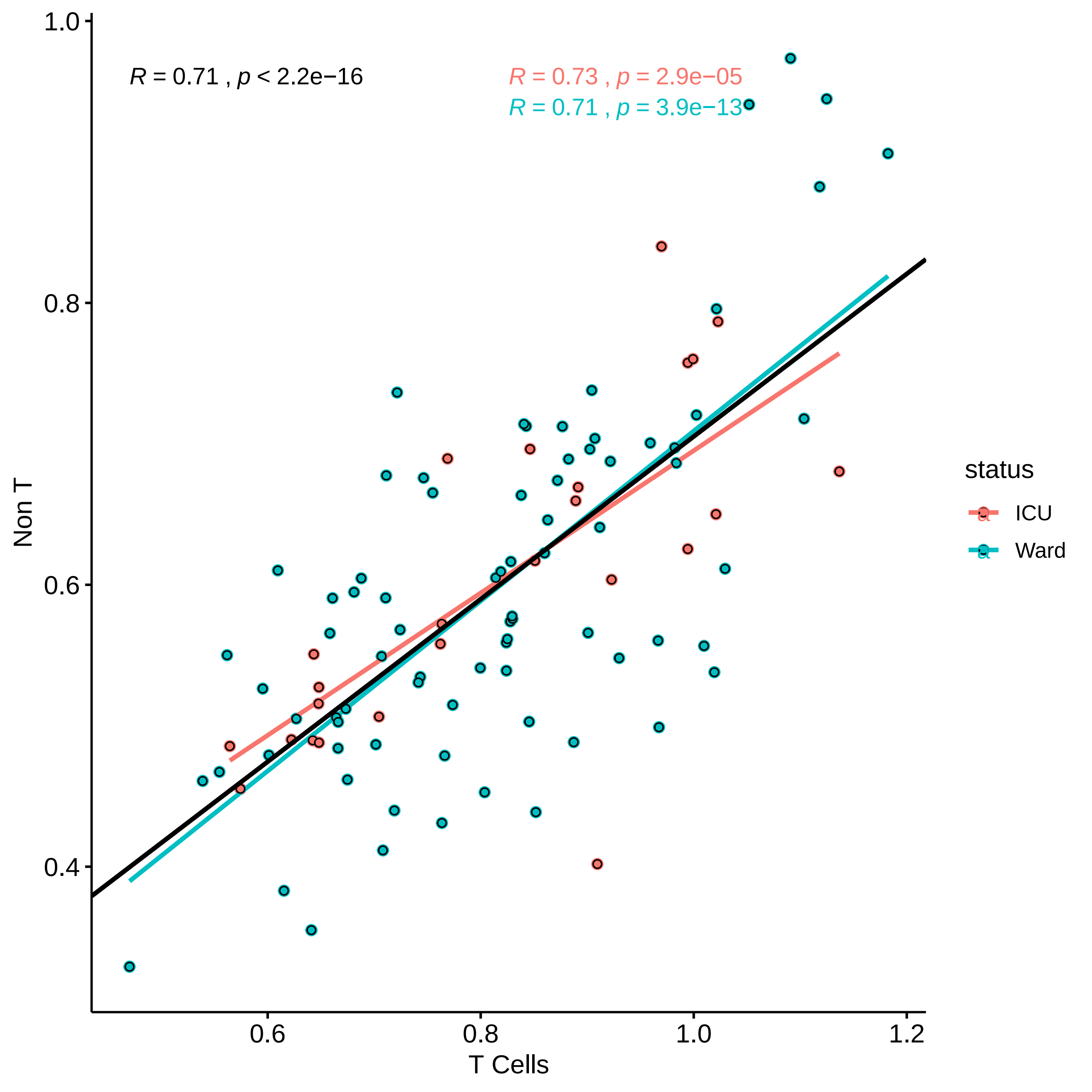

R = 0.71 , p < 2.2e−16
R = 0.73 , p = 2.9e−05
R = 0.71 , p = 3.9e−13
Non T
T Cells
status
ICU
Ward
1.0
0.8
0.6
0.4
0.6
0.8
1.0
1.2

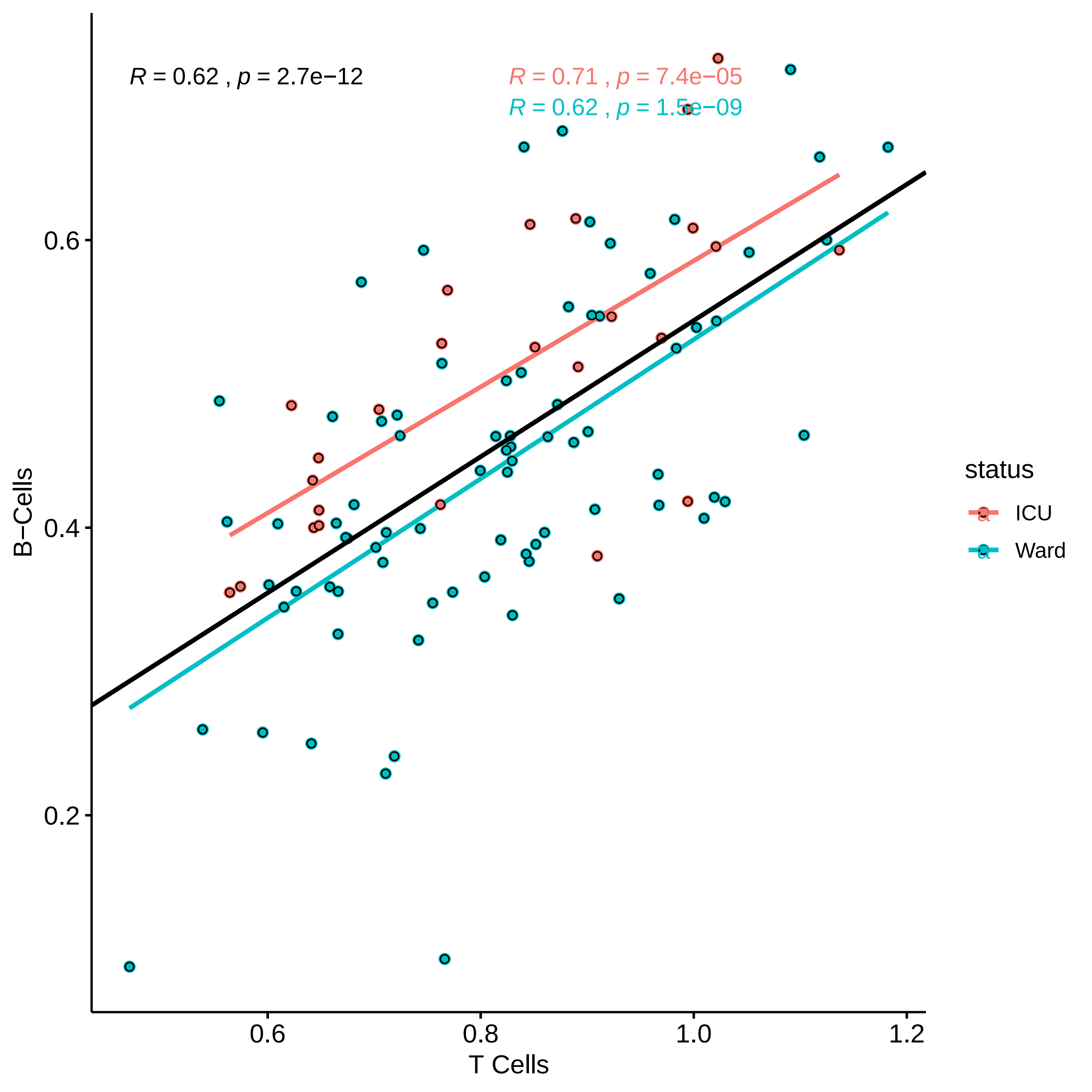

R = 0.62 , p = 2.7e−12
R = 0.71 , p = 7.4e−05
R = 0.62 , p = 1.5e−09
B−Cells
0.6
0.4
0.2
0.6
0.8
1.0
1.2
T Cells
status
ICU
Ward

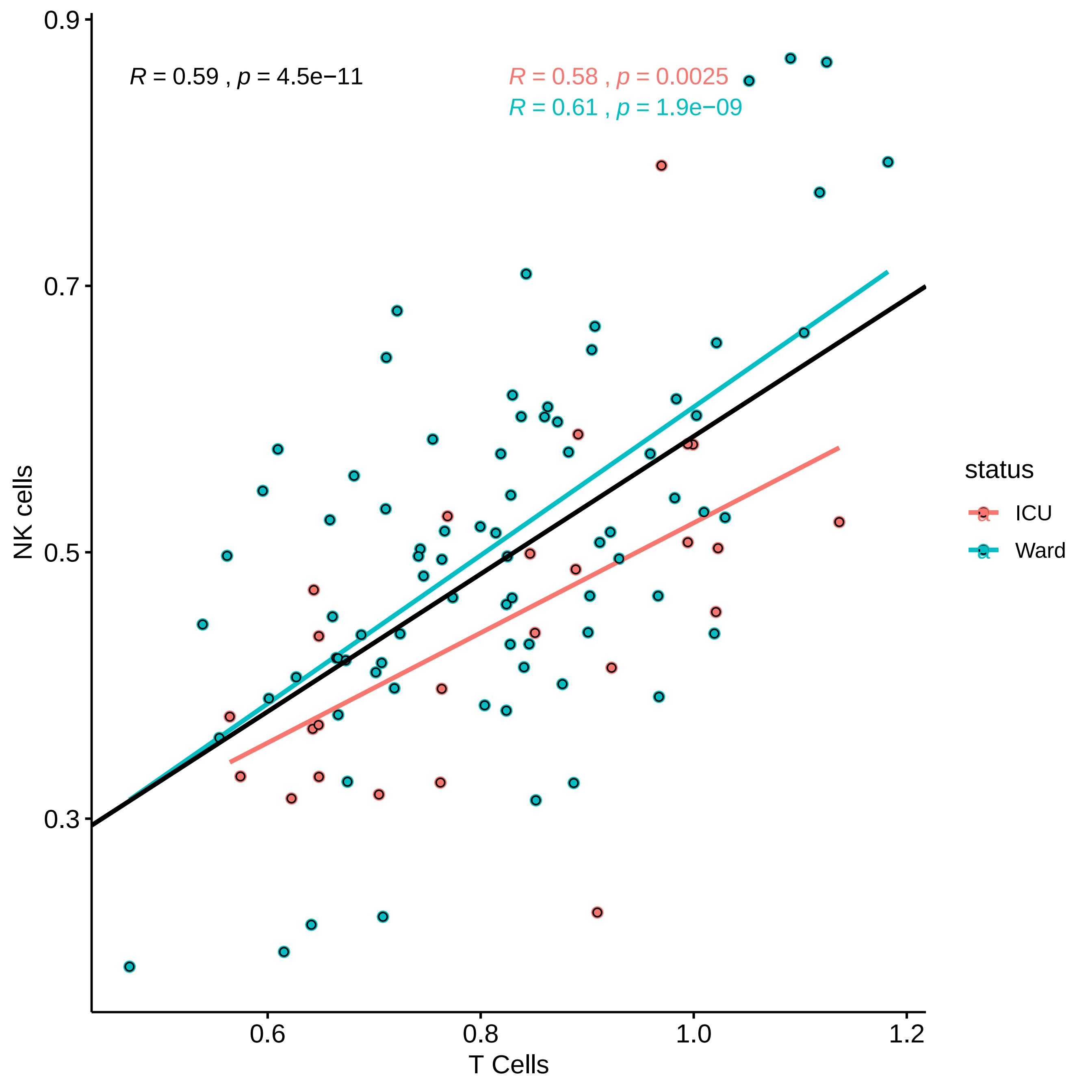

R = 0.59 , p = 4.5e−11
R = 0.58 , p = 0.0025
R = 0.61 , p = 1.9e−09
NK cells
0.9
0.7
0.5
0.3
0.6
0.8
1.0
1.2
T Cells
status
ICU
Ward

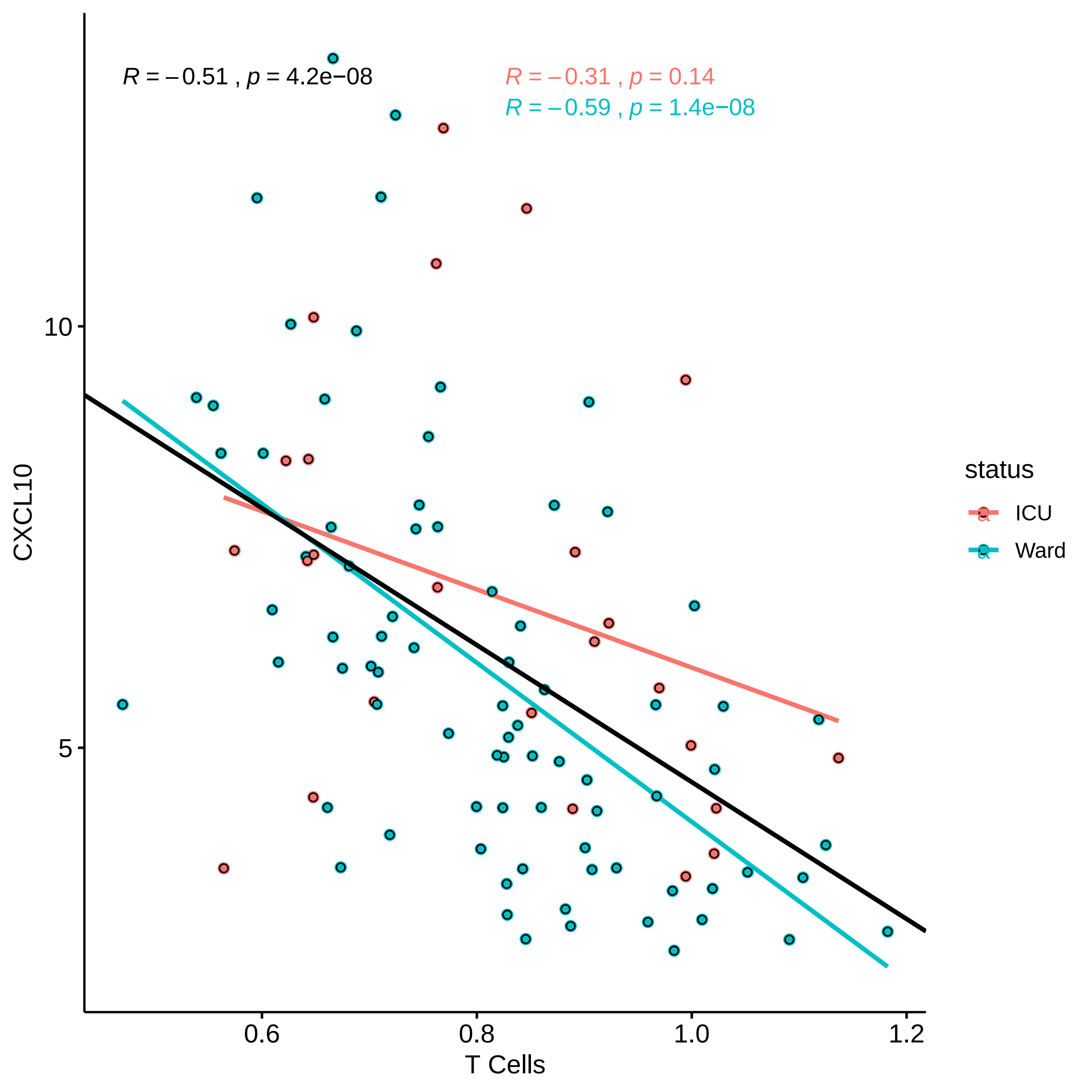

R = −0.51 , p = 4.2e−08
R = −0.31 , p = 0.14
R = −0.59 , p = 1.4e−08
CXCL10
10
5
0.6
0.8
1.0
1.2
T Cells
status
ICU
Ward

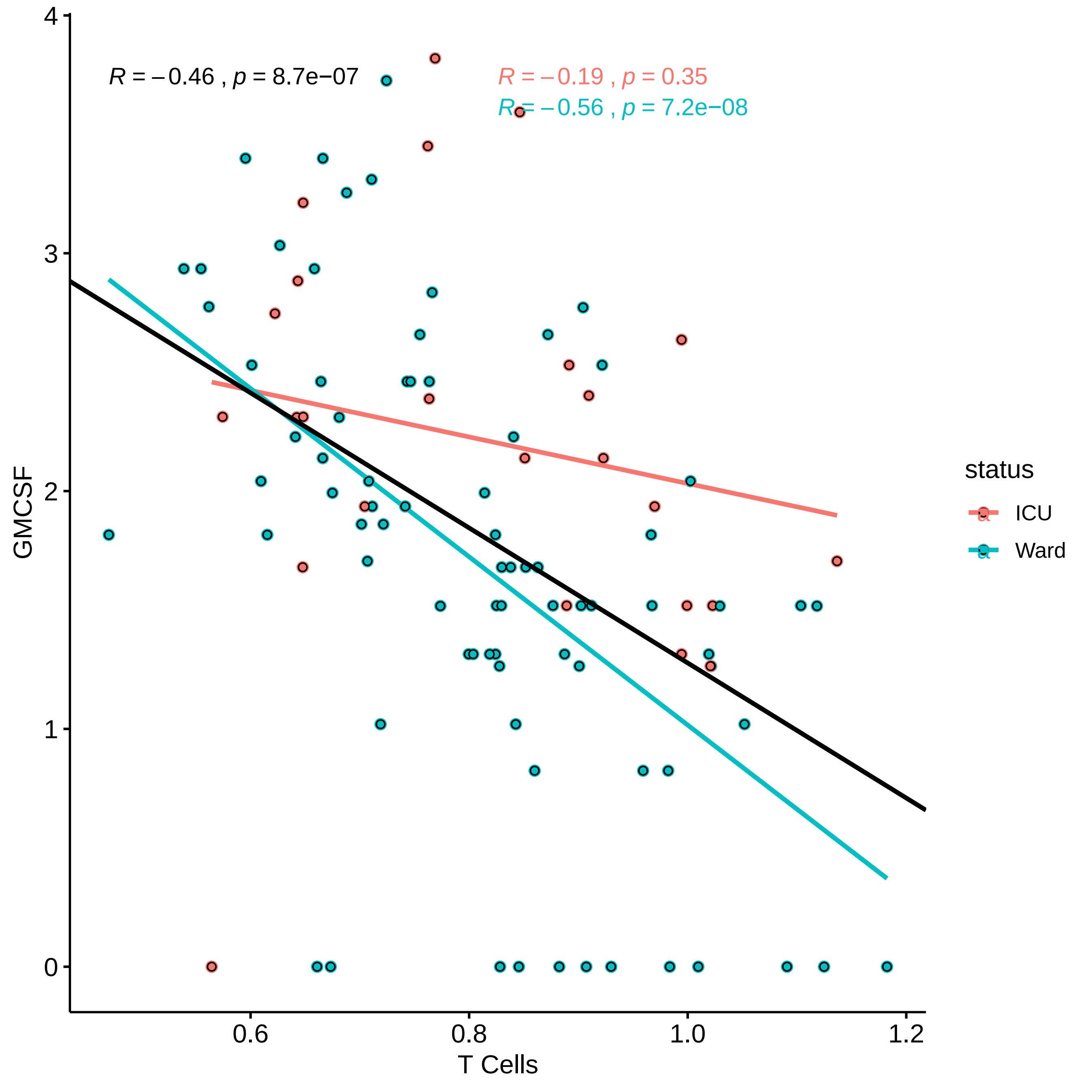

R = −0.46 , p = 8.7e−07
R = −0.19 , p = 0.35
R = −0.56 , p = 7.2e−08
GMCSF
T Cells
0
1
2
3
4
0.6
0.8
1.0
1.2
status
ICU
Ward

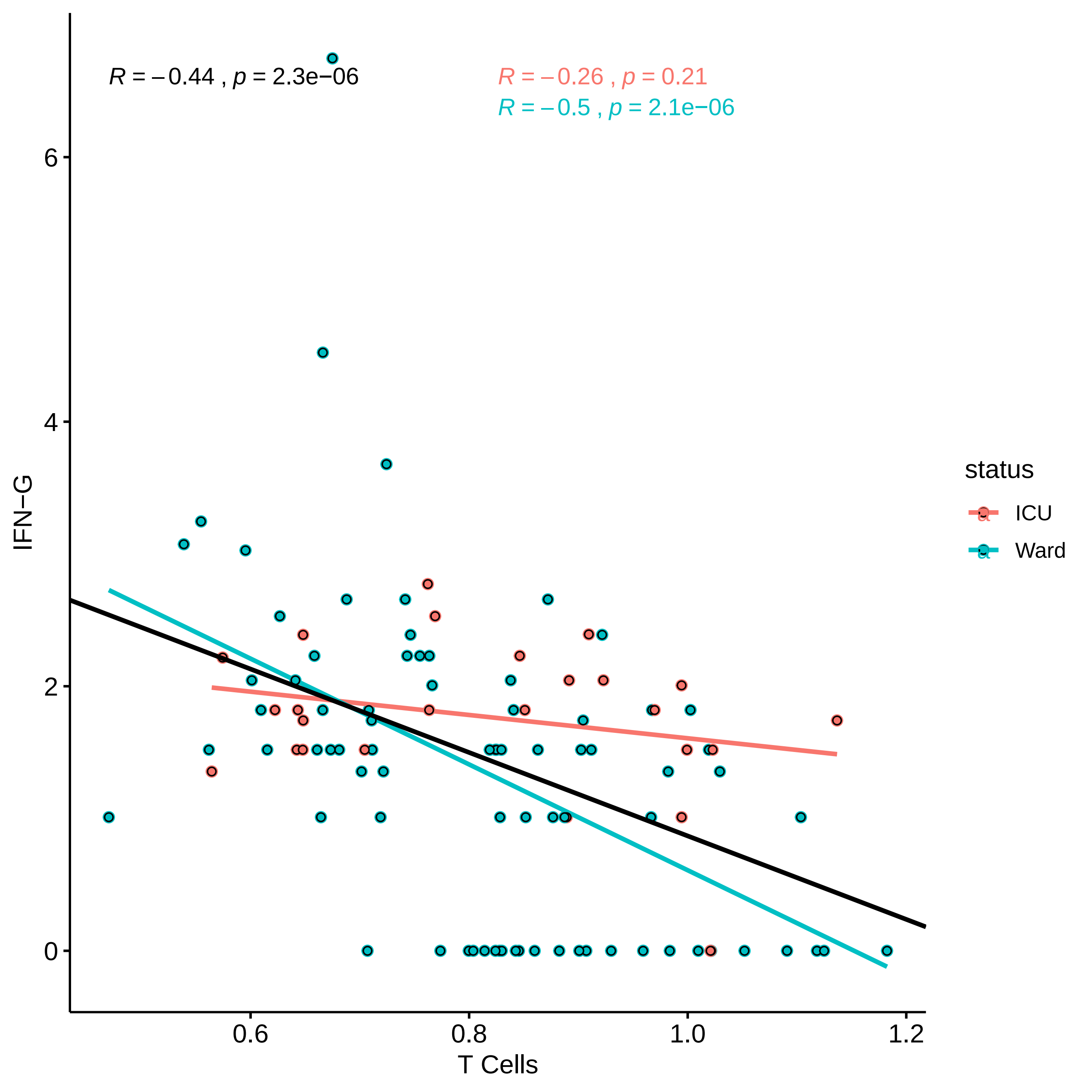

R = −0.44 , p = 2.3e−06
R = −0.26 , p = 0.21
R = −0.5 , p = 2.1e−06
IFN−G
T Cells
0
2
4
6
0.6
0.8
1.0
1.2
status
ICU
Ward

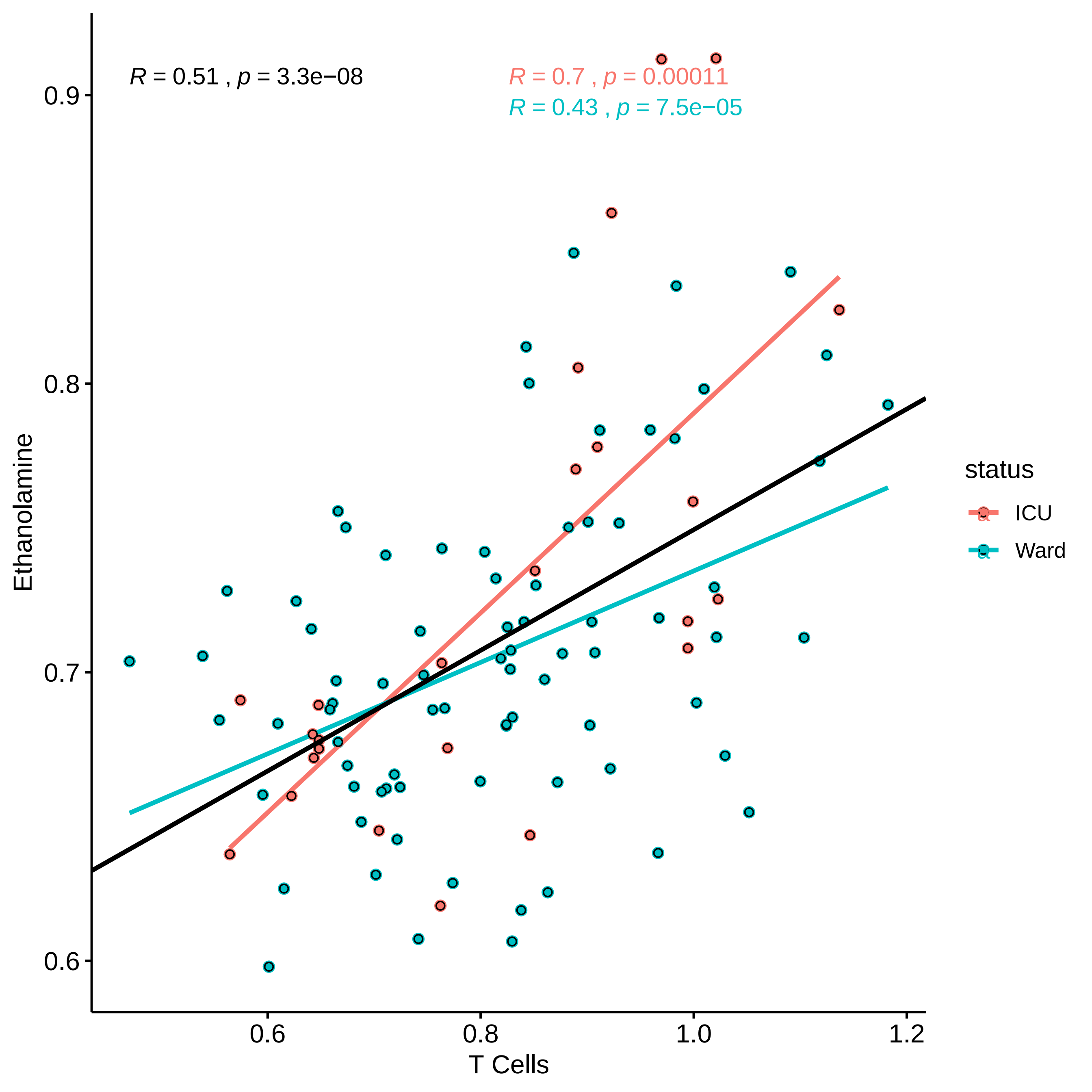

R = 0.51 , p = 3.3e−08
R = 0.7 , p = 0.00011
R = 0.43 , p = 7.5e−05
Ethanolamine
0.9
0.8
0.7
0.6
0.6
0.8
1.0
1.2
T Cells
status
ICU
Ward

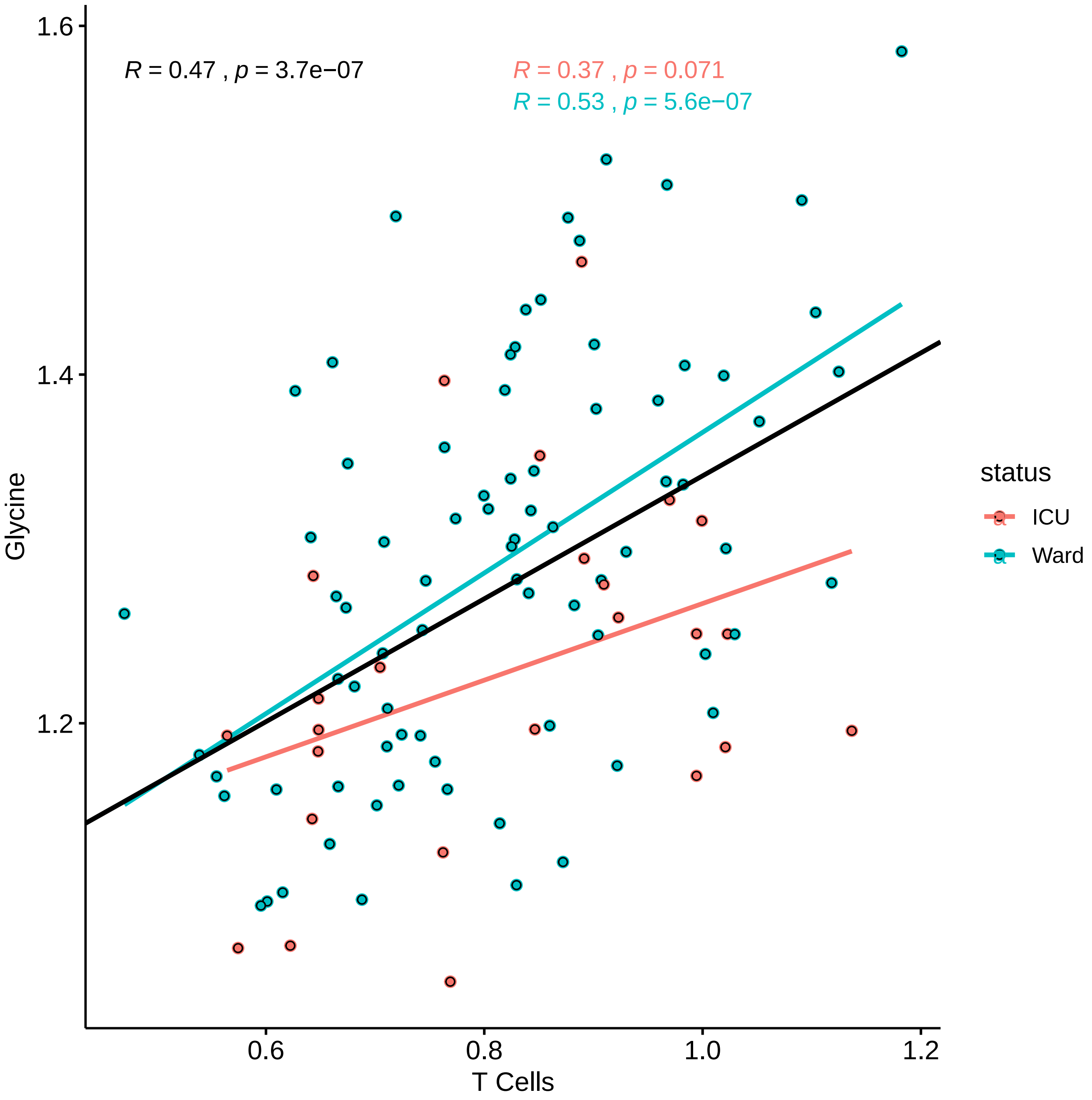

R = 0.47 , p = 3.7e−07
R = 0.37 , p = 0.071
R = 0.53 , p = 5.6e−07
1.6
1.4
1.2
Glycine
0.6
0.8
1.0
1.2
T Cells
status
ICU
Ward

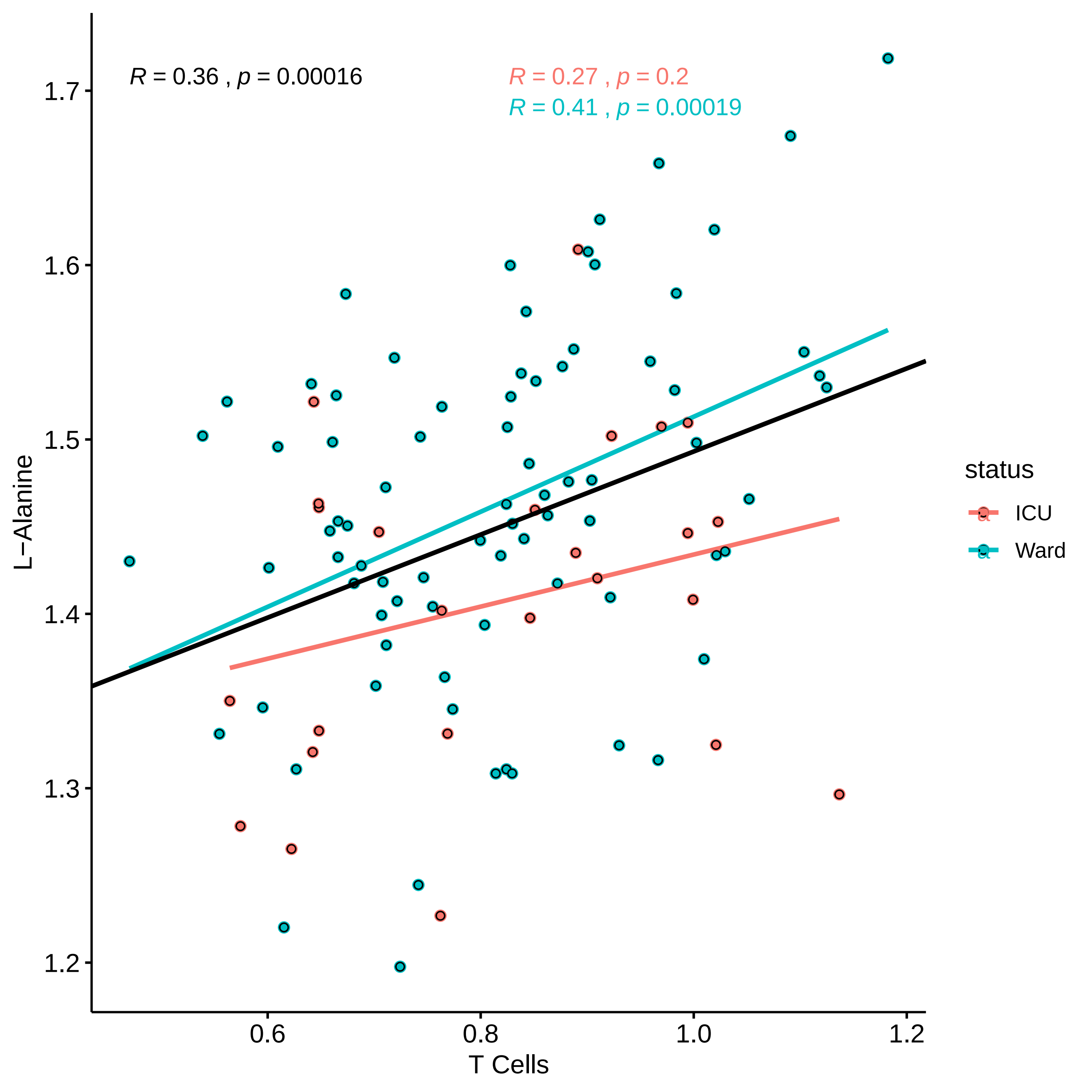

R = 0.36 , p = 0.00016
R = 0.27 , p = 0.2
R = 0.41 , p = 0.00019
L−Alanine
1.7
1.6
1.5
1.4
1.3
1.2
0.6
0.8
1.0
1.2
T Cells
status
ICU
Ward

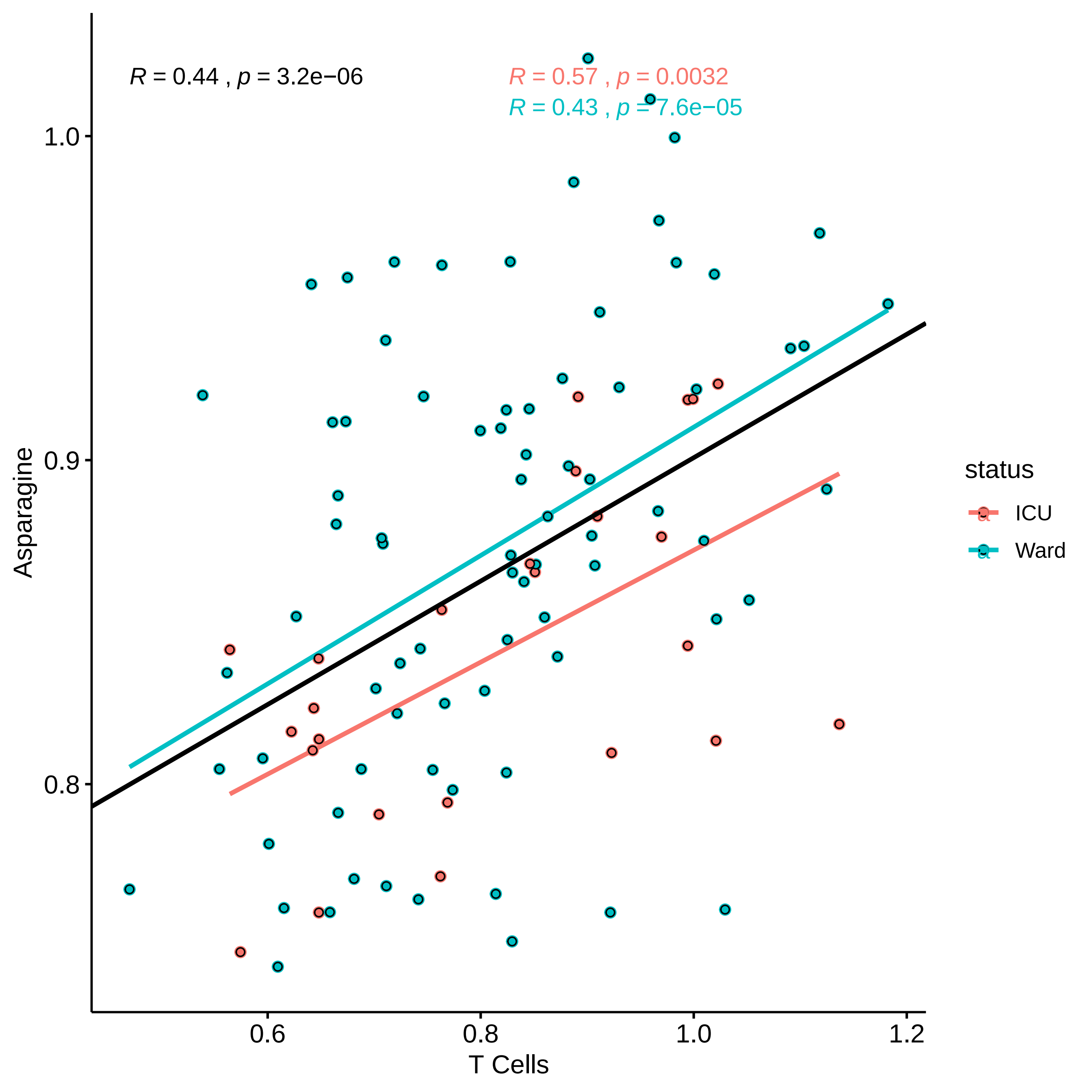
R = 0.44 , p = 3.2e−06
R = 0.57 , p = 0.0032
R = 0.43 , p = 7.6e−05
Asparagine
1.0
0.9
0.8
0.6
0.8
1.0
1.2
T Cells
status
ICU
Ward

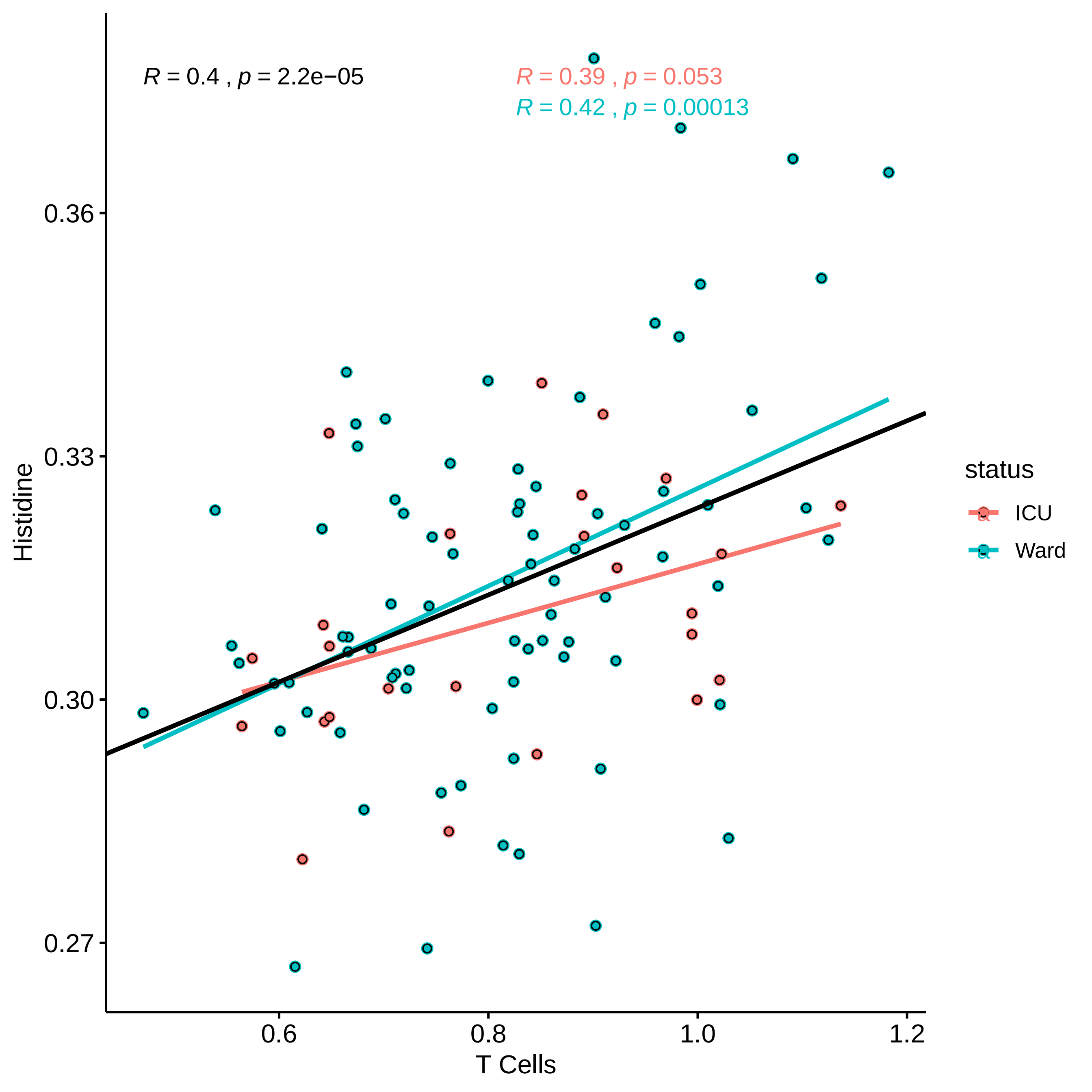

R = 0.4 , p = 2.2e−05
R = 0.39 , p = 0.053
R = 0.42 , p = 0.00013
Histidine
0.36
0.33
0.30
0.27
0.6
0.8
1.0
1.2
T Cells
status
ICU
Ward

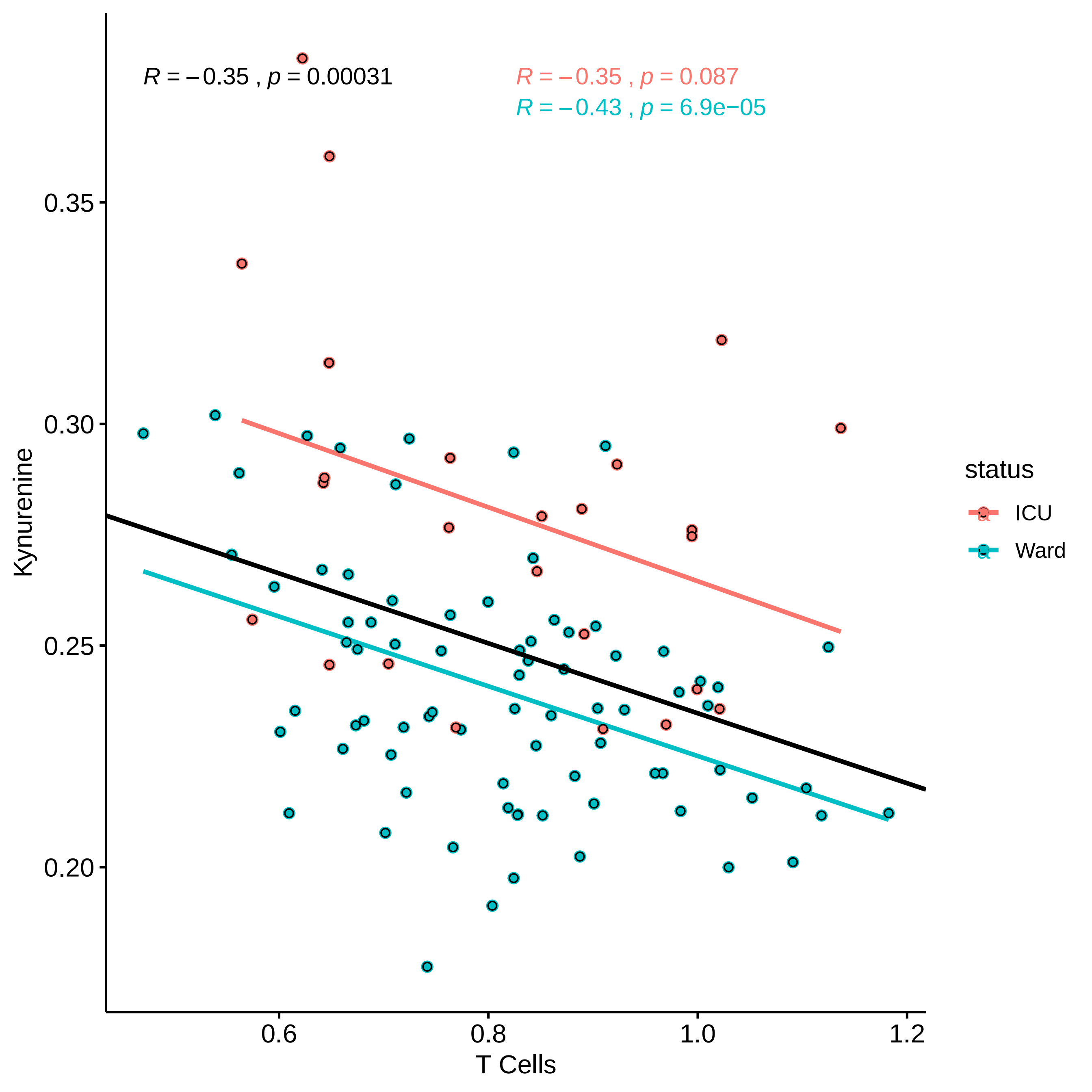
R = −0.35 , p = 0.00031
R = −0.35 , p = 0.087
R = −0.43 , p = 6.9e−05
0.35
0.30
0.25
0.20
Kynurenine
0.6
0.8
1.0
1.2
T Cells
status
ICU
Ward

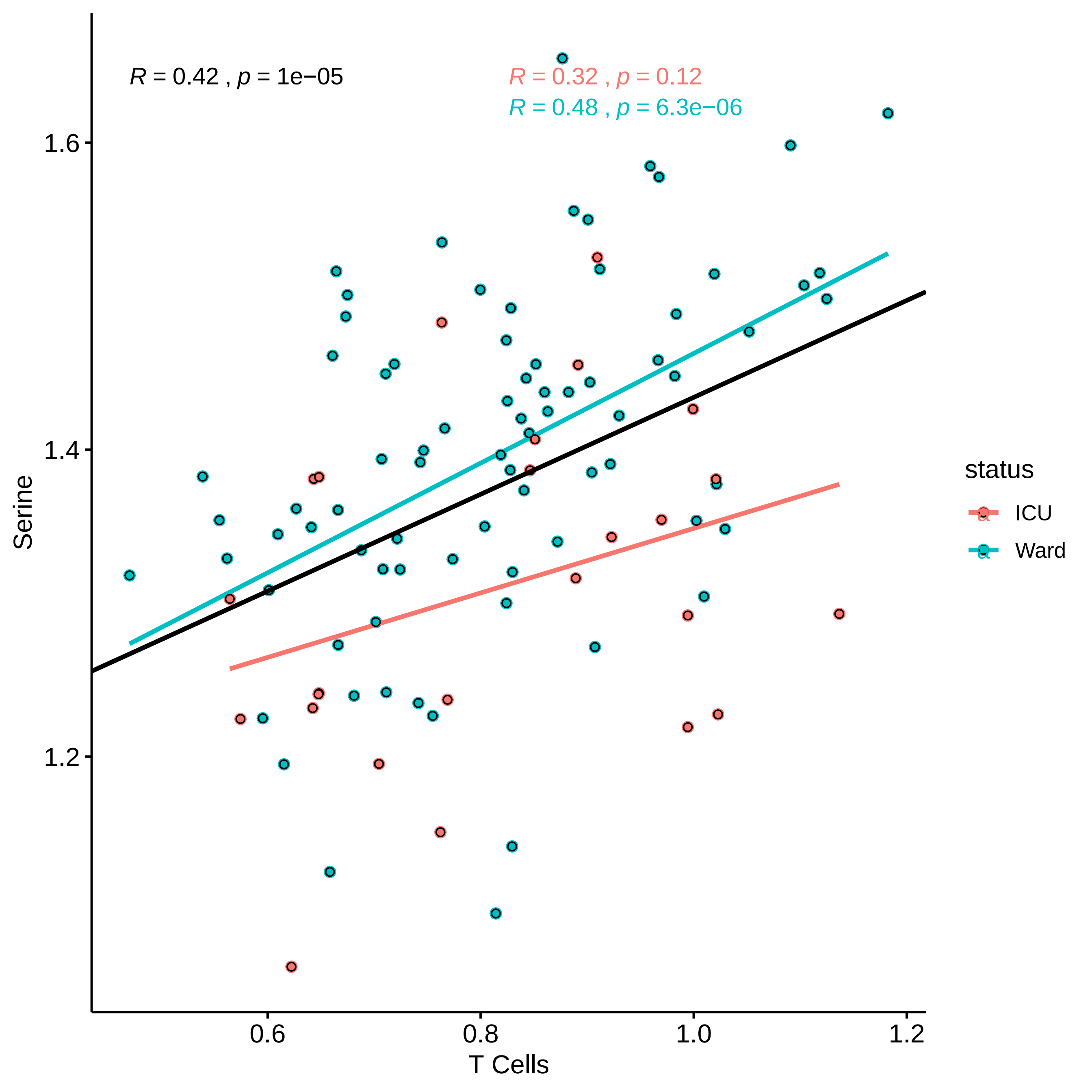

R = 0.42 , p = 1e−05
R = 0.32 , p = 0.12
R = 0.48 , p = 6.3e−06
1.6
1.4
1.2
Serine
0.6
0.8
1.0
1.2
T Cells
status
ICU
Ward

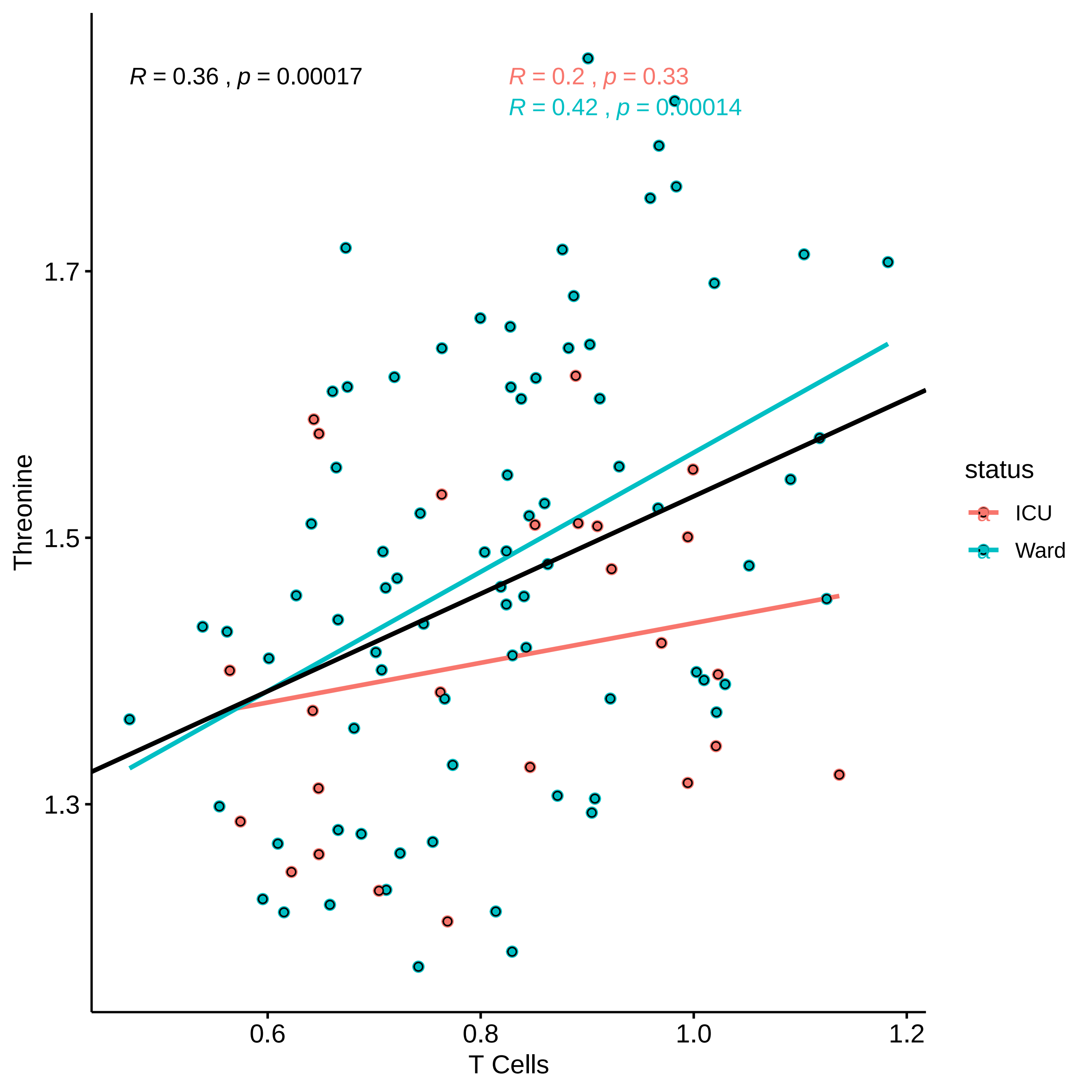

R = 0.36 , p = 0.00017
R = 0.2 , p = 0.33
R = 0.42 , p = 0.00014
Threonine
1.7
1.5
1.3
0.6
0.8
1.0
1.2
T Cells
status
ICU
Ward

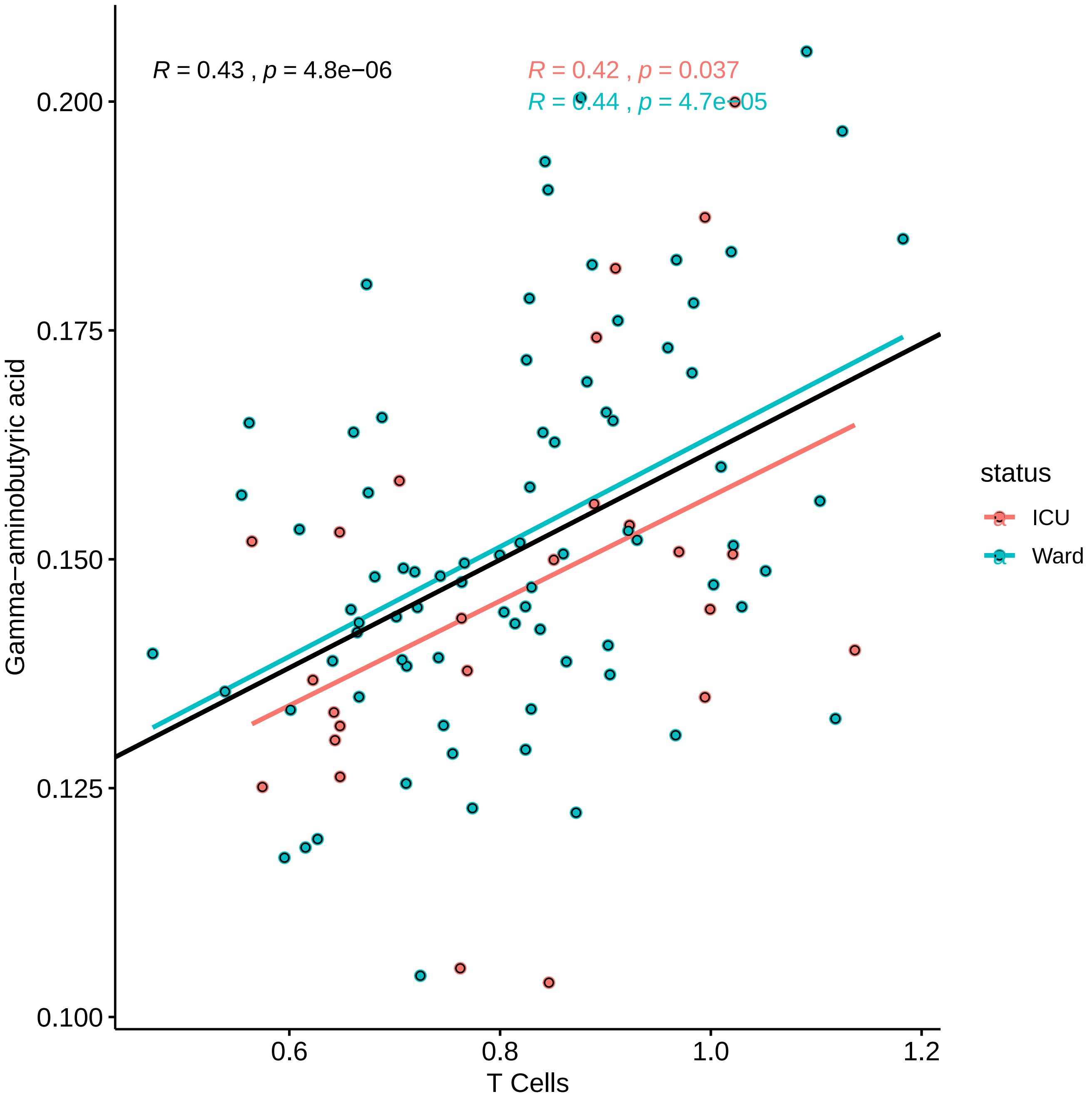
R = 0.43 , p = 4.8e−06
R = 0.42 , p = 0.037
R = 0.44 , p = 4.7e−05
Gamma−aminobutyric acid
0.200
0.175
0.150
0.125
0.100
0.6
0.8
1.0
1.2
T Cells
status
ICU
Ward

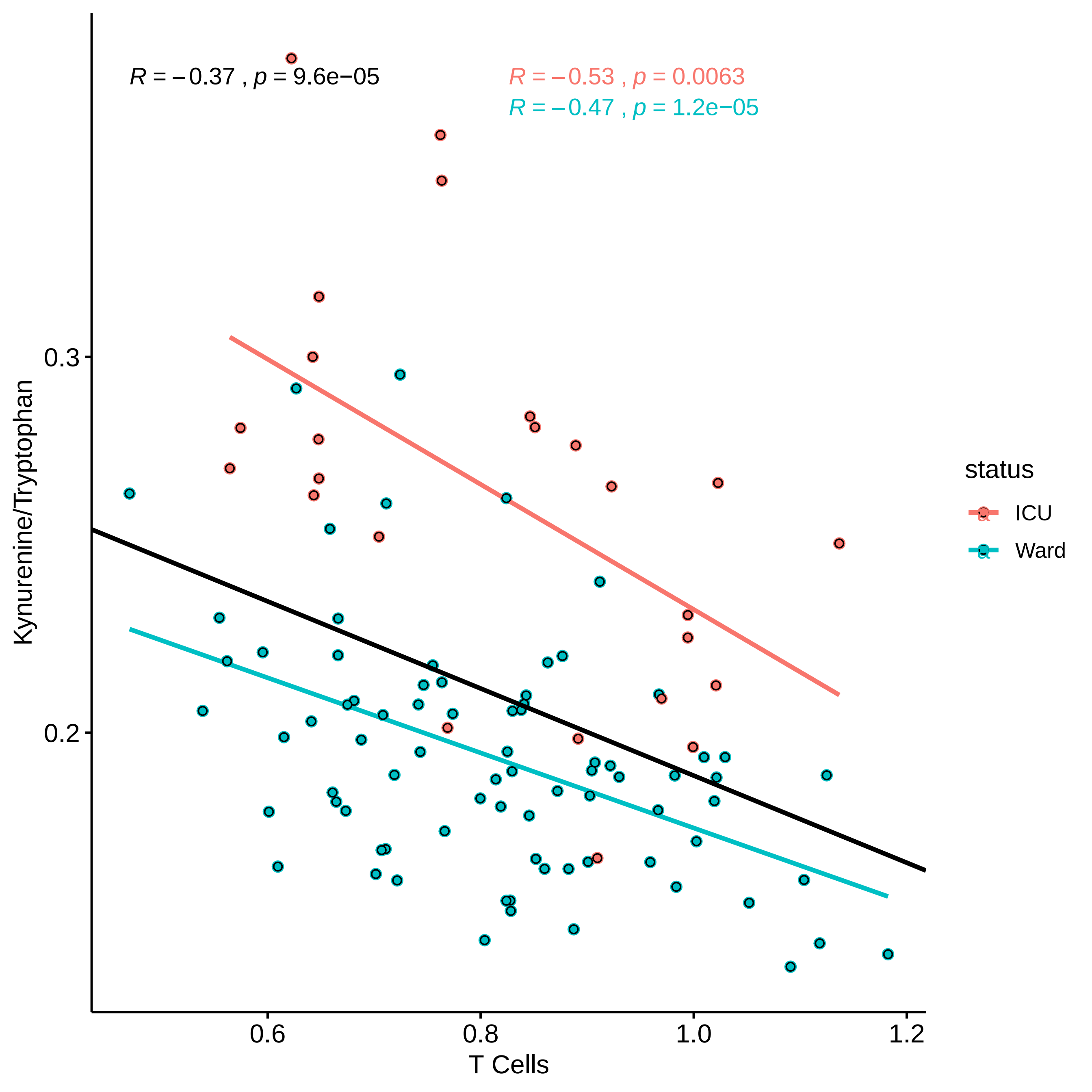

R = −0.37 , p = 9.6e−05
R = −0.53 , p = 0.0063
R = −0.47 , p = 1.2e−05
Kynurenine/Tryptophan
0.3
0.2
0.6
0.8
1.0
1.2
T Cells
status
ICU
Ward

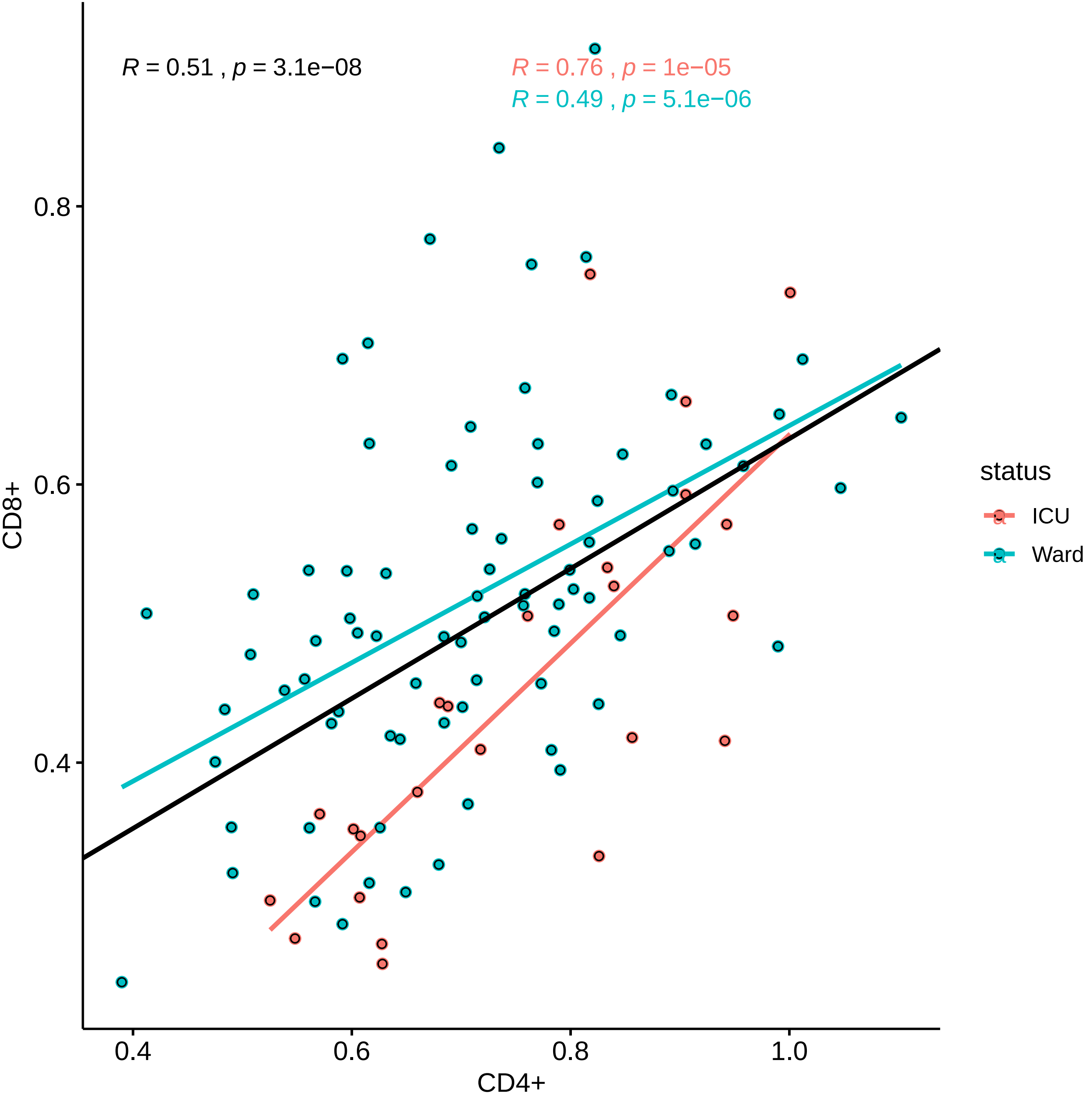

R = 0.51 , p = 3.1e−08
R = 0.76 , p = 1e−05
R = 0.49 , p = 5.1e−06
0.8
0.6
0.4
CD8+
0.4
0.6
0.8
1.0
CD4+
status
ICU
Ward

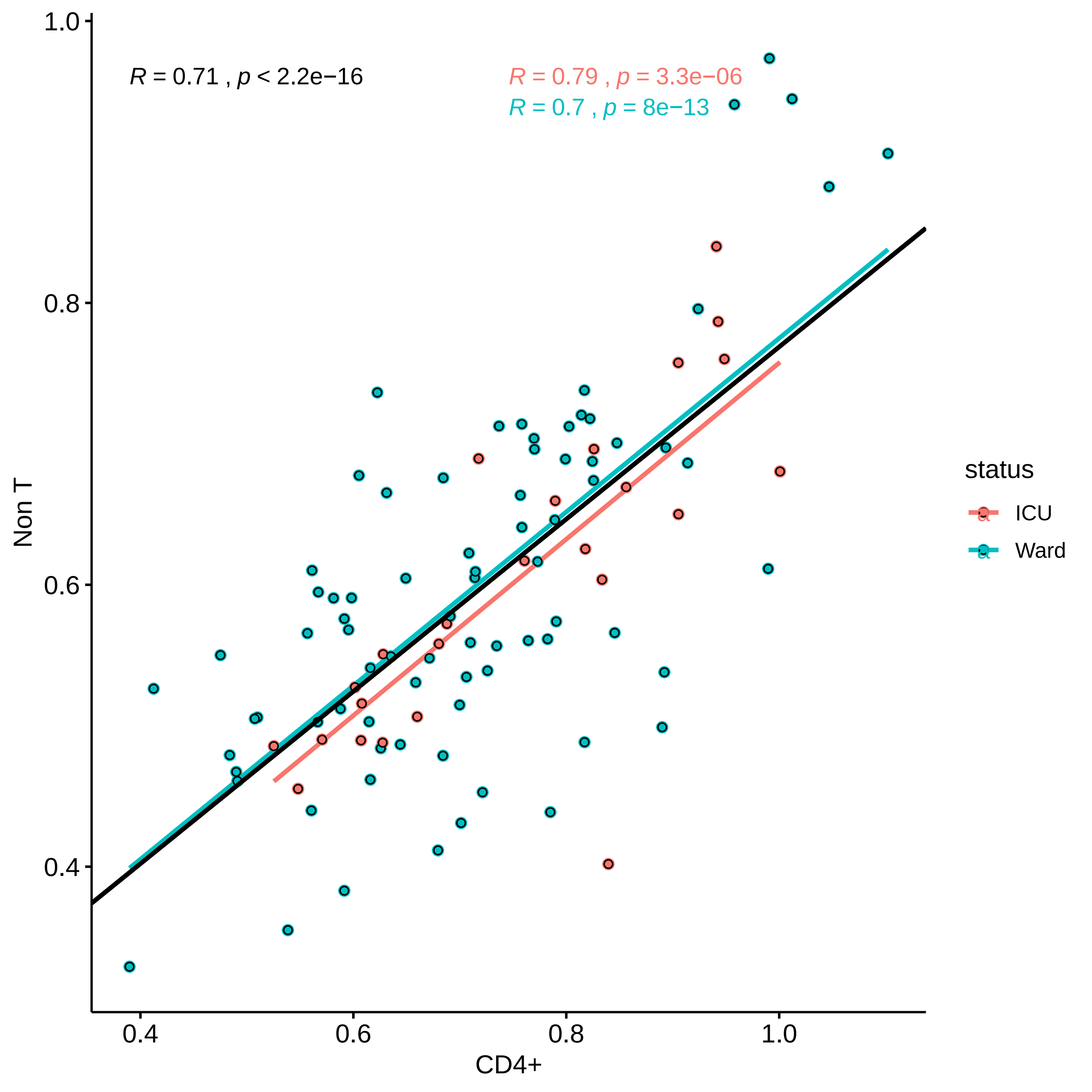

R = 0.71 , p < 2.2e−16
R = 0.79 , p = 3.3e−06
R = 0.7 , p = 8e−13
1.0
0.8
0.6
0.4
Non T
0.4
0.6
0.8
1.0
CD4+
status
ICU
Ward

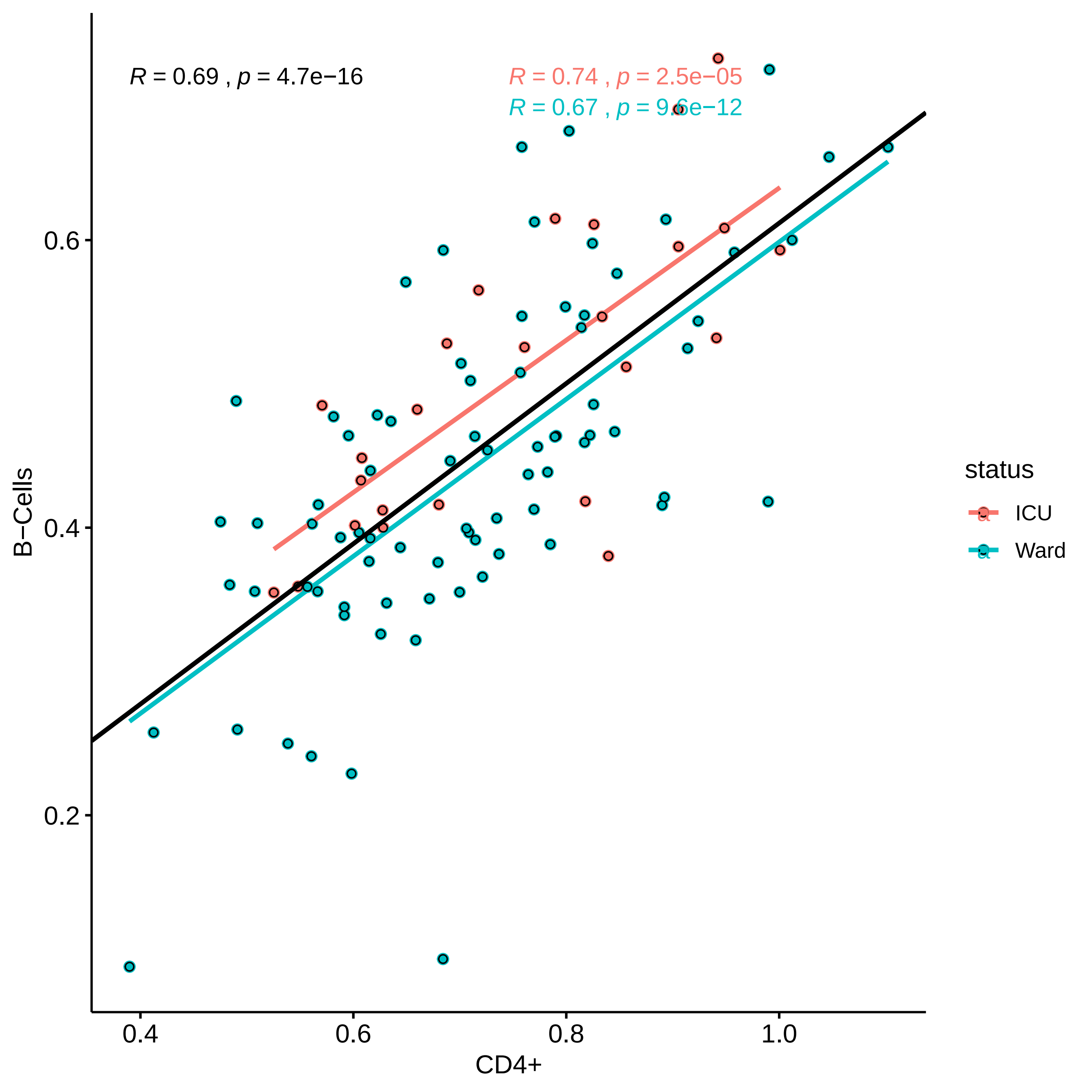

R = 0.69 , p = 4.7e−16
R = 0.74 , p = 2.5e−05
R = 0.67 , p = 9.6e−12
B−Cells
0.6
0.4
0.2
0.4
0.6
0.8
1.0
CD4+
status
ICU
Ward

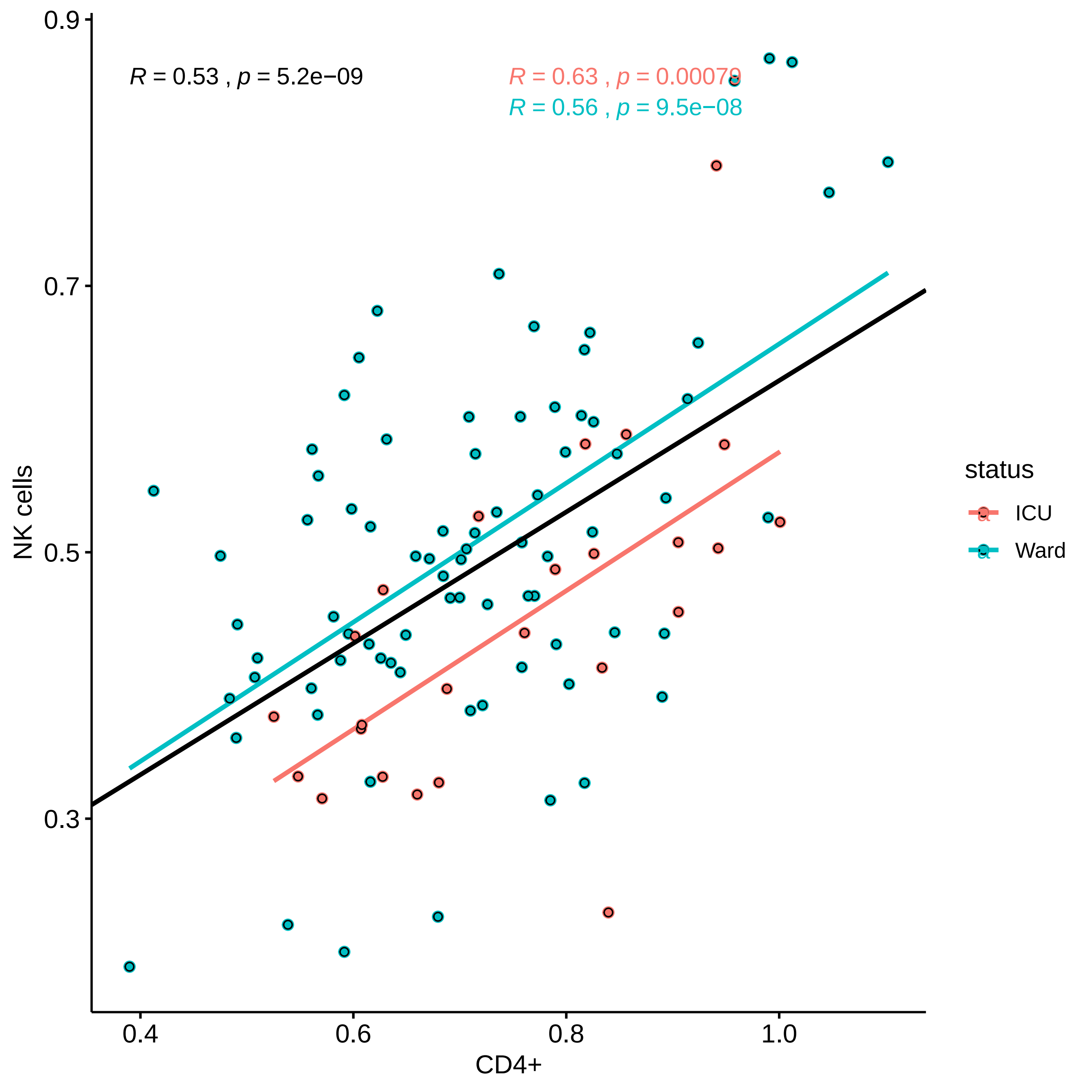
R = 0.53 , p = 5.2e−09
R = 0.63 , p = 0.00079
R = 0.56 , p = 9.5e−08
0.9
0.7
0.5
0.3
NK cells
0.4
0.6
0.8
1.0
CD4+
status
ICU
Ward

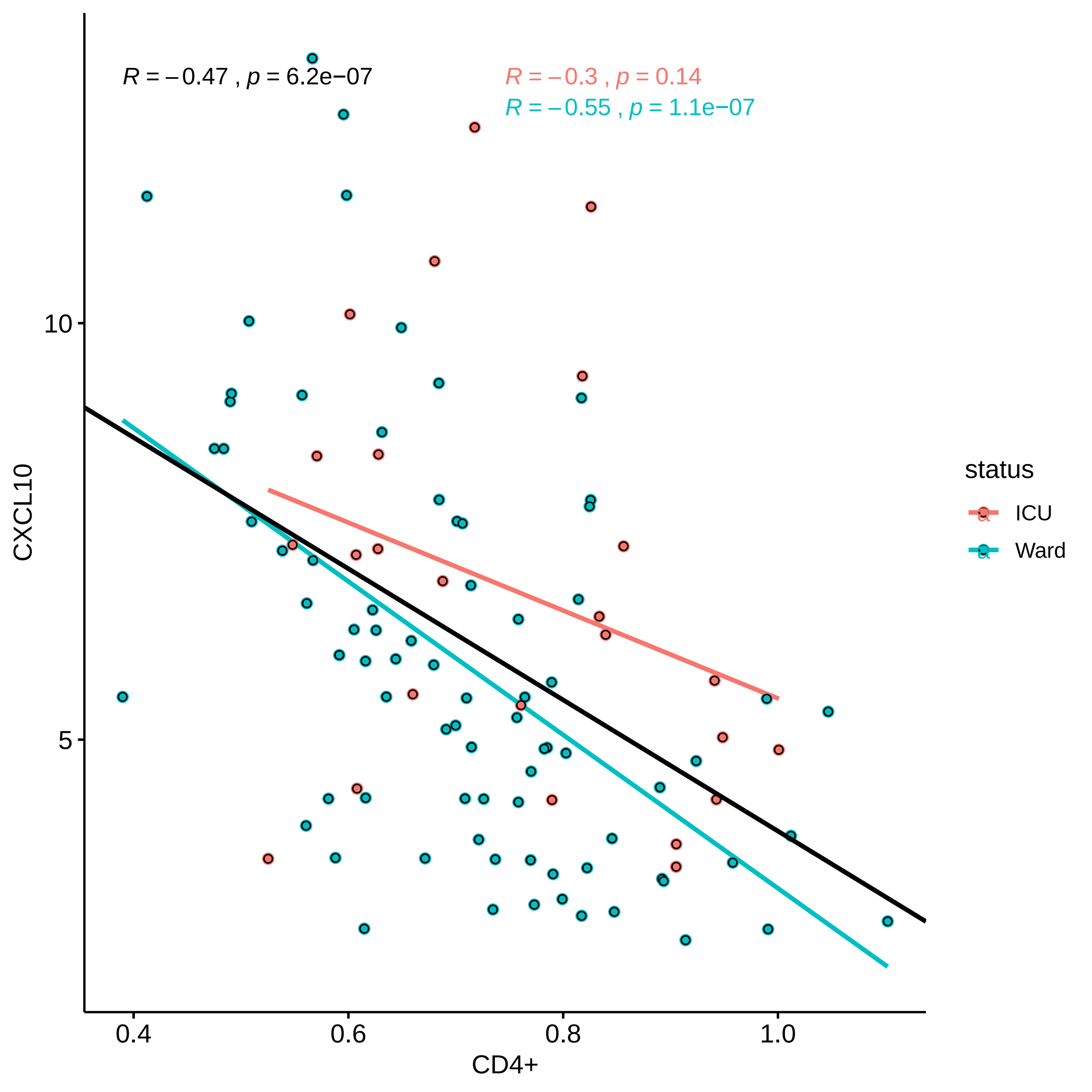

R = −0.47 , p = 6.2e−07
R = −0.3 , p = 0.14
R = −0.55 , p = 1.1e−07
CXCL10
10
5
0.4
0.6
0.8
1.0
CD4+
status
ICU
Ward

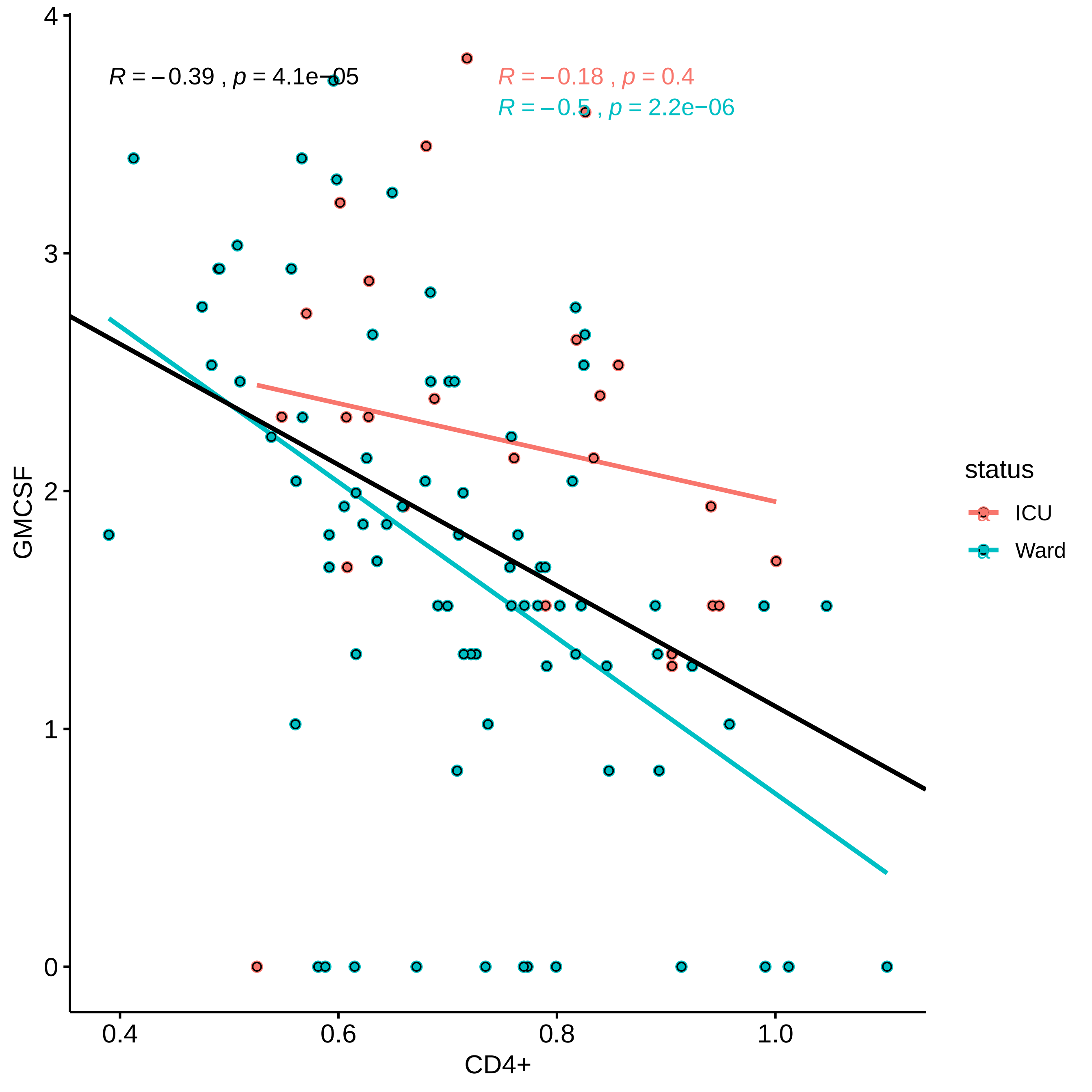
R = −0.39 , p = 4.1e−05
R = −0.18 , p = 0.4
R = −0.5 , p = 2.2e−06
GMCSF
CD4+
status
ICU
Ward
0
1
2
3
4
0.4
0.6
0.8
1.0

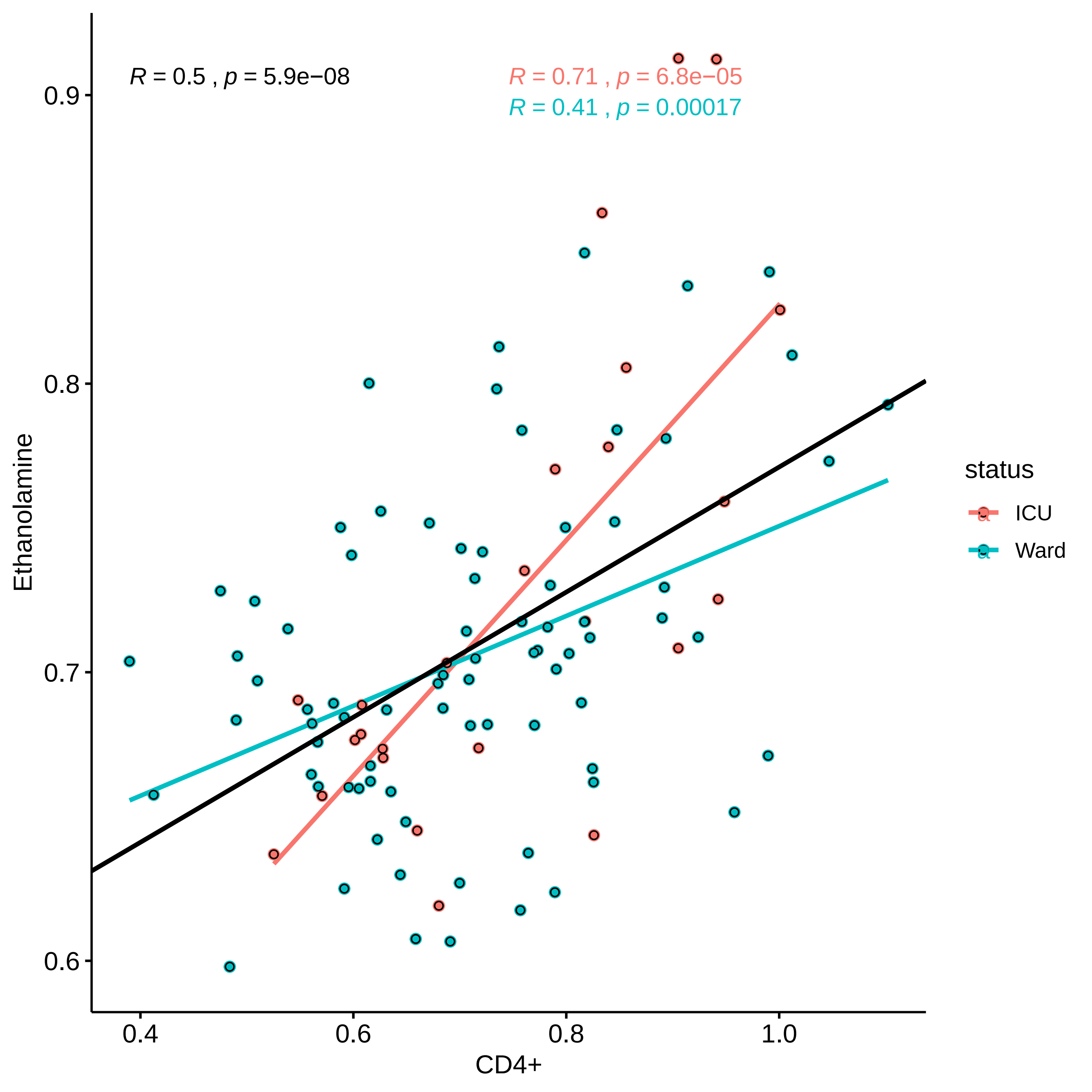

R = 0.5 , p = 5.9e−08
R = 0.71 , p = 6.8e−05
R = 0.41 , p = 0.00017
0.9
0.8
0.7
0.6
Ethanolamine
0.4
0.6
0.8
1.0
CD4+
status
ICU
Ward

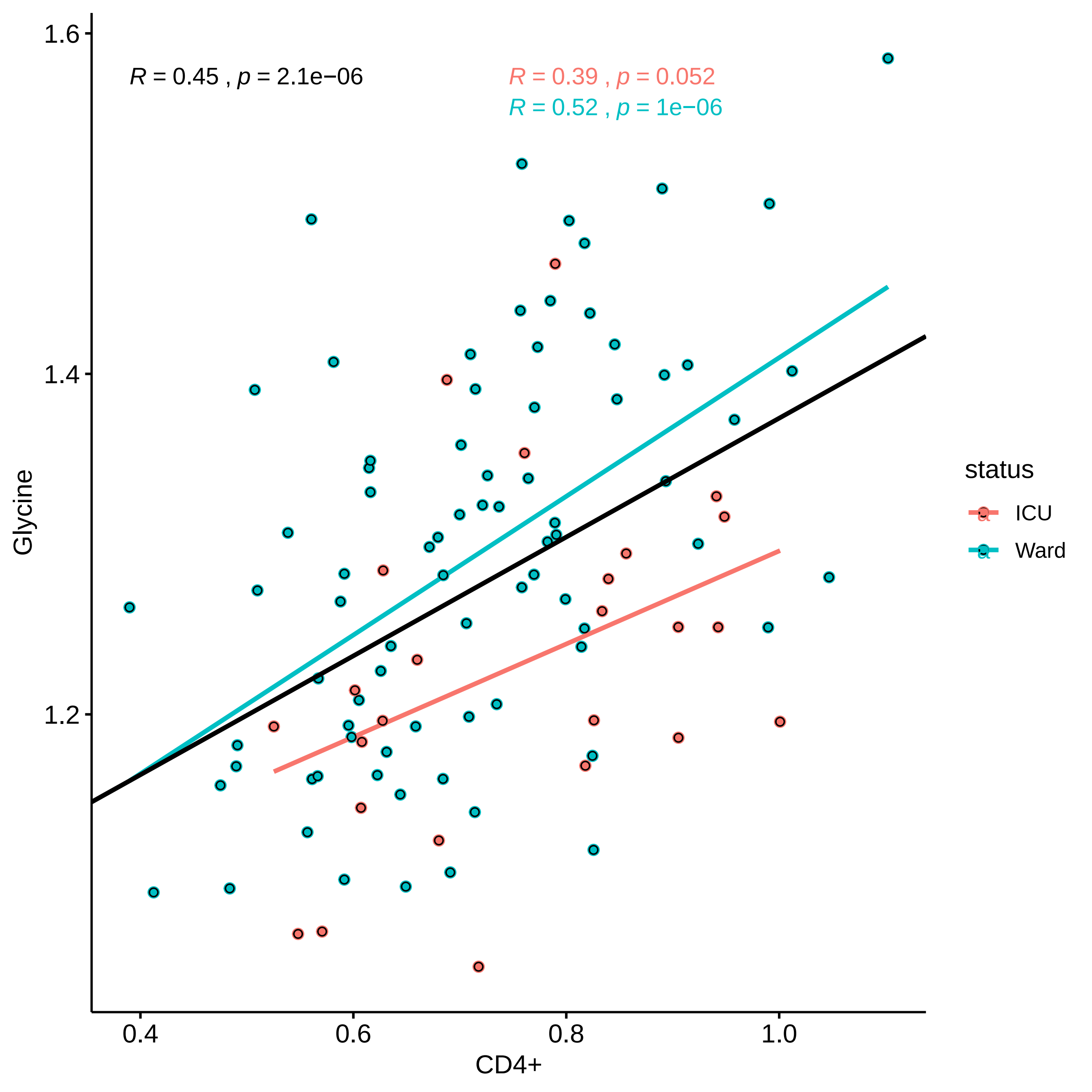

R = 0.45 , p = 2.1e−06
R = 0.39 , p = 0.052
R = 0.52 , p = 1e−06
1.6
1.4
1.2
Glycine
0.4
0.6
0.8
1.0
CD4+
status
ICU
Ward

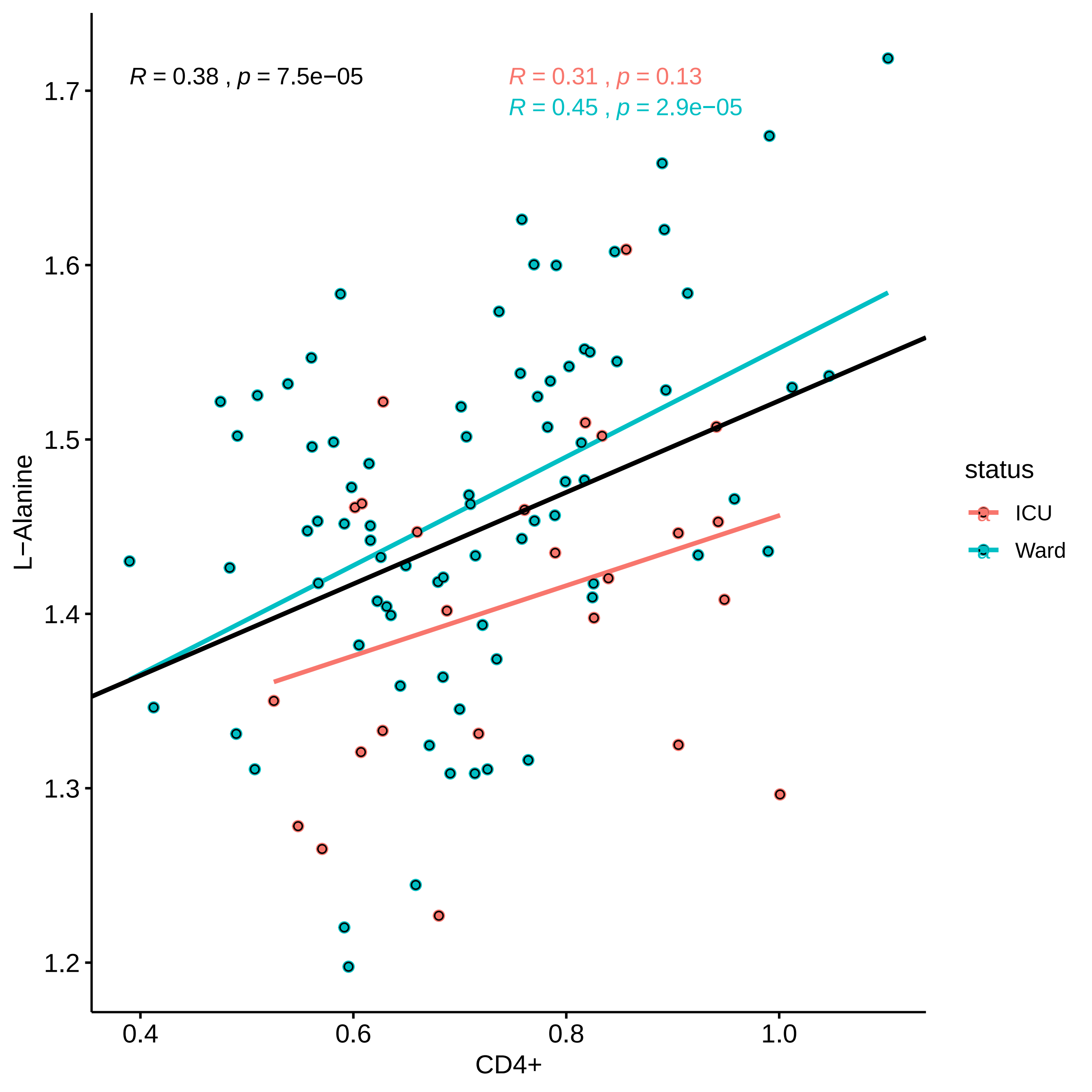

R = 0.38 , p = 7.5e−05
R = 0.31 , p = 0.13
R = 0.45 , p = 2.9e−05
L−Alanine
1.7
1.6
1.5
1.4
1.3
1.2
0.4
0.6
0.8
1.0
CD4+
status
ICU
Ward

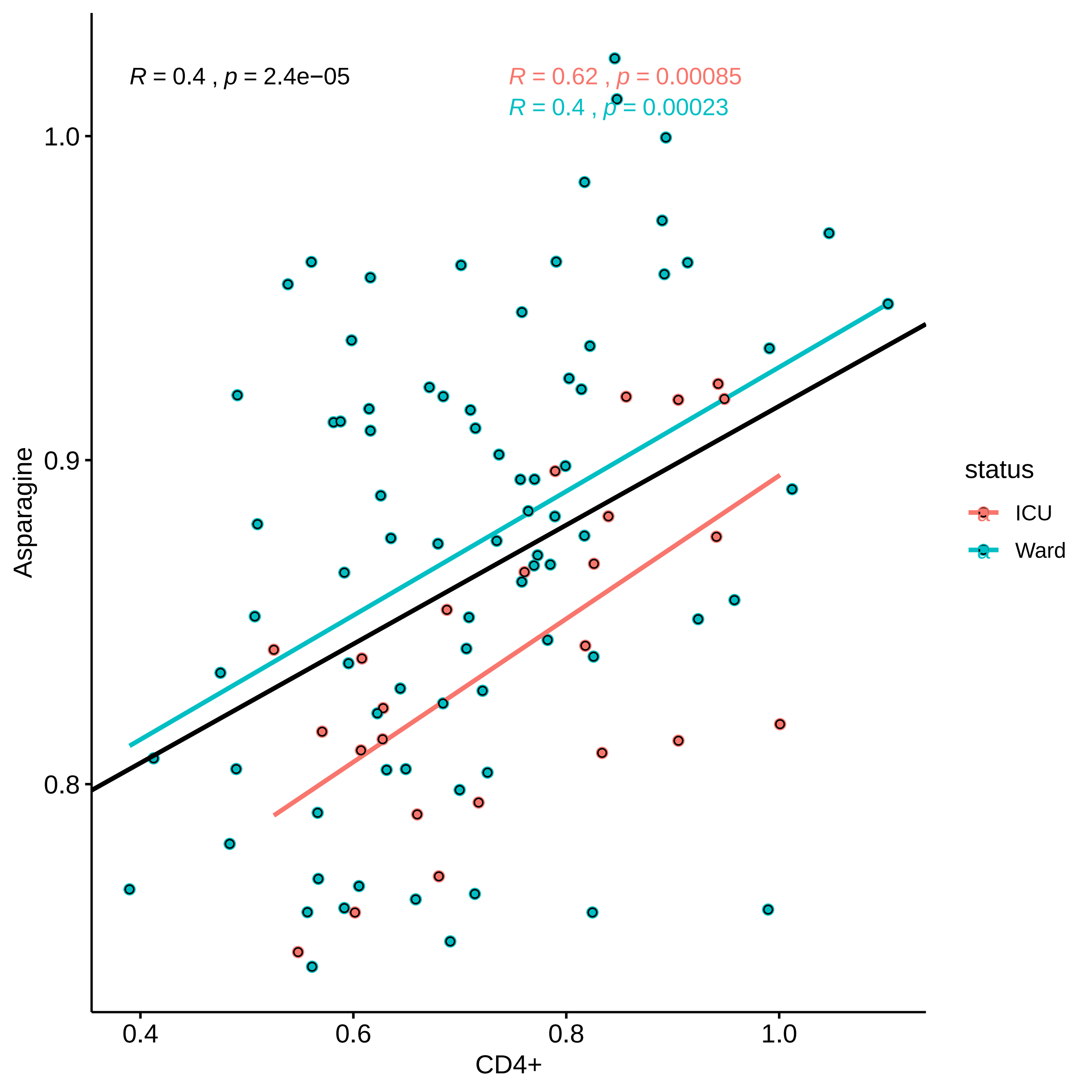

R = 0.4 , p = 2.4e−05
R = 0.62 , p = 0.00085
R = 0.4 , p = 0.00023
1.0
0.9
0.8
Asparagine
0.4
0.6
0.8
1.0
CD4+
status
ICU
Ward

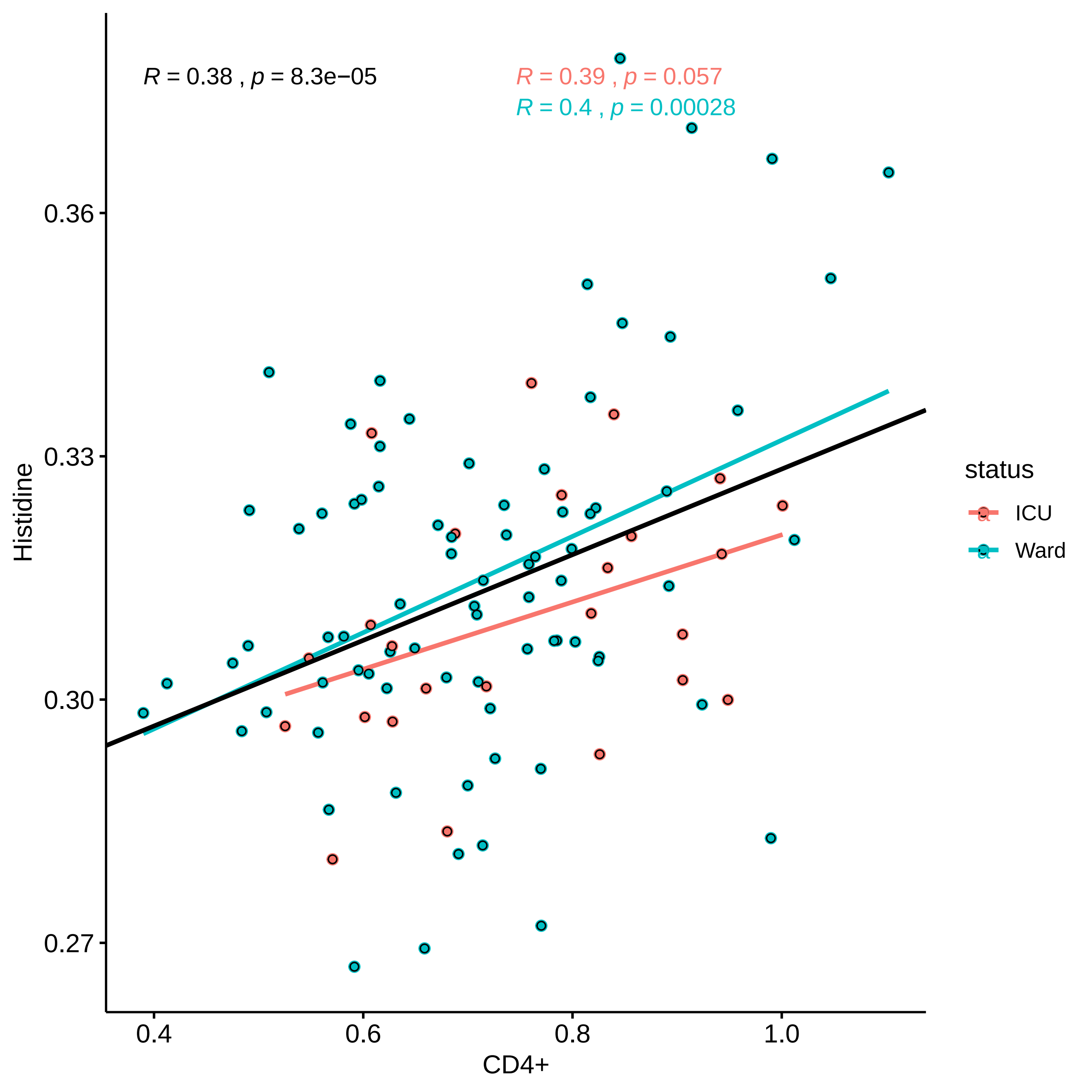
R = 0.38 , p = 8.3e−05
R = 0.39 , p = 0.057
R = 0.4 , p = 0.00028
Histidine
0.36
0.33
0.30
0.27
0.4
0.6
0.8
1.0
CD4+
status
ICU
Ward

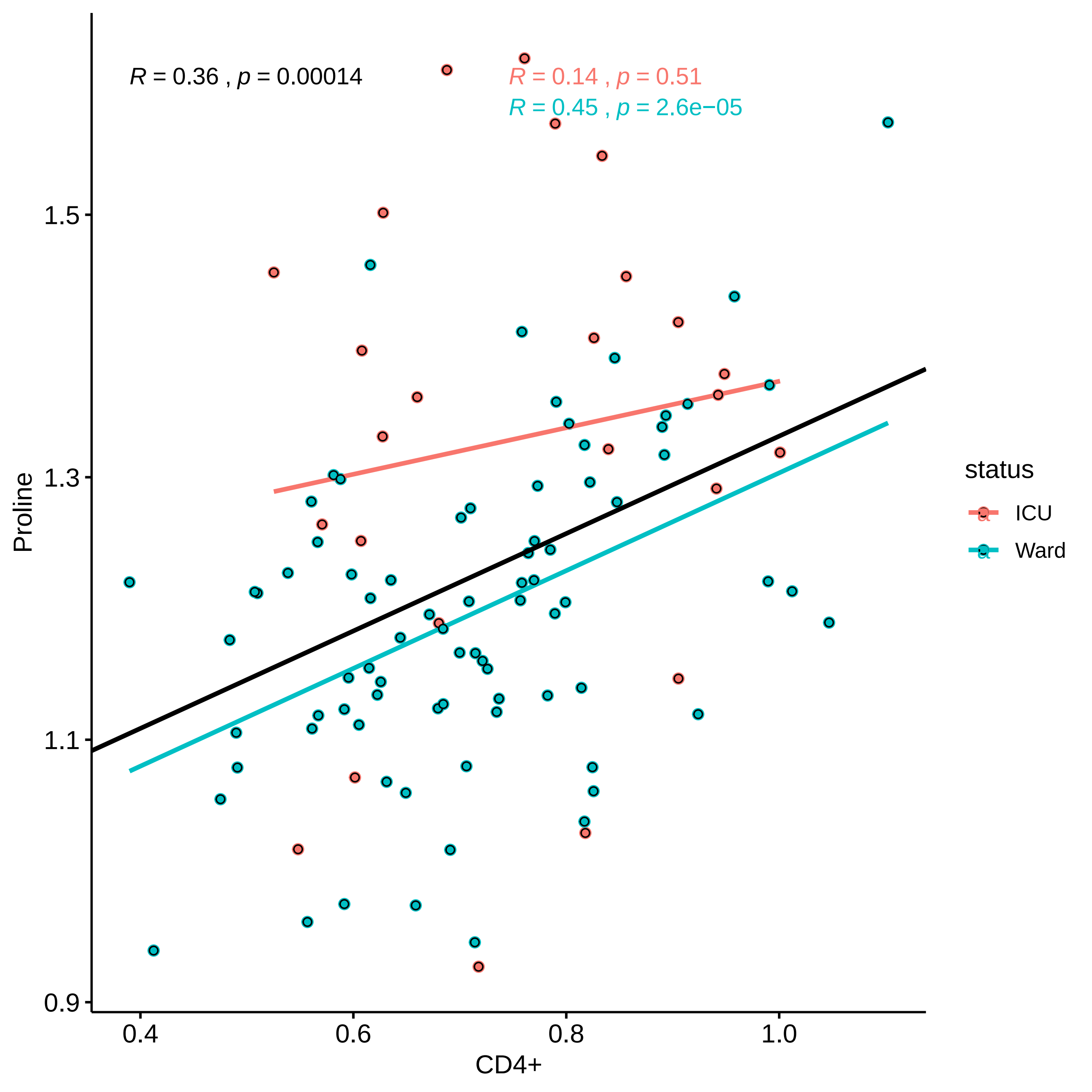

R = 0.36 , p = 0.00014
R = 0.14 , p = 0.51
R = 0.45 , p = 2.6e−05
Proline
1.5
1.3
1.1
0.9
0.4
0.6
0.8
1.0
CD4+
status
ICU
Ward

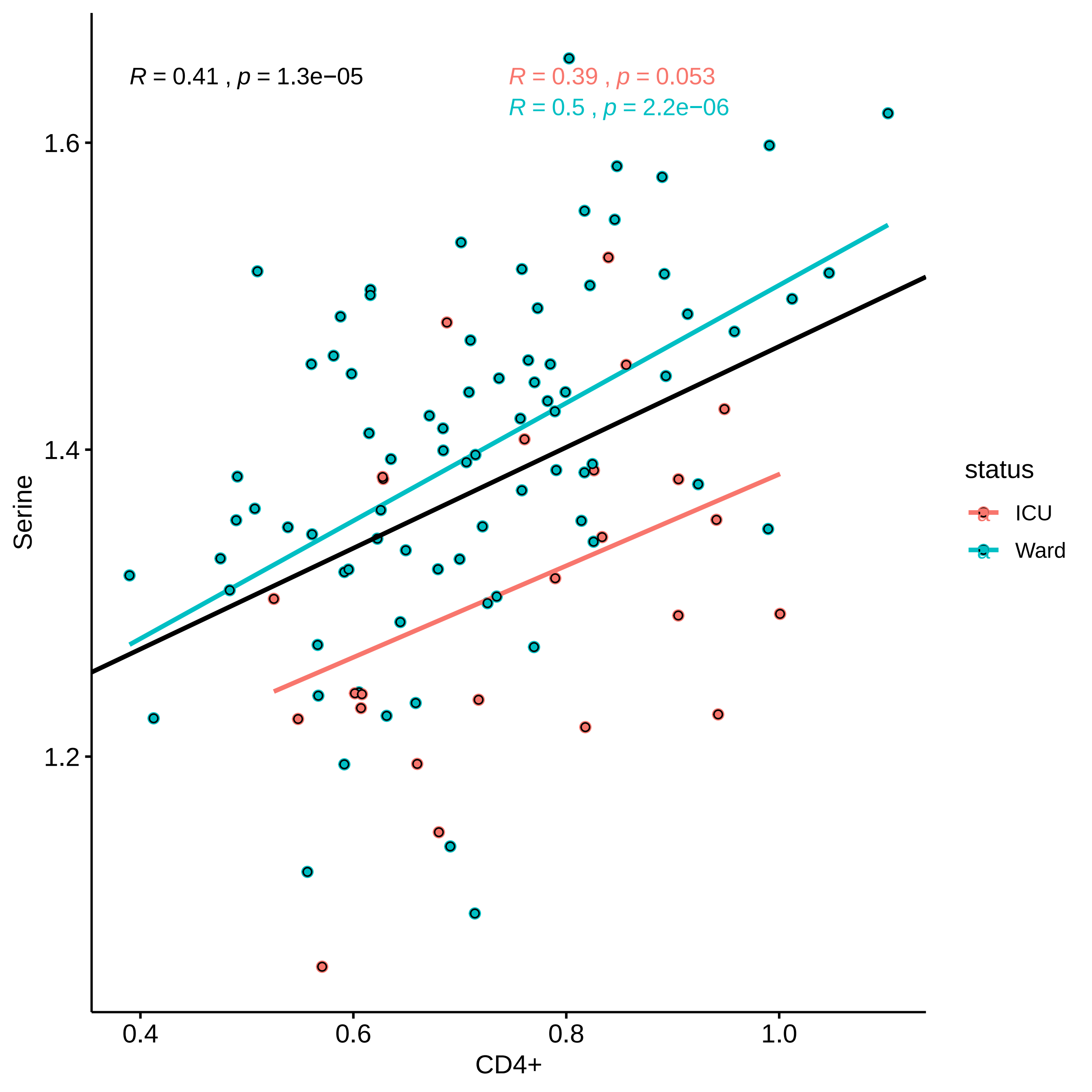

R = 0.41 , p = 1.3e−05
R = 0.39 , p = 0.053
R = 0.5 , p = 2.2e−06
1.6
1.4
1.2
Serine
0.4
0.6
0.8
1.0
CD4+
status
ICU
Ward

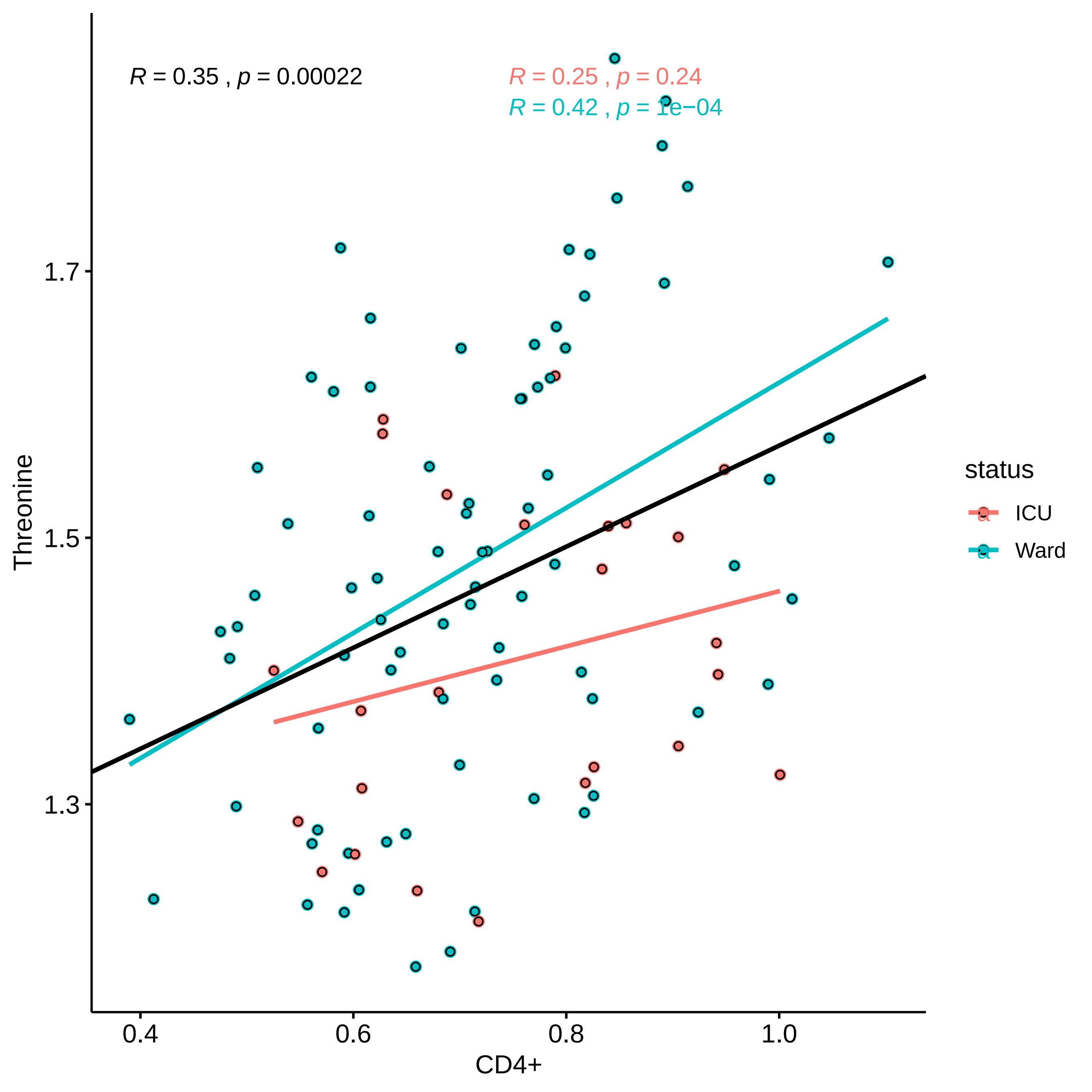
R = 0.35 , p = 0.00022
R = 0.25 , p = 0.24
R = 0.42 , p = 1e−04
1.7
1.5
1.3
Threonine
0.4
0.6
0.8
1.0
CD4+
status
ICU
Ward

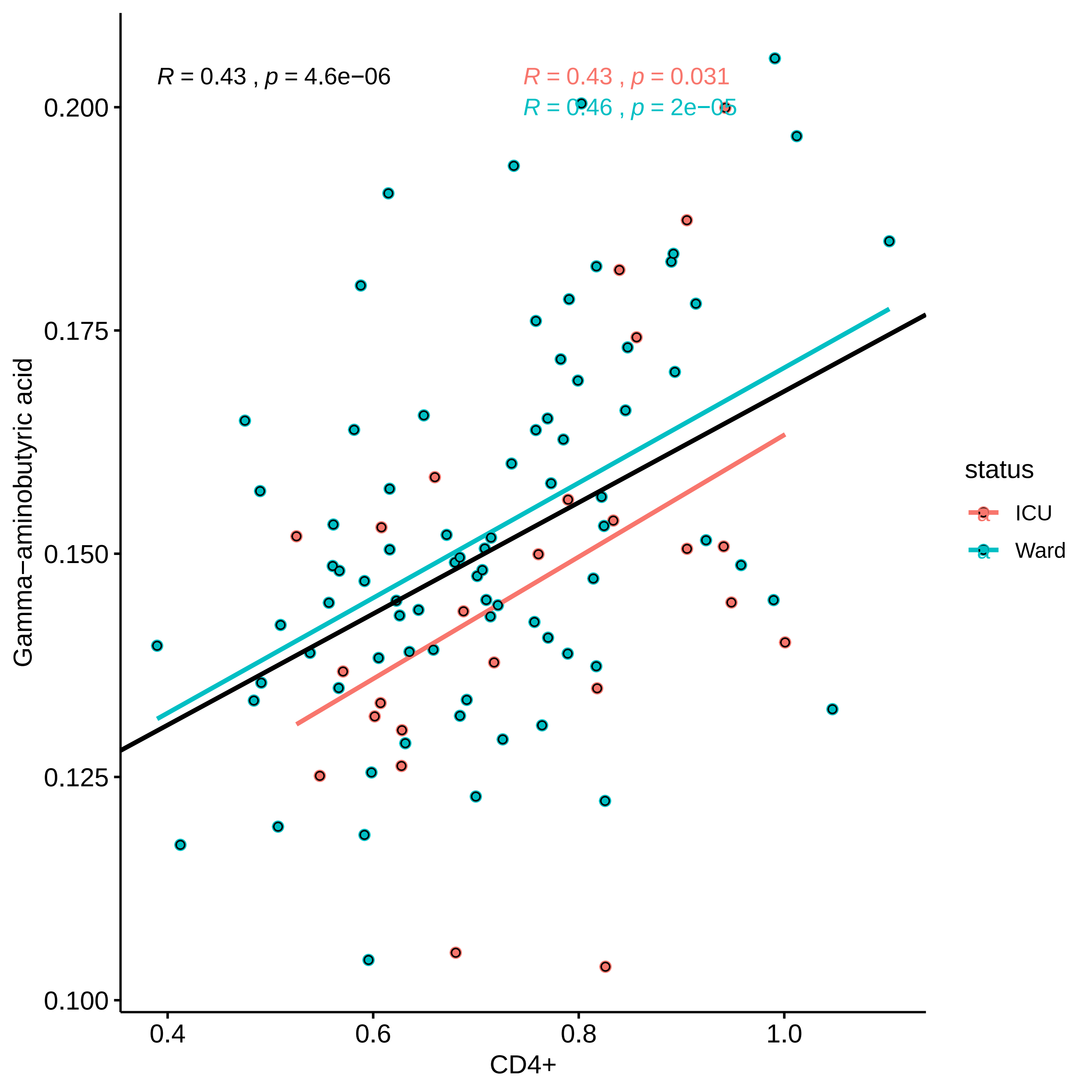
R = 0.43 , p = 4.6e−06
R = 0.43 , p = 0.031
R = 0.46 , p = 2e−05
Gamma−aminobutyric acid
0.200
0.175
0.150
0.125
0.100
0.4
0.6
0.8
1.0
CD4+
status
ICU
Ward

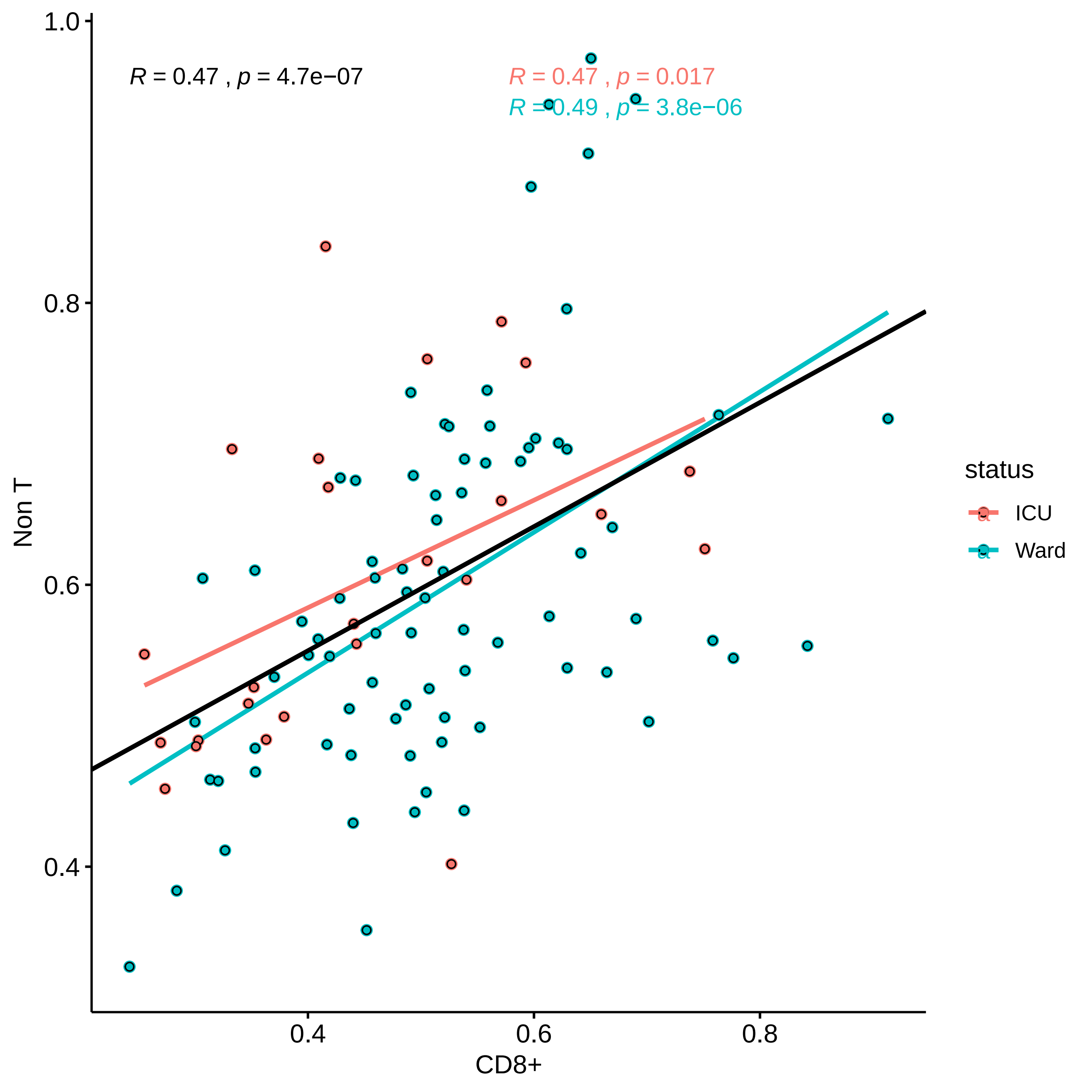

R = 0.47 , p = 4.7e−07
R = 0.47 , p = 0.017
R = 0.49 , p = 3.8e−06
1.0
0.8
0.6
0.4
Non T
0.4
0.6
0.8
CD8+
status
ICU
Ward

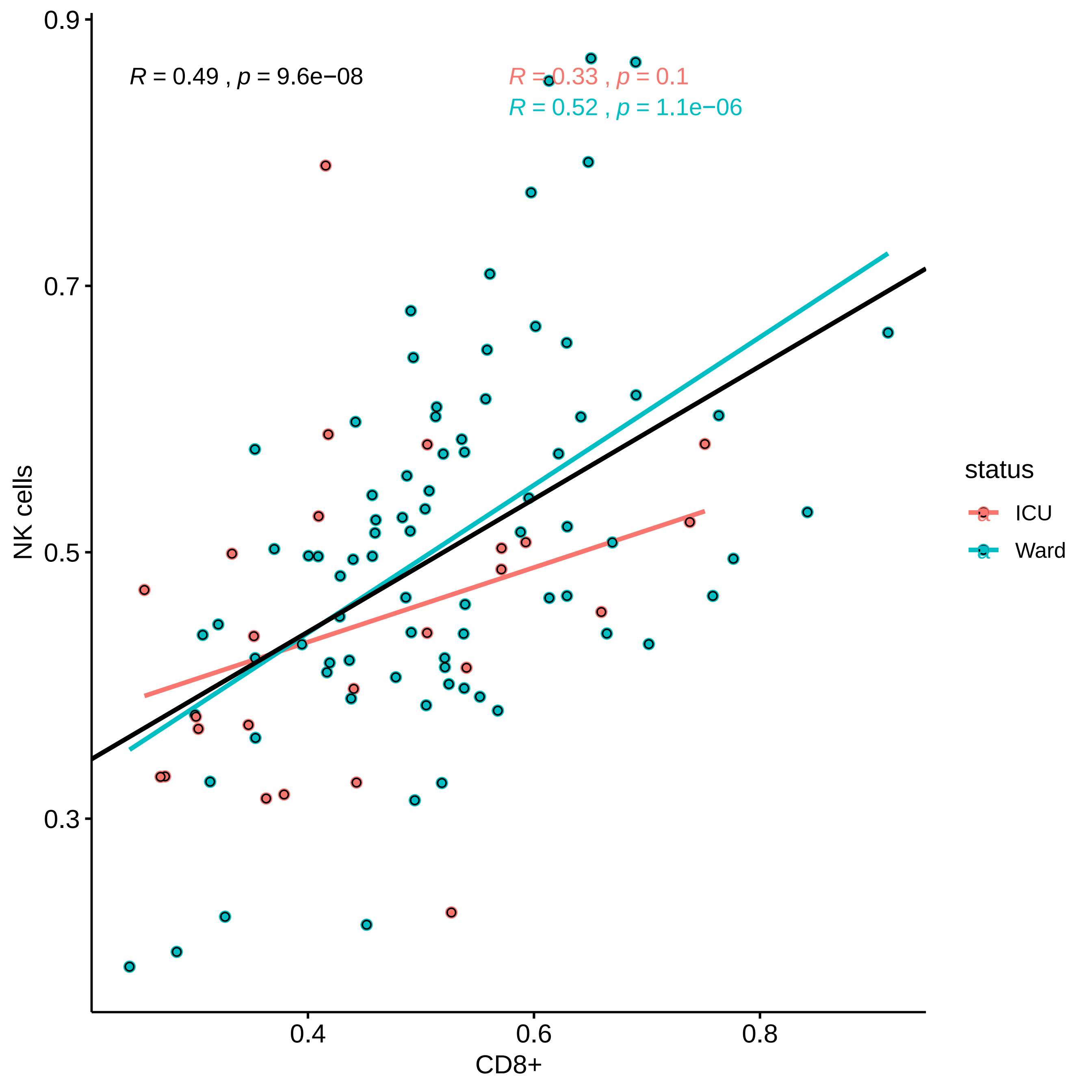

R = 0.49 , p = 9.6e−08
R = 0.33 , p = 0.1
R = 0.52 , p = 1.1e−06
0.9
0.7
0.5
0.3
NK cells
0.4
0.6
0.8
CD8+
status
ICU
Ward

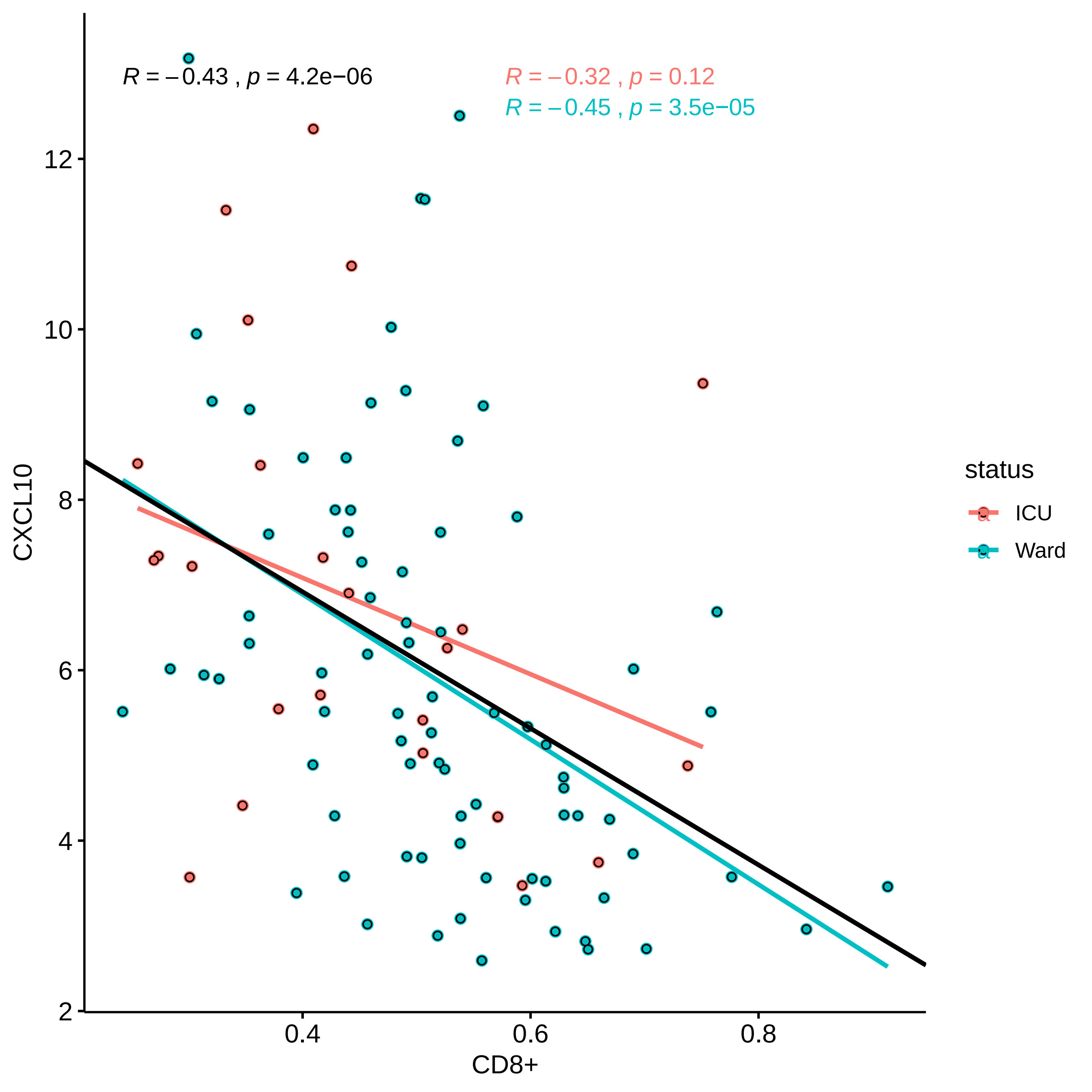
R = −0.43 , p = 4.2e−06
R = −0.32 , p = 0.12
R = −0.45 , p = 3.5e−05
CXCL10
CD8+
2
4
6
8
10
12
0.4
0.6
0.8
status
ICU
Ward

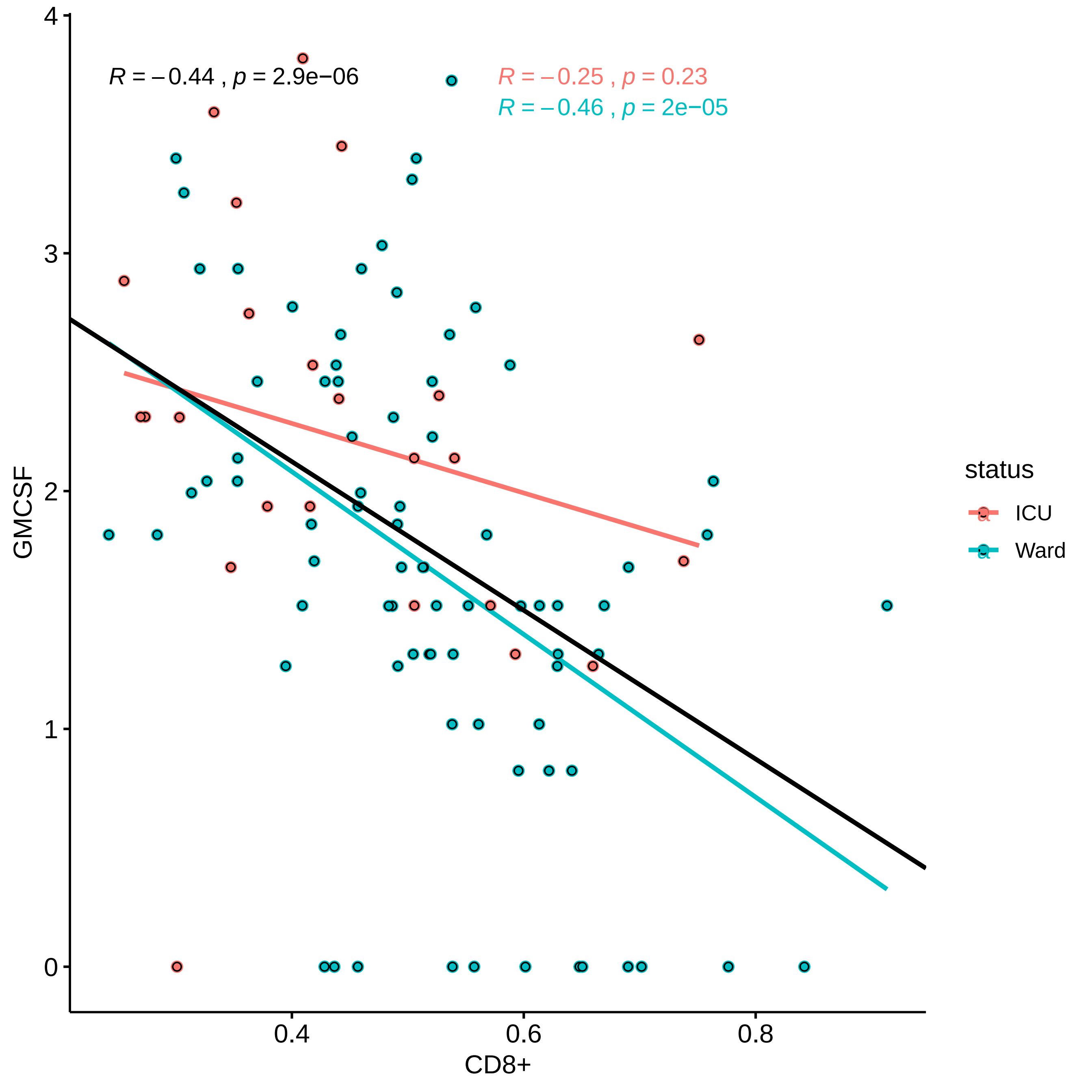

R = −0.44 , p = 2.9e−06
R = −0.25 , p = 0.23
R = −0.46 , p = 2e−05
GMCSF
CD8+
status
ICU
Ward
0
1
2
3
4
0.4
0.6
0.8

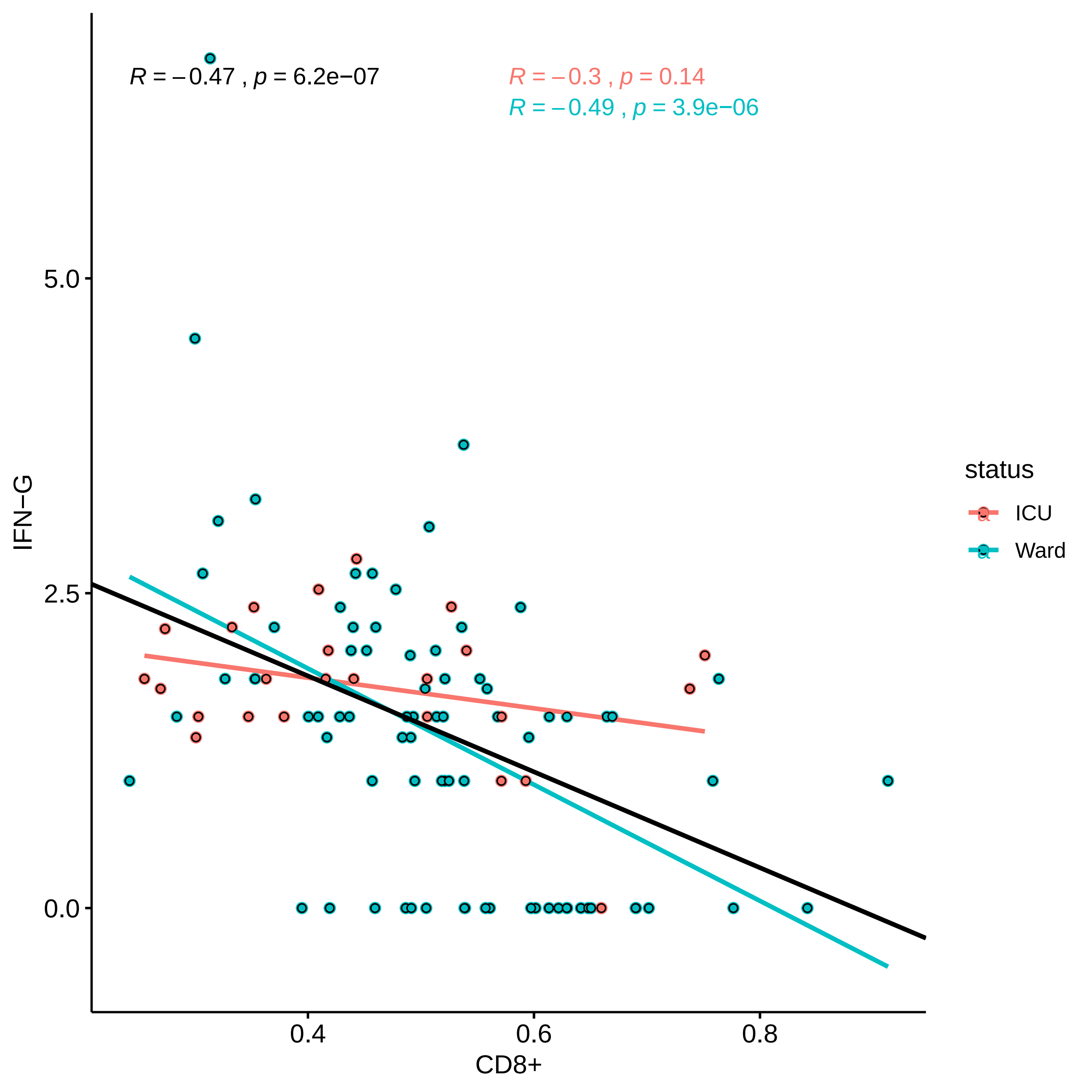

R = −0.47 , p = 6.2e−07
R = −0.3 , p = 0.14
R = −0.49 , p = 3.9e−06
IFN−G
5.0
2.5
0.0
0.4
0.6
0.8
CD8+
status
ICU
Ward

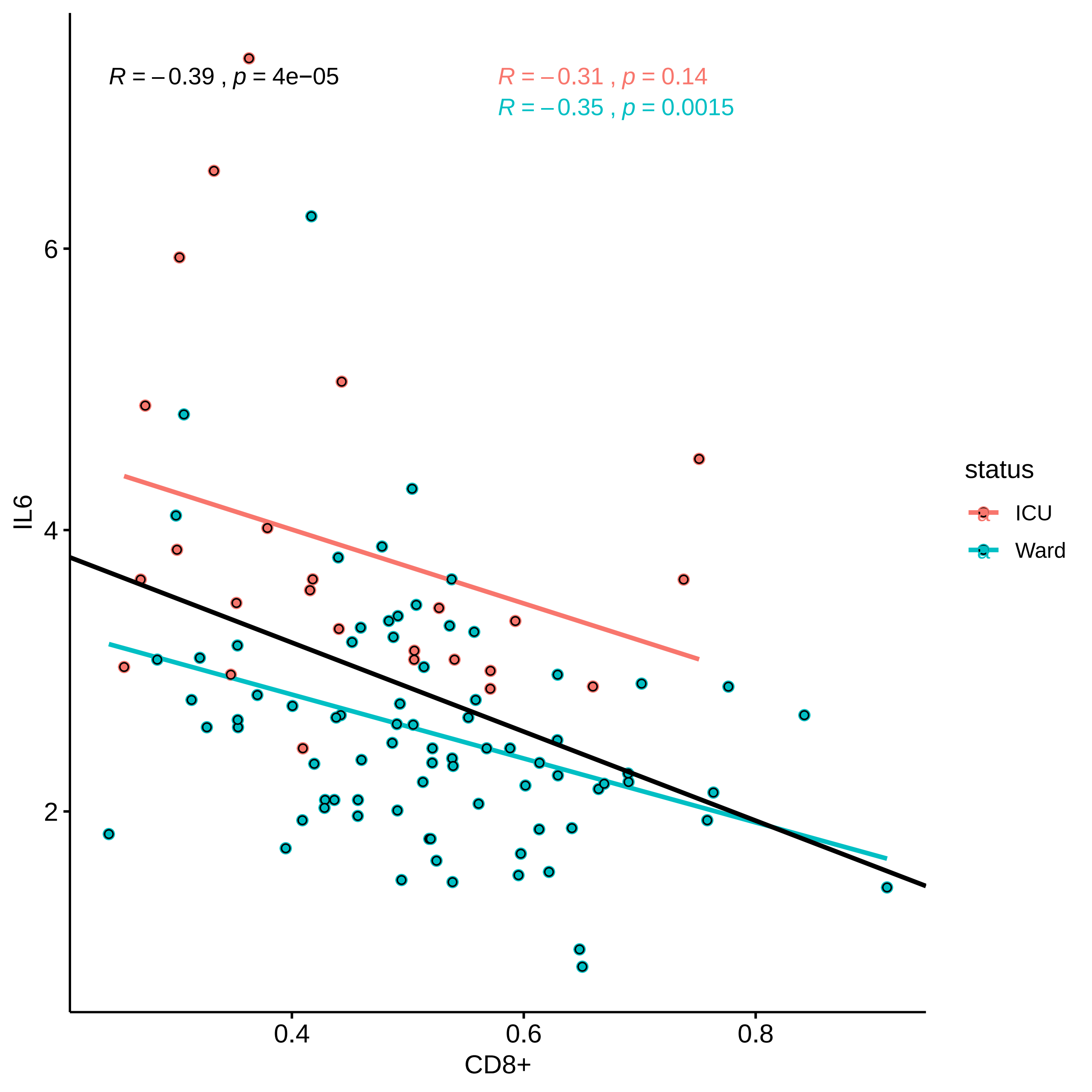

R = −0.39 , p = 4e−05
R = −0.31 , p = 0.14
R = −0.35 , p = 0.0015
IL6
6
4
2
0.4
0.6
0.8
CD8+
status
ICU
Ward

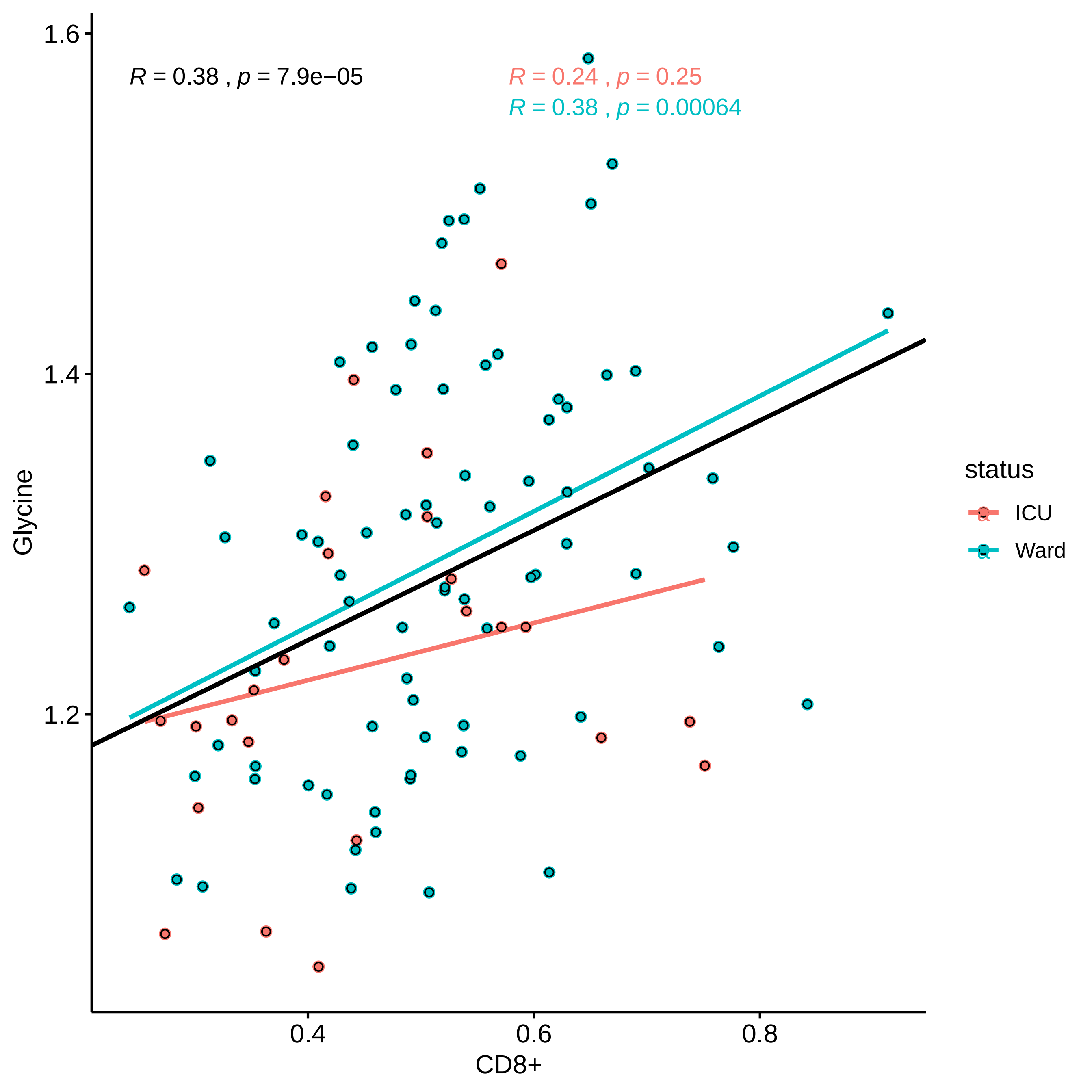

R = 0.38 , p = 7.9e−05
R = 0.24 , p = 0.25
R = 0.38 , p = 0.00064
1.6
1.4
1.2
Glycine
0.4
0.6
0.8
CD8+
status
ICU
Ward

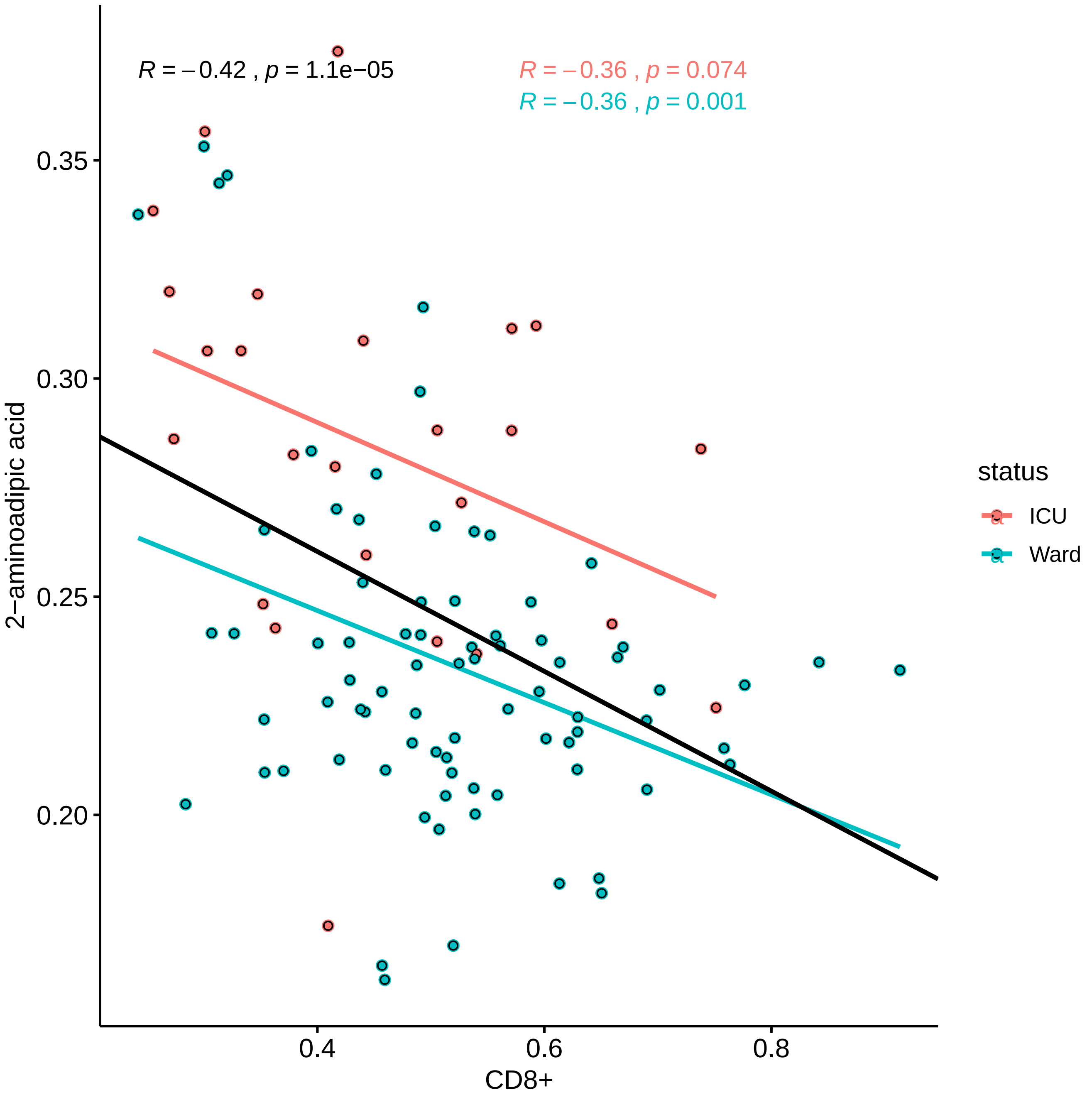

R = −0.42 , p = 1.1e−05
R = −0.36 , p = 0.074
R = −0.36 , p = 0.001
0.35
0.30
0.25
0.20
2−aminoadipic acid
0.4
0.6
0.8
CD8+
status
ICU
Ward

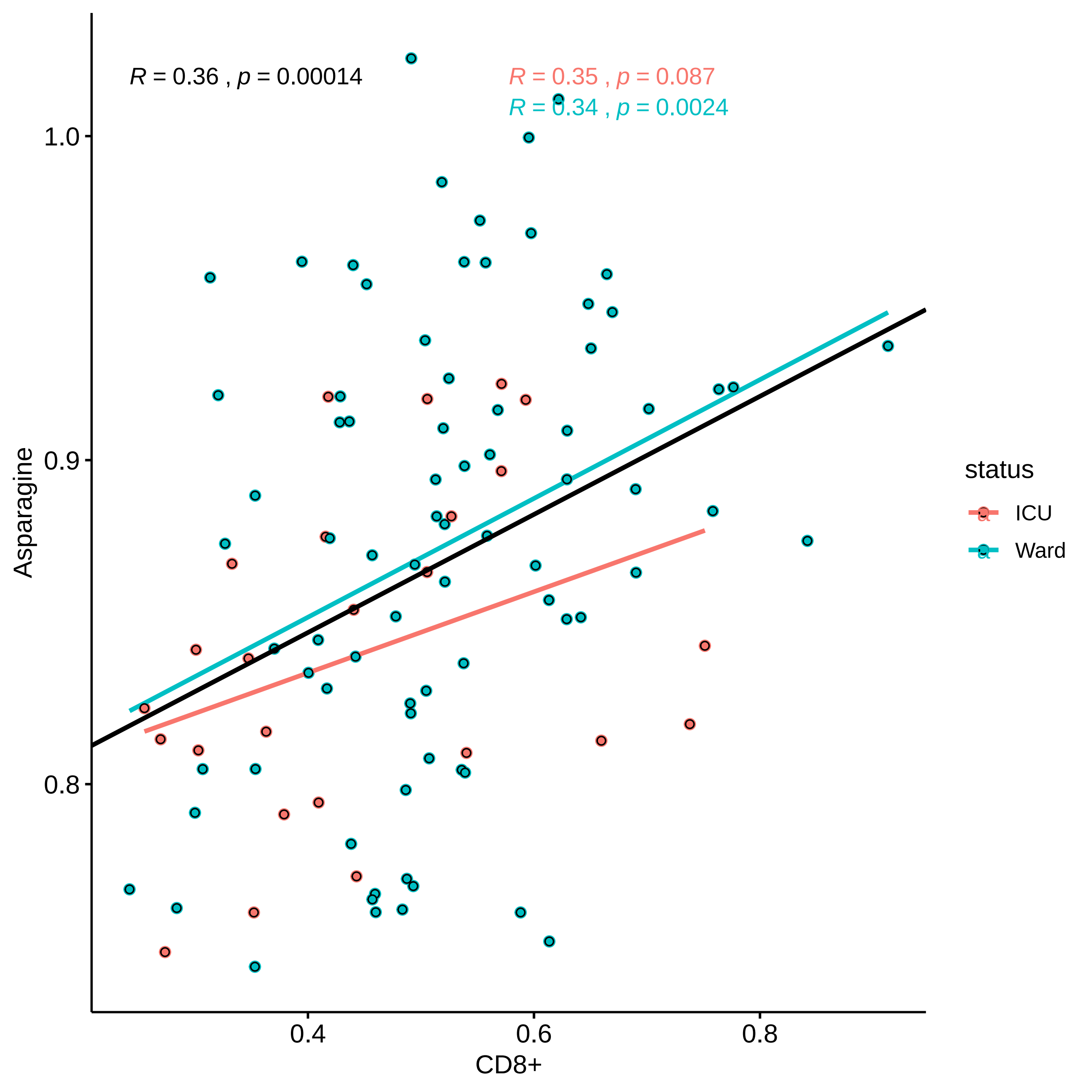

R = 0.36 , p = 0.00014
R = 0.35 , p = 0.087
R = 0.34 , p = 0.0024
1.0
0.9
0.8
Asparagine
0.4
0.6
0.8
CD8+
status
ICU
Ward

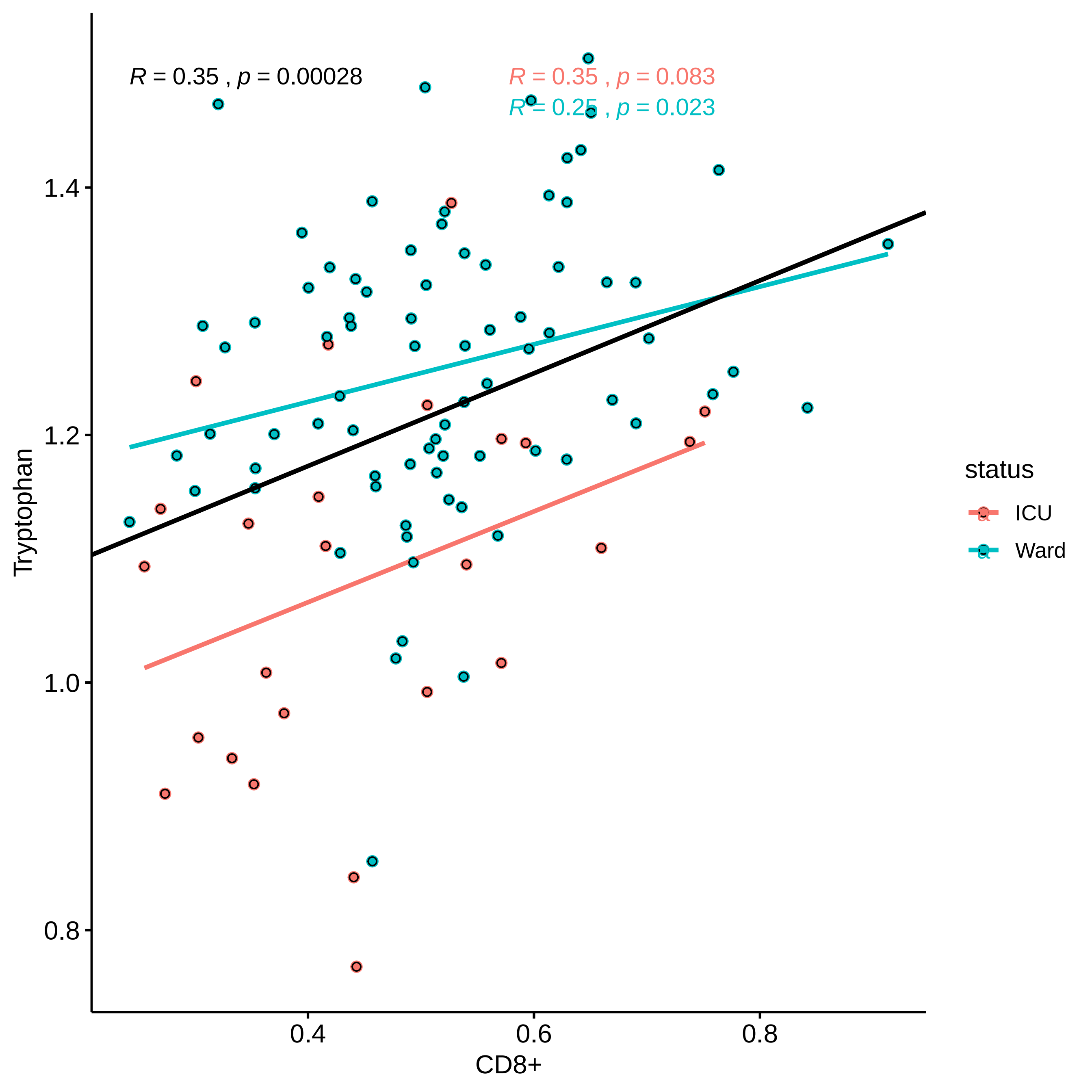

R = 0.35 , p = 0.00028
R = 0.35 , p = 0.083
R = 0.25 , p = 0.023
Tryptophan
1.4
1.2
1.0
0.8
CD8+
0.4
0.6
0.8
status
ICU
Ward

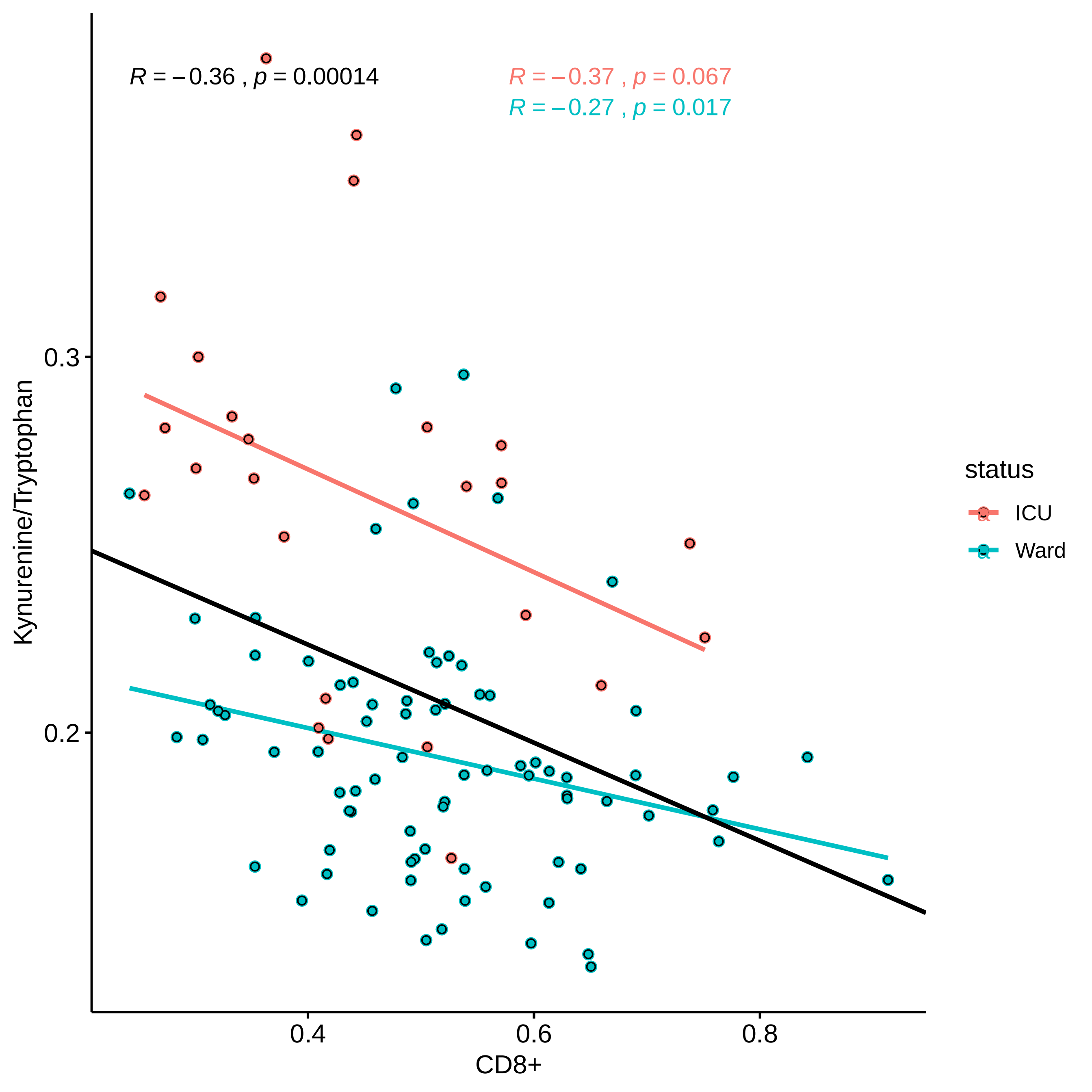

R = −0.36 , p = 0.00014
R = −0.37 , p = 0.067
R = −0.27 , p = 0.017
Kynurenine/Tryptophan
0.3
0.2
0.4
0.6
0.8
CD8+
status
ICU
Ward

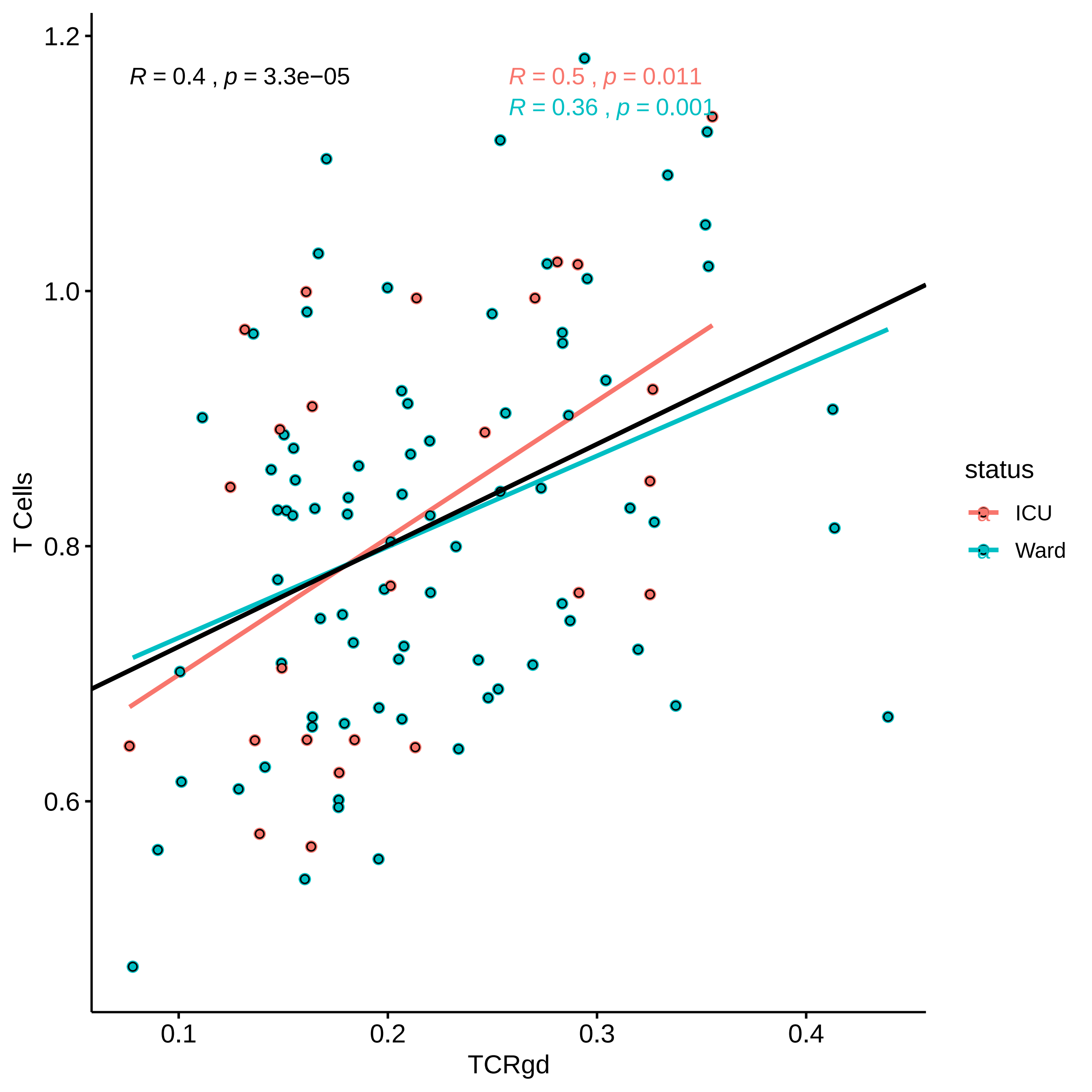

R = 0.4 , p = 3.3e−05
R = 0.5 , p = 0.011
R = 0.36 , p = 0.001
T Cells
1.2
1.0
0.8
0.6
0.1
0.2
0.3
0.4
TCRgd
status
ICU
Ward

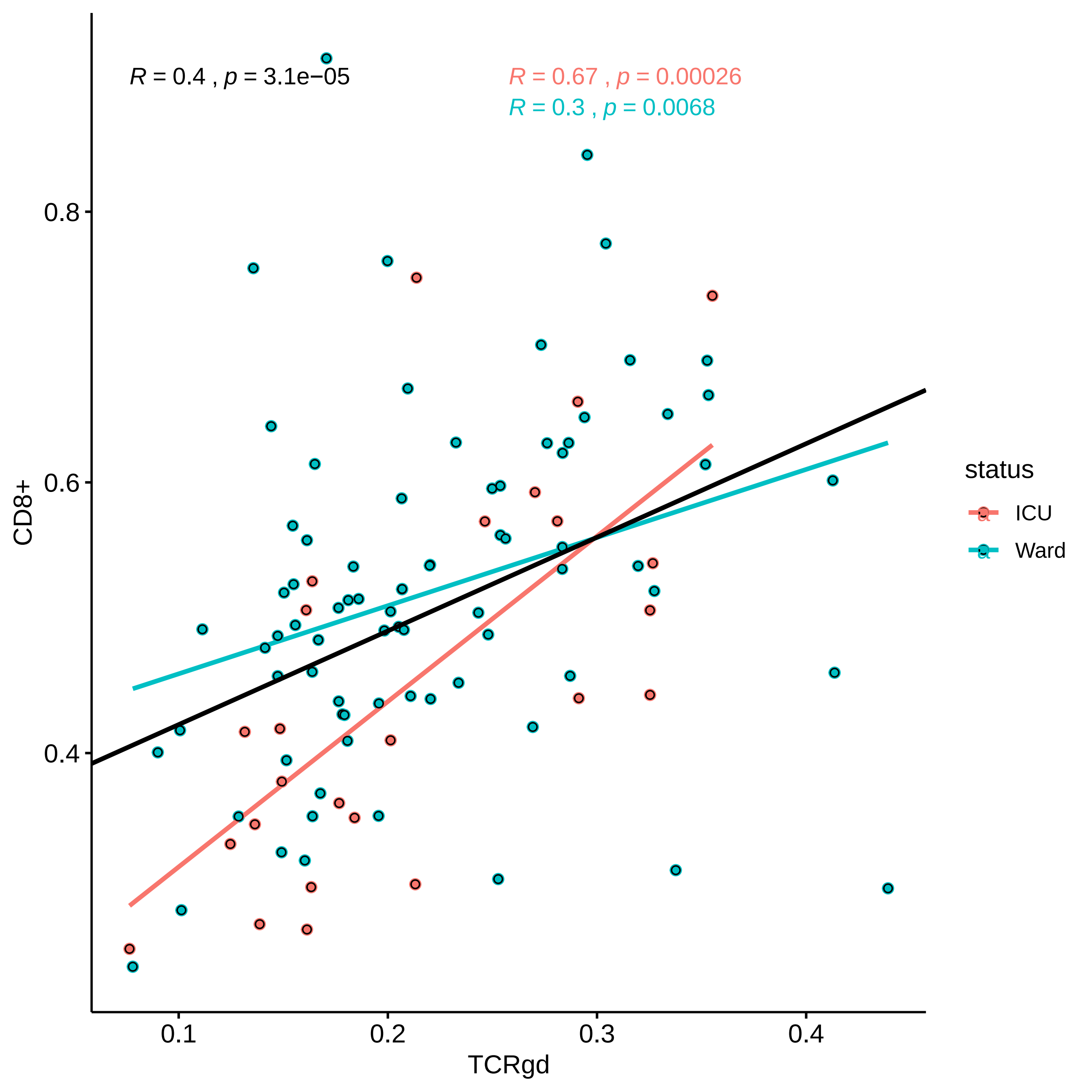

R = 0.4 , p = 3.1e−05
R = 0.67 , p = 0.00026
R = 0.3 , p = 0.0068
0.8
0.6
0.4
CD8+
0.1
0.2
0.3
0.4
TCRgd
status
ICU
Ward

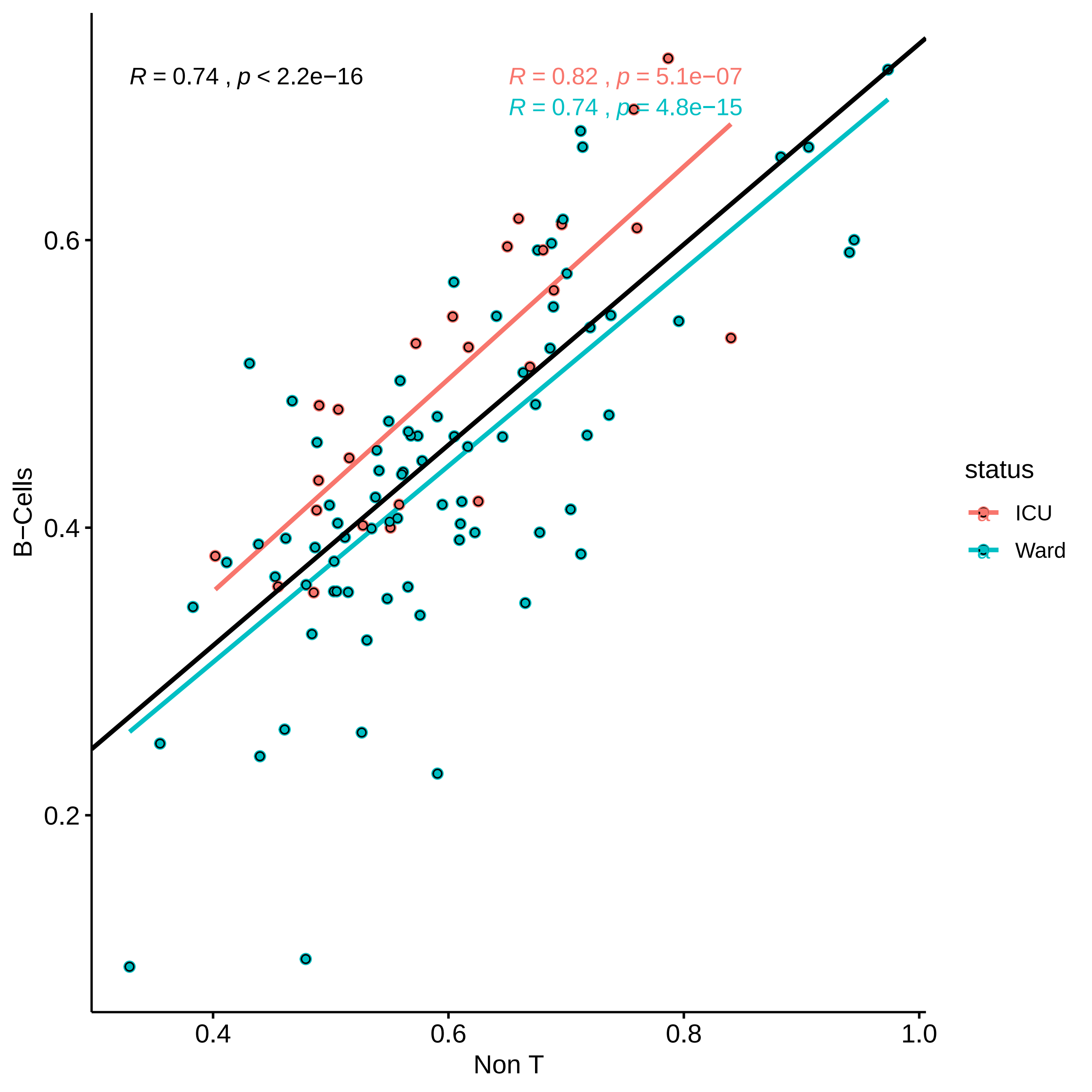

R = 0.74 , p < 2.2e−16
R = 0.82 , p = 5.1e−07
R = 0.74 , p = 4.8e−15
B−Cells
0.6
0.4
0.2
0.4
0.6
0.8
1.0
Non T
status
ICU
Ward

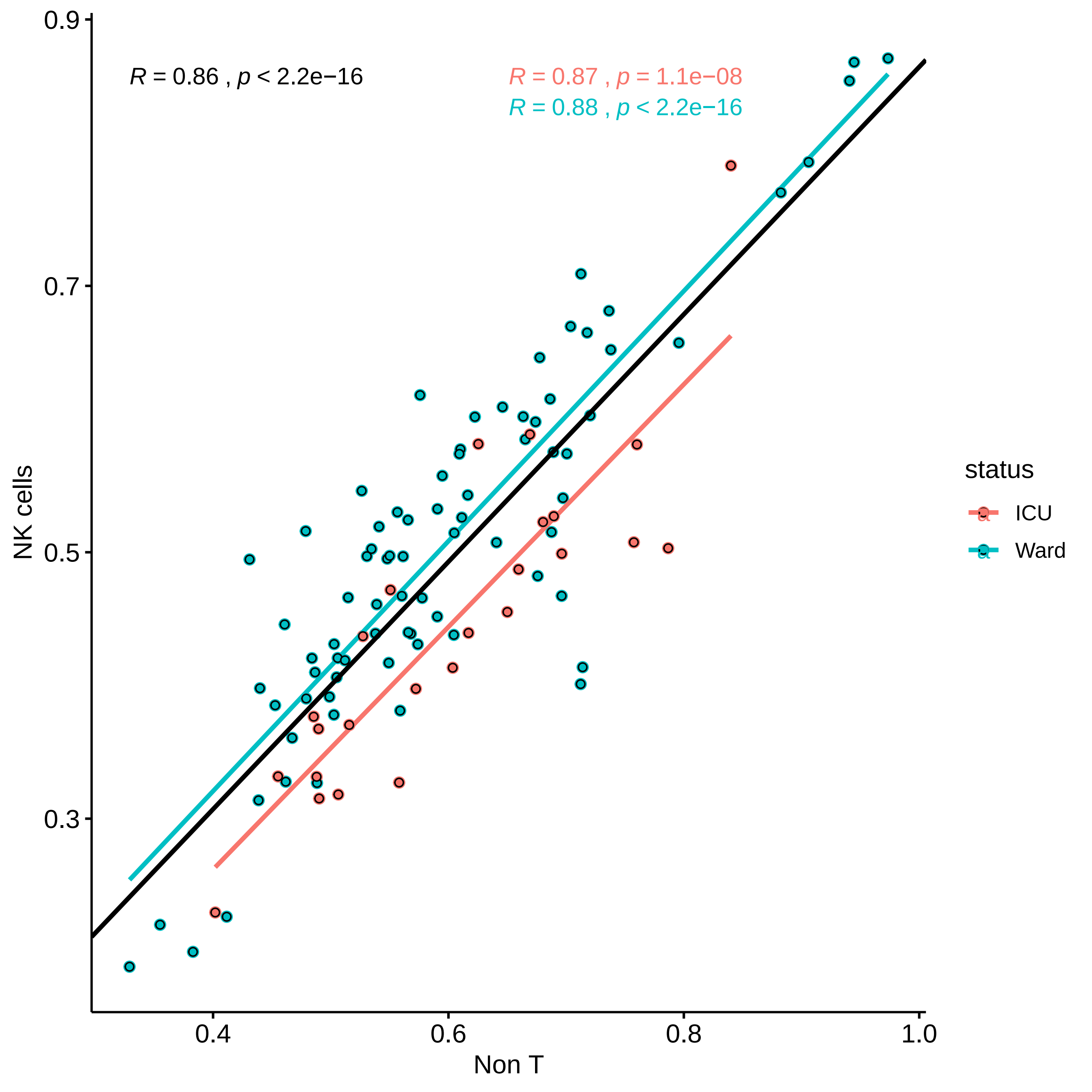

R = 0.86 , p < 2.2e−16
R = 0.87 , p = 1.1e−08
R = 0.88 , p < 2.2e−16
NK cells
Non T
0.9
0.7
0.5
0.3
0.4
0.6
0.8
1.0
status
ICU
Ward

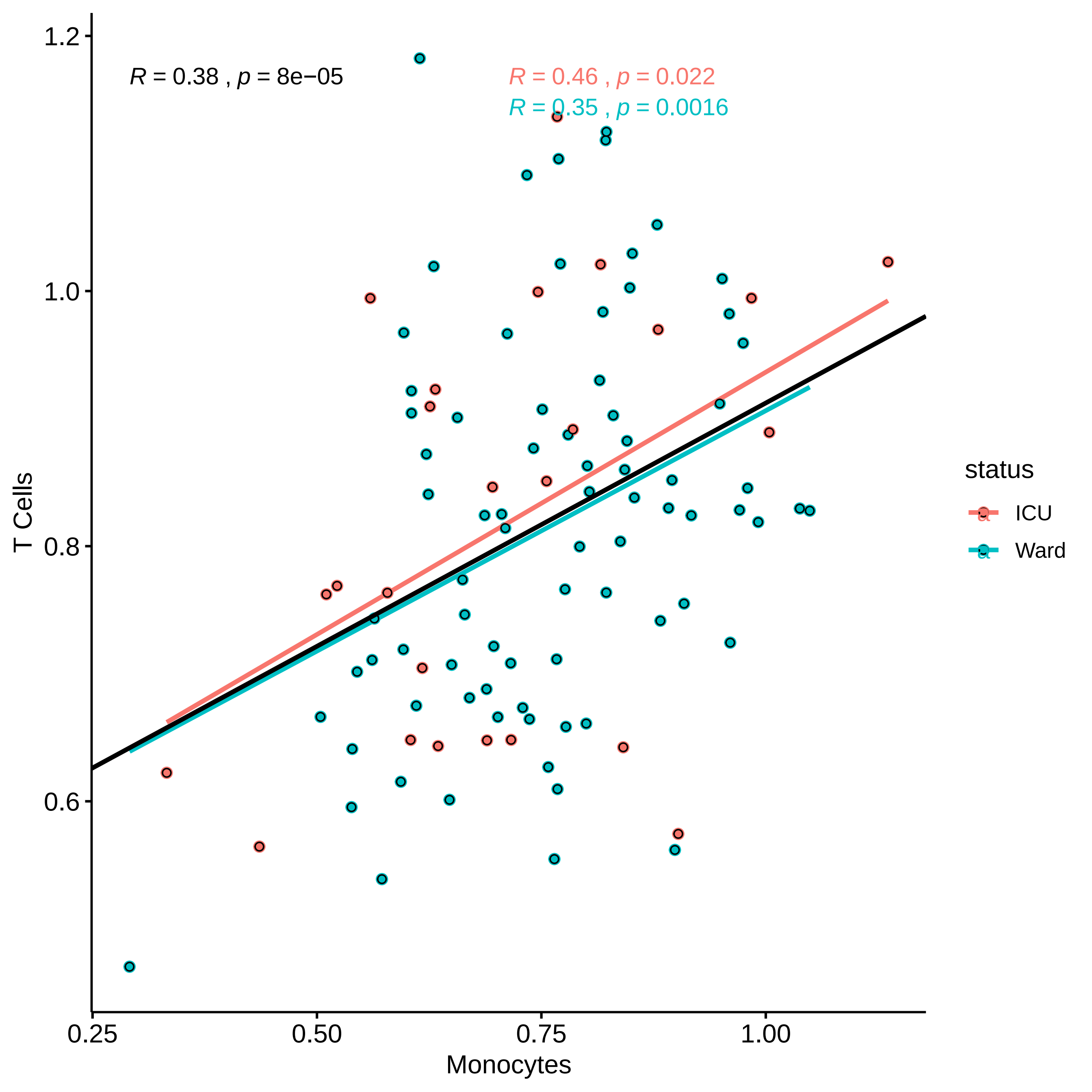

R = 0.38 , p = 8e−05
R = 0.46 , p = 0.022
R = 0.35 , p = 0.0016
1.2
1.0
0.8
0.6
T Cells
0.25
0.50
0.75
1.00
Monocytes
status
ICU
Ward

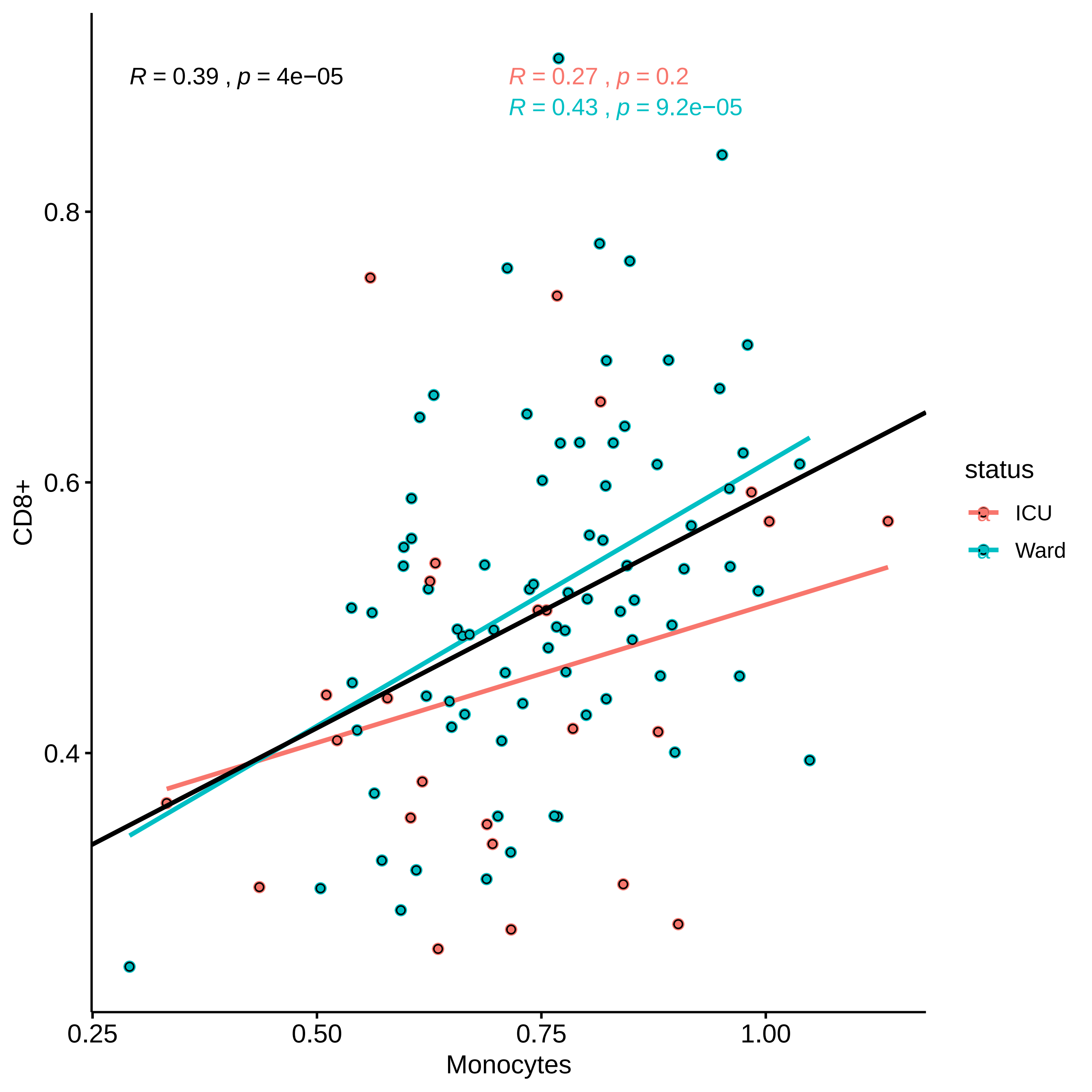

R = 0.39 , p = 4e−05
R = 0.27 , p = 0.2
R = 0.43 , p = 9.2e−05
CD8+
0.8
0.6
0.4
0.25
0.50
0.75
1.00
Monocytes
status
ICU
Ward

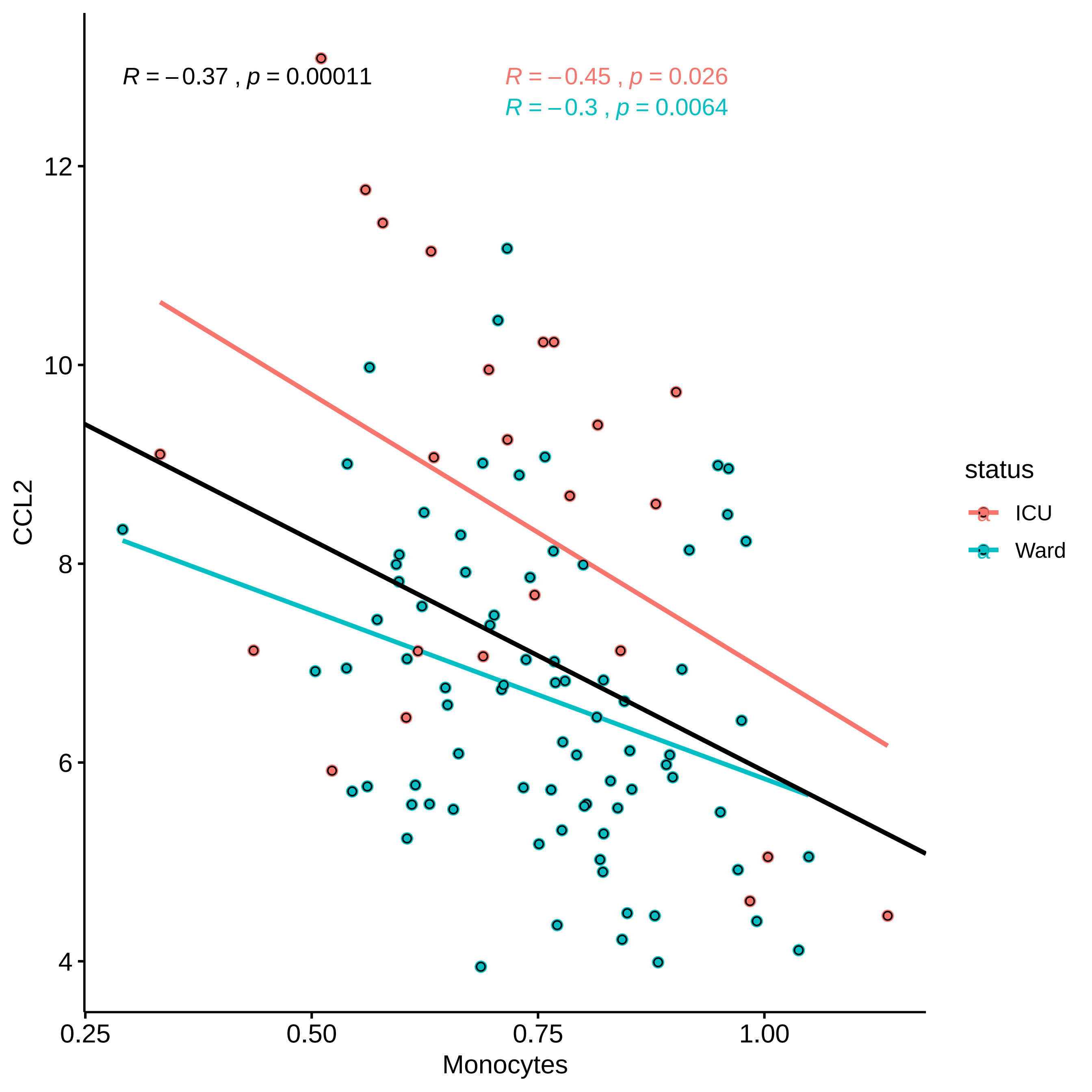

R = −0.37 , p = 0.00011
R = −0.45 , p = 0.026
R = −0.3 , p = 0.0064
12
10
8
6
4
CCL2
0.25
0.50
0.75
1.00
Monocytes
status
ICU
Ward

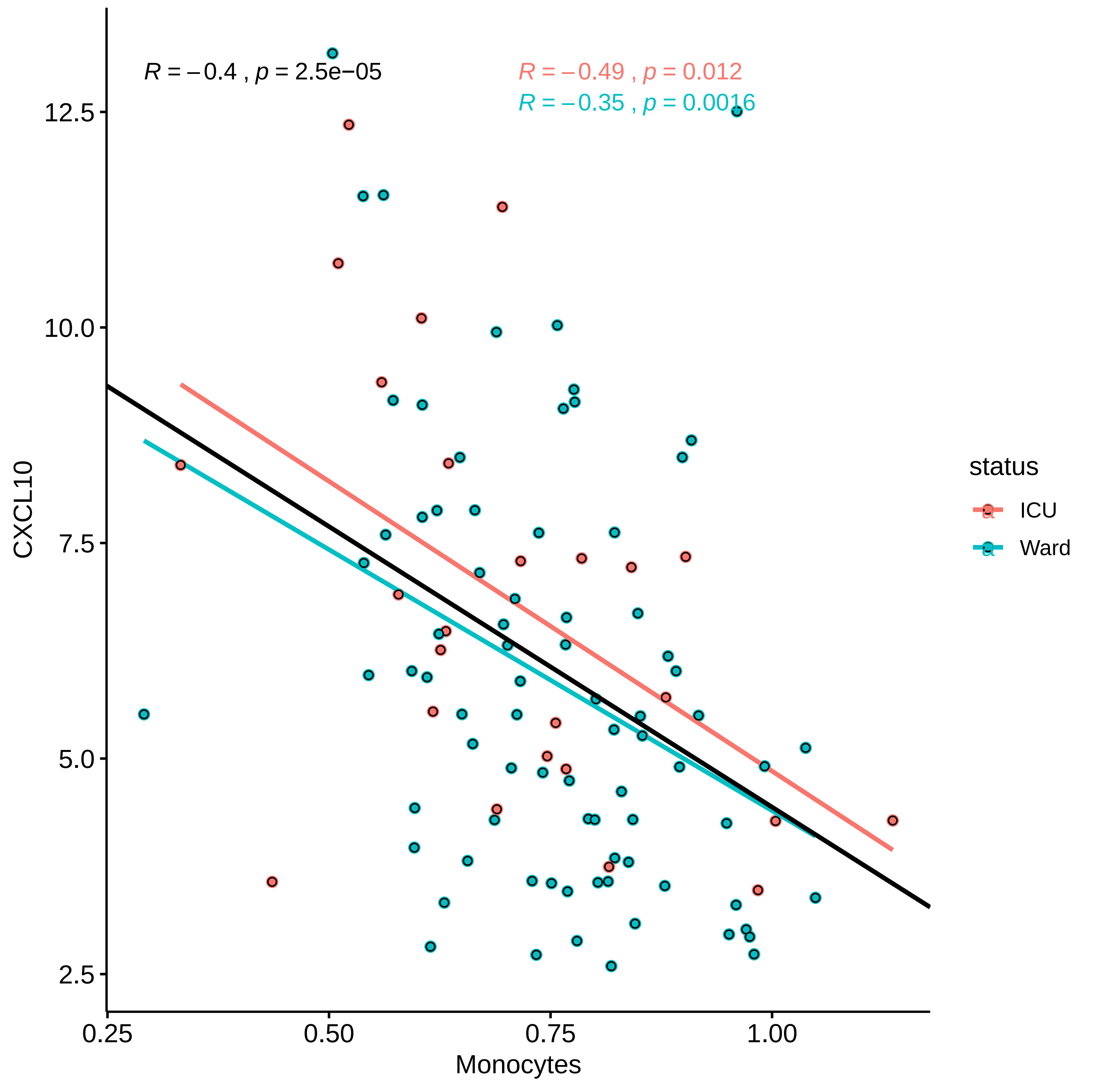

R = −0.4 , p = 2.5e−05
R = −0.49 , p = 0.012
R = −0.35 , p = 0.0016
12.5
10.0
7.5
5.0
2.5
CXCL10
0.25
0.50
0.75
1.00
Monocytes
status
ICU
Ward

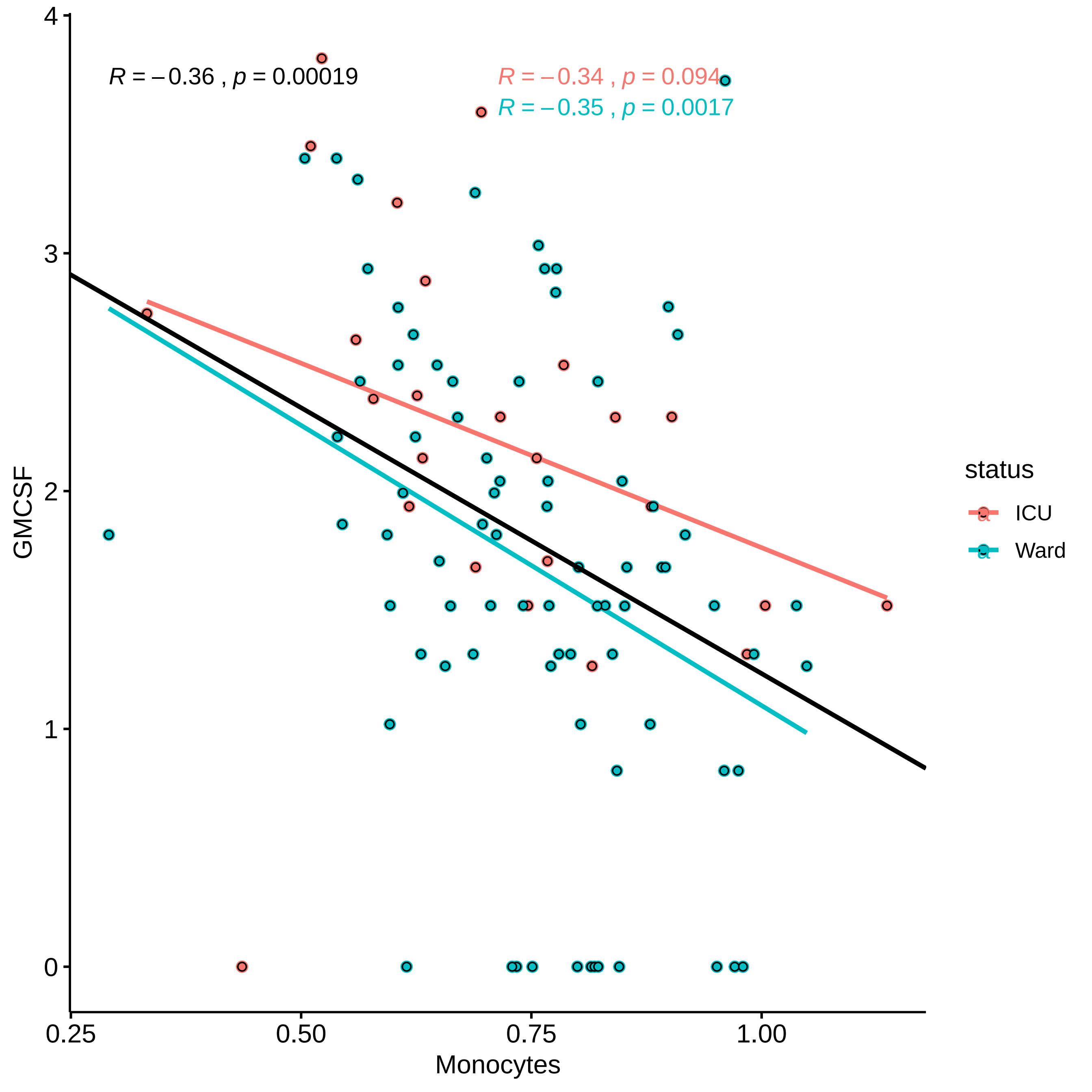
R = −0.36 , p = 0.00019
R = −0.34 , p = 0.094
R = −0.35 , p = 0.0017
GMCSF
Monocytes
0
1
2
3
4
0.25
0.50
0.75
1.00
status
ICU
Ward

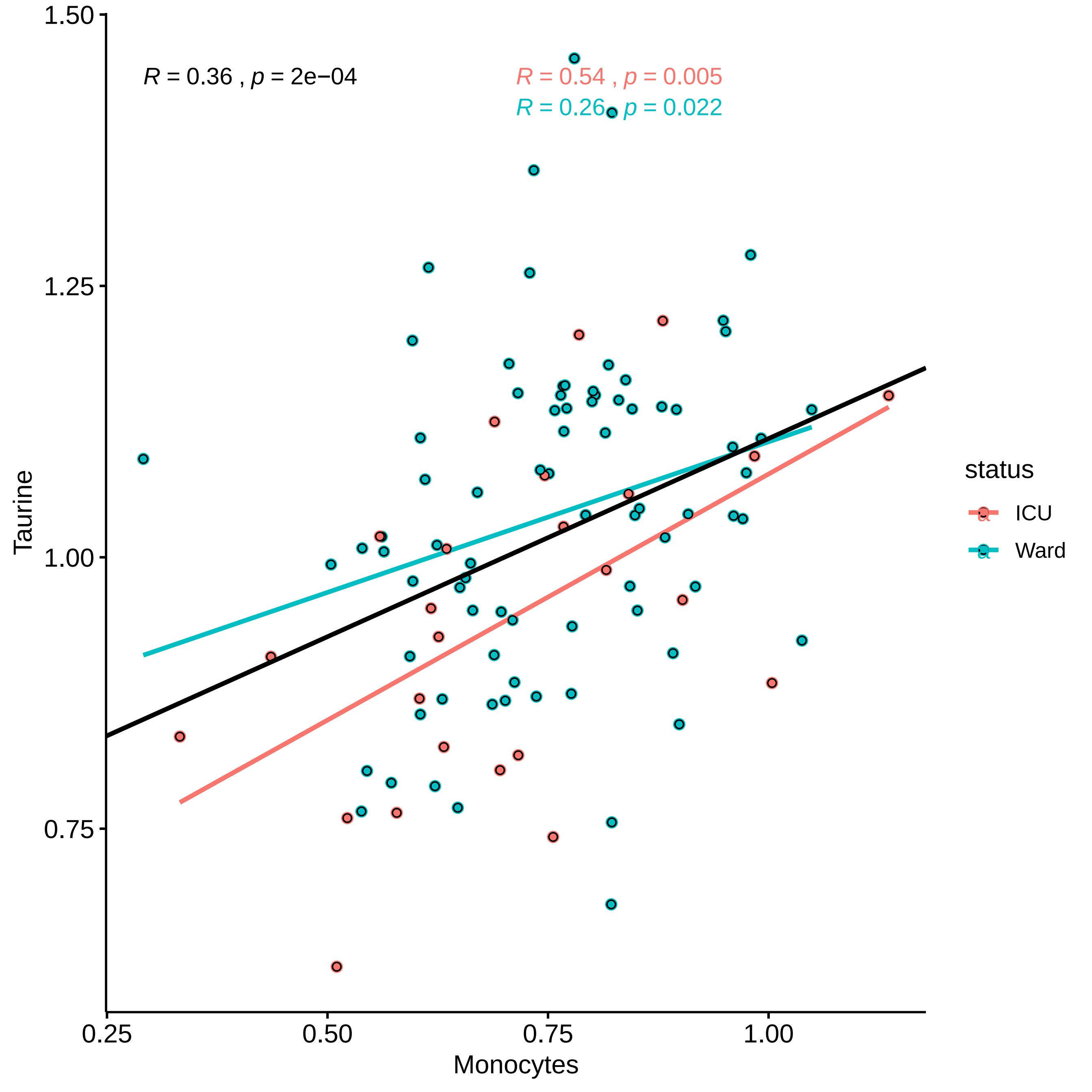

R = 0.36 , p = 2e−04
R = 0.54 , p = 0.005
R = 0.26 , p = 0.022
1.50
1.25
1.00
0.75
Taurine
0.25
0.50
0.75
1.00
Monocytes
status
ICU
Ward

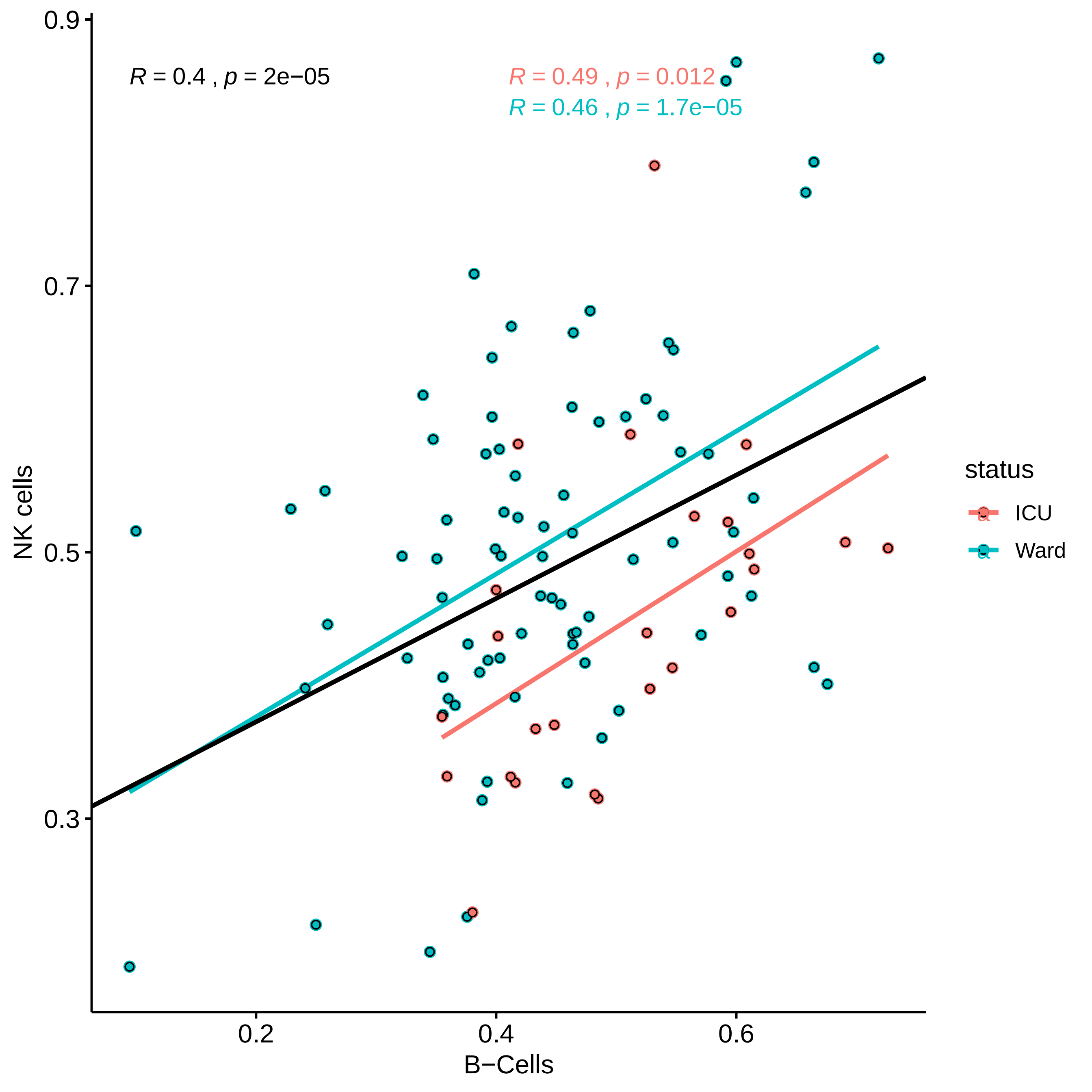
R = 0.4 , p = 2e−05
R = 0.49 , p = 0.012
R = 0.46 , p = 1.7e−05
0.9
0.7
0.5
0.3
0.2
0.4
0.6
NK cells
B−Cells
status
ICU
Ward

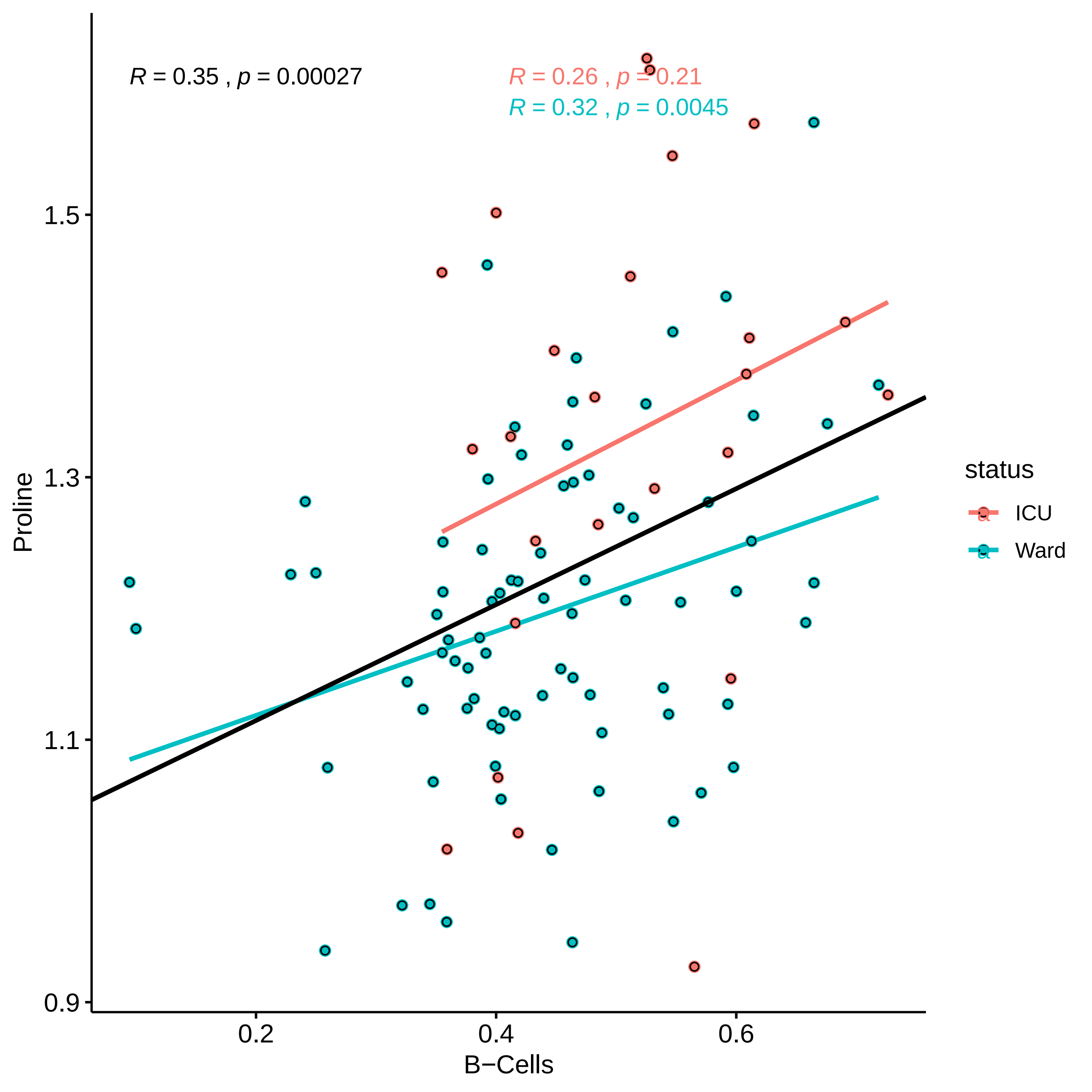

R = 0.35 , p = 0.00027
R = 0.26 , p = 0.21
R = 0.32 , p = 0.0045
Proline
1.5
1.3
1.1
0.9
0.2
0.4
0.6
B−Cells
status
ICU
Ward

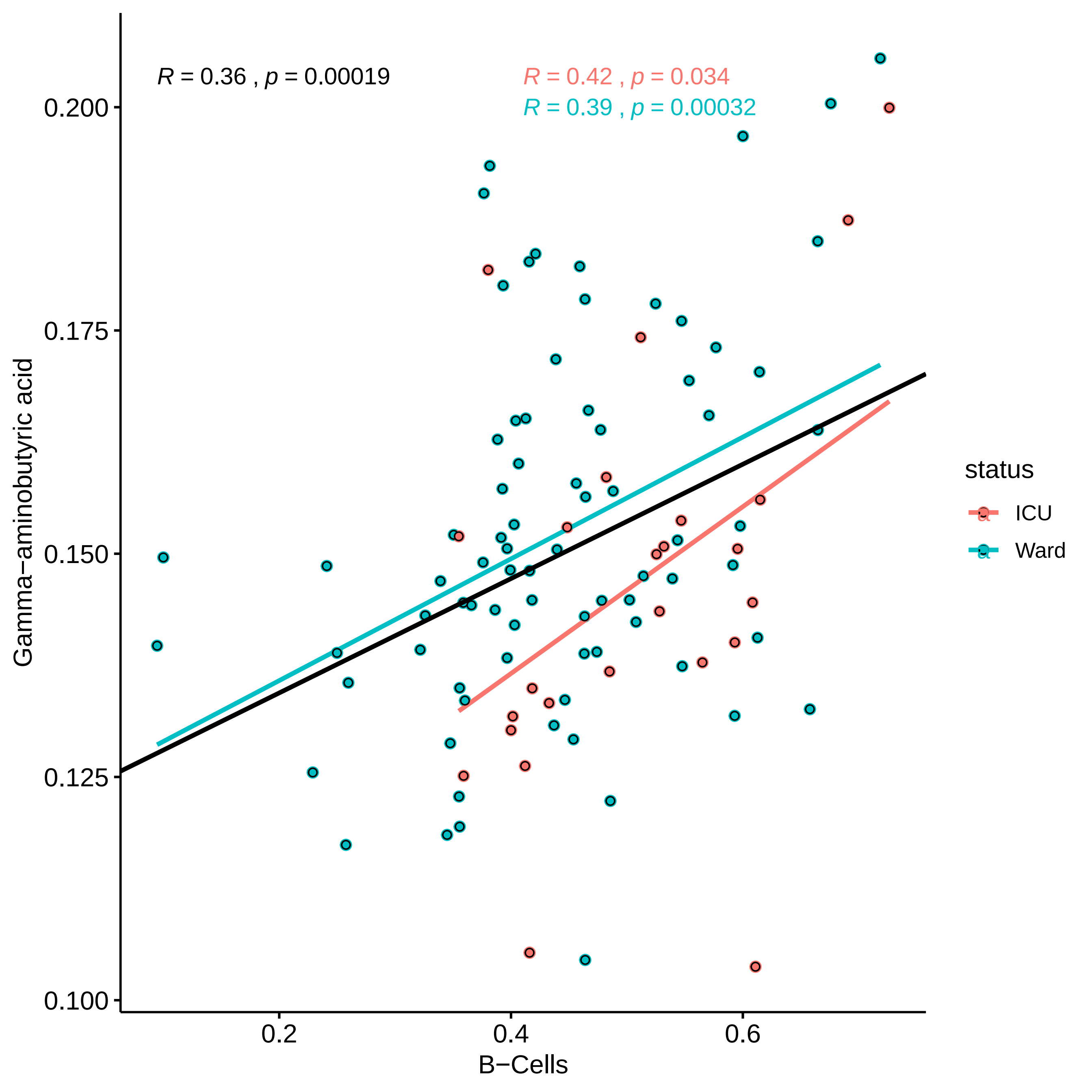

R = 0.36 , p = 0.00019
R = 0.42 , p = 0.034
R = 0.39 , p = 0.00032
0.200
0.175
0.150
0.125
0.100
Gamma−aminobutyric acid
0.2
0.4
0.6
B−Cells
status
ICU
Ward

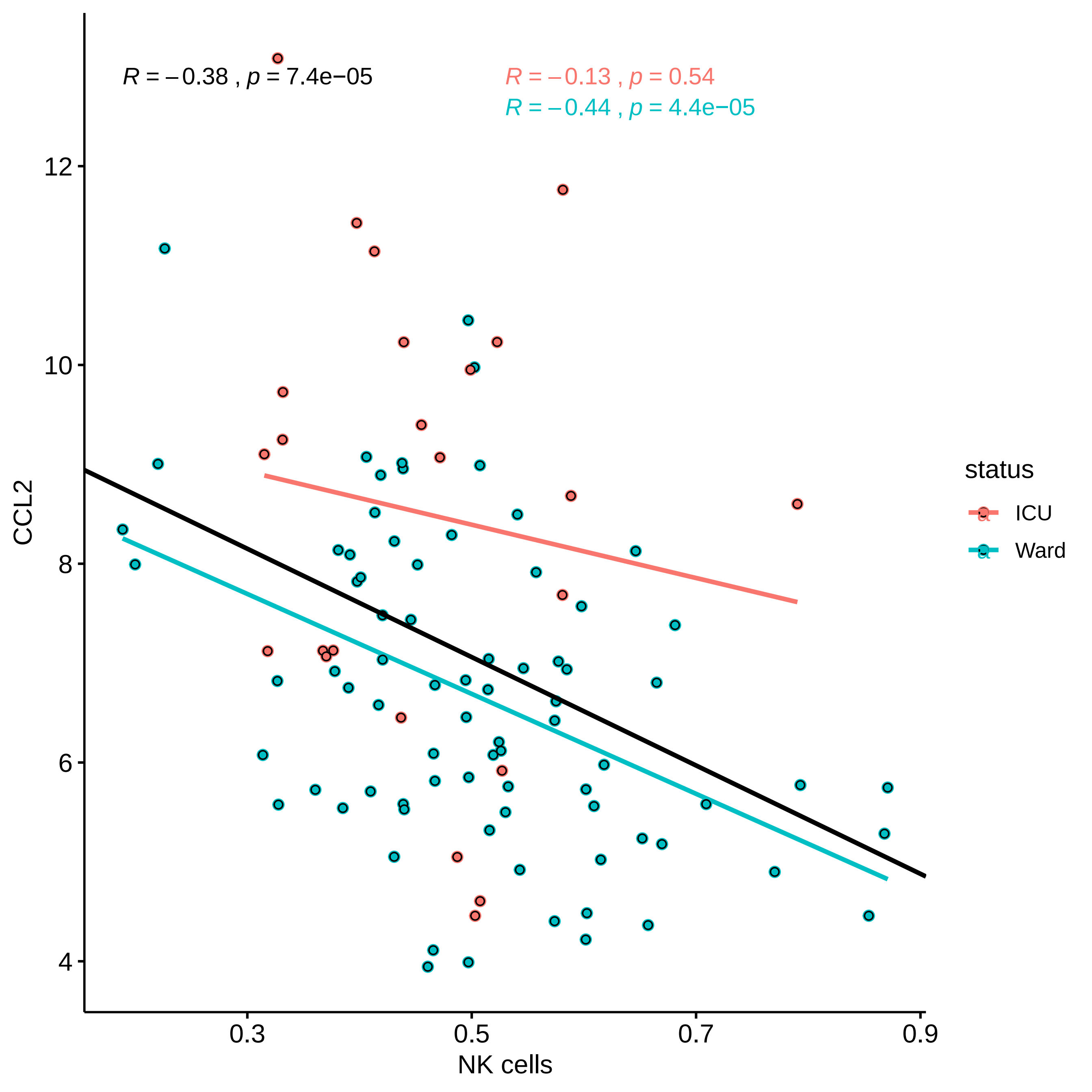

R = −0.38 , p = 7.4e−05
R = −0.13 , p = 0.54
R = −0.44 , p = 4.4e−05
CCL2
12
10
8
6
4
NK cells
0.3
0.5
0.7
0.9
status
ICU
Ward

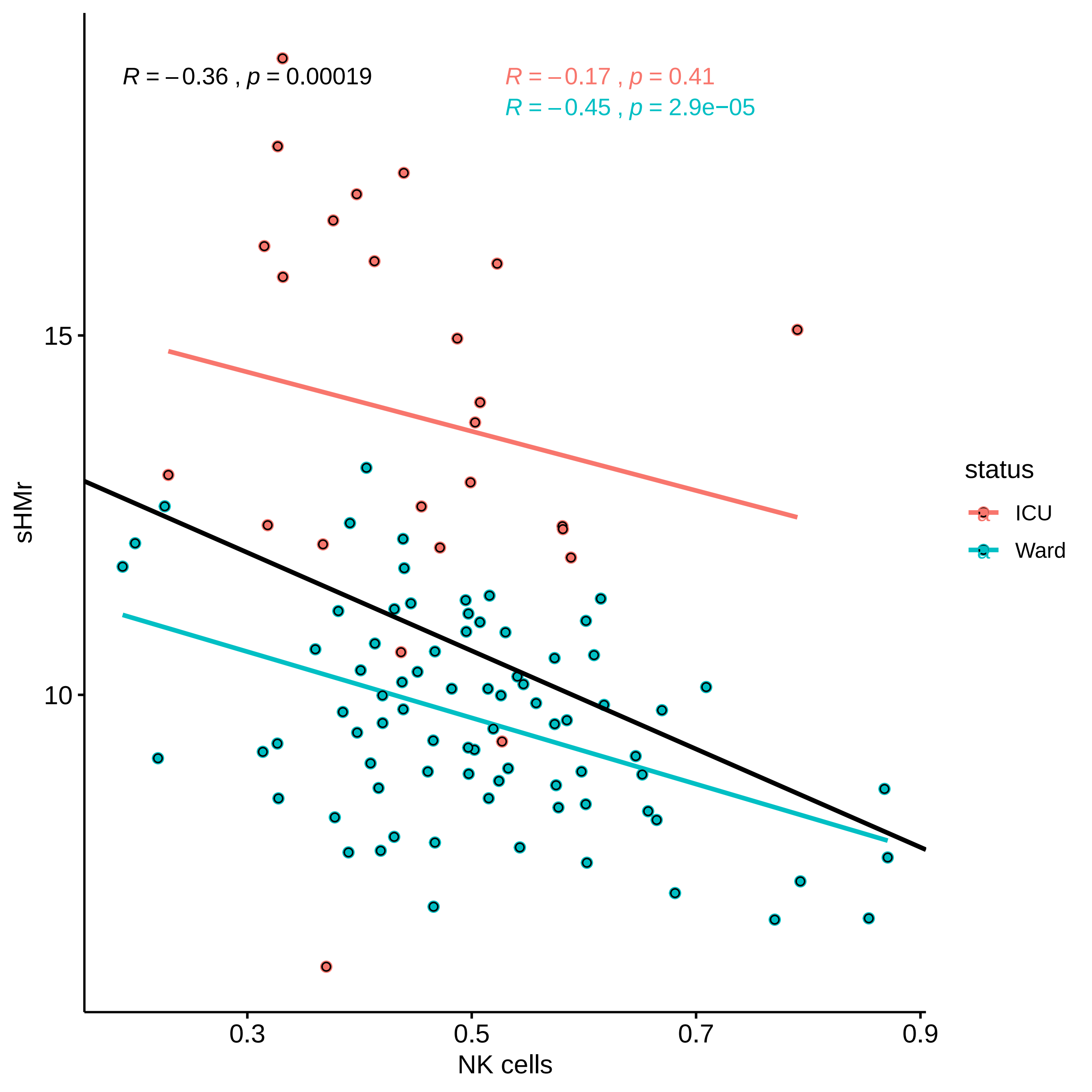

R = −0.36 , p = 0.00019
R = −0.17 , p = 0.41
R = −0.45 , p = 2.9e−05
sHMr
15
10
0.3
0.5
0.7
0.9
NK cells
status
ICU
Ward

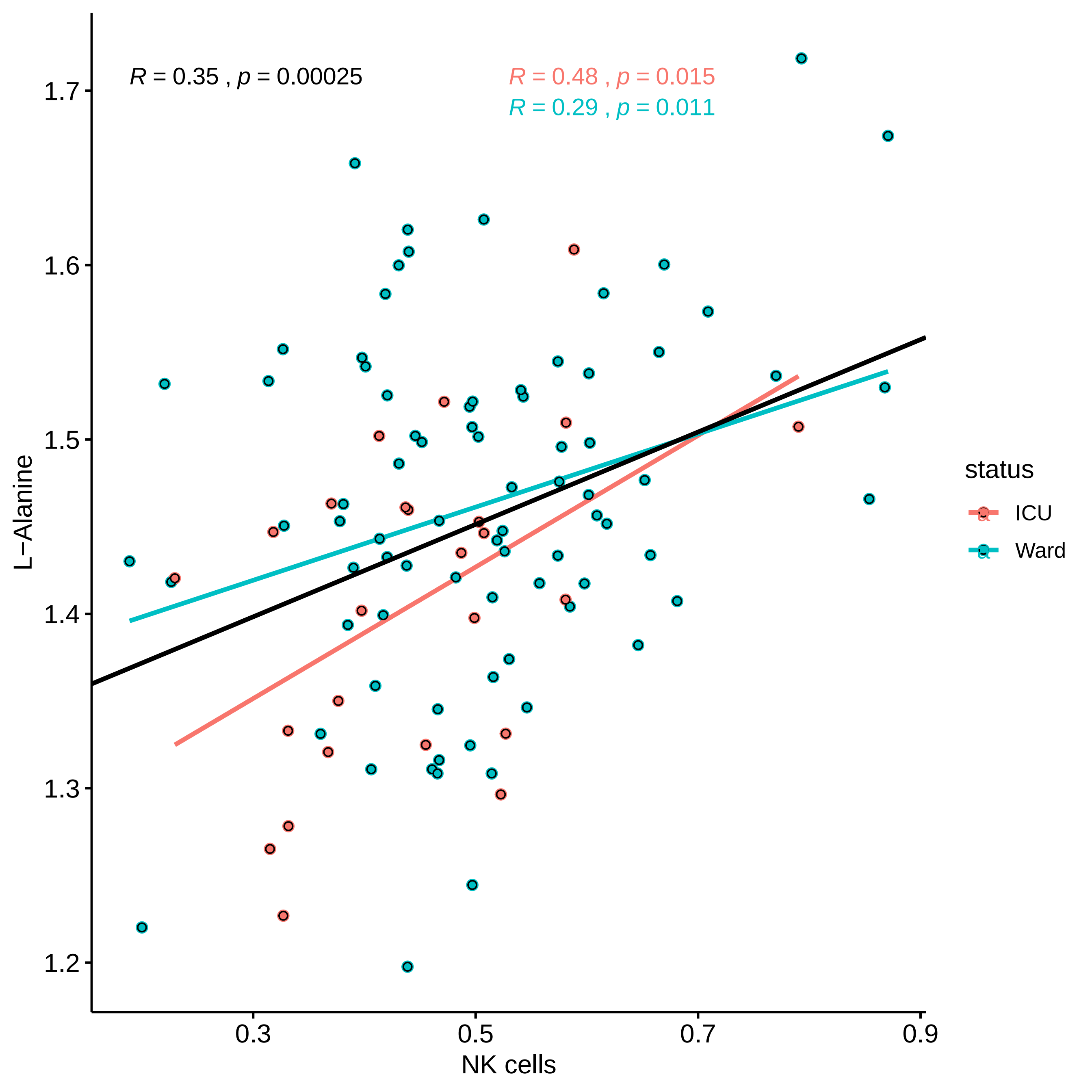

R = 0.35 , p = 0.00025
R = 0.48 , p = 0.015
R = 0.29 , p = 0.011
1.7
1.6
1.5
1.4
1.3
1.2
L−Alanine
0.3
0.5
0.7
0.9
NK cells
status
ICU
Ward

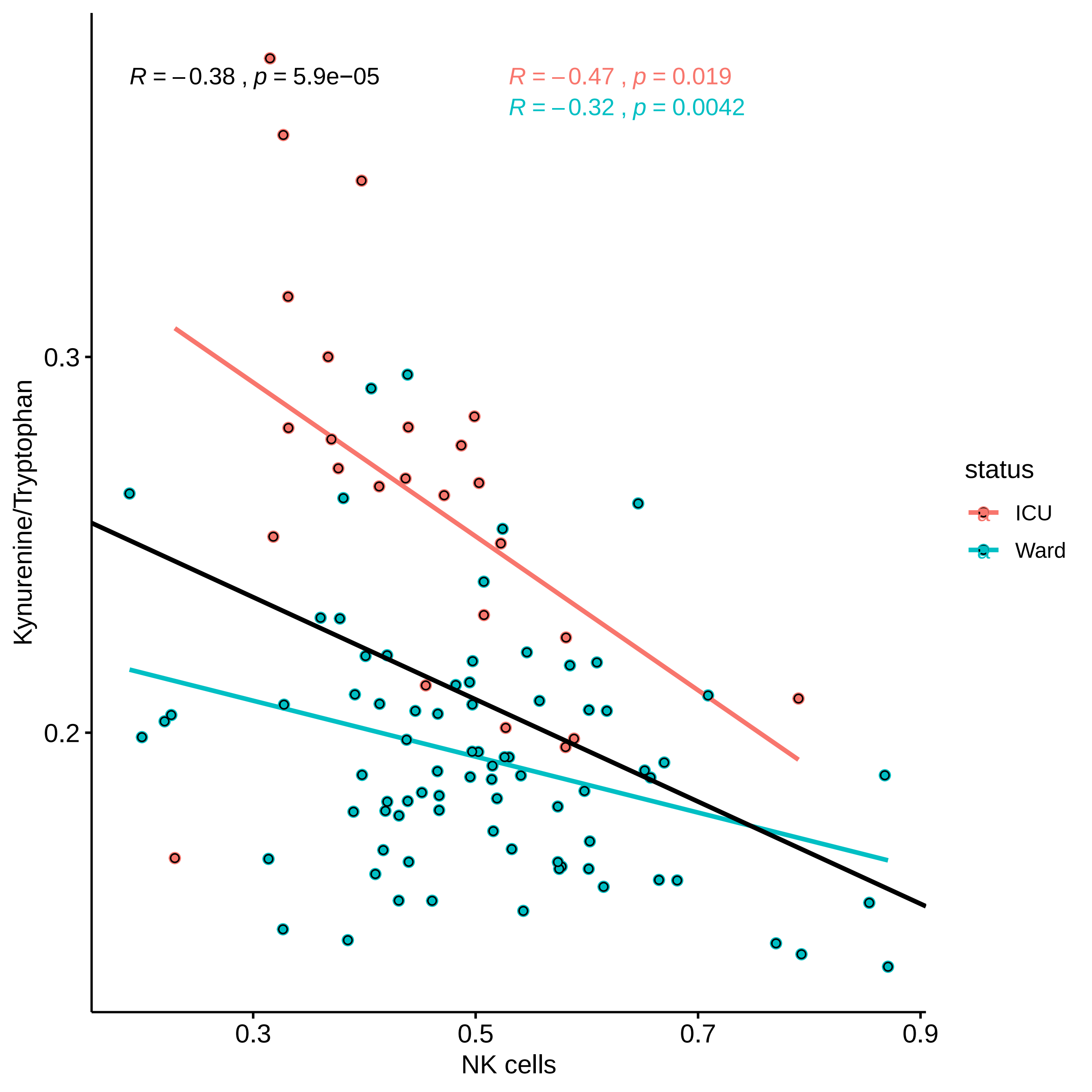

R = −0.38 , p = 5.9e−05
R = −0.47 , p = 0.019
R = −0.32 , p = 0.0042
Kynurenine/Tryptophan
0.3
0.2
0.3
0.5
0.7
0.9
NK cells
status
ICU
Ward

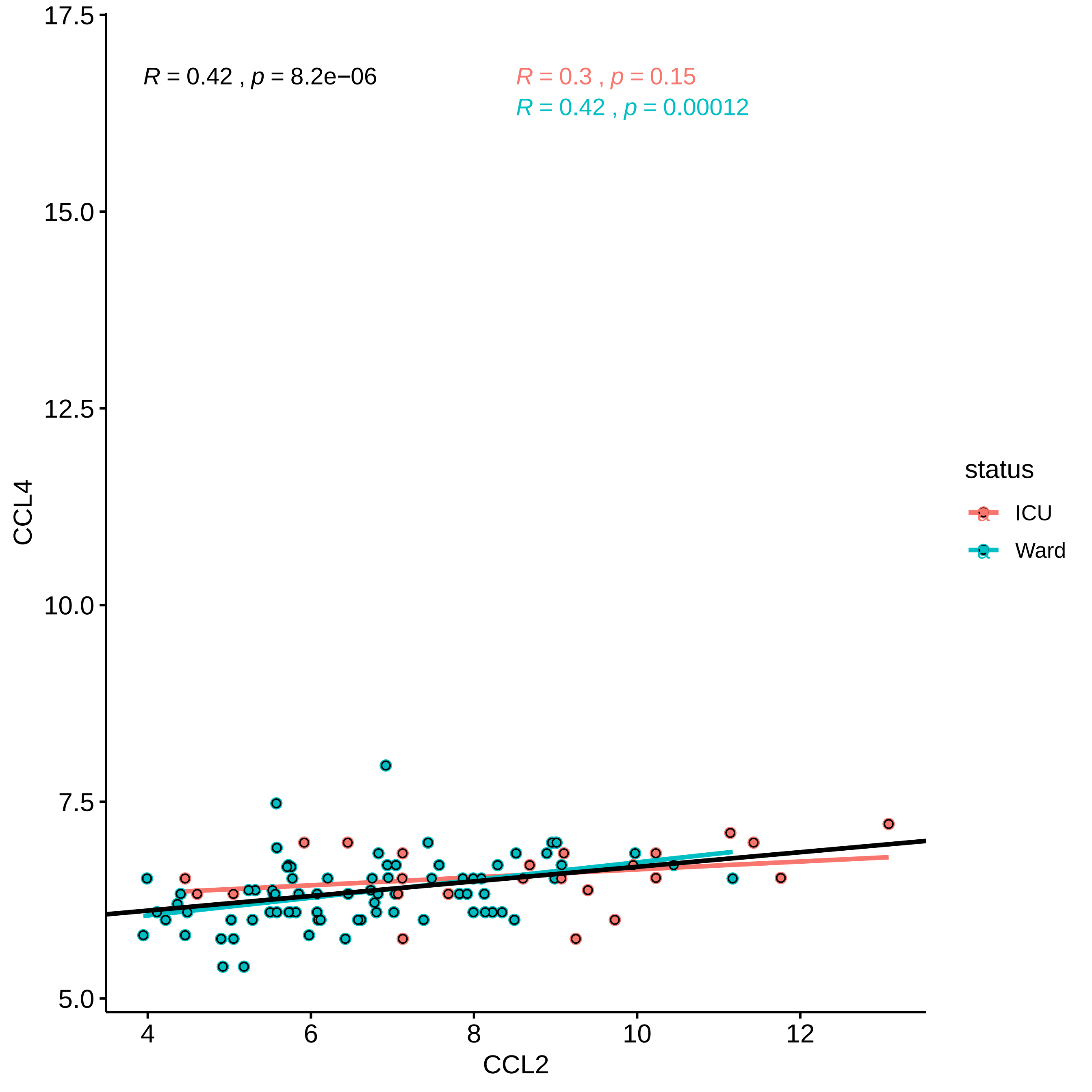
R = 0.42 , p = 8.2e−06
R = 0.3 , p = 0.15
R = 0.42 , p = 0.00012
CCL4
17.5
15.0
12.5
10.0
7.5
5.0
4
6
8
10
12
CCL2
status
ICU
Ward

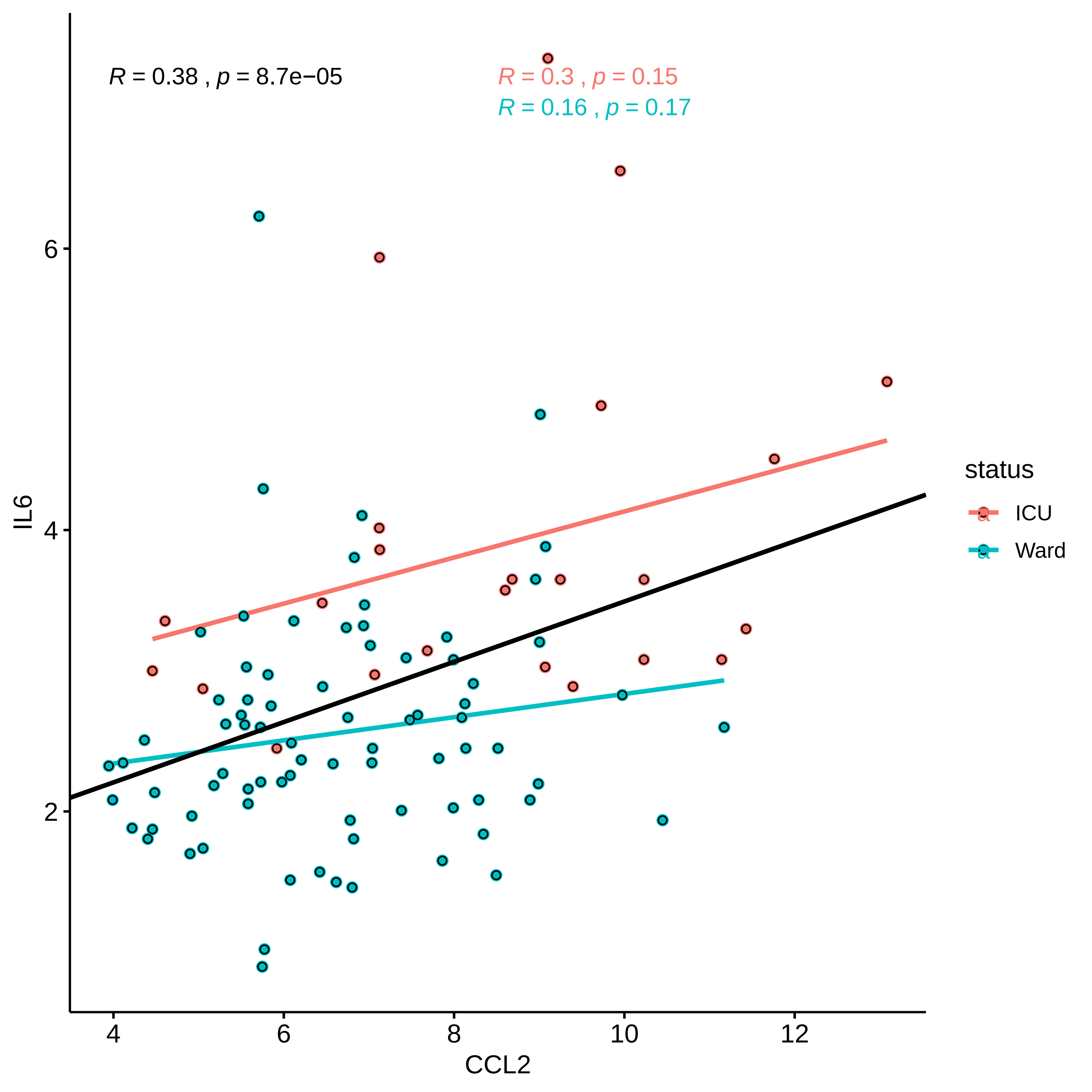

R = 0.38 , p = 8.7e−05
R = 0.3 , p = 0.15
R = 0.16 , p = 0.17
IL6
6
4
2
4
6
8
10
12
CCL2
status
ICU
Ward

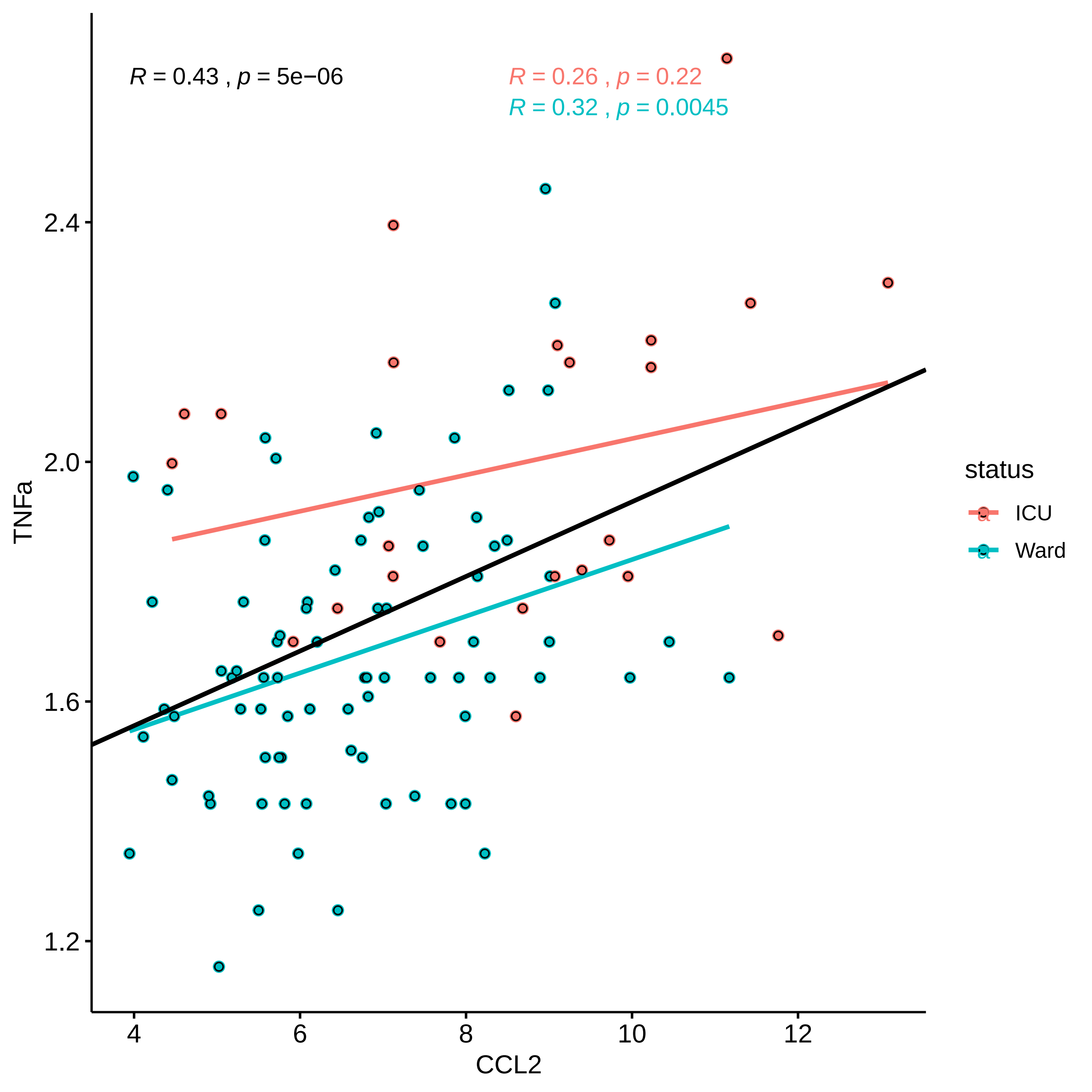
R = 0.43 , p = 5e−06
R = 0.26 , p = 0.22
R = 0.32 , p = 0.0045
TNFa
CCL2
2.4
2.0
1.6
1.2
4
6
8
10
12
status
ICU
Ward

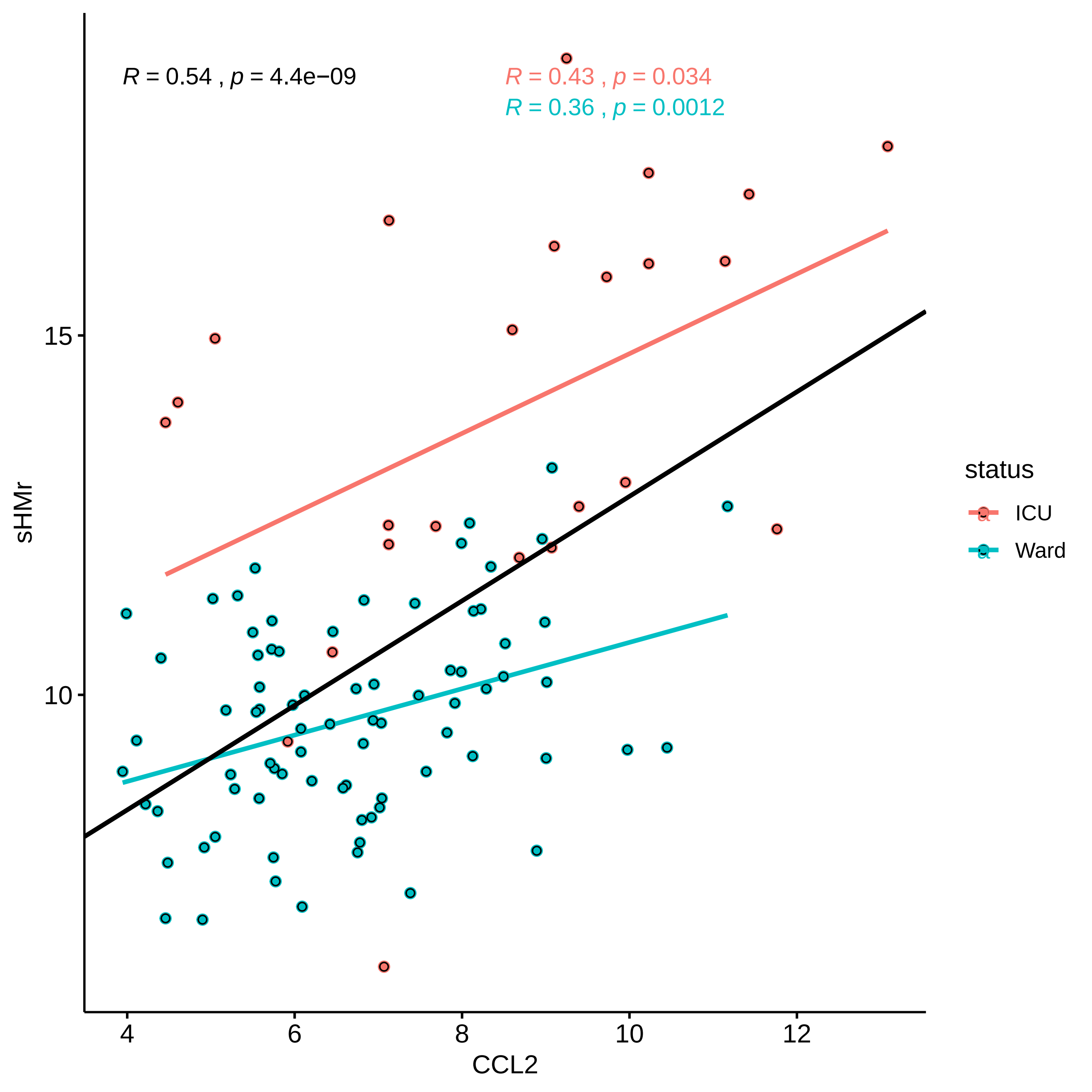

R = 0.54 , p = 4.4e−09
R = 0.43 , p = 0.034
R = 0.36 , p = 0.0012
sHMr
15
10
4
6
8
10
12
CCL2
status
ICU
Ward

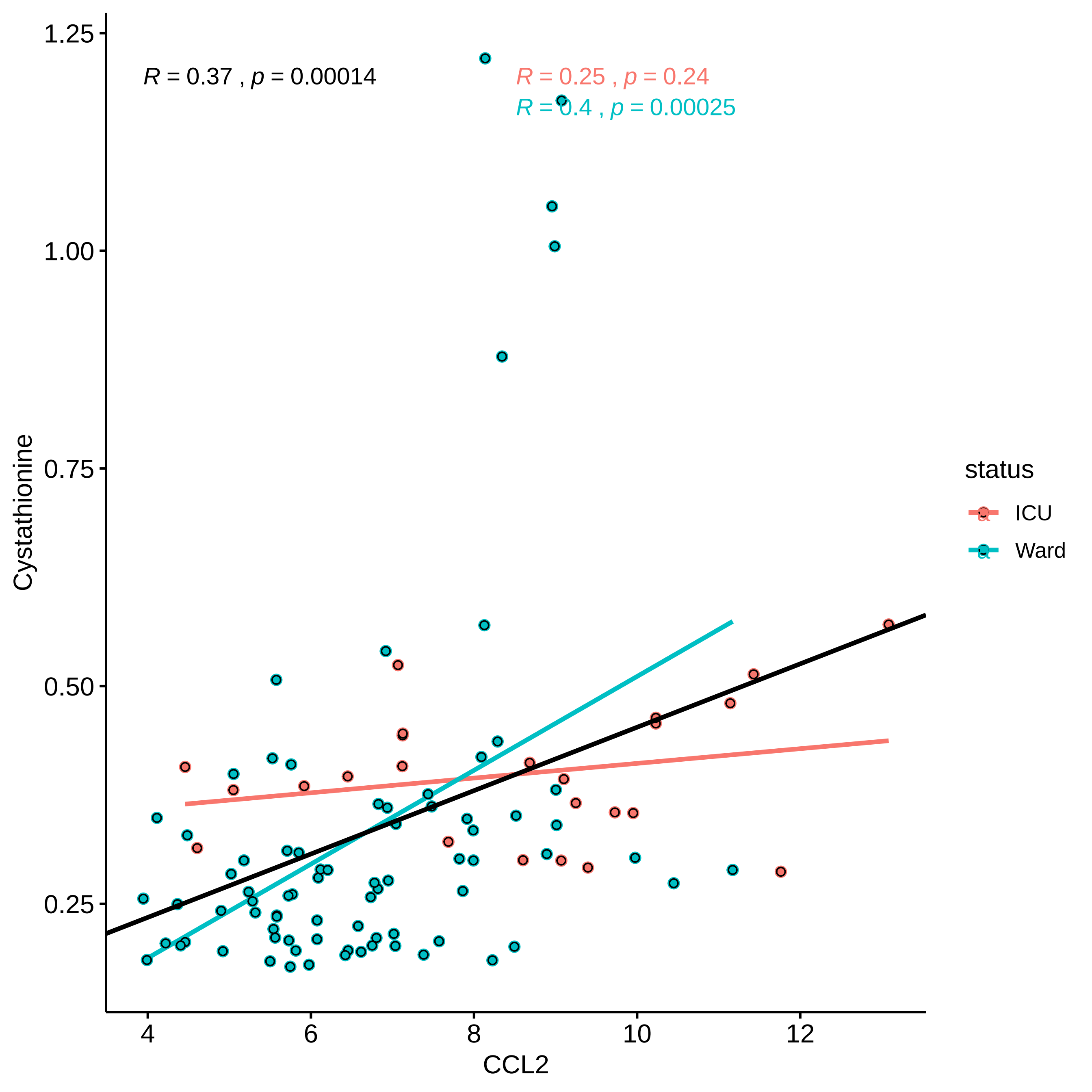

R = 0.37 , p = 0.00014
R = 0.25 , p = 0.24
R = 0.4 , p = 0.00025
1.25
1.00
0.75
0.50
0.25
Cystathionine
4
6
8
10
12
CCL2
status
ICU
Ward

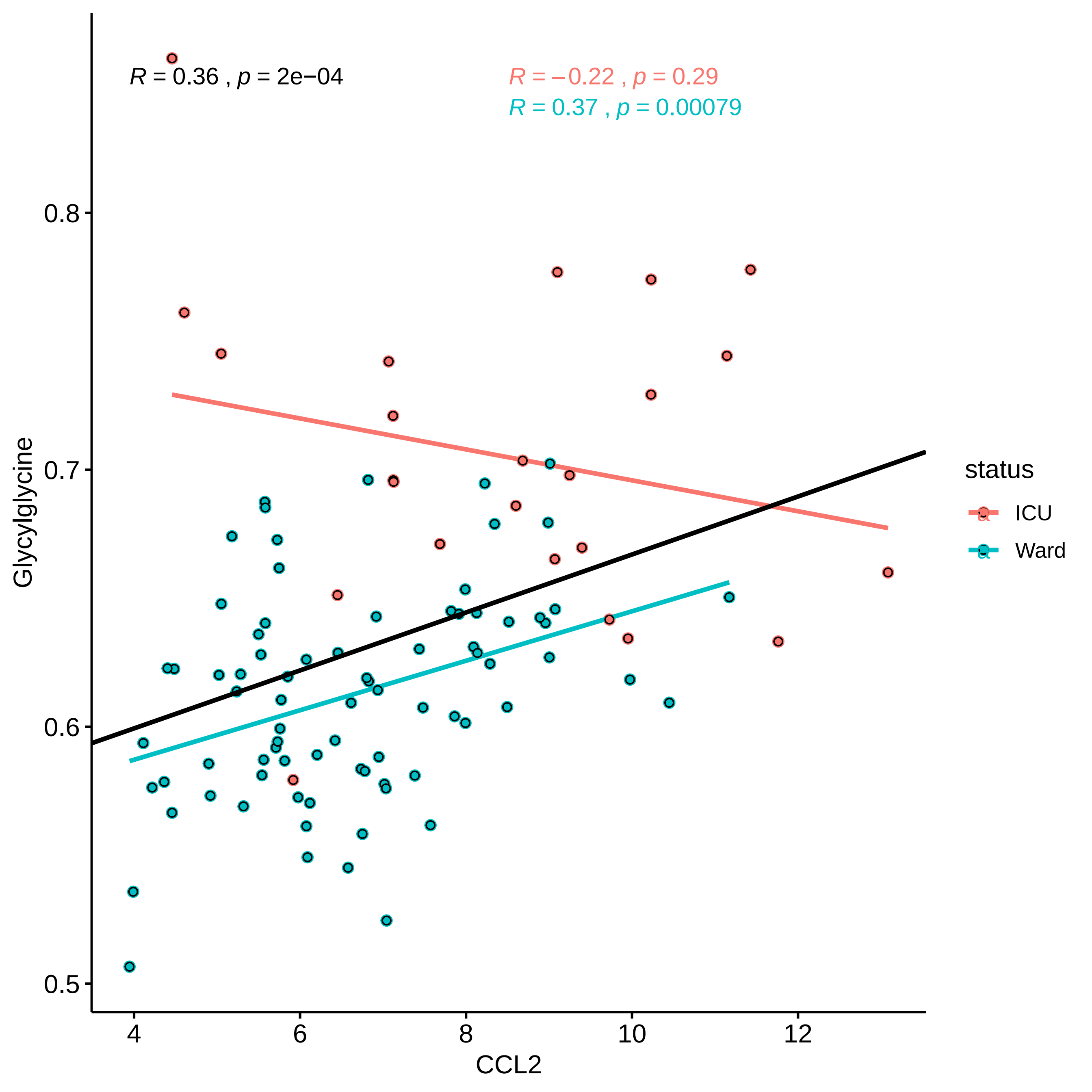

R = 0.36 , p = 2e−04
R = −0.22 , p = 0.29
R = 0.37 , p = 0.00079
Glycylglycine
0.8
0.7
0.6
0.5
4
6
8
10
12
CCL2
status
ICU
Ward

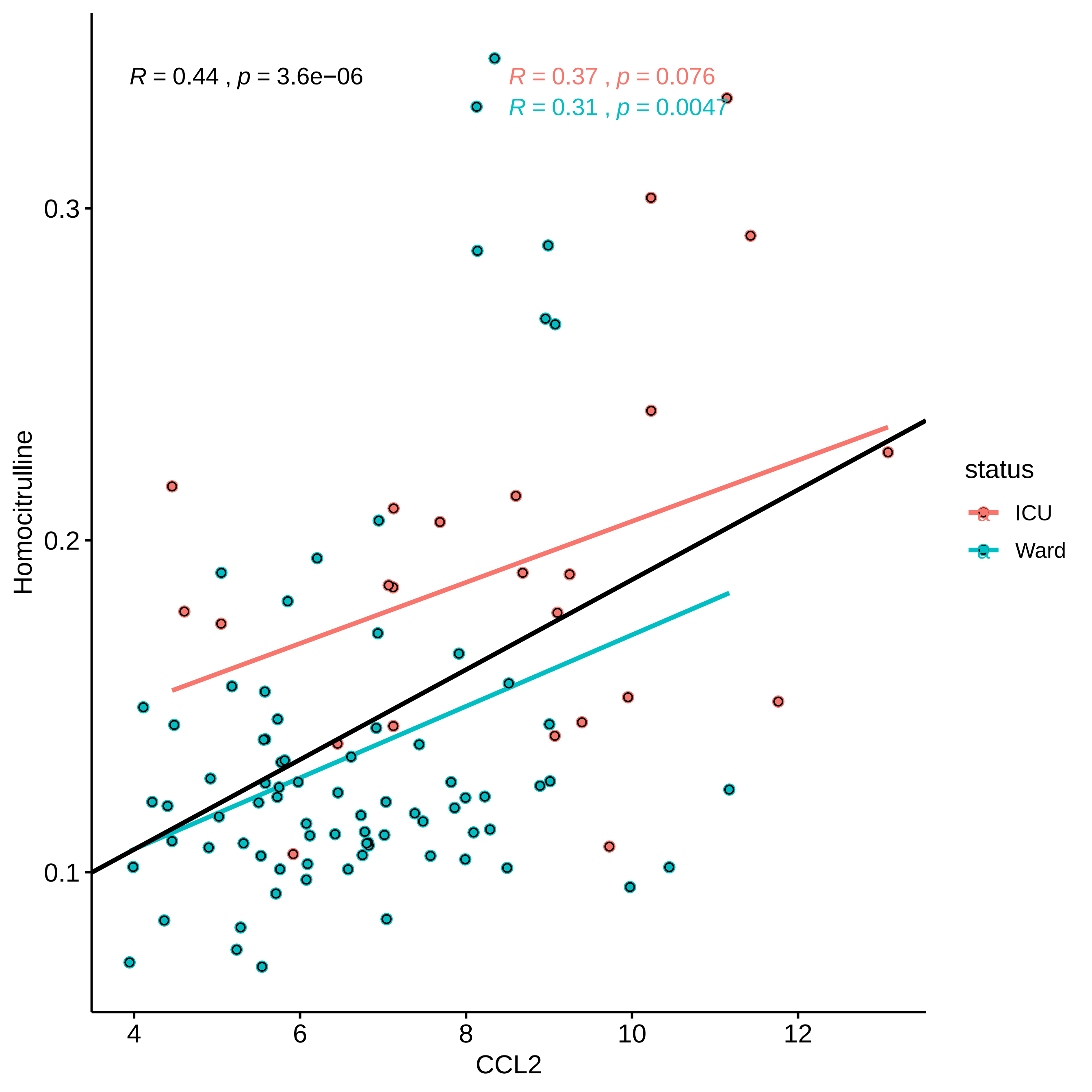

R = 0.44 , p = 3.6e−06
R = 0.37 , p = 0.076
R = 0.31 , p = 0.0047
Homocitrulline
0.3
0.2
0.1
4
6
8
10
12
CCL2
status
ICU
Ward

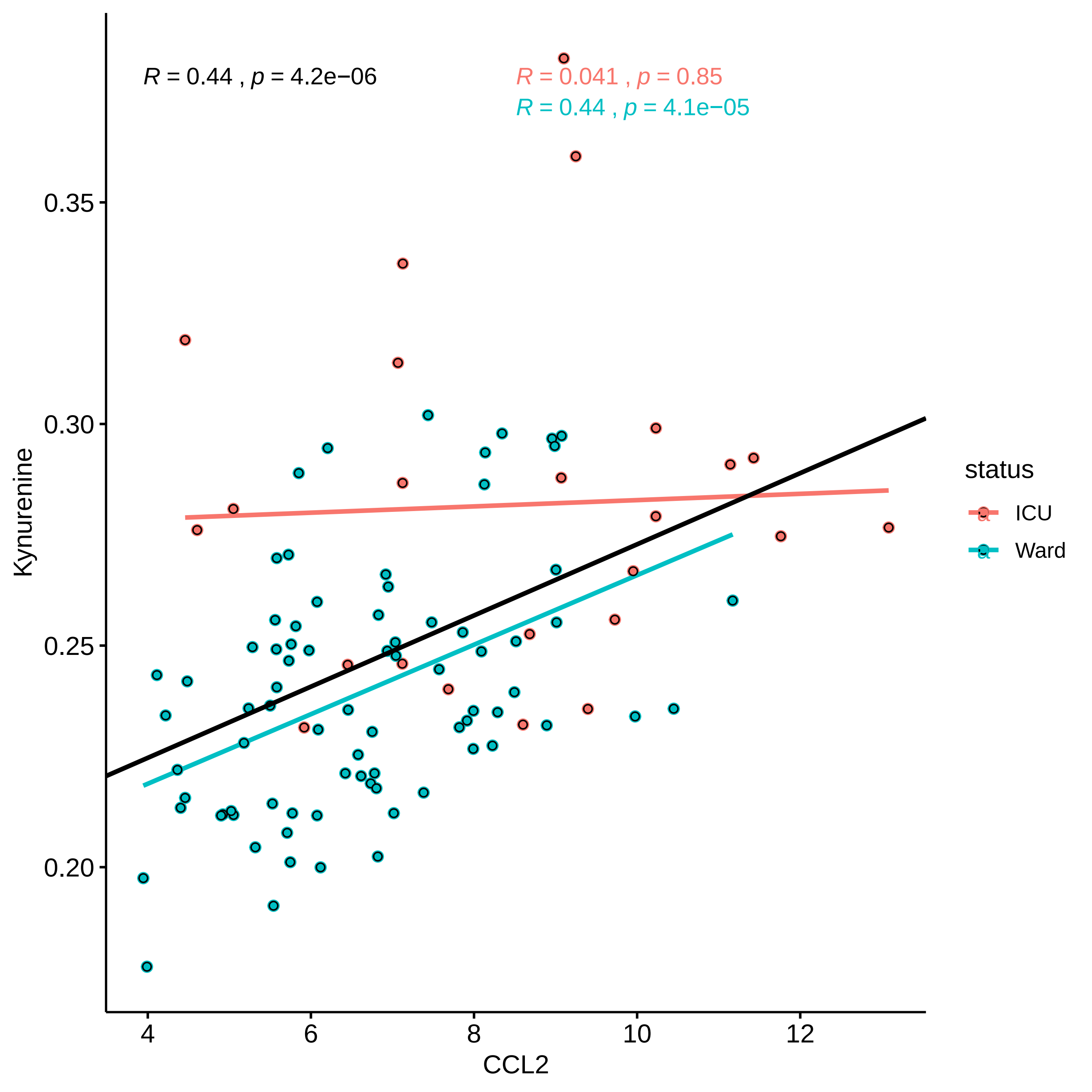

R = 0.44 , p = 4.2e−06
R = 0.041 , p = 0.85
R = 0.44 , p = 4.1e−05
0.35
0.30
0.25
0.20
Kynurenine
4
6
8
10
12
CCL2
status
ICU
Ward

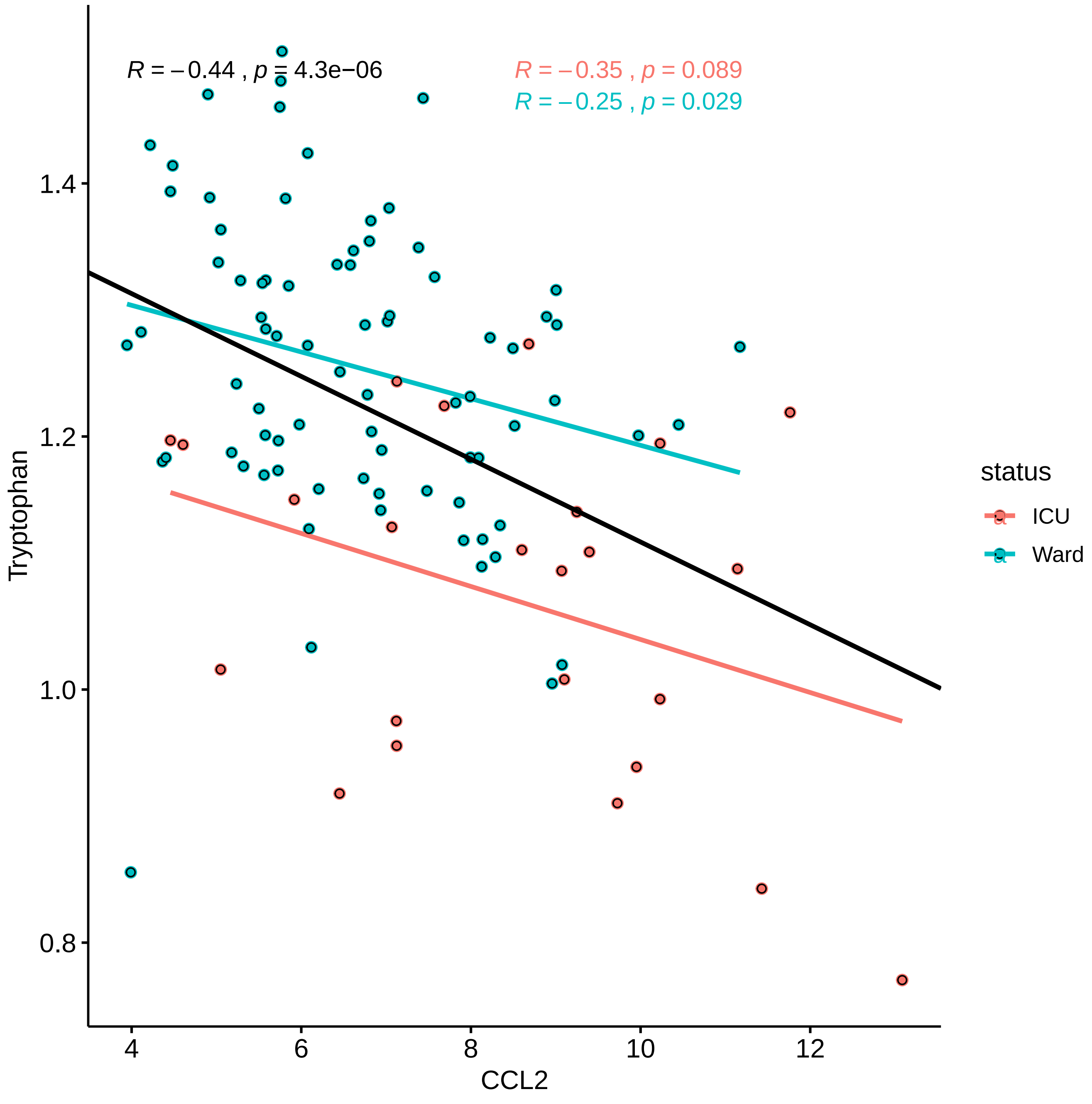

R = −0.44 , p = 4.3e−06
R = −0.35 , p = 0.089
R = −0.25 , p = 0.029
Tryptophan
1.4
1.2
1.0
0.8
CCL2
4
6
8
10
12
status
ICU
Ward

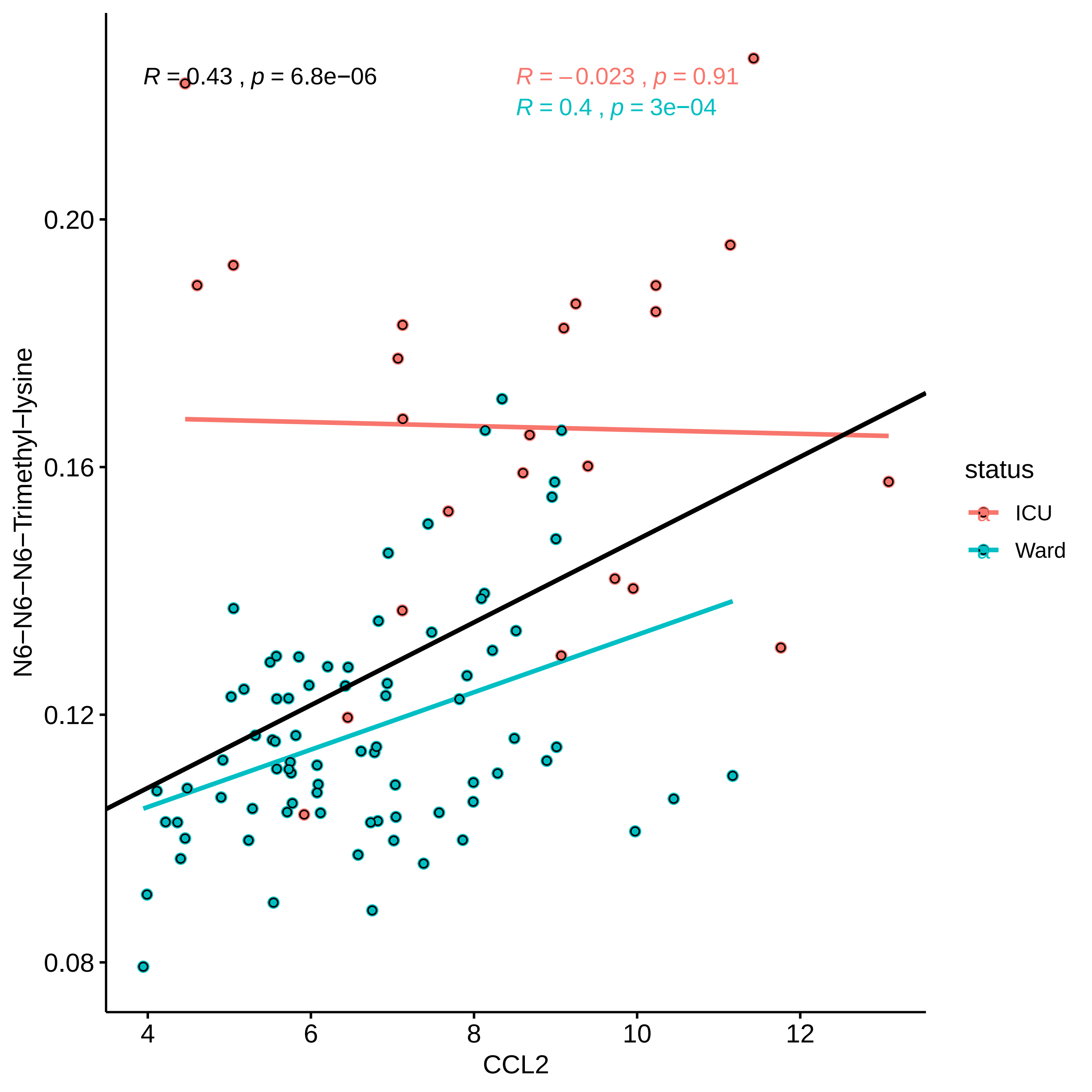

R = 0.43 , p = 6.8e−06
R = −0.023 , p = 0.91
R = 0.4 , p = 3e−04
N6−N6−N6−Trimethyl−lysine
0.08
0.12
0.16
0.20
4
6
8
10
12
CCL2
status
ICU
Ward

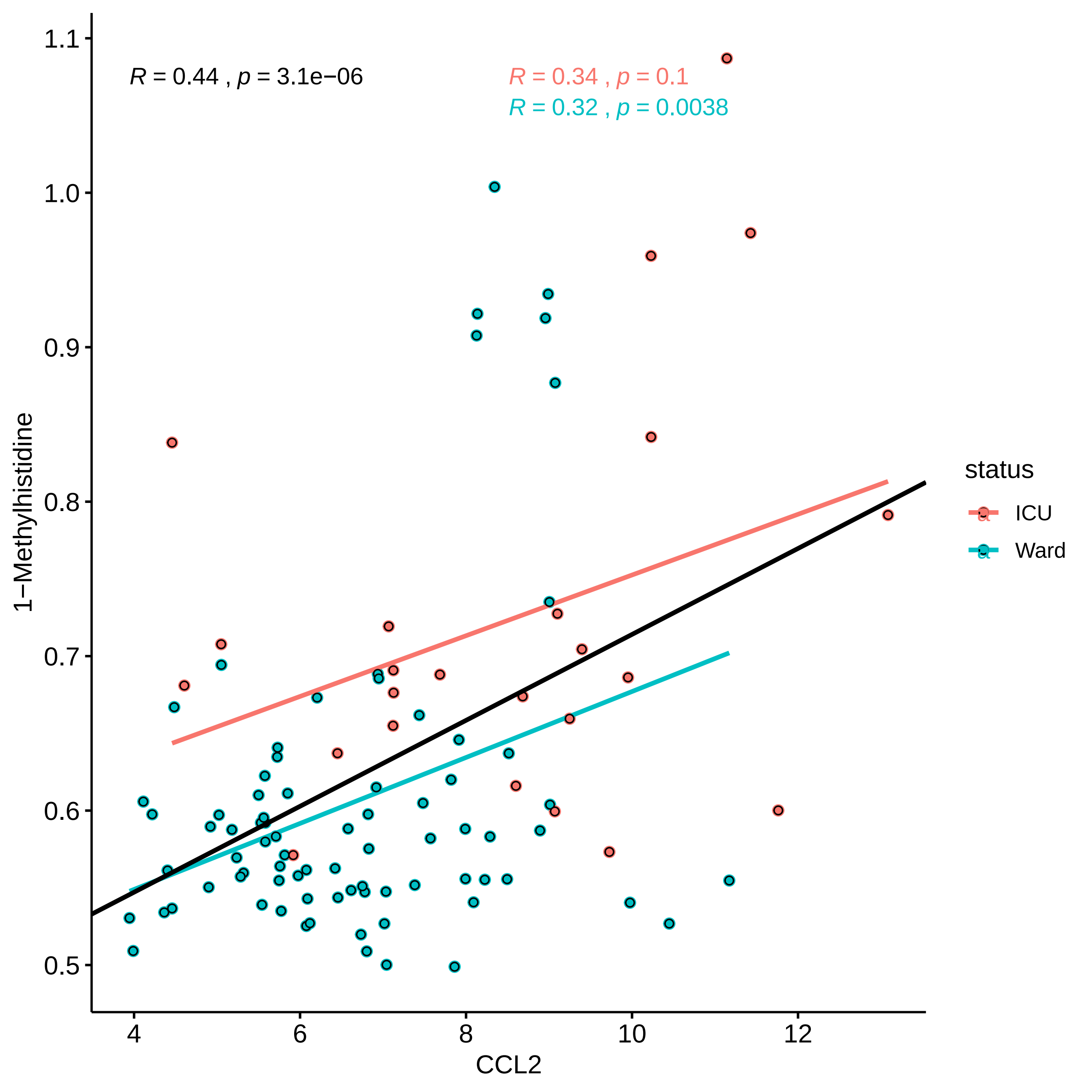

R = 0.44 , p = 3.1e−06
R = 0.34 , p = 0.1
R = 0.32 , p = 0.0038
1.1
1.0
0.9
0.8
0.7
0.6
0.5
1−Methylhistidine
4
6
8
10
12
CCL2
status
ICU
Ward

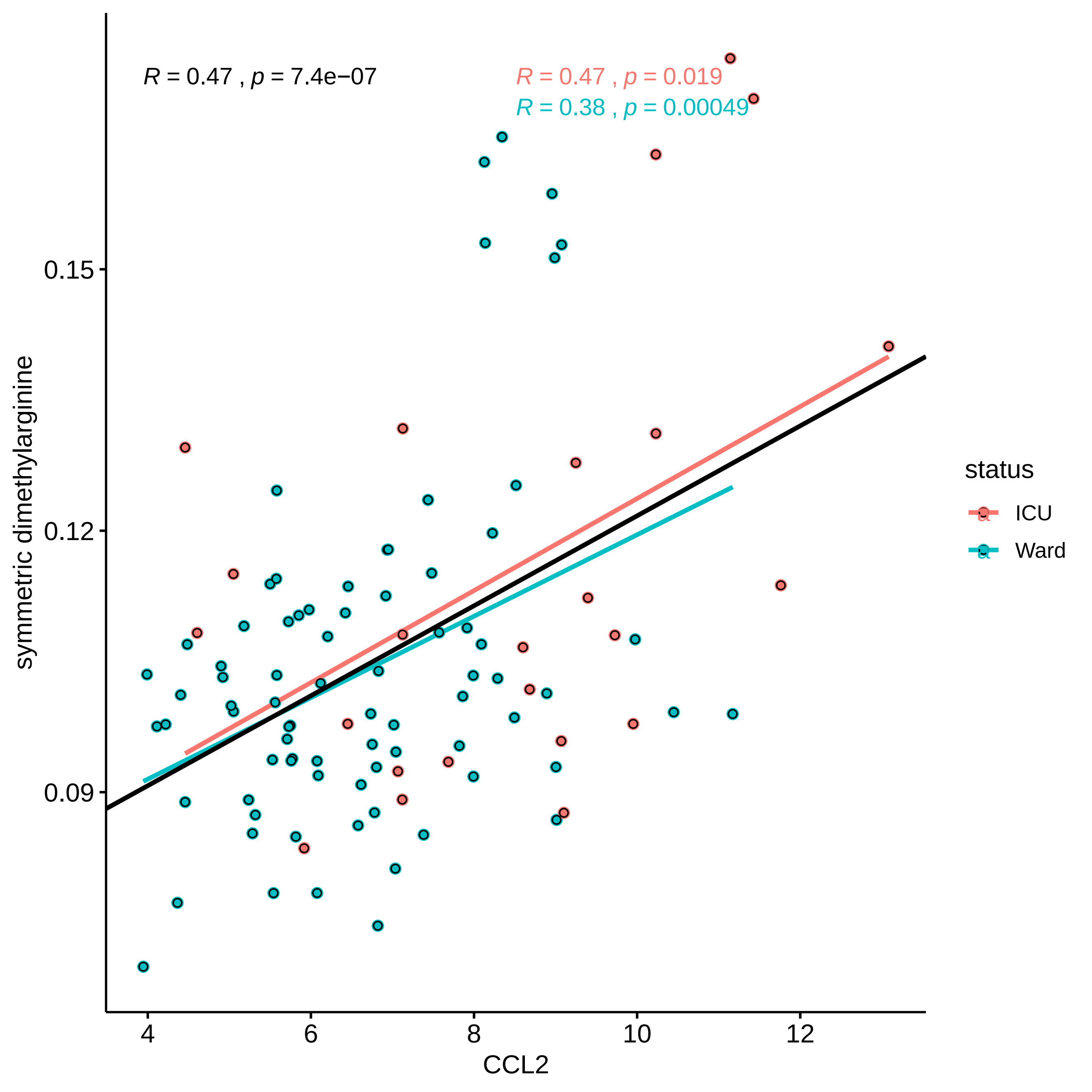

R = 0.47 , p = 7.4e−07
R = 0.47 , p = 0.019
R = 0.38 , p = 0.00049
symmetric dimethylarginine
0.15
0.12
0.09
4
6
8
10
12
CCL2
status
ICU
Ward

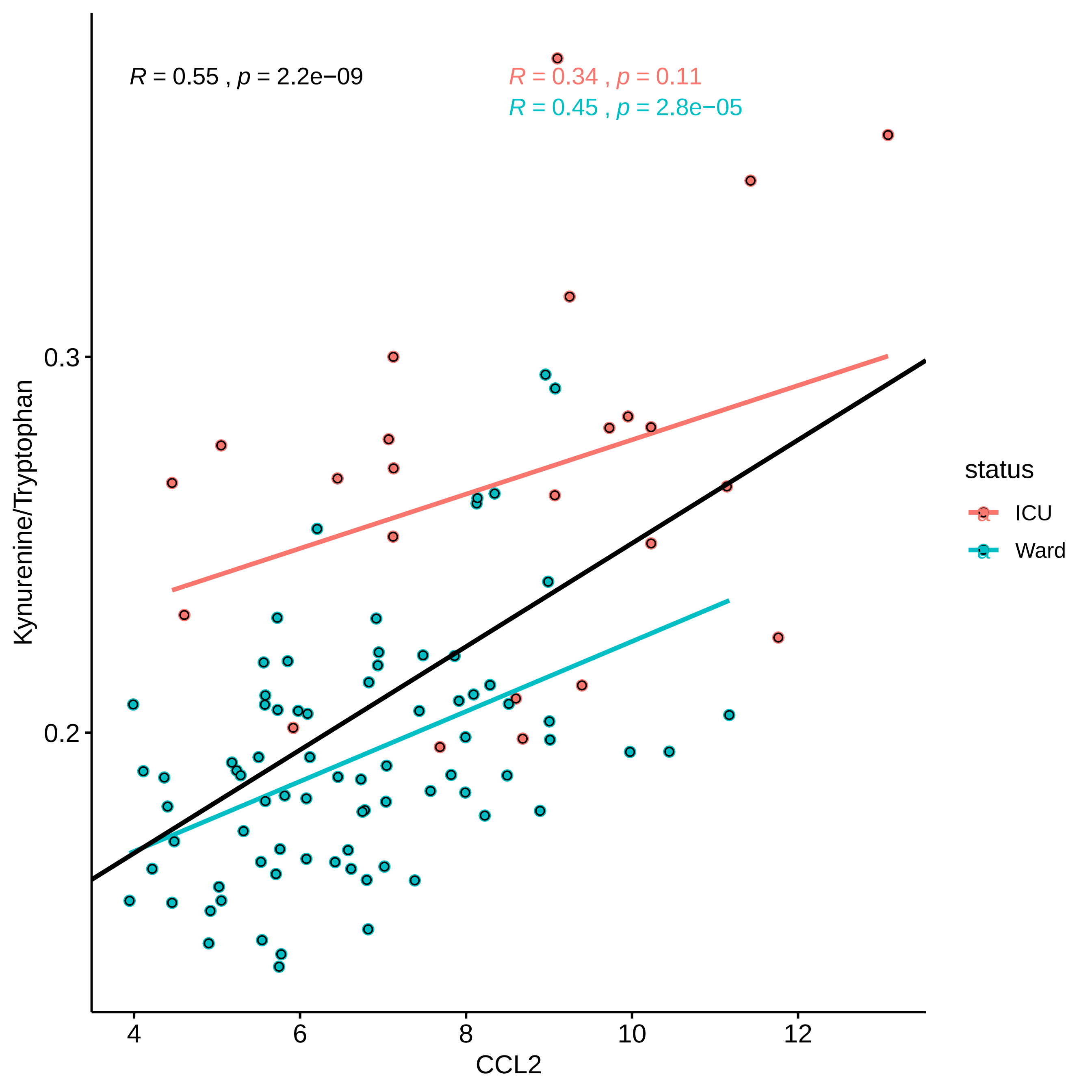

R = 0.55 , p = 2.2e−09
R = 0.34 , p = 0.11
R = 0.45 , p = 2.8e−05
Kynurenine/Tryptophan
0.3
0.2
4
6
8
10
12
CCL2
status
ICU
Ward

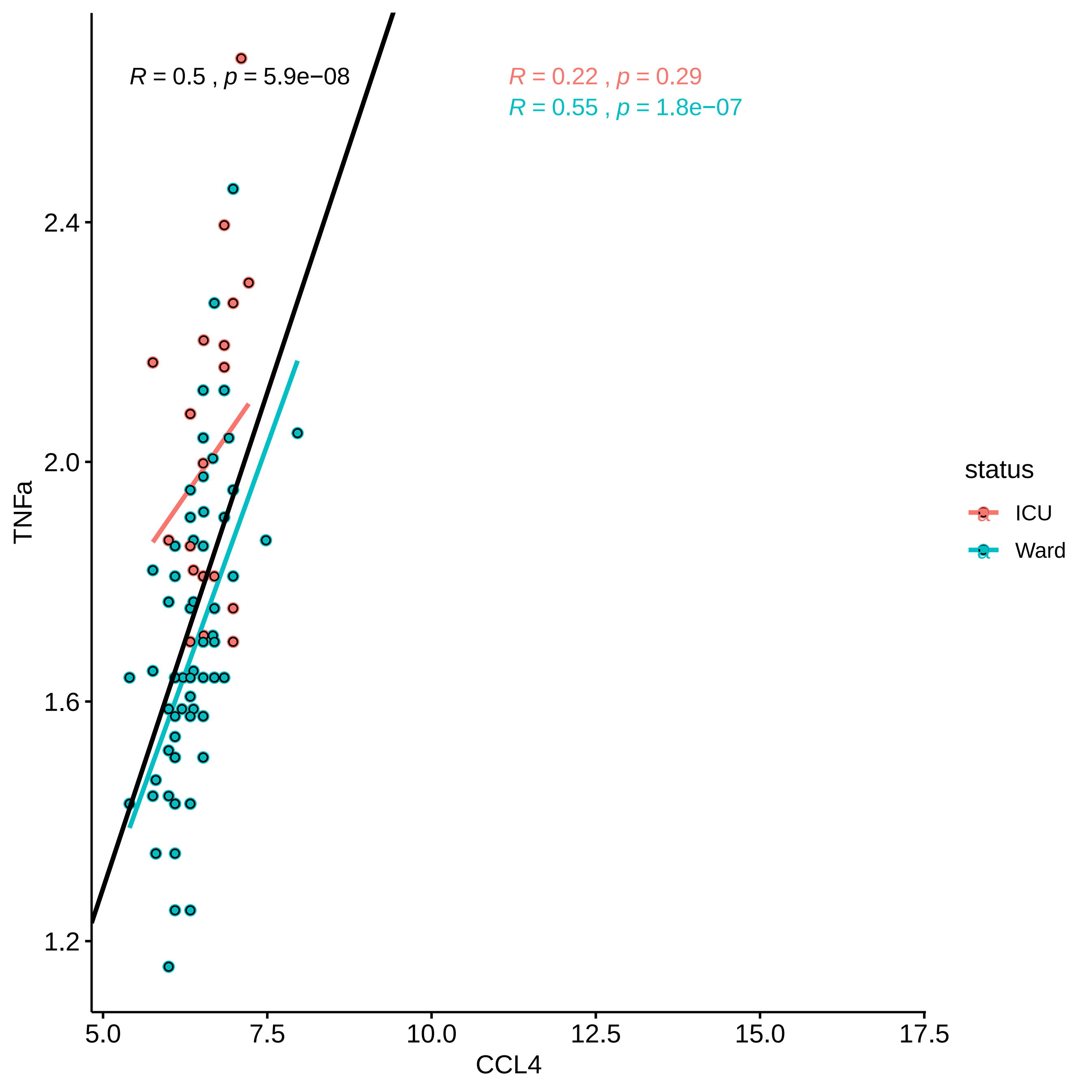

R = 0.5 , p = 5.9e−08
R = 0.22 , p = 0.29
R = 0.55 , p = 1.8e−07
TNFa
2.4
2.0
1.6
1.2
5.0
7.5
10.0
12.5
15.0
17.5
CCL4
status
ICU
Ward

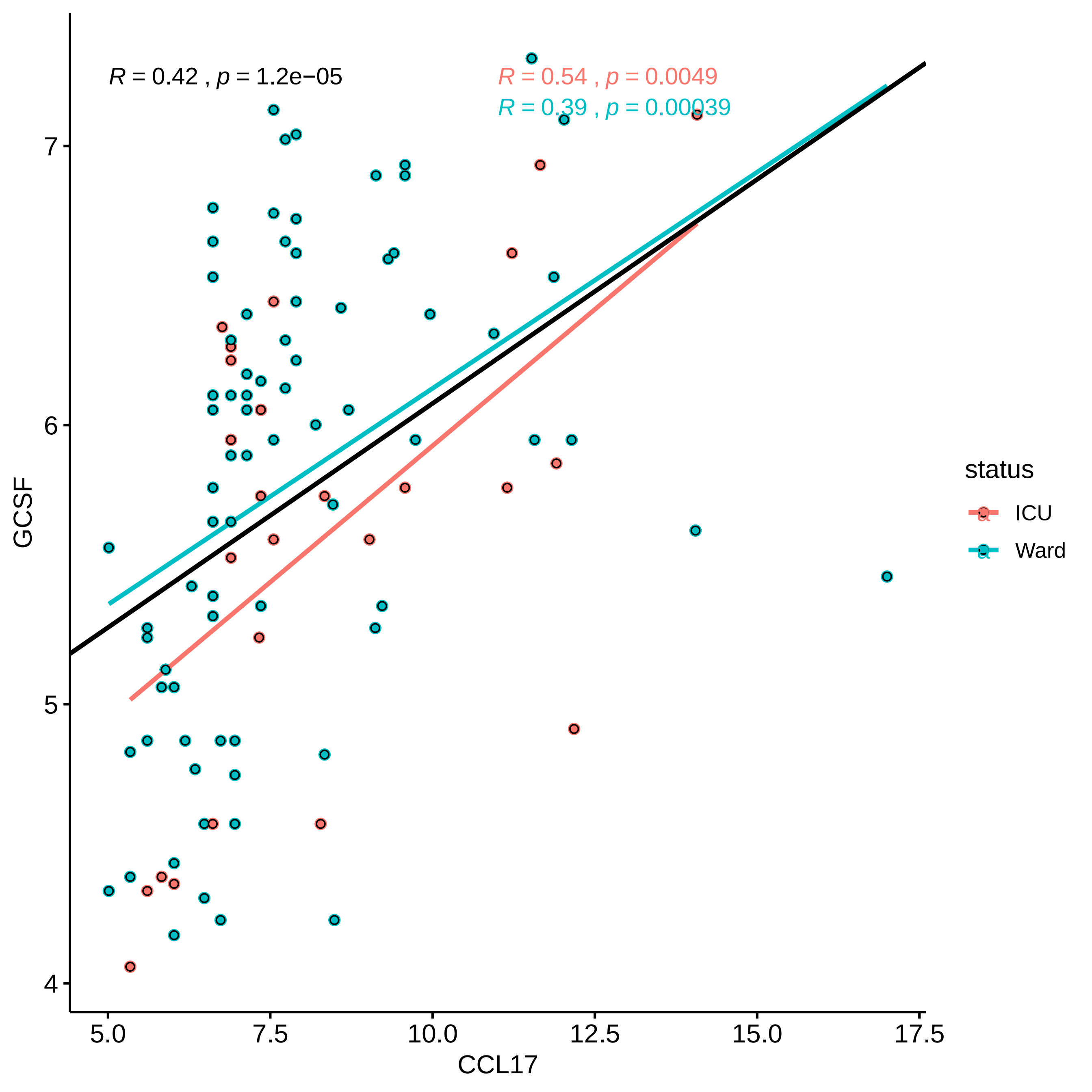

R = 0.42 , p = 1.2e−05
R = 0.54 , p = 0.0049
R = 0.39 , p = 0.00039
GCSF
CCL17
7
6
5
4
5.0
7.5
10.0
12.5
15.0
17.5
status
ICU
Ward

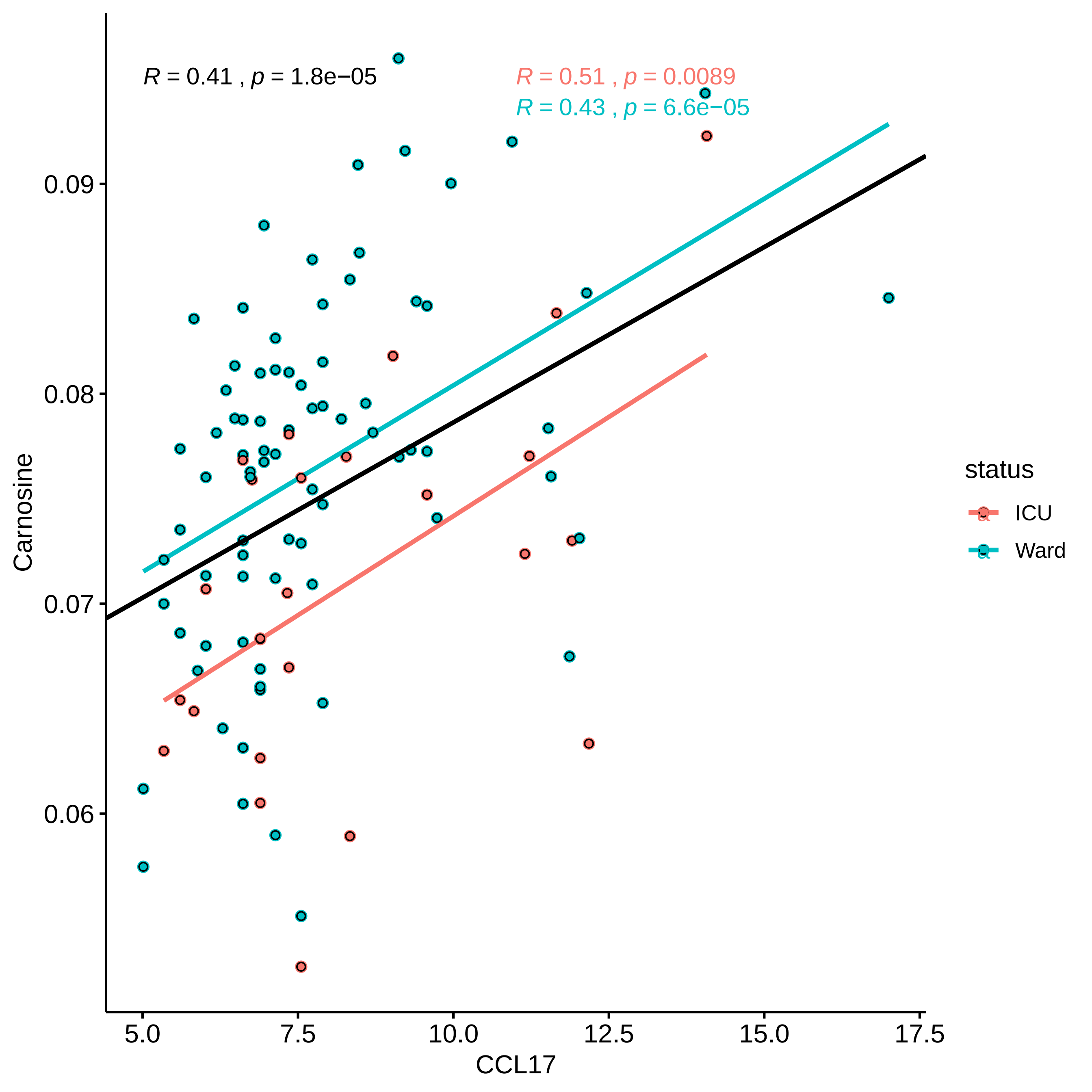

R = 0.41 , p = 1.8e−05
R = 0.51 , p = 0.0089
R = 0.43 , p = 6.6e−05
Carnosine
0.09
0.08
0.07
0.06
5.0
7.5
10.0
12.5
15.0
17.5
CCL17
status
ICU
Ward

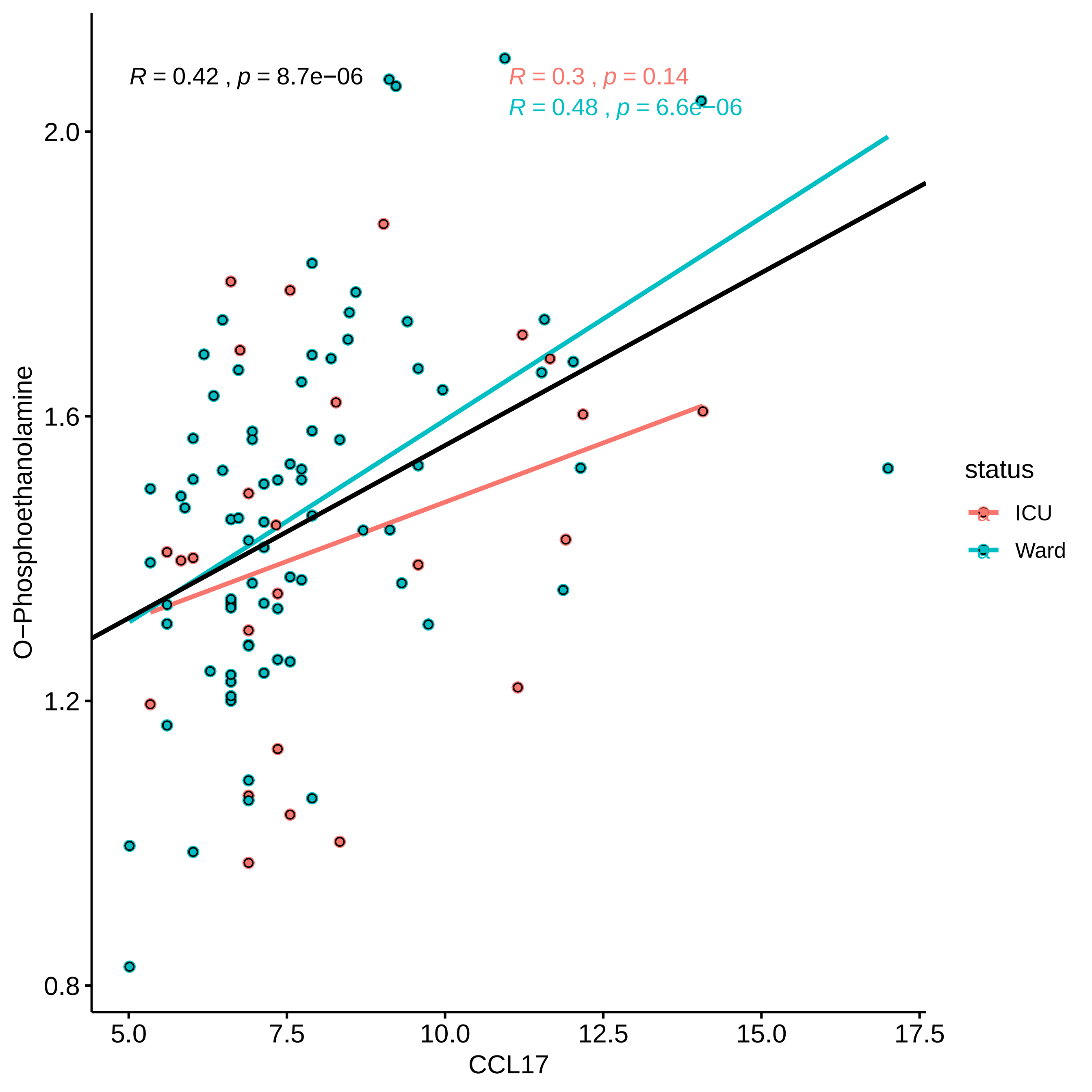

R = 0.42 , p = 8.7e−06
R = 0.3 , p = 0.14
R = 0.48 , p = 6.6e−06
2.0
1.6
1.2
0.8
O−Phosphoethanolamine
5.0
7.5
10.0
12.5
15.0
17.5
CCL17
status
ICU
Ward

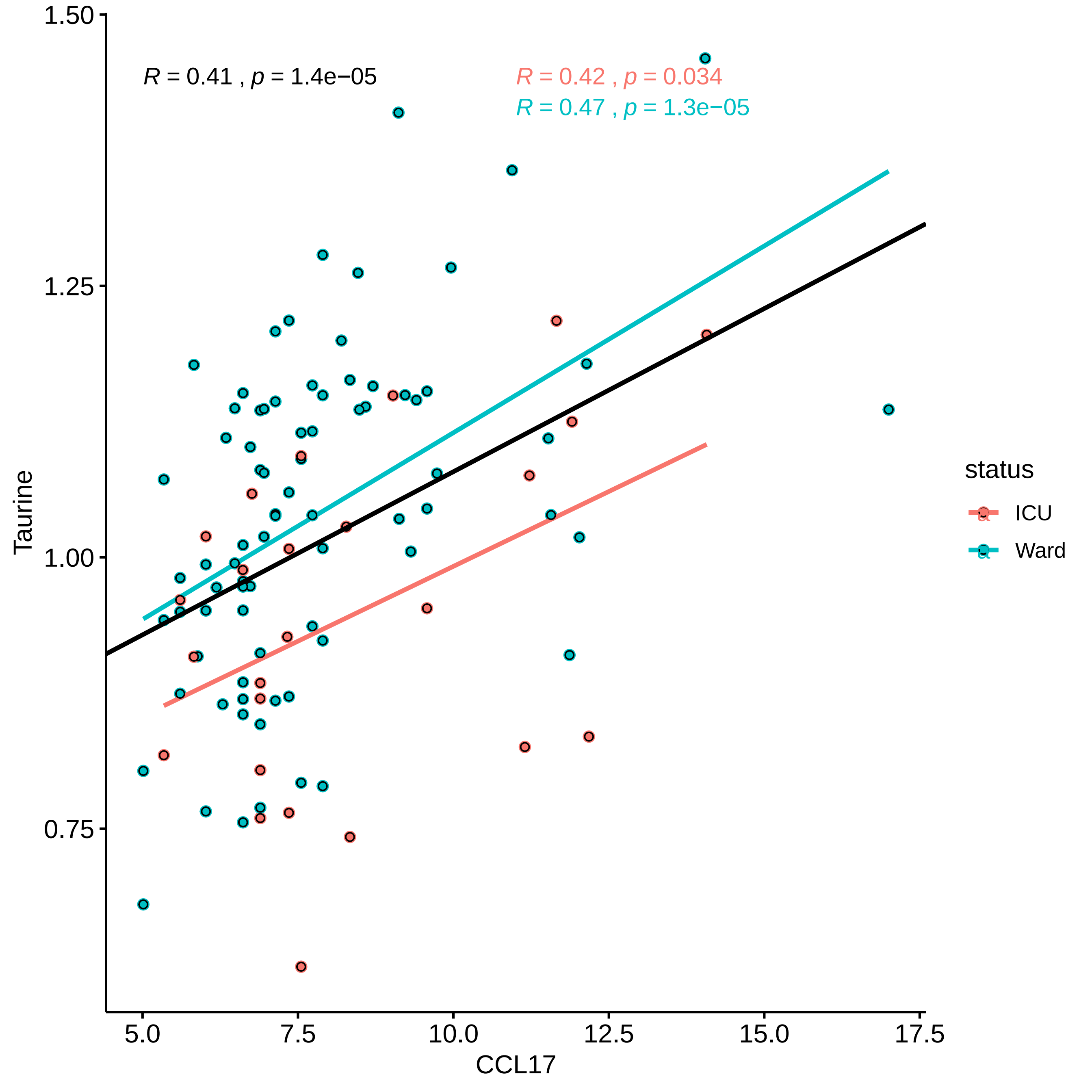

R = 0.41 , p = 1.4e−05
R = 0.42 , p = 0.034
R = 0.47 , p = 1.3e−05
Taurine
CCL17
1.50
1.25
1.00
0.75
5.0
7.5
10.0
12.5
15.0
17.5
status
ICU
Ward

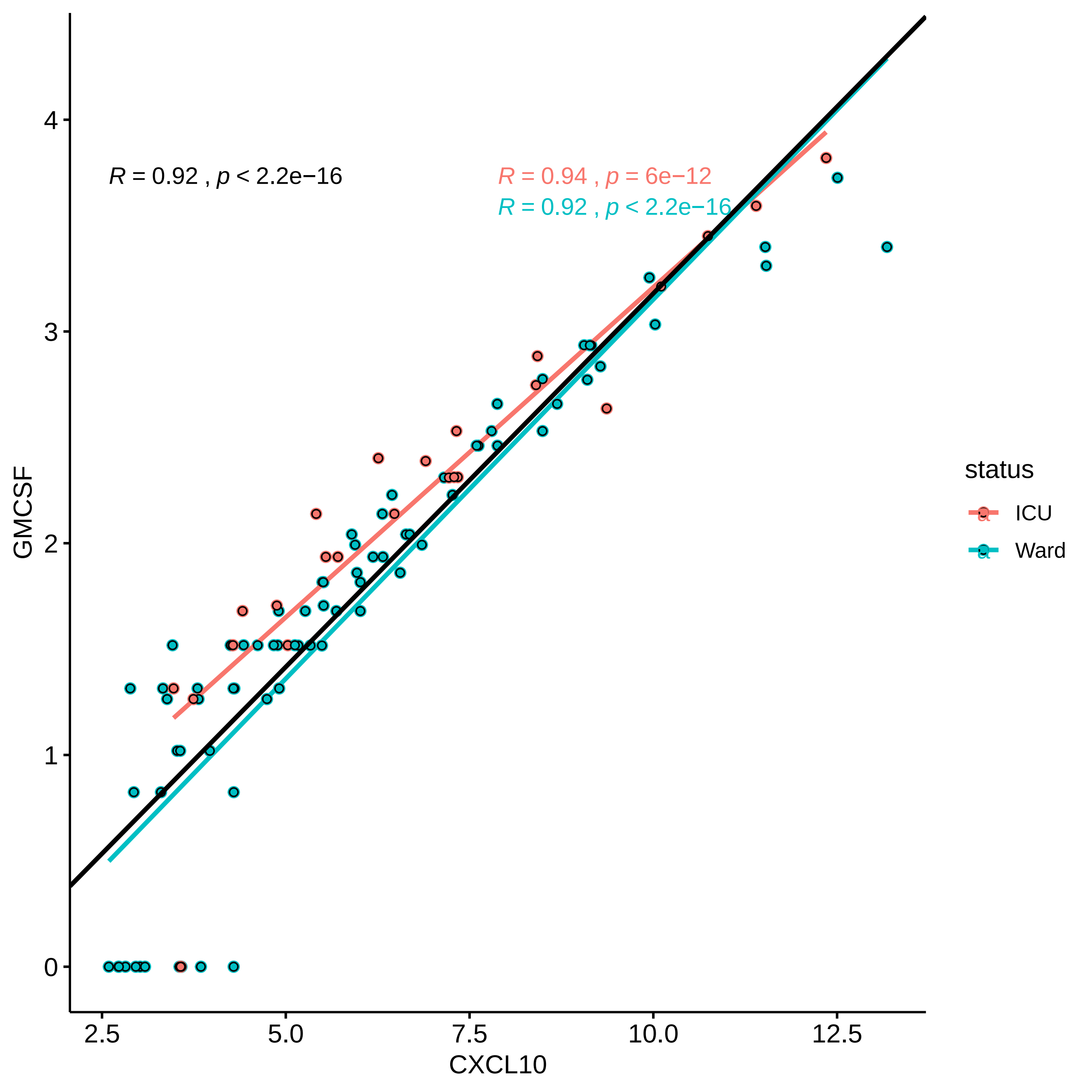

R = 0.92 , p < 2.2e−16
R = 0.94 , p = 6e−12
R = 0.92 , p < 2.2e−16
GMCSF
CXCL10
0
1
2
3
4
2.5
5.0
7.5
10.0
12.5
status
ICU
Ward

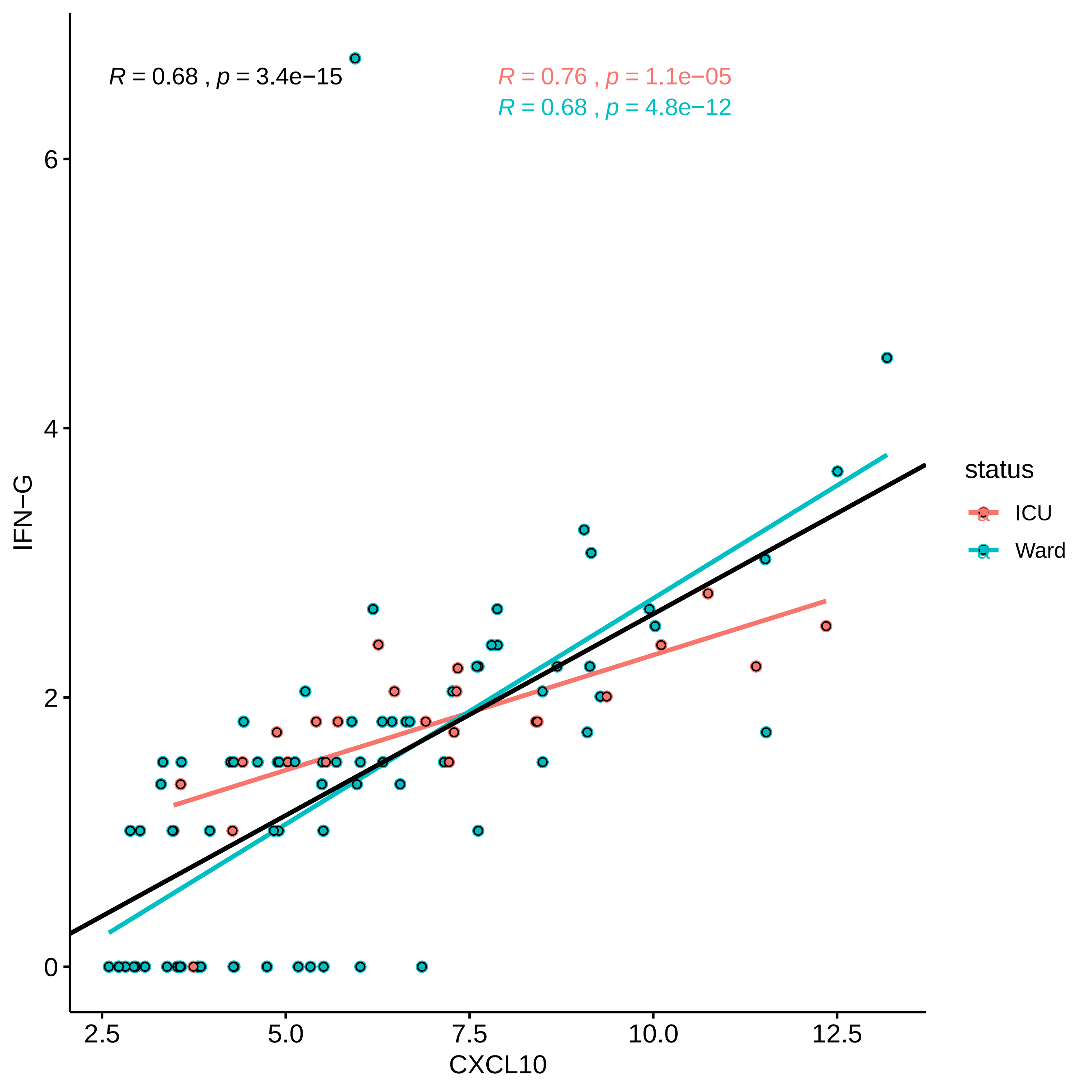
R = 0.68 , p = 3.4e−15
R = 0.76 , p = 1.1e−05
R = 0.68 , p = 4.8e−12
IFN−G
CXCL10
0
2
4
6
2.5
5.0
7.5
10.0
12.5
status
ICU
Ward

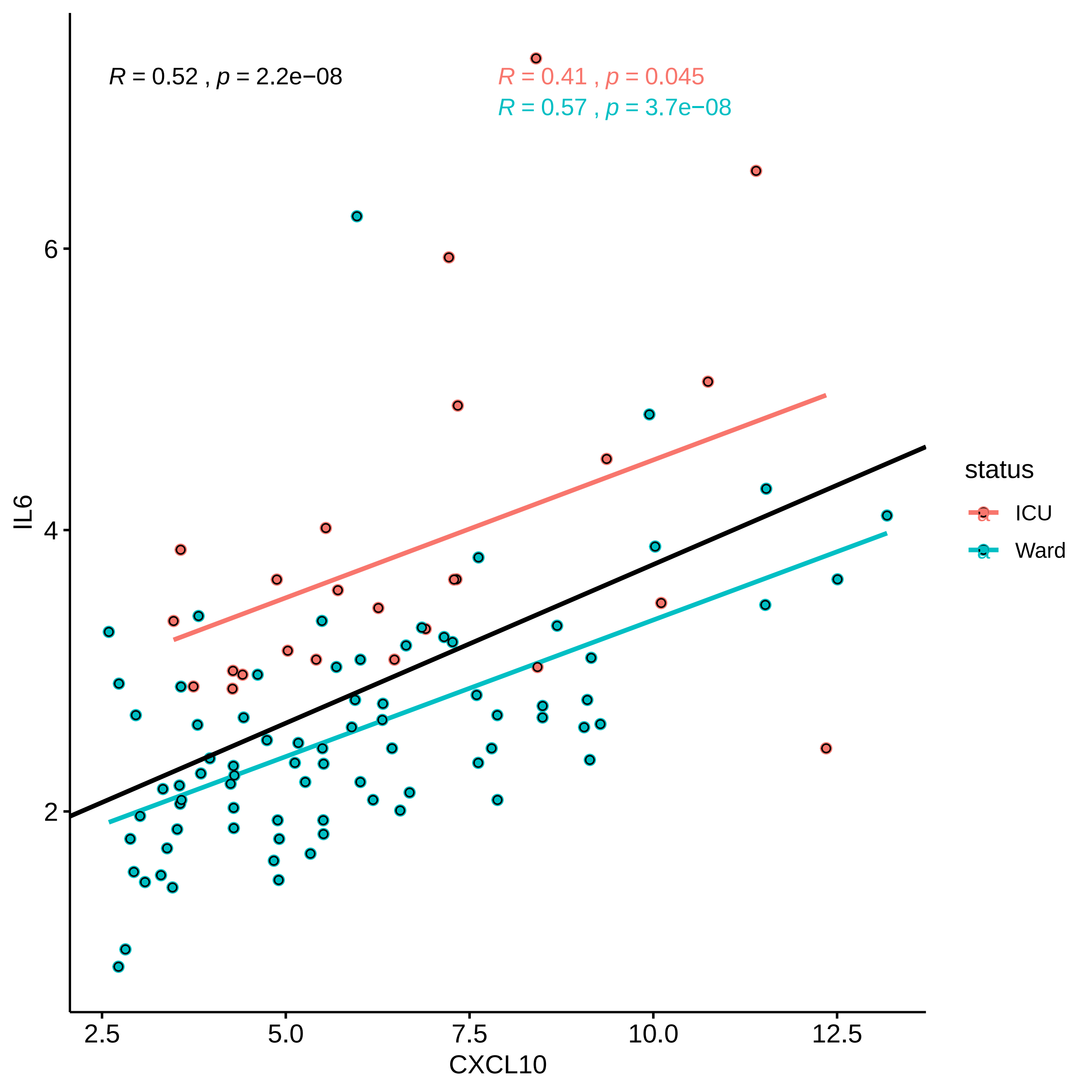

R = 0.52 , p = 2.2e−08
R = 0.41 , p = 0.045
R = 0.57 , p = 3.7e−08
IL6
6
4
2
2.5
5.0
7.5
10.0
12.5
CXCL10
status
ICU
Ward

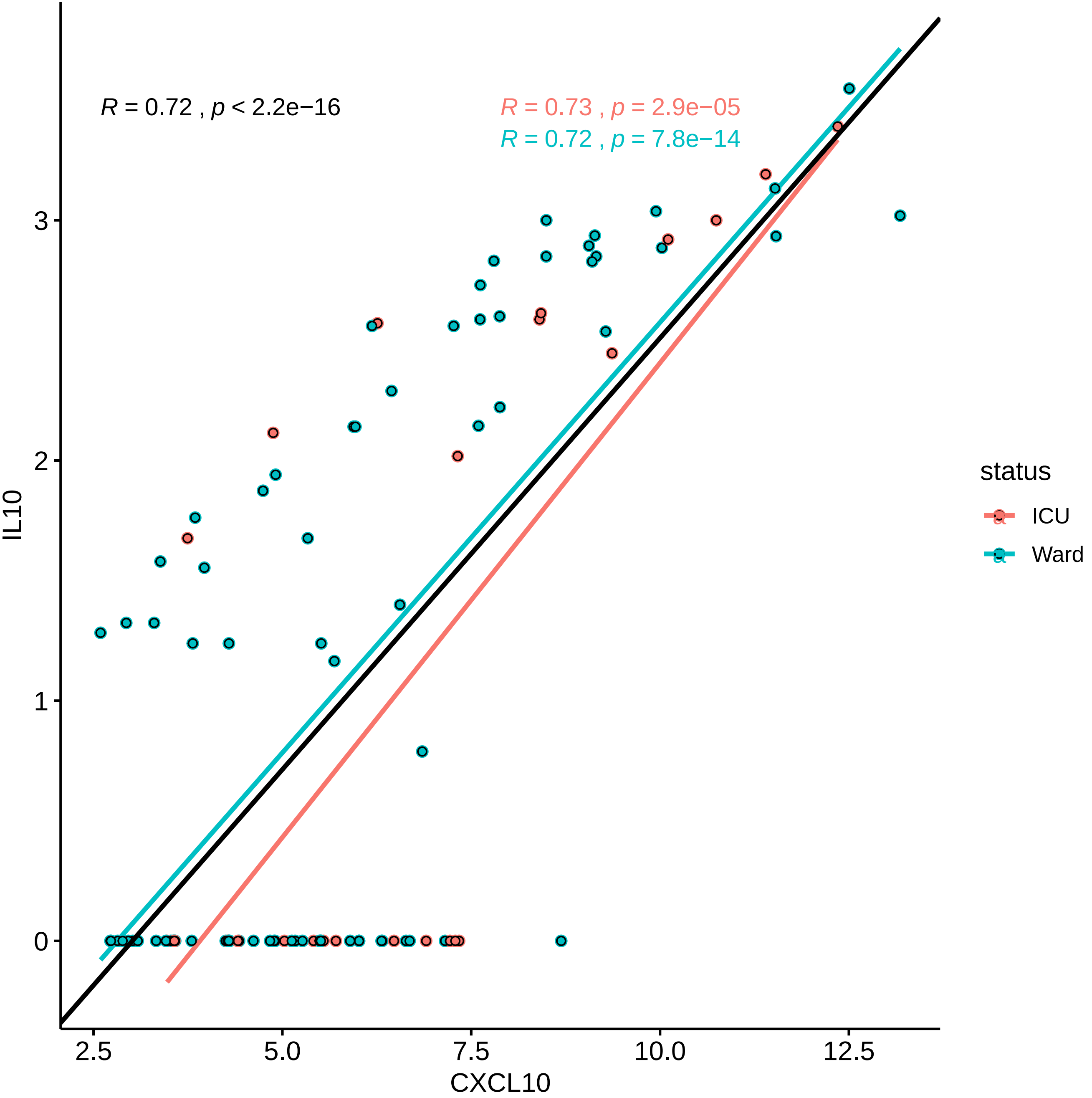

R = 0.72 , p < 2.2e−16
R = 0.73 , p = 2.9e−05
R = 0.72 , p = 7.8e−14
IL10
0
1
2
3
2.5
5.0
7.5
10.0
12.5
CXCL10
status
ICU
Ward

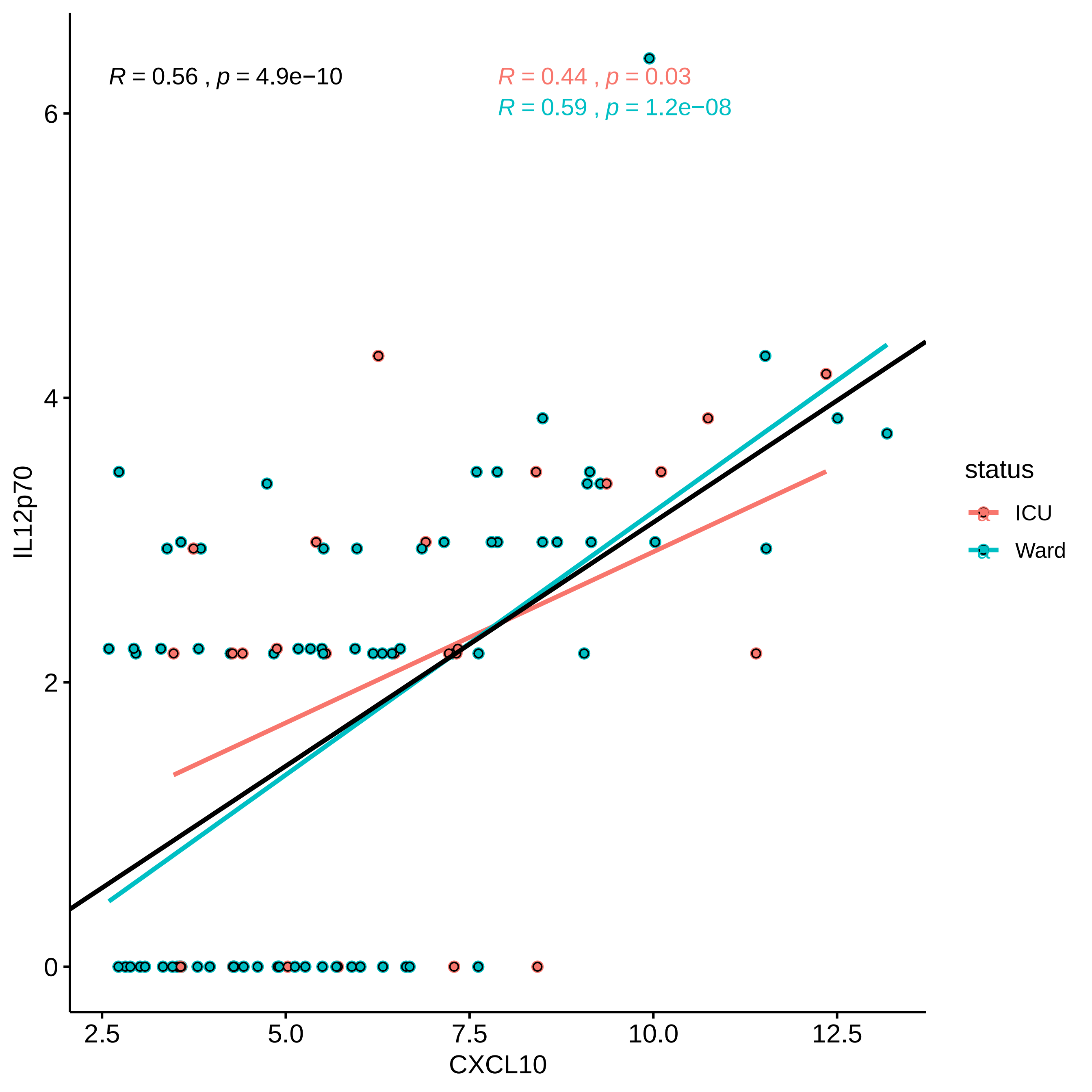

R = 0.56 , p = 4.9e−10
R = 0.44 , p = 0.03
R = 0.59 , p = 1.2e−08
IL12p70
6
4
2
0
2.5
5.0
7.5
10.0
12.5
CXCL10
status
ICU
Ward

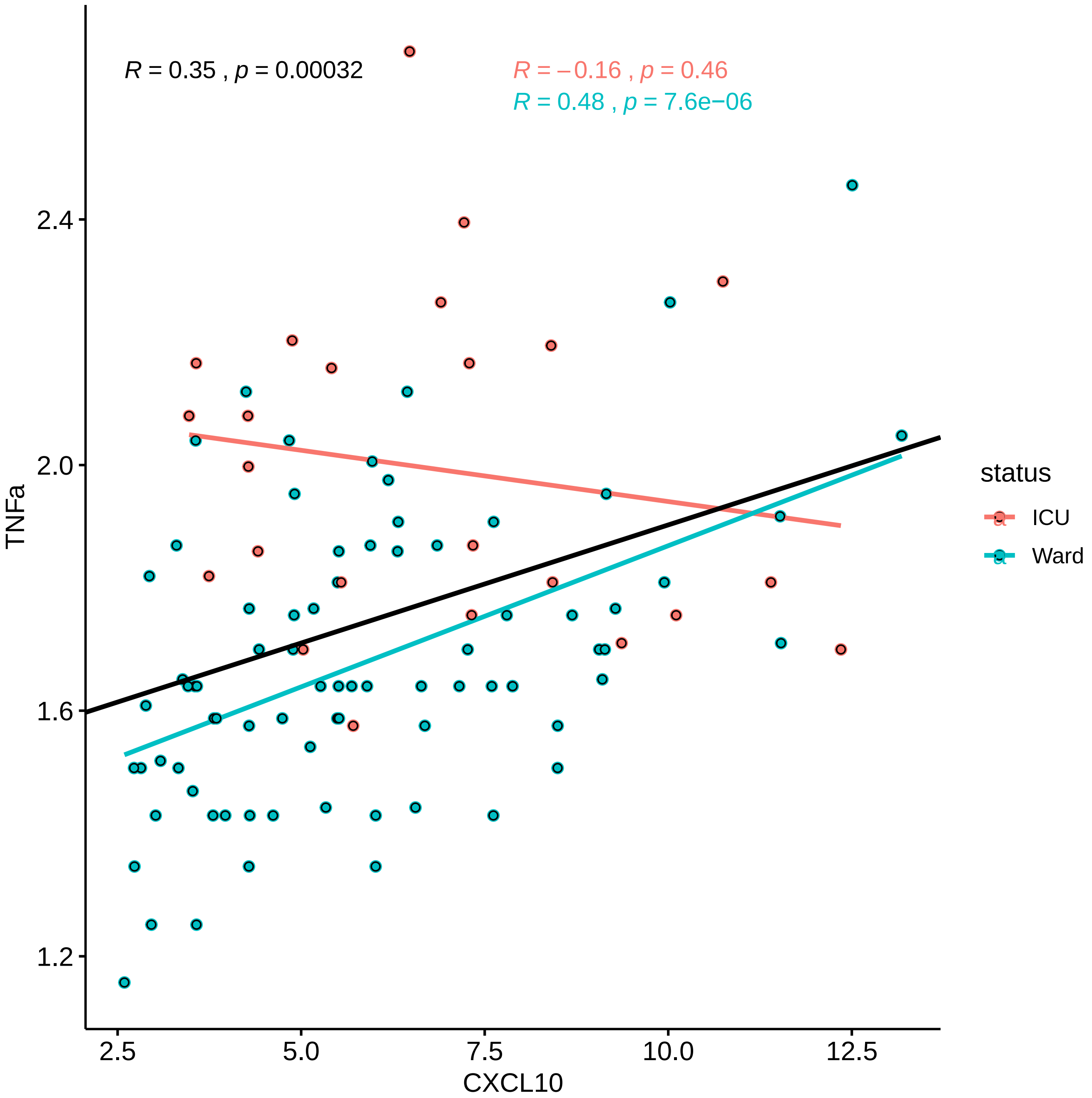

R = 0.35 , p = 0.00032
R = −0.16 , p = 0.46
R = 0.48 , p = 7.6e−06
TNFa
2.4
2.0
1.6
1.2
CXCL10
2.5
5.0
7.5
10.0
12.5
status
ICU
Ward

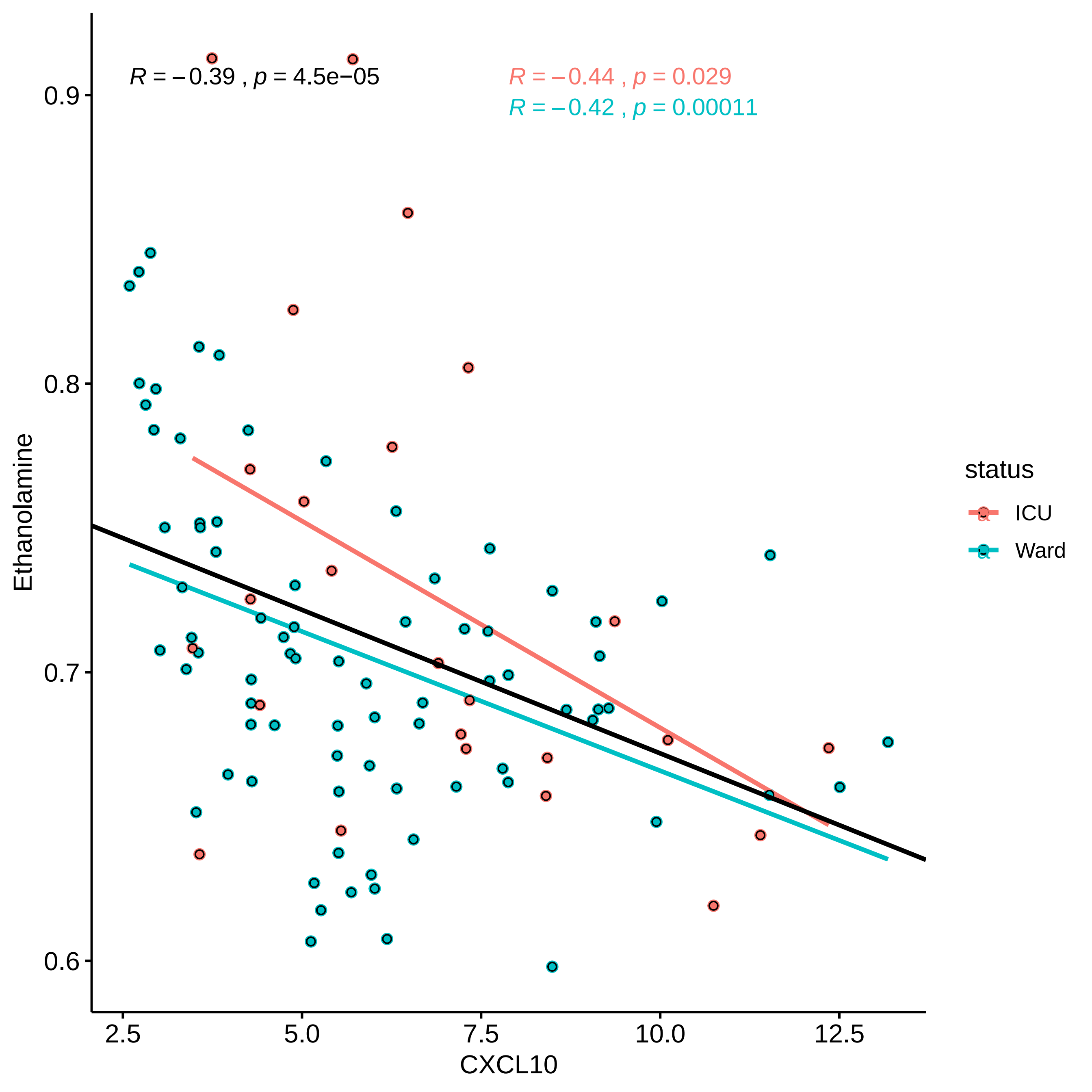

R = −0.39 , p = 4.5e−05
R = −0.44 , p = 0.029
R = −0.42 , p = 0.00011
Ethanolamine
0.9
0.8
0.7
0.6
2.5
5.0
7.5
10.0
12.5
CXCL10
status
ICU
Ward

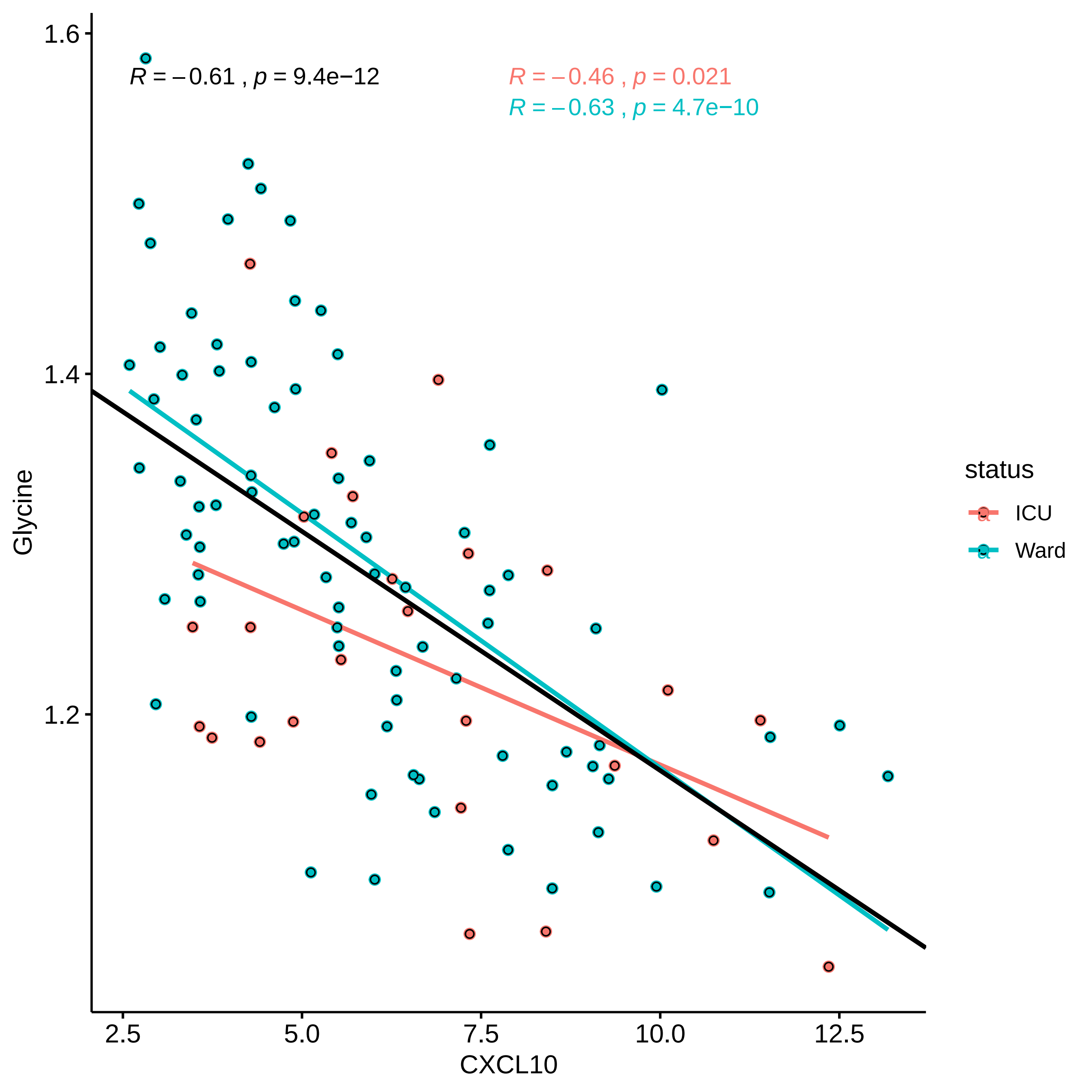
R = −0.61 , p = 9.4e−12
R = −0.46 , p = 0.021
R = −0.63 , p = 4.7e−10
1.6
1.4
1.2
Glycine
2.5
5.0
7.5
10.0
12.5
CXCL10
status
ICU
Ward

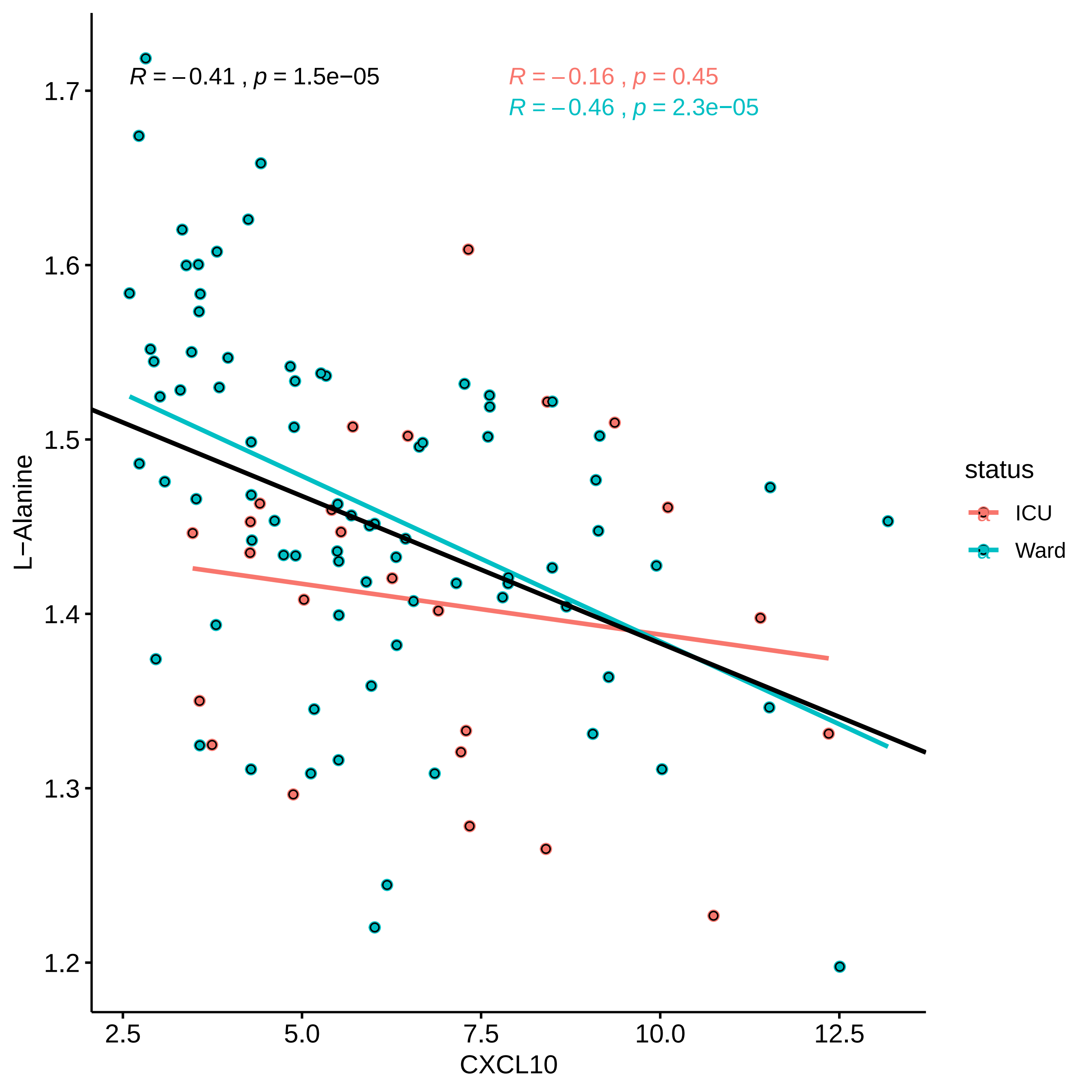
R = −0.41 , p = 1.5e−05
R = −0.16 , p = 0.45
R = −0.46 , p = 2.3e−05
L−Alanine
1.7
1.6
1.5
1.4
1.3
1.2
2.5
5.0
7.5
10.0
12.5
CXCL10
status
ICU
Ward

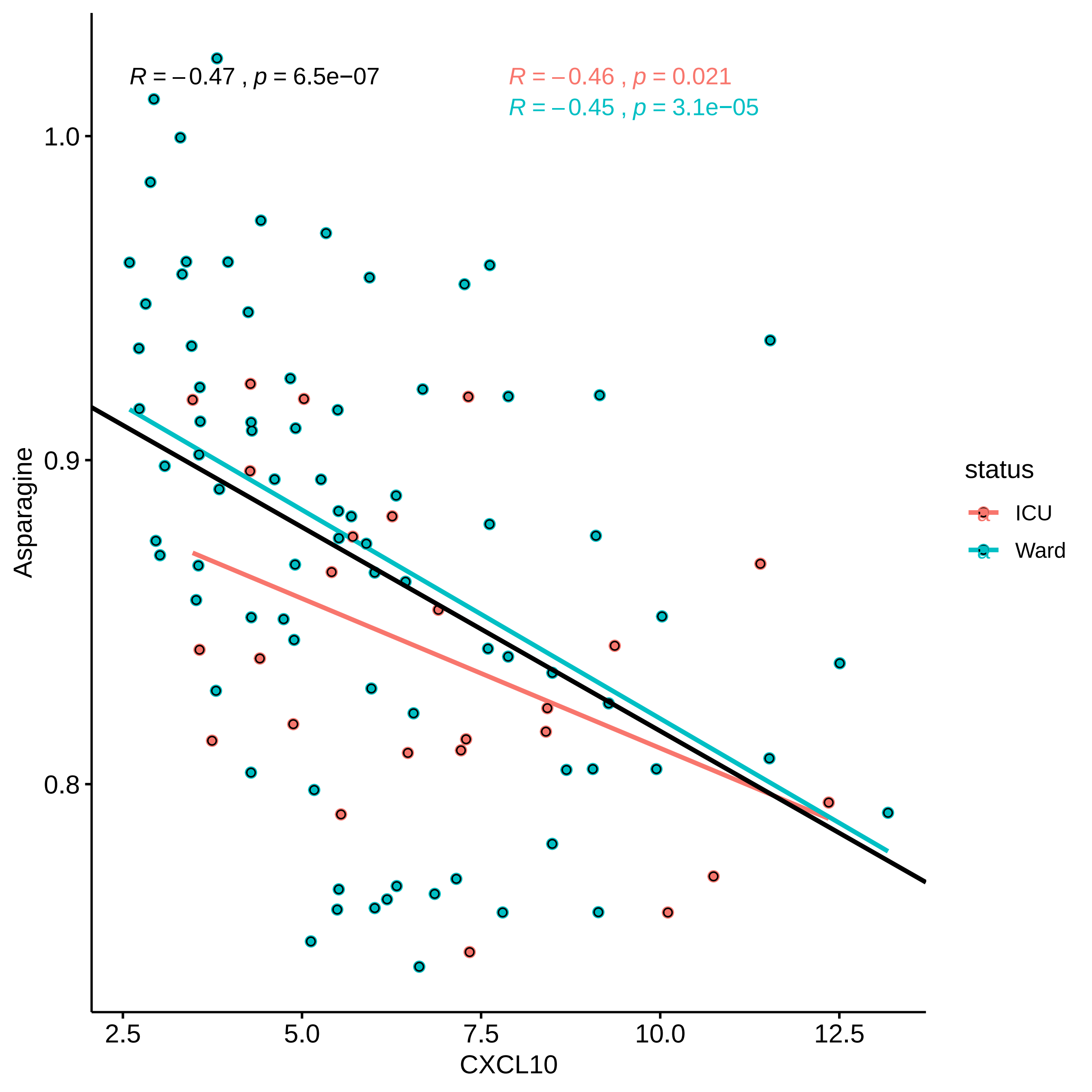

R = −0.47 , p = 6.5e−07
R = −0.46 , p = 0.021
R = −0.45 , p = 3.1e−05
1.0
0.9
0.8
Asparagine
2.5
5.0
7.5
10.0
12.5
CXCL10
status
ICU
Ward

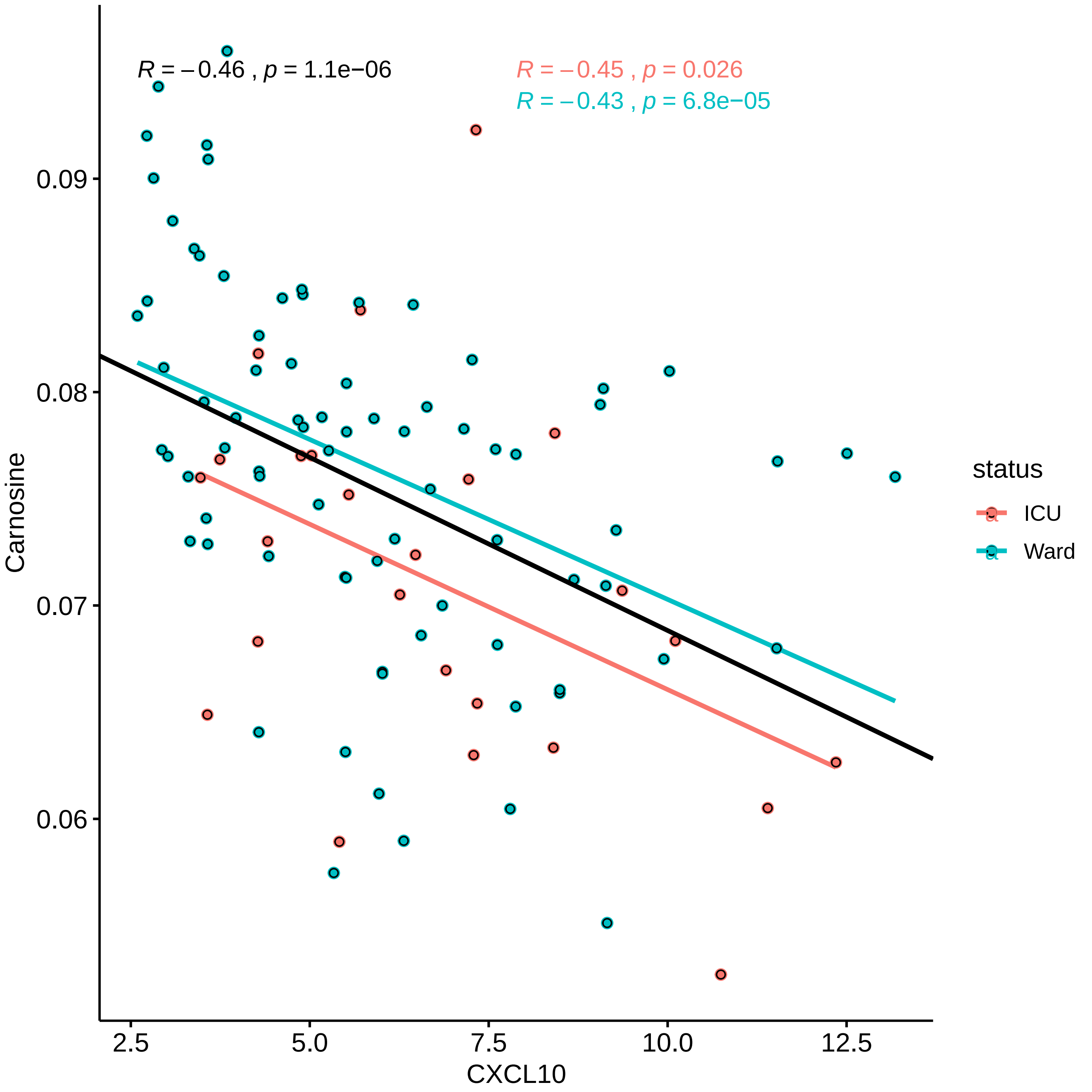

R = −0.46 , p = 1.1e−06
R = −0.45 , p = 0.026
R = −0.43 , p = 6.8e−05
0.09
0.08
0.07
0.06
Carnosine
2.5
5.0
7.5
10.0
12.5
CXCL10
status
ICU
Ward

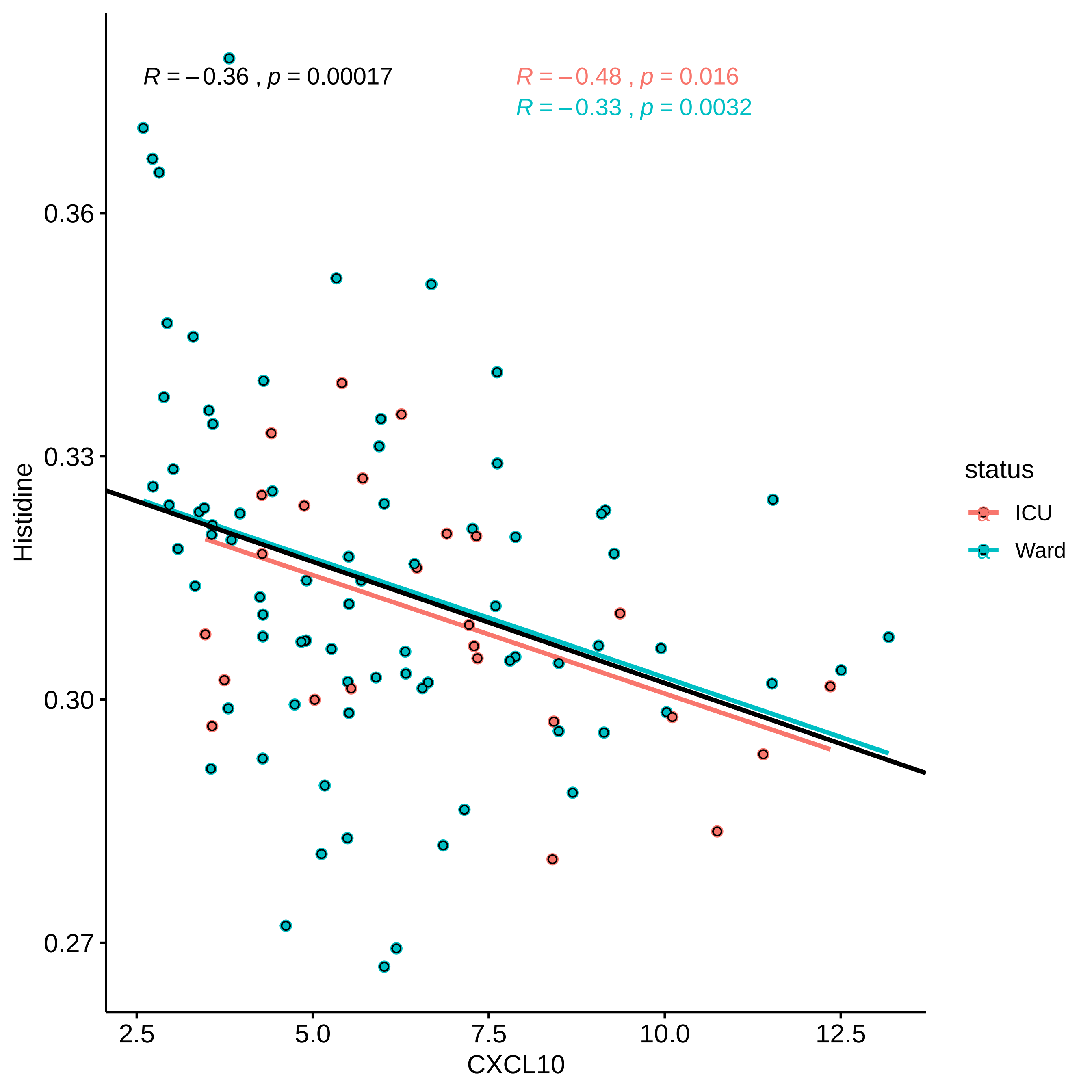

R = −0.36 , p = 0.00017
R = −0.48 , p = 0.016
R = −0.33 , p = 0.0032
Histidine
0.36
0.33
0.30
0.27
2.5
5.0
7.5
10.0
12.5
CXCL10
status
ICU
Ward

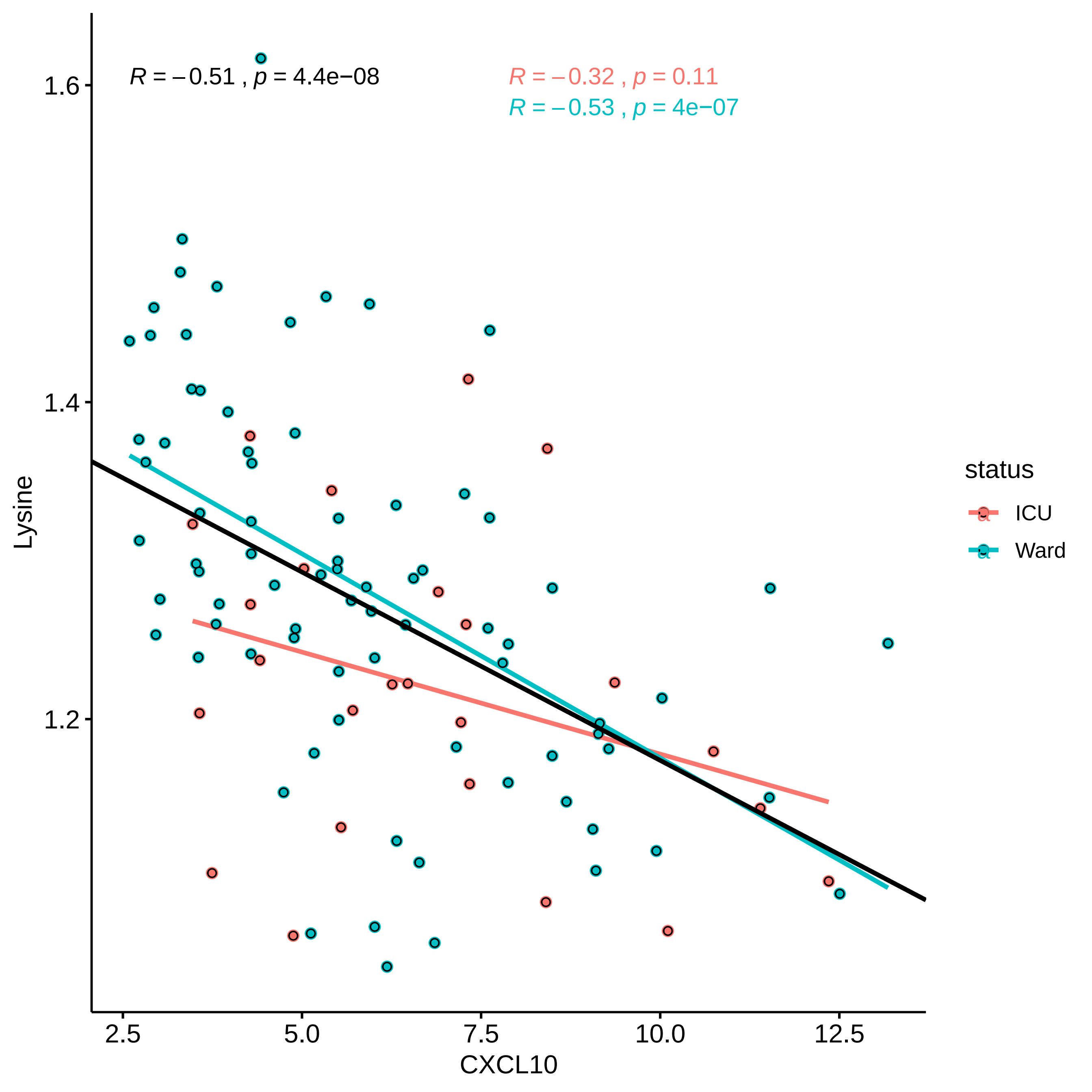

R = −0.51 , p = 4.4e−08
R = −0.32 , p = 0.11
R = −0.53 , p = 4e−07
Lysine
1.6
1.4
1.2
CXCL10
2.5
5.0
7.5
10.0
12.5
status
ICU
Ward

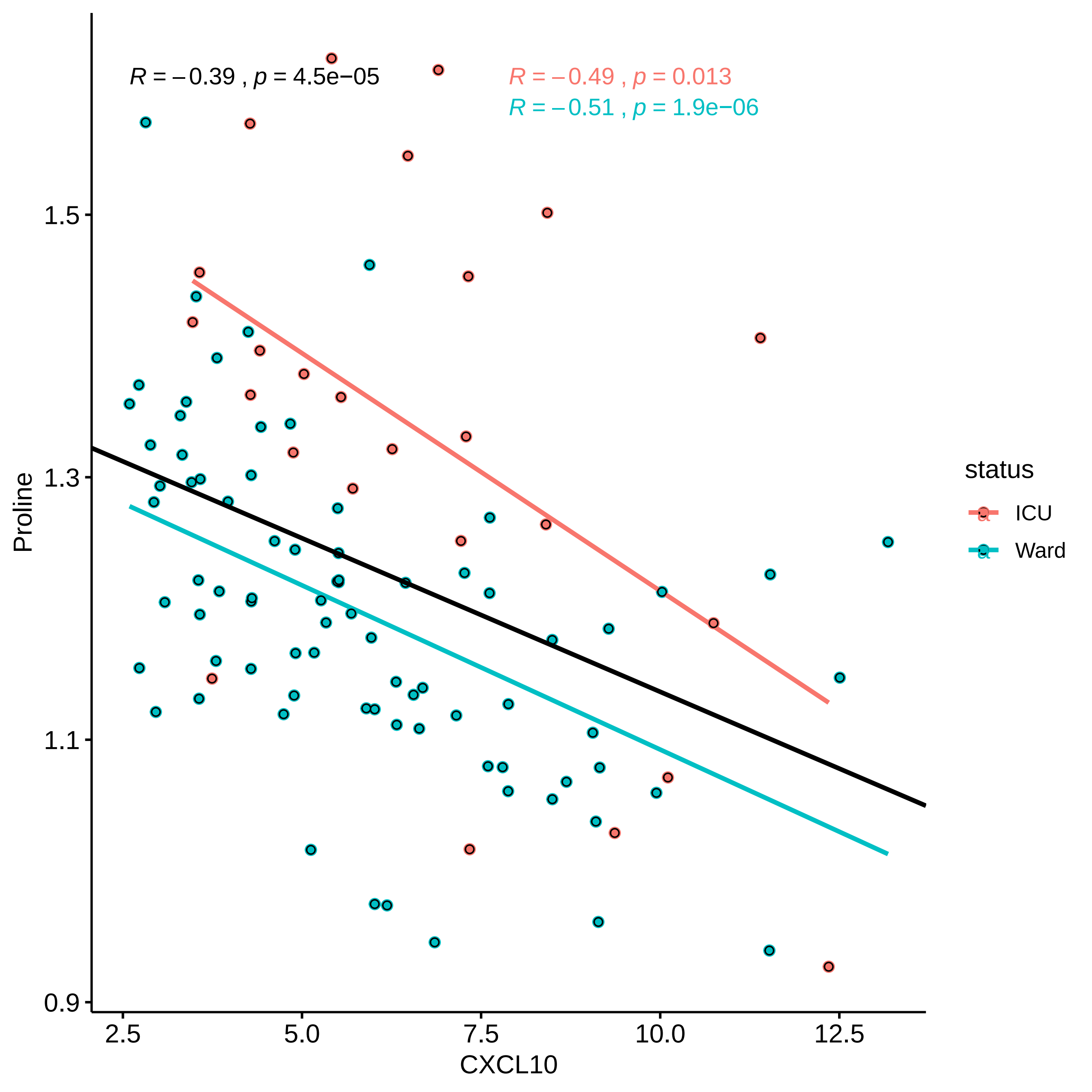

R = −0.39 , p = 4.5e−05
R = −0.49 , p = 0.013
R = −0.51 , p = 1.9e−06
Proline
1.5
1.3
1.1
0.9
2.5
5.0
7.5
10.0
12.5
CXCL10
status
ICU
Ward

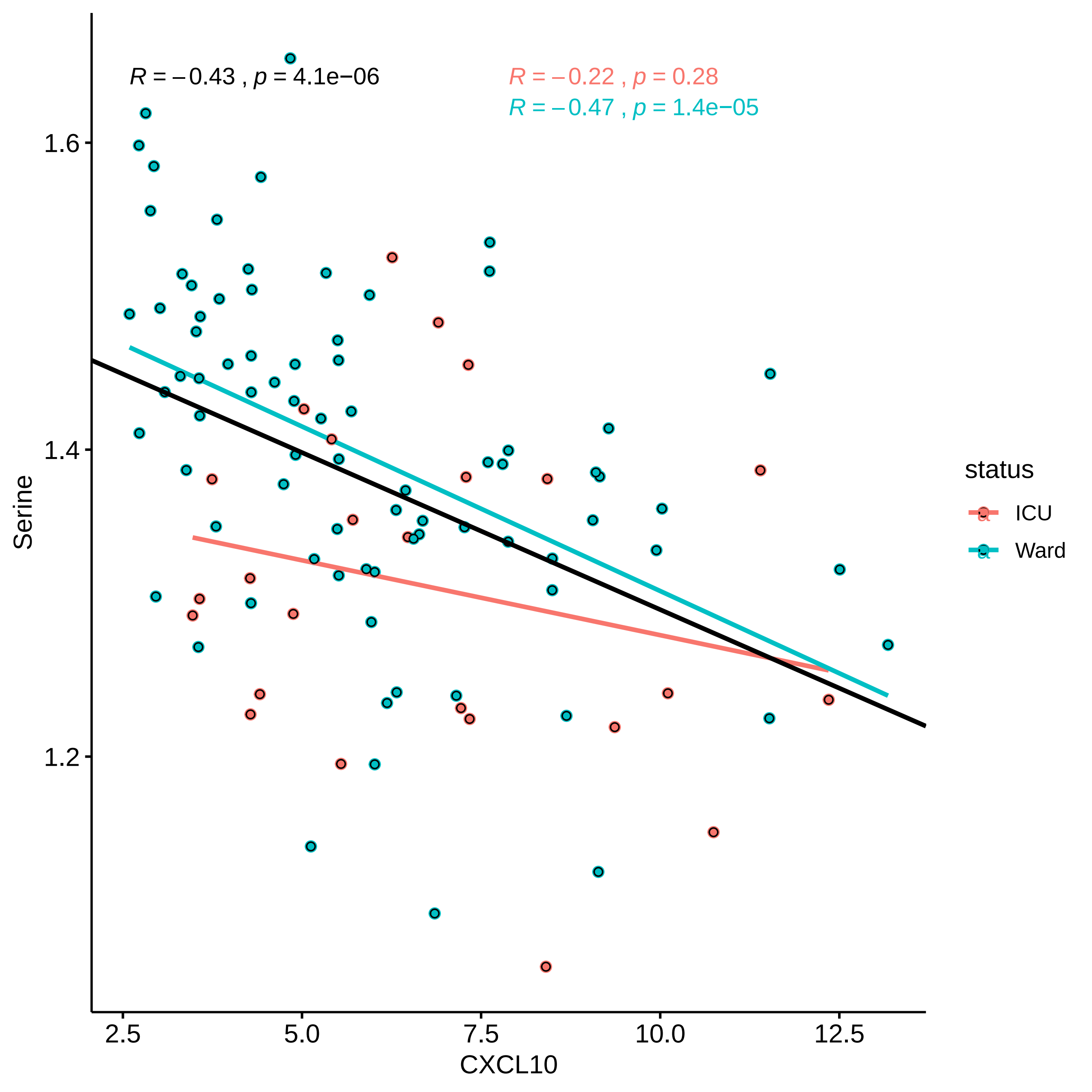

R = −0.43 , p = 4.1e−06
R = −0.22 , p = 0.28
R = −0.47 , p = 1.4e−05
Serine
1.6
1.4
1.2
CXCL10
2.5
5.0
7.5
10.0
12.5
status
ICU
Ward

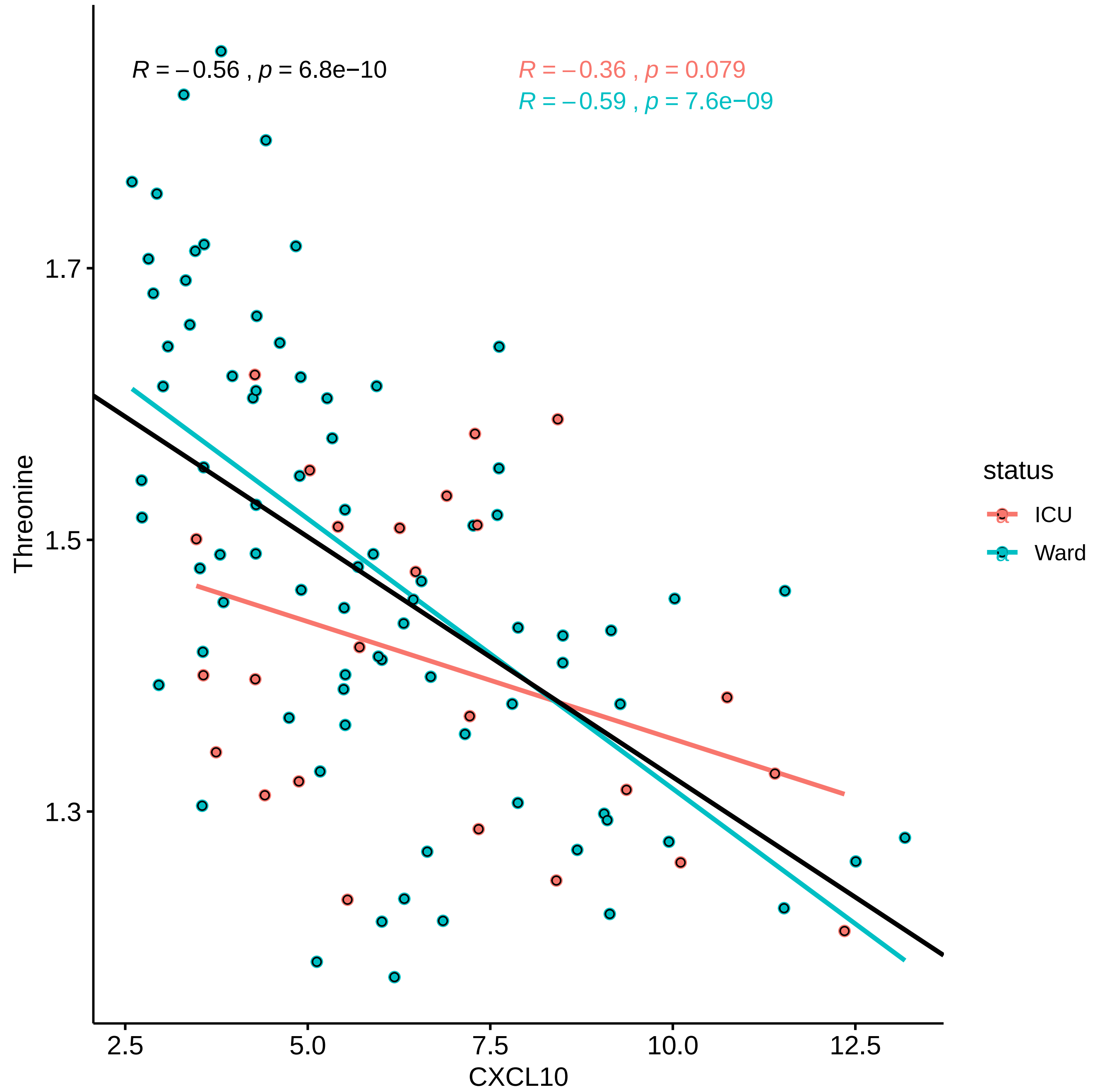

R = −0.56 , p = 6.8e−10
R = −0.36 , p = 0.079
R = −0.59 , p = 7.6e−09
Threonine
1.7
1.5
1.3
CXCL10
2.5
5.0
7.5
10.0
12.5
status
ICU
Ward

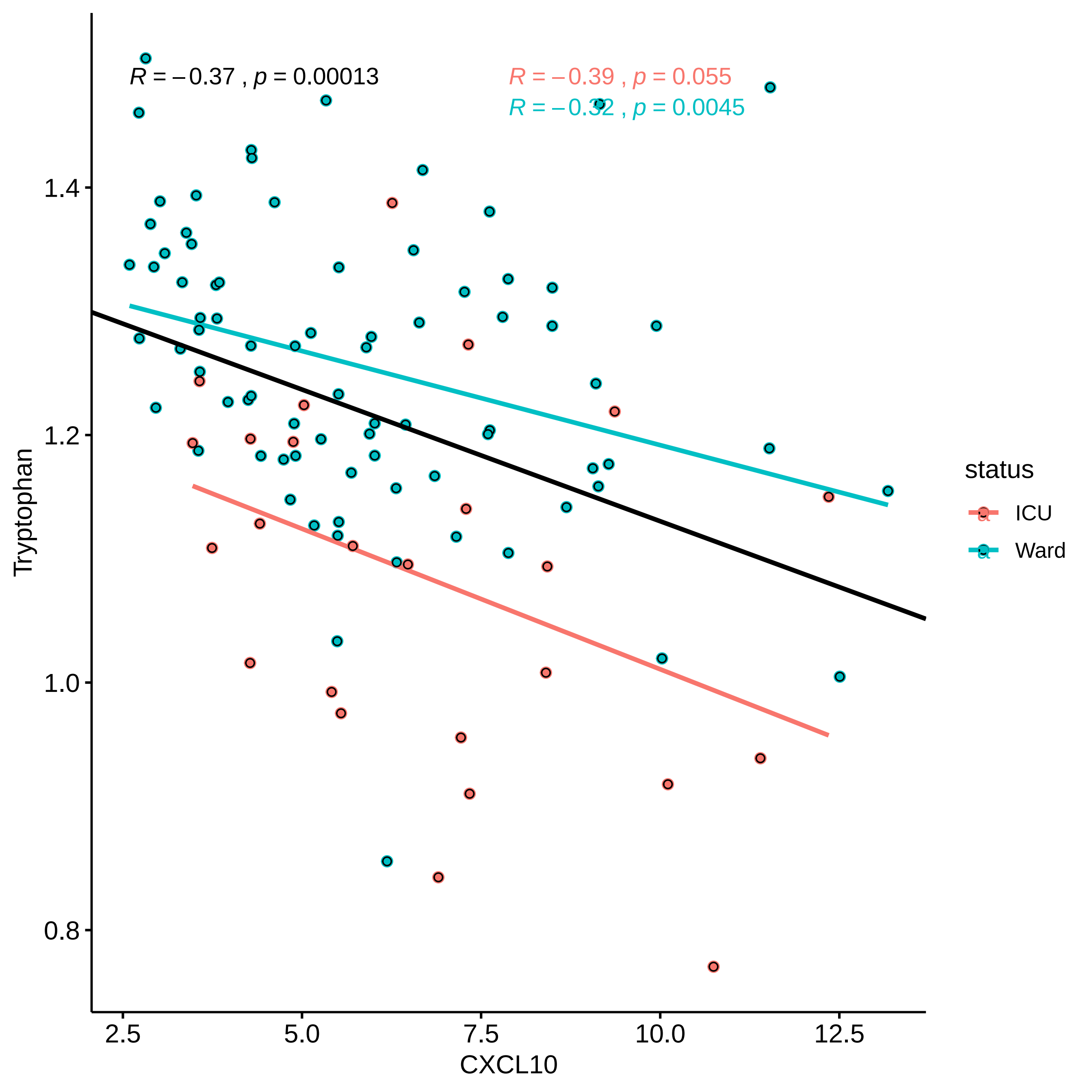

R = −0.37 , p = 0.00013
R = −0.39 , p = 0.055
R = −0.32 , p = 0.0045
1.4
1.2
1.0
0.8
Tryptophan
2.5
5.0
7.5
10.0
12.5
CXCL10
status
ICU
Ward

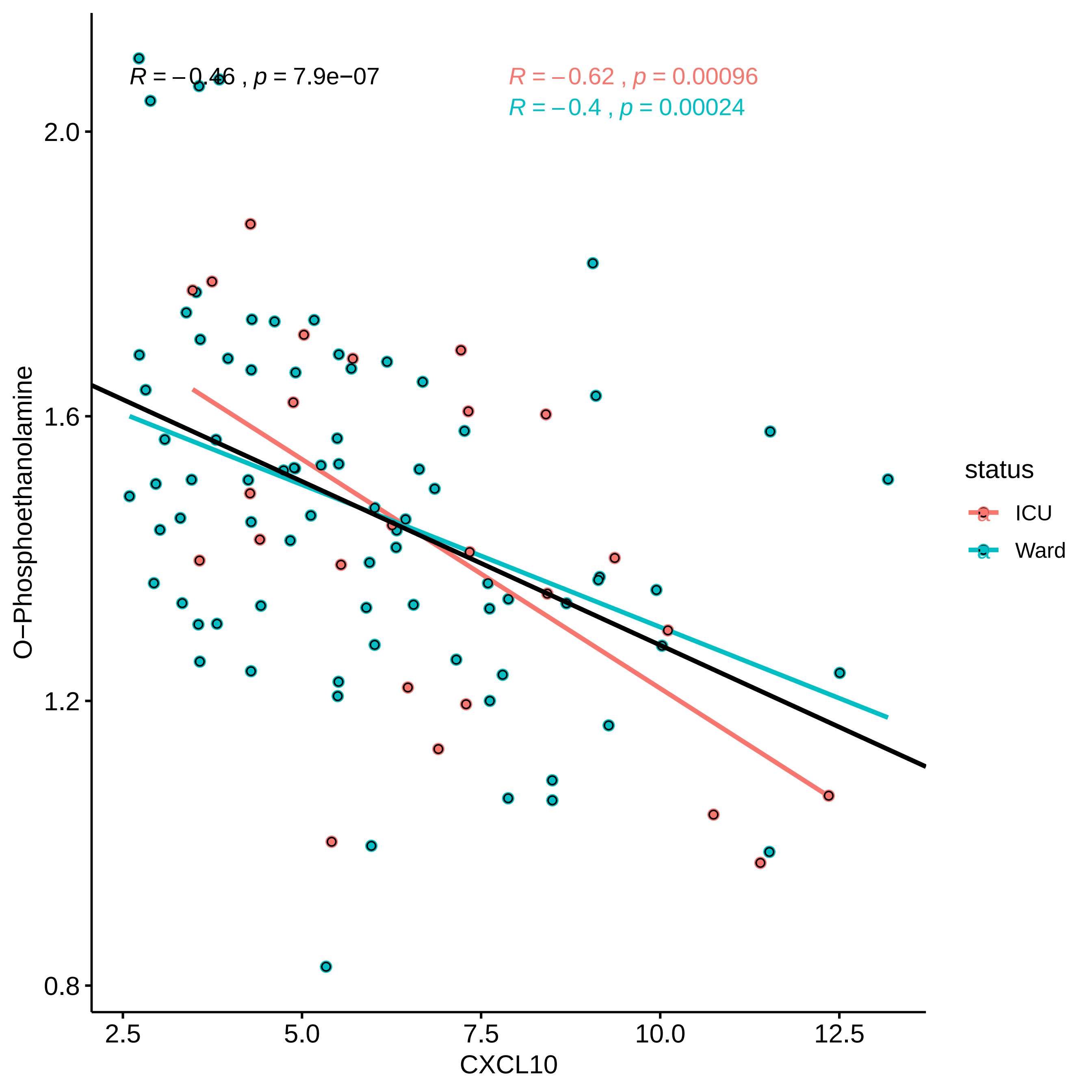

R = −0.46 , p = 7.9e−07
R = −0.62 , p = 0.00096
R = −0.4 , p = 0.00024
O−Phosphoethanolamine
2.0
1.6
1.2
0.8
2.5
5.0
7.5
10.0
12.5
CXCL10
status
ICU
Ward

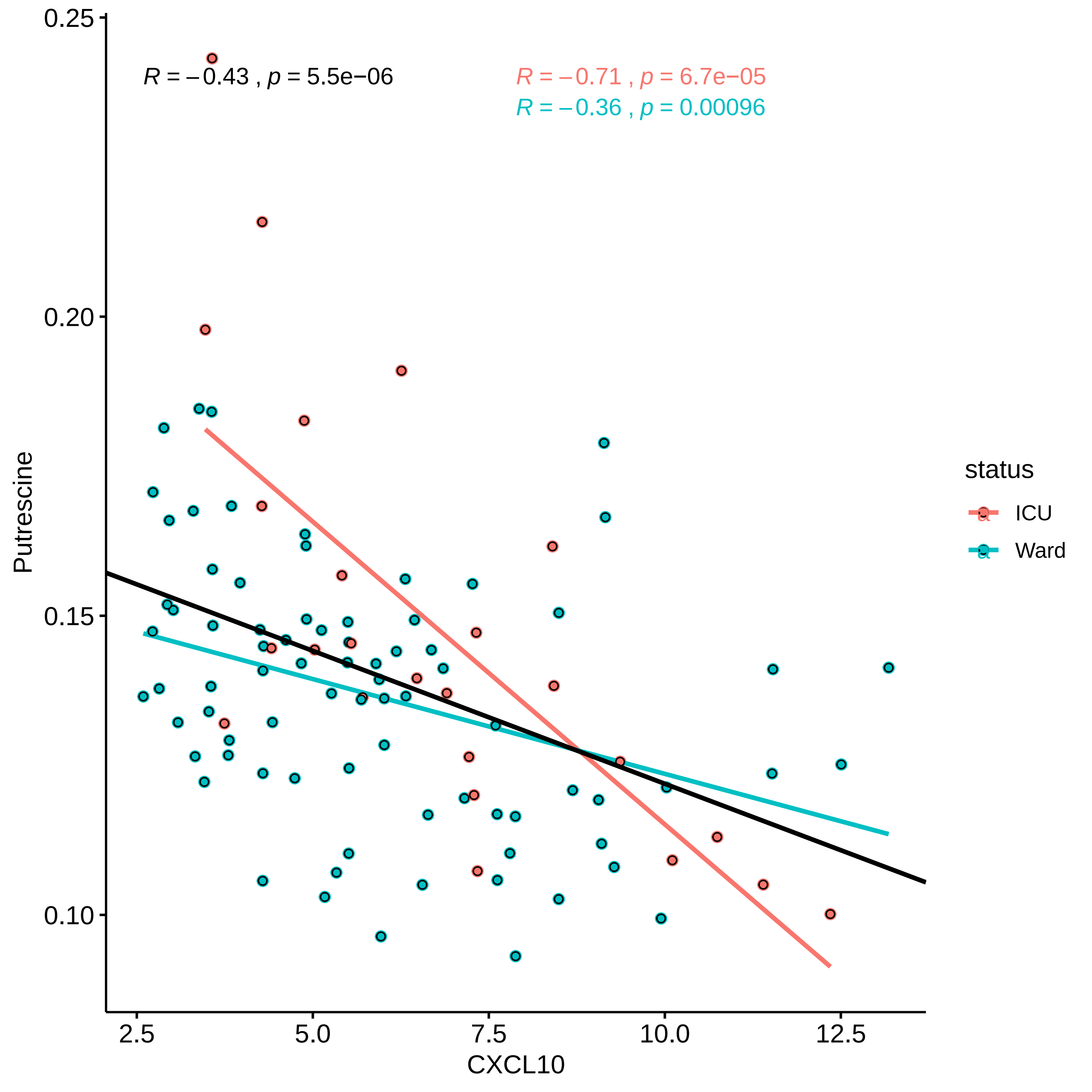

R = −0.43 , p = 5.5e−06
R = −0.71 , p = 6.7e−05
R = −0.36 , p = 0.00096
0.25
0.20
0.15
0.10
Putrescine
2.5
5.0
7.5
10.0
12.5
CXCL10
status
ICU
Ward

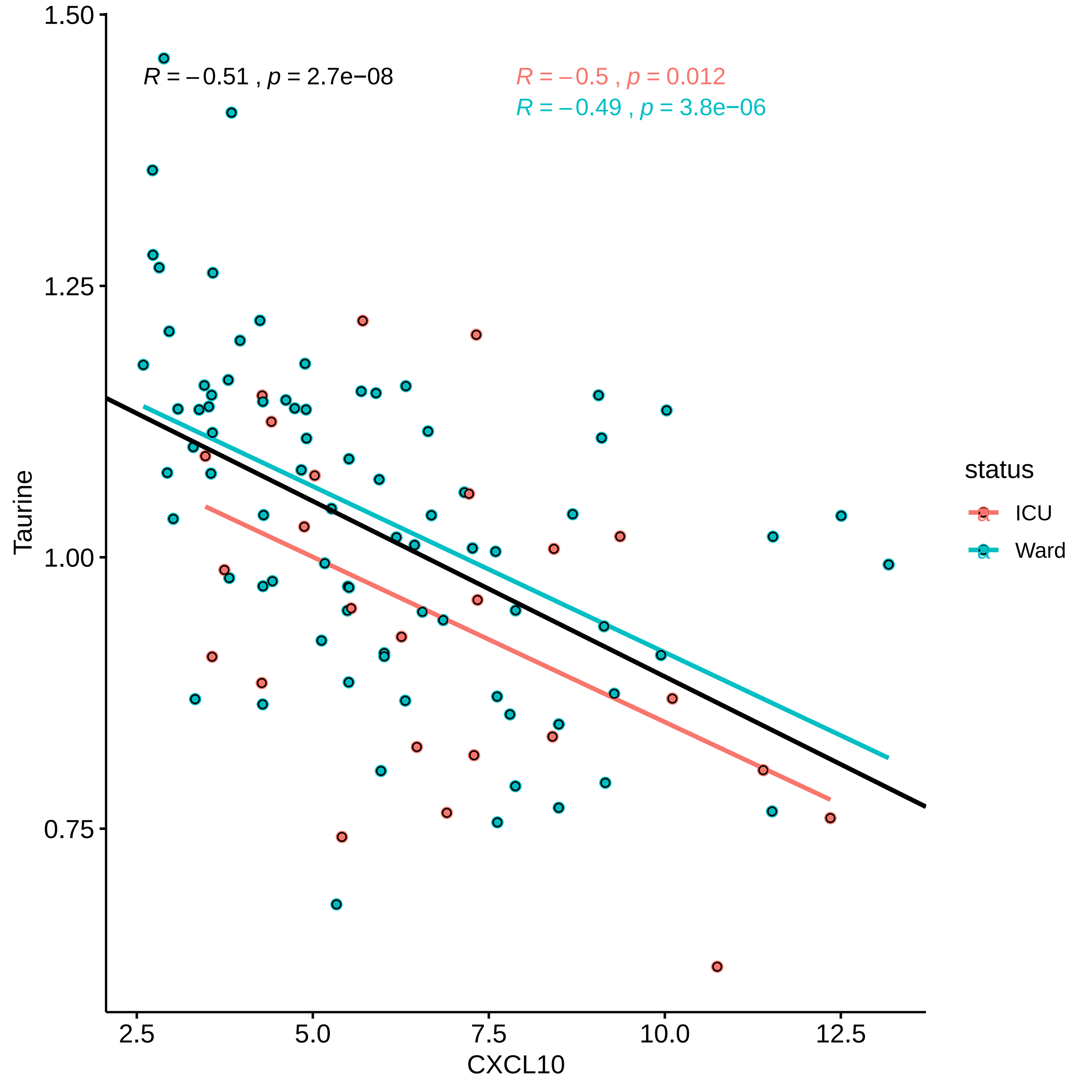

R = −0.51 , p = 2.7e−08
R = −0.5 , p = 0.012
R = −0.49 , p = 3.8e−06
Taurine
1.50
1.25
1.00
0.75
2.5
5.0
7.5
10.0
12.5
CXCL10
status
ICU
Ward

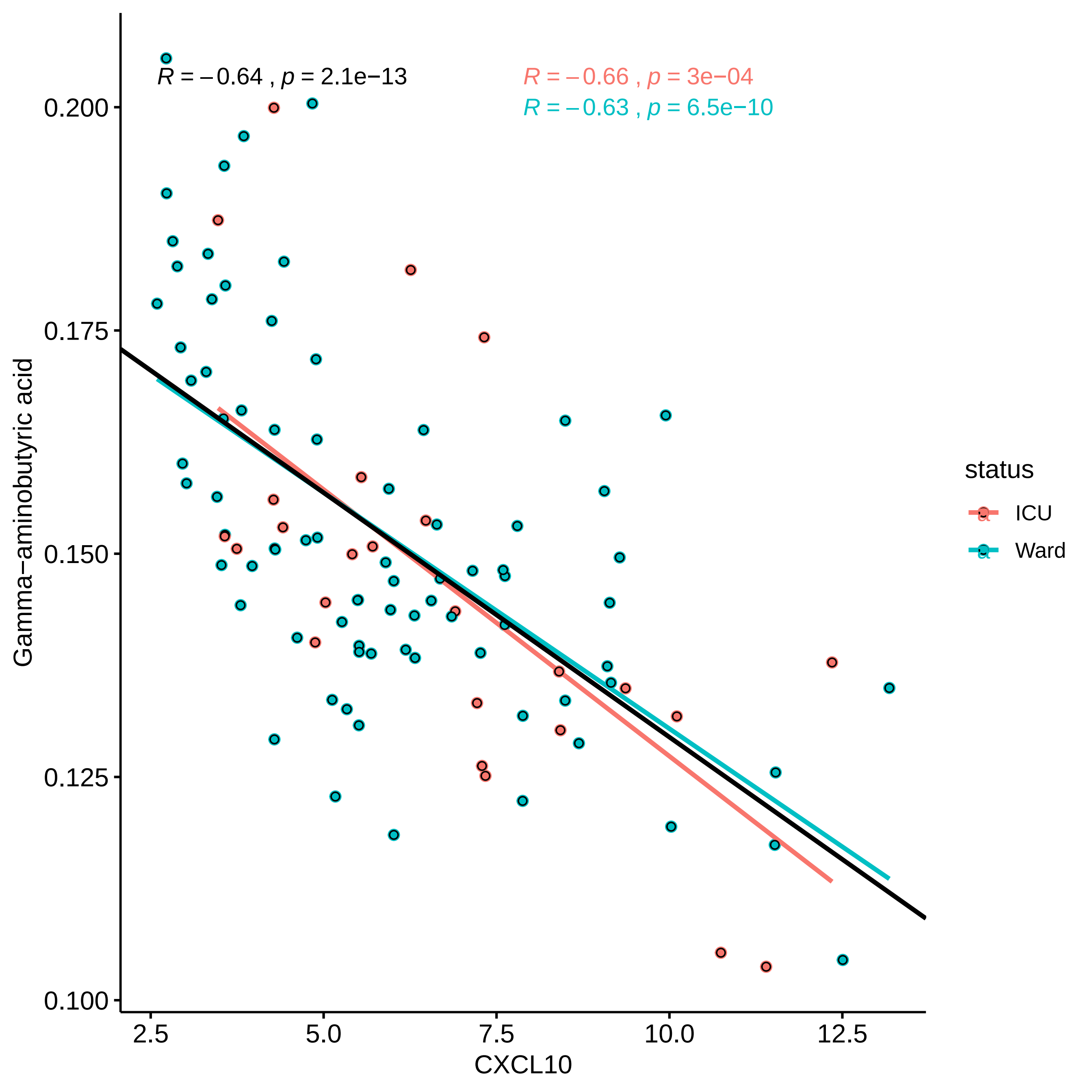

R = −0.64 , p = 2.1e−13
R = −0.66 , p = 3e−04
R = −0.63 , p = 6.5e−10
Gamma−aminobutyric acid
0.200
0.175
0.150
0.125
0.100
2.5
5.0
7.5
10.0
12.5
CXCL10
status
ICU
Ward

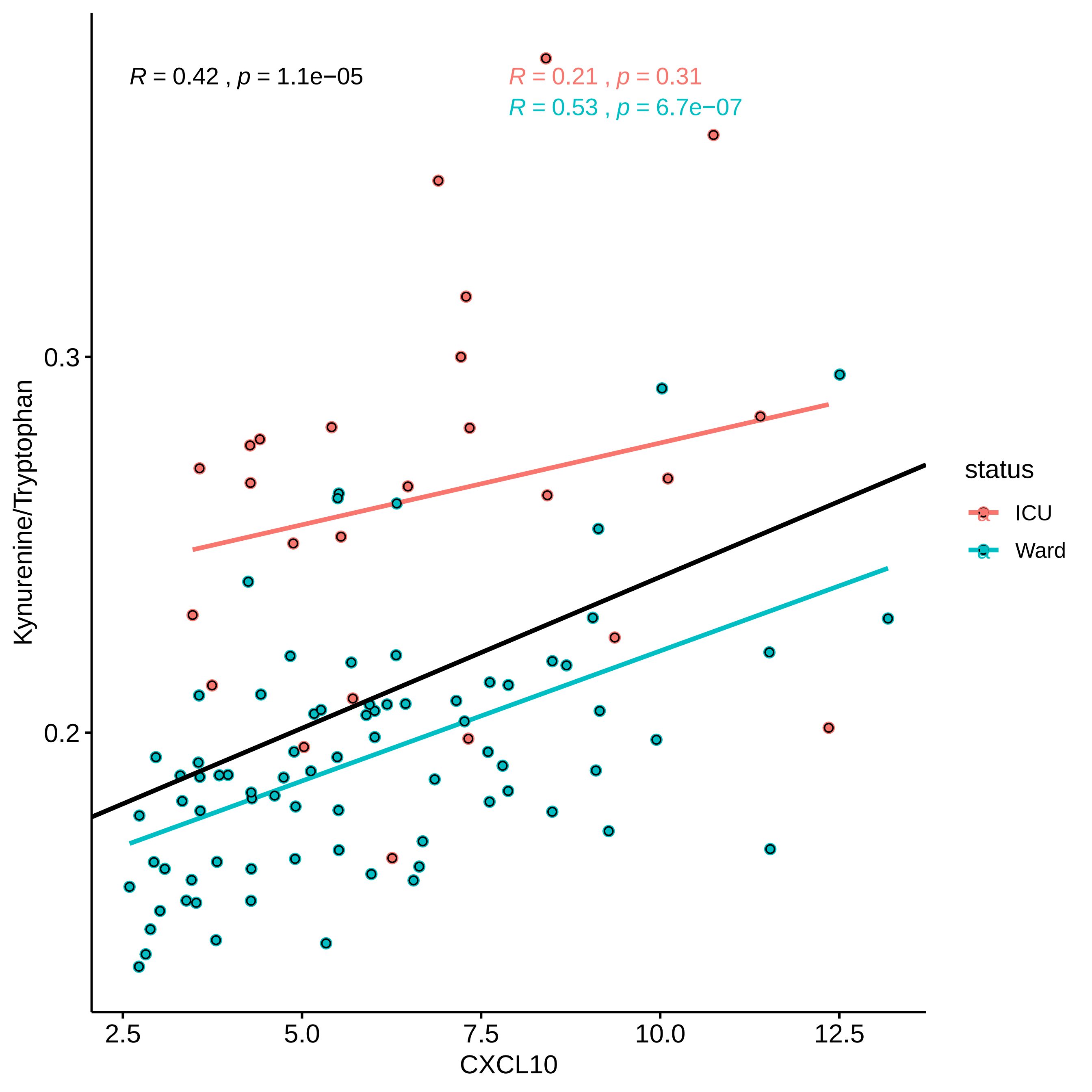

R = 0.42 , p = 1.1e−05
R = 0.21 , p = 0.31
R = 0.53 , p = 6.7e−07
Kynurenine/Tryptophan
0.3
0.2
2.5
5.0
7.5
10.0
12.5
CXCL10
status
ICU
Ward

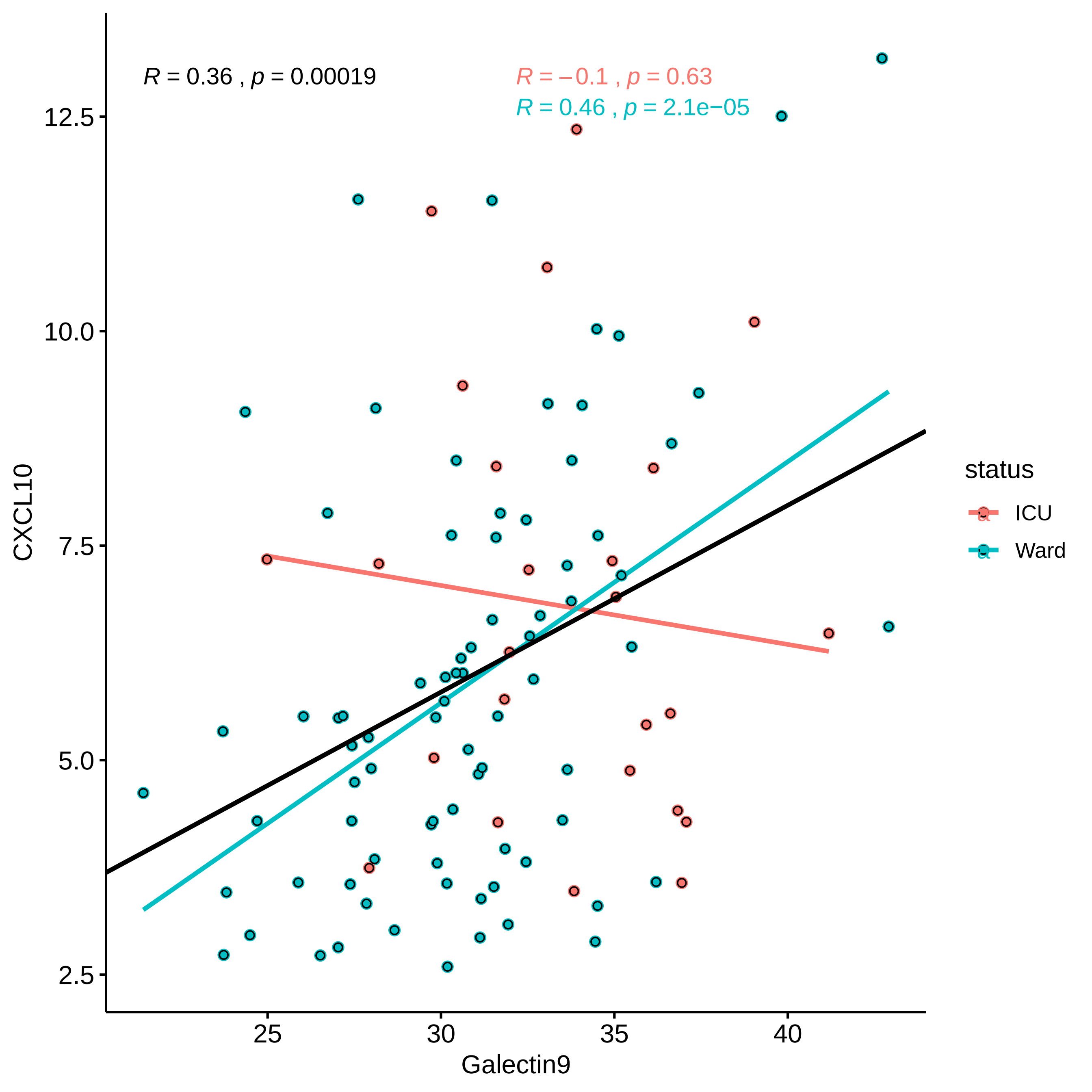

R = 0.36 , p = 0.00019
R = −0.1 , p = 0.63
R = 0.46 , p = 2.1e−05
CXCL10
12.5
10.0
7.5
5.0
2.5
25
30
35
40
Galectin9
status
ICU
Ward

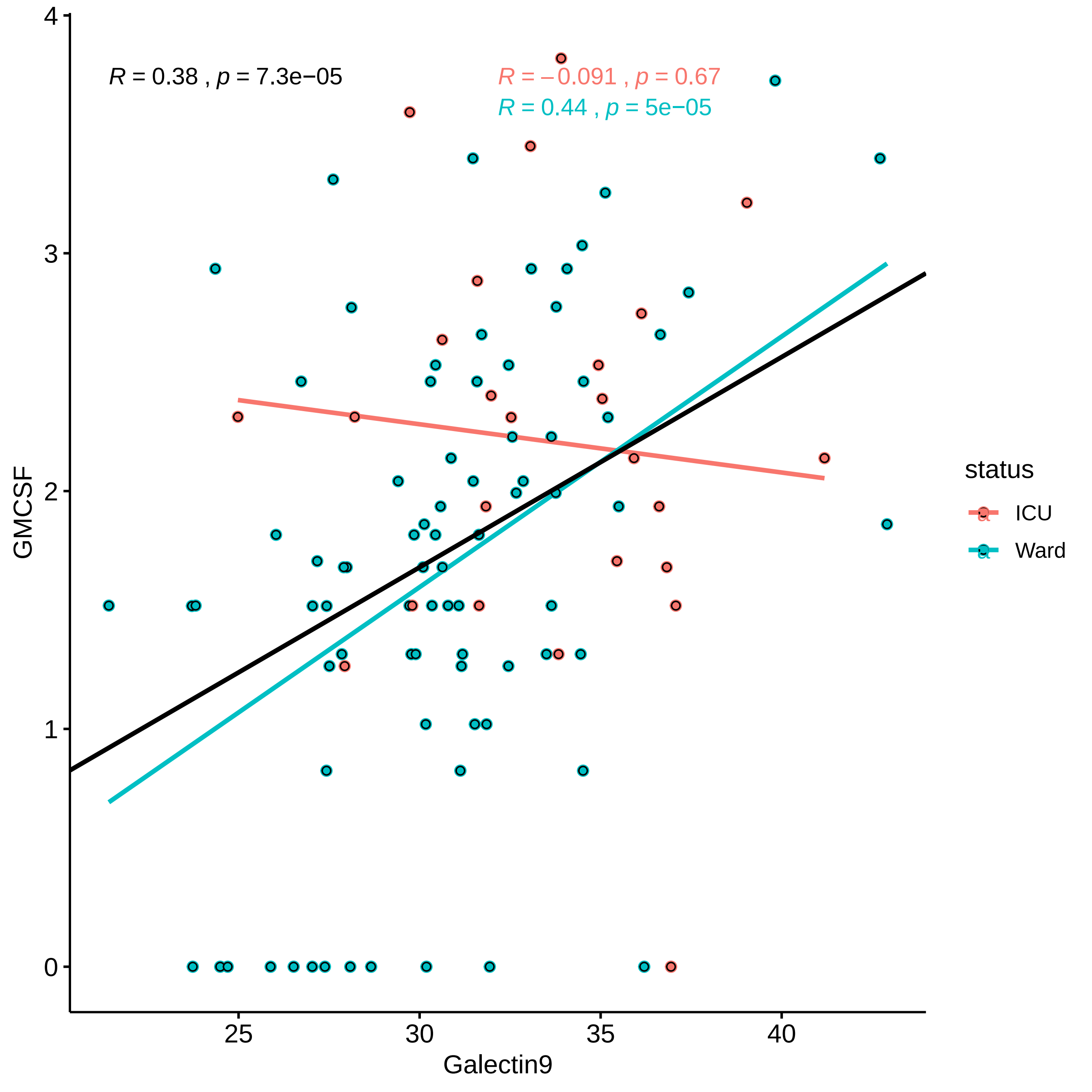
R = 0.38 , p = 7.3e−05
R = −0.091 , p = 0.67
R = 0.44 , p = 5e−05
GMCSF
Galectin9
0
1
2
3
4
25
30
35
40
status
ICU
Ward

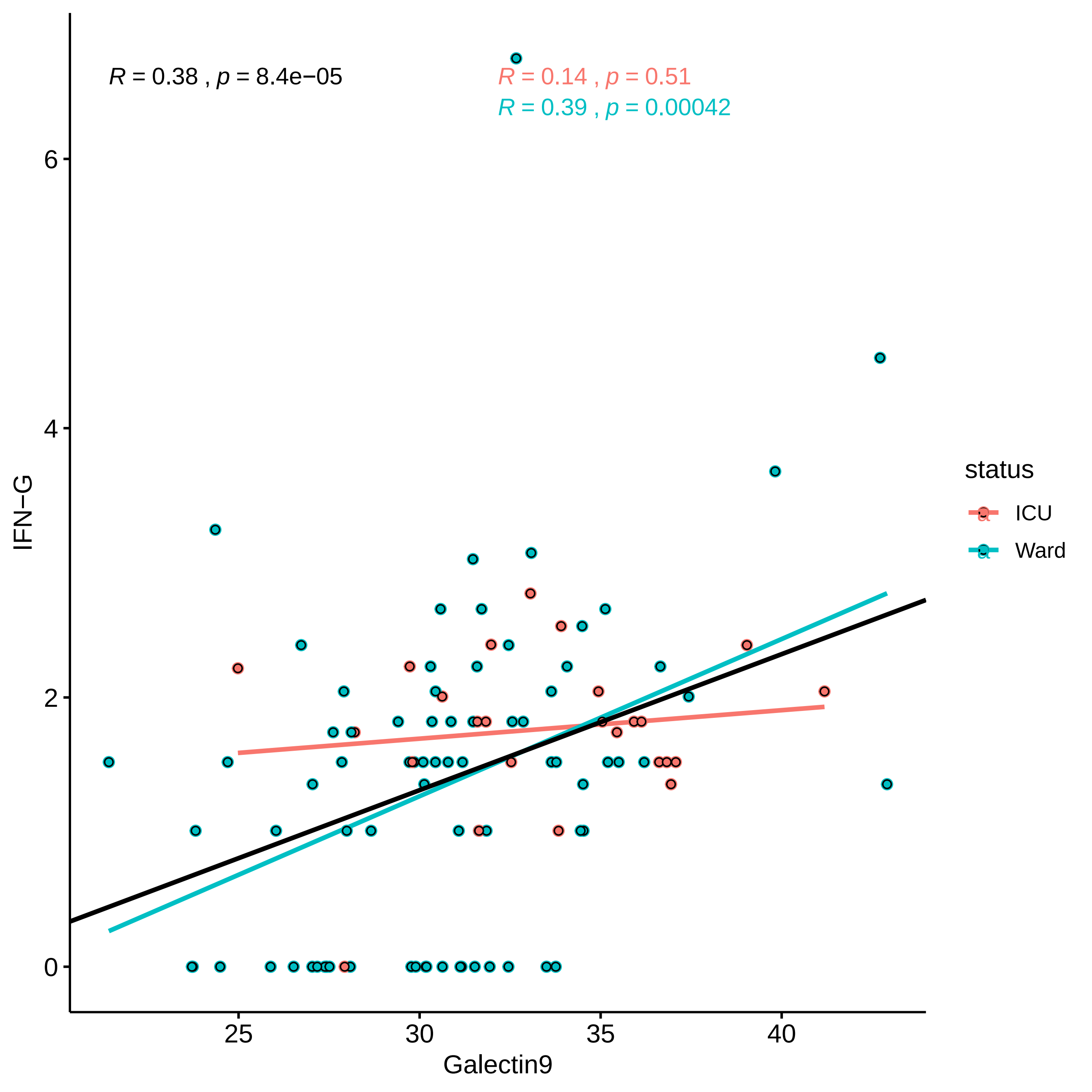

R = 0.38 , p = 8.4e−05
R = 0.14 , p = 0.51
R = 0.39 , p = 0.00042
IFN−G
6
4
2
0
25
30
35
40
Galectin9
status
ICU
Ward

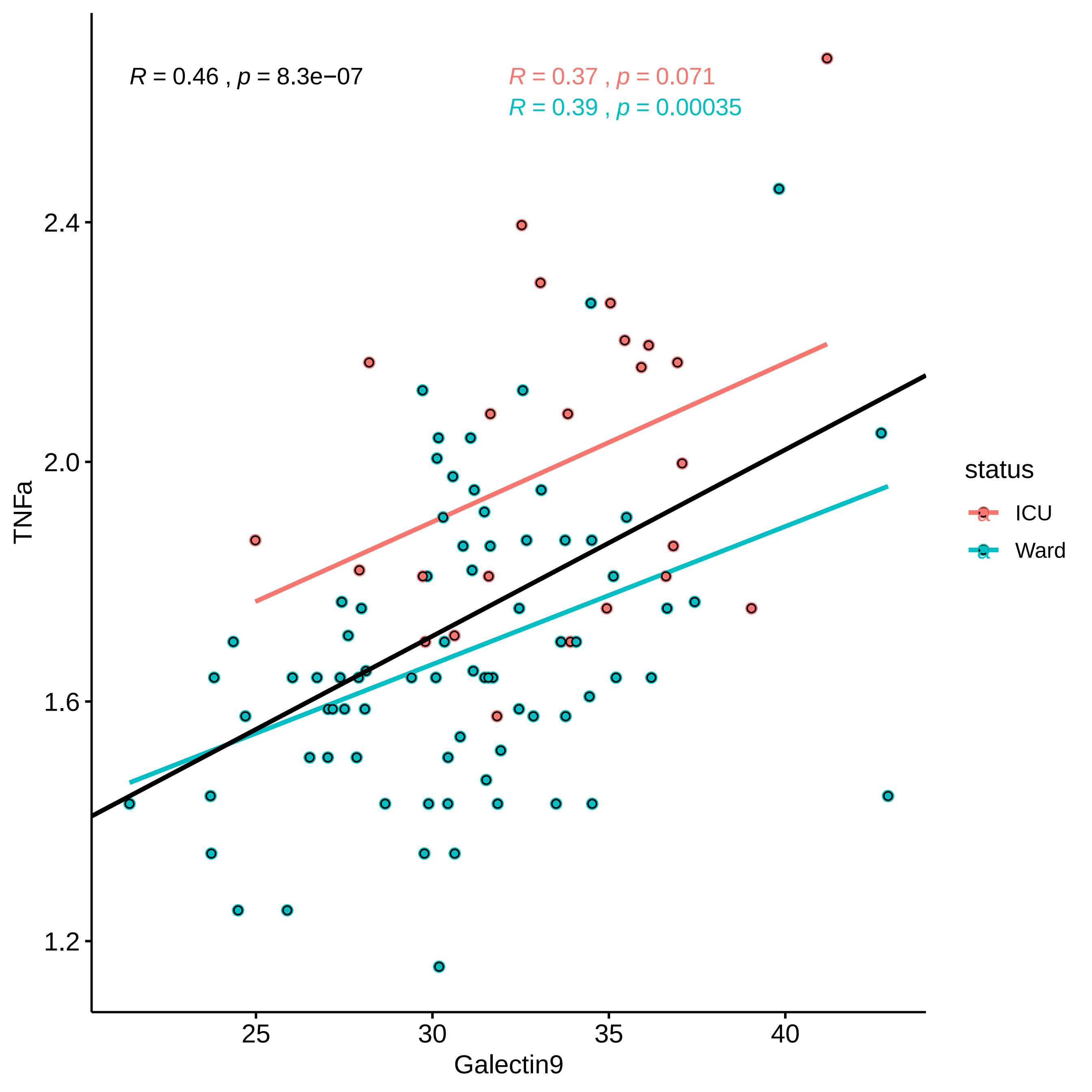

R = 0.46 , p = 8.3e−07
R = 0.37 , p = 0.071
R = 0.39 , p = 0.00035
TNFa
2.4
2.0
1.6
1.2
25
30
35
40
Galectin9
status
ICU
Ward

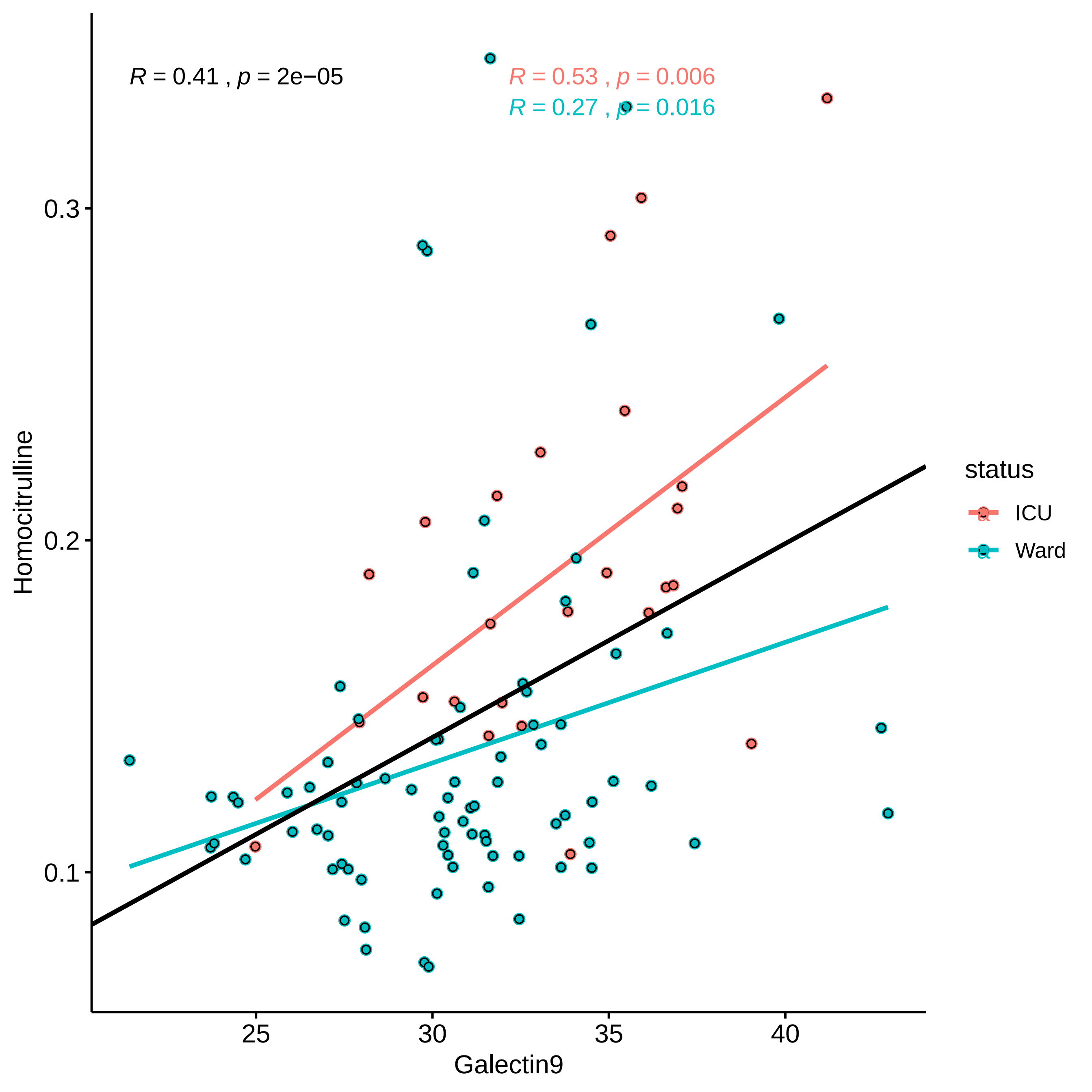

R = 0.41 , p = 2e−05
R = 0.53 , p = 0.006
R = 0.27 , p = 0.016
Homocitrulline
0.3
0.2
0.1
Galectin9
25
30
35
40
status
ICU
Ward

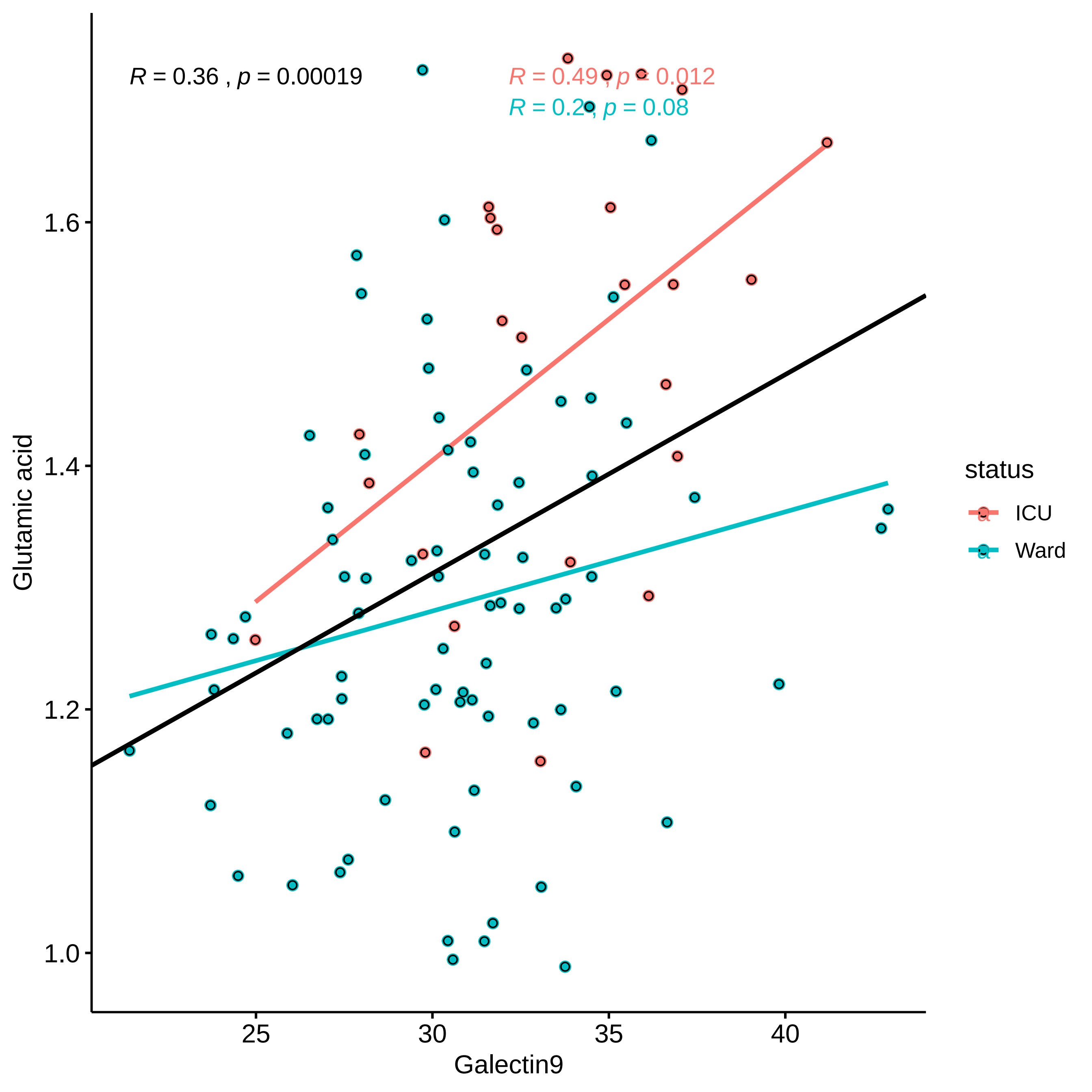
R = 0.36 , p = 0.00019
R = 0.49 , p = 0.012
R = 0.2 , p = 0.08
Glutamic acid
1.6
1.4
1.2
1.0
25
30
35
40
Galectin9
status
ICU
Ward

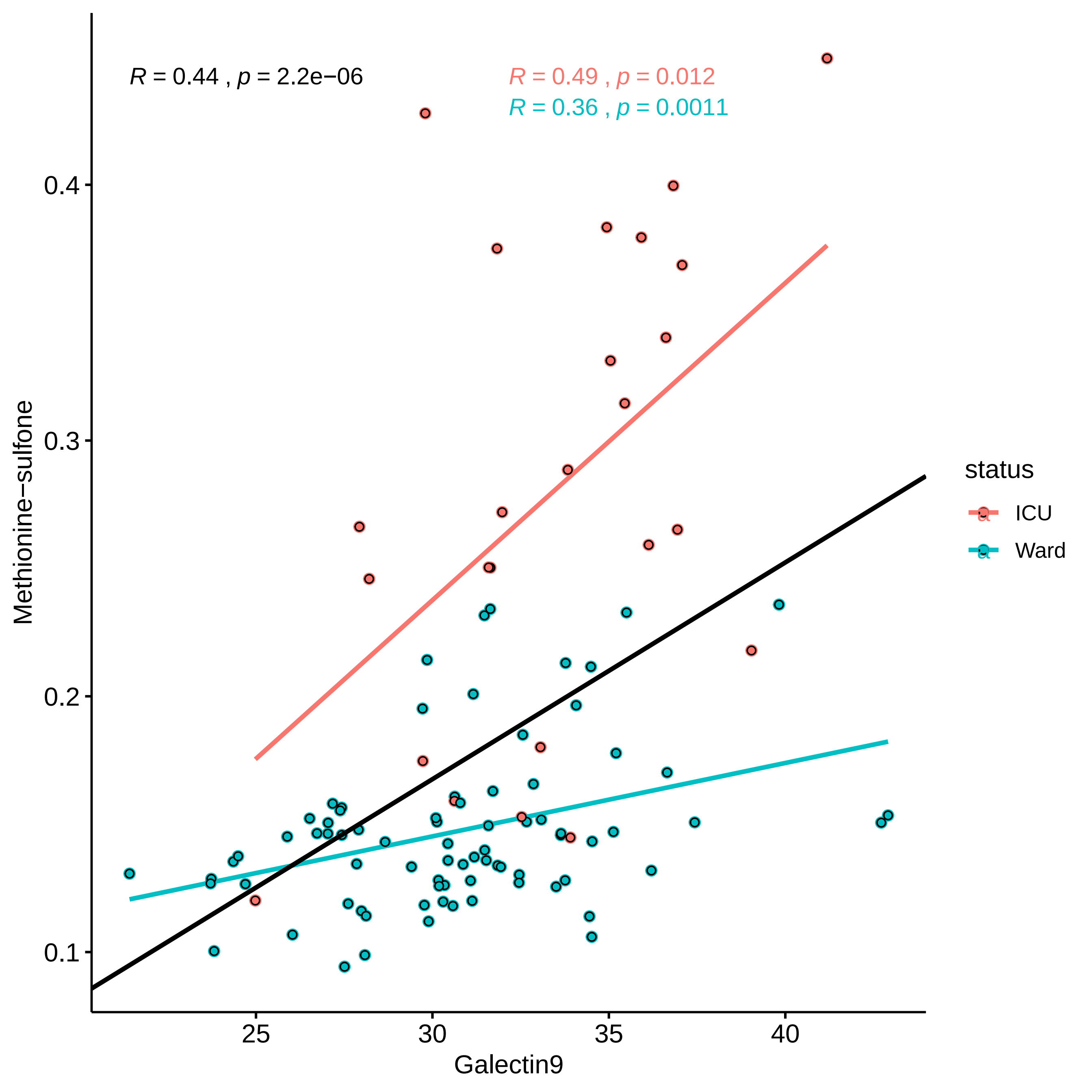

R = 0.44 , p = 2.2e−06
R = 0.49 , p = 0.012
R = 0.36 , p = 0.0011
Methionine−sulfone
0.1
0.2
0.3
0.4
25
30
35
40
Galectin9
status
ICU
Ward

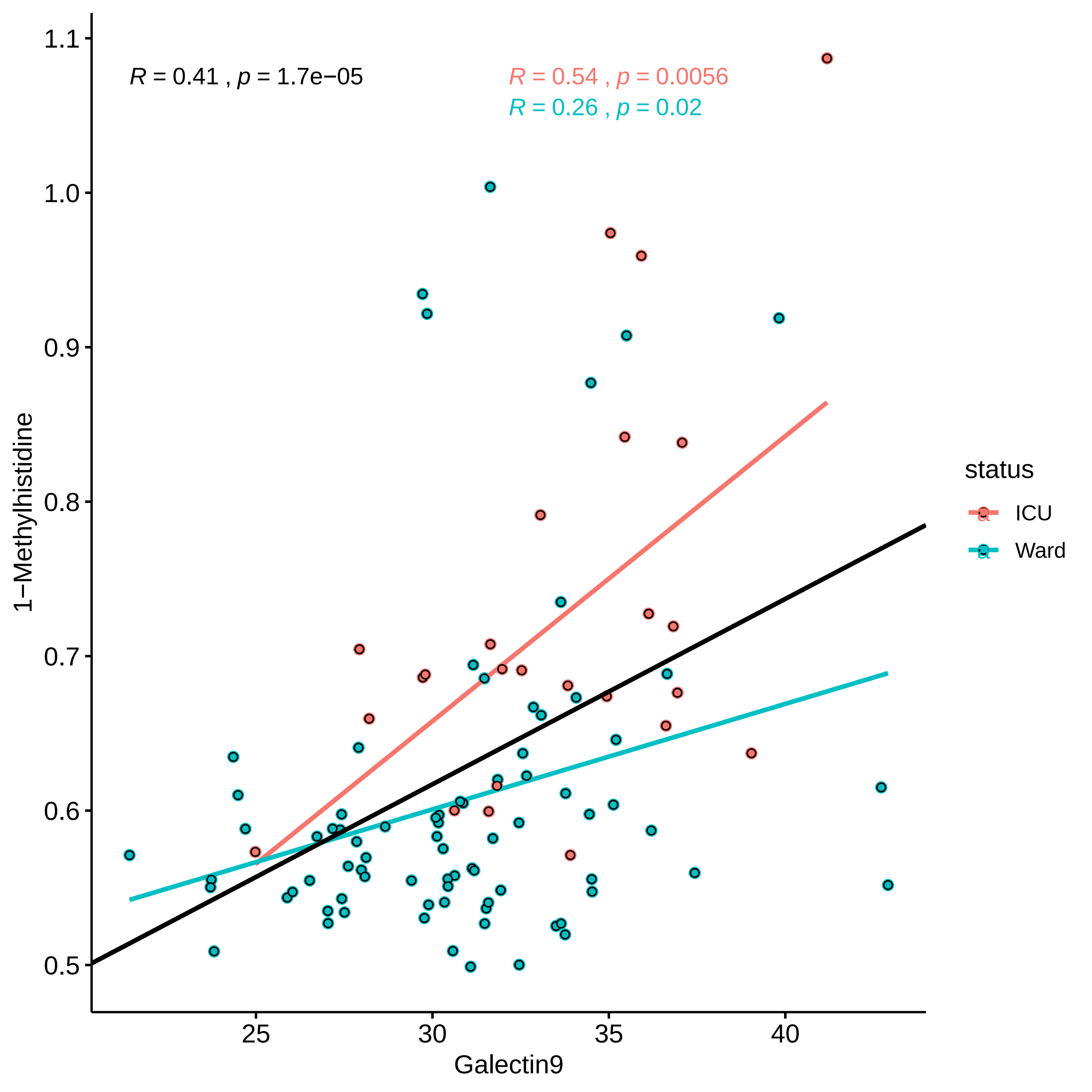

R = 0.41 , p = 1.7e−05
R = 0.54 , p = 0.0056
R = 0.26 , p = 0.02
1.1
1.0
0.9
0.8
0.7
0.6
0.5
1−Methylhistidine
25
30
35
40
Galectin9
status
ICU
Ward

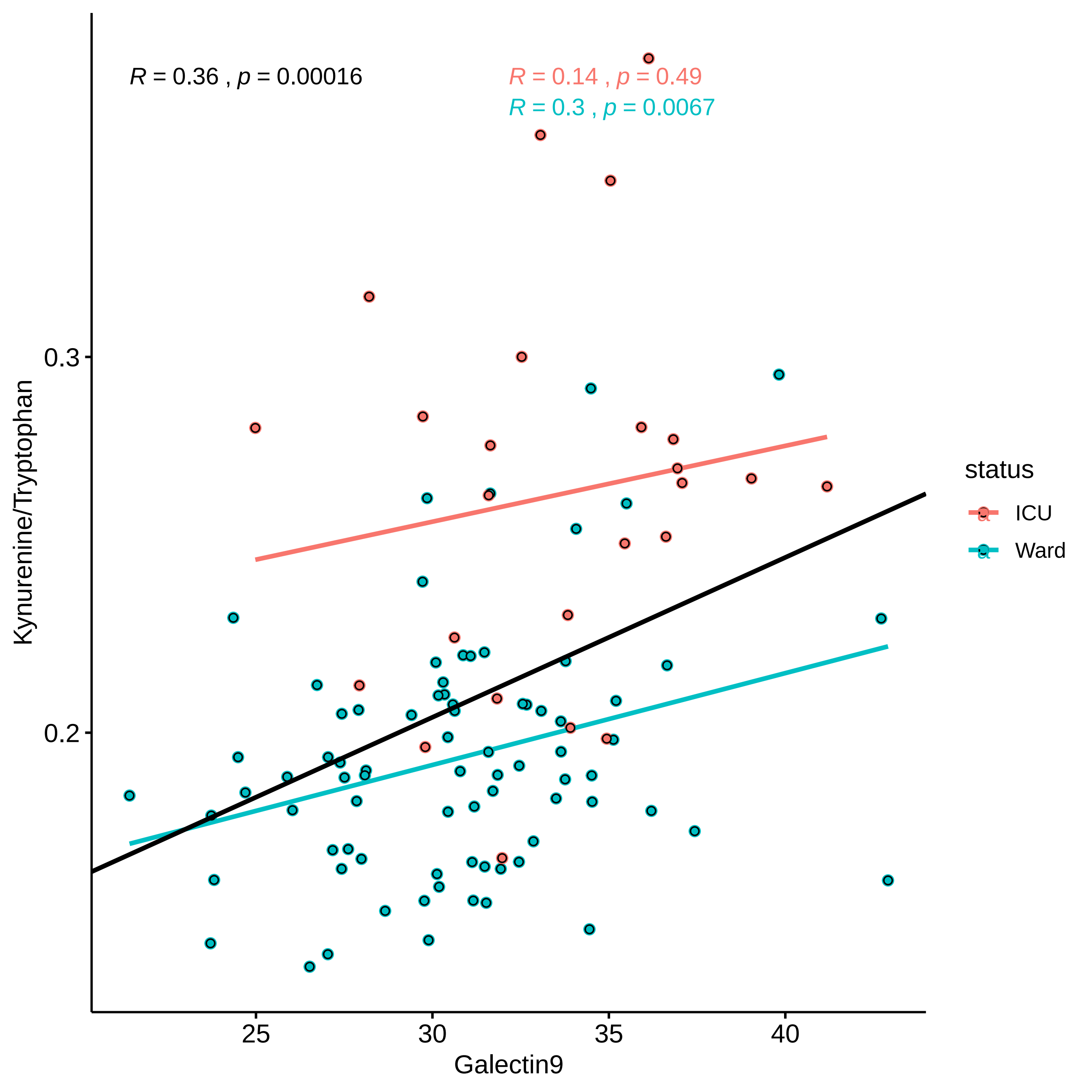

R = 0.36 , p = 0.00016
R = 0.14 , p = 0.49
R = 0.3 , p = 0.0067
Kynurenine/Tryptophan
0.3
0.2
25
30
35
40
Galectin9
status
ICU
Ward

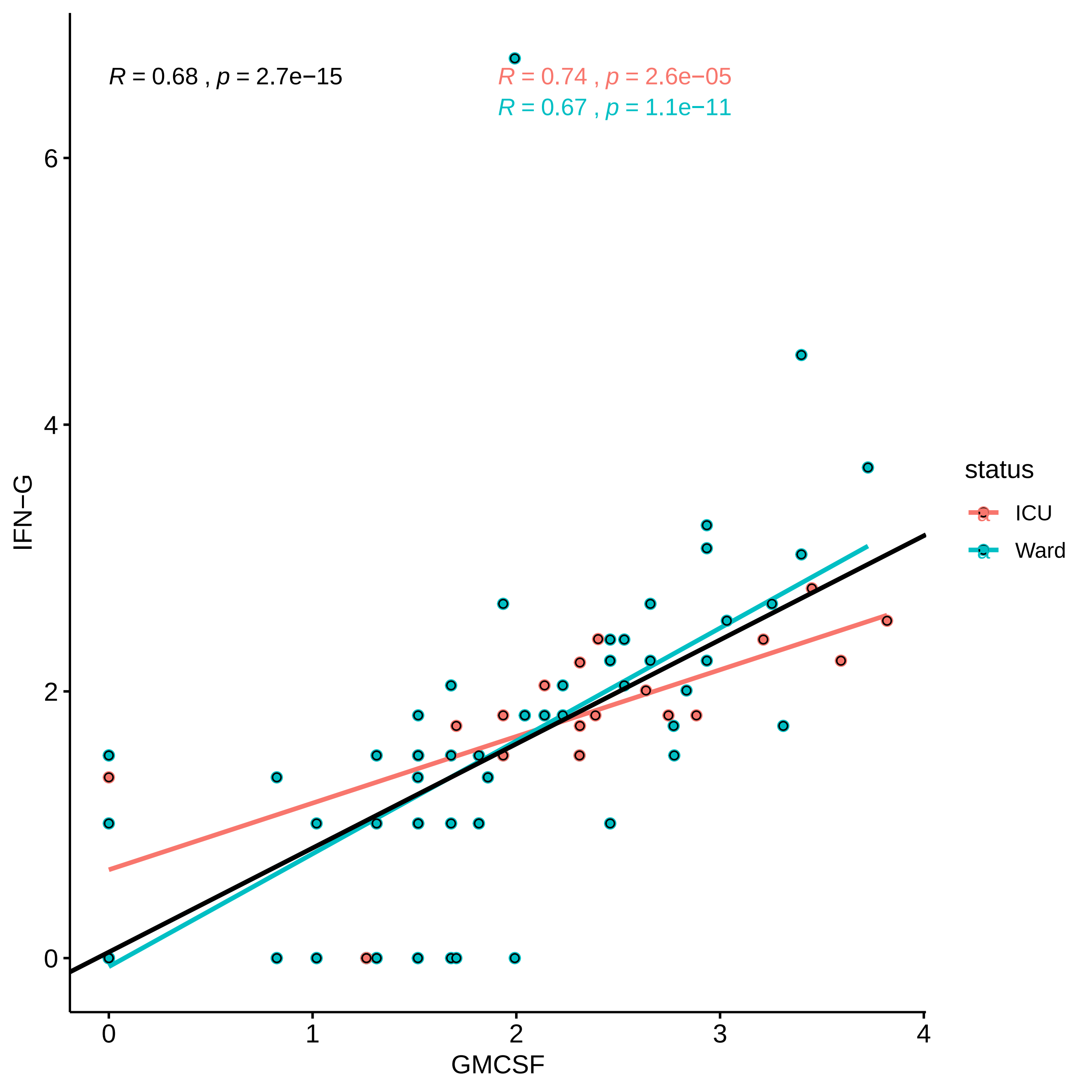

R = 0.68 , p = 2.7e−15
R = 0.74 , p = 2.6e−05
R = 0.67 , p = 1.1e−11
IFN−G
GMCSF
0
2
4
6
0
1
2
3
4
status
ICU
Ward

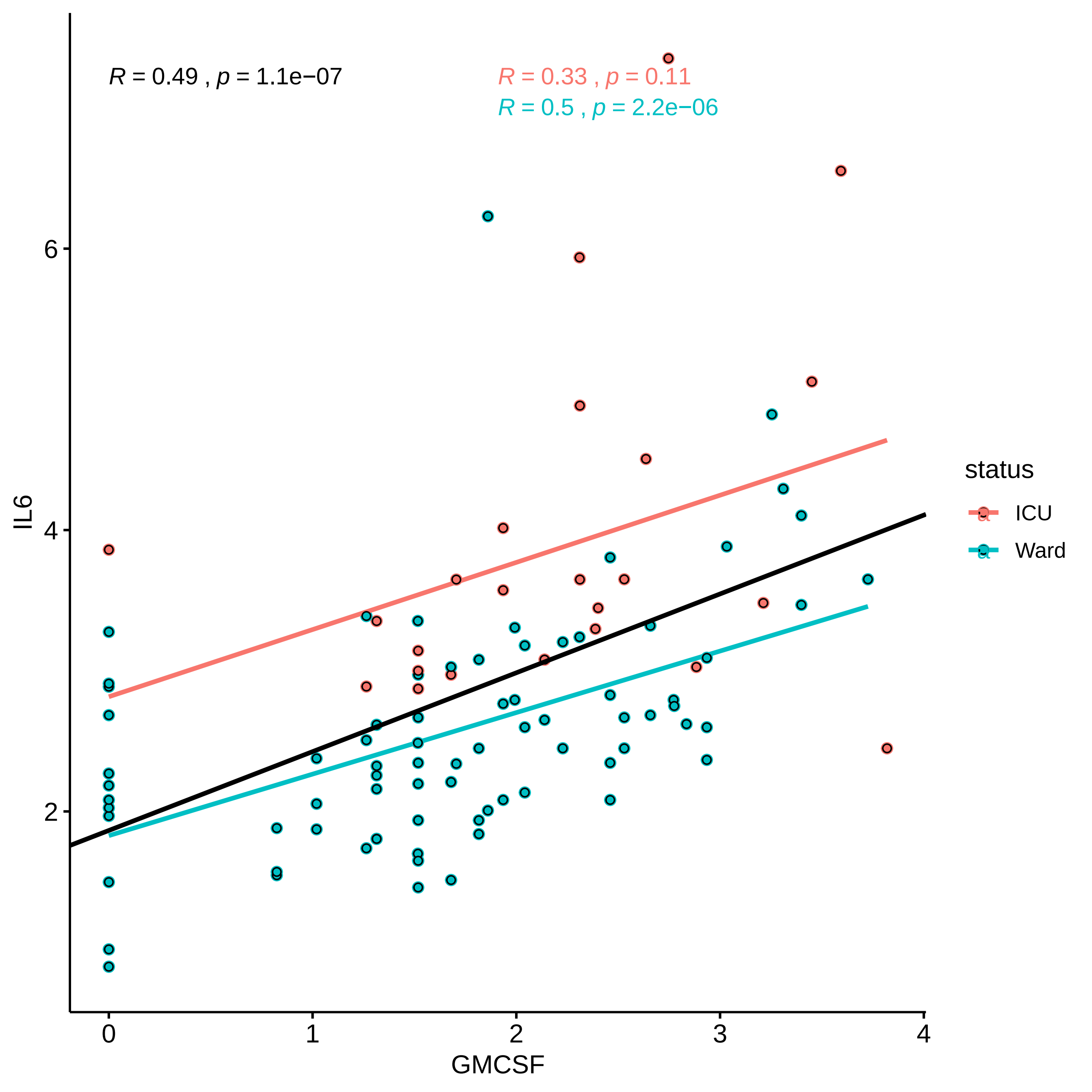

R = 0.49 , p = 1.1e−07
R = 0.33 , p = 0.11
R = 0.5 , p = 2.2e−06
IL6
6
4
2
0
1
2
3
4
GMCSF
status
ICU
Ward

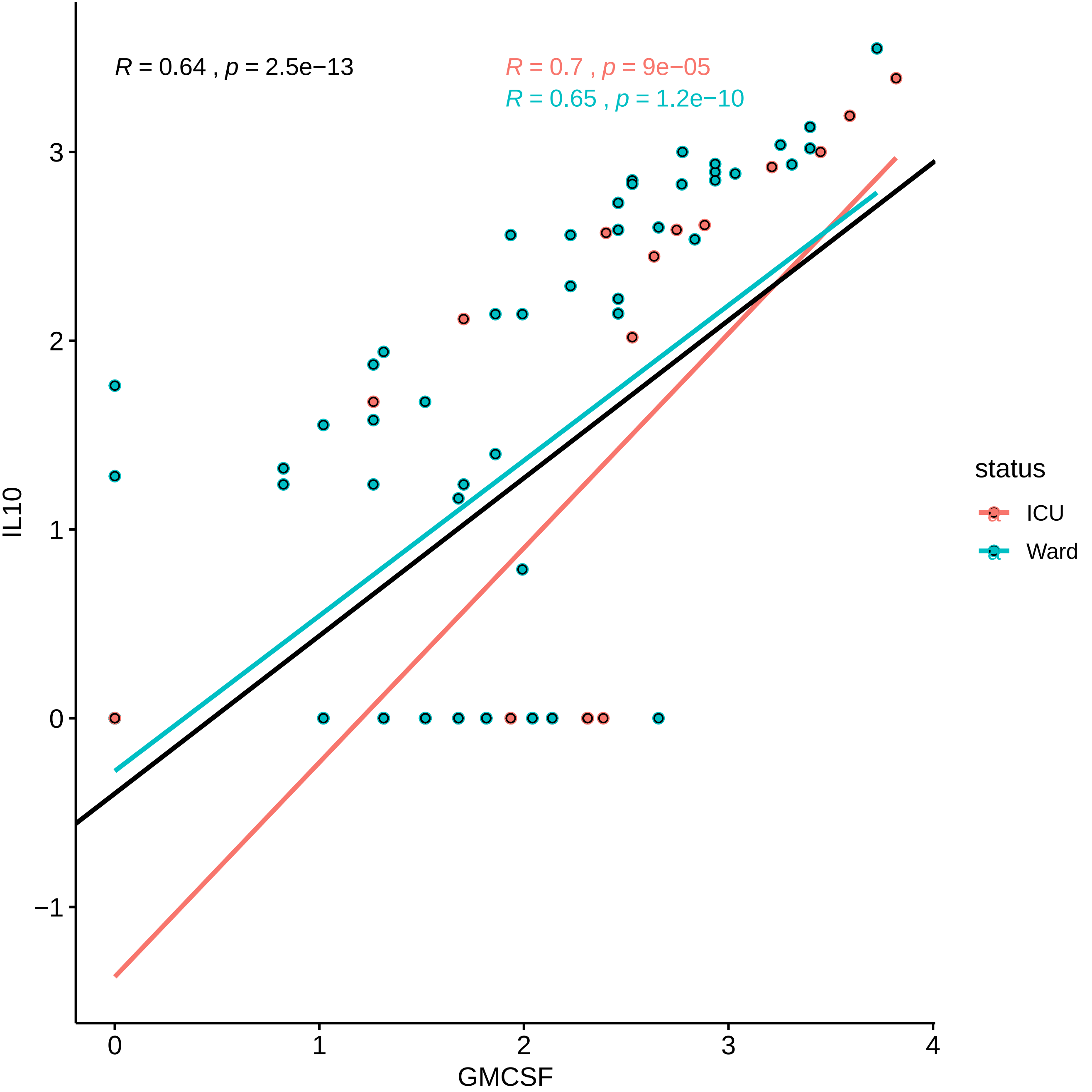
R = 0.64 , p = 2.5e−13
R = 0.7 , p = 9e−05
R = 0.65 , p = 1.2e−10
IL10
GMCSF
−1
0
1
2
3
0
1
2
3
4
status
ICU
Ward

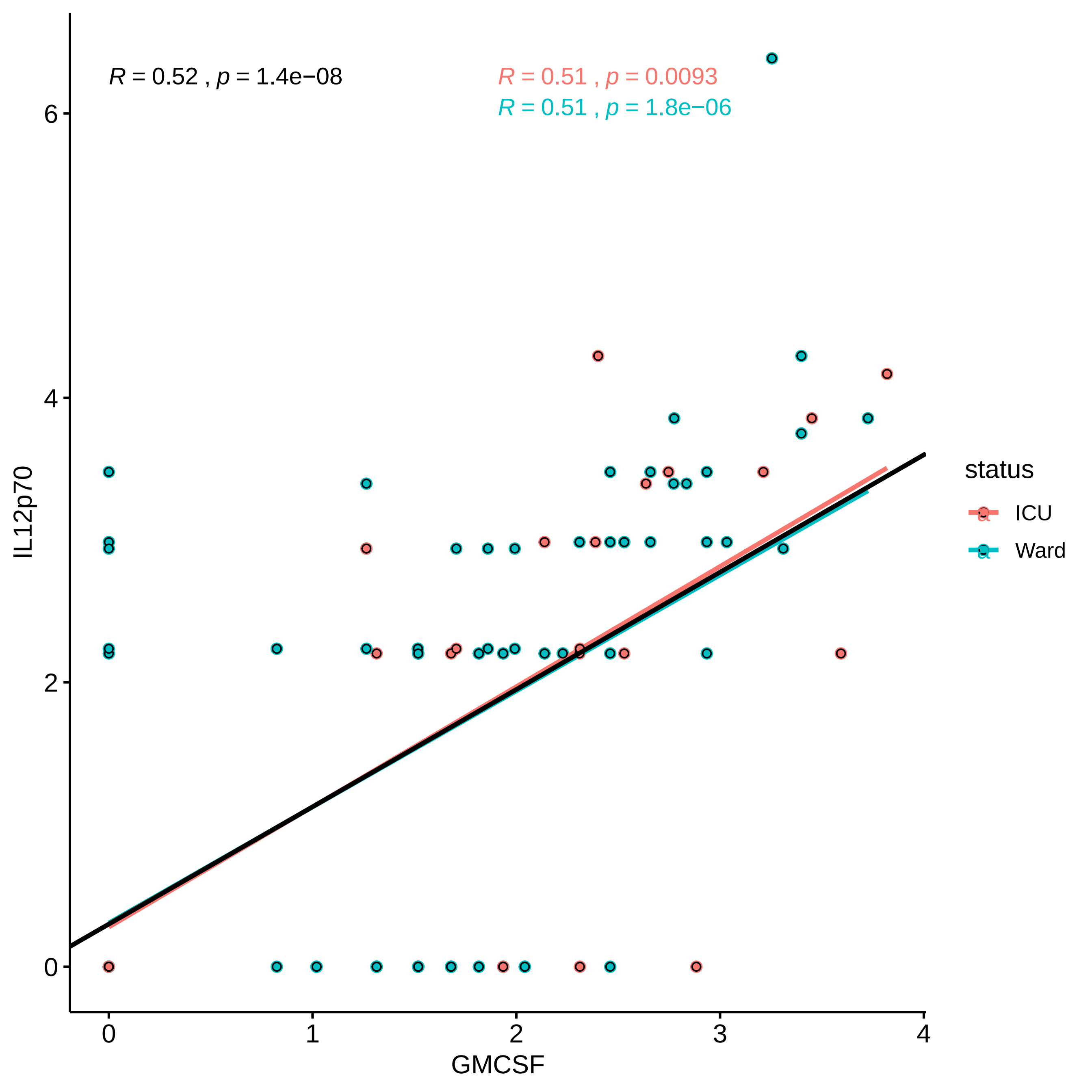

R = 0.52 , p = 1.4e−08
R = 0.51 , p = 0.0093
R = 0.51 , p = 1.8e−06
IL12p70
GMCSF
status
ICU
Ward
0
2
4
6
0
1
2
3
4

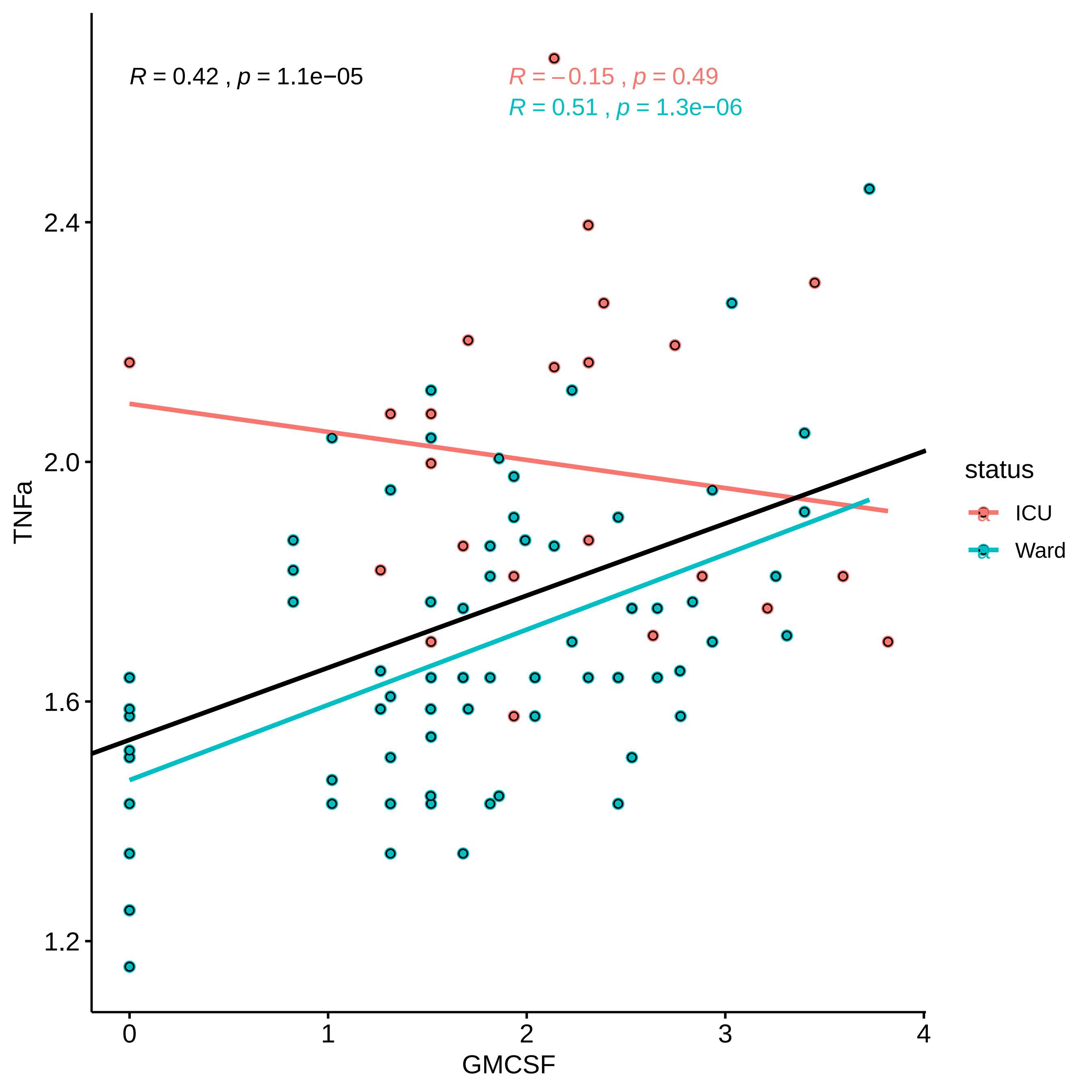

R = 0.42 , p = 1.1e−05
R = −0.15 , p = 0.49
R = 0.51 , p = 1.3e−06
2.4
2.0
1.6
1.2
TNFa
0
1
2
3
4
GMCSF
status
ICU
Ward

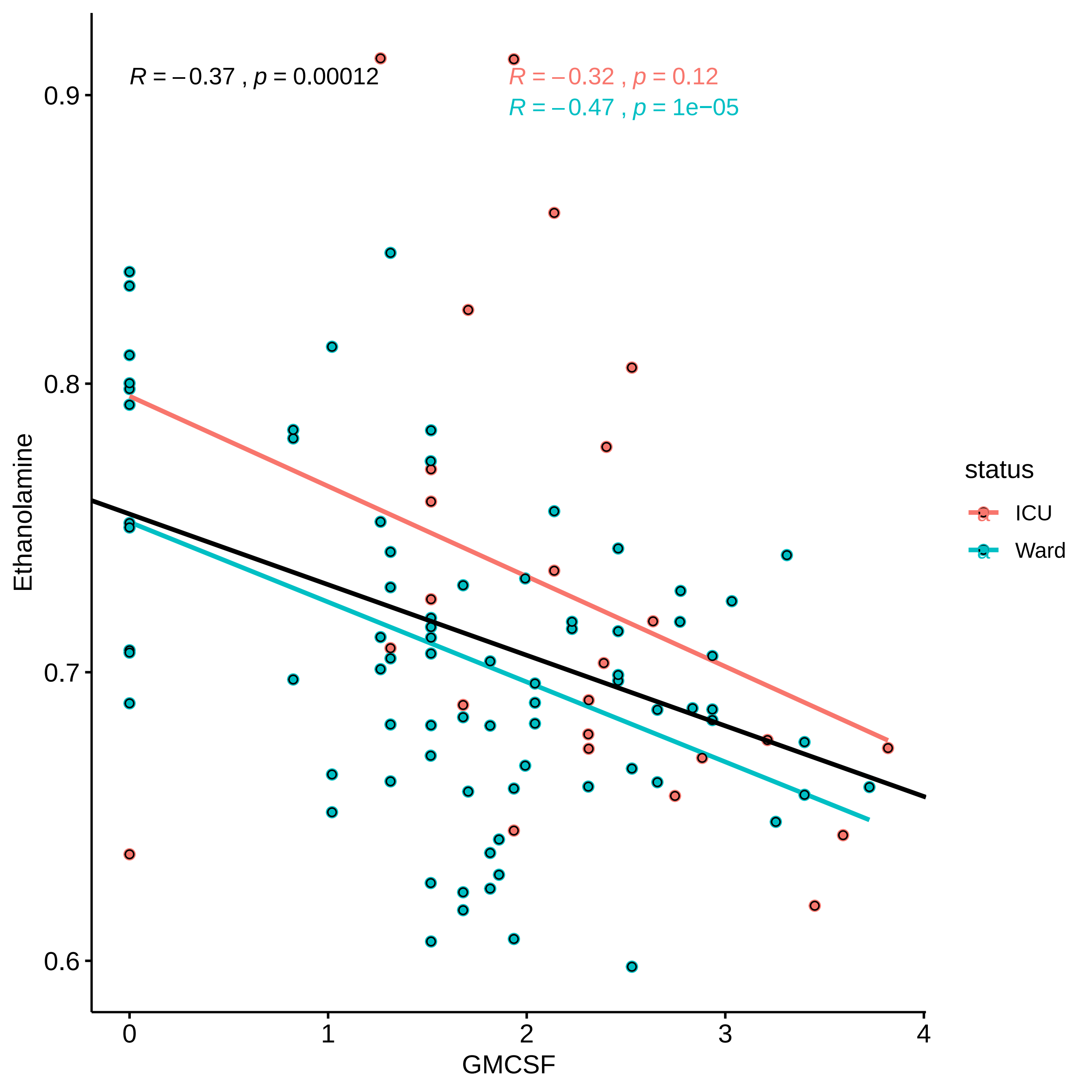

R = −0.37 , p = 0.00012
R = −0.32 , p = 0.12
R = −0.47 , p = 1e−05
Ethanolamine
0.9
0.8
0.7
0.6
0
1
2
3
4
GMCSF
status
ICU
Ward

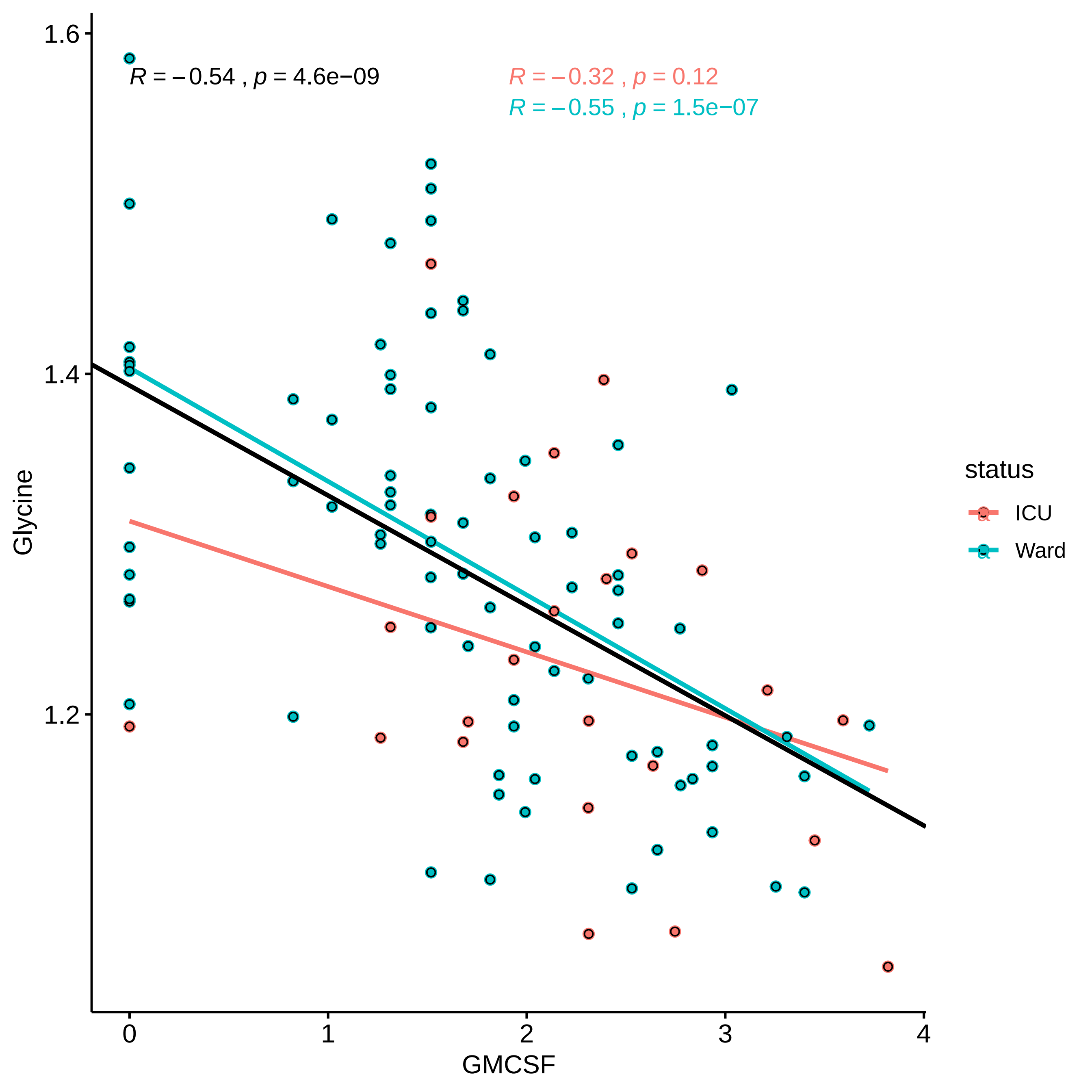
R = −0.54 , p = 4.6e−09
R = −0.32 , p = 0.12
R = −0.55 , p = 1.5e−07
1.6
1.4
1.2
Glycine
0
1
2
3
4
GMCSF
status
ICU
Ward

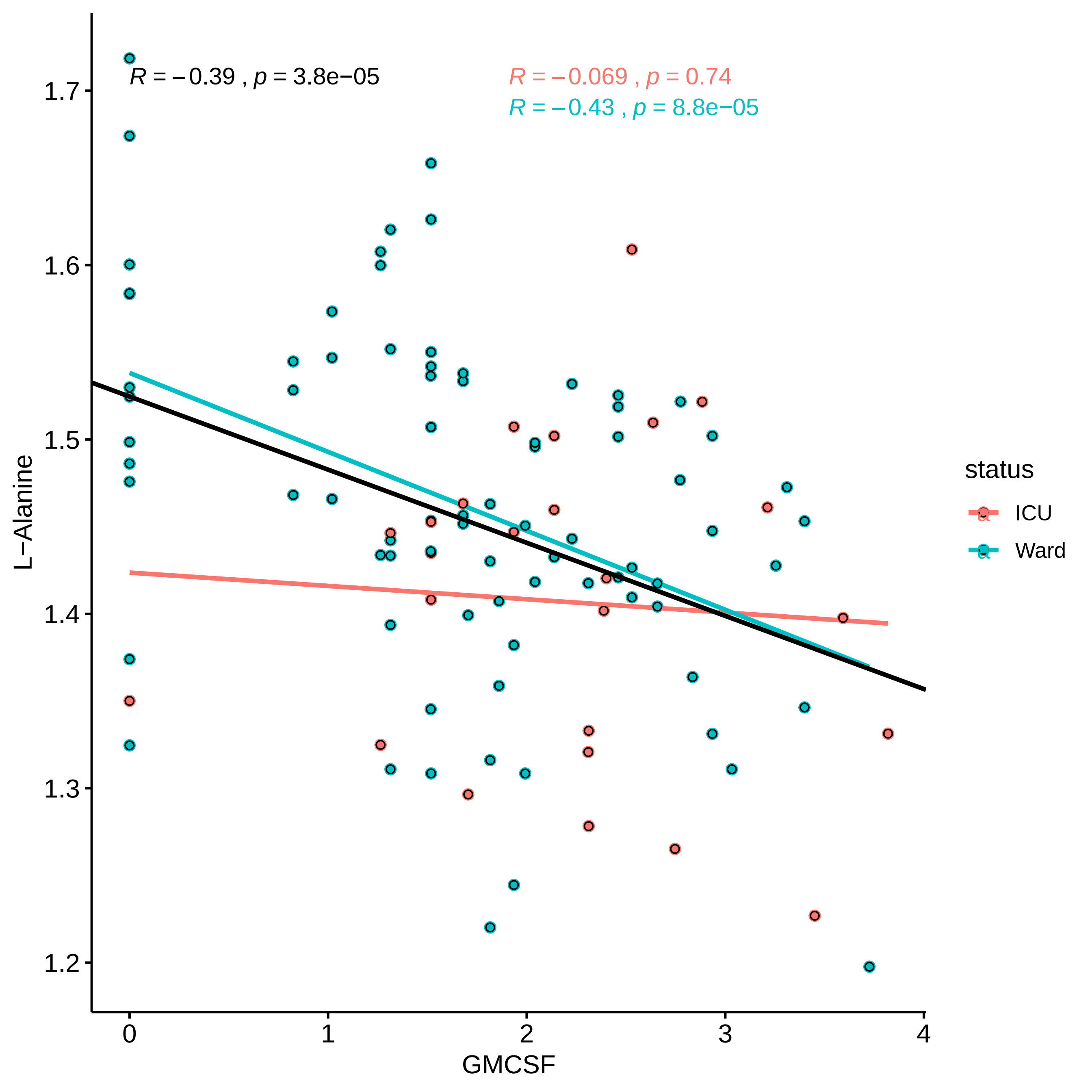

R = −0.39 , p = 3.8e−05
R = −0.069 , p = 0.74
R = −0.43 , p = 8.8e−05
L−Alanine
1.7
1.6
1.5
1.4
1.3
1.2
0
1
2
3
4
GMCSF
status
ICU
Ward

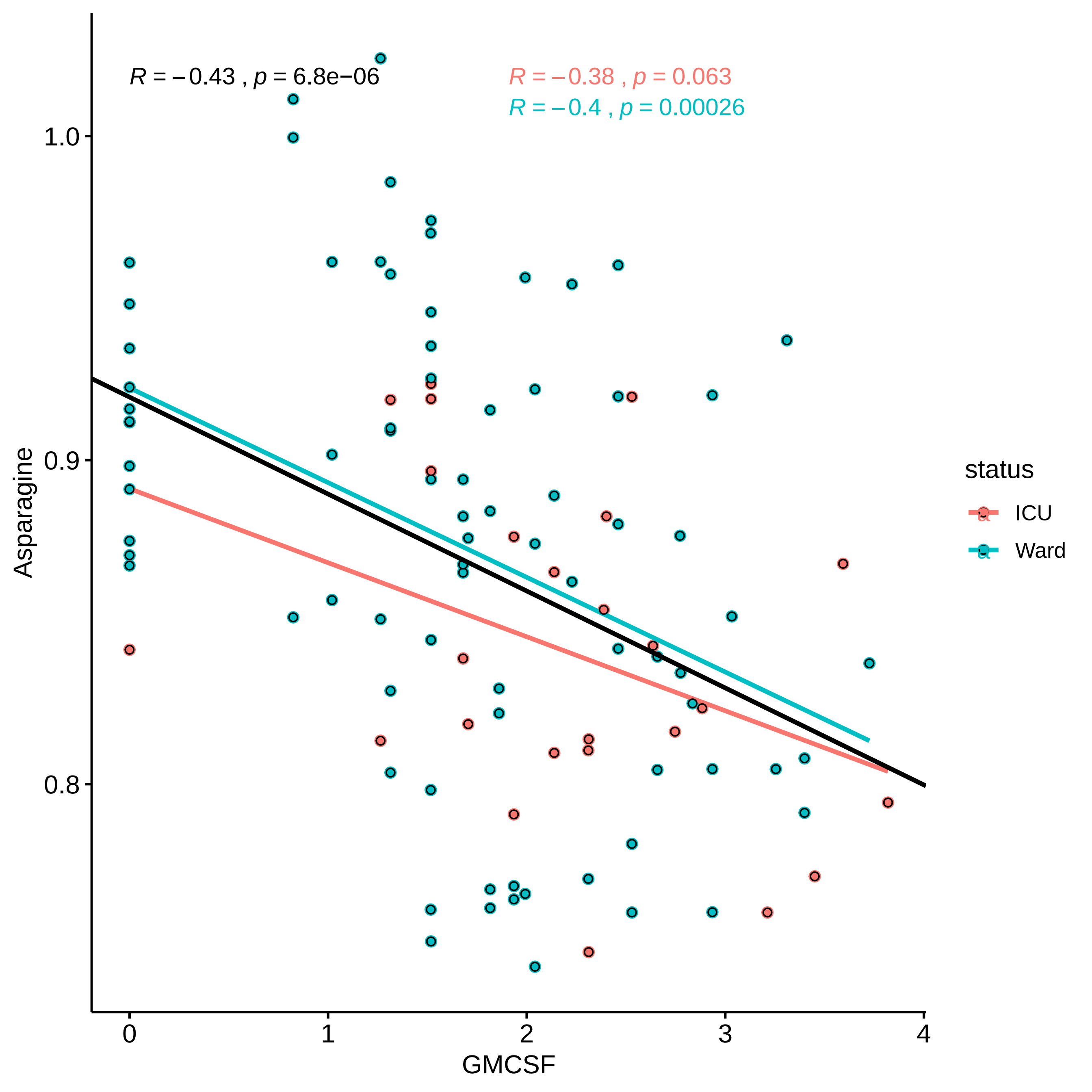

R = −0.43 , p = 6.8e−06
R = −0.38 , p = 0.063
R = −0.4 , p = 0.00026
1.0
0.9
0.8
Asparagine
0
1
2
3
4
GMCSF
status
ICU
Ward

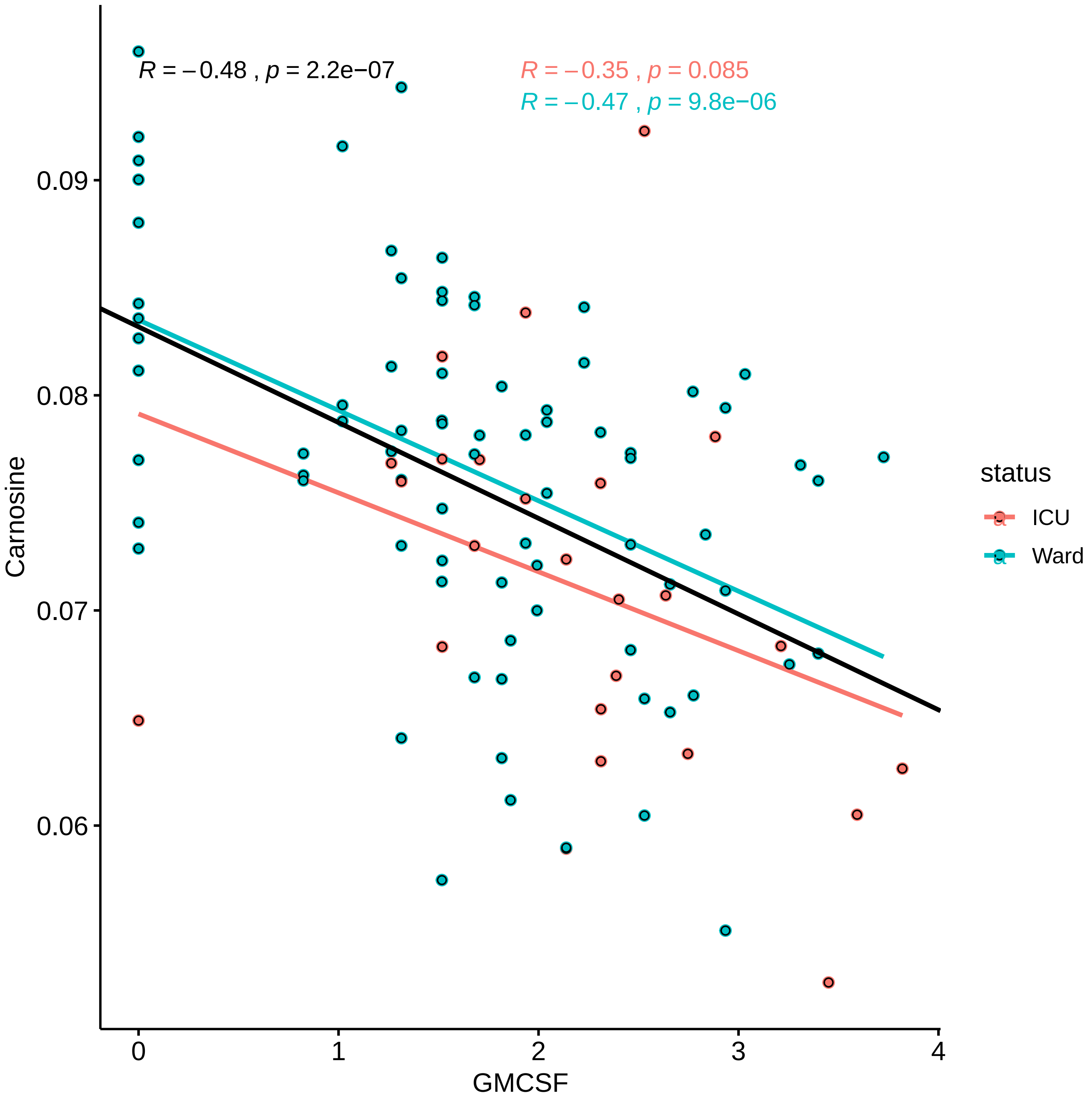

R = −0.48 , p = 2.2e−07
R = −0.35 , p = 0.085
R = −0.47 , p = 9.8e−06
0.09
0.08
0.07
0.06
Carnosine
0
1
2
3
4
GMCSF
status
ICU
Ward

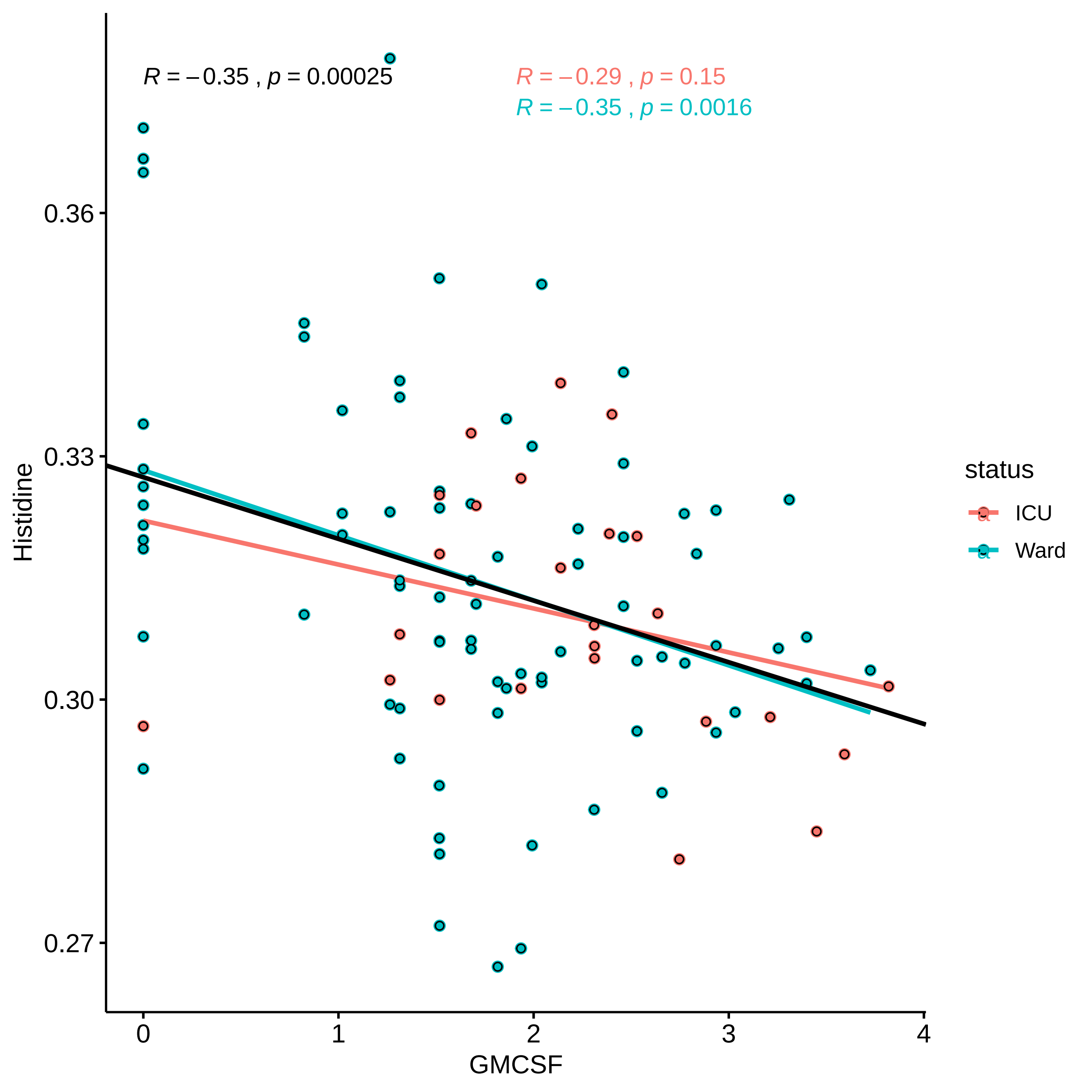
R = −0.35 , p = 0.00025
R = −0.29 , p = 0.15
R = −0.35 , p = 0.0016
Histidine
0.36
0.33
0.30
0.27
0
1
2
3
4
GMCSF
status
ICU
Ward

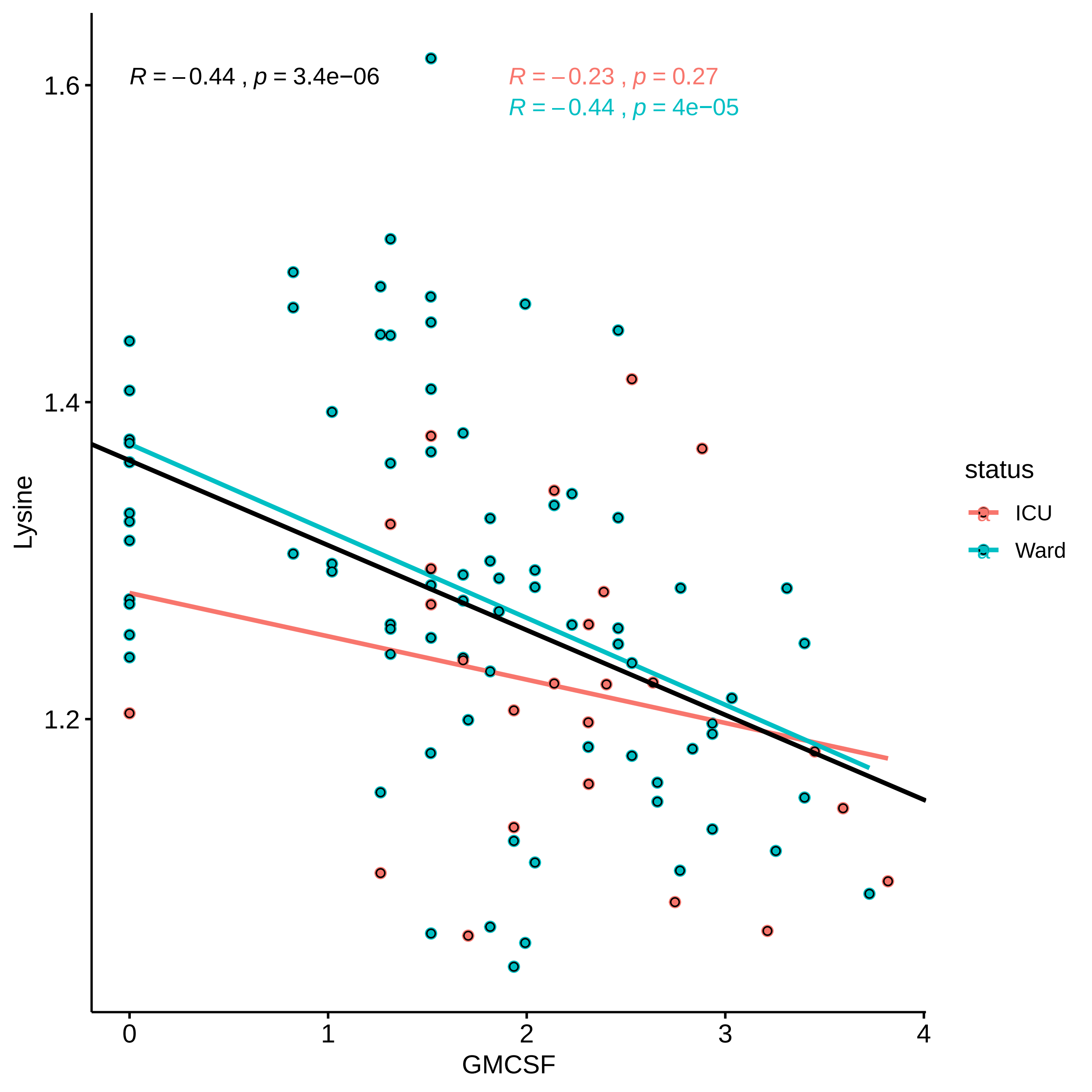

R = −0.44 , p = 3.4e−06
R = −0.23 , p = 0.27
R = −0.44 , p = 4e−05
1.6
1.4
1.2
Lysine
0
1
2
3
4
GMCSF
status
ICU
Ward

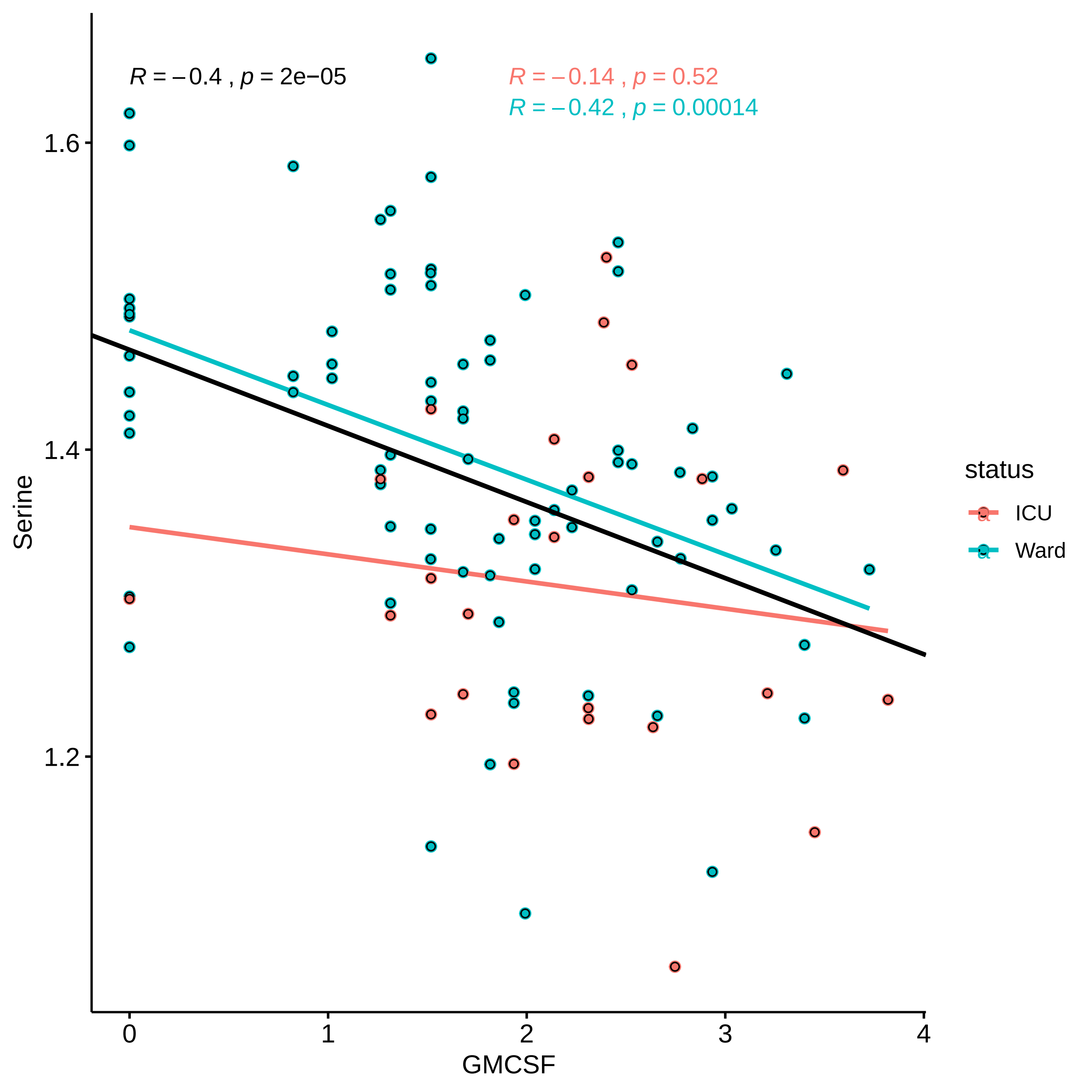

R = −0.4 , p = 2e−05
R = −0.14 , p = 0.52
R = −0.42 , p = 0.00014
1.6
1.4
1.2
Serine
0
1
2
3
4
GMCSF
status
ICU
Ward

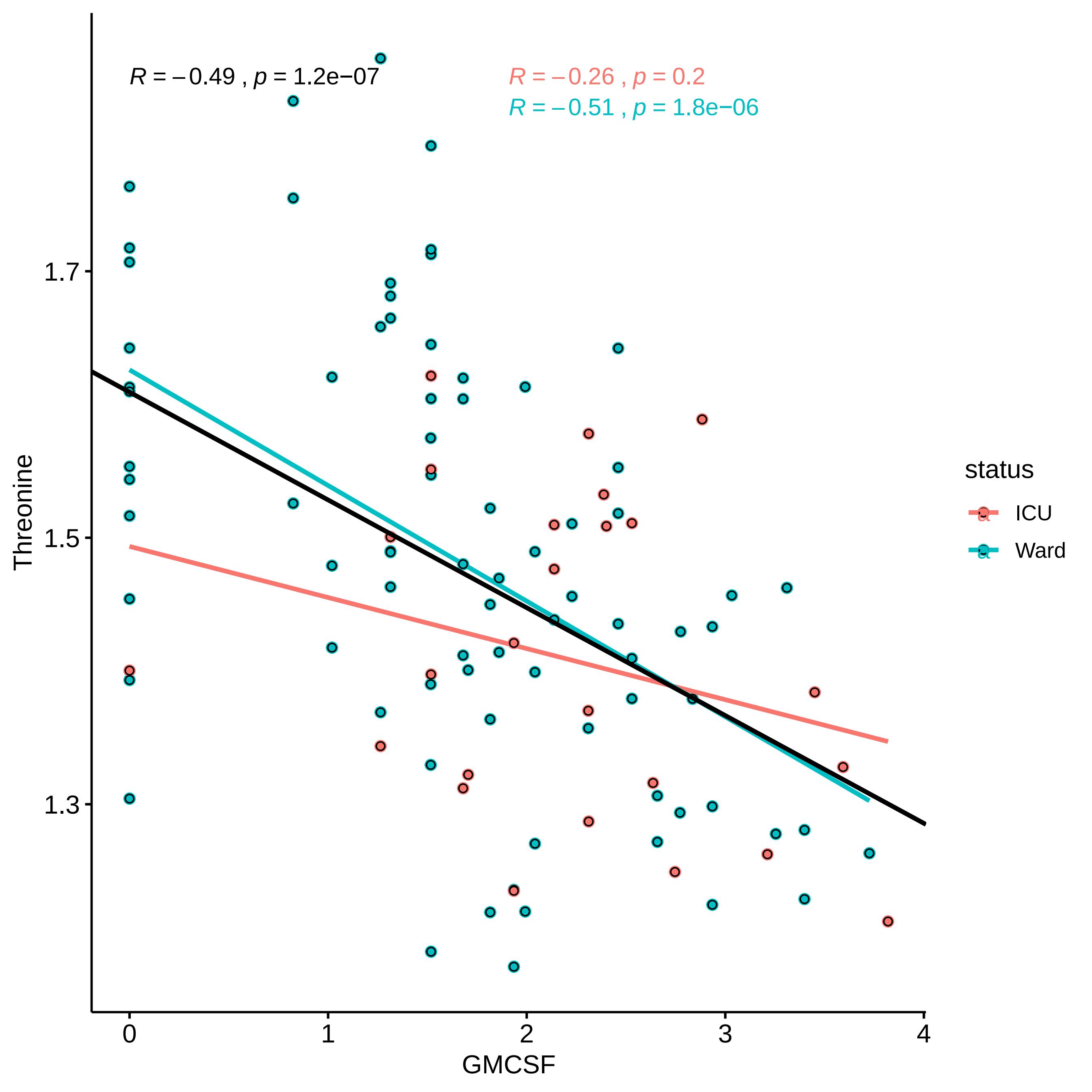

R = −0.49 , p = 1.2e−07
R = −0.26 , p = 0.2
R = −0.51 , p = 1.8e−06
Threonine
1.7
1.5
1.3
0
1
2
3
4
GMCSF
status
ICU
Ward

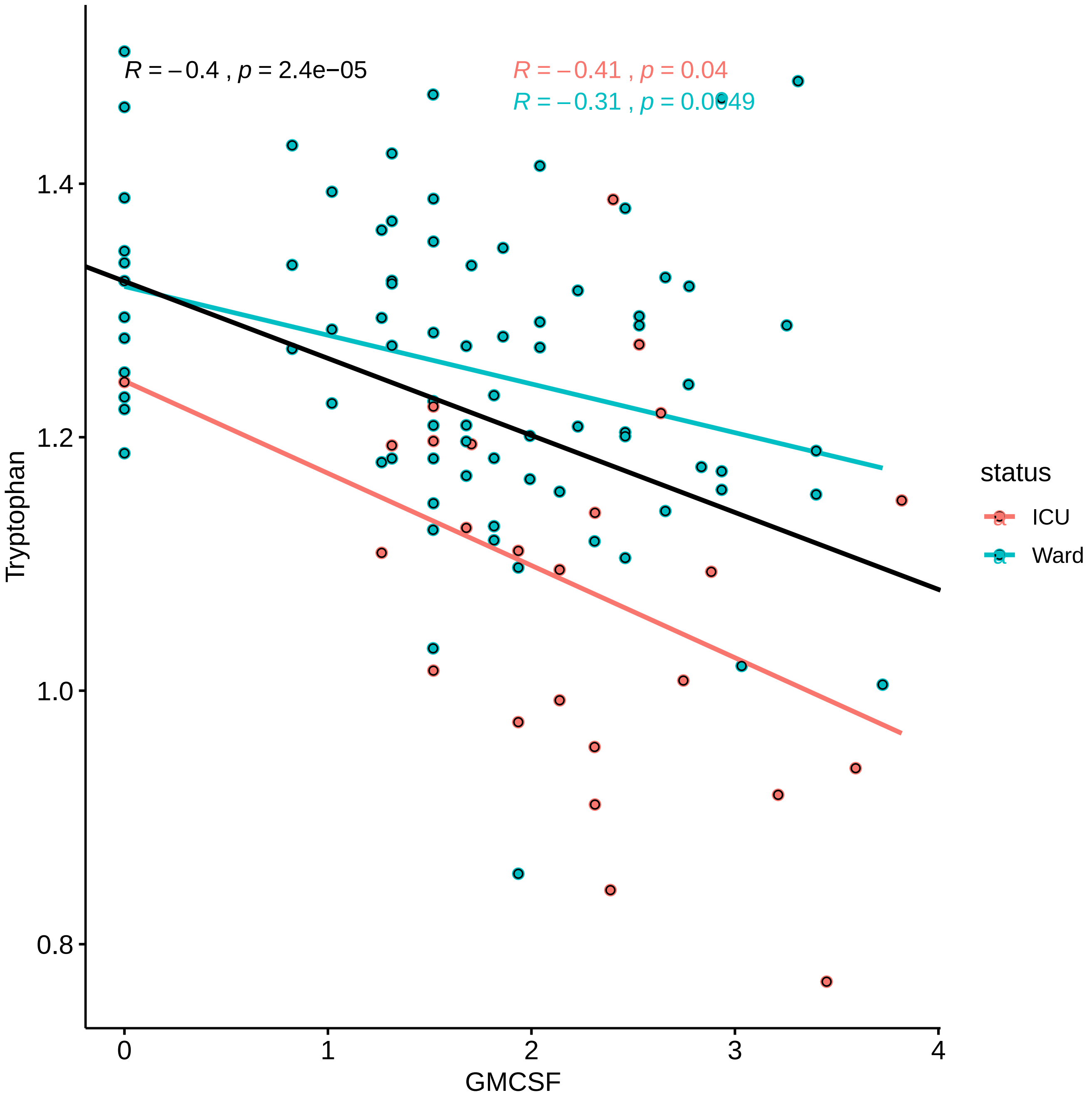

R = −0.4 , p = 2.4e−05
R = −0.41 , p = 0.04
R = −0.31 , p = 0.0049
Tryptophan
1.4
1.2
1.0
0.8
0
1
2
3
4
GMCSF
status
ICU
Ward

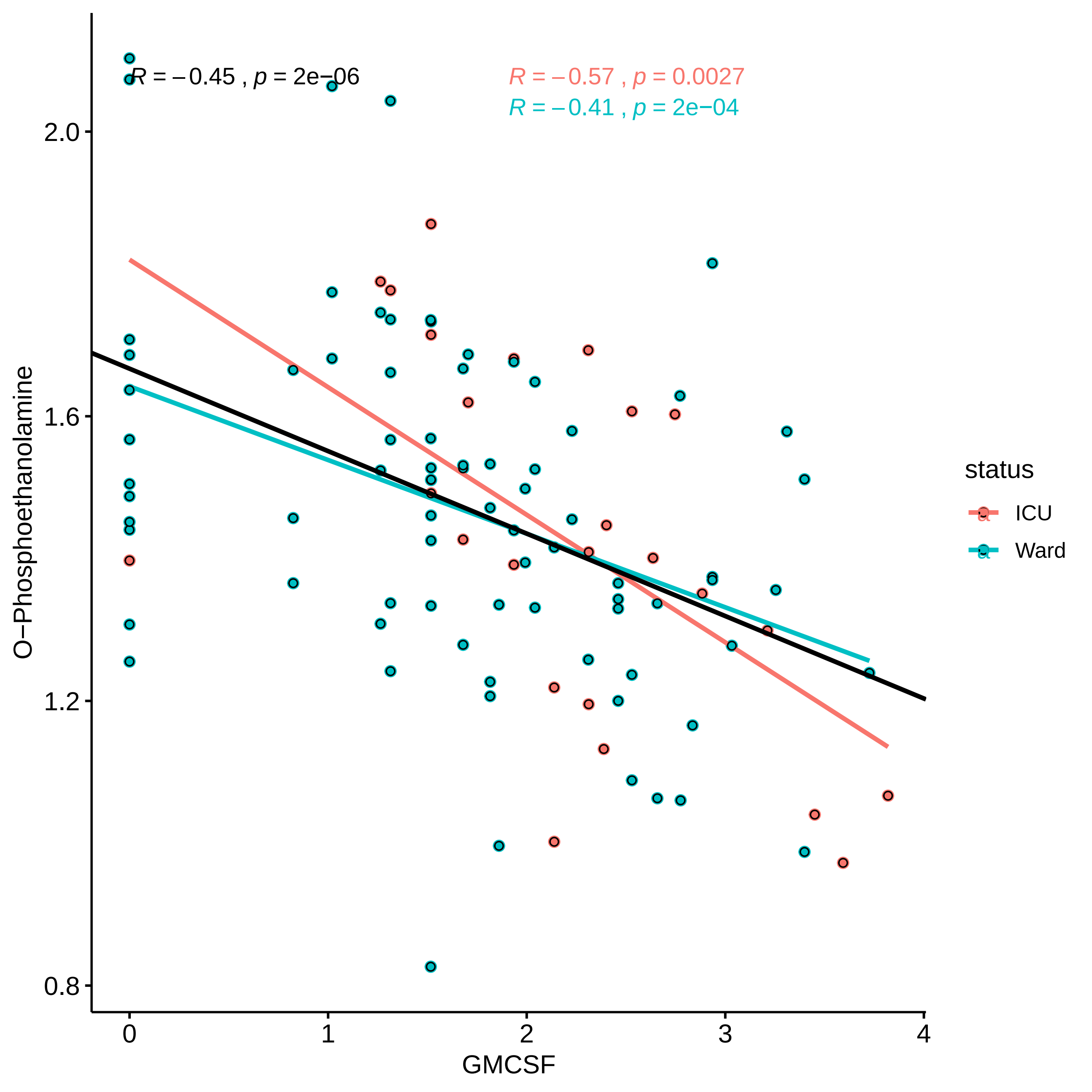

R = −0.45 , p = 2e−06
R = −0.57 , p = 0.0027
R = −0.41 , p = 2e−04
O−Phosphoethanolamine
GMCSF
2.0
1.6
1.2
0.8
0
1
2
3
4
status
ICU
Ward

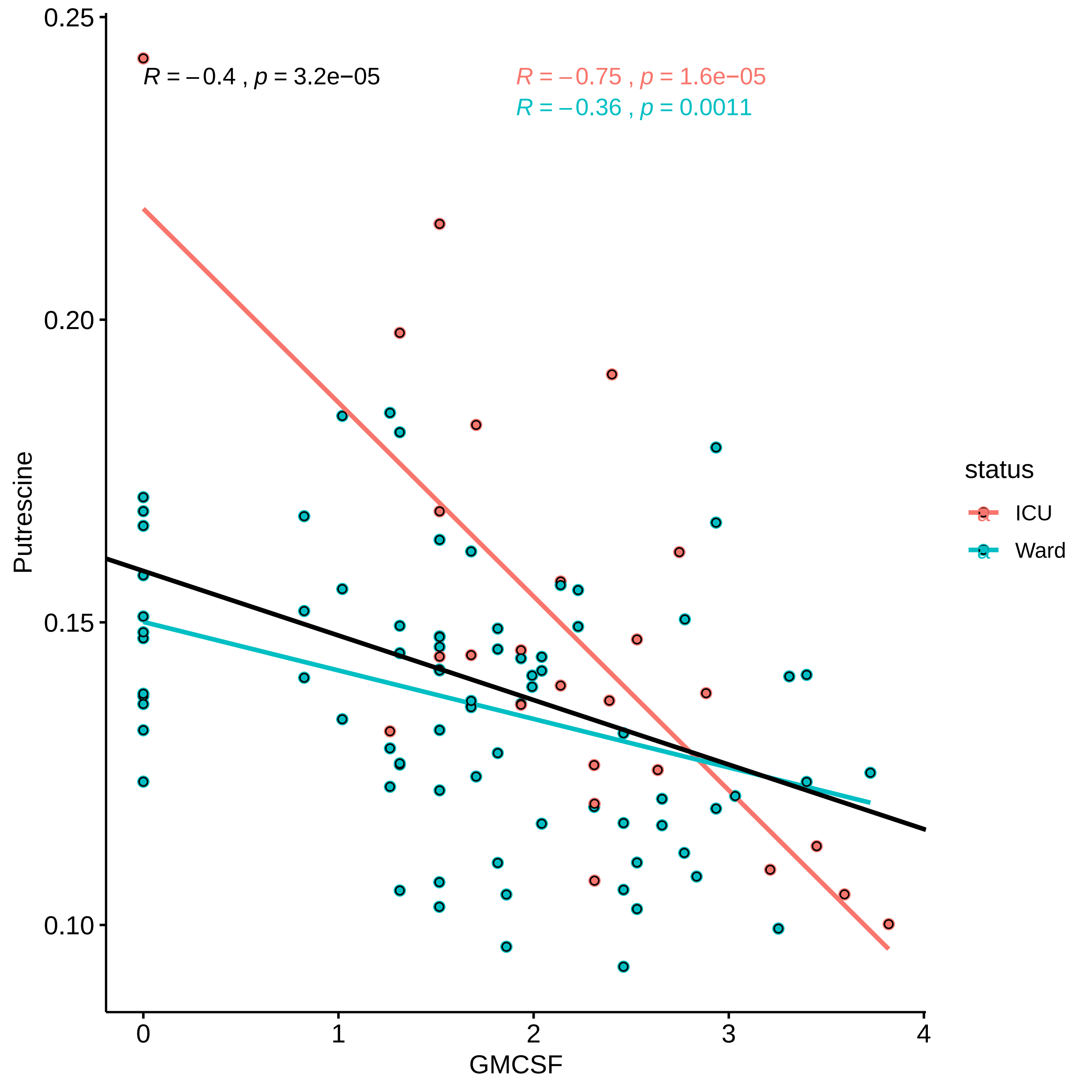

R = −0.4 , p = 3.2e−05
R = −0.75 , p = 1.6e−05
R = −0.36 , p = 0.0011
0.25
0.20
0.15
0.10
Putrescine
0
1
2
3
4
GMCSF
status
ICU
Ward

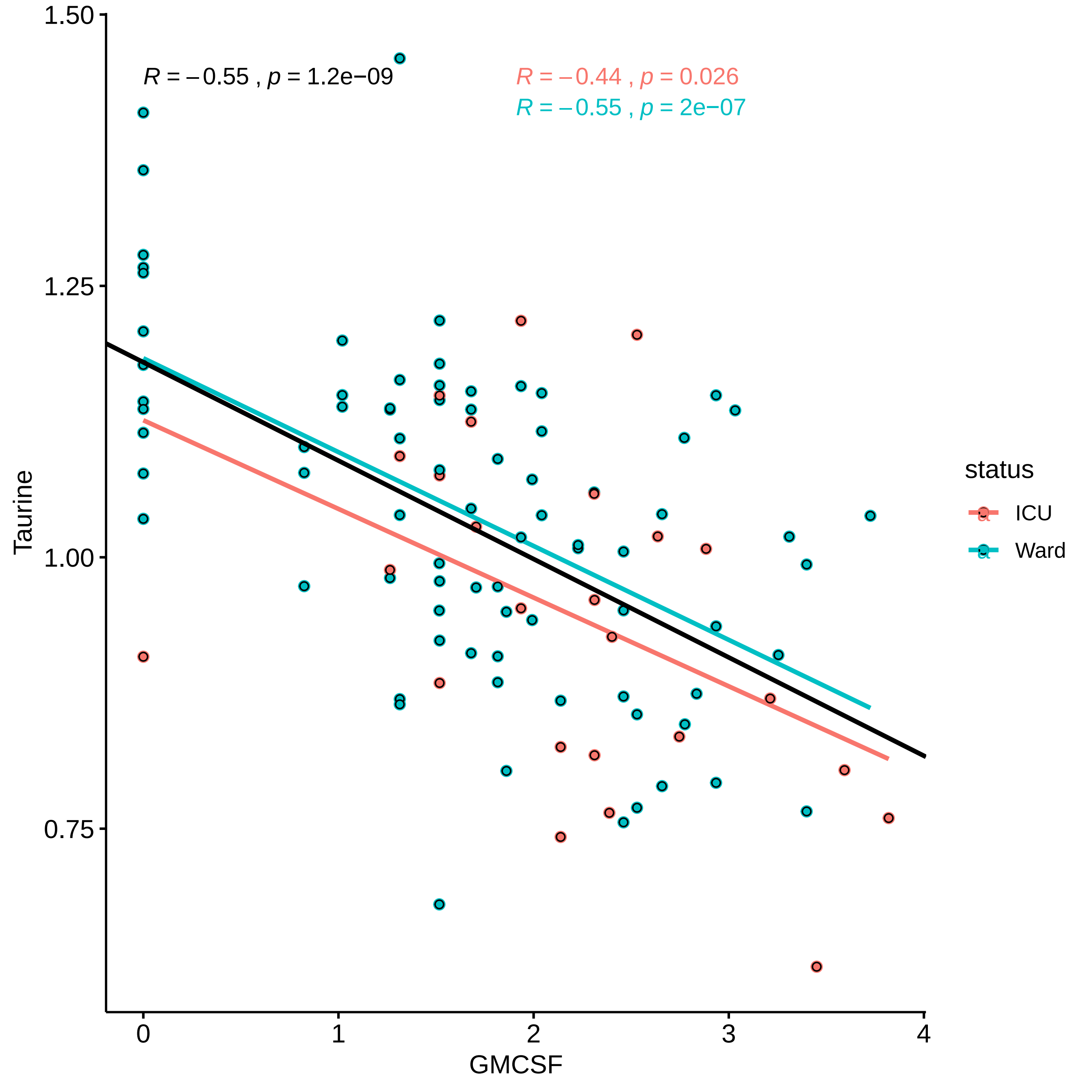

R = −0.55 , p = 1.2e−09
R = −0.44 , p = 0.026
R = −0.55 , p = 2e−07
1.50
1.25
1.00
0.75
Taurine
0
1
2
3
4
GMCSF
status
ICU
Ward

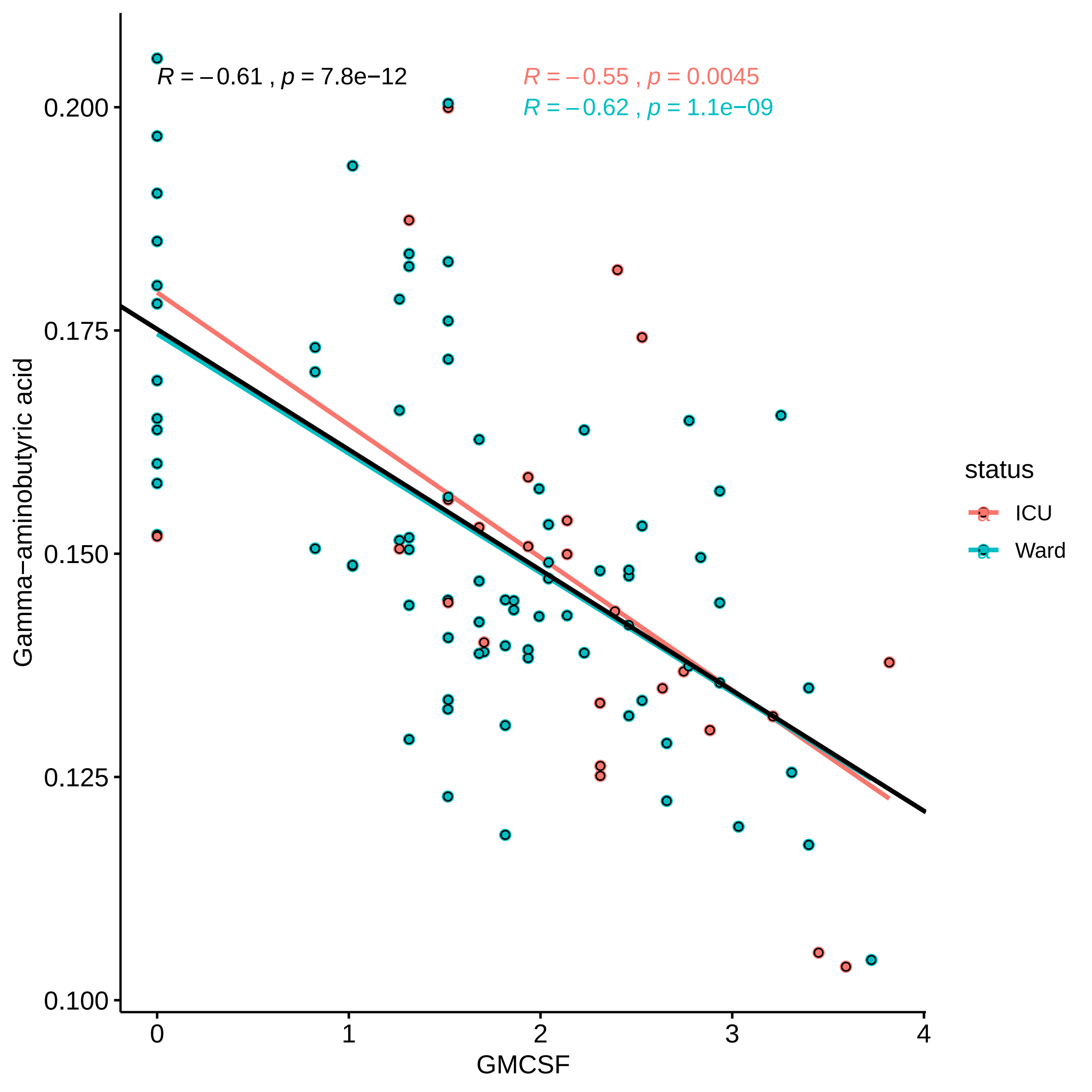

R = −0.61 , p = 7.8e−12
R = −0.55 , p = 0.0045
R = −0.62 , p = 1.1e−09
0.200
0.175
0.150
0.125
0.100
Gamma−aminobutyric acid
0
1
2
3
4
GMCSF
status
ICU
Ward

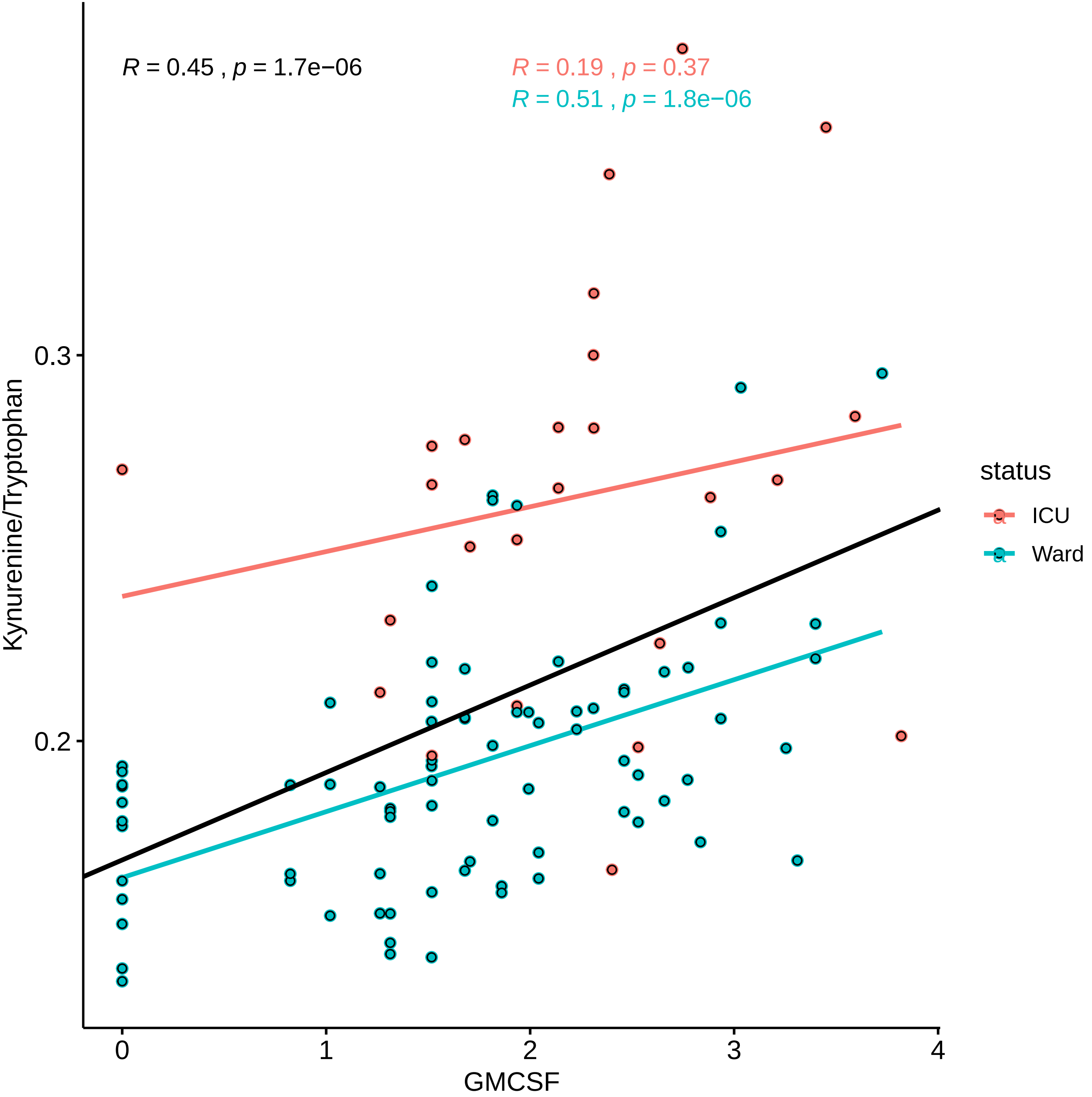

R = 0.45 , p = 1.7e−06
R = 0.19 , p = 0.37
R = 0.51 , p = 1.8e−06
Kynurenine/Tryptophan
0.3
0.2
0
1
2
3
4
GMCSF
status
ICU
Ward

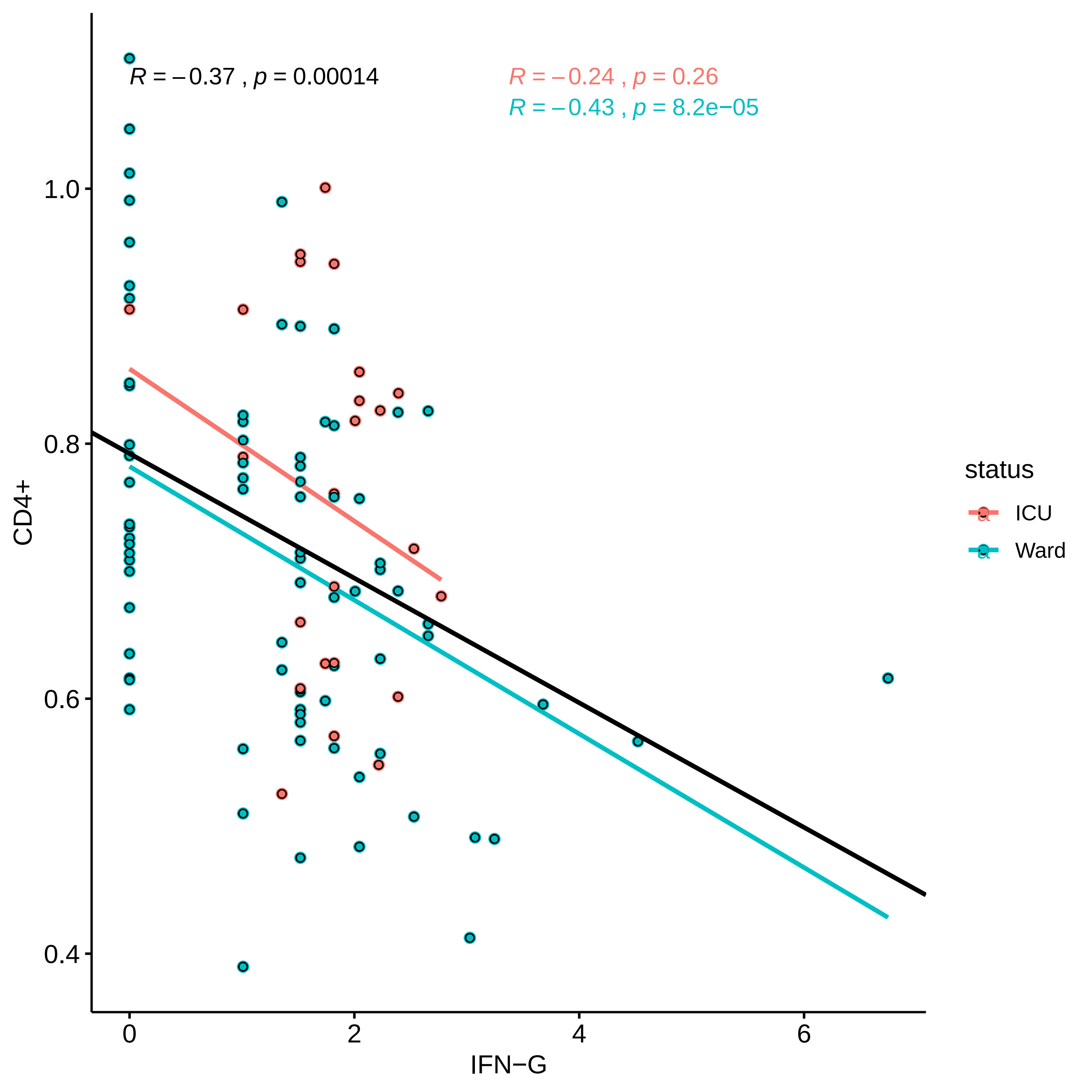

R = −0.37 , p = 0.00014
R = −0.24 , p = 0.26
R = −0.43 , p = 8.2e−05
CD4+
1.0
0.8
0.6
0.4
0
2
4
6
IFN−G
status
ICU
Ward

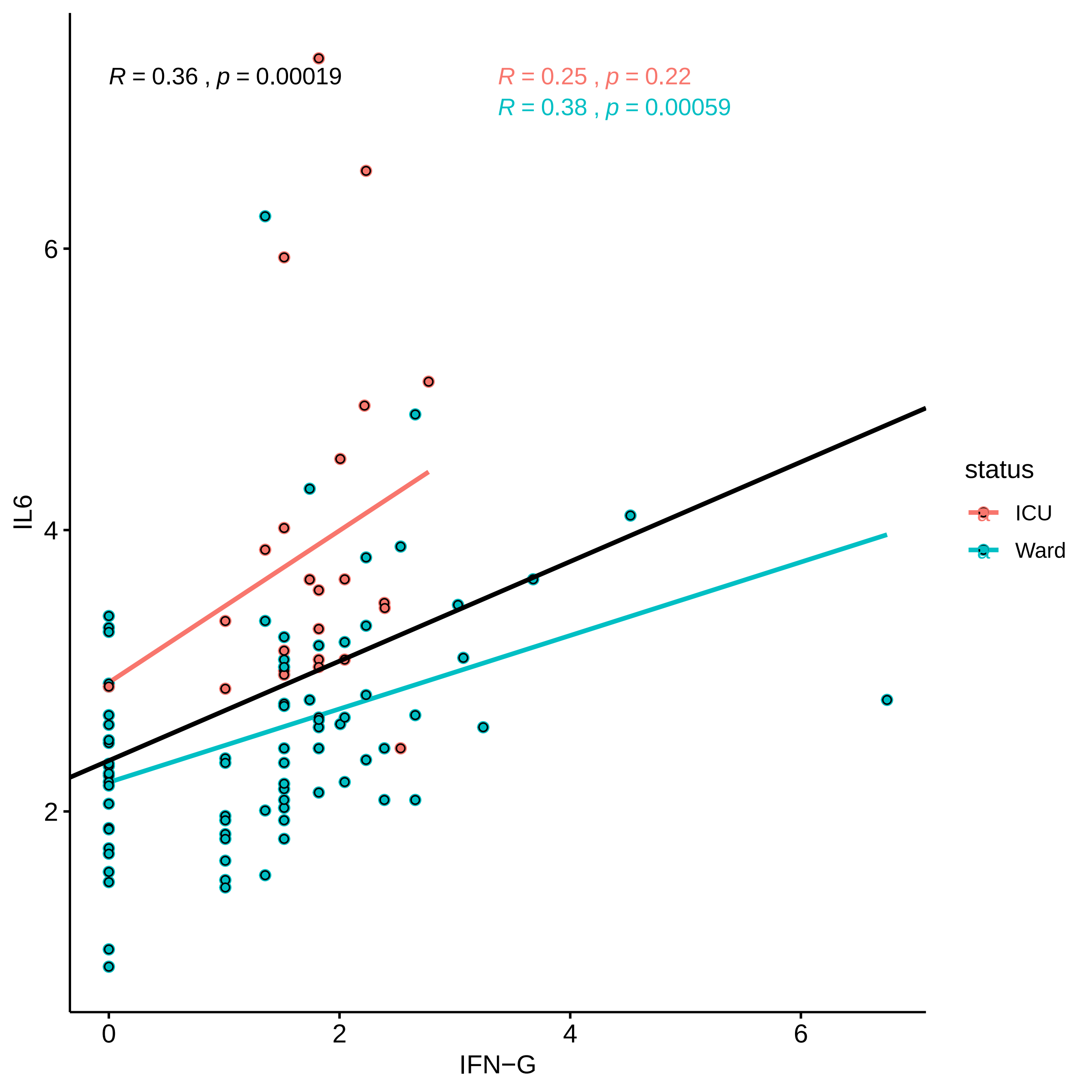

R = 0.36 , p = 0.00019
R = 0.25 , p = 0.22
R = 0.38 , p = 0.00059
IL6
6
4
2
0
2
4
6
IFN−G
status
ICU
Ward

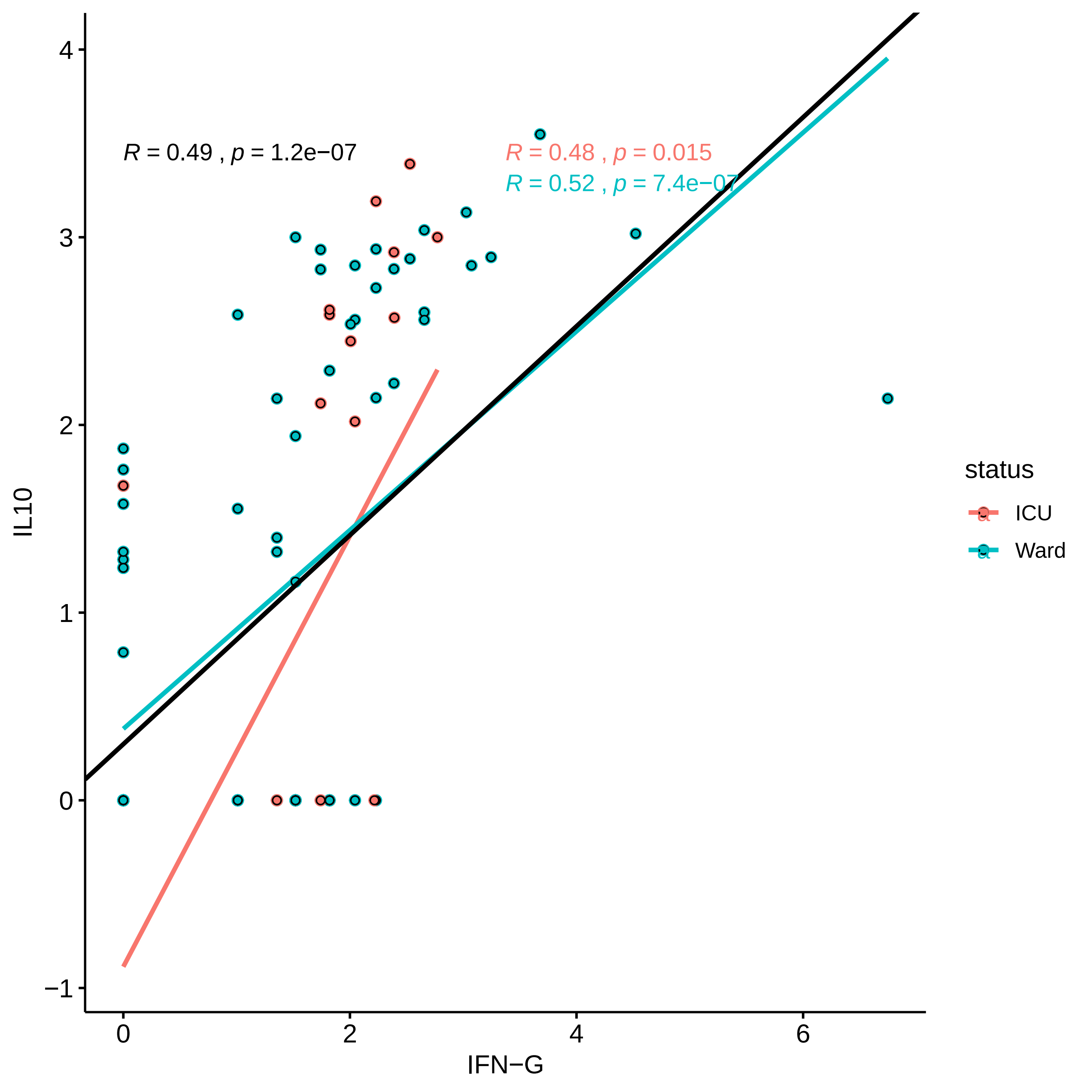

R = 0.49 , p = 1.2e−07
R = 0.48 , p = 0.015
R = 0.52 , p = 7.4e−07
IL10
IFN−G
status
ICU
Ward

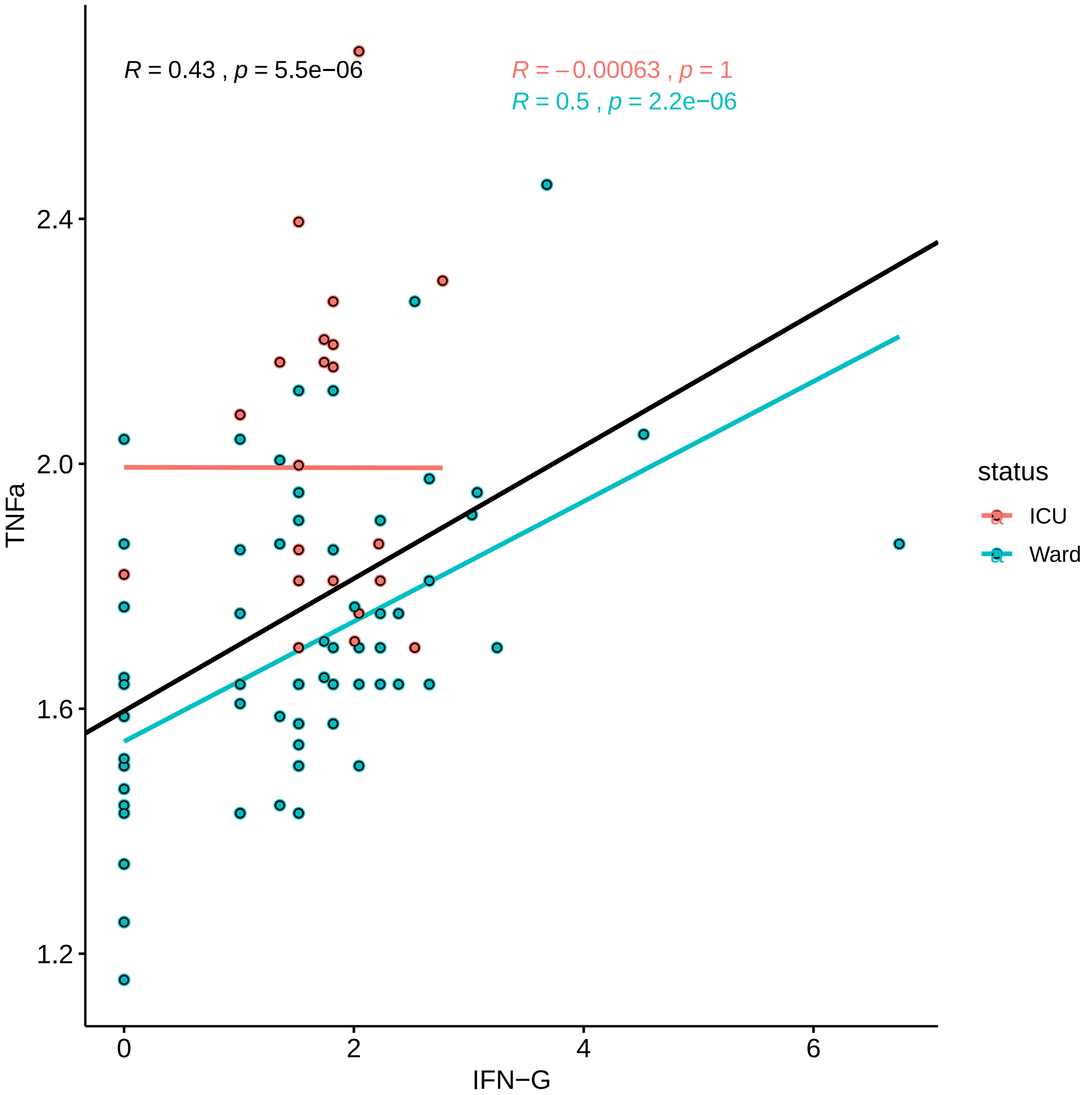

R = 0.43 , p = 5.5e−06
R = −0.00063 , p = 1
R = 0.5 , p = 2.2e−06
TNFa
2.4
2.0
1.6
1.2
0
2
4
6
IFN−G
status
ICU
Ward

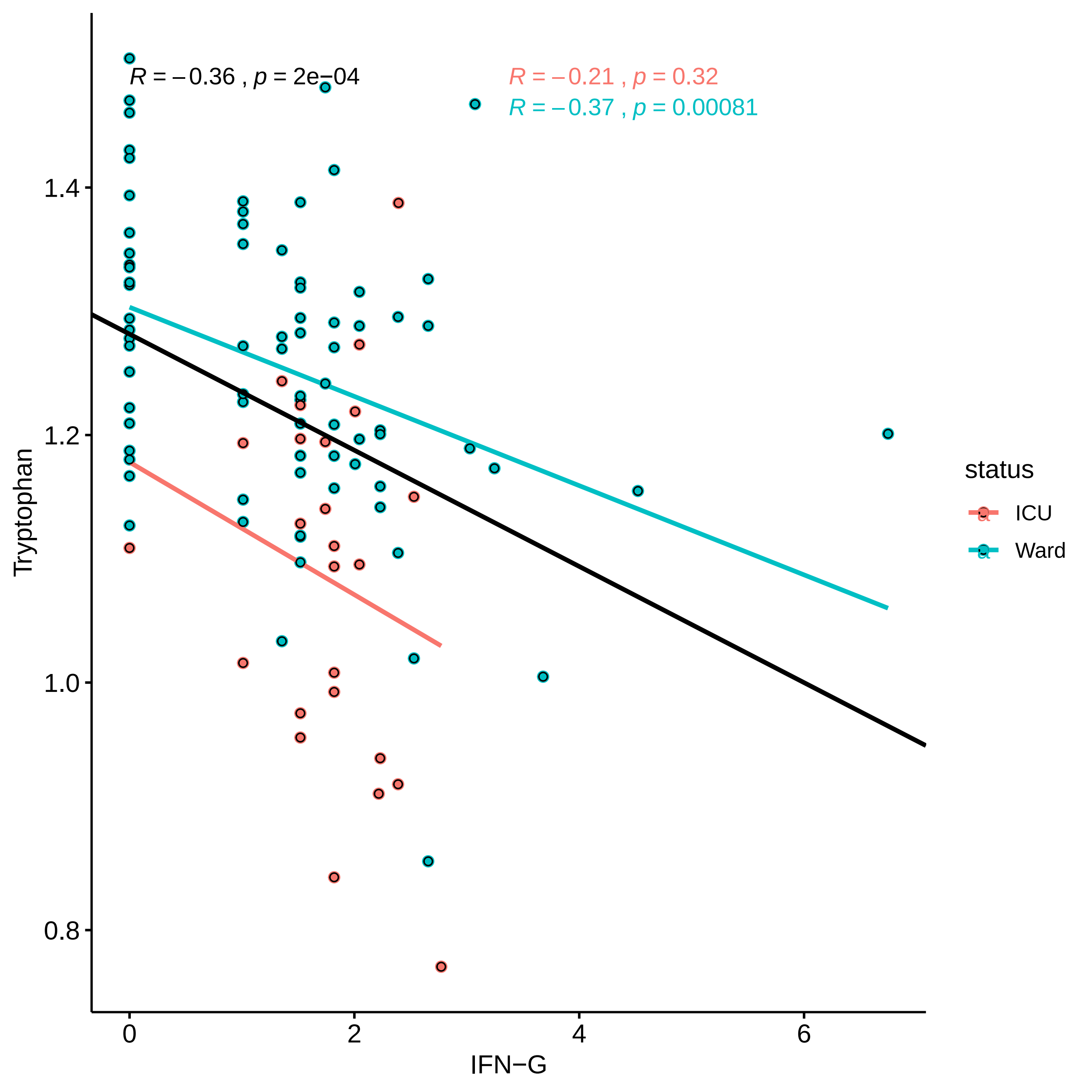

R = −0.36 , p = 2e−04
R = −0.21 , p = 0.32
R = −0.37 , p = 0.00081
1.4
1.2
1.0
0.8
Tryptophan
0
2
4
6
IFN−G
status
ICU
Ward

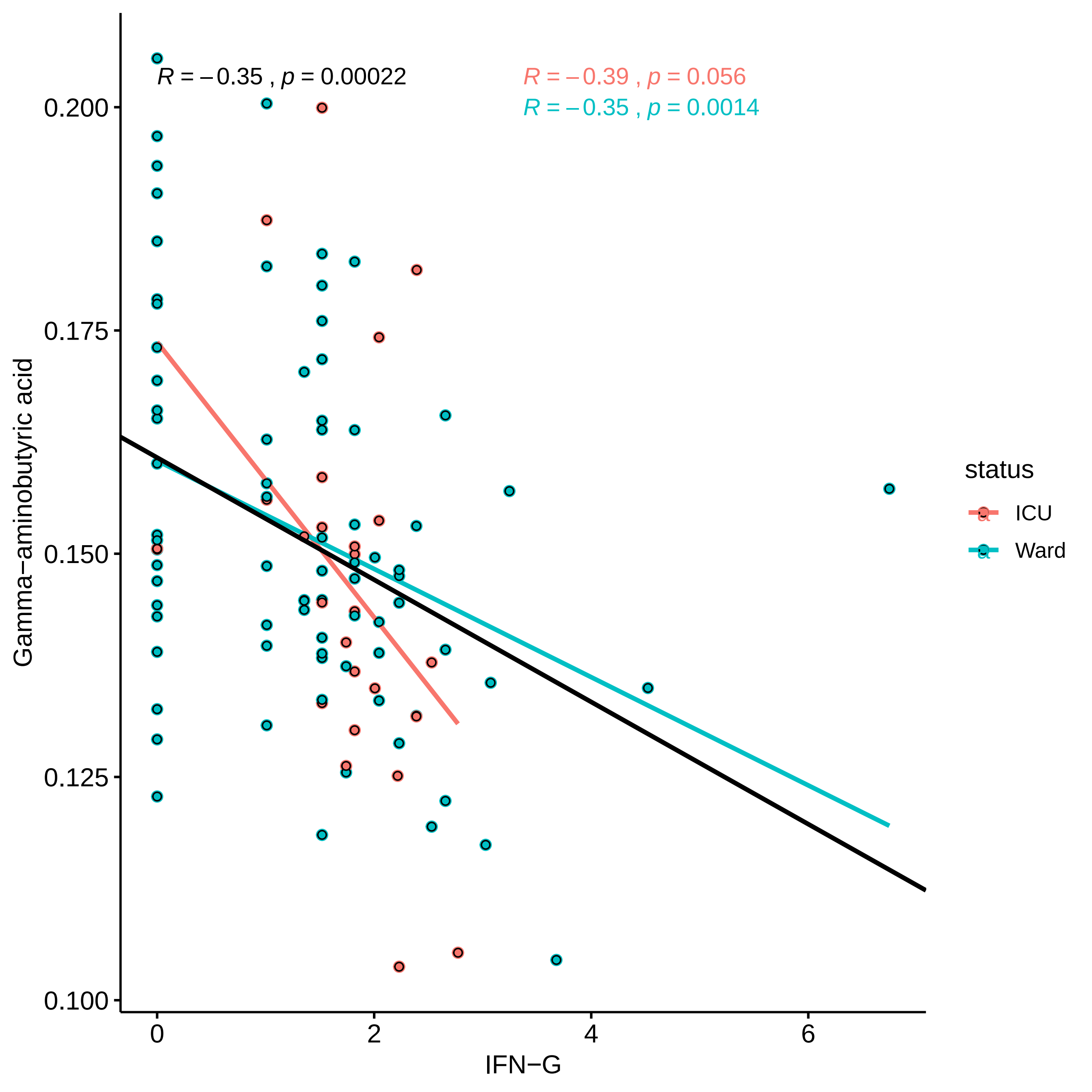

R = −0.35 , p = 0.00022
R = −0.39 , p = 0.056
R = −0.35 , p = 0.0014
0.200
0.175
0.150
0.125
0.100
Gamma−aminobutyric acid
0
2
4
6
IFN−G
status
ICU
Ward

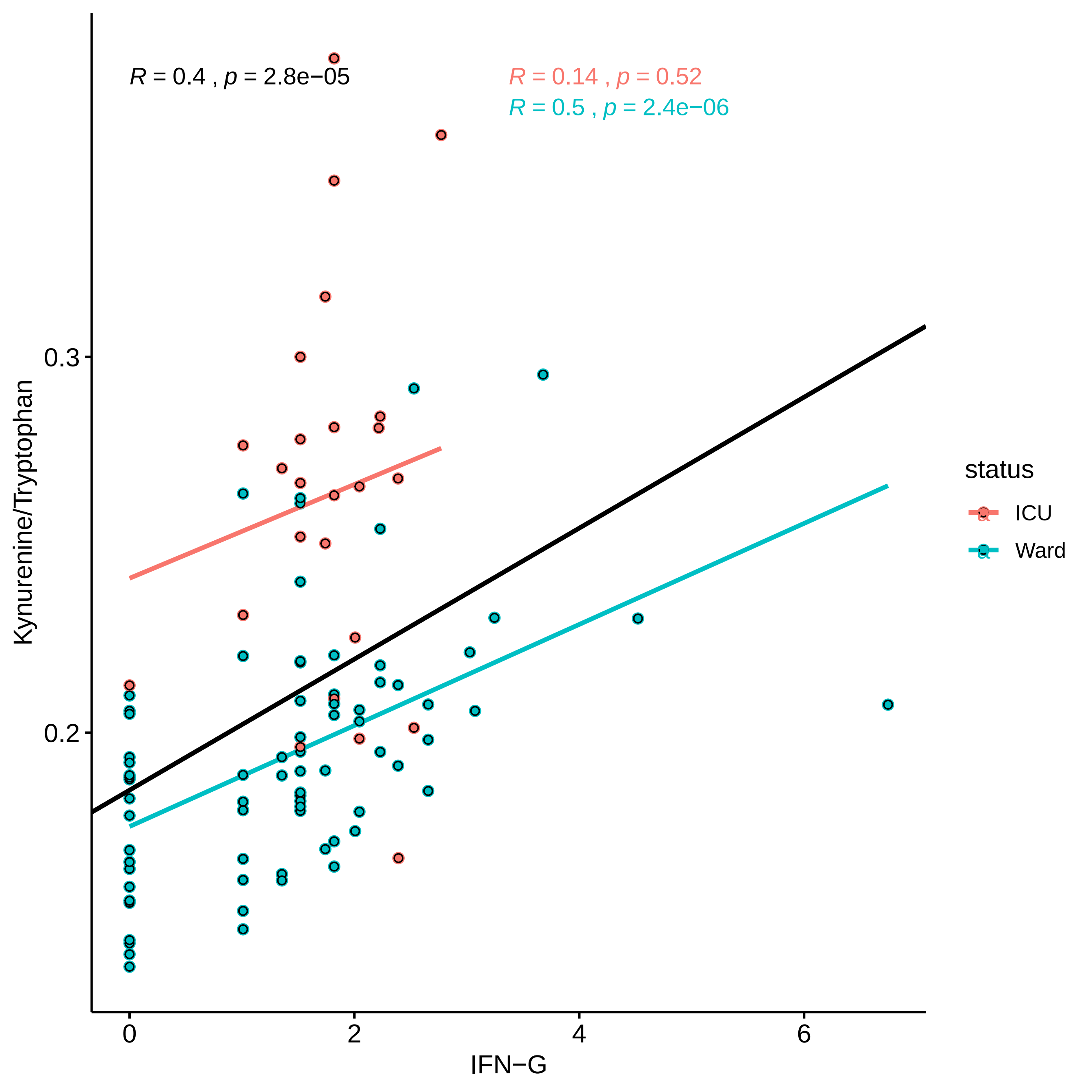

R = 0.4 , p = 2.8e−05
R = 0.14 , p = 0.52
R = 0.5 , p = 2.4e−06
Kynurenine/Tryptophan
0.3
0.2
0
2
4
6
IFN−G
status
ICU
Ward

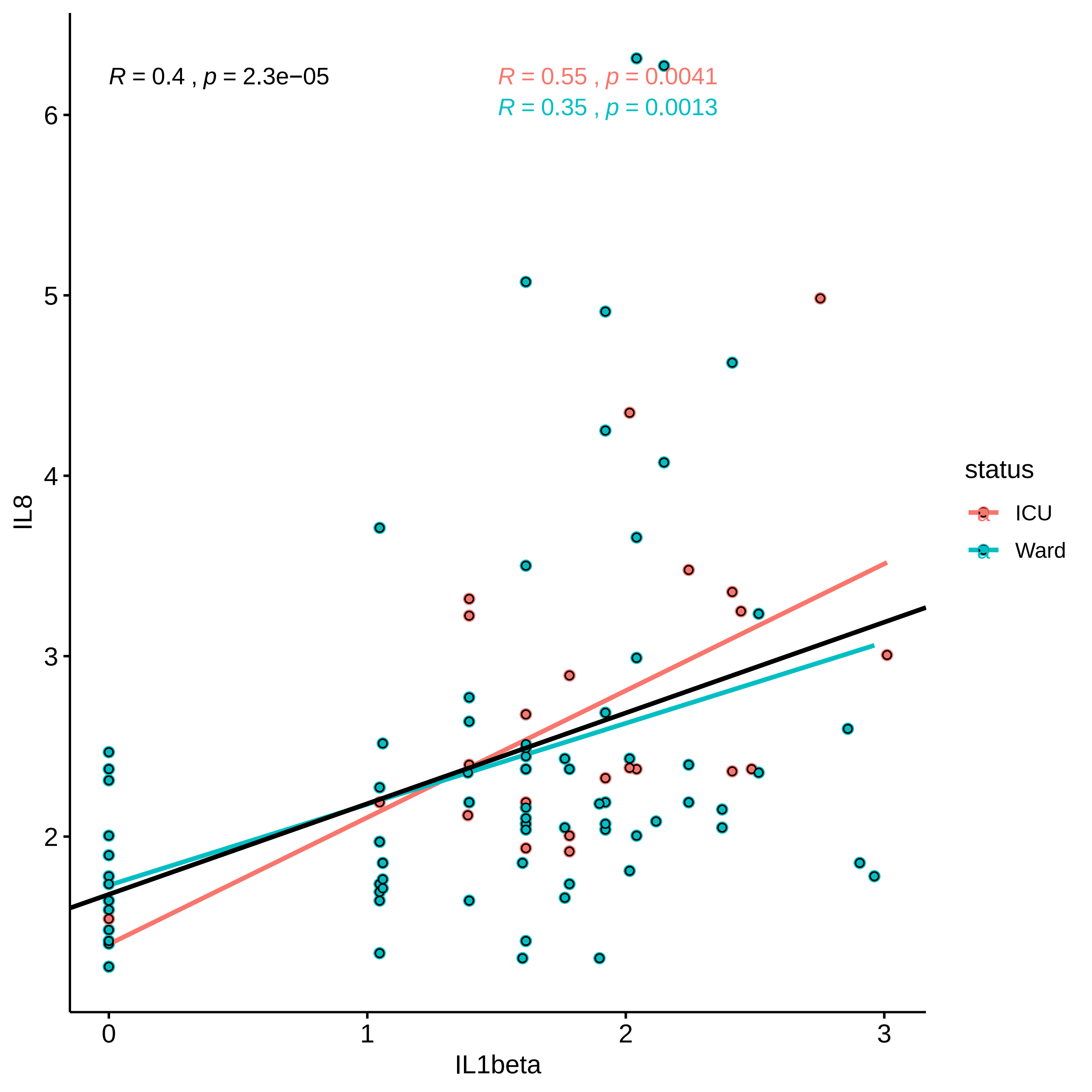
R = 0.4 , p = 2.3e−05
R = 0.55 , p = 0.0041
R = 0.35 , p = 0.0013
IL8
6
5
4
3
2
0
1
2
3
IL1beta
status
ICU
Ward

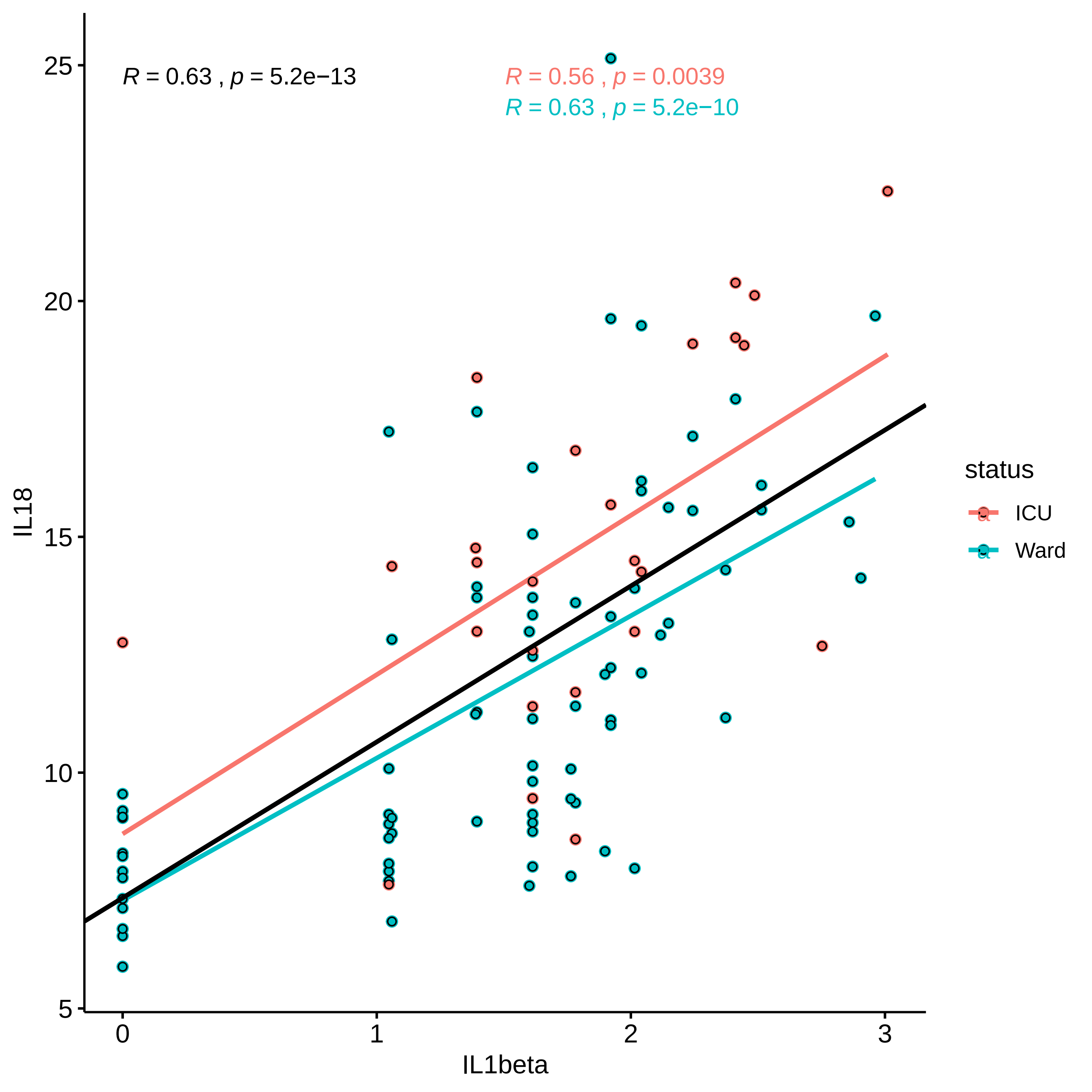

R = 0.63 , p = 5.2e−13
R = 0.56 , p = 0.0039
R = 0.63 , p = 5.2e−10
IL18
25
20
15
10
5
0
1
2
3
IL1beta
status
ICU
Ward

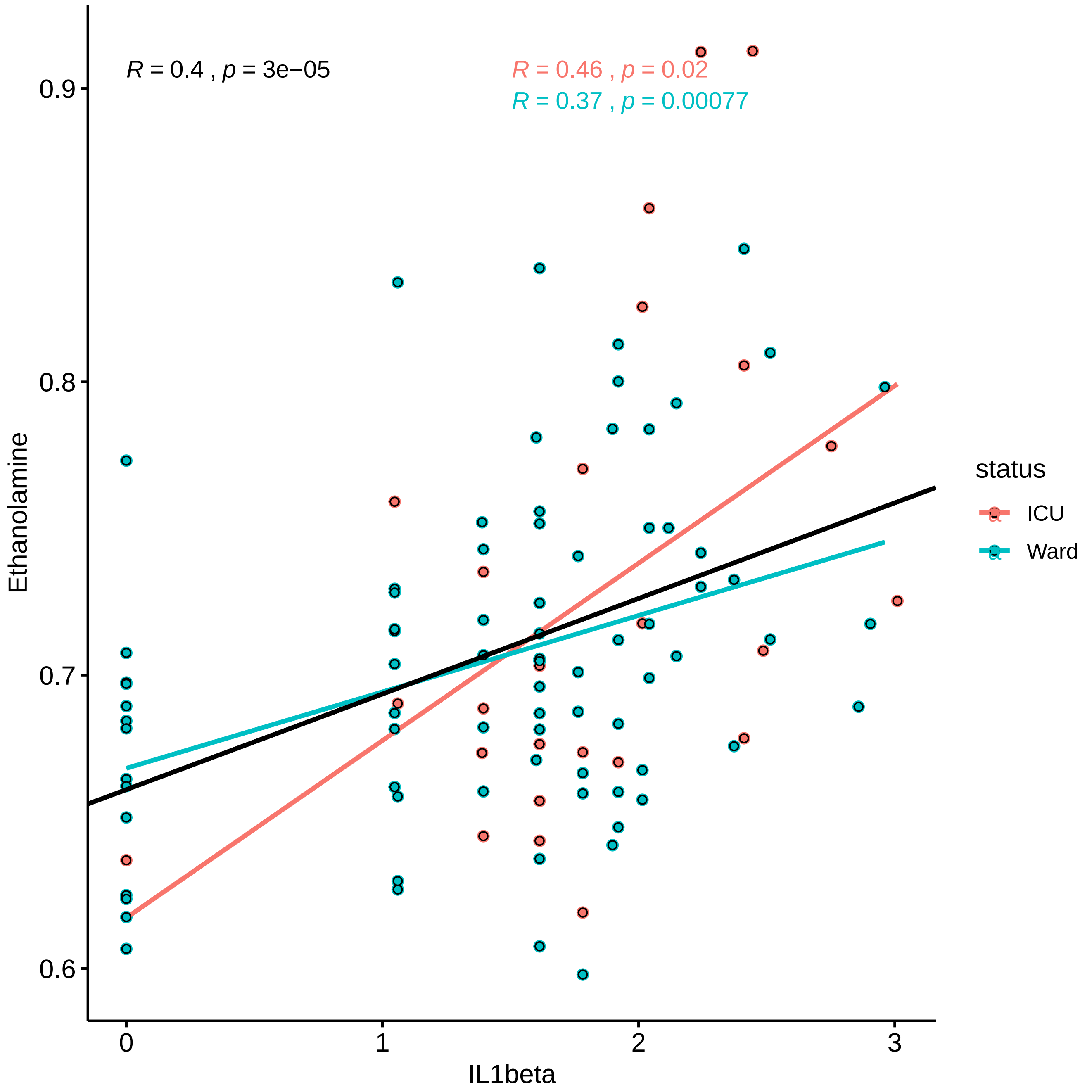

R = 0.4 , p = 3e−05
R = 0.46 , p = 0.02
R = 0.37 , p = 0.00077
0.9
0.8
0.7
0.6
Ethanolamine
0
1
2
3
IL1beta
status
ICU
Ward

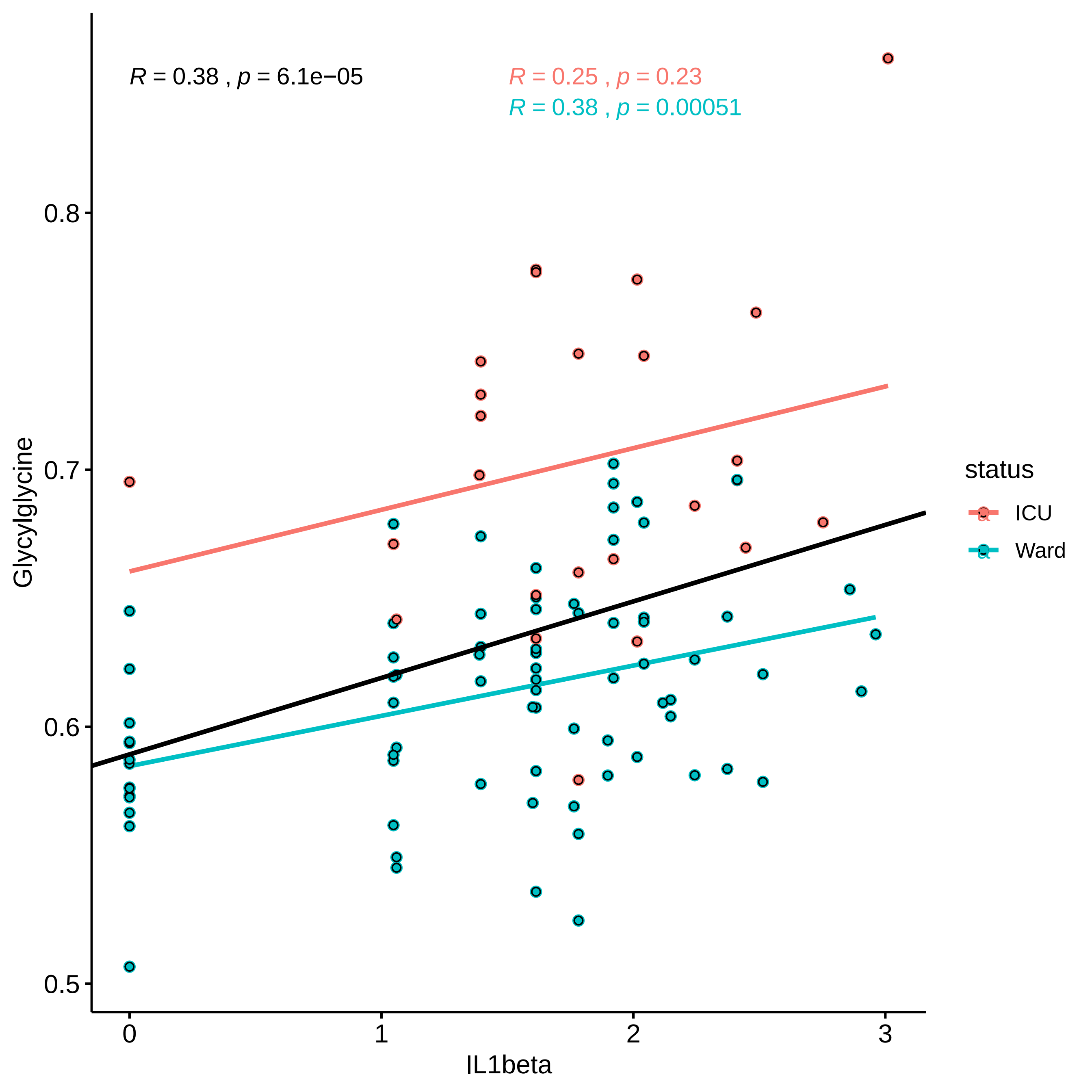

R = 0.38 , p = 6.1e−05
R = 0.25 , p = 0.23
R = 0.38 , p = 0.00051
Glycylglycine
0.8
0.7
0.6
0.5
0
1
2
3
IL1beta
status
ICU
Ward

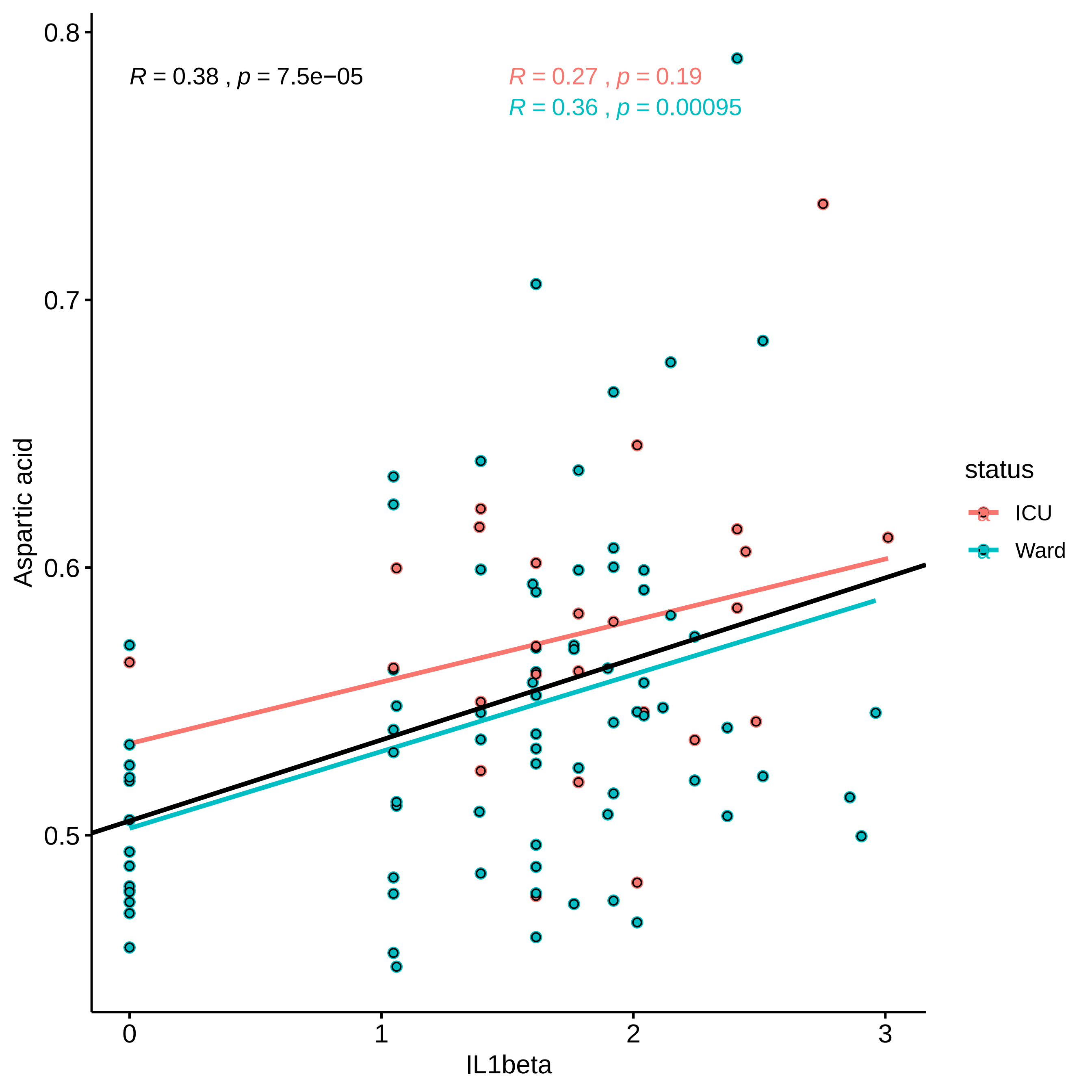

R = 0.38 , p = 7.5e−05
R = 0.27 , p = 0.19
R = 0.36 , p = 0.00095
0.8
0.7
0.6
0.5
Aspartic acid
0
1
2
3
IL1beta
status
ICU
Ward

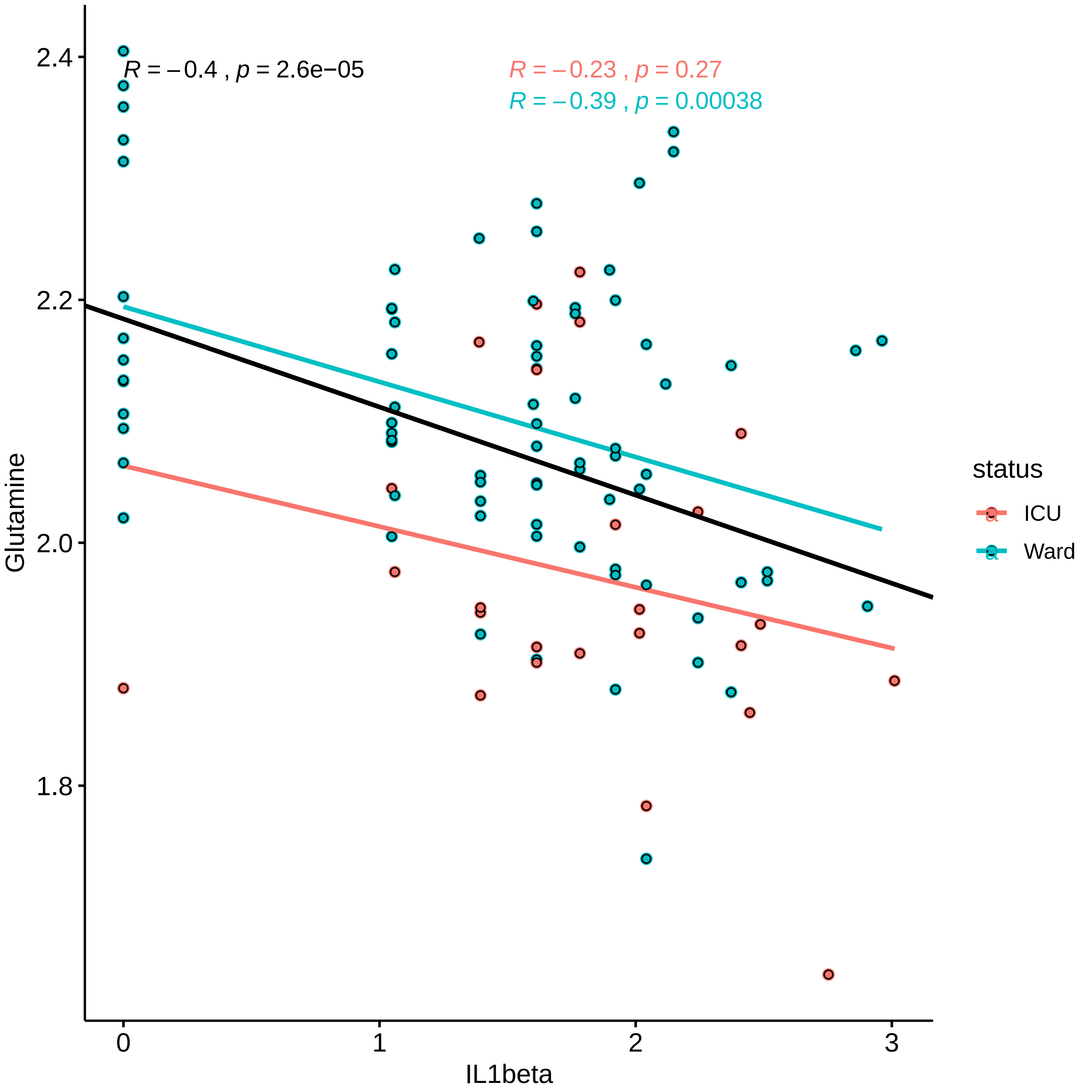

R = −0.4 , p = 2.6e−05
R = −0.23 , p = 0.27
R = −0.39 , p = 0.00038
2.4
2.2
2.0
1.8
Glutamine
0
1
2
3
IL1beta
status
ICU
Ward

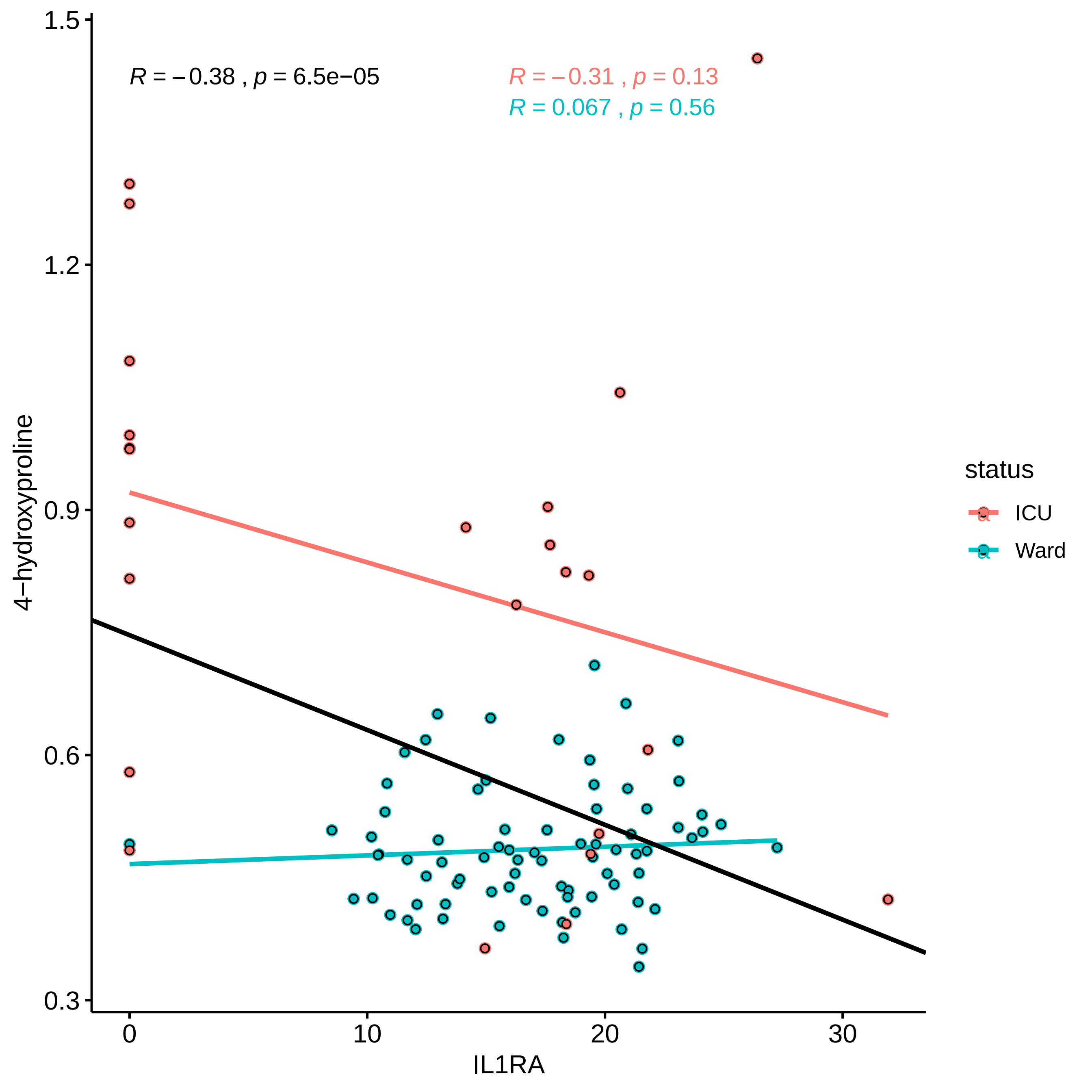

R = −0.38 , p = 6.5e−05
R = −0.31 , p = 0.13
R = 0.067 , p = 0.56
1.5
1.2
0.9
0.6
0.3
4−hydroxyproline
0
10
20
30
IL1RA
status
ICU
Ward

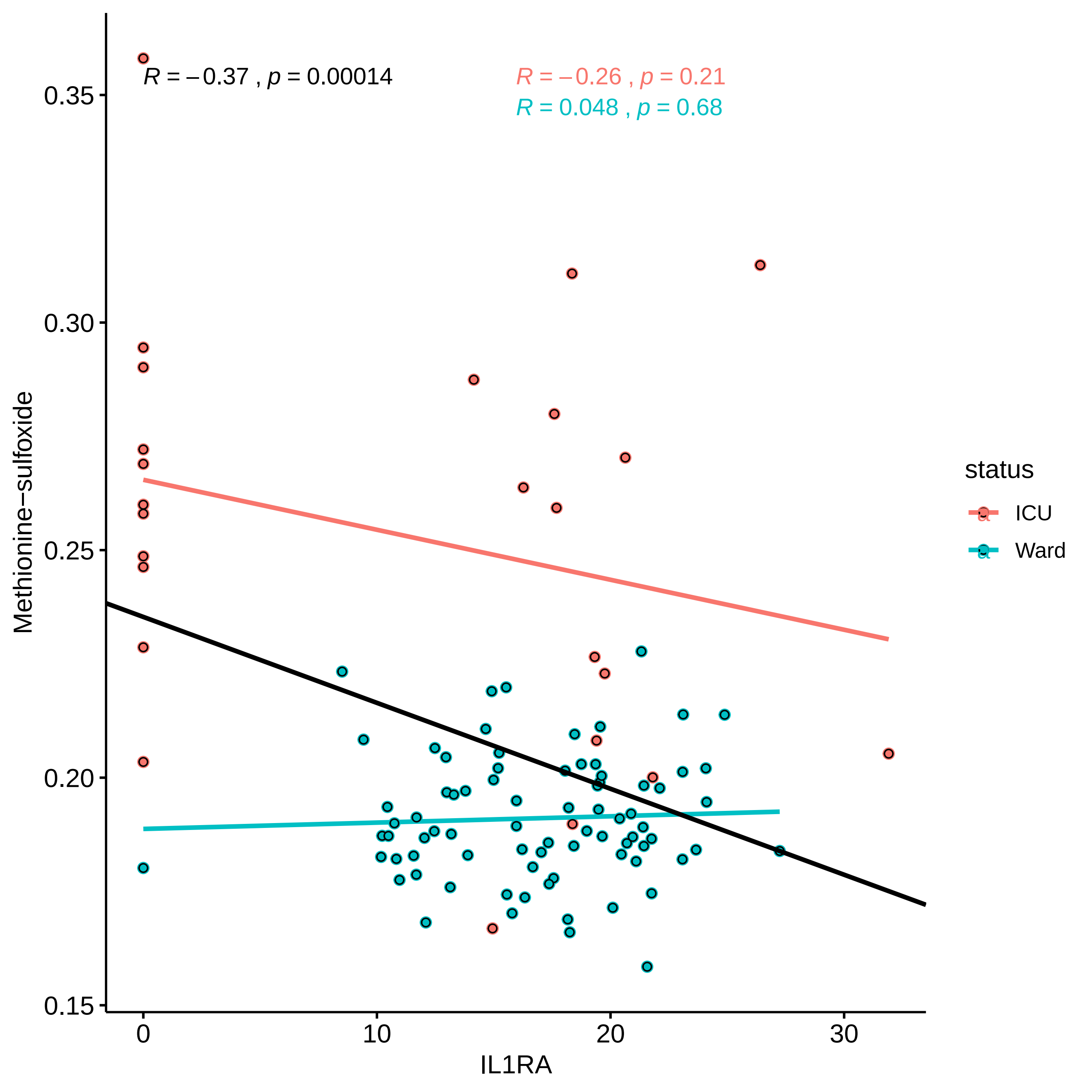
R = −0.37 , p = 0.00014
R = −0.26 , p = 0.21
R = 0.048 , p = 0.68
0.35
0.30
0.25
0.20
0.15
Methionine−sulfoxide
0
10
20
30
IL1RA
status
ICU
Ward

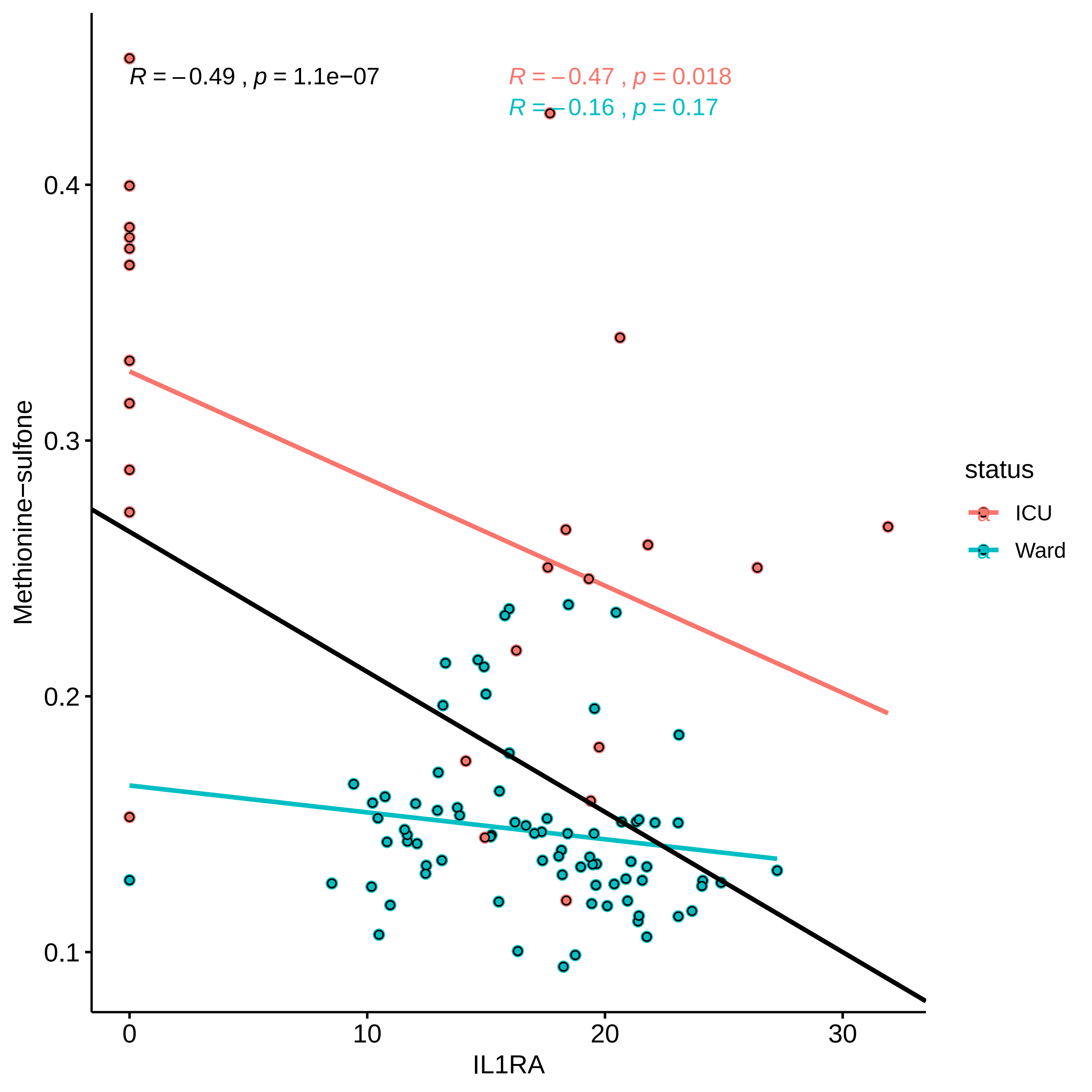

R = −0.49 , p = 1.1e−07
R = −0.47 , p = 0.018
R = −0.16 , p = 0.17
Methionine−sulfone
0.1
0.2
0.3
0.4
0
10
20
30
IL1RA
status
ICU
Ward

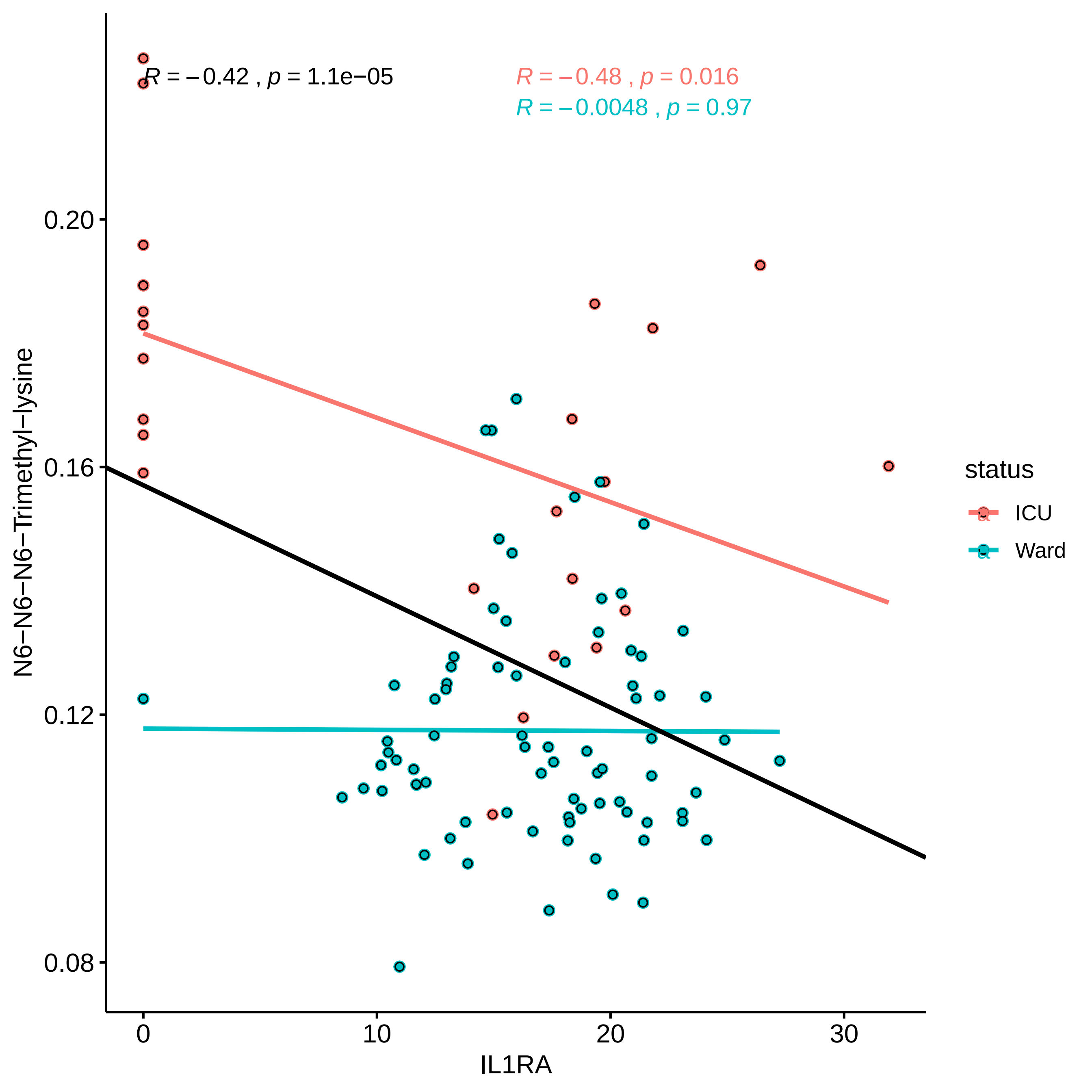

R = −0.42 , p = 1.1e−05
R = −0.48 , p = 0.016
R = −0.0048 , p = 0.97
N6−N6−N6−Trimethyl−lysine
0.20
0.16
0.12
0.08
0
10
20
30
IL1RA
status
ICU
Ward

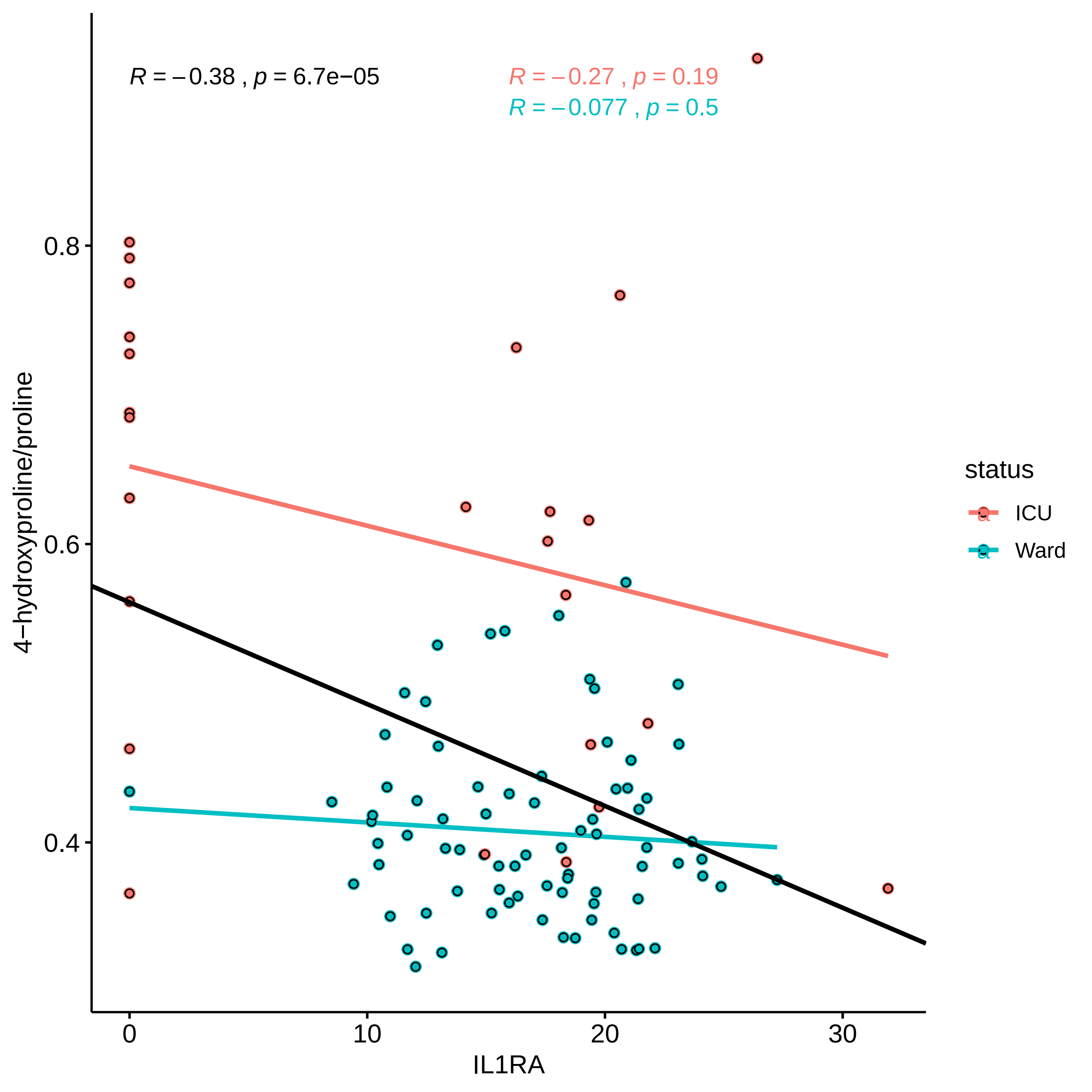

R = −0.38 , p = 6.7e−05
R = −0.27 , p = 0.19
R = −0.077 , p = 0.5
4−hydroxyproline/proline
0.8
0.6
0.4
0
10
20
30
IL1RA
status
ICU
Ward

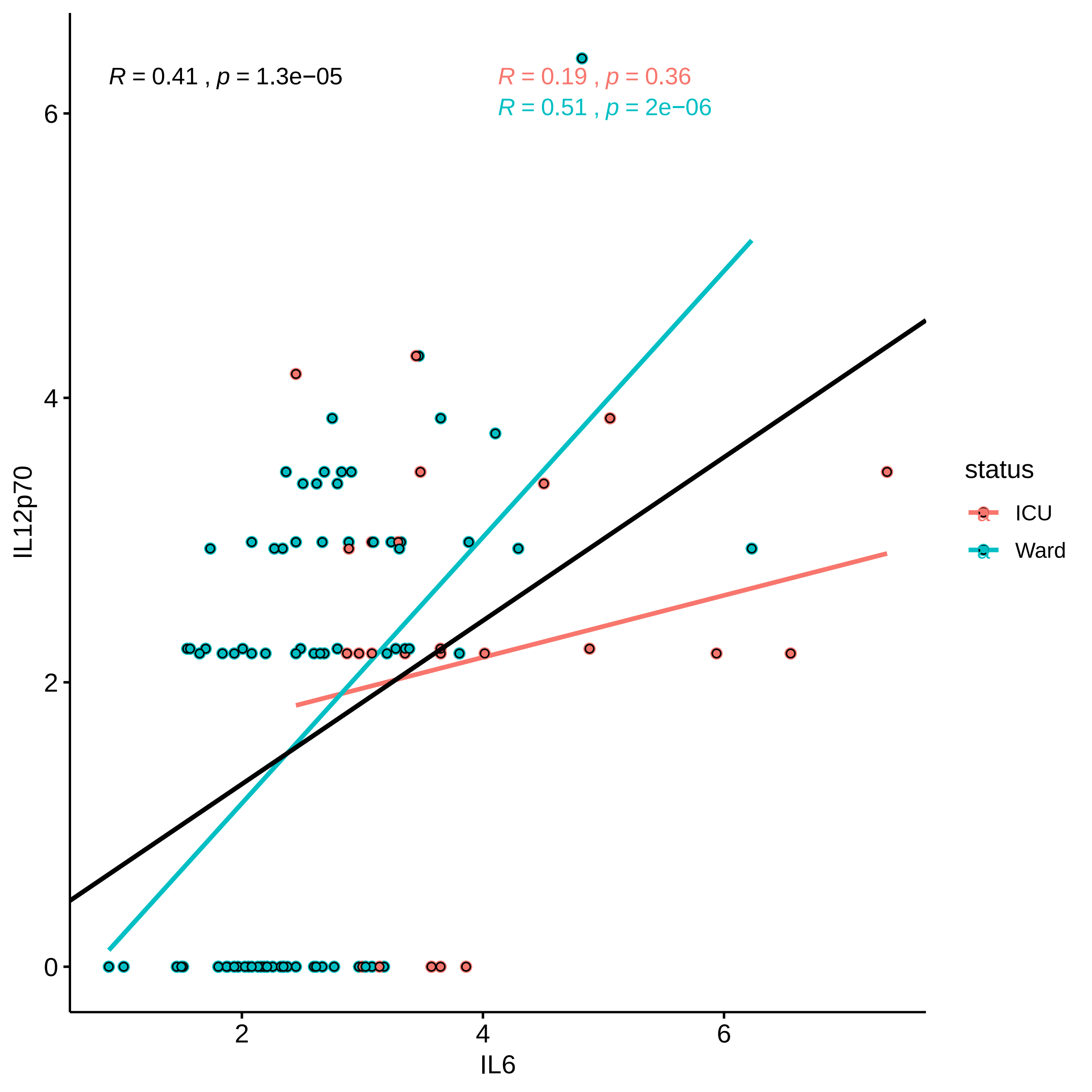

R = 0.41 , p = 1.3e−05
R = 0.19 , p = 0.36
R = 0.51 , p = 2e−06
IL12p70
IL6
status
ICU
Ward
0
2
4
6
2
4
6

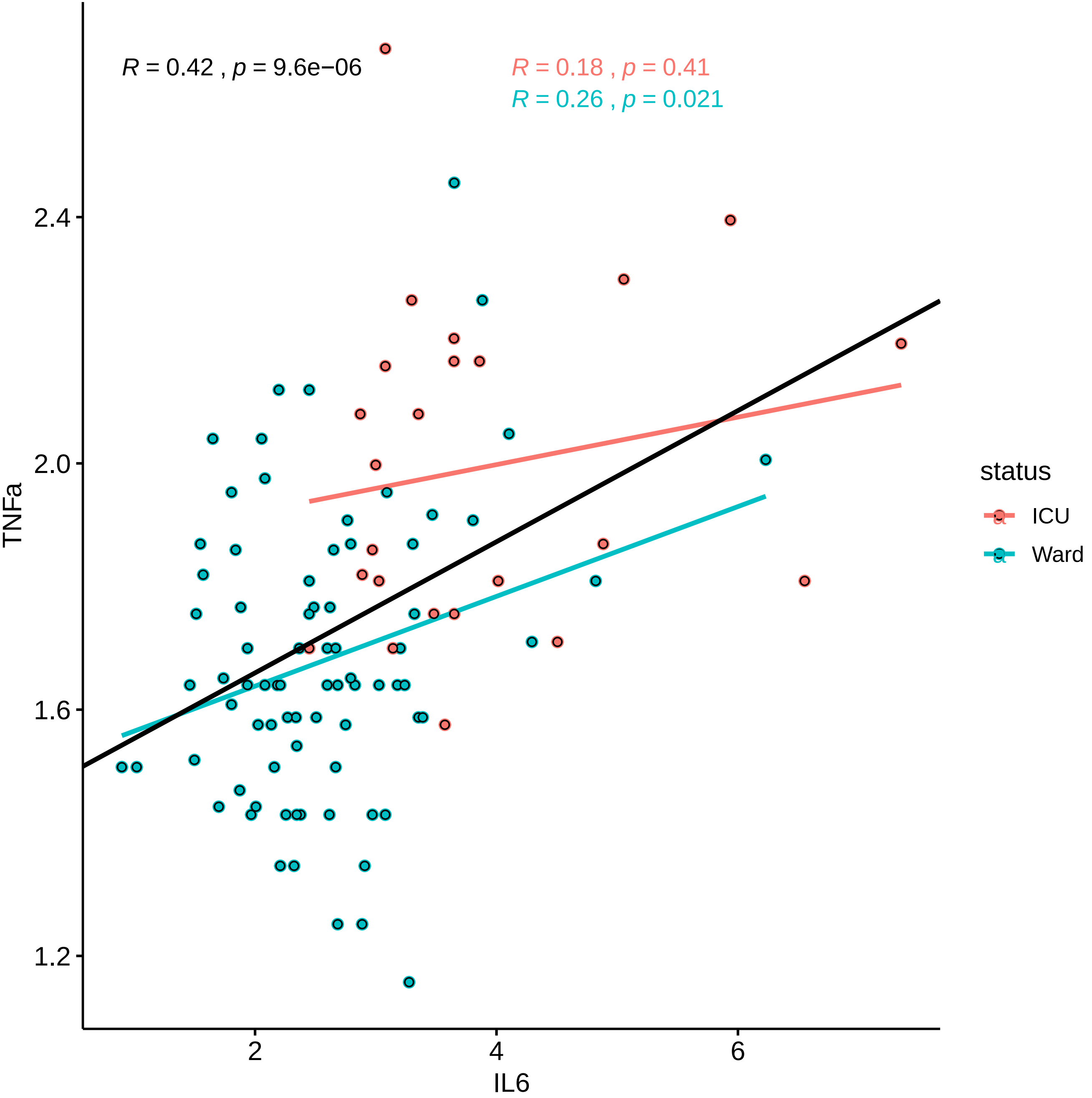

R = 0.42 , p = 9.6e−06
R = 0.18 , p = 0.41
R = 0.26 , p = 0.021
2.4
2.0
1.6
1.2
TNFa
2
4
6
IL6
status
ICU
Ward

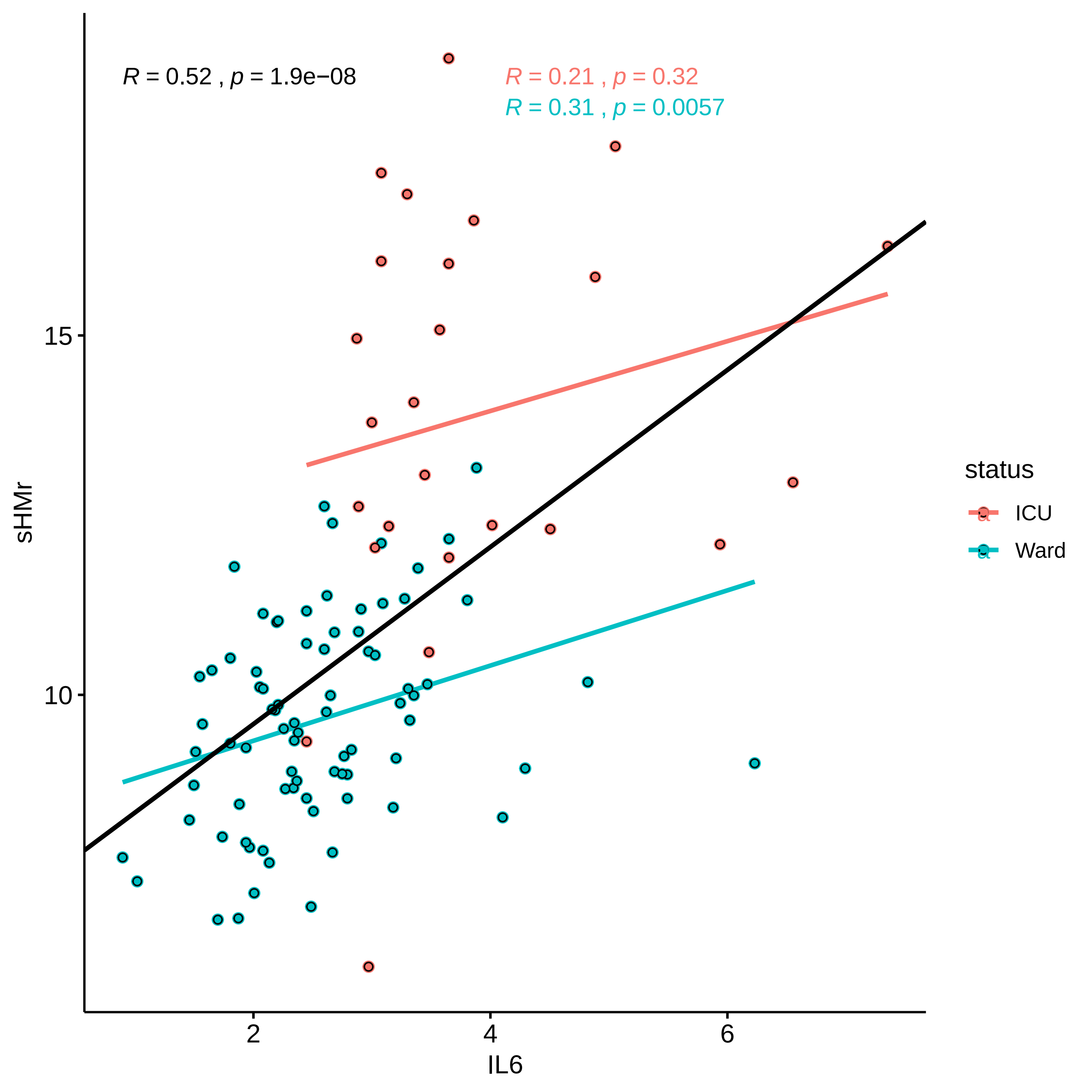

R = 0.52 , p = 1.9e−08
R = 0.21 , p = 0.32
R = 0.31 , p = 0.0057
15
10
sHMr
2
4
6
IL6
status
ICU
Ward

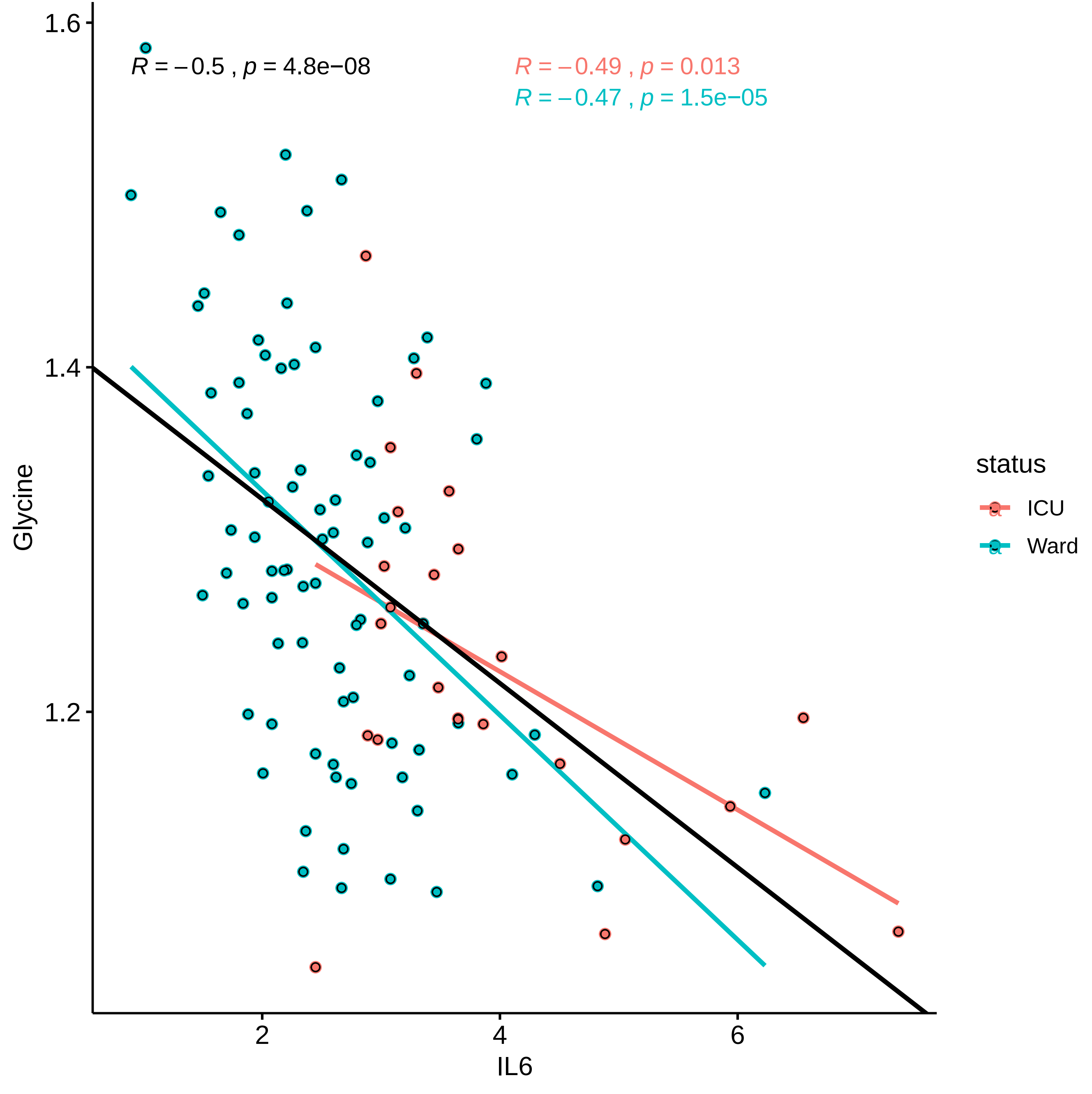

R = −0.5 , p = 4.8e−08
R = −0.49 , p = 0.013
R = −0.47 , p = 1.5e−05
1.6
1.4
1.2
2
4
6
Glycine
IL6
status
ICU
Ward

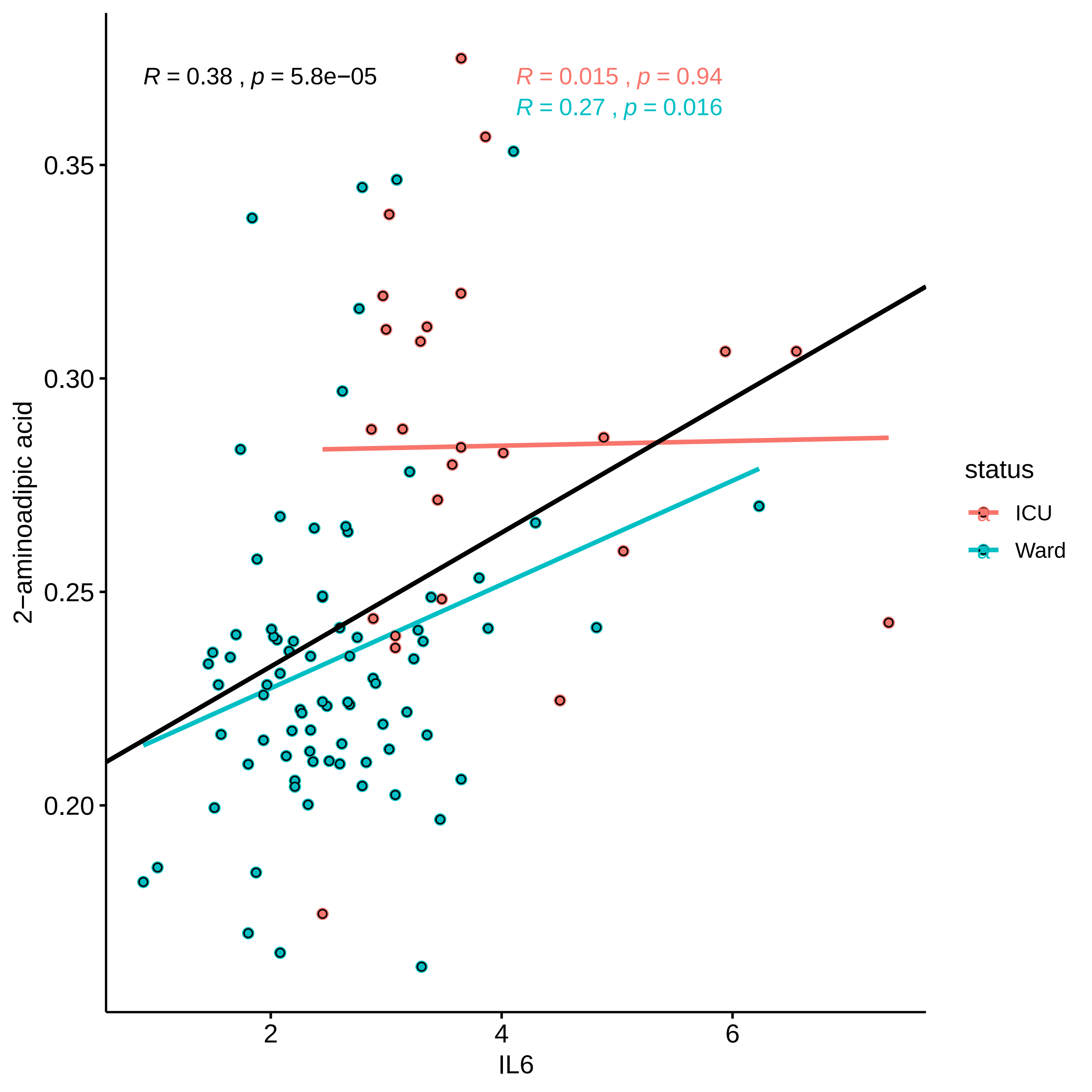

R = 0.38 , p = 5.8e−05
R = 0.015 , p = 0.94
R = 0.27 , p = 0.016
2−aminoadipic acid
0.35
0.30
0.25
0.20
2
4
6
IL6
status
ICU
Ward

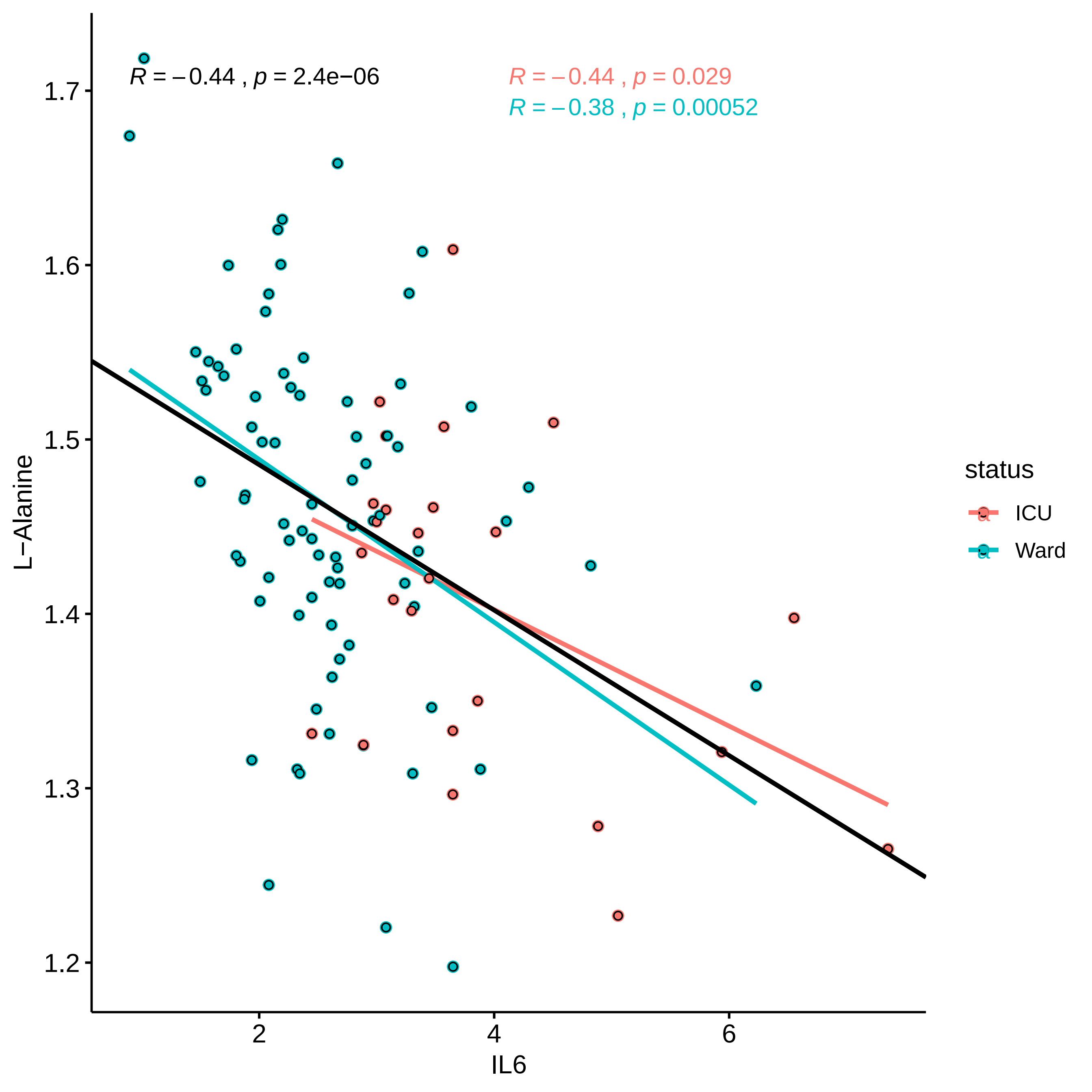

R = −0.44 , p = 2.4e−06
R = −0.44 , p = 0.029
R = −0.38 , p = 0.00052
L−Alanine
IL6
1.7
1.6
1.5
1.4
1.3
1.2
2
4
6
status
ICU
Ward

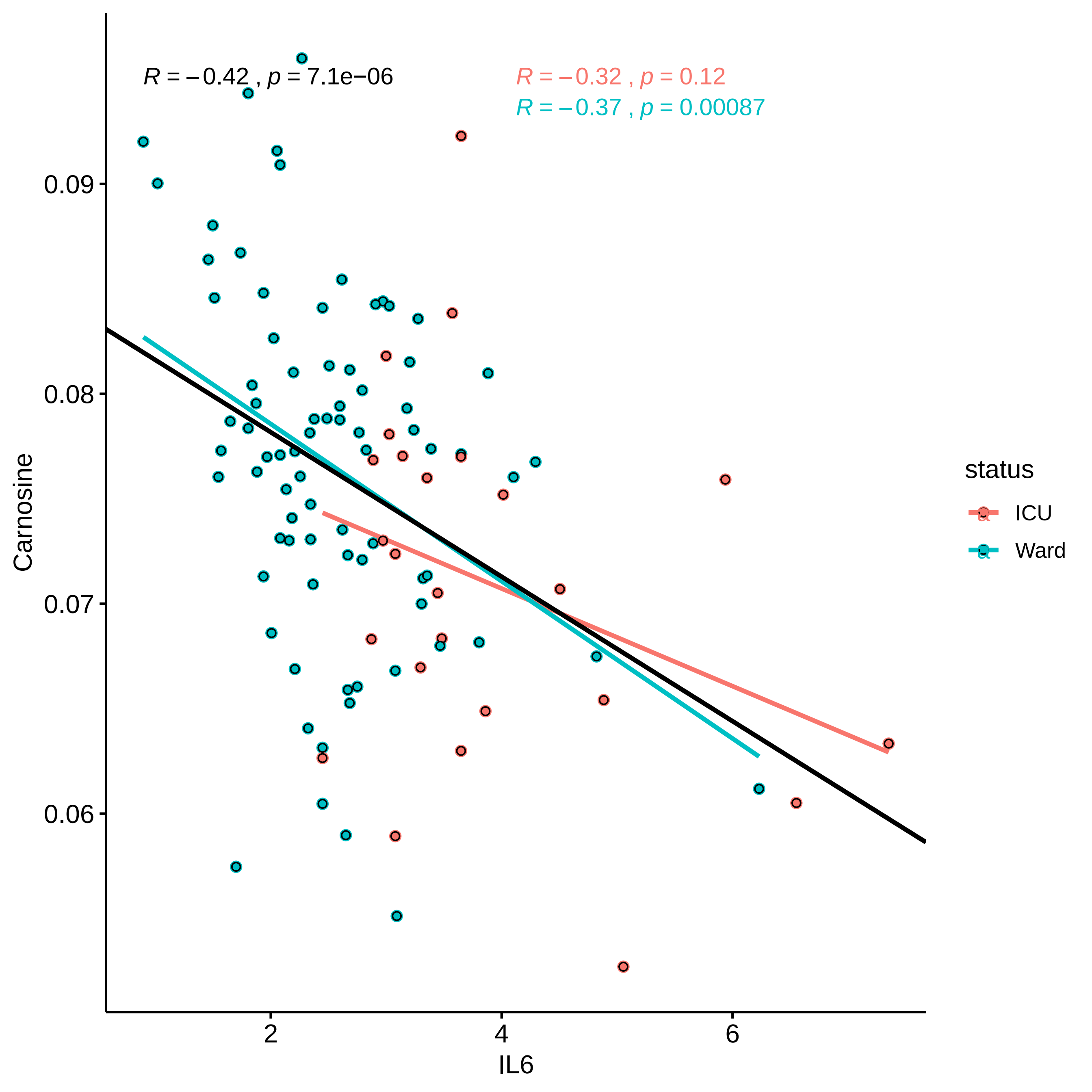

R = −0.42 , p = 7.1e−06
R = −0.32 , p = 0.12
R = −0.37 , p = 0.00087
Carnosine
0.09
0.08
0.07
0.06
2
4
6
IL6
status
ICU
Ward

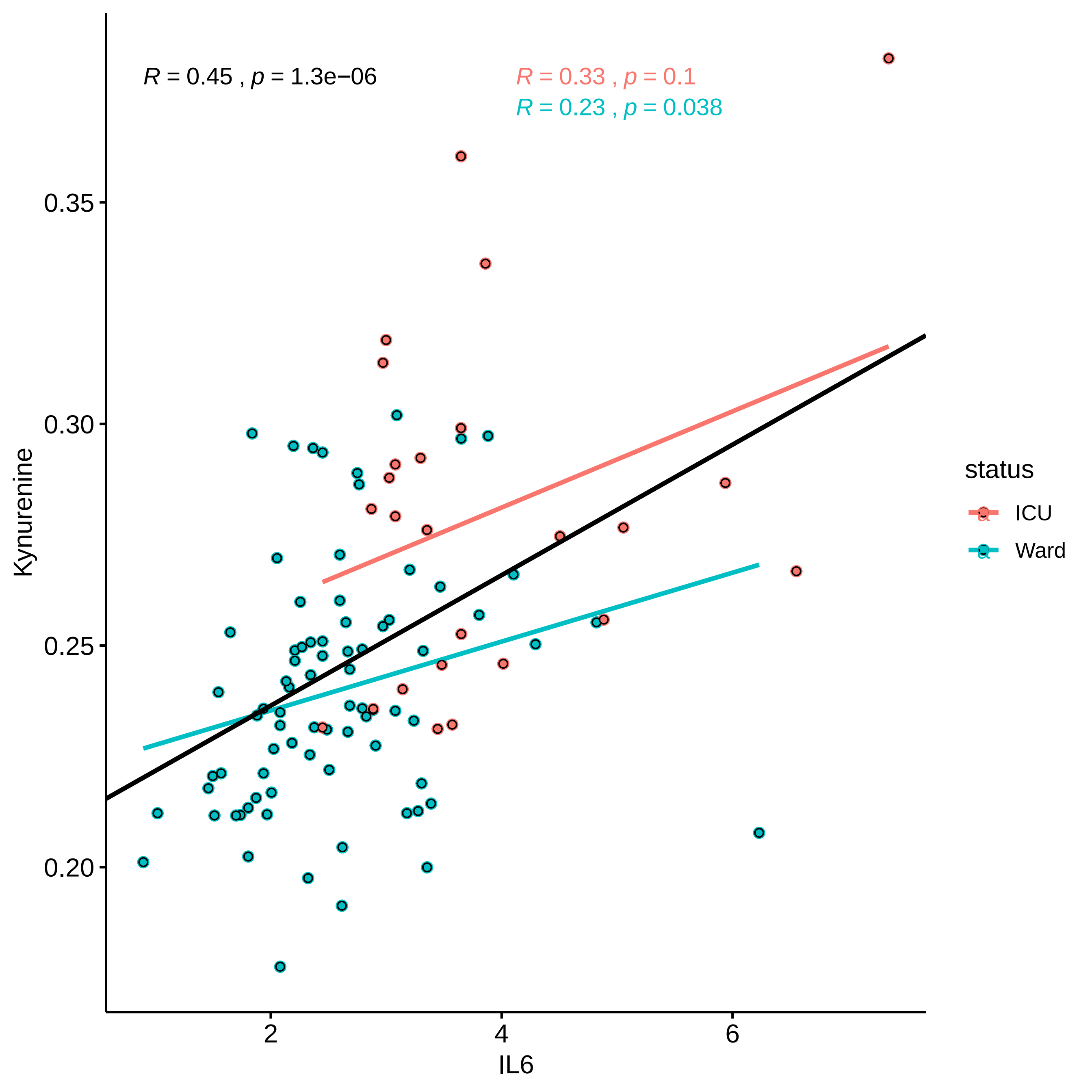

R = 0.45 , p = 1.3e−06
R = 0.33 , p = 0.1
R = 0.23 , p = 0.038
Kynurenine
0.35
0.30
0.25
0.20
2
4
6
IL6
status
ICU
Ward

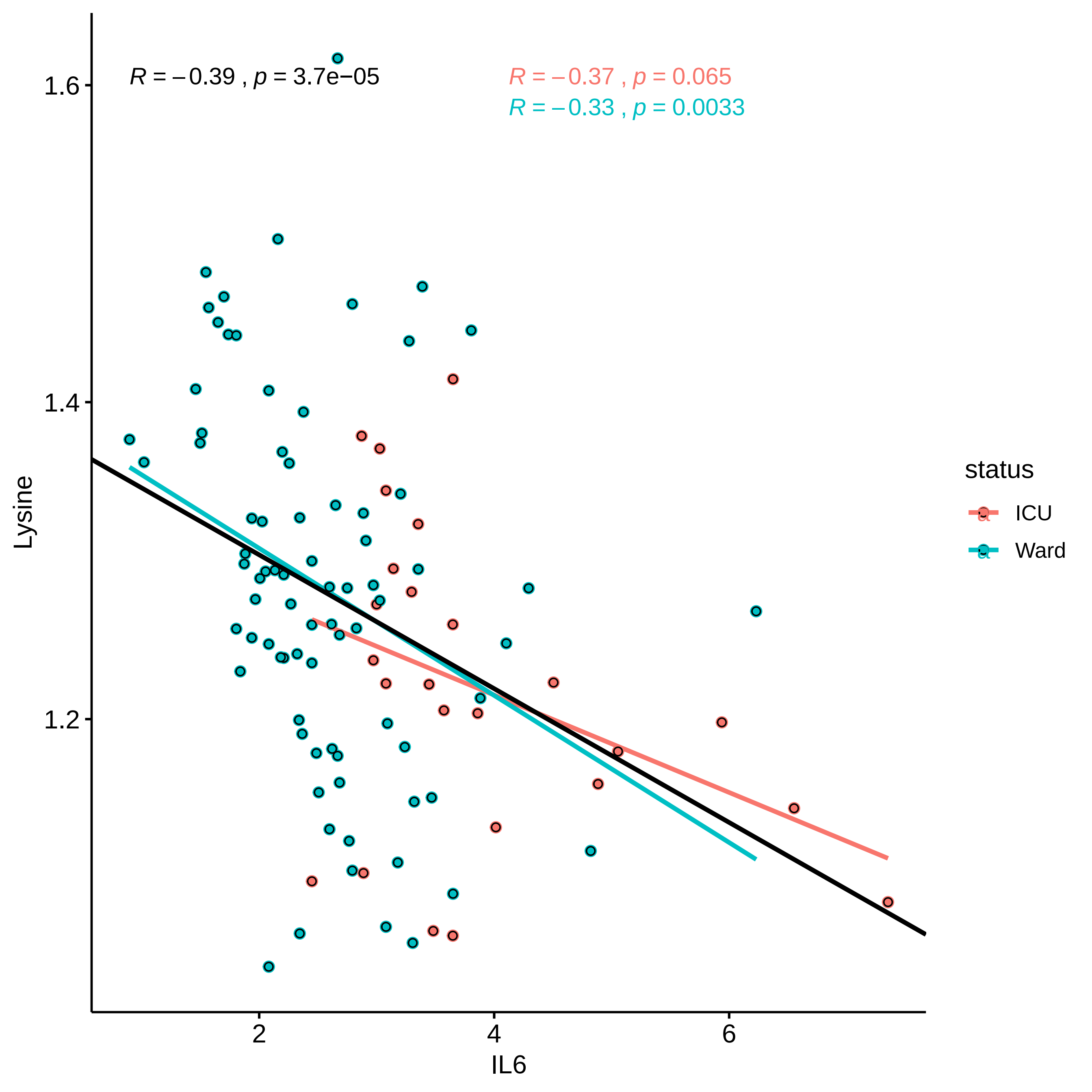

R = −0.39 , p = 3.7e−05
R = −0.37 , p = 0.065
R = −0.33 , p = 0.0033
1.6
1.4
1.2
Lysine
2
4
6
IL6
status
ICU
Ward

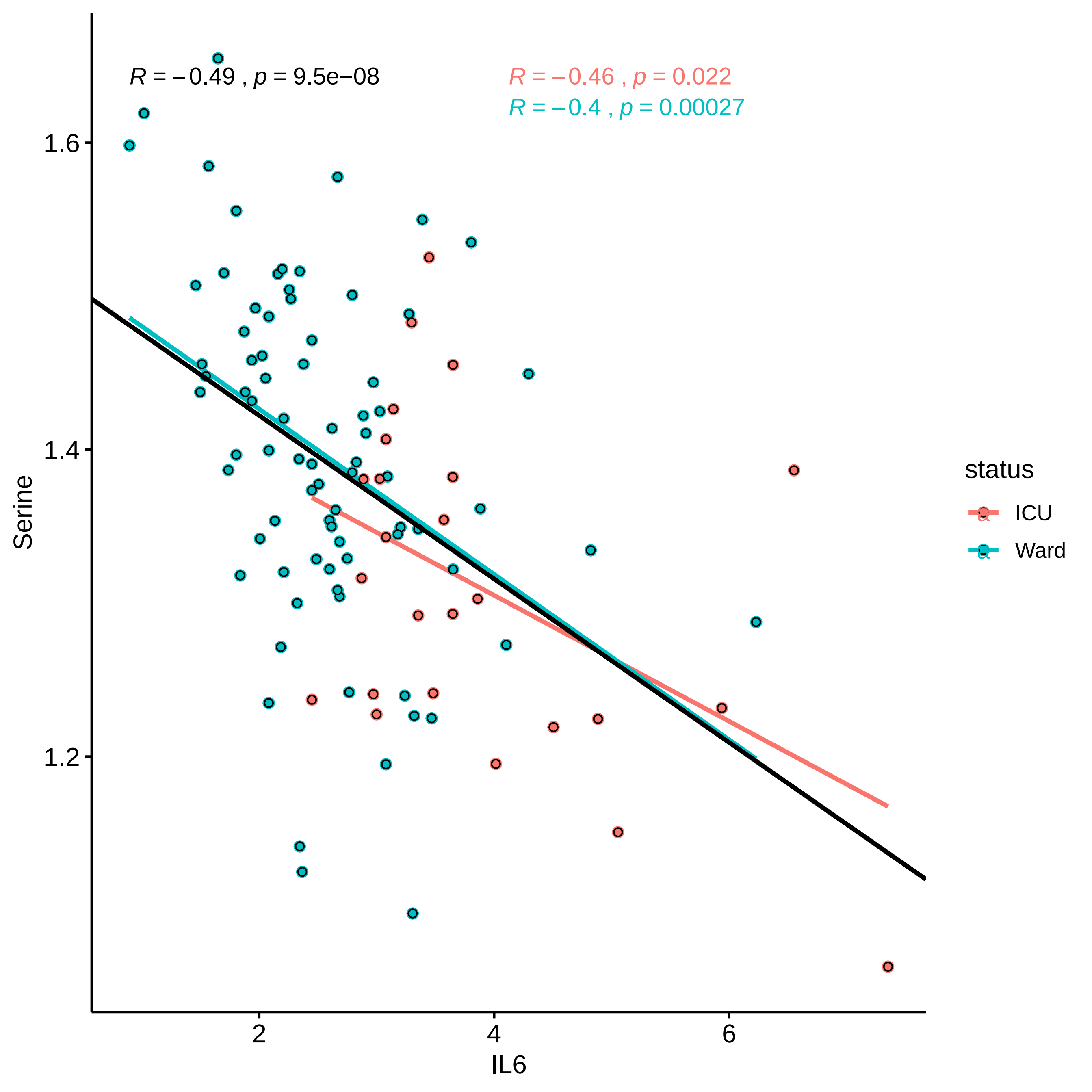

R = −0.49 , p = 9.5e−08
R = −0.46 , p = 0.022
R = −0.4 , p = 0.00027
1.6
1.4
1.2
2
4
6
IL6
Serine
status
ICU
Ward

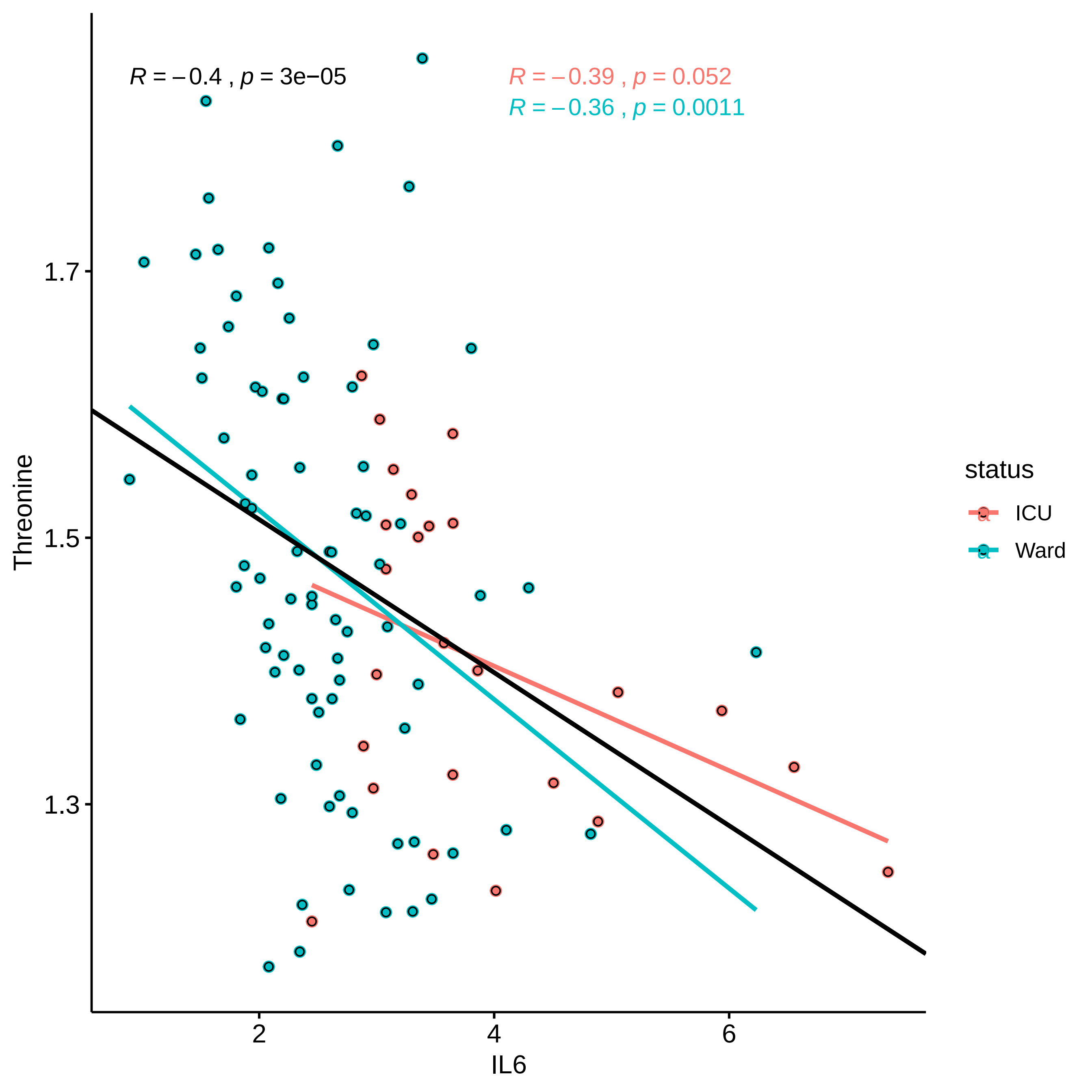
R = −0.4 , p = 3e−05
R = −0.39 , p = 0.052
R = −0.36 , p = 0.0011
1.7
1.5
1.3
Threonine
2
4
6
IL6
status
ICU
Ward

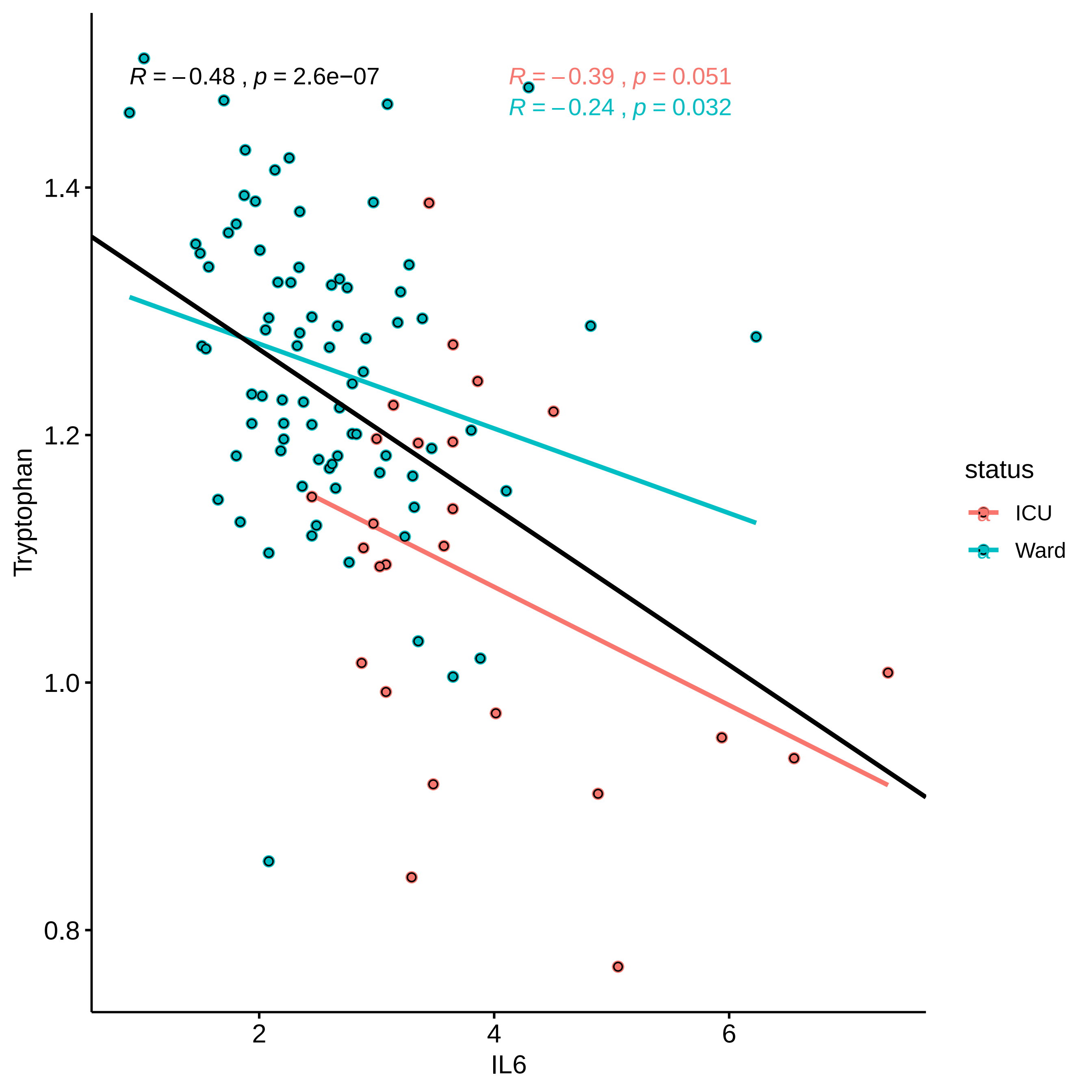

R = −0.48 , p = 2.6e−07
R = −0.39 , p = 0.051
R = −0.24 , p = 0.032
1.4
1.2
1.0
0.8
Tryptophan
2
4
6
IL6
status
ICU
Ward

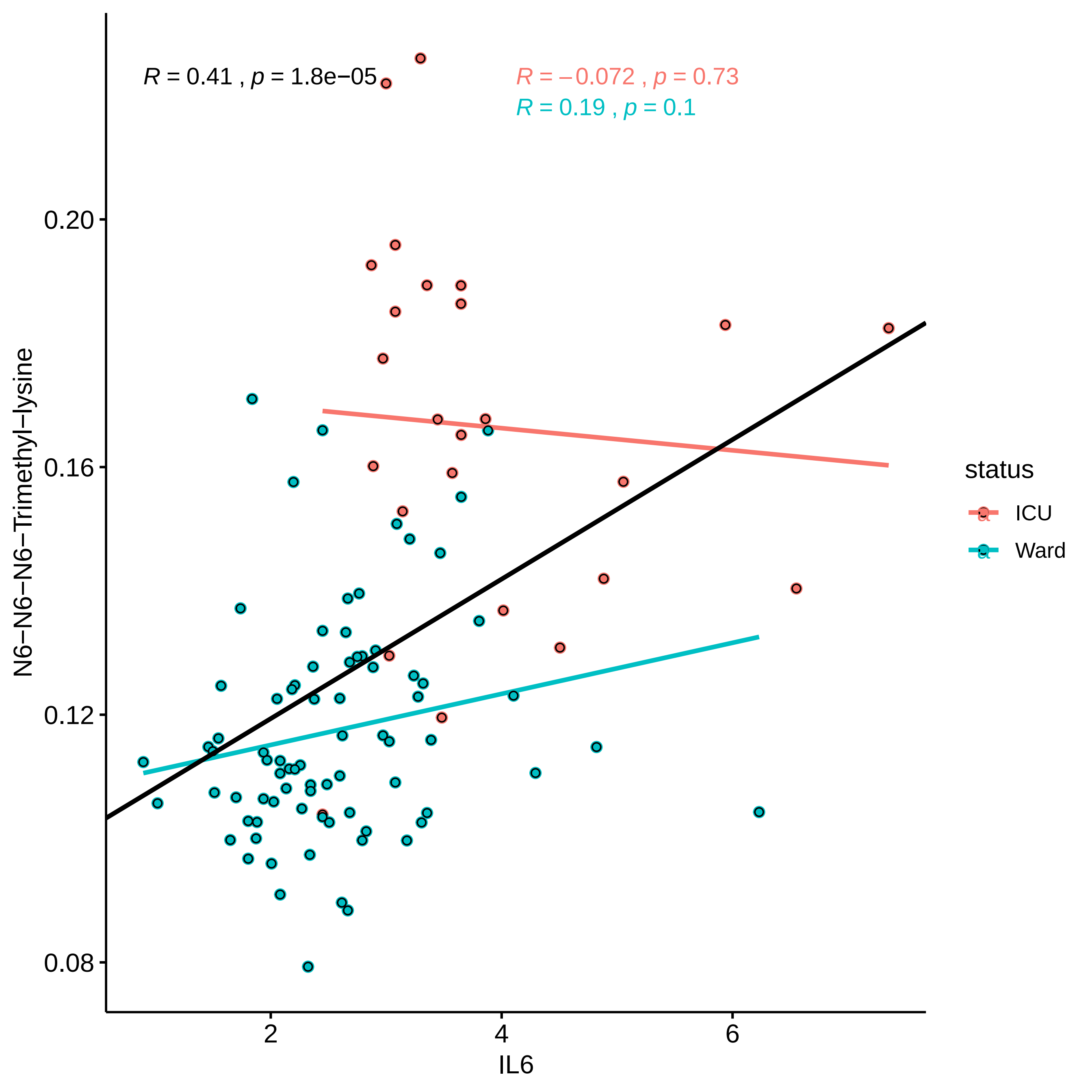

R = 0.41 , p = 1.8e−05
R = −0.072 , p = 0.73
R = 0.19 , p = 0.1
N6−N6−N6−Trimethyl−lysine
0.20
0.16
0.12
0.08
2
4
6
IL6
status
ICU
Ward

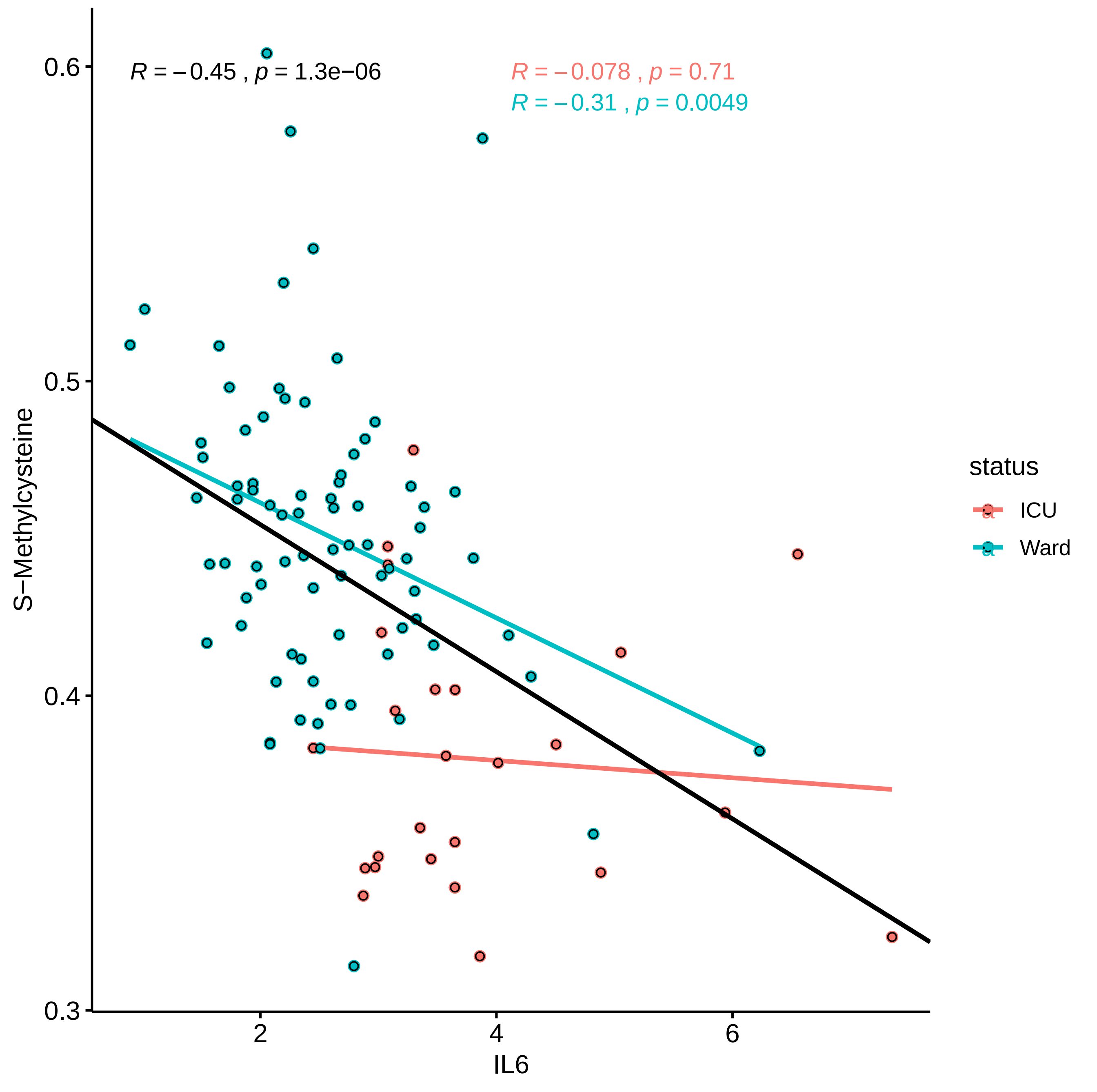

R = −0.45 , p = 1.3e−06
R = −0.078 , p = 0.71
R = −0.31 , p = 0.0049
S−Methylcysteine
IL6
0.6
0.5
0.4
0.3
2
4
6
status
ICU
Ward

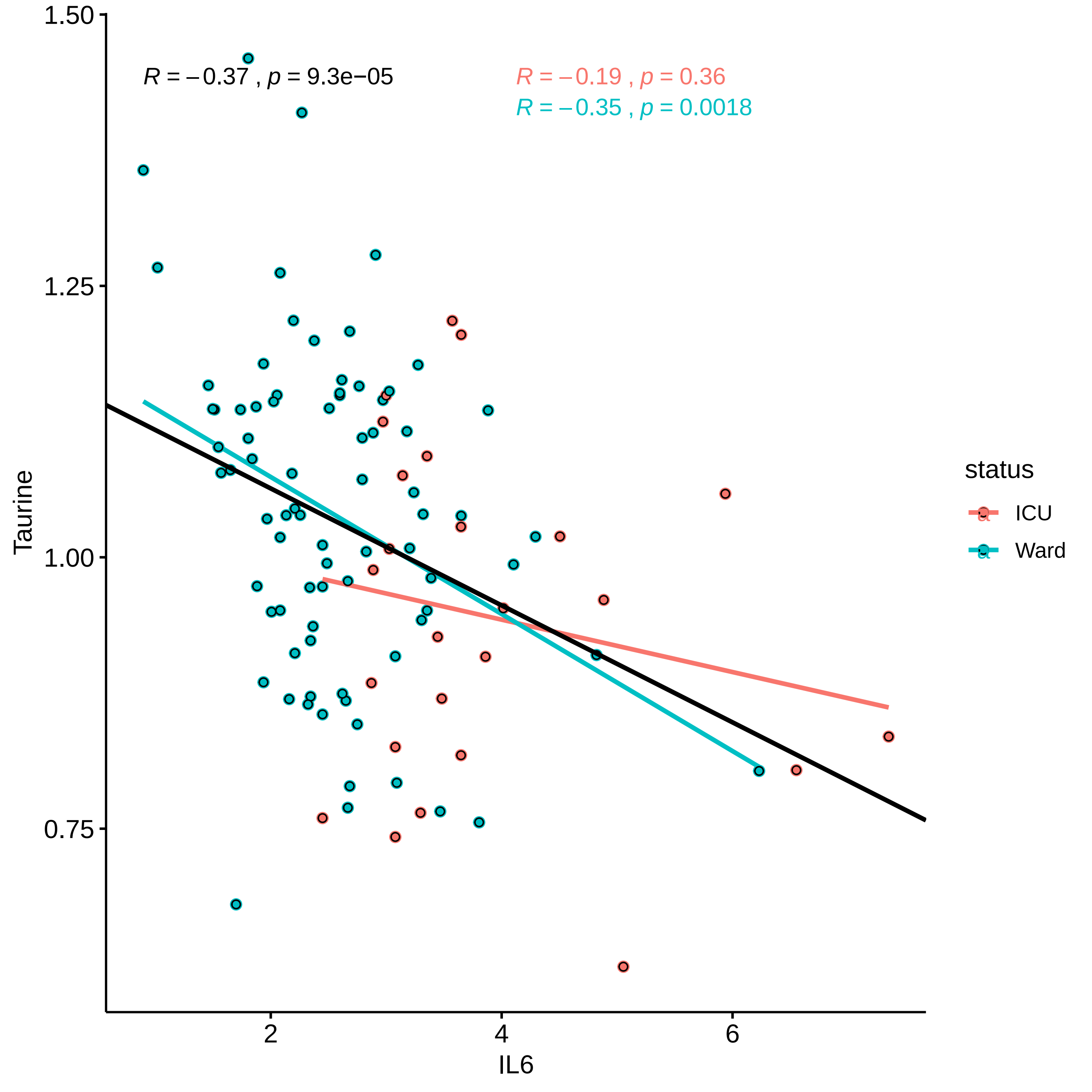

R = −0.37 , p = 9.3e−05
R = −0.19 , p = 0.36
R = −0.35 , p = 0.0018
1.50
1.25
1.00
0.75
Taurine
2
4
6
IL6
status
ICU
Ward

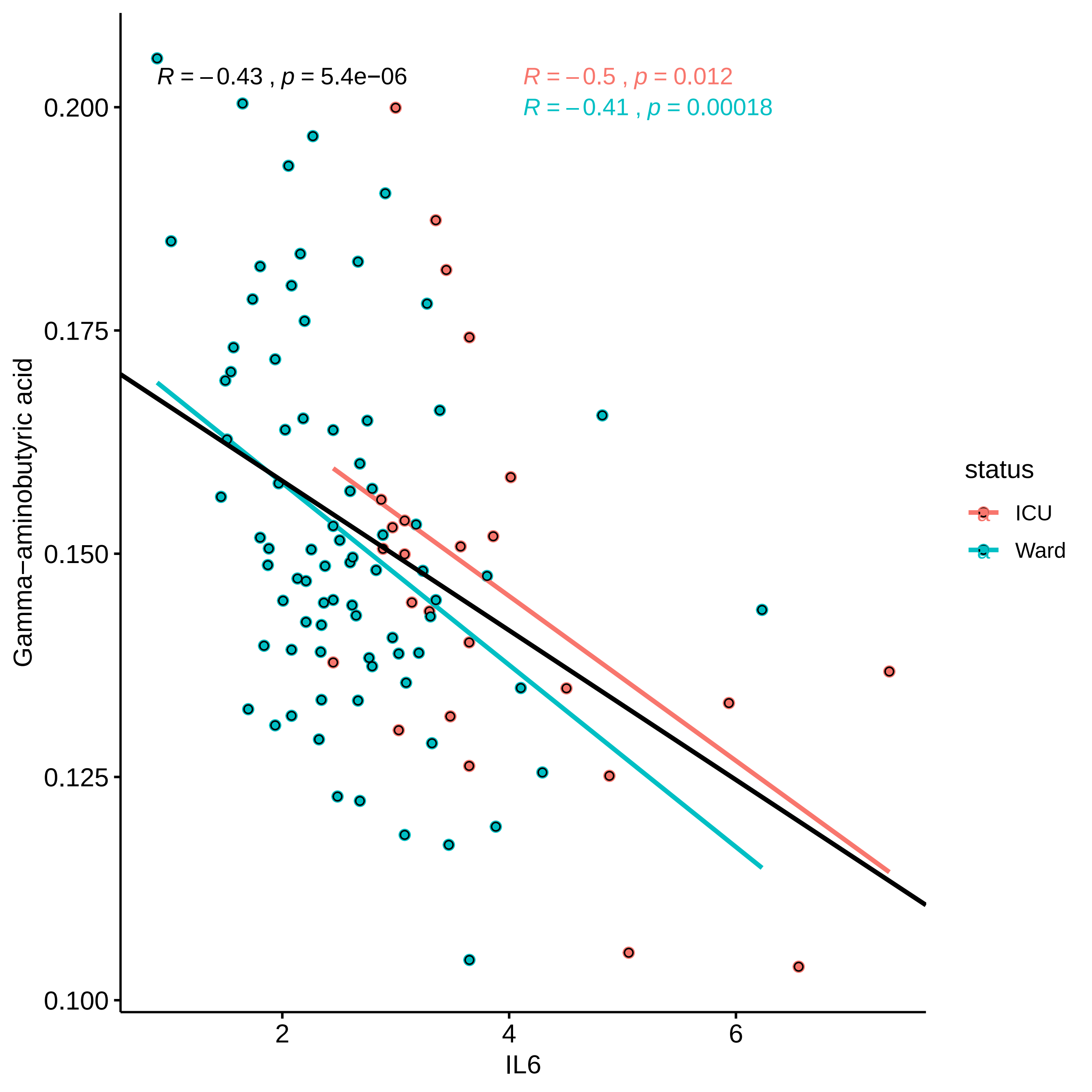

R = −0.43 , p = 5.4e−06
R = −0.5 , p = 0.012
R = −0.41 , p = 0.00018
0.200
0.175
0.150
0.125
0.100
Gamma−aminobutyric acid
2
4
6
IL6
status
ICU
Ward

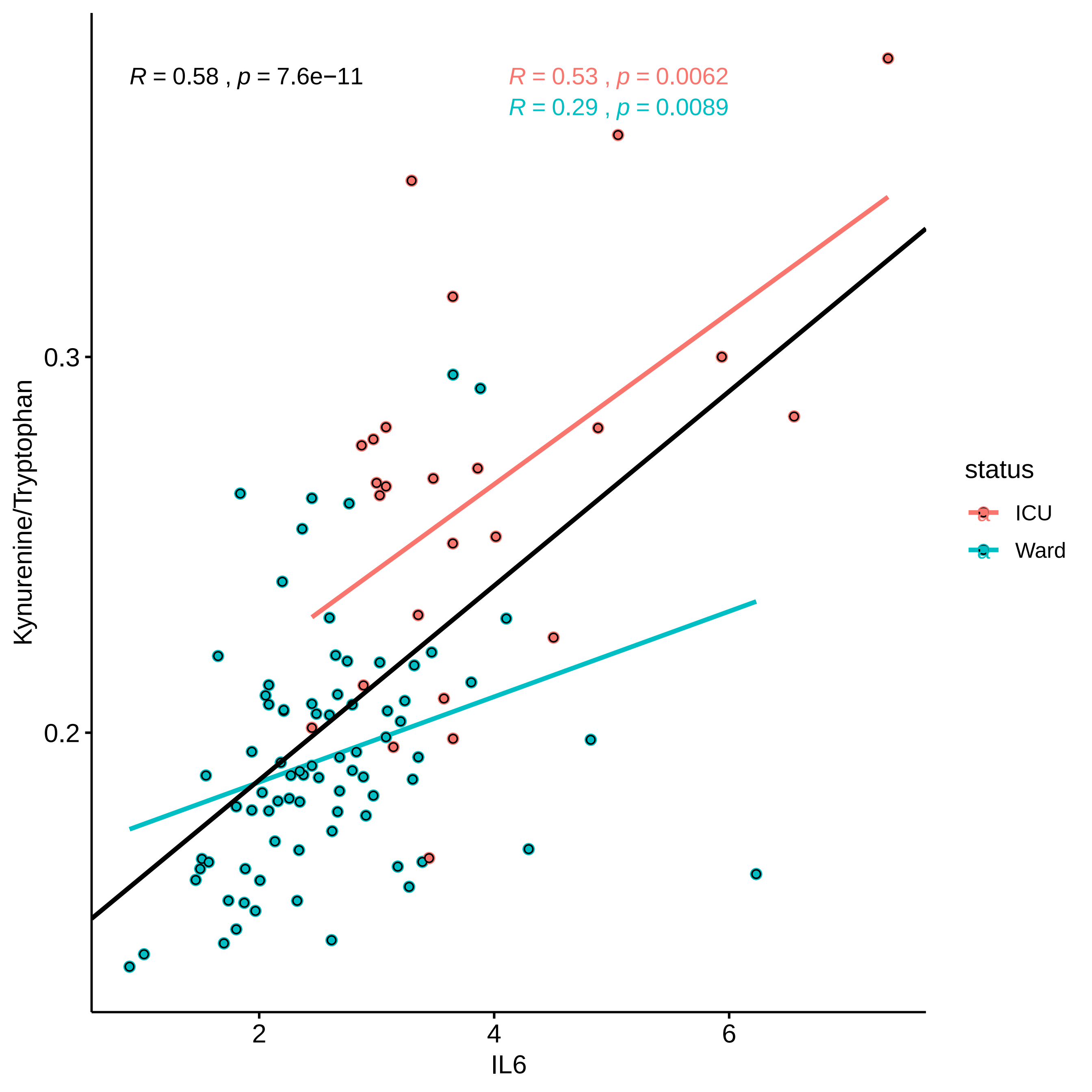

R = 0.58 , p = 7.6e−11
R = 0.53 , p = 0.0062
R = 0.29 , p = 0.0089
Kynurenine/Tryptophan
0.3
0.2
2
4
6
IL6
status
ICU
Ward

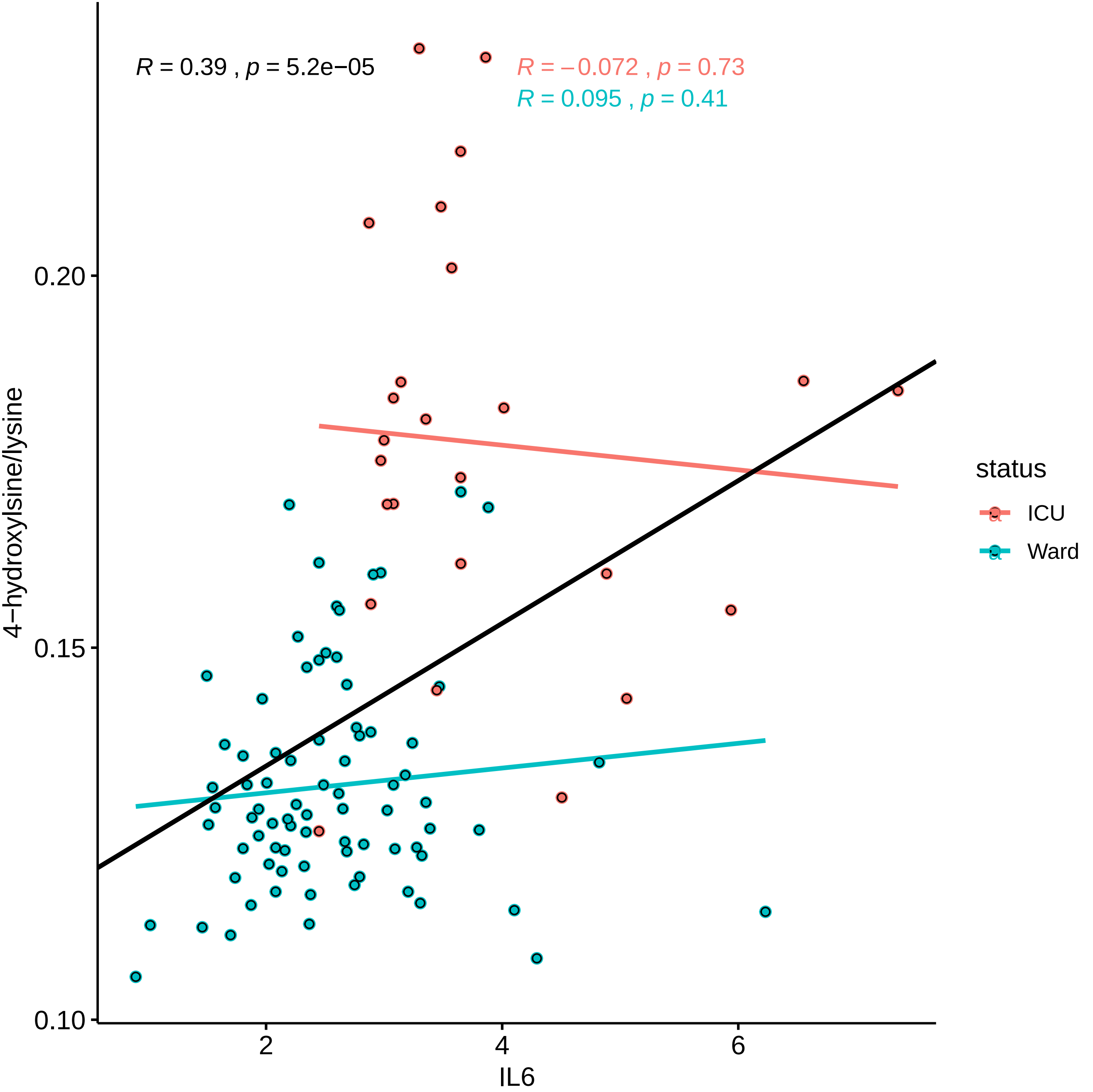

R = 0.39 , p = 5.2e−05
R = −0.072 , p = 0.73
R = 0.095 , p = 0.41
4−hydroxylsine/lysine
0.20
0.15
0.10
2
4
6
IL6
status
ICU
Ward

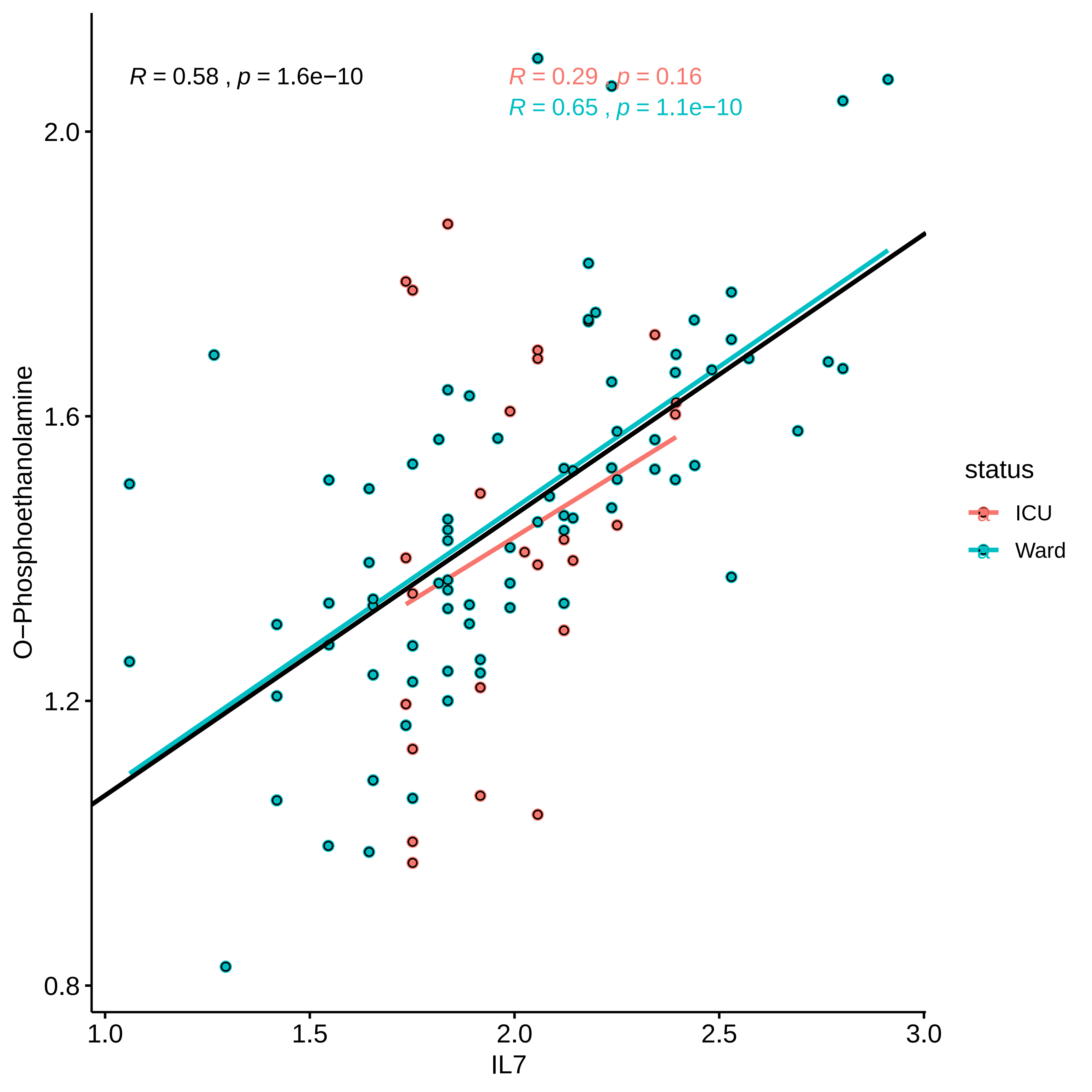

R = 0.58 , p = 1.6e−10
R = 0.29 , p = 0.16
R = 0.65 , p = 1.1e−10
O−Phosphoethanolamine
IL7
status
ICU
Ward
2.0
1.6
1.2
0.8
1.0
1.5
2.0
2.5
3.0

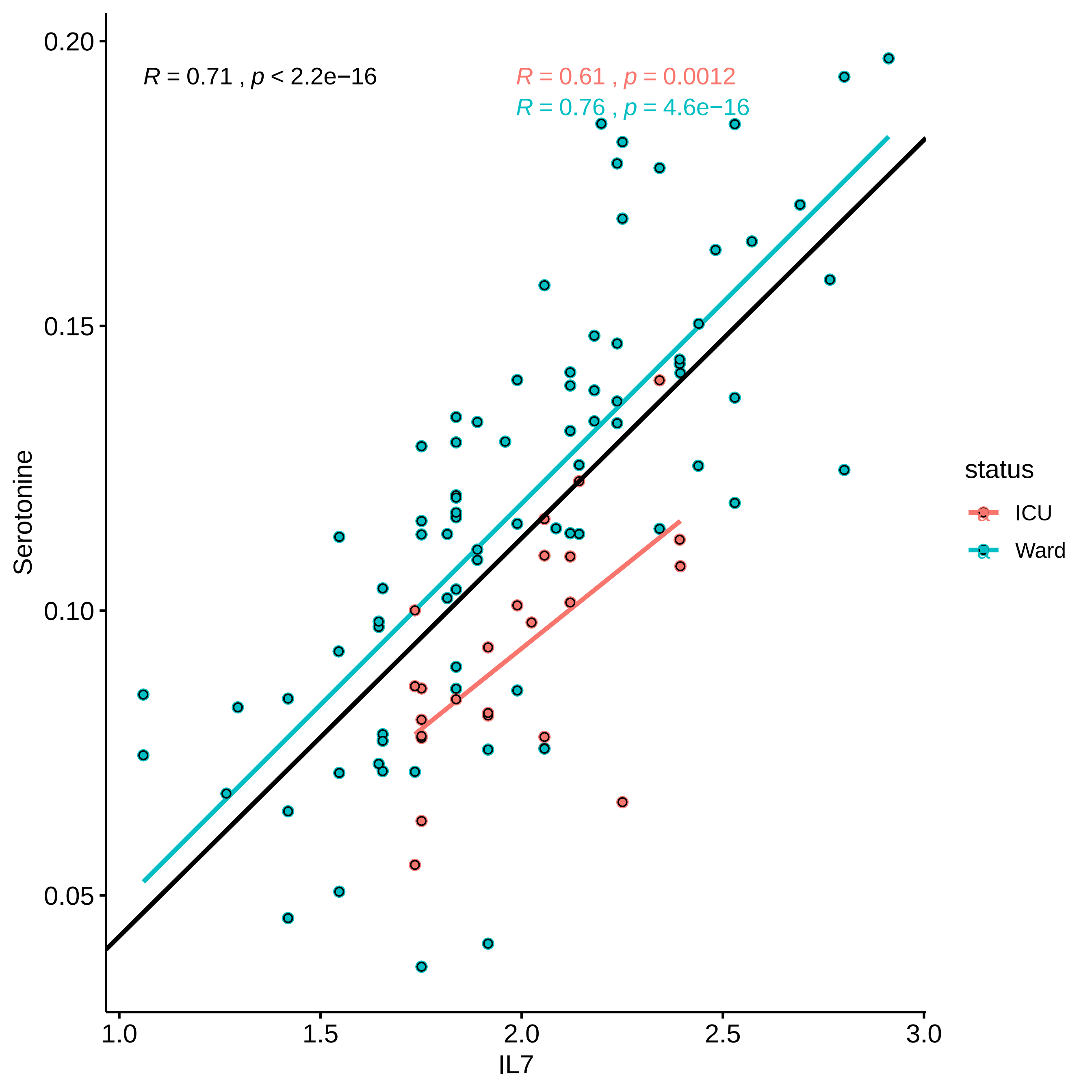
R = 0.71 , p < 2.2e−16
R = 0.61 , p = 0.0012
R = 0.76 , p = 4.6e−16
Serotonine
0.20
0.15
0.10
0.05
1.0
1.5
2.0
2.5
3.0
IL7
status
ICU
Ward

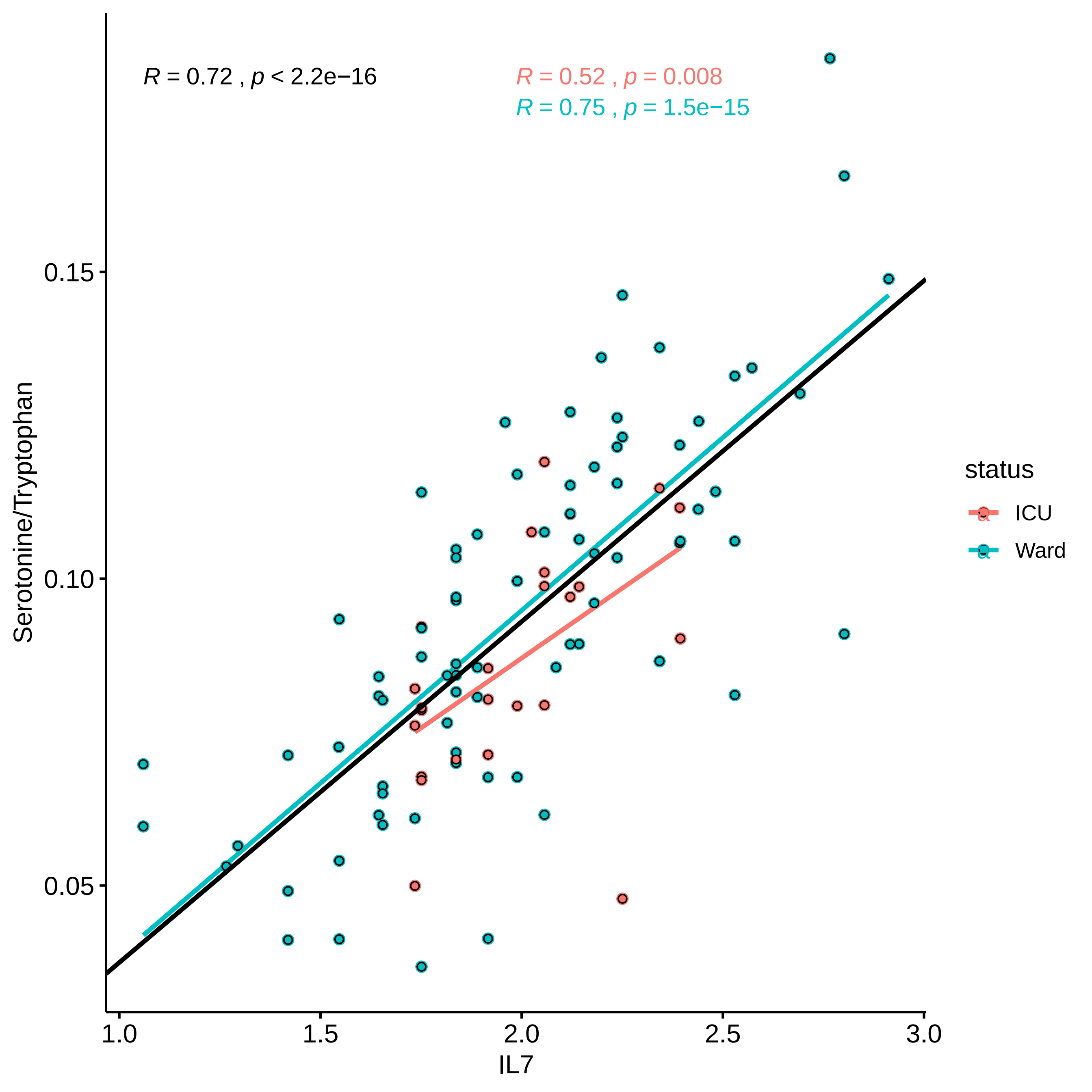

R = 0.72 , p < 2.2e−16
R = 0.52 , p = 0.008
R = 0.75 , p = 1.5e−15
Serotonine/Tryptophan
0.15
0.10
0.05
1.0
1.5
2.0
2.5
3.0
IL7
status
ICU
Ward

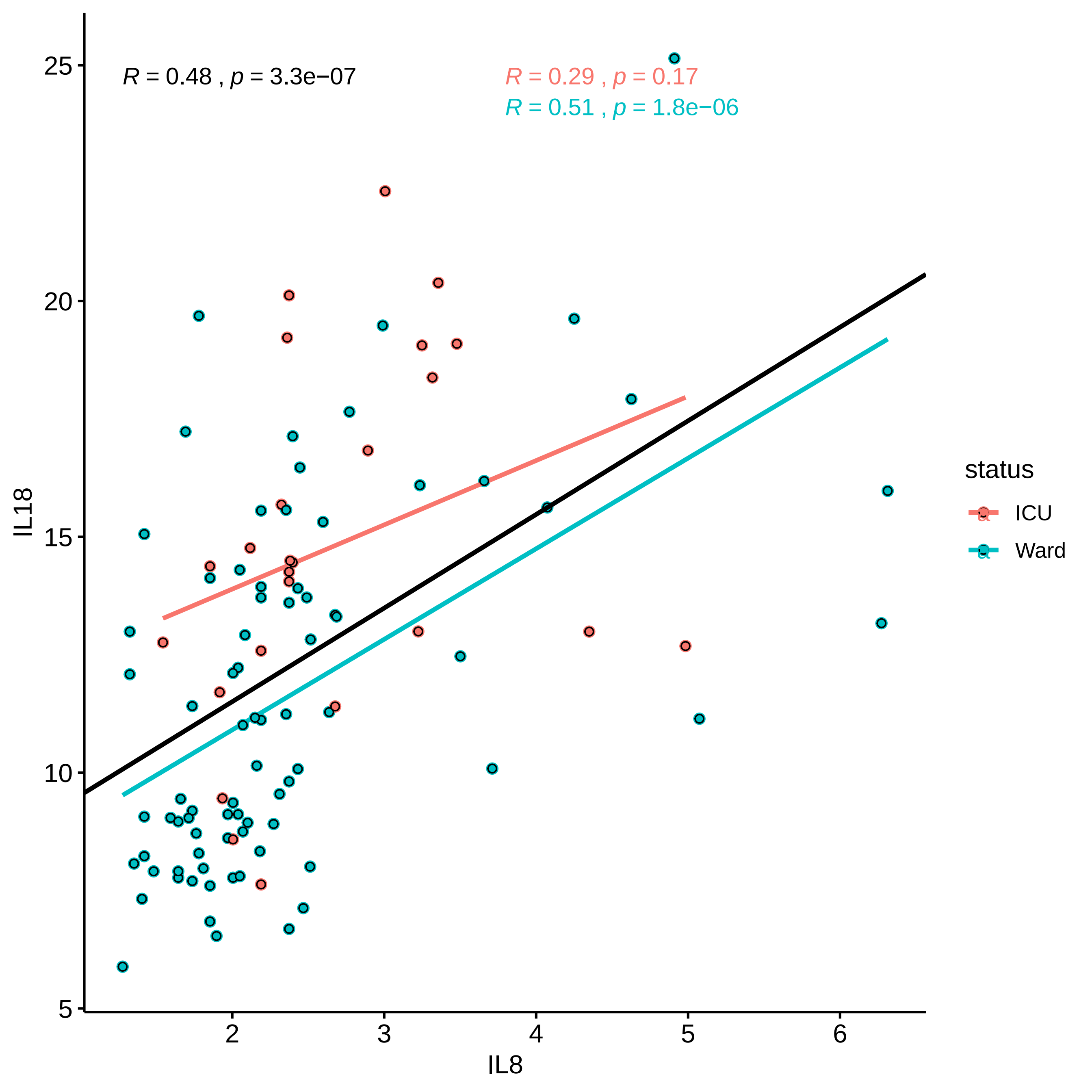

R = 0.48 , p = 3.3e−07
R = 0.29 , p = 0.17
R = 0.51 , p = 1.8e−06
25
20
15
10
5
IL18
2
3
4
5
6
IL8
status
ICU
Ward

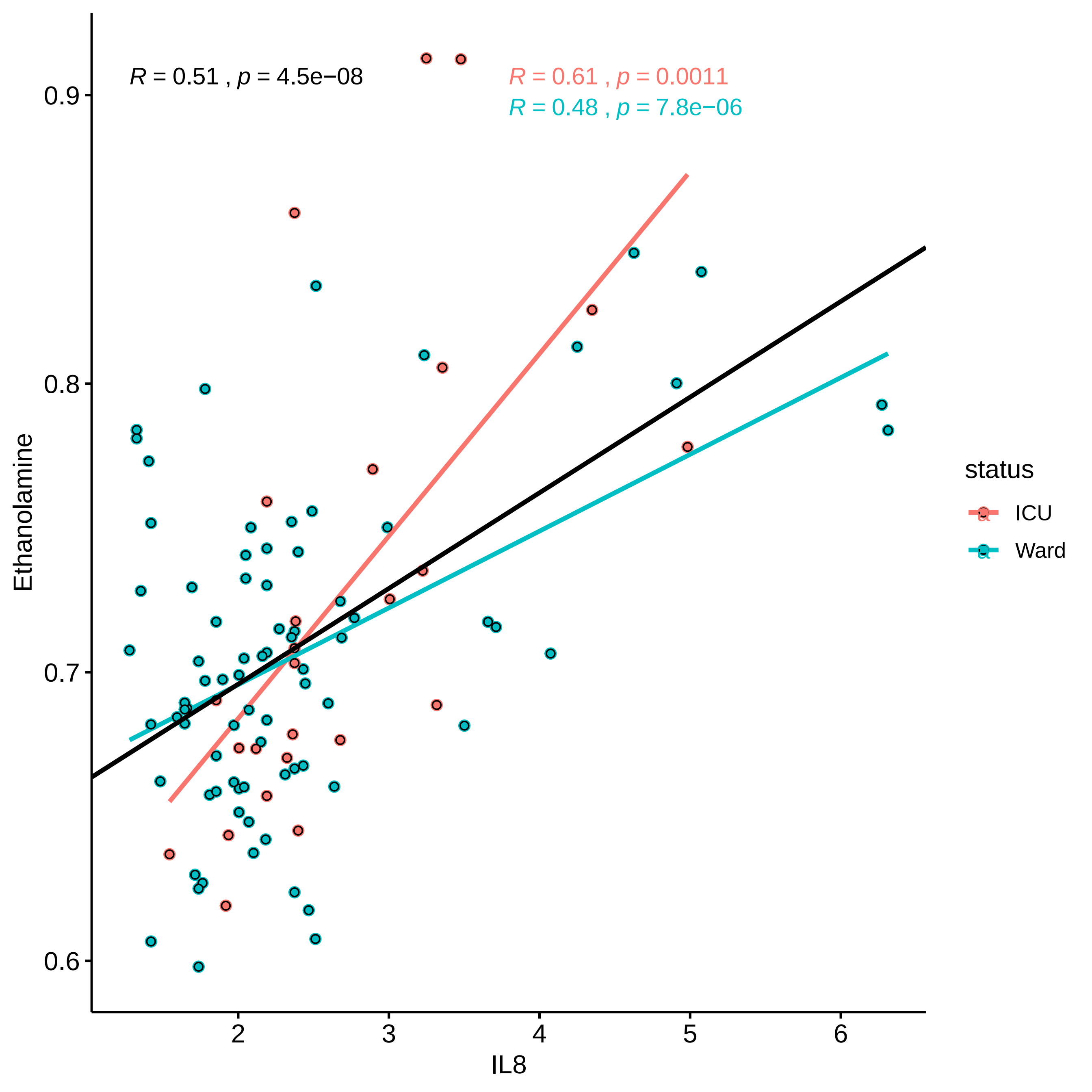

R = 0.51 , p = 4.5e−08
R = 0.61 , p = 0.0011
R = 0.48 , p = 7.8e−06
0.9
0.8
0.7
0.6
Ethanolamine
2
3
4
5
6
IL8
status
ICU
Ward

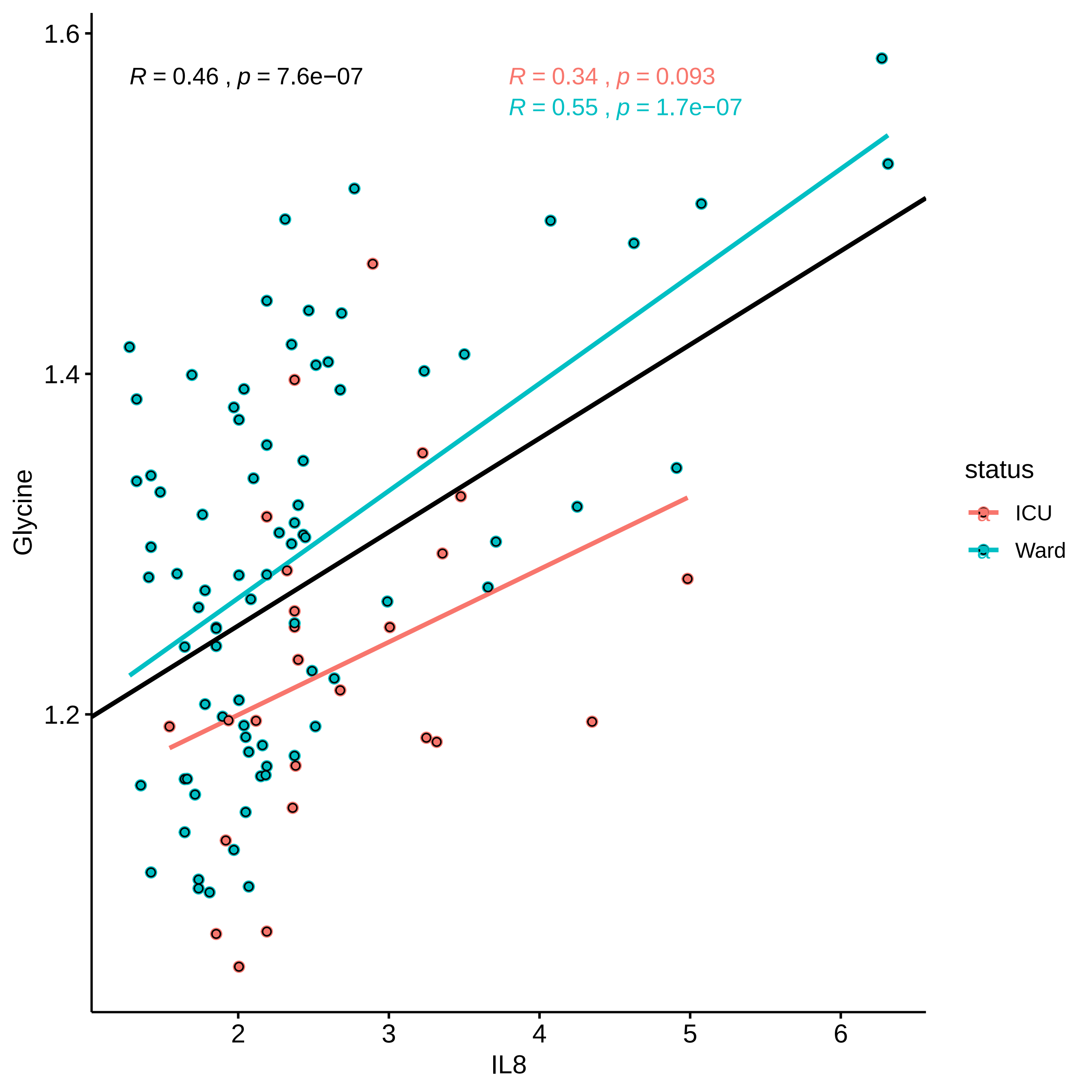

R = 0.46 , p = 7.6e−07
R = 0.34 , p = 0.093
R = 0.55 , p = 1.7e−07
Glycine
IL8
1.6
1.4
1.2
2
3
4
5
6
status
ICU
Ward

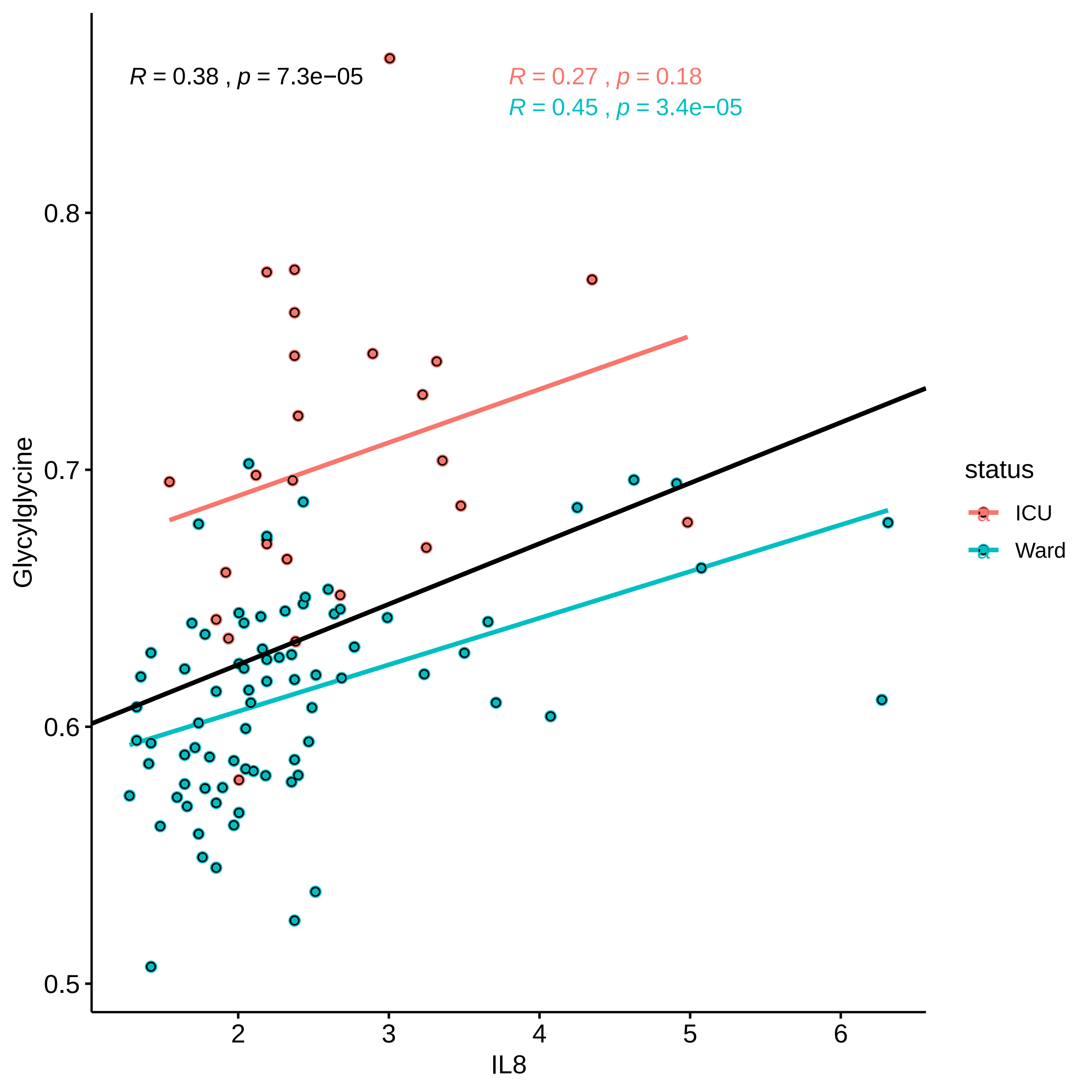

R = 0.38 , p = 7.3e−05
R = 0.27 , p = 0.18
R = 0.45 , p = 3.4e−05
Glycylglycine
0.8
0.7
0.6
0.5
2
3
4
5
6
IL8
status
ICU
Ward

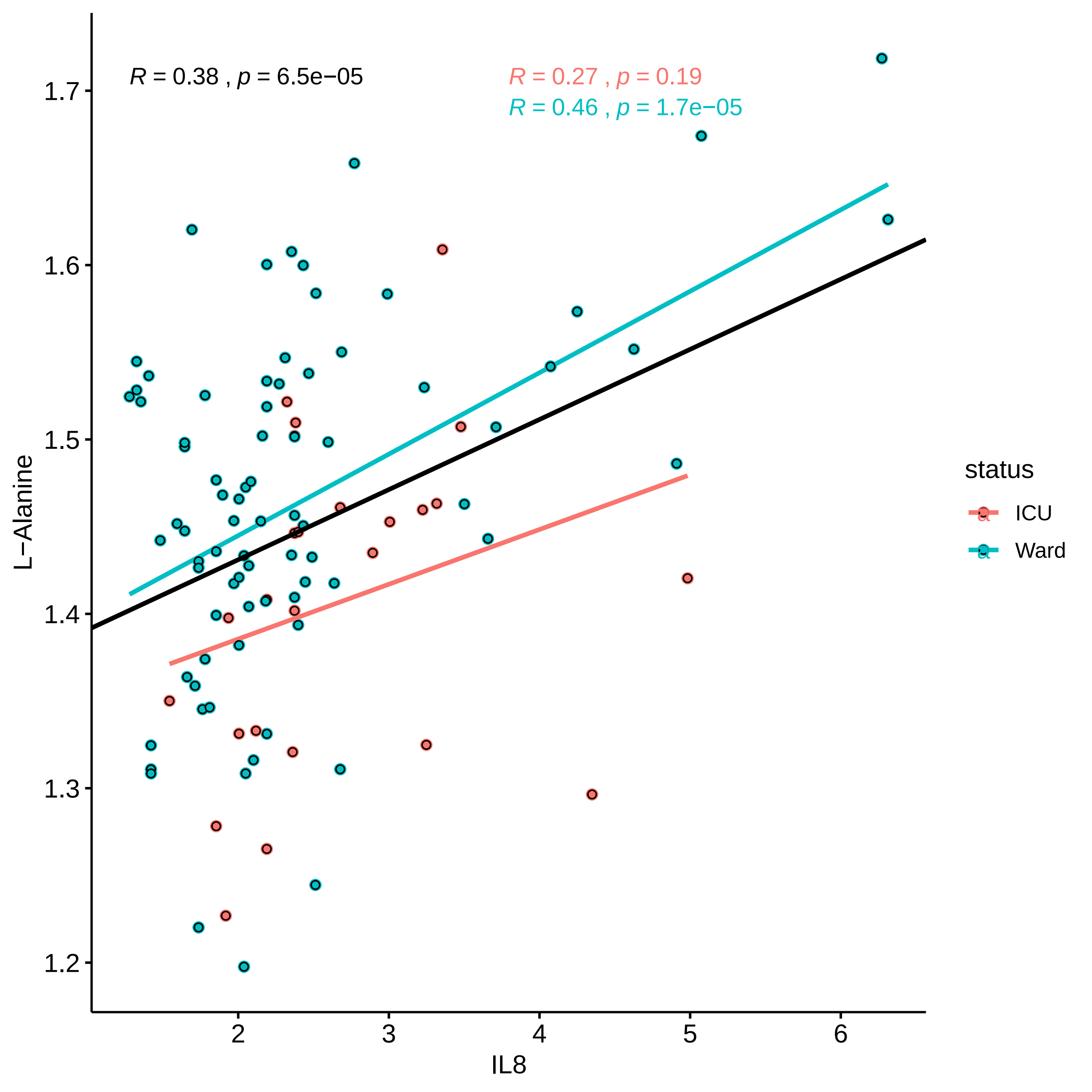
R = 0.38 , p = 6.5e−05
R = 0.27 , p = 0.19
R = 0.46 , p = 1.7e−05
L−Alanine
1.7
1.6
1.5
1.4
1.3
1.2
2
3
4
5
6
IL8
status
ICU
Ward

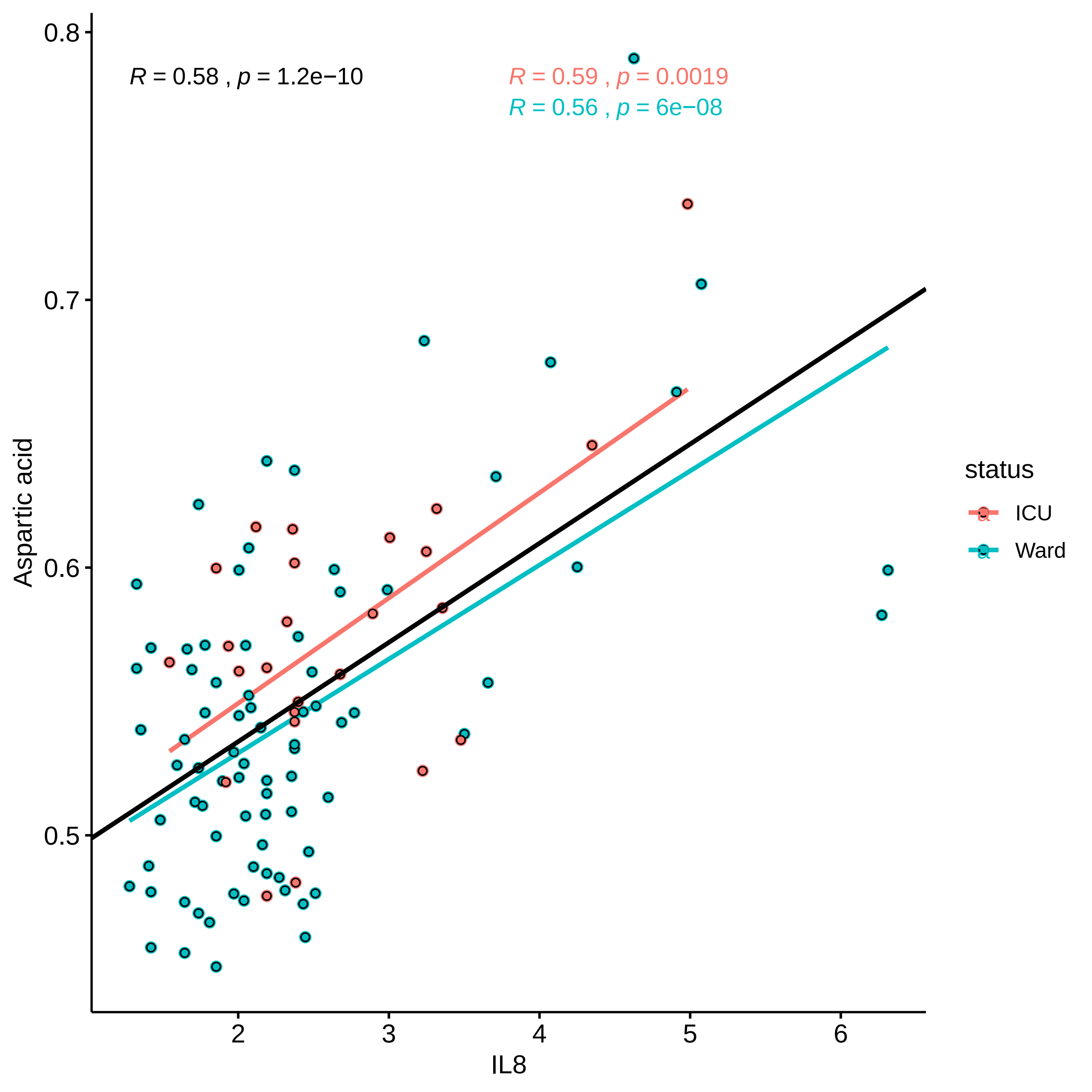

R = 0.58 , p = 1.2e−10
R = 0.59 , p = 0.0019
R = 0.56 , p = 6e−08
0.8
0.7
0.6
0.5
Aspartic acid
2
3
4
5
6
IL8
status
ICU
Ward

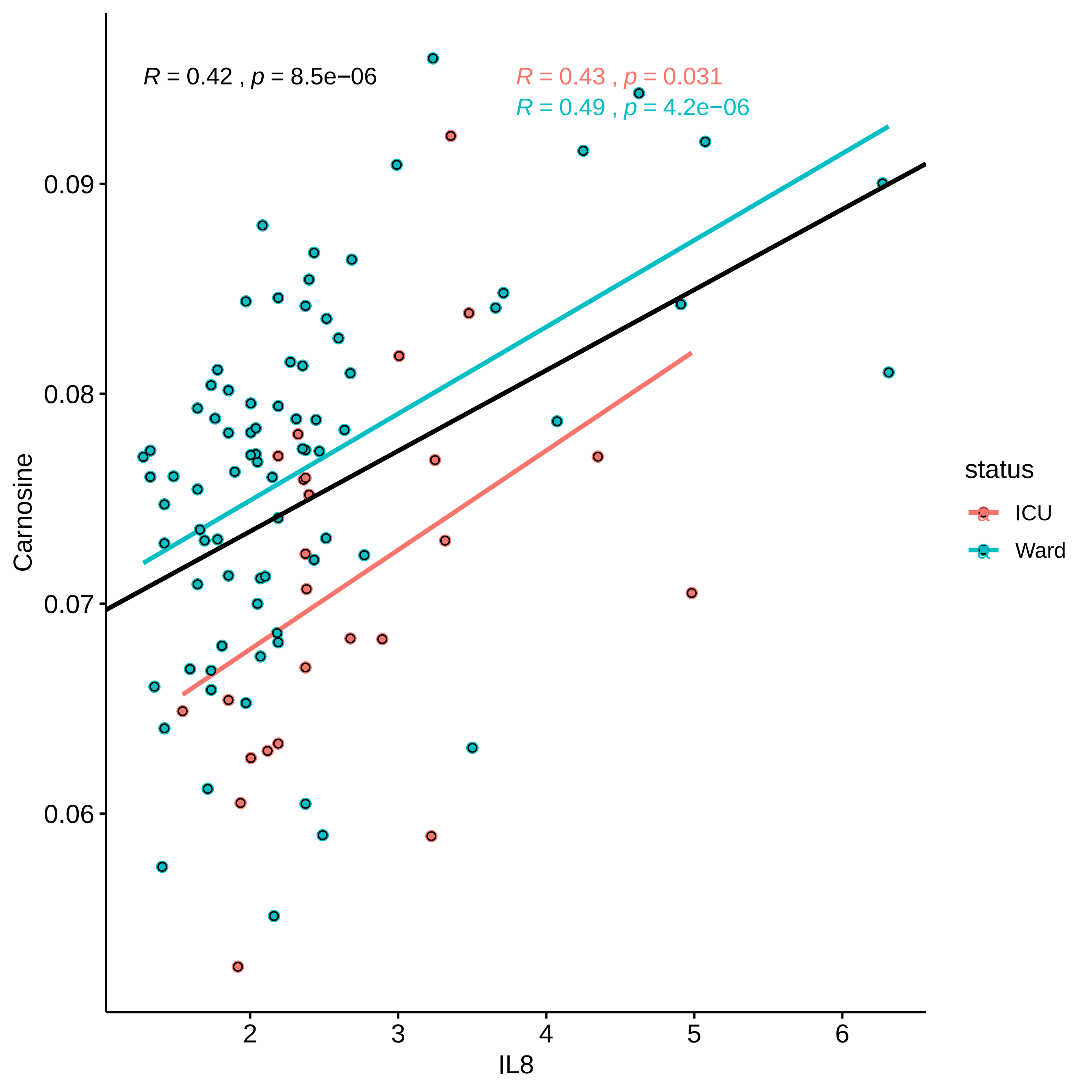

R = 0.42 , p = 8.5e−06
R = 0.43 , p = 0.031
R = 0.49 , p = 4.2e−06
0.09
0.08
0.07
0.06
Carnosine
2
3
4
5
6
IL8
status
ICU
Ward

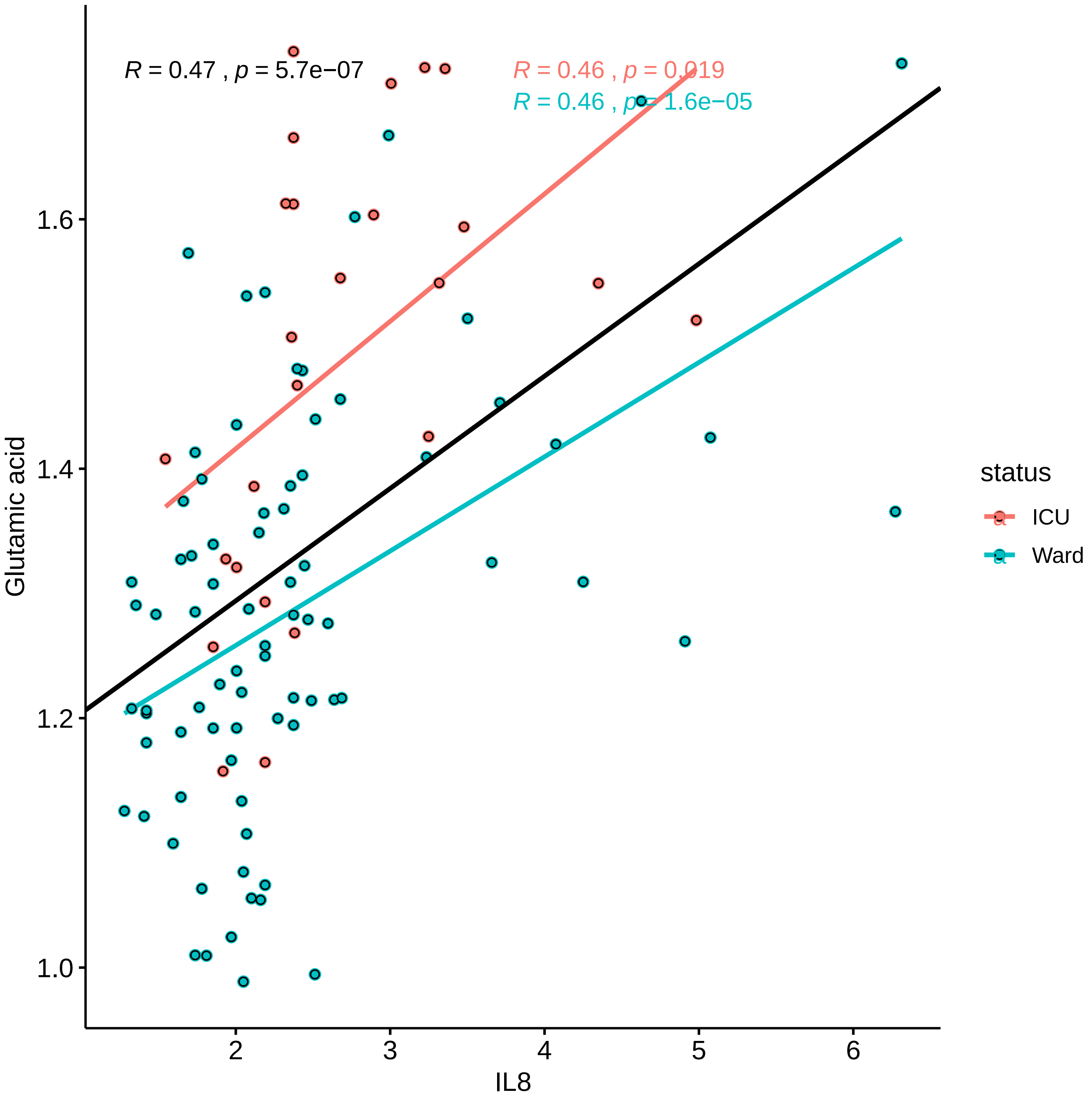

R = 0.47 , p = 5.7e−07
R = 0.46 , p = 0.019
R = 0.46 , p = 1.6e−05
Glutamic acid
1.0
1.2
1.4
1.6
IL8
2
3
4
5
6
status
ICU
Ward

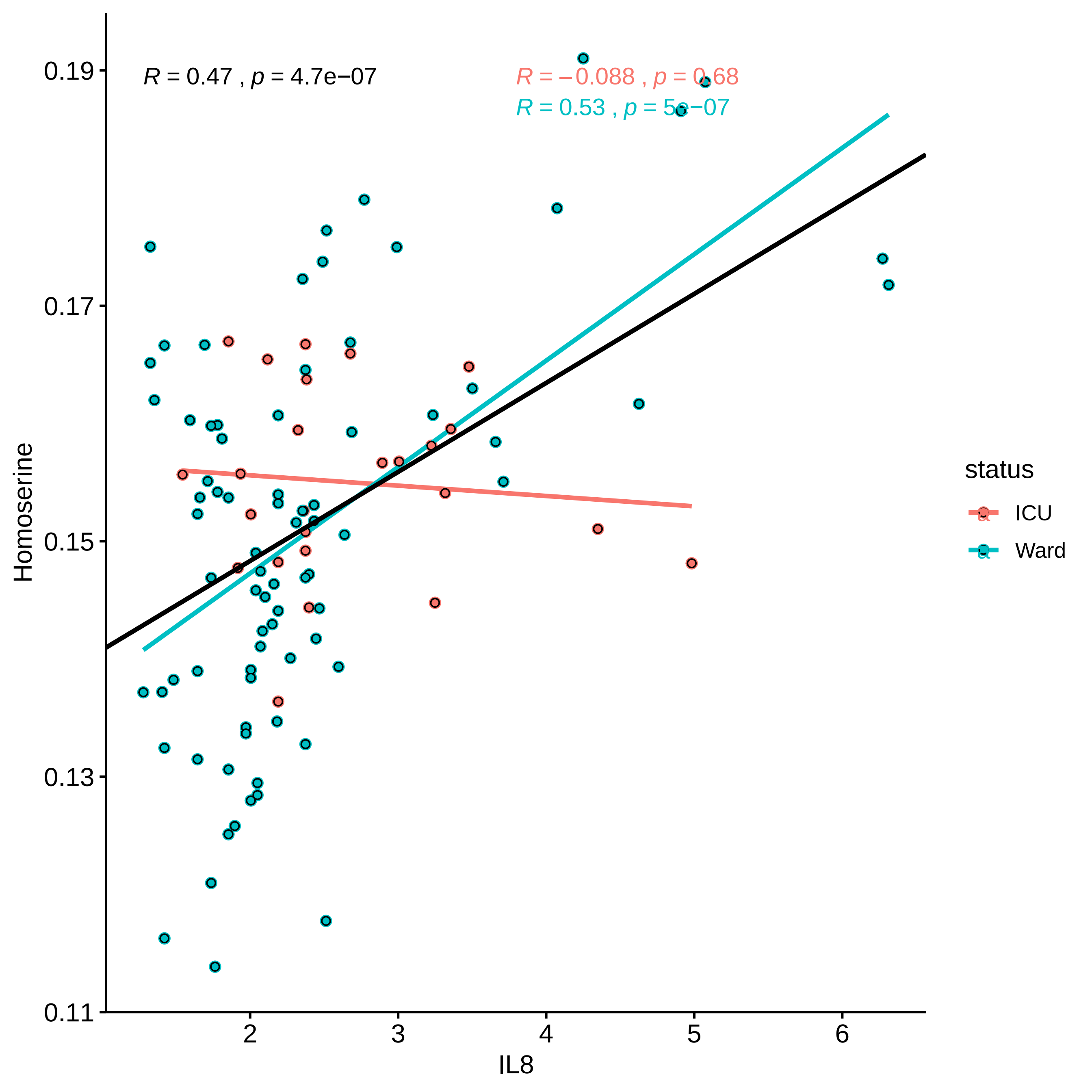

R = 0.47 , p = 4.7e−07
R = −0.088 , p = 0.68
R = 0.53 , p = 5e−07
Homoserine
0.19
0.17
0.15
0.13
0.11
2
3
4
5
6
IL8
status
ICU
Ward

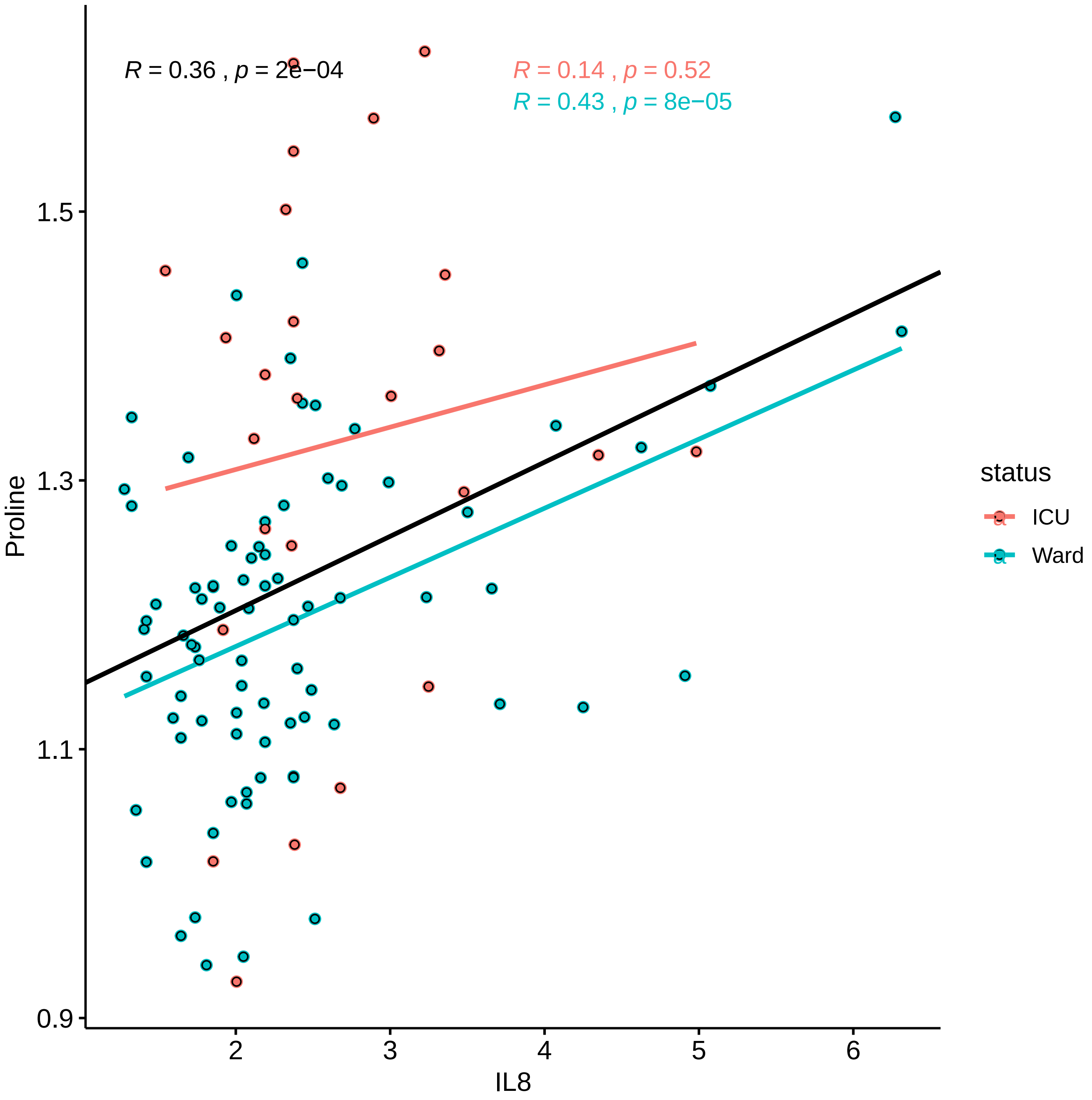

R = 0.36 , p = 2e−04
R = 0.14 , p = 0.52
R = 0.43 , p = 8e−05
Proline
1.5
1.3
1.1
0.9
2
3
4
5
6
IL8
status
ICU
Ward

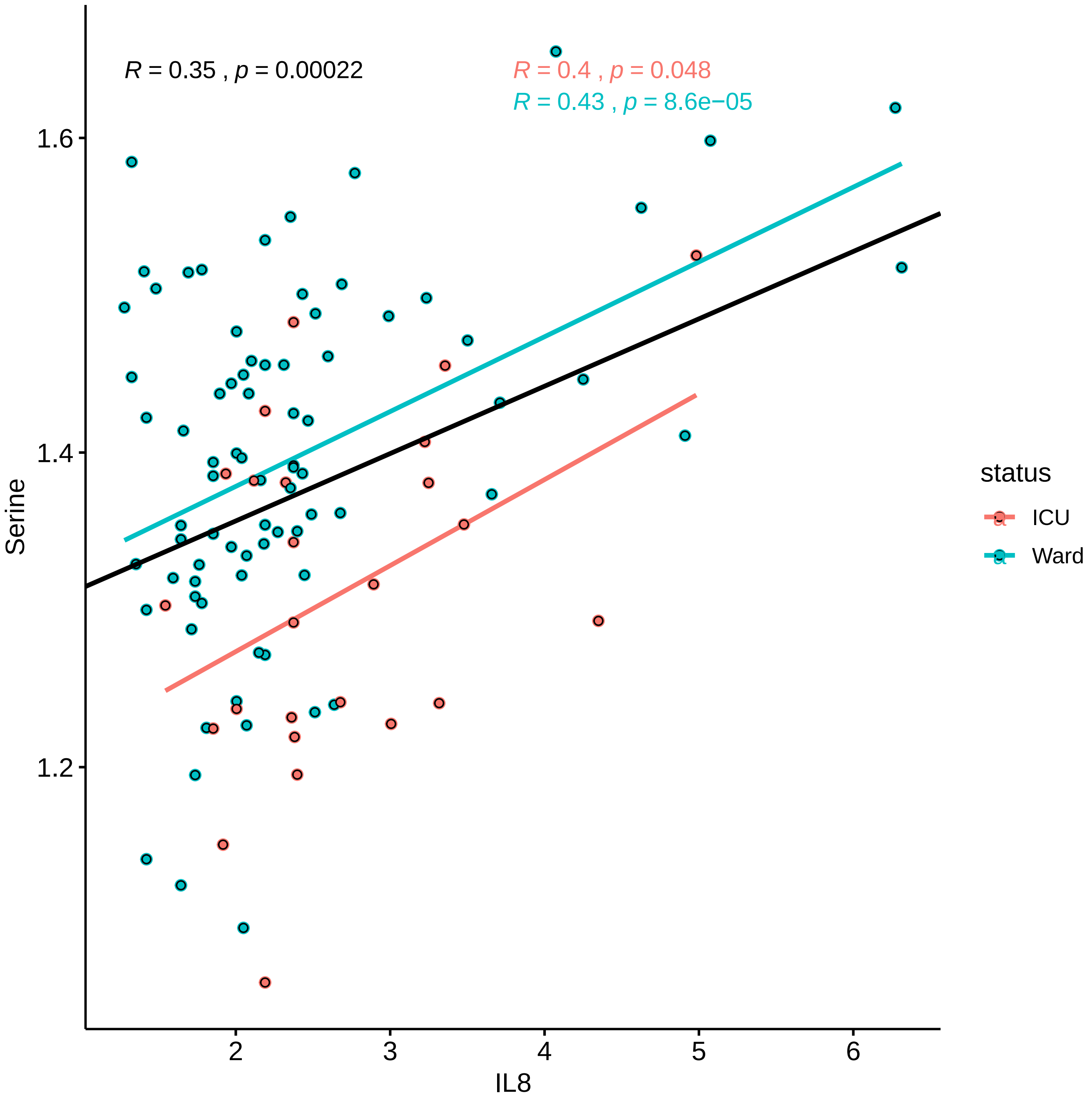

R = 0.35 , p = 0.00022
R = 0.4 , p = 0.048
R = 0.43 , p = 8.6e−05
1.6
1.4
1.2
Serine
2
3
4
5
6
IL8
status
ICU
Ward

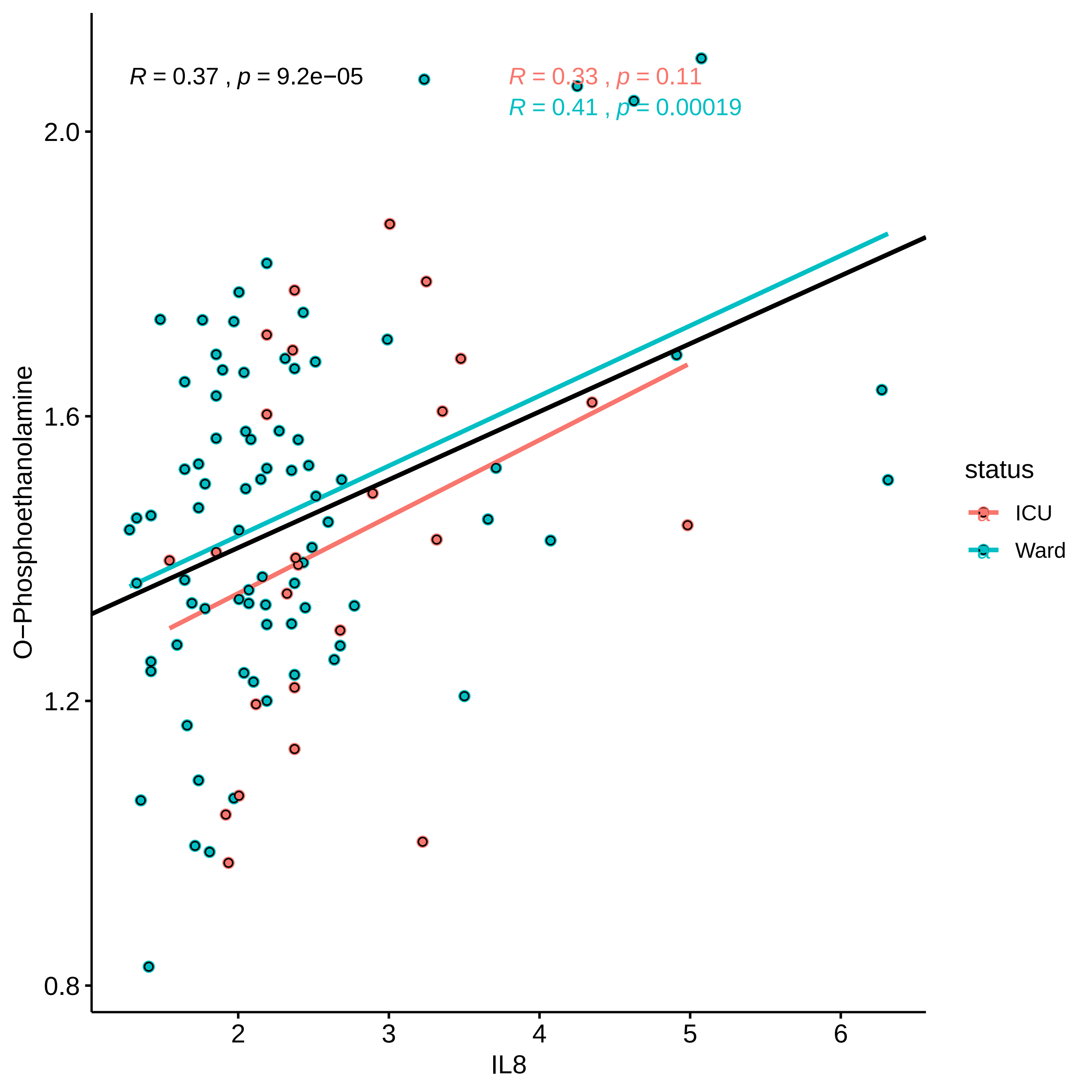

R = 0.37 , p = 9.2e−05
R = 0.33 , p = 0.11
R = 0.41 , p = 0.00019
2.0
1.6
1.2
0.8
O−Phosphoethanolamine
2
3
4
5
6
IL8
status
ICU
Ward

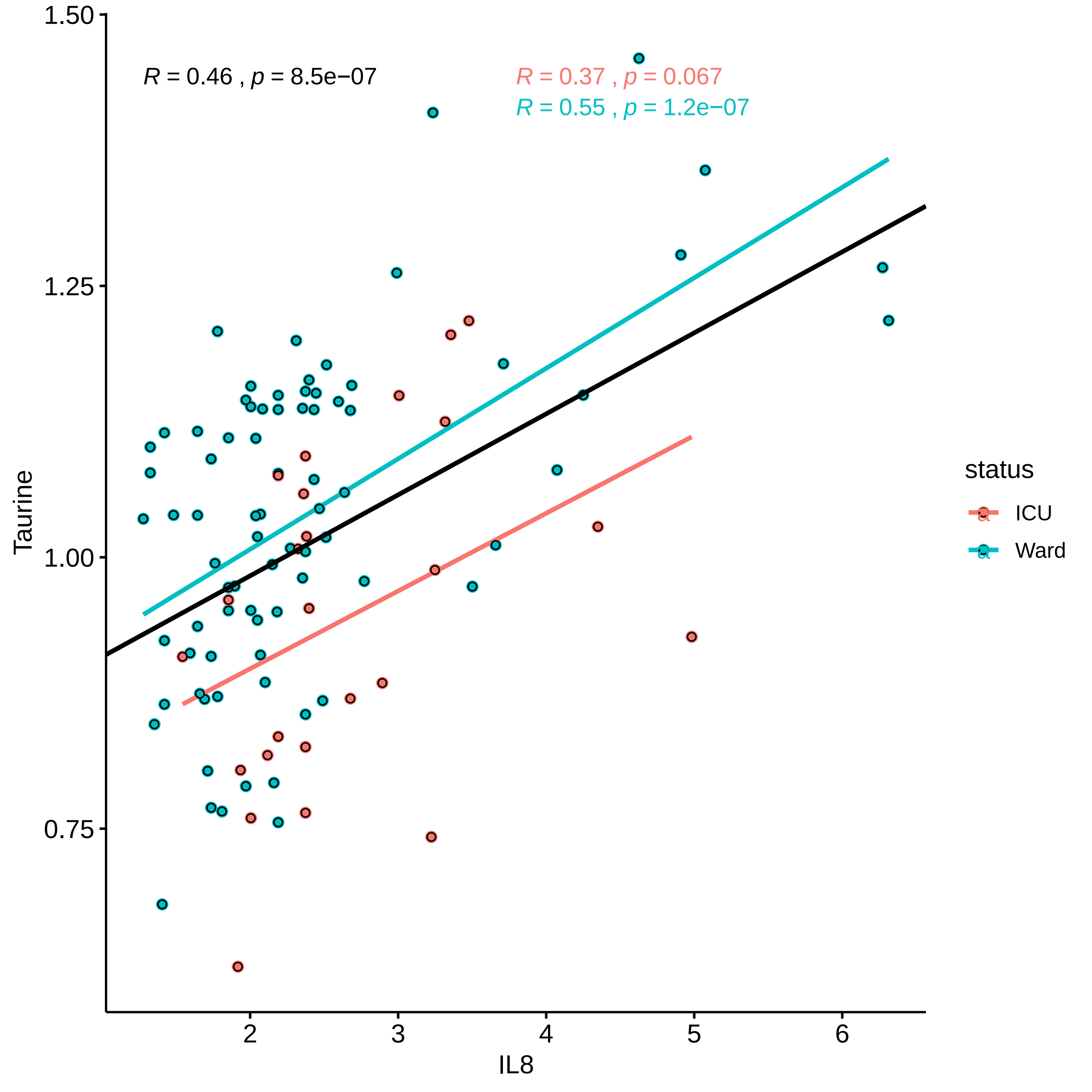

R = 0.46 , p = 8.5e−07
R = 0.37 , p = 0.067
R = 0.55 , p = 1.2e−07
Taurine
1.50
1.25
1.00
0.75
IL8
2
3
4
5
6
status
ICU
Ward

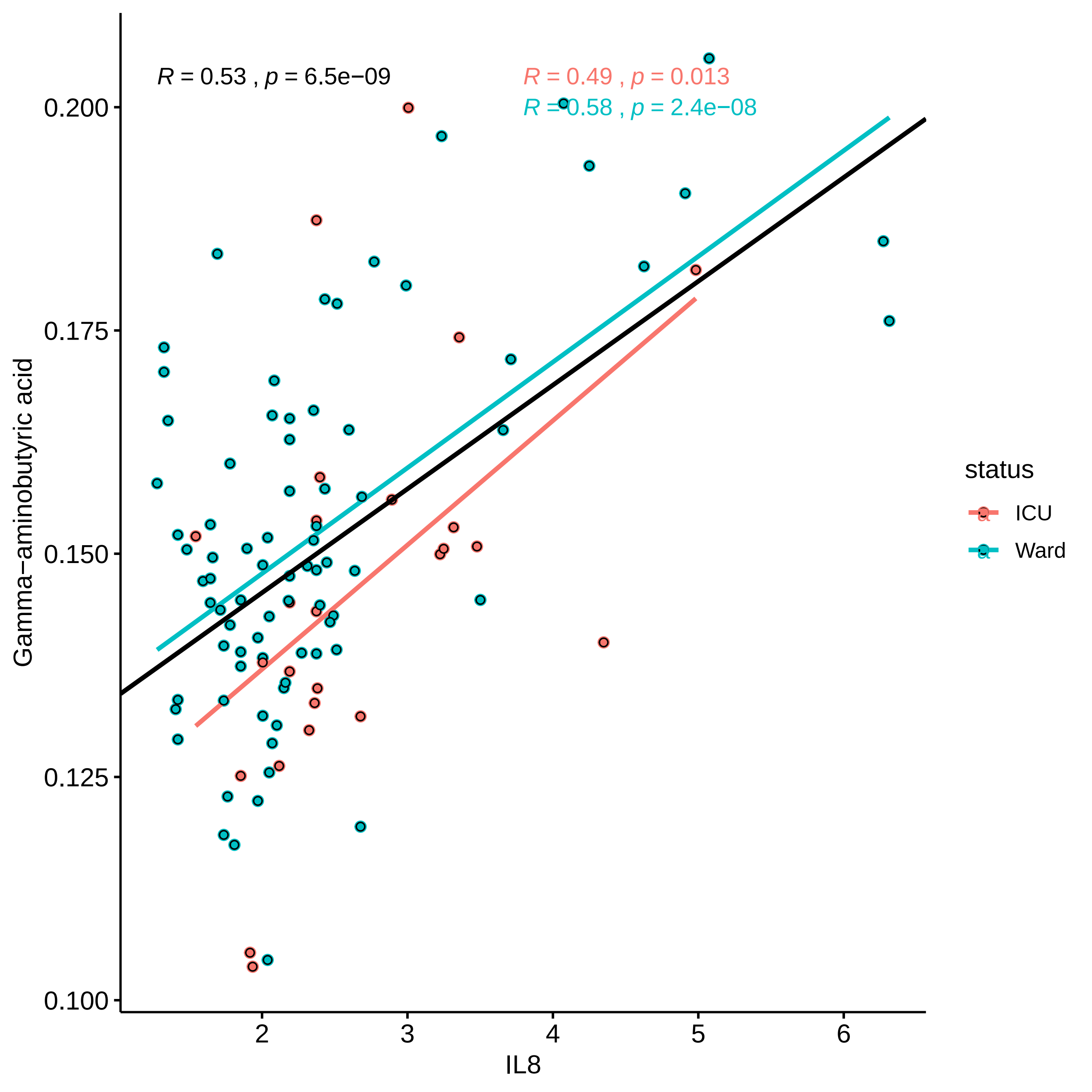

R = 0.53 , p = 6.5e−09
R = 0.49 , p = 0.013
R = 0.58 , p = 2.4e−08
Gamma−aminobutyric acid
0.200
0.175
0.150
0.125
0.100
2
3
4
5
6
IL8
status
ICU
Ward

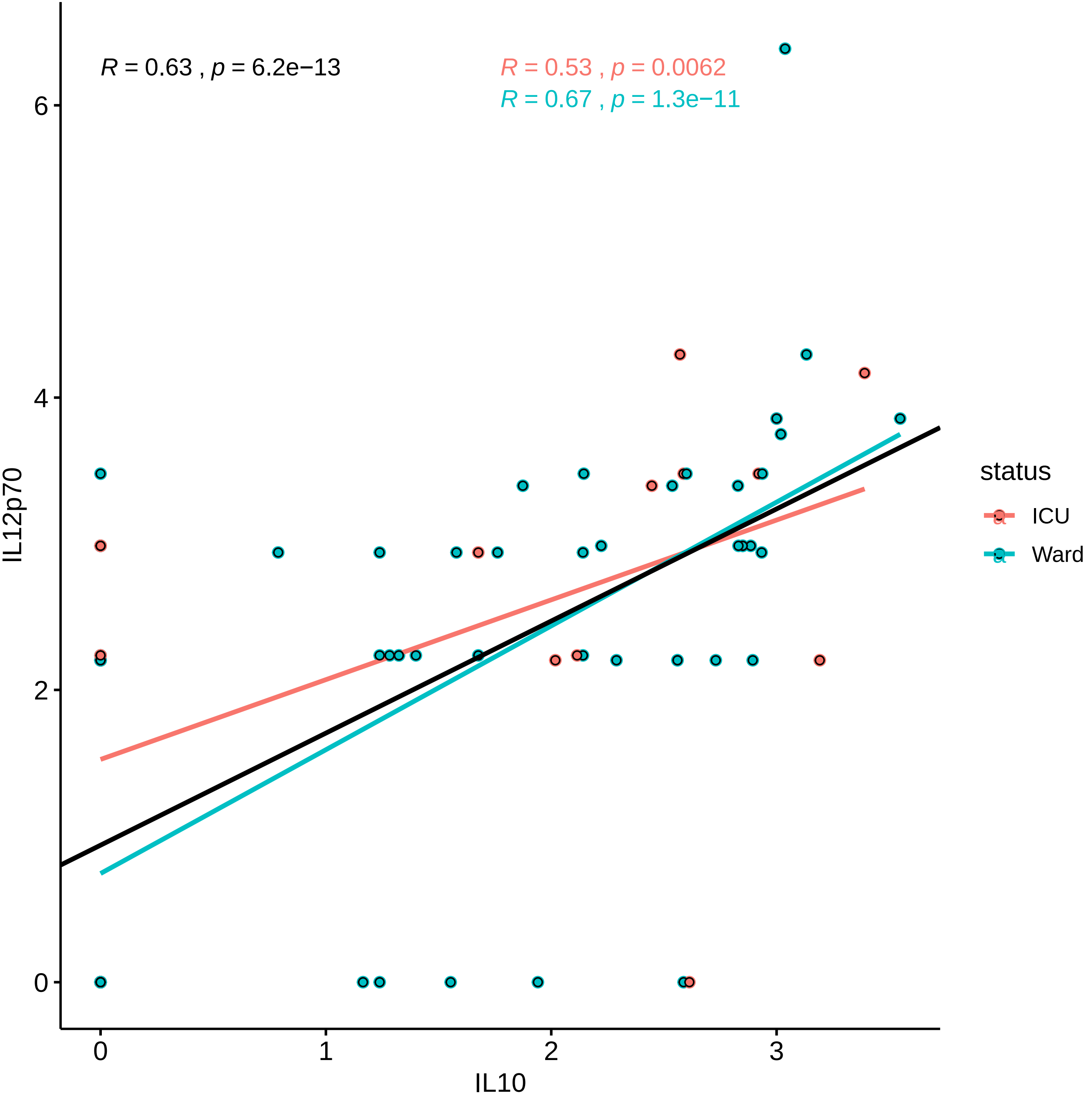

R = 0.63 , p = 6.2e−13
R = 0.53 , p = 0.0062
R = 0.67 , p = 1.3e−11
IL12p70
IL10
0
1
2
3
0
2
4
6
status
ICU
Ward

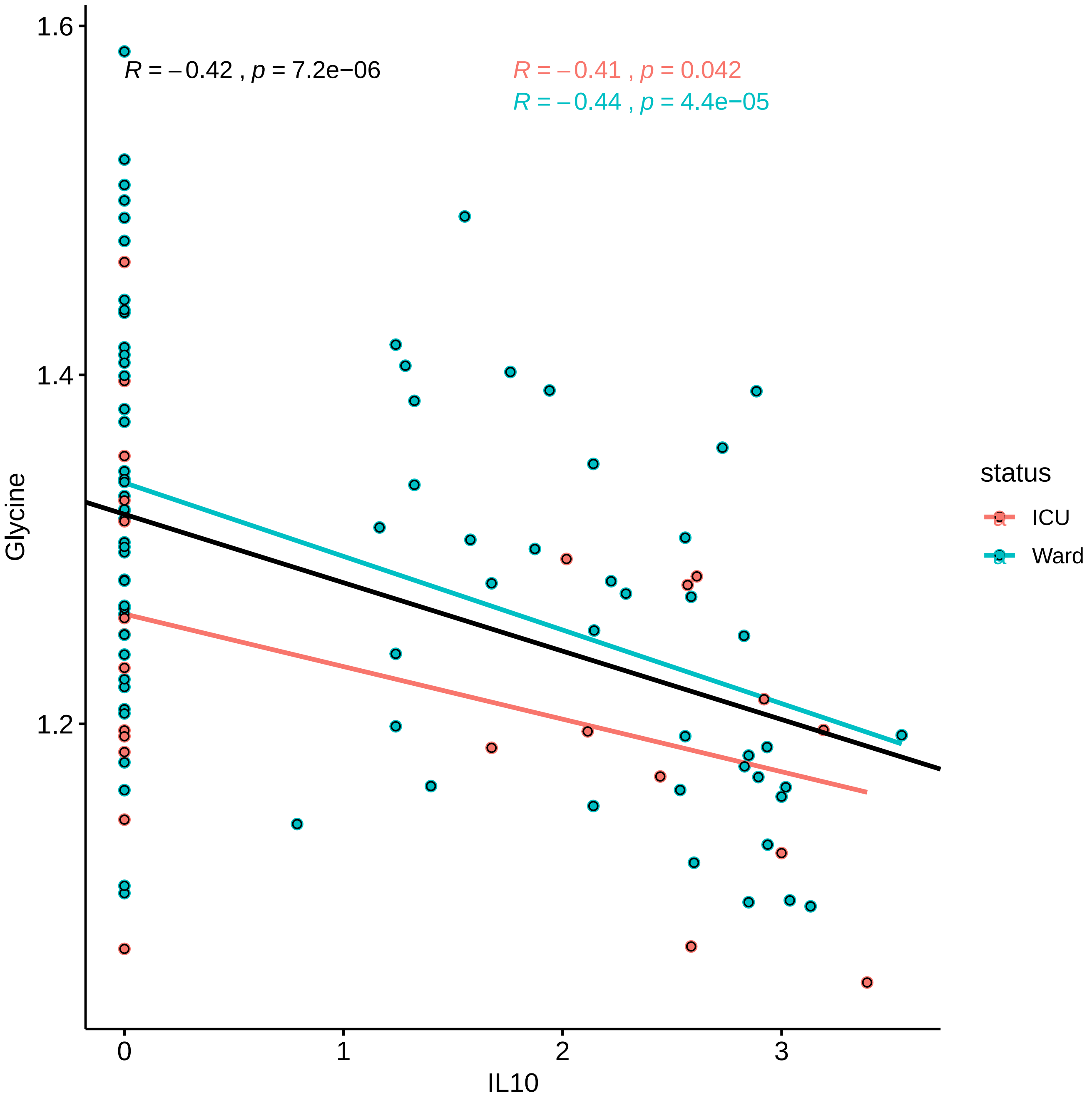

R = −0.42 , p = 7.2e−06
R = −0.41 , p = 0.042
R = −0.44 , p = 4.4e−05
1.6
1.4
1.2
Glycine
0
1
2
3
IL10
status
ICU
Ward

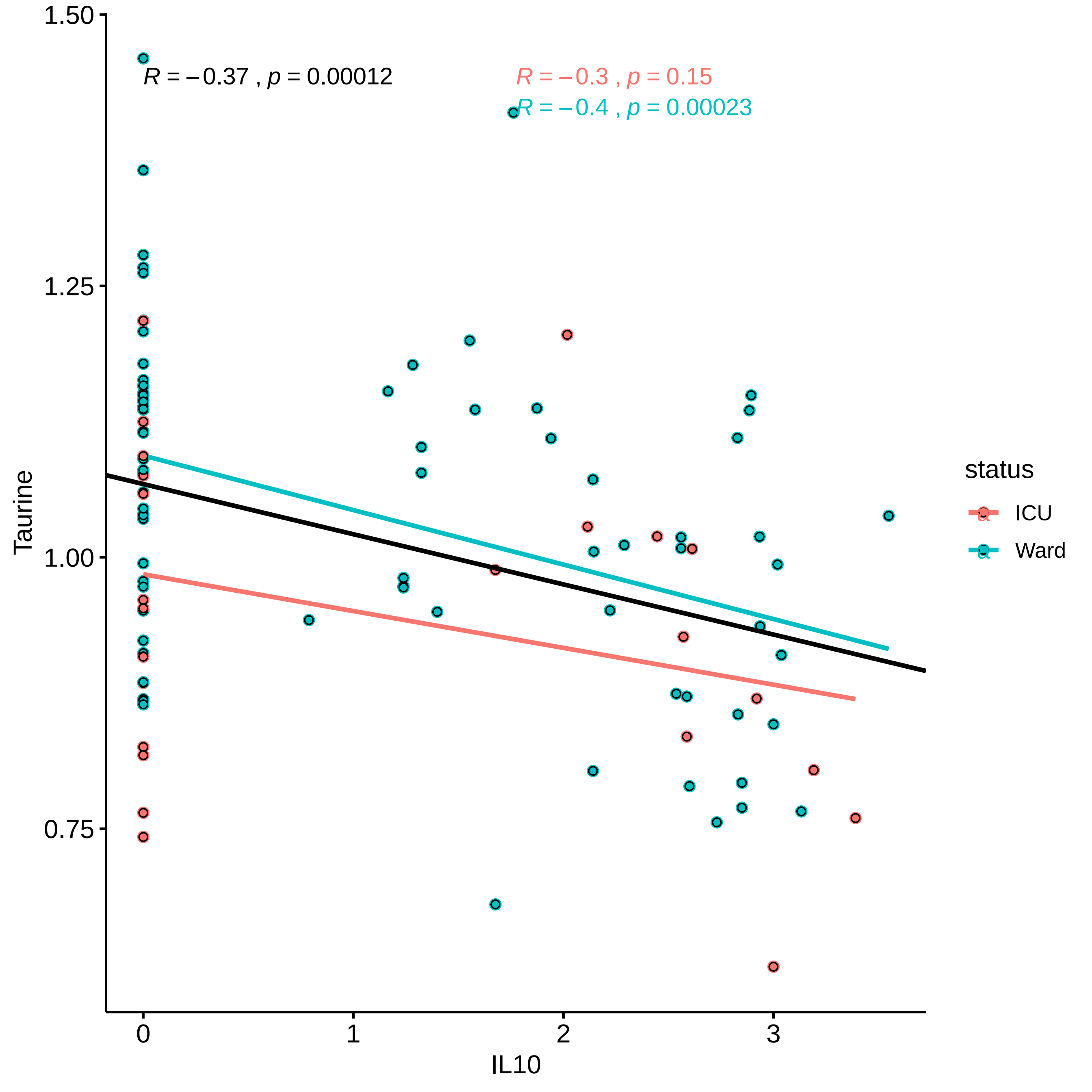

R = −0.37 , p = 0.00012
R = −0.3 , p = 0.15
R = −0.4 , p = 0.00023
1.50
1.25
1.00
0.75
Taurine
0
1
2
3
IL10
status
ICU
Ward

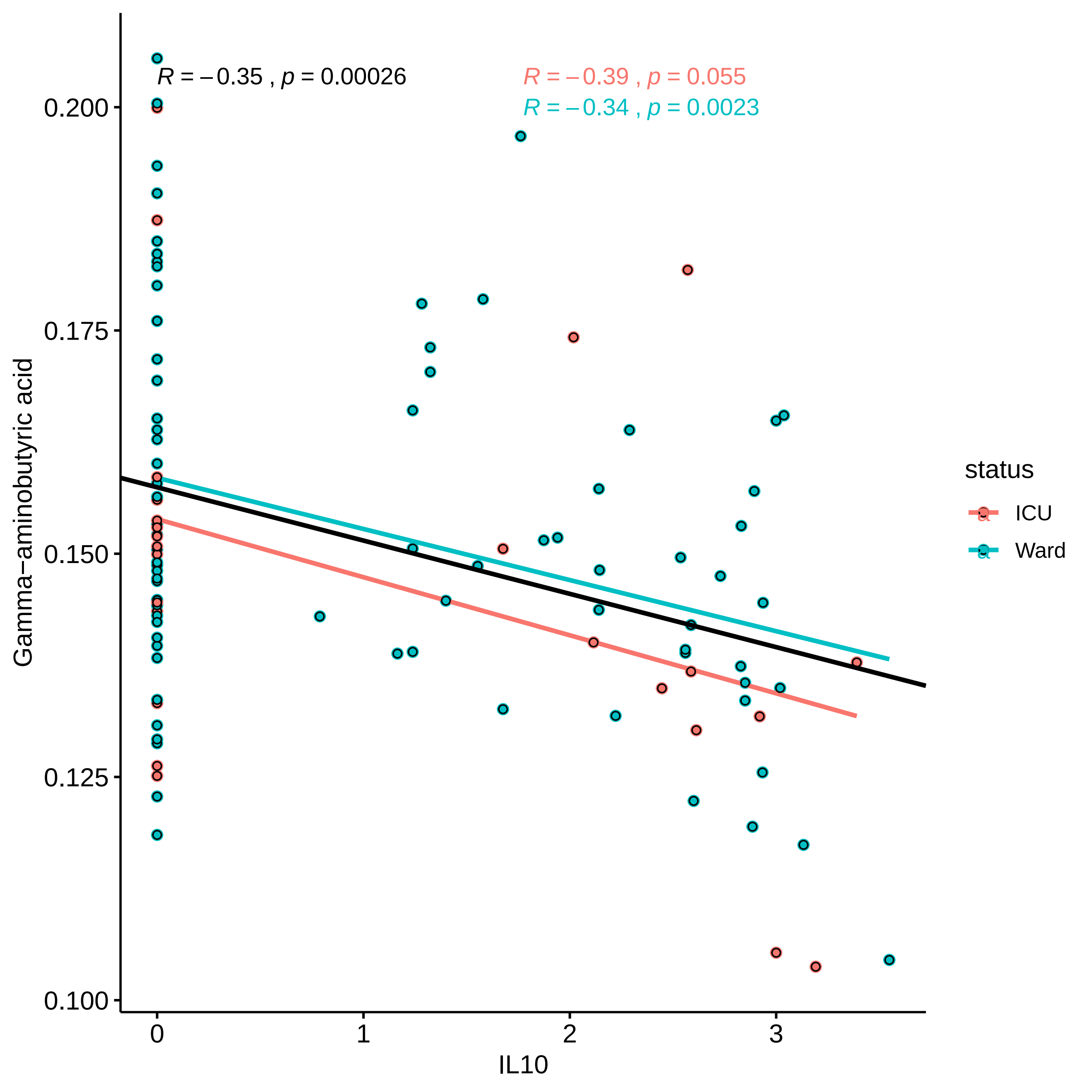

R = −0.35 , p = 0.00026
R = −0.39 , p = 0.055
R = −0.34 , p = 0.0023
0.200
0.175
0.150
0.125
0.100
Gamma−aminobutyric acid
0
1
2
3
IL10
status
ICU
Ward

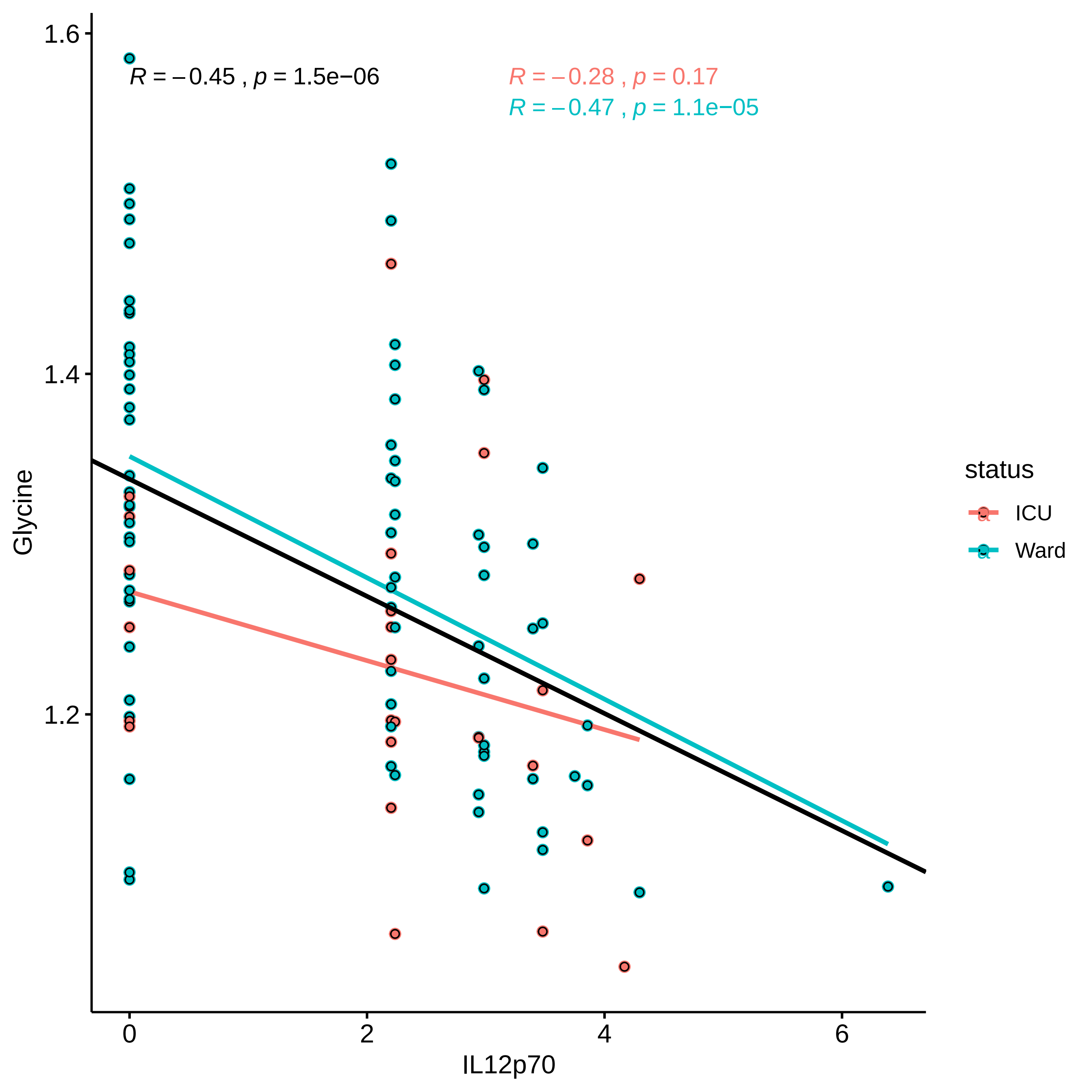

R = −0.45 , p = 1.5e−06
R = −0.28 , p = 0.17
R = −0.47 , p = 1.1e−05
Glycine
1.6
1.4
1.2
0
2
4
6
IL12p70
status
ICU
Ward

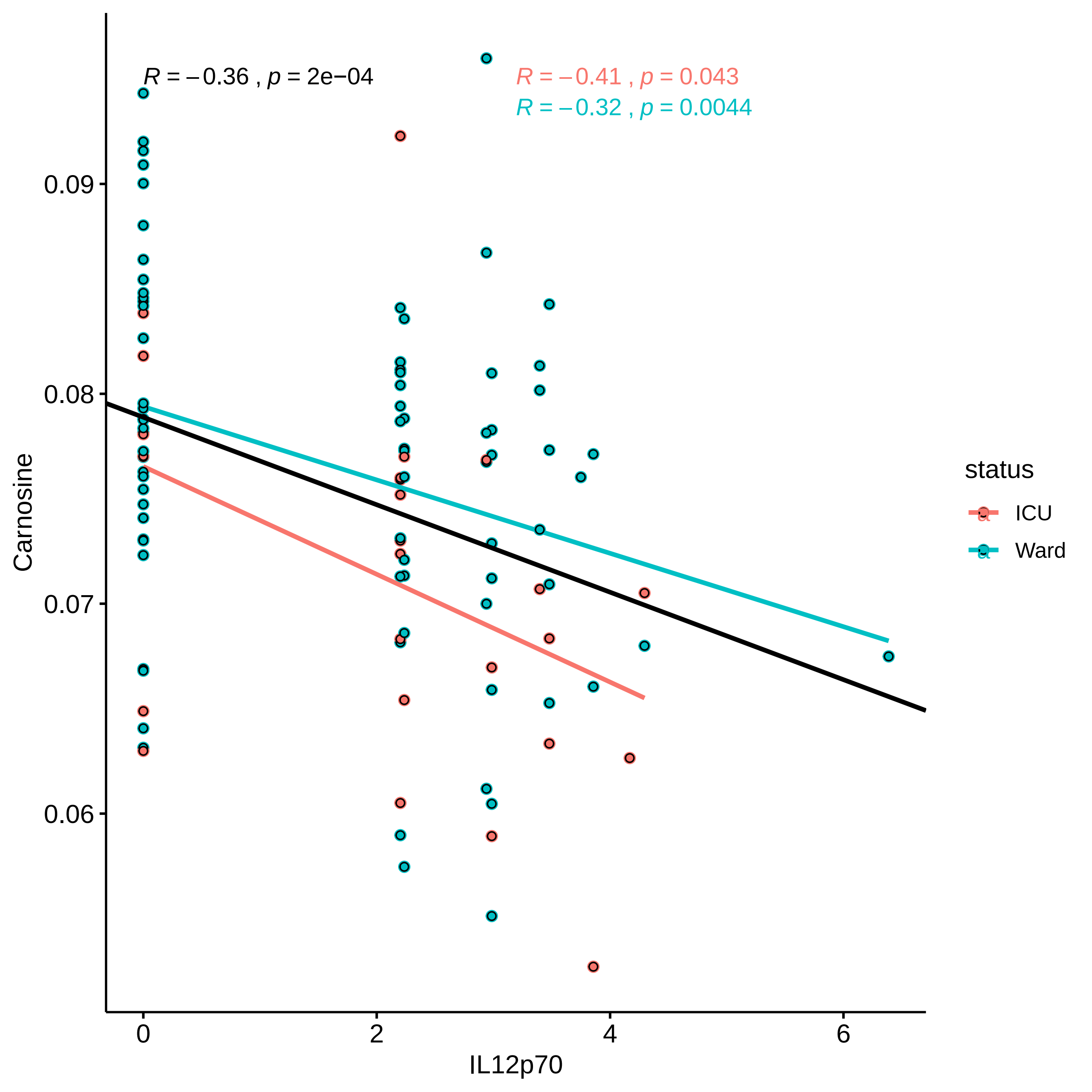

R = −0.36 , p = 2e−04
R = −0.41 , p = 0.043
R = −0.32 , p = 0.0044
Carnosine
0.09
0.08
0.07
0.06
0
2
4
6
IL12p70
status
ICU
Ward

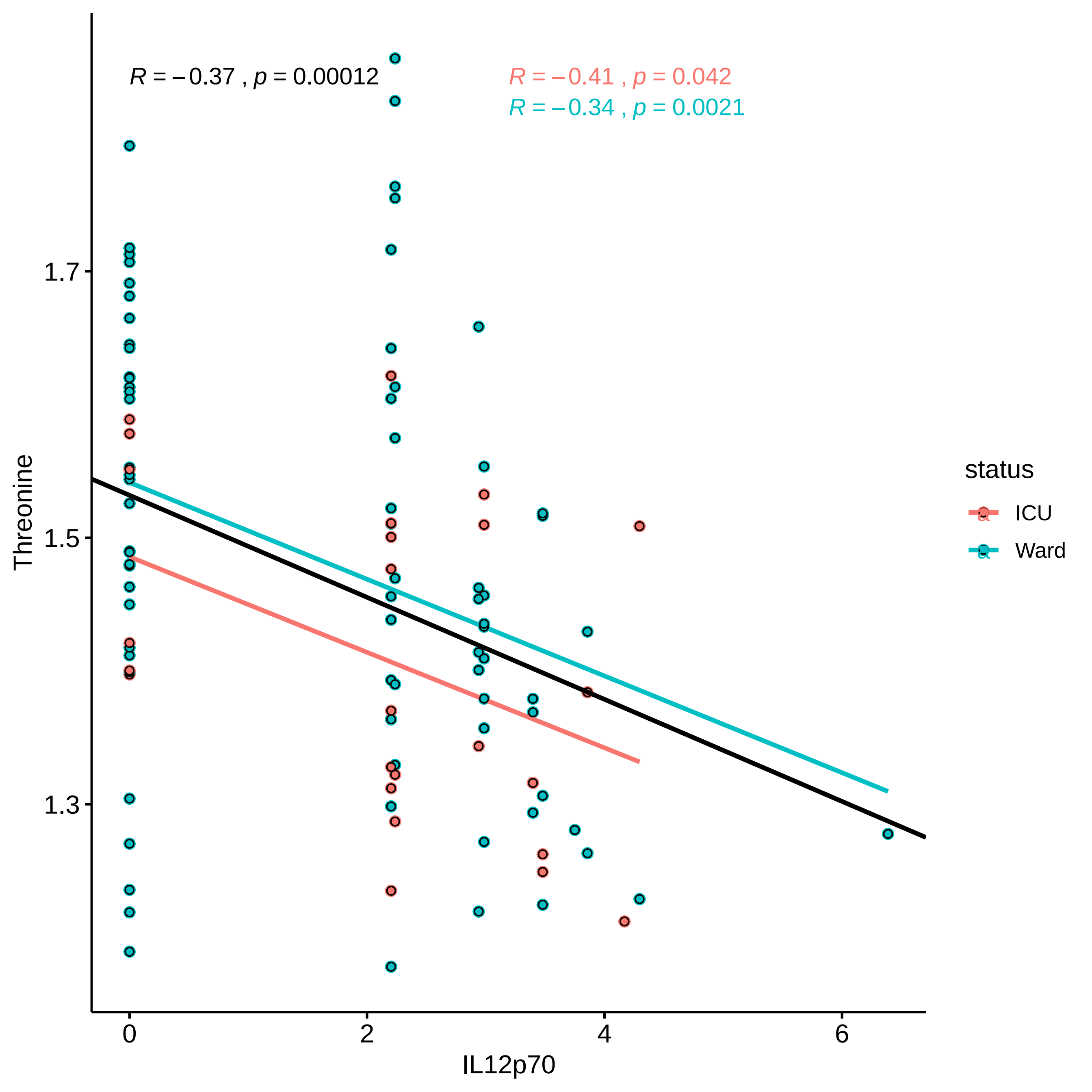

R = −0.37 , p = 0.00012
R = −0.41 , p = 0.042
R = −0.34 , p = 0.0021
Threonine
1.7
1.5
1.3
0
2
4
6
IL12p70
status
ICU
Ward

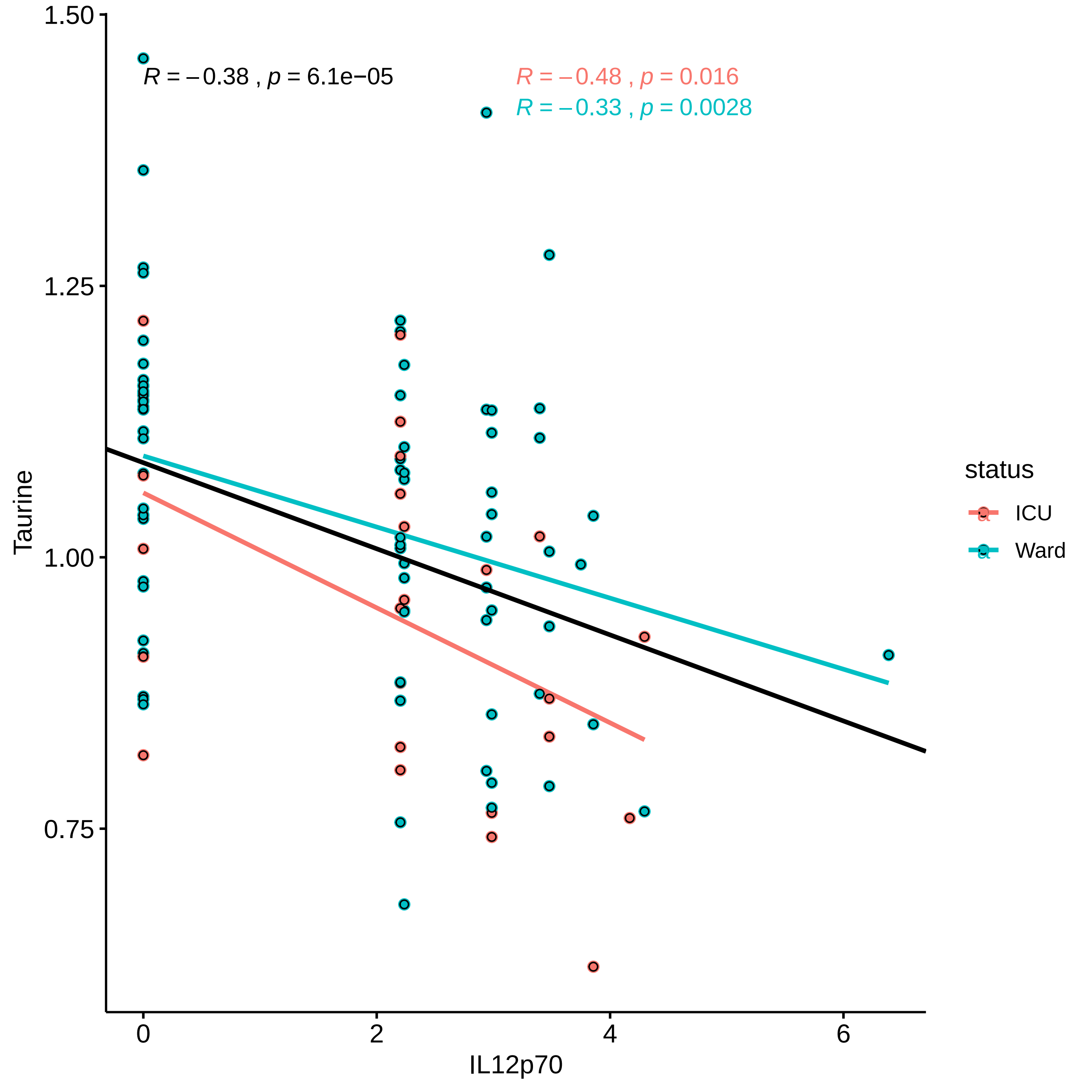

R = −0.38 , p = 6.1e−05
R = −0.48 , p = 0.016
R = −0.33 , p = 0.0028
Taurine
1.50
1.25
1.00
0.75
0
2
4
6
IL12p70
status
ICU
Ward

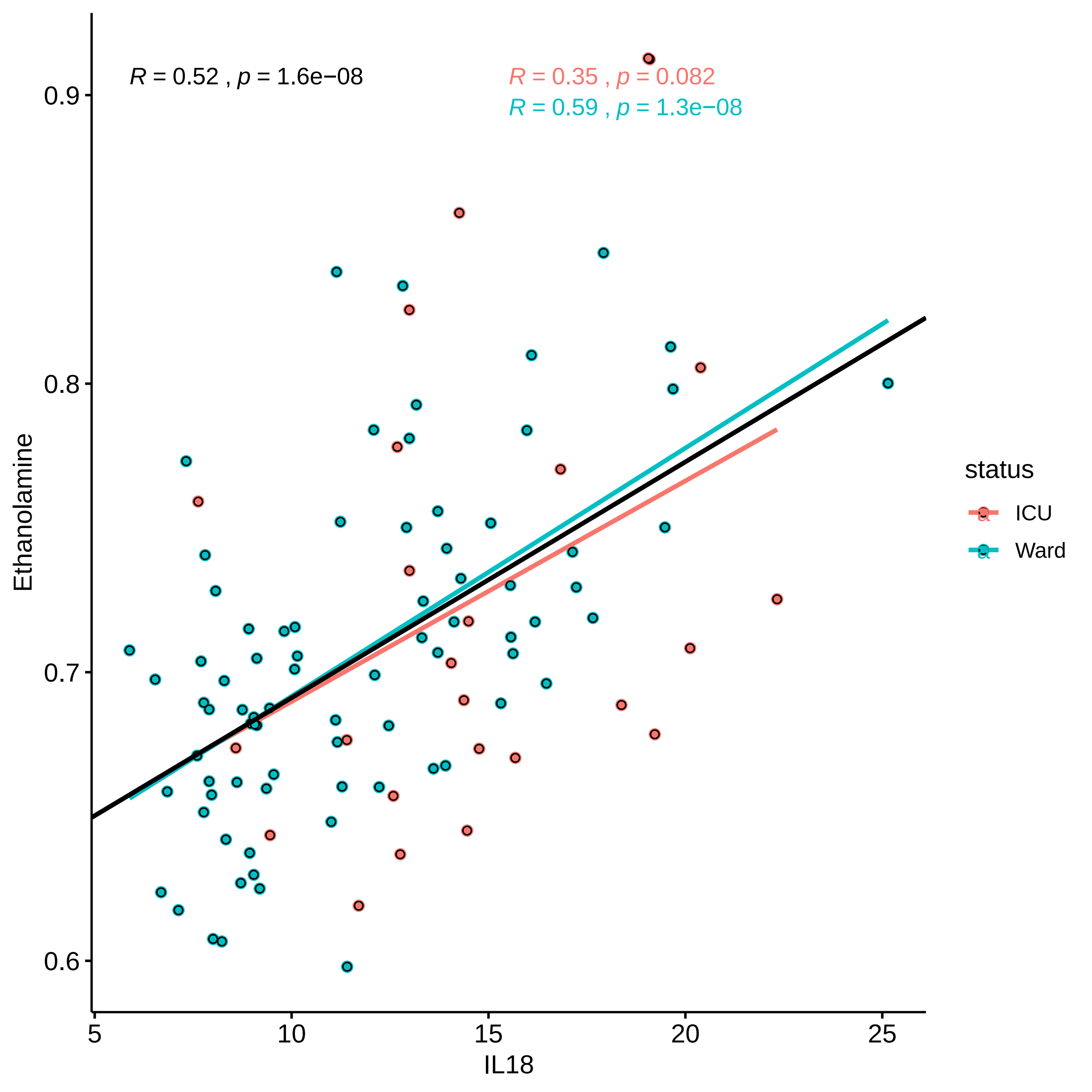

R = 0.52 , p = 1.6e−08
R = 0.35 , p = 0.082
R = 0.59 , p = 1.3e−08
0.9
0.8
0.7
0.6
Ethanolamine
5
10
15
20
25
IL18
status
ICU
Ward

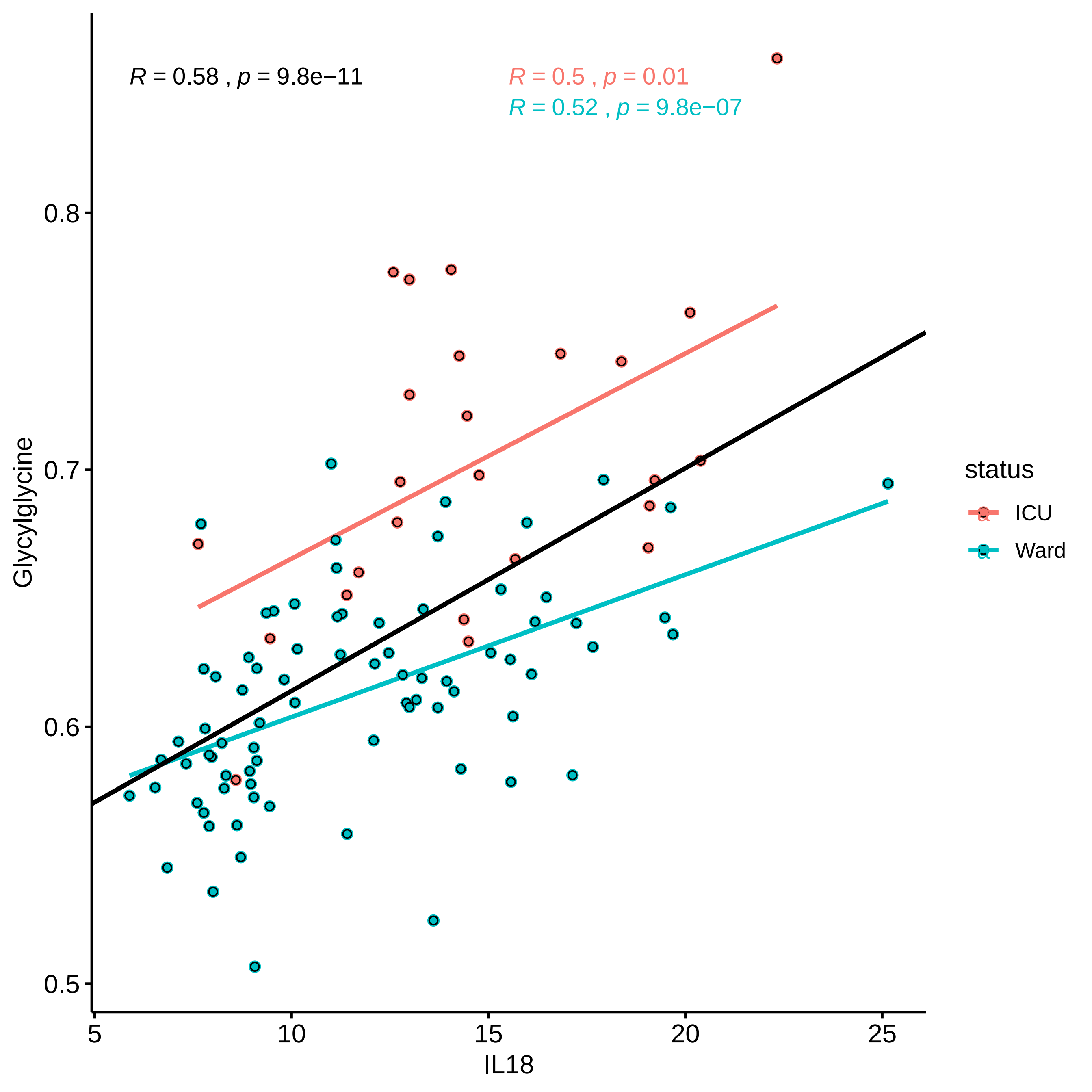

R = 0.58 , p = 9.8e−11
R = 0.5 , p = 0.01
R = 0.52 , p = 9.8e−07
Glycylglycine
0.8
0.7
0.6
0.5
5
10
15
20
25
IL18
status
ICU
Ward

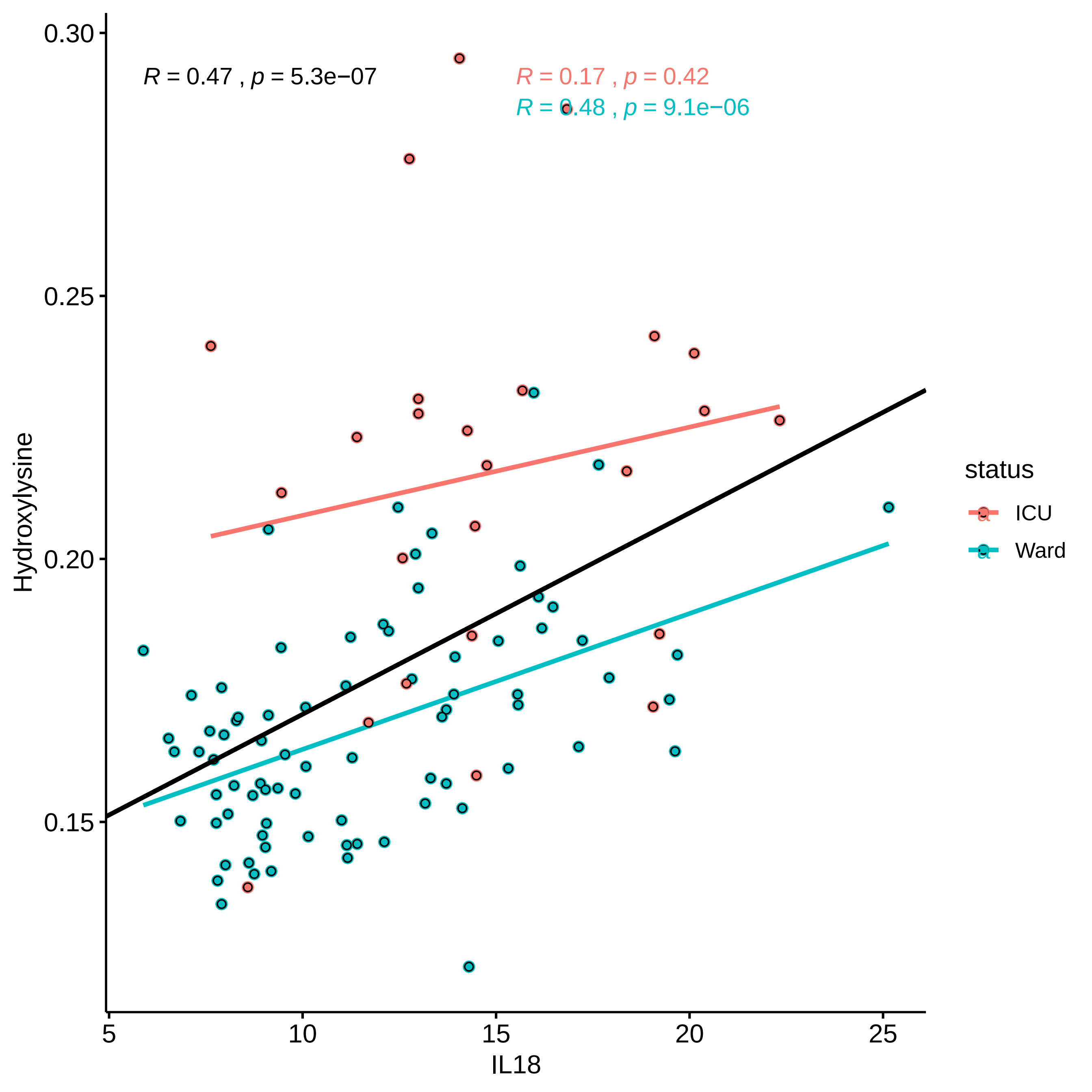

R = 0.47 , p = 5.3e−07
R = 0.17 , p = 0.42
R = 0.48 , p = 9.1e−06
Hydroxylysine
0.30
0.25
0.20
0.15
5
10
15
20
25
IL18
status
ICU
Ward

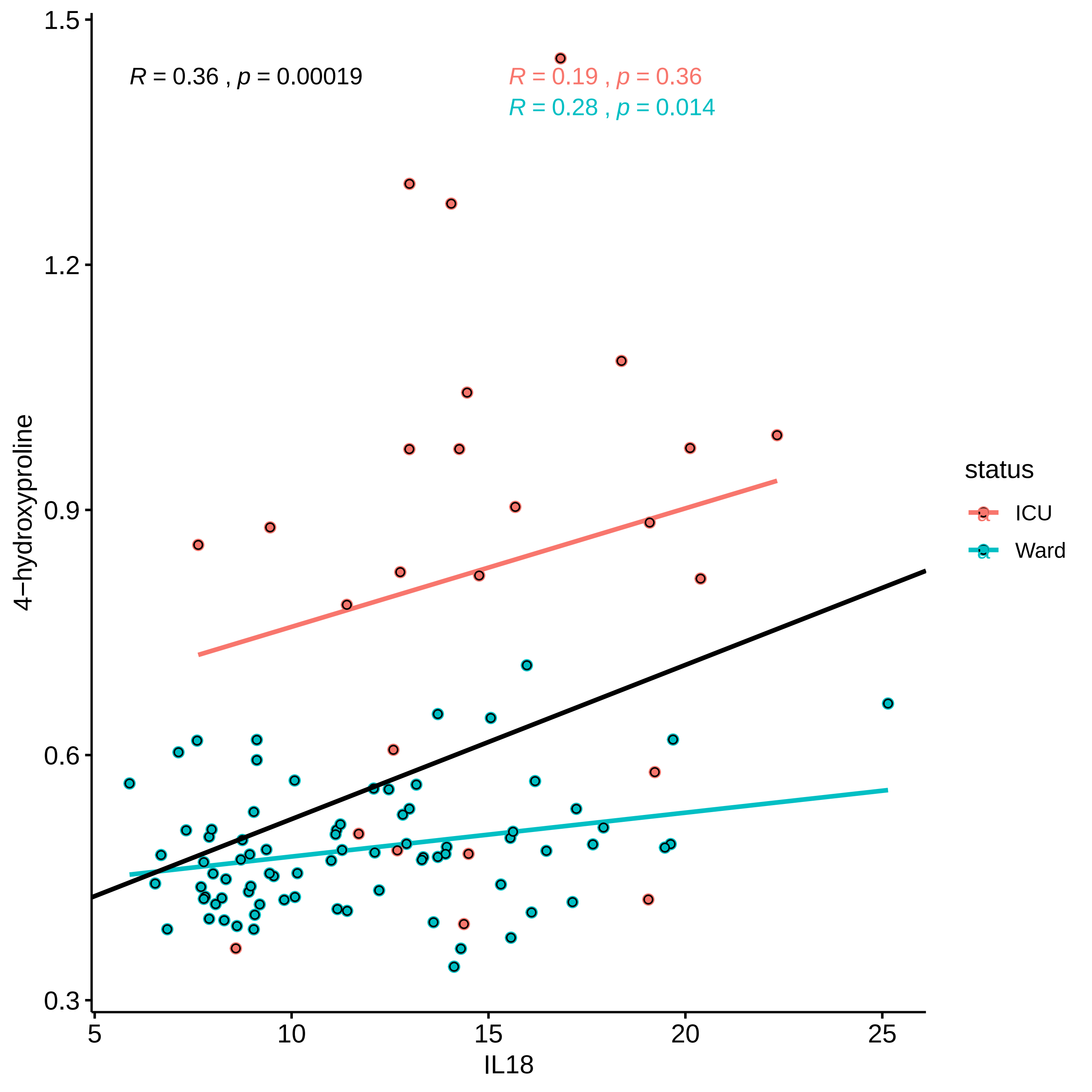

R = 0.36 , p = 0.00019
R = 0.19 , p = 0.36
R = 0.28 , p = 0.014
4−hydroxyproline
1.5
1.2
0.9
0.6
0.3
5
10
15
20
25
IL18
status
ICU
Ward

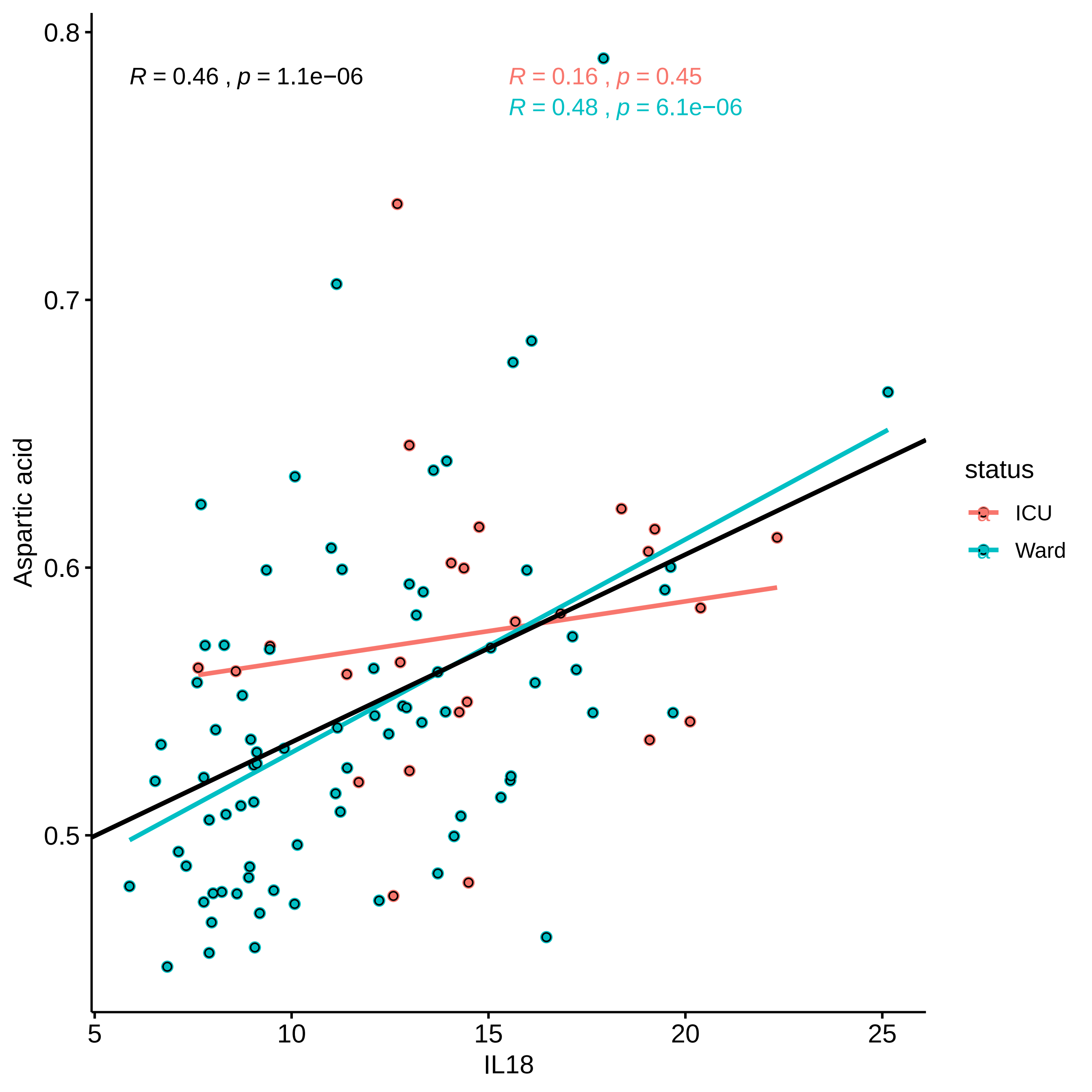

R = 0.46 , p = 1.1e−06
R = 0.16 , p = 0.45
R = 0.48 , p = 6.1e−06
0.8
0.7
0.6
0.5
Aspartic acid
5
10
15
20
25
IL18
status
ICU
Ward

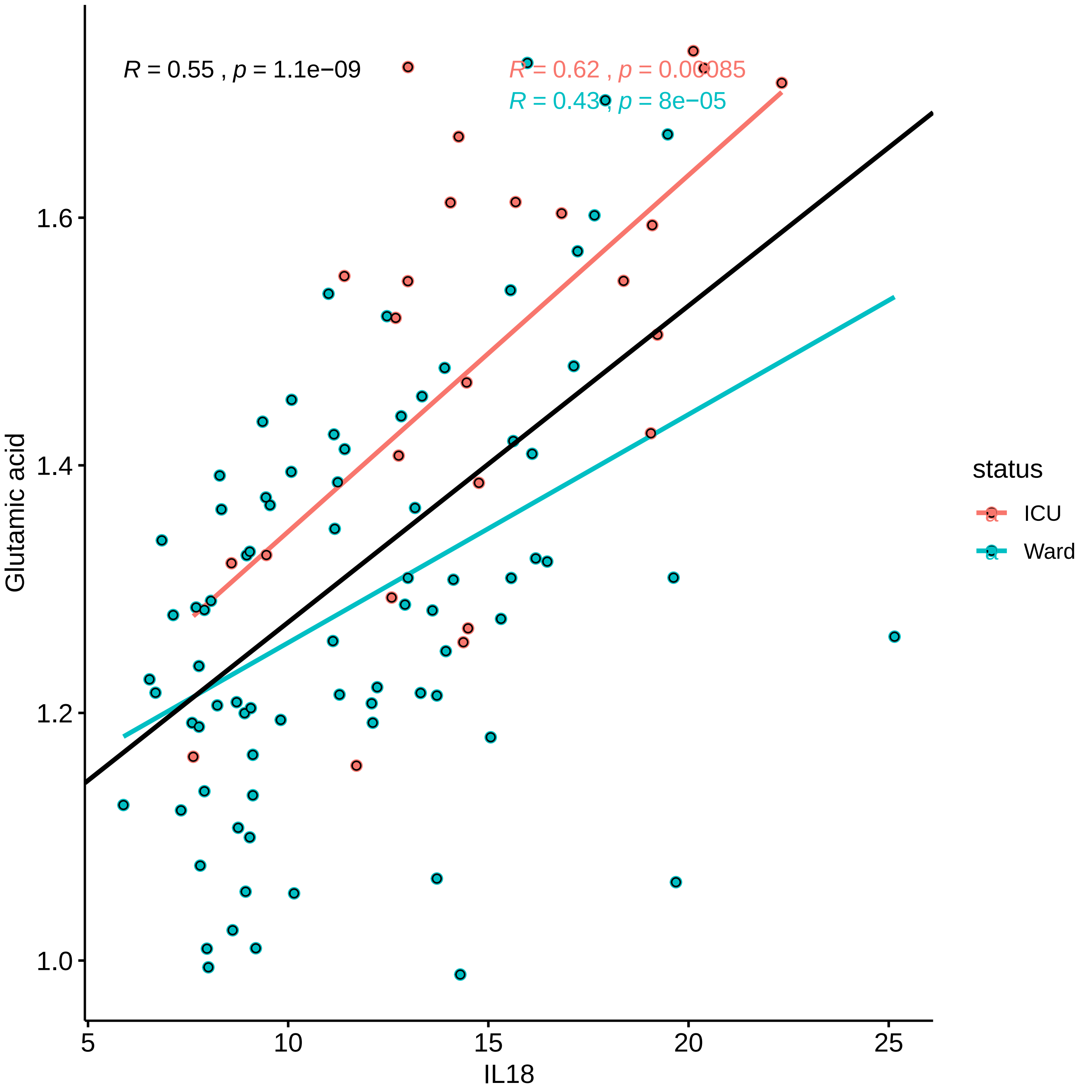

R = 0.55 , p = 1.1e−09
R = 0.62 , p = 0.00085
R = 0.43 , p = 8e−05
Glutamic acid
1.0
1.2
1.4
1.6
IL18
5
10
15
20
25
status
ICU
Ward

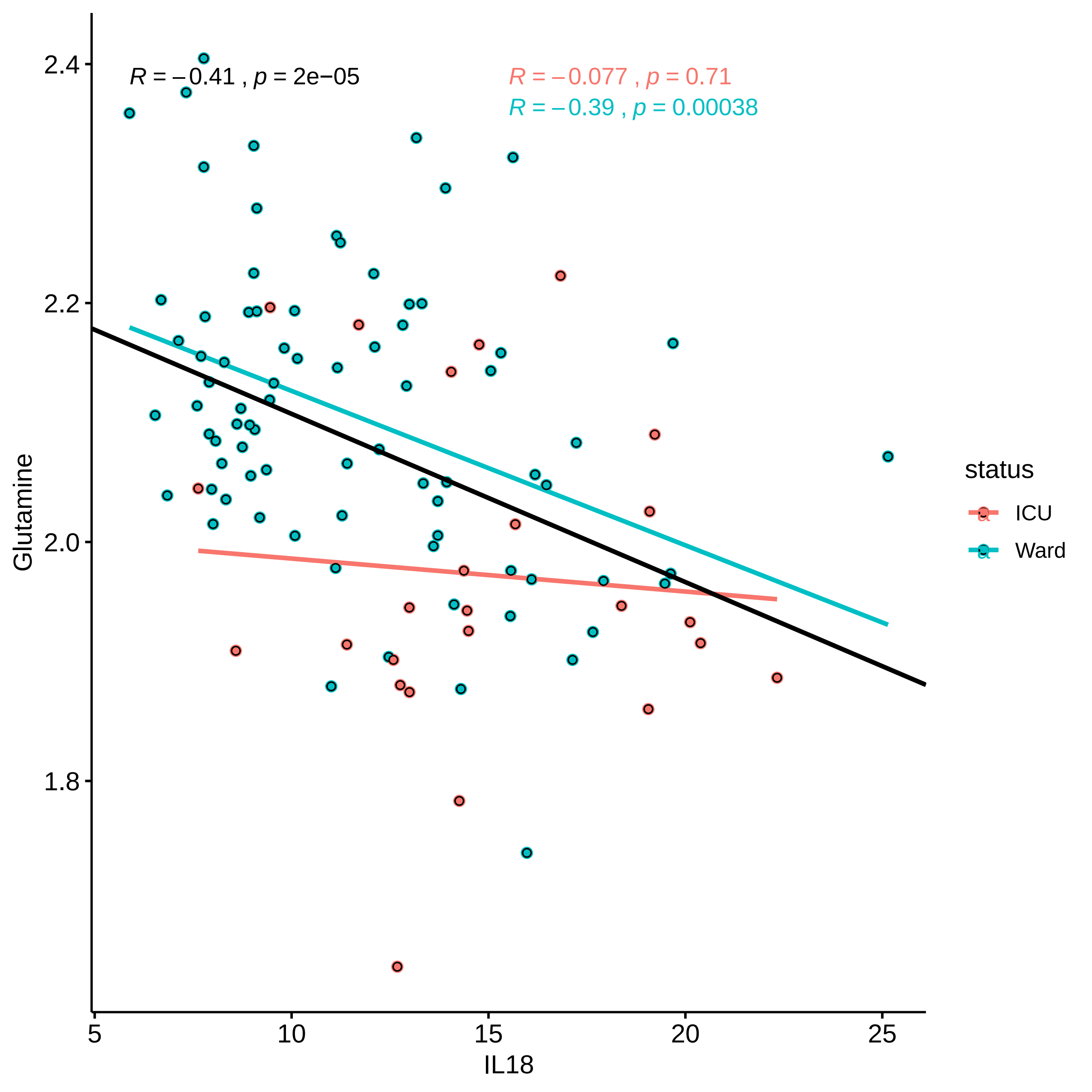

R = −0.41 , p = 2e−05
R = −0.077 , p = 0.71
R = −0.39 , p = 0.00038
Glutamine
2.4
2.2
2.0
1.8
5
10
15
20
25
IL18
status
ICU
Ward

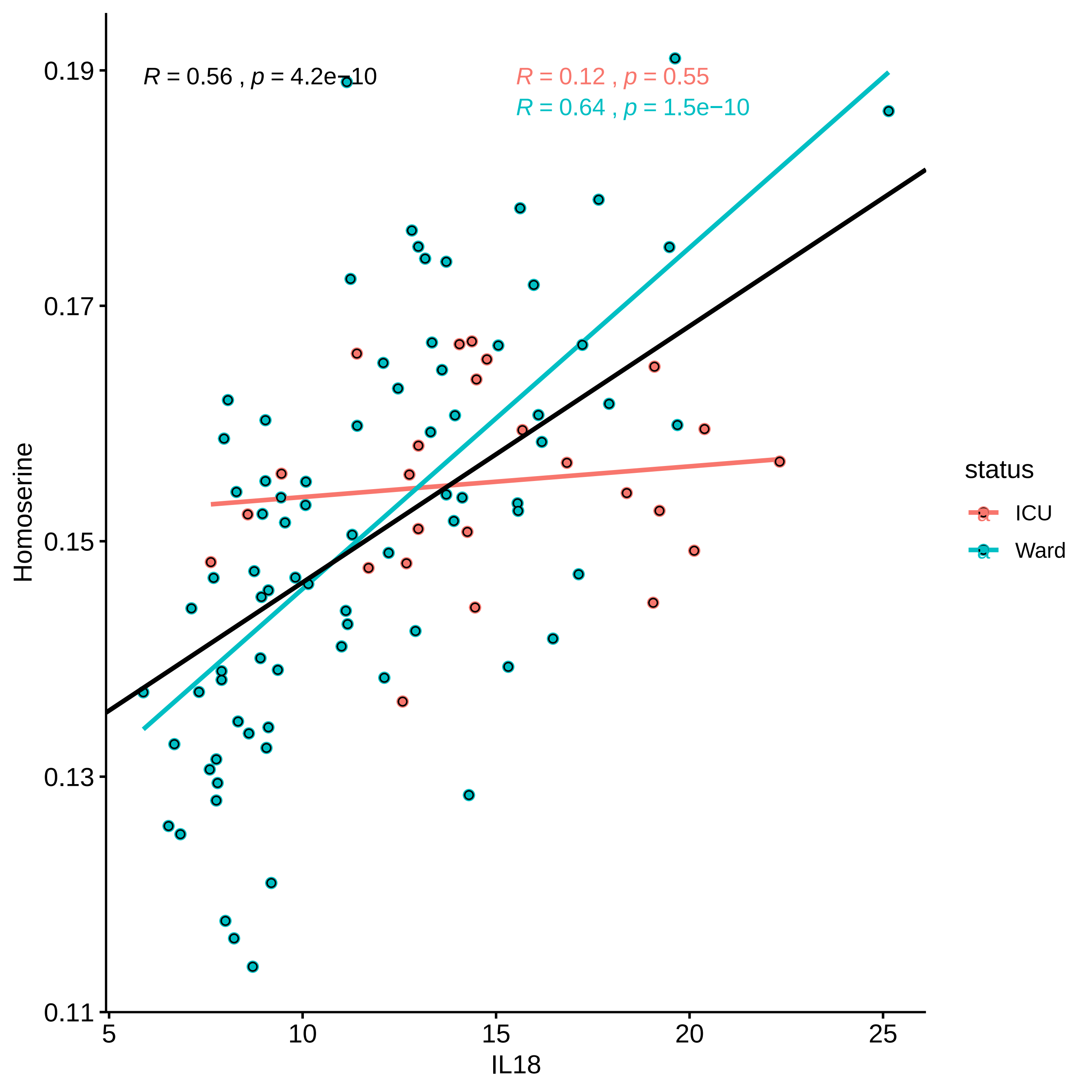

R = 0.56 , p = 4.2e−10
R = 0.12 , p = 0.55
R = 0.64 , p = 1.5e−10
Homoserine
IL18
0.19
0.17
0.15
0.13
0.11
5
10
15
20
25
status
ICU
Ward

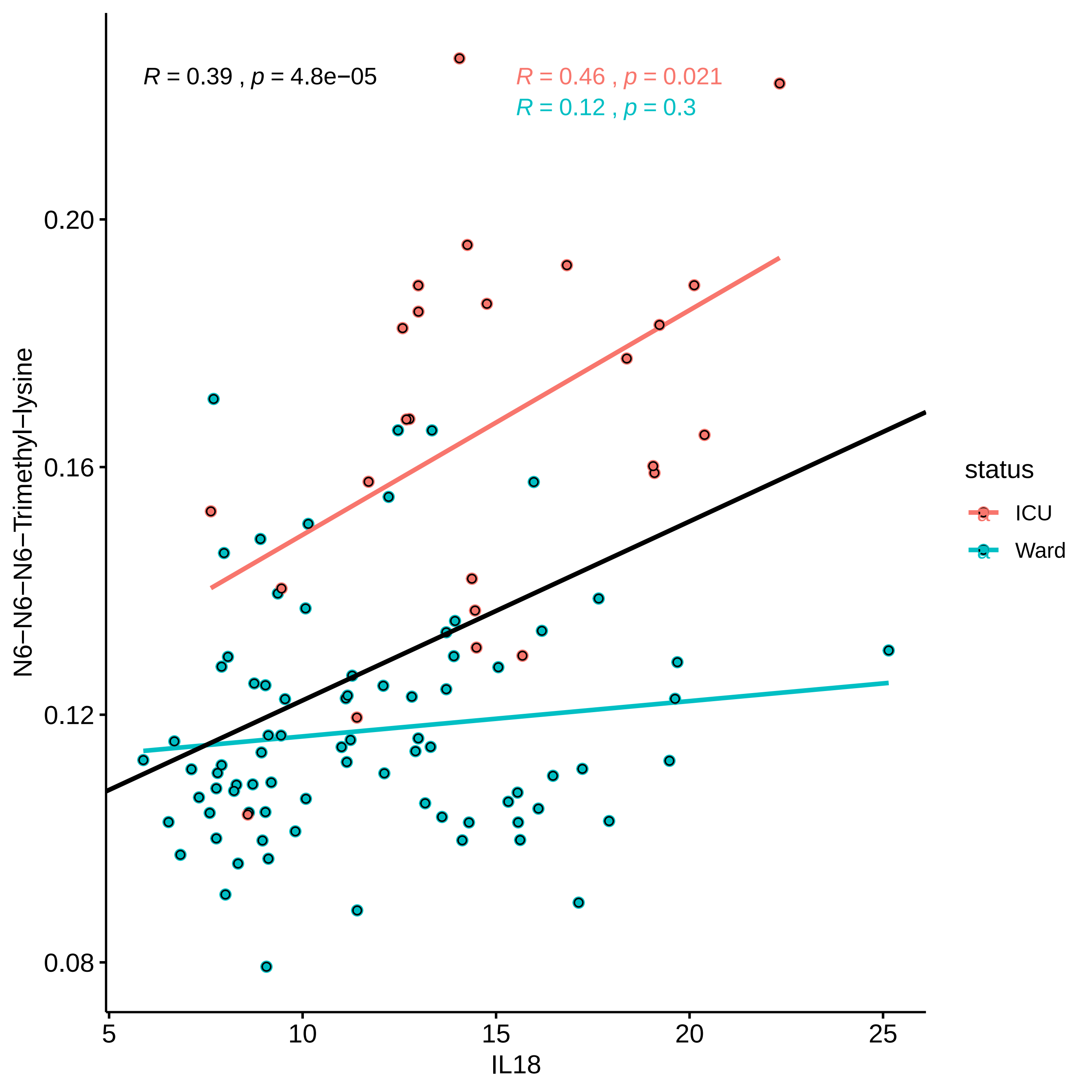
R = 0.39 , p = 4.8e−05
R = 0.46 , p = 0.021
R = 0.12 , p = 0.3
0.20
0.16
0.12
0.08
N6−N6−N6−Trimethyl−lysine
5
10
15
20
25
IL18
status
ICU
Ward

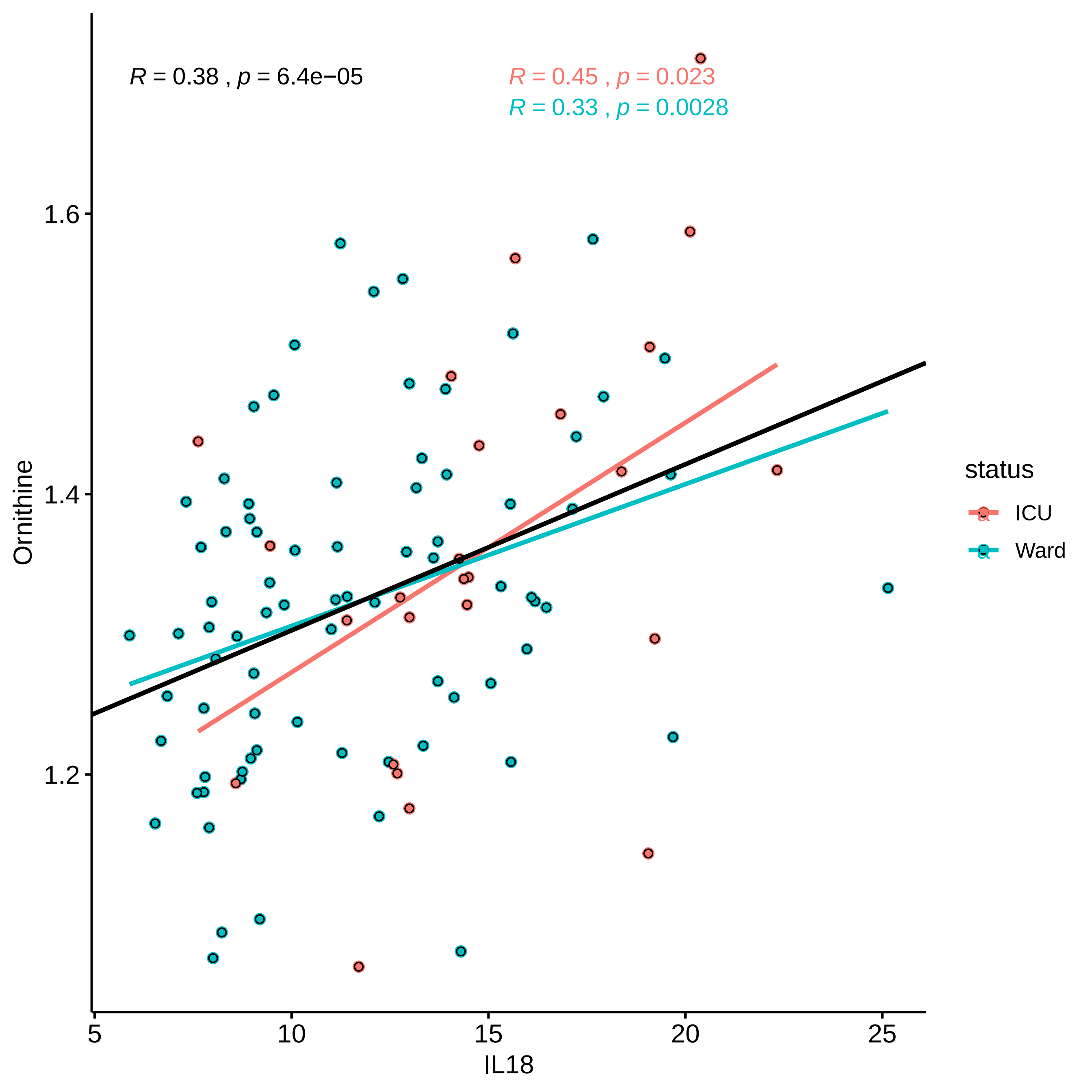

R = 0.38 , p = 6.4e−05
R = 0.45 , p = 0.023
R = 0.33 , p = 0.0028
Ornithine
1.6
1.4
1.2
5
10
15
20
25
IL18
status
ICU
Ward

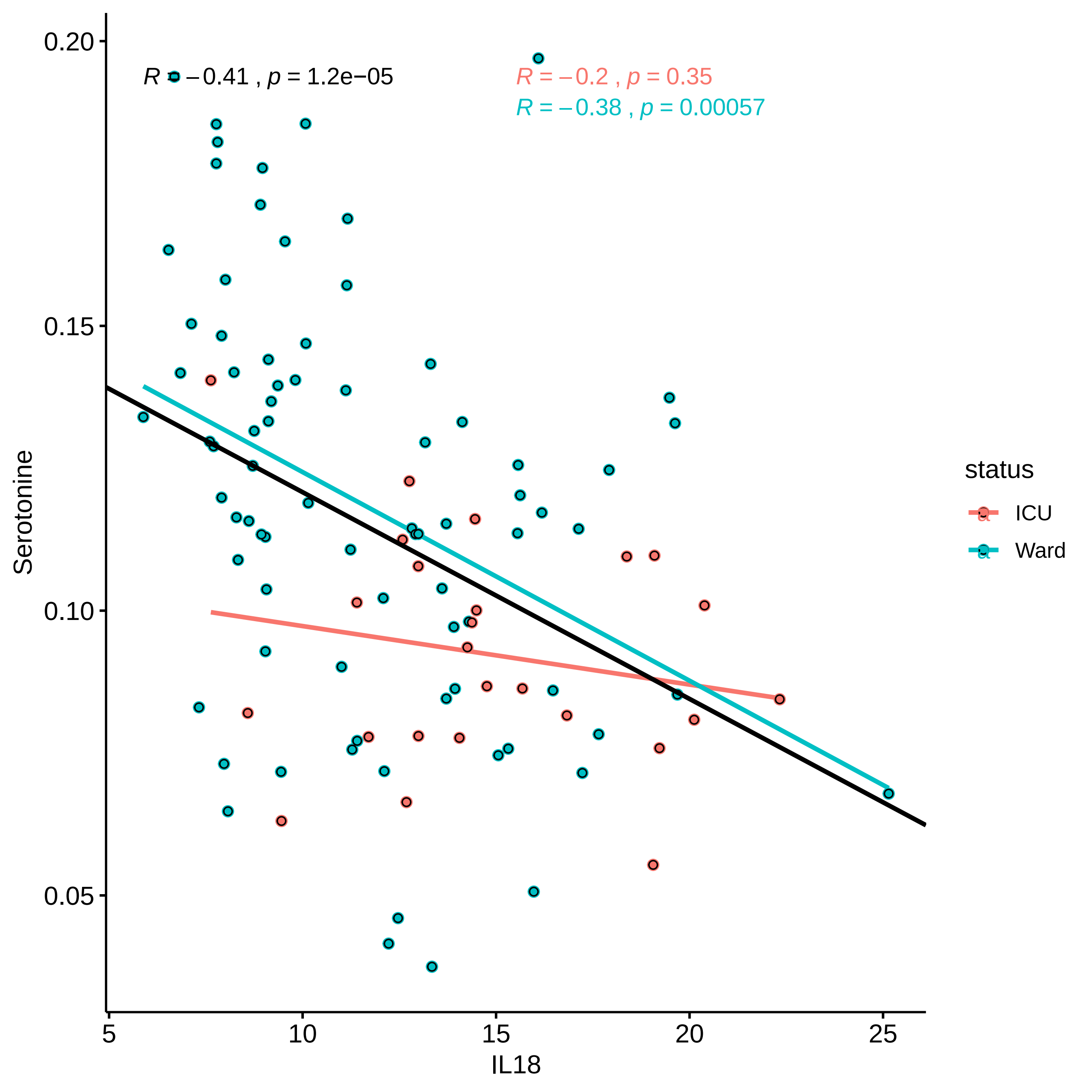

R = −0.41 , p = 1.2e−05
R = −0.2 , p = 0.35
R = −0.38 , p = 0.00057
Serotonine
0.20
0.15
0.10
0.05
IL18
5
10
15
20
25
status
ICU
Ward

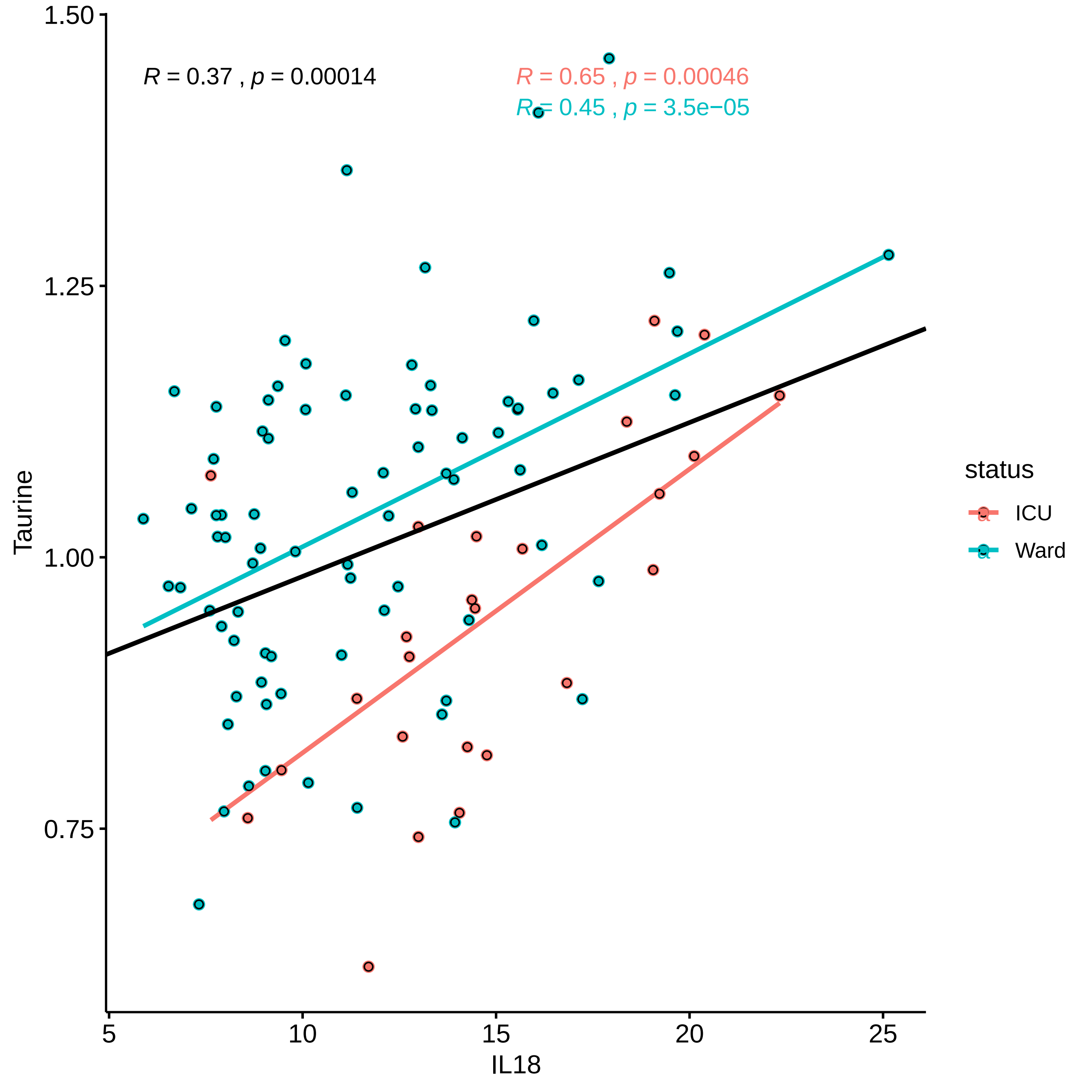

R = 0.37 , p = 0.00014
R = 0.65 , p = 0.00046
R = 0.45 , p = 3.5e−05
Taurine
1.50
1.25
1.00
0.75
IL18
5
10
15
20
25
status
ICU
Ward

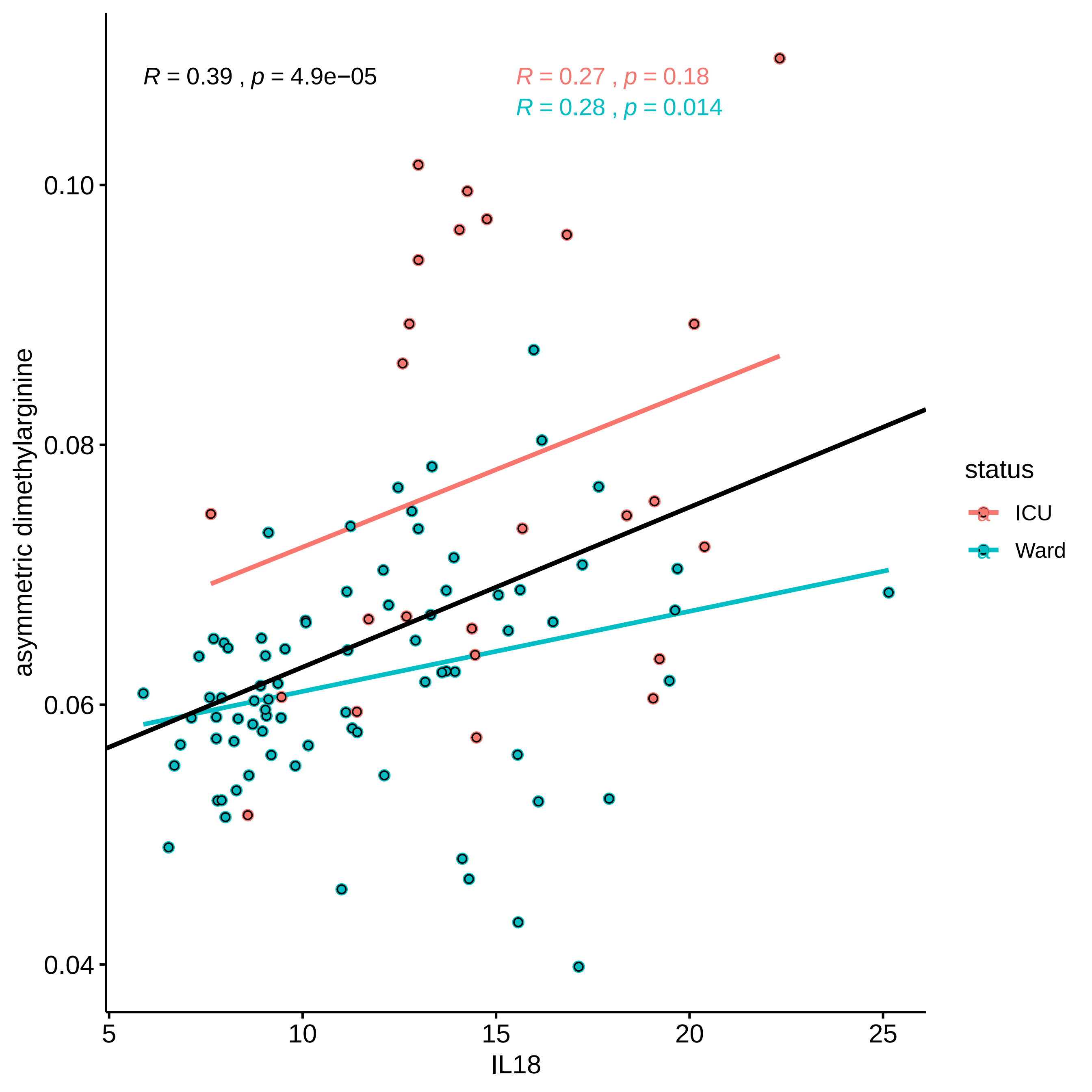

R = 0.39 , p = 4.9e−05
R = 0.27 , p = 0.18
R = 0.28 , p = 0.014
0.10
0.08
0.06
0.04
asymmetric dimethylarginine
5
10
15
20
25
IL18
status
ICU
Ward

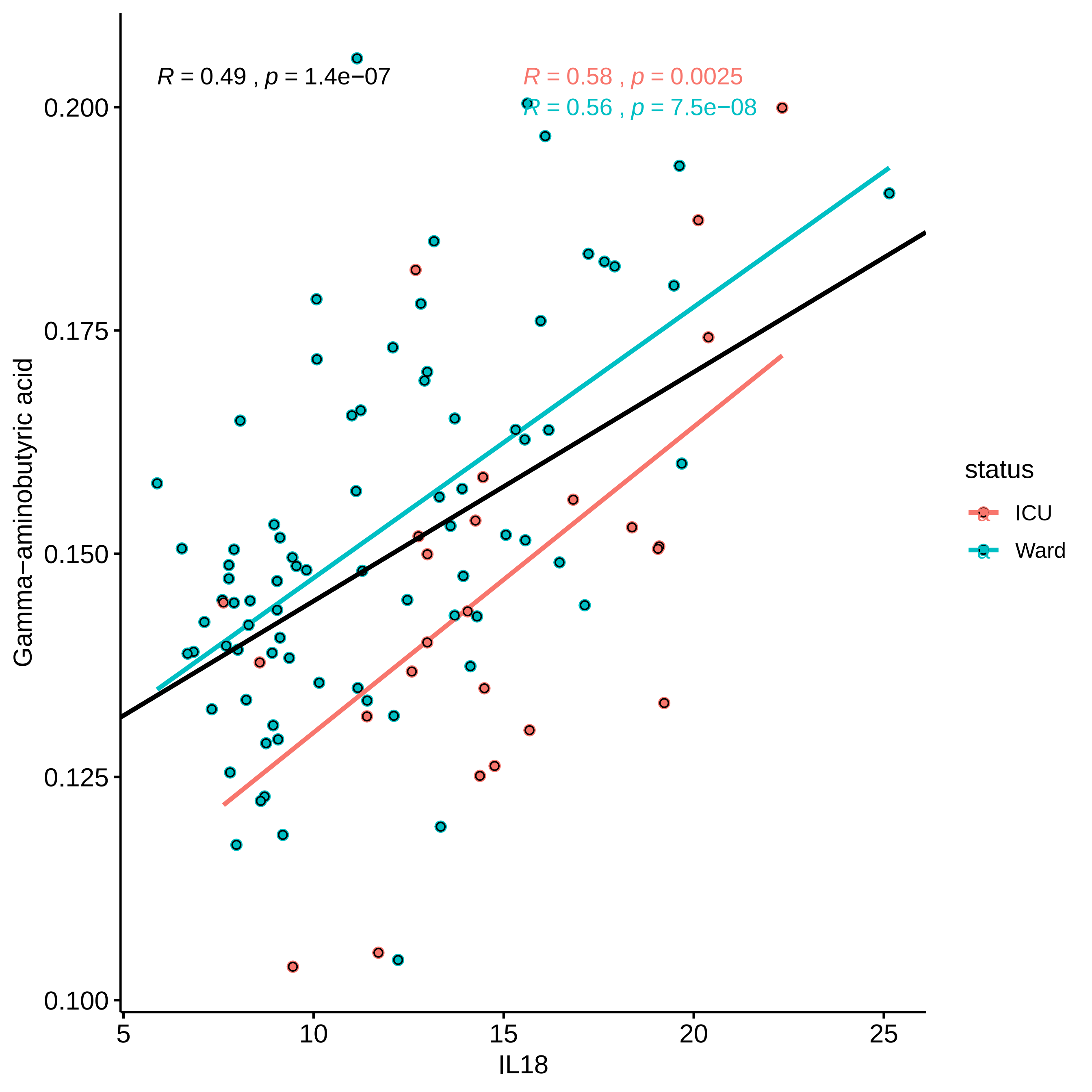

R = 0.49 , p = 1.4e−07
R = 0.58 , p = 0.0025
R = 0.56 , p = 7.5e−08
Gamma−aminobutyric acid
0.200
0.175
0.150
0.125
0.100
5
10
15
20
25
IL18
status
ICU
Ward

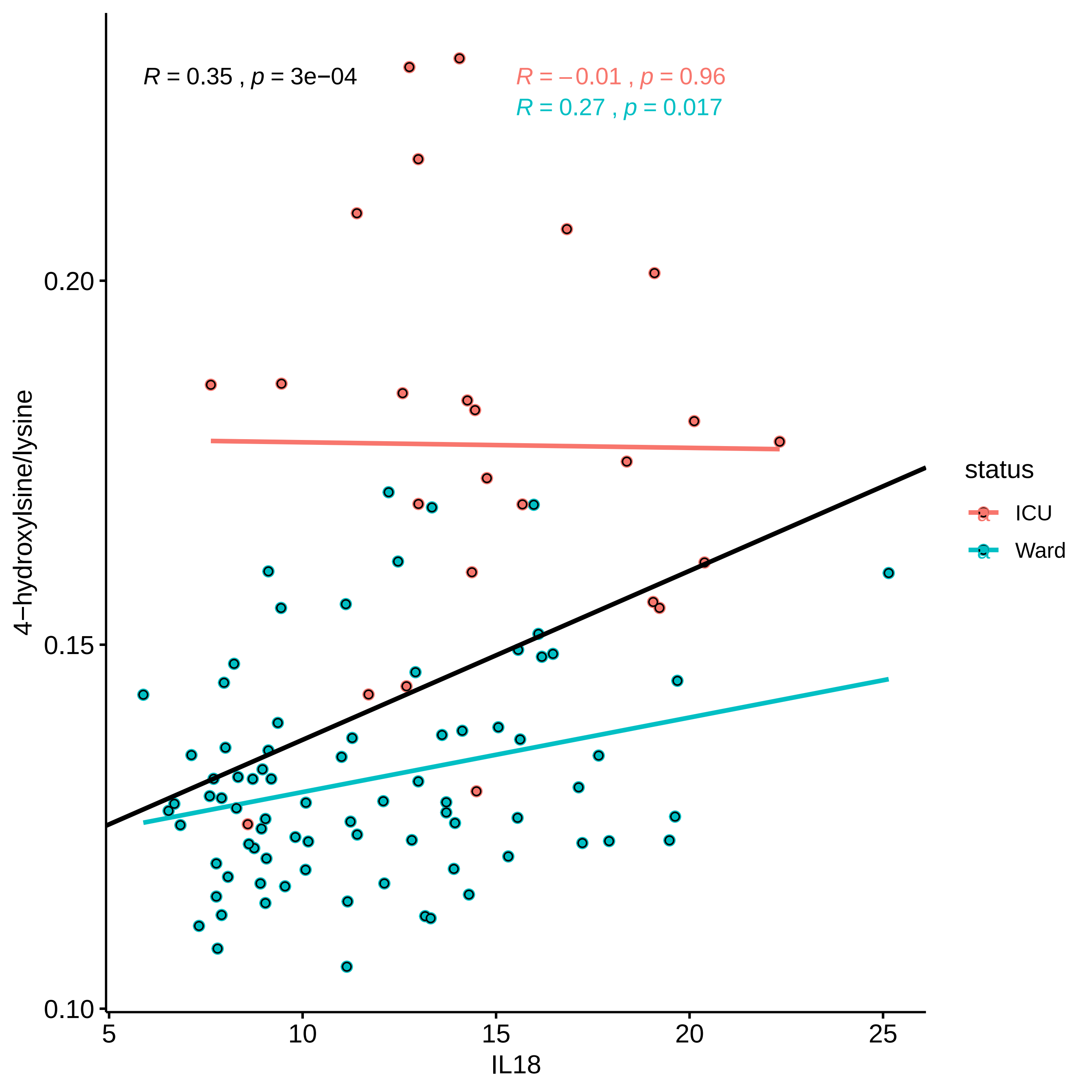

R = 0.35 , p = 3e−04
R = −0.01 , p = 0.96
R = 0.27 , p = 0.017
4−hydroxylsine/lysine
0.20
0.15
0.10
5
10
15
20
25
IL18
status
ICU
Ward

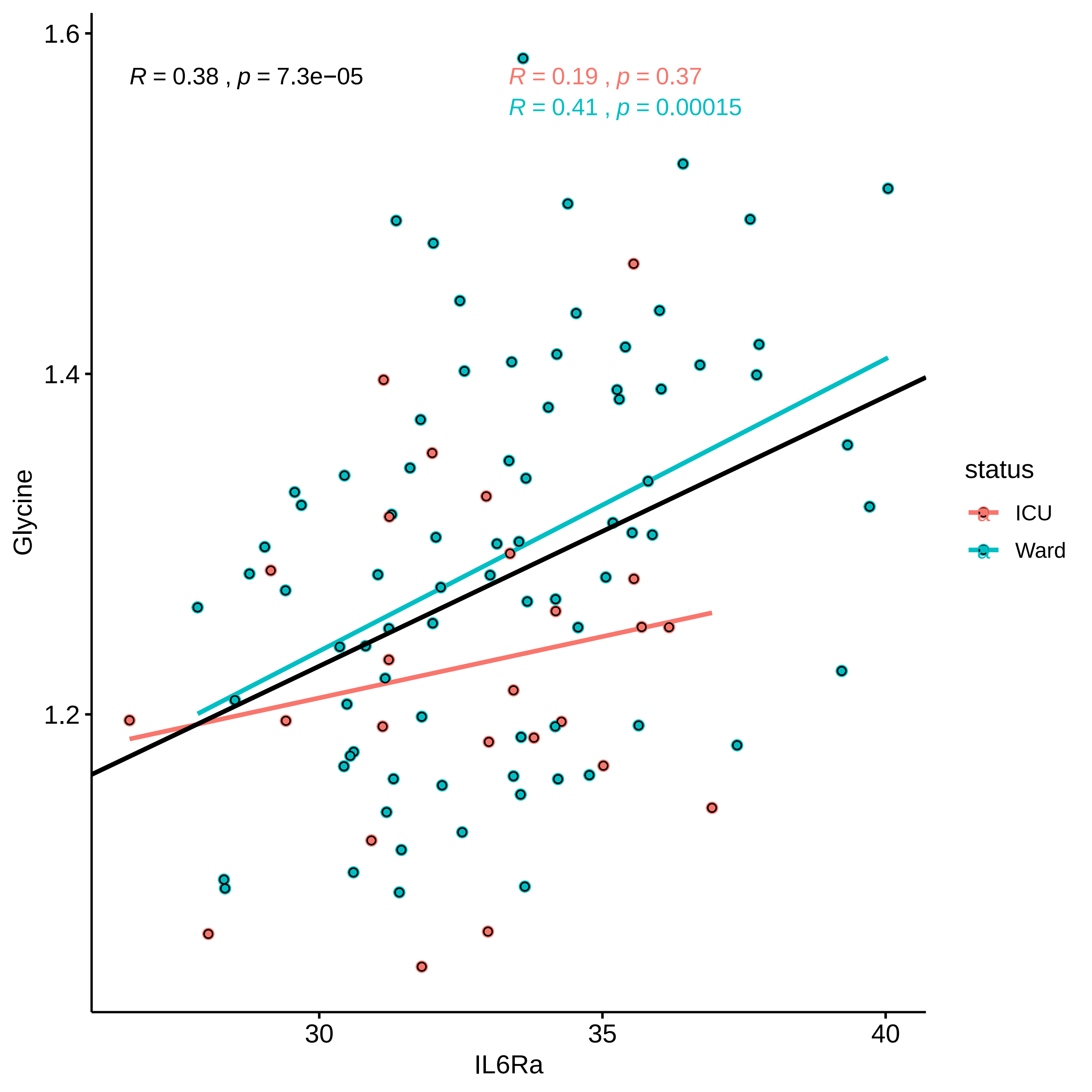

R = 0.38 , p = 7.3e−05
R = 0.19 , p = 0.37
R = 0.41 , p = 0.00015
1.6
1.4
1.2
30
35
40
Glycine
IL6Ra
status
ICU
Ward

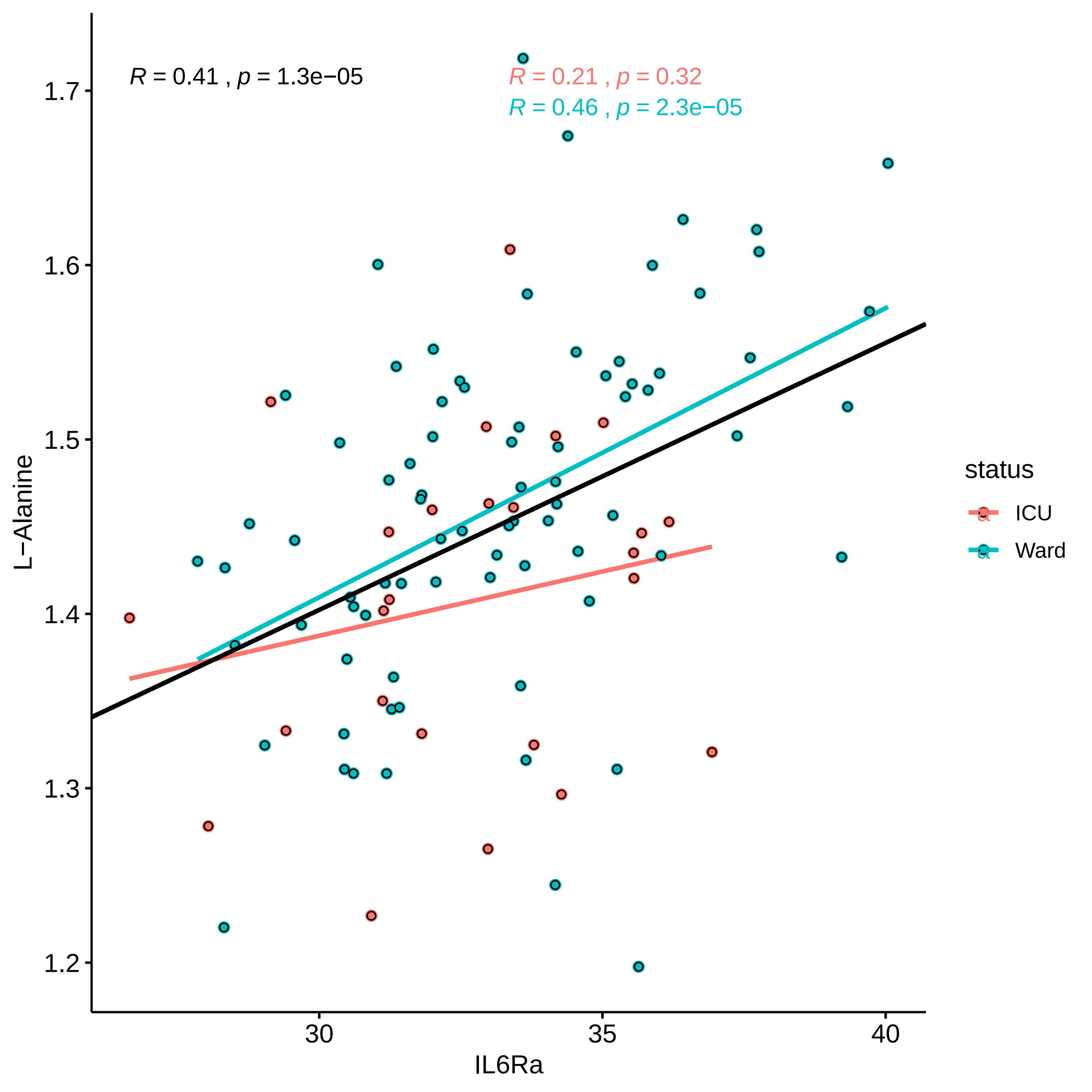

R = 0.41 , p = 1.3e−05
R = 0.21 , p = 0.32
R = 0.46 , p = 2.3e−05
L−Alanine
1.7
1.6
1.5
1.4
1.3
1.2
30
35
40
IL6Ra
status
ICU
Ward

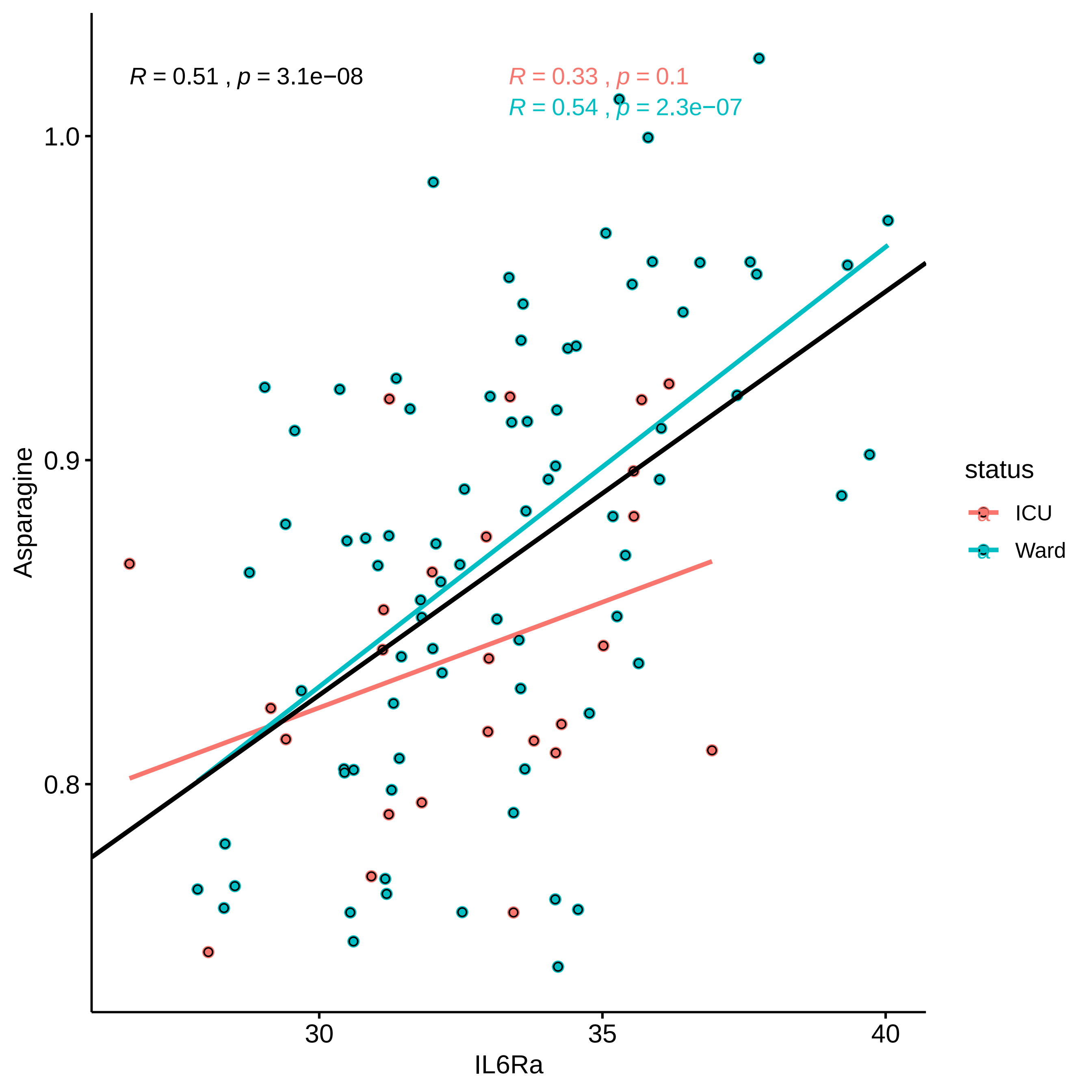

R = 0.51 , p = 3.1e−08
R = 0.33 , p = 0.1
R = 0.54 , p = 2.3e−07
1.0
0.9
0.8
Asparagine
30
35
40
IL6Ra
status
ICU
Ward

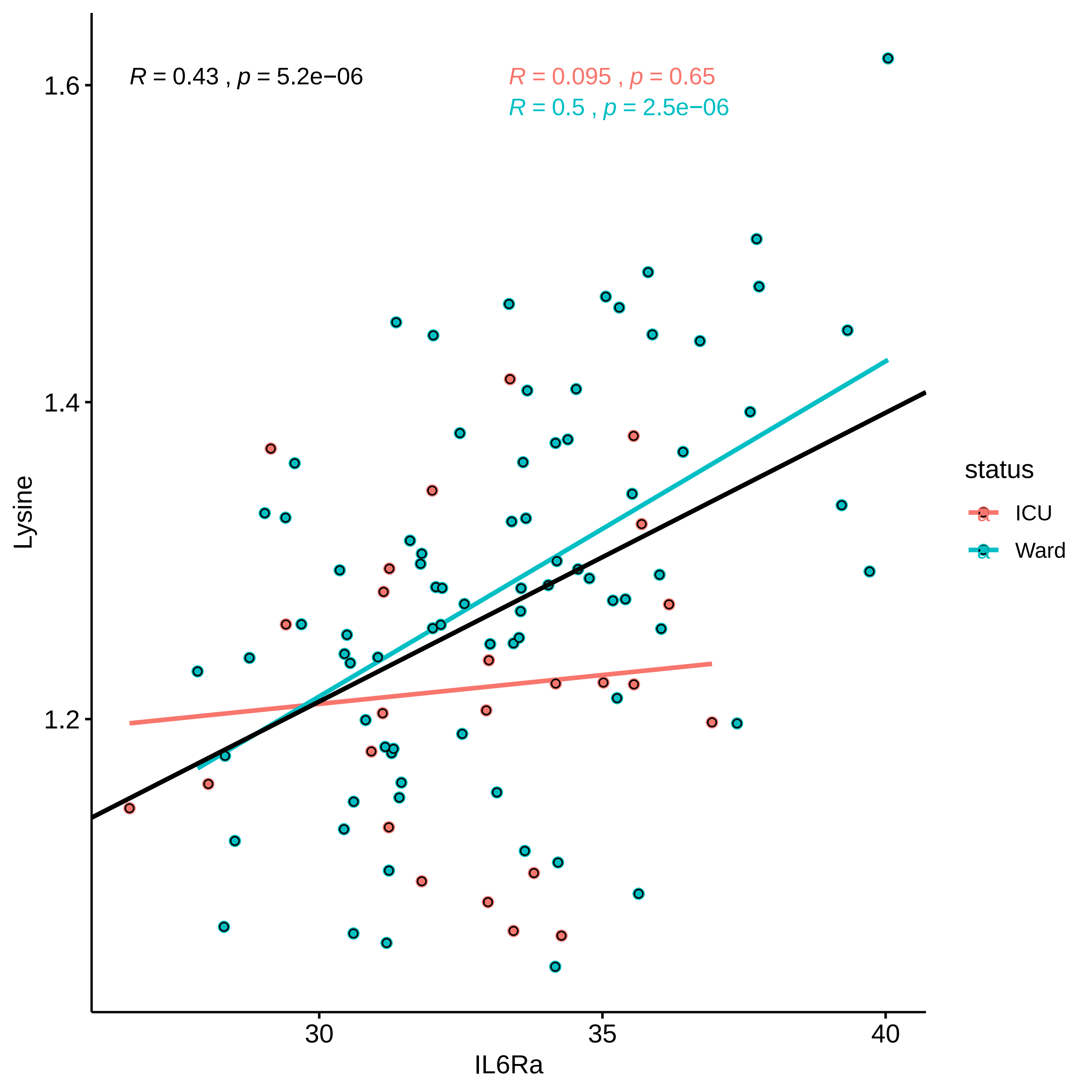

R = 0.43 , p = 5.2e−06
R = 0.095 , p = 0.65
R = 0.5 , p = 2.5e−06
Lysine
1.6
1.4
1.2
30
35
40
IL6Ra
status
ICU
Ward

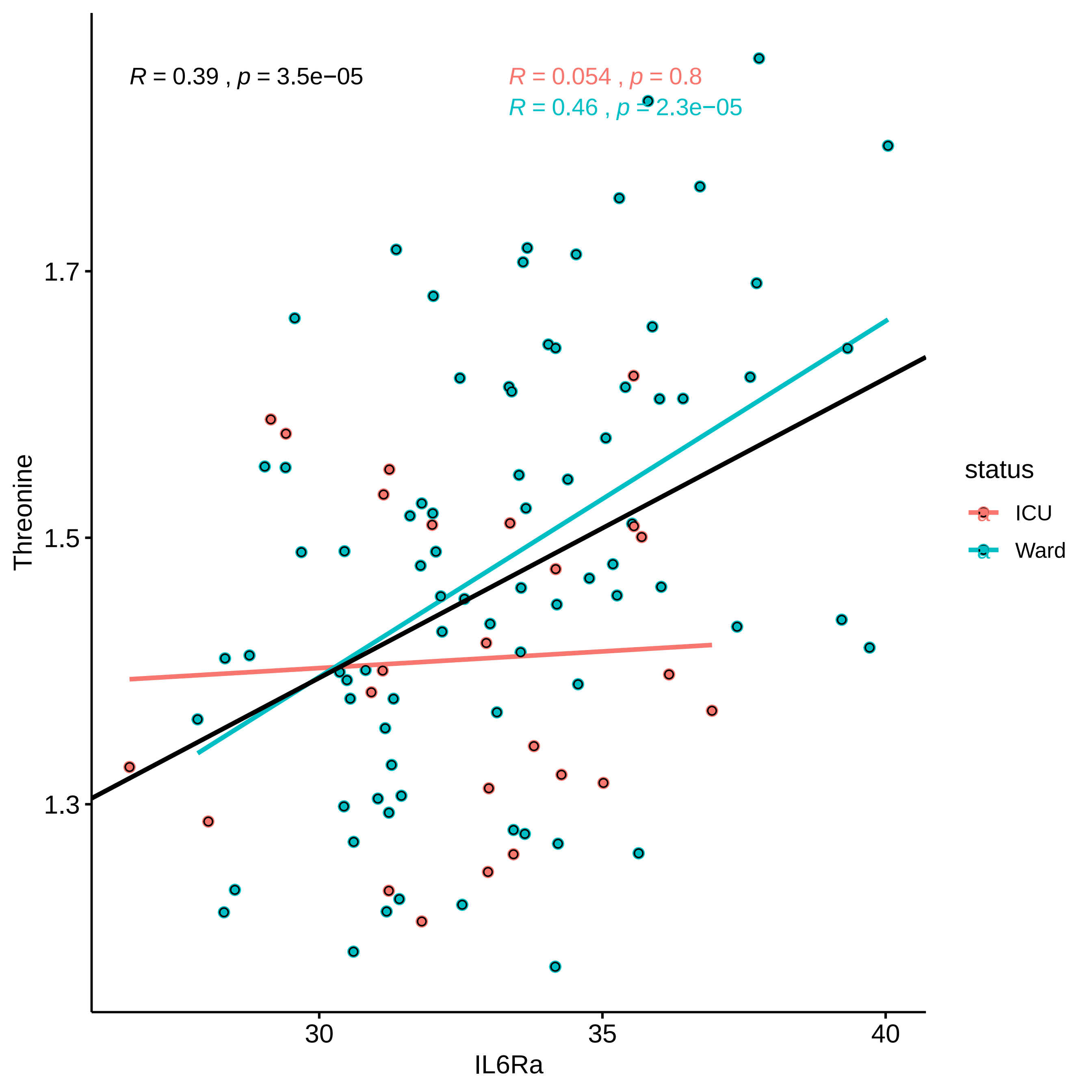

R = 0.39 , p = 3.5e−05
R = 0.054 , p = 0.8
R = 0.46 , p = 2.3e−05
Threonine
1.7
1.5
1.3
IL6Ra
30
35
40
status
ICU
Ward

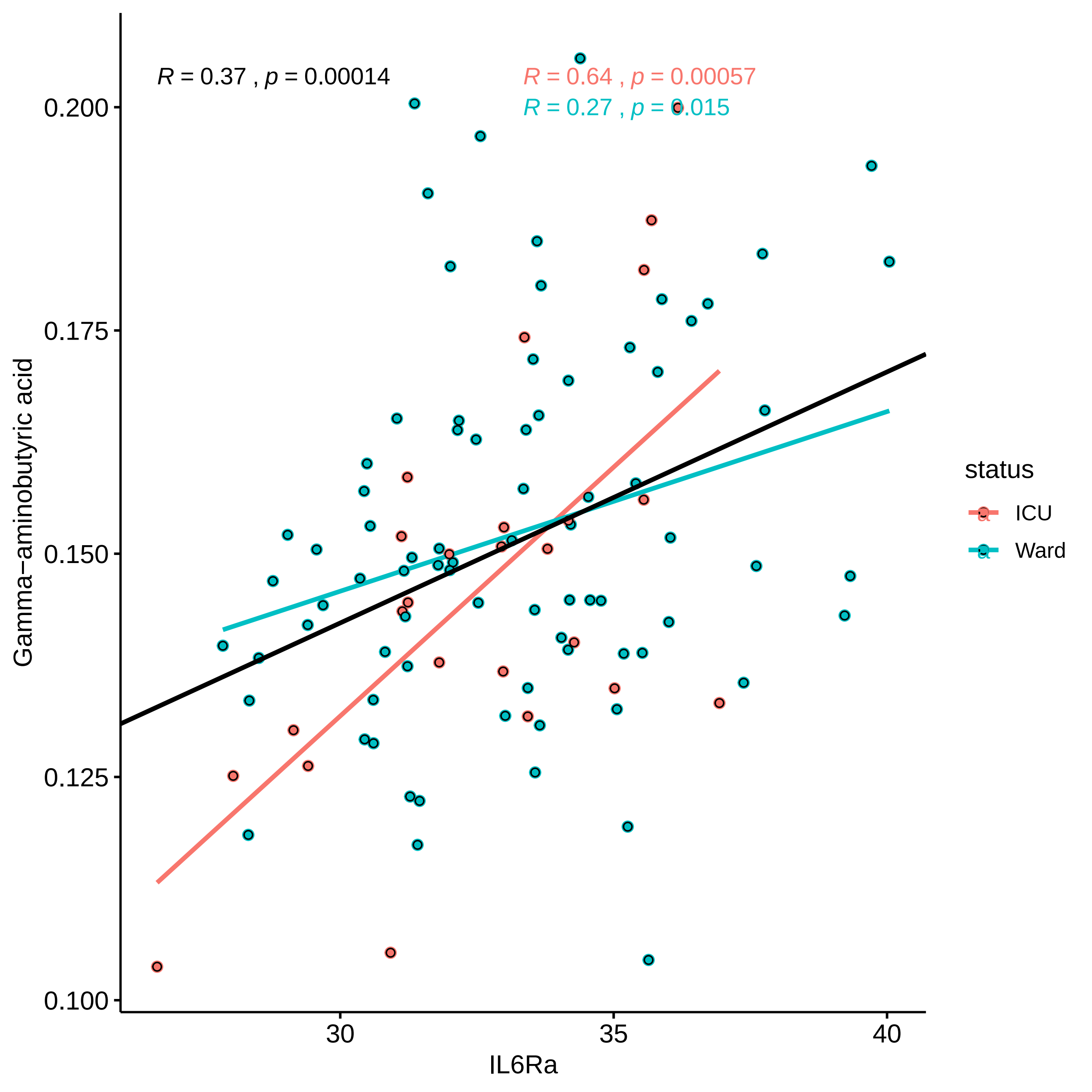

R = 0.37 , p = 0.00014
R = 0.64 , p = 0.00057
R = 0.27 , p = 0.015
0.200
0.175
0.150
0.125
0.100
Gamma−aminobutyric acid
30
35
40
IL6Ra
status
ICU
Ward

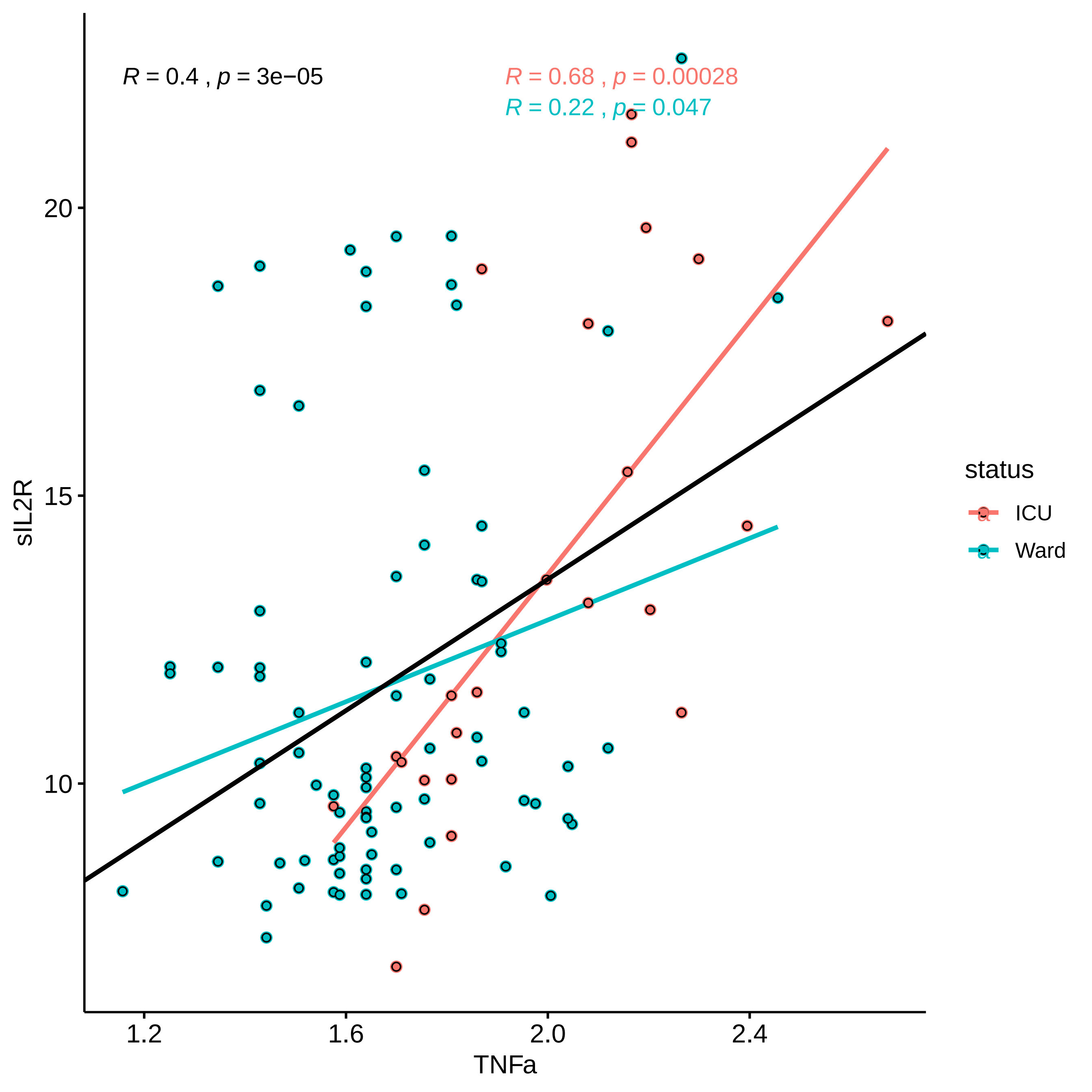

R = 0.4 , p = 3e−05
R = 0.68 , p = 0.00028
R = 0.22 , p = 0.047
sIL2R
20
15
10
1.2
1.6
2.0
2.4
TNFa
status
ICU
Ward

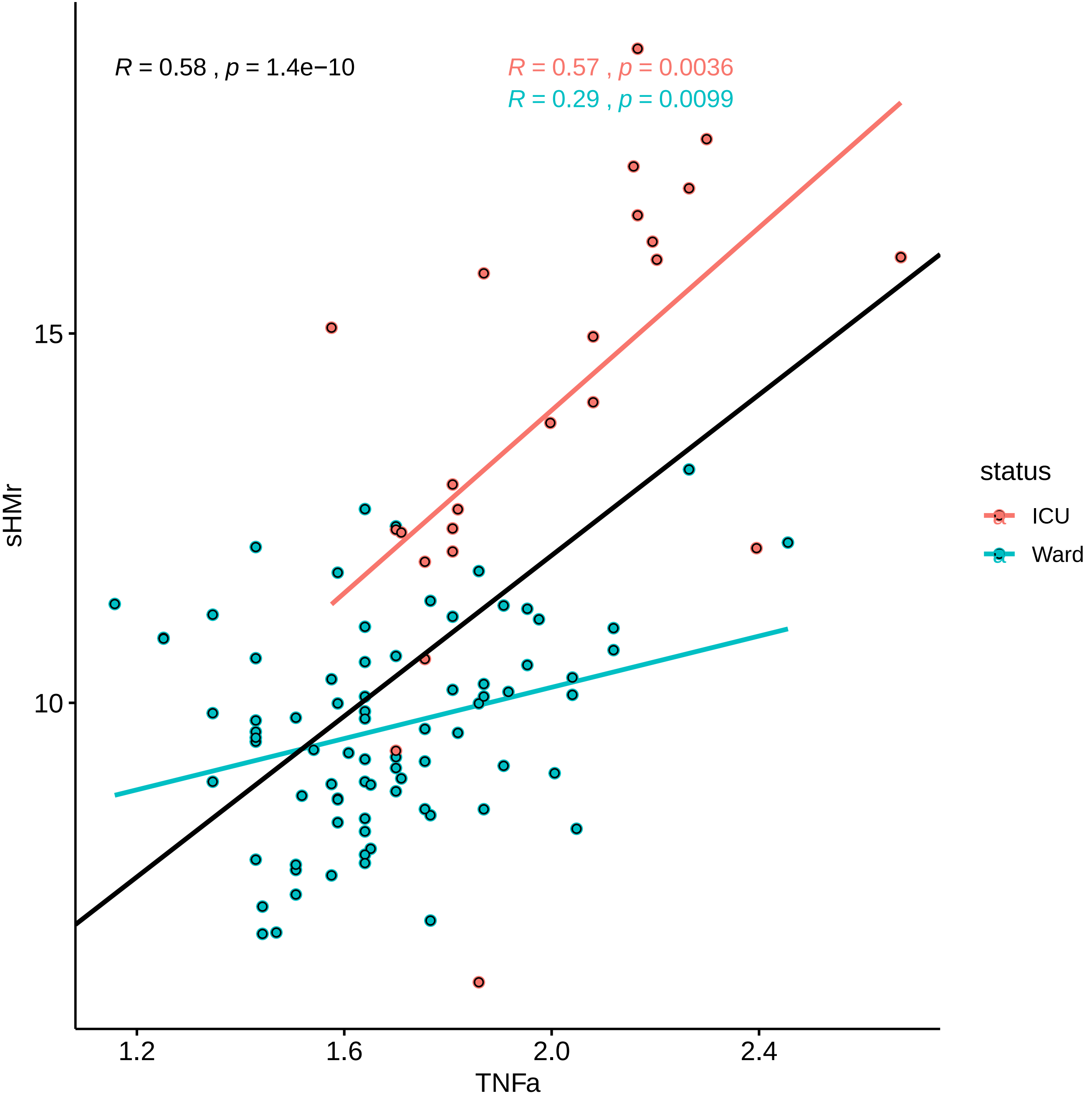

R = 0.58 , p = 1.4e−10
R = 0.57 , p = 0.0036
R = 0.29 , p = 0.0099
sHMr
15
10
1.2
1.6
2.0
2.4
TNFa
status
ICU
Ward

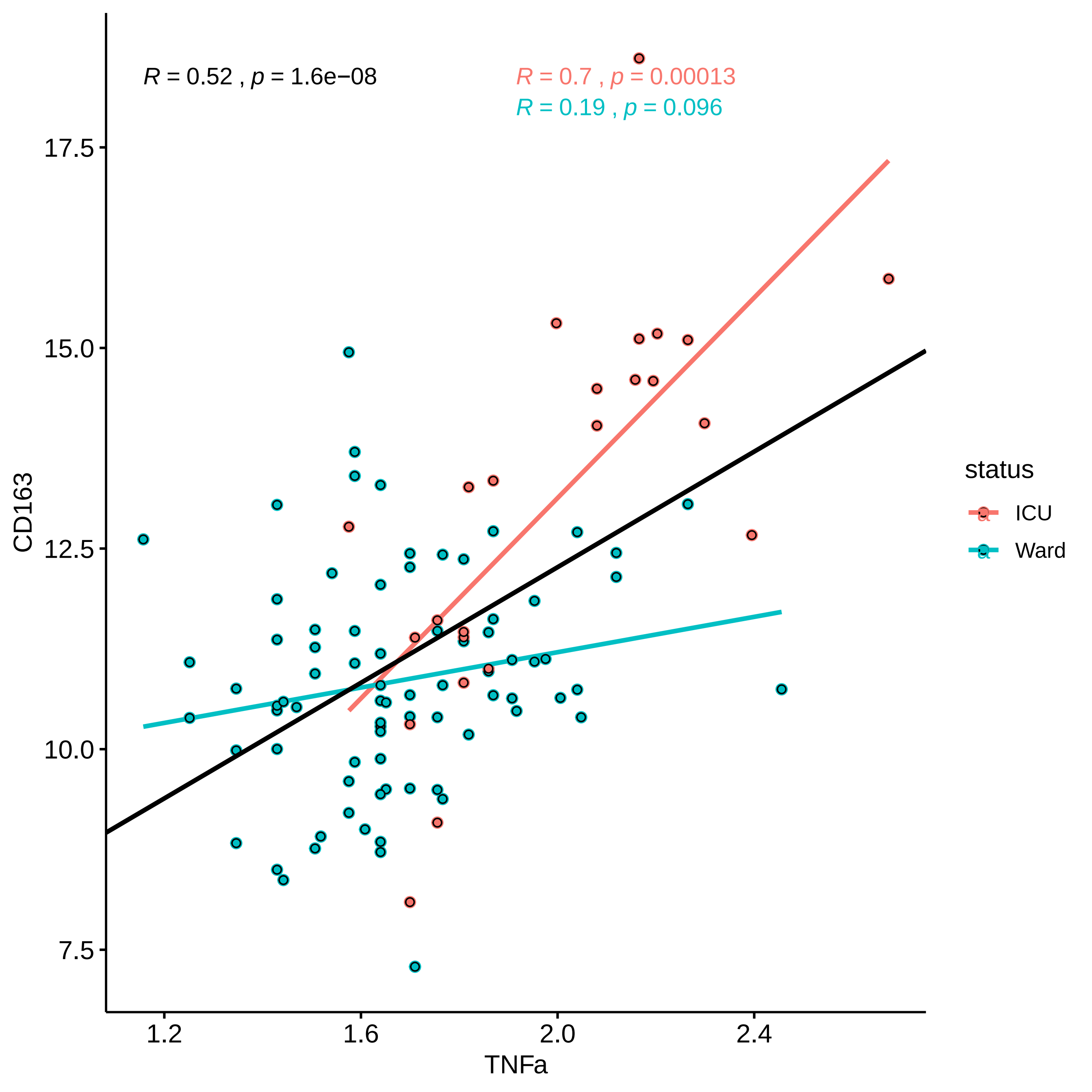

R = 0.52 , p = 1.6e−08
R = 0.7 , p = 0.00013
R = 0.19 , p = 0.096
17.5
15.0
12.5
10.0
7.5
CD163
1.2
1.6
2.0
2.4
TNFa
status
ICU
Ward

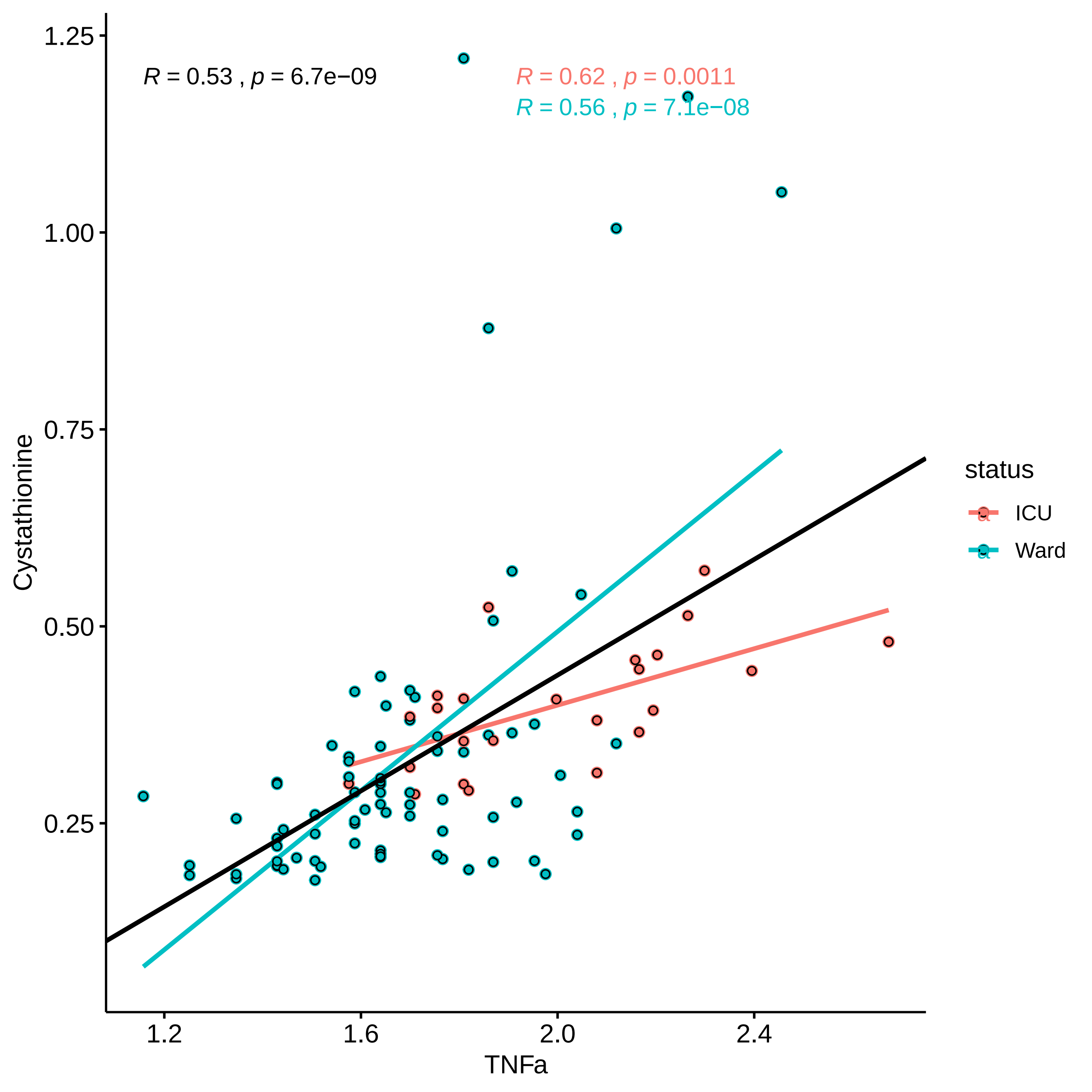

R = 0.53 , p = 6.7e−09
R = 0.62 , p = 0.0011
R = 0.56 , p = 7.1e−08
Cystathionine
TNFa
1.25
1.00
0.75
0.50
0.25
1.2
1.6
2.0
2.4
status
ICU
Ward

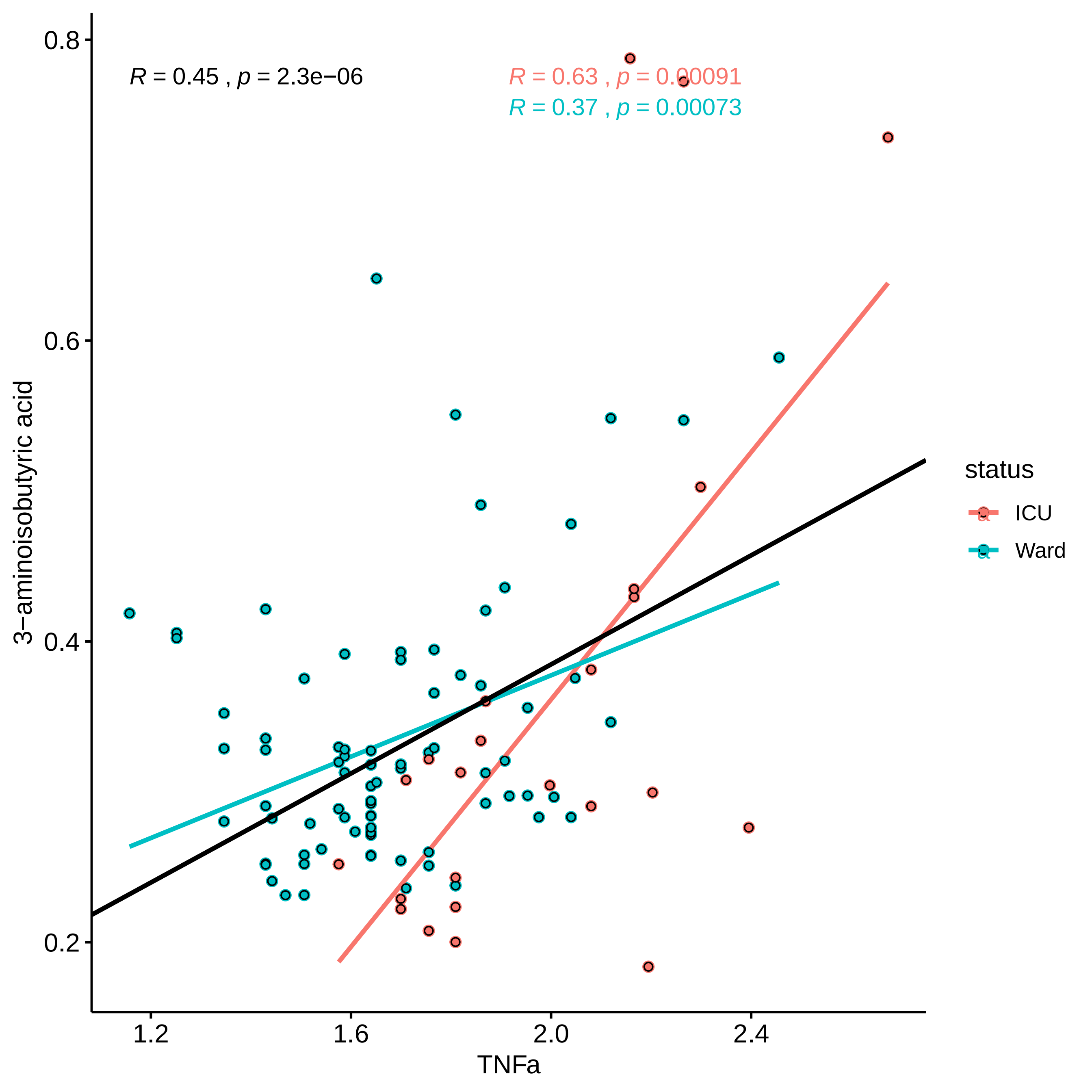

R = 0.45 , p = 2.3e−06
R = 0.63 , p = 0.00091
R = 0.37 , p = 0.00073
0.8
0.6
0.4
0.2
3−aminoisobutyric acid
1.2
1.6
2.0
2.4
TNFa
status
ICU
Ward

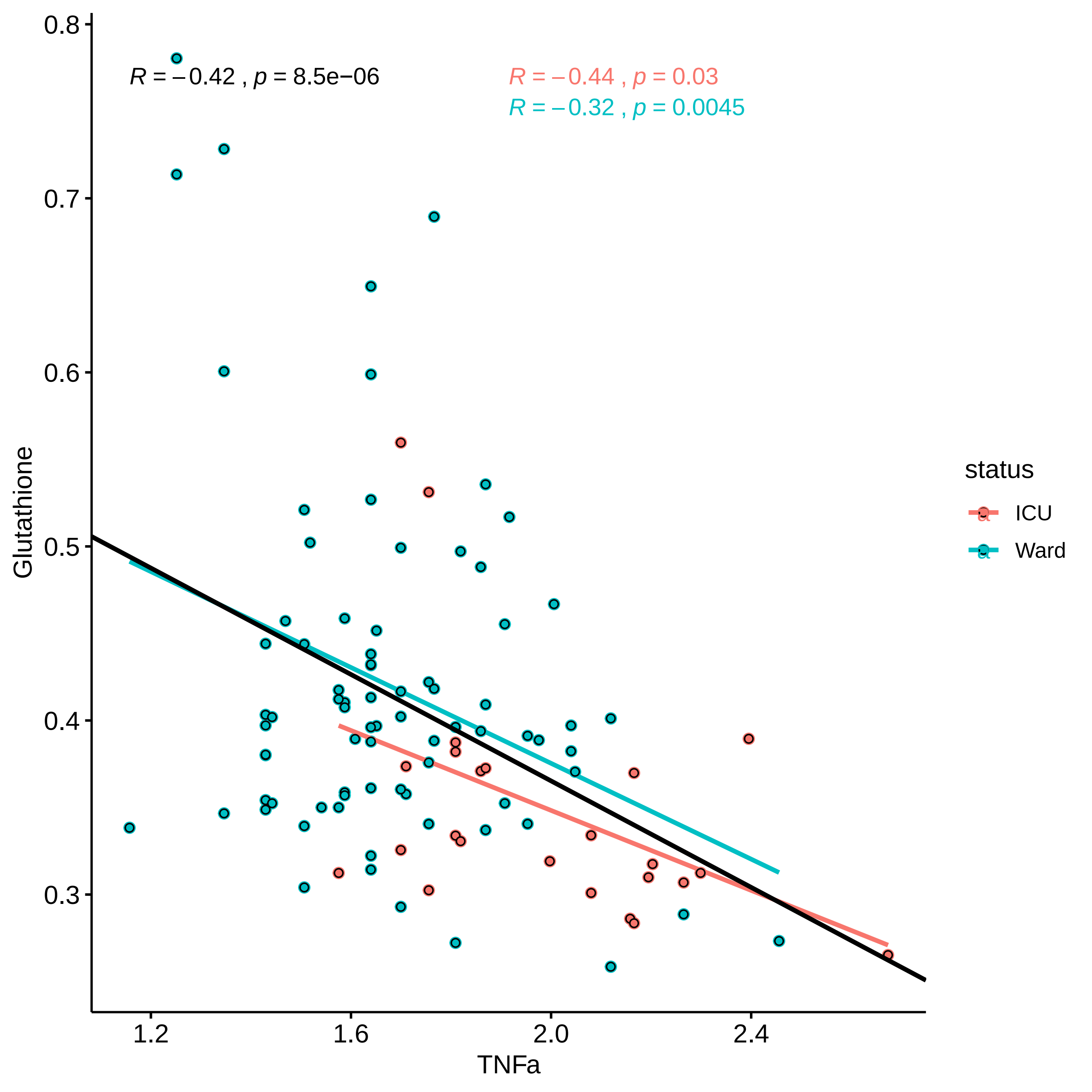

R = −0.42 , p = 8.5e−06
R = −0.44 , p = 0.03
R = −0.32 , p = 0.0045
Glutathione
0.8
0.7
0.6
0.5
0.4
0.3
1.2
1.6
2.0
2.4
TNFa
status
ICU
Ward

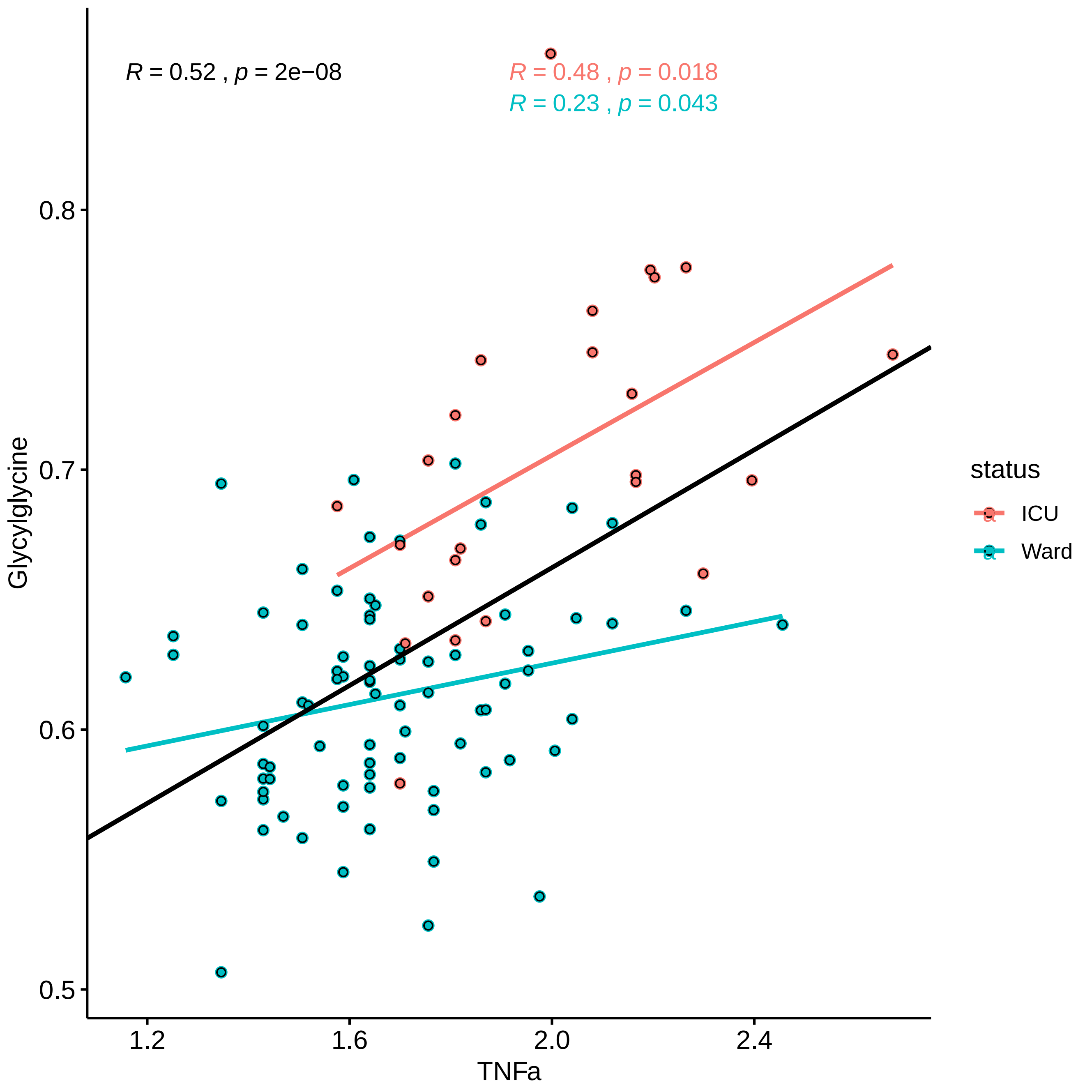

R = 0.52 , p = 2e−08
R = 0.48 , p = 0.018
R = 0.23 , p = 0.043
Glycylglycine
0.8
0.7
0.6
0.5
1.2
1.6
2.0
2.4
TNFa
status
ICU
Ward

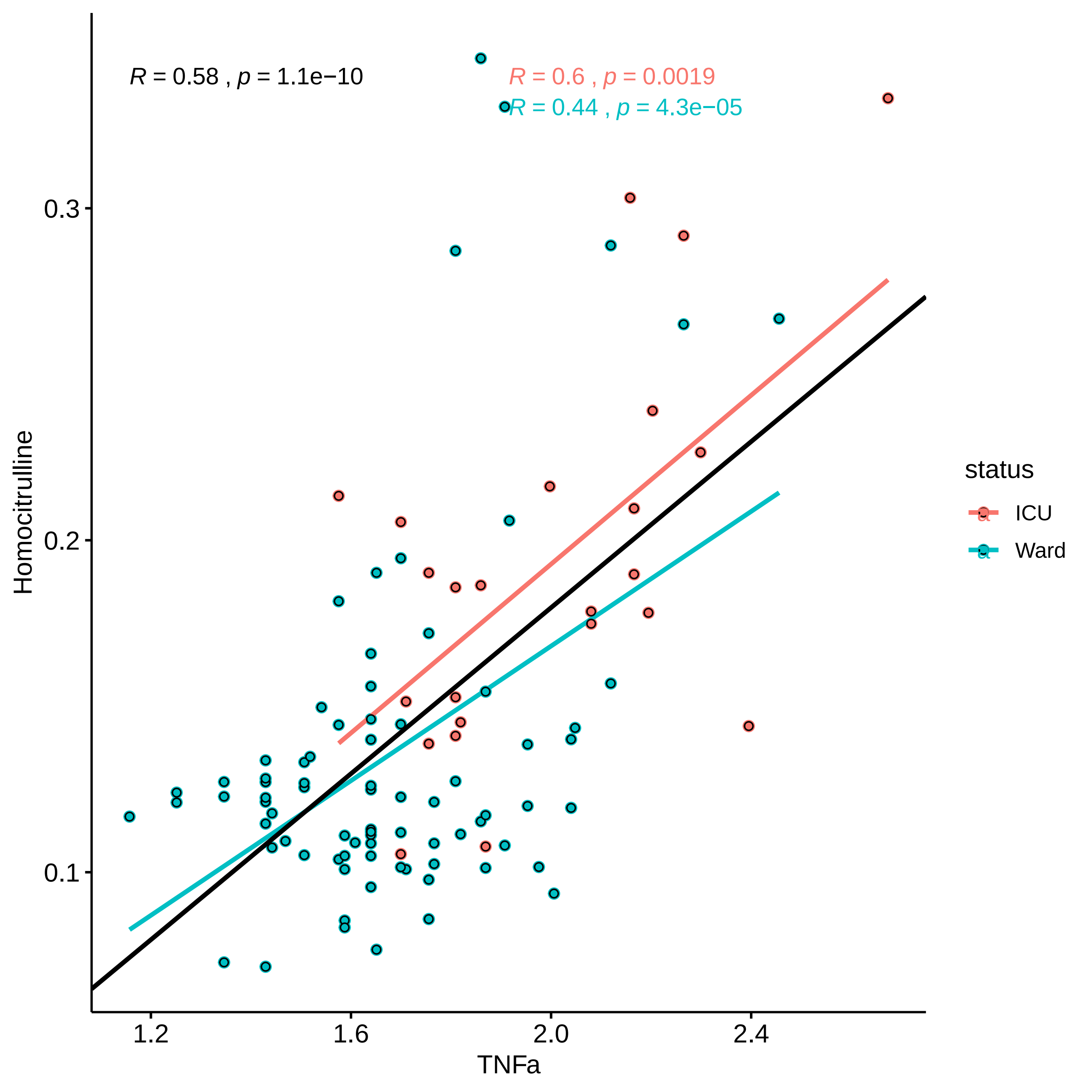

R = 0.58 , p = 1.1e−10
R = 0.6 , p = 0.0019
R = 0.44 , p = 4.3e−05
Homocitrulline
0.3
0.2
0.1
1.2
1.6
2.0
2.4
TNFa
status
ICU
Ward

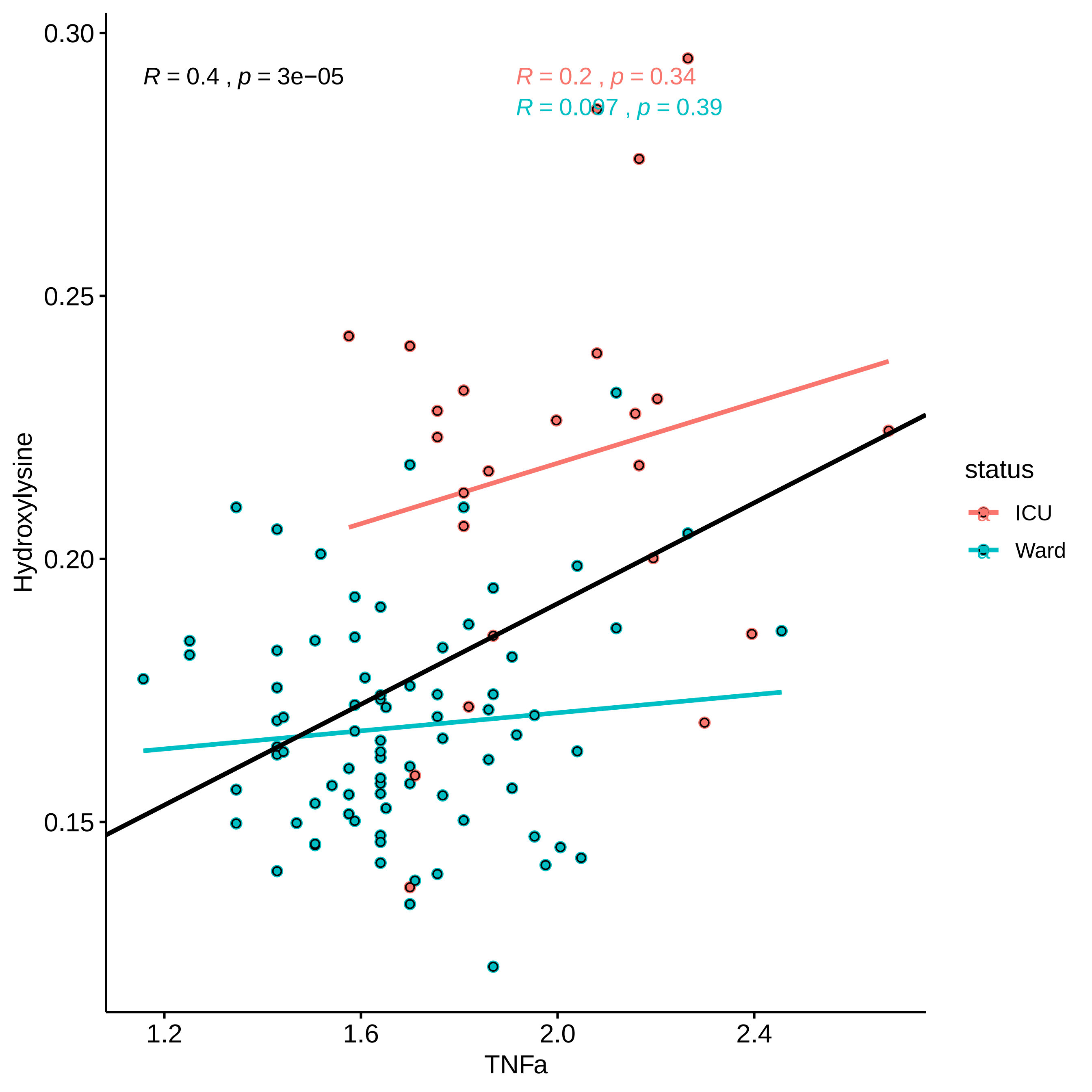

R = 0.4 , p = 3e−05
R = 0.2 , p = 0.34
R = 0.097 , p = 0.39
Hydroxylysine
0.30
0.25
0.20
0.15
1.2
1.6
2.0
2.4
TNFa
status
ICU
Ward

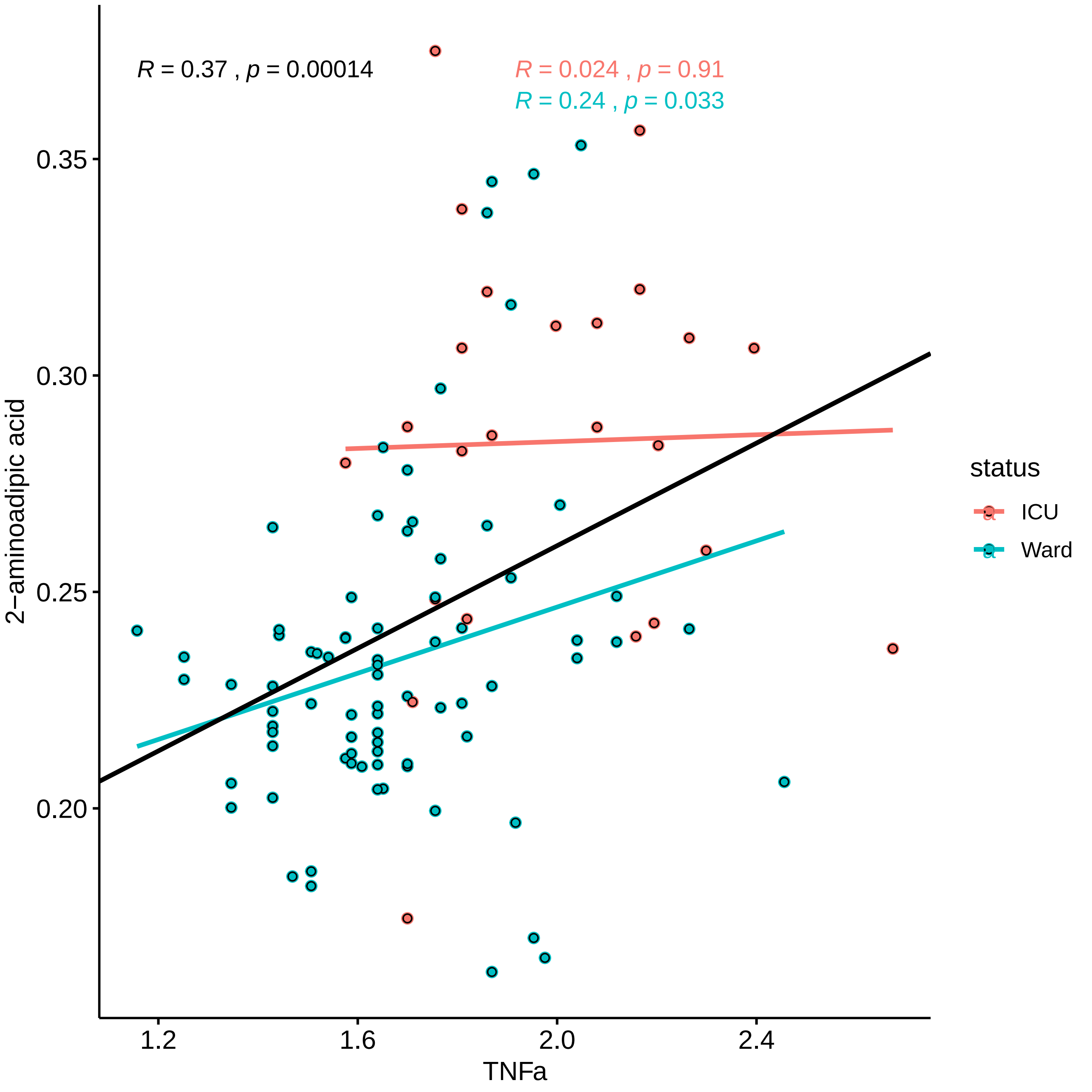

R = 0.37 , p = 0.00014
R = 0.024 , p = 0.91
R = 0.24 , p = 0.033
0.35
0.30
0.25
0.20
2−aminoadipic acid
1.2
1.6
2.0
2.4
TNFa
status
ICU
Ward

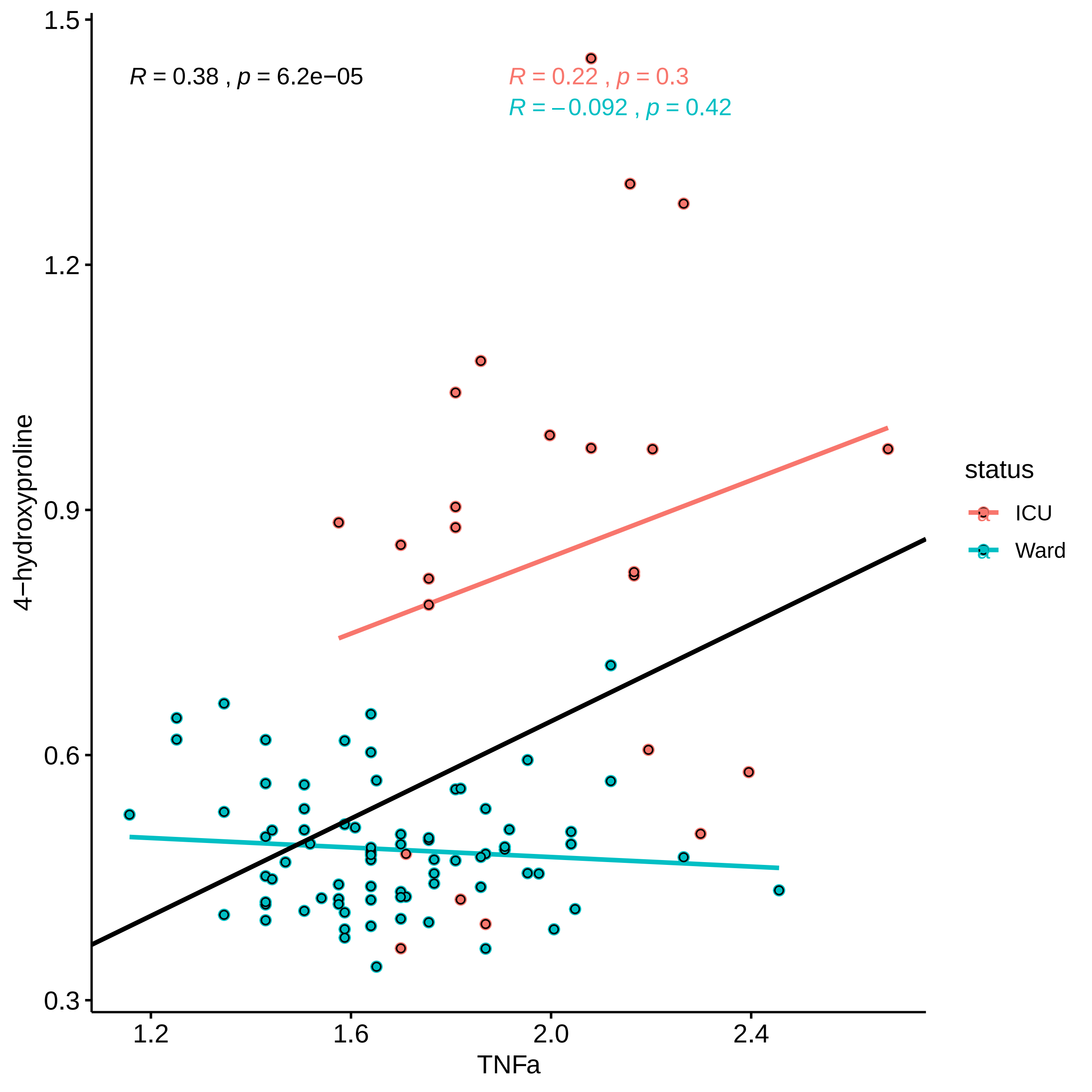

R = 0.38 , p = 6.2e−05
R = 0.22 , p = 0.3
R = −0.092 , p = 0.42
4−hydroxyproline
1.5
1.2
0.9
0.6
0.3
1.2
1.6
2.0
2.4
TNFa
status
ICU
Ward

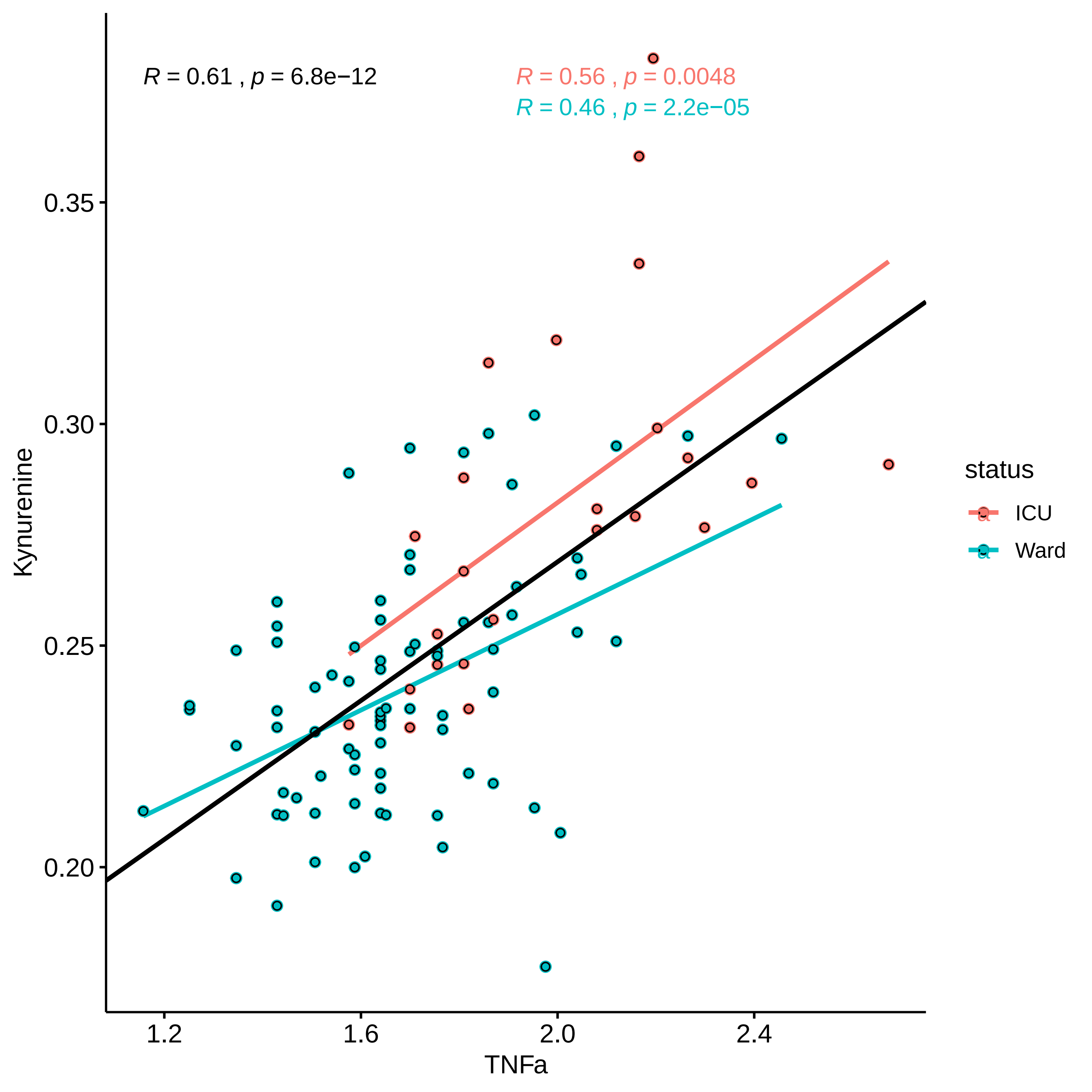

R = 0.61 , p = 6.8e−12
R = 0.56 , p = 0.0048
R = 0.46 , p = 2.2e−05
Kynurenine
0.35
0.30
0.25
0.20
TNFa
1.2
1.6
2.0
2.4
status
ICU
Ward

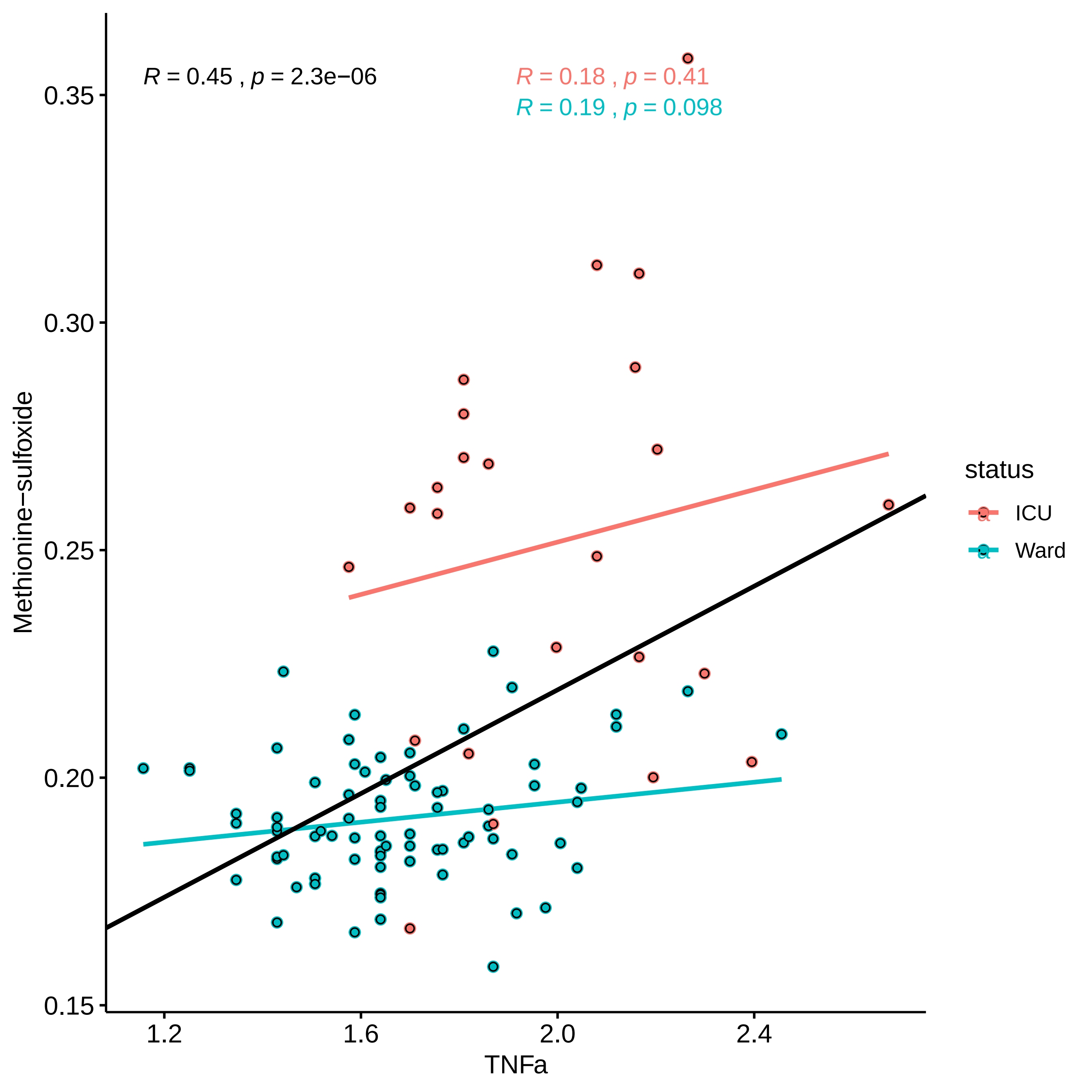

R = 0.45 , p = 2.3e−06
R = 0.18 , p = 0.41
R = 0.19 , p = 0.098
Methionine−sulfoxide
0.35
0.30
0.25
0.20
0.15
1.2
1.6
2.0
2.4
TNFa
status
ICU
Ward

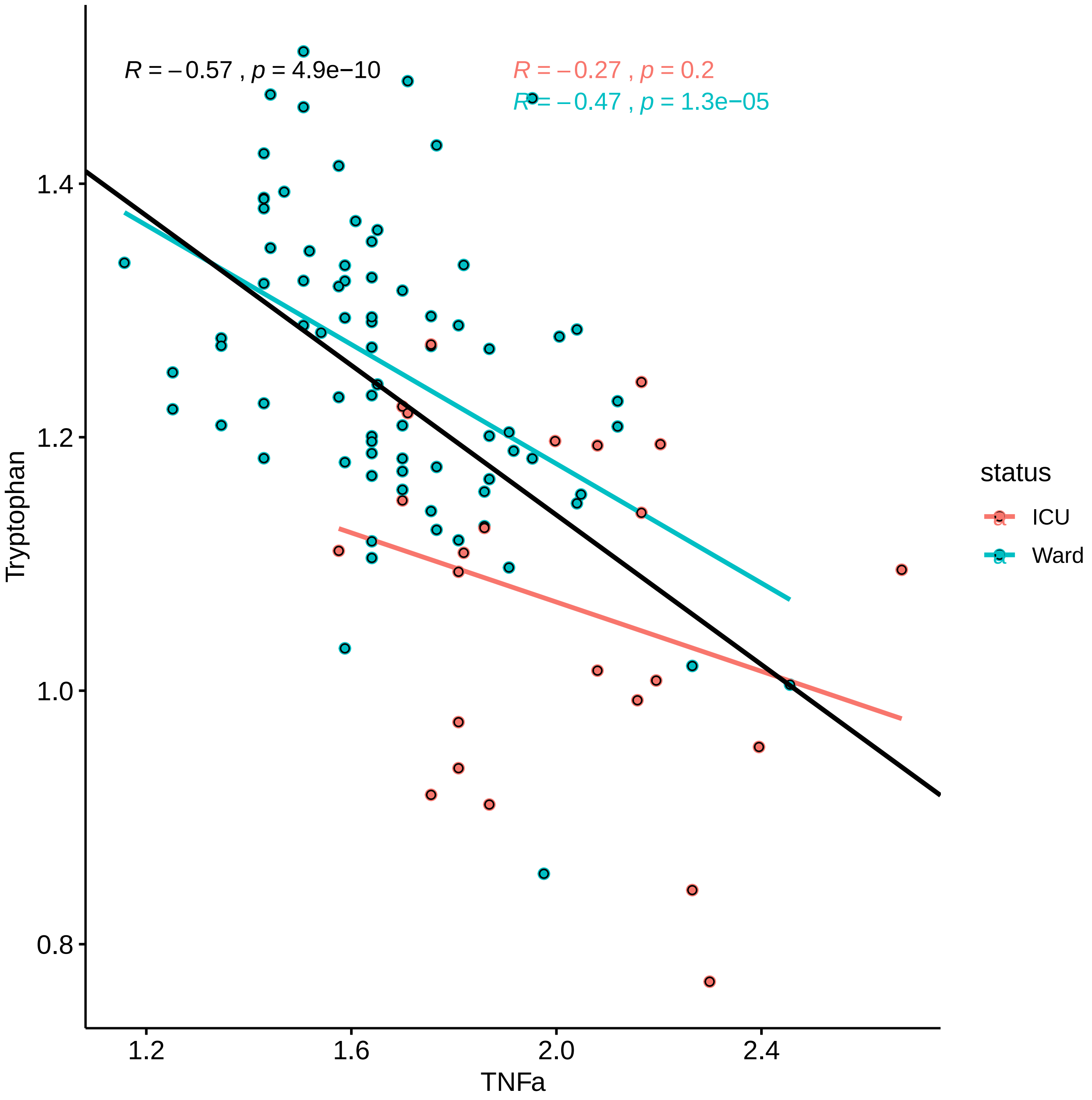

R = −0.57 , p = 4.9e−10
R = −0.27 , p = 0.2
R = −0.47 , p = 1.3e−05
Tryptophan
1.4
1.2
1.0
0.8
TNFa
1.2
1.6
2.0
2.4
status
ICU
Ward

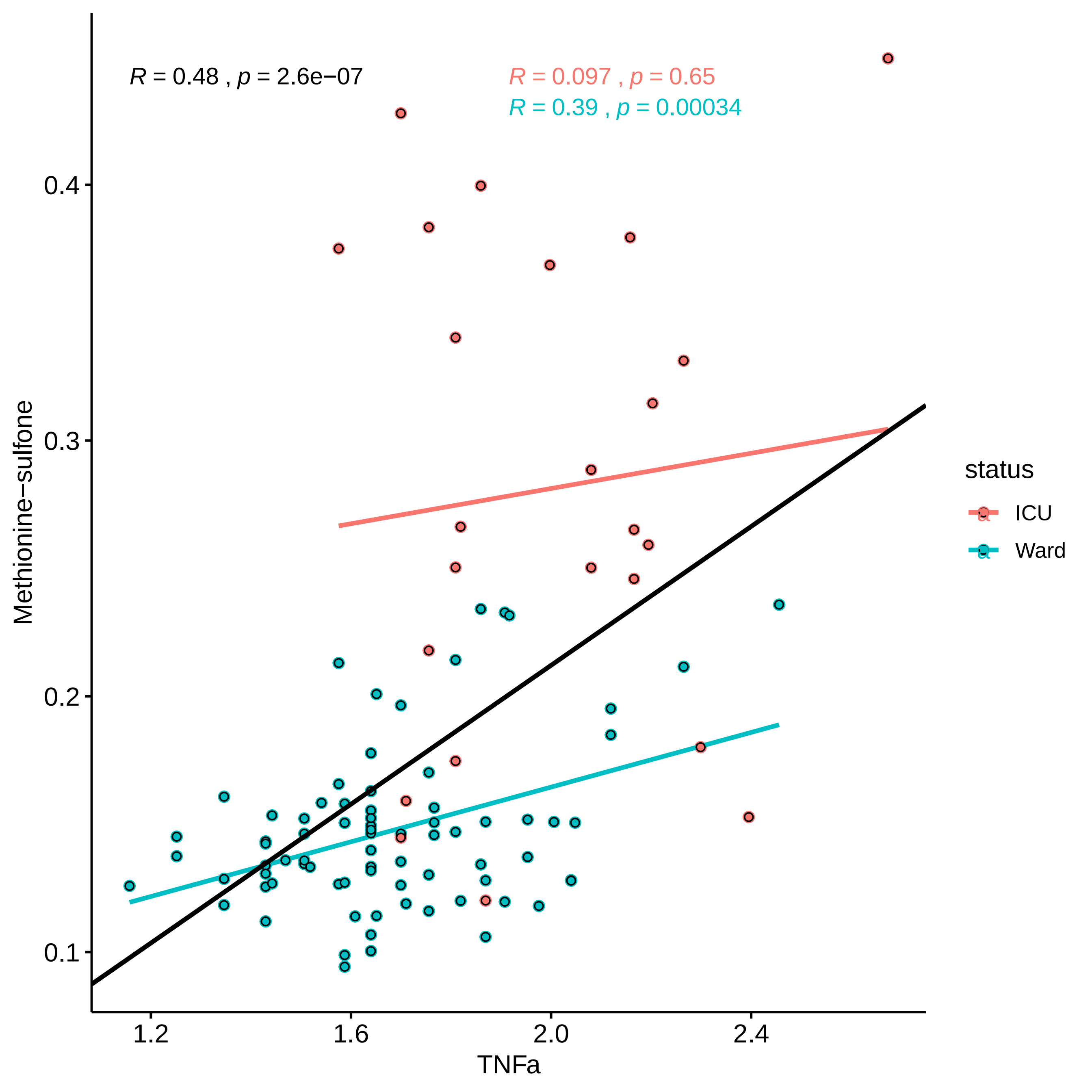

R = 0.48 , p = 2.6e−07
R = 0.097 , p = 0.65
R = 0.39 , p = 0.00034
Methionine−sulfone
0.1
0.2
0.3
0.4
1.2
1.6
2.0
2.4
TNFa
status
ICU
Ward

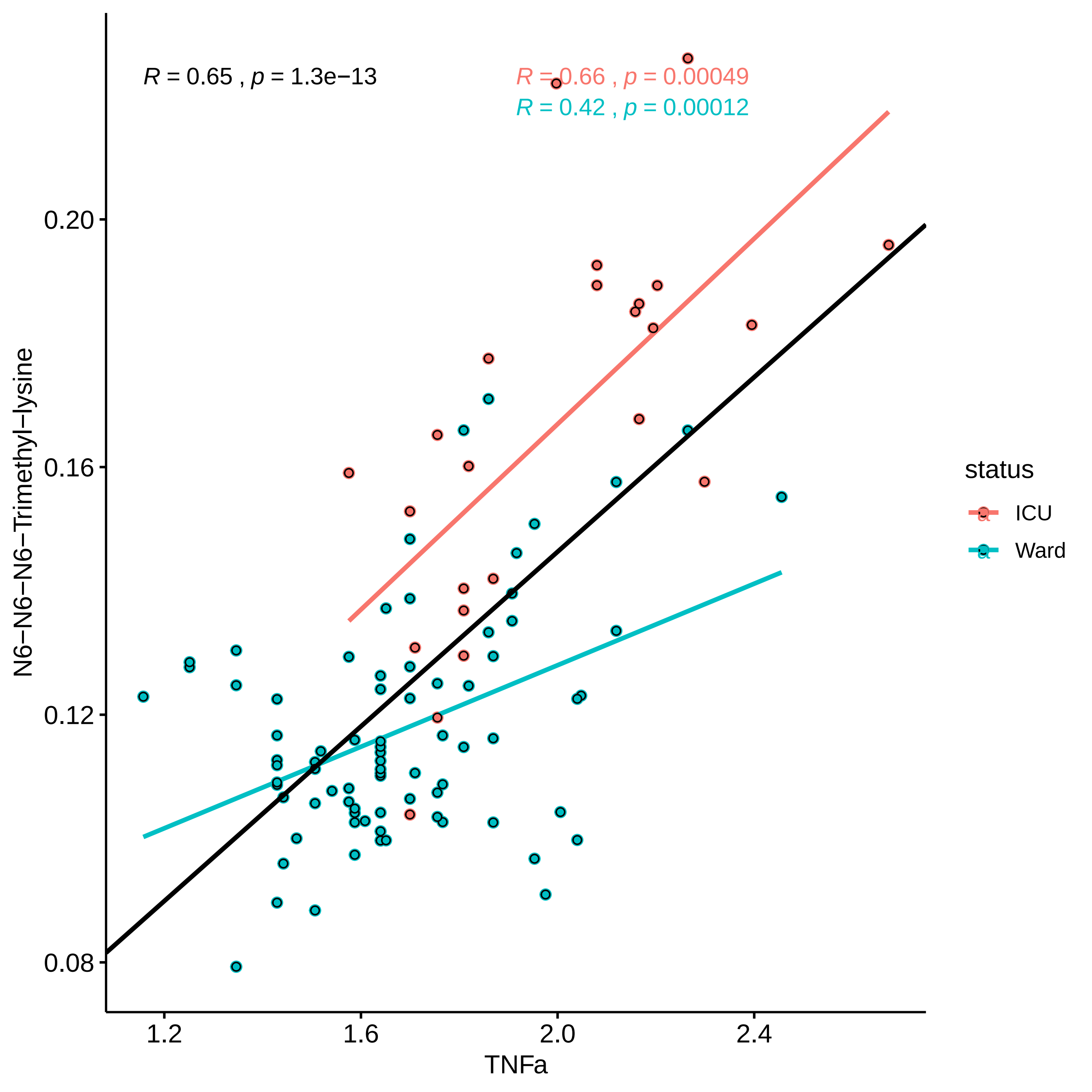

R = 0.65 , p = 1.3e−13
R = 0.66 , p = 0.00049
R = 0.42 , p = 0.00012
N6−N6−N6−Trimethyl−lysine
0.08
0.12
0.16
0.20
TNFa
1.2
1.6
2.0
2.4
status
ICU
Ward

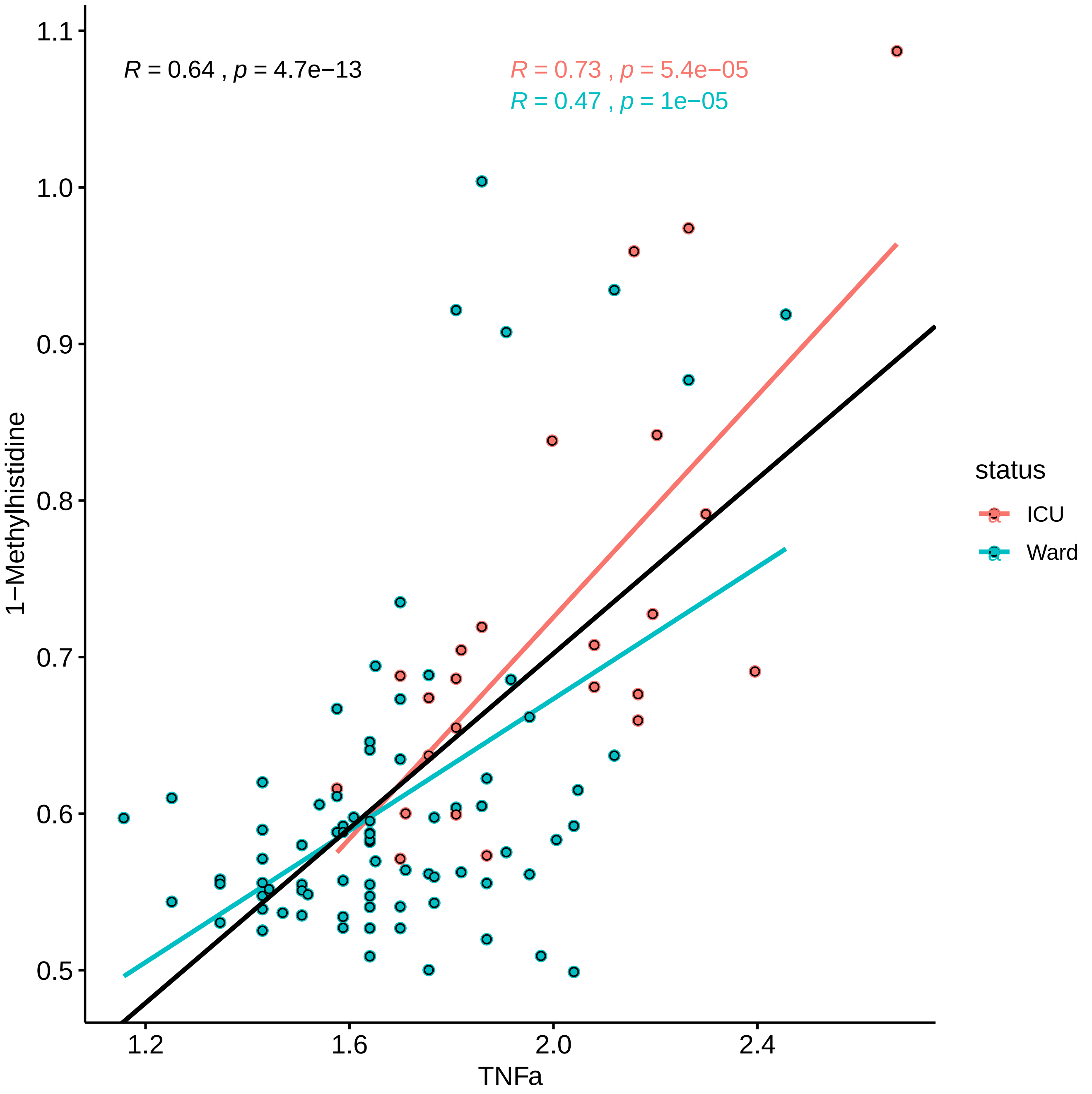
R = 0.64 , p = 4.7e−13
R = 0.73 , p = 5.4e−05
R = 0.47 , p = 1e−05
1−Methylhistidine
TNFa
status
ICU
Ward
1.1
1.0
0.9
0.8
0.7
0.6
0.5
1.2
1.6
2.0
2.4

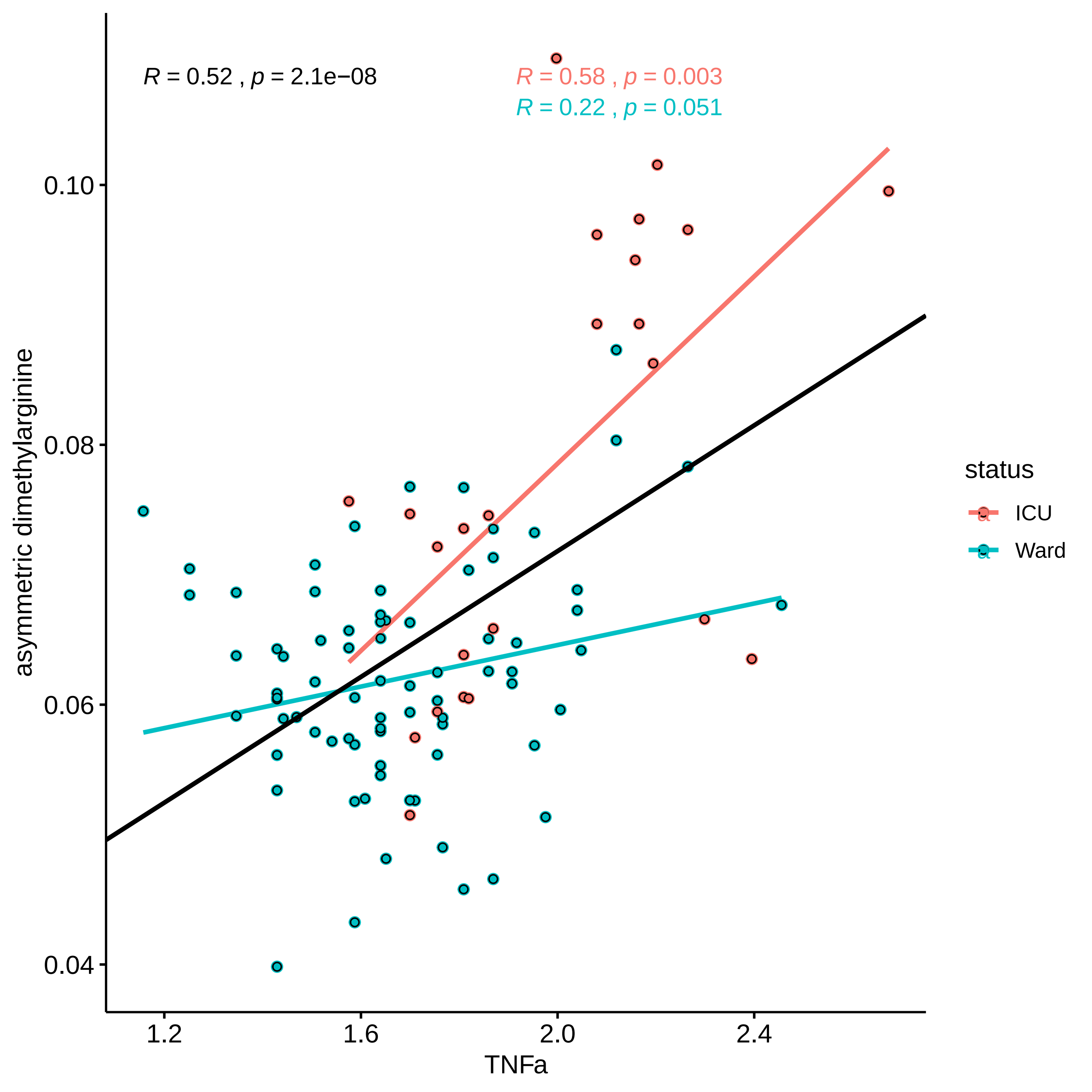

R = 0.52 , p = 2.1e−08
R = 0.58 , p = 0.003
R = 0.22 , p = 0.051
asymmetric dimethylarginine
0.10
0.08
0.06
0.04
1.2
1.6
2.0
2.4
TNFa
status
ICU
Ward

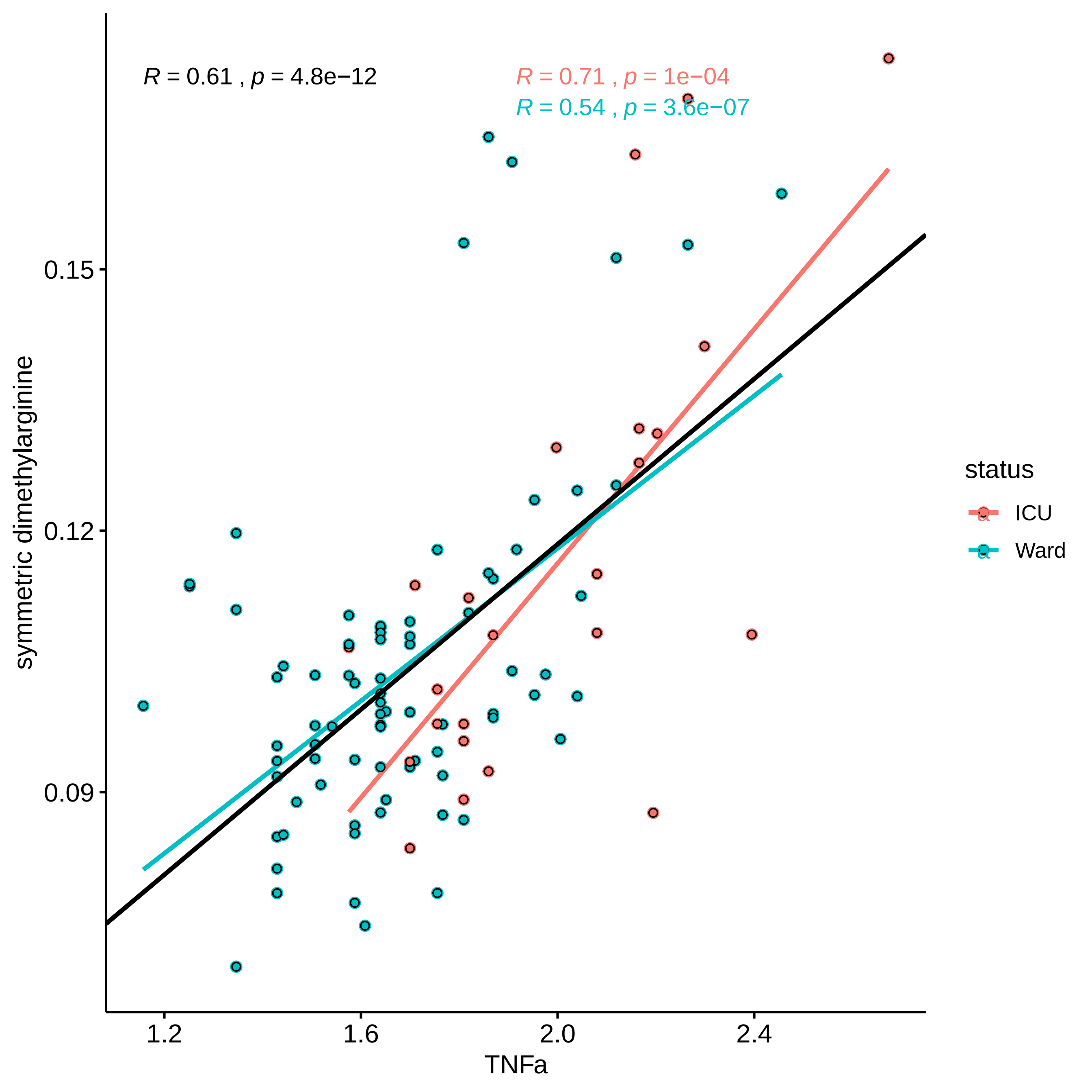

R = 0.61 , p = 4.8e−12
R = 0.71 , p = 1e−04
R = 0.54 , p = 3.6e−07
symmetric dimethylarginine
0.15
0.12
0.09
1.2
1.6
2.0
2.4
TNFa
status
ICU
Ward

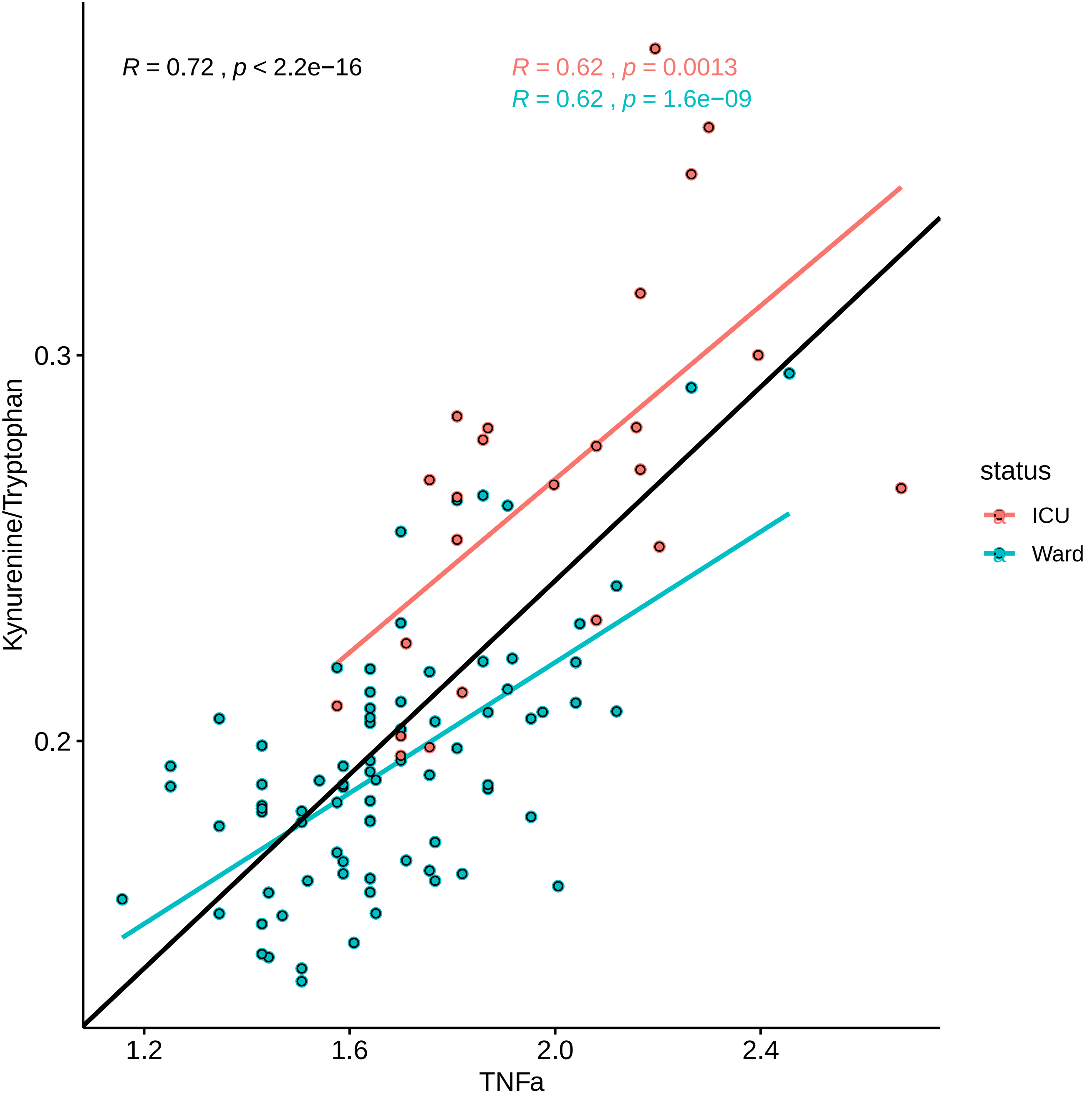

R = 0.72 , p < 2.2e−16
R = 0.62 , p = 0.0013
R = 0.62 , p = 1.6e−09
Kynurenine/Tryptophan
0.3
0.2
1.2
1.6
2.0
2.4
TNFa
status
ICU
Ward

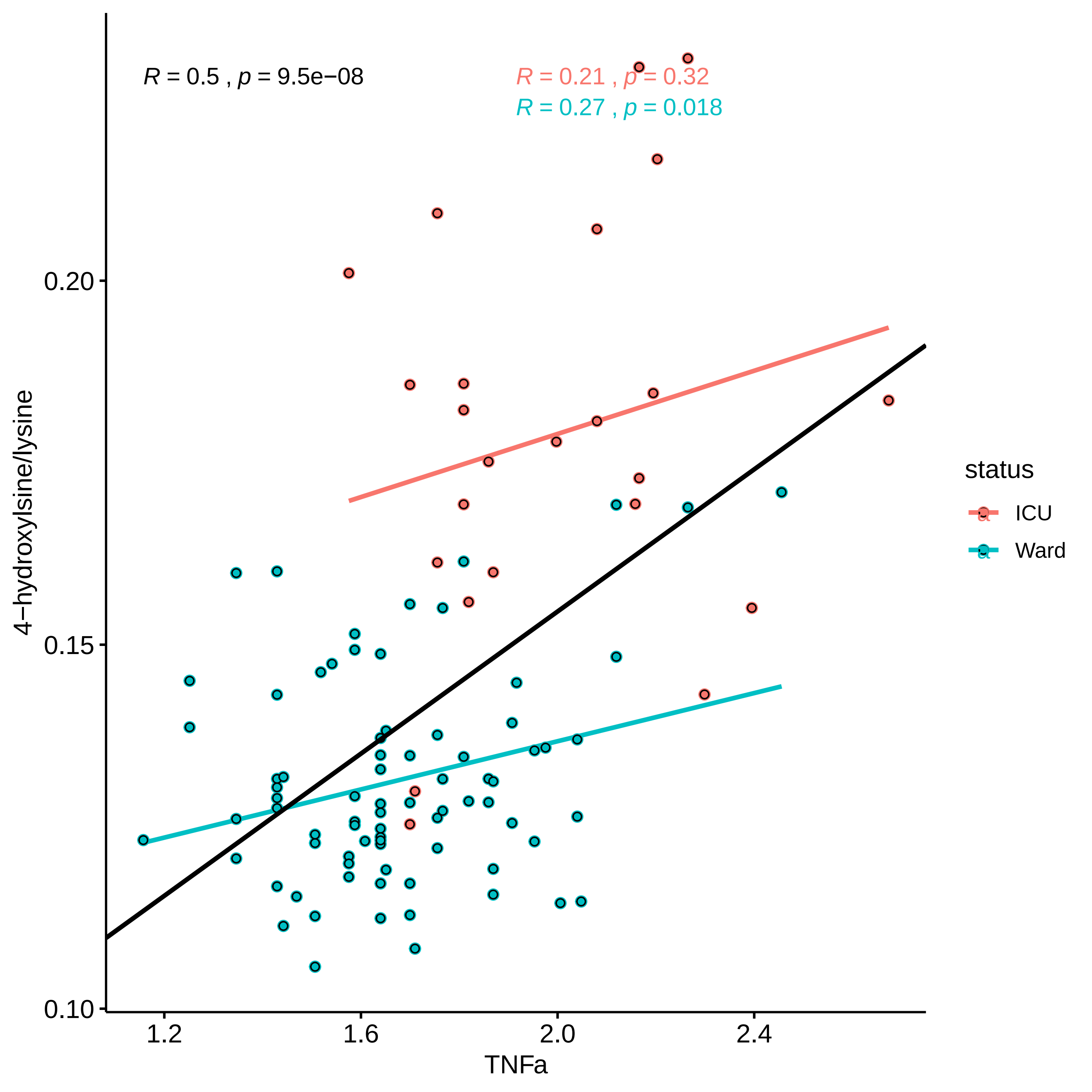

R = 0.5 , p = 9.5e−08
R = 0.21 , p = 0.32
R = 0.27 , p = 0.018
4−hydroxylsine/lysine
0.20
0.15
0.10
1.2
1.6
2.0
2.4
TNFa
status
ICU
Ward

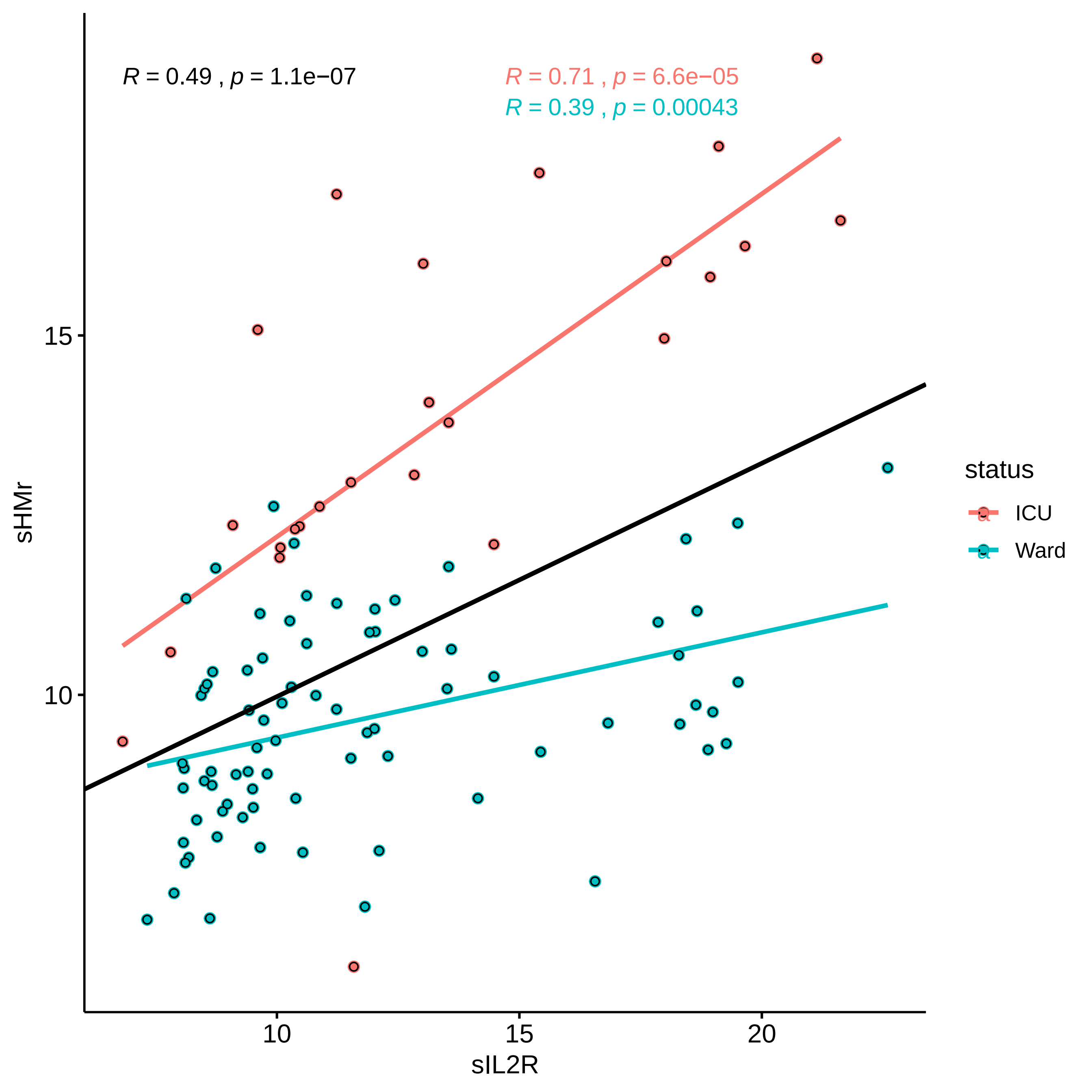

R = 0.49 , p = 1.1e−07
R = 0.71 , p = 6.6e−05
R = 0.39 , p = 0.00043
sHMr
15
10
10
15
20
sIL2R
status
ICU
Ward

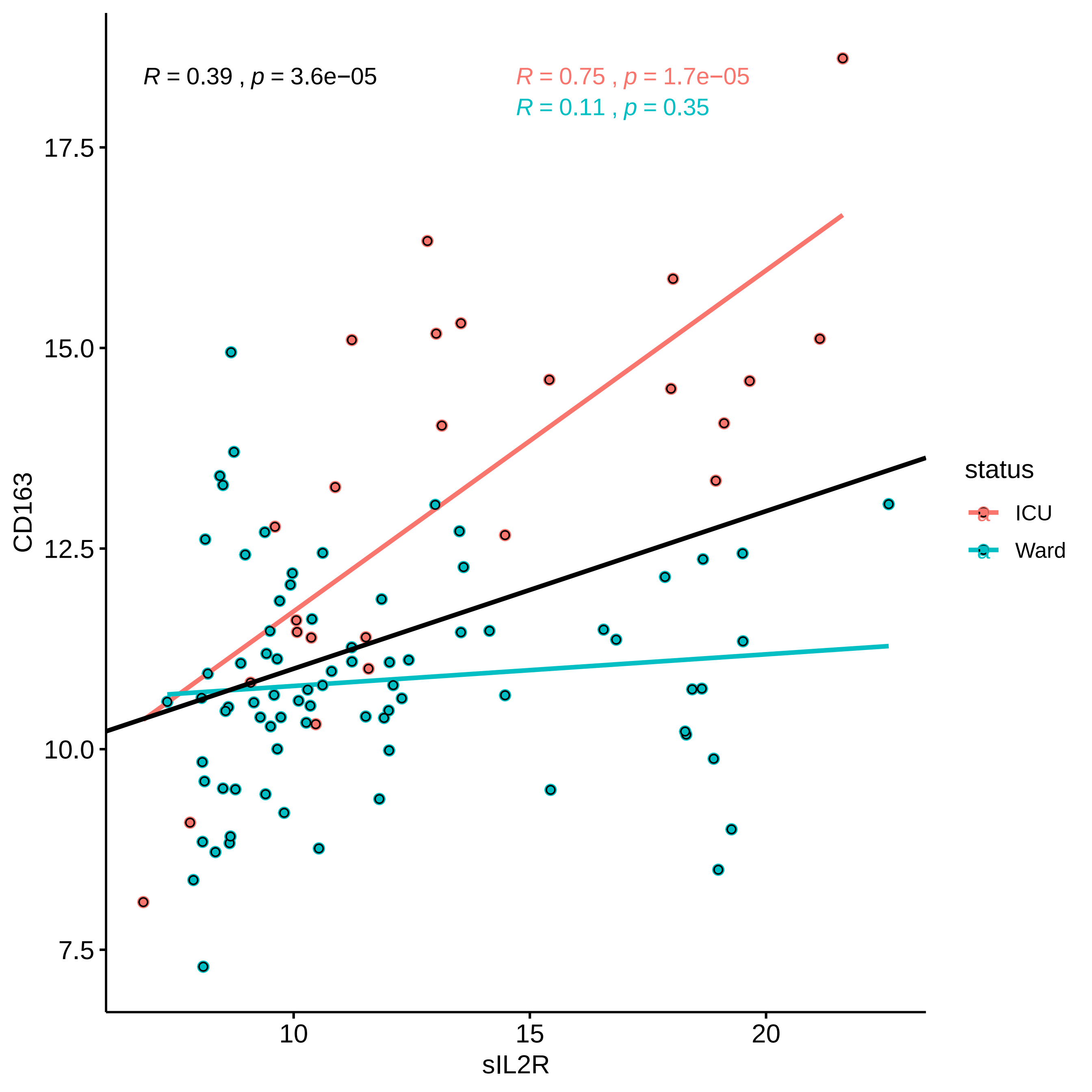

R = 0.39 , p = 3.6e−05
R = 0.75 , p = 1.7e−05
R = 0.11 , p = 0.35
17.5
15.0
12.5
10.0
7.5
CD163
10
15
20
sIL2R
status
ICU
Ward

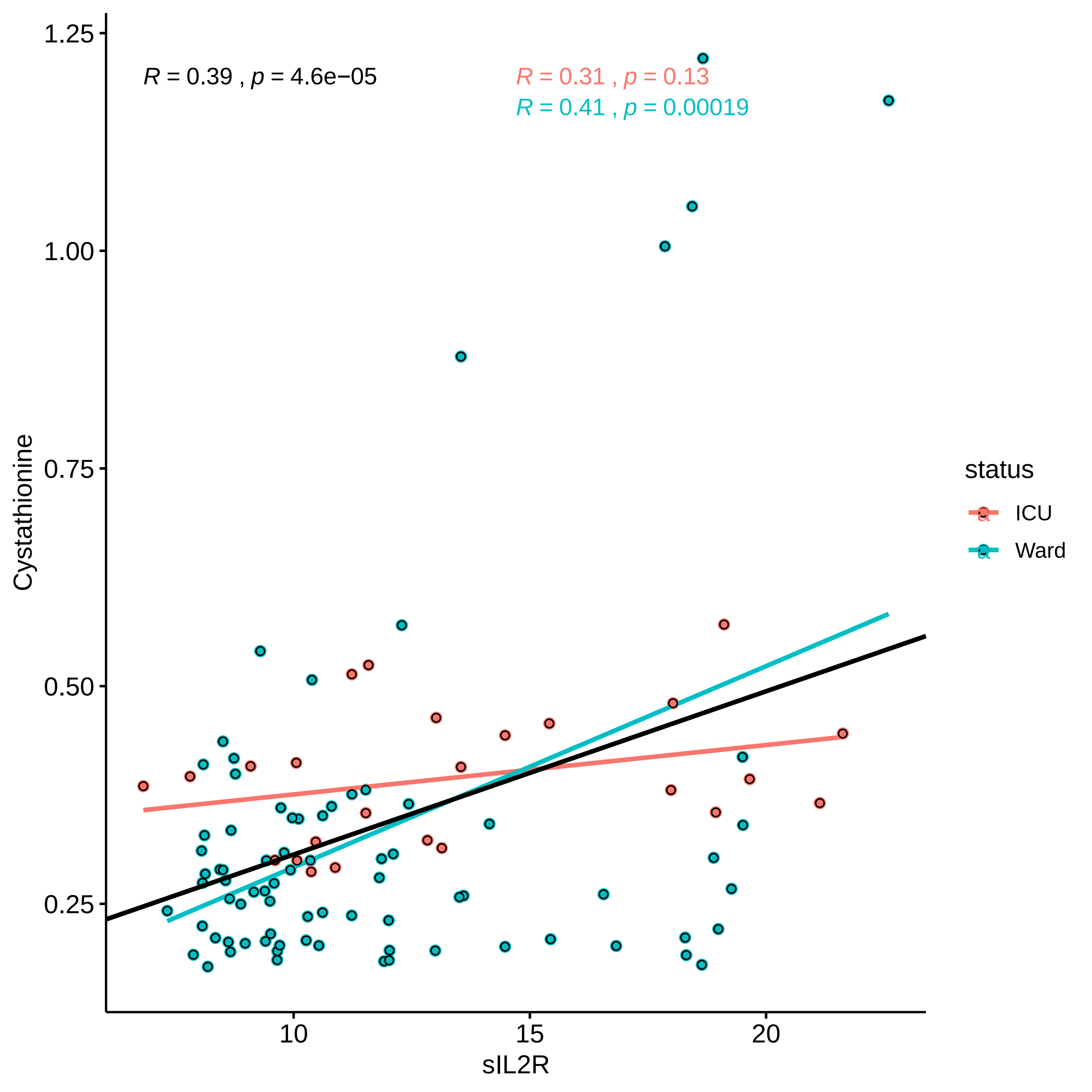
R = 0.39 , p = 4.6e−05
R = 0.31 , p = 0.13
R = 0.41 , p = 0.00019
1.25
1.00
0.75
0.50
0.25
Cystathionine
10
15
20
sIL2R
status
ICU
Ward

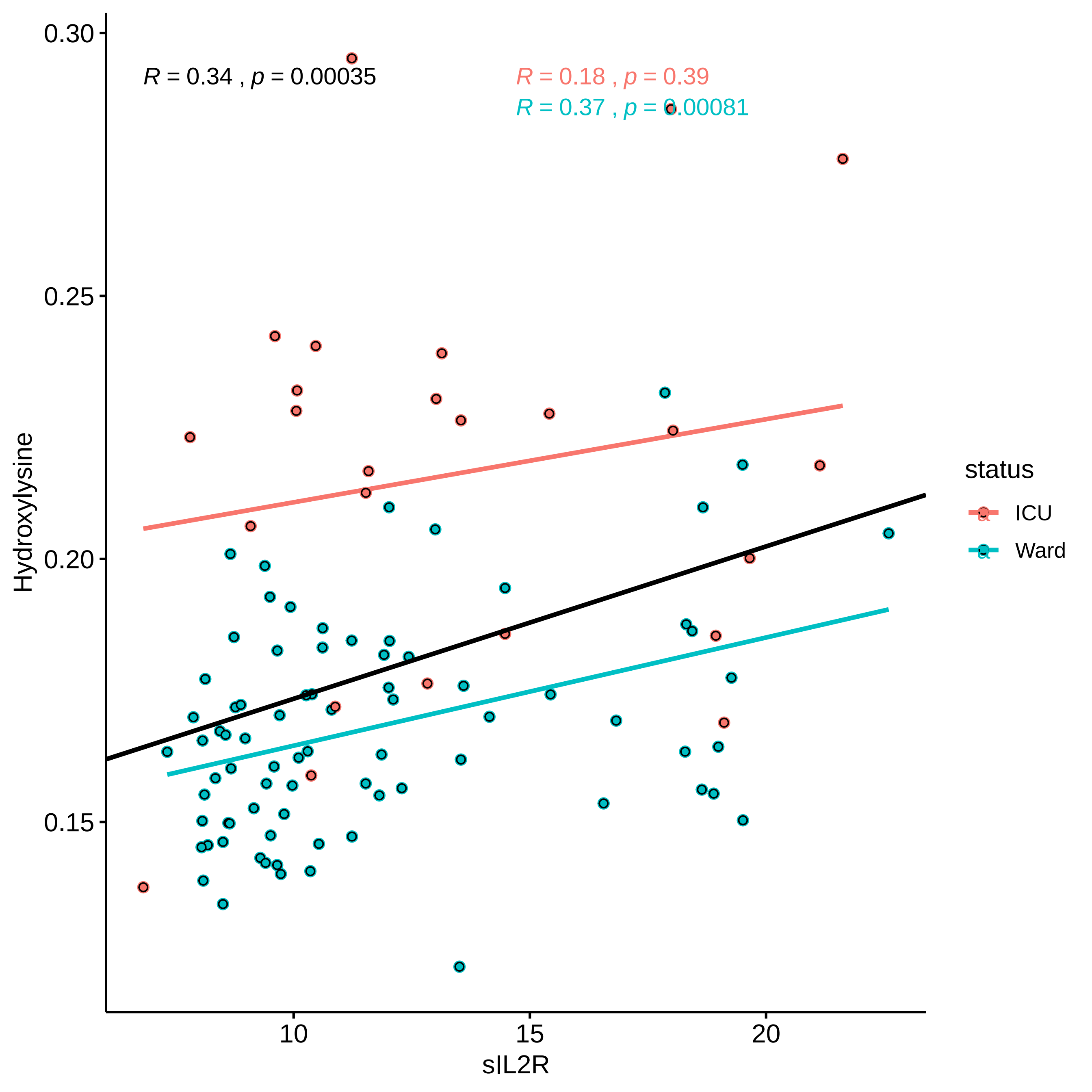
R = 0.34 , p = 0.00035
R = 0.18 , p = 0.39
R = 0.37 , p = 0.00081
Hydroxylysine
0.30
0.25
0.20
0.15
10
15
20
sIL2R
status
ICU
Ward

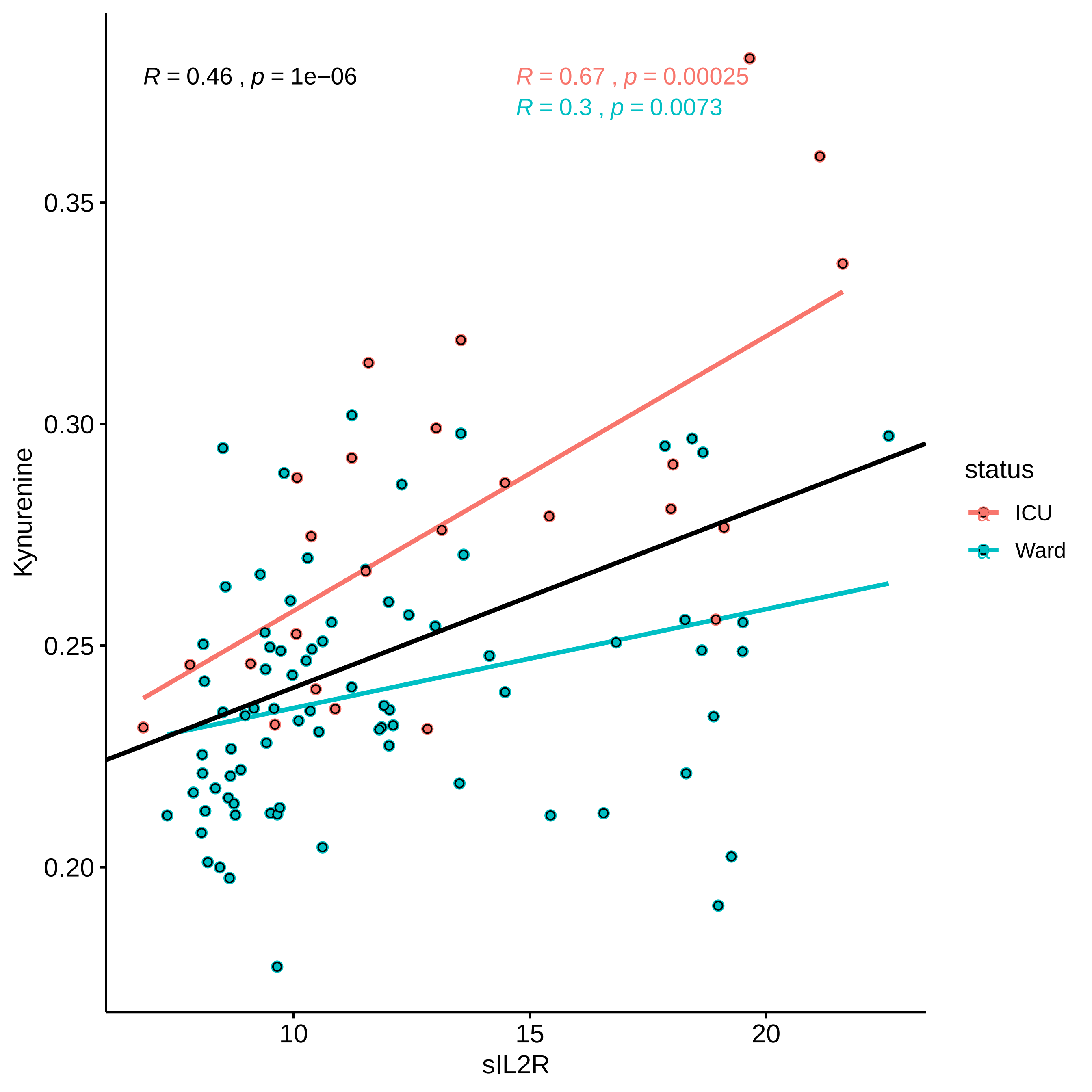

R = 0.46 , p = 1e−06
R = 0.67 , p = 0.00025
R = 0.3 , p = 0.0073
0.35
0.30
0.25
0.20
Kynurenine
10
15
20
sIL2R
status
ICU
Ward

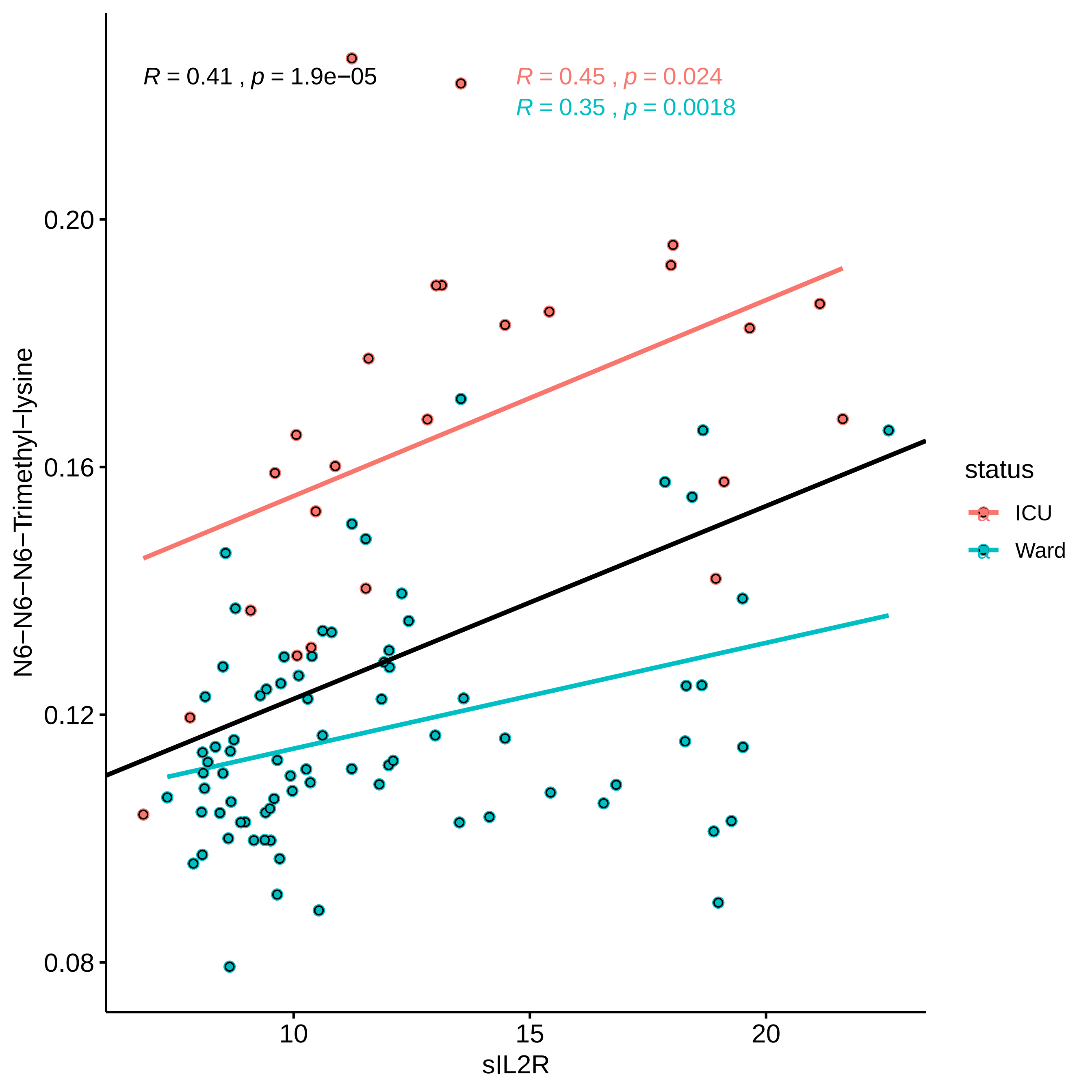

R = 0.41 , p = 1.9e−05
R = 0.45 , p = 0.024
R = 0.35 , p = 0.0018
0.20
0.16
0.12
0.08
N6−N6−N6−Trimethyl−lysine
10
15
20
sIL2R
status
ICU
Ward

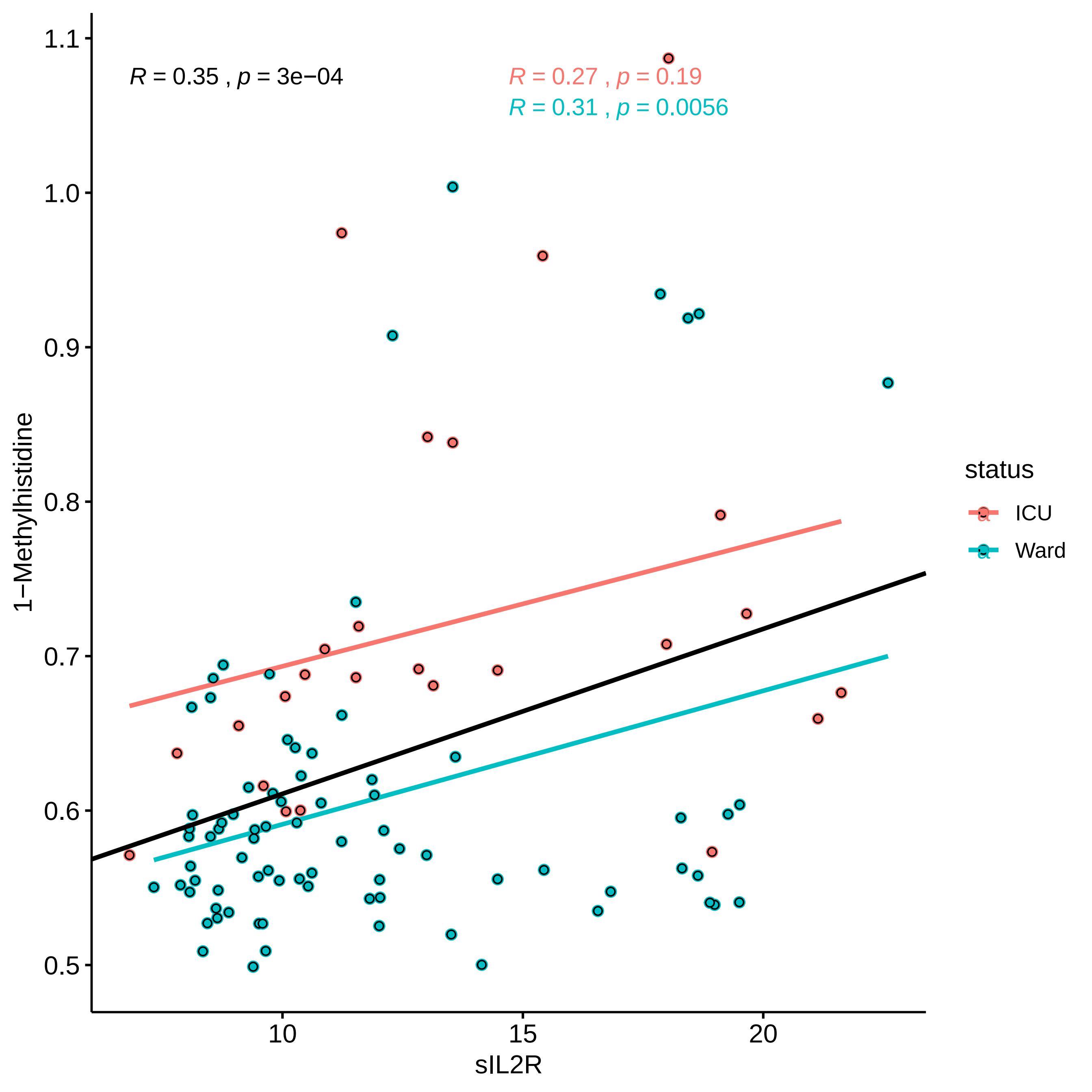

R = 0.35 , p = 3e−04
R = 0.27 , p = 0.19
R = 0.31 , p = 0.0056
1.1
1.0
0.9
0.8
0.7
0.6
0.5
1−Methylhistidine
10
15
20
sIL2R
status
ICU
Ward

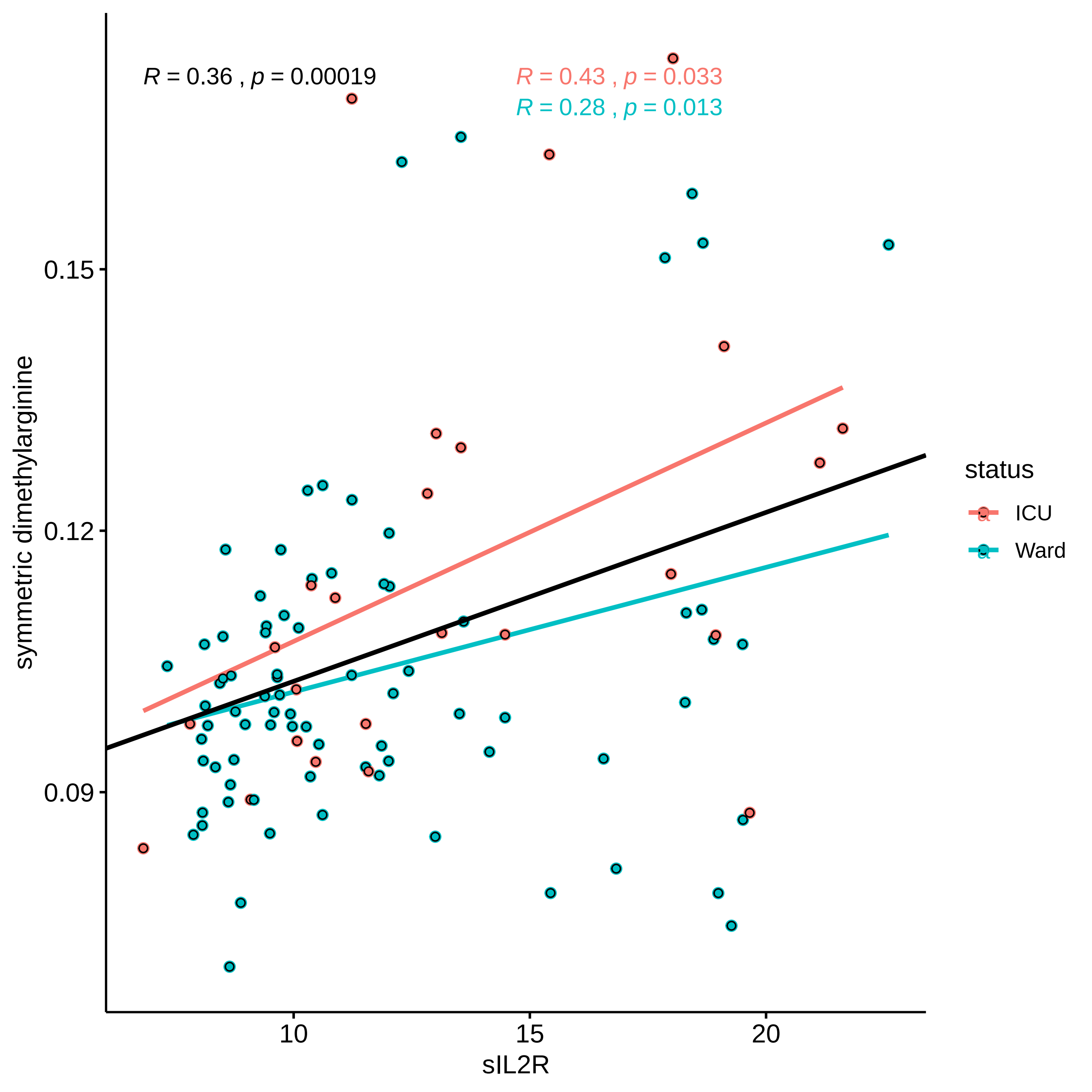

R = 0.36 , p = 0.00019
R = 0.43 , p = 0.033
R = 0.28 , p = 0.013
symmetric dimethylarginine
0.15
0.12
0.09
10
15
20
sIL2R
status
ICU
Ward

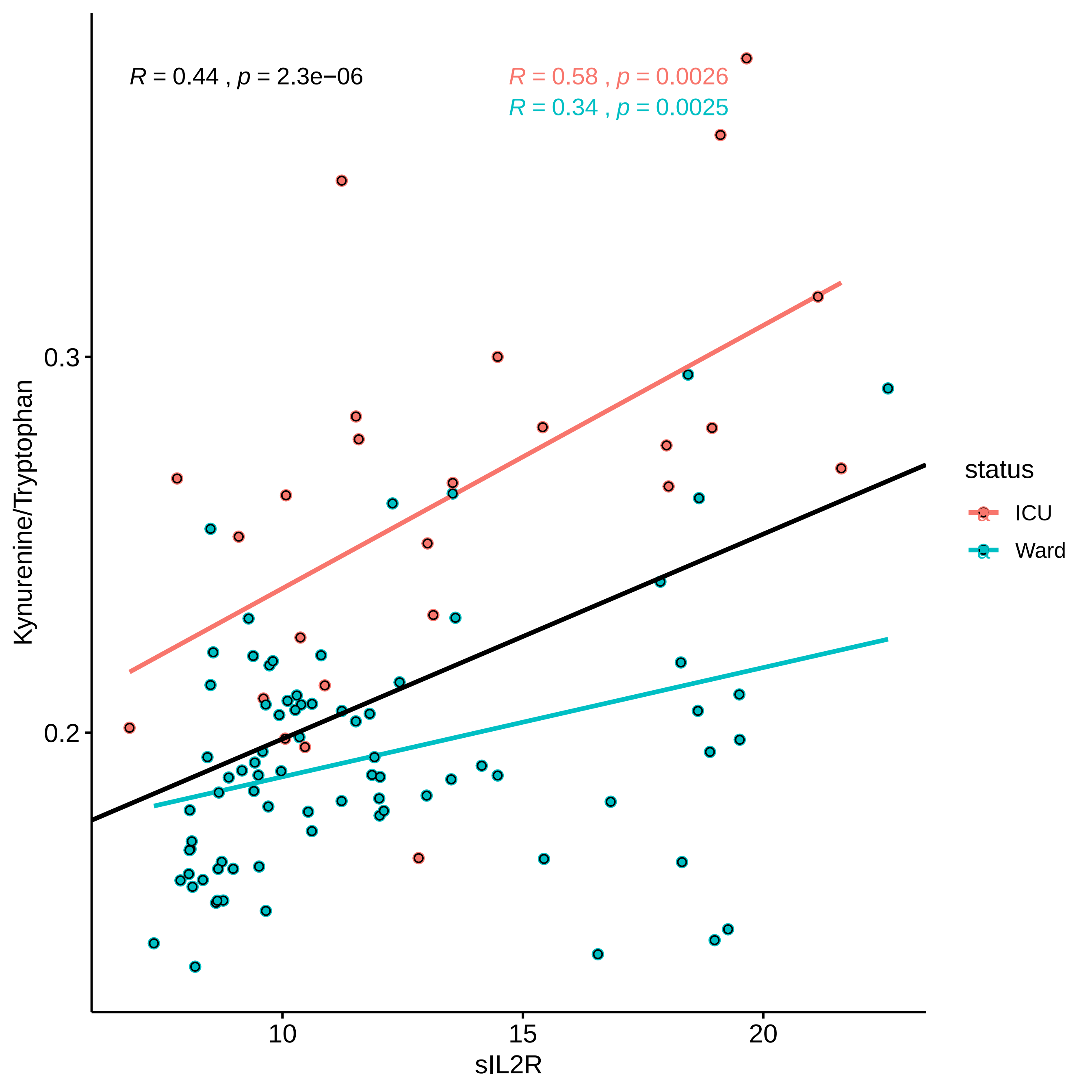
R = 0.44 , p = 2.3e−06
R = 0.58 , p = 0.0026
R = 0.34 , p = 0.0025
Kynurenine/Tryptophan
0.3
0.2
10
15
20
sIL2R
status
ICU
Ward

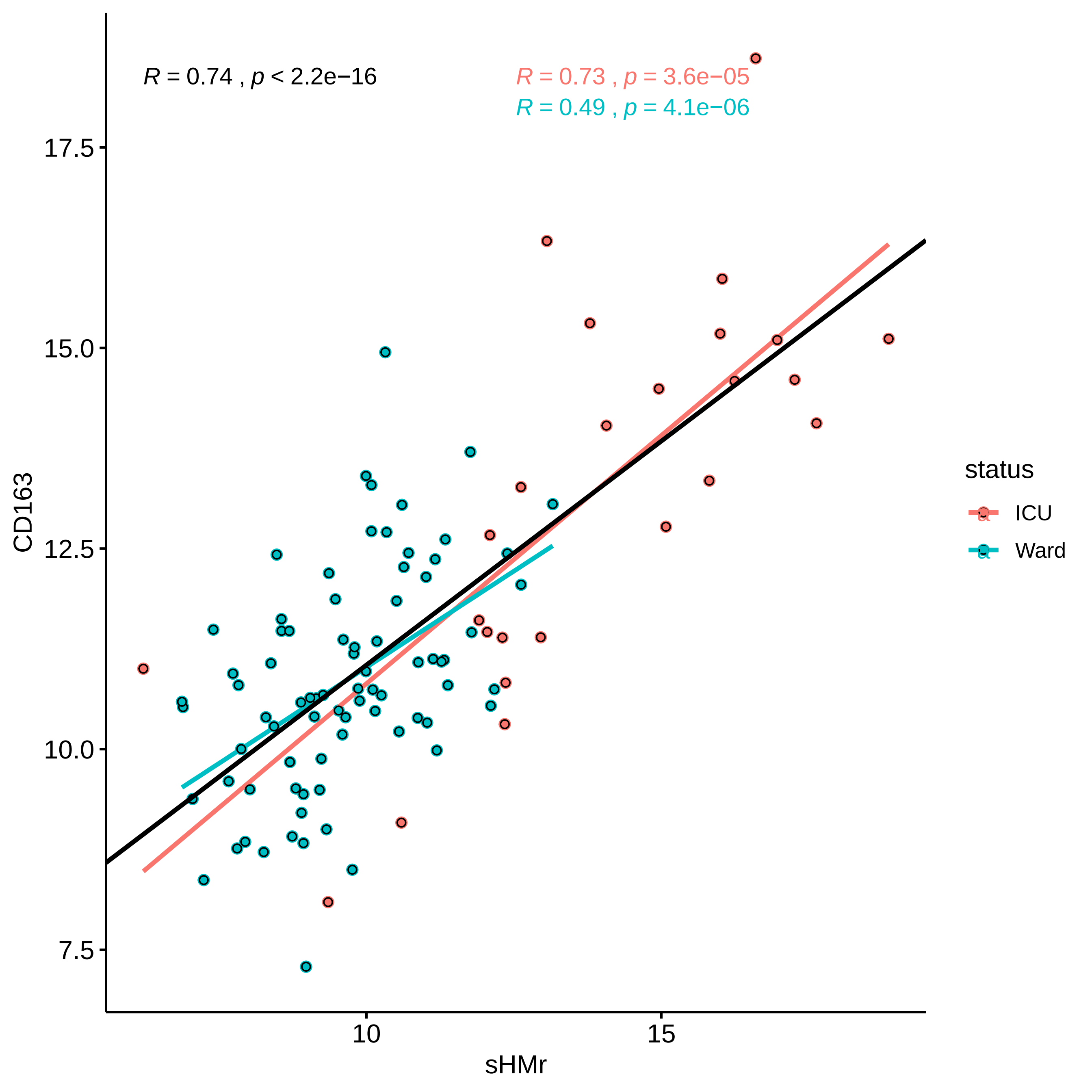

R = 0.74 , p < 2.2e−16
R = 0.73 , p = 3.6e−05
R = 0.49 , p = 4.1e−06
17.5
15.0
12.5
10.0
7.5
CD163
10
15
sHMr
status
ICU
Ward

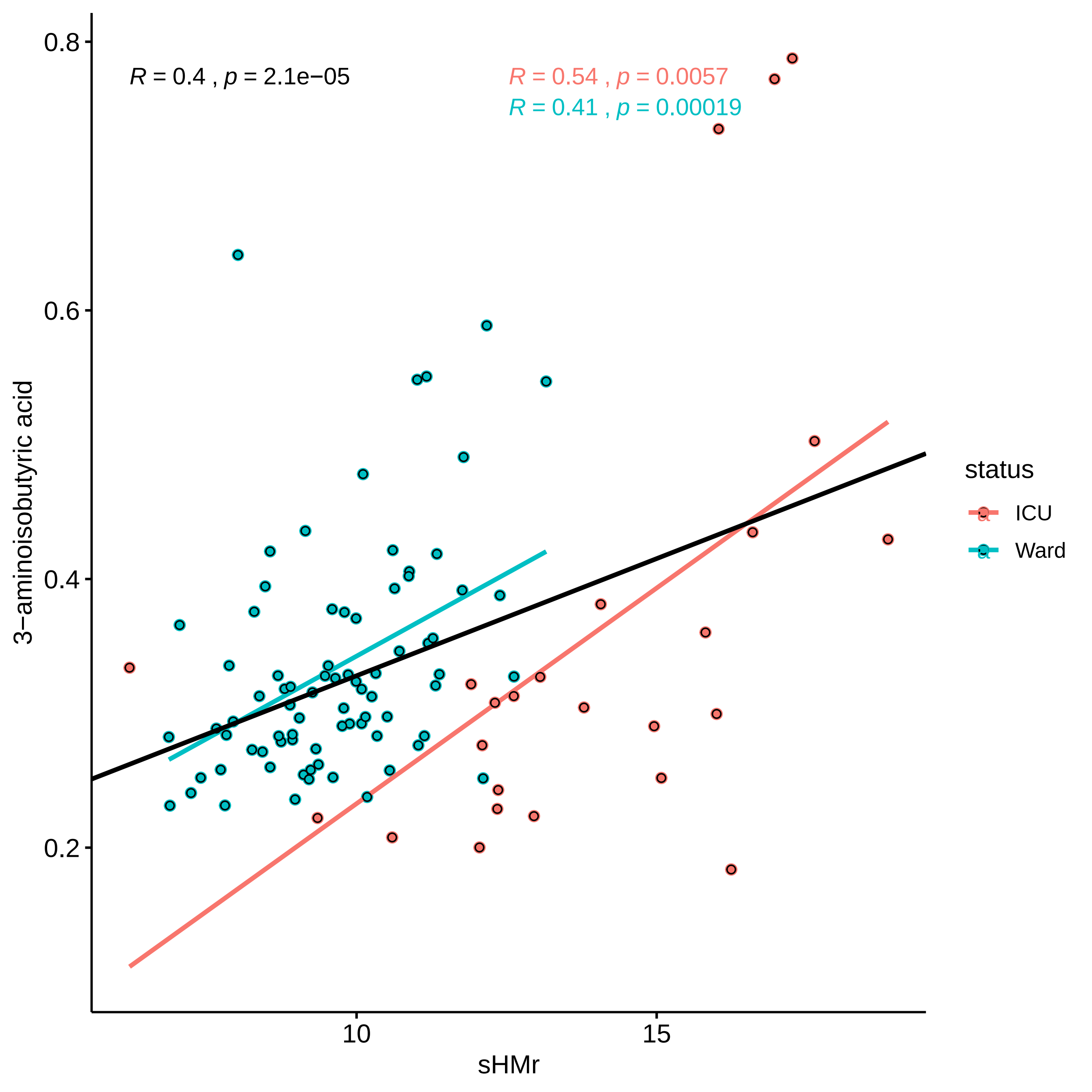

R = 0.4 , p = 2.1e−05
R = 0.54 , p = 0.0057
R = 0.41 , p = 0.00019
0.8
0.6
0.4
0.2
3−aminoisobutyric acid
10
15
sHMr
status
ICU
Ward

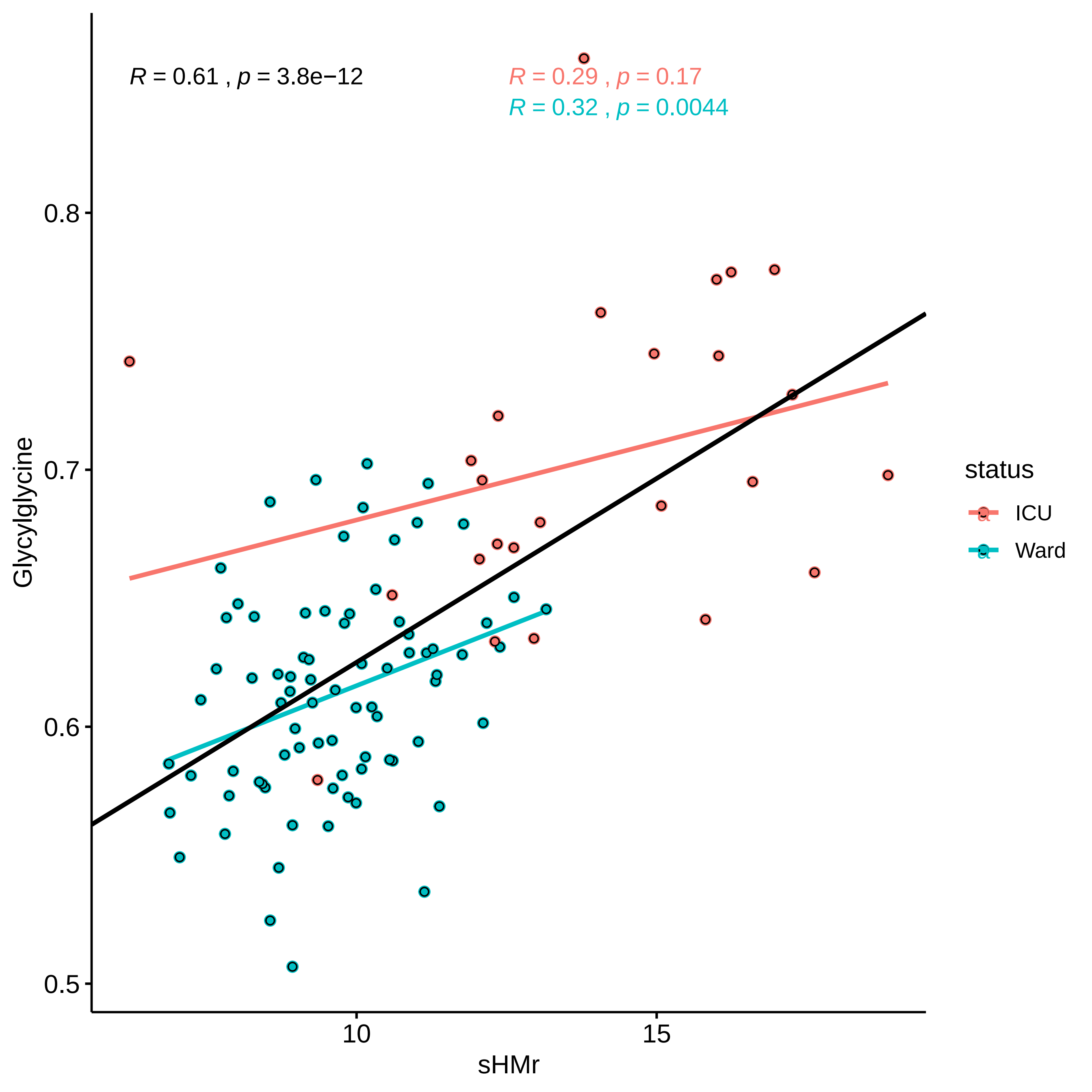

R = 0.61 , p = 3.8e−12
R = 0.29 , p = 0.17
R = 0.32 , p = 0.0044
0.8
0.7
0.6
0.5
Glycylglycine
10
15
sHMr
status
ICU
Ward

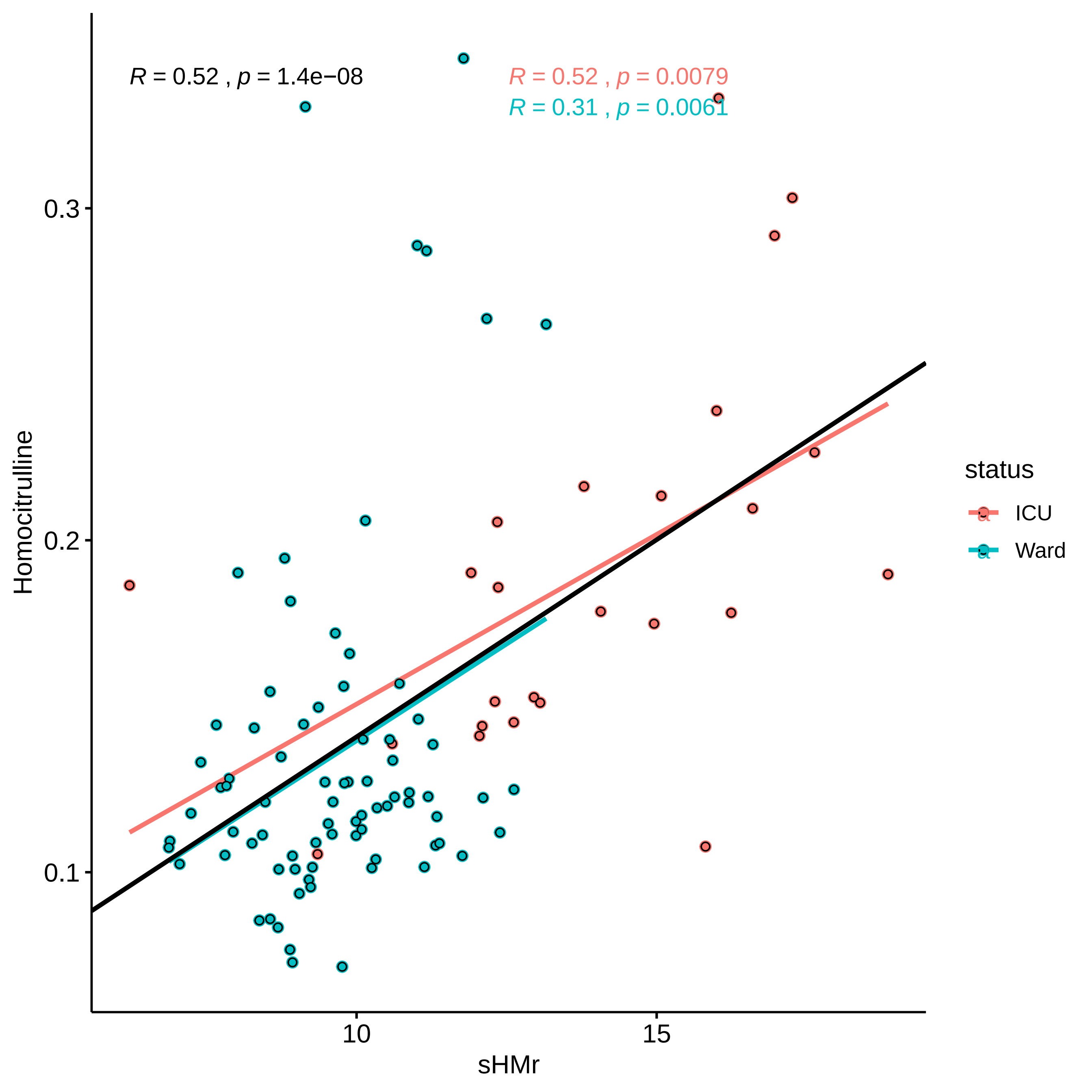

R = 0.52 , p = 1.4e−08
R = 0.52 , p = 0.0079
R = 0.31 , p = 0.0061
Homocitrulline
0.3
0.2
0.1
10
15
sHMr
status
ICU
Ward

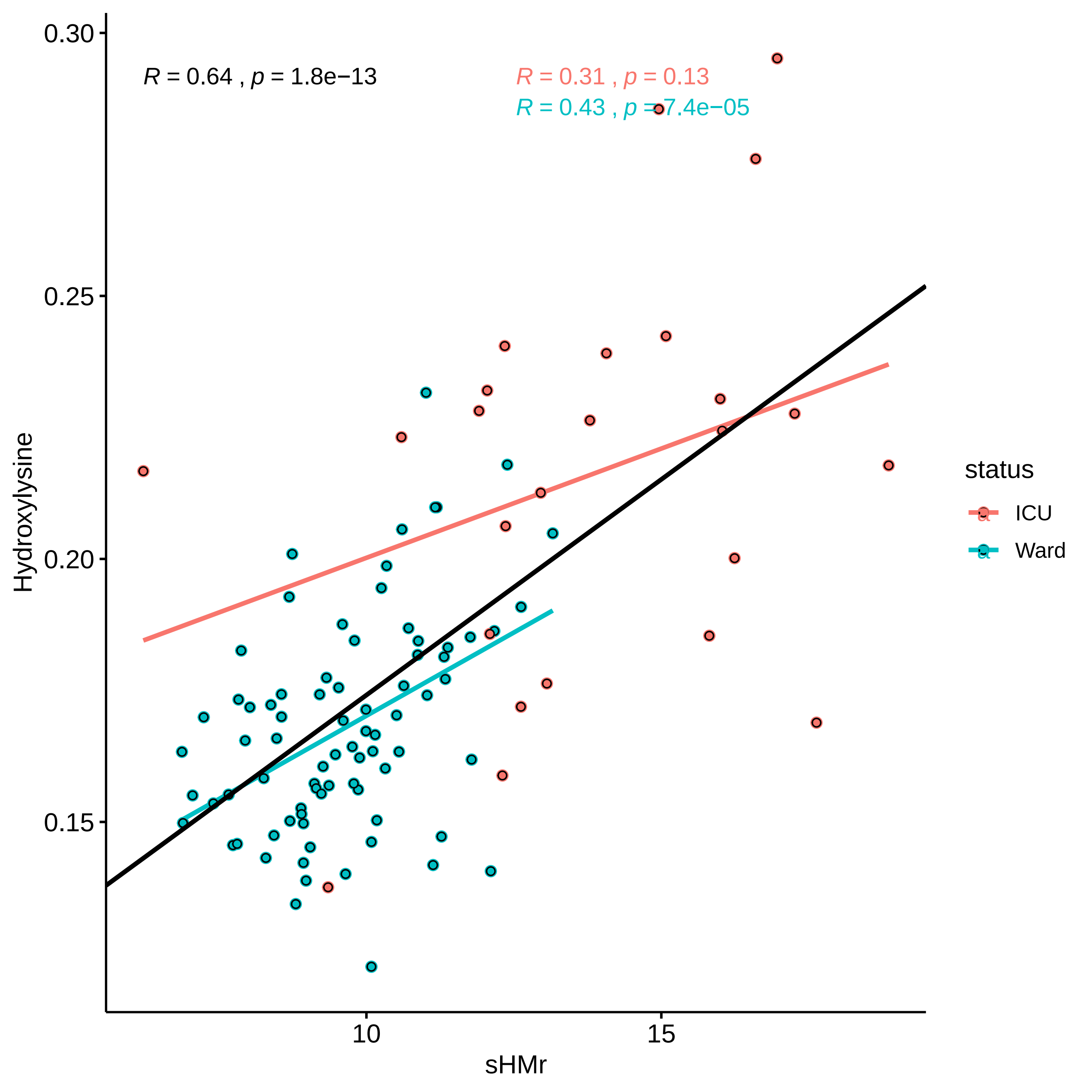

R = 0.64 , p = 1.8e−13
R = 0.31 , p = 0.13
R = 0.43 , p = 7.4e−05
Hydroxylysine
0.30
0.25
0.20
0.15
10
15
sHMr
status
ICU
Ward

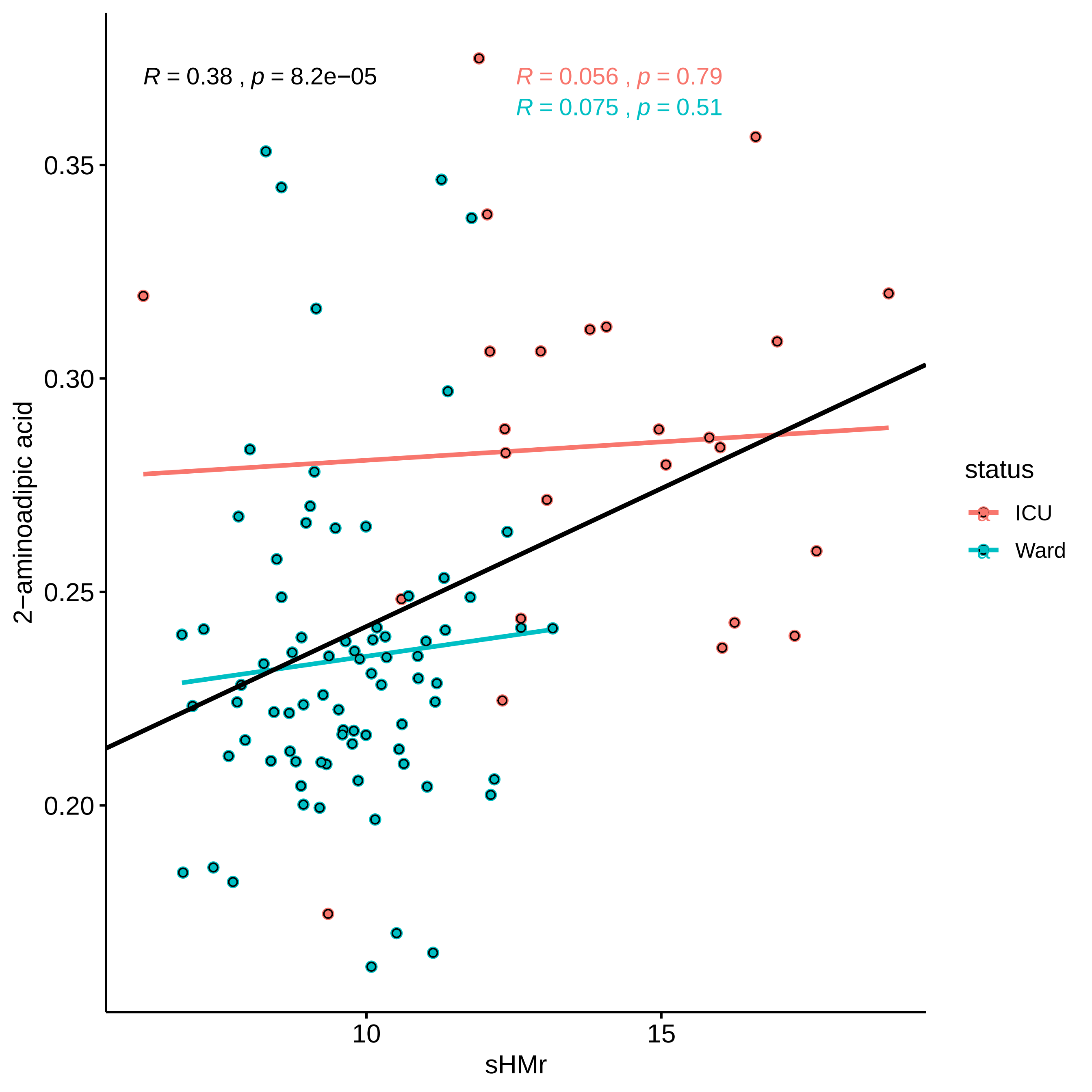

R = 0.38 , p = 8.2e−05
R = 0.056 , p = 0.79
R = 0.075 , p = 0.51
0.35
0.30
0.25
0.20
2−aminoadipic acid
10
15
sHMr
status
ICU
Ward

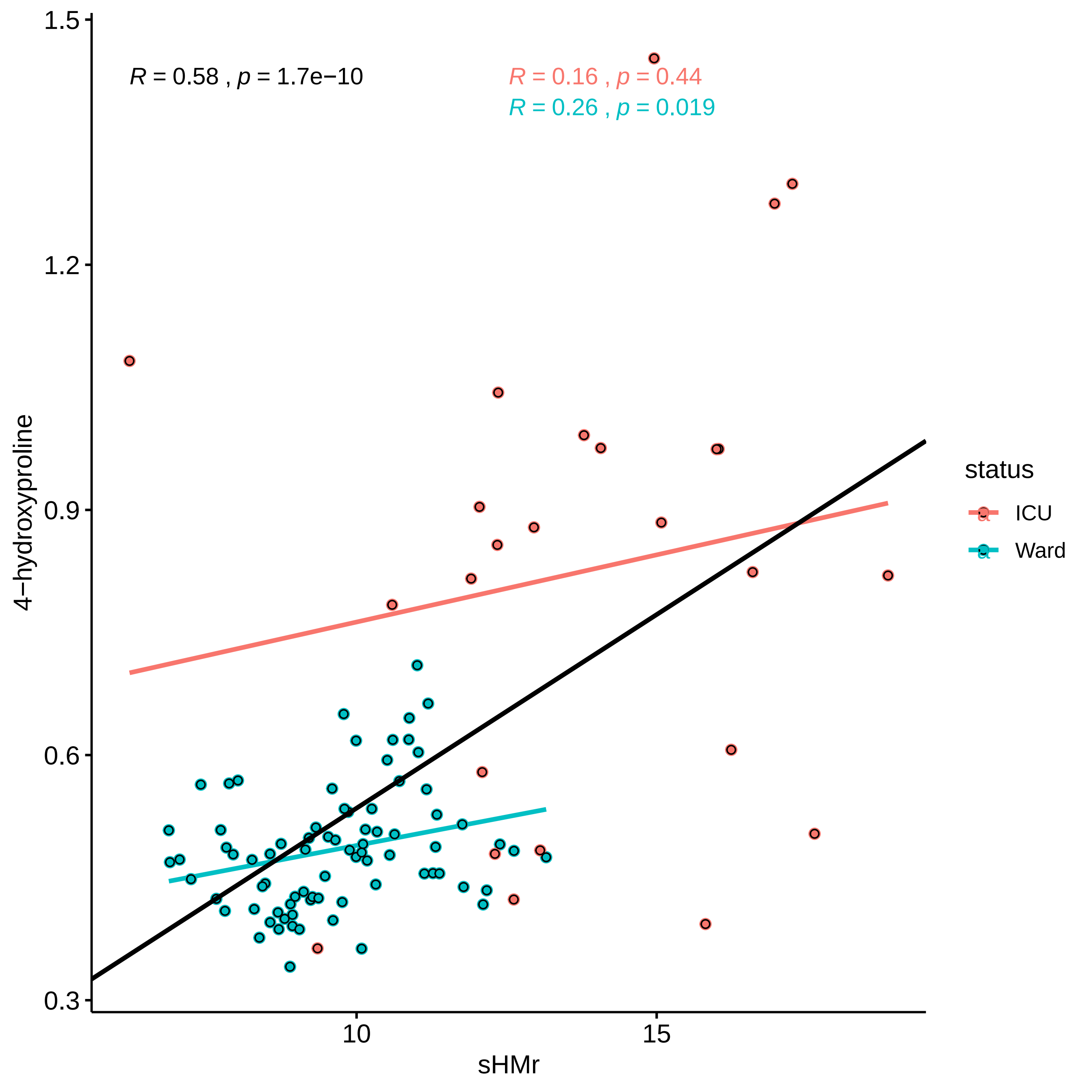

R = 0.58 , p = 1.7e−10
R = 0.16 , p = 0.44
R = 0.26 , p = 0.019
1.5
1.2
0.9
0.6
0.3
4−hydroxyproline
10
15
sHMr
status
ICU
Ward

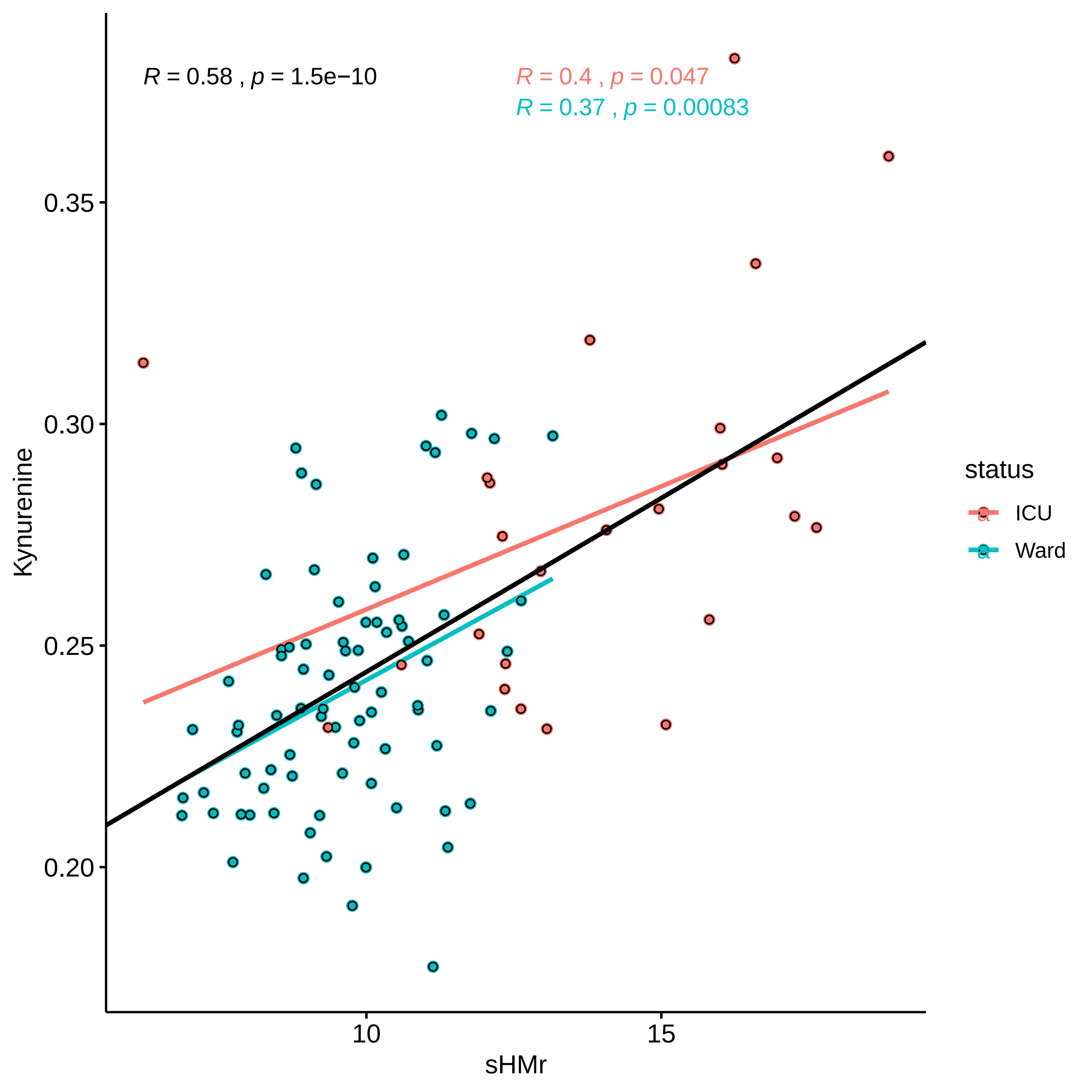

R = 0.58 , p = 1.5e−10
R = 0.4 , p = 0.047
R = 0.37 , p = 0.00083
0.35
0.30
0.25
0.20
Kynurenine
10
15
sHMr
status
ICU
Ward

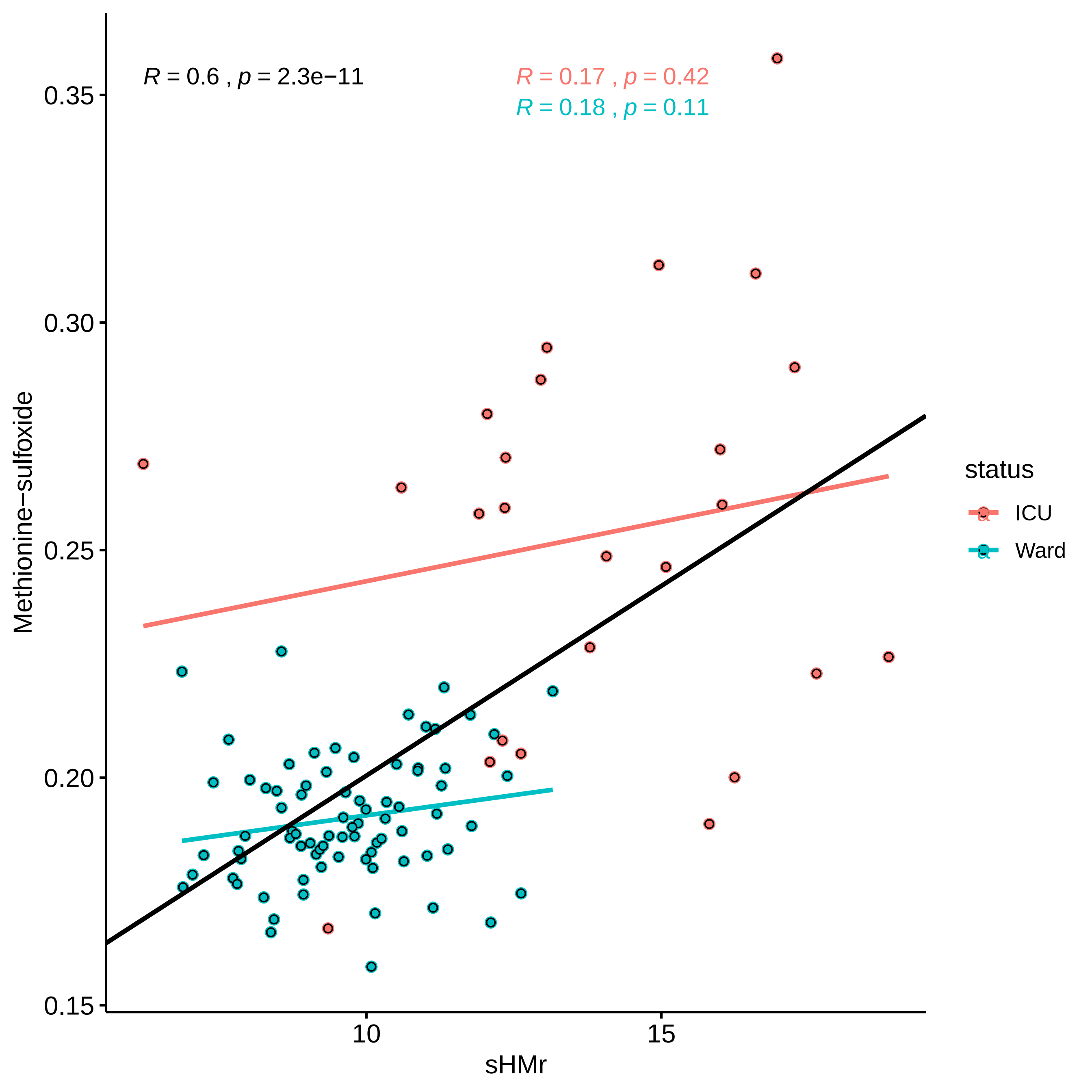

R = 0.6 , p = 2.3e−11
R = 0.17 , p = 0.42
R = 0.18 , p = 0.11
Methionine−sulfoxide
0.35
0.30
0.25
0.20
0.15
10
15
sHMr
status
ICU
Ward

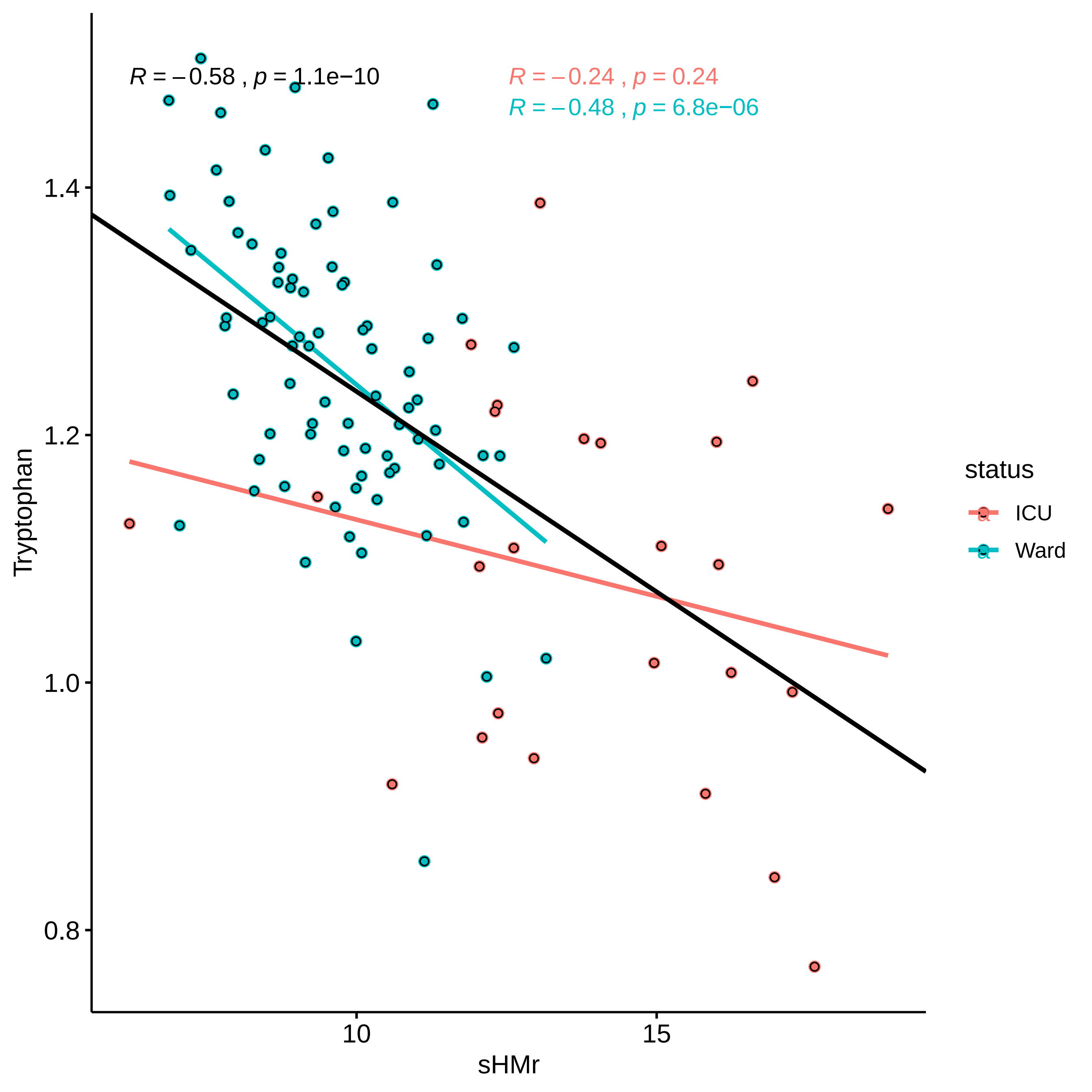

R = −0.58 , p = 1.1e−10
R = −0.24 , p = 0.24
R = −0.48 , p = 6.8e−06
Tryptophan
1.4
1.2
1.0
0.8
10
15
sHMr
status
ICU
Ward

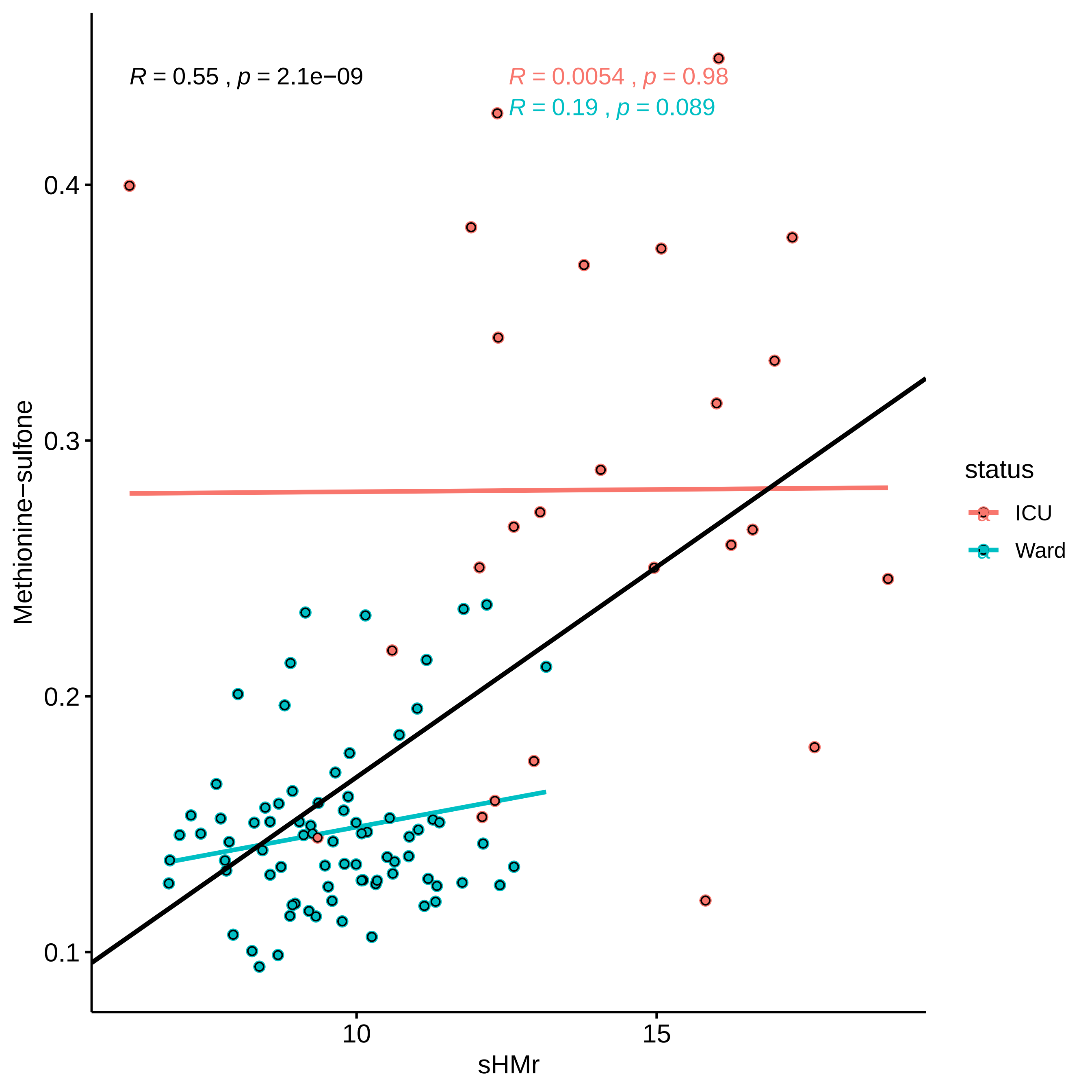
R = 0.55 , p = 2.1e−09
R = 0.0054 , p = 0.98
R = 0.19 , p = 0.089
Methionine−sulfone
0.1
0.2
0.3
0.4
10
15
sHMr
status
ICU
Ward

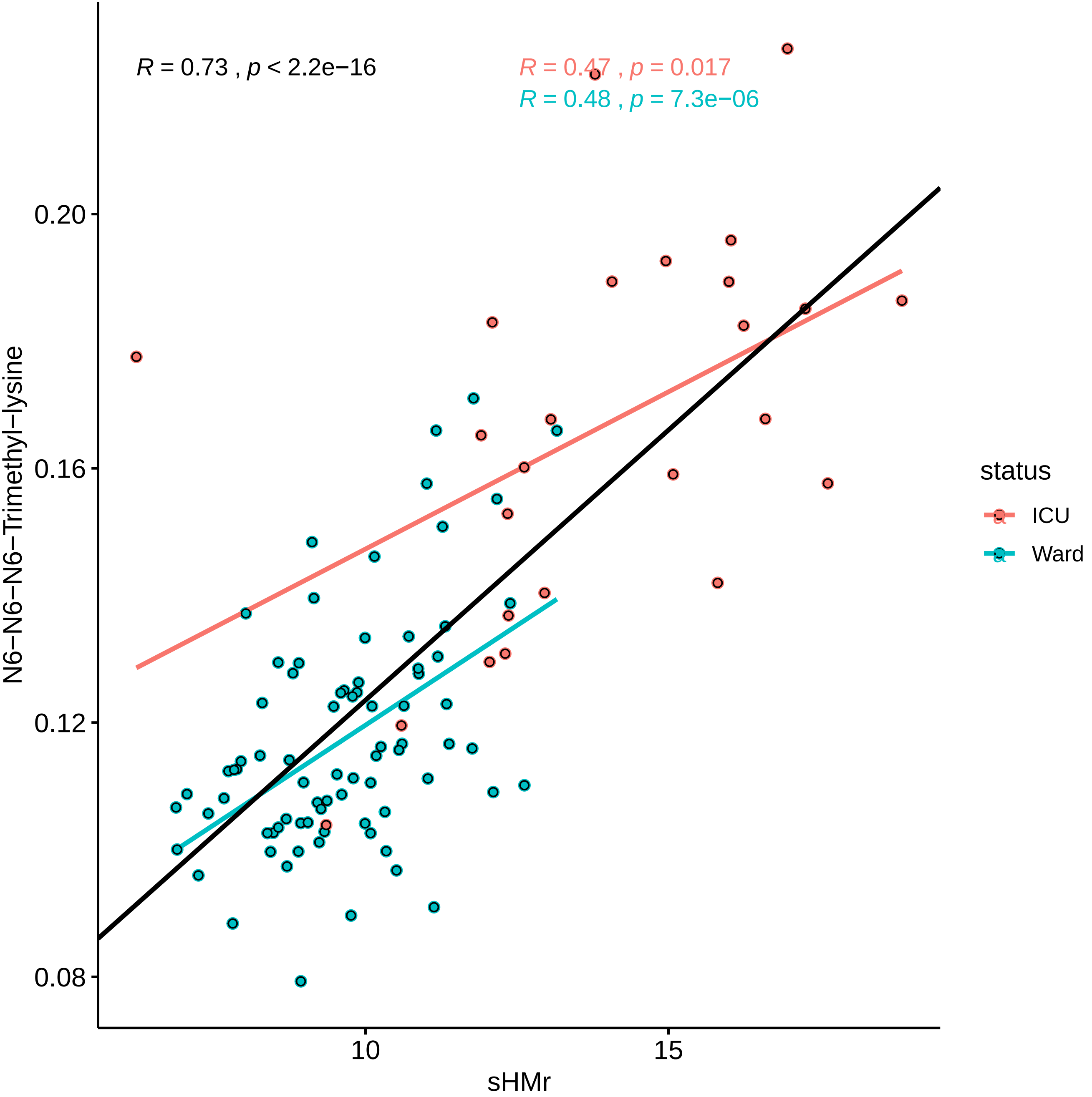

R = 0.73 , p < 2.2e−16
R = 0.47 , p = 0.017
R = 0.48 , p = 7.3e−06
N6−N6−N6−Trimethyl−lysine
0.20
0.16
0.12
0.08
10
15
sHMr
status
ICU
Ward

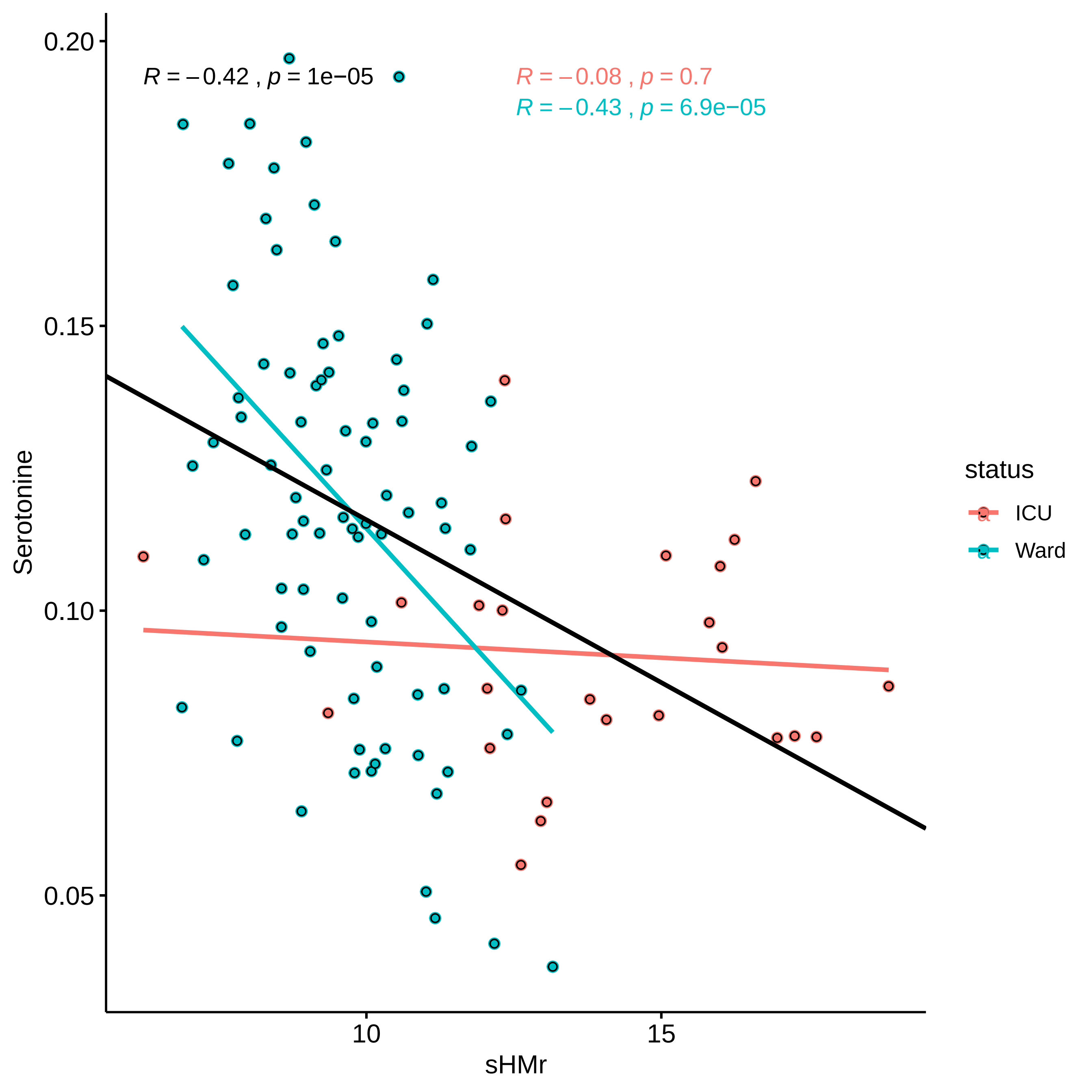

R = −0.42 , p = 1e−05
R = −0.08 , p = 0.7
R = −0.43 , p = 6.9e−05
0.20
0.15
0.10
0.05
Serotonine
10
15
sHMr
status
ICU
Ward

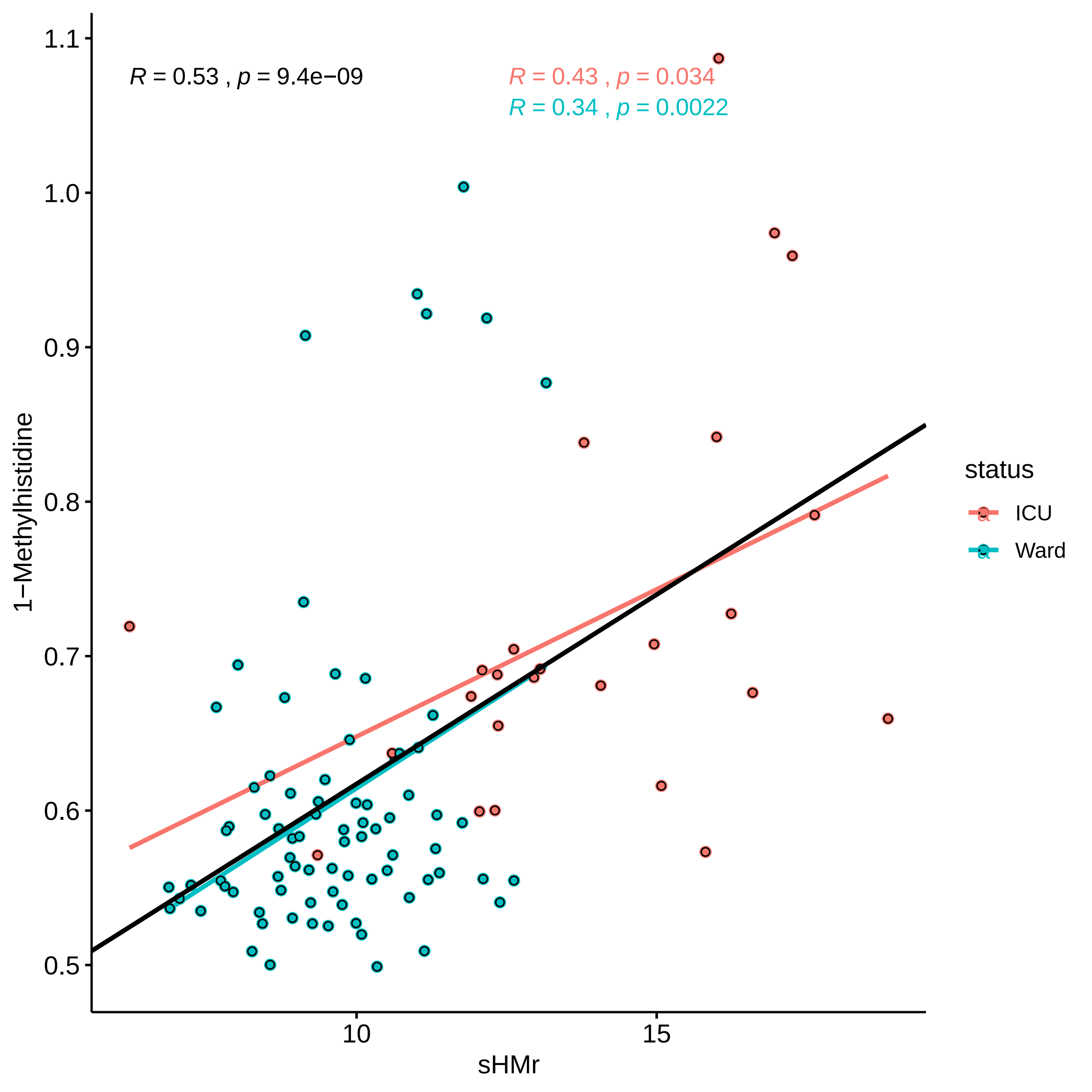

R = 0.53 , p = 9.4e−09
R = 0.43 , p = 0.034
R = 0.34 , p = 0.0022
1.1
1.0
0.9
0.8
0.7
0.6
0.5
1−Methylhistidine
10
15
sHMr
status
ICU
Ward

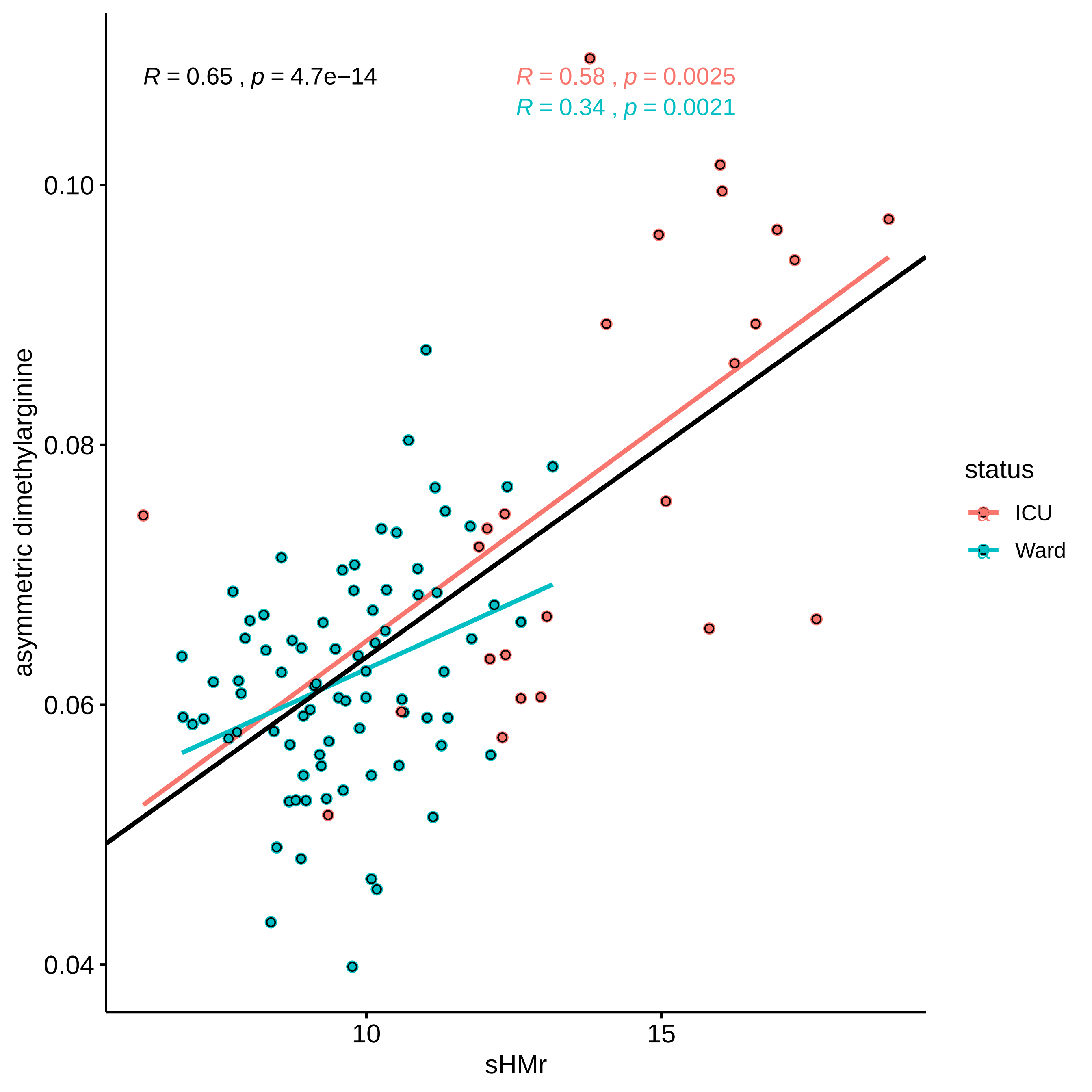

R = 0.65 , p = 4.7e−14
R = 0.58 , p = 0.0025
R = 0.34 , p = 0.0021
asymmetric dimethylarginine
0.10
0.08
0.06
0.04
10
15
sHMr
status
ICU
Ward

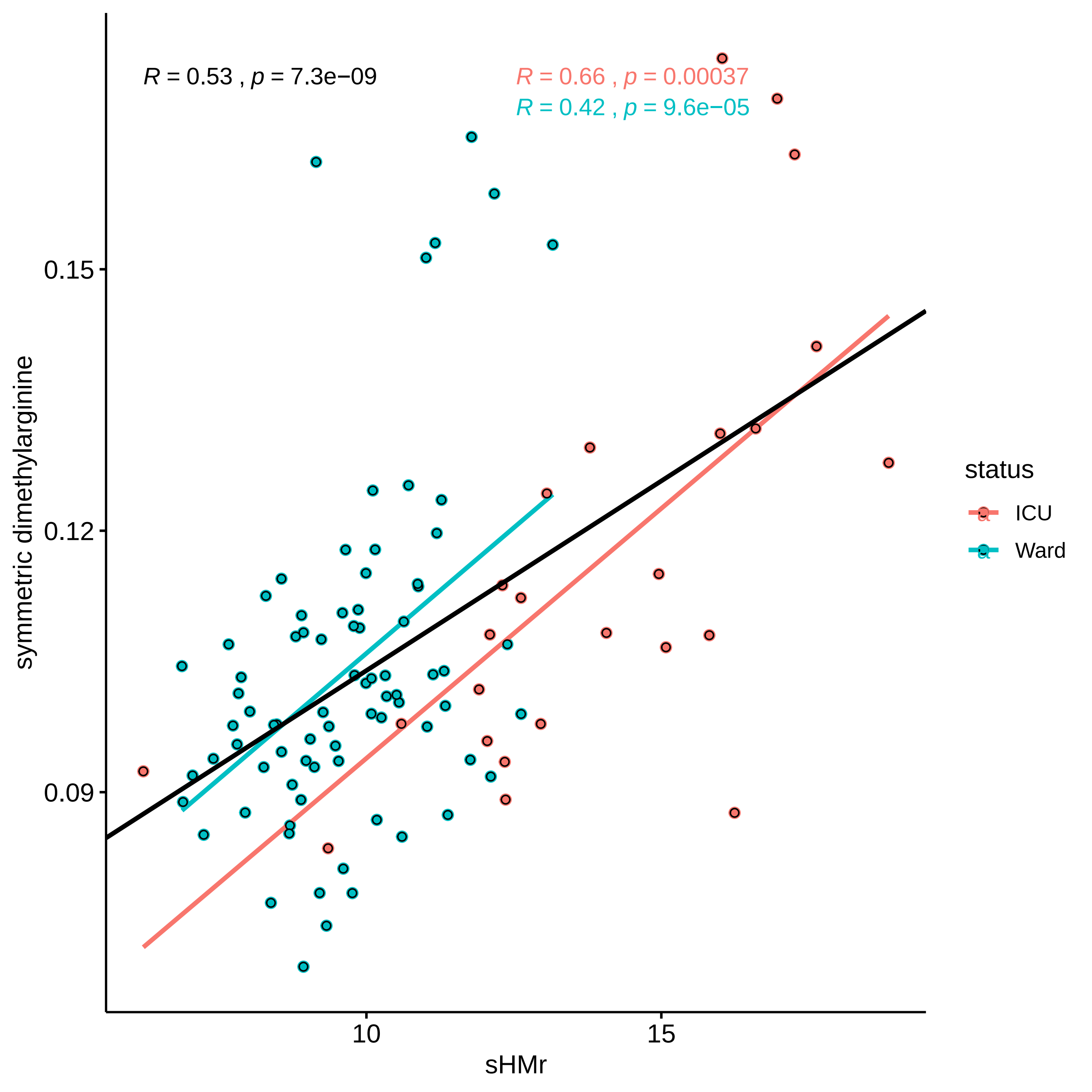

R = 0.53 , p = 7.3e−09
R = 0.66 , p = 0.00037
R = 0.42 , p = 9.6e−05
symmetric dimethylarginine
0.15
0.12
0.09
10
15
sHMr
status
ICU
Ward

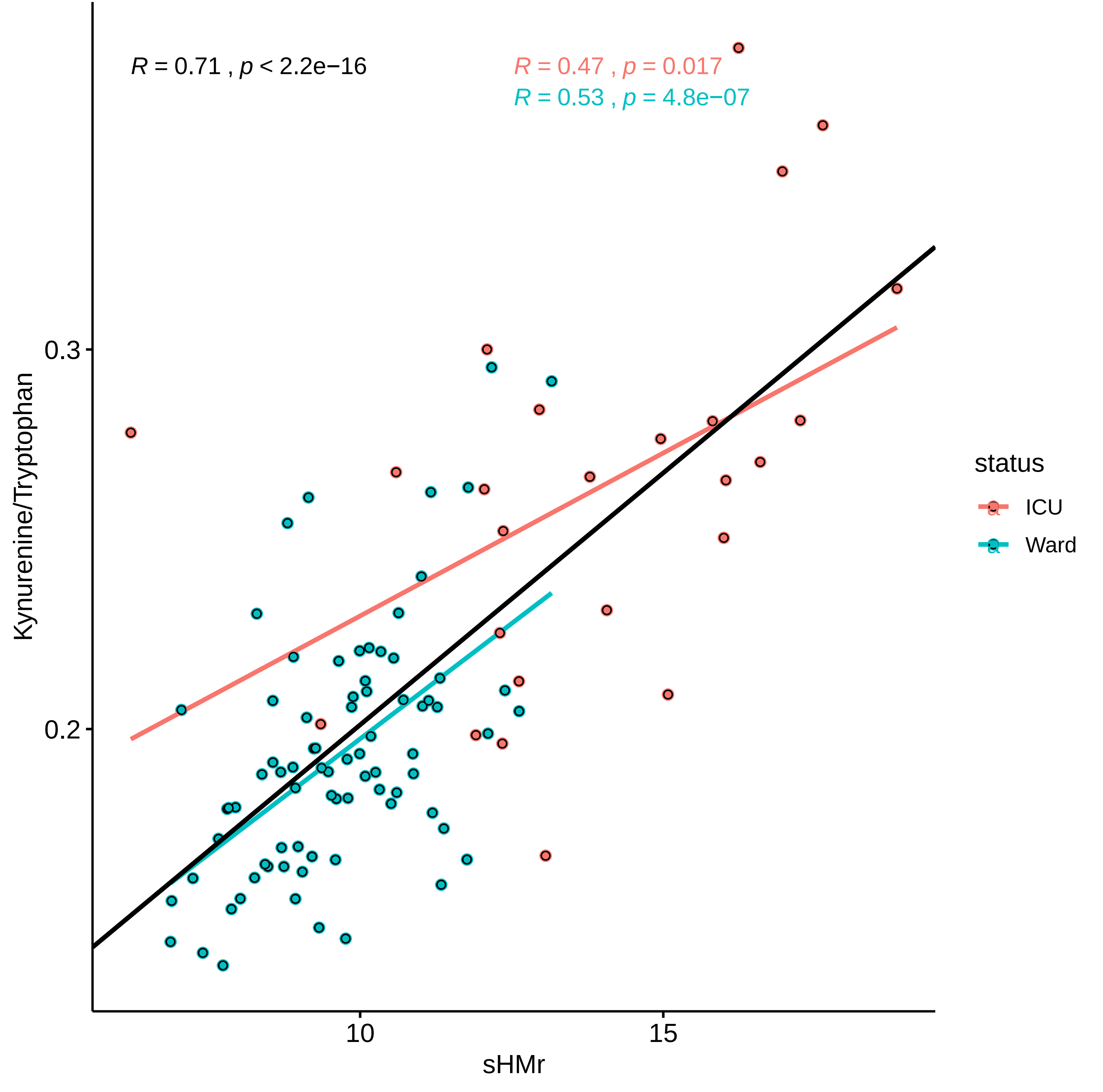

R = 0.71 , p < 2.2e−16
R = 0.47 , p = 0.017
R = 0.53 , p = 4.8e−07
Kynurenine/Tryptophan
0.3
0.2
10
15
sHMr
status
ICU
Ward

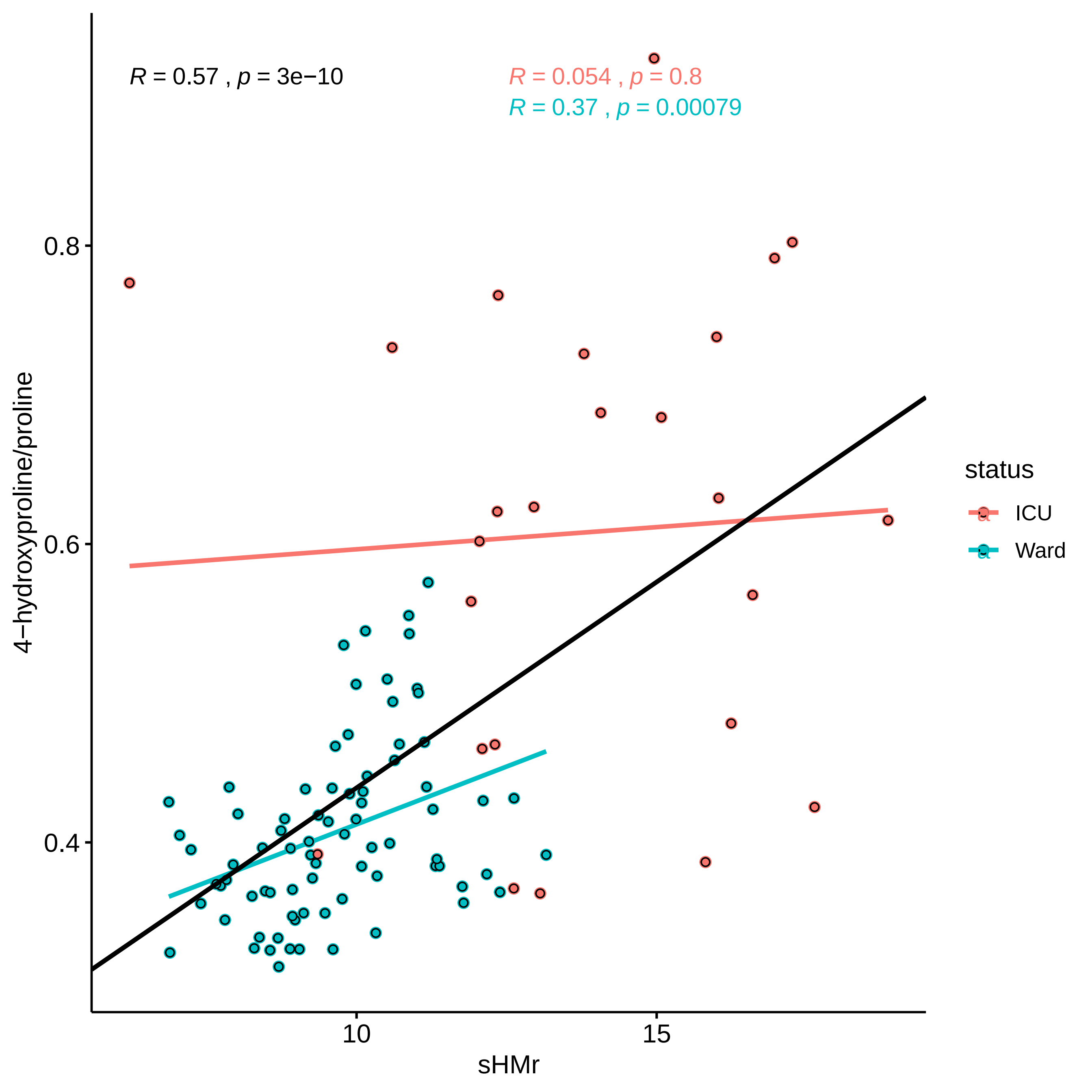

R = 0.57 , p = 3e−10
R = 0.054 , p = 0.8
R = 0.37 , p = 0.00079
0.8
0.6
0.4
10
15
sHMr
4−hydroxyproline/proline
status
ICU
Ward

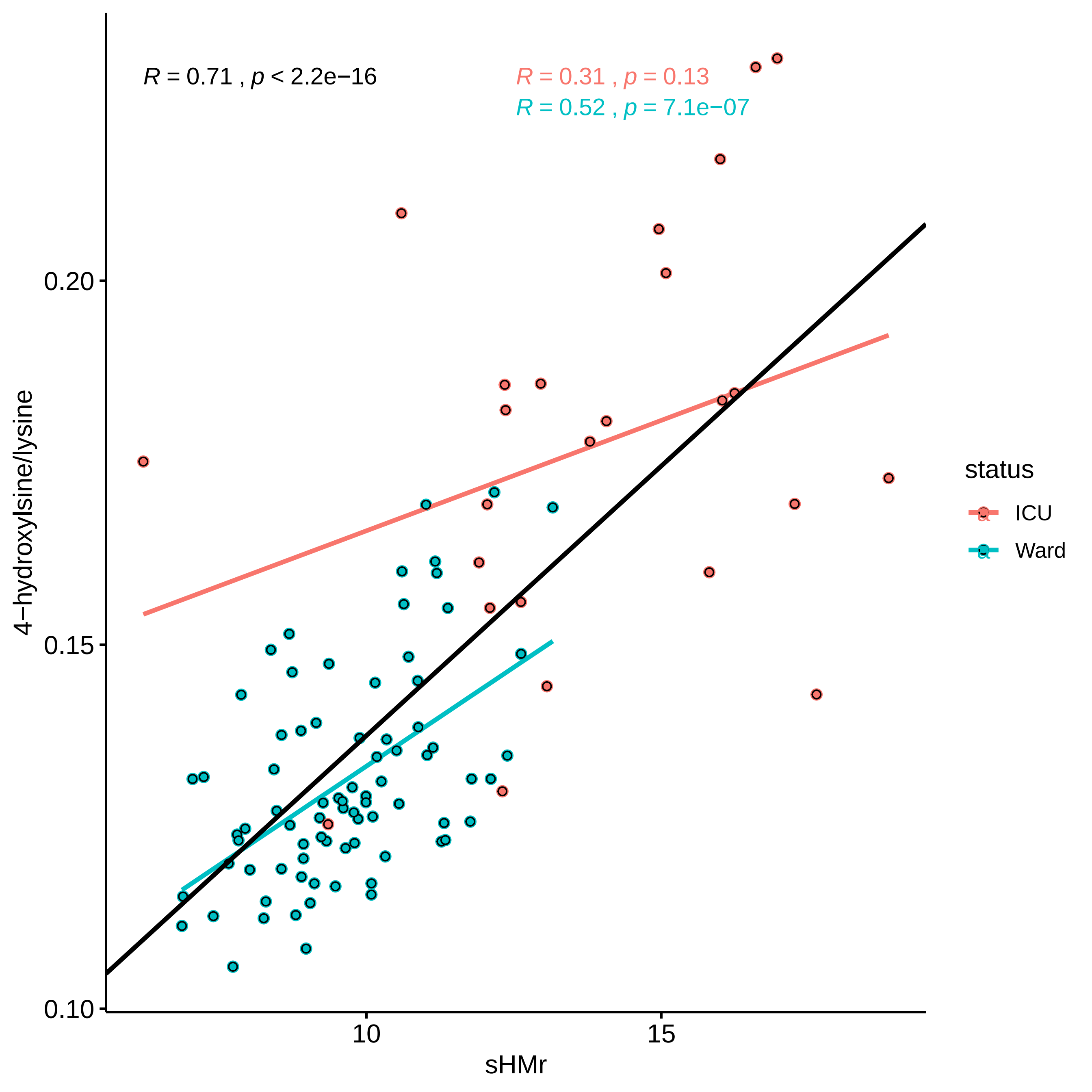

R = 0.71 , p < 2.2e−16
R = 0.31 , p = 0.13
R = 0.52 , p = 7.1e−07
4−hydroxylsine/lysine
0.20
0.15
0.10
10
15
sHMr
status
ICU
Ward

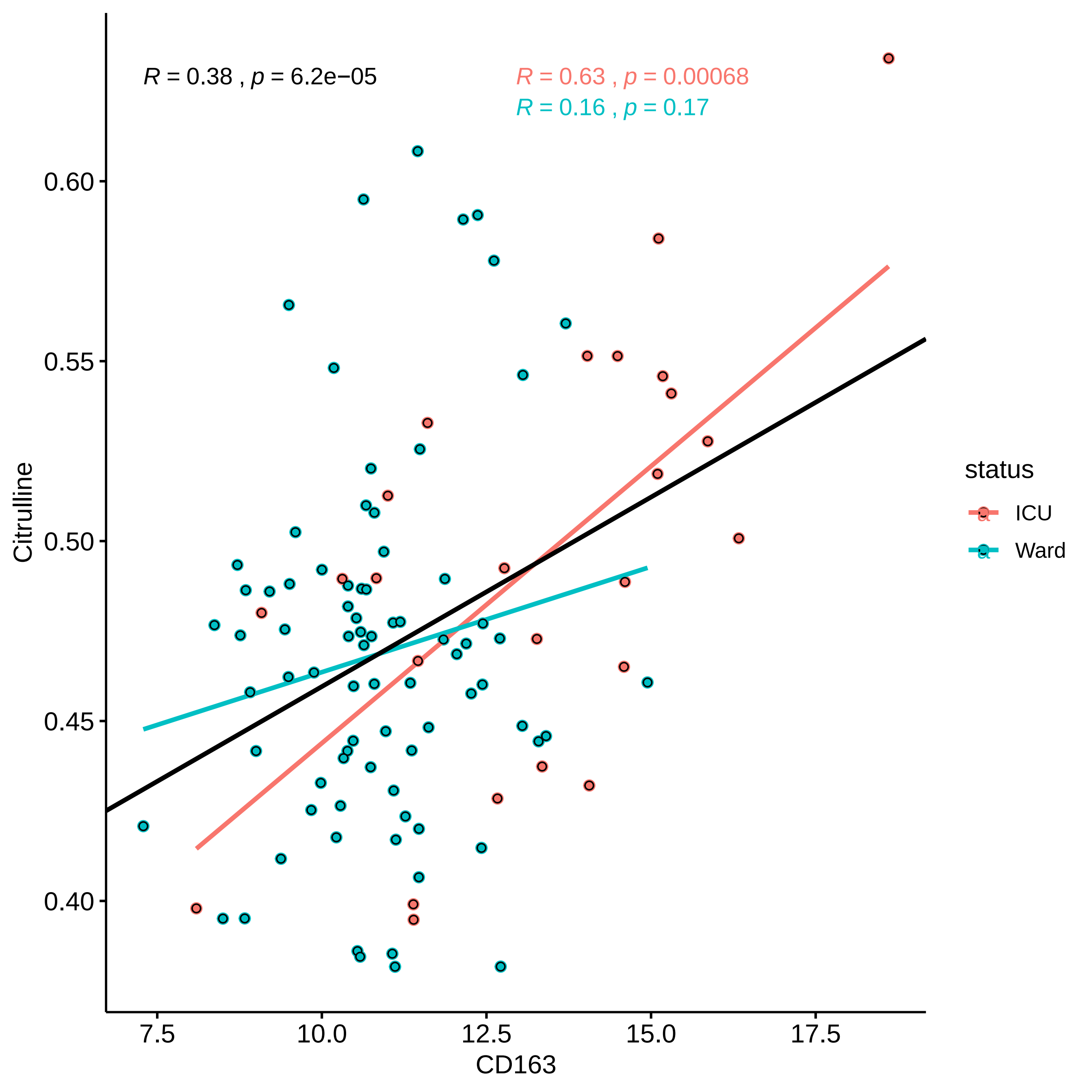

R = 0.38 , p = 6.2e−05
R = 0.63 , p = 0.00068
R = 0.16 , p = 0.17
Citrulline
0.60
0.55
0.50
0.45
0.40
7.5
10.0
12.5
15.0
17.5
CD163
status
ICU
Ward

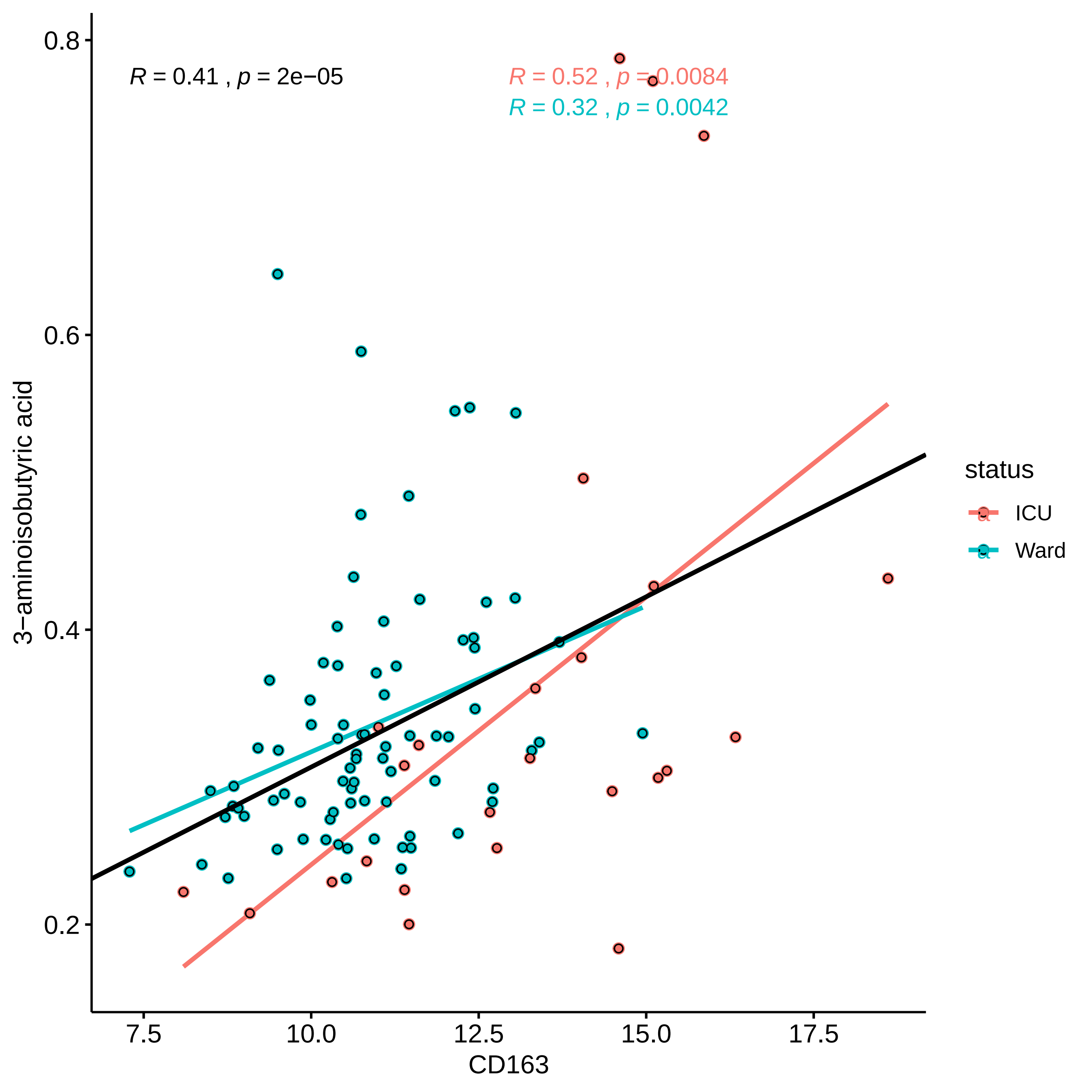

R = 0.41 , p = 2e−05
R = 0.52 , p = 0.0084
R = 0.32 , p = 0.0042
3−aminoisobutyric acid
0.8
0.6
0.4
0.2
7.5
10.0
12.5
15.0
17.5
CD163
status
ICU
Ward

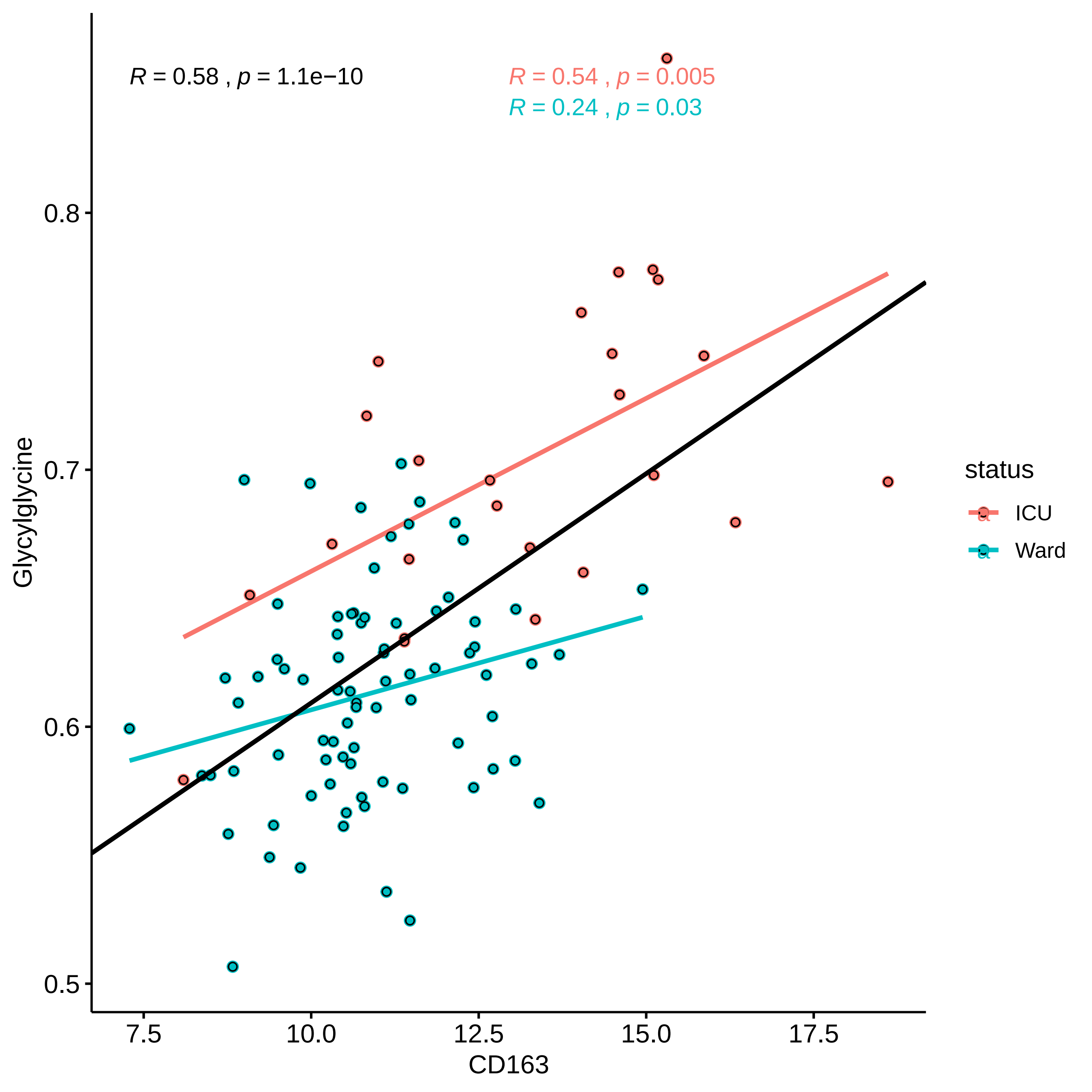

R = 0.58 , p = 1.1e−10
R = 0.54 , p = 0.005
R = 0.24 , p = 0.03
Glycylglycine
0.8
0.7
0.6
0.5
7.5
10.0
12.5
15.0
17.5
CD163
status
ICU
Ward

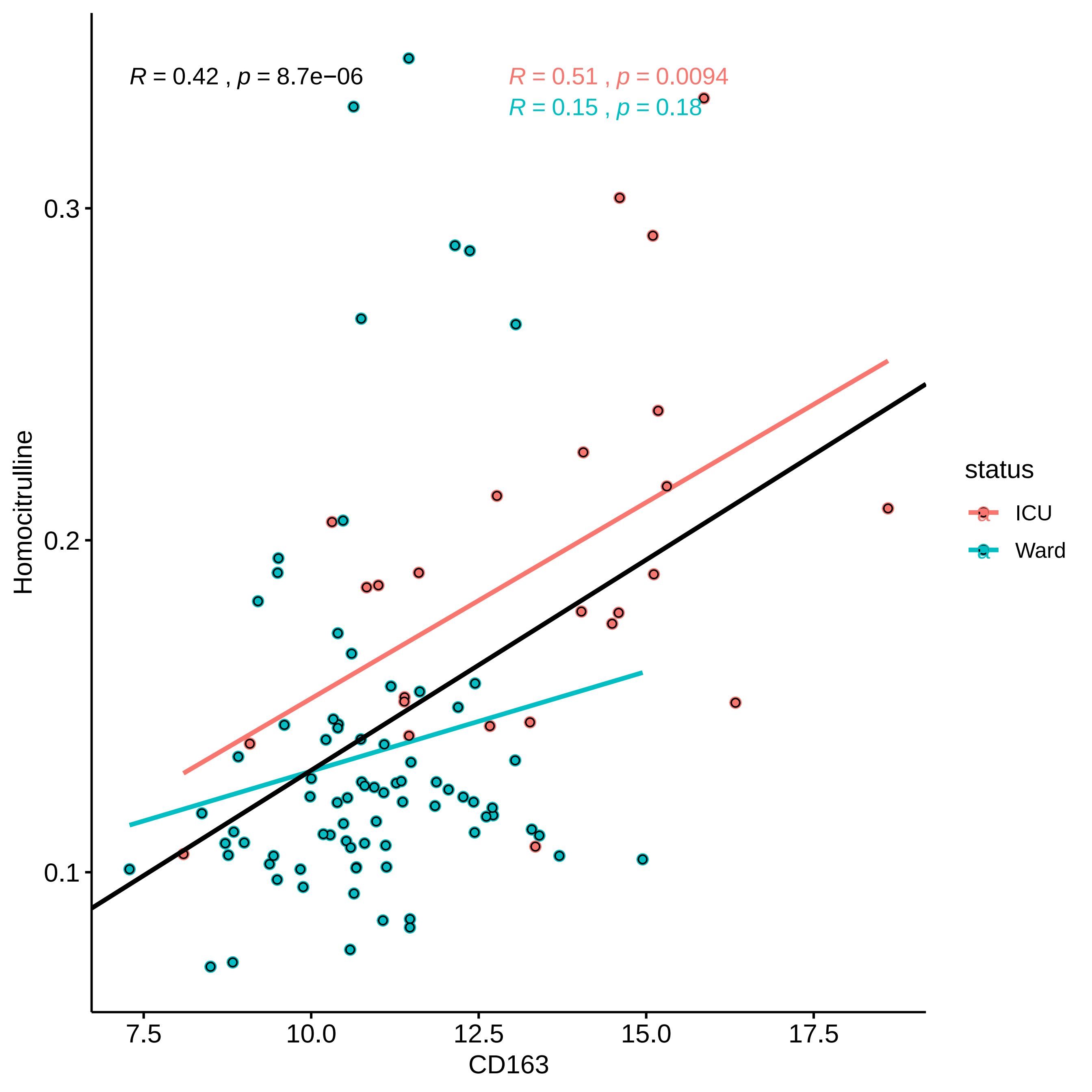

R = 0.42 , p = 8.7e−06
R = 0.51 , p = 0.0094
R = 0.15 , p = 0.18
Homocitrulline
0.3
0.2
0.1
7.5
10.0
12.5
15.0
17.5
CD163
status
ICU
Ward

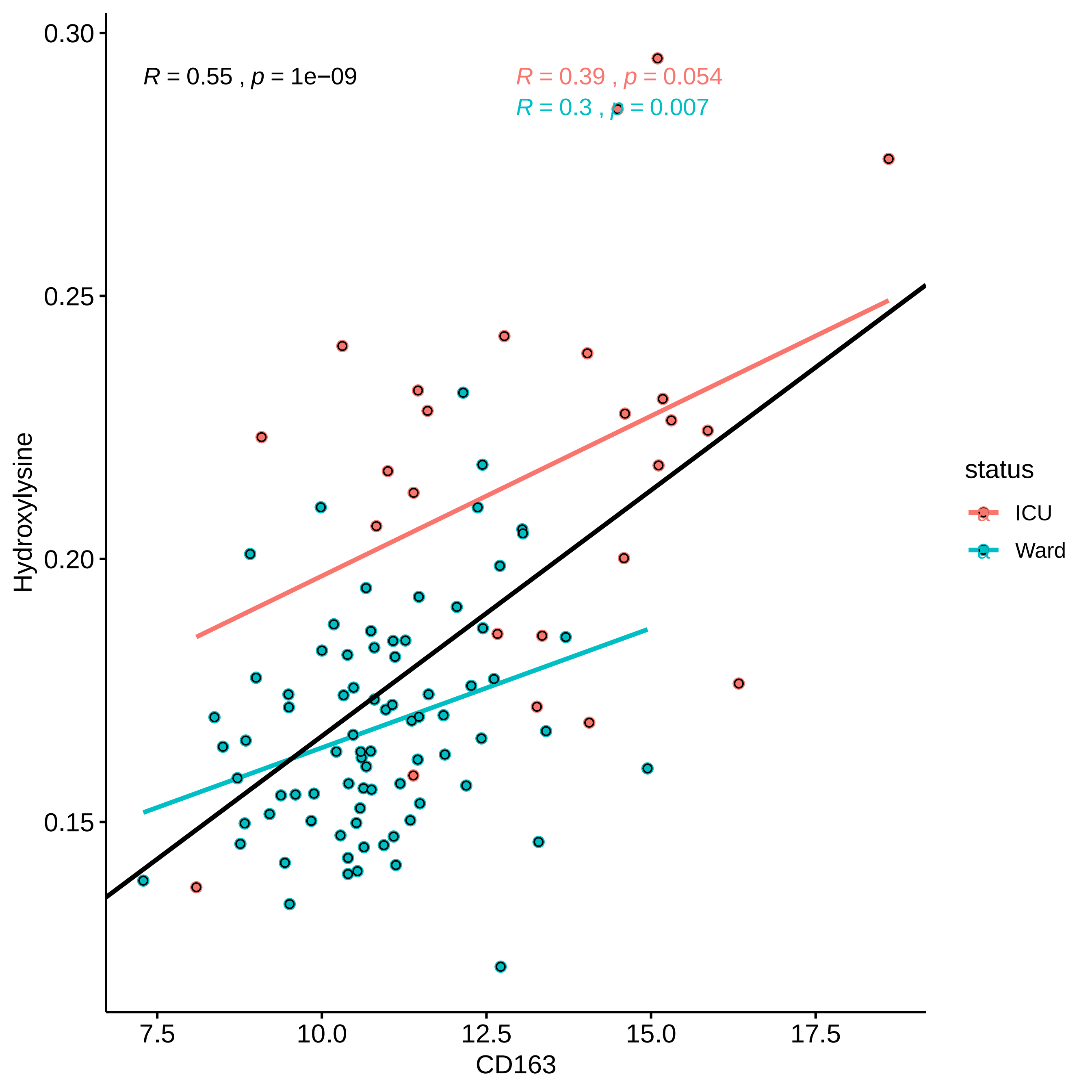

R = 0.55 , p = 1e−09
R = 0.39 , p = 0.054
R = 0.3 , p = 0.007
0.30
0.25
0.20
0.15
Hydroxylysine
7.5
10.0
12.5
15.0
17.5
CD163
status
ICU
Ward

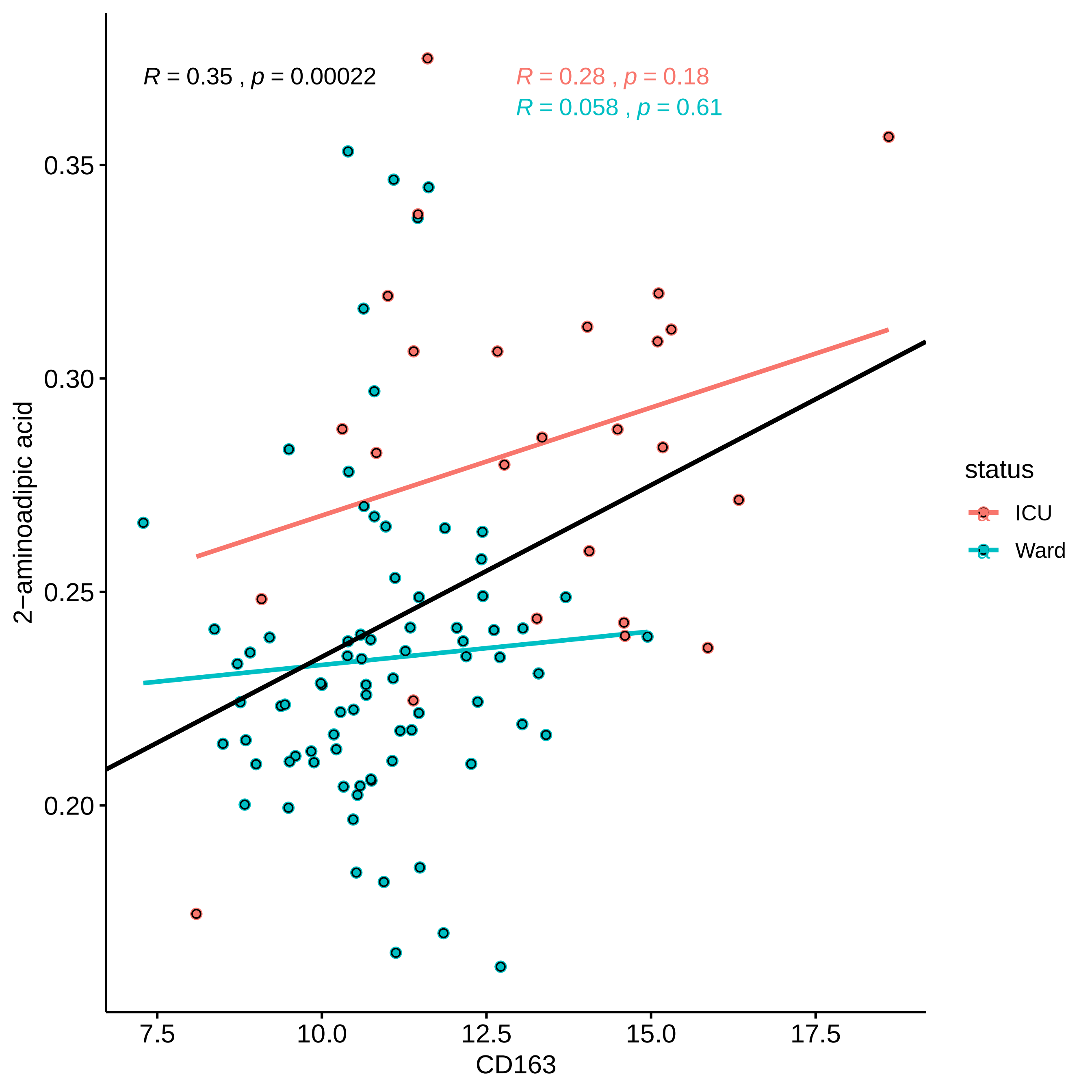

R = 0.35 , p = 0.00022
R = 0.28 , p = 0.18
R = 0.058 , p = 0.61
2−aminoadipic acid
0.35
0.30
0.25
0.20
7.5
10.0
12.5
15.0
17.5
CD163
status
ICU
Ward

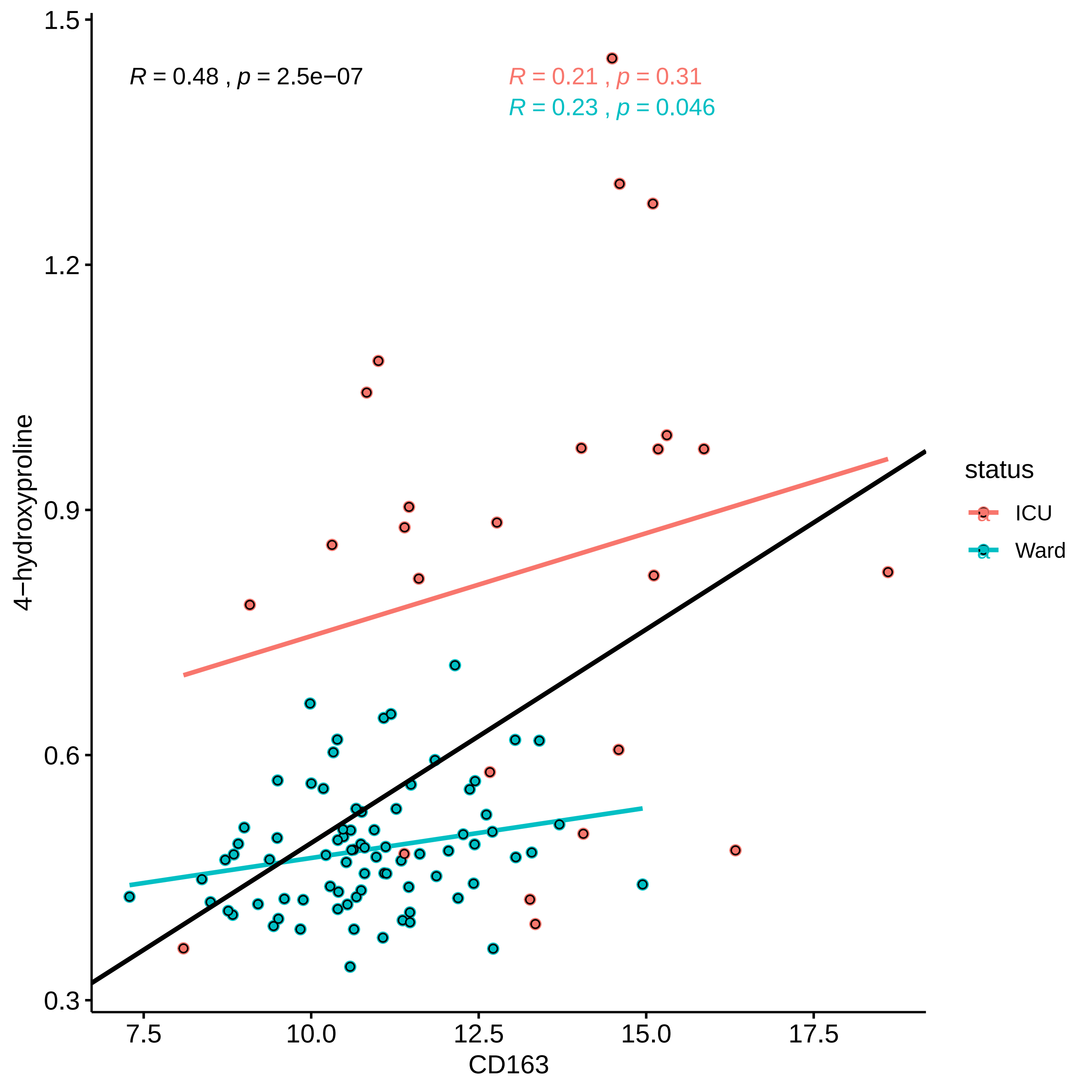

R = 0.48 , p = 2.5e−07
R = 0.21 , p = 0.31
R = 0.23 , p = 0.046
1.5
1.2
0.9
0.6
0.3
4−hydroxyproline
7.5
10.0
12.5
15.0
17.5
CD163
status
ICU
Ward

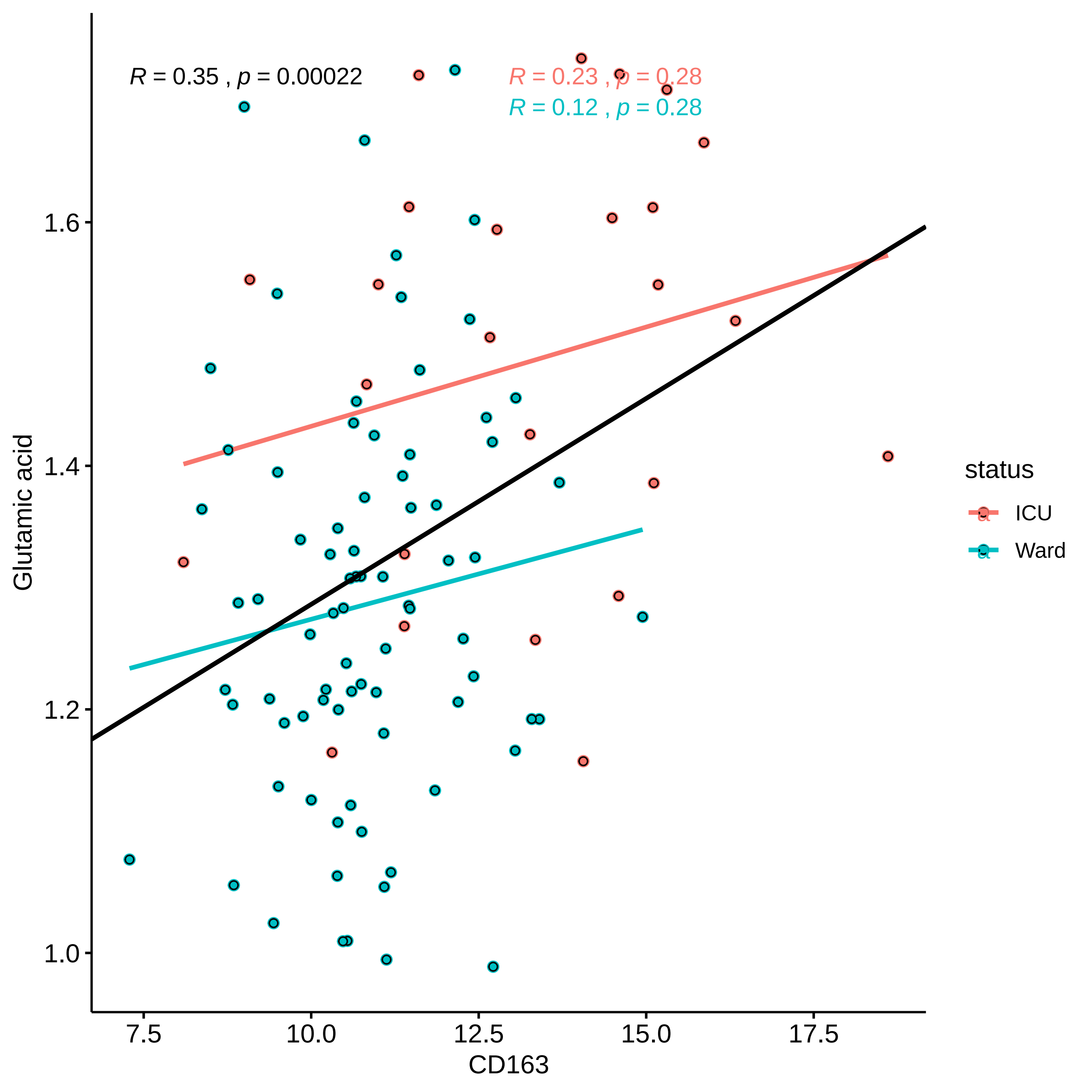

R = 0.35 , p = 0.00022
R = 0.23 , p = 0.28
R = 0.12 , p = 0.28
Glutamic acid
1.6
1.4
1.2
1.0
7.5
10.0
12.5
15.0
17.5
CD163
status
ICU
Ward

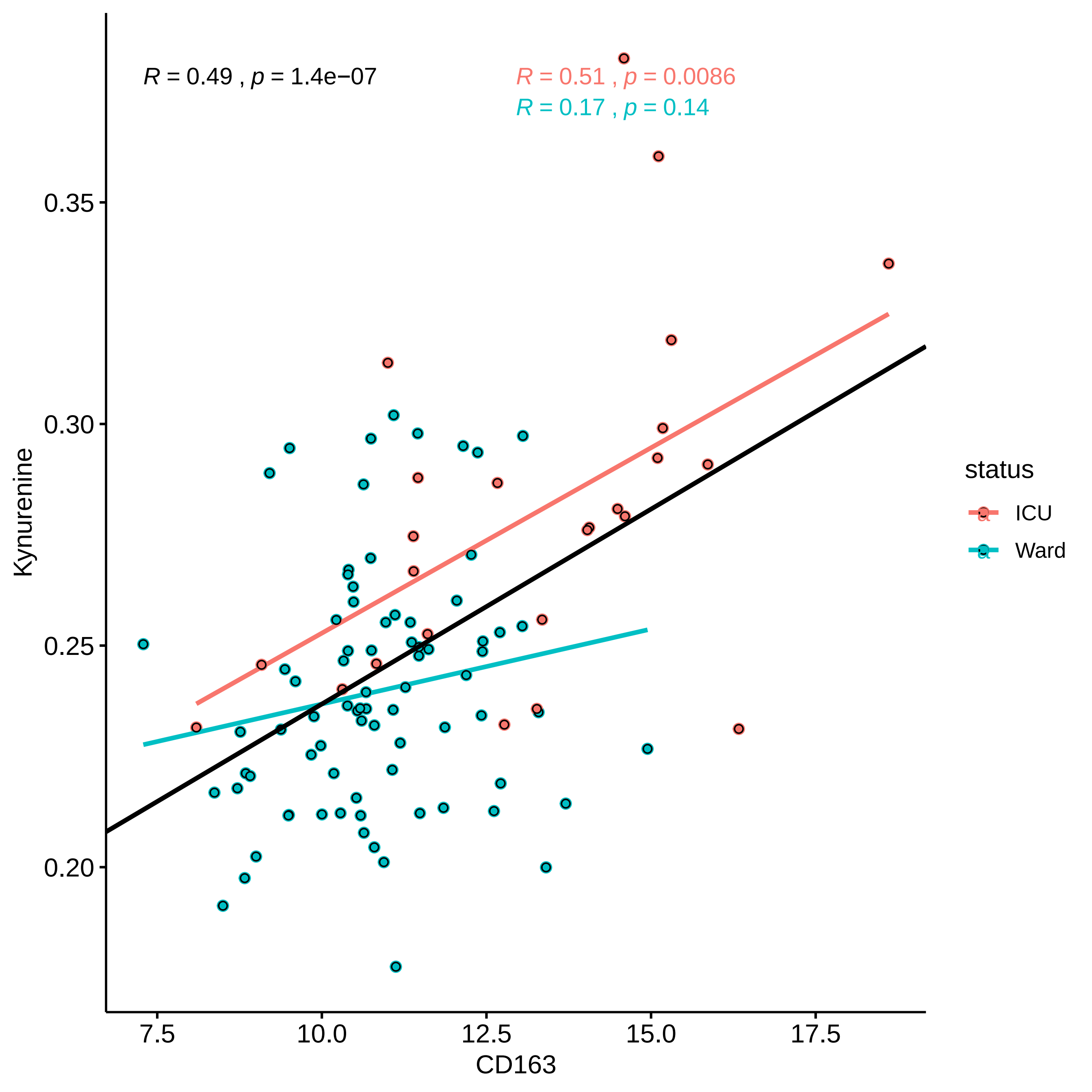

R = 0.49 , p = 1.4e−07
R = 0.51 , p = 0.0086
R = 0.17 , p = 0.14
Kynurenine
0.35
0.30
0.25
0.20
7.5
10.0
12.5
15.0
17.5
CD163
status
ICU
Ward

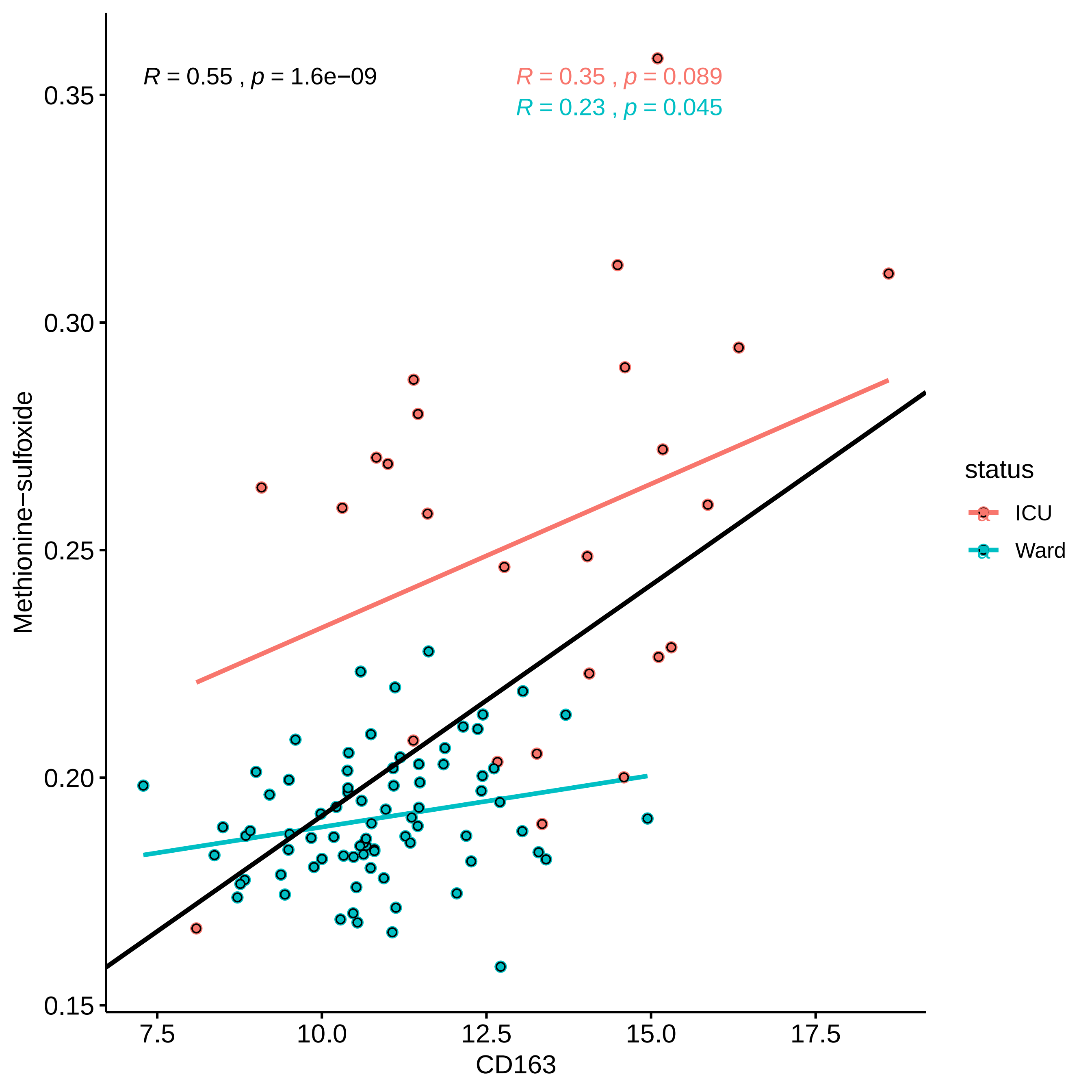

R = 0.55 , p = 1.6e−09
R = 0.35 , p = 0.089
R = 0.23 , p = 0.045
0.35
0.30
0.25
0.20
0.15
Methionine−sulfoxide
7.5
10.0
12.5
15.0
17.5
CD163
status
ICU
Ward

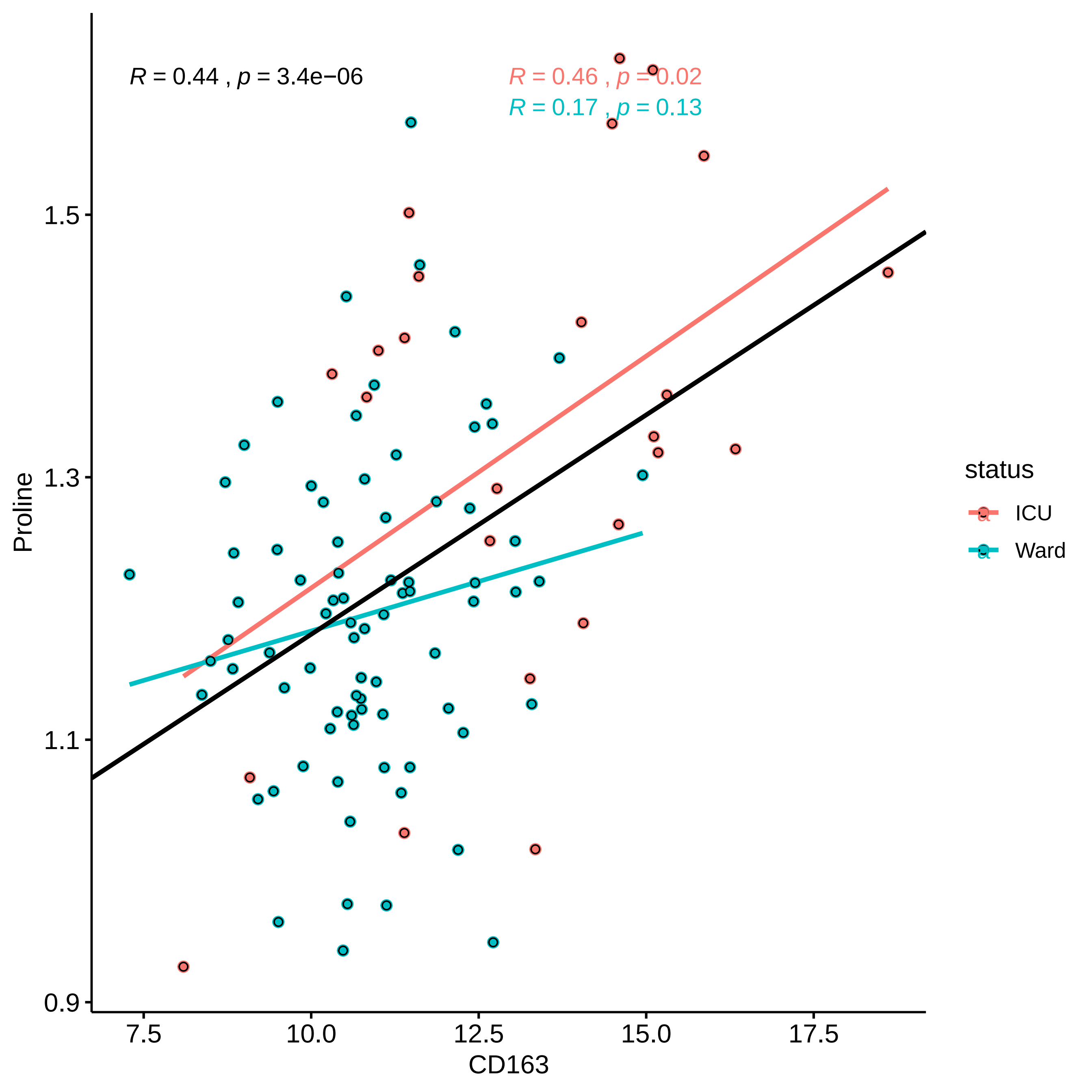

R = 0.44 , p = 3.4e−06
R = 0.46 , p = 0.02
R = 0.17 , p = 0.13
Proline
1.5
1.3
1.1
0.9
7.5
10.0
12.5
15.0
17.5
CD163
status
ICU
Ward

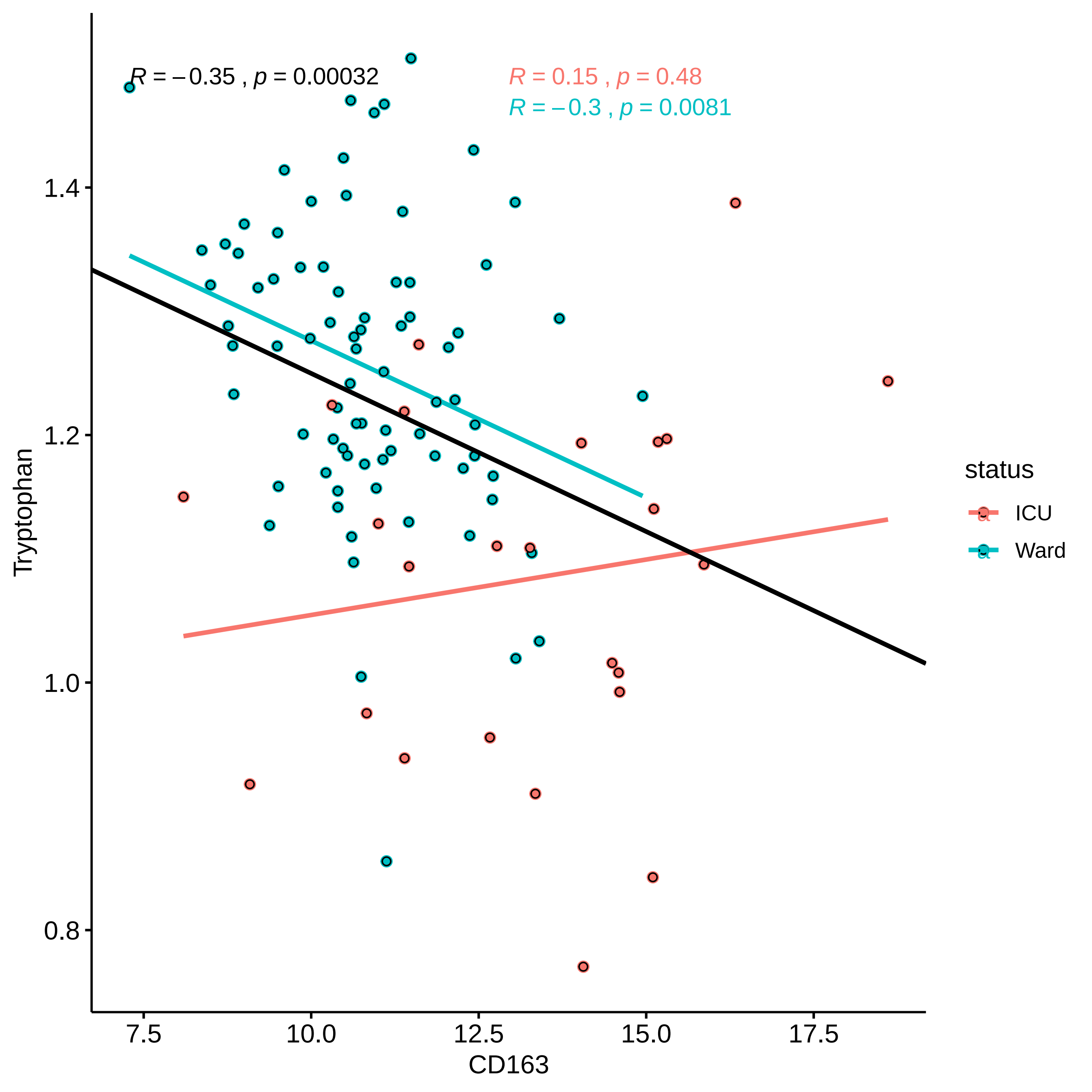

R = −0.35 , p = 0.00032
R = 0.15 , p = 0.48
R = −0.3 , p = 0.0081
Tryptophan
1.4
1.2
1.0
0.8
7.5
10.0
12.5
15.0
17.5
CD163
status
ICU
Ward

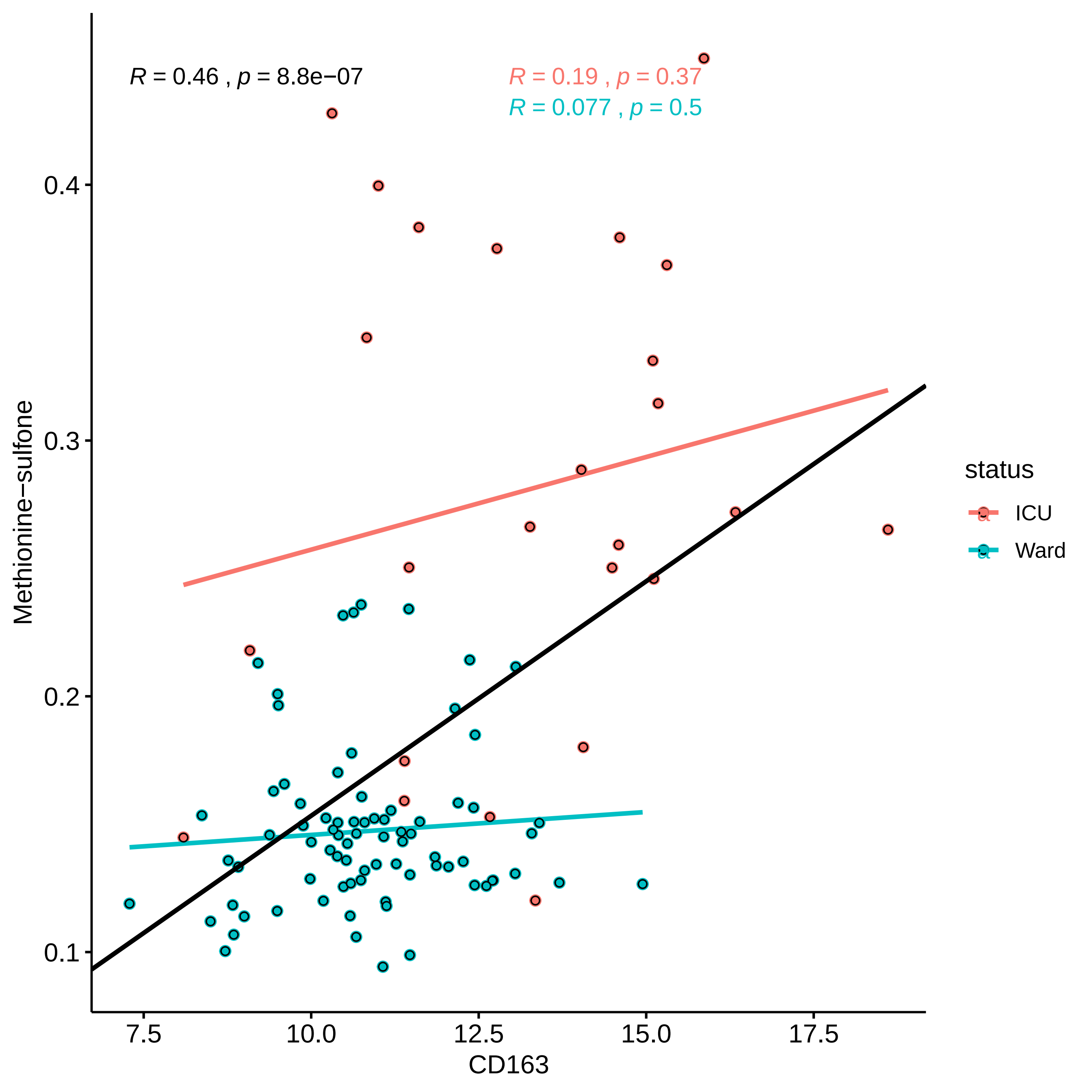
R = 0.46 , p = 8.8e−07
R = 0.19 , p = 0.37
R = 0.077 , p = 0.5
Methionine−sulfone
0.4
0.3
0.2
0.1
7.5
10.0
12.5
15.0
17.5
CD163
status
ICU
Ward

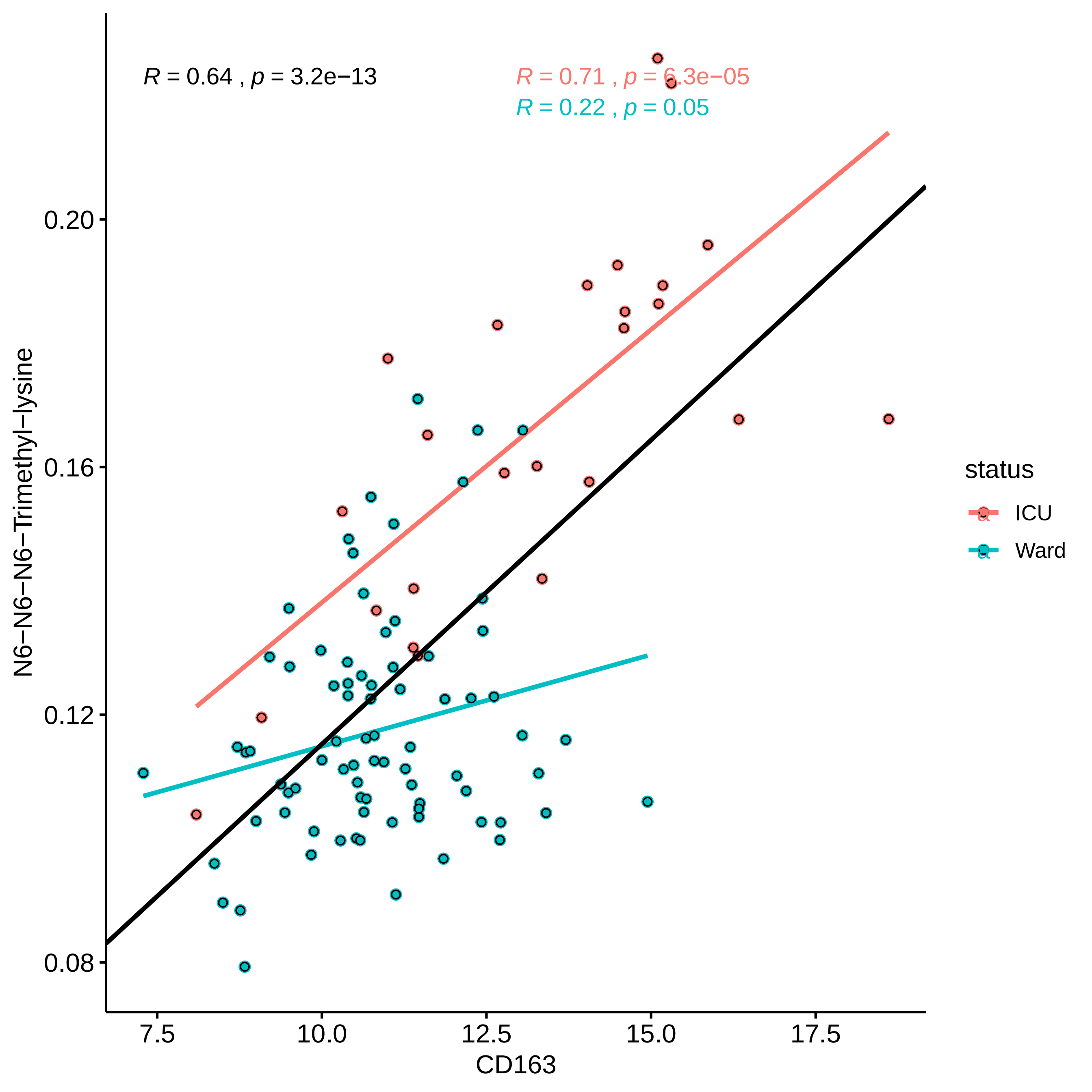

R = 0.64 , p = 3.2e−13
R = 0.71 , p = 6.3e−05
R = 0.22 , p = 0.05
N6−N6−N6−Trimethyl−lysine
0.20
0.16
0.12
0.08
7.5
10.0
12.5
15.0
17.5
CD163
status
ICU
Ward

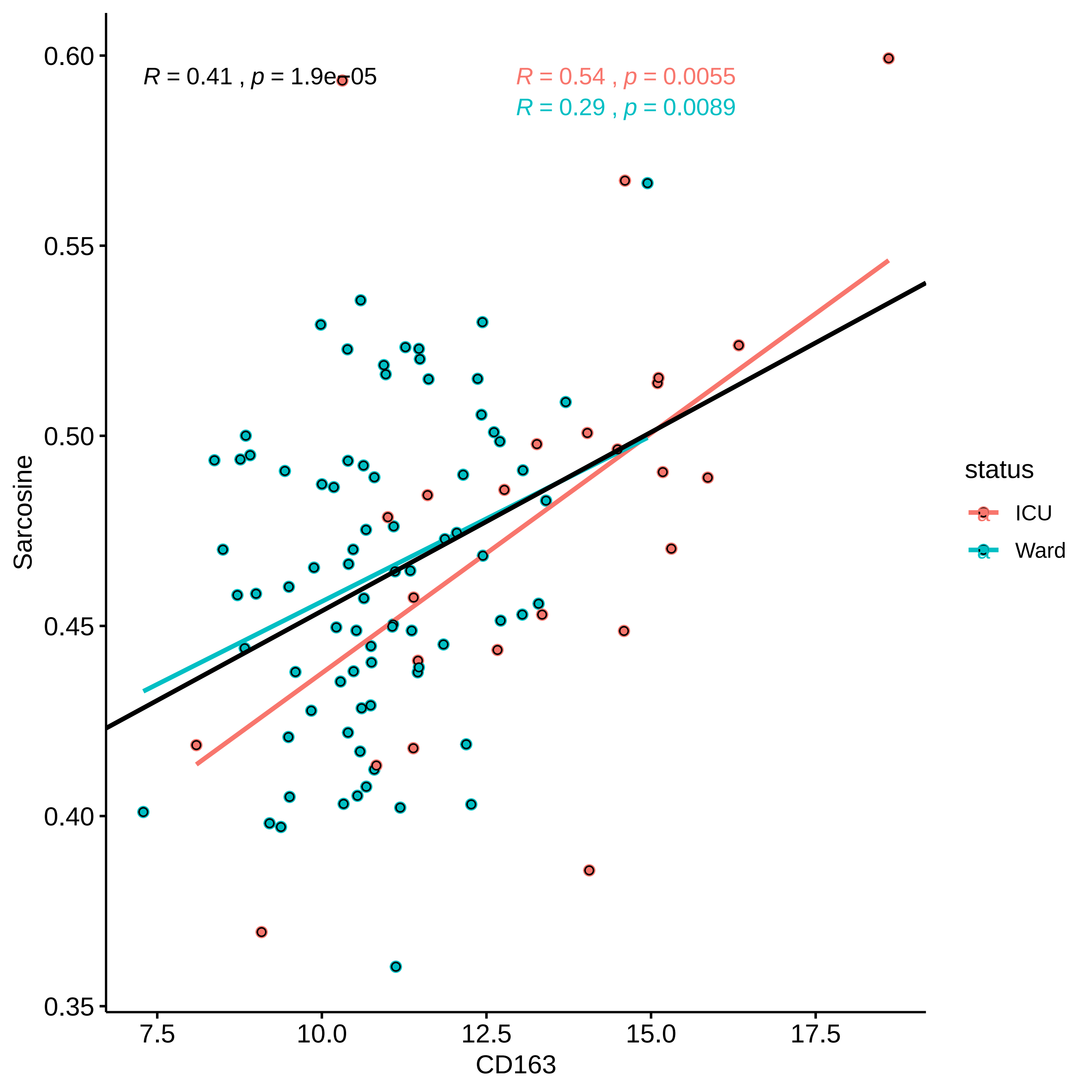

R = 0.41 , p = 1.9e−05
R = 0.54 , p = 0.0055
R = 0.29 , p = 0.0089
Sarcosine
CD163
0.60
0.55
0.50
0.45
0.40
0.35
7.5
10.0
12.5
15.0
17.5
status
ICU
Ward

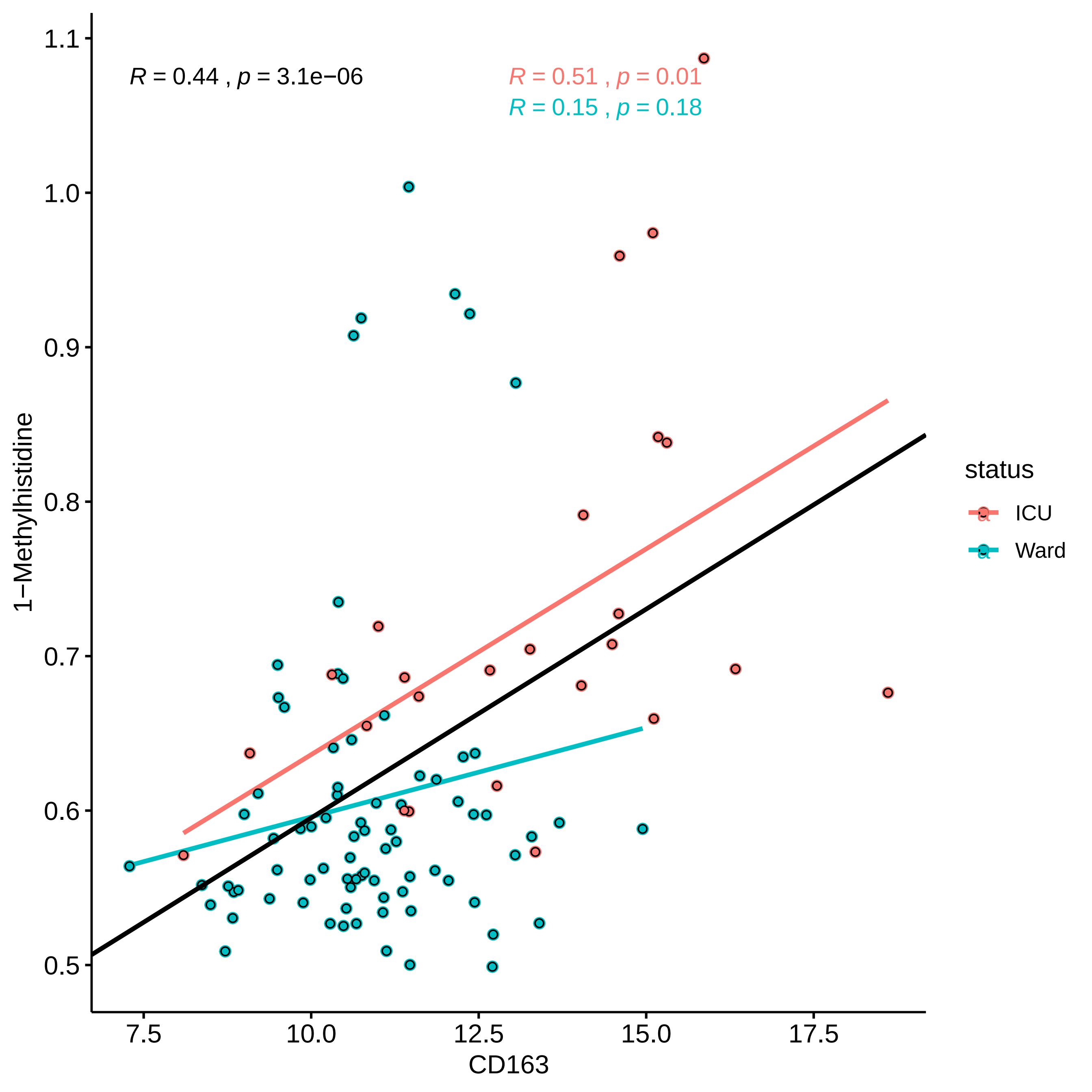
R = 0.44 , p = 3.1e−06
R = 0.51 , p = 0.01
R = 0.15 , p = 0.18
1−Methylhistidine
CD163
status
ICU
Ward
1.1
1.0
0.9
0.8
0.7
0.6
0.5
7.5
10.0
12.5
15.0
17.5

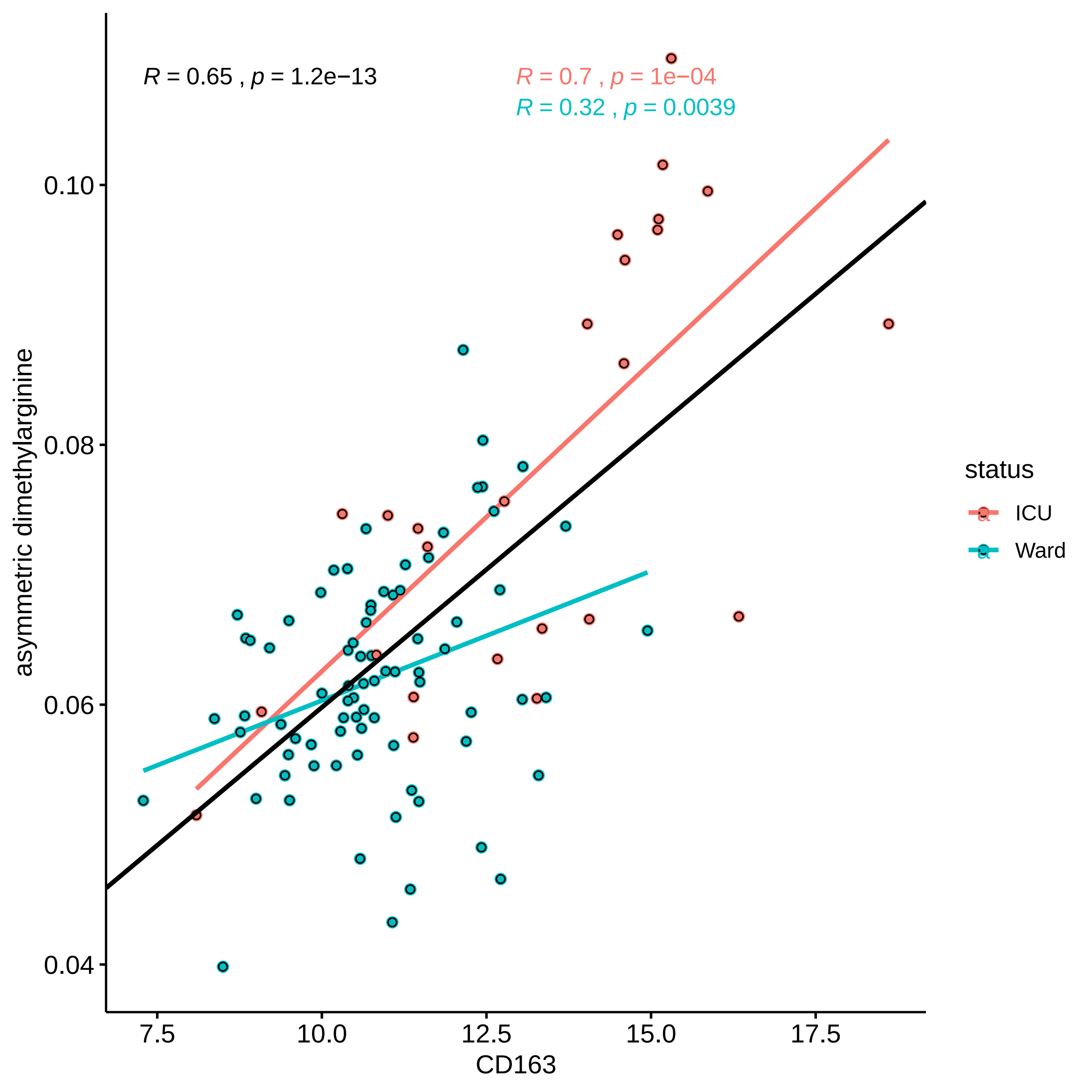
R = 0.65 , p = 1.2e−13
R = 0.7 , p = 1e−04
R = 0.32 , p = 0.0039
asymmetric dimethylarginine
0.10
0.08
0.06
0.04
7.5
10.0
12.5
15.0
17.5
CD163
status
ICU
Ward

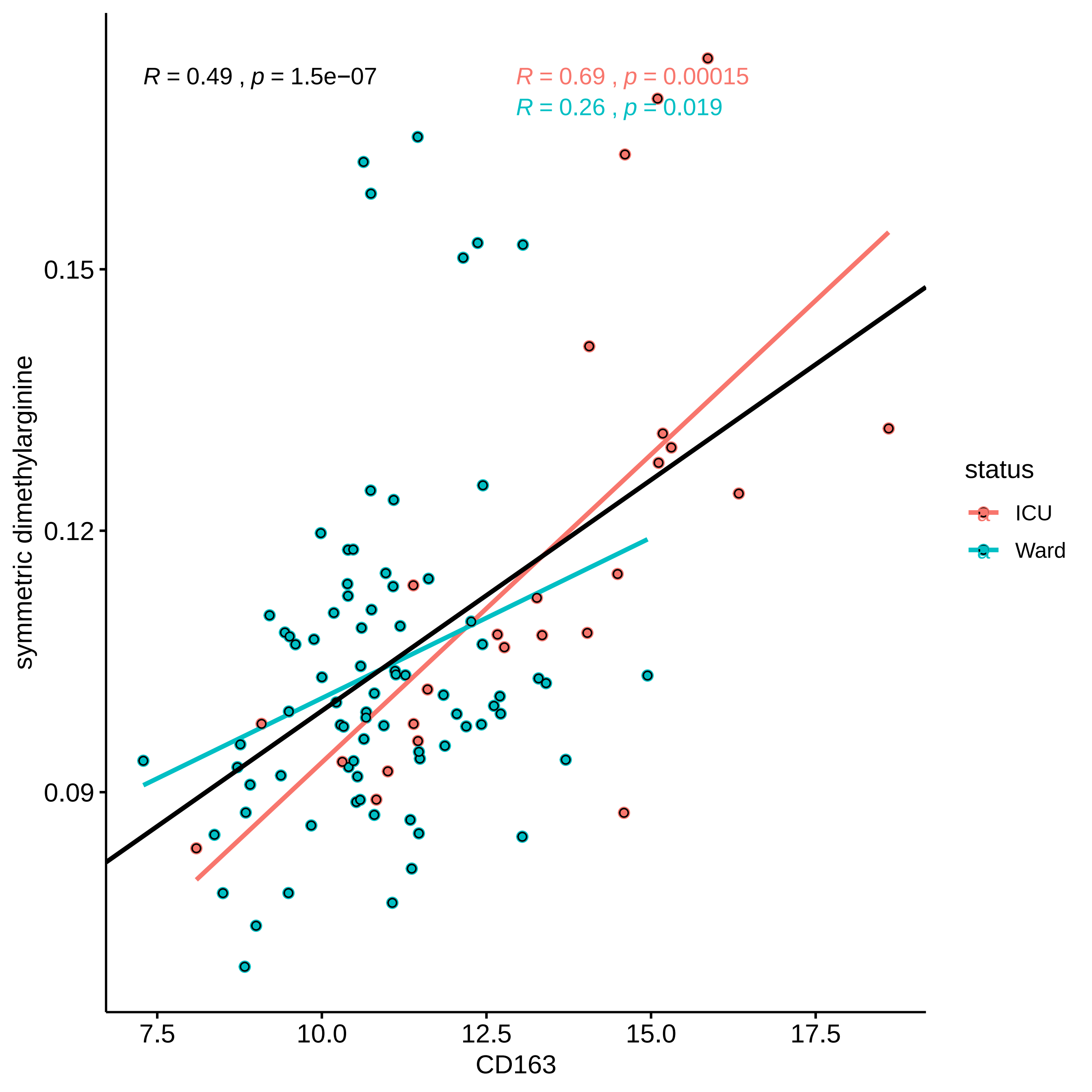

R = 0.49 , p = 1.5e−07
R = 0.69 , p = 0.00015
R = 0.26 , p = 0.019
symmetric dimethylarginine
0.15
0.12
0.09
7.5
10.0
12.5
15.0
17.5
CD163
status
ICU
Ward

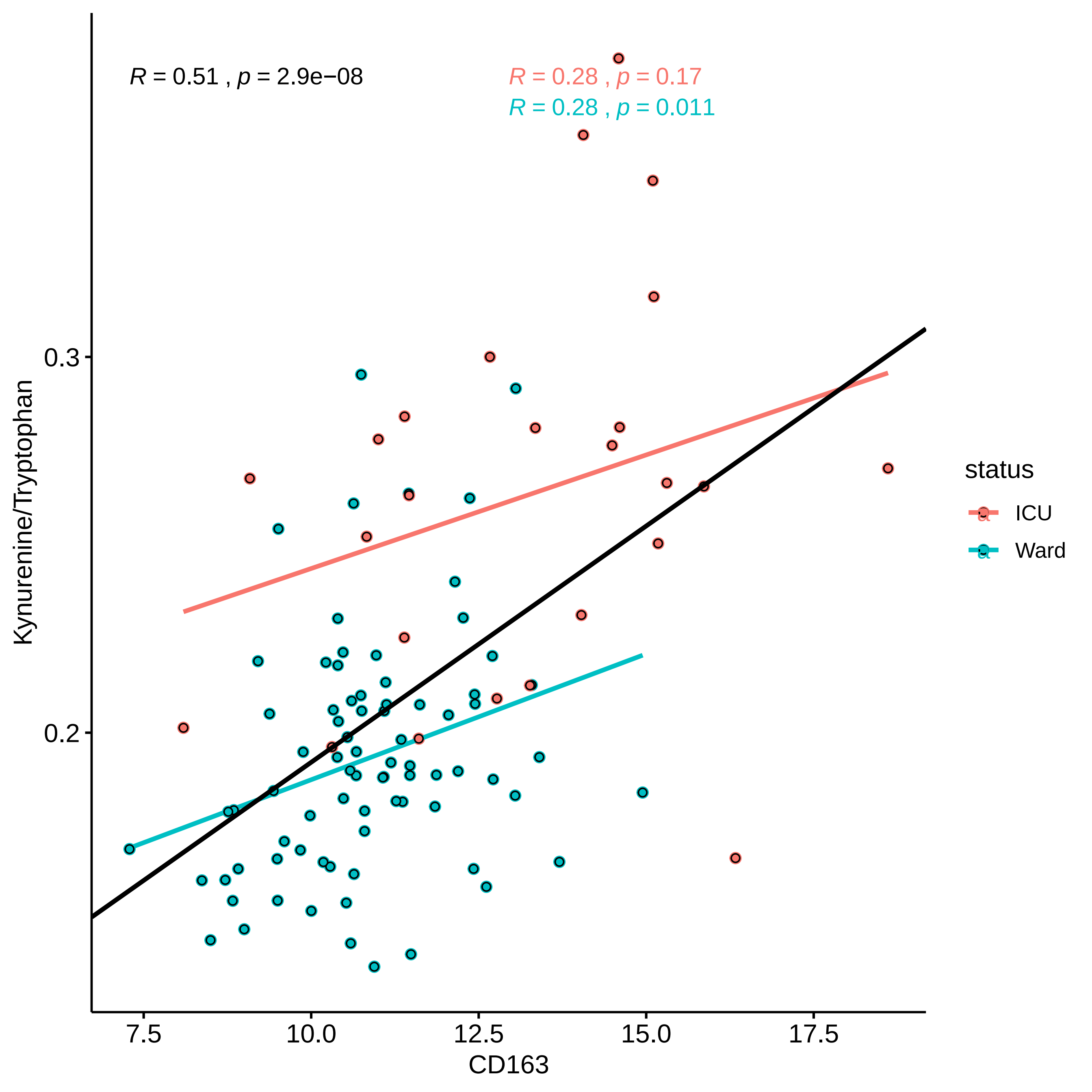

R = 0.51 , p = 2.9e−08
R = 0.28 , p = 0.17
R = 0.28 , p = 0.011
Kynurenine/Tryptophan
0.3
0.2
7.5
10.0
12.5
15.0
17.5
CD163
status
ICU
Ward

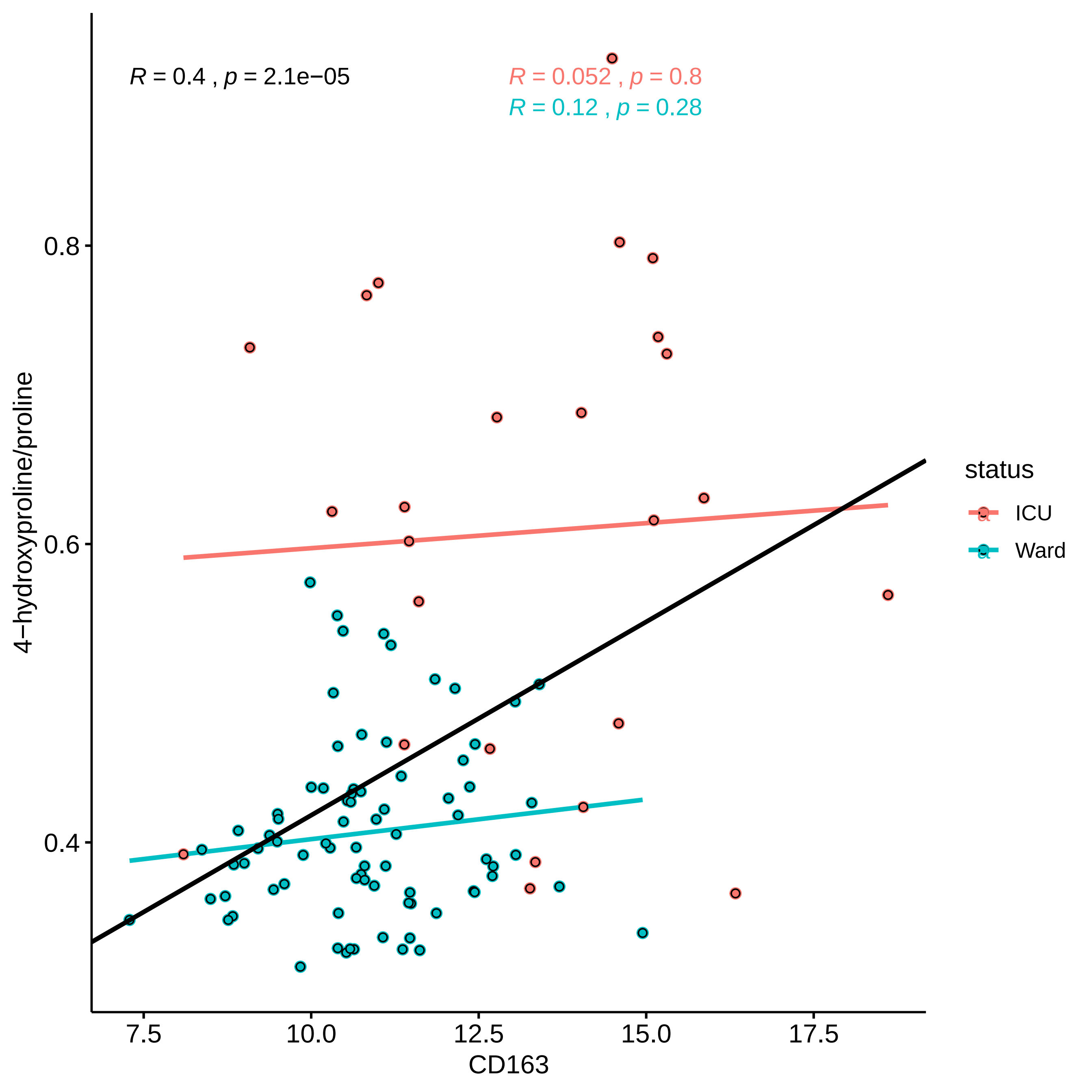

R = 0.4 , p = 2.1e−05
R = 0.052 , p = 0.8
R = 0.12 , p = 0.28
4−hydroxyproline/proline
0.4
0.6
0.8
7.5
10.0
12.5
15.0
17.5
CD163
status
ICU
Ward

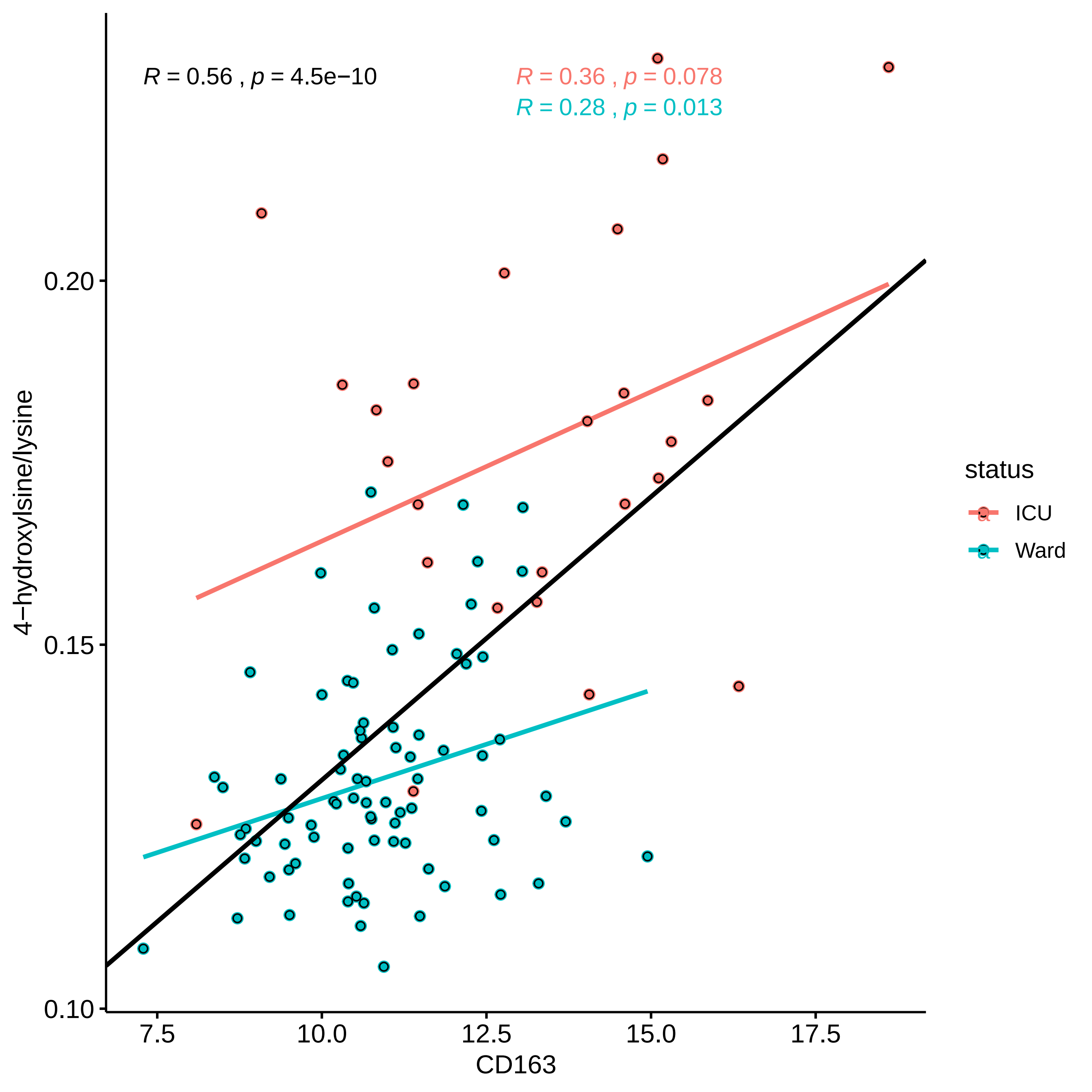

R = 0.56 , p = 4.5e−10
R = 0.36 , p = 0.078
R = 0.28 , p = 0.013
4−hydroxylsine/lysine
0.20
0.15
0.10
7.5
10.0
12.5
15.0
17.5
CD163
status
ICU
Ward

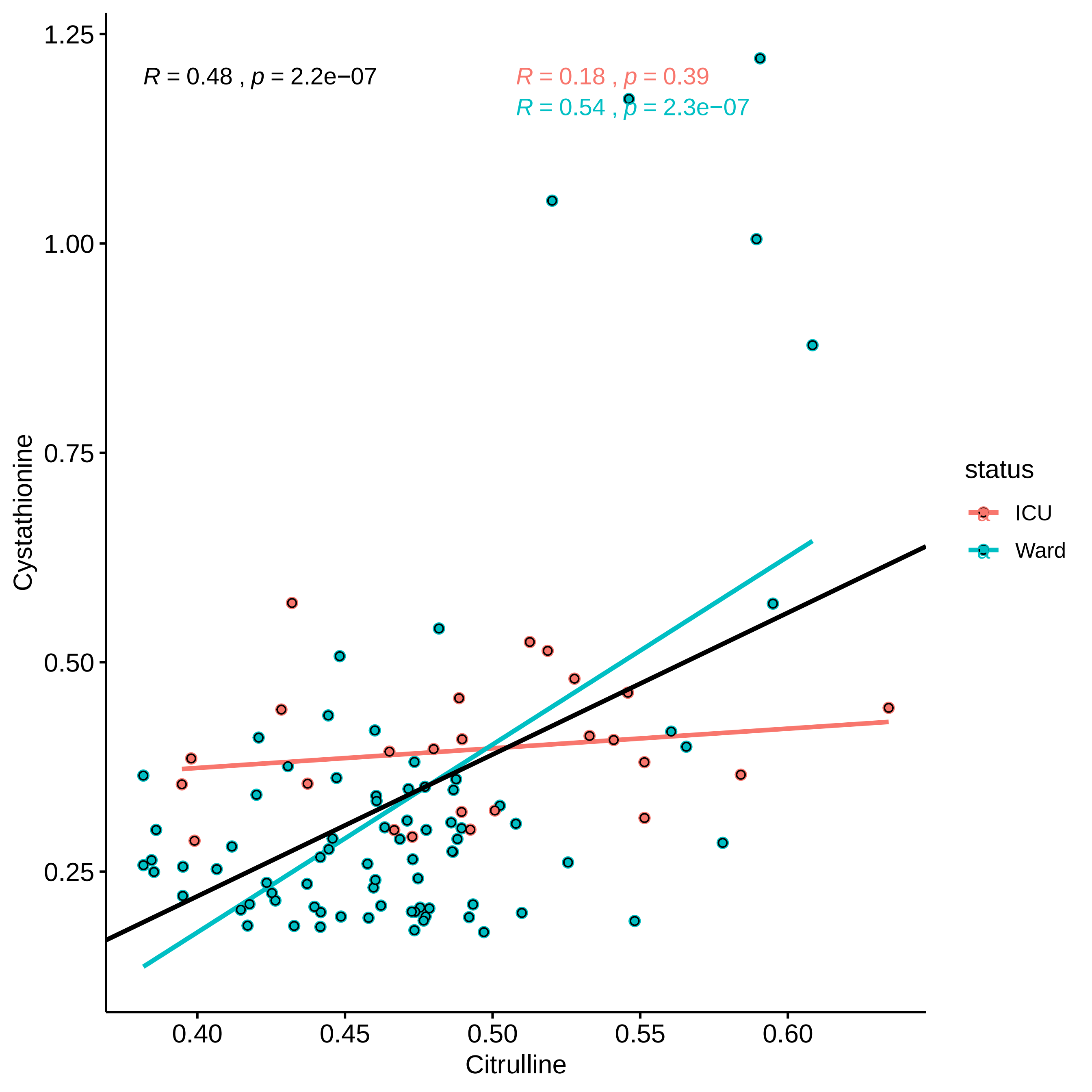

R = 0.48 , p = 2.2e−07
R = 0.18 , p = 0.39
R = 0.54 , p = 2.3e−07
1.25
1.00
0.75
0.50
0.25
Cystathionine
0.40
0.45
0.50
0.55
0.60
Citrulline
status
ICU
Ward

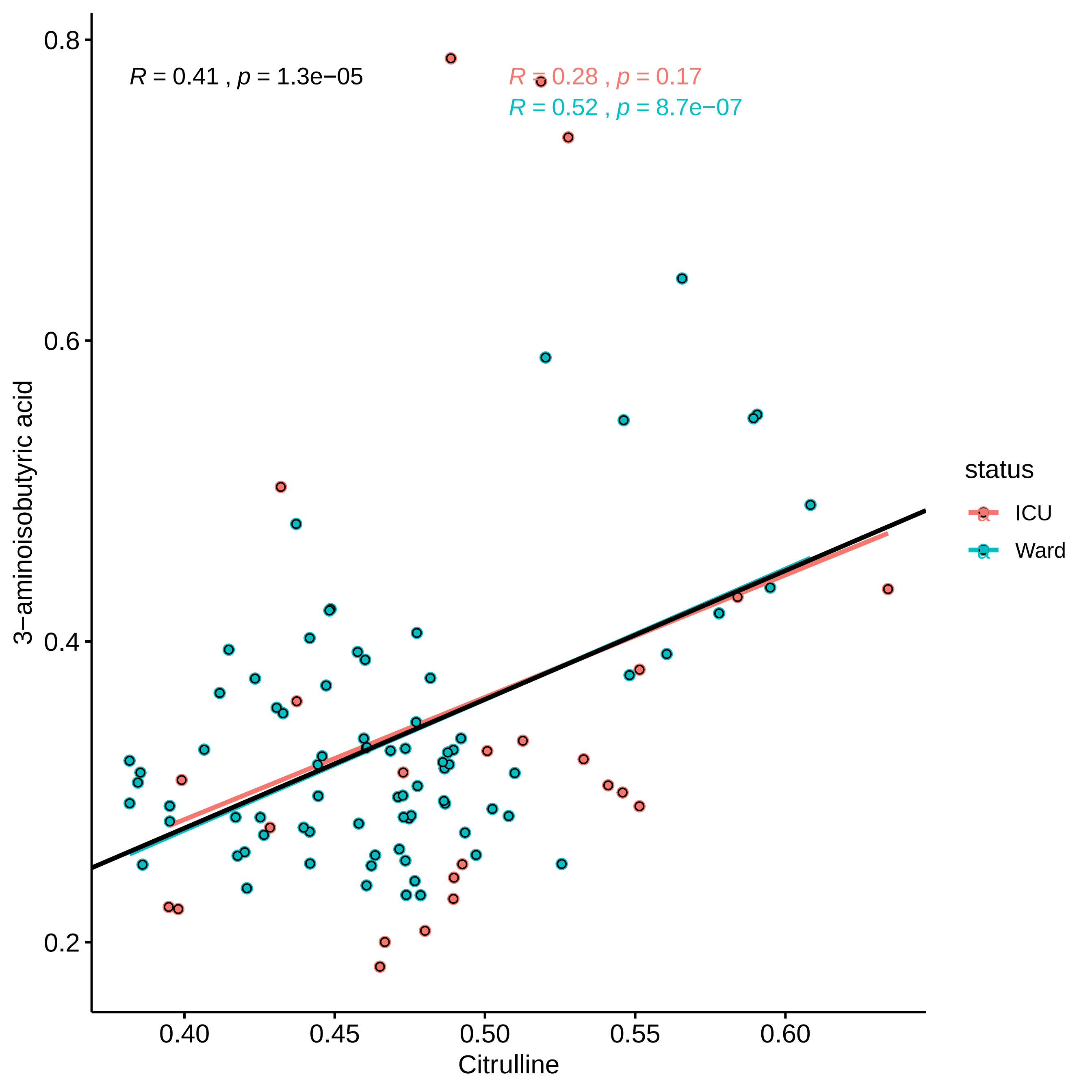

R = 0.41 , p = 1.3e−05
R = 0.28 , p = 0.17
R = 0.52 , p = 8.7e−07
0.8
0.6
0.4
0.2
3−aminoisobutyric acid
0.40
0.45
0.50
0.55
0.60
Citrulline
status
ICU
Ward

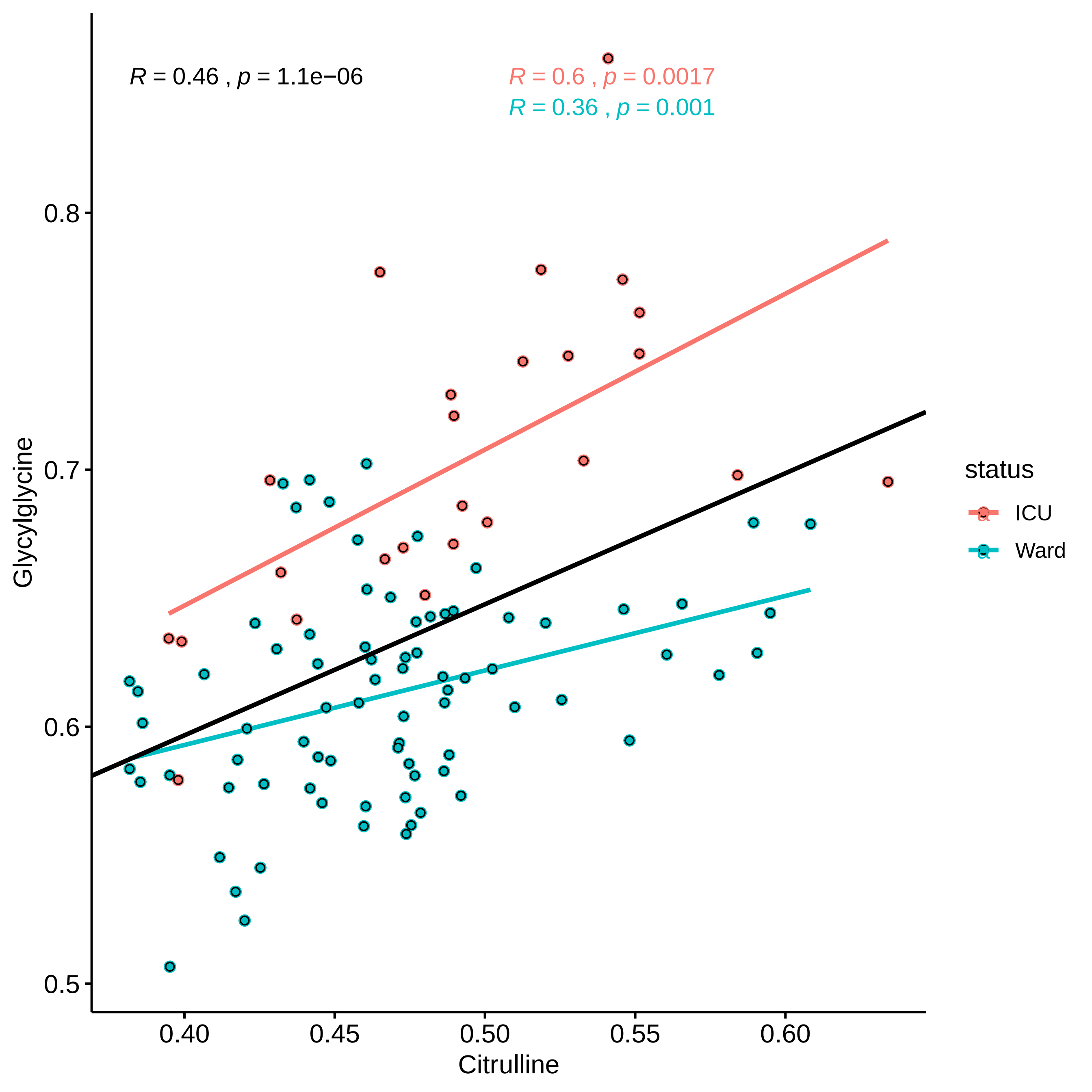

R = 0.46 , p = 1.1e−06
R = 0.6 , p = 0.0017
R = 0.36 , p = 0.001
0.8
0.7
0.6
0.5
Glycylglycine
0.40
0.45
0.50
0.55
0.60
Citrulline
status
ICU
Ward

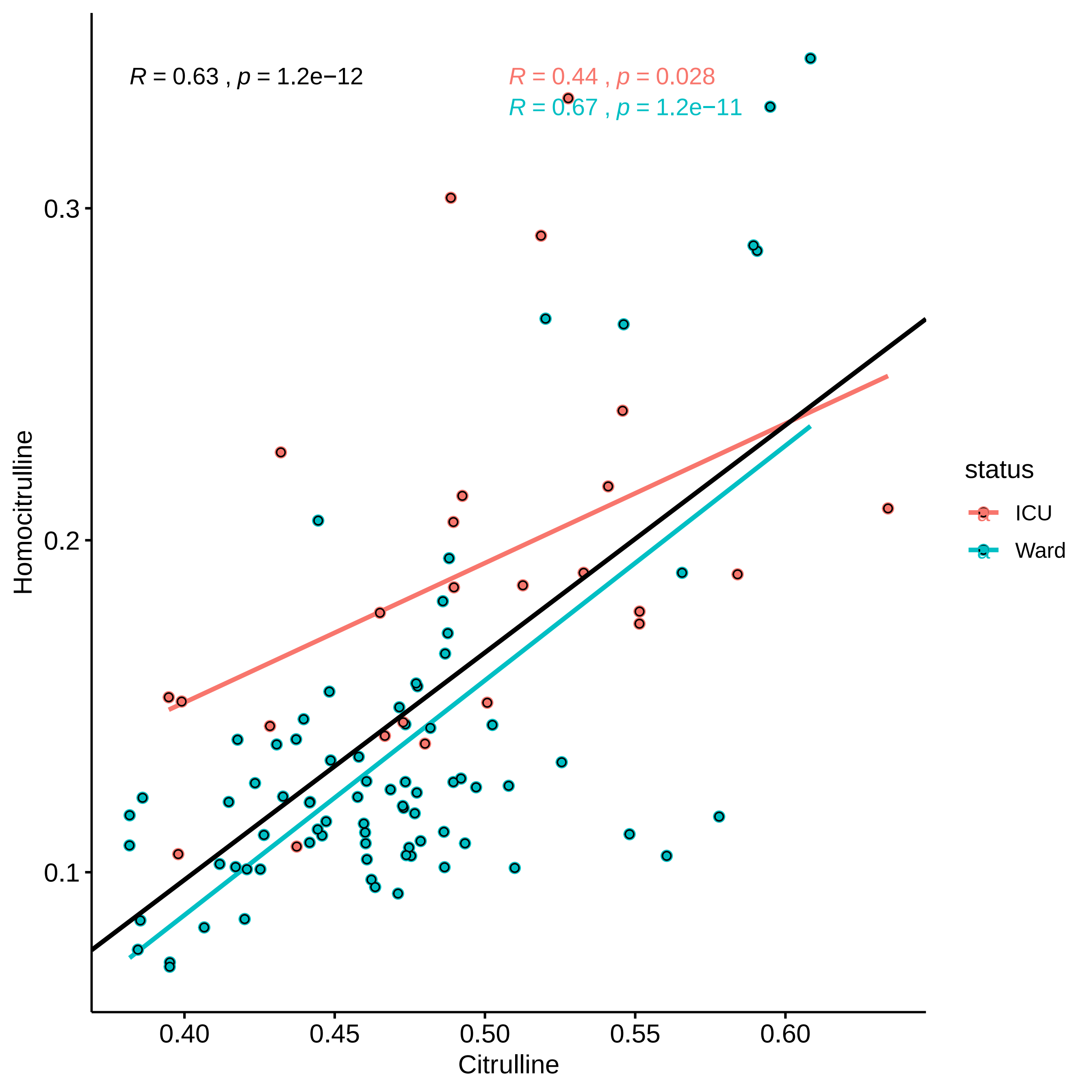

R = 0.63 , p = 1.2e−12
R = 0.44 , p = 0.028
R = 0.67 , p = 1.2e−11
0.3
0.2
0.1
Homocitrulline
0.40
0.45
0.50
0.55
0.60
Citrulline
status
ICU
Ward

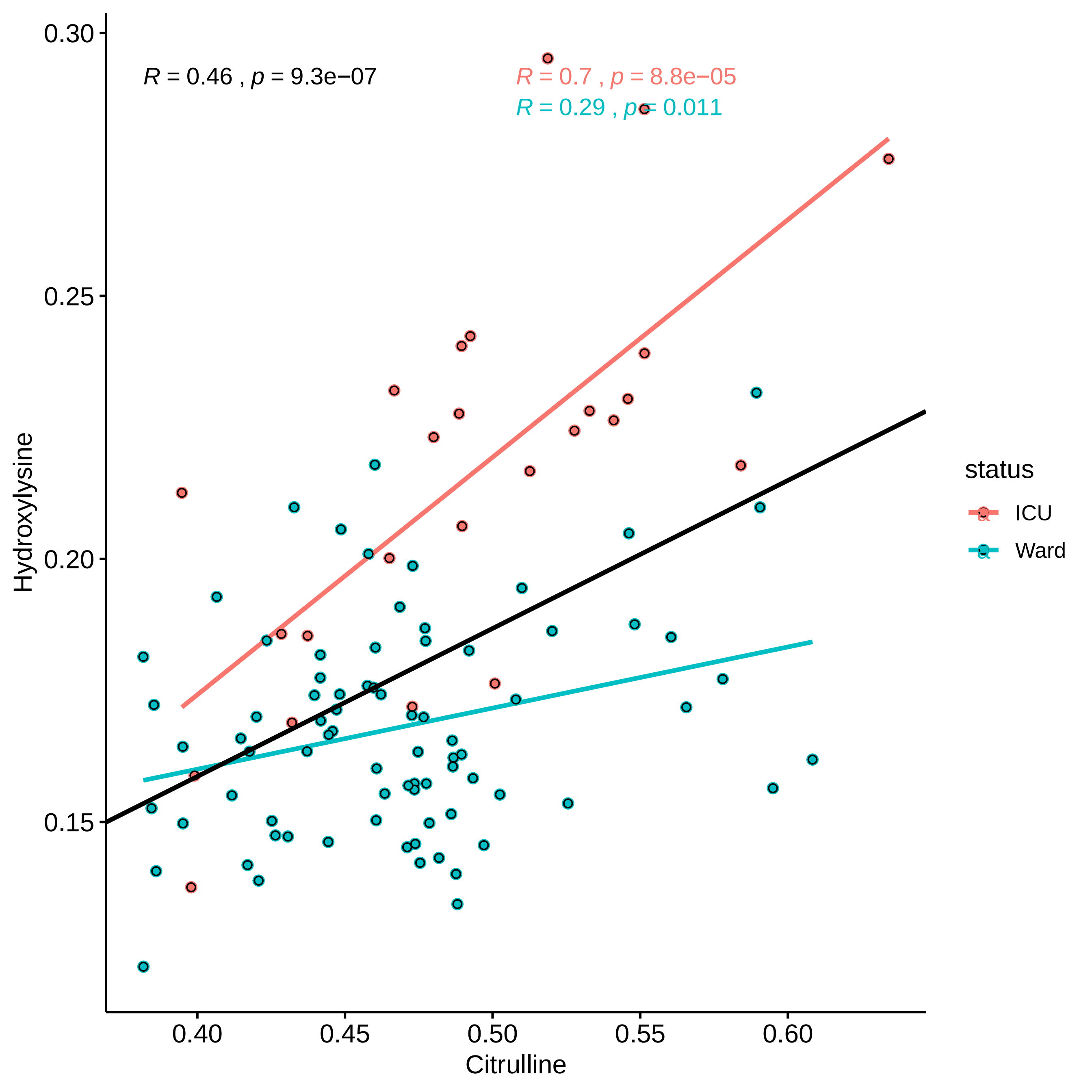

R = 0.46 , p = 9.3e−07
R = 0.7 , p = 8.8e−05
R = 0.29 , p = 0.011
Hydroxylysine
Citrulline
0.30
0.25
0.20
0.15
0.40
0.45
0.50
0.55
0.60
status
ICU
Ward

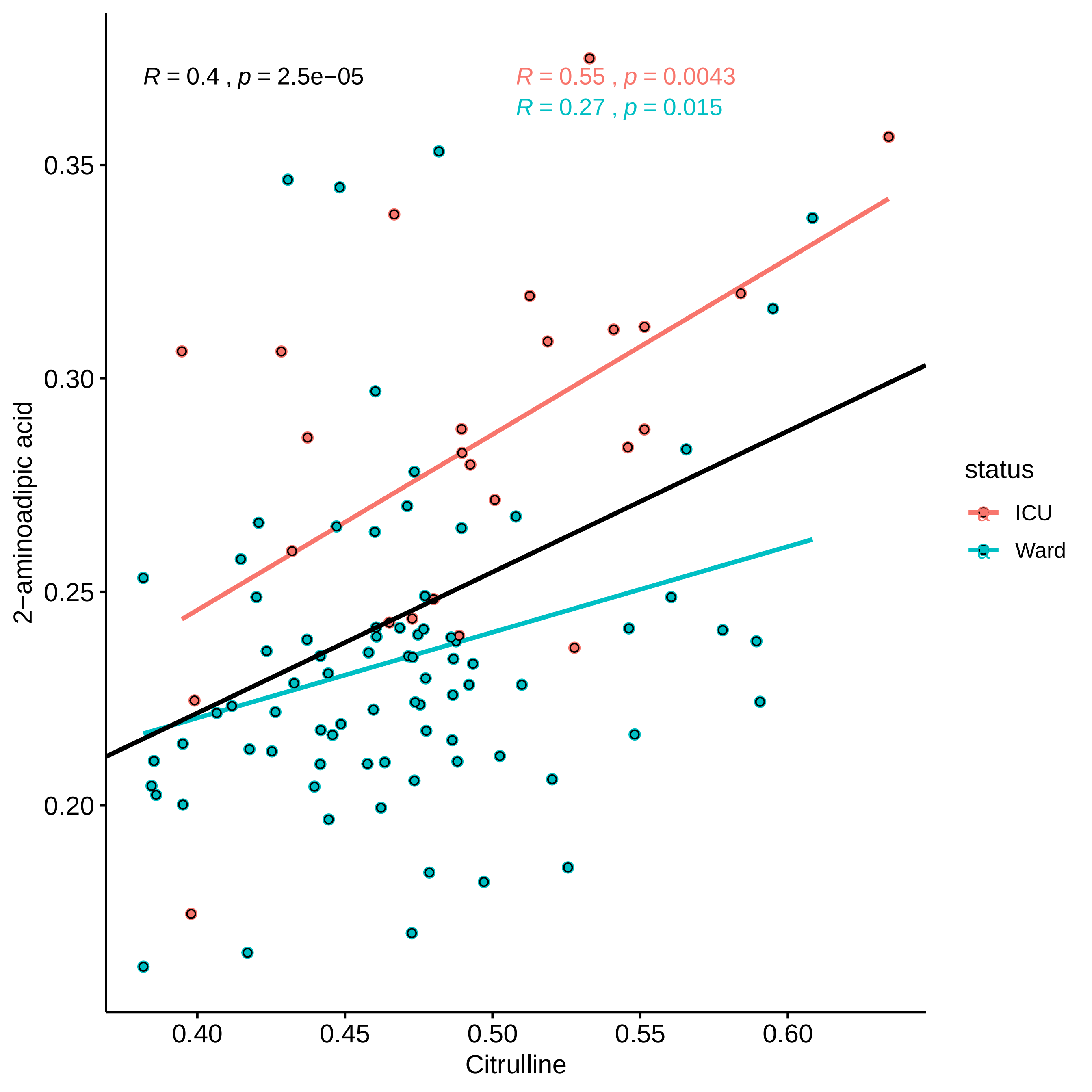

R = 0.4 , p = 2.5e−05
R = 0.55 , p = 0.0043
R = 0.27 , p = 0.015
0.35
0.30
0.25
0.20
2−aminoadipic acid
0.40
0.45
0.50
0.55
0.60
Citrulline
status
ICU
Ward

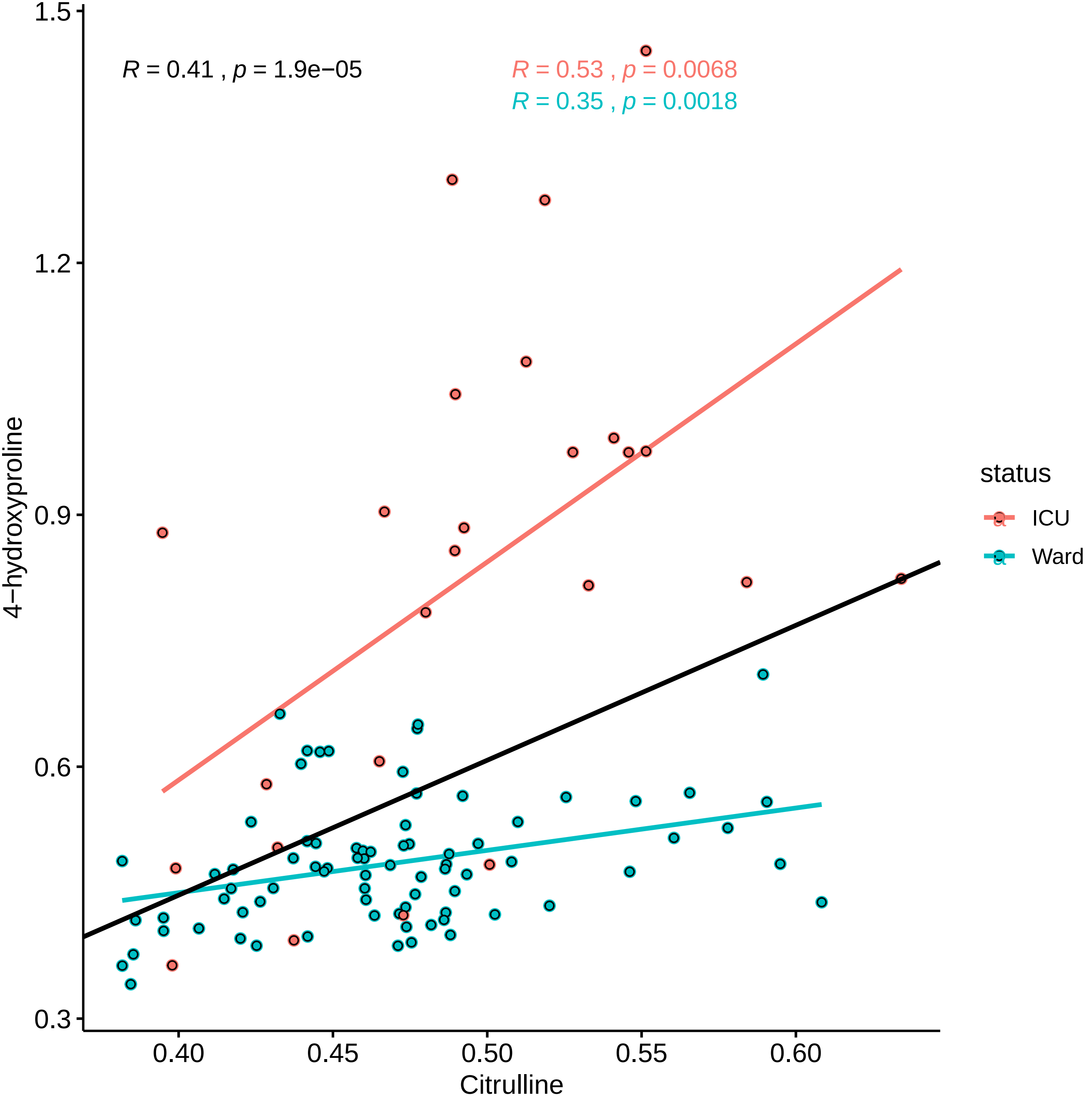

$R = 0.41$, $p = 1.9e{-}05$
$R = 0.53$, $p = 0.0068$
$R = 0.35$, $p = 0.0018$
4−hydroxyproline
1.5
1.2
0.9
0.6
0.3
0.40
0.45
0.50
0.55
0.60
Citrulline
status
ICU
Ward

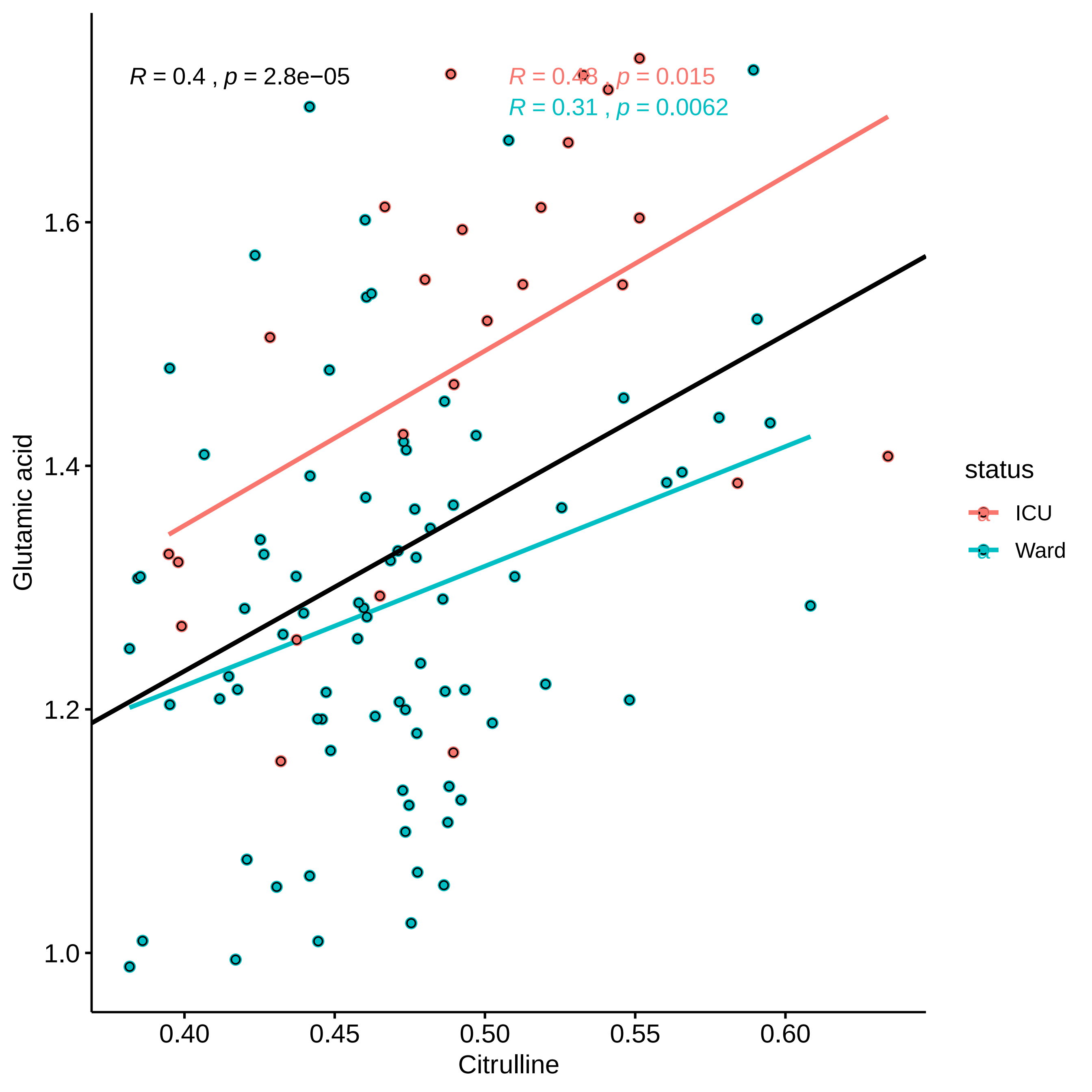

R = 0.4 , p = 2.8e−05
R = 0.48 , p = 0.015
R = 0.31 , p = 0.0062
Glutamic acid
1.6
1.4
1.2
1.0
0.40
0.45
0.50
0.55
0.60
Citrulline
status
ICU
Ward

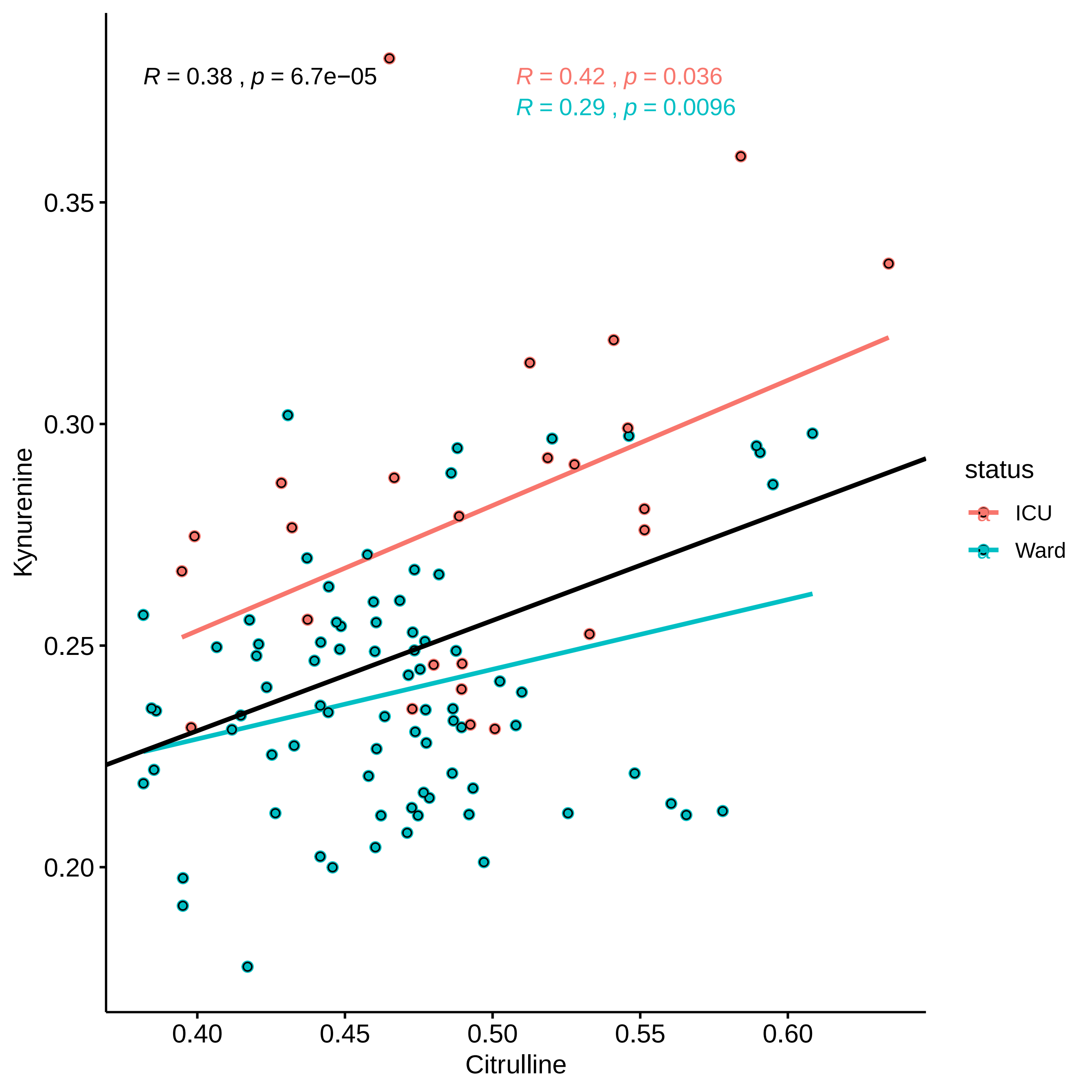

R = 0.38 , p = 6.7e−05
R = 0.42 , p = 0.036
R = 0.29 , p = 0.0096
0.35
0.30
0.25
0.20
Kynurenine
0.40
0.45
0.50
0.55
0.60
Citrulline
status
ICU
Ward

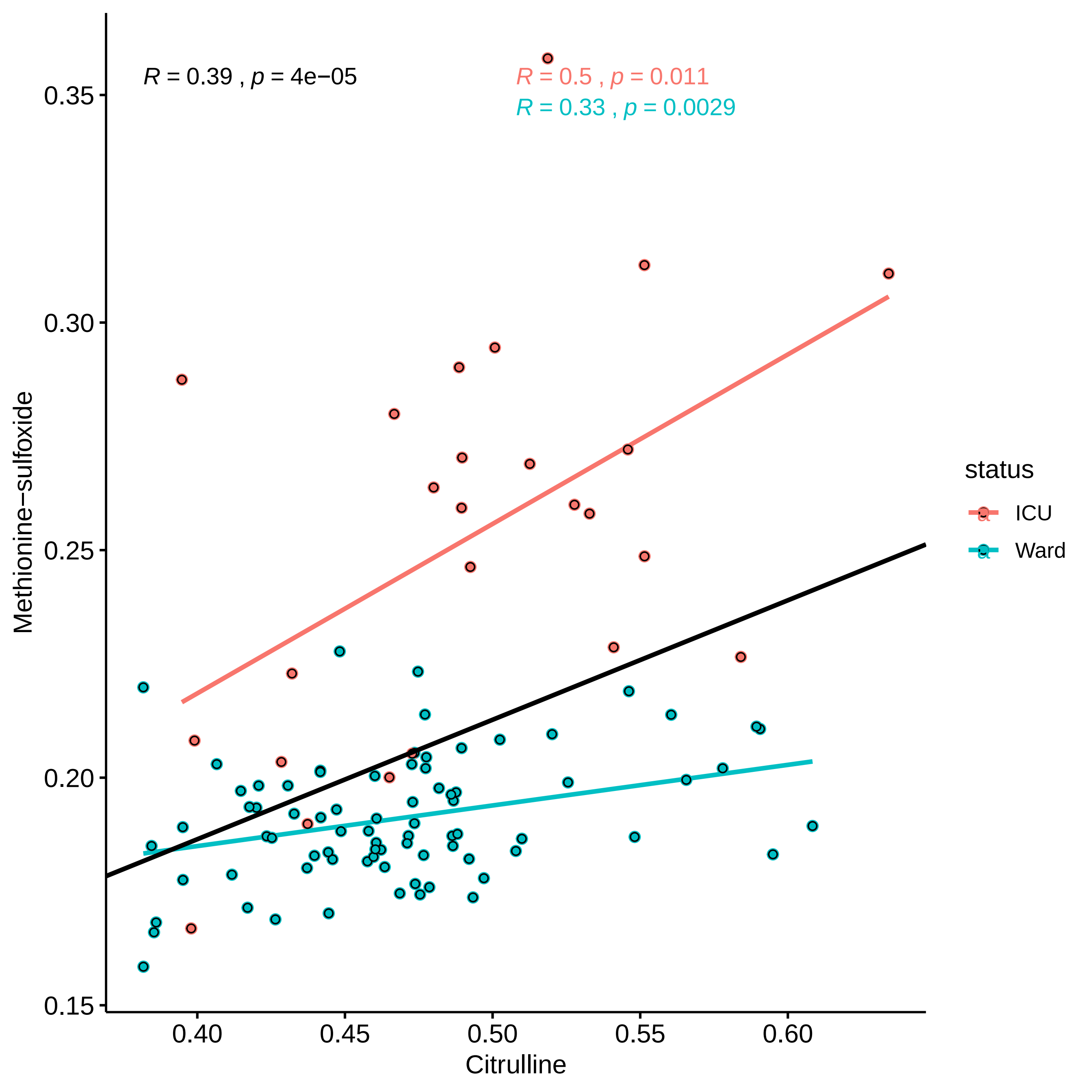

R = 0.39 , p = 4e−05
R = 0.5 , p = 0.011
R = 0.33 , p = 0.0029
Methionine−sulfoxide
Citrulline
0.35
0.30
0.25
0.20
0.15
0.40
0.45
0.50
0.55
0.60
status
ICU
Ward

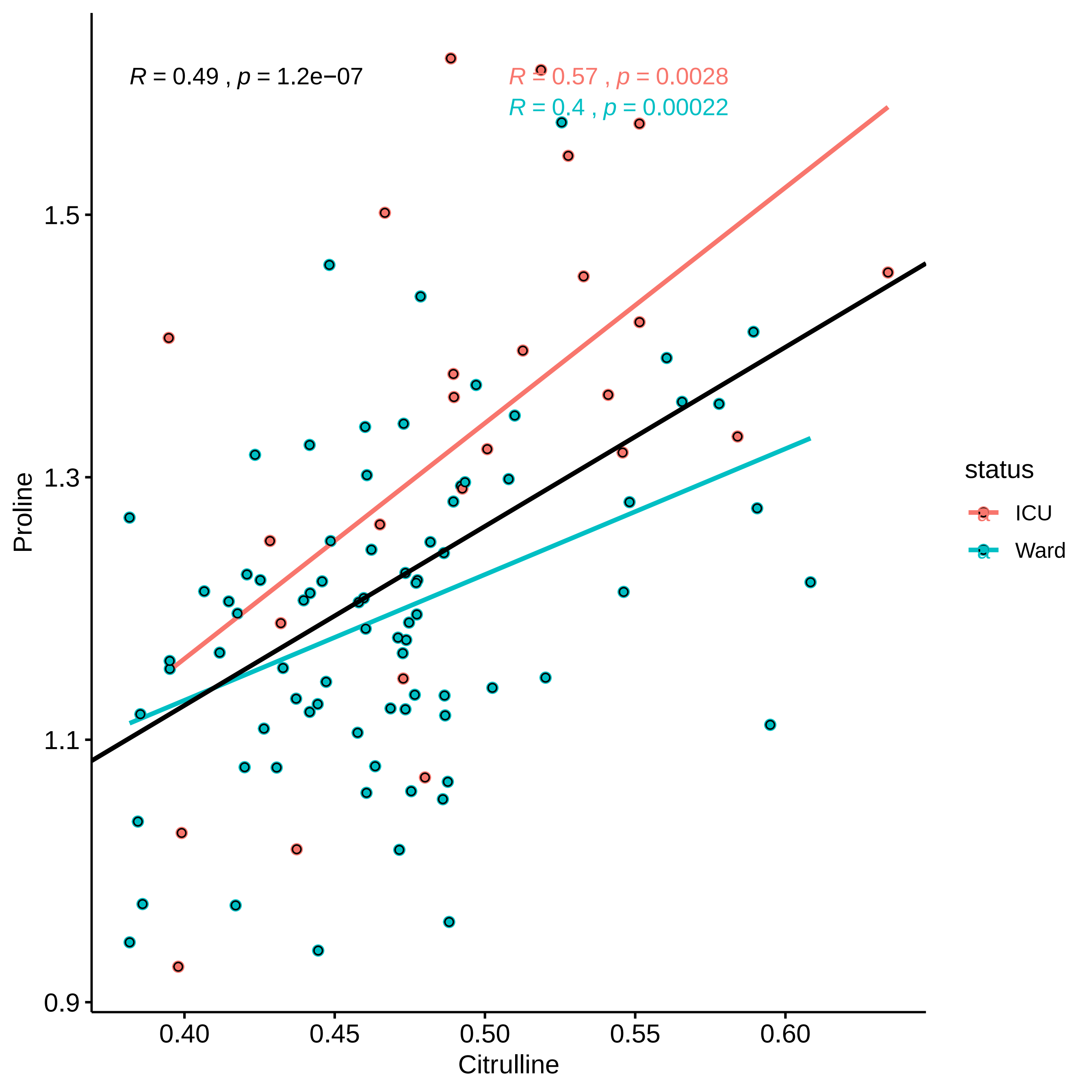

R = 0.49 , p = 1.2e−07
R = 0.57 , p = 0.0028
R = 0.4 , p = 0.00022
Proline
1.5
1.3
1.1
0.9
0.40
0.45
0.50
0.55
0.60
Citrulline
status
ICU
Ward

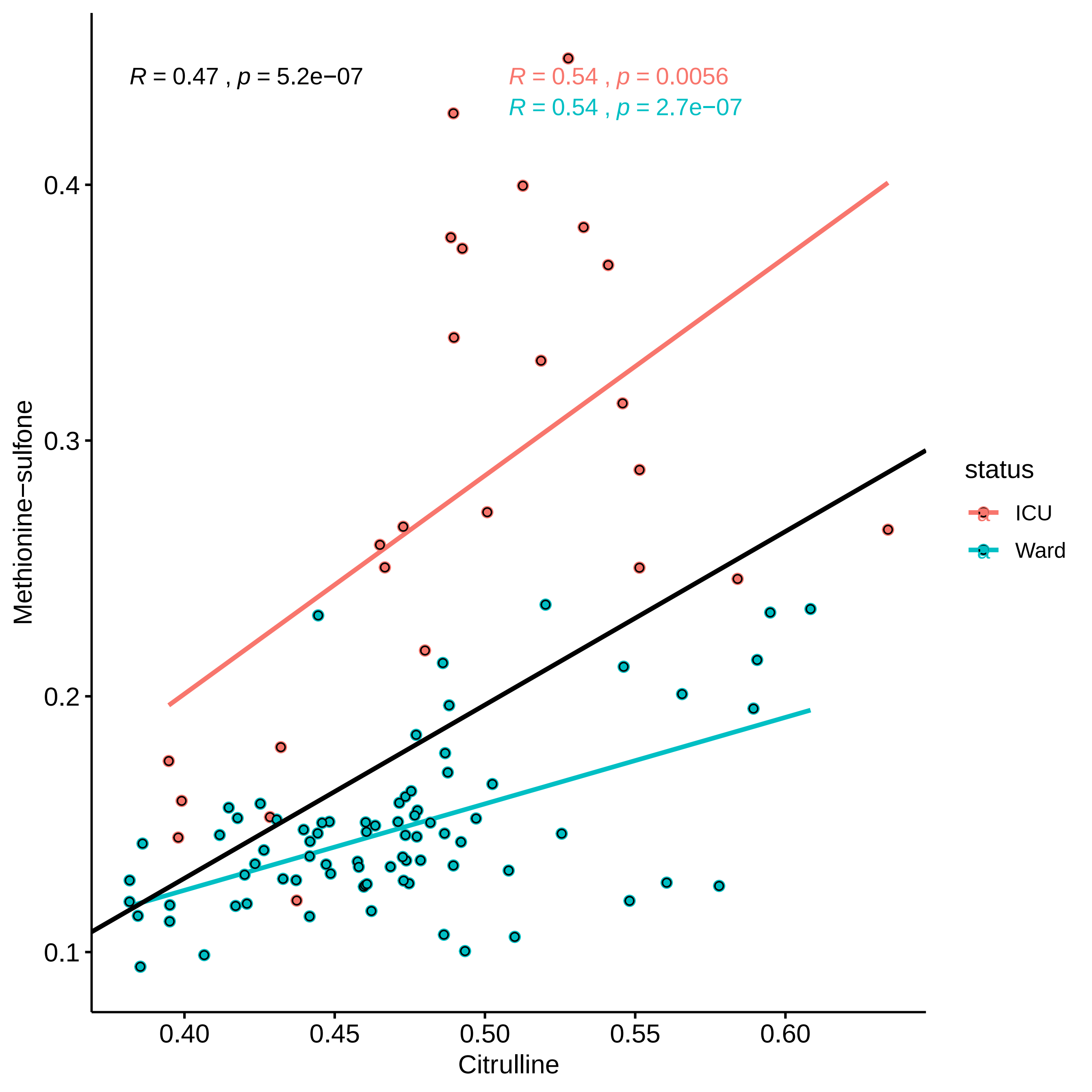
R = 0.47 , p = 5.2e−07
R = 0.54 , p = 0.0056
R = 0.54 , p = 2.7e−07
Methionine−sulfone
0.4
0.3
0.2
0.1
0.40
0.45
0.50
0.55
0.60
Citrulline
status
ICU
Ward

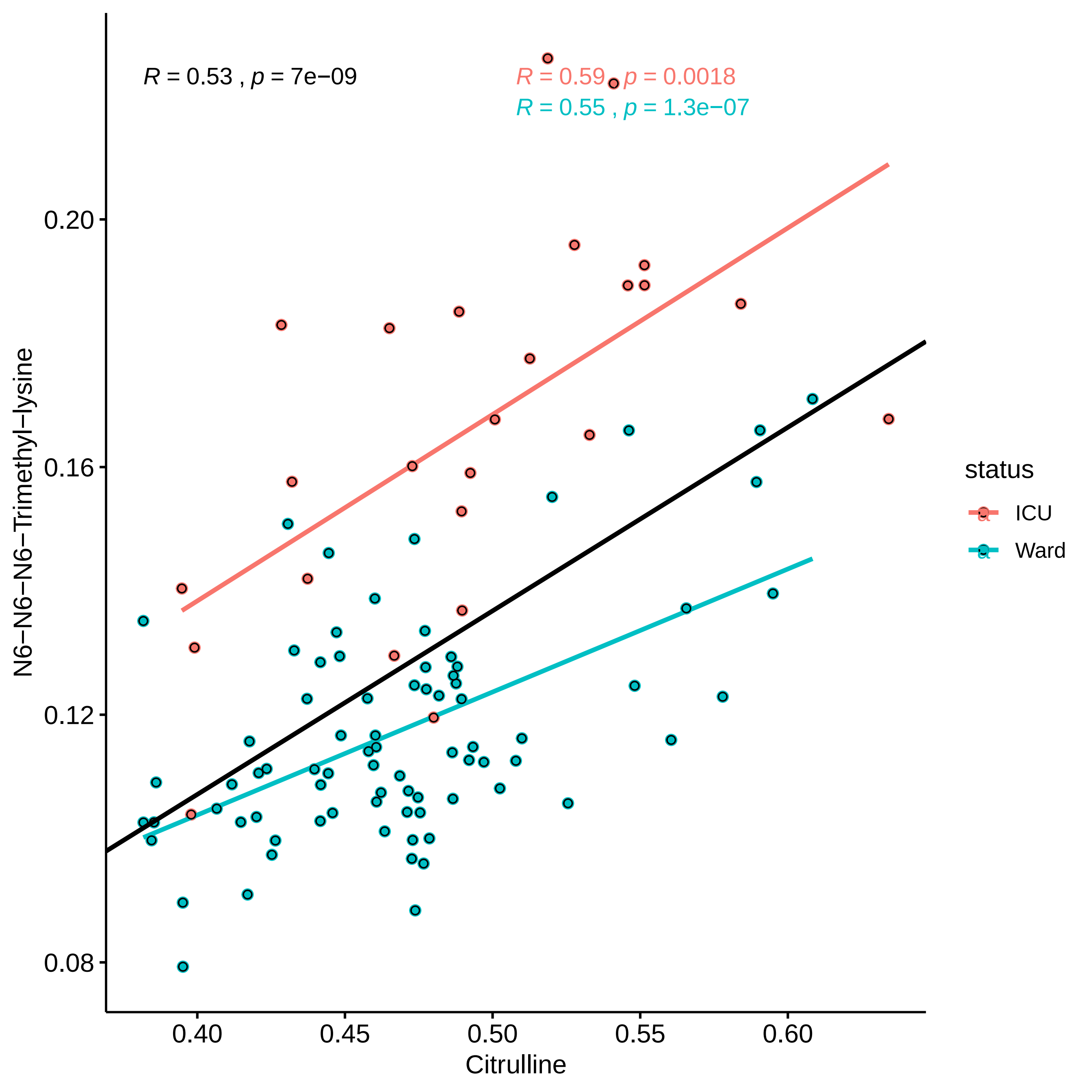

R = 0.53 , p = 7e−09
R = 0.59 , p = 0.0018
R = 0.55 , p = 1.3e−07
N6−N6−N6−Trimethyl−lysine
0.20
0.16
0.12
0.08
0.40
0.45
0.50
0.55
0.60
Citrulline
status
ICU
Ward

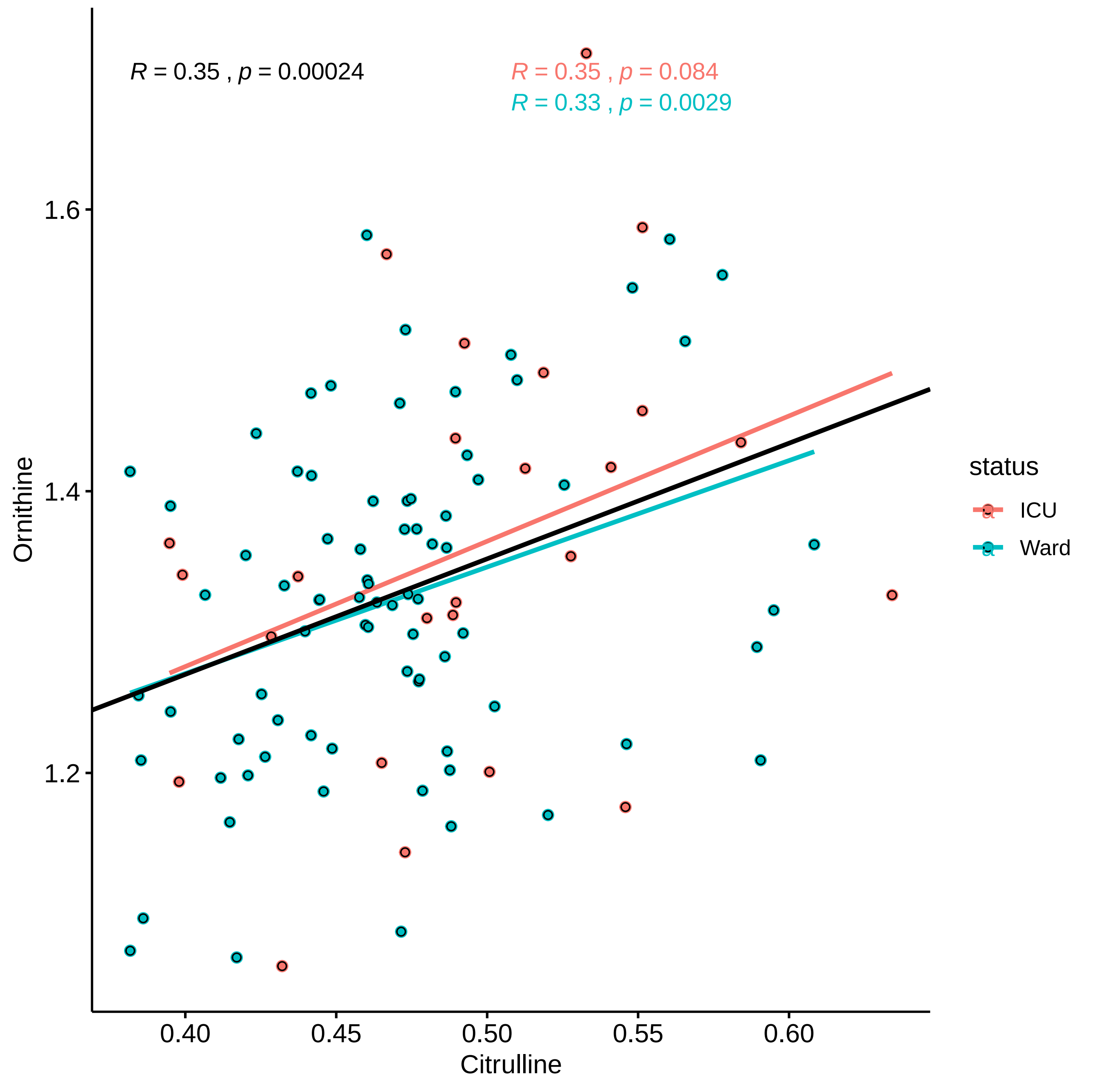
R = 0.35 , p = 0.00024
R = 0.35 , p = 0.084
R = 0.33 , p = 0.0029
1.6
1.4
1.2
Ornithine
0.40
0.45
0.50
0.55
0.60
Citrulline
status
ICU
Ward

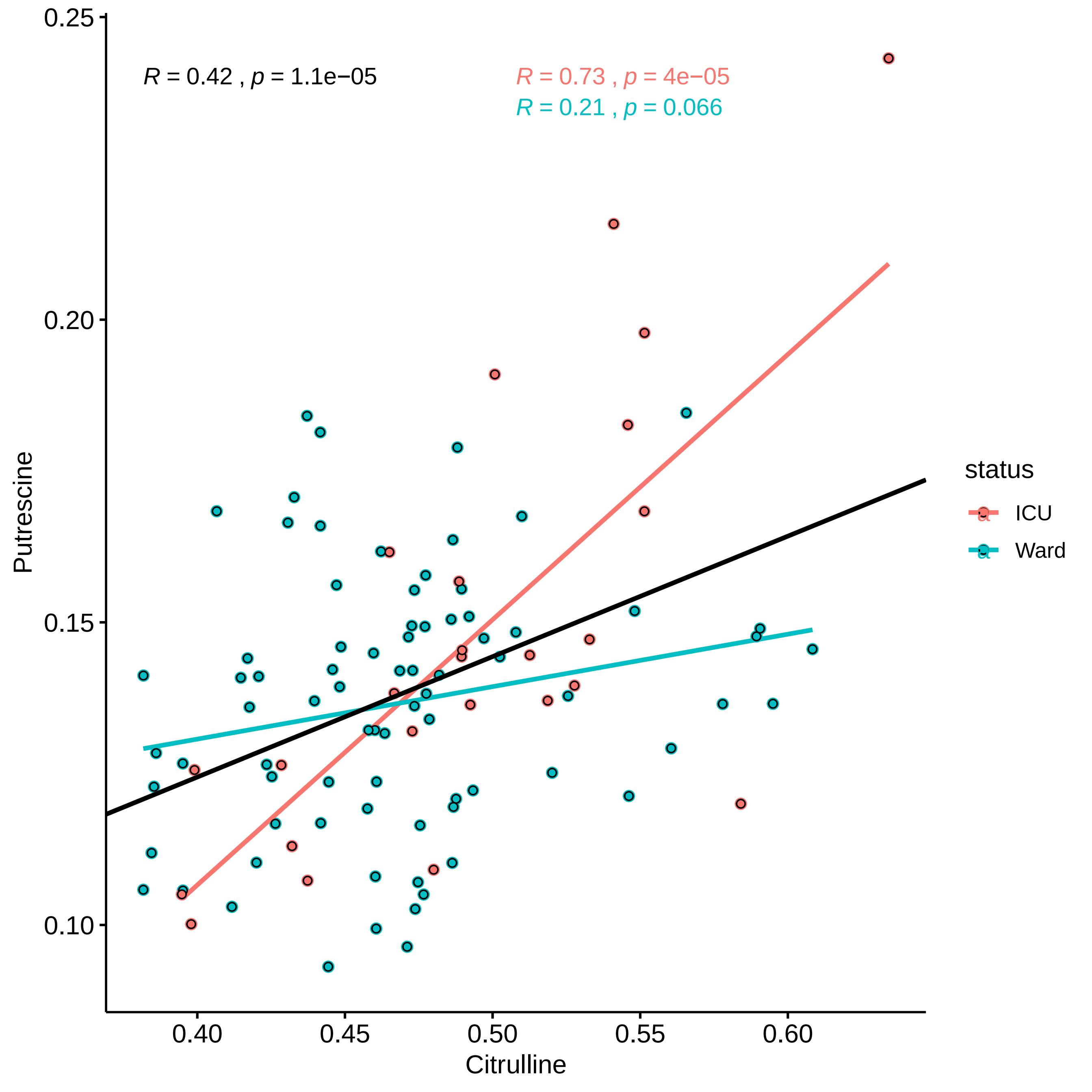

R = 0.42 , p = 1.1e−05
R = 0.73 , p = 4e−05
R = 0.21 , p = 0.066
0.25
0.20
0.15
0.10
Putrescine
0.40
0.45
0.50
0.55
0.60
Citrulline
status
ICU
Ward

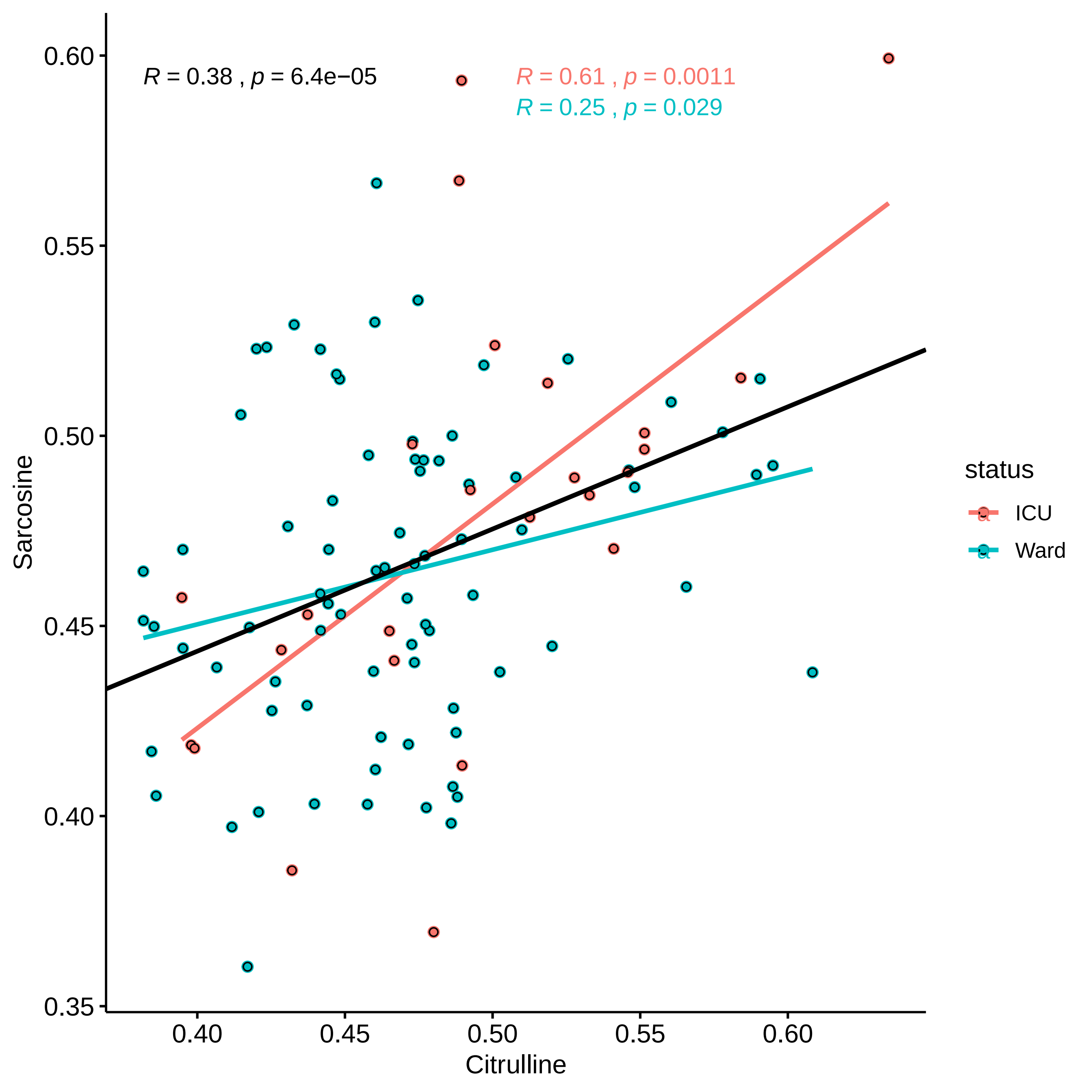

R = 0.38 , p = 6.4e−05
R = 0.61 , p = 0.0011
R = 0.25 , p = 0.029
Sarcosine
Citrulline
0.35
0.40
0.45
0.50
0.55
0.60
status
ICU
Ward

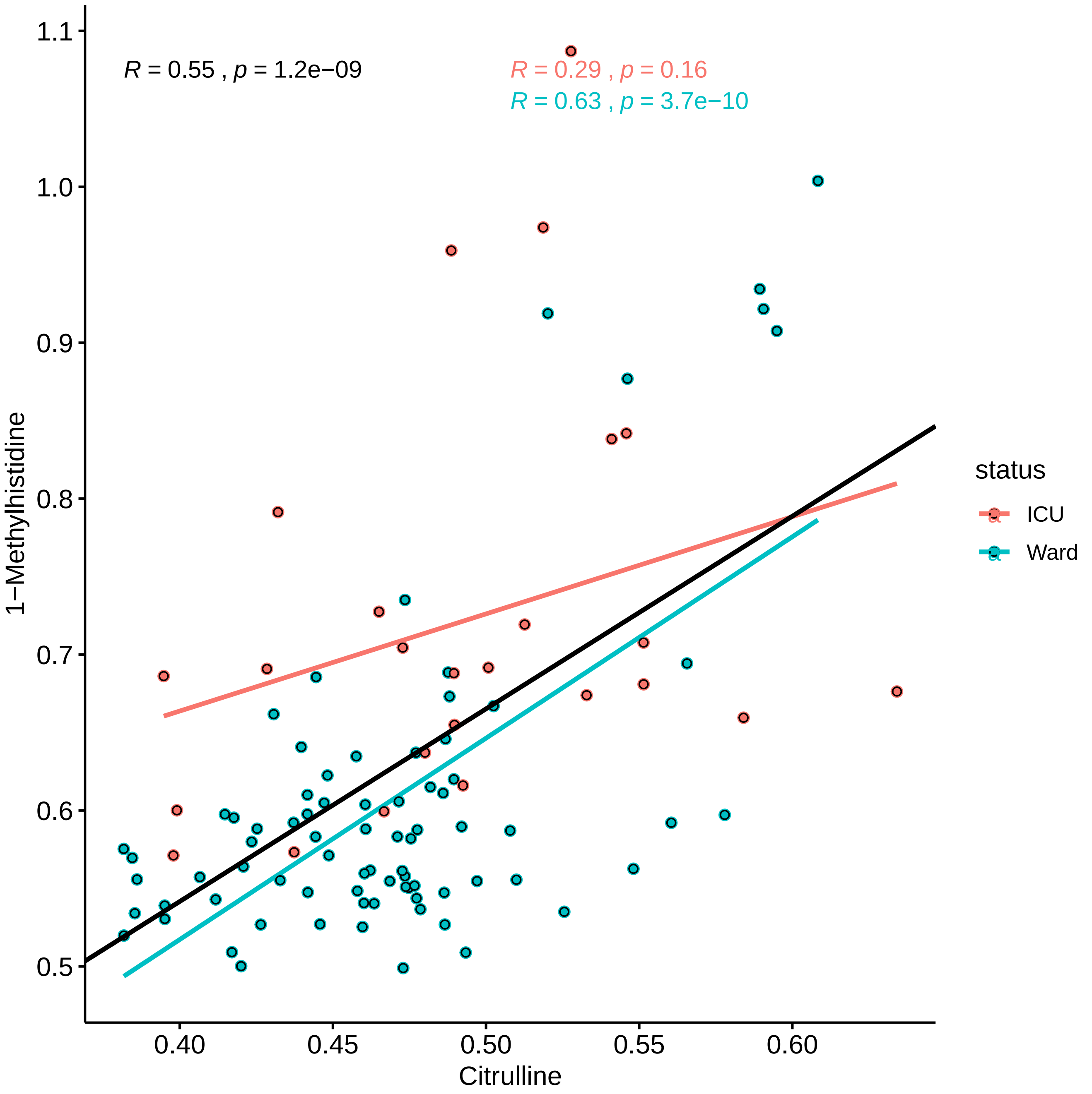

R = 0.55 , p = 1.2e−09
R = 0.29 , p = 0.16
R = 0.63 , p = 3.7e−10
1−Methylhistidine
1.1
1.0
0.9
0.8
0.7
0.6
0.5
0.40
0.45
0.50
0.55
0.60
Citrulline
status
ICU
Ward

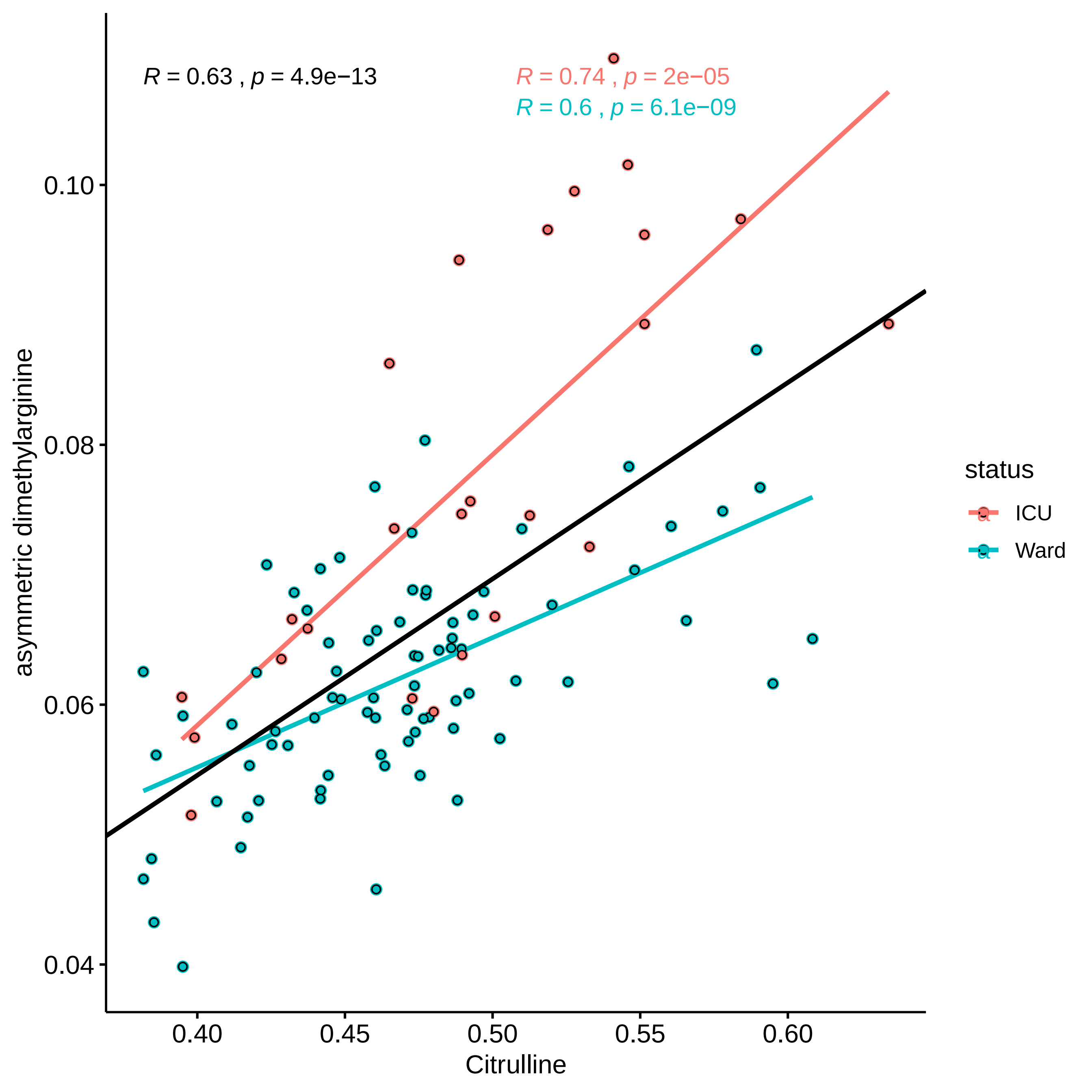
R = 0.63 , p = 4.9e−13
R = 0.74 , p = 2e−05
R = 0.6 , p = 6.1e−09
0.10
0.08
0.06
0.04
asymmetric dimethylarginine
0.40
0.45
0.50
0.55
0.60
Citrulline
status
ICU
Ward

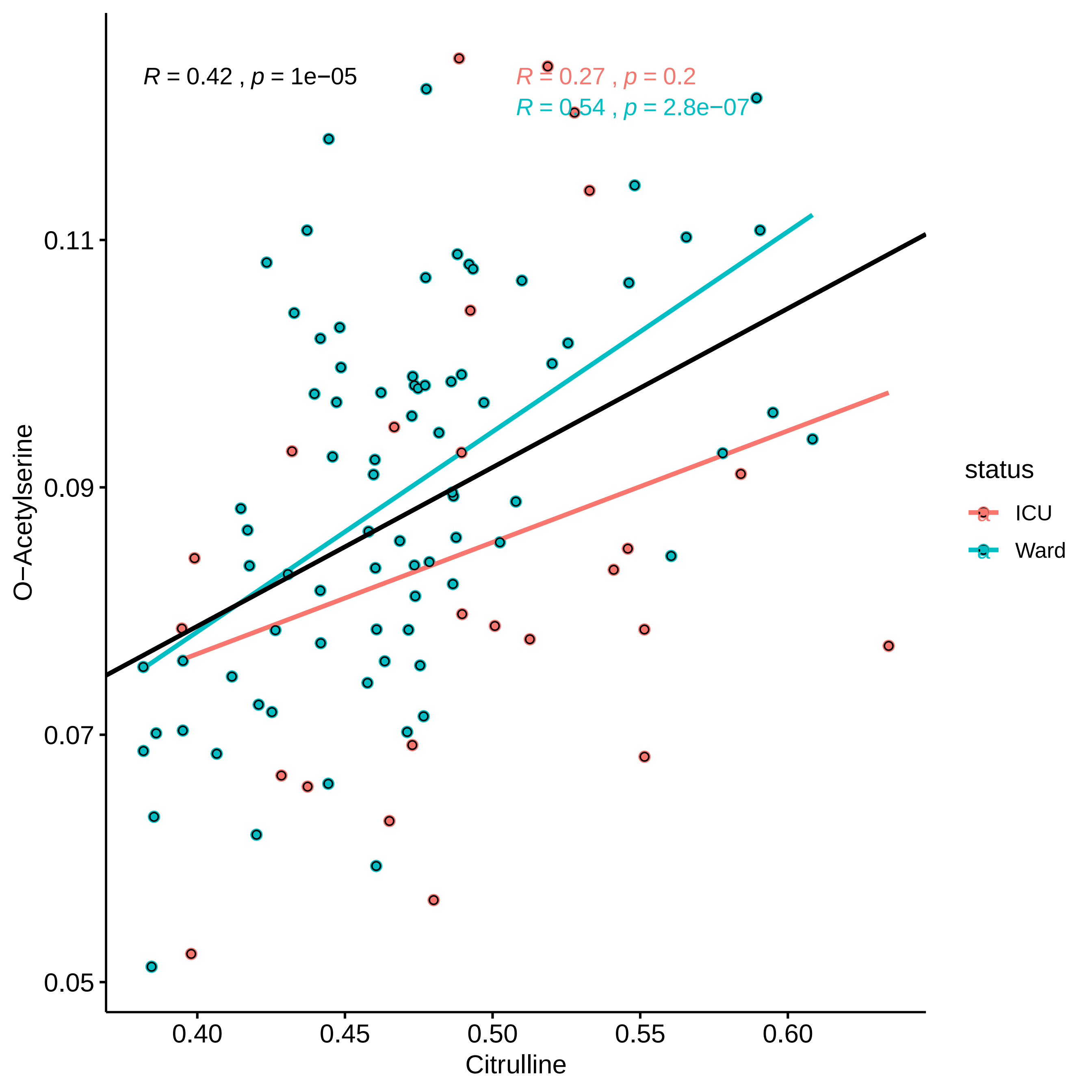

R = 0.42 , p = 1e−05
R = 0.27 , p = 0.2
R = 0.54 , p = 2.8e−07
O−Acetylserine
0.11
0.09
0.07
0.05
0.40
0.45
0.50
0.55
0.60
Citrulline
status
ICU
Ward

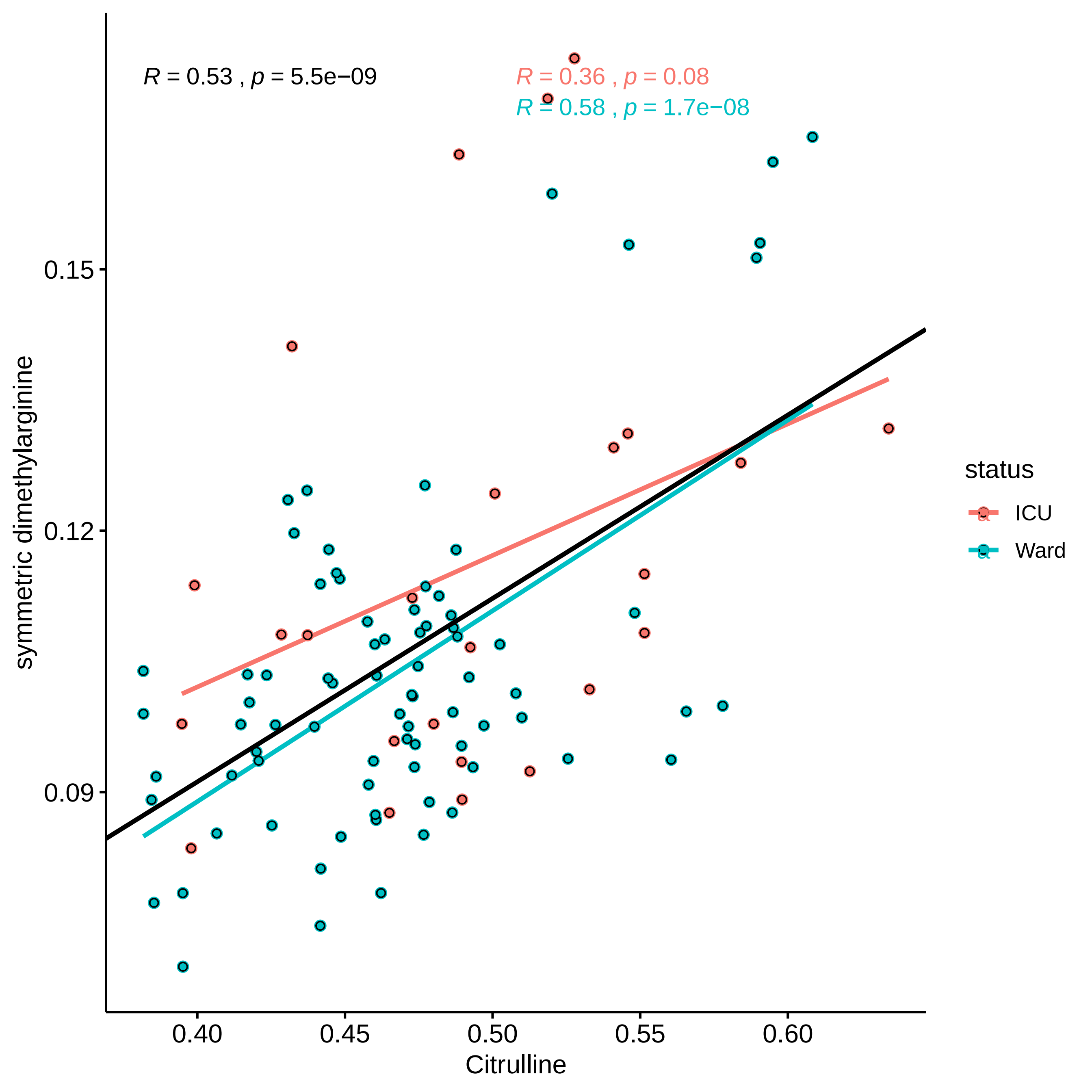

R = 0.53 , p = 5.5e−09
R = 0.36 , p = 0.08
R = 0.58 , p = 1.7e−08
symmetric dimethylarginine
0.15
0.12
0.09
0.40
0.45
0.50
0.55
0.60
Citrulline
status
ICU
Ward

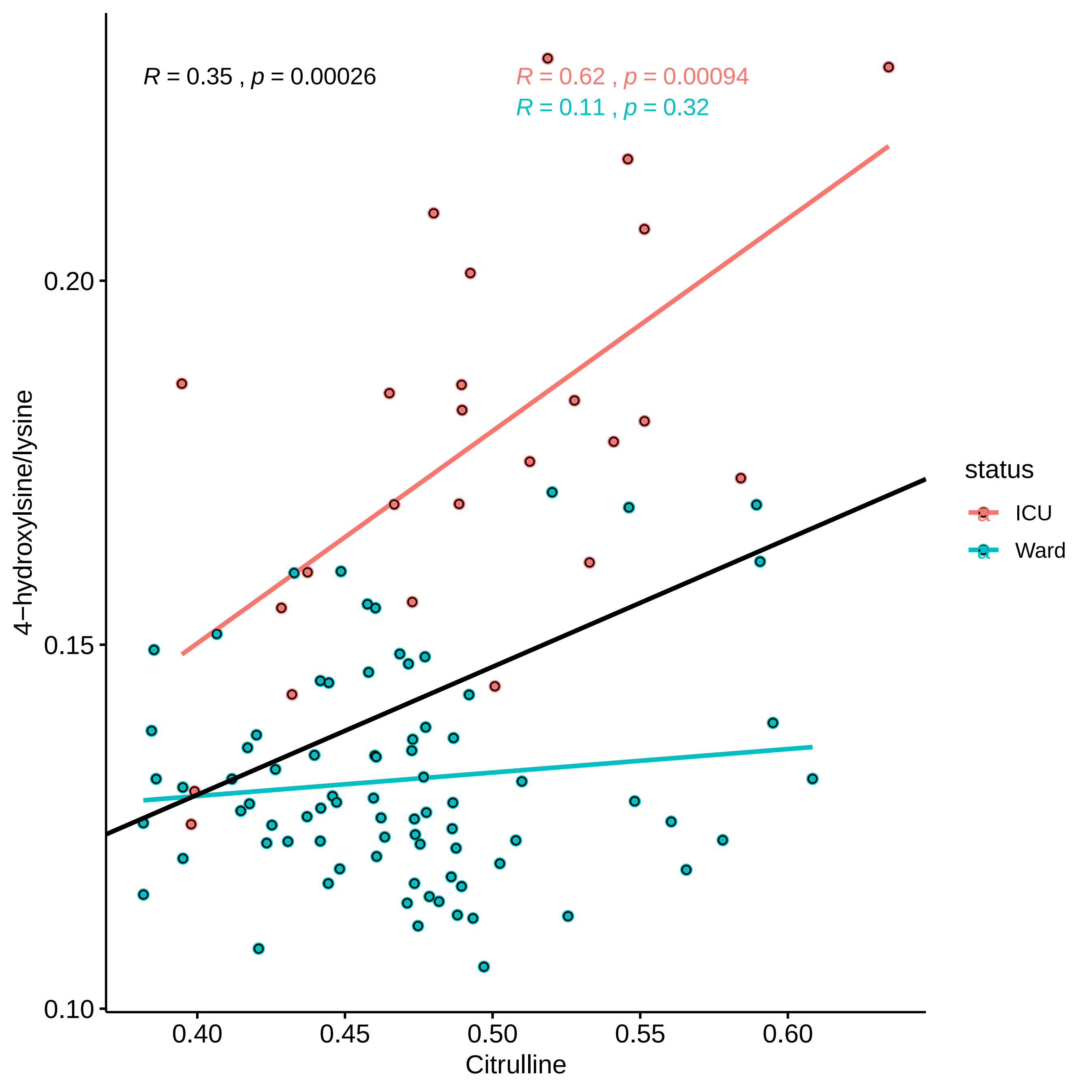

R = 0.35 , p = 0.00026
R = 0.62 , p = 0.00094
R = 0.11 , p = 0.32
4−hydroxylsine/lysine
0.20
0.15
0.10
0.40
0.45
0.50
0.55
0.60
Citrulline
status
ICU
Ward

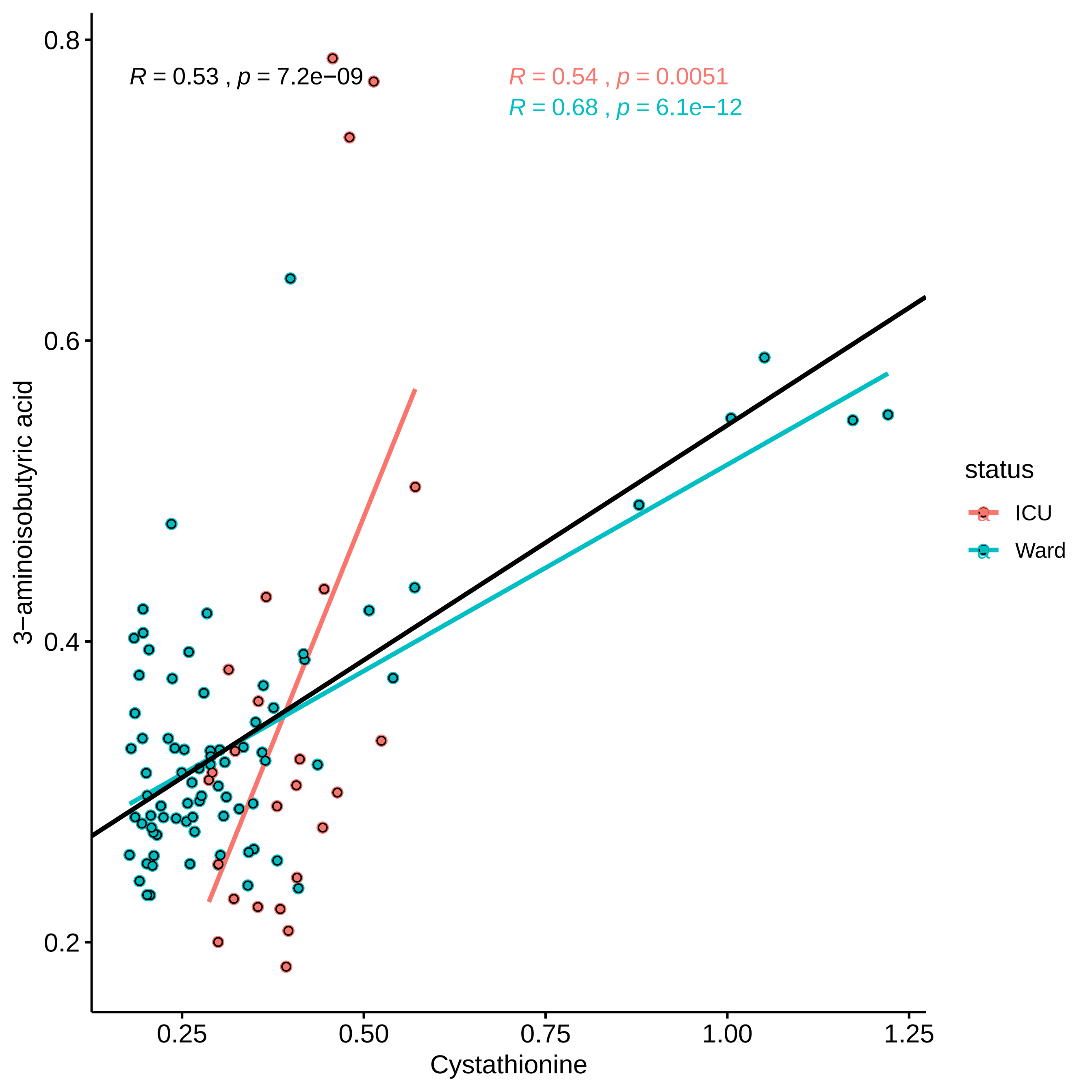

R = 0.53 , p = 7.2e−09
R = 0.54 , p = 0.0051
R = 0.68 , p = 6.1e−12
3−aminoisobutyric acid
0.8
0.6
0.4
0.2
0.25
0.50
0.75
1.00
1.25
Cystathionine
status
ICU
Ward

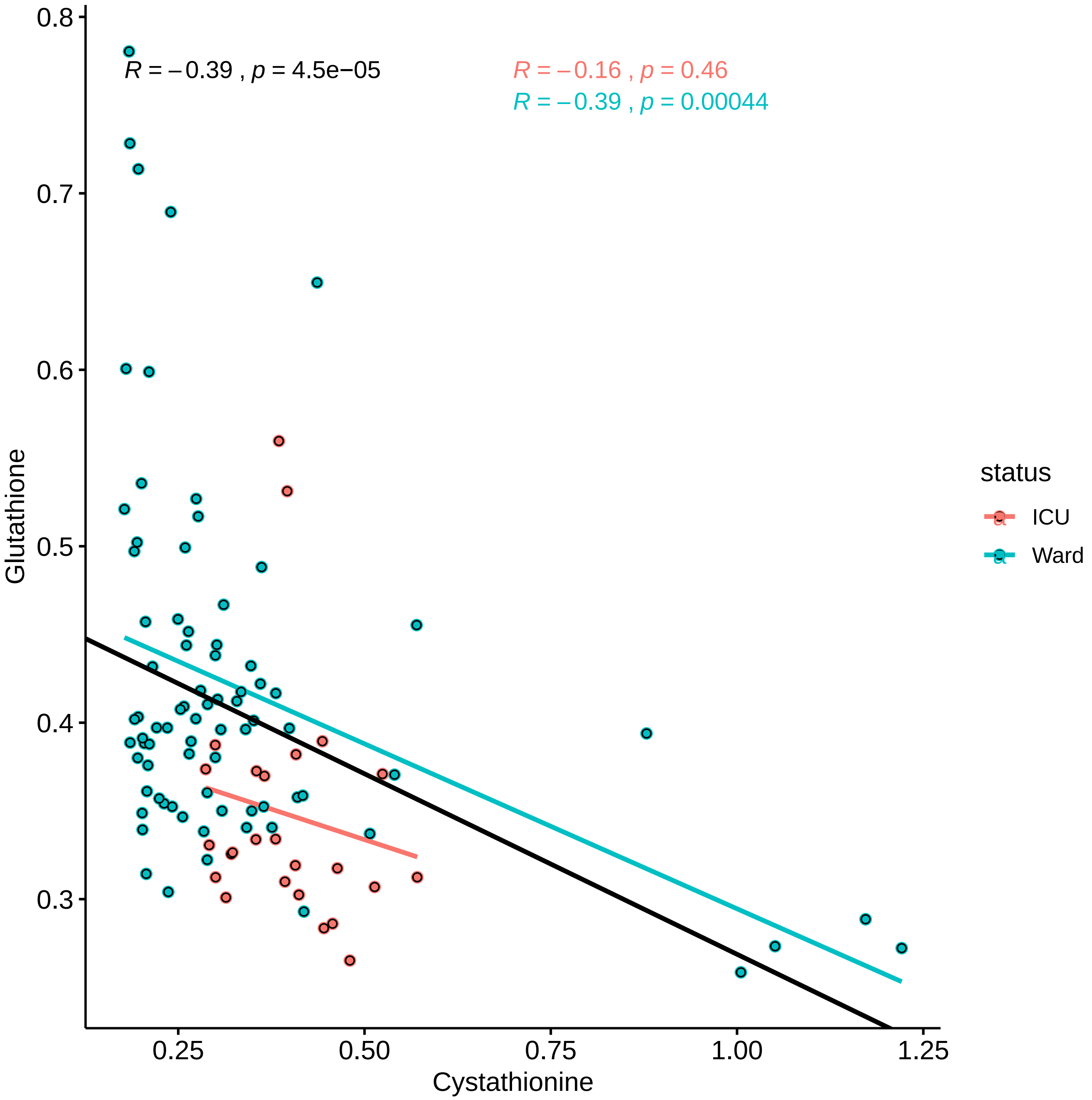

R = −0.39 , p = 4.5e−05
R = −0.16 , p = 0.46
R = −0.39 , p = 0.00044
Glutathione
Cystathionine
0.8
0.7
0.6
0.5
0.4
0.3
0.25
0.50
0.75
1.00
1.25
status
ICU
Ward

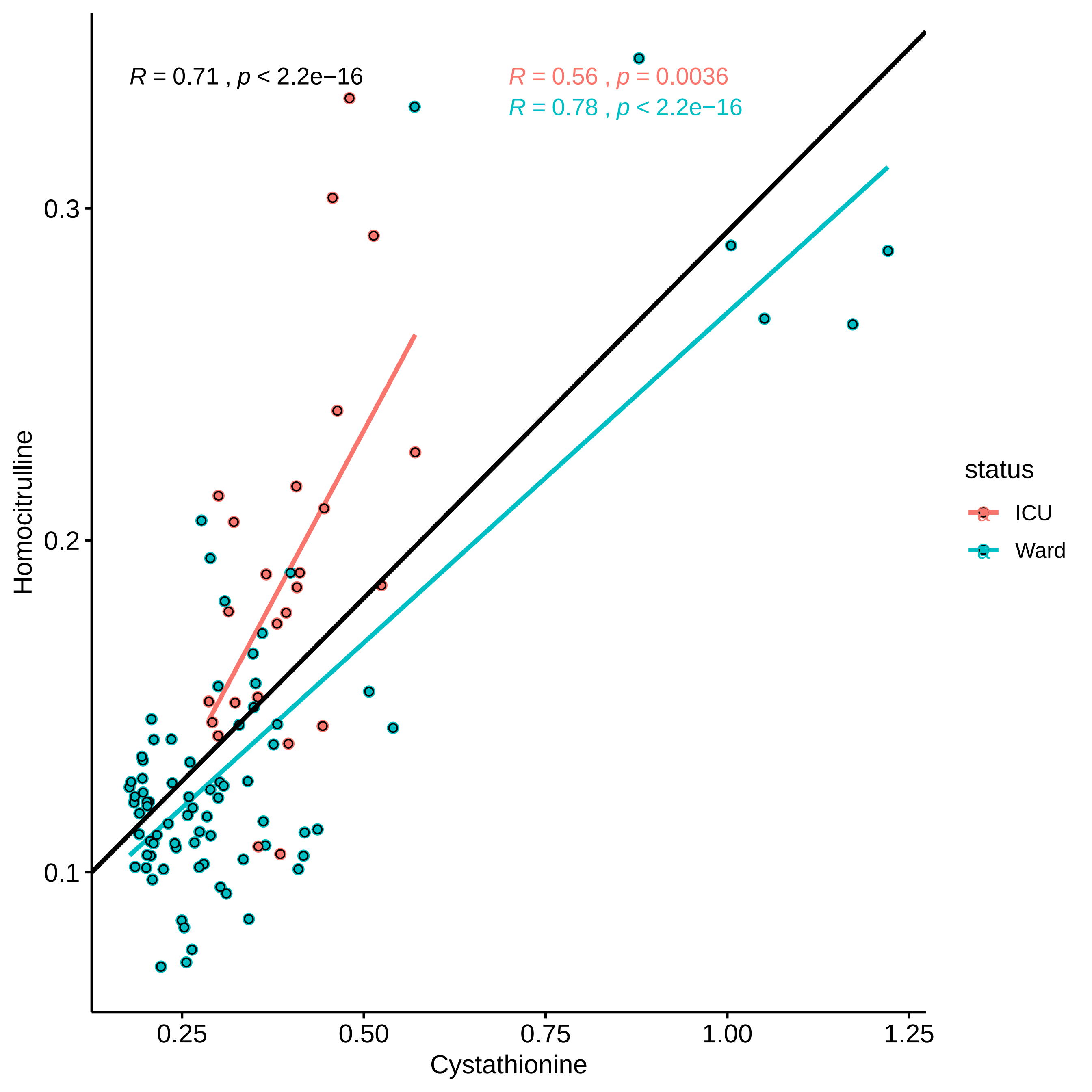

R = 0.71 , p < 2.2e−16
R = 0.56 , p = 0.0036
R = 0.78 , p < 2.2e−16
Homocitrulline
0.3
0.2
0.1
0.25
0.50
0.75
1.00
1.25
Cystathionine
status
ICU
Ward

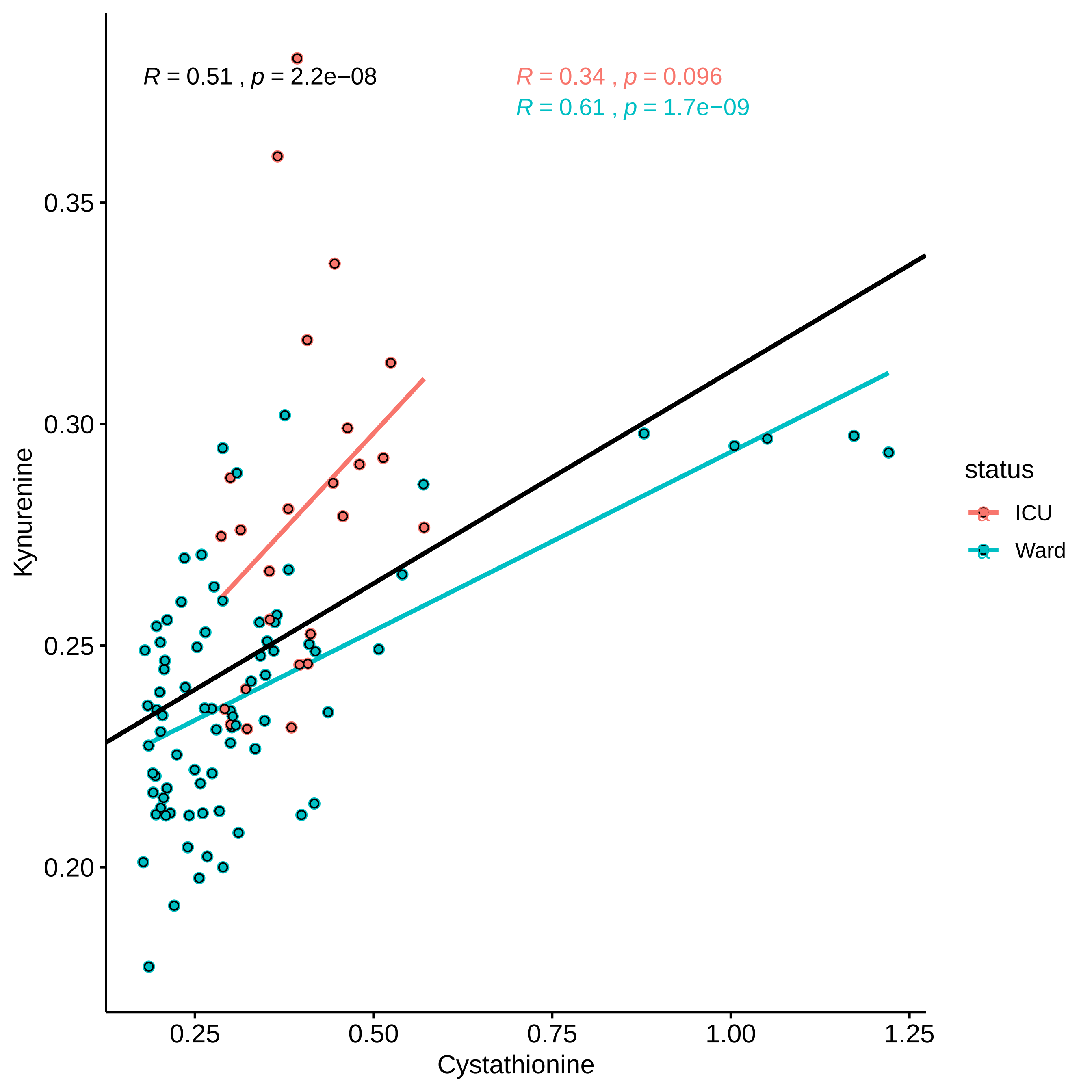

R = 0.51 , p = 2.2e−08
R = 0.34 , p = 0.096
R = 0.61 , p = 1.7e−09
0.35
0.30
0.25
0.20
Kynurenine
0.25
0.50
0.75
1.00
1.25
Cystathionine
status
ICU
Ward

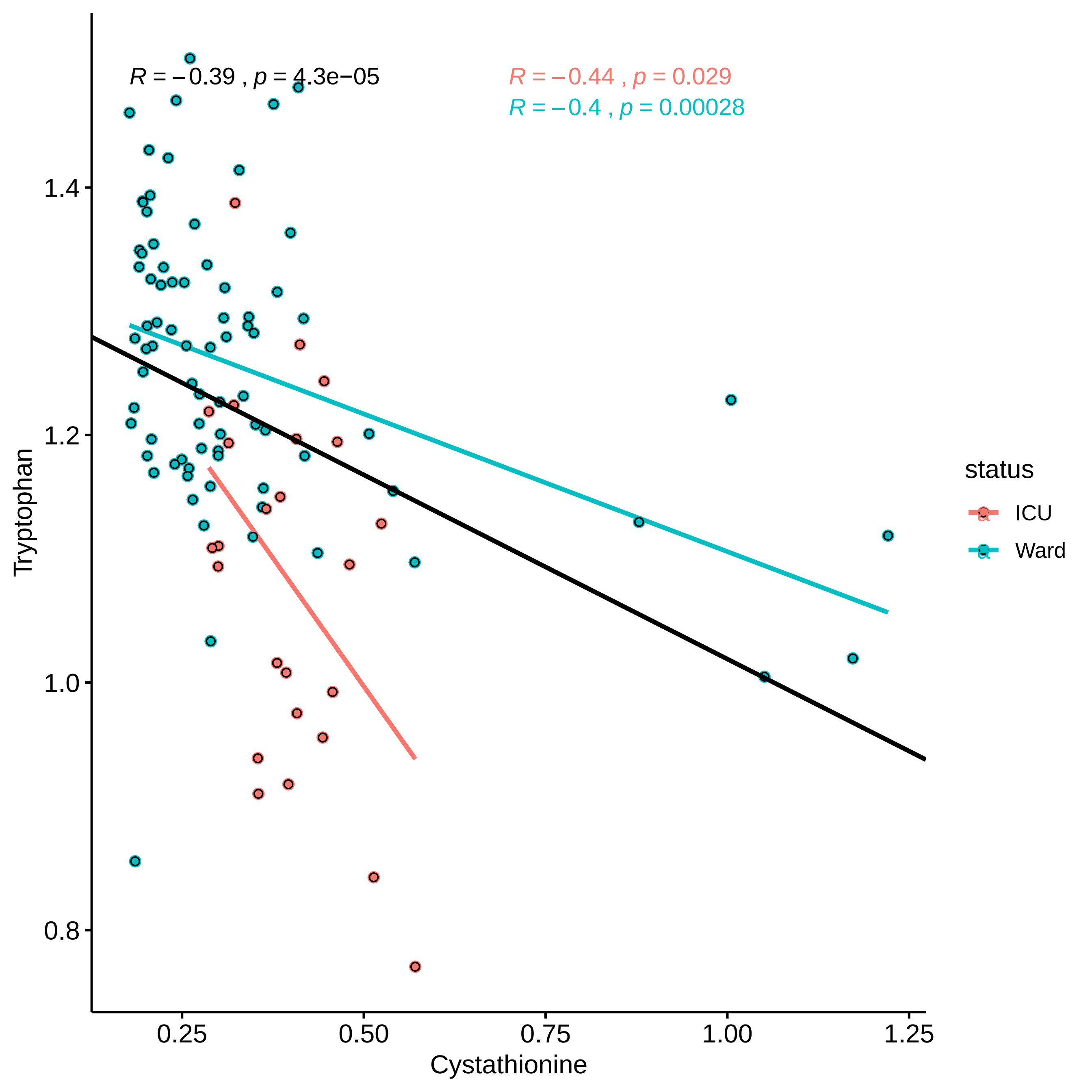

R = −0.39 , p = 4.3e−05
R = −0.44 , p = 0.029
R = −0.4 , p = 0.00028
Tryptophan
1.4
1.2
1.0
0.8
0.25
0.50
0.75
1.00
1.25
Cystathionine
status
ICU
Ward

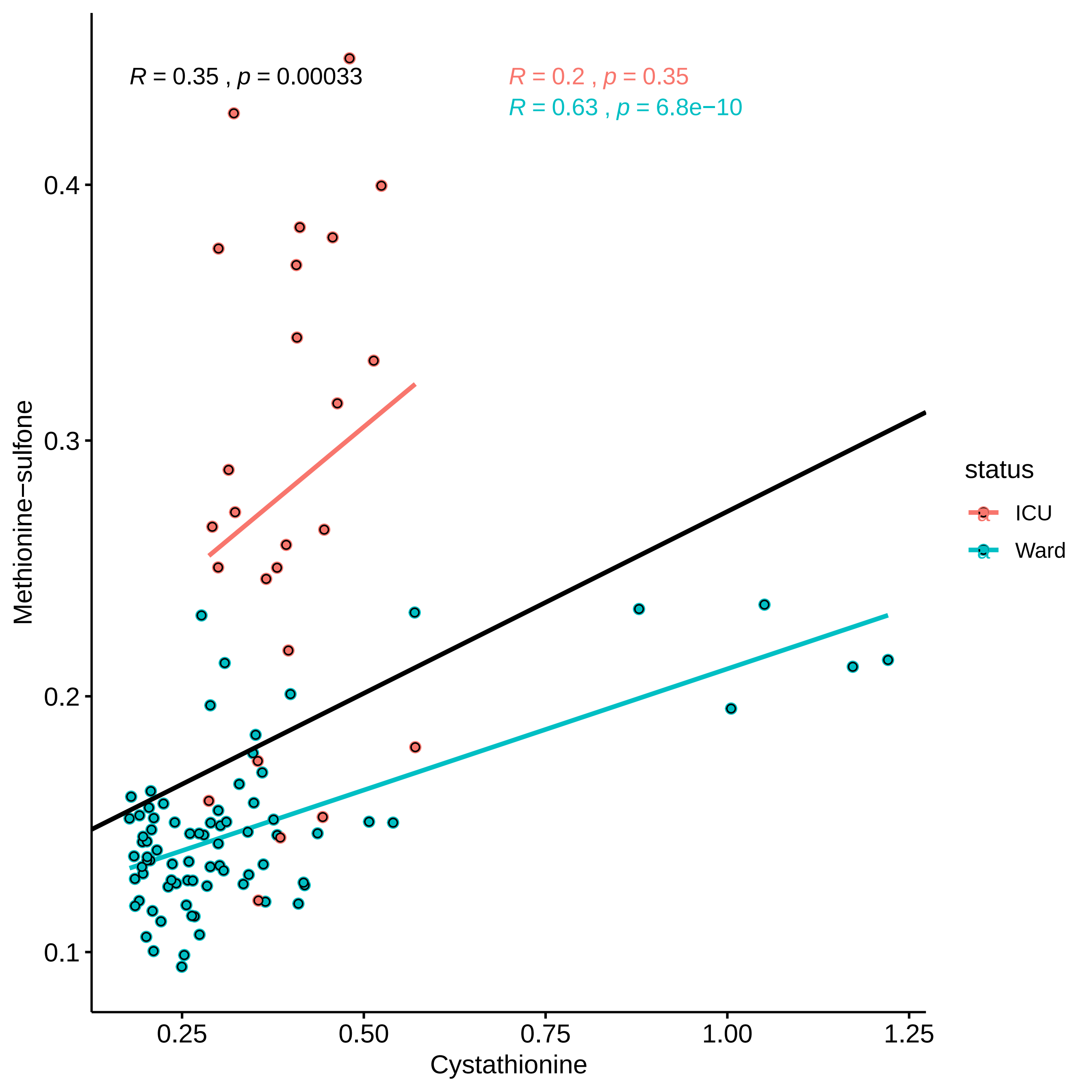

R = 0.35 , p = 0.00033
R = 0.2 , p = 0.35
R = 0.63 , p = 6.8e−10
Methionine−sulfone
0.4
0.3
0.2
0.1
Cystathionine
0.25
0.50
0.75
1.00
1.25
status
ICU
Ward

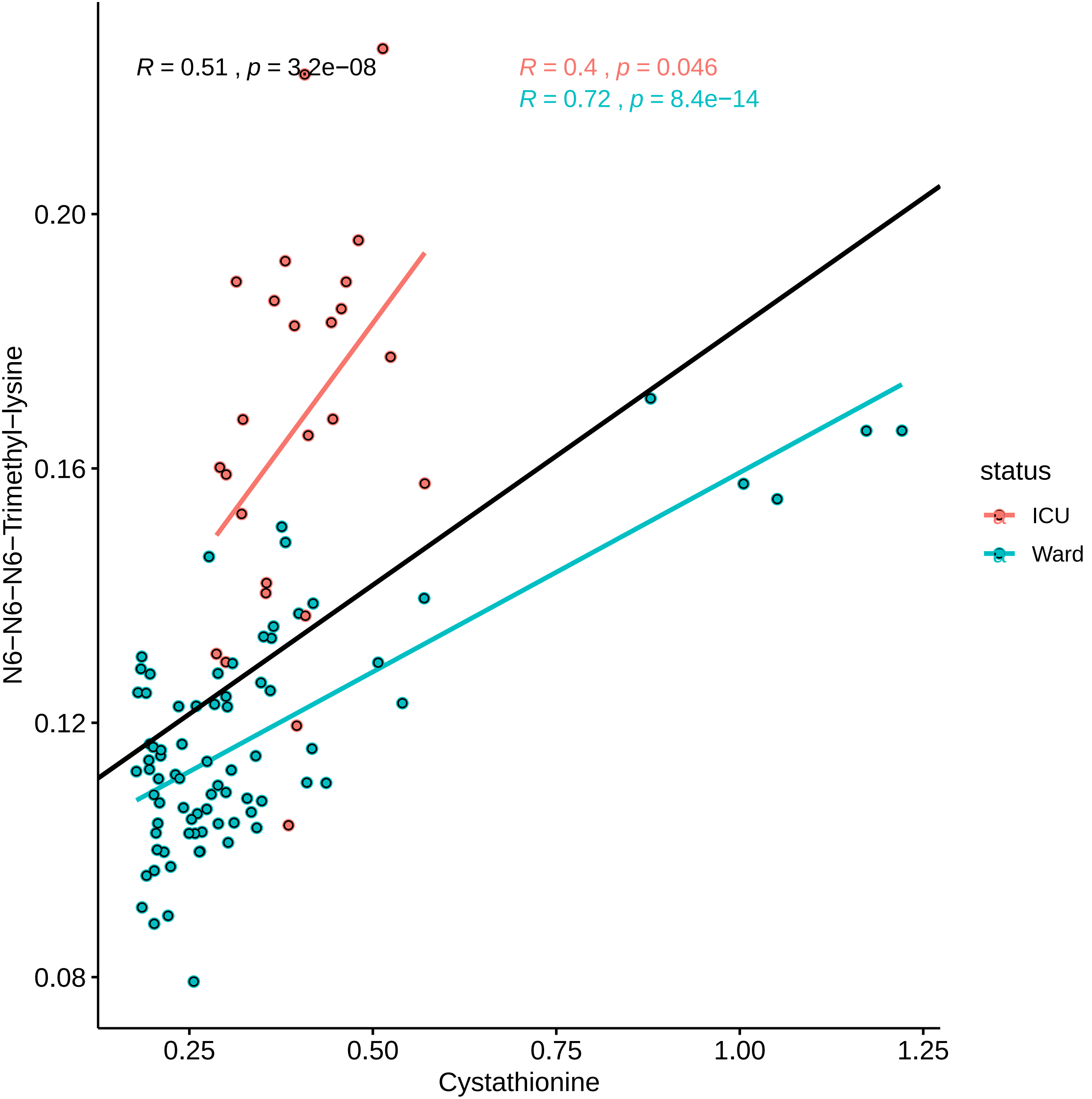

R = 0.51 , p = 3.2e−08
R = 0.4 , p = 0.046
R = 0.72 , p = 8.4e−14
N6−N6−N6−Trimethyl−lysine
0.20
0.16
0.12
0.08
0.25
0.50
0.75
1.00
1.25
Cystathionine
status
ICU
Ward

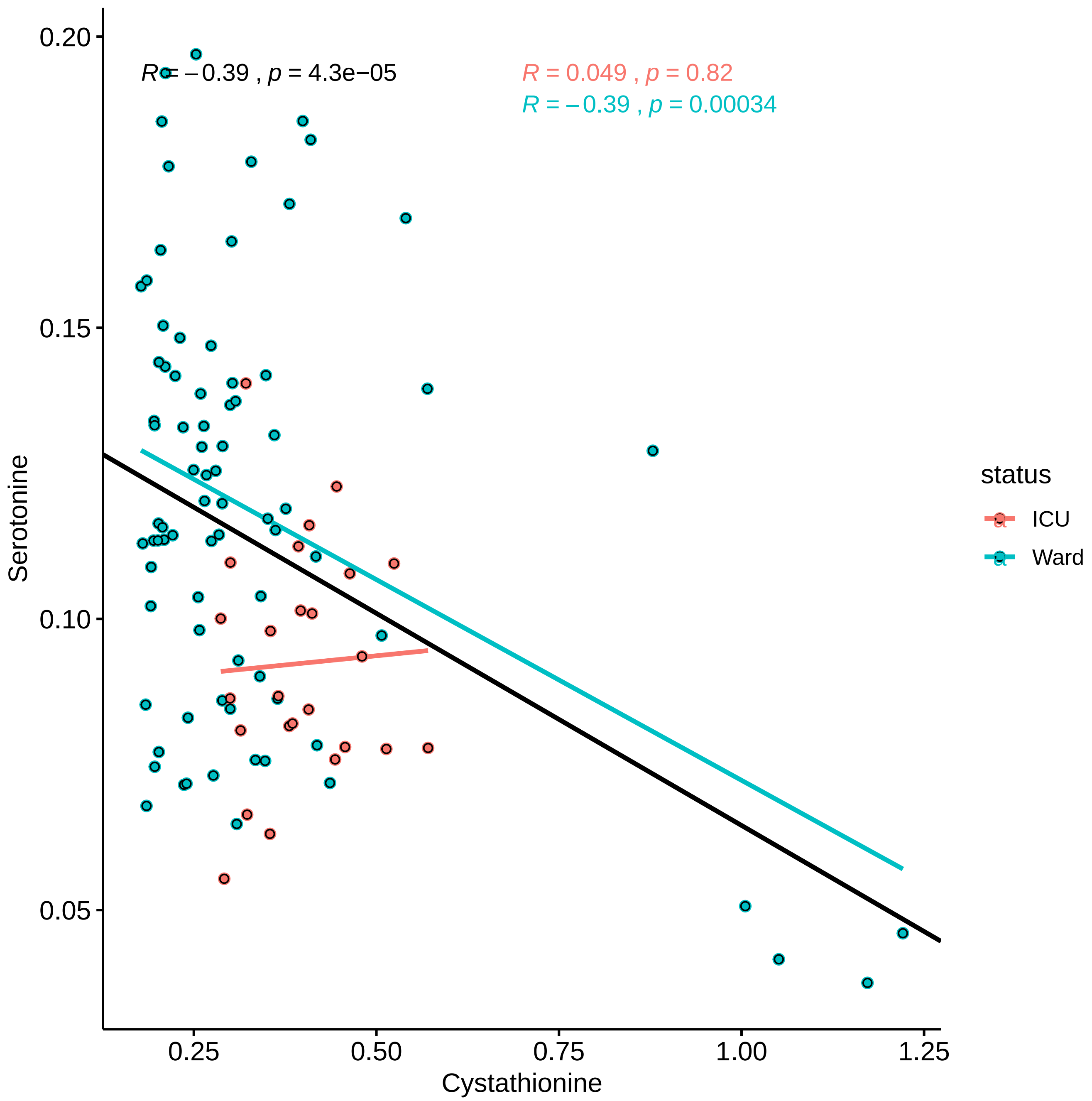
R = −0.39 , p = 4.3e−05
R = 0.049 , p = 0.82
R = −0.39 , p = 0.00034
Serotonine
0.20
0.15
0.10
0.05
0.25
0.50
0.75
1.00
1.25
Cystathionine
status
ICU
Ward

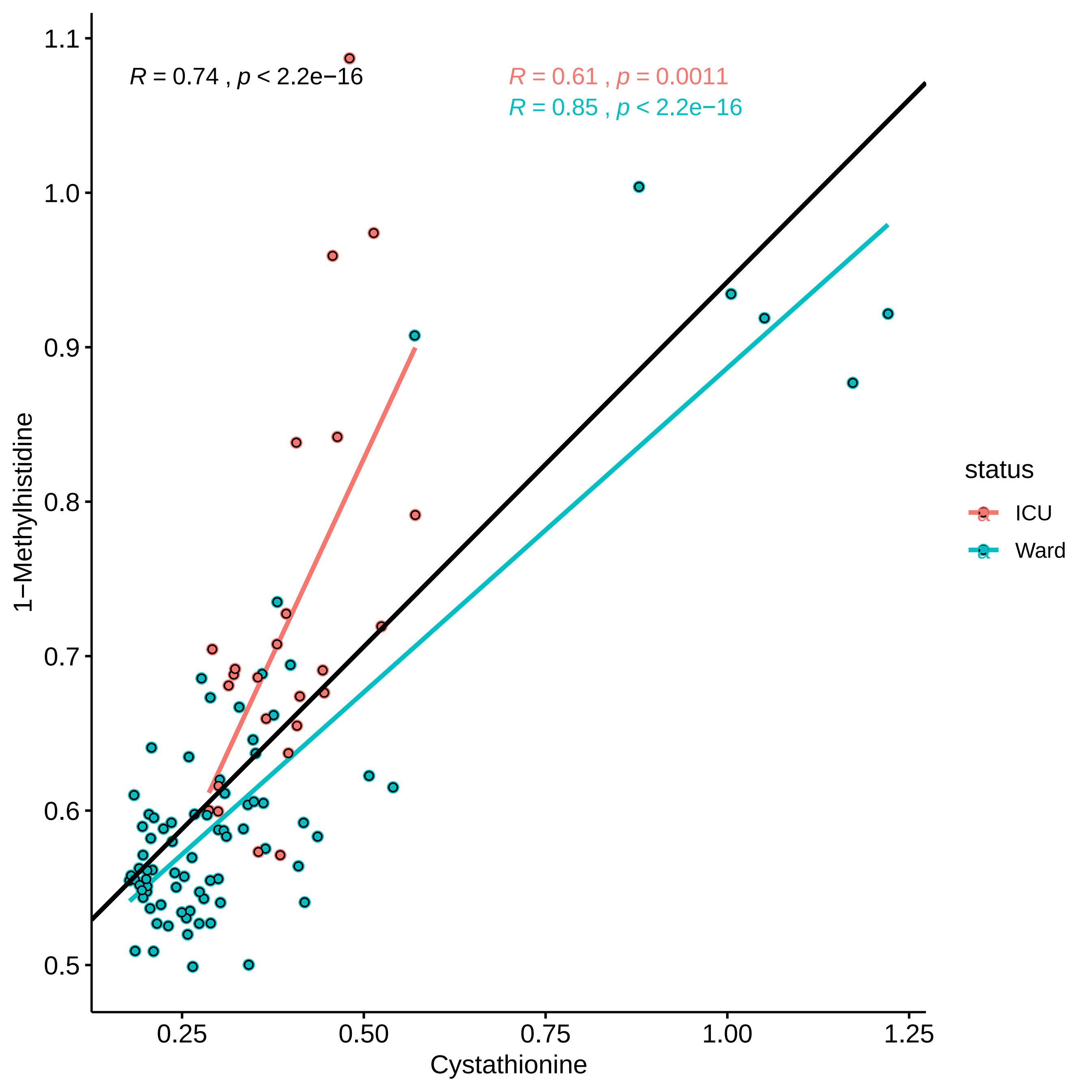

R = 0.74 , p < 2.2e−16
R = 0.61 , p = 0.0011
R = 0.85 , p < 2.2e−16
1−Methylhistidine
1.1
1.0
0.9
0.8
0.7
0.6
0.5
0.25
0.50
0.75
1.00
1.25
Cystathionine
status
ICU
Ward

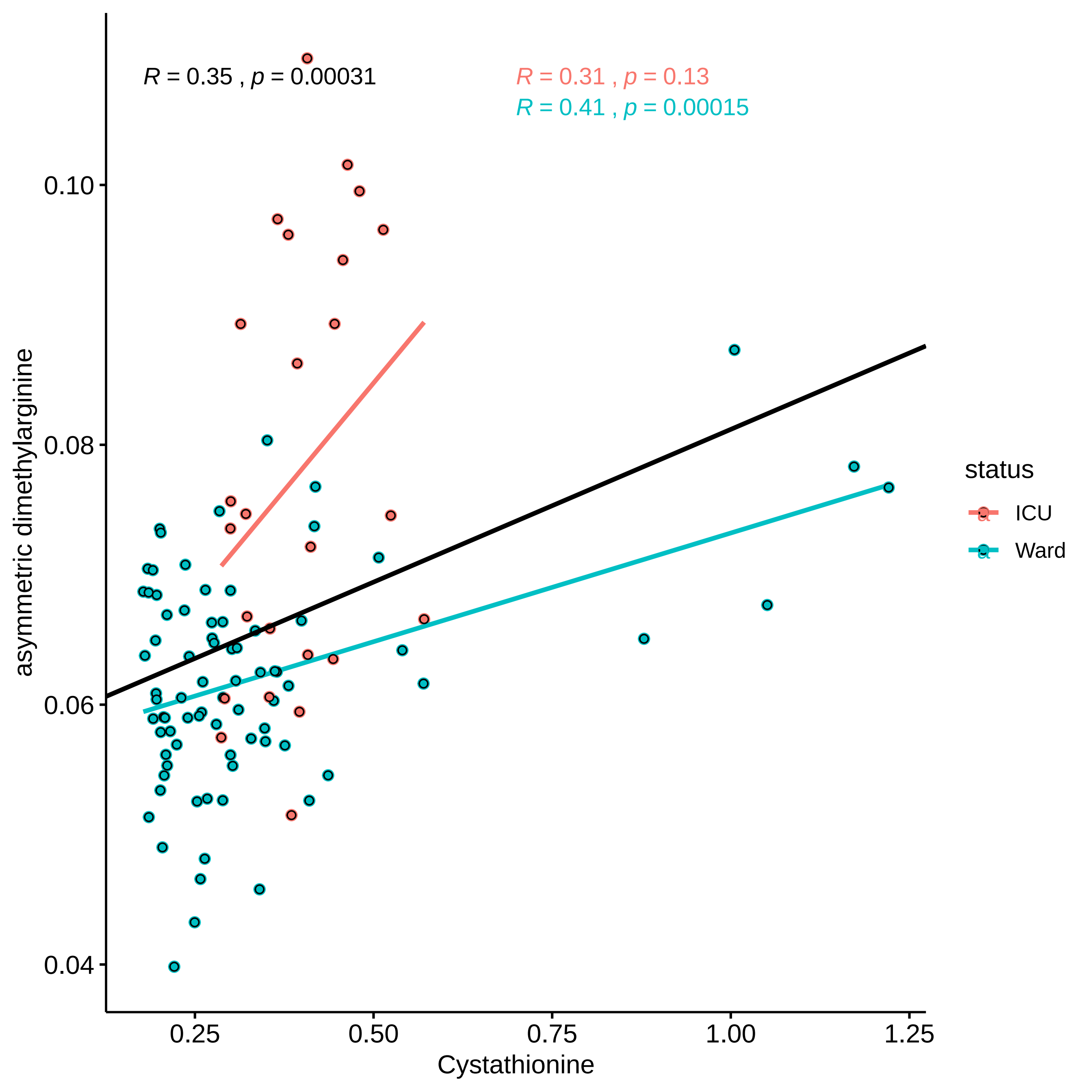

R = 0.35 , p = 0.00031
R = 0.31 , p = 0.13
R = 0.41 , p = 0.00015
0.10
0.08
0.06
0.04
asymmetric dimethylarginine
0.25
0.50
0.75
1.00
1.25
Cystathionine
status
ICU
Ward

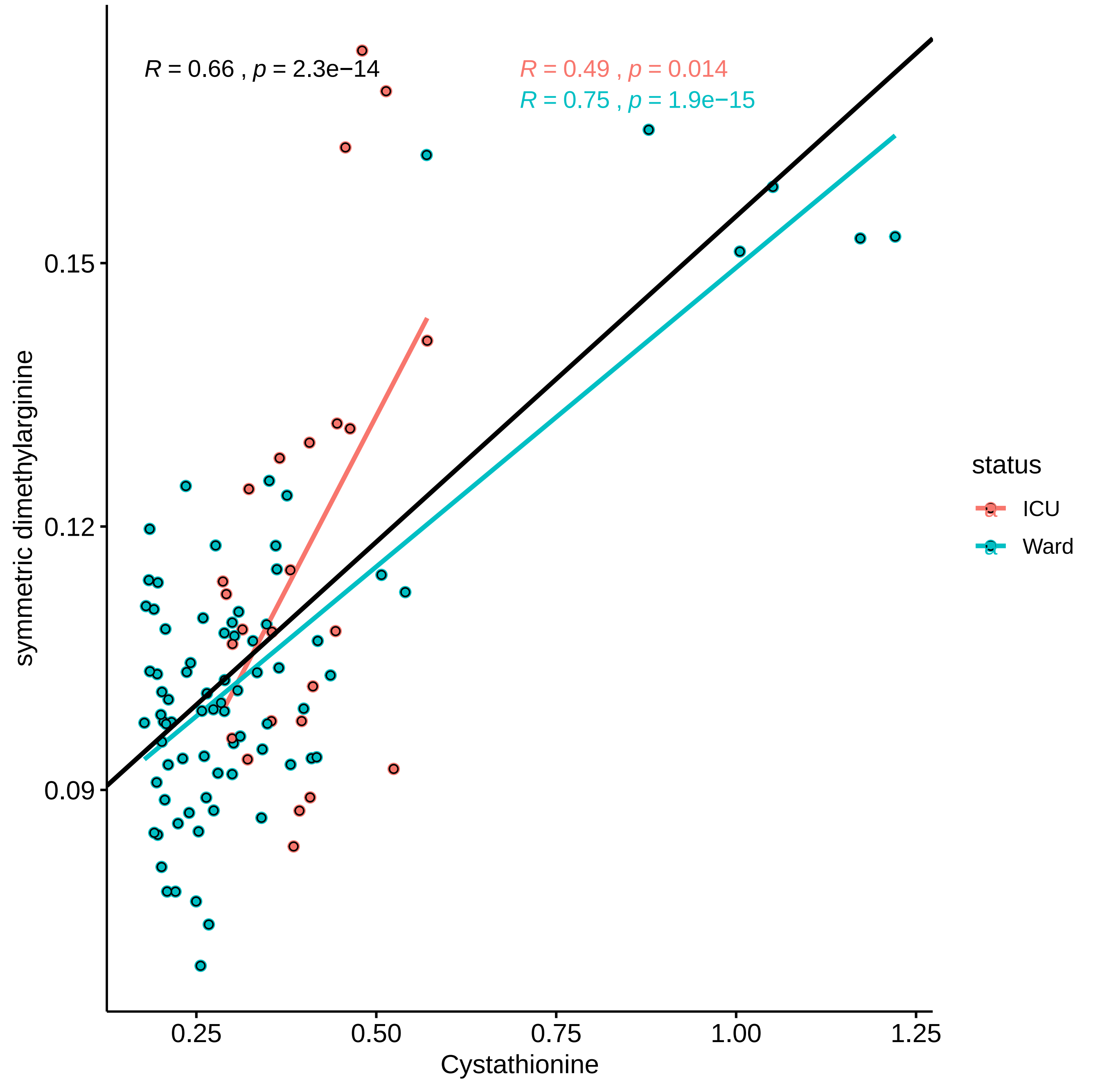

R = 0.66 , p = 2.3e−14
R = 0.49 , p = 0.014
R = 0.75 , p = 1.9e−15
symmetric dimethylarginine
0.15
0.12
0.09
Cystathionine
0.25
0.50
0.75
1.00
1.25
status
ICU
Ward

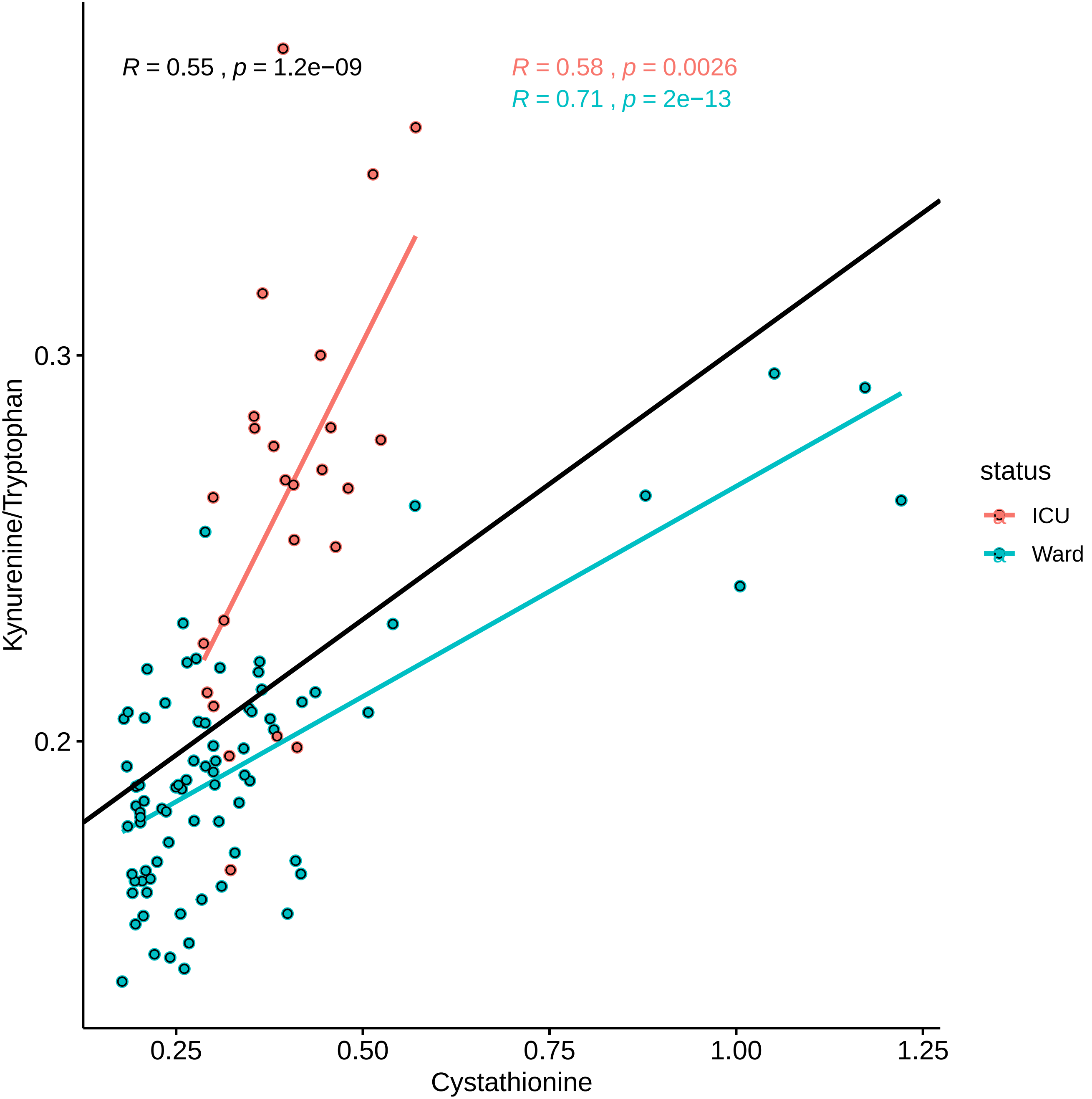

R = 0.55 , p = 1.2e−09
R = 0.58 , p = 0.0026
R = 0.71 , p = 2e−13
0.3
0.2
Kynurenine/Tryptophan
0.25
0.50
0.75
1.00
1.25
Cystathionine
status
ICU
Ward

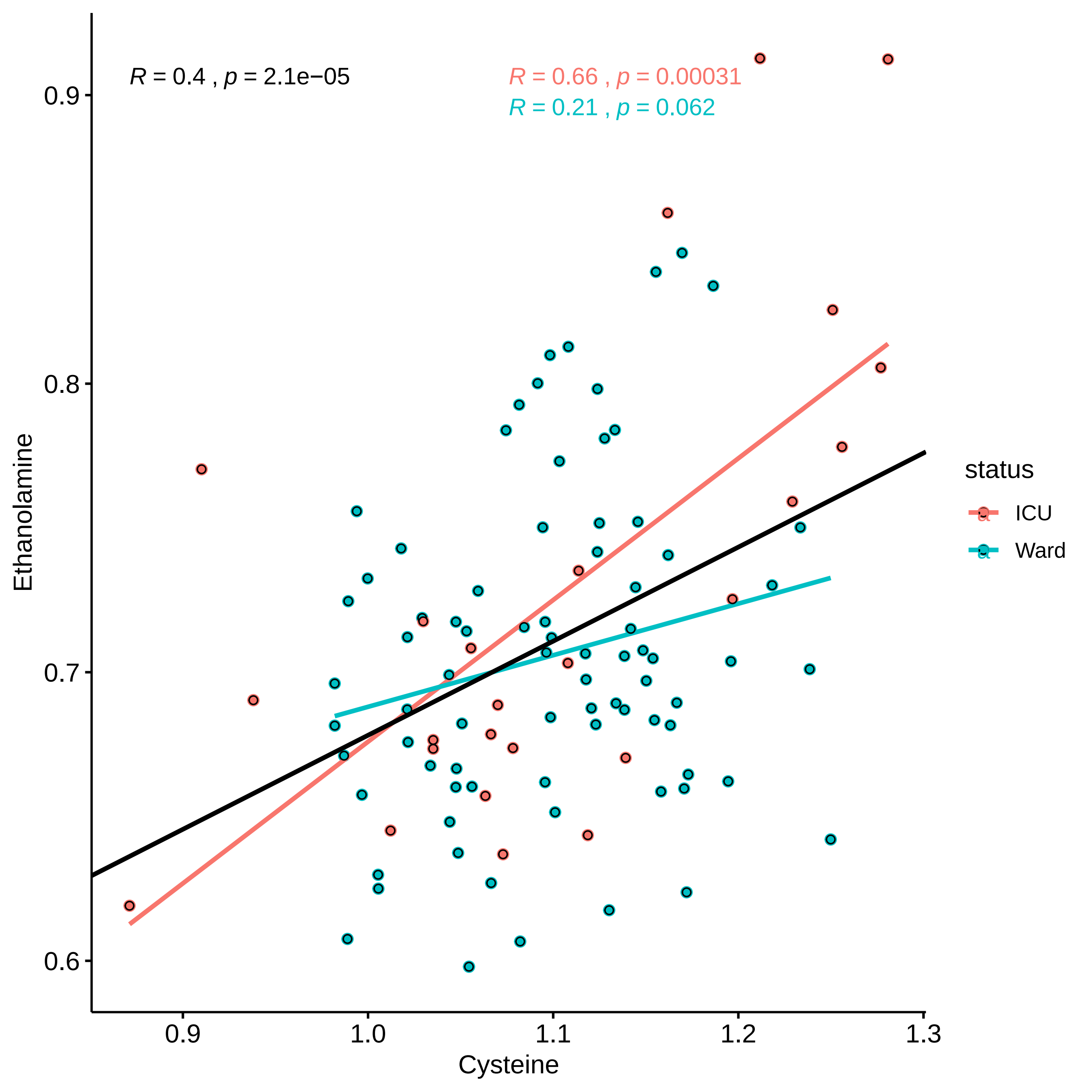

R = 0.4 , p = 2.1e−05
R = 0.66 , p = 0.00031
R = 0.21 , p = 0.062
Ethanolamine
0.9
0.8
0.7
0.6
0.9
1.0
1.1
1.2
1.3
Cysteine
status
ICU
Ward

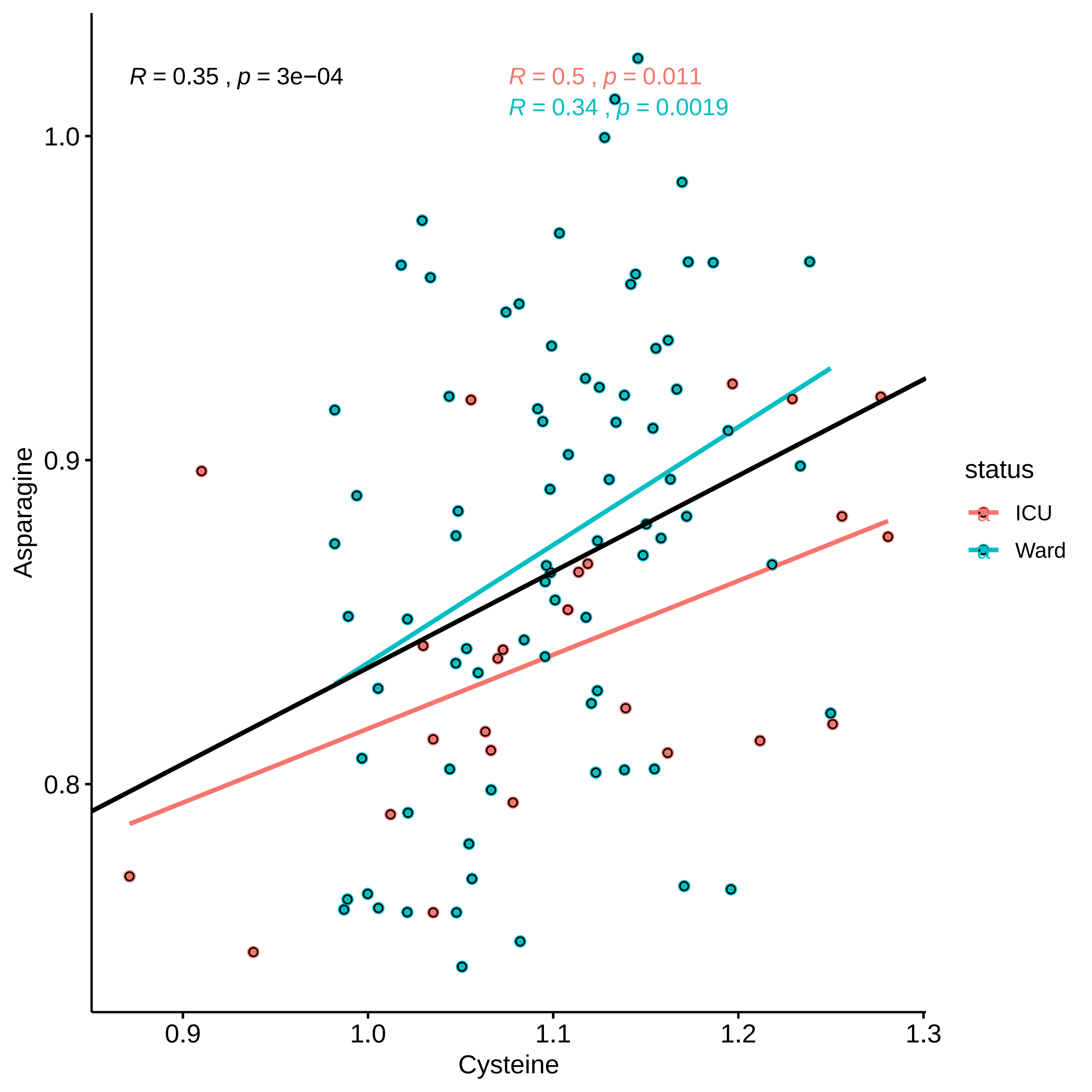

R = 0.35 , p = 3e−04
R = 0.5 , p = 0.011
R = 0.34 , p = 0.0019
1.0
0.9
0.8
Asparagine
0.9
1.0
1.1
1.2
1.3
Cysteine
status
ICU
Ward

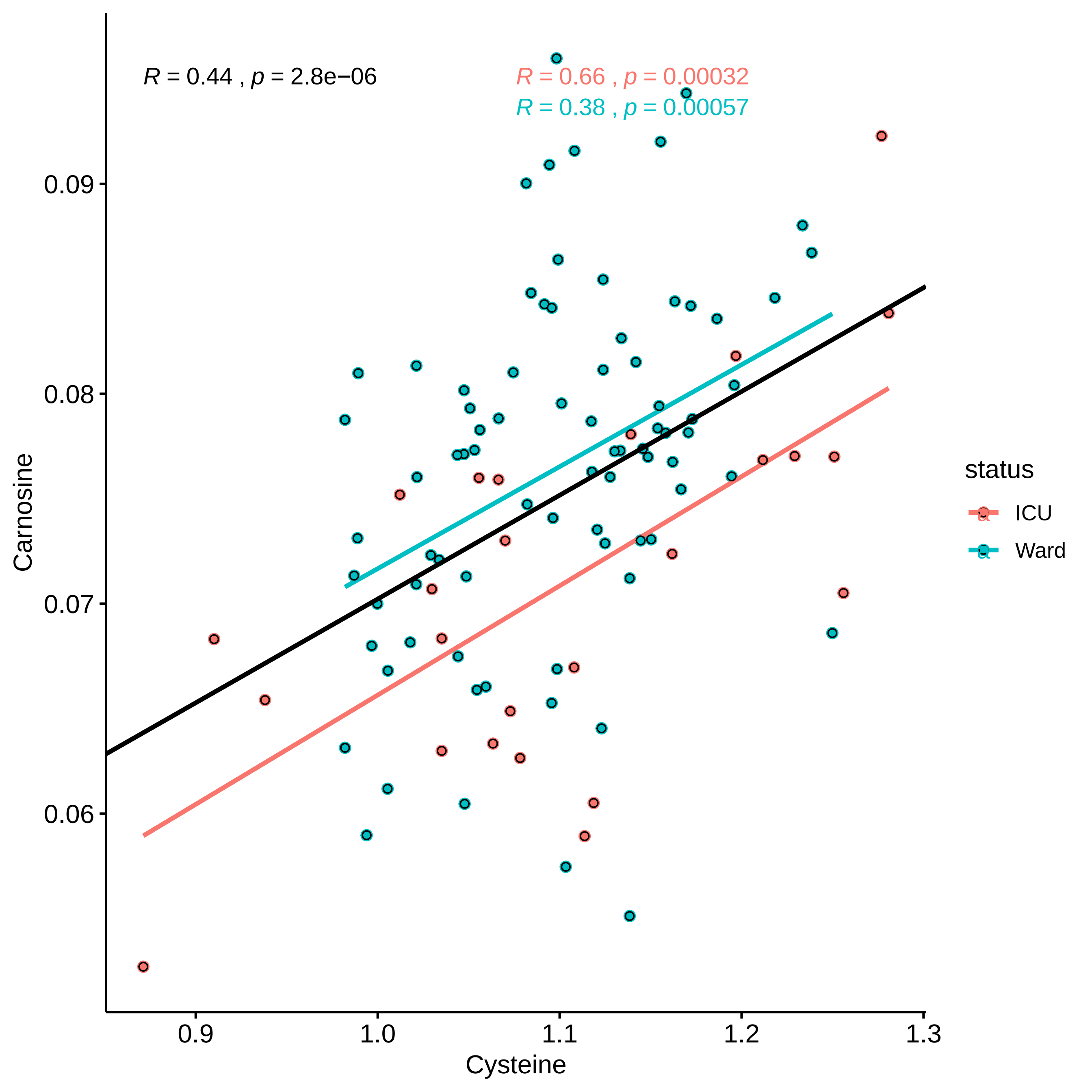

R = 0.44 , p = 2.8e−06
R = 0.66 , p = 0.00032
R = 0.38 , p = 0.00057
0.09
0.08
0.07
0.06
Carnosine
0.9
1.0
1.1
1.2
1.3
Cysteine
status
ICU
Ward

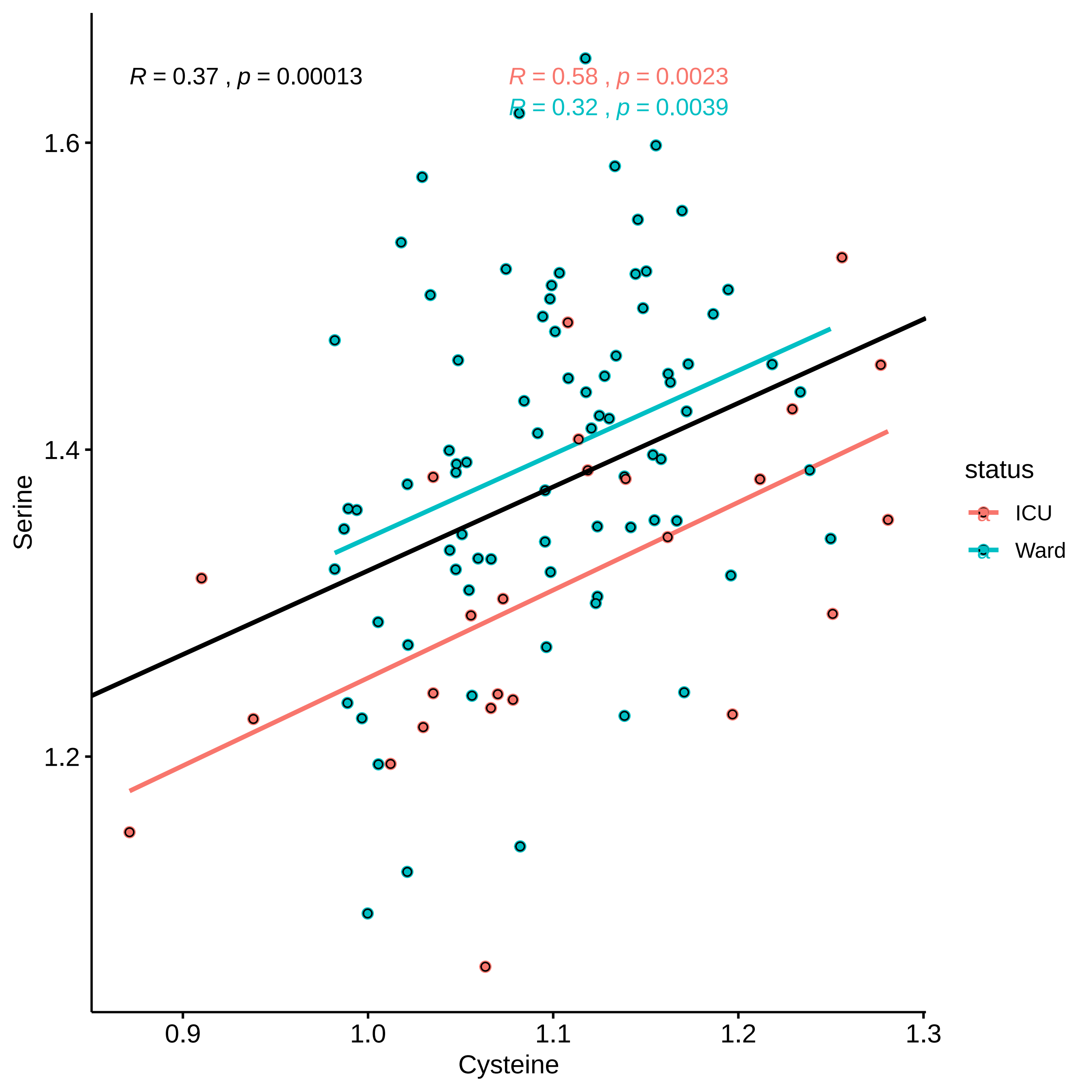

R = 0.37 , p = 0.00013
R = 0.58 , p = 0.0023
R = 0.32 , p = 0.0039
1.6
1.4
1.2
Serine
0.9
1.0
1.1
1.2
1.3
Cysteine
status
ICU
Ward

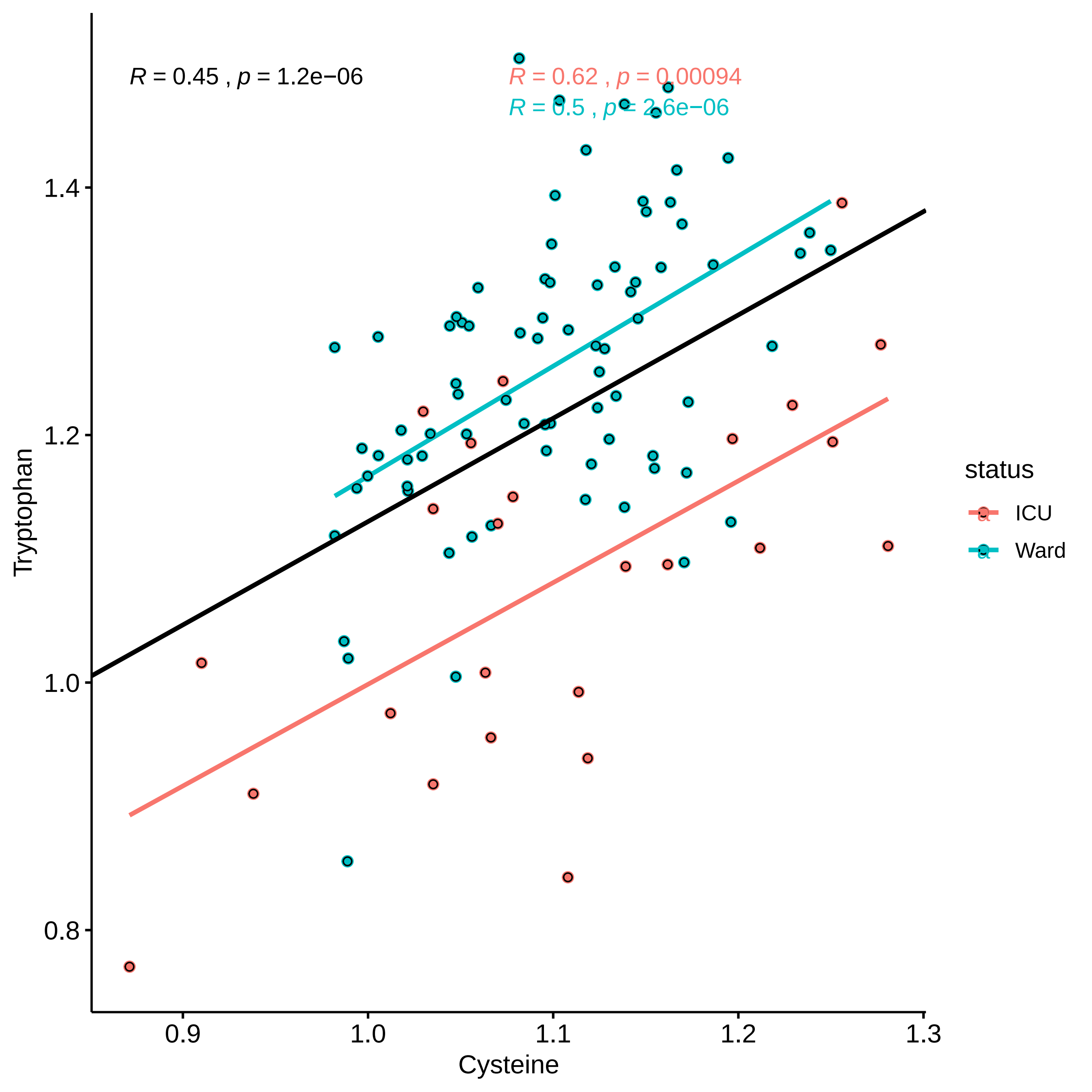

R = 0.45 , p = 1.2e−06
R = 0.62 , p = 0.00094
R = 0.5 , p = 2.6e−06
1.4
1.2
1.0
0.8
Tryptophan
0.9
1.0
1.1
1.2
1.3
Cysteine
status
ICU
Ward

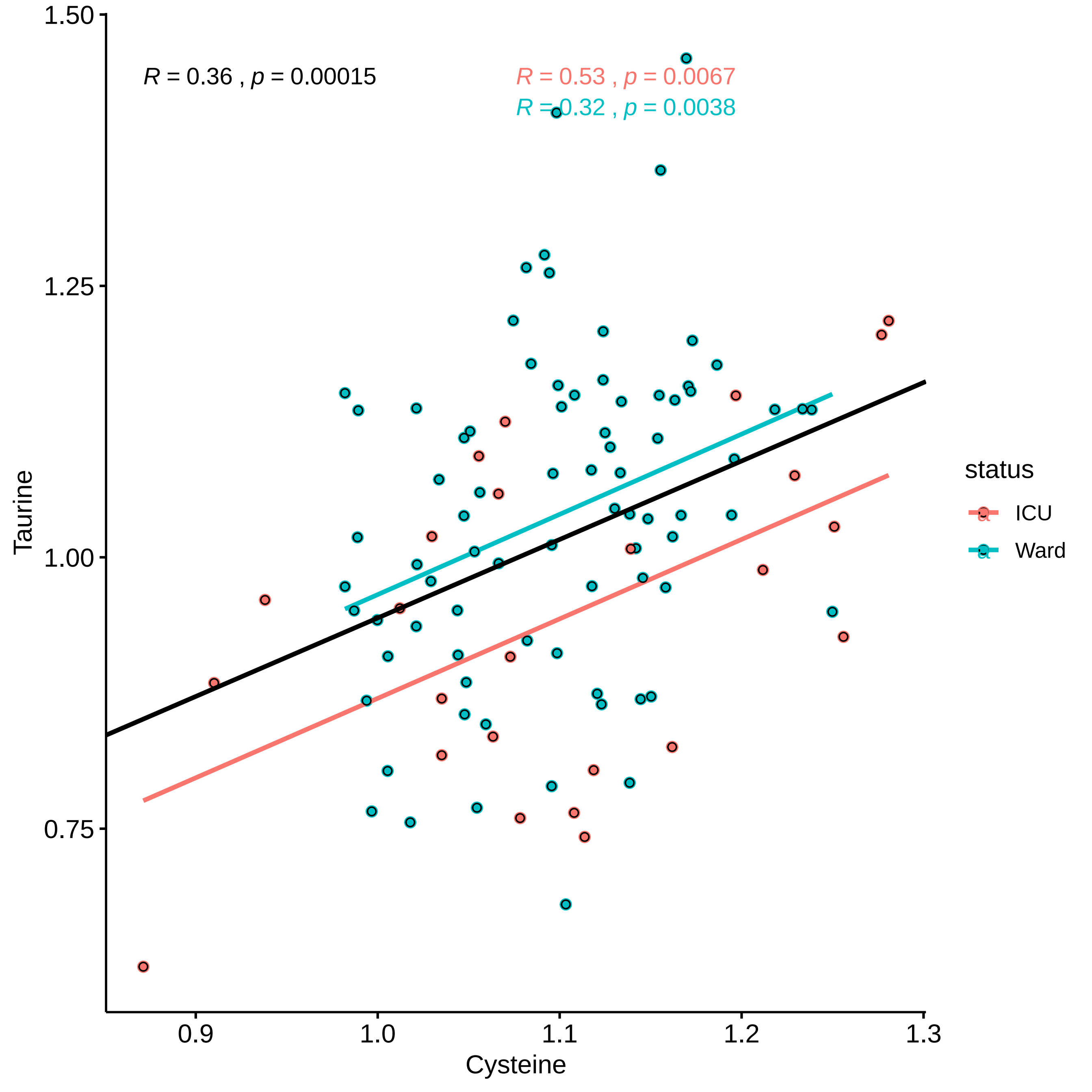

R = 0.36 , p = 0.00015
R = 0.53 , p = 0.0067
R = 0.32 , p = 0.0038
1.50
1.25
1.00
0.75
Taurine
0.9
1.0
1.1
1.2
1.3
Cysteine
status
ICU
Ward

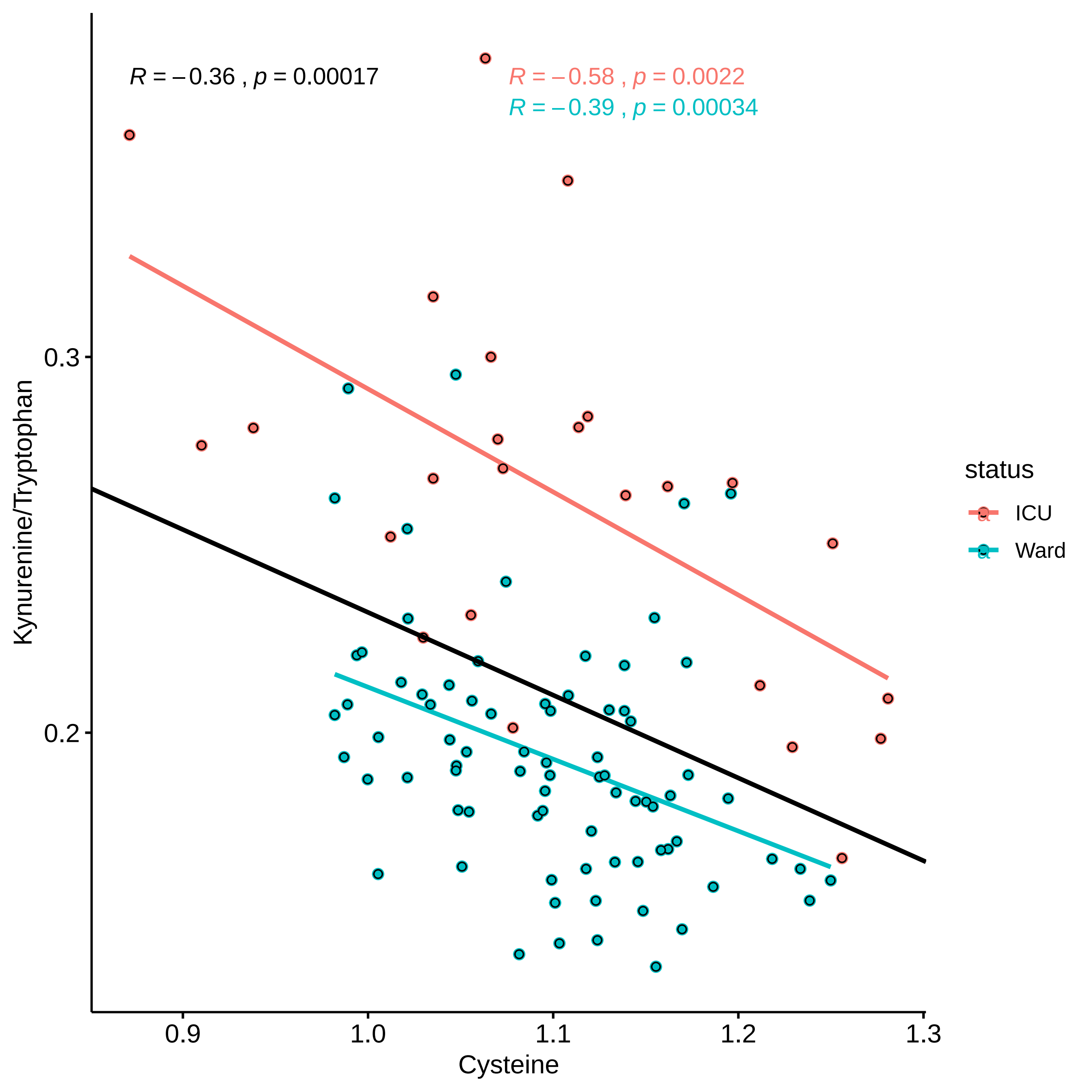

R = −0.36 , p = 0.00017
R = −0.58 , p = 0.0022
R = −0.39 , p = 0.00034
Kynurenine/Tryptophan
0.3
0.2
0.9
1.0
1.1
1.2
1.3
Cysteine
status
ICU
Ward

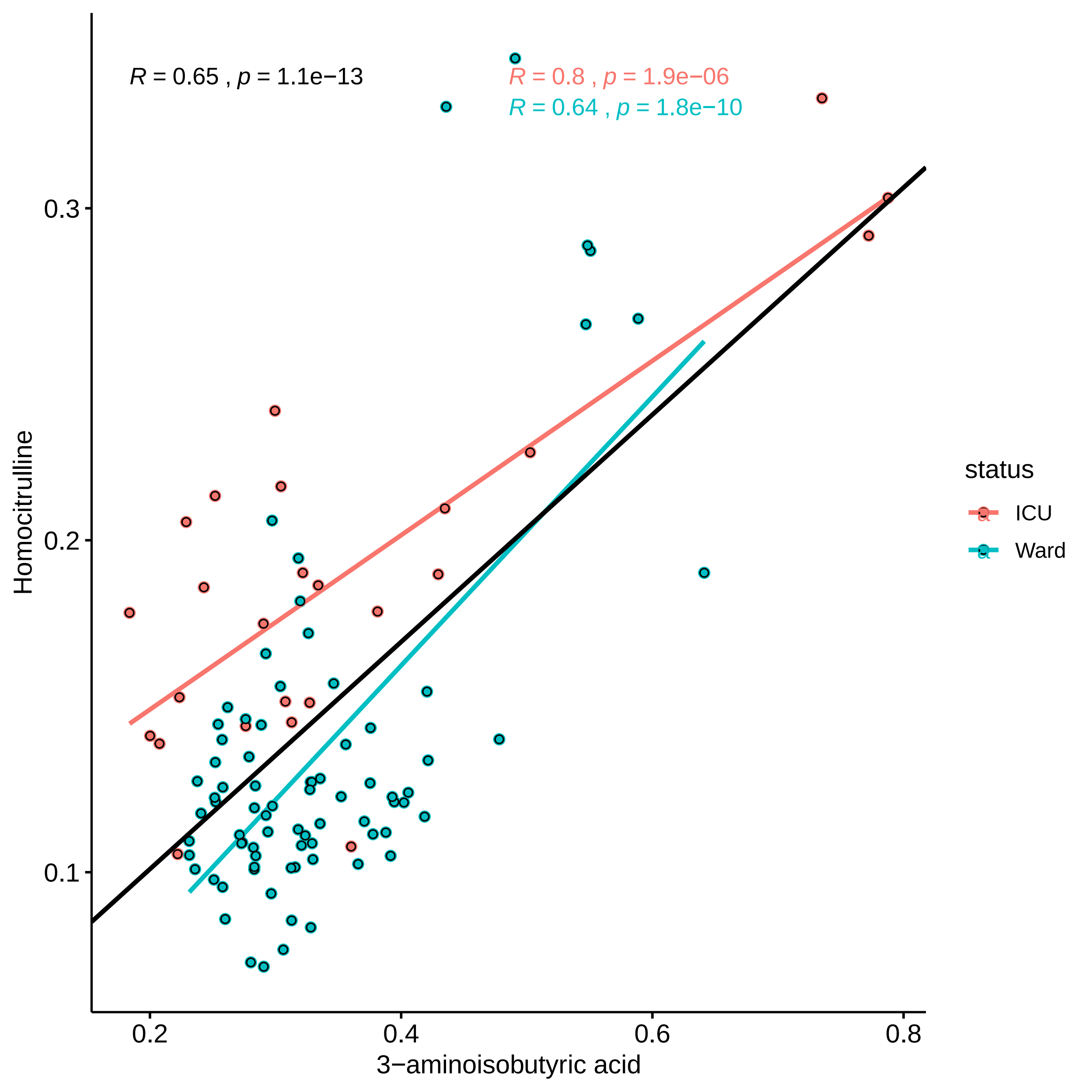

R = 0.65 , p = 1.1e−13
R = 0.8 , p = 1.9e−06
R = 0.64 , p = 1.8e−10
Homocitrulline
0.3
0.2
0.1
0.2
0.4
0.6
0.8
3−aminoisobutyric acid
status
ICU
Ward

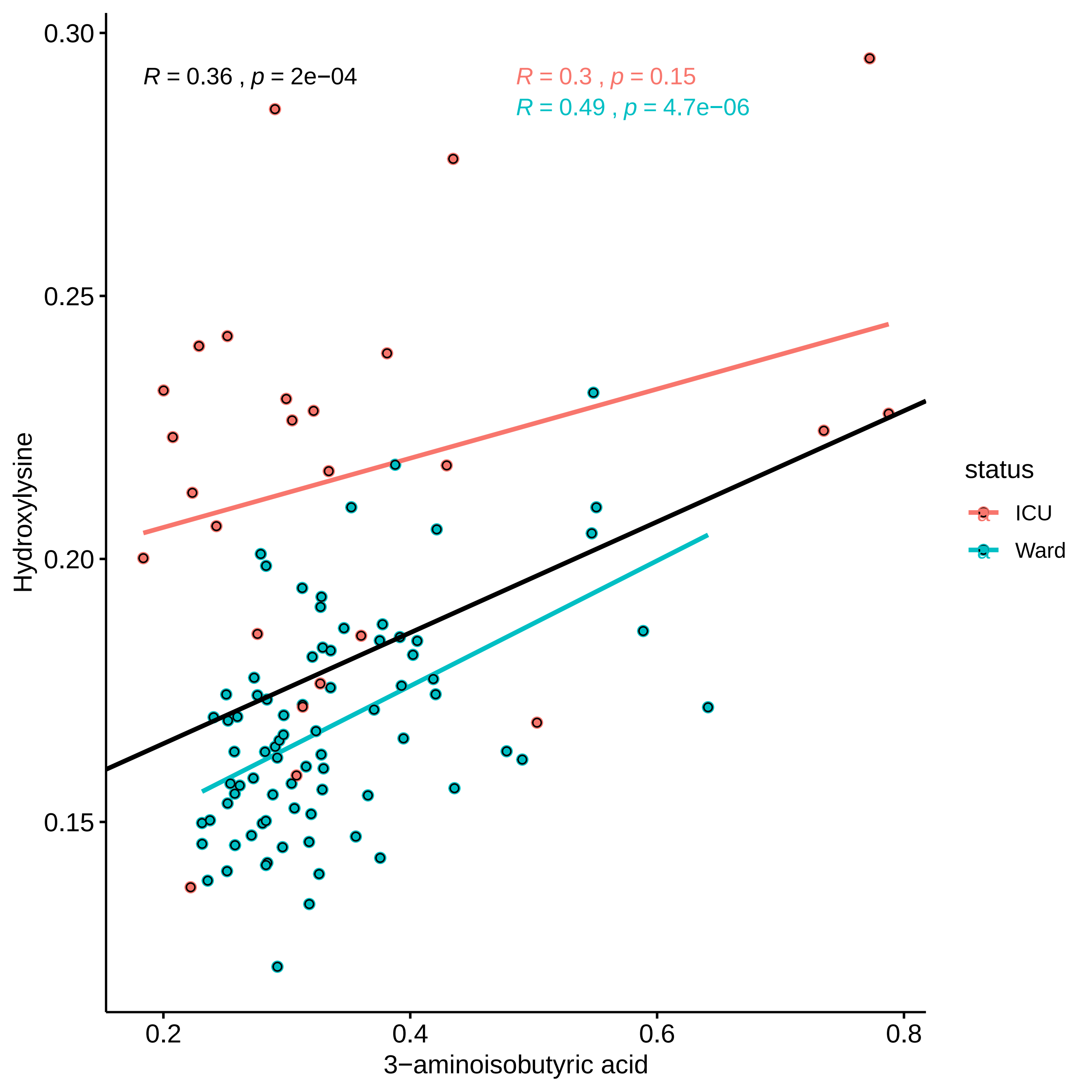

R = 0.36 , p = 2e−04
R = 0.3 , p = 0.15
R = 0.49 , p = 4.7e−06
0.30
0.25
0.20
0.15
Hydroxylysine
0.2
0.4
0.6
0.8
3−aminoisobutyric acid
status
ICU
Ward

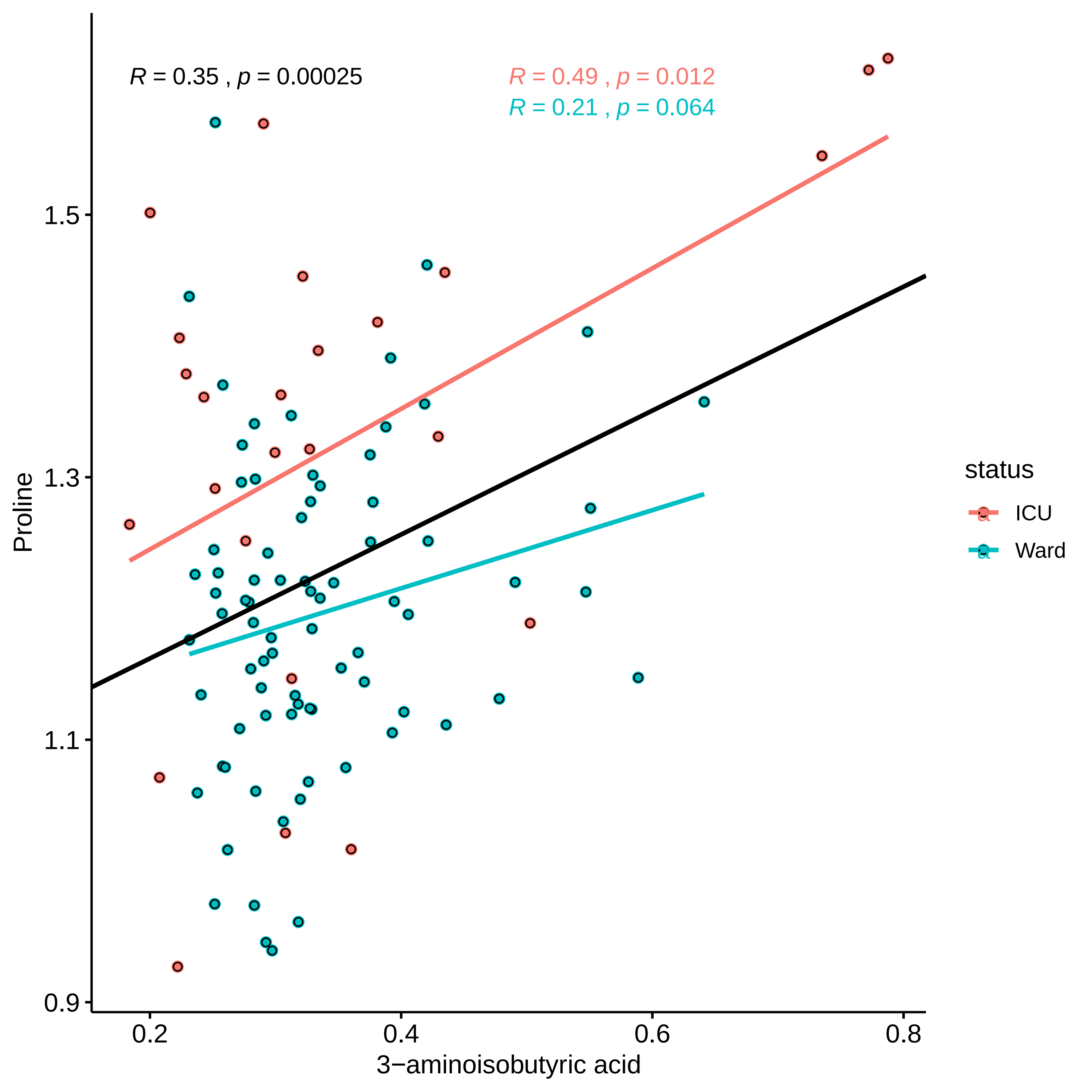

R = 0.35 , p = 0.00025
R = 0.49 , p = 0.012
R = 0.21 , p = 0.064
Proline
1.5
1.3
1.1
0.9
0.2
0.4
0.6
0.8
3−aminoisobutyric acid
status
ICU
Ward

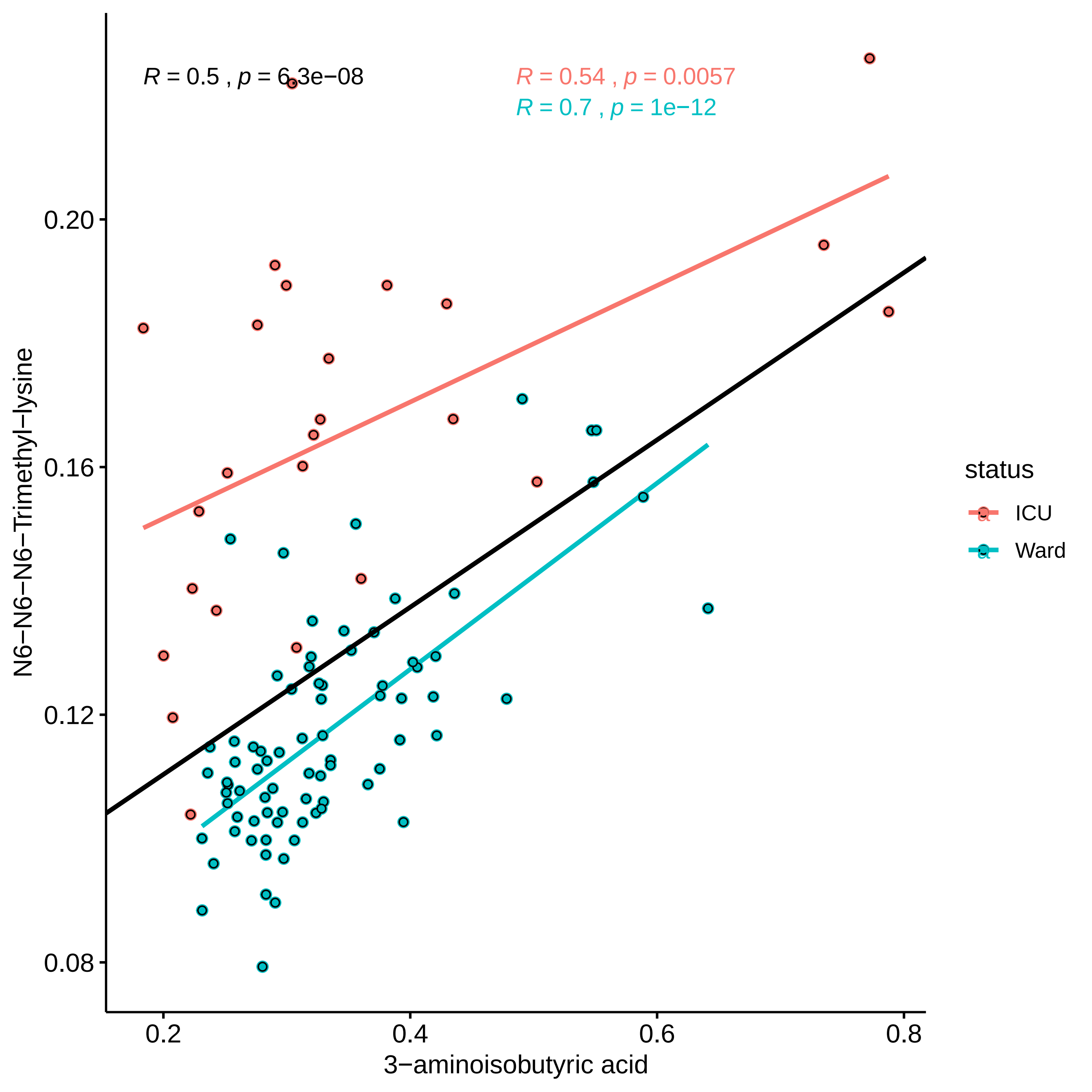

R = 0.5 , p = 6.3e−08
R = 0.54 , p = 0.0057
R = 0.7 , p = 1e−12
0.20
0.16
0.12
0.08
N6−N6−N6−Trimethyl−lysine
0.2
0.4
0.6
0.8
3−aminoisobutyric acid
status
ICU
Ward

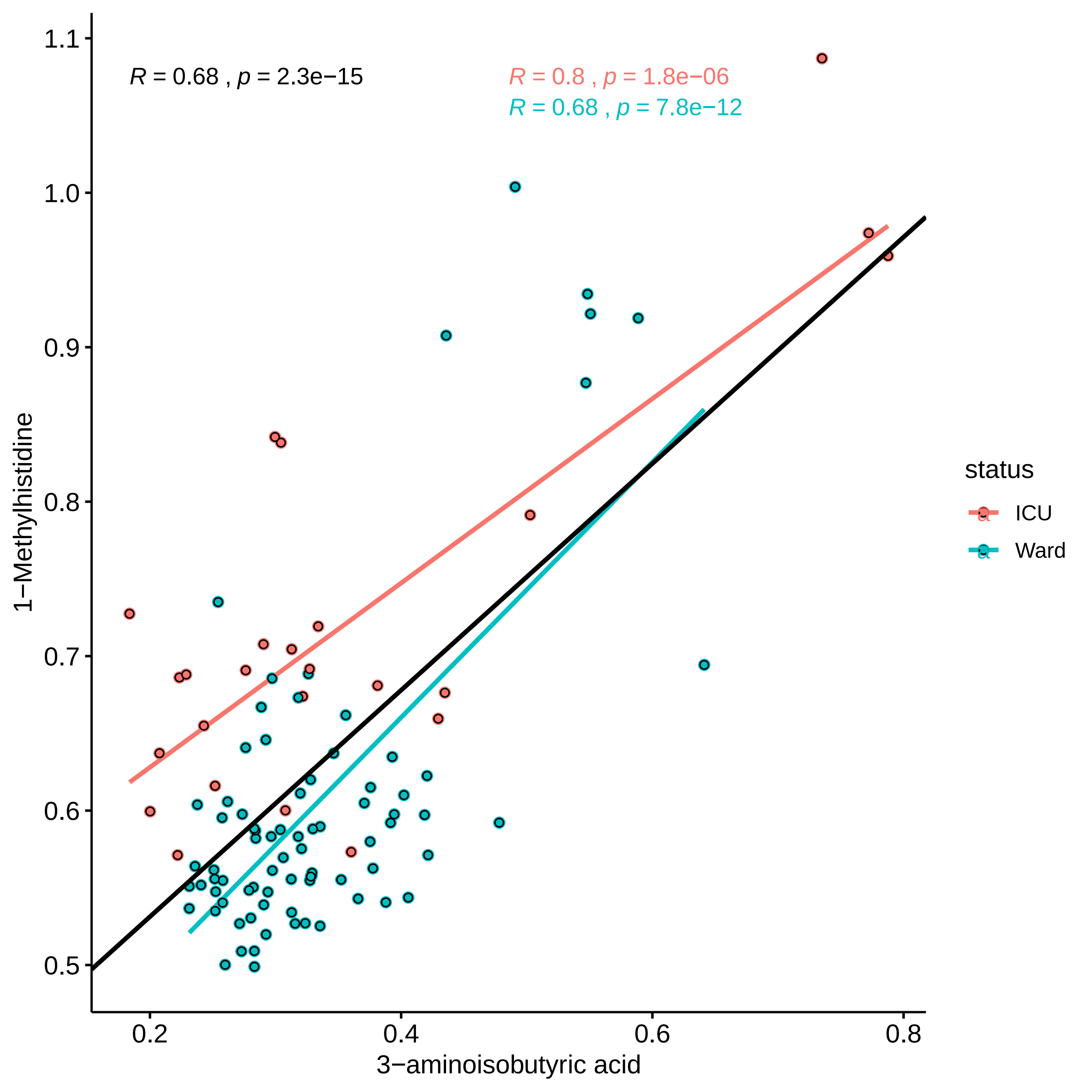

R = 0.68 , p = 2.3e−15
R = 0.8 , p = 1.8e−06
R = 0.68 , p = 7.8e−12
1−Methylhistidine
3−aminoisobutyric acid
1.1
1.0
0.9
0.8
0.7
0.6
0.5
0.2
0.4
0.6
0.8
status
ICU
Ward

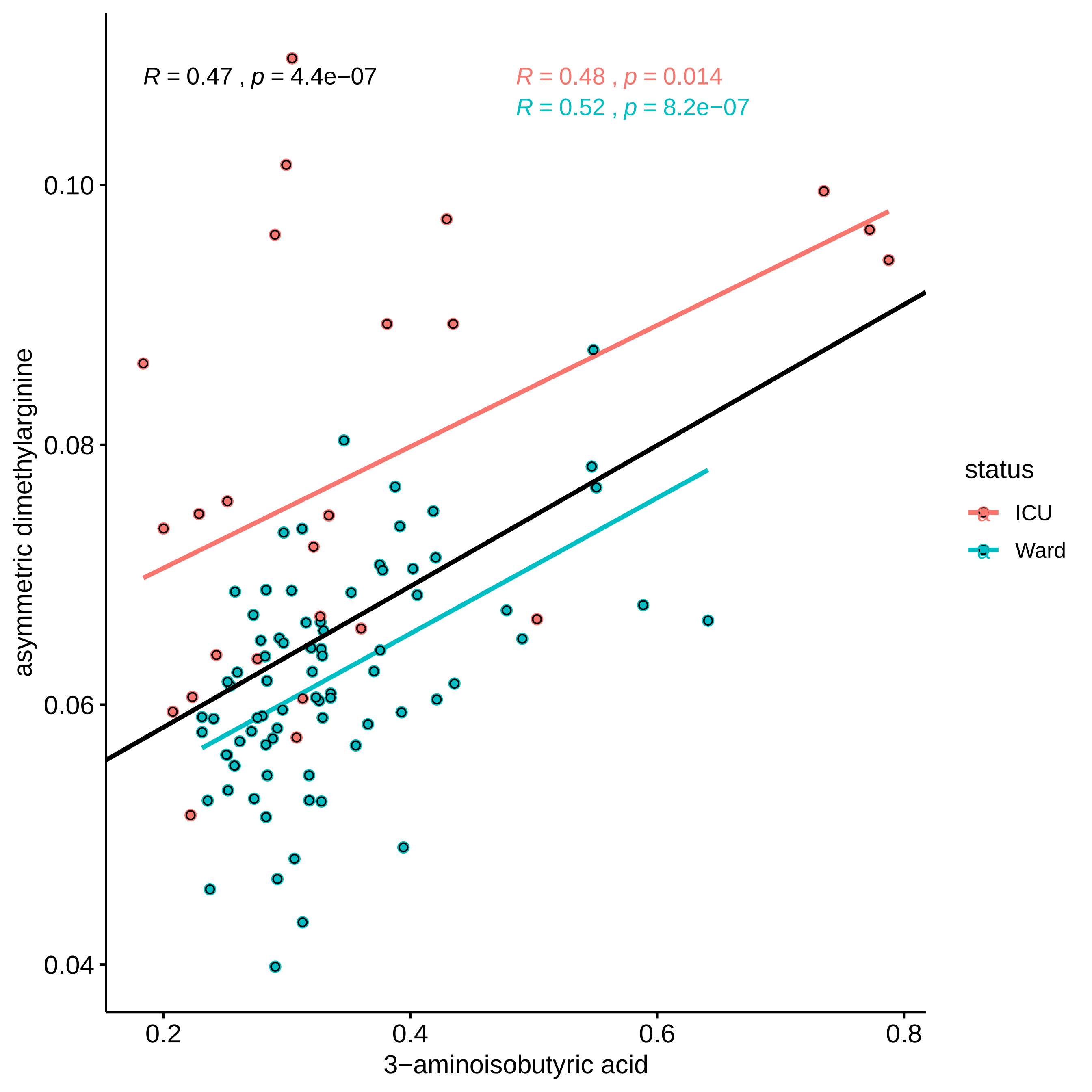

R = 0.47 , p = 4.4e−07
R = 0.48 , p = 0.014
R = 0.52 , p = 8.2e−07
0.10
0.08
0.06
0.04
asymmetric dimethylarginine
0.2
0.4
0.6
0.8
3−aminoisobutyric acid
status
ICU
Ward

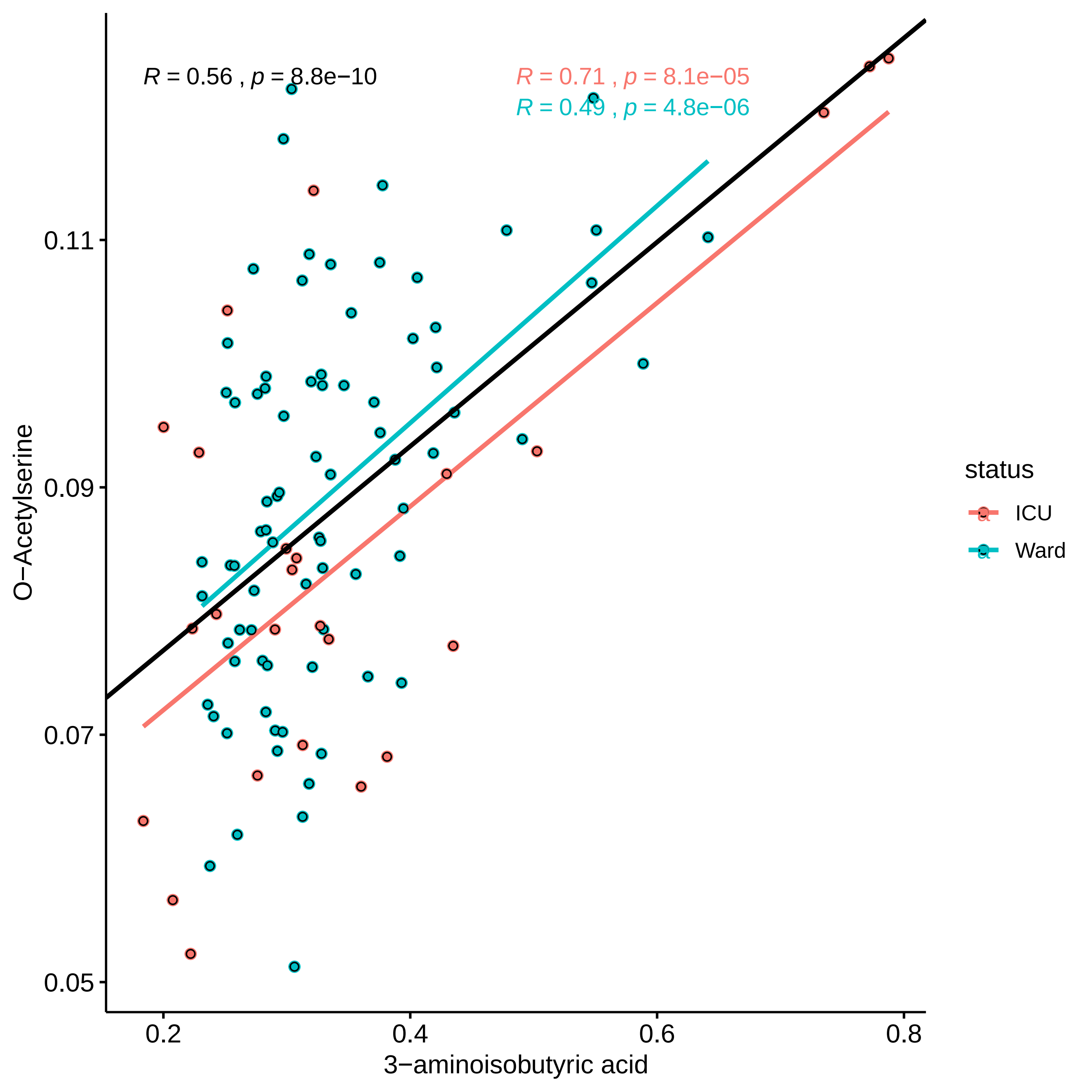

R = 0.56 , p = 8.8e−10
R = 0.71 , p = 8.1e−05
R = 0.49 , p = 4.8e−06
O−Acetylserine
0.11
0.09
0.07
0.05
0.2
0.4
0.6
0.8
3−aminoisobutyric acid
status
ICU
Ward

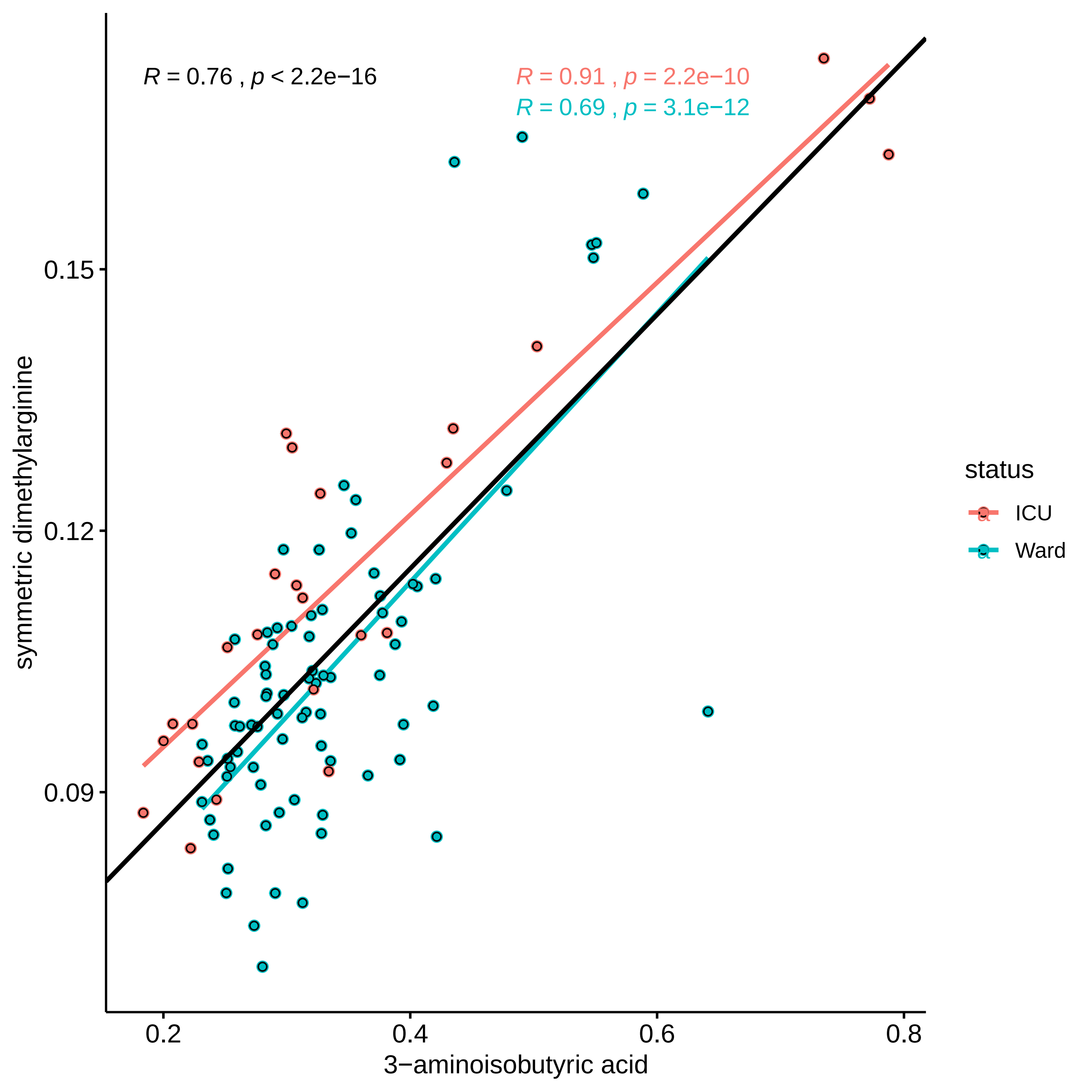
R = 0.76 , p < 2.2e−16
R = 0.91 , p = 2.2e−10
R = 0.69 , p = 3.1e−12
symmetric dimethylarginine
0.15
0.12
0.09
0.2
0.4
0.6
0.8
3−aminoisobutyric acid
status
ICU
Ward

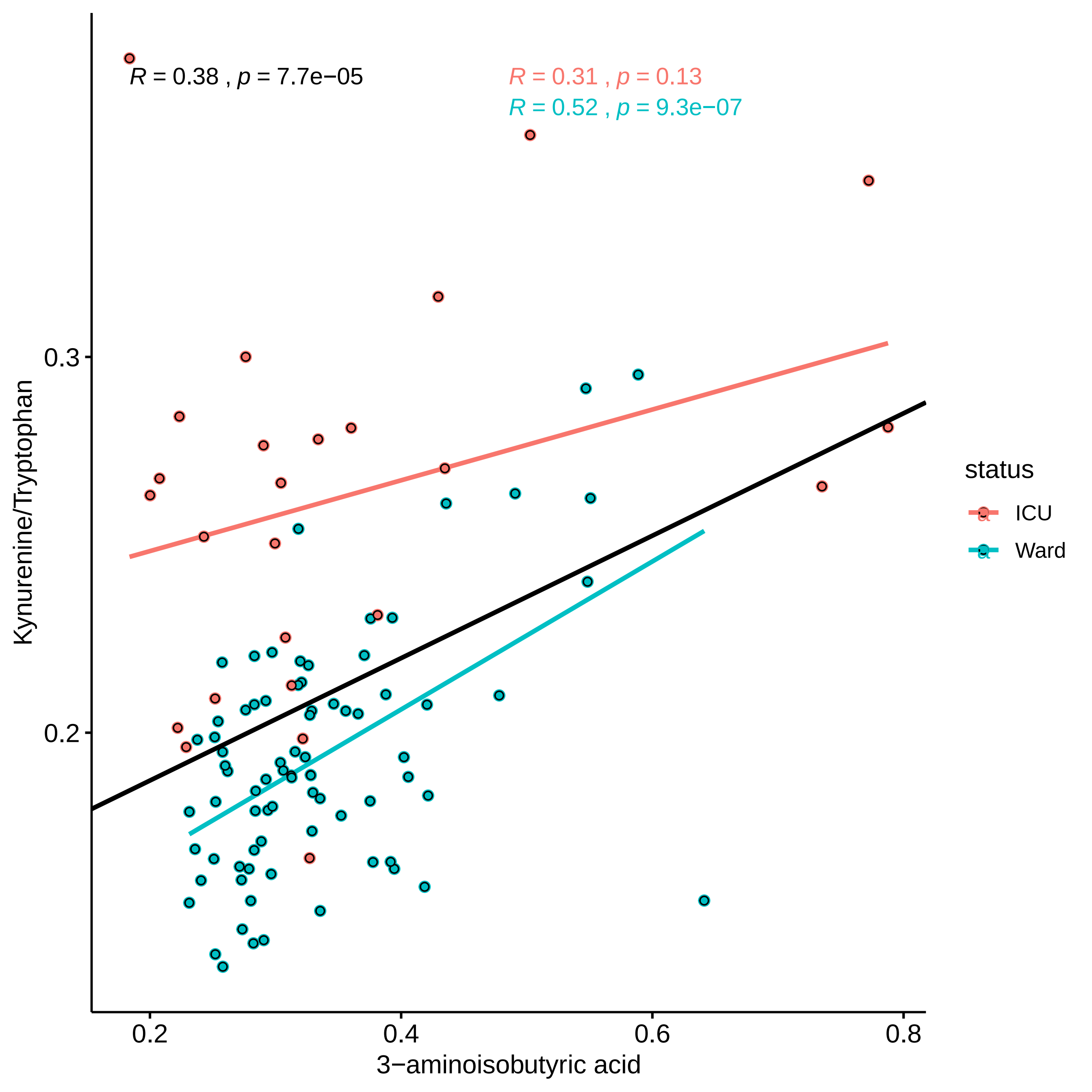
R = 0.38 , p = 7.7e−05
R = 0.31 , p = 0.13
R = 0.52 , p = 9.3e−07
Kynurenine/Tryptophan
0.3
0.2
0.2
0.4
0.6
0.8
3−aminoisobutyric acid
status
ICU
Ward

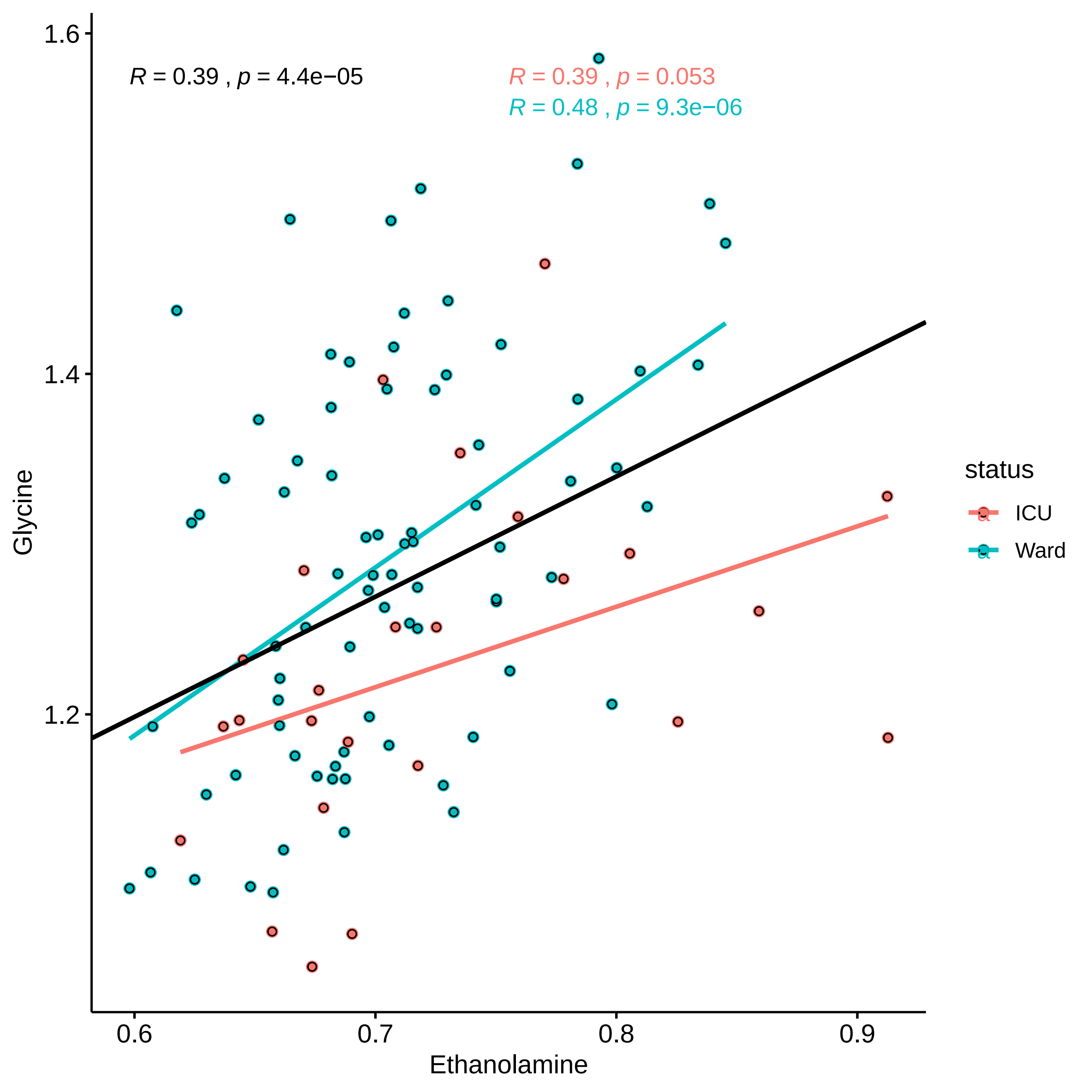

R = 0.39 , p = 4.4e−05
R = 0.39 , p = 0.053
R = 0.48 , p = 9.3e−06
Glycine
Ethanolamine
1.6
1.4
1.2
0.6
0.7
0.8
0.9
status
ICU
Ward

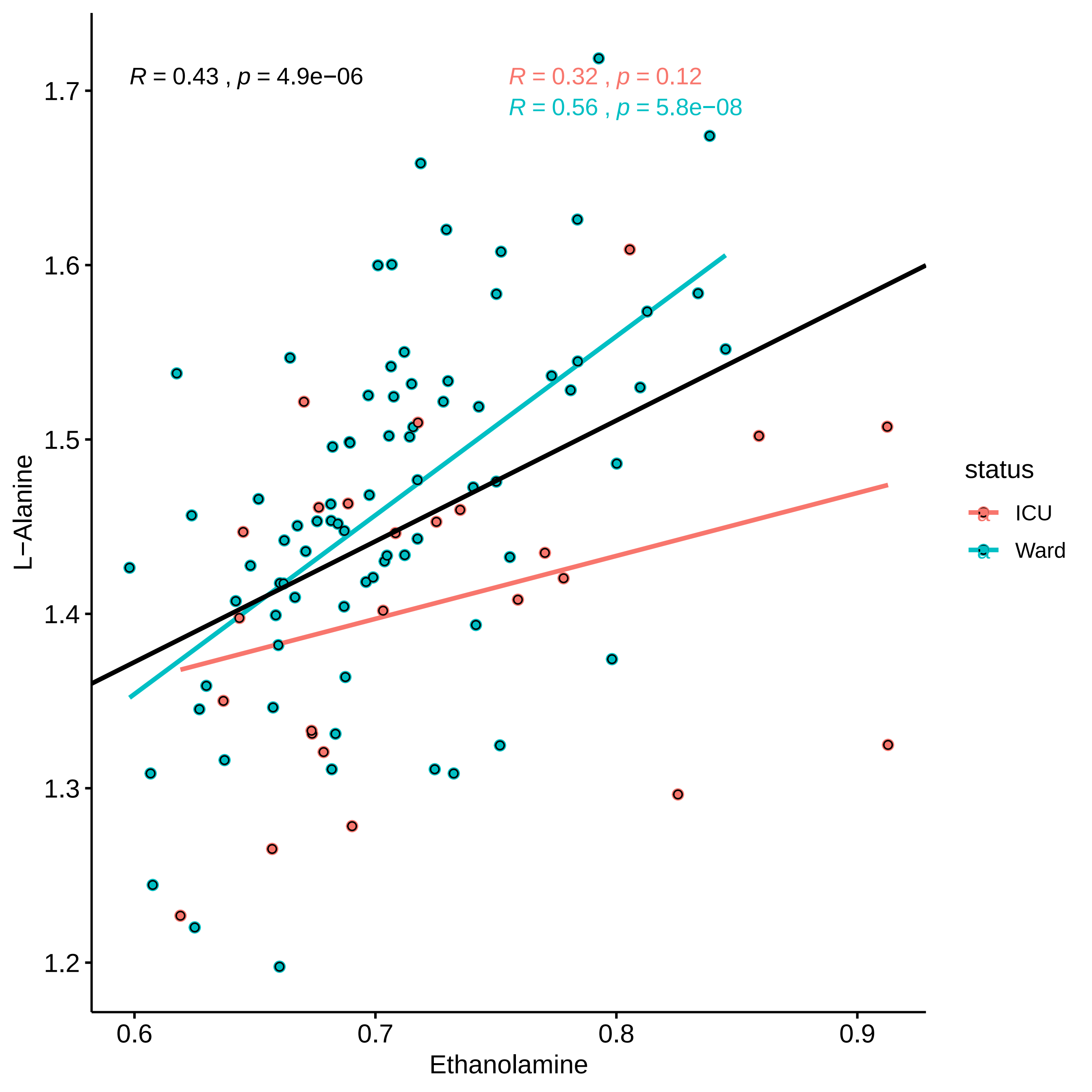

R = 0.43 , p = 4.9e−06
R = 0.32 , p = 0.12
R = 0.56 , p = 5.8e−08
L−Alanine
1.2
1.3
1.4
1.5
1.6
1.7
0.6
0.7
0.8
0.9
Ethanolamine
status
ICU
Ward

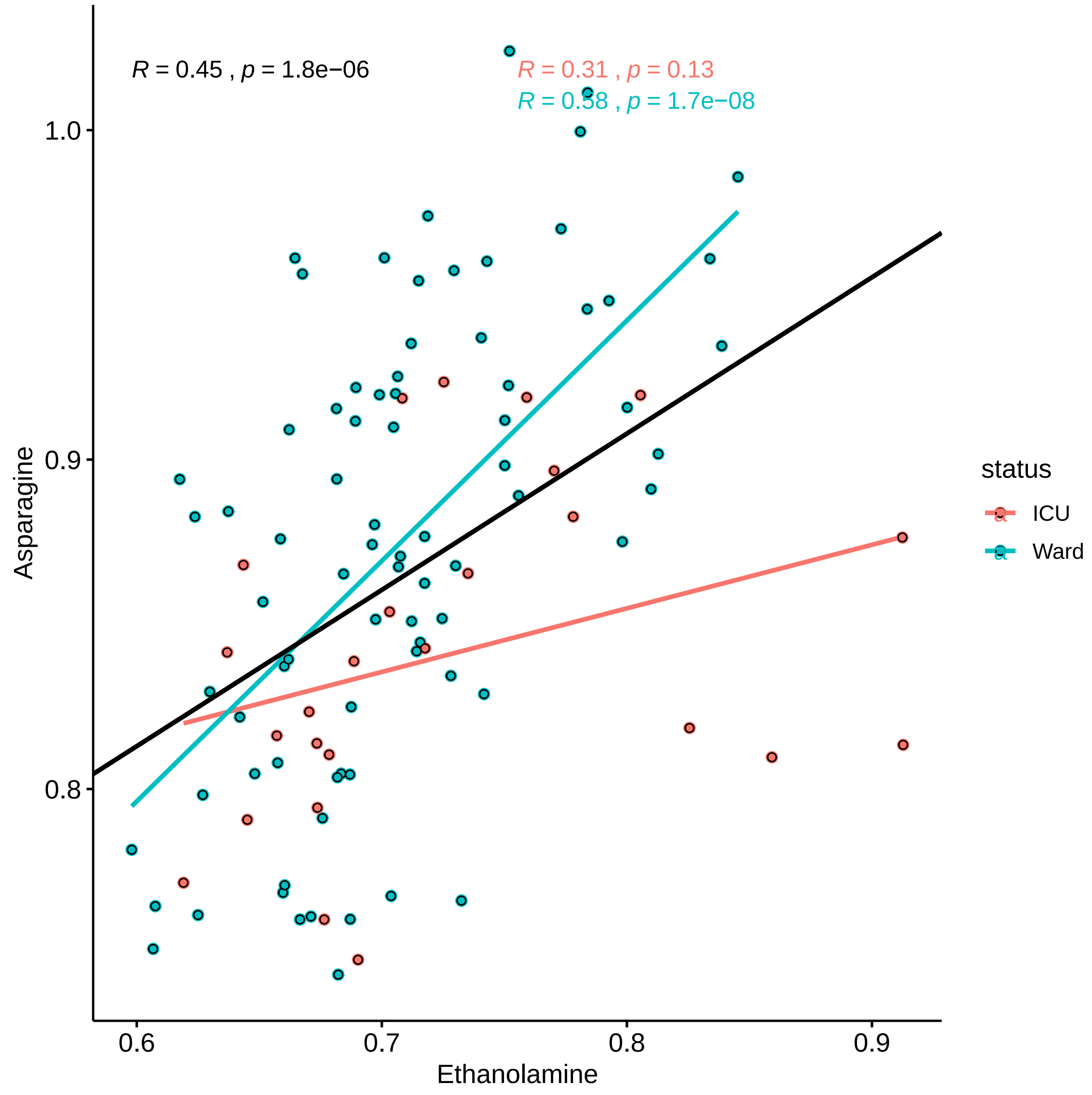
R = 0.45 , p = 1.8e−06
R = 0.31 , p = 0.13
R = 0.58 , p = 1.7e−08
1.0
0.9
0.8
Asparagine
0.6
0.7
0.8
0.9
Ethanolamine
status
ICU
Ward

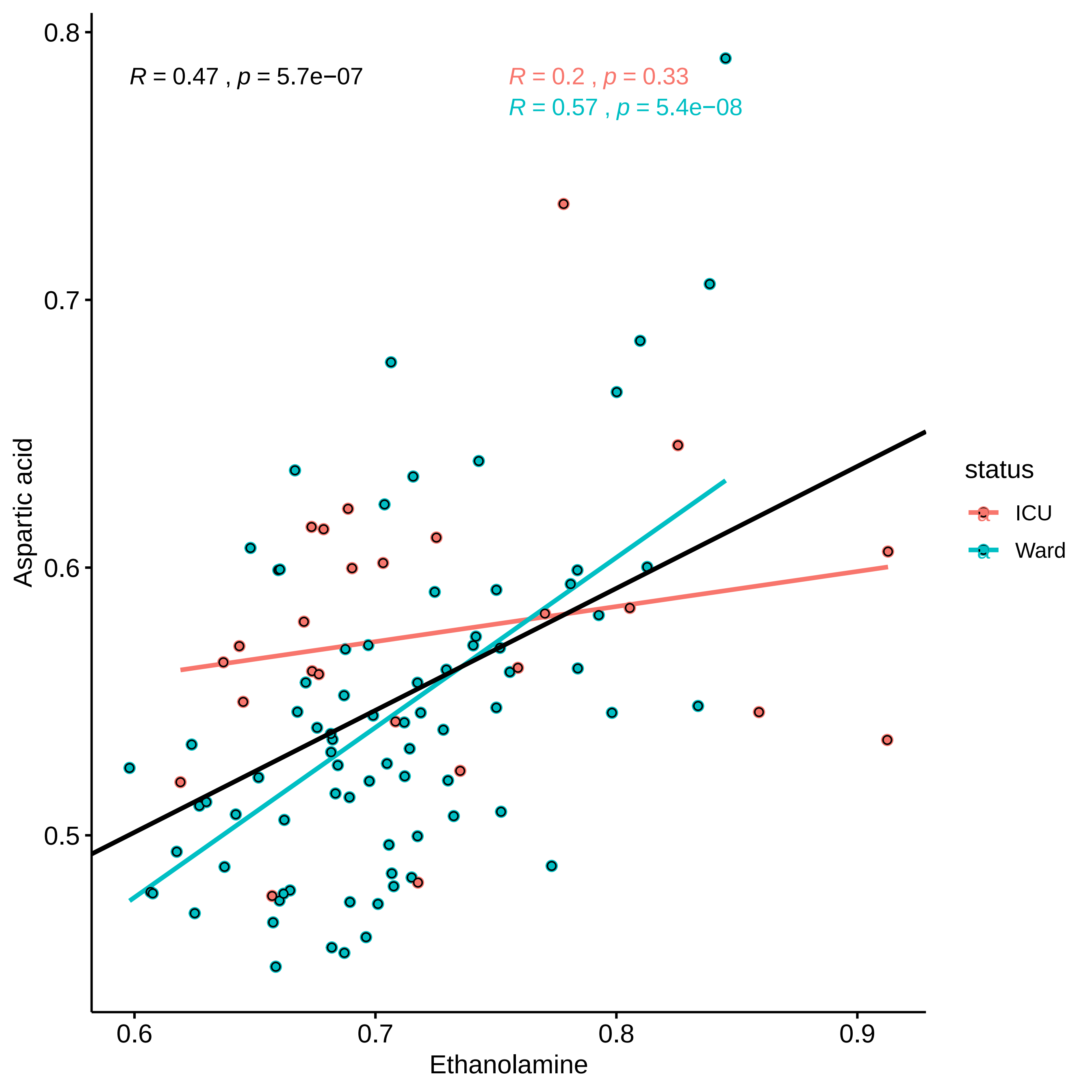

R = 0.47 , p = 5.7e−07
R = 0.2 , p = 0.33
R = 0.57 , p = 5.4e−08
Aspartic acid
0.8
0.7
0.6
0.5
0.6
0.7
0.8
0.9
Ethanolamine
status
ICU
Ward

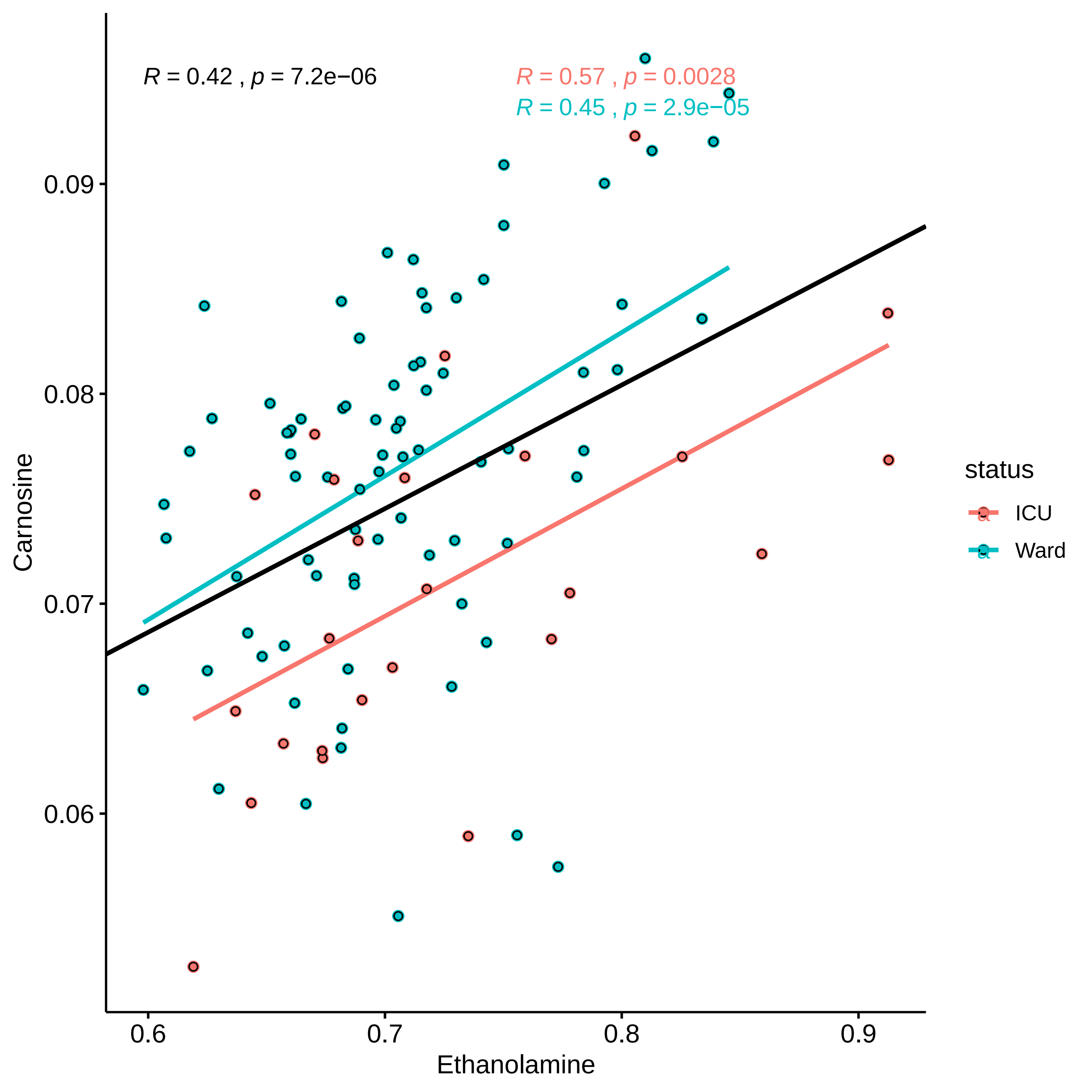

R = 0.42 , p = 7.2e−06
R = 0.57 , p = 0.0028
R = 0.45 , p = 2.9e−05
Carnosine
0.09
0.08
0.07
0.06
0.6
0.7
0.8
0.9
Ethanolamine
status
ICU
Ward

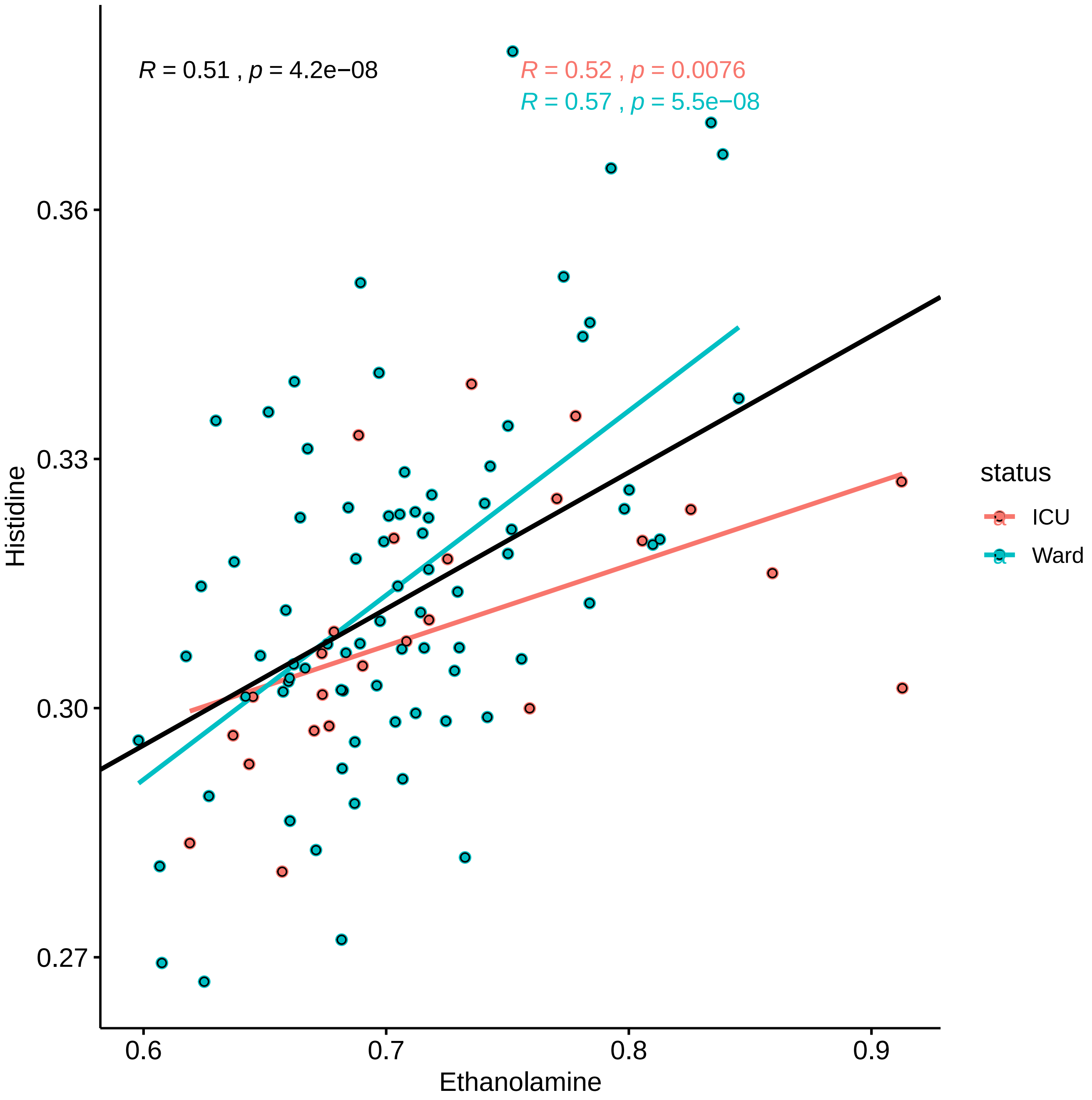

R = 0.51 , p = 4.2e−08
R = 0.52 , p = 0.0076
R = 0.57 , p = 5.5e−08
0.36
0.33
0.30
0.27
Histidine
0.6
0.7
0.8
0.9
Ethanolamine
status
ICU
Ward

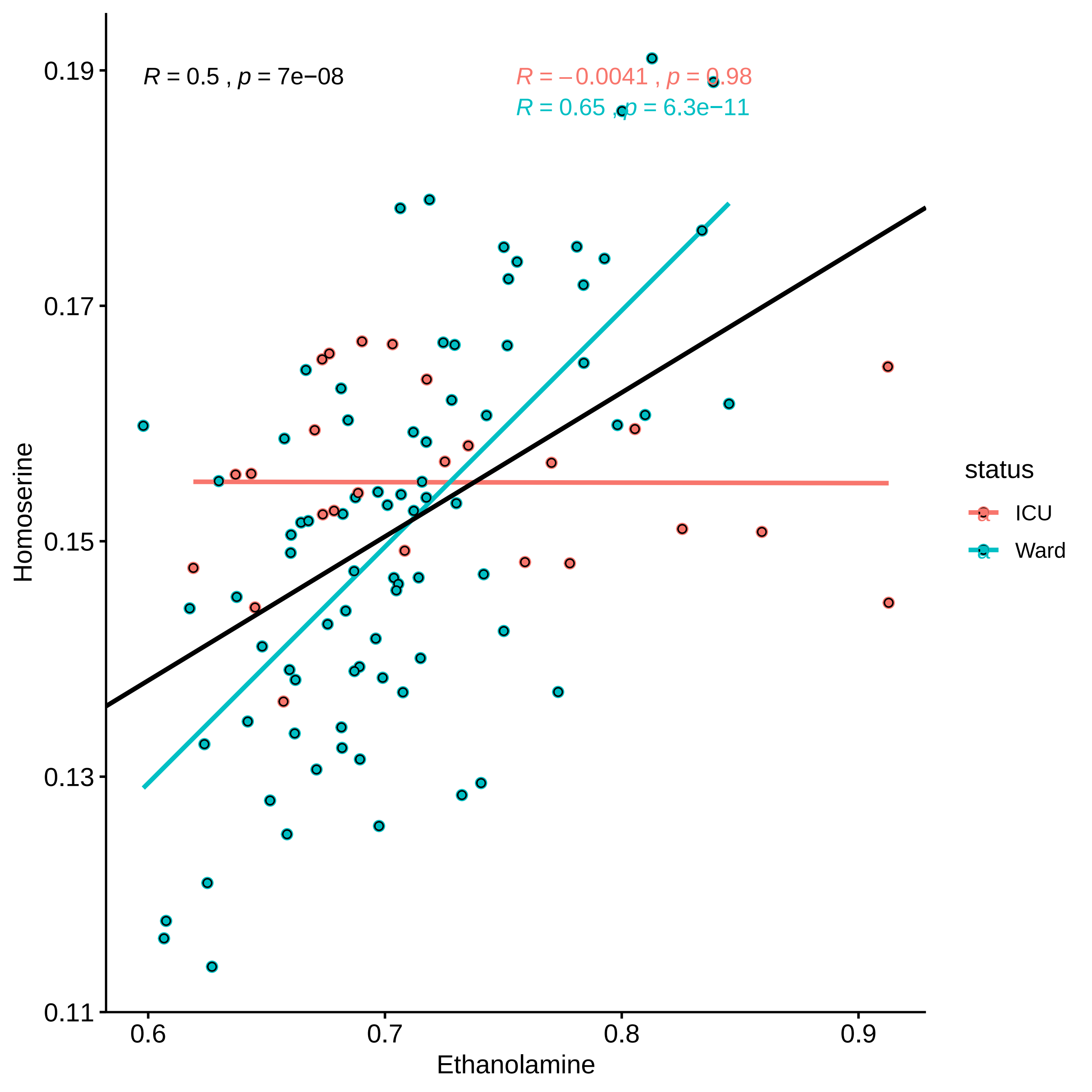

R = 0.5 , p = 7e−08
R = −0.0041 , p = 0.98
R = 0.65 , p = 6.3e−11
0.19
0.17
0.15
0.13
0.11
Homoserine
0.6
0.7
0.8
0.9
Ethanolamine
status
ICU
Ward

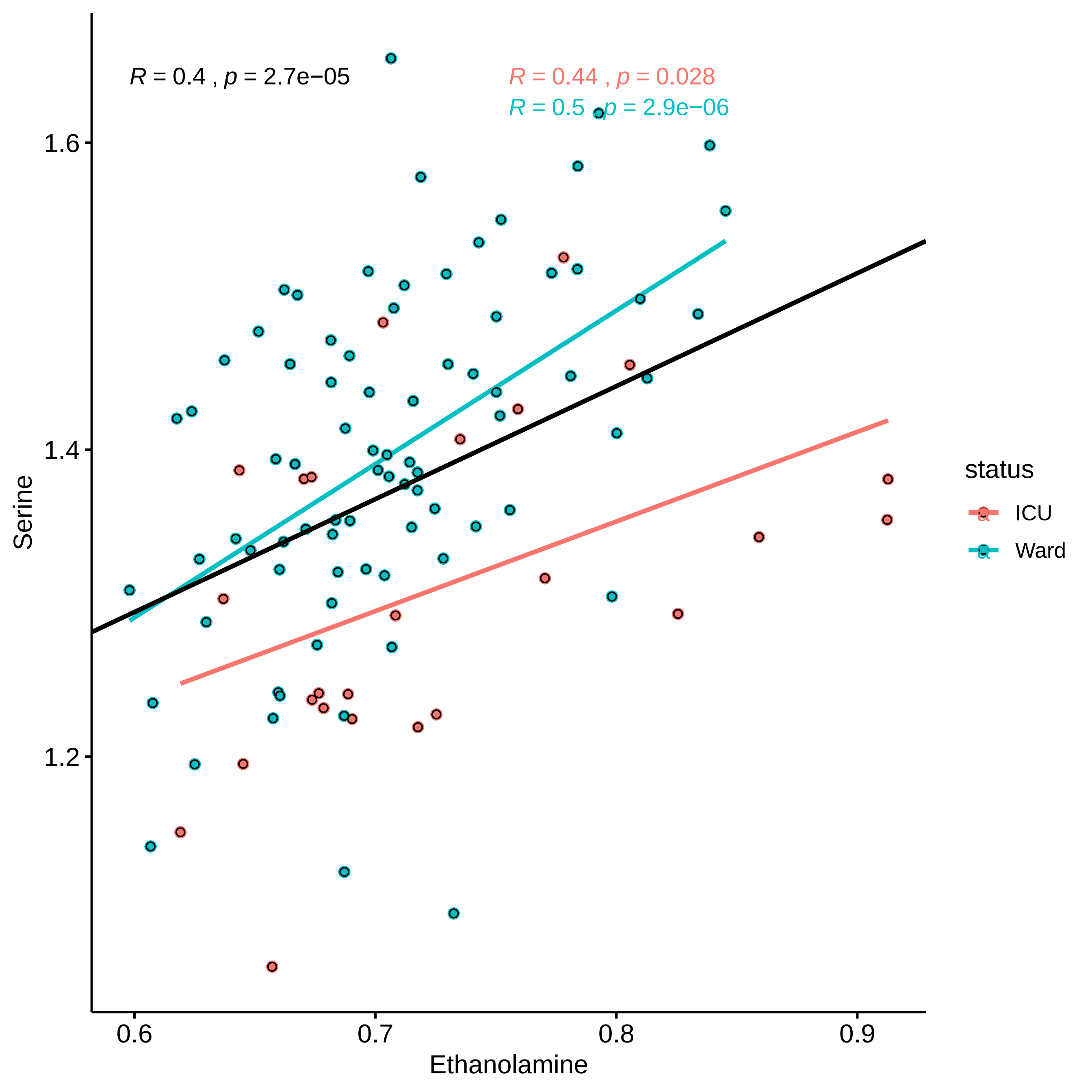

R = 0.4 , p = 2.7e−05
R = 0.44 , p = 0.028
R = 0.5 , p = 2.9e−06
1.6
1.4
1.2
Serine
0.6
0.7
0.8
0.9
Ethanolamine
status
ICU
Ward

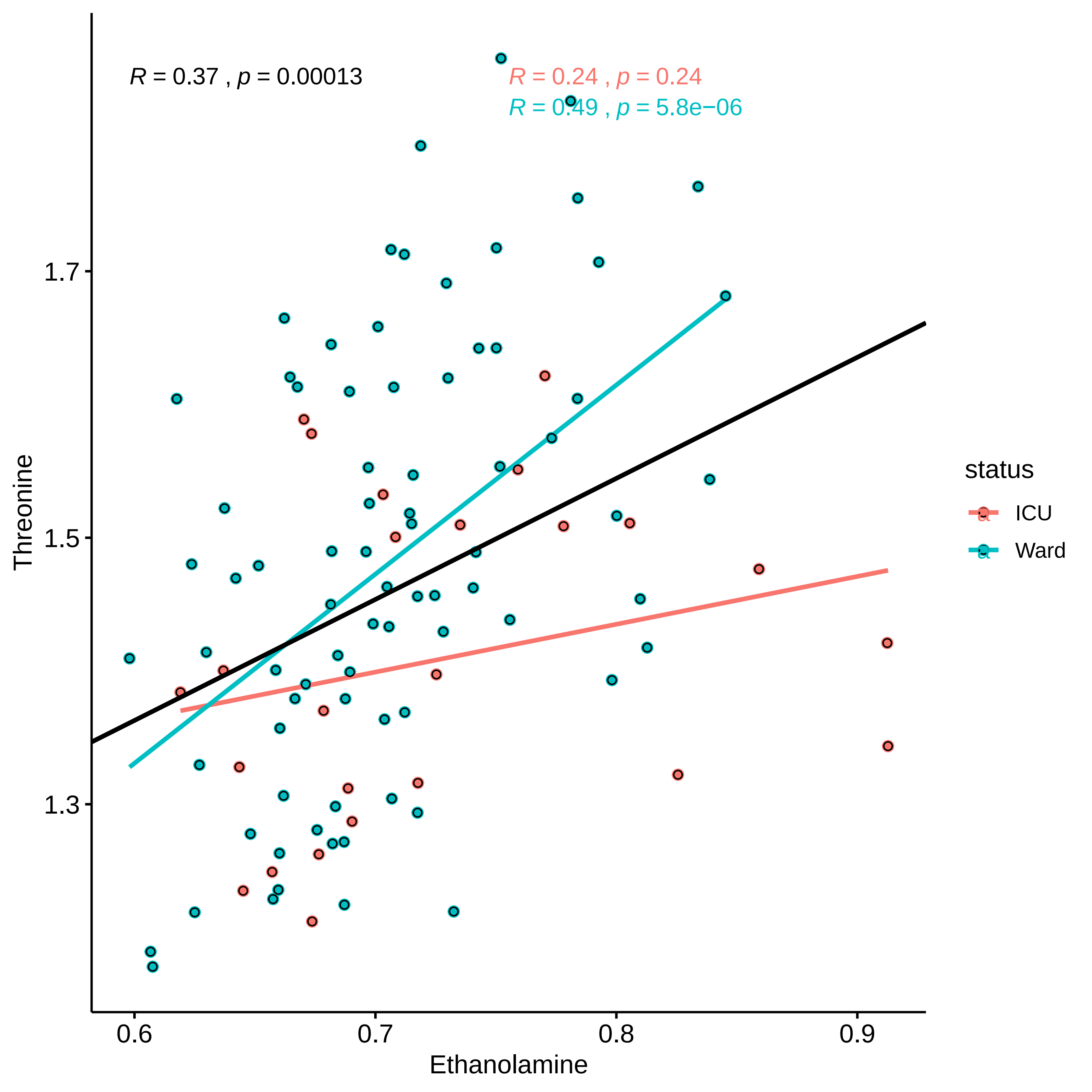

R = 0.37 , p = 0.00013
R = 0.24 , p = 0.24
R = 0.49 , p = 5.8e−06
Threonine
1.7
1.5
1.3
0.6
0.7
0.8
0.9
Ethanolamine
status
ICU
Ward

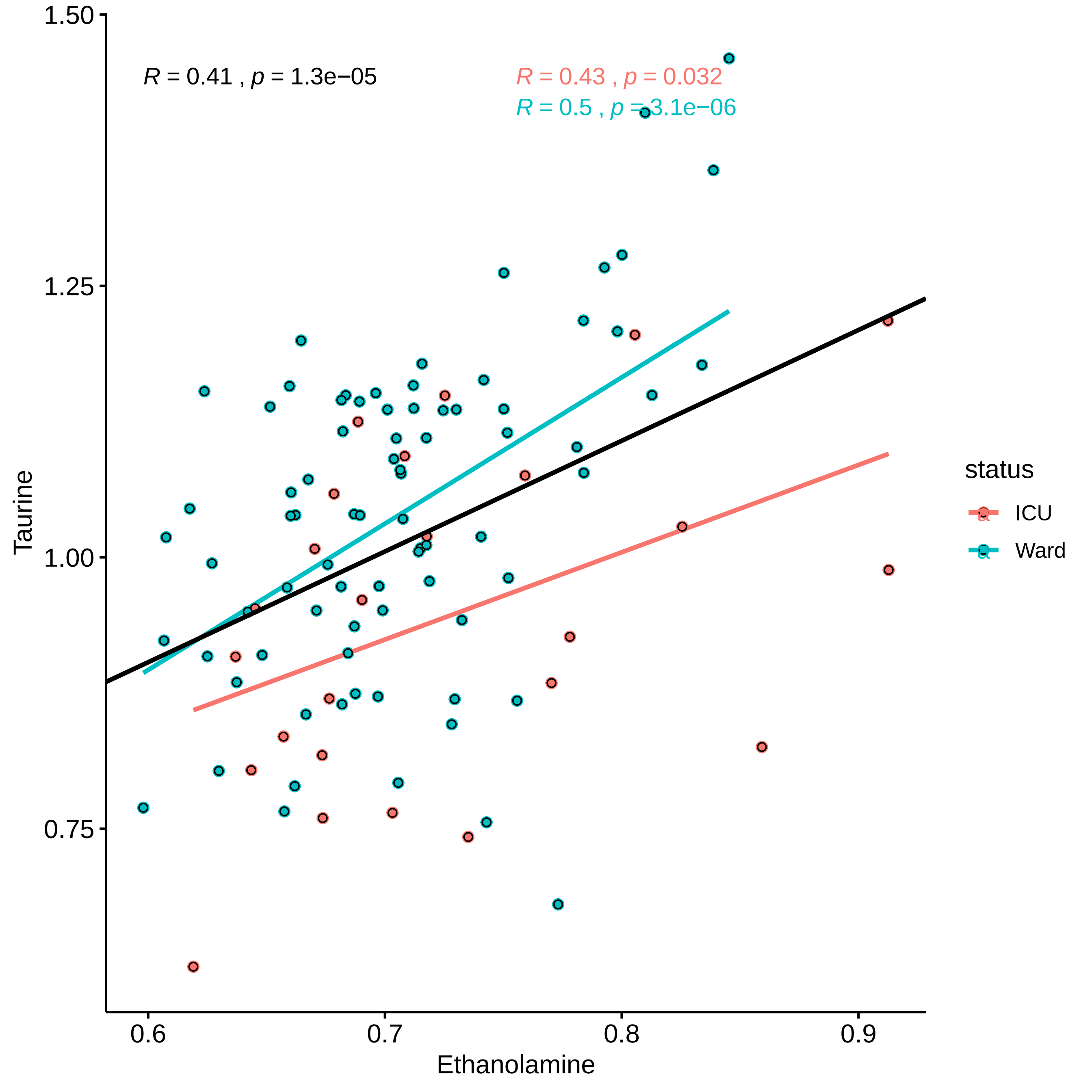

R = 0.41 , p = 1.3e−05
R = 0.43 , p = 0.032
R = 0.5 , p = 3.1e−06
Taurine
1.50
1.25
1.00
0.75
0.6
0.7
0.8
0.9
Ethanolamine
status
ICU
Ward

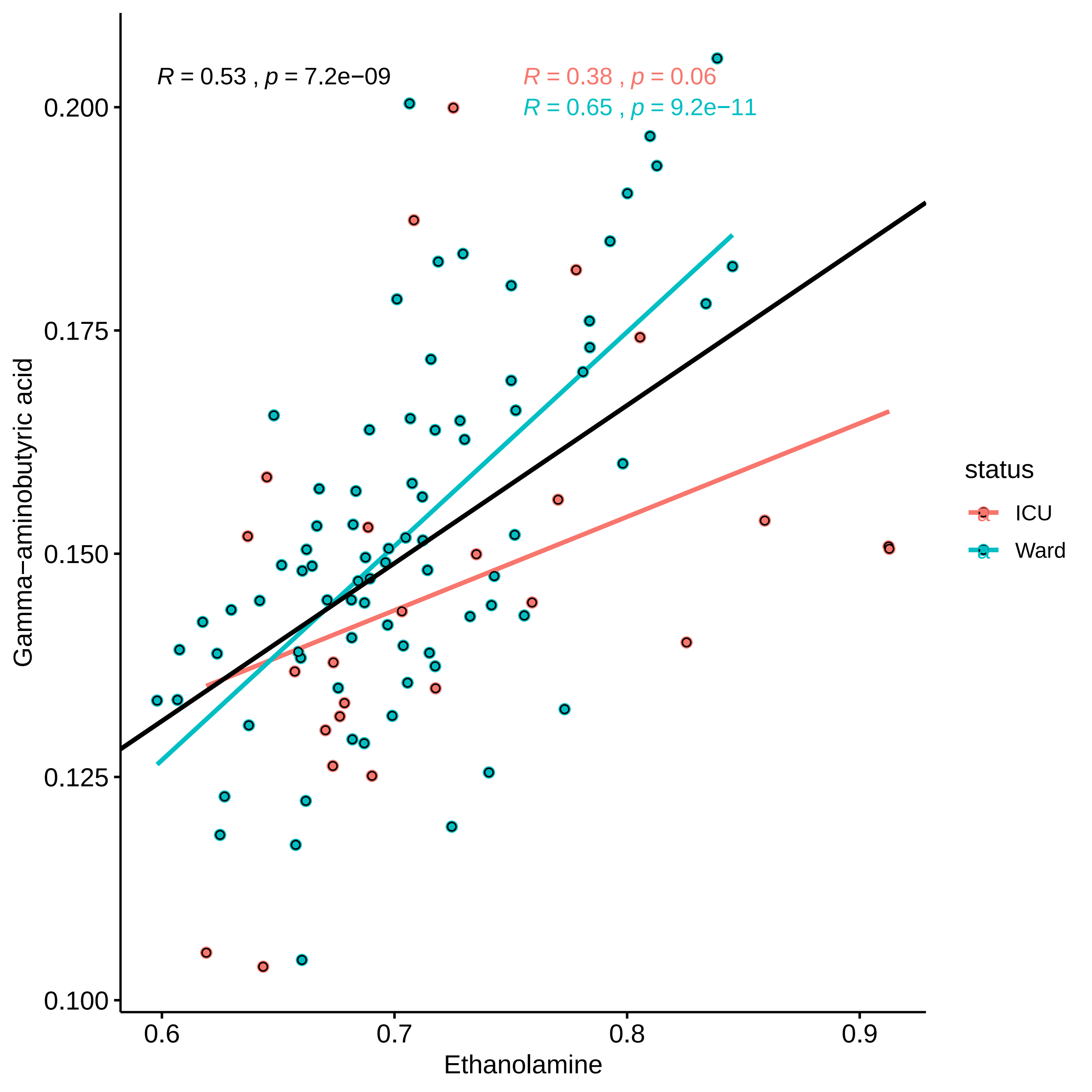

R = 0.53 , p = 7.2e−09
R = 0.38 , p = 0.06
R = 0.65 , p = 9.2e−11
0.200
0.175
0.150
0.125
0.100
Gamma−aminobutyric acid
0.6
0.7
0.8
0.9
Ethanolamine
status
ICU
Ward

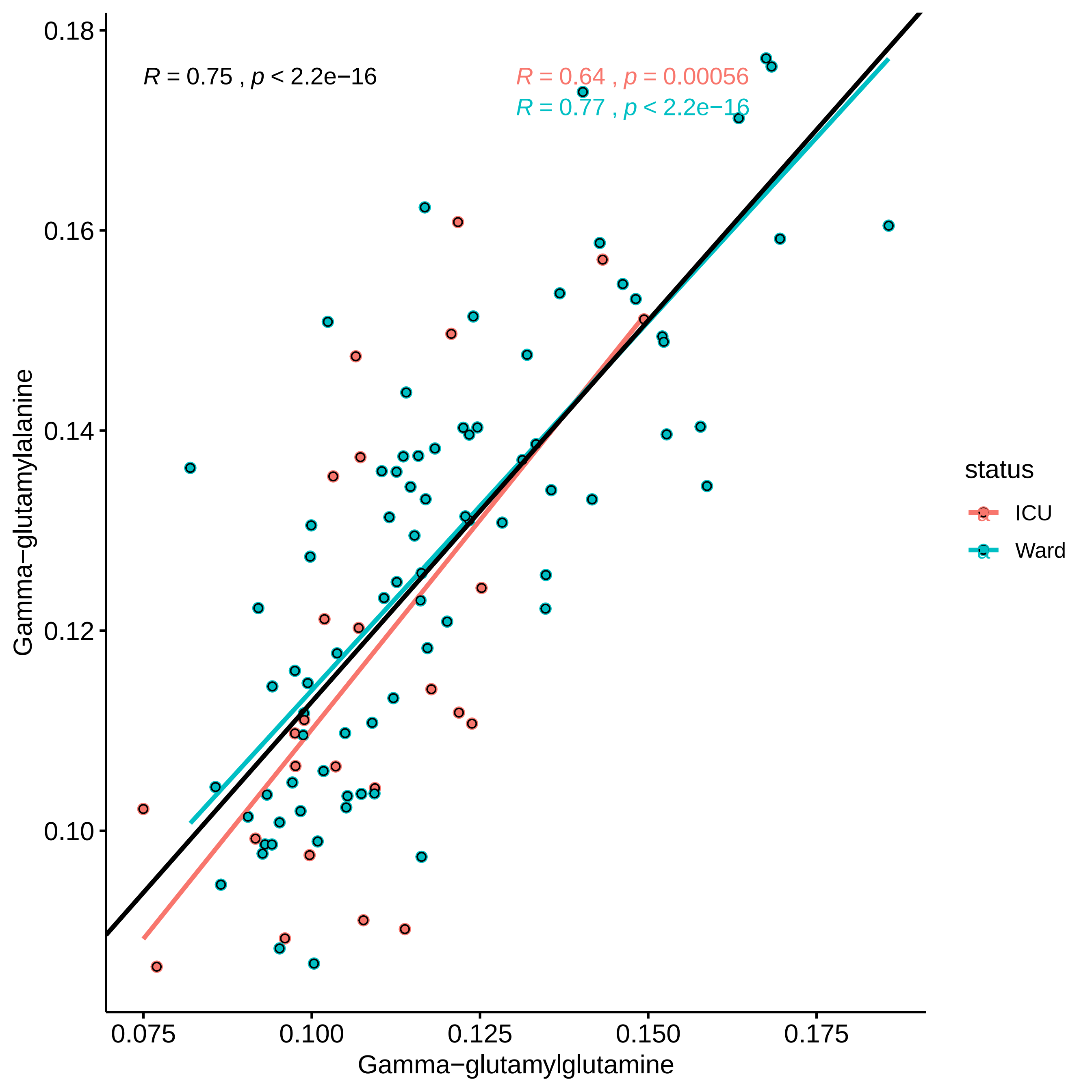

R = 0.75 , p < 2.2e−16
R = 0.64 , p = 0.00056
R = 0.77 , p < 2.2e−16
0.18
0.16
0.14
0.12
0.10
0.075
0.100
0.125
0.150
0.175
Gamma−glutamylalanine
Gamma−glutamylglutamine
status
ICU
Ward

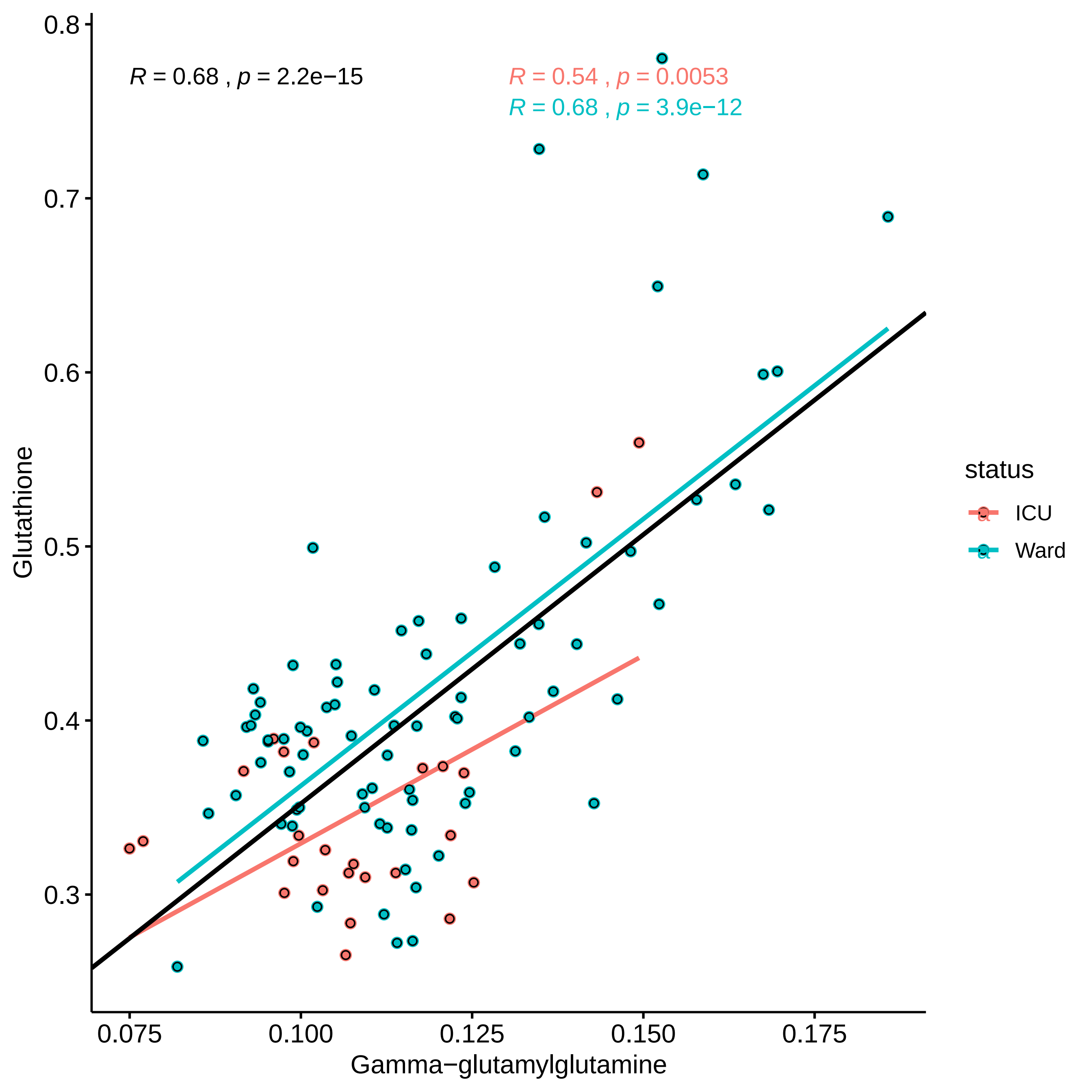

R = 0.68 , p = 2.2e−15
R = 0.54 , p = 0.0053
R = 0.68 , p = 3.9e−12
Glutathione
0.8
0.7
0.6
0.5
0.4
0.3
0.075
0.100
0.125
0.150
0.175
Gamma−glutamylglutamine
status
ICU
Ward

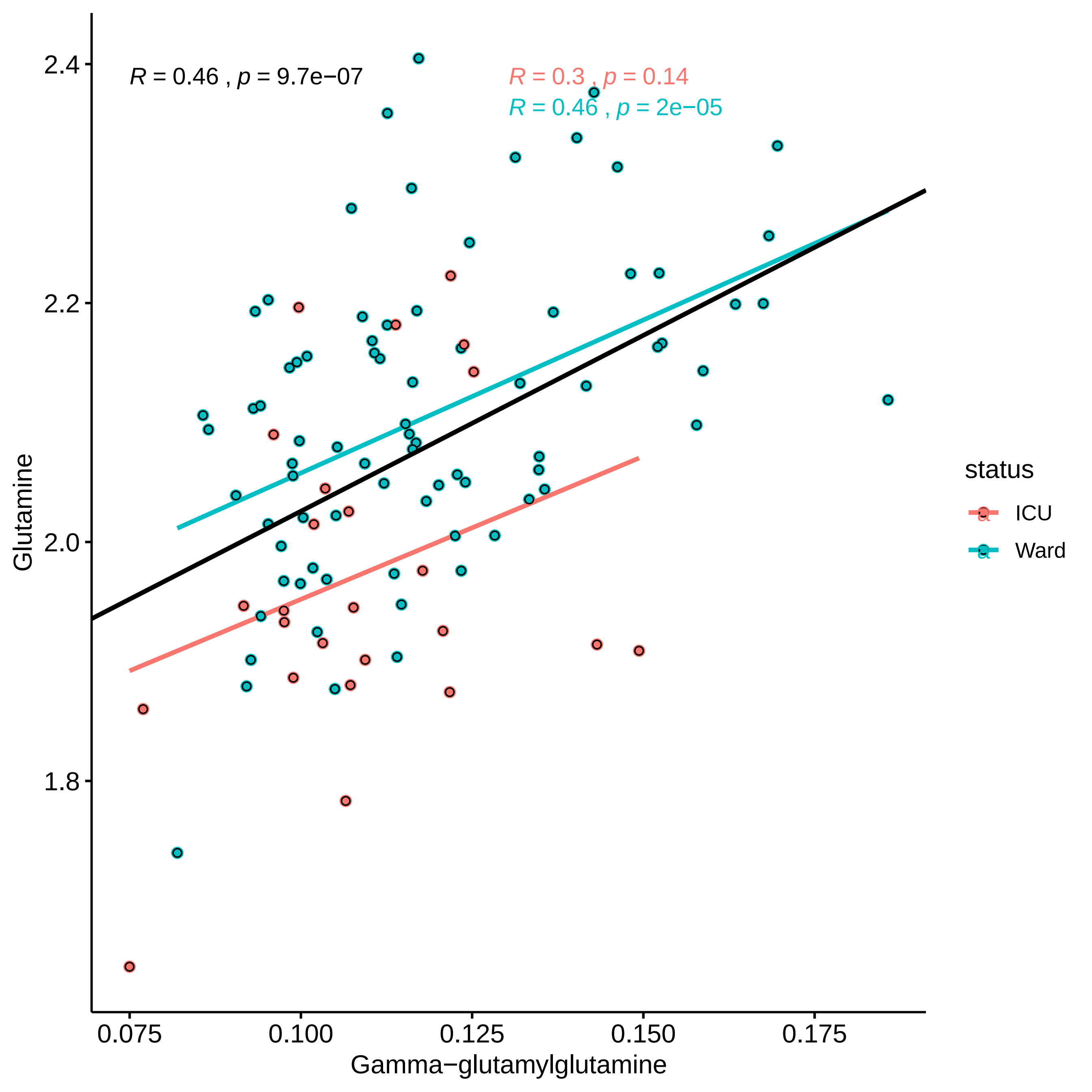

R = 0.46 , p = 9.7e−07
R = 0.3 , p = 0.14
R = 0.46 , p = 2e−05
2.4
2.2
2.0
1.8
Glutamine
0.075
0.100
0.125
0.150
0.175
Gamma−glutamylglutamine
status
ICU
Ward

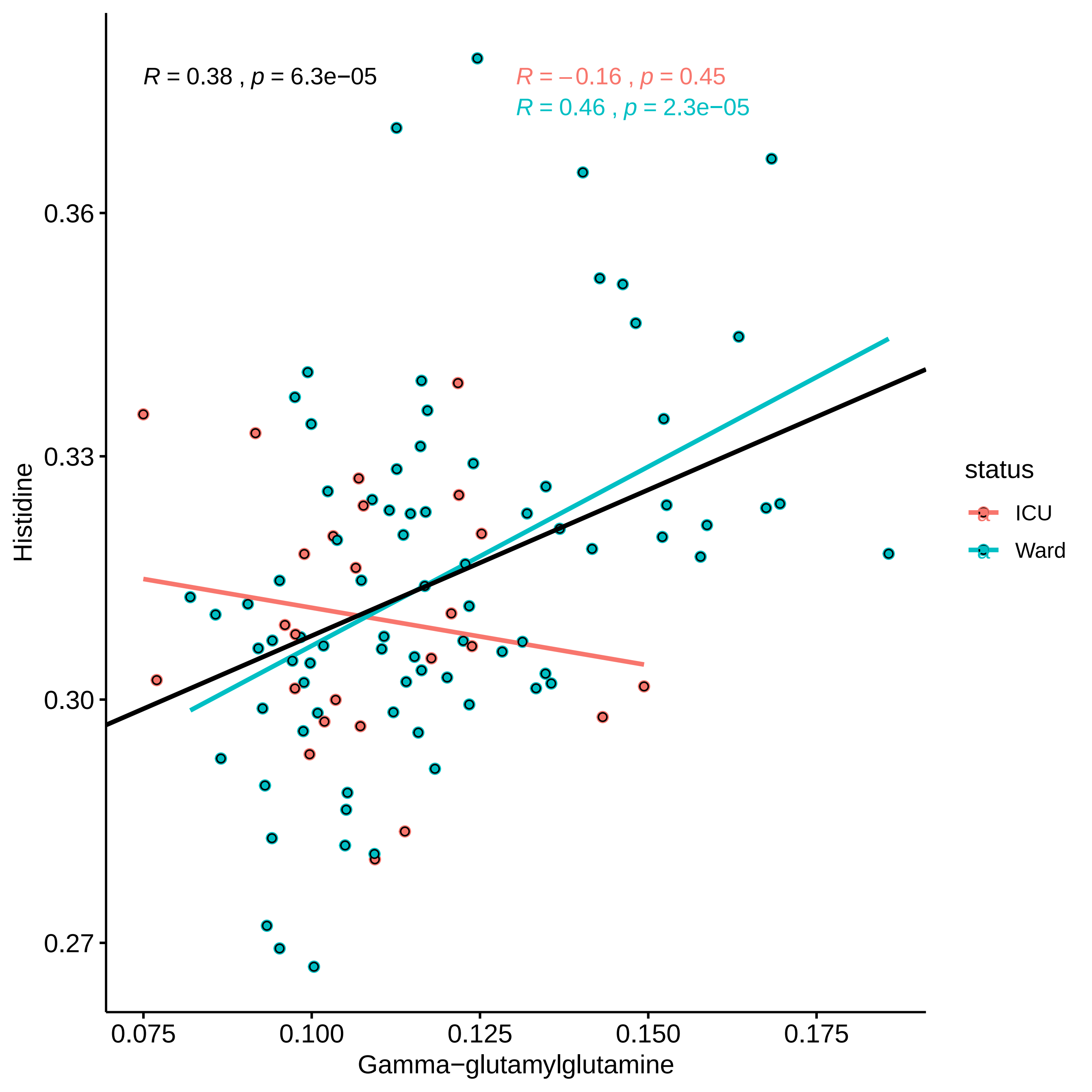

R = 0.38 , p = 6.3e−05
R = −0.16 , p = 0.45
R = 0.46 , p = 2.3e−05
Histidine
0.36
0.33
0.30
0.27
0.075
0.100
0.125
0.150
0.175
Gamma−glutamylglutamine
status
ICU
Ward

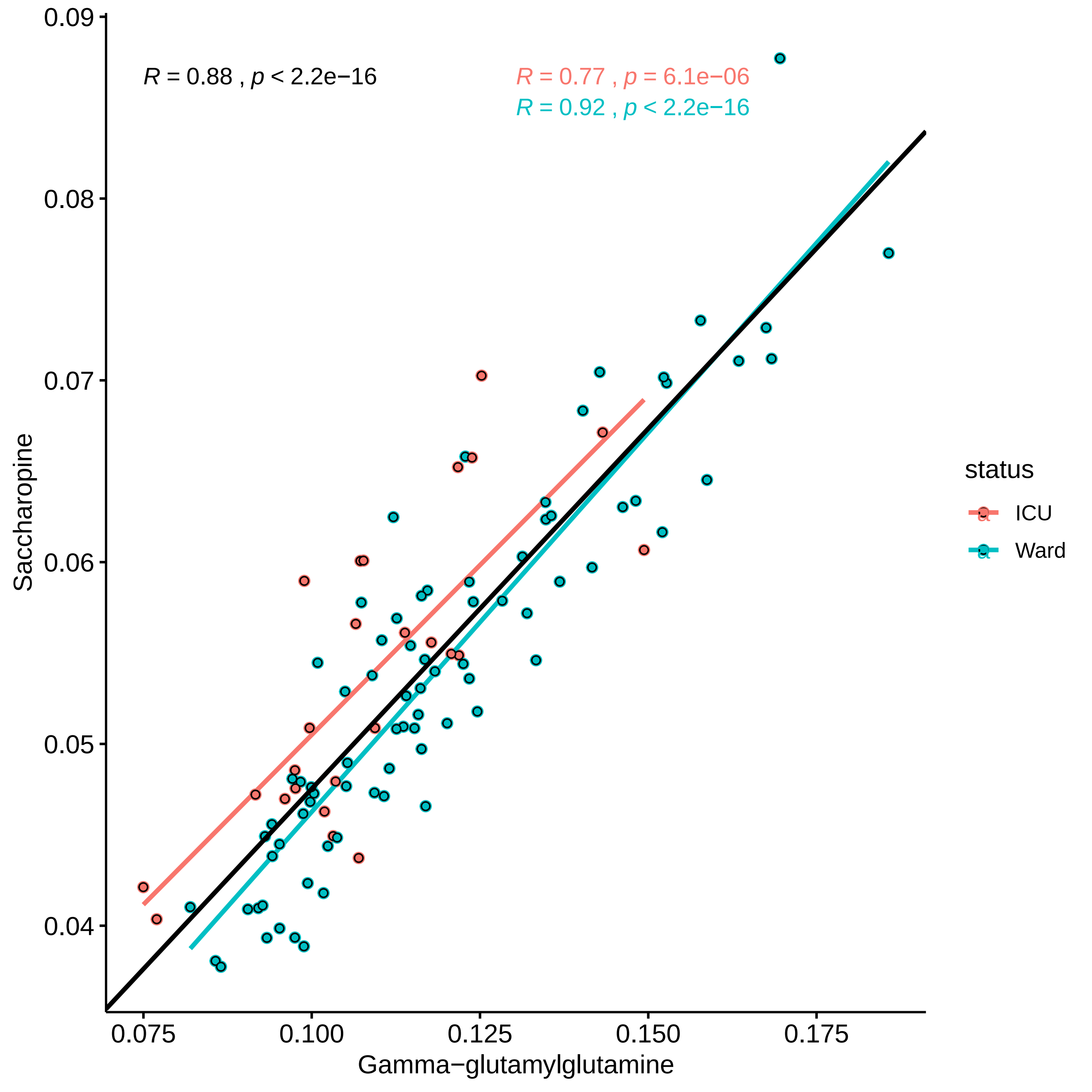

R = 0.88 , p < 2.2e−16
R = 0.77 , p = 6.1e−06
R = 0.92 , p < 2.2e−16
0.09
0.08
0.07
0.06
0.05
0.04
Saccharopine
0.075
0.100
0.125
0.150
0.175
Gamma−glutamylglutamine
status
ICU
Ward

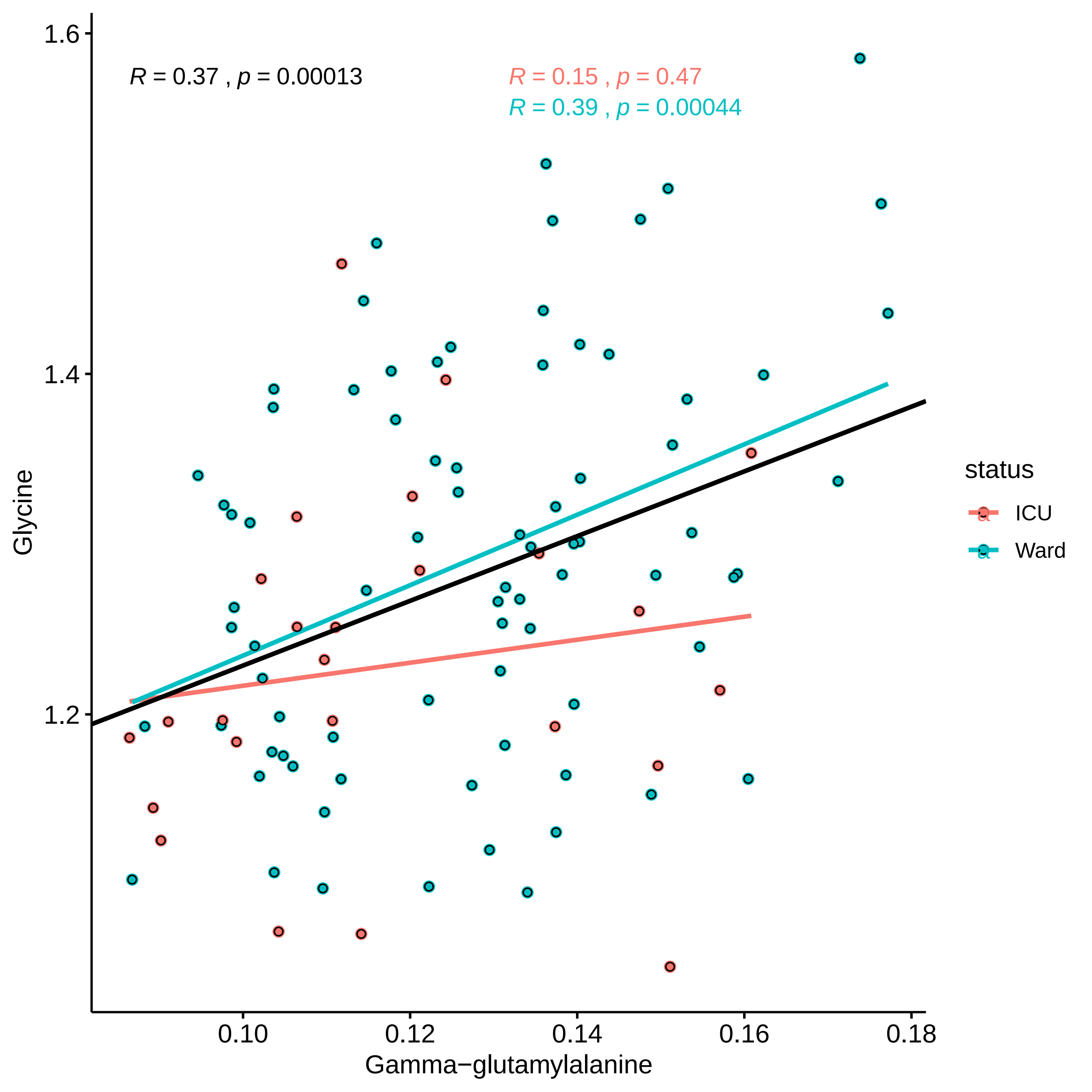

R = 0.37 , p = 0.00013
R = 0.15 , p = 0.47
R = 0.39 , p = 0.00044
1.6
1.4
1.2
Glycine
0.10
0.12
0.14
0.16
0.18
Gamma−glutamylalanine
status
ICU
Ward

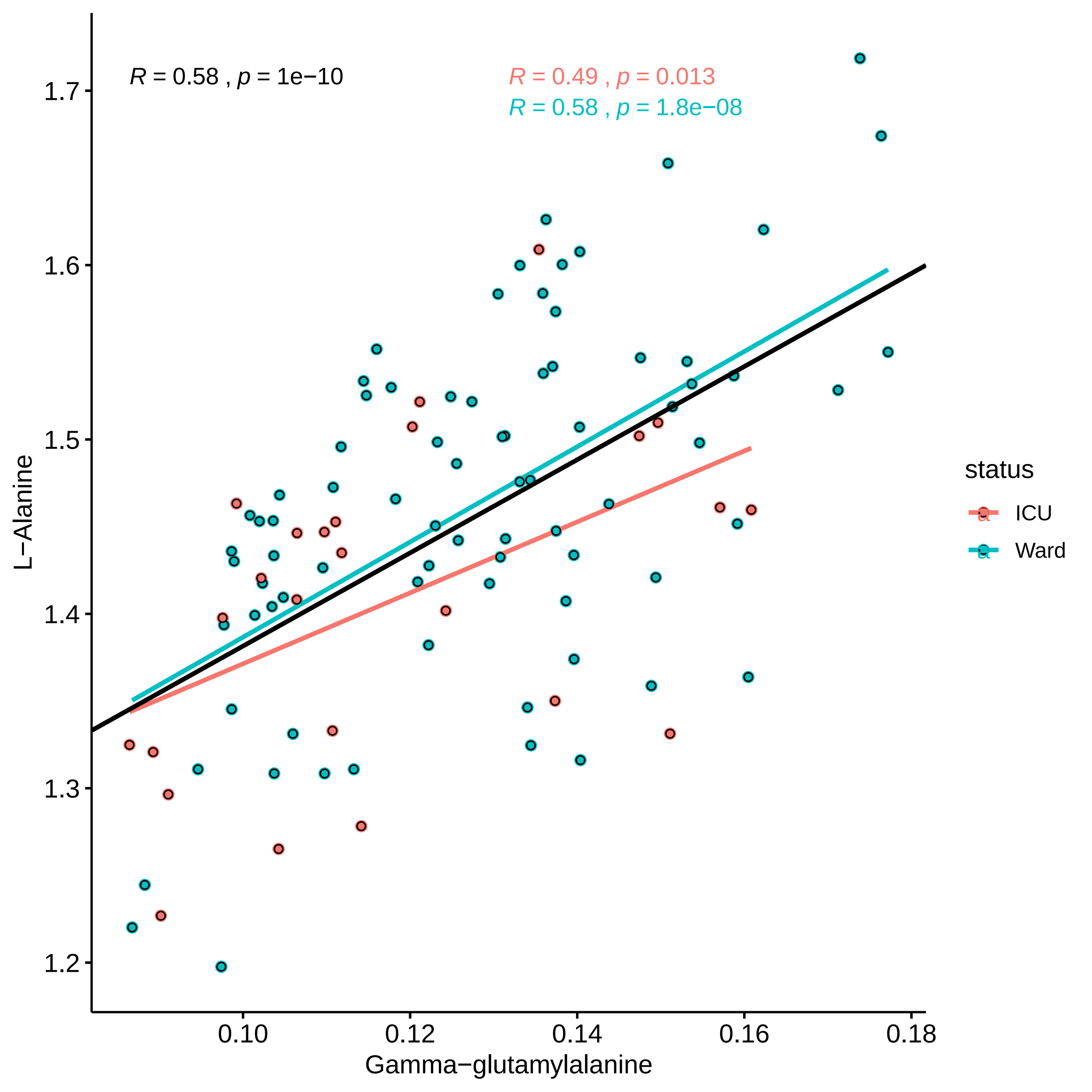

R = 0.58 , p = 1e−10
R = 0.49 , p = 0.013
R = 0.58 , p = 1.8e−08
1.7
1.6
1.5
1.4
1.3
1.2
L−Alanine
0.10
0.12
0.14
0.16
0.18
Gamma−glutamylalanine
status
ICU
Ward

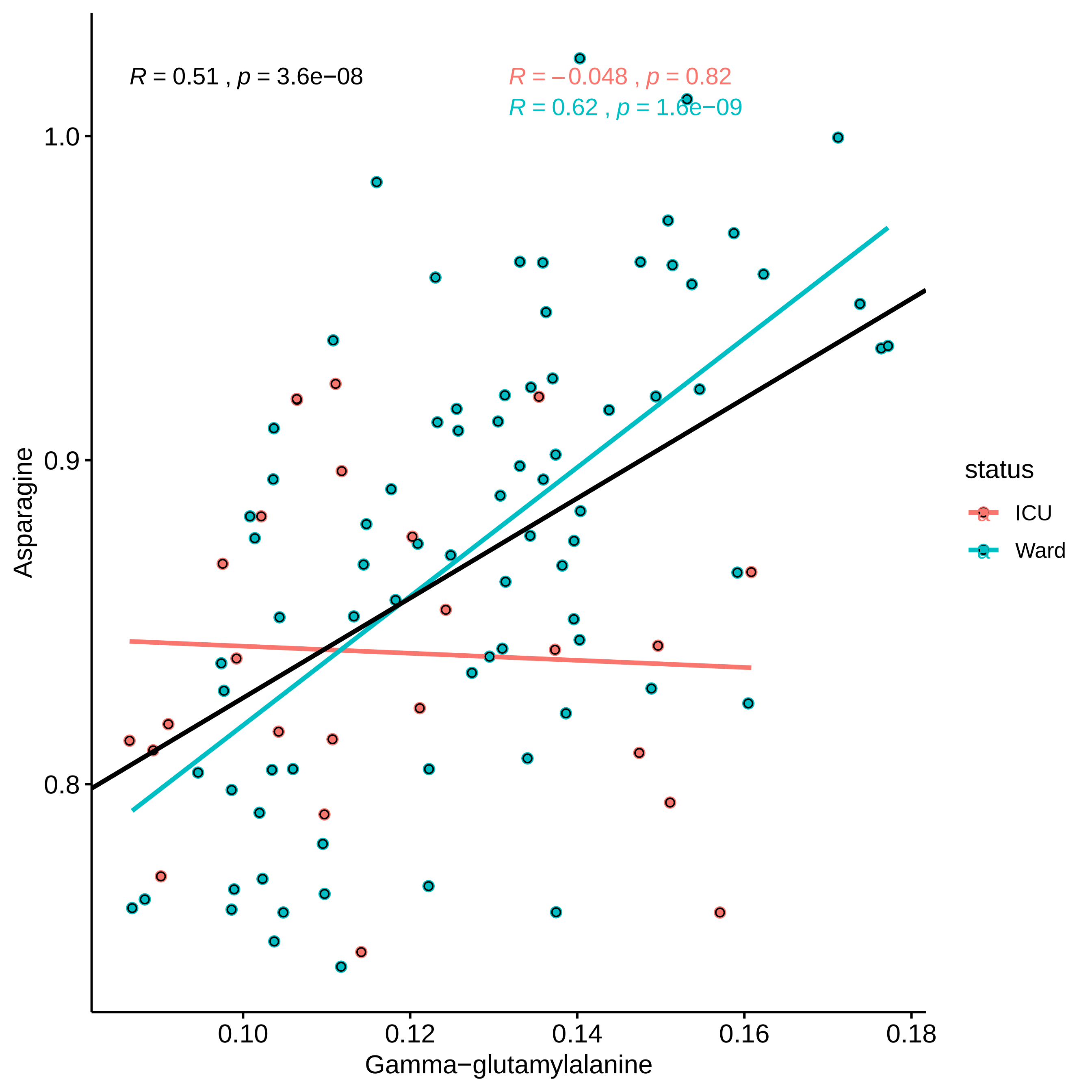

R = 0.51 , p = 3.6e−08
R = −0.048 , p = 0.82
R = 0.62 , p = 1.6e−09
1.0
0.9
0.8
Asparagine
0.10
0.12
0.14
0.16
0.18
Gamma−glutamylalanine
status
ICU
Ward

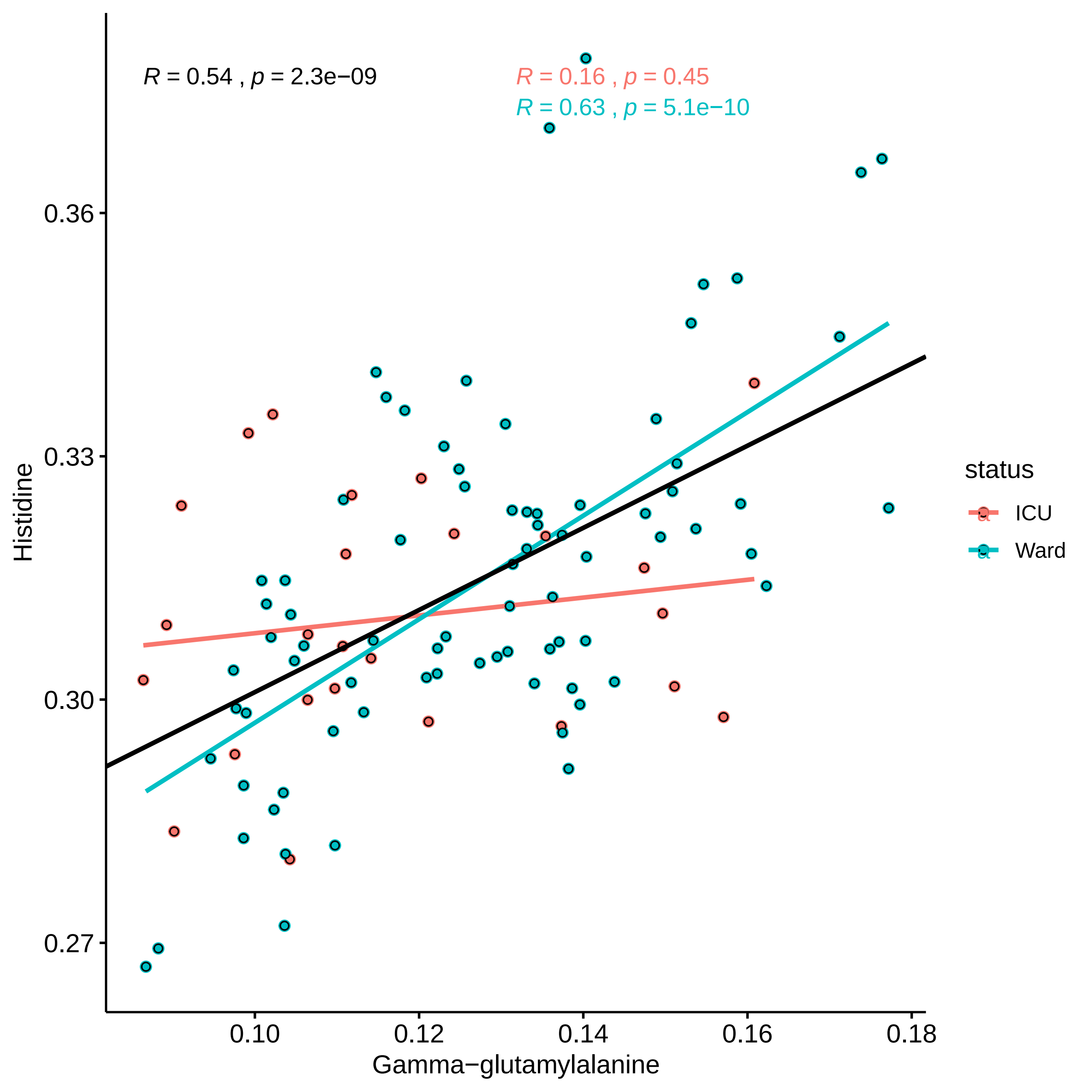

R = 0.54 , p = 2.3e−09
R = 0.16 , p = 0.45
R = 0.63 , p = 5.1e−10
Histidine
0.36
0.33
0.30
0.27
0.10
0.12
0.14
0.16
0.18
Gamma−glutamylalanine
status
ICU
Ward

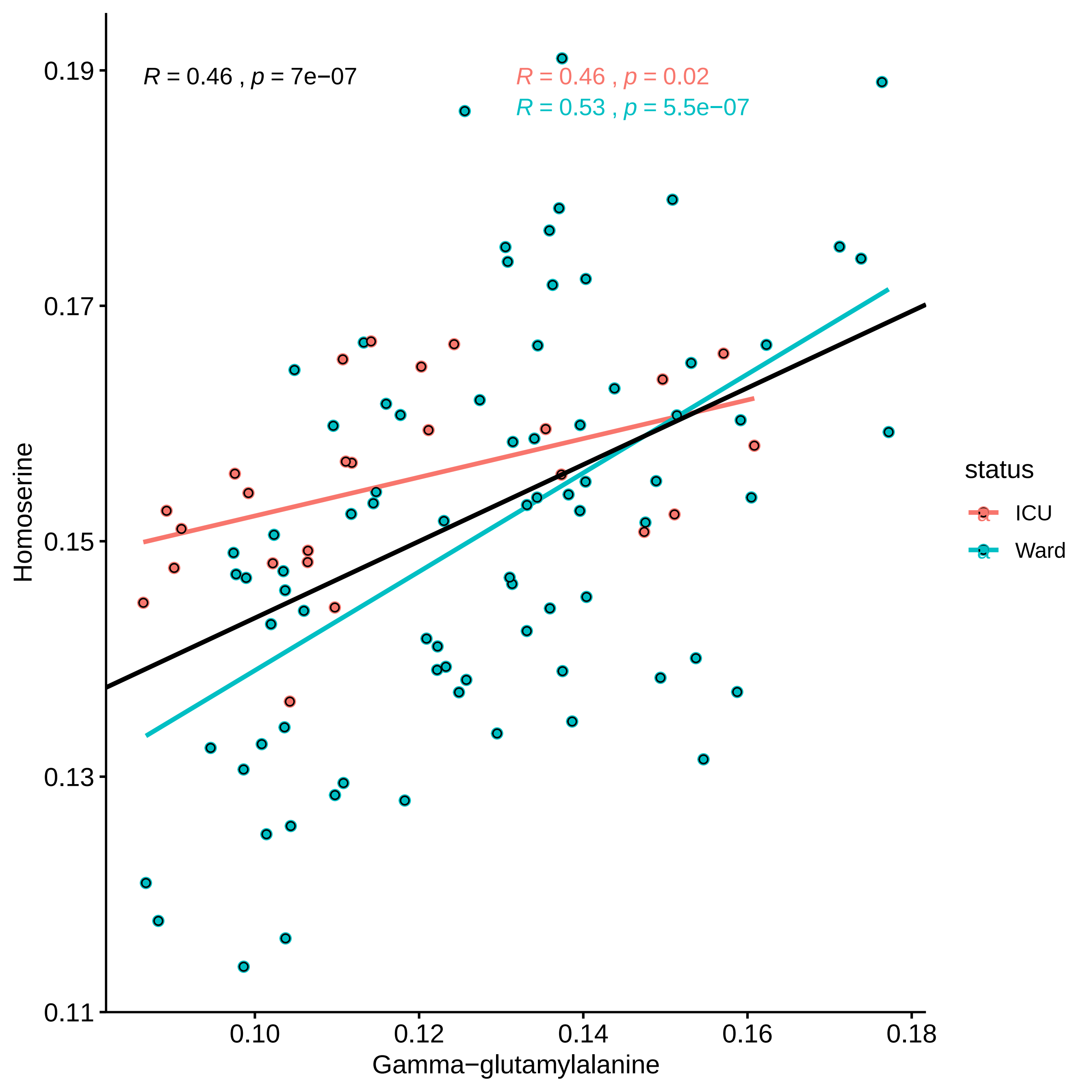

R = 0.46 , p = 7e−07
R = 0.46 , p = 0.02
R = 0.53 , p = 5.5e−07
Homoserine
Gamma−glutamylalanine
0.19
0.17
0.15
0.13
0.11
0.10
0.12
0.14
0.16
0.18
status
ICU
Ward

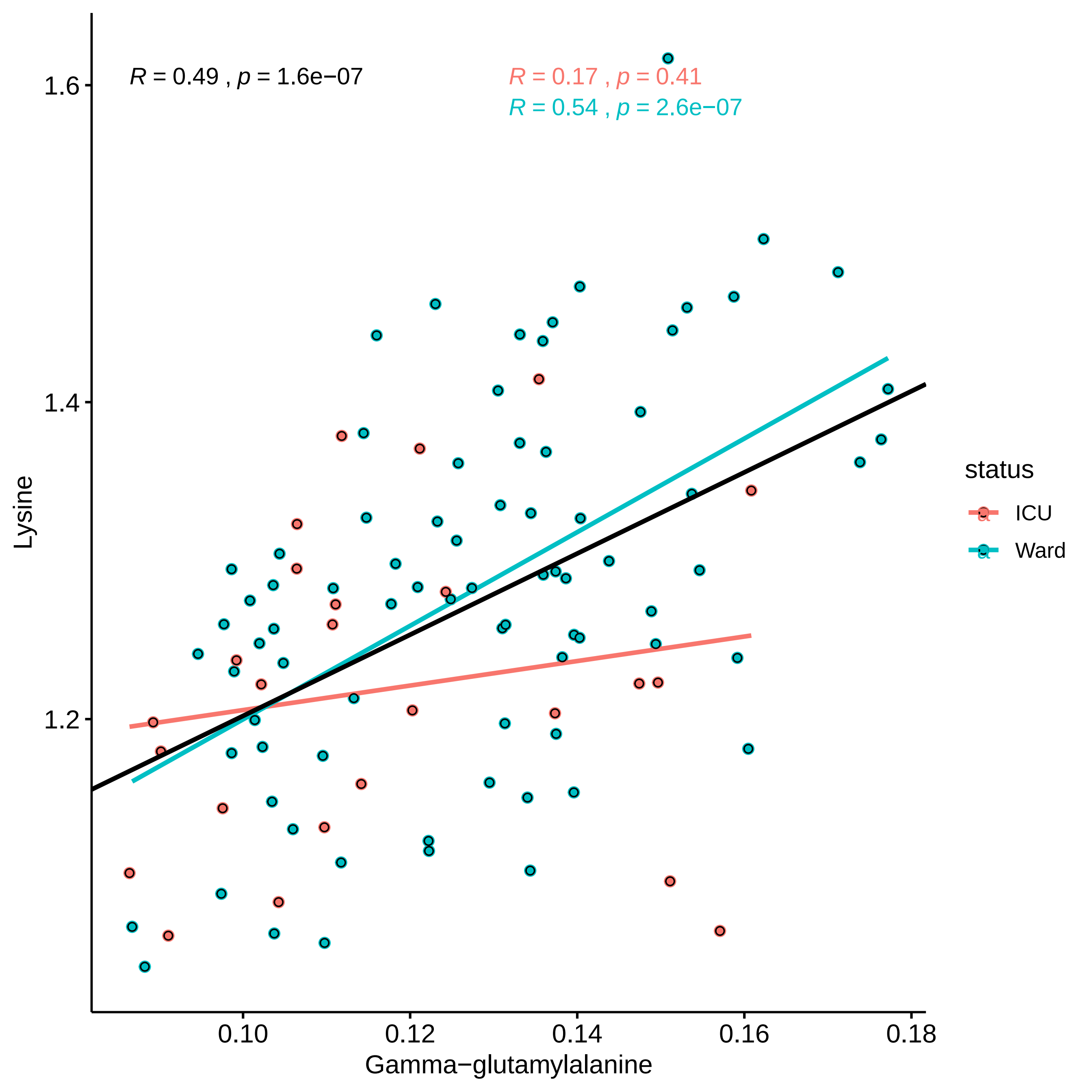

R = 0.49 , p = 1.6e−07
R = 0.17 , p = 0.41
R = 0.54 , p = 2.6e−07
Lysine
1.6
1.4
1.2
0.10
0.12
0.14
0.16
0.18
Gamma−glutamylalanine
status
ICU
Ward

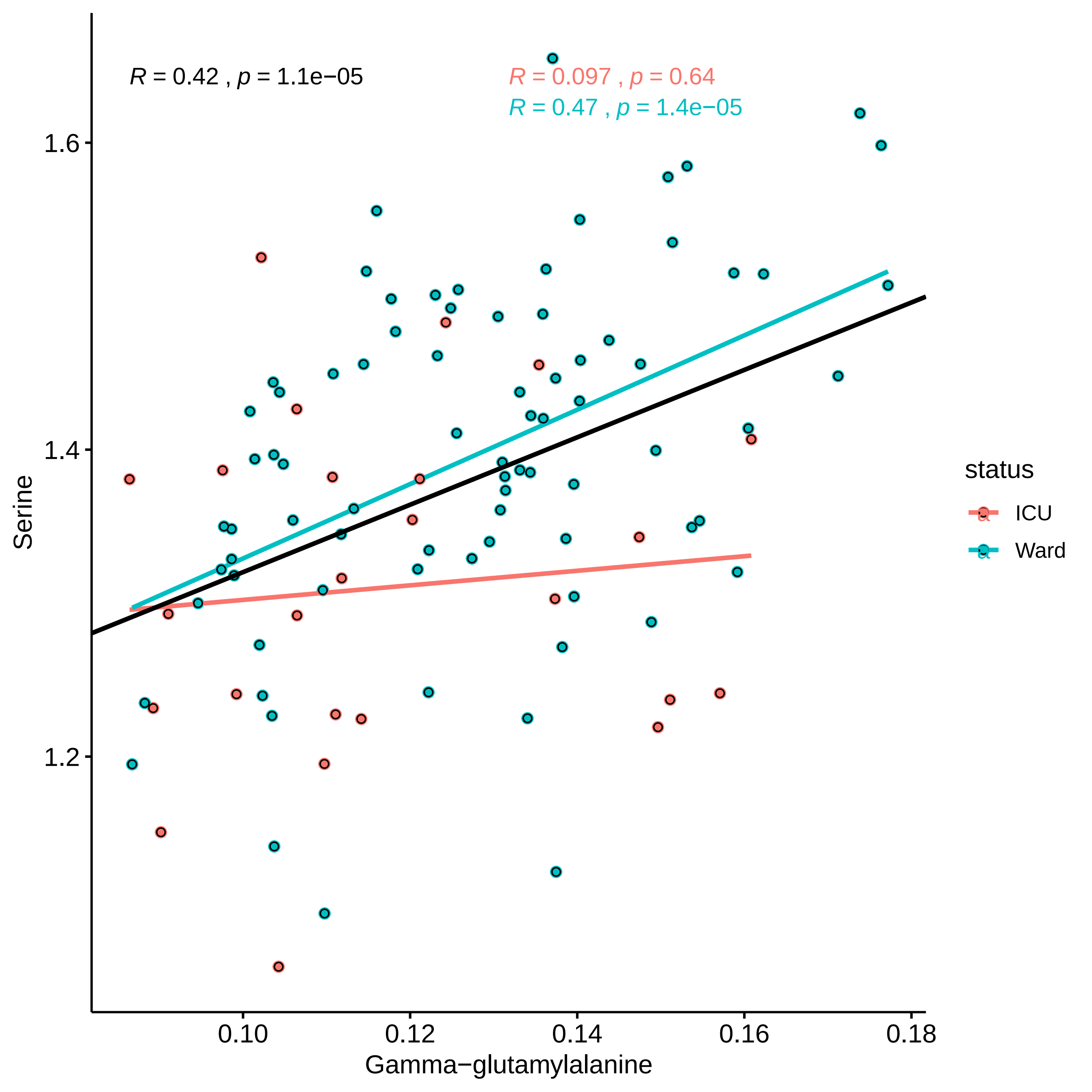

R = 0.42 , p = 1.1e−05
R = 0.097 , p = 0.64
R = 0.47 , p = 1.4e−05
1.6
1.4
1.2
Serine
0.10
0.12
0.14
0.16
0.18
Gamma−glutamylalanine
status
ICU
Ward

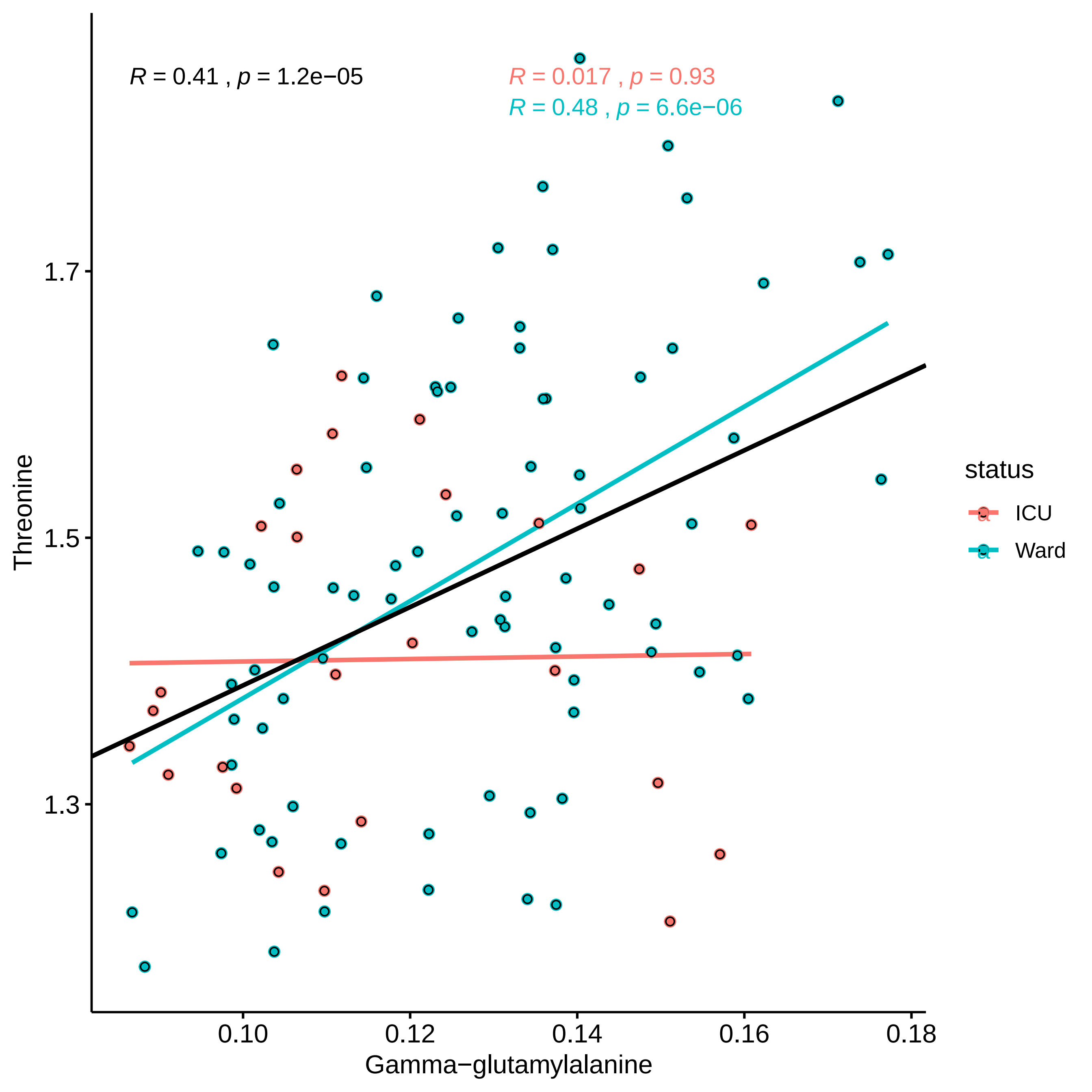

R = 0.41 , p = 1.2e−05
R = 0.017 , p = 0.93
R = 0.48 , p = 6.6e−06
Threonine
1.7
1.5
1.3
0.10
0.12
0.14
0.16
0.18
Gamma−glutamylalanine
status
ICU
Ward

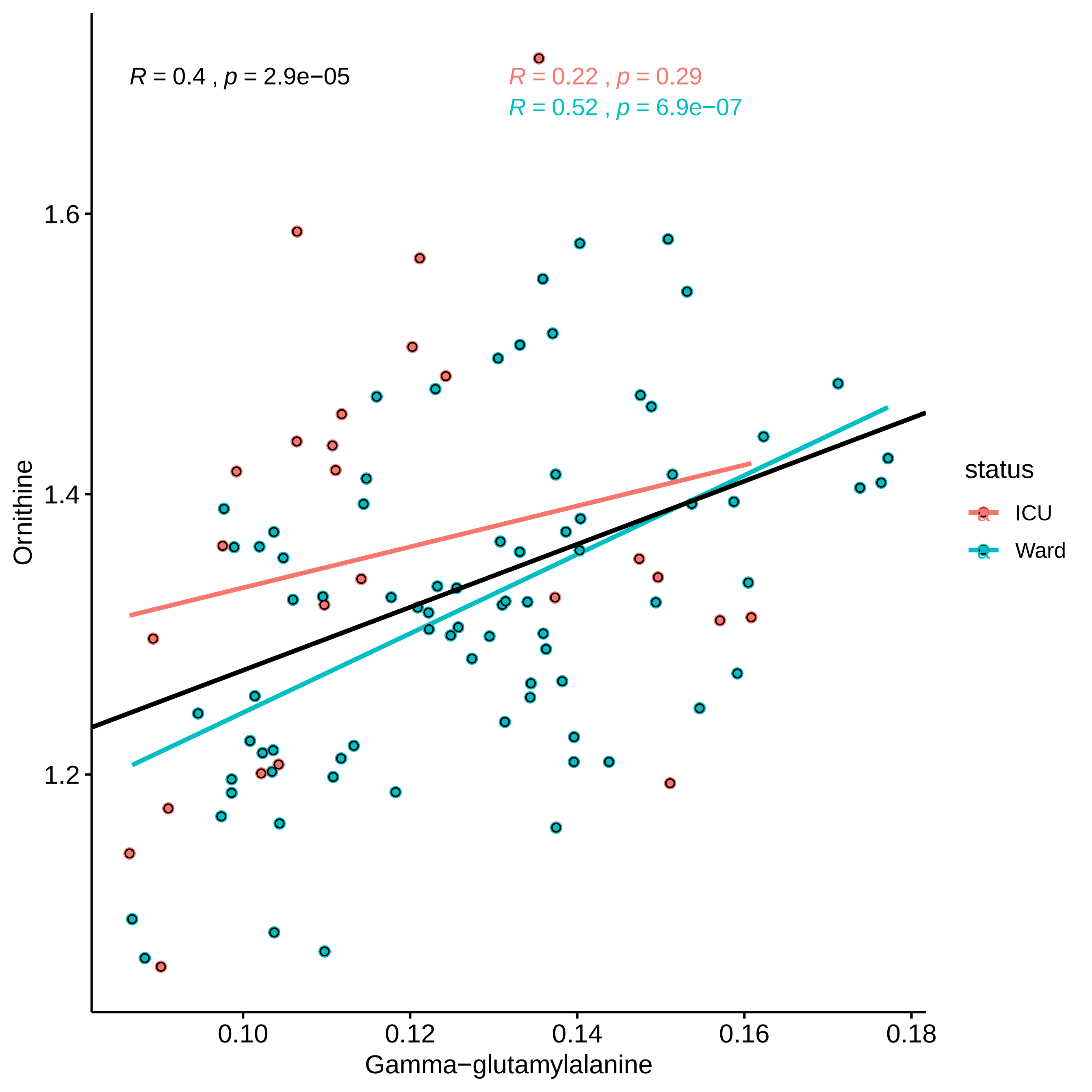

R = 0.4 , p = 2.9e−05
R = 0.22 , p = 0.29
R = 0.52 , p = 6.9e−07
1.6
1.4
1.2
Ornithine
0.10
0.12
0.14
0.16
0.18
Gamma−glutamylalanine
status
ICU
Ward

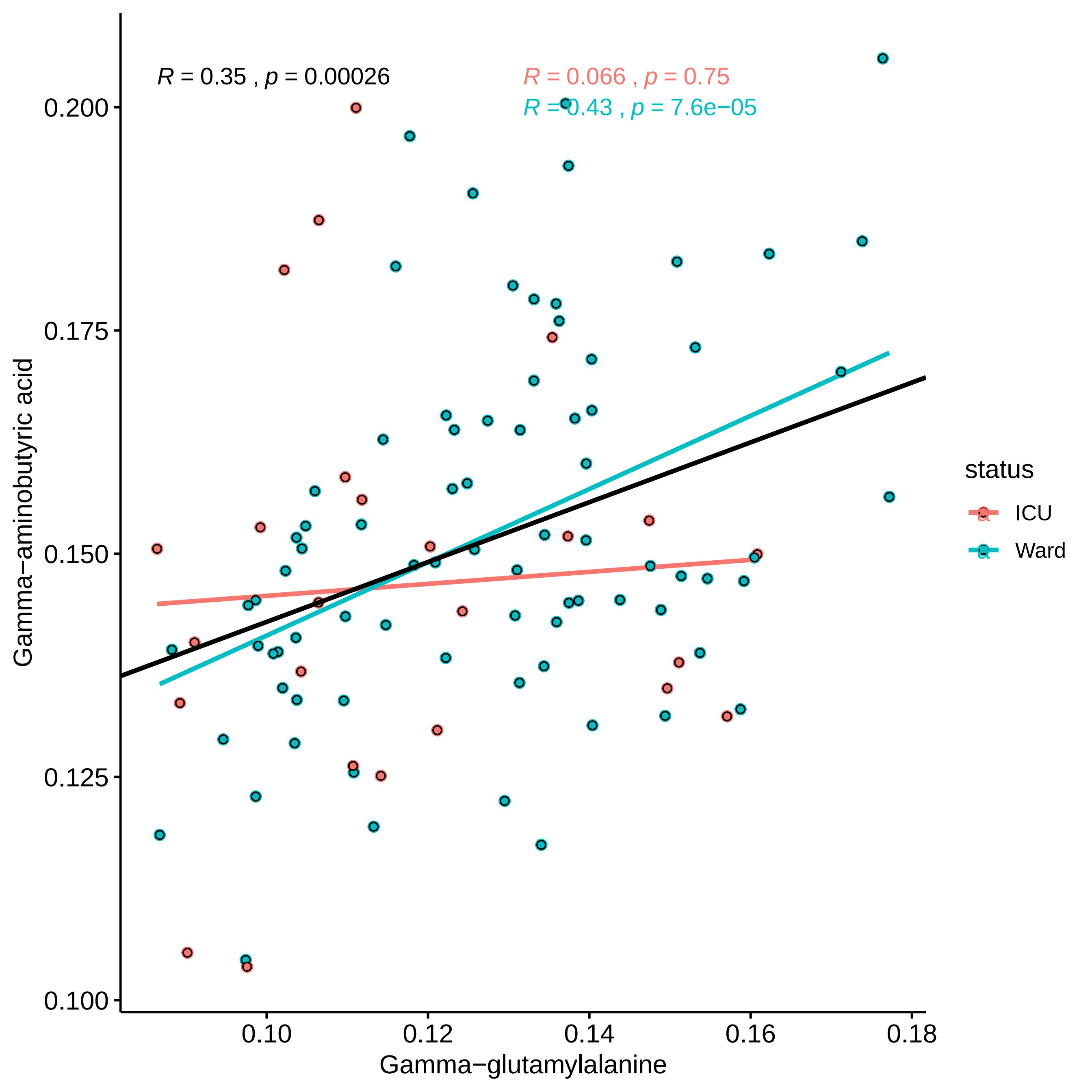

R = 0.35 , p = 0.00026
R = 0.066 , p = 0.75
R = 0.43 , p = 7.6e−05
Gamma−aminobutyric acid
0.200
0.175
0.150
0.125
0.100
0.10
0.12
0.14
0.16
0.18
Gamma−glutamylalanine
status
ICU
Ward

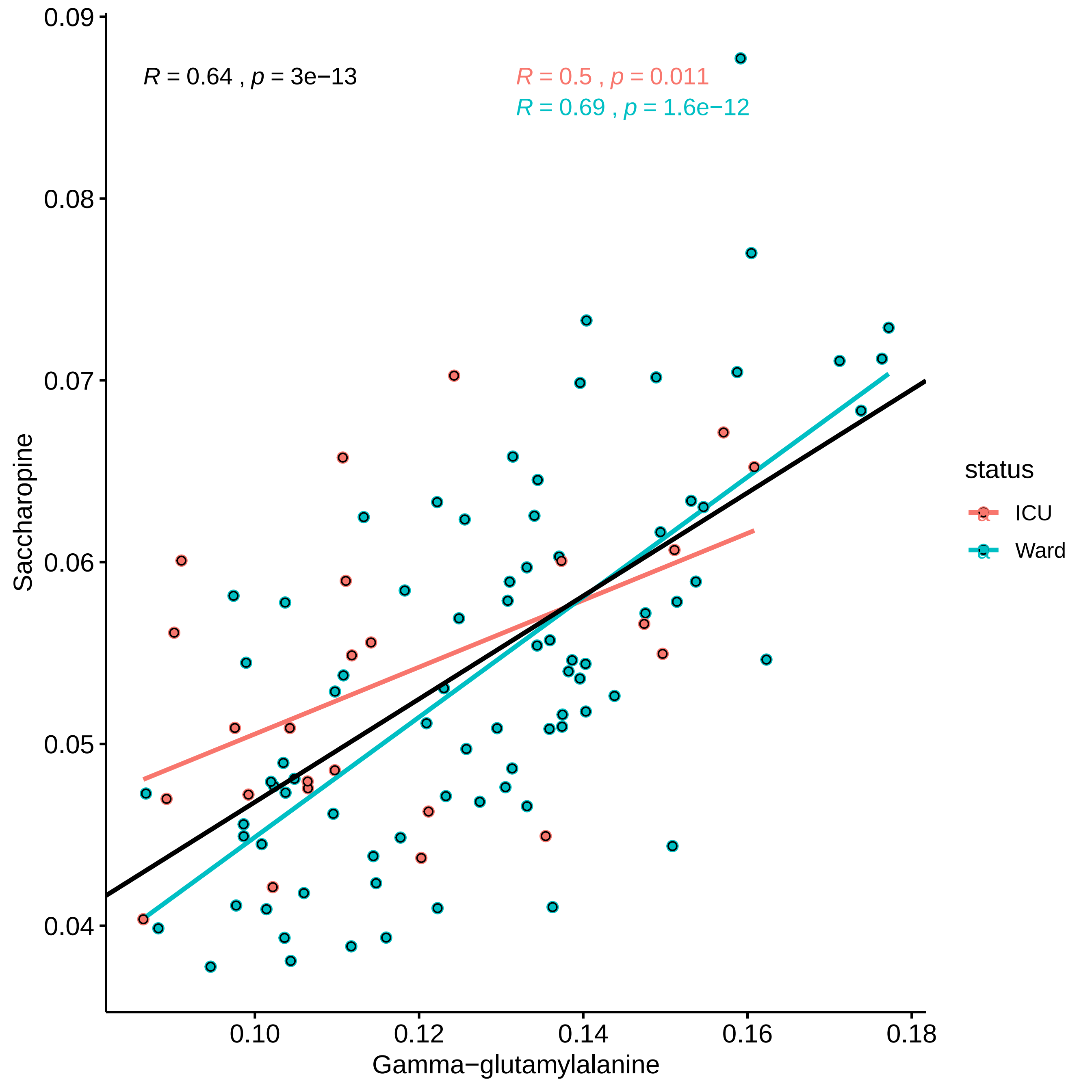

R = 0.64 , p = 3e−13
R = 0.5 , p = 0.011
R = 0.69 , p = 1.6e−12
Saccharopine
0.09
0.08
0.07
0.06
0.05
0.04
0.10
0.12
0.14
0.16
0.18
Gamma−glutamylalanine
status
ICU
Ward

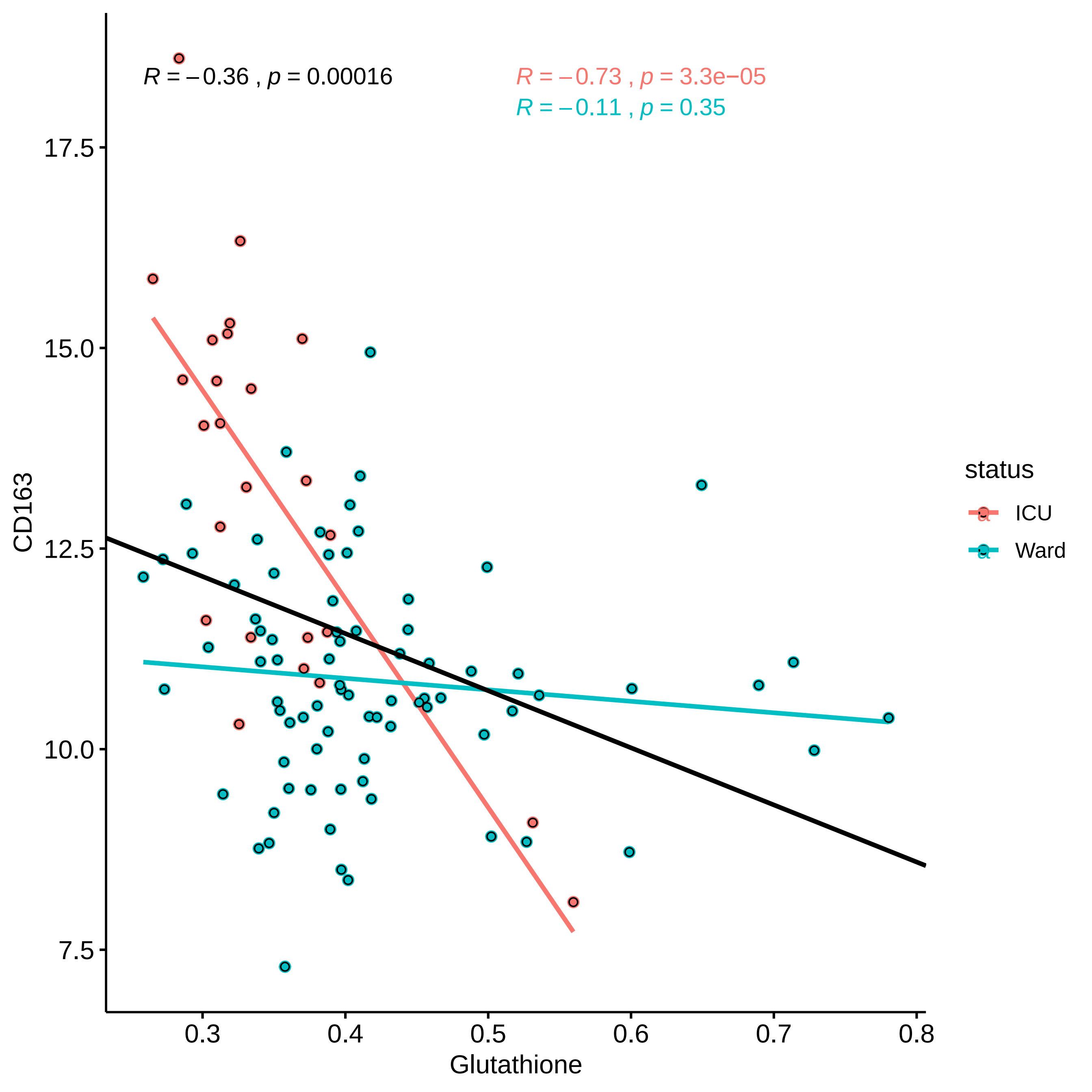

R = −0.36 , p = 0.00016
R = −0.73 , p = 3.3e−05
R = −0.11 , p = 0.35
CD163
17.5
15.0
12.5
10.0
7.5
0.3
0.4
0.5
0.6
0.7
0.8
Glutathione
status
ICU
Ward

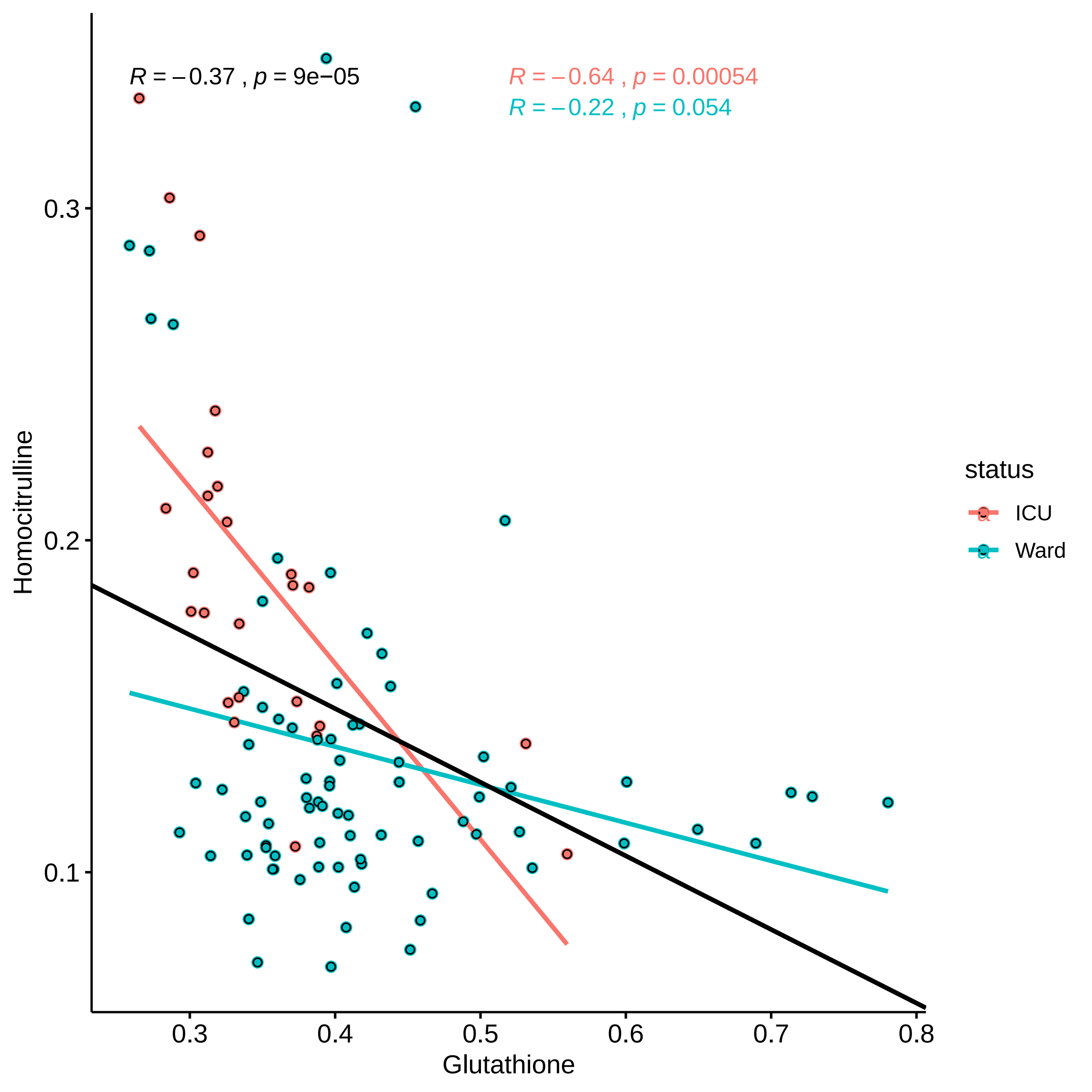

R = −0.37 , p = 9e−05
R = −0.64 , p = 0.00054
R = −0.22 , p = 0.054
0.3
0.2
0.1
Homocitrulline
0.3
0.4
0.5
0.6
0.7
0.8
Glutathione
status
ICU
Ward

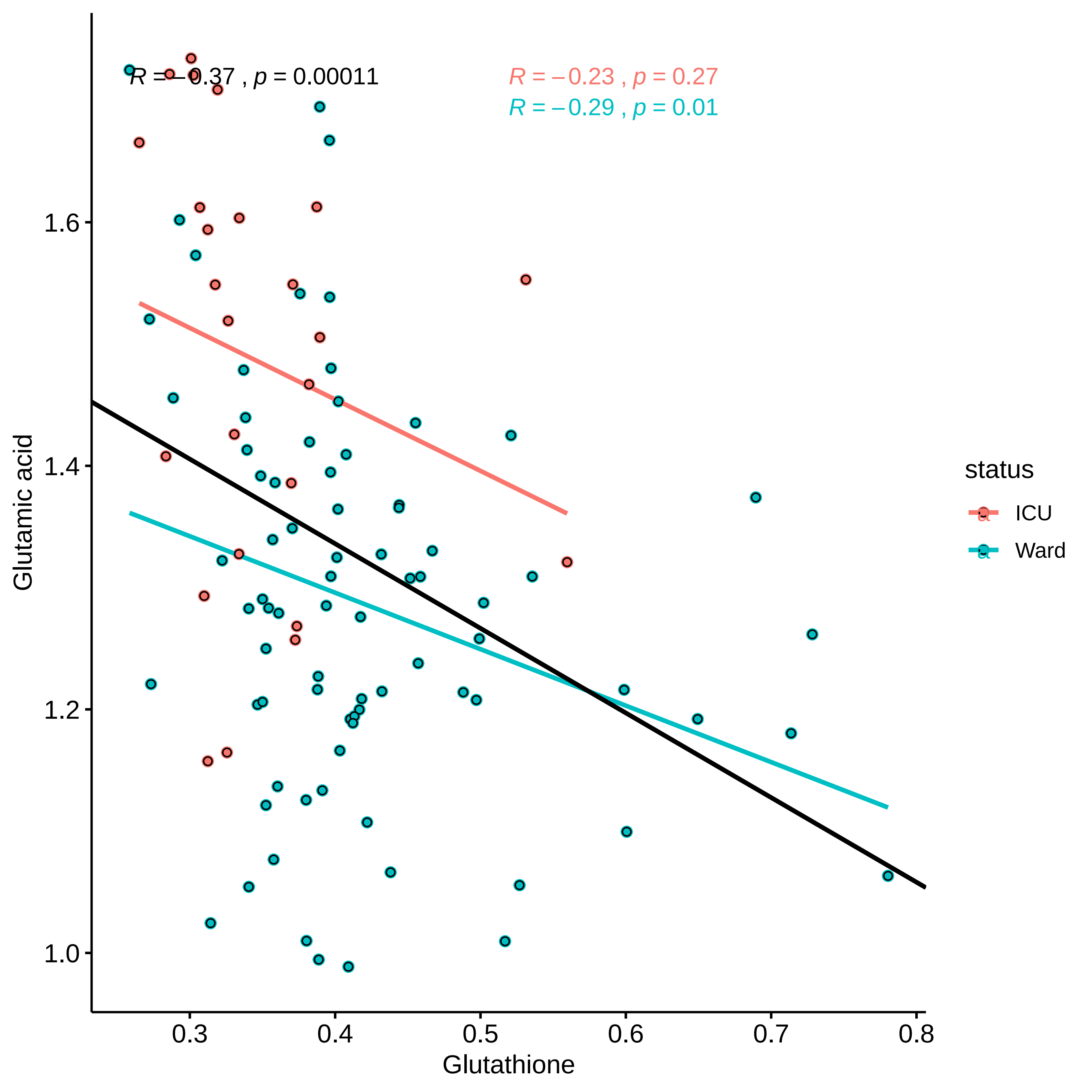

R = −0.37 , p = 0.00011
R = −0.23 , p = 0.27
R = −0.29 , p = 0.01
Glutamic acid
1.6
1.4
1.2
1.0
0.3
0.4
0.5
0.6
0.7
0.8
Glutathione
status
ICU
Ward

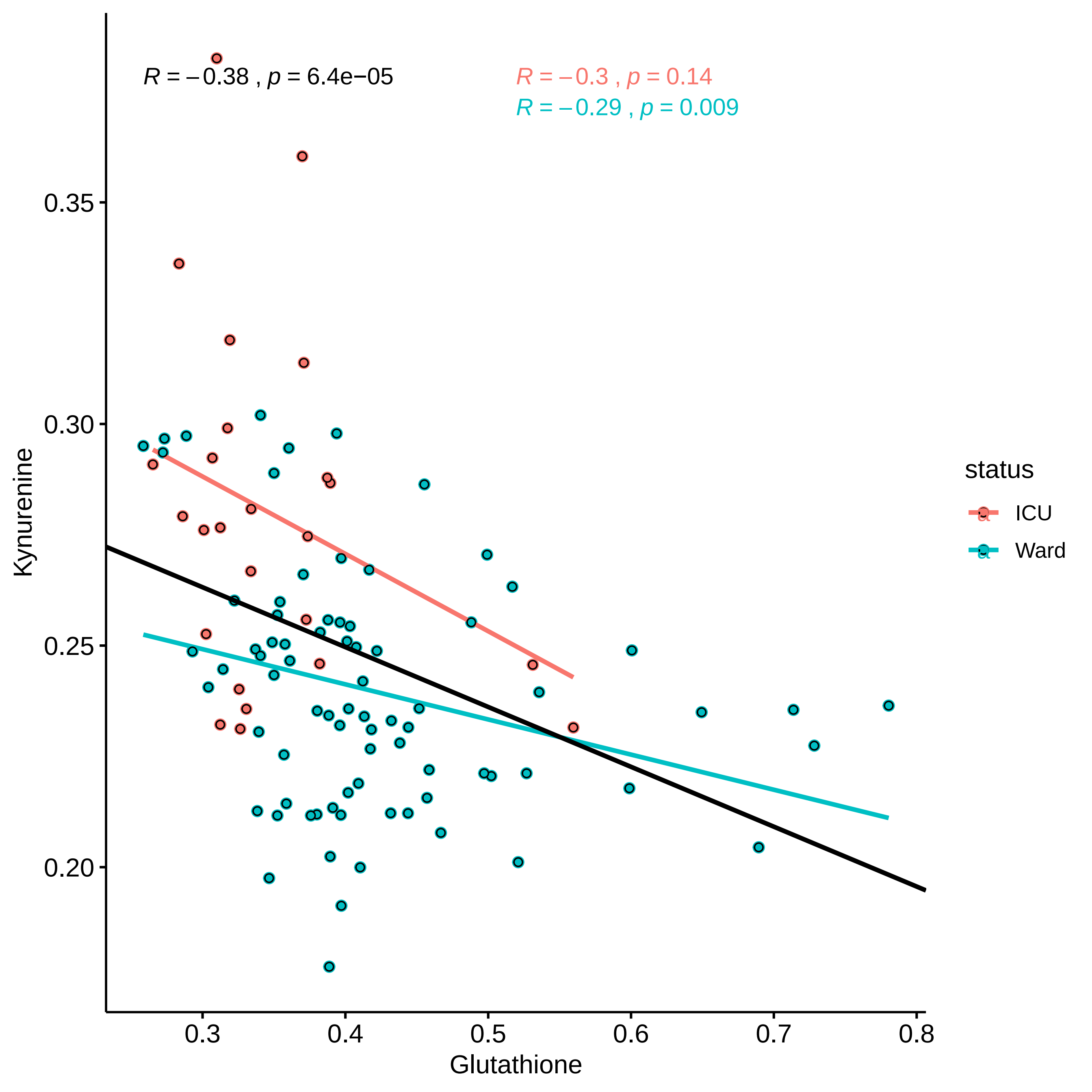
R = −0.38 , p = 6.4e−05
R = −0.3 , p = 0.14
R = −0.29 , p = 0.009
0.35
0.30
0.25
0.20
Kynurenine
0.3
0.4
0.5
0.6
0.7
0.8
Glutathione
status
ICU
Ward

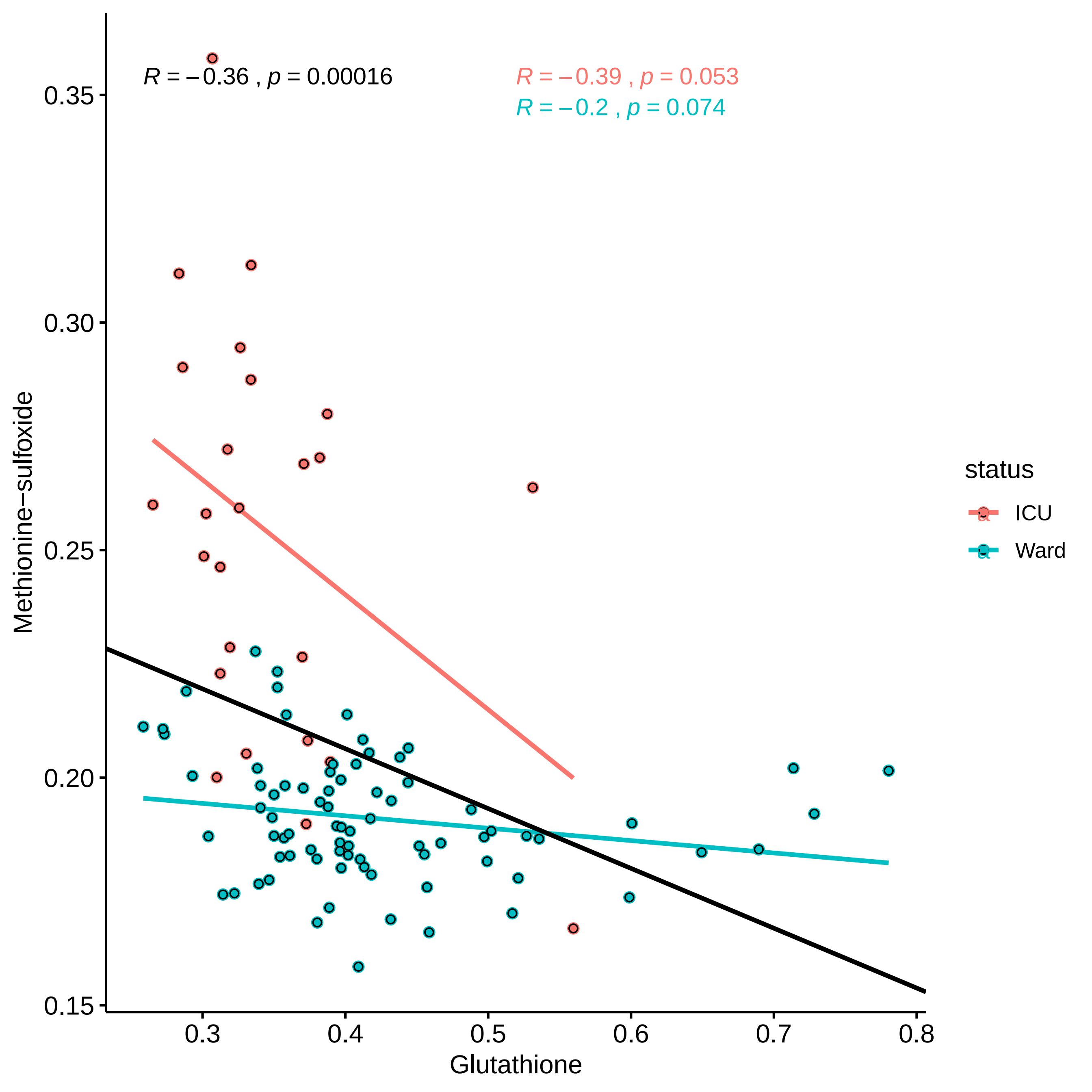

R = −0.36 , p = 0.00016
R = −0.39 , p = 0.053
R = −0.2 , p = 0.074
Methionine−sulfoxide
0.35
0.30
0.25
0.20
0.15
0.3
0.4
0.5
0.6
0.7
0.8
Glutathione
status
ICU
Ward

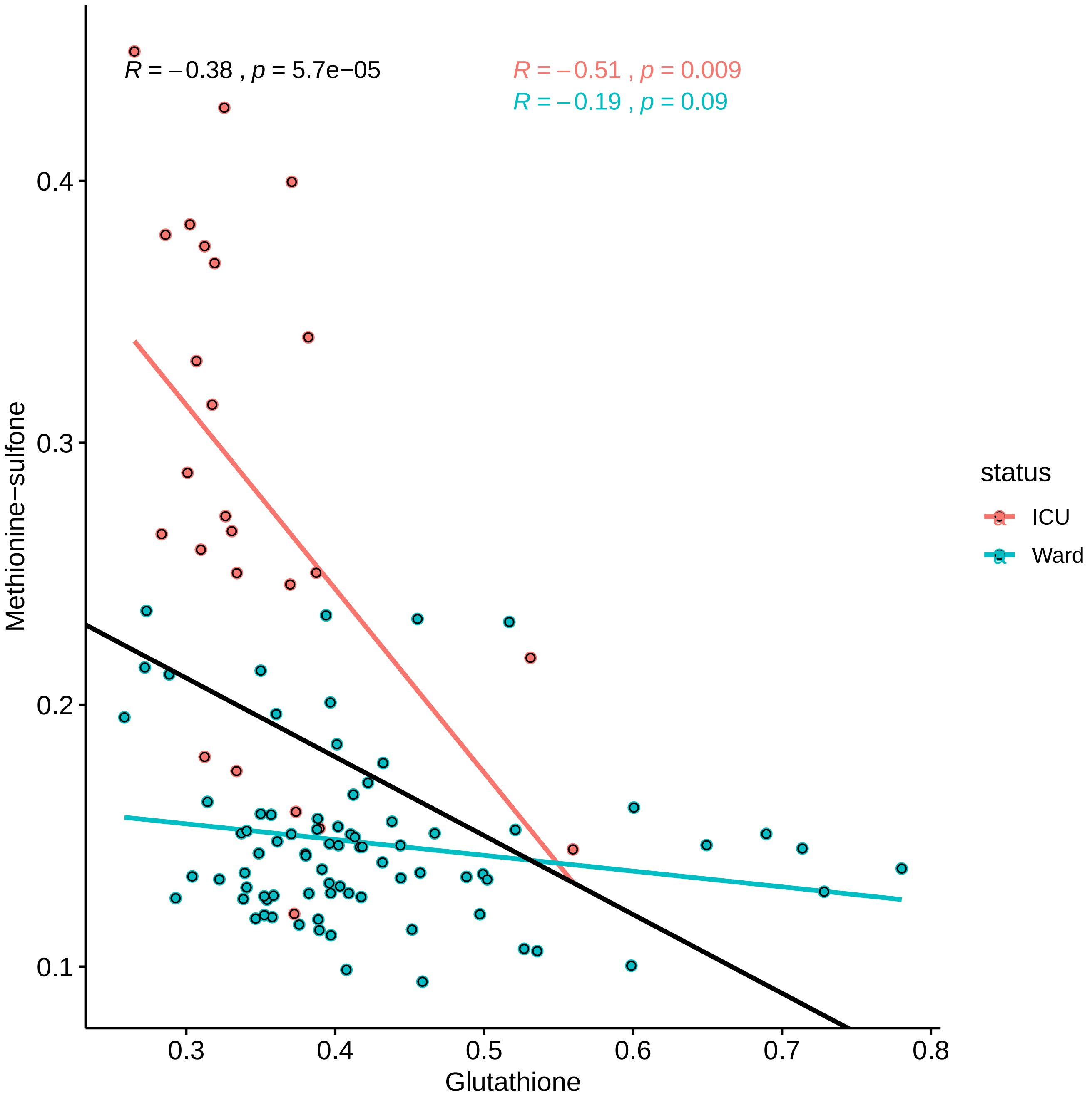

R = −0.38 , p = 5.7e−05
R = −0.51 , p = 0.009
R = −0.19 , p = 0.09
Methionine−sulfone
0.4
0.3
0.2
0.1
0.3
0.4
0.5
0.6
0.7
0.8
Glutathione
status
ICU
Ward

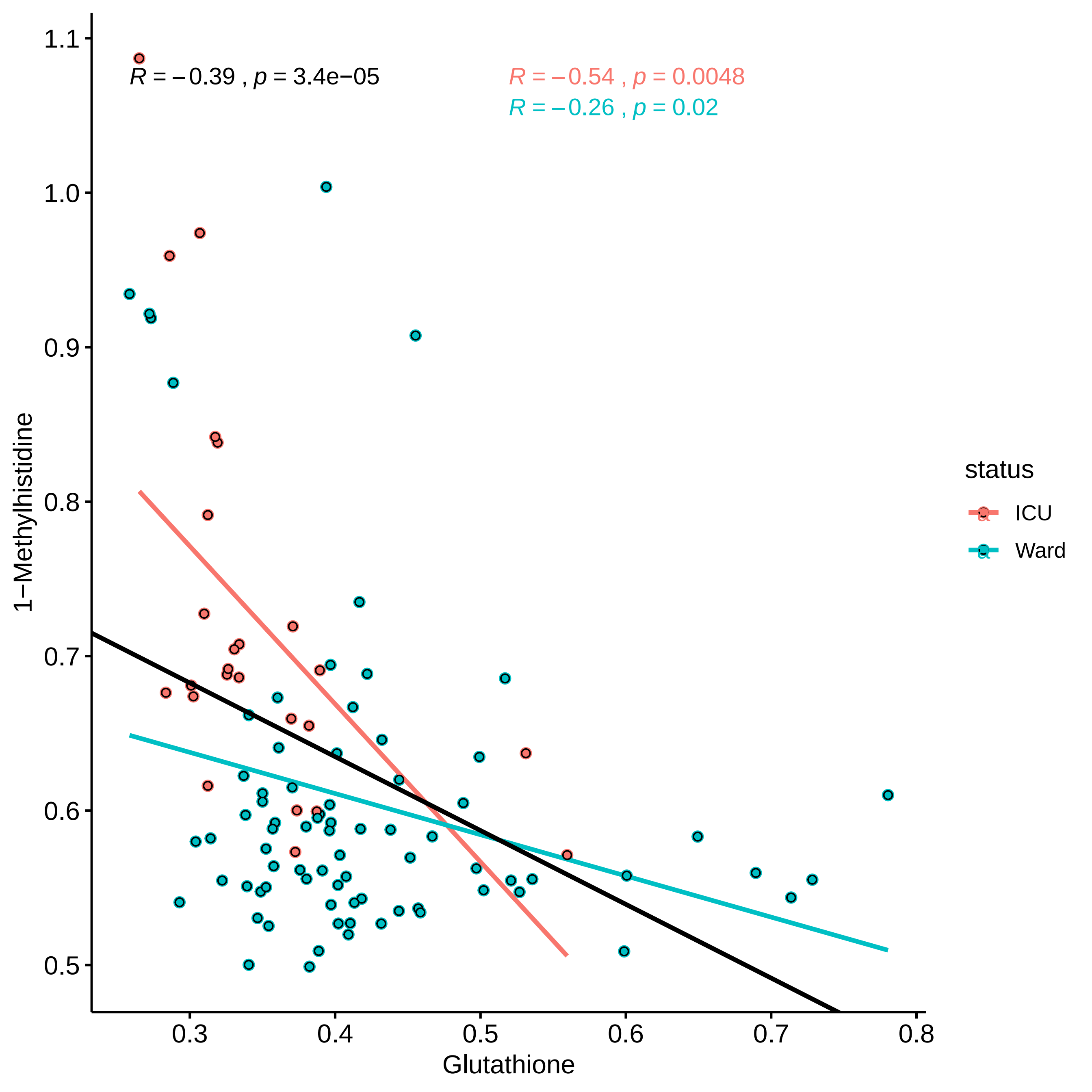

R = −0.39 , p = 3.4e−05
R = −0.54 , p = 0.0048
R = −0.26 , p = 0.02
1−Methylhistidine
Glutathione
status
ICU
Ward
1.1
1.0
0.9
0.8
0.7
0.6
0.5
0.3
0.4
0.5
0.6
0.7
0.8

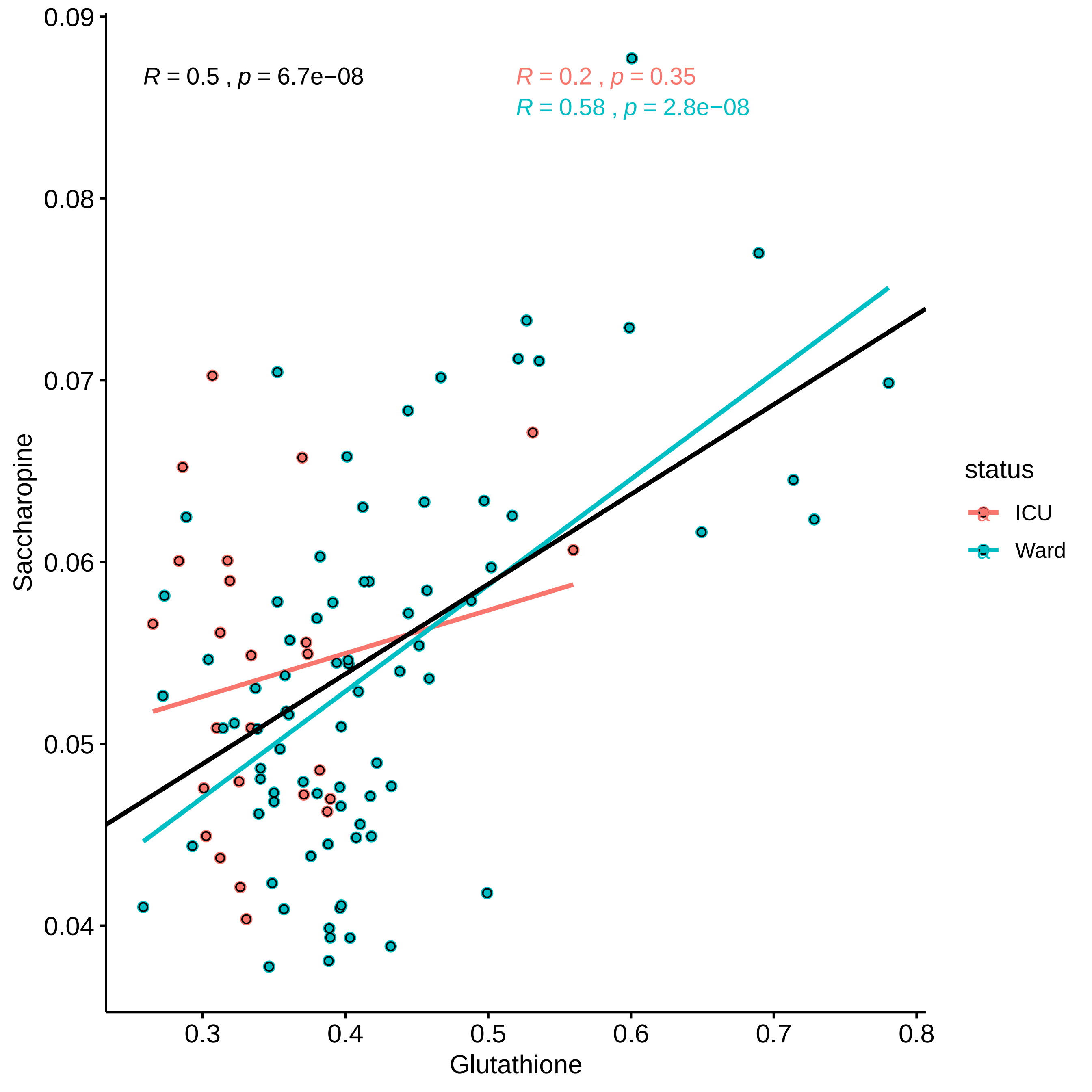

R = 0.5 , p = 6.7e−08
R = 0.2 , p = 0.35
R = 0.58 , p = 2.8e−08
0.09
0.08
0.07
0.06
0.05
0.04
Saccharopine
0.3
0.4
0.5
0.6
0.7
0.8
Glutathione
status
ICU
Ward

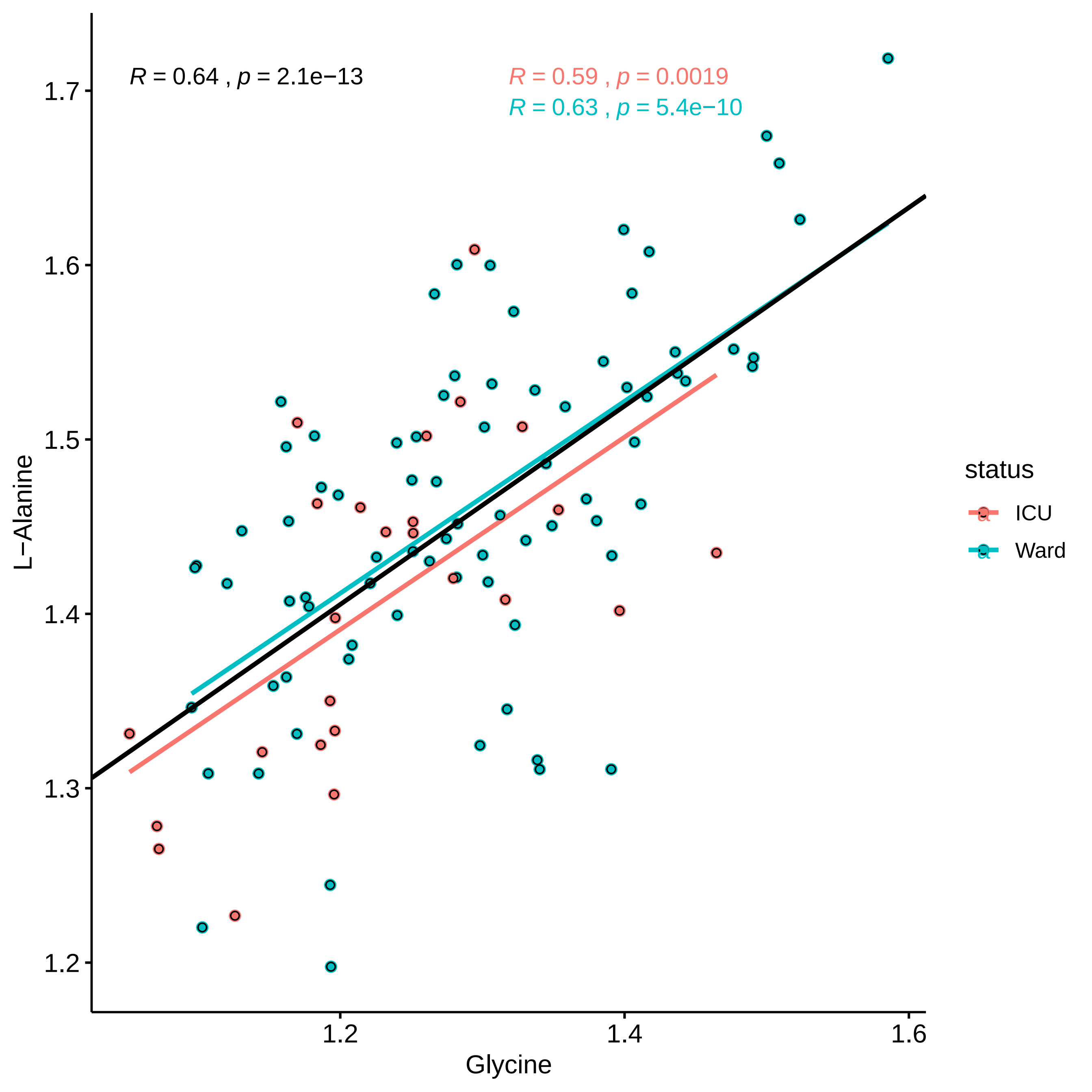

R = 0.64 , p = 2.1e−13
R = 0.59 , p = 0.0019
R = 0.63 , p = 5.4e−10
1.7
1.6
1.5
1.4
1.3
1.2
L−Alanine
1.2
1.4
1.6
Glycine
status
ICU
Ward

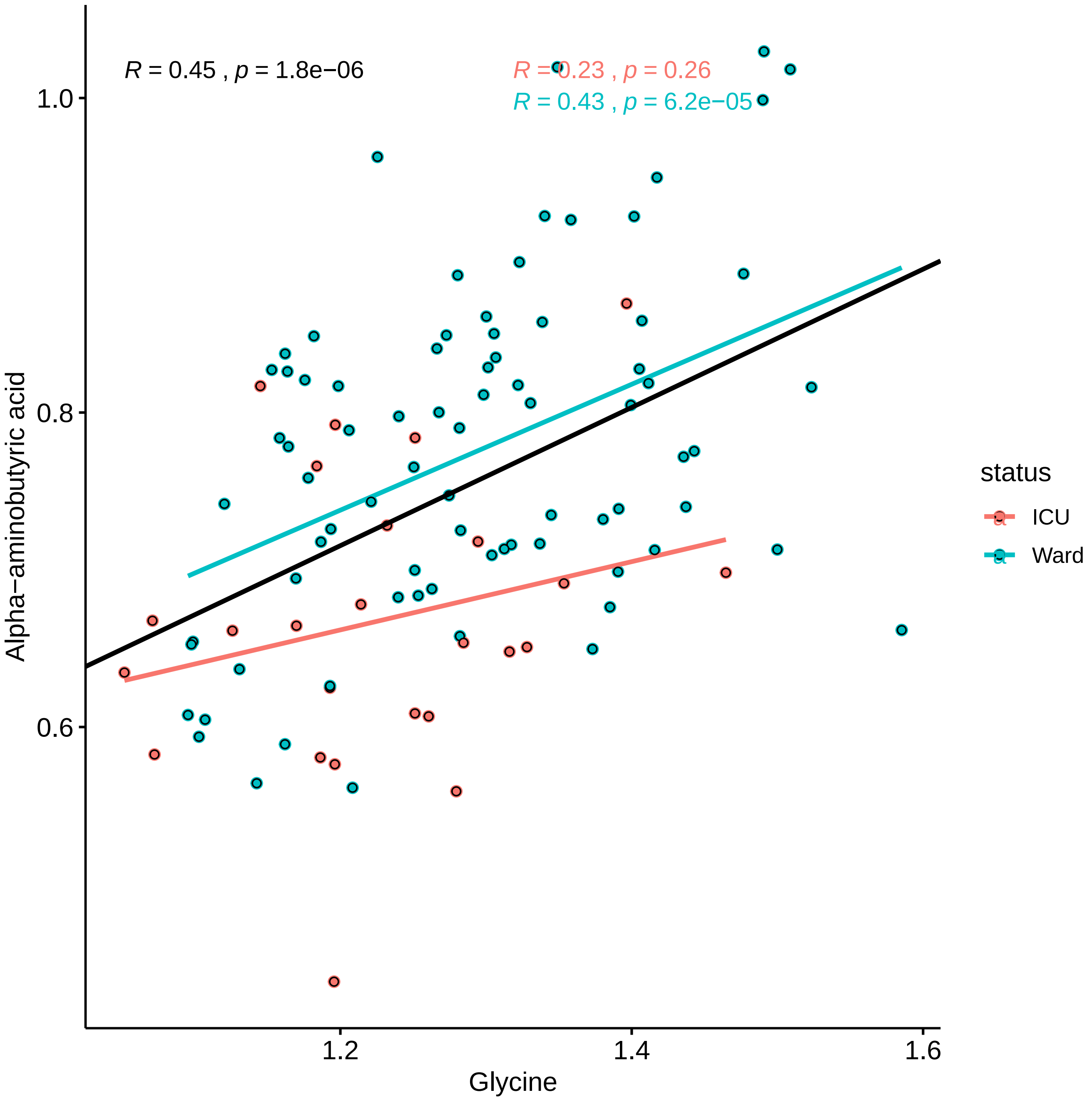

R = 0.45 , p = 1.8e−06
R = 0.23 , p = 0.26
R = 0.43 , p = 6.2e−05
1.0
0.8
0.6
1.2
1.4
1.6
Glycine
Alpha−aminobutyric acid
status
ICU
Ward

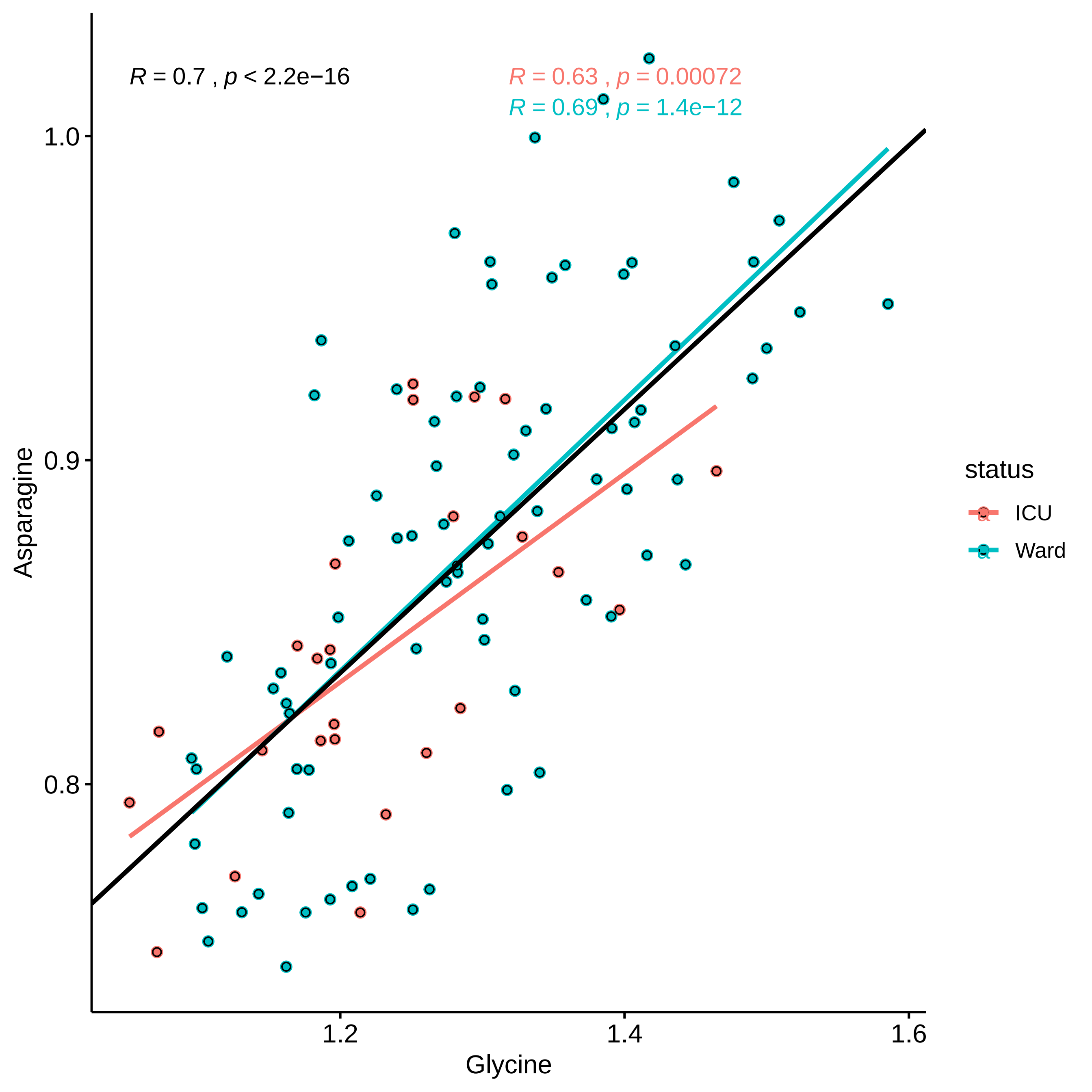

R = 0.7 , p < 2.2e−16
R = 0.63 , p = 0.00072
R = 0.69 , p = 1.4e−12
1.0
0.9
0.8
Asparagine
1.2
1.4
1.6
Glycine
status
ICU
Ward

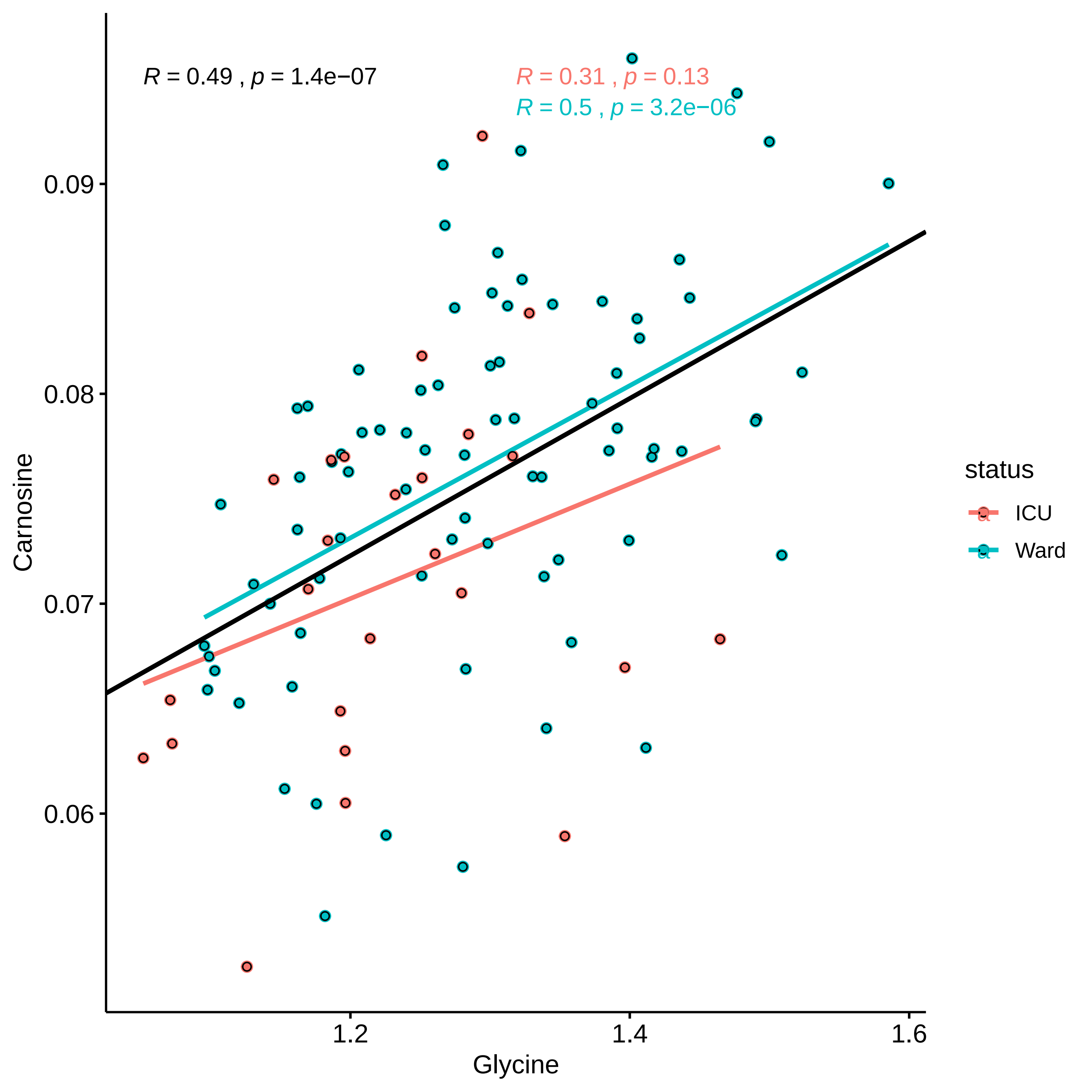

R = 0.49 , p = 1.4e−07
R = 0.31 , p = 0.13
R = 0.5 , p = 3.2e−06
Carnosine
0.09
0.08
0.07
0.06
1.2
1.4
1.6
Glycine
status
ICU
Ward

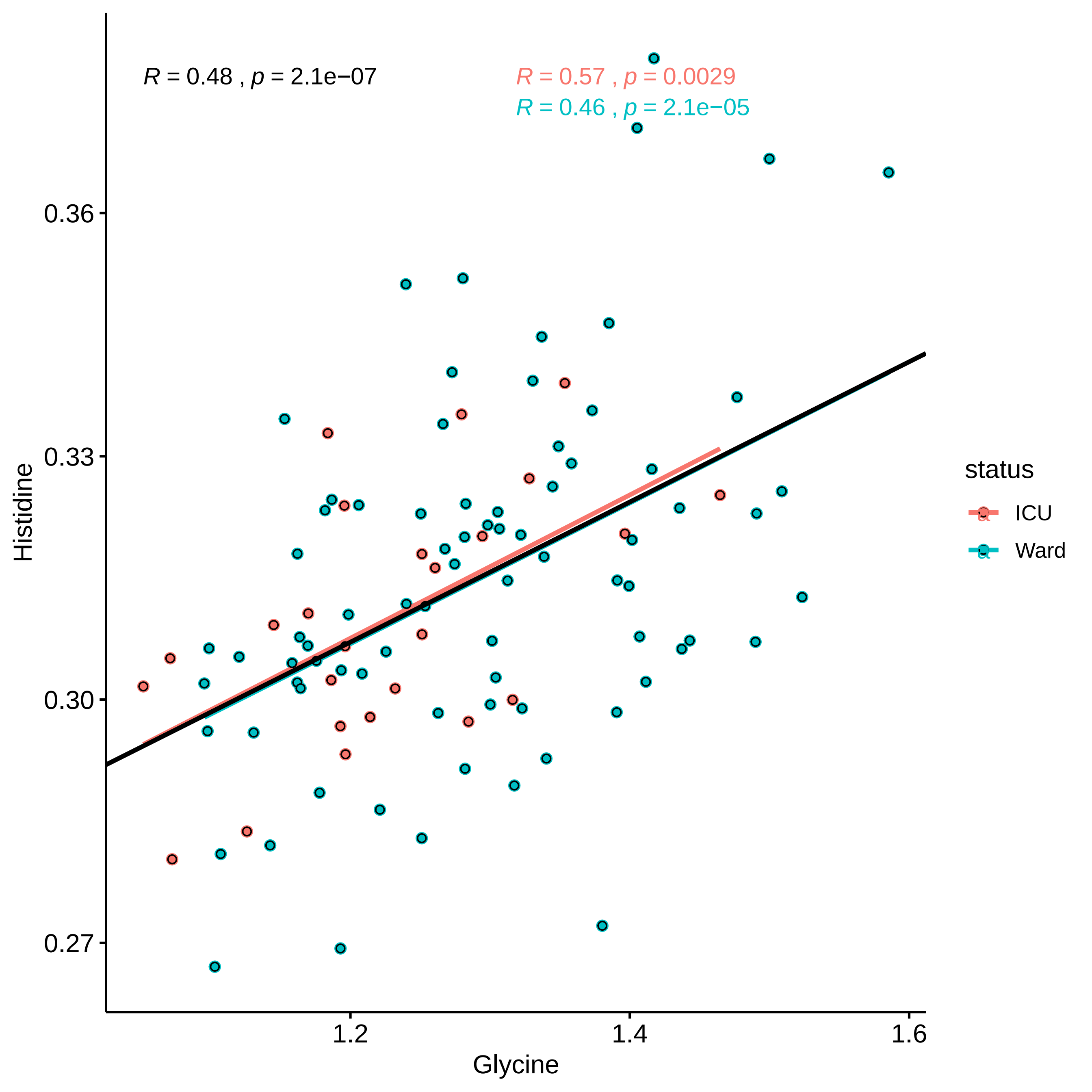
R = 0.48 , p = 2.1e−07
R = 0.57 , p = 0.0029
R = 0.46 , p = 2.1e−05
Histidine
0.36
0.33
0.30
0.27
1.2
1.4
1.6
Glycine
status
ICU
Ward

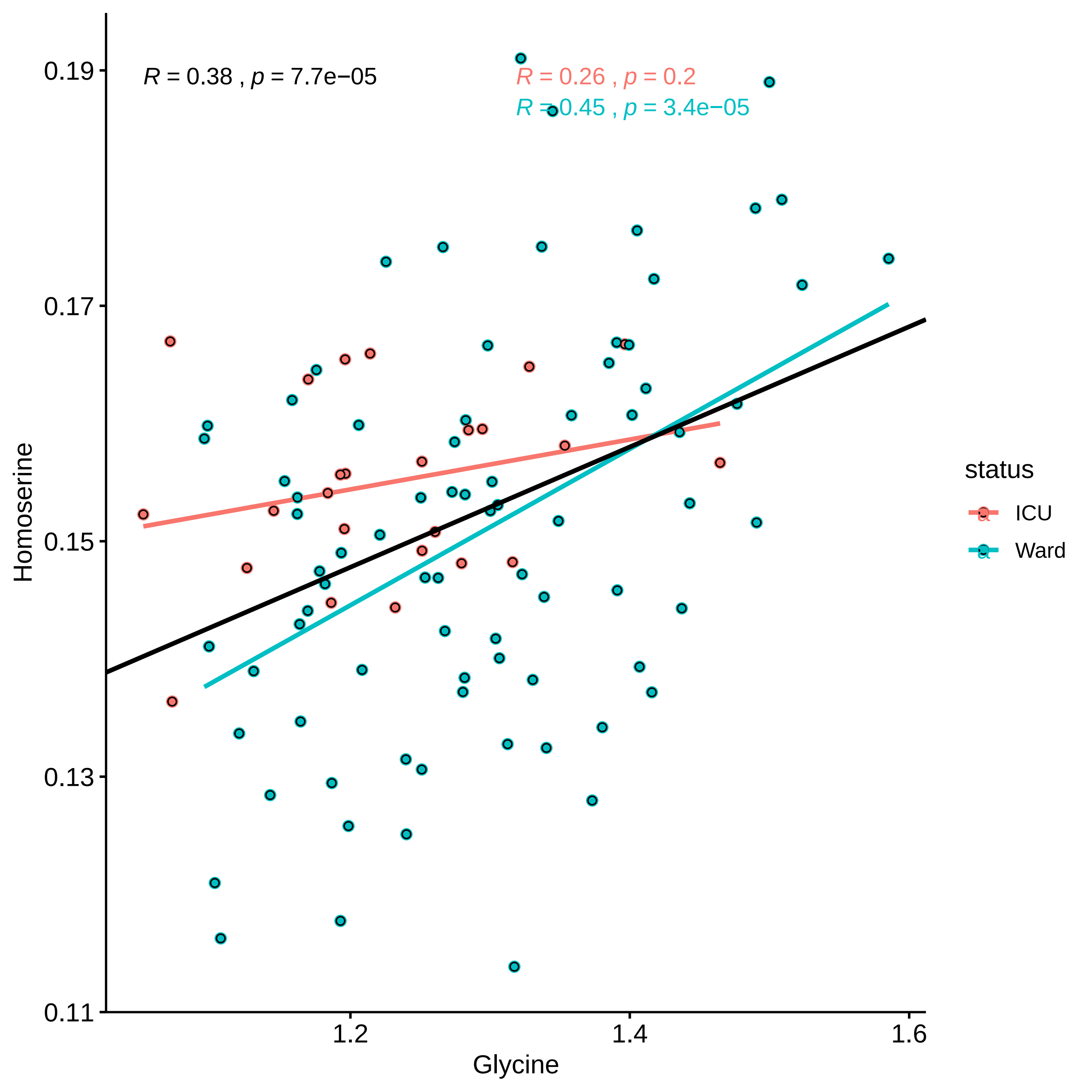

R = 0.38 , p = 7.7e−05
R = 0.26 , p = 0.2
R = 0.45 , p = 3.4e−05
Homoserine
Glycine
0.19
0.17
0.15
0.13
0.11
1.2
1.4
1.6
status
ICU
Ward

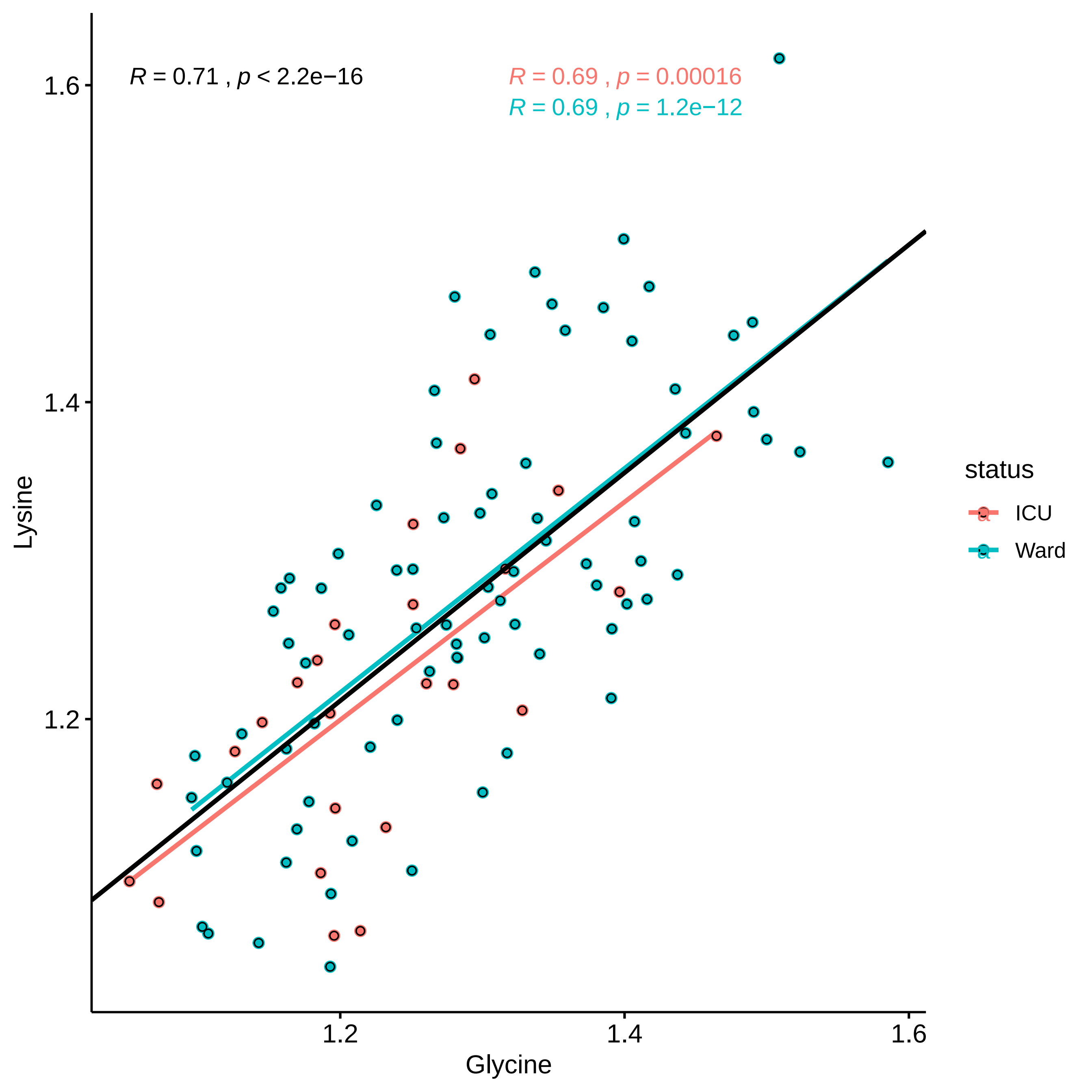

R = 0.71 , p < 2.2e−16
R = 0.69 , p = 0.00016
R = 0.69 , p = 1.2e−12
Lysine
Glycine
1.6
1.4
1.2
1.2
1.4
1.6
status
ICU
Ward

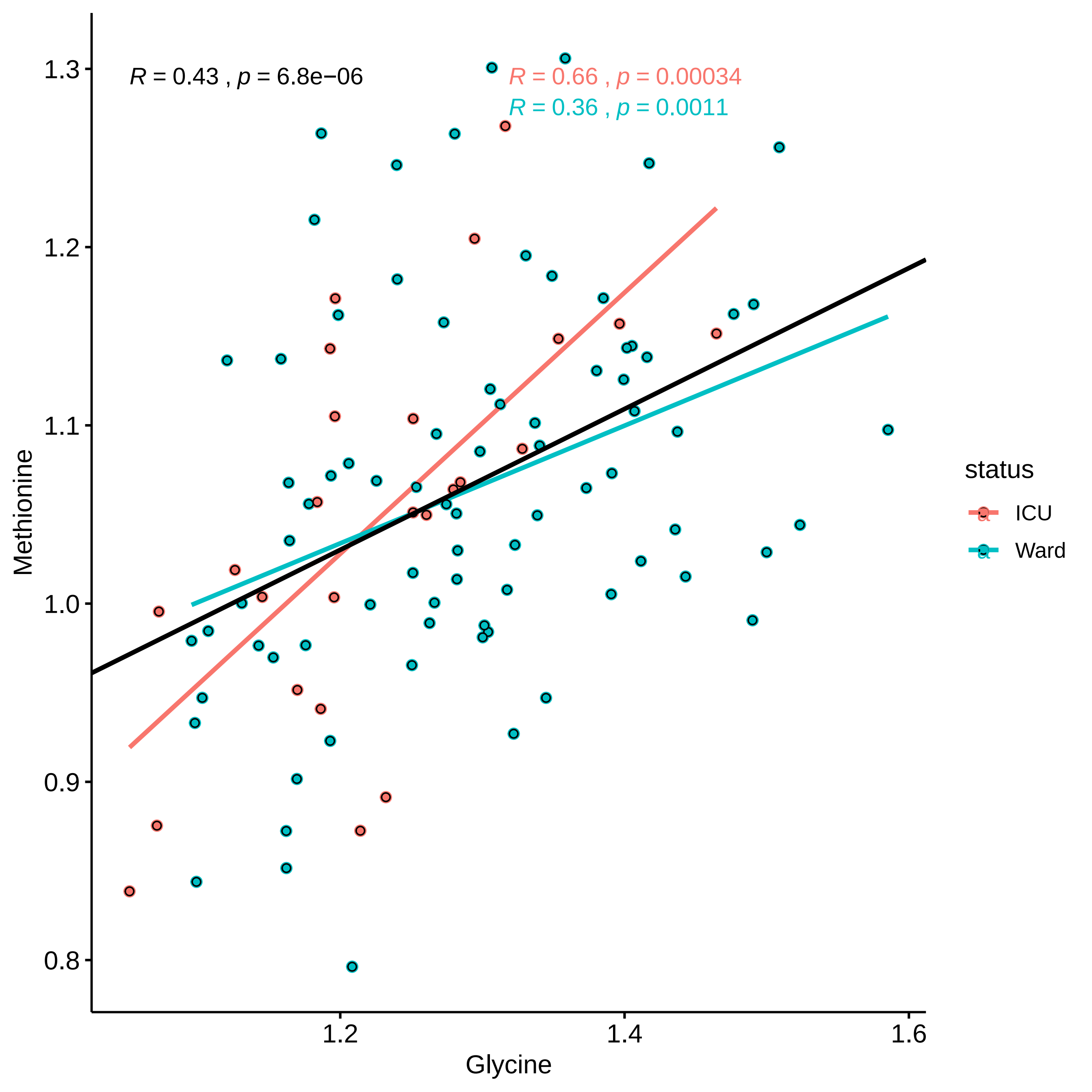

R = 0.43 , p = 6.8e−06
R = 0.66 , p = 0.00034
R = 0.36 , p = 0.0011
1.3
1.2
1.1
1.0
0.9
0.8
Methionine
1.2
1.4
1.6
Glycine
status
ICU
Ward

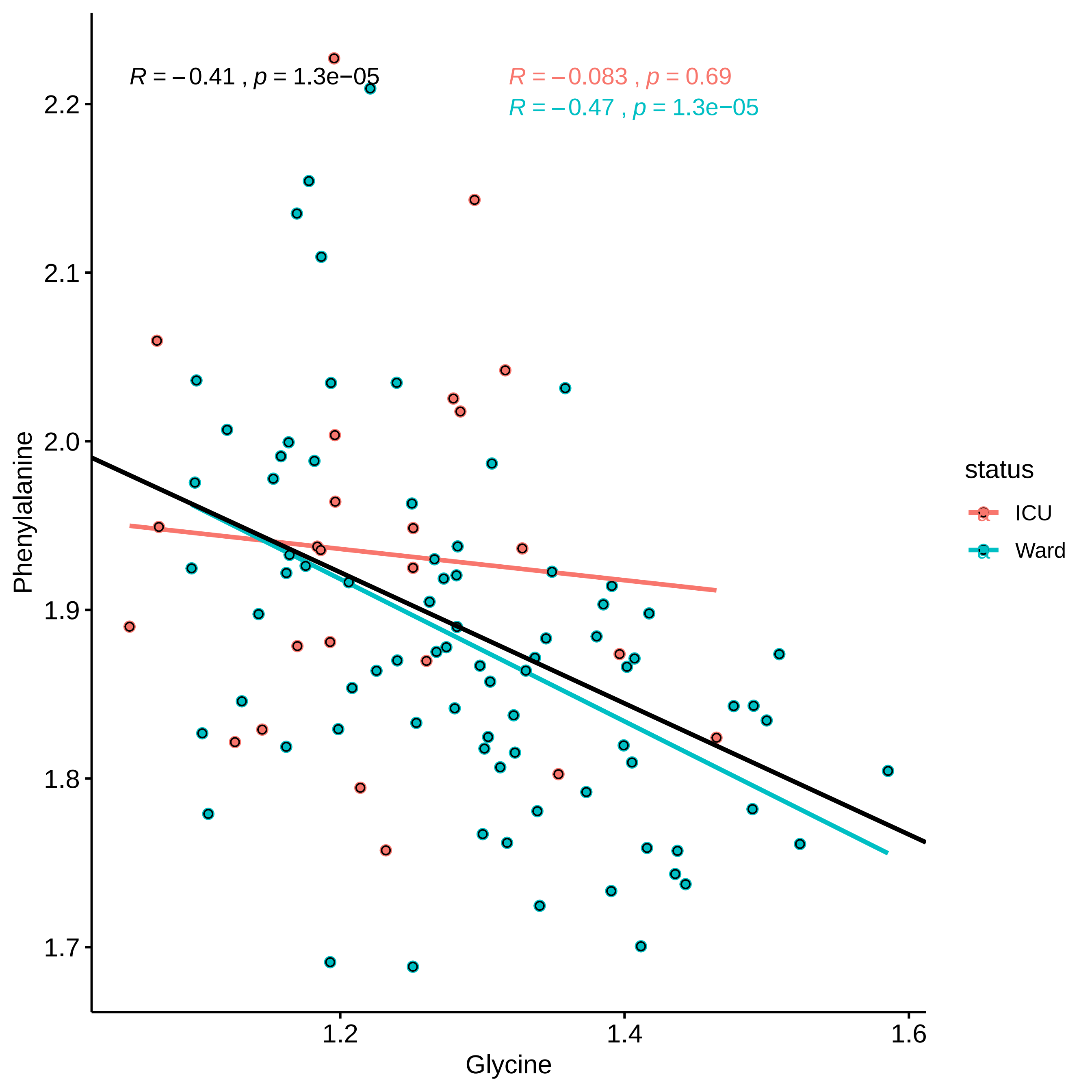

R = −0.41 , p = 1.3e−05
R = −0.083 , p = 0.69
R = −0.47 , p = 1.3e−05
2.2
2.1
2.0
1.9
1.8
1.7
Phenylalanine
1.2
1.4
1.6
Glycine
status
ICU
Ward

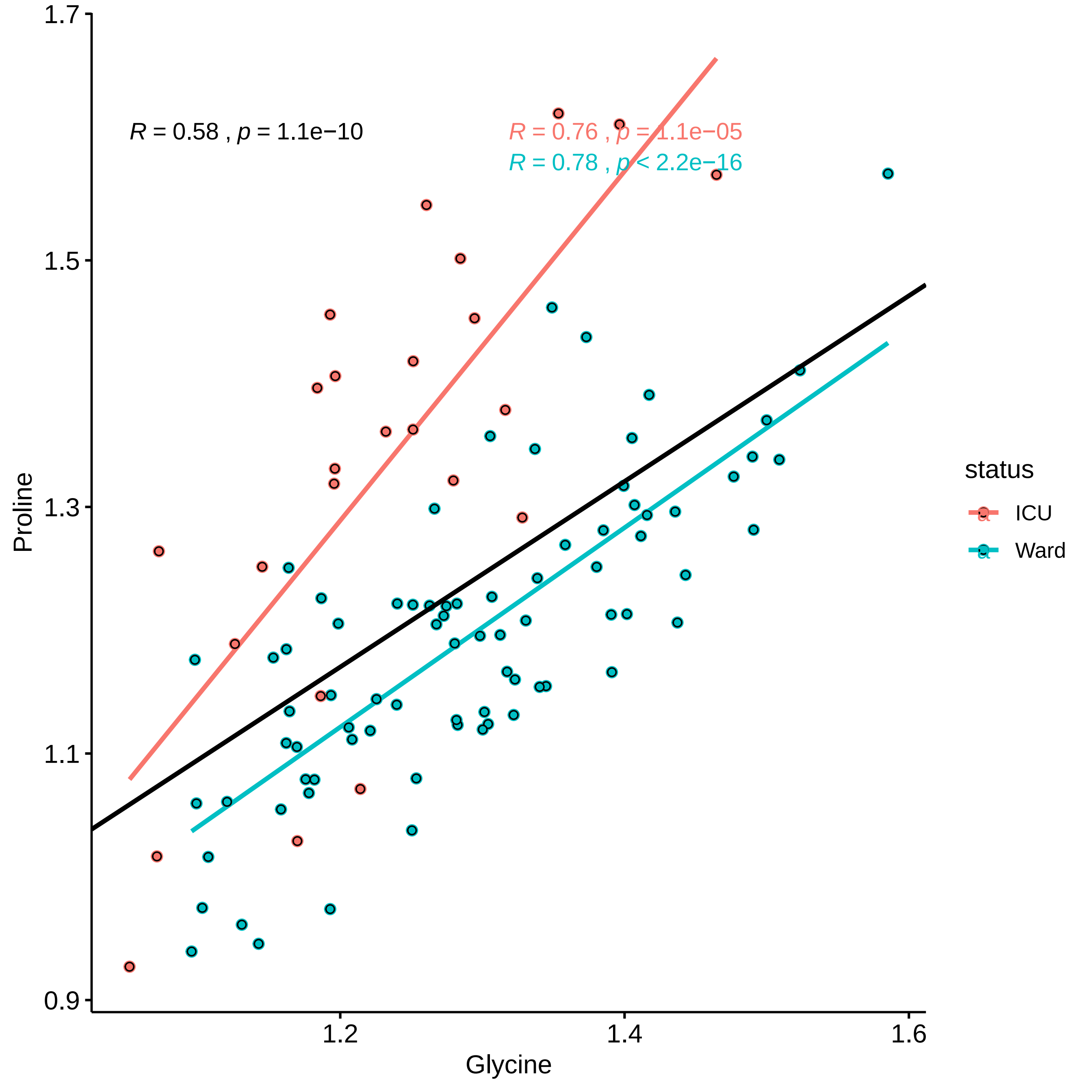

R = 0.58 , p = 1.1e−10
R = 0.76 , p = 1.1e−05
R = 0.78 , p < 2.2e−16
Proline
Glycine
1.7
1.5
1.3
1.1
0.9
1.2
1.4
1.6
status
ICU
Ward

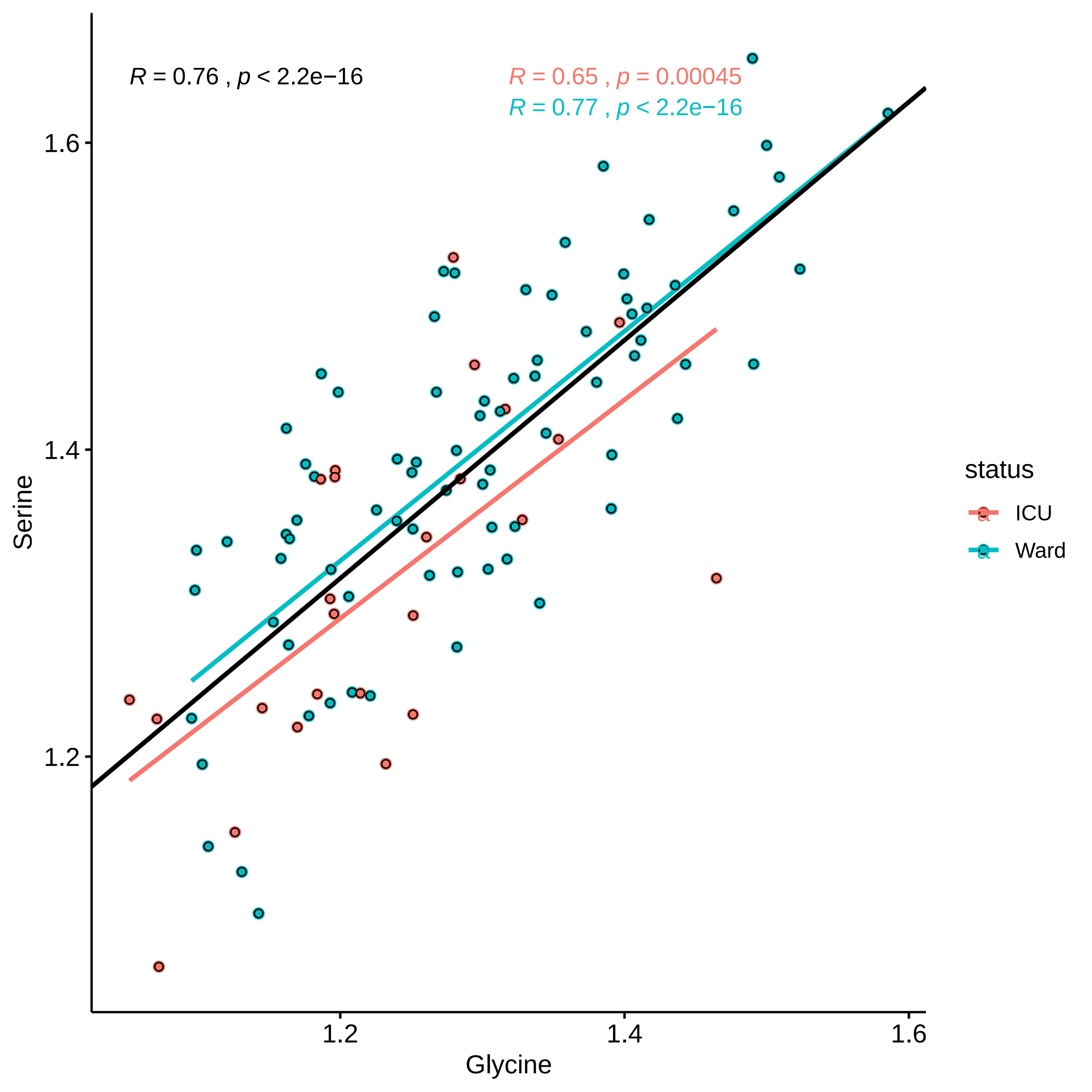

R = 0.76 , p < 2.2e−16
R = 0.65 , p = 0.00045
R = 0.77 , p < 2.2e−16
1.6
1.4
1.2
Serine
1.2
1.4
1.6
Glycine
status
ICU
Ward

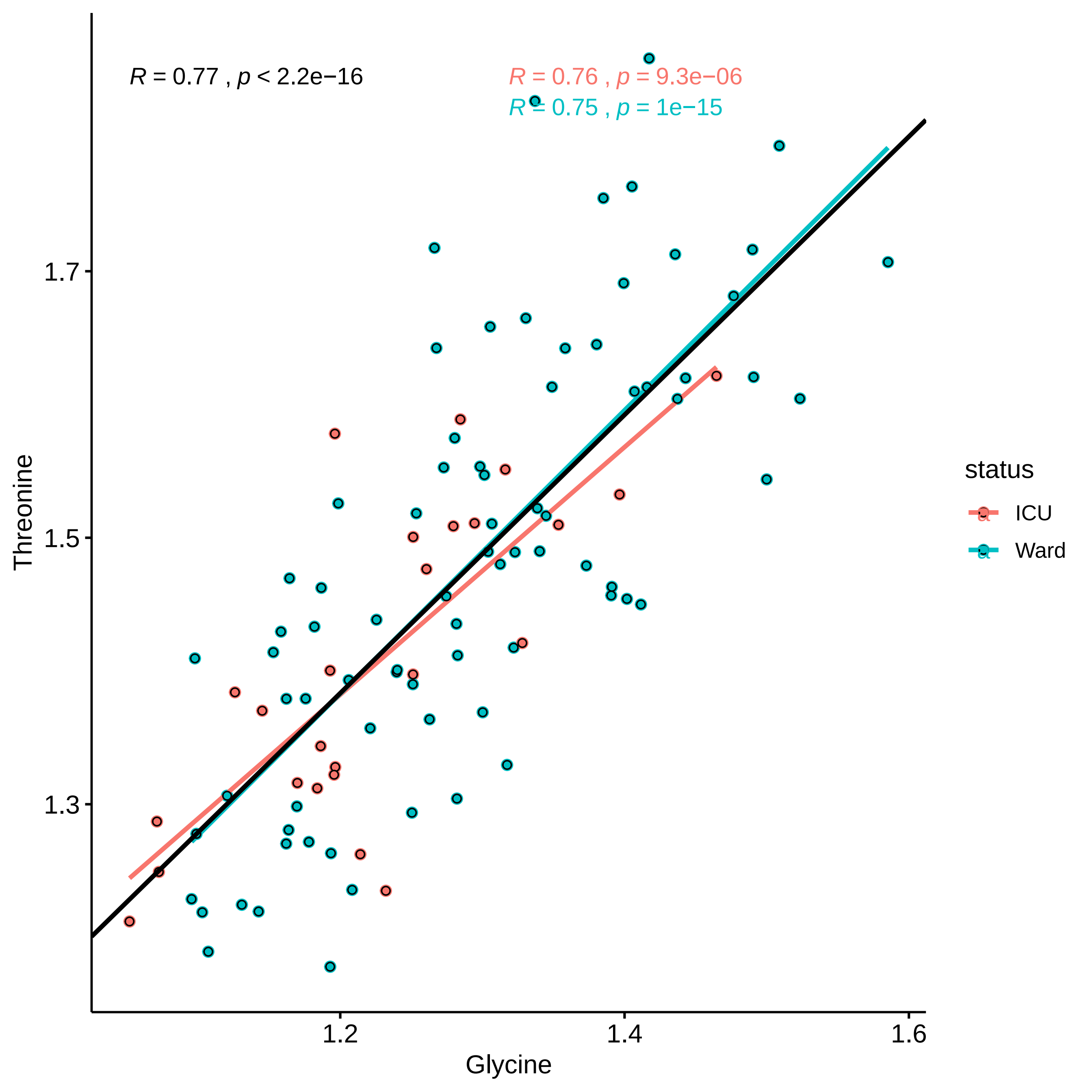

R = 0.77 , p < 2.2e−16
R = 0.76 , p = 9.3e−06
R = 0.75 , p = 1e−15
Threonine
1.7
1.5
1.3
1.2
1.4
1.6
Glycine
status
ICU
Ward

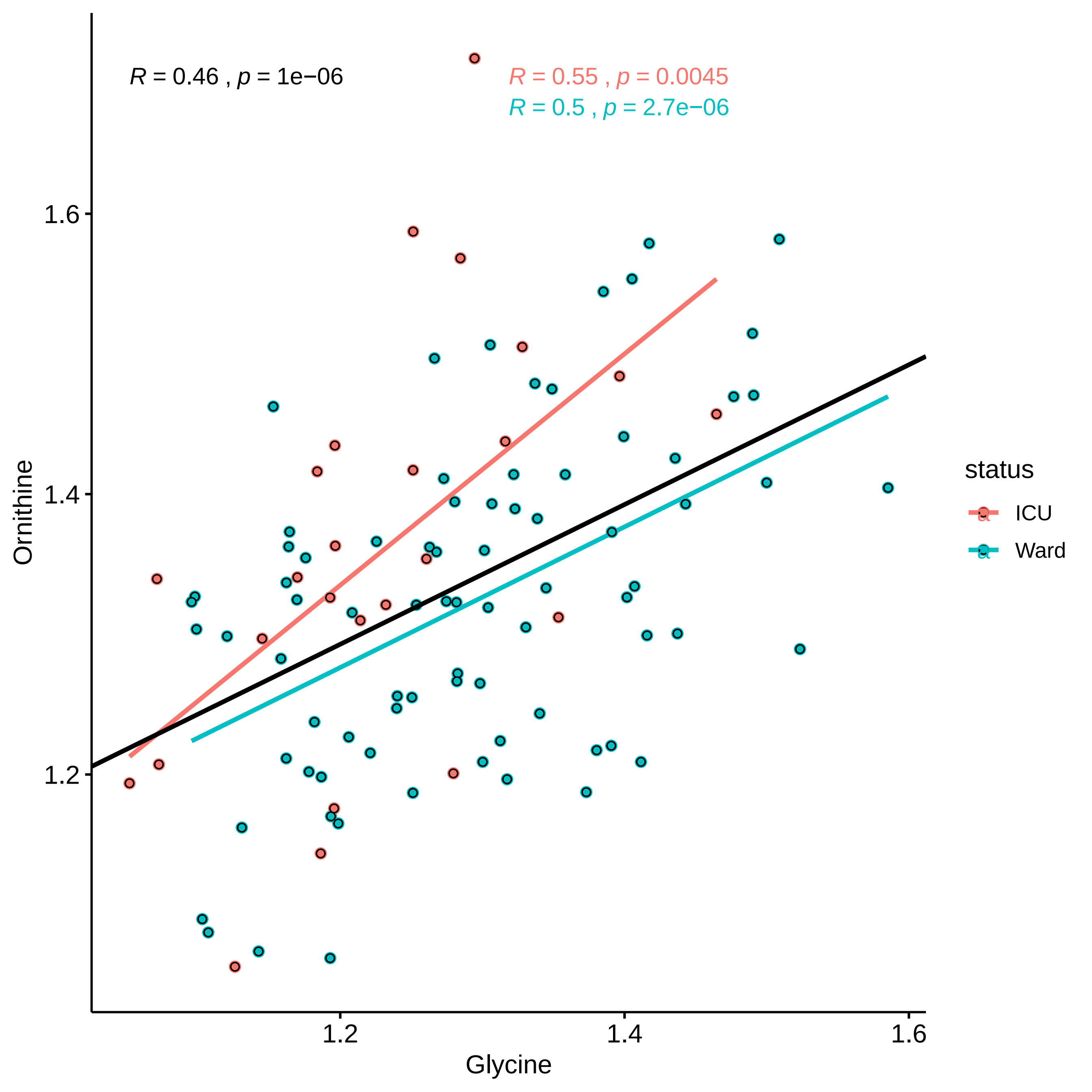

R = 0.46 , p = 1e−06
R = 0.55 , p = 0.0045
R = 0.5 , p = 2.7e−06
Ornithine
1.6
1.4
1.2
1.2
1.4
1.6
Glycine
status
ICU
Ward

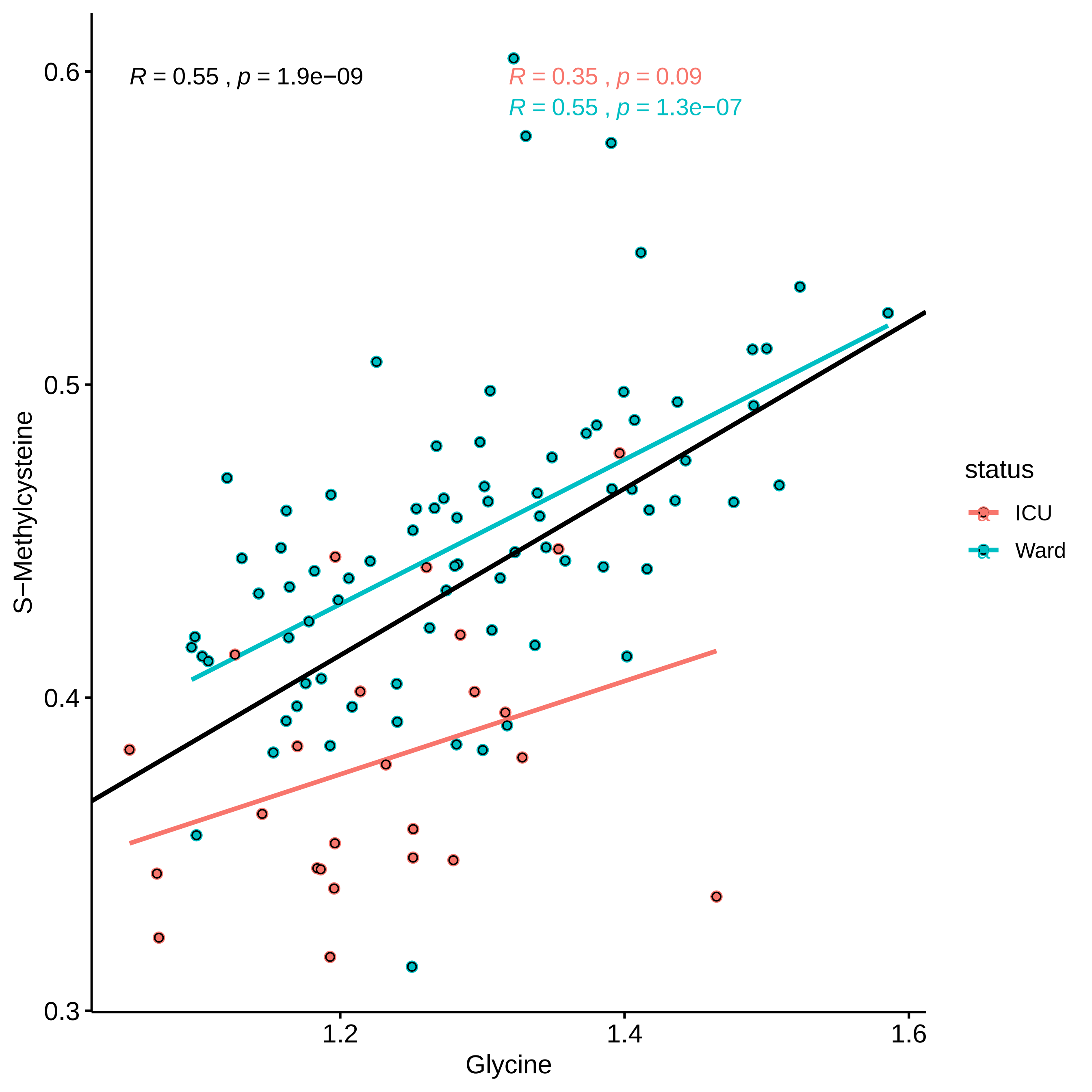

R = 0.55 , p = 1.9e−09
R = 0.35 , p = 0.09
R = 0.55 , p = 1.3e−07
S−Methylcysteine
0.6
0.5
0.4
0.3
1.2
1.4
1.6
Glycine
status
ICU
Ward

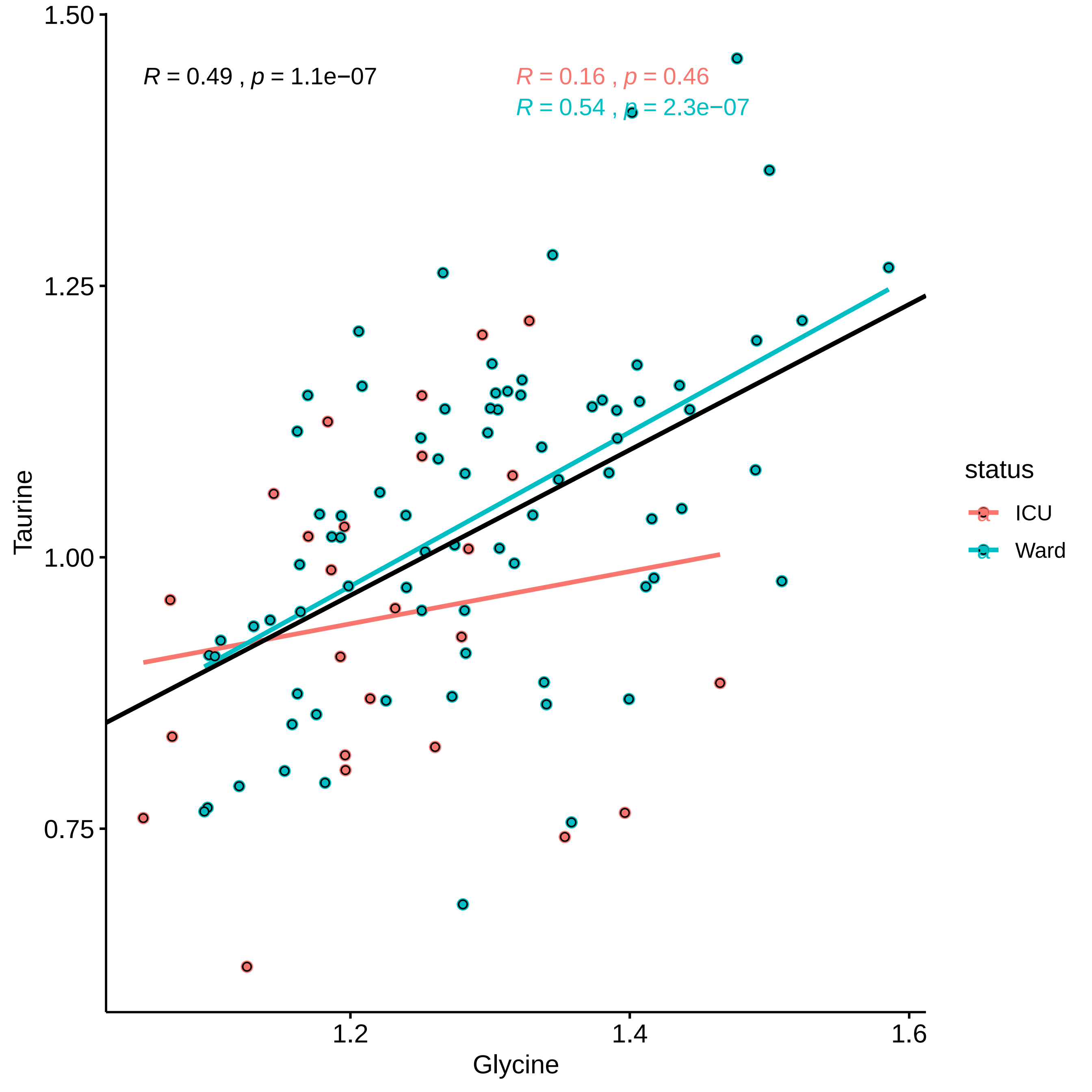

R = 0.49 , p = 1.1e−07
R = 0.16 , p = 0.46
R = 0.54 , p = 2.3e−07
1.50
1.25
1.00
0.75
Taurine
1.2
1.4
1.6
Glycine
status
ICU
Ward

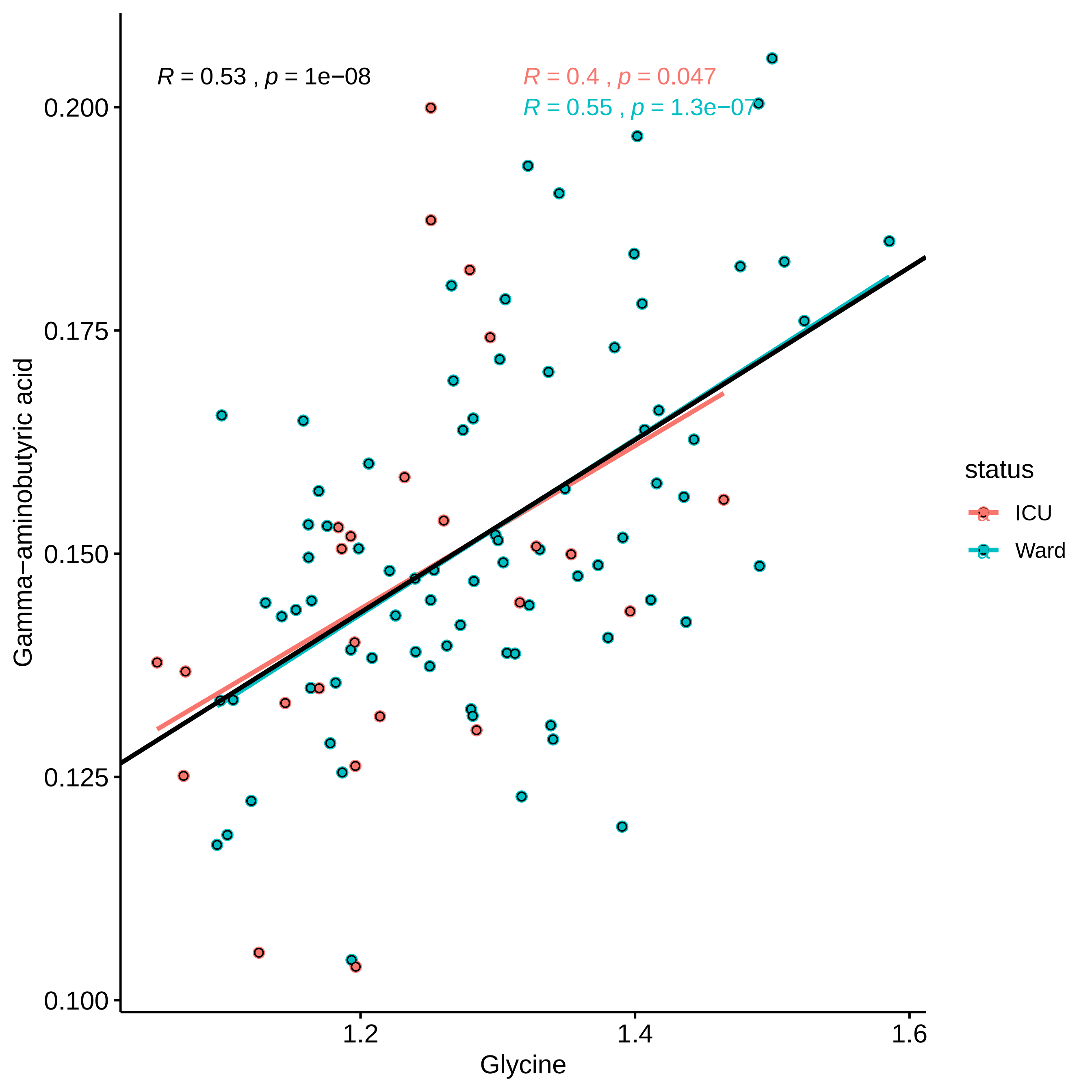

R = 0.53 , p = 1e−08
R = 0.4 , p = 0.047
R = 0.55 , p = 1.3e−07
0.200
0.175
0.150
0.125
0.100
Gamma−aminobutyric acid
1.2
1.4
1.6
Glycine
status
ICU
Ward

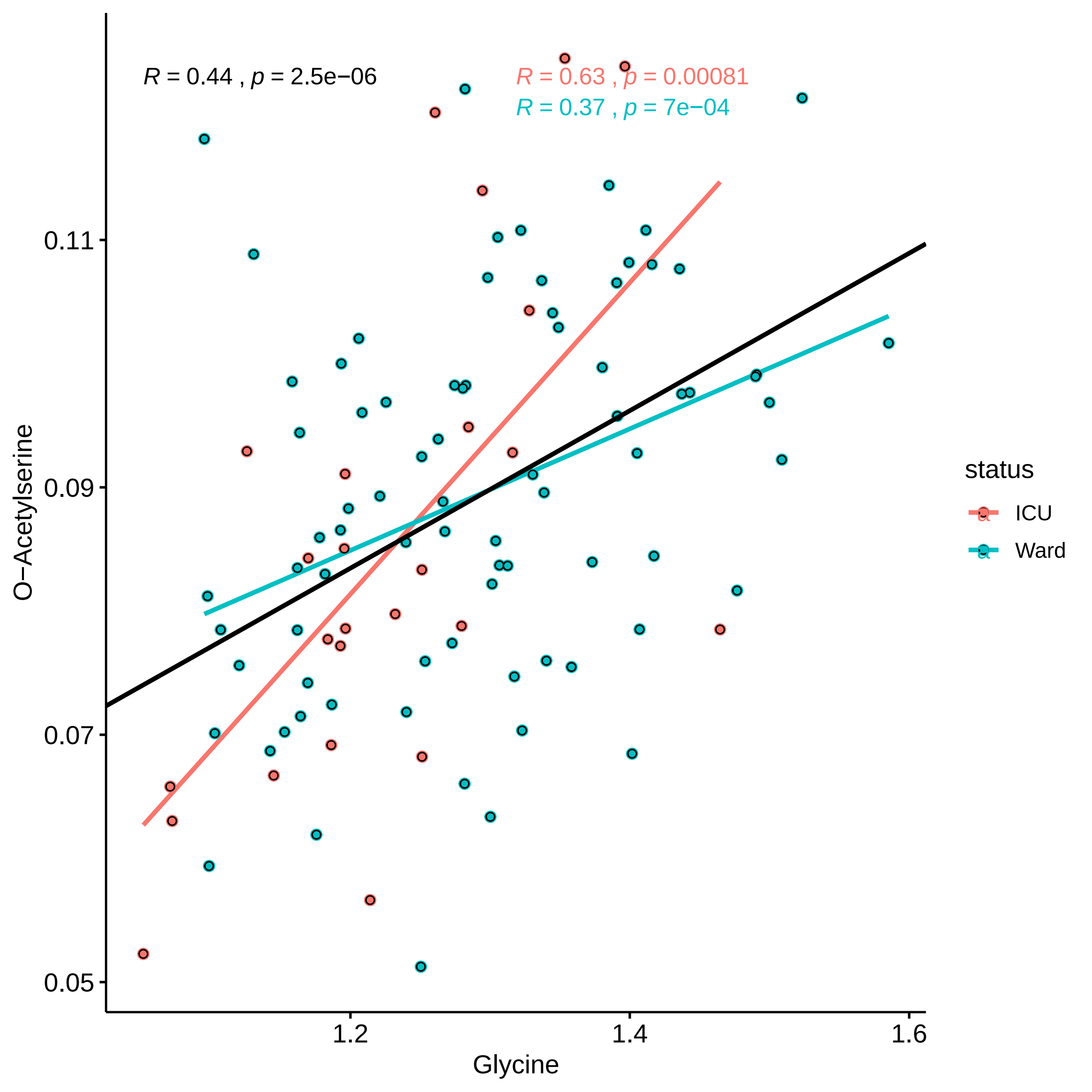

R = 0.44 , p = 2.5e−06
R = 0.63 , p = 0.00081
R = 0.37 , p = 7e−04
O−Acetylserine
0.11
0.09
0.07
0.05
1.2
1.4
1.6
Glycine
status
ICU
Ward

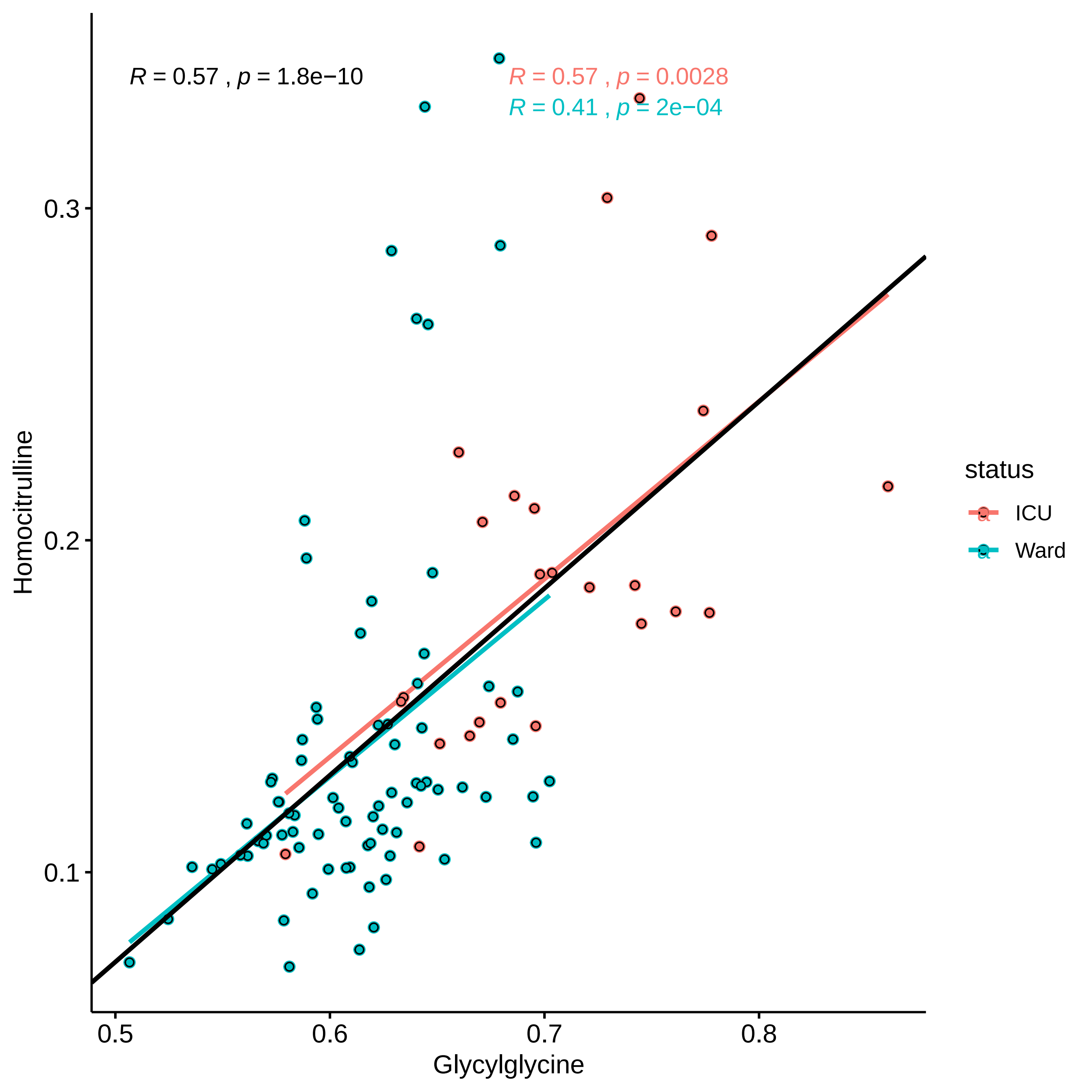

R = 0.57 , p = 1.8e−10
R = 0.57 , p = 0.0028
R = 0.41 , p = 2e−04
0.3
0.2
0.1
Homocitrulline
0.5
0.6
0.7
0.8
Glycylglycine
status
ICU
Ward

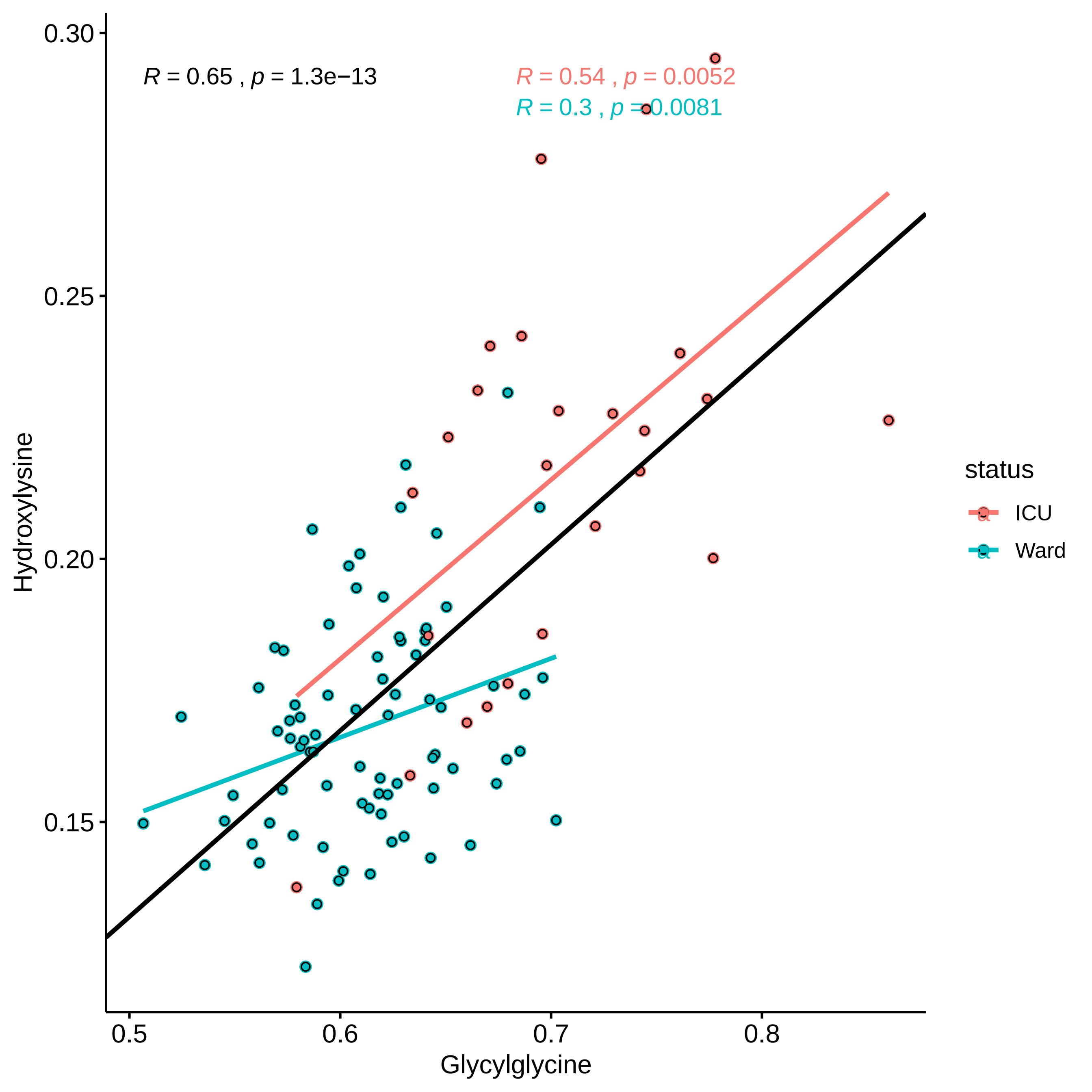

R = 0.65 , p = 1.3e−13
R = 0.54 , p = 0.0052
R = 0.3 , p = 0.0081
0.30
0.25
0.20
0.15
Hydroxylysine
0.5
0.6
0.7
0.8
Glycylglycine
status
ICU
Ward

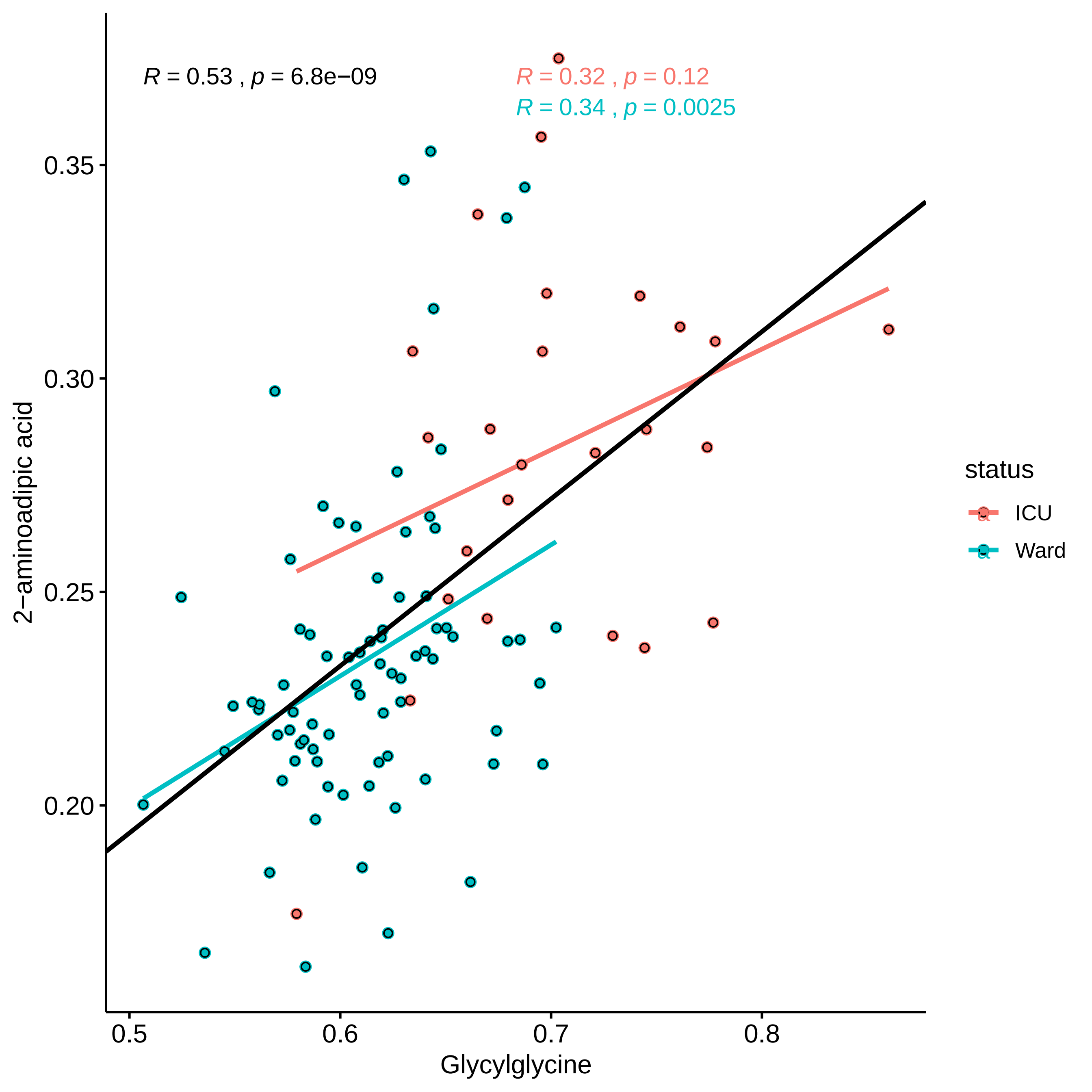

R = 0.53 , p = 6.8e−09
R = 0.32 , p = 0.12
R = 0.34 , p = 0.0025
2−aminoadipic acid
0.35
0.30
0.25
0.20
0.5
0.6
0.7
0.8
Glycylglycine
status
ICU
Ward

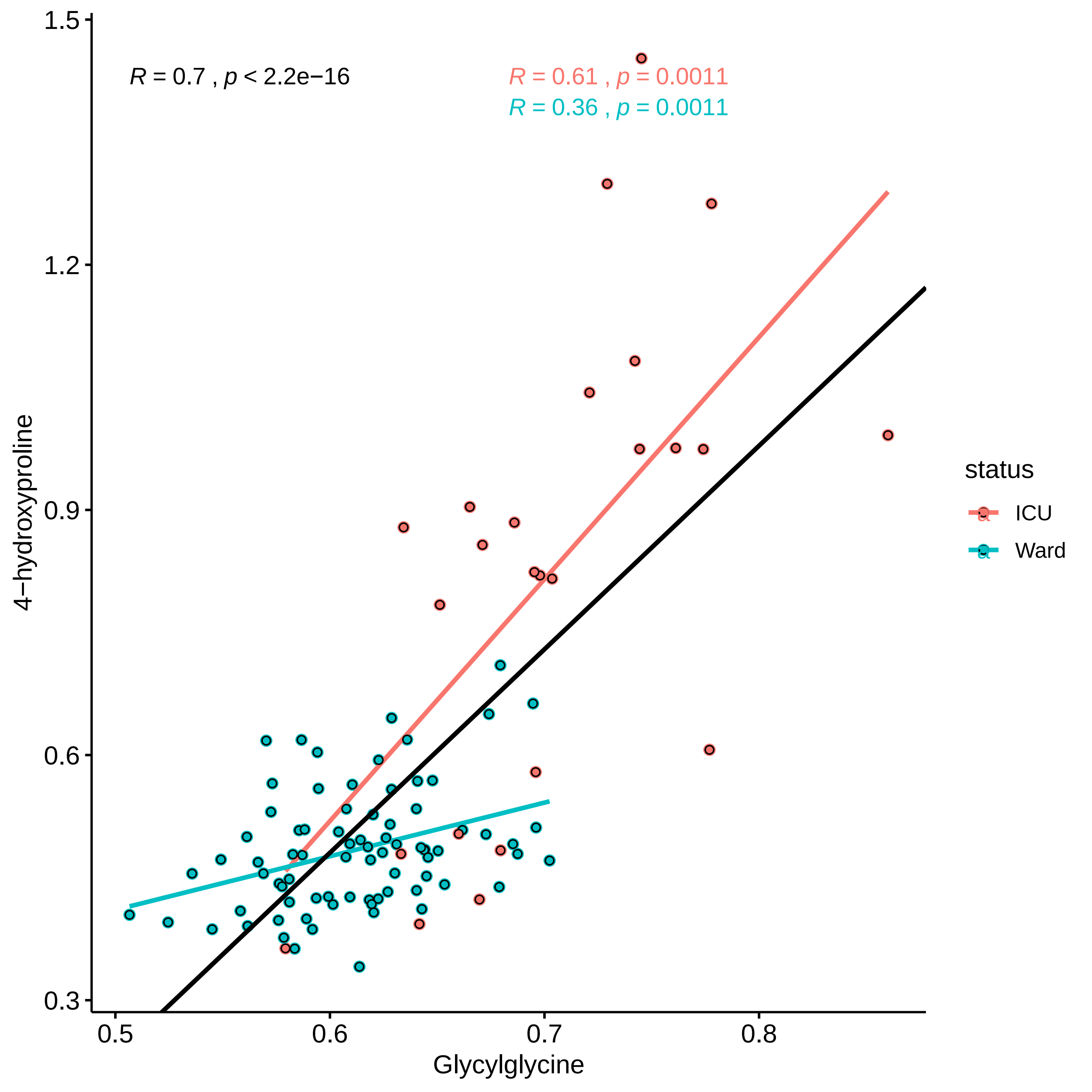

R = 0.7 , p < 2.2e−16
R = 0.61 , p = 0.0011
R = 0.36 , p = 0.0011
4−hydroxyproline
Glycylglycine
1.5
1.2
0.9
0.6
0.3
0.5
0.6
0.7
0.8
status
ICU
Ward

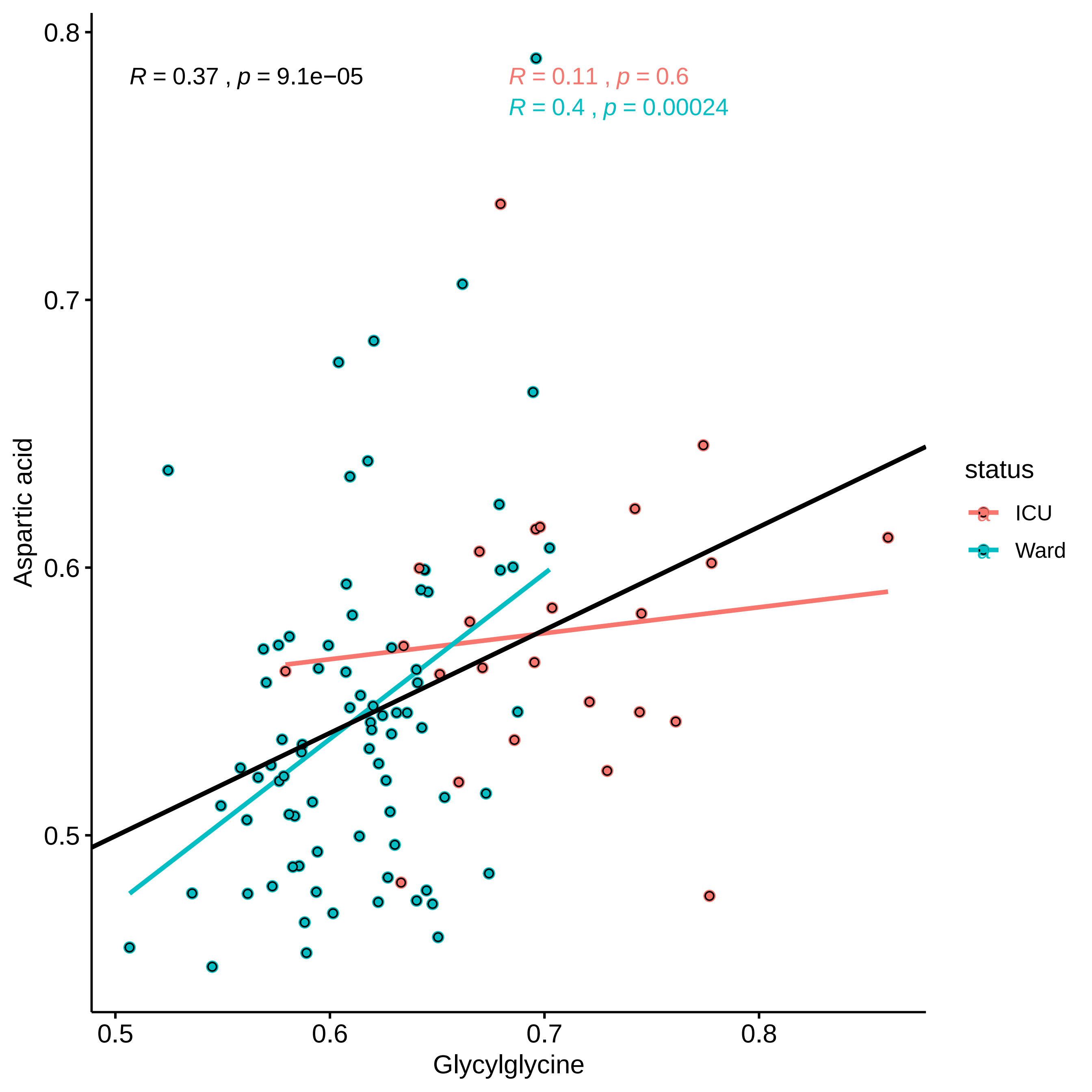

R = 0.37 , p = 9.1e−05
R = 0.11 , p = 0.6
R = 0.4 , p = 0.00024
Aspartic acid
0.8
0.7
0.6
0.5
Glycylglycine
0.5
0.6
0.7
0.8
status
ICU
Ward

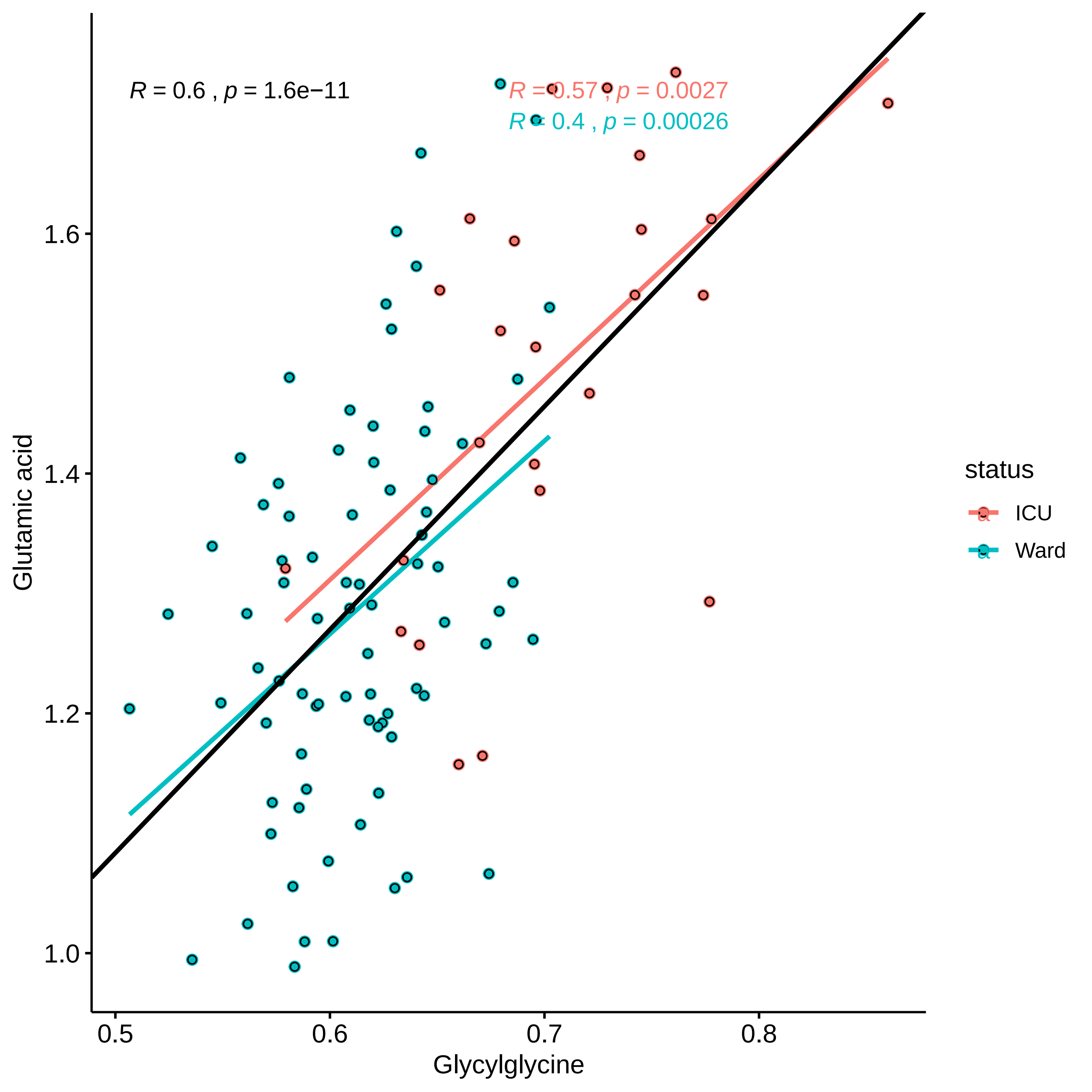

R = 0.6 , p = 1.6e−11
R = 0.57 , p = 0.0027
R = 0.4 , p = 0.00026
Glutamic acid
1.0
1.2
1.4
1.6
0.5
0.6
0.7
0.8
Glycylglycine
status
ICU
Ward

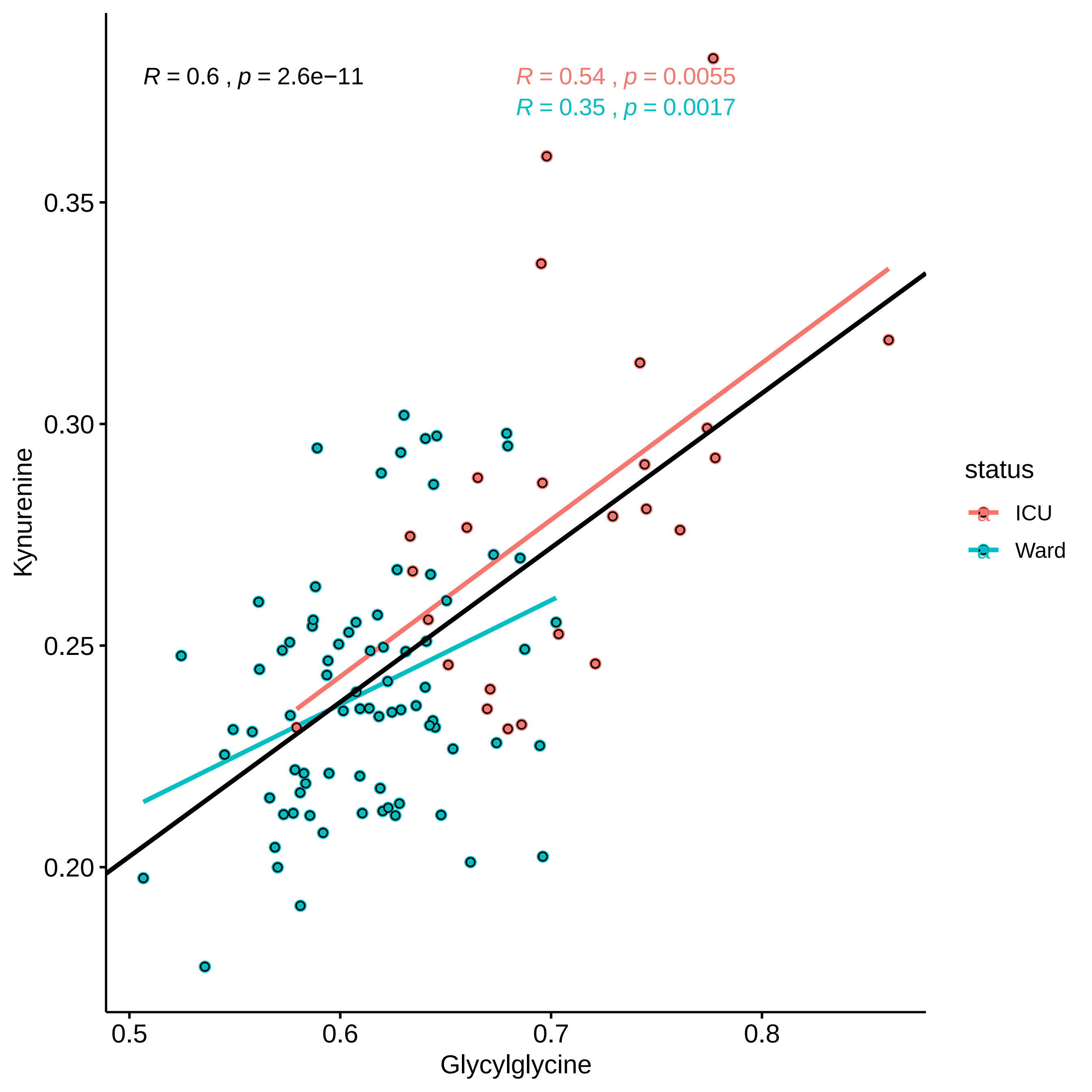

R = 0.6 , p = 2.6e−11
R = 0.54 , p = 0.0055
R = 0.35 , p = 0.0017
0.35
0.30
0.25
0.20
Kynurenine
0.5
0.6
0.7
0.8
Glycylglycine
status
ICU
Ward

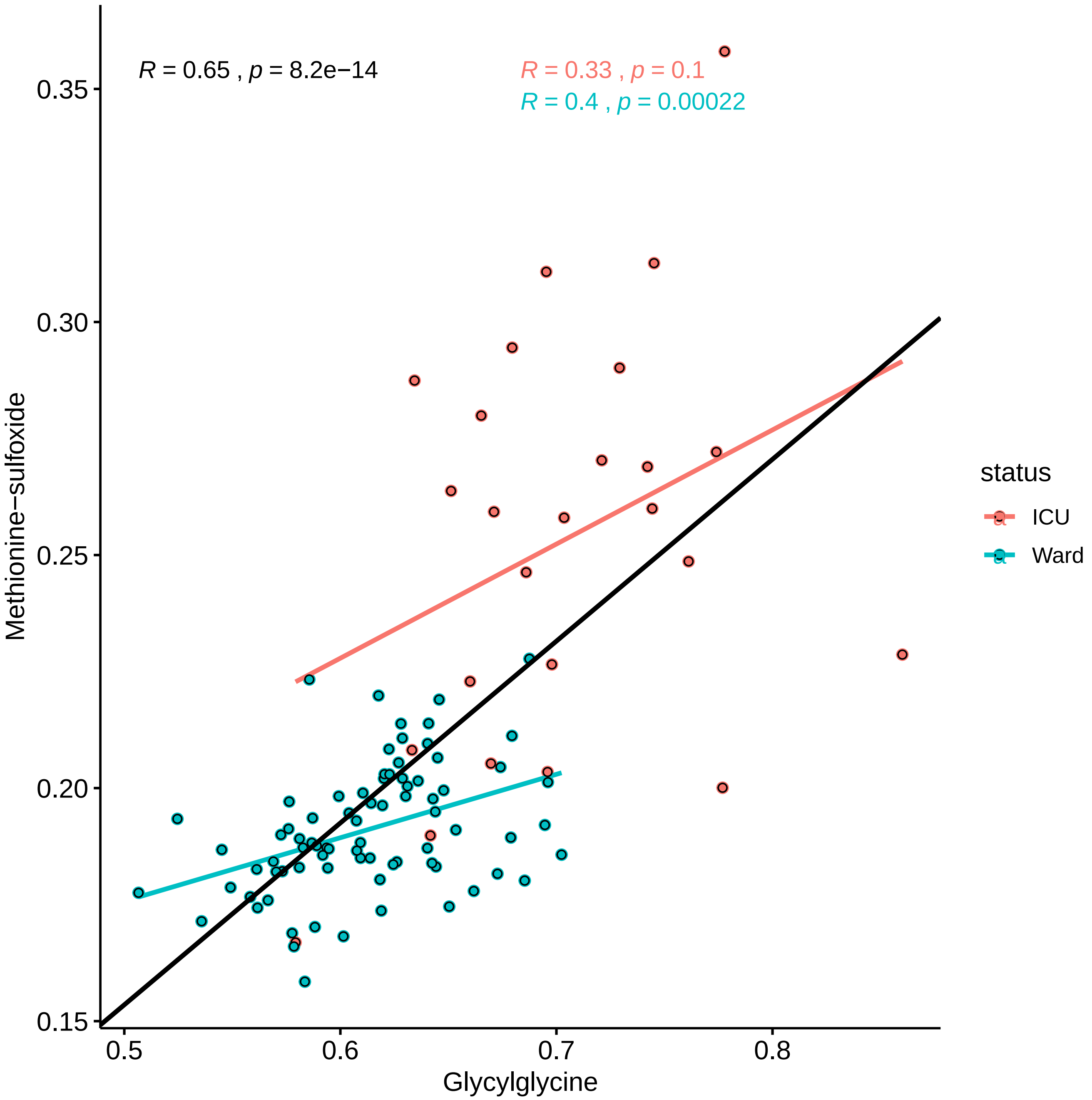

R = 0.65 , p = 8.2e−14
R = 0.33 , p = 0.1
R = 0.4 , p = 0.00022
0.35
0.30
0.25
0.20
0.15
Methionine−sulfoxide
0.5
0.6
0.7
0.8
Glycylglycine
status
ICU
Ward

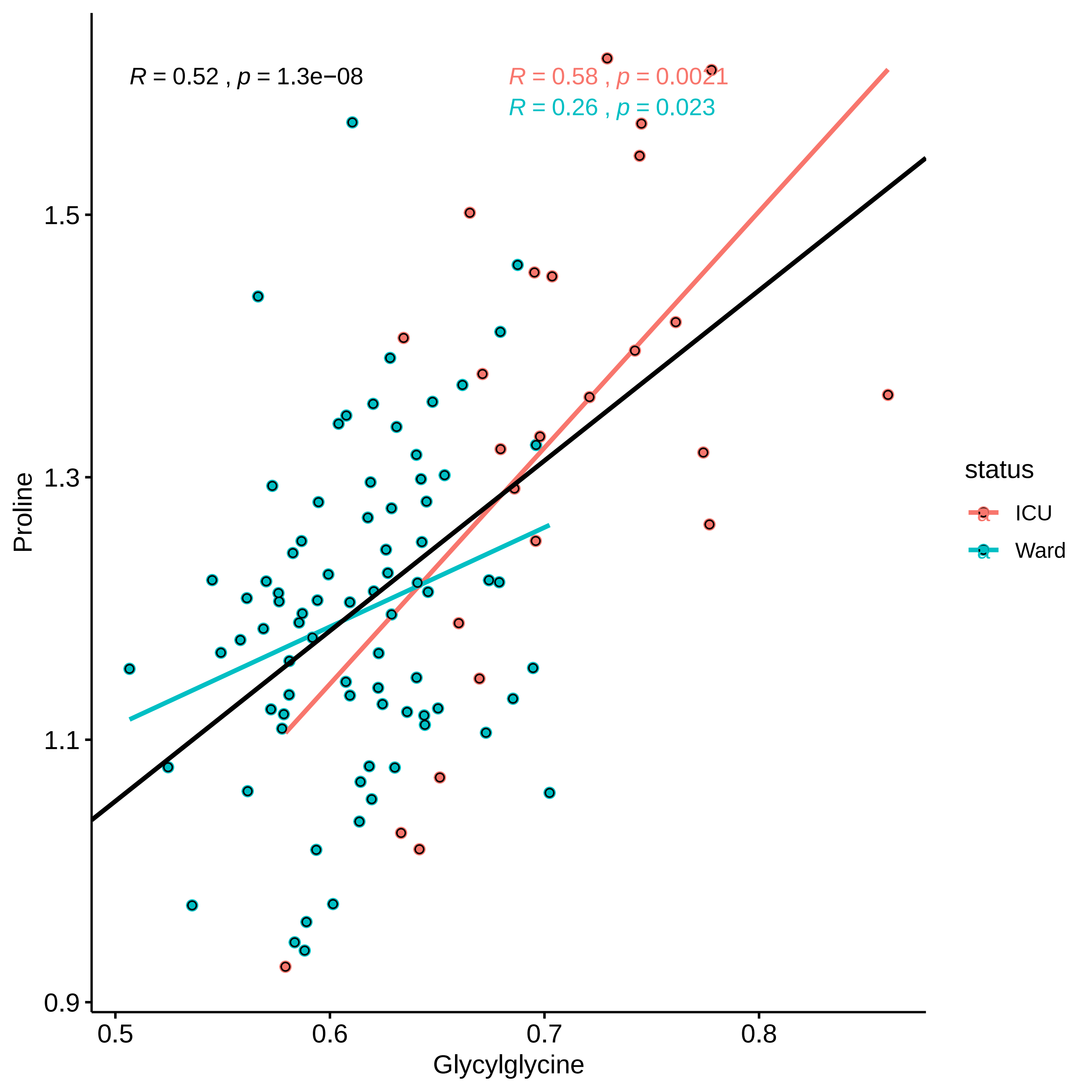

R = 0.52 , p = 1.3e−08
R = 0.58 , p = 0.0021
R = 0.26 , p = 0.023
Proline
1.5
1.3
1.1
0.9
0.5
0.6
0.7
0.8
Glycylglycine
status
ICU
Ward

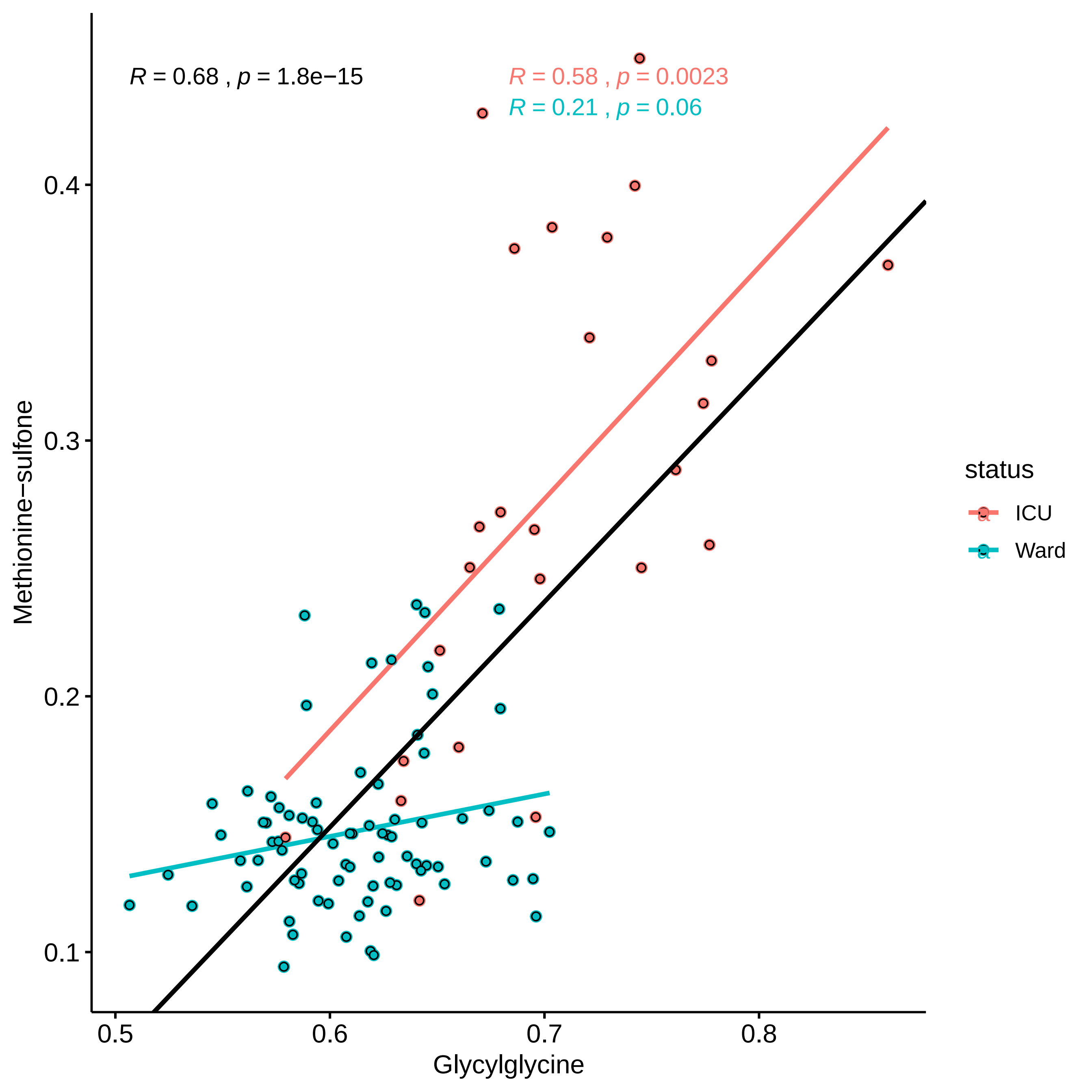

R = 0.68 , p = 1.8e−15
R = 0.58 , p = 0.0023
R = 0.21 , p = 0.06
Methionine−sulfone
0.4
0.3
0.2
0.1
0.5
0.6
0.7
0.8
Glycylglycine
status
ICU
Ward

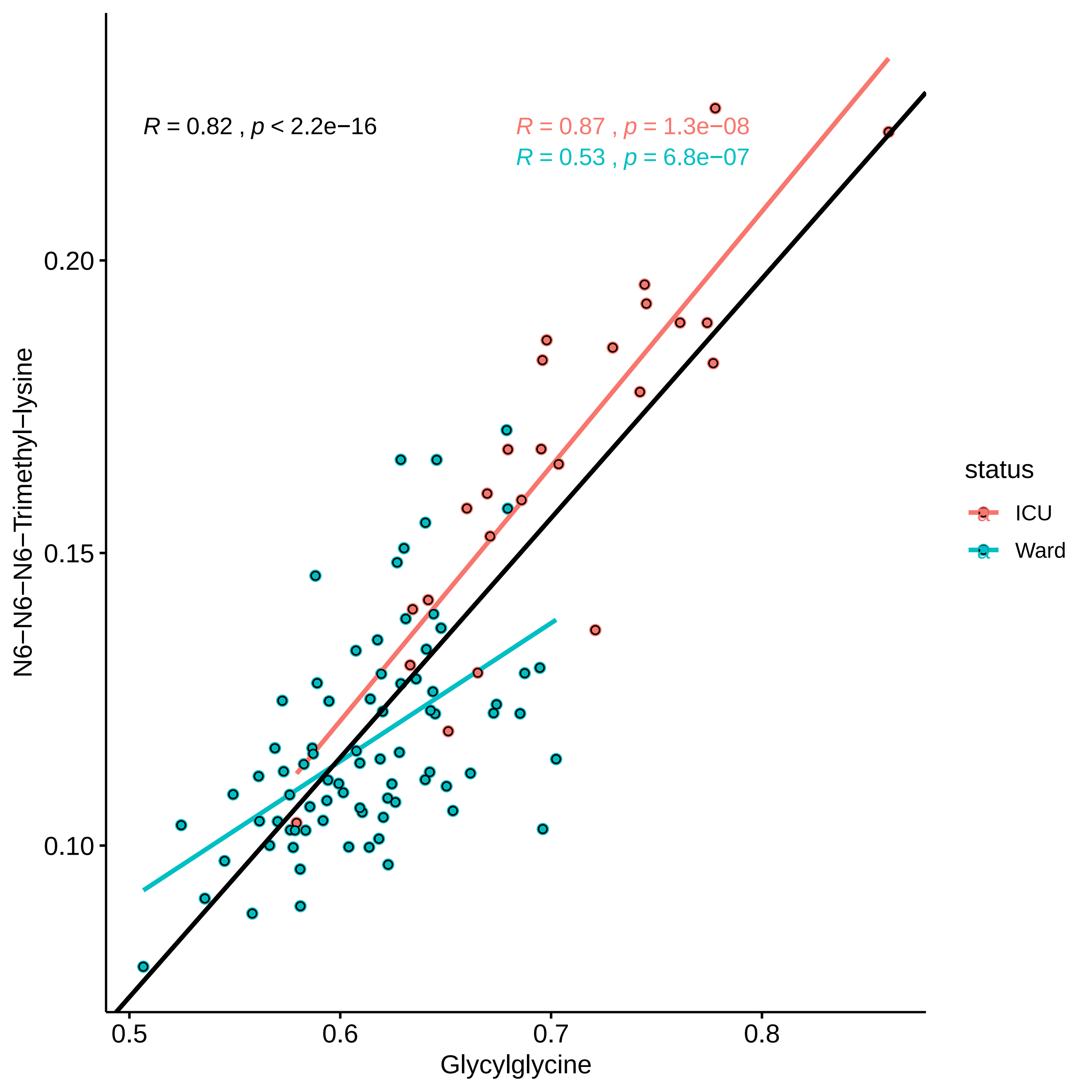

R = 0.82 , p < 2.2e−16
R = 0.87 , p = 1.3e−08
R = 0.53 , p = 6.8e−07
N6−N6−N6−Trimethyl−lysine
0.20
0.15
0.10
0.5
0.6
0.7
0.8
Glycylglycine
status
ICU
Ward

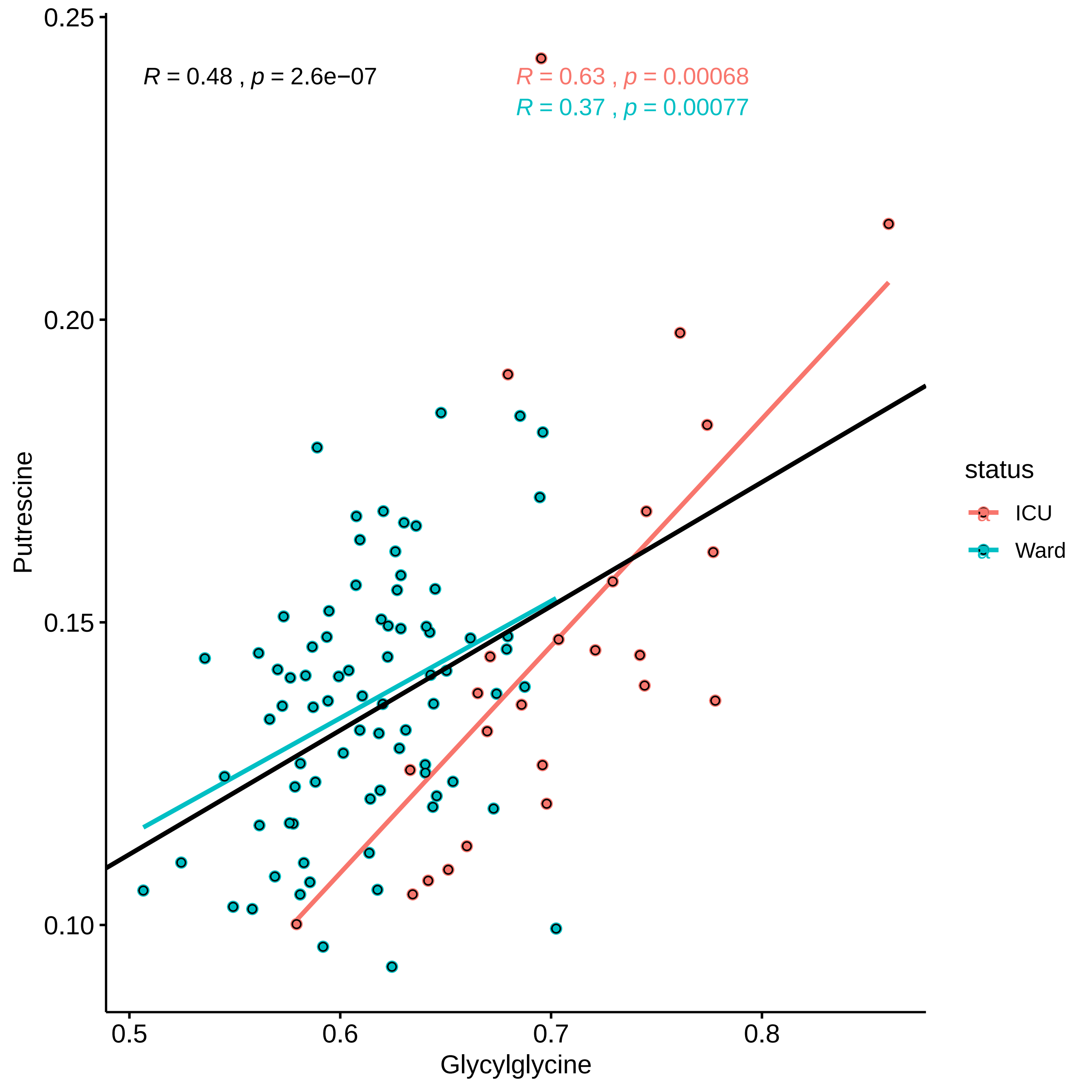

R = 0.48 , p = 2.6e−07
R = 0.63 , p = 0.00068
R = 0.37 , p = 0.00077
Putrescine
Glycylglycine
0.25
0.20
0.15
0.10
0.5
0.6
0.7
0.8
status
ICU
Ward

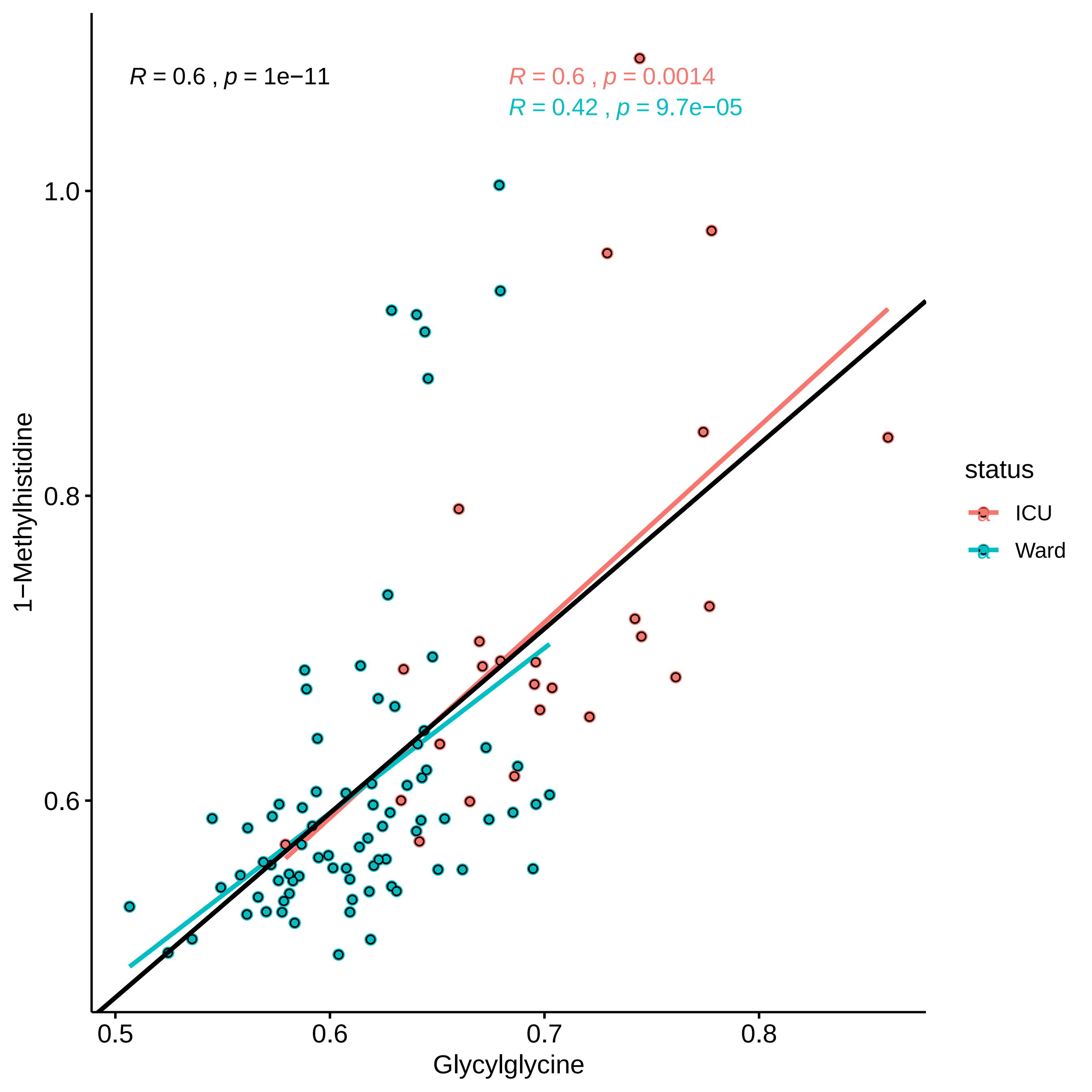

R = 0.6 , p = 1e−11
R = 0.6 , p = 0.0014
R = 0.42 , p = 9.7e−05
1.0
0.8
0.6
1−Methylhistidine
0.5
0.6
0.7
0.8
Glycylglycine
status
ICU
Ward

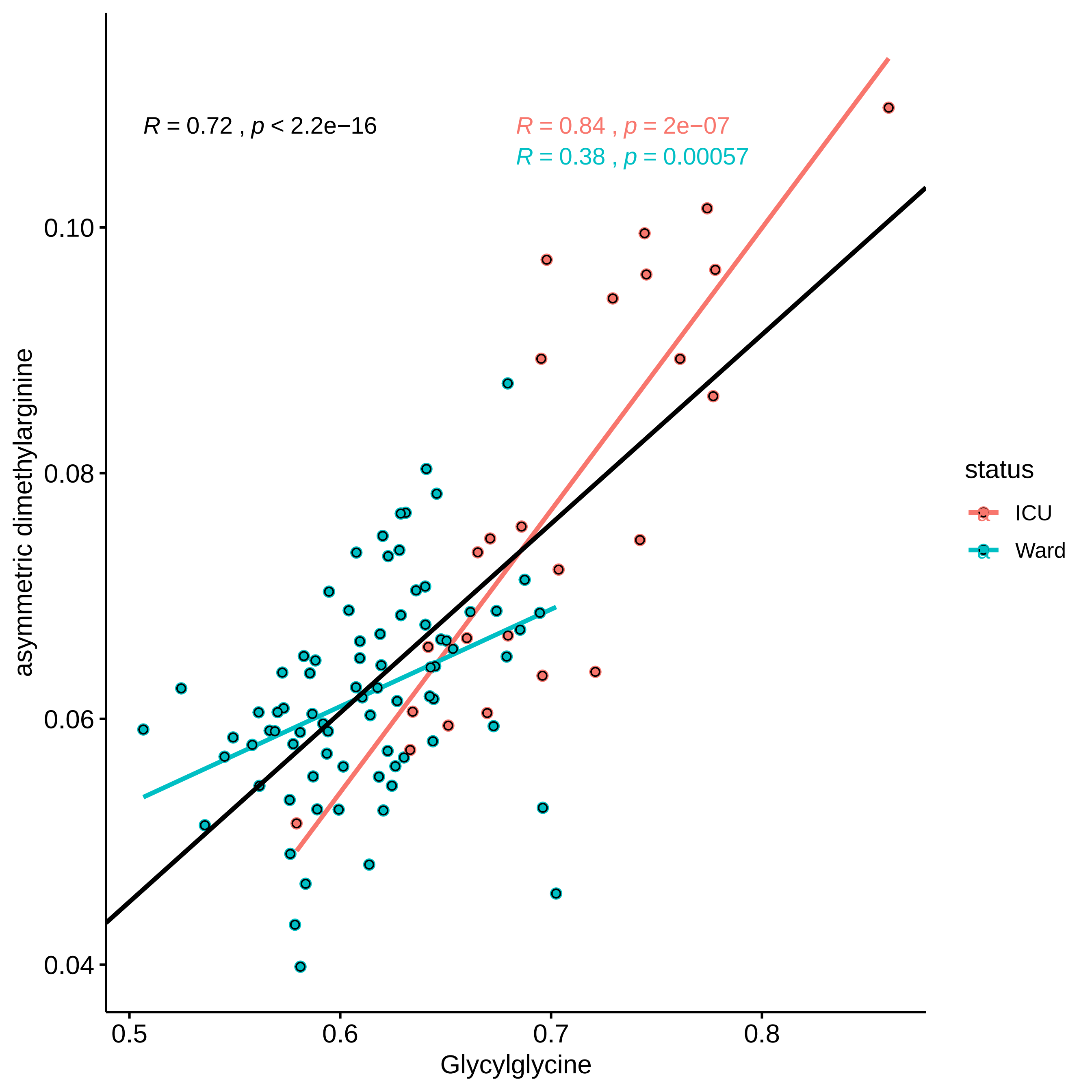

R = 0.72 , p < 2.2e−16
R = 0.84 , p = 2e−07
R = 0.38 , p = 0.00057
0.10
0.08
0.06
0.04
0.5
0.6
0.7
0.8
asymmetric dimethylarginine
Glycylglycine
status
ICU
Ward

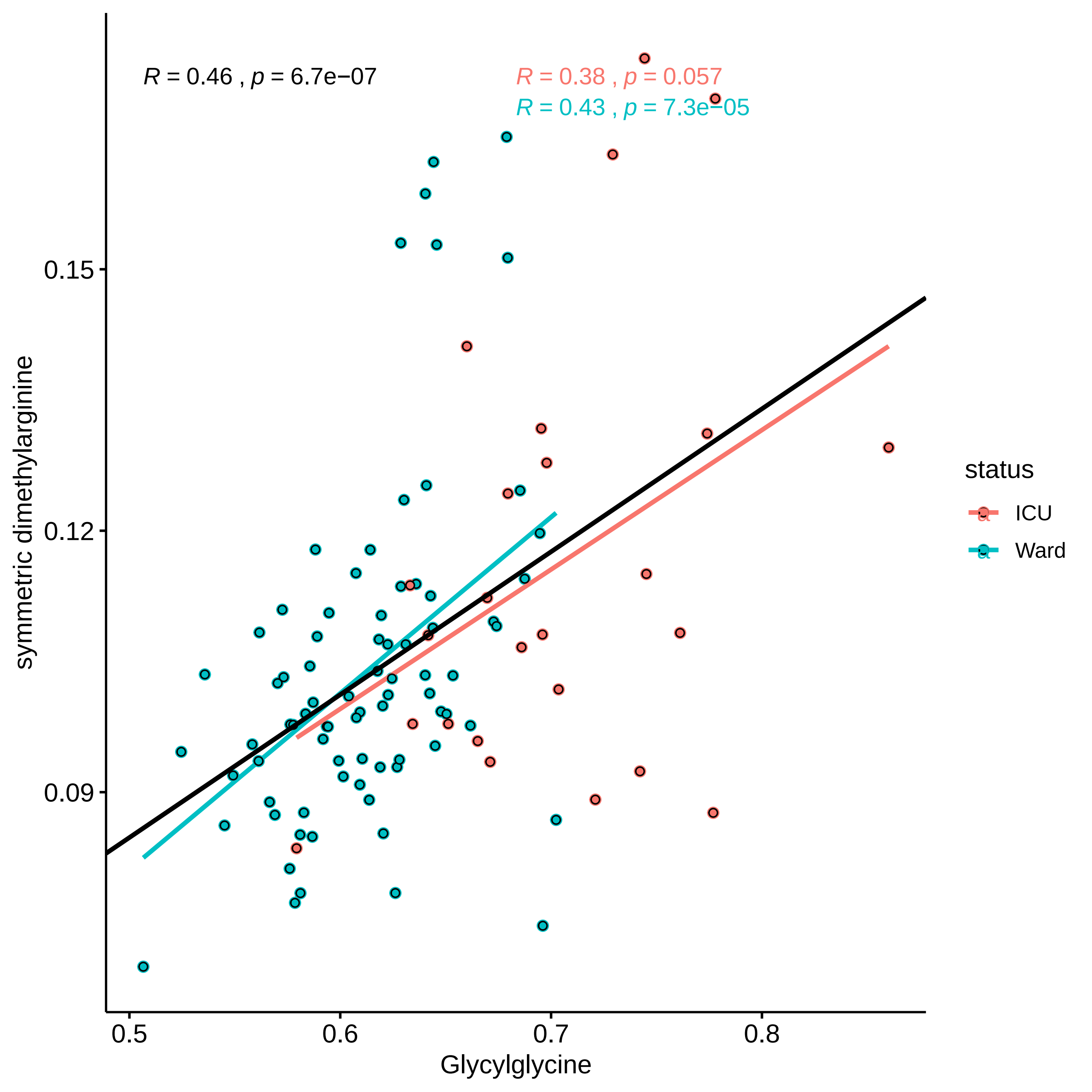

R = 0.46 , p = 6.7e−07
R = 0.38 , p = 0.057
R = 0.43 , p = 7.3e−05
symmetric dimethylarginine
0.15
0.12
0.09
0.5
0.6
0.7
0.8
Glycylglycine
status
ICU
Ward

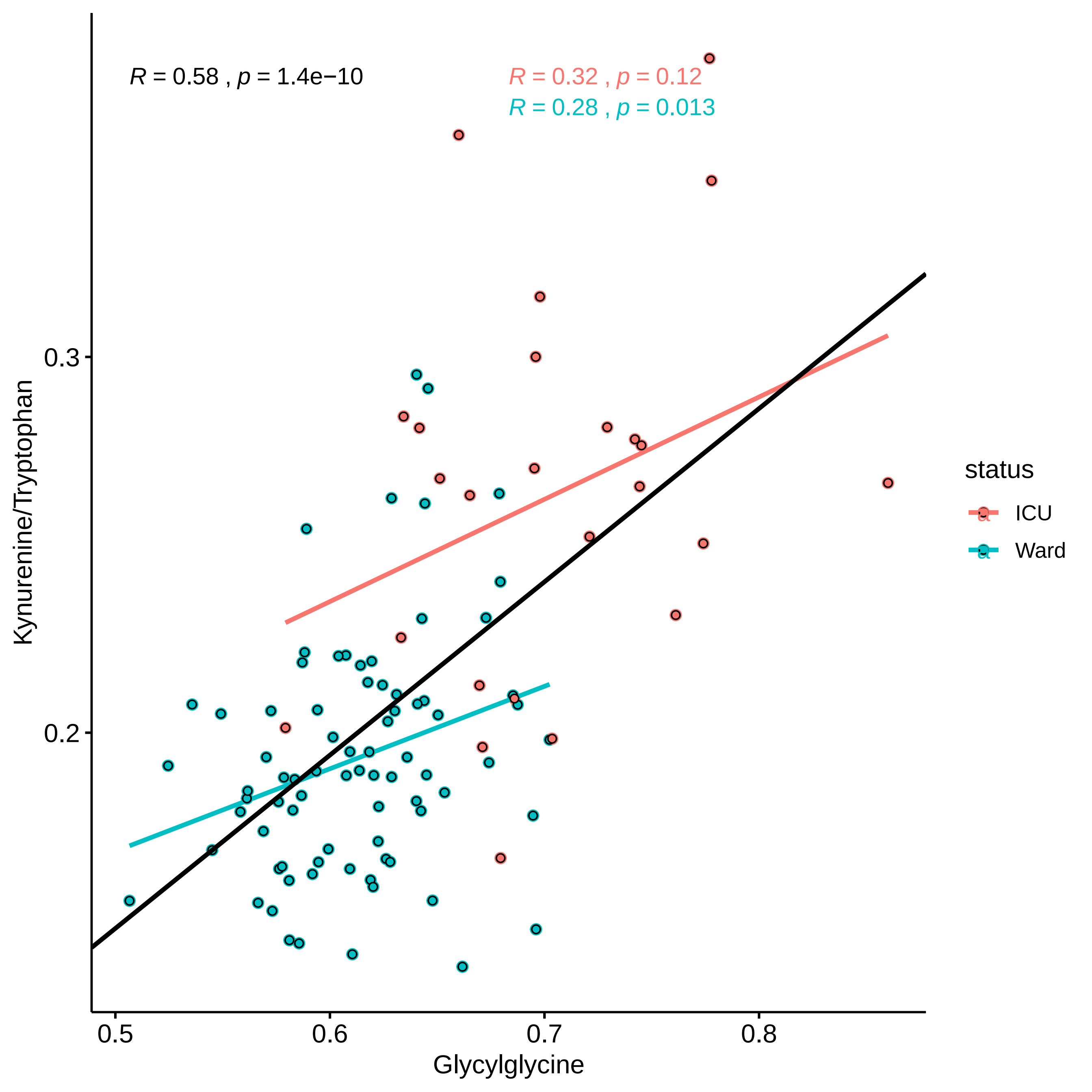

R = 0.58 , p = 1.4e−10
R = 0.32 , p = 0.12
R = 0.28 , p = 0.013
Kynurenine/Tryptophan
0.3
0.2
0.5
0.6
0.7
0.8
Glycylglycine
status
ICU
Ward

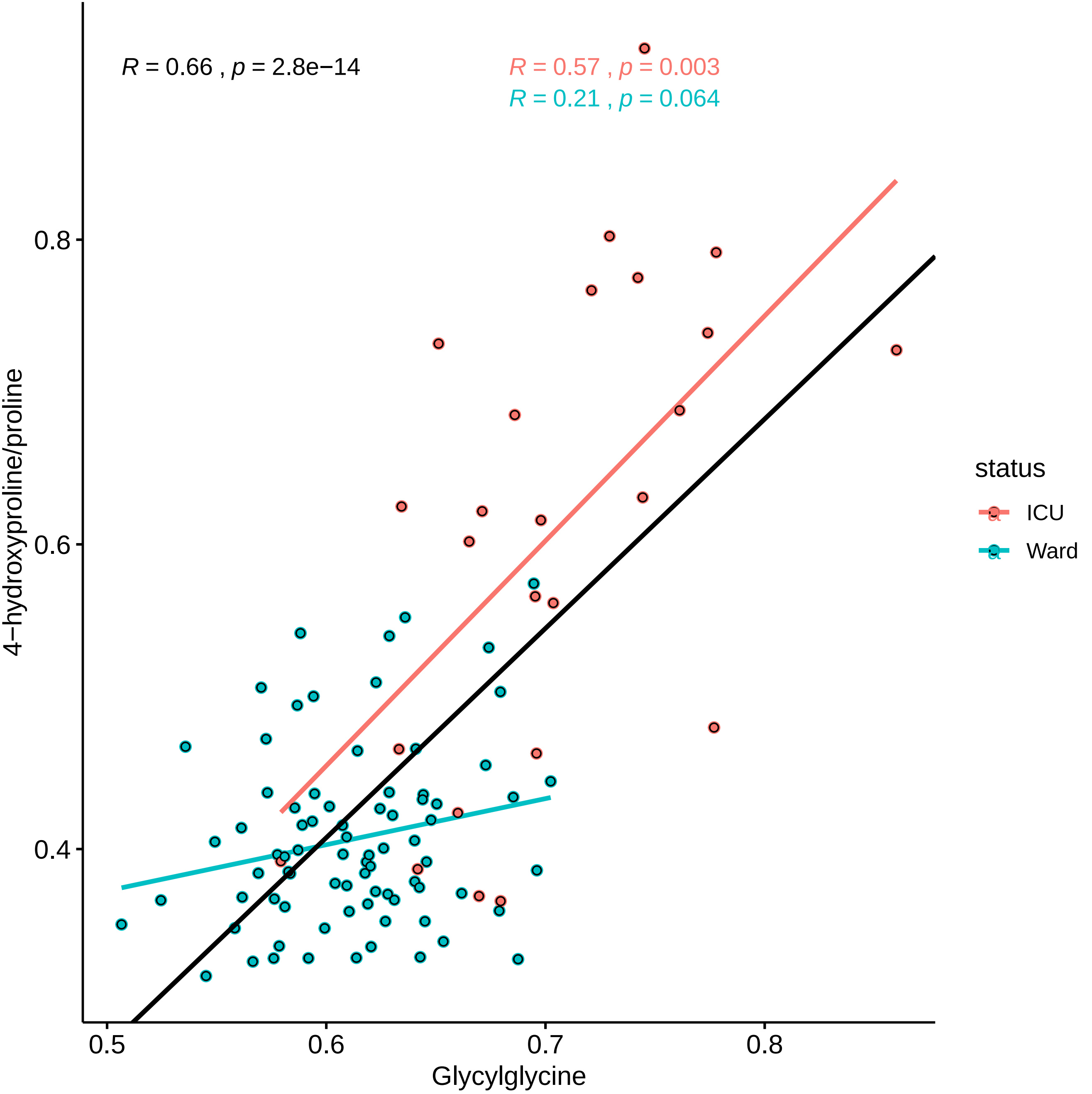

R = 0.66 , p = 2.8e−14
R = 0.57 , p = 0.003
R = 0.21 , p = 0.064
4−hydroxyproline/proline
0.8
0.6
0.4
0.5
0.6
0.7
0.8
Glycylglycine
status
ICU
Ward

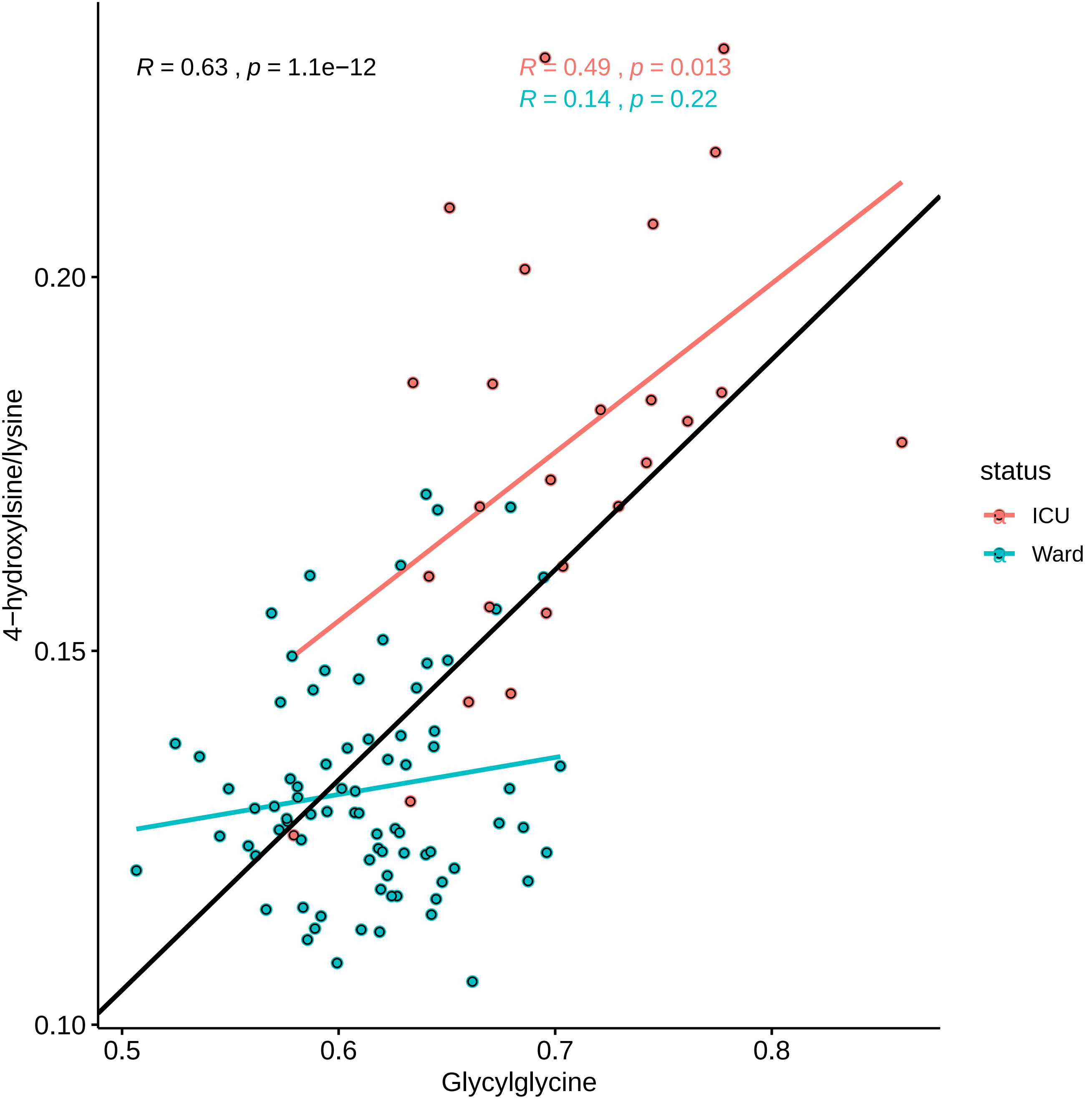

R = 0.63 , p = 1.1e−12
R = 0.49 , p = 0.013
R = 0.14 , p = 0.22
4−hydroxylsine/lysine
0.20
0.15
0.10
0.5
0.6
0.7
0.8
Glycylglycine
status
ICU
Ward

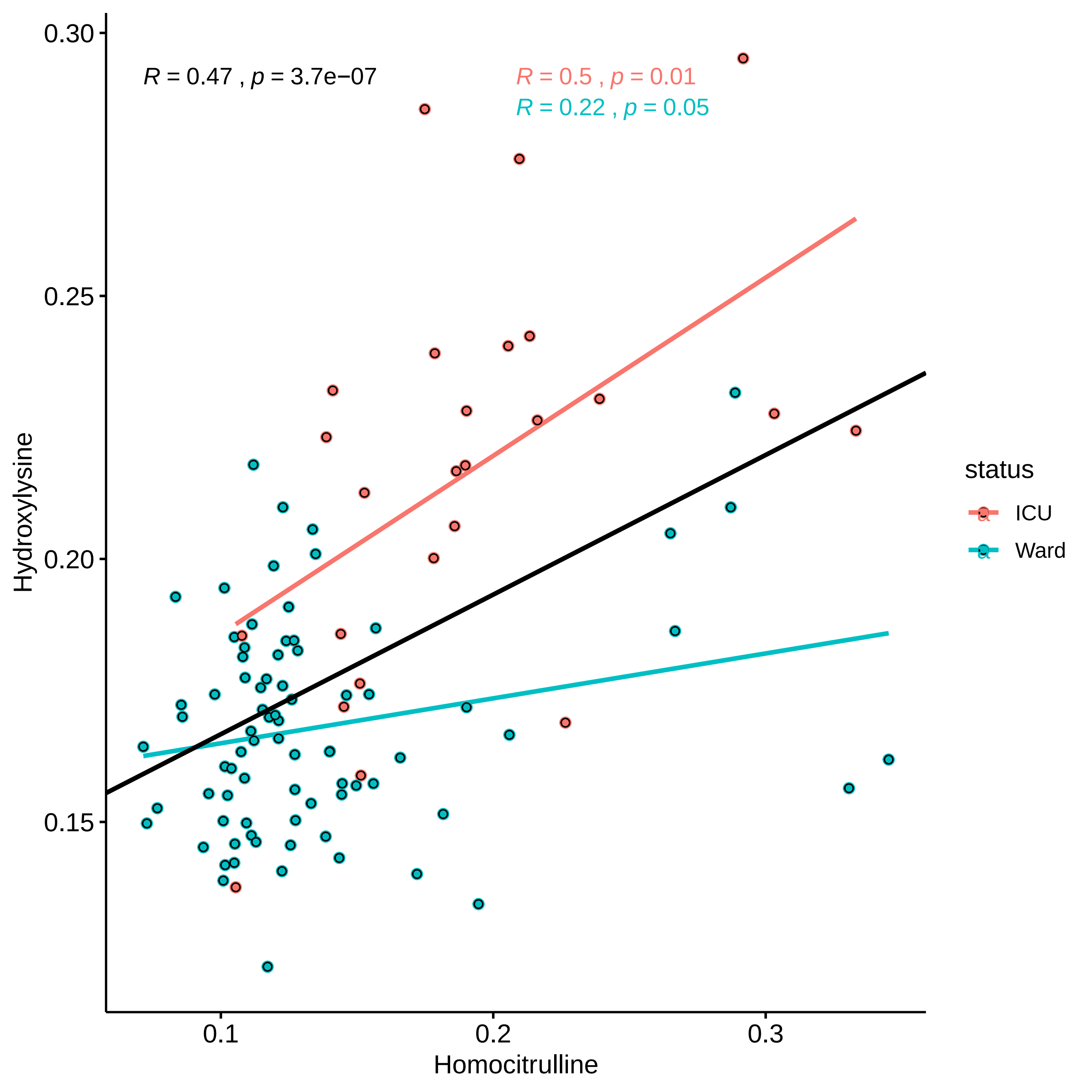

R = 0.47 , p = 3.7e−07
R = 0.5 , p = 0.01
R = 0.22 , p = 0.05
0.30
0.25
0.20
0.15
Hydroxylysine
0.1
0.2
0.3
Homocitrulline
status
ICU
Ward

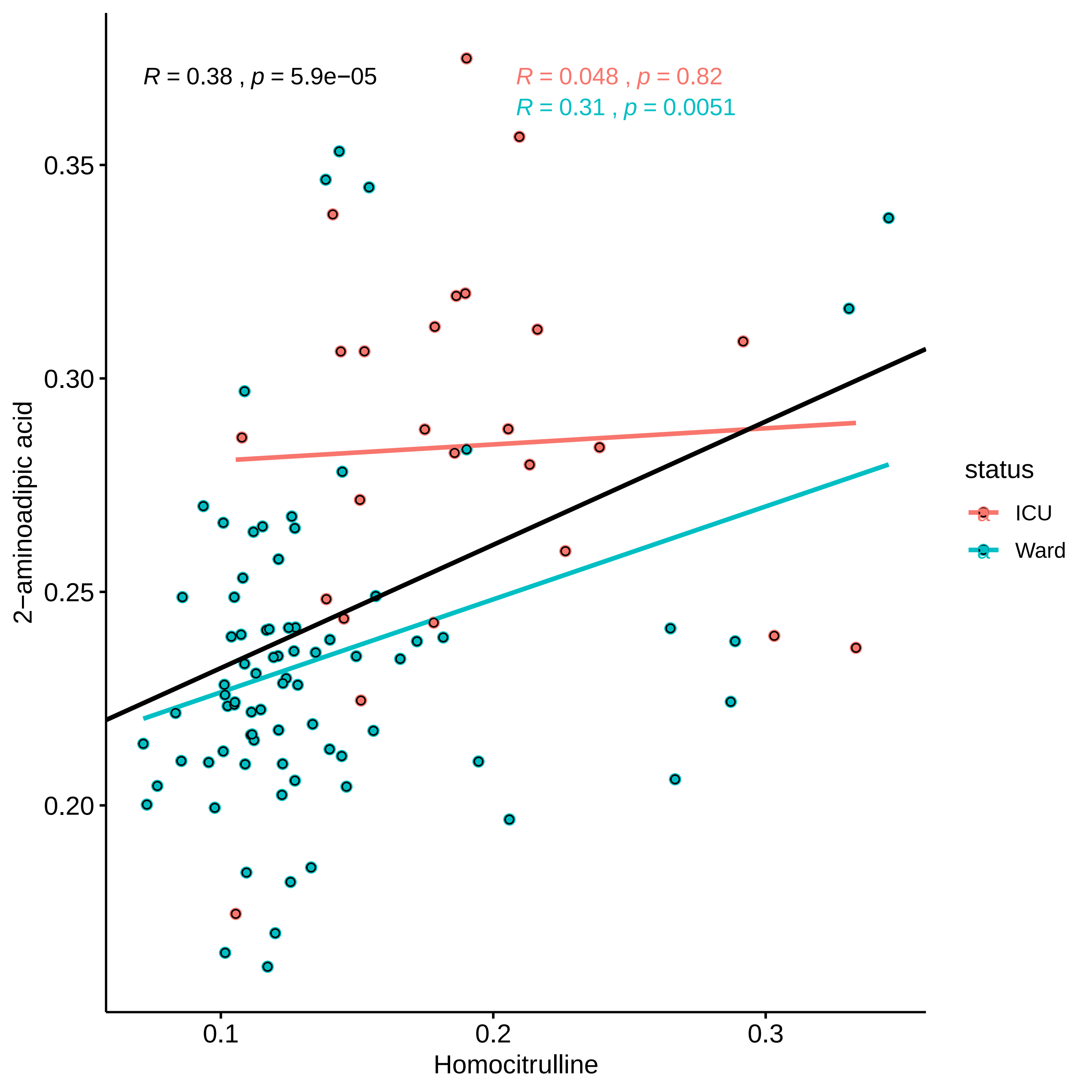
R = 0.38 , p = 5.9e−05
R = 0.048 , p = 0.82
R = 0.31 , p = 0.0051
0.35
0.30
0.25
0.20
2−aminoadipic acid
0.1
0.2
0.3
Homocitrulline
status
ICU
Ward

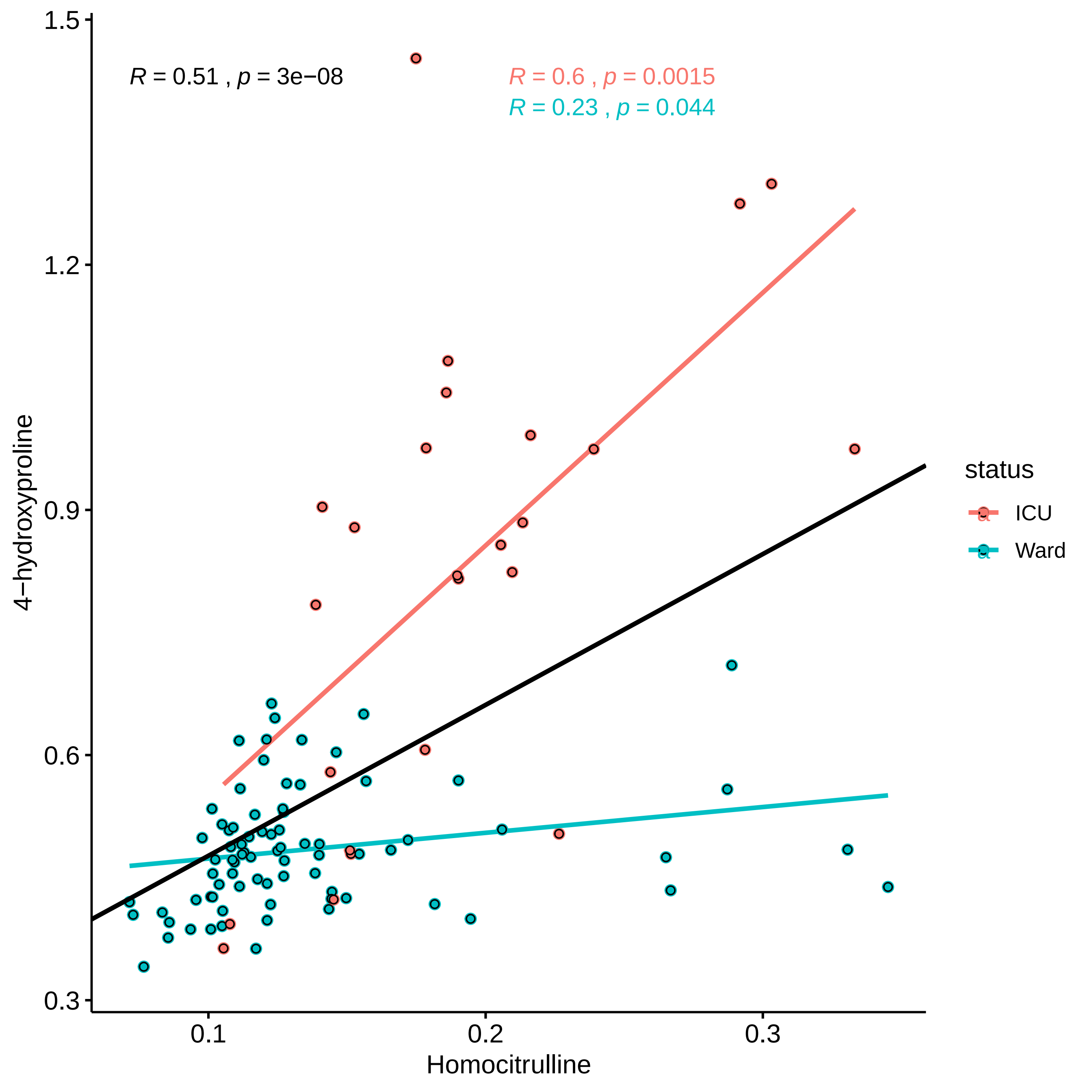

R = 0.51 , p = 3e−08
R = 0.6 , p = 0.0015
R = 0.23 , p = 0.044
4−hydroxyproline
1.5
1.2
0.9
0.6
0.3
0.1
0.2
0.3
Homocitrulline
status
ICU
Ward

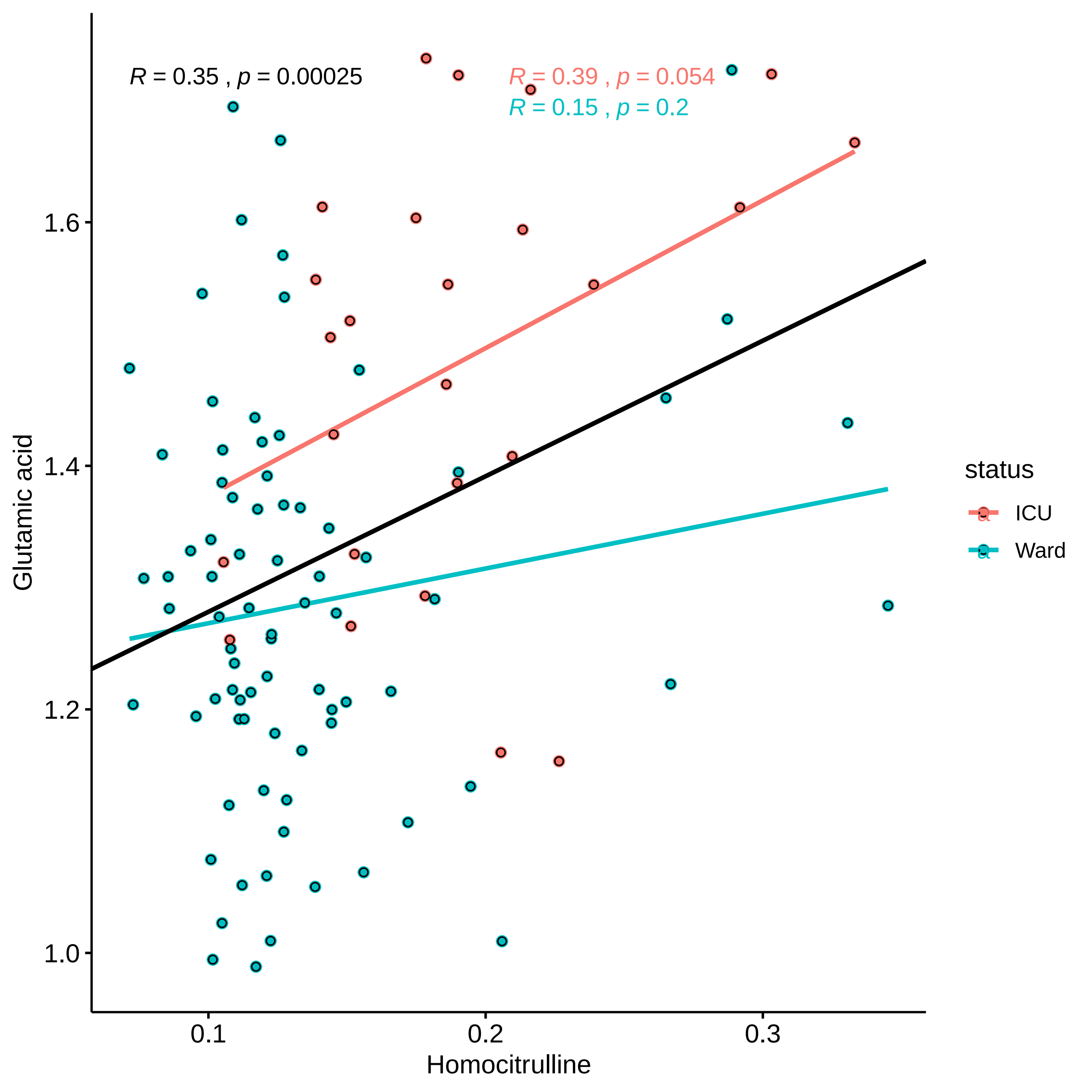

R = 0.35 , p = 0.00025
R = 0.39 , p = 0.054
R = 0.15 , p = 0.2
Glutamic acid
1.6
1.4
1.2
1.0
0.1
0.2
0.3
Homocitrulline
status
ICU
Ward

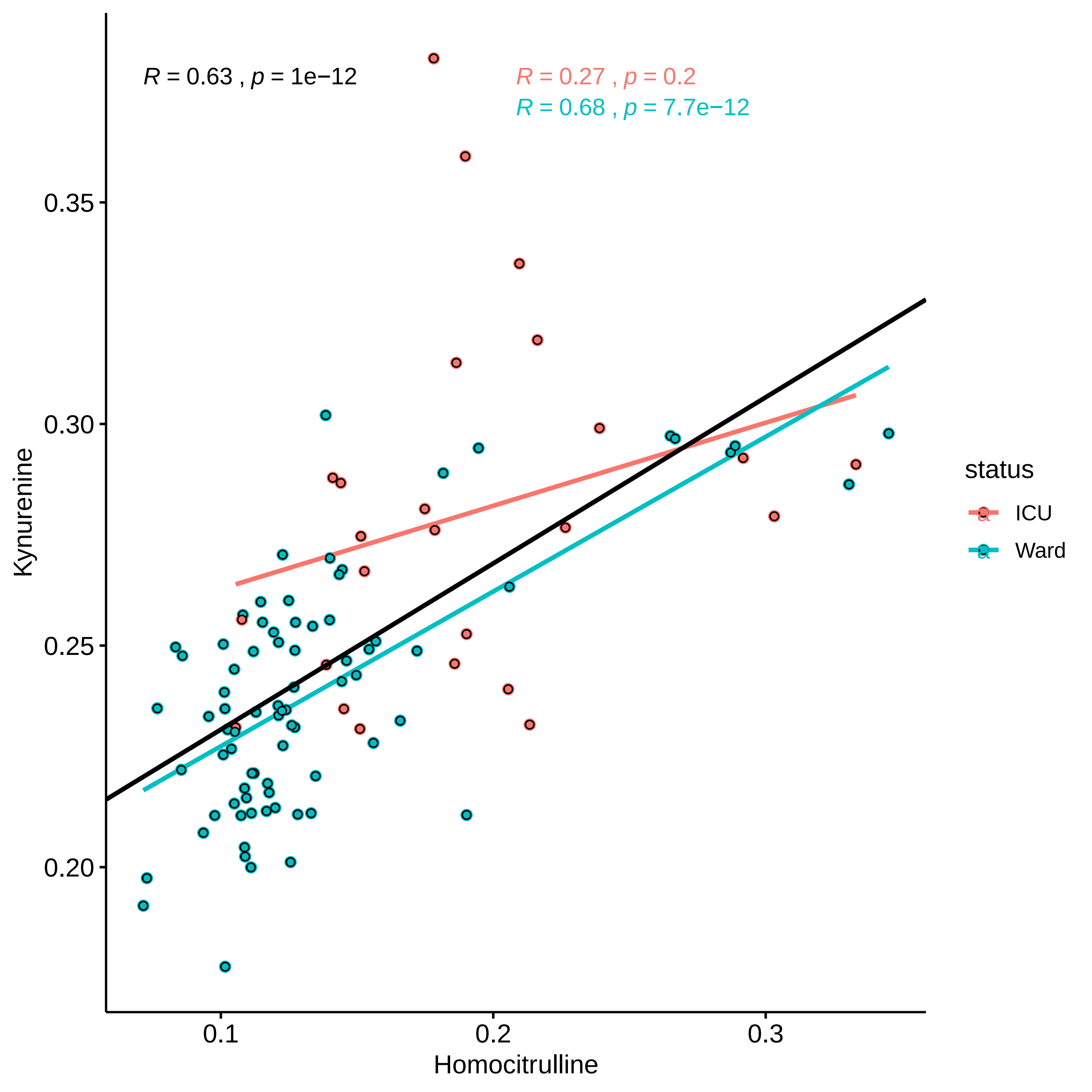

R = 0.63 , p = 1e−12
R = 0.27 , p = 0.2
R = 0.68 , p = 7.7e−12
Kynurenine
0.35
0.30
0.25
0.20
Homocitrulline
0.1
0.2
0.3
status
ICU
Ward

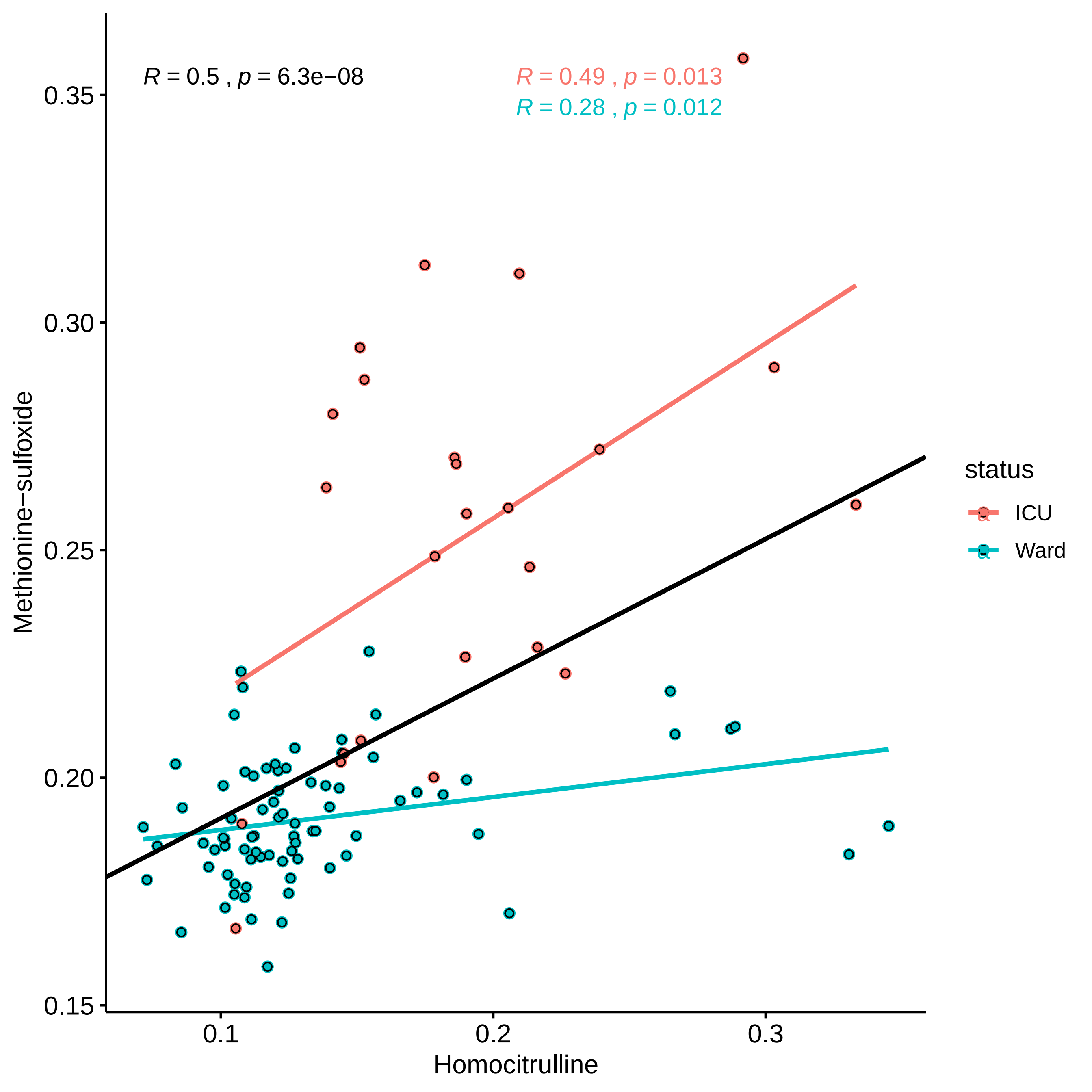

R = 0.5 , p = 6.3e−08
R = 0.49 , p = 0.013
R = 0.28 , p = 0.012
0.35
0.30
0.25
0.20
0.15
Methionine−sulfoxide
0.1
0.2
0.3
Homocitrulline
status
ICU
Ward

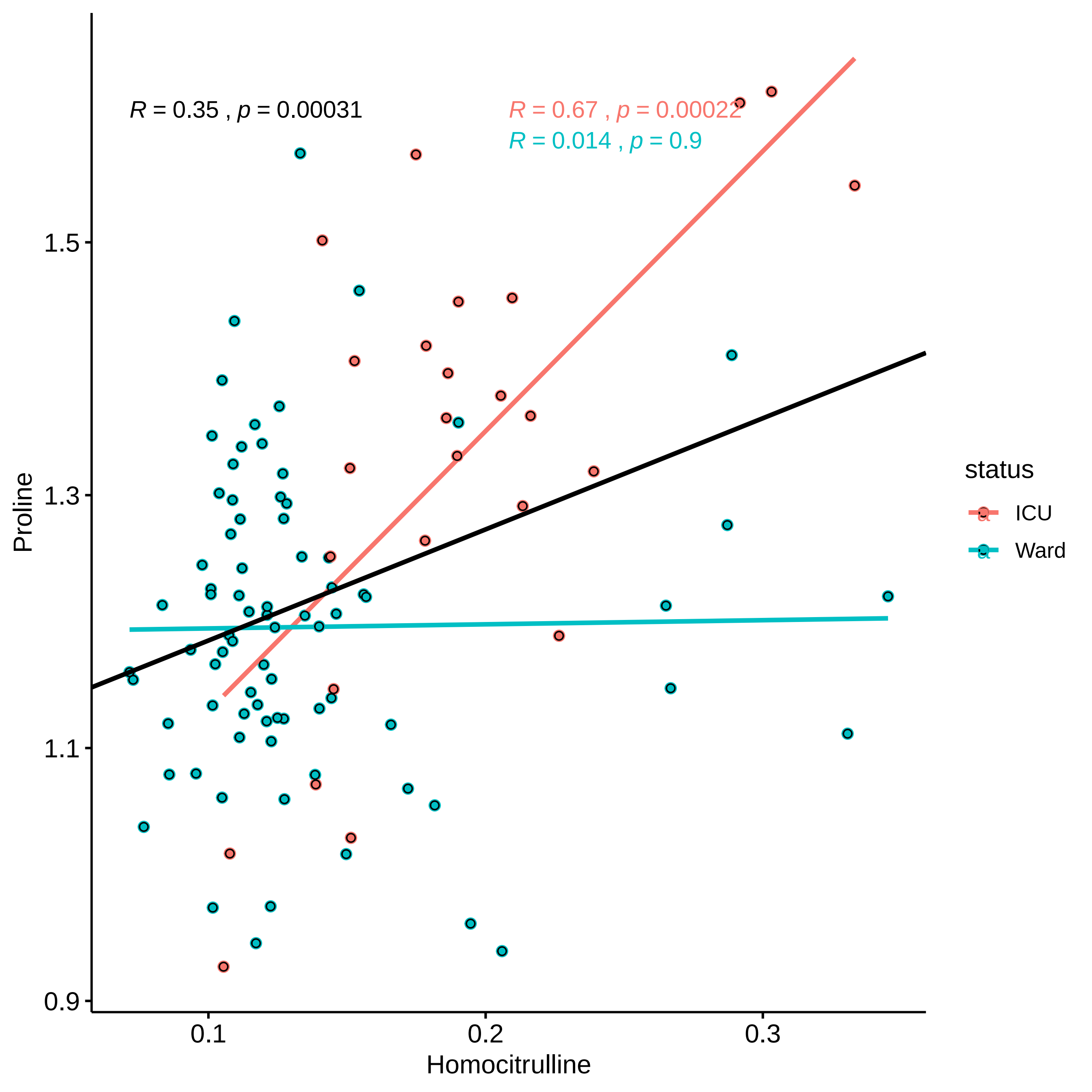

R = 0.35 , p = 0.00031
R = 0.67 , p = 0.00022
R = 0.014 , p = 0.9
Proline
1.5
1.3
1.1
0.9
0.1
0.2
0.3
Homocitrulline
status
ICU
Ward

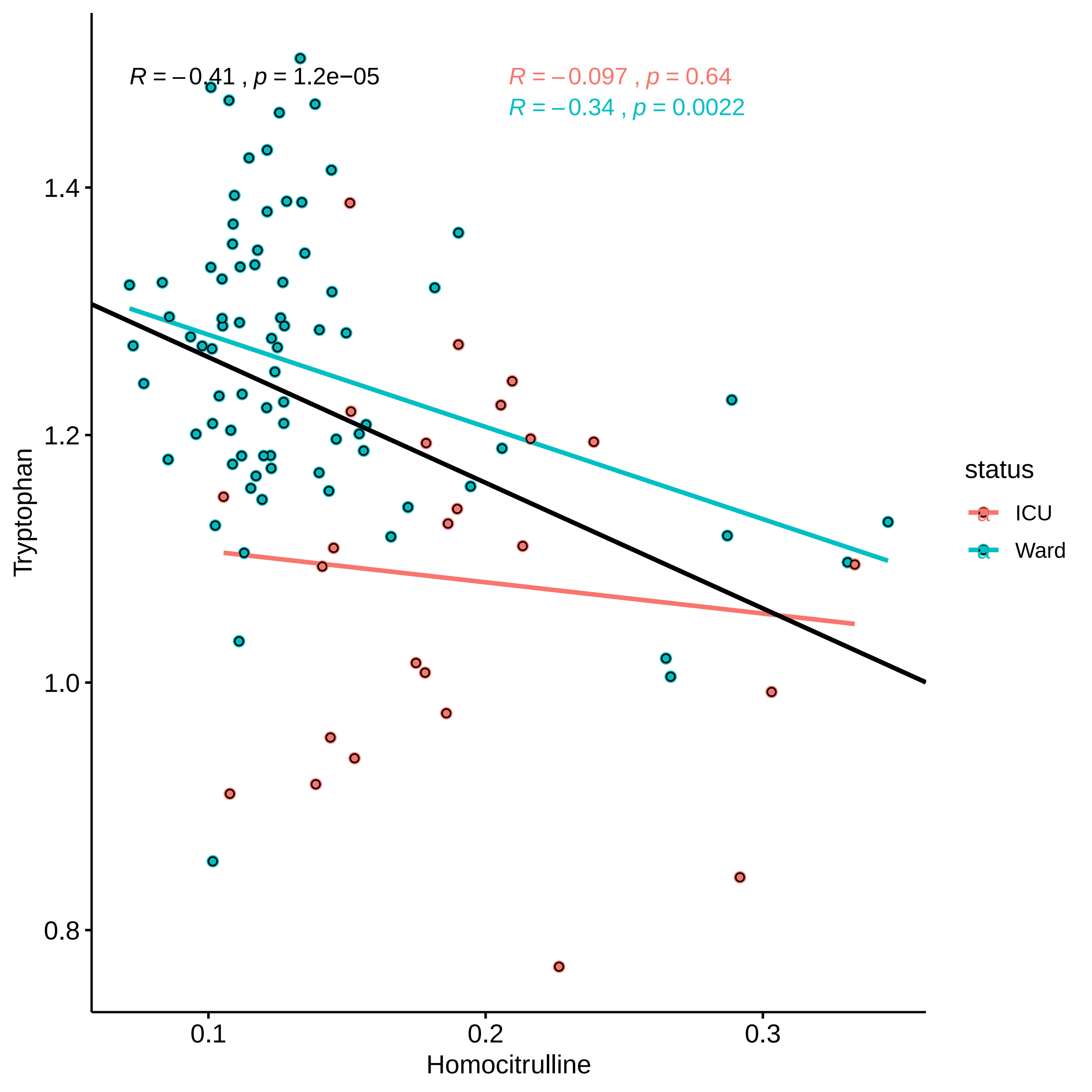

R = −0.41 , p = 1.2e−05
R = −0.097 , p = 0.64
R = −0.34 , p = 0.0022
1.4
1.2
1.0
0.8
Tryptophan
0.1
0.2
0.3
Homocitrulline
status
ICU
Ward

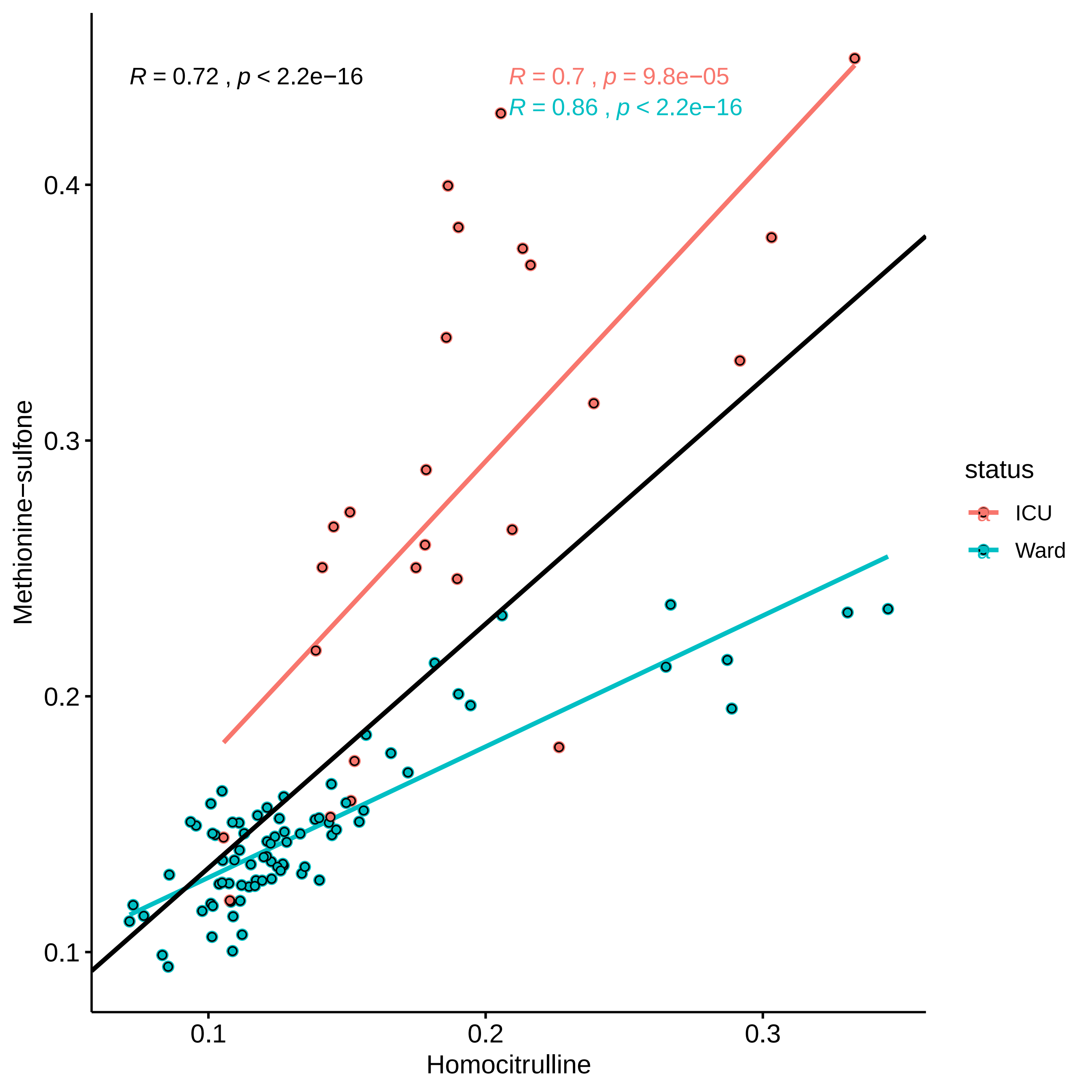

R = 0.72 , p < 2.2e−16
R = 0.7 , p = 9.8e−05
R = 0.86 , p < 2.2e−16
0.4
0.3
0.2
0.1
Methionine−sulfone
0.1
0.2
0.3
Homocitrulline
status
ICU
Ward

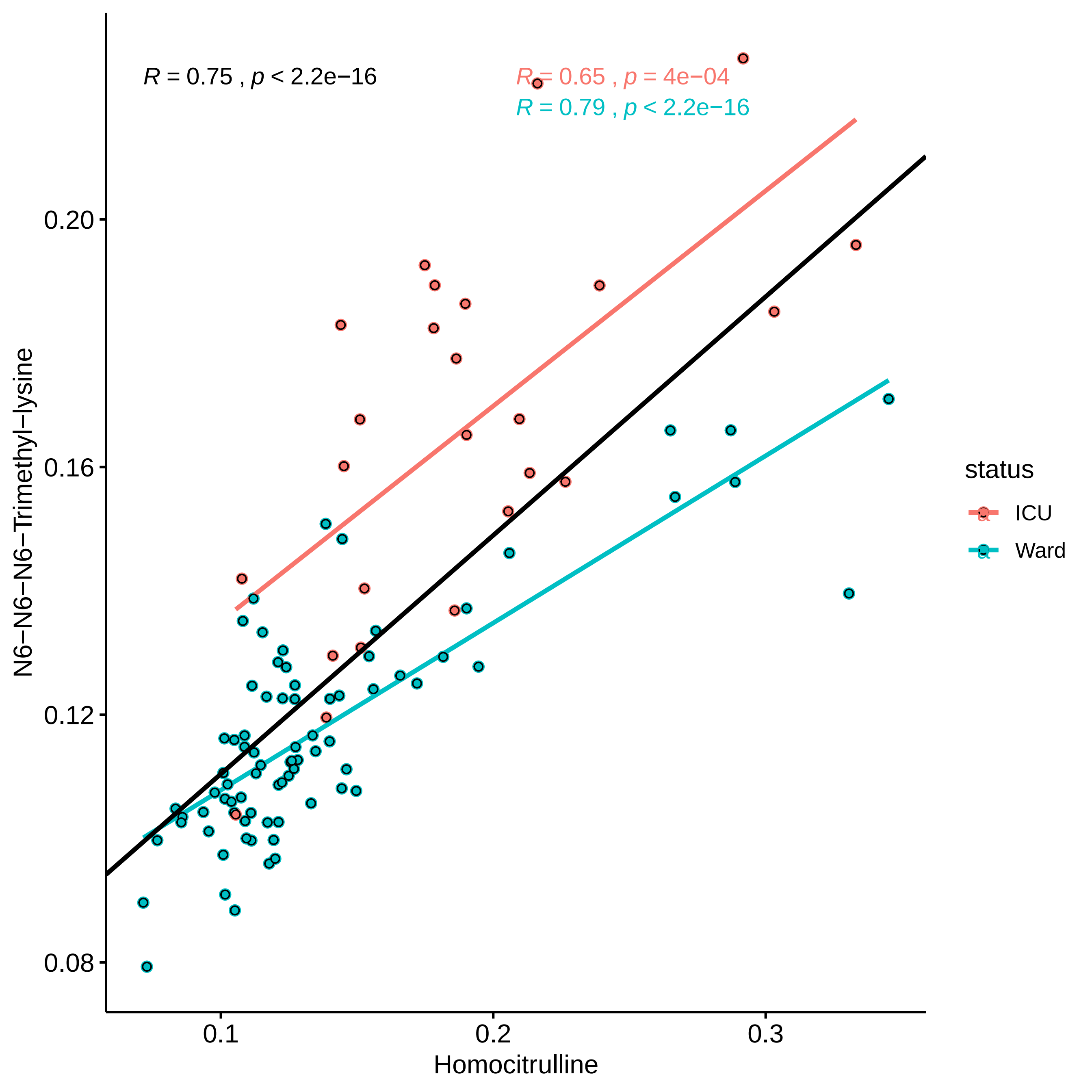

R = 0.75 , p < 2.2e−16
R = 0.65 , p = 4e−04
R = 0.79 , p < 2.2e−16
N6−N6−N6−Trimethyl−lysine
0.20
0.16
0.12
0.08
0.1
0.2
0.3
Homocitrulline
status
ICU
Ward

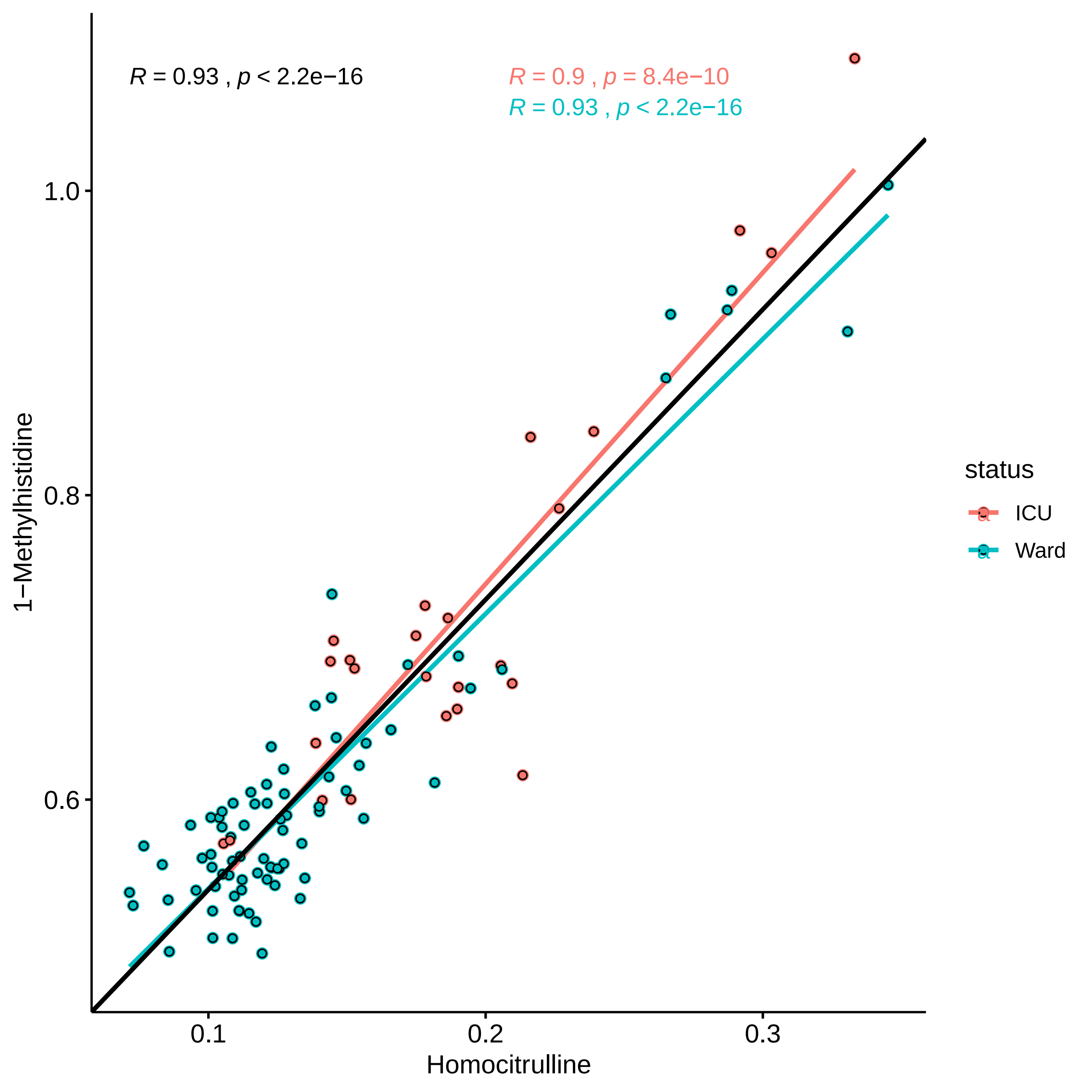

R = 0.93 , p < 2.2e−16
R = 0.9 , p = 8.4e−10
R = 0.93 , p < 2.2e−16
1.0
0.8
0.6
0.1
0.2
0.3
1−Methylhistidine
Homocitrulline
status
ICU
Ward

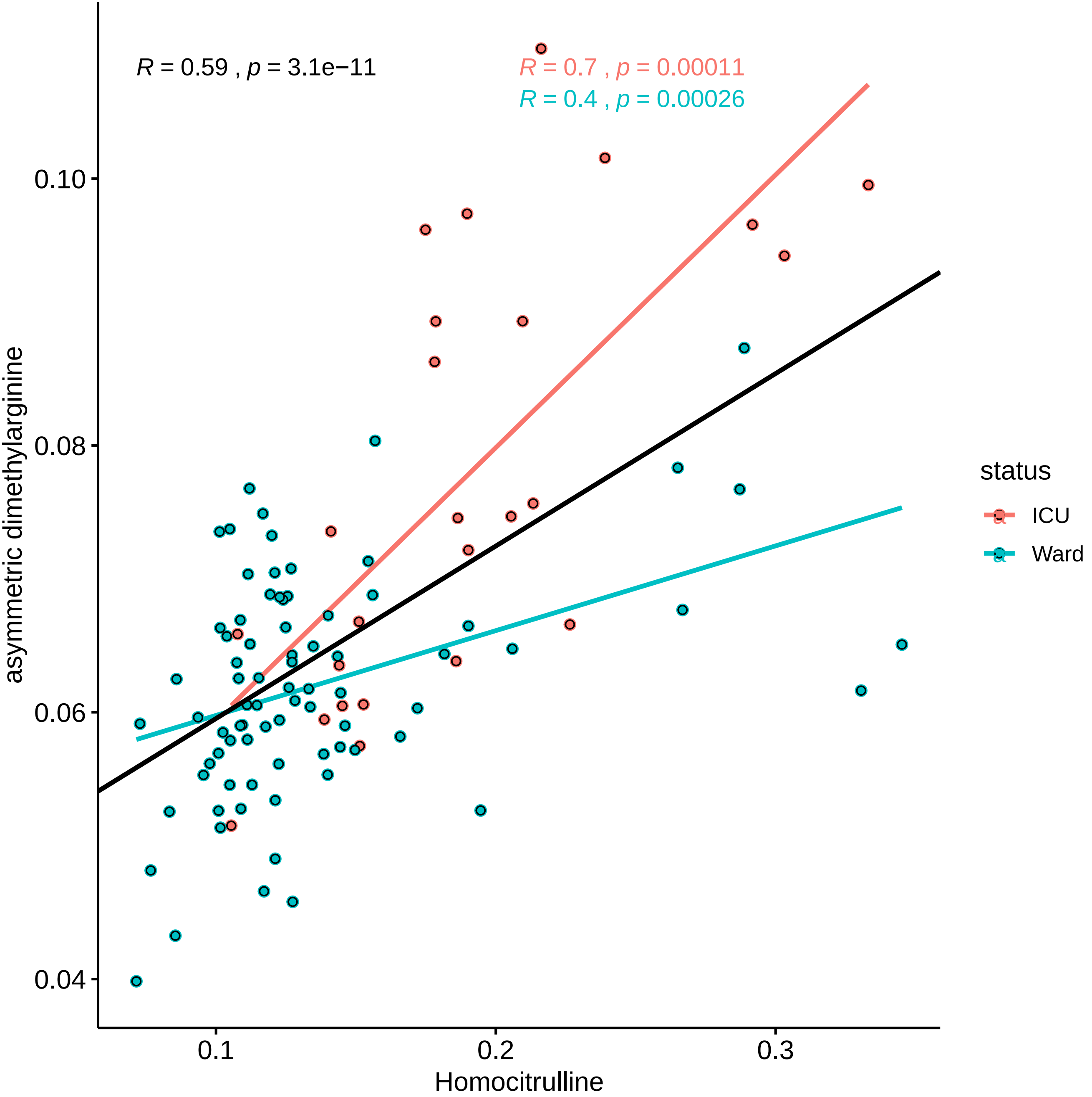

R = 0.59 , p = 3.1e−11
R = 0.7 , p = 0.00011
R = 0.4 , p = 0.00026
asymmetric dimethylarginine
0.10
0.08
0.06
0.04
0.1
0.2
0.3
Homocitrulline
status
ICU
Ward

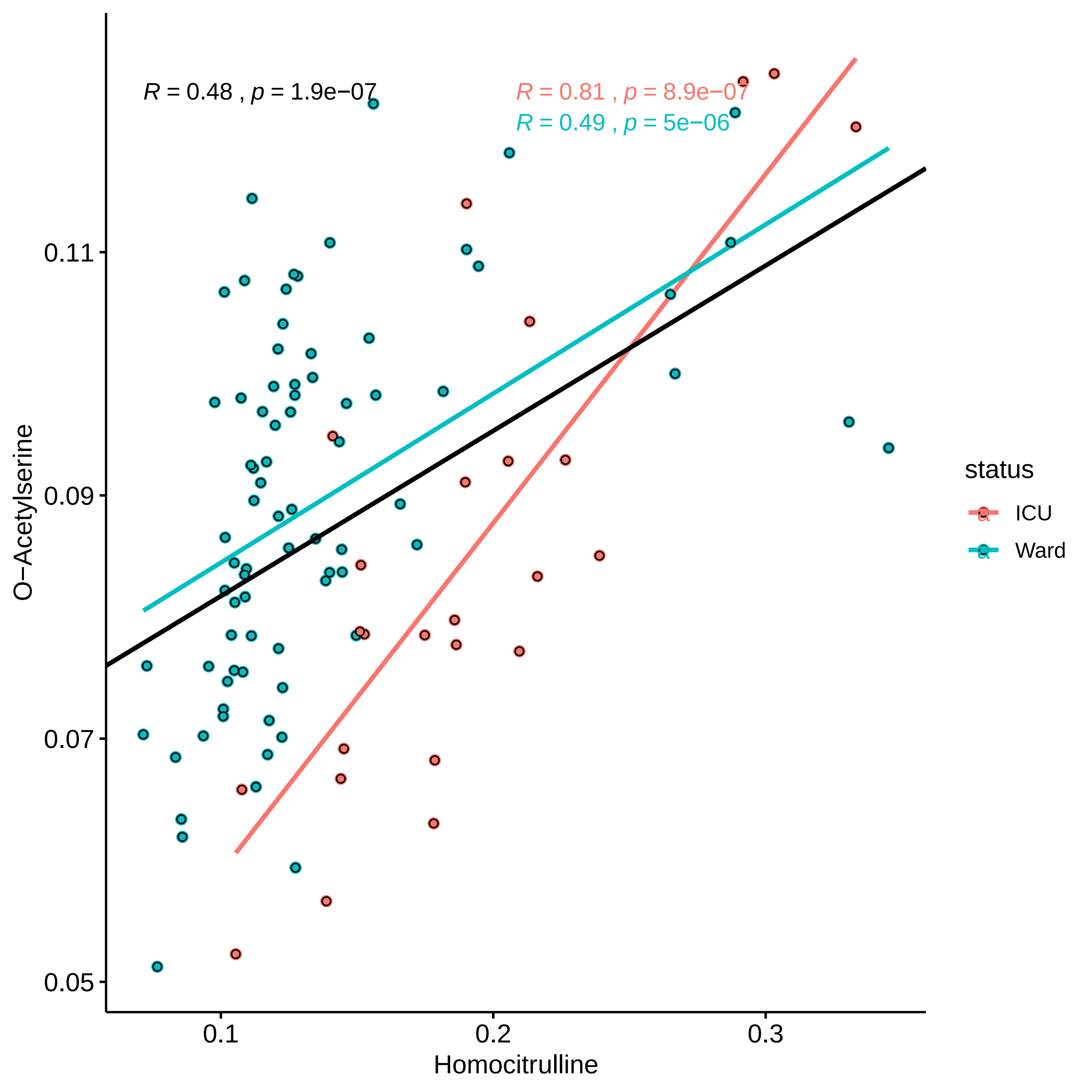

R = 0.48 , p = 1.9e−07
R = 0.81 , p = 8.9e−07
R = 0.49 , p = 5e−06
0.11
0.09
0.07
0.05
O−Acetylserine
0.1
0.2
0.3
Homocitrulline
status
ICU
Ward

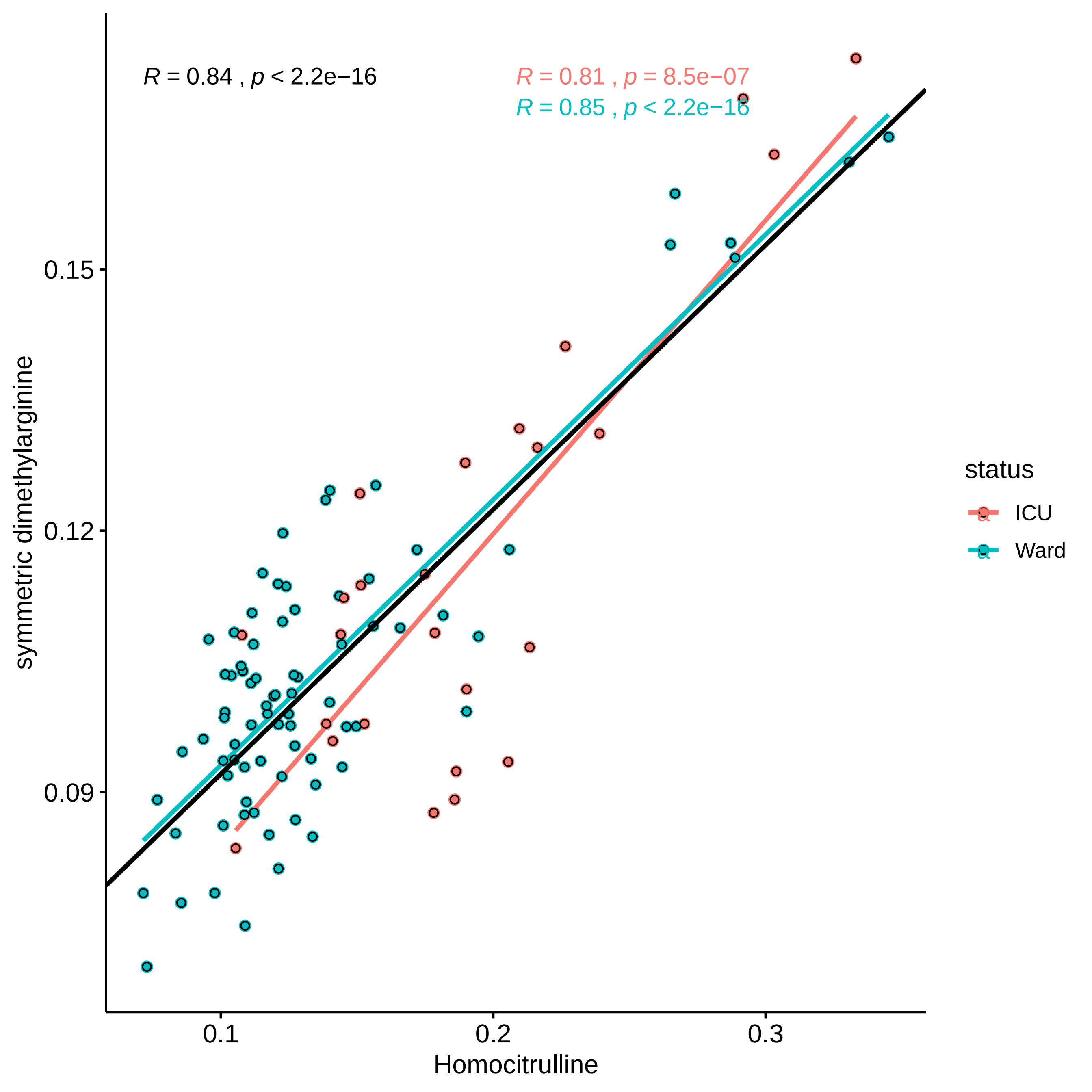

R = 0.84 , p < 2.2e−16
R = 0.81 , p = 8.5e−07
R = 0.85 , p < 2.2e−16
0.15
0.12
0.09
0.1
0.2
0.3
symmetric dimethylarginine
Homocitrulline
status
ICU
Ward

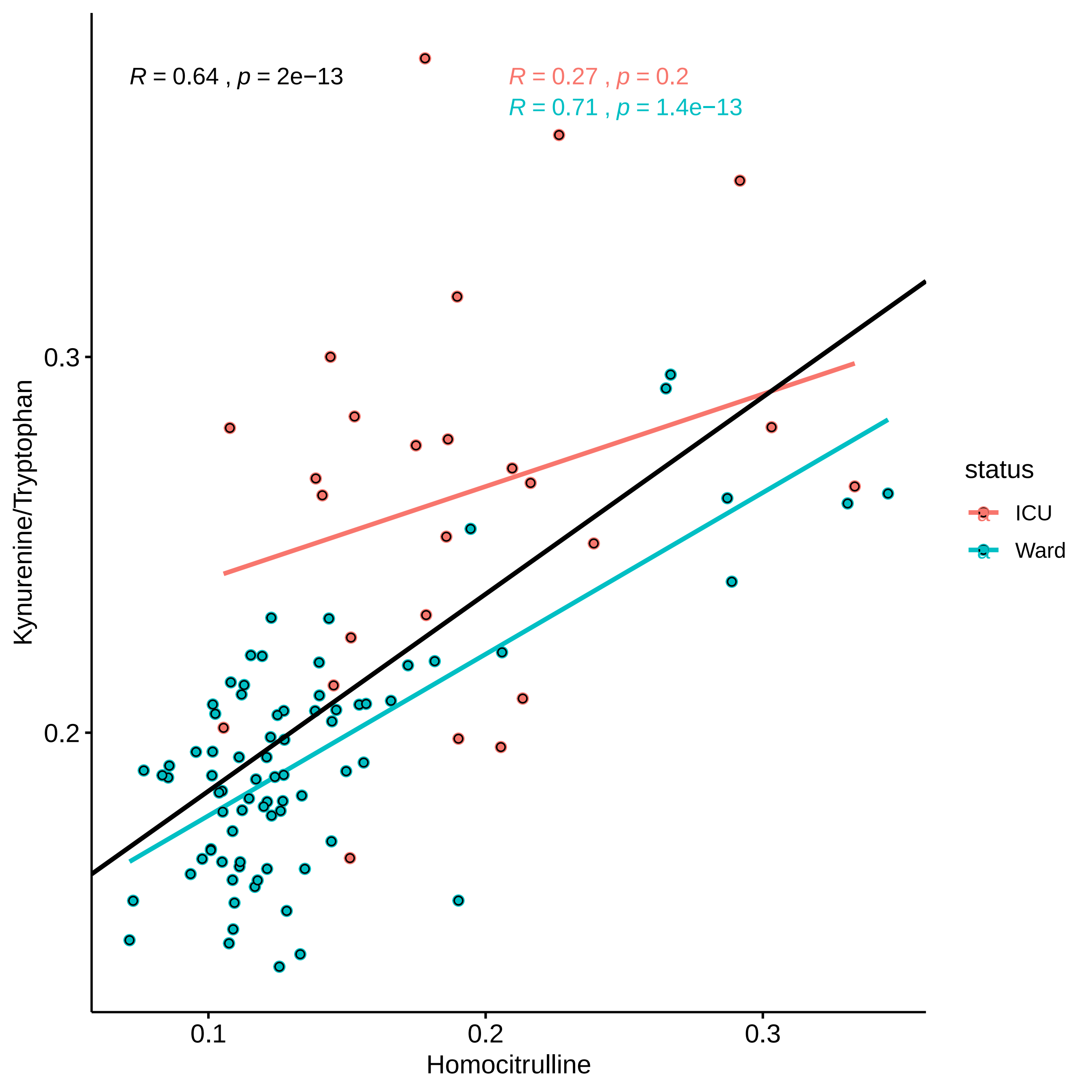

R = 0.64 , p = 2e−13
R = 0.27 , p = 0.2
R = 0.71 , p = 1.4e−13
Kynurenine/Tryptophan
0.3
0.2
0.1
0.2
0.3
Homocitrulline
status
ICU
Ward

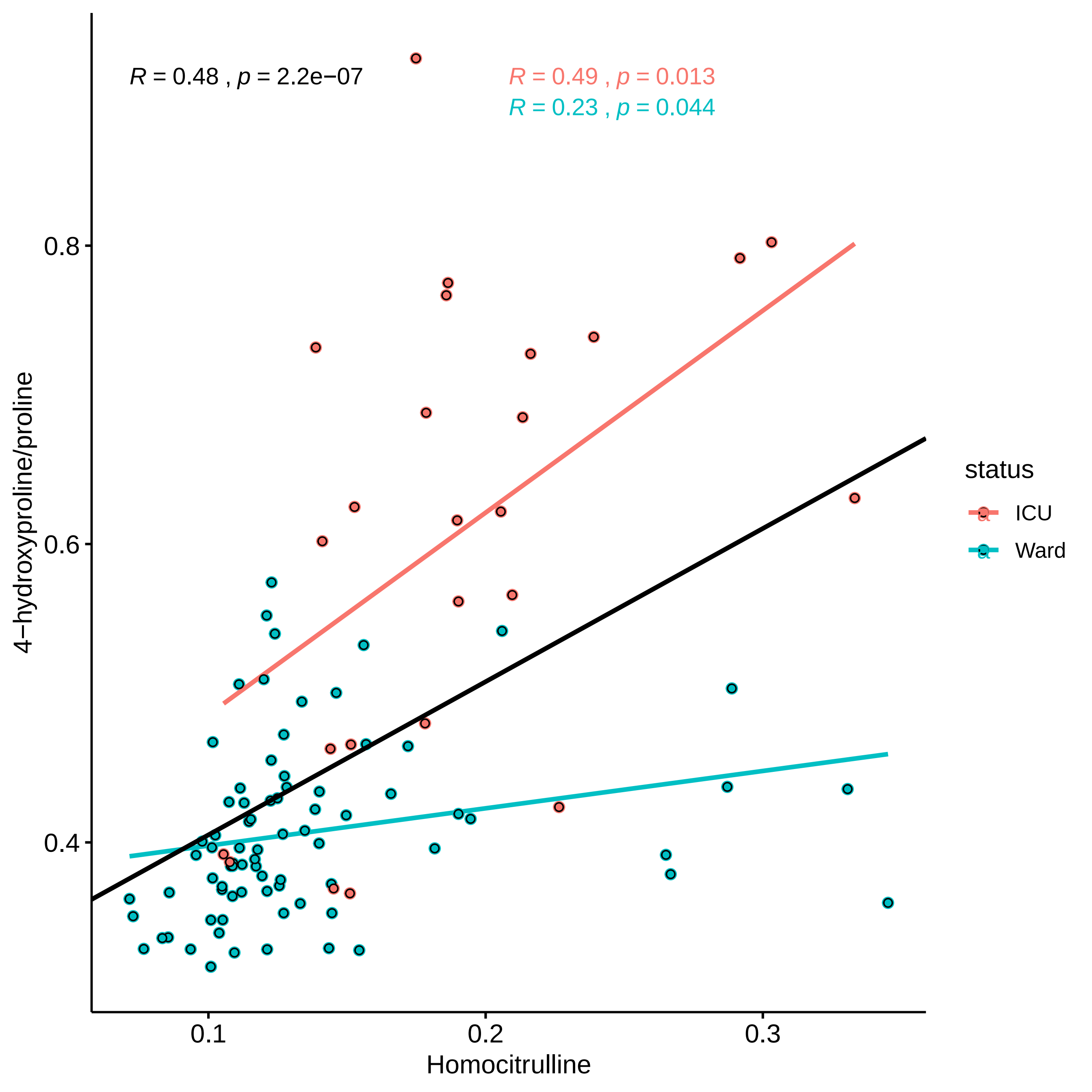
R = 0.48 , p = 2.2e−07
R = 0.49 , p = 0.013
R = 0.23 , p = 0.044
4−hydroxyproline/proline
0.8
0.6
0.4
0.1
0.2
0.3
Homocitrulline
status
ICU
Ward

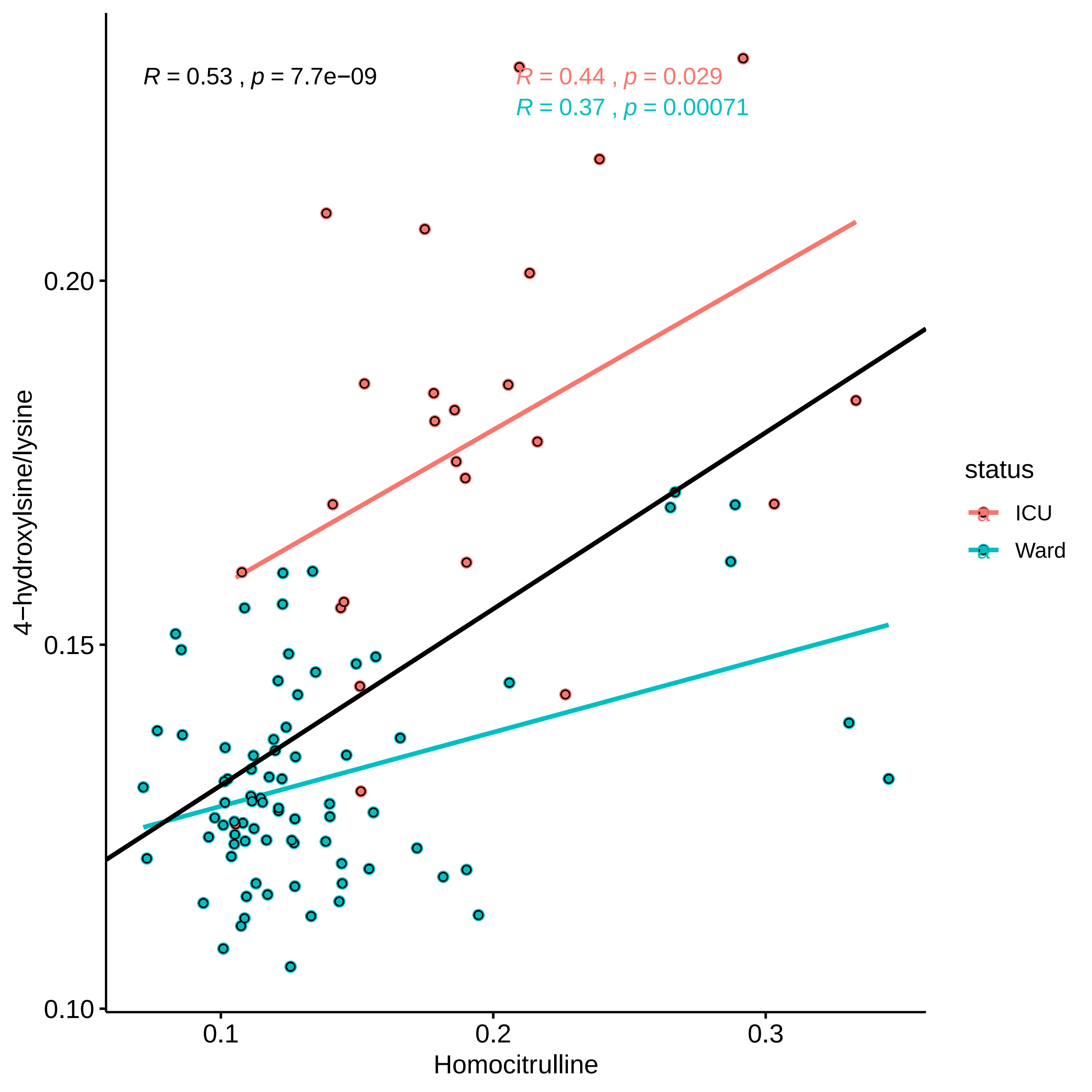

R = 0.53 , p = 7.7e−09
R = 0.44 , p = 0.029
R = 0.37 , p = 0.00071
4−hydroxylysine/lysine
0.20
0.15
0.10
0.1
0.2
0.3
Homocitrulline
status
ICU
Ward

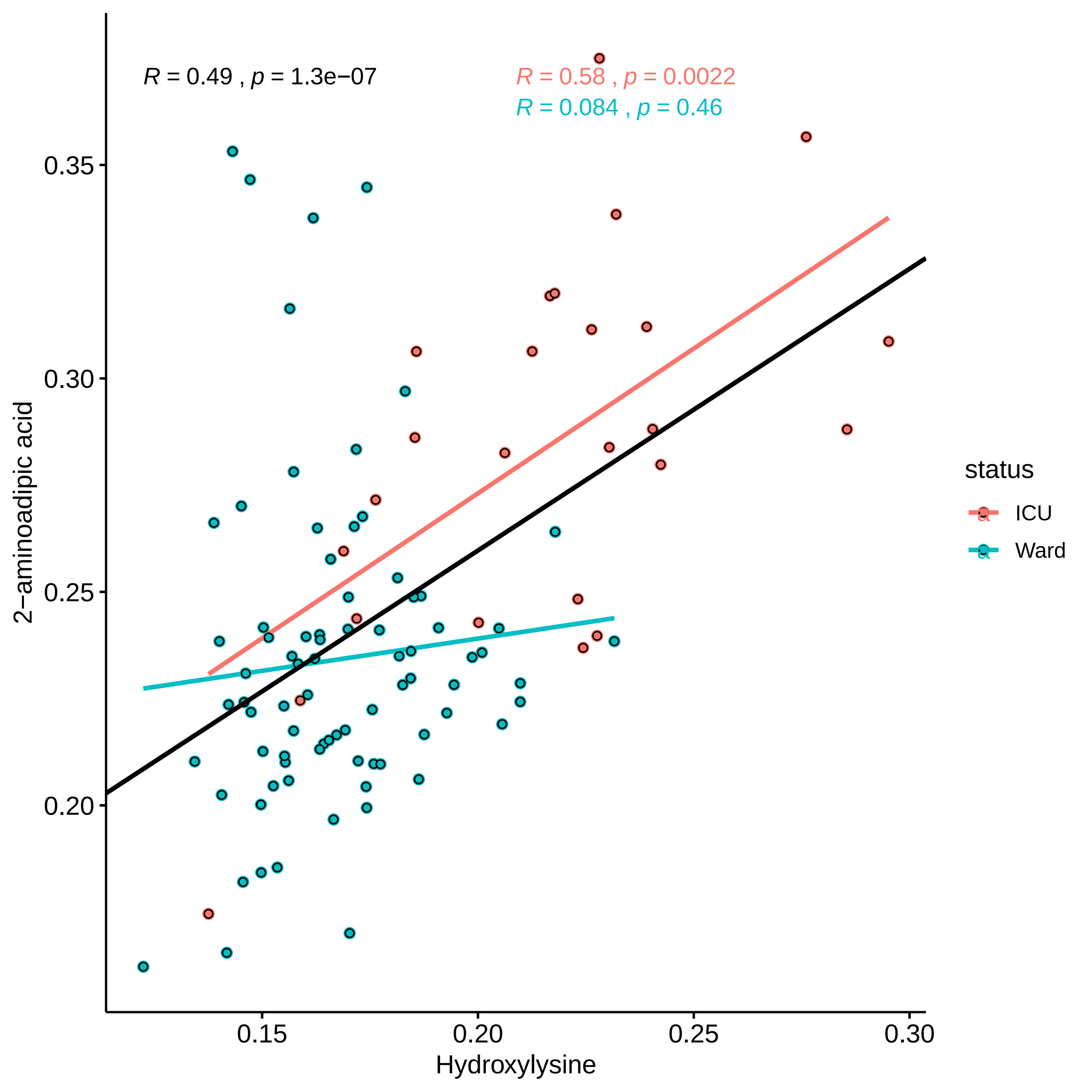

R = 0.49 , p = 1.3e−07
R = 0.58 , p = 0.0022
R = 0.084 , p = 0.46
0.35
0.30
0.25
0.20
2−aminoadipic acid
0.15
0.20
0.25
0.30
Hydroxylysine
status
ICU
Ward

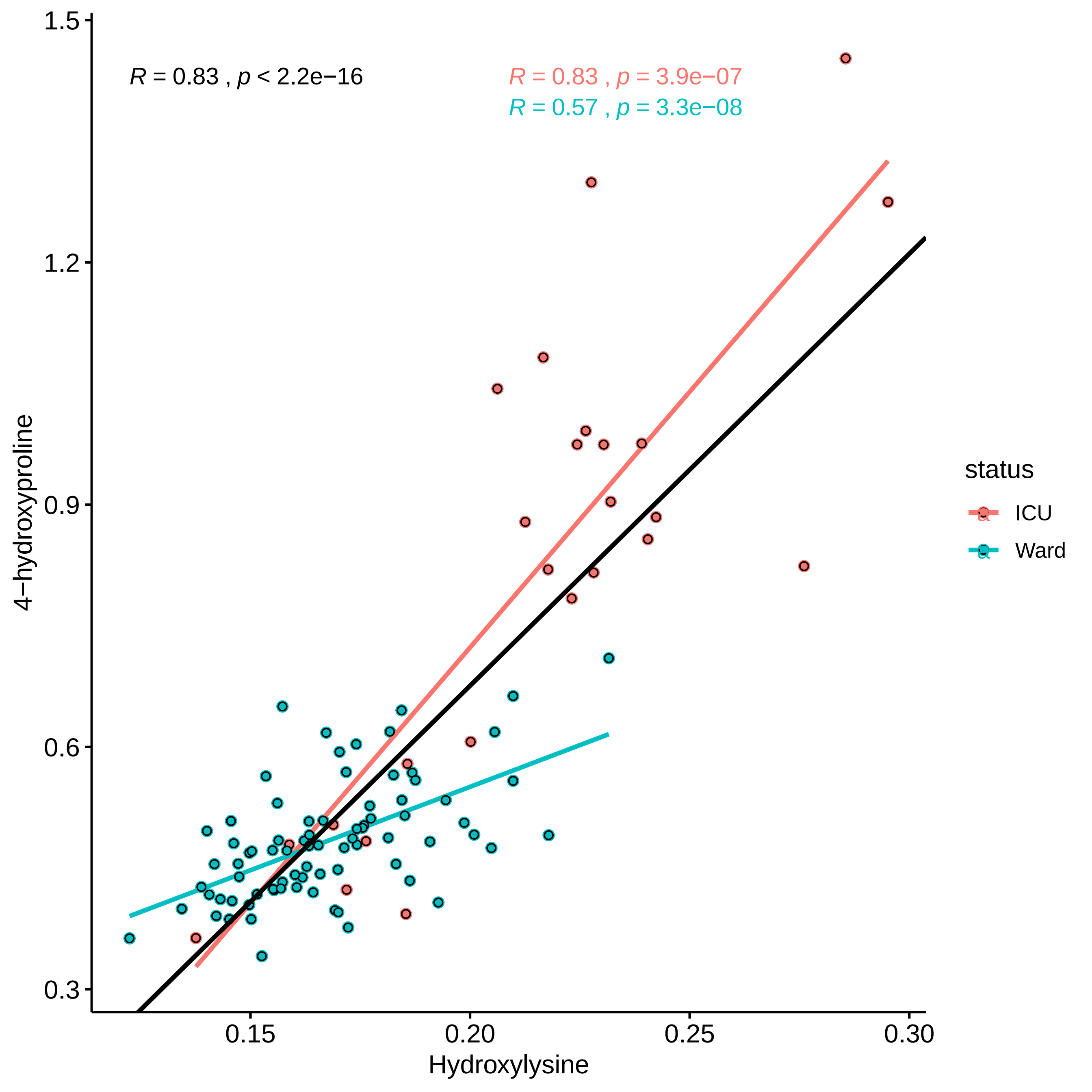

R = 0.83 , p < 2.2e−16
R = 0.83 , p = 3.9e−07
R = 0.57 , p = 3.3e−08
1.5
1.2
0.9
0.6
0.3
4−hydroxyproline
0.15
0.20
0.25
0.30
Hydroxylysine
status
ICU
Ward

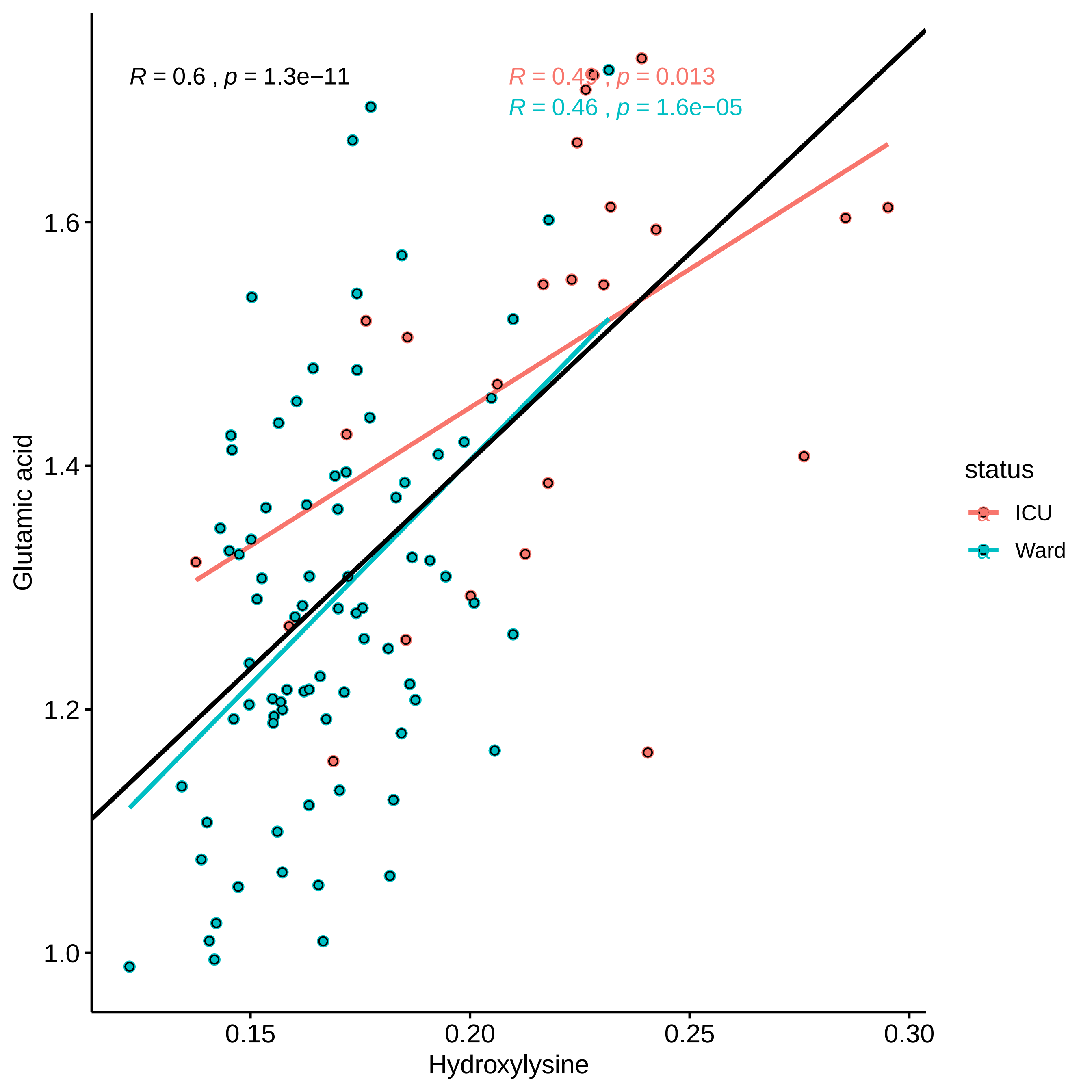

R = 0.6 , p = 1.3e−11
R = 0.49 , p = 0.013
R = 0.46 , p = 1.6e−05
Glutamic acid
1.6
1.4
1.2
1.0
0.15
0.20
0.25
0.30
Hydroxylysine
status
ICU
Ward

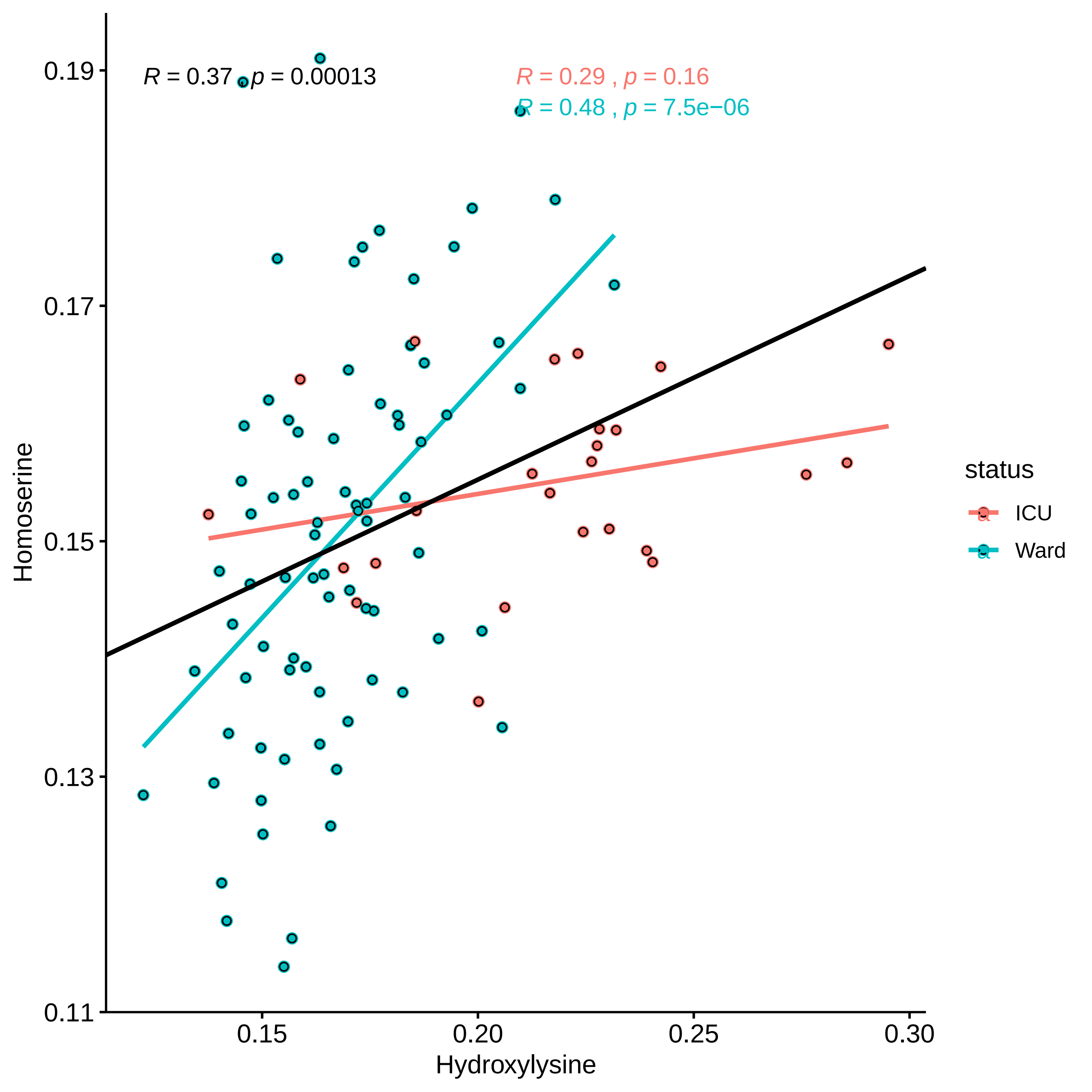

R = 0.37 , p = 0.00013
R = 0.29 , p = 0.16
R = 0.48 , p = 7.5e−06
Homoserine
0.19
0.17
0.15
0.13
0.11
Hydroxylysine
0.15
0.20
0.25
0.30
status
ICU
Ward

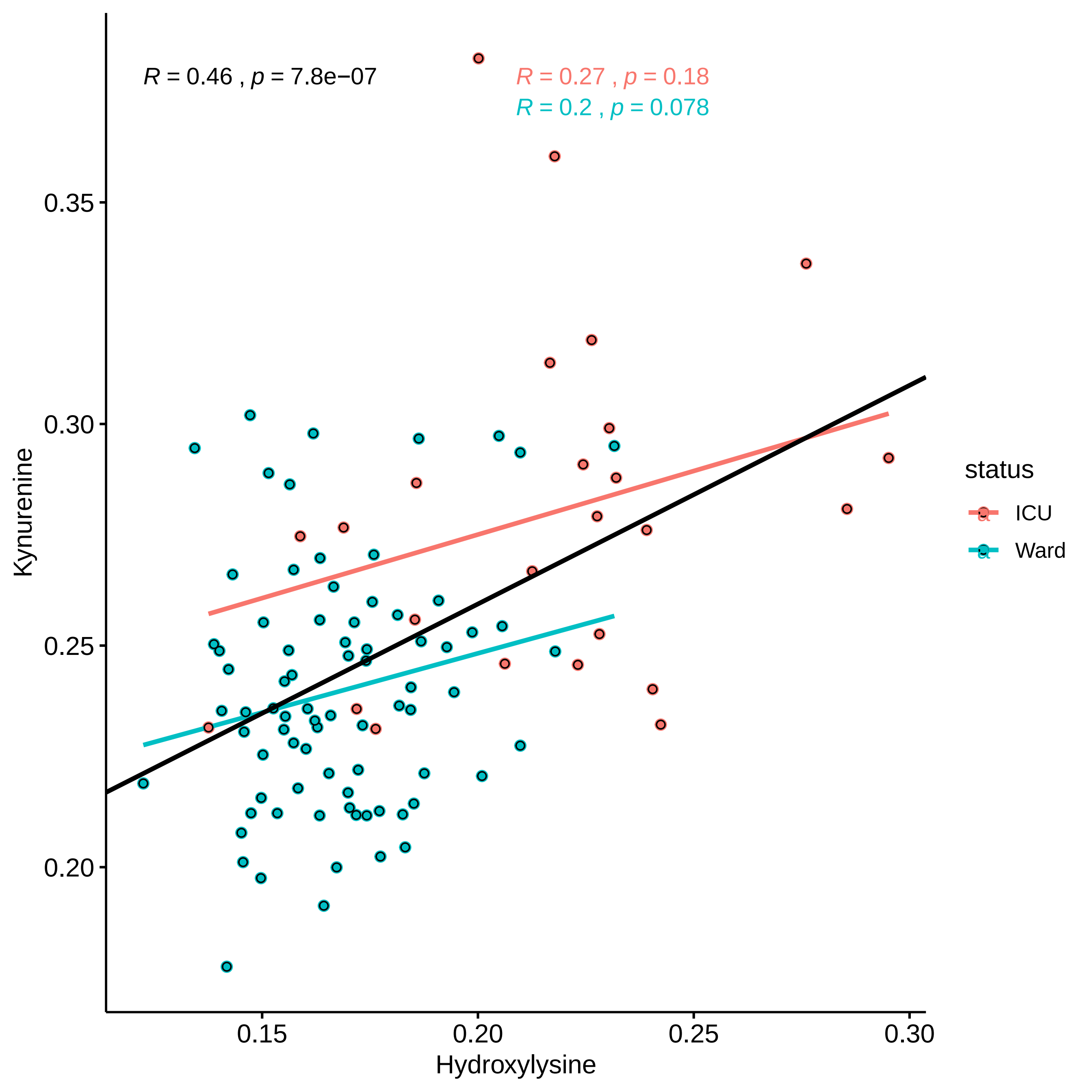

R = 0.46 , p = 7.8e−07
R = 0.27 , p = 0.18
R = 0.2 , p = 0.078
0.35
0.30
0.25
0.20
Kynurenine
0.15
0.20
0.25
0.30
Hydroxylysine
status
ICU
Ward

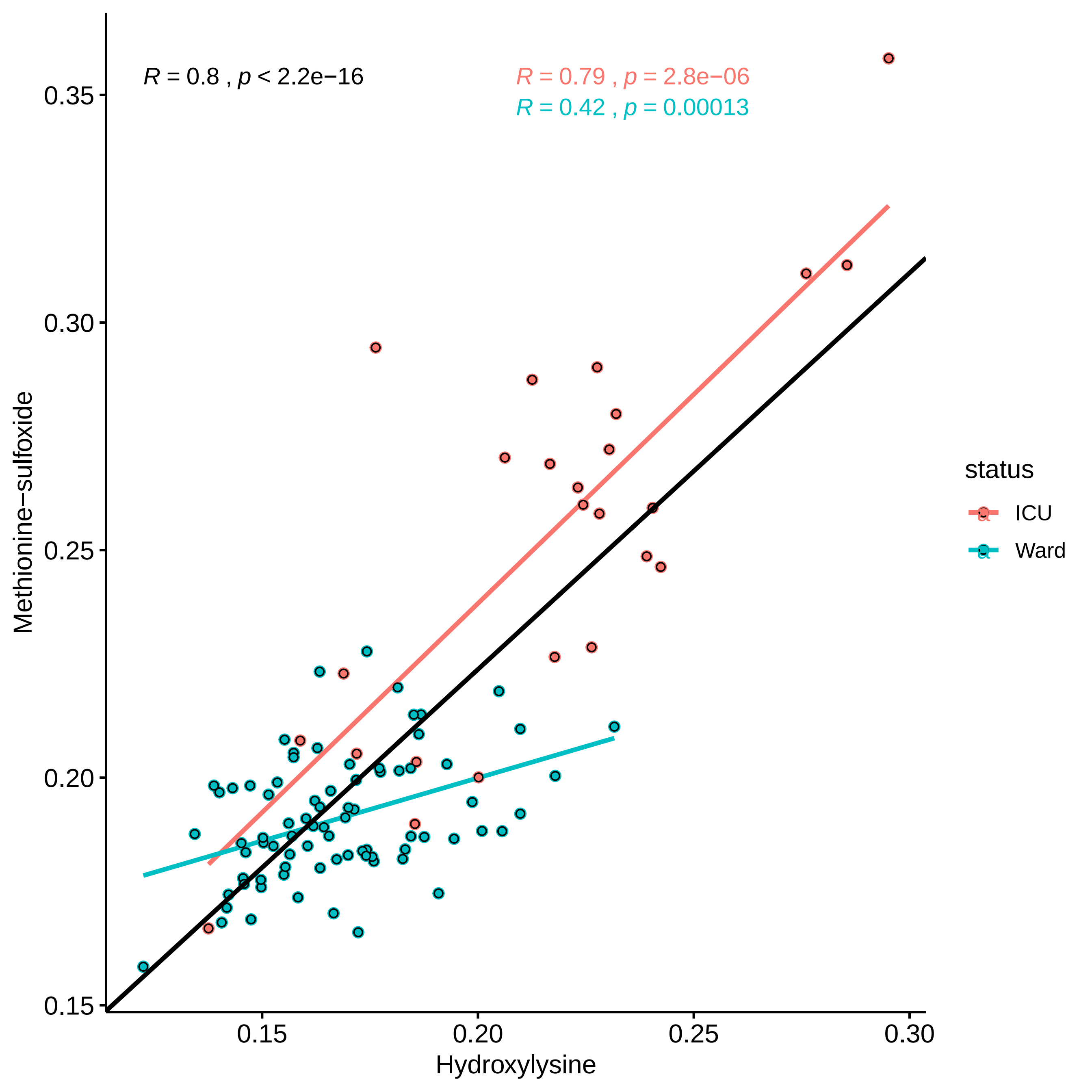
R = 0.8 , p < 2.2e−16
R = 0.79 , p = 2.8e−06
R = 0.42 , p = 0.00013
Methionine−sulfoxide
Hydroxylysine
0.15
0.20
0.25
0.30
0.35
0.15
0.20
0.25
0.30
status
ICU
Ward

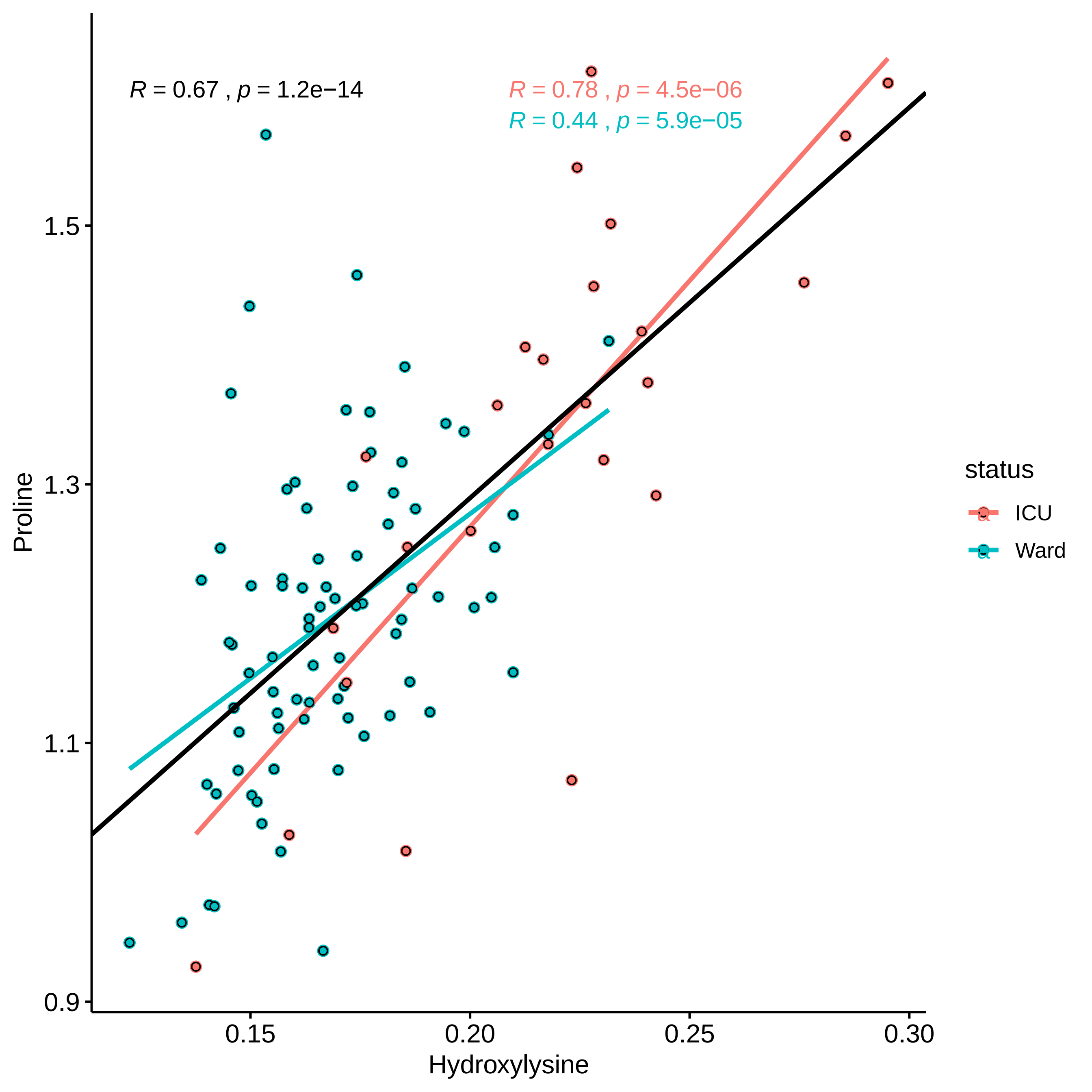

R = 0.67 , p = 1.2e−14
R = 0.78 , p = 4.5e−06
R = 0.44 , p = 5.9e−05
Proline
1.5
1.3
1.1
0.9
0.15
0.20
0.25
0.30
Hydroxylysine
status
ICU
Ward

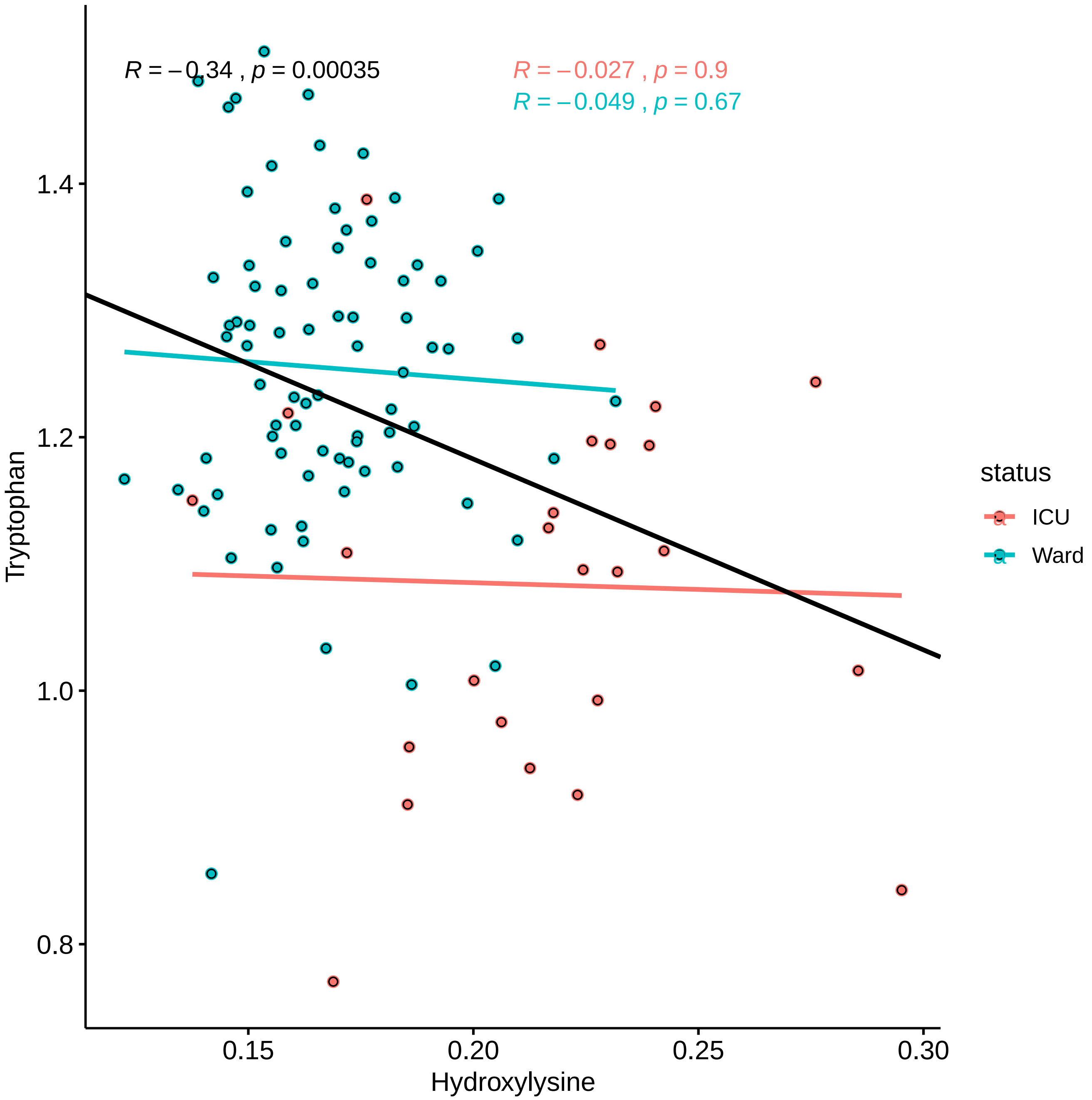

R = −0.34 , p = 0.00035
R = −0.027 , p = 0.9
R = −0.049 , p = 0.67
Tryptophan
1.4
1.2
1.0
0.8
0.15
0.20
0.25
0.30
Hydroxylysine
status
ICU
Ward

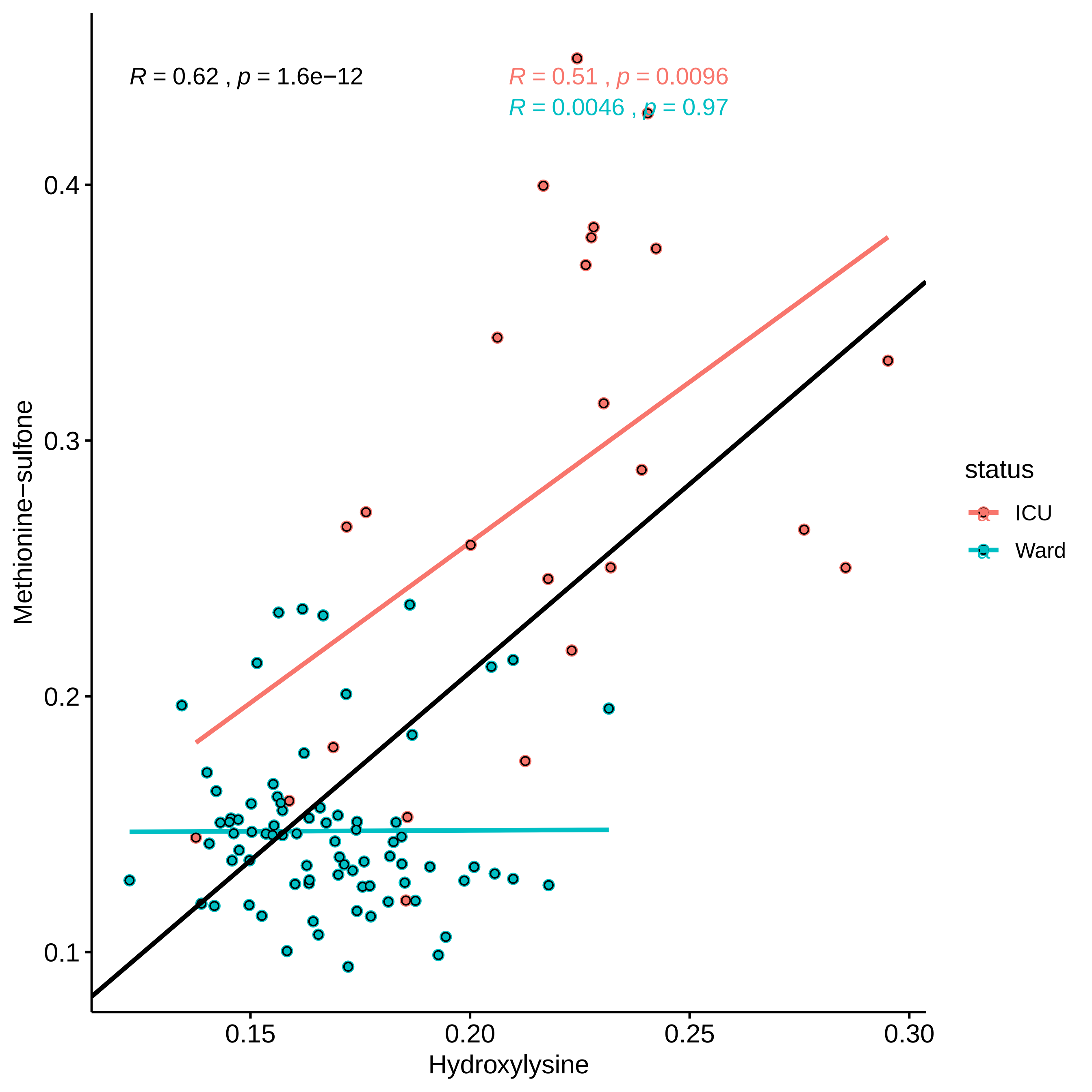

R = 0.62 , p = 1.6e−12
R = 0.51 , p = 0.0096
R = 0.0046 , p = 0.97
0.4
0.3
0.2
0.1
Methionine−sulfone
0.15
0.20
0.25
0.30
Hydroxylysine
status
ICU
Ward

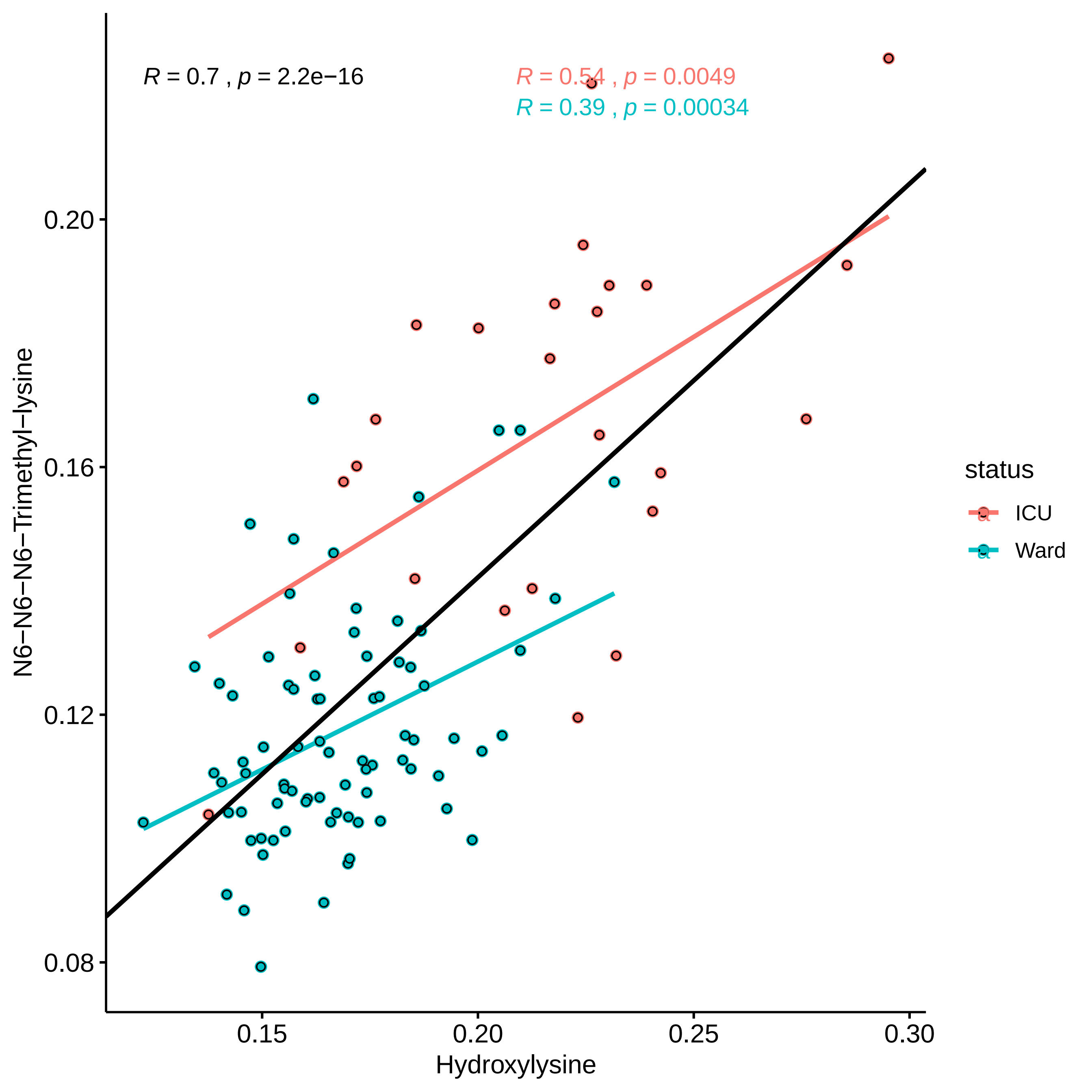

R = 0.7 , p = 2.2e−16
R = 0.54 , p = 0.0049
R = 0.39 , p = 0.00034
N6−N6−N6−Trimethyl−lysine
0.20
0.16
0.12
0.08
0.15
0.20
0.25
0.30
Hydroxylysine
status
ICU
Ward

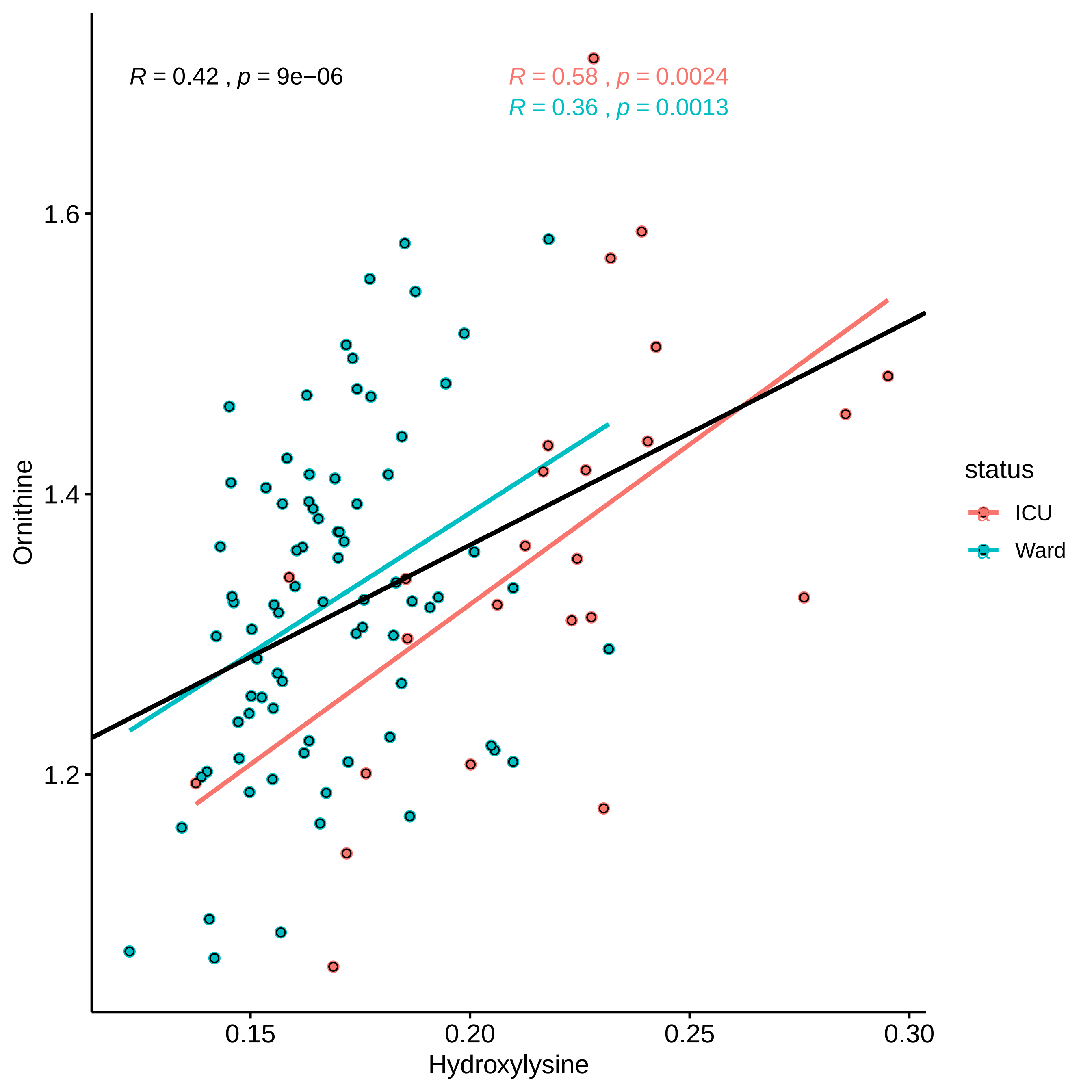

R = 0.42 , p = 9e−06
R = 0.58 , p = 0.0024
R = 0.36 , p = 0.0013
1.6
1.4
1.2
Ornithine
0.15
0.20
0.25
0.30
Hydroxylysine
status
ICU
Ward

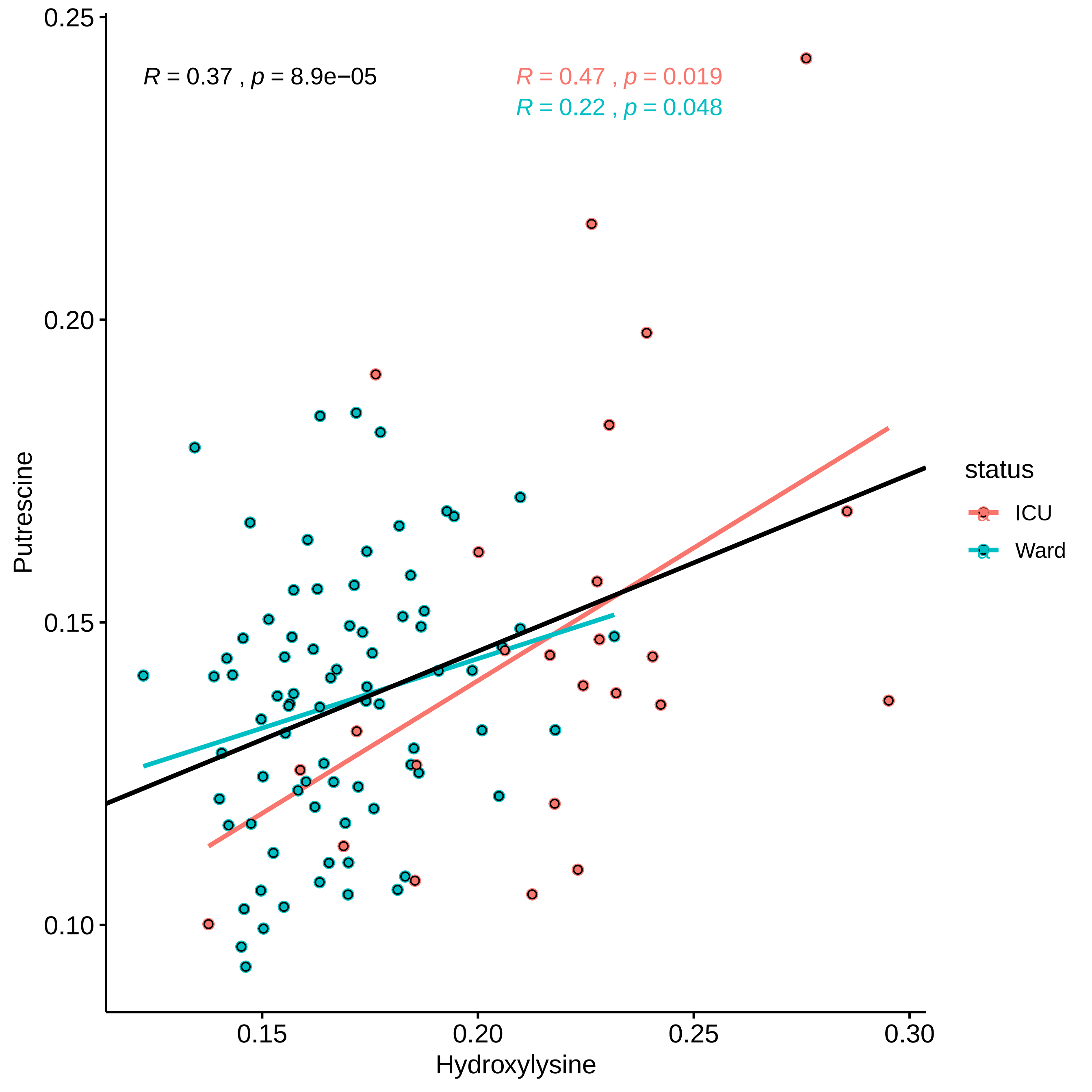

R = 0.37 , p = 8.9e−05
R = 0.47 , p = 0.019
R = 0.22 , p = 0.048
0.25
0.20
0.15
0.10
Putrescine
0.15
0.20
0.25
0.30
Hydroxylysine
status
ICU
Ward

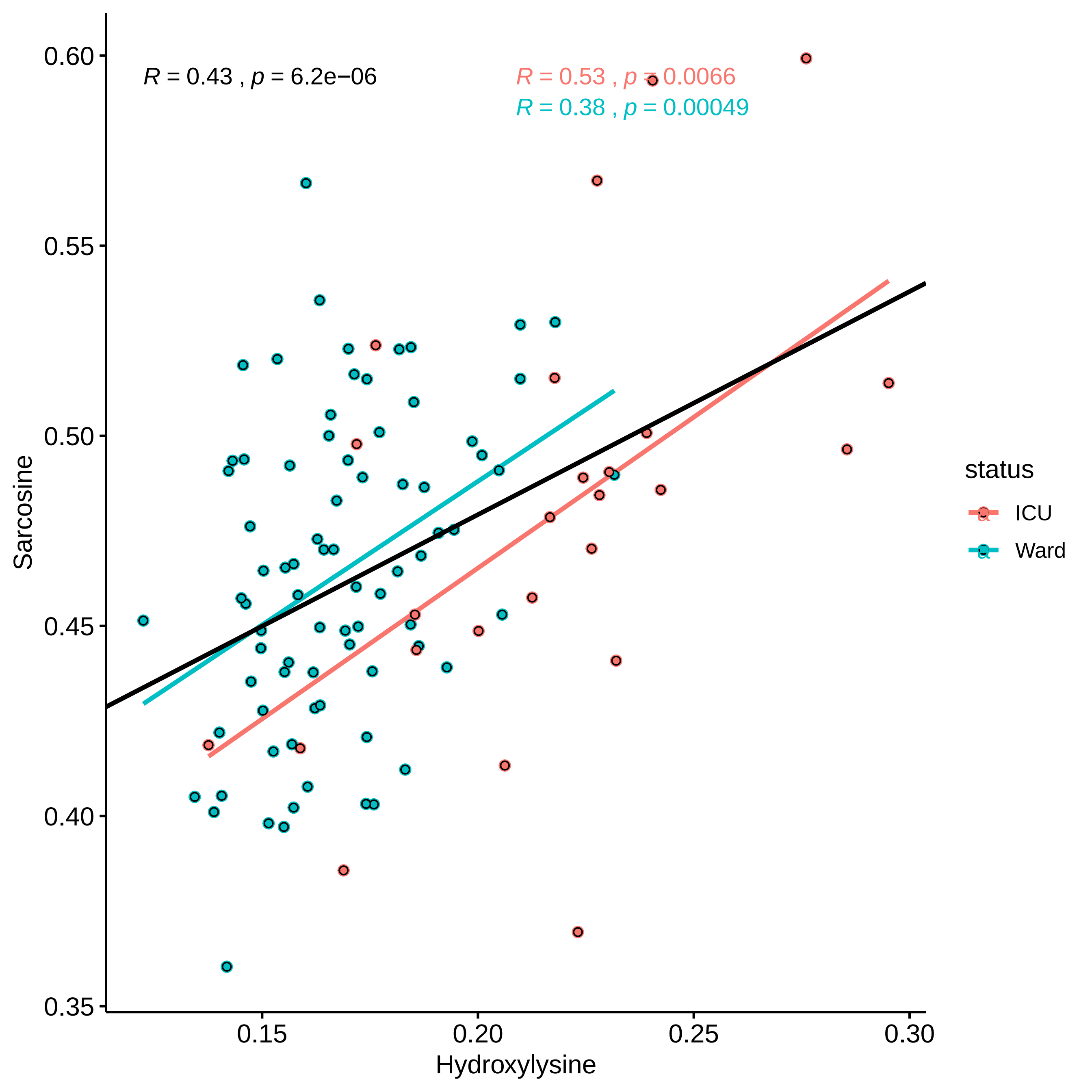

R = 0.43 , p = 6.2e−06
R = 0.53 , p = 0.0066
R = 0.38 , p = 0.00049
Sarcosine
Hydroxylysine
0.60
0.55
0.50
0.45
0.40
0.35
0.15
0.20
0.25
0.30
status
ICU
Ward

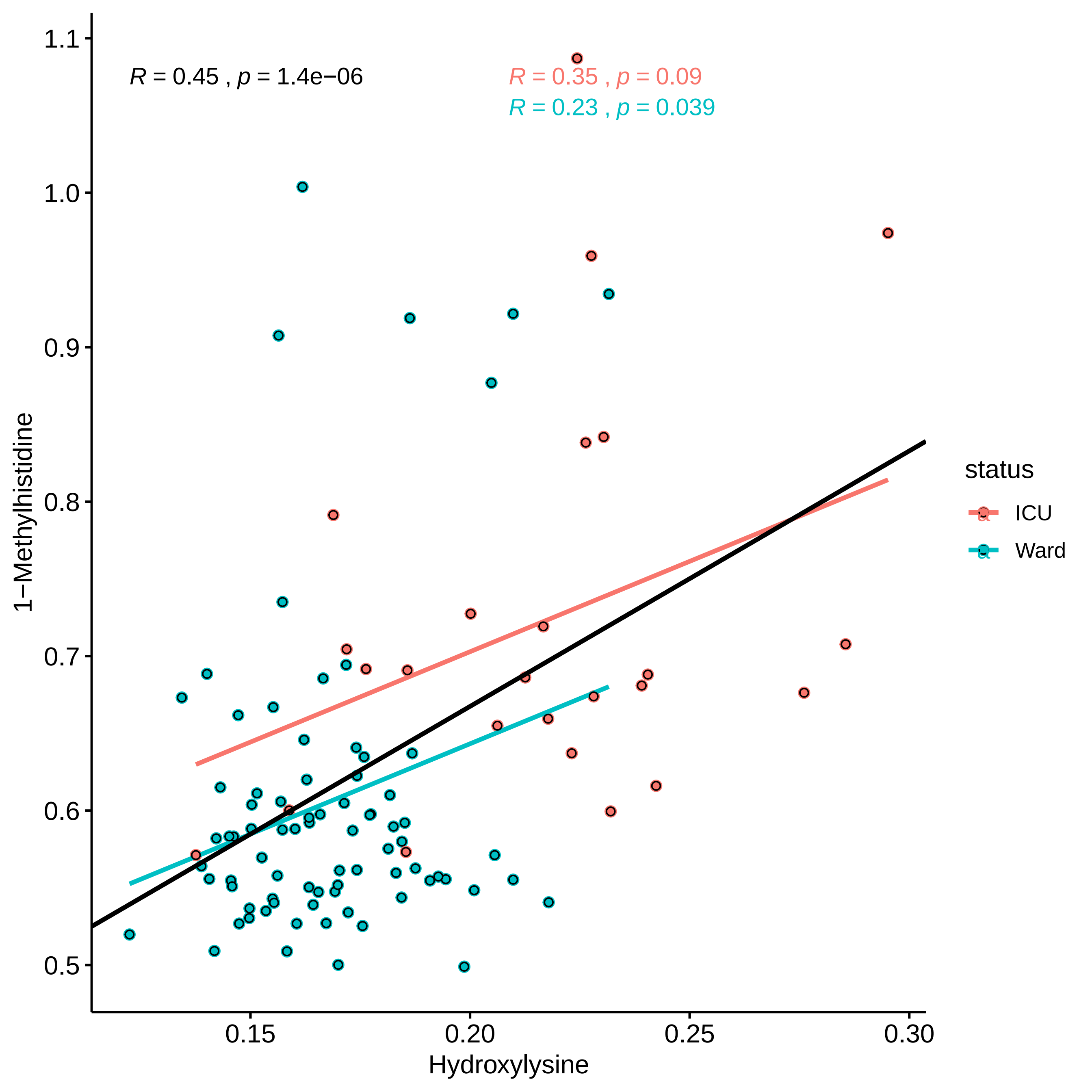

R = 0.45 , p = 1.4e−06
R = 0.35 , p = 0.09
R = 0.23 , p = 0.039
1.1
1.0
0.9
0.8
0.7
0.6
0.5
1−Methylhistidine
0.15
0.20
0.25
0.30
Hydroxylysine
status
ICU
Ward

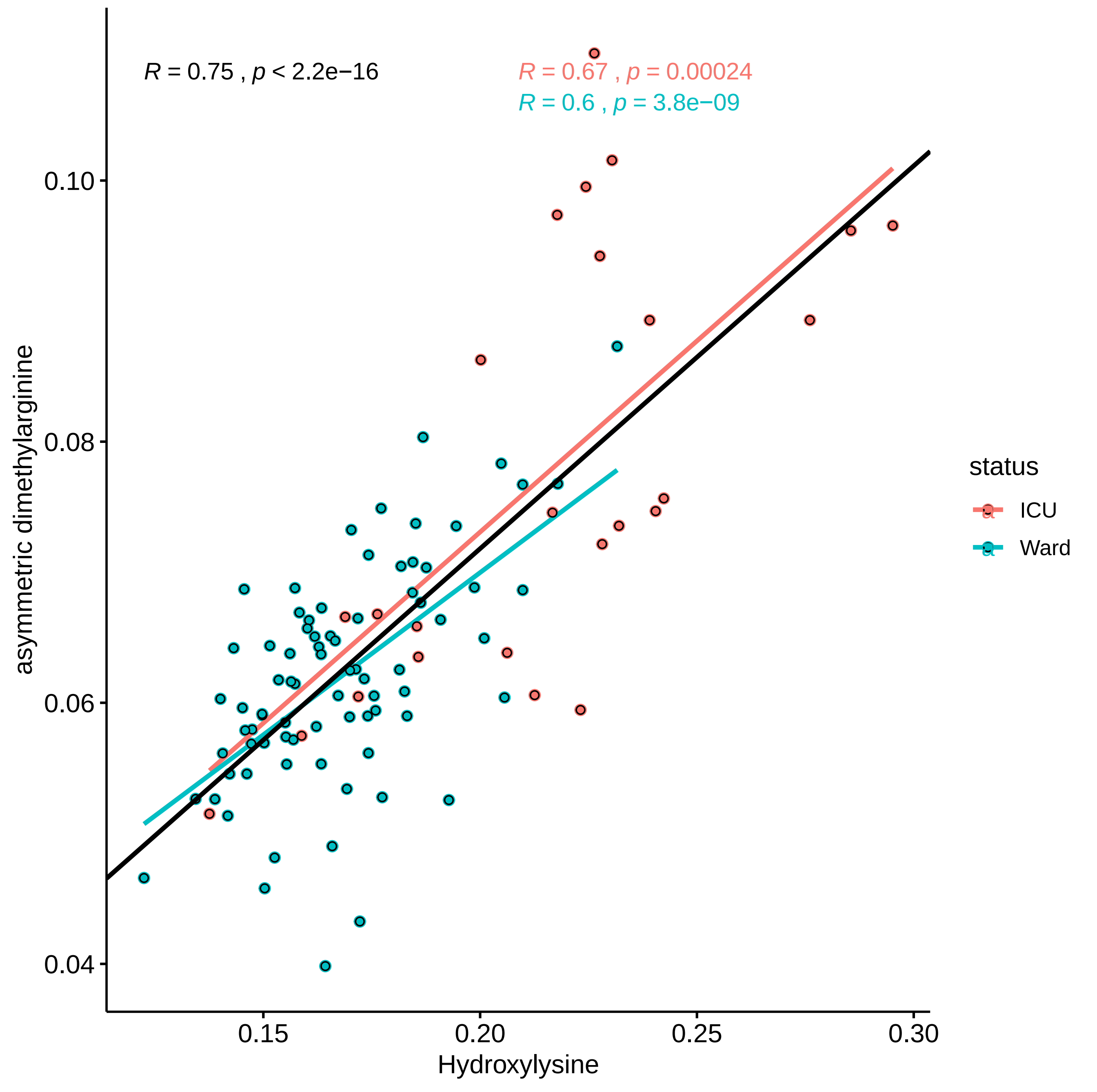

R = 0.75 , p < 2.2e−16
R = 0.67 , p = 0.00024
R = 0.6 , p = 3.8e−09
asymmetric dimethylarginine
0.10
0.08
0.06
0.04
0.15
0.20
0.25
0.30
Hydroxylysine
status
ICU
Ward

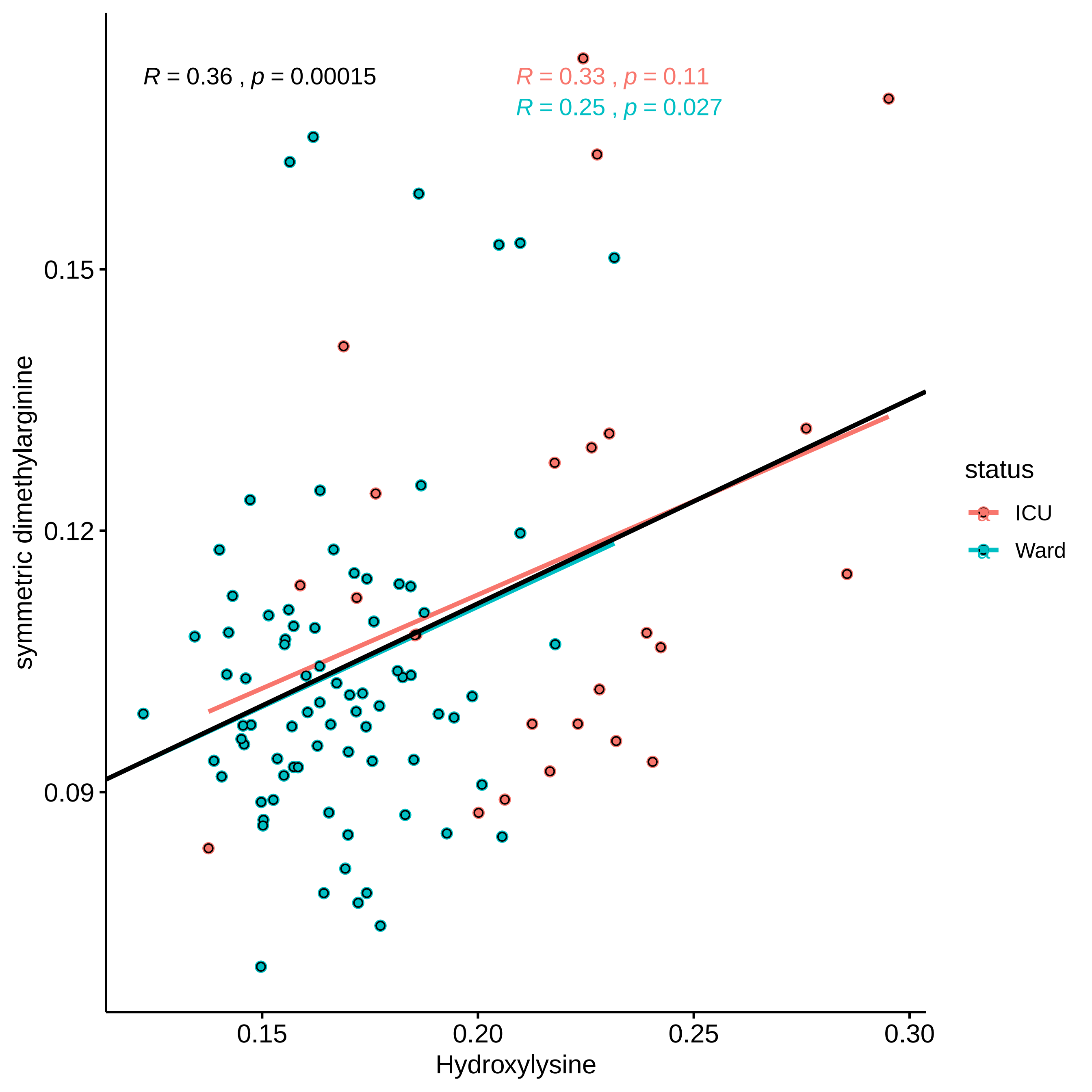

R = 0.36 , p = 0.00015
R = 0.33 , p = 0.11
R = 0.25 , p = 0.027
0.15
0.12
0.09
symmetric dimethylarginine
0.15
0.20
0.25
0.30
Hydroxylysine
status
ICU
Ward

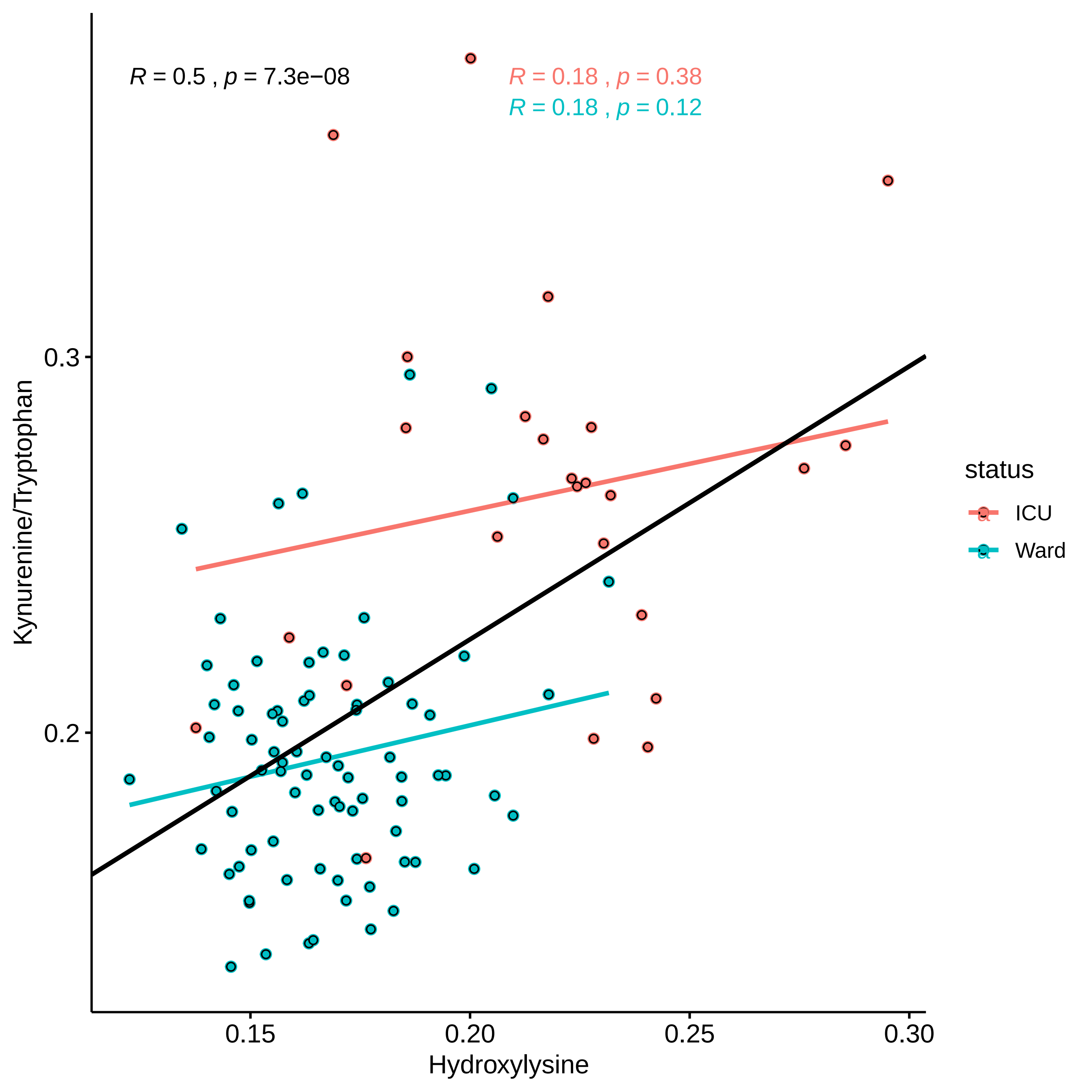

R = 0.5 , p = 7.3e−08
R = 0.18 , p = 0.38
R = 0.18 , p = 0.12
Kynurenine/Tryptophan
0.3
0.2
0.15
0.20
0.25
0.30
Hydroxylysine
status
ICU
Ward

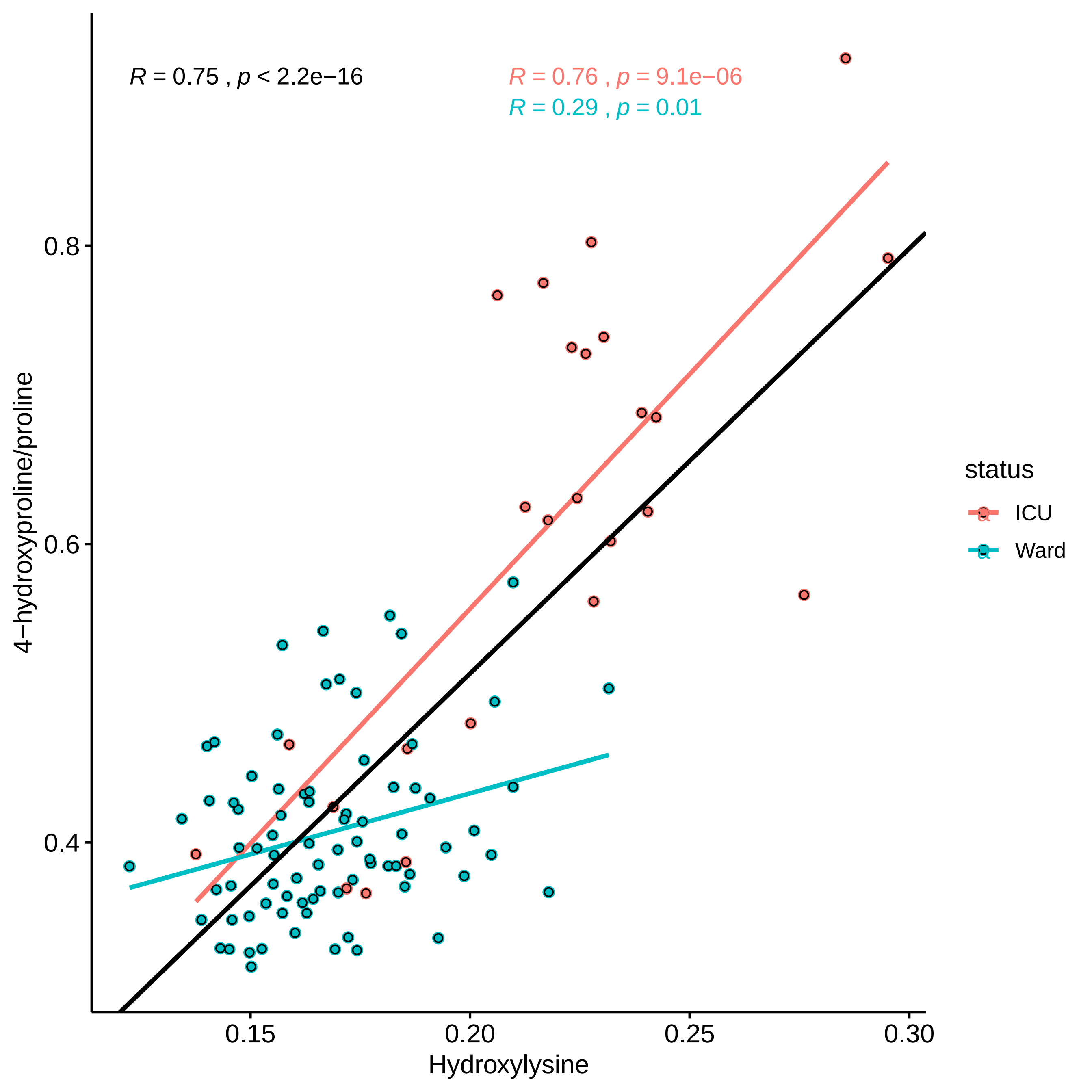
R = 0.75 , p < 2.2e−16
R = 0.76 , p = 9.1e−06
R = 0.29 , p = 0.01
4−hydroxyproline/proline
0.8
0.6
0.4
0.15
0.20
0.25
0.30
Hydroxylysine
status
ICU
Ward

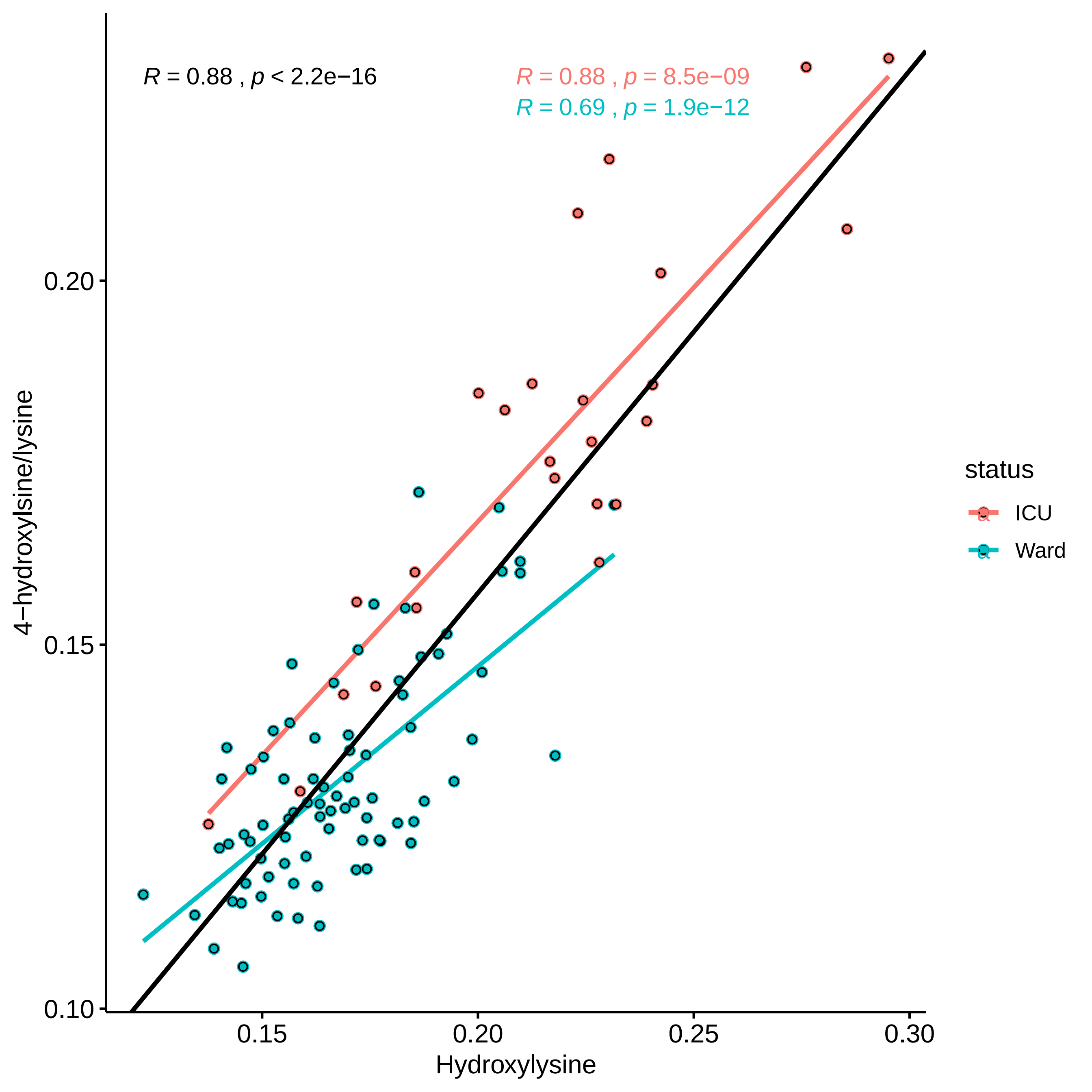
R = 0.88 , p < 2.2e−16
R = 0.88 , p = 8.5e−09
R = 0.69 , p = 1.9e−12
4−hydroxylsine/lysine
0.20
0.15
0.10
0.15
0.20
0.25
0.30
Hydroxylysine
status
ICU
Ward

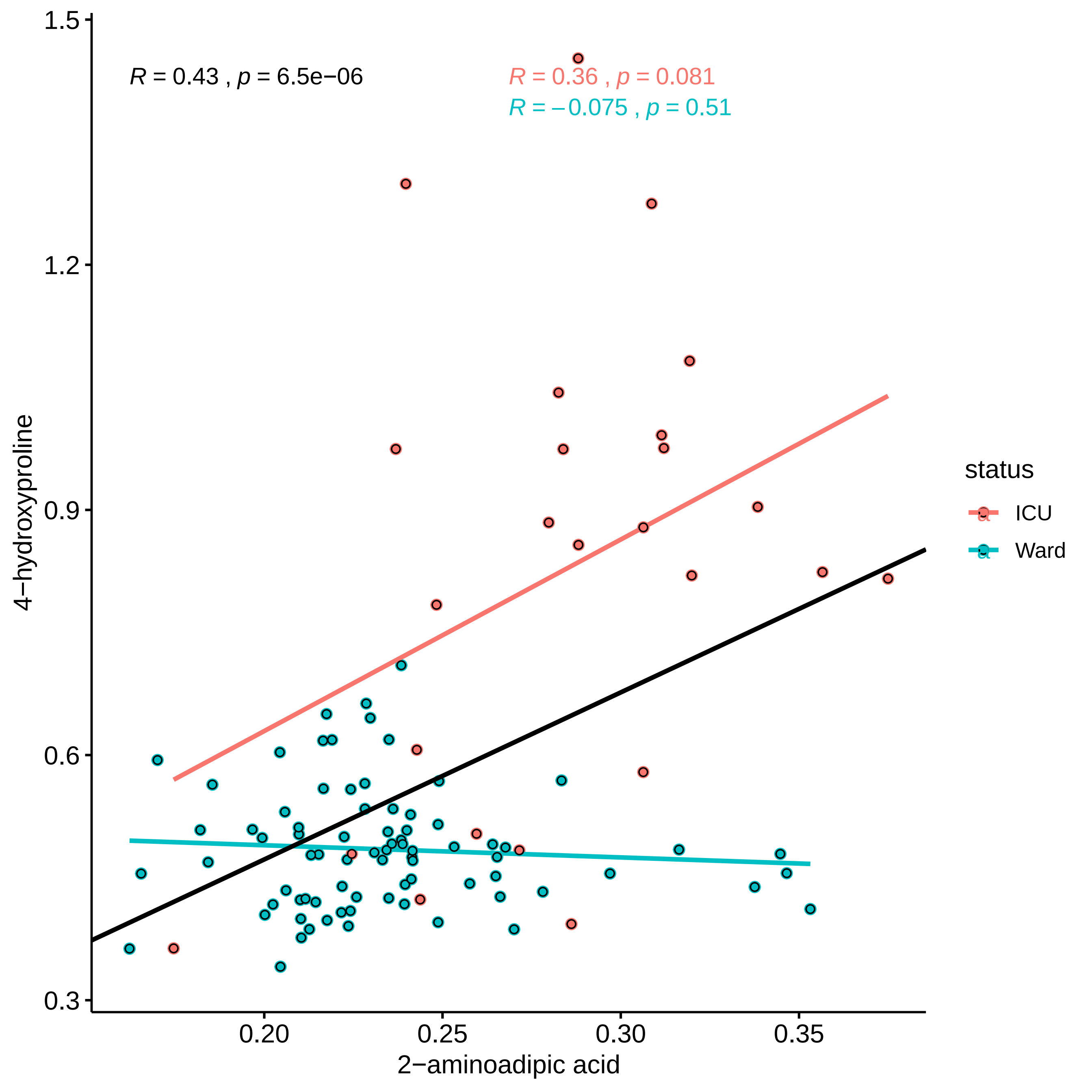

R = 0.43 , p = 6.5e−06
R = 0.36 , p = 0.081
R = −0.075 , p = 0.51
4−hydroxyproline
1.5
1.2
0.9
0.6
0.3
0.20
0.25
0.30
0.35
2−aminoadipic acid
status
ICU
Ward

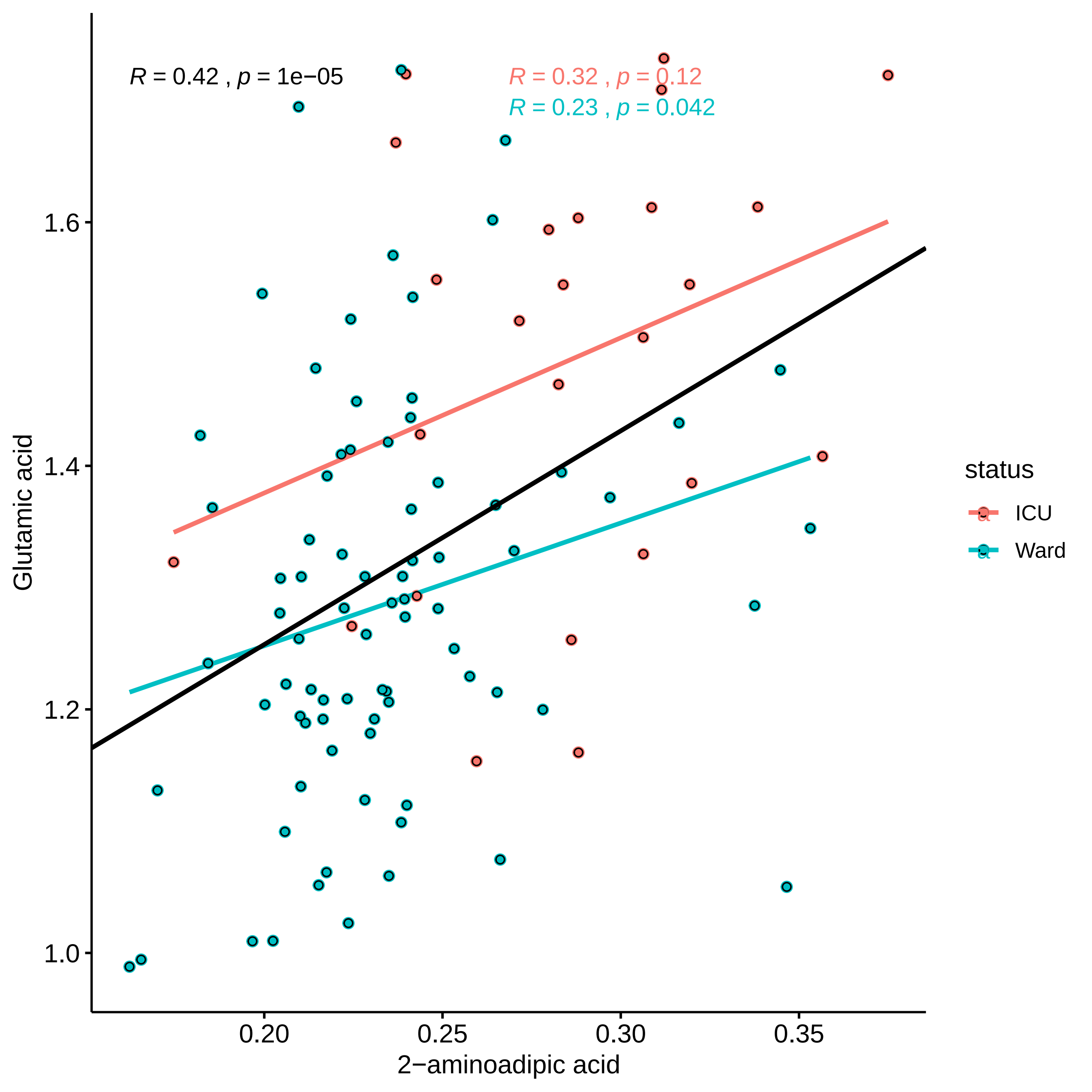
R = 0.42 , p = 1e−05
R = 0.32 , p = 0.12
R = 0.23 , p = 0.042
Glutamic acid
1.6
1.4
1.2
1.0
0.20
0.25
0.30
0.35
2−aminoadipic acid
status
ICU
Ward

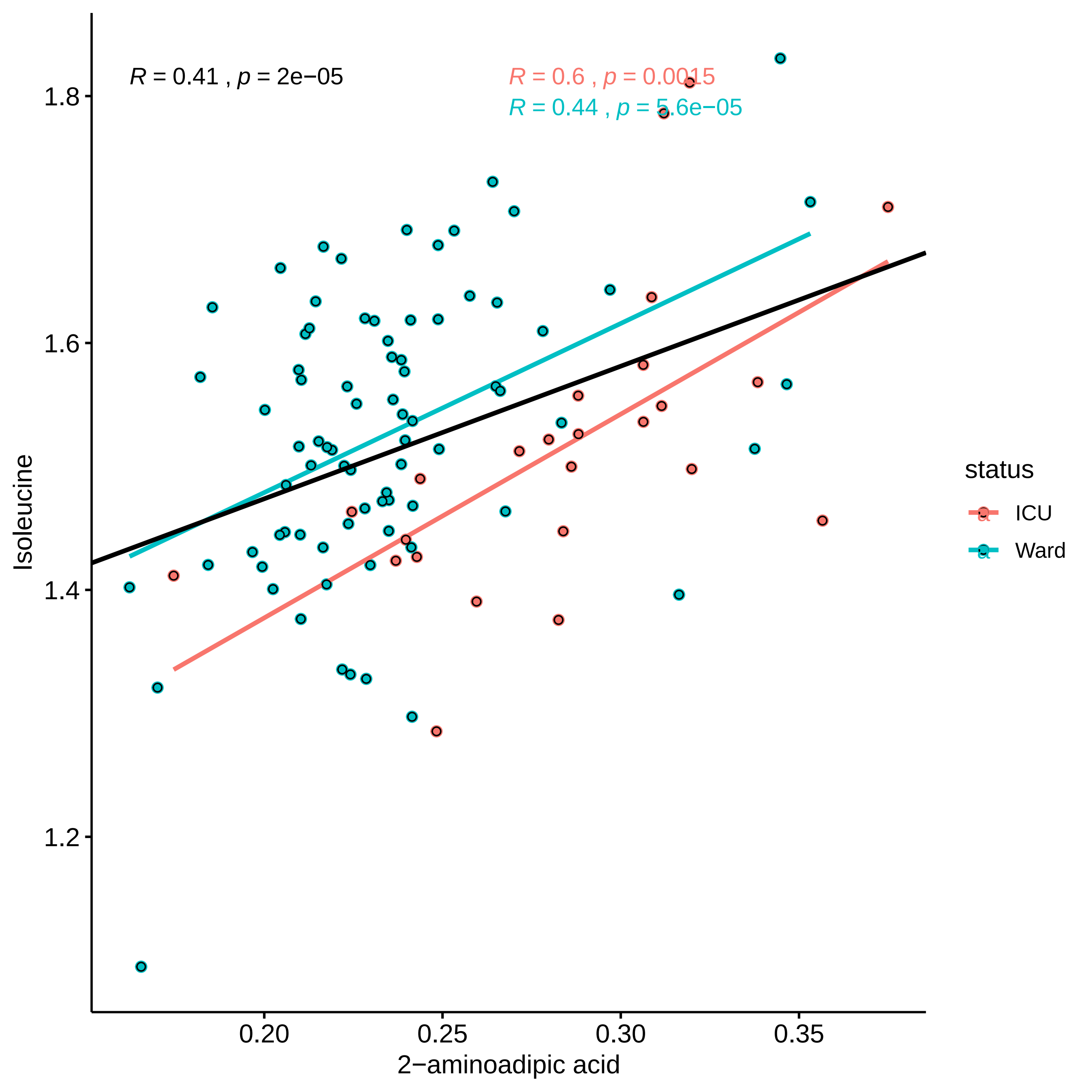

R = 0.41 , p = 2e−05
R = 0.6 , p = 0.0015
R = 0.44 , p = 5.6e−05
1.8
1.6
1.4
1.2
Isoleucine
0.20
0.25
0.30
0.35
2−aminoadipic acid
status
ICU
Ward

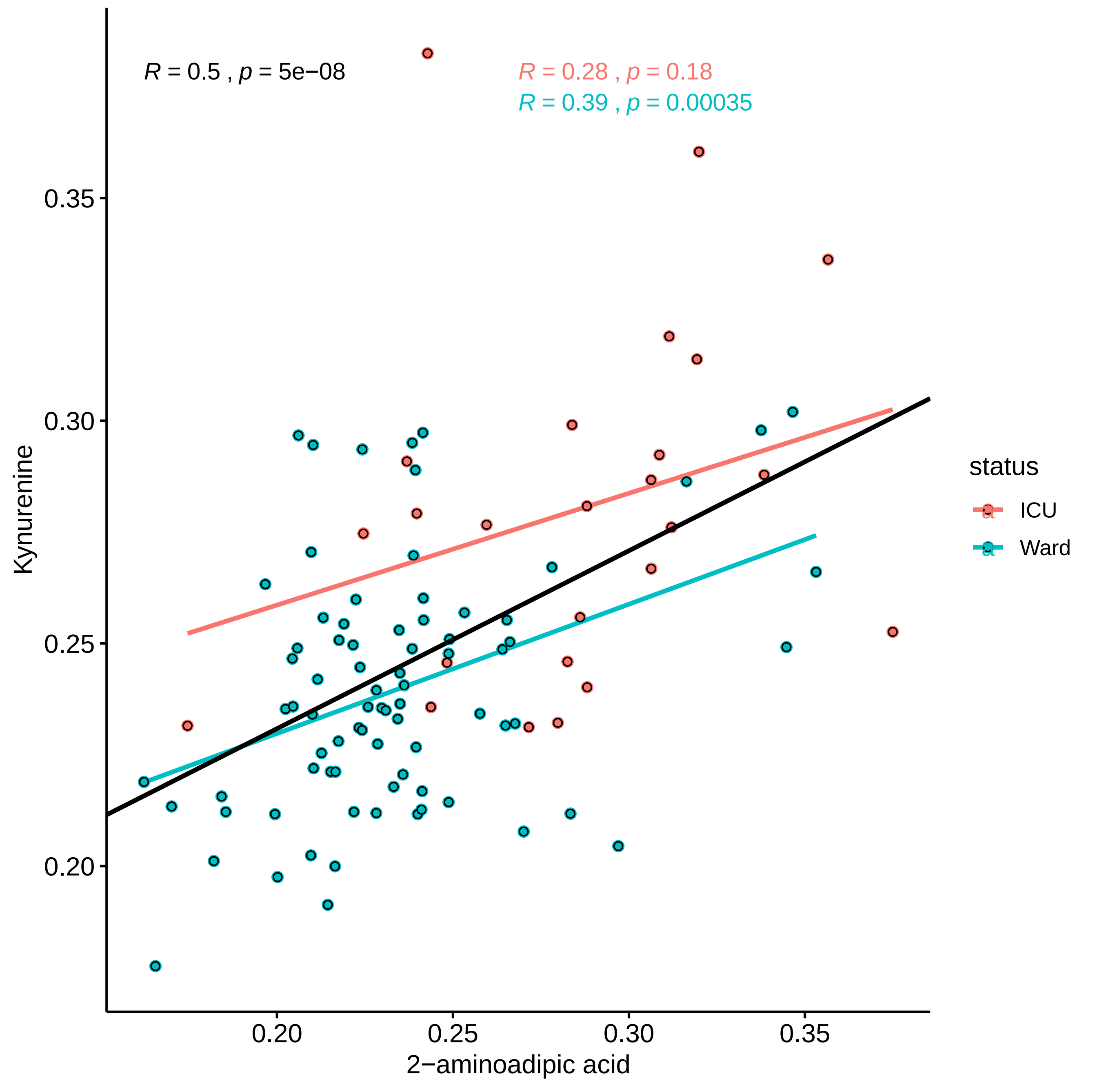

R = 0.5 , p = 5e−08
R = 0.28 , p = 0.18
R = 0.39 , p = 0.00035
0.35
0.30
0.25
0.20
Kynurenine
0.20
0.25
0.30
0.35
2−aminoadipic acid
status
ICU
Ward

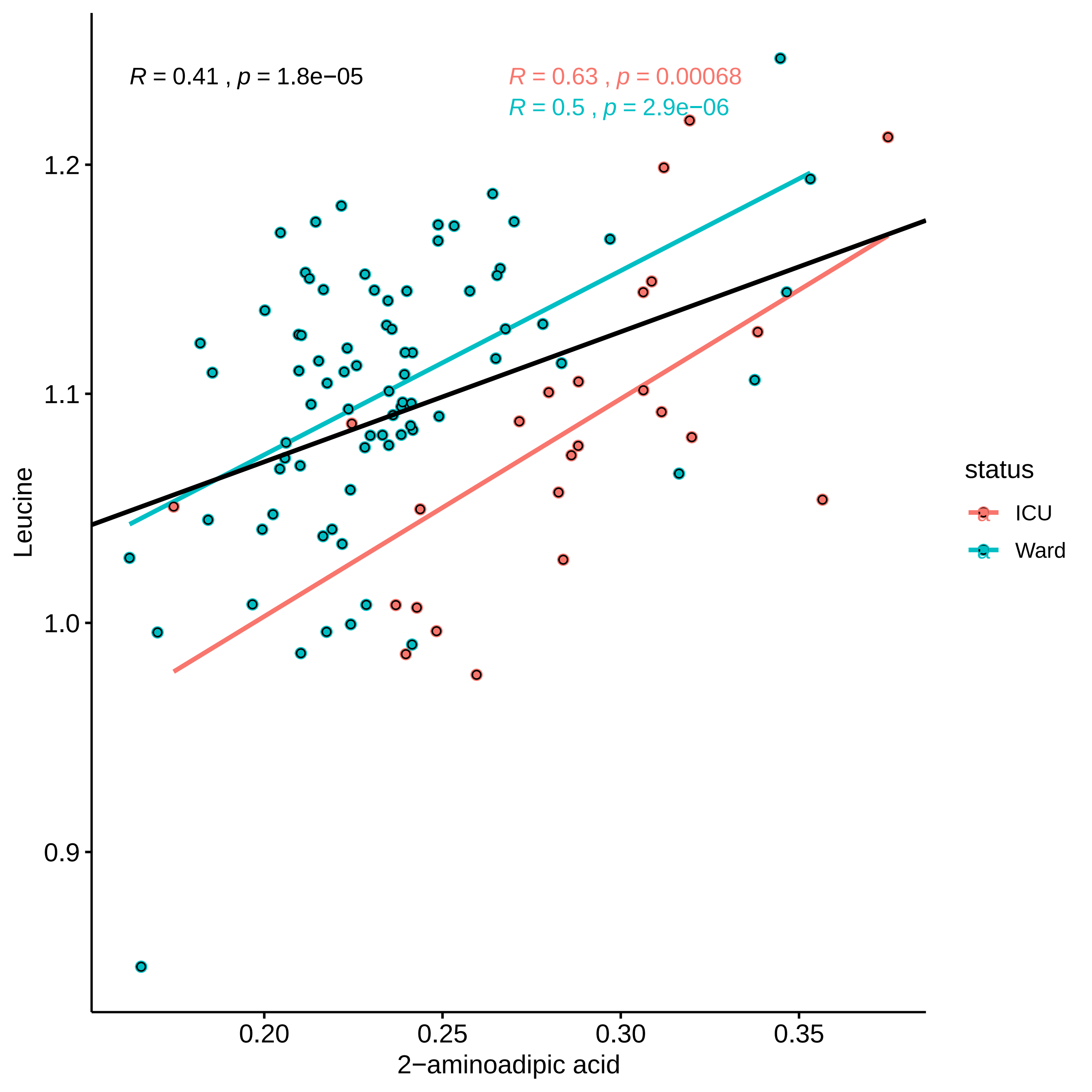
R = 0.41 , p = 1.8e−05
R = 0.63 , p = 0.00068
R = 0.5 , p = 2.9e−06
1.2
1.1
1.0
0.9
Leucine
0.20
0.25
0.30
0.35
2−aminoadipic acid
status
ICU
Ward

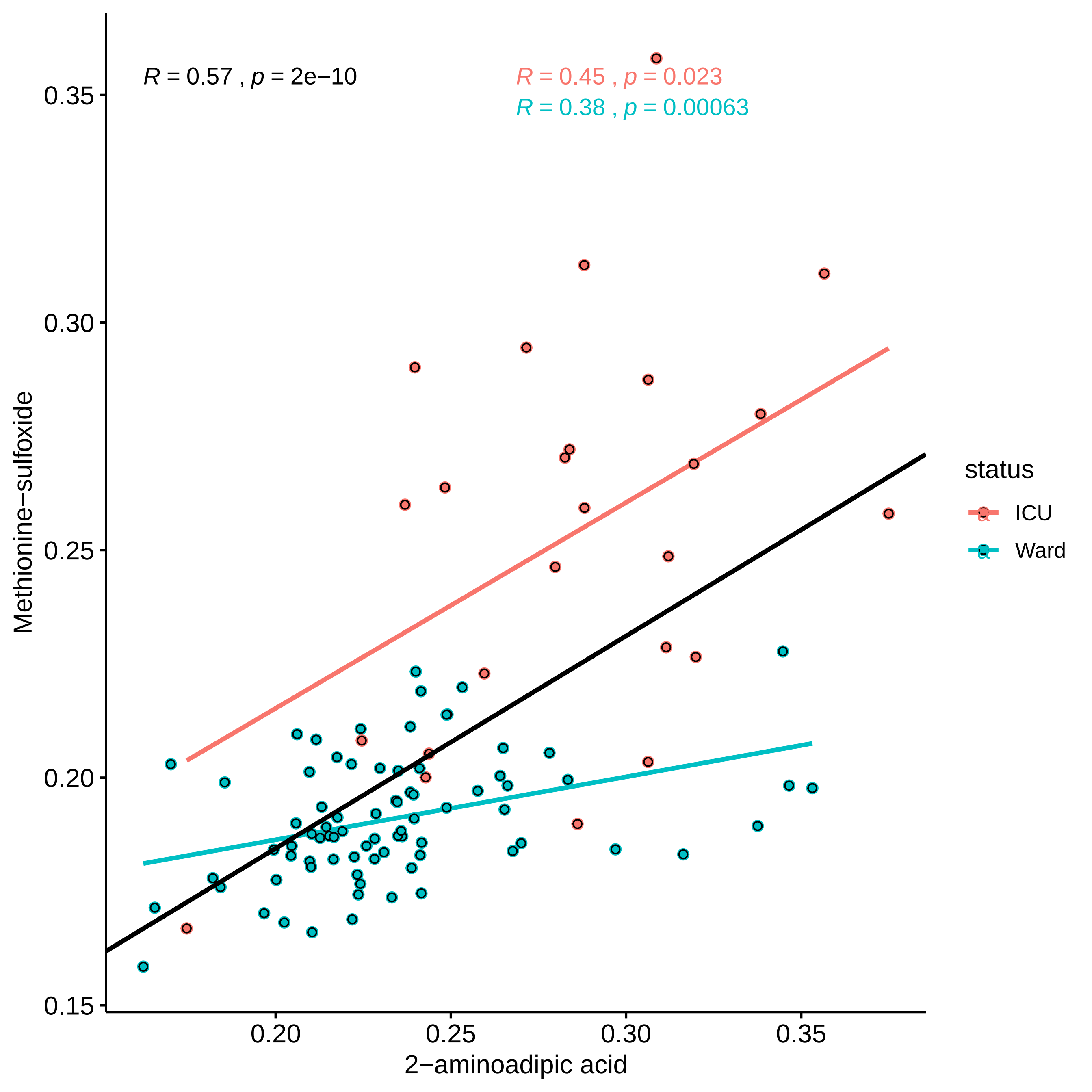

R = 0.57 , p = 2e−10
R = 0.45 , p = 0.023
R = 0.38 , p = 0.00063
Methionine−sulfoxide
0.35
0.30
0.25
0.20
0.15
0.20
0.25
0.30
0.35
2−aminoadipic acid
status
ICU
Ward

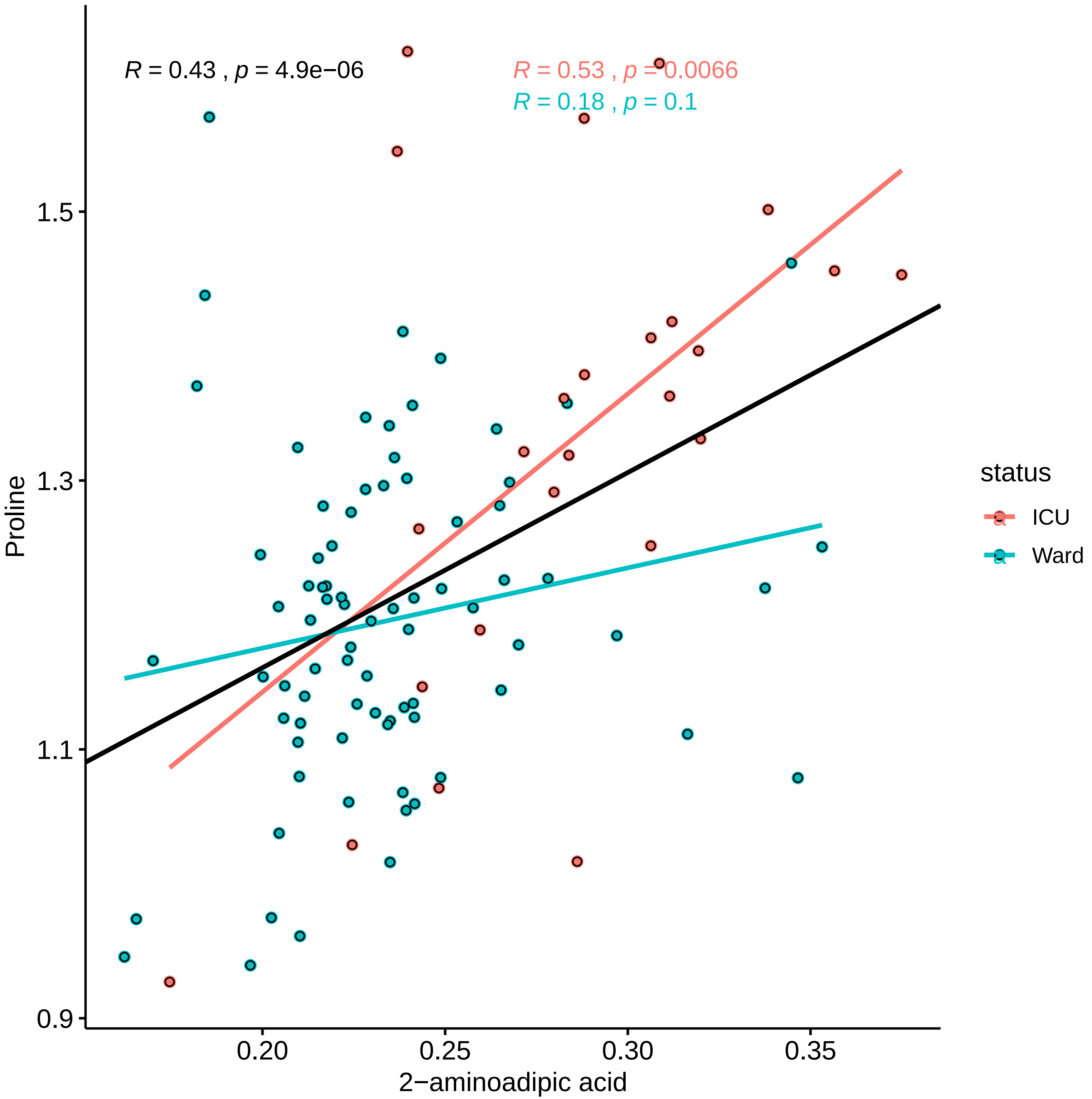

R = 0.43 , p = 4.9e−06
R = 0.53 , p = 0.0066
R = 0.18 , p = 0.1
Proline
1.5
1.3
1.1
0.9
0.20
0.25
0.30
0.35
2−aminoadipic acid
status
ICU
Ward

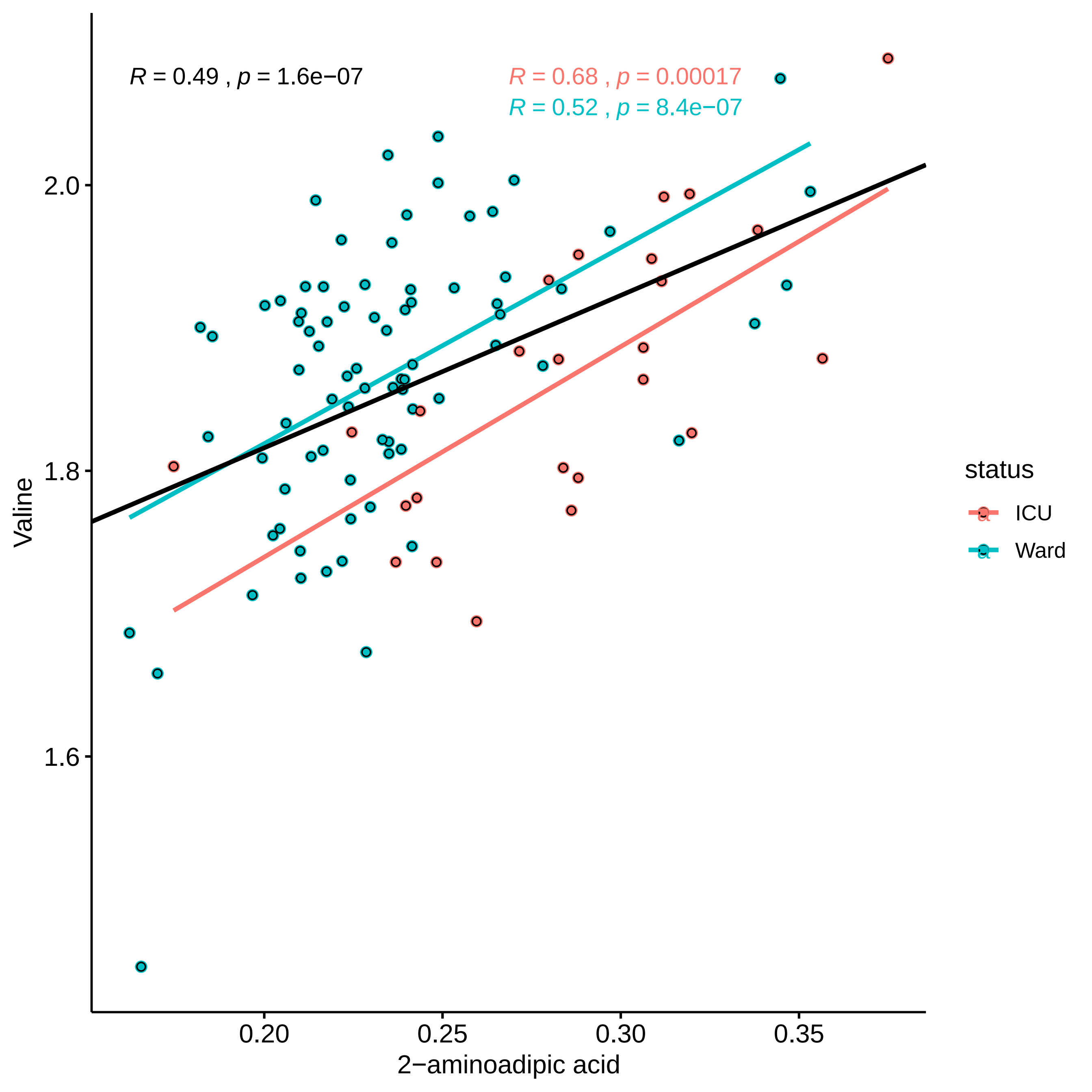

R = 0.49 , p = 1.6e−07
R = 0.68 , p = 0.00017
R = 0.52 , p = 8.4e−07
2.0
1.8
1.6
Valine
0.20
0.25
0.30
0.35
2−aminoadipic acid
status
ICU
Ward

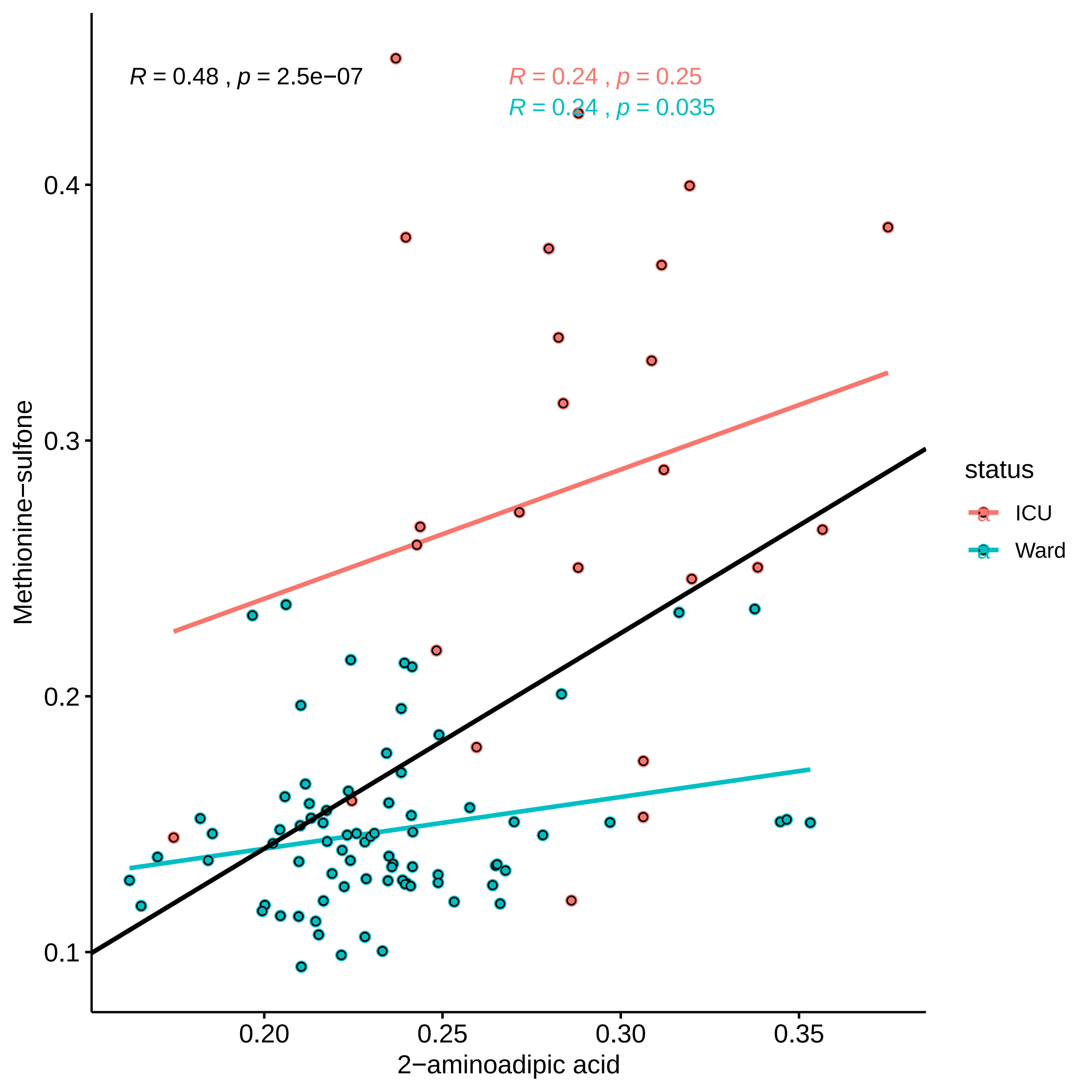

R = 0.48 , p = 2.5e−07
R = 0.24 , p = 0.25
R = 0.24 , p = 0.035
Methionine−sulfone
0.4
0.3
0.2
0.1
0.20
0.25
0.30
0.35
2−aminoadipic acid
status
ICU
Ward

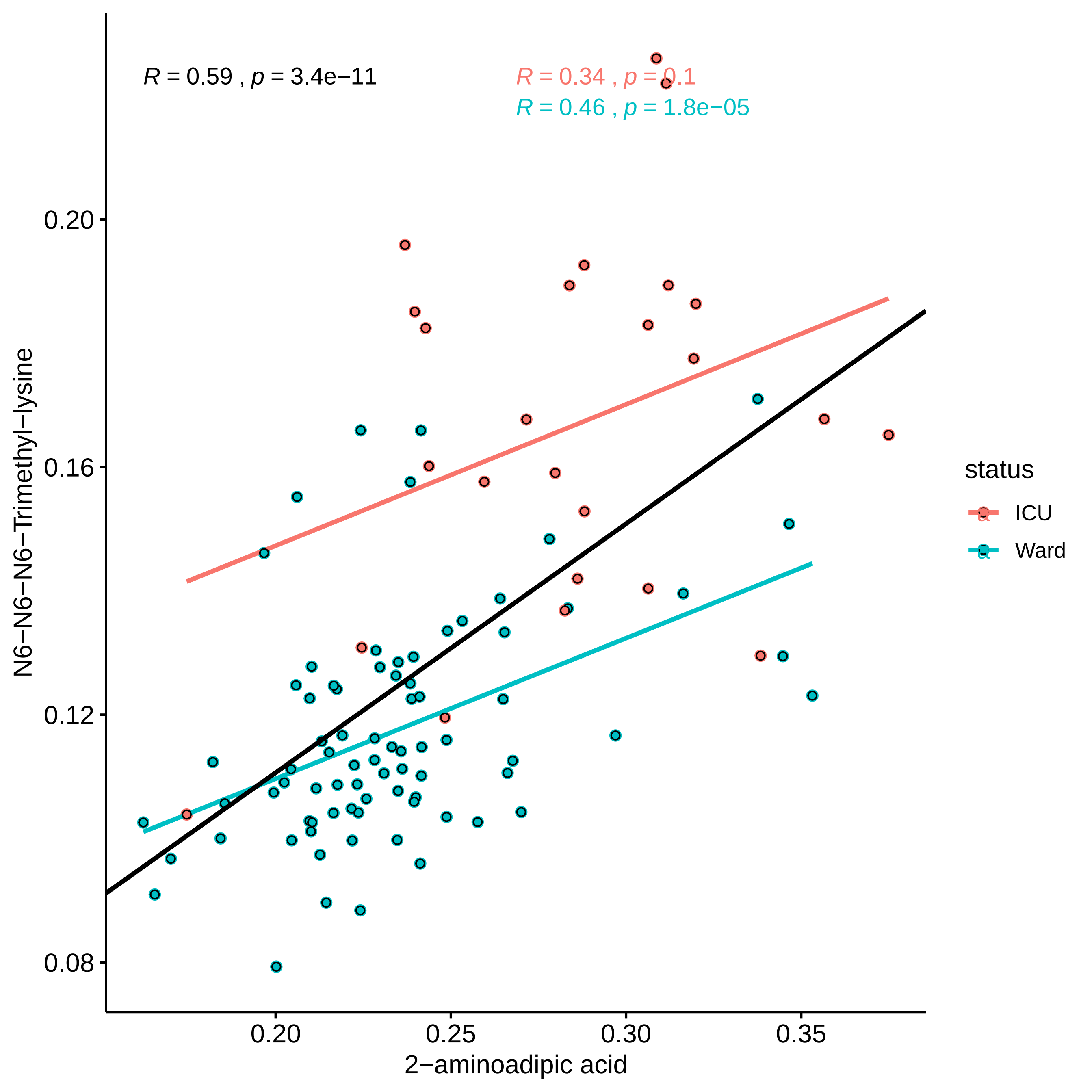

R = 0.59 , p = 3.4e−11
R = 0.34 , p = 0.1
R = 0.46 , p = 1.8e−05
N6−N6−N6−Trimethyl−lysine
0.20
0.16
0.12
0.08
0.20
0.25
0.30
0.35
2−aminoadipic acid
status
ICU
Ward

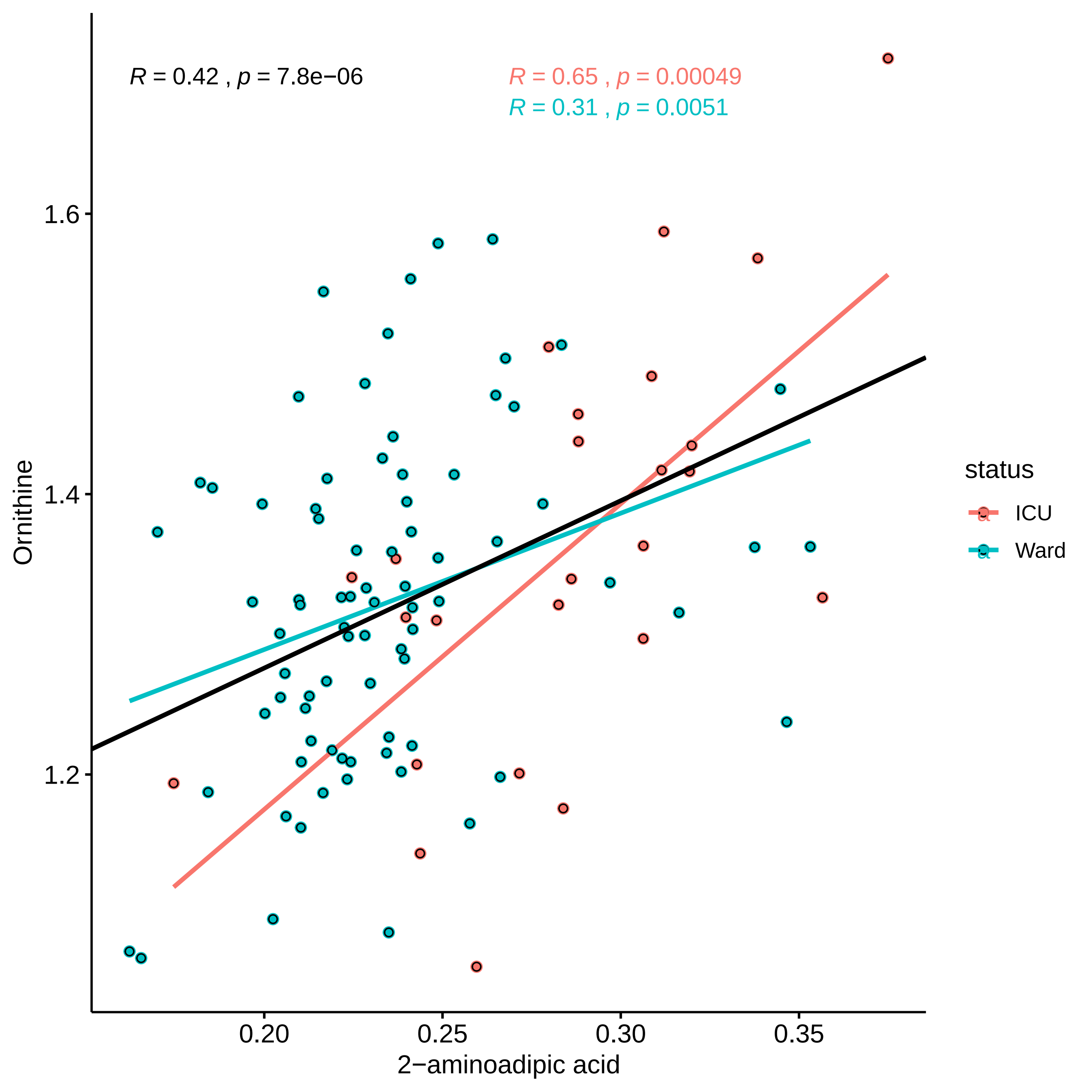

R = 0.42 , p = 7.8e−06
R = 0.65 , p = 0.00049
R = 0.31 , p = 0.0051
Ornithine
1.6
1.4
1.2
0.20
0.25
0.30
0.35
2−aminoadipic acid
status
ICU
Ward

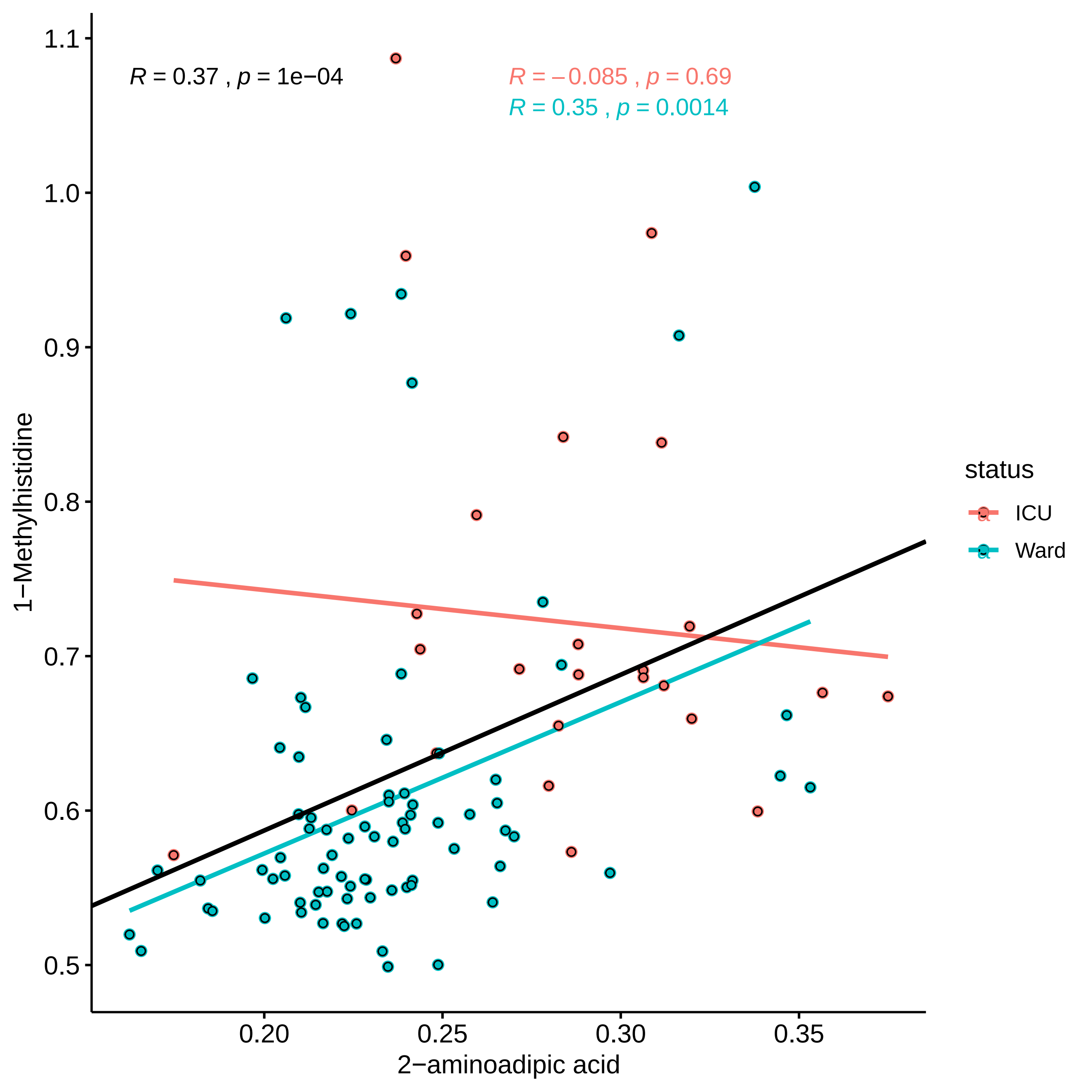

R = 0.37 , p = 1e−04
R = −0.085 , p = 0.69
R = 0.35 , p = 0.0014
1.1
1.0
0.9
0.8
0.7
0.6
0.5
1−Methylhistidine
0.20
0.25
0.30
0.35
2−aminoadipic acid
status
ICU
Ward

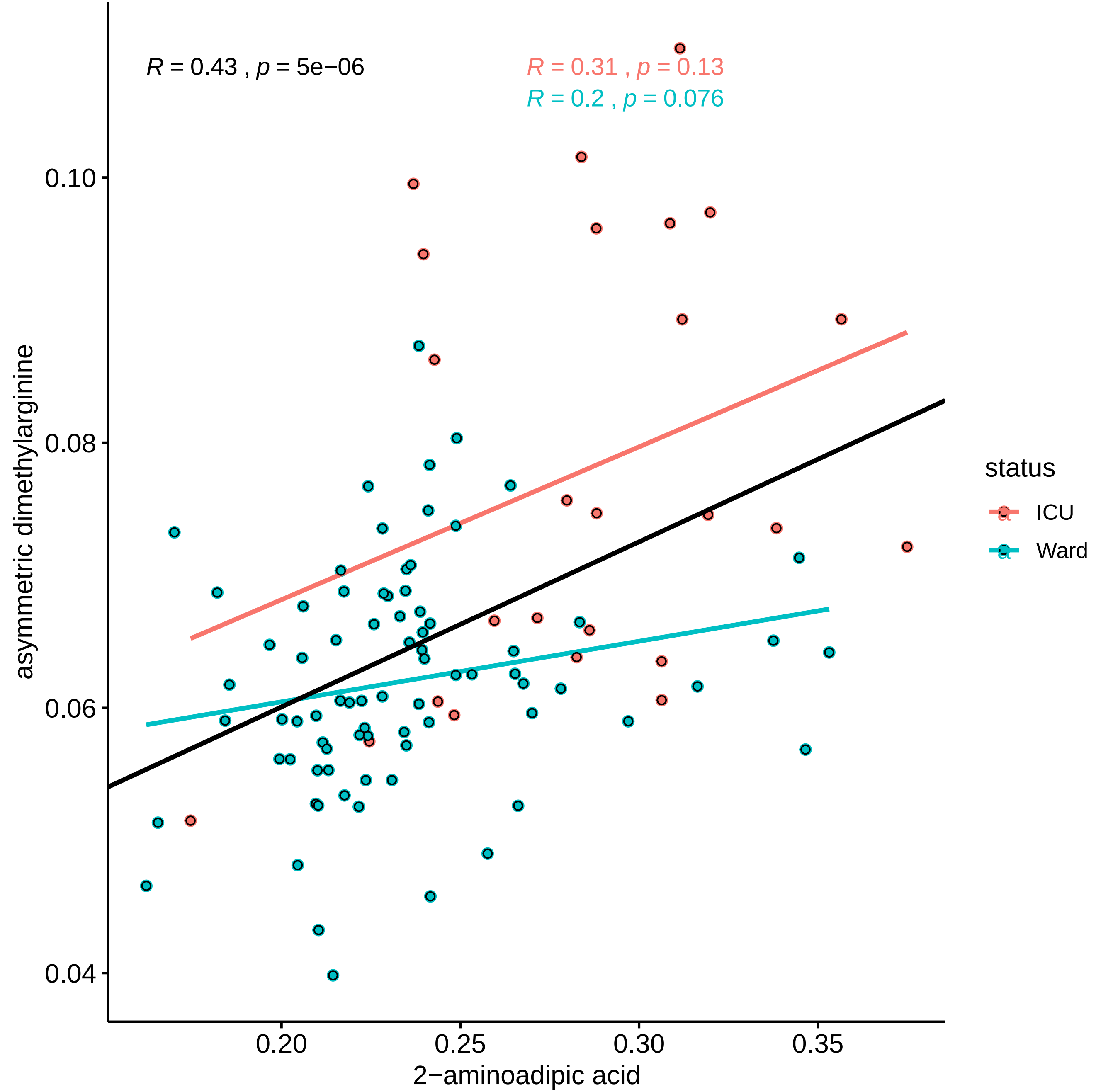

R = 0.43 , p = 5e−06
R = 0.31 , p = 0.13
R = 0.2 , p = 0.076
asymmetric dimethylarginine
0.10
0.08
0.06
0.04
0.20
0.25
0.30
0.35
2−aminoadipic acid
status
ICU
Ward

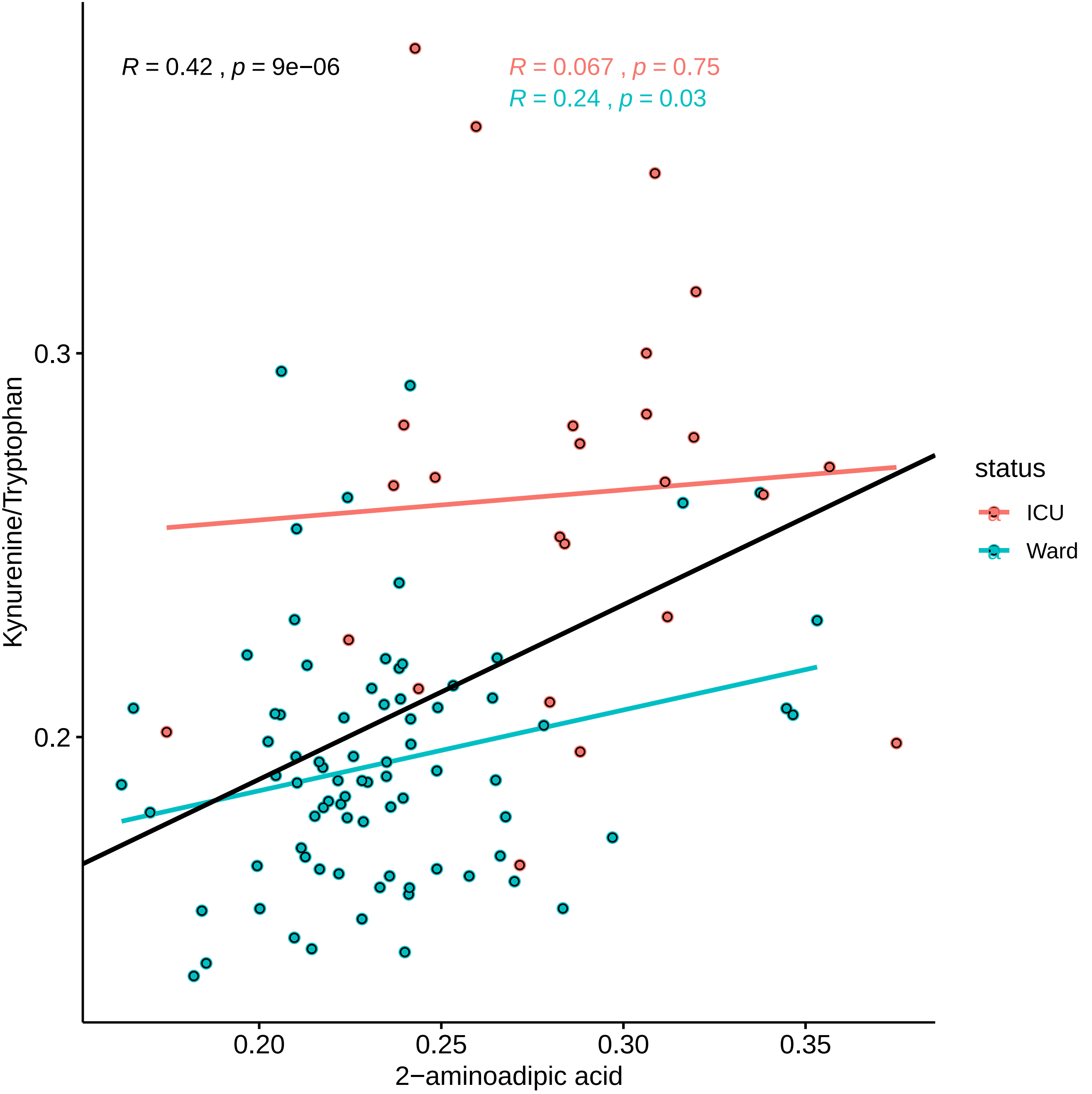

R = 0.42 , p = 9e−06
R = 0.067 , p = 0.75
R = 0.24 , p = 0.03
0.3
0.2
Kynurenine/Tryptophan
0.20
0.25
0.30
0.35
2−aminoadipic acid
status
ICU
Ward

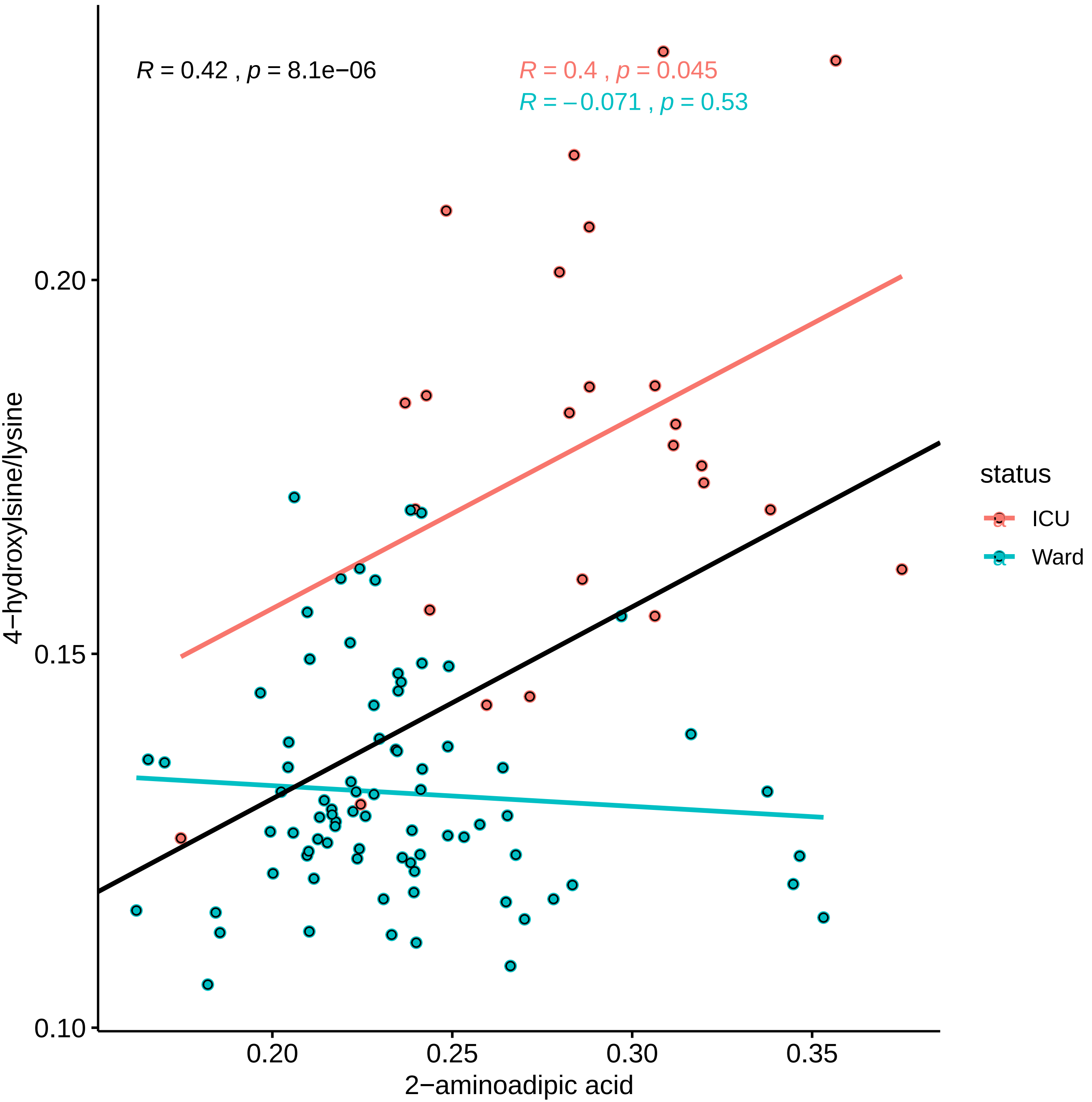

R = 0.42 , p = 8.1e−06
R = 0.4 , p = 0.045
R = −0.071 , p = 0.53
4−hydroxylsine/lysine
0.20
0.15
0.10
0.20
0.25
0.30
0.35
2−aminoadipic acid
status
ICU
Ward

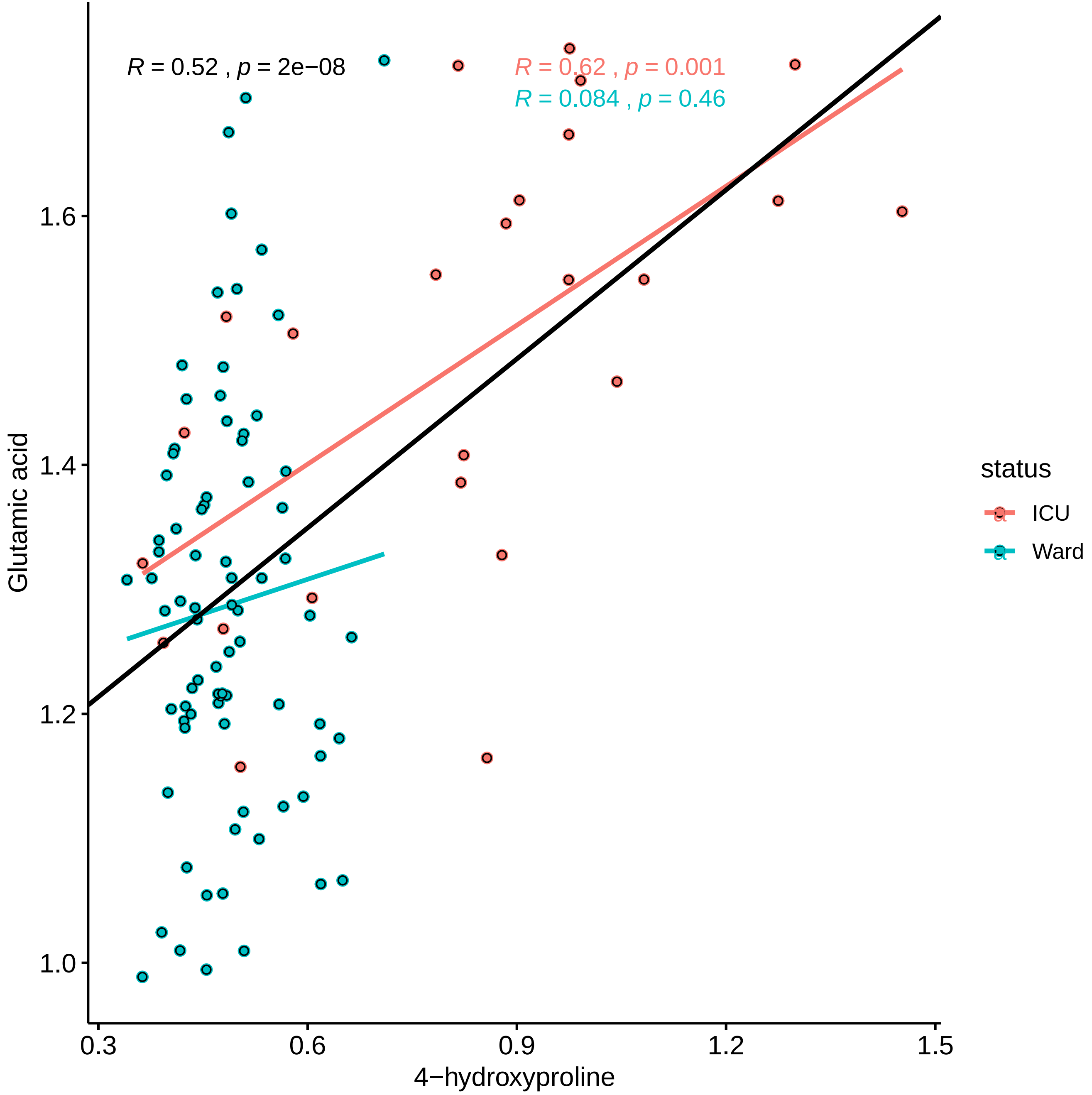
R = 0.52 , p = 2e−08
R = 0.62 , p = 0.001
R = 0.084 , p = 0.46
Glutamic acid
1.0
1.2
1.4
1.6
0.3
0.6
0.9
1.2
1.5
4−hydroxyproline
status
ICU
Ward

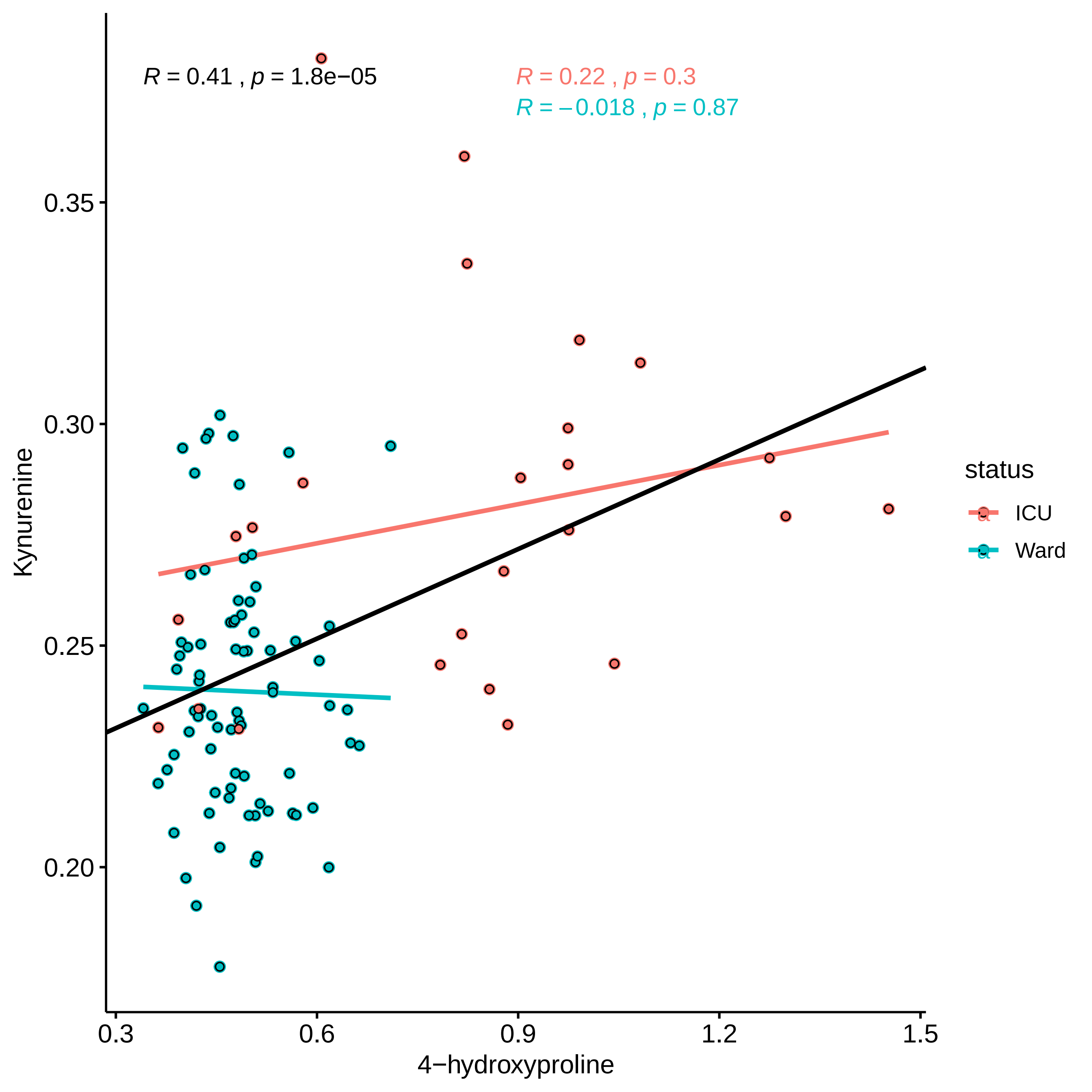

R = 0.41 , p = 1.8e−05
R = 0.22 , p = 0.3
R = −0.018 , p = 0.87
Kynurenine
0.35
0.30
0.25
0.20
0.3
0.6
0.9
1.2
1.5
4−hydroxyproline
status
ICU
Ward

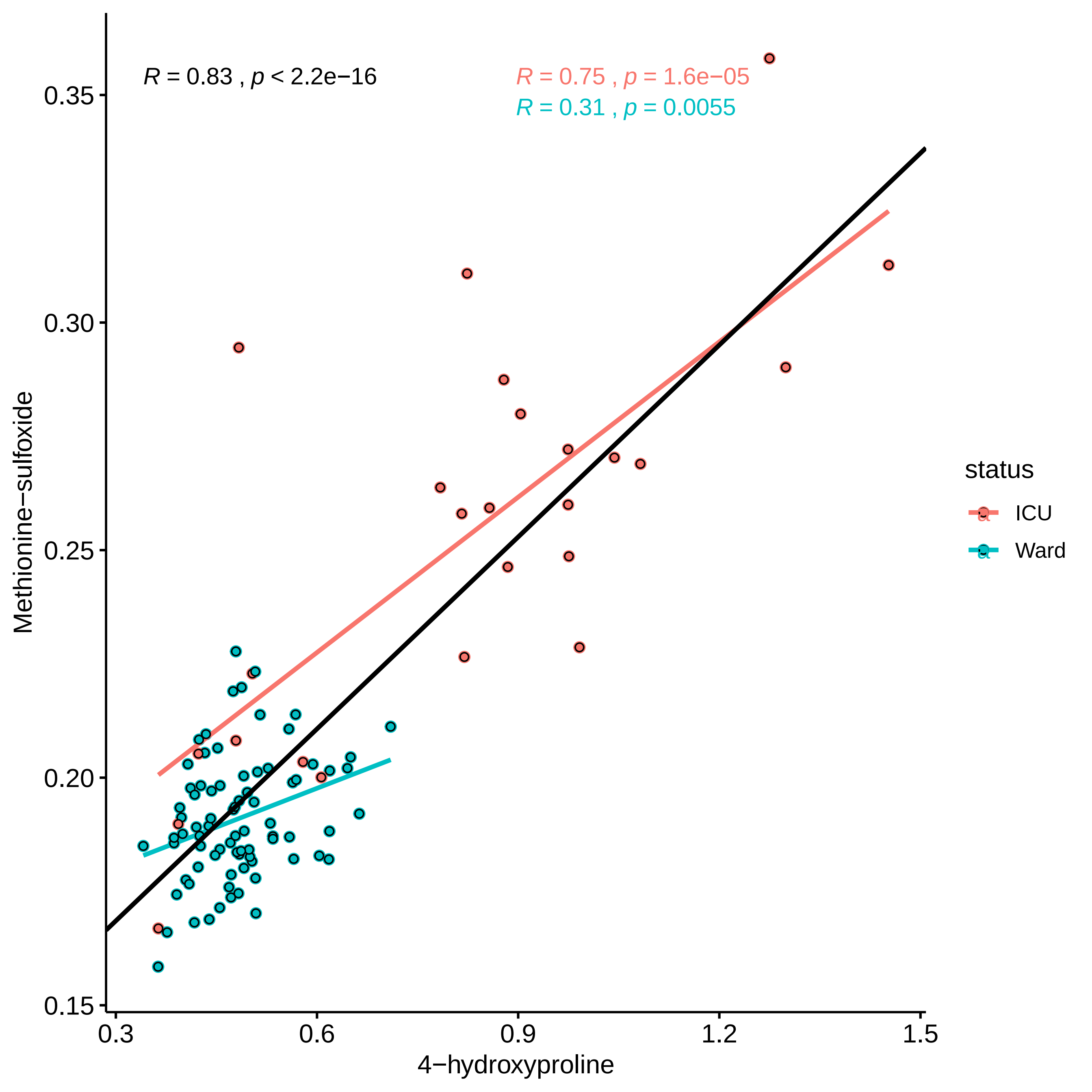
R = 0.83 , p < 2.2e−16
R = 0.75 , p = 1.6e−05
R = 0.31 , p = 0.0055
Methionine−sulfoxide
0.35
0.30
0.25
0.20
0.15
0.3
0.6
0.9
1.2
1.5
4−hydroxyproline
status
ICU
Ward

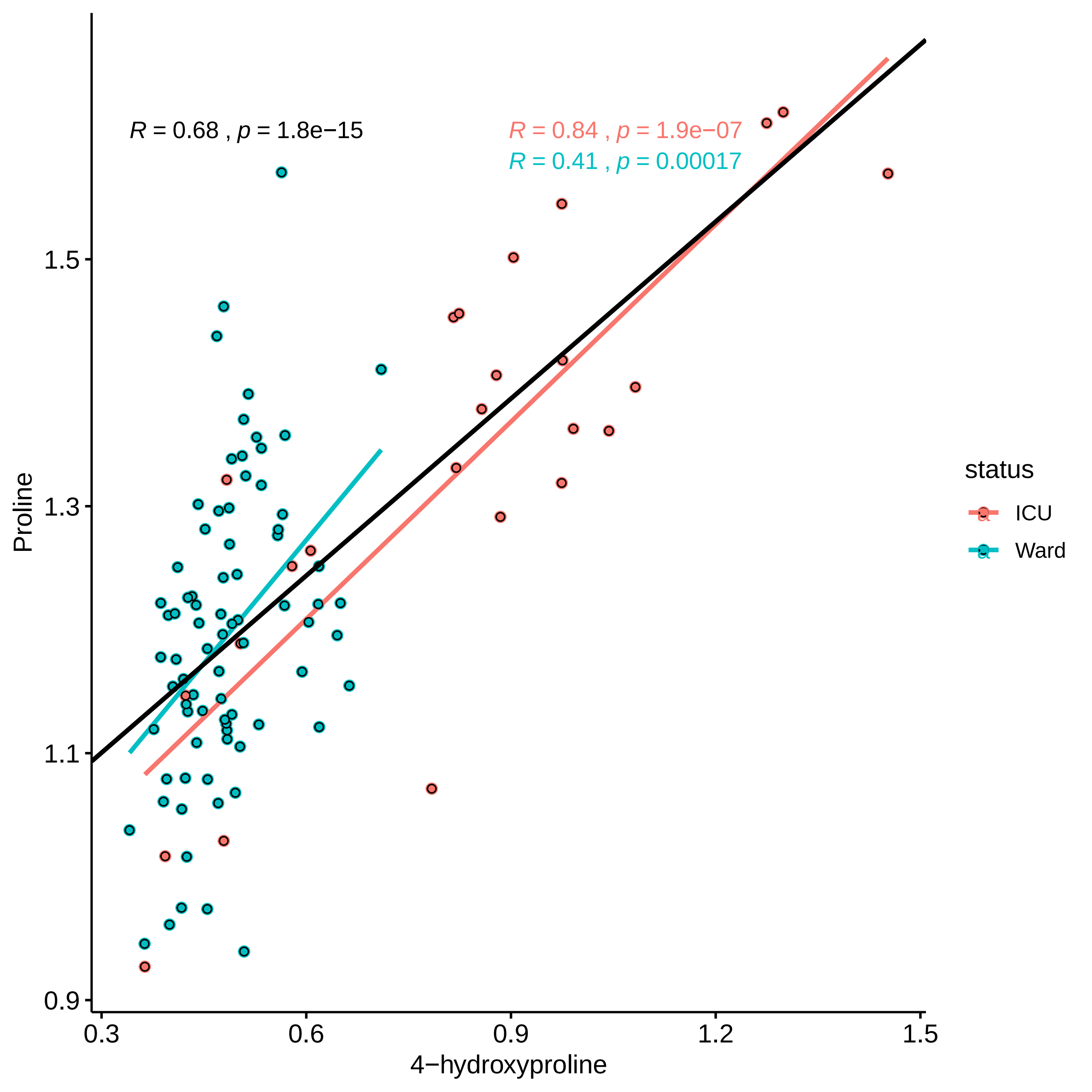

R = 0.68 , p = 1.8e−15
R = 0.84 , p = 1.9e−07
R = 0.41 , p = 0.00017
Proline
4−hydroxyproline
1.5
1.3
1.1
0.9
0.3
0.6
0.9
1.2
1.5
status
ICU
Ward

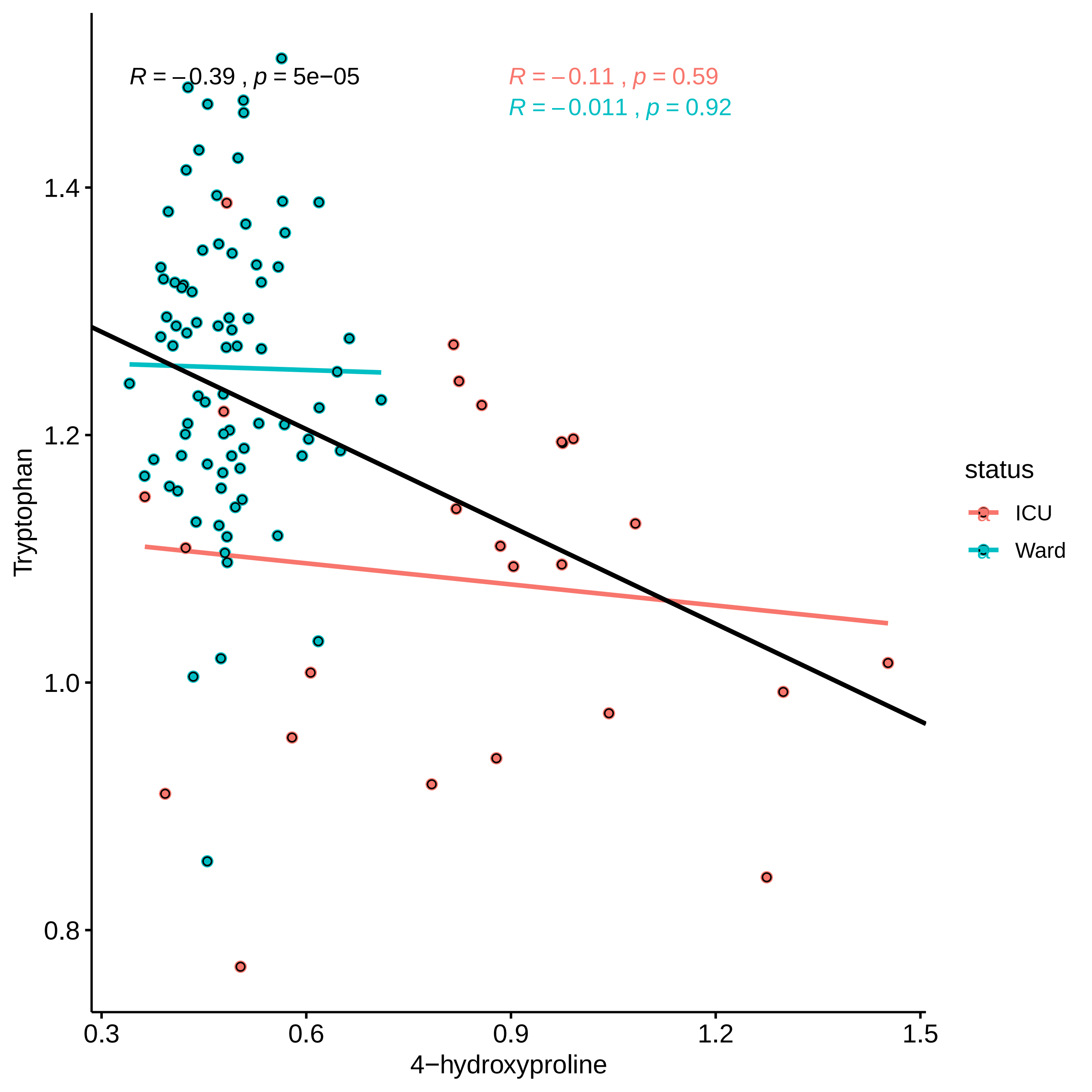
R = −0.39 , p = 5e−05
R = −0.11 , p = 0.59
R = −0.011 , p = 0.92
1.4
1.2
1.0
0.8
Tryptophan
0.3
0.6
0.9
1.2
1.5
4−hydroxyproline
status
ICU
Ward

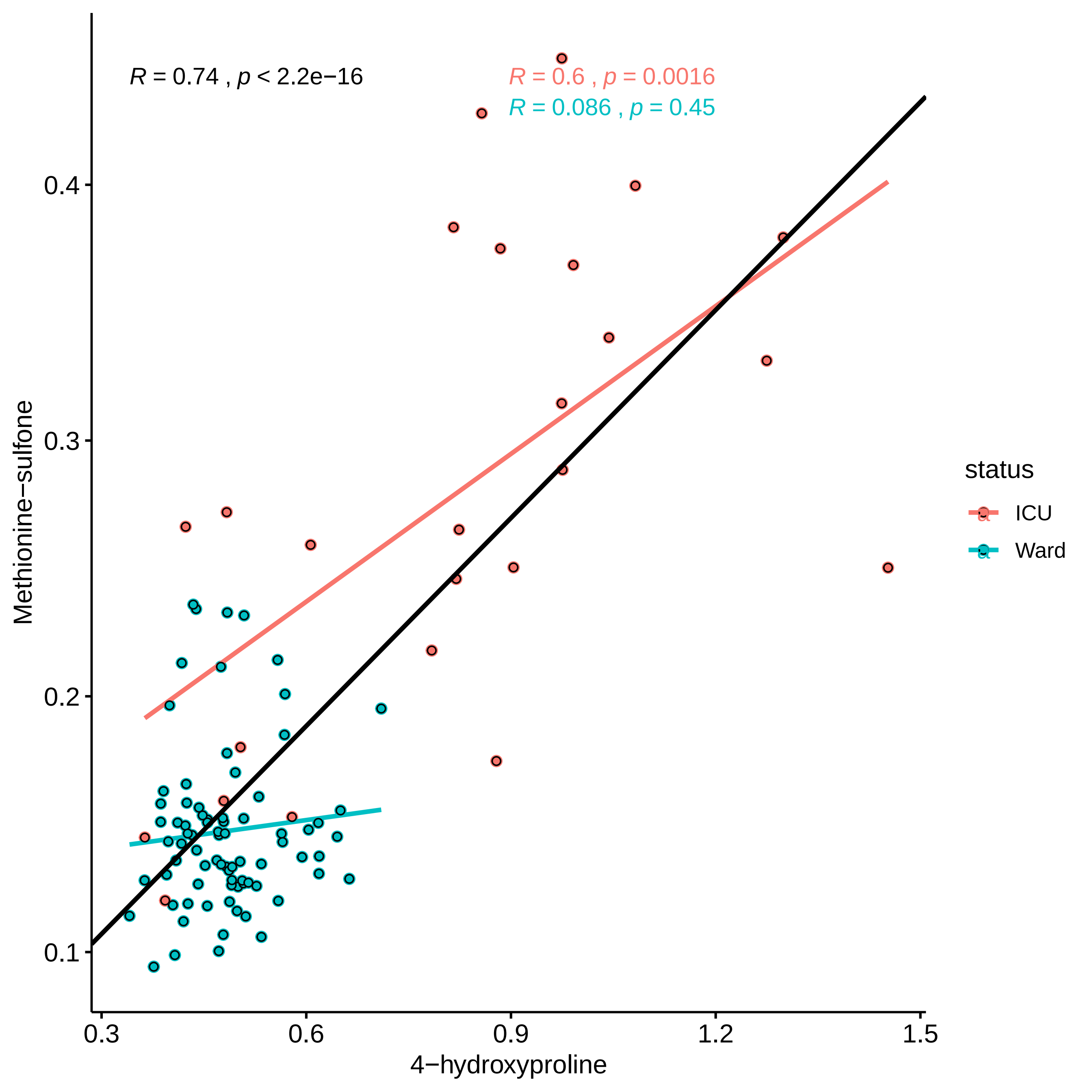

R = 0.74 , p < 2.2e−16
R = 0.6 , p = 0.0016
R = 0.086 , p = 0.45
Methionine−sulfone
0.4
0.3
0.2
0.1
0.3
0.6
0.9
1.2
1.5
4−hydroxyproline
status
ICU
Ward

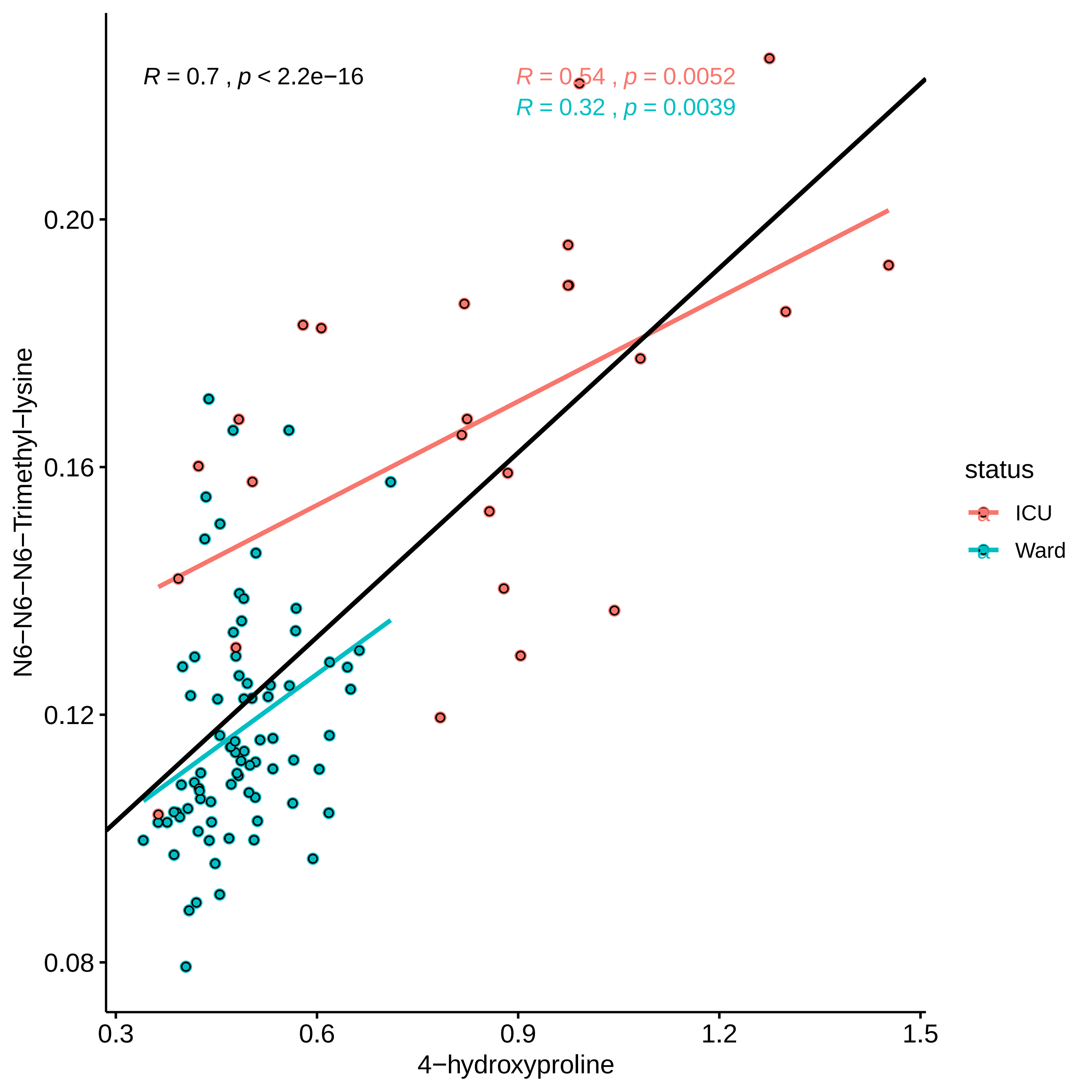

R = 0.7 , p < 2.2e−16
R = 0.54 , p = 0.0052
R = 0.32 , p = 0.0039
N6−N6−N6−Trimethyl−lysine
0.20
0.16
0.12
0.08
0.3
0.6
0.9
1.2
1.5
4−hydroxyproline
status
ICU
Ward

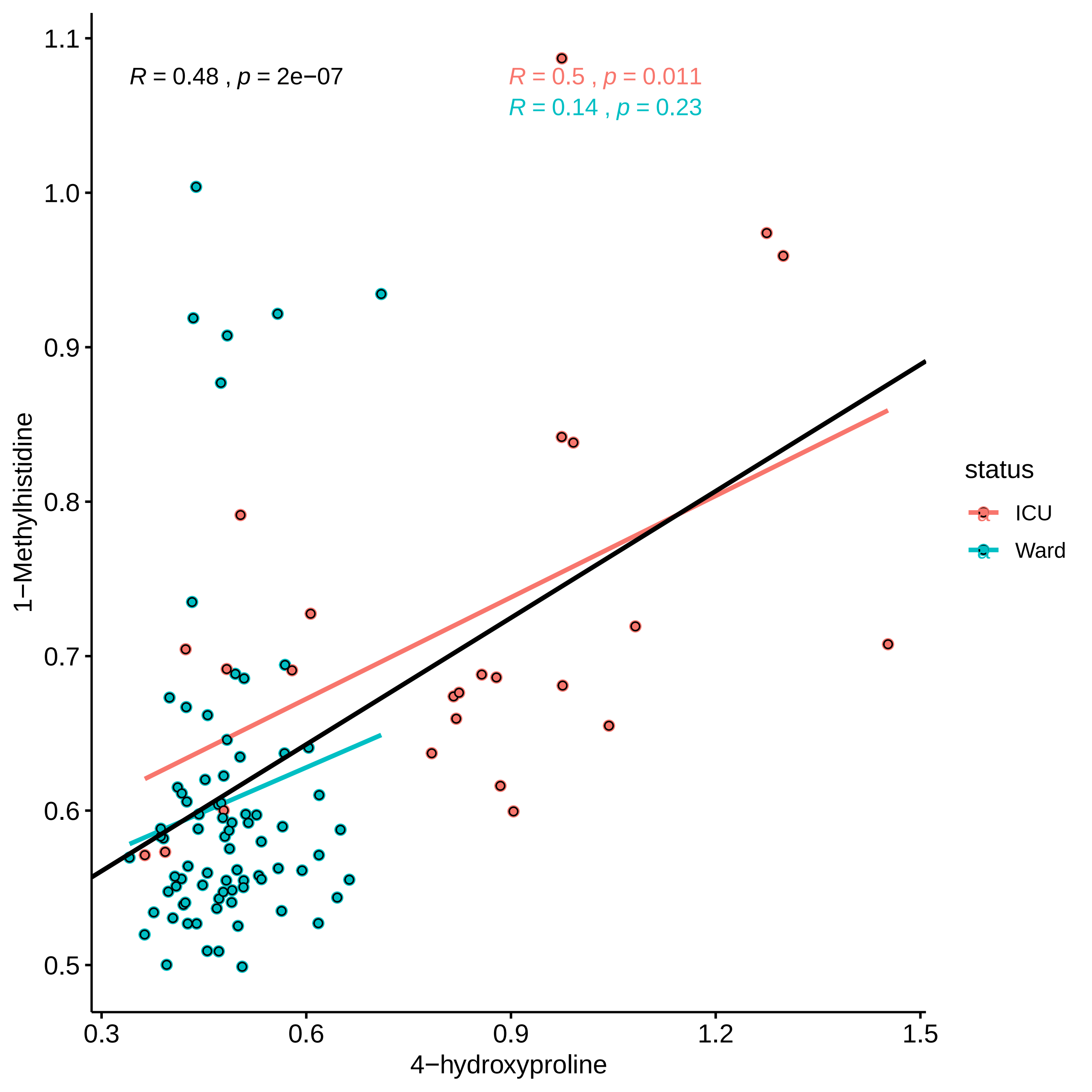
R = 0.48 , p = 2e−07
R = 0.5 , p = 0.011
R = 0.14 , p = 0.23
1.1
1.0
0.9
0.8
0.7
0.6
0.5
1−Methylhistidine
0.3
0.6
0.9
1.2
1.5
4−hydroxyproline
status
ICU
Ward

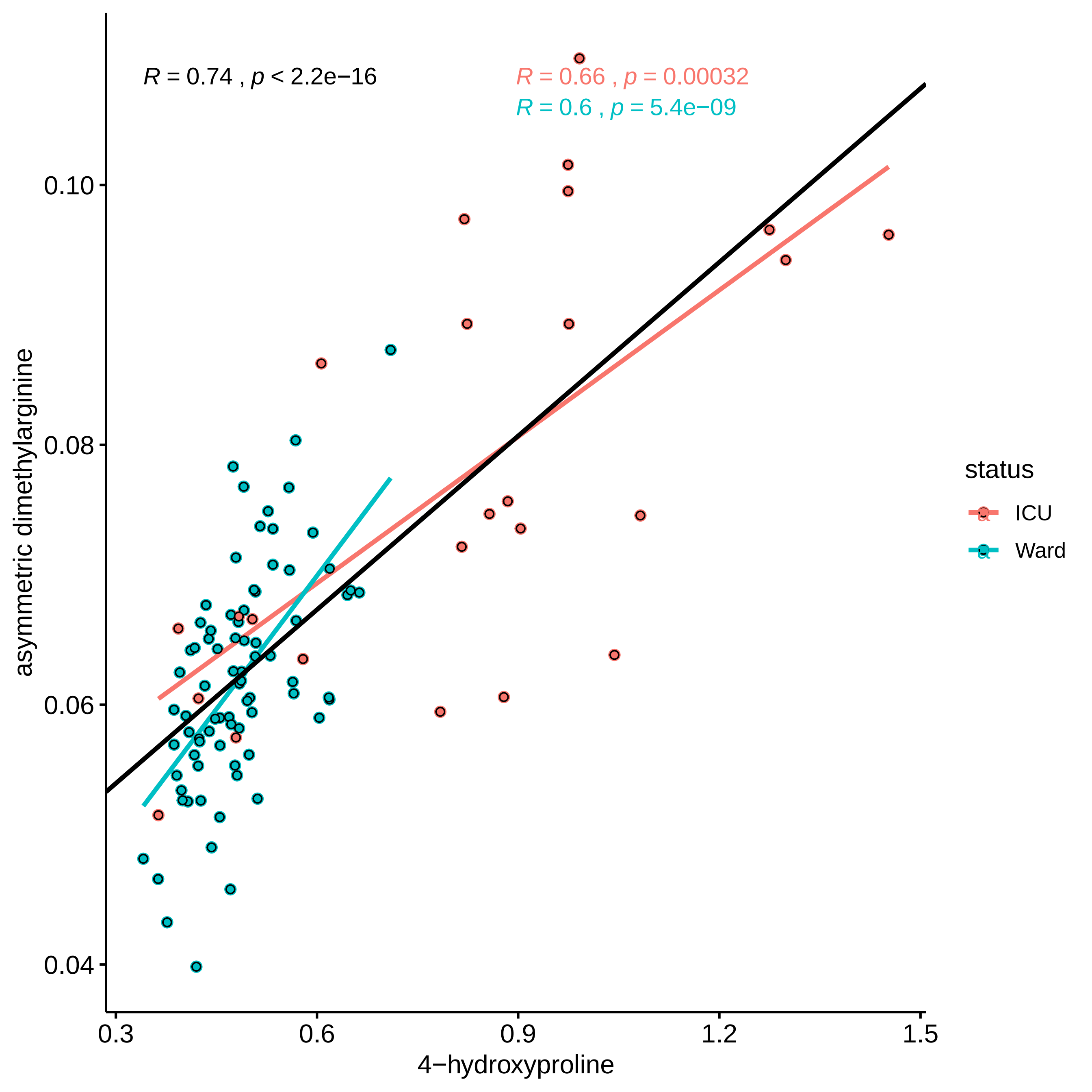

R = 0.74 , p < 2.2e−16
R = 0.66 , p = 0.00032
R = 0.6 , p = 5.4e−09
asymmetric dimethylarginine
0.10
0.08
0.06
0.04
0.3
0.6
0.9
1.2
1.5
4−hydroxyproline
status
ICU
Ward

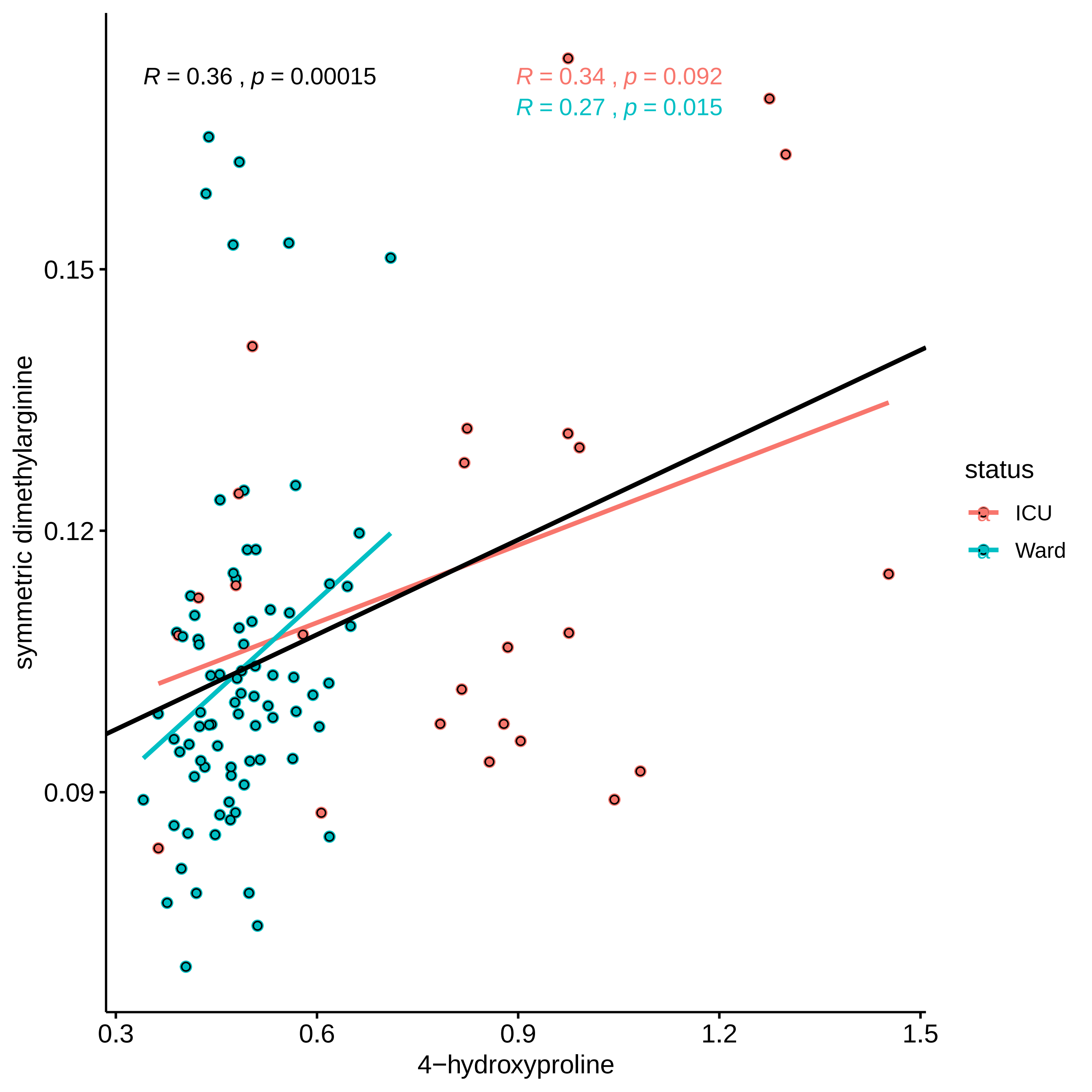

R = 0.36 , p = 0.00015
R = 0.34 , p = 0.092
R = 0.27 , p = 0.015
0.15
0.12
0.09
0.3
0.6
0.9
1.2
1.5
symmetric dimethylarginine
4−hydroxyproline
status
ICU
Ward

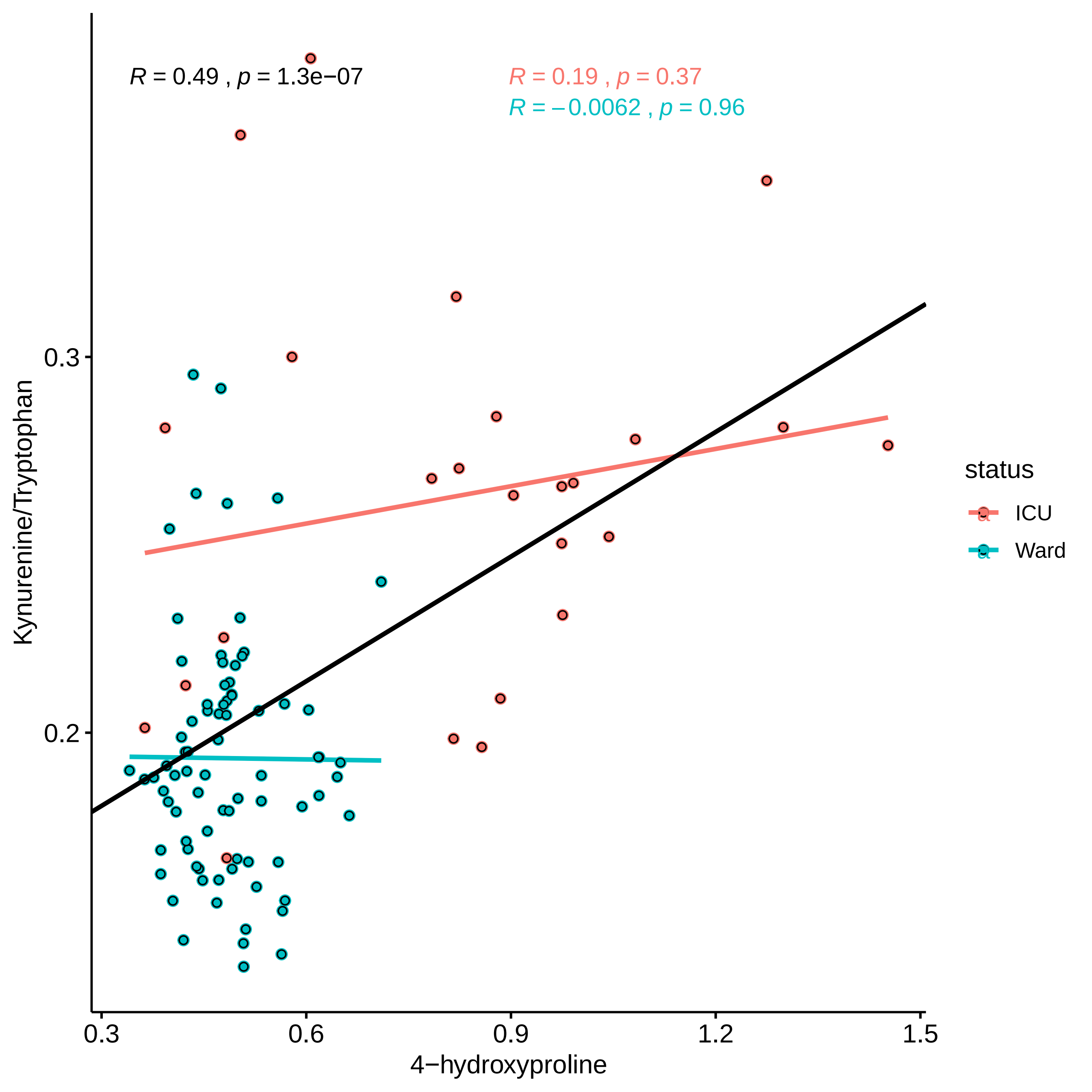

R = 0.49 , p = 1.3e−07
R = 0.19 , p = 0.37
R = −0.0062 , p = 0.96
Kynurenine/Tryptophan
0.3
0.2
0.3
0.6
0.9
1.2
1.5
4−hydroxyproline
status
ICU
Ward

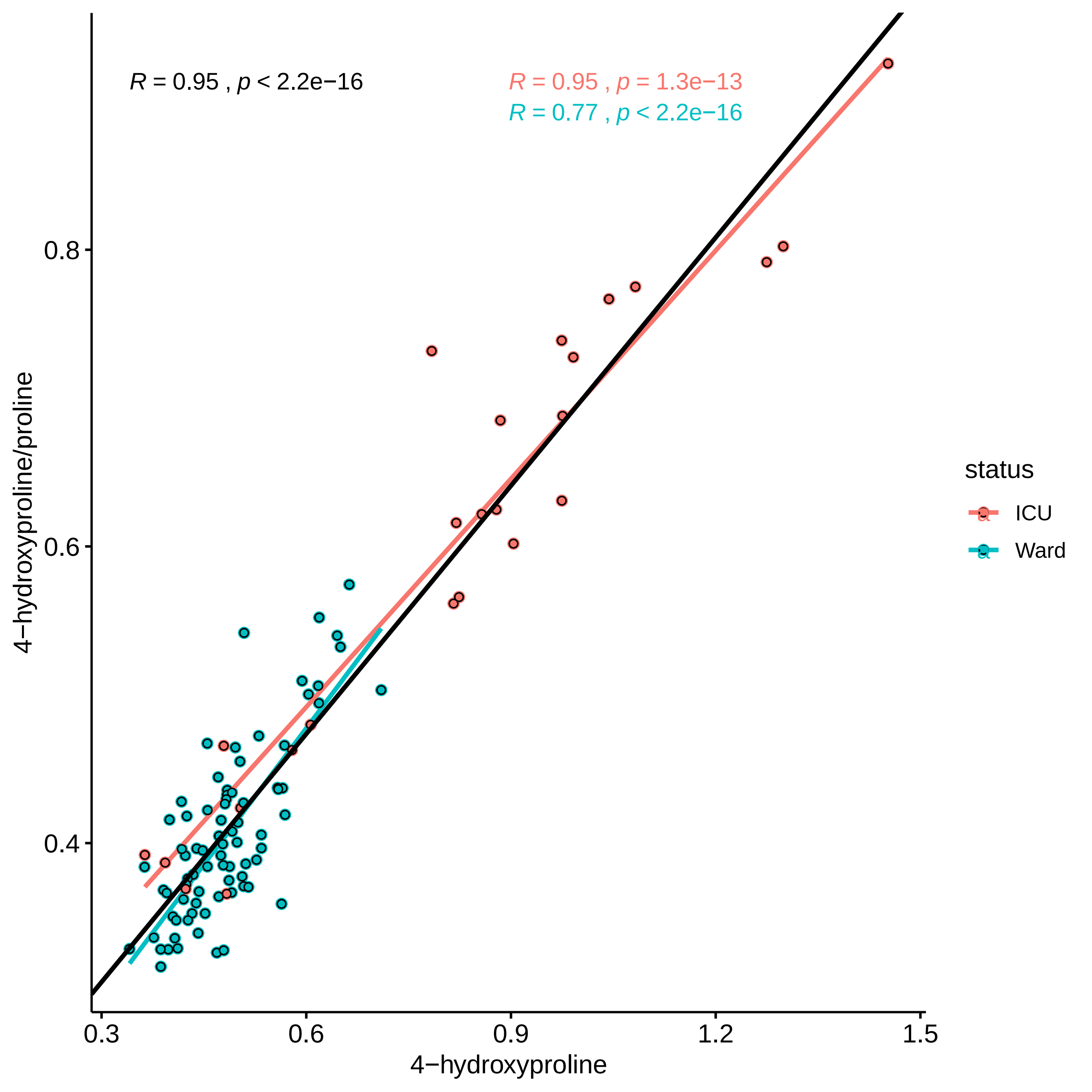

R = 0.95 , p < 2.2e−16
R = 0.95 , p = 1.3e−13
R = 0.77 , p < 2.2e−16
0.8
0.6
0.4
4−hydroxyproline/proline
0.3
0.6
0.9
1.2
1.5
4−hydroxyproline
status
ICU
Ward

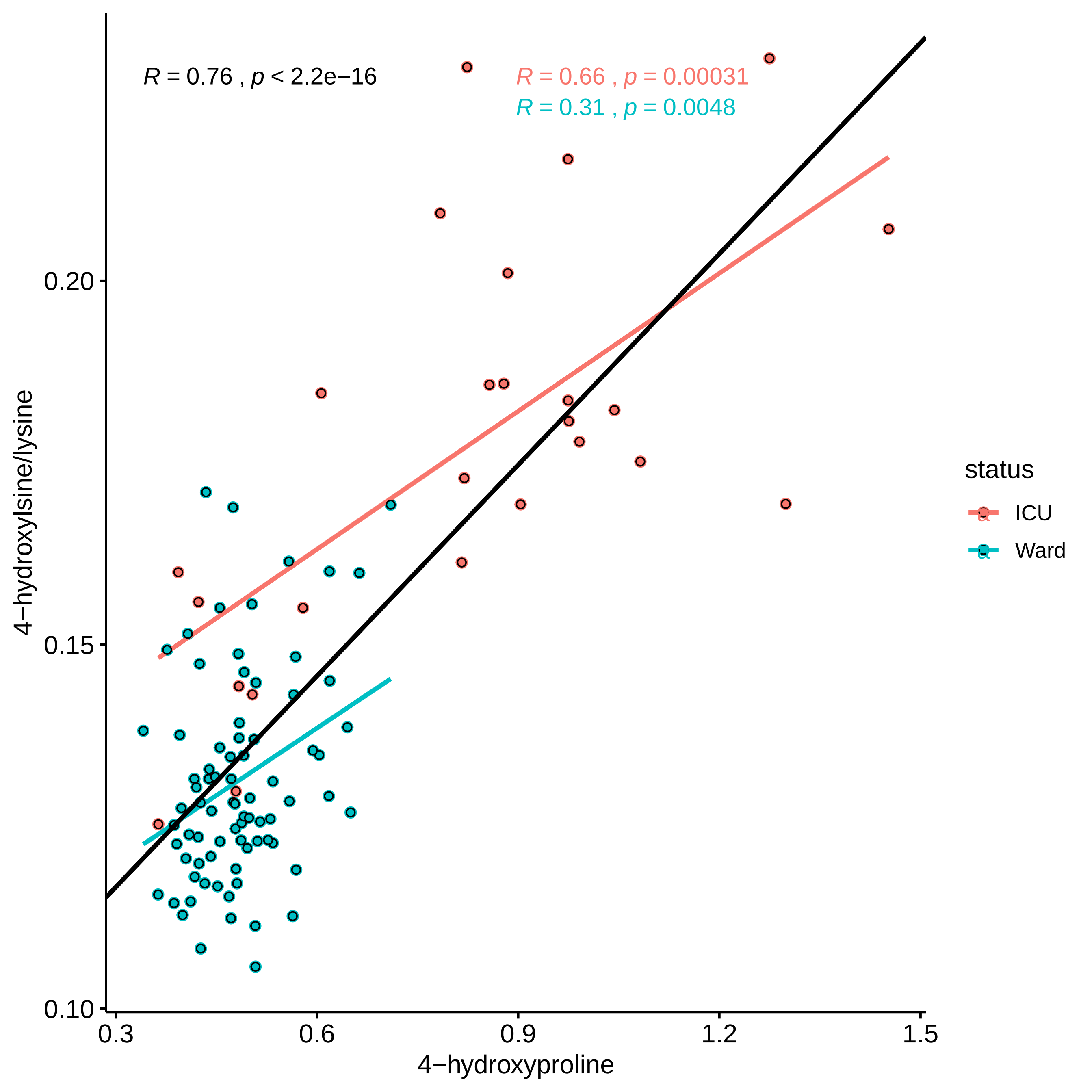

R = 0.76 , p < 2.2e−16
R = 0.66 , p = 0.00031
R = 0.31 , p = 0.0048
4−hydroxylysine/lysine
0.10
0.15
0.20
0.3
0.6
0.9
1.2
1.5
4−hydroxyproline
status
ICU
Ward

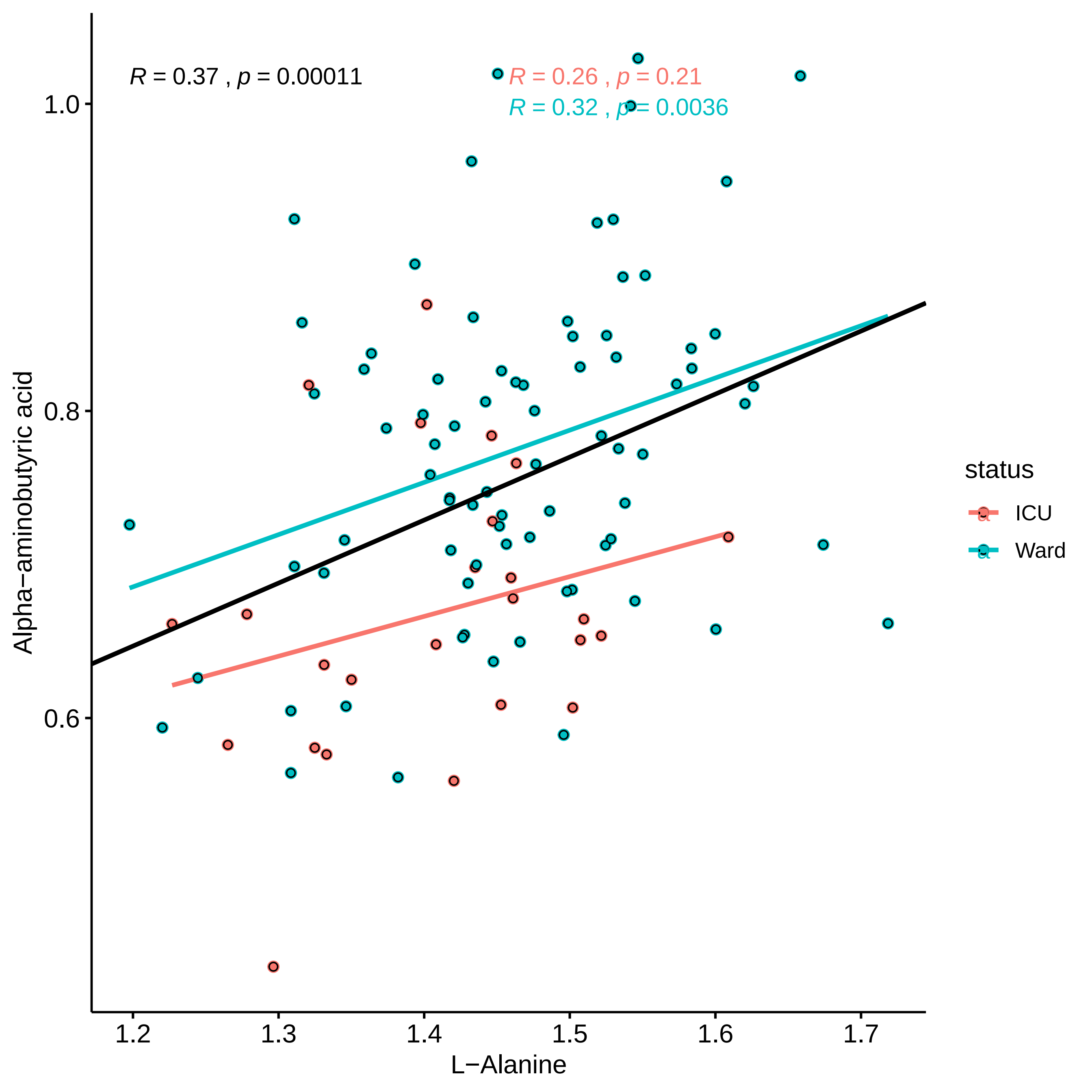

R = 0.37 , p = 0.00011
R = 0.26 , p = 0.21
R = 0.32 , p = 0.0036
1.0
0.8
0.6
Alpha−aminobutyric acid
1.2
1.3
1.4
1.5
1.6
1.7
L−Alanine
status
ICU
Ward

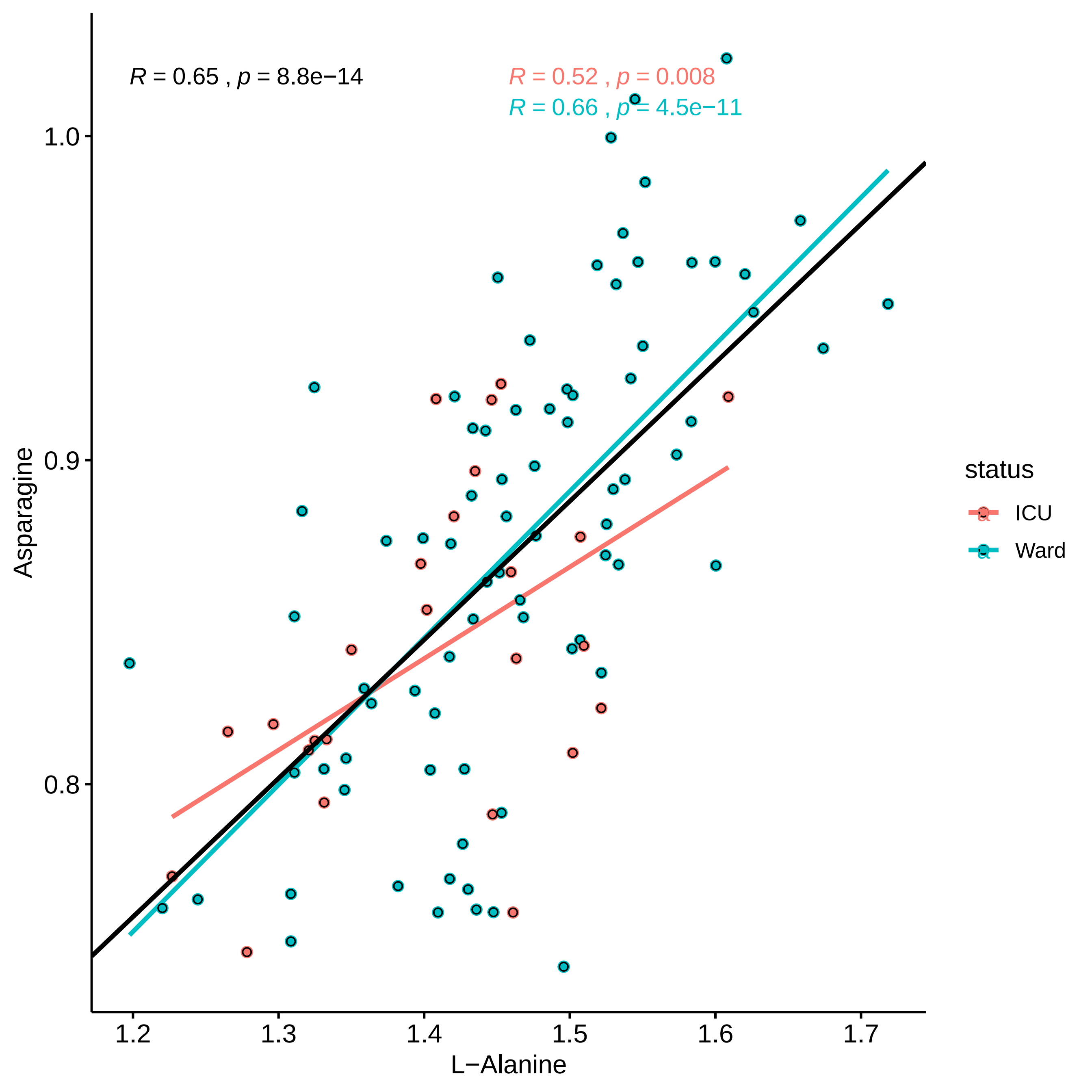

R = 0.65 , p = 8.8e−14
R = 0.52 , p = 0.008
R = 0.66 , p = 4.5e−11
Asparagine
L−Alanine
1.0
0.9
0.8
1.2
1.3
1.4
1.5
1.6
1.7
status
ICU
Ward

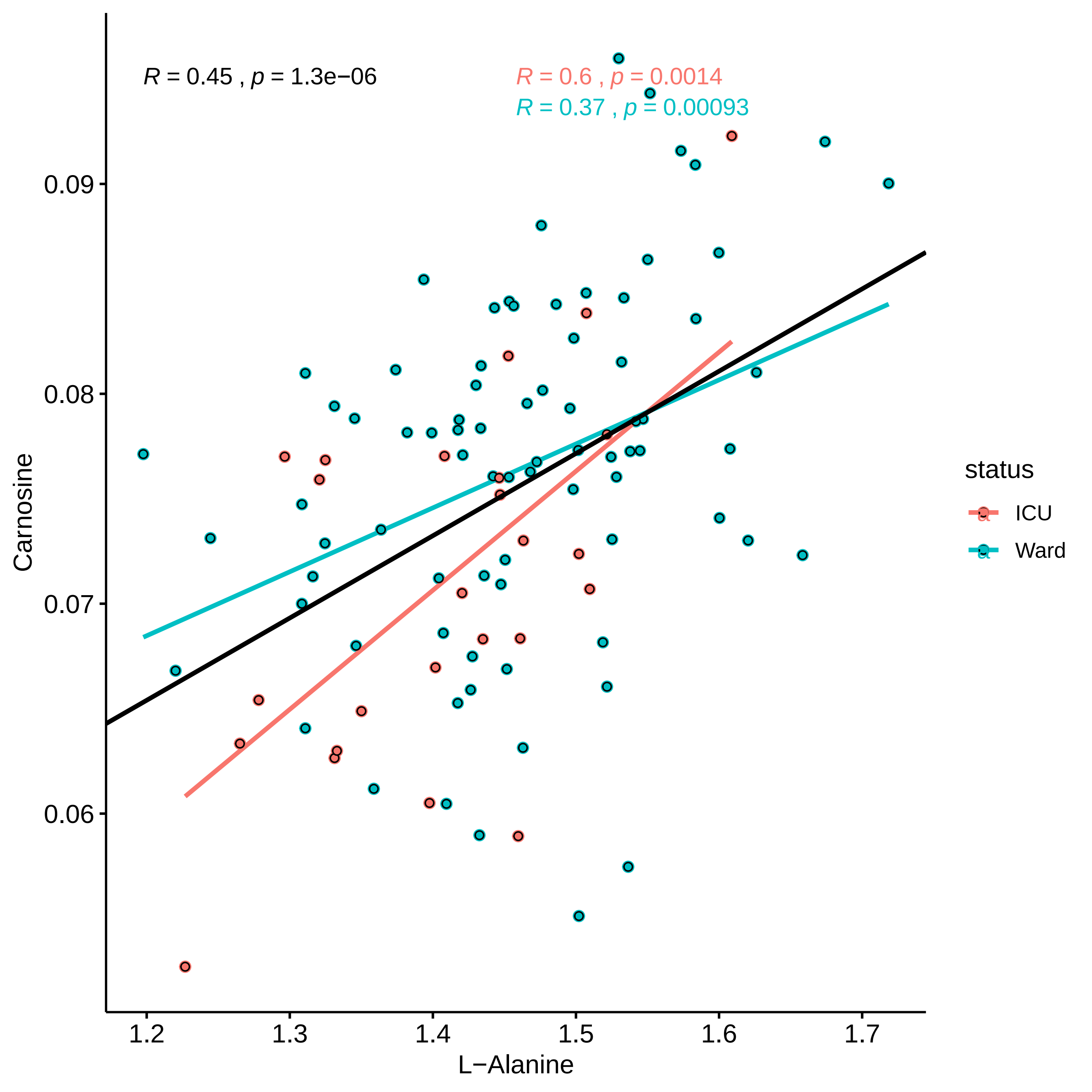

R = 0.45 , p = 1.3e−06
R = 0.6 , p = 0.0014
R = 0.37 , p = 0.00093
Carnosine
0.09
0.08
0.07
0.06
1.2
1.3
1.4
1.5
1.6
1.7
L−Alanine
status
ICU
Ward

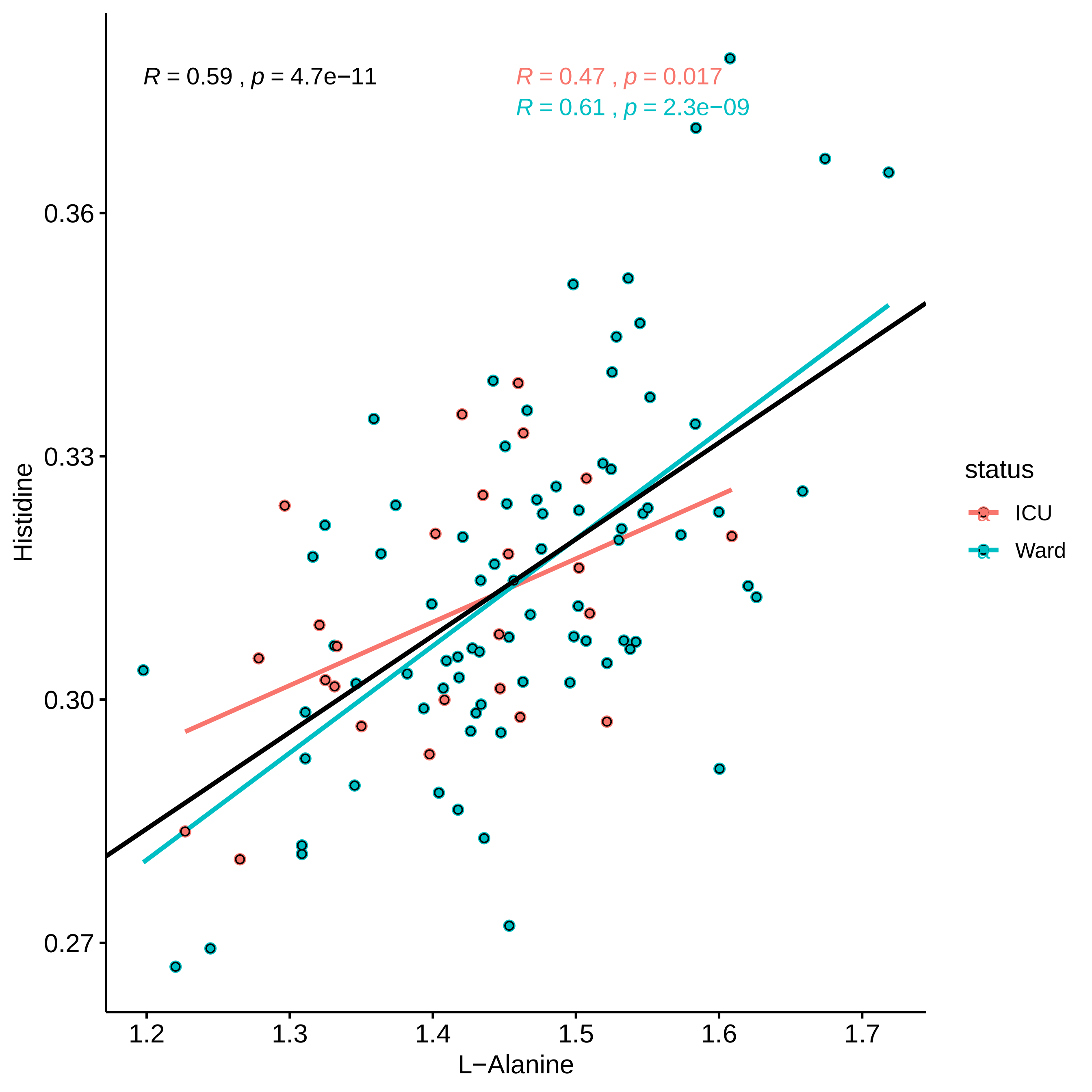

R = 0.59 , p = 4.7e−11
R = 0.47 , p = 0.017
R = 0.61 , p = 2.3e−09
Histidine
0.36
0.33
0.30
0.27
1.2
1.3
1.4
1.5
1.6
1.7
L−Alanine
status
ICU
Ward

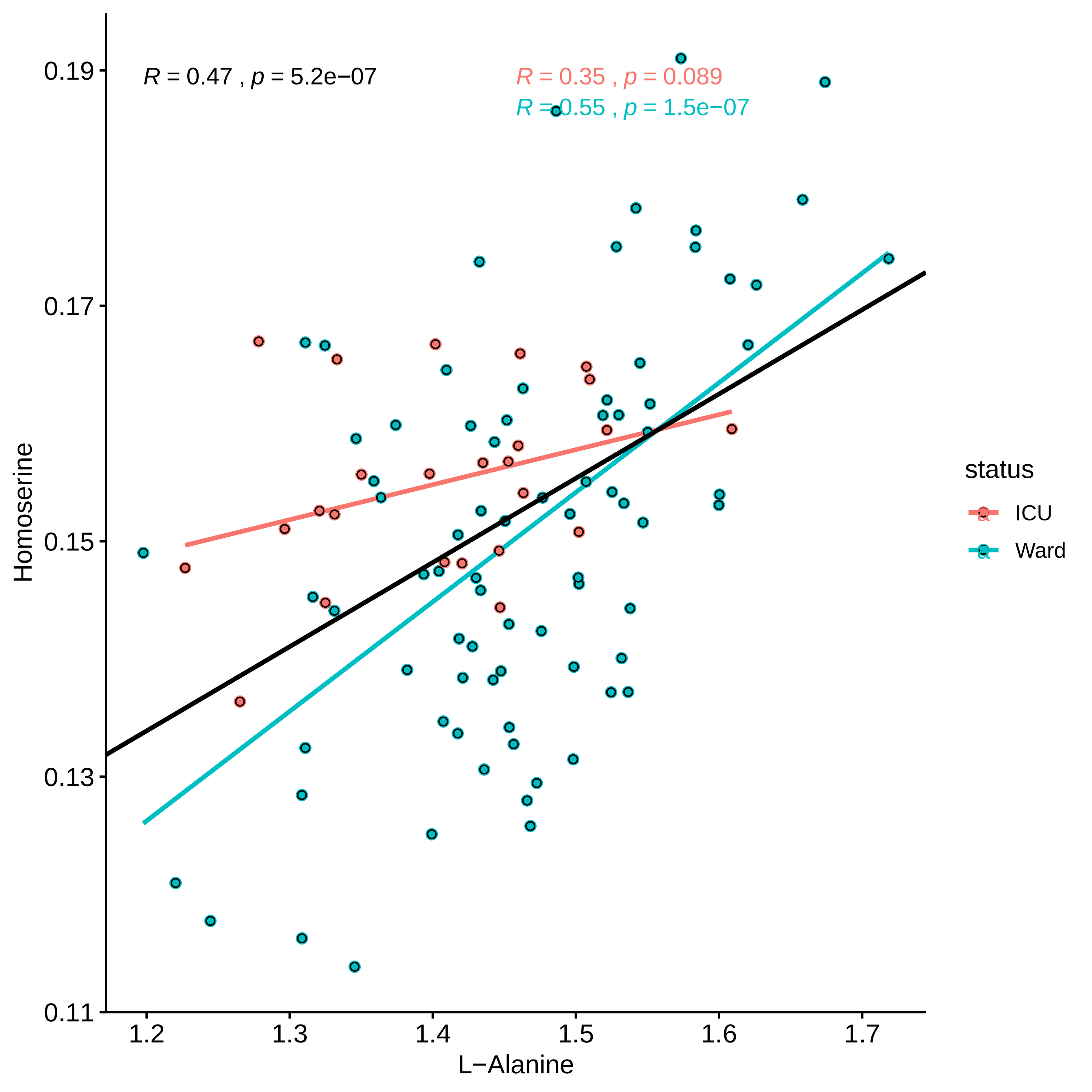

R = 0.47 , p = 5.2e−07
R = 0.35 , p = 0.089
R = 0.55 , p = 1.5e−07
Homoserine
0.19
0.17
0.15
0.13
0.11
1.2
1.3
1.4
1.5
1.6
1.7
L−Alanine
status
ICU
Ward

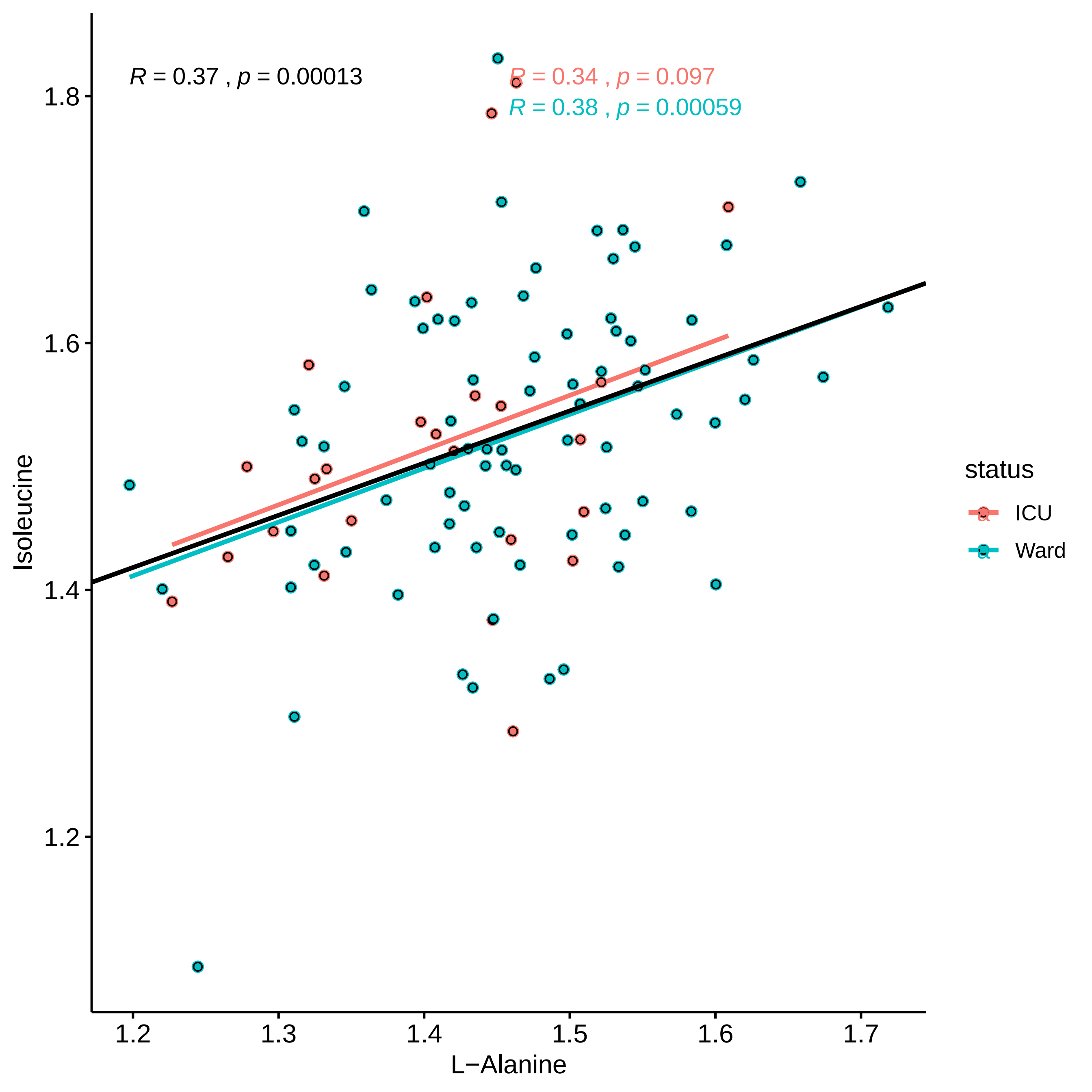

R = 0.37 , p = 0.00013
R = 0.34 , p = 0.097
R = 0.38 , p = 0.00059
1.8
1.6
1.4
1.2
Isoleucine
1.2
1.3
1.4
1.5
1.6
1.7
L−Alanine
status
ICU
Ward

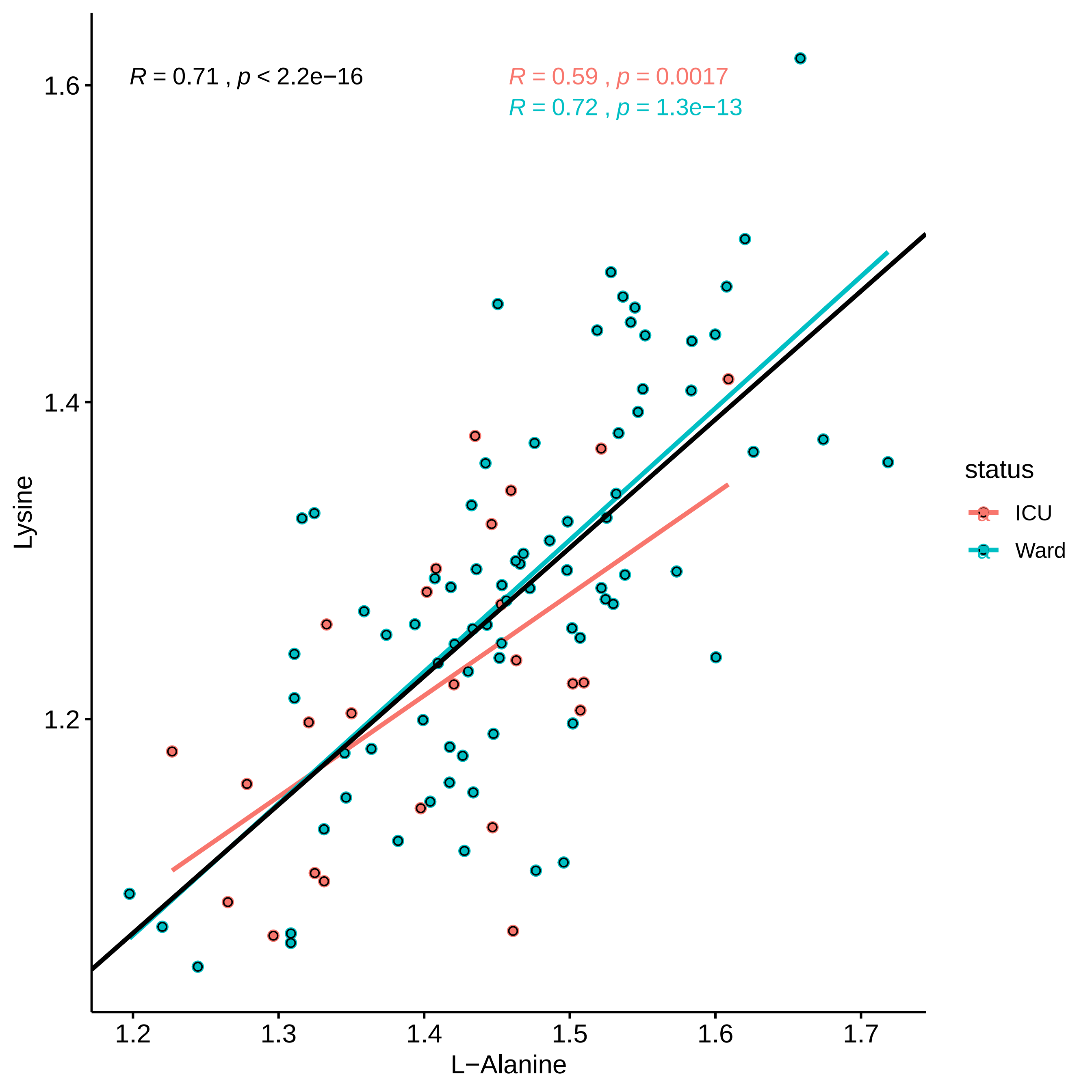

R = 0.71 , p < 2.2e−16
R = 0.59 , p = 0.0017
R = 0.72 , p = 1.3e−13
Lysine
1.6
1.4
1.2
1.2
1.3
1.4
1.5
1.6
1.7
L−Alanine
status
ICU
Ward

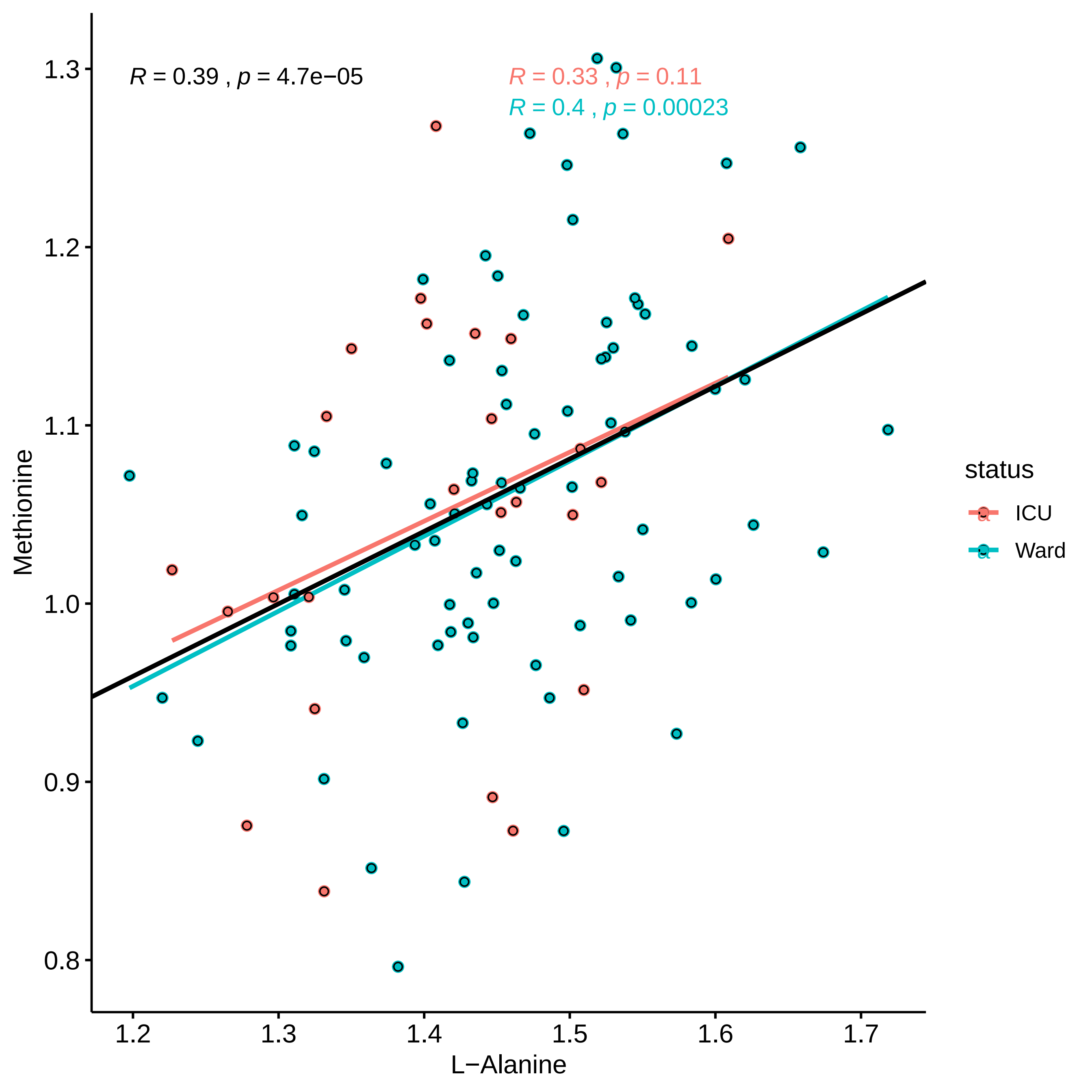

R = 0.39 , p = 4.7e−05
R = 0.33 , p = 0.11
R = 0.4 , p = 0.00023
1.3
1.2
1.1
1.0
0.9
0.8
Methionine
1.2
1.3
1.4
1.5
1.6
1.7
L−Alanine
status
ICU
Ward

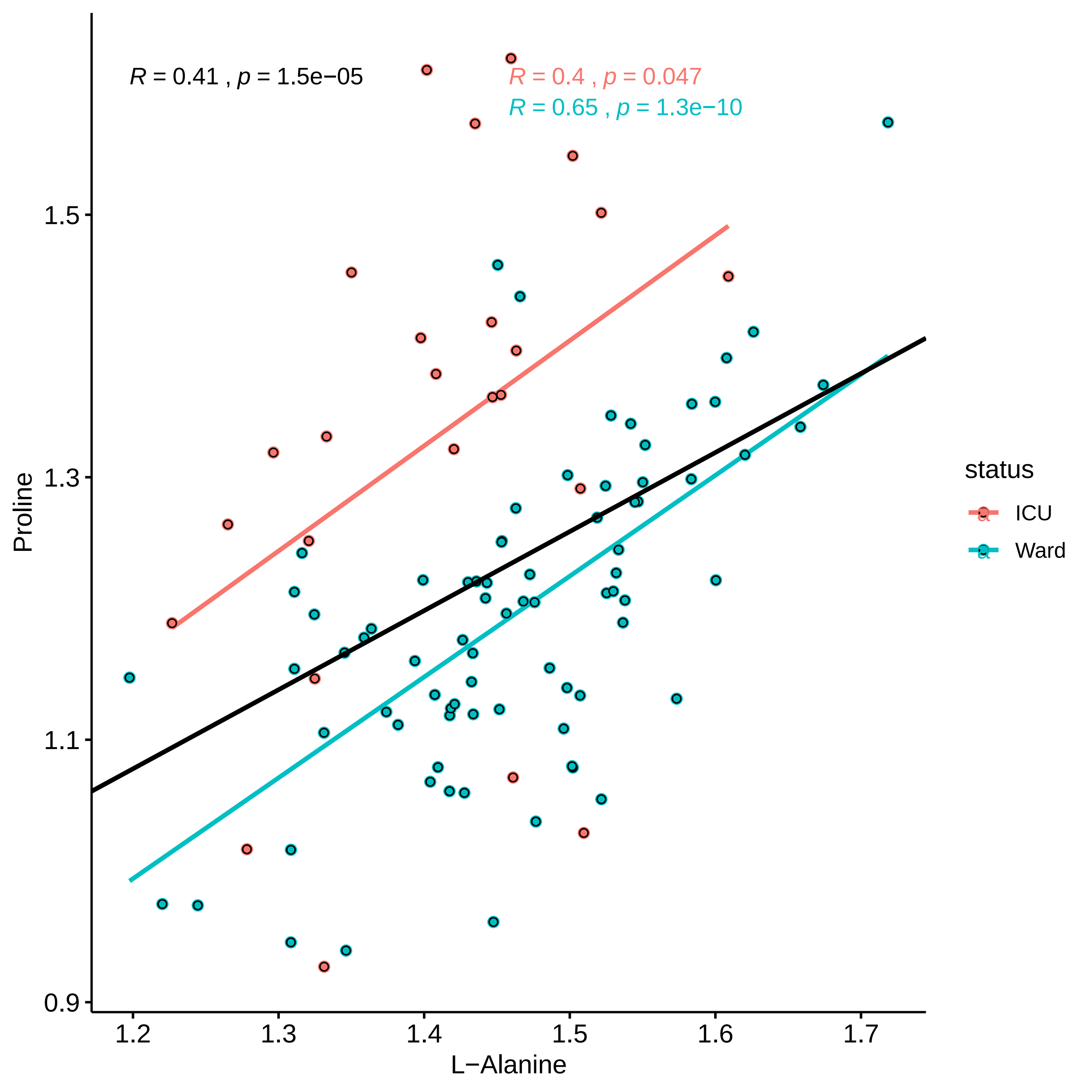

R = 0.41 , p = 1.5e−05
R = 0.4 , p = 0.047
R = 0.65 , p = 1.3e−10
Proline
1.5
1.3
1.1
0.9
1.2
1.3
1.4
1.5
1.6
1.7
L−Alanine
status
ICU
Ward

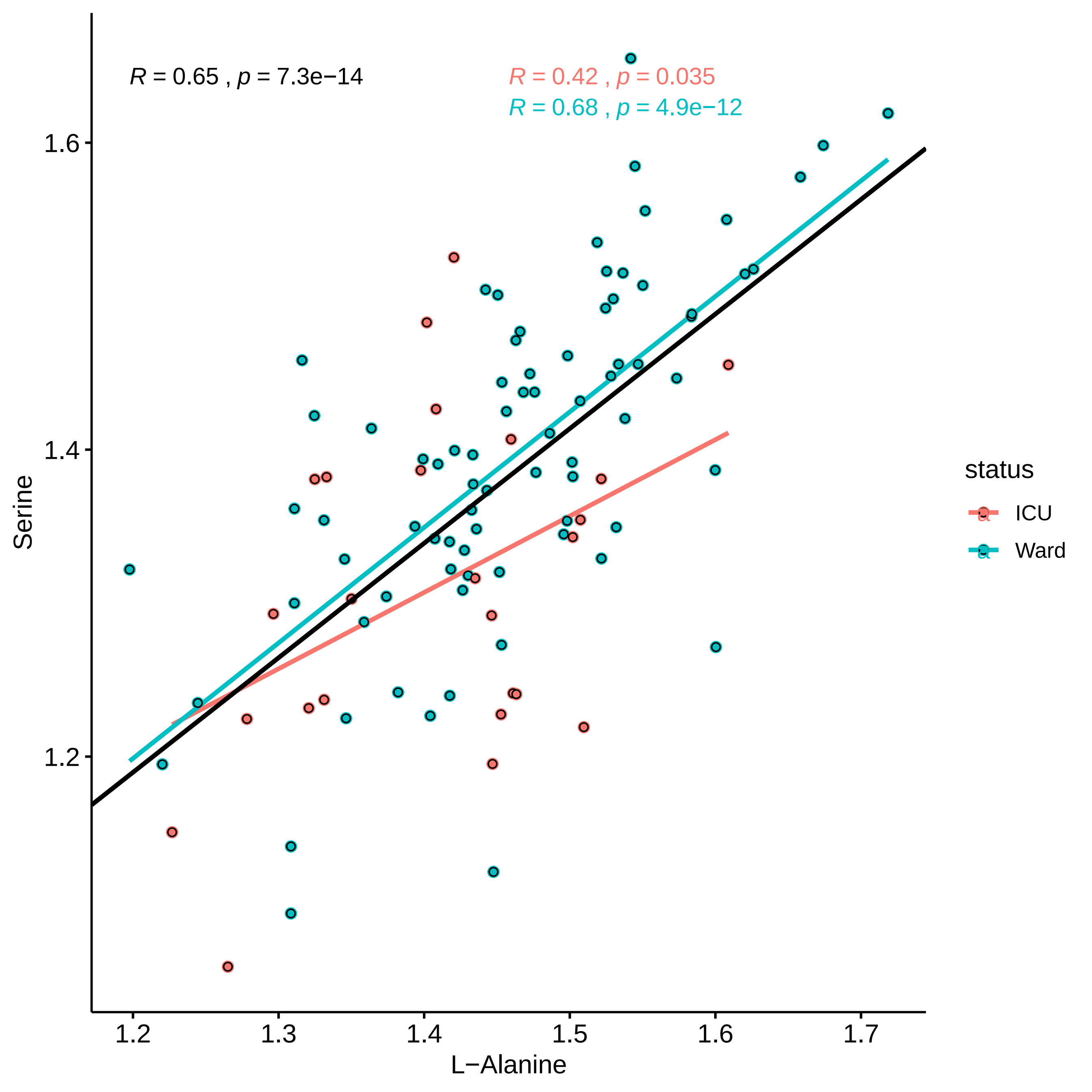

R = 0.65 , p = 7.3e−14
R = 0.42 , p = 0.035
R = 0.68 , p = 4.9e−12
1.6
1.4
1.2
Serine
1.2
1.3
1.4
1.5
1.6
1.7
L−Alanine
status
ICU
Ward

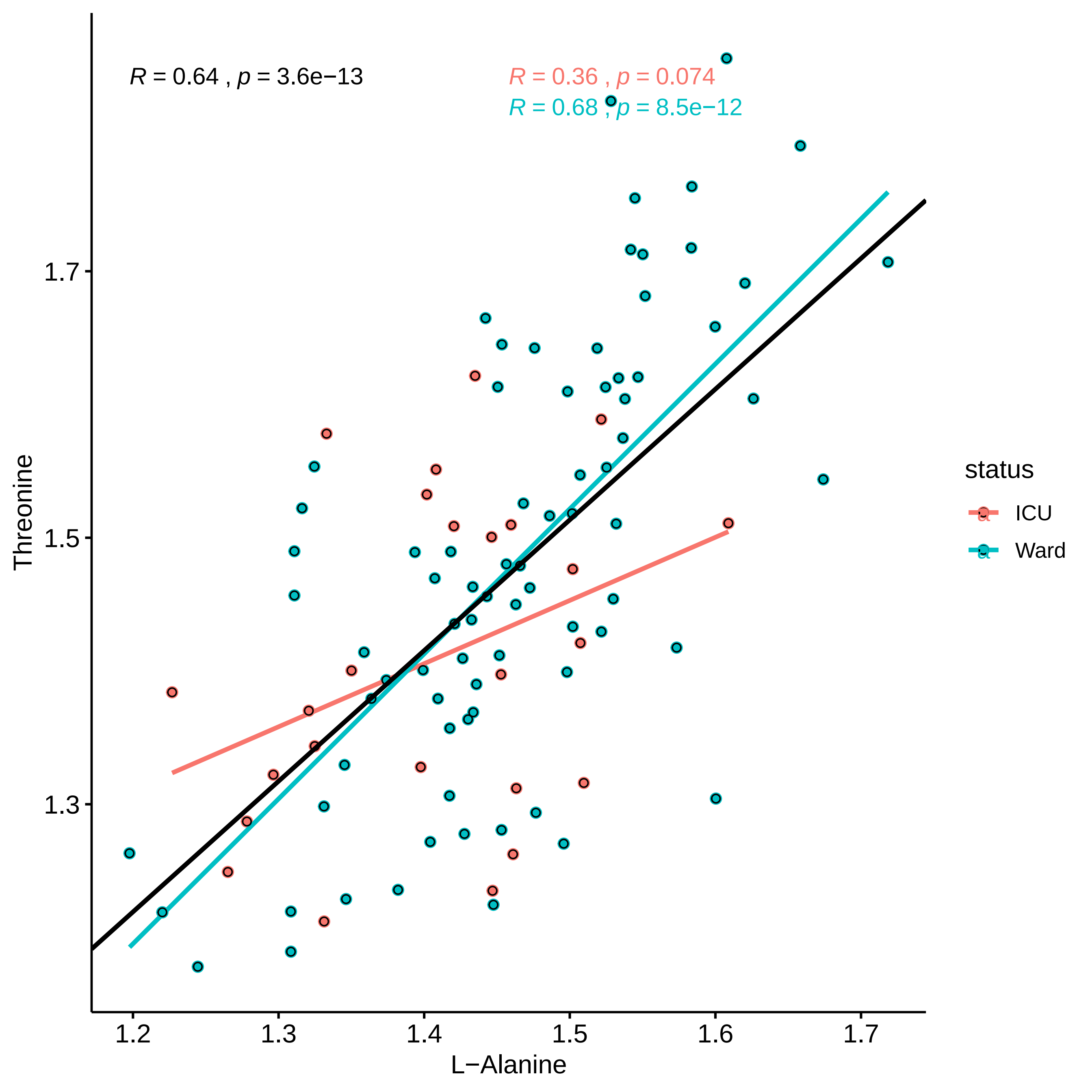

R = 0.64 , p = 3.6e−13
R = 0.36 , p = 0.074
R = 0.68 , p = 8.5e−12
Threonine
1.3
1.5
1.7
L−Alanine
1.2
1.3
1.4
1.5
1.6
1.7
status
ICU
Ward

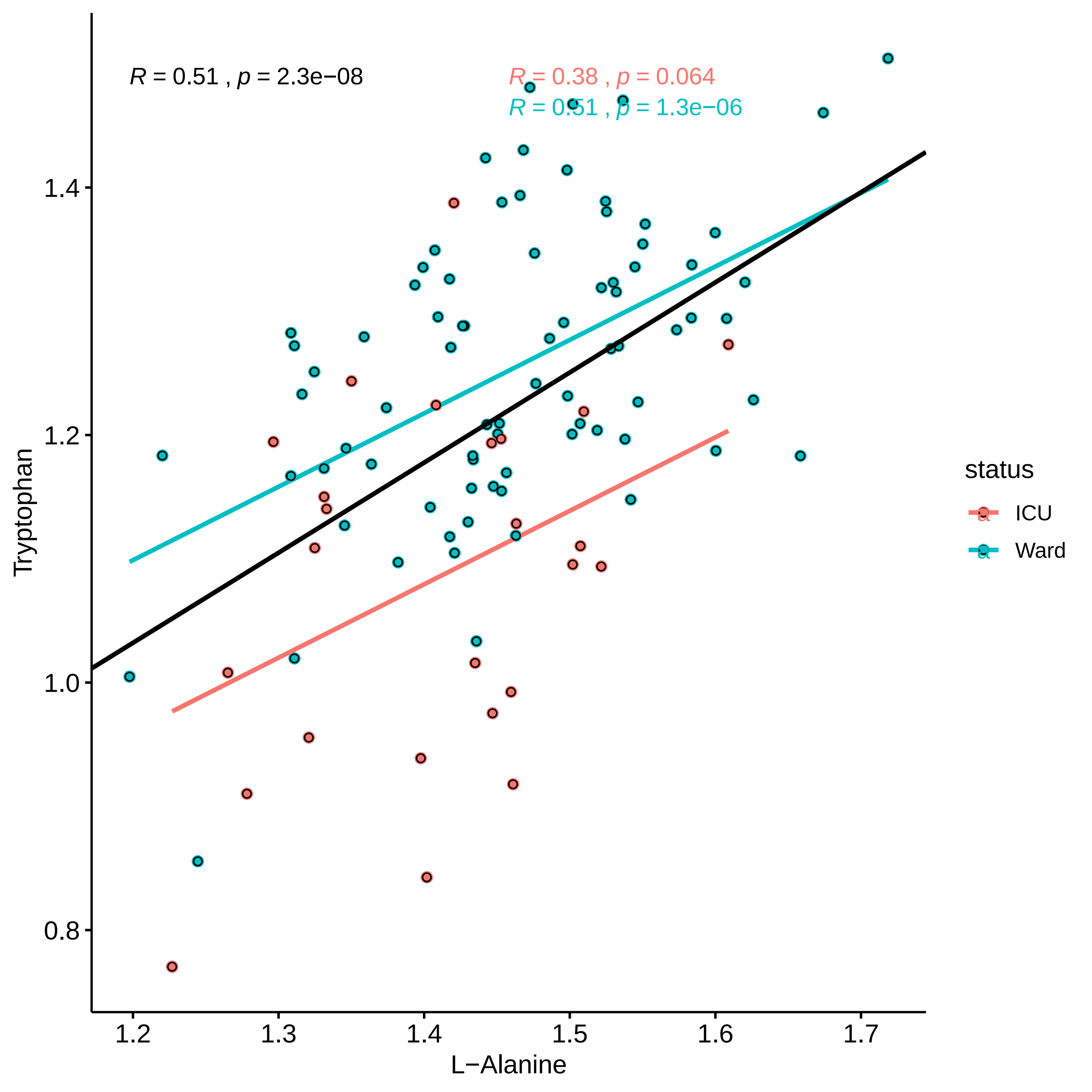

R = 0.51 , p = 2.3e−08
R = 0.38 , p = 0.064
R = 0.51 , p = 1.3e−06
Tryptophan
1.4
1.2
1.0
0.8
1.2
1.3
1.4
1.5
1.6
1.7
L−Alanine
status
ICU
Ward

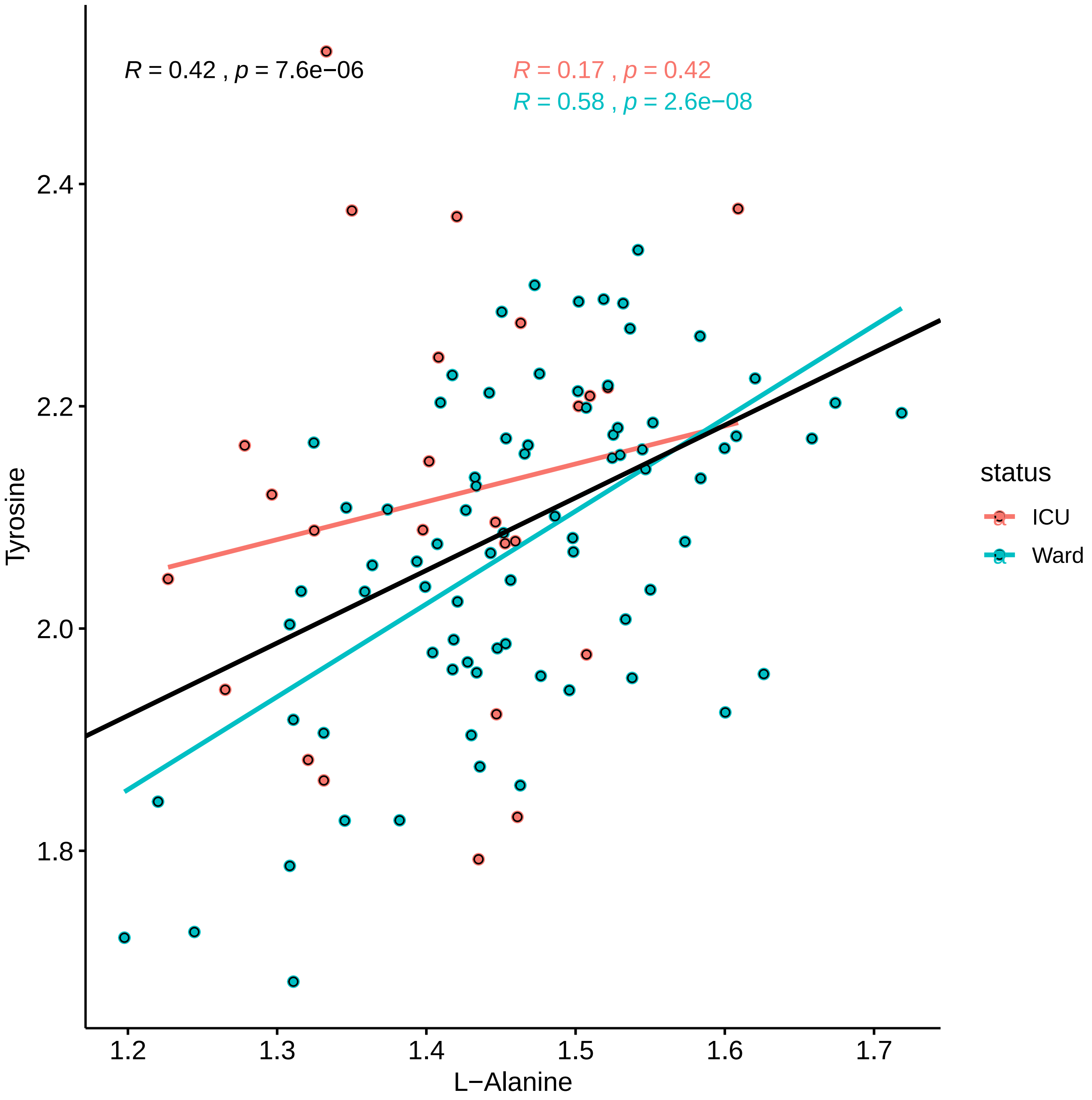

R = 0.42 , p = 7.6e−06
R = 0.17 , p = 0.42
R = 0.58 , p = 2.6e−08
Tyrosine
2.4
2.2
2.0
1.8
1.2
1.3
1.4
1.5
1.6
1.7
L−Alanine
status
ICU
Ward

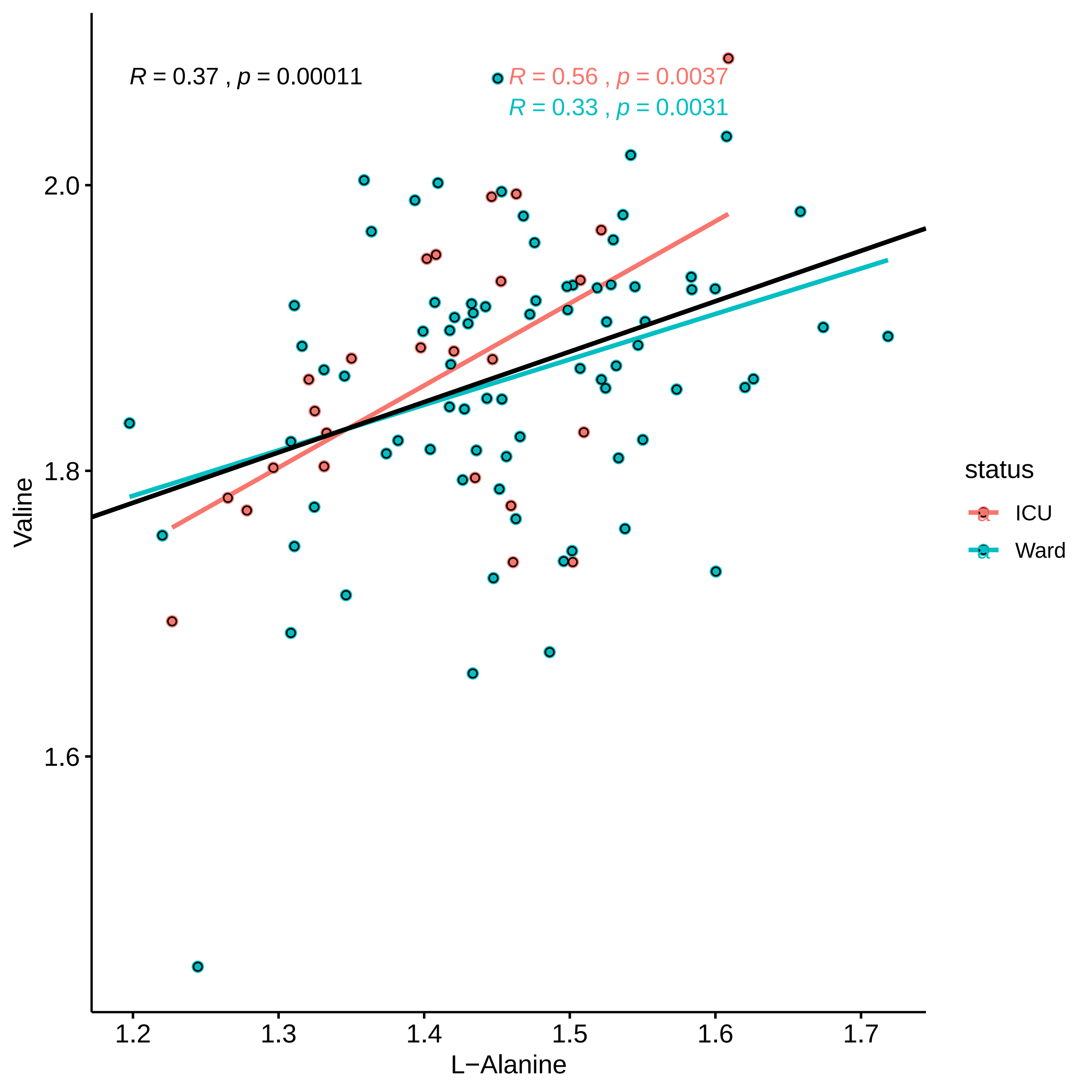

R = 0.37 , p = 0.00011
R = 0.56 , p = 0.0037
R = 0.33 , p = 0.0031
2.0
1.8
1.6
Valine
1.2
1.3
1.4
1.5
1.6
1.7
L−Alanine
status
ICU
Ward

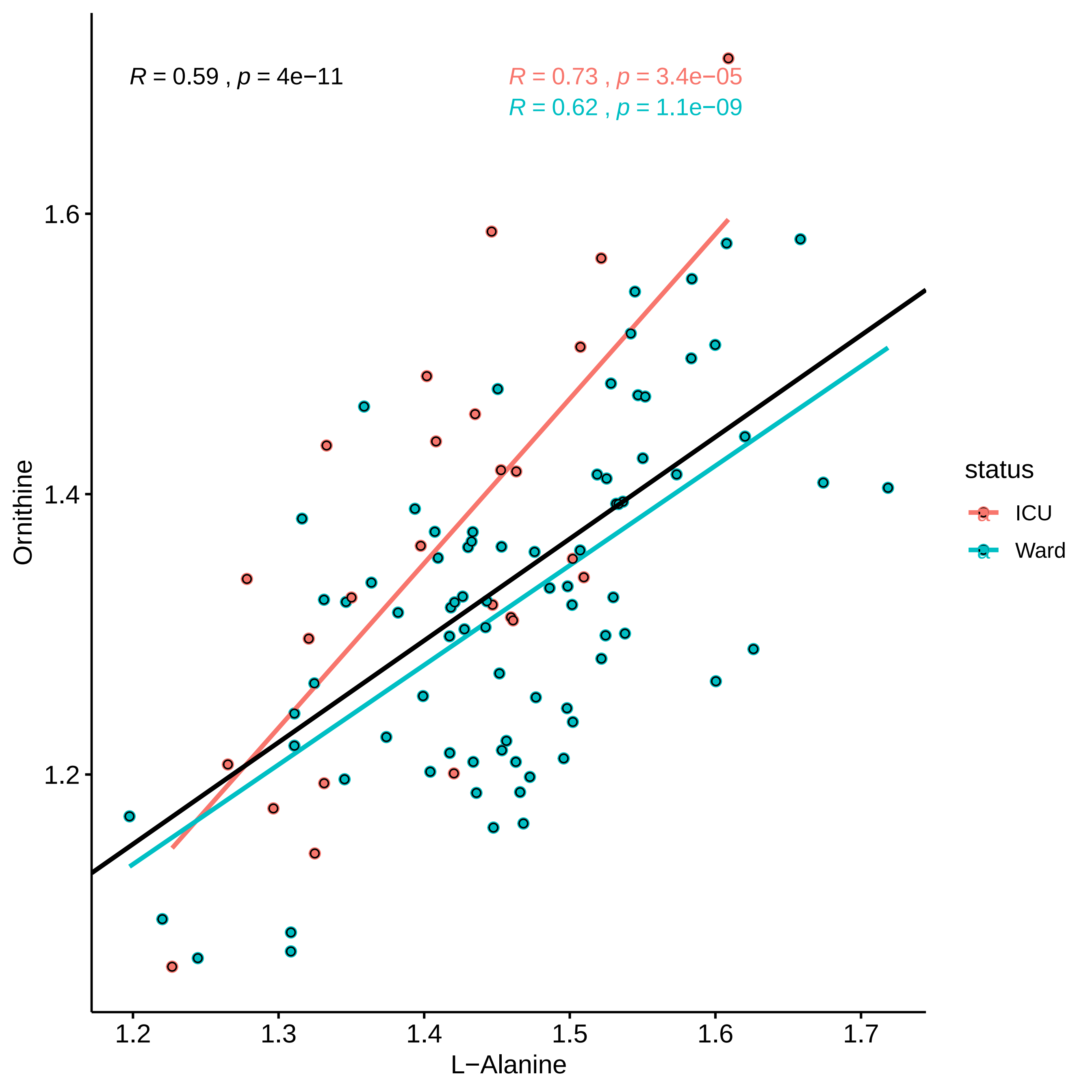
R = 0.59 , p = 4e−11
R = 0.73 , p = 3.4e−05
R = 0.62 , p = 1.1e−09
Ornithine
1.6
1.4
1.2
L−Alanine
1.2
1.3
1.4
1.5
1.6
1.7
status
ICU
Ward

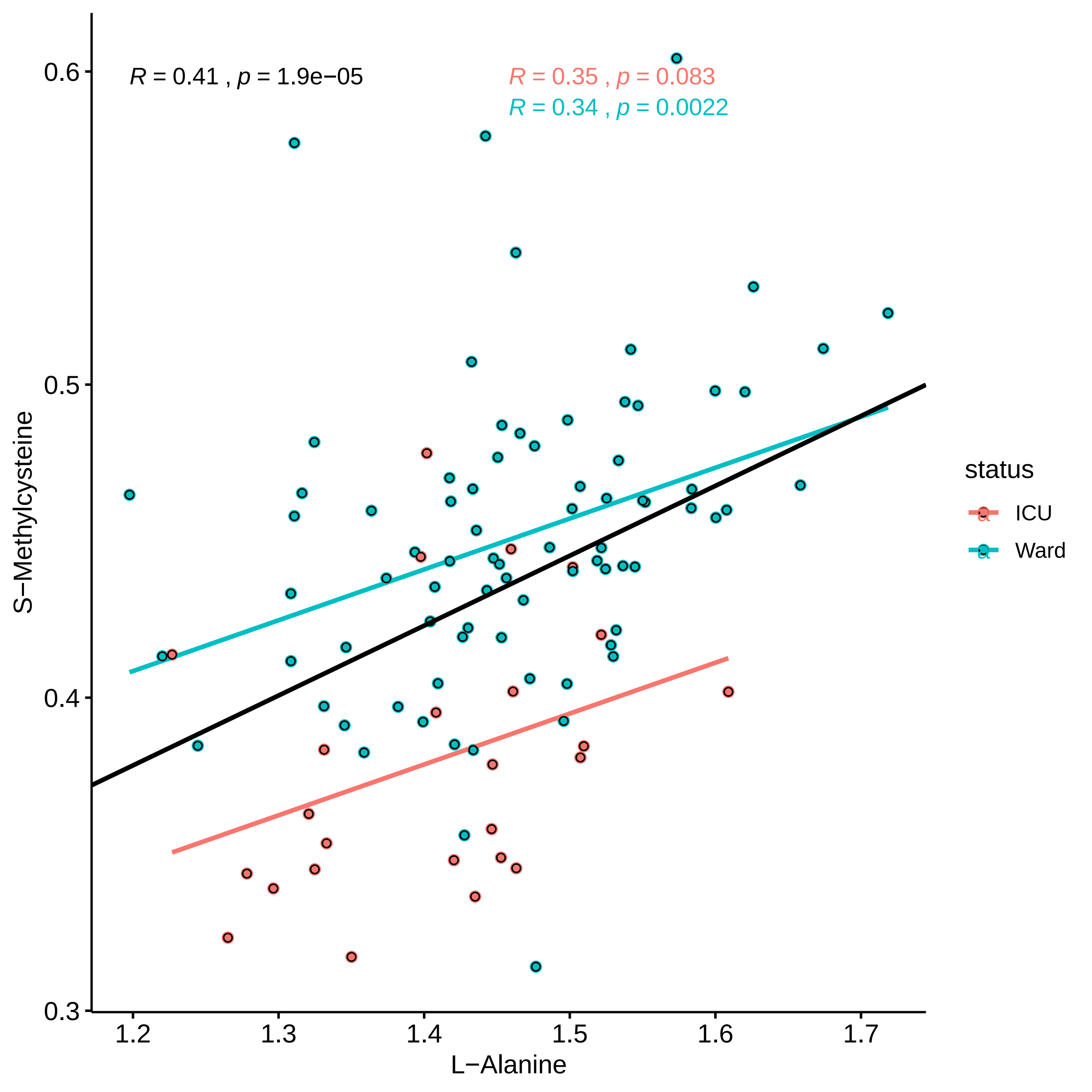

R = 0.41 , p = 1.9e−05
R = 0.35 , p = 0.083
R = 0.34 , p = 0.0022
S−Methylcysteine
L−Alanine
0.3
0.4
0.5
0.6
1.2
1.3
1.4
1.5
1.6
1.7
status
ICU
Ward

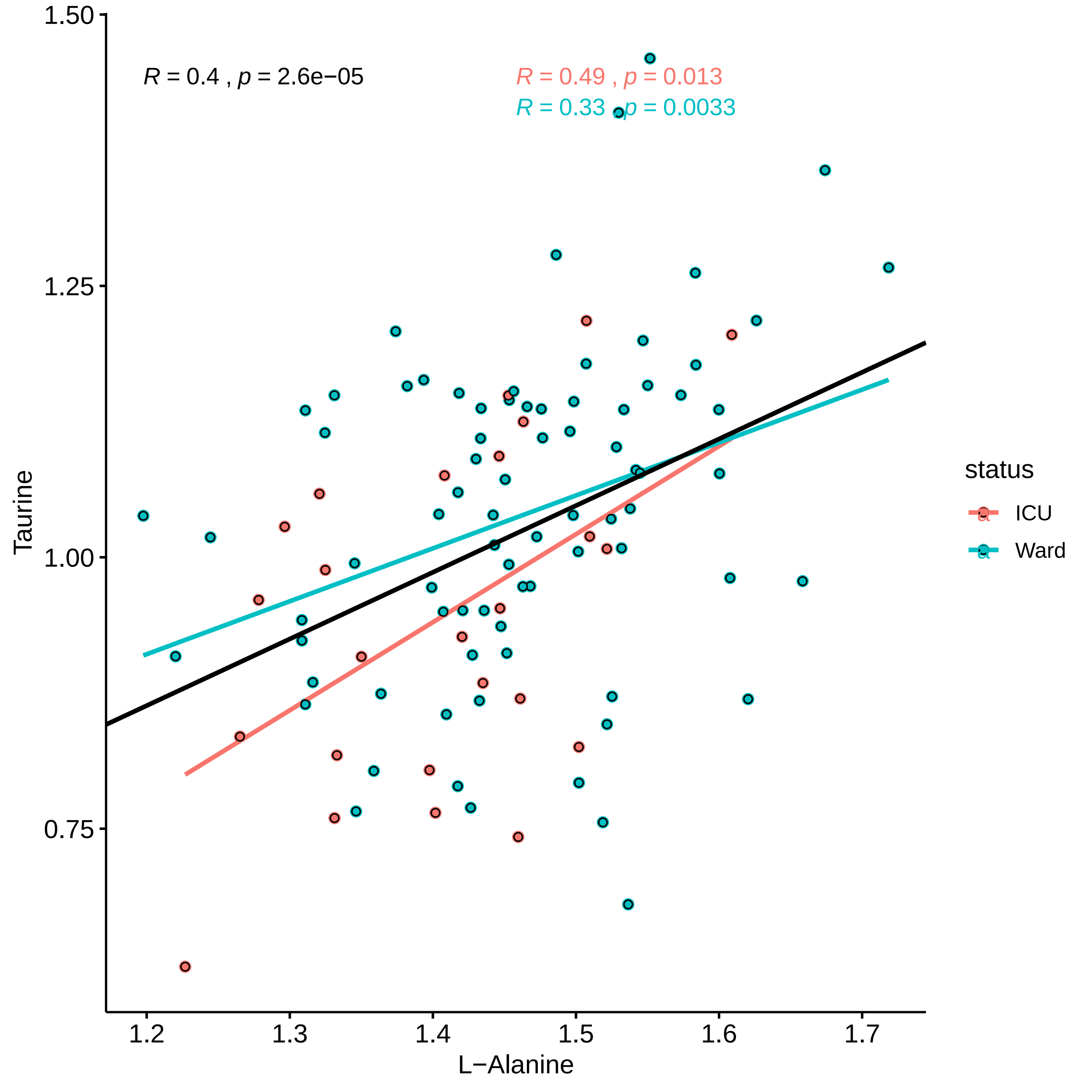
R = 0.4 , p = 2.6e−05
R = 0.49 , p = 0.013
R = 0.33 , p = 0.0033
Taurine
L−Alanine
1.50
1.25
1.00
0.75
1.2
1.3
1.4
1.5
1.6
1.7
status
ICU
Ward

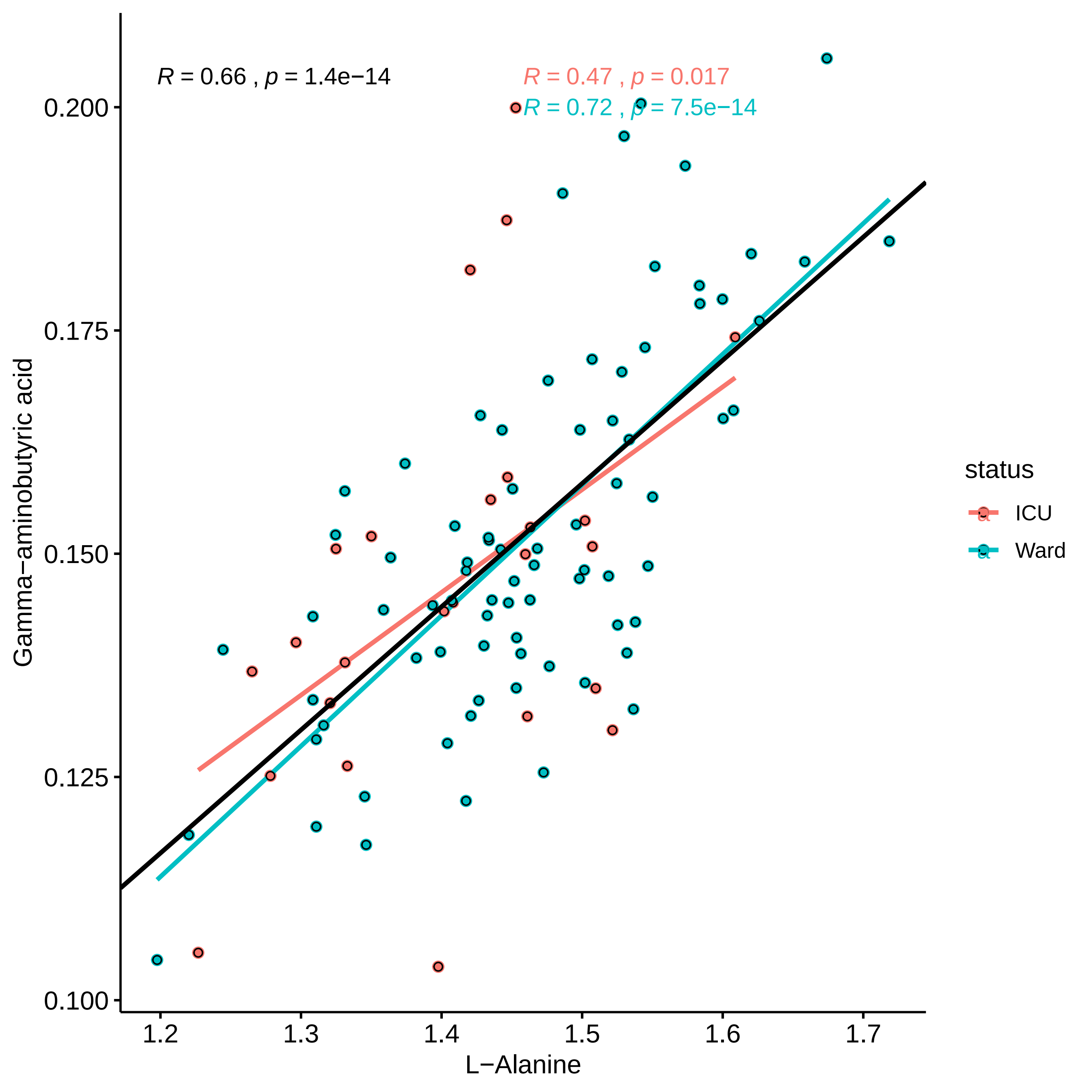

R = 0.66 , p = 1.4e−14
R = 0.47 , p = 0.017
R = 0.72 , p = 7.5e−14
Gamma−aminobutyric acid
0.200
0.175
0.150
0.125
0.100
1.2
1.3
1.4
1.5
1.6
1.7
L−Alanine
status
ICU
Ward

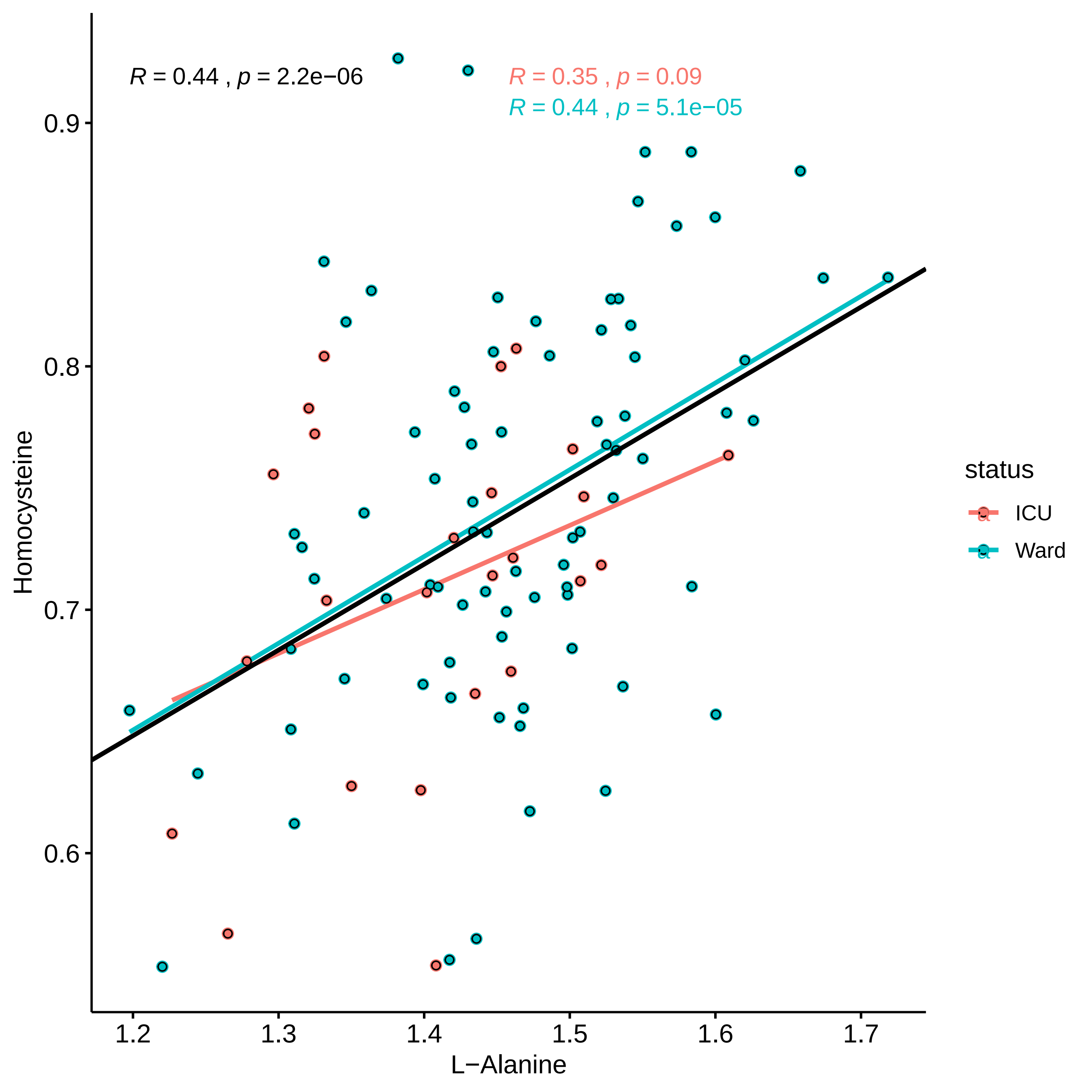

R = 0.44 , p = 2.2e−06
R = 0.35 , p = 0.09
R = 0.44 , p = 5.1e−05
0.9
0.8
0.7
0.6
Homocysteine
1.2
1.3
1.4
1.5
1.6
1.7
L−Alanine
status
ICU
Ward

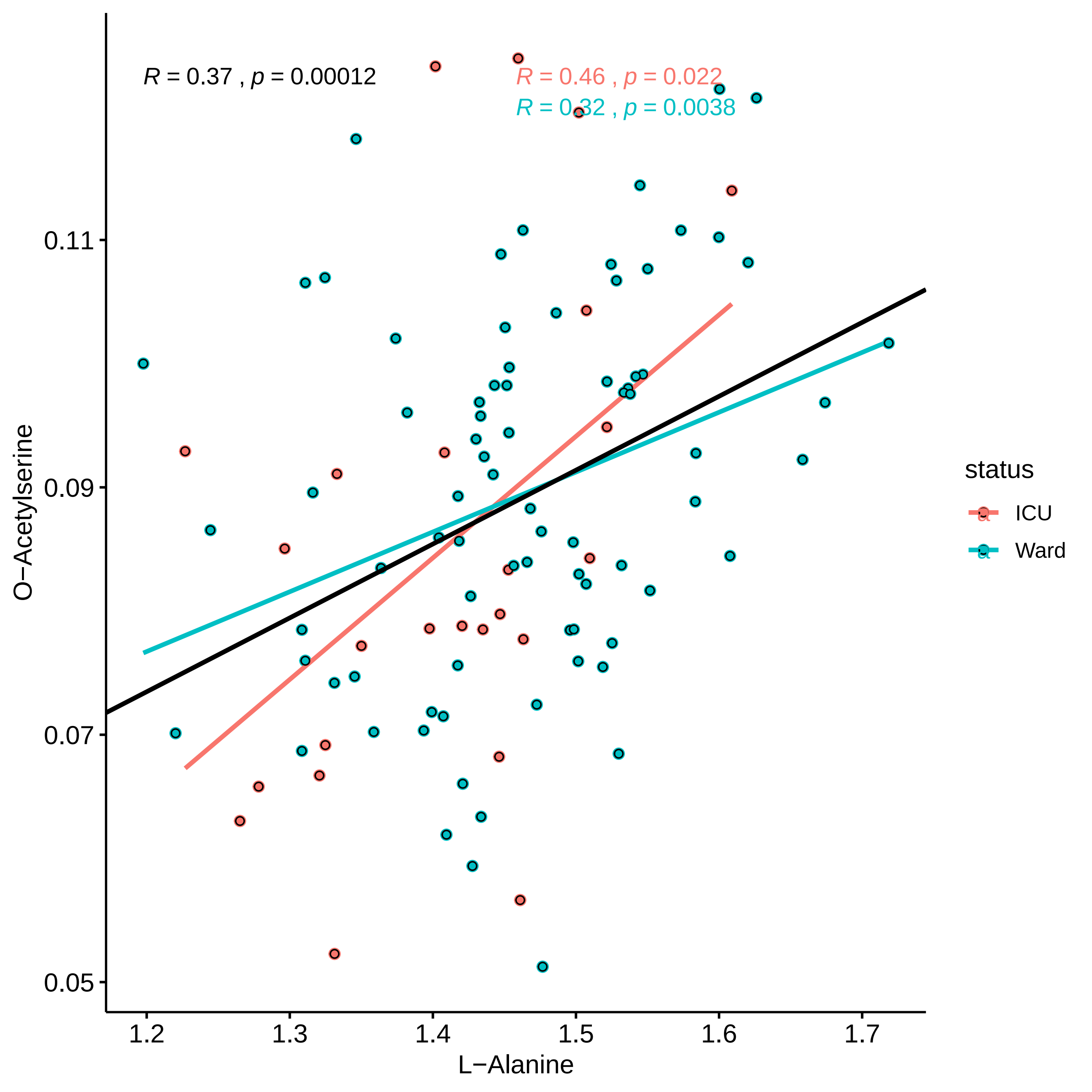

R = 0.37 , p = 0.00012
R = 0.46 , p = 0.022
R = 0.32 , p = 0.0038
O−Acetylserine
0.11
0.09
0.07
0.05
1.2
1.3
1.4
1.5
1.6
1.7
L−Alanine
status
ICU
Ward

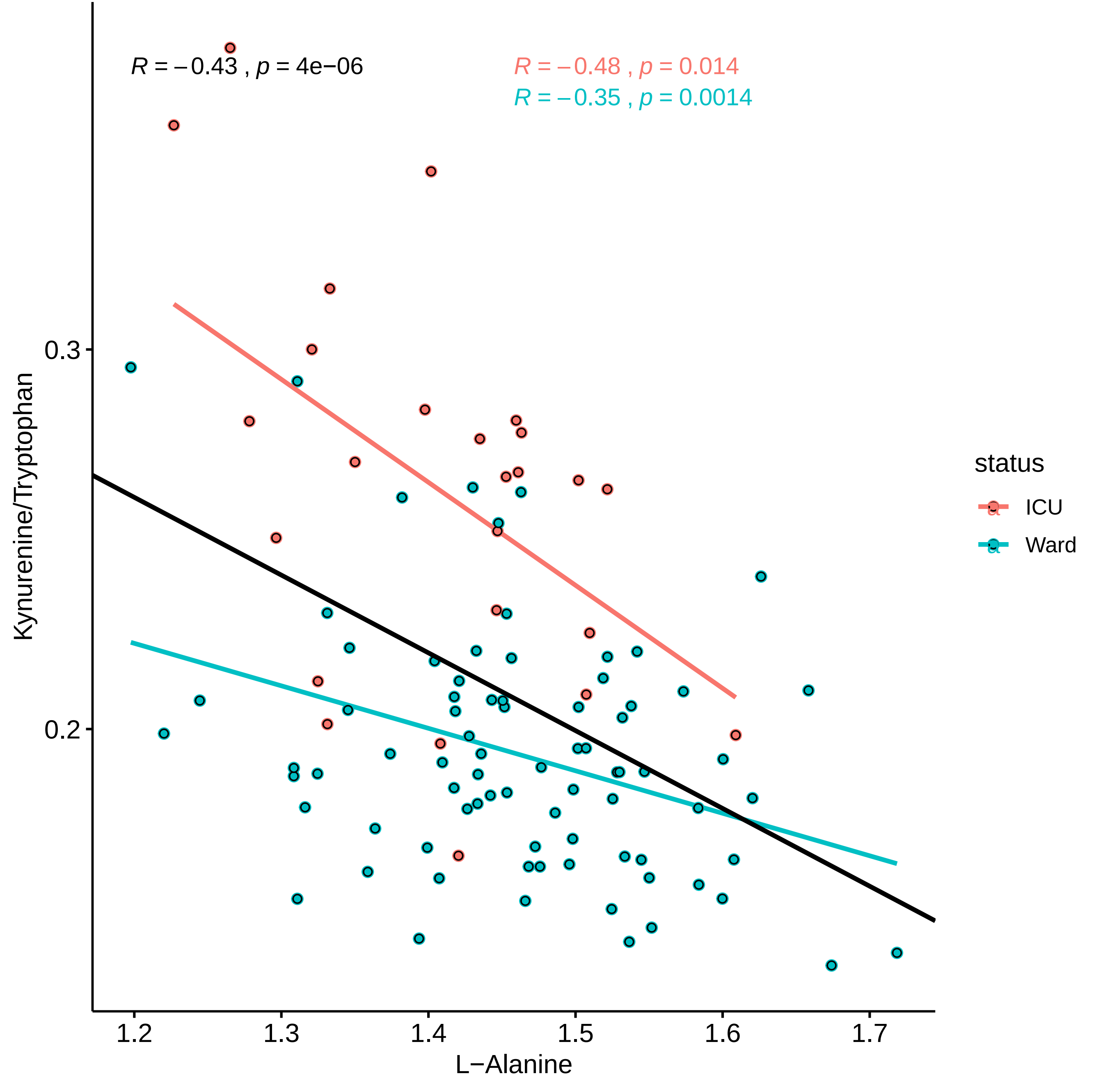

R = −0.43 , p = 4e−06
R = −0.48 , p = 0.014
R = −0.35 , p = 0.0014
Kynurenine/Tryptophan
0.3
0.2
1.2
1.3
1.4
1.5
1.6
1.7
L−Alanine
status
ICU
Ward

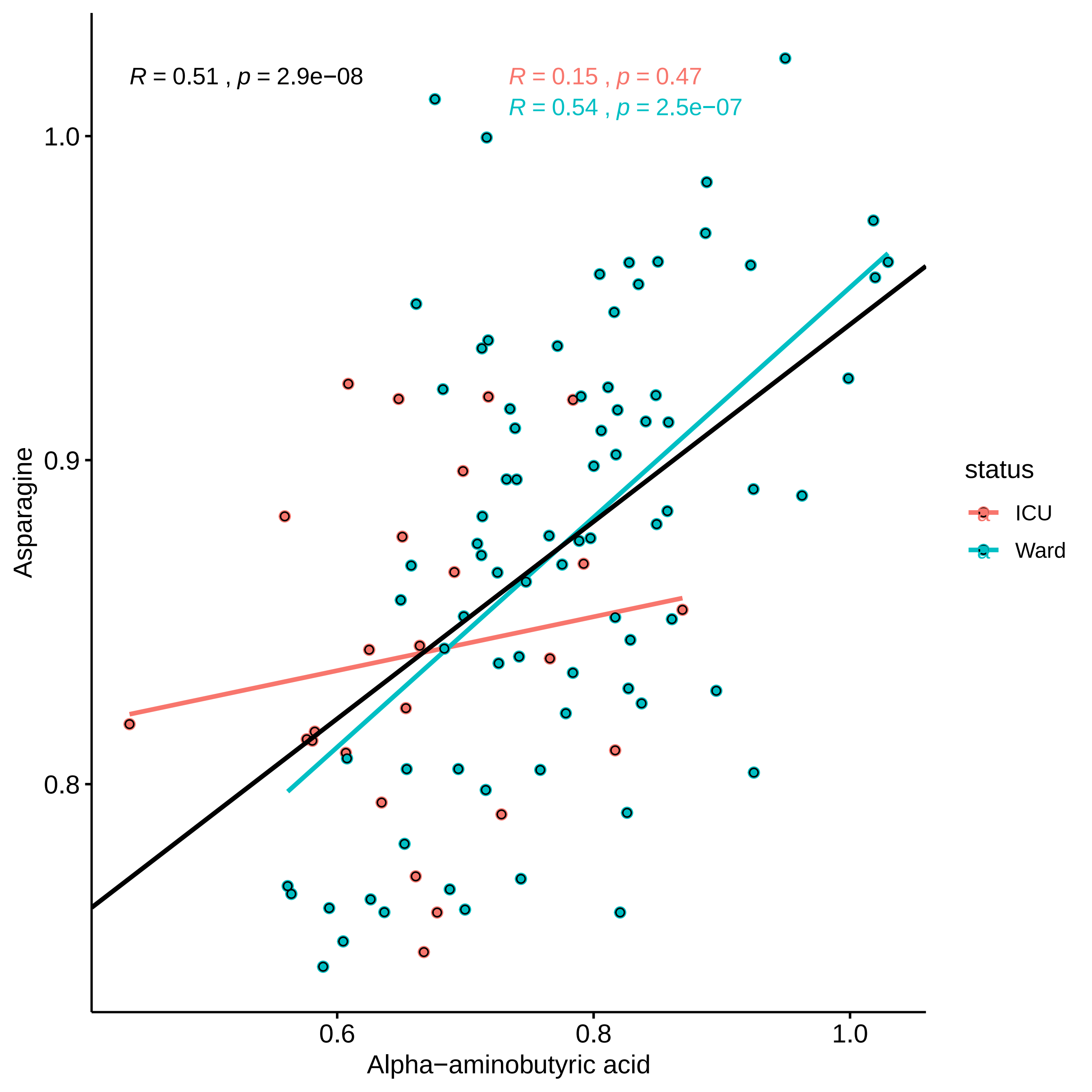

R = 0.51 , p = 2.9e−08
R = 0.15 , p = 0.47
R = 0.54 , p = 2.5e−07
Asparagine
1.0
0.9
0.8
0.6
0.8
1.0
Alpha−aminobutyric acid
status
ICU
Ward

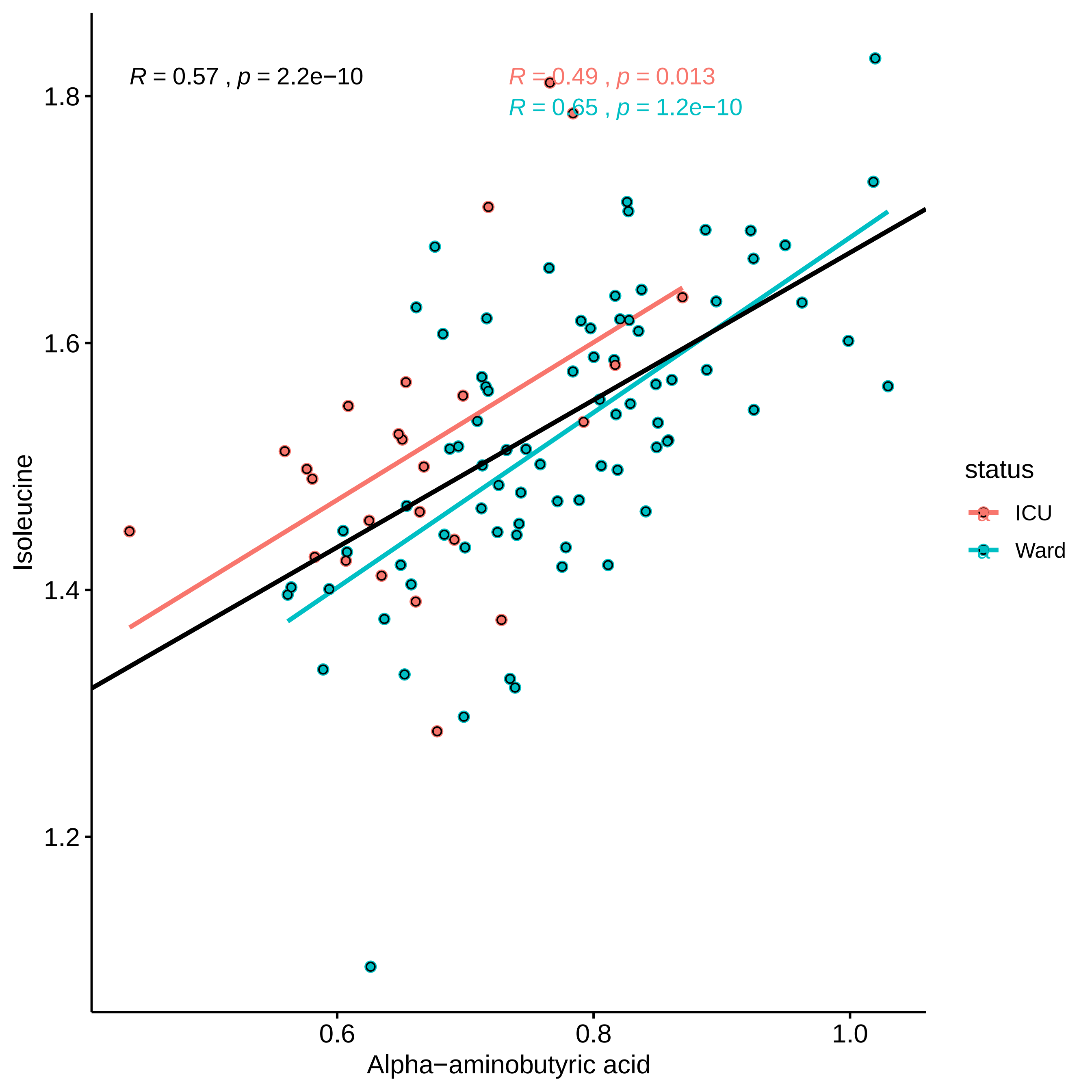

R = 0.57 , p = 2.2e−10
R = 0.49 , p = 0.013
R = 0.65 , p = 1.2e−10
1.8
1.6
1.4
1.2
0.6
0.8
1.0
Isoleucine
Alpha−aminobutyric acid
status
ICU
Ward

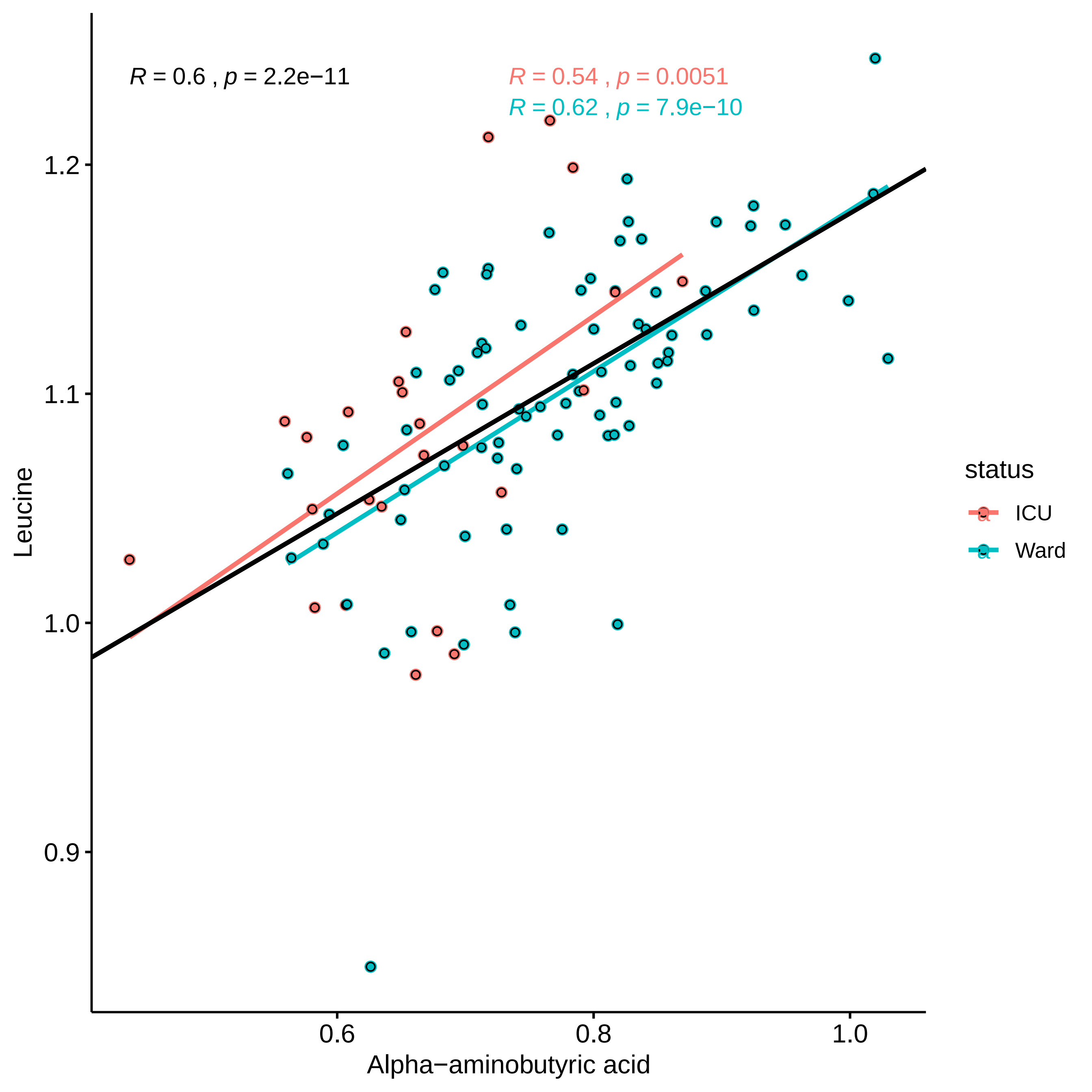
R = 0.6 , p = 2.2e−11
R = 0.54 , p = 0.0051
R = 0.62 , p = 7.9e−10
Leucine
1.2
1.1
1.0
0.9
0.6
0.8
1.0
Alpha−aminobutyric acid
status
ICU
Ward

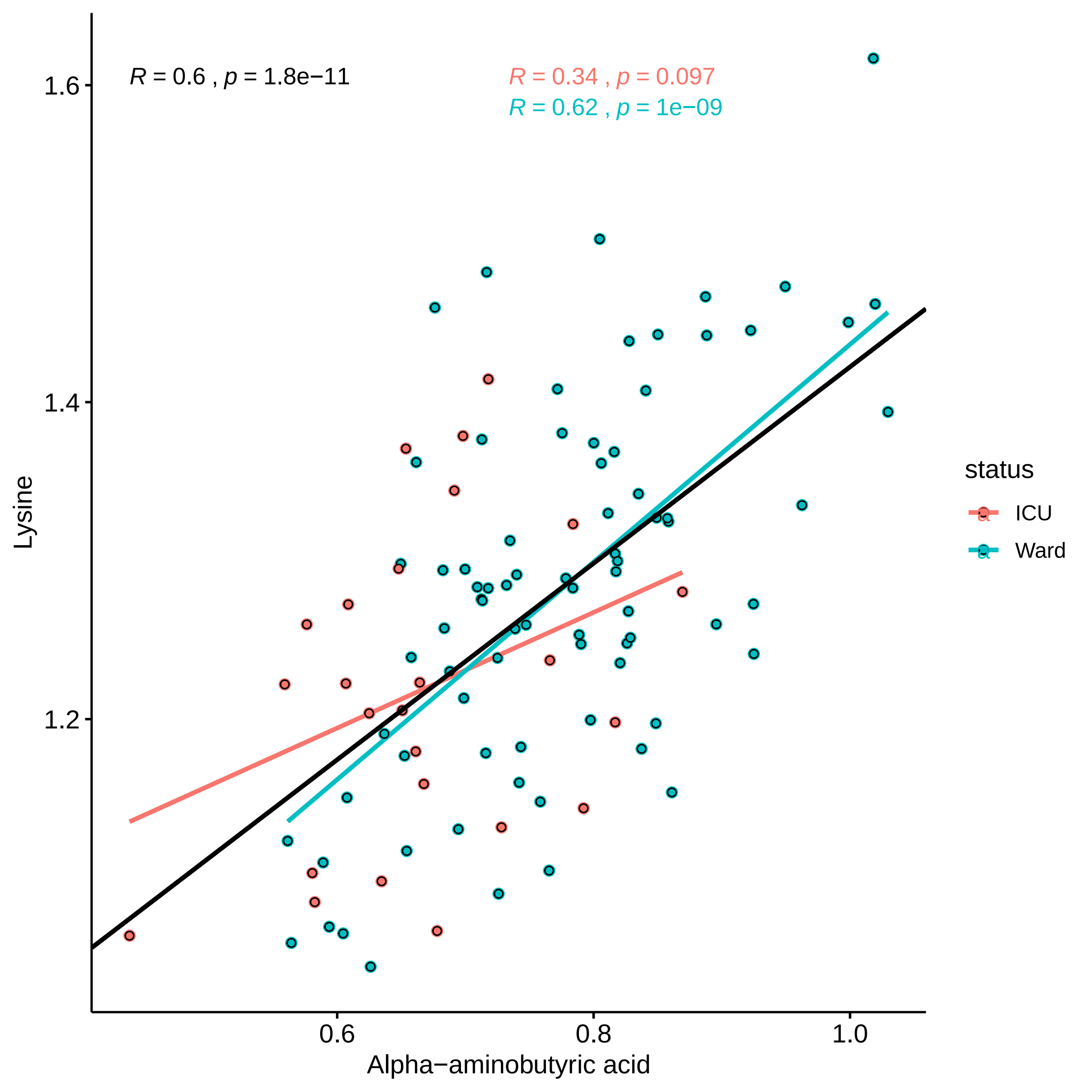

R = 0.6 , p = 1.8e−11
R = 0.34 , p = 0.097
R = 0.62 , p = 1e−09
1.6
1.4
1.2
Lysine
0.6
0.8
1.0
Alpha−aminobutyric acid
status
ICU
Ward

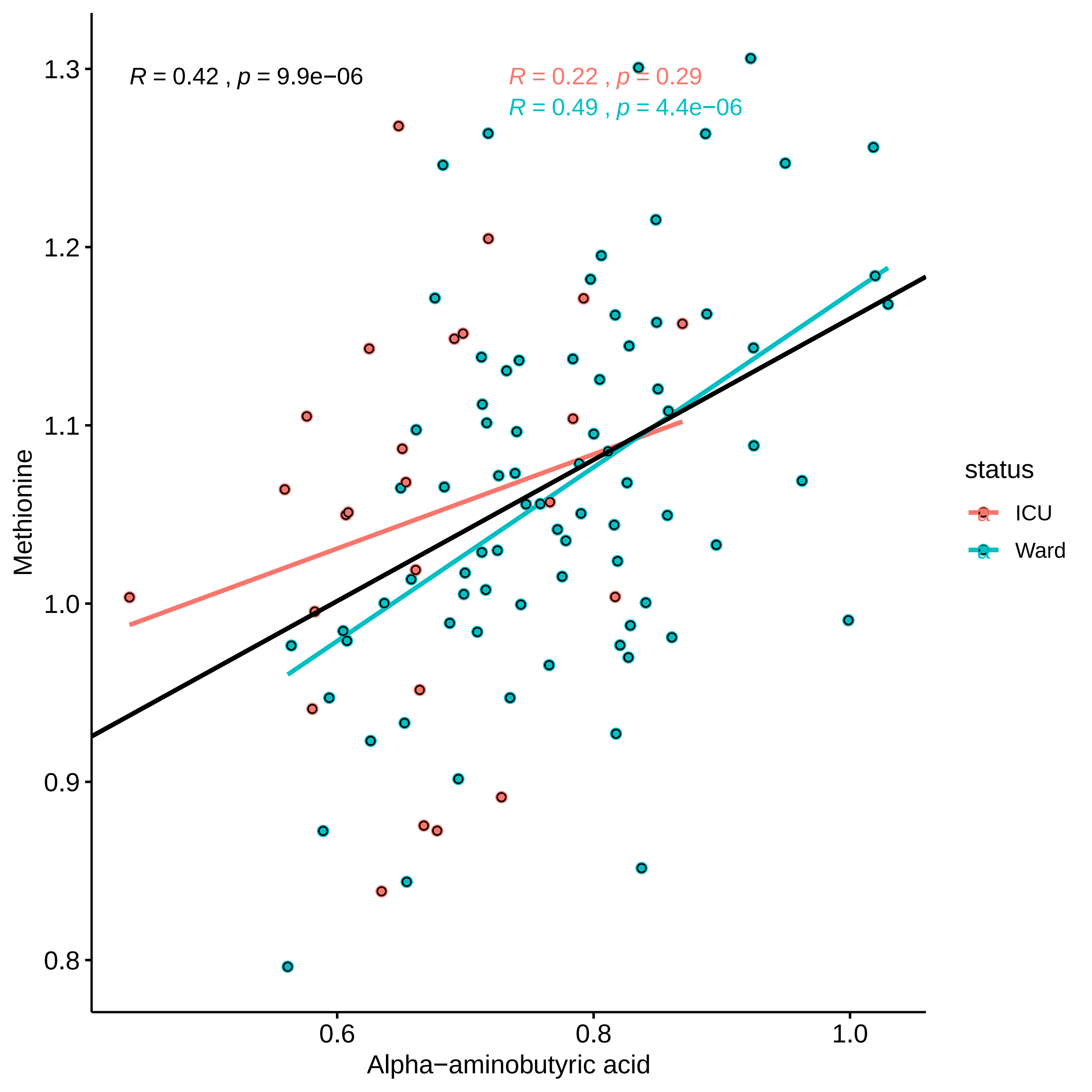

R = 0.42 , p = 9.9e−06
R = 0.22 , p = 0.29
R = 0.49 , p = 4.4e−06
1.3
1.2
1.1
1.0
0.9
0.8
Methionine
0.6
0.8
1.0
Alpha−aminobutyric acid
status
ICU
Ward

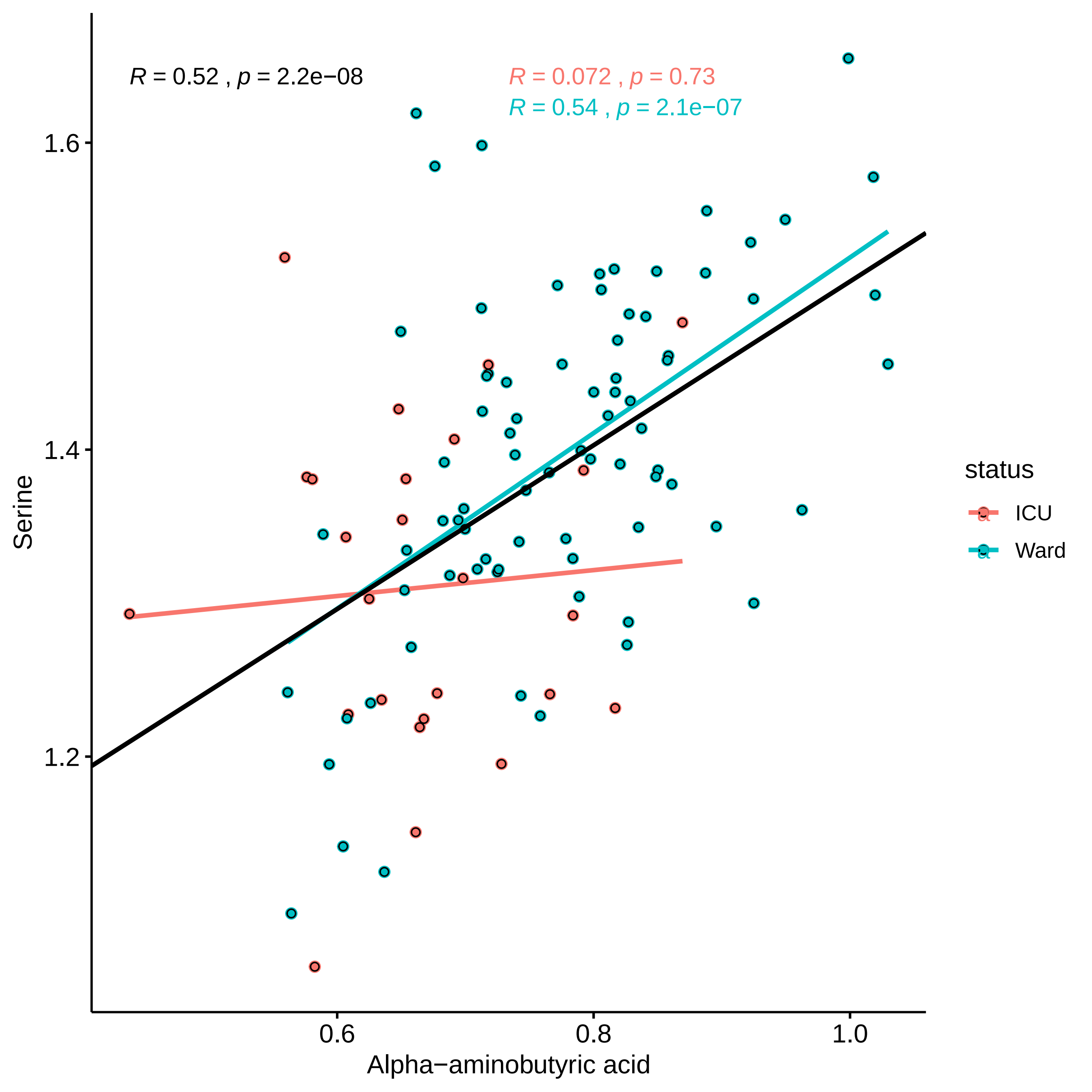
R = 0.52 , p = 2.2e−08
R = 0.072 , p = 0.73
R = 0.54 , p = 2.1e−07
1.6
1.4
1.2
Serine
0.6
0.8
1.0
Alpha−aminobutyric acid
status
ICU
Ward

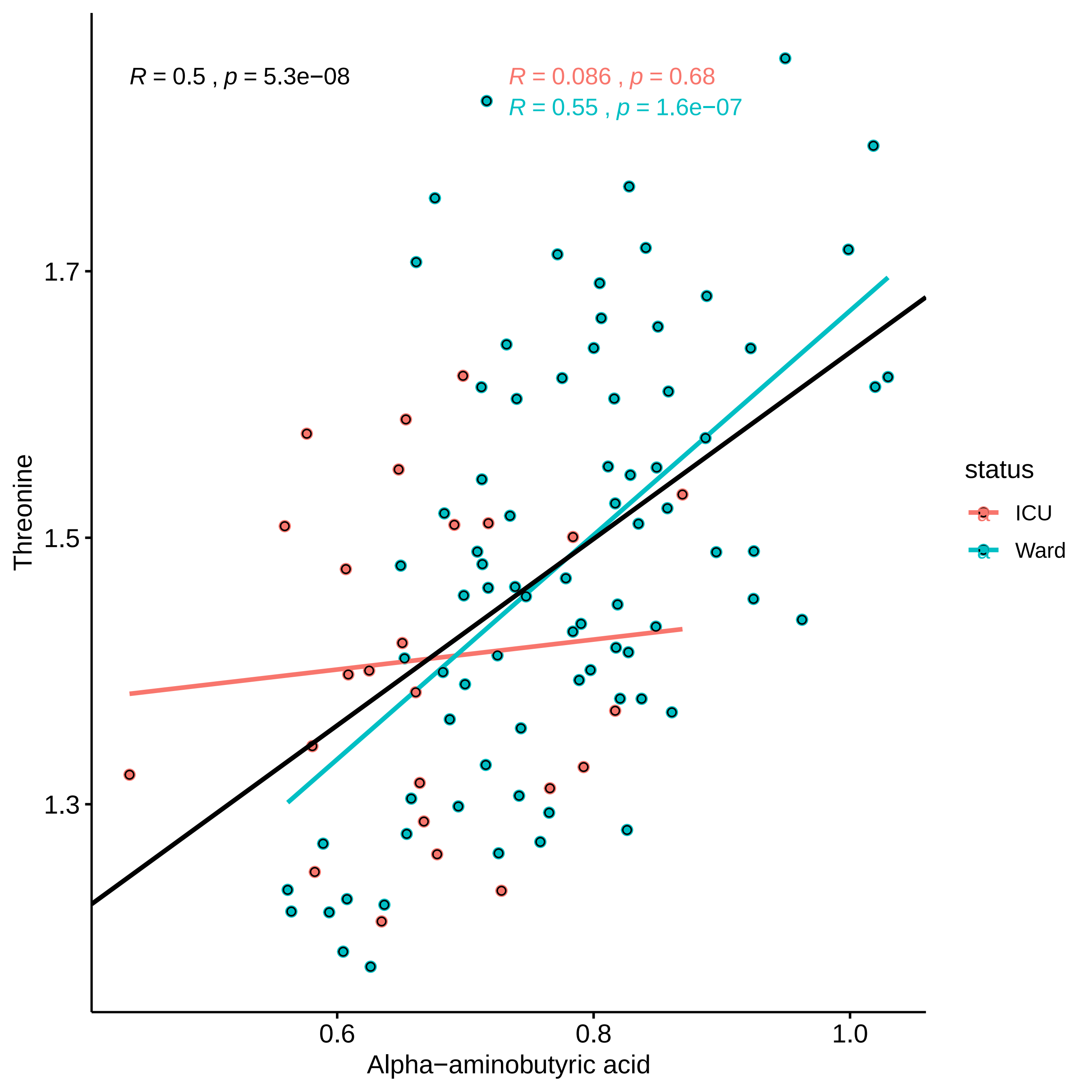

R = 0.5 , p = 5.3e−08
R = 0.086 , p = 0.68
R = 0.55 , p = 1.6e−07
Threonine
1.7
1.5
1.3
0.6
0.8
1.0
Alpha−aminobutyric acid
status
ICU
Ward

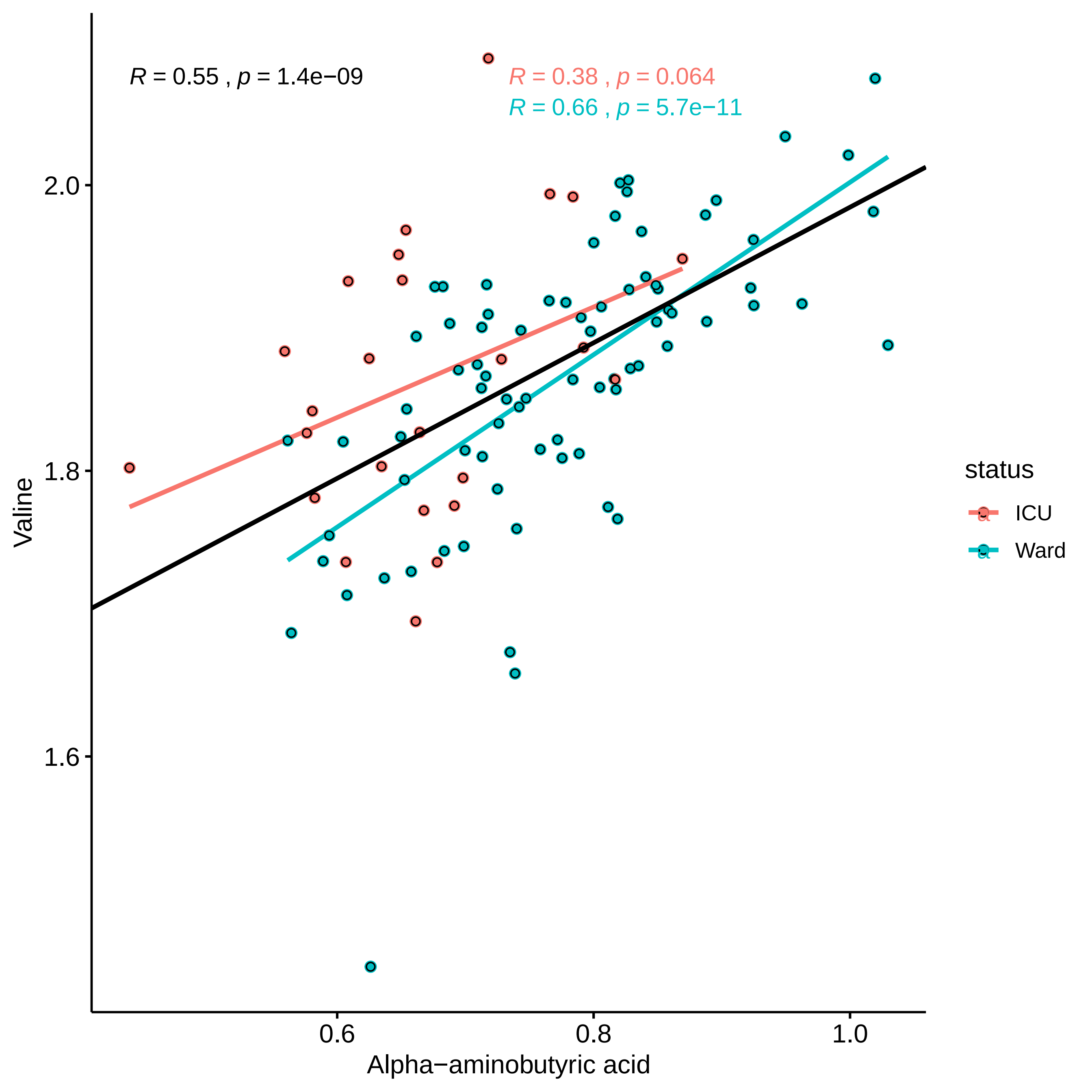

R = 0.55 , p = 1.4e−09
R = 0.38 , p = 0.064
R = 0.66 , p = 5.7e−11
2.0
1.8
1.6
Valine
0.6
0.8
1.0
Alpha−aminobutyric acid
status
ICU
Ward

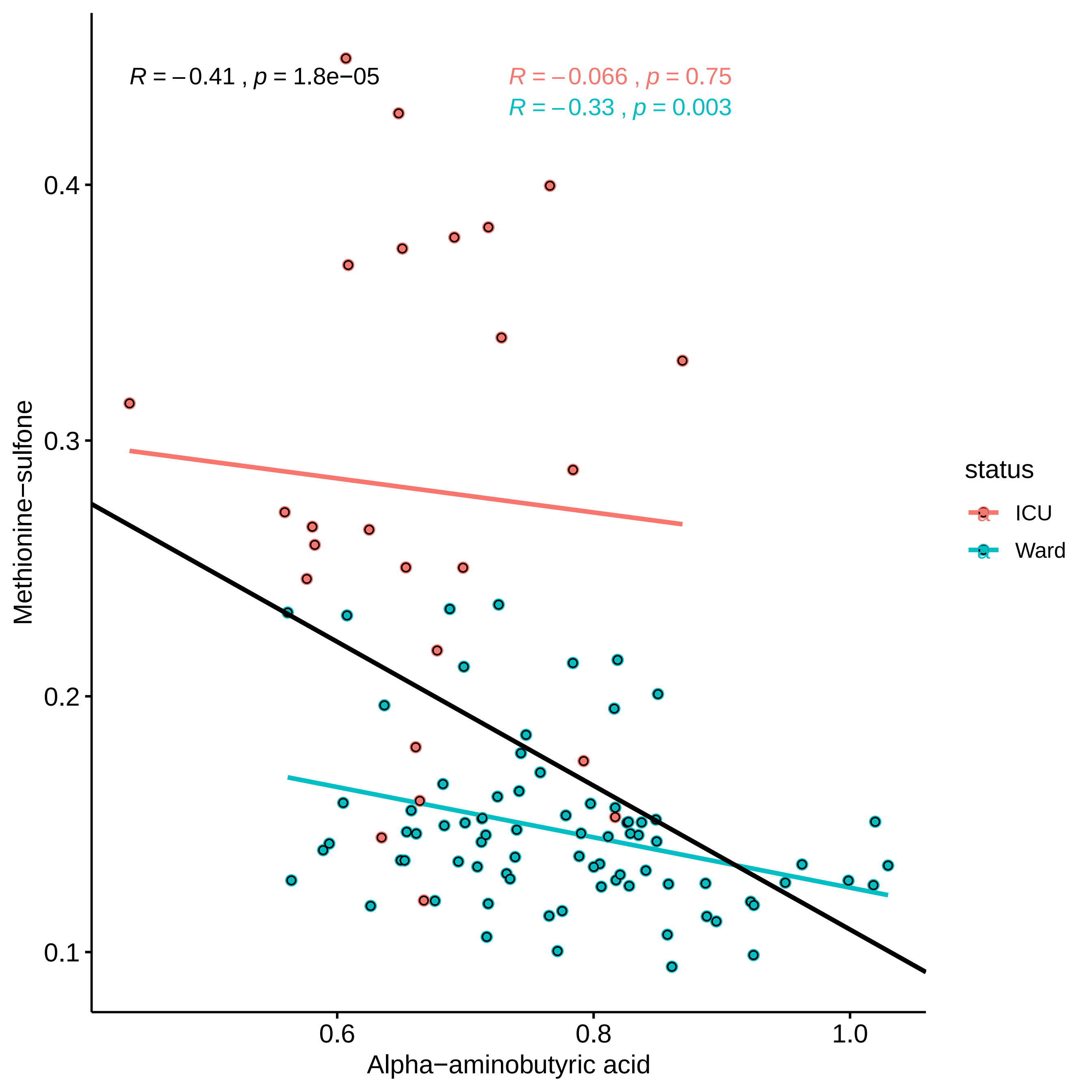

R = −0.41 , p = 1.8e−05
R = −0.066 , p = 0.75
R = −0.33 , p = 0.003
0.4
0.3
0.2
0.1
Methionine−sulfone
0.6
0.8
1.0
Alpha−aminobutyric acid
status
ICU
Ward

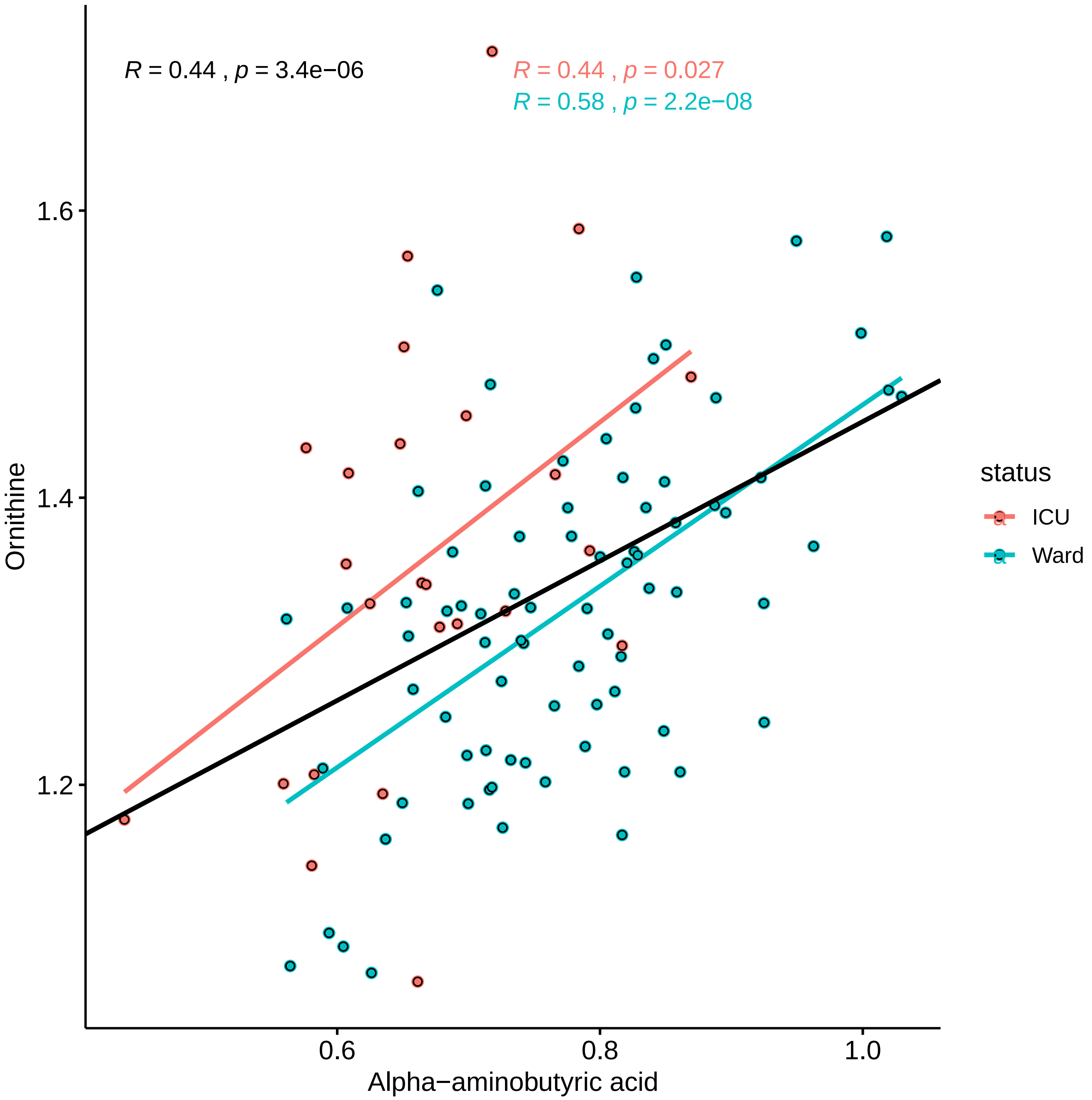

R = 0.44 , p = 3.4e−06
R = 0.44 , p = 0.027
R = 0.58 , p = 2.2e−08
1.6
1.4
1.2
0.6
0.8
1.0
Ornithine
Alpha−aminobutyric acid
status
ICU
Ward

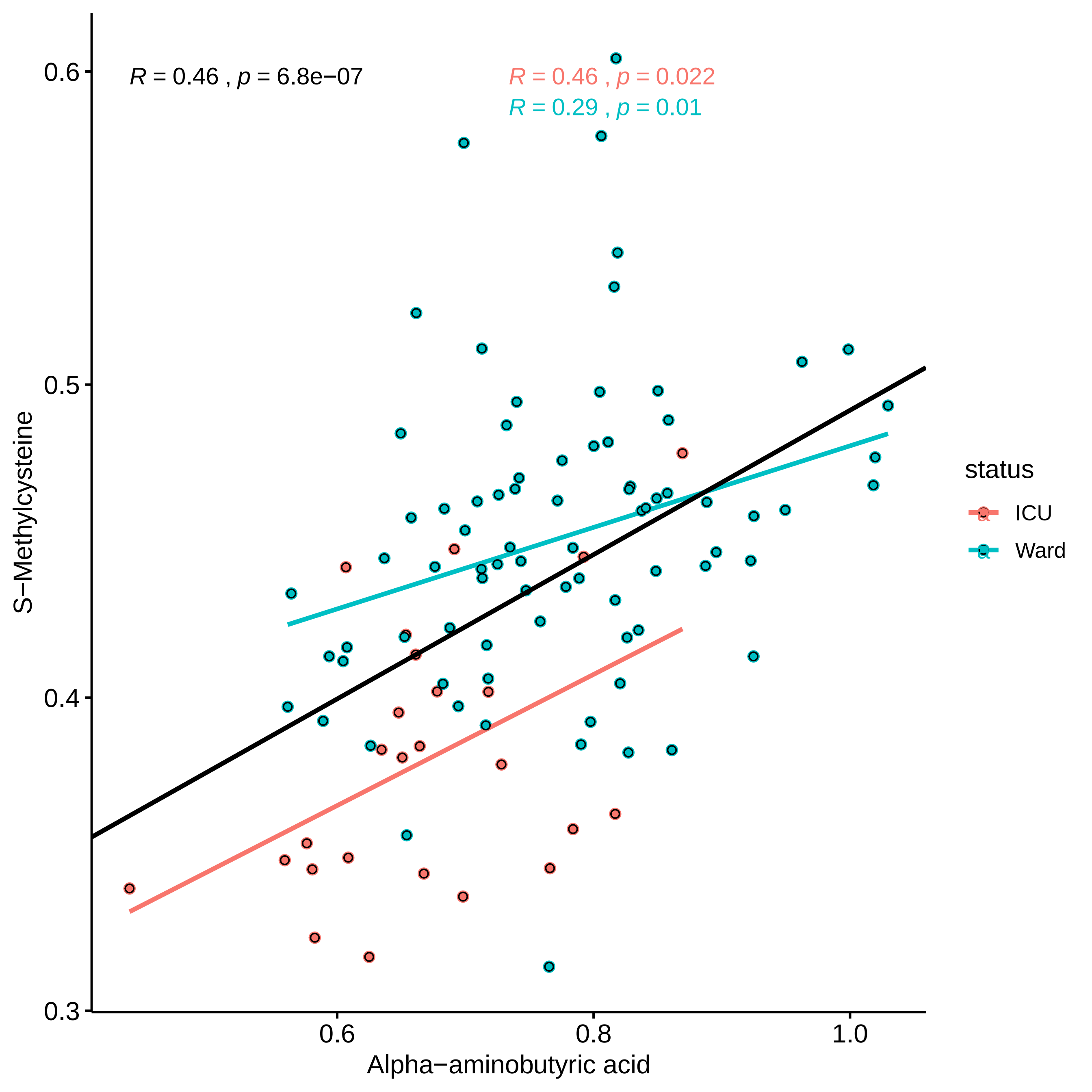
R = 0.46 , p = 6.8e−07
R = 0.46 , p = 0.022
R = 0.29 , p = 0.01
S−Methylcysteine
0.6
0.5
0.4
0.3
0.6
0.8
1.0
Alpha−aminobutyric acid
status
ICU
Ward

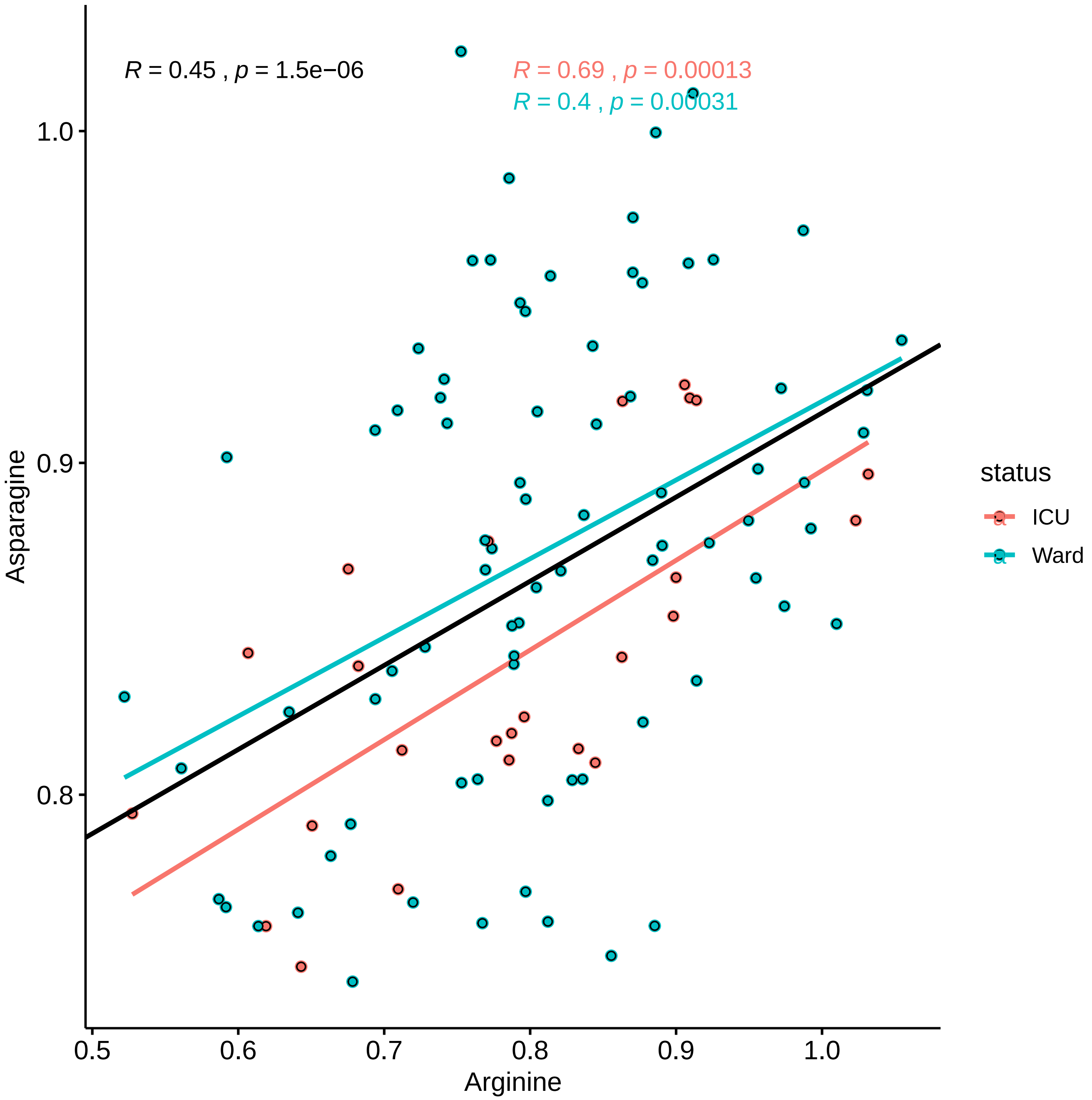

R = 0.45 , p = 1.5e−06
R = 0.69 , p = 0.00013
R = 0.4 , p = 0.00031
1.0
0.9
0.8
Asparagine
0.5
0.6
0.7
0.8
0.9
1.0
Arginine
status
ICU
Ward

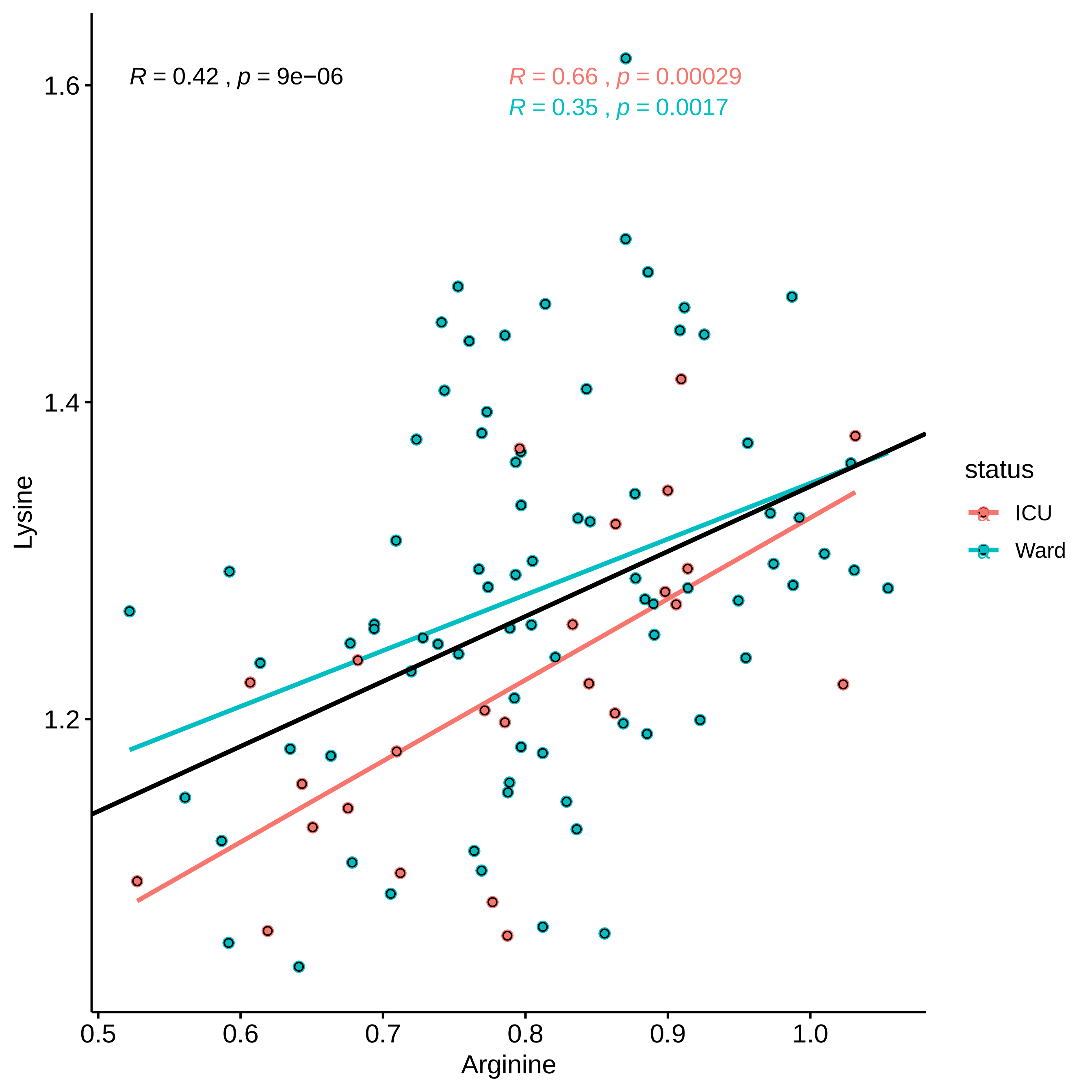

R = 0.42 , p = 9e−06
R = 0.66 , p = 0.00029
R = 0.35 , p = 0.0017
1.6
1.4
1.2
Lysine
0.5
0.6
0.7
0.8
0.9
1.0
Arginine
status
ICU
Ward

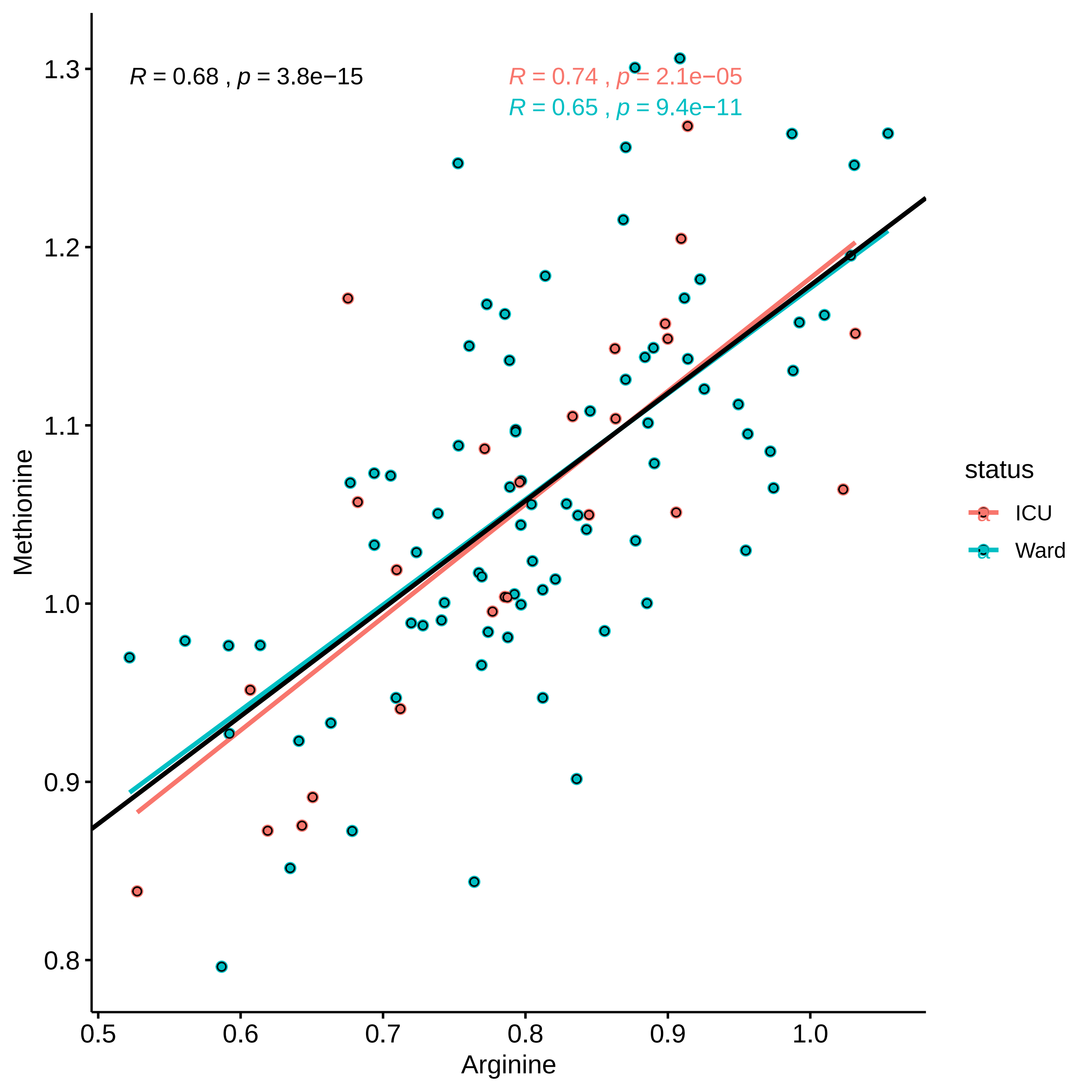

R = 0.68 , p = 3.8e−15
R = 0.74 , p = 2.1e−05
R = 0.65 , p = 9.4e−11
Methionine
1.3
1.2
1.1
1.0
0.9
0.8
0.5
0.6
0.7
0.8
0.9
1.0
Arginine
status
ICU
Ward

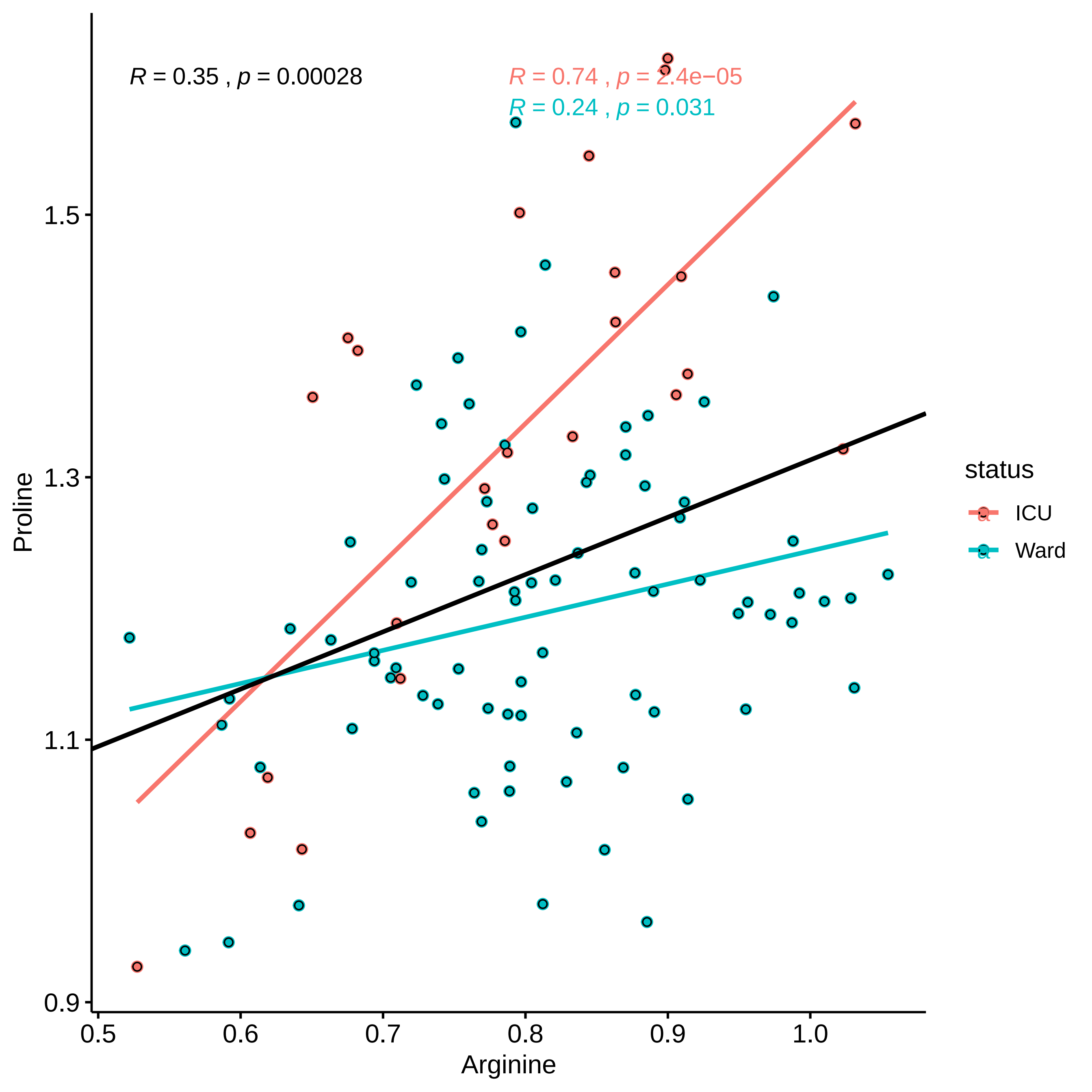

R = 0.35 , p = 0.00028
R = 0.74 , p = 2.4e−05
R = 0.24 , p = 0.031
Proline
1.5
1.3
1.1
0.9
0.5
0.6
0.7
0.8
0.9
1.0
Arginine
status
ICU
Ward

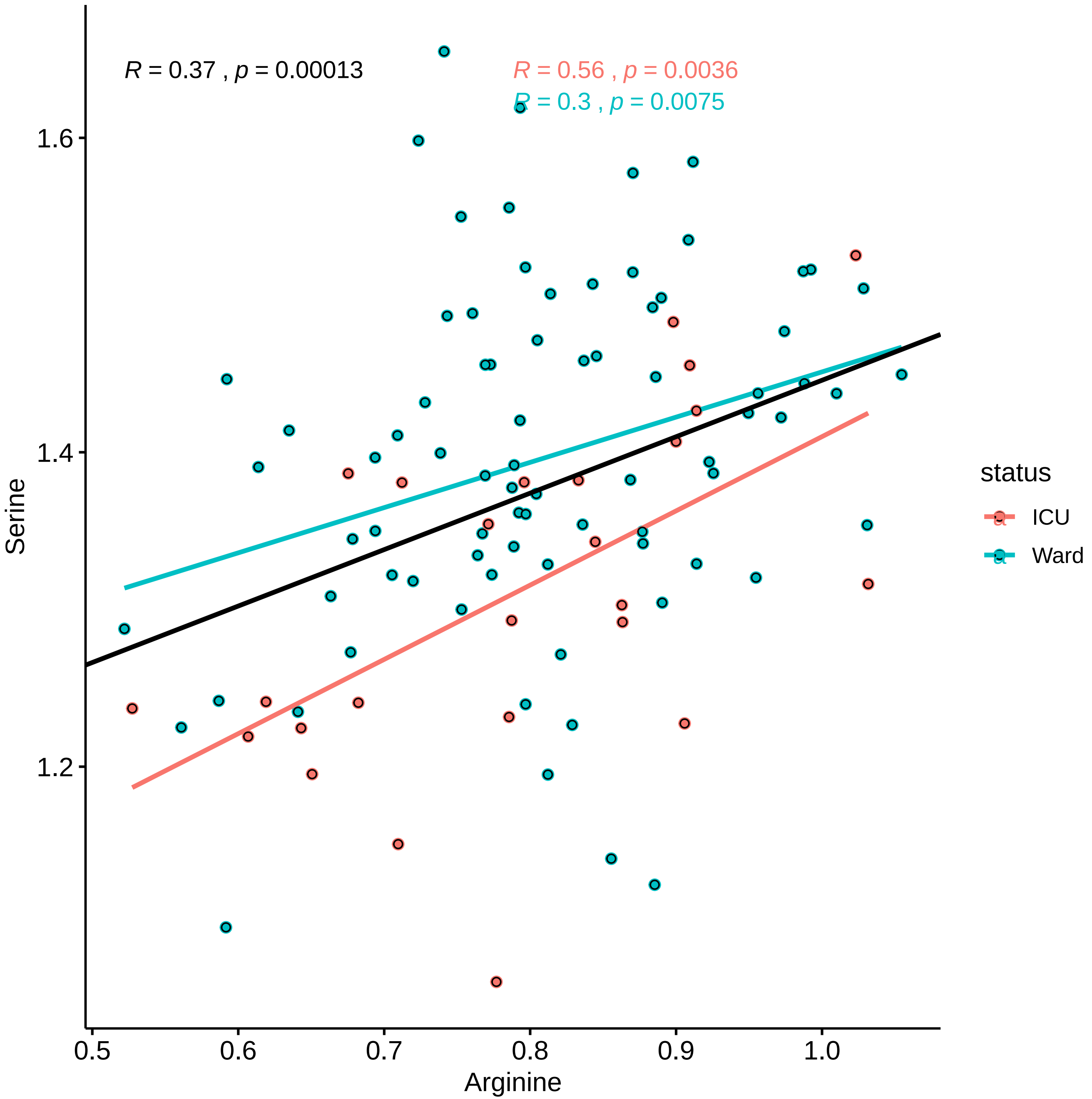

R = 0.37 , p = 0.00013
R = 0.56 , p = 0.0036
R = 0.3 , p = 0.0075
Serine
1.6
1.4
1.2
0.5
0.6
0.7
0.8
0.9
1.0
Arginine
status
ICU
Ward

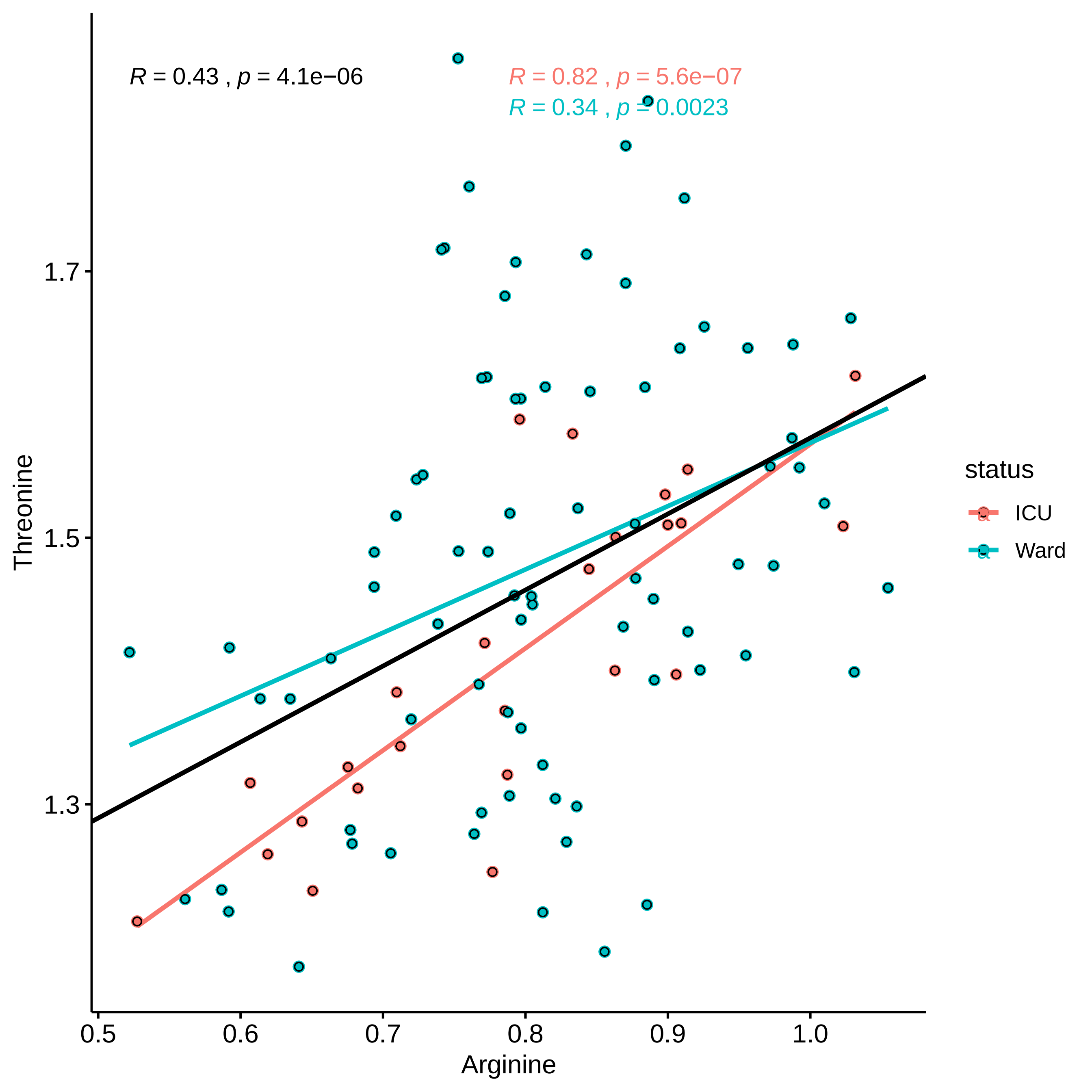

R = 0.43 , p = 4.1e−06
R = 0.82 , p = 5.6e−07
R = 0.34 , p = 0.0023
Threonine
1.7
1.5
1.3
0.5
0.6
0.7
0.8
0.9
1.0
Arginine
status
ICU
Ward

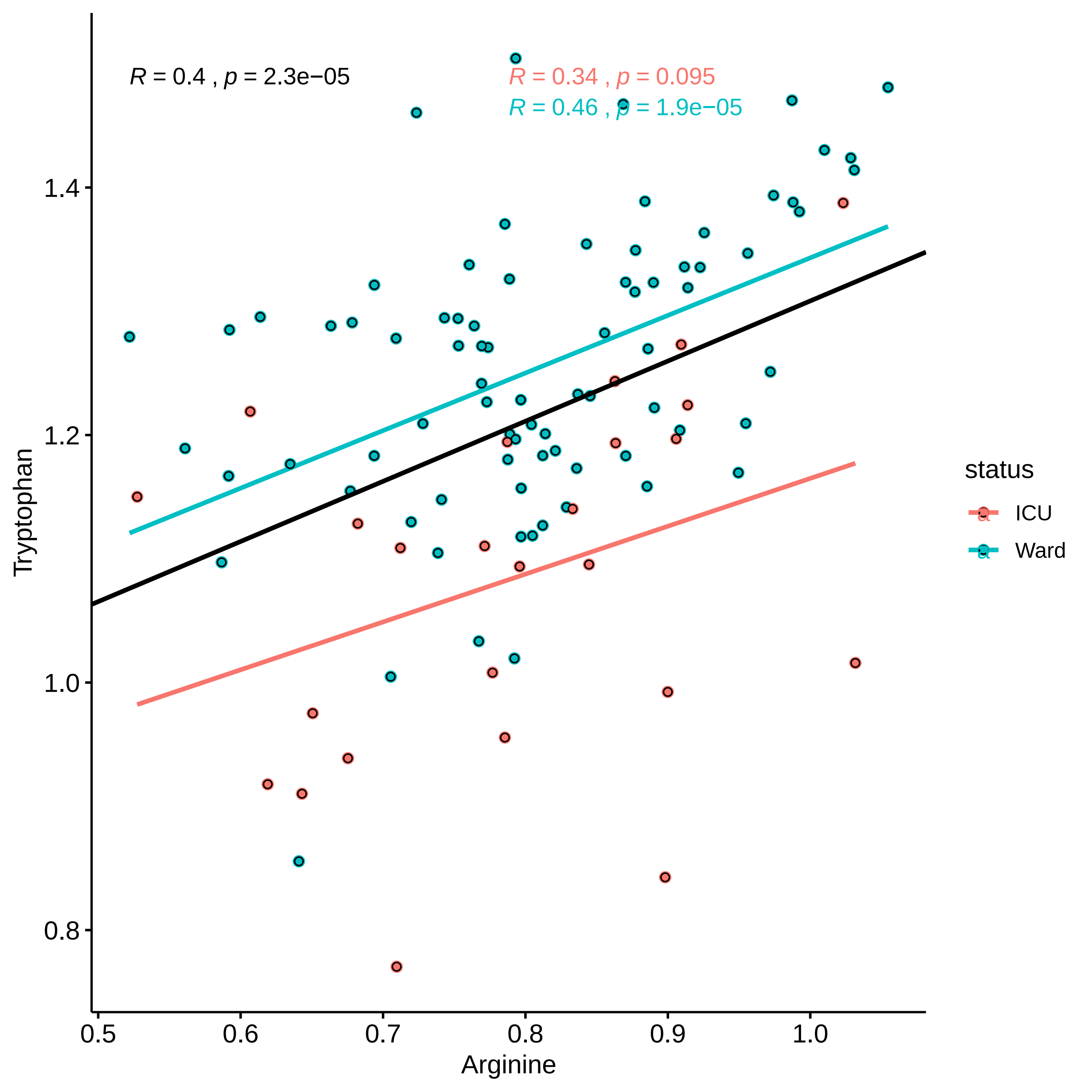

R = 0.4 , p = 2.3e−05
R = 0.34 , p = 0.095
R = 0.46 , p = 1.9e−05
1.4
1.2
1.0
0.8
Tryptophan
0.5
0.6
0.7
0.8
0.9
1.0
Arginine
status
ICU
Ward

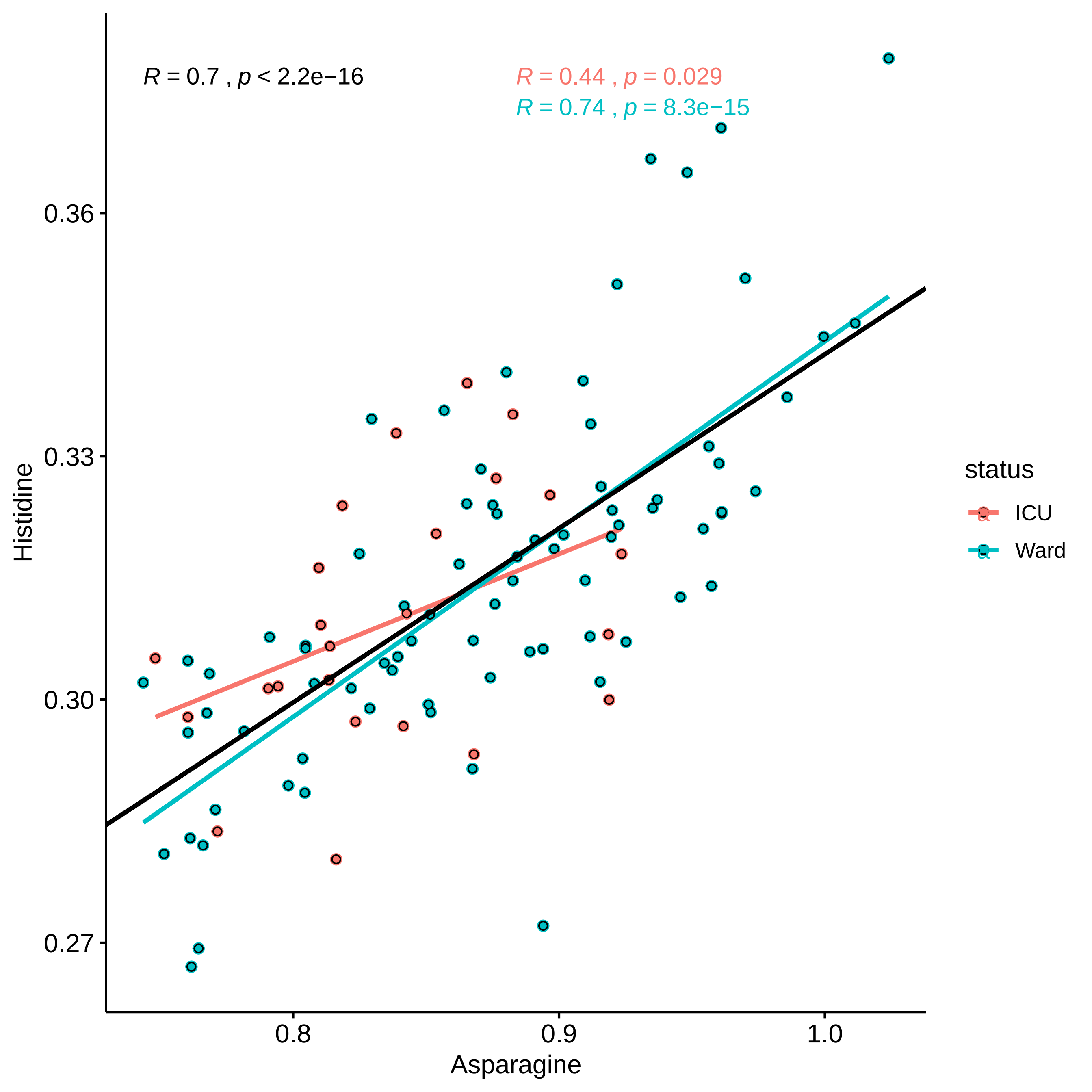

R = 0.7 , p < 2.2e−16
R = 0.44 , p = 0.029
R = 0.74 , p = 8.3e−15
Histidine
0.36
0.33
0.30
0.27
0.8
0.9
1.0
Asparagine
status
ICU
Ward

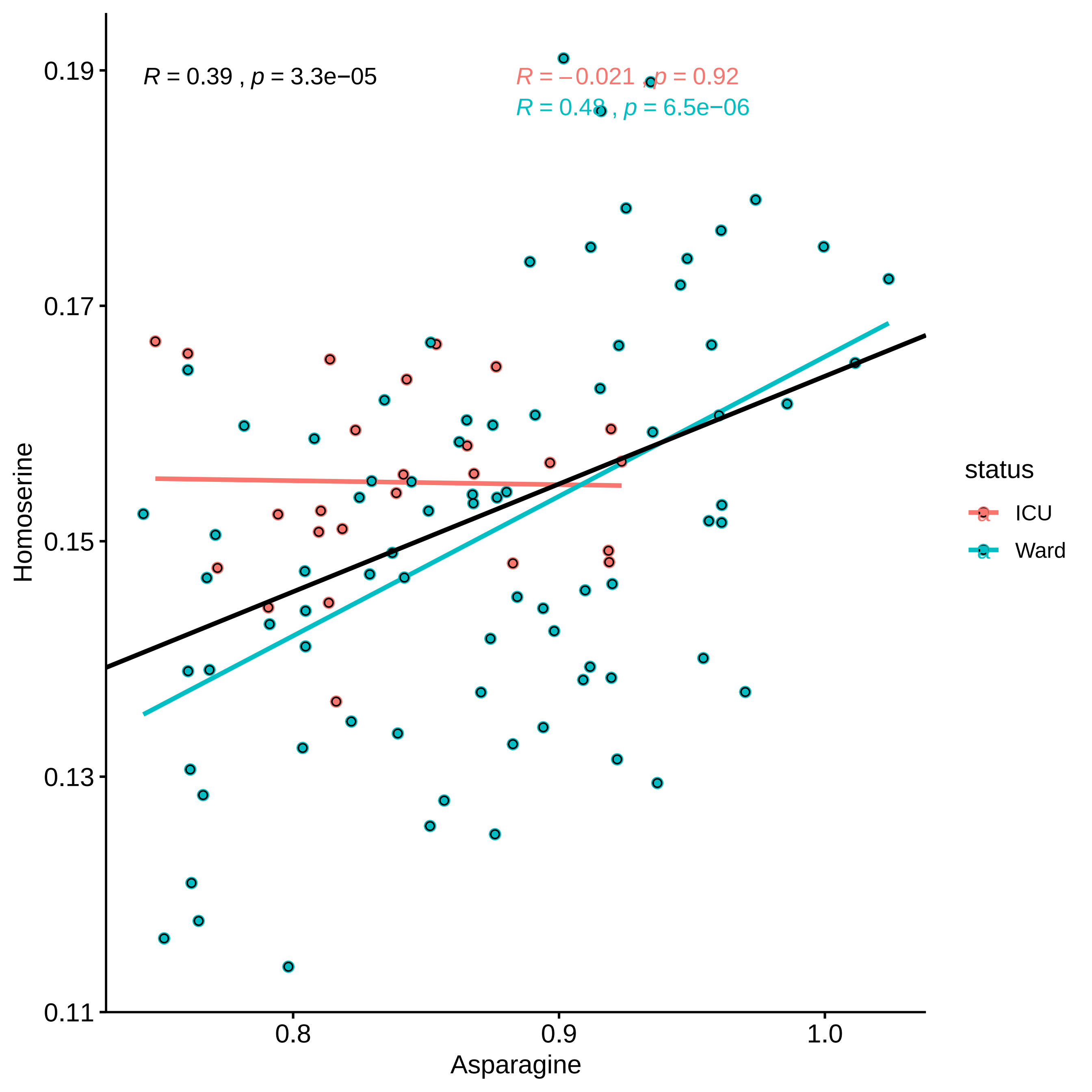

R = 0.39 , p = 3.3e−05
R = −0.021 , p = 0.92
R = 0.48 , p = 6.5e−06
0.19
0.17
0.15
0.13
0.11
Homoserine
0.8
0.9
1.0
Asparagine
status
ICU
Ward

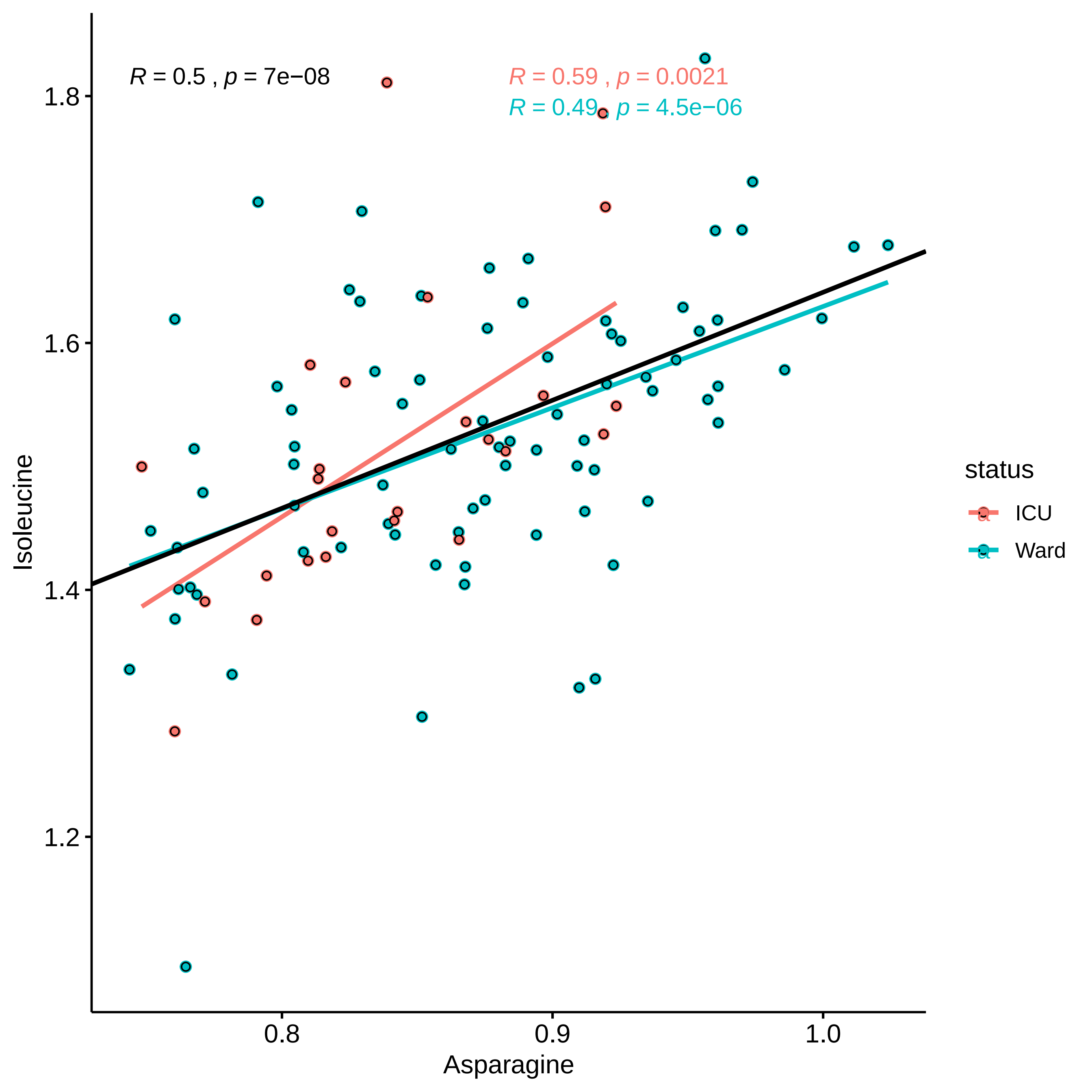

R = 0.5 , p = 7e−08
R = 0.59 , p = 0.0021
R = 0.49 , p = 4.5e−06
1.8
1.6
1.4
1.2
Isoleucine
0.8
0.9
1.0
Asparagine
status
ICU
Ward

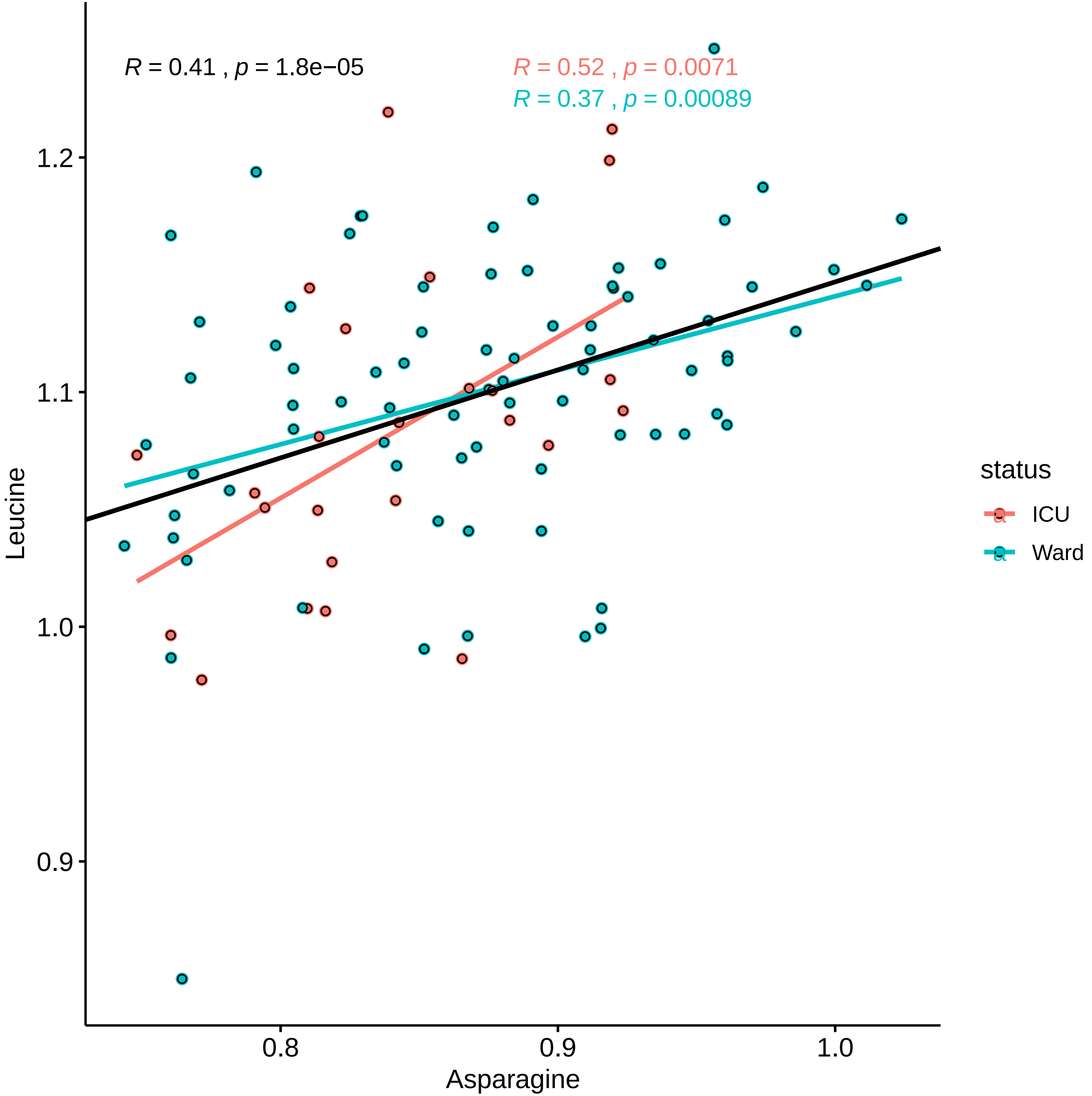

R = 0.41 , p = 1.8e−05
R = 0.52 , p = 0.0071
R = 0.37 , p = 0.00089
1.2
1.1
1.0
0.9
Leucine
0.8
0.9
1.0
Asparagine
status
ICU
Ward

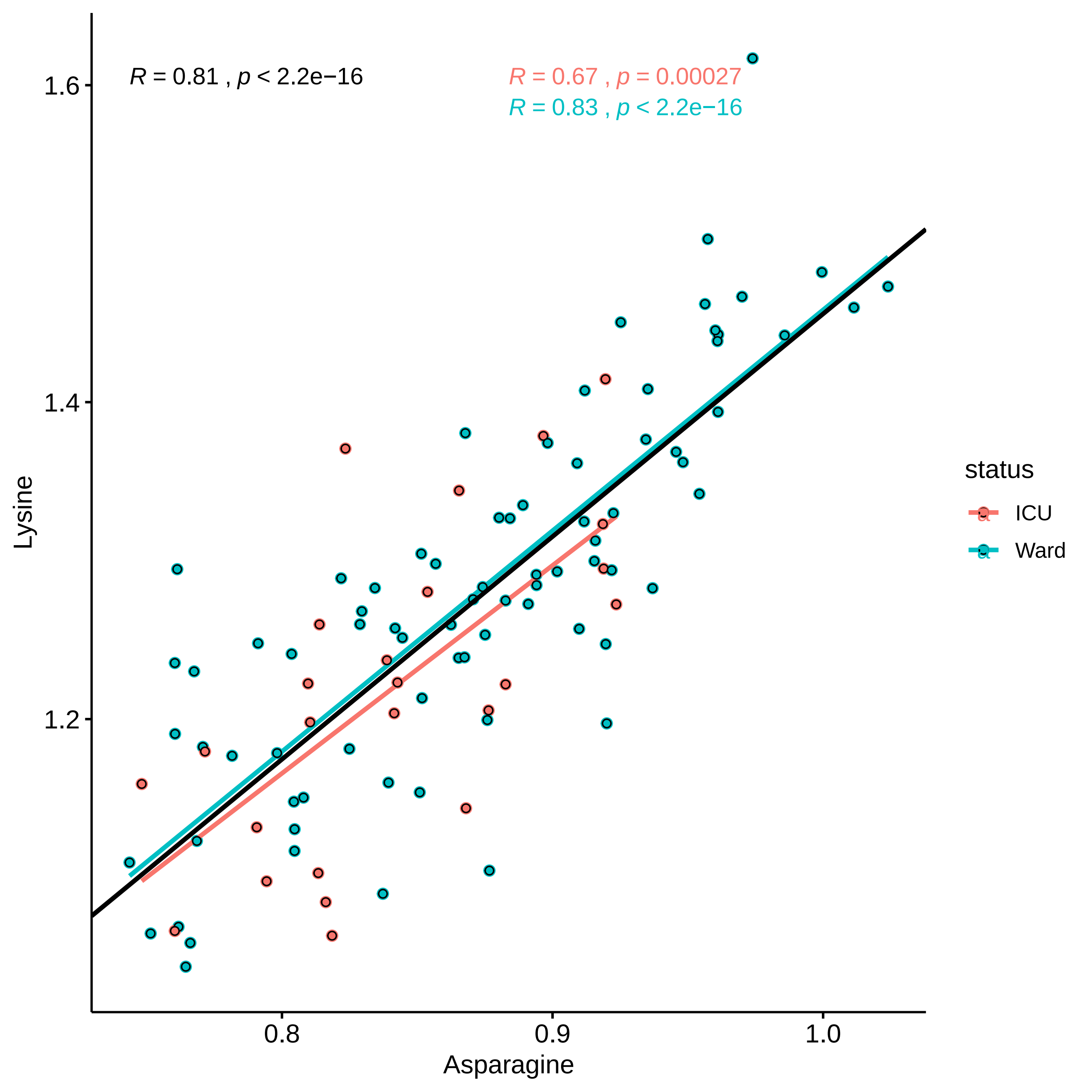

R = 0.81 , p < 2.2e−16
R = 0.67 , p = 0.00027
R = 0.83 , p < 2.2e−16
1.6
1.4
1.2
Lysine
0.8
0.9
1.0
Asparagine
status
ICU
Ward

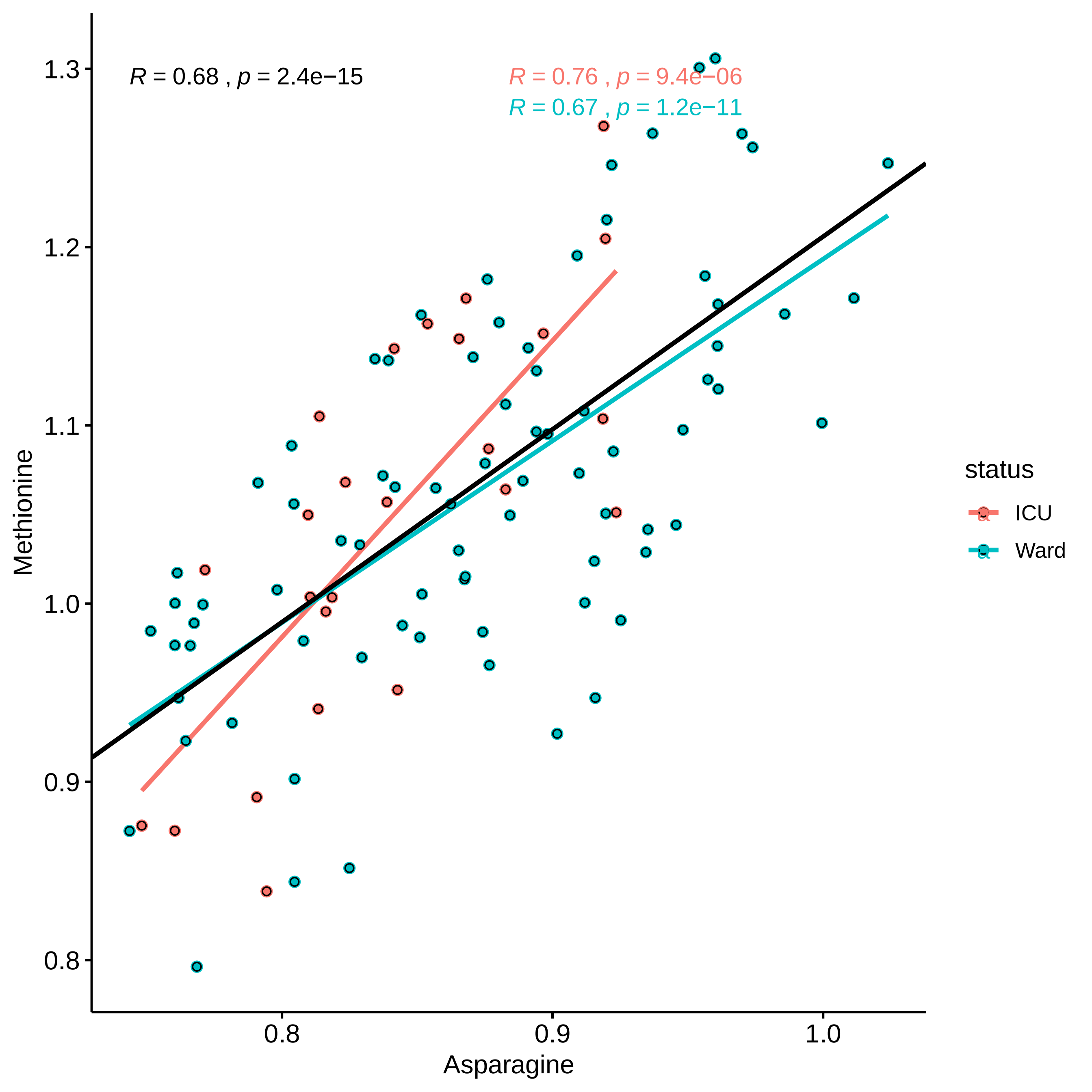

R = 0.68 , p = 2.4e−15
R = 0.76 , p = 9.4e−06
R = 0.67 , p = 1.2e−11
Methionine
1.3
1.2
1.1
1.0
0.9
0.8
0.8
0.9
1.0
Asparagine
status
ICU
Ward

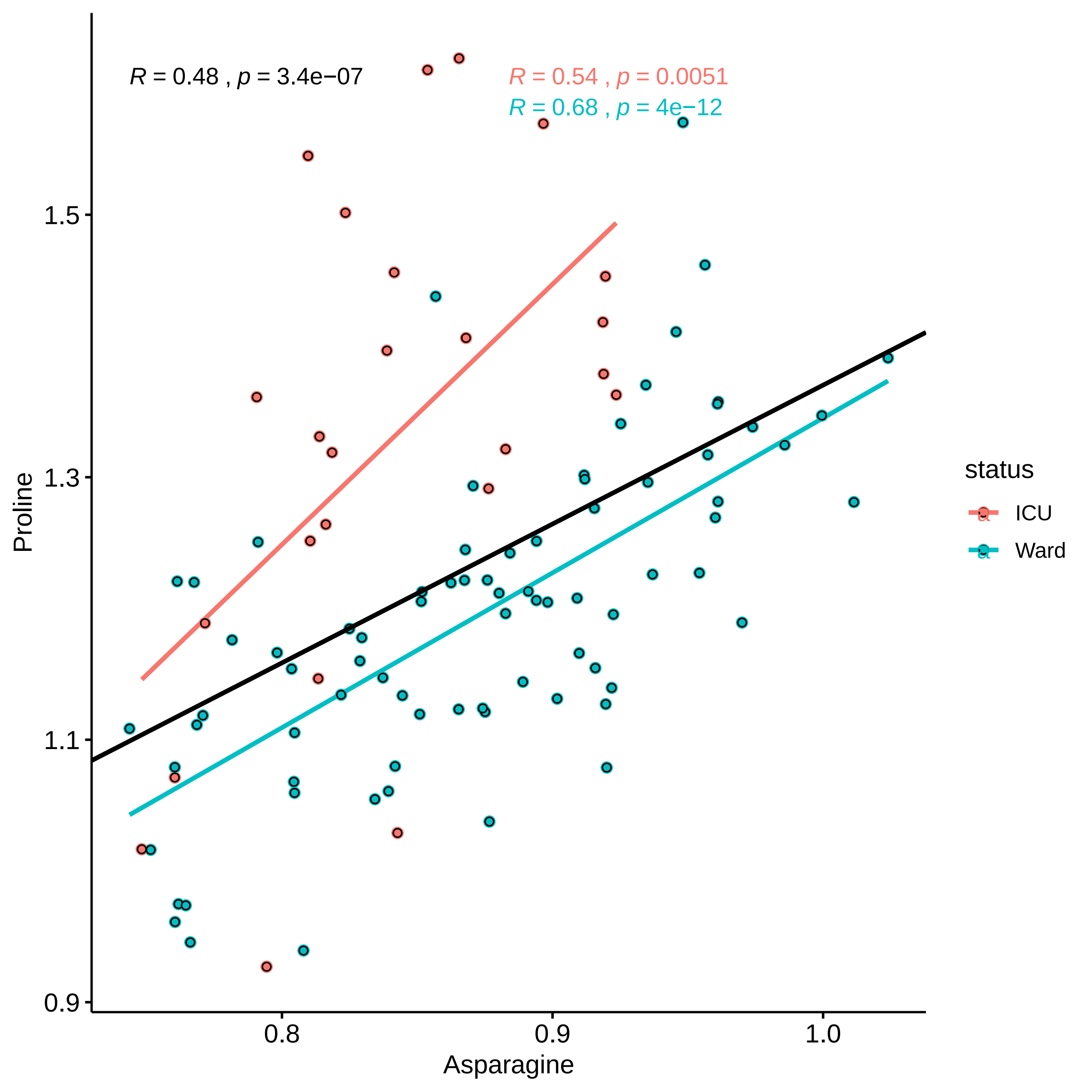
R = 0.48 , p = 3.4e−07
R = 0.54 , p = 0.0051
R = 0.68 , p = 4e−12
Proline
1.5
1.3
1.1
0.9
0.8
0.9
1.0
Asparagine
status
ICU
Ward

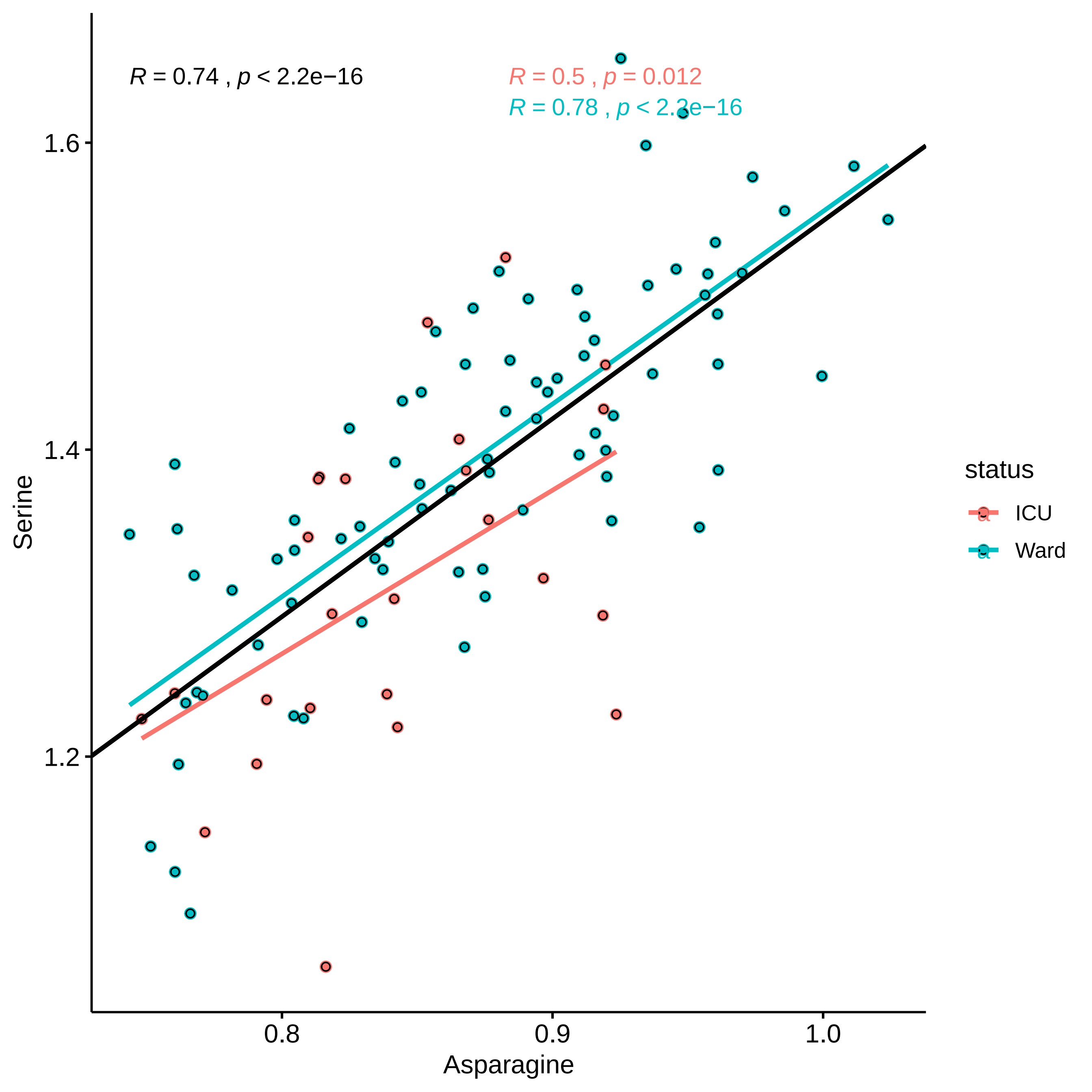

R = 0.74 , p < 2.2e−16
R = 0.5 , p = 0.012
R = 0.78 , p < 2.2e−16
1.6
1.4
1.2
Serine
0.8
0.9
1.0
Asparagine
status
ICU
Ward

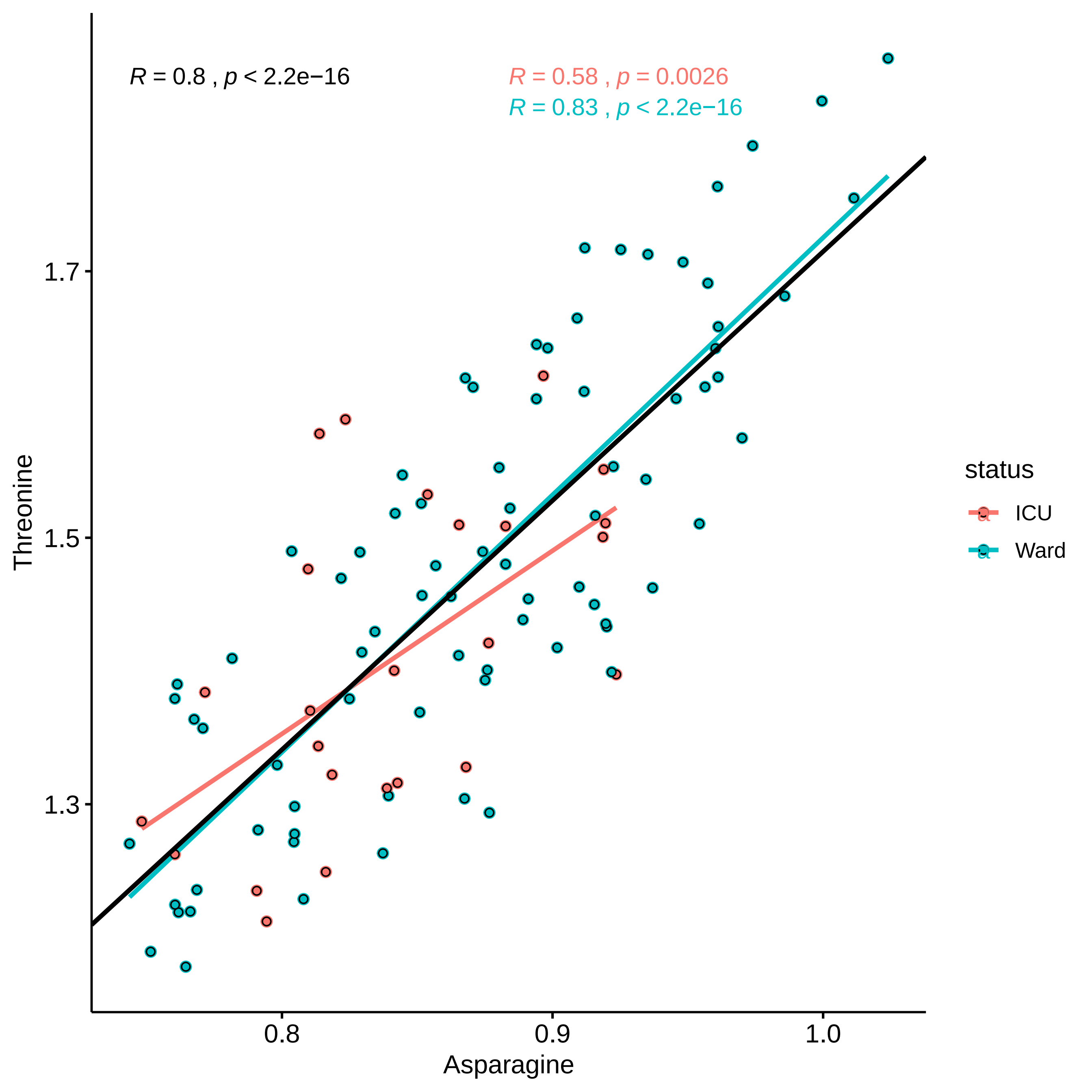

R = 0.8 , p < 2.2e−16
R = 0.58 , p = 0.0026
R = 0.83 , p < 2.2e−16
Threonine
1.7
1.5
1.3
0.8
0.9
1.0
Asparagine
status
ICU
Ward

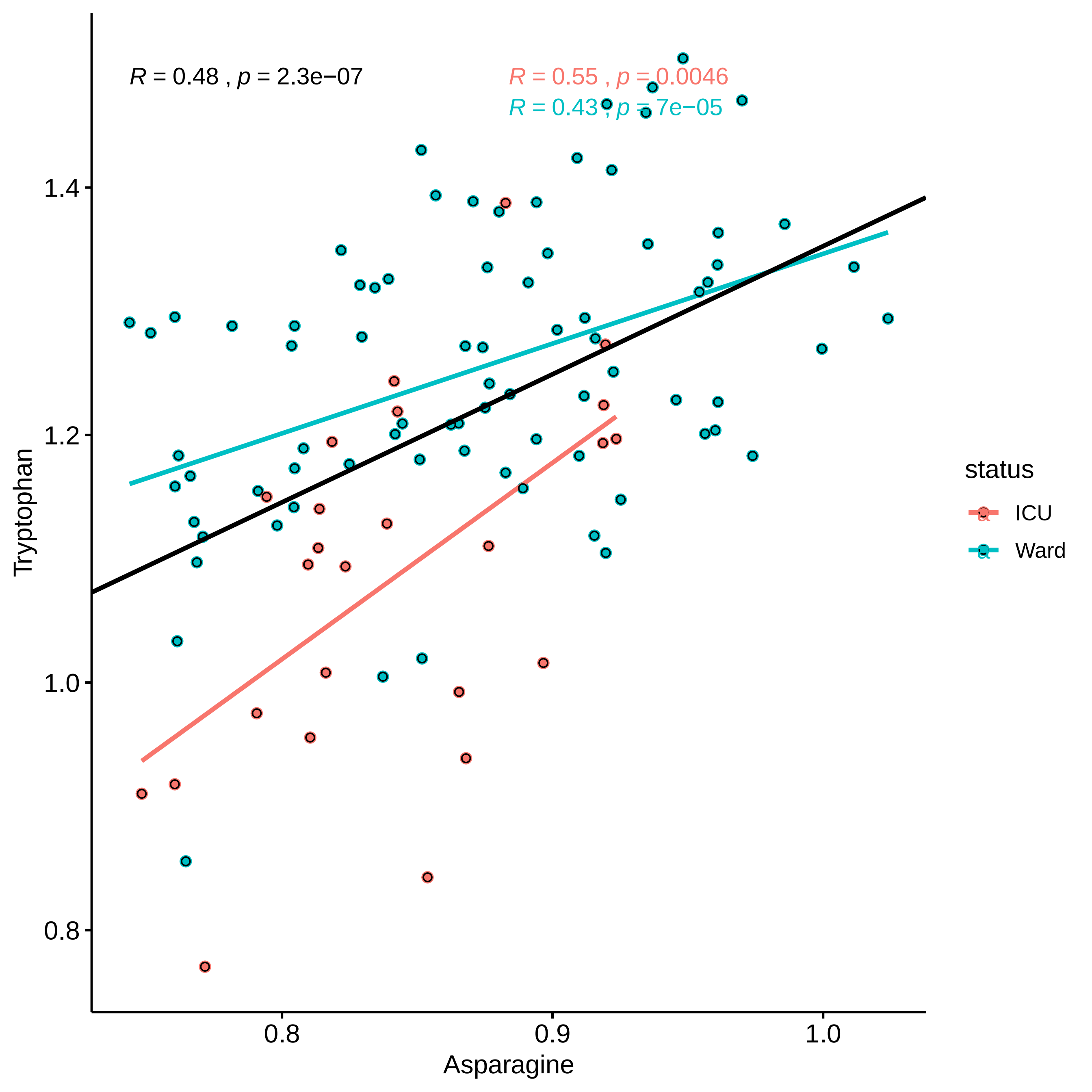

R = 0.48 , p = 2.3e−07
R = 0.55 , p = 0.0046
R = 0.43 , p = 7e−05
Tryptophan
1.4
1.2
1.0
0.8
0.8
0.9
1.0
Asparagine
status
ICU
Ward

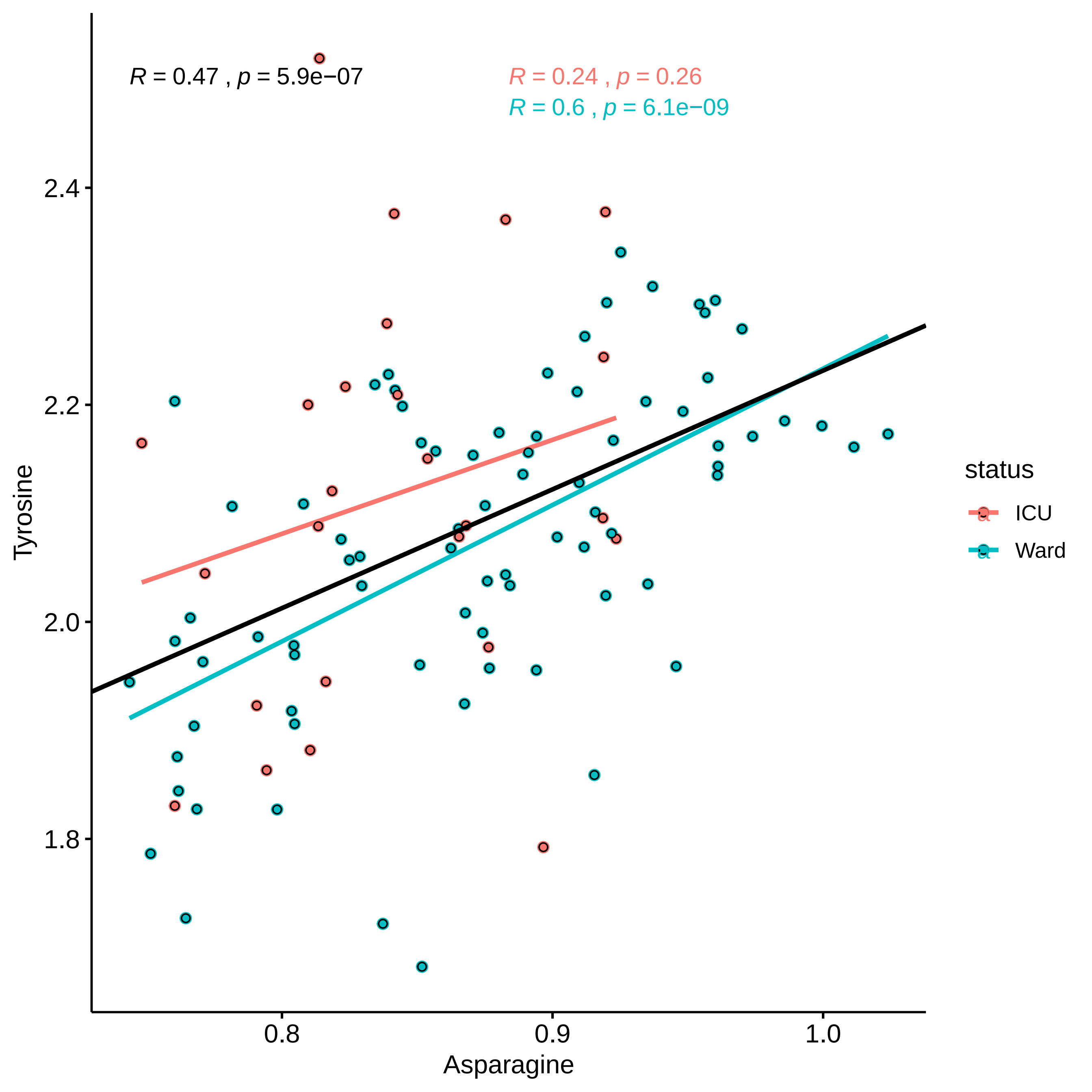
R = 0.47 , p = 5.9e−07
R = 0.24 , p = 0.26
R = 0.6 , p = 6.1e−09
Tyrosine
2.4
2.2
2.0
1.8
0.8
0.9
1.0
Asparagine
status
ICU
Ward

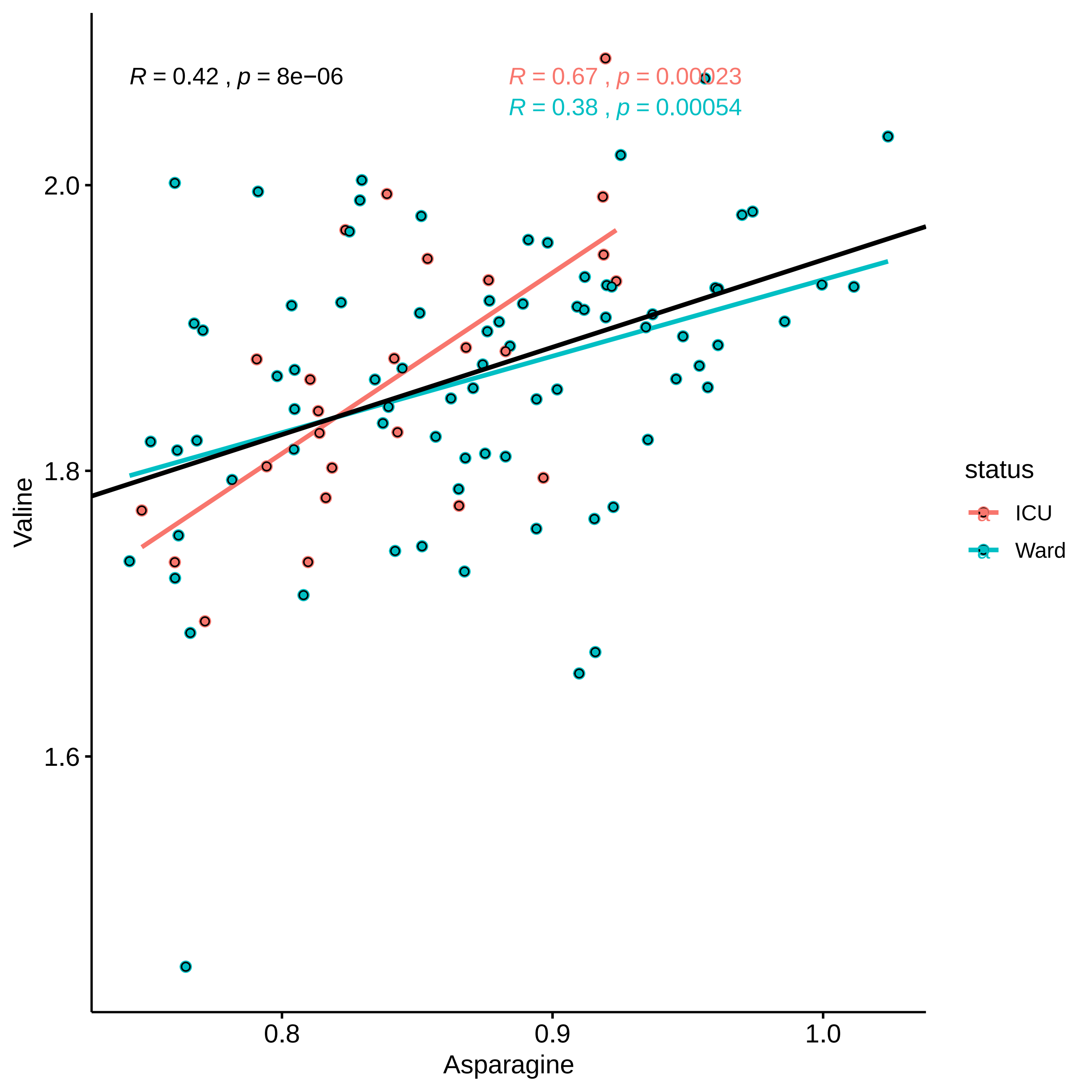
R = 0.42 , p = 8e−06
R = 0.67 , p = 0.00023
R = 0.38 , p = 0.00054
2.0
1.8
1.6
Valine
0.8
0.9
1.0
Asparagine
status
ICU
Ward

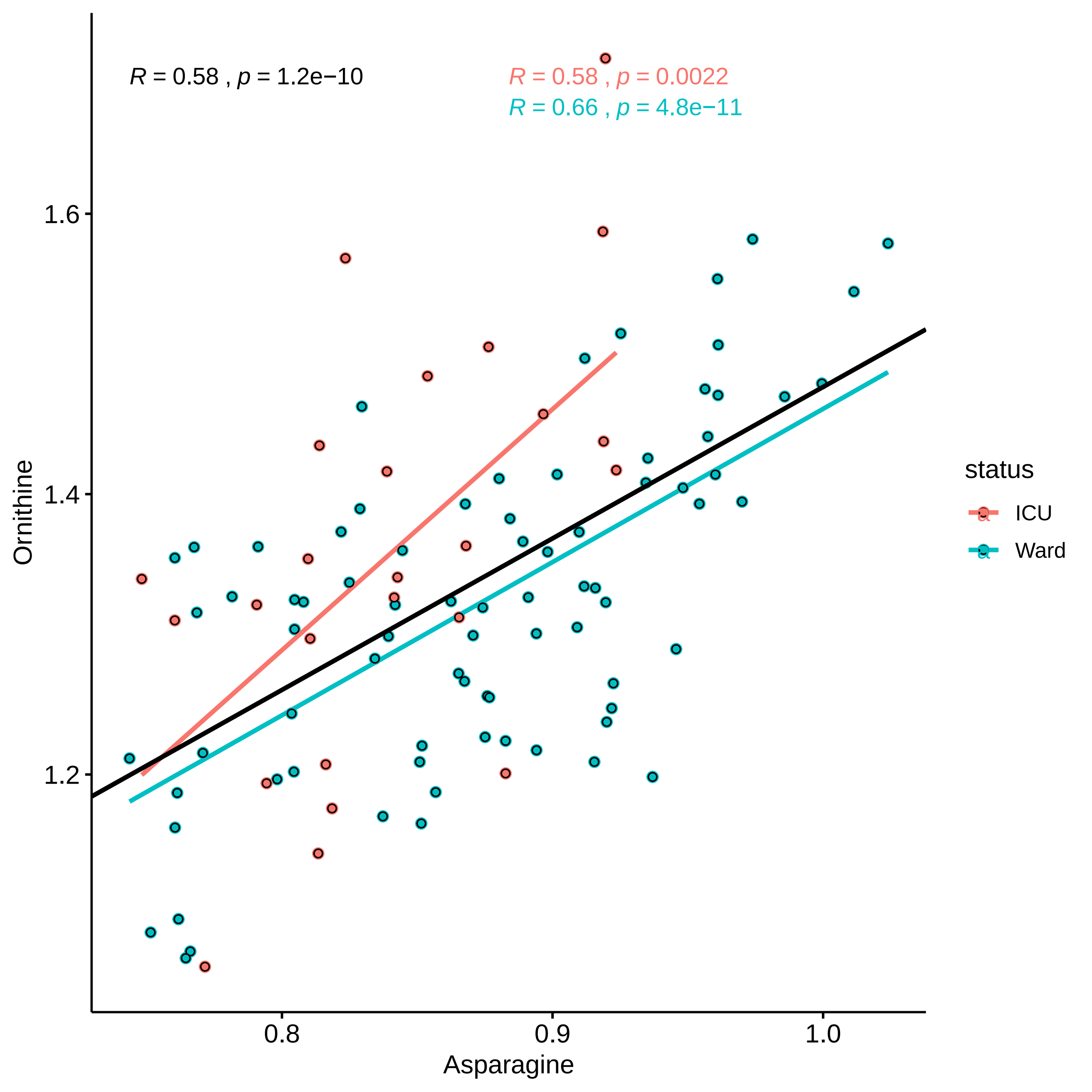

R = 0.58 , p = 1.2e−10
R = 0.58 , p = 0.0022
R = 0.66 , p = 4.8e−11
Ornithine
1.6
1.4
1.2
0.8
0.9
1.0
Asparagine
status
ICU
Ward

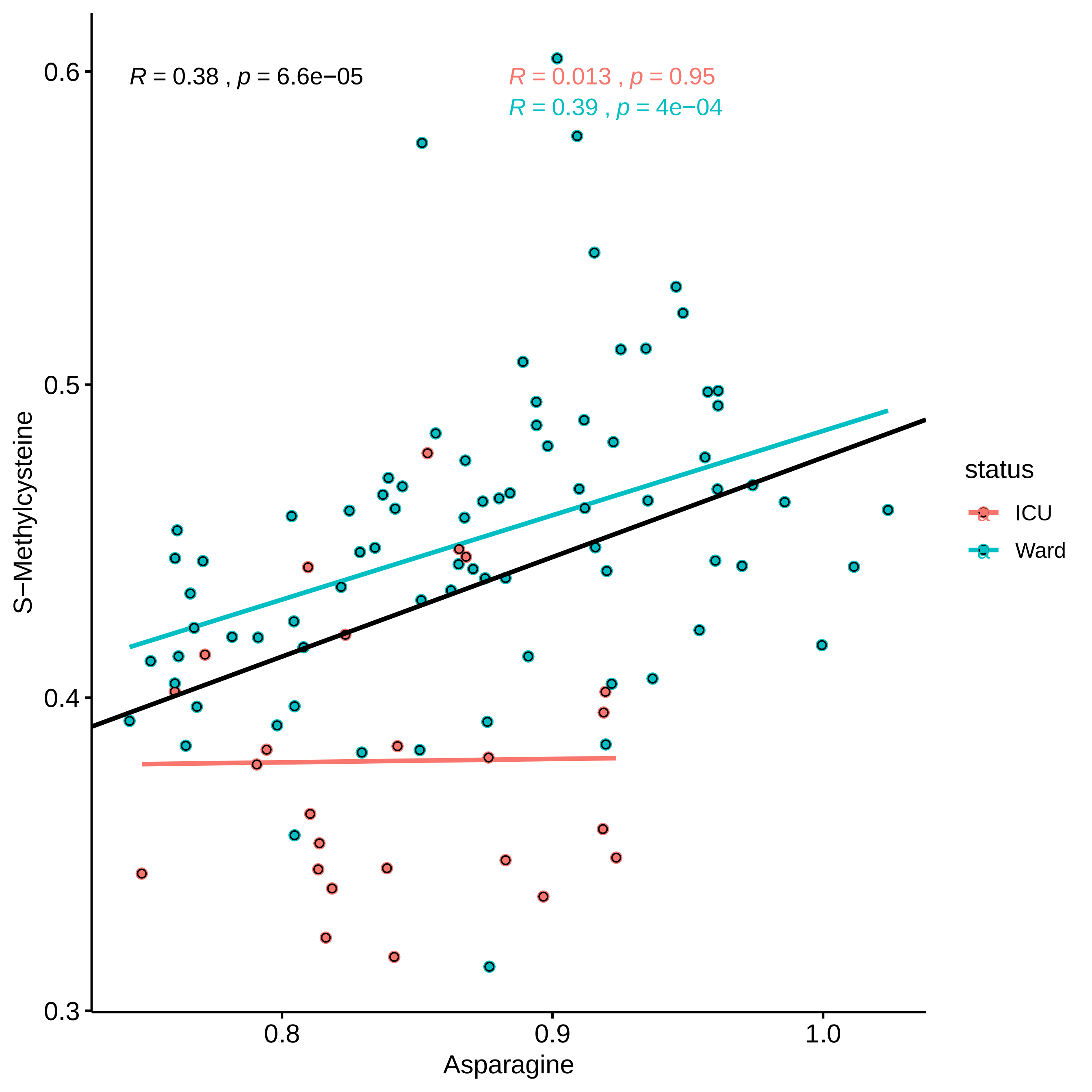

R = 0.38 , p = 6.6e−05
R = 0.013 , p = 0.95
R = 0.39 , p = 4e−04
0.6
0.5
0.4
0.3
S−Methylcysteine
0.8
0.9
1.0
Asparagine
status
ICU
Ward

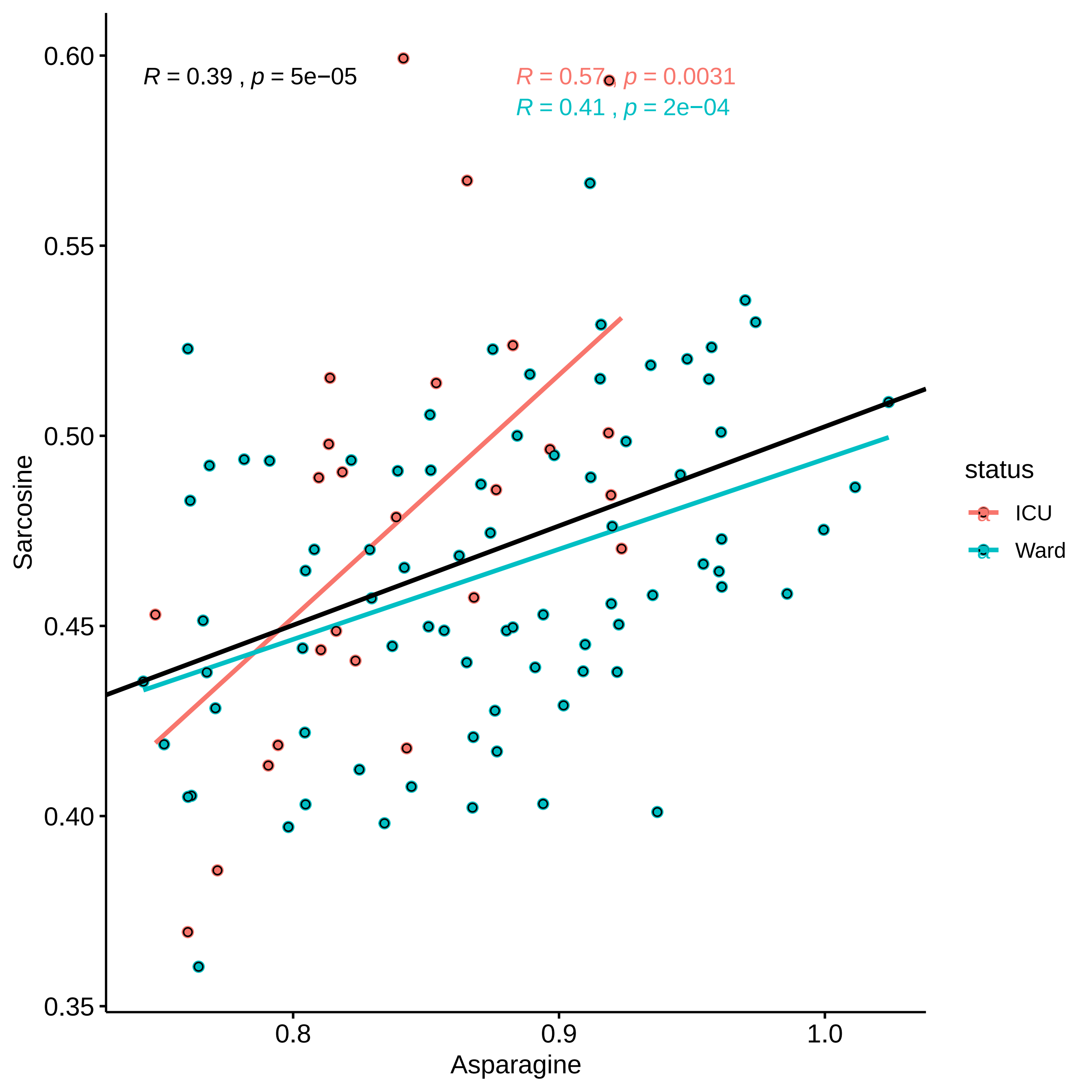

R = 0.39 , p = 5e−05
R = 0.57 , p = 0.0031
R = 0.41 , p = 2e−04
0.60
0.55
0.50
0.45
0.40
0.35
Sarcosine
0.8
0.9
1.0
Asparagine
status
ICU
Ward

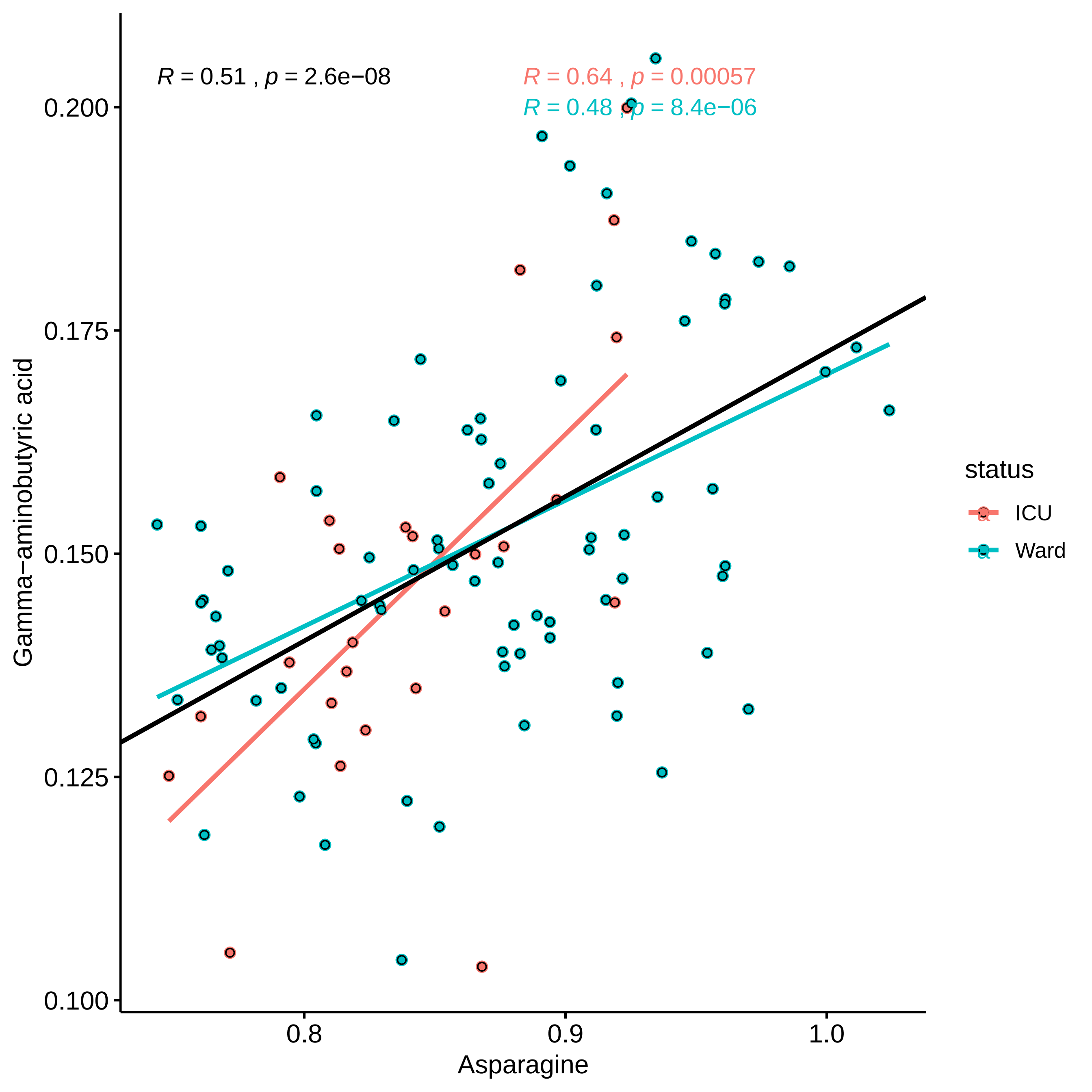
R = 0.51 , p = 2.6e−08
R = 0.64 , p = 0.00057
R = 0.48 , p = 8.4e−06
0.200
0.175
0.150
0.125
0.100
Gamma−aminobutyric acid
0.8
0.9
1.0
Asparagine
status
ICU
Ward

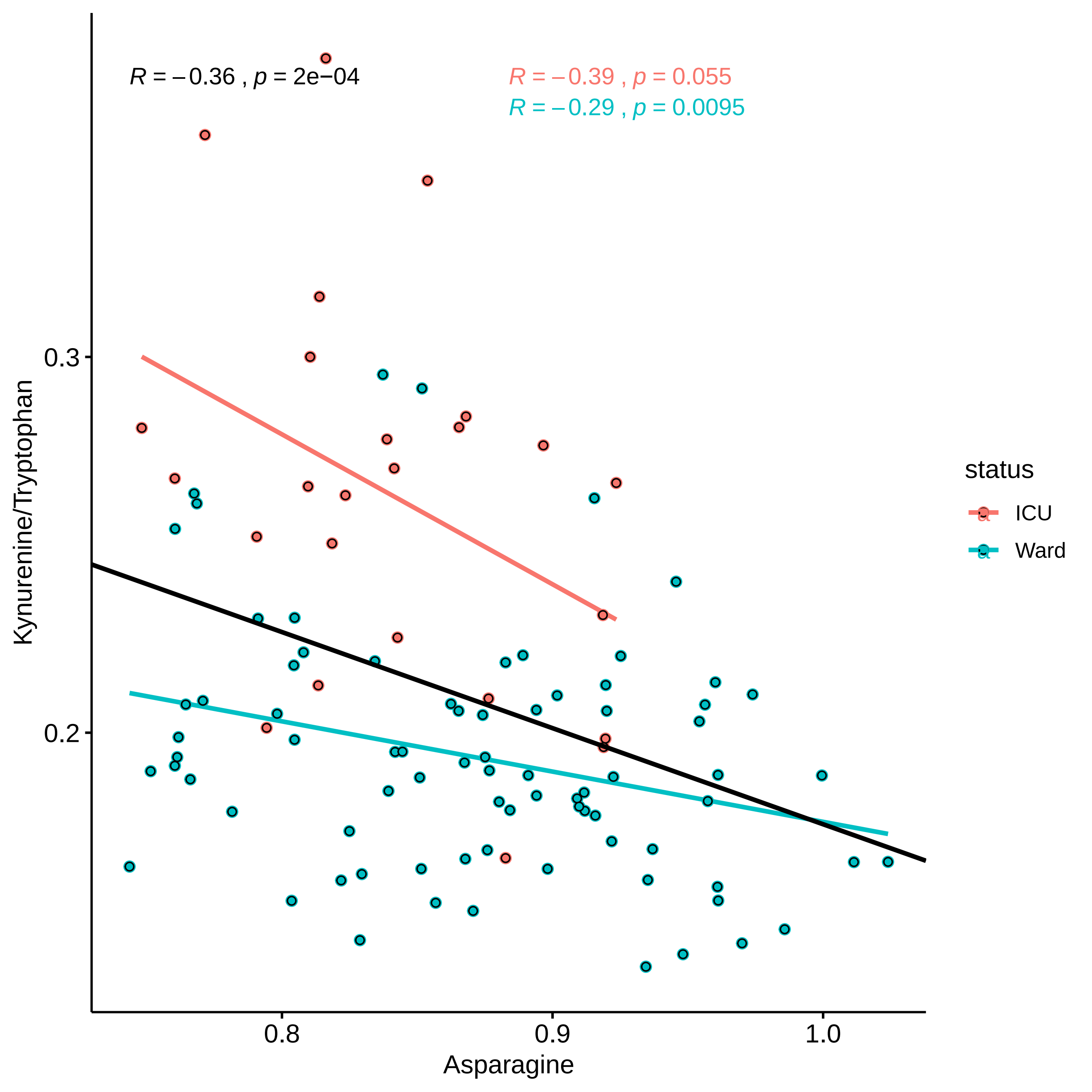

R = −0.36 , p = 2e−04
R = −0.39 , p = 0.055
R = −0.29 , p = 0.0095
Kynurenine/Tryptophan
0.3
0.2
0.8
0.9
1.0
Asparagine
status
ICU
Ward

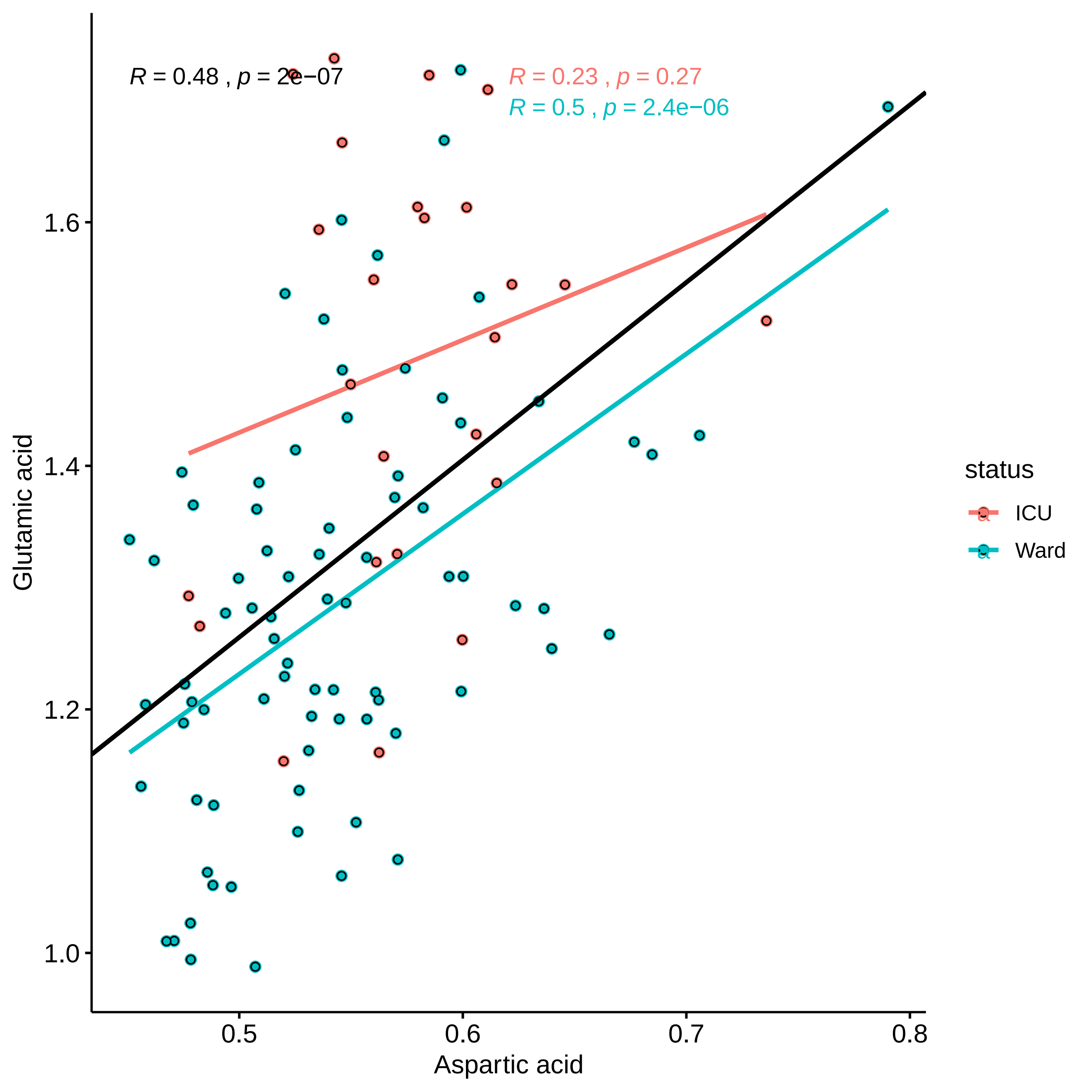

R = 0.48 , p = 2e−07
R = 0.23 , p = 0.27
R = 0.5 , p = 2.4e−06
Glutamic acid
1.0
1.2
1.4
1.6
Aspartic acid
0.5
0.6
0.7
0.8
status
ICU
Ward

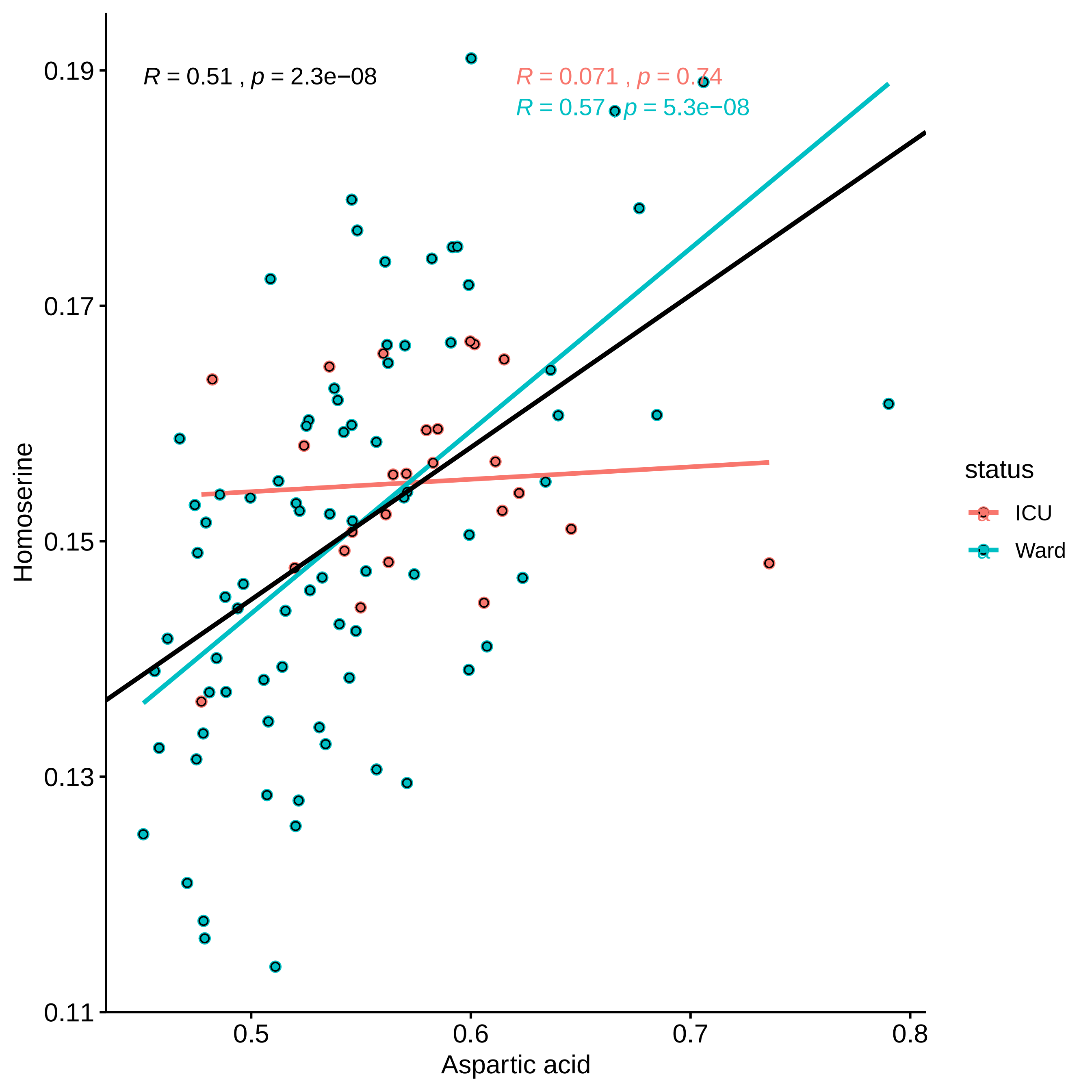

R = 0.51 , p = 2.3e−08
R = 0.071 , p = 0.74
R = 0.57 , p = 5.3e−08
Homoserine
0.19
0.17
0.15
0.13
0.11
0.5
0.6
0.7
0.8
Aspartic acid
status
ICU
Ward

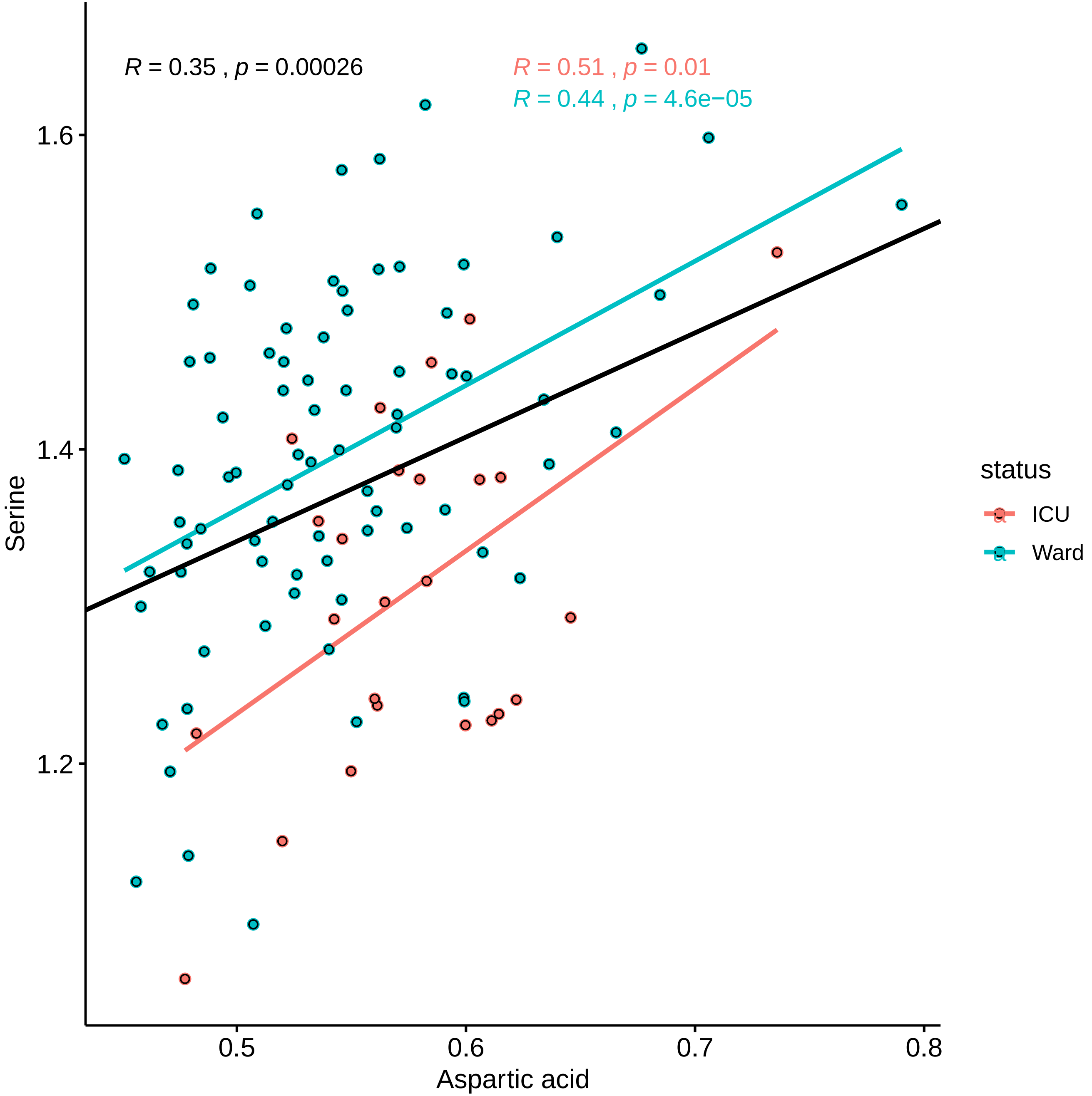
R = 0.35 , p = 0.00026
R = 0.51 , p = 0.01
R = 0.44 , p = 4.6e−05
1.6
1.4
1.2
0.5
0.6
0.7
0.8
Serine
Aspartic acid
status
ICU
Ward

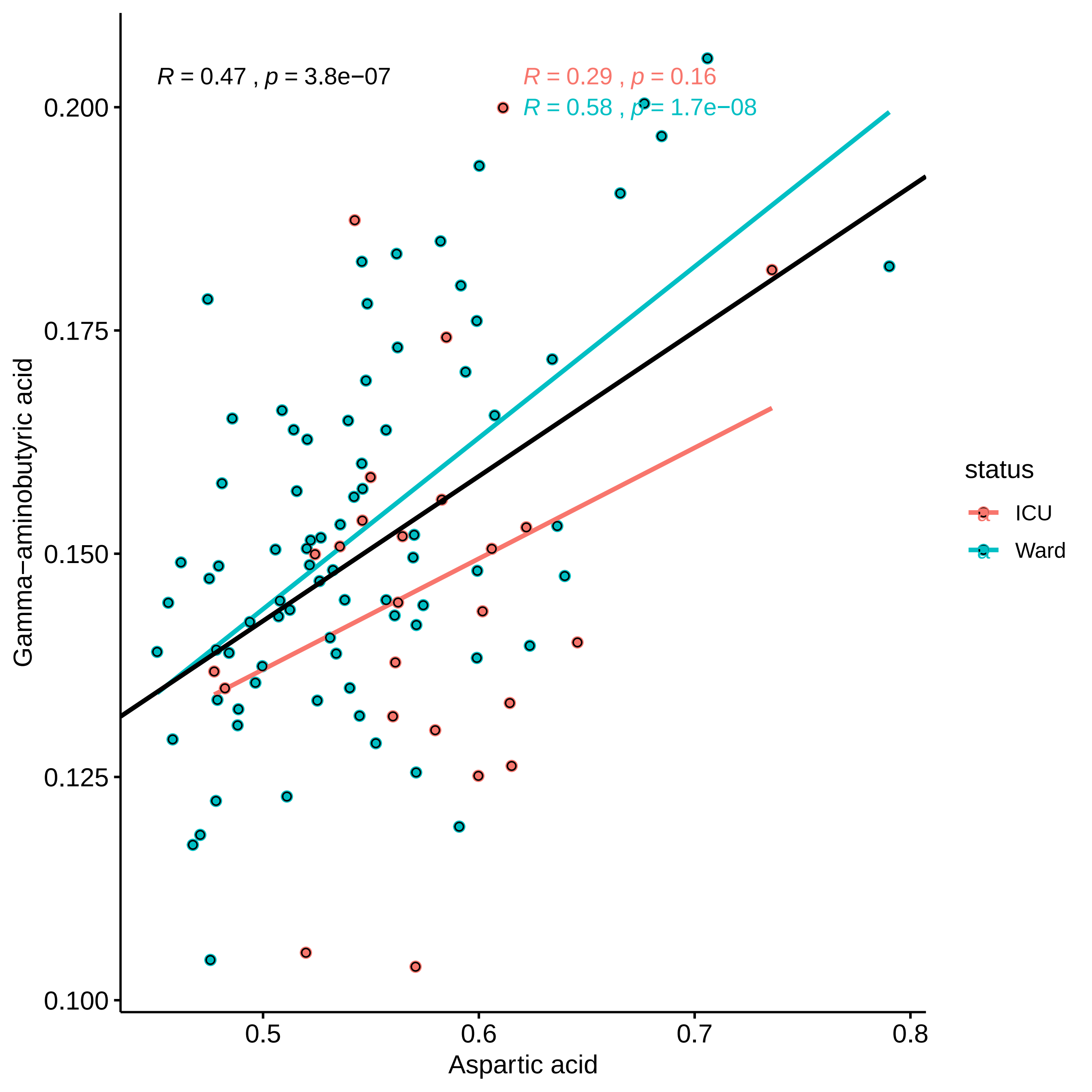

R = 0.47 , p = 3.8e−07
R = 0.29 , p = 0.16
R = 0.58 , p = 1.7e−08
0.200
0.175
0.150
0.125
0.100
Gamma−aminobutyric acid
0.5
0.6
0.7
0.8
Aspartic acid
status
ICU
Ward

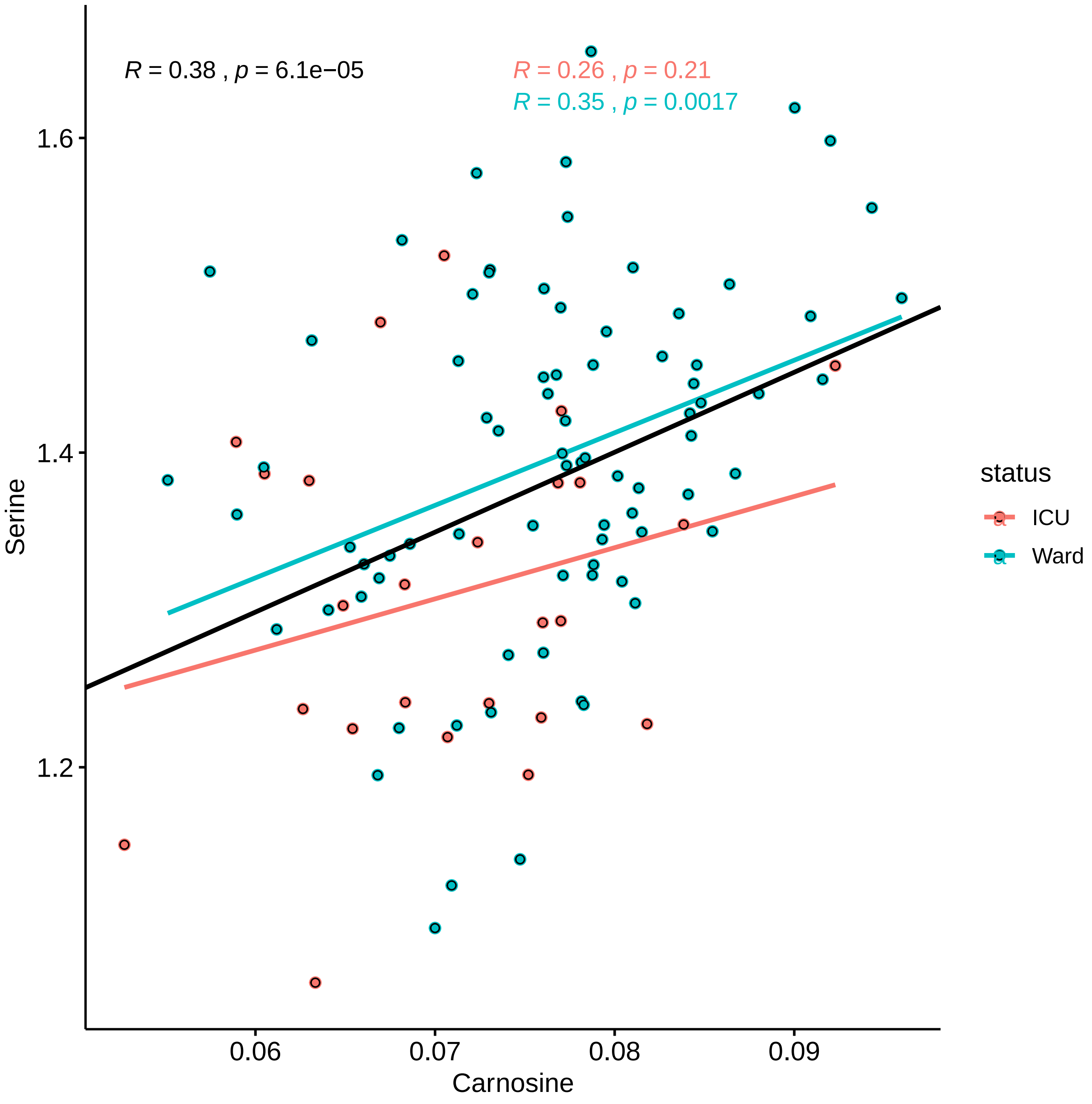

R = 0.38 , p = 6.1e−05
R = 0.26 , p = 0.21
R = 0.35 , p = 0.0017
1.6
1.4
1.2
Serine
0.06
0.07
0.08
0.09
Carnosine
status
ICU
Ward

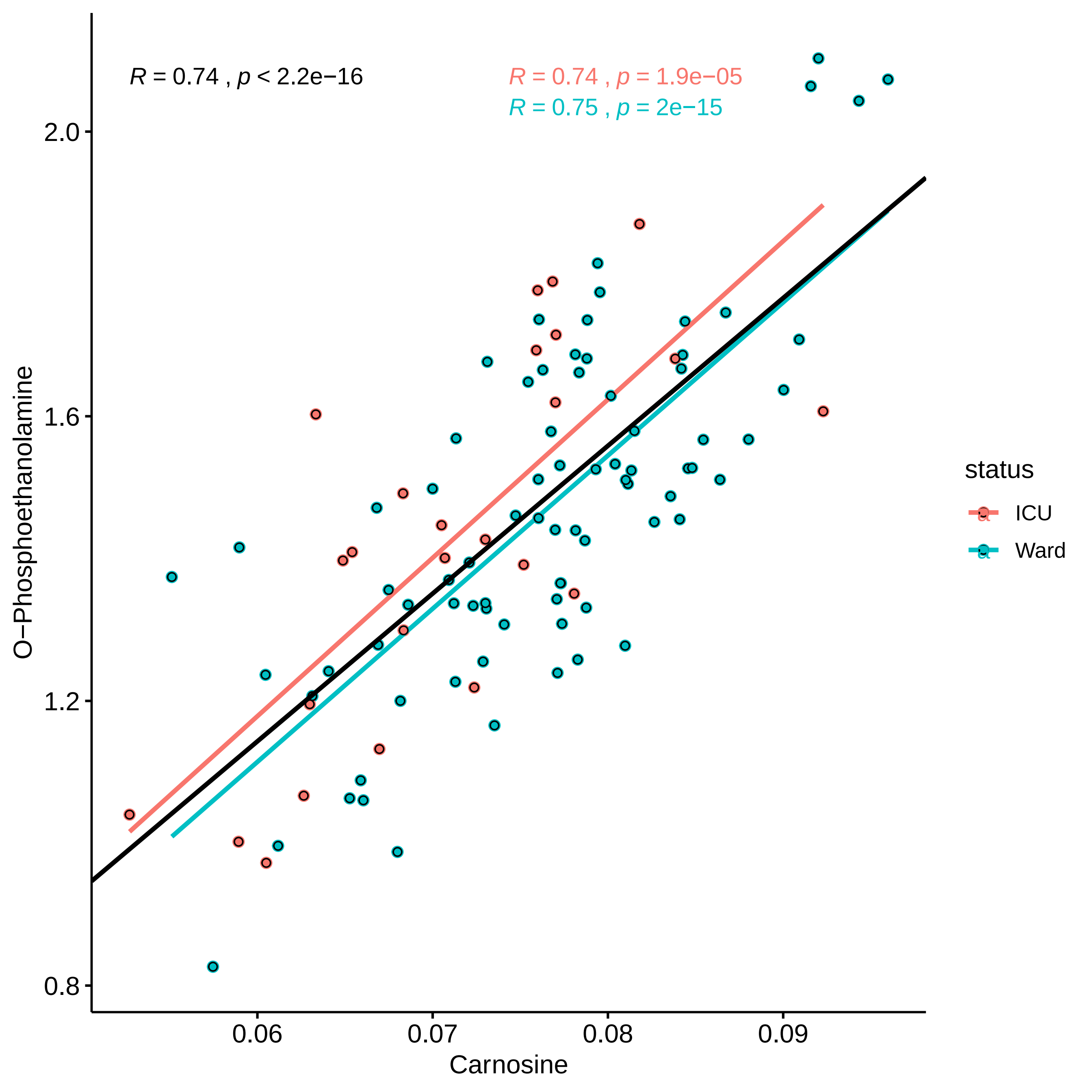

R = 0.74 , p < 2.2e−16
R = 0.74 , p = 1.9e−05
R = 0.75 , p = 2e−15
2.0
1.6
1.2
0.8
O−Phosphoethanolamine
0.06
0.07
0.08
0.09
Carnosine
status
ICU
Ward

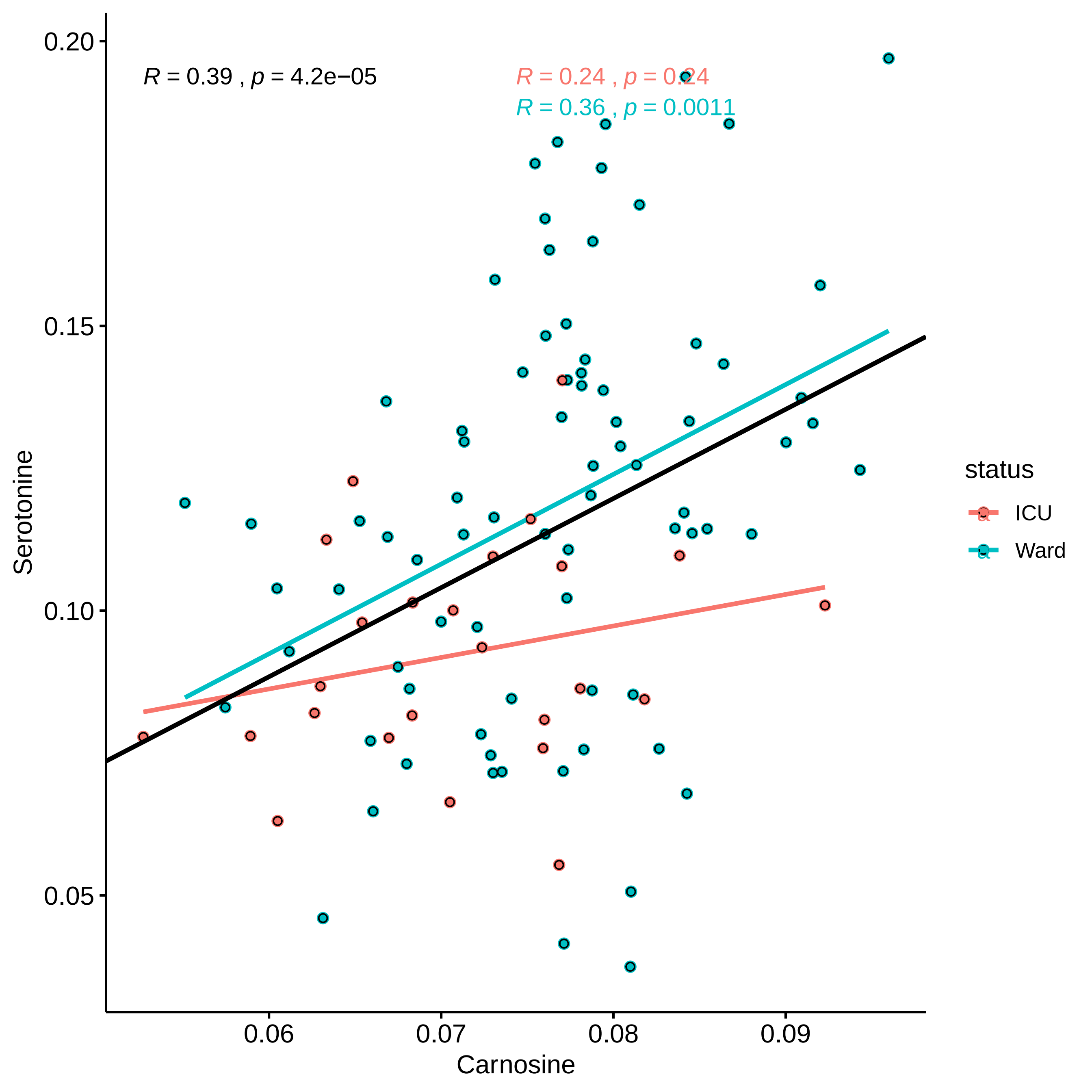

R = 0.39 , p = 4.2e−05
R = 0.24 , p = 0.24
R = 0.36 , p = 0.0011
0.20
0.15
0.10
0.05
Serotonine
0.06
0.07
0.08
0.09
Carnosine
status
ICU
Ward

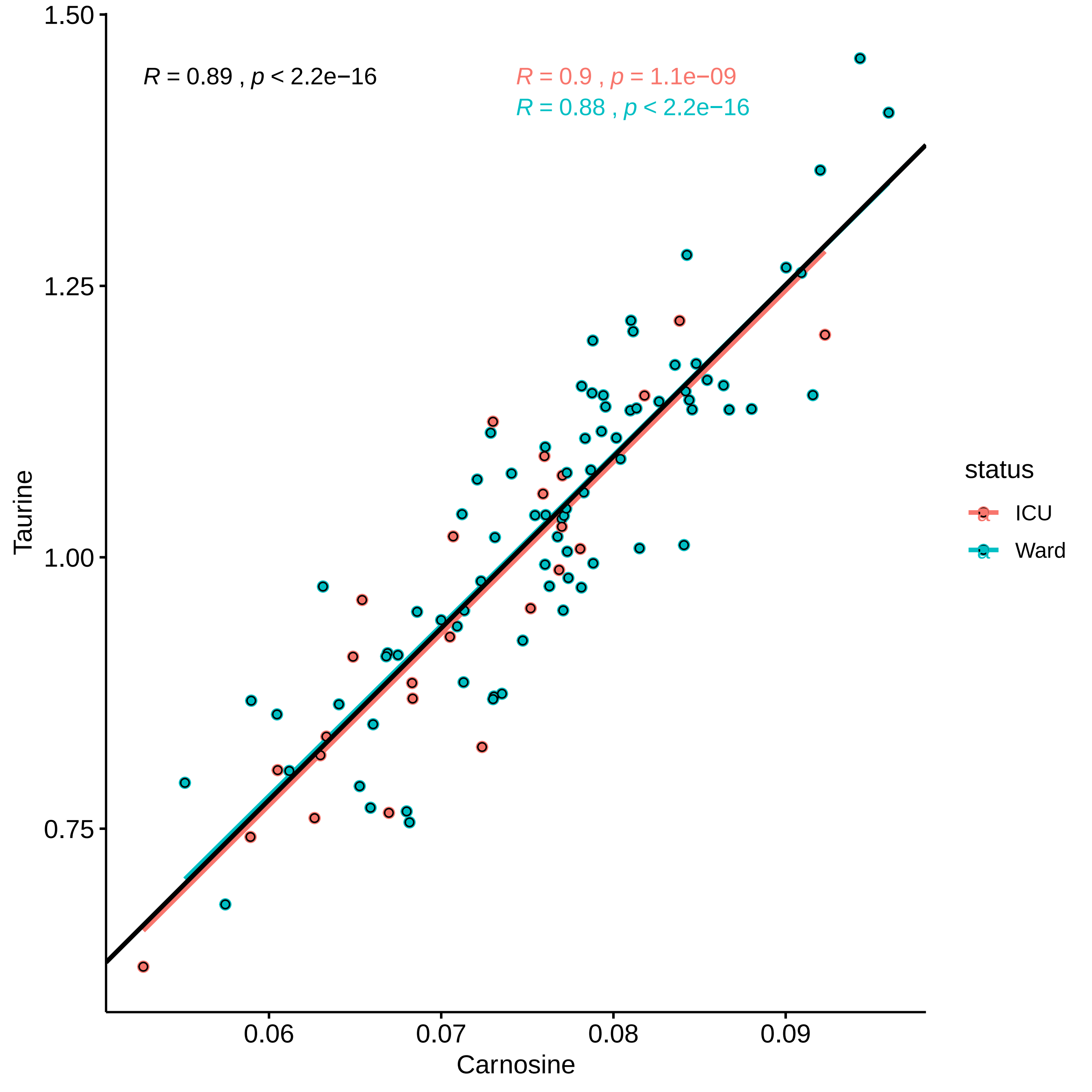

R = 0.89 , p < 2.2e−16
R = 0.9 , p = 1.1e−09
R = 0.88 , p < 2.2e−16
1.50
1.25
1.00
0.75
Taurine
0.06
0.07
0.08
0.09
Carnosine
status
ICU
Ward

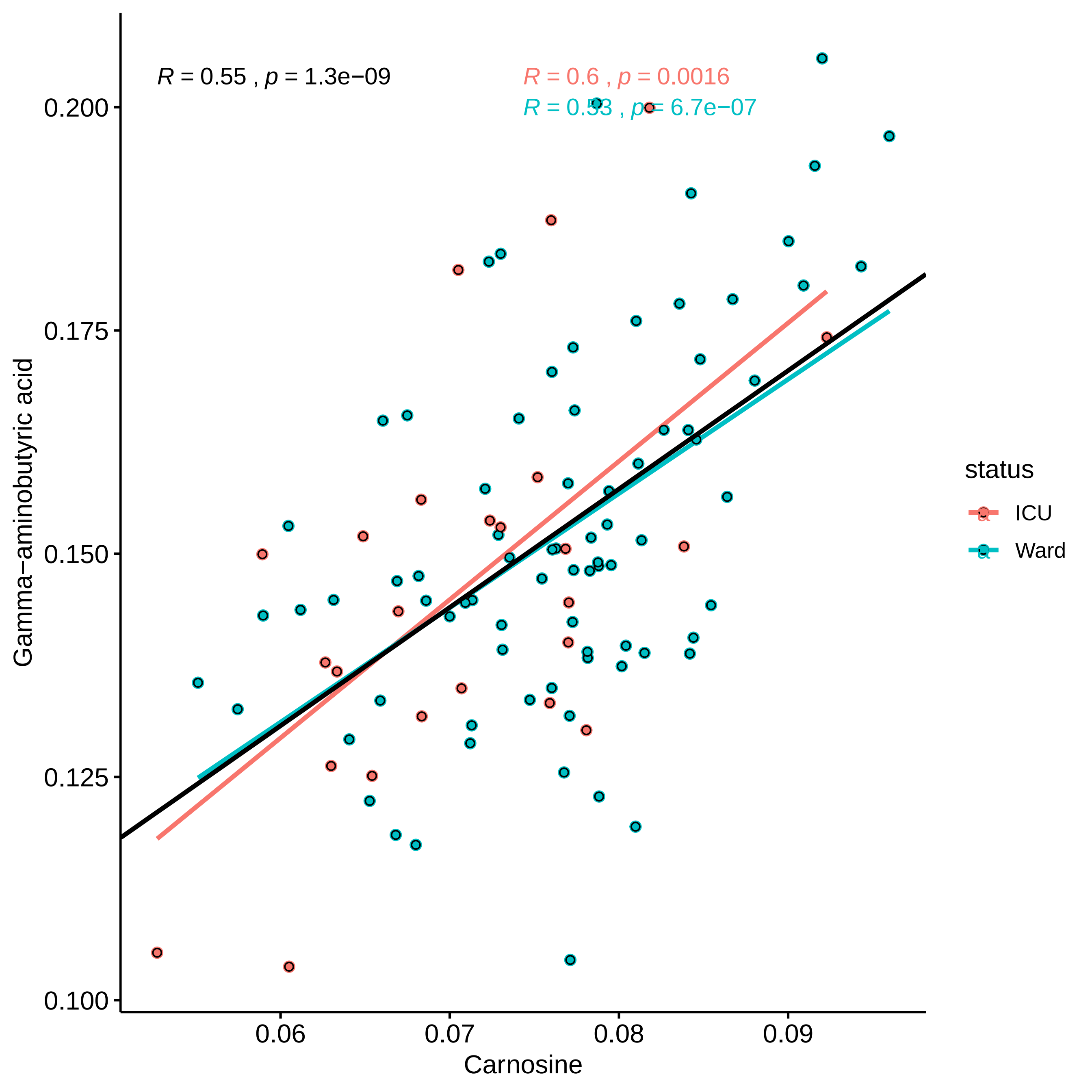

R = 0.55 , p = 1.3e−09
R = 0.6 , p = 0.0016
R = 0.53 , p = 6.7e−07
Gamma−aminobutyric acid
0.200
0.175
0.150
0.125
0.100
0.06
0.07
0.08
0.09
Carnosine
status
ICU
Ward

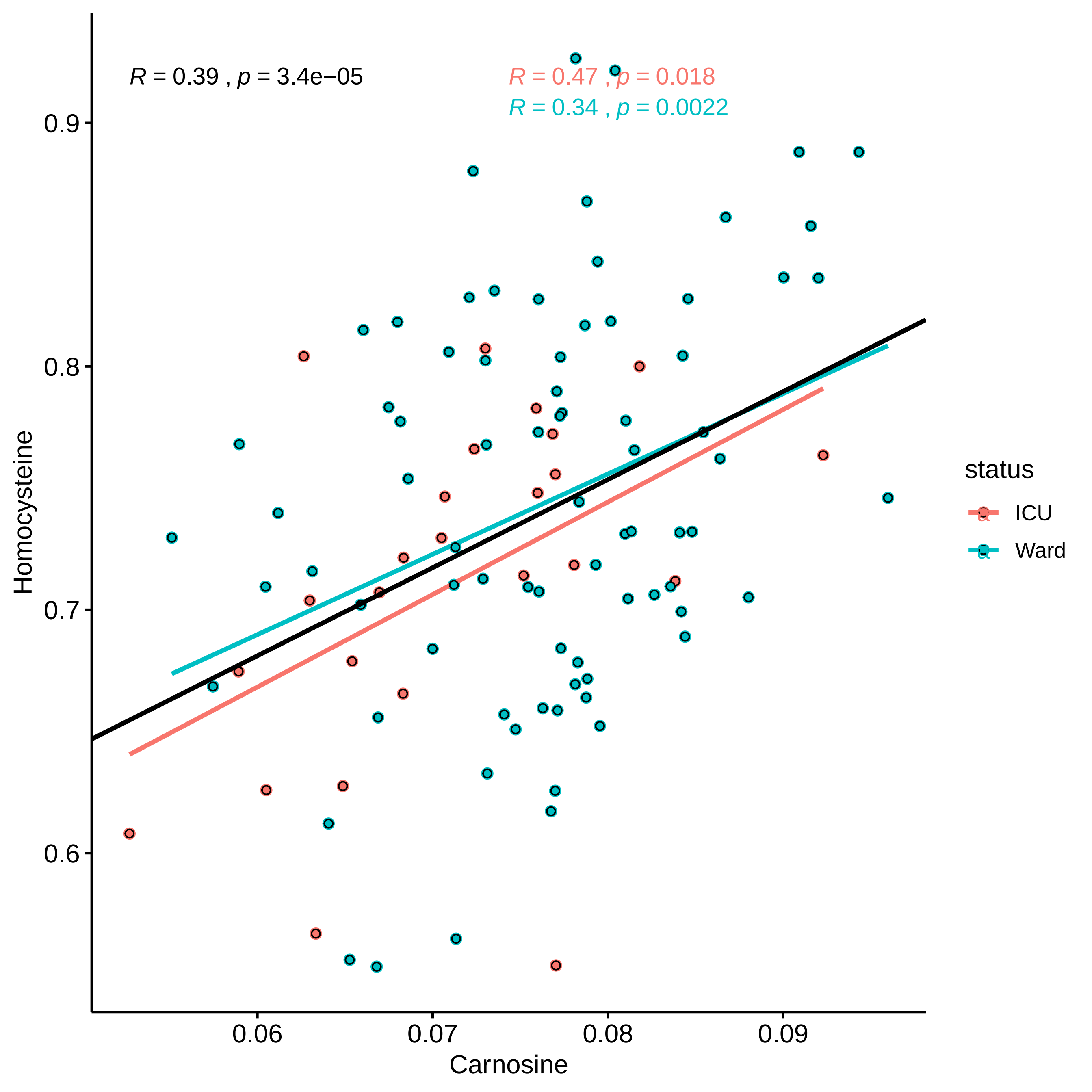
R = 0.39 , p = 3.4e−05
R = 0.47 , p = 0.018
R = 0.34 , p = 0.0022
0.9
0.8
0.7
0.6
Homocysteine
0.06
0.07
0.08
0.09
Carnosine
status
ICU
Ward

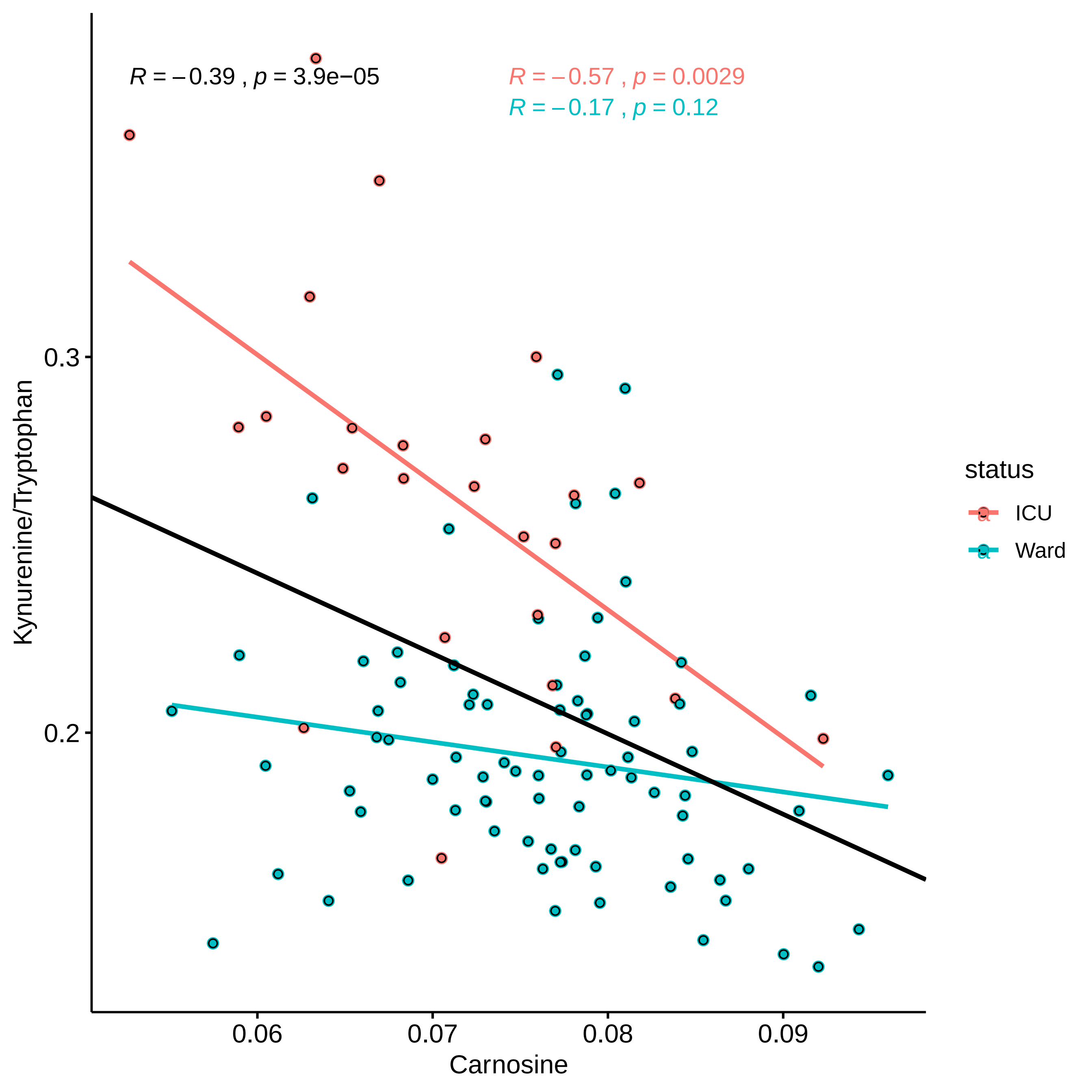

R = −0.39 , p = 3.9e−05
R = −0.57 , p = 0.0029
R = −0.17 , p = 0.12
Kynurenine/Tryptophan
0.3
0.2
0.06
0.07
0.08
0.09
Carnosine
status
ICU
Ward

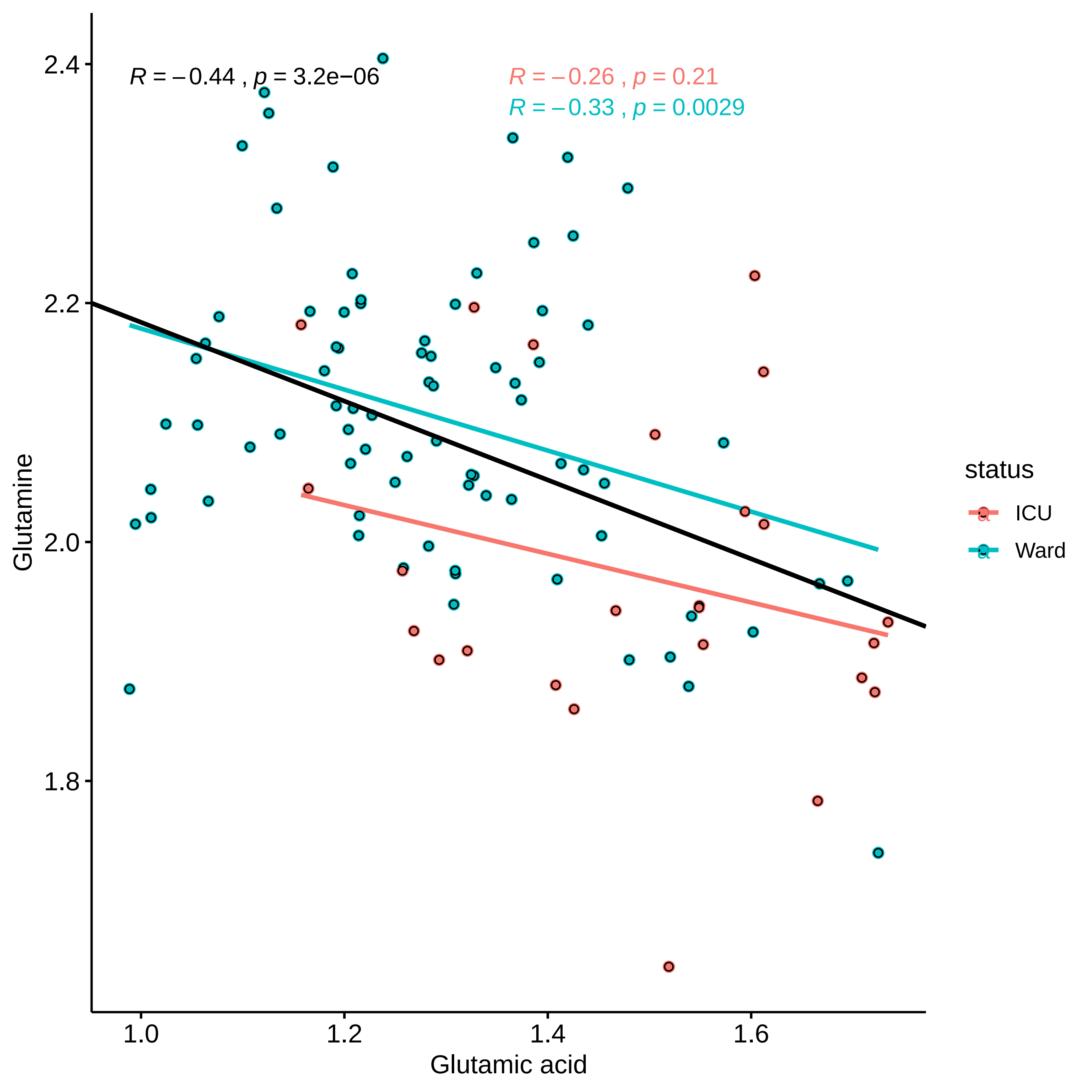

R = −0.44 , p = 3.2e−06
R = −0.26 , p = 0.21
R = −0.33 , p = 0.0029
2.4
2.2
2.0
1.8
Glutamine
1.0
1.2
1.4
1.6
Glutamic acid
status
ICU
Ward

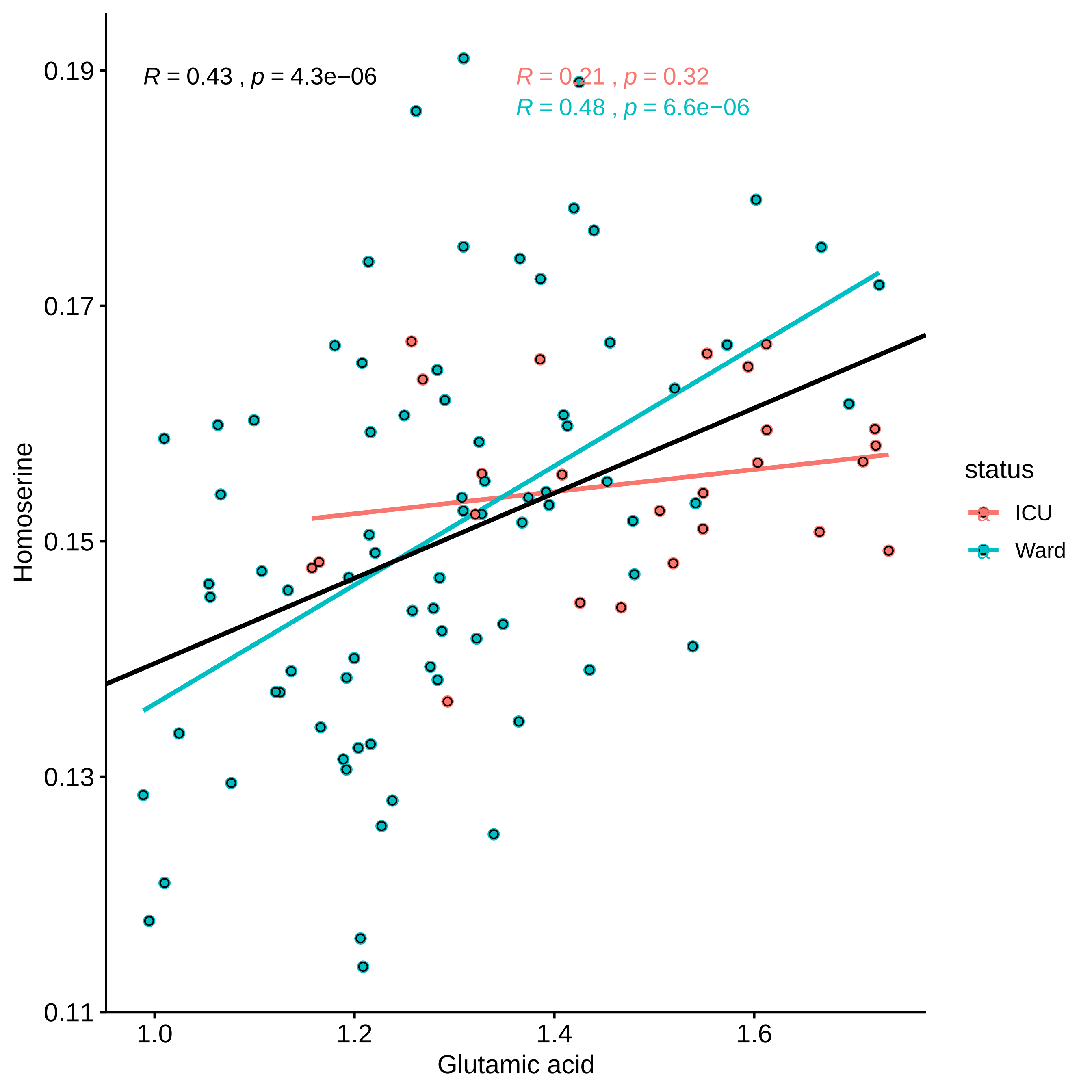

R = 0.43 , p = 4.3e−06
R = 0.21 , p = 0.32
R = 0.48 , p = 6.6e−06
Homoserine
0.19
0.17
0.15
0.13
0.11
1.0
1.2
1.4
1.6
Glutamic acid
status
ICU
Ward

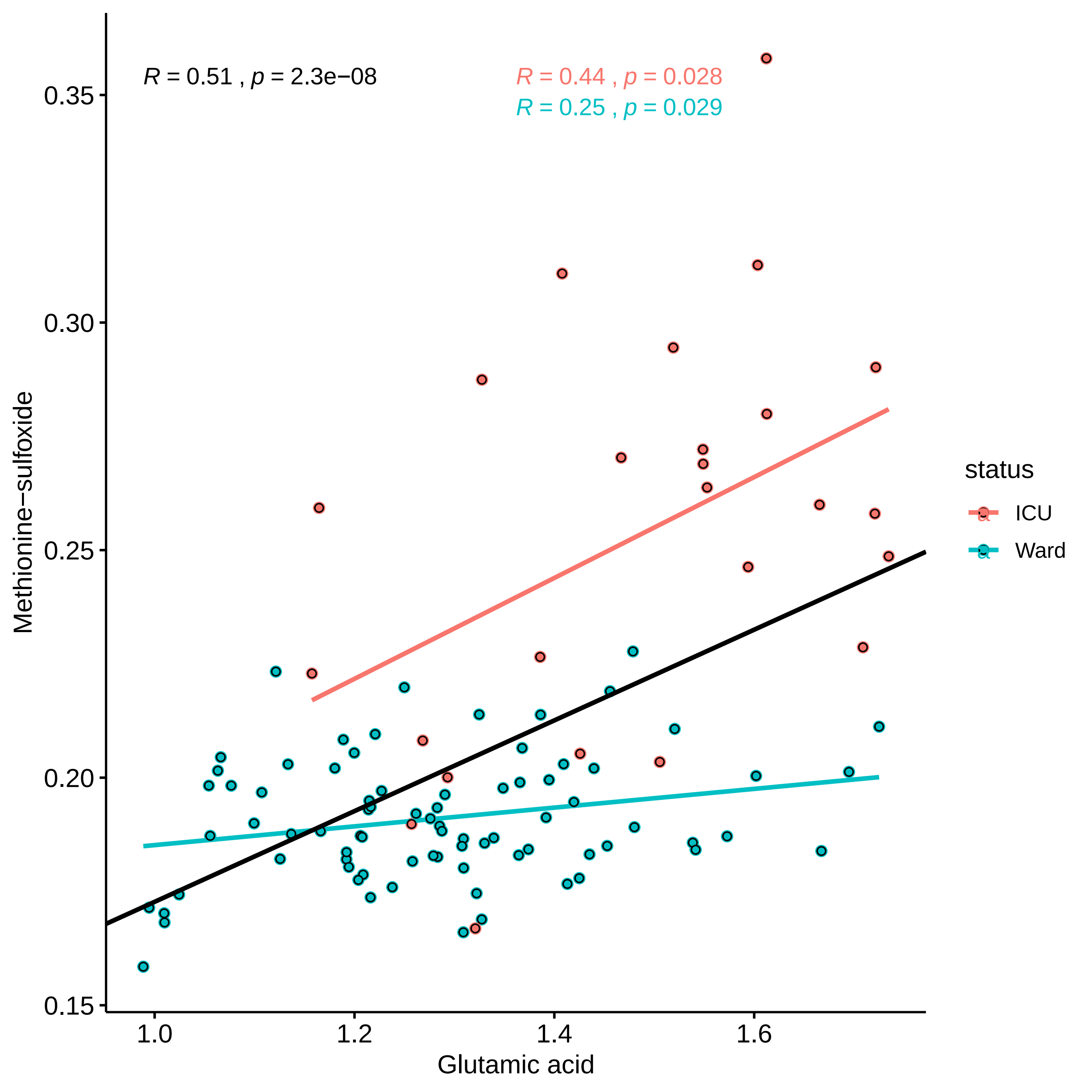

R = 0.51 , p = 2.3e−08
R = 0.44 , p = 0.028
R = 0.25 , p = 0.029
Methionine−sulfoxide
0.35
0.30
0.25
0.20
0.15
1.0
1.2
1.4
1.6
Glutamic acid
status
ICU
Ward

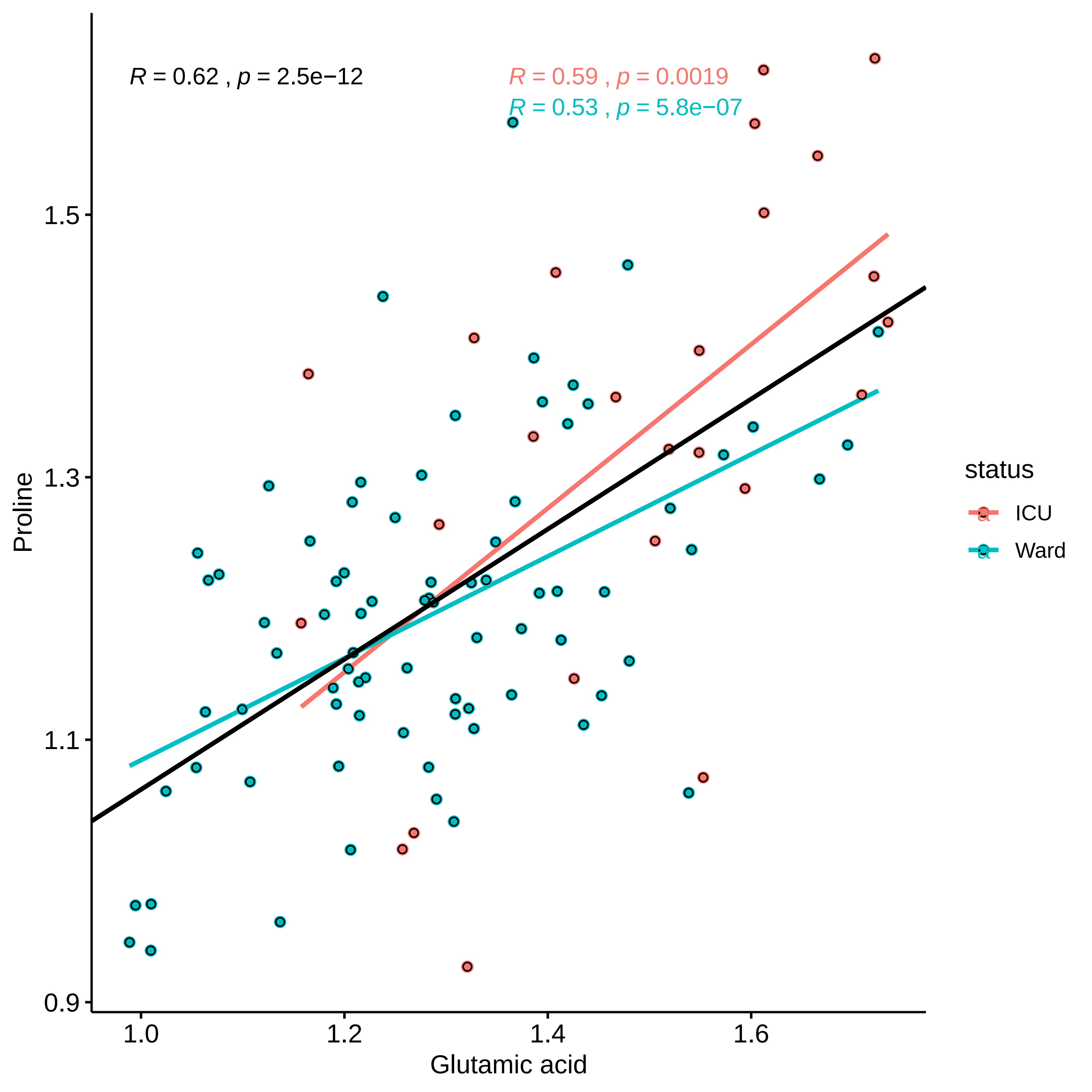

R = 0.62 , p = 2.5e−12
R = 0.59 , p = 0.0019
R = 0.53 , p = 5.8e−07
Proline
1.5
1.3
1.1
0.9
1.0
1.2
1.4
1.6
Glutamic acid
status
ICU
Ward

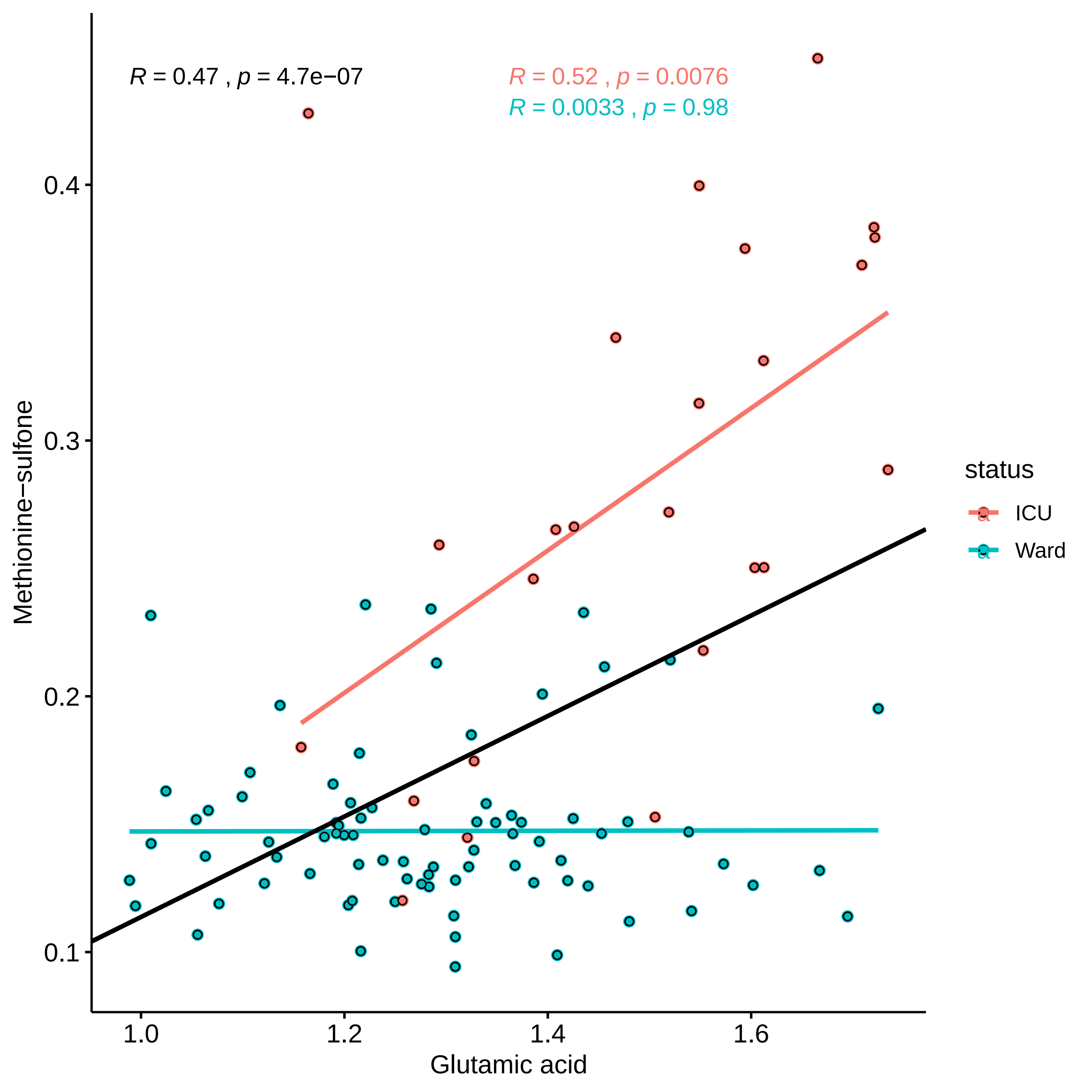

R = 0.47 , p = 4.7e−07
R = 0.52 , p = 0.0076
R = 0.0033 , p = 0.98
Methionine−sulfone
0.4
0.3
0.2
0.1
1.0
1.2
1.4
1.6
Glutamic acid
status
ICU
Ward

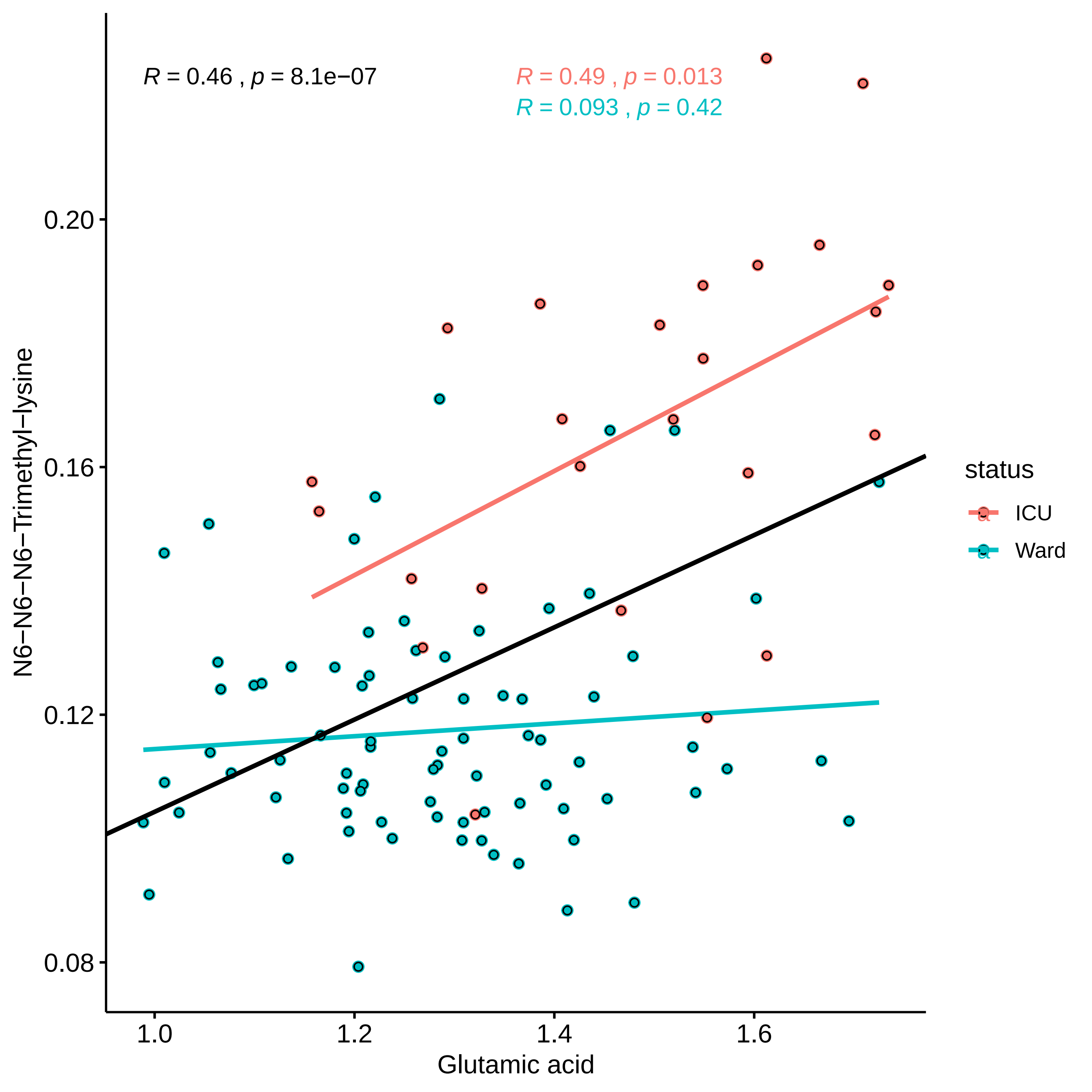
R = 0.46 , p = 8.1e−07
R = 0.49 , p = 0.013
R = 0.093 , p = 0.42
N6−N6−N6−Trimethyl−lysine
0.20
0.16
0.12
0.08
1.0
1.2
1.4
1.6
Glutamic acid
status
ICU
Ward

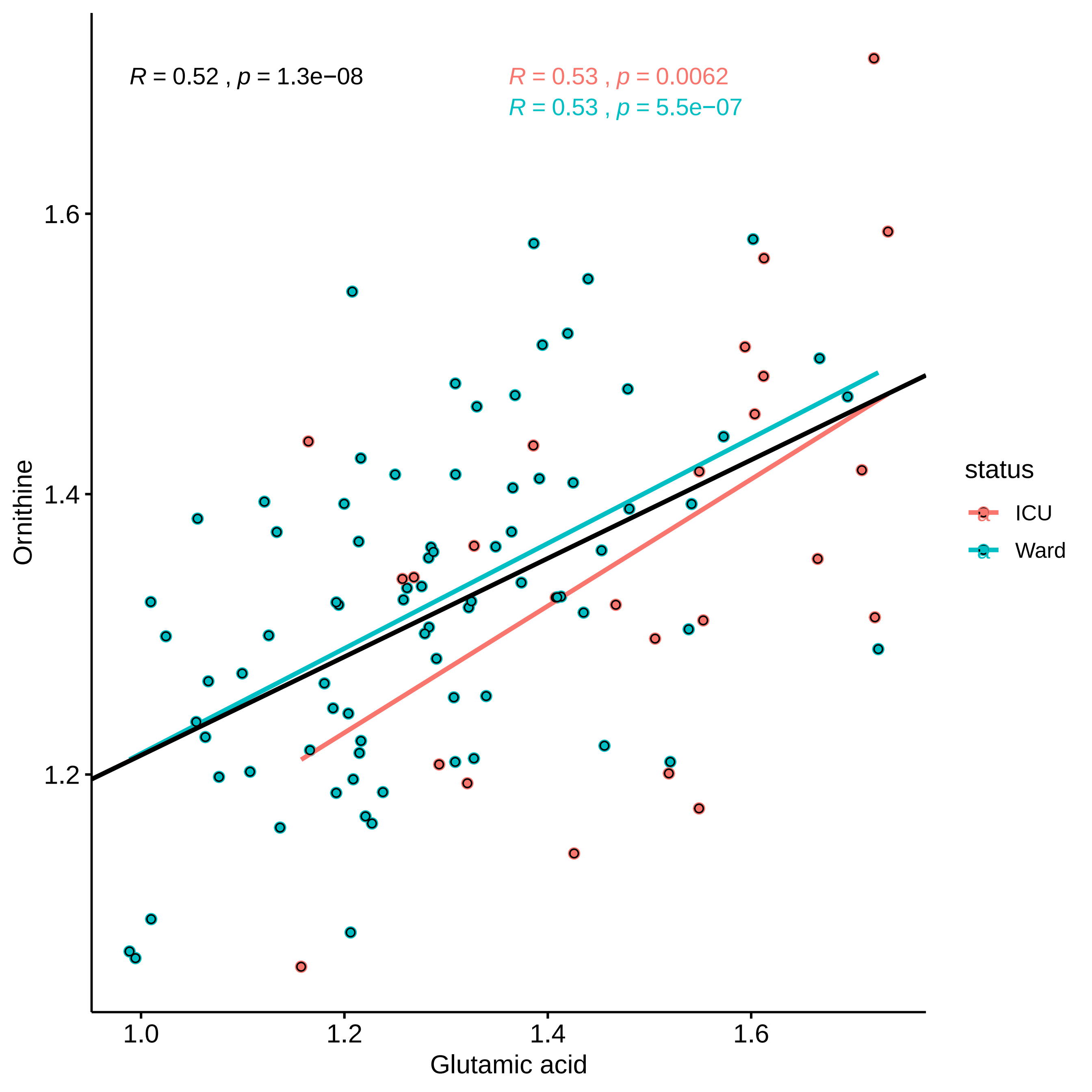

R = 0.52 , p = 1.3e−08
R = 0.53 , p = 0.0062
R = 0.53 , p = 5.5e−07
1.6
1.4
1.2
Ornithine
1.0
1.2
1.4
1.6
Glutamic acid
status
ICU
Ward

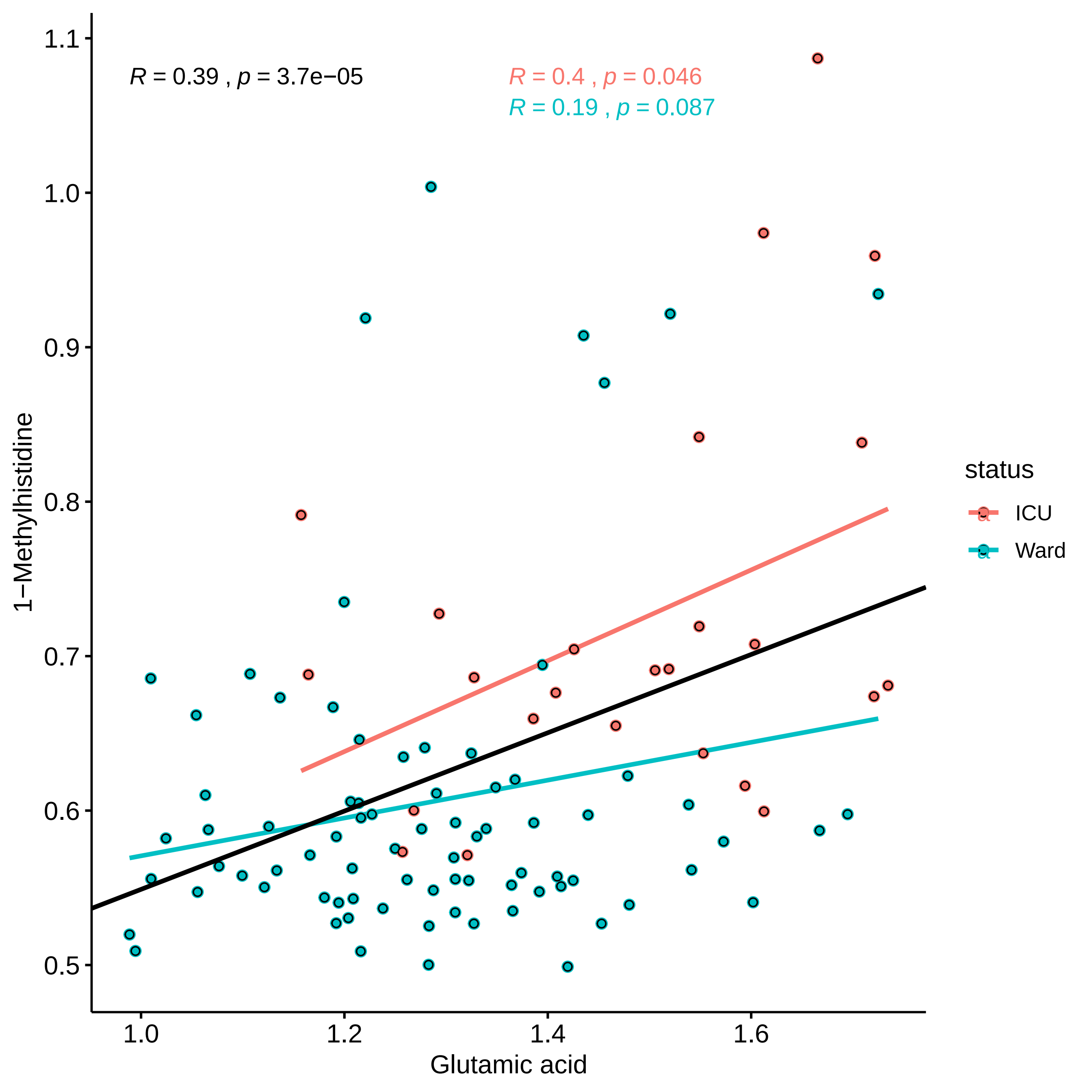

R = 0.39 , p = 3.7e−05
R = 0.4 , p = 0.046
R = 0.19 , p = 0.087
1.1
1.0
0.9
0.8
0.7
0.6
0.5
1−Methylhistidine
1.0
1.2
1.4
1.6
Glutamic acid
status
ICU
Ward

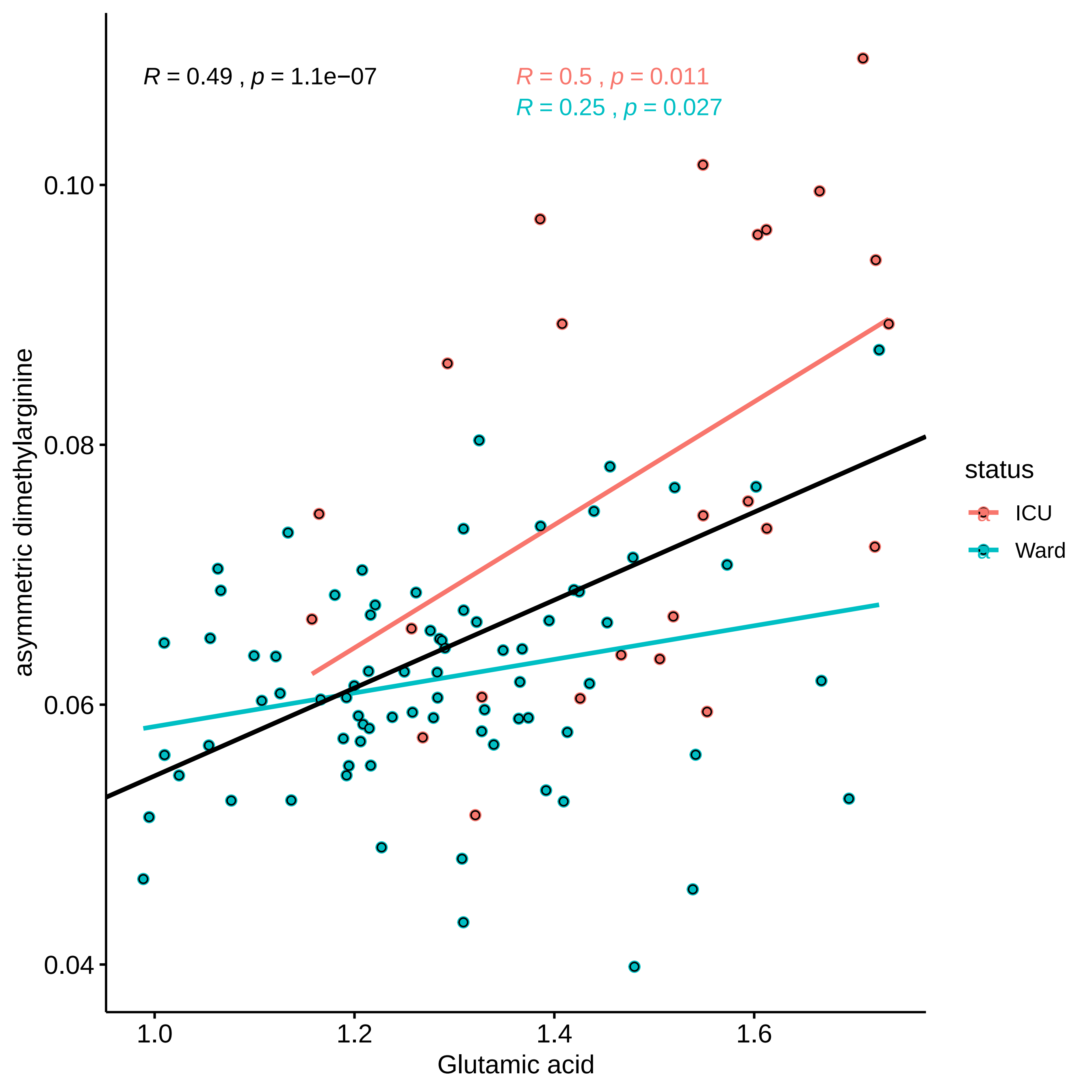

R = 0.49 , p = 1.1e−07
R = 0.5 , p = 0.011
R = 0.25 , p = 0.027
0.10
0.08
0.06
0.04
asymmetric dimethylarginine
1.0
1.2
1.4
1.6
Glutamic acid
status
ICU
Ward

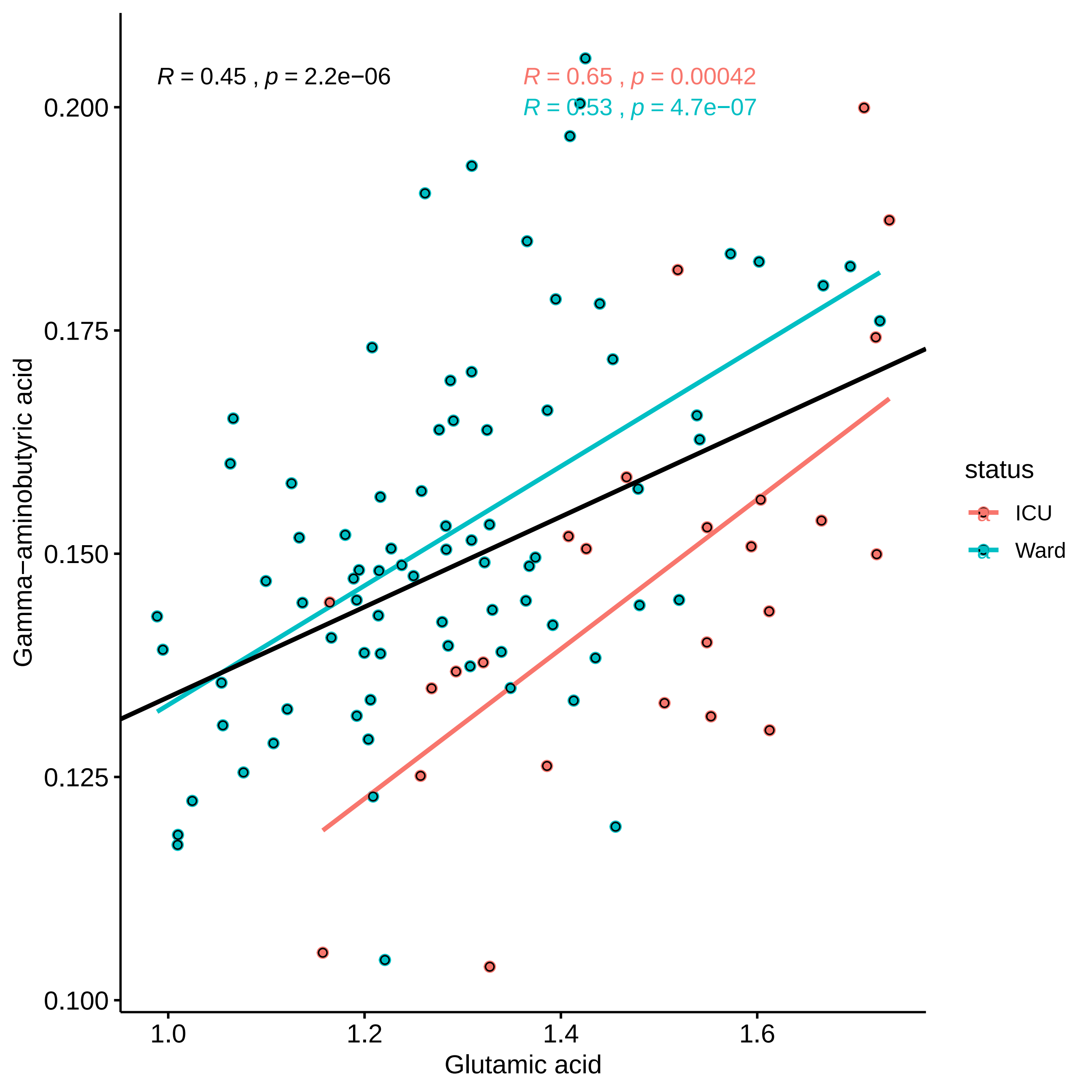

R = 0.45 , p = 2.2e−06
R = 0.65 , p = 0.00042
R = 0.53 , p = 4.7e−07
0.200
0.175
0.150
0.125
0.100
Gamma−aminobutyric acid
1.0
1.2
1.4
1.6
Glutamic acid
status
ICU
Ward

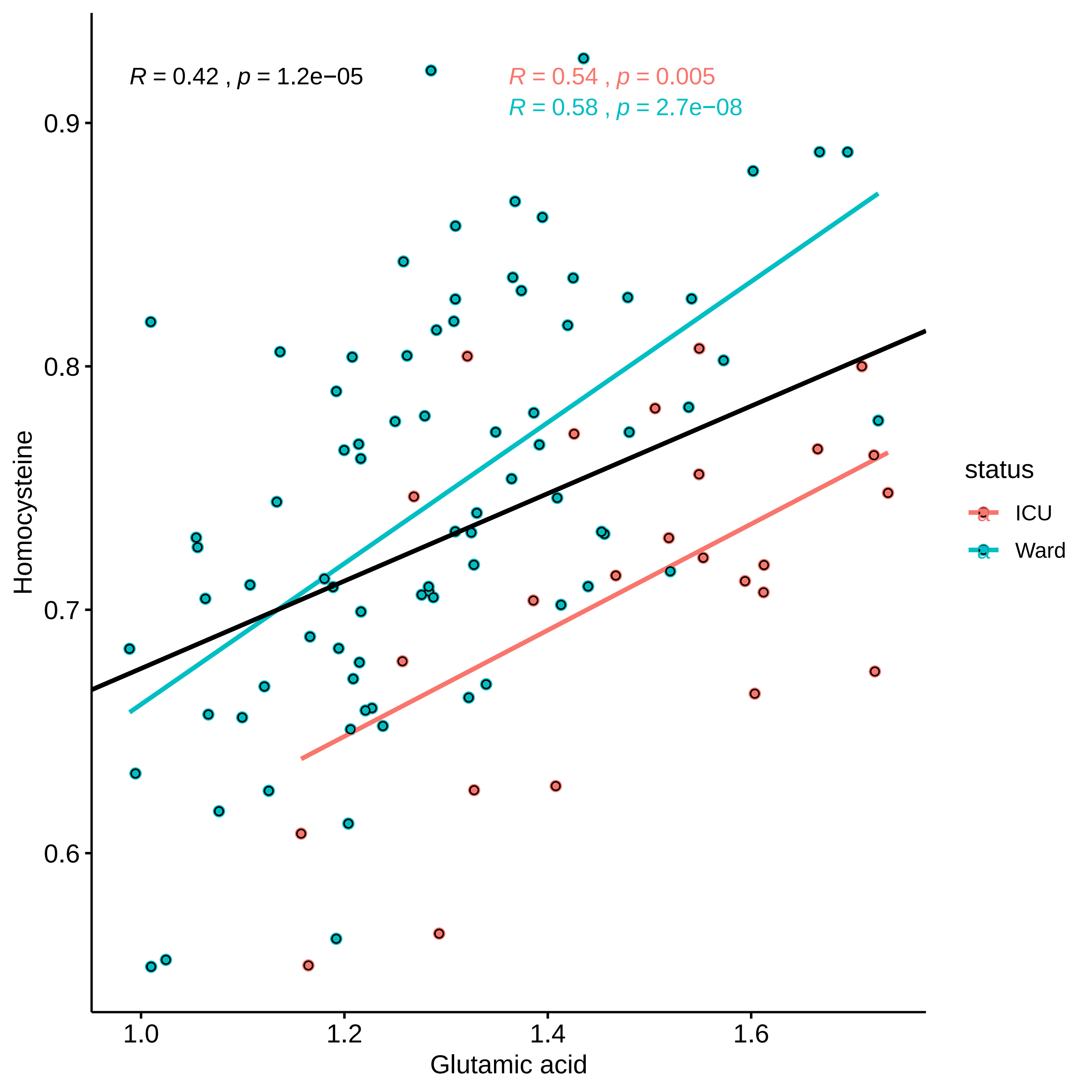

R = 0.42 , p = 1.2e−05
R = 0.54 , p = 0.005
R = 0.58 , p = 2.7e−08
0.9
0.8
0.7
0.6
Homocysteine
1.0
1.2
1.4
1.6
Glutamic acid
status
ICU
Ward

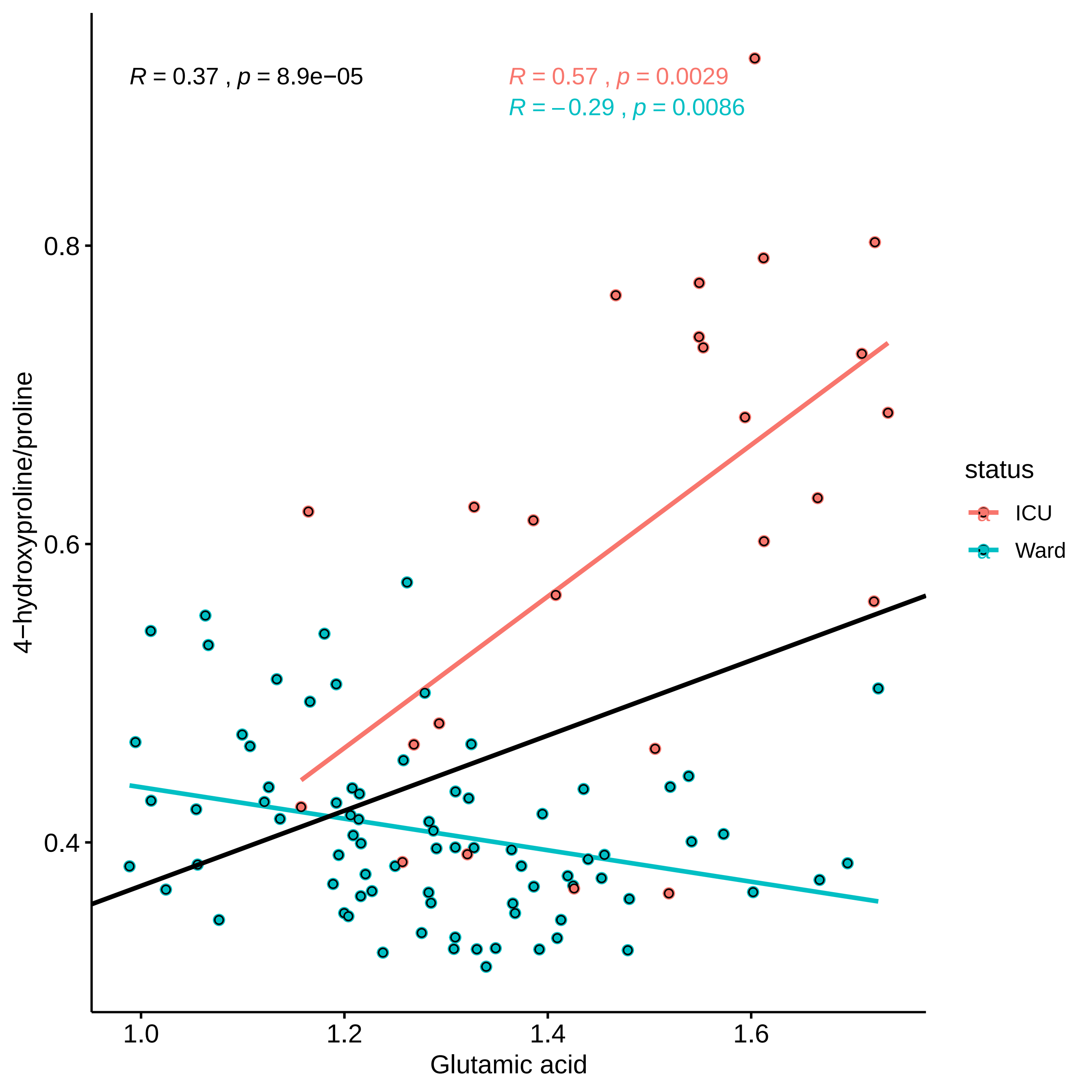

R = 0.37 , p = 8.9e−05
R = 0.57 , p = 0.0029
R = −0.29 , p = 0.0086
0.8
0.6
0.4
4−hydroxyproline/proline
1.0
1.2
1.4
1.6
Glutamic acid
status
ICU
Ward

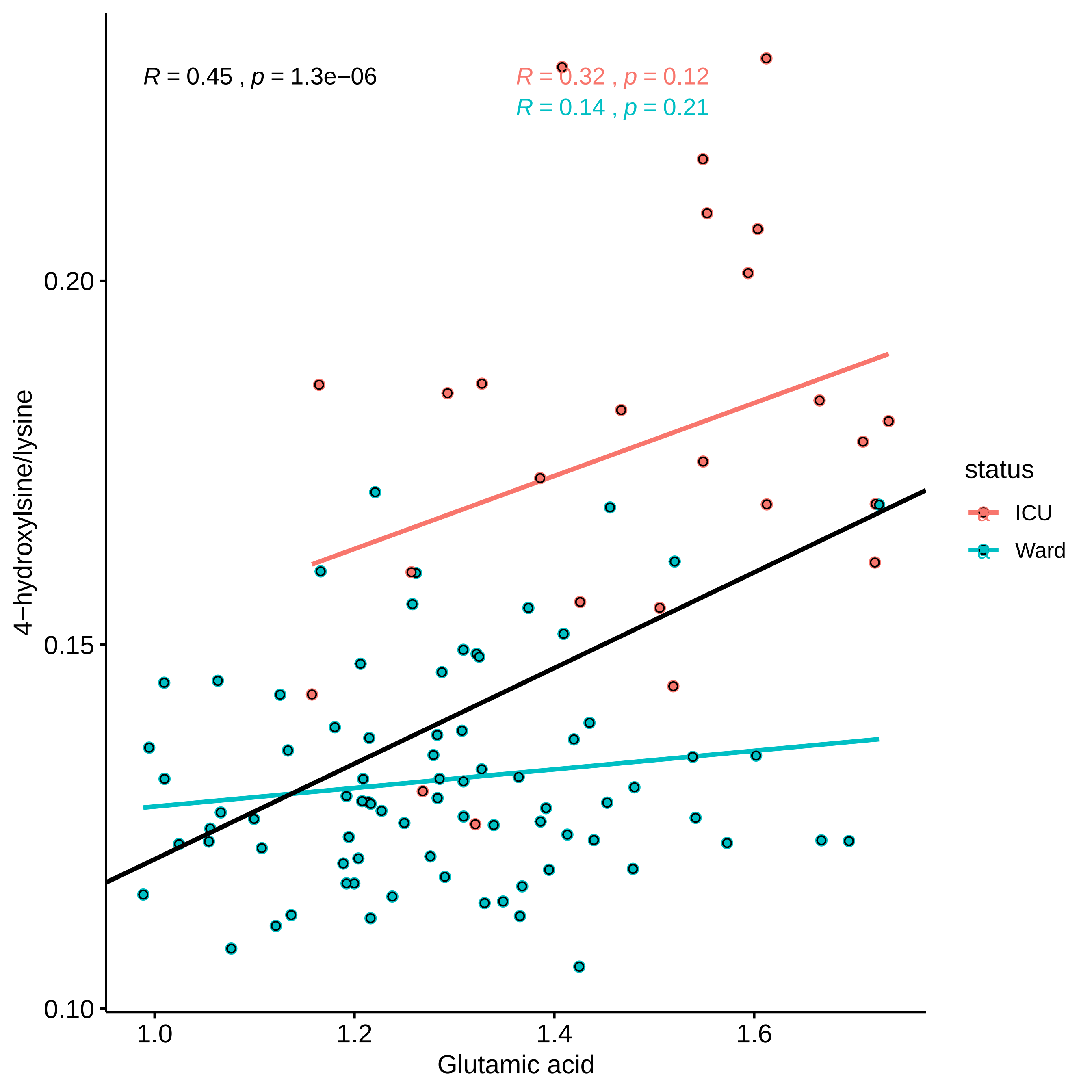
R = 0.45 , p = 1.3e−06
R = 0.32 , p = 0.12
R = 0.14 , p = 0.21
4−hydroxylsine/lysine
0.20
0.15
0.10
1.0
1.2
1.4
1.6
Glutamic acid
status
ICU
Ward

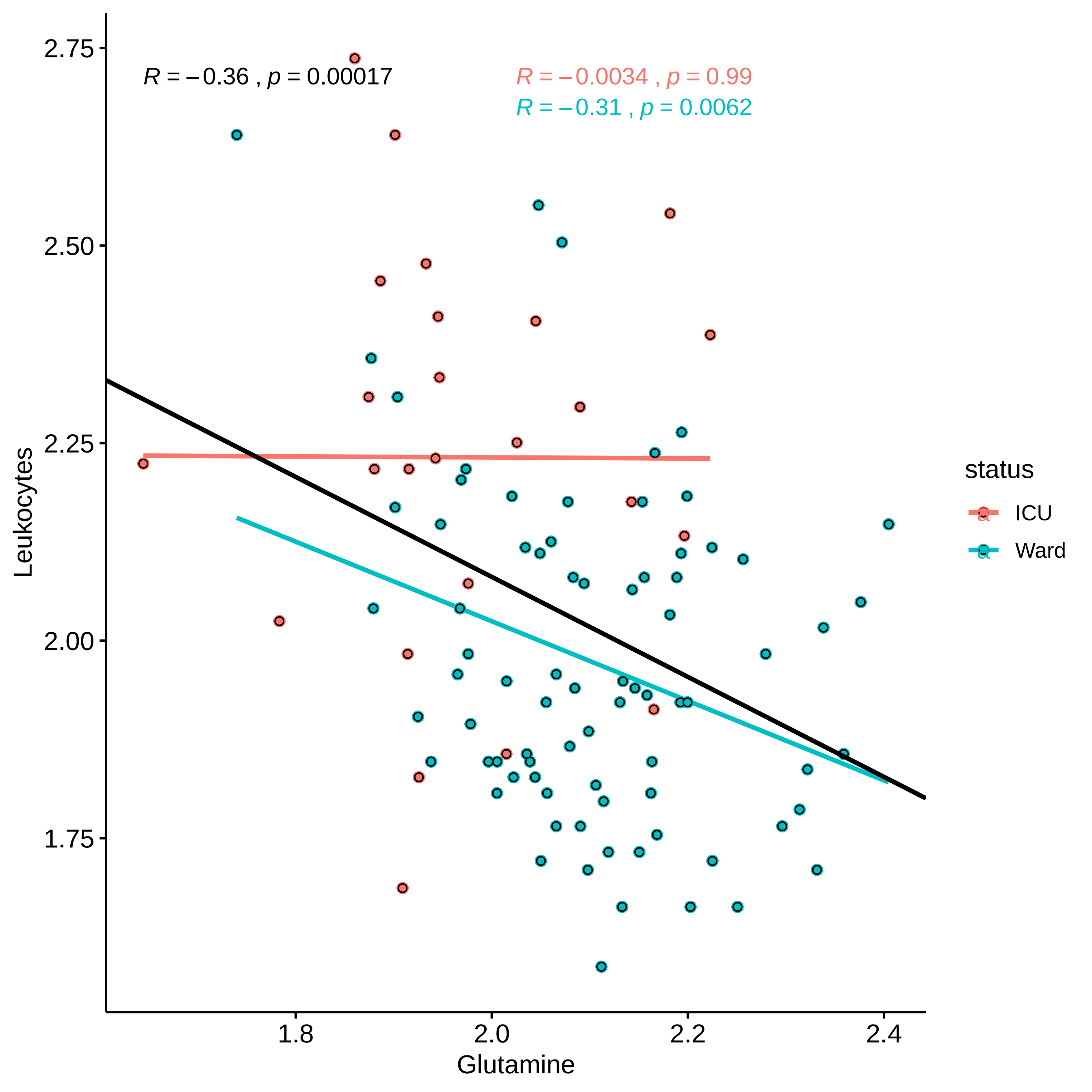

R = −0.36 , p = 0.00017
R = −0.0034 , p = 0.99
R = −0.31 , p = 0.0062
2.75
2.50
2.25
2.00
1.75
Leukocytes
1.8
2.0
2.2
2.4
Glutamine
status
ICU
Ward

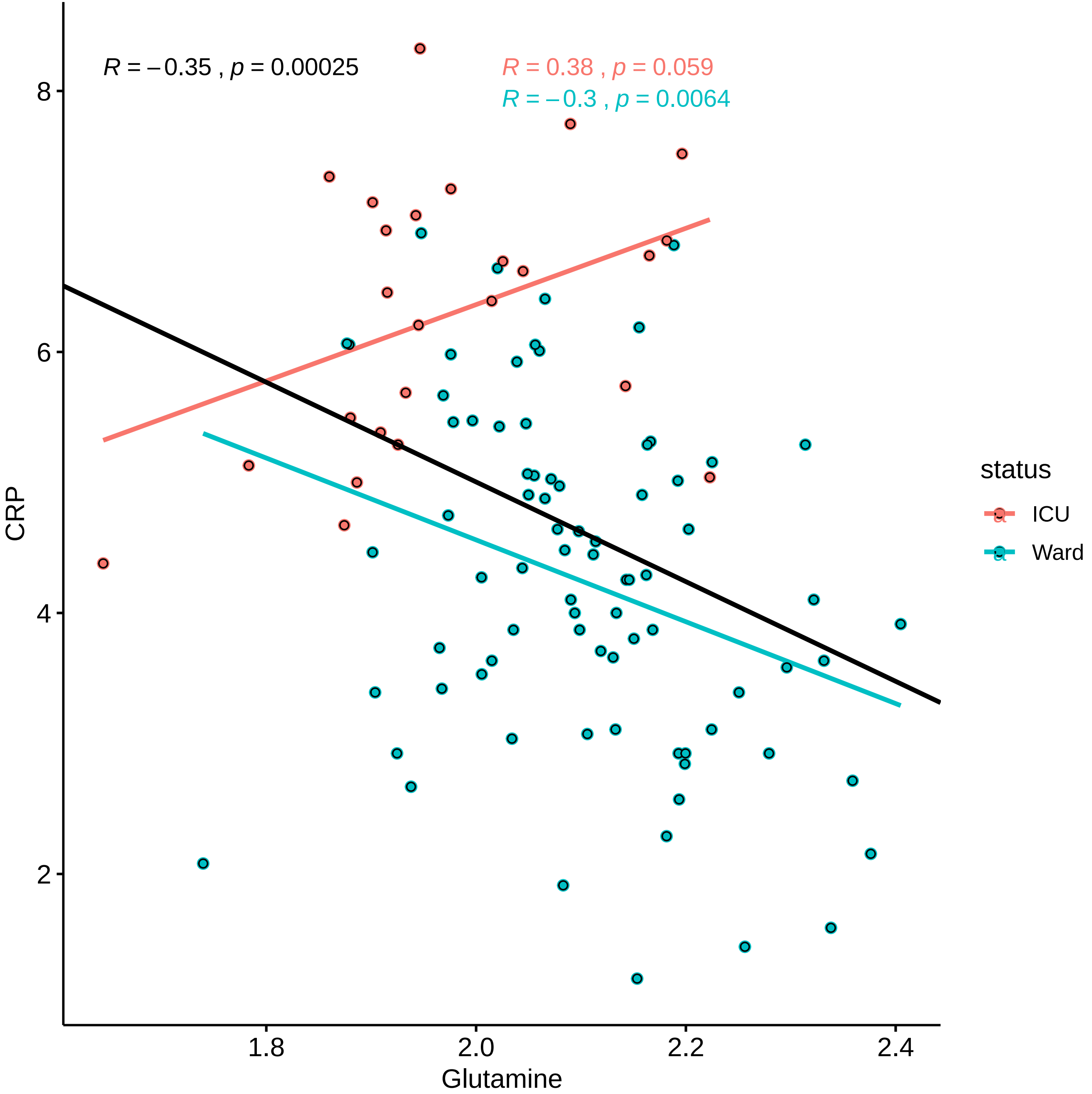

R = −0.35 , p = 0.00025
R = 0.38 , p = 0.059
R = −0.3 , p = 0.0064
CRP
8
6
4
2
1.8
2.0
2.2
2.4
Glutamine
status
ICU
Ward

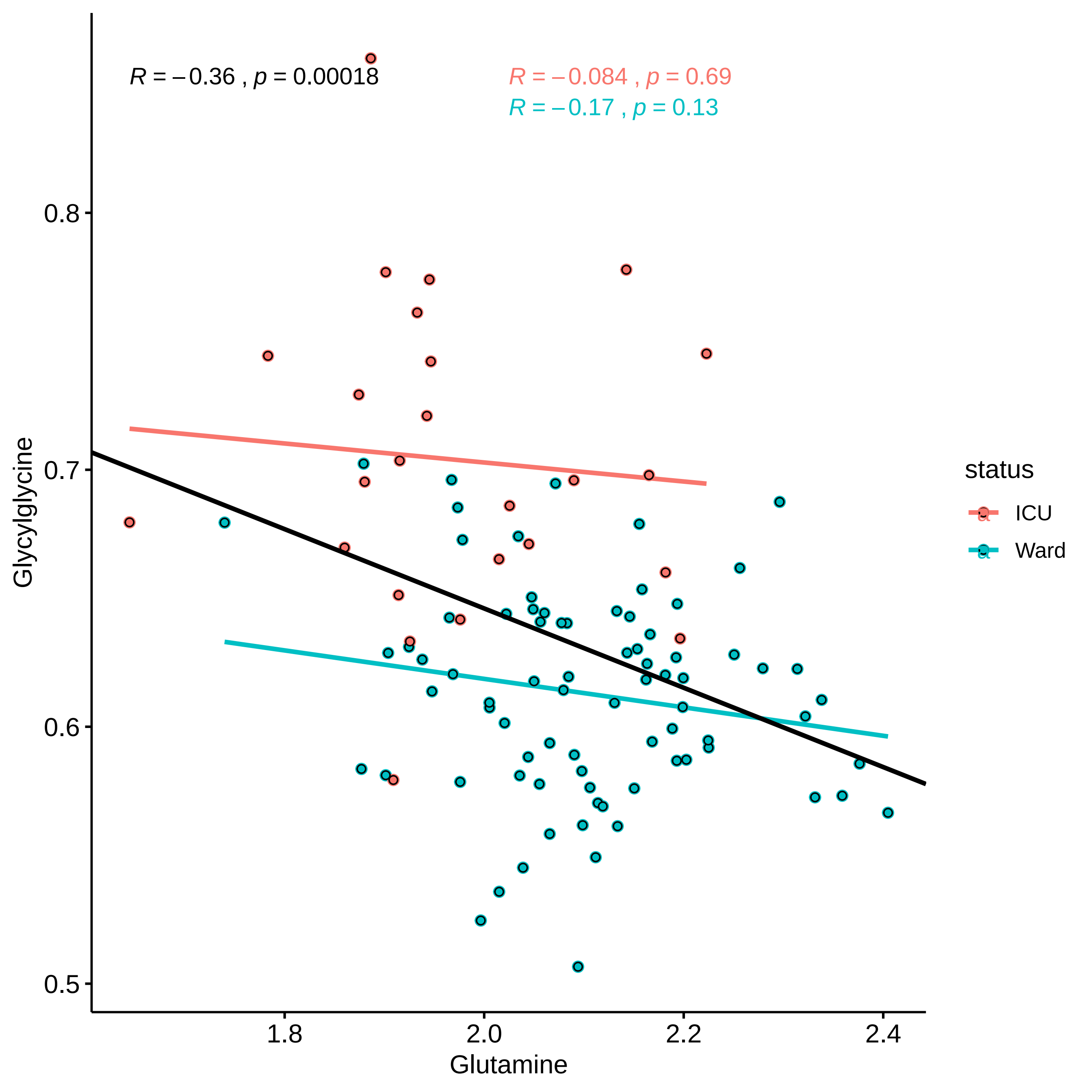
R = −0.36 , p = 0.00018
R = −0.084 , p = 0.69
R = −0.17 , p = 0.13
Glycylglycine
0.8
0.7
0.6
0.5
1.8
2.0
2.2
2.4
Glutamine
status
ICU
Ward

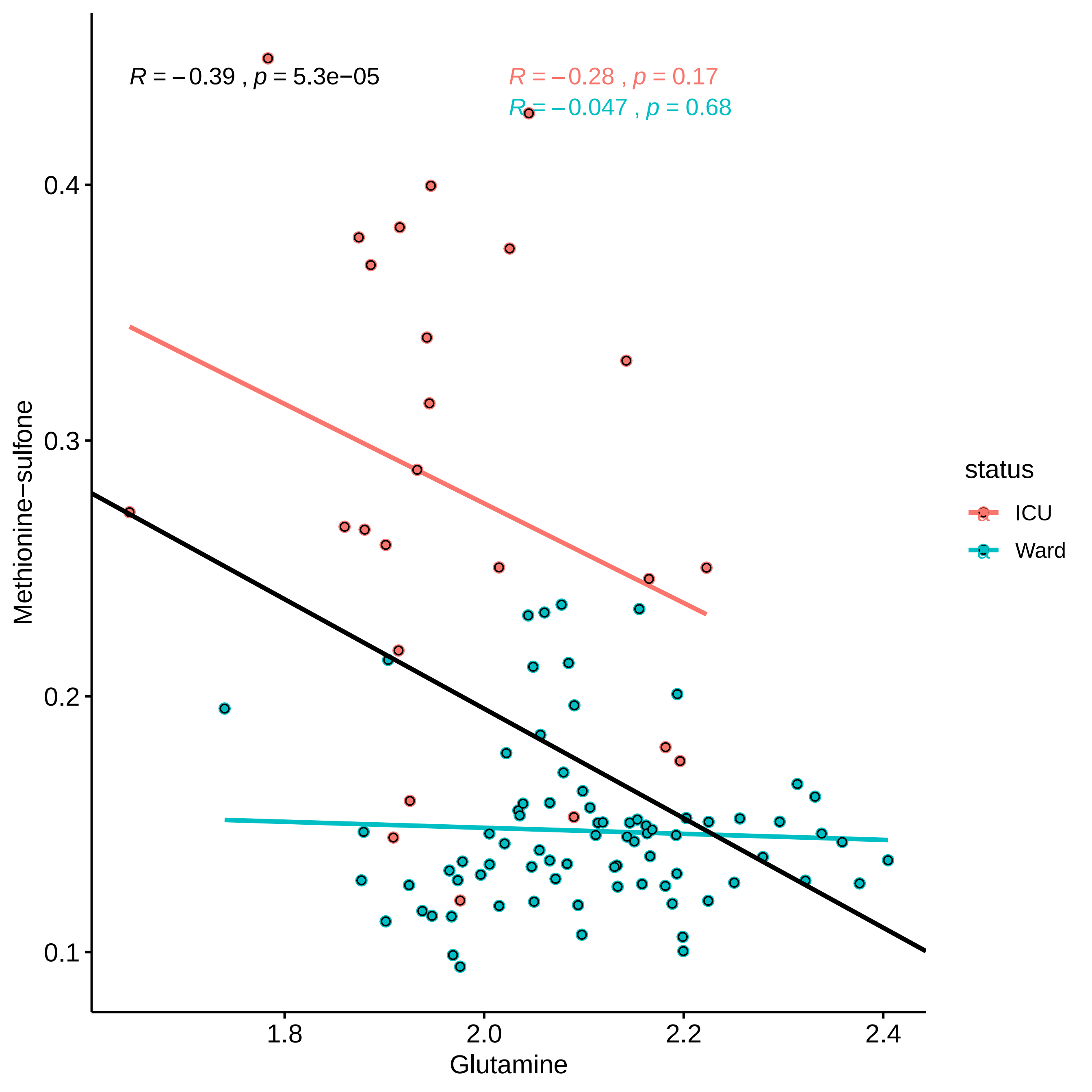
R = −0.39 , p = 5.3e−05
R = −0.28 , p = 0.17
R = −0.047 , p = 0.68
Methionine−sulfone
0.4
0.3
0.2
0.1
1.8
2.0
2.2
2.4
Glutamine
status
ICU
Ward

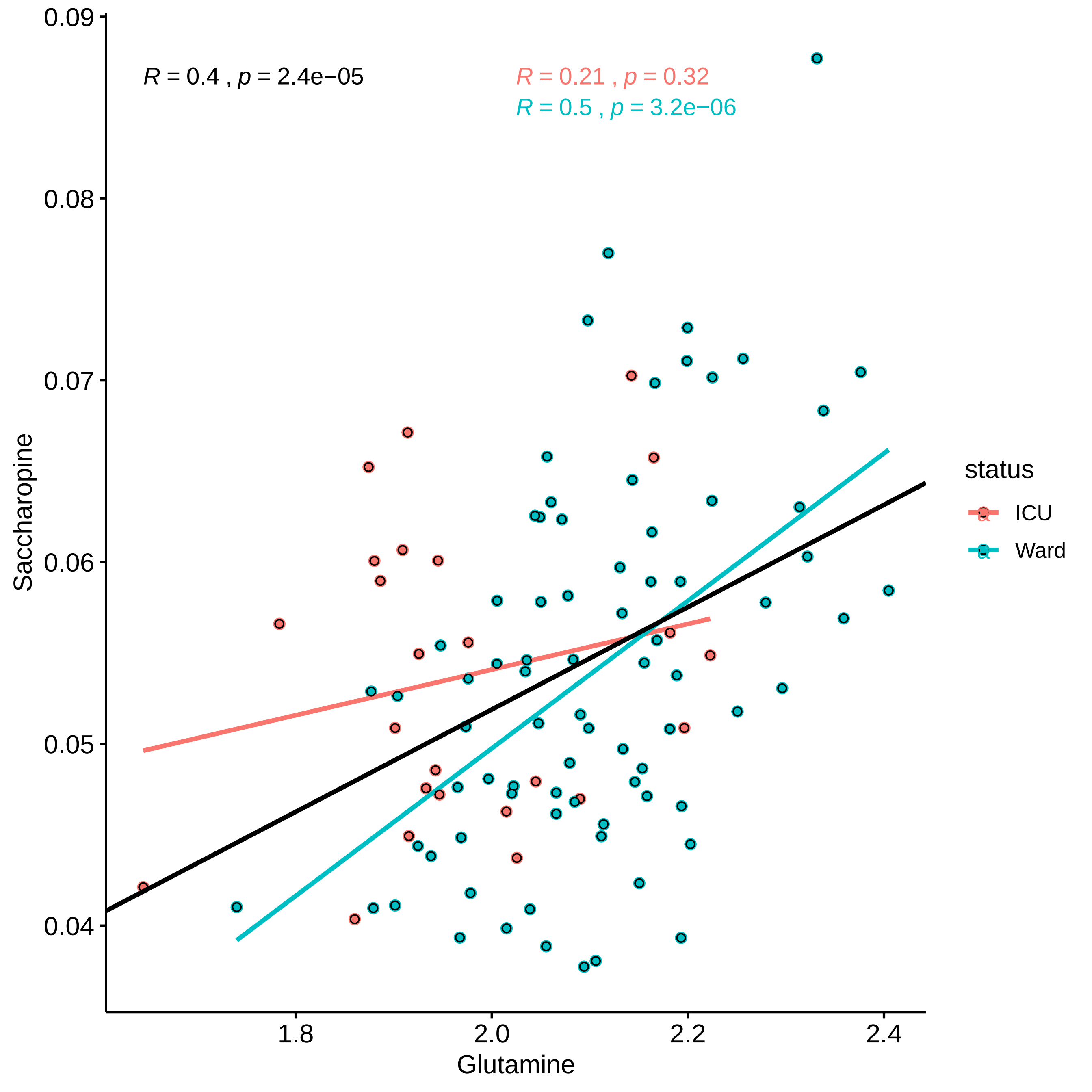

R = 0.4 , p = 2.4e−05
R = 0.21 , p = 0.32
R = 0.5 , p = 3.2e−06
0.09
0.08
0.07
0.06
0.05
0.04
Saccharopine
1.8
2.0
2.2
2.4
Glutamine
status
ICU
Ward

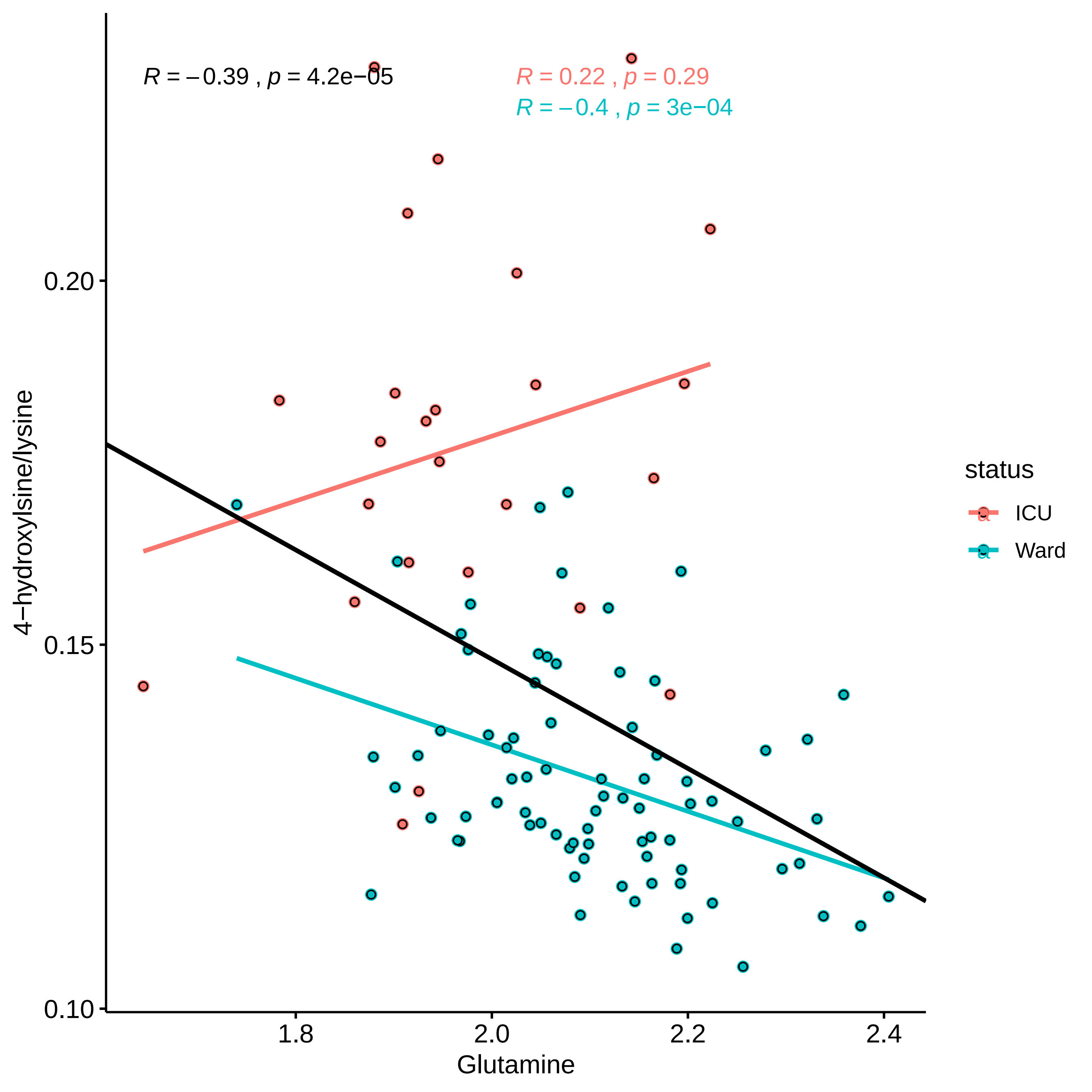
R = −0.39 , p = 4.2e−05
R = 0.22 , p = 0.29
R = −0.4 , p = 3e−04
4−hydroxylysine/lysine
0.20
0.15
0.10
1.8
2.0
2.2
2.4
Glutamine
status
ICU
Ward

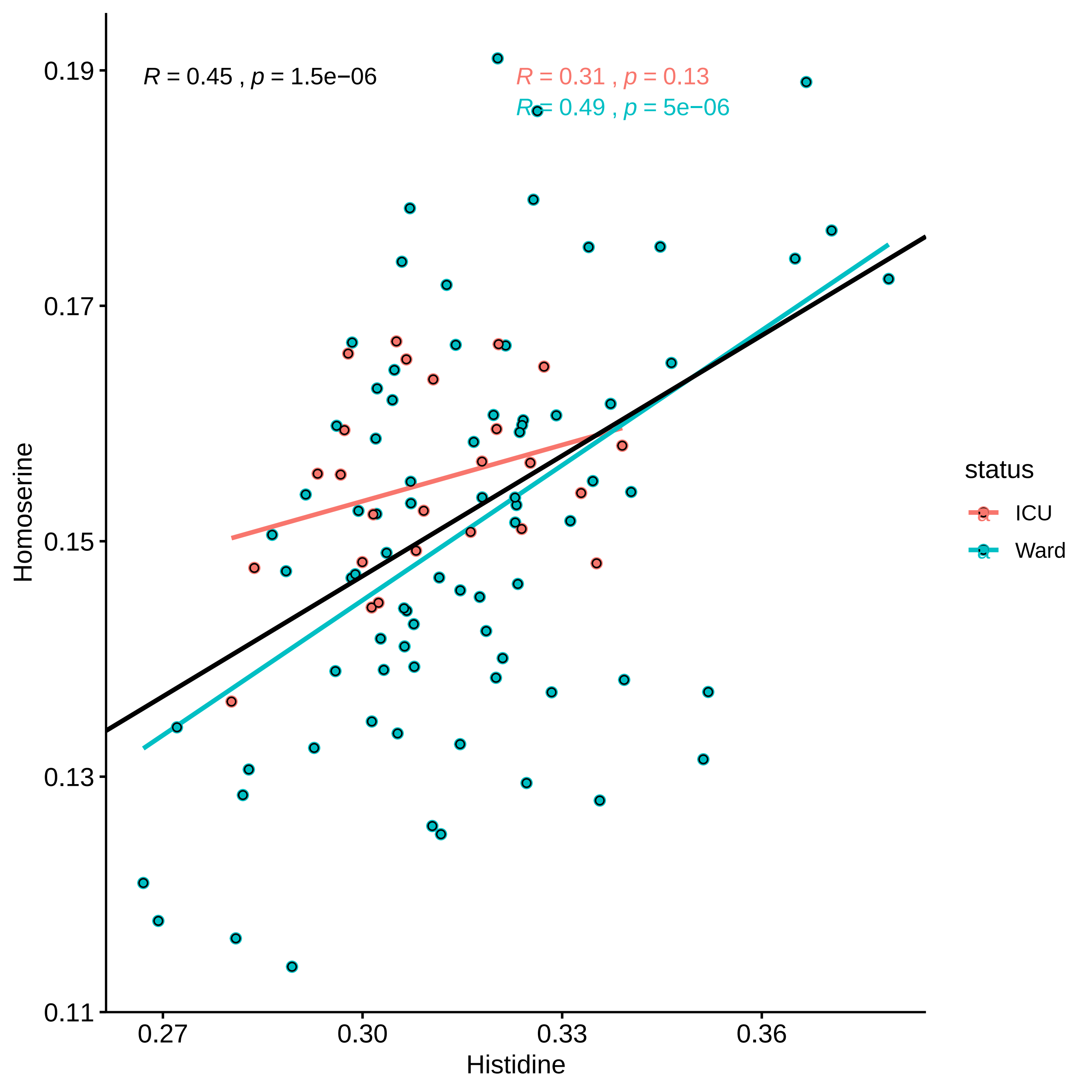

R = 0.45 , p = 1.5e−06
R = 0.31 , p = 0.13
R = 0.49 , p = 5e−06
0.19
0.17
0.15
0.13
0.11
Homoserine
0.27
0.30
0.33
0.36
Histidine
status
ICU
Ward

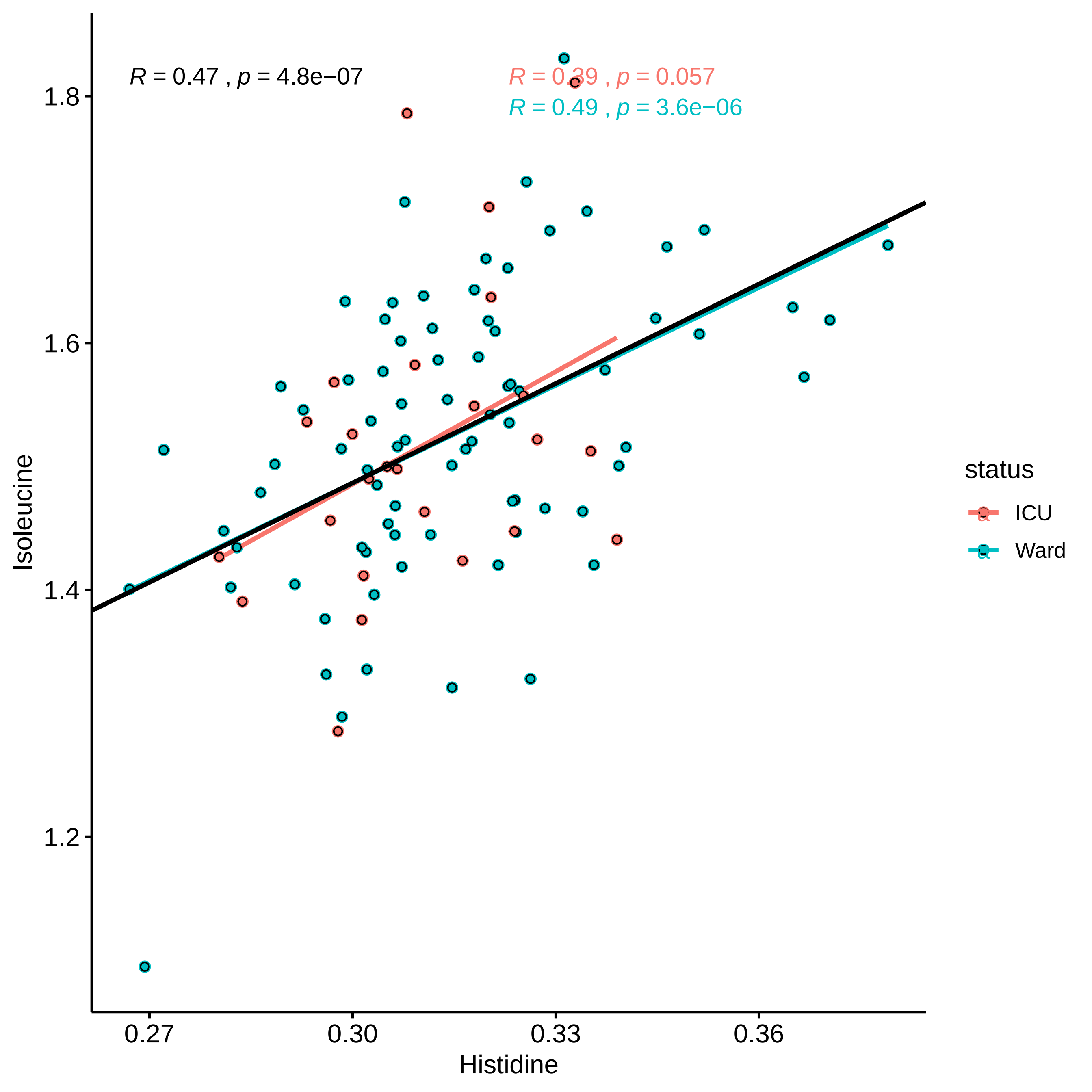

R = 0.47 , p = 4.8e−07
R = 0.39 , p = 0.057
R = 0.49 , p = 3.6e−06
1.8
1.6
1.4
1.2
Isoleucine
0.27
0.30
0.33
0.36
Histidine
status
ICU
Ward

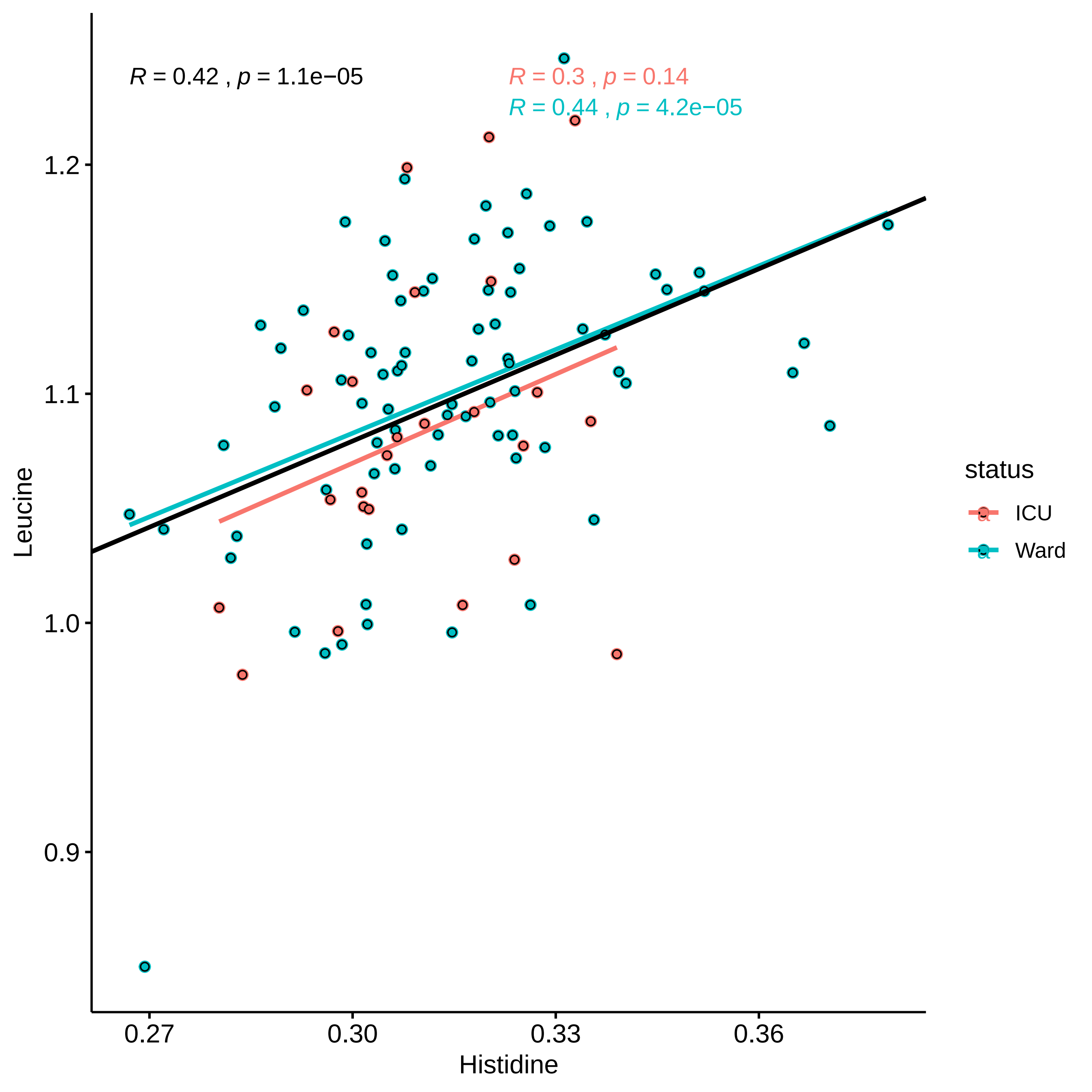
R = 0.42 , p = 1.1e−05
R = 0.3 , p = 0.14
R = 0.44 , p = 4.2e−05
Leucine
1.2
1.1
1.0
0.9
0.27
0.30
0.33
0.36
Histidine
status
ICU
Ward

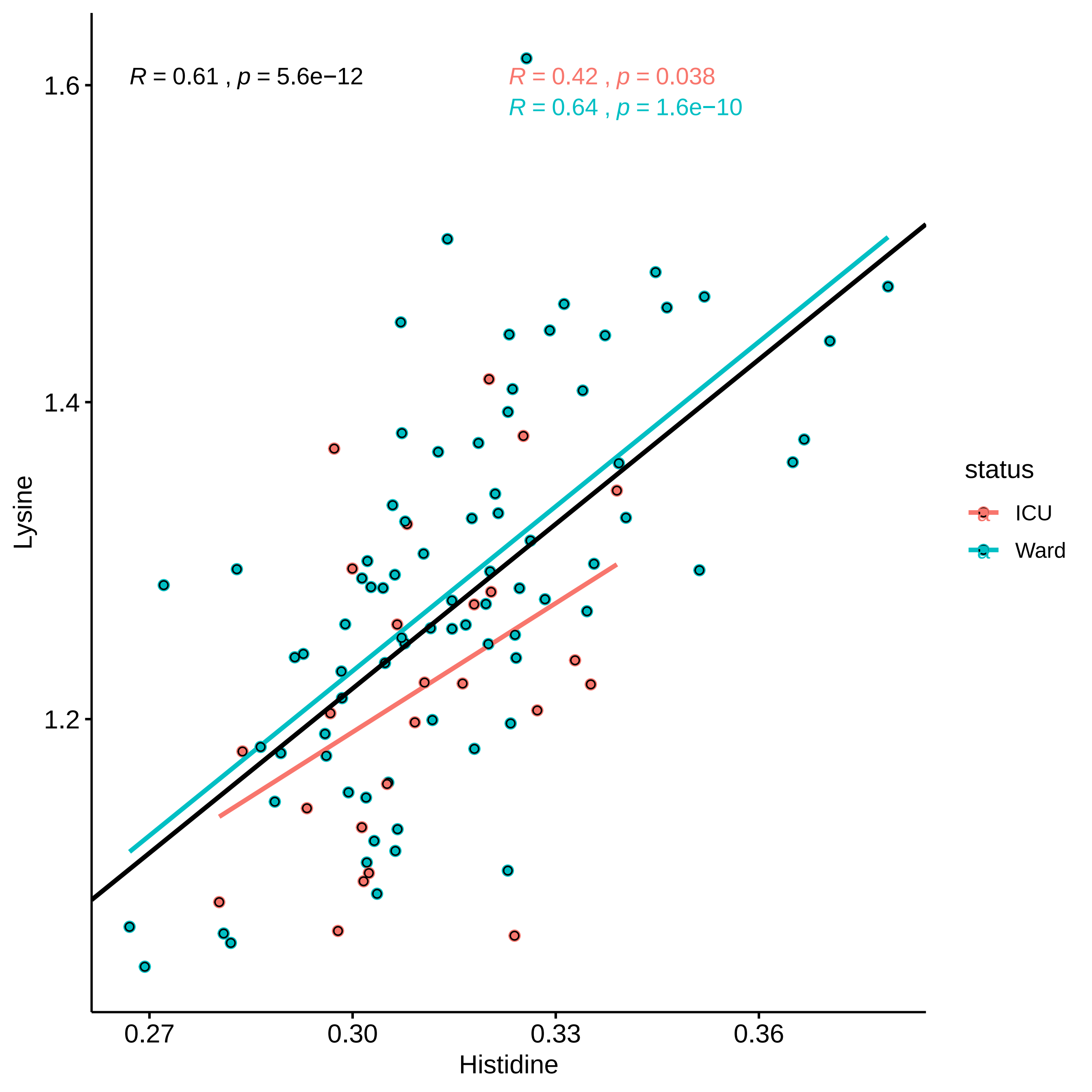
R = 0.61 , p = 5.6e−12
R = 0.42 , p = 0.038
R = 0.64 , p = 1.6e−10
1.6
1.4
1.2
0.27
0.30
0.33
0.36
Lysine
Histidine
status
ICU
Ward

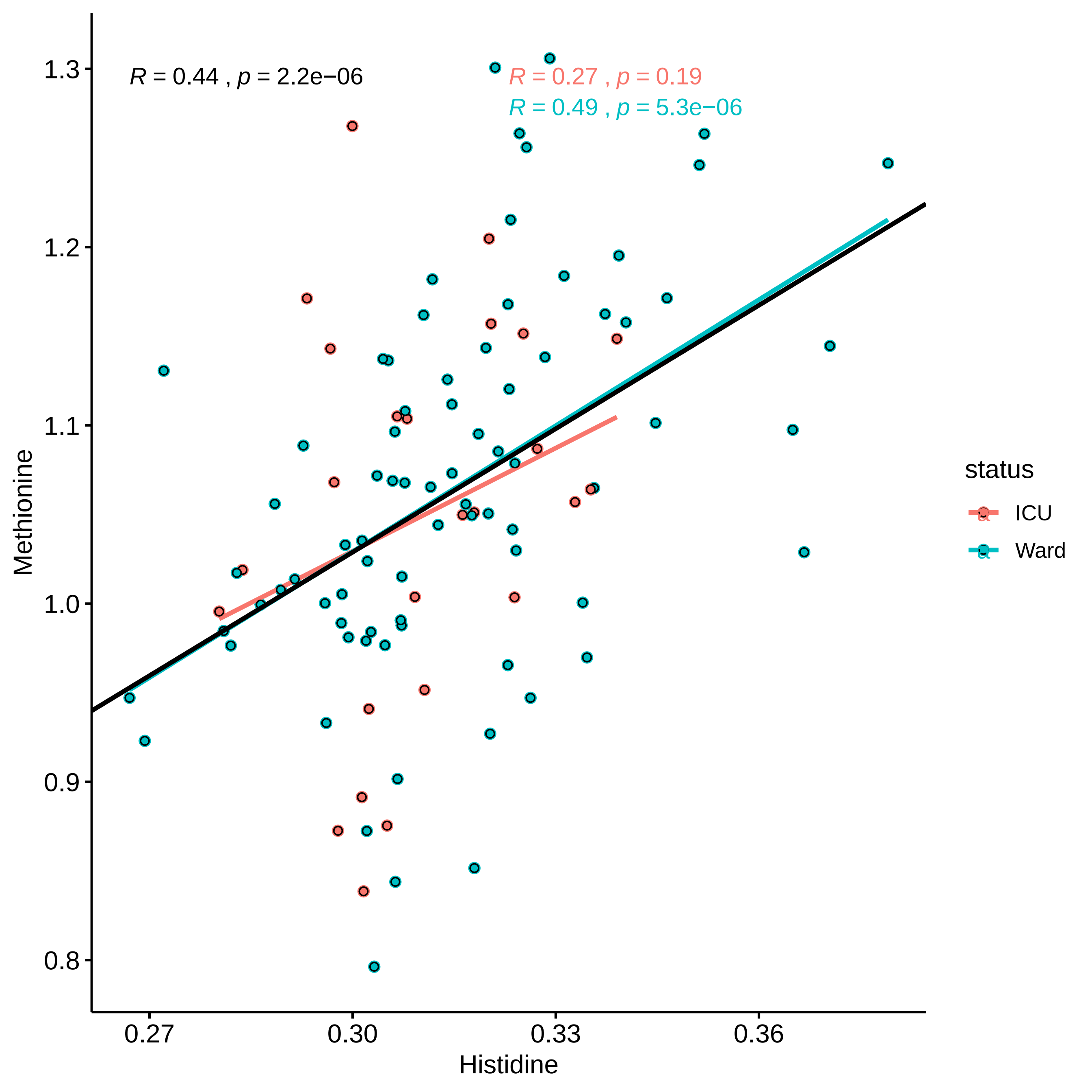

R = 0.44 , p = 2.2e−06
R = 0.27 , p = 0.19
R = 0.49 , p = 5.3e−06
Methionine
1.3
1.2
1.1
1.0
0.9
0.8
0.27
0.30
0.33
0.36
Histidine
status
ICU
Ward

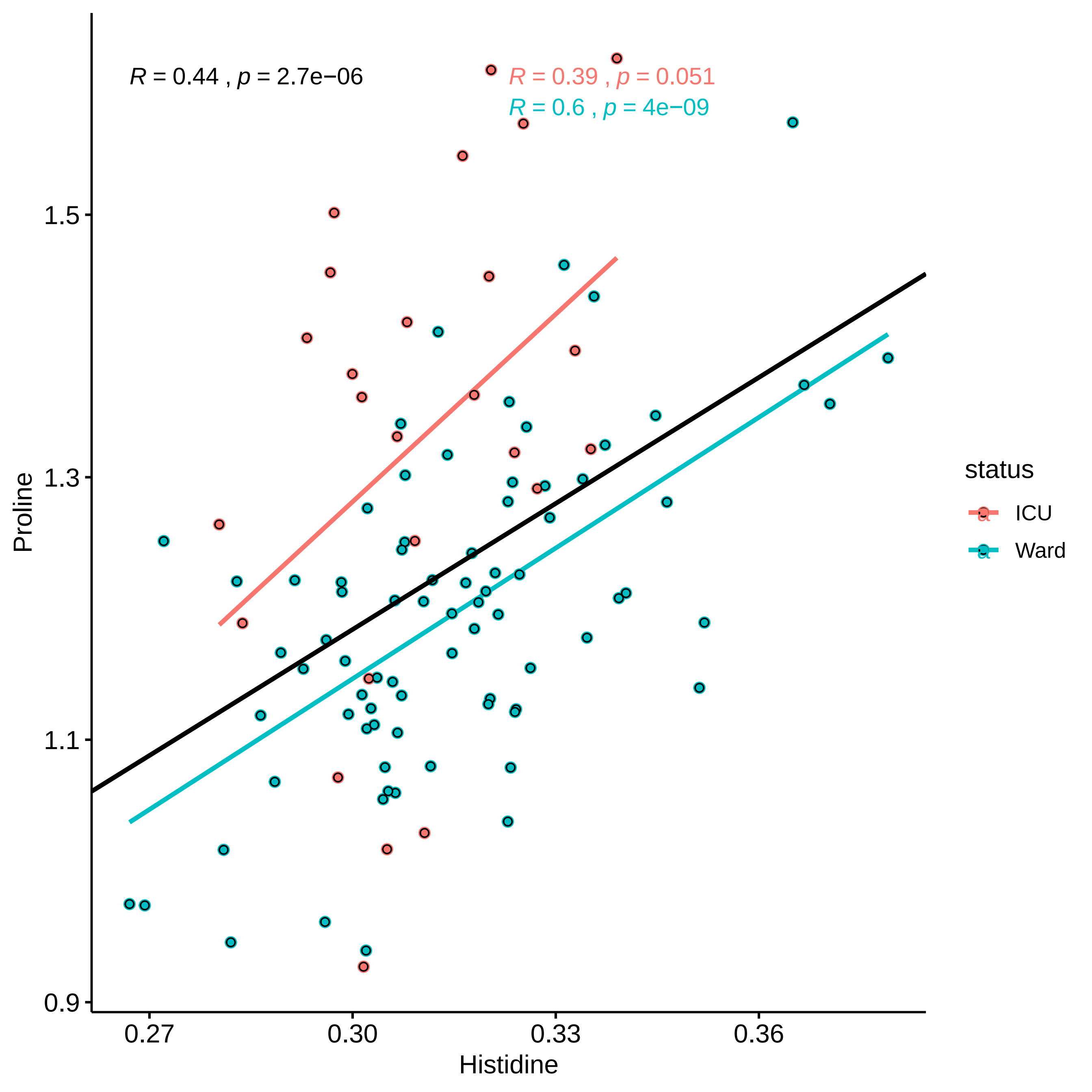

R = 0.44 , p = 2.7e−06
R = 0.39 , p = 0.051
R = 0.6 , p = 4e−09
Proline
1.5
1.3
1.1
0.9
0.27
0.30
0.33
0.36
Histidine
status
ICU
Ward

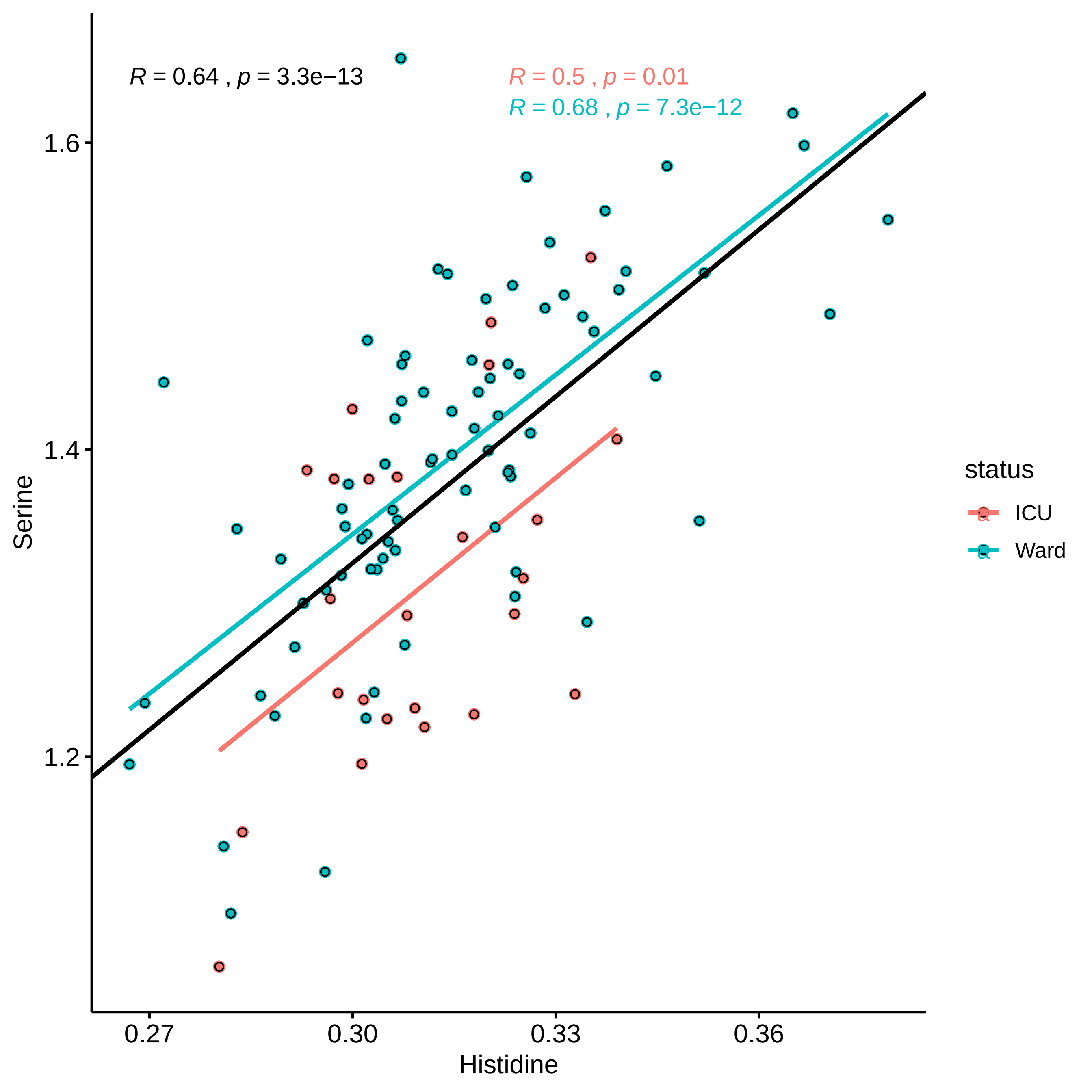

R = 0.64 , p = 3.3e−13
R = 0.5 , p = 0.01
R = 0.68 , p = 7.3e−12
1.6
1.4
1.2
Serine
0.27
0.30
0.33
0.36
Histidine
status
ICU
Ward

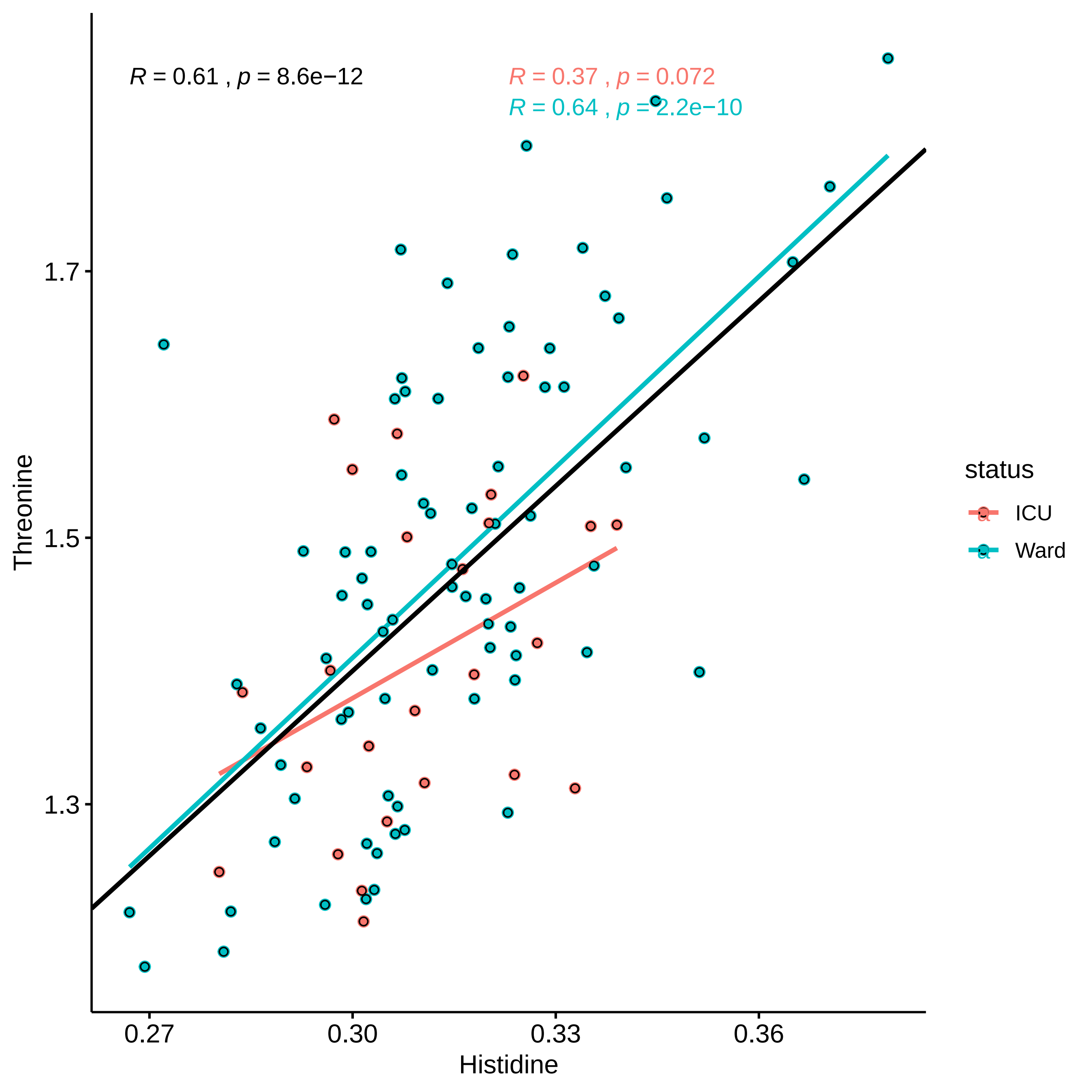

R = 0.61 , p = 8.6e−12
R = 0.37 , p = 0.072
R = 0.64 , p = 2.2e−10
Threonine
1.7
1.5
1.3
Histidine
0.27
0.30
0.33
0.36
status
ICU
Ward

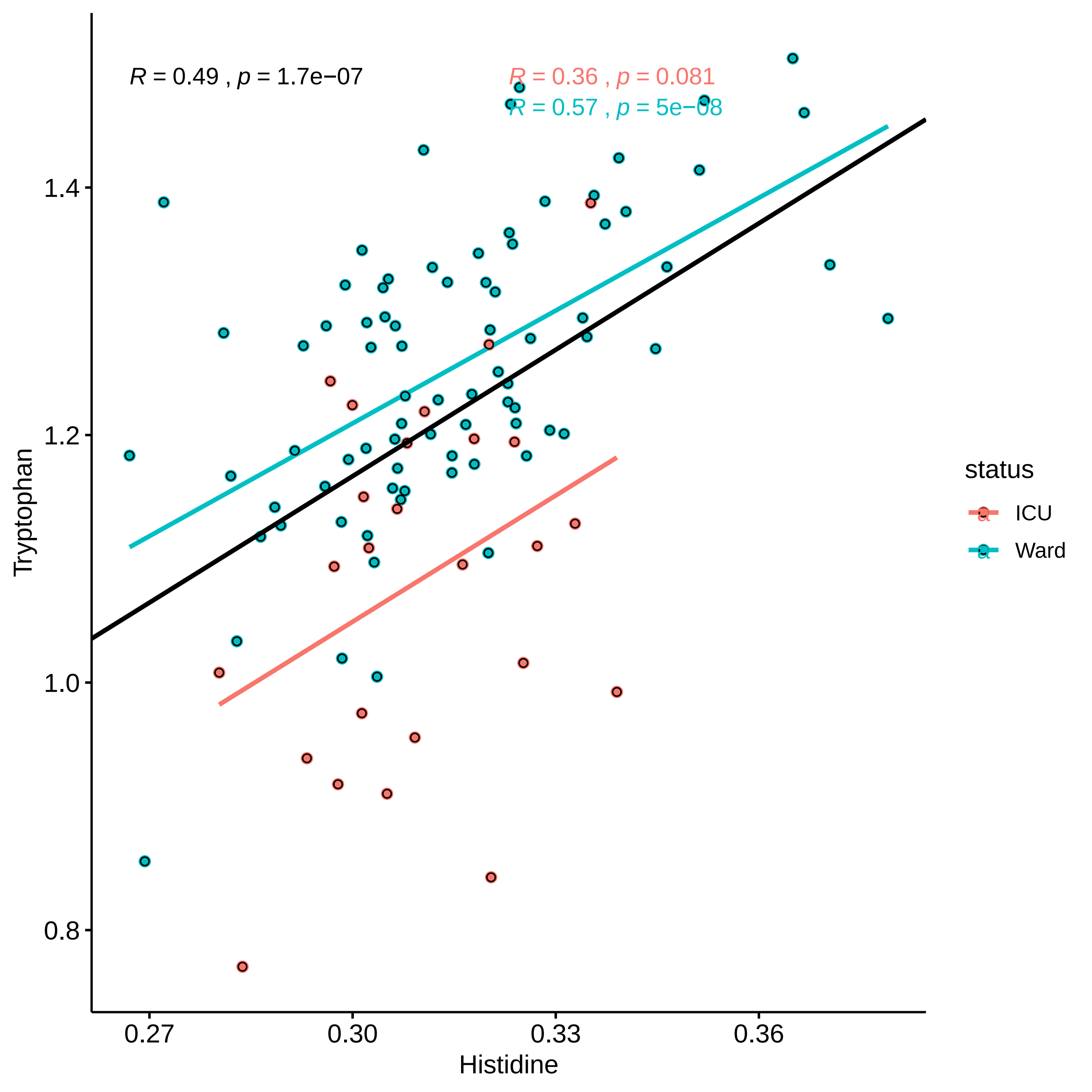

R = 0.49 , p = 1.7e−07
R = 0.36 , p = 0.081
R = 0.57 , p = 5e−08
Tryptophan
1.4
1.2
1.0
0.8
Histidine
0.27
0.30
0.33
0.36
status
ICU
Ward

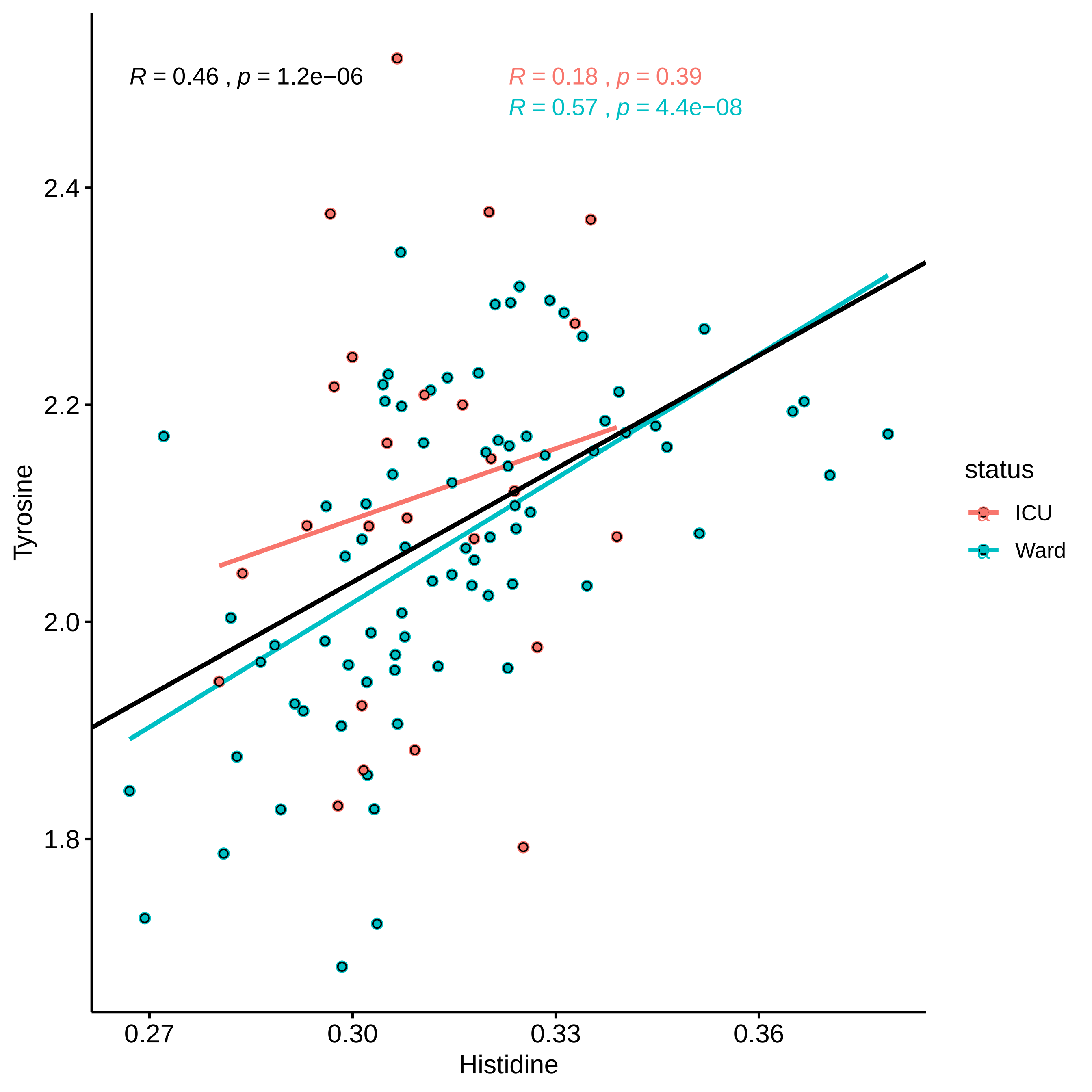

R = 0.46 , p = 1.2e−06
R = 0.18 , p = 0.39
R = 0.57 , p = 4.4e−08
2.4
2.2
2.0
1.8
Tyrosine
0.27
0.30
0.33
0.36
Histidine
status
ICU
Ward

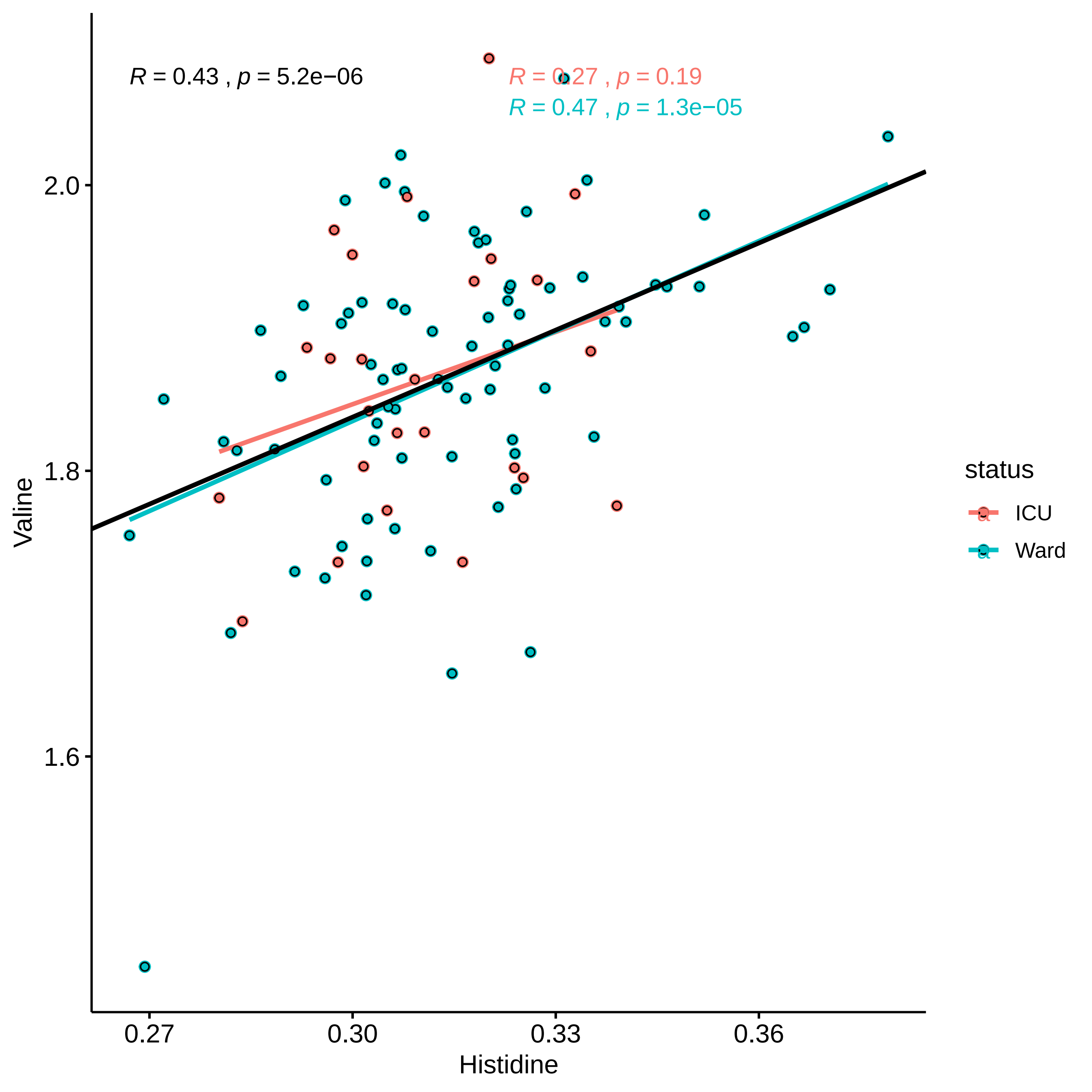

R = 0.43 , p = 5.2e−06
R = 0.27 , p = 0.19
R = 0.47 , p = 1.3e−05
2.0
1.8
1.6
Valine
0.27
0.30
0.33
0.36
Histidine
status
ICU
Ward

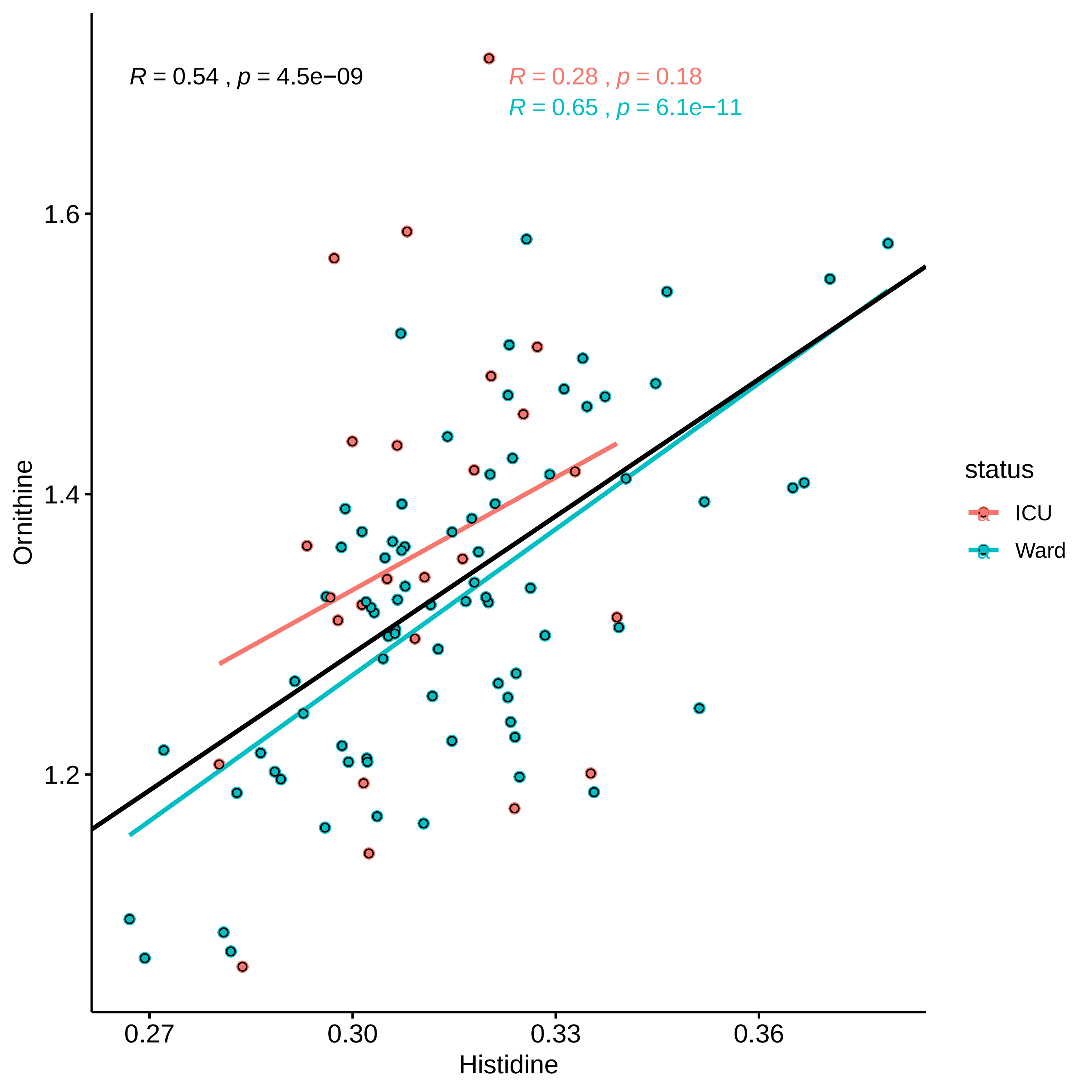

R = 0.54 , p = 4.5e−09
R = 0.28 , p = 0.18
R = 0.65 , p = 6.1e−11
1.6
1.4
1.2
Ornithine
0.27
0.30
0.33
0.36
Histidine
status
ICU
Ward

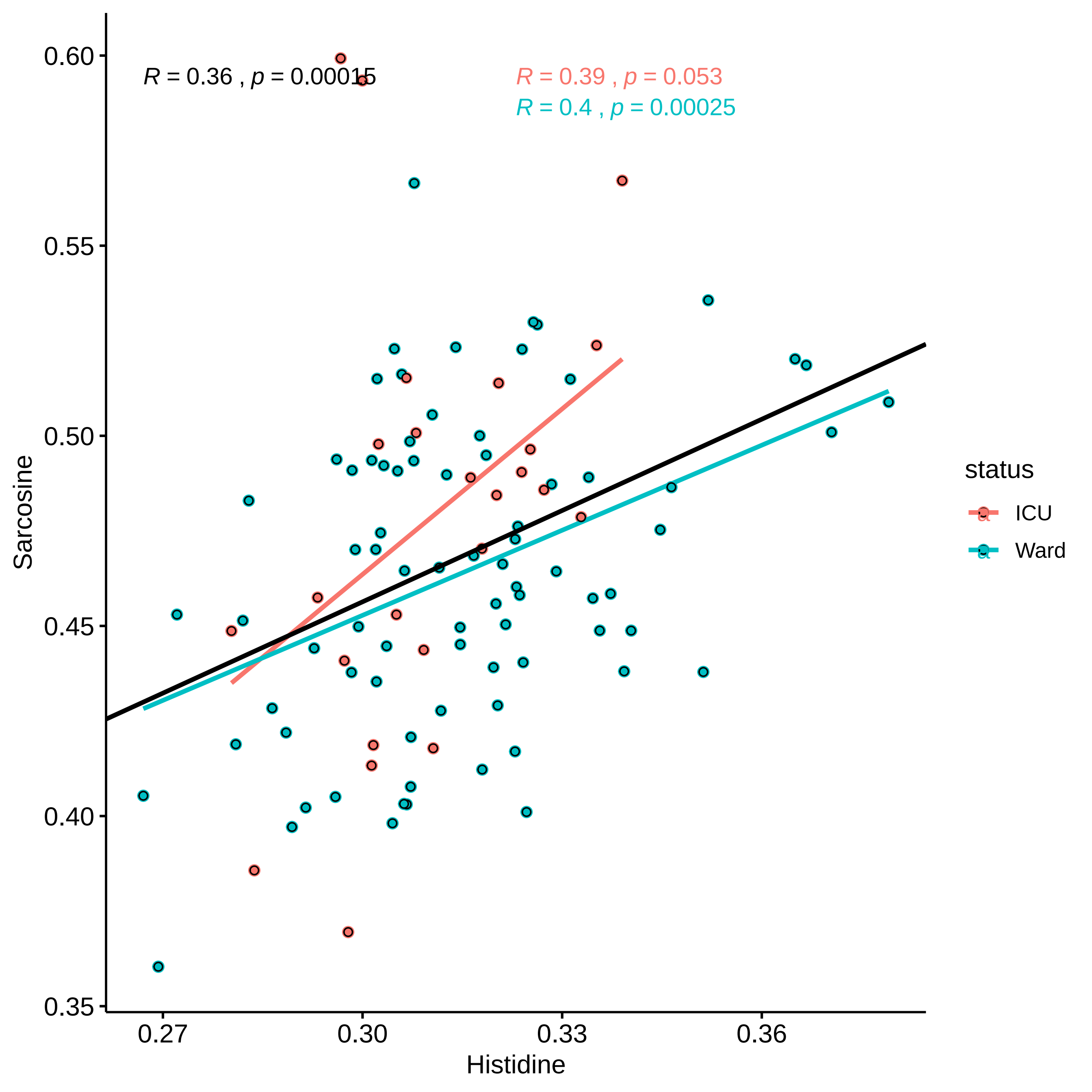

R = 0.36 , p = 0.00015
R = 0.39 , p = 0.053
R = 0.4 , p = 0.00025
Sarcosine
0.60
0.55
0.50
0.45
0.40
0.35
0.27
0.30
0.33
0.36
Histidine
status
ICU
Ward

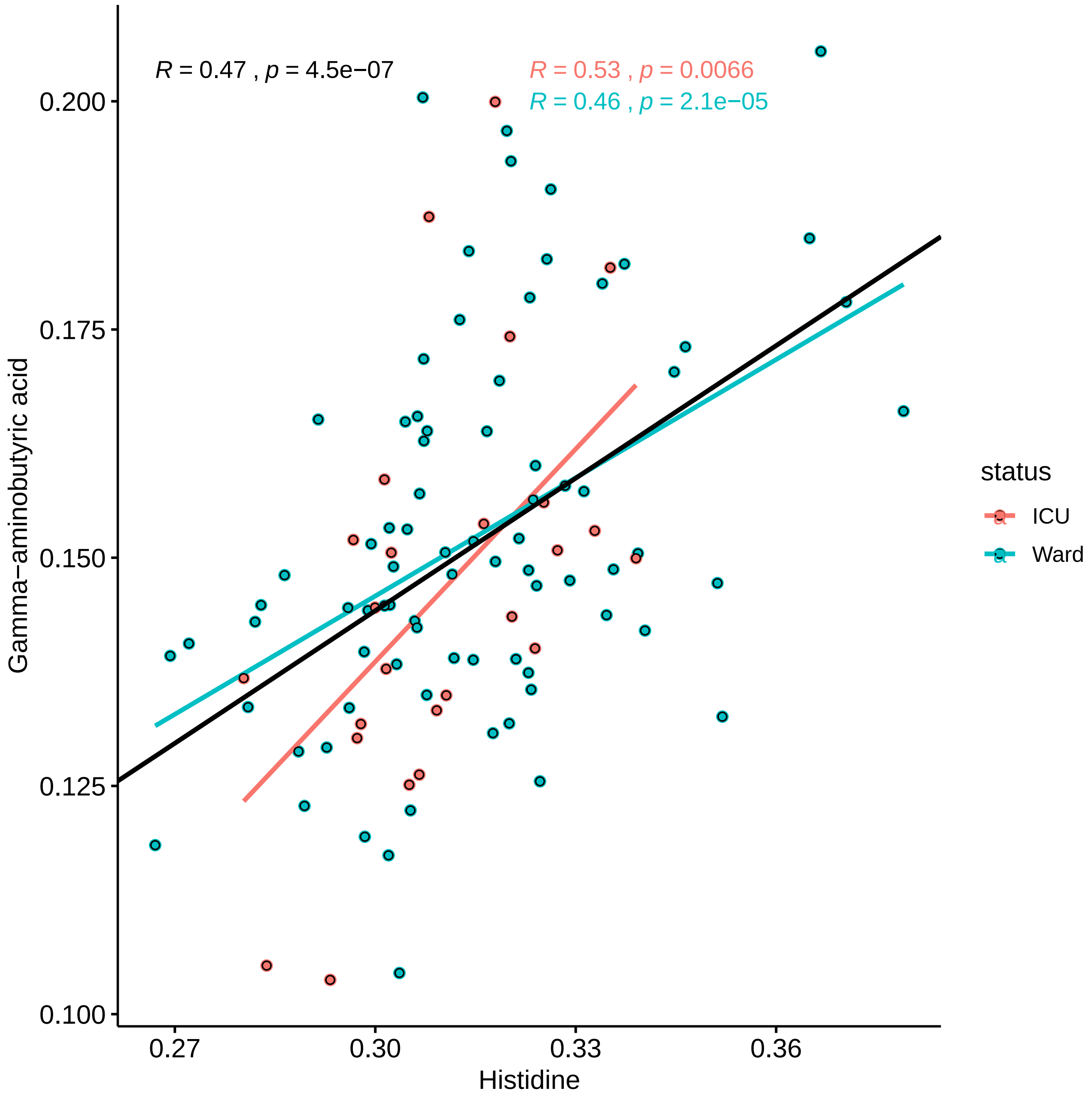

R = 0.47 , p = 4.5e−07
R = 0.53 , p = 0.0066
R = 0.46 , p = 2.1e−05
Gamma−aminobutyric acid
0.200
0.175
0.150
0.125
0.100
0.27
0.30
0.33
0.36
Histidine
status
ICU
Ward

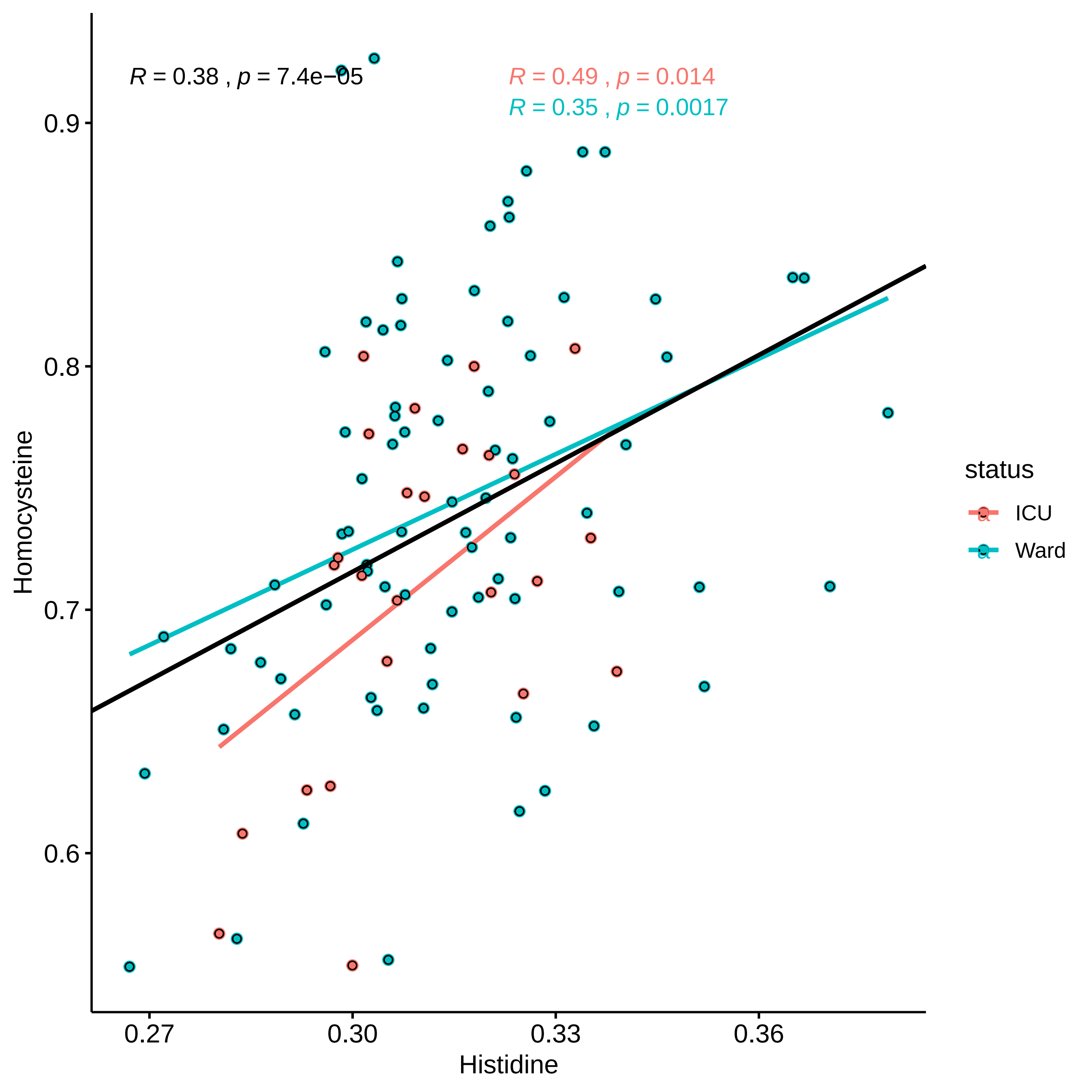

R = 0.38 , p = 7.4e−05
R = 0.49 , p = 0.014
R = 0.35 , p = 0.0017
0.9
0.8
0.7
0.6
Homocysteine
0.27
0.30
0.33
0.36
Histidine
status
ICU
Ward

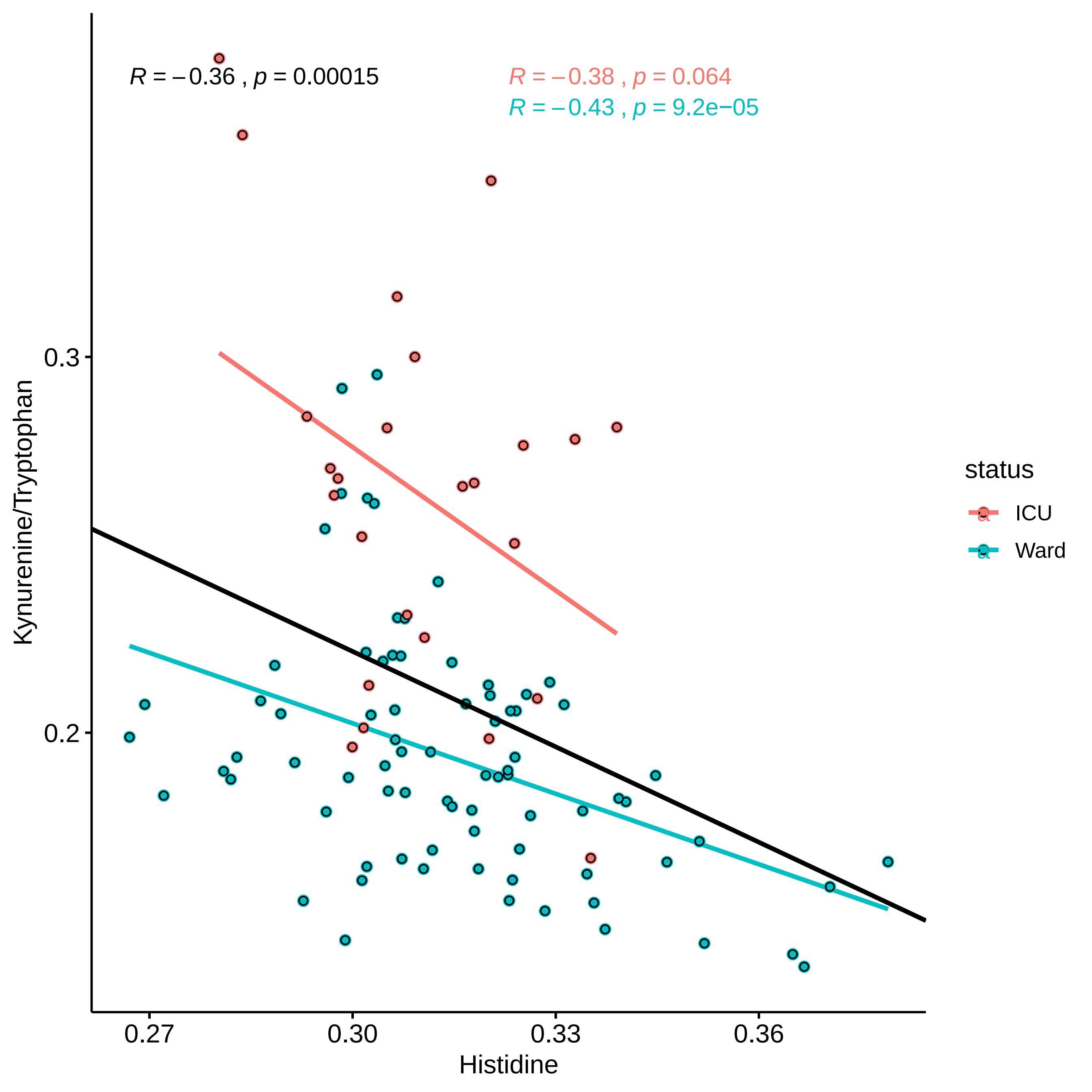

R = −0.36 , p = 0.00015
R = −0.38 , p = 0.064
R = −0.43 , p = 9.2e−05
Kynurenine/Tryptophan
0.3
0.2
0.27
0.30
0.33
0.36
Histidine
status
ICU
Ward

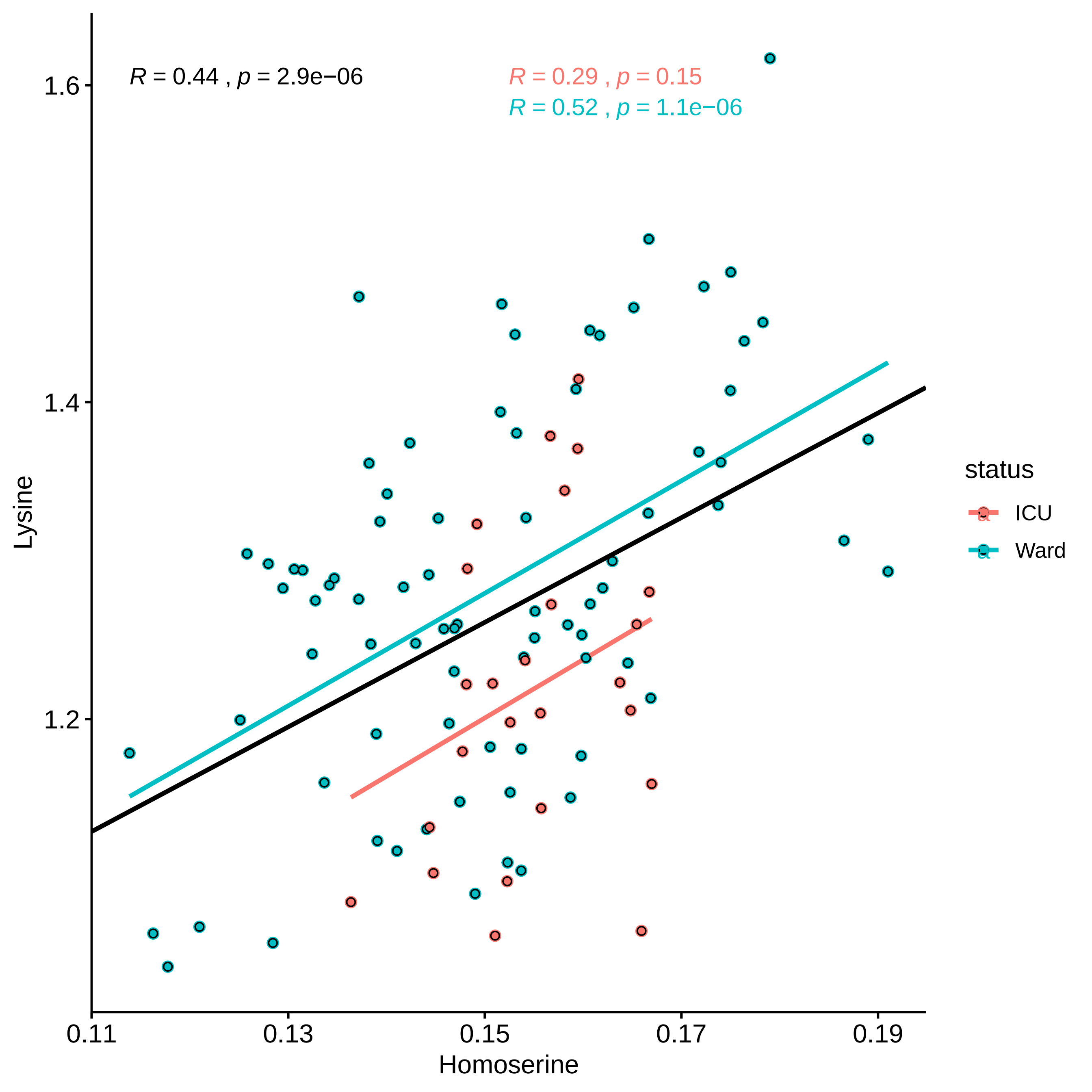

R = 0.44 , p = 2.9e−06
R = 0.29 , p = 0.15
R = 0.52 , p = 1.1e−06
1.6
1.4
1.2
Lysine
0.11
0.13
0.15
0.17
0.19
Homoserine
status
ICU
Ward

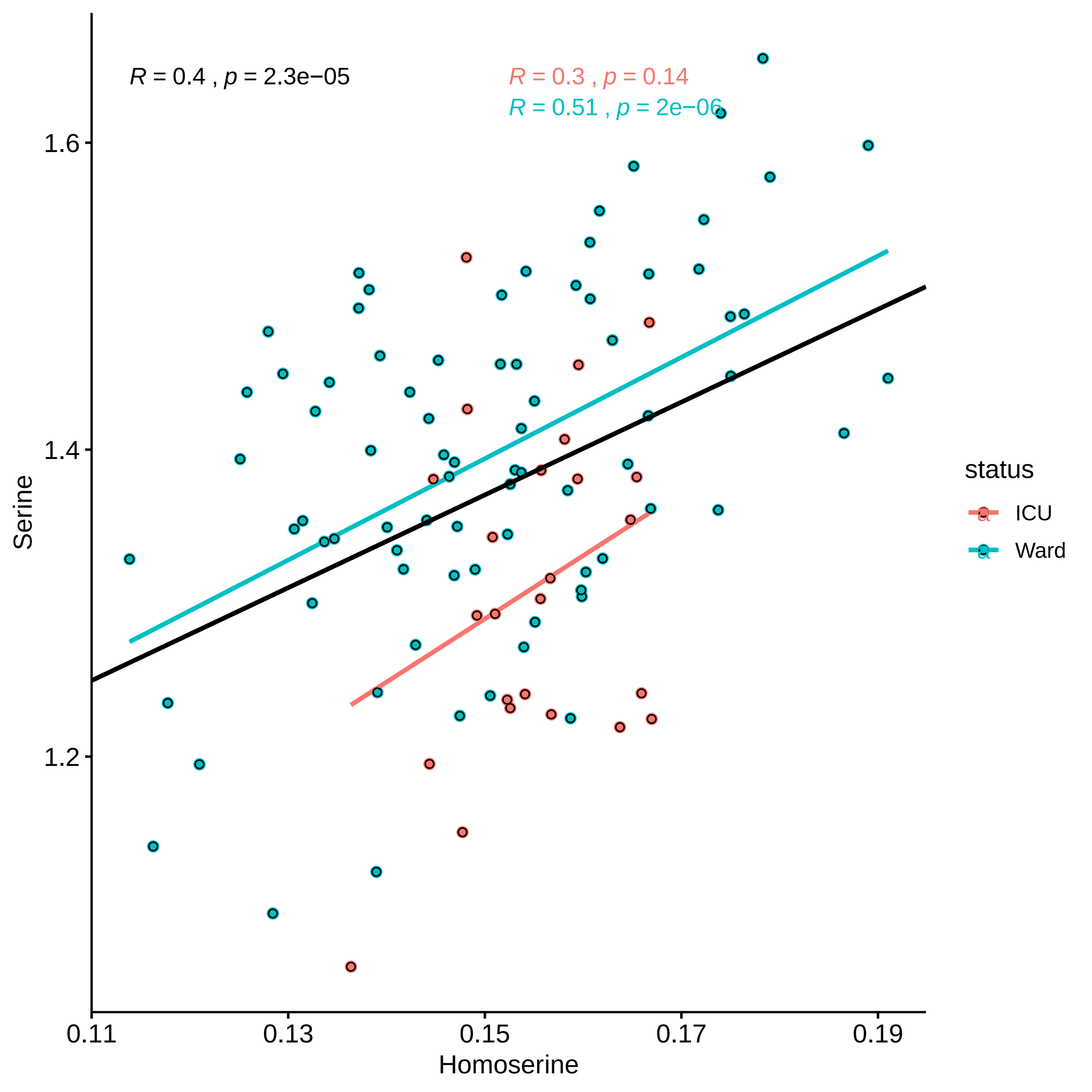

R = 0.4 , p = 2.3e−05
R = 0.3 , p = 0.14
R = 0.51 , p = 2e−06
1.6
1.4
1.2
Serine
0.11
0.13
0.15
0.17
0.19
Homoserine
status
ICU
Ward

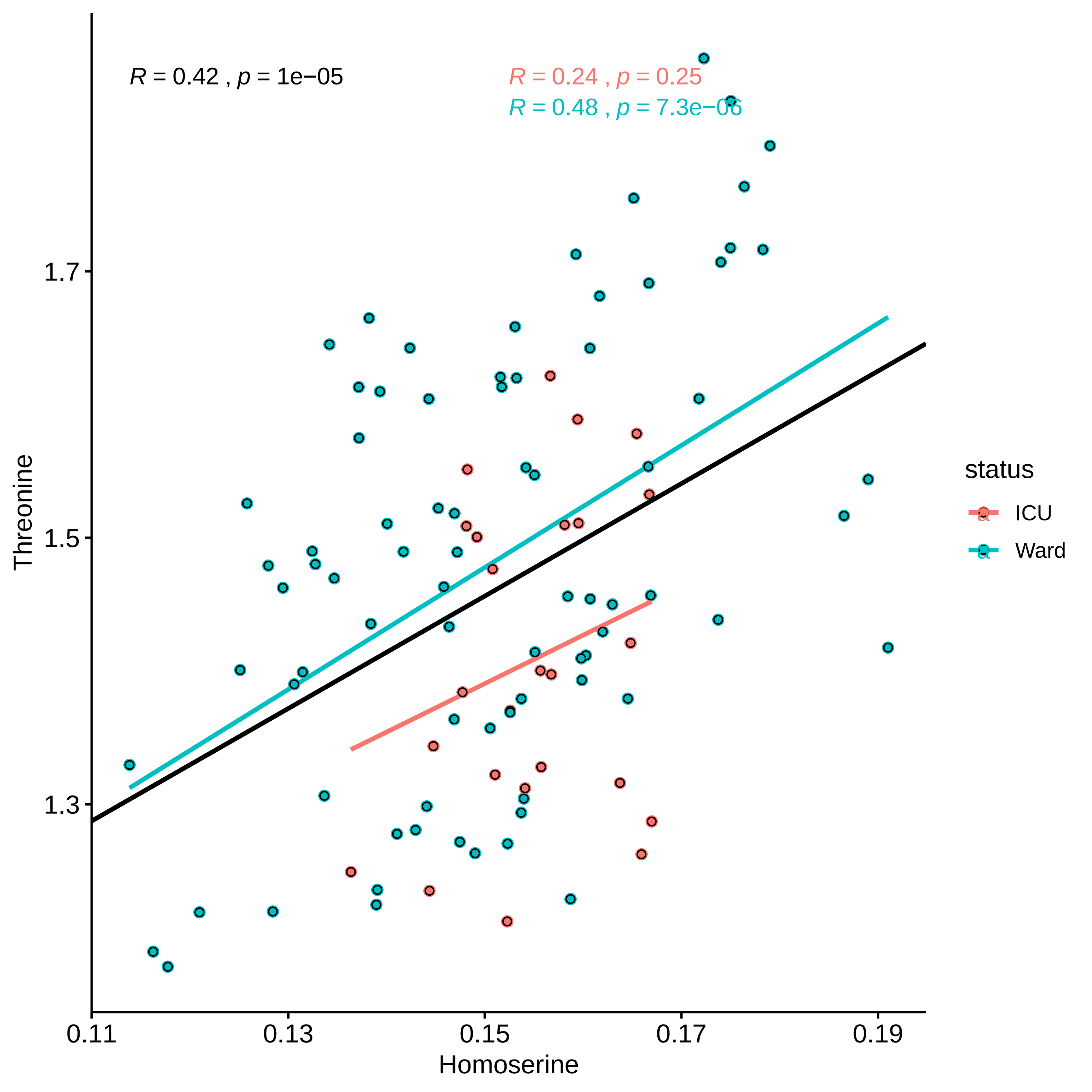

R = 0.42 , p = 1e−05
R = 0.24 , p = 0.25
R = 0.48 , p = 7.3e−06
Threonine
1.7
1.5
1.3
0.11
0.13
0.15
0.17
0.19
Homoserine
status
ICU
Ward

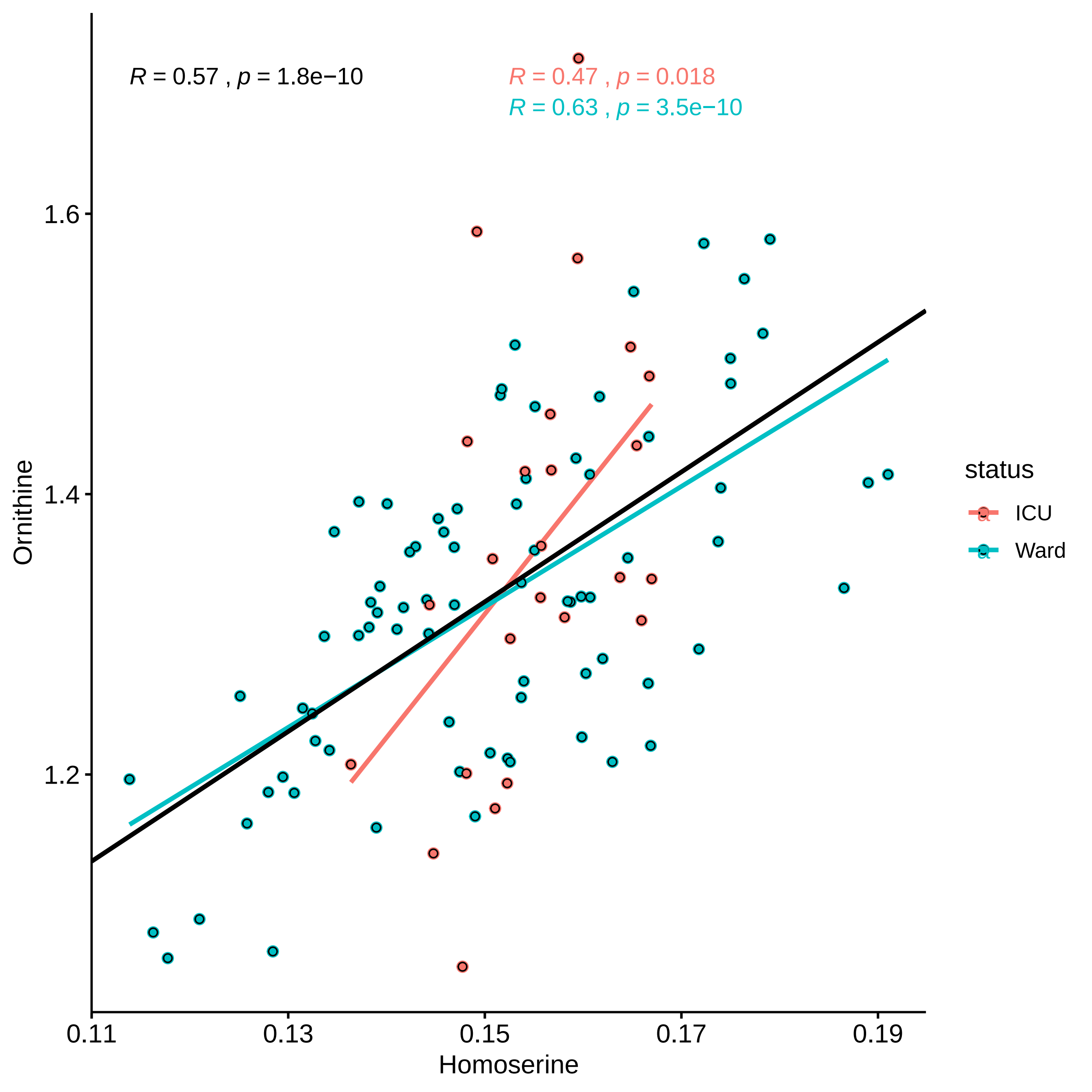

R = 0.57 , p = 1.8e−10
R = 0.47 , p = 0.018
R = 0.63 , p = 3.5e−10
1.6
1.4
1.2
Ornithine
0.11
0.13
0.15
0.17
0.19
Homoserine
status
ICU
Ward

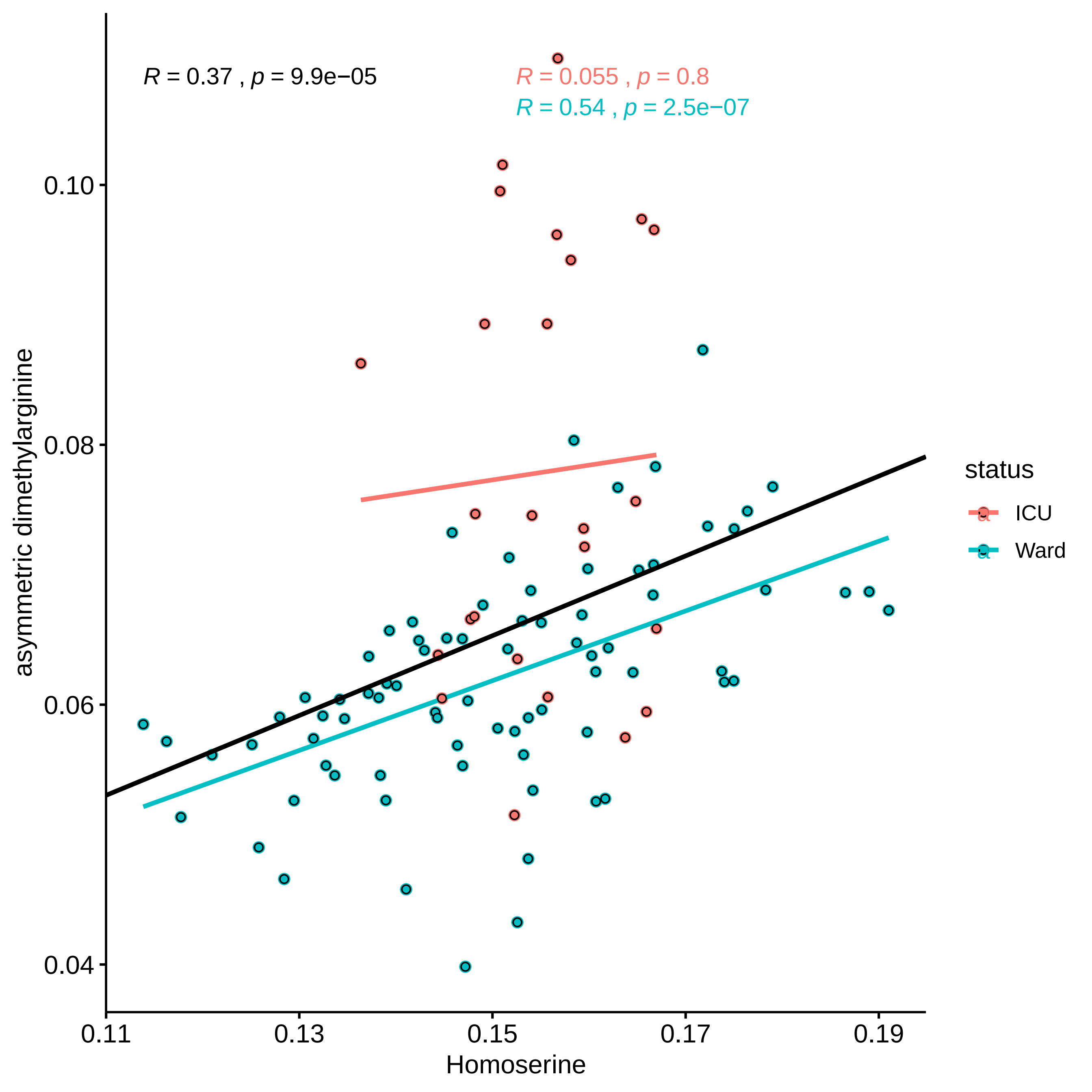

R = 0.37 , p = 9.9e−05
R = 0.055 , p = 0.8
R = 0.54 , p = 2.5e−07
asymmetric dimethylarginine
0.10
0.08
0.06
0.04
0.11
0.13
0.15
0.17
0.19
Homoserine
status
ICU
Ward

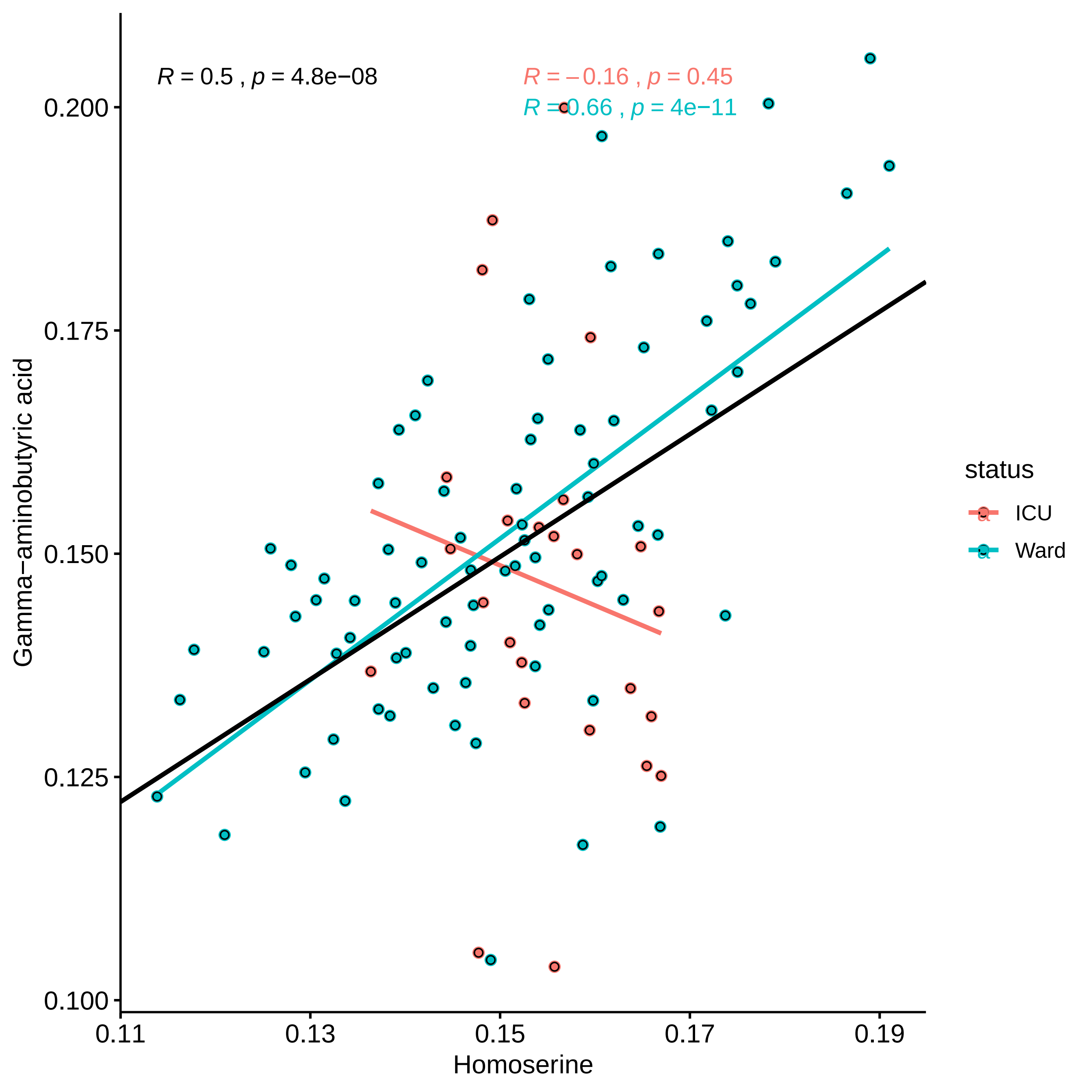

R = 0.5 , p = 4.8e−08
R = −0.16 , p = 0.45
R = 0.66 , p = 4e−11
Gamma−aminobutyric acid
0.200
0.175
0.150
0.125
0.100
0.11
0.13
0.15
0.17
0.19
Homoserine
status
ICU
Ward

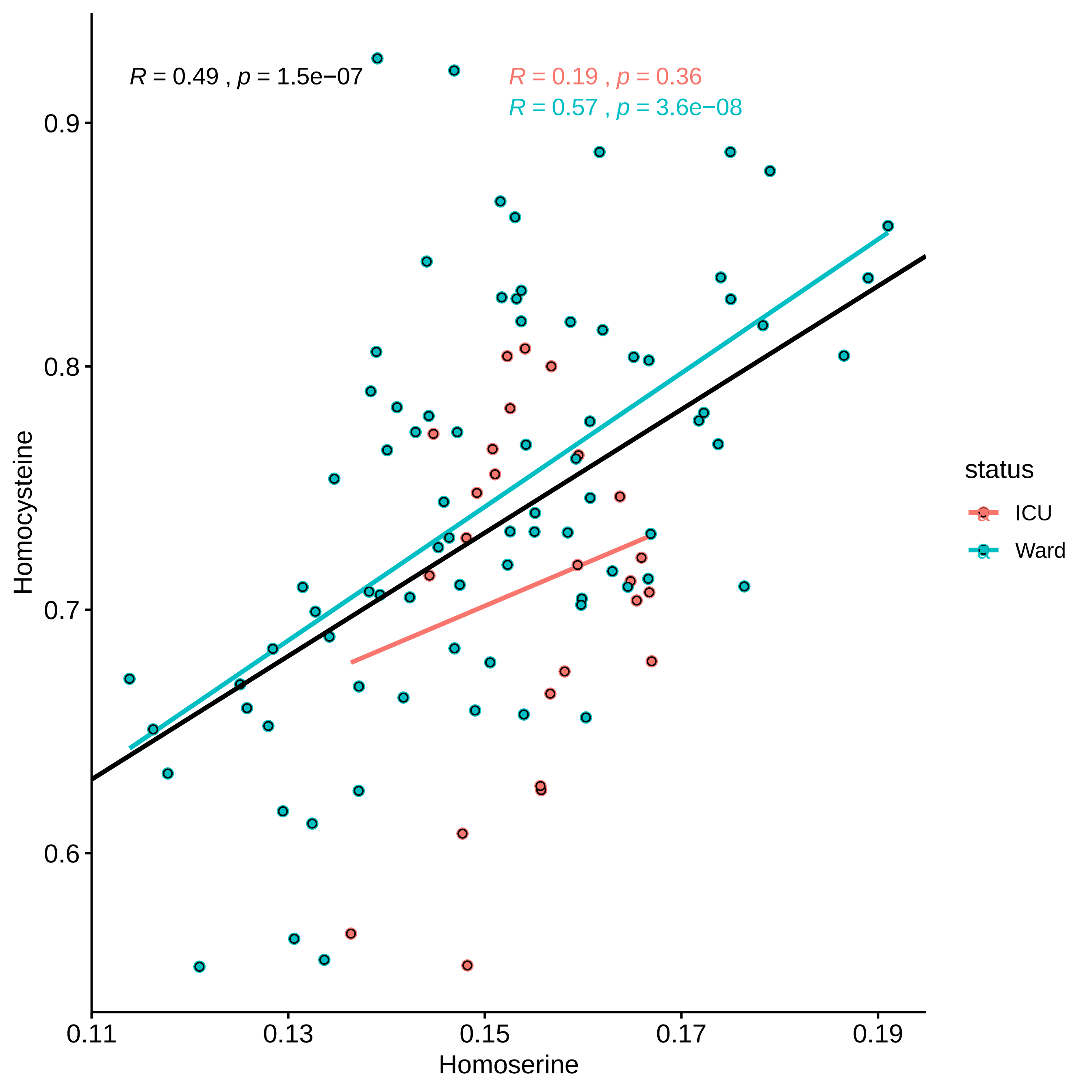

R = 0.49 , p = 1.5e−07
R = 0.19 , p = 0.36
R = 0.57 , p = 3.6e−08
0.9
0.8
0.7
0.6
Homocysteine
0.11
0.13
0.15
0.17
0.19
Homoserine
status
ICU
Ward

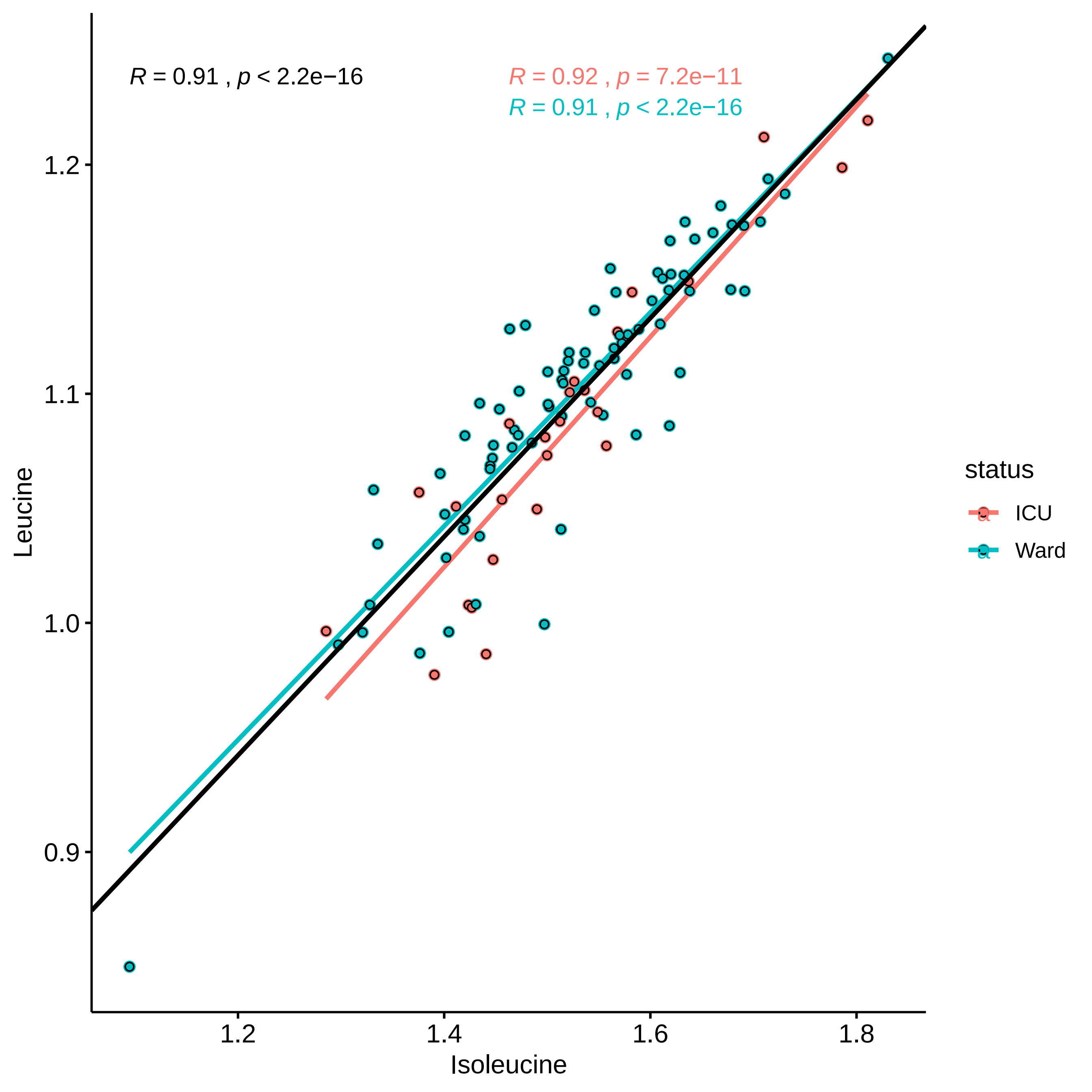

R = 0.91 , p < 2.2e−16
R = 0.92 , p = 7.2e−11
R = 0.91 , p < 2.2e−16
Leucine
1.2
1.1
1.0
0.9
1.2
1.4
1.6
1.8
Isoleucine
status
ICU
Ward

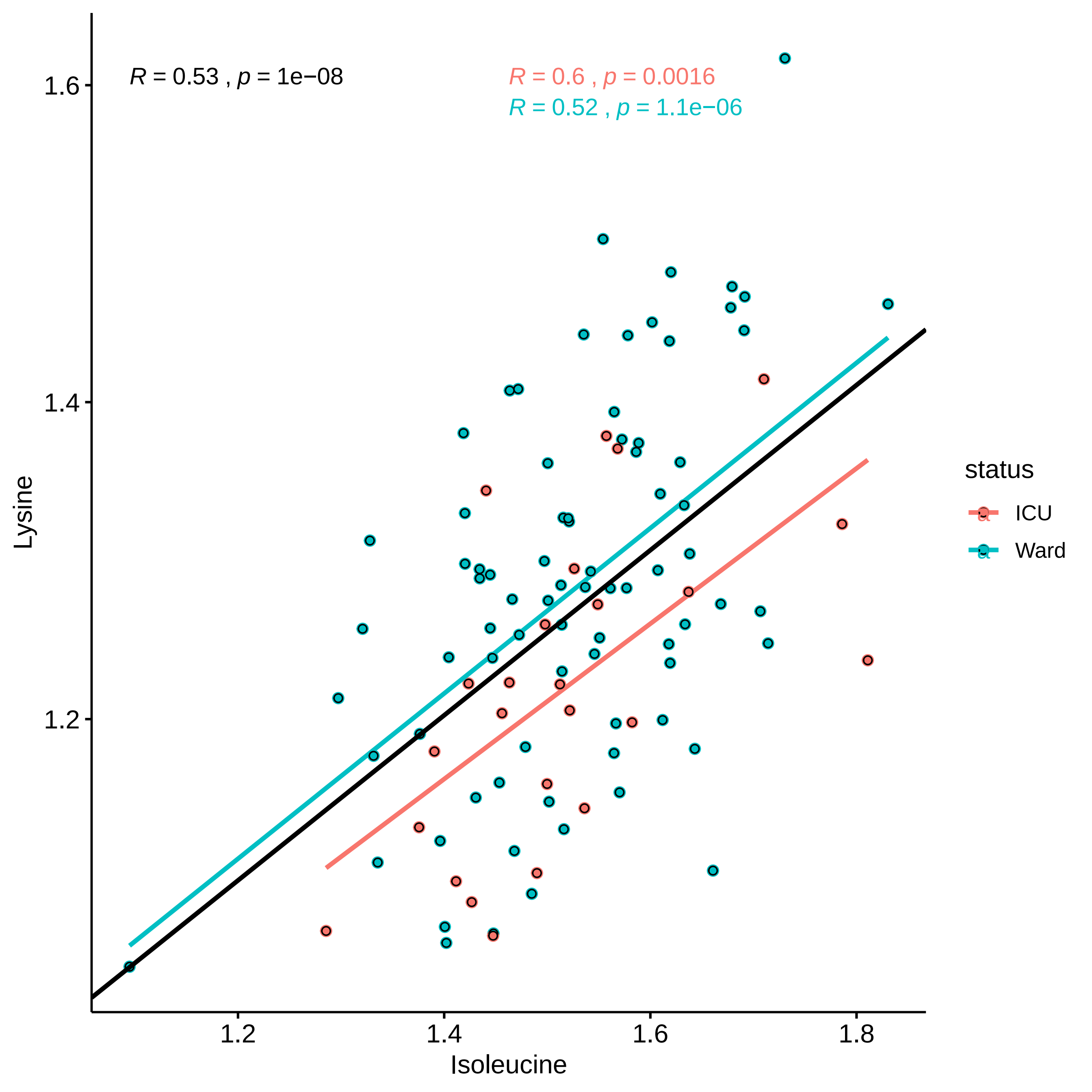

R = 0.53 , p = 1e−08
R = 0.6 , p = 0.0016
R = 0.52 , p = 1.1e−06
1.6
1.4
1.2
Lysine
1.2
1.4
1.6
1.8
Isoleucine
status
ICU
Ward

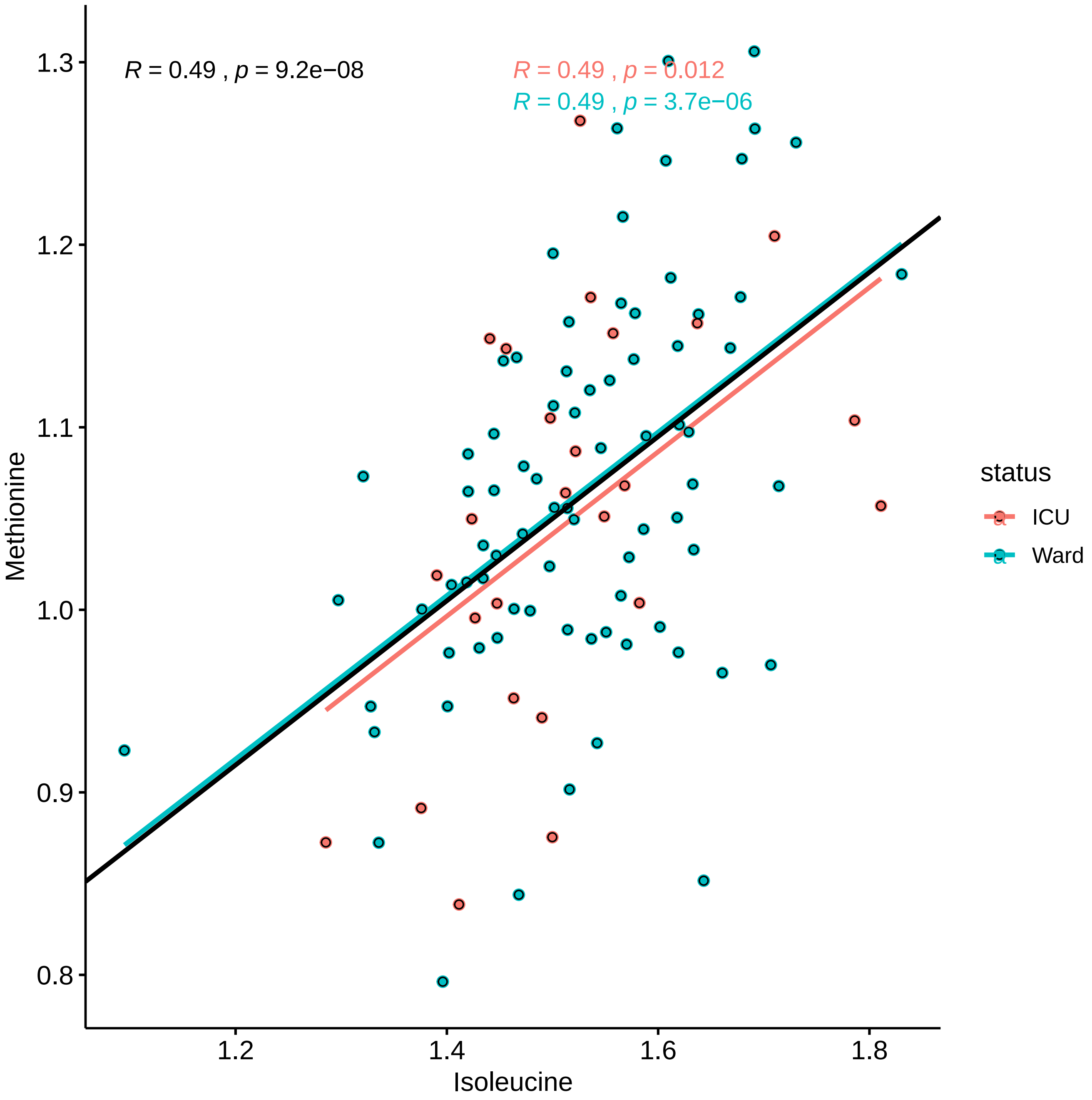

R = 0.49 , p = 9.2e−08
R = 0.49 , p = 0.012
R = 0.49 , p = 3.7e−06
Methionine
Isoleucine
1.3
1.2
1.1
1.0
0.9
0.8
1.2
1.4
1.6
1.8
status
ICU
Ward

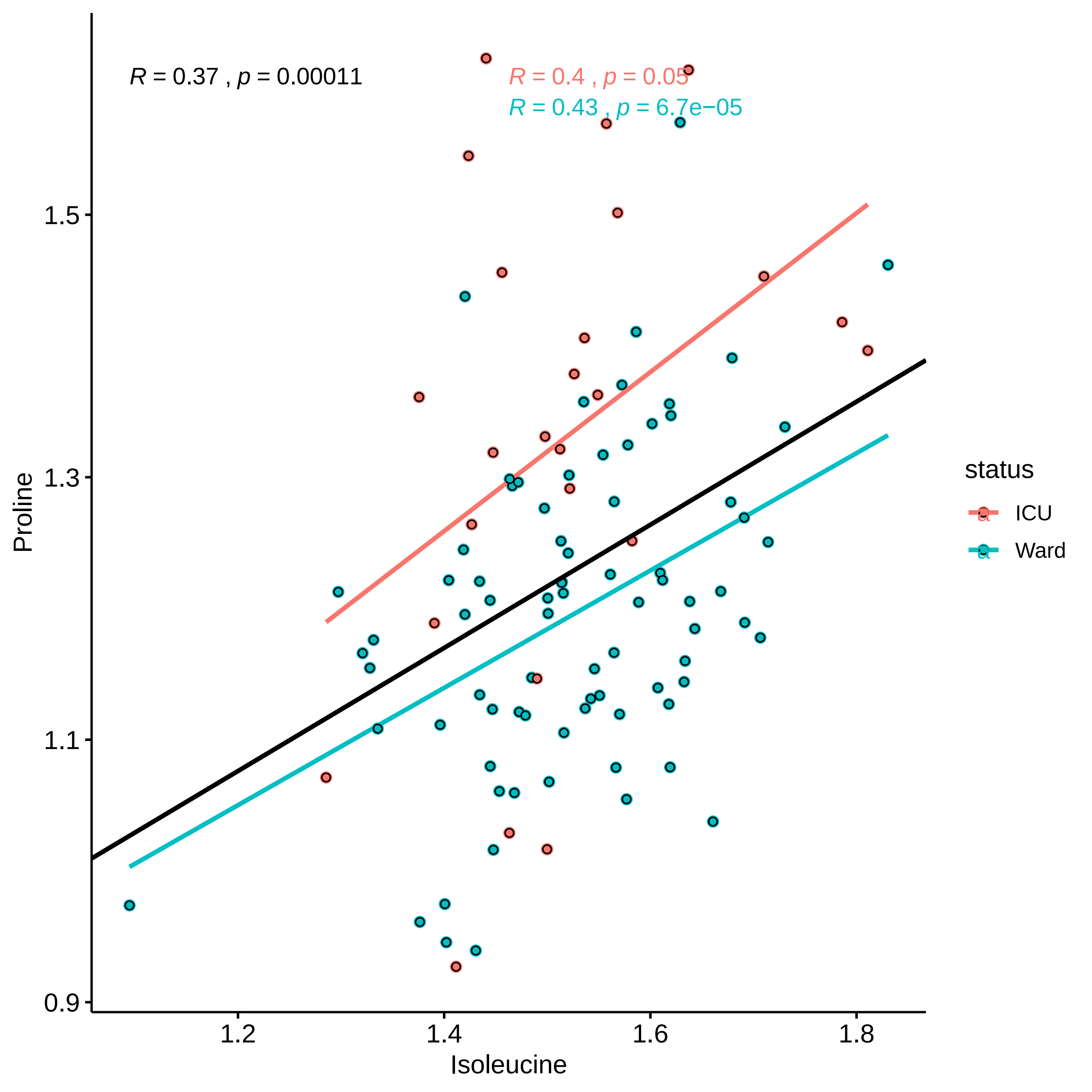

R = 0.37 , p = 0.00011
R = 0.4 , p = 0.05
R = 0.43 , p = 6.7e−05
Proline
1.5
1.3
1.1
0.9
1.2
1.4
1.6
1.8
Isoleucine
status
ICU
Ward

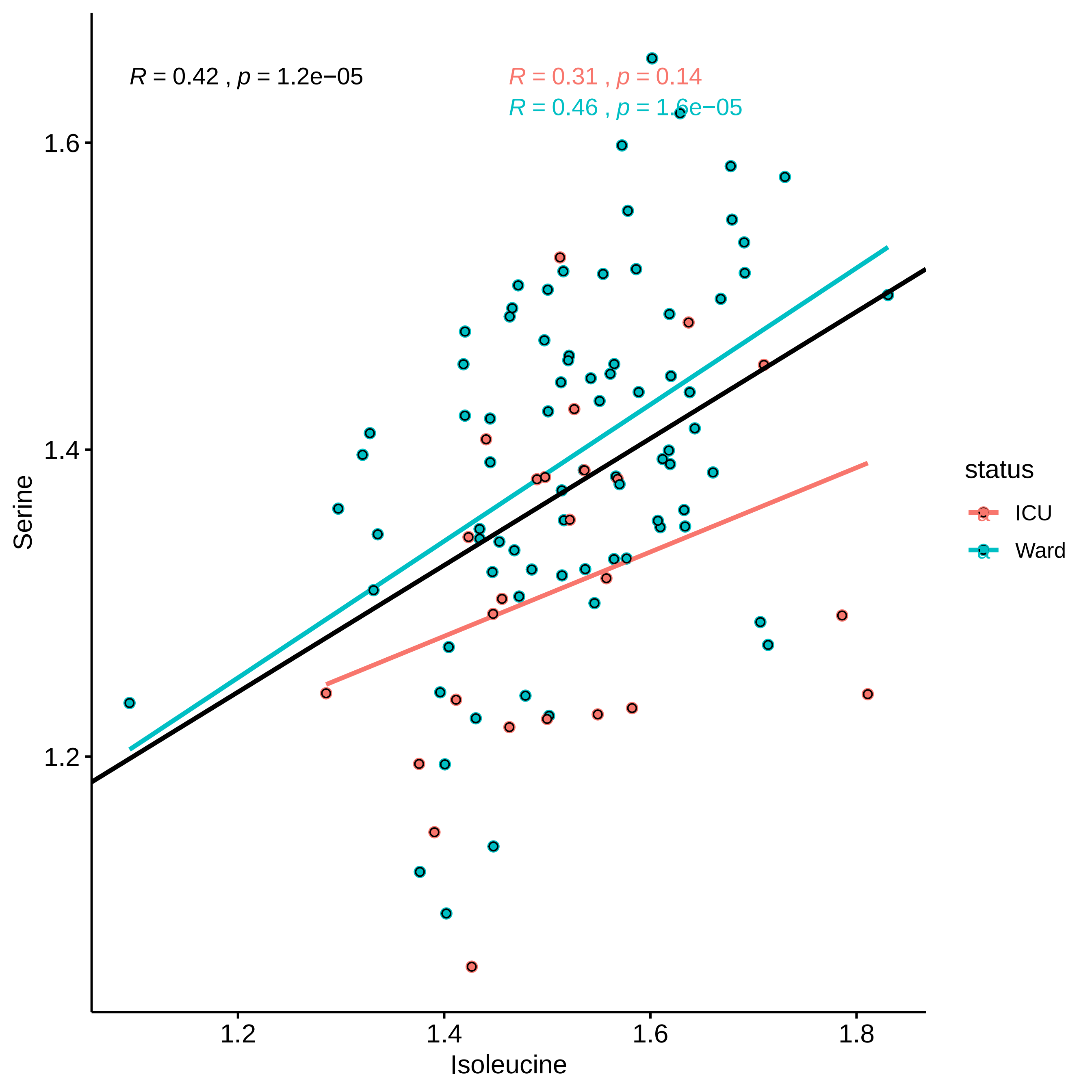

R = 0.42 , p = 1.2e−05
R = 0.31 , p = 0.14
R = 0.46 , p = 1.6e−05
1.6
1.4
1.2
Serine
1.2
1.4
1.6
1.8
Isoleucine
status
ICU
Ward

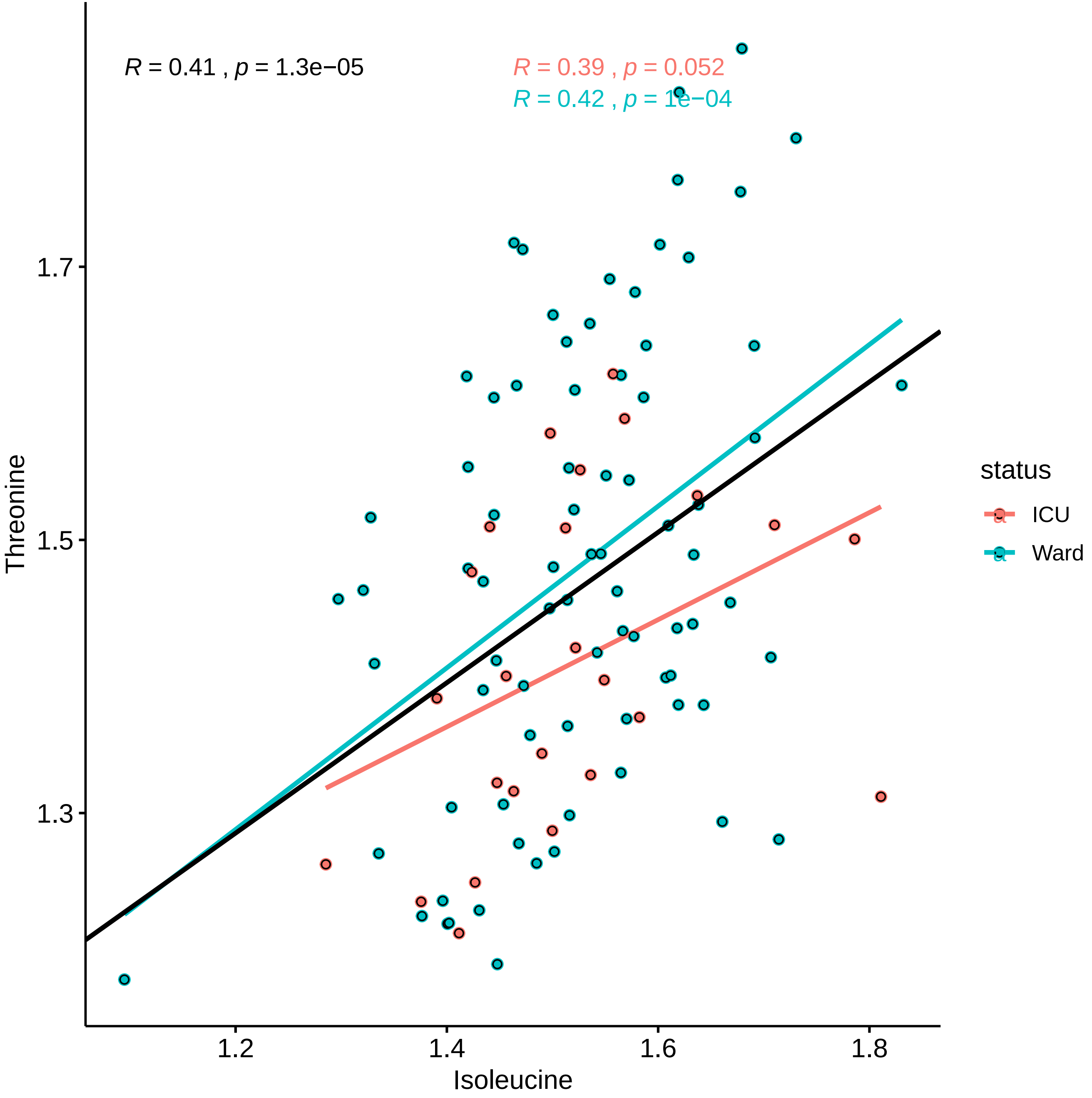

R = 0.41 , p = 1.3e−05
R = 0.39 , p = 0.052
R = 0.42 , p = 1e−04
1.7
1.5
1.3
Threonine
1.2
1.4
1.6
1.8
Isoleucine
status
ICU
Ward

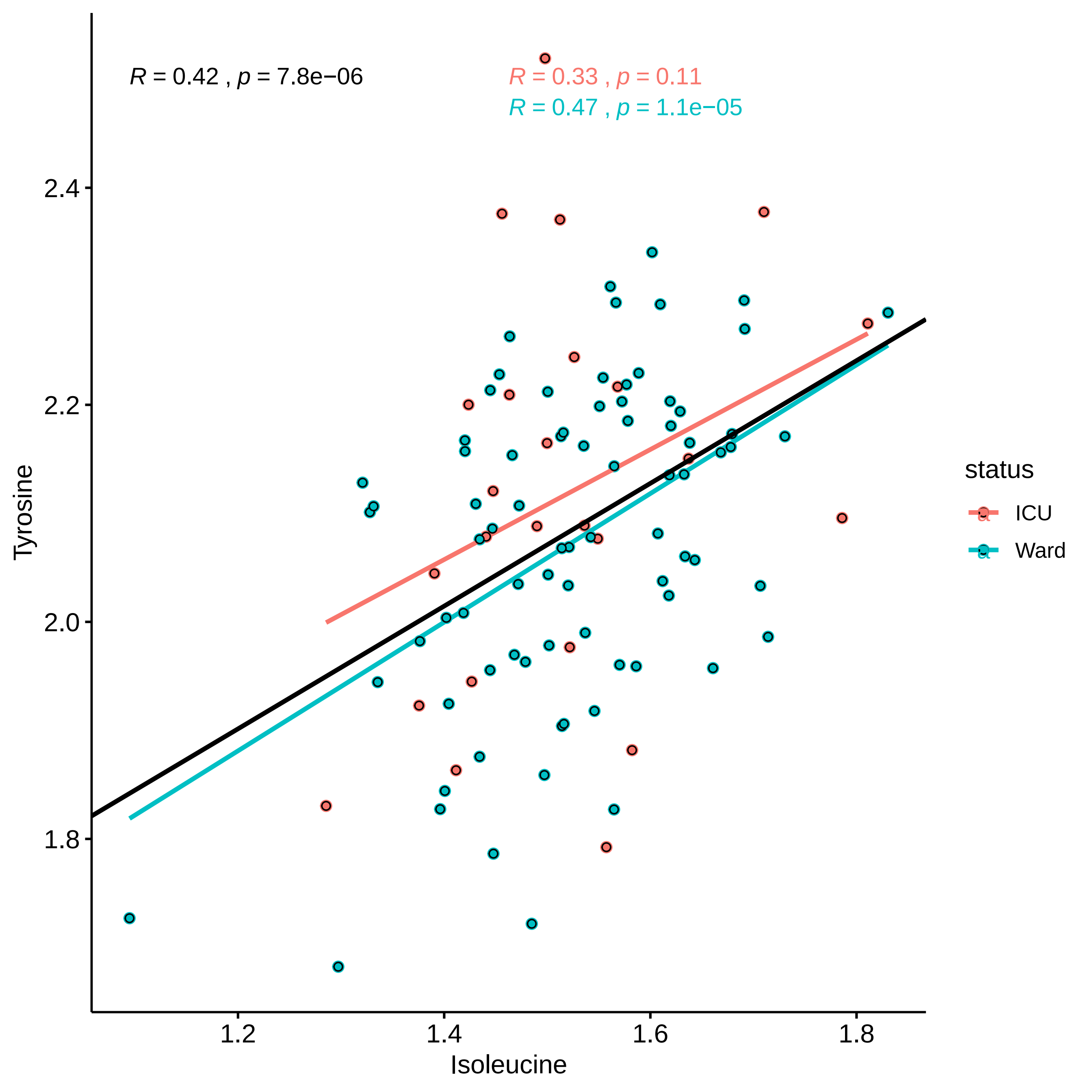

R = 0.42 , p = 7.8e−06
R = 0.33 , p = 0.11
R = 0.47 , p = 1.1e−05
2.4
2.2
2.0
1.8
Tyrosine
1.2
1.4
1.6
1.8
Isoleucine
status
ICU
Ward

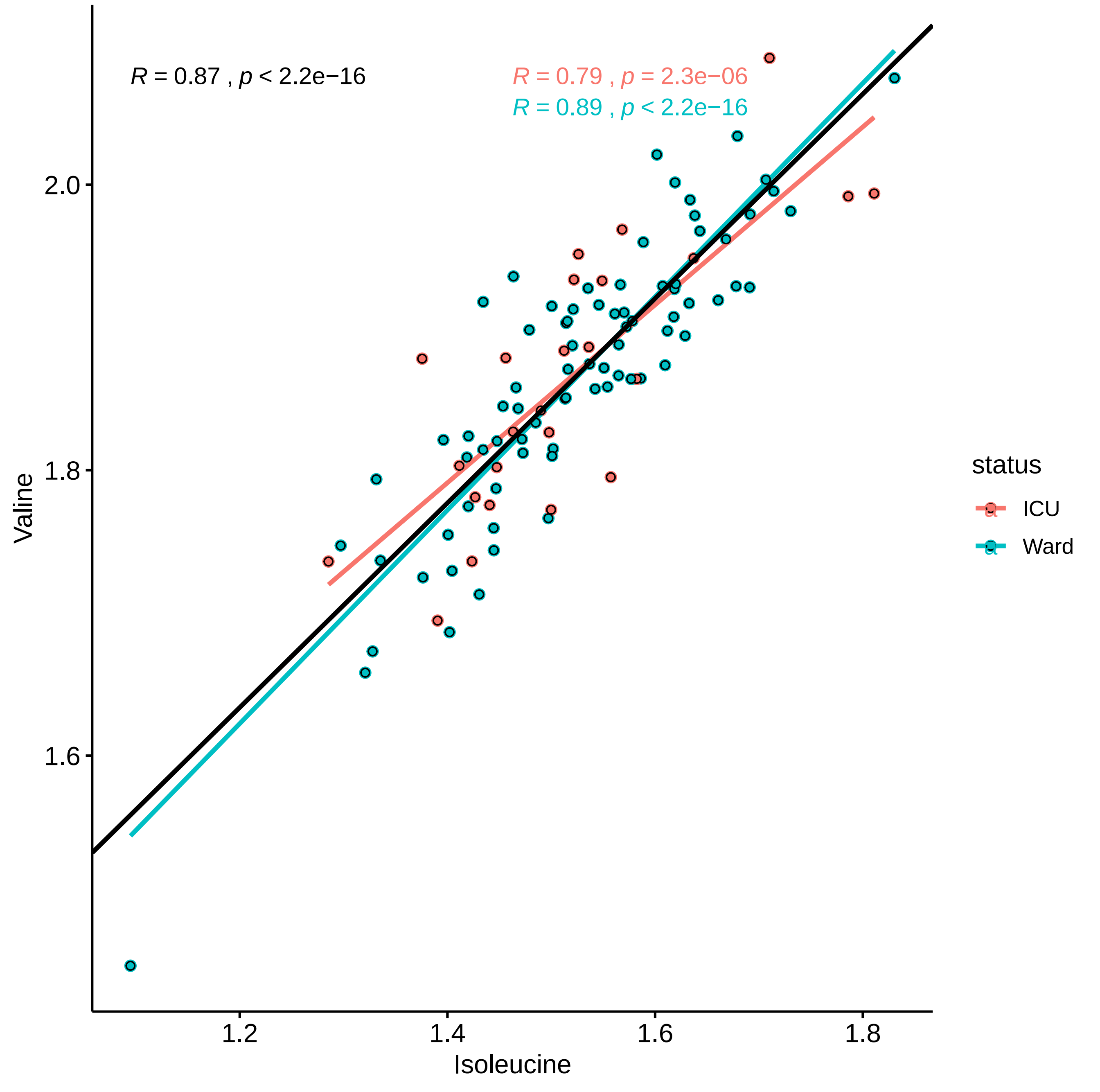

R = 0.87 , p < 2.2e−16
R = 0.79 , p = 2.3e−06
R = 0.89 , p < 2.2e−16
2.0
1.8
1.6
Valine
1.2
1.4
1.6
1.8
Isoleucine
status
ICU
Ward

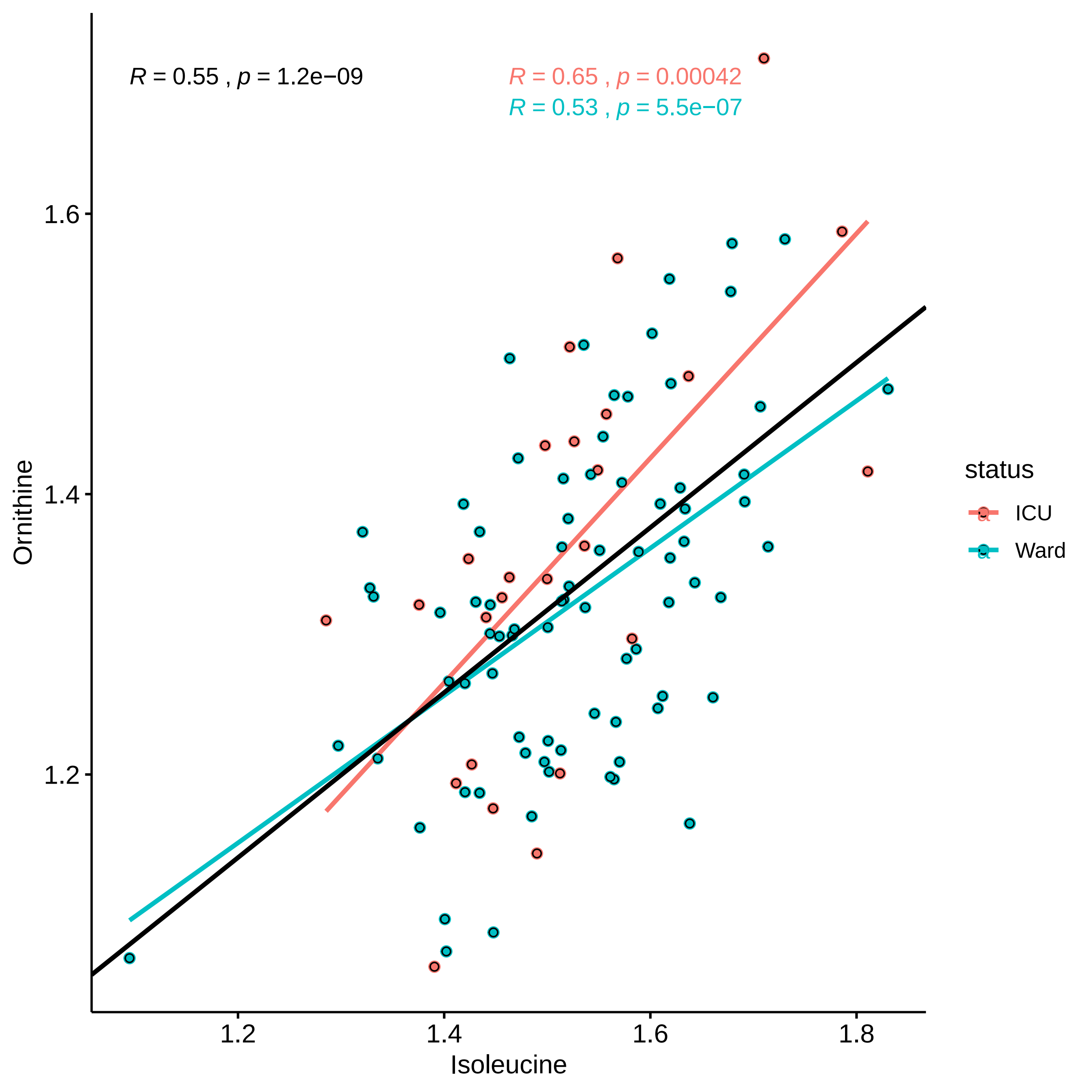
R = 0.55 , p = 1.2e−09
R = 0.65 , p = 0.00042
R = 0.53 , p = 5.5e−07
1.6
1.4
1.2
Ornithine
1.2
1.4
1.6
1.8
Isoleucine
status
ICU
Ward

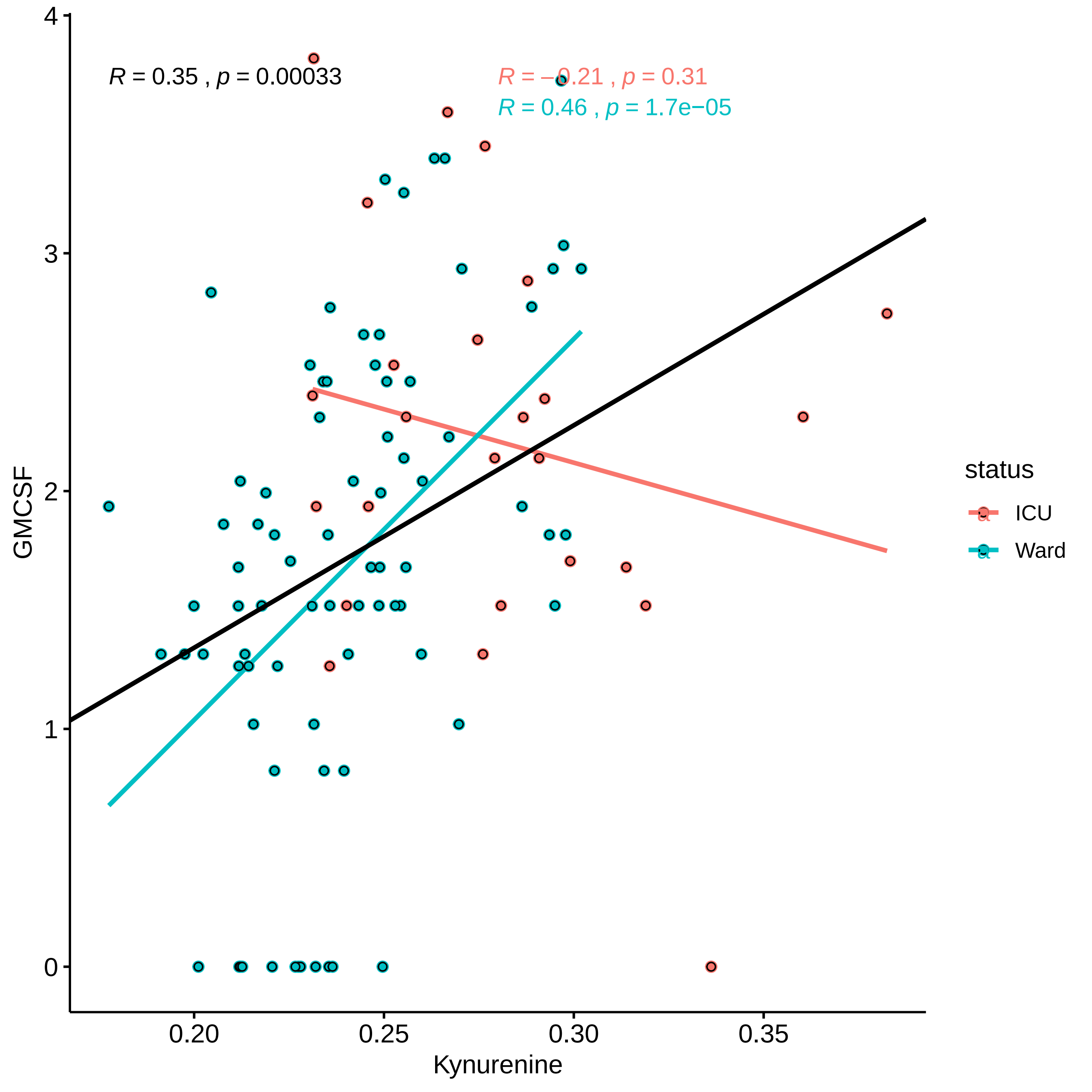

R = 0.35 , p = 0.00033
R = −0.21 , p = 0.31
R = 0.46 , p = 1.7e−05
GMCSF
0
1
2
3
4
0.20
0.25
0.30
0.35
Kynurenine
status
ICU
Ward

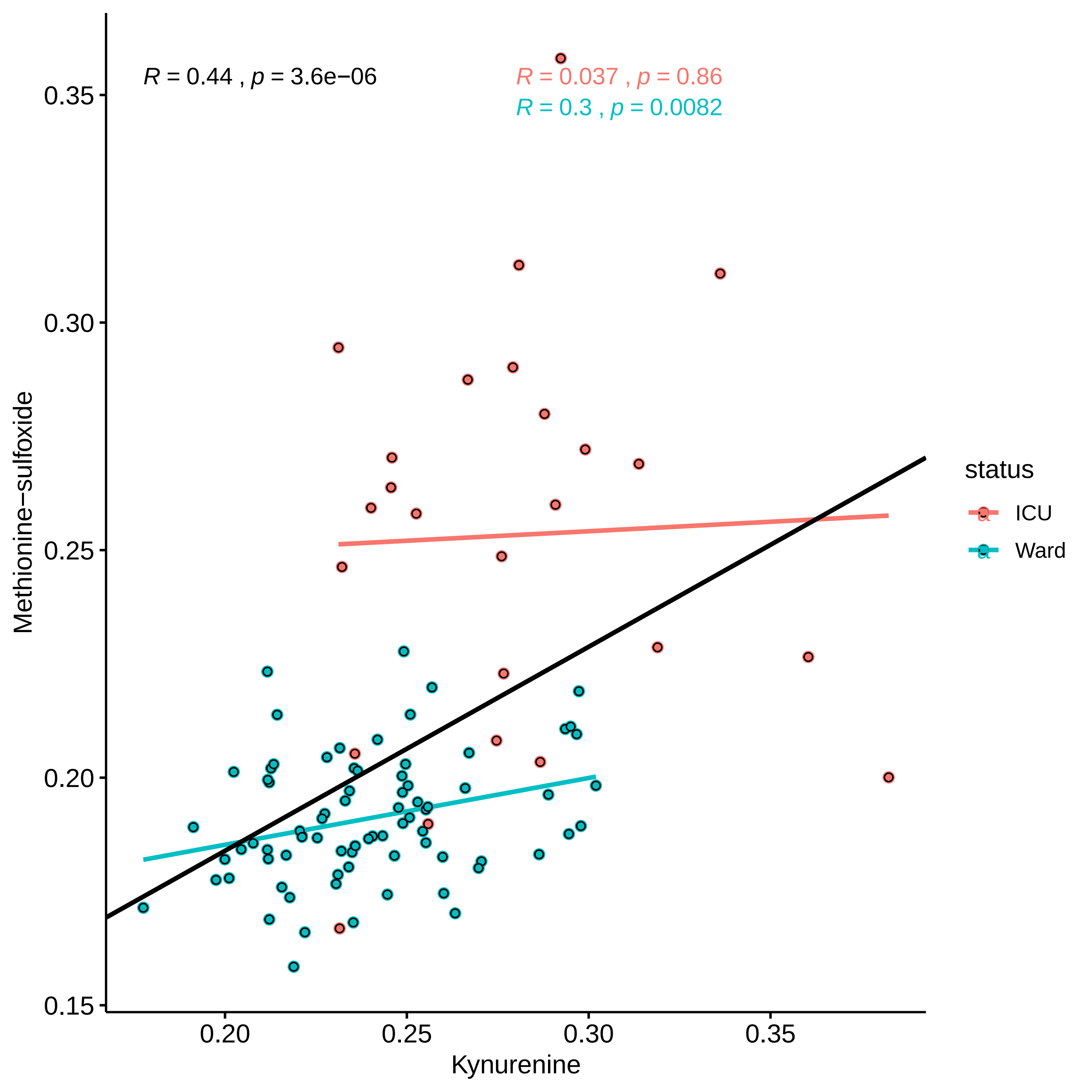

R = 0.44 , p = 3.6e−06
R = 0.037 , p = 0.86
R = 0.3 , p = 0.0082
Methionine−sulfoxide
Kynurenine
0.15
0.20
0.25
0.30
0.35
status
ICU
Ward

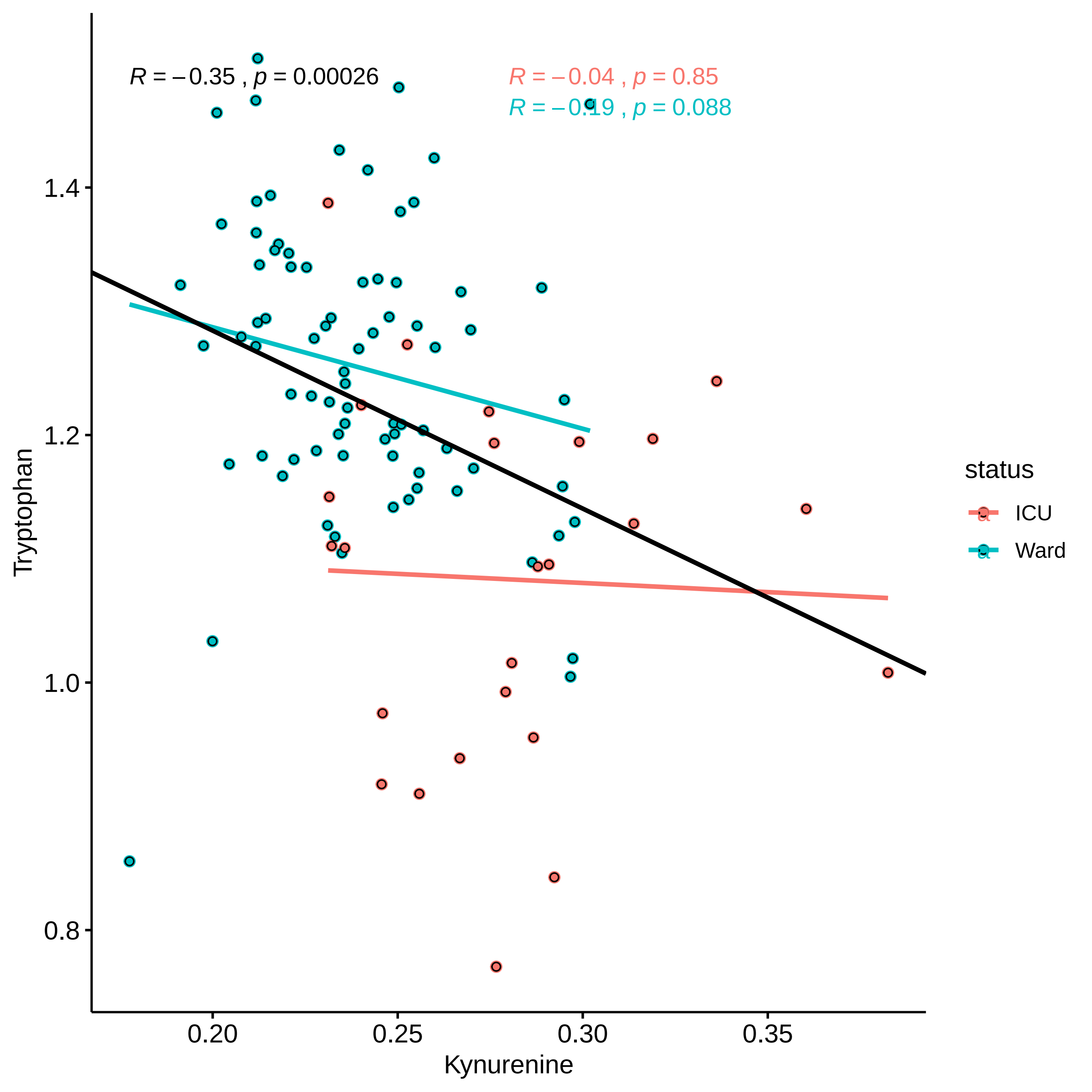

R = −0.35 , p = 0.00026
R = −0.04 , p = 0.85
R = −0.19 , p = 0.088
Tryptophan
1.4
1.2
1.0
0.8
0.20
0.25
0.30
0.35
Kynurenine
status
ICU
Ward

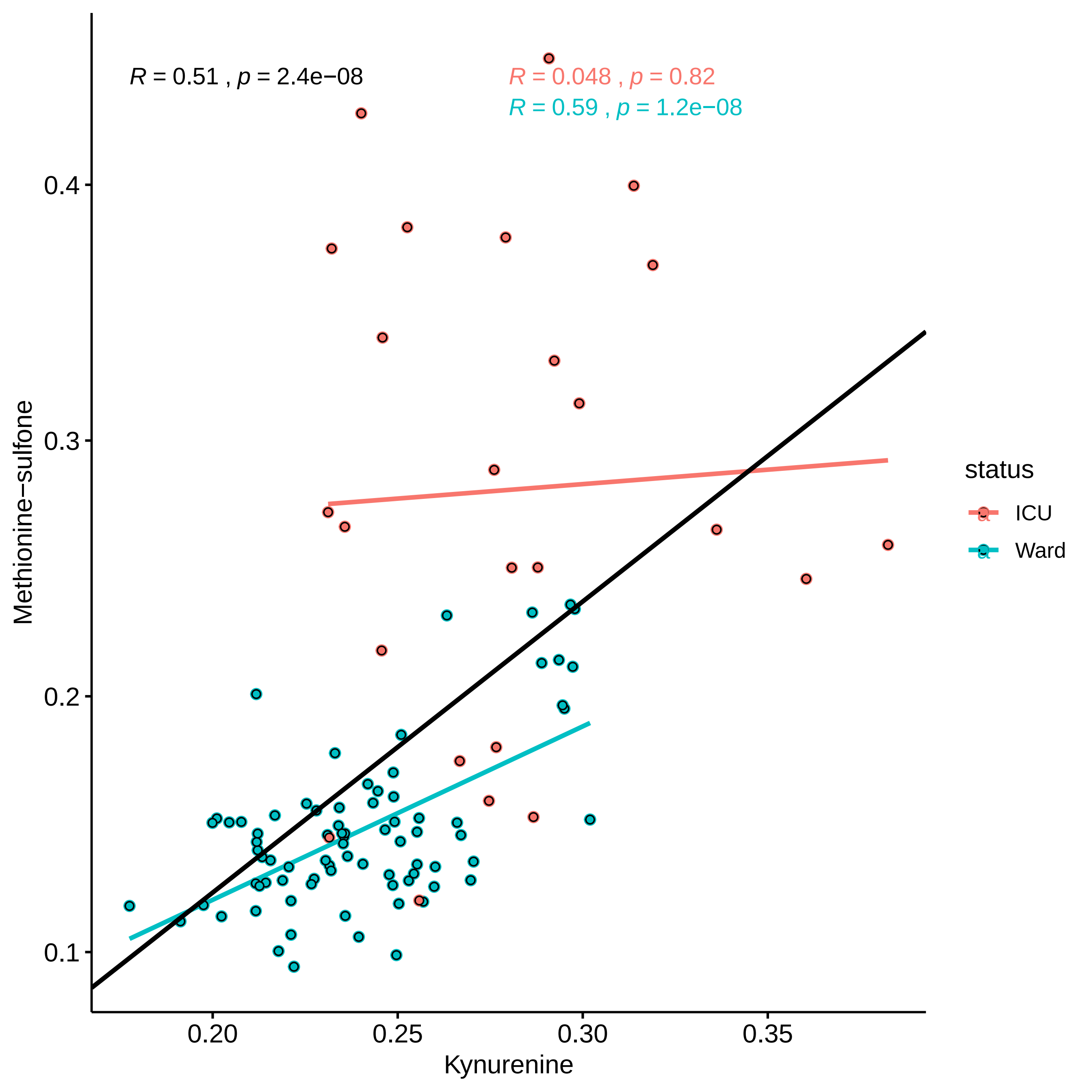

R = 0.51 , p = 2.4e−08
R = 0.048 , p = 0.82
R = 0.59 , p = 1.2e−08
Methionine−sulfone
0.4
0.3
0.2
0.1
0.20
0.25
0.30
0.35
Kynurenine
status
ICU
Ward

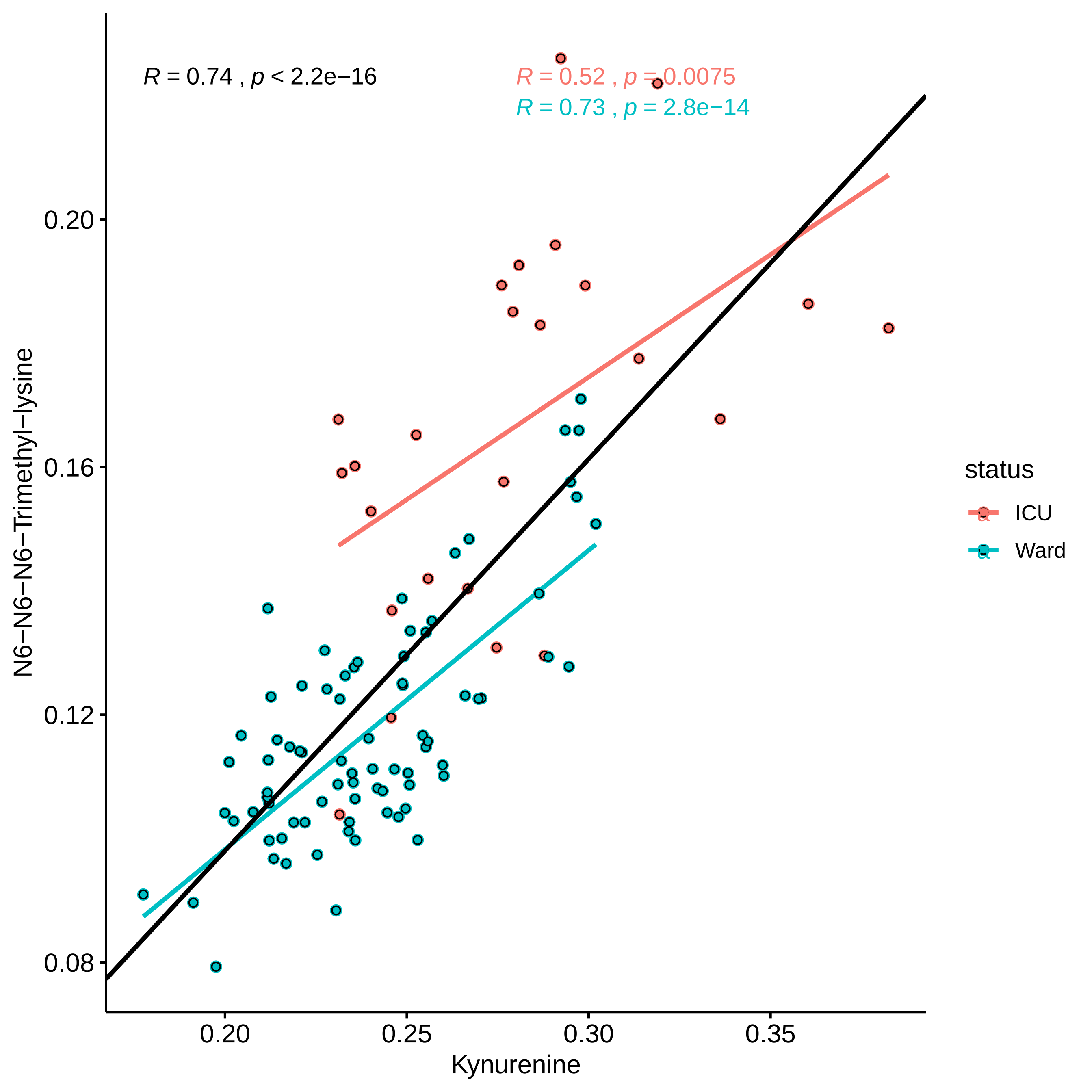

R = 0.74 , p < 2.2e−16
R = 0.52 , p = 0.0075
R = 0.73 , p = 2.8e−14
N6−N6−N6−Trimethyl−lysine
0.20
0.16
0.12
0.08
0.20
0.25
0.30
0.35
Kynurenine
status
ICU
Ward

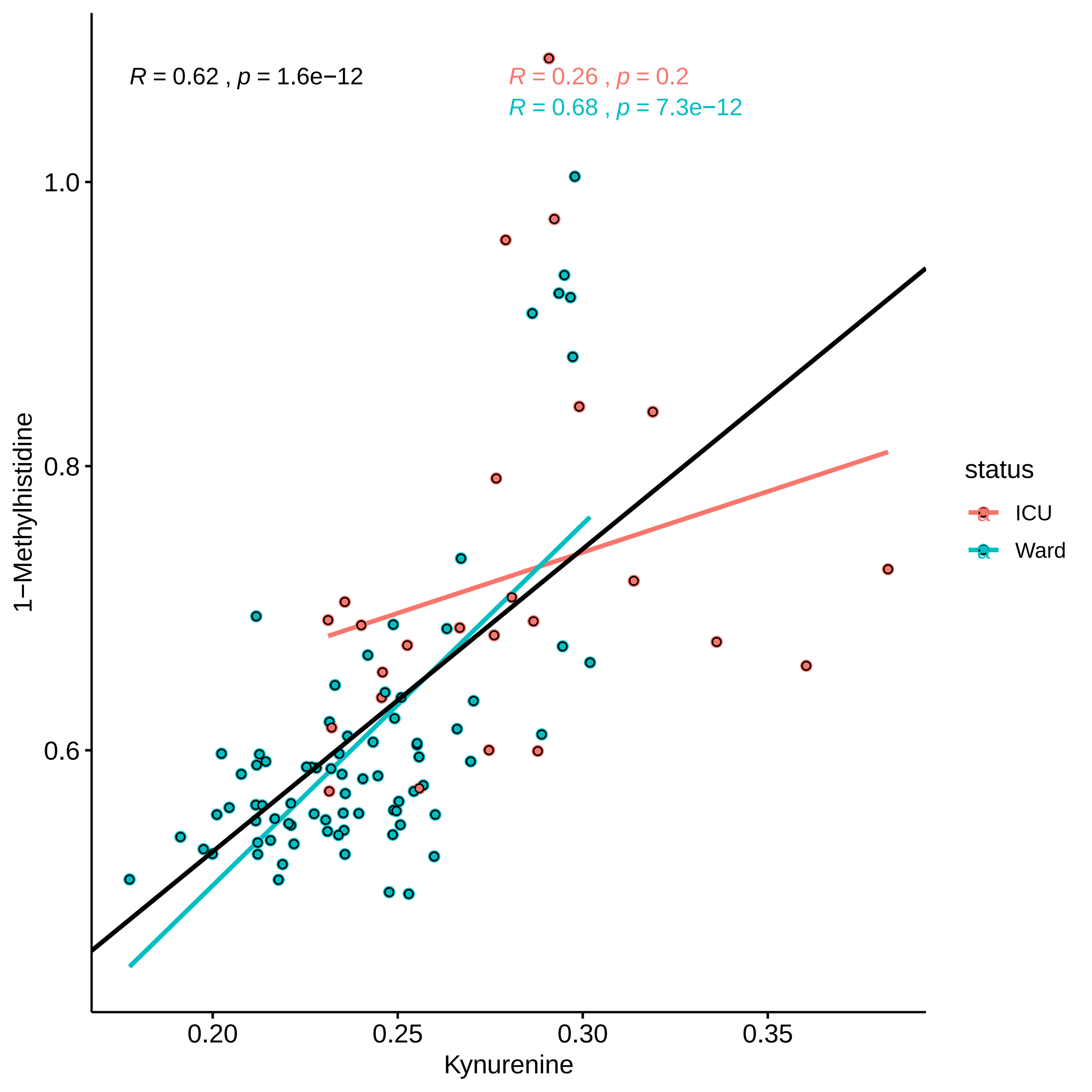

R = 0.62 , p = 1.6e−12
R = 0.26 , p = 0.2
R = 0.68 , p = 7.3e−12
1.0
0.8
0.6
1−Methylhistidine
0.20
0.25
0.30
0.35
Kynurenine
status
ICU
Ward

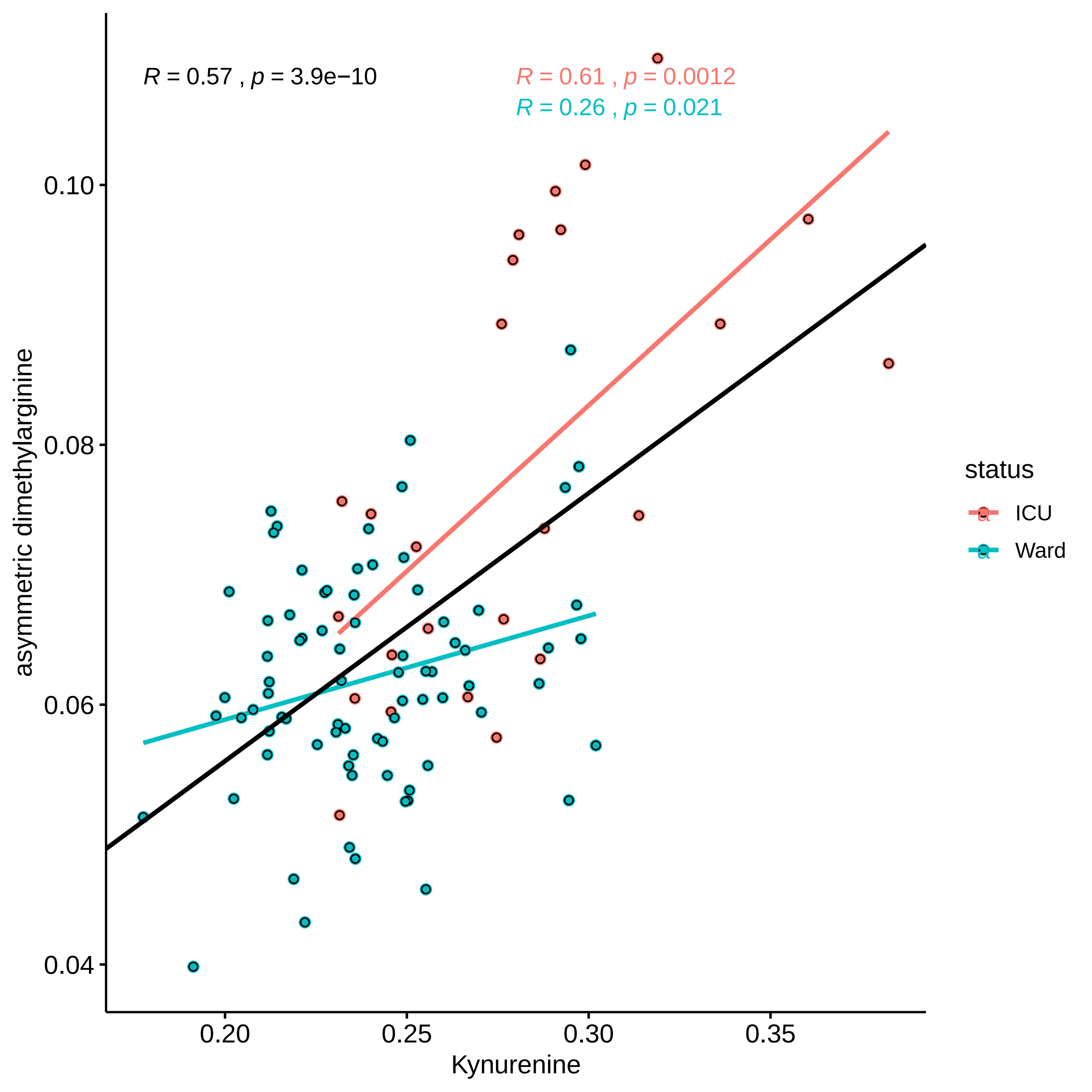

R = 0.57 , p = 3.9e−10
R = 0.61 , p = 0.0012
R = 0.26 , p = 0.021
0.10
0.08
0.06
0.04
asymmetric dimethylarginine
0.20
0.25
0.30
0.35
Kynurenine
status
ICU
Ward

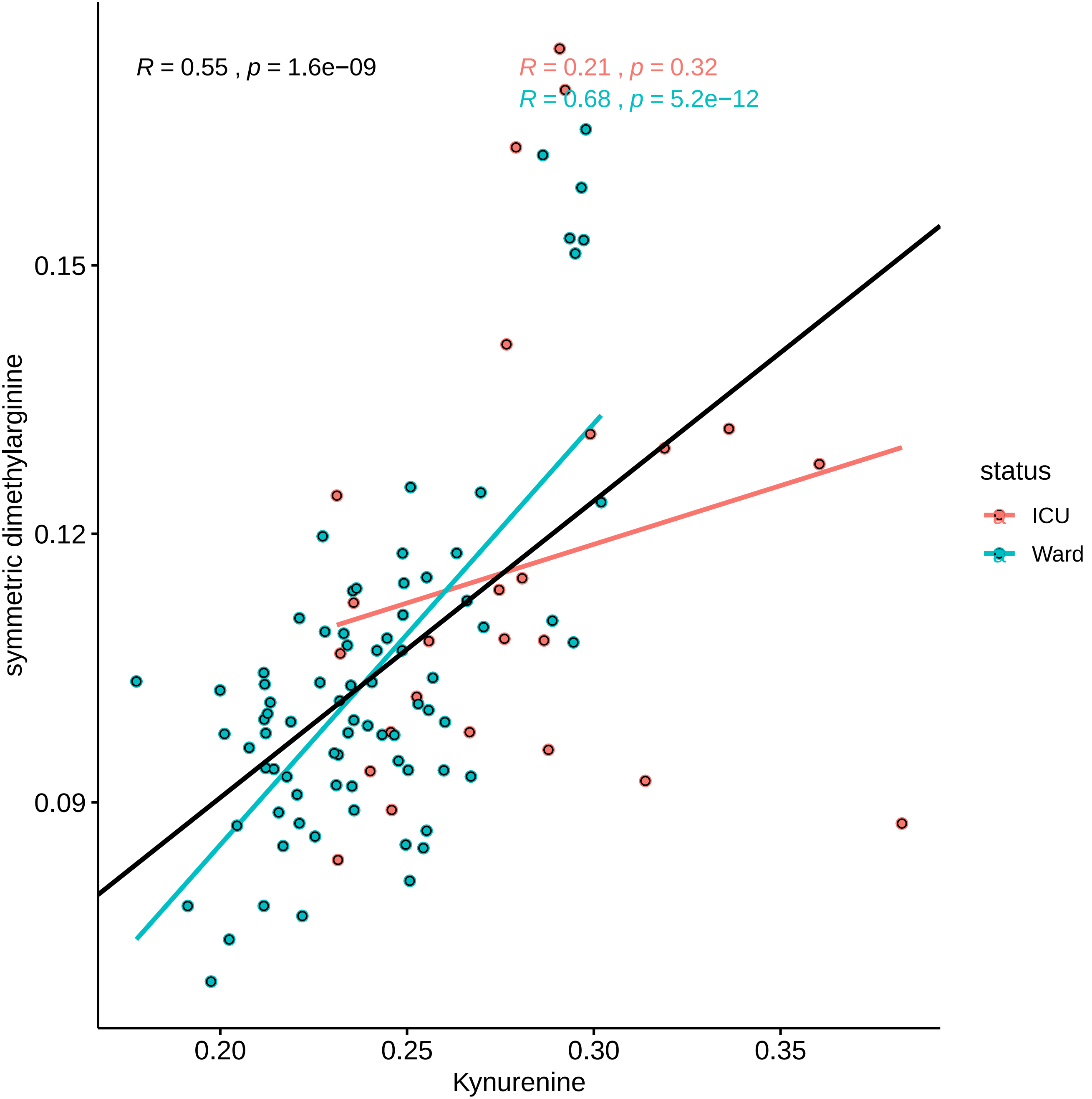

R = 0.55 , p = 1.6e−09
R = 0.21 , p = 0.32
R = 0.68 , p = 5.2e−12
0.15
0.12
0.09
symmetric dimethylarginine
0.20
0.25
0.30
0.35
Kynurenine
status
ICU
Ward

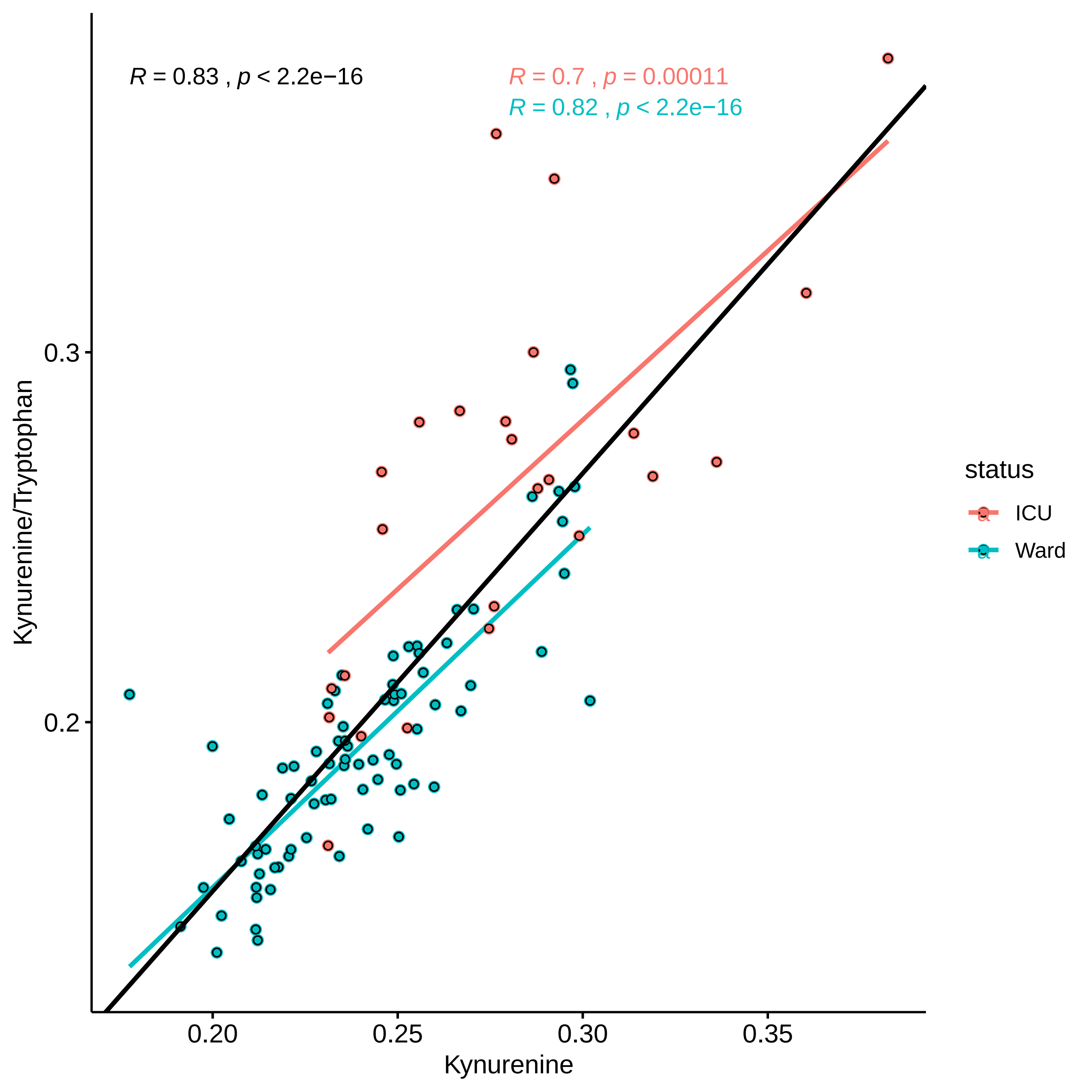

R = 0.83 , p < 2.2e−16
R = 0.7 , p = 0.00011
R = 0.82 , p < 2.2e−16
Kynurenine/Tryptophan
0.3
0.2
0.20
0.25
0.30
0.35
Kynurenine
status
ICU
Ward

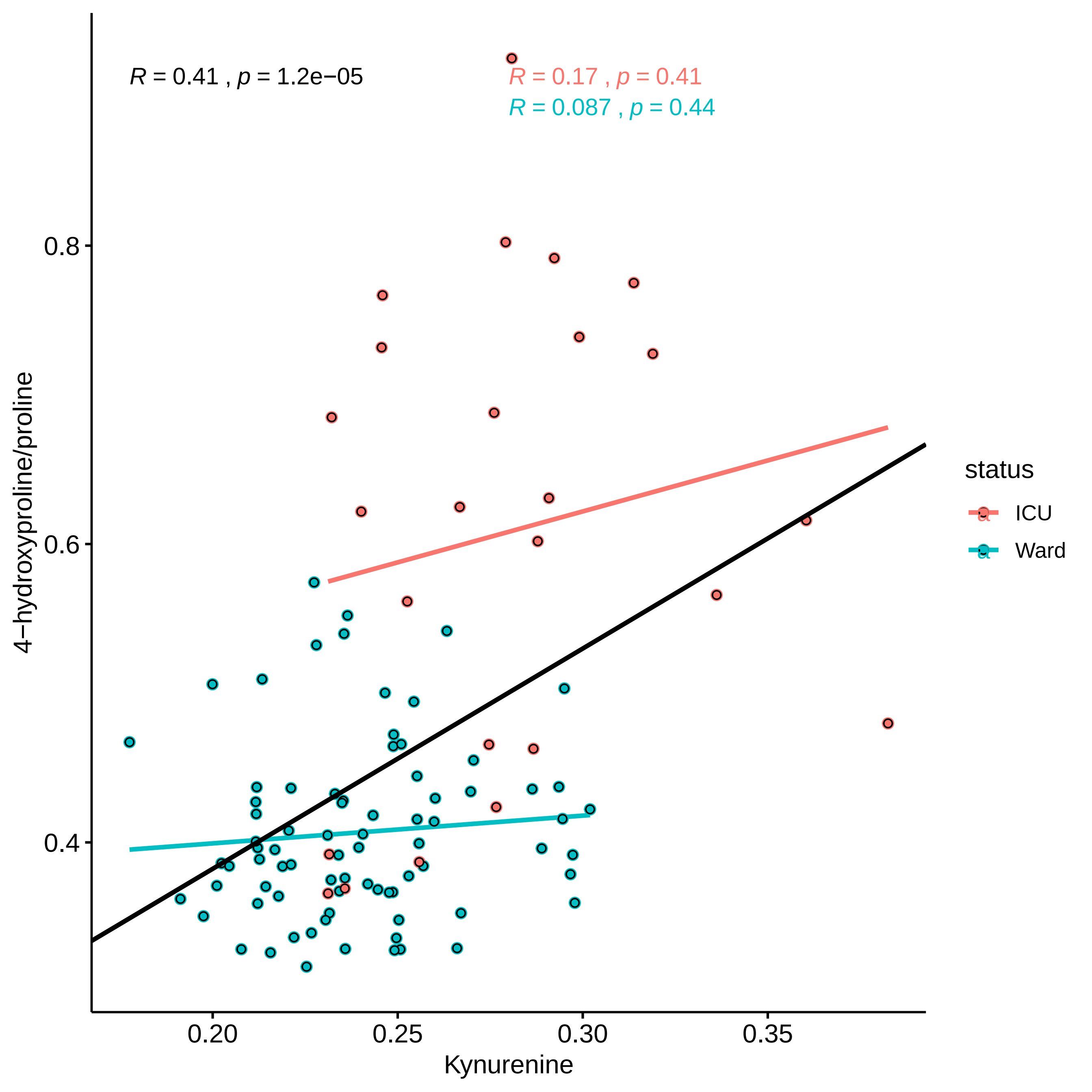
R = 0.41 , p = 1.2e−05
R = 0.17 , p = 0.41
R = 0.087 , p = 0.44
0.8
0.6
0.4
4−hydroxyproline/proline
0.20
0.25
0.30
0.35
Kynurenine
status
ICU
Ward

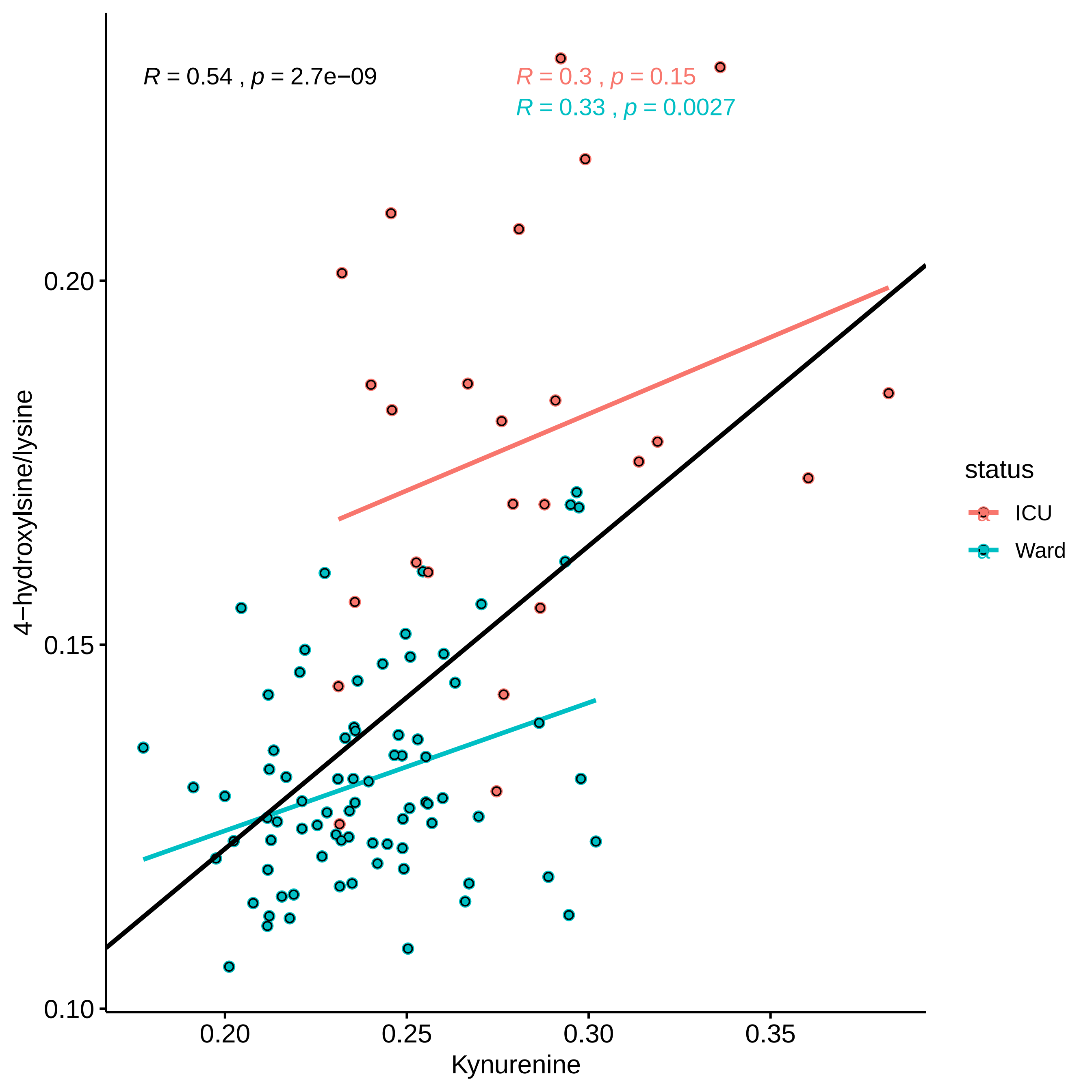
R = 0.54 , p = 2.7e−09
R = 0.3 , p = 0.15
R = 0.33 , p = 0.0027
4−hydroxylsine/lysine
0.20
0.15
0.10
0.20
0.25
0.30
0.35
Kynurenine
status
ICU
Ward

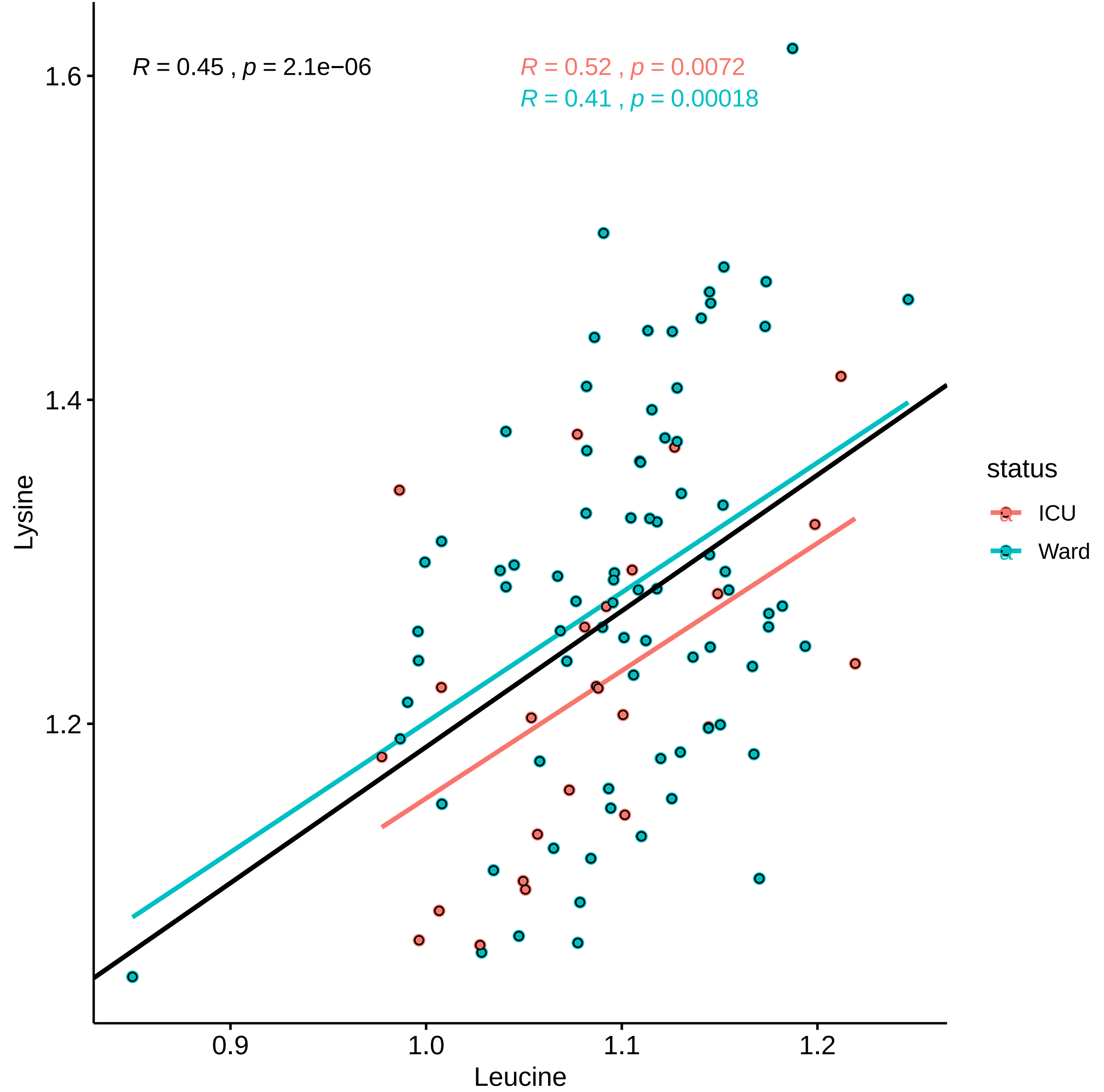

R = 0.45 , p = 2.1e−06
R = 0.52 , p = 0.0072
R = 0.41 , p = 0.00018
1.6
1.4
1.2
0.9
1.0
1.1
1.2
Lysine
Leucine
status
ICU
Ward

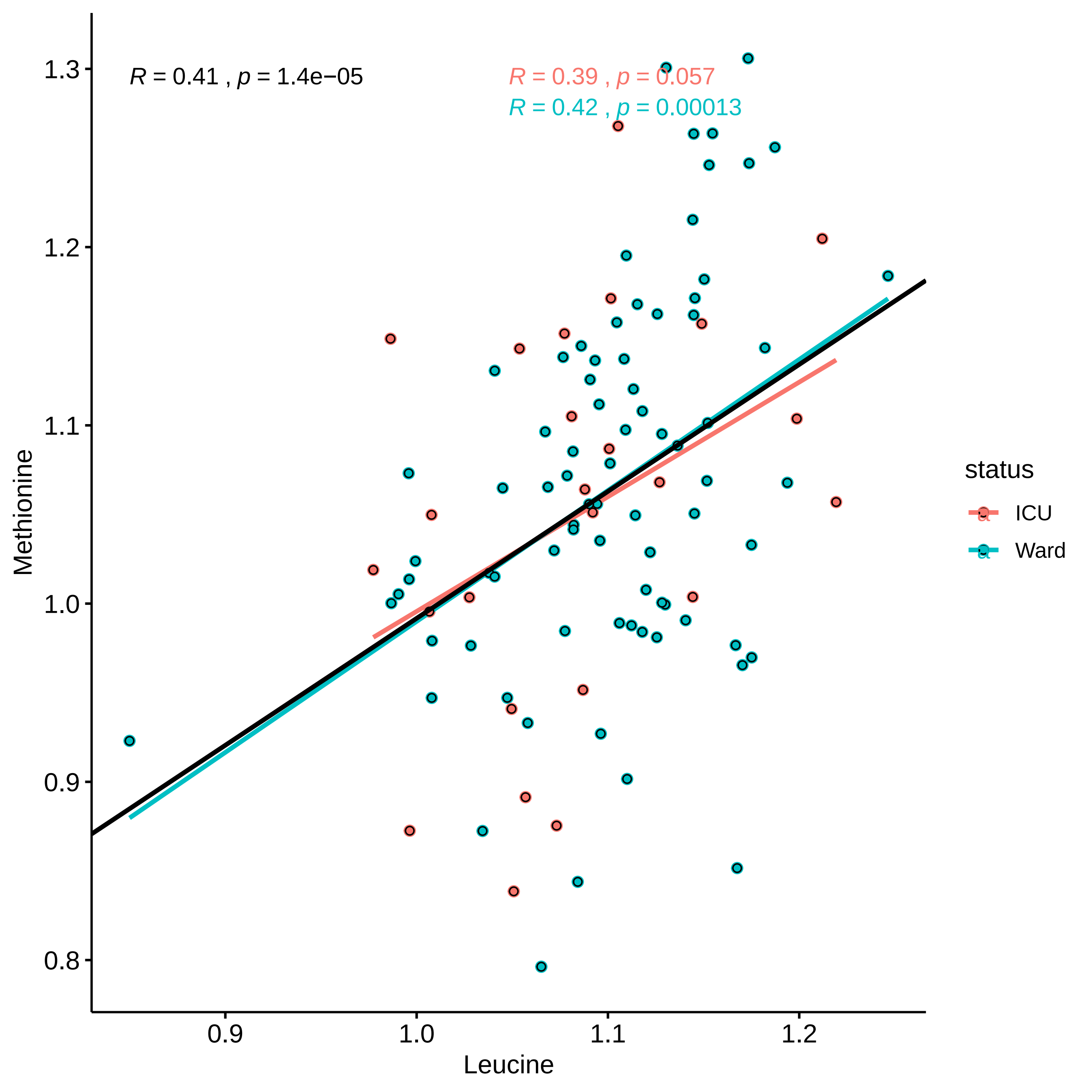

R = 0.41 , p = 1.4e−05
R = 0.39 , p = 0.057
R = 0.42 , p = 0.00013
1.3
1.2
1.1
1.0
0.9
0.8
Methionine
0.9
1.0
1.1
1.2
Leucine
status
ICU
Ward

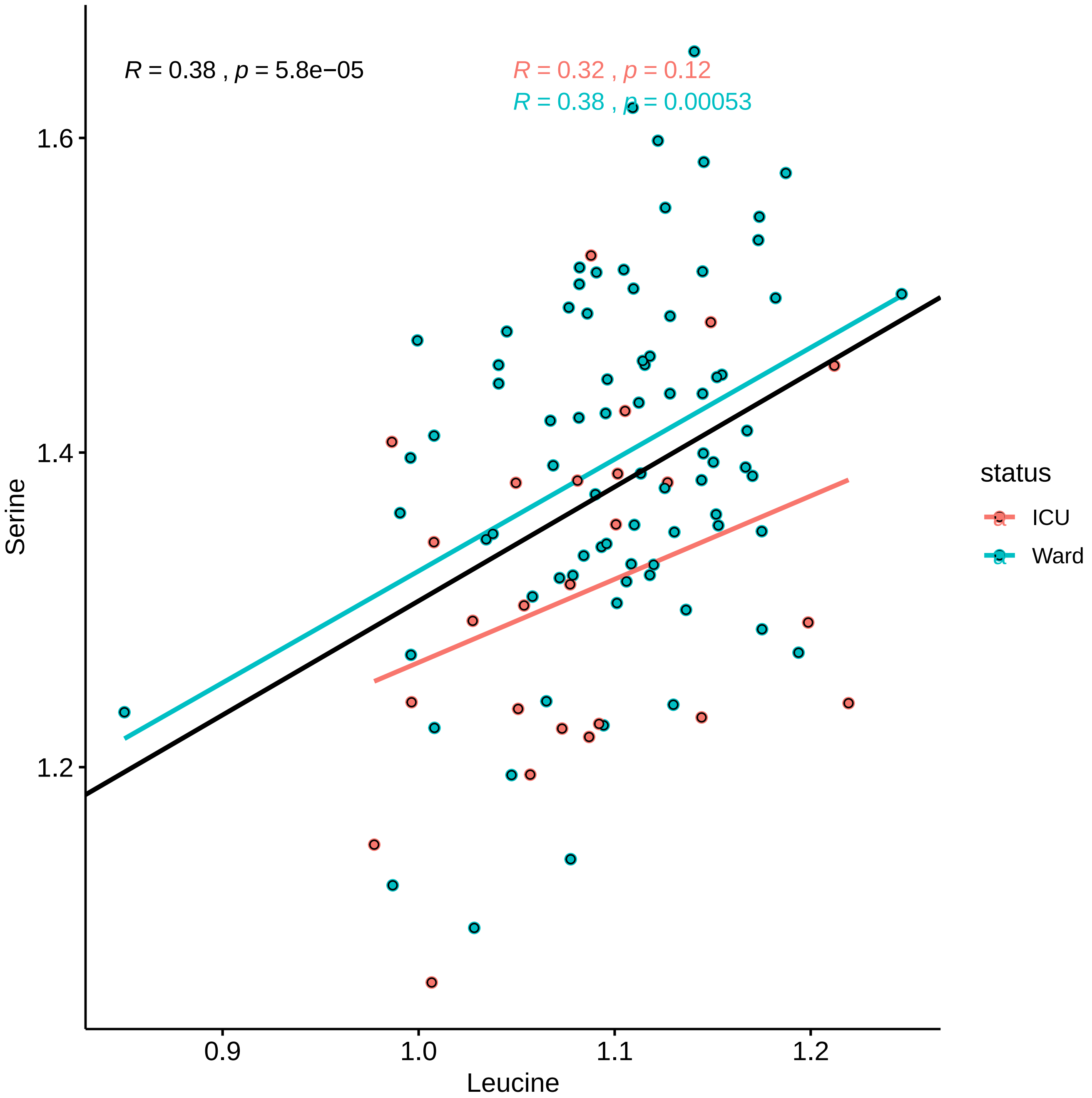

R = 0.38 , p = 5.8e−05
R = 0.32 , p = 0.12
R = 0.38 , p = 0.00053
1.6
1.4
1.2
Serine
0.9
1.0
1.1
1.2
Leucine
status
ICU
Ward

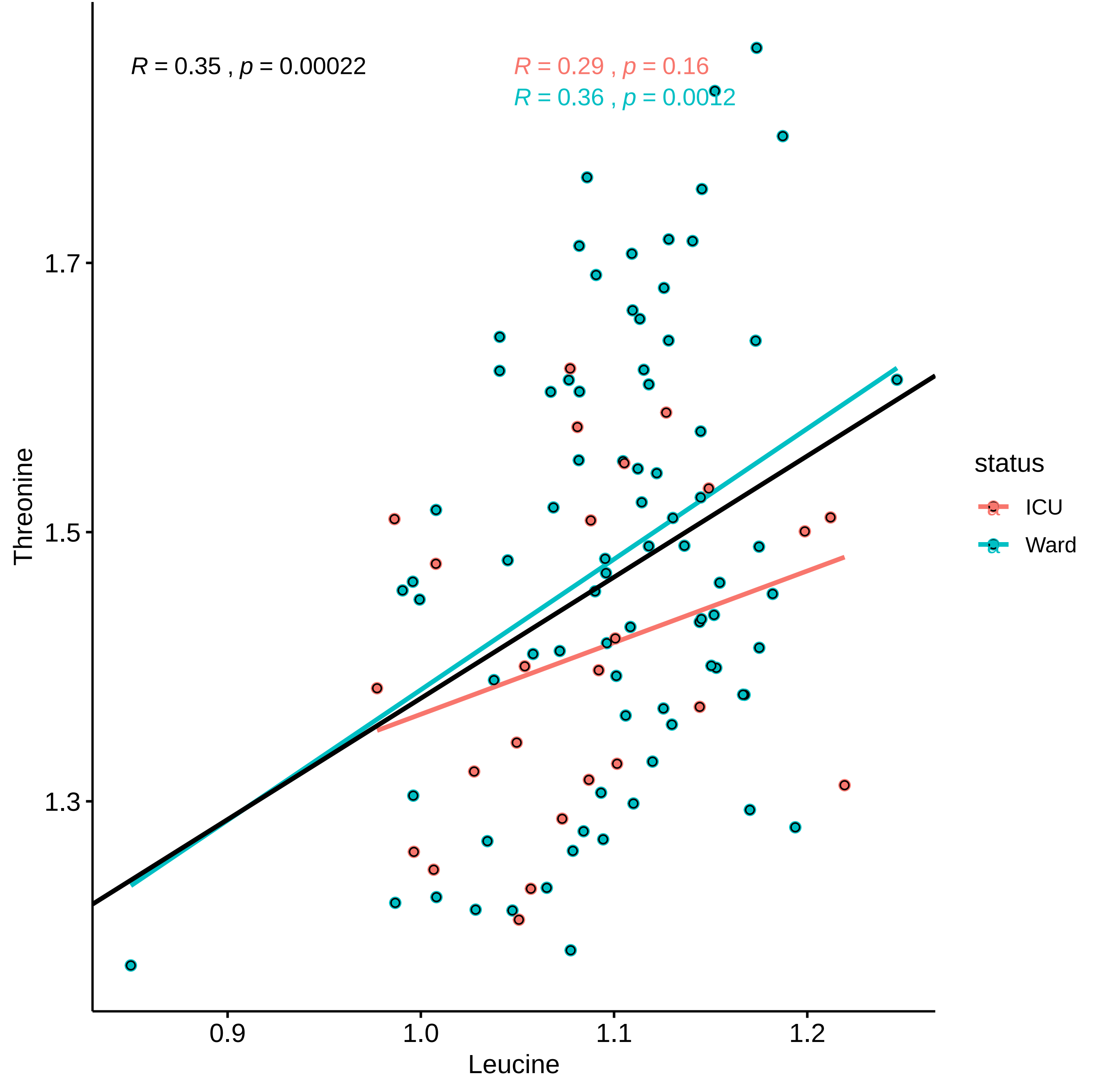

R = 0.35 , p = 0.00022
R = 0.29 , p = 0.16
R = 0.36 , p = 0.0012
1.7
1.5
1.3
Threonine
0.9
1.0
1.1
1.2
Leucine
status
ICU
Ward

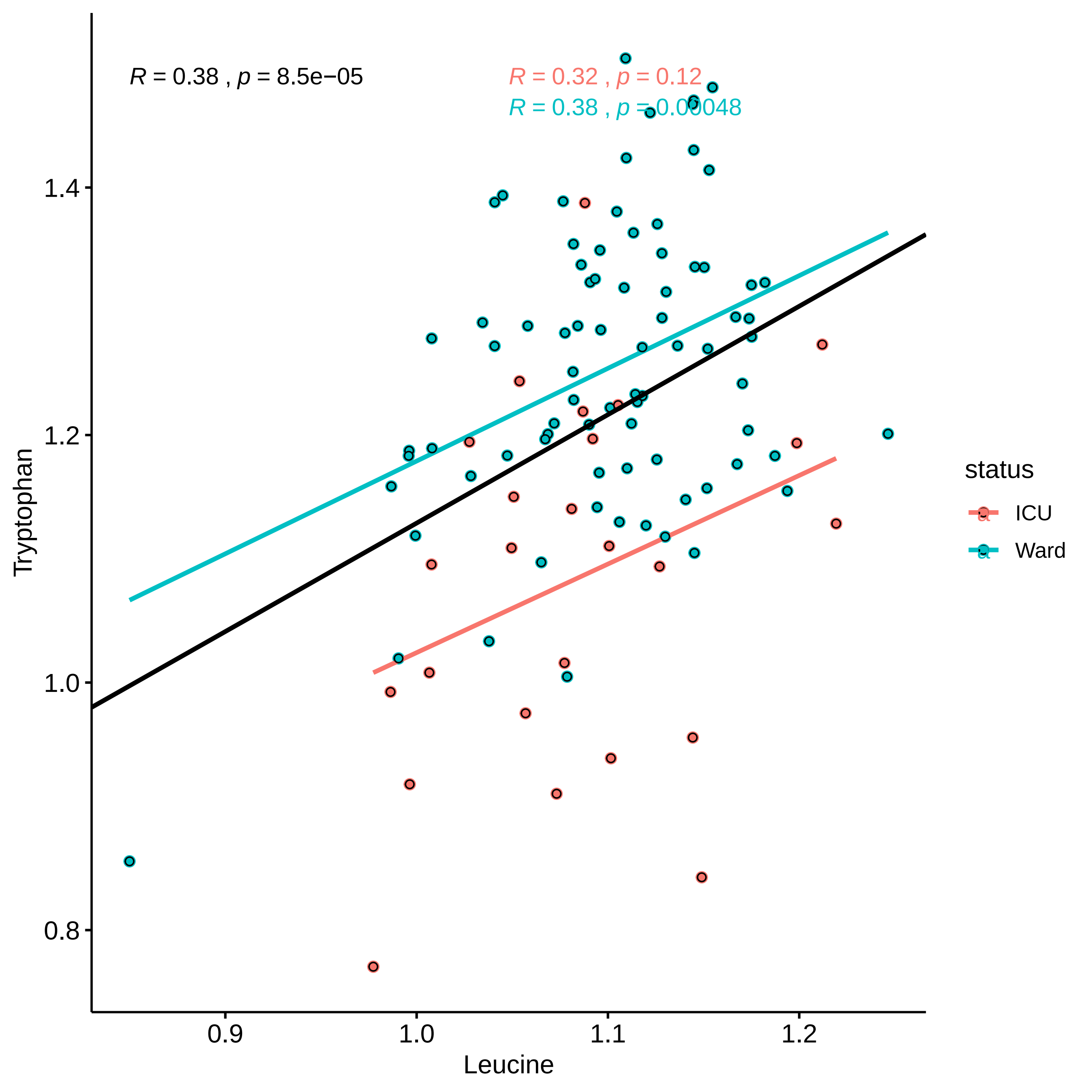

R = 0.38 , p = 8.5e−05
R = 0.32 , p = 0.12
R = 0.38 , p = 0.00048
Tryptophan
Leucine
status
ICU
Ward

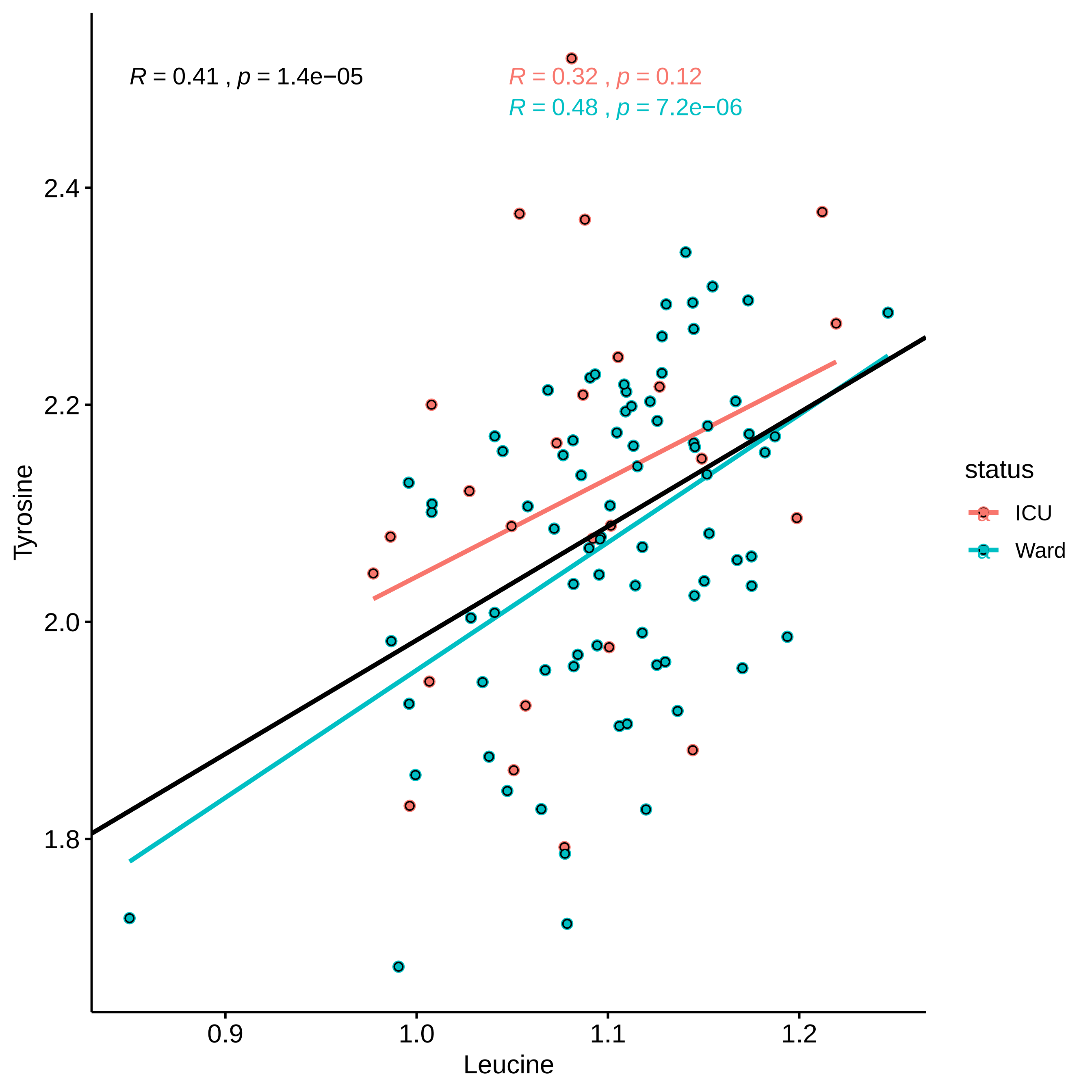

R = 0.41 , p = 1.4e−05
R = 0.32 , p = 0.12
R = 0.48 , p = 7.2e−06
2.4
2.2
2.0
1.8
Tyrosine
0.9
1.0
1.1
1.2
Leucine
status
ICU
Ward

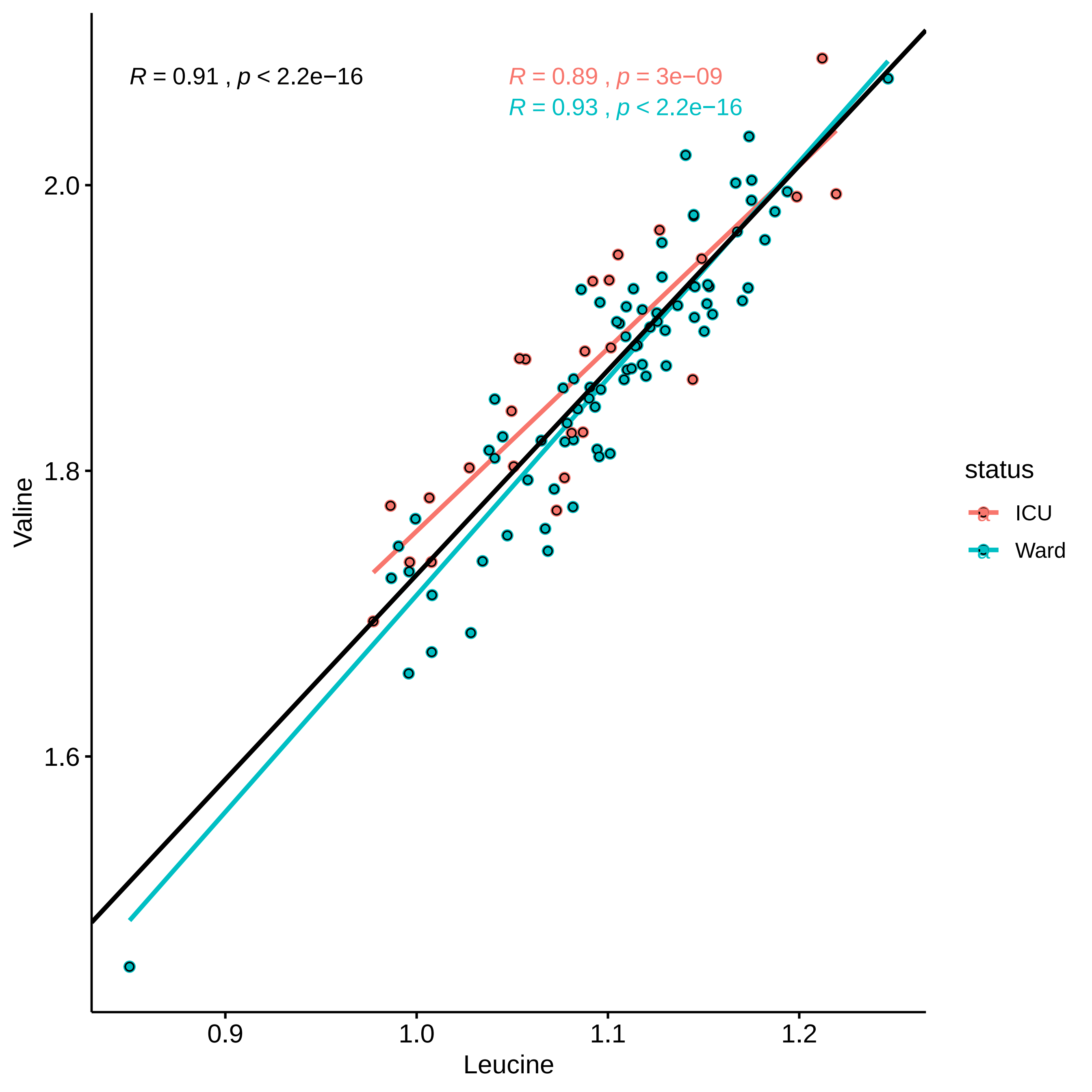

R = 0.91 , p < 2.2e−16
R = 0.89 , p = 3e−09
R = 0.93 , p < 2.2e−16
2.0
1.8
1.6
Valine
0.9
1.0
1.1
1.2
Leucine
status
ICU
Ward

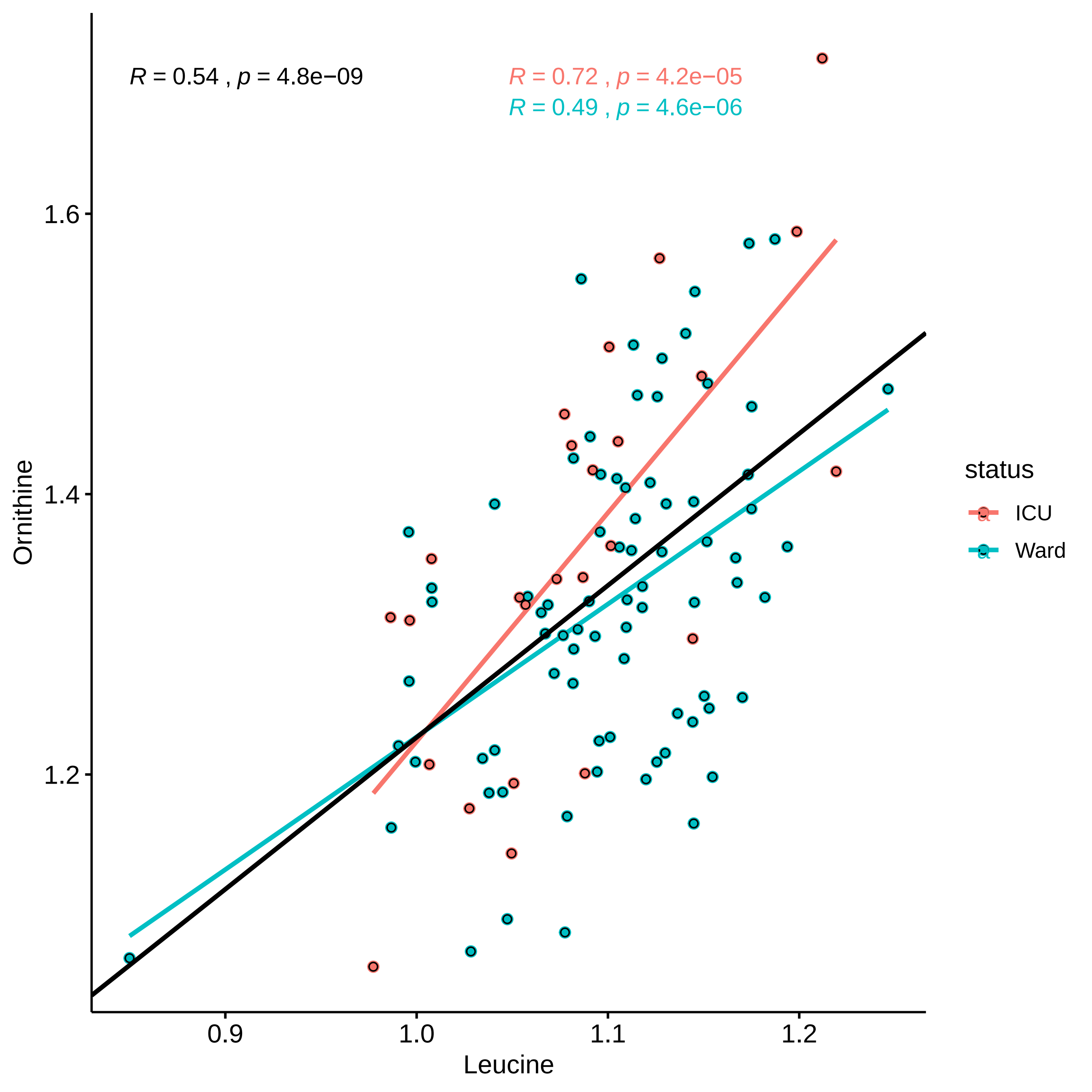

R = 0.54 , p = 4.8e−09
R = 0.72 , p = 4.2e−05
R = 0.49 , p = 4.6e−06
Ornithine
1.6
1.4
1.2
0.9
1.0
1.1
1.2
Leucine
status
ICU
Ward

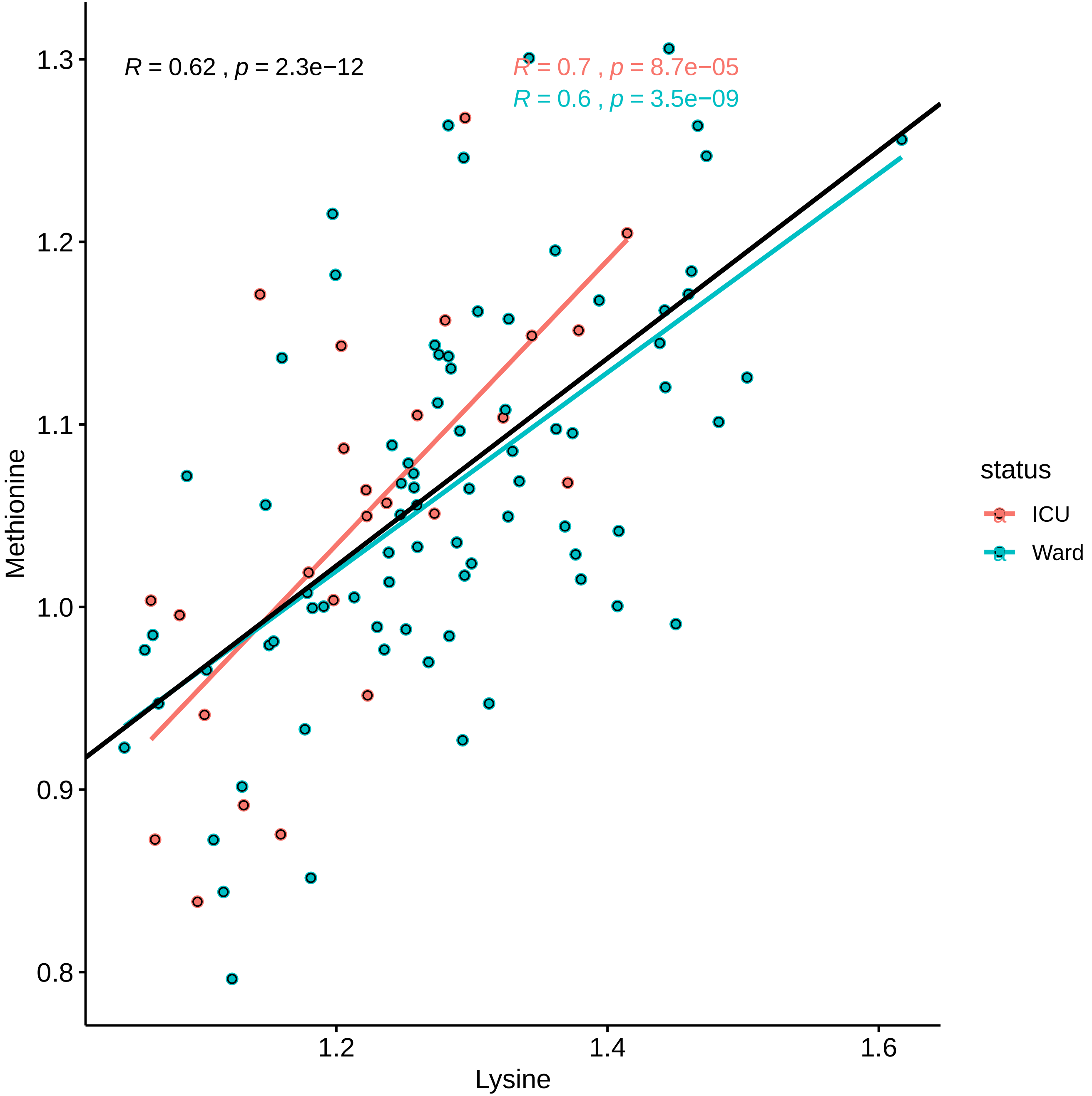

R = 0.62 , p = 2.3e−12
R = 0.7 , p = 8.7e−05
R = 0.6 , p = 3.5e−09
1.3
1.2
1.1
1.0
0.9
0.8
Methionine
1.2
1.4
1.6
Lysine
status
ICU
Ward

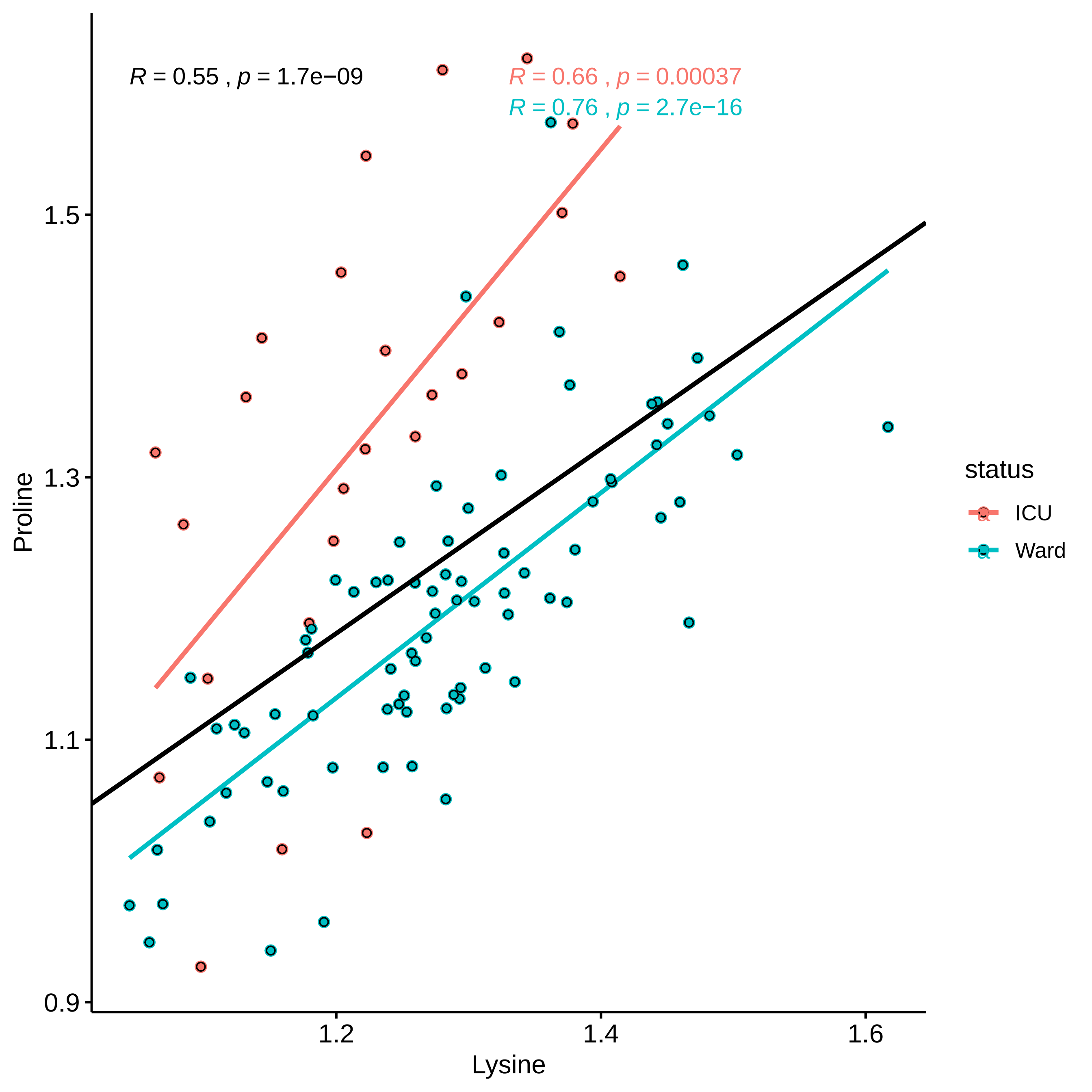

R = 0.55 , p = 1.7e−09
R = 0.66 , p = 0.00037
R = 0.76 , p = 2.7e−16
Proline
1.5
1.3
1.1
0.9
Lysine
1.2
1.4
1.6
status
ICU
Ward

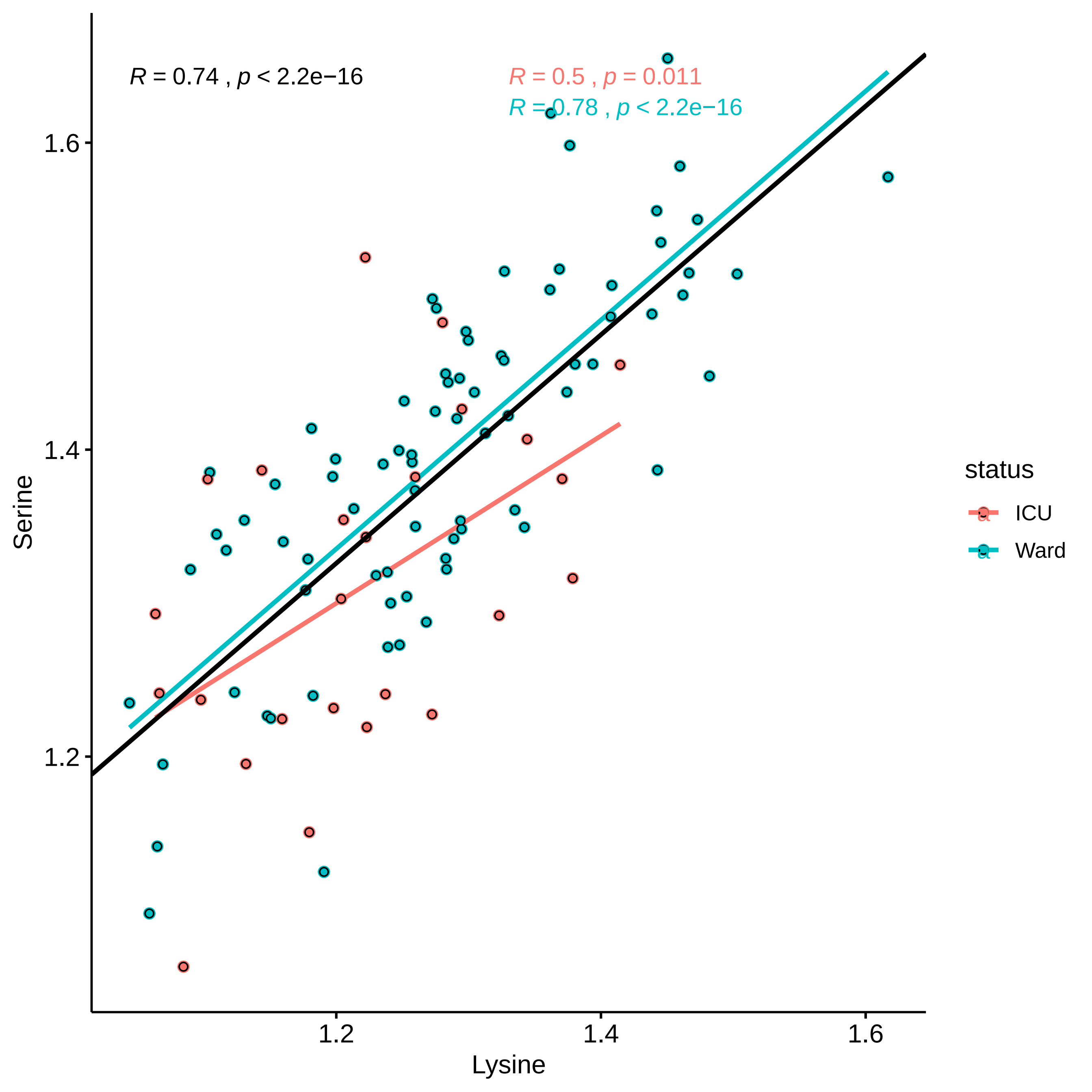

R = 0.74 , p < 2.2e−16
R = 0.5 , p = 0.011
R = 0.78 , p < 2.2e−16
Serine
1.2
1.4
1.6
Lysine
1.2
1.4
1.6
status
ICU
Ward

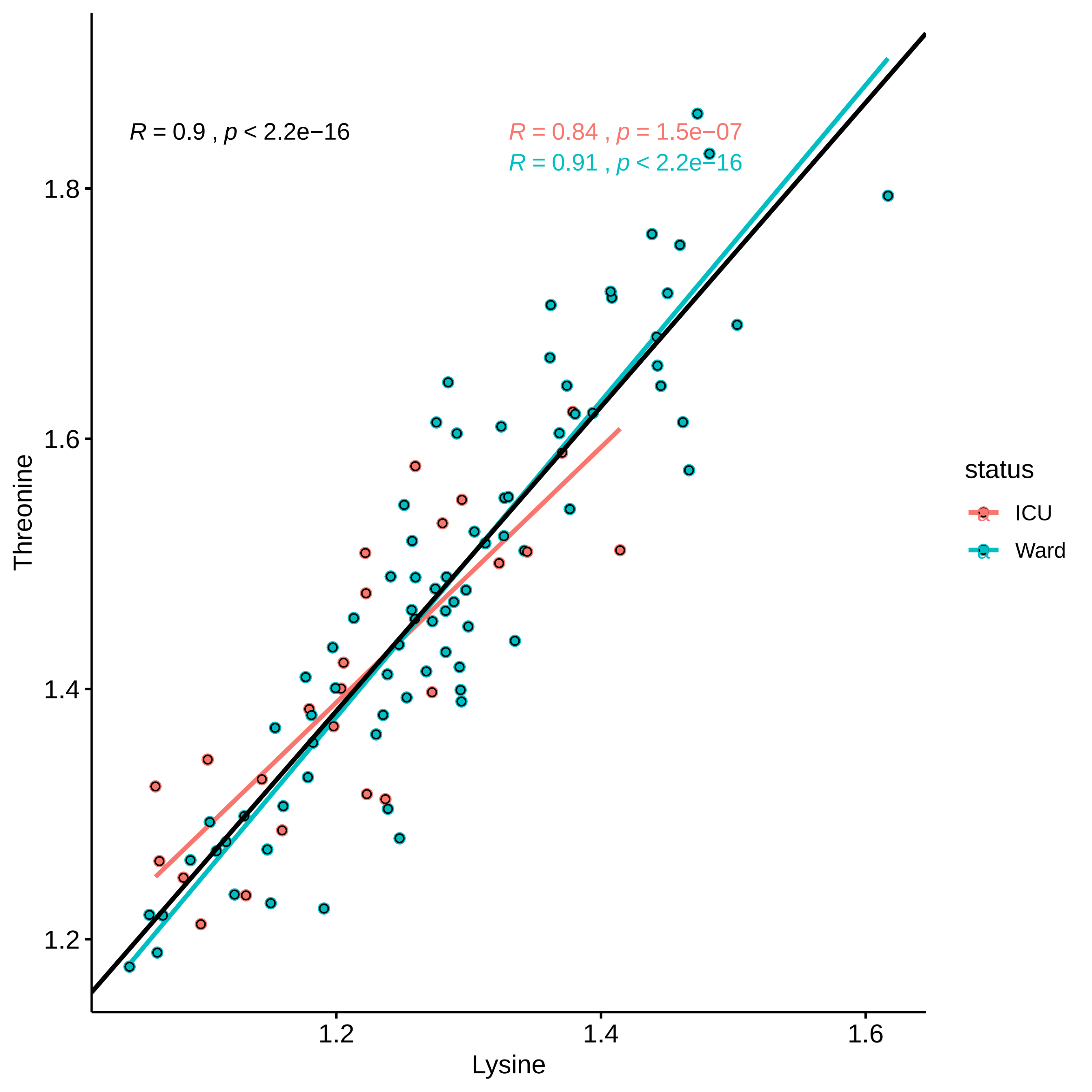
R = 0.9 , p < 2.2e−16
R = 0.84 , p = 1.5e−07
R = 0.91 , p < 2.2e−16
Threonine
1.8
1.6
1.4
1.2
Lysine
1.2
1.4
1.6
status
ICU
Ward

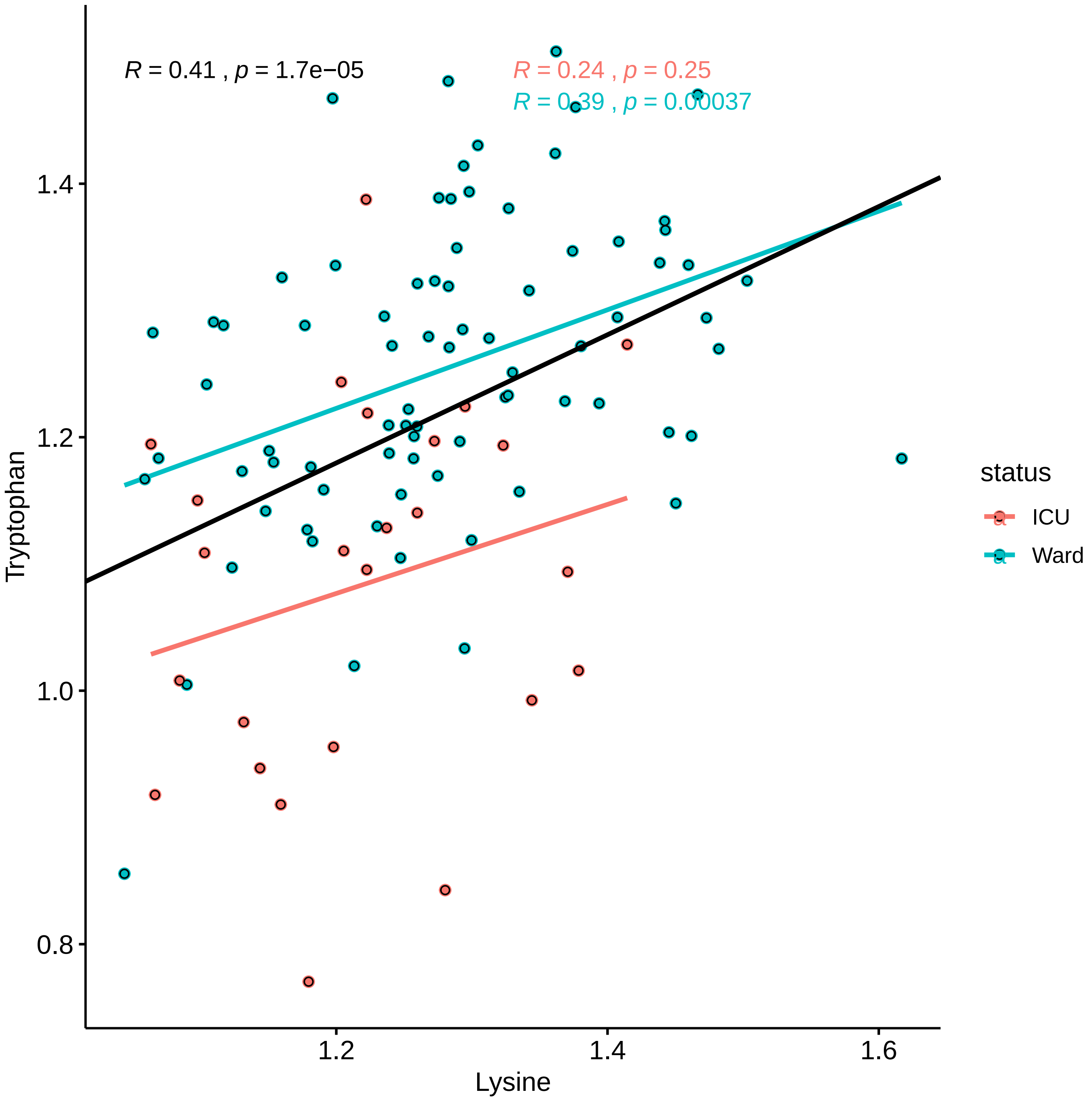

R = 0.41 , p = 1.7e−05
R = 0.24 , p = 0.25
R = 0.39 , p = 0.00037
Tryptophan
1.4
1.2
1.0
0.8
Lysine
1.2
1.4
1.6
status
ICU
Ward

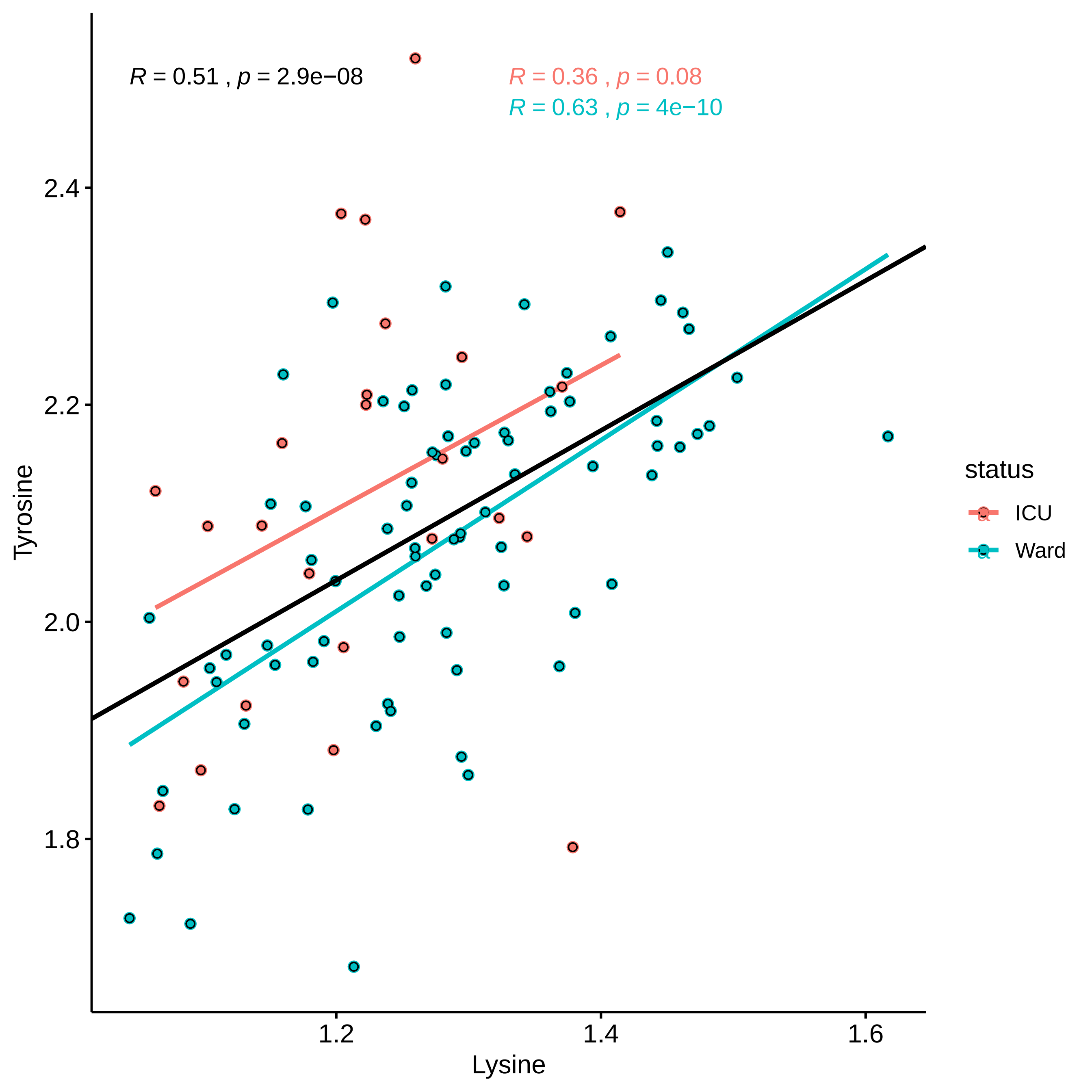

R = 0.51 , p = 2.9e−08
R = 0.36 , p = 0.08
R = 0.63 , p = 4e−10
2.4
2.2
2.0
1.8
Tyrosine
1.2
1.4
1.6
Lysine
status
ICU
Ward

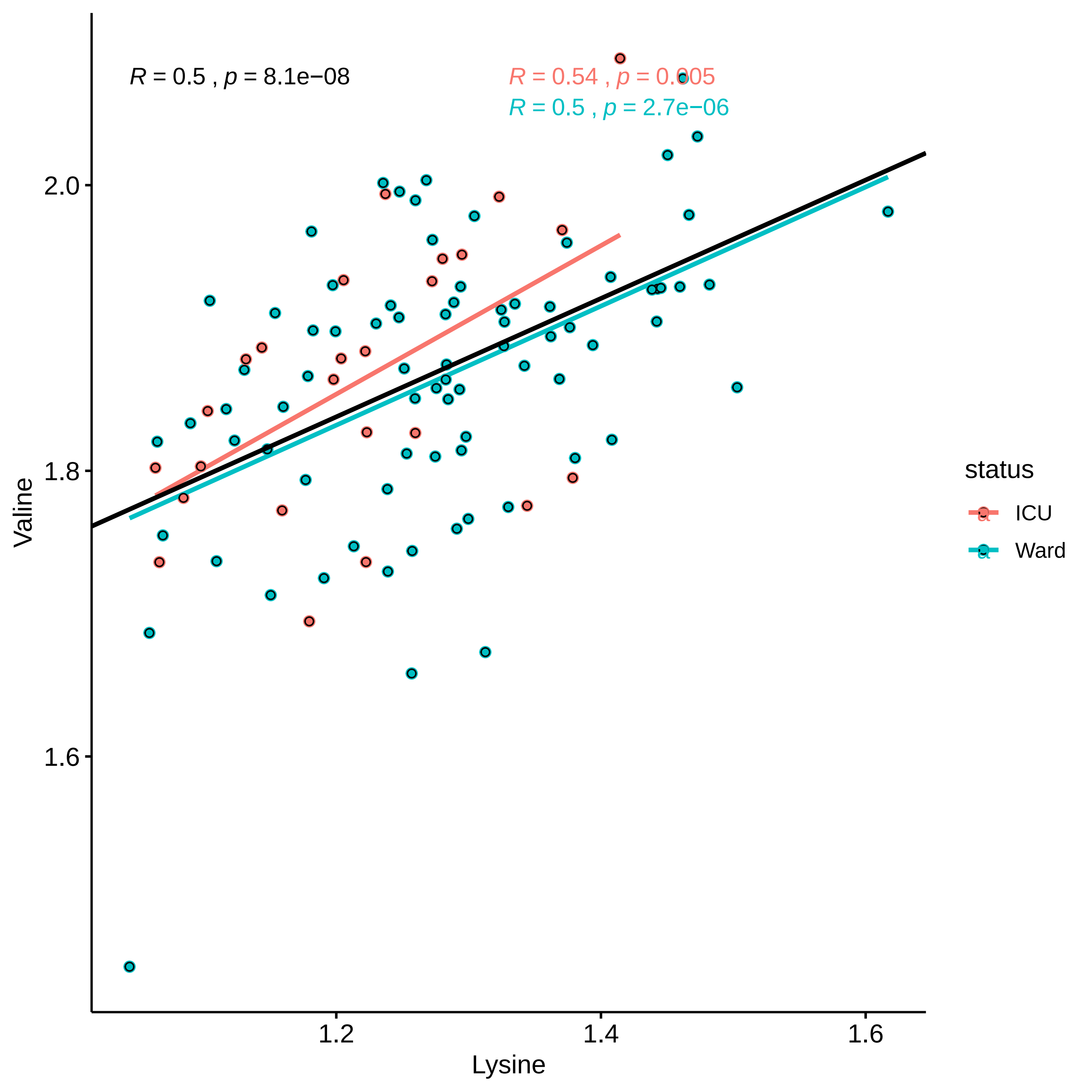

R = 0.5 , p = 8.1e−08
R = 0.54 , p = 0.005
R = 0.5 , p = 2.7e−06
2.0
1.8
1.6
Valine
1.2
1.4
1.6
Lysine
status
ICU
Ward

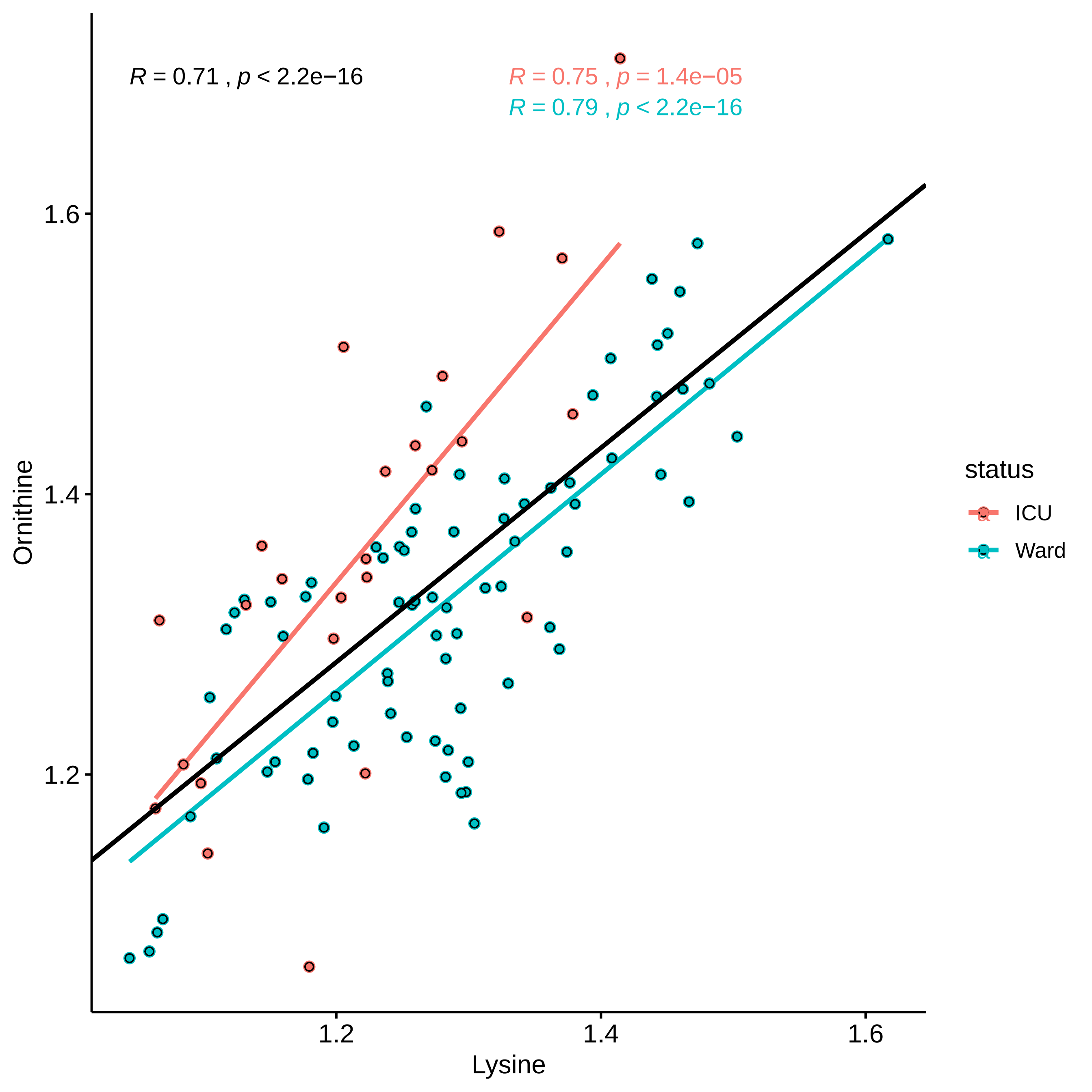

R = 0.71 , p < 2.2e−16
R = 0.75 , p = 1.4e−05
R = 0.79 , p < 2.2e−16
1.6
1.4
1.2
Ornithine
1.2
1.4
1.6
Lysine
status
ICU
Ward

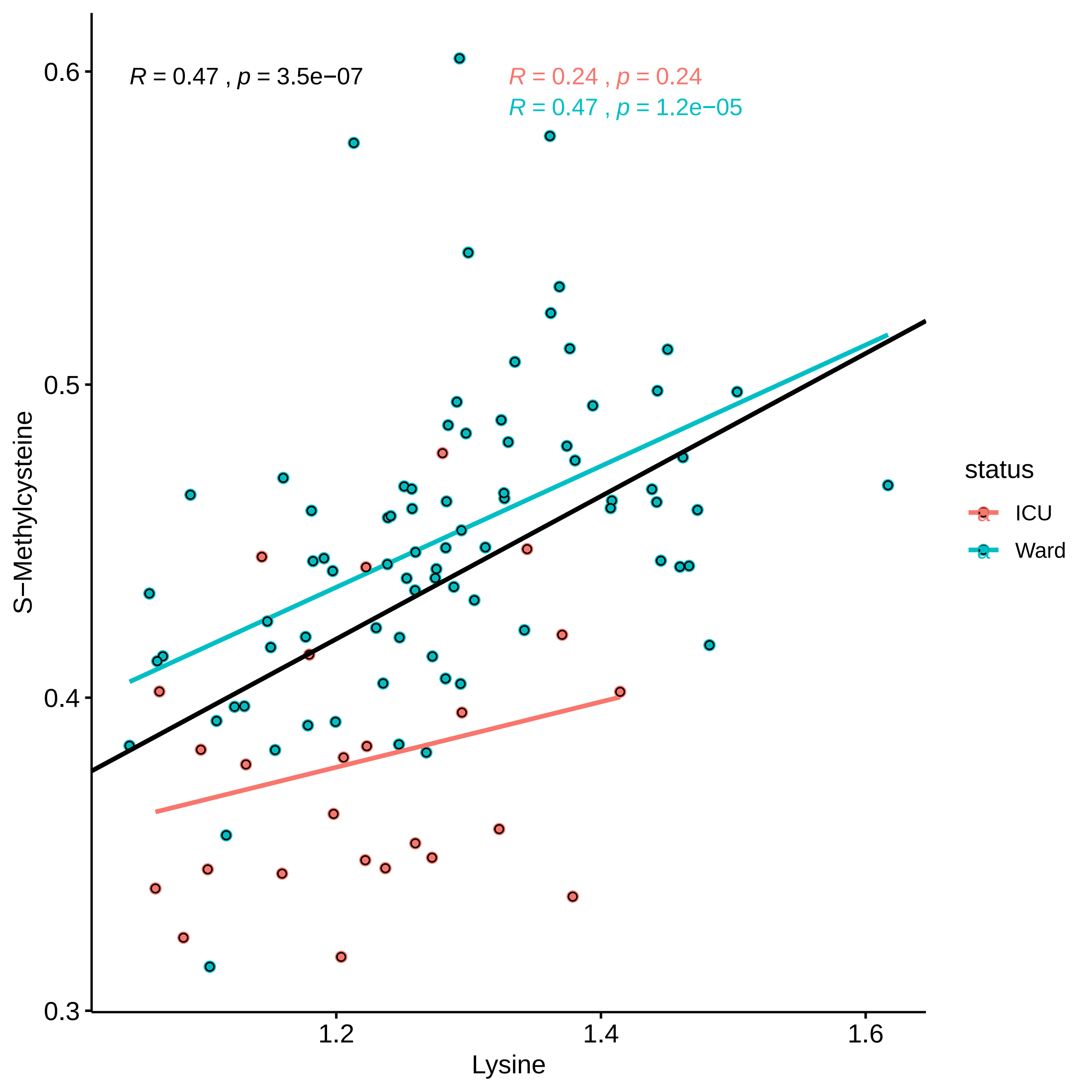

R = 0.47 , p = 3.5e−07
R = 0.24 , p = 0.24
R = 0.47 , p = 1.2e−05
S−Methylcysteine
Lysine
0.3
0.4
0.5
0.6
1.2
1.4
1.6
status
ICU
Ward

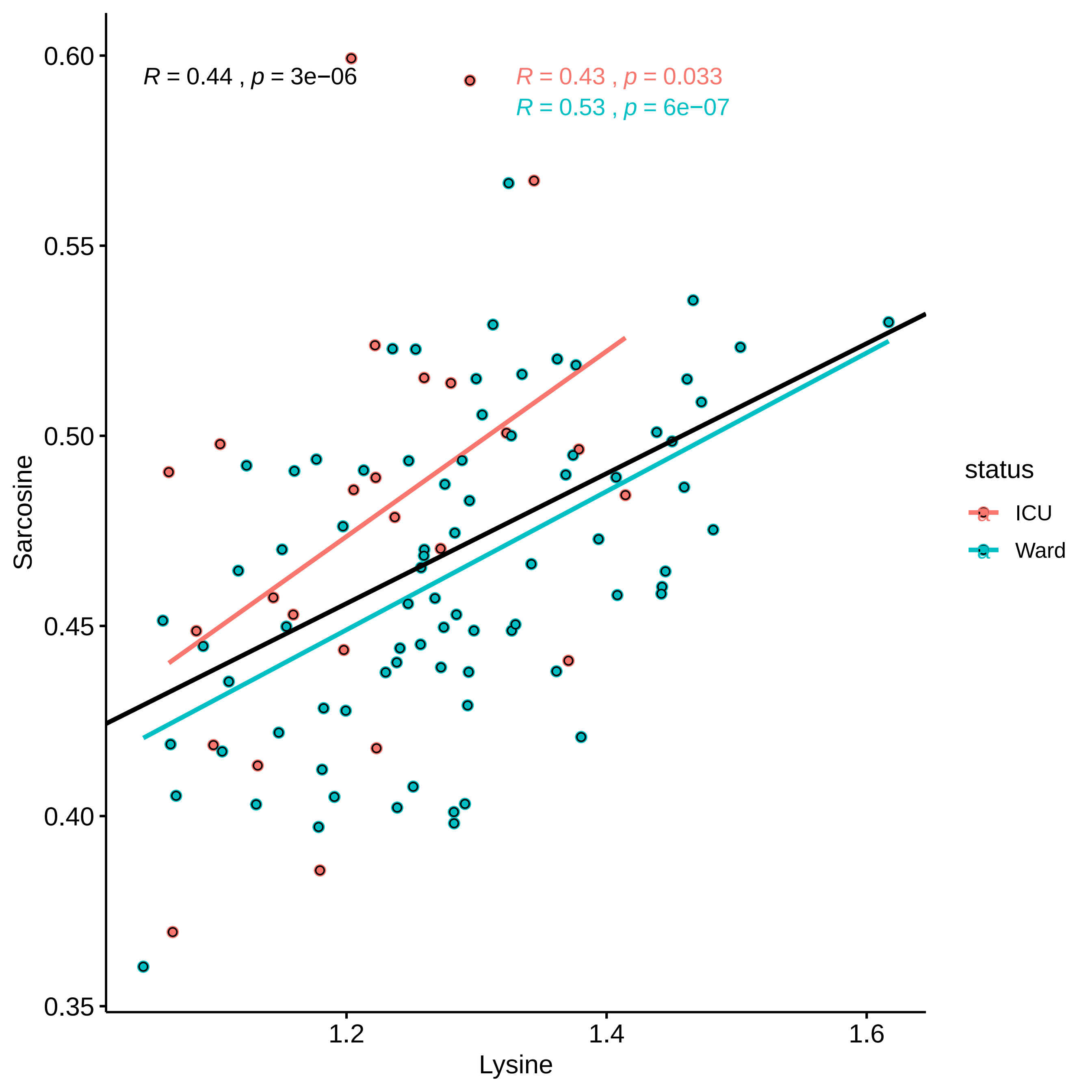

R = 0.44 , p = 3e−06
R = 0.43 , p = 0.033
R = 0.53 , p = 6e−07
Sarcosine
Lysine
0.60
0.55
0.50
0.45
0.40
0.35
1.2
1.4
1.6
status
ICU
Ward

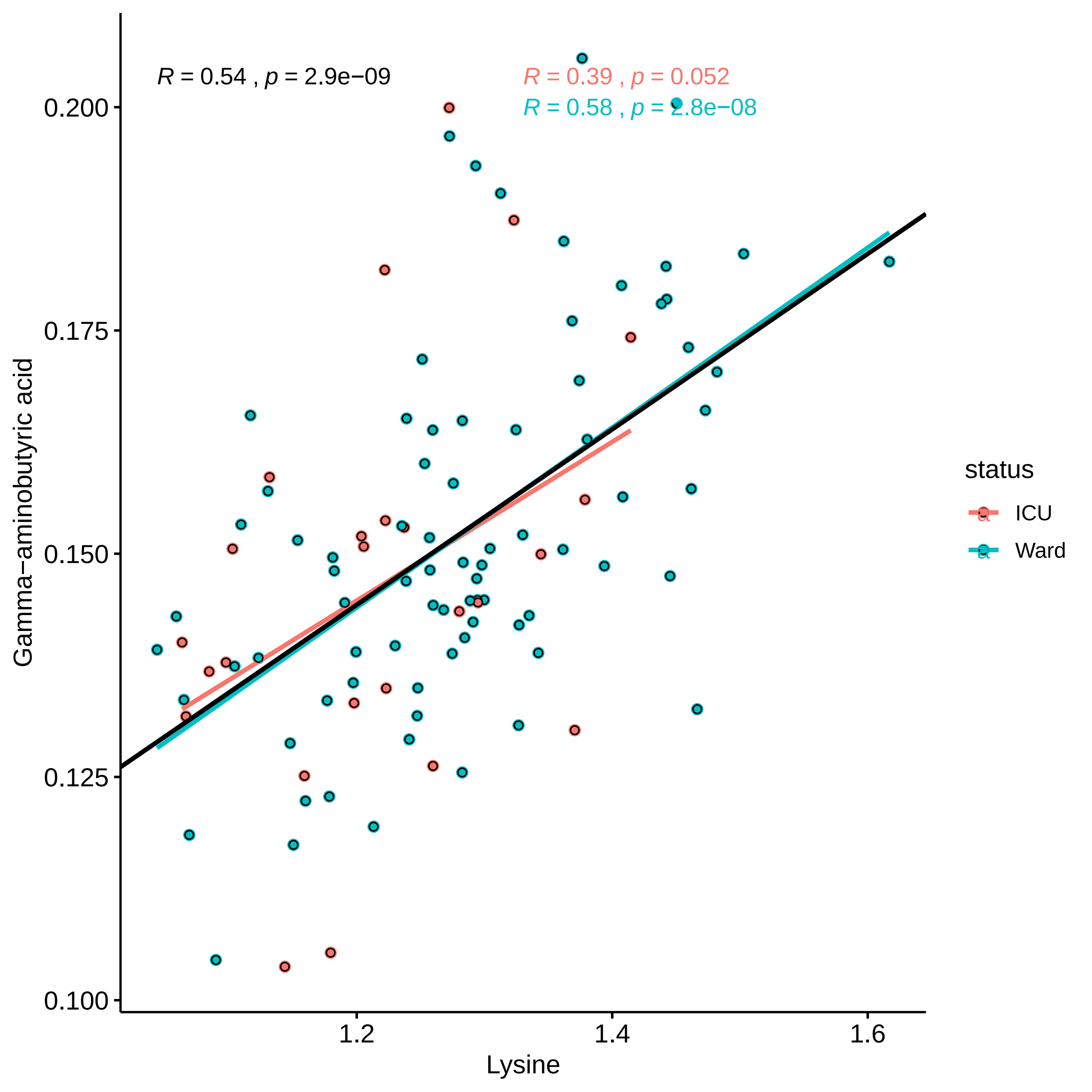

R = 0.54 , p = 2.9e−09
R = 0.39 , p = 0.052
R = 0.58 , p = 2.8e−08
Gamma−aminobutyric acid
0.200
0.175
0.150
0.125
0.100
1.2
1.4
1.6
Lysine
status
ICU
Ward

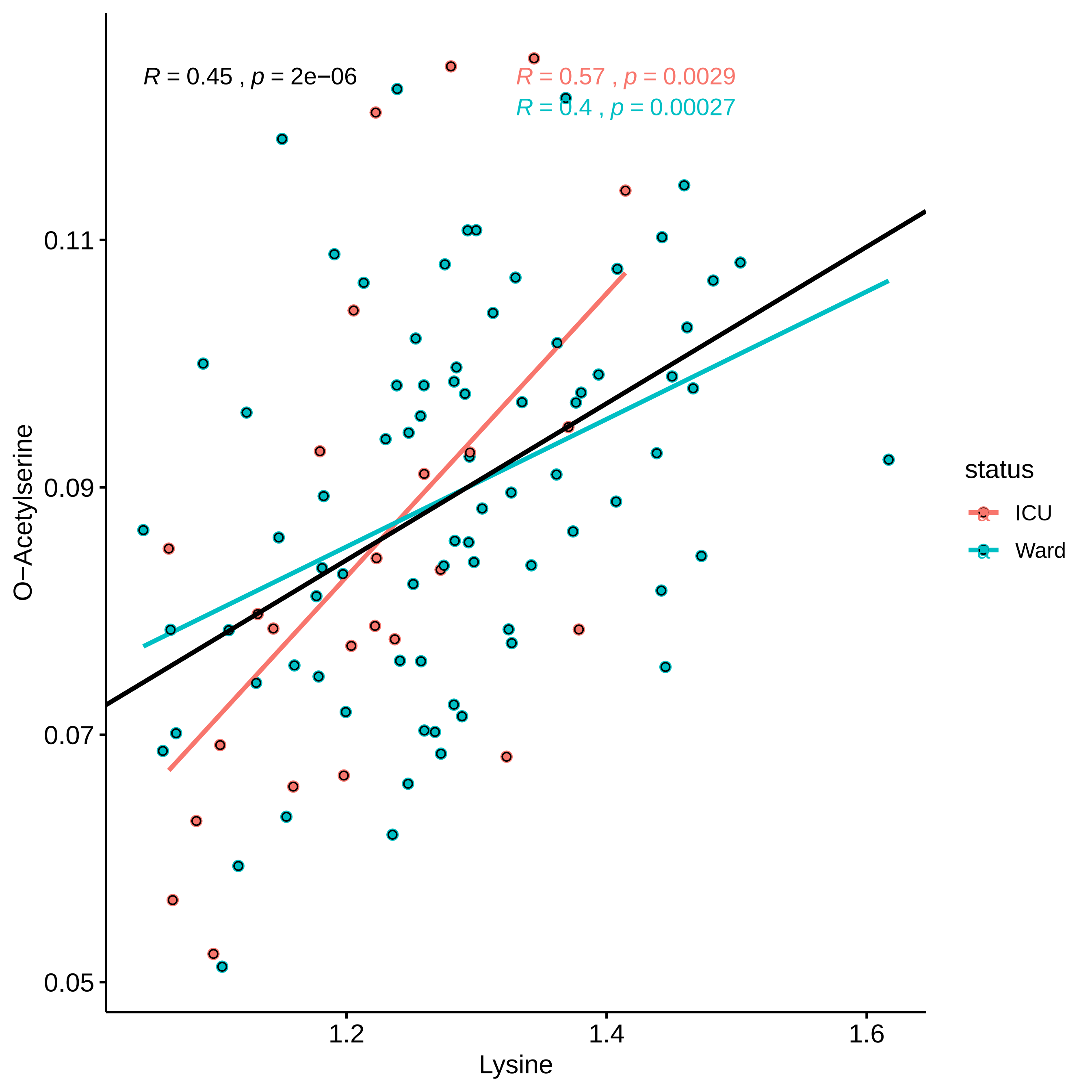

R = 0.45 , p = 2e−06
R = 0.57 , p = 0.0029
R = 0.4 , p = 0.00027
0.11
0.09
0.07
0.05
O−Acetylserine
1.2
1.4
1.6
Lysine
status
ICU
Ward

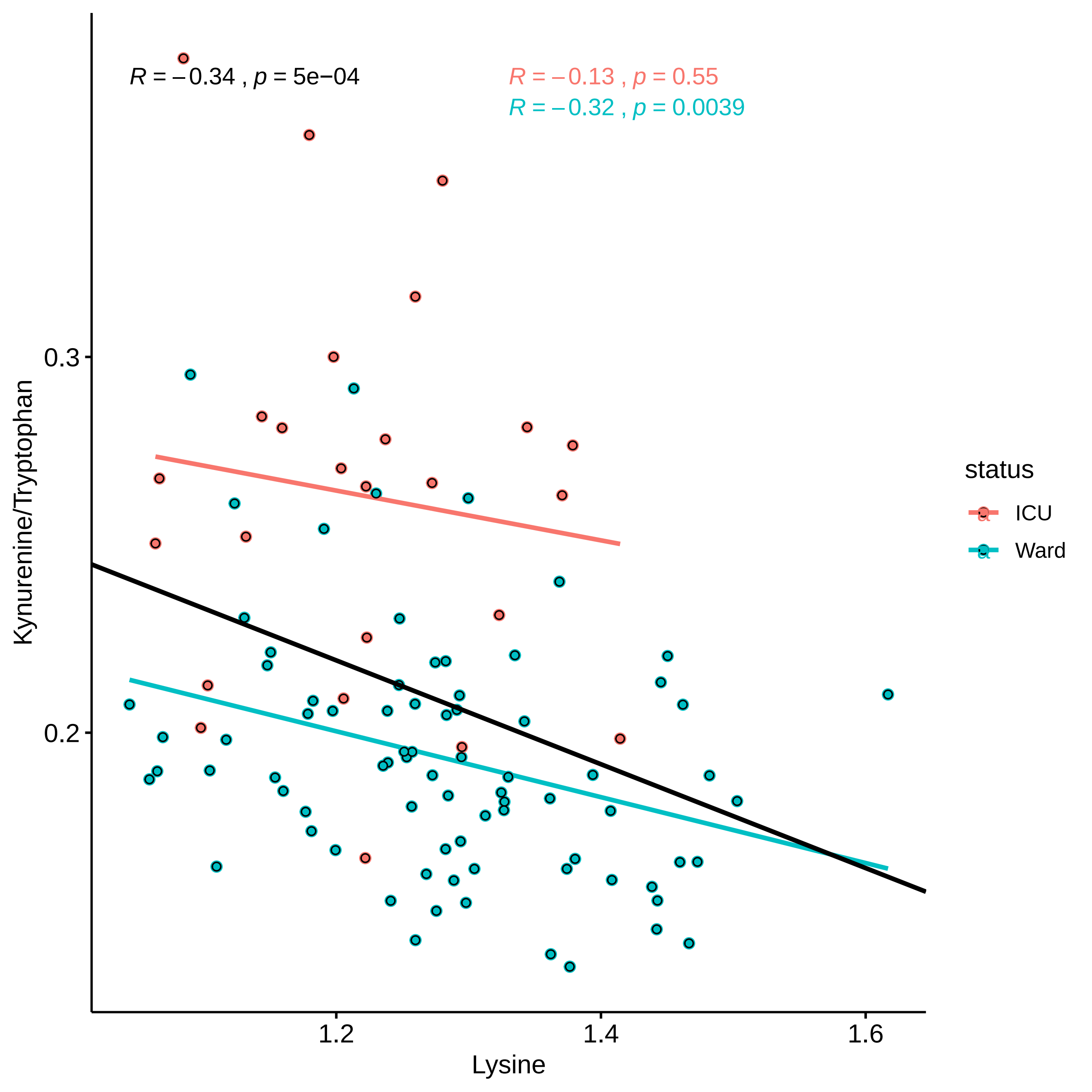

R = −0.34 , p = 5e−04
R = −0.13 , p = 0.55
R = −0.32 , p = 0.0039
0.3
0.2
Kynurenine/Tryptophan
1.2
1.4
1.6
Lysine
status
ICU
Ward

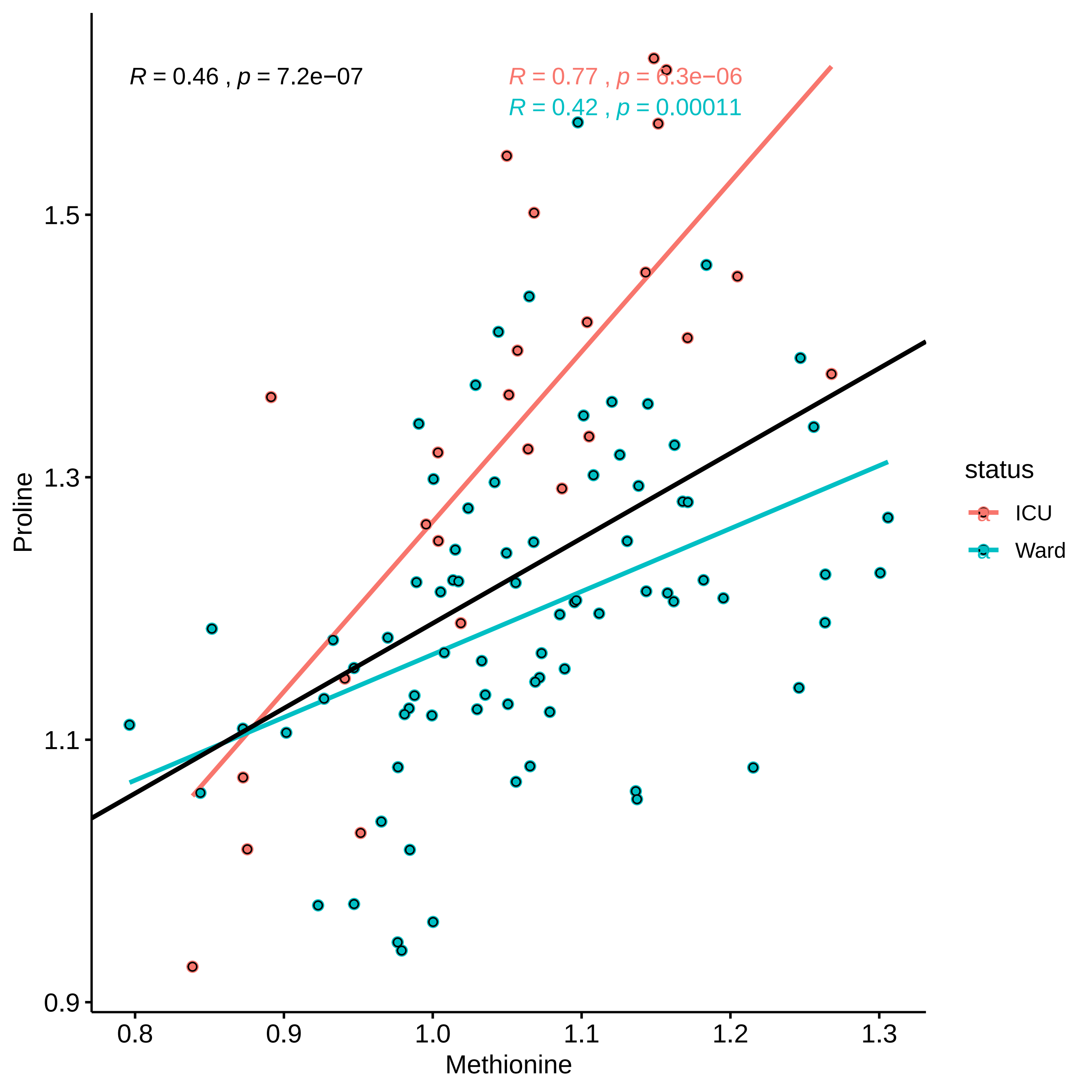

R = 0.46 , p = 7.2e−07
R = 0.77 , p = 6.3e−06
R = 0.42 , p = 0.00011
Proline
Methionine
1.5
1.3
1.1
0.9
0.8
0.9
1.0
1.1
1.2
1.3
status
ICU
Ward

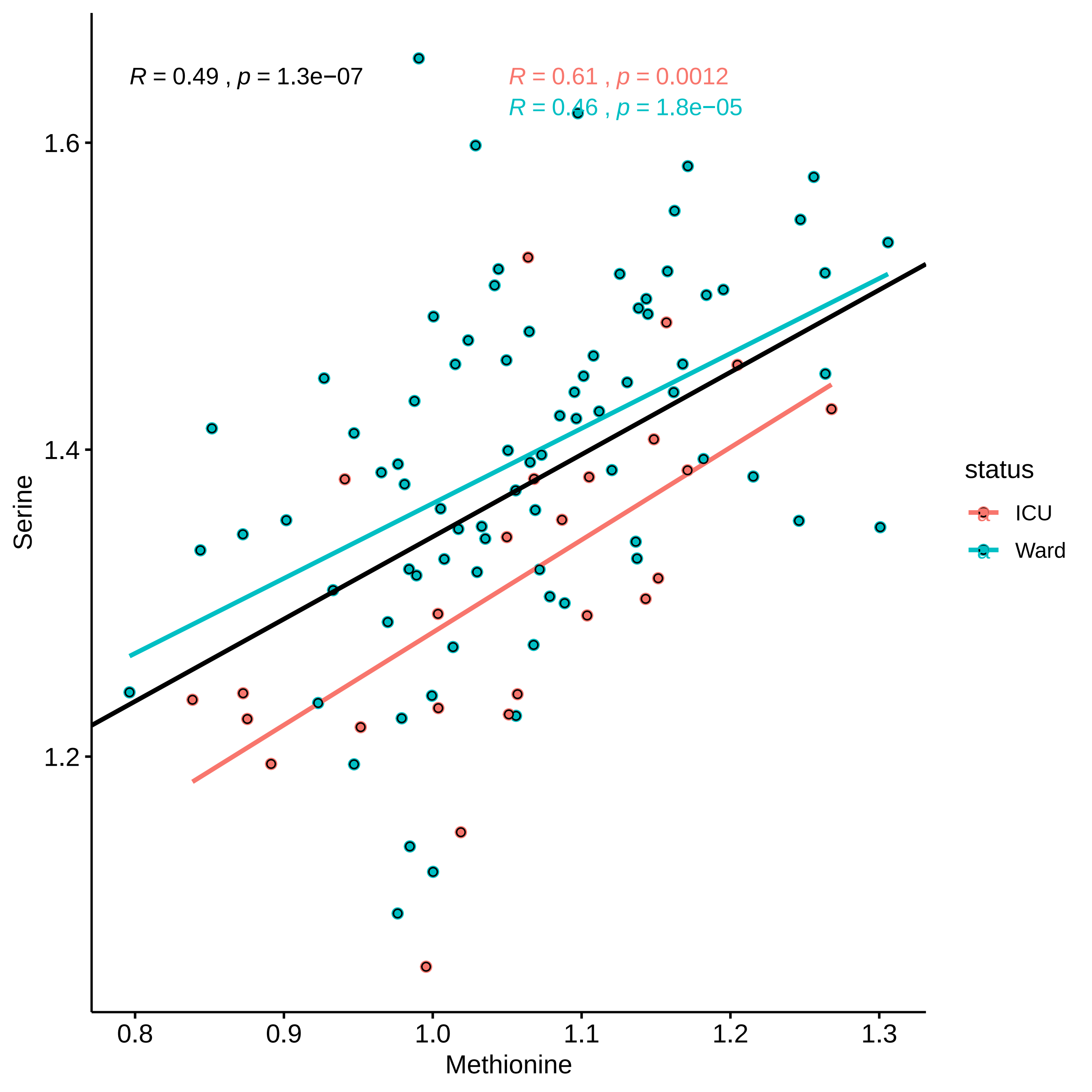

R = 0.49 , p = 1.3e−07
R = 0.61 , p = 0.0012
R = 0.46 , p = 1.8e−05
1.6
1.4
1.2
Serine
0.8
0.9
1.0
1.1
1.2
1.3
Methionine
status
ICU
Ward

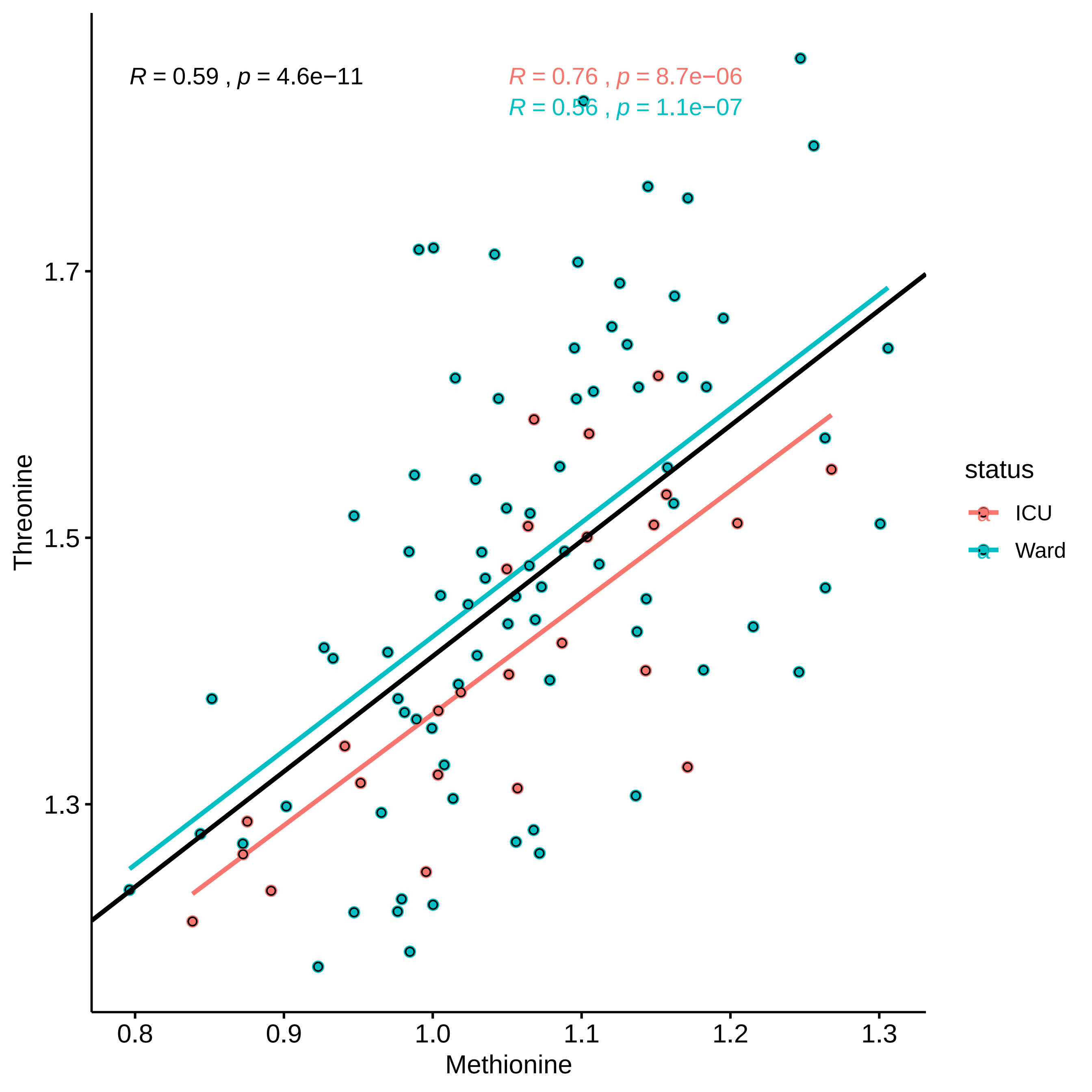

R = 0.59 , p = 4.6e−11
R = 0.76 , p = 8.7e−06
R = 0.56 , p = 1.1e−07
1.7
1.5
1.3
Threonine
0.8
0.9
1.0
1.1
1.2
1.3
Methionine
status
ICU
Ward

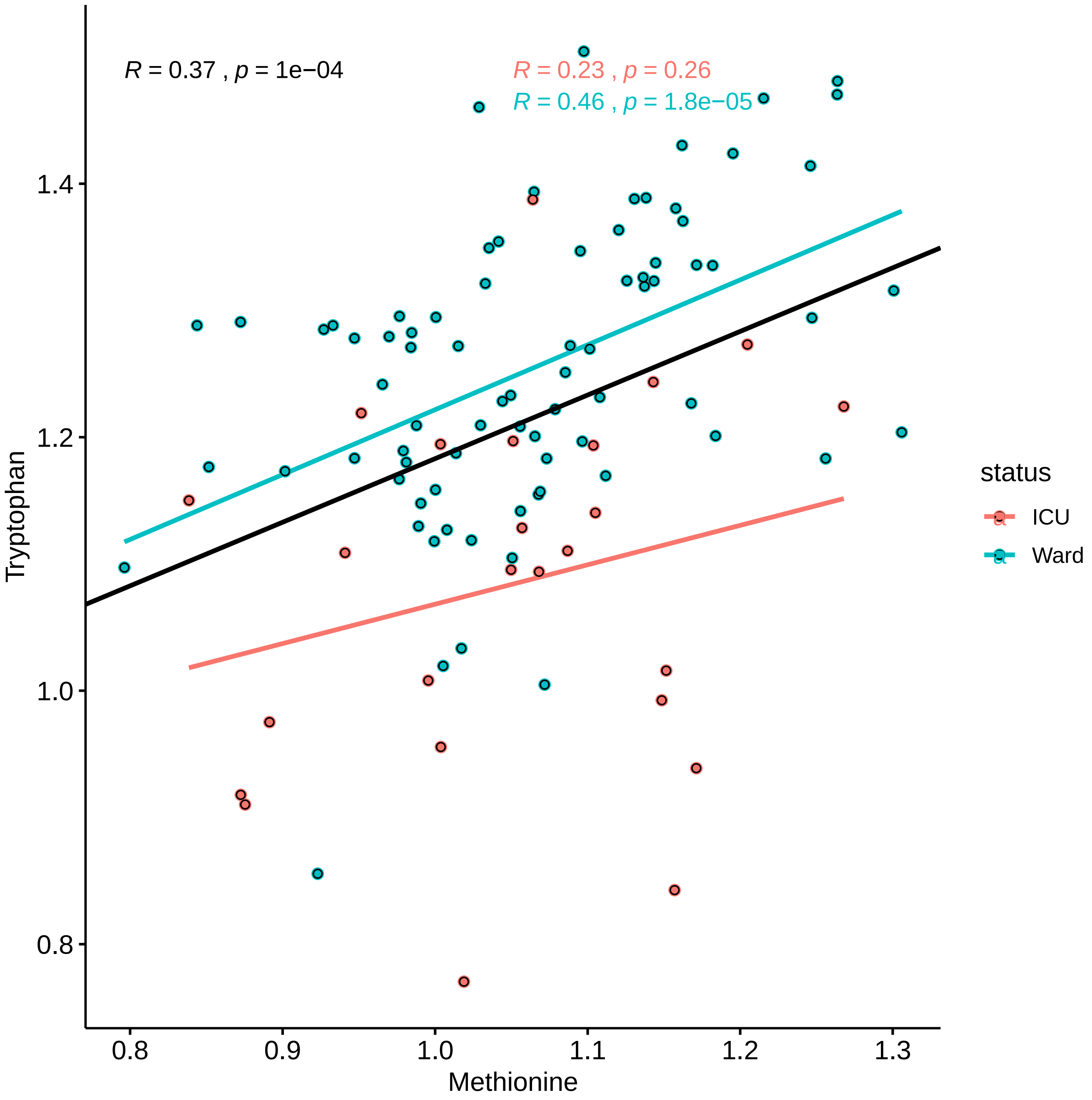

R = 0.37 , p = 1e−04
R = 0.23 , p = 0.26
R = 0.46 , p = 1.8e−05
Tryptophan
1.4
1.2
1.0
0.8
0.8
0.9
1.0
1.1
1.2
1.3
Methionine
status
ICU
Ward

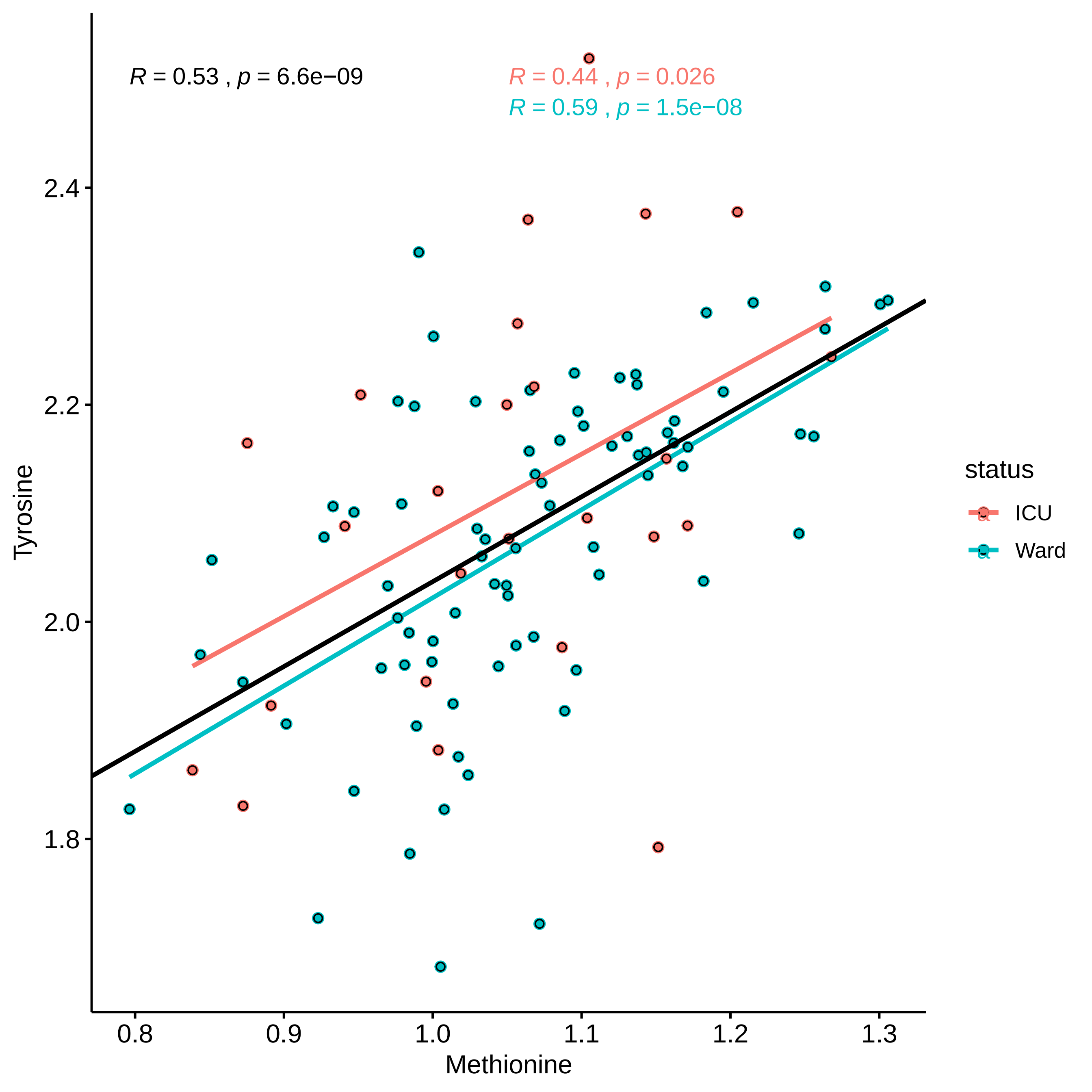

R = 0.53 , p = 6.6e−09
R = 0.44 , p = 0.026
R = 0.59 , p = 1.5e−08
Tyrosine
2.4
2.2
2.0
1.8
0.8
0.9
1.0
1.1
1.2
1.3
Methionine
status
ICU
Ward

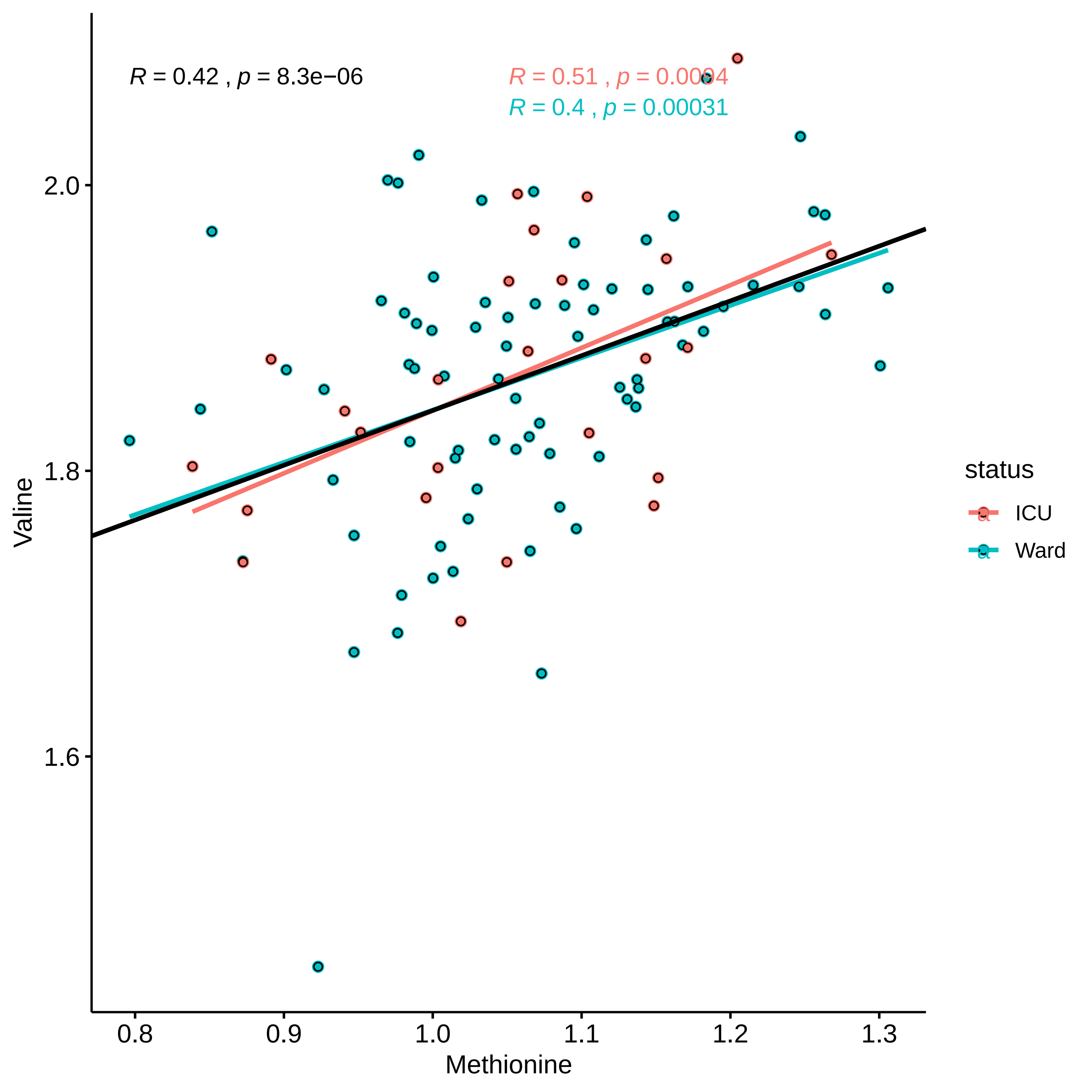

R = 0.42 , p = 8.3e−06
R = 0.51 , p = 0.0004
R = 0.4 , p = 0.00031
2.0
1.8
1.6
0.8
0.9
1.0
1.1
1.2
1.3
Valine
Methionine
status
ICU
Ward

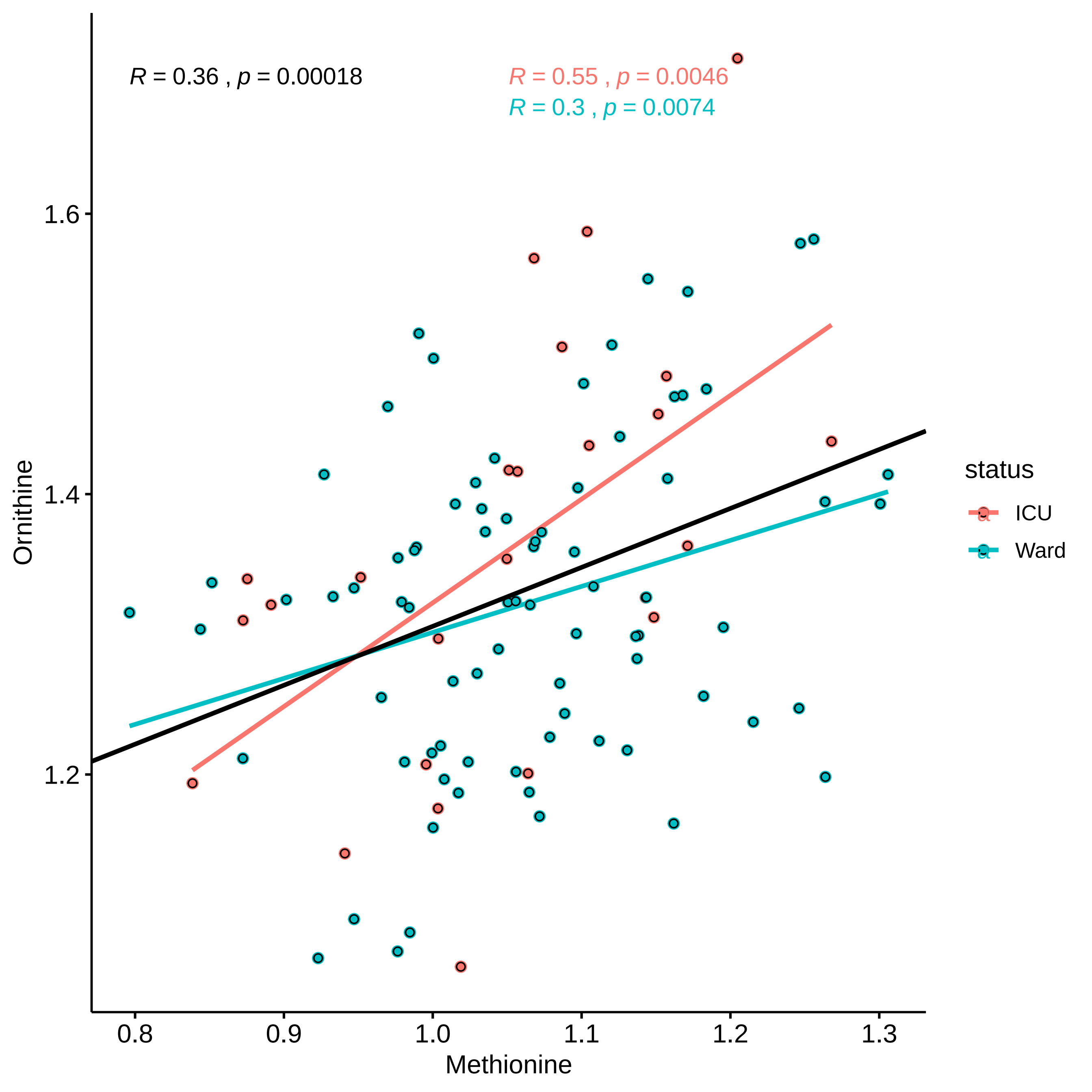

R = 0.36 , p = 0.00018
R = 0.55 , p = 0.0046
R = 0.3 , p = 0.0074
1.6
1.4
1.2
Ornithine
0.8
0.9
1.0
1.1
1.2
1.3
Methionine
status
ICU
Ward

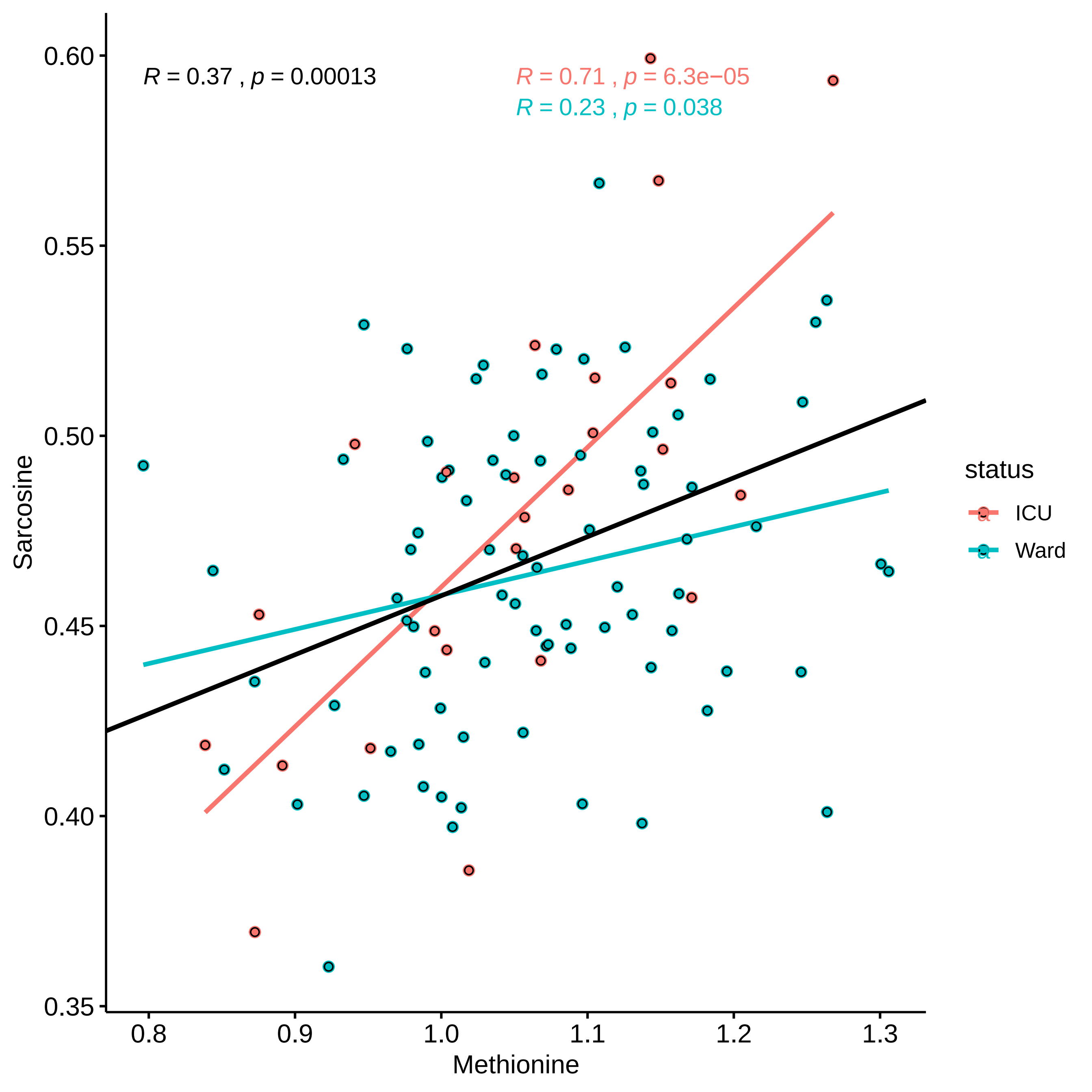

R = 0.37 , p = 0.00013
R = 0.71 , p = 6.3e−05
R = 0.23 , p = 0.038
Sarcosine
0.60
0.55
0.50
0.45
0.40
0.35
0.8
0.9
1.0
1.1
1.2
1.3
Methionine
status
ICU
Ward

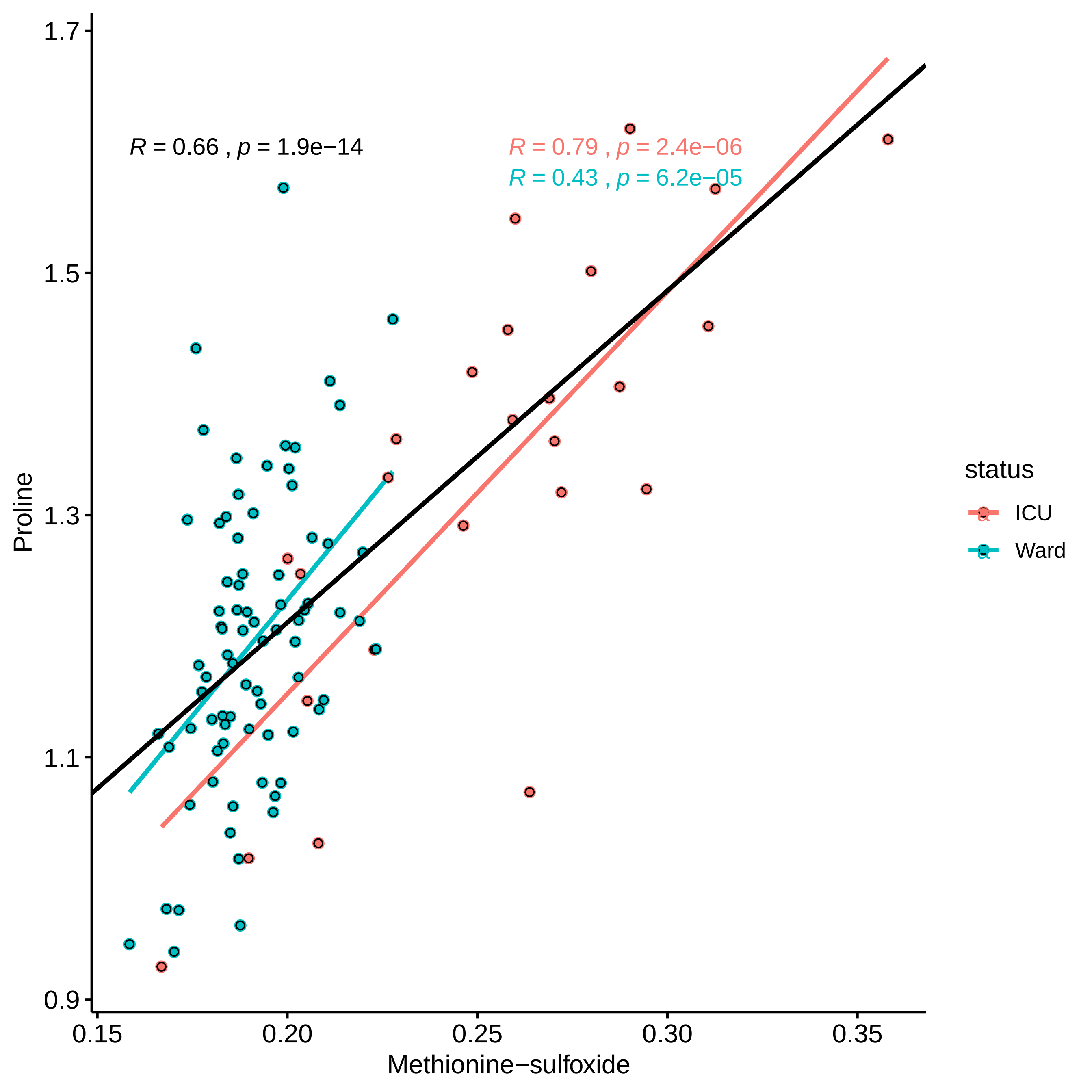

R = 0.66 , p = 1.9e−14
R = 0.79 , p = 2.4e−06
R = 0.43 , p = 6.2e−05
Proline
Methionine−sulfoxide
1.7
1.5
1.3
1.1
0.9
0.15
0.20
0.25
0.30
0.35
status
ICU
Ward

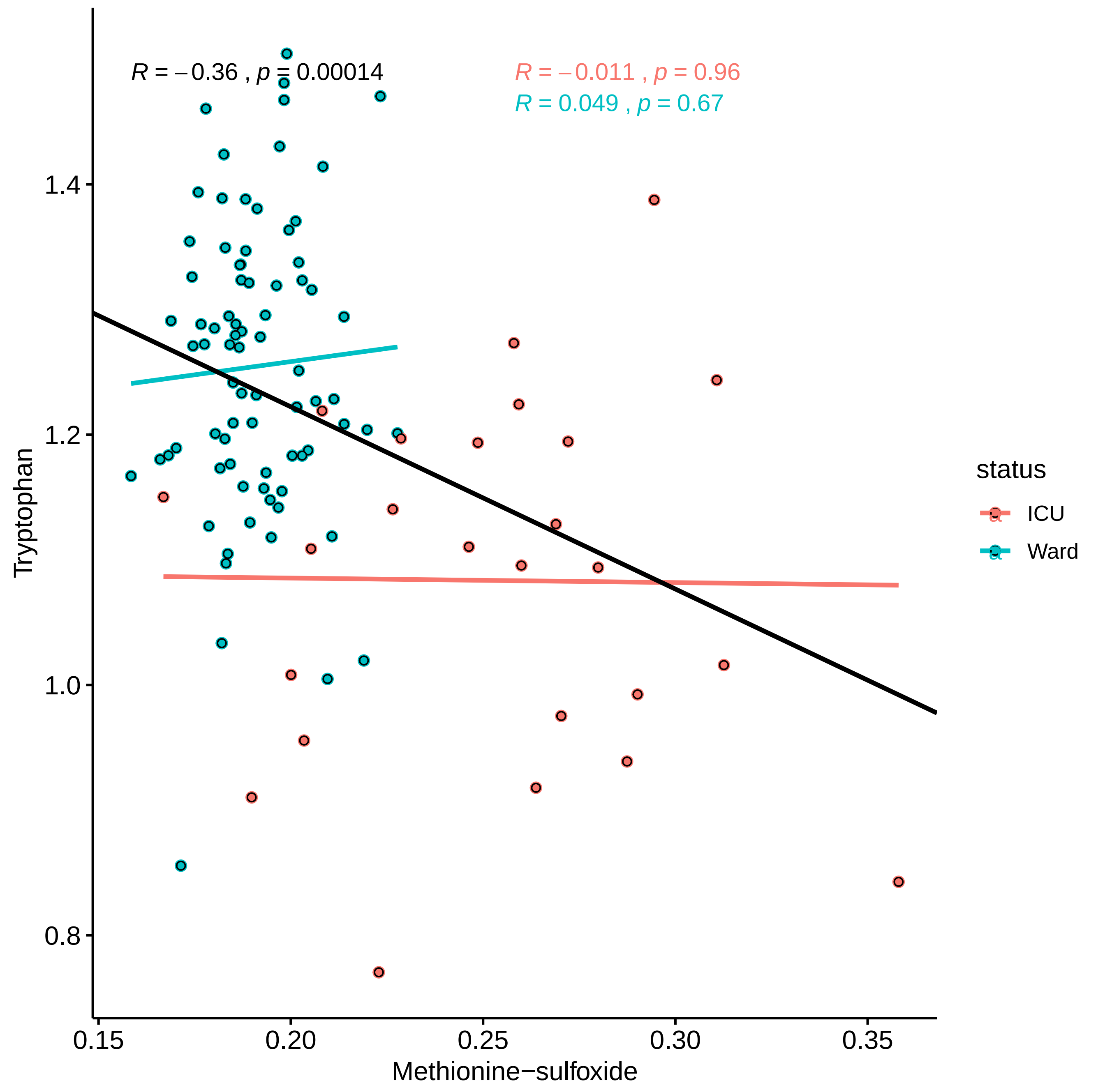

R = −0.36 , p = 0.00014
R = −0.011 , p = 0.96
R = 0.049 , p = 0.67
1.4
1.2
1.0
0.8
Tryptophan
0.15
0.20
0.25
0.30
0.35
Methionine−sulfoxide
status
ICU
Ward

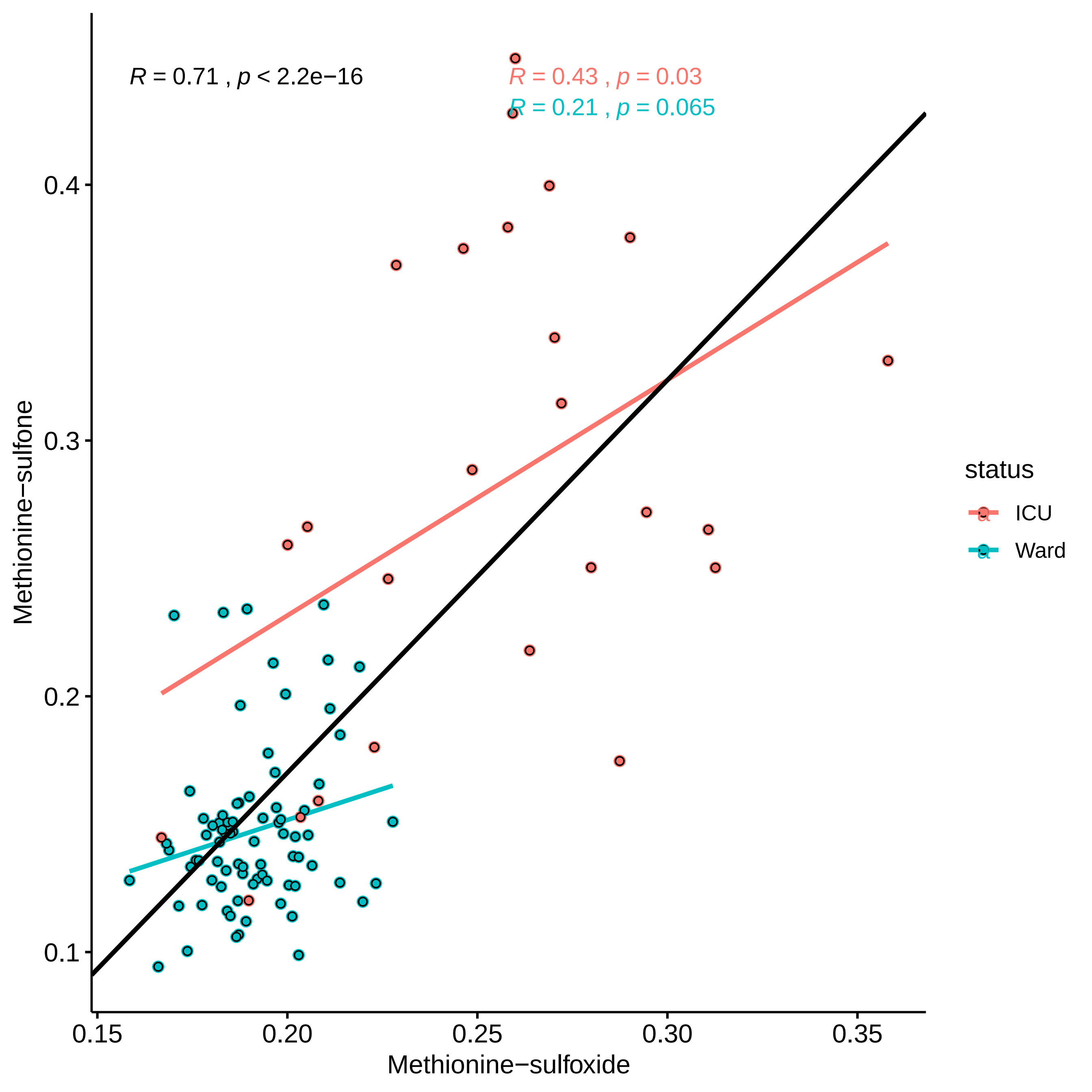

R = 0.71 , p < 2.2e−16
R = 0.43 , p = 0.03
R = 0.21 , p = 0.065
0.4
0.3
0.2
0.1
Methionine−sulfone
0.15
0.20
0.25
0.30
0.35
Methionine−sulfoxide
status
ICU
Ward

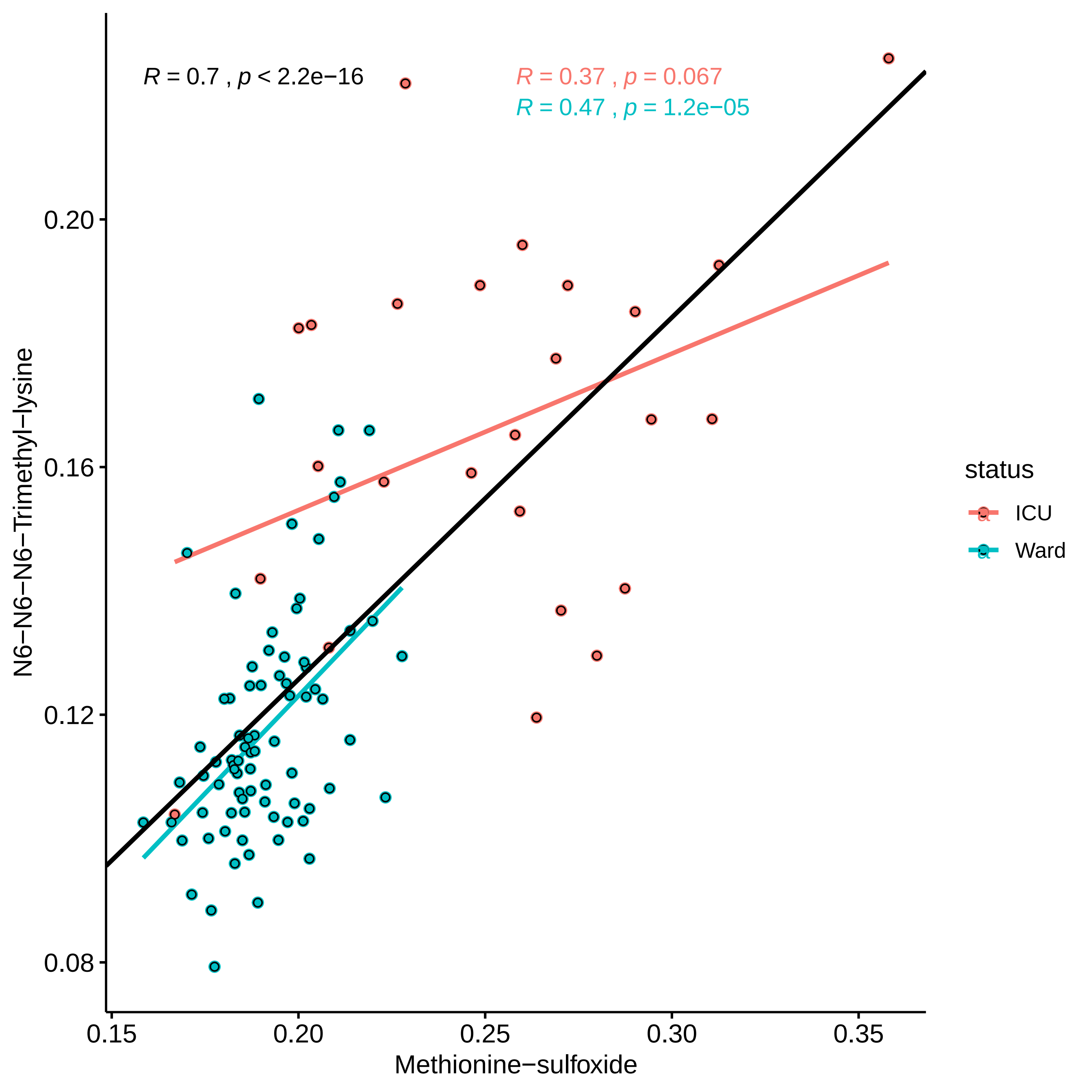

R = 0.7 , p < 2.2e−16
R = 0.37 , p = 0.067
R = 0.47 , p = 1.2e−05
N6−N6−N6−Trimethyl−lysine
0.20
0.16
0.12
0.08
0.15
0.20
0.25
0.30
0.35
Methionine−sulfoxide
status
ICU
Ward

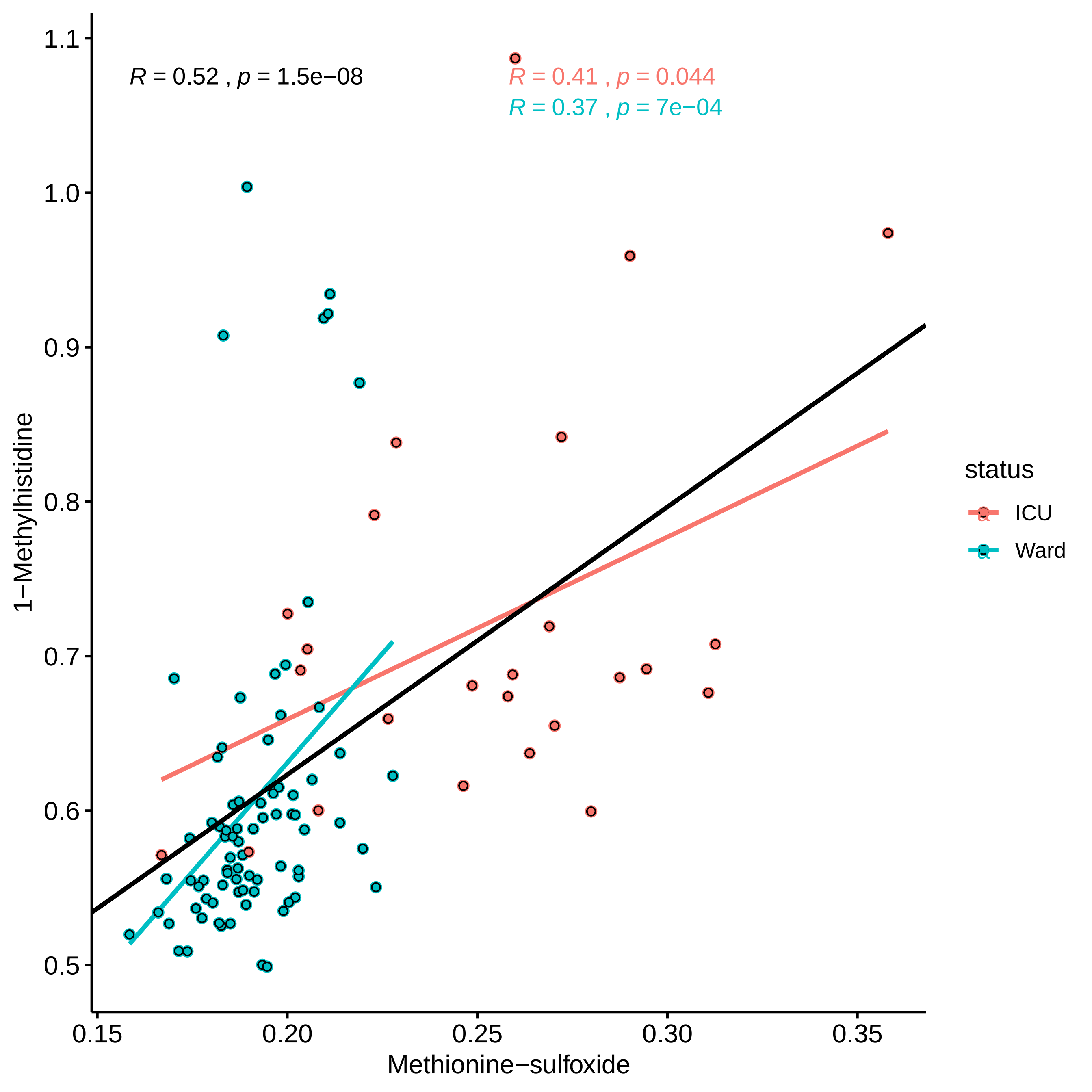

R = 0.52 , p = 1.5e−08
R = 0.41 , p = 0.044
R = 0.37 , p = 7e−04
1.1
1.0
0.9
0.8
0.7
0.6
0.5
1−Methylhistidine
0.15
0.20
0.25
0.30
0.35
Methionine−sulfoxide
status
ICU
Ward

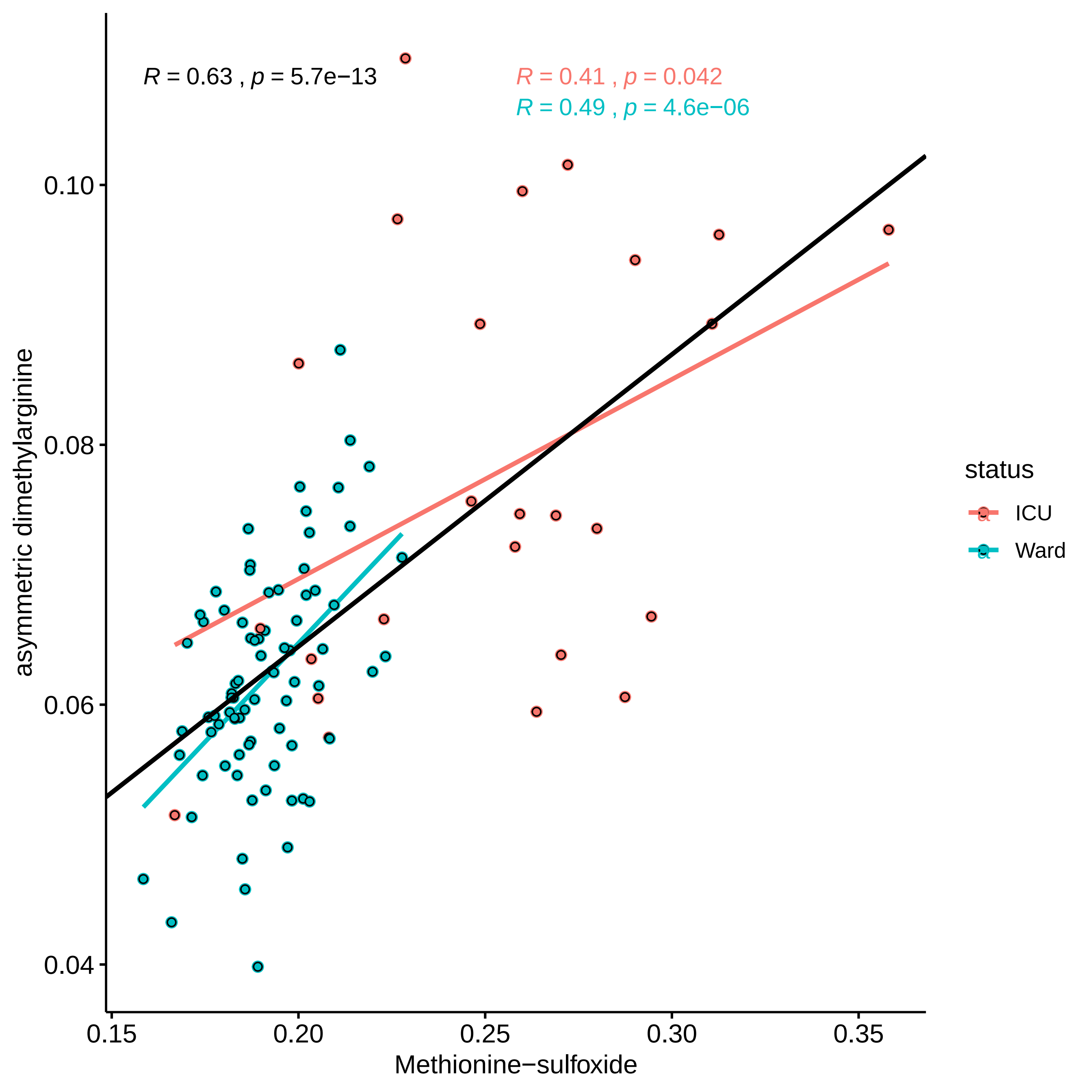
R = 0.63 , p = 5.7e−13
R = 0.41 , p = 0.042
R = 0.49 , p = 4.6e−06
asymmetric dimethylarginine
0.10
0.08
0.06
0.04
0.15
0.20
0.25
0.30
0.35
Methionine−sulfoxide
status
ICU
Ward

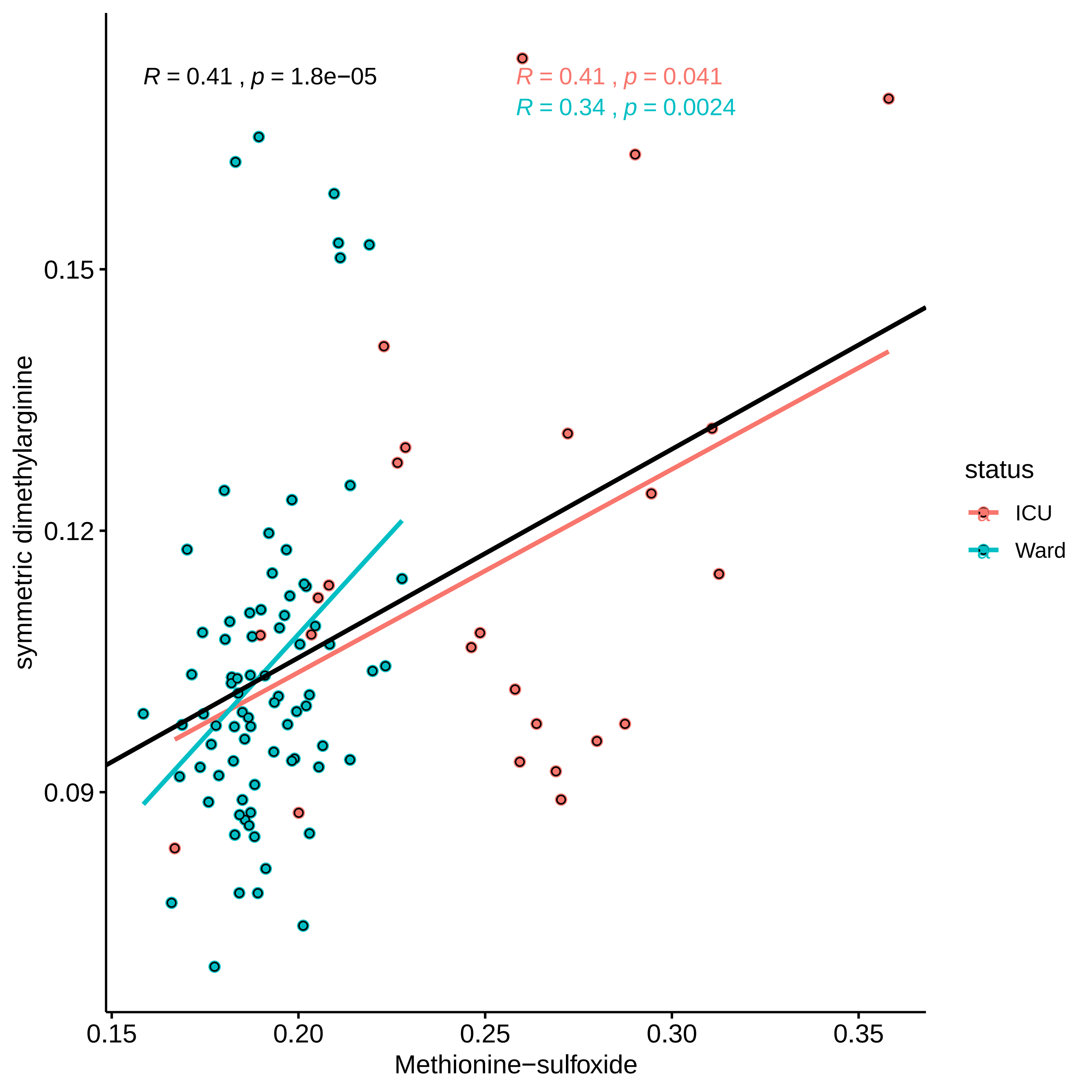

R = 0.41 , p = 1.8e−05
R = 0.41 , p = 0.041
R = 0.34 , p = 0.0024
0.15
0.12
0.09
symmetric dimethylarginine
0.15
0.20
0.25
0.30
0.35
Methionine−sulfoxide
status
ICU
Ward

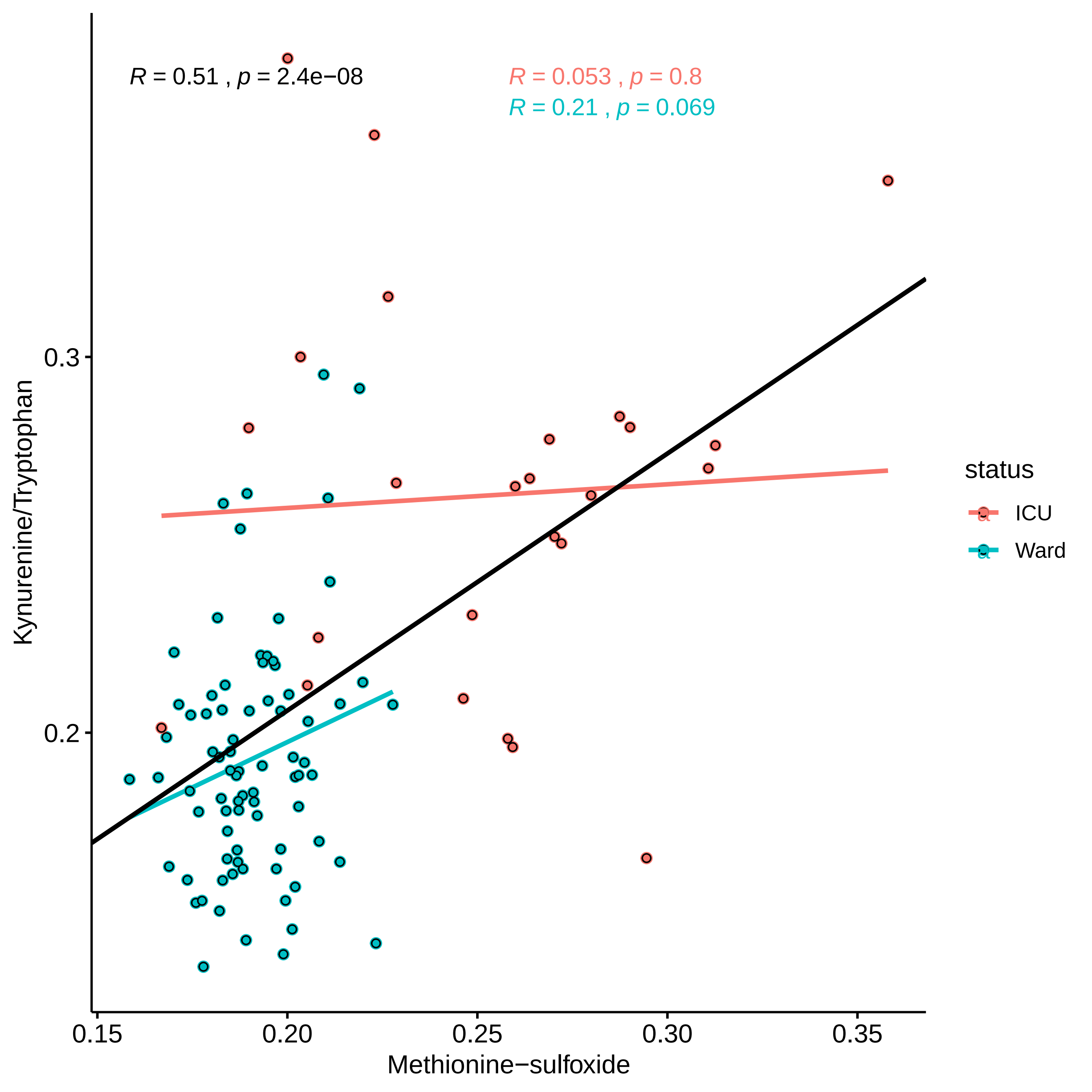

R = 0.51 , p = 2.4e−08
R = 0.053 , p = 0.8
R = 0.21 , p = 0.069
Kynurenine/Tryptophan
0.3
0.2
0.15
0.20
0.25
0.30
0.35
Methionine−sulfoxide
status
ICU
Ward

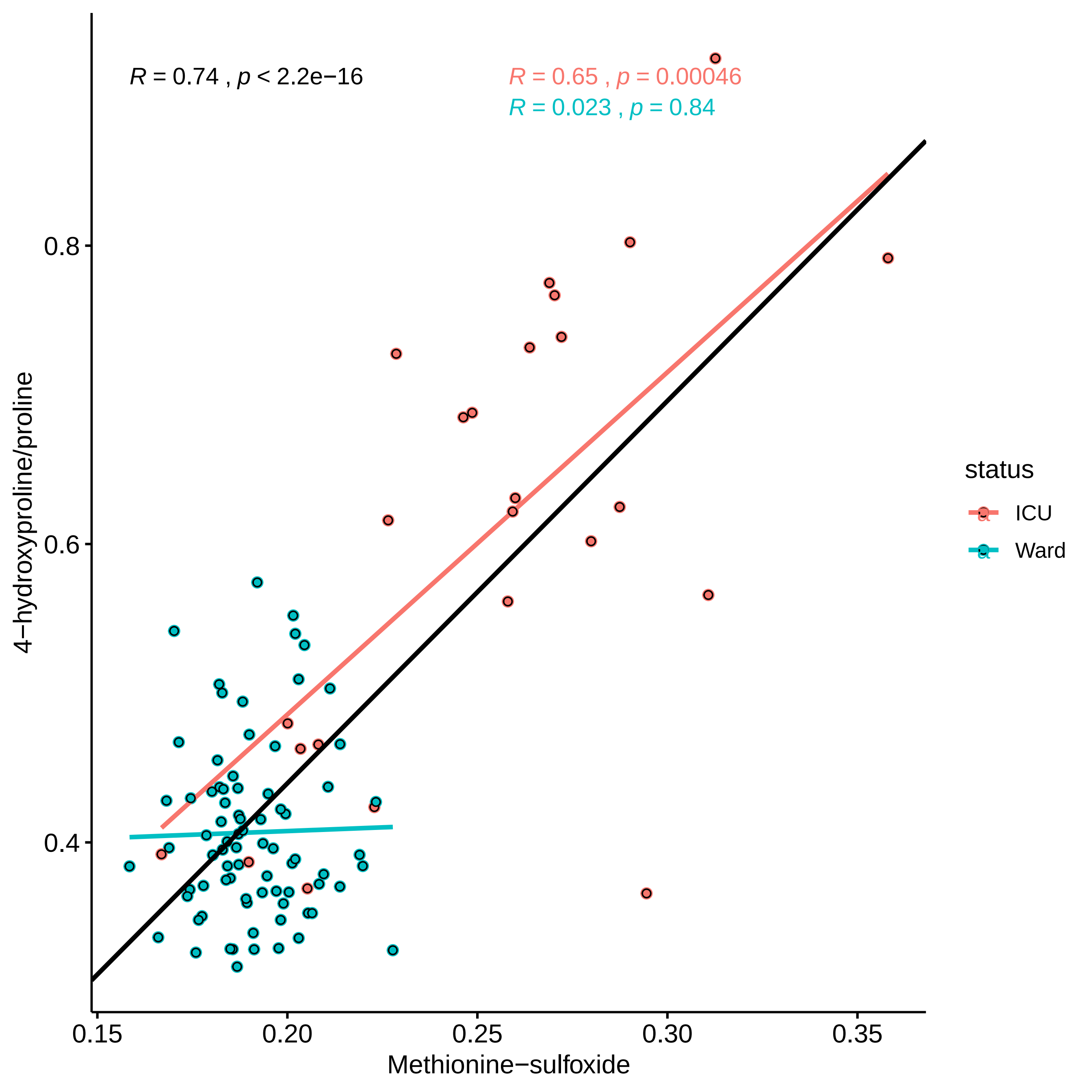

R = 0.74 , p < 2.2e−16
R = 0.65 , p = 0.00046
R = 0.023 , p = 0.84
4−hydroxyproline/proline
0.8
0.6
0.4
0.15
0.20
0.25
0.30
0.35
Methionine−sulfoxide
status
ICU
Ward

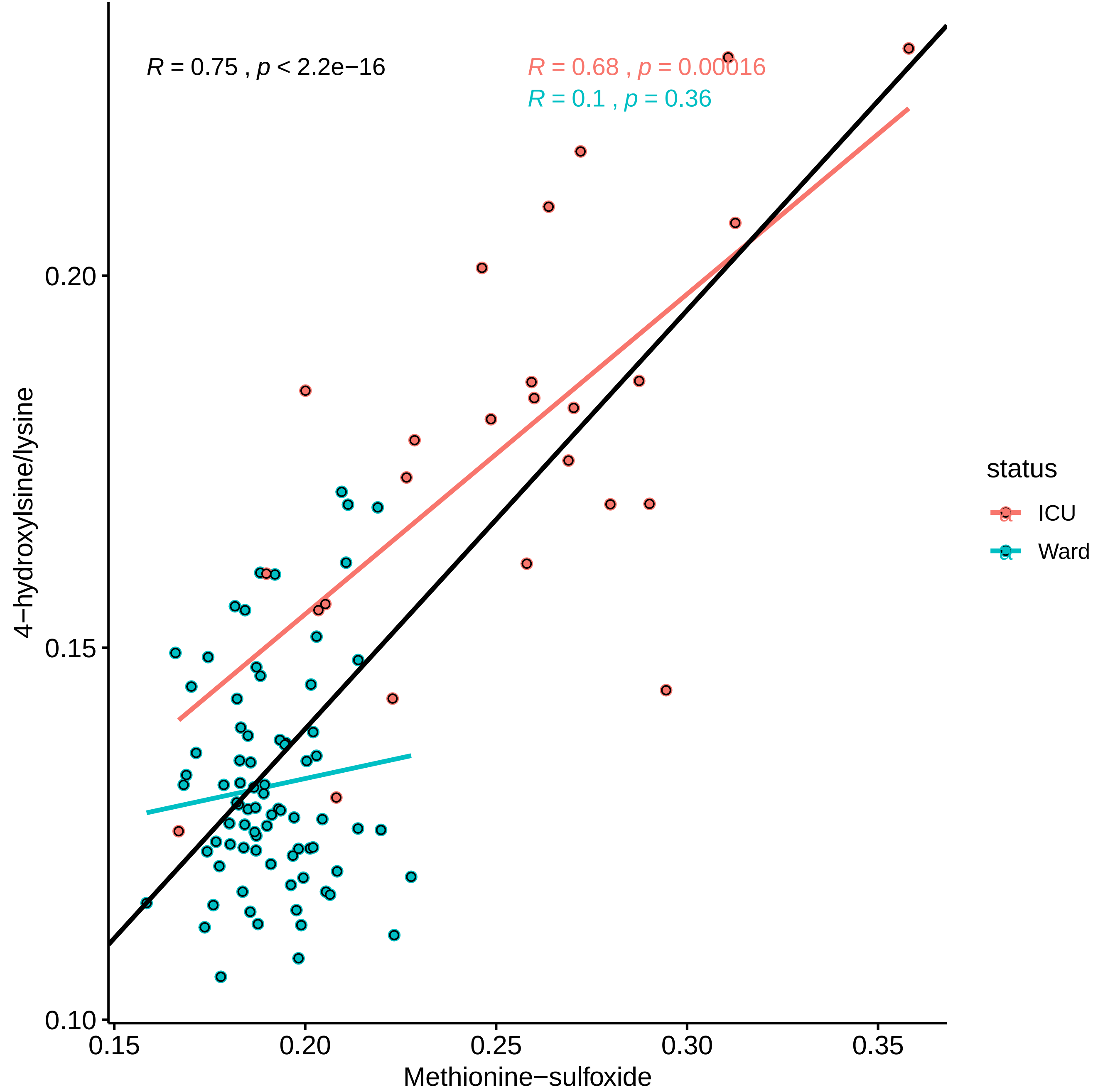

R = 0.75 , p < 2.2e−16
R = 0.68 , p = 0.00016
R = 0.1 , p = 0.36
4−hydroxylsine/lysine
0.20
0.15
0.10
0.15
0.20
0.25
0.30
0.35
Methionine−sulfoxide
status
ICU
Ward

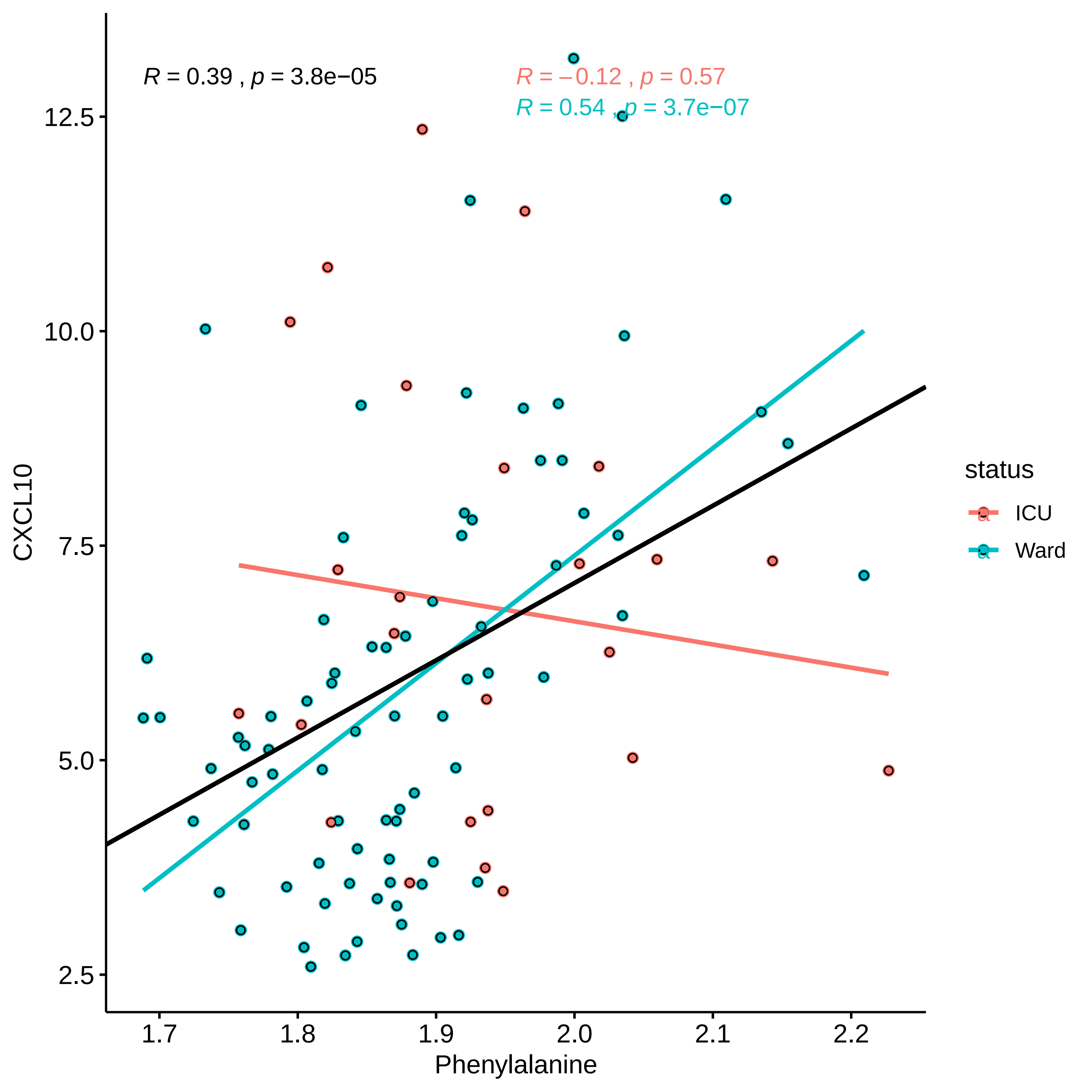
R = 0.39 , p = 3.8e−05
R = −0.12 , p = 0.57
R = 0.54 , p = 3.7e−07
CXCL10
Phenylalanine
status
ICU
Ward
12.5
10.0
7.5
5.0
2.5
1.7
1.8
1.9
2.0
2.1
2.2

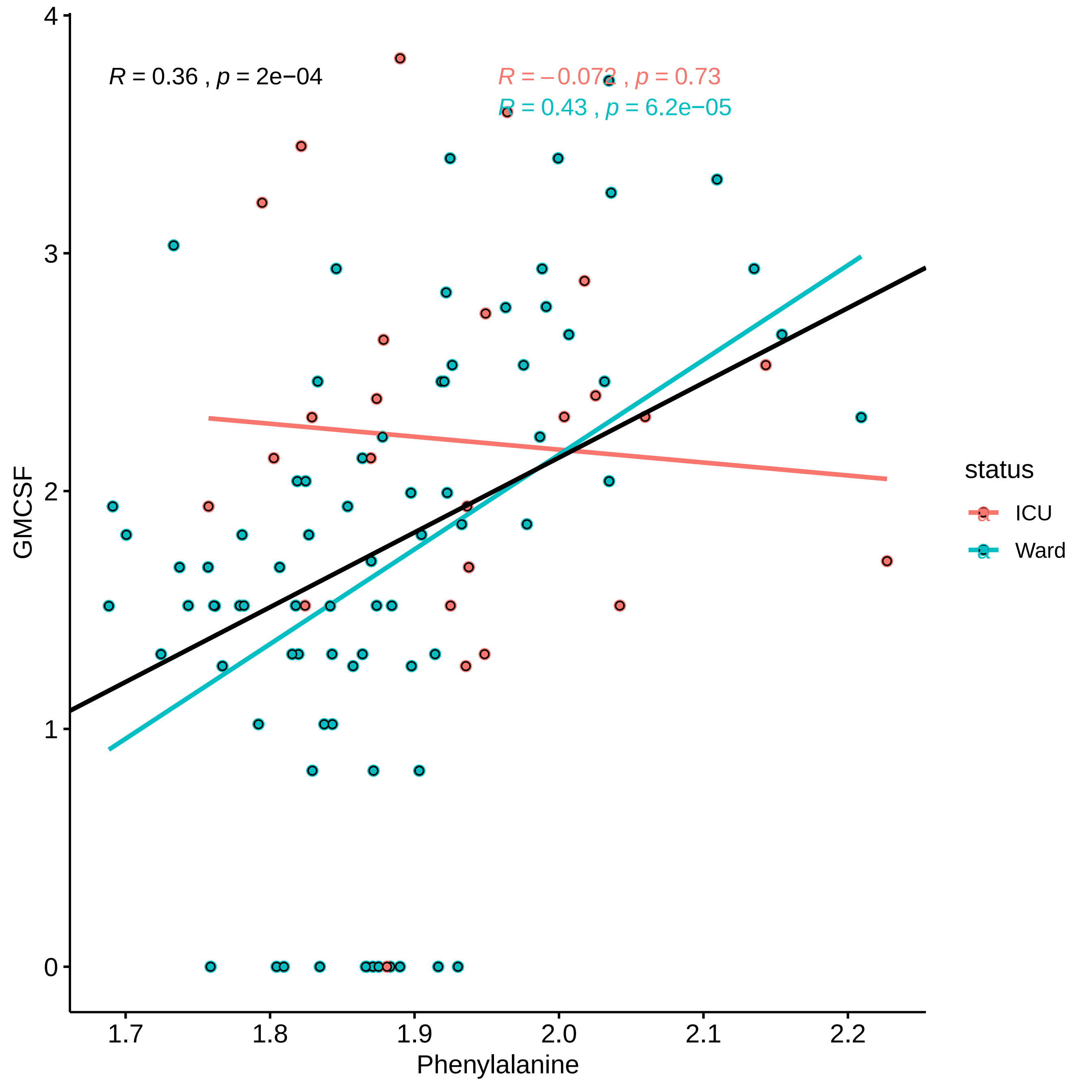

R = 0.36 , p = 2e−04
R = −0.072 , p = 0.73
R = 0.43 , p = 6.2e−05
GMCSF
Phenylalanine
status
ICU
Ward

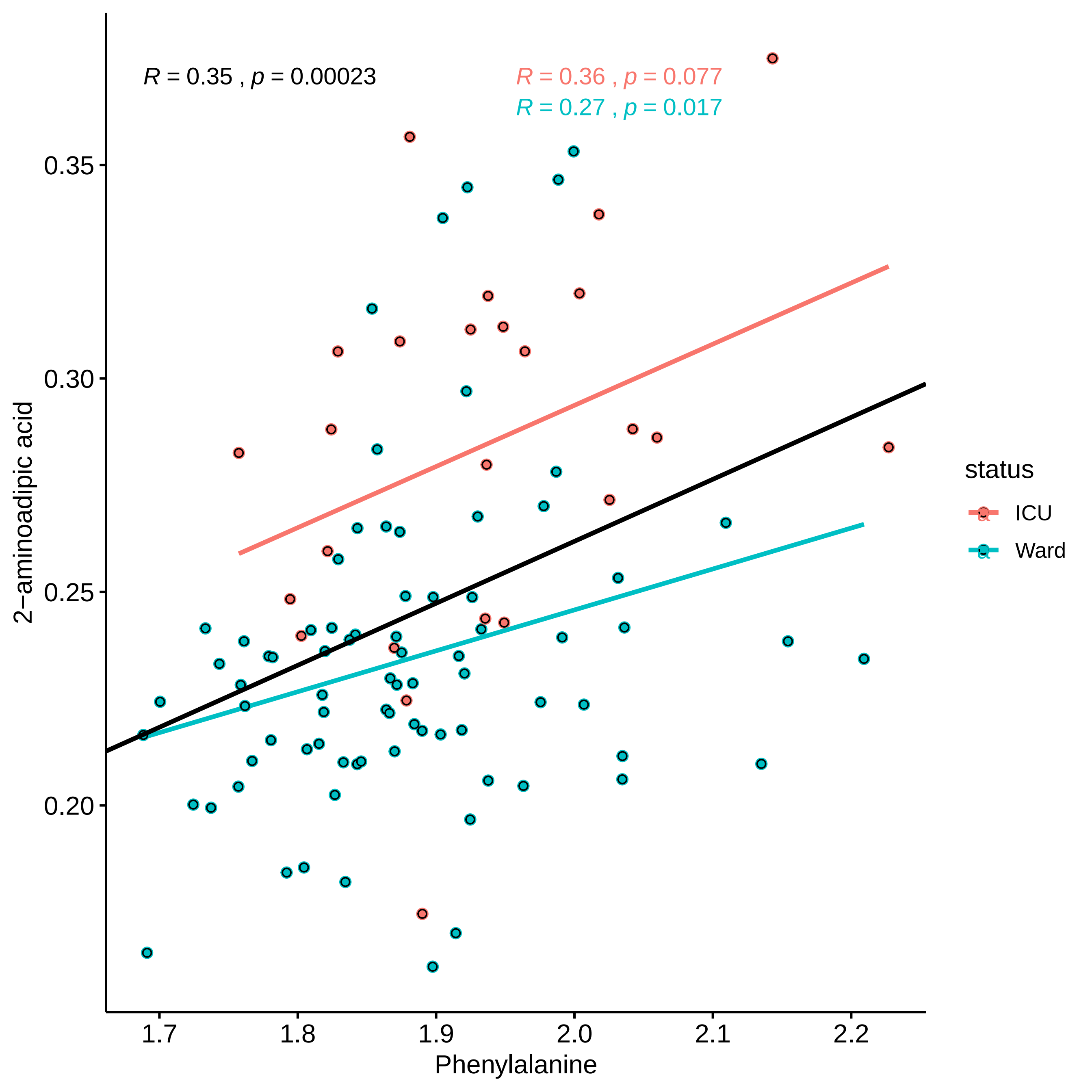

R = 0.35 , p = 0.00023
R = 0.36 , p = 0.077
R = 0.27 , p = 0.017
0.35
0.30
0.25
0.20
2−aminoadipic acid
1.7
1.8
1.9
2.0
2.1
2.2
Phenylalanine
status
ICU
Ward

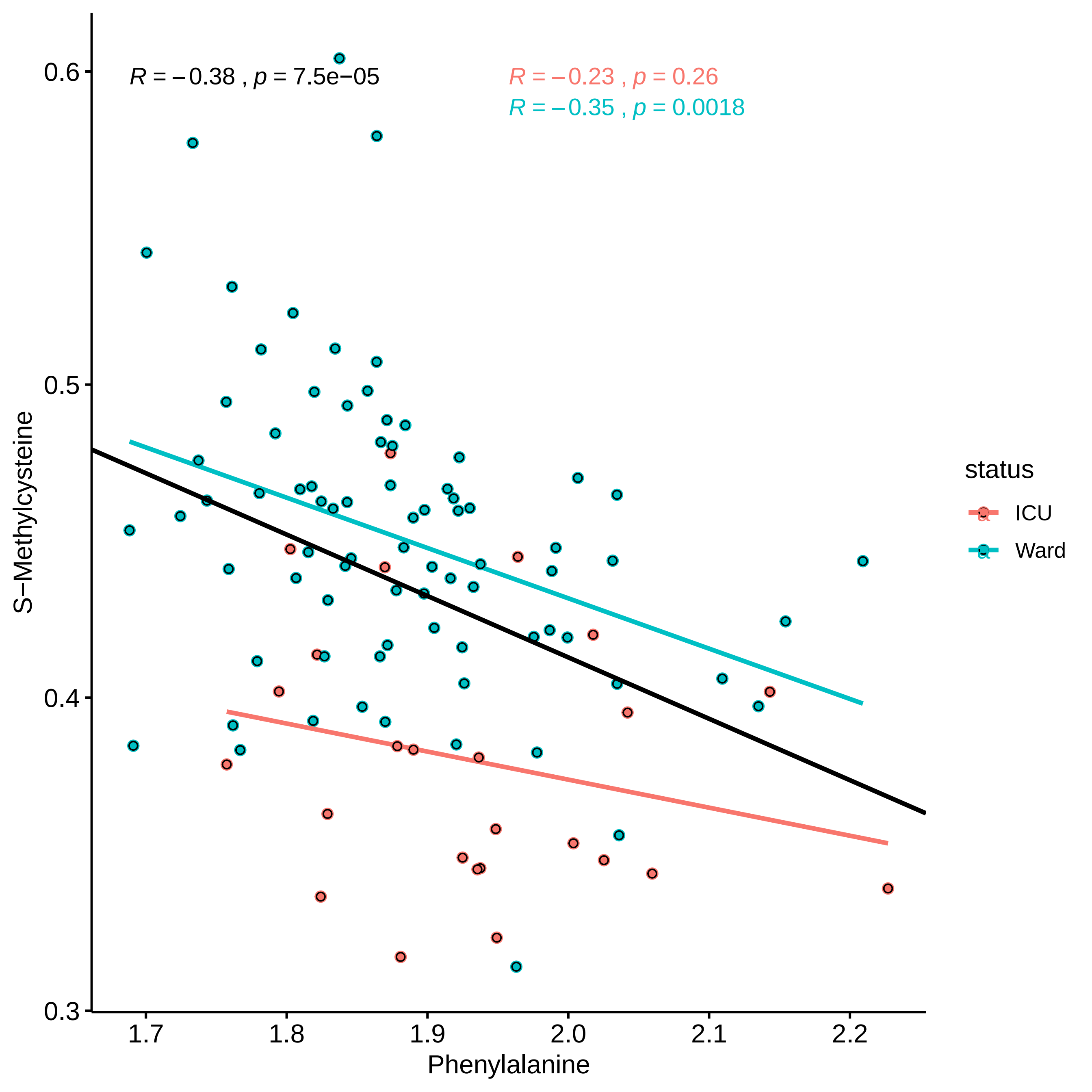

R = −0.38 , p = 7.5e−05
R = −0.23 , p = 0.26
R = −0.35 , p = 0.0018
0.6
0.5
0.4
0.3
S−Methylcysteine
1.7
1.8
1.9
2.0
2.1
2.2
Phenylalanine
status
ICU
Ward

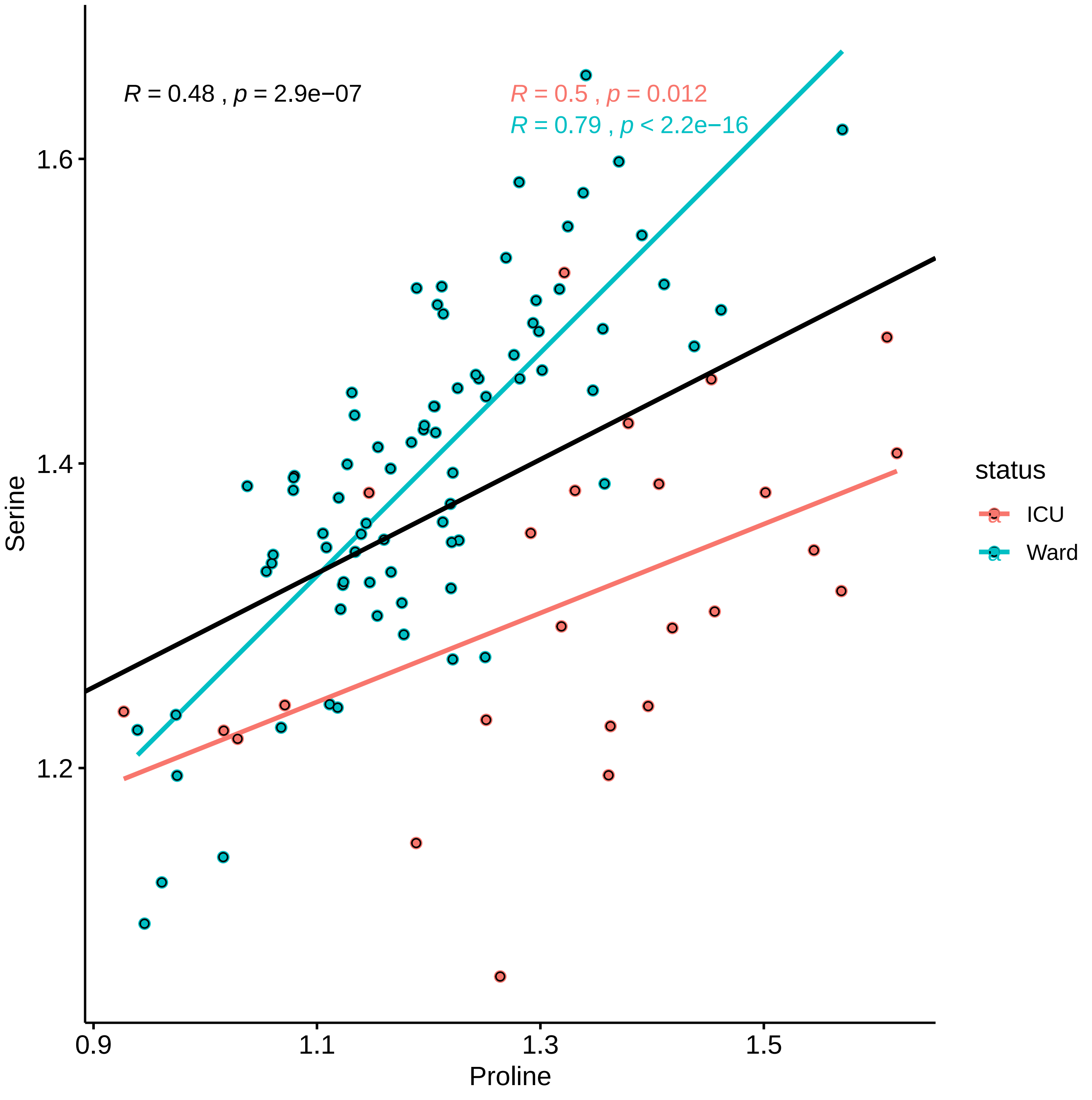

R = 0.48 , p = 2.9e−07
R = 0.5 , p = 0.012
R = 0.79 , p < 2.2e−16
1.6
1.4
1.2
Serine
0.9
1.1
1.3
1.5
Proline
status
ICU
Ward

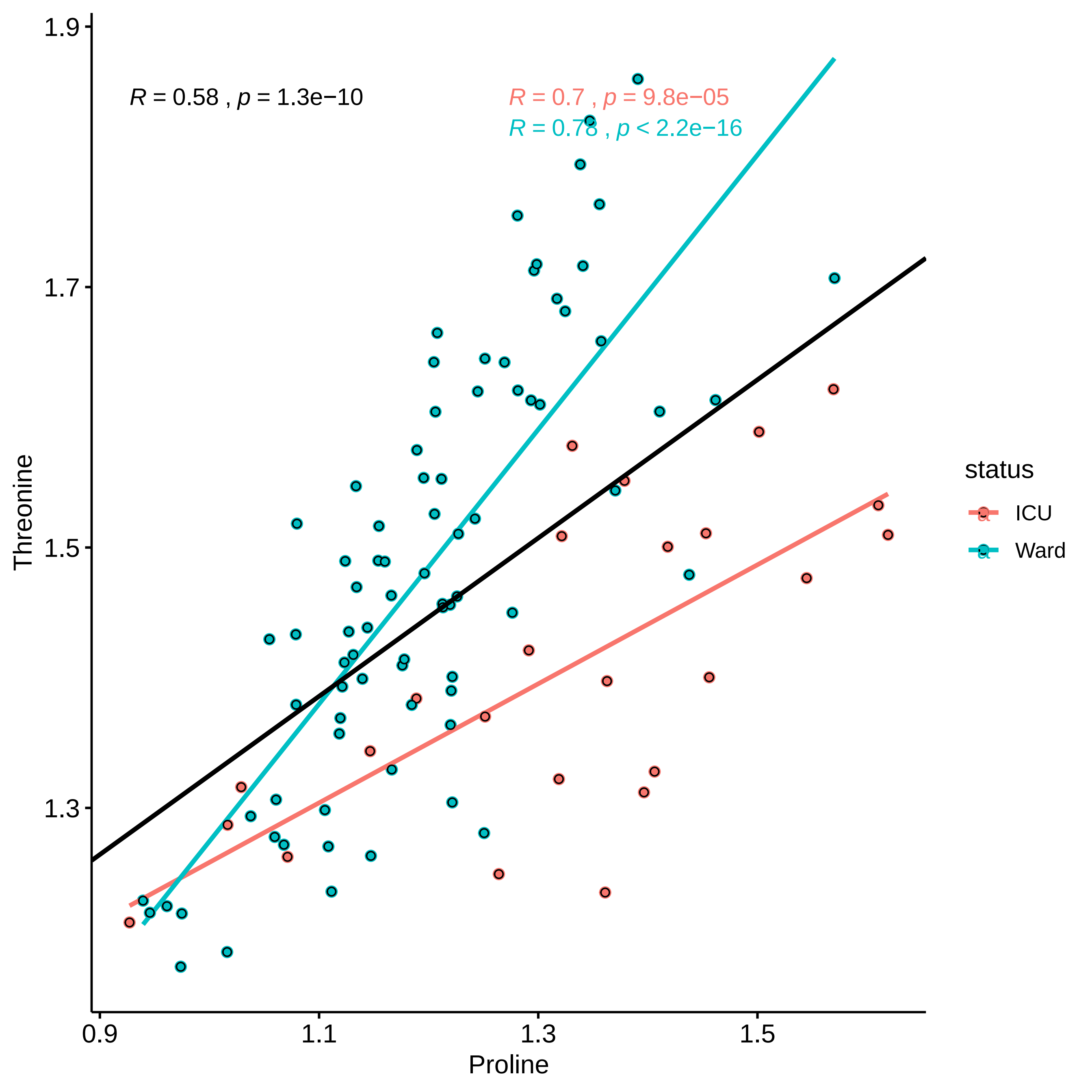

R = 0.58 , p = 1.3e−10
R = 0.7 , p = 9.8e−05
R = 0.78 , p < 2.2e−16
1.9
1.7
1.5
1.3
Threonine
0.9
1.1
1.3
1.5
Proline
status
ICU
Ward

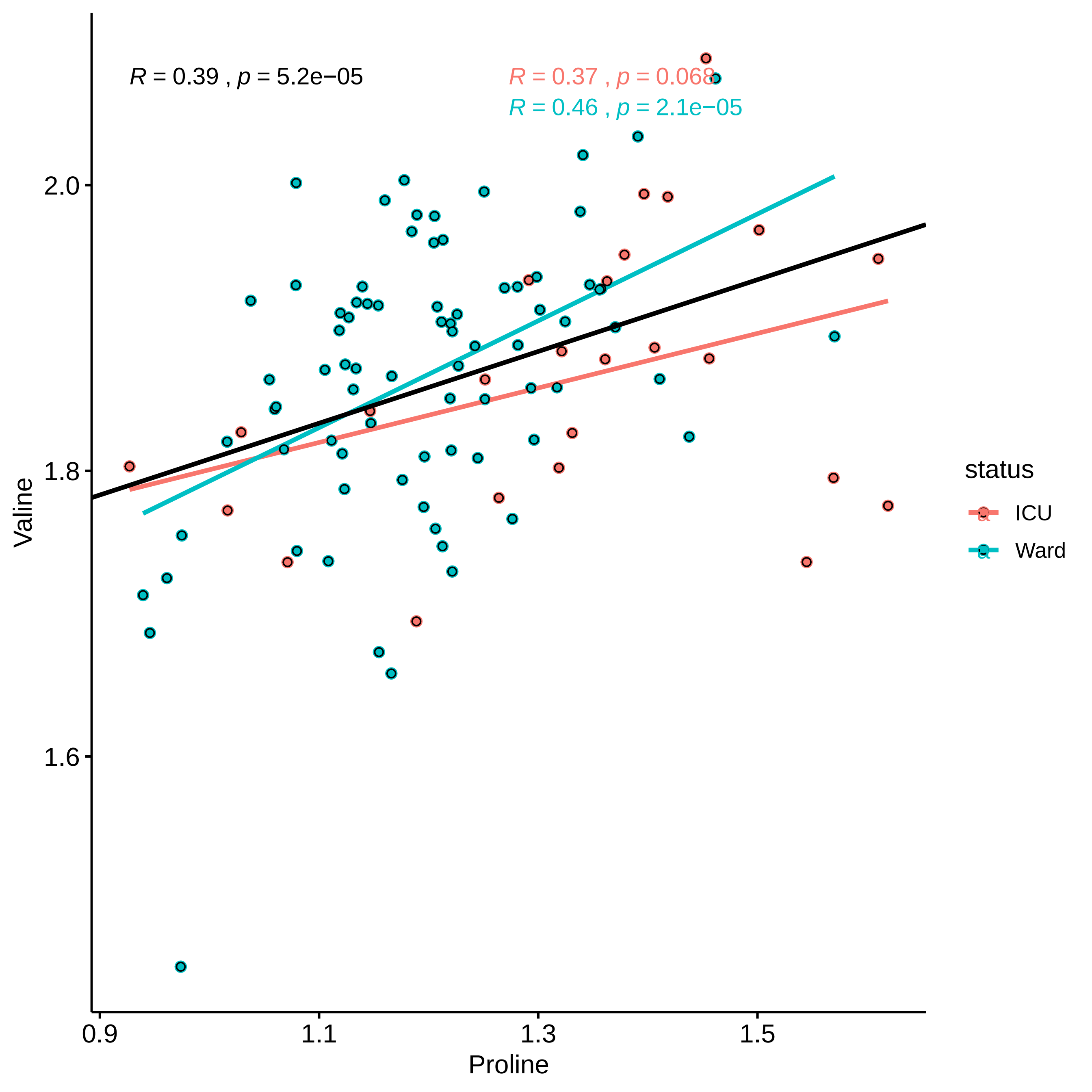
R = 0.39 , p = 5.2e−05
R = 0.37 , p = 0.068
R = 0.46 , p = 2.1e−05
2.0
1.8
1.6
Valine
0.9
1.1
1.3
1.5
Proline
status
ICU
Ward

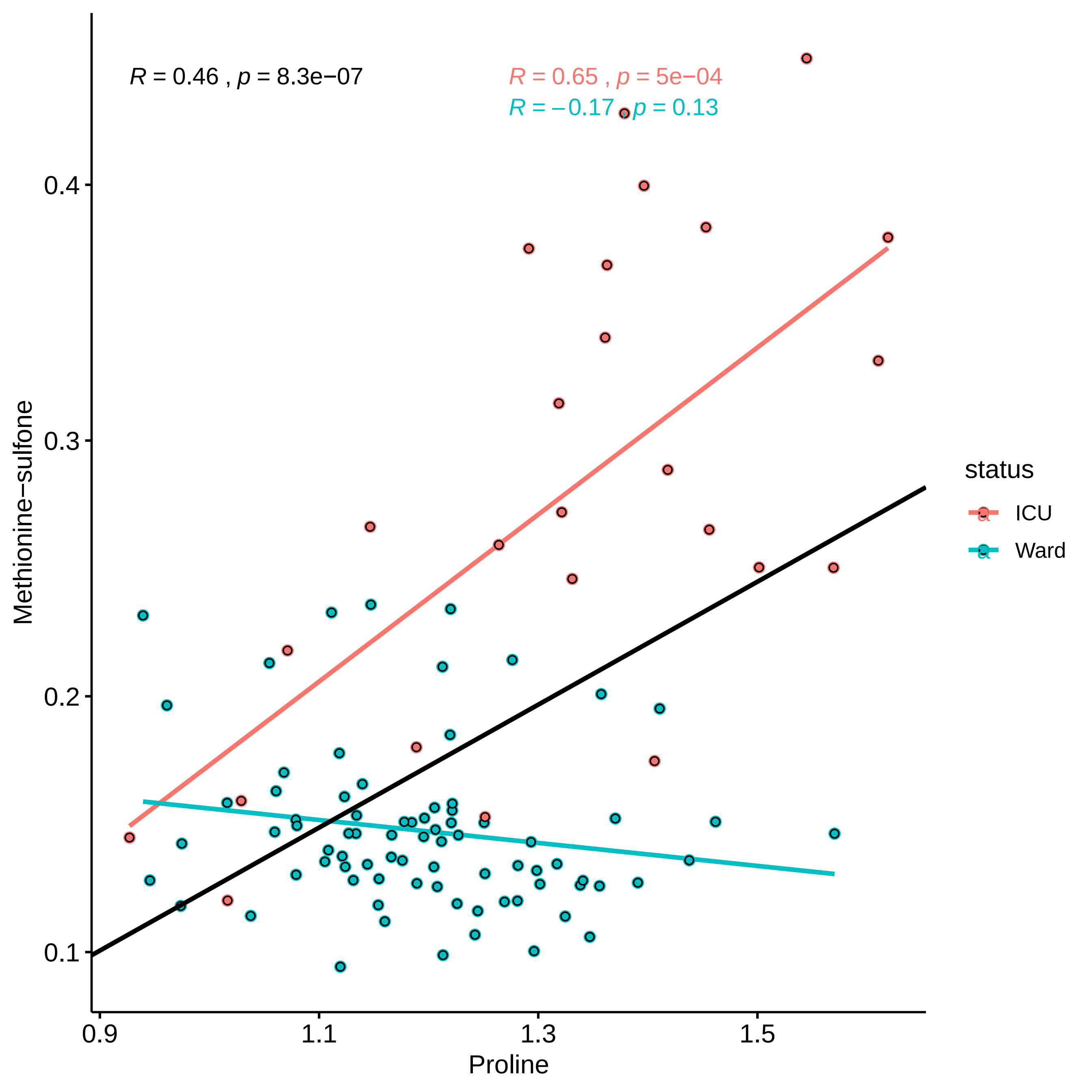

R = 0.46 , p = 8.3e−07
R = 0.65 , p = 5e−04
R = −0.17 , p = 0.13
Methionine−sulfone
0.4
0.3
0.2
0.1
0.9
1.1
1.3
1.5
Proline
status
ICU
Ward

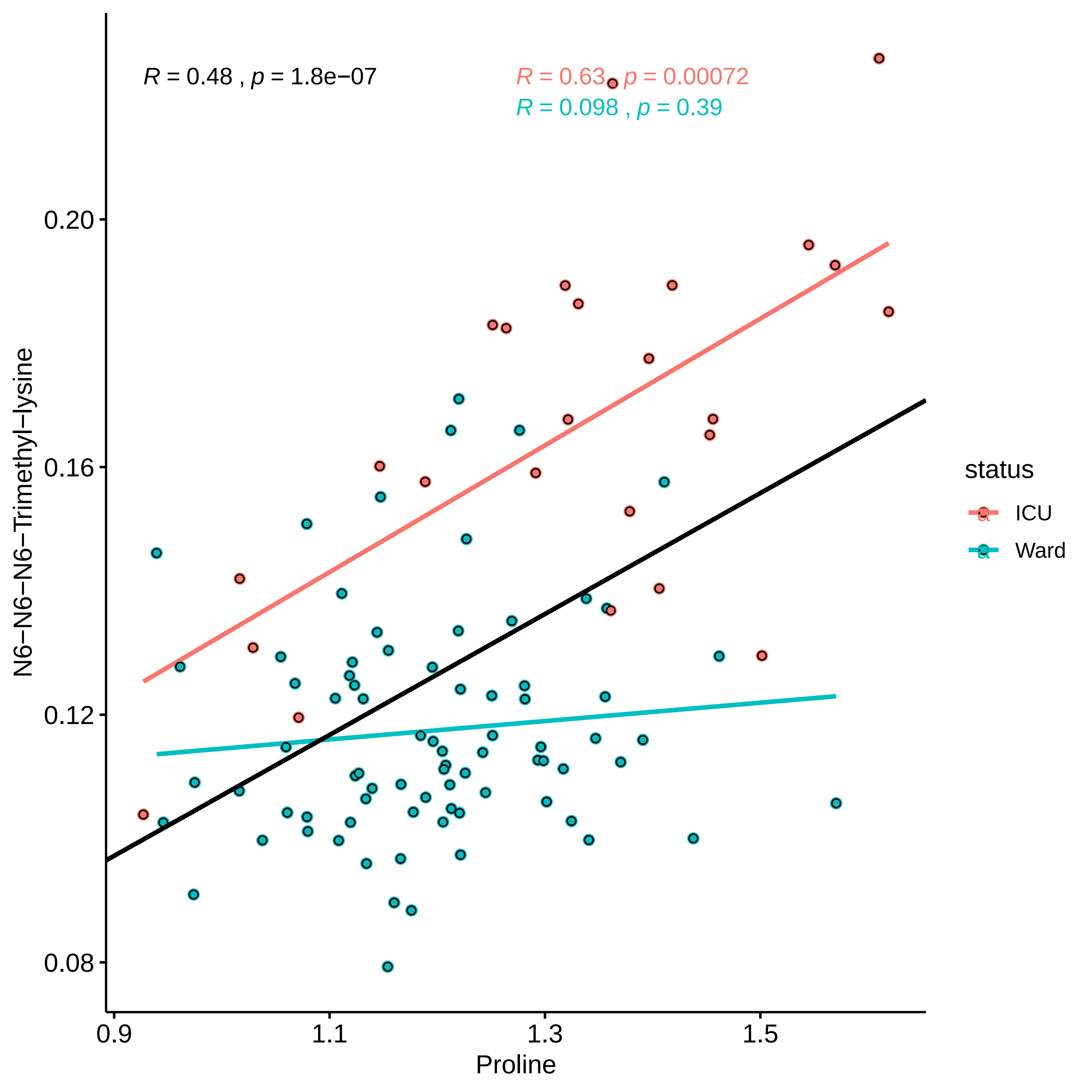
R = 0.48 , p = 1.8e−07
R = 0.63 , p = 0.00072
R = 0.098 , p = 0.39
N6−N6−N6−Trimethyl−lysine
0.20
0.16
0.12
0.08
0.9
1.1
1.3
1.5
Proline
status
ICU
Ward

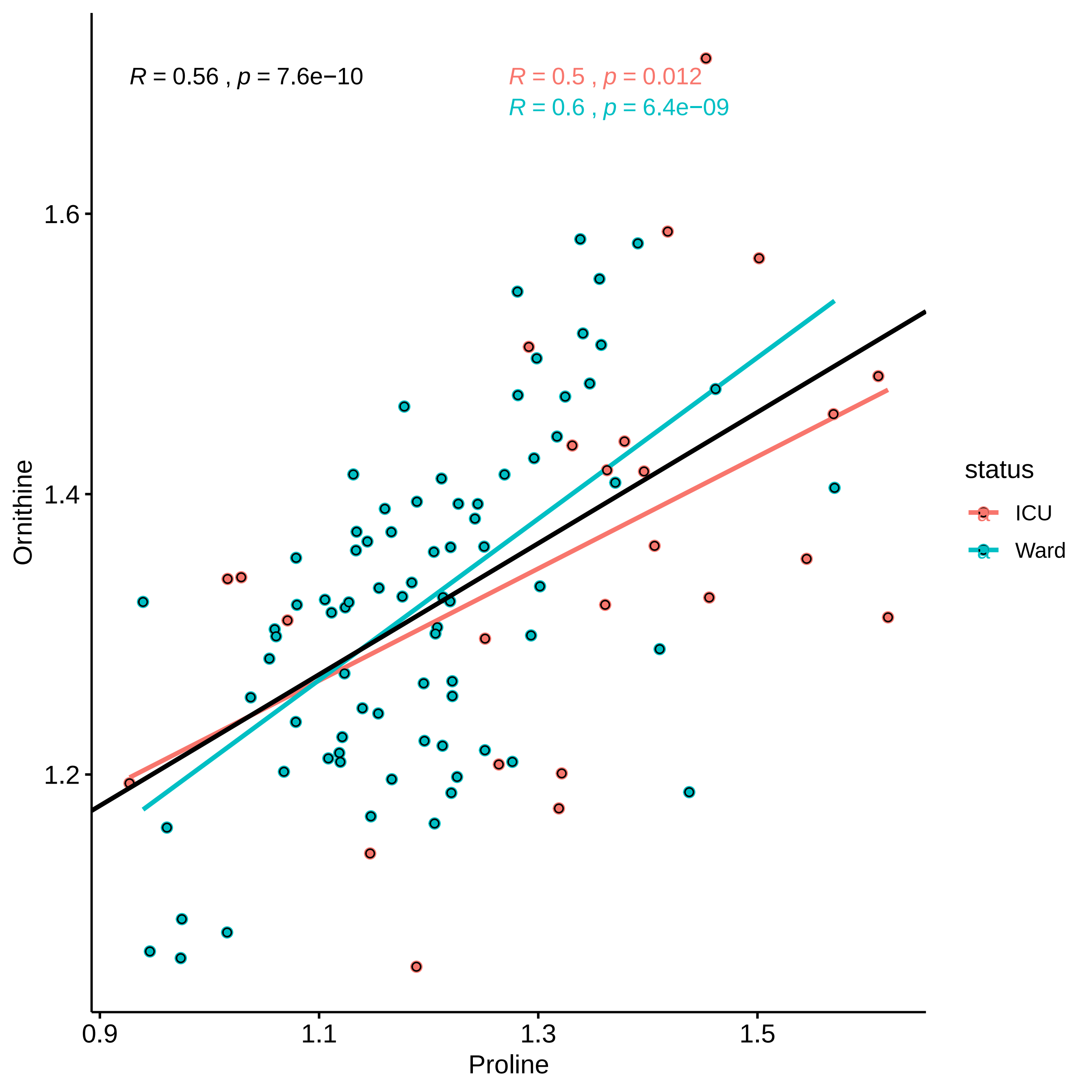

R = 0.56 , p = 7.6e−10
R = 0.5 , p = 0.012
R = 0.6 , p = 6.4e−09
1.6
1.4
1.2
Ornithine
0.9
1.1
1.3
1.5
Proline
status
ICU
Ward

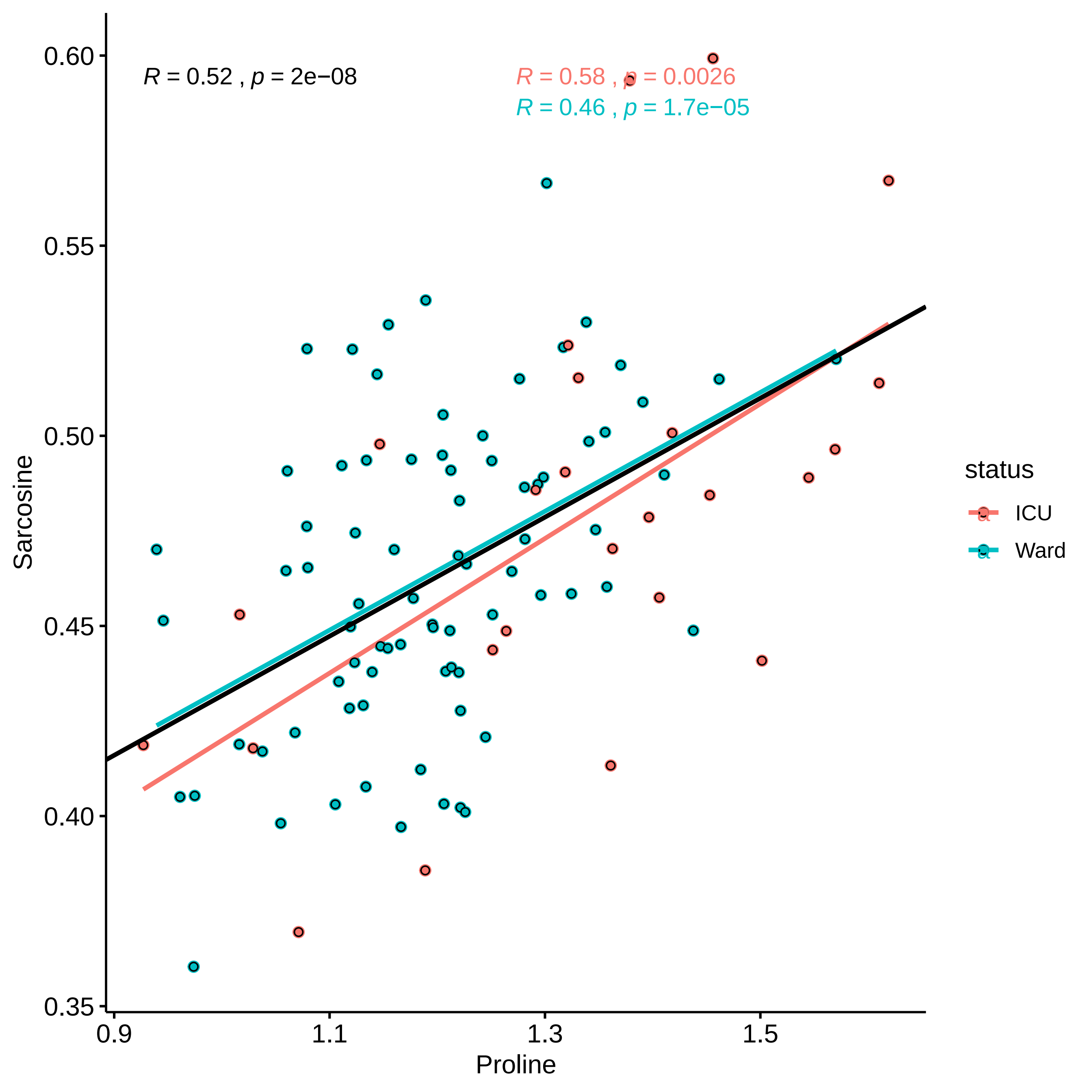

R = 0.52 , p = 2e−08
R = 0.58 , p = 0.0026
R = 0.46 , p = 1.7e−05
Sarcosine
0.60
0.55
0.50
0.45
0.40
0.35
0.9
1.1
1.3
1.5
Proline
status
ICU
Ward

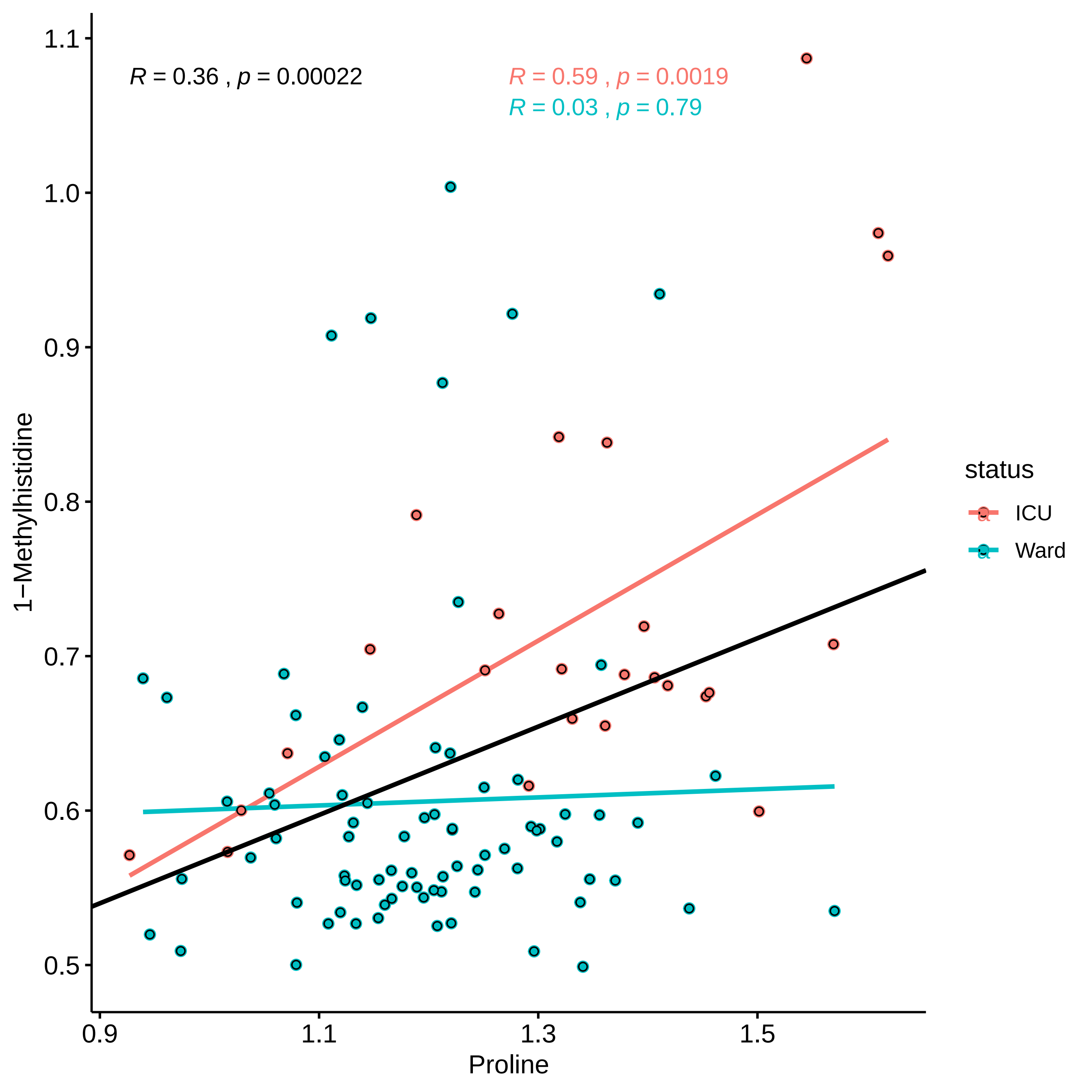

R = 0.36 , p = 0.00022
R = 0.59 , p = 0.0019
R = 0.03 , p = 0.79
1−Methylhistidine
1.1
1.0
0.9
0.8
0.7
0.6
0.5
0.9
1.1
1.3
1.5
Proline
status
ICU
Ward

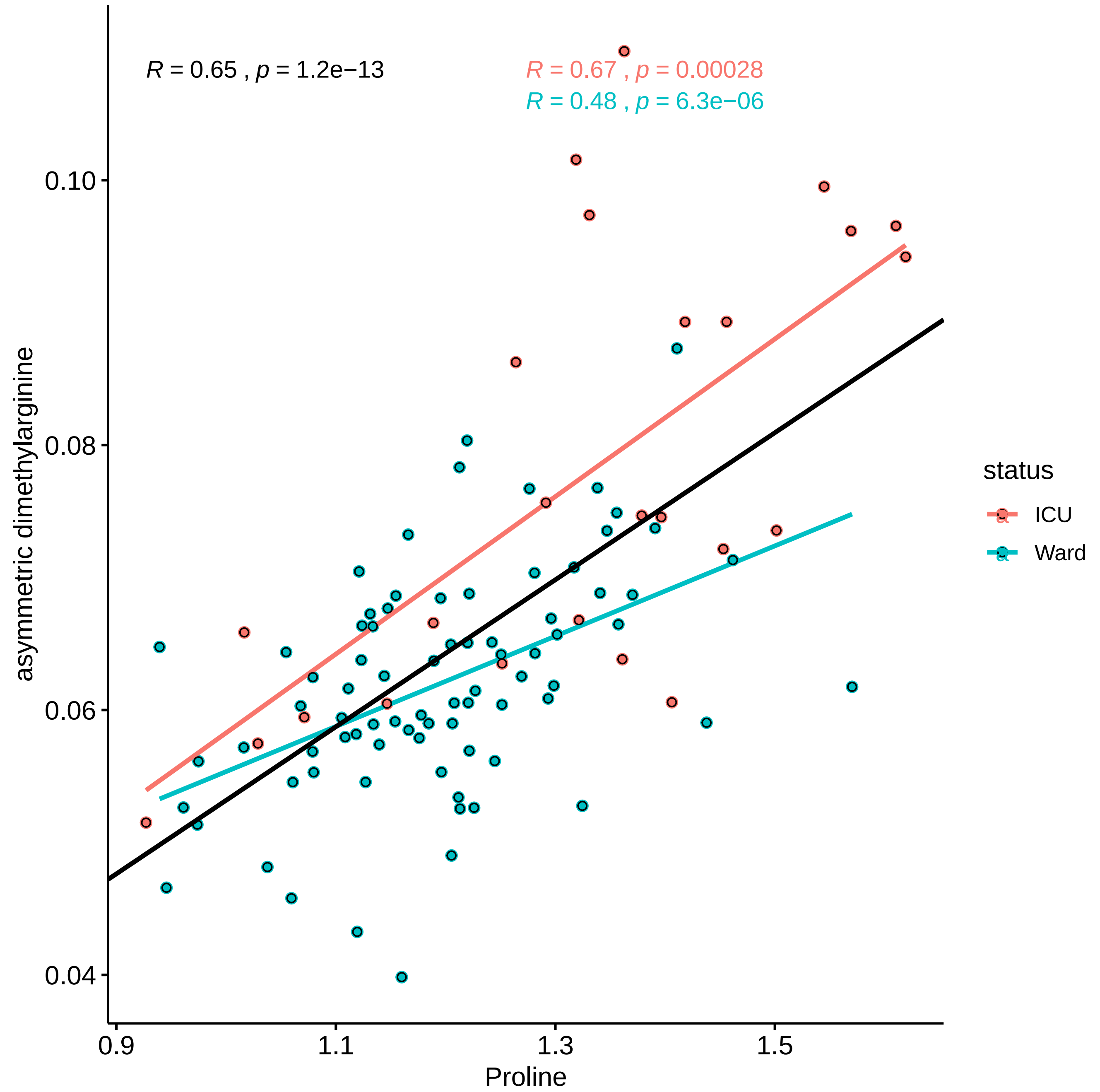

R = 0.65 , p = 1.2e−13
R = 0.67 , p = 0.00028
R = 0.48 , p = 6.3e−06
0.10
0.08
0.06
0.04
asymmetric dimethylarginine
0.9
1.1
1.3
1.5
Proline
status
ICU
Ward

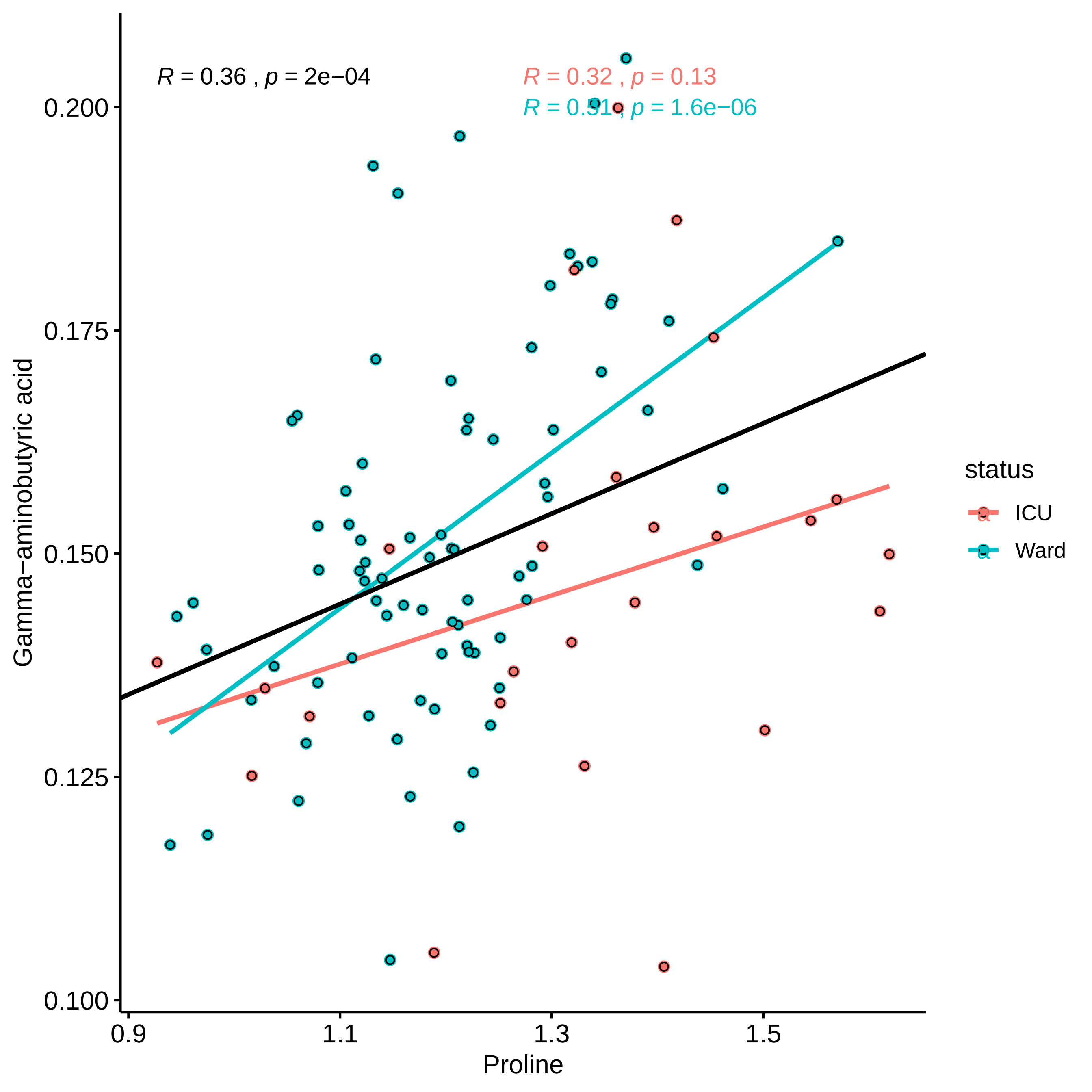

R = 0.36 , p = 2e−04
R = 0.32 , p = 0.13
R = 0.51 , p = 1.6e−06
Gamma−aminobutyric acid
0.200
0.175
0.150
0.125
0.100
0.9
1.1
1.3
1.5
Proline
status
ICU
Ward

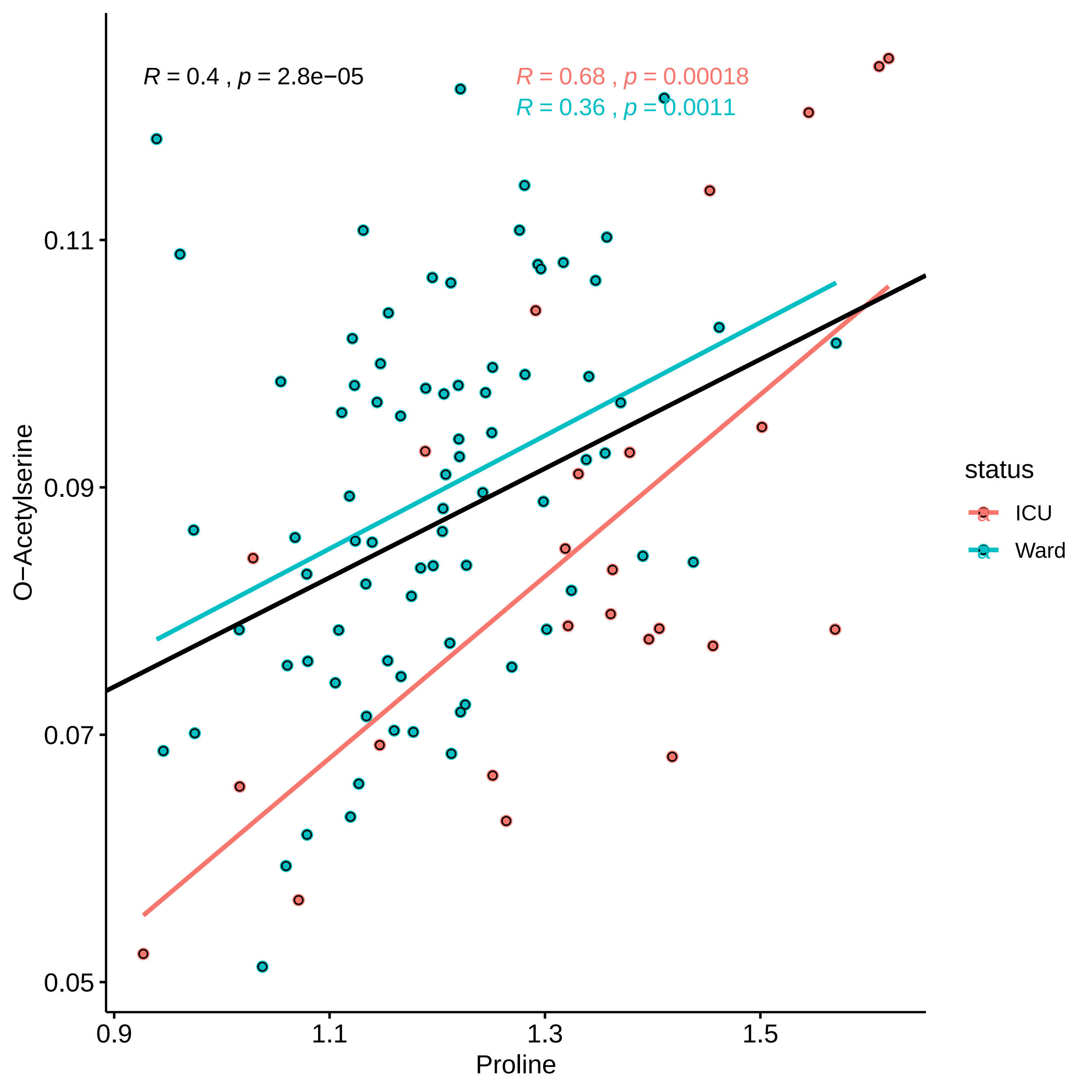

R = 0.4 , p = 2.8e−05
R = 0.68 , p = 0.00018
R = 0.36 , p = 0.0011
O−Acetylserine
0.11
0.09
0.07
0.05
0.9
1.1
1.3
1.5
Proline
status
ICU
Ward

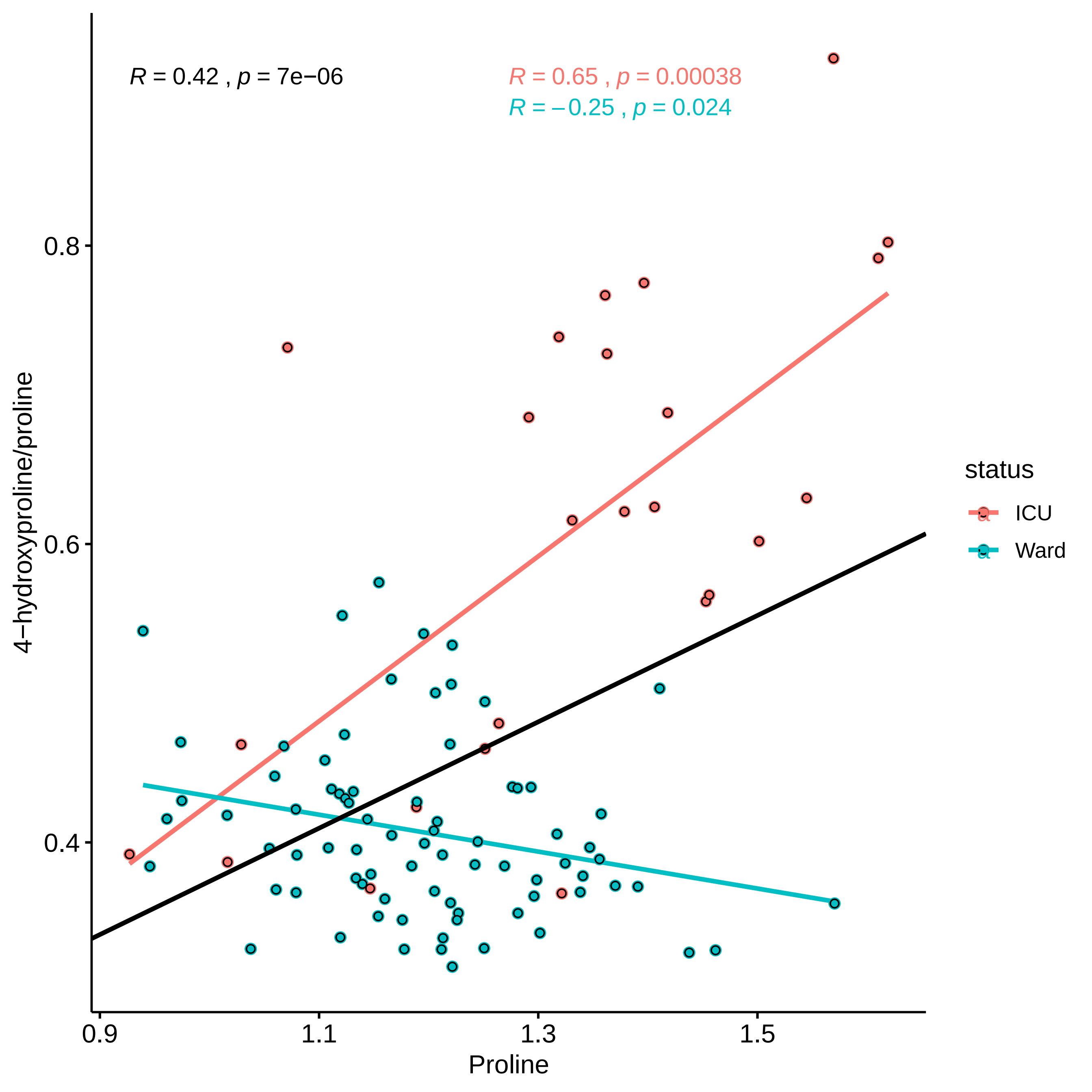

R = 0.42 , p = 7e−06
R = 0.65 , p = 0.00038
R = −0.25 , p = 0.024
0.8
0.6
0.4
4−hydroxyproline/proline
0.9
1.1
1.3
1.5
Proline
status
ICU
Ward

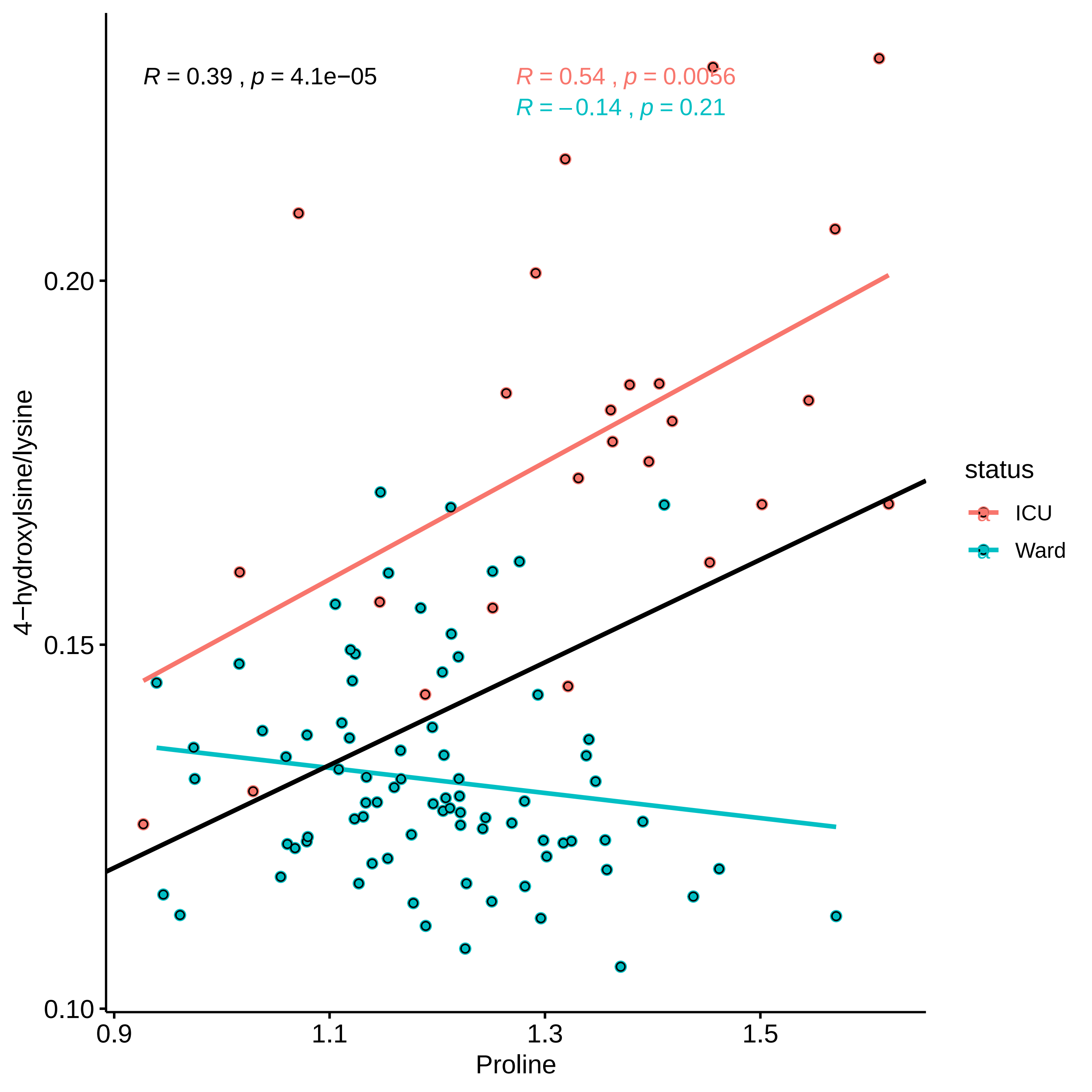

R = 0.39 , p = 4.1e−05
R = 0.54 , p = 0.0056
R = −0.14 , p = 0.21
4−hydroxylsine/lysine
0.20
0.15
0.10
0.9
1.1
1.3
1.5
Proline
status
ICU
Ward

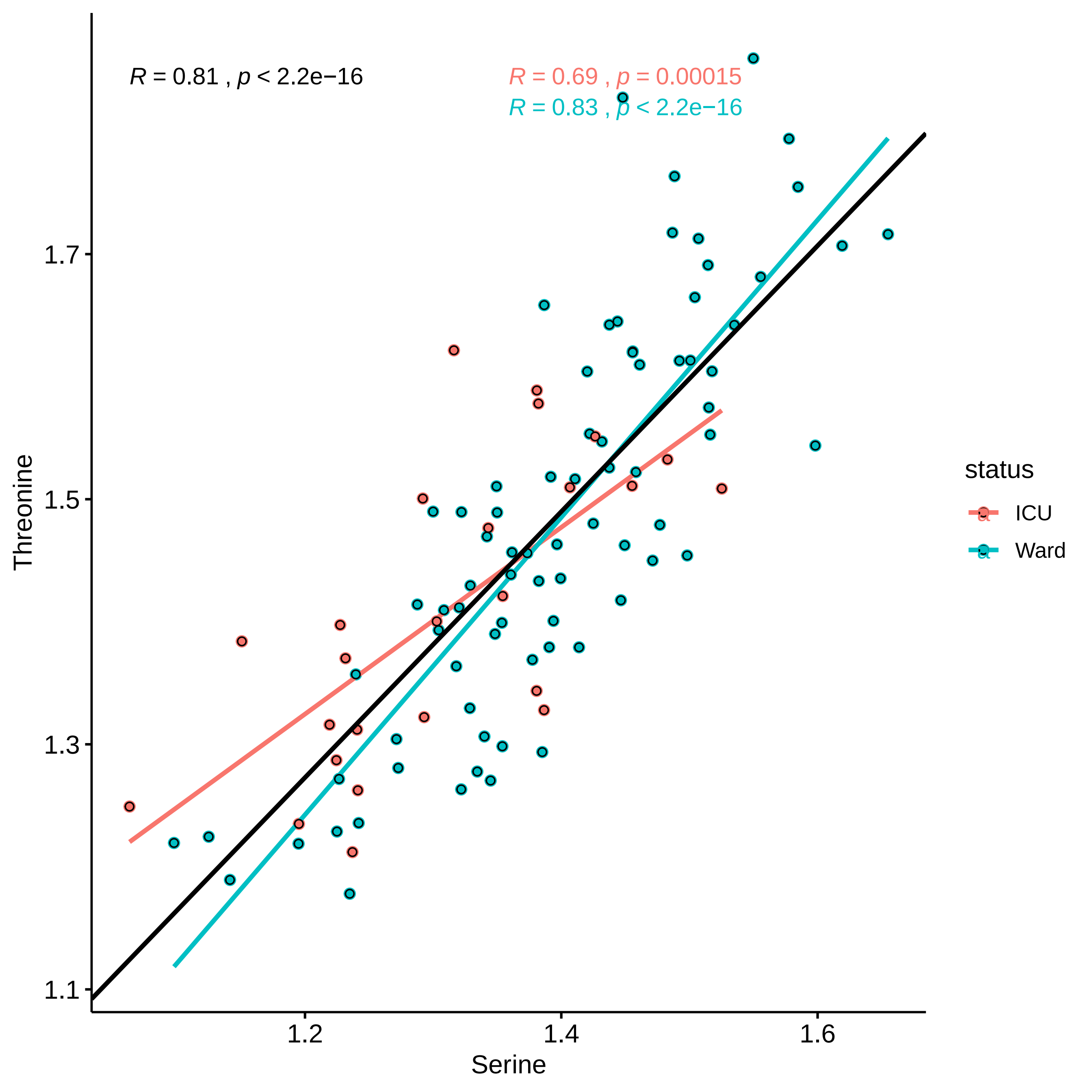

R = 0.81 , p < 2.2e−16
R = 0.69 , p = 0.00015
R = 0.83 , p < 2.2e−16
1.7
1.5
1.3
1.1
Threonine
1.2
1.4
1.6
Serine
status
ICU
Ward

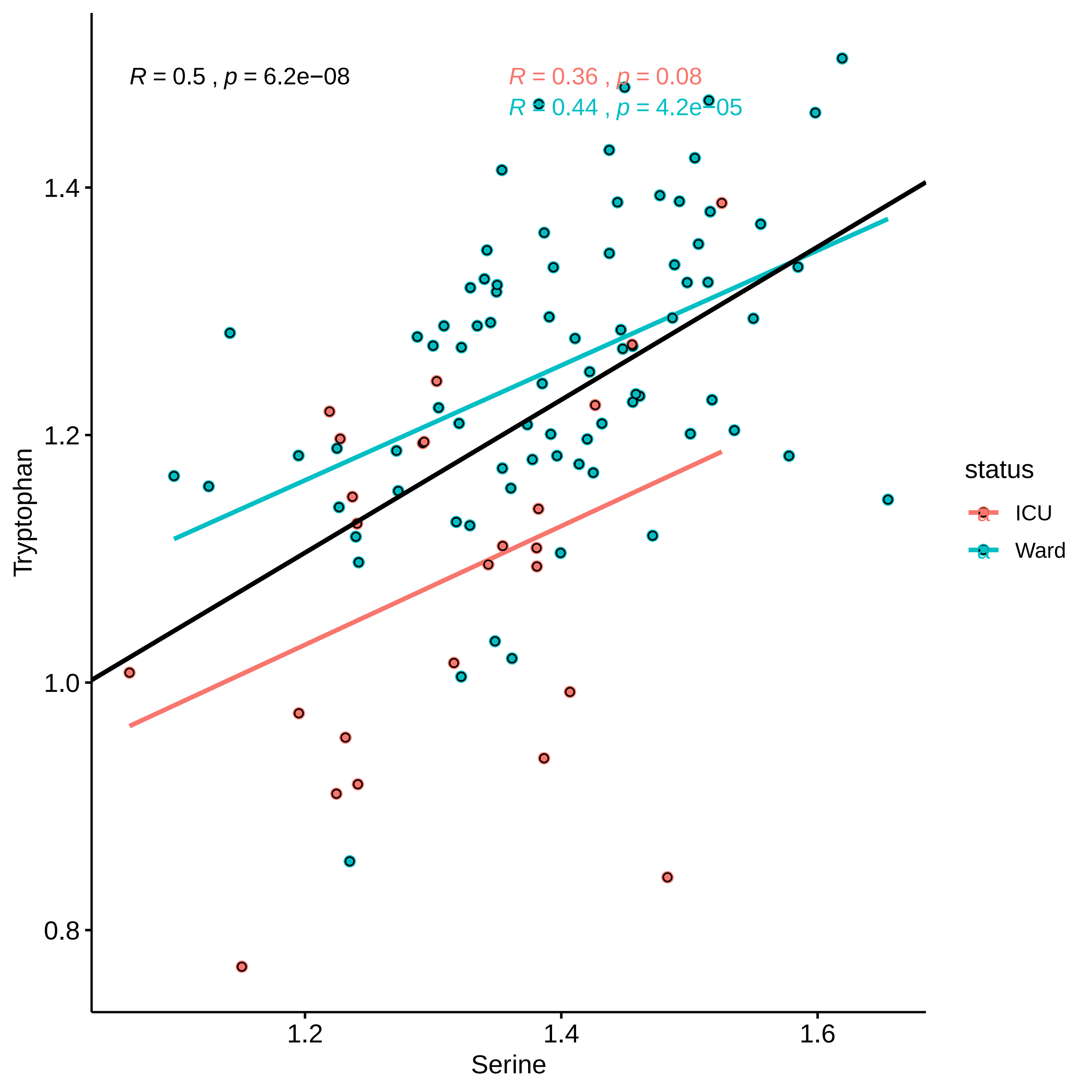

R = 0.5 , p = 6.2e−08
R = 0.36 , p = 0.08
R = 0.44 , p = 4.2e−05
Tryptophan
1.4
1.2
1.0
0.8
1.2
1.4
1.6
Serine
status
ICU
Ward

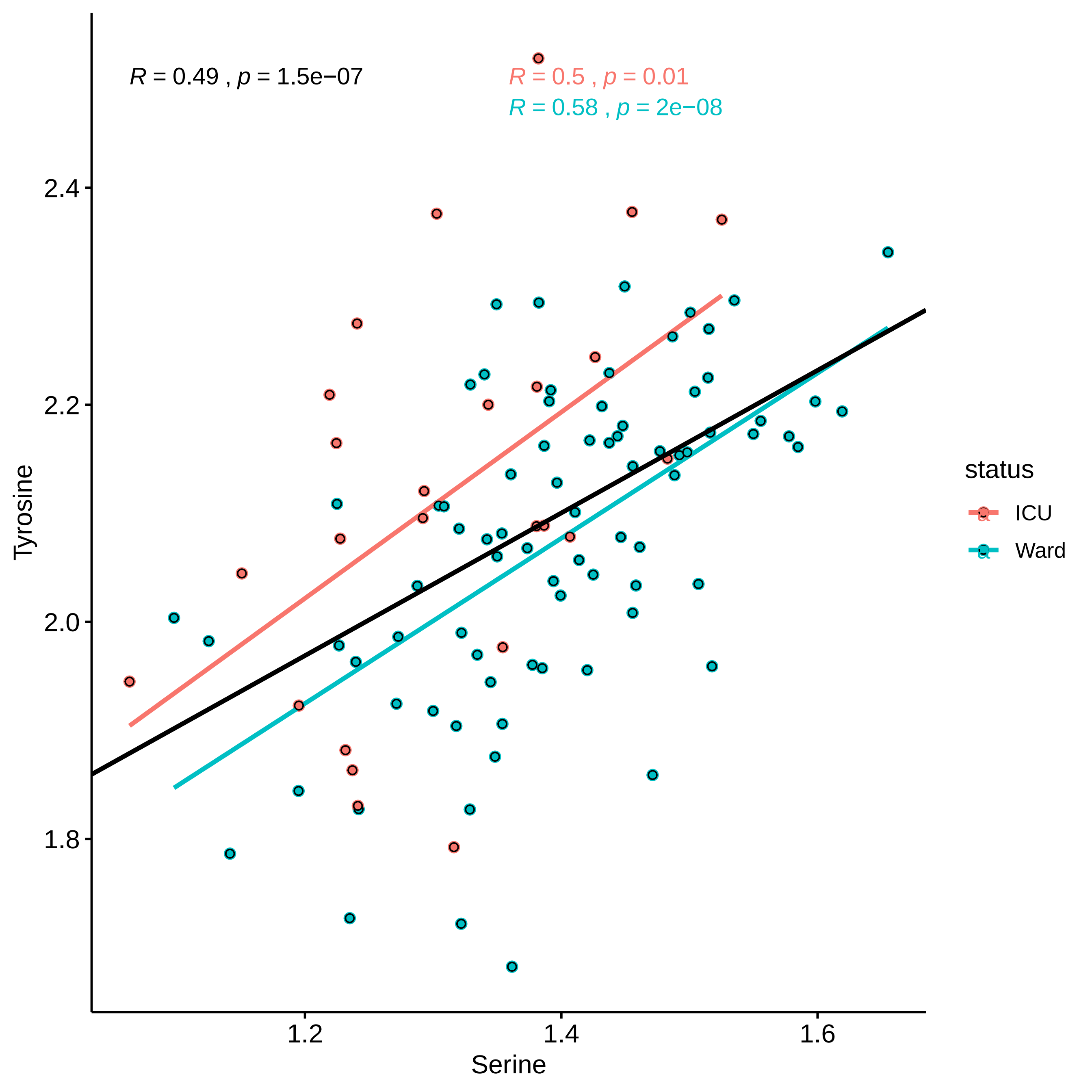

R = 0.49 , p = 1.5e−07
R = 0.5 , p = 0.01
R = 0.58 , p = 2e−08
Tyrosine
2.4
2.2
2.0
1.8
1.2
1.4
1.6
Serine
status
ICU
Ward

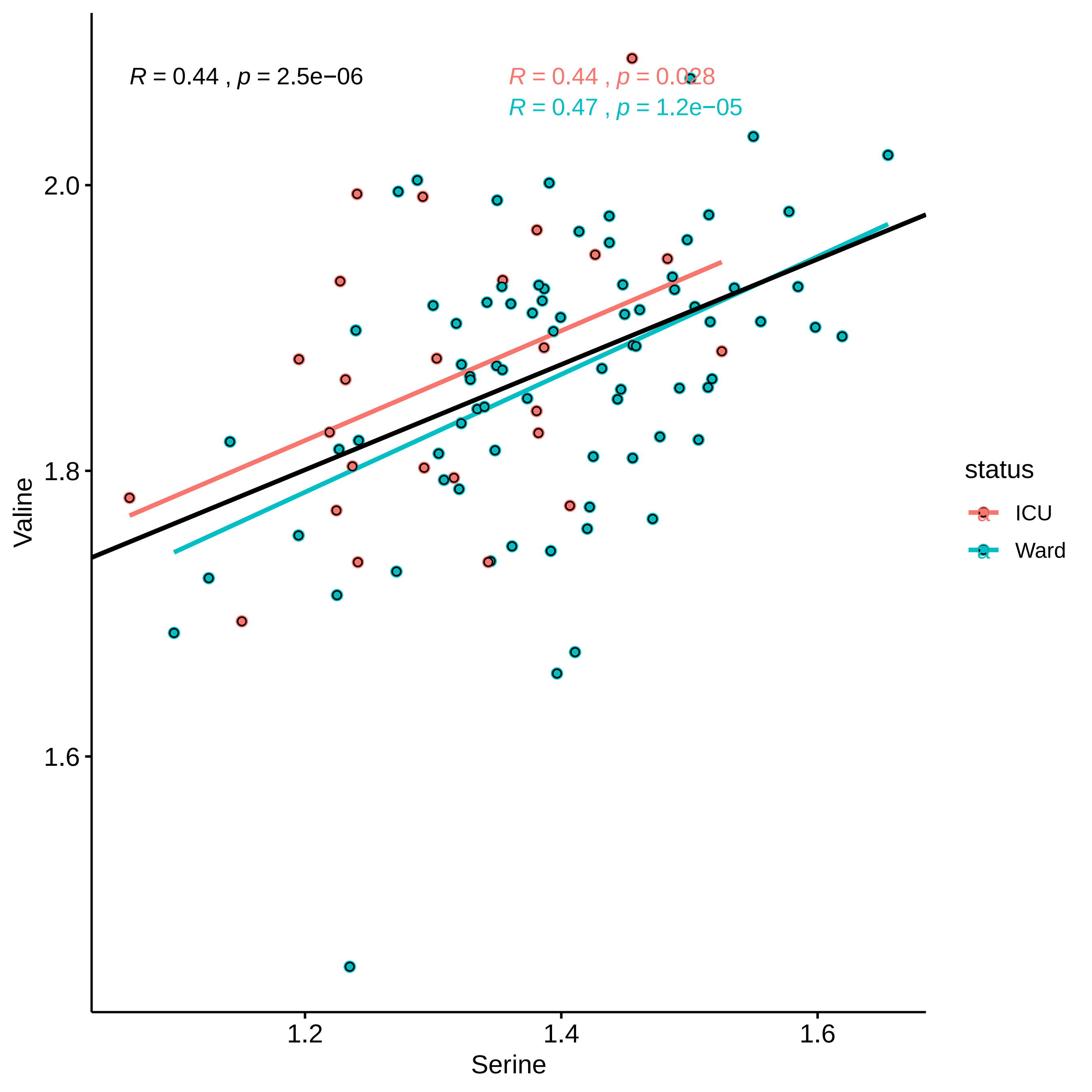

R = 0.44 , p = 2.5e−06
R = 0.44 , p = 0.028
R = 0.47 , p = 1.2e−05
2.0
1.8
1.6
Valine
1.2
1.4
1.6
Serine
status
ICU
Ward

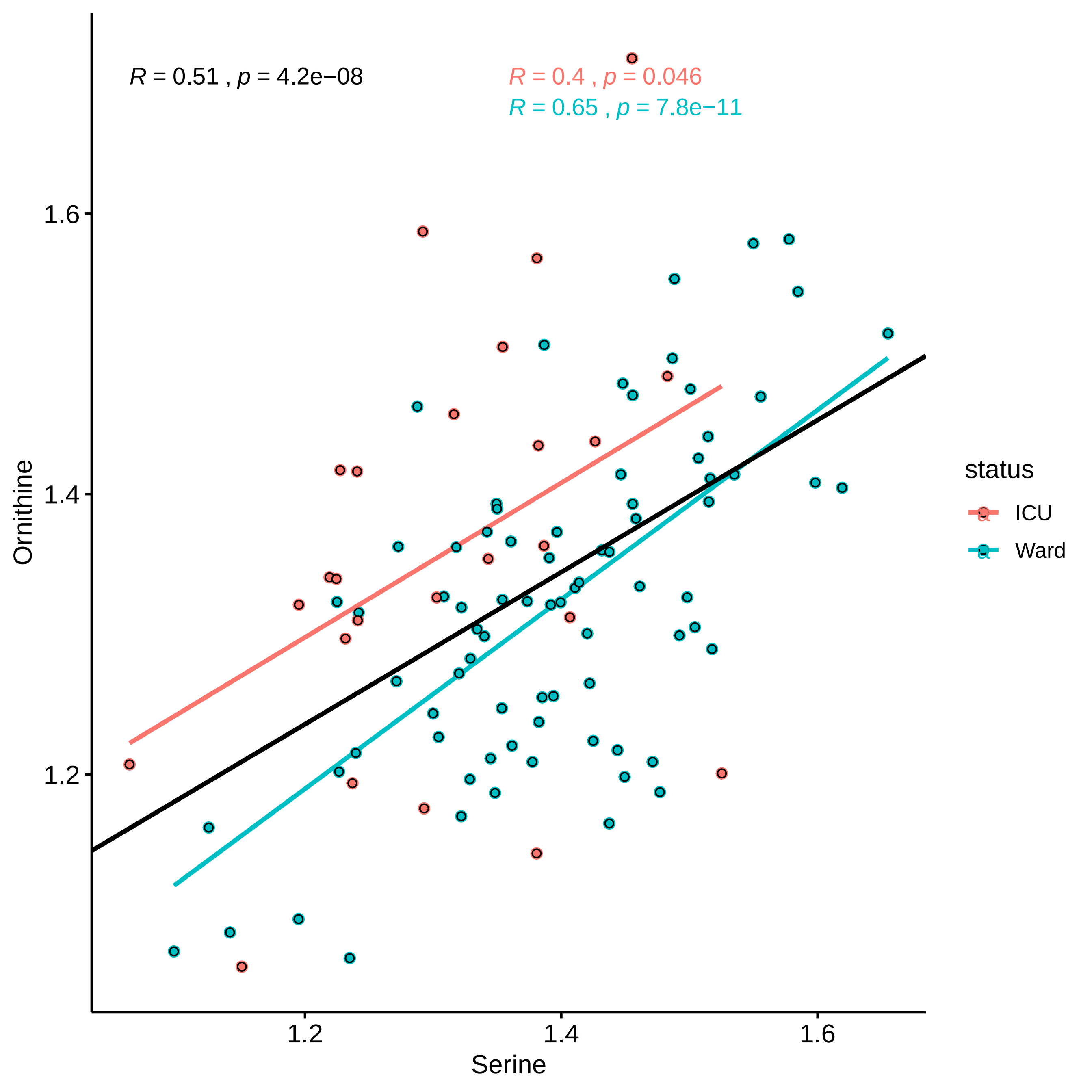

R = 0.51 , p = 4.2e−08
R = 0.4 , p = 0.046
R = 0.65 , p = 7.8e−11
Ornithine
1.6
1.4
1.2
Serine
1.2
1.4
1.6
status
ICU
Ward

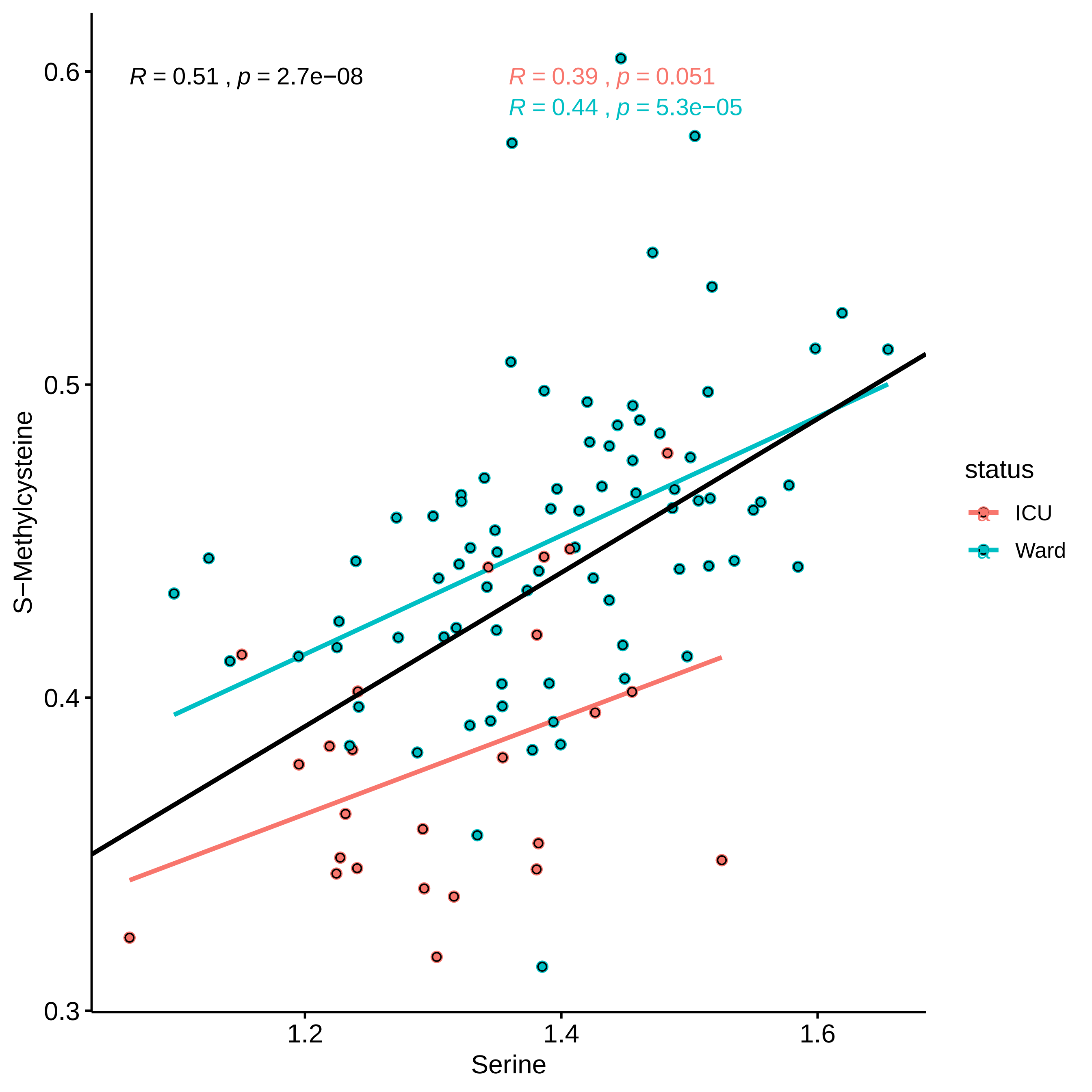

R = 0.51 , p = 2.7e−08
R = 0.39 , p = 0.051
R = 0.44 , p = 5.3e−05
S−Methylcysteine
Serine
0.3
0.4
0.5
0.6
1.2
1.4
1.6
status
ICU
Ward

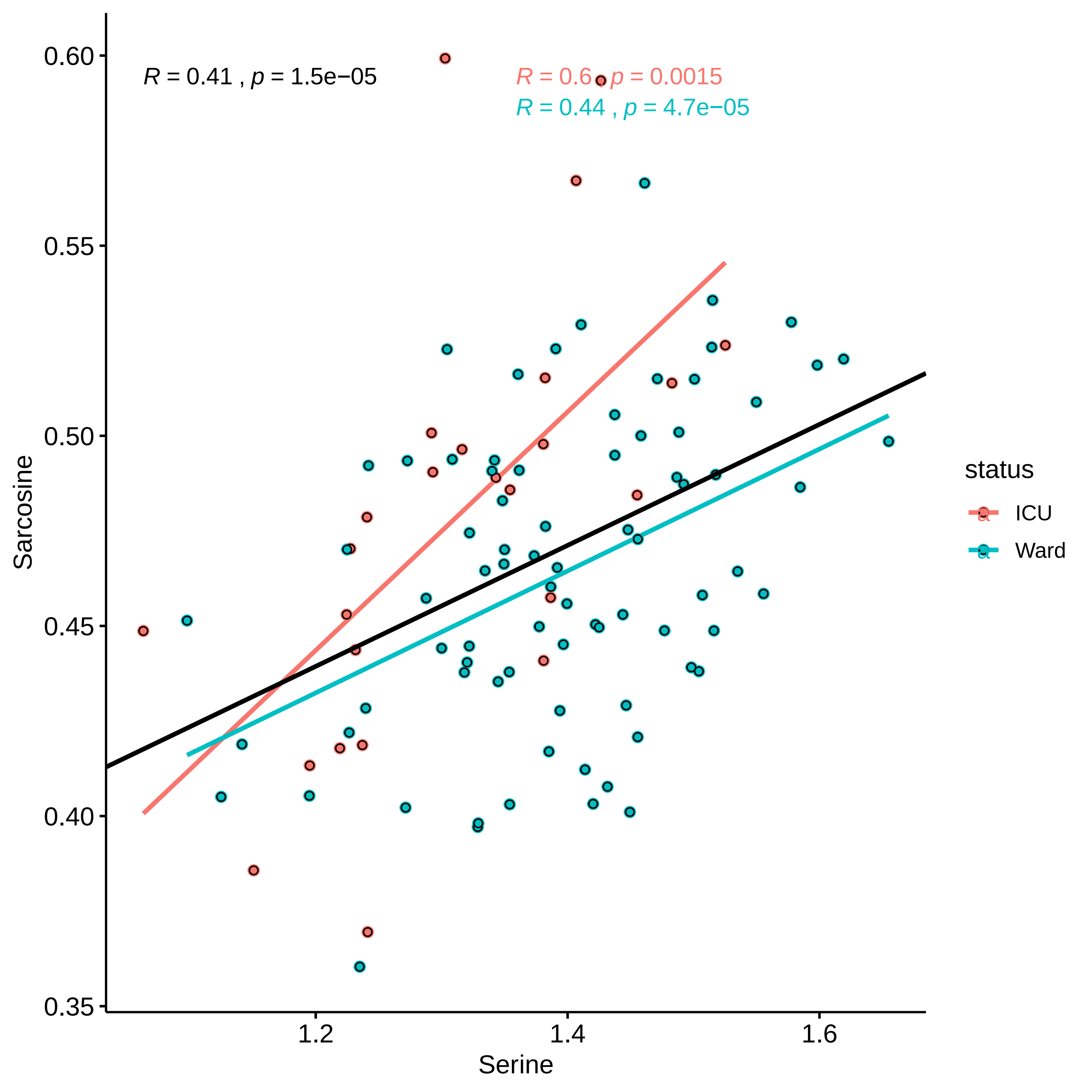
R = 0.41 , p = 1.5e−05
R = 0.6 , p = 0.0015
R = 0.44 , p = 4.7e−05
0.60
0.55
0.50
0.45
0.40
0.35
1.2
1.4
1.6
Serine
Sarcosine
status
ICU
Ward

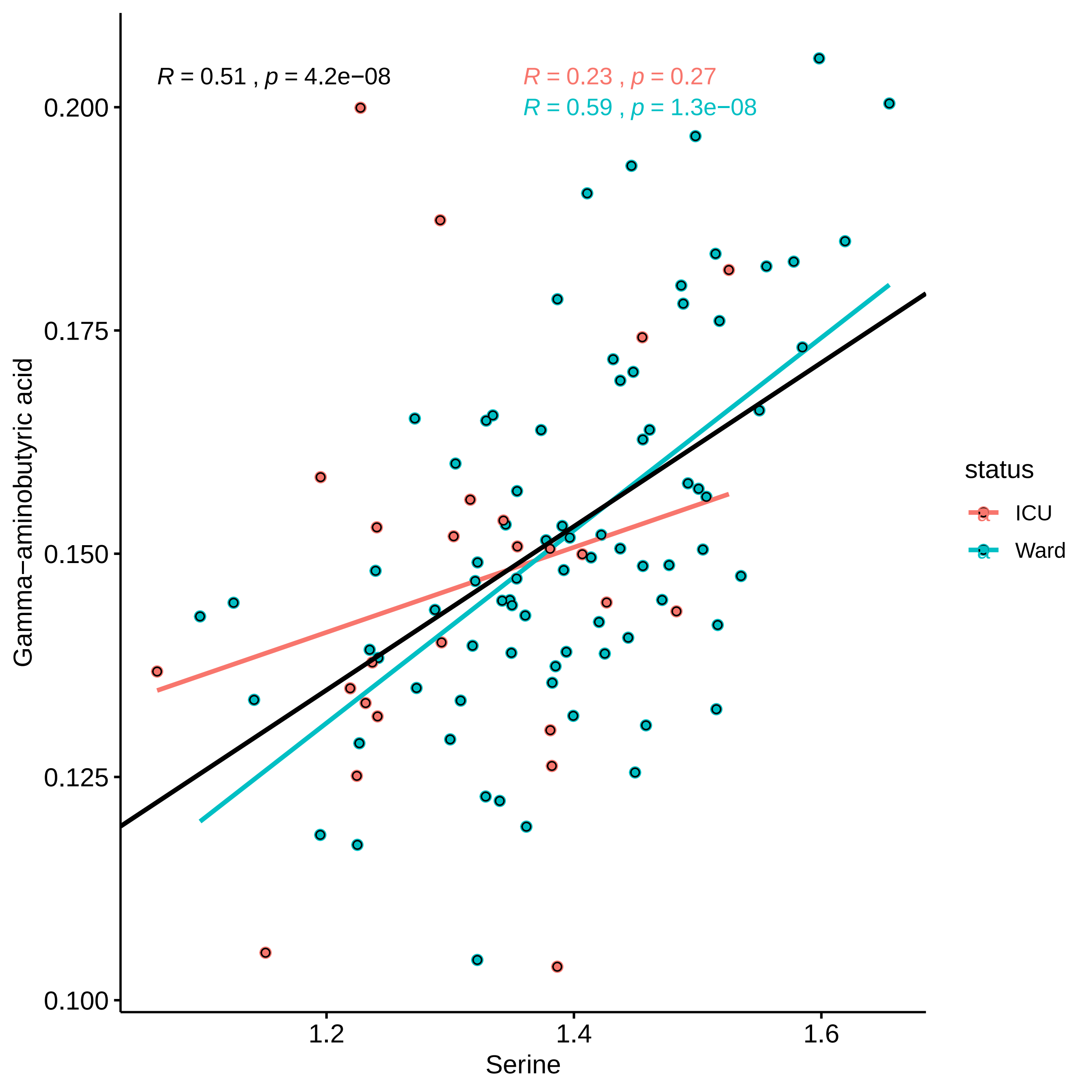

R = 0.51 , p = 4.2e−08
R = 0.23 , p = 0.27
R = 0.59 , p = 1.3e−08
Gamma−aminobutyric acid
0.200
0.175
0.150
0.125
0.100
1.2
1.4
1.6
Serine
status
ICU
Ward

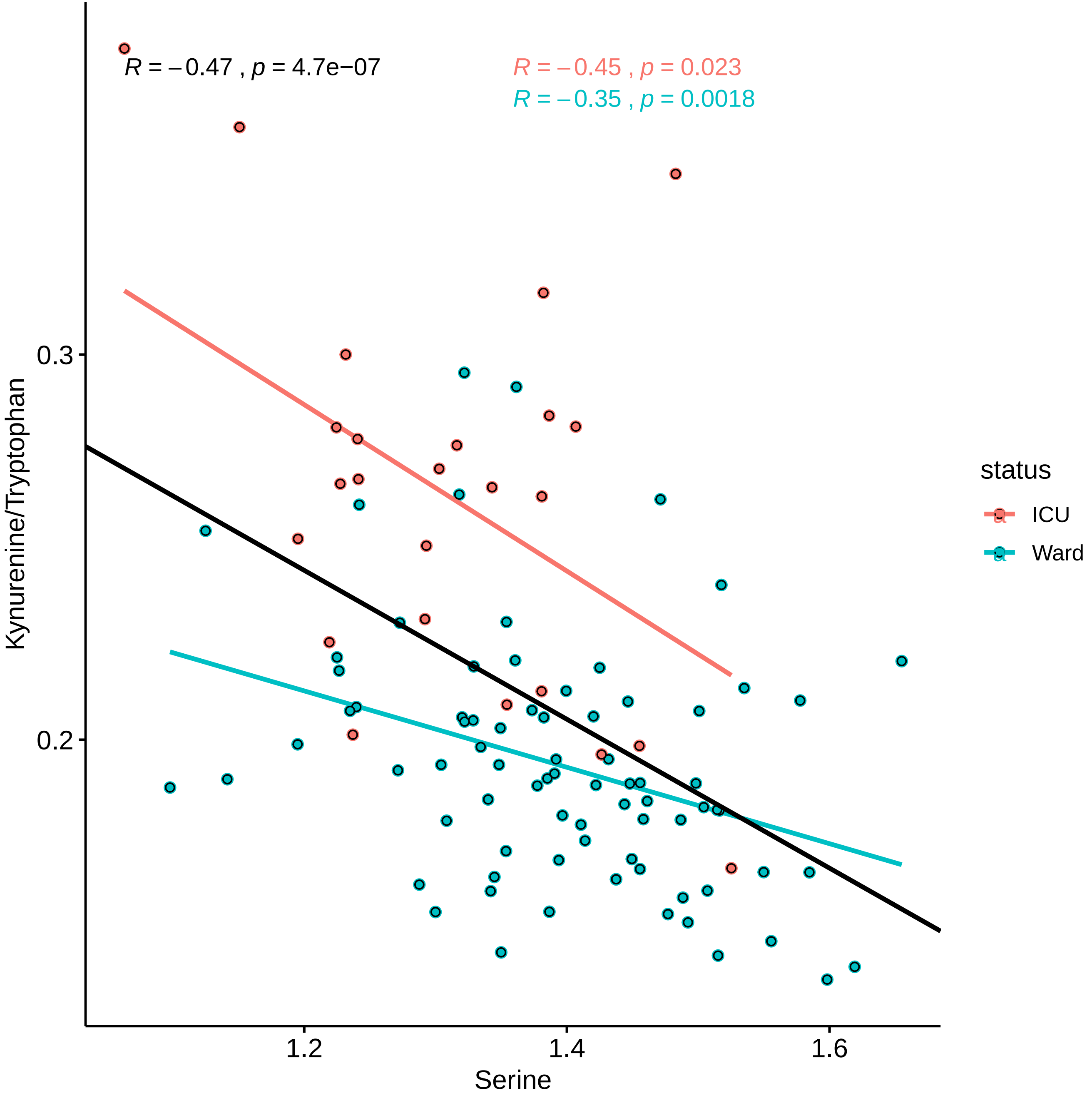
R = −0.47 , p = 4.7e−07
R = −0.45 , p = 0.023
R = −0.35 , p = 0.0018
0.3
0.2
Kynurenine/Tryptophan
1.2
1.4
1.6
Serine
status
ICU
Ward

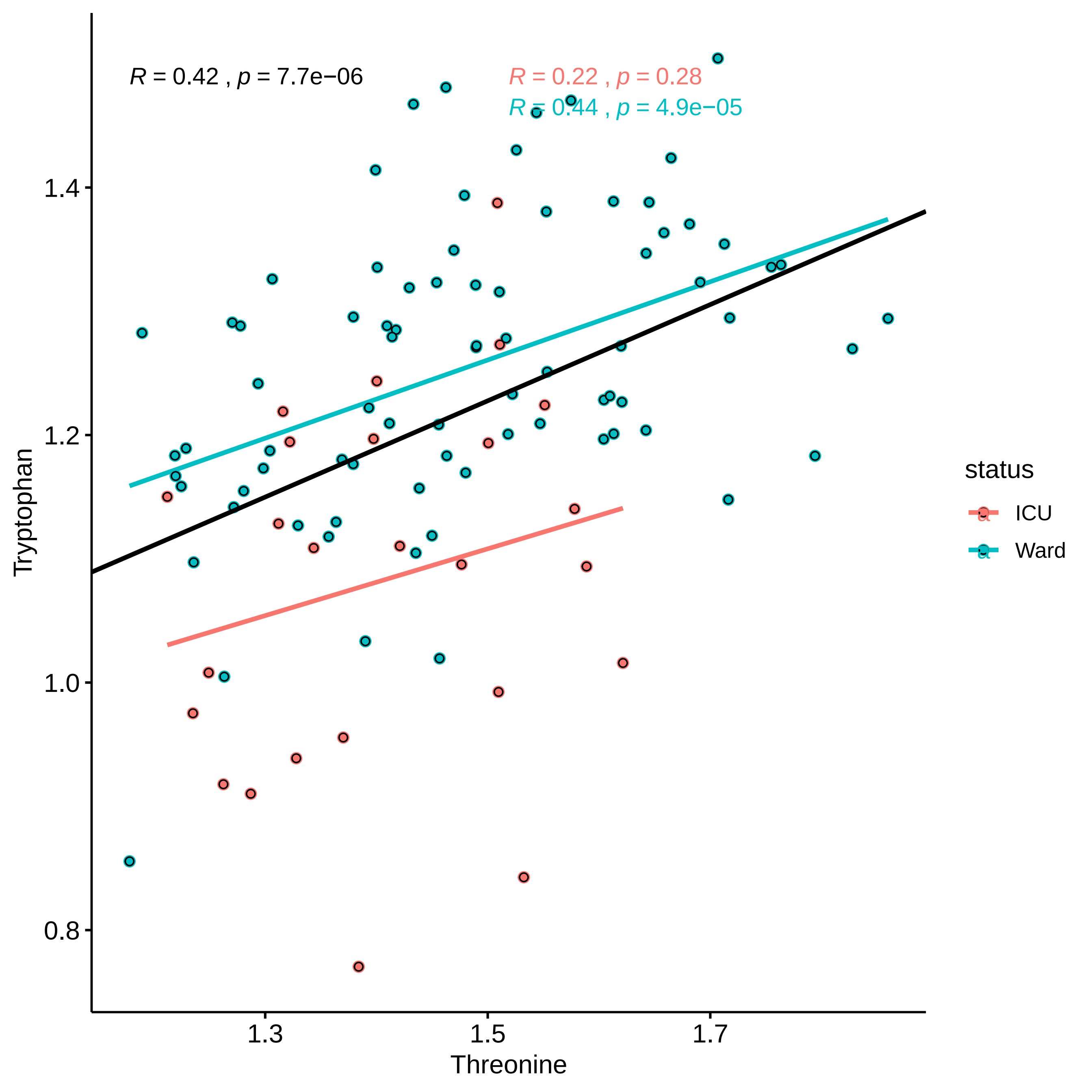
R = 0.42 , p = 7.7e−06
R = 0.22 , p = 0.28
R = 0.44 , p = 4.9e−05
Tryptophan
1.4
1.2
1.0
0.8
1.3
1.5
1.7
Threonine
status
ICU
Ward

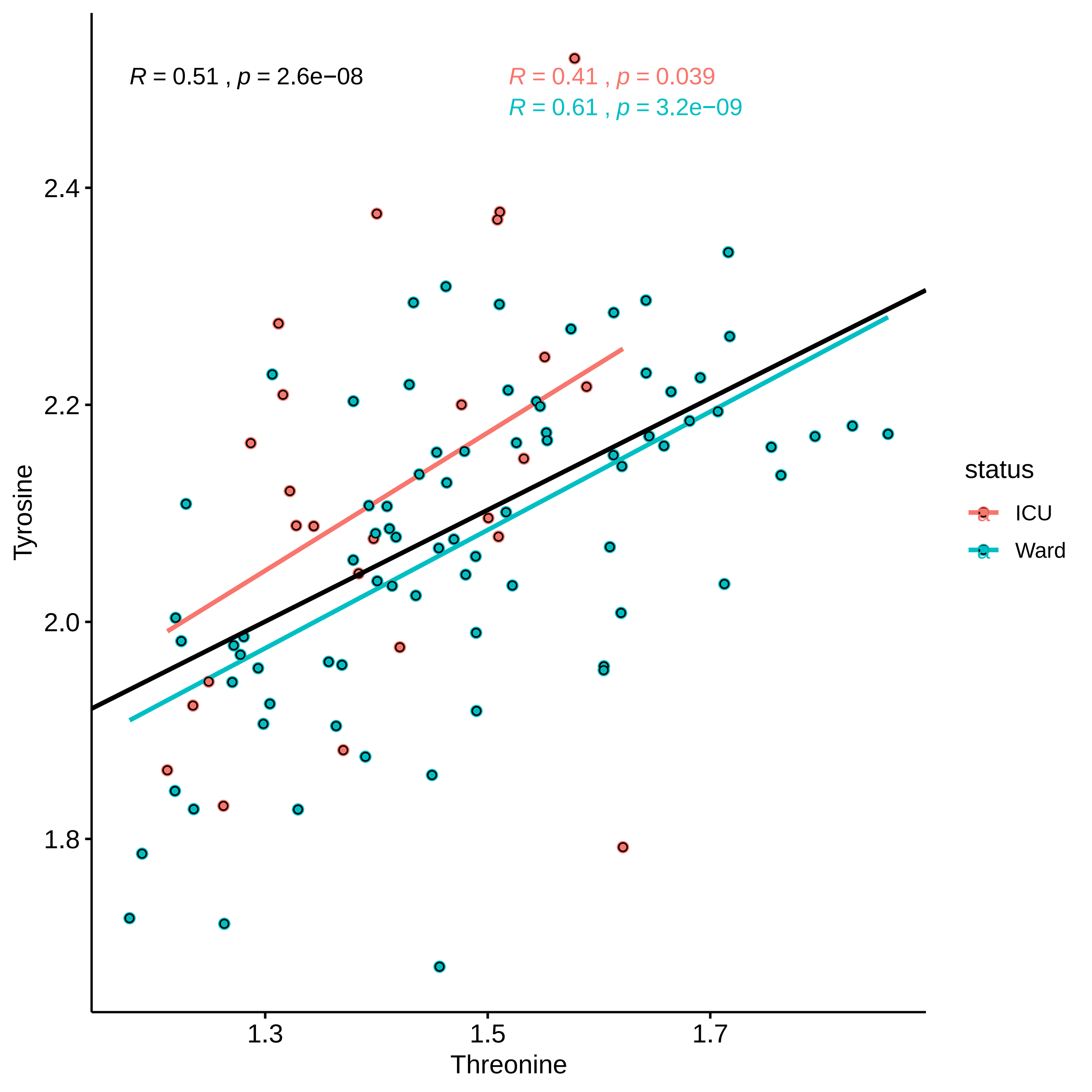

R = 0.51 , p = 2.6e−08
R = 0.41 , p = 0.039
R = 0.61 , p = 3.2e−09
Tyrosine
2.4
2.2
2.0
1.8
Threonine
1.3
1.5
1.7
status
ICU
Ward

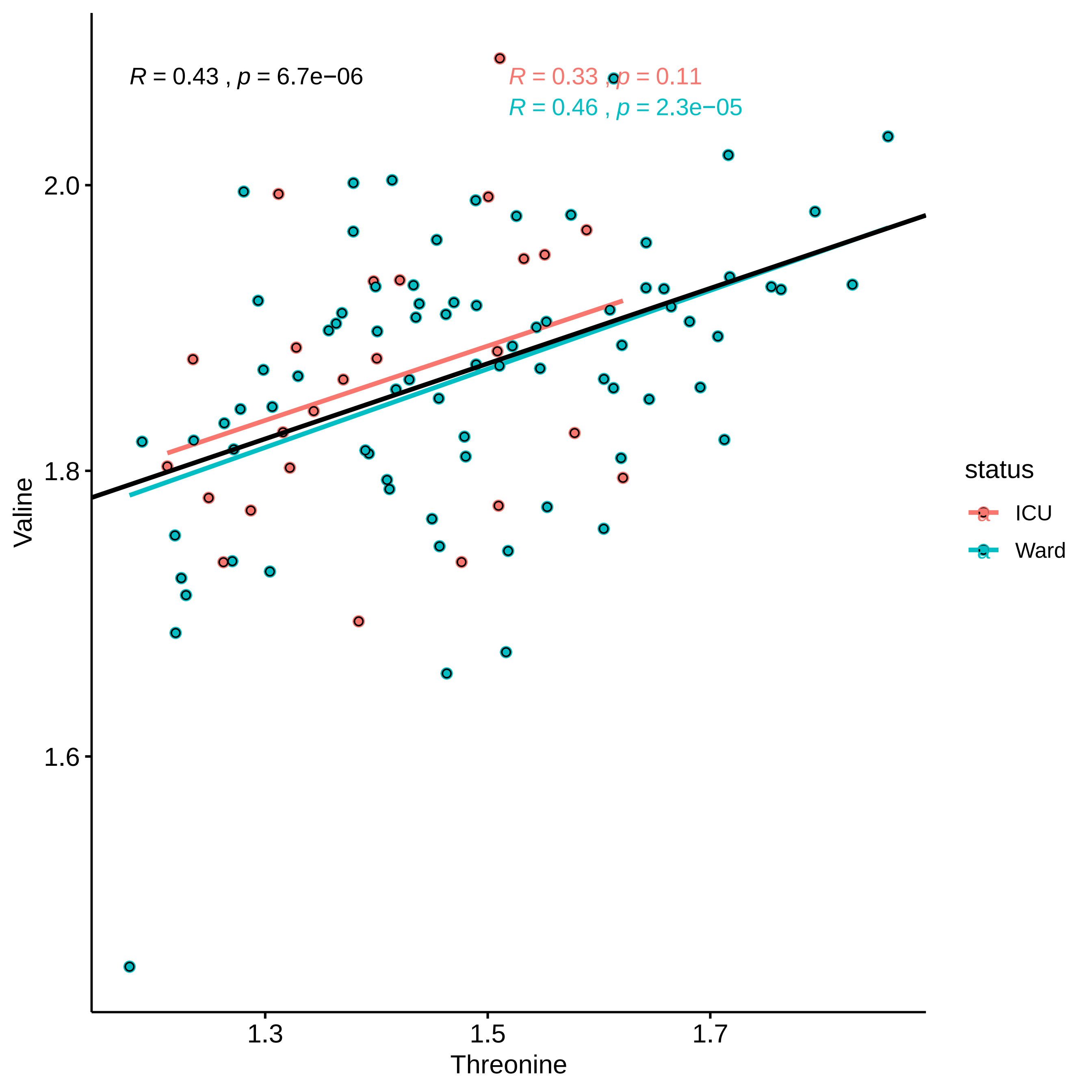

R = 0.43 , p = 6.7e−06
R = 0.33 , p = 0.11
R = 0.46 , p = 2.3e−05
2.0
1.8
1.6
Valine
1.3
1.5
1.7
Threonine
status
ICU
Ward

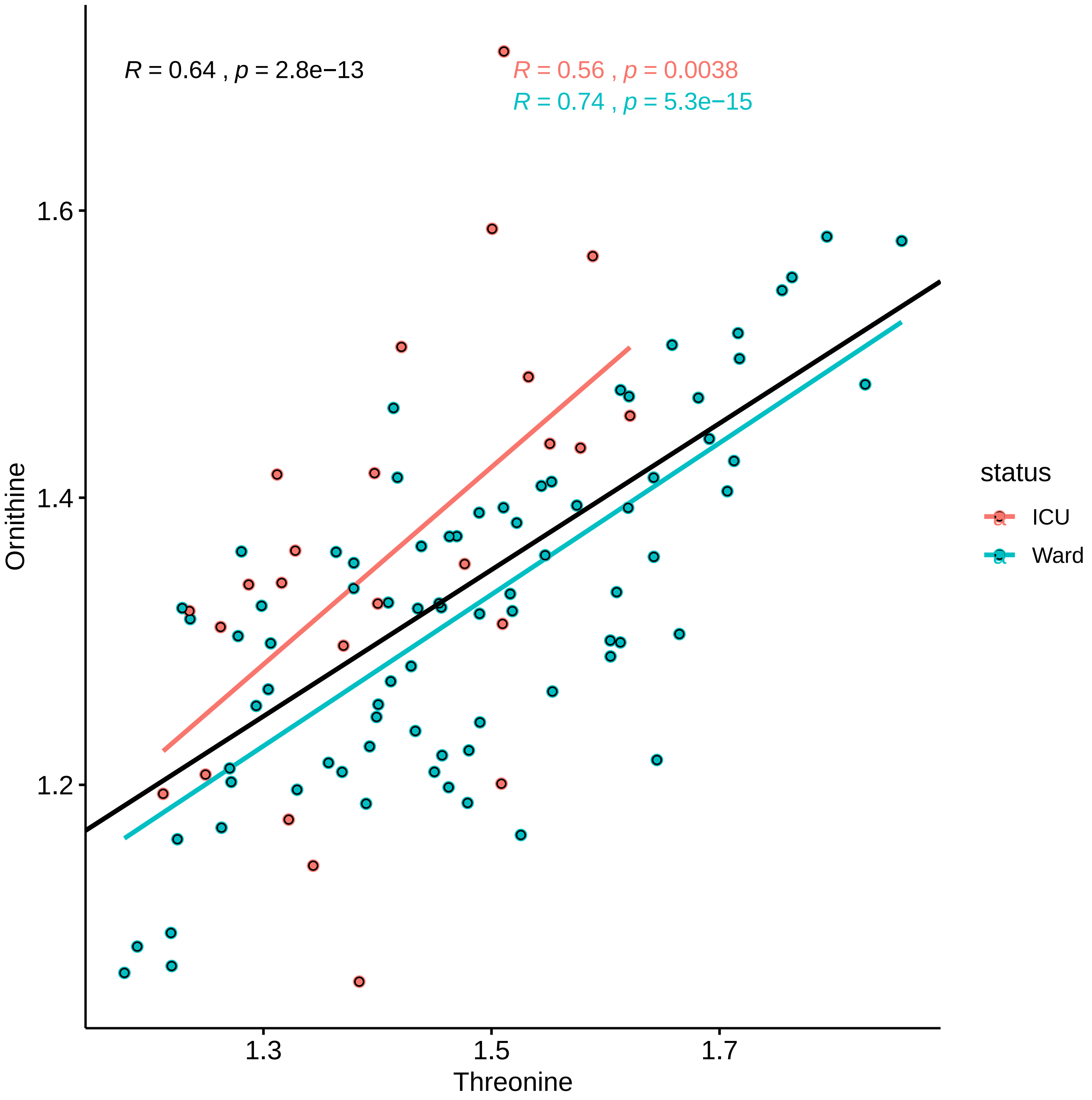
R = 0.64 , p = 2.8e−13
R = 0.56 , p = 0.0038
R = 0.74 , p = 5.3e−15
Ornithine
1.6
1.4
1.2
1.3
1.5
1.7
Threonine
status
ICU
Ward

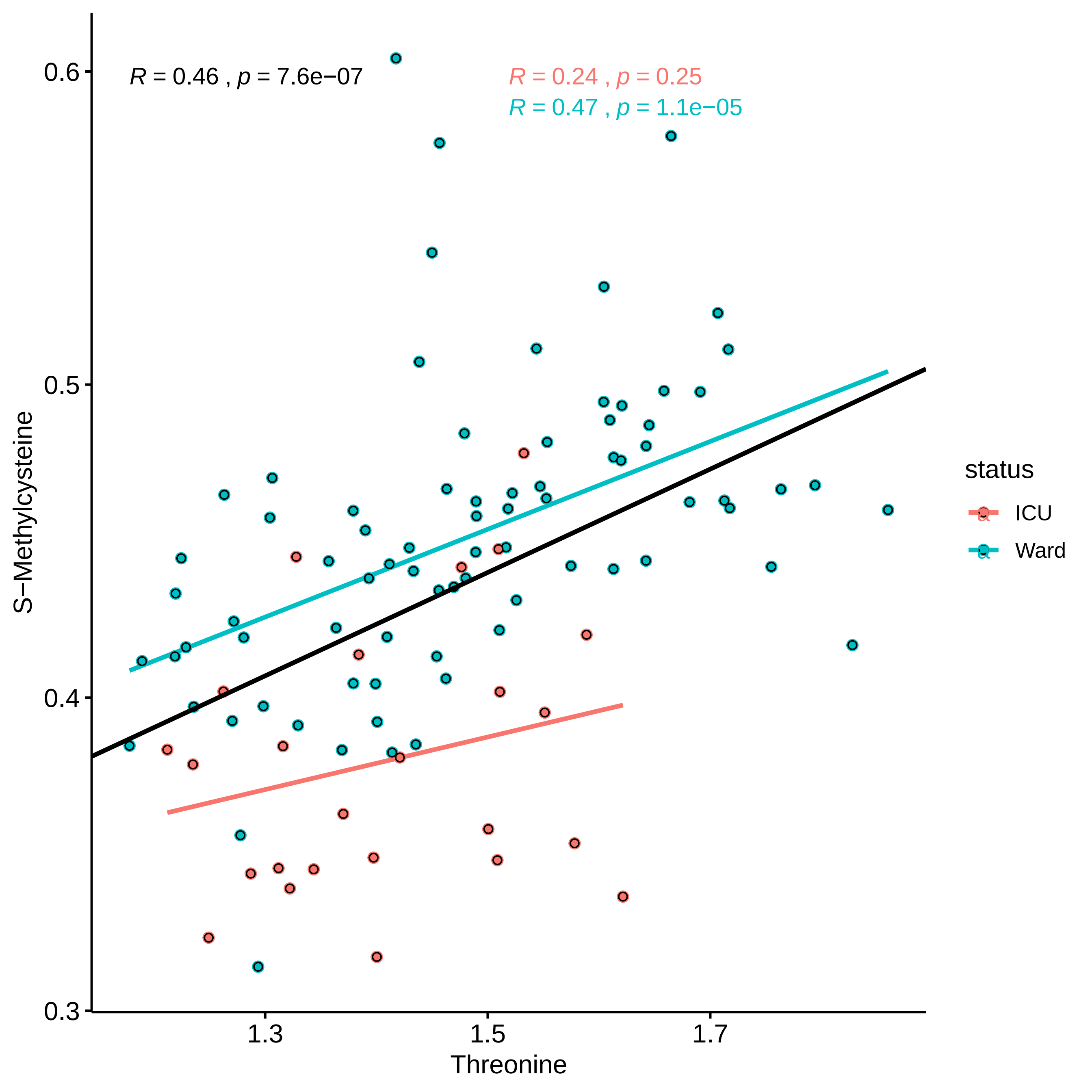
R = 0.46 , p = 7.6e−07
R = 0.24 , p = 0.25
R = 0.47 , p = 1.1e−05
S−Methylcysteine
Threonine
0.6
0.5
0.4
0.3
1.3
1.5
1.7
status
ICU
Ward

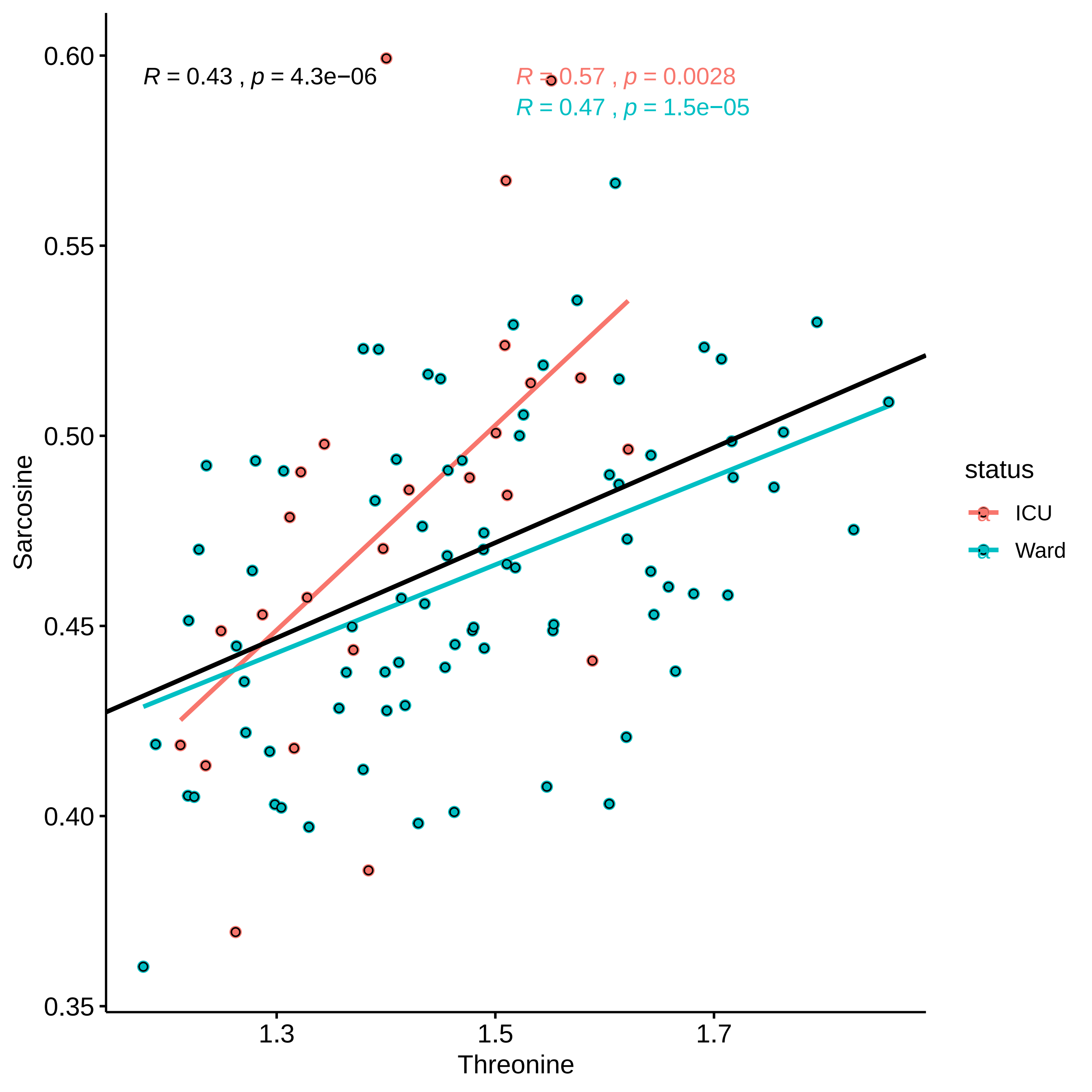

R = 0.43 , p = 4.3e−06
R = 0.57 , p = 0.0028
R = 0.47 , p = 1.5e−05
0.60
0.55
0.50
0.45
0.40
0.35
Sarcosine
1.3
1.5
1.7
Threonine
status
ICU
Ward

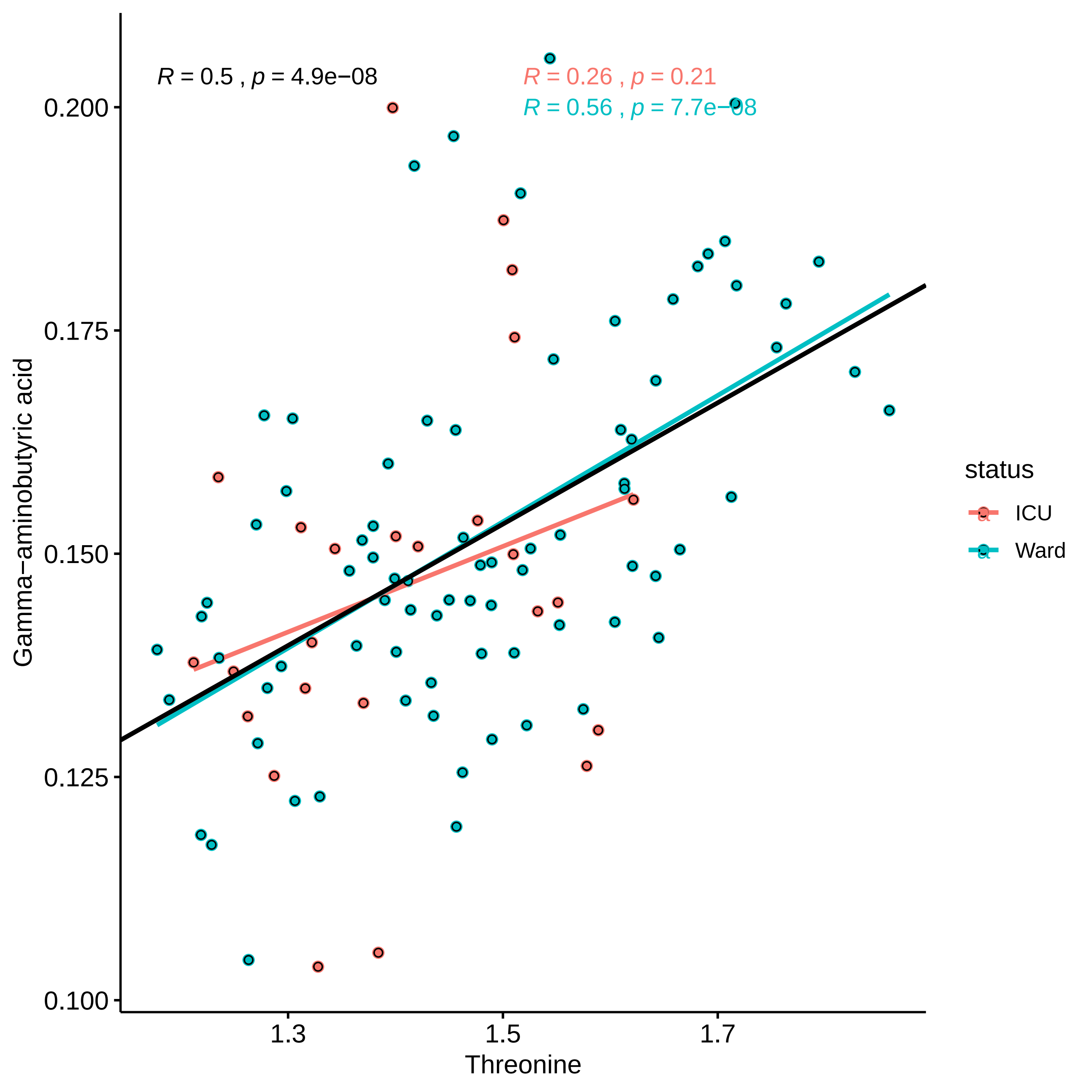

R = 0.5 , p = 4.9e−08
R = 0.26 , p = 0.21
R = 0.56 , p = 7.7e−08
Gamma−aminobutyric acid
0.200
0.175
0.150
0.125
0.100
1.3
1.5
1.7
Threonine
status
ICU
Ward

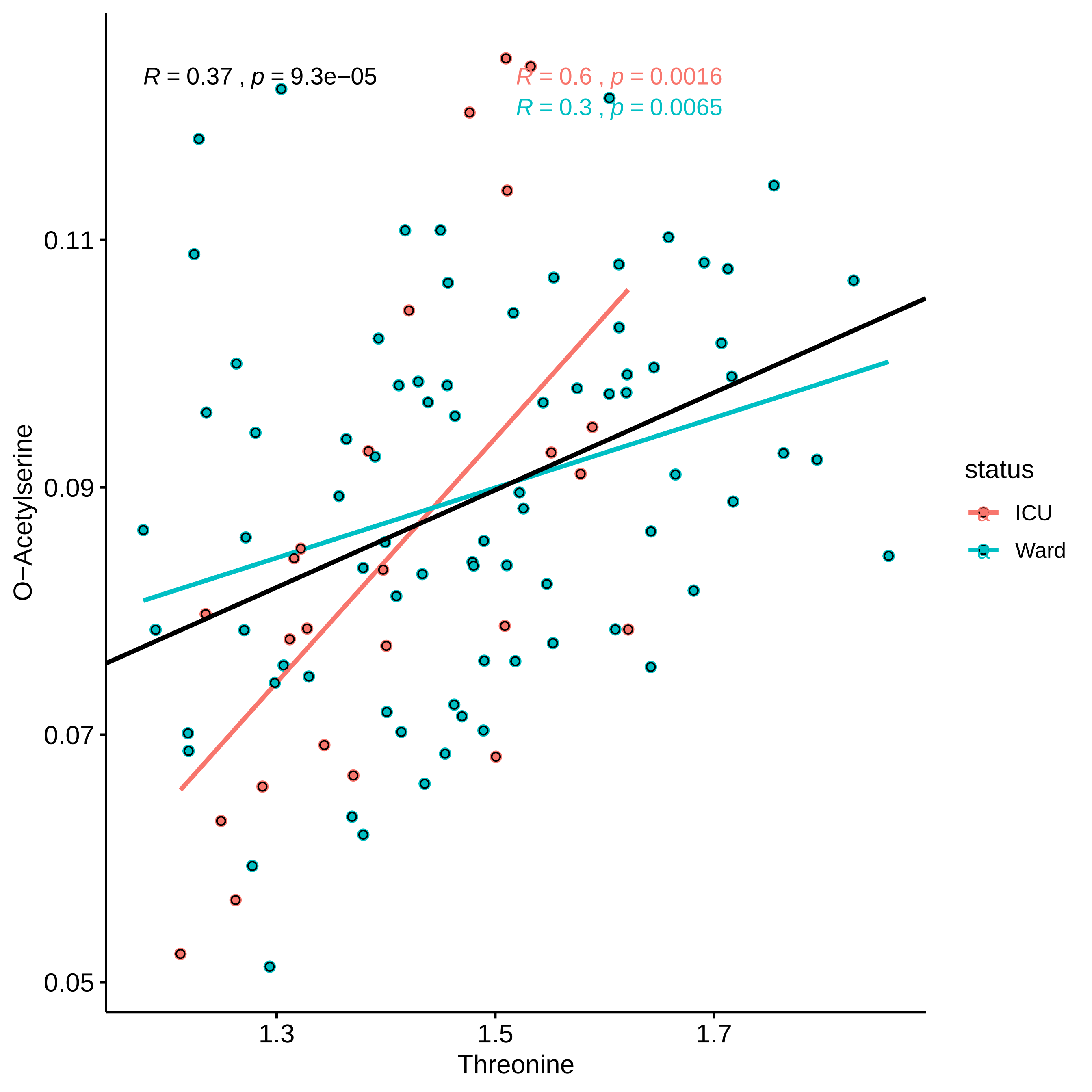

R = 0.37 , p = 9.3e−05
R = 0.6 , p = 0.0016
R = 0.3 , p = 0.0065
O−Acetylserine
0.11
0.09
0.07
0.05
1.3
1.5
1.7
Threonine
status
ICU
Ward

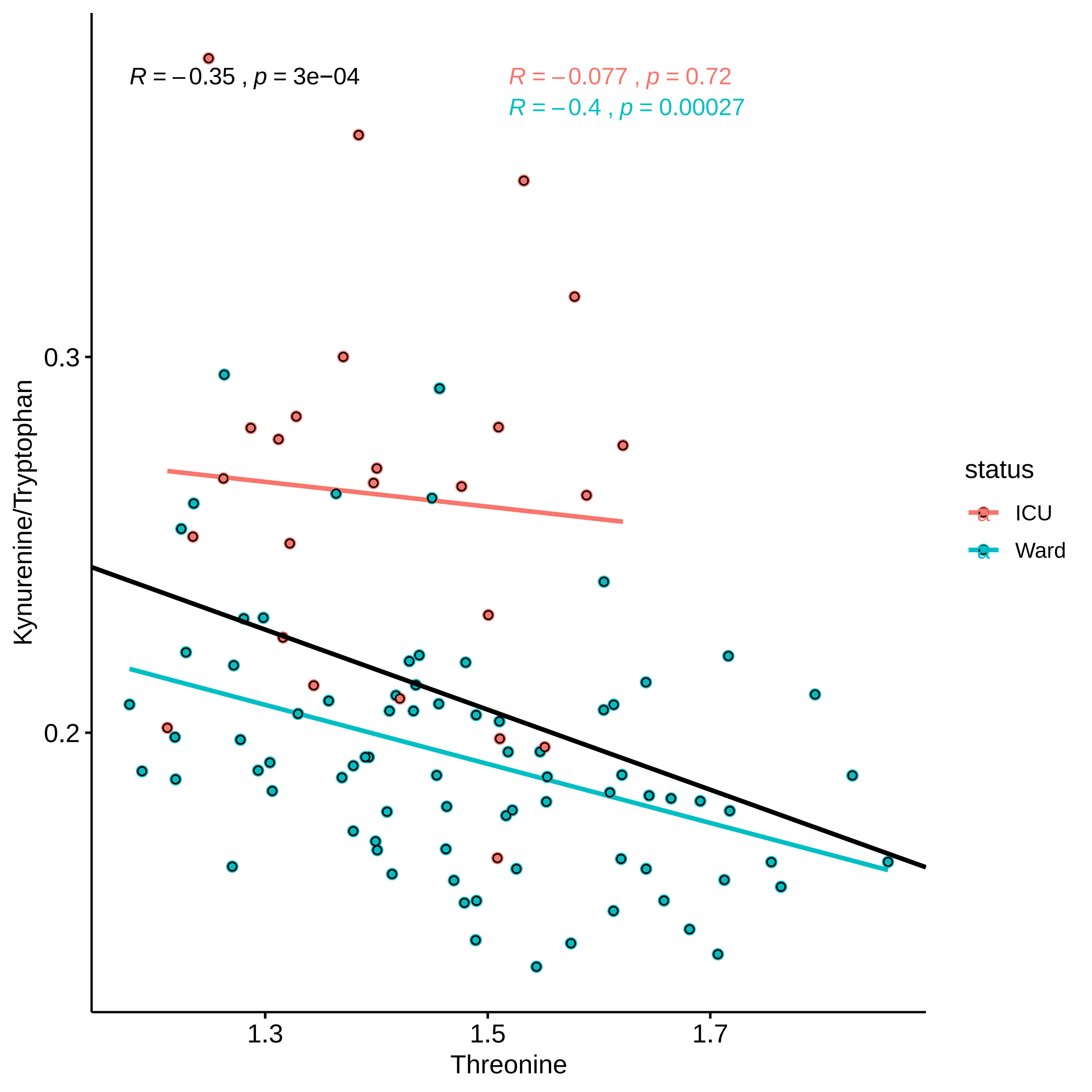

R = −0.35 , p = 3e−04
R = −0.077 , p = 0.72
R = −0.4 , p = 0.00027
Kynurenine/Tryptophan
0.3
0.2
1.3
1.5
1.7
Threonine
status
ICU
Ward

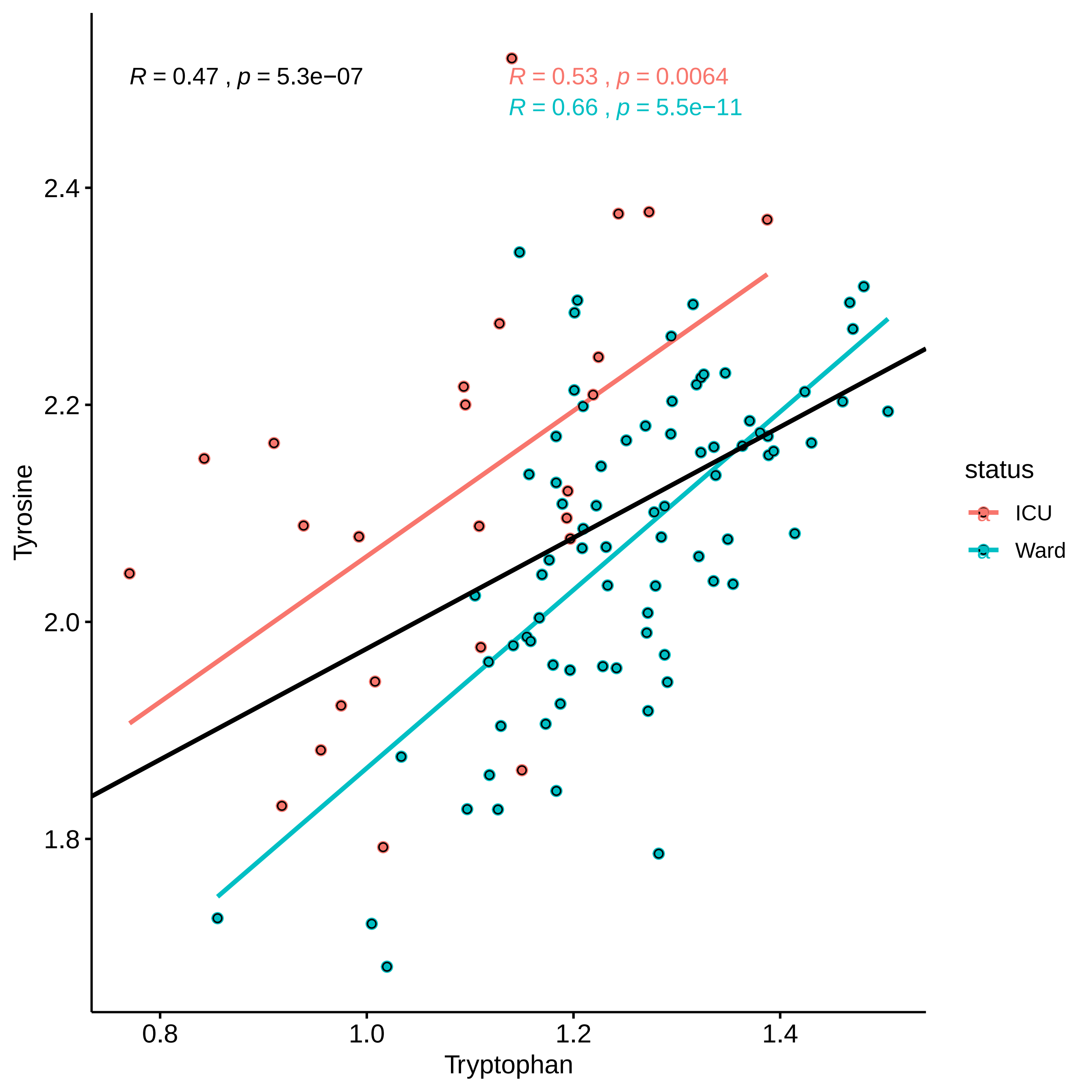

R = 0.47 , p = 5.3e−07
R = 0.53 , p = 0.0064
R = 0.66 , p = 5.5e−11
2.4
2.2
2.0
1.8
Tyrosine
0.8
1.0
1.2
1.4
Tryptophan
status
ICU
Ward

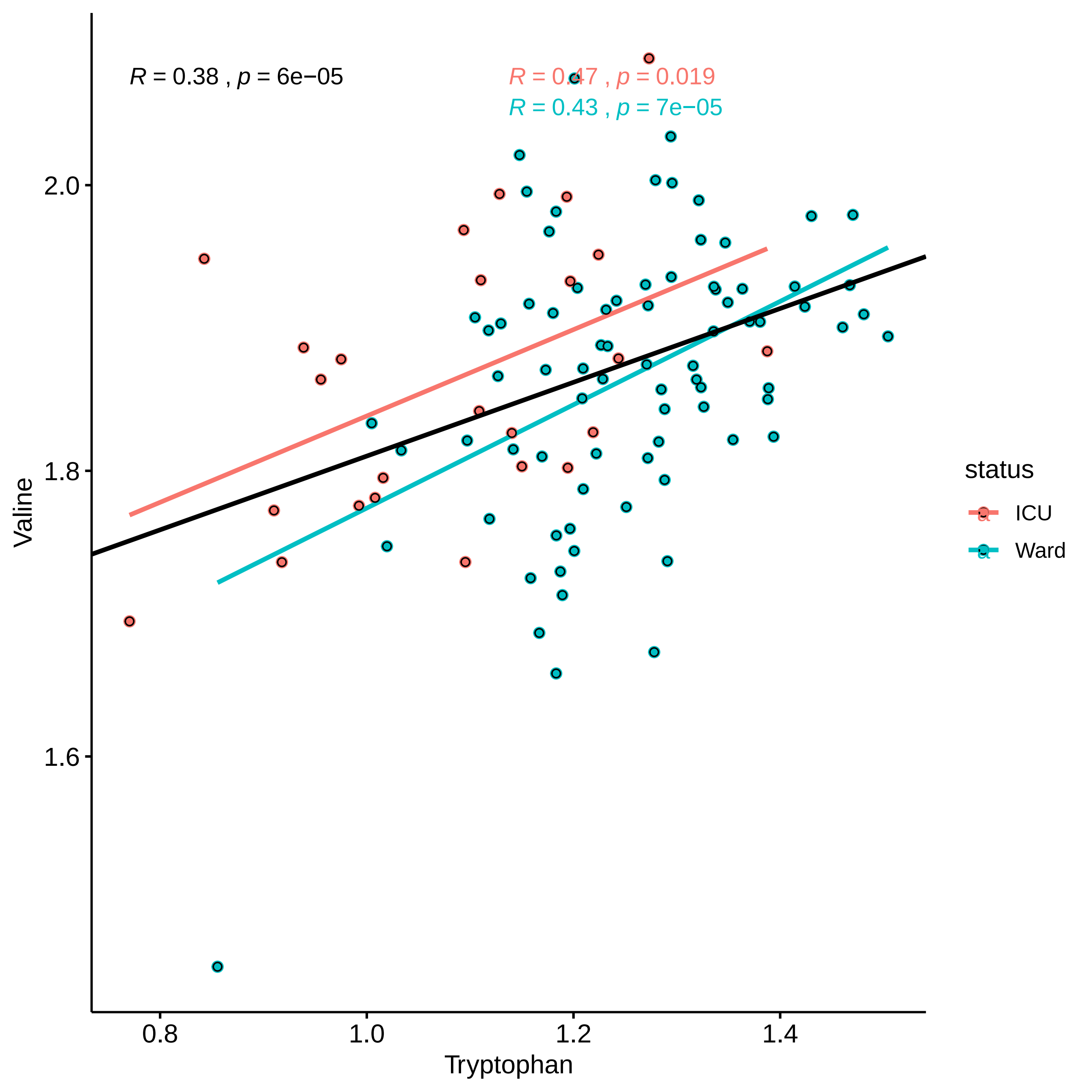

R = 0.38 , p = 6e−05
R = 0.47 , p = 0.019
R = 0.43 , p = 7e−05
Valine
2.0
1.8
1.6
0.8
1.0
1.2
1.4
Tryptophan
status
ICU
Ward

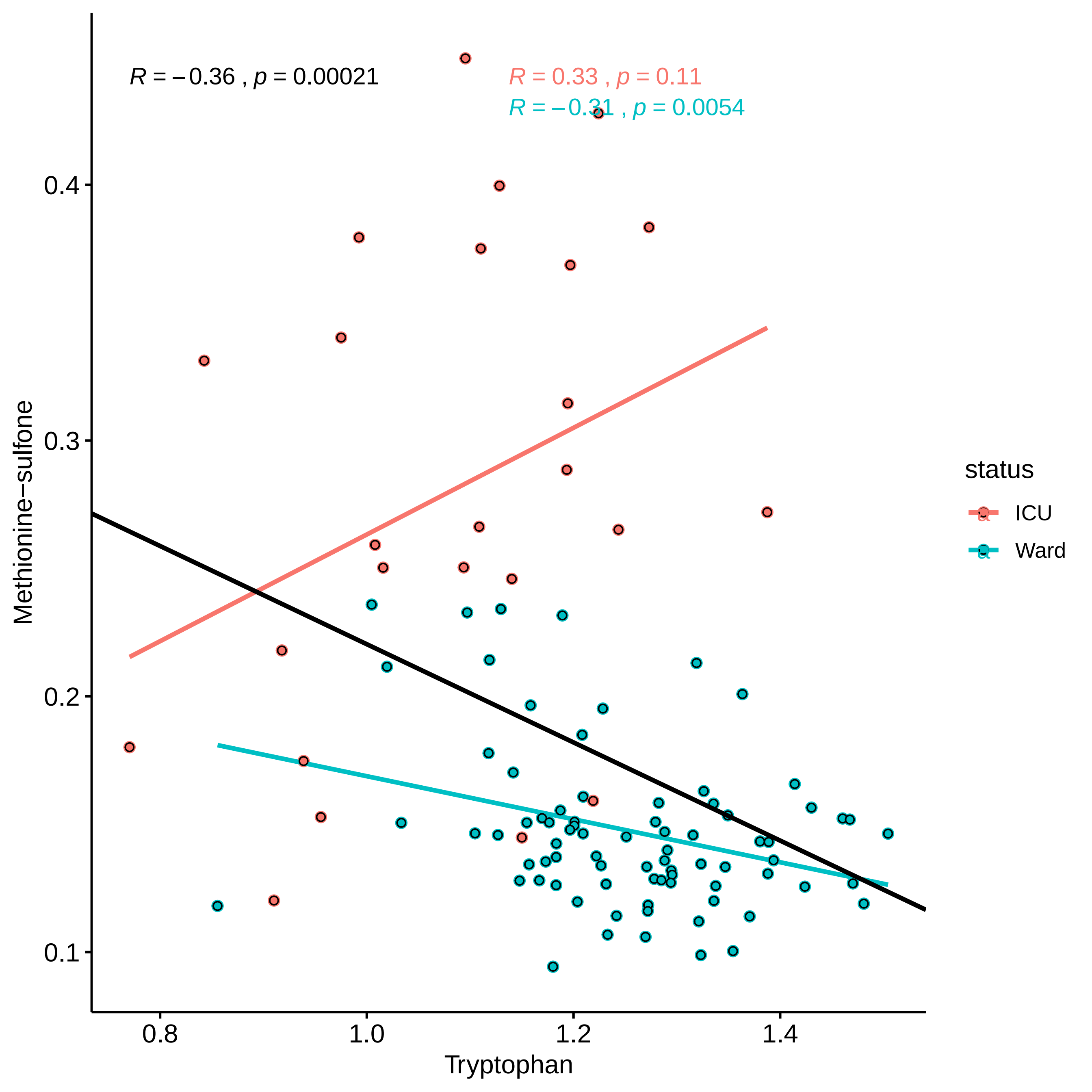
R = −0.36 , p = 0.00021
R = 0.33 , p = 0.11
R = −0.31 , p = 0.0054
Methionine−sulfone
0.4
0.3
0.2
0.1
0.8
1.0
1.2
1.4
Tryptophan
status
ICU
Ward

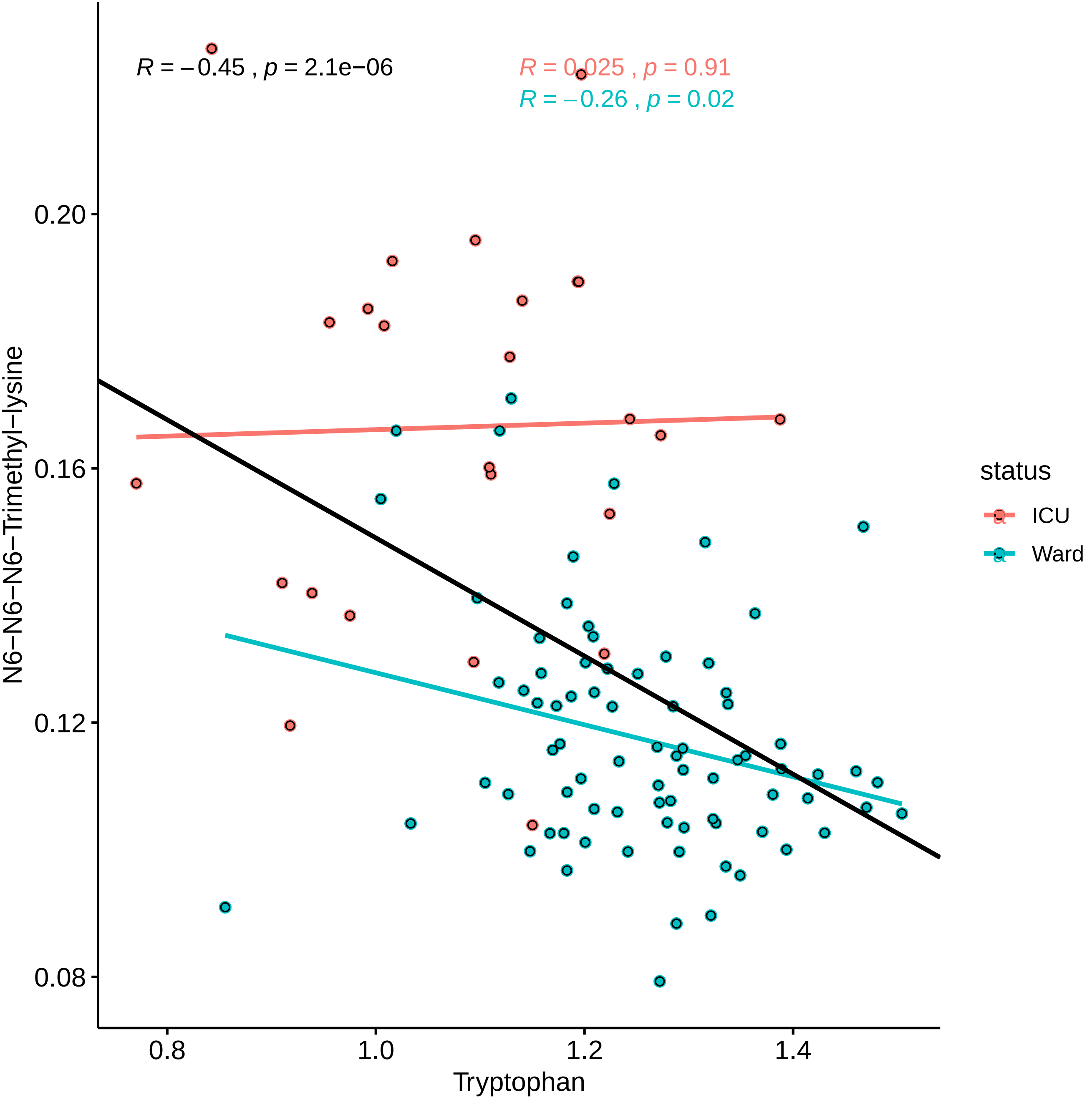

R = −0.45 , p = 2.1e−06
R = 0.025 , p = 0.91
R = −0.26 , p = 0.02
N6−N6−N6−Trimethyl−lysine
0.20
0.16
0.12
0.08
0.8
1.0
1.2
1.4
Tryptophan
status
ICU
Ward

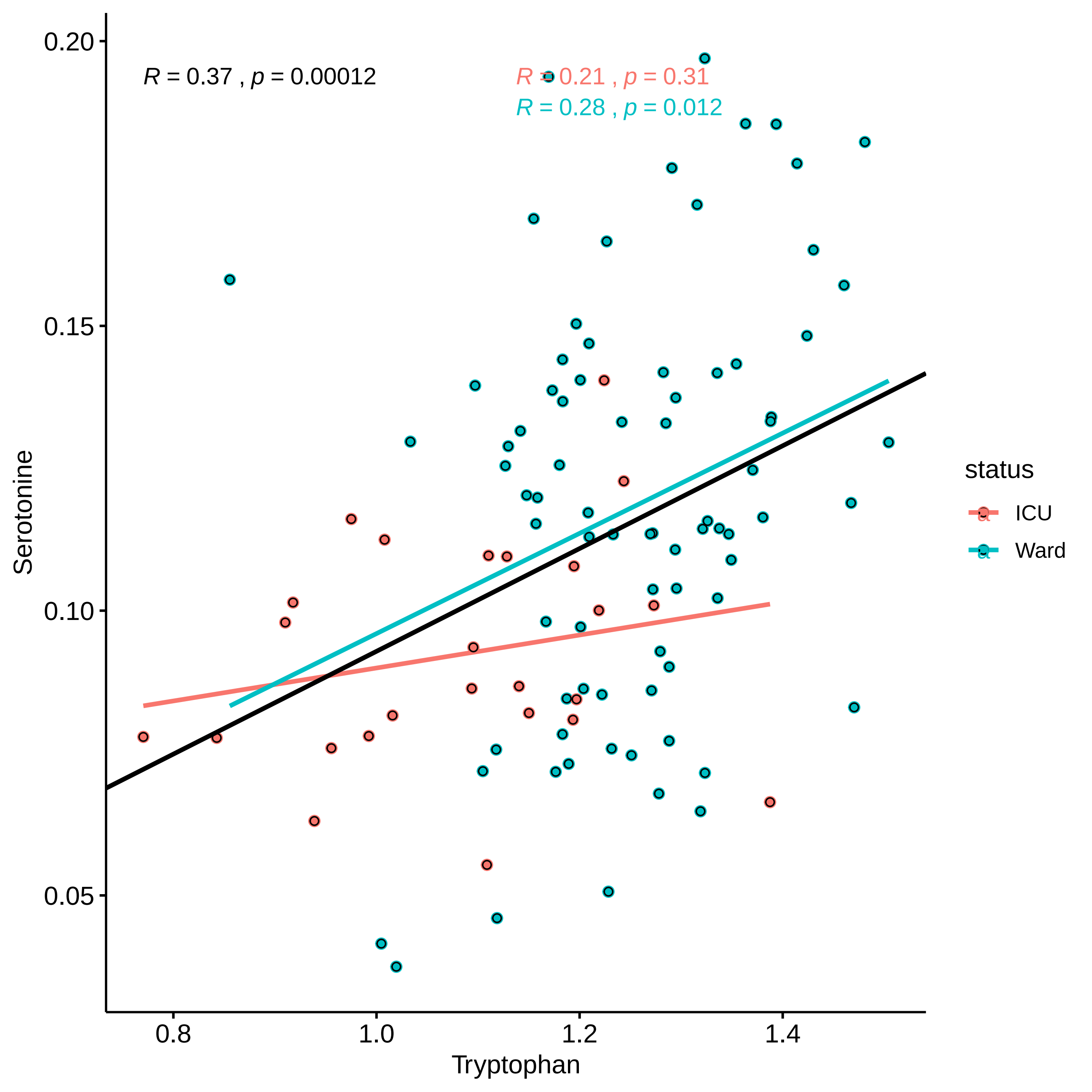

R = 0.37 , p = 0.00012
R = 0.21 , p = 0.31
R = 0.28 , p = 0.012
Serotonine
0.20
0.15
0.10
0.05
0.8
1.0
1.2
1.4
Tryptophan
status
ICU
Ward

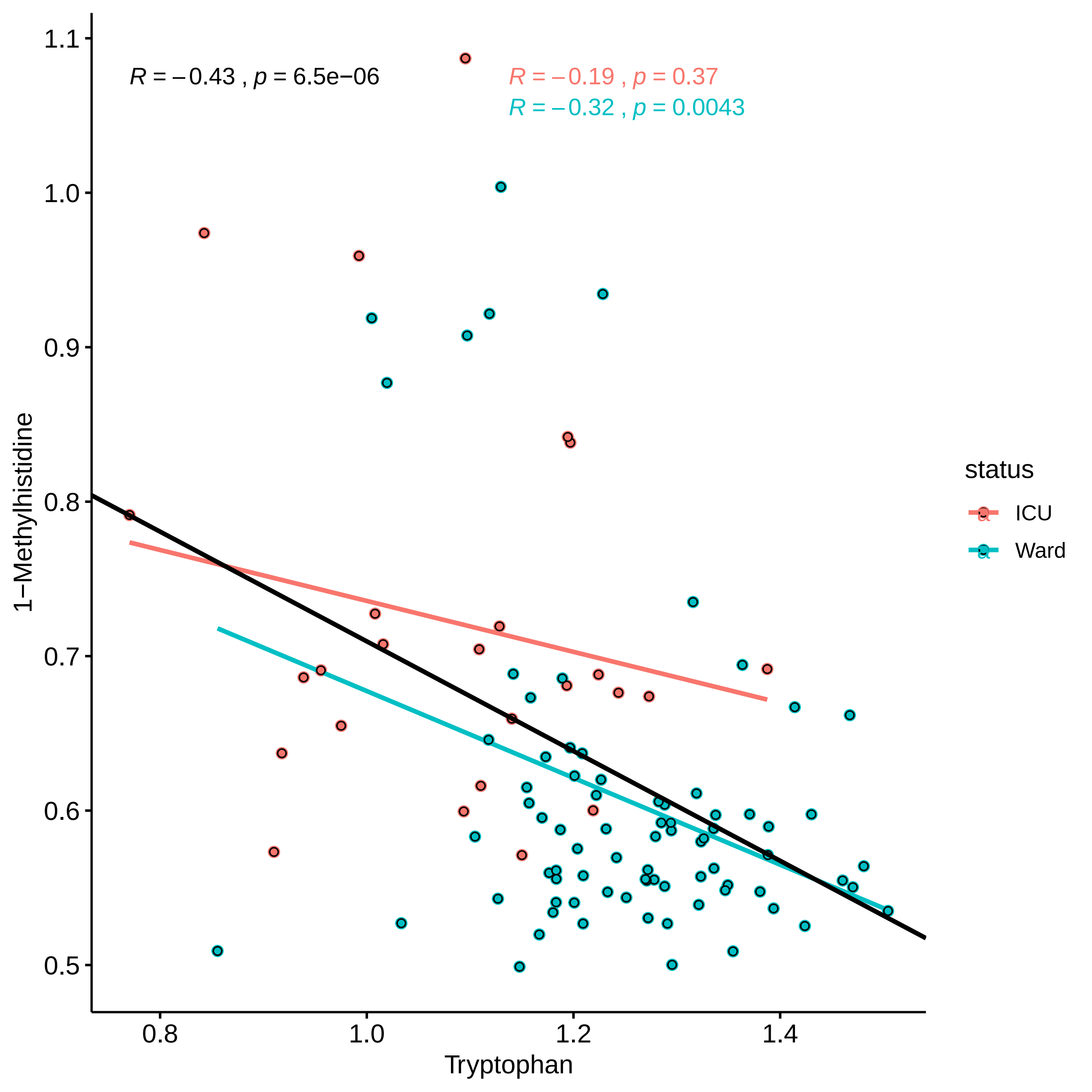

R = −0.43 , p = 6.5e−06
R = −0.19 , p = 0.37
R = −0.32 , p = 0.0043
1−Methylhistidine
1.1
1.0
0.9
0.8
0.7
0.6
0.5
0.8
1.0
1.2
1.4
Tryptophan
status
ICU
Ward

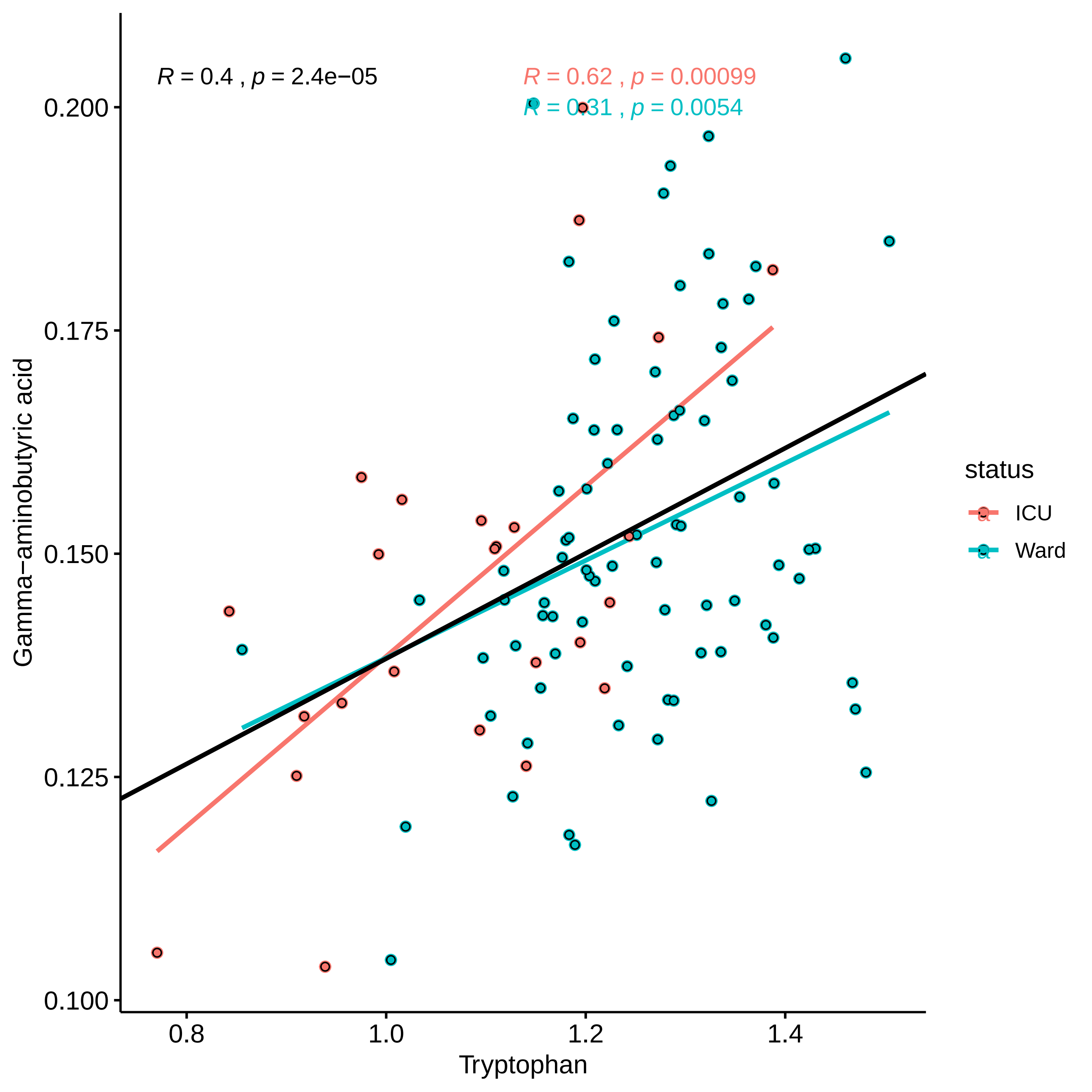

R = 0.4 , p = 2.4e−05
R = 0.62 , p = 0.00099
R = 0.31 , p = 0.0054
Gamma−aminobutyric acid
0.200
0.175
0.150
0.125
0.100
0.8
1.0
1.2
1.4
Tryptophan
status
ICU
Ward

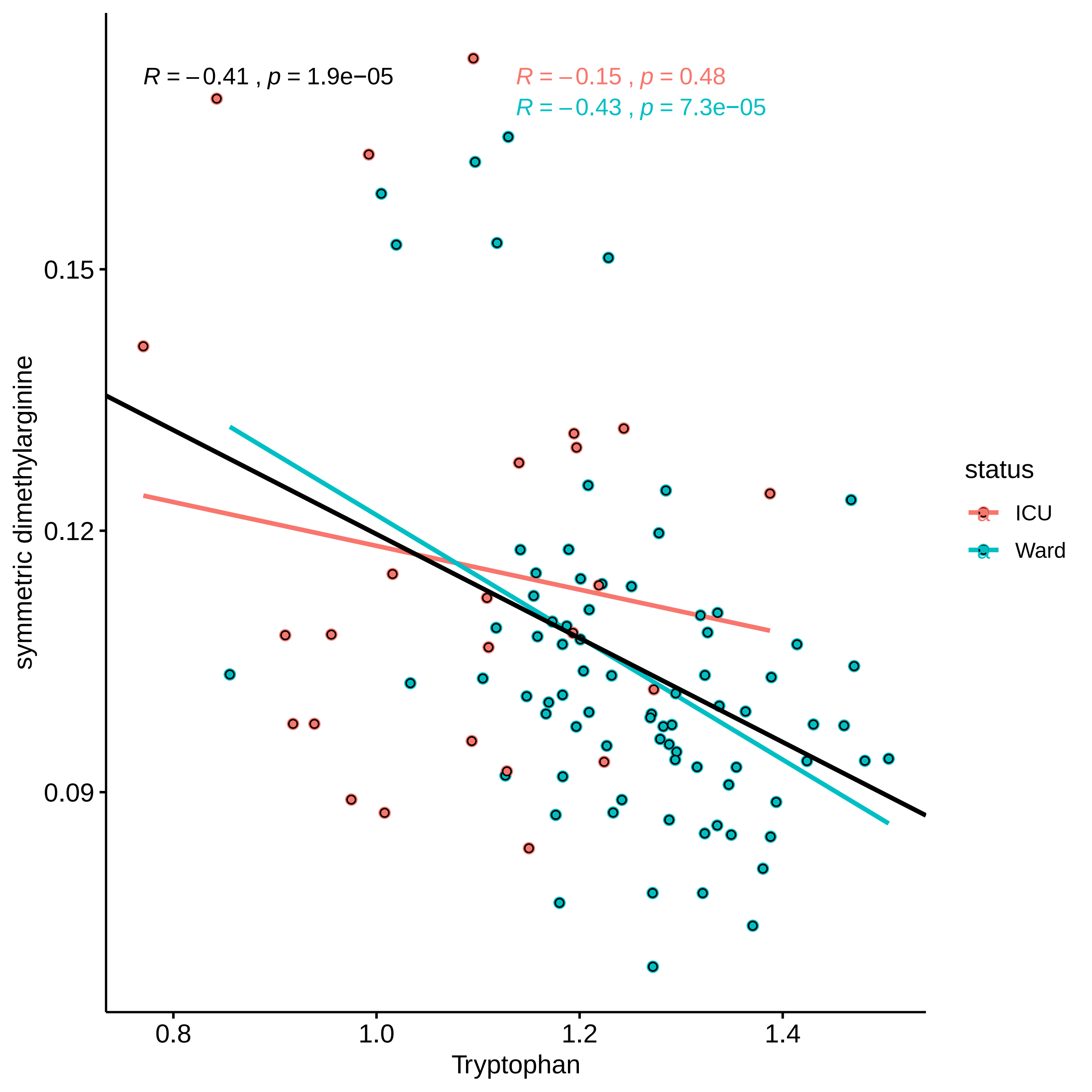

R = −0.41 , p = 1.9e−05
R = −0.15 , p = 0.48
R = −0.43 , p = 7.3e−05
0.15
0.12
0.09
symmetric dimethylarginine
0.8
1.0
1.2
1.4
Tryptophan
status
ICU
Ward

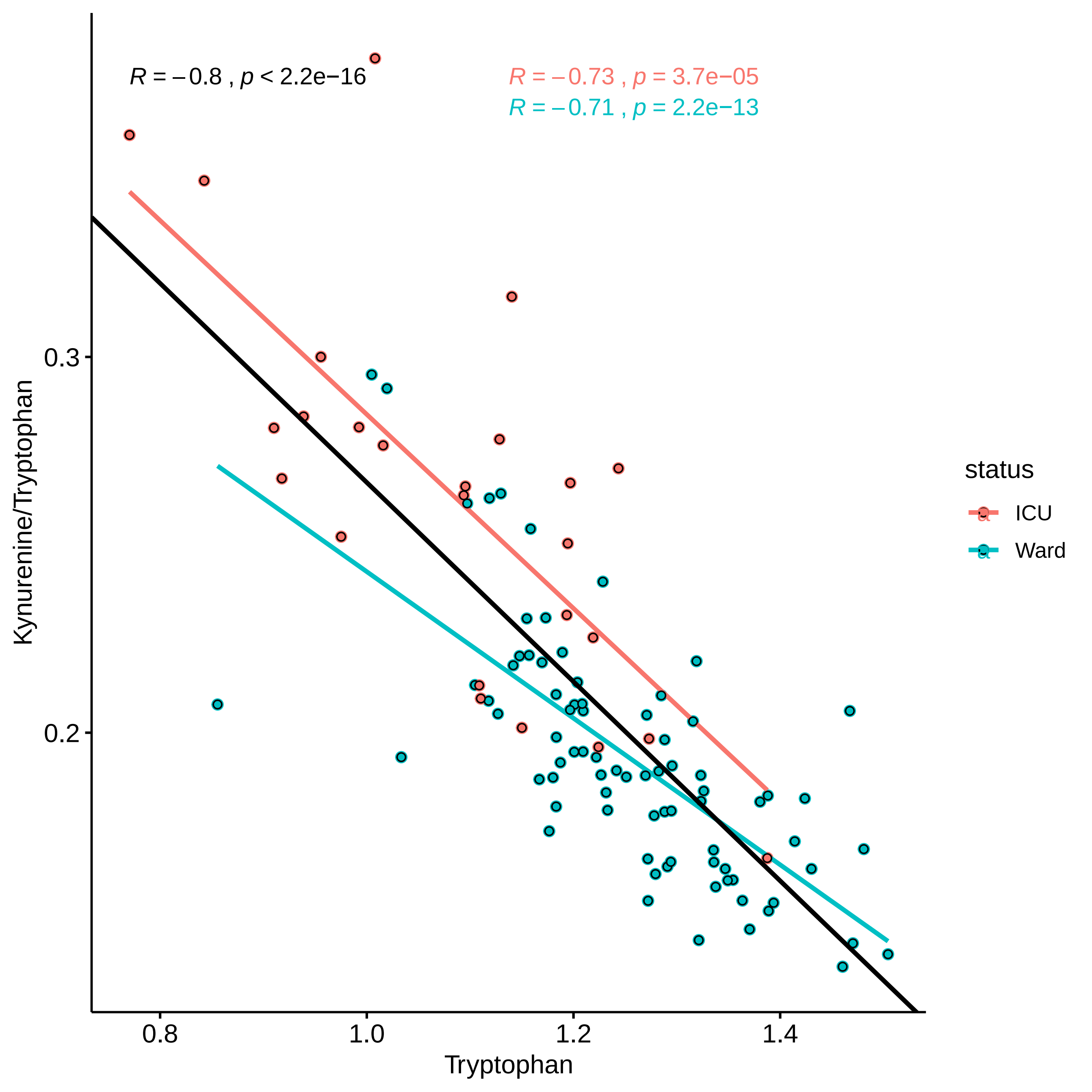

R = −0.8 , p < 2.2e−16
R = −0.73 , p = 3.7e−05
R = −0.71 , p = 2.2e−13
Kynurenine/Tryptophan
0.3
0.2
0.8
1.0
1.2
1.4
Tryptophan
status
ICU
Ward

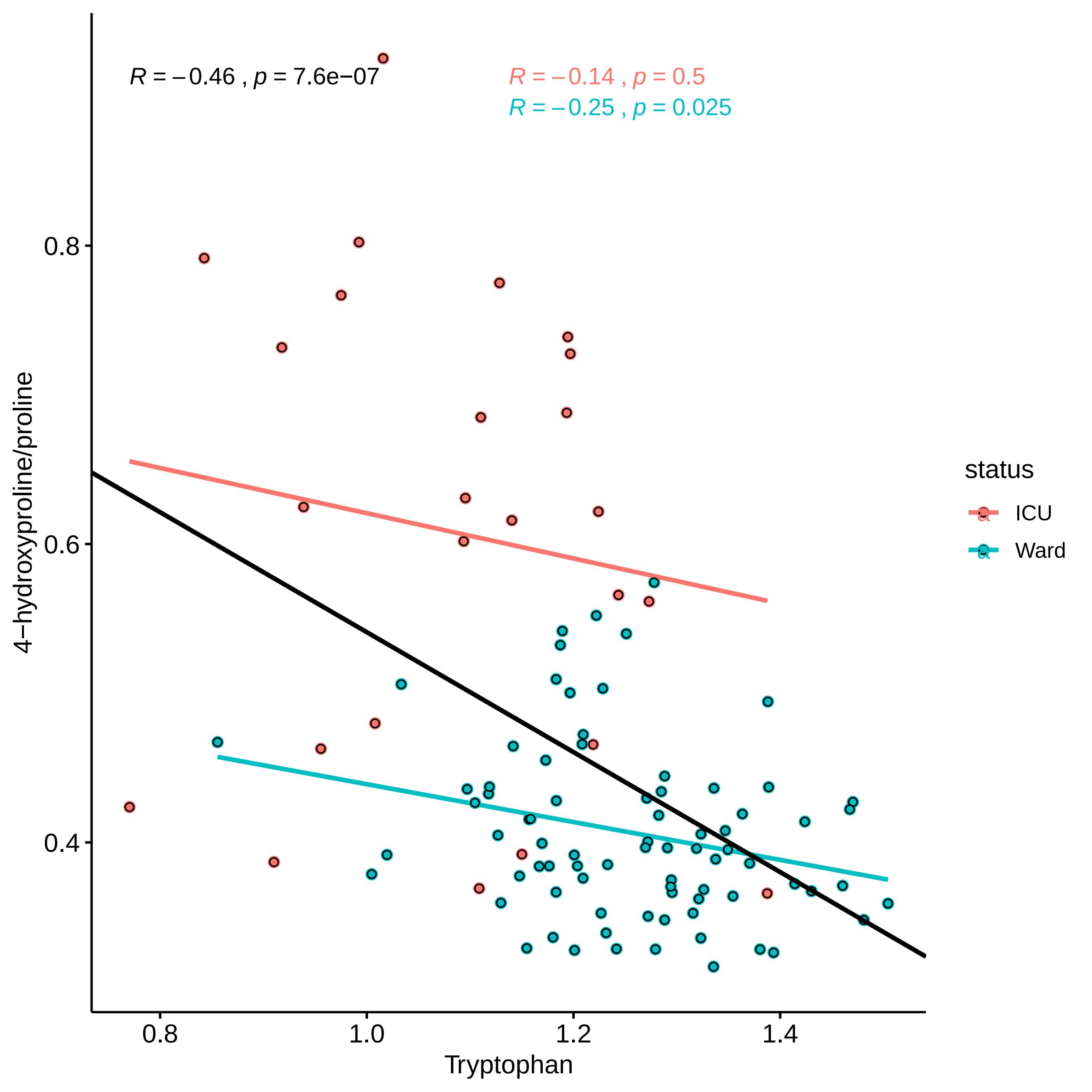
R = −0.46 , p = 7.6e−07
R = −0.14 , p = 0.5
R = −0.25 , p = 0.025
4−hydroxyproline/proline
0.8
0.6
0.4
0.8
1.0
1.2
1.4
Tryptophan
status
ICU
Ward

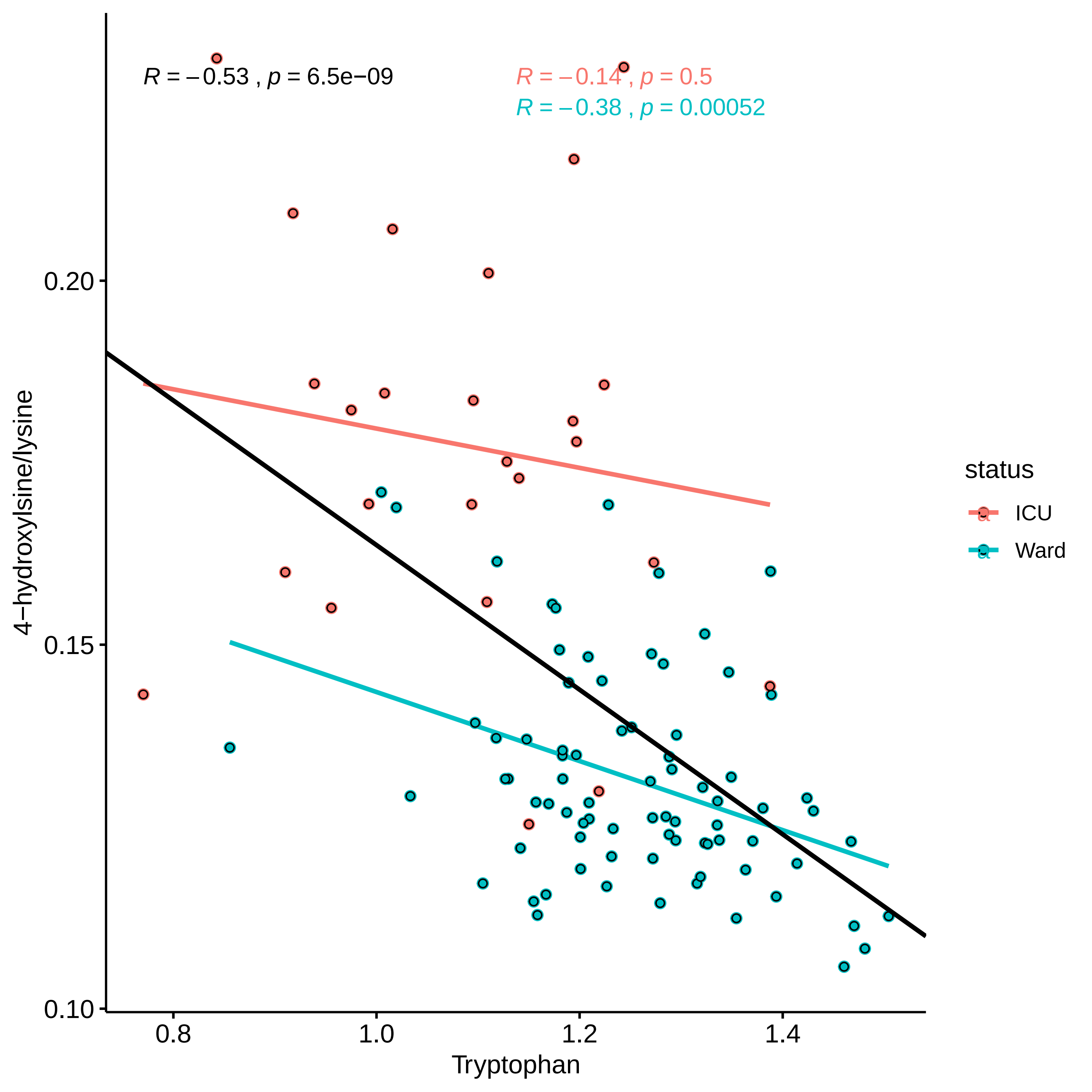

R = −0.53 , p = 6.5e−09
R = −0.14 , p = 0.5
R = −0.38 , p = 0.00052
4−hydroxylsine/lysine
0.20
0.15
0.10
0.8
1.0
1.2
1.4
Tryptophan
status
ICU
Ward

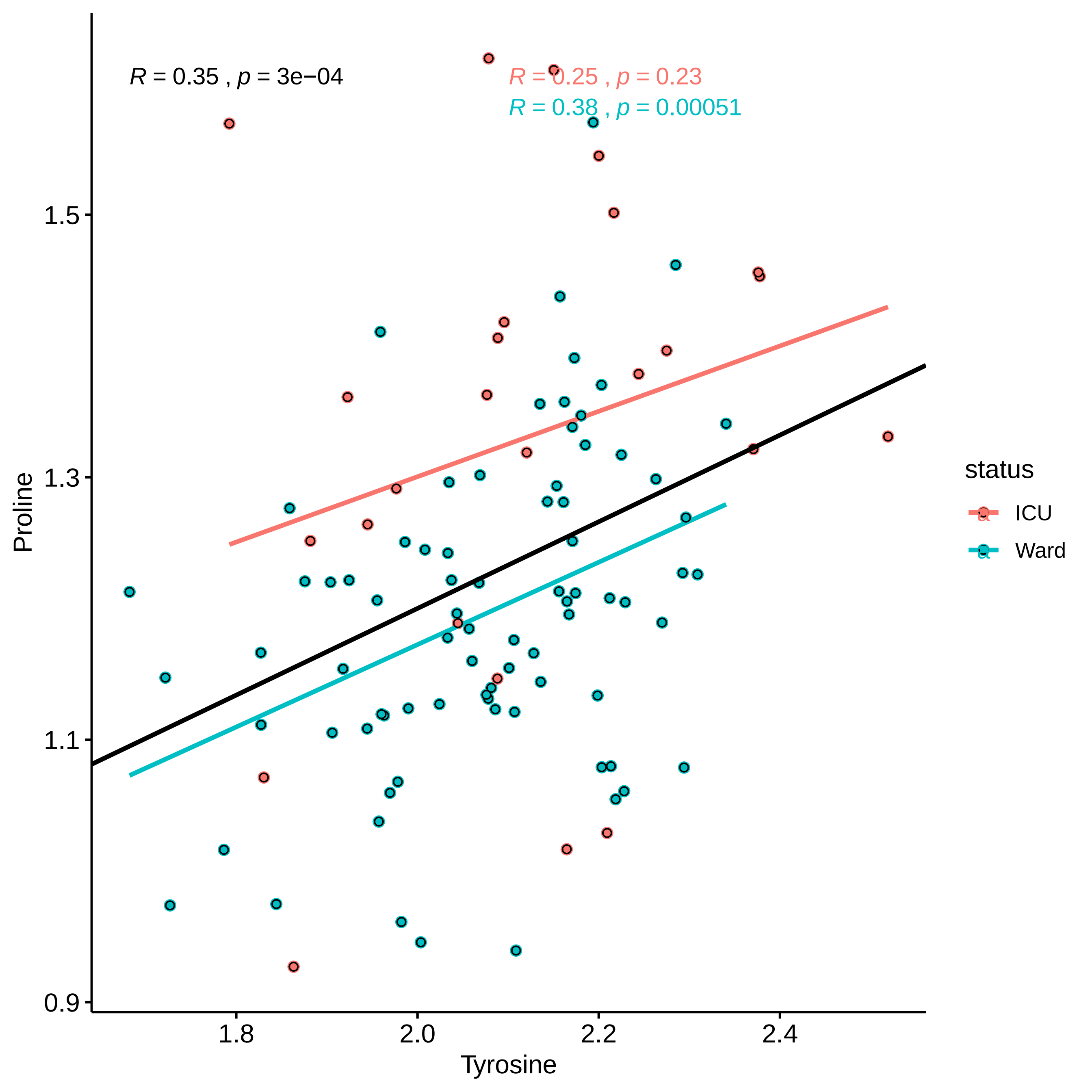

R = 0.35 , p = 3e−04
R = 0.25 , p = 0.23
R = 0.38 , p = 0.00051
Proline
1.5
1.3
1.1
0.9
1.8
2.0
2.2
2.4
Tyrosine
status
ICU
Ward

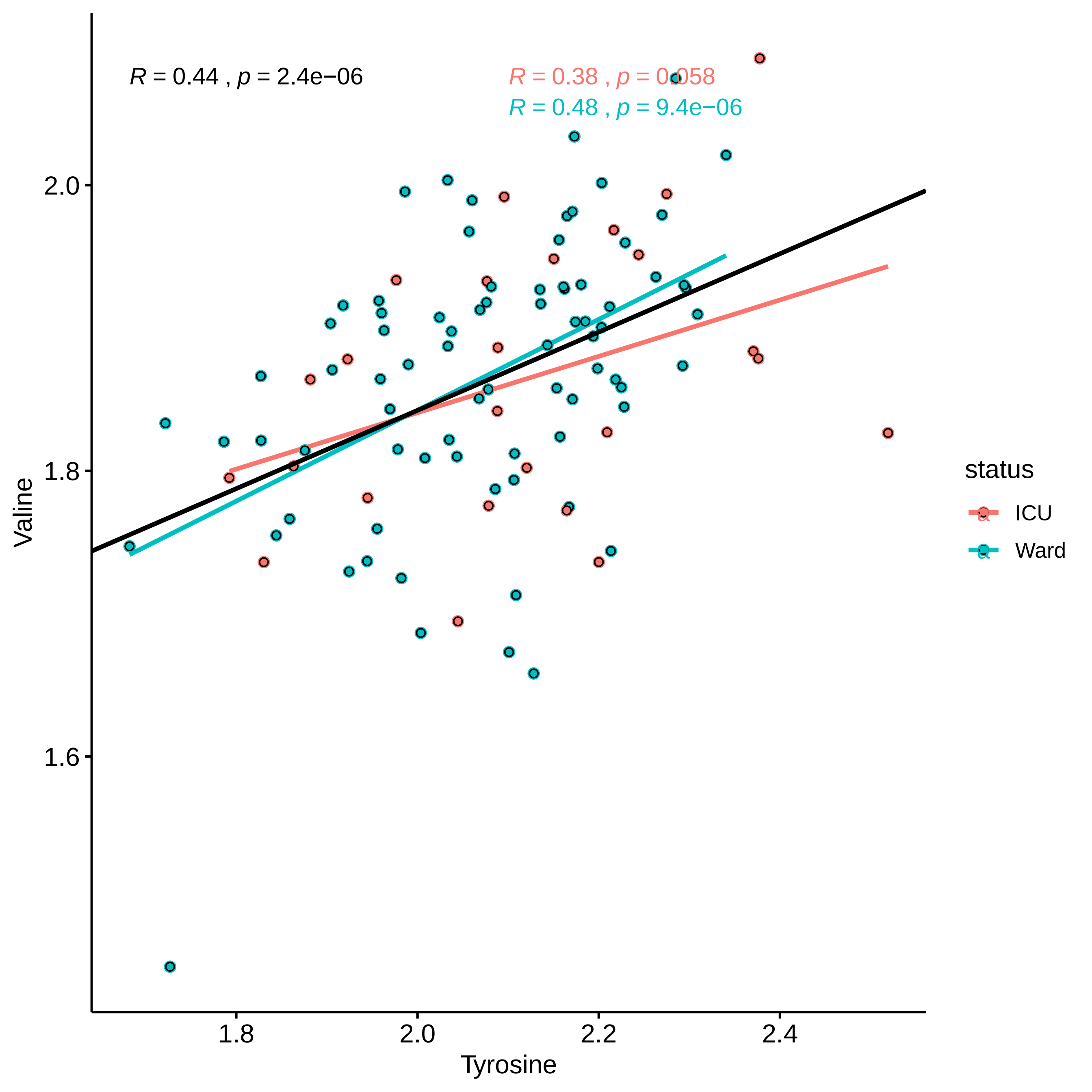

R = 0.44 , p = 2.4e−06
R = 0.38 , p = 0.058
R = 0.48 , p = 9.4e−06
2.0
1.8
1.6
Valine
1.8
2.0
2.2
2.4
Tyrosine
status
ICU
Ward

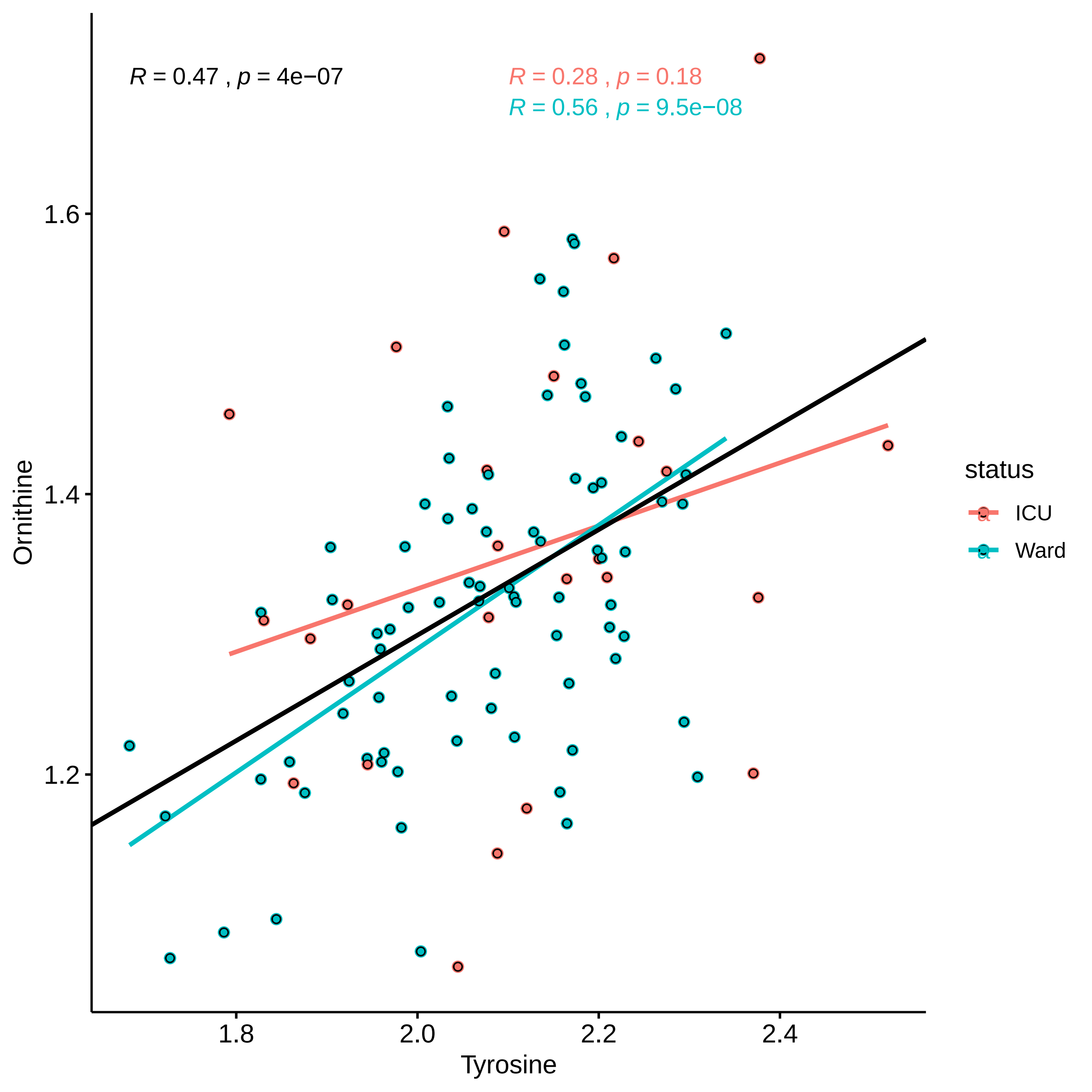

R = 0.47 , p = 4e−07
R = 0.28 , p = 0.18
R = 0.56 , p = 9.5e−08
Ornithine
Tyrosine
1.2
1.4
1.6
1.8
2.0
2.2
2.4
status
ICU
Ward

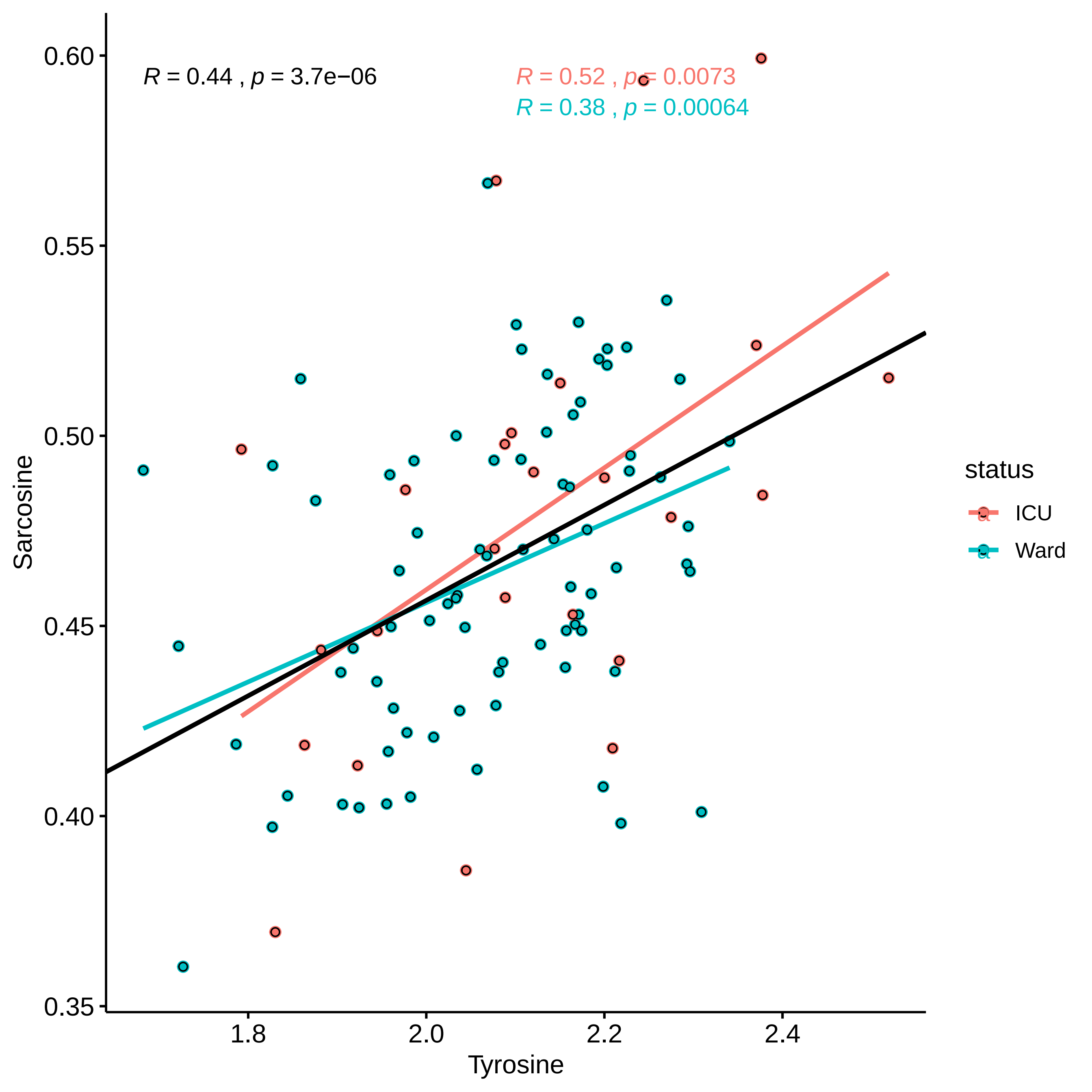

R = 0.44 , p = 3.7e−06
R = 0.52 , p = 0.0073
R = 0.38 , p = 0.00064
0.60
0.55
0.50
0.45
0.40
0.35
Sarcosine
1.8
2.0
2.2
2.4
Tyrosine
status
ICU
Ward

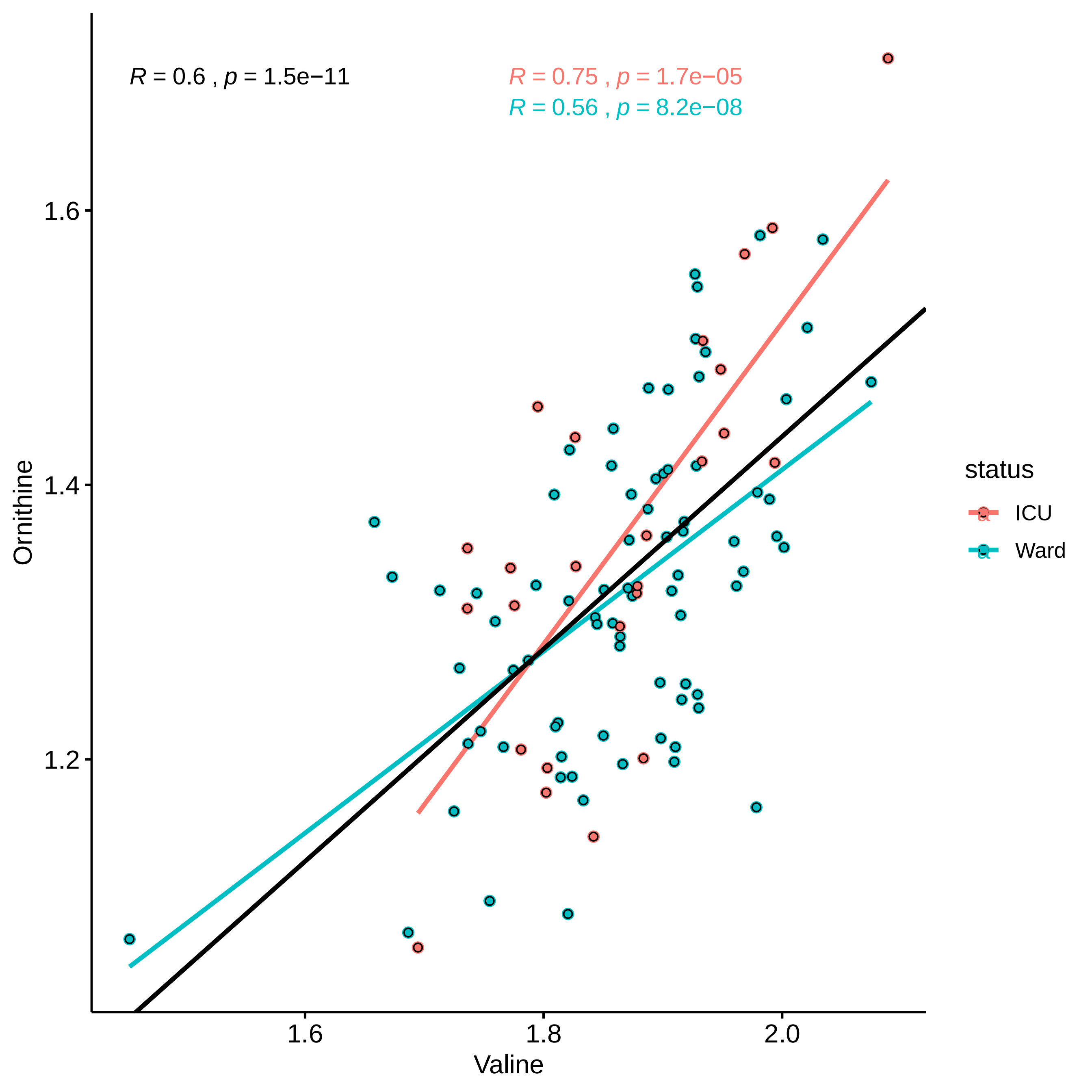

R = 0.6 , p = 1.5e−11
R = 0.75 , p = 1.7e−05
R = 0.56 , p = 8.2e−08
Ornithine
1.6
1.4
1.2
Valine
1.6
1.8
2.0
status
ICU
Ward

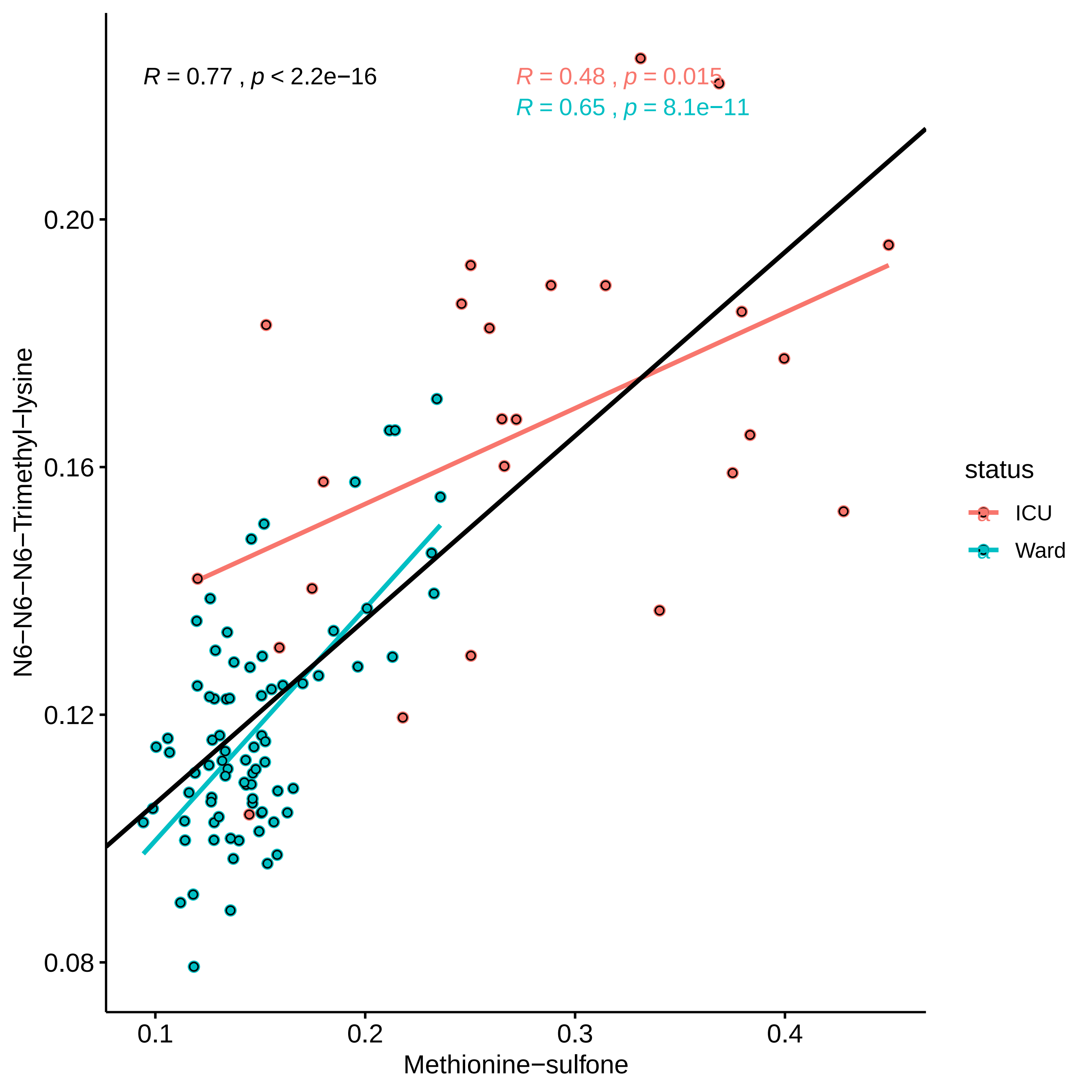

R = 0.77 , p < 2.2e−16
R = 0.48 , p = 0.015
R = 0.65 , p = 8.1e−11
N6−N6−N6−Trimethyl−lysine
0.20
0.16
0.12
0.08
0.1
0.2
0.3
0.4
Methionine−sulfone
status
ICU
Ward

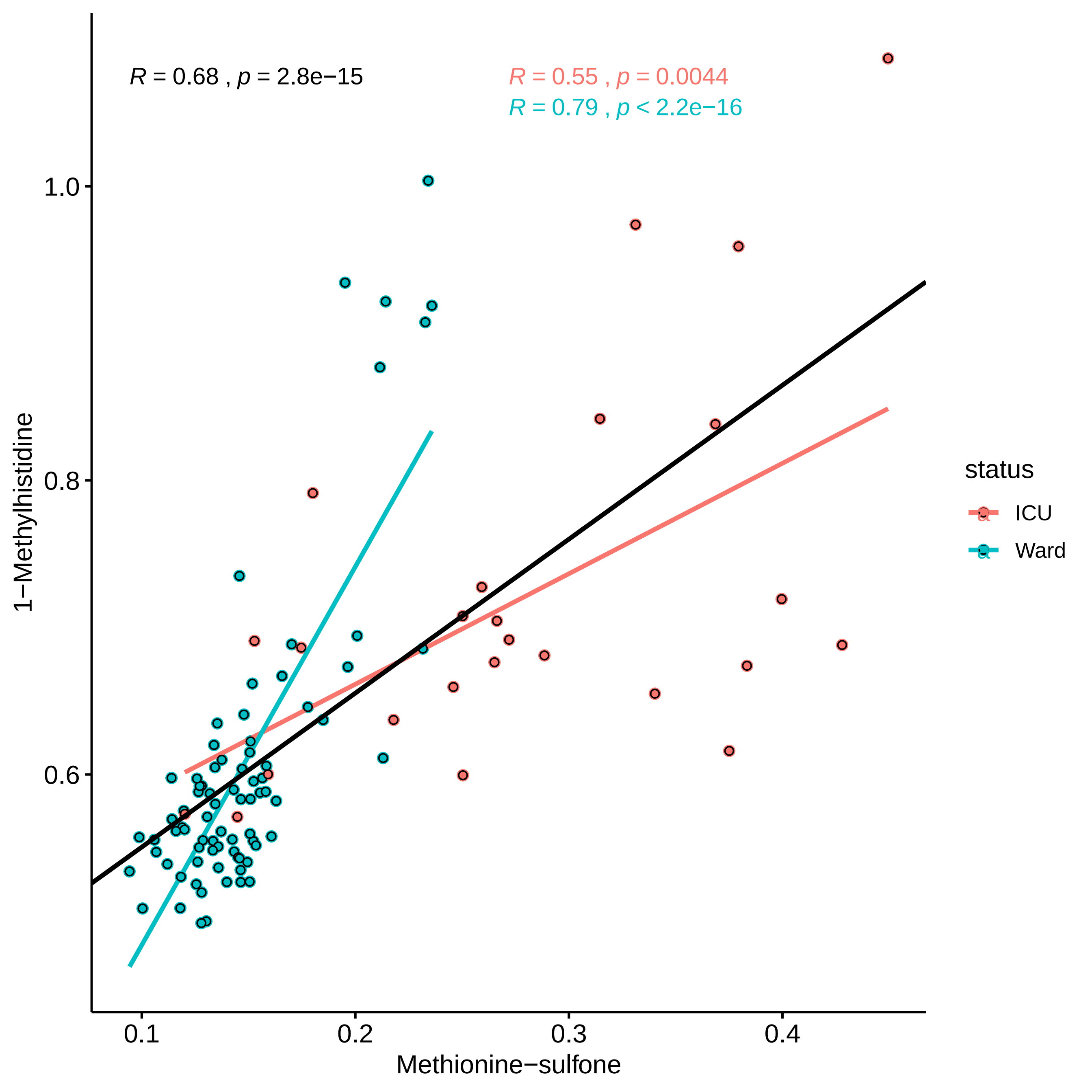

R = 0.68 , p = 2.8e−15
R = 0.55 , p = 0.0044
R = 0.79 , p < 2.2e−16
1.0
0.8
0.6
1−Methylhistidine
0.1
0.2
0.3
0.4
Methionine−sulfone
status
ICU
Ward

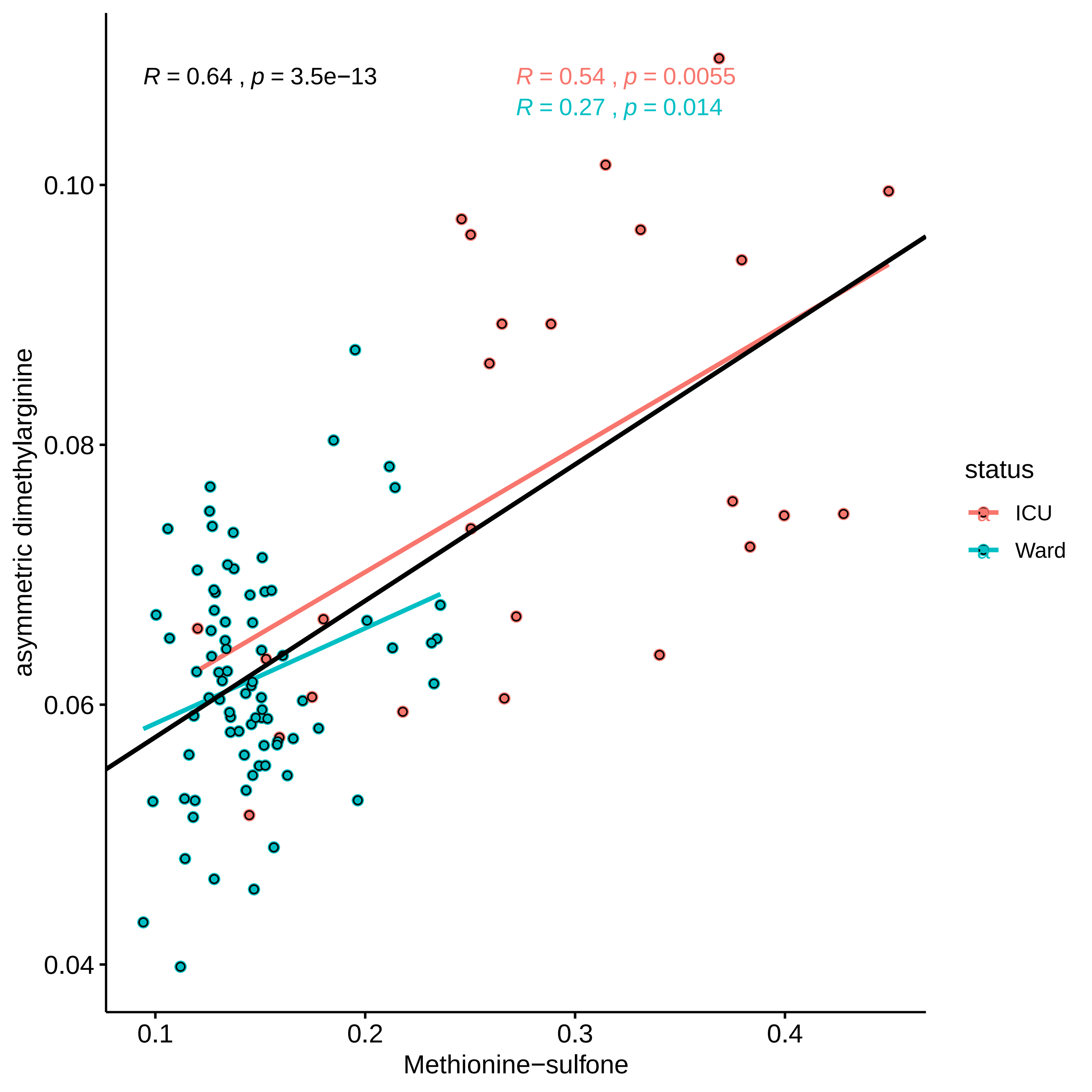

R = 0.64 , p = 3.5e−13
R = 0.54 , p = 0.0055
R = 0.27 , p = 0.014
0.10
0.08
0.06
0.04
asymmetric dimethylarginine
0.1
0.2
0.3
0.4
Methionine−sulfone
status
ICU
Ward

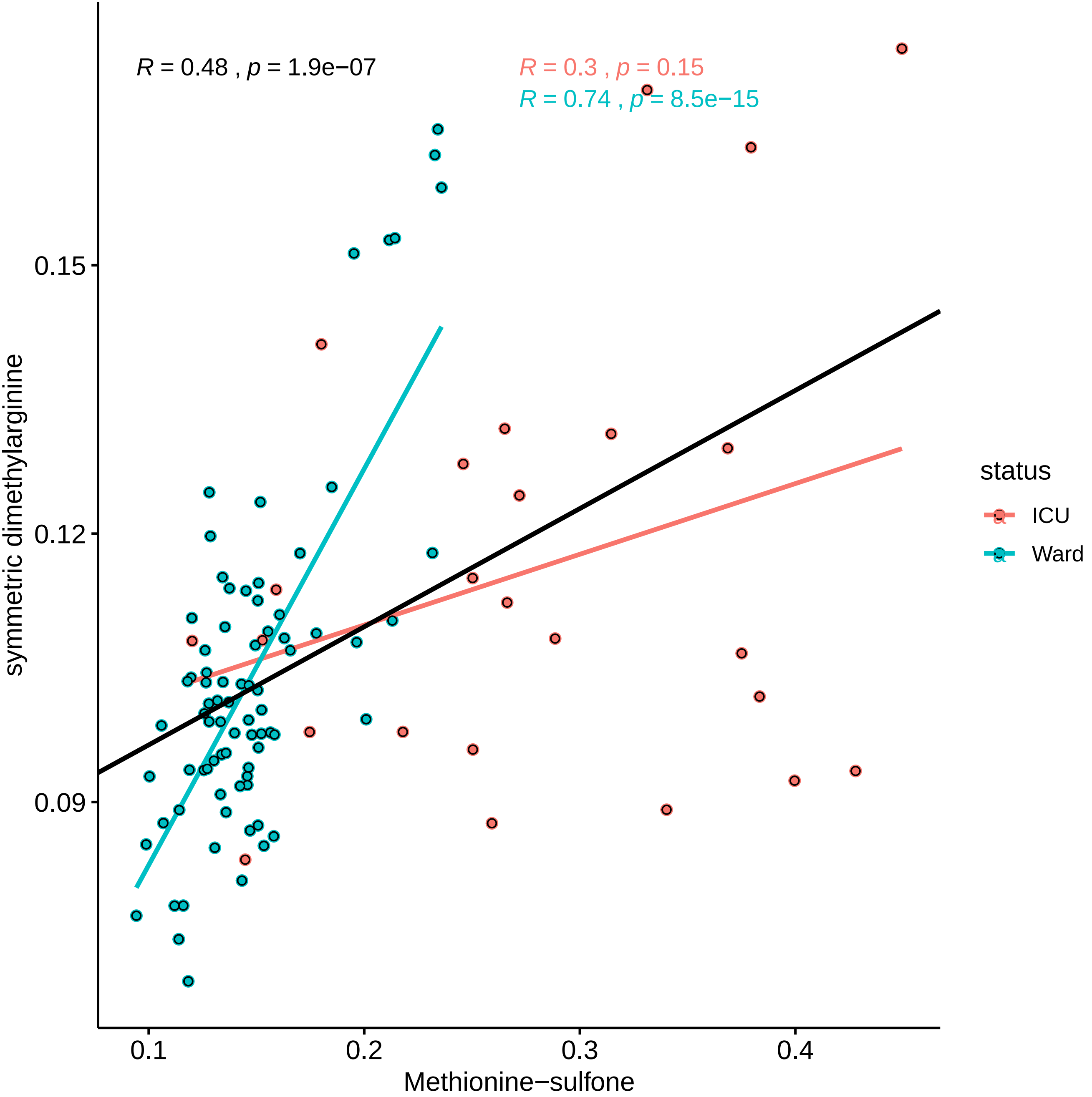

R = 0.48 , p = 1.9e−07
R = 0.3 , p = 0.15
R = 0.74 , p = 8.5e−15
0.15
0.12
0.09
symmetric dimethylarginine
0.1
0.2
0.3
0.4
Methionine−sulfone
status
ICU
Ward

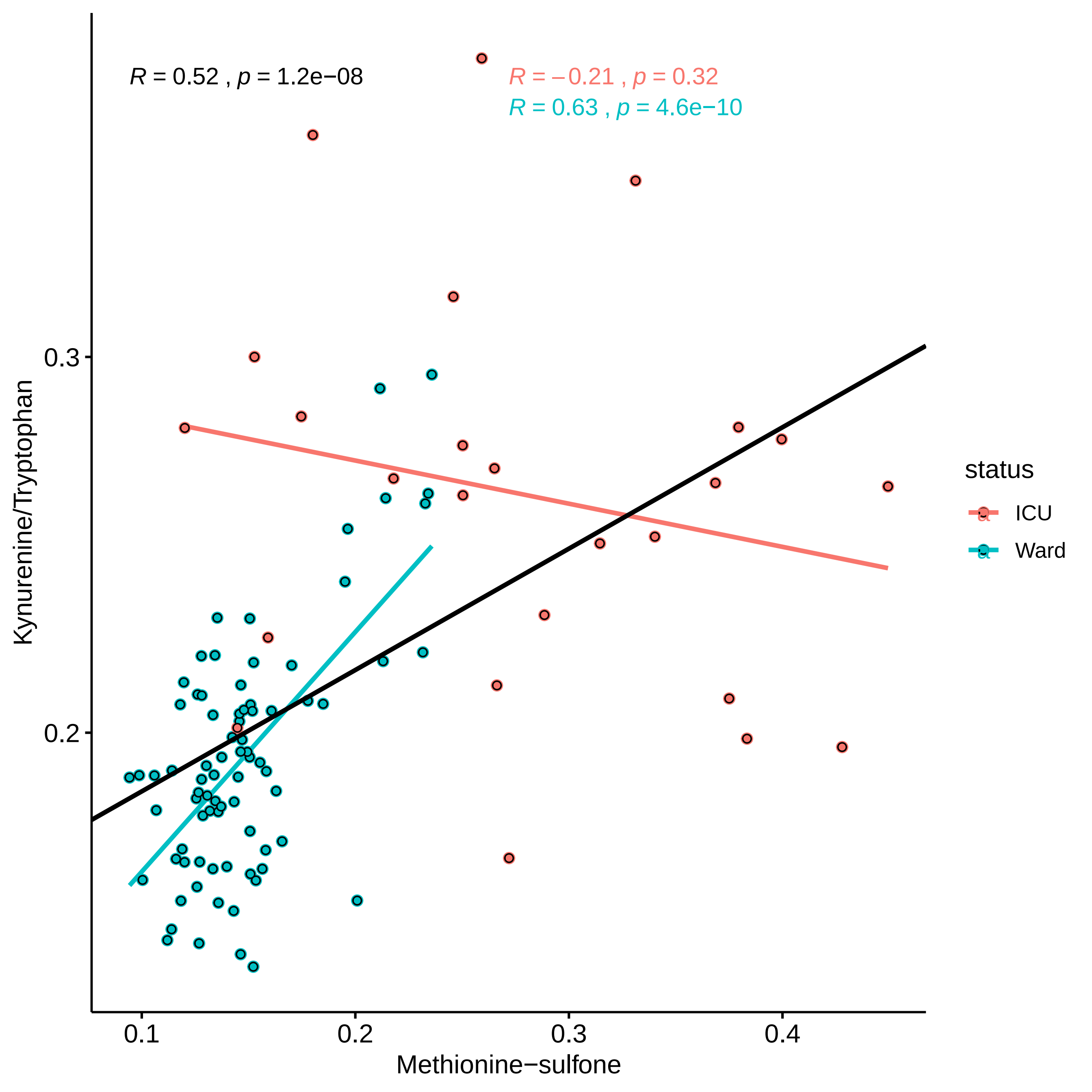

R = 0.52 , p = 1.2e−08
R = −0.21 , p = 0.32
R = 0.63 , p = 4.6e−10
0.3
0.2
Kynurenine/Tryptophan
0.1
0.2
0.3
0.4
Methionine−sulfone
status
ICU
Ward

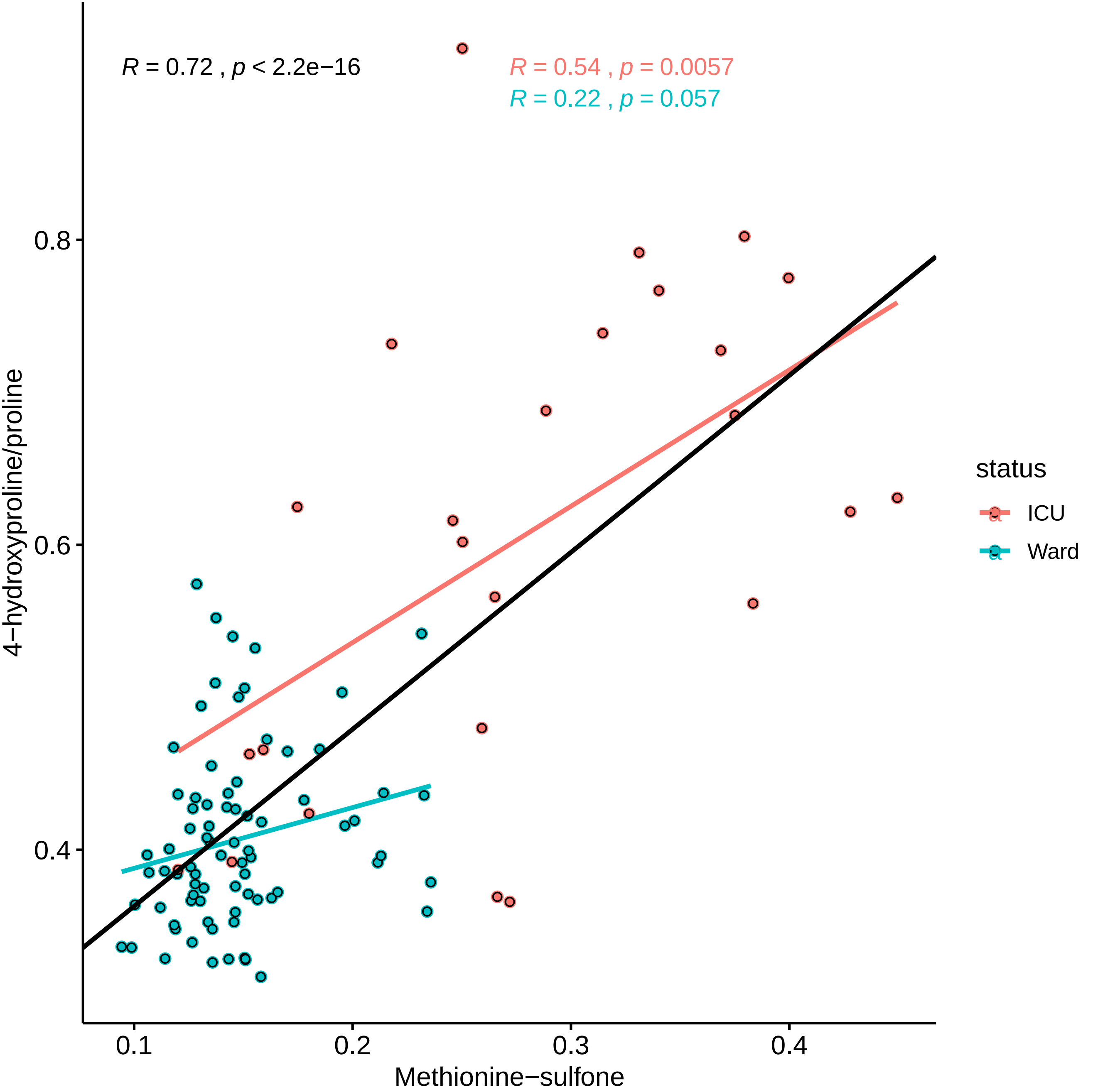

R = 0.72 , p < 2.2e−16
R = 0.54 , p = 0.0057
R = 0.22 , p = 0.057
0.8
0.6
0.4
4−hydroxyproline/proline
0.1
0.2
0.3
0.4
Methionine−sulfone
status
ICU
Ward

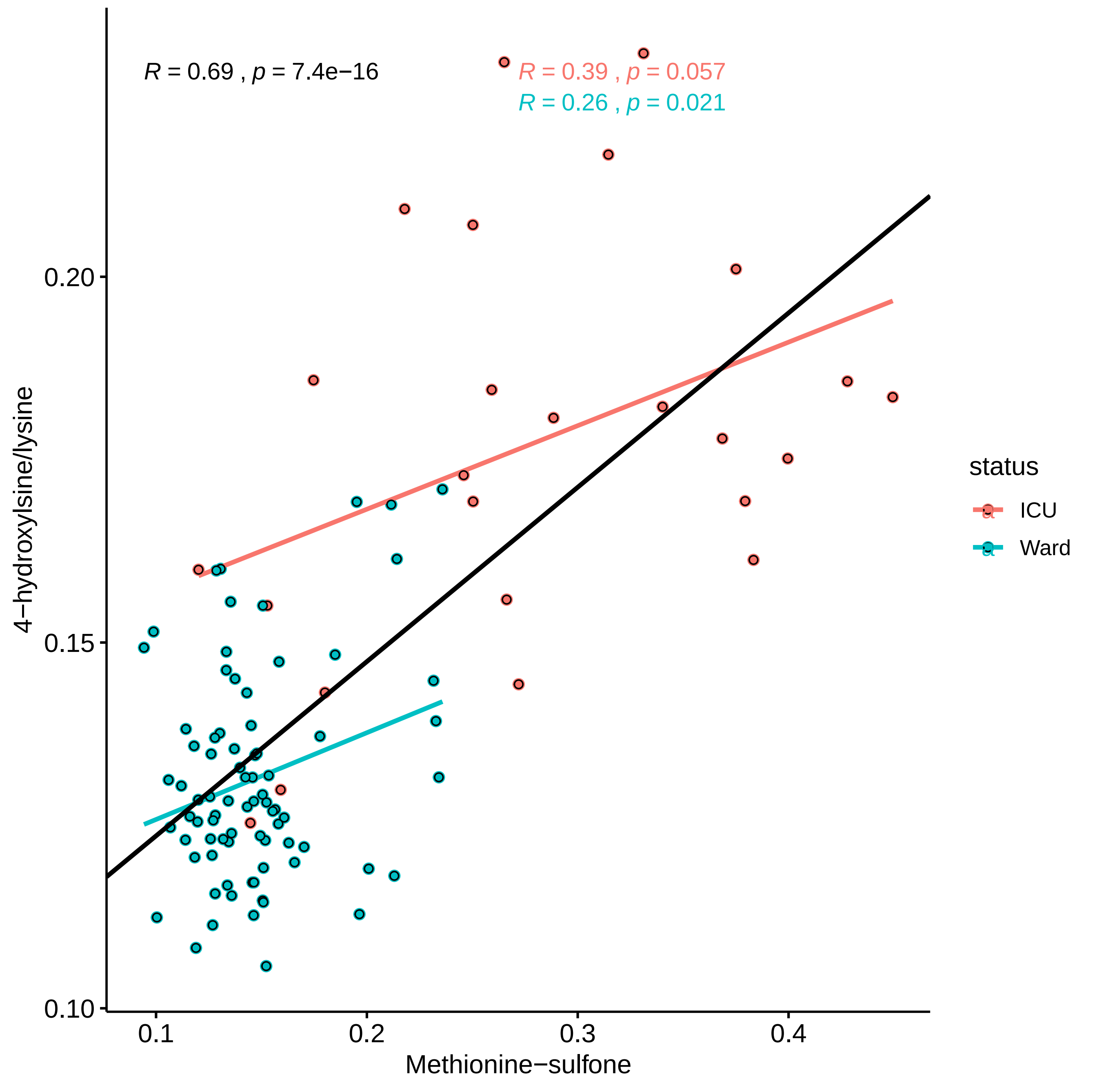

R = 0.69 , p = 7.4e−16
R = 0.39 , p = 0.057
R = 0.26 , p = 0.021
4−hydroxylsine/lysine
0.20
0.15
0.10
0.1
0.2
0.3
0.4
Methionine−sulfone
status
ICU
Ward

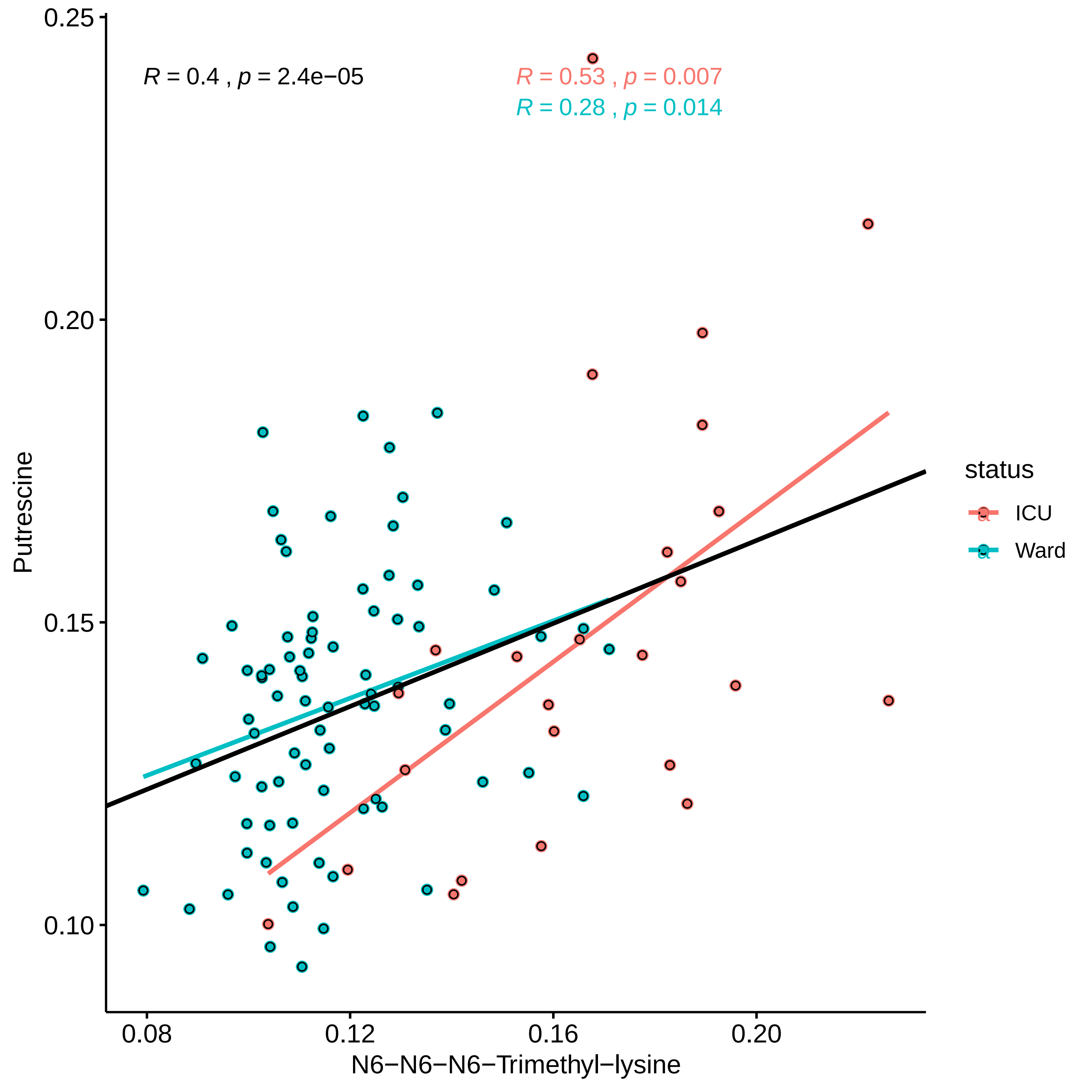

R = 0.4 , p = 2.4e−05
R = 0.53 , p = 0.007
R = 0.28 , p = 0.014
0.25
0.20
0.15
0.10
Putrescine
0.08
0.12
0.16
0.20
N6−N6−N6−Trimethyl−lysine
status
ICU
Ward

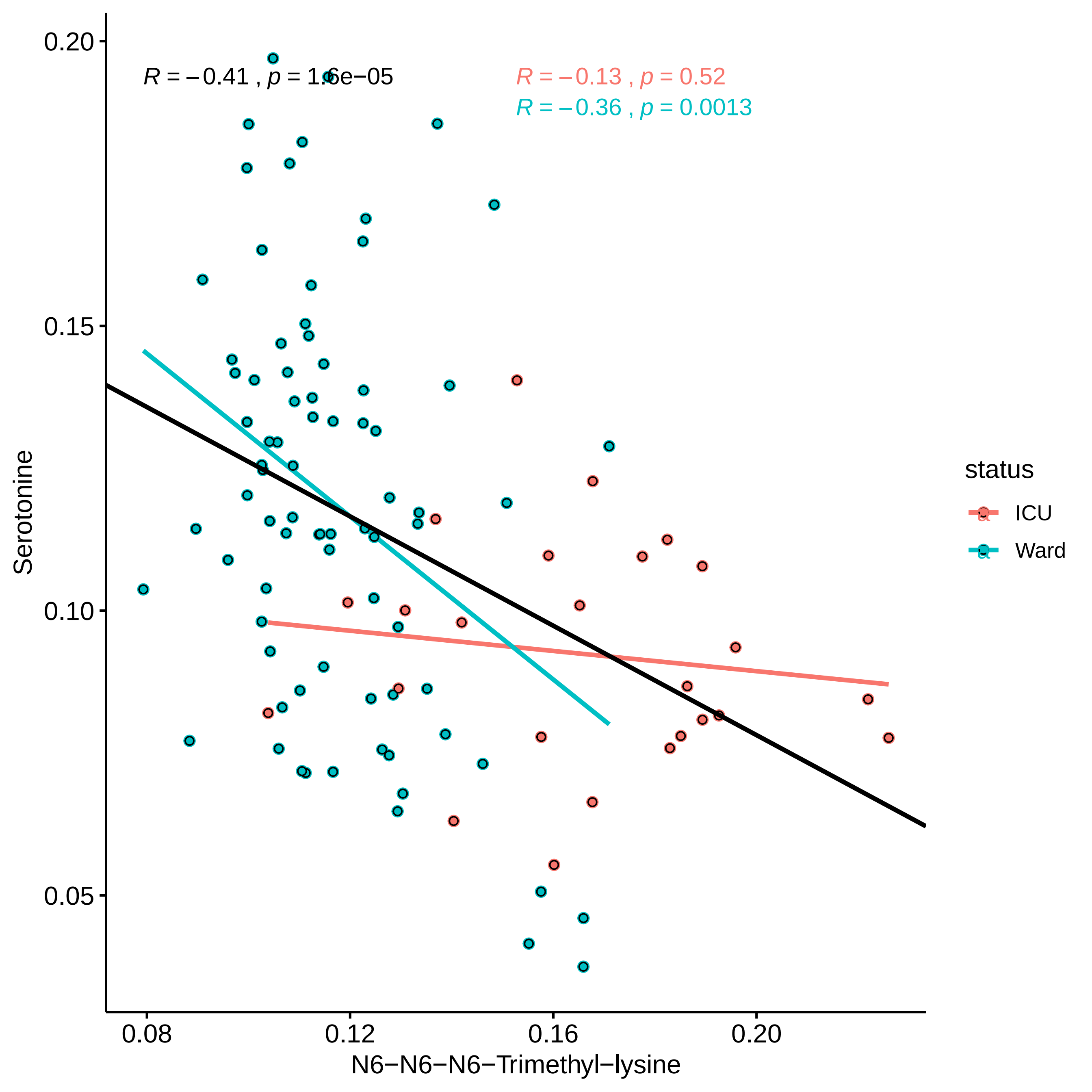

R = −0.41 , p = 1.6e−05
R = −0.13 , p = 0.52
R = −0.36 , p = 0.0013
0.20
0.15
0.10
0.05
Serotonine
0.08
0.12
0.16
0.20
N6−N6−N6−Trimethyl−lysine
status
ICU
Ward

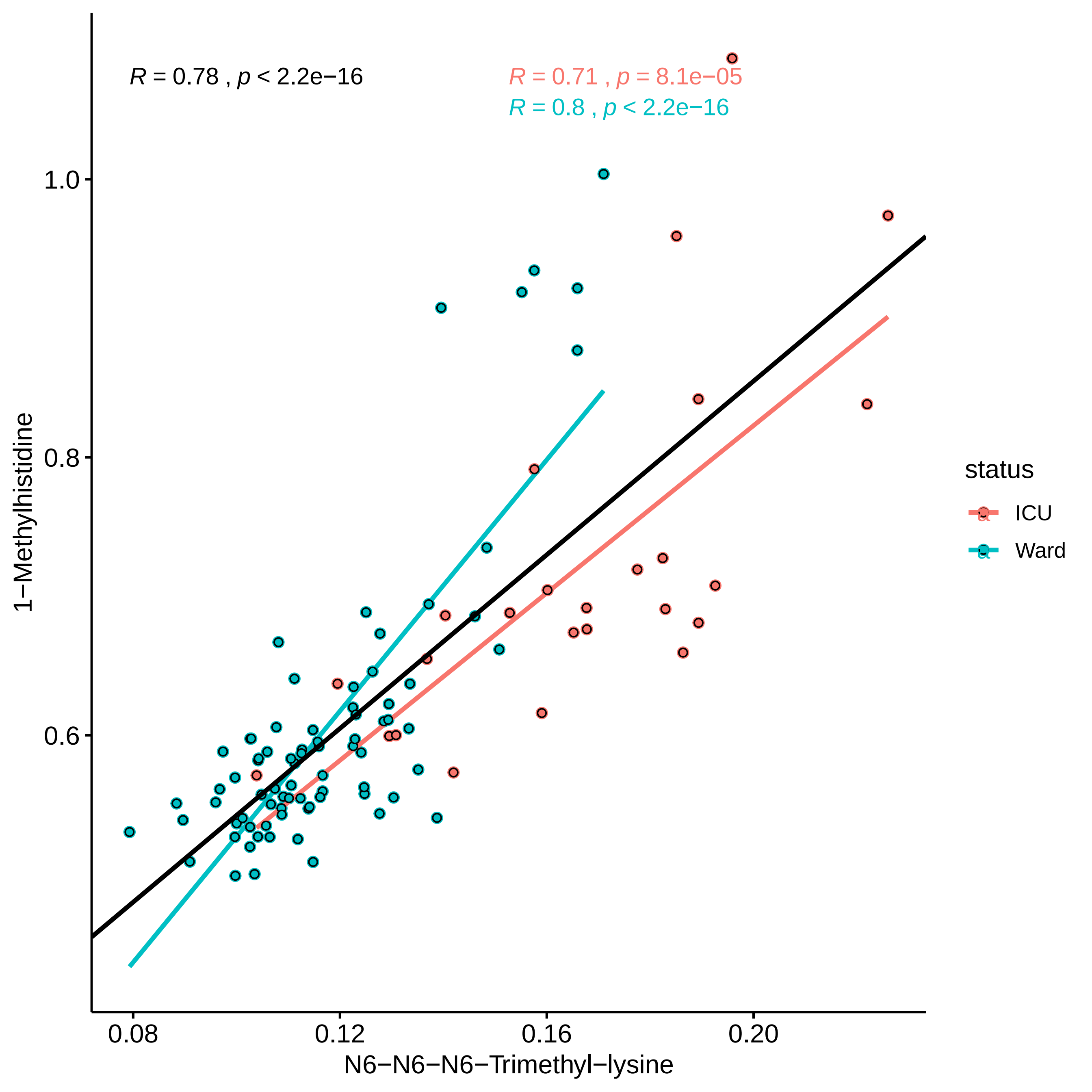
R = 0.78 , p < 2.2e−16
R = 0.71 , p = 8.1e−05
R = 0.8 , p < 2.2e−16
1.0
0.8
0.6
0.08
0.12
0.16
0.20
1−Methylhistidine
N6−N6−N6−Trimethyl−lysine
status
ICU
Ward

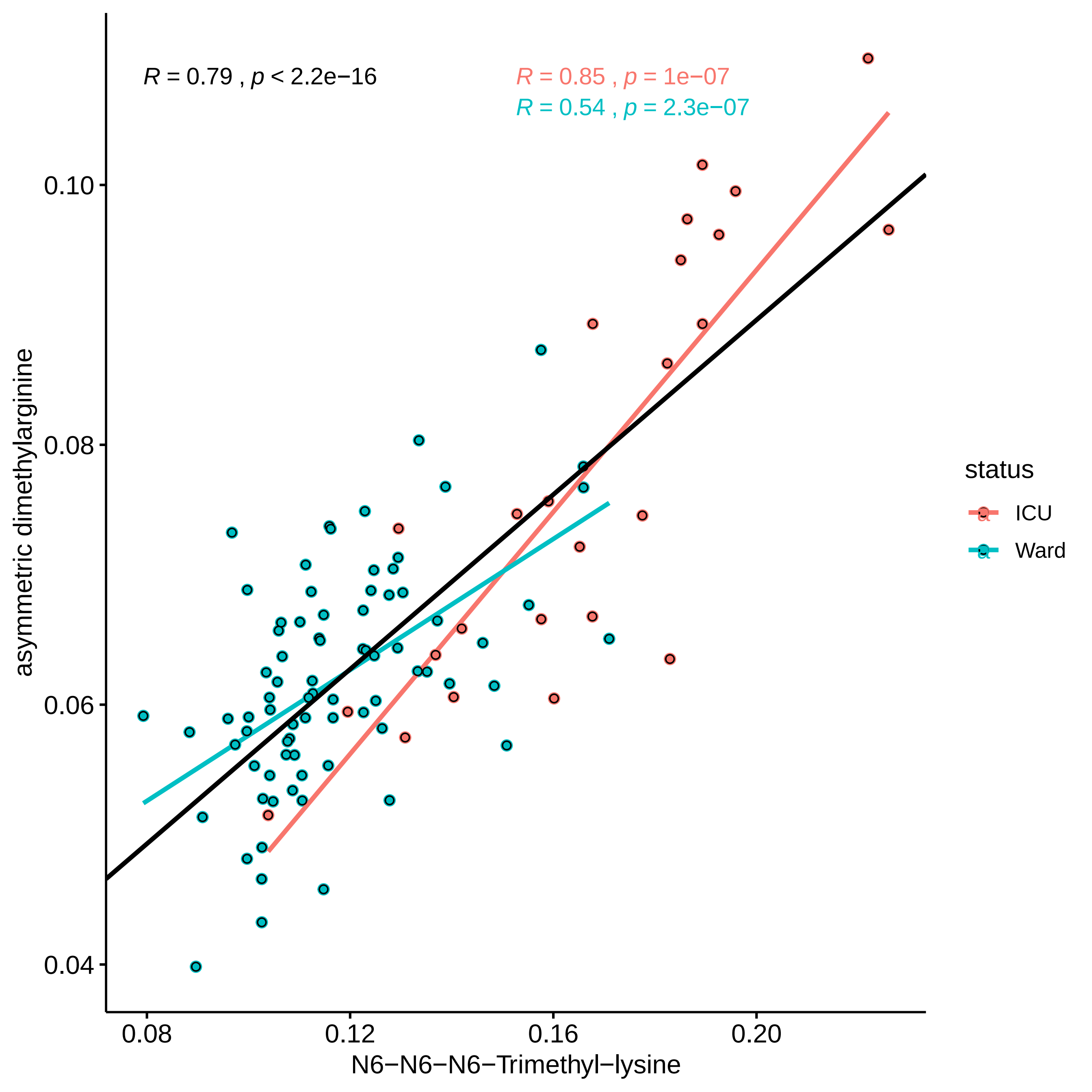

R = 0.79 , p < 2.2e−16
R = 0.85 , p = 1e−07
R = 0.54 , p = 2.3e−07
asymmetric dimethylarginine
0.10
0.08
0.06
0.04
0.08
0.12
0.16
0.20
N6−N6−N6−Trimethyl−lysine
status
ICU
Ward

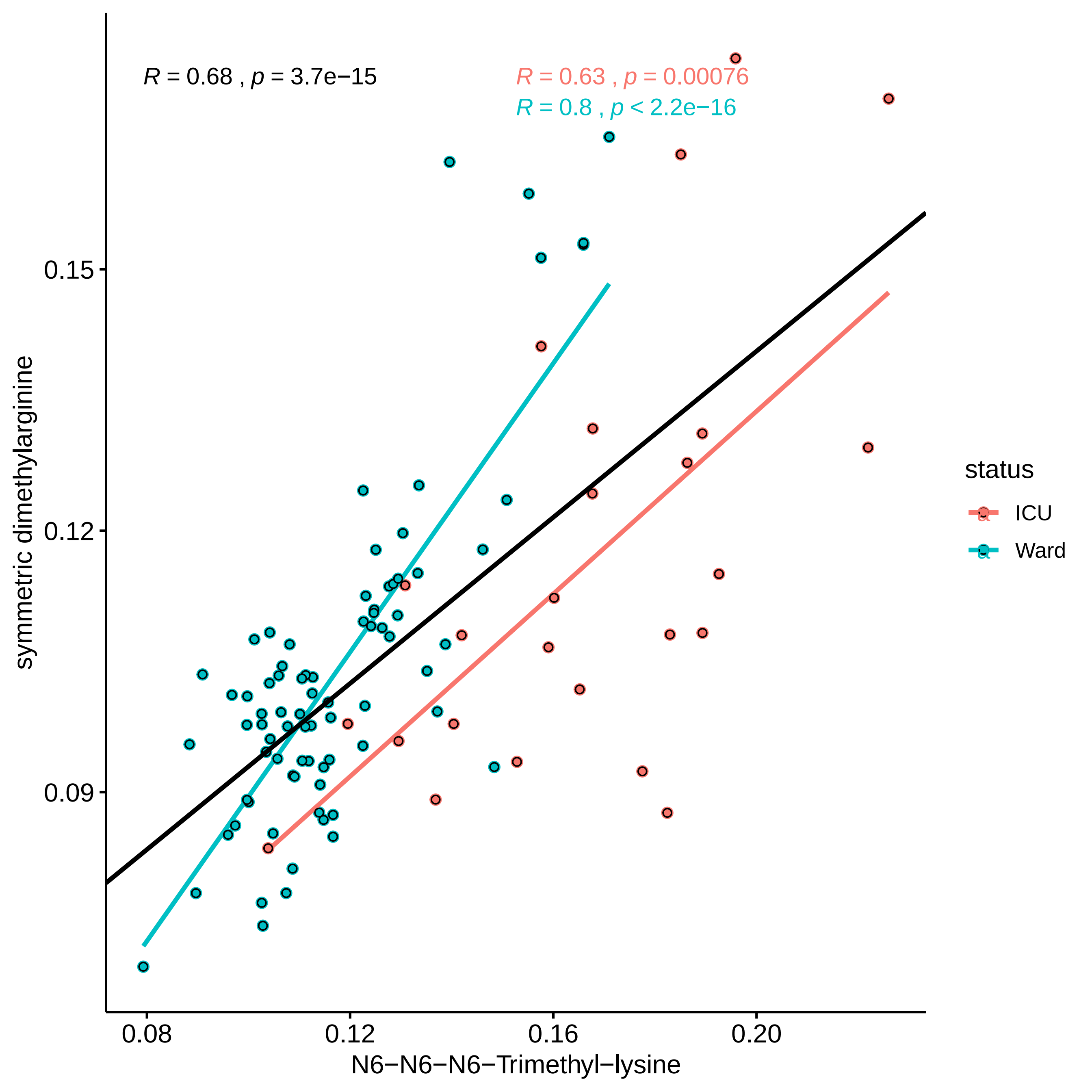

R = 0.68 , p = 3.7e−15
R = 0.63 , p = 0.00076
R = 0.8 , p < 2.2e−16
symmetric dimethylarginine
0.15
0.12
0.09
0.08
0.12
0.16
0.20
N6−N6−N6−Trimethyl−lysine
status
ICU
Ward

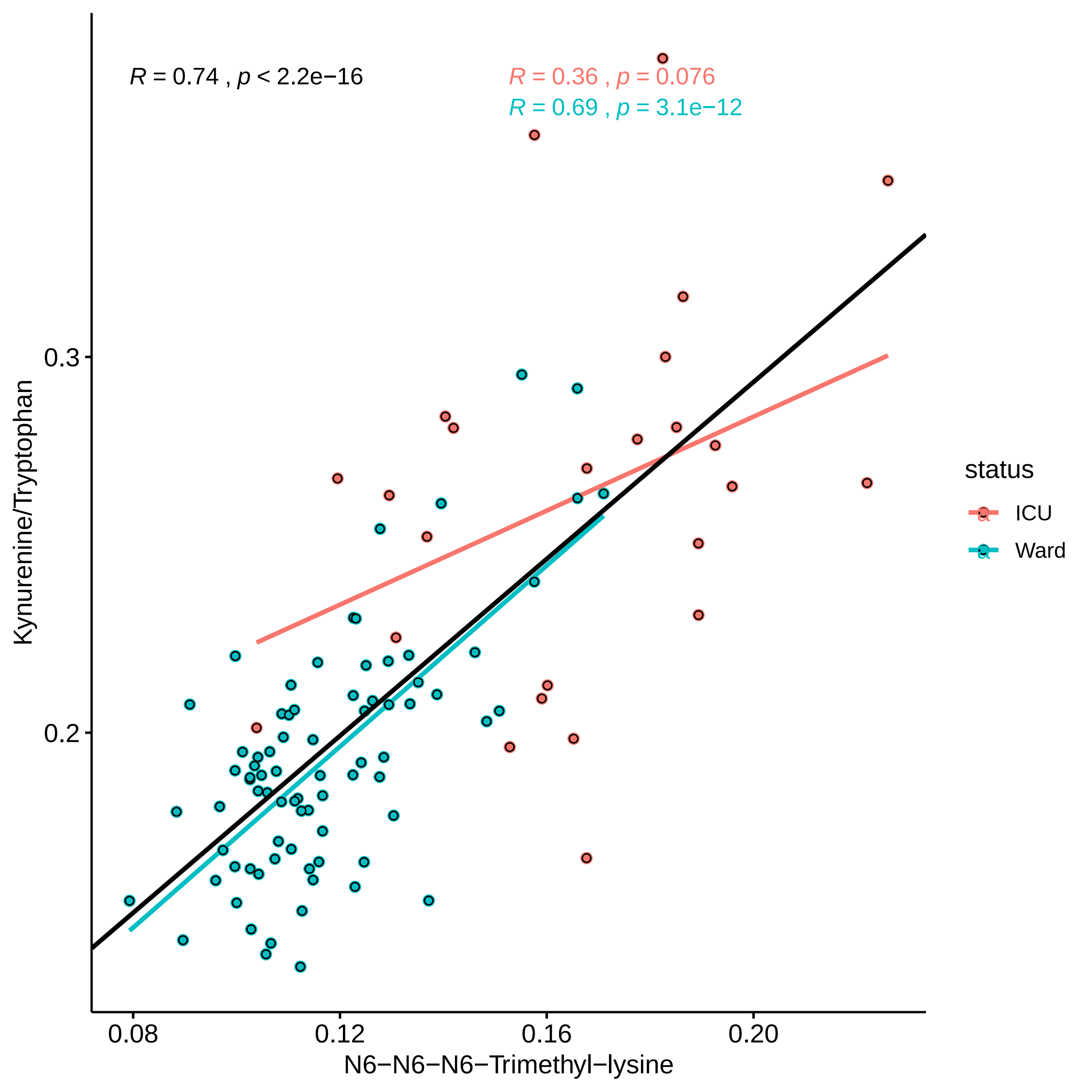

R = 0.74 , p < 2.2e−16
R = 0.36 , p = 0.076
R = 0.69 , p = 3.1e−12
Kynurenine/Tryptophan
0.3
0.2
0.08
0.12
0.16
0.20
N6−N6−N6−Trimethyl−lysine
status
ICU
Ward

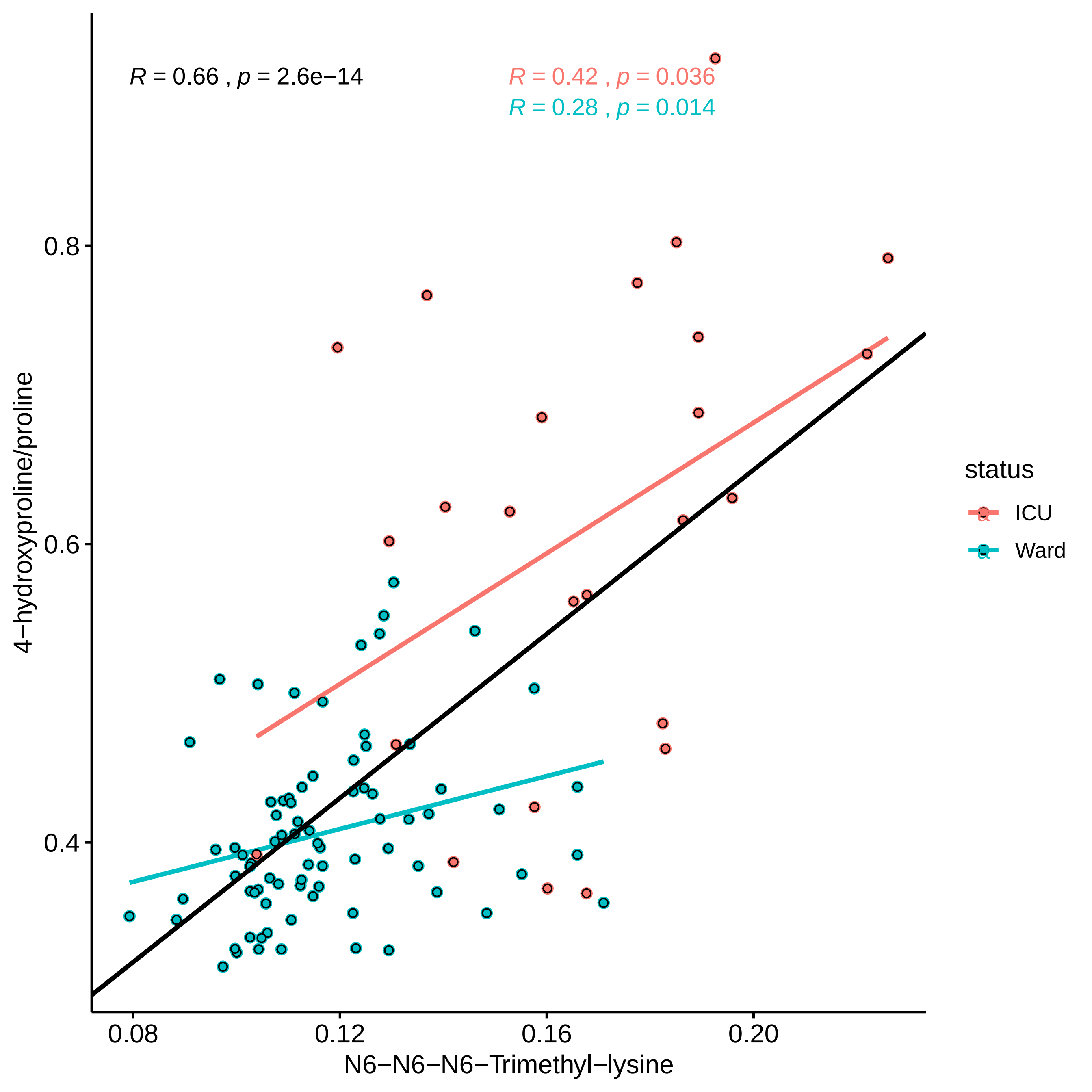
R = 0.66 , p = 2.6e−14
R = 0.42 , p = 0.036
R = 0.28 , p = 0.014
4−hydroxyproline/proline
0.8
0.6
0.4
0.08
0.12
0.16
0.20
N6−N6−N6−Trimethyl−lysine
status
ICU
Ward

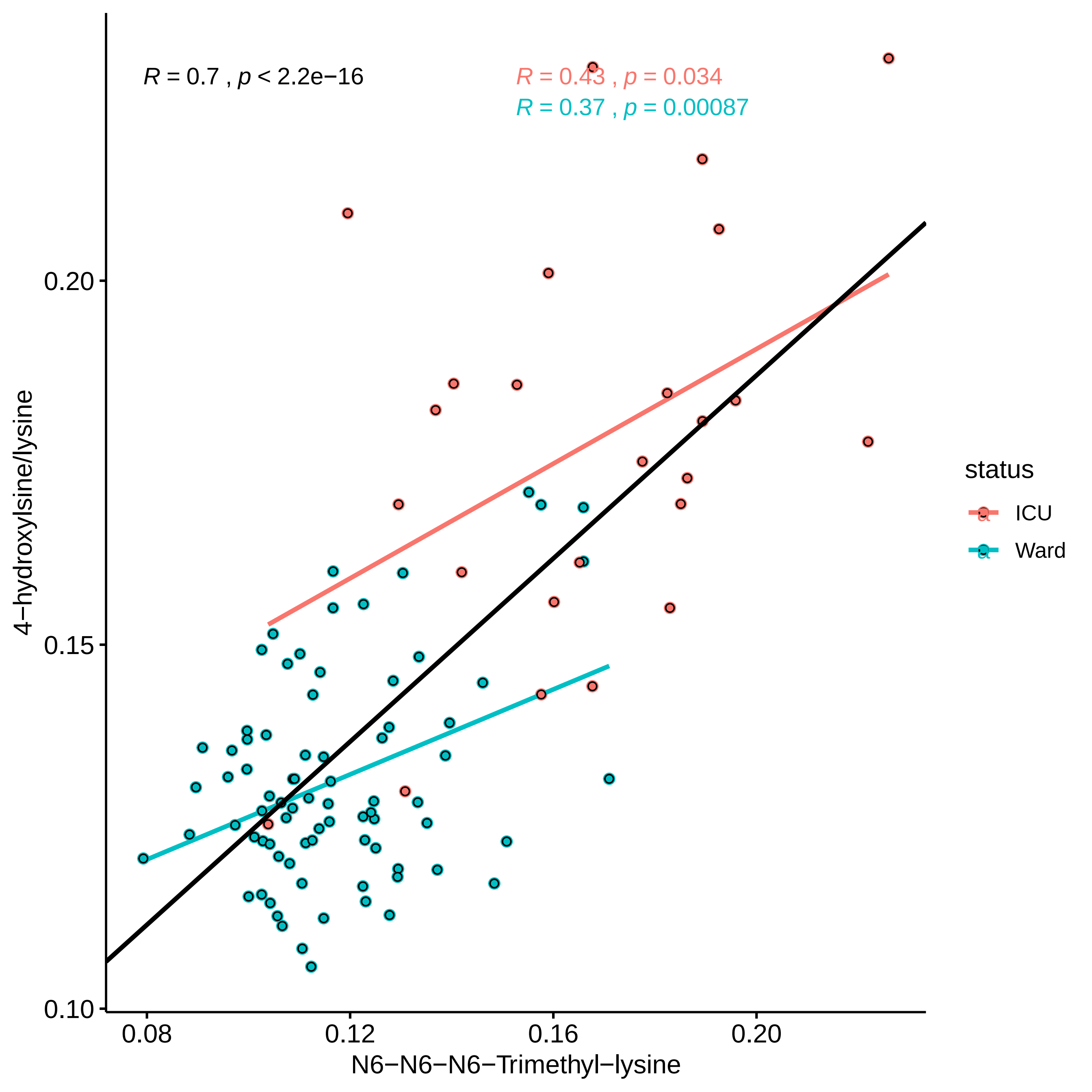

R = 0.7 , p < 2.2e−16
R = 0.43 , p = 0.034
R = 0.37 , p = 0.00087
0.20
0.15
0.10
0.08
0.12
0.16
0.20
4−hydroxylysine/lysine
N6−N6−N6−Trimethyl−lysine
status
ICU
Ward

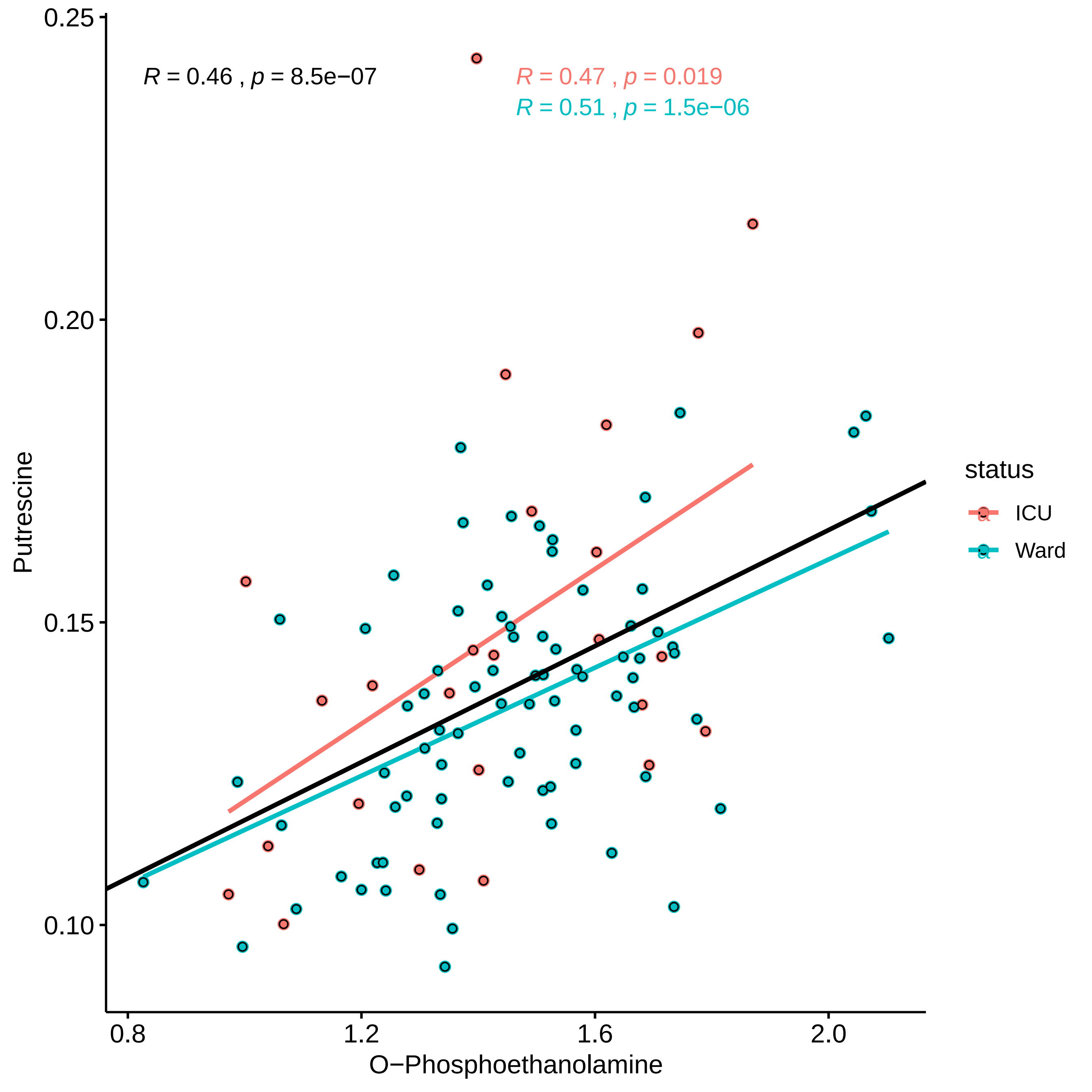

R = 0.46 , p = 8.5e−07
R = 0.47 , p = 0.019
R = 0.51 , p = 1.5e−06
0.25
0.20
0.15
0.10
0.8
1.2
1.6
2.0
Putrescine
O−Phosphoethanolamine
status
ICU
Ward

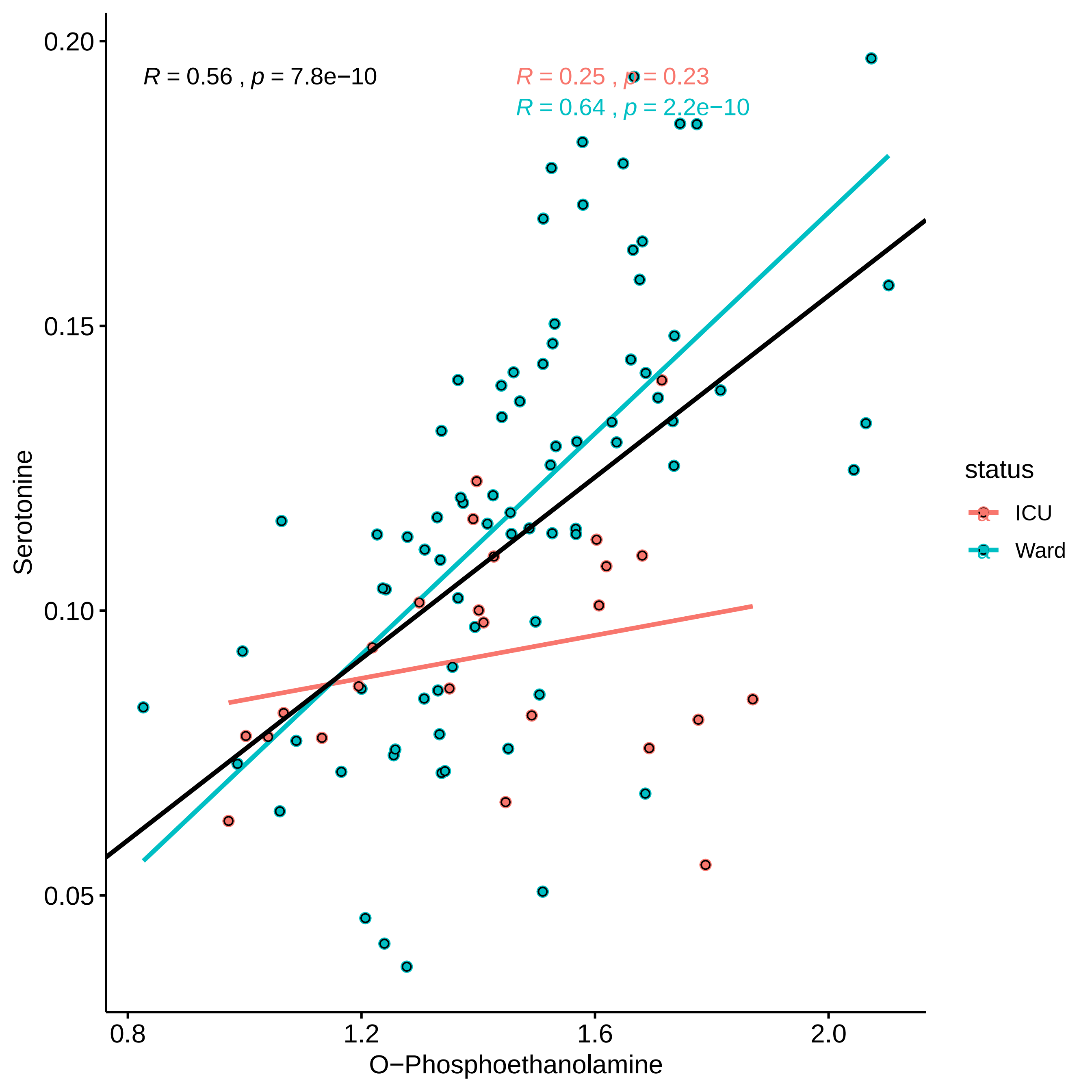

R = 0.56 , p = 7.8e−10
R = 0.25 , p = 0.23
R = 0.64 , p = 2.2e−10
Serotonine
O−Phosphoethanolamine
0.20
0.15
0.10
0.05
0.8
1.2
1.6
2.0
status
ICU
Ward

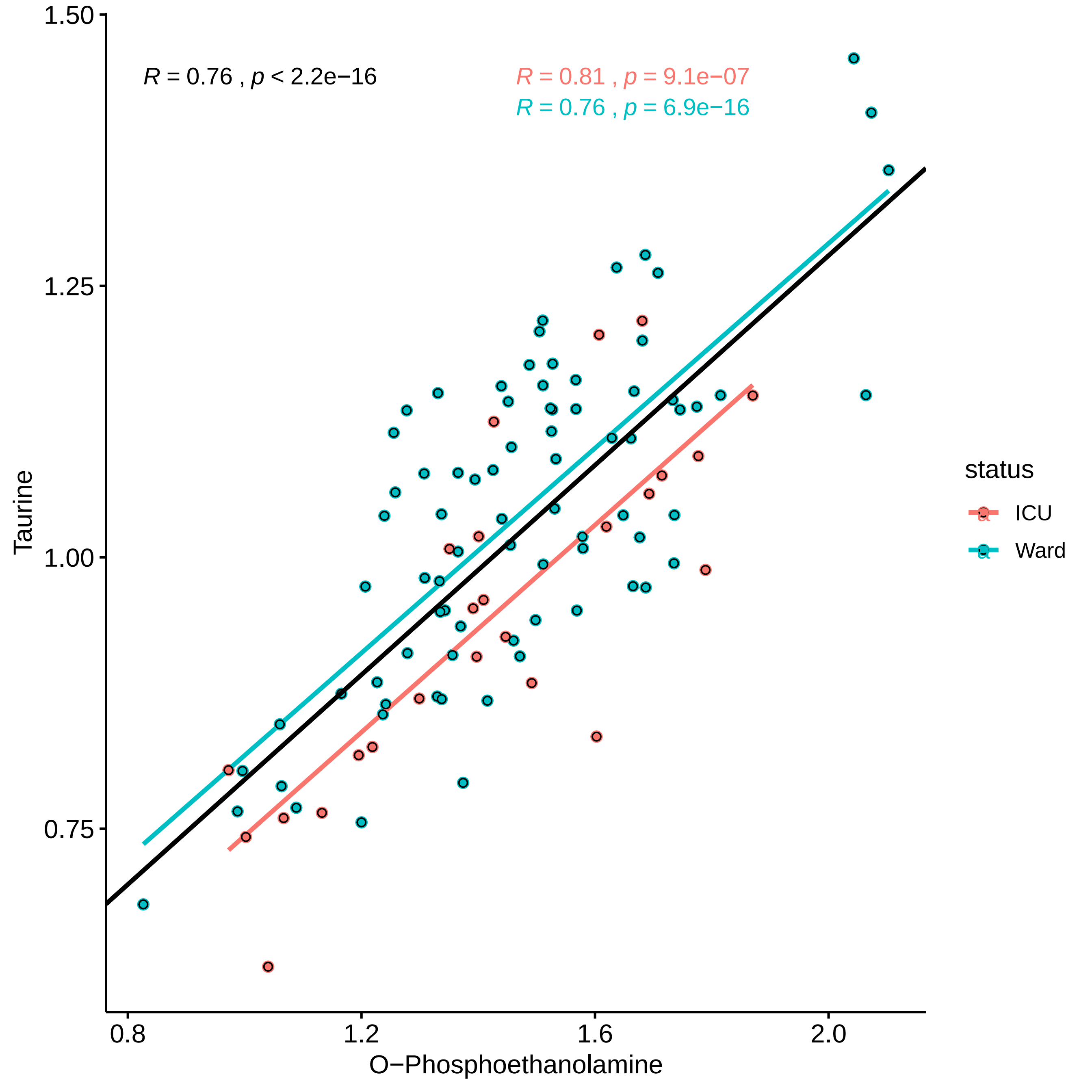

R = 0.76 , p < 2.2e−16
R = 0.81 , p = 9.1e−07
R = 0.76 , p = 6.9e−16
1.50
1.25
1.00
0.75
Taurine
0.8
1.2
1.6
2.0
O−Phosphoethanolamine
status
ICU
Ward

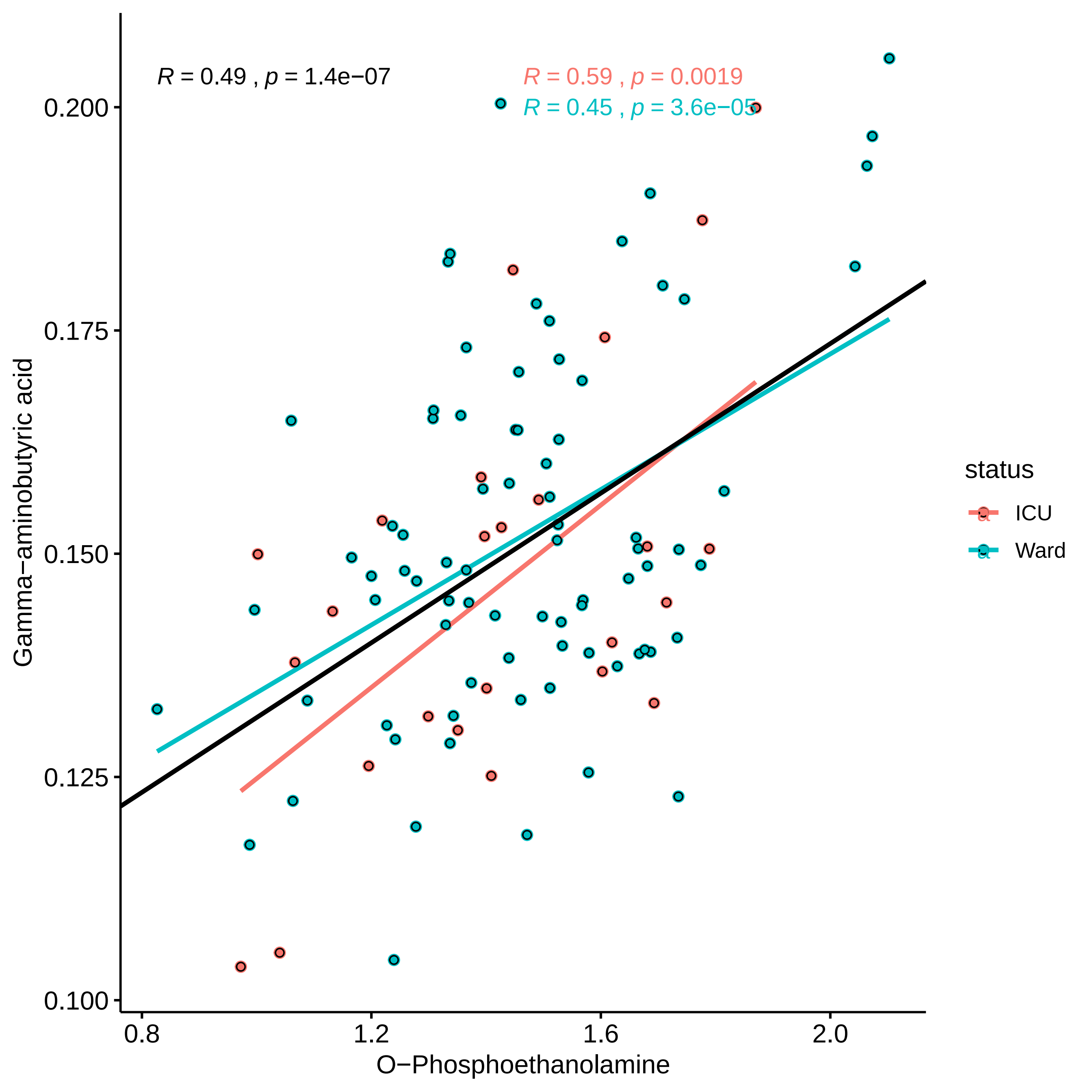

R = 0.49 , p = 1.4e−07
R = 0.59 , p = 0.0019
R = 0.45 , p = 3.6e−05
Gamma−aminobutyric acid
O−Phosphoethanolamine
0.100
0.125
0.150
0.175
0.200
0.8
1.2
1.6
2.0
status
ICU
Ward

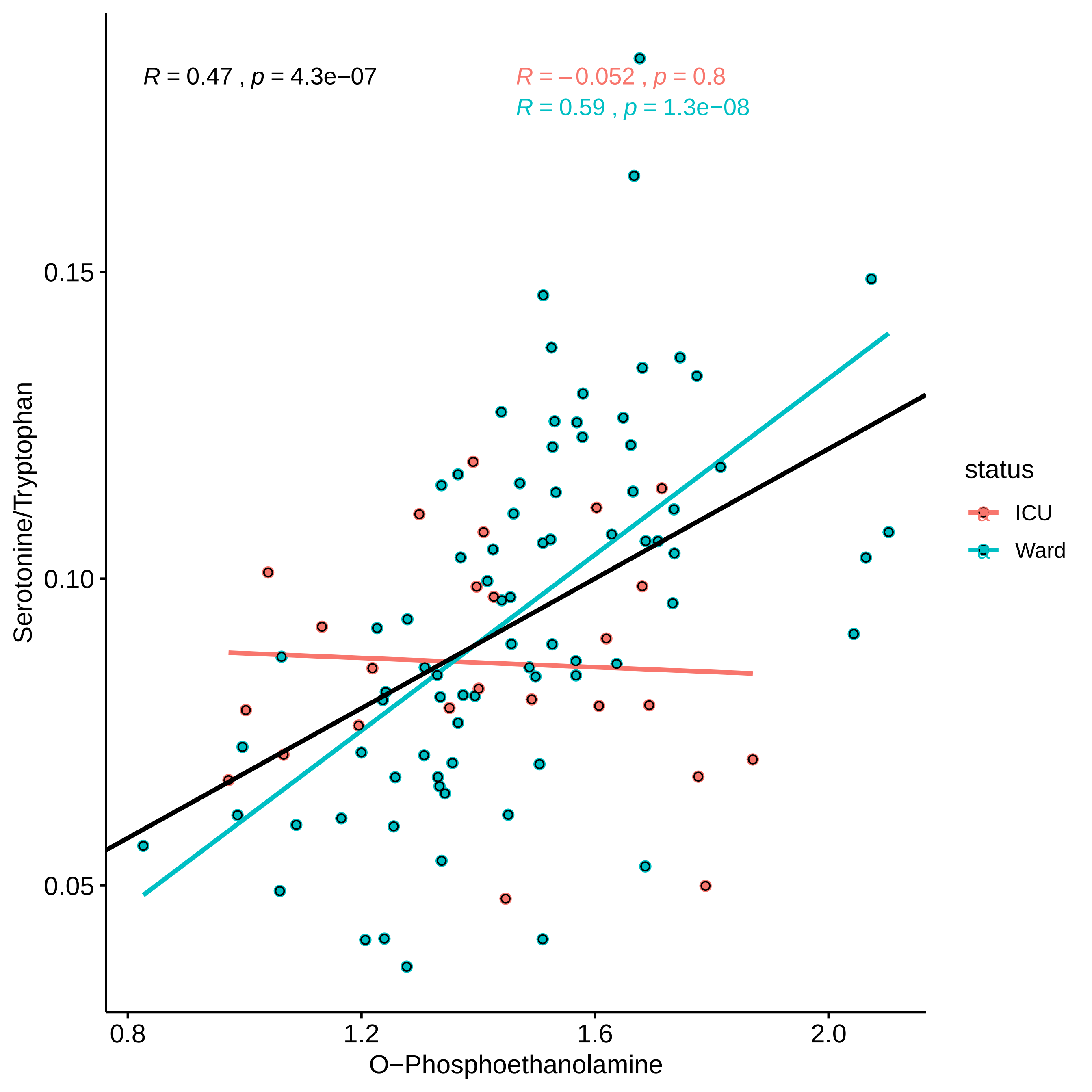

R = 0.47 , p = 4.3e−07
R = −0.052 , p = 0.8
R = 0.59 , p = 1.3e−08
Serotonine/Tryptophan
0.15
0.10
0.05
0.8
1.2
1.6
2.0
O−Phosphoethanolamine
status
ICU
Ward

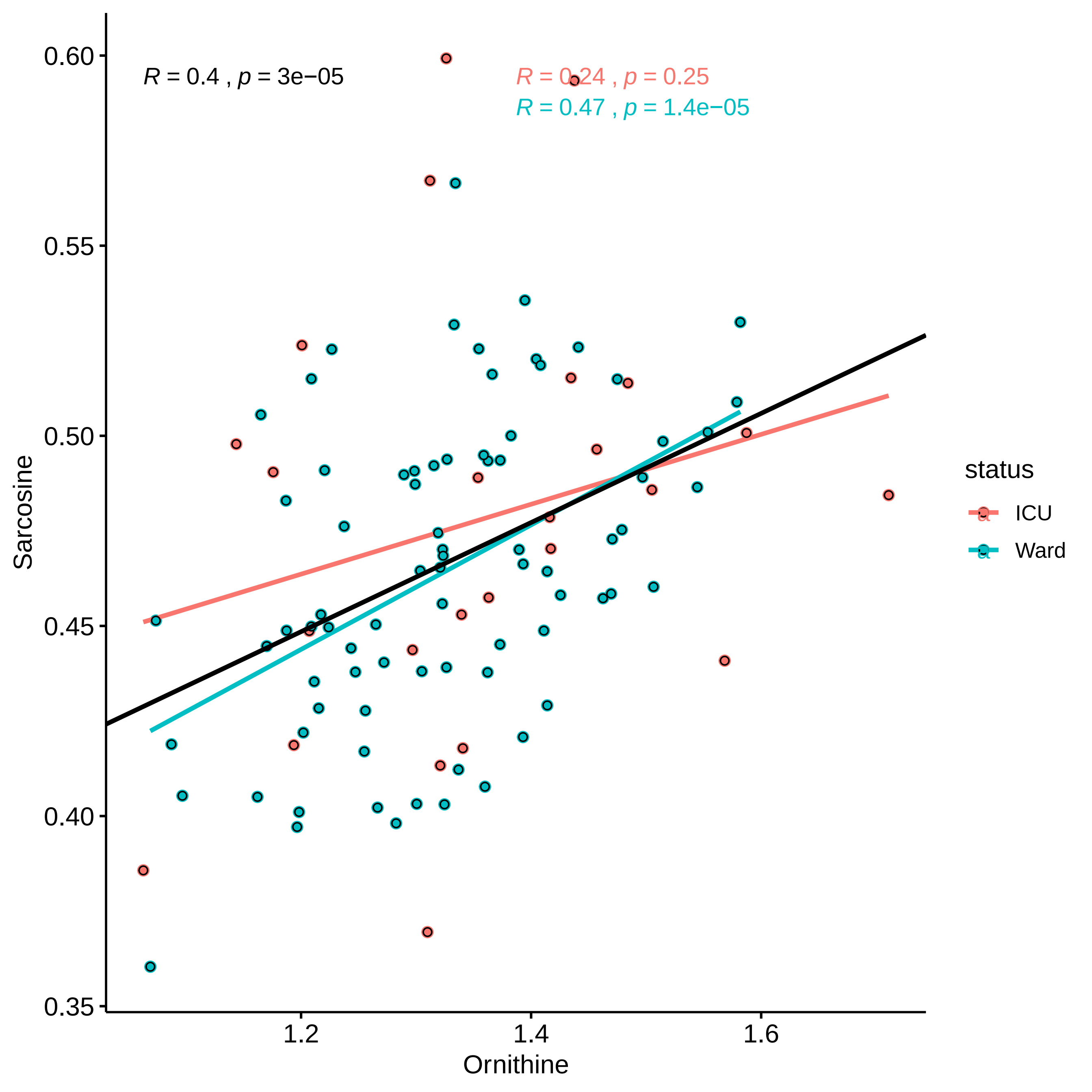

R = 0.4 , p = 3e−05
R = 0.24 , p = 0.25
R = 0.47 , p = 1.4e−05
0.60
0.55
0.50
0.45
0.40
0.35
Sarcosine
1.2
1.4
1.6
Ornithine
status
ICU
Ward

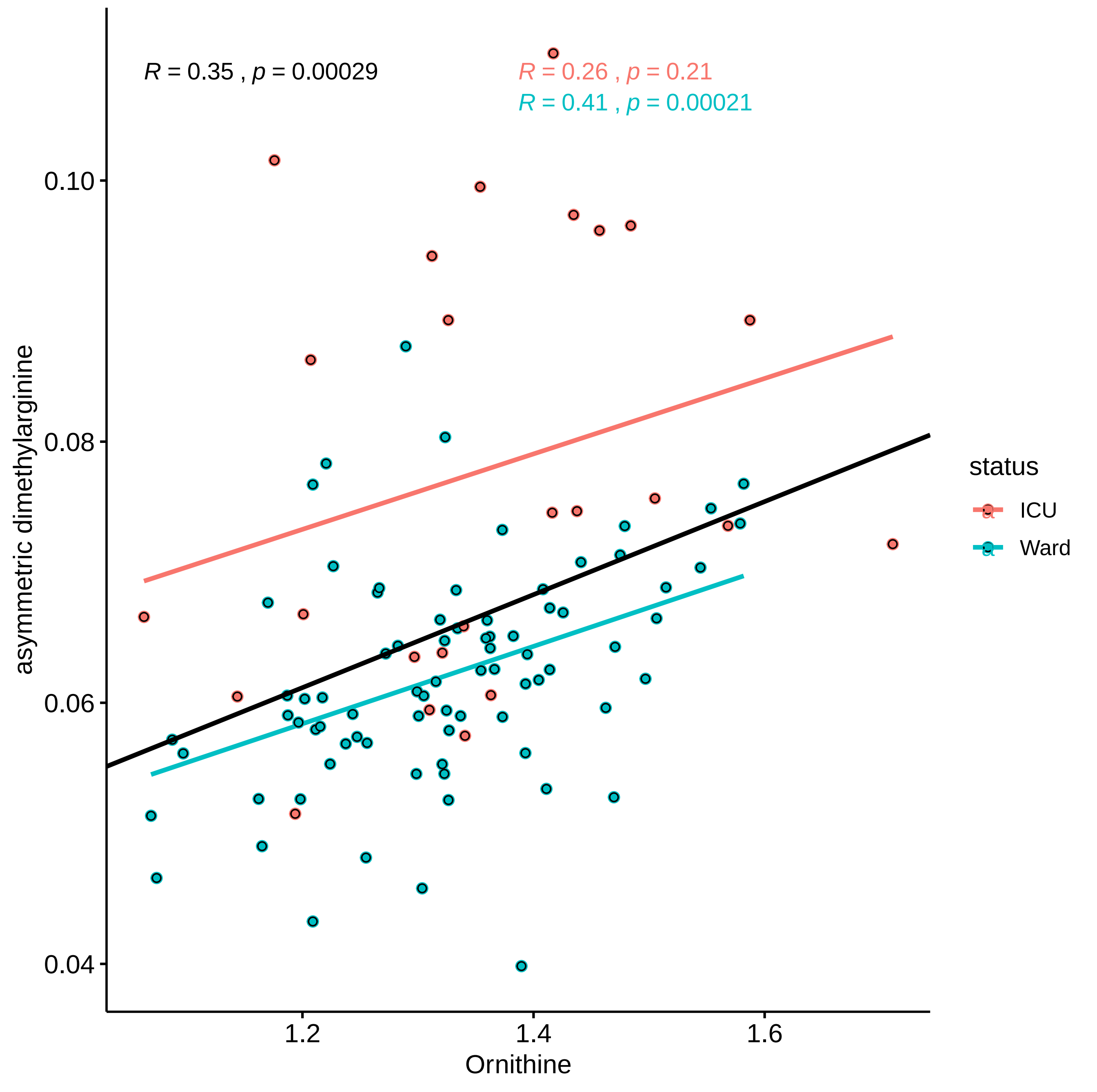

R = 0.35 , p = 0.00029
R = 0.26 , p = 0.21
R = 0.41 , p = 0.00021
asymmetric dimethylarginine
0.10
0.08
0.06
0.04
1.2
1.4
1.6
Ornithine
status
ICU
Ward

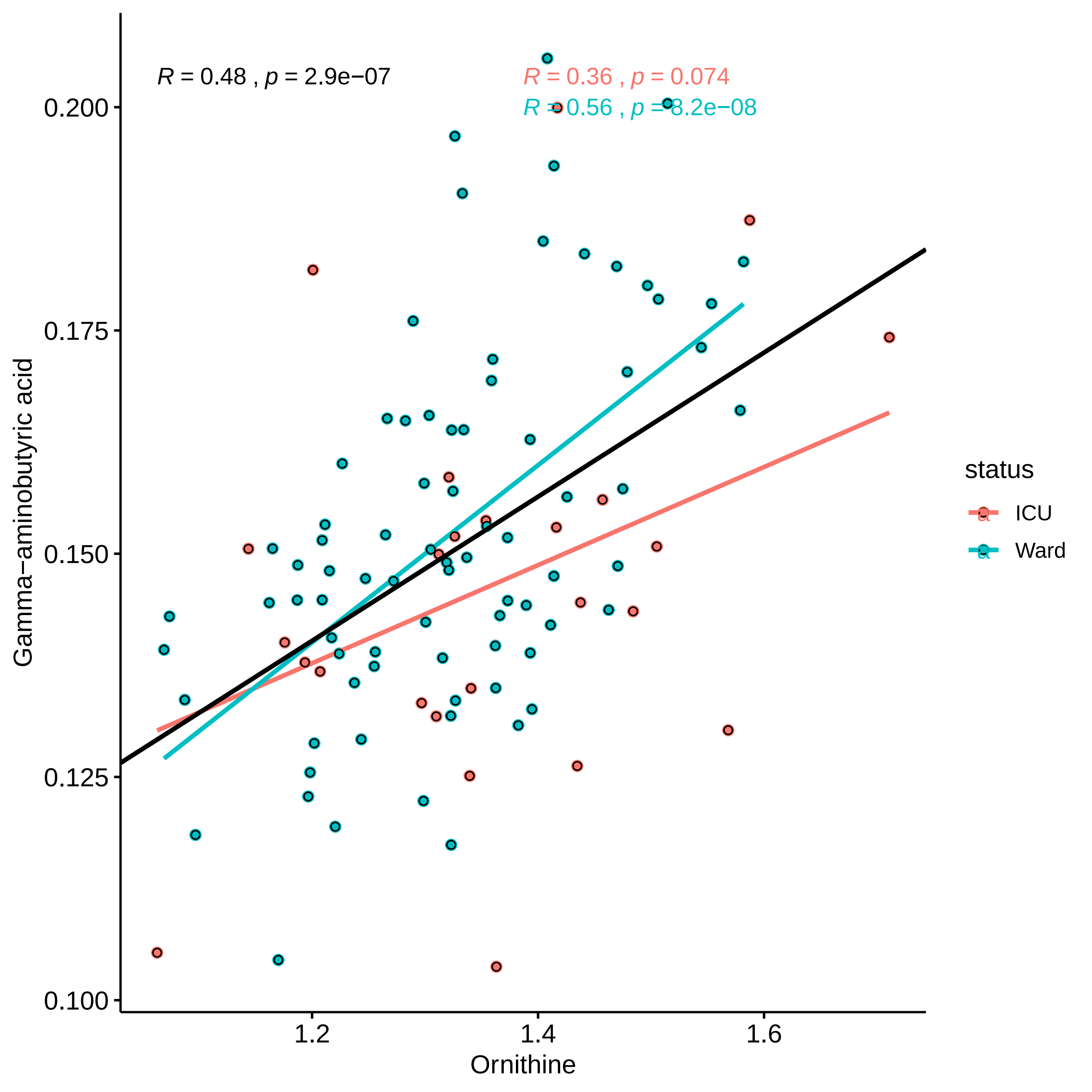

R = 0.48 , p = 2.9e−07
R = 0.36 , p = 0.074
R = 0.56 , p = 8.2e−08
0.200
0.175
0.150
0.125
0.100
Gamma−aminobutyric acid
1.2
1.4
1.6
Ornithine
status
ICU
Ward

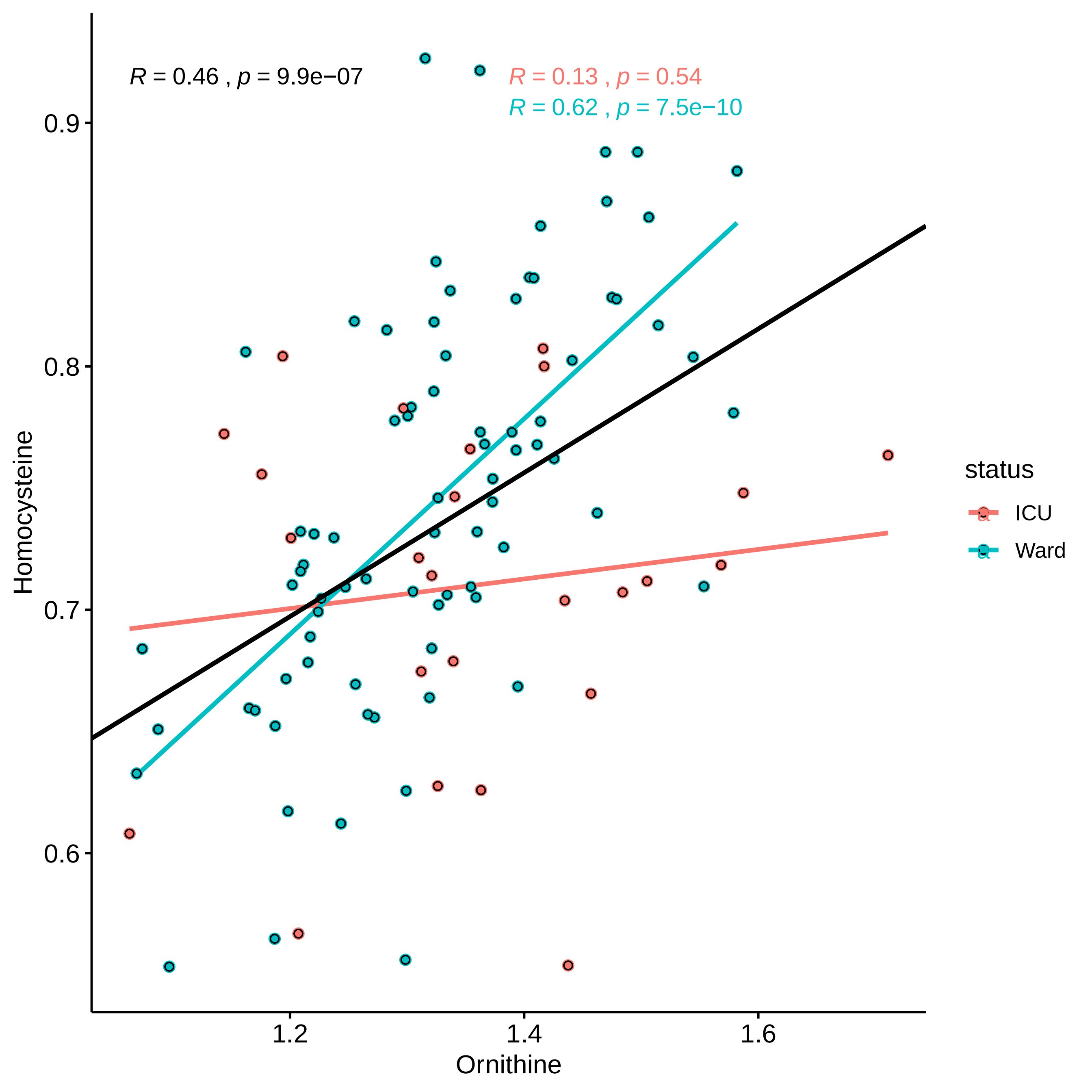

R = 0.46 , p = 9.9e−07
R = 0.13 , p = 0.54
R = 0.62 , p = 7.5e−10
0.9
0.8
0.7
0.6
Homocysteine
1.2
1.4
1.6
Ornithine
status
ICU
Ward

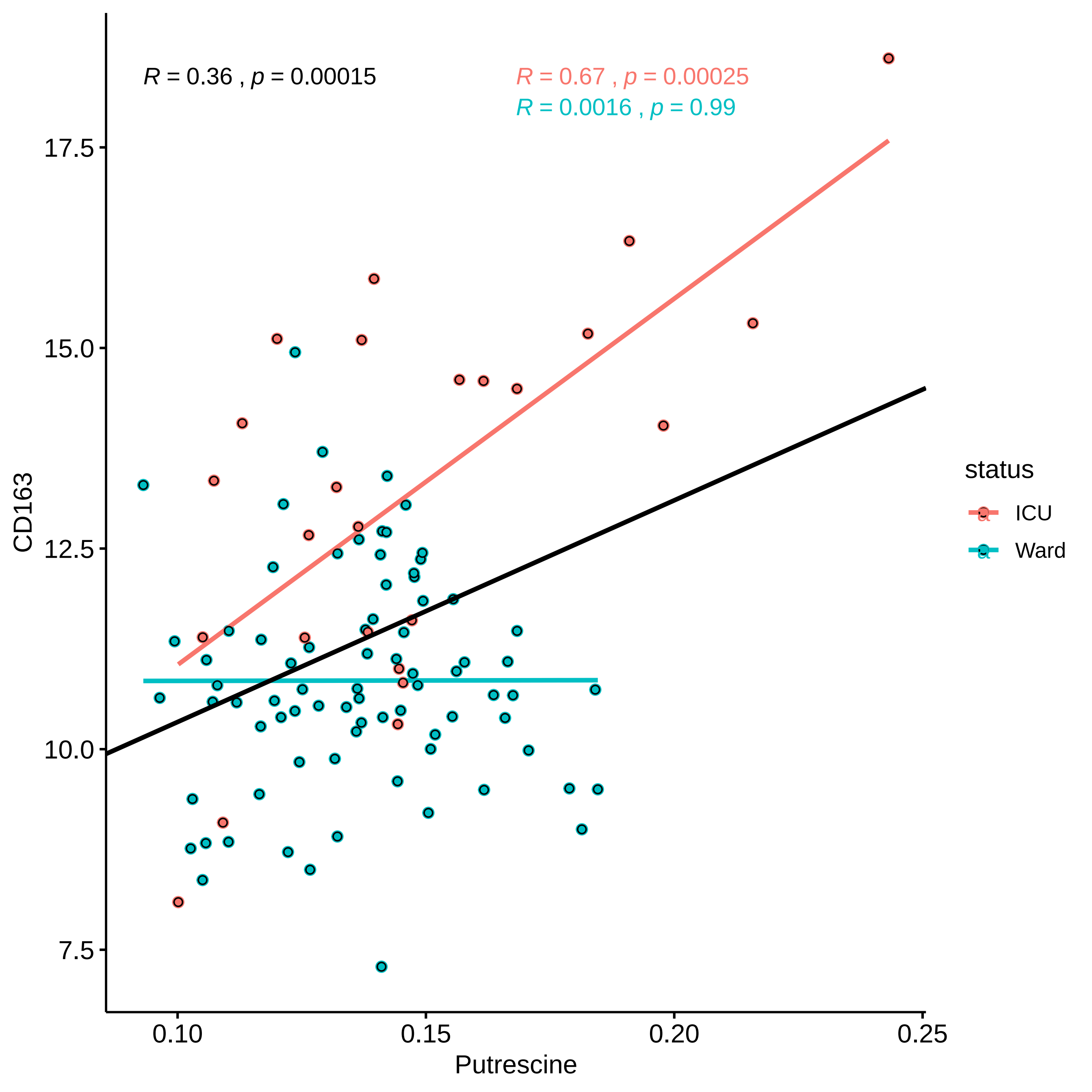

R = 0.36 , p = 0.00015
R = 0.67 , p = 0.00025
R = 0.0016 , p = 0.99
17.5
15.0
12.5
10.0
7.5
CD163
0.10
0.15
0.20
0.25
Putrescine
status
ICU
Ward

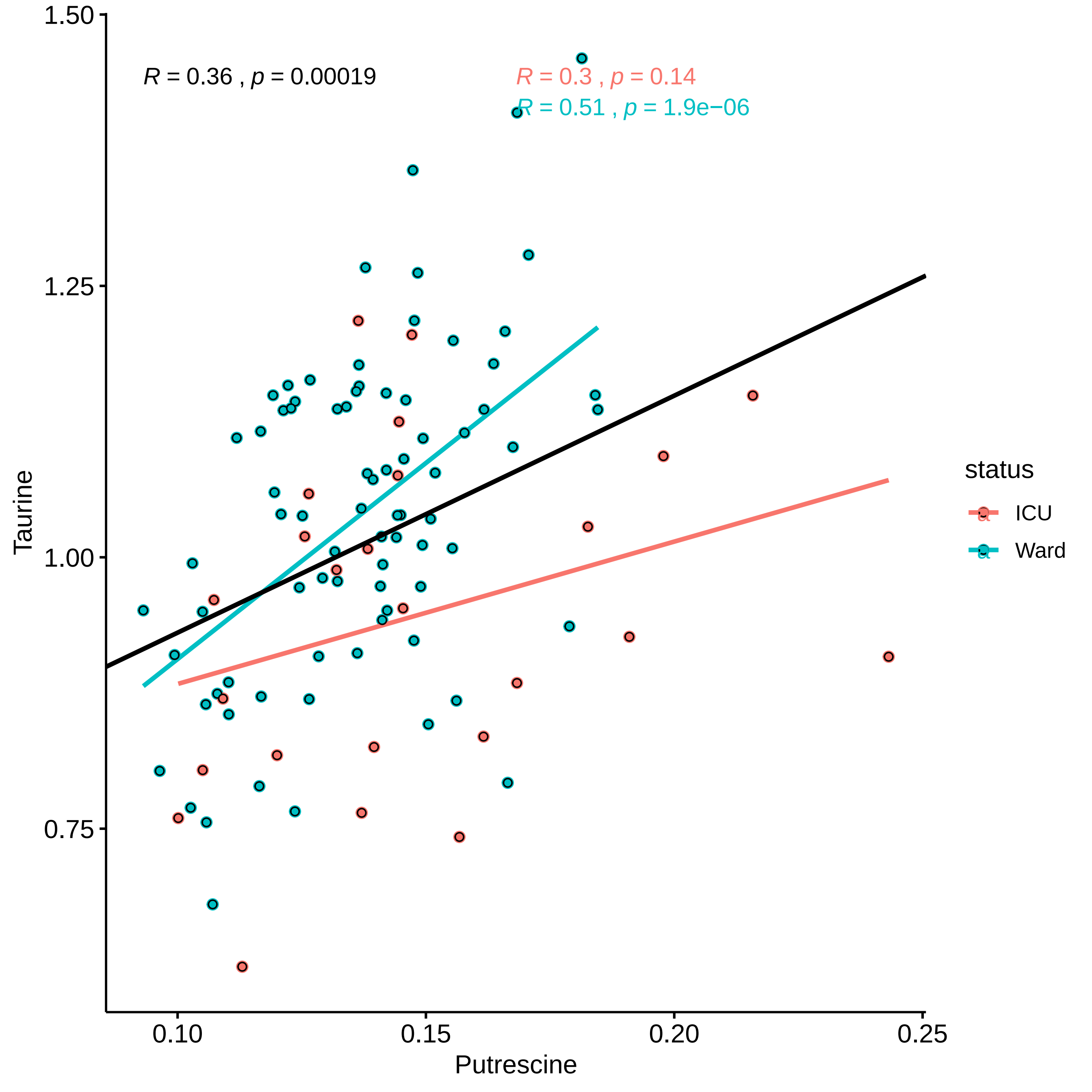

R = 0.36 , p = 0.00019
R = 0.3 , p = 0.14
R = 0.51 , p = 1.9e−06
1.50
1.25
1.00
0.75
Taurine
0.10
0.15
0.20
0.25
Putrescine
status
ICU
Ward

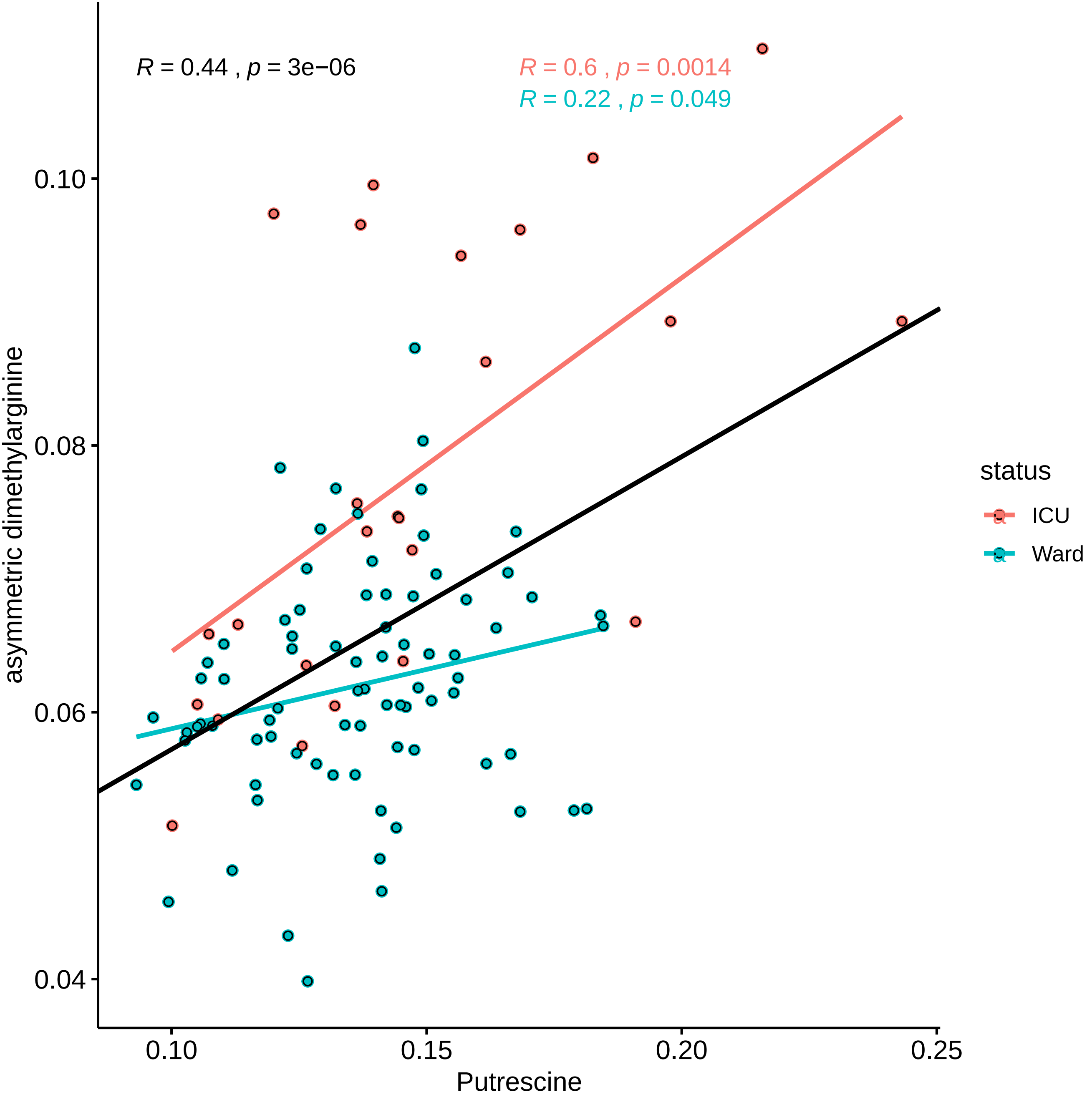

R = 0.44 , p = 3e−06
R = 0.6 , p = 0.0014
R = 0.22 , p = 0.049
asymmetric dimethylarginine
0.10
0.08
0.06
0.04
0.10
0.15
0.20
0.25
Putrescine
status
ICU
Ward

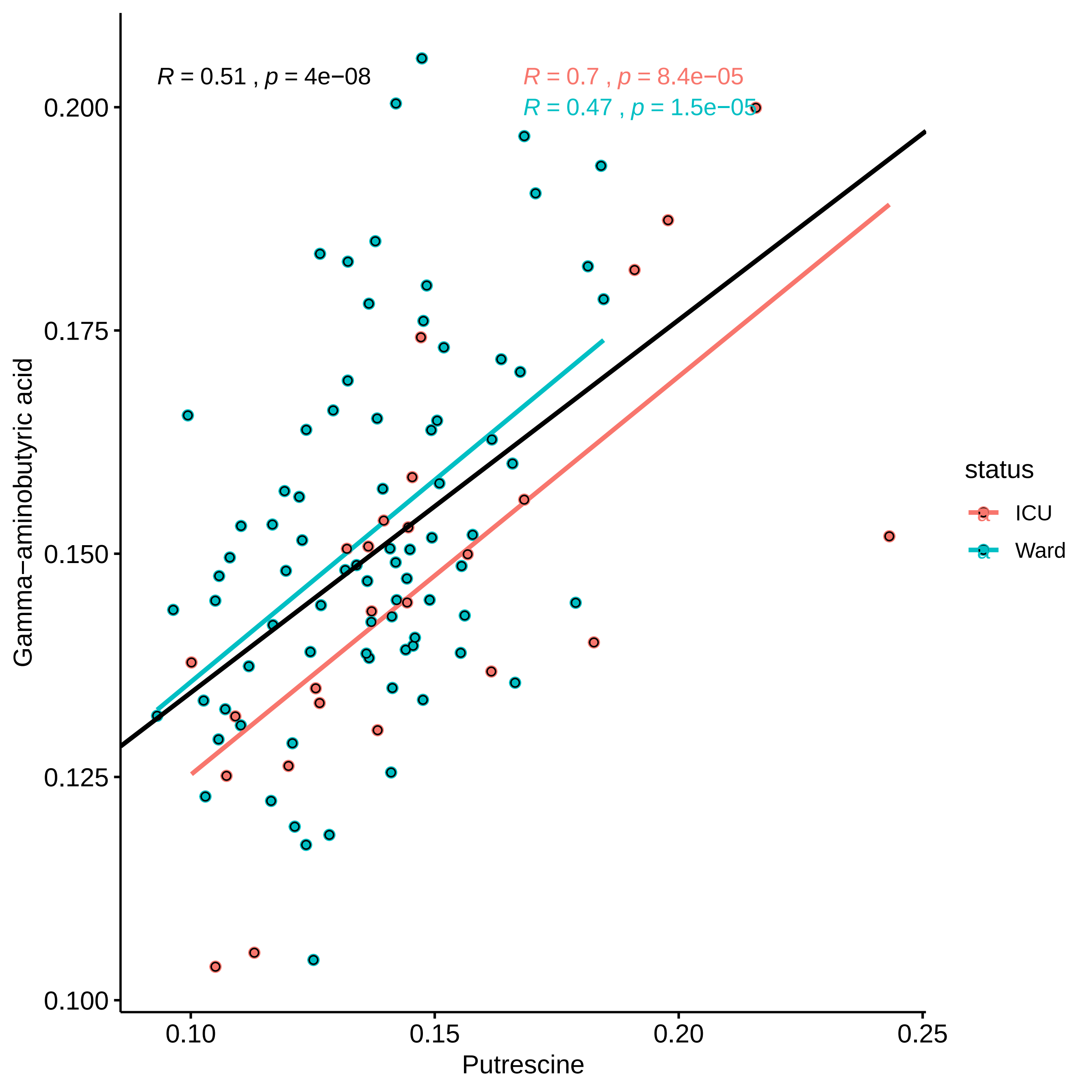

R = 0.51 , p = 4e−08
R = 0.7 , p = 8.4e−05
R = 0.47 , p = 1.5e−05
Gamma−aminobutyric acid
0.200
0.175
0.150
0.125
0.100
0.10
0.15
0.20
0.25
Putrescine
status
ICU
Ward

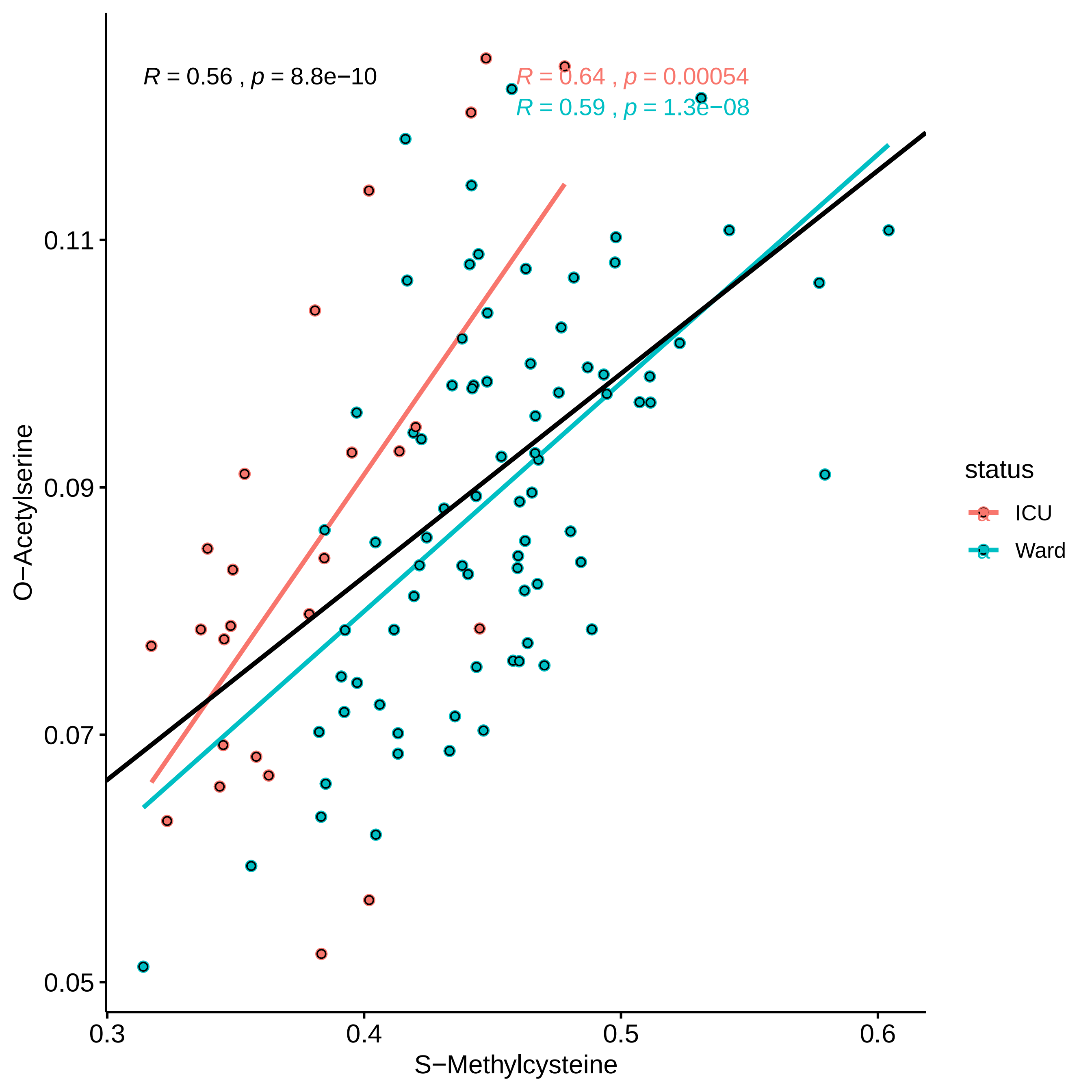

R = 0.56 , p = 8.8e−10
R = 0.64 , p = 0.00054
R = 0.59 , p = 1.3e−08
O−Acetylserine
S−Methylcysteine
0.11
0.09
0.07
0.05
0.3
0.4
0.5
0.6
status
ICU
Ward

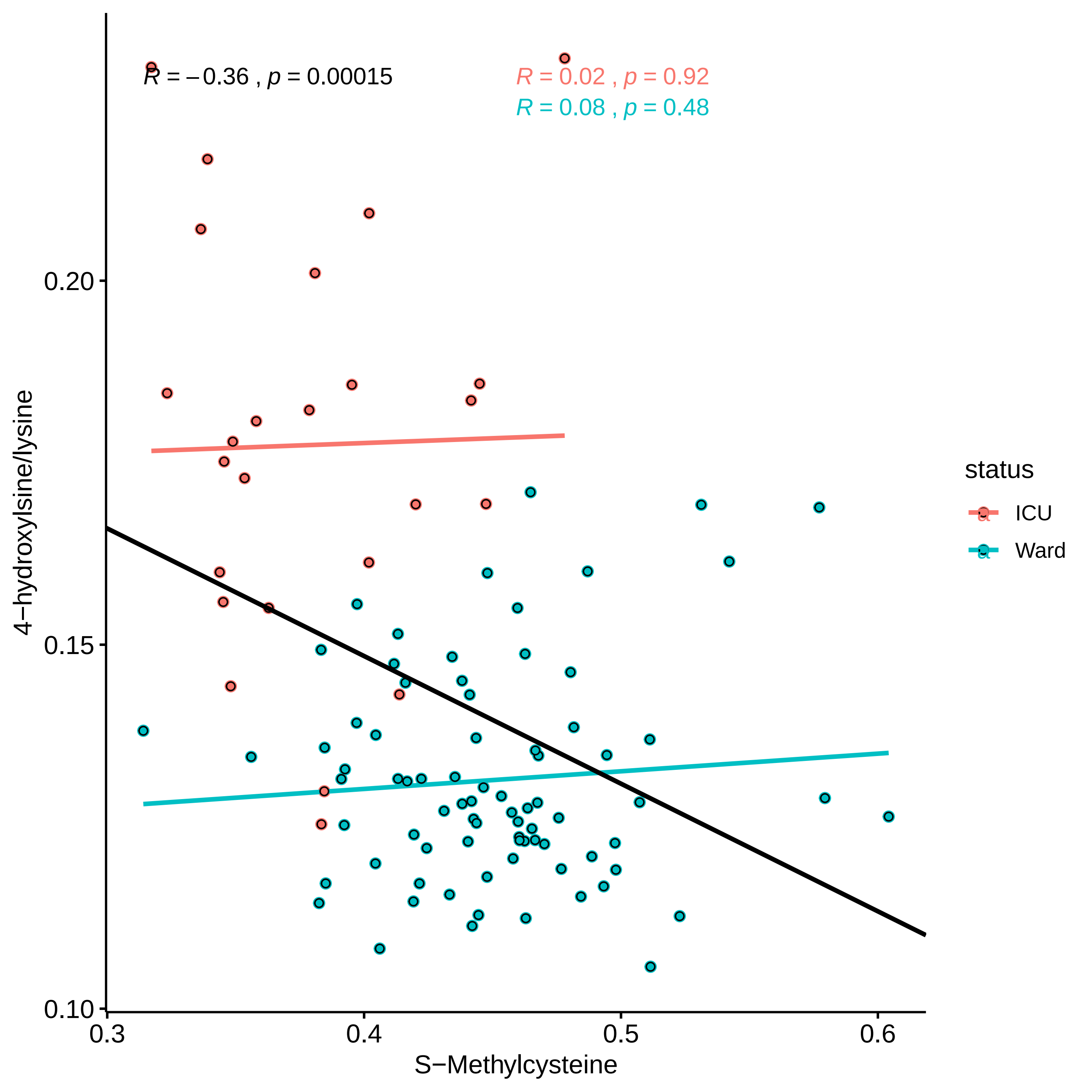

R = −0.36 , p = 0.00015
R = 0.02 , p = 0.92
R = 0.08 , p = 0.48
4−hydroxylsine/lysine
0.20
0.15
0.10
0.3
0.4
0.5
0.6
S−Methylcysteine
status
ICU
Ward

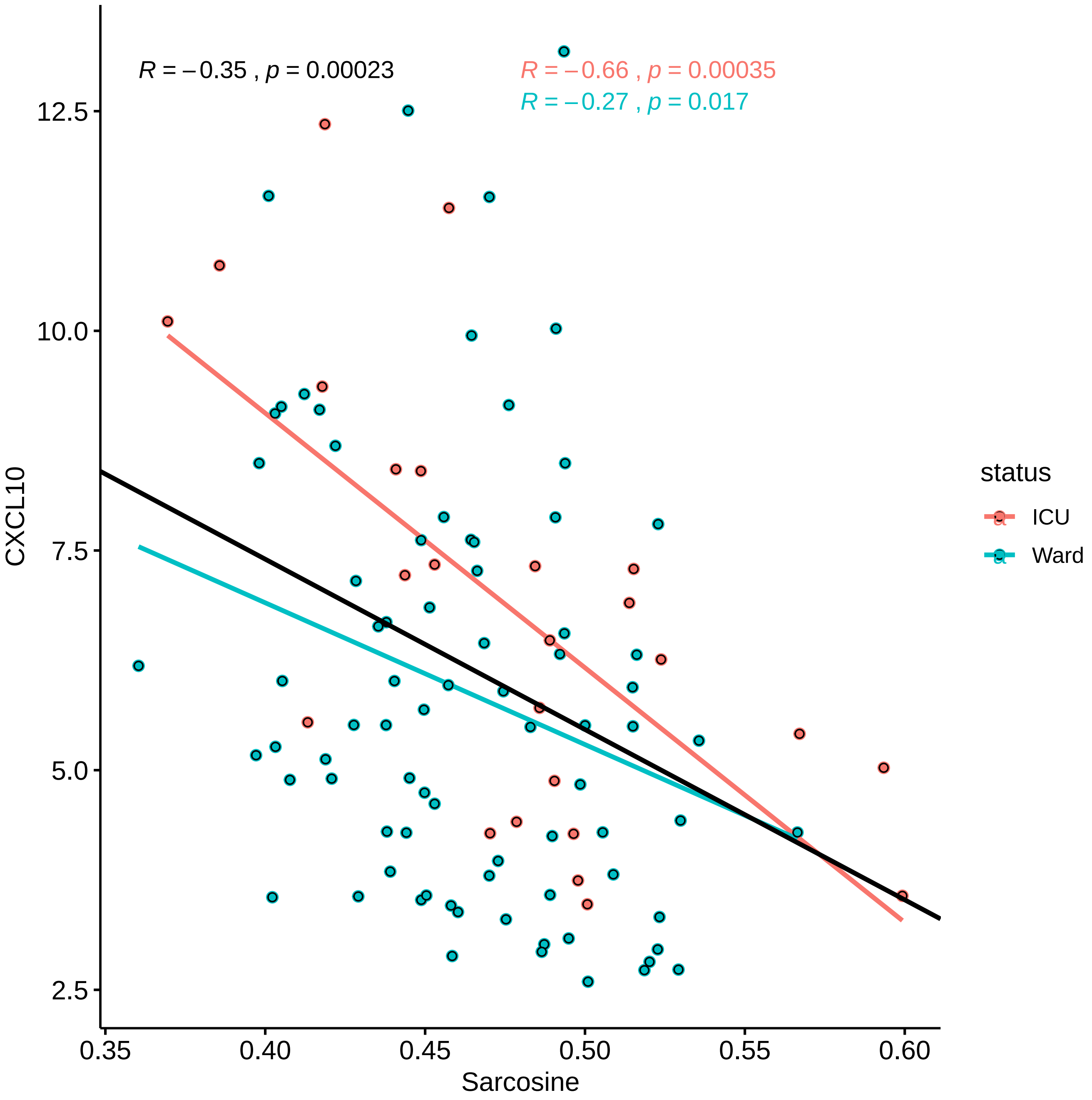

R = −0.35 , p = 0.00023
R = −0.66 , p = 0.00035
R = −0.27 , p = 0.017
CXCL10
12.5
10.0
7.5
5.0
2.5
0.35
0.40
0.45
0.50
0.55
0.60
Sarcosine
status
ICU
Ward

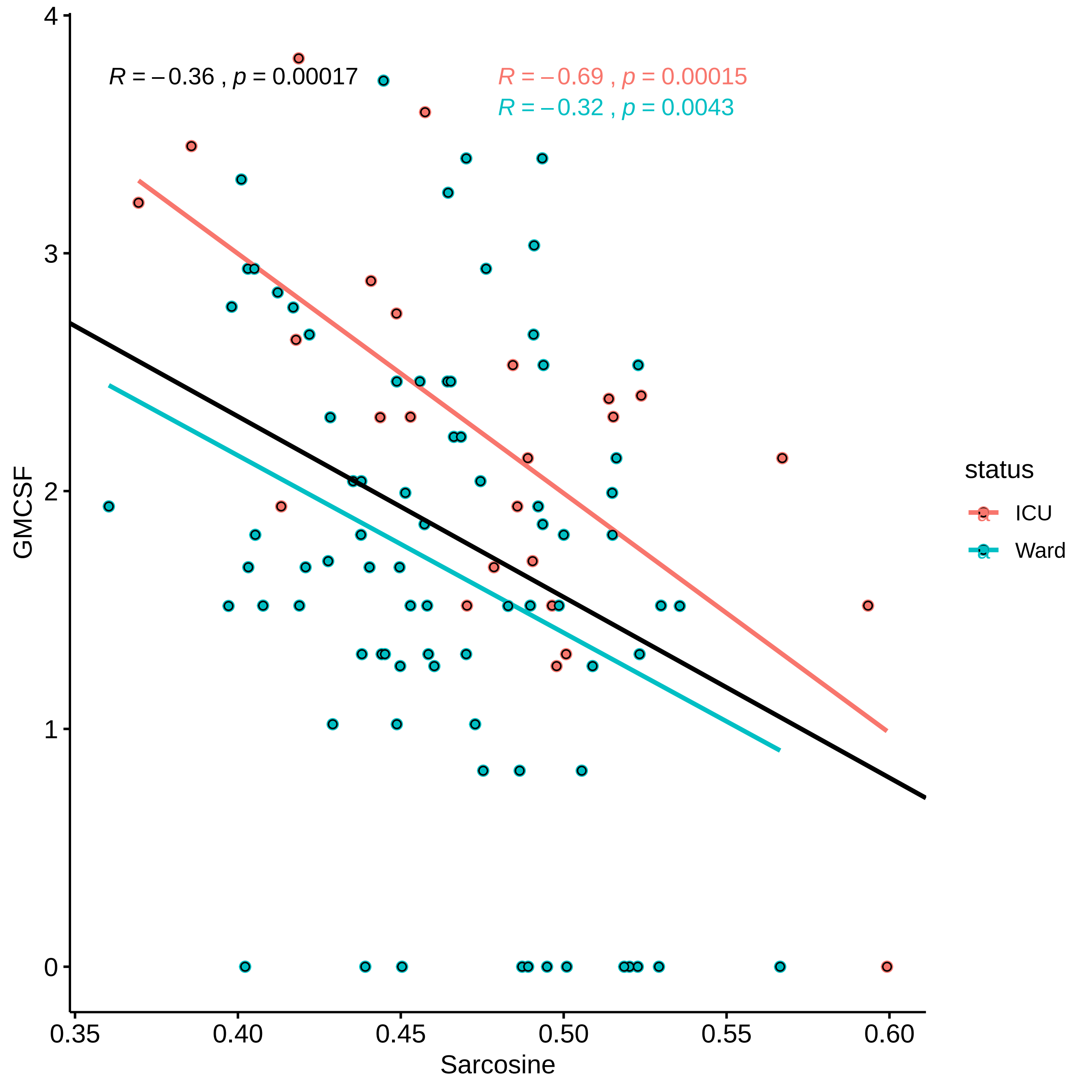

R = −0.36 , p = 0.00017
R = −0.69 , p = 0.00015
R = −0.32 , p = 0.0043
GMCSF
Sarcosine
status
ICU
Ward
0
1
2
3
4
0.35
0.40
0.45
0.50
0.55
0.60

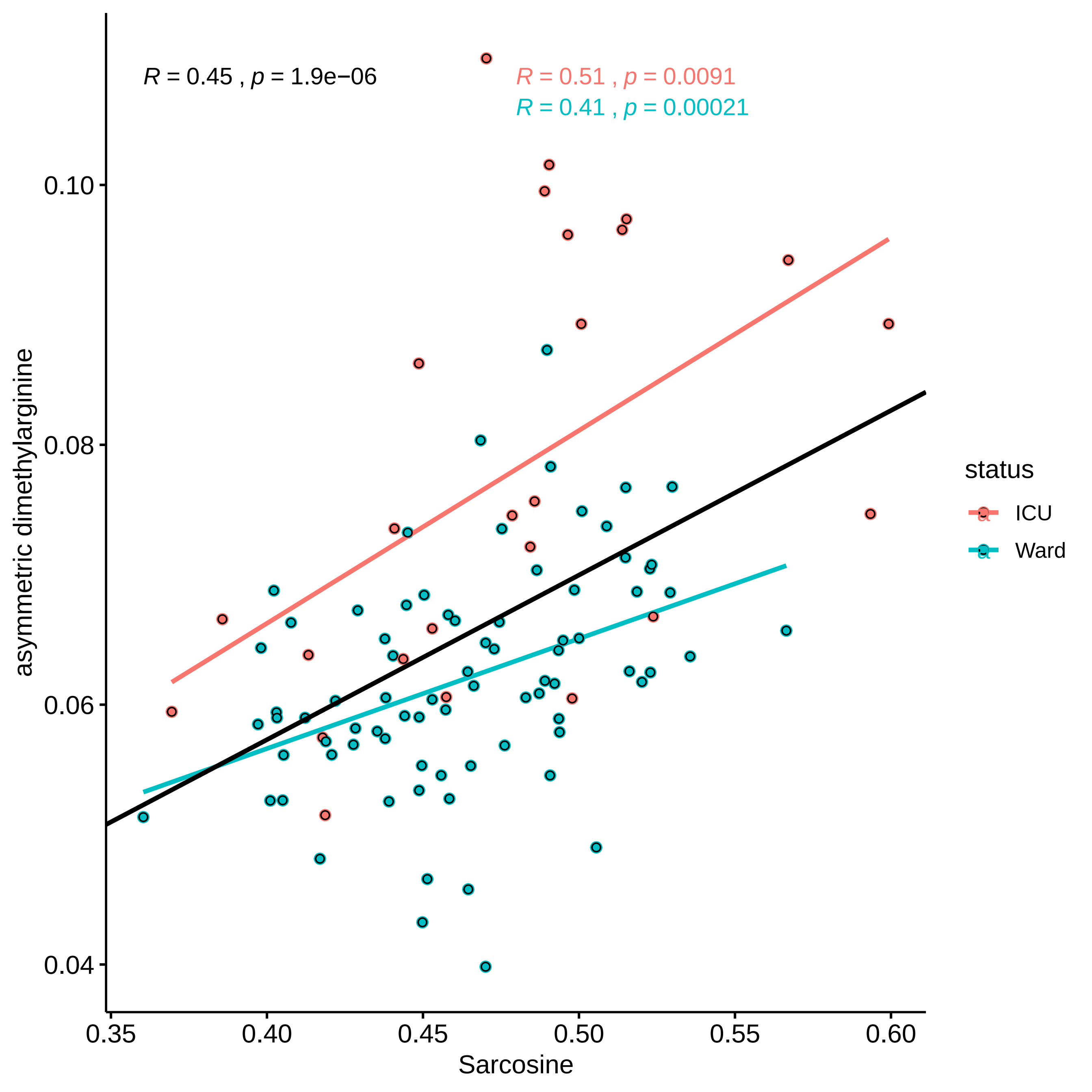

R = 0.45 , p = 1.9e−06
R = 0.51 , p = 0.0091
R = 0.41 , p = 0.00021
asymmetric dimethylarginine
0.10
0.08
0.06
0.04
0.35
0.40
0.45
0.50
0.55
0.60
Sarcosine
status
ICU
Ward

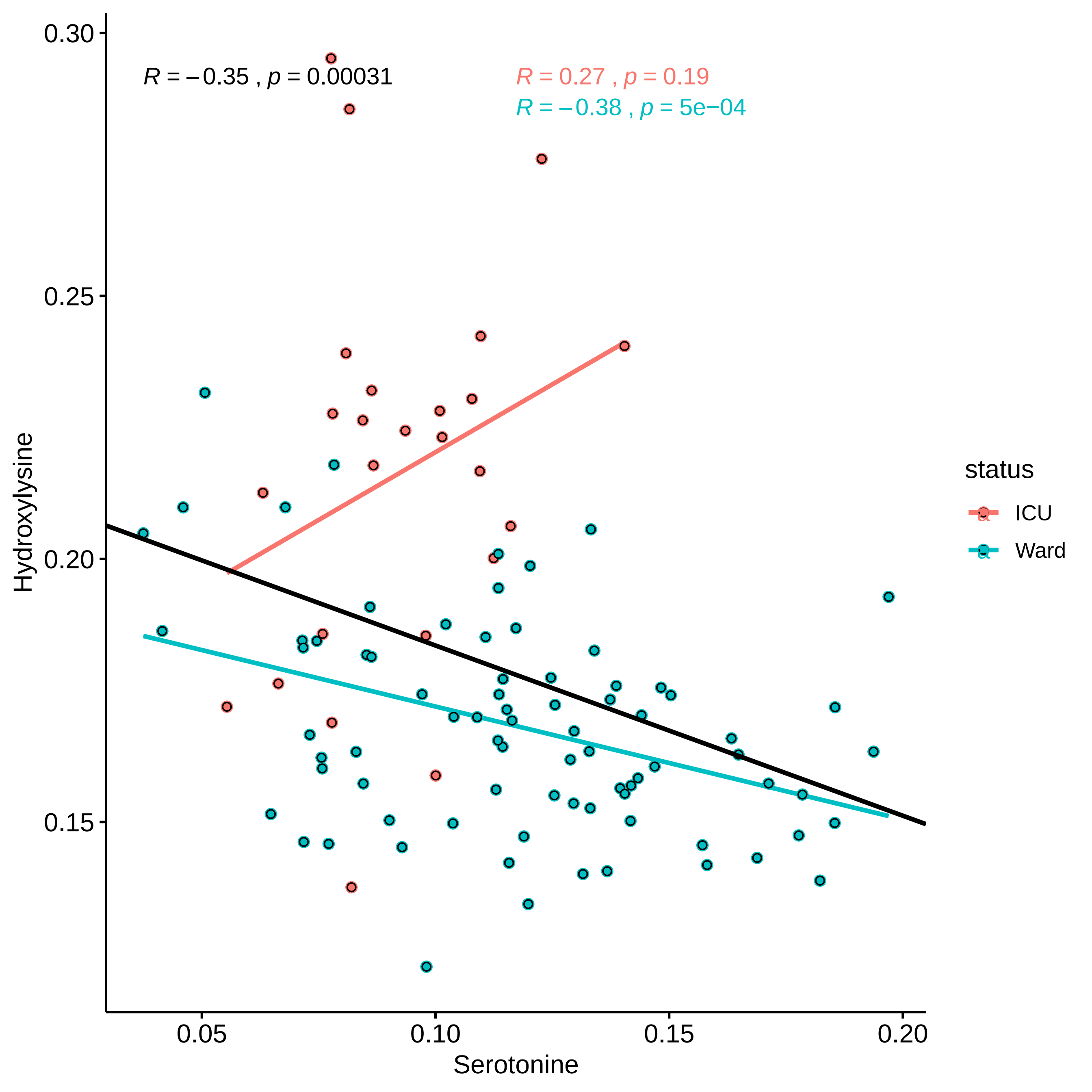

R = −0.35 , p = 0.00031
R = 0.27 , p = 0.19
R = −0.38 , p = 5e−04
Hydroxylysine
Serotonine
0.30
0.25
0.20
0.15
0.05
0.10
0.15
0.20
status
ICU
Ward

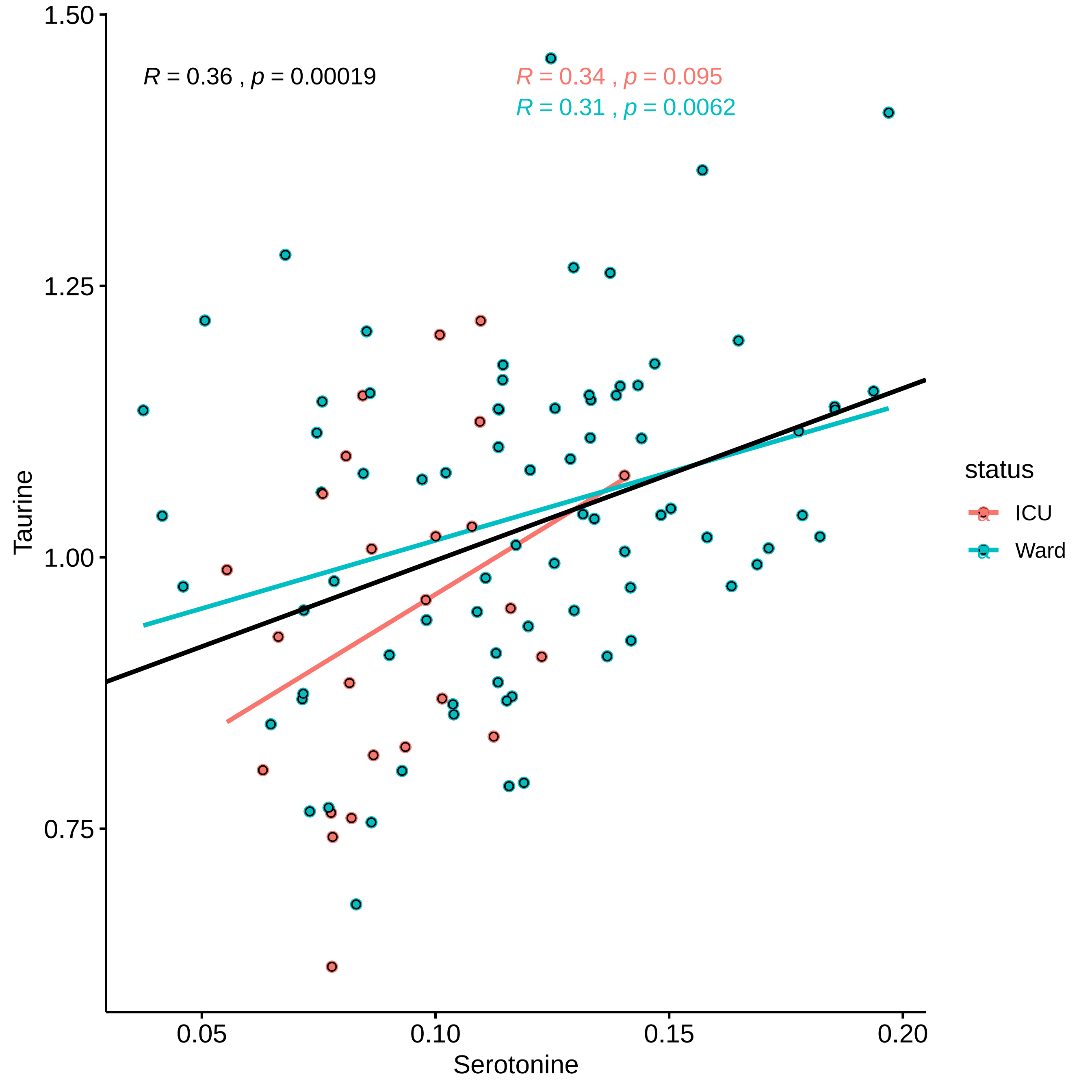

R = 0.36 , p = 0.00019
R = 0.34 , p = 0.095
R = 0.31 , p = 0.0062
1.50
1.25
1.00
0.75
Taurine
0.05
0.10
0.15
0.20
Serotonine
status
ICU
Ward

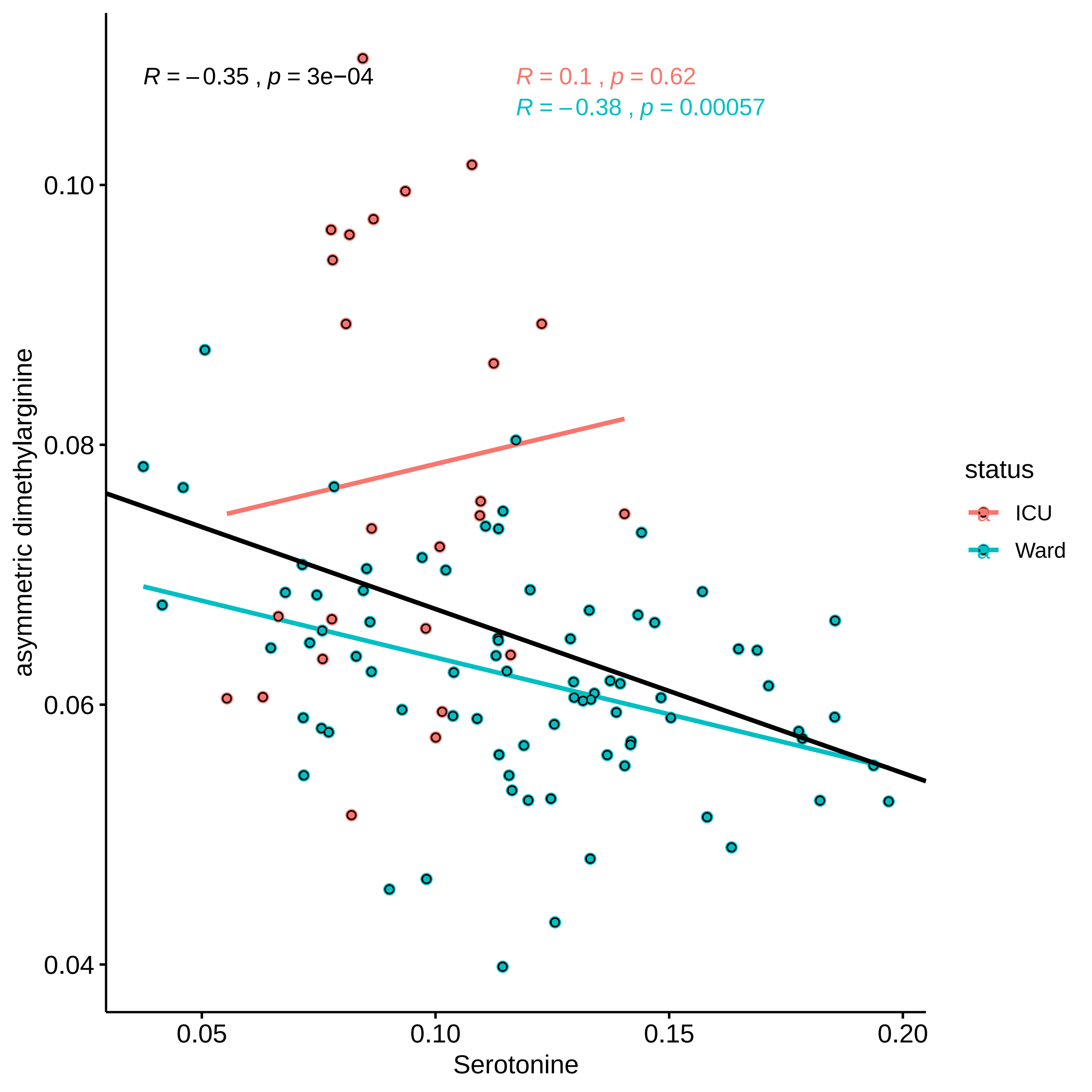
R = −0.35 , p = 3e−04
R = 0.1 , p = 0.62
R = −0.38 , p = 0.00057
asymmetric dimethylarginine
0.10
0.08
0.06
0.04
0.05
0.10
0.15
0.20
Serotonine
status
ICU
Ward

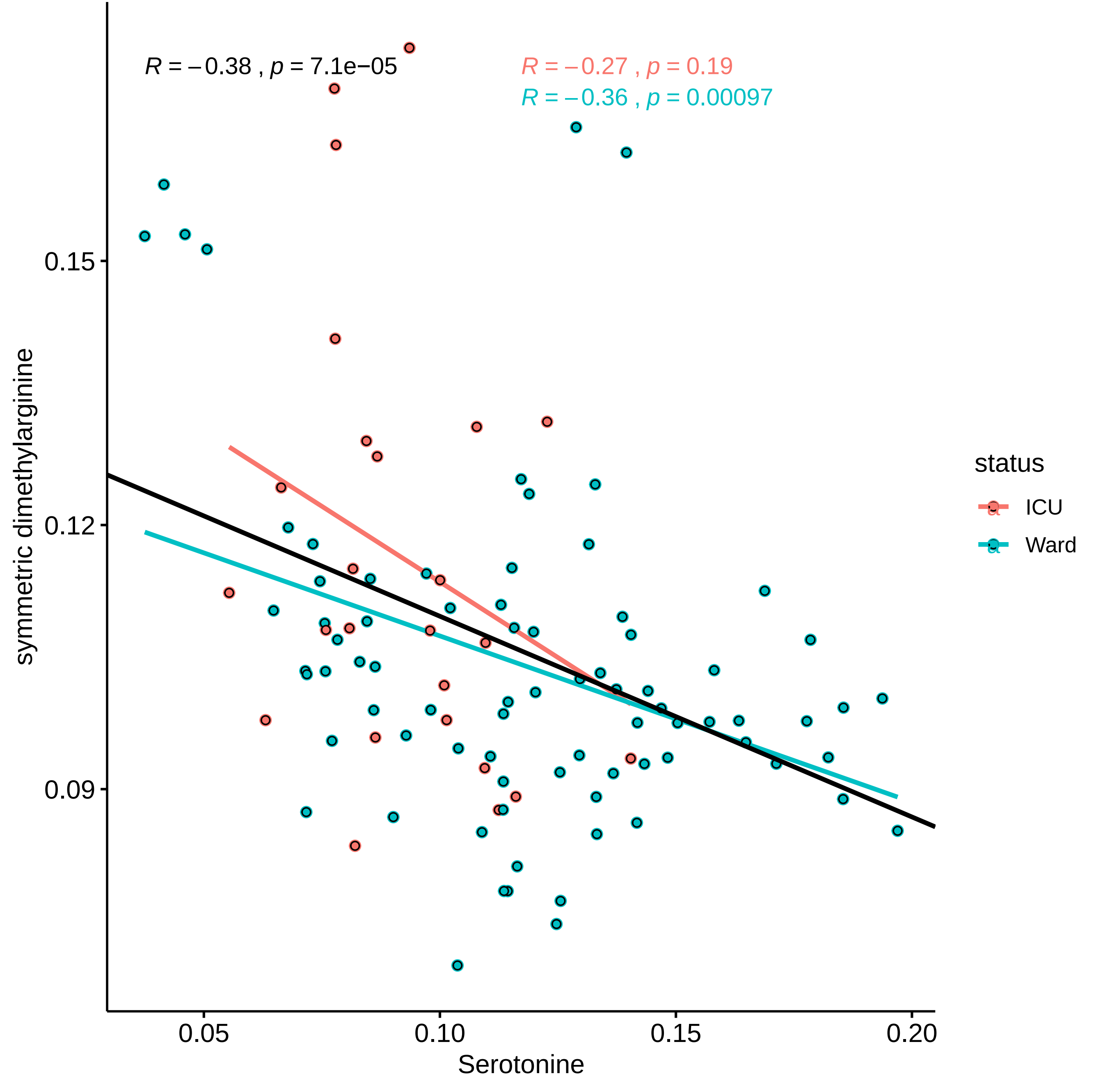

R = −0.38 , p = 7.1e−05
R = −0.27 , p = 0.19
R = −0.36 , p = 0.00097
0.15
0.12
0.09
symmetric dimethylarginine
0.05
0.10
0.15
0.20
Serotonine
status
ICU
Ward

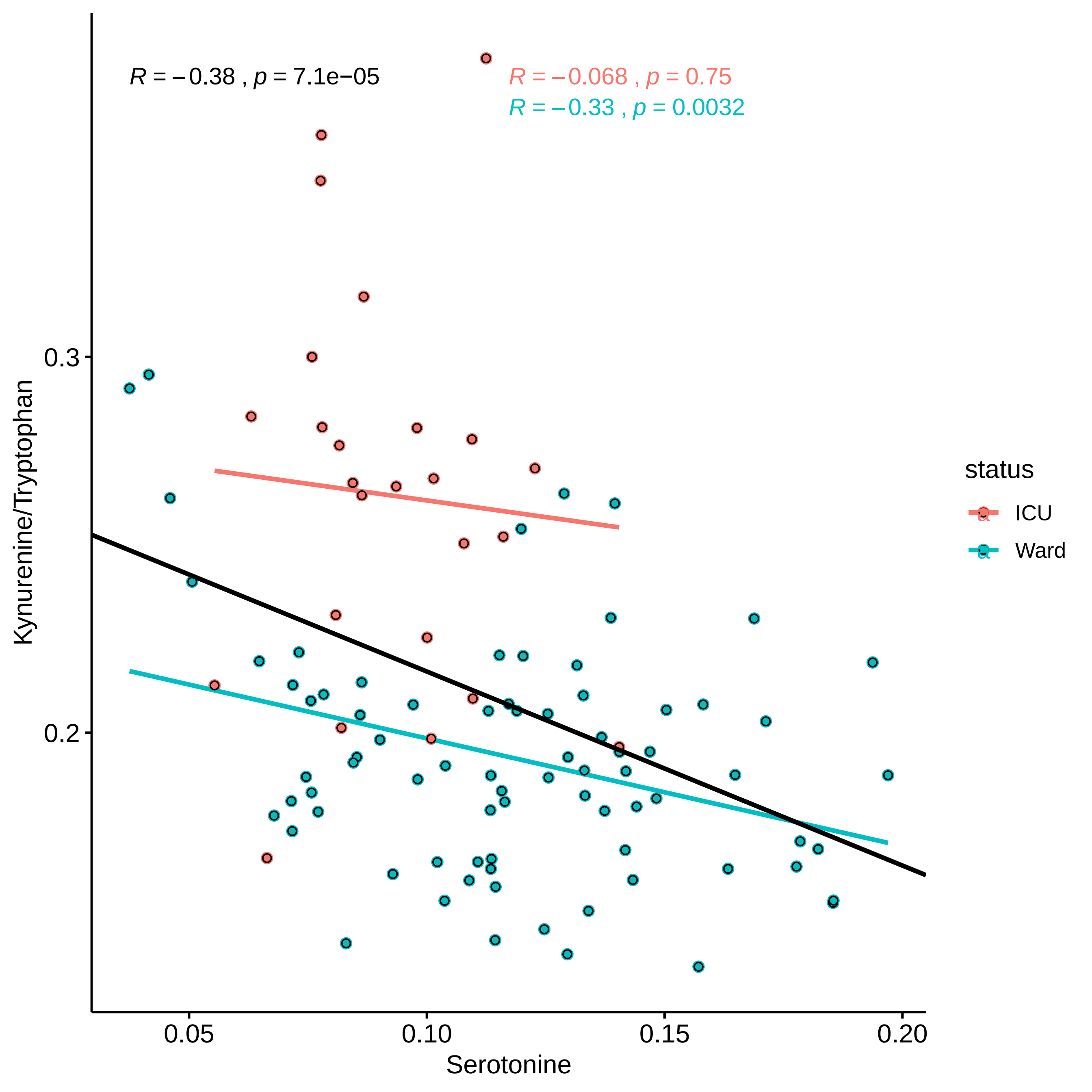

R = −0.38 , p = 7.1e−05
R = −0.068 , p = 0.75
R = −0.33 , p = 0.0032
0.3
0.2
Kynurenine/Tryptophan
0.05
0.10
0.15
0.20
Serotonine
status
ICU
Ward

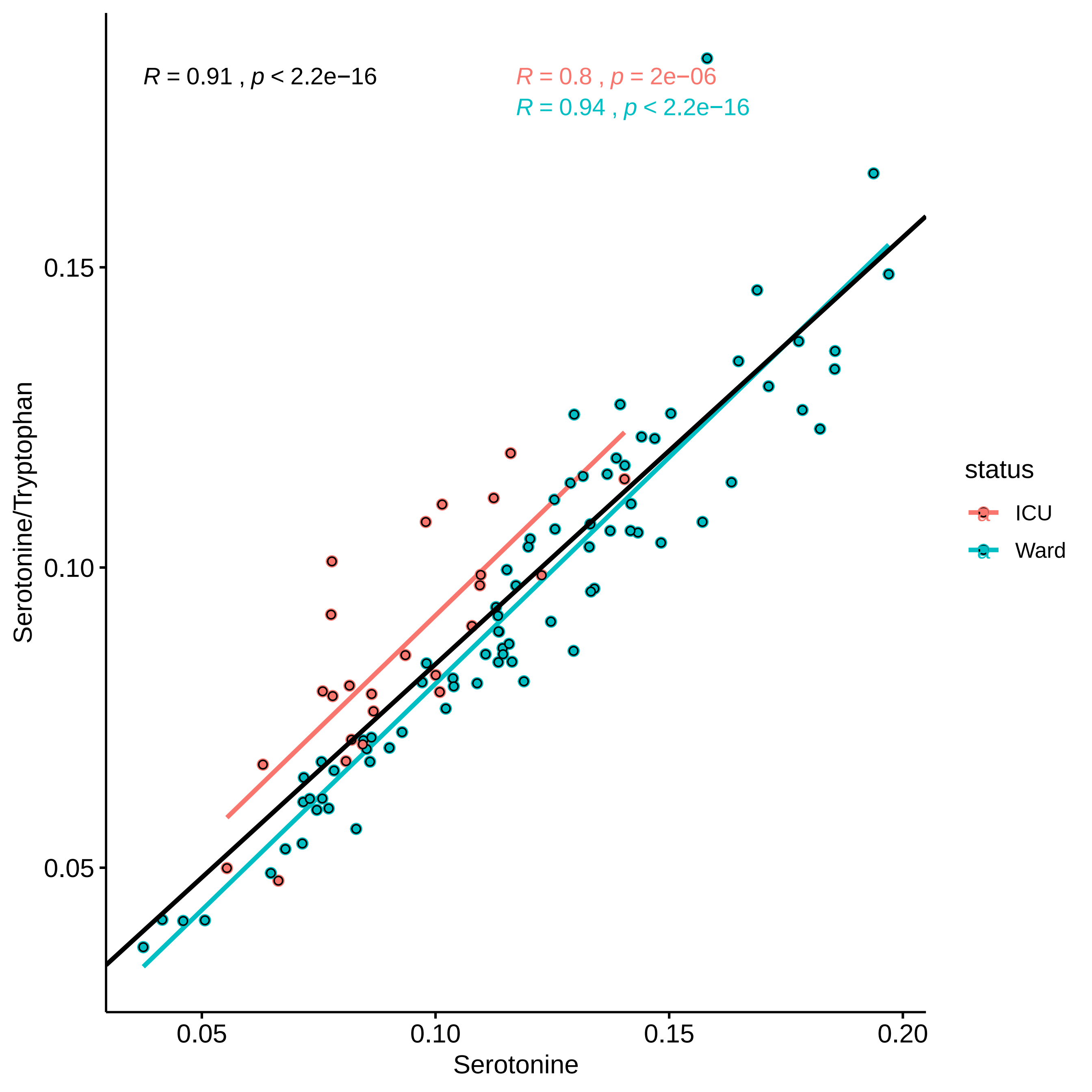

R = 0.91 , p < 2.2e−16
R = 0.8 , p = 2e−06
R = 0.94 , p < 2.2e−16
0.15
0.10
0.05
Serotonine/Tryptophan
0.05
0.10
0.15
0.20
Serotonine
status
ICU
Ward

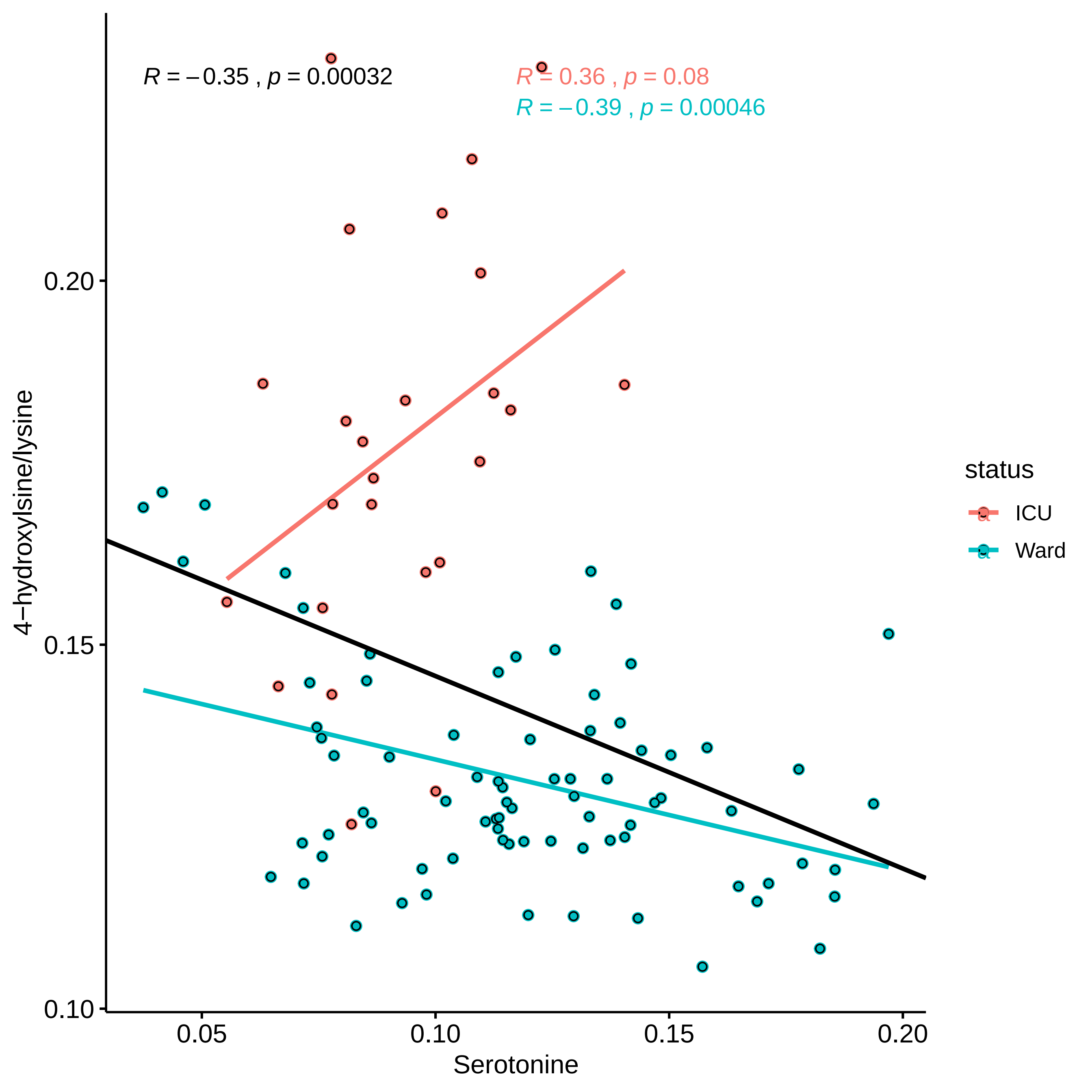

R = −0.35 , p = 0.00032
R = 0.36 , p = 0.08
R = −0.39 , p = 0.00046
4−hydroxylsine/lysine
0.20
0.15
0.10
0.05
0.10
0.15
0.20
Serotonine
status
ICU
Ward

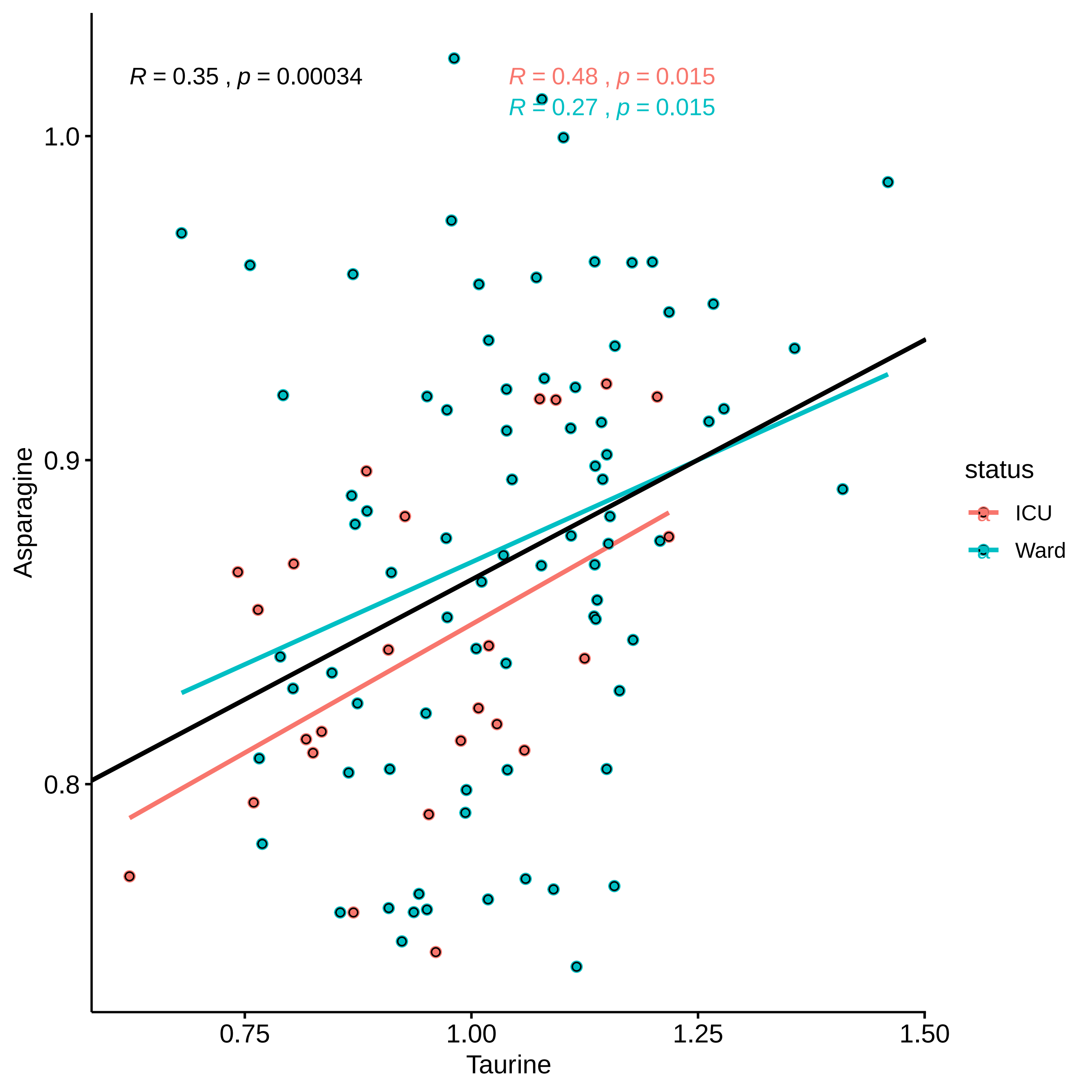

R = 0.35 , p = 0.00034
R = 0.48 , p = 0.015
R = 0.27 , p = 0.015
1.0
0.9
0.8
Asparagine
0.75
1.00
1.25
1.50
Taurine
status
ICU
Ward

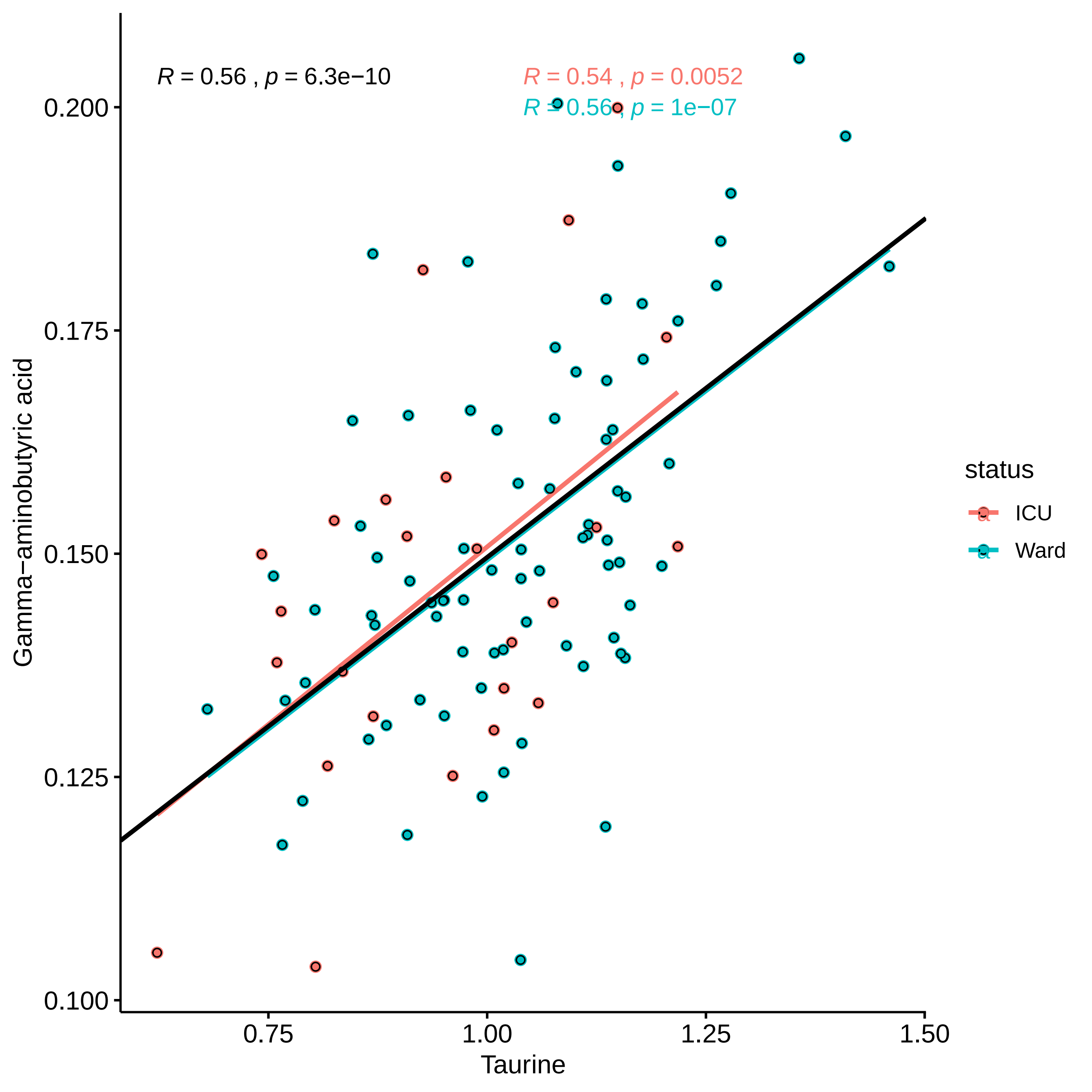

R = 0.56 , p = 6.3e−10
R = 0.54 , p = 0.0052
R = 0.56 , p = 1e−07
Gamma−aminobutyric acid
0.100
0.125
0.150
0.175
0.200
0.75
1.00
1.25
1.50
Taurine
status
ICU
Ward

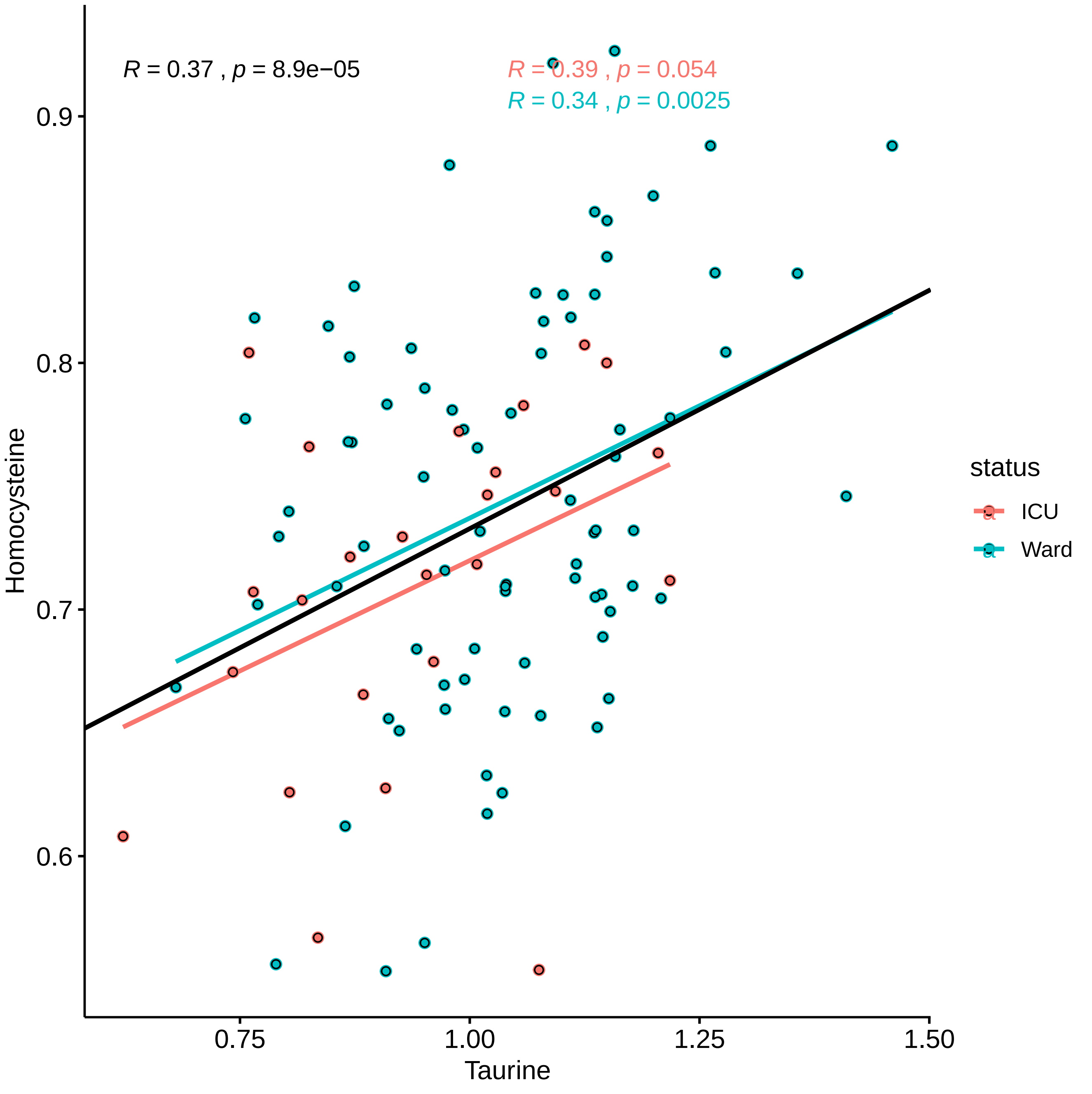

R = 0.37 , p = 8.9e−05
R = 0.39 , p = 0.054
R = 0.34 , p = 0.0025
0.9
0.8
0.7
0.6
Homocysteine
0.75
1.00
1.25
1.50
Taurine
status
ICU
Ward

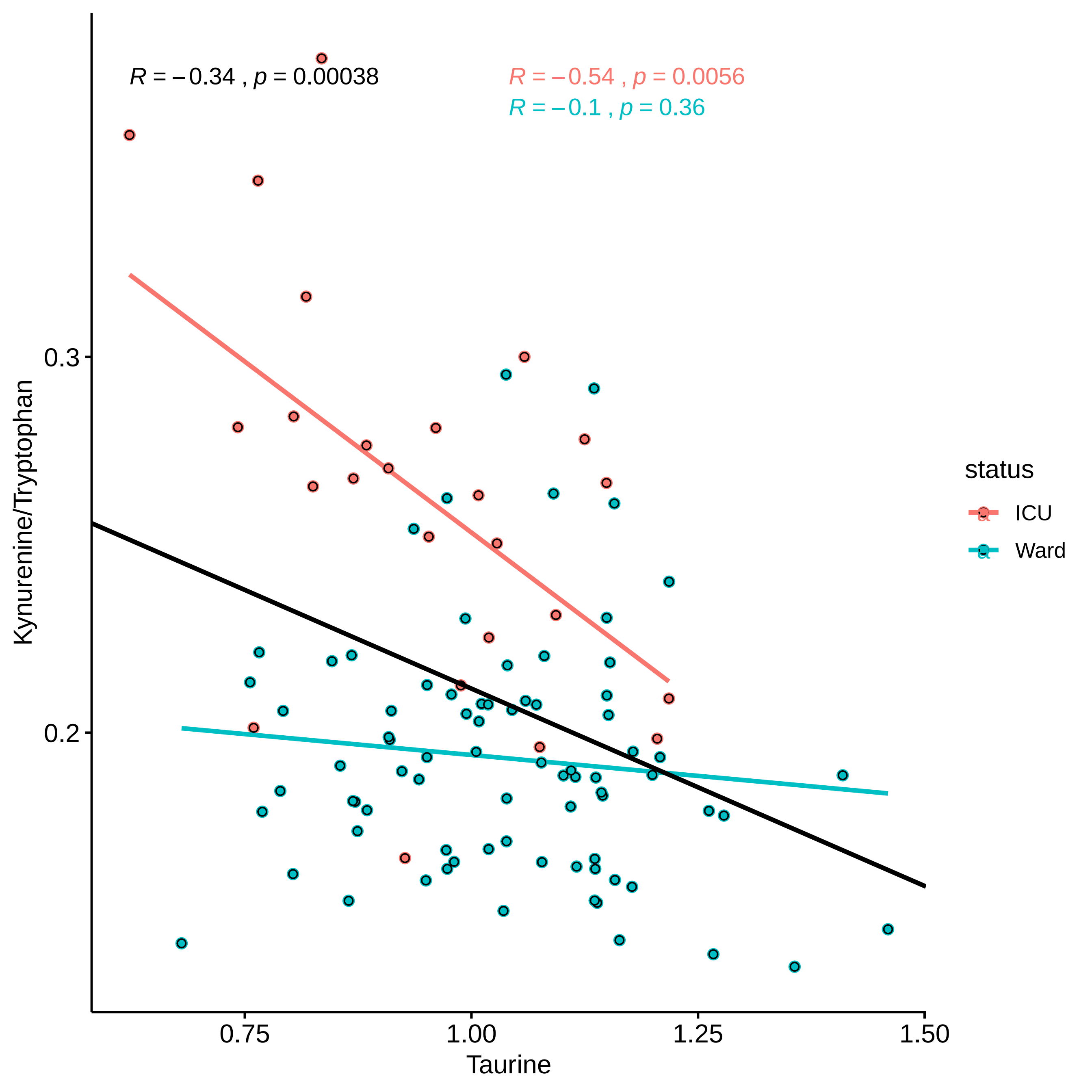

R = −0.34 , p = 0.00038
R = −0.54 , p = 0.0056
R = −0.1 , p = 0.36
0.3
0.2
Kynurenine/Tryptophan
0.75
1.00
1.25
1.50
Taurine
status
ICU
Ward

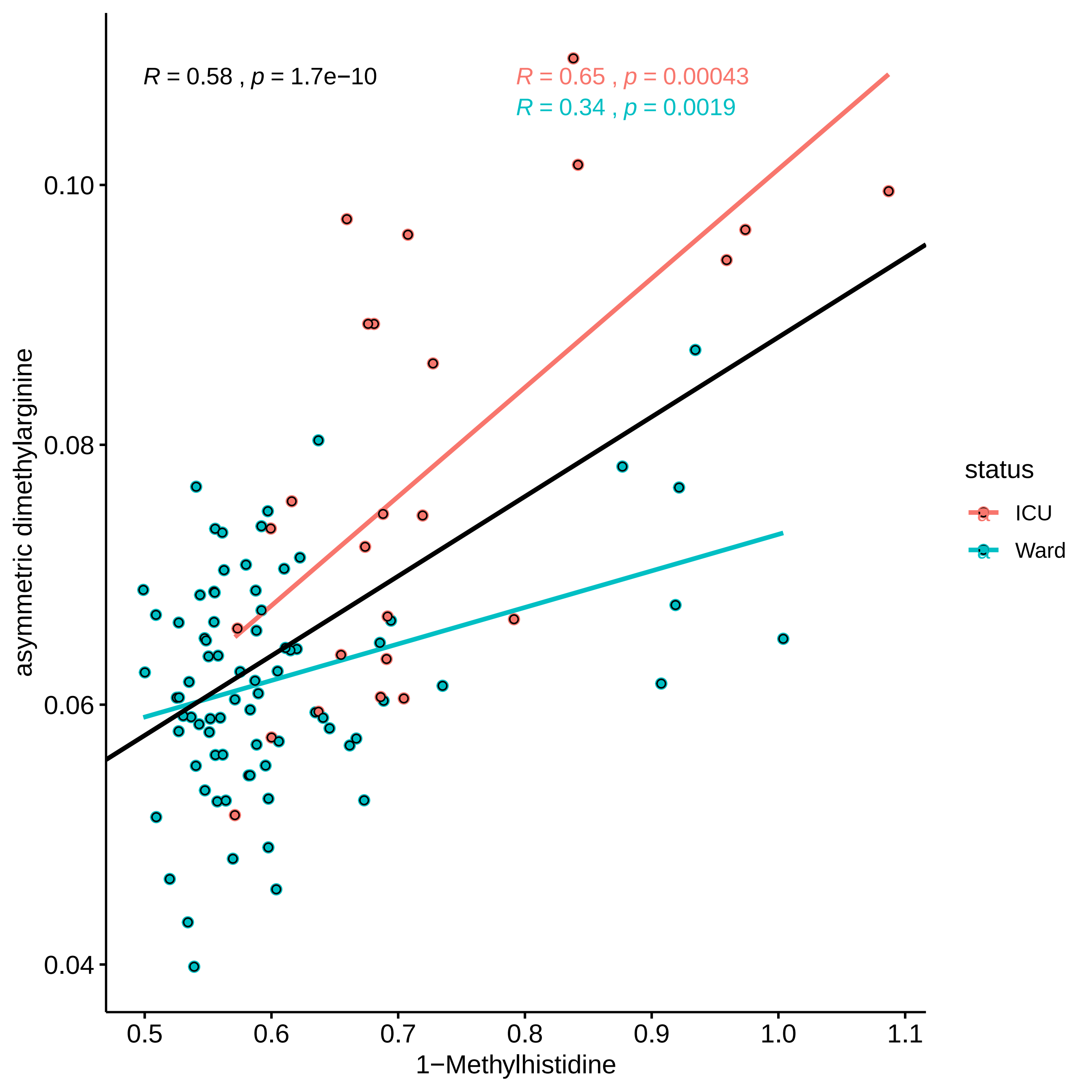

R = 0.58 , p = 1.7e−10
R = 0.65 , p = 0.00043
R = 0.34 , p = 0.0019
asymmetric dimethylarginine
0.10
0.08
0.06
0.04
0.5
0.6
0.7
0.8
0.9
1.0
1.1
1−Methylhistidine
status
ICU
Ward

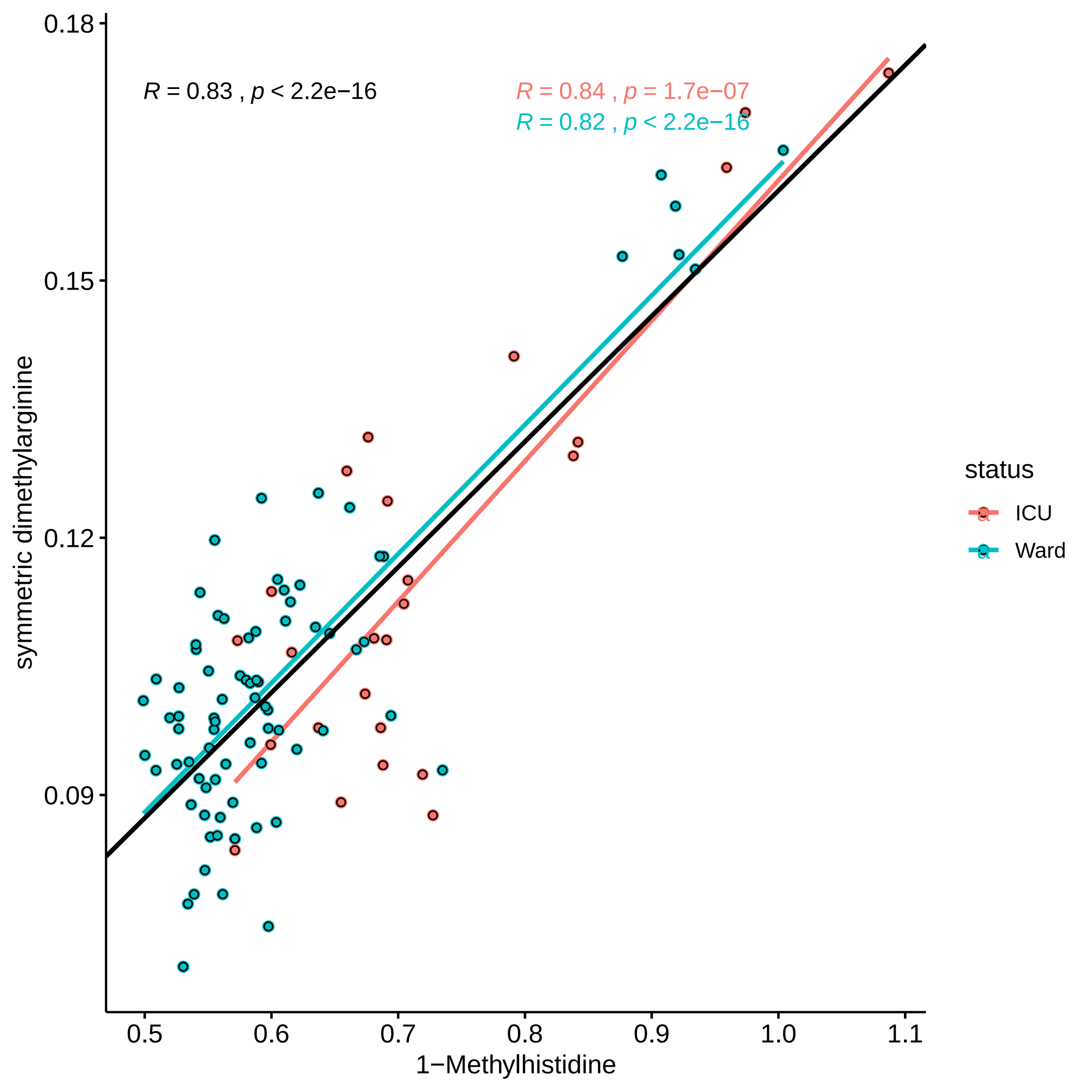

R = 0.83 , p < 2.2e−16
R = 0.84 , p = 1.7e−07
R = 0.82 , p < 2.2e−16
0.18
0.15
0.12
0.09
symmetric dimethylarginine
0.5
0.6
0.7
0.8
0.9
1.0
1.1
1−Methylhistidine
status
ICU
Ward

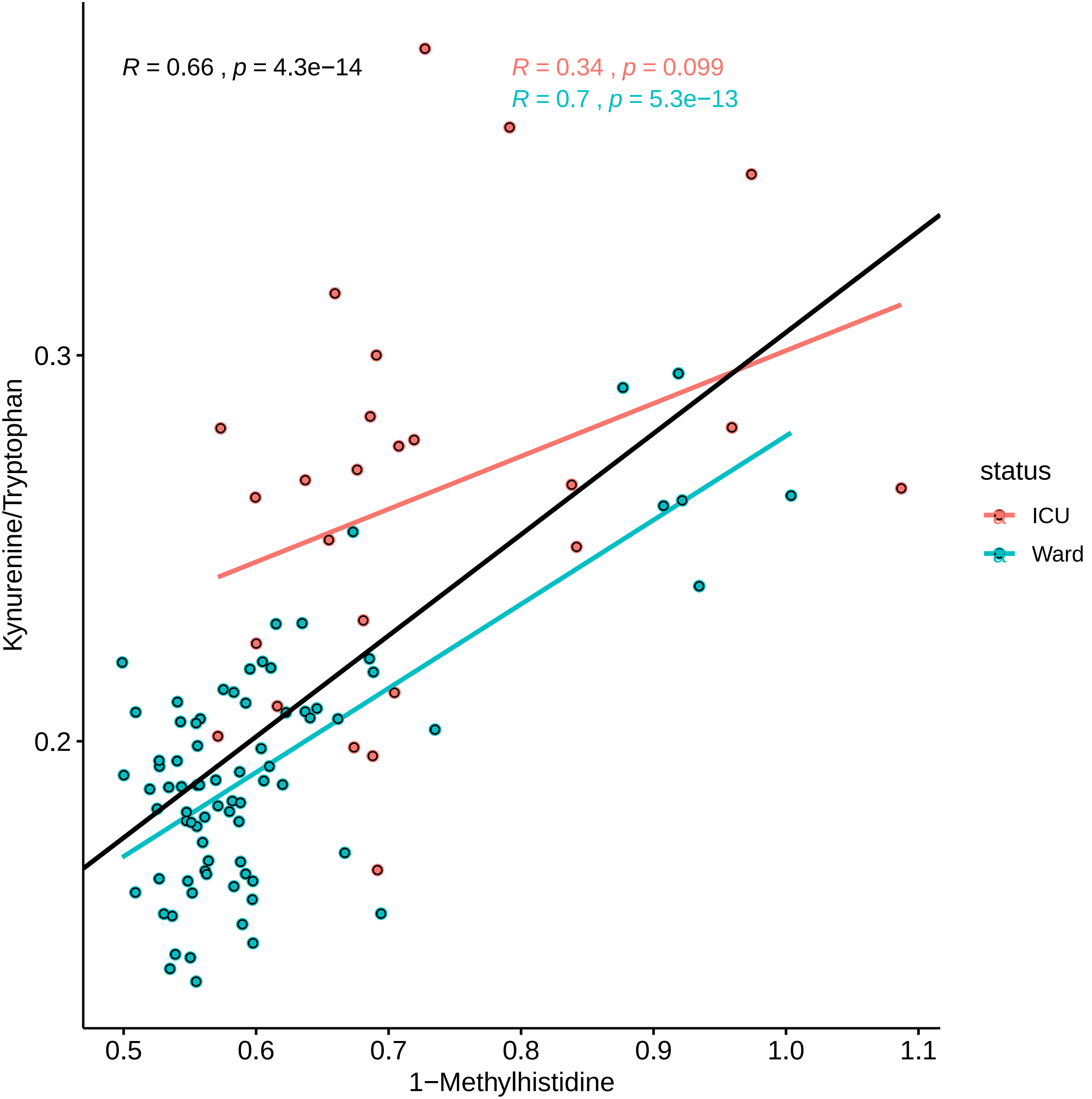

R = 0.66 , p = 4.3e−14
R = 0.34 , p = 0.099
R = 0.7 , p = 5.3e−13
Kynurenine/Tryptophan
0.3
0.2
0.5
0.6
0.7
0.8
0.9
1.0
1.1
1−Methylhistidine
status
ICU
Ward

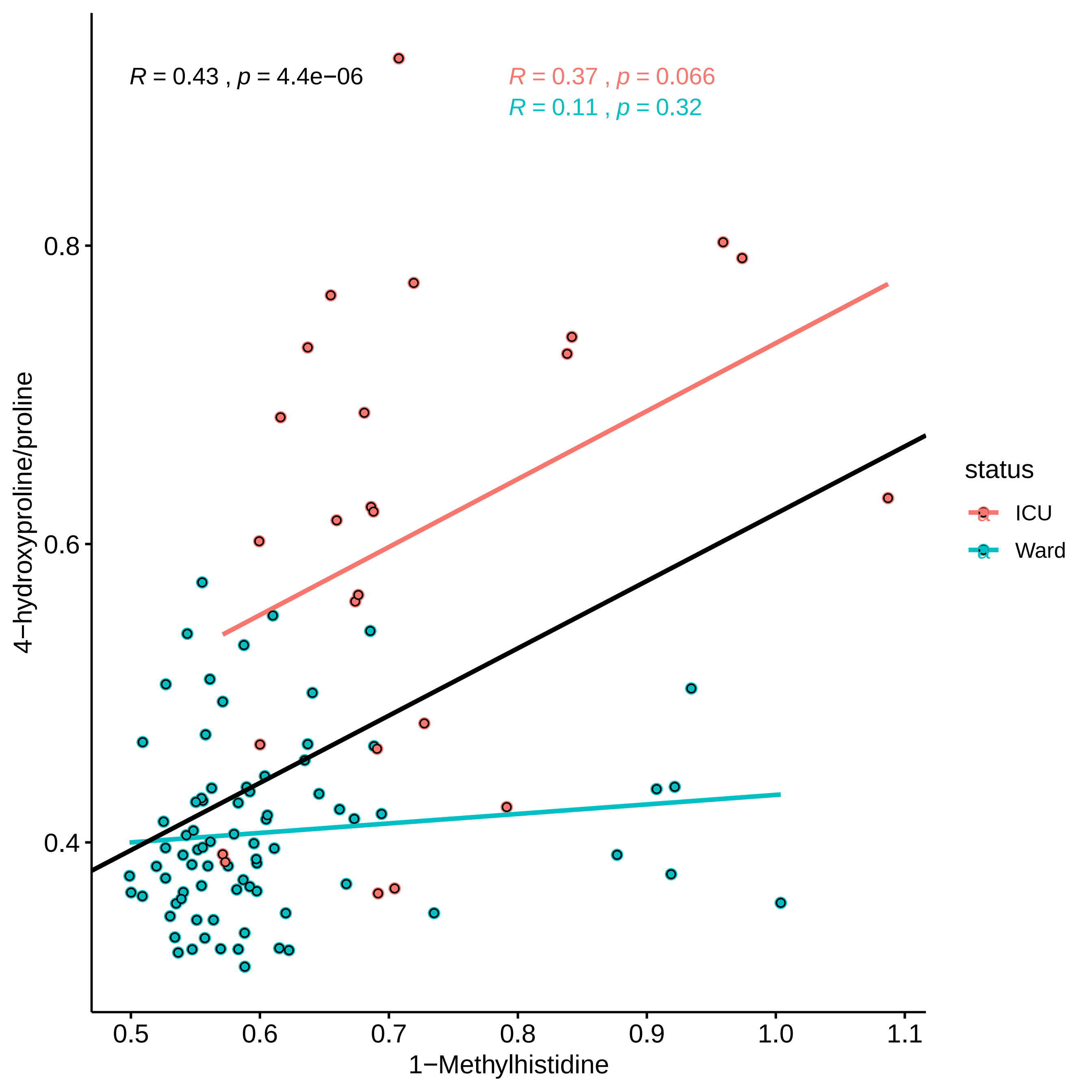

R = 0.43 , p = 4.4e−06
R = 0.37 , p = 0.066
R = 0.11 , p = 0.32
0.8
0.6
0.4
4−hydroxyproline/proline
0.5
0.6
0.7
0.8
0.9
1.0
1.1
1−Methylhistidine
status
ICU
Ward

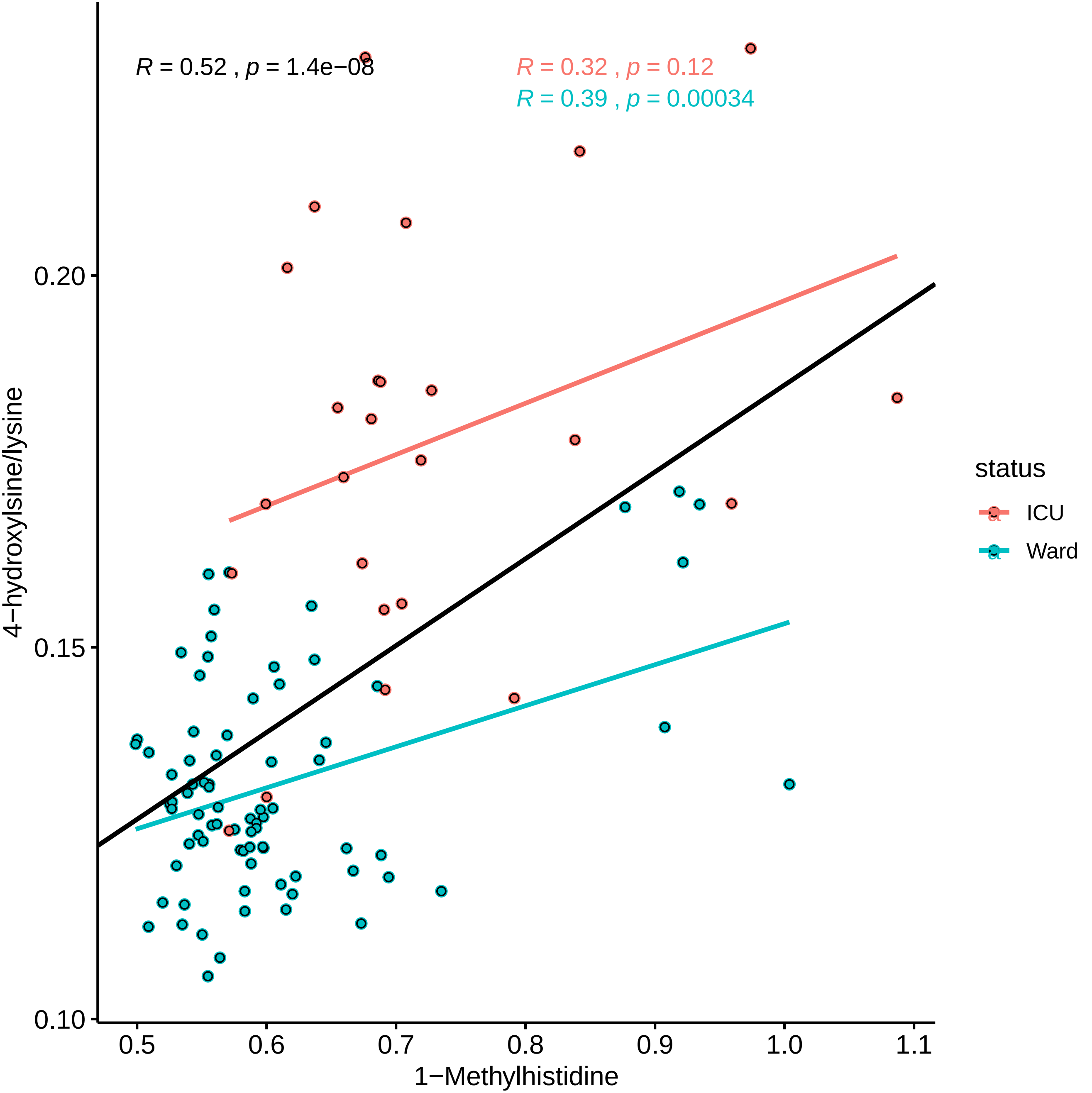

R = 0.52 , p = 1.4e−08
R = 0.32 , p = 0.12
R = 0.39 , p = 0.00034
4−hydroxylsine/lysine
0.20
0.15
0.10
0.5
0.6
0.7
0.8
0.9
1.0
1.1
1−Methylhistidine
status
ICU
Ward

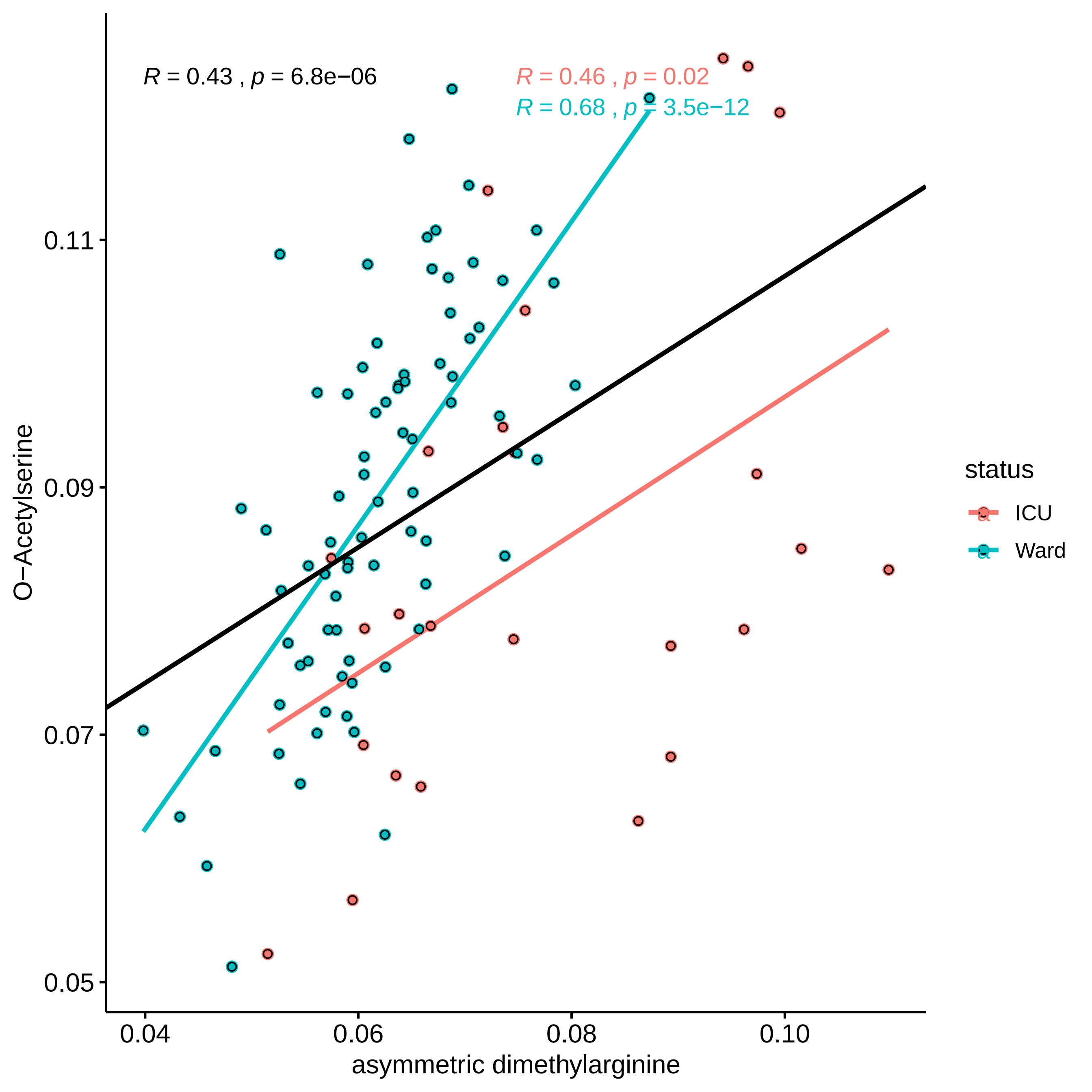

R = 0.43 , p = 6.8e−06
R = 0.46 , p = 0.02
R = 0.68 , p = 3.5e−12
O−Acetylserine
0.11
0.09
0.07
0.05
0.04
0.06
0.08
0.10
asymmetric dimethylarginine
status
ICU
Ward

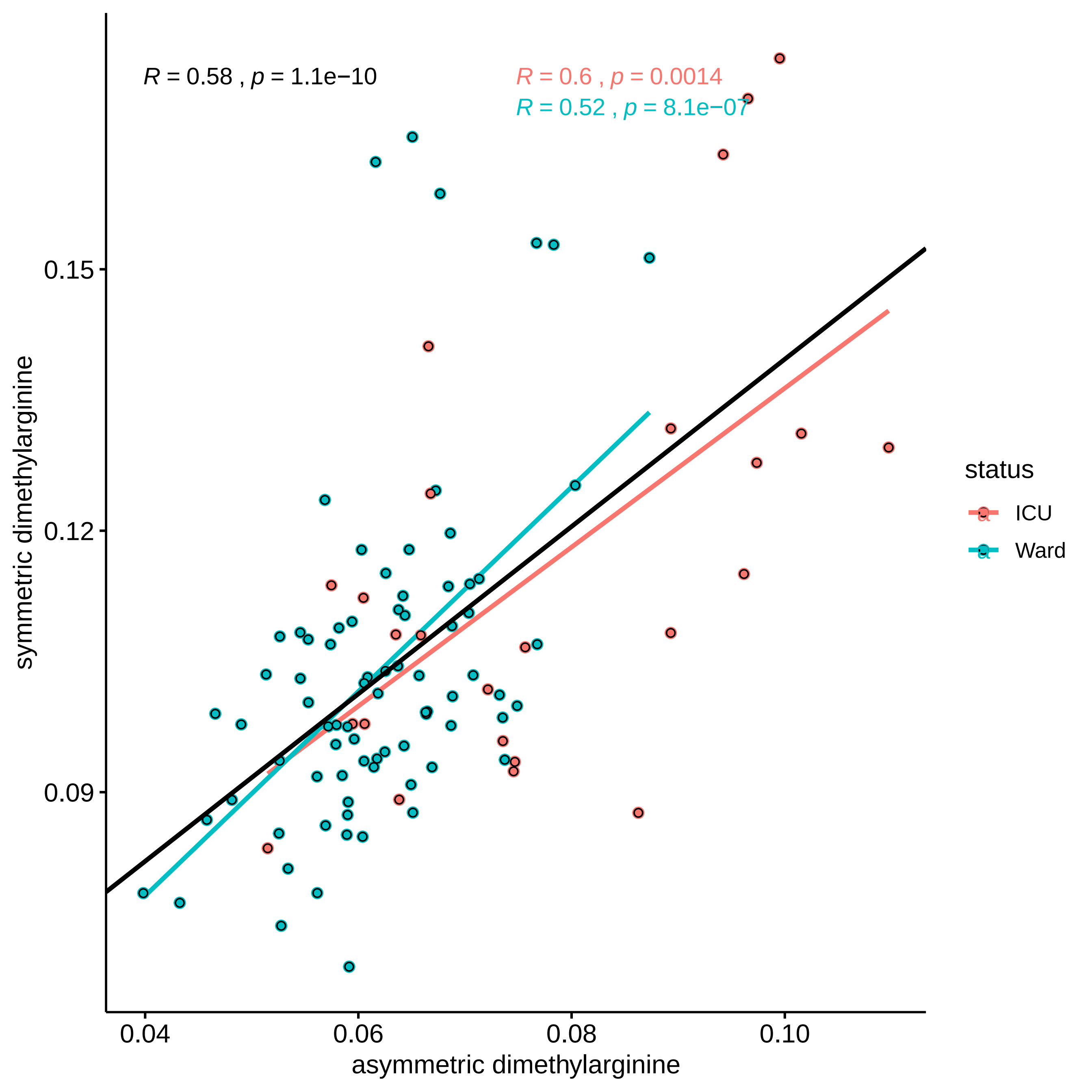

R = 0.58 , p = 1.1e−10
R = 0.6 , p = 0.0014
R = 0.52 , p = 8.1e−07
0.15
0.12
0.09
0.04
0.06
0.08
0.10
symmetric dimethylarginine
asymmetric dimethylarginine
status
ICU
Ward

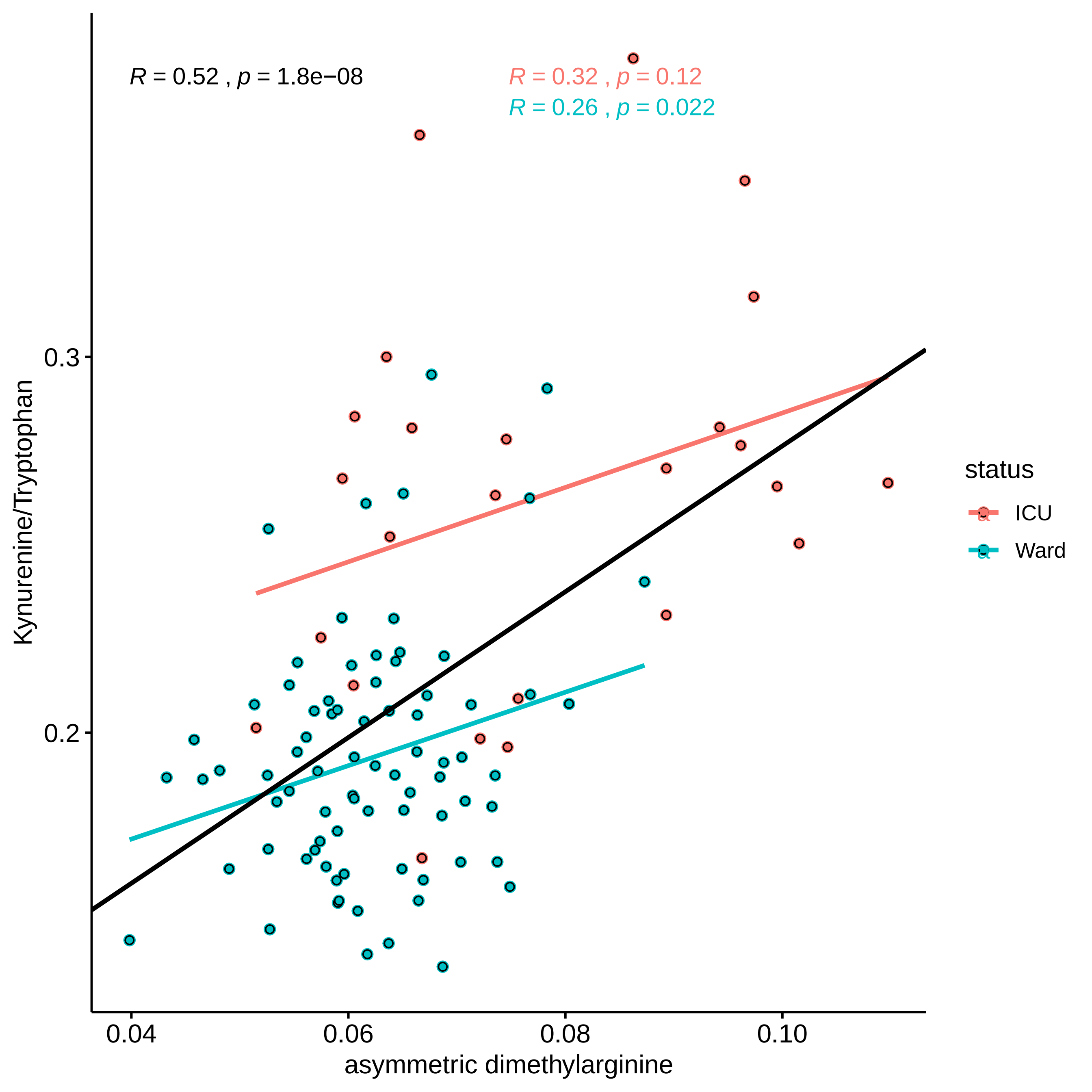

R = 0.52 , p = 1.8e−08
R = 0.32 , p = 0.12
R = 0.26 , p = 0.022
Kynurenine/Tryptophan
0.3
0.2
0.04
0.06
0.08
0.10
asymmetric dimethylarginine
status
ICU
Ward

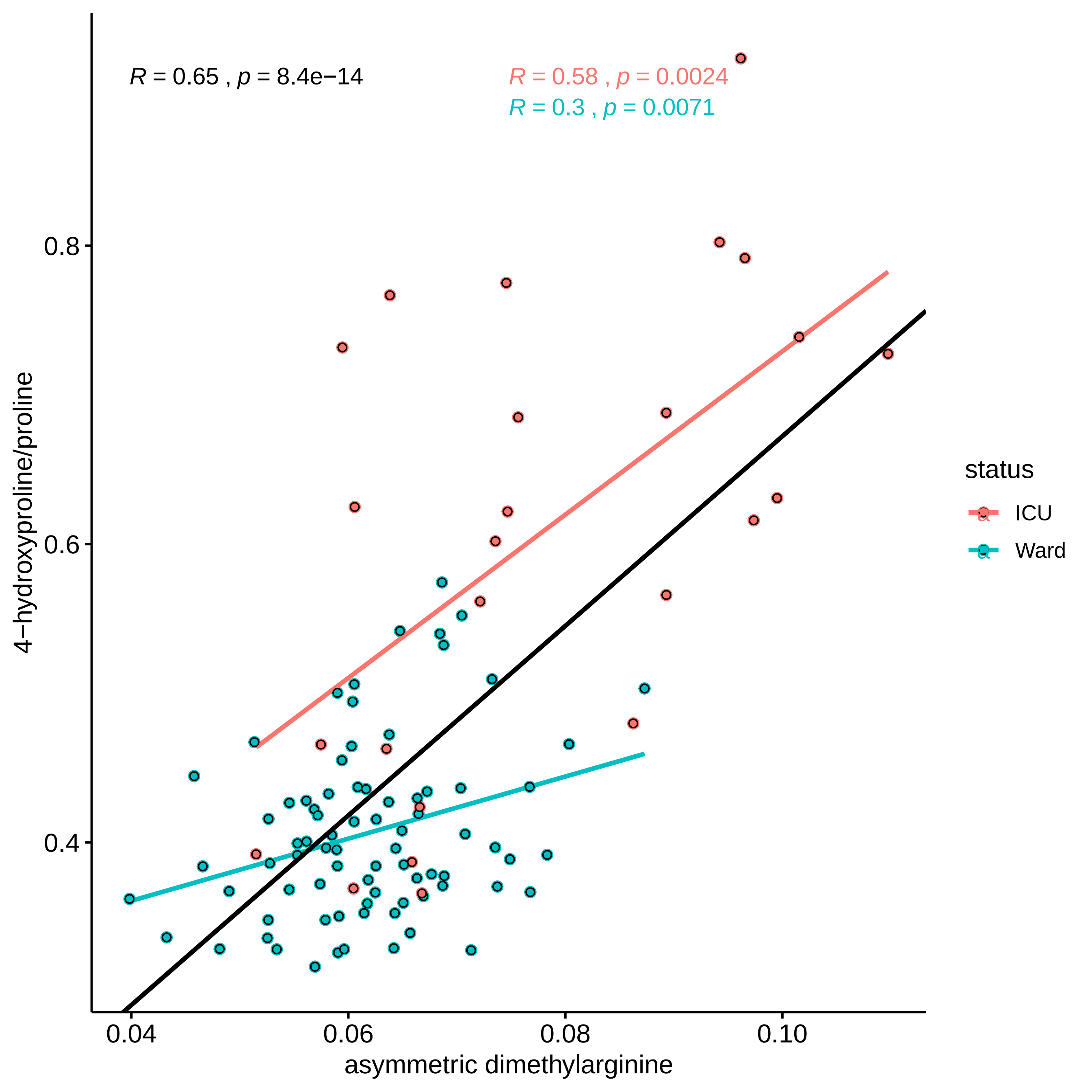

R = 0.65 , p = 8.4e−14
R = 0.58 , p = 0.0024
R = 0.3 , p = 0.0071
0.8
0.6
0.4
4−hydroxyproline/proline
0.04
0.06
0.08
0.10
asymmetric dimethylarginine
status
ICU
Ward

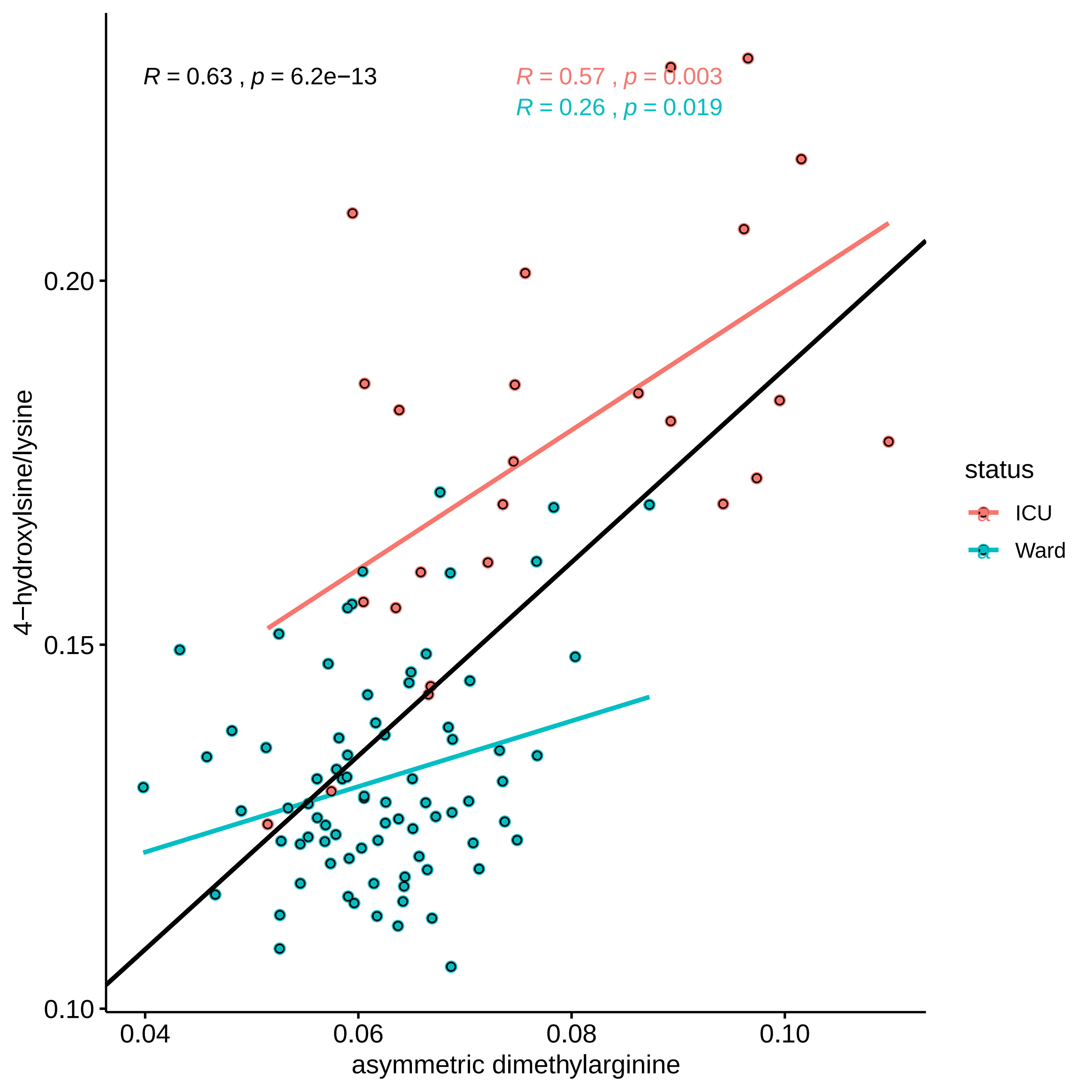

R = 0.63 , p = 6.2e−13
R = 0.57 , p = 0.003
R = 0.26 , p = 0.019
4−hydroxylysine/lysine
0.20
0.15
0.10
0.04
0.06
0.08
0.10
asymmetric dimethylarginine
status
ICU
Ward

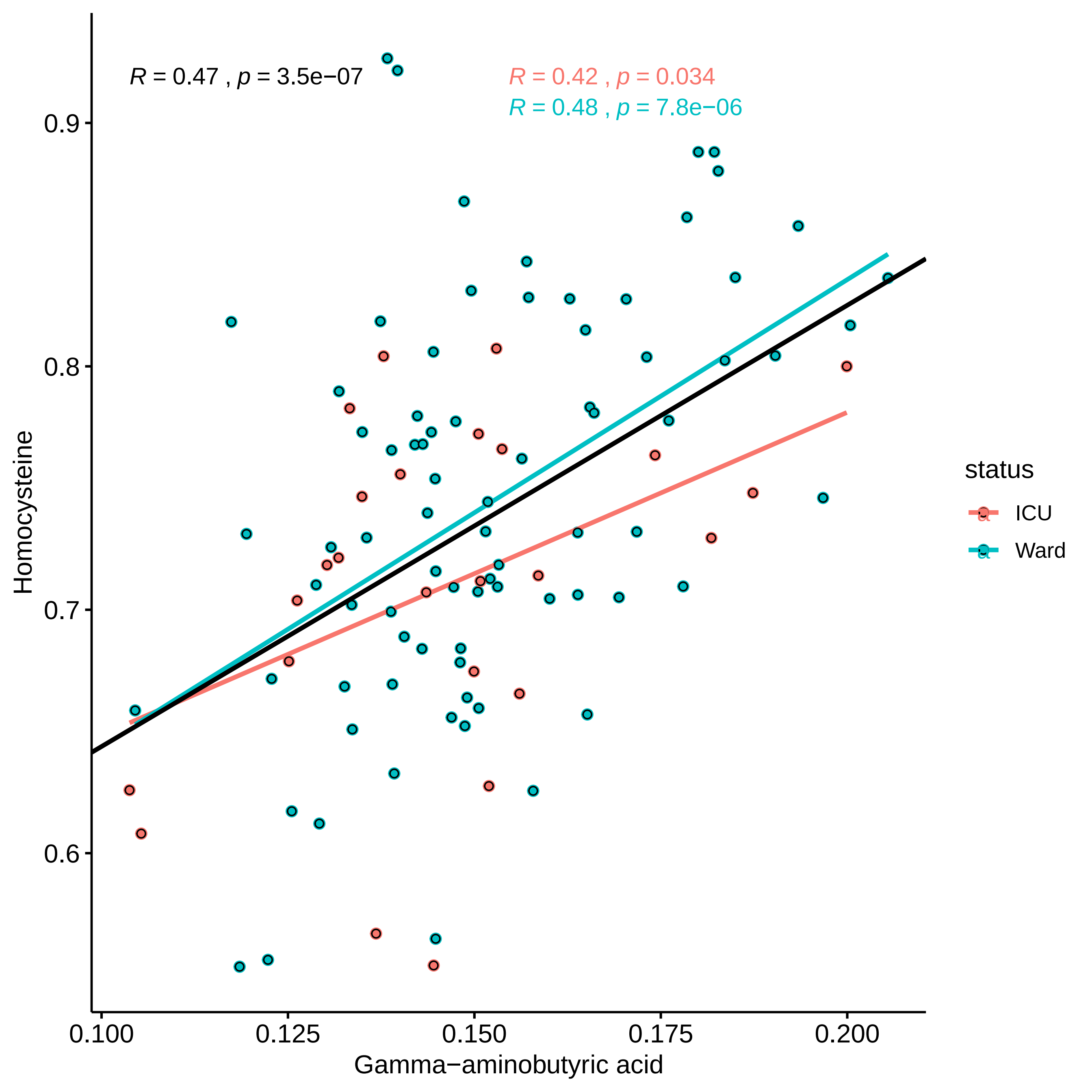

R = 0.47 , p = 3.5e−07
R = 0.42 , p = 0.034
R = 0.48 , p = 7.8e−06
0.9
0.8
0.7
0.6
Homocysteine
0.100
0.125
0.150
0.175
0.200
Gamma−aminobutyric acid
status
ICU
Ward

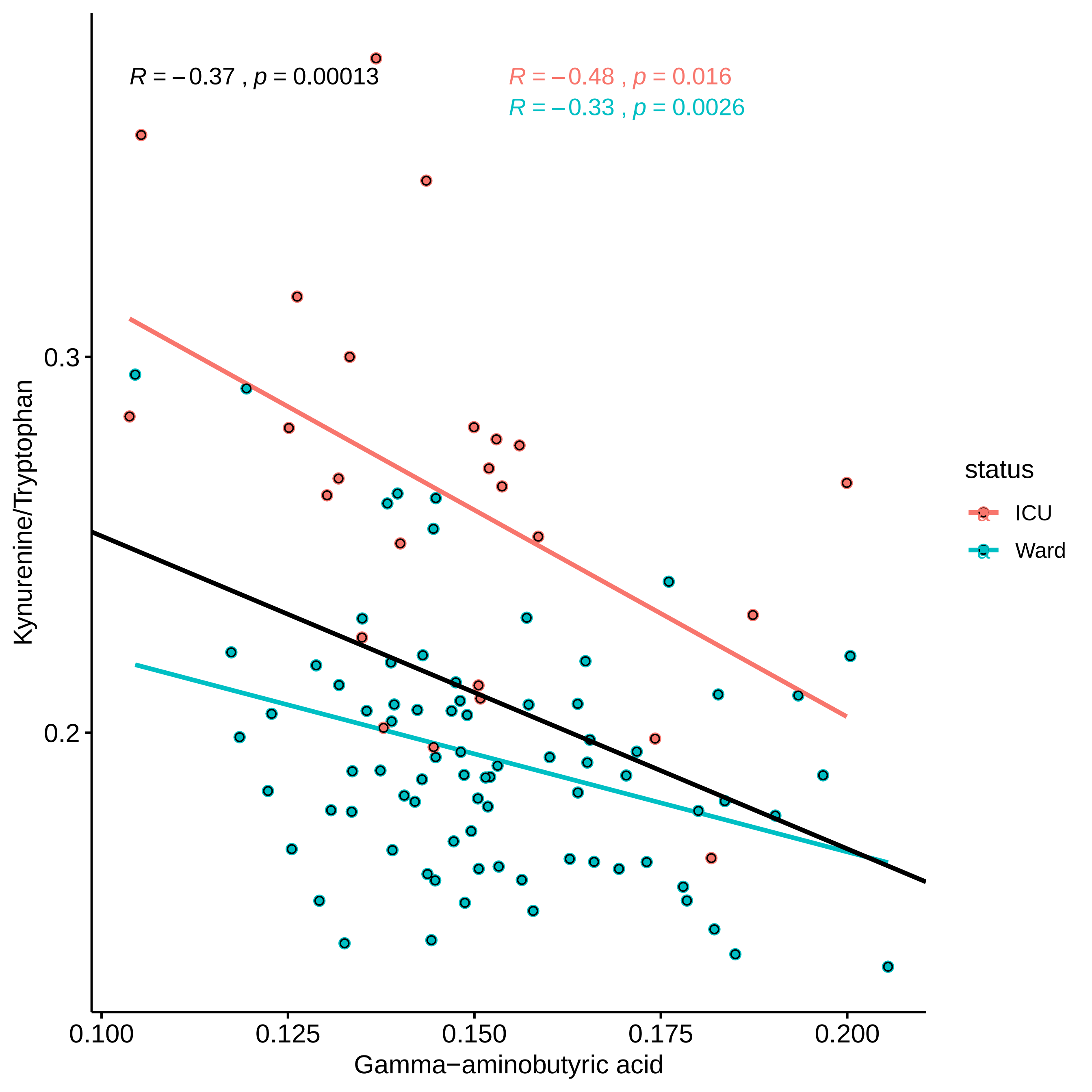

R = −0.37 , p = 0.00013
R = −0.48 , p = 0.016
R = −0.33 , p = 0.0026
0.3
0.2
Kynurenine/Tryptophan
0.100
0.125
0.150
0.175
0.200
Gamma−aminobutyric acid
status
ICU
Ward

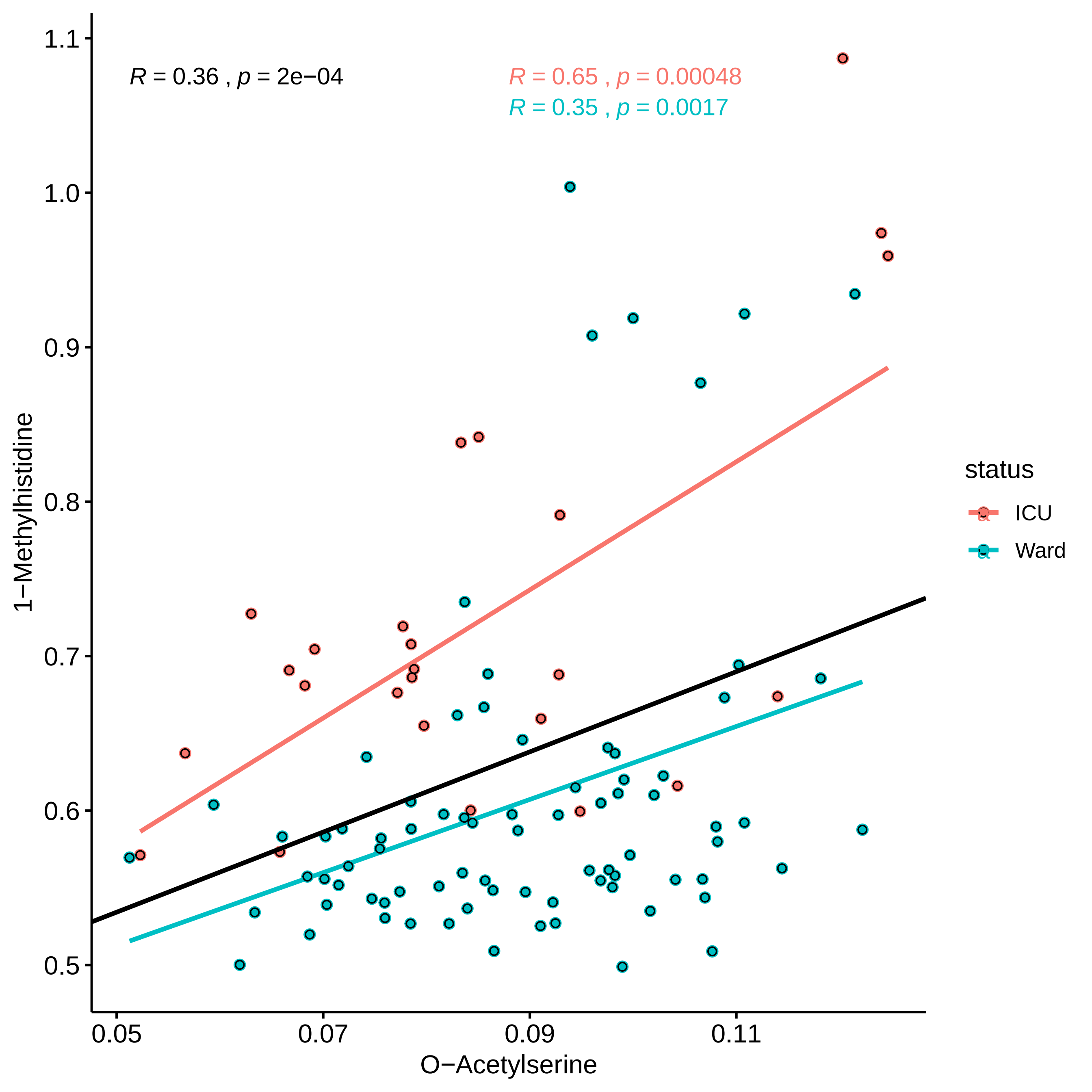

R = 0.36 , p = 2e−04
R = 0.65 , p = 0.00048
R = 0.35 , p = 0.0017
1.1
1.0
0.9
0.8
0.7
0.6
0.5
0.05
0.07
0.09
0.11
O−Acetylserine
1−Methylhistidine
status
ICU
Ward

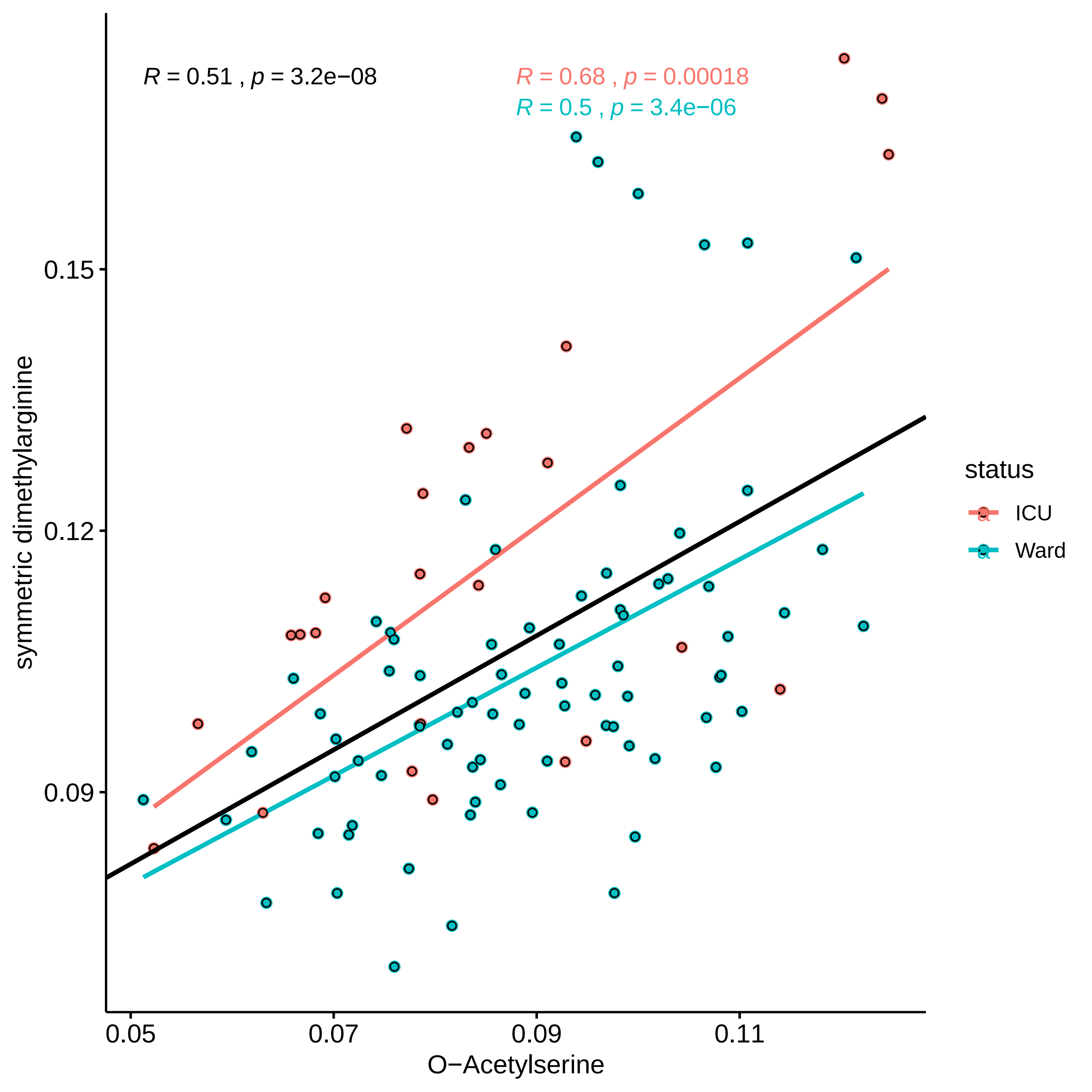

R = 0.51 , p = 3.2e−08
R = 0.68 , p = 0.00018
R = 0.5 , p = 3.4e−06
0.15
0.12
0.09
symmetric dimethylarginine
0.05
0.07
0.09
0.11
O−Acetylserine
status
ICU
Ward

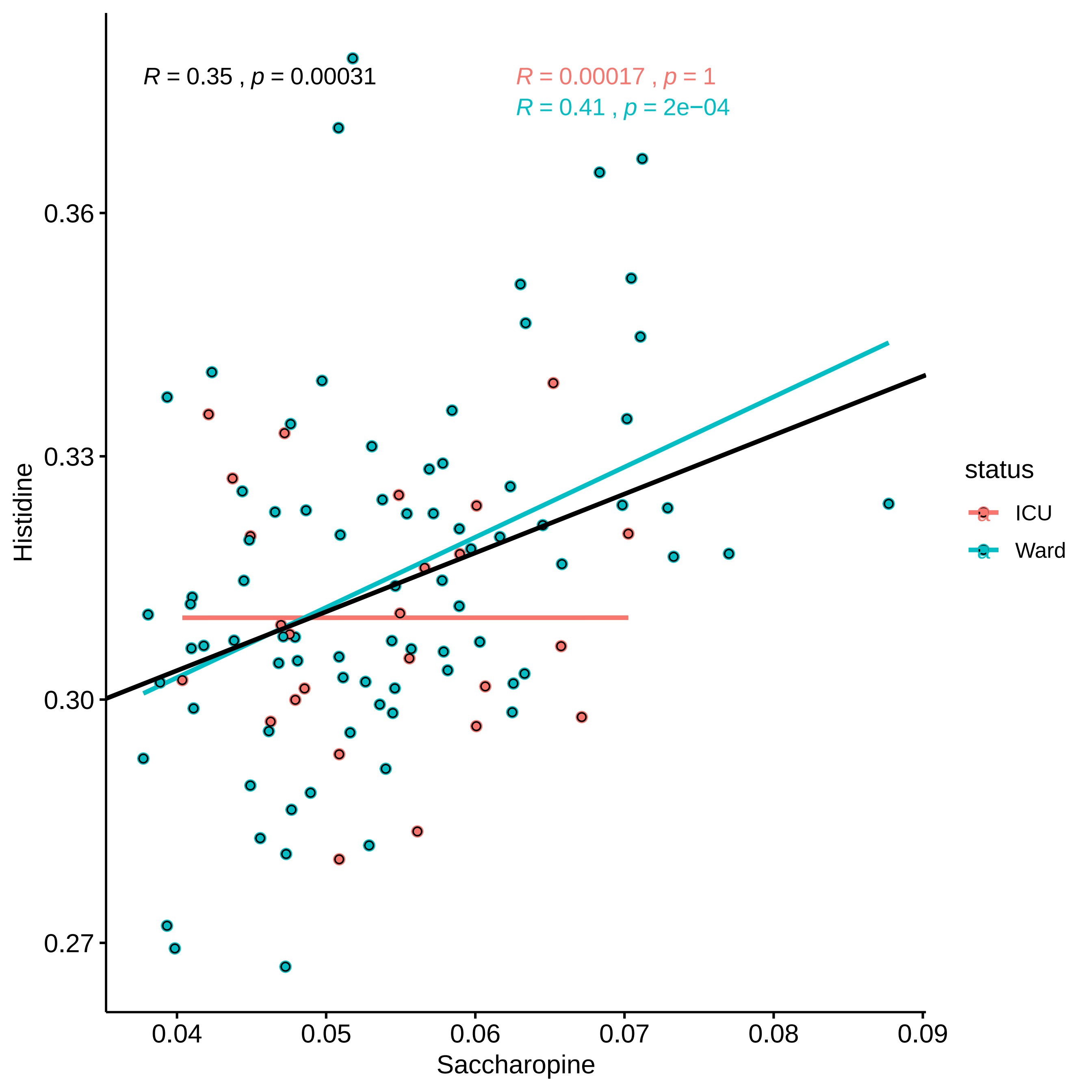

R = 0.35 , p = 0.00031
R = 0.00017 , p = 1
R = 0.41 , p = 2e−04
0.36
0.33
0.30
0.27
Histidine
0.04
0.05
0.06
0.07
0.08
0.09
Saccharopine
status
ICU
Ward

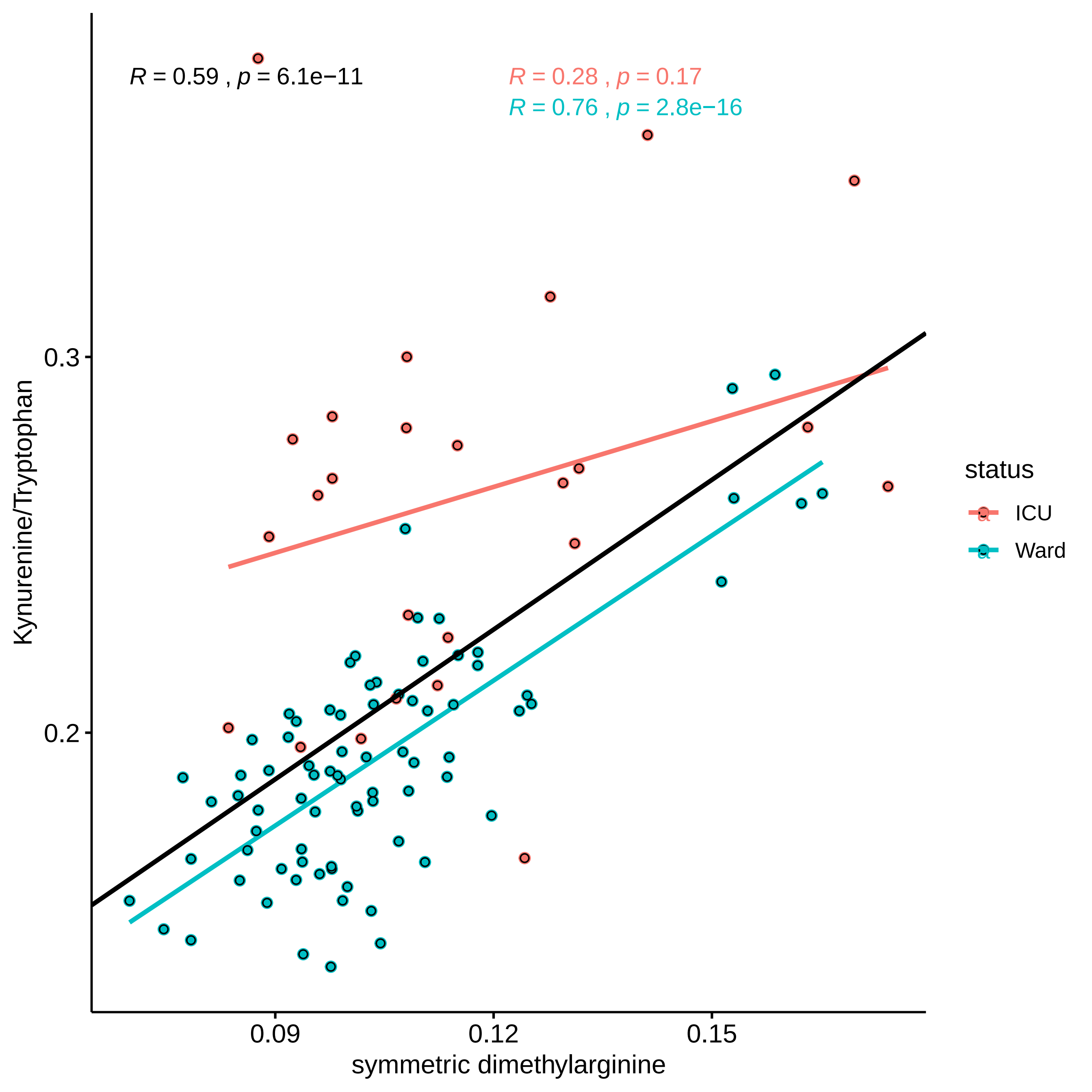

R = 0.59 , p = 6.1e−11
R = 0.28 , p = 0.17
R = 0.76 , p = 2.8e−16
Kynurenine/Tryptophan
0.3
0.2
0.09
0.12
0.15
symmetric dimethylarginine
status
ICU
Ward

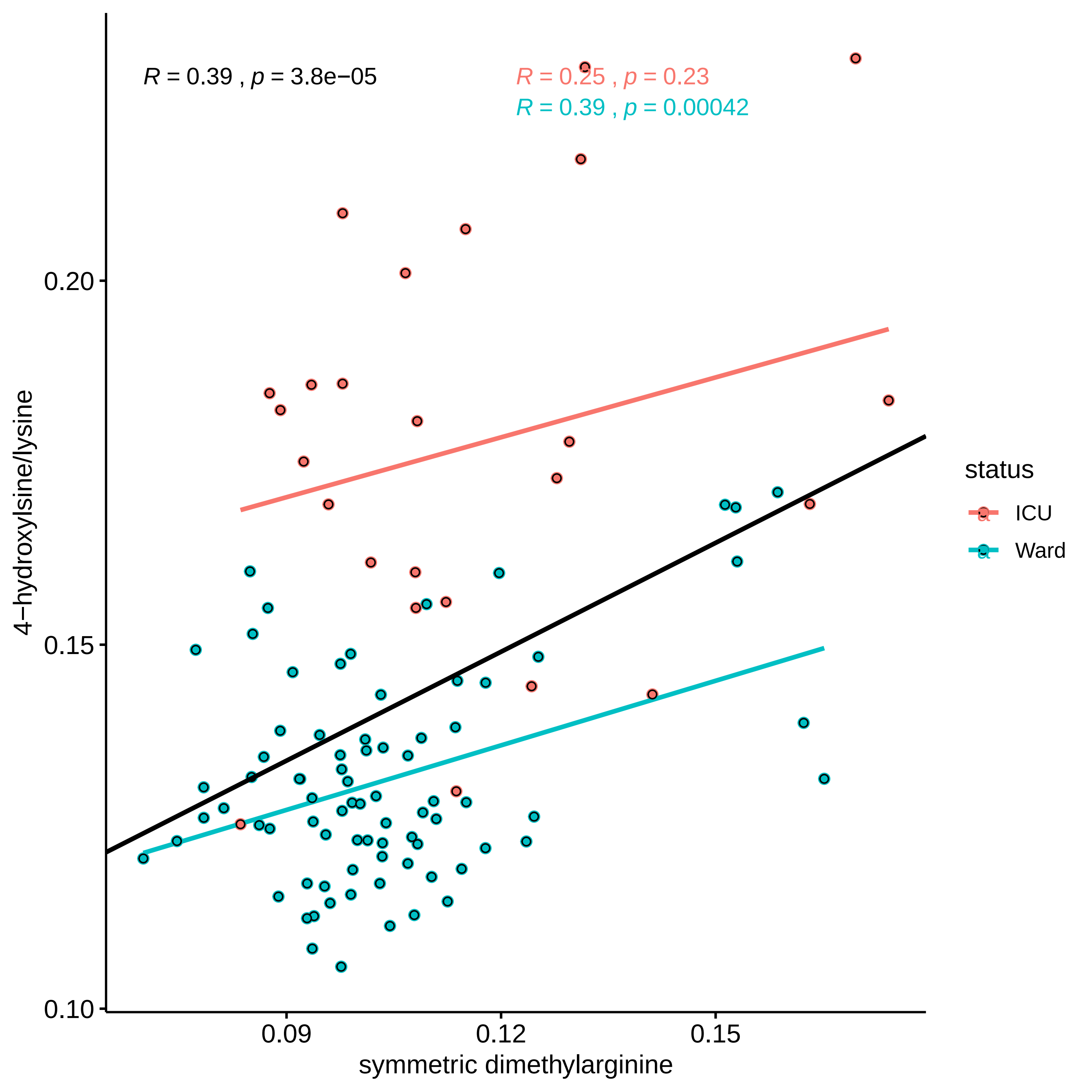

R = 0.39 , p = 3.8e−05
R = 0.25 , p = 0.23
R = 0.39 , p = 0.00042
4−hydroxylsine/lysine
0.20
0.15
0.10
0.09
0.12
0.15
symmetric dimethylarginine
status
ICU
Ward

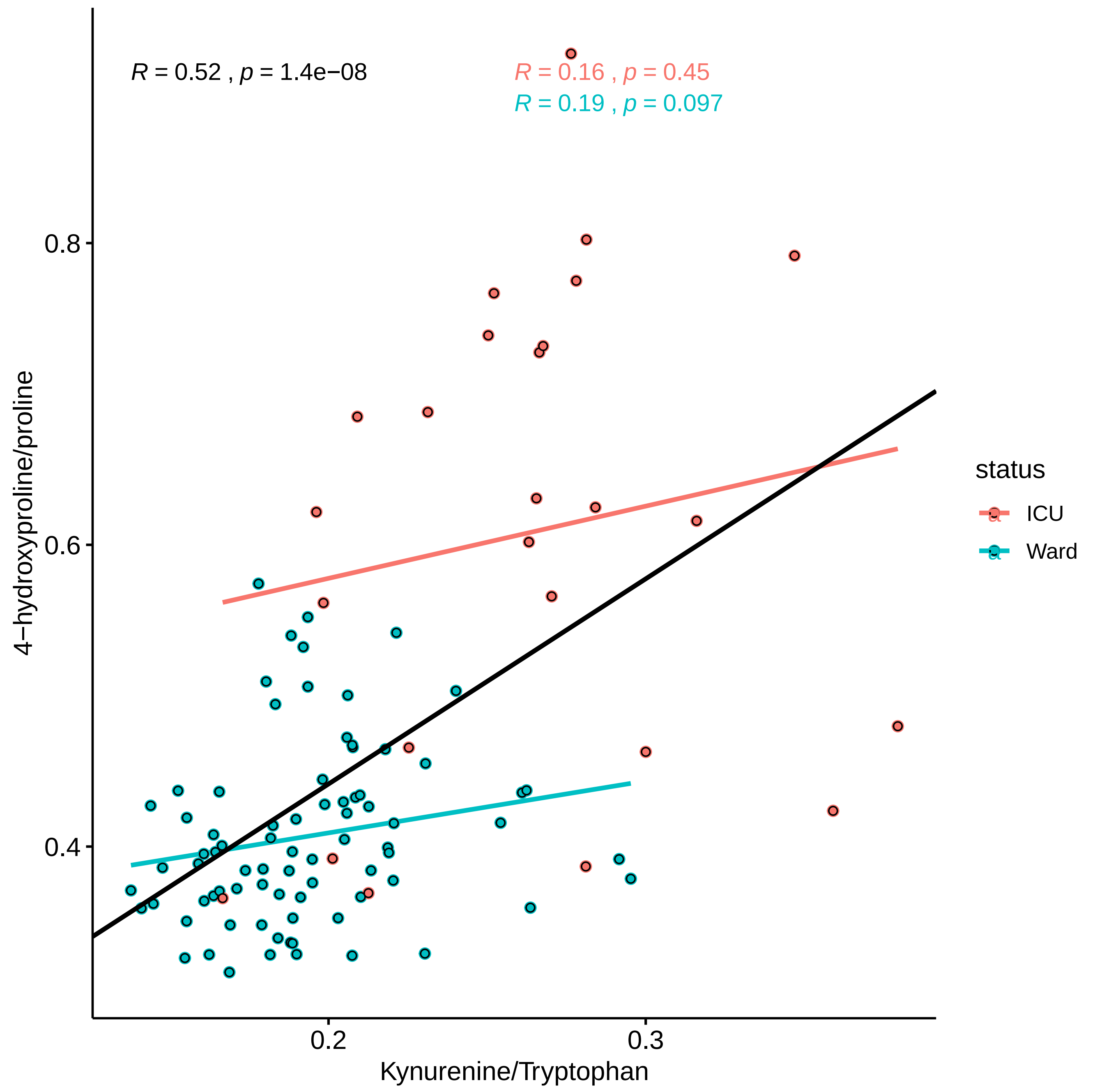

R = 0.52 , p = 1.4e−08
R = 0.16 , p = 0.45
R = 0.19 , p = 0.097
0.8
0.6
0.4
4−hydroxyproline/proline
0.2
0.3
Kynurenine/Tryptophan
status
ICU
Ward

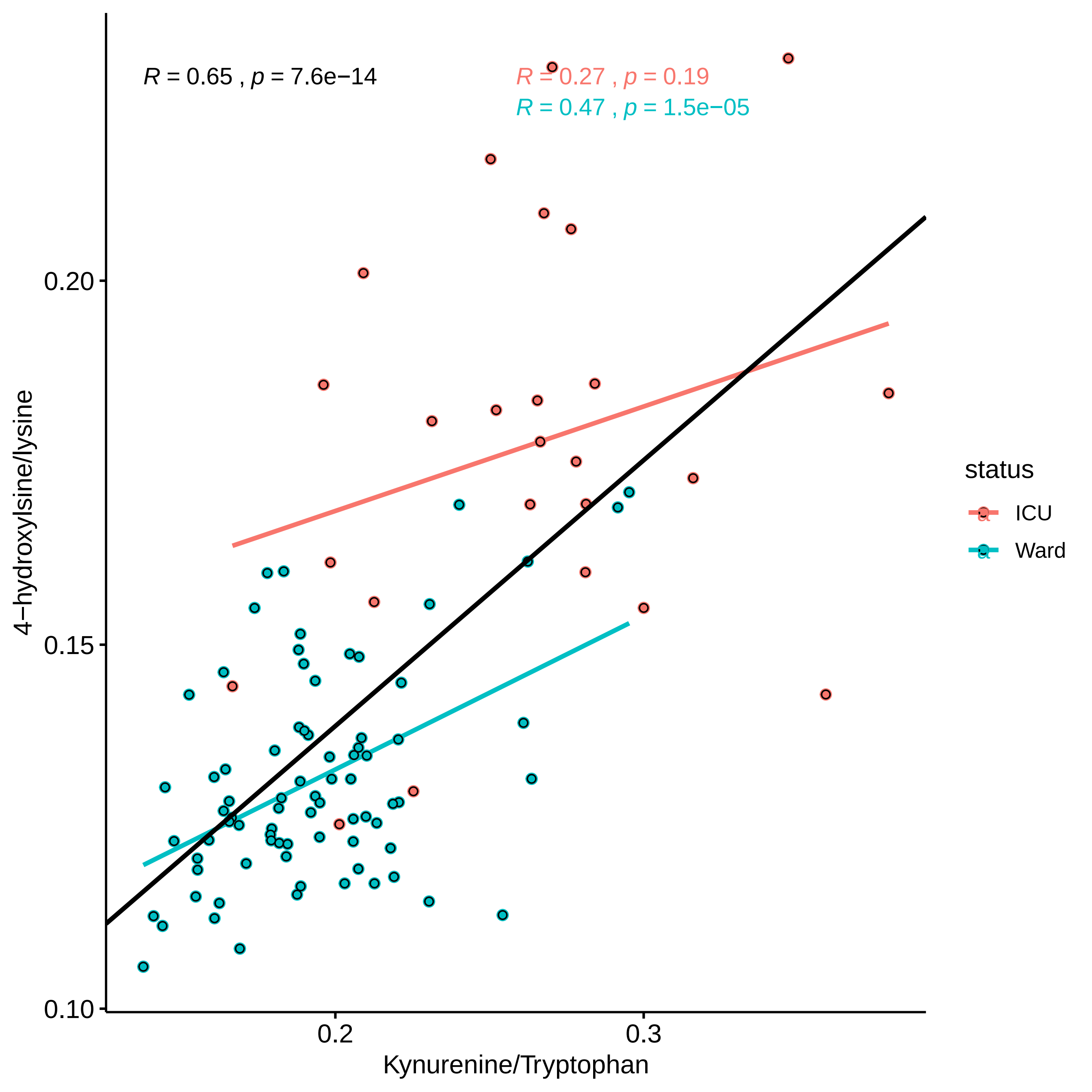

R = 0.65 , p = 7.6e−14
R = 0.27 , p = 0.19
R = 0.47 , p = 1.5e−05
4−hydroxylsine/lysine
0.20
0.15
0.10
0.2
0.3
Kynurenine/Tryptophan
status
ICU
Ward

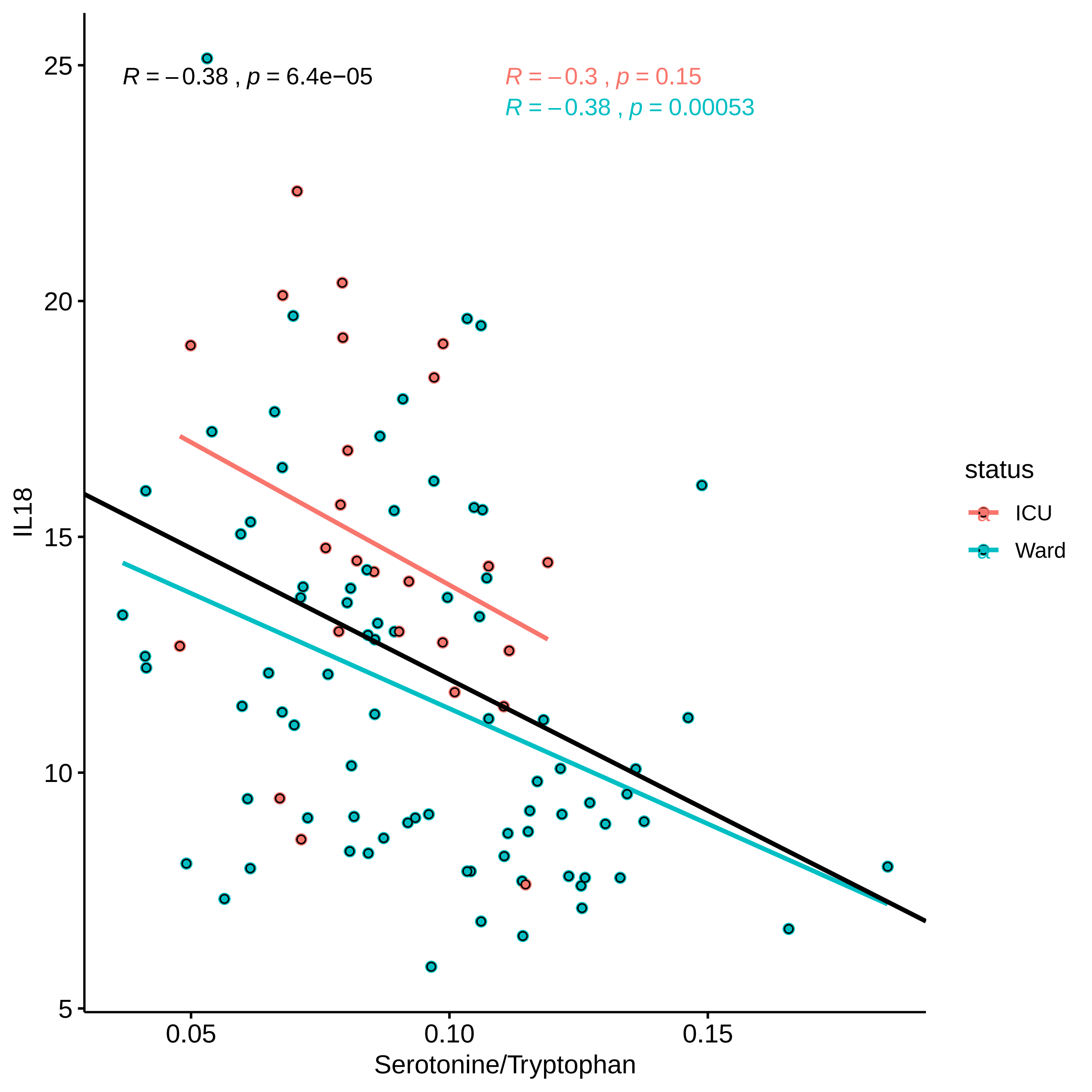

R = −0.38 , p = 6.4e−05
R = −0.3 , p = 0.15
R = −0.38 , p = 0.00053
IL18
25
20
15
10
5
0.05
0.10
0.15
Serotonine/Tryptophan
status
ICU
Ward

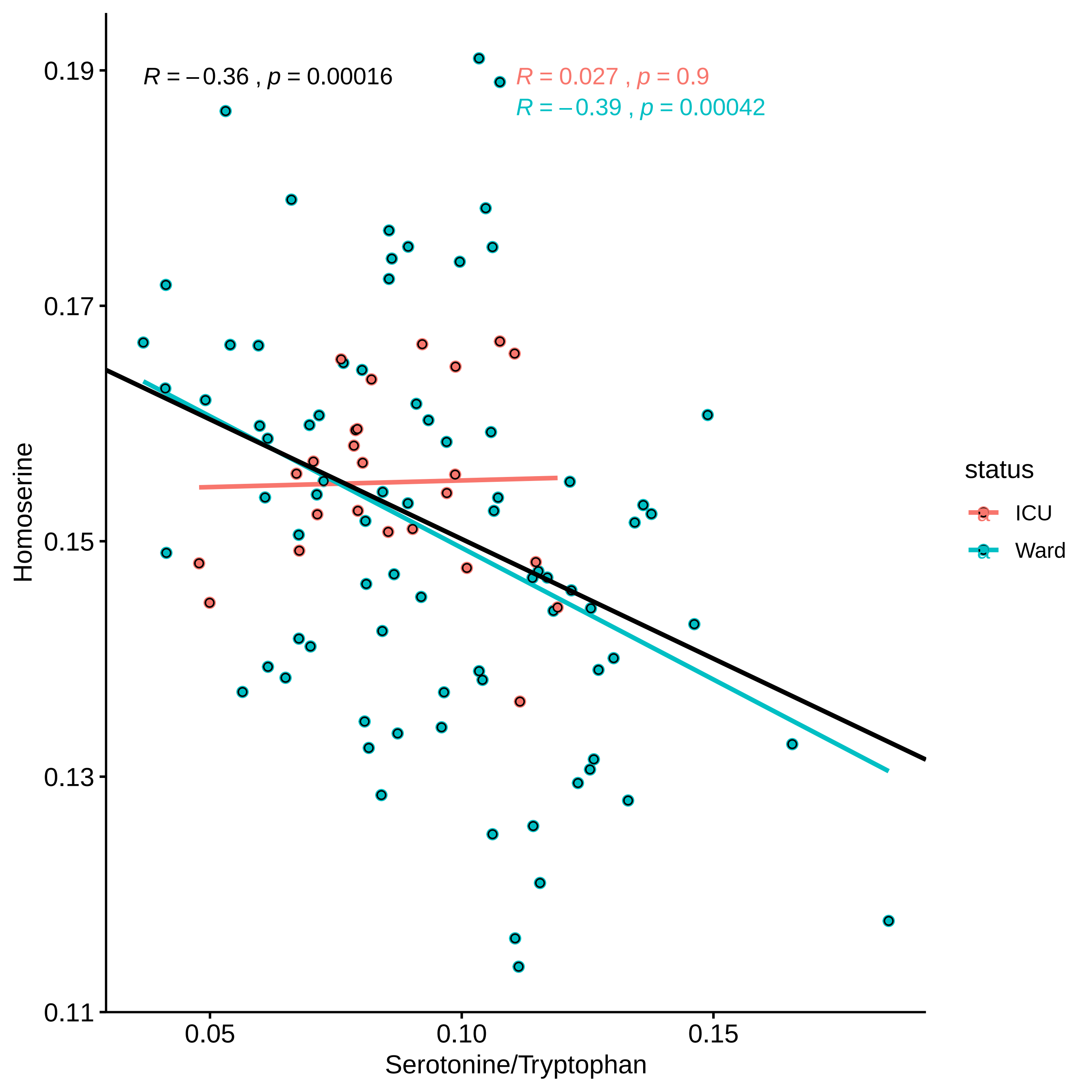

R = −0.36 , p = 0.00016
R = 0.027 , p = 0.9
R = −0.39 , p = 0.00042
0.19
0.17
0.15
0.13
0.11
Homoserine
0.05
0.10
0.15
Serotonine/Tryptophan
status
ICU
Ward

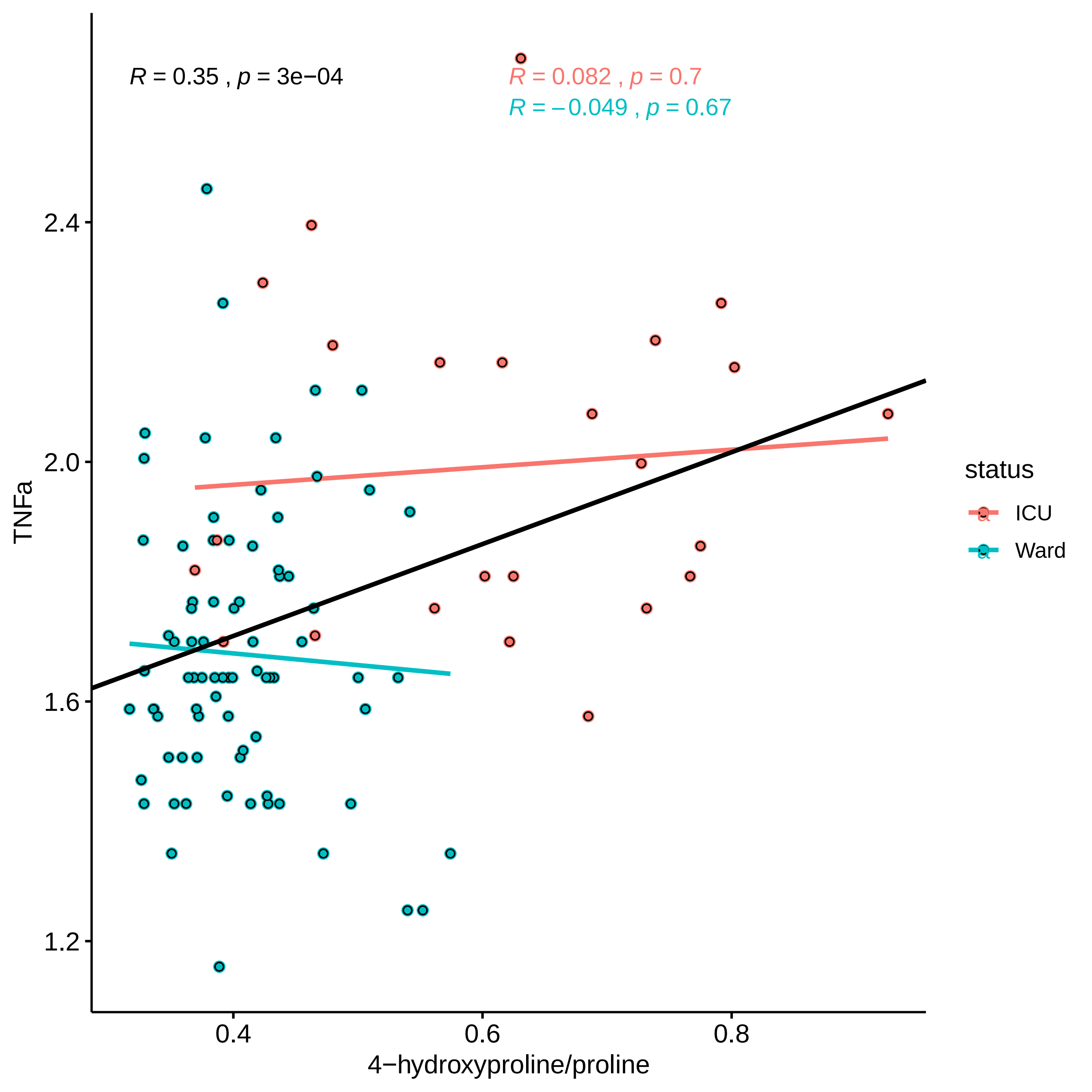

R = 0.35 , p = 3e−04
R = 0.082 , p = 0.7
R = −0.049 , p = 0.67
TNFa
2.4
2.0
1.6
1.2
0.4
0.6
0.8
4−hydroxyproline/proline
status
ICU
Ward

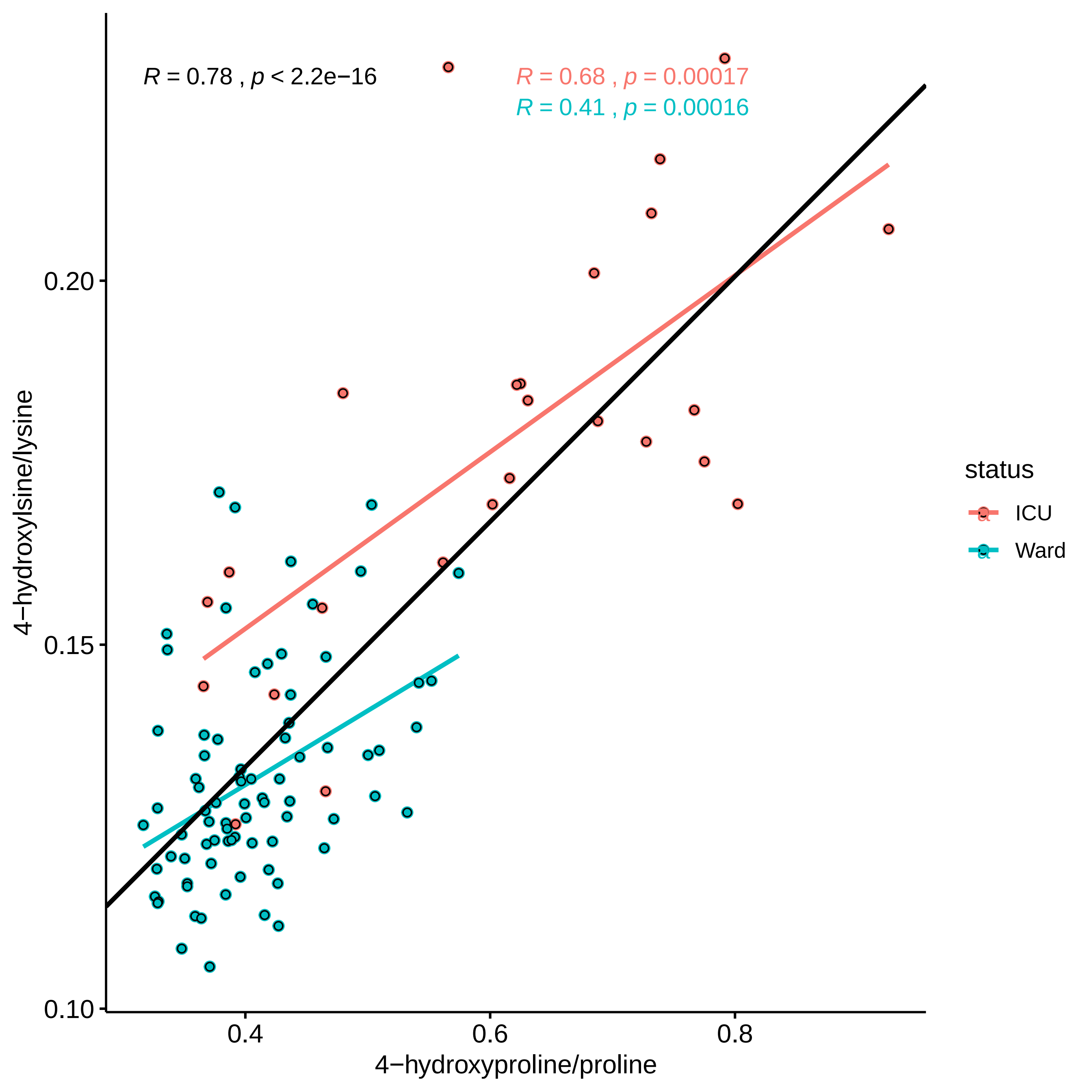

R = 0.78 , p < 2.2e−16
R = 0.68 , p = 0.00017
R = 0.41 , p = 0.00016
0.20
0.15
0.10
0.4
0.6
0.8
4−hydroxylsine/lysine
4−hydroxyproline/proline
status
ICU
Ward
